# The RNA-Binding Protein hnRNP K Mediates the Effect of BDNF on Dendritic mRNA Metabolism and Regulates Synaptic NMDA Receptors in Hippocampal Neurons

**DOI:** 10.1523/ENEURO.0268-17.2017

**Published:** 2017-12-12

**Authors:** Graciano Leal, Diogo Comprido, Pasqualino de Luca, Eduardo Morais, Luís Rodrigues, Miranda Mele, Ana R. Santos, Rui O. Costa, Maria Joana Pinto, Sudarshan Patil, Birgitte Berentsen, Pedro Afonso, Laura Carreto, Ka Wan Li, Paulo Pinheiro, Ramiro D. Almeida, Manuel A. S. Santos, Clive R. Bramham, Carlos B. Duarte

**Affiliations:** 1 CNC-Center for Neuroscience and Cell Biology, University of Coimbra, 3004-504 Coimbra, Portugal; 2 Institute for Interdisciplinary Research, University of Coimbra, 3030-789 Coimbra, Portugal; 3 Department of Biomedicine and KG Jebsen Centre for Neuropsychiatric Disorders, University of Bergen, 5020 Bergen, Norway; 4 Department of Medical Sciences and Institute of Biomedicine - iBiMED, University of Aveiro, 3810-193 Aveiro, Portugal; 5 Center for Neurogenomics and Cognitive Research, Amsterdam Neuroscience, Vrije Universiteit, 1081 Amsterdam, The Netherlands; 6 Department of Life Sciences, University of Coimbra, 3000-456 Coimbra, Portugal

**Keywords:** BDNF, local translation, long-term synaptic potentiation, neurotrophins, NMDA receptors, RNA transport

## Abstract

Brain-derived neurotrophic factor (BDNF) is an important mediator of long-term synaptic potentiation (LTP) in the hippocampus. The local effects of BDNF depend on the activation of translation activity, which requires the delivery of transcripts to the synapse. In this work, we found that neuronal activity regulates the dendritic localization of the RNA-binding protein heterogeneous nuclear ribonucleoprotein K (hnRNP K) in cultured rat hippocampal neurons by stimulating BDNF-Trk signaling. Microarray experiments identified a large number of transcripts that are coimmunoprecipitated with hnRNP K, and about 60% of these transcripts are dissociated from the protein upon stimulation of rat hippocampal neurons with BDNF. *In vivo* studies also showed a role for TrkB signaling in the dissociation of transcripts from hnRNP K upon high-frequency stimulation (HFS) of medial perforant path-granule cell synapses of male rat dentate gyrus (DG). Furthermore, treatment of rat hippocampal synaptoneurosomes with BDNF decreased the coimmunoprecipitation of hnRNP K with mRNAs coding for glutamate receptor subunits, Ca^2+^- and calmodulin-dependent protein kinase IIβ (CaMKIIβ) and BDNF. Downregulation of hnRNP K impaired the BDNF-induced enhancement of NMDA receptor (NMDAR)-mediated mEPSC, and similar results were obtained upon inhibition of protein synthesis with cycloheximide. The results demonstrate that BDNF regulates specific populations of hnRNP-associated mRNAs in neuronal dendrites and suggests an important role of hnRNP K in BDNF-dependent forms of synaptic plasticity.

## Significance Statement

Brain-derived neurotrophic factor (BDNF) is an important mediator of long-term synaptic potentiation (LTP) in the hippocampus, which is thought to underlie learning and memory formation. In this work we report the role of heterogeneous nuclear ribonucleoprotein K (hnRNP K) as a novel mediator of the effects of BDNF on RNA metabolism in the dendritic compartment of hippocampal neurons. We found that at excitatory synapses BDNF reduces the interaction of hnRNP K with transcripts coding for synaptic proteins, including glutamate receptor subunits. This is likely to play an important role in synaptic plasticity mechanisms since hnRNP K was found to mediate the BDNF-induced enhancement of the activity of synaptic NMDA receptors (NMDARs), an effect that is dependent on protein synthesis.

## Introduction

The neurotrophin brain-derived neurotrophic factor (BDNF) plays an important role on long-term synaptic potentiation (LTP) induced by high-frequency stimulation (HFS) of hippocampal Schaffer collateral-CA1 synapses (Korte et al., 1995, 1996; Kang et al., 1997; Minichiello et al., 1999) and at medial perforant path-granule cell synapses of the dentate gyrus (DG; Panja et al., 2014). Activation of BDNF-TrkB signaling also has a facilitatory effect on CA1 synapses, through stimulation of the protein synthesis machinery (Kang and Schuman, 1996; Schratt et al., 2004; Takei et al., 2004; Leal et al., 2014a; Panja and Bramham, 2014), and translation activity is required for the induction and consolidation of LTP following infusion of BDNF into the DG of anesthetized rats (Messaoudi et al., 2007; Panja et al., 2014). The BDNF-induced synthesis of proteins at the synapse relies on the local availability of the translation machinery (Steward and Levy, 1982), and on the presence of transcripts that are transported by motor proteins which travel along the microtubule tracks present in dendrites (Kanai et al., 2004; Santos et al., 2010; Leal et al., 2014a). The transport of RNAs along dendrites is made in large structures, the RNA granules, where the transcripts interact with specific proteins that stabilize them (Kanai et al., 2004; Elvira et al., 2006; Kosik, 2016). Studies performed in cultured hippocampal neurons showed that BDNF induces the dissociation of P-bodies, a class of RNA granules that may participate in the translational control of dendritically localized mRNAs, which may allow translation activity (Zeitelhofer et al., 2008). However, the identity of the extracellular signals and the downstream mechanisms that regulate RNA metabolism in dendrites are poorly understood.

The RNA-binding protein heterogeneous nuclear ribonucleoprotein K (hnRNP K) was identified as a component of RNA transport granules in neurons (Elvira et al., 2006), and was detected in synaptoneurosomal fractions (Liao et al., 2007) as well at the postsynaptic densities in hippocampal neurons (Proepper et al., 2011; Folci et al., 2014). hnRNP K has a modular structure with three K homology (KH) domains that interact with RNA and ssDNA, and a K interactive region (KI), which recruits a wide variety of factors, including kinases and regulators of splicing, mRNA stability and translation (Bomsztyk et al., 2004; Geuens et al., 2016). Given the presence of multiple domains for interaction with other molecules, it was proposed that hnRNP K may act as a docking platform to integrate signaling cascades by promoting the cross-talk between kinases and molecules involved in nucleic acid metabolism (Bomsztyk et al., 2004). At the postsynaptic density of hippocampal neurons hnRNP K interacts with Abelson-interacting protein 1 (Abi 1), a protein that plays an important role in the regulation of cytoskeleton reorganization and synaptic maturation (Courtney et al., 2000). Accordingly, hnRNP K was recently found to regulate the dendritic spine density in cultured hippocampal neurons (Folci et al., 2014). However, the role played by hnRNP K in the regulation of translation activity at the synapse remains to be characterized.

In this work we report a previously undescribed role of hnRNP K as a mediator of the effects of BDNF in RNA metabolism in the dendritic compartment of hippocampal neurons. We found that neuronal activity induces the punctate accumulation of hnRNP K in dendrites by a mechanism dependent of BDNF. At the synapse, BDNF reduces the interaction of hnRNP K with transcripts coding for synaptic proteins, including glutamate receptor subunits. This may be relevant for synaptic plasticity mechanisms since hnRNP K was found to play an important role in BDNF-induced enhancement of the activity of synaptic NMDA receptors (NMDARs).

## Materials and Methods

### Hippocampal cultures

High-density hippocampal cultures were prepared from the hippocampi of embryonic day E18–E19 Wistar rat embryos, after treatment with trypsin (0.06%; 15-min incubation at 37°C; Gibco, Life Technologies) and DNase I (5.36 mg/ml) in Ca^2+^- and Mg^2+^-free HBSS (5.36 mМ KCl, 0.44 mM KH_2_PO_4_, 137 mM NaCl, 4.16 mM NaHCO_3_, 0.34 mM Na_2_HPO_4_·2H_2_O, 5 mM glucose, 1 mM sodium pyruvate, 10 mM HEPES, and 0.001% phenol red). The hippocampi were then washed with HBSS containing 10% fetal bovine serum (Gibco, Life Technologies), to stop trypsin activity, and transferred to Neurobasal medium (Gibco, Life Technologies) supplemented with SM1 supplement (1:50 dilution, STEMCELL Technologies), 25 µM glutamate, 0.5 mM glutamine, and 0.12 mg/ml gentamycin (Gibco, Life Technologies). The cells were dissociated in this solution and then plated in six-well plates (90 × 10^3^ cells/cm^2^) coated with poly-D-lysine (0.1 mg/ml) for biochemical purposes (Western blotting and RNA coimmunoprecipitation), or on poly-D-lysine-coated coverslips (80 × 10^3^ cells/cm^2^) for the analysis of NMDAR-mediated miniature EPSCs (mEPSCs). Cultures were maintained in a humidified incubator of 5% CO_2_/95% air at 37°C for 14–17 d and then stimulated with 50 ng/ml BDNF (Peprotech) for the indicated periods of time.

Low-density hippocampal cultures were prepared as previously described (Kaech and Banker, 2006). Briefly, hippocampi were dissected from E18 rat embryos, and the cells were dissociated using trypsin (0.02%) before plating in neuronal plating medium (MEM supplemented with 10% horse serum, 0.6% glucose, and 1 mM pyruvic acid), at a final density of 1.43 × 10^4^ cells/cm^2^ on poly-D-lysine-coated glass coverslips. After 2–4 h, coverslips were flipped over an astroglial feeder layer in Neurobasal medium (Invitrogen) supplemented with SM1 supplement (1:50 dilution, STEMCELL Technologies), 25 μM glutamate, 0.5 mM glutamine, and 0.12 mg/ml gentamycin (Gibco, Life Technologies). The neurons grew face down over the feeder layer but were kept separate from the glia by wax dots on the neuronal side of the coverslips. To prevent overgrowth of glial cells, neuron cultures were treated with 5 μM cytosine arabinoside (Sigma-Aldrich) after 3 days in vitro (DIV). Cultures were maintained in a humidified incubator with 5% CO_2_/95% air, at 37°C, for up to two weeks, feeding the cells once per week. At DIV14–DIV15, neurons were stimulated for 30 min with 100 ng/ml BDNF (Peprotech) or with 50 µM bicuculline (Tocris), 2.5 mM 4-AP (Tocris), and 10 µM glycine (Sigma-Aldrich) to stimulate glutamate release and to increase synaptic activity. Where indicated, cells were pretreated for 30 min with the Trk receptor inhibitor SHN722 (1 μM; Martin et al., 2011; Gomes et al., 2012) or with the scavenger of extracellular ligands of TrkB receptors TrkB-Fc (1 μg/ml; R&D Systems) before stimulation with 100 ng/ml BDNF (Peprotech) or with the cocktail solution containing bicuculline (50 µM bicuculline, 2.5 mM 4-AP, and 10 µM glycine), respectively. Experiments were performed in a basal saline solution (132 mM NaCl, 4 mM KCl, 1.4 mM MgCl_2,_ 2.5 mM CaCl_2_, 6 mM glucose, and 10 mM HEPES at a final pH 7.4). Cells were then processed for immunocytochemistry.

### Immunocytochemistry

Hippocampal neurons were fixed in 4% sucrose/paraformaldehyde (in PBS) for 15 min at room temperature and permeabilized with 0.3% Triton X-100 in PBS. Neurons were then incubated with 10% BSA in PBS, for 30 min at 37°C, to block nonspecific staining, and incubated overnight at 4°C with the primary antibodies diluted in 3% BSA in PBS. The following primary antibodies and dilutions were used: anti-hnRNP K (sc-28380, 1:200; Santa Cruz Biotechnology), anti-GFP (598, 1:500, MBL International), and anti-MAP2 (ab5392, 1:10.000, Abcam). The cells were washed six times with PBS for 2 min and incubated with Alexa Fluor 568 (1:500, Invitrogen), Alexa Fluor 488 (1:500; Invitrogen), and aminomethylcoumarin (AMCA) (1:200; Jackson ImmunoResearch) conjugated secondary antibodies, for 45 min at 37°C. After washing the cells six times with PBS for 2 min, the coverslips were mounted with a fluorescence mounting medium (DAKO).

### Microscopy and quantitative fluorescence analysis

Imaging was performed on a Zeiss Observer Z.1 microscope using a 63 × 1.4 NA oil objective. Images were quantified using the ImageJ image analysis software. For quantitation, sets of cells were cultured and stained simultaneously, and imaged using identical settings. The protein signals were analyzed after setting the thresholds, and the recognizable clusters under those conditions were included in the analysis. Regions of interest (ROI) were drawn in secondary dendrites with no segmentation performed. The integrated intensity, area, and number of hnRNP K particles was determined only within the ROI and represented per dendritic area (as assessed by MAP2 staining). All analyses were performed blind to the experimental condition.

### Preparation of hippocampal culture extracts

Hippocampal cultures with 15 DIV (90 × 10^3^ cells/cm^2^) were washed twice with ice-cold PBS, and once more with PBS buffer supplemented with 1 mM dithiothreitol (DTT) and a cocktail of protease inhibitors [0.1 mM phenylmethylsulfonyl fluoride (PMSF) and CLAP (1 μg/ml chymostatin, 1 μg/ml leupeptin, 1 μg/ml antipain, and 1 μg/ml pepstatin; Sigma)]. The cells were then lysed with RIPA (150 mM NaCl, 50 mM Tris-HCl, pH 7.4, 5 mM EGTA, 1% Triton, 0.5% DOC, and 0.1% SDS at a final pH 7.5) supplemented with 50 mM sodium fluoride (NaF), 1.5 mM sodium ortovanadate (Na_3_VO_4_), and the cocktail of protease inhibitors. After sonication and centrifugation at 16,100 × *g* for 10 min at 4°C, protein in the supernatants was quantified using the bicinchoninic acid (BCA) assay kit (Pierce). Samples were then denaturated with 2× concentrated denaturating buffer (125 mM Tris, pH 6.8, 100 mM glycine, 4% SDS, 200 mM DTT, 40% glycerol, 3 mM Na_3_VO_4_, and 0.01% bromophenol blue) for 5 min at 95°C, and proteins of interest were analyzed by Western blotting. Alternatively, extracts were performed in a lysis buffer supplemented with 50 U/ml of RNase inhibitor (SUPERaseIn, Ambion Applied Biosystems) and samples processed for RNA coimmunoprecipitation experiments.

### Synaptoneurosome preparation

Synaptoneurosomes were prepared as previously described with slight modifications (Yin et al., 2002). Briefly, six to eight hippocampi were dissected from adult male and female Wistar rats, and the tissue was minced with scissors and homogenized with a glass homogenizer in a buffer containing 0.32 M sucrose, 10 mM HEPES-Tris, pH 7.4, and 0.1 mM EGTA. After centrifugation for 3 min at 1000 × *g*, the supernatant was collected and passed initially through nylon membranes (150 and 50 µm, VWR) and finally through an 8-µm pore size filter (Millipore). The flow-through was centrifuged for 15 min at 10,000 × *g*, and the pellet was resuspended in incubation buffer (8 mM KCl, 3 mM CaCl_2_, 5 mM Na_2_HPO_4_, 2 mM MgCl_2_, 33 mM Tris, 72 mM NaCl, and 100 mM sucrose). All the procedure was done at 4°C. Synaptoneurosomes were incubated or not with 50 ng/ml BDNF (Peprotech) or 20 ng/ml PDGF (Peprotech) for 10 min at 30°C and were then centrifuged at maximum speed, in a Minispin microcentrifuge for 30 s. For each time point considered a control experiment was also performed in the absence of the neurotrophic factors. The pellet was resuspended in RIPA buffer supplemented as indicated for the hippocampal culture extract preparation, followed by sonication and protein quantification using the BCA method. Proteins of interest were analyzed by Western blotting. For RNA coimmunoprecipitation experiments, RIPA buffer was supplemented with 50 U/ml of the RNase inhibitor SUPERase.In (Ambion Applied Biosystems).

### Lactate dehydrogenase (LDH) activity

Synaptoneurosomes were centrifuged at top speed for 30 s to separate the pellet containing the synaptic fraction and the “extracellular” fraction, and the pellet was lysed in 15 mM Tris-HCl, pH 7.1 (*t* = 0 min). Alternatively, synaptoneurosomes were maintained in incubation buffer for 45 min at 30°C and processed in the same way. The concentration of protein in the synaptoneurosomes extract was quantified using the Bio-Rad method and 25 μg of total protein were used to assay LDH activity. LDH activity was also measured in the extracellular fraction. The activity of the enzyme was measured at 340 nm in 100 mM Tris-HCl, pH 7.1, supplemented with 0.3 mM NADH (Sigma) and 10 mM pyruvate (Sigma). The absorbance of NAD^+^ was measured at 37°C during 5 min, with intervals of 50 s. A negative control was performed in the absence of pyruvate. LDH activity in each sample was calculated by subtracting the slope of the negative control. LDH activity for each fraction was calculated as the ratio to the total LDH activity.

### Western blotting

Samples were resolved by SDS-PAGE in 10% polyacrylamide gels. For Western blot analysis, proteins were transferred onto a PVDF membrane (Millipore) by electroblotting (40 V, overnight at 4°C). The membranes were blocked for 1 h with skin milk and 0.1% Tween 20 in TBS (20 mM Tris, 137 mM NaCl, pH 7.6; TBS-T), and probed with the primary antibody overnight at 4°C. Following several washes with TBS-T, the membranes were incubated with an alkaline phosphatase-conjugated IgG secondary antibody (anti-mouse or anti-rabbit, depending on the primary antibody host-species) for 1 h at room temperature. The membranes were then washed again and immunostaining was visualized by the enhanced chemifluorescence method (ECF) on a Storm 860 Gel and Blot Imaging System (GE Health Care). For the analysis of total extracts from DG homogenates, horseradish peroxidase (HRP)-conjugated secondary antibodies were used and immunostaining was developed using chemiluminescence reagents (ECL, GE Healthcare). In this case the blots were scanned using Gel DOC EQ (Bio-Rad). Antibodies used in Western blotting experiments were the following: anti-hnRNP K (sc-28380, 1:1000; Santa Cruz Biotechnology), anti-p-Akt S304 (1:1000; Cell Signaling), anti-pERK1/2 Thr202/Tyr204 (1:500; Promega), anti-HR3 (1:1000; Millipore), anti-PSD-95 (1:2000; Cell Signaling Technology), anti-vesicular GABA transporter (VGAT; 1:2000; Synaptic Systems), anti-GFAP (1:1000; Oncogene), and anti-synaptophysin (1:1000; Abcam). When indicated, anti-β-tubulin (T7816, 1:30,000; Sigma-Aldrich), anti-GAPDH (sc32233, 1:5000; Santa Cruz Biotechnology), or anti-β-actin (A5441, 1:5000; Sigma-Aldrich) antibodies were used as loading control.

### Immunoprecipitation and mRNA extraction

Antibody-immobilized beads were prepared by incubating 6 μg of hnRNP K or mouse IgG antibodies with 100 μl of Protein G PLUS-Agarose beads (Santa Cruz Biotechnology), overnight at 4°C in NT_2_ 2× buffer containing 100 mM Tris-HCl, 300 mM NaCl, 2 mM MgCl_2_, 0.1% IGEPAL, pH 7.4, and supplemented with 1 mM DTT and a cocktail of protease inhibitors [0.1 mM PMSF and CLAP (Sigma)]. The immobilized antibodies were incubated with 1 mg of protein for 1 h at 4°C, and the beads were washed four times (2-min centrifugations, 2000 × *g*) with NT_2_ 1x buffer at 4°C. The supernatant was discarded and the final pellet, containing the immunoprecipitated hnRNP bound to the antibody-immobilized beads, was used for further analysis. For RNA coimmuprecipitations, NT_2_ buffer was supplemented with 50 U/ml of RNase inhibitor (SUPERaseIn, Ambion Applied Biosystems), and the TRIzol Reagent (Invitrogen) was immediately added to the pellet and the RNA extracted according to manufacturer’s instructions.

For the RNA coimmunoprecipitations performed using total extracts from DG homogenates, the same procedure was performed with minor changes: 50 μl of Protein G Sepharose 4 Fast Flow (GE Health Care) beads and 500 μg of protein were used for the immunoprecipitations and the NT_2_ buffer was supplemented with cOmplete, Mini EDTA-free protease inhibitors cocktail (Roche) and 40 U/ml RiboLockRNase inhibitor (Thermo Fischer).

For the microarray analysis, RNA coimmunoprecipitations were performed with the following modifications: cellular extracts were prepared using the RiboCluster Profiler RIP-Assay kit (MBL International Corporation) supplemented with 50 mM NaF, 1:200 Protease Inhibitor Cocktail Set III (Calbiochem, Merck), 1 mM DTT, and 80 U of RNase inhibitor (SUPERaseIn, Ambion Applied Biosystems). Immunoprecipitated mRNAs were isolated using the RiboCluster Profiler RIP-Assay kit (MBL International Corporation).

In all cases, parallel experiments were performed in which the nonspecific binding of RNAs to Protein G PLUS-Agarose beads (or Protein G Sepharose 4 Fast Flow beads) was determined and the RNAs were resuspended in the same volume of RNase-free water. The RNA concentration was determined using NanoDrop (Thermo Scientific), and samples were stored at −80°C until further use.

### Gene expression microarray

The RNA isolated from the hnRNP/IgG immunoprecipitates was subjected to microarray analysis using the One-Color Microarray-Based Gene Expression Protocol v6.0. The Low Input Quick Amp Labeling (Agilent Technologies) protocol was used for the preparation and labeling of the biological targets, hybridization, washing, scanning, and data analysis, as recommended by the manufacturer. The Whole Rat Genome Microarray kit (4 × 44K, Agilent Technologies) was used and analyzed with a high-resolution microarray scanner (G2565AA, Agilent Technologies). hnRNP K-bound mRNAs were identified by setting a cutoff value of the fold variation between the hnRNP K and IgG samples. For this identification, we have used a cutoff value of 5, i.e., only transcripts showing at least 5-fold variation in their abundance in the hnRNP K immunoprecipitates when compared with the IgG controls were considered specifically associated with the RNP. Transcripts regulated by BDNF were identified after subtracting the results obtained in extracts incubated with mouse IgG, and then by comparing the hnRNP K immunoprecipitates microarray data obtained for control and BDNF-treated hippocampal neurons. Calculation of the fold variation in RNA-hnRNP K interaction induced by BDNF showed significant changes (*p* < 0.05) for 9509 transcripts.

The list of all mRNAs that coimmunoprecipitated with hnRNP K (16,015) and those that were significantly regulated by BDNF (9509) are provided in Extended Data Tables 1-1, 1-5, respectively. The PANTHER classification system (Mi et al., 2013a) was used to evaluate functional categories present and enriched in the list of mRNAs that associate with hnRNP K and are regulated by BDNF. The Gene Ontology (GO) analysis included the most enriched biological processes associated with transcripts bound to hnRNP K and those that were regulated by BDNF. Only categories showing at least a 2-fold increase, when comparing the number of mRNAs obtained in each category with the expected number considering the size of our lists, were considered for analysis. Categories were then ordered according to the highest -log (*p* value).

### Reverse transcription and quantitative PCR (qPCR)

For mRNA measurement, 500 ng to 1 μg of total RNA was reverse transcribed using a blend of oligo (dT) and random hexamer primers, and iScript Reverse Transcriptase (iScriptcDNA Synthesis kit, 170-8891; Bio-Rad). Primers for qRT-PCR were designed by Beacon Designer 7 software (Premier Biosoft International). The following considerations were taken: (1) GC content ∼50%; (2) annealing temperature (Ta) between 55 ± 5°C; (3) secondary structures and primer dimers were avoided; (4) primer length 18-24 bp; and (5) final product length 100-200 bp.

Primer sequences were as follows: *Gria1* (GluA1), FW-ACTACATCCTCGCCAATCTG; REV-AGTCACTTGTCCTCCATTGC; *Gria2* (GluA2), FW-TCTCTTCTAACAGCATACA; REV-AAACTGAACCATCCCTAC; *Grin1* (GluN1), FW-CGGCTCTTGGAAGATACAG; REV-GAGTGAAGTGGTCGTTGG; *Camk2b* (CaMKIIβ), FW-GCTATACGAGGATATTGG; REV-TCTTGGTGTTAATGATCT; *Hnrnpk* (hnRNP K), FW-AACACTCAGACAACAATCA; REV-TCCTCCAATAAGAACAACTC; *Hprt1* (Hprt), FW-CCTTGACTATAATGAGCACTTC; REV-GCCACATCAACAGGACTC; *Npas4* (NPAS4), FW-AATGGAGATATTCAGGCT; REV-TAGTTATTGGCAGTAATAGG; *Ntrk2* (TrkB), FW-GATCTTCACCTACGGCAAGC; REV-TCGCCAAGTTCTGAAGGAGT; and *Bdnf* (BDNF), FW-TAACCTCGCTCATTCATTA; REV-TCAACTCTCATCCACCTT. An additional set of primers was used for mRNA measurements from DG total homogenates: *bdnf* (BDNF), FW-TGGGACTCTGGAGAGCGTGAATGG; REV-CGGGACTTTCTCCAGGACTGTGAC.

qPCR was performed using SsoFast EvaGreen Supermix (172-5201; Bio-Rad). A total of 2 μl of 1:5 diluted cDNA (1:4 for microarray analysis) was used and the final concentration of each primer was 250 nM in a 20 μl final volume. The thermocycling reaction was initiated with activation of Taq DNA polymerase by heating at 95°C during 30 s, followed by 45 cycles of a 10s denaturation step at 95°C, a 30-s annealing step at the optimal primer temperature of annealing and a 30-s elongation step at 72°C. The fluorescence was measured after the extension step by the iQ5 Multicolor Real-Time PCR Detection System (Bio-Rad). After the thermocycling reaction, the melting step was performed with slow heating, starting at 55°C and with a rate of 0.5°C per 10 s, up to 95°C, with continuous measurement of fluorescence allowing detection of nonspecific products. To analyze the mRNA coimmunoprecipitated with hnRNP K from DG total homogenates, the qRT-PCR was performed in a final volume of 8 μl with 2× SYBR Green Master Mix (Bio-Rad), and using a LightCycler 480 (Roche). The reaction was initiated with a preamplification step of 3 min at 95°C, followed by 45 cycles of a 10-s denaturation step at 95°C, a 10-s annealing step at the optimal primer temperature of annealing, a 10-s elongation step at 72°C, and warming from 65°C to 95°C for the melting curve.

### qPCR data analysis

The comparative threshold cycle (Ct) method was used to quantitate the relative gene expression across the experimental conditions. The Ct represents the detectable fluorescence signal above background resulting from the accumulation of amplified product, and is a proportional measure of the starting target sequence concentration. Ct was measured on the exponential phase and, for every run, Ct was set at the same fluorescence value. Data analysis of the log-transformed expression data were performed using GenEx (MultiD Analysis) software for real-time PCR expression profiling.

### Animals and presurgical treatment

Animals used in the *in vivo* experiments were Sprague Dawley outbreed strain (M&B A/S) weighing 250-300 g at the time of use. Animals were housed in a temperature- and light-controlled vivarium (21 ± 1°C; 12/12 h light/dark artificial circadian rhythm) and supplied with a high protein diet type MR1 (Special Diet Services) and water for at least one week before surgery. Animals were retrieved from the animal facility in a separate cage into the laboratory where they were anesthetized with Urethane (250 mg/ml; 1.4-1.8 mg/kg) according to their individual weight. Urethane was administered via intraperitoneal injection. The first injection contained 1/3 of the total dosage and after 5 min the animal was weighed for accurate measurements, and the final 2/3 of the anesthetic was given.

### Stereotaxic surgery and electrode positioning

Male Sprague Dawley rats were positioned in a stereotaxic frame (David Kopfs Instruments) with the upper incisor bar 2 mm below the interaural line (skull flat position), the ear bars placed at the side of the head in the natural jaw sockets and a nose-and-tooth bar supported by the upper jaw of the animal. If required, supplemental doses of urethane were given to maintain a surgical level of anesthesia. Rectal temperature was maintained at 36°C with a thermostatically controlled electric heating pad. A scalpel was used to make a 1.5-cm longitudinal cut on the top of the animal’s head. Four bulldog clamps (FST) were used to reflect the skin giving open access to the scalp. The surface of the scalp was kept dry and free from blood. Burr holes were drilled in the appropriate location for the insertion of the stimulating and recording electrodes. A sharp needle was used to incise the dura to facilitate the penetration of the electrodes. Two holes were drilled anterior to bregma in the frontal bone to attach the ground and reference electrodes. The stimulation electrodes were bipolar, concentric, stainless steel, and with a vertical tip separation of 500 μm (SNEX 100; Rhodes Medical Instruments). The recording electrodes were Teflon-coated seven-strand stainless steel (or tungsten) wires (#7955/7960, A-M System Inc) of ∼8–10 cm in length. The ground and reference electrodes were Teflon-coated seven-strand stainless steel wires (#7925/7935, A-M System Inc). Stereotaxic coordinates for the unilateral stimulation of the medial perforant path fibers in the angular bundle were as follows (in mm, relative to bregma): 7.9 posterior and 4.2 lateral. Stereotaxic coordinates for recording in the hilar region of the DG were as follows (in mm, relative to bregma): 3.9 posterior and 2.2 lateral. When positioned on the accurate coordinates, both stimulation and recording electrodes were gradually lowered down to their final position. The final depth for the stimulation and recording electrodes was 1.8–2.4 and 3.3 mm below dura, respectively.

### *In vivo* electrophysiology and intrahippocampal infusion

After the correct positioning of the electrodes, the medial perforant path fibers in the angular bundle were unilaterally stimulated and the evoked field potentials (fEPSPs) recorded in the hilar region of the DG. After stabilization, baseline was recorded for 20 min. Test pulses were applied at 0.033 Hz throughout the experiment except during the period of HFS. The intensity of the stimulus for test pulses and for HFS was set to the intensity that evoked 1/3 of maximum population spike. The HFS paradigm to induce long-term potentiation (LTP) consisted of eight pulses of 400 Hz, repeated four times at 10-s intervals. Three sessions of HFS were given at intervals of five minutes. Intrahippocampal infusions were made using a stainless steel cannula system (Plastics One) consisting of an outer guide tube (24 gauge) and an inner infusion tube (31 gauge). The guide cannula was beveled sharp at the tip to facilitate brain insertion. The recording electrode was attached to the guide cannula and cut so that the distance between the electrode and the tip of the inner infusion cannula was 0.8–0.9 mm. The guide cannula–electrode assembly was slowly lowered until a positive-going field EPSP (fEPSP) of maximum slope was obtained in the dentate hilus. The infusion cannula was then inserted so that the tip protruded 300 μm below the end of the guide cannula. The infusion site was located 700 μm above the hilar recording site (corresponding to deep CA1 stratum lacunosum-moleculare), and 300–400 μm above the medial perforant path-granule cell synapses. The response was allowed to stabilize for 1 h. After baseline recordings (20 min) the infusions were performed with a pump that ensures the gradual infusion of 1 μl TrkB-Fc (100 μg/μl, 688-TK) or IgG-Fc (100 μg/μl,110-HG; R&D Systems) over a 12.5-min time period. HFS was performed 18 min after the infusion.

### Dissection and preparation of DG homogenates

At the end of the electrophysiological recording, the rats were decapitated and the brain rapidly removed and transferred to a glass plate covered with ice-cold saline-soaked filter paper. The DG was dissected under ice-cold conditions. After the hippocampus has been removed and placed ventral side up, the DG was gently rolled out and cut along the fissure separating it from CA3. The fimbria was also cut away and blood vessels removed. The DG and the hippocampal CA1 and CA3 regions were then stored in microtubes, and were instantly frozen in a mixture of 96% methanol and dry ice. Samples were kept at −80°C until further use. The dentate gyri were homogenized in 400 μl ice-cold RIPA buffer (150 mM NaCl, 50 mM Tris–HCl, pH 7.4, 5 mM EGTA, 1% Triton, 0.5% DOC, and 0.1% SDS at a final pH 7.5) containing 50 mM NaF and supplemented with with cOmplete, Mini EDTA-free protease inhibitors cocktail (Roche), and 40 U/ml RiboLock RNase inhibitor (Thermo Fischer). A fraction of the homogenate sample was set aside for Western blot analysis and the remaining homogenate used for the RNA coimmunoprecipitation experiments.

### Field potential analysis

The fEPSP was analyzed using the software Datawave Experimental Workbench (DataWave Systems). Between two points on the fEPSP, five points were randomly selected to calculate the steepness of the slope. Data files were coverted to ASCII format and further analyzed in the Microsoft Office Excel 2010 (Microsoft Corporation). fEPSP is presented as percentage change from baseline.

### hnRNP K knockdown in neuronal cultures

#### Constructs

TRIPΔU3-E1a-EGFP (pTRIP) lentiviral vectors (Sirven et al., 2001) were used to deliver double-stranded hairpin RNA sequences (shRNA) for hnRNP K knockdown in neuronal primary cultures. To obtain shRNA templates, the sense and antisense strands were designed to contain 19-22 nt duplex connected by a short loop-structure (5’-TTCAAGAGA-3’), and flanked by 5’-BglII and 3’-HindIII restriction site. The templates used were 5’-gatgaacgctctggatgcg-3’ for the nontargeting (control) shRNA template and 5’-gtaactattcccaaagatt-3’ for hnRNP K (Table 1).

**Table 1.** Templates used for nontargeting (control) shRNA and hnRNP K

	Target sequence	Sense oligo	Anti-sense oligo
sh-scramble	none	GATCCCC **GATGAACGCTCTGGATGCG** TTCAAGAGA **CGCATCCAGAGCGTTCATC** TTTTTGGAAA	AGCTTTTCCAAAAA **GATGAACGCTCTGGATGCG** TCTCTTGAA **CGCATCCAGAGCGTTCATC** GGG
sh-hnRNP K	1201-1219 GUAACUAUUCCCAAAGAUU	GATCCCC **GTAACTATTCCCAAAGATT** TTCAAGAGA **AATCTTTGGGAATAGTTAC** TTTTTGGAAA	AGCTTTTCCAAAAA **GTAACTATTCCCAAAGATT** TCTCTTGAA **AATCTTTGGGAATAGTTAC** GGG

10.1523/ENEURO.0268-17.2017.t1-1 Table 1-1 Download Table 1-1, PDF file.

10.1523/ENEURO.0268-17.2017.t1-2 Table 1-2 Download Table 1-2, PDF file.

10.1523/ENEURO.0268-17.2017.t1-3 Table 1-3 Download Table 1-3, PDF file.

10.1523/ENEURO.0268-17.2017.t1-4 Table 1-4 Download Table 1-4, PDF file.

10.1523/ENEURO.0268-17.2017.t1-5 Table 1-5 Download Table 1-5, PDF file.

10.1523/ENEURO.0268-17.2017.t1-6 Table 1-6 Download Table 1-6, PDF file.

10.1523/ENEURO.0268-17.2017.t1-7 Table 1-7 Download Table 1-7, PDF file.

10.1523/ENEURO.0268-17.2017.t1-8 Table 1-8 Download Table 1-8, PDF file.

After annealing, oligonucleotides were cloned into the BglII and HindIII digested pSuper (EcoRI) intermediate vector. Then, a fragment containing the H1 promoter and hairpin sequences were obtained from EcoRI-digested pSuper, and subcloned into the EcoRI site of the pTRIP lentiviral vector.

### Lentiviruses construction and transduction of neuronal cultures

Lentiviruses were generated by triple calcium-phosphate transfection of pTRIPshRNA (coding also GFP), pCMV-ΔR8.91, and pMD (VSVG; which encode the VSVG envelope glycoprotein gene and the gag/pol/tat genes, respectively) into HEK293T cells. HEK293T cells were grown for 2 d in 10-cm Petri dishes until they reached ∼60% confluence. A solution of CaCl_2_ and DNA (Helper plasmids: 10 μg pCMV-ΔR8.91, 6 μg pMD.G(VSVG); plasmid with the specific constructs: 5 μg pTrip -shRNA) was added drop-wise to a solution of 2× HEPES-buffered saline (HBS; 50 mM HEPES, 280 mM NaCl, and 1.5 mM Na_2_HPO_4_, pH 7.0). The solution of calcium-DNA was dropwise added to 2× HBS and rested for 10 min to form the precipitates. The precipitates were then distributed evenly over the HEK293T cultures. The cells were allowed to incorporate the precipitates for 6 h and were further incubated for ∼60 h to express the plasmid content. During these periods cells were maintained at 37°C, with saturating humidity and 5% CO_2_/95% air. The supernatant containing viral particles was then collected and concentrated by centrifugation at 60,000 × *g* for 2 h at 22°C (Pinto et al., 2016). Viral particles were resuspended in 0.1% BSA in PBS and stored at −80°C. Viral titer was calculated as previously described (Janas et al., 2006).

Neuronal cultures were transduced at DIV11 with a multiplicity of infection (MOI) equal to 5, which represents ∼80% of neuronal infection. Coverslips with low-density hippocampal neuronal cultures growing over a layer of astroglia cells were transferred to sterile 12-multiwell plates where the cells were transduced for 6 h in 500 µl of conditioned media. After that period, the coverslips were gently washed in sterile PBS and then transferred to the wells containing the astroglia cell layer. Neurons were allowed to express the shRNA for 3 d. At 14 DIV, the neurons were processed for immunocytochemistry. The shRNA constructs used in Western blotting experiments were designed to carry a mCherry tag instead of GFP.

### Transfection of shRNA constructs

Hippocampal cultures were transfected using the calcium phosphate coprecipitation method with constructs carrying shRNAs targeting hnRNP K or a scramble sequence. Briefly, hippocampal neurons were incubated with cultured-conditioned medium with 2 mM kynurenic acid (Sigma) for 15 min. Two μg of plasmid DNA was diluted in Tris-EDTA (TE), pH 7.3, and mixed with 2.5 M CaCl_2_. This DNA/TE/calcium mix was added to 10 mM HBS solution (270 mM NaCl, 10 mM KCl, 1.4 mM Na_2_HPO_4_, 11 mM dextrose, and 42 mM HEPES, pH 7.2). The precipitates were added drop-wise to each well and incubated for 1 h 30 min at 37°C, in a humidified incubator with 95% air/5% CO_2_. The cells were then washed with acidic culture medium containing 2 mM kynurenic acid and returned to the 95% air/5% CO_2_ incubator for 20 min at 37°C. Finally, the medium was replaced with the initial culture-conditioned medium, and the cells were further incubated in a 95% air/5% CO_2_ incubator for 72 h at 37°C. Hippocampal neurons transfected with sh-scramble or sh-hnRNP K were stimulated or not with BDNF (50 ng/ml) during at least 30 min before recording the NMDAR-mediated mEPSCs.

### Analysis of NMDAR-mediated mEPSCs

Cultured hippocampal neurons (2.71 × 10^5^ cells/well) with pyramidal morphology (15–17 DIV), were whole-cell voltage-clamped to −60 mV, at room temperature, in a MgCl_2_-free Tyrode’s solution containing: 150 mM NaCl, 4 mM KCl, 10 mM glucose, 10 mM HEPES, and 2 mM CaCl_2_, pH 7.35 (310 mOsm). To record and isolate NMDAR-mediated mEPSCs, 6-cyano-7-nitroquinoxaline-2,3-dione (CNQX; 10 µM; Tocris; AMPA/kainate receptor antagonist), bicuculline (10 µM; Tocris; GABA_A_ receptor antagonist), tetrodotoxin (TTX; 500 nM; Tocris; blocker of voltage gated Na^+^ channels), and glycine (15 µM; Sigma-Aldrich; coagonist of NMDARs) were added to the bath solution (Atasoy et al., 2008). The electrode solution had the following composition: 115 mM Cs-MeSO_3_, 20 mM CsCl, 2.5 mM MgCl_2_, 10 mM HEPES, 0.6 mM EGTA, 4 mM Na_2_-ATP, and 0.4 mM Na-GTP, pH 7.3 (300 mOsm; Sigma; Kessels et al., 2013). Where indicated, hippocampal neurons were preincubated with cycloheximide (CHX; 50 μg/ml) or with vehicle (DMSO; 1:1000 dilution) for 15 min before recording the NMDAR-mediated mEPSC. When the effect of BDNF was tested, hippocampal neurons were preincubated with the neurotrophin (50 ng/ml) for at least 30 min before recording the NMDAR-mediated mEPSCs. Recording electrodes were made of borosilicate glass capillaries and pulled on a horizontal stage Sutter Instrument P-97 puller (resistances: 3-4 MΩ). Recordings were made without series resistance compensation. Cells were held for a period of 5 min and the baseline for the analysis of NMDAR-mediated mEPSCs was manually determined as the average current level of silent episodes during a recording. Whole-cell recordings from hippocampal neurons were performed using an Axon CNS, Multiclamp 700B amplifier, an Axon Digidata 1550 A acquisition board, and pClamp software (version 10.5; Molecular Devices). Signals were filtered at 2.8 Hz, sampled at 25 kHz and the amplitude of NMDAR-mediated currents was analyzed offline with pClamp software (version 10.5; Molecular Devices).

### Statistical analysis

Data are presented as mean ± SEM of at least three different experiments, performed in independent preparations. Statistical analysis of the results was performed using one-way ANOVA analysis followed by the Dunnett or Bonferroni multiple comparison tests, as indicated in the figure captions. Where indicated, comparison between two experimental groups was performed using the Student’s *t*-test.

## Results

### Neuronal activity regulates the dendritic expression of hnRNP K through activation of TrkB receptors

**Figure 1.**
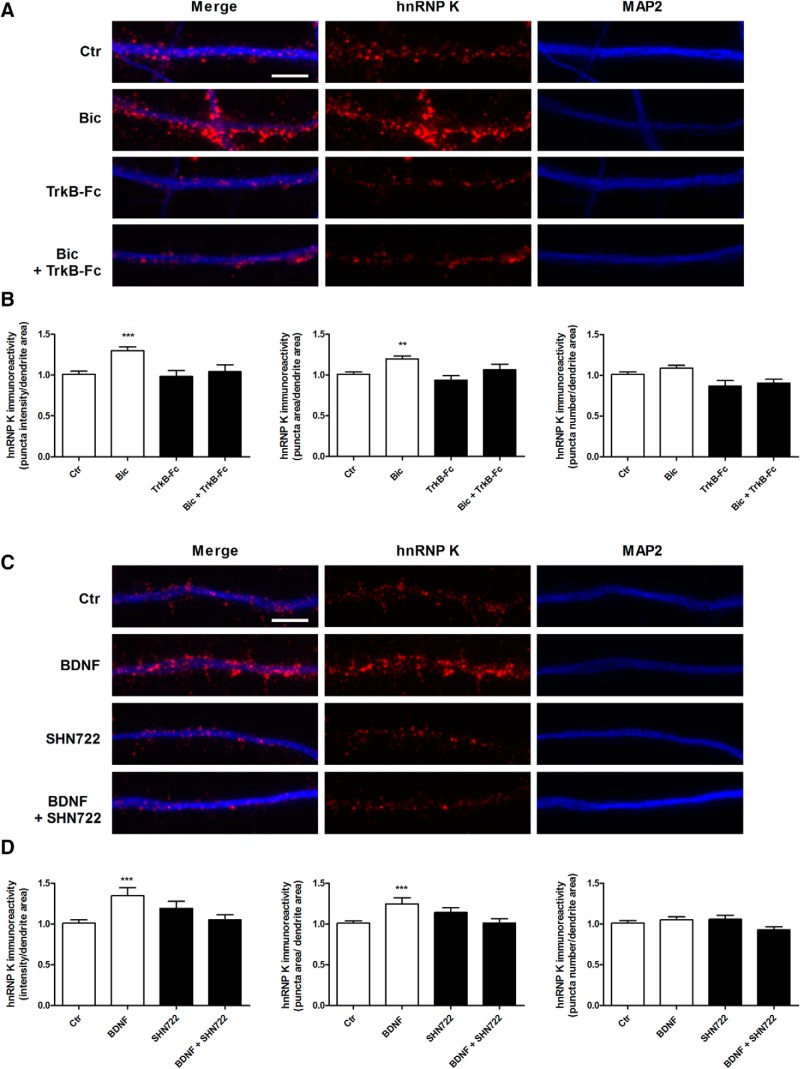
Activity-induced BDNF-dependent dendritic accumulation of hnRNP K in hippocampal neurons. ***A***, ***B***, Synaptic activity induces the accumulation of hnRNP K in dendrites of hippocampal neurons. Cultured hippocampal neurons (14-15 DIV) were stimulated or not with bicuculline (50 μM), 4-AP (2.5 mM), and glycine (10 μM), for 30 min. Where indicated, neurons were treated with the extracellular scavenger of TrkB ligands TrkB-Fc (1 μg/ml) for 30 min and were then stimulated or not with bicuculline in the presence of the scavenger. Cells were then fixed and immunostained for hnRNP K (red) and MAP2 (blue) (***A***). The integrated fluorescence intensity, area, and number of hnRNP K puncta in dendrites was analyzed using ImageJ software and represented per dendritic area (***B***). Results are normalized to control and are the mean ± SEM of 4–10 different experiments performed in independent preparations. Ctr, *n* = 108 cells; Bic, *n* = 97 cells; TrkB-Fc, *n* = 49 cells; Bic + TrkB-Fc, *n* = 52 cells. ***C***, ***D***, BDNF increases hnRNP K levels in neuronal dendrites. Cultured hippocampal neurons (14–15 DIV) were stimulated or not with BDNF (100 ng/ml) for 30 min. Where indicated, neurons were preincubated with the Trk receptor inhibitor SHN722 for 30 min, and the inhibitor was also present during the period of incubation with the neurotrophin. Cells were then fixed and immunostained for hnRNP K (red) and MAP 2 (blue) (***C***). The integrated fluorescence intensity, area, and number of hnRNP K puncta in dendrites was analyzed using ImageJ software and represented per dendritic area (***D***). Results are normalized to control and are the mean ± SEM of 4–10 different experiments performed in independent preparations. Ctr, *n* = 108 cells; BDNF, *n* = 53 cells; SHN722, *n* = 41 cells; BDNF + SHN722, *n* = 51 cells. Statistical analysis was performed by one-way ANOVA, followed by the Bonferroni’s multiple comparison test. **p < 0.01; ***p < 0.001. Scale bars: 5 μm. Extended Data Figure 1-1 shows that neuronal activity does not change total hnRNP K protein levels in cultured hippocampal neurons.

Several transcripts are transported to dendrites on neuronal activation, including the mRNAs encoding for Arc (Link et al., 1995; Lyford et al., 1995; Steward et al., 1998; Steward and Worley, 2001), β-actin (Tiruchinapalli et al., 2003), CaMKIIα (Thomas et al., 1994; Rook et al., 2000; Håvik et al., 2003), TrkB, and BDNF (Tongiorgi et al., 1997). To understand whether neuronal activity regulates hnRNP K protein levels in dendrites, we stimulated cultured hippocampal neurons (14–15 DIV) with a cocktail solution containing bicuculline to increase the excitatory activity of the neuronal network (Hardingham et al., 2002). The dendritic distribution of hnRNP K was evaluated by immunocytochemistry, through colocalization with the dendritic marker MAP2. hnRNP K exhibited a punctate distribution along dendrites, as expected for a component of neuronal mRNP involved in dendritic mRNA transport. Bicuculline treatment significantly increased the integrated intensity, as well as the area of hnRNP K puncta in dendrites (Fig. 1*A*,*B*). However, no alteration was observed in the total number of hnRNP K puncta (Fig. 1*A*,*B*).

10.1523/ENEURO.0268-17.2017.f1-1 Figure 1-1 Neuronal activity does not change total hnRNP K protein levels in cultured hippocampal neurons. Cultured hippocampal neurons (14-15 DIV) were stimulated or not with bicuculline (50 μM), 4-AP (2.5 mM), and glycine (10 μM), for 30 min. Total hnRNP K protein levels were assessed by Western blotting and β-actin was used as loading control. The results are the average ± SEM of three independent experiments, performed in different preparations. Statistical analysis was performed using the Student's *t test*. Download Figure 1-1, TIF file.

Since BDNF is released in an activity-dependent manner (Edelmann et al., 2014), we hypothesized that the neurotrophin could play a role in bicuculline-induced dendritic accumulation of hnRNP K. To address this hypothesis, hippocampal neurons were stimulated with bicuculline in the presence of an extracellular scavenger of TrkB ligands, TrkB-Fc. Buffering of extracellular BDNF with TrkB-Fc abrogated the effect of neuronal activity on the dendritic distribution of hnRNP K without affecting the basal levels of the protein in the same compartment, suggesting an important role for BDNF in the activity-induced accumulation of hnRNP K in dendrites. Accordingly, stimulation of hippocampal neurons with BDNF also upregulated the intensity and area of dendritic hnRNP K puncta, and this effect was abrogated by the Trk receptor inhibitor SHN722 (Martin et al., 2011; Fig. 1*C*,*D*). Furthermore, no effect of BDNF was observed in the number of hnRNP K puncta (Fig. 1*C*,*D*), in accordance with the results obtained on bicuculline treatment. Together, these results show that BDNF plays an important role in the activity-dependent expression pattern of dendritic hnRNP K protein. Since bicuculline stimulation was without effect on total hnRNP K protein levels, as determined by Western blotting (Extended Data Fig. 1-1), the activity-dependent alteration in the localization of the RNA-binding protein in hippocampal neurons is likely to result from its redistribution.

### Characterization of the hnRNP K-binding transcripts

The presence of hnRNP K in dendrites suggests that this RNP plays a role in the delivery of transcripts away from the soma. Therefore, we characterized the transcripts that (directly or indirectly) interact with hnRNP K using a RNP immunoprecipitation (RIP) assay. hnRNP K was immunoprecipitated from cultured hippocampal neurons (DIV15) under control conditions, and the coimmunoprecipitated RNA fractions were subjected to whole-rat genome Agilent microarray analysis. Control experiments using a mouse IgG antibody showed no hnRNP K immunoprecipitation confirming the specificity of the method (not shown). The RNA transcripts that specifically coimmunoprecipitated with hnRNP K were identified by setting a cutoff value of 5 in the fold change of transcript coprecipitation when comparing hnRNP K and IgG samples. Thus, only transcripts showing at least 5-fold variation, when their abundance in the hnRNP K immunoprecipitates was compared with the IgG controls, were considered specifically associated with the RNP.

**Figure 2.**
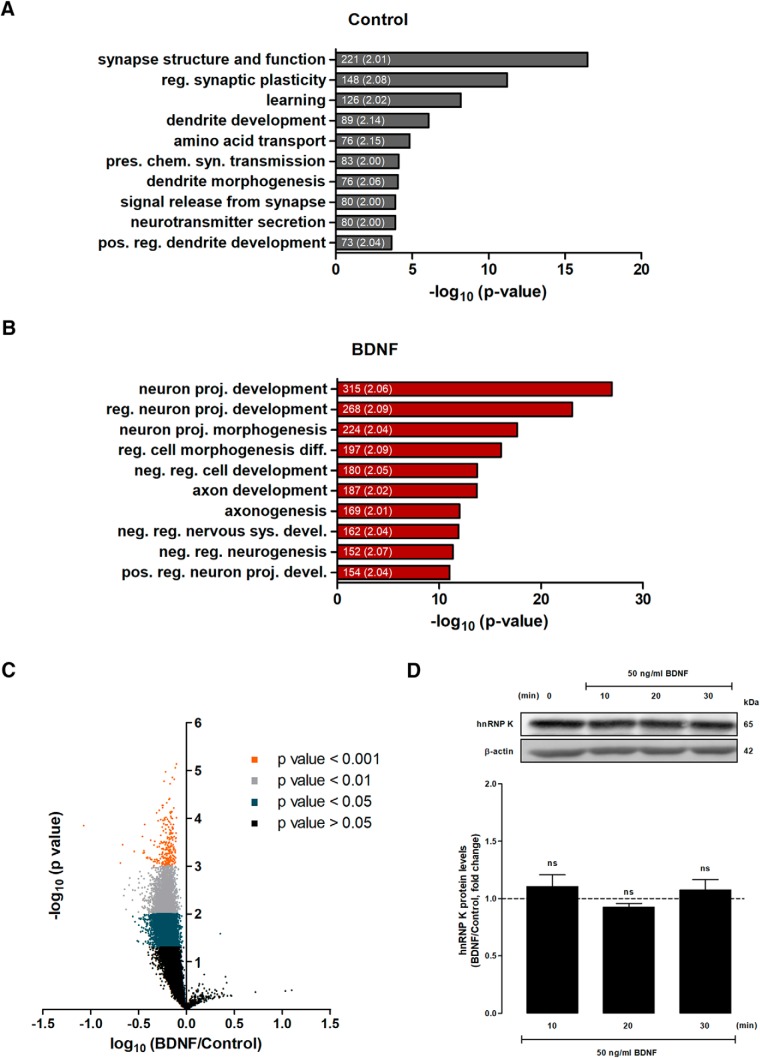
Stimulation of hippocampal neurons with BDNF decreases the interaction of hnRNP K with a large number of transcripts. ***A***, ***B***, List of the 10 most significantly enriched biological processes associated with mRNAs bound to hnRNP K (***A***) and those that were regulated by BDNF (50 ng/ml for 10 min) (***B***), identified with the PANTHER classification system. Only categories showing at least a 2-fold enrichment (considering the size of our lists) were analyzed and the 10 categories displaying the highest –log_10_ (*p* value) are shown. The number of transcripts belonging to each category and the fold change (designated in parenthesis) are indicated within graph bars. reg., regulation; pres., presynaptic; chem, chemical; syn., synaptic; pos., positive; proj., projection; diff., differentiation; neg., negative; sys., system; devel., development. ***C***, Cultured hippocampal neurons were stimulated or not with BDNF (50 ng/ml) for 10 min before preparation of cellular extracts. hnRNP K was immunoprecipitated from control and BDNF-treated hippocampal neuron homogenates, and the associated transcripts were identified by microarray analysis. The specificity of transcripts associated with hnRNP K was assessed by subtracting the levels of correspondent mRNAs pulled down together with mouse IgG antibodies. hnRNP K-associated mRNAs were then compared between control and BDNF treated neurons. The results were obtained from the quantification of four different experiments performed in independent preparations, and are expressed as -log (*p* value) and log fold change (BDNF vs control). A total of 9509 transcripts showed a decrease in the interaction with hnRNP K in cells stimulated with BDNF; *p* < 0.05 (gray dots) as determined by the paired Student’s *t*-test. ***D***, Stimulation of cultured hippocampal neurons with BDNF does not affect total hnRNP K protein levels. Hippocampal neurons were stimulated with BDNF for 10, 20, and 30 min, and the cellular extracts were analyzed by Western blotting. β-Actin was used as loading control. The results represent quantification of three independent experiments, and are expressed as percentage (mean ± SEM) of control. ns, nonsignificant as determined by ANOVA followed by Dunnett's multiple comparison test.

The RNAs that coimmunoprecipitated with hnRNP K, a total of 16,015 transcripts (Extended Data Table 1-1), were analyzed using the PANTHER classification system that uses the GO algorithm (Mi et al., 2013a). Classification of these transcripts based on their role in biological processes showed that the most enriched categories were related with excitatory synapse plasticity (Table 2; Fig. 2*A*). The mRNAs coimmunoprecipitated with hnRNP K are involved in diverse processes such as glutamate receptor signaling, regulation of synapse structure and function, regulation of dendrite development, synapse assembly, postsynaptic signaling, and learning (Fig. 2*A*; Table 2; Extended Data Table 1-2). Accordingly, when the coimmunoprecipitated transcripts were analyzed based on the cellular component, the identified mRNAs were found to be associated with postsynaptic membrane/postsynaptic density/dendritic spines, further suggesting that hnRNP K plays an important regulatory role at the postsynaptic level (Extended Data Table 1-3). Interestingly, a significant fraction of the transcripts identified code for proteins of the presynaptic active zone and synaptic vesicles (Extended Data Table 1-3). Together, these data suggest that hnRNP K may act as a modulator of mRNA metabolism, to regulate plastic changes at excitatory synapses. It is also important to highlight the fact that among the identified mRNAs there are transcripts related with a wide variety of functions, processes and compartments (e.g., related with cytoskeleton, Golgi apparatus, nucleus, cell development and migration, protein ubiquitination; Extended Data Tables 1-2–4), confirming the key role of hnRNP K in the functional regulation of cells.

**Table 2.** List of the most enriched biological processes associated with hnRNP K target mRNAs

GO biological process	*Rattus novergicus* (reference list)	Uploaded list	(Expected)	(Over/under)	fold enrichment	(*p* value)
Glutamate receptor signaling pathway	44	39	17.26	+	2.26	3.81E-02
Amino acid transport	90	76	35.31	+	2.15	1.46E-05
Synapse assembly	51	43	20.01	+	2.15	4.35E-02
Dendrite morphogenesis	57	48	22.36	+	2.15	1.34E-02
Dendrite development	106	89	41.58	+	2.14	8.56E-07
Regulation of neuronal synaptic plasticity	71	59	27.85	+	2.12	1.46E-03
Regulation of synaptic plasticity	181	148	71.00	+	2.08	6.28E-12
Forebrain cell migration	70	57	27.46	+	2.08	4.35E-03
Regulation of dendrite morphogenesis	94	76	36.88	+	2.06	8.66E-05
Telencephalon cell migration	67	54	26.28	+	2.05	1.14E-02
Positive regulation of dendrite development	91	73	35.70	+	2.04	2.24E-04
Negative regulation of synaptic transmission	64	51	25.11	+	2.03	2.96E-02
Learning	159	126	62.37	+	2.02	6.53E-09
Regulation of synapse structure or activity	280	221	109.84	+	2.01	3.29E-17
Positive regulation of synapse assembly	66	52	25.89	+	2.01	3.27E-02
Synaptic vesicle cycle	94	74	36.88	+	2.01	3.75E-04
Regulation of postsynaptic membrane potential	65	51	25.50	+	2.00	4.48E-02
Neurotransmitter secretion	102	80	40.01	+	2.00	1.29E-04
Signal release from synapse	102	80	40.01	+	2.00	1.29E-04
Regulation of synapse assembly	83	65	32.56	+	2.00	2.79E-03
Presynaptic process involved in chemical synaptic transmission	106	83	41.58	+	2.00	7.57E-05

More detailed information is provided in Extended Data Tables 1-1–1-8: Table 1-1, hnRNP K coimmunoprecipitated transcripts; Table 1-2, biological processes associated with mRNAs coimmunoprecipitated with hnRNP K as assessed by GO; Table 1-3, cellular components associated with mRNAs coimmunoprecipitated with hnRNP K as assessed by GO; Table 1-4, molecular functions associated with mRNAs coimmunoprecipitated with hnRNP K as assessed by GO; Table 1-5, hnRNP K coimmunoprecipitated transcripts regulated by BDNF; Table 1-6, biological processes associated with mRNAs coimmunoprecipitated with hnRNP K and regulated by BDNF as assessed by GO; 1-7, cellular components associated with mRNAs coimmunoprecipitated with hnRNP K and regulated by BDNF as assessed by GO; 1-8, molecular functions associated with mRNAs coimmunoprecipitated with hnRNP K and regulated by BDNF as assessed by GO.

### Effect of BDNF stimulation on the interaction of hnRNP K with mRNAs

In a gel-based proteome profiling of the effects of BDNF in cultured hippocampal neurons we observed a neurotrophin-induced upregulation of several protein spots identified as hnRNP K (Manadas et al., 2009), suggesting that this RNA-binding protein undergoes posttranslational modifications in neurons exposed to the neurotrophin. In particular, one of the up-regulated spots had a more acidic isoelectric point (pI) suggesting that hnRNP K may be phosphorylated on stimulation with BDNF.

Given the evidence pointing to posttranslational modifications in hnRNP K following stimulation of hippocampal neurons with BDNF, we investigated whether the neurotrophin-induced signaling regulates the interaction of the RNP with mRNAs using the RIP assay, as described above. Cultured hippocampal neurons were stimulated or not with BDNF (50 ng/ml; 10 min), and the transcripts that were regulated by the neurotrophin were identified by comparing microarray data from hnRNP K immunoprecipitates obtained from BDNF-treated and nonstimulated hippocampal neurons. This comparison was performed after subtraction of microarray data corresponding to the unspecific binding obtained from the immunoprecipitates with mouse IgG antibodies. Calculation of the fold variation in RNA-hnRNP K interaction induced by BDNF showed significant changes (*p* < 0.05) for 9509 transcripts. Remarkably, 9508 (99.9%) transcripts were negatively regulated by BDNF and only 1 (0.01%) of the mRNAs showed an increase in binding (Fig. 2*C*; Extended Data Table 1-5). This clearly shows a massive effect of BDNF on the dissociation of mRNAs from hnRNP K and/or hnRNP K-associated proteins. Control experiments showed no changes in total hnRNP K protein levels in hippocampal neurons stimulated with BDNF for 10-30 min, indicating that the effects of the neurotrophin on the interaction of RNP with the transcripts cannot be attributed to alterations in the total abundance of the protein (Fig. 2*D*).

The 9509 transcripts that were found to coimmunoprecipitate with hnRNP K by a mechanism sensitive to BDNF-stimulation were also analyzed and distributed in three categories using the PANTHER classification system: biological processes, cellular component and molecular function (Tables 1-6–8). We also compared the most enriched biological process categories to which mRNAs associated with hnRNP K (control), and those that were regulated by BDNF (BDNF), belong (Fig. 2*A*,*B*). In this analysis we found that BDNF regulates preferentially hnRNP K-bound mRNAs that are involved in neuronal development, morphogenesis and differentiation (Fig. 2*B*). These findings are in agreement with the prominent role of hnRNP K in the posttranscriptional regulation of mRNAs crucial for axon outgrowth and development in *Xenopus* (Hutchins and Szaro, 2013). The stringent criteria used in this analysis (see methods section) excludes other functional and relevant enriched categories regulated by BDNF [e.g., regulation of synapse structure or activity (GO:0050803); 148 transcripts; 2.05-fold increase; -log_10_ (*p* value) = 10.67572].

### BDNF modulates hnRNP K interaction with transcripts at the synapse

**Figure 3.**
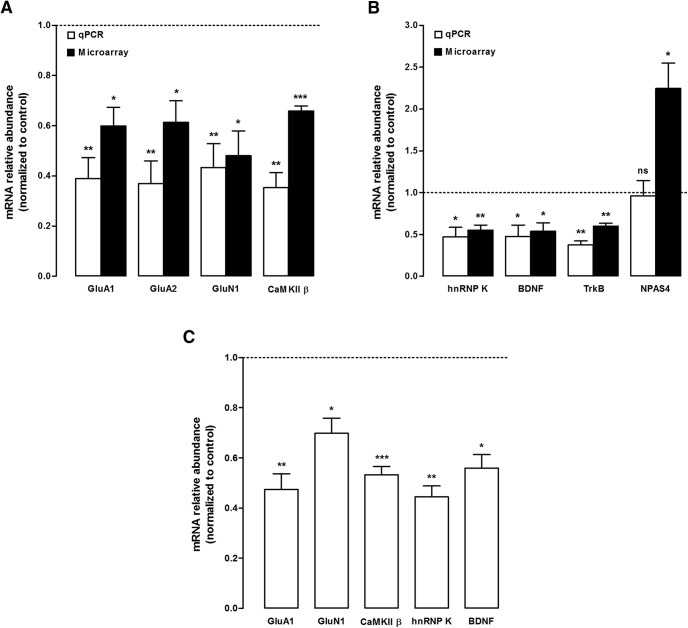
BDNF induces the dissociation of mRNA from hnRNP K at the synapse. ***A***, ***B***, Effect of BDNF stimulation (50 ng/ml; 10 min) on transcript coimmunoprecipitation with hnRNP K in cultured hippocampal neurons as assessed by microarray and qPCR. The specificity of transcripts associated with hnRNP K was assessed by subtracting the levels of correspondent mRNAs pulled down together with mouse IgG antibodies. hnRNP K-associated mRNAs were then compared between control and BDNF treated neurons. ***A***, Genes coding for synaptic proteins: GluA1, AMPA receptor subunit 1; GluA2, AMPA receptor subunit 2; GluN1, NMDAR subunit 1. ***B***, Other genes: TrkB, tropomyosin-related kinase B receptor; NPAS4, neuronal PAS domain protein 4. The results represent quantitation of four different experiments performed in independent preparations, and are expressed as percentage [mean ± SEM (qPCR) or SD (microarray)] of control; *p < 0.05, **p < 0.01, ***p < 0.001 as determined by ANOVA followed by Dunnett’s multiple comparison test (qPCR) or using the paired Student’s *t* test (microarray). ***C***, BDNF decreases the interaction of hnRNP K with mRNAs coding for synaptic proteins in hippocampal synaptoneurosomes. Synaptoneurosomes were stimulated or not with BDNF (50 ng/ml) for 10 min and the mRNA levels coimmunoprecipitated with hnRNP K (control and BDNF-treated synaptoneurosomes) were normalized to the correspondent IgG-pulled down mRNA to exclude any unspecific binding. Transcripts that were specifically associated with hnRNP K were then compared between control and BDNF-stimulated synaptoneurosomes. The relative abundance of each transcript was evaluated by qPCR. Results were normalized for mRNA expression under control conditions and are the average of at least four independent experiments. Statistical analysis was performed by one-way ANOVA followed by the Bonferroni’s multiple comparison test; *p < 0.05; **p < 0.01;***p < 0.001. Extended data Figure 3-1 shows the characterization of the synaptosomal preparation, using specific protein markers.

Since hnRNP K may be localized at synaptic sites under resting conditions (Folci et al., 2014), we next validated the results obtained in the microarray studies regarding the effects of BDNF on the interaction of the RNA-binding protein with transcripts coding for proteins that are relevant in synaptic plasticity. These studies were performed in cultured hippocampal neurons (Fig. 3*A*,*B*) and in rat brain synaptoneurosomes, a subcellular fraction containing resealed presynaptic structures with attached sealed postsynaptic entities (Troca-Marín et al., 2010; Fig. 3*C*). The results were first validated for eight genes by qPCR, using cultured hippocampal neurons: (1) four genes coding for proteins with synaptic functions [GluA1 and GluA2 (AMPA receptor subunits), GluN1 (NMDAR subunit), and CaMKIIβ; Fig. 3*A*); (2) two genes involved in BDNF signaling (BDNF and TrkB; Fig. 3*B*); (3) hnRNP K; and (4) a mRNA that showed a very robust increase in the interaction of hnRNP K following stimulation of hippocampal neurons with BDNF (NPAS4; Fig. 3*B*). The results obtained in the qPCR experiments confirmed a BDNF-induced decrease in the interaction of hnRNP K with the transcripts for GluA1, GluA2, GluN1, BDNF, TrkB, hnRNP K, and CaMKIIβ. The only result obtained in the microarray experiments that was not validated by qPCR was the increase in the NPAS4 transcripts coimmunoprecipitated with hnRNP K in cells stimulated with BDNF.

10.1523/ENEURO.0268-17.2017.f3-1 Figure 3-1 Characterization of purified synaptoneurosomes obtained from the hippocampus of adult rats. ***A***, Enrichment in synaptic proteins (PSD**-**95, synaptophysin and the VGAT) in the synaptoneurosome preparation when compared to the total lysate (homogenate). On the contrary, somatic proteins such as tubulin and the histone 3 are less abundant in the synaptic preparation, as well as the marker for astrocytes (GFAP). ***B***, The integrity of synaptoneurosomes throughout the experimental procedures used was assessed by measuring the activity of a cytoplasmic enzyme, LDH, in both the extracellular and synaptoneurosome fractions. The activity of LDH was much higher in the synaptoneurosome fraction than in the extracellular fraction, showing that the preparation contains resealed and nonleaky synaptoneurosomes. After an incubation period of 45 min at 30°C, the activity of LDH was unchanged, indicating that synaptoneurosomes are stable during the incubation period used. Analysis was done using ANOVA followed by Tukey's multiple comparisons test; *n* = 4 independent preparations. Download Figure 3-1, DOCX file.

To investigate whether BDNF induces the dissociation of transcripts from hnRNP K at the synapse, the interaction of the RNP with specific transcripts was investigated in hippocampal synaptoneurosomes, which are subcellular neuronal membrane fractions containing sealed presynaptic nerve endings attached to their corresponding postsynaptic counterparts (Extended Data Fig. 3-1). Stimulation of hippocampal synaptoneurosomes with BDNF (50 ng/ml; 10 min) significantly decreased the interaction of the RNP with transcripts coding for GluA1, GluN1, CaMKIIβ, hnRNP K, and BDNF (Fig. 3*C*). Since synaptoneurosomes are a closed system, one may conclude that these effects are due to a local effect of BDNF rather than a global effect of the neurotrophin on gene expression.

**Figure 4.**
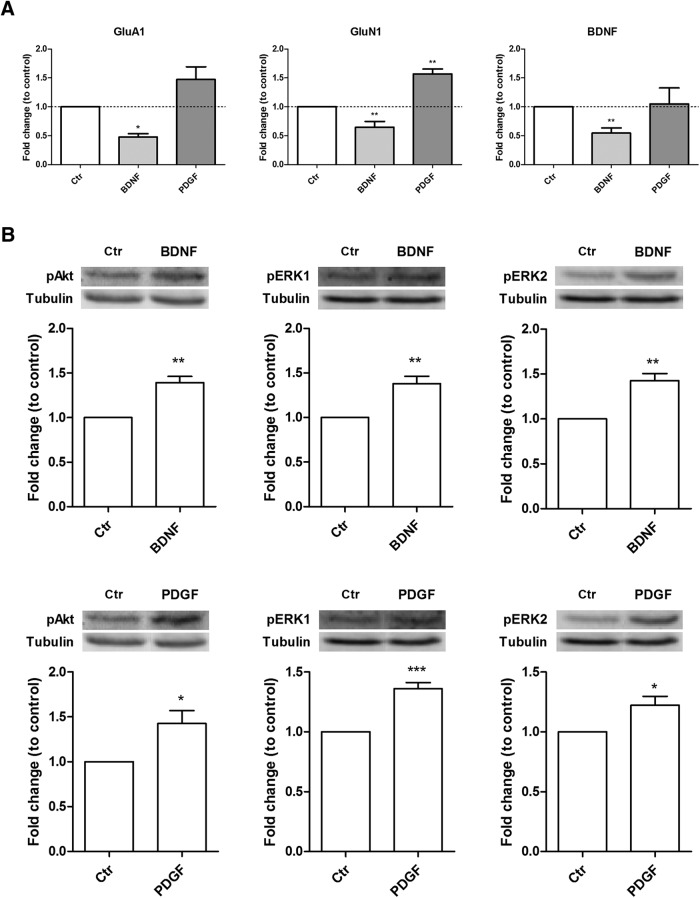
BDNF signaling specifically induces the release of mRNAs from hnRNP K-containing complexes at synaptic sites. ***A***, Effect of BDNF and PDGF on the interaction of GluA1, GluN1, and BDNF mRNAs with hnRNP K in synaptoneurosomes. The relative abundance of each mRNA coimmunoprecipitated with hnRNP K was evaluated by qPCR. Results are the mean ± SEM of four different experiments performed in independent preparations, and are expressed as fold change to control; *p < 0.05, **p < 0.01, as determined by one-way ANOVA followed by the Dunnett’s multiple comparison test. Activation of Akt and ERK1/2 by BDNF (***B***) and PDGF (***C***) in hippocampal synaptoneurosomes. Graphs represent the fold change in the levels of pAkt, pERK1, or pERK2 in hippocampal synaptoneurosomes stimulated with BDNF (50 ng/ml) or PDGF (20 ng/ml) for 10 min. Representative Western blotting images showing the results obtained for the phosphorylated proteins and tubulin under control conditions and following stimulation with BDNF or PDGF, as indicated. The results represent the quantification of six to nine experiments performed in independent preparations, and are expressed as fold change (mean ± SEM) to control; *p < 0.05, **p < 0.01, ***p < 0.001 as determined by the Student’s *t* test.

To determine whether the effects of BDNF on the interaction of hnRNP K with the transcripts at the synapse is shared by other ligands that activate receptor tyrosine kinases, we compared the effect of the neurotrophin and the response to PDGF. In these experiments the mRNAs pulled-down with hnRNP K were normalized to the levels of mRNA pulled-down with IgG under the same conditions to exclude any unspecific binding. The levels of hnRNP K-bound transcripts obtained from PDGF- and BDNF-treated synaptoneurosomes were then compared to the control. Controls were obtained by incubating synaptoneurosomes for 10 min in the same buffer but without BDNF or PDGF. The PDGF receptors (PDGFR-β) and TrkB receptors activate similar signaling mechanisms, and PDGFR-β receptors were shown to localize in pre- and postsynaptic sites in the hippocampus where they mediate LTP (Shioda et al., 2012). In contrast with the effect of BDNF (50 ng/ml; 10 min), which decreases the coimmunoprecipitation of hnRNP K with mRNA for GluA1, GluN1, and BDNF, stimulation of hippocampal synaptoneurosomes with PDGF did not change the interaction of the RNP with GluA1 and BDNF transcripts, and enhanced the amount of GluN1 transcripts pulled down together with hnRNP K (Fig. 4*A*). Control experiments showed that BDNF and PDGF activate the ERK and Akt signaling pathways under the experimental conditions used, as determined by Western blotting with antibodies against pAkt and pERK1/2 (Fig. 4*B*,*C*). Together, these results indicate that BDNF-induced signaling is specifically coupled to the regulation of hnRNP K interaction with transcripts coding for GluA1, GluN1, and BDNF.

### HFS-induced LTP in the DG differentially modulates the interaction of hnRNP K with the transcripts

**Figure 5.**
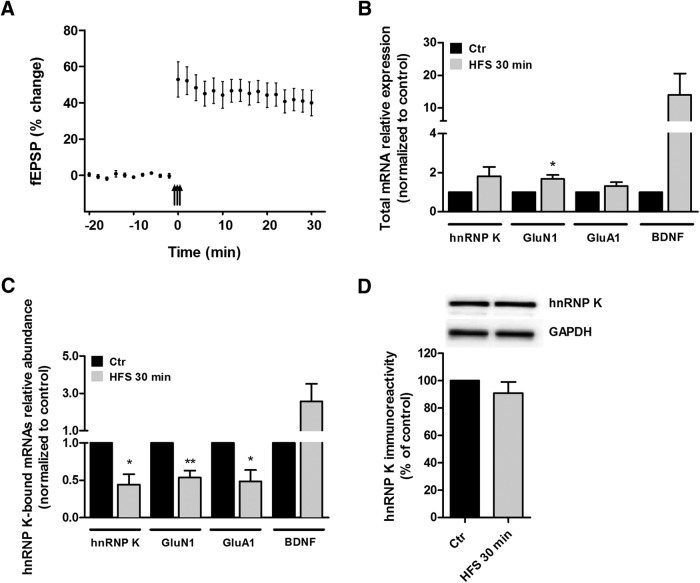
LTP-induced regulation of hnRNP K-associated mRNAs *in vivo*. Experiments were performed in live anesthetized rats. Electrodes were positioned for selective unilateral stimulation of the medial perforant path fibers in the angular bundle and recording of the evoked field potentials in the hilar region of the DG. ***A***, Time course plots showing changes in the medial perforant path-evoked fEPSP slope expressed as percentage of baseline. Values are means ± SEM. Test pulses were applied at 0.033 Hz. The HFS paradigm (indicated by arrows) consisted of eight pulses of 400 Hz, repeated four times at 10-s intervals. Three sessions of HFS were given at intervals of five min. *n* = 6 for each time point. ***B***, The variation of hnRNP K, GluN1, GluA1, and BDNF mRNA levels was assayed by qPCR using total RNA samples obtained from DG homogenates collected 30-min post-HFS and the nonstimulated contralateral control tissue. The results are presented as mean ± SEM normalized to the contralateral nonstimulated DG, and *Hprt1* (hypoxanthine guanine phosphoribosyl transferase 1) was used as internal control gene. Results are the average ± SEM of six experiments (*n* = 6 DG) analyzed in three independent preparations; *p < 0.05 as determined by Student’s *t* test. ***C***, The levels of hnRNP K, GluN1, GluA1, and BDNF transcripts coimmunoprecitated with hnRNP K were assayed by qPCR. hnRNP K protein was immunoprecipitated from equal amounts (500 μg) of total extracts from homogenized DG collected 30-min post-HFS and the contralateral tissue. Results are presented as mean ± SEM normalized to the contralateral DG and are the average ± SEM of six experiments (*n* = 6 DG) analyzed in three independent preparations; **p < 0.01; *p < 0.05 as determined by the Student’s *t* test. ***D***, hnRNP K protein levels were measured by Western blotting using DG homogenate samples collected 30-min post-HFS and the contralateral nonstimulated tissue. The results are the average ± SEM of six experiments (*n* = 6 DG) analyzed in three independent preparations and are presented as the percentage change in hnRNP K protein levels in the treated DG relative to the nonstimulated contralateral tissue. GAPDH was used as loading control.

**Figure 6.**
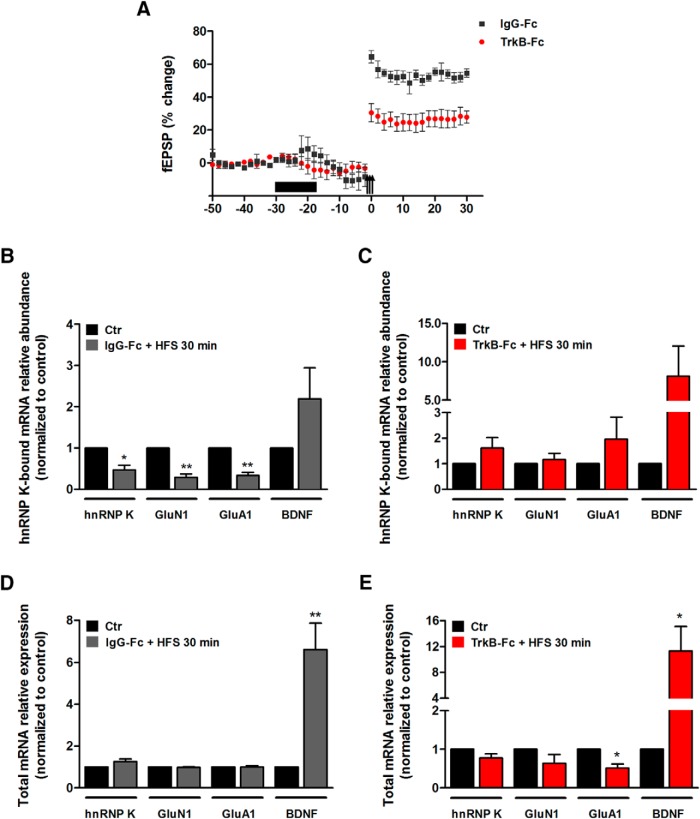
BDNF signaling is required for LTP maintenance and LTP-induced alterations in mRNA interaction with hnRNP K. ***A***, Time-course plots showing changes in the medial perforant path-evoked fEPSP in rats receiving TrkB-Fc (1 μl, 100 μg) or IgG-Fc (1 μl, 100 μg) infusion before HFS (arrows). Values are means ± SEM and are expressed in percentage of the baseline. Test pulses were applied at 0.033 Hz. HFS was applied in three series of 400-Hz bursts separated by 5 min. TrkB-Fc (1 μl, 100 μg; *n* = 6) or IgG-Fc (1 μl, 100 μg; *n* = 6) were infused in the dorsal DG at 0.08 μl/min during the period indicated by the black bar. Test pulses were not given during the HFS period. ***B***, ***C***, The levels of hnRNP K, GluN1, GluA1, and BDNF mRNAs coimmunoprecipitated with hnRNP K were assayed by qPCR in DG homogenates obtained from (***B***) IgG-Fc (*n* = 6) and (***C***) TrkB-Fc (*n* = 6) infused rats at 30-min post-HFS. Results are presented as mean ± SEM normalized to the correspondent contralateral DG. ***D***, ***E***, The variation of total hnRNP K, GluN1, GluA1, and BDNF mRNA levels in (***D***) IgG-Fc (*n* = 6) and (***E***) TrkB-Fc (*n* = 6) infused rats was assayed by qPCR in DG homogenates obtained at 30-min post-HFS. Results are presented as mean ± SEM normalized to the correspondent contralateral nonstimulated DG, and *Hprt1* was used as internal control gene; *p < 0.05; **p < 0.01, as determined by the paired Student’s *t* test.

To investigate whether hnRNP K-associated mRNAs are regulated by synaptic activity *in vivo*, we use a model of HFS-induced LTP in the DG of live anesthetized rats (Fig. 5*A*). The LTP at medial perforant path-granule cell synapses induced by HFS prompts the transport of different mRNAs into dendrites (Steward et al., 1998; Messaoudi et al., 2007; Panja et al., 2009; Dziembowska et al., 2012), and the maintenance of this form of LTP is sensitive to the BDNF scavenger TrkB-Fc (Fig. 6*A*; Panja et al., 2014). The medial perforant path fibers in the angular bundle were unilaterally stimulated and the evoked fEPSPs were recorded in the hilar region of the DG (Fig. 5*A*). LTP was induced by spaced stimulation consisting of three sessions of HFS (400 Hz, eight pulses) with 5 min between sessions. This paradigm induces a robust and sustained increase in the fEPSP (Fig. 5*A*). The DG was dissected immediately after the experiments (at *t* = 30 min after HFS) and the tissue was processed to analyze the changes in total abundance of transcripts coding for hnRNP K, GluA1, GluN1, and BDNF, as well as for their coimmunoprecipitation with hnRNP K. qPCR experiments showed that HFS induces a massive increase in the total abundance of BDNF mRNA, and significantly increased total GluN1 mRNA levels. No significant effect was observed for total GluA1 and hnRNP K total mRNA under the same conditions (Fig. 5*B*). HFS resulted in a decrease in hnRNP K and GluN1 mRNA levels associated with hnRNP K protein (Fig. 5*C*), indicating that synaptic activity induces the dissociation of hnRNPK-bound mRNAs *in vivo*. In contrast, BDNF mRNA levels were increased in the coimmunoprecipitates at 30-min post-HFS (Fig. 5*C*), possibly due to the massive increase in the total abundance of these transcripts induced by HFS. No differences in hnRNP K total protein levels were observed 30-min post-HFS (Fig. 5*D*) as determined by Western blotting, demonstrating that the observed alterations in the amount of mRNAs associated with the hnRNP K cannot be attributed to differences in the abundance of the RNP.

The HFS-induced LTP in the DG *in vivo* is partly mediated by TrkB signaling, as observed in experiments using TrkB-Fc, an effective extracellular scavenger of TrkB ligands. Intrahippocampal infusion of TrkB-Fc (1 μl, 100 μg, 12.5 min) before HFS abrogated the increase in the magnitude of the fEPSP slope when compared to IgG-Fc infused control and the noninfused control (Fig. 6*A*). In control experiments using IgG-Fc, HFS reduced the amount of hnRNP K, GluN1, and GluA1 transcripts coimmunoprecipiated with hnRNP K (Fig. 6*B*), in accordance to the results of Figure 5*A*. Importantly, on TrkB-Fc infusion, HFS did not affect the interaction of hnRNP K with the GluN1, GluA1, and hnRNP K mRNAs (Fig. 6*C*), showing a key role for TrkB signaling in the regulation of the RNP interaction with the transcripts after induction of LTP. The total levels of hnRNP K, GluA1, and GluN1 mRNA did not change in the DG on HFS in the presence of IgG-Fc, while an increase in BDNF transcripts was observed (Fig. 6*D*). Similar results were obtained after injection of TrkB-Fc, with the exception of a downregulation of GluA1 mRNA (Fig. 6*E*). Altogether these results indicate that the activity-induced regulation of hnRNP K-associated mRNAs requires TrkB signaling *in vivo*.

### Downregulation of hnRNP K decreases the amplitude of NMDAR-mediated mEPSC

**Figure 7.**
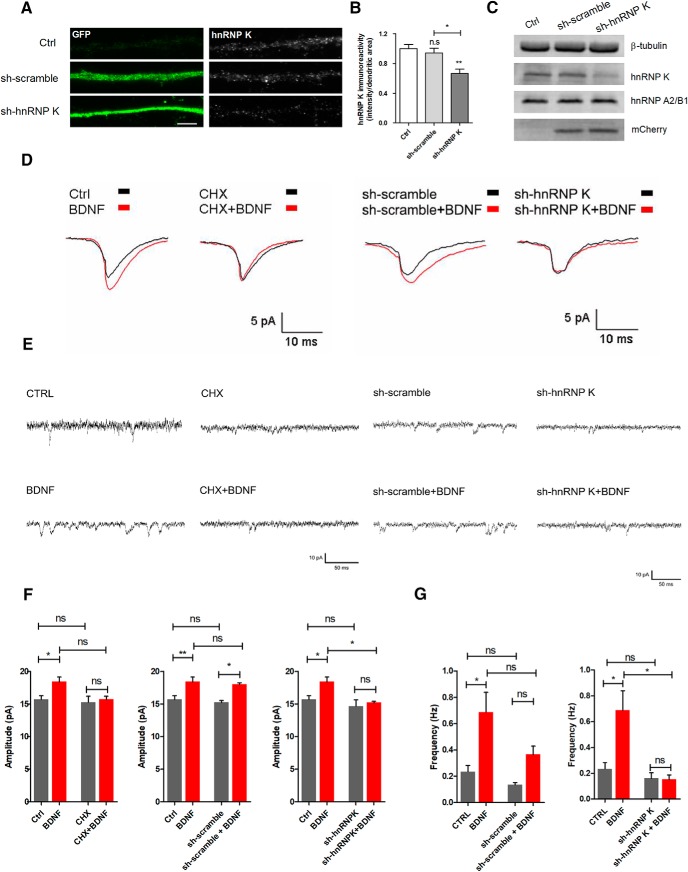
BDNF upregulates NMDAR-mediated mEPSC by a mechanism dependent on hnRNP K. ***A***, Low-density cultured hippocampal neurons were transduced with shRNA (sh-hnRNP K; sh-scramble, which does not target a specific sequence) constructs at DIV11. The cells were fixed at DIV14 and then immunostained for hnRNP K (gray), GFP (green), and MAP2 (not shown in the figure). The hnRNP K immunoreactivity was measured in dendrites (***A***, ***B***), using the ImageJ software. Results are normalized to control and are the average of three to four different experiments performed in independent preparations (19–34 cells analyzed in neurons expressing the shRNAs for 3 d). ***C***, Western blot analysis of hnRNP K, hnRNP A2/B1, β-tubulin (loading control), and mCherry (infection control), in cultured hippocampal neurons transduced or not with sh-hnRNP K and sh-scramble constructs. ***D***, ***E***, NMDAR-mediated mEPSC were recorded under control conditions (*n* = 13) and after 30 min of stimulation with BDNF (50 ng/ml; *n* = 14). Where indicated, the cells were transfected with sh-scramble (*n* = 6) or sh-hnRNP K (*n* = 6), or incubated with cycloheximide (50 µg/ml). The cells were preincubated with the translation inhibitor for 15 min before stimulation with BDNF. Cells transfected with sh-hnRNP K or incubated with cycloheximide showed no significant increase in the amplitude of NMDAR-mediated mEPSC on BDNF treatment, while the scramble shRNA was without effect (***F***). Analysis of NMDAR-mediated mEPSC frequency in nontransfected cells (*n* = 12) and cells transfected with sh-sramble (*n* = 8) or sh-hnRNP K (*n* = 6), under control conditions and following BDNF stimulation (***G***). The average mEPSC traces recorded are shown in ***D*** and representative traces are shown in ***E***. Results are presented as mean ± SEM for the indicated number of experiments. Statistical analysis was performed by one-way ANOVA, followed by Bonferroni’s multiple comparison test. n.s., not significant; *p < 0.05; **p < 0.01. Scale bar: 5 μm.

BDNF is known to enhance the activity of postsynaptic NMDAR in cortical and hippocampal pyramidal neurons (Kolb et al., 2005; Madara and Levine, 2008). Given the results showing the dissociation of transcripts bound to hnRNP K on stimulation of synaptoneurosomes with BDNF (e.g., GluN1; see Figure 3*C*), we hypothesized that the RNP may play a role in the modulation of postsynaptic NMDAR by the neurotrophin. To address this hypothesis, we tested the effect of BDNF on the amplitude of NMDAR-mediated mEPSCs, and hnRNP K was downregulated using a specific shRNA (sh-hnRNP K). Figure 7*A*,*B* show that sh-hnRNP K expression for 3 d decreases hnRNP K in the dendritic compartment by 33.3%, while a scramble shRNA which does not target a specific sequence (sh-scramble) was without effect. Importantly, the shRNA targeting hnRNP K decreases hnRNP K protein levels but does not affect the expression of another member of the hnRNP family of proteins, hnRNP A2/B1 (Fig. 7*C*), proving the specificity of the knockdown.

NMDAR-mediated mEPSC were recorded in the absence of presynaptic stimulation and in the presence of TTX, resulting from the spontaneous release of glutamate from nerve terminals. Furthermore, NMDAR-dependent synaptic responses were pharmacologically isolated by blocking AMPA and GABA receptors, and by supplementing the salt solution with the NMDAR coagonist glycine. The postsynaptic NMDAR-mediated component was expressed by using a salt solution lacking Mg^2+^, which allowed recording the mEPSC activity at a physiological holding potential of −60 mV. Alterations in the number of NMDAR at the synapse are expected to correlate with changes in the amplitude of mEPSC. Incubation of cultured hippocampal neurons with BDNF (50 ng/ml; *t* > 30 min) increased the amplitude of mEPSC, and this effect was abrogated by transfection with sh-hnRNP K (Fig. 7*D*–*F*). In contrast, an increase in the mEPSC amplitude was observed in cells transfected with the control shRNA (sh-scramble) when stimulated with BDNF (Fig. 7*D*–*F*). Interestingly, downregulation of hnRNP K was without effect on the NMDAR-mediated mEPSC measured in the absence of BDNF (Fig. 7*D*–*F*).

The results showing a role for hnRNP K in the regulation of NMDAR-mediated mEPSC by BDNF may be due to the release of transcripts which will become available for translation. This hypothesis was tested by evaluating the effect of cycloheximide, an inhibitor of protein synthesis, on the NMDAR-mediated mEPSC, under control and in hippocampal neurons stimulated with BDNF. Inhibition of protein synthesis abrogated the BDNF-induced increase in the amplitude of mEPSC mediated by NMDAR. However, it was without effect when the NMDAR currents were measured under resting conditions (Fig. 7*D*–*F*).

Several lines of evidence now demonstrate that BDNF may increase mESPCs frequency via presynaptic mechanisms (Carvalho et al., 2008). In agreement with these findings, we also observed a significant increase on NMDAR-mediated mESPSCs frequency following BDNF treatment (Fig. 7*G*) which was not observed after hnRNP K downregulation (Fig. 7*G*). Neurons transfected with a scramble construct showed a modest increase in NMDAR-mediated mESPC frequency on exogenous application of BDNF (Fig. 7*G*).

## Discussion

In this work, we identified a large number of mRNAs, belonging to different categories, including transcripts coding for synaptic proteins, in hnRNP K immunoprecipitates from cultured hippocampal neurons. Stimulation with BDNF induced a massive dissociation of a subpopulation of transcripts that coimmunoprecipitate with hnRNP K, suggesting that this RNP is a major mediator of the effects of BDNF on translation activity in hippocampal neurons. This hypothesis is also supported by the results showing a BDNF-dependent dissociation of several transcripts from hnRNP K following HFS of the DG synapses. Some of the regulated transcripts code for proteins relevant for the plasticity of glutamatergic synapses (GluN1, GluA1, BDNF, and CamKIIβ; Santos et al., 2010; Huganir and Nicoll, 2013; Fan et al., 2014; Leal et al., 2014a; Kim et al., 2016), and BDNF had a similar effect in hippocampal synaptoneurosomes, suggesting a role for these regulatory mechanisms in the modulation of local protein synthesis required for LTP maintenance. Accordingly, downregulation of hnRNP K impaired the effects of BDNF in enhancing NMDAR-mediated mEPSC, and similar effects were obtained on inhibition of translation activity.

Under resting conditions, we identified 16,015 transcripts present in the hnRNP K immunoprecipitates, coding for proteins involved in different biological processes, and with distinct molecular functions and cellular localization, as described with GO. It is remarkable that genes related with excitatory synaptic plasticity are highly represented in the group of transcripts coimmunoprecipitated with hnRNP K in extracts prepared from cultured hippocampal neurons. From the list of mRNAs that were specifically pulled-down together with hnRNP K in extracts prepared from hippocampal neurons under resting conditions, only 59.4% showed a significant change in the interaction with the RNP following stimulation with BDNF. This shows that the signaling mechanisms activated by the neurotrophin target a specific subpopulation of transcripts, rather than having a global effect. Furthermore, with the stringent analysis here performed, we found that mRNAs regulated by BDNF belong preferentially to GO categories related with neuronal development and morphogenesis, although other functional relevant categories are enriched to a similar extent. Importantly, all transcripts showing a change in interaction with hnRNP K following stimulation with BDNF were dissociated from the RNP, and no mRNA showed an increased binding in hippocampal neurons stimulated with the neurotrophin. hnRNP K has numerous putative phosphorylation sites, some of them located in the KH domains responsible for RNA and DNA binding (Bomsztyk et al., 2004). Since previous studies have shown that the interaction of hnRNP K with mRNAs is regulated by the phosphorylation state of the protein (Ostareck-Lederer et al., 2002; Iwasaki et al., 2008; Laursen et al., 2011; Tahir et al., 2014), it may be hypothesized that the phosphorylation of specific amino acid residues accounts for the observed BDNF-evoked dissociation of transcripts from the RNP. Phosphorylation of hnRNP K at an ERK phosphorylation site located within the KI domain was shown to promote the translation of mRNA of axonal cytoskeleton proteins that interact with the RNP (Hutchins et al., 2015). The transcripts that remain associated with hnRNP K following stimulation of hippocampal neurons with BDNF may interact with a different region of the RNA-binding protein, which may account for a differential regulation.

The group of transcripts dissociated from hnRNP K following stimulation of hippocampal synaptoneurosomes with BDNF included mRNAs coding for AMPA (GluA1) and NMDA (GluN1) receptor subunits, in addition to CaMKIIβ and BDNF, which play important roles in LTP of glutamatergic synapses. Furthermore, HFS-induced LTP in the DG was also accompanied by a dissociation of the GluA1 and GluN1 mRNA from hnRNP K through a BDNF-dependent mechanism, providing evidence for a similar role of BDNF *in vivo*. It may be hypothesized that after being released from hnRNP K, the transcripts will become available for local translation at the synapse thereby contributing to synaptic potentiation. Accordingly, stimulation with BDNF was shown to up-regulate GluA1 (Schratt et al., 2004), CaMKII β (Liao et al., 2007), and Homer2 (Schratt et al., 2004) protein levels in synaptoneurosomes. GluN1 mRNA is also among the RNAs that are present in both soma and dendrites (Benson, 1997; Gazzaley et al., 1997; Pal et al., 2003), and is associated with RNA granules (Krichevsky and Kosik, 2001), but the effects of BDNF on the expression levels of this receptor subunit at the synapse has not been yet reported. A similar role for hnRNP K in the release of transcripts used for local translation may be hypothesized in the BDNF-induced dendritic synthesis of BDNF and TrkB receptors (Shiina et al., 2005; Baj et al., 2016), and in the translation of the hnRNP K mRNAs at the synapse (Liao et al., 2007). The local synthesis of RNA-binding proteins at the synapse, including hnRNP K, following stimulation with BDNF is an intriguing observation, but it may contribute to increasing the local buffering capacity of the transcripts released after the disassembly of the RNA granules, thereby contributing to the stabilization of the mRNAs. The newly synthesized protein may also play a role in cytoskeleton regulation (Yoo et al., 2006).

In contrast with the results obtained in cultured neurons and in hippocampal synaptoneurosomes, *in vivo* HFS of medial perforant path fibers enhanced the interaction of BDNF mRNA with the RNA-binding protein. This may be attributed to the massive increase in the total amount of transcripts for BDNF, as observed in the qPCR experiments from total DG extracts, due to the activity-dependent upregulation of the BDNF gene (Patterson et al., 1992; Bramham et al., 1996). Our findings in synaptoneurosomes indicate that BDNF triggers the release of *BDNF* mRNA from the hnRNP K complex, making the *BDNF* mRNA available for local translation (Vanevski and Xu, 2015; Baj et al., 2016). During DG LTP, sustained translation of BDNF mRNA is observed in the synaptoneurosome fraction (Panja et al., 2014). This release of BDNF mRNA from hnRNP K may provide a continuous supply of BDNF transcripts, and a consequent sustained (hours) activation of the BDNF-TrkB signaling, which is required for the consolidation of LTP in the DG of live rodents (Panja et al., 2014) as well as in CA1 synapses (Kang et al., 1997).

The mRNA for the NMDAR subunit GluN1 was among the transcripts that showed a decrease in coimmunoprecipitation with hnRNP K following stimulation of hippocampal neurons or synaptoneurosomes with BDNF, and similar results were obtained in the DG after HFS of medial perforant path fibers. Since the mRNAs coding for other subunits of NMDAR were also dissociated from the hnRNP K following stimulation with BDNF, this may contribute to the local synthesis of receptors. According to this hypothesis, stimulation of cultured hippocampal neurons with BDNF upregulated the NMDAR-mediated mEPSCs by a hnRNP K-dependent mechanism, which was also sensitive to the protein synthesis inhibitor cycloheximide. This effect is likely to result from an upregulation in the synaptic expression of NMDAR, and may account for synaptic facilitation on incubation of hippocampal neurons with BDNF (Kang and Schuman, 1996; Santos et al., 2015) as well as for LTP following HFS (Korte et al., 1995; Korte et al., 1996; Kang et al., 1997; Minichiello et al., 1999; Panja et al., 2014). Together, our data points to a key role of hnRNP K in BDNF-induced synaptic plasticity events which is likely related with effects of the neurotrophin on hnRNP K-associated mRNAs, although we cannot not exclude any other putative functions of hnRNP K (e.g., nuclear effects). Interestingly, downregulation of hnRNP K selectively impaired the BDNF-induced enhancement of the NMDAR-mediated mEPSC, as it was without effect on the currents measured under resting conditions. These results suggest that BDNF recruits a distinct pool of receptors, which are synthesized *de novo* as indicated by the results obtained in the presence of cycloheximide. A similar mechanism may be involved in the delivery of GluA1-containing AMPA receptors to the membrane on stimulation with BDNF, since (1) it is a newly synthesized pool of receptors that is readily incorporated on the plasma membrane (Fortin et al., 2012) and (2) our results showed a dissociation of GluA1 mRNA from hnRNP K in synaptoneurosomes incubated with the neurotrophin. Similarly, protein synthesis may also be involved in the increase in GluA1 surface expression under conditions that induce chemical LTP (Folci et al., 2014).

BDNF was also observed to enhance the frequency of mEPSC in accordance with previous reports (Carvalho et al., 2008), and this effect was abrogated on knockdown of hnRNP K. Although this RNA-binding protein is present and plays a role at presynaptic sites (Liu and Szaro, 2011; Hutchins et al., 2015), the effect of hnRNP K knockdown on the frequency of NMDAR-mediated mRPSC is likely to involve a postsynaptic mechanism as transfected neurons receive inputs from untransfected cells.

In contrast with the effect of BDNF, activation of the receptors for PDGF (PDGFR-β), which also belong to the receptor tyrosine kinase family, did not decrease the interaction of hnRNP K with GluA1, GluN1, and BDNF mRNAs. This shows the specificity of the effects triggered by BDNF, and may result from a differential distribution of the receptors for the two ligands in distinct synapses. In addition to the KH domains responsible for binding mRNAs, hnRNP K also possesses a KI region that is responsible for the interaction with other proteins (Bomsztyk et al., 2004; Leal G., Duarte C.B. and Li K.W. unpublished observations). Since some of these binding partners may also interact with specific mRNAs (Mikula et al., 2006), it is not possible to determine whether each of the mRNAs that coimmunoprecipitate with hnRNP K bind directly or indirectly to the RNP.

BDNF was found to have a dual role in the regulation of hnRNP K in hippocampal neurons: it regulates the interaction of the protein with a large number of transcripts as discussed above and induces an accumulation of the RNP in dendrites following an increase in neuronal activity. The activity-dependent accumulation of hnRNP K in dendrites is expected to enhance the role of this RNP in synaptic regulation by BDNF, as the protein clustered in granules travel near active synapses. The effect of BDNF-TrkB signaling on the distribution of hnRNP K may be partly mediated by activation of the Ras/ERK signaling pathway, since phosphorylation of the protein by ERK1/2 enhances its nuclear export (Habelhah et al., 2001; Mikula et al., 2006). Similarly to the effects on the dendritic distribution of hnRNP K, BDNF was shown to induce the synaptic delivery of hnRNP A2/B1, a protein belonging to the same family of RNA-binding proteins (Leal et al., 2014b). hnRNP A/B (also known as CBF-A) was also shown to transport the *Arc*, *CaMKIIα*, and the *BDNF* mRNAs along dendrites, and this transport in enhanced by neuronal activity (Raju et al., 2011). Activation of NMDA and AMPA receptors was found to enhance the binding of hnRNP A/B with the *Arc*, *CaMKIIα*, and the *BDNF* mRNAs (Raju et al., 2011), suggesting a role for the protein in the long range transport of the transcripts from the soma to the distal region of the dendrites.

In conclusion, in this work, we show that synaptic activity and BDNF regulate hnRNP K and hnRNP K-bound mRNAs *in vitro* and *in vivo*. The large number of transcripts showing a decreased coimmunoprecipitation with hnRNP K on stimulation with BDNF suggests a role for this RNP in the regulation of neuronal function in general and, in particular, in the BDNF-mediated plasticity events.

## Synthesis

Reviewing Editor: Pablo Castillo, Albert Einstein College of Medicine Decisions are customarily a result of the Reviewing Editor and the peer reviewers coming together and discussing their recommendations until a consensus is reached. When revisions are invited, a fact-based synthesis statement explaining their decision and outlining what is needed to prepare a revision will be listed below. The following reviewer(s) agreed to reveal their identity: Young Yoon, Eric Levine

Your manuscript has been evaluated by two reviewers. They both agree on the significance, novelty and good quality of your study. However, they also raised a number of concerns that you should thoroughly address in the revision. In particular, you must address whether hnRNP K is a key mediator of BDNF effects on dendritic RNA metabolism. Below please find the reviewers' specific comments on your manuscirpt.

REVIEWER #1

In the manuscript, “The RNA binding protein hnRNP K mediates the effect of BDNF on dendritic mRNA metabolism and regulates the activity of synaptic NMDA receptors in hippocampal neurons,” the authors present data showing that BDNF can lead to the dissociation of hnRNP K from dendritically localized mRNAs as indication of translation. Although there is no direct evidence that the dissociated mRNAs undergo translation, the idea that post-synaptic stimulation can result in displacement of hnRNP K from transcripts is an intriguing and exciting hypothesis.

Current understanding of the role of heterogeneous nuclear ribonucleoproteins (hnRNPs) on dendritically localized mRNA has been for the assembly and transport of RNA granules-where hnRNPs remain bound to the mRNA (Leal et al, 2014 and others). Moreover, hnRNP K has been implicated as having a role in dendritic and spine development (Proepper et al, 2011; Folci et al, 2014). The novelty of the work presented here is that hnRNP K can be “bumped-off” of mRNAs by translating ribosomes in a BDNF-dependent manner and could potentially serve as an indicator of mRNAs that are translated upon stimulation. If the authors can address the concerns below, the manuscript should be sufficient for publication.

Major concerns:

- If hnRNP K is indeed dissociating from mRNA in dendrites, the authors must address why the effect is less pronounced in synaptosomes relative to total lysate. For example, GluN1 in synaptosomes (Fig 3C) remains bound to hnRNP K compared to total lysate (Fig 3A), which contradicts the hypothesis. Such results suggest that the effect may be dominated by events occurring at the soma rather than in dendrites as BDNF is bath-applied.

- In Figures 3, 5 and 6, the calculation of “relative abundance” should be normalized to the total amount of RNA detected for each sample (total or synaptosome) rather than to control RNA IP. This is important as the authors did not show what fraction of mRNAs are bound to hnRNP K in steady state or post-BDNF stimulation. For example, if only 5-10% of a given RNA species is bound to hnRNP K at any given time, the results may not be representative.

- The use of cycloheximide (CHX), a translation inhibitor, in Figure 7 is very indirect. To determine whether hnRNP K dissociation from mRNA is due to translation, it would be necessary to include a data set of RNA IP performed in the presence of a translation inhibitor.

Minor concerns:

- The authors should consider changing the title, as “regulates the activity of synaptic NMDA receptors” is not addressed in the manuscript.

- For Figure 1B&D, use of histograms would better represent the data than averages; averaging per puncta requires more detail on how image thresholding and segmentation was performed for particle analyses.

- The figure panels should follow the order in which they are first mentioned in the text (Fig panels 2C&D come before 2A&B; and 6C before 6B).

- Figure 7B should include a representative western blot of the knock-down (Folci et al, 2014)

- Figure 7B shows only a ~30% knock-down of hnRNP K. Include explanation as to how such a modest reduction in hnRNP K can lead to reduction of mEPSC peak amplitude.

- Figure 7 should include analysis of mini frequency and not just amplitude.

- In Figure 7E, all 3 panels seem to show identical Ctrl and BDNF results. If so, why is the p-value between Ctrl and BDNF different in the center panel?

REVIEWER #2

This paper addresses an important question regarding the mechanisms underlying the effects of BDNF at synapses, specifically related to activation of translational activity in dendrites. There is a well carried out series of studies describing novel interactions between BDNF and hnRNP K and its role in regulating synaptic plasticity and NMDA receptor modulation.

The authors demonstrate that activity-induced hnRNP accumulation in dendrites depends on BDNF signaling, and BDNF decreases the interaction of hnRNP with numerous transcripts. This BDNF - hnRNP K interaction is well documented. However, in the title and elsewhere in the text, the claim is made that hnRNP K is a key mediator of BDNF effects on dendritic RNA metabolism. However, no other hnRNP's were examined, so there is no specific evidence that hnRNP is the key mediator. Some comparison with other hnRNP's is needed to support this claim.

The paper (including title) also claims that hnRNP K regulates the activity of NMDA receptors, but the hnRNP knockdown by itself did not affect NMDA receptors. This should be addressed in more detail, and it would be more accurate to say that hnRNP may mediate the effect of BDNF on NMDA receptors.

Other concerns:

- SHN722, which is used to block the effect of BDNF, is a tyrosine kinase inhibitor which may be better than K252a, but is still not selective for trk receptors. It would be preferable to attempt to block BDNF effects with the trkB receptor antagonists ANA-12 or cyclotraxin-B.

- It is not clear if the shRNA against hnRNP K is selective or if it affects other hnRNPs.

- Details should be provided for dendritic analysis of hnRNP immunoreactivity, how were the dendritic areas chosen, number of areas and mean length per cell, etc.

- For the bicuculline effect on total hnRNP levels, quantification should be provided in the text and /or a supplementary figure.

- Is there any effect of BDNF on mEPSC frequency? Previous reports suggest that BDNF should increase mEPSC frequency via presynaptic mechanisms, it would be important to see if these effects are also hnRNP-dependent.

## Author Response

Manuscript #eNeuro eN-TNWR-0268-17X

We are very grateful to the Reviewers for the constructive comments that helped us to improve the manuscript. Below we address all comments (in blue) raised by the Reviewers. The revised text in the manuscript is highlighted in yellow.

REVIEWER #1

In the manuscript, “The RNA binding protein hnRNP K mediates the effect of BDNF on dendritic mRNA metabolism and regulates the activity of synaptic NMDA receptors in hippocampal neurons,” the authors present data showing that BDNF can lead to the dissociation of hnRNP K from dendritically localized mRNAs as indication of translation. Although there is no direct evidence that the dissociated mRNAs undergo translation, the idea that post-synaptic stimulation can result in displacement of hnRNP K from transcripts is an intriguing and exciting hypothesis.

Current understanding of the role of heterogeneous nuclear ribonucleoproteins (hnRNPs) on dendritically localized mRNA has been for the assembly and transport of RNA granules-where hnRNPs remain bound to the mRNA (Leal et al, 2014 and others). Moreover, hnRNP K has been implicated as having a role in dendritic and spine development (Proepper et al, 2011; Folci et al, 2014). The novelty of the work presented here is that hnRNP K can be “bumped-off” of mRNAs by translating ribosomes in a BDNF-dependent manner and could potentially serve as an indicator of mRNAs that are translated upon stimulation. If the authors can address the concerns below, the manuscript should be sufficient for publication.

Major concerns:

- If hnRNP K is indeed dissociating from mRNA in dendrites, the authors must address why the effect is less pronounced in synaptosomes relative to total lysate. For example, GluN1 in synaptosomes (Fig 3C) remains bound to hnRNP K compared to total lysate (Fig 3A), which contradicts the hypothesis. Such results suggest that the effect may be dominated by events occurring at the soma rather than in dendrites as BDNF is bath-applied.

We do agree with the reviewer when stating that some of the effects shown on Fig. 3A are likely events occurring at the cell soma. Nonetheless, since synaptoneurosomes are a closed system, the results obtained using this prepararation do not reflect any changes at the transcriptional and/or at the soma level but rather local actions of BDNF at the synapse. The difference between the effects of BDNF detected in synaptoneurosomes when compared with total cell extracts (results from panel C and panel A on figure 3) may be due to activation of distinct signaling pathways downstream of TrkB receptors localized at the soma- and synaptic- compartments.

- In Figures 3, 5 and 6, the calculation of “relative abundance” should be normalized to the total amount of RNA detected for each sample (total or synaptosome) rather than to control RNA IP. This is important as the authors did not show what fraction of mRNAs are bound to hnRNP K in steady state or post-BDNF stimulation. For example, if only 5-10% of a given RNA species is bound to hnRNP K at any given time, the results may not be representative.

Indeed we do understand the question raised by the reviewer but we think that calculating the percentage of enrichment over inputs does not allow comparing directly the effects of BDNF on hnRNP K-associated mRNAs. Moreover, even if only 20 % of a certain RNA species is bound to hnRNP K (which may be the case since mRNAs are known to be transported and regulated by several RNPs) it does not mean that the dissociation from hnRNP K complexes following BDNF is not relevant from the functional point of view.

- The use of cycloheximide (CHX), a translation inhibitor, in Figure 7 is very indirect. To determine whether hnRNP K dissociation from mRNA is due to translation, it would be necessary to include a data set of RNA IP performed in the presence of a translation inhibitor.

We would like to thank the reviewer for this comment. However, according to the model tested in this work, translation is likely to occur upon release of mRNAs from hnRNP K following BDNF treatment [as recently shown for the disassembly of ribonucleoprotein complexes containing hnRNP K and MENA (Vidaki et al., Neuron 95, 608-622 [2017]). Therefore, inhibition of translation per se is not expected to affect the release of transcripts from hnRNP K, as assessed by RNA-co-IP experiments.

Minor concerns:

- The authors should consider changing the title, as “regulates the activity of synaptic NMDA receptors” is not addressed in the manuscript.

The title was changed as suggested by the reviewer. The title of the revised manuscript is 'The RNA binding protein hnRNP K mediates the effect of BDNF on dendritic mRNA metabolism and regulates synaptic NMDA receptors in hippocampal neurons'.

- For Figure 1B&D, use of histograms would better represent the data than averages; averaging per puncta requires more detail on how image thresholding and segmentation was performed for particle analyses.

One additional paragraph was added to the methods section providing more details about the dendritic areas chosen and overall image processing and analysis.

- The figure panels should follow the order in which they are first mentioned in the text (Fig panels 2C&D come before 2A&B; and 6C before 6B).

Panels from Figure 2 were changed as suggested. The GO analysis of transcripts bound to hnRNP K and regulated by BDNF (former panels C and D, respectively) are now panels 2A and 2B. Furthermore, in the revised manuscript Figure 6B is mentioned before Figure 6C.

- Figure 7B should include a representative western blot of the knock-down (Folci et al, 2014)

A representative Western blot of hnRNP K knockdown is now included in Figure 7C as suggested by the reviewer. The figure caption was changed accordingly.

- Figure 7B shows only a ~30% knock-down of hnRNP K. Include explanation as to how such a modest reduction in hnRNP K can lead to reduction of mEPSC peak amplitude.

Since hnRNP K is far less abundant in the cytoplasm, one can hypothesize that 30% reduction on its levels may have a higher impact in dendrites when comparing to its nuclear/soma functions. Importantly, the conditions used to knock-down hnRNP K abrogated the synaptic NMDA receptor mediated mEPSC.

- Figure 7 should include analysis of mini frequency and not just amplitude.

As suggested by the reviewer, analysis of mEPSCs frequency is now included on Figure 7 (panel G). The figure caption in the revised manuscript also describes the new panel.

- In Figure 7E, all 3 panels seem to show identical Ctrl and BDNF results. If so, why is the p-value between Ctrl and BDNF different in the center panel?

These changes in p-value can be explained by the fact that we used a multiple comparison test which takes into consideration the amplitude values obtained in other experimental conditions.

REVIEWER #2

This paper addresses an important question regarding the mechanisms underlying the effects of BDNF at synapses, specifically related to activation of translational activity in dendrites. There is a well carried out series of studies describing novel interactions between BDNF and hnRNP K and its role in regulating synaptic plasticity and NMDA receptor modulation.

The authors demonstrate that activity-induced hnRNP accumulation in dendrites depends on BDNF signaling, and BDNF decreases the interaction of hnRNP with numerous transcripts. This BDNF - hnRNP K interaction is well documented. However, in the title and elsewhere in the text, the claim is made that hnRNP K is a key mediator of BDNF effects on dendritic RNA metabolism. However, no other hnRNP's were examined, so there is no specific evidence that hnRNP is the key mediator. Some comparison with other hnRNP's is needed to support this claim.

We are grateful for this valuable comment. In fact, we cannot exclude other putative players involved in the regulation of mRNA metabolism by BDNF. Indeed, it is well known that other trans-acting factors may be regulated by BDNF and contribute to local changes on synaptic proteome induced by this neurotrophin (Leal et al 2014). We did alter the manuscript in order to make clear that hnRNP K is not the only RNA binding protein acting downstream of TrkB signaling but rather a new and prominent mechanism by which BDNF may affect translation at excitatory synapses (Significance Statement, line 3; Introduction, first line of the last paragraph).

The paper (including title) also claims that hnRNP K regulates the activity of NMDA receptors, but the hnRNP knockdown by itself did not affect NMDA receptors. This should be addressed in more detail, and it would be more accurate to say that hnRNP may mediate the effect of BDNF on NMDA receptors.

The title and all the manuscript was revised as suggested by the reviewer.

Other concerns:

- SHN722, which is used to block the effect of BDNF, is a tyrosine kinase inhibitor which may be better than K252a, but is still not selective for trk receptors. It would be preferable to attempt to block BDNF effects with the trkB receptor antagonists ANA-12 or cyclotraxin-B.

We do agree with the reviewer when stating that SHN722 is not selective for TrkB receptors. Nonetheless, since (i) in such experiments we are blocking effects of exogenous BDNF, and (ii) in parallel experiments we show that TrkB-Fc scavenger blocks activity-induced hnRNP K accumulation in dendrites, we think this clearly demonstrates that TrkB receptors mediate the actions of both exogenous and endogenous BDNF on hnRNP K distribution.

- It is not clear if the shRNA against hnRNP K is selective or if it affects other hnRNPs.

We include on Figure 7 (panel C) of the revised manuscript a representative Western blot showing that the shRNA targeting hnRNP K effectively decreases hnRNP K protein levels but is without effect on the levels of hnRNP A2/B1. This further suggests that the shRNA tested is specific for hnRNP K.

- Details should be provided for dendritic analysis of hnRNP immunoreactivity, how were the dendritic areas chosen, number of areas and mean length per cell, etc.

One additional paragraph was added to the methods section providing more details about the dendritic areas chosen and overall image processing and analysis.

- For the bicuculline effect on total hnRNP levels, quantification should be provided in the text and /or a supplementary figure.

One supplementary figure was added with this data, as suggested by the reviewer (Figure 1-1). A figure caption was also included in the revised manuscript.

- Is there any effect of BDNF on mEPSC frequency? Previous reports suggest that BDNF should increase mEPSC frequency via presynaptic mechanisms, it would be important to see if these effects are also hnRNP-dependent.

We appreciate this valuable comment by the reviewer. Figure 7 (panel G) now contains the analysis of mESPCs frequency measured following BDNF treatment, under control conditions and when hnRNP K is reduced. The figure caption in the revised manuscript also describes the new panel.

## References

[B1] Atasoy D, Ertunc M, Moulder KL, Blackwell J, Chung C, Su J, Kavalali ET (2008) Spontaneous and evoked glutamate release activates two populations of NMDA receptors with limited overlap. J Neurosci 28:10151–10166. 10.1523/JNEUROSCI.2432-08.2008 18829973 PMC2578837

[B2] Baj G, Pinhero V, Vaghi V, Tongiorgi E (2016) Signaling pathways controlling activity-dependent local translation of BDNF and their localization in dendritic arbors. J Cell Sci 129:2852–2864. 10.1242/jcs.177626 27270670

[B3] Benson DL (1997) Dendritic compartmentation of NMDA receptor mRNA in cultured hippocampal neurons. Neuroreport 8:823–828. 9141046 10.1097/00001756-199703030-00004

[B4] Bomsztyk K, Denisenko O, Ostrowski J (2004) hnRNP K: one protein multiple processes. Bioessays 26:629–638. 10.1002/bies.20048 15170860

[B5] Bramham CR, Southard T, Sarvey JM, Herkenham M, Brady LS (1996) Unilateral LTP triggers bilateral increases in hippocampal neurotrophin and trk receptor mRNA expression in behaving rats: evidence for interhemispheric communication. J Comp Neur 368:371–382. 10.1002/(SICI)1096-9861(19960506)368:3&amp;lt;371::AID-CNE4&amp;gt;3.0.CO;2-2 8725345

[B6] Carvalho AL, Caldeira MV, Santos SD, Duarte CB (2008) Role of the brain-derived neurotrophic factor at glutamatergic synapses. Br J Pharmacol 153 [Suppl 1 ]:S310–S324. 10.1038/sj.bjp.0707509 18059328 PMC2268077

[B7] Courtney KD, Grove M, Vandongen H, Vandongen A, LaMantia AS, Pendergast AM (2000) Localization and phosphorylation of Abl-interactor proteins, Abi-1 and Abi-2, in the developing nervous system. Mol Cell Neurosci 16:244–257. 10.1006/mcne.2000.0865 10995551

[B8] Dziembowska M, Milek J, Janusz A, Rejmak E, Romanowska E, Gorkiewicz T, Tiron A, Bramham CR, Kaczmarek L (2012) Activity-dependent local translation of matrix metalloproteinase-9. J Neurosci 32:14538–14547. 10.1523/JNEUROSCI.6028-11.2012 23077039 PMC6621441

[B9] Edelmann E, Lessmann V, Brigadski T (2014) Pre- and postsynaptic twists in BDNF secretion and action in synaptic plasticity. Neuropharmacol 76 [Pt C]:610–627. 10.1016/j.neuropharm.2013.05.043 23791959

[B10] Elvira G, Wasiak S, Blandford V, Tong XK, Serrano A, Fan X, del Rayo Sánchez-Carbente M, Servant F, Bell AW, Boismenu D, Lacaille JC, McPherson PS, DesGroseillers L, Sossin WS (2006) Characterization of an RNA granule from developing brain. Mol Cell Proteomics 5:635–651. 10.1074/mcp.M500255-MCP200 16352523

[B11] Fan X, Jin WY, Wang YT (2014) The NMDA receptor complex: a multifunctional machine at the glutamatergic synapse. Front Cell Neurosci 8:160. 10.3389/fncel.2014.00160 24959120 PMC4051310

[B12] Folci A, Mapelli L, Sassone J, Prestori F, D'Angelo E, Bassani S, Passafaro M (2014) Loss of hnRNP K impairs synaptic plasticity in hippocampal neurons. J Neurosci 34:9088–9095. 10.1523/JNEUROSCI.0303-14.2014 24990929 PMC6608249

[B13] Fortin DA, Srivastava T, Dwarakanath D, Pierre P, Nygaard S, Derkach VA, Soderling TR (2012) Brain-derived neurotrophic factor activation of CaM-kinase kinase via transient receptor potential canonical channels induces the translation and synaptic incorporation of GluA1-containing calcium-permeable AMPA receptors. J Neurosci 32:8127–8137. 10.1523/JNEUROSCI.6034-11.2012 22699894 PMC3390208

[B14] Gazzaley AH, Benson DL, Huntley GW, Morrison JH (1997) Differential subcellular regulation of NMDAR1 protein and mRNA in dendrites of dentate gyrus granule cells after perforant path transection. J Neurosci 17:2006–2017. 9045729 10.1523/JNEUROSCI.17-06-02006.1997 PMC6793768

[B15] Geuens T, Bouhy D, Timmerman V (2016) The hnRNP family: insights into their role in health and disease. Hum Genet 135:851–867. 10.1007/s00439-016-1683-5 27215579 PMC4947485

[B16] Gomes JR, Costa JT, Melo CV, Felizzi F, Monteiro P, Pinto MJ, Inácio AR, Wieloch T, Almeida RD, Grãos M, Duarte CB (2012) Excitotoxicity downregulates TrkB.FL signaling and upregulates the neuroprotective truncated TrkB receptors in cultured hippocampal and striatal neurons. J Neurosci 32:4610–4622. 10.1523/JNEUROSCI.0374-12.2012 22457507 PMC6622054

[B17] Habelhah H, Shah K, Huang L, Ostareck-Lederer A, Burlingame AL, Shokat KM, Hentze MW, Ronai Z (2001) ERK phosphorylation drives cytoplasmic accumulation of hnRNP-K and inhibition of mRNA translation. Nat Cell Biol 3:325–330. 10.1038/35060131 11231586

[B18] Hardingham GE, Fukunaga Y, Bading H (2002) Extrasynaptic NMDARs oppose synaptic NMDARs by triggering CREB shut-off and cell death pathways. Nat Neurosci 5:405–414. 10.1038/nn835 11953750

[B19] Håvik B, Røkke H, Bårdsen K, Davanger S, Bramham CR (2003) Bursts of high-frequency stimulation trigger rapid delivery of pre-existing alpha-CaMKII mRNA to synapses: a mechanism in dendritic protein synthesis during long-term potentiation in adult awake rats. Eur J Neurosci 17:2679–2689. 12823475 10.1046/j.1460-9568.2003.02712.x

[B20] Huganir RL, Nicoll RA (2013) AMPARs and synaptic plasticity: the last 25 years. Neuron 80:704–717. 10.1016/j.neuron.2013.10.025 24183021 PMC4195488

[B21] Hutchins EJ, Szaro BG (2013) c-Jun N-terminal kinase phosphorylation of heterogeneous nuclear ribonucleoprotein K regulates vertebrate axon outgrowth via a posttranscriptional mechanism. J Neurosci 33:14666–14680. 10.1523/JNEUROSCI.4821-12.2013 24027268 PMC6705169

[B22] Hutchins EJ, Belrose JL, Szaro BG (2015) Phosphorylation of heterogeneous nuclear ribonucleoprotein K at an extracellular signal-regulated kinase phosphorylation site promotes neurofilament-medium protein expression and axon outgrowth in *Xenopus* . Neurosci Lett 607:59–65. 10.1016/j.neulet.2015.09.027 26409787

[B23] Iwasaki T, Koretomo Y, Fukuda T, Paronetto MP, Sette C, Fukami Y, Sato K (2008) Expression, phosphorylation, and mRNA-binding of heterogeneous nuclear ribonucleoprotein K in *Xenopus* oocytes, eggs, and early embryos. Dev Growth Differ 50:23–40. 10.1111/j.1440-169X.2007.00974.x 18042150

[B24] Janas J, Skowronski J, Van Aelst L (2006) Lentiviral delivery of RNAi in hippocampal neurons. Meth Enzymol 406:593–605. 10.1016/S0076-6879(06)06046-0 16472690

[B25] Kaech S, Banker G (2006) Culturing hippocampal neurons. Nat Protoc 1:2406–2415. 10.1038/nprot.2006.356 17406484

[B26] Kanai Y, Dohmae N, Hirokawa N (2004) Kinesin transports RNA: isolation and characterization of an RNA-transporting granule. Neuron 43:513–525. 10.1016/j.neuron.2004.07.022 15312650

[B27] Kang H, Schuman EM (1996) A requirement for local protein synthesis in neurotrophin-induced hippocampal synaptic plasticity. Science 273:1402–1406. 8703078 10.1126/science.273.5280.1402

[B28] Kang H, Welcher AA, Shelton D, Schuman EM (1997) Neurotrophins and time: different roles for TrkB signaling in hippocampal long-term potentiation. Neuron 19:653–664. 9331355 10.1016/s0896-6273(00)80378-5

[B29] Kessels HW, Nabavi S, Malinow R (2013) Metabotropic NMDA receptor function is required for β-amyloid-induced synaptic depression. Proc Natl Acad Sci USA 110:4033–4038. 10.1073/pnas.1219605110 23431156 PMC3593880

[B30] Kim K, Saneyoshi T, Hosokawa T, Okamoto K, Hayashi Y (2016) Interplay of enzymatic and structural functions of CaMKII in long-term potentiation. J Neurochem 139:959–972. 10.1111/jnc.13672 27207106

[B31] Kolb JE, Trettel J, Levine ES (2005) BDNF enhancement of postsynaptic NMDA receptors is blocked by ethanol. Synapse 55:52–57. 10.1002/syn.20090 15515007

[B32] Korte M, Carroll P, Wolf E, Brem G, Thoenen H, Bonhoeffer T (1995) Hippocampal long-term potentiation is impaired in mice lacking brain-derived neurotrophic factor. Proc Natl Acad Sci USA 92:8856–8860. 7568031 10.1073/pnas.92.19.8856 PMC41066

[B33] Korte M, Staiger V, Griesbeck O, Thoenen H, Bonhoeffer T (1996) The involvement of brain-derived neurotrophic factor in hippocampal long-term potentiation revealed by gene targeting experiments. J Physiol Paris 90:157–164. 9116659 10.1016/s0928-4257(97)81415-5

[B34] Kosik KS (2016) Life at low copy number: how dendrites manage with so few mRNAs. Neuron 92:1168–1180. 10.1016/j.neuron.2016.11.002 28009273

[B35] Krichevsky AM, Kosik KS (2001) Neuronal RNA granules: a link between RNA localization and stimulation-dependent translation. Neuron 32:683–696. 11719208 10.1016/s0896-6273(01)00508-6

[B36] Laursen LS, Chan CW, Ffrench-Constant C (2011) Translation of myelin basic protein mRNA in oligodendrocytes is regulated by integrin activation and hnRNP-K. J Cell Biol 192 797–811. 10.1083/jcb.201007014 21357748 PMC3051817

[B37] Leal G, Comprido D, Duarte CB (2014a) BDNF-induced local protein synthesis and synaptic plasticity. Neuropharmacol 76 [Pt C]:639–656. 10.1016/j.neuropharm.2013.04.005 23602987

[B38] Leal G, Afonso PM, Duarte CB (2014b) Neuronal activity induces synaptic delivery of hnRNP A2/B1 by a BDNF-dependent mechanism in cultured hippocampal neurons. PLoS One 9:e108175. 25286195 10.1371/journal.pone.0108175 PMC4186808

[B39] Liao L, Pilotte J, Xu T, Wong CC, Edelman GM, Vanderklish P, Yates JR 3rd (2007) BDNF induces widespread changes in synaptic protein content and up-regulates components of the translation machinery: an analysis using high-throughput proteomics. J Proteome Res 6:1059–1071. 10.1021/pr060358f 17330943

[B40] Link W, Konietzko U, Kauselmann G, Krug M, Schwanke B, Frey U, Kuhl D (1995) Somatodendritic expression of an immediate early gene is regulated by synaptic activity. Proc Natl Acad Sci USA 92:5734–5738. 7777577 10.1073/pnas.92.12.5734 PMC41771

[B41] Liu Y, Szaro BG (2011) hnRNP K post-transcriptionally co-regulates multiple cytoskeletal genes needed for axonogenesis. Development 138:3079–3090. 10.1242/dev.066993 21693523

[B42] Lyford GL, Yamagata K, Kaufmann WE, Barnes CA, Sanders LK, Copeland NG, Gilbert DJ, Jenkins NA, Lanahan AA, Worley PF (1995) Arc, a growth factor and activity-regulated gene, encodes a novel cytoskeleton-associated protein that is enriched in neuronal dendrites. Neuron 14:433–445. 7857651 10.1016/0896-6273(95)90299-6

[B43] Madara JC, Levine ES (2008) Presynaptic and postsynaptic NMDA receptors mediate distinct effects of brain-derived neurotrophic factor on synaptic transmission. J Neurophysiol 100:3175–3184. 10.1152/jn.90880.2008 18922945 PMC2604859

[B44] Manadas B, Santos AR, Szabadfi K, Gomes JR, Garbis SD, Fountoulakis M, Duarte CB (2009) BDNF-induced changes in the expression of the translation machinery in hippocampal neurons: protein levels and dendritic mRNA. J Proteome Res 8:4536–4552. 10.1021/pr900366x 19702335

[B45] Martin KJ, Shpiro N, Traynor R, Elliott M, Arthur JS (2011) Comparison of the specificity of Trk inhibitors in recombinant and neuronal assays. Neuropharmacol 61:148–155. 10.1016/j.neuropharm.2011.03.021 21466816

[B46] Messaoudi E, Kanhema T, Soulé J, Tiron A, Dagyte G, da Silva B, Bramham CR (2007) Sustained Arc/Arg3.1 synthesis controls long-term potentiation consolidation through regulation of local actin polymerization in the dentate gyrus in vivo. J Neurosci 27:10445–10455. 10.1523/JNEUROSCI.2883-07.2007 17898216 PMC6673172

[B47] Mi H, Muruganujan A, Casagrande JT, Thomas PD (2013a) Large-scale gene function analysis with the PANTHER classification system. Nat Protoc 8:1551–1566. 10.1038/nprot.2013.092 23868073 PMC6519453

[B48] Mikula M, Dzwonek A, Karczmarski J, Rubel T, Dadlez M, Wyrwicz LS, Bomsztyk K, Ostrowski J (2006) Landscape of the hnRNP K protein-protein interactome. Proteomics 6:2395–2406. 10.1002/pmic.200500632 16518874

[B49] Minichiello L, Korte M, Wolfer D, Kühn R, Unsicker K, Cestari V, Rossi-Arnaud C, Lipp HP, Bonhoeffer T, Klein R (1999) Essential role for TrkB receptors in hippocampus-mediated learning. Neuron 24:401–414. 10571233 10.1016/s0896-6273(00)80853-3

[B50] Ostareck-Lederer A, Ostareck DH, Cans C, Neubauer G, Bomsztyk K, Superti-Furga G, Hentze MW (2002) c-Src-mediated phosphorylation of hnRNP K drives translational activation of specifically silenced mRNAs. Mol Cell Biol 22:4535–4543. 12052863 10.1128/MCB.22.13.4535-4543.2002 PMC133888

[B51] Pal R, Agbas A, Bao X, Hui D, Leary C, Hunt J, Naniwadekar A, Michaelis ML, Kumar KN, Michaelis EK (2003) Selective dendrite-targeting of mRNAs of NR1 splice variants without exon 5: identification of a cis-acting sequence and isolation of sequence-binding proteins. Brain Res 994:1–18. 14642443 10.1016/j.brainres.2003.08.046

[B52] Panja D, Bramham CR (2014) BDNF mechanisms in late LTP formation: a synthesis and breakdown. Neuropharmacol 76 [Pt C]:664–676. 10.1016/j.neuropharm.2013.06.024 23831365

[B53] Panja D, Dagyte G, Bidinosti M, Wibrand K, Kristiansen AM, Sonenberg N, Bramham CR (2009) Novel translational control in Arc-dependent long term potentiation consolidation in vivo. J Biol Chem 284:31498–31511. 10.1074/jbc.M109.056077 19755425 PMC2797219

[B54] Panja D, Kenney JW, D'Andrea L, Zalfa F, Vedeler A, Wibrand K, Fukunaga R, Bagni C, Proud CG, Bramham CR (2014) Two-stage translational control of dentate gyrus LTP consolidation is mediated by sustained BDNF-TrkB signaling to MNK. Cell Rep 9:1430–1445. 10.1016/j.celrep.2014.10.016 25453757

[B55] Patterson SL, Grover LM, Schwartzkroin PA, Bothwell M (1992) Neurotrophin expression in rat hippocampal slices: a stimulus paradigm inducing LTP in CA1 evokes increases in BDNF and NT-3 mRNAs. Neuron 9:1081–1088. 1463608 10.1016/0896-6273(92)90067-n

[B56] Pinto MJ, Alves PL, Martins L, Pedro JR, Ryu HR, Jeon NL, Taylor AM, Almeida RD (2016) The proteasome controls presynaptic differentiation through modulation of an on-site pool of polyubiquitinated conjugates. J Cell Biol 212:789–801. 10.1083/jcb.201509039 27022091 PMC4810304

[B57] Proepper C, Steinestel K, Schmeisser MJ, Heinrich J, Steinestel J, Bockmann J, Liebau S, Boeckers TM (2011) Heterogeneous nuclear ribonucleoprotein k interacts with Abi-1 at postsynaptic sites and modulates dendritic spine morphology. PLoS One 6:e27045. 10.1371/journal.pone.0027045 22102872 PMC3216941

[B58] Raju CS, Fukuda N, López-Iglesias C, López C, Visa N, Percipalle P (2011) In neurons, activity-dependent association of dendritically transported mRNA transcripts with the transacting factor CBF-A is mediated by A2RE/RTS elements. Mol Biol Cell 22:1864–1877. 10.1091/mbc.E10-11-0904 21471000 PMC3103402

[B59] Rook MS, Lu M, Kosik KS (2000) CaMKIIalpha 3' untranslated region-directed mRNA translocation in living neurons: visualization by GFP linkage. J Neurosci 20:6385–6393. 10964944 10.1523/JNEUROSCI.20-17-06385.2000 PMC6772957

[B60] Santos AR, Comprido D, Duarte CB (2010) Regulation of local translation at the synapse by BDNF. Prog Neurobiol 92:505–516. 10.1016/j.pneurobio.2010.08.004 20713125

[B61] Santos AR, Mele M, Vaz SH, Kellermayer B, Grimaldi M, Colino-Oliveira M, Rombo DM, Comprido D, Sebastião AM, Duarte CB (2015) Differential role of the proteasome in the early and late phases of BDNF-induced facilitation of LTP. J Neurosci 35:3319–3329. 10.1523/JNEUROSCI.4521-14.2015 25716833 PMC6605551

[B62] Schratt GM, Nigh EA, Chen WG, Hu L, Greenberg ME (2004) BDNF regulates the translation of a select group of mRNAs by a mammalian target of rapamycin-phosphatidylinositol 3-kinase-dependent pathway during neuronal development. J Neurosci 24:7366–7377. 10.1523/JNEUROSCI.1739-04.2004 15317862 PMC6729778

[B63] Shiina N, Shinkura K, Tokunaga M (2005) A novel RNA-binding protein in neuronal RNA granules: regulatory machinery for local translation. J Neurosci 25:4420–4434. 10.1523/JNEUROSCI.0382-05.2005 15858068 PMC6725113

[B64] Shioda N, Moriguchi S, Oya T, Ishii Y, Shen J, Matsushima T, Nishijo H, Sasahara M, Fukunaga K (2012) Aberrant hippocampal spine morphology and impaired memory formation in neuronal platelet-derived growth factor β-receptor lacking mice. Hippocampus 22:1371–1378. 10.1002/hipo.20973 21997856

[B65] Sirven A, Ravet E, Charneau P, Zennou V, Coulombel L, Guétard D, Pflumio F, Dubart-Kupperschmitt A (2001) Enhanced transgene expression in cord blood CD34^+^-derived hematopoietic cells, including developing T cells and NOD/SCID mouse repopulating cells, following transduction with modified trip lentiviral vectors. Mol Ther 3:438–448. 10.1006/mthe.2001.0282 11319904

[B66] Steward O, Levy WB (1982) Preferential localization of polyribosomes under the base of dendritic spines in granule cells of the dentate gyrus. J Neurosci 2:284–291. 7062109 10.1523/JNEUROSCI.02-03-00284.1982 PMC6564334

[B67] Steward O, Worley PF (2001) Selective targeting of newly synthesized Arc mRNA to active synapses requires NMDA receptor activation. Neuron 30:227–240. 11343657 10.1016/s0896-6273(01)00275-6

[B68] Steward O, Wallace CS, Lyford GL, Worley PF (1998) Synaptic activation causes the mRNA for the IEG Arc to localize selectively near activated postsynaptic sites on dendrites. Neuron 21:741–751. 9808461 10.1016/s0896-6273(00)80591-7

[B69] Tahir TA, Singh H, Brindle NP (2014) The RNA binding protein hnRNP-K mediates post-transcriptional regulation of uncoupling protein-2 by angiopoietin-1. Cell Signal 26:1379–1384. 10.1016/j.cellsig.2014.03.005 24642125 PMC4039131

[B70] Takei N, Inamura N, Kawamura M, Namba H, Hara K, Yonezawa K, Nawa H (2004) Brain-derived neurotrophic factor induces mammalian target of rapamycin-dependent local activation of translation machinery and protein synthesis in neuronal dendrites. J Neurosci 24:9760–9769. 10.1523/JNEUROSCI.1427-04.2004 15525761 PMC6730227

[B71] Thomas KL, Laroche S, Errington ML, Bliss TV, Hunt SP (1994) Spatial and temporal changes in signal transduction pathways during LTP. Neuron 13:737–745. 7917303 10.1016/0896-6273(94)90040-x

[B72] Tiruchinapalli DM, Oleynikov Y, Kelic S, Shenoy SM, Hartley A, Stanton PK, Singer RH, Bassell GJ (2003) Activity-dependent trafficking and dynamic localization of zipcode binding protein 1 and beta-actin mRNA in dendrites and spines of hippocampal neurons. J Neurosci 23:3251–3261. 12716932 10.1523/JNEUROSCI.23-08-03251.2003 PMC6742306

[B73] Tongiorgi E, Righi M, Cattaneo A (1997) Activity-dependent dendritic targeting of BDNF and TrkB mRNAs in hippocampal neurons. J Neurosci 17:9492–9505. 9391005 10.1523/JNEUROSCI.17-24-09492.1997 PMC6573421

[B74] Troca-Marín JA, Alves-Sampaio A, Tejedor FJ, Montesinos ML (2010) Local translation of dendritic RhoA revealed by an improved synaptoneurosome preparation. Mol Cell Neurosci 43:308–314. 10.1016/j.mcn.2009.12.004 20035871

[B75] Vanevski F, Xu B (2015) HuD interacts with Bdnf mRNA and is essential for activity-induced BDNF synthesis in dendrites. PLoS One 10:e0117264. 10.1371/journal.pone.0117264 25692578 PMC4332865

[B76] Yin Y, Edelman GM, Vanderklish PW (2002) The brain-derived neurotrophic factor enhances synthesis of Arc in synaptoneurosomes. Proc Natl Acad Sci USA 99:2368–2373. 10.1073/pnas.042693699 11842217 PMC122371

[B77] Yoo Y, Wu X, Egile C, Li R, Guan JL (2006) Interaction of N-WASP with hnRNPK and its role in filopodia formation and cell spreading. J Biol Chem 281:15352–15360. 10.1074/jbc.M511825200 16574661

[B78] Zeitelhofer M, Karra D, Macchi P, Tolino M, Thomas S, Schwarz M, Kiebler M, Dahm R (2008) Dynamic interaction between P-bodies and transport ribonucleoprotein particles in dendrites of mature hippocampal neurons. J Neurosci 28:7555–7562. 10.1523/JNEUROSCI.0104-08.2008 18650333 PMC6670838

