# Supplementary material for: The RNA-Binding Protein hnRNP K Mediates the Effect of BDNF on Dendritic mRNA Metabolism and Regulates Synaptic NMDA Receptors in Hippocampal Neurons
Source: eNeuro. 2017 Dec 12;4(6):ENEURO.0268-17.2017. doi: 10.1523/ENEURO.0268-17.2017 (PMC5732018; doi:10.1523/ENEURO.0268-17.2017)
Supplement: Table 1-1 — Download Table 1-1, PDF file. [file sup_enu-eN-NWR-0268-17-s04.pdf]

Table 1-1 - hnRNP K co-immunoprecipitated transcripts

Gene Symbol	Description	Fold change
LOC290876	Rattus norvegicus similar to RIKEN cDNA 1700029H14 (LOC290876), mRNA [NM_001037182]	**468,618**
Lmx1b	Homeodomain protein LMX1b [Source:UniProtKB/TrEMBL;Acc:Q811V1] [ENSRNOT00000022953]	**330,314**
0	Unknown	**212,281**
Sod3	Rattus norvegicus superoxide dismutase 3, extracellular (Sod3), mRNA [NM_012880]	**197,456**
Kcnc3	Rattus norvegicus mRNA sequence. [M84210]	**194,736**
Ctnnd1	Rattus norvegicus catenin (cadherin associated protein), delta 1 (Ctnnd1), mRNA [NM_001107740]	**188,589**
Pcdhgc5	Rattus norvegicus protocadherin gamma c5 (Pcdhgc5), mRNA [NM_001164288]	**187,617**
LOC688314	PREDICTED: Rattus norvegicus similar to stimulated by retinoic acid 13 (LOC688314), mRNA [XM_001081840]	**170,099**
Antxr1	Rattus norvegicus anthrax toxin receptor 1 (Antxr1), mRNA [NM_001044249]	**169,160**
Hspb6	Rattus norvegicus heat shock protein, alpha-crystallin-related, B6 (Hspb6), mRNA [NM_138887]	**169,062**
Iglon5	PREDICTED: Rattus norvegicus IgLON family member 5 (Iglon5), mRNA [XM_218634]	**168,063**
0	Unknown	**160,431**
Nrgn	Rattus norvegicus neurogranin (Nrgn), mRNA [NM_024140]	**158,519**
RGD1561016	Uncharacterized protein [Source:UniProtKB/TrEMBL;Acc:D3ZDR7] [ENSRNOT00000017340]	**153,143**
RGD1564163	Rattus norvegicus similar to RIKEN cDNA 1700025K23 (RGD1564163), mRNA [NM_001109266]	**152,681**
0	Rattus norvegicus TL0AEA67YN06 mRNA sequence. [FQ231880]	**149,071**
Gpr37l1	Rattus norvegicus G protein-coupled receptor 37-like 1 (Gpr37l1), mRNA [NM_145784]	**148,764**
Nlgn3	Rattus norvegicus neuroligin 3 (Nlgn3), mRNA [NM_134336]	**147,096**
Gatc	Rattus norvegicus glutamyl-tRNA(Gln) amidotransferase, subunit C homolog (bacterial) (Gatc), mRNA [NM_001108339]	**138,415**
Gstm7	Rattus norvegicus glutathione S-transferase, mu 7 (Gstm7), mRNA [NM_031154]	**136,907**
Gm2a	Rattus norvegicus GM2 ganglioside activator (Gm2a), mRNA [NM_172335]	**135,988**
Slc9a3r1	Rattus norvegicus solute carrier family 9 (sodium/hydrogen exchanger), member 3 regulator 1 (Slc9a3r1), mRNA [NM_021594]	**134,266**
Slc13a3	Rattus norvegicus solute carrier family 13 (sodium-dependent dicarboxylate transporter), member 3 (Slc13a3), mRNA [NM_022866]	**132,498**
Tcf3	Rattus norvegicus transcription factor 3 (Tcf3), mRNA [NM_001107865]	**129,071**
Prrt1	Rattus norvegicus proline-rich transmembrane protein 1 (Prrt1), mRNA [NM_001032285]	**128,651**
Ocln	Rattus norvegicus occludin (Ocln), mRNA [NM_031329]	**127,667**
Znf575	Rattus norvegicus zinc finger protein 575 (Znf575), mRNA [NM_001107489]	**126,828**
Syn1	Rattus norvegicus synapsin I (Syn1), transcript variant a, mRNA [NM_019133]	**125,695**
Lrrc8a	Rattus norvegicus leucine rich repeat containing 8 family, member A (Lrrc8a), mRNA [NM_001024782]	**124,763**
Dpysl5	Rattus norvegicus dihydropyrimidinase-like 5 (Dpysl5), mRNA [NM_023023]	**124,181**

Cldnd1	Rattus norvegicus claudin domain containing 1 (Cldnd1), mRNA [NM_001006955]	**122,625**
Ncam1	Rattus norvegicus neural cell adhesion molecule 1 (Ncam1), mRNA [NM_031521]	**122,287**
Bcan	Rattus norvegicus brevican (Bcan), transcript variant 2, mRNA [NM_012916]	**121,479**
Tbx6	Rattus norvegicus T-box 6 (Tbx6), mRNA [NM_001108920]	**120,419**
Vangl2	Rattus norvegicus vang-like 2 (van gogh, Drosophila) (Vangl2), mRNA [NM_001105969]	**120,172**
0	Unknown	**118,895**
Ly6e	Rattus norvegicus lymphocyte antigen 6 complex, locus E (Ly6e), mRNA [NM_001017467]	**118,666**
Eln	Rattus norvegicus elastin (Eln), mRNA [NM_012722]	**117,300**
Clip3	Rattus norvegicus CAP-GLY domain containing linker protein 3 (Clip3), mRNA [NM_001107501]	**116,712**
Apba1	Rattus norvegicus amyloid beta (A4) precursor protein-binding, family A, member 1 (Apba1), mRNA [NM_031779]	**116,398**
Sepp1	Rattus norvegicus selenoprotein P, plasma, 1 (Sepp1), transcript variant 2, mRNA [NM_001083911]	**116,206**
Olr857	Rattus norvegicus olfactory receptor 857 (Olr857), mRNA [NM_001000583]	**113,176**
Fam163b	Rattus norvegicus family with sequence similarity 163, member B (Fam163b), mRNA [NM_001109458]	**113,048**
Ppap2b	Rattus norvegicus phosphatidic acid phosphatase type 2B (Ppap2b), mRNA [NM_138905]	**110,887**
Ina	Rattus norvegicus internexin neuronal intermediate filament protein, alpha (Ina), mRNA [NM_019128]	**110,483**
Uba1	Rattus norvegicus ubiquitin-like modifier activating enzyme 1 (Uba1), mRNA [NM_001014080]	**109,022**
Dlg4	Rattus norvegicus discs, large homolog 4 (Drosophila) (Dlg4), mRNA [NM_019621]	**108,950**
Fa2h	Rattus norvegicus fatty acid 2-hydroxylase (Fa2h), mRNA [NM_001135583]	**108,842**
Kcnc3	Rattus norvegicus potassium voltage gated channel, Shaw-related subfamily, member 3 (Kcnc3), mRNA [NM_053997]	**107,429**
Nnat	Rattus norvegicus neuronatin (Nnat), transcript variant 1, mRNA [NM_053601]	**106,863**
Aes	Rattus norvegicus amino-terminal enhancer of split (Aes), mRNA [NM_019220]	**106,196**
Mrpl52	Rattus norvegicus mitochondrial ribosomal protein L52 (Mrpl52), nuclear gene encoding mitochondrial protein, mRNA [NM_001108375]	**105,588**
0	Unknown	**104,046**
Prrt1	Rattus norvegicus proline-rich transmembrane protein 1 (Prrt1), mRNA [NM_001032285]	**103,917**
Wbp2	Rattus norvegicus WW domain binding protein 2 (Wbp2), mRNA [NM_138975]	**103,817**
Ntrk2	Rattus norvegicus neurotrophic tyrosine kinase, receptor, type 2 (Ntrk2), transcript variant 2, mRNA [NM_001163168]	**103,273**
Stk32b	Rattus norvegicus serine/threonine kinase 32B (Stk32b), mRNA [NM_001107224]	**101,830**
Zmat2	Rattus norvegicus zinc finger, matrin type 2 (Zmat2), mRNA [NM_001135582]	**101,750**
Eny2	Rattus norvegicus enhancer of yellow 2 homolog (Drosophila) (Eny2), mRNA [NM_001130580]	**101,561**
Dynlrb1	Rattus norvegicus dynein light chain roadblock-type 1 (Dynlrb1), mRNA [NM_131910]	**101,527**
Mon1b	Rattus norvegicus MON1 homolog b (yeast) (Mon1b), mRNA [NM_001107433]	**100,633**
Prkaca	Rattus norvegicus protein kinase, cAMP-dependent, catalytic, alpha (Prkaca), mRNA [NM_001100922]	**99,762**
Elmo1	Rattus norvegicus engulfment and cell motility 1 (Elmo1), mRNA [NM_001108415]	**99,642**
Sipa1l3	Rattus norvegicus signal-induced proliferation-associated 1 like 3 (Sipa1l3), mRNA [NM_001013066]	**99,295**

Rab23	Rattus norvegicus RAB23, member RAS oncogene family (Rab23), mRNA [NM_001109005]	**99,288**
Accn2	Rattus norvegicus amiloride-sensitive cation channel 2, neuronal (Accn2), mRNA [NM_024154]	**98,677**
LOC306766	Rattus norvegicus hypothetical LOC306766 (LOC306766), mRNA [NM_001014007]	**98,595**
Zfp398	Rattus norvegicus zinc finger protein 398 (Zfp398), mRNA [NM_001109230]	**98,298**
Ccnk	Rattus norvegicus cyclin K (Ccnk), mRNA [NM_001109672]	**98,278**
RGD1565095	Rattus norvegicus similar to hypothetical protein MGC52110 (RGD1565095), mRNA [NM_001195488]	**98,067**
Nptx1	Rattus norvegicus neuronal pentraxin 1 (Nptx1), mRNA [NM_153735]	**97,874**
Scrn2	Rattus norvegicus secernin 2 (Scrn2), mRNA [NM_001012142]	**97,427**
Fem1c	Rattus norvegicus fem-1 homolog c (C. elegans) (Fem1c), mRNA [NM_001106932]	**97,067**
RGD1565920	PREDICTED: Rattus norvegicus similar to OTTMUSP00000000438 (RGD1565920), mRNA [XM_344713]	**96,552**
Coro2b	Coro2b protein [Source:UniProtKB/TrEMBL;Acc:Q5EB67] [ENSRNOT00000020951]	**96,245**
Dlc1	Rattus norvegicus deleted in liver cancer 1 (Dlc1), mRNA [NM_001127446]	**96,153**
Sox8	Rattus norvegicus SRY (sex determining region Y)-box 8 (Sox8), mRNA [NM_001106989]	**96,082**
LOC100188932	Rattus norvegicus dolichyl-diphosphooligosaccharide--protein glycosyltransferase subunit 4 (LOC100188932), transcript variant 2, mRNA [NM_001134690]	**95,839**
Cxcl16	Rattus norvegicus chemokine (C-X-C motif) ligand 16 (Cxcl16), mRNA [NM_001017478]	**95,602**
Copz2	Rattus norvegicus coatomer protein complex, subunit zeta 2 (Copz2), mRNA [NM_001108294]	**95,214**
Dab2	Rattus norvegicus disabled homolog 2 (Drosophila) (Dab2), mRNA [NM_024159]	**94,146**
Zbed3	Rattus norvegicus zinc finger, BED-type containing 3 (Zbed3), mRNA [NM_001025729]	**93,809**
Hspb2	Rattus norvegicus heat shock protein beta 2 (Hspb2), mRNA [NM_130431]	**93,693**
Fn3krp	Rattus norvegicus fructosamine-3-kinase-related protein (Fn3krp), mRNA [NM_001107077]	**93,575**
Ctdsp1	Rattus norvegicus CTD (carboxy-terminal domain, RNA polymerase II, polypeptide A) small phosphatase 1 (Ctdsp1), mRNA [NM_001128079]	**93,471**
RGD1308772	Uncharacterized protein [Source:UniProtKB/TrEMBL;Acc:D3ZIN5] [ENSRNOT00000013780]	**93,309**
Bcl9l	Rattus norvegicus B-cell CLL/lymphoma 9-like (Bcl9l), mRNA [NM_001106817]	**92,888**
Nrarp	Rattus norvegicus Notch-regulated ankyrin repeat protein (Nrarp), mRNA [NM_001143750]	**92,119**
Bak1	Rattus norvegicus BCL2-antagonist/killer 1 (Bak1), mRNA [NM_053812]	**92,047**
Ganc	Rattus norvegicus glucosidase, alpha; neutral C (Ganc), mRNA [NM_001145840]	**91,864**
Col6a2	Rattus norvegicus collagen, type VI, alpha 2 (Col6a2), mRNA [NM_001100741]	**91,744**
LOC689574	Rattus norvegicus hypothetical protein LOC689574 (LOC689574), nuclear gene encoding mitochondrial protein, transcript variant 1, mRNA [NM_001195503]	**90,699**
Tmem69	Rattus norvegicus transmembrane protein 69 (Tmem69), mRNA [NM_001035001]	**90,639**
Paqr4	Rattus norvegicus progestin and adipoQ receptor family member IV (Paqr4), mRNA [NM_001017377]	**90,260**
Tubb4	Rattus norvegicus tubulin, beta 4 (Tubb4), mRNA [NM_080882]	**90,205**
Grlf1	PREDICTED: Rattus norvegicus glucocorticoid receptor DNA binding factor 1 (Grlf1), mRNA [XM_001053554]	**90,070**

Ppp1r9b	Rattus norvegicus protein phosphatase 1, regulatory subunit 9B (Ppp1r9b), mRNA [NM_053474]	**89,856**
Slc25a23	Rattus norvegicus solute carrier family 25 (mitochondrial carrier; phosphate carrier), member 23 (Slc25a23), nuclear gene encoding mitochondrial protein, mRNA [NM_001106873]	**89,771**
Tet3	Uncharacterized protein [Source:UniProtKB/TrEMBL;Acc:D3ZES0] [ENSRNOT00000031312]	**89,707**
Vom1r61	Rattus norvegicus vomeronasal 1 receptor 61 (Vom1r61), mRNA [NM_001008935]	**89,667**
Opa3	Rattus norvegicus optic atrophy 3 (human) (Opa3), nuclear gene encoding mitochondrial protein, mRNA [NM_001107486]	**89,617**
LOC691024	Rattus norvegicus similar to Protein C9orf25 homolog (LOC691024), mRNA [NM_001109616]	**89,522**
0	Q8VEL4_MOUSE (Q8VEL4) Dullard homolog, partial (91%) [TC579581]	**89,371**
0	Rattus norvegicus similar to Spindlin homolog (Protein DXF34) (LOC367474), mRNA [XM_346153]	**89,237**
0	Uncharacterized protein [Source:UniProtKB/TrEMBL;Acc:D4A5I5] [ENSRNOT00000039250]	**89,137**
Sema6d	Rattus norvegicus sema domain, transmembrane domain (TM), and cytoplasmic domain, (semaphorin) 6D (Sema6d), mRNA [NM_001107768]	**88,268**
Rph3a	Rattus norvegicus rabphilin 3A (Rph3a), mRNA [NM_133518]	**88,198**
Fam100a	Rattus norvegicus family with sequence similarity 100, member A (Fam100a), mRNA [NM_001007668]	**87,396**
Map1a	Rattus norvegicus microtubule-associated protein 1A (Map1a), mRNA [NM_030995]	**86,845**
LOC652956	Rattus norvegicus p55 protein (LOC652956), mRNA [NM_001037659]	**86,513**
Cdc7	Rattus norvegicus cell division cycle 7 homolog (S. cerevisiae) (Cdc7), mRNA [NM_001108352]	**86,196**
Pip4k2a	Rattus norvegicus phosphatidylinositol-5-phosphate 4-kinase, type II, alpha (Pip4k2a), mRNA [NM_053926]	**86,132**
Tpp1	Rattus norvegicus tripeptidyl peptidase I (Tpp1), mRNA [NM_031357]	**85,951**
Sfxn3	Rattus norvegicus sideroflexin 3 (Sfxn3), mRNA [NM_022948]	**85,826**
Tmem169	Rattus norvegicus transmembrane protein 169 (Tmem169), mRNA [NM_001109574]	**85,567**
Znf580	PREDICTED: Rattus norvegicus zinc finger protein 580 (Znf580), mRNA [XM_001072400]	**85,553**
Myl12b	Rattus norvegicus myosin, light chain 12B, regulatory (Myl12b), mRNA [NM_017343]	**85,448**
Slc26a1	Rattus norvegicus solute carrier family 26 (sulfate transporter), member 1 (Slc26a1), mRNA [NM_022287]	**85,162**
Ube2q2l	Rattus norvegicus ubiquitin-conjugating enzyme E2Q family member 2-like (Ube2q2l), mRNA [NM_001135997]	**85,147**
Nfia	Rattus norvegicus nuclear factor I/A (Nfia), mRNA [NM_012988]	**84,714**
Cxcl14	Rattus norvegicus chemokine (C-X-C motif) ligand 14 (Cxcl14), mRNA [NM_001013137]	**84,680**
Ndufs4	Rattus norvegicus NADH dehydrogenase (ubiquinone) Fe-S protein 4 (Ndufs4), nuclear gene encoding mitochondrial protein, mRNA [NM_001025146]	**84,427**
Stxbp1	Rattus norvegicus syntaxin binding protein 1 (Stxbp1), mRNA [NM_013038]	**84,419**
Creb3l1	Rattus norvegicus cAMP responsive element binding protein 3-like 1 (Creb3l1), mRNA [NM_001005562]	**84,403**
Copz1	Rattus norvegicus coatomer protein complex, subunit zeta 1 (Copz1), mRNA [NM_001108117]	**84,393**
Fam163a	Rattus norvegicus family with sequence similarity 163, member A (Fam163a), mRNA [NM_001109072]	**84,047**
0	Unknown	**83,828**
LOC691024	Rattus norvegicus similar to Protein C9orf25 homolog (LOC691024), mRNA [NM_001109616]	**83,421**

Lrp5	Rattus norvegicus low density lipoprotein receptor-related protein 5 (Lrp5), mRNA [NM_001106321]	**83,320**
RGD1565164	PREDICTED: Rattus norvegicus similar to associated molecule with the SH3 domain of STAM (RGD1565164), mRNA [XM_001053795]	**83,272**
0	Unknown	**83,003**
Fktn	Rattus norvegicus fukutin (Fktn), mRNA [NM_001108667]	**82,970**
Sprr1al	PREDICTED: Rattus norvegicus small proline-rich protein 1A-like (Sprr1al), mRNA [XM_001056859]	**82,926**
0	Unknown	**82,773**
LOC313672	Rattus norvegicus kazrin (LOC313672), mRNA [NM_001014070]	**82,459**
Lhpp	Rattus norvegicus phospholysine phosphohistidine inorganic pyrophosphate phosphatase (Lhpp), mRNA [NM_001009706]	**82,143**
0	Putative bHLH transcription factor [Source:UniProtKB/TrEMBL;Acc:Q8VD56] [ENSRNOT00000018309]	**81,656**
Kcnj10	Rattus norvegicus potassium inwardly-rectifying channel, subfamily J, member 10 (Kcnj10), mRNA [NM_031602]	**81,627**
Aif1l	Rattus norvegicus allograft inflammatory factor 1-like (Aif1l), mRNA [NM_001108578]	**81,552**
Igfbp5	Rattus norvegicus insulin-like growth factor binding protein 5 (Igfbp5), mRNA [NM_012817]	**81,322**
RGD1560691	Rattus norvegicus similar to calcium/calmodulin-dependent protein kinase 1D (RGD1560691), mRNA [NM_001107365]	**81,284**
Mmachc	Rattus norvegicus methylmalonic aciduria (cobalamin deficiency) cblC type, with homocystinuria (Mmachc), mRNA [NM_001107962]	**81,156**
LOC681292	PREDICTED: Rattus norvegicus hypothetical protein LOC681292 (LOC681292), mRNA [XM_001061110]	**80,991**
Mtss1l	Rattus norvegicus metastasis suppressor 1-like (Mtss1l), mRNA [NM_001191558]	**80,966**
LOC684327	PREDICTED: Rattus norvegicus similar to inter-alpha (globulin) inhibitor H5 (LOC684327), mRNA [XM_001069890]	**80,543**
Cyb5r3	Rattus norvegicus cytochrome b5 reductase 3 (Cyb5r3), mRNA [NM_138877]	**80,499**
Smg6	Rattus norvegicus Smg-6 homolog, nonsense mediated mRNA decay factor (C. elegans) (Smg6), mRNA [NM_001105808]	**80,443**
Zfp278	Rattus norvegicus zinc finger protein 278 (Zfp278), mRNA [NM_001107231]	**80,132**
Mapre3	Rattus norvegicus microtubule-associated protein, RP/EB family, member 3 (Mapre3), mRNA [NM_001007656]	**79,978**
G6pd	Rattus norvegicus glucose-6-phosphate dehydrogenase (G6pd), mRNA [NM_017006]	**79,881**
0	Unknown	**79,657**
Prkcg	Rattus norvegicus protein kinase C, gamma (Prkcg), mRNA [NM_012628]	**79,476**
Cplx1	Rattus norvegicus complexin 1 (Cplx1), mRNA [NM_022864]	**79,348**
Sox10	Rattus norvegicus SRY (sex determining region Y)-box 10 (Sox10), mRNA [NM_019193]	**79,193**
Spata2L	Rattus norvegicus spermatogenesis associated 2-like (Spata2L), mRNA [NM_001109133]	**79,173**
Slc25a35	Rattus norvegicus solute carrier family 25, member 35 (Slc25a35), mRNA [NM_001109033]	**78,903**
Shank1	Rattus norvegicus SH3 and multiple ankyrin repeat domains 1 (Shank1), mRNA [NM_031751]	**78,681**
Ttyh3	Rattus norvegicus tweety homolog 3 (Drosophila) (Ttyh3), mRNA [NM_001107124]	**78,648**
Pdk2	Rattus norvegicus pyruvate dehydrogenase kinase, isozyme 2 (Pdk2), nuclear gene encoding mitochondrial protein, mRNA [NM_030872]	**78,567**
Dnm3	Rattus norvegicus dynamin 3 (Dnm3), mRNA [NM_138538]	**78,455**
Samd4b	Rattus norvegicus sterile alpha motif domain containing 4B (Samd4b), mRNA [NM_001107498]	**77,795**
Git1	Rattus norvegicus G protein-coupled receptor kinase interacting ArfGAP 1 (Git1), mRNA [NM_031814]	**77,735**

RGD1559892	PREDICTED: Rattus norvegicus similar to 60S ribosomal protein L29 (P23) (RGD1559892), mRNA [XM_001071693]	**77,630**
Cyfip2	Rattus norvegicus cytoplasmic FMR1 interacting protein 2 (Cyfip2), mRNA [NM_001106996]	**77,511**
Adcyap1r1	Rattus norvegicus adenylate cyclase activating polypeptide 1 receptor 1 (Adcyap1r1), mRNA [NM_133511]	**77,342**
Atxn2l	Rattus norvegicus ataxin 2-like (Atxn2l), mRNA [NM_001130097]	**76,964**
Aplp1	Rattus norvegicus amyloid beta (A4) precursor-like protein 1 (Aplp1), mRNA [NM_001100802]	**76,904**
Fntb	Rattus norvegicus farnesyltransferase, CAAX box, beta (Fntb), mRNA [NM_172034]	**76,822**
0	Uncharacterized protein [Source:UniProtKB/TrEMBL;Acc:D3ZCL1] [ENSRNOT00000032257]	**76,709**
Bcorl1	Rattus norvegicus BCL6 co-repressor-like 1 (Bcorl1), mRNA [NM_001191587]	**76,672**
0	Unknown	**76,124**
Gas7	Rattus norvegicus growth arrest specific 7 (Gas7), mRNA [NM_053484]	**75,999**
Fam113b	Rattus norvegicus family with sequence similarity 113, member B (Fam113b), mRNA [NM_001039454]	**75,642**
Slc29a4	Rattus norvegicus solute carrier family 29 (nucleoside transporters), member 4 (Slc29a4), mRNA [NM_001105911]	**75,553**
0	Unknown	**75,347**
Mtss1l	Rattus norvegicus metastasis suppressor 1-like (Mtss1l), mRNA [NM_001191558]	**75,194**
Gpr75	Rattus norvegicus G protein-coupled receptor 75 (Gpr75), mRNA [NM_001109096]	**75,108**
LOC685385	PREDICTED: Rattus norvegicus similar to S100 calcium binding protein A14 (LOC685385), mRNA [XM_002725995]	**75,020**
Opcml	Rattus norvegicus opioid binding protein/cell adhesion molecule-like (Opcml), mRNA [NM_053848]	**74,830**
Zmpste24	Rattus norvegicus zinc metallopeptidase, STE24 homolog (S. cerevisiae) (Zmpste24), mRNA [NM_001107974]	**74,244**
Ppme1	Rattus norvegicus protein phosphatase methylesterase 1 (Ppme1), mRNA [NM_001191838]	**74,169**
Csrp1	Rattus norvegicus cysteine and glycine-rich protein 1 (Csrp1), mRNA [NM_017148]	**74,131**
Slc27a1	Rattus norvegicus solute carrier family 27 (fatty acid transporter), member 1 (Slc27a1), mRNA [NM_053580]	**74,119**
Ssbp3	Rattus norvegicus single stranded DNA binding protein 3 (Ssbp3), mRNA [NM_053358]	**74,018**
0	Unknown	**73,788**
Cdkn2aipnl	Rattus norvegicus CDKN2A interacting protein N-terminal like (Cdkn2aipnl), mRNA [NM_001008278]	**73,704**
LOC691984	PREDICTED: Rattus norvegicus similar to Glypican-6 precursor (LOC691984), mRNA [XM_002725130]	**73,692**
RGD1565772	Rattus norvegicus similar to hypothetical protein A430110N23 (RGD1565772), mRNA [NM_001134545]	**73,544**
Sf3a2	Rattus norvegicus splicing factor 3a, subunit 2 (Sf3a2), mRNA [NM_001011986]	**73,527**
Ece1	Rattus norvegicus endothelin converting enzyme 1 (Ece1), mRNA [NM_053596]	**73,439**
Carm1	Rattus norvegicus coactivator-associated arginine methyltransferase 1 (Carm1), transcript variant 1, mRNA [NM_001030041]	**73,060**
Grm4	Rattus norvegicus glutamate receptor, metabotropic 4 (Grm4), mRNA [NM_022666]	**73,043**
Cd248	Rattus norvegicus CD248 molecule, endosialin (Cd248), mRNA [NM_001106325]	**72,951**
Pip5k1c	Rattus norvegicus phosphatidylinositol-4-phosphate 5-kinase, type I, gamma (Pip5k1c), transcript variant c, mRNA [NM_001009967]	**72,467**
Adcyap1r1	Rattus norvegicus adenylate cyclase activating polypeptide 1 receptor 1 (Adcyap1r1), mRNA [NM_133511]	**72,378**
Arsb	Rattus norvegicus arylsulfatase B (Arsb), mRNA [NM_033443]	**72,356**

Wipf3	Rattus norvegicus WAS/WASL interacting protein family, member 3 (Wipf3), mRNA [NM_147211]	**72,237**
Znf703	Rattus norvegicus zinc finger protein 703 (Znf703), mRNA [NM_001109425]	**71,872**
Elmo3	Rattus norvegicus engulfment and cell motility 3 (Elmo3), mRNA [NM_001030028]	**71,618**
B3gnt7	Rattus norvegicus UDP-GlcNAc:betaGal beta-1,3-N-acetylglucosaminyltransferase 7 (B3gnt7), mRNA [NM_001012134]	**71,514**
0	Unknown	**71,460**
Igf2	Rattus norvegicus insulin-like growth factor 2 (Igf2), transcript variant 1, mRNA [NM_031511]	**71,400**
Rab5b	Rattus norvegicus RAB5B, member RAS oncogene family (Rab5b), mRNA [NM_001079936]	**71,078**
Eef1a2	Rattus norvegicus eukaryotic translation elongation factor 1 alpha 2 (Eef1a2), mRNA [NM_012660]	**71,025**
Brunol5	Rattus norvegicus bruno-like 5, RNA binding protein (Drosophila) (Brunol5), mRNA [NM_001135603]	**70,841**
Coq10a	Rattus norvegicus coenzyme Q10 homolog A (S. cerevisiae) (Coq10a), nuclear gene encoding mitochondrial protein, mRNA [NM_001108727]	**70,436**
Cblb	Rattus norvegicus Cas-Br-M (murine) ecotropic retroviral transforming sequence b (Cblb), mRNA [NM_133601]	**70,336**
Creb3l2	Rattus norvegicus cAMP responsive element binding protein 3-like 2 (Creb3l2), mRNA [NM_001012188]	**70,306**
Shisa7	Rattus norvegicus shisa homolog 7 (Xenopus laevis) (Shisa7), mRNA [NM_001145175]	**70,281**
0	LAMBV_CHICK (Q01636) Laminin beta-1 chain variant (Laminin beta-1-2 chain) (Fragment), partial (11%) [TC599571]	**70,253**
Tmem214	Rattus norvegicus transmembrane protein 214 (Tmem214), mRNA [NM_001014195]	**70,045**
Rab1b	Rattus norvegicus RAB1B, member RAS oncogene family (Rab1b), mRNA [NM_001109979]	**69,993**
Cbll1	Rattus norvegicus Cas-Br-M (murine) ecotropic retroviral transforming sequence-like 1 (Cbll1), mRNA [NM_001108018]	**69,808**
Cacng4	Rattus norvegicus calcium channel, voltage-dependent, gamma subunit 4 (Cacng4), mRNA [NM_080692]	**69,568**
0	Unknown	**69,336**
Midn	Rattus norvegicus midnolin (Midn), mRNA [NM_001191577]	**69,223**
RGD1309823	PREDICTED: Rattus norvegicus similar to hypothetical protein FLJ21156 (RGD1309823), miscRNA [XR_085840]	**69,195**
0	Rattus norvegicus, 25 clones, strain BN/SsNHsdMCW RNOR03324481, whole genome shotgun sequence [AABR03128056]	**69,193**
RGD1562814	Rattus norvegicus similar to Peptidyl-prolyl cis-trans isomerase A (PPIase) (Rotamase) (Cyclophilin A) (Cyclosporin A-binding protein) (SP18) (LOC366866), mRNA [XM_345810]	**69,178**
0	Rattus norvegicus clone UI-R-FJ0-cpy-I-05-0-UI unknown mRNA. [AY724520]	**69,019**
Sarm1	Rattus norvegicus sterile alpha and TIR motif containing 1 (Sarm1), mRNA [NM_001105817]	**69,018**
Dap	Rattus norvegicus death-associated protein (Dap), mRNA [NM_022526]	**69,007**
Nrxn2	Rattus norvegicus neurexin 2 (Nrxn2), mRNA [NM_053846]	**68,994**
Ryr2	Rattus norvegicus ryanodine receptor 2, cardiac (Ryr2), transcript variant 1, mRNA [NM_032078]	**68,987**
Mapre2	Rattus norvegicus microtubule-associated protein, RP/EB family, member 2 (Mapre2), mRNA [NM_001101000]	**68,915**
RGD1309228	Rattus norvegicus similar to putative protein, with at least 9 transmembrane domains, of eukaryotic origin (43.9 kD) (2G415) (RGD1309228), mRNA [NM_001017451]	**68,760**
Med15	Rattus norvegicus mediator complex subunit 15 (Med15), mRNA [NM_001108325]	**68,709**
LOC314140	Rattus norvegicus ribose-phosphate pyrophosphokinase I -like (LOC314140), mRNA [NM_001009694]	**68,657**

0	Unknown	**68,550**
Cnot4	Rattus norvegicus CCR4-NOT transcription complex, subunit 4 (Cnot4), mRNA [NM_001037782]	**68,445**
Sema4b	Rattus norvegicus sema domain, immunoglobulin domain (Ig), transmembrane domain (TM) and short cytoplasmic domain, (semaphorin) 4B (Sema4b), mRNA [NM_001170462]	**68,400**
Cacng7	Rattus norvegicus calcium channel, voltage-dependent, gamma subunit 7 (Cacng7), mRNA [NM_080695]	**68,390**
0	Somatoliberin [Source:UniProtKB/Swiss-Prot;Acc:P09916] [ENSRNOT00000010298]	**68,314**
Zfp278	Rattus norvegicus zinc finger protein 278 (Zfp278), mRNA [NM_001107231]	**68,303**
Spon1	Rattus norvegicus spondin 1, extracellular matrix protein (Spon1), mRNA [NM_172067]	**68,199**
Rph3al	Rattus norvegicus rabphilin 3A-like (without C2 domains) (Rph3al), mRNA [NM_133591]	**68,119**
0	Unknown	**67,923**
Klrk1	Rattus norvegicus killer cell lectin-like receptor subfamily K, member 1 (Klrk1), mRNA [NM_133512]	**67,769**
Krt1	Rattus norvegicus keratin 1 (Krt1), mRNA [NM_001008802]	**67,680**
0	AGENCOURT_109872229 NIH_MGC_418 Rattus norvegicus cDNA clone IMAGE:9024004 5', mRNA sequence [EV766865]	**67,586**
Cntnap1	Rattus norvegicus contactin associated protein 1 (Cntnap1), mRNA [NM_032061]	**67,481**
Raver1	Rattus norvegicus ribonucleoprotein, PTB-binding 1 (Raver1), mRNA [NM_001013939]	**67,345**
Sos2	Rattus norvegicus son of sevenless homolog 2 (Drosophila) (Sos2), mRNA [NM_001135561]	**67,293**
Ctdsp1	Rattus norvegicus CTD (carboxy-terminal domain, RNA polymerase II, polypeptide A) small phosphatase 1 (Ctdsp1), mRNA [NM_001128079]	**67,074**
Prr13	Rattus norvegicus proline rich 13 (Prr13), mRNA [NM_001008379]	**67,007**
RGD1562890	Rattus norvegicus RGD1562890 (RGD1562890), non-coding RNA [NR_037704]	**66,820**
Tgfb2	Rattus norvegicus transforming growth factor, beta 2 (Tgfb2), mRNA [NM_031131]	**66,785**
0	RGS6_HUMAN (P49758) Regulator of G-protein signaling 6 (RGS6) (S914), partial (11%) [TC592366]	**66,700**
Mudeng	Rattus norvegicus MU-2/AP1M2 domain containing, death-inducing (Mudeng), mRNA [NM_001030036]	**66,689**
0	RATMTCYTOC Rattus norvegicus tRNA-Trp, tRNA-Ala, tRNA-Asn, tRNA-Cys, and tRNA-Tyr genes, complete sequence; cytochrome c oxidase subunit I (Co I) gene, complete cds; tRNA-Ser and tRNA-Asp genes, complete sequence; cytochrome c oxidase subunit II (Co II) g	**66,509**
Dcakd	Rattus norvegicus dephospho-CoA kinase domain containing (Dcakd), mRNA [NM_001007724]	**66,477**
Smap2	Rattus norvegicus small ArfGAP2 (Smap2), mRNA [NM_001100669]	**66,419**
Rab6b	Rattus norvegicus RAB6B, member RAS oncogene family (Rab6b), mRNA [NM_001108775]	**66,407**
Myo1e	Rattus norvegicus myosin IE (Myo1e), mRNA [NM_173101]	**66,373**
Nrep	Rattus norvegicus neuronal regeneration related protein (Nrep), mRNA [NM_178096]	**66,207**
Rere	Rattus norvegicus arginine-glutamic acid dipeptide (RE) repeats (Rere), mRNA [NM_053885]	**66,181**
Egr1	Rattus norvegicus early growth response 1 (Egr1), mRNA [NM_012551]	**66,176**
0	Unknown	**66,172**
Pitpnm2	Rattus norvegicus phosphatidylinositol transfer protein, membrane-associated 2 (Pitpnm2), mRNA [NM_001107139]	**66,144**
Zc4h2	Rattus norvegicus zinc finger, C4H2 domain containing (Zc4h2), mRNA [NM_001126374]	**66,101**

Plekhm1	Rattus norvegicus pleckstrin homology domain containing, family M (with RUN domain) member 1 (Plekhm1), mRNA [NM_001009677]	**66,081**
Cugbp1	Rattus norvegicus CUG triplet repeat, RNA binding protein 1 (Cugbp1), mRNA [NM_001025421]	**66,008**
Nlgn2	Rattus norvegicus neuroligin 2 (Nlgn2), mRNA [NM_053992]	**65,983**
RGD1563516	PREDICTED: Rattus norvegicus similar to histone protein Hist2h3c1 (RGD1563516), miscRNA [XR_006811]	**65,907**
Atoh1	Rattus norvegicus atonal homolog 1 (Drosophila) (Atoh1), mRNA [NM_001109238]	**65,885**
Bend6	Rattus norvegicus BEN domain containing 6 (Bend6), mRNA [NM_001108792]	**65,788**
0	Unknown	**65,764**
Ube2l3	Rattus norvegicus ubiquitin-conjugating enzyme E2L 3 (Ube2l3), mRNA [NM_001108847]	**65,706**
Cpt1a	Rattus norvegicus carnitine palmitoyltransferase 1a, liver (Cpt1a), nuclear gene encoding mitochondrial protein, mRNA [NM_031559]	**65,597**
0	Unknown	**65,530**
Kdm2a	Rattus norvegicus lysine (K)-specific demethylase 2A (Kdm2a), mRNA [NM_001108515]	**65,433**
Med25	Rattus norvegicus mediator complex subunit 25 (Med25), mRNA [NM_001170426]	**65,428**
Camk2d	Rattus norvegicus calcium/calmodulin-dependent protein kinase II delta (Camk2d), mRNA [NM_012519]	**65,348**
0	BF285957 EST450548 Rat Gene Index, normalized rat, Rattus norvegicus cDNA Rattus norvegicus cDNA clone RGIFL30, mRNA sequence [BF285957]	**65,345**
RGD1304952	Rattus norvegicus similar to RIKEN cDNA C530028O21 gene (RGD1304952), mRNA [NM_001014059]	**65,340**
Gng7	Rattus norvegicus guanine nucleotide binding protein (G protein), gamma 7 (Gng7), mRNA [NM_024138]	**65,295**
Nrsn1	Rattus norvegicus neurensin 1 (Nrsn1), mRNA [NM_001106109]	**65,271**
Plekhb1	Rattus norvegicus pleckstrin homology domain containing, family B (evectins) member 1 (Plekhb1), mRNA [NM_172033]	**65,242**
Ube2d1	Rattus norvegicus ubiquitin-conjugating enzyme E2D 1, UBC4/5 homolog (yeast) (Ube2d1), mRNA [NM_001108530]	**64,957**
Hic1	Rattus norvegicus hypermethylated in cancer 1 (Hic1), mRNA [NM_001107021]	**64,894**
Prkaca	Rattus norvegicus protein kinase, cAMP-dependent, catalytic, alpha (Prkaca), mRNA [NM_001100922]	**64,862**
Ppard	Rattus norvegicus peroxisome proliferator-activated receptor delta (Ppard), mRNA [NM_013141]	**64,821**
Il13ra1	Rattus norvegicus interleukin 13 receptor, alpha 1 (Il13ra1), mRNA [NM_145789]	**64,543**
Bbs1	Rattus norvegicus Bardet-Biedl syndrome 1 (Bbs1), mRNA [NM_001107569]	**64,496**
Megf11	PREDICTED: Rattus norvegicus multiple EGF-like-domains 11 (Megf11), mRNA [XM_001078620]	**64,480**
Cfl1	Rattus norvegicus cofilin 1, non-muscle (Cfl1), mRNA [NM_017147]	**64,387**
0	Unknown	**64,283**
Hcfc1r1	Rattus norvegicus host cell factor C1 regulator 1 (XPO1-dependent) (Hcfc1r1), transcript variant 1, mRNA [NM_001185047]	**64,278**
Hmgcs2	Rattus norvegicus 3-hydroxy-3-methylglutaryl-Coenzyme A synthase 2 (mitochondrial) (Hmgcs2), nuclear gene encoding mitochondrial protein, mRNA [NM_173094]	**64,277**
LOC100364559	PREDICTED: Rattus norvegicus gene model 691, (NCBI)-like (LOC100364559), mRNA [XM_002726287]	**64,173**
Nrxn2	Rattus norvegicus neurexin 2 (Nrxn2), mRNA [NM_053846]	**64,044**
Pllp	Rattus norvegicus plasma membrane proteolipid (plasmolipin) (Pllp), mRNA [NM_022533]	**63,933**

Dullard	Rattus norvegicus Dullard homolog (Xenopus laevis) (Dullard), mRNA [NM_001100494]	**63,875**
39692	Rattus norvegicus septin 8 (Sept8), mRNA [NM_001107002]	**63,656**
Fxyd7	Rattus norvegicus FXYD domain-containing ion transport regulator 7 (Fxyd7), mRNA [NM_022008]	**63,544**
Zc3h7b	Rattus norvegicus zinc finger CCCH-type containing 7B (Zc3h7b), mRNA [NM_001130695]	**63,359**
Wiz	Rattus norvegicus widely-interspaced zinc finger motifs (Wiz), mRNA [NM_001108064]	**63,332**
0	Palmitoyl-protein thioesterase 1 [Source:UniProtKB/Swiss-Prot;Acc:P45479] [ENSRNOT00000017998]	**63,067**
H2afx	Rattus norvegicus H2A histone family, member X (H2afx), mRNA [NM_001109291]	**62,915**
Apln	Rattus norvegicus apelin (Apln), mRNA [NM_031612]	**62,878**
Banf1	Rattus norvegicus barrier to autointegration factor 1 (Banf1), mRNA [NM_053631]	**62,836**
0	Unknown	**62,789**
Slc41a1	Rattus norvegicus solute carrier family 41, member 1 (Slc41a1), mRNA [NM_001108855]	**62,752**
0	Unknown	**62,724**
0	Unknown	**62,719**
Atxn2l	Rattus norvegicus ataxin 2-like (Atxn2l), mRNA [NM_001130097]	**62,580**
Hist1h2bh	Rattus norvegicus histone cluster 1, H2bh (Hist1h2bh), mRNA [NM_001107352]	**62,546**
Men1	Rattus norvegicus multiple endocrine neoplasia 1 (Men1), mRNA [NM_019208]	**62,536**
0	PREDICTED: Rattus norvegicus similar to mKIAA0574 protein (RGD1559470), mRNA [XM_001056143]	**62,422**
Slc38a7	Rattus norvegicus solute carrier family 38, member 7 (Slc38a7), mRNA [NM_001003705]	**62,234**
LOC313641	PREDICTED: Rattus norvegicus perlecan (LOC313641), miscRNA [XR_009630]	**62,209**
Kdm6b	Rattus norvegicus lysine (K)-specific demethylase 6B (Kdm6b), mRNA [NM_001108829]	**62,121**
Pkp2	Rattus norvegicus plakophilin 2 (Pkp2), mRNA [NM_001100499]	**62,093**
0	Unknown	**62,026**
LOC686506	Rattus norvegicus hypothetical protein LOC686506 (LOC686506), mRNA [NM_001134729]	**61,975**
Jam2	Rattus norvegicus junctional adhesion molecule 2 (Jam2), mRNA [NM_001034004]	**61,972**
Zfp39	Rattus norvegicus zinc finger protein 39 (Zfp39), mRNA [NM_001107004]	**61,953**
Emp3	Rattus norvegicus epithelial membrane protein 3 (Emp3), mRNA [NM_030847]	**61,628**
Prrg3	PREDICTED: Rattus norvegicus proline rich Gla (G-carboxyglutamic acid) 3 (transmembrane) (Prrg3), mRNA [XM_219845]	**61,600**
Sox13	Rattus norvegicus SRY (sex determining region Y)-box 13 (Sox13), mRNA [NM_001105952]	**61,482**
Slc7a5	Rattus norvegicus solute carrier family 7 (cationic amino acid transporter, y+ system), member 5 (Slc7a5), mRNA [NM_017353]	**61,445**
Sap25	PREDICTED: Rattus norvegicus similar to FLJ00248 protein (RGD1562406), mRNA [XM_001076431]	**61,427**
Il1f5	Rattus norvegicus interleukin 1 family, member 5 (delta) (Il1f5), mRNA [NM_001107814]	**61,300**
RGD1308124	PREDICTED: Rattus norvegicus similar to KIAA1357 protein, transcript variant 2 (RGD1308124), mRNA [XM_002725450]	**61,168**
Mrps2	Rattus norvegicus mitochondrial ribosomal protein S2 (Mrps2), nuclear gene encoding mitochondrial protein, mRNA [NM_001108576]	**61,089**
0	additional sex combs like 3 (Drosophila) Gene [Source:MGI Symbol;Acc:MGI:2685175] [ENSRNOT00000020661]	**61,012**

Glrx1	Rattus norvegicus glutaredoxin 1 (Glrx1), mRNA [NM_022278]	**60,980**
Vcl	Rattus norvegicus vinculin (Vcl), mRNA [NM_001107248]	**60,936**
Wiz	Rattus norvegicus widely-interspaced zinc finger motifs (Wiz), mRNA [NM_001108064]	**60,846**
S100b	Rattus norvegicus S100 calcium binding protein B (S100b), mRNA [NM_013191]	**60,719**
Dock1	Rattus norvegicus dedicator of cyto-kinesis 1 (Dock1), mRNA [NM_001143858]	**60,686**
Fam107a	Rattus norvegicus family with sequence similarity 107, member A (Fam107a), mRNA [NM_001025129]	**60,653**
Irf2bp1	Rattus norvegicus interferon regulatory factor 2 binding protein 1 (Irf2bp1), mRNA [NM_001107483]	**60,605**
Alk	Rattus norvegicus anaplastic lymphoma kinase (Alk), mRNA [NM_001169101]	**60,529**
Sdc3	Rattus norvegicus syndecan 3 (Sdc3), mRNA [NM_053893]	**60,480**
Cd47	Rattus norvegicus Cd47 molecule (Cd47), mRNA [NM_019195]	**60,383**
0	Unknown	**60,343**
0	Unknown	**60,261**
Mcart1l	Rattus norvegicus mitochondrial carrier triple repeat 1-like (Mcart1l), nuclear gene encoding mitochondrial protein, mRNA [NM_001127602]	**60,109**
Vom1r-ps39	Rattus norvegicus vomeronasal 1 receptor pseudogene 39 (Vom1r-ps39), mRNA [NM_001009509]	**60,046**
0	Uncharacterized protein [Source:UniProtKB/TrEMBL;Acc:D4A0J6] [ENSRNOT00000051194]	**60,027**
Fmn2	PREDICTED: Rattus norvegicus formin 2 (Fmn2), mRNA [XM_001058601]	**60,016**
Mesdc1	Rattus norvegicus mesoderm development candidate 1 (Mesdc1), mRNA [NM_001013149]	**59,993**
Notch2	Rattus norvegicus Notch homolog 2 (Drosophila) (Notch2), mRNA [NM_024358]	**59,875**
RGD1563319	Uncharacterized protein [Source:UniProtKB/TrEMBL;Acc:D3ZMZ8] [ENSRNOT00000027344]	**59,827**
Nrm	Rattus norvegicus nurim (nuclear envelope membrane protein) (Nrm), mRNA [NM_212508]	**59,824**
Eif4ebp2	Rattus norvegicus eukaryotic translation initiation factor 4E binding protein 2 (Eif4ebp2), mRNA [NM_001033069]	**59,754**
Ube2d2	Rattus norvegicus ubiquitin-conjugating enzyme E2D 2 (UBC4/5 homolog, yeast) (Ube2d2), mRNA [NM_001037292]	**59,693**
0	Unknown	**59,522**
Gstm6l	Rattus norvegicus glutathione S-transferase, mu 6-like (Gstm6l), mRNA [NM_001106464]	**59,494**
0	Rattus norvegicus similar to 60S RIBOSOMAL PROTEIN L29 (P23) (LOC293697), mRNA [XM_219533]	**59,428**
Dazap2	Rattus norvegicus DAZ associated protein 2 (Dazap2), mRNA [NM_001013107]	**59,421**
Rbms2	Rattus norvegicus RNA binding motif, single stranded interacting protein 2 (Rbms2), mRNA [NM_001025403]	**59,396**
0	Unknown	**59,370**
Calcoco1	Rattus norvegicus calcium binding and coiled coil domain 1 (Calcoco1), mRNA [NM_139190]	**59,307**
0	Q8QQ04_CAMPS (Q8QQ04) CMP190R, partial (13%) [TC605838]	**59,234**
Atxn7l1	Uncharacterized protein [Source:UniProtKB/TrEMBL;Acc:D3ZHW8] [ENSRNOT00000014054]	**59,173**
LOC680207	PREDICTED: Rattus norvegicus hypothetical protein LOC680207 (LOC680207), mRNA [XM_001056119]	**59,036**
RGD1305215	Rattus norvegicus similar to expressed sequence AA960436 (RGD1305215), mRNA [NM_001014013]	**58,819**
Mll5	Mll5 protein [Source:UniProtKB/TrEMBL;Acc:B1WBR7] [ENSRNOT00000038978]	**58,779**

Abhd15	Rattus norvegicus abhydrolase domain containing 15 (Abhd15), mRNA [NM_001107025]	**58,747**
Kcna7	Rattus norvegicus potassium voltage-gated channel, shaker-related subfamily, member 7 (Kcna7), mRNA [NM_001108914]	**58,743**
0	Kinesin light chain 2 (Predicted), isoform CRA_bKlc2 protein [Source:UniProtKB/TrEMBL;Acc:B2GV74] [ENSRNOT00000041238]	**58,536**
Abhd4	Rattus norvegicus abhydrolase domain containing 4 (Abhd4), mRNA [NM_001108866]	**58,501**
0	Unknown	**58,485**
Ptpn23	Rattus norvegicus protein tyrosine phosphatase, non-receptor type 23 (Ptpn23), mRNA [NM_057204]	**58,467**
Pcdh15	Unknown	**58,450**
0	AW914971 EST346275 Normalized rat ovary, Bento Soares Rattus sp. cDNA clone RGIBN04 5' end, mRNA sequence [AW914971]	**58,444**
Myd88	Rattus norvegicus myeloid differentiation primary response gene 88 (Myd88), mRNA [NM_198130]	**58,379**
Copz1	Rattus norvegicus coatomer protein complex, subunit zeta 1 (Copz1), mRNA [NM_001108117]	**58,366**
Grm4	Rattus norvegicus glutamate receptor, metabotropic 4 (Grm4), mRNA [NM_022666]	**58,346**
Rsu1	Rattus norvegicus Ras suppressor protein 1 (Rsu1), mRNA [NM_001109404]	**58,328**
Pnpla2	Rattus norvegicus patatin-like phospholipase domain containing 2 (Pnpla2), mRNA [NM_001108509]	**58,290**
Urm1	Rattus norvegicus ubiquitin related modifier 1 homolog (S. cerevisiae) (Urm1), mRNA [NM_001137562]	**58,276**
Enah	Rattus norvegicus enabled homolog (Drosophila) (Enah), mRNA [NM_001012150]	**58,271**
Ube2n	Rattus norvegicus ubiquitin-conjugating enzyme E2N (UBC13 homolog, yeast) (Ube2n), mRNA [NM_053928]	**58,212**
0	Ketohexokinase [Source:UniProtKB/Swiss-Prot;Acc:Q02974] [ENSRNOT00000043572]	**58,094**
C1qtnf6	Rattus norvegicus C1q and tumor necrosis factor related protein 6 (C1qtnf6), mRNA [NM_001034932]	**58,087**
RGD1562674	PREDICTED: Rattus norvegicus similar to kinase suppressor of ras 2 (RGD1562674), mRNA [XM_222212]	**57,944**
Stard6	Rattus norvegicus StAR-related lipid transfer (START) domain containing 6 (Stard6), mRNA [NM_001007627]	**57,843**
0	Uncharacterized protein [Source:UniProtKB/TrEMBL;Acc:D3Z7Z0] [ENSRNOT00000033201]	**57,839**
0	Unknown	**57,827**
Cybrd1	Rattus norvegicus cytochrome b reductase 1 (Cybrd1), mRNA [NM_001011954]	**57,750**
Gdf6	Rattus norvegicus growth differentiation factor 6 (Gdf6), mRNA [NM_001013038]	**57,715**
Arl8a	Rattus norvegicus ADP-ribosylation factor-like 8A (Arl8a), mRNA [NM_001109071]	**57,694**
Elf3	Rattus norvegicus E74-like factor 3 (Elf3), mRNA [NM_001024768]	**57,608**
Nr1d1	Rattus norvegicus nuclear receptor subfamily 1, group D, member 1 (Nr1d1), transcript variant 2, mRNA [NM_145775]	**57,508**
Slc39a1	Rattus norvegicus solute carrier family 39 (zinc transporter), member 1 (Slc39a1), mRNA [NM_001134577]	**57,485**
0	Unknown	**57,214**
R3hdm2	Rattus norvegicus R3H domain containing 2 (R3hdm2), mRNA [NM_001130557]	**57,198**
Arhgef9	Rattus norvegicus Cdc42 guanine nucleotide exchange factor (GEF) 9 (Arhgef9), mRNA [NM_023957]	**57,172**
0	Unknown	**57,146**
Psmf1	Rattus norvegicus proteasome inhibitor subunit 1 (Psmf1), mRNA [NM_001101005]	**57,133**
Lpl	Rattus norvegicus lipoprotein lipase (Lpl), mRNA [NM_012598]	**56,974**

Prkca	Rattus norvegicus protein kinase C, alpha (Prkca), mRNA [NM_001105713]	**56,964**
Atn1	Rattus norvegicus atrophin 1 (Atn1), mRNA [NM_017228]	**56,886**
0	Unknown	**56,886**
Col1a1	Rattus norvegicus collagen, type I, alpha 1 (Col1a1), mRNA [NM_053304]	**56,683**
LOC682105	Rattus norvegicus similar to receptor expression enhancing protein 2 (LOC682105), mRNA [NM_001048047]	**56,613**
Gjd2	Rattus norvegicus gap junction protein, delta 2 (Gjd2), mRNA [NM_019281]	**56,517**
Cugbp2	Rattus norvegicus CUG triplet repeat, RNA binding protein 2 (Cugbp2), transcript variant 2, mRNA [NM_001083586]	**56,515**
Actn4	Rattus norvegicus actinin alpha 4 (Actn4), mRNA [NM_031675]	**56,476**
Slc22a23	Rattus norvegicus solute carrier family 22, member 23 (Slc22a23), mRNA [NM_022624]	**56,337**
Olfml3	Rattus norvegicus olfactomedin-like 3 (Olfml3), mRNA [NM_001107708]	**56,309**
0	Unknown	**56,298**
RGD1565591	PREDICTED: Rattus norvegicus similar to Ski protein (RGD1565591), partial mRNA [XM_001077382]	**56,256**
Tax1bp3	Rattus norvegicus Tax1 (human T-cell leukemia virus type I) binding protein 3 (Tax1bp3), mRNA [NM_001025419]	**56,240**
0	Rattus norvegicus similar to RIKEN cDNA 4930415K17 (LOC302803), mRNA [XM_217625]	**56,211**
0	C5AR_RABIT (Q9TUE1) C5a anaphylatoxin chemotactic receptor (C5a-R) (C5aR) (CD88 antigen) (Fragment), partial (7%) [TC623897]	**56,191**
Efnb1	Rattus norvegicus ephrin B1 (Efnb1), mRNA [NM_017089]	**56,174**
Gna14	Rattus norvegicus guanine nucleotide binding protein, alpha 14 (Gna14), mRNA [NM_001013151]	**56,171**
Syt5	Rattus norvegicus synaptotagmin V (Syt5), mRNA [NM_019350]	**56,018**
Plvap	Rattus norvegicus plasmalemma vesicle associated protein (Plvap), mRNA [NM_020086]	**55,927**
0	Unknown	**55,907**
Abhd2	Rattus norvegicus abhydrolase domain containing 2 (Abhd2), mRNA [NM_001106275]	**55,881**
Atg13	Putative uncharacterized protein RGD1310685_predictedUncharacterized protein [Source:UniProtKB/TrEMBL;Acc:D3ZA45] [ENSRNOT00000023237]	**55,875**
Nptxr	Rattus norvegicus neuronal pentraxin receptor (Nptxr), mRNA [NM_030841]	**55,874**
Lypd6b	Rattus norvegicus LY6/PLAUR domain containing 6B (Lypd6b), mRNA [NM_001134580]	**55,824**
Rab8a	Rattus norvegicus RAB8A, member RAS oncogene family (Rab8a), mRNA [NM_053998]	**55,664**
Dusp18	Rattus norvegicus dual specificity phosphatase 18 (Dusp18), mRNA [NM_001013128]	**55,649**
Mospd3	Rattus norvegicus motile sperm domain containing 3 (Mospd3), mRNA [NM_001025629]	**55,606**
Sv2a	Rattus norvegicus synaptic vesicle glycoprotein 2a (Sv2a), mRNA [NM_057210]	**55,552**
Sirt6	Rattus norvegicus sirtuin (silent mating type information regulation 2 homolog) 6 (S. cerevisiae) (Sirt6), mRNA [NM_001031649]	**55,530**
0	Rattus norvegicus TL0ACA70YE02 mRNA sequence. [FQ224293]	**55,499**
LOC690120	PREDICTED: Rattus norvegicus hypothetical protein LOC690120 (LOC690120), miscRNA [XR_085881]	**55,392**
Kif1c	Rattus norvegicus kinesin family member 1C (Kif1c), mRNA [NM_145877]	**55,381**
Hdgf	Rattus norvegicus hepatoma-derived growth factor (Hdgf), mRNA [NM_053707]	**55,368**

Vwf	von Willebrand factor [Source:UniProtKB/Swiss-Prot;Acc:Q62935] [ENSRNOT00000026643]	**55,281**
Slc48a1	Rattus norvegicus solute carrier family 48 (heme transporter), member 1 (Slc48a1), mRNA [NM_001127456]	**55,266**
RGD1564387	PREDICTED: Rattus norvegicus similar to RIKEN cDNA C030014K22 gene (RGD1564387), mRNA [XM_002728030]	**55,251**
Lphn3	Rattus norvegicus latrophilin 3 (Lphn3), mRNA [NM_130822]	**55,196**
0	CITE4_MOUSE (Q9WUL8) Cbp/p300-interacting transactivator 4 (MSG1-related protein 2) (MRG-2), partial (8%) [TC620332]	**55,114**
Abpa	Rattus norvegicus androgen binding protein, alpha (Abpa), mRNA [NM_001100859]	**55,004**
0	Uncharacterized protein [Source:UniProtKB/TrEMBL;Acc:D3ZTW2] [ENSRNOT00000036540]	**54,909**
Dad1	Rattus norvegicus defender against cell death 1 (Dad1), mRNA [NM_138910]	**54,892**
Tbl1x	Rattus norvegicus transducin (beta)-like 1 X-linked (Tbl1x), mRNA [NM_001106964]	**54,883**
0	Unknown	**54,878**
Ntsr2	Rattus norvegicus neurotensin receptor 2 (Ntsr2), mRNA [NM_022695]	**54,862**
Papss2	Rattus norvegicus 3'-phosphoadenosine 5'-phosphosulfate synthase 2 (Papss2), mRNA [NM_001106375]	**54,787**
LOC686590	PREDICTED: Rattus norvegicus similar to IQ motif and Sec7 domain 1, transcript variant 2 (LOC686590), mRNA [XM_001073009]	**54,783**
LOC689226	Rattus norvegicus similar to ubiquitin-conjugating enzyme E2R 2 (LOC689226), mRNA [NM_001127573]	**54,778**
Epas1	Rattus norvegicus endothelial PAS domain protein 1 (Epas1), mRNA [NM_023090]	**54,738**
Olr200	Rattus norvegicus olfactory receptor 200 (Olr200), mRNA [NM_001000189]	**54,732**
Klhl7	Rattus norvegicus kelch-like 7 (Drosophila) (Klhl7), mRNA [NM_001012187]	**54,631**
LOC688966	Rattus norvegicus similar to K11B4.2 (LOC688966), mRNA [NM_001145726]	**54,583**
LOC498750	Rattus norvegicus similar to cDNA sequence BC005537 (LOC498750), mRNA [NM_001017510]	**54,571**
Hecw1	Rattus norvegicus HECT, C2 and WW domain containing E3 ubiquitin protein ligase 1 (Hecw1), mRNA [NM_001106117]	**54,525**
Slc6a1	Rattus norvegicus solute carrier family 6 (neurotransmitter transporter, GABA), member 1 (Slc6a1), mRNA [NM_024371]	**54,467**
0	Q8R3U9_MOUSE (Q8R3U9) Dlgap4 protein, partial (34%) [TC609401]	**54,320**
0	LOC100125365 protein [Source:UniProtKB/TrEMBL;Acc:Q5U2N5] [ENSRNOT00000051777]	**54,291**
0	Unknown	**54,283**
Tshz3	Rattus norvegicus teashirt zinc finger homeobox 3 (Tshz3), mRNA [NM_001107506]	**54,258**
Sorcs2	Rattus norvegicus sortilin-related VPS10 domain containing receptor 2 (Sorcs2), mRNA [NM_001107225]	**54,178**
Rab5b	Rattus norvegicus RAB5B, member RAS oncogene family (Rab5b), mRNA [NM_001079936]	**54,176**
Gltp	Rattus norvegicus glycolipid transfer protein (Gltp), mRNA [NM_001134413]	**54,153**
Crkrs	Rattus norvegicus Cdc2-related kinase, arginine/serine-rich (Crkrs), transcript variant 1, mRNA [NM_001033867]	**54,040**
Cdc14b	Rattus norvegicus CDC14 cell division cycle 14 homolog B (S. cerevisiae) (Cdc14b), mRNA [NM_001108404]	**53,839**
Sh2b2	Rattus norvegicus SH2B adaptor protein 2 (Sh2b2), mRNA [NM_053669]	**53,829**
0	Rattus norvegicus TL0ADA49YG04 mRNA sequence. [FQ229104]	**53,811**
Gatsl2	Rattus norvegicus GATS protein-like 2 (Gatsl2), mRNA [NM_001100561]	**53,800**
Vom1r28	Rattus norvegicus vomeronasal 1 receptor 28 (Vom1r28), mRNA [NM_001008966]	**53,768**

Cend1	Rattus norvegicus cell cycle exit and neuronal differentiation 1 (Cend1), mRNA [NM_001014163]	**53,712**
0	Ataxin 2 (Predicted)Uncharacterized protein [Source:UniProtKB/TrEMBL;Acc:D4A2N8] [ENSRNOT00000001691]	**53,703**
Cplx1	Rattus norvegicus complexin 1 (Cplx1), mRNA [NM_022864]	**53,654**
0	Q4T0B3_TETNG (Q4T0B3) Chromosome undetermined SCAF11265, whole genome shotgun sequence. (Fragment), partial (15%) [TC606852]	**53,440**
0	Unknown	**53,429**
Eif2ak1	Rattus norvegicus eukaryotic translation initiation factor 2 alpha kinase 1 (Eif2ak1), mRNA [NM_013223]	**53,428**
RGD1564114	Rattus norvegicus similar to FLJ46082 protein (RGD1564114), mRNA [NM_001127563]	**53,423**
Sort1	Rattus norvegicus sortilin 1 (Sort1), mRNA [NM_031767]	**53,412**
Acpl2	Rattus norvegicus acid phosphatase-like 2 (Acpl2), mRNA [NM_001007710]	**53,395**
LOC684841	PREDICTED: Rattus norvegicus similar to CG31613-PA (LOC684841), mRNA [XM_001072155]	**53,377**
Map3k13	Rattus norvegicus similar to mitogen-activated protein kinase kinase kinase 13; leucine zipper-bearing kinase, mRNA (cDNA clone MGC:94134 IMAGE:7127060), complete cds. [BC081976]	**53,342**
Sh3kbp1	Rattus norvegicus SH3-domain kinase binding protein 1 (Sh3kbp1), mRNA [NM_053360]	**53,292**
Mylk	Rattus norvegicus myosin light chain kinase (Mylk), mRNA [NM_001105874]	**53,243**
0	Cobl-like 1 Gene [Source:MGI Symbol;Acc:MGI:2442894] [ENSRNOT00000001606]	**53,096**
0	Ras association (RalGDS/AF-6) and pleckstrin homology domains 1 (Predicted), isoform CRA_aUncharacterized protein [Source:UniProtKB/TrEMBL;Acc:D4ADX8] [ENSRNOT00000036194]	**53,070**
LOC690286	PREDICTED: Rattus norvegicus similar to hepatic leukemia factor (LOC690286), mRNA [XM_001073964]	**53,046**
0	Uncharacterized protein [Source:UniProtKB/TrEMBL;Acc:D3ZK71] [ENSRNOT00000041732]	**52,903**
Nfix	Rattus norvegicus nuclear factor I/X (CCAAT-binding transcription factor) (Nfix), mRNA [NM_030866]	**52,838**
Vps37c	Rattus norvegicus vacuolar protein sorting 37 homolog C (S. cerevisiae) (Vps37c), mRNA [NM_001107463]	**52,802**
Apba2	Rattus norvegicus amyloid beta (A4) precursor protein-binding, family A, member 2 (Apba2), mRNA [NM_031780]	**52,786**
Lgals3bp	Rattus norvegicus lectin, galactoside-binding, soluble, 3 binding protein (Lgals3bp), mRNA [NM_139096]	**52,643**
Atg9a	Rattus norvegicus ATG9 autophagy related 9 homolog A (S. cerevisiae) (Atg9a), mRNA [NM_001014218]	**52,632**
LOC682920	PREDICTED: Rattus norvegicus similar to phosphatase and actin regulator 4 (LOC682920), mRNA [XM_001063709]	**52,615**
LOC686841	Rattus norvegicus similar to Protein EAN57 (LOC686841), mRNA [NM_001044289]	**52,602**
Zfp385b	Rattus norvegicus zinc finger protein 385B (Zfp385b), mRNA [NM_001107736]	**52,577**
0	Unknown	**52,567**
Prp2l1	Rat proline-rich protein (PRP-1) [Source:UniProtKB/TrEMBL;Acc:Q63455] [ENSRNOT00000004642]	**52,563**
Mect1	Rattus norvegicus mucoepidermoid carcinoma translocated 1 (Mect1), mRNA [NM_001047115]	**52,529**
Atn1	Rattus norvegicus atrophin 1 (Atn1), mRNA [NM_017228]	**52,474**
0	Uncharacterized protein [Source:UniProtKB/TrEMBL;Acc:D4ABJ0] [ENSRNOT00000028944]	**52,442**
Gng3	Rattus norvegicus guanine nucleotide binding protein (G protein), gamma 3 (Gng3), mRNA [NM_053658]	**52,405**
Fxyd1	Rattus norvegicus FXYD domain-containing ion transport regulator 1 (Fxyd1), mRNA [NM_031648]	**52,311**

Jup	Rattus norvegicus junction plakoglobin (Jup), mRNA [NM_031047]	**52,283**
Rab1b	Rattus norvegicus RAB1B, member RAS oncogene family (Rab1b), mRNA [NM_001109979]	**52,279**
LOC691918	PREDICTED: Rattus norvegicus similar to Centrosomal protein of 27 kDa (Cep27 protein), transcript variant 2 (LOC691918), mRNA [XM_002726165]	**52,258**
RGD1564677	PREDICTED: Rattus norvegicus similar to transcription factor ONECUT2 (RGD1564677), partial mRNA [XM_002725360]	**52,255**
Muc1	Rattus norvegicus mucin 1, cell surface associated (Muc1), mRNA [NM_012602]	**52,243**
0	Unknown	**52,234**
Brd4	Rattus norvegicus bromodomain containing 4 (Brd4), mRNA [NM_001100903]	**52,229**
Repin1	Rattus norvegicus replication initiator 1 (Repin1), mRNA [NM_001005893]	**52,180**
0	Unknown	**52,164**
RGD1564969	PREDICTED: Rattus norvegicus similar to 60S ribosomal protein L29 (P23) (RGD1564969), mRNA [XM_002726901]	**52,157**
0	Neurexin-3-alpha [Source:UniProtKB/Swiss-Prot;Acc:Q07310] [ENSRNOT00000032331]	**52,148**
0	host cell factor C1 regulator 1 [Source:RefSeq peptide;Acc:NP_001093962] [ENSRNOT00000004702]	**52,032**
Atpbd4	Rattus norvegicus ATP binding domain 4 (Atpbd4), mRNA [NM_001014181]	**51,925**
Crebbp	Rattus norvegicus CREB binding protein (Crebbp), mRNA [NM_133381]	**51,915**
0	Unknown	**51,861**
Idh3a	Rattus norvegicus isocitrate dehydrogenase 3 (NAD+) alpha (Idh3a), nuclear gene encoding mitochondrial protein, mRNA [NM_053638]	**51,857**
Arid1a	Rattus norvegicus AT rich interactive domain 1A (SWI-like) (Arid1a), mRNA [NM_001106635]	**51,837**
Zfp385a	Rattus norvegicus zinc finger protein 385A (Zfp385a), transcript variant 1, mRNA [NM_001135088]	**51,781**
Wnk1	Rattus norvegicus WNK lysine deficient protein kinase 1 (Wnk1), transcript variant 3, mRNA [NM_053794]	**51,697**
Nol4	Rattus norvegicus nucleolar protein 4 (Nol4), mRNA [NM_001107401]	**51,670**
Manbal	Rattus norvegicus mannosidase, beta A, lysosomal-like (Manbal), mRNA [NM_001173380]	**51,663**
Olig1	Rattus norvegicus oligodendrocyte transcription factor 1 (Olig1), mRNA [NM_021770]	**51,620**
Hs1bp3	PREDICTED: Rattus norvegicus HCLS1 binding protein 3, transcript variant 2 (Hs1bp3), mRNA [XM_233975]	**51,580**
RGD1559896	Rattus norvegicus similar to RIKEN cDNA 2310022B05 (RGD1559896), mRNA [NM_001109134]	**51,545**
Rhob	Rattus norvegicus ras homolog gene family, member B (Rhob), mRNA [NM_022542]	**51,542**
Crtap	cartilage-associated protein [Source:RefSeq peptide;Acc:NP_001102255] [ENSRNOT00000013519]	**51,538**
0	Rattus norvegicus similar to ubiquitin-conjugating enzyme E2N (homologous to yeast UBC13); bendless protein (LOC301282), mRNA [XM_236973]	**51,513**
Gnat1	Rattus norvegicus guanine nucleotide binding protein (G protein), alpha transducing 1 (Gnat1), mRNA [NM_001108780]	**51,511**
Tnks1bp1	Uncharacterized protein [Source:UniProtKB/TrEMBL;Acc:D3ZF26] [ENSRNOT00000012209]	**51,500**
Ei24	Rattus norvegicus etoposide induced 2.4 mRNA (Ei24), mRNA [NM_001025660]	**51,460**
Mgat5b	Rattus norvegicus mannosyl (alpha-1,6-)-glycoprotein beta-1,6-N-acetyl-glucosaminyltransferase, isozyme B (Mgat5b), mRNA [NM_001107068]	**51,348**
Tln1	Rattus norvegicus talin 1 (Tln1), mRNA [NM_001039025]	**51,310**

Mlf2	Rattus norvegicus myeloid leukemia factor 2 (Mlf2), mRNA [NM_001107889]	**51,263**
Gchfr	Rattus norvegicus GTP cyclohydrolase I feedback regulator (Gchfr), mRNA [NM_133595]	**51,222**
Tacr3	Rattus norvegicus tachykinin receptor 3 (Tacr3), mRNA [NM_017053]	**51,151**
Olr922	Rattus norvegicus olfactory receptor 922 (Olr922), mRNA [NM_001000705]	**51,126**
0	Unknown	**51,123**
RGD1565591	PREDICTED: Rattus norvegicus similar to Ski protein (RGD1565591), partial mRNA [XM_001077382]	**51,081**
Ak3l1	Rattus norvegicus adenylate kinase 3-like 1 (Ak3l1), nuclear gene encoding mitochondrial protein, mRNA [NM_017135]	**51,014**
Nkain1	PREDICTED: Rattus norvegicus similar to RIKEN cDNA 2610200G18 (RGD1561205), mRNA [XM_575920]	**50,983**
Mark2	Rattus norvegicus MAP/microtubule affinity-regulating kinase 2 (Mark2), mRNA [NM_021699]	**50,859**
Mpped1	Rattus norvegicus metallophosphoesterase domain containing 1 (Mpped1), mRNA [NM_001130569]	**50,852**
Rarg	Rattus norvegicus retinoic acid receptor, gamma (Rarg), transcript variant 1, mRNA [NM_001135249]	**50,841**
LOC685244	PREDICTED: Rattus norvegicus similar to IQ motif and Sec7 domain 2, transcript variant 2 (LOC685244), mRNA [XM_002727538]	**50,812**
Ccdc40	Rattus norvegicus coiled-coil domain containing 40 (Ccdc40), mRNA [NM_001134688]	**50,803**
Mrpl40	Rattus norvegicus mitochondrial ribosomal protein L40 (Mrpl40), nuclear gene encoding mitochondrial protein, mRNA [NM_001024865]	**50,791**
Tnr	Rattus norvegicus tenascin R (Tnr), mRNA [NM_013045]	**50,768**
0	Unknown	**50,746**
Reep3	Rattus norvegicus receptor accessory protein 3 (Reep3), mRNA [NM_001106386]	**50,706**
Slc9a3r2	Rattus norvegicus solute carrier family 9 (sodium/hydrogen exchanger), member 3 regulator 2 (Slc9a3r2), mRNA [NM_053811]	**50,688**
Kcnip2	Rattus norvegicus Kv channel-interacting protein 2 (Kcnip2), transcript variant a, mRNA [NM_020094]	**50,673**
0	Rattus norvegicus similar to 60S RIBOSOMAL PROTEIN L29 (P23) (LOC294697), mRNA [XM_226719]	**50,670**
Rnasek	Rattus norvegicus ribonuclease, RNase K (Rnasek), mRNA [NM_001137561]	**50,631**
Sike	Rattus norvegicus suppressor of IKK epsilon (Sike), mRNA [NM_001012182]	**50,622**
Dnali1	Rattus norvegicus dynein, axonemal, light intermediate chain 1 (Dnali1), mRNA [NM_001031647]	**50,617**
Disp2	Rattus norvegicus dispatched homolog 2 (Drosophila) (Disp2), mRNA [NM_001107759]	**50,607**
Sharpin	Rattus norvegicus SHANK-associated RH domain interactor (Sharpin), mRNA [NM_031153]	**50,603**
0	Unknown	**50,597**
0	Uncharacterized protein [Source:UniProtKB/TrEMBL;Acc:D4A3C4] [ENSRNOT00000067627]	**50,595**
LOC100359980	PREDICTED: Rattus norvegicus smooth muscle and non-muscle myosin alkali light chain 6B-like (LOC100359980), mRNA [XM_002726853]	**50,554**
Oasl2	Rattus norvegicus 2'-5' oligoadenylate synthetase-like 2 (Oasl2), mRNA [NM_001009682]	**50,550**
Kcnj6	Rattus norvegicus potassium inwardly-rectifying channel, subfamily J, member 6 (Kcnj6), mRNA [NM_013192]	**50,547**
Cnnm1	Rattus norvegicus cyclin M1 (Cnnm1), mRNA [NM_001107593]	**50,545**
RGD1305938	Rattus norvegicus similar to expressed sequence AW549877 (RGD1305938), mRNA [NM_001134548]	**50,525**
Apob48r	Rattus norvegicus apolipoprotein B48 receptor (Apob48r), mRNA [NM_001109154]	**50,515**
Pcdh1	PREDICTED: Rattus norvegicus protocadherin 1 (Pcdh1), partial mRNA [XM_225997]	**50,515**

Epb49	Rattus norvegicus erythrocyte membrane protein band 4.9 (dematin) (Epb49), mRNA [NM_001108385]	**50,459**
Guca2b	Rattus norvegicus guanylate cyclase activator 2B (Guca2b), mRNA [NM_022284]	**50,418**
Nrep	Rattus norvegicus neuronal regeneration related protein (Nrep), mRNA [NM_178096]	**50,413**
Zdhhc9	Rattus norvegicus zinc finger, DHHC-type containing 9 (Zdhhc9), mRNA [NM_001039016]	**50,374**
RGD1310571	Rattus norvegicus similar to hypothetical protein (RGD1310571), mRNA [NM_001014147]	**50,363**
Cdkn1a	Rattus norvegicus cyclin-dependent kinase inhibitor 1A (Cdkn1a), mRNA [NM_080782]	**50,323**
Gpr137	Rattus norvegicus G protein-coupled receptor 137 (Gpr137), mRNA [NM_001109562]	**50,289**
0	Unknown	**50,276**
Pea15a	Rattus norvegicus phosphoprotein enriched in astrocytes 15A (Pea15a), mRNA [NM_001013231]	**50,218**
LOC100125364	Rattus norvegicus hypothetical protein LOC100125364 (LOC100125364), mRNA [NM_001103356]	**50,190**
RGD1311564	Rattus norvegicus LOC360590 (RGD1311564), mRNA [NM_001108286]	**50,184**
Tspan18	Rattus norvegicus tetraspanin 18 (Tspan18), mRNA [NM_001107750]	**50,180**
Arf4	Rattus norvegicus ADP-ribosylation factor 4 (Arf4), mRNA [NM_024151]	**50,177**
Cited2	Rattus norvegicus Cbp/p300-interacting transactivator, with Glu/Asp-rich carboxy-terminal domain, 2 (Cited2), mRNA [NM_053698]	**50,152**
0	Uncharacterized protein [Source:UniProtKB/TrEMBL;Acc:D3ZC15] [ENSRNOT00000041970]	**50,139**
Ptprt	Rattus norvegicus protein tyrosine phosphatase, receptor type, T (Ptprt), mRNA [NM_001108603]	**50,126**
Sbk1	Rattus norvegicus SH3-binding domain kinase 1 (Sbk1), mRNA [NM_147135]	**50,116**
0	Unknown	**50,106**
Mink1	PREDICTED: Rattus norvegicus similar to Map4k6-pending protein (LOC303259), mRNA [XM_001079459]	**50,080**
Papola	Rattus norvegicus poly (A) polymerase alpha (Papola), mRNA [NM_001108056]	**50,041**
0	Unknown	**50,036**
RGD1565685	Rattus norvegicus similar to RIKEN cDNA 1810030O07 (RGD1565685), mRNA [NM_001126286]	**50,030**
Cckbr	Rattus norvegicus cholecystokinin B receptor (Cckbr), mRNA [NM_013165]	**49,906**
Kctd13	Rattus norvegicus potassium channel tetramerisation domain containing 13 (Kctd13), mRNA [NM_198736]	**49,849**
Phf21b	Rattus norvegicus PHD finger protein 21B (Phf21b), mRNA [NM_001130680]	**49,837**
0	Unknown	**49,795**
Trak1	Rattus norvegicus trafficking protein, kinesin binding 1 (Trak1), mRNA [NM_001134565]	**49,745**
Lsm12	Rattus norvegicus LSM12 homolog (S. cerevisiae) (Lsm12), mRNA [NM_001105843]	**49,744**
Igf2bp2	PREDICTED: Rattus norvegicus insulin-like growth factor 2 mRNA binding protein 2, transcript variant 2 (Igf2bp2), mRNA [XM_221343]	**49,731**
Atxn2l	Rattus norvegicus ataxin 2-like (Atxn2l), mRNA [NM_001130097]	**49,689**
Tpd52	Rattus norvegicus tumor protein D52 (Tpd52), mRNA [NM_001106421]	**49,665**
Astn1	Rattus norvegicus astrotactin 1 (Astn1), mRNA [NM_001170603]	**49,598**
Sema4g	Rattus norvegicus sema domain, immunoglobulin domain (Ig), transmembrane domain (TM) and short cytoplasmic domain, (semaphorin) 4G (Sema4g), mRNA [NM_001108526]	**49,583**

0	Unknown	**49,569**
LOC683788	Rattus norvegicus similar to Fascin (Singed-like protein) (LOC683788), mRNA [NM_001100806]	**49,516**
Bgn	Rattus norvegicus biglycan (Bgn), mRNA [NM_017087]	**49,493**
Slc39a1	Rattus norvegicus solute carrier family 39 (zinc transporter), member 1 (Slc39a1), mRNA [NM_001134577]	**49,447**
Alg3	Rattus norvegicus asparagine-linked glycosylation 3, alpha-1,3- mannosyltransferase homolog (S. cerevisiae) (Alg3), transcript variant 1, mRNA [NM_001142363]	**49,442**
Tessp1	Rattus norvegicus testis serine protease 1 (Tessp1), mRNA [NM_001135087]	**49,421**
Bmpr1a	Rattus norvegicus bone morphogenetic protein receptor, type IA (Bmpr1a), mRNA [NM_030849]	**49,419**
Cntn3	Rattus norvegicus contactin 3 (plasmacytoma associated) (Cntn3), mRNA [NM_019329]	**49,358**
Sirpa	Rattus norvegicus signal-regulatory protein alpha (Sirpa), mRNA [NM_013016]	**49,348**
0	Unknown	**49,323**
RGD1310656	RCG21340Uncharacterized protein [Source:UniProtKB/TrEMBL;Acc:D3ZL52] [ENSRNOT00000030600]	**49,321**
0	Unknown	**49,318**
Ttyh3	Rattus norvegicus tweety homolog 3 (Drosophila) (Ttyh3), mRNA [NM_001107124]	**49,306**
Fam134a	PREDICTED: Rattus norvegicus family with sequence similarity 134, member A (Fam134a), mRNA [XM_001057799]	**49,293**
LOC298018	AGENCOURT_113679838 NIH_MGC_431 Rattus norvegicus cDNA clone IMAGE:9092772 5', mRNA sequence [EV770462]	**49,232**
Arid3a	Rattus norvegicus AT rich interactive domain 3A (Bright like) (Arid3a), mRNA [NM_001108066]	**49,173**
0	Potassium voltage-gated channel subfamily D member 3 [Source:UniProtKB/Swiss-Prot;Acc:Q62897] [ENSRNOT00000051835]	**49,158**
Olfm1	Rattus norvegicus olfactomedin 1 (Olfm1), mRNA [NM_053573]	**49,056**
Dnajb5	Rattus norvegicus DnaJ (Hsp40) homolog, subfamily B, member 5 (Dnajb5), mRNA [NM_001108004]	**49,051**
Cpeb4	Rattus norvegicus cytoplasmic polyadenylation element binding protein 4 (Cpeb4), mRNA [NM_001106992]	**49,011**
0	Q6NUI3_HUMAN (Q6NUI3) Beta 3-glycosyltransferase-like, partial (23%) [TC629324]	**48,971**
Srd5a3	Rattus norvegicus steroid 5 alpha-reductase 3 (Srd5a3), mRNA [NM_001013990]	**48,962**
Notch1	Rattus norvegicus Notch homolog 1, translocation-associated (Drosophila) (Notch1), mRNA [NM_001105721]	**48,932**
Slc35d1	Rattus norvegicus solute carrier family 35 (UDP-glucuronic acid/UDP-N-acetylgalactosamine dual transporter), member D1 (Slc35d1), mRNA [NM_001106668]	**48,930**
0	Unknown	**48,897**
0	Uncharacterized protein [Source:UniProtKB/TrEMBL;Acc:D4A1N7] [ENSRNOT00000056097]	**48,808**
0	Unknown	**48,782**
Cald1	Rattus norvegicus caldesmon 1 (Cald1), mRNA [NM_013146]	**48,721**
Pde4a	Rattus norvegicus phosphodiesterase 4A, cAMP-specific (phosphodiesterase E2 dunce homolog, Drosophila) (Pde4a), mRNA [NM_013101]	**48,704**
0	Unknown	**48,700**
Brunol5	Rattus norvegicus bruno-like 5, RNA binding protein (Drosophila) (Brunol5), mRNA [NM_001135603]	**48,696**
Fam64a	Rattus norvegicus family with sequence similarity 64, member A (Fam64a), mRNA [NM_001113781]	**48,694**

Lig3	Rattus norvegicus ligase III, DNA, ATP-dependent (Lig3), nuclear gene encoding mitochondrial protein, mRNA [NM_001012011]	**48,692**
Fam57b	Rattus norvegicus family with sequence similarity 57, member B (Fam57b), mRNA [NM_001106296]	**48,679**
LOC680802	Rattus norvegicus TL0AAA71YE16 mRNA sequence. [FQ212058]	**48,656**
Susd3	Rattus norvegicus sushi domain containing 3 (Susd3), mRNA [NM_001107341]	**48,654**
RGD1309403	Rattus norvegicus similar to hypothetical protein FLJ12661 (RGD1309403), mRNA [NM_001107676]	**48,541**
Wasf1	Rattus norvegicus WAS protein family, member 1 (Wasf1), mRNA [NM_001025114]	**48,529**
B4galnt1	Rattus norvegicus beta-1,4-N-acetyl-galactosaminyl transferase 1 (B4galnt1), mRNA [NM_022860]	**48,526**
LOC292722	Uncharacterized protein [Source:UniProtKB/TrEMBL;Acc:D3Z9I0] [ENSRNOT00000027783]	**48,477**
Golph3l	Rattus norvegicus golgi phosphoprotein 3-like (Golph3l), mRNA [NM_001007698]	**48,455**
Shank1	Rattus norvegicus SH3 and multiple ankyrin repeat domains 1 (Shank1), mRNA [NM_031751]	**48,294**
0	PREDICTED: Rattus norvegicus similar to 60S ribosomal protein L29 (P23) (RGD1562489), mRNA [XM_001071419]	**48,199**
Lynx1	Rattus norvegicus Ly6/neurotoxin 1 (Lynx1), mRNA [NM_001130546]	**48,060**
Fbxo46	Rattus norvegicus F-box protein 46 (Fbxo46), mRNA [NM_001025642]	**48,005**
Cd24	Rattus norvegicus CD24 molecule (Cd24), mRNA [NM_012752]	**47,963**
Dennd1a	Rattus norvegicus DENN/MADD domain containing 1A (Dennd1a), mRNA [NM_001191747]	**47,962**
Olr371	Rattus norvegicus olfactory receptor 371 (Olr371), mRNA [NM_001000258]	**47,950**
Xpr1	Rattus norvegicus xenotropic and polytropic retrovirus receptor 1 (Xpr1), mRNA [NM_001105992]	**47,950**
Hcfc1	Rattus norvegicus host cell factor C1 (Hcfc1), mRNA [NM_001139507]	**47,941**
Cotl1	Rattus norvegicus coactosin-like 1 (Dictyostelium) (Cotl1), mRNA [NM_001108452]	**47,917**
Tmem71	PREDICTED: Rattus norvegicus transmembrane protein 71 (Tmem71), mRNA [XM_001075116]	**47,902**
Coro1c	Rattus norvegicus coronin, actin binding protein 1C (Coro1c), mRNA [NM_001109327]	**47,900**
Wipf3	Rattus norvegicus WAS/WASL interacting protein family, member 3 (Wipf3), mRNA [NM_147211]	**47,900**
Col5a2	Rattus norvegicus collagen, type V, alpha 2 (Col5a2), mRNA [NM_053488]	**47,883**
RGD1562582	Rattus norvegicus similar to KIAA0406-like protein (RGD1562582), mRNA [NM_001134619]	**47,879**
RGD1562865	Rattus norvegicus similar to BTB and CNC homology 1, basic leucine zipper transcription factor 2 (RGD1562865), mRNA [NM_001135754]	**47,862**
Wdr41	Rattus norvegicus WD repeat domain 41 (Wdr41), mRNA [NM_001108541]	**47,810**
RGD1359158	Rattus norvegicus similar to RIKEN cDNA 1110059E24 (RGD1359158), mRNA [NM_001007737]	**47,801**
0	Unknown	**47,780**
Pbx1	Rattus norvegicus pre-B-cell leukemia homeobox 1 (Pbx1), transcript variant 2, mRNA [NM_001100681]	**47,778**
Sirt2	Rattus norvegicus sirtuin (silent mating type information regulation 2 homolog) 2 (S. cerevisiae) (Sirt2), mRNA [NM_001008368]	**47,765**
Vamp2	Rattus norvegicus vesicle-associated membrane protein 2 (Vamp2), mRNA [NM_012663]	**47,748**
0	Unknown	**47,726**
Aplp2	PREDICTED: Rattus norvegicus amyloid beta (A4) precursor-like protein 2 (Aplp2), mRNA [XM_343513]	**47,721**
Rilp	Rattus norvegicus Rab interacting lysosomal protein (Rilp), mRNA [NM_001105811]	**47,668**

Neurod2	Rattus norvegicus neurogenic differentiation 2 (Neurod2), mRNA [NM_019326]	**47,649**
Csgalnact1	Rattus norvegicus chondroitin sulfate N-acetylgalactosaminyltransferase 1 (Csgalnact1), mRNA [NM_001107309]	**47,646**
Mras	Rattus norvegicus muscle RAS oncogene homolog (Mras), mRNA [NM_012981]	**47,636**
0	Sema domain, transmembrane domain (TM), and cytoplasmic domain, (Semaphorin) 6A (Predicted), isoform CRA_aUncharacterized protein [Source:UniProtKB/TrEMBL;Acc:D3ZAG0] [ENSRNOT00000005476]	**47,590**
Tox	Rattus norvegicus thymocyte selection-associated high mobility group box (Tox), mRNA [NM_001108654]	**47,589**
Ssh2	Rattus norvegicus slingshot homolog 2 (Drosophila) (Ssh2), mRNA [NM_001107024]	**47,509**
Tmem131	Uncharacterized protein [Source:UniProtKB/TrEMBL;Acc:D4A2I0] [ENSRNOT00000066288]	**47,503**
Tbc1d9	Rattus norvegicus TBC1 domain family, member 9 (Tbc1d9), mRNA [NM_001134539]	**47,496**
Cmbl	Rattus norvegicus carboxymethylenebutenolidase homolog (Pseudomonas) (Cmbl), mRNA [NM_001008770]	**47,480**
Zbtb7a	Rattus norvegicus zinc finger and BTB domain containing 7a (Zbtb7a), mRNA [NM_054002]	**47,474**
Ccdc92	Rattus norvegicus coiled-coil domain containing 92 (Ccdc92), mRNA [NM_001083898]	**47,461**
Dnajb12	Rattus norvegicus DnaJ (Hsp40) homolog, subfamily B, member 12 (Dnajb12), mRNA [NM_001013907]	**47,453**
Reep5	Rattus norvegicus receptor accessory protein 5 (Reep5), mRNA [NM_001108888]	**47,428**
0	Uncharacterized protein [Source:UniProtKB/TrEMBL;Acc:D3ZXW1] [ENSRNOT00000030613]	**47,406**
Icam1	Rattus norvegicus intercellular adhesion molecule 1 (Icam1), mRNA [NM_012967]	**47,395**
0	Q8VDS2_MOUSE (Q8VDS2) Ttll3 protein, partial (38%) [TC587171]	**47,348**
Fhl1	Rattus norvegicus four and a half LIM domains 1 (Fhl1), transcript variant 1, mRNA [NM_001033926]	**47,336**
0	Unknown	**47,291**
Sorbs3	Rattus norvegicus sorbin and SH3 domain containing 3 (Sorbs3), mRNA [NM_001005762]	**47,276**
0	Unknown	**47,252**
Pole3	Rattus norvegicus polymerase (DNA directed), epsilon 3 (p17 subunit) (Pole3), mRNA [NM_001007652]	**47,245**
0	Uncharacterized protein [Source:UniProtKB/TrEMBL;Acc:D3ZJX9] [ENSRNOT00000057090]	**47,236**
Shank3	Rattus norvegicus SH3 and multiple ankyrin repeat domains 3 (Shank3), mRNA [NM_021676]	**47,225**
Pmepa1	prostate transmembrane protein, androgen induced 1 [Source:RefSeq peptide;Acc:NP_001101277] [ENSRNOT00000008261]	**47,216**
Ipo9	Rattus norvegicus importin 9 (Ipo9), mRNA [NM_001107180]	**47,204**
Kcnh4	Rattus norvegicus potassium voltage-gated channel, subfamily H (eag-related), member 4 (Kcnh4), mRNA [NM_053630]	**47,185**
RGD1564943	Rattus norvegicus similar to 4930429A08Rik protein (RGD1564943), mRNA [NM_001134628]	**47,183**
Mobkl1a	Rattus norvegicus MOB1, Mps One Binder kinase activator-like 1A (yeast) (Mobkl1a), mRNA [NM_001108357]	**47,166**
RGD1305899	PREDICTED: Rattus norvegicus similar to Protein C20orf158 (RGD1305899), mRNA [XM_001058977]	**47,164**
Fam168a	Rattus norvegicus family with sequence similarity 168, member A (Fam168a), mRNA [NM_001108494]	**47,144**
Slc7a8	Rattus norvegicus solute carrier family 7 (cationic amino acid transporter, y+ system), member 8 (Slc7a8), mRNA [NM_053442]	**47,115**
Atp6v0e2	Rattus norvegicus ATPase, H+ transporting V0 subunit e2 (Atp6v0e2), mRNA [NM_001002253]	**47,067**
0	Q14610_HUMAN (Q14610) Interferon-gamma variant, partial (12%) [TC620342]	**47,051**

Gosr1	Rattus norvegicus golgi SNAP receptor complex member 1 (Gosr1), mRNA [NM_053584]	**47,048**
0	Q3QX63_9RHOB (Q3QX63) Phage baseplate assembly protein V, partial (10%) [TC626448]	**47,040**
0	Centrosomal protein 1 (Predicted)Uncharacterized protein [Source:UniProtKB/TrEMBL;Acc:D3ZRZ3] [ENSRNOT00000007906]	**47,021**
Gjb2	Rattus norvegicus gap junction protein, beta 2 (Gjb2), mRNA [NM_001004099]	**46,975**
Dolpp1	Rattus norvegicus dolichyl pyrophosphate phosphatase 1 (Dolpp1), mRNA [NM_001106567]	**46,908**
0	Unknown	**46,904**
Rab33b	Rattus norvegicus RAB33B, member of RAS oncogene family (Rab33b), mRNA [NM_001108944]	**46,867**
Crip2	Rattus norvegicus cysteine-rich protein 2 (Crip2), mRNA [NM_022501]	**46,837**
Brsk2	PREDICTED: Rattus norvegicus brain serine/threonine kinase 2 (Brsk2), mRNA [XM_219498]	**46,830**
Tmem151a	Rattus norvegicus transmembrane protein 151A (Tmem151a), mRNA [NM_001107570]	**46,808**
Olig2	Rattus norvegicus oligodendrocyte lineage transcription factor 2 (Olig2), mRNA [NM_001100557]	**46,780**
0	DNA-directed RNA polymerase [Source:UniProtKB/TrEMBL;Acc:D3ZGF8] [ENSRNOT00000046484]	**46,749**
RGD1561113	Uncharacterized protein [Source:UniProtKB/TrEMBL;Acc:D3ZBT2] [ENSRNOT00000034930]	**46,736**
Arf2	Rattus norvegicus ADP-ribosylation factor 2 (Arf2), mRNA [NM_024150]	**46,727**
Stx1b	Syntaxin-1B [Source:UniProtKB/Swiss-Prot;Acc:P61265] [ENSRNOT00000026063]	**46,671**
Efnb3	Rattus norvegicus ephrin B3 (Efnb3), mRNA [NM_001100980]	**46,627**
Pou2f1	Rattus norvegicus POU class 2 homeobox 1 (Pou2f1), mRNA [NM_001100639]	**46,592**
0	Nope protein [Source:UniProtKB/TrEMBL;Acc:B5DFA9] [ENSRNOT00000040854]	**46,583**
Srgap2	Rattus norvegicus TL0AAA55YK04 mRNA sequence. [FQ212420]	**46,569**
Ino80d	Rattus norvegicus INO80 complex subunit D (Ino80d), mRNA [NM_001191809]	**46,569**
Fam117a	Rattus norvegicus family with sequence similarity 117, member A (Fam117a), mRNA [NM_001109039]	**46,542**
LOC679462	Rattus norvegicus similar to Tetraspanin-15 (Tspan-15) (Transmembrane 4 superfamily member 15) (Tetraspan NET-7) (LOC679462), mRNA [NM_001115032]	**46,346**
Ino80c	Rattus norvegicus INO80 complex subunit C (Ino80c), mRNA [NM_001017446]	**46,221**
Map1b	Rattus norvegicus microtubule-associated protein 1B (Map1b), mRNA [NM_019217]	**46,200**
RGD1310127	Rattus norvegicus similar to cDNA sequence BC017158 (RGD1310127), mRNA [NM_001035517]	**46,155**
0	Unknown	**46,126**
0	Unknown	**46,114**
Olr569	Rattus norvegicus olfactory receptor 569 (Olr569), mRNA [NM_001000664]	**46,079**
Cxxc5	Rattus norvegicus CXXC finger 5 (Cxxc5), mRNA [NM_001007628]	**46,040**
Arhgdia	Rattus norvegicus Rho GDP dissociation inhibitor (GDI) alpha (Arhgdia), mRNA [NM_001007005]	**46,023**
Tmem9	Rattus norvegicus transmembrane protein 9 (Tmem9), mRNA [NM_001105953]	**46,023**
Ubl7	Rattus norvegicus ubiquitin-like 7 (bone marrow stromal cell-derived) (Ubl7), mRNA [NM_001004247]	**45,997**
Scly	Rattus norvegicus selenocysteine lyase (Scly), mRNA [NM_001007755]	**45,994**

0	Unknown	**45,992**
Efhd1	Rattus norvegicus EF-hand domain family, member D1 (Efhd1), mRNA [NM_001109310]	**45,978**
Atf6	Rattus norvegicus activating transcription factor 6 (Atf6), mRNA [NM_001107196]	**45,898**
0	NADH-ubiquinone oxidoreductase chain 4L [Source:UniProtKB/Swiss-Prot;Acc:P05507] [ENSRNOT00000044582]	**45,881**
Ypel4	Rattus norvegicus yippee-like 4 (Drosophila) (Ypel4), mRNA [NM_001024369]	**45,868**
Slc38a3	Rattus norvegicus solute carrier family 38, member 3 (Slc38a3), mRNA [NM_145776]	**45,807**
Rnf208	Rattus norvegicus ring finger protein 208 (Rnf208), mRNA [NM_001109195]	**45,725**
RGD1564836	Uncharacterized protein [Source:UniProtKB/TrEMBL;Acc:D3ZAX1] [ENSRNOT00000038940]	**45,716**
Pip4k2a	Rattus norvegicus phosphatidylinositol-5-phosphate 4-kinase, type II, alpha (Pip4k2a), mRNA [NM_053926]	**45,686**
Ly6g6c	Rattus norvegicus lymphocyte antigen 6 complex, locus G6C (Ly6g6c), mRNA [NM_001001969]	**45,679**
Ube2v1	Rattus norvegicus ubiquitin-conjugating enzyme E2 variant 1 (Ube2v1), mRNA [NM_001110345]	**45,673**
Nfasc	Rattus norvegicus neurofascin (Nfasc), transcript variant 1, mRNA [NM_001160314]	**45,668**
Tom1	Rattus norvegicus target of myb1 homolog (chicken) (Tom1), mRNA [NM_001008365]	**45,652**
Klf3	Rattus norvegicus Kruppel-like factor 3 (basic) (Klf3), mRNA [NM_001105742]	**45,637**
Hnrnpul1	Rattus norvegicus heterogeneous nuclear ribonucleoprotein U-like 1 (Hnrnpul1), mRNA [NM_001108477]	**45,637**
Shroom2	Rattus norvegicus shroom family member 2 (Shroom2), mRNA [NM_001047893]	**45,634**
LOC100366259	PREDICTED: Rattus norvegicus rCG52099-like (LOC100366259), mRNA [XM_002725118]	**45,630**
0	Rattus norvegicus polycystic kidney disease 1 homolog (Pkd1), mRNA [XM_340765]	**45,619**
0	Unknown	**45,594**
Prps2	Rattus norvegicus phosphoribosyl pyrophosphate synthetase 2 (Prps2), mRNA [NM_012634]	**45,576**
Ugt1a6	Rattus norvegicus UDP glucuronosyltransferase 1 family, polypeptide A6 (Ugt1a6), transcript variant 1, mRNA [NM_001039691]	**45,573**
Ino80e	Rattus norvegicus INO80 complex subunit E (Ino80e), mRNA [NM_001013900]	**45,501**
Praf2	Rattus norvegicus PRA1 domain family, member 2 (Praf2), mRNA [NM_001109013]	**45,491**
0	Rattus norvegicus similar to 60S RIBOSOMAL PROTEIN L29 (P23) (LOC292938), mRNA [XM_218668]	**45,449**
Slc13a2	Rattus norvegicus solute carrier family 13 (sodium-dependent dicarboxylate transporter), member 2 (Slc13a2), mRNA [NM_031746]	**45,444**
Ddah1	Rattus norvegicus dimethylarginine dimethylaminohydrolase 1 (Ddah1), mRNA [NM_022297]	**45,433**
Fam100b	PREDICTED: Rattus norvegicus family with sequence similarity 100, member B (Fam100b), mRNA [XM_213532]	**45,418**
0	Uncharacterized protein [Source:UniProtKB/TrEMBL;Acc:D3ZRD4] [ENSRNOT00000054998]	**45,414**
Zfp36l1	Rattus norvegicus zinc finger protein 36, C3H type-like 1 (Zfp36l1), mRNA [NM_017172]	**45,361**
Reep6	Rattus norvegicus receptor accessory protein 6 (Reep6), mRNA [NM_001013218]	**45,355**
Optn	Rattus norvegicus optineurin (Optn), mRNA [NM_145081]	**45,248**
Ubqln2	Rattus norvegicus ubiquilin 2 (Ubqln2), mRNA [NM_001108251]	**45,246**
0	Unknown	**45,242**
Tob2	Rattus norvegicus transducer of ERBB2, 2 (Tob2), mRNA [NM_001007146]	**45,236**

Zfp385a	Rattus norvegicus zinc finger protein 385A (Zfp385a), transcript variant 1, mRNA [NM_001135088]	**45,176**
Kctd4	Rattus norvegicus potassium channel tetramerisation domain containing 4 (Kctd4), mRNA [NM_001109650]	**45,171**
Npepps	Rattus norvegicus aminopeptidase puromycin sensitive (Npepps), mRNA [NM_080395]	**45,152**
Rnf165	Rattus norvegicus ring finger protein 165 (Rnf165), mRNA [NM_001164505]	**45,150**
Ap3m1	Rattus norvegicus adaptor-related protein complex 3, mu 1 subunit (Ap3m1), mRNA [NM_133593]	**45,133**
Ncstn	Rattus norvegicus nicastrin (Ncstn), mRNA [NM_174864]	**45,101**
Scn3b	Rattus norvegicus sodium channel, voltage-gated, type III, beta (Scn3b), mRNA [NM_139097]	**45,060**
Fam128b	Rattus norvegicus family with sequence similarity 128, member B (Fam128b), mRNA [NM_001105860]	**45,046**
Mknk1	Rattus norvegicus MAP kinase-interacting serine/threonine kinase 1 (Mknk1), mRNA [NM_001044267]	**45,040**
0	Q2I375_HUMAN (Q2I375) QKI, partial (64%) [TC627221]	**45,027**
Kcng1	Rattus norvegicus potassium voltage-gated channel, subfamily G, member 1 (Kcng1), mRNA [NM_001106545]	**44,999**
Spata2L	Rattus norvegicus spermatogenesis associated 2-like (Spata2L), mRNA [NM_001109133]	**44,991**
Mrpl10	Rattus norvegicus mitochondrial ribosomal protein L10 (Mrpl10), nuclear gene encoding mitochondrial protein, mRNA [NM_001109620]	**44,976**
0	Unknown	**44,969**
LOC304558	PREDICTED: Rattus norvegicus similar to TPR repeat-containing protein KIAA1043 (LOC304558), mRNA [XM_222260]	**44,909**
Sepw1	Rattus norvegicus selenoprotein W, 1 (Sepw1), mRNA [NM_013027]	**44,901**
Cbara1	Rattus norvegicus calcium binding atopy-related autoantigen 1 (Cbara1), nuclear gene encoding mitochondrial protein, mRNA [NM_199412]	**44,859**
Ube2g1	Rattus norvegicus ubiquitin-conjugating enzyme E2G 1 (UBC7 homolog, yeast) (Ube2g1), mRNA [NM_022690]	**44,847**
Gsk3a	Rattus norvegicus glycogen synthase kinase 3 alpha (Gsk3a), mRNA [NM_017344]	**44,819**
Vcl	Rattus norvegicus vinculin (Vcl), mRNA [NM_001107248]	**44,811**
Pa2g4	Rattus norvegicus proliferation-associated 2G4 (Pa2g4), mRNA [NM_001004206]	**44,808**
RGD621098	Rattus norvegicus similar to RIKEN cDNA D230025D16Rik (RGD621098), mRNA [NM_139040]	**44,775**
Lnx2	Rattus norvegicus ligand of numb-protein X 2 (Lnx2), mRNA [NM_001108329]	**44,726**
Rnf166	Rattus norvegicus ring finger protein 166 (Rnf166), mRNA [NM_001002279]	**44,718**
Tp53inp2	PREDICTED: Rattus norvegicus tumor protein p53 inducible nuclear protein 2 (Trp53inp2), mRNA [XM_001074013]	**44,681**
RGD1560471	Rattus norvegicus similar to hypothetical protein 4933429F08 (RGD1560471), mRNA [NM_001100974]	**44,665**
Gatad2a	Rattus norvegicus GATA zinc finger domain containing 2A (Gatad2a), mRNA [NM_001013881]	**44,650**
Zfp358	Rattus norvegicus zinc finger protein 358 (Zfp358), mRNA [NM_001108328]	**44,643**
Spred2	Rattus norvegicus sprouty-related, EVH1 domain containing 2 (Spred2), mRNA [NM_001047094]	**44,629**
Lppr5	Rattus norvegicus lipid phosphate phosphatase-related protein type 5 (Lppr5), mRNA [NM_001107720]	**44,558**
0	Rattus norvegicus cDNA clone IMAGE:7367251. [BC167759]	**44,536**
Atp6v0a1	Rattus norvegicus ATPase, H+ transporting, lysosomal V0 subunit A1 (Atp6v0a1), mRNA [NM_031604]	**44,482**
Zyx	Rattus norvegicus zyxin (Zyx), mRNA [NM_053761]	**44,468**
Cacng3	Rattus norvegicus calcium channel, voltage-dependent, gamma subunit 3 (Cacng3), mRNA [NM_080691]	**44,415**

Lppr4	Rattus norvegicus lipid phosphate phosphatase-related protein type 4 (Lppr4), mRNA [NM_001001508]	**44,406**
Arf3	Rattus norvegicus ADP-ribosylation factor 3 (Arf3), mRNA [NM_080904]	**44,403**
Nbl1	Rattus norvegicus neuroblastoma, suppression of tumorigenicity 1 (Nbl1), mRNA [NM_031609]	**44,396**
Phlda1	Rattus norvegicus pleckstrin homology-like domain, family A, member 1 (Phlda1), mRNA [NM_017180]	**44,365**
Megf9	Rattus norvegicus multiple EGF-like-domains 9 (Megf9), mRNA [NM_001107940]	**44,360**
0	Unknown	**44,336**
0	Unknown	**44,312**
Neo1	PREDICTED: Rattus norvegicus neogenin homolog 1 (chicken) (Neo1), mRNA [XM_001074913]	**44,279**
Rtkn	Rattus norvegicus rhotekin (Rtkn), mRNA [NM_184046]	**44,260**
Cacnb4	Rattus norvegicus calcium channel, voltage-dependent, beta 4 subunit (Cacnb4), mRNA [NM_001105733]	**44,235**
Shank1	Rattus norvegicus SH3 and multiple ankyrin repeat domains 1 (Shank1), mRNA [NM_031751]	**44,223**
0	abl interactor 2 [Source:RefSeq peptide;Acc:NP_775166] [ENSRNOT00000058341]	**44,213**
Sfxn1	Rattus norvegicus sideroflexin 1 (Sfxn1), mRNA [NM_001012213]	**44,209**
Layn	Rattus norvegicus layilin (Layn), mRNA [NM_001191997]	**44,164**
Limk1	Rattus norvegicus LIM domain kinase 1 (Limk1), mRNA [NM_031727]	**44,164**
Aldh9a1	Rattus norvegicus aldehyde dehydrogenase 9 family, member A1 (Aldh9a1), mRNA [NM_022273]	**44,141**
Fam70b	Rattus norvegicus family with sequence similarity 70, member B (Fam70b), mRNA [NM_001106094]	**44,140**
Cbfa2t3	Rattus norvegicus core-binding factor, runt domain, alpha subunit 2; translocated to, 3 (Cbfa2t3), mRNA [NM_001108453]	**44,136**
Asb13	Rattus norvegicus ankyrin repeat and SOCS box-containing 13 (Asb13), mRNA [NM_001108420]	**44,123**
0	Rattus norvegicus similar to 60S RIBOSOMAL PROTEIN L29 (P23) (LOC364407), mRNA [XM_344423]	**44,041**
Pcsk4	Rattus norvegicus proprotein convertase subtilisin/kexin type 4 (Pcsk4), mRNA [NM_133559]	**44,026**
Nova1	RNA-binding protein Nova-1 [Source:UniProtKB/Swiss-Prot;Acc:Q80WA4] [ENSRNOT00000050372]	**44,024**
Mmp24	Rattus norvegicus matrix metallopeptidase 24 (Mmp24), mRNA [NM_031757]	**44,001**
LOC685179	PREDICTED: Rattus norvegicus similar to SWI/SNF-related matrix-associated actin-dependent regulator of chromatin c2, transcript variant 4 (LOC685179), mRNA [XM_001055795]	**43,995**
Pdpn	Rattus norvegicus podoplanin (Pdpn), mRNA [NM_019358]	**43,971**
Pus7l	PREDICTED: Rattus norvegicus pseudouridylate synthase 7 homolog (S. cerevisiae)-like (Pus7l), mRNA [XM_001058268]	**43,962**
0	Unknown	**43,907**
Foxj2	Rattus norvegicus forkhead box J2 (Foxj2), mRNA [NM_001109352]	**43,873**
Prkca	Rattus norvegicus protein kinase C, alpha (Prkca), mRNA [NM_001105713]	**43,859**
Calhm3	Rattus norvegicus calcium homeostasis modulator 3 (Calhm3), mRNA [NM_001191956]	**43,820**
Hapln1	Rattus norvegicus hyaluronan and proteoglycan link protein 1 (Hapln1), mRNA [NM_019189]	**43,800**
Eefsec	Rattus norvegicus eukaryotic elongation factor, selenocysteine-tRNA-specific (Eefsec), mRNA [NM_001109249]	**43,792**
Trim46	Rattus norvegicus tripartite motif-containing 46 (Trim46), mRNA [NM_001107691]	**43,791**

S100a16	Rattus norvegicus S100 calcium binding protein A16 (S100a16), mRNA [NM_001108557]	**43,764**
0	Unknown	**43,707**
Ccdc91	Rattus norvegicus coiled-coil domain containing 91 (Ccdc91), mRNA [NM_001014061]	**43,687**
Cbs	Rattus norvegicus cystathionine beta synthase (Cbs), mRNA [NM_012522]	**43,670**
Zswim4	Rattus norvegicus zinc finger, SWIM-type containing 4 (Zswim4), mRNA [NM_001107163]	**43,662**
Ncoa4	Rattus norvegicus nuclear receptor coactivator 4 (Ncoa4), transcript variant 2, mRNA [NM_001034008]	**43,653**
Nudt2	Rattus norvegicus nudix (nucleoside diphosphate linked moiety X)-type motif 2 (Nudt2), mRNA [NM_207596]	**43,619**
Zfp583	Rattus norvegicus zinc finger protein 583 (Zfp583), mRNA [NM_001134609]	**43,597**
0	Unknown	**43,587**
LOC685249	PREDICTED: Rattus norvegicus hypothetical protein LOC685249 (LOC685249), mRNA [XM_001063008]	**43,561**
Atf5	Rattus norvegicus activating transcription factor 5 (Atf5), mRNA [NM_172336]	**43,550**
Pclo	Rattus norvegicus piccolo (presynaptic cytomatrix protein) (Pclo), transcript variant 2, mRNA [NM_001110797]	**43,519**
Tcf19	Rattus norvegicus transcription factor 19 (Tcf19), mRNA [NM_213561]	**43,517**
Pcsk4	Rattus norvegicus proprotein convertase subtilisin/kexin type 4 (Pcsk4), mRNA [NM_133559]	**43,404**
37865	Rattus norvegicus septin 3 (Sept3), mRNA [NM_019375]	**43,380**
Ube2d4	Rattus norvegicus ubiquitin-conjugating enzyme E2D 4 (Ube2d4), mRNA [NM_031001]	**43,368**
Csdc2	Rattus norvegicus cold shock domain containing C2, RNA binding (Csdc2), mRNA [NM_001170542]	**43,357**
Xylb	Rattus norvegicus xylulokinase homolog (H. influenzae) (Xylb), mRNA [NM_001033704]	**43,334**
Capzb	Rattus norvegicus capping protein (actin filament) muscle Z-line, beta (Capzb), mRNA [NM_001005903]	**43,325**
0	Unknown	**43,312**
Tanc2	Rattus norvegicus tetratricopeptide repeat, ankyrin repeat and coiled-coil containing 2 (Tanc2), mRNA [NM_001191653]	**43,282**
Fhl1	Rattus norvegicus four and a half LIM domains 1 (Fhl1), transcript variant 2, mRNA [NM_145669]	**43,247**
Rbm4b	Rattus norvegicus RNA binding motif protein 4B (Rbm4b), mRNA [NM_001007014]	**43,236**
Helz	Rattus norvegicus helicase with zinc finger (Helz), mRNA [NM_001105848]	**43,179**
Cyp4f1	Rattus norvegicus cytochrome P450, family 4, subfamily f, polypeptide 1 (Cyp4f1), mRNA [NM_019623]	**43,172**
Dhrs11	Rattus norvegicus dehydrogenase/reductase (SDR family) member 11 (Dhrs11), mRNA [NM_001014119]	**43,158**
Sdc2	Rattus norvegicus syndecan 2 (Sdc2), mRNA [NM_013082]	**43,154**
0	DRNBZC09 Rat DRG Library Rattus norvegicus cDNA clone DRNBZC09 5', mRNA sequence [BG671896]	**43,141**
Olr192	Rattus norvegicus olfactory receptor 192 (Olr192), mRNA [NM_001000549]	**43,124**
Afap1	Rattus norvegicus actin filament associated protein 1 (Afap1), mRNA [NM_080900]	**43,121**
Pvrl1	Nectin-1 [Source:UniProtKB/TrEMBL;Acc:Q9WTY6] [ENSRNOT00000008614]	**43,113**
Cdk5r2	Rattus norvegicus cyclin-dependent kinase 5, regulatory subunit 2 (p39) (Cdk5r2), mRNA [NM_001109309]	**43,102**
Tnfrsf1a	Rattus norvegicus tumor necrosis factor receptor superfamily, member 1a (Tnfrsf1a), mRNA [NM_013091]	**43,061**
Sdk2	Rattus norvegicus sidekick homolog 2 (chicken) (Sdk2), mRNA [NM_001108303]	**43,053**

Solh	Rattus norvegicus small optic lobes homolog (Drosophila) (Solh), mRNA [NM_001106990]	**43,048**
Thpo	Rattus norvegicus thrombopoietin (Thpo), mRNA [NM_031133]	**42,989**
Tanc2	Rattus norvegicus tetratricopeptide repeat, ankyrin repeat and coiled-coil containing 2 (Tanc2), mRNA [NM_001191653]	**42,984**
Jund	Rattus norvegicus jun D proto-oncogene (Jund), mRNA [NM_138875]	**42,983**
Zic4	Rattus norvegicus Zic family member 4 (Zic4), mRNA [NM_001108176]	**42,978**
Kcnj3	G protein-activated inward rectifier potassium channel 1 [Source:UniProtKB/Swiss-Prot;Acc:P63251] [ENSRNOT00000007335]	**42,955**
Adamts3	Rattus norvegicus ADAM metallopeptidase with thrombospondin type 1, motif 3 (Adamts3), mRNA [NM_001107212]	**42,954**
Litaf	Rattus norvegicus lipopolysaccharide-induced TNF factor (Litaf), mRNA [NM_001105735]	**42,951**
RGD1563300	Uncharacterized protein [Source:UniProtKB/TrEMBL;Acc:D3Z8Q4] [ENSRNOT00000032995]	**42,941**
0	Uncharacterized protein [Source:UniProtKB/TrEMBL;Acc:D3ZF37] [ENSRNOT00000047805]	**42,905**
0	Unknown	**42,892**
RGD1312026	Rattus norvegicus similar to RIKEN cDNA C230081A13 (RGD1312026), mRNA [NM_001108149]	**42,883**
0	Unknown	**42,863**
Ap1g1	Rattus norvegicus adaptor-related protein complex 1, gamma 1 subunit (Ap1g1), mRNA [NM_134460]	**42,860**
Epb4.1l2	PREDICTED: Rattus norvegicus erythrocyte membrane protein band 4.1-like 2 (Epb4.1l2), mRNA [XM_001053351]	**42,845**
Gltpd1	Rattus norvegicus glycolipid transfer protein domain containing 1 (Gltpd1), mRNA [NM_001007703]	**42,845**
0	Unknown	**42,831**
Nrg3	PREDICTED: Rattus norvegicus neuregulin 3, transcript variant 2 (Nrg3), mRNA [XM_573873]	**42,811**
Myl12b	Rattus norvegicus myosin, light chain 12B, regulatory (Myl12b), mRNA [NM_017343]	**42,792**
0	PREDICTED: Rattus norvegicus similar to Tubby-related protein 3 (Tubby-like protein 3) (LOC688749), mRNA [XM_001071995]	**42,767**
Fam172a	Rattus norvegicus family with sequence similarity 172, member A (Fam172a), mRNA [NM_001106401]	**42,759**
Wdyhv1	Rattus norvegicus WDYHV motif containing 1 (Wdyhv1), mRNA [NM_001025024]	**42,735**
Ankrd49	Rattus norvegicus ankyrin repeat domain 49 (Ankrd49), mRNA [NM_001126283]	**42,709**
Pdgfra	Rattus norvegicus platelet derived growth factor receptor, alpha polypeptide (Pdgfra), mRNA [NM_012802]	**42,690**
Slc6a9	Rattus norvegicus solute carrier family 6 (neurotransmitter transporter, glycine), member 9 (Slc6a9), mRNA [NM_053818]	**42,638**
Mxd1	Rattus norvegicus max dimerization protein 1 (Mxd1), mRNA [NM_001100749]	**42,622**
S1pr1	Rattus norvegicus sphingosine-1-phosphate receptor 1 (S1pr1), mRNA [NM_017301]	**42,614**
Cacnb1	Rattus norvegicus calcium channel, voltage-dependent, beta 1 subunit (Cacnb1), mRNA [NM_017346]	**42,613**
Usp4	Rattus norvegicus ubiquitin specific peptidase 4 (proto-oncogene) (Usp4), transcript variant 1, mRNA [NM_001135012]	**42,613**
Slc25a10	Rattus norvegicus solute carrier family 25 (mitochondrial carrier; dicarboxylate transporter), member 10 (Slc25a10), nuclear gene encoding mitochondrial protein, mRNA [NM_133418]	**42,607**
Gnai2	Rattus norvegicus guanine nucleotide binding protein (G protein), alpha inhibiting 2 (Gnai2), mRNA [NM_031035]	**42,602**
Lsm2	Rattus norvegicus LSM2 homolog, U6 small nuclear RNA associated (S. cerevisiae) (Lsm2), transcript variant 2, mRNA [NM_001165922]	**42,586**
Cyp2j3	Rattus norvegicus cytochrome P450, family 2, subfamily j, polypeptide 3 (Cyp2j3), mRNA [NM_175766]	**42,582**

Agpat1	Rattus norvegicus 1-acylglycerol-3-phosphate O-acyltransferase 1 (lysophosphatidic acid acyltransferase, alpha) (Agpat1), mRNA [NM_212458]	**42,577**
Gnao1	Rattus norvegicus guanine nucleotide binding protein (G protein), alpha activating activity polypeptide O (Gnao1), mRNA [NM_017327]	**42,576**
0	Unknown	**42,516**
Slc15a2	Rattus norvegicus solute carrier family 15 (H+/peptide transporter), member 2 (Slc15a2), mRNA [NM_031672]	**42,512**
Atp6v1g2	Rattus norvegicus ATPase, H+ transporting, lysosomal V1 subunit G2 (Atp6v1g2), mRNA [NM_212490]	**42,507**
0	AGENCOURT_109870703 NIH_MGC_418 Rattus norvegicus cDNA clone IMAGE:9022754 5', mRNA sequence [EV768930]	**42,484**
0	PREDICTED: Rattus norvegicus similar to 60S ribosomal protein L29 (P23) (LOC690662), mRNA [XM_001075132]	**42,455**
Lss	Rattus norvegicus lanosterol synthase (2,3-oxidosqualene-lanosterol cyclase) (Lss), mRNA [NM_031049]	**42,428**
Sst	Rattus norvegicus somatostatin (Sst), mRNA [NM_012659]	**42,422**
Cdk2ap1	Rattus norvegicus CDK2-associated protein 1 (Cdk2ap1), mRNA [NM_001113751]	**42,421**
Dagla	Rattus norvegicus diacylglycerol lipase, alpha (Dagla), mRNA [NM_001005886]	**42,412**
Cmpk2	Rattus norvegicus cytidine monophosphate (UMP-CMP) kinase 2, mitochondrial (Cmpk2), nuclear gene encoding mitochondrial protein, mRNA [NM_001108017]	**42,409**
LOC100365983	abl interactor 2 [Source:RefSeq peptide;Acc:NP_775166] [ENSRNOT00000058340]	**42,401**
En1	PREDICTED: Rattus norvegicus engrailed homeobox 1, transcript variant 2 (En1), mRNA [XM_001056772]	**42,396**
Znf295	Rattus norvegicus zinc finger protein 295 (Znf295), mRNA [NM_001107105]	**42,393**
Lppr2	Rattus norvegicus lipid phosphate phosphatase-related protein type 2 (Lppr2), mRNA [NM_001005881]	**42,382**
Olfml1	Rattus norvegicus olfactomedin-like 1 (Olfml1), mRNA [NM_001013192]	**42,346**
Mmgt1	Rattus norvegicus membrane magnesium transporter 1 (Mmgt1), mRNA [NM_001106970]	**42,334**
M6pr	Rattus norvegicus mannose-6-phosphate receptor, cation dependent (M6pr), mRNA [NM_001007700]	**42,323**
Cdkn1c	Rattus norvegicus cyclin-dependent kinase inhibitor 1C (Cdkn1c), transcript variant 1, mRNA [NM_001033757]	**42,262**
0	LRRGT00107 [Source:UniProtKB/TrEMBL;Acc:Q6TUD7] [ENSRNOT00000035353]	**42,243**
Sgcd	Rattus norvegicus sarcoglycan, delta (dystrophin-associated glycoprotein) (Sgcd), transcript variant 1, mRNA [NM_001134826]	**42,233**
Phyhip	Rattus norvegicus phytanoyl-CoA 2-hydroxylase interacting protein (Phyhip), mRNA [NM_001017376]	**42,211**
Ugcg	Rattus norvegicus UDP-glucose ceramide glucosyltransferase (Ugcg), mRNA [NM_031795]	**42,142**
Stk4	Rattus norvegicus serine/threonine kinase 4 (Stk4), mRNA [NM_001107800]	**42,136**
Mef2c	PREDICTED: Rattus norvegicus myocyte enhancer factor 2C (Mef2c), mRNA [XM_001056692]	**42,084**
Tmem86b	Rattus norvegicus transmembrane protein 86B (Tmem86b), mRNA [NM_001109604]	**42,079**
Ppp1r11	Rattus norvegicus protein phosphatase 1, regulatory (inhibitor) subunit 11 (Ppp1r11), mRNA [NM_212542]	**42,010**
Begain	Rattus norvegicus brain-enriched guanylate kinase-associated (Begain), transcript variant 2, mRNA [NM_024163]	**42,001**
Prox1	Rattus norvegicus prospero homeobox 1 (Prox1), mRNA [NM_001107201]	**41,988**
Bhlhe40	Rattus norvegicus basic helix-loop-helix family, member e40 (Bhlhe40), mRNA [NM_053328]	**41,982**
Hist1h1t	Rattus norvegicus histone cluster 1, H1t (Hist1h1t), mRNA [NM_012579]	**41,954**
Nicn1	Rattus norvegicus nicolin 1 (Nicn1), mRNA [NM_001034999]	**41,925**

FAM120C	Uncharacterized protein [Source:UniProtKB/TrEMBL;Acc:D3ZNI4] [ENSRNOT00000003565]	**41,906**
Ckap4	Rattus norvegicus cytoskeleton-associated protein 4 (Ckap4), mRNA [NM_001108740]	**41,868**
Olr278	Rattus norvegicus olfactory receptor 278 (Olr278), mRNA [NM_001000730]	**41,854**
RGD1305587	Rattus norvegicus similar to RIKEN cDNA 2010107G23 (RGD1305587), mRNA [NM_001127452]	**41,838**
0	Unknown	**41,820**
0	Rattus norvegicus chromosome 1, 5 clones, strain BN/SsNHsdMCW RNOR03207166, whole genome shotgun sequence [AABR03000122]	**41,812**
0	Q5DTV9_MOUSE (Q5DTV9) MKIAA1671 protein (Fragment), partial (75%) [TC598280]	**41,799**
Sema6d	Rattus norvegicus sema domain, transmembrane domain (TM), and cytoplasmic domain, (semaphorin) 6D (Sema6d), mRNA [NM_001107768]	**41,785**
Fam73b	Rattus norvegicus family with sequence similarity 73, member B (Fam73b), mRNA [NM_001106566]	**41,782**
Ankrd56	Uncharacterized protein [Source:UniProtKB/TrEMBL;Acc:D4A4Q1] [ENSRNOT00000002995]	**41,765**
Plcxd2	Rattus norvegicus phosphatidylinositol-specific phospholipase C, X domain containing 2 (Plcxd2), mRNA [NM_001134481]	**41,754**
LOC311352	Rattus norvegicus similar to Adenosine deaminase CG11994-PA (LOC311352), mRNA [NM_001014047]	**41,750**
Cops7b	Rattus norvegicus COP9 constitutive photomorphogenic homolog subunit 7B (Arabidopsis) (Cops7b), mRNA [NM_001108807]	**41,732**
Ninj1	Rattus norvegicus ninjurin 1 (Ninj1), mRNA [NM_012867]	**41,728**
Tcp11l1	Rattus norvegicus t-complex 11 like 1 (Tcp11l1), mRNA [NM_001109202]	**41,694**
Camk2n1	Rattus norvegicus calcium/calmodulin-dependent protein kinase II inhibitor 1 (Camk2n1), mRNA [NM_173337]	**41,692**
Sgta	Rattus norvegicus small glutamine-rich tetratricopeptide repeat (TPR)-containing, alpha (Sgta), mRNA [NM_022703]	**41,682**
0	PREDICTED: Rattus norvegicus similar to 60S ribosomal protein L29 (P23) (RGD1559865), mRNA [XM_346093]	**41,629**
Grin3a	Rattus norvegicus glutamate receptor, ionotropic, N-methyl-D-aspartate 3A (Grin3a), transcript variant 1, mRNA [NM_138546]	**41,601**
Ctsb	Rattus norvegicus cathepsin B (Ctsb), mRNA [NM_022597]	**41,588**
Psrc1	Rattus norvegicus proline/serine-rich coiled-coil 1 (Psrc1), mRNA [NM_001044302]	**41,588**
Arhgap21	Rattus norvegicus Rho GTPase activating protein 21 (Arhgap21), mRNA [NM_001191693]	**41,524**
LOC500956	Rattus norvegicus hypothetical protein LOC500956 (LOC500956), mRNA [NM_001025054]	**41,511**
Klf7	Rattus norvegicus Kruppel-like factor 7 (ubiquitous) (Klf7), mRNA [NM_001108800]	**41,510**
0	Unknown	**41,503**
0	Unknown	**41,463**
LOC499330	Rattus norvegicus similar to Nicotinamide riboside kinase 1 (LOC499330), mRNA [NM_001024292]	**41,457**
Gtlf3b	Rattus norvegicus gene trap locus F3b (Gtlf3b), mRNA [NM_001170541]	**41,447**
LOC691477	PREDICTED: Rattus norvegicus similar to 60S ribosomal protein L29 (P23) (LOC691477), mRNA [XM_001078461]	**41,445**
0	PREDICTED: Rattus norvegicus similar to 60S ribosomal protein L29 (P23) (RGD1566186), mRNA [XM_001070155]	**41,403**
Sema5a	Rattus norvegicus sema domain, seven thrombospondin repeats (type 1 and type 1-like), transmembrane domain (TM) and short cytoplasmic domain, (semaphorin) 5A (Sema5a), mRNA [NM_001107659]	**41,394**
0	UI-R-BJ1-atd-d-01-0-UI.s1 UI-R-BJ1 Rattus norvegicus cDNA clone UI-R-BJ1-atd-d-01-0-UI 3', mRNA sequence [BE098737]	**41,390**
0	Unknown	**41,328**

Slc35f1	Rattus norvegicus solute carrier family 35, member F1 (Slc35f1), mRNA [NM_001109338]	**41,326**
0	Unknown	**41,318**
0	UI-R-BO1-asq-d-09-0-UI.s1 UI-R-BO1 Rattus norvegicus cDNA clone UI-R-BO1-asq-d-09-0-UI 3', mRNA sequence [BE106644]	**41,291**
Prr14	Uncharacterized protein [Source:UniProtKB/TrEMBL;Acc:D3ZWF4] [ENSRNOT00000055002]	**41,290**
Rab7a	Rattus norvegicus RAB7A, member RAS oncogene family (Rab7a), mRNA [NM_023950]	**41,279**
Cpsf7	Rattus norvegicus cleavage and polyadenylation specific factor 7 (Cpsf7), mRNA [NM_001014245]	**41,247**
0	Unknown	**41,230**
0	Unknown	**41,210**
Bmp1	Rattus norvegicus bone morphogenetic protein 1 (Bmp1), mRNA [NM_031323]	**41,173**
Dph3	Rattus norvegicus DPH3, KTI11 homolog (S. cerevisiae) (Dph3), mRNA [NM_001134850]	**41,164**
LOC683470	PREDICTED: Rattus norvegicus similar to growth arrest specific 1 (LOC683470), mRNA [XM_001066058]	**41,158**
Auts2	Uncharacterized protein [Source:UniProtKB/TrEMBL;Acc:D3ZZY1] [ENSRNOT00000044800]	**41,155**
Coro2b	Coro2b protein [Source:UniProtKB/TrEMBL;Acc:Q5EB67] [ENSRNOT00000020951]	**41,151**
Zdhhc9	Rattus norvegicus zinc finger, DHHC-type containing 9 (Zdhhc9), mRNA [NM_001039016]	**41,150**
St6galnac3	Rattus norvegicus ST6 (alpha-N-acetyl-neuraminyl-2,3-beta-galactosyl-1,3)-N-acetylgalactosaminide alpha-2,6-sialyltransferase 3 (St6galnac3), mRNA [NM_019123]	**41,144**
Nrxn2	Rattus norvegicus neurexin 2 (Nrxn2), mRNA [NM_053846]	**41,068**
0	Rattus norvegicus TL0ADA15YN18 mRNA sequence. [FQ223062]	**41,051**
Fcrla	Rattus norvegicus Fc receptor-like A (Fcrla), mRNA [NM_001100682]	**41,044**
Cbln1	Rattus norvegicus cerebellin 1 precursor (Cbln1), mRNA [NM_001109127]	**41,034**
Lta	Rattus norvegicus lymphotoxin alpha (TNF superfamily, member 1) (Lta), mRNA [NM_080769]	**40,982**
Fam53b	Rattus norvegicus family with sequence similarity 53, member B (Fam53b), mRNA [NM_001107556]	**40,975**
Wdtc1	Rattus norvegicus WD and tetratricopeptide repeats 1 (Wdtc1), mRNA [NM_001107908]	**40,921**
RGD1560833	PREDICTED: Rattus norvegicus similar to myocardin-related transcription factor B (RGD1560833), mRNA [XM_001075589]	**40,899**
Derl3	Rattus norvegicus Der1-like domain family, member 3 (Derl3), mRNA [NM_001109577]	**40,882**
Tanc1	Rattus norvegicus tetratricopeptide repeat, ankyrin repeat and coiled-coil containing 1 (Tanc1), mRNA [NM_001002854]	**40,879**
Tcf7l2	Rattus norvegicus transcription factor 7-like 2 (T-cell specific, HMG-box) (Tcf7l2), mRNA [NM_001191052]	**40,877**
Parva	Rattus norvegicus parvin, alpha (Parva), mRNA [NM_020656]	**40,874**
Cplx2	Rattus norvegicus complexin 2 (Cplx2), mRNA [NM_053878]	**40,853**
Cbln4	Rattus norvegicus cerebellin 4 precursor (Cbln4), mRNA [NM_001109210]	**40,795**
39873	Rattus norvegicus membrane-associated ring finger (C3HC4) 9 (March9), mRNA [NM_001100601]	**40,776**
LOC361414	PREDICTED: Rattus norvegicus similar to Synaptic vesicle membrane protein VAT-1 homolog (LOC361414), miscRNA [XR_006986]	**40,746**
Ywhab	Rattus norvegicus tyrosine 3-monooxygenase/tryptophan 5-monooxygenase activation protein, beta polypeptide (Ywhab), mRNA [NM_019377]	**40,730**
0	Uncharacterized protein [Source:UniProtKB/TrEMBL;Acc:D3ZXW1] [ENSRNOT00000030613]	**40,728**

Kdm2a	Rattus norvegicus lysine (K)-specific demethylase 2A (Kdm2a), mRNA [NM_001108515]	**40,680**
Rnf144a	Rattus norvegicus ring finger protein 144A (Rnf144a), mRNA [NM_001082410]	**40,670**
0	Unknown	**40,639**
Rsu1	Rattus norvegicus Ras suppressor protein 1 (Rsu1), mRNA [NM_001109404]	**40,623**
Epn1	Rattus norvegicus Epsin 1 (Epn1), mRNA [NM_057136]	**40,550**
Scamp4	Rattus norvegicus secretory carrier membrane protein 4 (Scamp4), mRNA [NM_031725]	**40,540**
LOC684112	PREDICTED: Rattus norvegicus similar to KIAA0999 protein (LOC684112), mRNA [XM_001068984]	**40,539**
Sh3bgrl3	Rattus norvegicus SH3 domain binding glutamic acid-rich protein-like 3 (Sh3bgrl3), mRNA [NM_001106688]	**40,536**
Sars2	Rattus norvegicus seryl-tRNA synthetase 2, mitochondrial (Sars2), nuclear gene encoding mitochondrial protein, mRNA [NM_001106240]	**40,528**
Pitpnb	Rattus norvegicus phosphatidylinositol transfer protein, beta (Pitpnb), mRNA [NM_053742]	**40,524**
Mk1	Rattus norvegicus Mk1 protein (Mk1), mRNA [NM_134399]	**40,496**
Fam134c	Rattus norvegicus family with sequence similarity 134, member C (Fam134c), mRNA [NM_001135804]	**40,461**
Fiz1	Rattus norvegicus FLT3-interacting zinc finger 1 (Fiz1), mRNA [NM_001106223]	**40,384**
Atp2b4	Rattus norvegicus ATPase, Ca++ transporting, plasma membrane 4 (Atp2b4), mRNA [NM_001005871]	**40,368**
0	Unknown	**40,342**
Rtn3	Rattus norvegicus reticulon 3 (Rtn3), transcript variant 1, mRNA [NM_080909]	**40,340**
Upk1b	Rattus norvegicus uroplakin 1B (Upk1b), mRNA [NM_001024253]	**40,328**
LOC688430	PREDICTED: Rattus norvegicus similar to Cofilin-1 (Cofilin, non-muscle isoform), transcript variant 1 (LOC688430), mRNA [XM_001067293]	**40,316**
0	Unknown	**40,313**
0	Unknown	**40,308**
Nutf2	Rattus norvegicus nuclear transport factor 2 (Nutf2), mRNA [NM_001007629]	**40,300**
Gstm1	Rattus norvegicus glutathione S-transferase mu 1 (Gstm1), mRNA [NM_017014]	**40,290**
0	AW917568 EST348872 Rat gene index, normalized rat, norvegicus, Bento Soares Rattus norvegicus cDNA clone RGIEF42 5' end, mRNA sequence [AW917568]	**40,253**
Ank2	Uncharacterized protein [Source:UniProtKB/TrEMBL;Acc:D4A4Q9] [ENSRNOT00000055615]	**40,223**
Taf6	Rattus norvegicus TAF6 RNA polymerase II, TATA box binding protein (TBP)-associated factor (Taf6), mRNA [NM_001044225]	**40,204**
0	Unknown	**40,202**
Rbm22	Rattus norvegicus RNA binding motif protein 22 (Rbm22), mRNA [NM_001025676]	**40,201**
Mapkbp1	Rattus norvegicus mitogen activated protein kinase binding protein 1 (Mapkbp1), mRNA [NM_001108589]	**40,200**
Tgfb1i1	Rattus norvegicus transforming growth factor beta 1 induced transcript 1 (Tgfb1i1), mRNA [NM_001191840]	**40,198**
Cd300a	Rattus norvegicus CD300A molecule (Cd300a), mRNA [NM_001205348]	**40,173**
Mbd6	Rattus norvegicus methyl-CpG binding domain protein 6 (Mbd6), mRNA [NM_001170566]	**40,099**
Akt1s1	Rattus norvegicus AKT1 substrate 1 (proline-rich) (Akt1s1), mRNA [NM_001106259]	**40,090**
Sncb	Rattus norvegicus synuclein, beta (Sncb), mRNA [NM_080777]	**40,078**

Lhx2	Rattus norvegicus LIM homeobox 2 (Lhx2), mRNA [NM_001106571]	**40,063**
Iiig9	Rattus norvegicus IIIG9 protein (Iiig9), mRNA [NM_145786]	**40,050**
Mrpl43	Rattus norvegicus mitochondrial ribosomal protein L43 (Mrpl43), nuclear gene encoding mitochondrial protein, mRNA [NM_001107598]	**40,040**
Abcc4	Rattus norvegicus ATP-binding cassette, subfamily C (CFTR/MRP), member 4 (Abcc4), mRNA [NM_133411]	**39,986**
Rabl2b	Rattus norvegicus RAB, member of RAS oncogene family-like 2B (Rabl2b), mRNA [NM_001013221]	**39,984**
40057	Rattus norvegicus septin 9 (Sept9), transcript variant 2, mRNA [NM_176856]	**39,963**
Slc6a8	Rattus norvegicus solute carrier family 6 (neurotransmitter transporter, creatine), member 8 (Slc6a8), mRNA [NM_017348]	**39,960**
Zfhx2	Rattus norvegicus zinc finger homeobox 2 (Zfhx2), mRNA [NM_001098803]	**39,913**
0	Unknown	**39,881**
0	Unknown	**39,876**
Wdfy3	Rattus norvegicus WD repeat and FYVE domain containing 3 (Wdfy3), mRNA [NM_001170551]	**39,867**
Sf1	Rattus norvegicus splicing factor 1 (Sf1), transcript variant 1, mRNA [NM_001110793]	**39,848**
Wdr7	Rattus norvegicus WD repeat domain 7 (Wdr7), mRNA [NM_023975]	**39,845**
Pcyt1b	Rattus norvegicus phosphate cytidylyltransferase 1, choline, beta (Pcyt1b), mRNA [NM_173151]	**39,825**
C2cd3	Rattus norvegicus C2 calcium-dependent domain containing 3 (C2cd3), mRNA [NM_001191602]	**39,818**
Man1a2	Rattus norvegicus mannosidase, alpha, class 1A, member 2 (Man1a2), mRNA [NM_001106452]	**39,811**
Znf609	Rattus norvegicus zinc finger protein 609 (Znf609), mRNA [NM_001173371]	**39,803**
Gdf11	Growth/differentiation factor 11 [Source:UniProtKB/Swiss-Prot;Acc:Q9Z217] [ENSRNOT00000010035]	**39,787**
Trak1	Rattus norvegicus trafficking protein, kinesin binding 1 (Trak1), mRNA [NM_001134565]	**39,786**
Atp5sl	Rattus norvegicus ATP5S-like (Atp5sl), mRNA [NM_001009705]	**39,780**
0	Q9UH66_HUMAN (Q9UH66) 7h3 protein (Fragment), partial (4%) [TC581403]	**39,771**
0	Rattus norvegicus TL0AEA68YE13 mRNA sequence. [FQ231763]	**39,768**
Epn2	Rattus norvegicus epsin 2 (Epn2), transcript variant 2, mRNA [NM_001033914]	**39,759**
0	Uncharacterized protein [Source:UniProtKB/TrEMBL;Acc:D4A0X2] [ENSRNOT00000026785]	**39,757**
Cd276	Rattus norvegicus Cd276 molecule (Cd276), mRNA [NM_182824]	**39,747**
Atp1b2	Rattus norvegicus ATPase, Na+/K+ transporting, beta 2 polypeptide (Atp1b2), mRNA [NM_012507]	**39,745**
Rfx7	Rattus norvegicus regulatory factor X, 7 (Rfx7), mRNA [NM_001127490]	**39,737**
Sfrp5	Rattus norvegicus secreted frizzled-related protein 5 (Sfrp5), mRNA [NM_001107591]	**39,730**
0	Unknown	**39,729**
0	Unknown	**39,702**
0	Unknown	**39,660**
Dyrk1b	Rattus norvegicus dual-specificity tyrosine-(Y)-phosphorylation regulated kinase 1b (Dyrk1b), mRNA [NM_001107496]	**39,654**
Tmem189	Rattus norvegicus transmembrane protein 189 (Tmem189), mRNA [NM_001113752]	**39,652**
Cdk2ap2	Rattus norvegicus CDK2-associated protein 2 (Cdk2ap2), mRNA [NM_001109498]	**39,638**

Tnrc4	Rattus norvegicus trinucleotide repeat containing 4 (Tnrc4), mRNA [NM_001109190]	**39,568**
Pcbp2	Rattus norvegicus poly(rC) binding protein 2 (Pcbp2), mRNA [NM_001013223]	**39,556**
Nrsn2	Rattus norvegicus neurensin 2 (Nrsn2), mRNA [NM_001109561]	**39,535**
Gpt2	Rattus norvegicus Cc2-5 mRNA, complete cds. [AY325245]	**39,531**
0	Syntaxin-binding protein 1 [Source:UniProtKB/Swiss-Prot;Acc:P61765] [ENSRNOT00000021189]	**39,530**
Fgfr2	Rattus norvegicus fibroblast growth factor receptor 2 (Fgfr2), transcript variant a, mRNA [NM_012712]	**39,509**
Vamp3	Rattus norvegicus vesicle-associated membrane protein 3 (Vamp3), mRNA [NM_057097]	**39,492**
Atxn3	Rattus norvegicus ataxin 3 (Atxn3), mRNA [NM_021702]	**39,462**
Hcn1	Rattus norvegicus hyperpolarization-activated cyclic nucleotide-gated potassium channel 1 (Hcn1), mRNA [NM_053375]	**39,461**
Stc1	Rattus norvegicus stanniocalcin 1 (Stc1), mRNA [NM_031123]	**39,456**
Pak4	Rattus norvegicus p21 protein (Cdc42/Rac)-activated kinase 4 (Pak4), mRNA [NM_001106238]	**39,453**
0	HEG homolog 1 (zebrafish) Gene [Source:MGI Symbol;Acc:MGI:1924696] [ENSRNOT00000002443]	**39,435**
Slc39a13	Rattus norvegicus solute carrier family 39 (zinc transporter), member 13 (Slc39a13), mRNA [NM_001039196]	**39,432**
Epb41l3	Rattus norvegicus erythrocyte membrane protein band 4.1-like 3 (Epb41l3), mRNA [NM_053927]	**39,425**
RGD1563579	Uncharacterized protein [Source:UniProtKB/TrEMBL;Acc:D4A888] [ENSRNOT00000057412]	**39,356**
Il13ra1	Rattus norvegicus interleukin 13 receptor, alpha 1 (Il13ra1), mRNA [NM_145789]	**39,354**
Yipf6	Rattus norvegicus Yip1 domain family, member 6 (Yipf6), mRNA [NM_001025747]	**39,336**
Zbtb7c	Rattus norvegicus zinc finger and BTB domain containing 7C (Zbtb7c), mRNA [NM_001127375]	**39,295**
Scn5a	Rattus norvegicus sodium channel, voltage-gated, type V, alpha subunit (Scn5a), transcript variant 1, mRNA [NM_013125]	**39,287**
Tspan5	Rattus norvegicus tetraspanin 5 (Tspan5), mRNA [NM_001004090]	**39,271**
Ndst1	Rattus norvegicus N-deacetylase/N-sulfotransferase (heparan glucosaminyl) 1 (Ndst1), mRNA [NM_024361]	**39,266**
Kcnd3	Rattus norvegicus potassium voltage-gated channel, Shal-related subfamily, member 3 (Kcnd3), mRNA [NM_031739]	**39,236**
Hmga1	Rattus norvegicus high mobility group AT-hook 1 (Hmga1), mRNA [NM_139327]	**39,217**
LOC691918	PREDICTED: Rattus norvegicus similar to Centrosomal protein of 27 kDa (Cep27 protein), transcript variant 2 (LOC691918), mRNA [XM_002726165]	**39,211**
Tcfcp2	Rattus norvegicus transcription factor CP2 (Tcfcp2), mRNA [NM_001134714]	**39,204**
Hey2	Rattus norvegicus hairy/enhancer-of-split related with YRPW motif 2 (Hey2), mRNA [NM_130417]	**39,182**
0	PREDICTED: Rattus norvegicus thymic stromal lymphopoietin (Tslp), mRNA [XM_001067649]	**39,150**
Eif4e3	Rattus norvegicus eukaryotic translation initiation factor 4E family member 3 (Eif4e3), mRNA [NM_001106612]	**39,134**
Pkmyt1	Rattus norvegicus protein kinase, membrane associated tyrosine/threonine 1 (Pkmyt1), mRNA [NM_001105766]	**39,132**
0	Uncharacterized protein [Source:UniProtKB/TrEMBL;Acc:D3Z808] [ENSRNOT00000020428]	**39,131**
Dand5	Uncharacterized protein [Source:UniProtKB/TrEMBL;Acc:D3ZGN3] [ENSRNOT00000044133]	**39,127**
0	PREDICTED: Rattus norvegicus similar to 60S ribosomal protein L29 (P23) (RGD1562139), mRNA [XM_235395]	**39,117**
Pdcd6ip	Rattus norvegicus programmed cell death 6 interacting protein (Pdcd6ip), mRNA [NM_001029910]	**39,096**

Dzip1	Dzip1 protein [Source:UniProtKB/TrEMBL;Acc:Q5EB75] [ENSRNOT00000038596]	**39,005**
Ubap2	Rattus norvegicus ubiquitin-associated protein 2 (Ubap2), mRNA [NM_001107928]	**39,004**
Zkscan1	Rattus norvegicus zinc finger with KRAB and SCAN domains 1 (Zkscan1), mRNA [NM_001025760]	**38,997**
Mobkl2b	Rattus norvegicus MOB1, Mps One Binder kinase activator-like 2B (yeast) (Mobkl2b), mRNA [NM_001108970]	**38,990**
Otop1	Rattus norvegicus otopetrin 1 (Otop1), mRNA [NM_181433]	**38,967**
Fads1	Rattus norvegicus fatty acid desaturase 1 (Fads1), mRNA [NM_053445]	**38,954**
Prkx	Rattus norvegicus protein kinase, X-linked (Prkx), mRNA [NM_001033963]	**38,949**
Bhlhb9	Rattus norvegicus basic helix-loop-helix domain containing, class B, 9 (Bhlhb9), mRNA [NM_207611]	**38,938**
Dlgap4	Rattus norvegicus discs, large homolog-associated protein 4 (Drosophila) (Dlgap4), mRNA [NM_173145]	**38,929**
Irs4	PREDICTED: Rattus norvegicus insulin receptor substrate 4 (Irs4), mRNA [XM_001056753]	**38,918**
Abcd1	Rattus norvegicus ATP-binding cassette, subfamily D (ALD), member 1 (Abcd1), mRNA [NM_001108821]	**38,909**
Adamts2	Rattus norvegicus ADAM metallopeptidase with thrombospondin type 1 motif, 2 (Adamts2), mRNA [NM_001137622]	**38,895**
Dlgap1	Rattus norvegicus discs, large (Drosophila) homolog-associated protein 1 (Dlgap1), mRNA [NM_022946]	**38,861**
Olr1530	Rattus norvegicus olfactory receptor 1530 (Olr1530), mRNA [NM_001001101]	**38,860**
Npy	Rattus norvegicus neuropeptide Y (Npy), mRNA [NM_012614]	**38,847**
Sulf2	Rattus norvegicus sulfatase 2 (Sulf2), mRNA [NM_001034927]	**38,846**
Map3k3	Rattus norvegicus mitogen activated protein kinase kinase kinase 3 (Map3k3), mRNA [NM_001107058]	**38,842**
Gabbr2	Rattus norvegicus gamma-aminobutyric acid (GABA) B receptor 2 (Gabbr2), mRNA [NM_031802]	**38,833**
Gdi1	Rattus norvegicus GDP dissociation inhibitor 1 (Gdi1), mRNA [NM_017088]	**38,806**
0	Unknown	**38,805**
Phf12	Rattus norvegicus PHD finger protein 12 (Phf12), mRNA [NM_001013117]	**38,774**
Tmem80	Rattus norvegicus transmembrane protein 80 (Tmem80), mRNA [NM_001017455]	**38,741**
Fam53c	Uncharacterized protein [Source:UniProtKB/TrEMBL;Acc:D3ZH55] [ENSRNOT00000027499]	**38,723**
0	Uncharacterized protein [Source:UniProtKB/TrEMBL;Acc:D3ZEI4] [ENSRNOT00000012248]	**38,711**
Zbtb39	Rattus norvegicus zinc finger and BTB domain containing 39 (Zbtb39), mRNA [NM_001130537]	**38,710**
Osbp	Rattus norvegicus oxysterol binding protein (Osbp), mRNA [NM_001108927]	**38,706**
Wdr62	Rattus norvegicus WD repeat domain 62 (Wdr62), mRNA [NM_001191623]	**38,657**
Ccdc90a	Putative uncharacterized protein RGD1307673_predictedUncharacterized protein [Source:UniProtKB/TrEMBL;Acc:D3ZEJ2] [ENSRNOT00000024206]	**38,618**
Zfhx3	PREDICTED: Rattus norvegicus zinc finger homeobox 3, transcript variant 2 (Zfhx3), mRNA [XM_226464]	**38,590**
Zcchc14	Uncharacterized protein [Source:UniProtKB/TrEMBL;Acc:D3ZB77] [ENSRNOT00000058486]	**38,566**
RGD1305680	Rattus norvegicus similar to KIAA0240 (RGD1305680), mRNA [NM_001106888]	**38,565**
Plp1	Rattus norvegicus proteolipid protein 1 (Plp1), mRNA [NM_030990]	**38,548**

Srd5a1	Rattus norvegicus steroid-5-alpha-reductase, alpha polypeptide 1 (3-oxo-5 alpha-steroid delta 4-dehydrogenase alpha 1) (Srd5a1), mRNA [NM_017070]	**38,548**
Cdk2ap2	Rattus norvegicus CDK2-associated protein 2 (Cdk2ap2), mRNA [NM_001109498]	**38,538**
0	Rattus norvegicus TL0AEA61YM21 mRNA sequence. [FQ233449]	**38,536**
Cldn19	Rattus norvegicus claudin 19 (Cldn19), mRNA [NM_001008514]	**38,526**
0	Transforming growth factor beta-2 [Source:UniProtKB/Swiss-Prot;Acc:Q07257] [ENSRNOT00000057338]	**38,522**
0	Q52LS7_HUMAN (Q52LS7) SCRT1 protein, partial (45%) [TC630587]	**38,488**
Jak3	Rattus norvegicus Janus kinase 3 (Jak3), mRNA [NM_012855]	**38,463**
Olr402	Rattus norvegicus olfactory receptor 402 (Olr402), mRNA [NM_001001014]	**38,436**
0	Unknown	**38,435**
Prdm2	Rattus norvegicus PR domain containing 2, with ZNF domain (Prdm2), mRNA [NM_001077648]	**38,431**
Clic4	Rattus norvegicus chloride intracellular channel 4 (Clic4), nuclear gene encoding mitochondrial protein, mRNA [NM_031818]	**38,424**
Prelp	Rattus norvegicus proline/arginine-rich end leucine-rich repeat protein (Prelp), mRNA [NM_053385]	**38,397**
Eif1ad	Rattus norvegicus eukaryotic translation initiation factor 1A domain containing (Eif1ad), mRNA [NM_001008305]	**38,383**
Mmp2	Rattus norvegicus matrix metallopeptidase 2 (Mmp2), mRNA [NM_031054]	**38,365**
0	Unknown	**38,362**
Cyfip2	Rattus norvegicus cytoplasmic FMR1 interacting protein 2 (Cyfip2), mRNA [NM_001106996]	**38,337**
Tmx4	Rattus norvegicus thioredoxin-related transmembrane protein 4 (Tmx4), mRNA [NM_001100529]	**38,323**
Zmiz1	Rattus norvegicus zinc finger, MIZ-type containing 1 (Zmiz1), mRNA [NM_001108393]	**38,317**
Atp1a2	Rattus norvegicus ATPase, Na+/K+ transporting, alpha 2 polypeptide (Atp1a2), mRNA [NM_012505]	**38,317**
0	PREDICTED: Rattus norvegicus similar to 60S ribosomal protein L29 (P23) (RGD1560076), mRNA [XM_001073916]	**38,285**
Cln5	Rattus norvegicus ceroid-lipofuscinosis, neuronal 5 (Cln5), mRNA [NM_001191689]	**38,281**
Meis2	Rattus norvegicus Meis homeobox 2 (Meis2), mRNA [NM_001107758]	**38,266**
Pbx1	Rattus norvegicus pre-B-cell leukemia homeobox 1 (Pbx1), transcript variant 2, mRNA [NM_001100681]	**38,235**
Shroom2	Rattus norvegicus shroom family member 2 (Shroom2), mRNA [NM_001047893]	**38,201**
0	WW domain containing adaptor with coiled-coil Gene [Source:MGI Symbol;Acc:MGI:2387357] [ENSRNOT00000058850]	**38,189**
Sfrs15	Rattus norvegicus splicing factor, arginine/serine-rich 15 (Sfrs15), mRNA [NM_001037347]	**38,181**
Gpr152	PREDICTED: Rattus norvegicus G protein-coupled receptor 152 (Gpr152), mRNA [XM_002725764]	**38,119**
Gja1	Rattus norvegicus gap junction protein, alpha 1 (Gja1), mRNA [NM_012567]	**38,092**
0	Unknown	**38,056**
E2f3	Rattus norvegicus E2F transcription factor 3 (E2f3), mRNA [NM_001137626]	**38,027**
Pdpn	Rattus norvegicus podoplanin (Pdpn), mRNA [NM_019358]	**38,024**
Atp6ap1	Rattus norvegicus ATPase, H+ transporting, lysosomal accessory protein 1 (Atp6ap1), mRNA [NM_031785]	**38,017**
Tmem179b	Rattus norvegicus transmembrane protein 179B (Tmem179b), mRNA [NM_001109572]	**38,004**

Fgf18	Rattus norvegicus fibroblast growth factor 18 (Fgf18), mRNA [NM_019199]	**37,971**
Cib2	Rattus norvegicus calcium and integrin binding family member 2 (Cib2), mRNA [NM_001015010]	**37,964**
Cntfr	Rattus norvegicus ciliary neurotrophic factor receptor (Cntfr), mRNA [NM_001003929]	**37,959**
0	Transcription initiation factor TFIID subunit 6 [Source:UniProtKB/Swiss-Prot;Acc:Q63801] [ENSRNOT00000001829]	**37,945**
RGD1559613	Rattus norvegicus RGD1559613 (RGD1559613), mRNA [NM_001109138]	**37,939**
0	Unknown	**37,934**
Neurod1	Rattus norvegicus neurogenic differentiation 1 (Neurod1), mRNA [NM_019218]	**37,911**
LOC100365551	PREDICTED: Rattus norvegicus hypothetical protein LOC100365551 (LOC100365551), mRNA [XM_002727224]	**37,902**
0	Protein phosphatase 1 regulatory subunit 11 [Source:UniProtKB/Swiss-Prot;Acc:Q6MFY6] [ENSRNOT00000045664]	**37,899**
0	Unknown	**37,885**
Scarb1	Rattus norvegicus scavenger receptor class B, member 1 (Scarb1), mRNA [NM_031541]	**37,885**
LOC691221	PREDICTED: Rattus norvegicus similar to CG1998-PA (LOC691221), mRNA [XM_001077260]	**37,852**
Atp2b3	Rattus norvegicus ATPase, Ca++ transporting, plasma membrane 3 (Atp2b3), mRNA [NM_133288]	**37,826**
LOC305806	Rattus norvegicus similar to glutaredoxin 1 (thioltransferase); glutaredoxin (LOC305806), mRNA [NM_001013993]	**37,821**
Sf3a1	Rattus norvegicus splicing factor 3a, subunit 1 (Sf3a1), mRNA [NM_001107235]	**37,801**
Ddx58	Rattus norvegicus DEAD (Asp-Glu-Ala-Asp) box polypeptide 58 (Ddx58), mRNA [NM_001106645]	**37,800**
Zap70	Rattus norvegicus zeta-chain (TCR) associated protein kinase (Zap70), mRNA [NM_001012002]	**37,784**
Gatm	Rattus norvegicus glycine amidinotransferase (L-arginine:glycine amidinotransferase) (Gatm), nuclear gene encoding mitochondrial protein, mRNA [NM_031031]	**37,757**
0	Unknown	**37,730**
Fam149a	PREDICTED: Rattus norvegicus family with sequence similarity 149, member A (Fam149a), mRNA [XM_001061521]	**37,727**
LOC100362849	PREDICTED: Rattus norvegicus hypothetical protein LOC100362849 (LOC100362849), partial mRNA [XM_002729412]	**37,716**
Sh3bp1	Rattus norvegicus SH3-domain binding protein 1 (Sh3bp1), mRNA [NM_001171981]	**37,702**
Otub1	Rattus norvegicus OTU domain, ubiquitin aldehyde binding 1 (Otub1), mRNA [NM_001106332]	**37,667**
0	Unknown	**37,662**
Rfx1	Rattus norvegicus regulatory factor X, 1 (influences HLA class II expression) (Rfx1), mRNA [NM_001105944]	**37,636**
0	Potassium voltage-gated channel subfamily C member 3 [Source:UniProtKB/Swiss-Prot;Acc:Q01956] [ENSRNOT00000027043]	**37,611**
Col1a2	Rattus norvegicus collagen, type I, alpha 2 (Col1a2), mRNA [NM_053356]	**37,557**
Usp54	Rattus norvegicus ubiquitin specific peptidase 54 (Usp54), mRNA [NM_001008863]	**37,552**
Lrrtm2	Rattus norvegicus leucine rich repeat transmembrane neuronal 2 (Lrrtm2), mRNA [NM_001109469]	**37,543**
Parvb	Rattus norvegicus parvin, beta (Parvb), mRNA [NM_001134780]	**37,540**
Pofut2	Rattus norvegicus protein O-fucosyltransferase 2 (Pofut2), mRNA [NM_001107621]	**37,540**
Fam168a	Rattus norvegicus family with sequence similarity 168, member A (Fam168a), mRNA [NM_001108494]	**37,539**
RGD1565033	Rattus norvegicus similar to hypothetical protein LOC284018 isoform b (RGD1565033), mRNA [NM_001109050]	**37,486**

0	NADH-ubiquinone oxidoreductase chain 5 [Source:UniProtKB/Swiss-Prot;Acc:P11661] [ENSRNOT00000048767]	**37,486**
Lrrc56	Rattus norvegicus leucine rich repeat containing 56 (Lrrc56), mRNA [NM_001024902]	**37,485**
0	Unknown	**37,477**
LOC497952	PREDICTED: Rattus norvegicus similar to Ubiquitin-associated protein 2-like (LOC497952), miscRNA [XR_005763]	**37,459**
0	Unknown	**37,450**
FAM120C	PREDICTED: Rattus norvegicus family with sequence similarity 120C (FAM120C), mRNA [XM_001068090]	**37,449**
0	ROBO1_RAT (O55005) Roundabout homolog 1 precursor, complete [TC573812]	**37,440**
Csf1	Rattus norvegicus colony stimulating factor 1 (macrophage) (Csf1), mRNA [NM_023981]	**37,419**
RGD1562699	Rattus norvegicus RGD1562699 (RGD1562699), mRNA [NM_001106141]	**37,414**
Chchd10	Rattus norvegicus coiled-coil-helix-coiled-coil-helix domain containing 10 (Chchd10), mRNA [NM_001007008]	**37,407**
Hs6st1	Rattus norvegicus heparan sulfate 6-O-sulfotransferase 1 (Hs6st1), mRNA [NM_001108210]	**37,396**
Diras1	Rattus norvegicus DIRAS family, GTP-binding RAS-like 1 (Diras1), mRNA [NM_001108987]	**37,393**
Shisa7	Rattus norvegicus shisa homolog 7 (Xenopus laevis) (Shisa7), mRNA [NM_001145175]	**37,379**
Csk	Rattus norvegicus c-src tyrosine kinase (Csk), mRNA [NM_001030039]	**37,370**
0	Uncharacterized protein [Source:UniProtKB/TrEMBL;Acc:D3ZV82] [ENSRNOT00000037181]	**37,360**
Slc1a3	Rattus norvegicus solute carrier family 1 (glial high affinity glutamate transporter), member 3 (Slc1a3), mRNA [NM_019225]	**37,355**
Cul3	Rattus norvegicus cullin 3 (Cul3), mRNA [NM_001106923]	**37,333**
Ankrd34a	Rattus norvegicus ankyrin repeat domain 34A (Ankrd34a), mRNA [NM_001024980]	**37,326**
Prom1	Rattus norvegicus prominin 1 (Prom1), transcript variant 1, mRNA [NM_021751]	**37,325**
0	Unknown	**37,318**
Kif13b	Rattus norvegicus kinesin family member 13B (Kif13b), mRNA [NM_213626]	**37,315**
Galm	Rattus norvegicus galactose mutarotase (aldose 1-epimerase) (Galm), mRNA [NM_001007704]	**37,315**
Gnaq	Rattus norvegicus guanine nucleotide binding protein (G protein), q polypeptide (Gnaq), mRNA [NM_031036]	**37,314**
0	ATP synthase protein 8 [Source:UniProtKB/Swiss-Prot;Acc:P11608] [ENSRNOT00000046201]	**37,304**
LOC690079	Rattus norvegicus hypothetical protein LOC690079 (LOC690079), mRNA [NM_001109566]	**37,298**
0	Unknown	**37,283**
Trim17	Rattus norvegicus tripartite motif-containing 17 (Trim17), mRNA [NM_022798]	**37,276**
Vhl	Rattus norvegicus von Hippel-Lindau tumor suppressor (Vhl), mRNA [NM_052801]	**37,253**
Aplnr	Rattus norvegicus apelin receptor (Aplnr), mRNA [NM_031349]	**37,240**
0	Rattus norvegicus clone UI-R-FJ0-cpy-l-05-0-UI unknown mRNA. [AY724520]	**37,228**
Olr727	Rattus norvegicus olfactory receptor 727 (Olr727), mRNA [NM_001000619]	**37,193**
Fgf14	Rattus norvegicus fibroblast growth factor-like factor 4D mRNA, partial cds. [AF348523]	**37,181**
Rab4a	Rattus norvegicus RAB4A, member RAS oncogene family (Rab4a), mRNA [NM_013019]	**37,154**
Gli2	Rattus norvegicus GLI family zinc finger 2 (Gli2), mRNA [NM_001107169]	**37,152**

LOC306766	Rattus norvegicus hypothetical LOC306766 (LOC306766), mRNA [NM_001014007]	**37,152**
Plekhg1	Rattus norvegicus pleckstrin homology domain containing, family G (with RhoGef domain) member 1 (Plekhg1), mRNA [NM_001190999]	**37,152**
RGD1566149	Rattus norvegicus similar to CDNA sequence BC017647 (RGD1566149), mRNA [NM_001109038]	**37,149**
Zc4h2	Rattus norvegicus zinc finger, C4H2 domain containing (Zc4h2), mRNA [NM_001126374]	**37,144**
0	Uncharacterized protein [Source:UniProtKB/TrEMBL;Acc:D4A7M0] [ENSRNOT00000011178]	**37,143**
Fgf20	Rattus norvegicus fibroblast growth factor 20 (Fgf20), mRNA [NM_023961]	**37,143**
Kcna3	Rattus norvegicus potassium voltage-gated channel, shaker-related subfamily, member 3 (Kcna3), mRNA [NM_019270]	**37,142**
Nfix	Rattus norvegicus nuclear factor I/X (CCAAT-binding transcription factor) (Nfix), mRNA [NM_030866]	**37,136**
LOC292199	Rattus norvegicus hypothetical LOC292199 (LOC292199), mRNA [NM_001144859]	**37,126**
0	Unknown	**37,113**
Sez6	Rattus norvegicus seizure related 6 homolog (mouse) (Sez6), mRNA [NM_001105754]	**37,080**
Ctxn1	Rattus norvegicus cortexin 1 (Ctxn1), mRNA [NM_001109935]	**37,066**
Plagl1	Rattus norvegicus pleiomorphic adenoma gene-like 1 (Plagl1), mRNA [NM_012760]	**37,065**
Cpne4	Rattus norvegicus copine IV (Cpne4), mRNA [NM_001109003]	**37,064**
0	Unknown	**37,050**
Mrpl4	Rattus norvegicus mitochondrial ribosomal protein L4 (Mrpl4), nuclear gene encoding mitochondrial protein, mRNA [NM_001108754]	**37,047**
Lrrc3b	PREDICTED: Rattus norvegicus leucine rich repeat containing 3B (Lrrc3b), mRNA [XM_002725036]	**37,036**
Mpp2	Rattus norvegicus membrane protein, palmitoylated 2 (MAGUK p55 subfamily member 2) (Mpp2), mRNA [NM_053513]	**37,031**
RGD1562310	Rattus norvegicus similar to hypothetical protein FLJ21415 (RGD1562310), mRNA [NM_001109066]	**37,006**
Stk40	Rattus norvegicus serine/threonine kinase 40 (Stk40), mRNA [NM_183056]	**37,003**
Ppdpf	Rattus norvegicus pancreatic progenitor cell differentiation and proliferation factor homolog (zebrafish) (Ppdpf), mRNA [NM_001009316]	**36,997**
Eif5a	Rattus norvegicus eukaryotic translation initiation factor 5A (Eif5a), mRNA [NM_001033681]	**36,992**
Rtn3	Rattus norvegicus reticulon 3 (Rtn3), transcript variant 1, mRNA [NM_080909]	**36,990**
Tex15	Rattus norvegicus testis expressed 15 (Tex15), mRNA [NM_001106087]	**36,980**
Sec14l2	Rattus norvegicus SEC14-like 2 (S. cerevisiae) (Sec14l2), mRNA [NM_053801]	**36,970**
Zfhx4	Rattus norvegicus zinc finger homeobox 4 (Zfhx4), mRNA [NM_001191702]	**36,967**
0	Rattus norvegicus similar to 60S RIBOSOMAL PROTEIN L29 (P23) (LOC291920), mRNA [XM_226340]	**36,966**
Pkig	Rattus norvegicus protein kinase inhibitor, gamma (Pkig), mRNA [NM_153469]	**36,939**
Txnl4b	Rattus norvegicus thioredoxin-like 4B (Txnl4b), mRNA [NM_001013891]	**36,932**
Cyb5b	Rattus norvegicus cytochrome b5 type B (outer mitochondrial membrane) (Cyb5b), nuclear gene encoding mitochondrial protein, mRNA [NM_030586]	**36,930**
0	RGD1305664 protein [Source:UniProtKB/TrEMBL;Acc:Q5EB79] [ENSRNOT00000004829]	**36,910**
Snai2	Rattus norvegicus snail homolog 2 (Drosophila) (Snai2), mRNA [NM_013035]	**36,905**
0	Q96FH0_HUMAN (Q96FH0) MEF2B protein, complete [TC593056]	**36,902**

Defb52	Rattus norvegicus defensin beta 52 (Defb52), mRNA [NM_001037524]	**36,902**
Hyou1	Rattus norvegicus hypoxia up-regulated 1 (Hyou1), transcript variant 1, mRNA [NM_138867]	**36,900**
Dcc	Rattus norvegicus deleted in colorectal carcinoma (Dcc), mRNA [NM_012841]	**36,899**
Alpl	Rattus norvegicus alkaline phosphatase, liver/bone/kidney (Alpl), mRNA [NM_013059]	**36,890**
Rnf185	Rattus norvegicus ring finger protein 185 (Rnf185), mRNA [NM_001024271]	**36,882**
Solh	Rattus norvegicus small optic lobes homolog (Drosophila) (Solh), mRNA [NM_001106990]	**36,834**
0	KCNA3_RAT (P15384) Potassium voltage-gated channel subfamily A member 3 (Voltage-gated potassium channel subunit Kv1.3) (RGK5) (RCK3) (KV3), complete [TC574813]	**36,834**
Lrpap1	Alpha-2-macroglobulin receptor-associated protein [Source:UniProtKB/Swiss-Prot;Acc:Q99068] [ENSRNOT00000012665]	**36,822**
Prickle3	Rattus norvegicus prickle homolog 3 (Drosophila) (Prickle3), mRNA [NM_001014110]	**36,797**
LOC687609	PREDICTED: Rattus norvegicus similar to ras homolog gene family, member f (LOC687609), mRNA [XM_001079372]	**36,781**
Mllt1	Rattus norvegicus myeloid/lymphoid or mixed-lineage leukemia (trithorax homolog, Drosophila); translocated to, 1 (Mllt1), mRNA [NM_001106876]	**36,770**
Rlbp1	Rattus norvegicus retinaldehyde binding protein 1 (Rlbp1), mRNA [NM_001106274]	**36,754**
Kcnma1	Rattus norvegicus potassium large conductance calcium-activated channel, subfamily M, alpha member 1 (Kcnma1), mRNA [NM_031828]	**36,726**
RGD1563155	Rattus norvegicus similar to RIKEN cDNA 1700054O13 (RGD1563155), mRNA [NM_001134598]	**36,712**
Trpm3	Rattus norvegicus transient receptor potential cation channel, subfamily M, member 3 (Trpm3), mRNA [NM_001191562]	**36,689**
0	slingshot homolog 1 (Drosophila) Gene [Source:MGI Symbol;Acc:MGI:2686240] [ENSRNOT00000044751]	**36,682**
Atg12	Rattus norvegicus ATG12 autophagy related 12 homolog (S. cerevisiae) (Atg12), mRNA [NM_001038495]	**36,674**
Slitrk3	Rattus norvegicus SLIT and NTRK-like family, member 3 (Slitrk3), mRNA [NM_001107683]	**36,649**
0	Unknown	**36,645**
Fnbp1	Rattus norvegicus formin binding protein 1 (Fnbp1), mRNA [NM_138914]	**36,643**
Nme4	Rattus norvegicus non-metastatic cells 4, protein expressed in (Nme4), nuclear gene encoding mitochondrial protein, mRNA [NM_001109478]	**36,614**
Cand2	Rattus norvegicus cullin-associated and neddylation-dissociated 2 (putative) (Cand2), mRNA [NM_181362]	**36,606**
Surf4	Rattus norvegicus surfeit 4 (Surf4), mRNA [NM_001033868]	**36,594**
Chrna4	Rattus norvegicus cholinergic receptor, nicotinic, alpha 4 (Chrna4), mRNA [NM_024354]	**36,576**
Efna2	Rattus norvegicus ephrin A2 (Efna2), mRNA [NM_001168670]	**36,576**
Olr651	Rattus norvegicus olfactory receptor 651 (Olr651), mRNA [NM_001000341]	**36,568**
Plec	Rattus norvegicus plectin (Plec), transcript variant 6, mRNA [NM_022401]	**36,547**
0	Unknown	**36,539**
Sec31a	Rattus norvegicus SEC31 homolog A (S. cerevisiae) (Sec31a), mRNA [NM_033021]	**36,516**
Grin3a	Rattus norvegicus glutamate receptor, ionotropic, N-methyl-D-aspartate 3A (Grin3a), transcript variant 1, mRNA [NM_138546]	**36,509**
Nkx2-1	Rattus norvegicus NK2 homeobox 1 (Nkx2-1), mRNA [NM_013093]	**36,477**
Maml1	Rattus norvegicus mastermind like 1 (Drosophila) (Maml1), mRNA [NM_001106997]	**36,473**

Pcbd1	Rattus norvegicus pterin-4 alpha-carbinolamine dehydratase/dimerization cofactor of hepatocyte nuclear factor 1 alpha (Pcbd1), mRNA [NM_001007601]	**36,469**
Fmod	Rattus norvegicus fibromodulin (Fmod), mRNA [NM_080698]	**36,465**
LOC499330	Rattus norvegicus similar to Nicotinamide riboside kinase 1 (LOC499330), mRNA [NM_001024292]	**36,454**
0	Similar to HLA-B associated transcript-2 isoform aUncharacterized protein [Source:UniProtKB/TrEMBL;Acc:D3ZUK2] [ENSRNOT00000013546]	**36,435**
Hccs	Rattus norvegicus holocytochrome c synthetase (Hccs), mRNA [NM_001191732]	**36,427**
Capn1	Rattus norvegicus calpain 1 (Capn1), mRNA [NM_019152]	**36,404**
Usp9x	Rattus norvegicus ubiquitin specific peptidase 9, X-linked (Usp9x), transcript variant 1, mRNA [NM_001135893]	**36,401**
Mettl7a	Rattus norvegicus methyltransferase like 7A (Mettl7a), mRNA [NM_001037355]	**36,400**
Tead2	Rattus norvegicus TEA domain family member 2 (Tead2), mRNA [NM_001107512]	**36,393**
0	BP474671 Rattus norvegicus pancreatic islet Rattus norvegicus cDNA clone RBC11656 3', mRNA sequence [BP474671]	**36,392**
Gbf1	Rattus norvegicus golgi-specific brefeldin A resistant guanine nucleotide exchange factor 1 (Gbf1), mRNA [NM_001191634]	**36,388**
Cdc42ep4	Rattus norvegicus CDC42 effector protein (Rho GTPase binding) 4 (Cdc42ep4), mRNA [NM_001107063]	**36,369**
Nr4a3	Rattus norvegicus nuclear receptor subfamily 4, group A, member 3 (Nr4a3), transcript variant 2, mRNA [NM_017352]	**36,358**
Kcne1l	Rattus norvegicus potassium voltage-gated channel, Isk-related family, member 1-like (Kcne1l), mRNA [NM_001101003]	**36,318**
Diras2	Rattus norvegicus DIRAS family, GTP-binding RAS-like 2 (Diras2), mRNA [NM_001169578]	**36,270**
Rprd1b	Rattus norvegicus regulation of nuclear pre-mRNA domain containing 1B (Rprd1b), mRNA [NM_001098727]	**36,269**
Hipk3	Rattus norvegicus homeodomain interacting protein kinase 3 (Hipk3), mRNA [NM_031787]	**36,268**
RGD1562533	Rattus norvegicus similar to mKIAA0774 protein (RGD1562533), mRNA [NM_001100989]	**36,251**
LOC686326	PREDICTED: Rattus norvegicus similar to Interferon-alpha/beta receptor beta chain precursor (IFN-alpha-REC) (Type I interferon receptor) (IFN-R) (Interferon alpha/beta receptor-2) (LOC686326), mRNA [XM_001073550]	**36,247**
Htr5b	Rattus norvegicus 5-hydroxytryptamine (serotonin) receptor 5B (Htr5b), mRNA [NM_024395]	**36,244**
RGD1559644	Uncharacterized protein [Source:UniProtKB/TrEMBL;Acc:D3ZBJ1] [ENSRNOT00000068281]	**36,231**
38961	Rattus norvegicus septin 6 (Sept6), mRNA [NM_001173429]	**36,226**
Prkar1b	Rattus norvegicus protein kinase, cAMP dependent regulatory, type I, beta (Prkar1b), mRNA [NM_001033679]	**36,225**
Npr1	Rattus norvegicus natriuretic peptide receptor A/guanylate cyclase A (atrionatriuretic peptide receptor A) (Npr1), mRNA [NM_012613]	**36,216**
0	Unknown	**36,205**
Efhd2	Rattus norvegicus EF-hand domain family, member D2 (Efhd2), mRNA [NM_001031648]	**36,198**
Hmox2	Rattus norvegicus heme oxygenase (decycling) 2 (Hmox2), mRNA [NM_024387]	**36,155**
Med19	Rattus norvegicus mediator complex subunit 19 (Med19), mRNA [NM_001107741]	**36,142**
Dpp8	Rattus norvegicus dipeptidylpeptidase 8 (Dpp8), mRNA [NM_001108159]	**36,141**
Hip1	Rattus norvegicus huntingtin interacting protein 1 (Hip1), mRNA [NM_001100475]	**36,133**
RGD1311429	PREDICTED: Rattus norvegicus similar to KIAA1267 protein (RGD1311429), miscRNA [XR_006336]	**36,111**
Ccni	Rattus norvegicus cyclin I (Ccni), mRNA [NM_001105998]	**36,109**

Stat2	Rattus norvegicus signal transducer and activator of transcription 2 (Stat2), mRNA [NM_001011905]	**36,082**
Mink1	Uncharacterized protein [Source:UniProtKB/TrEMBL;Acc:D3ZMP1] [ENSRNOT00000043732]	**36,073**
0	Unknown	**36,054**
Pfn1	Rattus norvegicus profilin 1 (Pfn1), mRNA [NM_022511]	**36,051**
Paip2b	Putative uncharacterized protein RGD1307930_predictedUncharacterized protein [Source:UniProtKB/TrEMBL;Acc:D4AAB9] [ENSRNOT00000019399]	**36,014**
Shkbp1	SH3KBP1-binding protein 1 [Source:UniProtKB/Swiss-Prot;Acc:P0C5J9] [ENSRNOT00000028348]	**36,013**
Pan3	Uncharacterized protein [Source:UniProtKB/TrEMBL;Acc:D3ZTP2] [ENSRNOT00000044134]	**35,975**
0	Unknown	**35,971**
Akap12	Rattus norvegicus A kinase (PRKA) anchor protein 12 (Akap12), transcript variant 1, mRNA [NM_057103]	**35,959**
Aqp4	Rattus norvegicus aquaporin 4 (Aqp4), transcript variant 2, mRNA [NM_001142366]	**35,939**
Pole3	Rattus norvegicus polymerase (DNA directed), epsilon 3 (p17 subunit) (Pole3), mRNA [NM_001007652]	**35,939**
Zic1	Rattus norvegicus Zic family member 1 (odd-paired homolog, Drosophila) (Zic1), mRNA [NM_022677]	**35,920**
Lsm10	Rattus norvegicus LSM10, U7 small nuclear RNA associated (Lsm10), mRNA [NM_001108976]	**35,899**
0	Unknown	**35,893**
Ergic1	PREDICTED: Rattus norvegicus endoplasmic reticulum-golgi intermediate compartment (ERGIC) 1 (Ergic1), mRNA [XM_001066818]	**35,890**
Trip12	Rattus norvegicus thyroid hormone receptor interactor 12 (Trip12), mRNA [NM_001031659]	**35,874**
Pnkd	Rattus norvegicus paroxysmal nonkinesiogenic dyskinesia (Pnkd), nuclear gene encoding mitochondrial protein, transcript variant 2, mRNA [NM_001134751]	**35,829**
Saps1	Rattus norvegicus SAPS domain family, member 1 (Saps1), mRNA [NM_001135849]	**35,826**
Adad2	PREDICTED: Rattus norvegicus similar to testis nuclear RNA-binding protein (LOC691275), mRNA [XM_002725395]	**35,824**
Nrcam	Rattus norvegicus neuronal cell adhesion molecule (Nrcam), mRNA [NM_013150]	**35,809**
Znf524	Rattus norvegicus zinc finger protein 524 (Znf524), mRNA [NM_001108905]	**35,790**
Pttg1ip	Rattus norvegicus pituitary tumor-transforming 1 interacting protein (Pttg1ip), mRNA [NM_001013238]	**35,769**
Hrnbp3	similar to ataxin 2-binding protein 1 isoform 2 (RGD1560070), mRNA [Source:RefSeq DNA;Acc:NM_001134498] [ENSRNOT00000004524]	**35,769**
Ccdc92	Rattus norvegicus coiled-coil domain containing 92 (Ccdc92), mRNA [NM_001083898]	**35,757**
Rhog	Rattus norvegicus ras homolog gene family, member G (rho G) (Rhog), mRNA [NM_001037195]	**35,744**
Mllt11	Rattus norvegicus myeloid/lymphoid or mixed-lineage leukemia (trithorax homolog, Drosophila); translocated to, 11 (Mllt11), mRNA [NM_001013912]	**35,735**
Pcdh21	Rattus norvegicus protocadherin 21 (Pcdh21), mRNA [NM_053572]	**35,696**
Nudt3	Rattus norvegicus nudix (nucleoside diphosphate linked moiety X)-type motif 3 (Nudt3), mRNA [NM_001024243]	**35,663**
Sgsm2	Rattus norvegicus small G protein signaling modulator 2 (Sgsm2), mRNA [NM_001107020]	**35,660**
Mad1l1	Rattus norvegicus MAD1 mitotic arrest deficient-like 1 (yeast) (Mad1l1), mRNA [NM_001109387]	**35,644**
Cntn4	Rattus norvegicus contactin 4 (Cntn4), mRNA [NM_053879]	**35,628**

0	RVL20318 Wackym-Soares normalized rat vestibular cDNA library Rattus norvegicus cDNA 5', mRNA sequence [DV727767]	**35,620**
Dcp1a	Rattus norvegicus DCP1 decapping enzyme homolog A (S. cerevisiae) (Dcp1a), mRNA [NM_001191831]	**35,573**
Tcf7l2	Rattus norvegicus transcription factor 7-like 2 (T-cell specific, HMG-box) (Tcf7l2), mRNA [NM_001191052]	**35,544**
Bche	Rattus norvegicus butyrylcholinesterase (Bche), mRNA [NM_022942]	**35,541**
Zbtb6	Rattus norvegicus zinc finger and BTB domain containing 6 (Zbtb6), mRNA [NM_001108953]	**35,540**
Foxo3	Rattus norvegicus forkhead box O3 (Foxo3), mRNA [NM_001106395]	**35,535**
Mro	PREDICTED: Rattus norvegicus maestro (Mro), mRNA [XM_001053368]	**35,500**
Ell2	Ell2 protein [Source:UniProtKB/TrEMBL;Acc:Q2NL49] [ENSRNOT00000032974]	**35,474**
RGD1566084	Uncharacterized protein [Source:UniProtKB/TrEMBL;Acc:D3ZPT0] [ENSRNOT00000043197]	**35,471**
Fgd3	Rattus norvegicus FYVE, RhoGEF and PH domain containing 3 (Fgd3), mRNA [NM_001108409]	**35,460**
LOC100365546	PREDICTED: Rattus norvegicus rCG30673-like (LOC100365546), mRNA [XM_002726646]	**35,448**
Entpd3	Rattus norvegicus ectonucleoside triphosphate diphosphohydrolase 3 (Entpd3), mRNA [NM_178106]	**35,446**
0	Q4SB82_TETNG (Q4SB82) Chromosome undetermined SCAF14677, whole genome shotgun sequence, partial (5%) [TC590476]	**35,436**
Zfp418	Rattus norvegicus zinc finger protein 418 (Zfp418), mRNA [NM_001191620]	**35,433**
Dhdpsl	Rattus norvegicus dihydrodipicolinate synthase-like, mitochondrial (Dhdpsl), nuclear gene encoding mitochondrial protein, mRNA [NM_001106355]	**35,408**
Rnf24	Rattus norvegicus ring finger protein 24 (Rnf24), mRNA [NM_001108591]	**35,408**
Acsl1	Rattus norvegicus acyl-CoA synthetase long-chain family member 1 (Acsl1), mRNA [NM_012820]	**35,392**
Meis1	Rattus norvegicus Meis homeobox 1 (Meis1), mRNA [NM_001134702]	**35,384**
0	Unknown	**35,379**
RGD1564285	PREDICTED: Rattus norvegicus similar to hypothetical protein 4930509O22 (RGD1564285), mRNA [XM_001079282]	**35,378**
Spert	Rattus norvegicus spermatid associated (Spert), mRNA [NM_001017506]	**35,374**
Gabra1	Rattus norvegicus gamma-aminobutyric acid (GABA) A receptor, alpha 1 (Gabra1), mRNA [NM_183326]	**35,365**
0	Q62262_MOUSE (Q62262) Spermatid perinuclear RNA binding protein, partial (12%) [TC595714]	**35,362**
Dhcr7	Rattus norvegicus 7-dehydrocholesterol reductase (Dhcr7), mRNA [NM_022389]	**35,349**
Slc39a9	Rattus norvegicus solute carrier family 39 (zinc transporter), member 9 (Slc39a9), mRNA [NM_001034929]	**35,322**
0	Unknown	**35,319**
0	Rattus norvegicus similar to 60S RIBOSOMAL PROTEIN L29 (P23) (LOC291290), mRNA [XM_225531]	**35,297**
Deadc1	Rattus norvegicus deaminase domain containing 1 (Deadc1), mRNA [NM_001115028]	**35,276**
0	Unknown	**35,268**
LOC367084	PREDICTED: Rattus norvegicus similar to 60S ribosomal protein L29 (P23) (LOC367084), mRNA [XM_002729903]	**35,250**
Tmigd1	Rattus norvegicus transmembrane and immunoglobulin domain containing 1 (Tmigd1), mRNA [NM_001135029]	**35,244**
Kit	Rattus norvegicus v-kit Hardy-Zuckerman 4 feline sarcoma viral oncogene homolog (Kit), mRNA [NM_022264]	**35,217**
RGD1305090	Rattus norvegicus similar to CD2-associated protein (RGD1305090), mRNA [NM_001162535]	**35,214**
Rbmx	Rattus norvegicus RNA binding motif protein, X-linked (Rbmx), mRNA [NM_001025663]	**35,210**

Naf1	Rattus norvegicus nuclear assembly factor 1 homolog (S. cerevisiae) (Naf1), mRNA [NM_001024772]	**35,207**
Elk1	Rattus norvegicus ELK1, member of ETS oncogene family (Elk1), mRNA [NM_001108059]	**35,186**
Vps39	Rattus norvegicus vacuolar protein sorting 39 homolog (S. cerevisiae) (Vps39), mRNA [NM_001012186]	**35,184**
Pacs1	Rattus norvegicus phosphofurin acidic cluster sorting protein 1 (Pacs1), mRNA [NM_134406]	**35,174**
0	PREDICTED: Rattus norvegicus similar to zinc finger and SCAN domain containing 4 (RGD1561748), mRNA [XM_001078969]	**35,159**
Gstt3	Rattus norvegicus glutathione S-transferase, theta 3 (Gstt3), mRNA [NM_001137643]	**35,157**
Mutyh	Rattus norvegicus mutY homolog (E. coli) (Mutyh), nuclear gene encoding mitochondrial protein, mRNA [NM_133316]	**35,145**
0	Unknown	**35,134**
Creg1	Rattus norvegicus cellular repressor of E1A-stimulated genes 1 (Creg1), mRNA [NM_001105966]	**35,123**
Sp2	Rattus norvegicus Sp2 transcription factor (Sp2), mRNA [NM_001107045]	**35,120**
Ntrk2	Rattus norvegicus neurotrophic tyrosine kinase, receptor, type 2 (Ntrk2), transcript variant 1, mRNA [NM_012731]	**35,105**
Atp2a2	Rattus norvegicus ATPase, Ca++ transporting, cardiac muscle, slow twitch 2 (Atp2a2), transcript variant 3, mRNA [NM_001110823]	**35,098**
Bmyc	Rattus norvegicus brain expressed myelocytomatosis oncogene (Bmyc), mRNA [NM_001013163]	**35,089**
RGD1565947	Rattus norvegicus similar to netrin 4 (RGD1565947), mRNA [NM_001106780]	**35,084**
Alg2	Rattus norvegicus asparagine-linked glycosylation 2, alpha-1,3-mannosyltransferase homolog (S. cerevisiae) (Alg2), mRNA [NM_001100710]	**35,046**
Olr403	Rattus norvegicus olfactory receptor 403 (Olr403), mRNA [NM_001000381]	**35,034**
Fbrs	PREDICTED: Rattus norvegicus fibrosin (Fbrs), mRNA [XM_002725689]	**35,025**
Plp1	Rattus norvegicus proteolipid protein 1 (Plp1), mRNA [NM_030990]	**35,018**
Pcif1	Rattus norvegicus PDX1 C-terminal inhibiting factor 1 (Pcif1), mRNA [NM_001108605]	**35,012**
Nmnat2	Rattus norvegicus nicotinamide nucleotide adenylyltransferase 2 (Nmnat2), mRNA [NM_001048042]	**34,984**
0	Unknown	**34,983**
Ppp2r1a	Rattus norvegicus protein phosphatase 2 (formerly 2A), regulatory subunit A, alpha isoform (Ppp2r1a), mRNA [NM_057140]	**34,983**
RGD1565498	Rattus norvegicus similar to Hypothetical protein LOC270802 (RGD1565498), mRNA [NM_001109287]	**34,977**
RGD1566264	Uncharacterized protein [Source:UniProtKB/TrEMBL;Acc:D3ZFF1] [ENSRNOT00000051929]	**34,935**
Stx17	Rattus norvegicus syntaxin 17 (Stx17), mRNA [NM_145723]	**34,931**
Axin2	Rattus norvegicus axin 2 (Axin2), mRNA [NM_024355]	**34,917**
LOC689226	Rattus norvegicus similar to ubiquitin-conjugating enzyme E2R 2 (LOC689226), mRNA [NM_001127573]	**34,901**
Ccdc97	Rattus norvegicus coiled-coil domain containing 97 (Ccdc97), mRNA [NM_001106235]	**34,892**
Htr3a	Rattus norvegicus 5-hydroxytryptamine (serotonin) receptor 3a (Htr3a), mRNA [NM_024394]	**34,860**
0	Glutamate receptor subunit GluR1 [Source:UniProtKB/TrEMBL;Acc:Q924I5] [ENSRNOT00000003279]	**34,852**
0	Unknown	**34,845**
0	Unknown	**34,844**
Dnajc10	Rattus norvegicus DnaJ (Hsp40) homolog, subfamily C, member 10 (Dnajc10), mRNA [NM_001106486]	**34,829**
Pkig	Rattus norvegicus protein kinase inhibitor, gamma (Pkig), mRNA [NM_153469]	**34,819**

Nr2f2	Rattus norvegicus nuclear receptor subfamily 2, group F, member 2 (Nr2f2), mRNA [NM_080778]	**34,799**
Gnao1	Rattus norvegicus guanine nucleotide binding protein (G protein), alpha activating activity polypeptide O (Gnao1), mRNA [NM_017327]	**34,797**
Tmed4	Rattus norvegicus transmembrane emp24 protein transport domain containing 4 (Tmed4), mRNA [NM_001107238]	**34,756**
Unc93b1	Rattus norvegicus unc-93 homolog B1 (C. elegans) (Unc93b1), mRNA [NM_001108513]	**34,755**
Pou2f1	Rattus norvegicus POU class 2 homeobox 1 (Pou2f1), mRNA [NM_001100639]	**34,748**
Aff3	Rattus norvegicus AF4/FMR2 family, member 3 (Aff3), mRNA [NM_001191887]	**34,746**
Oprm1	Rattus norvegicus opioid receptor, mu 1 (Oprm1), transcript variant 1, mRNA [NM_013071]	**34,731**
Prkaa2	Rattus norvegicus protein kinase, AMP-activated, alpha 2 catalytic subunit (Prkaa2), mRNA [NM_023991]	**34,712**
Bcl9	Rattus norvegicus B-cell CLL/lymphoma 9 (Bcl9), mRNA [NM_001107703]	**34,699**
0	Uncharacterized protein [Source:UniProtKB/TrEMBL;Acc:D3ZAQ3] [ENSRNOT00000024129]	**34,692**
0	SWI/SNF complex subunit SMARCC1 [Source:RefSeq peptide;Acc:NP_001100331] [ENSRNOT00000056153]	**34,687**
Dbp	Rattus norvegicus D site of albumin promoter (albumin D-box) binding protein (Dbp), mRNA [NM_012543]	**34,681**
Cmip	Rattus norvegicus c-Maf-inducing protein (Cmip), mRNA [NM_001163273]	**34,674**
Atp2b2	Rattus norvegicus ATPase, Ca++ transporting, plasma membrane 2 (Atp2b2), mRNA [NM_012508]	**34,670**
Grik3	Rattus norvegicus glutamate receptor, ionotropic, kainate 3 (Grik3), transcript variant 2, mRNA [NM_181373]	**34,661**
Usp6nl	Rattus norvegicus USP6 N-terminal like (Usp6nl), mRNA [NM_001106120]	**34,660**
LOC294154	similar to chromosome 6 open reading frame 106 isoform a (LOC294154), mRNA [Source:RefSeq DNA;Acc:NM_001039607] [ENSRNOT00000059426]	**34,655**
Lrrc61	Rattus norvegicus leucine rich repeat containing 61 (Lrrc61), mRNA [NM_001109231]	**34,645**
Pqcp	Rattus norvegicus polyglutamine-containing protein (Pqcp), mRNA [NM_001012470]	**34,638**
Vps26b	Rattus norvegicus vacuolar protein sorting 26 homolog B (S. pombe) (Vps26b), mRNA [NM_001106809]	**34,634**
0	Uncharacterized protein [Source:UniProtKB/TrEMBL;Acc:D3ZS88] [ENSRNOT00000011803]	**34,620**
Supt4h1	Rattus norvegicus suppressor of Ty 4 homolog 1 (S. cerevisiae) (Supt4h1), mRNA [NM_001105828]	**34,618**
0	Unknown	**34,605**
Nkiras2	Rattus norvegicus NFKB inhibitor interacting Ras-like 2 (Nkiras2), mRNA [NM_001105839]	**34,603**
Mecp2	Rattus norvegicus methyl CpG binding protein 2 (Mecp2), mRNA [NM_022673]	**34,598**
Ube2v1	Rattus norvegicus ubiquitin-conjugating enzyme E2 variant 1 (Ube2v1), mRNA [NM_001110345]	**34,558**
Kcnc2	Rattus norvegicus potassium voltage gated channel, Shaw-related subfamily, member 2 (Kcnc2), transcript variant b, mRNA [NM_139217]	**34,525**
Mmgt1	Rattus norvegicus membrane magnesium transporter 1 (Mmgt1), mRNA [NM_001106970]	**34,502**
Map1lc3b	Rattus norvegicus microtubule-associated protein 1 light chain 3 beta (Map1lc3b), mRNA [NM_022867]	**34,496**
RGD1302996	Rattus norvegicus hypothetical protein MGC:15854 (RGD1302996), mRNA [NM_213610]	**34,466**
0	LOC100125377 proteinRCG58522 [Source:UniProtKB/TrEMBL;Acc:Q5HZD9] [ENSRNOT00000039741]	**34,464**
0	predicted pseudogene 8325 Pseudogene [Source:MGI Symbol;Acc:MGI:3644852] [ENSRNOT00000037392]	**34,451**
0	Unknown	**34,450**

Samd4a	Rattus norvegicus sterile alpha motif domain containing 4A (Samd4a), mRNA [NM_001107254]	**34,445**
Mmgt2	Rattus norvegicus membrane magnesium transporter 2 (Mmgt2), mRNA [NM_001013967]	**34,430**
0	Unknown	**34,424**
Spock2	Rattus norvegicus sparc/osteonectin, cwcv and kazal-like domains proteoglycan 2 (Spock2), mRNA [NM_001108533]	**34,419**
Rgma	Rattus norvegicus RGM domain family, member A (Rgma), mRNA [NM_001107524]	**34,404**
Cx3cl1	Rattus norvegicus chemokine (C-X3-C motif) ligand 1 (Cx3cl1), mRNA [NM_134455]	**34,404**
Ncdn	Rattus norvegicus neurochondrin (Ncdn), mRNA [NM_053543]	**34,402**
Pigg	Uncharacterized protein [Source:UniProtKB/TrEMBL;Acc:D3ZDH0] [ENSRNOT00000033973]	**34,400**
Pax1	Rattus norvegicus paired box 1 (Pax1), mRNA [NM_001107787]	**34,376**
Smtnl2	Rattus norvegicus smoothelin-like 2 (Smtnl2), mRNA [NM_001190998]	**34,371**
Pcbp3	Rattus norvegicus poly(rC) binding protein 3 (Pcbp3), mRNA [NM_001011945]	**34,371**
Prkce	Rattus norvegicus protein kinase C, epsilon (Prkce), mRNA [NM_017171]	**34,368**
Zfp407	PREDICTED: Rattus norvegicus zinc finger protein 407 (Zfp407), mRNA [XM_225679]	**34,365**
Bmp7	Rattus norvegicus bone morphogenetic protein 7 (Bmp7), mRNA [NM_001191856]	**34,359**
Git1	Rattus norvegicus G protein-coupled receptor kinase interacting ArfGAP 1 (Git1), mRNA [NM_031814]	**34,328**
0	NADH-ubiquinone oxidoreductase chain 2 [Source:UniProtKB/Swiss-Prot;Acc:P11662] [ENSRNOT00000040993]	**34,313**
Slc22a17	Rattus norvegicus solute carrier family 22, member 17 (Slc22a17), mRNA [NM_177421]	**34,303**
0	Rattus norvegicus similar to 60S RIBOSOMAL PROTEIN L29 (P23) (LOC290219), mRNA [XM_224186]	**34,268**
Fam125b	Uncharacterized protein [Source:UniProtKB/TrEMBL;Acc:D4A732] [ENSRNOT00000023172]	**34,260**
Mat2a	Rattus norvegicus methionine adenosyltransferase II, alpha (Mat2a), mRNA [NM_134351]	**34,250**
Sall2	Rattus norvegicus sal-like 2 (Drosophila) (Sall2), mRNA [NM_001107262]	**34,224**
Slc35d3	Rattus norvegicus solute carrier family 35, member D3 (Slc35d3), mRNA [NM_001107522]	**34,205**
0	Q53FN7_HUMAN (Q53FN7) BZW1 protein variant (Fragment), complete [TC583097]	**34,161**
Ctnna2	Rattus norvegicus catenin (cadherin associated protein), alpha 2 (Ctnna2), mRNA [NM_001106598]	**34,148**
Ccrk	Rattus norvegicus cell cycle related kinase (Ccrk), mRNA [NM_001025752]	**34,095**
Mthfs	Rattus norvegicus 5,10-methenyltetrahydrofolate synthetase (5-formyltetrahydrofolate cyclo-ligase) (Mthfs), mRNA [NM_001009349]	**34,093**
Exoc6b	Rattus norvegicus exocyst complex component 6B (Exoc6b), mRNA [NM_001109246]	**34,080**
RGD1563224	Rattus norvegicus similar to 4930438D12Rik protein (RGD1563224), mRNA [NM_001100995]	**34,069**
Pde4a	Rattus norvegicus phosphodiesterase 4A, cAMP-specific (phosphodiesterase E2 dunce homolog, Drosophila) (Pde4a), mRNA [NM_013101]	**34,049**
Btrc	Rattus norvegicus beta-transducin repeat containing (Btrc), mRNA [NM_001007148]	**34,035**
0	Unknown	**34,034**
0	Potassium voltage-gated channel subfamily D member 3 [Source:UniProtKB/Swiss-Prot;Acc:Q62897] [ENSRNOT00000051835]	**34,030**
Olr1128	Rattus norvegicus olfactory receptor 1128 (Olr1128), mRNA [NM_001000987]	**34,027**
Sptan1	Rattus norvegicus spectrin, alpha, non-erythrocytic 1 (Sptan1), mRNA [NM_171983]	**34,006**

Tspan31	Rattus norvegicus tetraspanin 31 (Tspan31), mRNA [NM_001008378]	**34,003**
Numb	Rattus norvegicus numb homolog (Drosophila) (Numb), mRNA [NM_133287]	**34,000**
Frmd8	Rattus norvegicus FERM domain containing 8 (Frmd8), mRNA [NM_001008348]	**33,995**
Sf1	Rattus norvegicus splicing factor 1 (Sf1), transcript variant 2, mRNA [NM_058210]	**33,982**
Haus2	PREDICTED: Rattus norvegicus HAUS augmin-like complex, subunit 2, transcript variant 1 (Haus2), mRNA [XM_001053441]	**33,971**
0	Unknown	**33,960**
Rab36	Rattus norvegicus RAB36, member RAS oncogene family (Rab36), mRNA [NM_001109589]	**33,947**
RGD1563216	Rattus norvegicus similar to HESB like domain containing 1 (RGD1563216), mRNA [NM_001109278]	**33,936**
Vamp1	Rattus norvegicus vesicle-associated membrane protein 1 (Vamp1), mRNA [NM_013090]	**33,917**
0	E3 ubiquitin-protein ligase HUWE1 [Source:UniProtKB/Swiss-Prot;Acc:P51593] [ENSRNOT00000068719]	**33,914**
0	Rattus norvegicus similar to 60S RIBOSOMAL PROTEIN L29 (P23) (LOC302428), mRNA [XM_228586]	**33,910**
Oxsr1	Rattus norvegicus oxidative-stress responsive 1 (Oxsr1), mRNA [NM_001108194]	**33,906**
Lin52	Similar to CG15929-PA (Predicted), isoform CRA_aUncharacterized protein [Source:UniProtKB/TrEMBL;Acc:D4A6T8] [ENSRNOT00000064426]	**33,894**
Tsc2	Rattus norvegicus tuberous sclerosis 2 (Tsc2), mRNA [NM_012680]	**33,880**
Prkaa1	Rattus norvegicus protein kinase, AMP-activated, alpha 1 catalytic subunit (Prkaa1), mRNA [NM_019142]	**33,864**
0	Unknown	**33,864**
0	MMU64033 Tera {Mus musculus} (exp=-1; wgp=0; cg=0), partial (13%) [TC591393]	**33,862**
Klf12	Rattus norvegicus Kruppel-like factor 12 (Klf12), mRNA [NM_001107281]	**33,857**
Pbx2	Rattus norvegicus pre-B-cell leukemia homeobox 2 (Pbx2), mRNA [NM_001002828]	**33,842**
Plcb4	Rattus norvegicus phospholipase C, beta 4 (Plcb4), mRNA [NM_024353]	**33,840**
Olr1694	Rattus norvegicus olfactory receptor 1694 (Olr1694), mRNA [NM_001001110]	**33,838**
Wdfy3	Rattus norvegicus WD repeat and FYVE domain containing 3 (Wdfy3), mRNA [NM_001170551]	**33,829**
Rgma	Rattus norvegicus RGM domain family, member A (Rgma), mRNA [NM_001107524]	**33,819**
Kirrel3	Rattus norvegicus kin of IRRE like 3 (Drosophila) (Kirrel3), mRNA [NM_001048215]	**33,795**
Dyrk1a	Rattus norvegicus dual-specificity tyrosine-(Y)-phosphorylation regulated kinase 1A (Dyrk1a), mRNA [NM_012791]	**33,734**
Map4	Rattus norvegicus microtubule-associated protein 4 (Map4), mRNA [NM_001024278]	**33,726**
LOC100270669	Rattus norvegicus hypothetical protein LOC100270669 (LOC100270669), mRNA [NM_001144957]	**33,718**
0	Unknown	**33,717**
Gtf3c5	Rattus norvegicus general transcription factor IIIC, polypeptide 5 (Gtf3c5), mRNA [NM_001079941]	**33,691**
Dnmt3a	Rattus norvegicus DNA (cytosine-5-)-methyltransferase 3 alpha (Dnmt3a), transcript variant 1, mRNA [NM_001003958]	**33,682**
Rnf32	Rattus norvegicus ring finger protein 32 (Rnf32), mRNA [NM_001012095]	**33,677**
Fam70a	Rattus norvegicus family with sequence similarity 70, member A (Fam70a), mRNA [NM_182822]	**33,677**
Slc17a7	Rattus norvegicus solute carrier family 17 (sodium-dependent inorganic phosphate cotransporter), member 7 (Slc17a7), mRNA [NM_053859]	**33,661**
Dnajc16	Rattus norvegicus DnaJ (Hsp40) homolog, subfamily C, member 16 (Dnajc16), mRNA [NM_001014194]	**33,654**

Tbp	Rattus norvegicus TATA box binding protein (Tbp), mRNA [NM_001004198]	**33,645**
Cdc42bpa	Rattus norvegicus CDC42 binding protein kinase alpha (Cdc42bpa), mRNA [NM_053657]	**33,631**
Ube2v1	Rattus norvegicus ubiquitin-conjugating enzyme E2 variant 1 (Ube2v1), mRNA [NM_001110345]	**33,614**
Acbd3	Rattus norvegicus acyl-Coenzyme A binding domain containing 3 (Acbd3), mRNA [NM_182843]	**33,594**
Lsm7	Rattus norvegicus LSM7 homolog, U6 small nuclear RNA associated (S. cerevisiae) (Lsm7), mRNA [NM_001108732]	**33,577**
Lrrc57	Rattus norvegicus leucine rich repeat containing 57 (Lrrc57), mRNA [NM_001012354]	**33,576**
Pgc	Rattus norvegicus progastricsin (pepsinogen C) (Pgc), mRNA [NM_133284]	**33,564**
Gca	Rattus norvegicus grancalcin (Gca), mRNA [NM_001106483]	**33,559**
0	Uncharacterized protein [Source:UniProtKB/TrEMBL;Acc:D3ZIJ6] [ENSRNOT00000041124]	**33,553**
Slc4a4	Rattus norvegicus solute carrier family 4, sodium bicarbonate cotransporter, member 4 (Slc4a4), mRNA [NM_053424]	**33,535**
0	PREDICTED: Rattus norvegicus WAP four-disulfide core domain 6A (Wfdc6a), mRNA [XM_001062561]	**33,534**
0	Unknown	**33,533**
0	Unknown	**33,515**
Hmgn2	Rattus norvegicus high mobility group nucleosomal binding domain 2 (Hmgn2), mRNA [NM_001025624]	**33,500**
LOC498606	Rattus norvegicus hypothetical protein LOC498606 (LOC498606), mRNA [NM_001025143]	**33,496**
Wdr48	Rattus norvegicus WD repeat domain 48 (Wdr48), mRNA [NM_001135895]	**33,486**
0	PREDICTED: Rattus norvegicus extracellular leucine-rich repeat and fibronectin type III domain containing 2 (Elfn2), miscRNA [XR_086301]	**33,480**
Gng8	Rattus norvegicus guanine nucleotide binding protein (G protein), gamma 8 (Gng8), mRNA [NM_139185]	**33,477**
0	AGENCOURT_31543064 NIH_MGC_269 Rattus norvegicus cDNA clone IMAGE:7462807 5', mRNA sequence [CV121387]	**33,473**
Papola	Rattus norvegicus poly (A) polymerase alpha (Papola), mRNA [NM_001108056]	**33,468**
Shpk	Rattus norvegicus sedoheptulokinase (Shpk), mRNA [NM_001033682]	**33,468**
0	Ab1-334 [Source:UniProtKB/TrEMBL;Acc:Q7TP85] [ENSRNOT00000032968]	**33,462**
Cetn4	Unknown	**33,445**
Pcsk4	Rattus norvegicus proprotein convertase subtilisin/kexin type 4 (Pcsk4), mRNA [NM_133559]	**33,439**
0	UI-R-FS0-crv-o-03-0-UI.s1 UI-R-FS0 Rattus norvegicus cDNA clone UI-R-FS0-crv-o-03-0-UI 3', mRNA sequence [CB328432]	**33,418**
Fbxo44	Rattus norvegicus TL0AAA48YG12 mRNA sequence. [FQ213337]	**33,416**
RGD1311575	PREDICTED: Rattus norvegicus hypothetical LOC289568 (RGD1311575), mRNA [XM_002724955]	**33,400**
Chrm4	Rattus norvegicus cholinergic receptor, muscarinic 4 (Chrm4), mRNA [NM_031547]	**33,347**
Pctk1	Rattus norvegicus PCTAIRE protein kinase 1 (Pctk1), transcript variant 1, mRNA [NM_001004132]	**33,321**
0	Unknown	**33,287**
Foxn3	Rattus norvegicus forkhead box N3 (Foxn3), mRNA [NM_001108047]	**33,271**
0	Unknown	**33,267**
0	Rattus norvegicus TL0AAA46YN14 mRNA sequence. [FQ213594]	**33,265**
Slc9a1	Rattus norvegicus solute carrier family 9 (sodium/hydrogen exchanger), member 1 (Slc9a1), mRNA [NM_012652]	**33,251**

Rai14	Rattus norvegicus retinoic acid induced 14 (Rai14), mRNA [NM_001011947]	**33,242**
Nacc2	Rattus norvegicus nucleus accumbens associated 2, BEN and BTB (POZ) domain containing (Nacc2), mRNA [NM_001100533]	**33,240**
0	Unknown	**33,231**
LOC678880	PREDICTED: Rattus norvegicus similar to mammalian retrotransposon derived 8b (LOC678880), mRNA [XM_001053139]	**33,224**
Abcd2	Rattus norvegicus ATP-binding cassette, subfamily D (ALD), member 2 (Abcd2), mRNA [NM_033352]	**33,199**
Fiz1	Rattus norvegicus FLT3-interacting zinc finger 1 (Fiz1), mRNA [NM_001106223]	**33,193**
Pi16	Rattus norvegicus peptidase inhibitor 16 (Pi16), mRNA [NM_001170481]	**33,146**
Prr12	Uncharacterized protein [Source:UniProtKB/TrEMBL;Acc:D4A6M5] [ENSRNOT00000038067]	**33,141**
Ptp4a1	Rattus norvegicus protein tyrosine phosphatase type IVA, member 1 (Ptp4a1), mRNA [NM_031579]	**33,101**
Psme4	Rattus norvegicus proteasome (prosome, macropain) activator subunit 4 (Psme4), mRNA [NM_001025140]	**33,100**
Tomm40b	Rattus norvegicus translocase of outer mitochondrial membrane 40 homolog B (yeast) (Tomm40b), nuclear gene encoding mitochondrial protein, mRNA [NM_001083338]	**33,085**
0	BT006320 At1g66470 {Arabidopsis thaliana} (exp=-1; wgp=0; cg=0), partial (5%) [TC589845]	**33,077**
0	Q87VI1_PSESM (Q87VI1) Rhodanese domain protein/phosphatidylserine decarboxylase, partial (3%) [TC610837]	**33,056**
Cdh13	Rattus norvegicus cadherin 13 (Cdh13), mRNA [NM_138889]	**33,055**
Bsn	Rattus norvegicus bassoon (Bsn), mRNA [NM_019146]	**33,047**
Ncan	Rattus norvegicus neurocan (Ncan), mRNA [NM_031653]	**33,046**
Cnot3	Rattus norvegicus CCR4-NOT transcription complex, subunit 3 (Cnot3), mRNA [NM_001107471]	**33,045**
Eps15	Rattus norvegicus epidermal growth factor receptor pathway substrate 15 (Eps15), mRNA [NM_001009424]	**33,042**
Lgi3	Rattus norvegicus leucine-rich repeat LGI family, member 3 (Lgi3), mRNA [NM_001107277]	**33,034**
Uvrag	Rattus norvegicus UV radiation resistance associated gene (Uvrag), mRNA [NM_001107536]	**33,020**
LOC684399	PREDICTED: Rattus norvegicus similar to 60S ribosomal protein L29 (P23) (LOC684399), mRNA [XM_001070213]	**33,006**
Tmem108	PREDICTED: Rattus norvegicus transmembrane protein 108 (Tmem108), mRNA [XM_217247]	**32,988**
Tmco4	Rattus norvegicus transmembrane and coiled-coil domains 4 (Tmco4), mRNA [NM_001034949]	**32,971**
RGD1304884	Rattus norvegicus similar to RIKEN cDNA 6430548M08 (RGD1304884), mRNA [NM_001107436]	**32,964**
Snap25	Rattus norvegicus synaptosomal-associated protein 25 (Snap25), mRNA [NM_030991]	**32,960**
Pcbp2	Rattus norvegicus poly(rC) binding protein 2 (Pcbp2), mRNA [NM_001013223]	**32,951**
Slc35c1	Rattus norvegicus solute carrier family 35, member C1 (Slc35c1), mRNA [NM_001107748]	**32,935**
Rgl1	Rattus norvegicus ral guanine nucleotide dissociation stimulator,-like 1 (Rgl1), mRNA [NM_001105957]	**32,921**
Dazap2	Rattus norvegicus DAZ associated protein 2 (Dazap2), mRNA [NM_001013107]	**32,920**
Fto	Rattus norvegicus fat mass and obesity associated (Fto), mRNA [NM_001039713]	**32,911**
Nfyc	Rattus norvegicus nuclear transcription factor-Y gamma (Nfyc), mRNA [NM_012866]	**32,908**
Ttc9c	Rattus norvegicus tetratricopeptide repeat domain 9C (Ttc9c), mRNA [NM_001007693]	**32,900**
0	Potassium voltage-gated channel subfamily D member 3 [Source:UniProtKB/Swiss-Prot;Acc:Q62897] [ENSRNOT00000019997]	**32,896**

Cnnm3	Rattus norvegicus cyclin M3 (Cnnm3), mRNA [NM_001106901]	**32,885**
Rb1	Rattus norvegicus retinoblastoma 1 (Rb1), mRNA [NM_017045]	**32,884**
Tuba3b	Rattus norvegicus tubulin, alpha 3B (Tuba3b), mRNA [NM_001024336]	**32,879**
Palm	Rattus norvegicus paralemmin (Palm), mRNA [NM_130829]	**32,865**
Rimklb	Uncharacterized protein [Source:UniProtKB/TrEMBL;Acc:D3ZJ79] [ENSRNOT00000032690]	**32,863**
Dvl3	Rattus norvegicus dishevelled, dsh homolog 3 (Drosophila) (Dvl3), mRNA [NM_001107081]	**32,860**
Rqcd1	Rattus norvegicus rcd1 (required for cell differentiation) homolog 1 (S. pombe) (Rqcd1), mRNA [NM_001009357]	**32,858**
Eif4e	Rattus norvegicus eukaryotic translation initiation factor 4E (Eif4e), mRNA [NM_053974]	**32,841**
0	Unknown	**32,821**
Sec14l5	Rattus norvegicus SEC14-like 5 (S. cerevisiae) (Sec14l5), mRNA [NM_001135710]	**32,810**
Stim1	Rattus norvegicus stromal interaction molecule 1 (Stim1), mRNA [NM_001108496]	**32,805**
Nop2	Rattus norvegicus NOP2 nucleolar protein homolog (yeast) (Nop2), mRNA [NM_001191785]	**32,801**
Zc3h10	Rattus norvegicus zinc finger CCCH type containing 10 (Zc3h10), mRNA [NM_001191090]	**32,800**
Prrx1	Rattus norvegicus paired related homeobox 1 (Prrx1), mRNA [NM_153821]	**32,796**
Tbc1d9	Rattus norvegicus TBC1 domain family, member 9 (Tbc1d9), mRNA [NM_001134539]	**32,778**
Napepld	Rattus norvegicus N-acyl phosphatidylethanolamine phospholipase D (Napepld), mRNA [NM_199381]	**32,769**
Fndc5	PREDICTED: Rattus norvegicus fibronectin type III domain containing 5 (Fndc5), mRNA [XM_002729542]	**32,769**
Padi2	Rattus norvegicus peptidyl arginine deiminase, type II (Padi2), mRNA [NM_017226]	**32,763**
0	Uncharacterized protein [Source:UniProtKB/TrEMBL;Acc:D3ZGV8] [ENSRNOT00000004410]	**32,744**
Slc16a3	Rattus norvegicus solute carrier family 16, member 3 (monocarboxylic acid transporter 4) (Slc16a3), mRNA [NM_030834]	**32,725**
LOC294154	Rattus norvegicus similar to chromosome 6 open reading frame 106 isoform a (LOC294154), mRNA [NM_001039607]	**32,712**
Tsc2	Rattus norvegicus tuberous sclerosis 2 (Tsc2), mRNA [NM_012680]	**32,652**
Vom2r15	Rattus norvegicus vomeronasal 2 receptor, 15 (Vom2r15), mRNA [NM_001099490]	**32,646**
Rab11b	Rattus norvegicus RAB11B, member RAS oncogene family (Rab11b), mRNA [NM_032617]	**32,636**
0	PREDICTED: Rattus norvegicus similar to procollagen, type VI, alpha 3 isoform 4 (RGD1564060), mRNA [XM_002727099]	**32,614**
Vps25	Rattus norvegicus vacuolar protein sorting 25 homolog (S. cerevisiae) (Vps25), mRNA [NM_001173451]	**32,610**
Tmem164	Rattus norvegicus transmembrane protein 164 (Tmem164), mRNA [NM_001109014]	**32,600**
0	Unknown	**32,597**
Hpcal1	Rattus norvegicus hippocalcin-like 1 (Hpcal1), mRNA [NM_017356]	**32,591**
Med15	Rattus norvegicus mediator complex subunit 15 (Med15), mRNA [NM_001108325]	**32,590**
Hspa12a	Rattus norvegicus heat shock protein 12A (Hspa12a), mRNA [NM_001107445]	**32,590**
LOC499573	Rattus norvegicus LRRGT00056 (LOC499573), mRNA [NM_001047947]	**32,556**
0	Unknown	**32,540**
Vps37b	Rattus norvegicus vacuolar protein sorting 37 homolog B (S. cerevisiae) (Vps37b), mRNA [NM_001105928]	**32,536**

Chst12	Rattus norvegicus carbohydrate (chondroitin 4) sulfotransferase 12 (Chst12), mRNA [NM_001037775]	**32,530**
Otog	Rattus norvegicus otogelin (Otog), mRNA [NM_001106262]	**32,502**
Cav1	Rattus norvegicus caveolin 1, caveolae protein (Cav1), transcript variant 2, mRNA [NM_133651]	**32,492**
Pctk3	Rattus norvegicus PCTAIRE protein kinase 3 (Pctk3), mRNA [NM_001100506]	**32,492**
Tsga10	Rattus norvegicus testis specific 10 (Tsga10), mRNA [NM_001030022]	**32,492**
Lmf1	Uncharacterized protein [Source:UniProtKB/TrEMBL;Acc:D3ZF16] [ENSRNOT00000000246]	**32,476**
Mast3	Rattus norvegicus microtubule associated serine/threonine kinase 3 (Mast3), mRNA [NM_001134796]	**32,468**
Aldh4a1	Rattus norvegicus aldehyde dehydrogenase 4 family, member A1 (Aldh4a1), nuclear gene encoding mitochondrial protein, mRNA [NM_001134698]	**32,453**
Lrrn2	Rattus norvegicus leucine rich repeat neuronal 2 (Lrrn2), mRNA [NM_001177368]	**32,447**
Pcgf2	Rattus norvegicus polycomb group ring finger 2 (Pcgf2), mRNA [NM_001105836]	**32,435**
Uxt	Rattus norvegicus ubiquitously expressed transcript (Uxt), mRNA [NM_001006982]	**32,433**
Gab1	Rattus norvegicus GRB2-associated binding protein 1 (Gab1), mRNA [NM_001108444]	**32,428**
Mocs1	Rattus norvegicus molybdenum cofactor synthesis 1 (Mocs1), mRNA [NM_001106881]	**32,417**
Rac1	Rattus norvegicus ras-related C3 botulinum toxin substrate 1 (Rac1), mRNA [NM_134366]	**32,383**
0	BTB/POZ domain-containing protein KCTD1 [Source:UniProtKB/Swiss-Prot;Acc:Q8R4G8] [ENSRNOT00000061504]	**32,366**
Adap1	Rattus norvegicus ArfGAP with dual PH domains 1 (Adap1), mRNA [NM_133567]	**32,352**
Prkce	Rattus norvegicus protein kinase C, epsilon (Prkce), mRNA [NM_017171]	**32,342**
Ogdh	Rattus norvegicus TL0ADA19YE17 mRNA sequence. [FQ222842]	**32,339**
Olr791	Rattus norvegicus olfactory receptor 791 (Olr791), mRNA [NM_001000580]	**32,329**
Nrxn3	Rattus norvegicus neurexin 3 (Nrxn3), mRNA [NM_053817]	**32,313**
Ccr1l1	Rattus norvegicus chemokine (C-C motif) receptor 1-like 1 (Ccr1l1), mRNA [NM_001106872]	**32,307**
Ncapd2	Condensin complex subunit 1 [Source:UniProtKB/TrEMBL;Acc:D3ZHP6] [ENSRNOT00000025889]	**32,306**
Vac14	Rattus norvegicus Vac14 homolog (S. cerevisiae) (Vac14), mRNA [NM_177930]	**32,292**
Sema4a	Rattus norvegicus sema domain, immunoglobulin domain (Ig), transmembrane domain (TM) and short cytoplasmic domain, (semaphorin) 4A (Sema4a), mRNA [NM_001012078]	**32,291**
P2rx3	Rattus norvegicus purinergic receptor P2X, ligand-gated ion channel, 3 (P2rx3), mRNA [NM_031075]	**32,290**
Grm2	Rattus norvegicus glutamate receptor, metabotropic 2 (Grm2), mRNA [NM_001105711]	**32,285**
Mgrn1	Rattus norvegicus mahogunin, ring finger 1 (Mgrn1), mRNA [NM_001013964]	**32,281**
Foxp4	Rattus norvegicus forkhead box P4 (Foxp4), mRNA [NM_001108788]	**32,262**
Gatad2b	Rattus norvegicus GATA zinc finger domain containing 2B (Gatad2b), mRNA [NM_001024888]	**32,231**
Rab1b	Rattus norvegicus RAB1B, member RAS oncogene family (Rab1b), mRNA [NM_001109979]	**32,228**
Grip1	Rattus norvegicus glutamate receptor interacting protein 1 (Grip1), mRNA [NM_032069]	**32,216**
Mll5	PREDICTED: Rattus norvegicus myeloid/lymphoid or mixed-lineage leukemia 5 (trithorax homolog, Drosophila) (Mll5), mRNA [XM_231287]	**32,216**
0	Uncharacterized protein [Source:UniProtKB/TrEMBL;Acc:D4A5I4] [ENSRNOT00000037100]	**32,215**

0	Uncharacterized protein [Source:UniProtKB/TrEMBL;Acc:D3ZM70] [ENSRNOT00000006897]	**32,214**
RGD1310453	Rattus norvegicus similar to hypothetical protein FLJ23451 (RGD1310453), mRNA [NM_001134463]	**32,202**
Pcdhga7	Rattus norvegicus protocadherin gamma subfamily A, 7 (Pcdhga7), mRNA [NM_001014773]	**32,195**
0	Unknown	**32,184**
RGD1305733	Rattus norvegicus similar to RIKEN cDNA 2900011O08 (RGD1305733), mRNA [NM_001014114]	**32,178**
Dkk3	Rattus norvegicus dickkopf homolog 3 (Xenopus laevis) (Dkk3), mRNA [NM_138519]	**32,176**
Trib1	Rattus norvegicus tribbles homolog 1 (Drosophila) (Trib1), mRNA [NM_023985]	**32,159**
Ccdc116	Rattus norvegicus coiled-coil domain containing 116 (Ccdc116), mRNA [NM_001024740]	**32,150**
Dpp8	Rattus norvegicus dipeptidylpeptidase 8 (Dpp8), mRNA [NM_001108159]	**32,124**
RGD1311493	Uncharacterized protein [Source:UniProtKB/TrEMBL;Acc:D4A5H7] [ENSRNOT00000028407]	**32,119**
Ikzf5	Rattus norvegicus IKAROS family zinc finger 5 (Ikzf5), mRNA [NM_001107555]	**32,095**
0	Unknown	**32,092**
Patl1	Rattus norvegicus protein associated with topoisomerase II homolog 1 (yeast) (Patl1), mRNA [NM_001108520]	**32,090**
Sema4b	Rattus norvegicus sema domain, immunoglobulin domain (Ig), transmembrane domain (TM) and short cytoplasmic domain, (semaphorin) 4B (Sema4b), mRNA [NM_001170462]	**32,087**
Plekhg1	Rattus norvegicus pleckstrin homology domain containing, family G (with RhoGef domain) member 1 (Plekhg1), mRNA [NM_001190999]	**32,085**
Srrm1	Rattus norvegicus serine/arginine repetitive matrix 1 (Srrm1), mRNA [NM_001107986]	**32,082**
Prkcb	Rattus norvegicus protein kinase C, beta (Prkcb), transcript variant 1, mRNA [NM_012713]	**32,074**
0	Unknown	**32,066**
Clip2	Rattus norvegicus CAP-GLY domain containing linker protein 2 (Clip2), mRNA [NM_021997]	**32,062**
Tbl1x	Rattus norvegicus transducin (beta)-like 1 X-linked (Tbl1x), mRNA [NM_001106964]	**32,042**
Tmub2	Rattus norvegicus transmembrane and ubiquitin-like domain containing 2 (Tmub2), mRNA [NM_001031651]	**32,038**
Cic	Rattus norvegicus capicua homolog (Drosophila) (Cic), mRNA [NM_001107490]	**32,034**
Meis1	Rattus norvegicus Meis homeobox 1 (Meis1), mRNA [NM_001134702]	**32,030**
Spock2	Rattus norvegicus sparc/osteonectin, cwcv and kazal-like domains proteoglycan 2 (Spock2), mRNA [NM_001108533]	**32,028**
Shroom2	Rattus norvegicus shroom family member 2 (Shroom2), mRNA [NM_001047893]	**32,027**
0	Uncharacterized protein [Source:UniProtKB/TrEMBL;Acc:D3ZGM0] [ENSRNOT00000056197]	**31,989**
Lrrc41	Rattus norvegicus leucine rich repeat containing 41 (Lrrc41), mRNA [NM_001009710]	**31,973**
Sv2b	Rattus norvegicus synaptic vesicle glycoprotein 2b (Sv2b), mRNA [NM_057207]	**31,966**
LOC690402	Rattus norvegicus similar to Short palate, lung and nasal epithelium carcinoma-associated protein 3 homolog precursor (LOC690402), mRNA [NM_001109588]	**31,964**
LOC685179	Uncharacterized protein [Source:UniProtKB/TrEMBL;Acc:D3ZPF5] [ENSRNOT00000028244]	**31,940**
0	Unknown	**31,925**
0	Uncharacterized protein [Source:UniProtKB/TrEMBL;Acc:D3ZGV8] [ENSRNOT00000004410]	**31,925**

Lef1	Rattus norvegicus lymphoid enhancer binding factor 1 (Lef1), mRNA [NM_130429]	**31,918**
Car15	Rattus norvegicus carbonic anhydrase 15 (Car15), mRNA [NM_001105901]	**31,902**
LOC305806	Rattus norvegicus similar to glutaredoxin 1 (thioltransferase); glutaredoxin (LOC305806), mRNA [NM_001013993]	**31,870**
Slc41a2	Rattus norvegicus solute carrier family 41, member 2 (Slc41a2), mRNA [NM_001108742]	**31,856**
Cldn19	Rattus norvegicus claudin 19 (Cldn19), mRNA [NM_001008514]	**31,855**
Sobpl	Rattus norvegicus sine oculis-binding protein homolog-like (Drosophila) (Sobpl), mRNA [NM_001104640]	**31,838**
Col4a2	PREDICTED: Rattus norvegicus collagen, type IV, alpha 2 (Col4a2), mRNA [XM_001076134]	**31,829**
Sipa1l1	Rattus norvegicus signal-induced proliferation-associated 1 like 1 (Sipa1l1), mRNA [NM_139330]	**31,829**
0	RVL8997 Wackym-Soares normalized rat vestibular cDNA library Rattus norvegicus cDNA 5', mRNA sequence [DV718414]	**31,821**
0	Unknown	**31,820**
Srd5a1	Rattus norvegicus steroid-5-alpha-reductase, alpha polypeptide 1 (3-oxo-5 alpha-steroid delta 4-dehydrogenase alpha 1) (Srd5a1), mRNA [NM_017070]	**31,819**
LOC683844	PREDICTED: Rattus norvegicus similar to RING finger protein 11 (NEDD4 WW domain-binding protein 2) (Sid 1669) (LOC683844), mRNA [XM_001067720]	**31,817**
Rims2	Rattus norvegicus regulating synaptic membrane exocytosis 2 (Rims2), transcript variant 1, mRNA [NM_053945]	**31,811**
0	Unknown	**31,785**
Dact3	Rattus norvegicus dapper, antagonist of beta-catenin, homolog 3 (Xenopus laevis) (Dact3), mRNA [NM_001191947]	**31,763**
LOC678741	PREDICTED: Rattus norvegicus similar to Zinc finger CCCH-type domain containing protein 6 (LOC678741), mRNA [XM_001053214]	**31,760**
Chrna5	Rattus norvegicus cholinergic receptor, nicotinic, alpha 5 (Chrna5), mRNA [NM_017078]	**31,748**
0	Rattus norvegicus similar to ubiquitin-conjugating enzyme E2 variant 1 isoform b; DNA-binding protein (LOC296390), mRNA [XM_215948]	**31,735**
0	Unknown	**31,719**
LOC682988	Rattus norvegicus similar to mediator of RNA polymerase II transcription, subunit 18 homolog (LOC682988), mRNA [NM_001115037]	**31,695**
0	Unknown	**31,690**
B3gnt2	Rattus norvegicus UDP-GlcNAc:betaGal beta-1,3-N-acetylglucosaminyltransferase 2 (B3gnt2), mRNA [NM_001107240]	**31,679**
Zmiz2	Rattus norvegicus zinc finger, MIZ-type containing 2 (Zmiz2), mRNA [NM_001100507]	**31,679**
Abca1	Rattus norvegicus ATP-binding cassette, subfamily A (ABC1), member 1 (Abca1), mRNA [NM_178095]	**31,677**
Rprd1b	Rattus norvegicus regulation of nuclear pre-mRNA domain containing 1B (Rprd1b), mRNA [NM_001098727]	**31,676**
LOC689927	Rattus norvegicus similar to keratin associated protein 10-10 (LOC689927), mRNA [NM_001109555]	**31,675**
Dpy19l3	Rattus norvegicus dpy-19-like 3 (C. elegans) (Dpy19l3), mRNA [NM_001135835]	**31,641**
Pmp22	Rattus norvegicus peripheral myelin protein 22 (Pmp22), mRNA [NM_017037]	**31,624**
0	salt inducible kinase 2 Gene [Source:MGI Symbol;Acc:MGI:2445031] [ENSRNOT00000066893]	**31,623**
Nfic	Rattus norvegicus nuclear factor I/C (Nfic), mRNA [NM_031567]	**31,618**
Arpp21	Rattus norvegicus cAMP-regulated phosphoprotein 21 (Arpp21), transcript variant 1, mRNA [NM_001135046]	**31,616**
Cfl2	Rattus norvegicus cofilin 2, muscle (Cfl2), mRNA [NM_001108982]	**31,613**

Mybbp1a	Rattus norvegicus MYB binding protein (P160) 1a (Mybbp1a), mRNA [NM_031668]	**31,611**
Nfu1	Rattus norvegicus NFU1 iron-sulfur cluster scaffold homolog (S. cerevisiae) (Nfu1), nuclear gene encoding mitochondrial protein, mRNA [NM_001106606]	**31,608**
Ptp4a2	Rattus norvegicus protein tyrosine phosphatase 4a2 (Ptp4a2), mRNA [NM_053475]	**31,571**
Plod1	Rattus norvegicus procollagen-lysine 1, 2-oxoglutarate 5-dioxygenase 1 (Plod1), mRNA [NM_053827]	**31,566**
RGD1560601	PREDICTED: Rattus norvegicus similar to Jumonji/ARID domain-containing protein 1C (SmcX protein) (RGD1560601), mRNA [XM_001064297]	**31,560**
Pcdh7	Rattus norvegicus protocadherin 7 (Pcdh7), mRNA [NM_001004087]	**31,556**
Pank3	Rattus norvegicus pantothenate kinase 3 (Pank3), mRNA [NM_001108272]	**31,548**
Nab2	Rattus norvegicus Ngfi-A binding protein 2 (Nab2), mRNA [NM_001134874]	**31,534**
0	Unknown	**31,523**
Pygm	Rattus norvegicus phosphorylase, glycogen, muscle (Pygm), mRNA [NM_012638]	**31,518**
Ogt	Rattus norvegicus O-linked N-acetylglucosamine (GlcNAc) transferase (UDP-N-acetylglucosamine:polypeptide-N-acetylglucosaminyl transferase) (Ogt), mRNA [NM_017107]	**31,504**
C1ql3	Rattus norvegicus complement component 1, q subcomponent-like 3 (C1ql3), mRNA [NM_001109403]	**31,490**
Tmem138	Rattus norvegicus transmembrane protein 138 (Tmem138), mRNA [NM_198777]	**31,484**
LOC691293	PREDICTED: Rattus norvegicus similar to reproductive homeobox on chromosome X, 7 (LOC691293), mRNA [XM_001077586]	**31,479**
Apoc4	Rattus norvegicus apolipoprotein C-IV (Apoc4), mRNA [NM_001109419]	**31,472**
Msto1	Rattus norvegicus misato homolog 1 (Drosophila) (Msto1), mRNA [NM_001106443]	**31,463**
Rara	Rattus norvegicus retinoic acid receptor, alpha (Rara), mRNA [NM_031528]	**31,455**
LOC301128	Rattus norvegicus similar to jumonji domain containing 2B (LOC301128), mRNA [NM_001044236]	**31,447**
Marcksl1	Rattus norvegicus MARCKS-like 1 (Marcksl1), mRNA [NM_030862]	**31,432**
Steap2	Rattus norvegicus six transmembrane epithelial antigen of the prostate 2 (Steap2), mRNA [NM_001107846]	**31,421**
Btbd2	PREDICTED: Rattus norvegicus BTB (POZ) domain containing 2 (Btbd2), mRNA [XM_576181]	**31,411**
Pex19	Rattus norvegicus peroxisomal biogenesis factor 19 (Pex19), transcript variant 1, mRNA [NM_001107375]	**31,410**
Zmym4	Rattus norvegicus zinc finger, MYM-type 4 (Zmym4), mRNA [NM_001107982]	**31,408**
Ankrd45	Uncharacterized protein [Source:UniProtKB/TrEMBL;Acc:D3Z8M5] [ENSRNOT00000003857]	**31,394**
Gosr2	Rattus norvegicus golgi SNAP receptor complex member 2 (Gosr2), mRNA [NM_031685]	**31,381**
Coro1c	Rattus norvegicus coronin, actin binding protein 1C (Coro1c), mRNA [NM_001109327]	**31,368**
Atf7	Rattus norvegicus activating transcription factor 7 (Atf7), mRNA [NM_001108115]	**31,368**
Hdac7	PREDICTED: Rattus norvegicus histone deacetylase 7 (Hdac7), mRNA [XM_001059057]	**31,366**
0	Unknown	**31,338**
Hs3st2	Rattus norvegicus heparan sulfate (glucosamine) 3-O-sulfotransferase 2 (Hs3st2), mRNA [NM_181370]	**31,334**
Sez6l	Rattus norvegicus seizure related 6 homolog (mouse)-like (Sez6l), mRNA [NM_001134538]	**31,328**
Spcs2	Rattus norvegicus signal peptidase complex subunit 2 homolog (S. cerevisiae) (Spcs2), mRNA [NM_001191601]	**31,320**

Elk3	Rattus norvegicus ELK3, ETS-domain protein (Elk3), mRNA [NM_001108743]	**31,319**
Ehd1	Rattus norvegicus EH-domain containing 1 (Ehd1), mRNA [NM_001011939]	**31,311**
Pla2g6	Rattus norvegicus phospholipase A2, group VI (cytosolic, calcium-independent) (Pla2g6), mRNA [NM_001005560]	**31,295**
0	Unknown	**31,282**
Lppr5	Rattus norvegicus lipid phosphate phosphatase-related protein type 5 (Lppr5), mRNA [NM_001107720]	**31,260**
RGD1310717	Rattus norvegicus similar to RIKEN cDNA E030002O03 (RGD1310717), mRNA [NM_001106287]	**31,250**
Dpysl2	Rattus norvegicus dihydropyrimidinase-like 2 (Dpysl2), mRNA [NM_001105717]	**31,238**
Pex19	Rattus norvegicus peroxisomal biogenesis factor 19 (Pex19), transcript variant 2, mRNA [NM_001134777]	**31,218**
Mknk2	Rattus norvegicus MAP kinase-interacting serine/threonine kinase 2 (Mknk2), mRNA [NM_001011985]	**31,213**
Slc8a2	Rattus norvegicus solute carrier family 8 (sodium/calcium exchanger), member 2 (Slc8a2), mRNA [NM_078619]	**31,207**
Abcg4	Rattus norvegicus ATP-binding cassette, subfamily G (WHITE), member 4 (Abcg4), mRNA [NM_001106816]	**31,203**
Tspan7	Rattus norvegicus tetraspanin 7 (Tspan7), mRNA [NM_001108815]	**31,190**
Hip1	Rattus norvegicus huntingtin interacting protein 1 (Hip1), mRNA [NM_001100475]	**31,166**
Slc24a3	Rattus norvegicus solute carrier family 24 (sodium/potassium/calcium exchanger), member 3 (Slc24a3), mRNA [NM_053505]	**31,155**
Agfg2	Rattus norvegicus ArfGAP with FG repeats 2 (Agfg2), mRNA [NM_001107131]	**31,150**
Necab1	Rattus norvegicus N-terminal EF-hand calcium binding protein 1 (Necab1), mRNA [NM_022302]	**31,149**
0	Uncharacterized protein [Source:UniProtKB/TrEMBL;Acc:D3ZWV1] [ENSRNOT00000048860]	**31,131**
Pnpo	Rattus norvegicus pyridoxine 5'-phosphate oxidase (Pnpo), mRNA [NM_022601]	**31,116**
Slc25a44	Rattus norvegicus solute carrier family 25, member 44 (Slc25a44), mRNA [NM_001108947]	**31,108**
RGD1309079	Rattus norvegicus similar to Ab2-095 (RGD1309079), mRNA [NM_001134472]	**31,101**
MGC114440	Rattus norvegicus similar to RIKEN cDNA 4930555I21 (MGC114440), mRNA [NM_001025772]	**31,084**
RGD1307067	Rattus norvegicus LOC362840 (RGD1307067), mRNA [NM_001173974]	**31,080**
Smad7	Rattus norvegicus SMAD family member 7 (Smad7), mRNA [NM_030858]	**31,074**
Ksr1	Rattus norvegicus kinase suppressor of ras 1 (Ksr1), mRNA [NM_001108284]	**31,056**
Snx19	Rattus norvegicus sorting nexin 19 (Snx19), mRNA [NM_001108131]	**31,045**
Tor1aip1	Rattus norvegicus torsin A interacting protein 1 (Tor1aip1), mRNA [NM_145092]	**31,043**
Dusp8	Rattus norvegicus dual specificity phosphatase 8 (Dusp8), mRNA [NM_001108510]	**31,041**
Eif4a1	Rattus norvegicus eukaryotic translation initiation factor 4A, isoform 1 (Eif4a1), mRNA [NM_199372]	**31,039**
Lamtor1	Rattus norvegicus RhoA activator C11orf59 homolog (Lamtor1), mRNA [NM_199102]	**31,033**
Slc3a1	Rattus norvegicus solute carrier family 3, member 1 (Slc3a1), mRNA [NM_017216]	**31,029**
Tpm3	Rattus norvegicus tropomyosin 3, gamma (Tpm3), transcript variant 2, mRNA [NM_173111]	**31,021**
0	Rattus norvegicus cDNA clone IMAGE:7459750. [BC099100]	**31,010**
Ptk2b	Rattus norvegicus PTK2B protein tyrosine kinase 2 beta (Ptk2b), mRNA [NM_017318]	**31,005**
0	Unknown	**30,978**

Caskin1	Rattus norvegicus CASK interacting protein 1 (Caskin1), mRNA [NM_080690]	**30,976**
Foxm1	Rattus norvegicus forkhead box M1 (Foxm1), mRNA [NM_031633]	**30,975**
0	Uncharacterized protein [Source:UniProtKB/TrEMBL;Acc:D3ZW02] [ENSRNOT00000036647]	**30,951**
Chst11	Rattus norvegicus carbohydrate (chondroitin 4) sulfotransferase 11 (Chst11), mRNA [NM_001108079]	**30,940**
Tpbg	Rattus norvegicus trophoblast glycoprotein (Tpbg), mRNA [NM_031807]	**30,938**
Apoa5	Rattus norvegicus apolipoprotein A-V (Apoa5), mRNA [NM_080576]	**30,929**
Fam5b	Rattus norvegicus family with sequence similarity 5, member B (Fam5b), mRNA [NM_173115]	**30,925**
Add2	Rattus norvegicus adducin 2 (beta) (Add2), transcript variant 1, mRNA [NM_001109880]	**30,923**
Dgki	Rattus norvegicus diacylglycerol kinase, iota (Dgki), mRNA [NM_198782]	**30,880**
Hmg20b	Rattus norvegicus high mobility group 20 B (Hmg20b), mRNA [NM_001108731]	**30,880**
Clasp2	Rattus norvegicus cytoplasmic linker associated protein 2 (Clasp2), mRNA [NM_053722]	**30,866**
0	Q69114_EBVG (Q69114) BZLF2 (Fragment), partial (21%) [TC636606]	**30,862**
Nsdhl	Rattus norvegicus NAD(P) dependent steroid dehydrogenase-like (Nsdhl), mRNA [NM_001009399]	**30,860**
Mef2d	Rattus norvegicus myocyte enhancer factor 2D (Mef2d), mRNA [NM_030860]	**30,849**
0	Q8R111_MOUSE (Q8R111) Plekha6 protein (Fragment), partial (70%) [TC597504]	**30,849**
Zcchc24	Rattus norvegicus zinc finger, CCHC domain containing 24 (Zcchc24), mRNA [NM_001108394]	**30,840**
B3gat1	Rattus norvegicus beta-1,3-glucuronyltransferase 1 (glucuronosyltransferase P) (B3gat1), mRNA [NM_054003]	**30,828**
0	Q8IVU8_HUMAN (Q8IVU8) LOC340529 protein (Fragment), partial (54%) [TC642990]	**30,795**
Whsc1	Rattus norvegicus Wolf-Hirschhorn syndrome candidate 1 (human) (Whsc1), mRNA [NM_001191552]	**30,794**
Pppde1	Rattus norvegicus PPPDE peptidase domain containing 1 (Pppde1), mRNA [NM_001013873]	**30,785**
Psme3	Rattus norvegicus proteasome (prosome, macropain) activator subunit 3 (Psme3), mRNA [NM_001011894]	**30,769**
Gapdh-ps1	Rattus norvegicus glyceraldehyde-3-phosphate dehydrogenase, pseudogene 1 (Gapdh-ps1), non-coding RNA [NR_003722]	**30,762**
LOC688163	PREDICTED: Rattus norvegicus hypothetical protein LOC688163, transcript variant 2 (LOC688163), mRNA [XM_002724542]	**30,760**
Sema5b	Rattus norvegicus sema domain, seven thrombospondin repeats (type 1 and type 1-like), transmembrane domain (TM) and short cytoplasmic domain, (semaphorin) 5B (Sema5b), mRNA [NM_001107091]	**30,751**
Zfp278	Rattus norvegicus zinc finger protein 278, mRNA (cDNA clone IMAGE:7376163), partial cds. [BC098801]	**30,744**
Phc3	Rattus norvegicus polyhomeotic homolog 3 (Drosophila) (Phc3), mRNA [NM_001107662]	**30,741**
Ralb	Rattus norvegicus v-ral simian leukemia viral oncogene homolog B (ras related; GTP binding protein) (Ralb), mRNA [NM_053821]	**30,726**
Uba1	Rattus norvegicus ubiquitin-like modifier activating enzyme 1 (Uba1), mRNA [NM_001014080]	**30,720**
C1H6orf35	Rattus norvegicus chromosome 1 open reading frame, human C6orf35 (C1H6orf35), mRNA [NM_001144860]	**30,705**
Sh3gl2	Endophilin-A1 [Source:UniProtKB/Swiss-Prot;Acc:O35179] [ENSRNOT00000008999]	**30,700**
0	Uncharacterized protein [Source:UniProtKB/TrEMBL;Acc:D3ZSV9] [ENSRNOT00000011770]	**30,700**
Zfp418	Rattus norvegicus zinc finger protein 418 (Zfp418), mRNA [NM_001191620]	**30,691**
0	Unknown	**30,684**

LOC680138	Unknown	**30,678**
0	Rattus norvegicus similar to 60S RIBOSOMAL PROTEIN L29 (P23) (LOC294697), mRNA [XM_226719]	**30,675**
Rad21	Rattus norvegicus RAD21 homolog (S. pombe) (Rad21), mRNA [NM_001025701]	**30,668**
RGD1566073	Uncharacterized protein [Source:UniProtKB/TrEMBL;Acc:D3ZY63] [ENSRNOT00000034681]	**30,644**
Nfia	Rattus norvegicus nuclear factor I/A (Nfia), mRNA [NM_012988]	**30,628**
Sec14l1	Rattus norvegicus SEC14-like 1 (S. cerevisiae) (Sec14l1), mRNA [NM_001108309]	**30,562**
0	Calcium/calmodulin-dependent protein kinase type 1G [Source:UniProtKB/Swiss-Prot;Acc:Q7TNJ7] [ENSRNOT00000044161]	**30,552**
Rabl3	Rattus norvegicus RAB, member of RAS oncogene family-like 3 (Rabl3), mRNA [NM_001108319]	**30,547**
LOC100364190	Similar to contactin associated protein-like 2 isoform a, isoform CRA_bUncharacterized protein [Source:UniProtKB/TrEMBL;Acc:D3ZA73] [ENSRNOT00000008688]	**30,538**
Theg	Rattus norvegicus testicular haploid expressed gene (Theg), mRNA [NM_001013102]	**30,523**
Cpne6	Rattus norvegicus copine VI (Cpne6), mRNA [NM_001191113]	**30,519**
0	Unknown	**30,509**
Ptbp1	Rattus norvegicus polypyrimidine tract binding protein 1 (Ptbp1), transcript variant 2, mRNA [NM_022516]	**30,506**
Mypop	Rattus norvegicus Myb-related transcription factor, partner of profilin (Mypop), mRNA [NM_001109139]	**30,494**
Npy	Rattus norvegicus neuropeptide Y (Npy), mRNA [NM_012614]	**30,480**
Dpf1	Rattus norvegicus D4, zinc and double PHD fingers family 1 (Dpf1), mRNA [NM_001105729]	**30,446**
Glul	Rattus norvegicus glutamate-ammonia ligase (glutamine synthetase) (Glul), mRNA [NM_017073]	**30,441**
0	Unknown	**30,425**
Shc1	Rattus norvegicus SHC (Src homology 2 domain containing) transforming protein 1 (Shc1), nuclear gene encoding mitochondrial protein, transcript variant 1, mRNA [NM_001164060]	**30,422**
0	Unknown	**30,403**
0	Unknown	**30,400**
Araf	Rattus norvegicus v-raf murine sarcoma 3611 viral oncogene homolog (Araf), transcript variant 1, mRNA [NM_022532]	**30,391**
RGD1310351	Rattus norvegicus similar to RIKEN cDNA 4732418C07 (RGD1310351), mRNA [NM_001106677]	**30,391**
Cyp2d4	Cytochrome P450 2D18 [Source:UniProtKB/Swiss-Prot;Acc:Q64680] [ENSRNOT00000011880]	**30,367**
Znf609	Rattus norvegicus zinc finger protein 609 (Znf609), mRNA [NM_001173371]	**30,343**
Rab2b	Rattus norvegicus RAB2B, member RAS oncogene family, mRNA (cDNA clone IMAGE:7316063), partial cds. [BC092636]	**30,342**
Prkar2b	Rattus norvegicus protein kinase, cAMP dependent regulatory, type II beta (Prkar2b), mRNA [NM_001030020]	**30,329**
Ppapdc2	Rattus norvegicus phosphatidic acid phosphatase type 2 domain containing 2 (Ppapdc2), mRNA [NM_001034854]	**30,328**
Rerg	PREDICTED: Rattus norvegicus RAS-like, estrogen-regulated, growth-inhibitor (Rerg), mRNA [XM_578417]	**30,328**
Foxp1	Rattus norvegicus forkhead box P1 (Foxp1), mRNA [NM_001034131]	**30,309**
Spag1	Rattus norvegicus sperm associated antigen 1 (Spag1), mRNA [NM_001012116]	**30,304**
0	RVL9282 Wackym-Soares normalized rat vestibular cDNA library Rattus norvegicus cDNA 5', mRNA sequence [DV718665]	**30,267**

Eps15l1	Rattus norvegicus epidermal growth factor receptor pathway substrate 15-like 1 (Eps15l1), mRNA [NM_001029921]	**30,253**
Cfl2	Rattus norvegicus cofilin 2, muscle (Cfl2), mRNA [NM_001108982]	**30,245**
Clcn7	Rattus norvegicus chloride channel 7 (Clcn7), mRNA [NM_031568]	**30,241**
Osr1	Rattus norvegicus odd-skipped related 1 (Drosophila) (Osr1), mRNA [NM_001106716]	**30,227**
Bcap31	Rattus norvegicus B-cell receptor-associated protein 31 (Bcap31), mRNA [NM_001004224]	**30,225**
Kptn	Rattus norvegicus kaptin (actin binding protein) (Kptn), mRNA [NM_001107457]	**30,220**
Aard	Rattus norvegicus alanine and arginine rich domain containing protein (Aard), mRNA [NM_145093]	**30,211**
0	Rattus norvegicus similar to 60S RIBOSOMAL PROTEIN L29 (P23) (LOC290219), mRNA [XM_224186]	**30,202**
Zfp275	Rattus norvegicus zinc finger protein 275 (Zfp275), mRNA [NM_001106343]	**30,201**
Lif	Rattus norvegicus leukemia inhibitory factor (Lif), mRNA [NM_022196]	**30,200**
Atg4c	Rattus norvegicus ATG4 autophagy related 4 homolog C (S. cerevisiae) (Atg4c), mRNA [NM_001107948]	**30,192**
Nubp2	Rattus norvegicus nucleotide binding protein 2 (Nubp2), mRNA [NM_001011891]	**30,170**
Hdgf	Rattus norvegicus hepatoma-derived growth factor (Hdgf), mRNA [NM_053707]	**30,166**
Rnf38	Rattus norvegicus ring finger protein 38 (Rnf38), mRNA [NM_134467]	**30,166**
Tp53	Rattus norvegicus tumor protein p53 (Tp53), mRNA [NM_030989]	**30,166**
Slc6a11	Rattus norvegicus solute carrier family 6 (neurotransmitter transporter, GABA), member 11 (Slc6a11), mRNA [NM_024372]	**30,162**
RGD1307554	Rattus norvegicus similar to CG16812-PA (RGD1307554), mRNA [NM_001109664]	**30,157**
Ak1	Rattus norvegicus adenylate kinase 1 (Ak1), mRNA [NM_024349]	**30,146**
0	Unknown	**30,141**
Otx2	Rattus norvegicus orthodenticle homeobox 2 (Otx2), mRNA [NM_001100566]	**30,120**
Gps2	Rattus norvegicus G protein pathway suppressor 2 (Gps2), mRNA [NM_001017477]	**30,118**
Gnl3l	Rattus norvegicus guanine nucleotide binding protein-like 3 (nucleolar)-like (Gnl3l), mRNA [NM_001081958]	**30,107**
Kcna6	Rattus norvegicus potassium voltage gated channel, shaker related subfamily, member 6 (Kcna6), mRNA [NM_023954]	**30,105**
Cflar	Rattus norvegicus CASP8 and FADD-like apoptosis regulator (Cflar), transcript variant 2, mRNA [NM_057138]	**30,103**
Dclk2	Rattus norvegicus doublecortin-like kinase 2 (Dclk2), transcript variant 2, mRNA [NM_001009691]	**30,098**
Msn	Rattus norvegicus moesin (Msn), mRNA [NM_030863]	**30,087**
Gclc	Rattus norvegicus glutamate-cysteine ligase, catalytic subunit (Gclc), mRNA [NM_012815]	**30,078**
Stx1b	Rattus norvegicus syntaxin 1B (Stx1b), mRNA [NM_012700]	**30,051**
Pde10a	Rattus norvegicus phosphodiesterase 10A (Pde10a), mRNA [NM_022236]	**30,024**
Olr1l	Rattus norvegicus olfactory receptor 1-like (Olr1l), mRNA [NM_001000538]	**30,013**
Pkp1	Rattus norvegicus plakophilin 1 (Pkp1), mRNA [NM_001107181]	**30,013**
RGD1564637	PREDICTED: Rattus norvegicus similar to interferon alpha 8/6 precursor; IFNa8/6 (B6) (RGD1564637), mRNA [XM_001054291]	**30,004**
0	Unknown	**30,000**
Pdlim7	Rattus norvegicus PDZ and LIM domain 7 (Pdlim7), mRNA [NM_173125]	**30,000**

0	Unknown	**29,997**
Emx2	Rattus norvegicus empty spiracles homeobox 2 (Emx2), mRNA [NM_001109169]	**29,997**
Sh3kbp1	Rattus norvegicus SH3-domain kinase binding protein 1 (Sh3kbp1), mRNA [NM_053360]	**29,987**
RGD1561849	Rattus norvegicus similar to RIKEN cDNA 3110035E14 (RGD1561849), mRNA [NM_001109260]	**29,985**
Sema3c	Rattus norvegicus sema domain, immunoglobulin domain (Ig), short basic domain, secreted, (semaphorin) 3C (Sema3c), mRNA [NM_001106578]	**29,972**
Faim2	Rattus norvegicus Fas apoptotic inhibitory molecule 2 (Faim2), mRNA [NM_144756]	**29,951**
LOC685233	PREDICTED: Rattus norvegicus hypothetical protein LOC685233 (LOC685233), mRNA [XM_001062937]	**29,936**
Cacna1g	Rattus norvegicus calcium channel, voltage-dependent, T type, alpha 1G subunit (Cacna1g), mRNA [NM_031601]	**29,934**
0	Q40555_TOBAC (Q40555) Peroxidase, partial (5%) [TC599198]	**29,910**
RGD1560902	PREDICTED: Rattus norvegicus similar to Cofilin, non-muscle isoform (Cofilin-1) (RGD1560902), mRNA [XM_002724509]	**29,907**
Znf689	Rattus norvegicus zinc finger protein 689 (Znf689), mRNA [NM_173330]	**29,904**
0	Q5DBE9_SCHJA (Q5DBE9) SJCHGC01974 protein, partial (22%) [TC633657]	**29,894**
LOC290595	Rattus norvegicus hypothetical gene supported by AF152002 (LOC290595), mRNA [NM_001106063]	**29,891**
Camta2	Rattus norvegicus calmodulin binding transcription activator 2 (Camta2), mRNA [NM_001105801]	**29,855**
LOC683460	PREDICTED: Rattus norvegicus hypothetical protein LOC683460, transcript variant 2 (LOC683460), mRNA [XM_001065403]	**29,837**
RGD1559747	Rattus norvegicus similar to Zinc finger and SCAN domain containing protein 2 (Zinc finger protein 29) (RGD1559747), non-coding RNA [NR_027235]	**29,836**
Tpd52	Rattus norvegicus tumor protein D52 (Tpd52), mRNA [NM_001106421]	**29,821**
Rnf181	Rattus norvegicus ring finger protein 181 (Rnf181), mRNA [NM_001007647]	**29,798**
Tnfsf12	Rattus norvegicus tumor necrosis factor ligand superfamily member 12 (Tnfsf12), mRNA [NM_001001513]	**29,793**
LOC687381	PREDICTED: Rattus norvegicus similar to COX10 homolog, cytochrome c oxidase assembly protein, heme A: farnesyltransferase (LOC687381), mRNA [XM_001077717]	**29,788**
Galntl1	Rattus norvegicus UDP-N-acetyl-alpha-D-galactosamine:polypeptide N-acetylgalactosaminyltransferase-like 1 (Galntl1), mRNA [NM_001100863]	**29,786**
0	Rattus norvegicus TL0AEA65YB02 mRNA sequence. [FQ232612]	**29,781**
Nptxr	Rattus norvegicus neuronal pentraxin receptor (Nptxr), mRNA [NM_030841]	**29,781**
Pcyox1l	Rattus norvegicus prenylcysteine oxidase 1 like (Pcyox1l), mRNA [NM_001134542]	**29,765**
Cyp2s1	Rattus norvegicus cytochrome P450, family 2, subfamily s, polypeptide 1 (Cyp2s1), mRNA [NM_001107495]	**29,756**
Naf1	Rattus norvegicus nuclear assembly factor 1 homolog (S. cerevisiae) (Naf1), mRNA [NM_001024772]	**29,752**
Irak1	Rattus norvegicus interleukin-1 receptor-associated kinase 1 (Irak1), mRNA [NM_001127555]	**29,743**
Zfp316	Rattus norvegicus zinc finger protein 316 (Zfp316), mRNA [NM_001107121]	**29,742**
Fnbp1	Rattus norvegicus formin binding protein 1 (Fnbp1), mRNA [NM_138914]	**29,734**
H6pd	Rattus norvegicus hexose-6-phosphate dehydrogenase (glucose 1-dehydrogenase) (H6pd), mRNA [NM_001106698]	**29,724**
Zfp469	Rattus norvegicus zinc finger protein 469 (Zfp469), mRNA [NM_001107123]	**29,720**
0	Rattus norvegicus similar to myosin regulatory light chain-like (LOC316125), mRNA [XM_236776]	**29,697**

Pex19	Rattus norvegicus peroxisomal biogenesis factor 19 (Pex19), transcript variant 1, mRNA [NM_001107375]	**29,691**
Snapc2	Rattus norvegicus small nuclear RNA activating complex, polypeptide 2 (Snapc2), mRNA [NM_001013121]	**29,685**
Pard3b	Rattus norvegicus par-3 partitioning defective 3 homolog B (C. elegans) (Pard3b), mRNA [NM_001191808]	**29,680**
LOC498350	Rattus norvegicus similar to testicular haploid expressed gene product isoform 2 (LOC498350), mRNA [NM_001017498]	**29,669**
Tbx19	Rattus norvegicus T-box 19 (Tbx19), mRNA [NM_001107193]	**29,667**
Runx1t1	Rattus norvegicus runt-related transcription factor 1; translocated to, 1 (cyclin D-related) (Runx1t1), mRNA [NM_001108657]	**29,661**
0	Unknown	**29,661**
Dnajc12	Rattus norvegicus DnaJ (Hsp40) homolog, subfamily C, member 12 (Dnajc12), mRNA [NM_001034032]	**29,661**
Srebf2	Rattus norvegicus sterol regulatory element binding transcription factor 2 (Srebf2), mRNA [NM_001033694]	**29,653**
Cxcl12	Rattus norvegicus chemokine (C-X-C motif) ligand 12 (stromal cell-derived factor 1) (Cxcl12), transcript variant 1, mRNA [NM_022177]	**29,643**
Syvn1	Rattus norvegicus synovial apoptosis inhibitor 1, synoviolin (Syvn1), mRNA [NM_001100739]	**29,638**
0	Unknown	**29,638**
Ccdc127	Rattus norvegicus coiled-coil domain containing 127 (Ccdc127), mRNA [NM_198766]	**29,637**
Arhgef18	Rattus norvegicus rho/rac guanine nucleotide exchange factor (GEF) 18 (Arhgef18), mRNA [NM_001107115]	**29,636**
Arhgef7	Rattus norvegicus Rho guanine nucleotide exchange factor (GEF7) (Arhgef7), transcript variant 1, mRNA [NM_001113521]	**29,614**
Nfib	Rattus norvegicus nuclear factor I/B (Nfib), mRNA [NM_031566]	**29,606**
Erlin1	Rattus norvegicus ER lipid raft associated 1 (Erlin1), mRNA [NM_001106353]	**29,603**
0	Rattus norvegicus similar to 60S RIBOSOMAL PROTEIN L29 (P23) (LOC295470), mRNA [XM_227709]	**29,599**
Mapre1	Rattus norvegicus microtubule-associated protein, RP/EB family, member 1 (Mapre1), mRNA [NM_138509]	**29,588**
Scn2a1	Rattus norvegicus sodium channel, voltage-gated, type II, alpha 1 (Scn2a1), mRNA [NM_012647]	**29,586**
0	Unknown	**29,579**
Wipf1	Rattus norvegicus WAS/WASL interacting protein family, member 1 (Wipf1), mRNA [NM_057192]	**29,577**
Xpnpep3	Rattus norvegicus X-prolyl aminopeptidase (aminopeptidase P) 3, putative (Xpnpep3), mRNA [NM_001130582]	**29,568**
LOC501618	Rattus norvegicus LRRGT00179 (LOC501618), mRNA [NM_001047970]	**29,564**
Pcdhb20	Rattus norvegicus protocadherin beta 20 (Pcdhb20), mRNA [NM_001109395]	**29,548**
40057	Rattus norvegicus septin 9 (Sept9), transcript variant 2, mRNA [NM_176856]	**29,548**
Csnk1e	Rattus norvegicus casein kinase 1, epsilon (Csnk1e), mRNA [NM_031617]	**29,545**
Nedd4l	Rattus norvegicus neural precursor cell expressed, developmentally down-regulated 4-like (Nedd4l), mRNA [NM_001008300]	**29,538**
0	Unknown	**29,515**
LOC680692	PREDICTED: Rattus norvegicus similar to Golgi phosphoprotein 2 (Golgi membrane protein GP73) (LOC680692), mRNA [XM_002725221]	**29,514**
0	Unknown	**29,508**
Carhsp1	Rattus norvegicus calcium regulated heat stable protein 1 (Carhsp1), mRNA [NM_152790]	**29,496**
Slc31a1	Rattus norvegicus solute carrier family 31 (copper transporters), member 1 (Slc31a1), mRNA [NM_133600]	**29,491**
0	Unknown	**29,487**

0	Unknown	**29,460**
Dnajb9	Rattus norvegicus DnaJ (Hsp40) homolog, subfamily B, member 9 (Dnajb9), mRNA [NM_012699]	**29,454**
Apc	Rattus norvegicus adenomatous polyposis coli (Apc), mRNA [NM_012499]	**29,452**
Rims1	Rattus norvegicus regulating synaptic membrane exocytosis 1 (Rims1), mRNA [NM_052829]	**29,441**
Tmem229b	Rattus norvegicus transmembrane protein 229B (Tmem229b), mRNA [NM_001109359]	**29,424**
Bmp4	Rattus norvegicus bone morphogenetic protein 4 (Bmp4), mRNA [NM_012827]	**29,411**
Mxi1	Rattus norvegicus MAX interactor 1 (Mxi1), mRNA [NM_013160]	**29,409**
Mtus1	Rattus norvegicus mitochondrial tumor suppressor 1 (Mtus1), nuclear gene encoding mitochondrial protein, mRNA [NM_178093]	**29,398**
LOC689103	Uncharacterized protein [Source:UniProtKB/TrEMBL;Acc:D3ZX44] [ENSRNOT00000025346]	**29,391**
LOC302495	Rattus norvegicus hypothetical LOC302495 (LOC302495), mRNA [NM_001106950]	**29,383**
Anxa11	Rattus norvegicus annexin A11 (Anxa11), mRNA [NM_001011918]	**29,380**
LOC500013	PREDICTED: Rattus norvegicus similar to sterile alpha motif domain containing 9-like (LOC500013), miscRNA [XR_005677]	**29,365**
0	Unknown	**29,360**
Ntsr1	Rattus norvegicus neurotensin receptor 1 (Ntsr1), mRNA [NM_001108967]	**29,359**
Popdc2	Rattus norvegicus popeye domain containing 2 (Popdc2), mRNA [NM_199113]	**29,353**
Atp2b4	Rattus norvegicus ATPase, Ca++ transporting, plasma membrane 4 (Atp2b4), mRNA [NM_001005871]	**29,346**
Sos1	Rattus norvegicus Son of sevenless homolog 1 (Drosophila) (Sos1), mRNA [NM_001100716]	**29,322**
Usp31	Rattus norvegicus ubiquitin specific peptidase 31 (Usp31), mRNA [NM_001107548]	**29,303**
Sigmar1	Rattus norvegicus sigma non-opioid intracellular receptor 1 (Sigmar1), mRNA [NM_030996]	**29,303**
Fam20b	Rattus norvegicus family with sequence similarity 20, member B (Fam20b), mRNA [NM_001107187]	**29,299**
Nudt7	Rattus norvegicus nudix (nucleoside diphosphate linked moiety X)-type motif 7 (Nudt7), mRNA [NM_001108450]	**29,279**
Il10rb	Rattus norvegicus interleukin 10 receptor, beta (Il10rb), mRNA [NM_001107111]	**29,257**
Srpr	Rattus norvegicus signal recognition particle receptor ('docking protein') (Srpr), mRNA [NM_001034150]	**29,241**
Cacna1h	Rattus norvegicus calcium channel, voltage-dependent, T type, alpha 1H subunit (Cacna1h), mRNA [NM_153814]	**29,237**
0	PREDICTED: Rattus norvegicus similar to Myosin light chain 1 slow a (RGD1560334), mRNA [XM_228900]	**29,224**
Tnfrsf21	Rattus norvegicus tumor necrosis factor receptor superfamily, member 21 (Tnfrsf21), mRNA [NM_001108207]	**29,215**
Ccdc94	Rattus norvegicus coiled-coil domain containing 94 (Ccdc94), mRNA [NM_001109673]	**29,213**
Dbn1	Rattus norvegicus drebrin 1 (Dbn1), mRNA [NM_031024]	**29,211**
Tmem25	Rattus norvegicus transmembrane protein 25 (Tmem25), mRNA [NM_001109528]	**29,205**
Rab6b	Rattus norvegicus RAB6B, member RAS oncogene family (Rab6b), mRNA [NM_001108775]	**29,181**
RGD1565959	Rattus norvegicus RGD1565959 (RGD1565959), mRNA [NM_001106886]	**29,177**
Cdh6	Rattus norvegicus cadherin 6 (Cdh6), mRNA [NM_012927]	**29,176**
Npb	Rattus norvegicus neuropeptide B (Npb), mRNA [NM_153293]	**29,171**
Pip5k1c	Rattus norvegicus phosphatidylinositol-4-phosphate 5-kinase, type I, gamma (Pip5k1c), transcript variant b, mRNA [NM_001033970]	**29,169**

Gpr4	Rattus norvegicus G protein-coupled receptor 4 (Gpr4), mRNA [NM_001025680]	**29,114**
Cadm1	Rattus norvegicus cell adhesion molecule 1 (Cadm1), mRNA [NM_001012201]	**29,111**
Treh	Rattus norvegicus trehalase (brush-border membrane glycoprotein) (Treh), mRNA [NM_001136141]	**29,108**
Tnr	Rattus norvegicus tenascin R (Tnr), mRNA [NM_013045]	**29,106**
0	Unknown	**29,105**
0	Uncharacterized protein [Source:UniProtKB/TrEMBL;Acc:D3ZEV1] [ENSRNOT00000041121]	**29,097**
Tp53	Rattus norvegicus tumor protein p53 (Tp53), mRNA [NM_030989]	**29,084**
Tgfb3	Rattus norvegicus transforming growth factor, beta 3 (Tgfb3), mRNA [NM_013174]	**29,081**
Zcchc11	Rattus norvegicus zinc finger, CCHC domain containing 11 (Zcchc11), mRNA [NM_001107953]	**29,072**
Csnk1e	Rattus norvegicus casein kinase 1, epsilon (Csnk1e), mRNA [NM_031617]	**29,057**
0	Unknown	**29,054**
0	Uncharacterized protein [Source:UniProtKB/TrEMBL;Acc:D3ZGV8] [ENSRNOT00000004410]	**29,045**
Itpripl2	Rattus norvegicus inositol 1,4,5-triphosphate receptor interacting protein-like 2 (Itpripl2), mRNA [NM_001127303]	**29,039**
Sssca1	Rattus norvegicus Sjogren's syndrome/scleroderma autoantigen 1 homolog (human) (Sssca1), mRNA [NM_001109537]	**29,035**
Sap30bp	Rattus norvegicus SAP30 binding protein (Sap30bp), mRNA [NM_001108305]	**29,025**
Pgrmc2	Rattus norvegicus progesterone receptor membrane component 2 (Pgrmc2), mRNA [NM_001008374]	**29,021**
Lsm16	PREDICTED: Rattus norvegicus LSM16 homolog (EDC3, S. cerevisiae), transcript variant 1 (Lsm16), mRNA [XM_001072079]	**29,021**
Bmp15	Rattus norvegicus bone morphogenetic protein 15 (Bmp15), mRNA [NM_021670]	**29,015**
Plekhh1	Rattus norvegicus pleckstrin homology domain containing, family H (with MyTH4 domain) member 1 (Plekhh1), mRNA [NM_001108036]	**29,014**
0	NRX3A_RAT (Q07310) Neurexin-3-alpha precursor (Neurexin III-alpha), complete [TC573962]	**29,002**
Zdhhc3	Rattus norvegicus zinc finger, DHHC-type containing 3 (Zdhhc3), mRNA [NM_001039014]	**28,990**
Lgals5	Rattus norvegicus lectin, galactose binding, soluble 5 (Lgals5), mRNA [NM_012976]	**28,990**
0	Unknown	**28,983**
LOC687483	PREDICTED: Rattus norvegicus hypothetical protein LOC687483 (LOC687483), mRNA [XM_001078767]	**28,981**
Gabbr1	Rattus norvegicus gamma-aminobutyric acid (GABA) B receptor 1 (Gabbr1), mRNA [NM_031028]	**28,977**
Scarb1	Rattus norvegicus scavenger receptor class B, member 1 (Scarb1), mRNA [NM_031541]	**28,963**
Fam184b	Uncharacterized protein [Source:UniProtKB/TrEMBL;Acc:D3ZTZ0] [ENSRNOT00000004923]	**28,962**
RGD1561962	Rattus norvegicus TL0ABA17YG18 mRNA sequence. [FQ211110]	**28,957**
LOC689656	Uncharacterized protein [Source:UniProtKB/TrEMBL;Acc:D3ZUC4] [ENSRNOT00000039322]	**28,954**
Kcnq3	Rattus norvegicus potassium voltage-gated channel, KQT-like subfamily, member 3 (Kcnq3), mRNA [NM_031597]	**28,921**
Ocel1	Rattus norvegicus occludin/ELL domain containing 1 (Ocel1), mRNA [NM_001106065]	**28,917**
Tex10	Rattus norvegicus testis expressed 10 (Tex10), mRNA [NM_001106653]	**28,902**
Ddx17	Rattus norvegicus DEAD (Asp-Glu-Ala-Asp) box polypeptide 17 (Ddx17), mRNA [NM_001015018]	**28,902**
Kcnk3	Rattus norvegicus potassium channel, subfamily K, member 3 (Kcnk3), mRNA [NM_033376]	**28,890**

Mbtps2	Rattus norvegicus membrane-bound transcription factor peptidase, site 2 (Mbtps2), mRNA [NM_001035007]	**28,878**
Tgfa	Rattus norvegicus transforming growth factor alpha (Tgfa), mRNA [NM_012671]	**28,856**
Glis2	Rattus norvegicus GLIS family zinc finger 2 (Glis2), mRNA [NM_001106978]	**28,840**
Gabpb1	Rattus norvegicus GA binding protein transcription factor, beta subunit 1 (Gabpb1), mRNA [NM_001039036]	**28,824**
Znf579	Rattus norvegicus zinc finger protein 579 (Znf579), mRNA [NM_001126276]	**28,807**
Fbxo16	Rattus norvegicus F-box protein 16 (Fbxo16), mRNA [NM_001013132]	**28,805**
0	Rattus norvegicus similar to splicing-related factor RNPS1 (LOC312994), mRNA [XM_232688]	**28,801**
Otud7b	Rattus norvegicus OTU domain containing 7B (Otud7b), mRNA [NM_001107697]	**28,800**
Tiam1	Rattus norvegicus T-cell lymphoma invasion and metastasis 1 (Tiam1), mRNA [NM_001100558]	**28,791**
0	Rattus norvegicus similar to peroxiredoxin 6 (LOC287416), mRNA [XM_220591]	**28,783**
0	Unknown	**28,783**
0	Uncharacterized protein [Source:UniProtKB/TrEMBL;Acc:D3ZDV6] [ENSRNOT00000011793]	**28,781**
RGD1560286	Rattus norvegicus similar to DNA segment, Chr 4, ERATO Doi 22, expressed (RGD1560286), transcript variant 1, mRNA [NM_001114599]	**28,772**
Lims2	Rattus norvegicus LIM and senescent cell antigen like domains 2 (Lims2), mRNA [NM_001012163]	**28,769**
0	Unknown	**28,761**
Abcc1	Rattus norvegicus ATP-binding cassette, subfamily C (CFTR/MRP), member 1 (Abcc1), mRNA [NM_022281]	**28,753**
RGD1565236	Rattus norvegicus similar to transcription factor (RGD1565236), mRNA [NM_001109261]	**28,750**
Gbp2	Rattus norvegicus guanylate binding protein 2 (Gbp2), mRNA [NM_133624]	**28,745**
0	Uncharacterized protein [Source:UniProtKB/TrEMBL;Acc:D3ZJB6] [ENSRNOT00000040584]	**28,739**
Cryab	Rattus norvegicus crystallin, alpha B (Cryab), mRNA [NM_012935]	**28,736**
0	Unknown	**28,723**
Neurod6	Rattus norvegicus neurogenic differentiation 6 (Neurod6), mRNA [NM_001109237]	**28,716**
Mxd1	Rattus norvegicus max dimerization protein 1 (Mxd1), mRNA [NM_001100749]	**28,712**
Rmnd5a	PREDICTED: Rattus norvegicus required for meiotic nuclear division 5 homolog A (S. cerevisiae) (Rmnd5a), mRNA [XM_002726419]	**28,692**
Adprhl1	Rattus norvegicus ADP-ribosylhydrolase like 1 (Adprhl1), mRNA [NM_001013054]	**28,687**
Clcn4-2	Rattus norvegicus chloride channel 4-2 (Clcn4-2), mRNA [NM_022198]	**28,685**
Ttc7b	Rattus norvegicus tetratricopeptide repeat domain 7B (Ttc7b), mRNA [NM_001108719]	**28,683**
Pcyt1a	Rattus norvegicus phosphate cytidylyltransferase 1, choline, alpha (Pcyt1a), mRNA [NM_078622]	**28,670**
Tnip1	Rattus norvegicus TNFAIP3 interacting protein 1 (Tnip1), mRNA [NM_001108826]	**28,646**
Dmwd	PREDICTED: Rattus norvegicus dystrophia myotonica, WD repeat containing (Dmwd), mRNA [XM_002725588]	**28,638**
Mxra7	PREDICTED: Rattus norvegicus matrix-remodelling associated 7 (Mxra7), mRNA [XM_002724653]	**28,637**
Cabin1	Rattus norvegicus calcineurin binding protein 1 (Cabin1), mRNA [NM_053575]	**28,634**
Mnt	Rattus norvegicus max binding protein (Mnt), mRNA [NM_001105807]	**28,632**
Gast	Rattus norvegicus gastrin (Gast), mRNA [NM_012849]	**28,631**

Shank3	Rattus norvegicus SH3 and multiple ankyrin repeat domains 3 (Shank3), mRNA [NM_021676]	**28,628**
0	Rattus norvegicus TL0ADA41YI21 mRNA sequence. [FQ220826]	**28,626**
0	Q4RY53_TETNG (Q4RY53) Chromosome 3 SCAF14978, whole genome shotgun sequence. (Fragment), partial (3%) [TC596344]	**28,611**
Rnf14	Rattus norvegicus ring finger protein 14 (Rnf14), mRNA [NM_001034995]	**28,611**
Nf1	Rattus norvegicus neurofibromin 1 (Nf1), mRNA [NM_012609]	**28,598**
Scamp5	Rattus norvegicus secretory carrier membrane protein 5 (Scamp5), mRNA [NM_031726]	**28,598**
Tp53i11	Rattus norvegicus tumor protein p53 inducible protein 11 (Tp53i11), mRNA [NM_001107749]	**28,591**
Rab1b	Rattus norvegicus RAB1B, member RAS oncogene family (Rab1b), mRNA [NM_001109979]	**28,590**
Naga	Rattus norvegicus N-acetyl galactosaminidase, alpha (Naga), mRNA [NM_001012120]	**28,589**
Hsf2	Rattus norvegicus heat shock transcription factor 2 (Hsf2), mRNA [NM_031694]	**28,560**
0	RCG55642Uncharacterized protein [Source:UniProtKB/TrEMBL;Acc:D4AB34] [ENSRNOT00000051810]	**28,550**
Ndufb4	Rattus norvegicus NADH dehydrogenase (ubiquinone) 1 beta subcomplex 4 (Ndufb4), nuclear gene encoding mitochondrial protein, mRNA [NM_001037338]	**28,547**
0	PREDICTED: Rattus norvegicus hypothetical protein LOC100364461 (LOC100364461), mRNA [XM_002726605]	**28,545**
Scai	PREDICTED: Rattus norvegicus similar to Protein C9orf126 homolog (LOC690538), mRNA [XM_001074743]	**28,540**
RGD1305572	Rattus norvegicus similar to hypothetical protein MGC30618 (RGD1305572), mRNA [NM_001024970]	**28,540**
Snai1	Rattus norvegicus snail homolog 1 (Drosophila) (Snai1), mRNA [NM_053805]	**28,531**
Casp2	Rattus norvegicus caspase 2 (Casp2), mRNA [NM_022522]	**28,490**
Gpr155	Rattus norvegicus G protein-coupled receptor 155 (Gpr155), mRNA [NM_001107811]	**28,484**
Bhlhe41	PREDICTED: Rattus norvegicus basic helix-loop-helix family, member e41 (Bhlhe41), mRNA [XM_002729454]	**28,479**
Atp6v0c	Rattus norvegicus ATPase, H+ transporting, lysosomal V0 subunit C (Atp6v0c), mRNA [NM_130823]	**28,444**
Perld1	Rattus norvegicus per1-like domain containing 1 (Perld1), mRNA [NM_001143895]	**28,443**
0	Unknown	**28,440**
Wdfy3	Rattus norvegicus WD repeat and FYVE domain containing 3 (Wdfy3), mRNA [NM_001170551]	**28,433**
Ncoa3	PREDICTED: Rattus norvegicus nuclear receptor coactivator 3 (Ncoa3), mRNA [XM_215947]	**28,428**
Tstd2	Rattus norvegicus thiosulfate sulfurtransferase (rhodanese)-like domain containing 2 (Tstd2), mRNA [NM_001108663]	**28,425**
Gcap14	PREDICTED: Rattus norvegicus granule cell antiserum positive 14 (Gcap14), mRNA [XM_224672]	**28,422**
0	PREDICTED: Rattus norvegicus ubiquitination factor E4B (Ube4b), mRNA [XM_233679]	**28,418**
0	Unknown	**28,416**
Napa	Rattus norvegicus N-ethylmaleimide-sensitive factor attachment protein, alpha (Napa), mRNA [NM_080585]	**28,391**
Clec16a	PREDICTED: Rattus norvegicus C-type lectin domain family 16, member A (Clec16a), mRNA [XM_213209]	**28,386**
0	Q7TQM0_MOUSE (Q7TQM0) Fbxo10 protein (Fragment), partial (7%) [TC646406]	**28,383**
Sumo3	Rattus norvegicus SMT3 suppressor of mif two 3 homolog 3 (S. cerevisiae) (Sumo3), mRNA [NM_001024295]	**28,382**
Scara3	Rattus norvegicus scavenger receptor class A, member 3 (Scara3), mRNA [NM_001108870]	**28,377**

Tnk2	Rattus norvegicus tyrosine kinase, non-receptor, 2 (Tnk2), mRNA [NM_001008336]	**28,377**
LOC680222	LOC680222 protein [Source:UniProtKB/TrEMBL;Acc:B2GNF2] [ENSRNOT00000047256]	**28,376**
Dchs1	Rattus norvegicus dachsous 1 (Drosophila) (Dchs1), mRNA [NM_001107544]	**28,376**
Smarcd1	Rattus norvegicus SWI/SNF related, matrix associated, actin dependent regulator of chromatin, subfamily d, member 1 (Smarcd1), mRNA [NM_001108752]	**28,355**
0	Unknown	**28,334**
St3gal2	Rattus norvegicus ST3 beta-galactoside alpha-2,3-sialyltransferase 2 (St3gal2), mRNA [NM_031695]	**28,324**
Scarf2	Rattus norvegicus scavenger receptor class F, member 2 (Scarf2), mRNA [NM_001105864]	**28,322**
Cnpy3	Rattus norvegicus canopy 3 homolog (zebrafish) (Cnpy3), mRNA [NM_001134710]	**28,318**
Xkr8	Rattus norvegicus XK, Kell blood group complex subunit-related family, member 8 (Xkr8), mRNA [NM_001012099]	**28,303**
Sspn	Rattus norvegicus sarcospan (Sspn), mRNA [NM_001109255]	**28,293**
Afmid	Rattus norvegicus arylformamidase (Afmid), mRNA [NM_001111366]	**28,285**
Golga1	Rattus norvegicus golgi autoantigen, golgin subfamily a, 1 (Golga1), mRNA [NM_001107842]	**28,265**
Cpsf2	Rattus norvegicus cleavage and polyadenylation specific factor 2 (Cpsf2), mRNA [NM_001106753]	**28,260**
LOC680643	PREDICTED: Rattus norvegicus similar to MIC2 like 1 (LOC680643), mRNA [XM_001058120]	**28,236**
Prl6a1	Rattus norvegicus prolactin family 6, subfamily a, member 1 (Prl6a1), mRNA [NM_022176]	**28,220**
Slc35f2	Rattus norvegicus solute carrier family 35, member F2 (Slc35f2), mRNA [NM_001106822]	**28,185**
LOC689158	PREDICTED: Rattus norvegicus similar to 60S ribosomal protein L29 (LOC689158), mRNA [XM_002727076]	**28,184**
0	Solute carrier family 39 (Zinc transporter), member 10 (Predicted), isoform CRA_aUncharacterized protein [Source:UniProtKB/TrEMBL;Acc:D4A517] [ENSRNOT00000016262]	**28,180**
S100a3	Rattus norvegicus S100 calcium binding protein A3 (S100a3), mRNA [NM_053681]	**28,178**
Baalc	Rattus norvegicus brain and acute leukemia, cytoplasmic (Baalc), mRNA [NM_144762]	**28,173**
Cyp1b1	Rattus norvegicus cytochrome P450, family 1, subfamily b, polypeptide 1 (Cyp1b1), mRNA [NM_012940]	**28,167**
Reep3	Rattus norvegicus receptor accessory protein 3 (Reep3), mRNA [NM_001106386]	**28,165**
Fkbp9	Rattus norvegicus FK506 binding protein 9 (Fkbp9), mRNA [NM_001007646]	**28,163**
Npas2	Rattus norvegicus neuronal PAS domain protein 2 (Npas2), mRNA [NM_001108214]	**28,159**
0	Uncharacterized protein [Source:UniProtKB/TrEMBL;Acc:D3Z8T0] [ENSRNOT00000041537]	**28,154**
Slc24a2	Rattus norvegicus solute carrier family 24 (sodium/potassium/calcium exchanger), member 2 (Slc24a2), mRNA [NM_031743]	**28,153**
0	Unknown	**28,151**
Cdc42se1	Rattus norvegicus CDC42 small effector 1 (Cdc42se1), mRNA [NM_001039044]	**28,149**
0	Uncharacterized protein [Source:UniProtKB/TrEMBL;Acc:D3ZCM5] [ENSRNOT00000024501]	**28,139**
Gan	Rattus norvegicus gigaxonin (Gan), mRNA [NM_001107434]	**28,135**
Iffo2	Rattus norvegicus intermediate filament family orphan 2 (Iffo2), mRNA [NM_001134703]	**28,127**
RGD1311575	Uncharacterized protein [Source:UniProtKB/TrEMBL;Acc:D4A5F4] [ENSRNOT00000002908]	**28,126**

Ppp2r3a	Rattus norvegicus protein phosphatase 2, regulatory subunit B'', alpha (Ppp2r3a), mRNA [NM_001012202]	**28,116**
Sfrs15	Rattus norvegicus splicing factor, arginine/serine-rich 15 (Sfrs15), mRNA [NM_001037347]	**28,103**
Cacna1c	Rattus norvegicus calcium channel, voltage-dependent, L type, alpha 1C subunit (Cacna1c), mRNA [NM_012517]	**28,096**
Dpf2	Rattus norvegicus D4, zinc and double PHD fingers family 2 (Dpf2), mRNA [NM_001108516]	**28,091**
Slc38a9	Rattus norvegicus solute carrier family 38, member 9 (Slc38a9), mRNA [NM_001035251]	**28,086**
Sp4	Rattus norvegicus Sp4 transcription factor (Sp4), mRNA [NM_012761]	**28,073**
Phyhipl	Rattus norvegicus phytanoyl-CoA 2-hydroxylase interacting protein-like (Phyhipl), mRNA [NM_001012076]	**28,050**
Kndc1	PREDICTED: Rattus norvegicus kinase non-catalytic C-lobe domain (KIND) containing 1 (Kndc1), mRNA [XM_002725727]	**28,050**
RGD1305823	Rattus norvegicus similar to RIKEN cDNA 0610037P05 (RGD1305823), mRNA [NM_001108261]	**28,046**
LOC685067	PREDICTED: Rattus norvegicus similar to guanylate binding protein family, member 6 (LOC685067), miscRNA [XR_006095]	**28,034**
0	reversion-inducing cysteine-rich protein with Kazal motifs [Source:RefSeq peptide;Acc:NP_001101424] [ENSRNOT00000020755]	**28,013**
Egr1	Early growth response protein 1 [Source:UniProtKB/Swiss-Prot;Acc:P08154] [ENSRNOT00000026303]	**27,990**
RGD1560891	RCG36219Uncharacterized protein [Source:UniProtKB/TrEMBL;Acc:D4ACQ4] [ENSRNOT00000044006]	**27,990**
Capza1	Rattus norvegicus capping protein (actin filament) muscle Z-line, alpha 1 (Capza1), mRNA [NM_001109625]	**27,984**
Tfcp2l1	Rattus norvegicus transcription factor CP2-like 1 (Tfcp2l1), mRNA [NM_001107170]	**27,966**
Gnai1	Rattus norvegicus guanine nucleotide binding protein (G protein), alpha inhibiting 1 (Gnai1), mRNA [NM_013145]	**27,960**
Elovl2	Rattus norvegicus elongation of very long chain fatty acids (FEN1/Elo2, SUR4/Elo3, yeast)-like 2 (Elovl2), mRNA [NM_001109118]	**27,957**
RGD1561472	PREDICTED: Rattus norvegicus similar to mKIAA2005 protein (RGD1561472), miscRNA [XR_008610]	**27,956**
Lima1	Rattus norvegicus LIM domain and actin binding 1 (Lima1), mRNA [NM_001191615]	**27,954**
Kcnip1	Rattus norvegicus Kv channel-interacting protein 1 (Kcnip1), mRNA [NM_022929]	**27,951**
Serpinh1	Rattus norvegicus serine (or cysteine) peptidase inhibitor, clade H, member 1 (Serpinh1), mRNA [NM_017173]	**27,943**
LOC685438	PREDICTED: Rattus norvegicus similar to paired immunoglobin-like type 2 receptor beta (LOC685438), mRNA [XM_001063789]	**27,942**
0	Rattus norvegicus TL0ADA47YE11 mRNA sequence. [FQ229630]	**27,923**
Ccdc6	Uncharacterized protein [Source:UniProtKB/TrEMBL;Acc:D4AEK9] [ENSRNOT00000032159]	**27,919**
Cyb5b	Rattus norvegicus cytochrome b5 type B (outer mitochondrial membrane) (Cyb5b), nuclear gene encoding mitochondrial protein, mRNA [NM_030586]	**27,916**
Sorbs2	Rattus norvegicus sorbin and SH3 domain containing 2 (Sorbs2), mRNA [NM_053770]	**27,916**
0	Unknown	**27,911**
Plch1	Rattus norvegicus TL0AAA86YN08 mRNA sequence. [FQ211591]	**27,894**
Agtrap	Rattus norvegicus angiotensin II receptor-associated protein (Agtrap), mRNA [NM_001007654]	**27,885**
Sorcs1	Rattus norvegicus sortilin-related VPS10 domain containing receptor 1 (Sorcs1), mRNA [NM_001191563]	**27,884**
0	Rapgefl1 protein [Source:UniProtKB/TrEMBL;Acc:Q5BJR9] [ENSRNOT00000030425]	**27,878**
Gspt1	Rattus norvegicus G1 to S phase transition 1 (Gspt1), mRNA [NM_001003978]	**27,871**
Wwox	Rattus norvegicus WW domain-containing oxidoreductase (Wwox), mRNA [NM_001106188]	**27,861**

Cul2	Rattus norvegicus cullin 2 (Cul2), mRNA [NM_001108417]	**27,853**
0	Q4RQ24_TETNG (Q4RQ24) Chromosome 17 SCAF15006, whole genome shotgun sequence, partial (4%) [TC582327]	**27,852**
0	Unknown	**27,844**
Dbn1	Rattus norvegicus drebrin 1 (Dbn1), mRNA [NM_031024]	**27,841**
Gpr158	Rattus norvegicus G protein-coupled receptor 158 (Gpr158), mRNA [NM_001170326]	**27,826**
Sfrp1	PREDICTED: Rattus norvegicus secreted frizzled-related protein 1 (Sfrp1), mRNA [XM_001072532]	**27,826**
Mrpl47	Rattus norvegicus mitochondrial ribosomal protein L47 (Mrpl47), nuclear gene encoding mitochondrial protein, mRNA [NM_001037183]	**27,819**
LOC305633	Rattus norvegicus similar to Antxr2 protein, mRNA (cDNA clone IMAGE:7322598). [BC088294]	**27,818**
Tm9sf1	Rattus norvegicus transmembrane 9 superfamily member 1 (Tm9sf1), mRNA [NM_001012155]	**27,801**
Angel1	Rattus norvegicus angel homolog 1 (Drosophila) (Angel1), mRNA [NM_001108717]	**27,801**
Ap1s2	Rattus norvegicus adaptor-related protein complex 1, sigma 2 subunit (Ap1s2), mRNA [NM_001127531]	**27,798**
0	AW143179 EST293475 Normalized rat brain, Bento Soares Rattus sp. cDNA clone RGIBF04 5' end, mRNA sequence [AW143179]	**27,791**
Entpd5	Rattus norvegicus ectonucleoside triphosphate diphosphohydrolase 5 (Entpd5), mRNA [NM_199394]	**27,772**
RGD1311345	Rattus norvegicus similar to CG9752-PA (RGD1311345), mRNA [NM_001173436]	**27,761**
0	Unknown	**27,731**
37865	Rattus norvegicus septin 3 (Sept3), mRNA [NM_019375]	**27,723**
RGD1560286	Rattus norvegicus similar to DNA segment, Chr 4, ERATO Doi 22, expressed (RGD1560286), transcript variant 1, mRNA [NM_001114599]	**27,714**
Appbp2	Rattus norvegicus amyloid beta precursor protein (cytoplasmic tail) binding protein 2 (Appbp2), mRNA [NM_001100969]	**27,709**
Sumo2	Rattus norvegicus SMT3 suppressor of mif two 3 homolog 2 (S. cerevisiae) (Sumo2), mRNA [NM_133594]	**27,706**
Gstm4	Rattus norvegicus glutathione S-transferase mu 4 (Gstm4), mRNA [NM_001024304]	**27,705**
Zzef1	Uncharacterized protein [Source:UniProtKB/TrEMBL;Acc:D3ZG78] [ENSRNOT00000036278]	**27,696**
Nr2e1	Rattus norvegicus nuclear receptor subfamily 2, group E, member 1 (Nr2e1), mRNA [NM_001113197]	**27,683**
0	Shultzomica03080 Rat lung airway and parenchyma cDNA libraries Rattus norvegicus cDNA clone Contig2706 5', mRNA sequence [CF109829]	**27,652**
0	Unknown	**27,650**
Brunol6	Rattus norvegicus bruno-like 6, RNA binding protein (Drosophila) (Brunol6), mRNA [NM_001106827]	**27,648**
Api5	Rattus norvegicus apoptosis inhibitor 5 (Api5), mRNA [NM_001127379]	**27,642**
Zcchc2	Rattus norvegicus zinc finger, CCHC domain containing 2 (Zcchc2), mRNA [NM_001122677]	**27,637**
RGD1307493	PREDICTED: Rattus norvegicus similar to membrane protein expressed in epithelial-like lung adenocarcinoma (RGD1307493), mRNA [XM_001080742]	**27,628**
Vma21	PREDICTED: Rattus norvegicus similar to 2610030H06Rik protein (RGD1566155), mRNA [XM_001067193]	**27,619**
0	Rattus norvegicus similar to glyceraldehyde-3-phosphate dehydrogenase (phosphorylating) (EC 1.2.1.12) - mouse (LOC363486), mRNA [XM_343809]	**27,617**
Pld1	Rattus norvegicus phospholipase D1 (Pld1), mRNA [NM_030992]	**27,603**
0	Unknown	**27,600**

Ace	Rattus norvegicus angiotensin I converting enzyme (peptidyl-dipeptidase A) 1 (Ace), mRNA [NM_012544]	**27,596**
RGD1560612	Uncharacterized protein [Source:UniProtKB/TrEMBL;Acc:D3ZKV2] [ENSRNOT00000041680]	**27,593**
RGD1308907	Rattus norvegicus similar to FLJ20689 (RGD1308907), mRNA [NM_001134559]	**27,588**
Nfat5	Rattus norvegicus nuclear factor of activated T-cells 5 (Nfat5), mRNA [NM_001107425]	**27,567**
Irs1	Rattus norvegicus insulin receptor substrate 1 (Irs1), mRNA [NM_012969]	**27,554**
Akap6	Rattus norvegicus A kinase (PRKA) anchor protein 6 (Akap6), mRNA [NM_022618]	**27,543**
0	Unknown	**27,536**
0	slingshot homolog 1 (Drosophila) Gene [Source:MGI Symbol;Acc:MGI:2686240] [ENSRNOT00000044751]	**27,536**
MGC94207	Rattus norvegicus similar to RIKEN cDNA C030006K11 (MGC94207), mRNA [NM_001007751]	**27,536**
Tbc1d10b	Rattus norvegicus TBC1 domain family, member 10b (Tbc1d10b), mRNA [NM_001108921]	**27,535**
0	Unknown	**27,529**
Tecr	Rattus norvegicus trans-2,3-enoyl-CoA reductase (Tecr), mRNA [NM_138549]	**27,524**
Bcl9	Rattus norvegicus B-cell CLL/lymphoma 9 (Bcl9), mRNA [NM_001107703]	**27,520**
Igfbp5	Insulin-like growth factor-binding protein 5 [Source:UniProtKB/Swiss-Prot;Acc:P24594] [ENSRNOT00000023530]	**27,512**
Pgap2	Rattus norvegicus post-GPI attachment to proteins 2 (Pgap2), mRNA [NM_053895]	**27,509**
Nfx1	Rattus norvegicus nuclear transcription factor, X-box binding 1 (Nfx1), mRNA [NM_001024784]	**27,502**
Hs6st1	Rattus norvegicus heparan sulfate 6-O-sulfotransferase 1 (Hs6st1), mRNA [NM_001108210]	**27,492**
0	Unknown	**27,483**
Dera	PREDICTED: Rattus norvegicus 2-deoxyribose-5-phosphate aldolase homolog (C. elegans) (Dera), miscRNA [XR_085824]	**27,482**
Pcdhb19	PREDICTED: Rattus norvegicus protocadherin beta 19 (Pcdhb19), mRNA [XM_001056051]	**27,481**
Zmat2	Rattus norvegicus zinc finger, matrin type 2 (Zmat2), mRNA [NM_001135582]	**27,473**
0	Sema domain, transmembrane domain (TM), and cytoplasmic domain, (Semaphorin) 6A (Predicted), isoform CRA_aUncharacterized protein [Source:UniProtKB/TrEMBL;Acc:D3ZAG0] [ENSRNOT00000005476]	**27,470**
LOC682102	Rattus norvegicus hypothetical protein LOC682102 (LOC682102), mRNA [NM_001134732]	**27,467**
Commd2	Rattus norvegicus COMM domain containing 2 (Commd2), mRNA [NM_001109503]	**27,465**
Cbll1	Rattus norvegicus Cas-Br-M (murine) ecotropic retroviral transforming sequence-like 1 (Cbll1), mRNA [NM_001108018]	**27,435**
0	Q6PG85_MOUSE (Q6PG85) NIPA-like domain containing 3, partial (75%) [TC618767]	**27,433**
Upk2	Rattus norvegicus uroplakin 2 (Upk2), mRNA [NM_001109523]	**27,410**
LOC679114	PREDICTED: Rattus norvegicus similar to sin3 associated polypeptide (LOC679114), mRNA [XM_001054754]	**27,398**
0	Unknown	**27,398**
0	Unknown	**27,393**
Fam114a1	PREDICTED: Rattus norvegicus family with sequence similarity 114, member A1 (Fam114a1), mRNA [XM_001078310]	**27,393**
Xkr6	Rattus norvegicus XK, Kell blood group complex subunit-related family, member 6 (Xkr6), mRNA [NM_001012042]	**27,392**
0	Unknown	**27,387**

Slmo1	Rattus norvegicus slowmo homolog 1 (Drosophila) (Slmo1), mRNA [NM_001109570]	**27,386**
0	Unknown	**27,382**
Mbd1	Rattus norvegicus methyl-CpG binding domain protein 1 (Mbd1), mRNA [NM_001011924]	**27,382**
Atp6v1g2	Rattus norvegicus ATPase, H+ transporting, lysosomal V1 subunit G2 (Atp6v1g2), mRNA [NM_212490]	**27,382**
0	Rattus norvegicus cDNA clone IMAGE:7374474. [BC091254]	**27,381**
Tk1	Rattus norvegicus thymidine kinase 1, soluble (Tk1), mRNA [NM_052800]	**27,358**
RGD1561065	Rattus norvegicus similar to mKIAA1111 protein (RGD1561065), mRNA [NM_001108253]	**27,358**
Gnat1	Rattus norvegicus guanine nucleotide binding protein (G protein), alpha transducing 1 (Gnat1), mRNA [NM_001108780]	**27,357**
0	Uncharacterized protein [Source:UniProtKB/TrEMBL;Acc:D3ZPG8] [ENSRNOT00000068110]	**27,355**
Fam189b	Rattus norvegicus family with sequence similarity 189, member B (Fam189b), mRNA [NM_001107690]	**27,351**
Smg7	Rattus norvegicus Smg-7 homolog, nonsense mediated mRNA decay factor (C. elegans) (Smg7), mRNA [NM_001191549]	**27,349**
Tspan9	Rattus norvegicus tetraspanin 9 (Tspan9), mRNA [NM_001107890]	**27,335**
Tmem150a	Rattus norvegicus transmembrane protein 150A (Tmem150a), mRNA [NM_139107]	**27,329**
0	Unknown	**27,328**
Prr5	Rattus norvegicus proline rich 5 (renal) (Prr5), mRNA [NM_001012121]	**27,322**
Slc22a23	Rattus norvegicus solute carrier family 22, member 23 (Slc22a23), mRNA [NM_022624]	**27,318**
Caln1	Rattus norvegicus calneuron 1 (Caln1), mRNA [NM_001077201]	**27,298**
Arl15	PREDICTED: Rattus norvegicus ADP-ribosylation factor-like 15 (Arl15), mRNA [XM_001069431]	**27,295**
Chrnb1	Rattus norvegicus cholinergic receptor, nicotinic, beta 1 (muscle) (Chrnb1), mRNA [NM_012528]	**27,294**
Bcl2l12	Rattus norvegicus BCL2-like 12 (proline rich) (Bcl2l12), mRNA [NM_001108480]	**27,284**
Zfp180	Rattus norvegicus zinc finger protein 180 (Zfp180), mRNA [NM_144757]	**27,283**
C2cd2	Rattus norvegicus C2 calcium-dependent domain containing 2 (C2cd2), mRNA [NM_199391]	**27,280**
MGC94282	Rattus norvegicus similar to 5930416I19Rik protein (MGC94282), transcript variant 1, non-coding RNA [NR_027366]	**27,280**
Mrpl38	Rattus norvegicus mitochondrial ribosomal protein L38 (Mrpl38), nuclear gene encoding mitochondrial protein, mRNA [NM_001009369]	**27,277**
Lrrc20	Rattus norvegicus leucine rich repeat containing 20 (Lrrc20), mRNA [NM_001109171]	**27,261**
Tmem109	Rattus norvegicus transmembrane protein 109 (Tmem109), mRNA [NM_001007736]	**27,257**
Rdh2	Rattus norvegicus retinol dehydrogenase 2 (Rdh2), mRNA [NM_199208]	**27,253**
Cdh13	Rattus norvegicus cadherin 13 (Cdh13), mRNA [NM_138889]	**27,253**
Terc	Rattus norvegicus telomerase RNA component (Terc), telomerase RNA [NR_001567]	**27,250**
Tor3a	Rattus norvegicus torsin family 3, member A (Tor3a), mRNA [NM_001009683]	**27,249**
Iqgap3	Rattus norvegicus IQ motif containing GTPase activating protein 3 (Iqgap3), mRNA [NM_001191709]	**27,241**
Dnajc27	Rattus norvegicus DnaJ (Hsp40) homolog, subfamily C, member 27 (Dnajc27), mRNA [NM_206845]	**27,238**
Ankrd13b	PREDICTED: Rattus norvegicus similar to novel protein (RGD1564005), mRNA [XM_001080794]	**27,232**
Stx7	Rattus norvegicus syntaxin 7 (Stx7), mRNA [NM_021869]	**27,202**

Lphn1	Rattus norvegicus latrophilin 1 (Lphn1), mRNA [NM_022962]	**27,184**
Lingo1	Rattus norvegicus leucine rich repeat and Ig domain containing 1 (Lingo1), mRNA [NM_001100722]	**27,182**
Crkl	Rattus norvegicus v-crk sarcoma virus CT10 oncogene homolog (avian)-like (Crkl), mRNA [NM_001008284]	**27,145**
Fbxl16	Rattus norvegicus F-box and leucine-rich repeat protein 16 (Fbxl16), mRNA [NM_001009504]	**27,143**
Narf	Rattus norvegicus nuclear prelamin A recognition factor (Narf), mRNA [NM_001039207]	**27,143**
Morn4	Rattus norvegicus MORN repeat containing 4 (Morn4), mRNA [NM_001024975]	**27,141**
Olfm3	Rattus norvegicus olfactomedin 3 (Olfm3), mRNA [NM_145777]	**27,141**
0	Unknown	**27,137**
Map4k4	Rattus norvegicus mitogen-activated protein kinase kinase kinase kinase 4 (Map4k4), mRNA [NM_001106904]	**27,125**
LOC688613	Rattus norvegicus hypothetical protein LOC688613 (LOC688613), mRNA [NM_001134845]	**27,121**
Ubap1	Rattus norvegicus ubiquitin-associated protein 1 (Ubap1), mRNA [NM_001012190]	**27,095**
Ppp1r7	Rattus norvegicus protein phosphatase 1, regulatory (inhibitor) subunit 7 (Ppp1r7), mRNA [NM_001009825]	**27,095**
Tmem231	PREDICTED: Rattus norvegicus similar to predicted CDS, putative protein of bilaterial origin (4J193) (RGD1306153), mRNA [XM_001075665]	**27,092**
Pdzd11	Rattus norvegicus PDZ domain containing 11 (Pdzd11), mRNA [NM_001106945]	**27,091**
Cacnb3	Rattus norvegicus calcium channel, voltage-dependent, beta 3 subunit (Cacnb3), mRNA [NM_012828]	**27,089**
Frs2	Rattus norvegicus fibroblast growth factor receptor substrate 2 (Frs2), mRNA [NM_001108097]	**27,086**
0	Unknown	**27,083**
Chst1	Rattus norvegicus carbohydrate (keratan sulfate Gal-6) sulfotransferase 1 (Chst1), mRNA [NM_001011955]	**27,083**
Igsf11	Rattus norvegicus immunoglobulin superfamily, member 11 (Igsf11), mRNA [NM_001013120]	**27,082**
0	Unknown	**27,078**
Htr2a	5-hydroxytryptamine receptor 2A [Source:UniProtKB/Swiss-Prot;Acc:P14842] [ENSRNOT00000013408]	**27,064**
Shisa6	Rattus norvegicus shisa homolog 6 (Xenopus laevis) (Shisa6), mRNA [NM_001191922]	**27,043**
Cables2	Q8R3U5_MOUSE (Q8R3U5) Cables2-pending protein (Fragment), partial (97%) [TC630600]	**27,041**
0	UI-R-FS1-cqi-l-02-0-UI.s1 UI-R-FS1 Rattus norvegicus cDNA clone UI-R-FS1-cqi-l-02-0-UI 3', mRNA sequence [BU760441]	**27,029**
Pctk1	Rattus norvegicus PCTAIRE protein kinase 1 (Pctk1), transcript variant 2, mRNA [NM_031077]	**27,013**
0	Unknown	**27,001**
0	Unknown	**26,992**
Lyrm2	Rattus norvegicus LYR motif containing 2 (Lyrm2), mRNA [NM_001126096]	**26,989**
Actb	Rattus norvegicus actin, beta (Actb), mRNA [NM_031144]	**26,989**
0	Unknown	**26,984**
Gpr155	Rattus norvegicus G protein-coupled receptor 155 (Gpr155), mRNA [NM_001107811]	**26,983**
0	Unknown	**26,976**
0	PREDICTED: Rattus norvegicus similar to putative pheromone receptor (Go-VN5) (LOC682835), mRNA [XM_001063328]	**26,974**
Helt	PREDICTED: Rattus norvegicus HES/HEY-like transcription factor (Helt), miscRNA [XR_085643]	**26,968**

Fam113a	Rattus norvegicus family with sequence similarity 113, member A (Fam113a), mRNA [NM_001012348]	**26,964**
RGD1309543	Rattus norvegicus similar to 2310014H01Rik protein (RGD1309543), mRNA [NM_001126287]	**26,961**
Cxxc4	Rattus norvegicus CXXC finger 4 (Cxxc4), mRNA [NM_053342]	**26,955**
0	Uncharacterized protein [Source:UniProtKB/TrEMBL;Acc:D4A9G6] [ENSRNOT00000034001]	**26,929**
Psen1	Rattus norvegicus presenilin 1 (Psen1), mRNA [NM_019163]	**26,926**
Sorl1	Rattus norvegicus sortilin-related receptor, LDLR class A repeats-containing (Sorl1), mRNA [NM_053519]	**26,925**
Sez6l2	Rattus norvegicus seizure related 6 homolog (mouse)-like 2 (Sez6l2), mRNA [NM_001107550]	**26,918**
Olr213	Rattus norvegicus olfactory receptor 213 (Olr213), mRNA [NM_001000735]	**26,909**
0	Unknown	**26,904**
0	Unknown	**26,903**
Zfp35	Rattus norvegicus zinc finger protein 35 (Zfp35), mRNA [NM_001013141]	**26,900**
RGD1564614	Rattus norvegicus similar to complement factor H-related protein (RGD1564614), mRNA [NM_001134792]	**26,895**
Vamp1	Rattus norvegicus vesicle-associated membrane protein 1 (Vamp1), mRNA [NM_013090]	**26,889**
0	Protein prune homolog 2 [Source:UniProtKB/Swiss-Prot;Acc:Q5BJR4] [ENSRNOT00000054794]	**26,864**
Cd59	Rattus norvegicus CD59 molecule, complement regulatory protein (Cd59), mRNA [NM_012925]	**26,847**
0	Rattus norvegicus similar to RIKEN cDNA 1700001F09 (LOC316932), mRNA [XM_229506]	**26,846**
Runx1	Rattus norvegicus runt-related transcription factor 1 (Runx1), mRNA [NM_017325]	**26,833**
Ggps1	Rattus norvegicus geranylgeranyl diphosphate synthase 1 (Ggps1), mRNA [NM_001007626]	**26,810**
Acat3	Rattus norvegicus acetyl-Coenzyme A acetyltransferase 3 (Acat3), mRNA [NM_001006995]	**26,808**
Mbp	Rattus norvegicus myelin basic protein (Mbp), transcript variant 1, mRNA [NM_001025291]	**26,807**
Stt3a	Rattus norvegicus STT3, subunit of the oligosaccharyltransferase complex, homolog A (S. cerevisiae) (Stt3a), mRNA [NM_001134749]	**26,804**
Prdm2	Rattus norvegicus PR domain containing 2, with ZNF domain (Prdm2), mRNA [NM_001077648]	**26,801**
Ddx41	Rattus norvegicus DEAD (Asp-Glu-Ala-Asp) box polypeptide 41 (Ddx41), mRNA [NM_001108046]	**26,797**
Tspan9	Rattus norvegicus tetraspanin 9 (Tspan9), mRNA [NM_001107890]	**26,792**
0	Rattus norvegicus TL0AEA59YG01 mRNA sequence. [FQ234328]	**26,788**
Hivep3	Rattus norvegicus human immunodeficiency virus type I enhancer binding protein 3 (Hivep3), mRNA [NM_001107972]	**26,788**
0	Uncharacterized protein [Source:UniProtKB/TrEMBL;Acc:D4A6V0] [ENSRNOT00000044039]	**26,775**
Rabgef1	Rattus norvegicus RAB guanine nucleotide exchange factor (GEF) 1 (Rabgef1), mRNA [NM_001108333]	**26,760**
Rbp2	Rattus norvegicus retinol binding protein 2, cellular (Rbp2), mRNA [NM_012640]	**26,739**
Rai2	Rattus norvegicus retinoic acid induced 2 (Rai2), mRNA [NM_001109316]	**26,739**
Slc36a1	Rattus norvegicus solute carrier family 36 (proton/amino acid symporter), member 1 (Slc36a1), mRNA [NM_130415]	**26,738**
Srm	Rattus norvegicus spermidine synthase (Srm), mRNA [NM_053464]	**26,732**
Ostm1	Rattus norvegicus osteopetrosis associated transmembrane protein 1 (Ostm1), mRNA [NM_001029925]	**26,726**
Aldh6a1	Rattus norvegicus aldehyde dehydrogenase 6 family, member A1 (Aldh6a1), nuclear gene encoding mitochondrial protein, mRNA [NM_031057]	**26,726**

Ccna2	Rattus norvegicus cyclin A2 (Ccna2), mRNA [NM_053702]	**26,725**
0	Unknown	**26,719**
LOC503175	PREDICTED: Rattus norvegicus similar to Protein KIAA0280 (LOC503175), mRNA [XM_578699]	**26,719**
Pex19	Rattus norvegicus peroxisomal biogenesis factor 19 (Pex19), transcript variant 1, mRNA [NM_001107375]	**26,714**
Mapt	Rattus norvegicus microtubule-associated protein tau (Mapt), mRNA [NM_017212]	**26,709**
0	RCG44206Uncharacterized protein [Source:UniProtKB/TrEMBL;Acc:D3ZR35] [ENSRNOT00000006790]	**26,705**
Hist1h2bb	Histone H2B [Source:UniProtKB/TrEMBL;Acc:D3ZWM5] [ENSRNOT00000030198]	**26,699**
Akr1b8	Rattus norvegicus aldo-keto reductase family 1, member B8 (Akr1b8), mRNA [NM_173136]	**26,695**
Tac2	Rattus norvegicus tachykinin 2 (Tac2), mRNA [NM_019162]	**26,694**
RGD1563020	Uncharacterized protein [Source:UniProtKB/TrEMBL;Acc:D3ZRK8] [ENSRNOT00000016940]	**26,691**
Tmed2	Rattus norvegicus transmembrane emp24 domain trafficking protein 2 (Tmed2), mRNA [NM_031722]	**26,661**
Trpt1	Rattus norvegicus tRNA phosphotransferase 1 (Trpt1), mRNA [NM_001106331]	**26,640**
Mdc1	Rattus norvegicus mediator of DNA damage checkpoint 1 (Mdc1), mRNA [NM_001166275]	**26,635**
Tjp1	Rattus norvegicus tight junction protein 1 (Tjp1), mRNA [NM_001106266]	**26,622**
Zdhhc19	Rattus norvegicus zinc finger, DHHC-type containing 19 (Zdhhc19), mRNA [NM_001039259]	**26,622**
0	Unknown	**26,616**
Zer1	Rattus norvegicus zer-1 homolog (C. elegans) (Zer1), mRNA [NM_001100707]	**26,613**
Sdc4	Rattus norvegicus syndecan 4 (Sdc4), mRNA [NM_012649]	**26,613**
Nudt12	Rattus norvegicus nudix (nucleoside diphosphate linked moiety X)-type motif 12 (Nudt12), mRNA [NM_001109010]	**26,590**
Pla2g2d	Rattus norvegicus phospholipase A2, group IID (Pla2g2d), mRNA [NM_001013428]	**26,585**
Mtmr1	Rattus norvegicus myotubularin related protein 1 (Mtmr1), mRNA [NM_001191725]	**26,581**
Adam10	PREDICTED: Rattus norvegicus ADAM metallopeptidase domain 10 (Adam10), mRNA [XM_001054737]	**26,576**
0	AGENCOURT_27851764 NIH_MGC_252 Rattus norvegicus cDNA clone IMAGE:7309973 5', mRNA sequence [CO400749]	**26,561**
Irf5	Rattus norvegicus interferon regulatory factor 5 (Irf5), mRNA [NM_001106586]	**26,554**
Samd12	Rattus norvegicus sterile alpha motif domain containing 12 (Samd12), mRNA [NM_001130562]	**26,552**
Snx19	Rattus norvegicus sorting nexin 19 (Snx19), mRNA [NM_001108131]	**26,552**
0	Unknown	**26,544**
Mxi1	Rattus norvegicus MAX interactor 1 (Mxi1), mRNA [NM_013160]	**26,537**
0	Unknown	**26,520**
Pip4k2b	Rattus norvegicus phosphatidylinositol-5-phosphate 4-kinase, type II, beta (Pip4k2b), mRNA [NM_053550]	**26,519**
RGD1566380	Rattus norvegicus hypothetical gene supported by NM_017187 (RGD1566380), mRNA [NM_001134593]	**26,518**
Gnpda1	Rattus norvegicus glucosamine-6-phosphate deaminase 1 (Gnpda1), mRNA [NM_001134995]	**26,517**
Lin7a	Rattus norvegicus lin-7 homolog a (C. elegans) (Lin7a), mRNA [NM_053514]	**26,508**
Cyp21a1	Rattus norvegicus cytochrome P450, family 21, subfamily a, polypeptide 1 (Cyp21a1), mRNA [NM_057101]	**26,506**

0	Unknown	**26,502**
0	Unknown	**26,492**
Arhgef9	Rattus norvegicus Cdc42 guanine nucleotide exchange factor (GEF) 9 (Arhgef9), mRNA [NM_023957]	**26,491**
Rab6a	Rattus norvegicus RAB6A, member RAS oncogene family (Rab6a), mRNA [NM_053366]	**26,485**
Tbc1d22b	Rattus norvegicus TBC1 domain family, member 22B (Tbc1d22b), mRNA [NM_001025059]	**26,484**
0	PREDICTED: Rattus norvegicus similar to 60S ribosomal protein L29 (P23) (RGD1564980), mRNA [XM_001053982]	**26,482**
Trim32	Rattus norvegicus tripartite motif-containing 32 (Trim32), mRNA [NM_001012103]	**26,472**
Ppp1r9b	Rattus norvegicus protein phosphatase 1, regulatory subunit 9B (Ppp1r9b), mRNA [NM_053474]	**26,463**
LOC690000	PREDICTED: Rattus norvegicus similar to CG3740-PA, transcript variant 2 (LOC690000), mRNA [XM_001073988]	**26,452**
0	Unknown	**26,447**
Filip1	Rattus norvegicus filamin A interacting protein 1 (Filip1), mRNA [NM_145682]	**26,442**
Dpp9	PREDICTED: Rattus norvegicus dipeptidyl peptidase 9 (Dpp9), mRNA [XM_001061312]	**26,441**
Gstt3	Rattus norvegicus glutathione S-transferase, theta 3 (Gstt3), mRNA [NM_001137643]	**26,441**
Plekhm3	Uncharacterized protein [Source:UniProtKB/TrEMBL;Acc:D3ZSZ9] [ENSRNOT00000032782]	**26,435**
Dysf	Rattus norvegicus dysferlin (Dysf), mRNA [NM_001107869]	**26,425**
Nucb1	Rattus norvegicus nucleobindin 1 (Nucb1), mRNA [NM_053463]	**26,418**
Zmynd11	Rattus norvegicus zinc finger, MYND domain containing 11 (Zmynd11), transcript variant 3, mRNA [NM_203369]	**26,414**
0	Unknown	**26,410**
Fyn	Rattus norvegicus FYN oncogene related to SRC, FGR, YES (Fyn), mRNA [NM_012755]	**26,409**
Nptn	Rattus norvegicus neuroplastin (Nptn), mRNA [NM_019380]	**26,408**
LOC686539	Rattus norvegicus similar to immunoglobulin superfamily containing leucine-rich repeat (LOC686539), mRNA [NM_001126300]	**26,400**
Ptprj	Rattus norvegicus protein tyrosine phosphatase, receptor type, J (Ptprj), mRNA [NM_017269]	**26,399**
Phf17	Rattus norvegicus PHD finger protein 17 (Phf17), mRNA [NM_001107670]	**26,395**
0	LRRGT00080 (LOC501233), mRNA [Source:RefSeq DNA;Acc:NM_001047967] [ENSRNOT00000066771]	**26,370**
Sp1	Rattus norvegicus Sp1 transcription factor (Sp1), mRNA [NM_012655]	**26,370**
Speg	Rattus norvegicus SPEG complex locus (Speg), transcript variant 1, mRNA [NM_001108802]	**26,366**
Gtpbp3	Rattus norvegicus GTP binding protein 3 (Gtpbp3), nuclear gene encoding mitochondrial protein, mRNA [NM_001011919]	**26,356**
Strada	Rattus norvegicus STE20-related kinase adaptor alpha (Strada), mRNA [NM_182820]	**26,356**
Trhde	Rattus norvegicus thyrotropin-releasing hormone degrading enzyme (Trhde), mRNA [NM_001108991]	**26,349**
0	Uncharacterized protein [Source:UniProtKB/TrEMBL;Acc:D4ACD7] [ENSRNOT00000014120]	**26,336**
Dio3	Rattus norvegicus deiodinase, iodothyronine, type III (Dio3), mRNA [NM_017210]	**26,333**
LOC690326	Rattus norvegicus hypothetical protein LOC690326 (LOC690326), mRNA [NM_001109578]	**26,326**
Hpca	Rattus norvegicus hippocalcin (Hpca), mRNA [NM_017122]	**26,322**
Impad1	Rattus norvegicus inositol monophosphatase domain containing 1 (Impad1), mRNA [NM_001008772]	**26,318**

Caly	Rattus norvegicus calcyon neuron-specific vesicular protein (Caly), transcript variant 1, mRNA [NM_138915]	**26,317**
Dlgap3	Rattus norvegicus discs, large (Drosophila) homolog-associated protein 3 (Dlgap3), mRNA [NM_173138]	**26,314**
RGD1566319	Rattus norvegicus similar to Sestrin 2 (Hi95) (RGD1566319), mRNA [NM_001109358]	**26,311**
Ddn	Rattus norvegicus dendrin (Ddn), mRNA [NM_030993]	**26,299**
0	Unknown	**26,296**
Ceacam19	Rattus norvegicus carcinoembryonic antigen-related cell adhesion molecule 19 (Ceacam19), mRNA [NM_001198970]	**26,294**
Ercc6	Rattus norvegicus excision repair cross-complementing rodent repair deficiency, complementation group 6 (Ercc6), mRNA [NM_001107296]	**26,287**
0	Uncharacterized protein [Source:UniProtKB/TrEMBL;Acc:D3ZD04] [ENSRNOT00000045918]	**26,281**
Mblac2	Rattus norvegicus metallo-beta-lactamase domain containing 2 (Mblac2), mRNA [NM_001108934]	**26,278**
0	Uncharacterized protein [Source:UniProtKB/TrEMBL;Acc:D4A1B0] [ENSRNOT00000045263]	**26,269**
Zfp385d	Rattus norvegicus zinc finger protein 385D (Zfp385d), mRNA [NM_001013992]	**26,263**
Kcnc1	Rattus norvegicus potassium voltage gated channel, Shaw-related subfamily, member 1 (Kcnc1), mRNA [NM_012856]	**26,257**
0	Unknown	**26,246**
Fbxl7	Rattus norvegicus F-box and leucine-rich repeat protein 7 (Fbxl7), mRNA [NM_001108545]	**26,242**
RGD1566112	Rattus norvegicus similar to pleckstrin homology domain protein (5V327) (RGD1566112), mRNA [NM_001127566]	**26,234**
Elmod1	Rattus norvegicus ELMO/CED-12 domain containing 1 (Elmod1), mRNA [NM_001191579]	**26,232**
Ankrd61	Rattus norvegicus ankyrin repeat domain 61 (Ankrd61), mRNA [NM_001044297]	**26,231**
0	D42151S6 Rattus norvegicus gene for Ad4BP, complete cds and exon 7 [D42156]	**26,228**
Atg13	Rattus norvegicus TL0ACA31YH23 mRNA sequence. [FQ217242]	**26,223**
Myo5a	Rattus norvegicus myosin VA (Myo5a), mRNA [NM_022178]	**26,220**
Stac2	Rattus norvegicus SH3 and cysteine rich domain 2 (Stac2), mRNA [NM_001108834]	**26,220**
Med1	Rattus norvegicus mediator complex subunit 1 (Med1), mRNA [NM_001134361]	**26,219**
Stx1a	Rattus norvegicus syntaxin 1A (brain) (Stx1a), mRNA [NM_053788]	**26,204**
Cpne4	Rattus norvegicus copine IV (Cpne4), mRNA [NM_001109003]	**26,197**
LOC688390	Rattus norvegicus hypothetical protein LOC688390 (LOC688390), mRNA [NM_001109496]	**26,196**
LOC688272	PREDICTED: Rattus norvegicus similar to ring finger protein 157 (LOC688272), mRNA [XM_001081716]	**26,193**
Wnk2	Rattus norvegicus WNK lysine deficient protein kinase 2 (Wnk2), mRNA [NM_001191556]	**26,186**
Slc1a4	Rattus norvegicus solute carrier family 1 (glutamate/neutral amino acid transporter), member 4 (Slc1a4), mRNA [NM_198763]	**26,176**
Hist1h2bl	Rattus norvegicus histone cluster 1, H2bl (Hist1h2bl), mRNA [NM_022647]	**26,175**
RGD1563049	Rattus norvegicus RGD1563049 (RGD1563049), mRNA [NM_001134521]	**26,168**
Pank1	Rattus norvegicus pantothenate kinase 1 (Pank1), mRNA [NM_001106373]	**26,158**
Gprc5b	Rattus norvegicus G protein-coupled receptor, family C, group 5, member B (Gprc5b), mRNA [NM_001106304]	**26,155**
Sft2d3	Rattus norvegicus SFT2 domain containing 3 (Sft2d3), mRNA [NM_001108887]	**26,150**
Sox9	PREDICTED: Rattus norvegicus SRY-box containing gene 9 (Sox9), mRNA [XM_343981]	**26,130**

Jazf1	PREDICTED: Rattus norvegicus JAZF zinc finger 1 (Jazf1), mRNA [XM_002726367]	**26,125**
Klf12	Rattus norvegicus Kruppel-like factor 12 (Klf12), mRNA [NM_001107281]	**26,111**
S1pr2	Rattus norvegicus sphingosine-1-phosphate receptor 2 (S1pr2), mRNA [NM_017192]	**26,111**
LOC305052	PREDICTED: Rattus norvegicus hypothetical LOC305052 (LOC305052), miscRNA [XR_008366]	**26,109**
Ptbp1	Rattus norvegicus polypyrimidine tract binding protein 1 (Ptbp1), transcript variant 2, mRNA [NM_022516]	**26,104**
RGD1309707	Rattus norvegicus similar to RIKEN cDNA 4930431E10 (RGD1309707), mRNA [NM_001109598]	**26,091**
RGD1309651	Uncharacterized protein [Source:UniProtKB/TrEMBL;Acc:D3ZSX5] [ENSRNOT00000023721]	**26,089**
Faah	Rattus norvegicus fatty acid amide hydrolase (Faah), mRNA [NM_024132]	**26,086**
Socs7	Uncharacterized protein [Source:UniProtKB/TrEMBL;Acc:D4A4T8] [ENSRNOT00000014943]	**26,083**
0	Unknown	**26,080**
RGD1305464	Rattus norvegicus similar to human chromosome 15 open reading frame 39 (RGD1305464), mRNA [NM_001025011]	**26,068**
Acvr1b	Rattus norvegicus activin A receptor, type IB (Acvr1b), mRNA [NM_199230]	**26,066**
Sfrp2	Rattus norvegicus secreted frizzled-related protein 2 (Sfrp2), mRNA [NM_001100700]	**26,055**
LOC100362040	Rattus norvegicus Ac2-143-like (LOC100362040), mRNA [NM_001177817]	**26,045**
Rcan2	Rattus norvegicus regulator of calcineurin 2 (Rcan2), mRNA [NM_175578]	**26,042**
Stam2	Rattus norvegicus signal transducing adaptor molecule (SH3 domain and ITAM motif) 2 (Stam2), mRNA [NM_001012085]	**26,038**
L1cam	Rattus norvegicus L1 cell adhesion molecule (L1cam), mRNA [NM_017345]	**26,036**
Bcas1	Rattus norvegicus breast carcinoma amplified sequence 1 (Bcas1), mRNA [NM_145670]	**26,034**
Timp4	Rattus norvegicus tissue inhibitor of metalloproteinase 4 (Timp4), mRNA [NM_001109393]	**26,033**
Spry4	Rattus norvegicus sprouty homolog 4 (Drosophila) (Spry4), mRNA [NM_001106150]	**26,029**
Atp10b	Uncharacterized protein [Source:UniProtKB/TrEMBL;Acc:D4A4W5] [ENSRNOT00000029118]	**26,027**
0	Unknown	**26,026**
Glra1	Rattus norvegicus glycine receptor, alpha 1 (Glra1), mRNA [NM_013133]	**26,019**
Ptgfrn	Rattus norvegicus prostaglandin F2 receptor negative regulator (Ptgfrn), mRNA [NM_019243]	**26,015**
Ncor1	PREDICTED: Rattus norvegicus nuclear receptor co-repressor 1 (Ncor1), mRNA [XM_001077495]	**26,007**
Pla2g4f	PREDICTED: Rattus norvegicus phospholipase A2, group IVF (Pla2g4f), mRNA [XM_001080057]	**26,003**
Klhl25	Rattus norvegicus kelch-like 25 (Drosophila) (Klhl25), mRNA [NM_001039006]	**25,996**
0	TGF-beta activated kinase 1/MAP3K7 binding protein 3 Gene [Source:MGI Symbol;Acc:MGI:1913974] [ENSRNOT00000004854]	**25,995**
Taf9b	Rattus norvegicus TAF9B RNA polymerase II, TATA box binding protein (TBP)-associated factor (Taf9b), mRNA [NM_133615]	**25,994**
0	MTA3_HUMAN (Q9BTC8) Metastasis-associated protein MTA3, partial (5%) [TC620956]	**25,992**
Ccnc	Rattus norvegicus cyclin C (Ccnc), mRNA [NM_001100472]	**25,991**
Opcml	Rattus norvegicus opioid binding protein/cell adhesion molecule-like (Opcml), mRNA [NM_053848]	**25,979**
Herv-frd	Rattus norvegicus HERV-FRD provirus ancestral Env polyprotein (Herv-frd), mRNA [NM_001024239]	**25,973**
Epb41l3	Rattus norvegicus erythrocyte membrane protein band 4.1-like 3 (Epb41l3), mRNA [NM_053927]	**25,971**

Des	Rattus norvegicus desmin (Des), mRNA [NM_022531]	**25,950**
0	Unknown	**25,947**
Hk1	Rattus norvegicus hexokinase 1 (Hk1), nuclear gene encoding mitochondrial protein, mRNA [NM_012734]	**25,945**
0	AF4/FMR2 family, member 2 Gene [Source:MGI Symbol;Acc:MGI:1202294] [ENSRNOT00000065537]	**25,944**
Fasn	Rattus norvegicus fatty acid synthase (Fasn), mRNA [NM_017332]	**25,938**
Uhrf1bp1l	Rattus norvegicus UHRF1 binding protein 1-like (Uhrf1bp1l), mRNA [NM_001108753]	**25,937**
Pik3r2	Rattus norvegicus phosphoinositide-3-kinase, regulatory subunit 2 (beta) (Pik3r2), mRNA [NM_022185]	**25,928**
Sema3f	Rattus norvegicus sema domain, immunoglobulin domain (Ig), short basic domain, secreted, (semaphorin) 3 F (Sema3f), mRNA [NM_001108185]	**25,927**
Snn	Rattus norvegicus stannin (Snn), mRNA [NM_001034083]	**25,927**
Chrna7	Rattus norvegicus cholinergic receptor, nicotinic, alpha 7 (Chrna7), mRNA [NM_012832]	**25,922**
Znf536	Uncharacterized protein [Source:UniProtKB/TrEMBL;Acc:D3ZJS6] [ENSRNOT00000018956]	**25,920**
RGD1562342	Rattus norvegicus similar to RIKEN cDNA 1110012D08 (RGD1562342), mRNA [NM_001109281]	**25,917**
Lrrtm4	Rattus norvegicus leucine rich repeat transmembrane neuronal 4 (Lrrtm4), mRNA [NM_001134746]	**25,916**
Ubxn4	Rattus norvegicus UBX domain protein 4 (Ubxn4), mRNA [NM_001012025]	**25,912**
RGD1308319	PREDICTED: Rattus norvegicus similar to KIAA0802 protein (RGD1308319), partial mRNA [XM_002727262]	**25,912**
Ccdc8	Rattus norvegicus coiled-coil domain containing 8 (Ccdc8), mRNA [NM_001009533]	**25,904**
Shisa5	Rattus norvegicus shisa homolog 5 (Xenopus laevis) (Shisa5), mRNA [NM_001006989]	**25,903**
0	Unknown	**25,897**
0	Unknown	**25,896**
Cd68	Rattus norvegicus Cd68 molecule (Cd68), mRNA [NM_001031638]	**25,874**
Rab22a	Rattus norvegicus RAB22A, member RAS oncogene family (Rab22a), mRNA [NM_001108966]	**25,865**
LOC691352	Rattus norvegicus similar to Robo-1 (LOC691352), mRNA [NM_001109638]	**25,864**
Col4a5	PREDICTED: Rattus norvegicus collagen, type IV, alpha 5 (Col4a5), mRNA [XM_001055156]	**25,845**
Slc12a3	Rattus norvegicus solute carrier family 12 (sodium/chloride transporters), member 3 (Slc12a3), mRNA [NM_019345]	**25,842**
0	Unknown	**25,840**
Vom2r65	Rattus norvegicus vomeronasal 2 receptor, 65 (Vom2r65), mRNA [NM_001099654]	**25,834**
Arl6ip5	Rattus norvegicus ADP-ribosylation-like factor 6 interacting protein 5 (Arl6ip5), mRNA [NM_023972]	**25,825**
RGD1564719	Rattus norvegicus similar to RIKEN cDNA C230052I12 (RGD1564719), mRNA [NM_001109335]	**25,819**
0	Rattus norvegicus hypothetical LOC100125371, mRNA (cDNA clone MGC:105701 IMAGE:7309421), complete cds. [BC089106]	**25,818**
Rabif	Rattus norvegicus RAB interacting factor (Rabif), mRNA [NM_001007678]	**25,817**
Sidt2	Rattus norvegicus SID1 transmembrane family, member 2 (Sidt2), mRNA [NM_001108142]	**25,814**
Col4a1	Rattus norvegicus collagen, type IV, alpha 1 (Col4a1), mRNA [NM_001135009]	**25,796**
Slc12a7	Rattus norvegicus solute carrier family 12 (potassium/chloride transporters), member 7 (Slc12a7), mRNA [NM_001013144]	**25,795**
Vat1	Rattus norvegicus vesicle amine transport protein 1 homolog (T californica) (Vat1), mRNA [NM_001033683]	**25,793**

Akap12	Rattus norvegicus A kinase (PRKA) anchor protein 12 (Akap12), transcript variant 1, mRNA [NM_057103]	**25,788**
0	Rattus norvegicus TL0AEA10YI10 mRNA sequence. [FQ228098]	**25,784**
Pkm2	Rattus norvegicus clone UI-R-FJ0-cpu-c-03-0-UI unknown mRNA. [AY724474]	**25,775**
Xbp1	Rattus norvegicus X-box binding protein 1 (Xbp1), mRNA [NM_001004210]	**25,774**
Rfesd	Rattus norvegicus Rieske (Fe-S) domain containing (Rfesd), mRNA [NM_001108540]	**25,767**
Rnf5	Rattus norvegicus ring finger protein 5 (Rnf5), mRNA [NM_001109025]	**25,765**
Chsy1	Rattus norvegicus chondroitin sulfate synthase 1 (Chsy1), mRNA [NM_001106268]	**25,764**
Gpr123	Rattus norvegicus G protein-coupled receptor 123 (Gpr123), mRNA [NM_001107559]	**25,759**
Dctd	Rattus norvegicus dCMP deaminase (Dctd), transcript variant 1, mRNA [NM_001013882]	**25,758**
Lsamp	Rattus norvegicus limbic system-associated membrane protein (Lsamp), mRNA [NM_017242]	**25,751**
Gsk3b	Rattus norvegicus glycogen synthase kinase 3 beta (Gsk3b), mRNA [NM_032080]	**25,742**
Myef2	Rattus norvegicus myelin expression factor 2 (Myef2), mRNA [NM_001013205]	**25,742**
Tcerg1l	Rattus norvegicus transcription elongation regulator 1-like (Tcerg1l), mRNA [NM_001130077]	**25,742**
Cs	Rattus norvegicus citrate synthase (Cs), nuclear gene encoding mitochondrial protein, mRNA [NM_130755]	**25,734**
Rnf4	Rattus norvegicus ring finger protein 4 (Rnf4), mRNA [NM_019182]	**25,730**
Stom	Rattus norvegicus stomatin (Stom), mRNA [NM_001011965]	**25,729**
0	Unknown	**25,726**
Grip1	Rattus norvegicus glutamate receptor interacting protein isoform c4-7 (GRIP1) mRNA, complete cds; alternatively spliced. [AY437398]	**25,724**
Ccl22	Rattus norvegicus chemokine (C-C motif) ligand 22 (Ccl22), mRNA [NM_057203]	**25,722**
Celsr3	Rattus norvegicus cadherin, EGF LAG seven-pass G-type receptor 3 (flamingo homolog, Drosophila) (Celsr3), mRNA [NM_031320]	**25,720**
0	Unknown	**25,717**
Tnpo2	Rattus norvegicus transportin 2 (Tnpo2), mRNA [NM_001107166]	**25,713**
Usp46	Rattus norvegicus ubiquitin specific peptidase 46 (Usp46), mRNA [NM_001191596]	**25,712**
Smn1	Rattus norvegicus survival motor neuron 1 (Smn1), mRNA [NM_022509]	**25,711**
Myst2	Rattus norvegicus MYST histone acetyltransferase 2 (Myst2), mRNA [NM_181081]	**25,698**
Tsc22d4	Rattus norvegicus TSC22 domain family, member 4 (Tsc22d4), mRNA [NM_001044284]	**25,671**
Uck1	Rattus norvegicus uridine-cytidine kinase 1 (Uck1), mRNA [NM_001107831]	**25,666**
Atp1a3	Rattus norvegicus ATPase, Na+/K+ transporting, alpha 3 polypeptide (Atp1a3), mRNA [NM_012506]	**25,665**
Ppt1	Rattus norvegicus palmitoyl-protein thioesterase 1 (Ppt1), mRNA [NM_022502]	**25,665**
Ppif	Rattus norvegicus peptidylprolyl isomerase F (Ppif), nuclear gene encoding mitochondrial protein, mRNA [NM_172243]	**25,647**
0	Rattus norvegicus similar to RIKEN cDNA 1200007D18 (LOC287177), mRNA [XM_213272]	**25,645**
Pias3	Rattus norvegicus protein inhibitor of activated STAT, 3 (Pias3), mRNA [NM_031784]	**25,635**
Nova2	PREDICTED: Rattus norvegicus neuro-oncological ventral antigen 2 (Nova2), mRNA [XM_001075246]	**25,629**
Ggct	Rattus norvegicus gamma-glutamyl cyclotransferase (Ggct), mRNA [NM_001108629]	**25,627**

Tgm3	Rattus norvegicus transglutaminase 3, E polypeptide (Tgm3), mRNA [NM_001108959]	**25,624**
Ptpdc1	Rattus norvegicus protein tyrosine phosphatase domain containing 1 (Ptpdc1), mRNA [NM_001106104]	**25,617**
RGD1564058	Rattus norvegicus similar to cDNA sequence BC056474 (RGD1564058), mRNA [NM_001105947]	**25,615**
Man2b2	Rattus norvegicus mannosidase, alpha, class 2B, member 2 (Man2b2), mRNA [NM_001134971]	**25,609**
Rassf3	Rattus norvegicus Ras association (RalGDS/AF-6) domain family member 3 (Rassf3), mRNA [NM_001108747]	**25,608**
0	Unknown	**25,601**
Slc16a7	Rattus norvegicus solute carrier family 16, member 7 (monocarboxylic acid transporter 2) (Slc16a7), mRNA [NM_017302]	**25,598**
Dpysl4	Rattus norvegicus dihydropyrimidinase-like 4 (Dpysl4), mRNA [NM_012933]	**25,595**
0	PREDICTED: Rattus norvegicus similar to 60S ribosomal protein L29 (P23) (RGD1563134), mRNA [XM_001080572]	**25,586**
Strada	Rattus norvegicus STE20-related kinase adaptor alpha (Strada), mRNA [NM_182820]	**25,583**
She	Uncharacterized protein [Source:UniProtKB/TrEMBL;Acc:D3ZSK0] [ENSRNOT00000028225]	**25,578**
Lifr	Rattus norvegicus leukemia inhibitory factor receptor alpha (Lifr), mRNA [NM_031048]	**25,577**
Plg	Rattus norvegicus plasminogen (Plg), mRNA [NM_053491]	**25,571**
Prosc	Rattus norvegicus TL0ACA41YL07 mRNA sequence. [FQ216663]	**25,560**
RGD1306446	Rattus norvegicus similar to RIKEN cDNA 9930032O22 gene (RGD1306446), mRNA [NM_001008554]	**25,556**
Rhbdd2	Rattus norvegicus rhomboid domain containing 2 (Rhbdd2), mRNA [NM_001191827]	**25,536**
Hist1h2bc	Rattus norvegicus histone cluster 1, H2bc (Hist1h2bc), mRNA [NM_001109400]	**25,525**
Rasd2	Rattus norvegicus RASD family, member 2 (Rasd2), mRNA [NM_133568]	**25,520**
RGD1306151	Rattus norvegicus similar to hypothetical protein DKFZp761D0211 (RGD1306151), mRNA [NM_001108652]	**25,507**
0	Unknown	**25,507**
Zic2	Rattus norvegicus Zic family member 2 (odd-paired homolog, Drosophila) (Zic2), mRNA [NM_001108392]	**25,499**
Arhgap17	Rattus norvegicus Rho GTPase activating protein 17 (Arhgap17), mRNA [NM_022244]	**25,477**
0	Unknown	**25,475**
Snap91	Rattus norvegicus synaptosomal-associated protein 91 (Snap91), mRNA [NM_031728]	**25,472**
0	Unknown	**25,461**
Susd4	Rattus norvegicus sushi domain containing 4 (Susd4), mRNA [NM_001105982]	**25,451**
Elavl1	Rattus norvegicus ELAV (embryonic lethal, abnormal vision, Drosophila)-like 1 (Hu antigen R) (Elavl1), mRNA [NM_001108848]	**25,447**
Slc25a34	Rattus norvegicus solute carrier family 25, member 34 (Slc25a34), mRNA [NM_001013936]	**25,431**
Cenpm	Rattus norvegicus centromere protein M (Cenpm), mRNA [NM_001130504]	**25,417**
Cdc42bpb	Rattus norvegicus CDC42 binding protein kinase beta (DMPK-like) (Cdc42bpb), mRNA [NM_053620]	**25,410**
Tbkbp1	Rattus norvegicus TBK1 binding protein 1 (Tbkbp1), mRNA [NM_172021]	**25,408**
Rffl	Rattus norvegicus ring finger and FYVE like domain containing protein (Rffl), mRNA [NM_001004068]	**25,401**
0	RNA binding motif protein 27 (Predicted), isoform CRA_aUncharacterized protein [Source:UniProtKB/TrEMBL;Acc:D3ZE52] [ENSRNOT00000060199]	**25,395**

Fut9	Rattus norvegicus fucosyltransferase 9 (alpha (1,3) fucosyltransferase) (Fut9), mRNA [NM_053465]	**25,387**
Ece1	Endothelin-converting enzyme 1 [Source:UniProtKB/Swiss-Prot;Acc:P42893] [ENSRNOT00000067616]	**25,384**
Trpc2	Rattus norvegicus transient receptor potential cation channel, subfamily C, member 2, mRNA (cDNA clone MGC:189397 IMAGE:9091675), complete cds. [BC169022]	**25,382**
Taok2	Rattus norvegicus TAO kinase 2 (Taok2), mRNA [NM_022702]	**25,379**
Phldb1	Rattus norvegicus pleckstrin homology-like domain, family B, member 1 (Phldb1), mRNA [NM_001191578]	**25,379**
Pcdhga1	Rattus norvegicus protocadherin gamma subfamily A, 1 (Pcdhga1), mRNA [NM_001037140]	**25,374**
0	YG01_MOUSE (Q9CQB7) LYR family protein A-211C6.1 homolog, partial (31%) [TC592130]	**25,373**
Abcc9	Rattus norvegicus ATP-binding cassette, subfamily C (CFTR/MRP), member 9 (Abcc9), mRNA [NM_013040]	**25,354**
Kif18b	Rattus norvegicus kinesin family member 18B (Kif18b), mRNA [NM_001039019]	**25,349**
Diablo	Rattus norvegicus diablo homolog (Drosophila) (Diablo), nuclear gene encoding mitochondrial protein, mRNA [NM_001008292]	**25,348**
Maz	Rattus norvegicus MYC-associated zinc finger protein (purine-binding transcription factor) (Maz), mRNA [NM_001110319]	**25,342**
Mier1	Rattus norvegicus mesoderm induction early response 1 homolog (Xenopus laevis) (Mier1), mRNA [NM_001131012]	**25,329**
Mansc1	Rattus norvegicus MANSC domain containing 1 (Mansc1), mRNA [NM_001109603]	**25,323**
Insl3	Rattus norvegicus insulin-like 3 (Insl3), mRNA [NM_053680]	**25,316**
Trim16	Rattus norvegicus tripartite motif-containing 16 (Trim16), mRNA [NM_001135033]	**25,305**
Grm5	Rattus norvegicus glutamate receptor, metabotropic 5 (Grm5), mRNA [NM_017012]	**25,304**
Rabl4	Rattus norvegicus RAB, member of RAS oncogene family-like 4 (Rabl4), mRNA [NM_001130495]	**25,301**
Timp4	Rattus norvegicus tissue inhibitor of metalloproteinase 4 (Timp4), mRNA [NM_001109393]	**25,298**
Wbscr17	Rattus norvegicus Williams-Beuren syndrome chromosome region 17 homolog (human) (Wbscr17), mRNA [NM_001025112]	**25,287**
RGD1566130	Rattus norvegicus similar to mKIAA1940 protein (RGD1566130), mRNA [NM_001107871]	**25,286**
0	Unknown	**25,281**
Slc12a6	Rattus norvegicus solute carrier family 12, member 6 (Slc12a6), mRNA [NM_001109630]	**25,275**
Adam19	Rattus norvegicus a disintegrin and metallopeptidase domain 19 (meltrin beta) (Adam19), mRNA [NM_001160228]	**25,257**
Sema5a	Rattus norvegicus sema domain, seven thrombospondin repeats (type 1 and type 1-like), transmembrane domain (TM) and short cytoplasmic domain, (semaphorin) 5A (Sema5a), mRNA [NM_001107659]	**25,257**
Bcr	PREDICTED: Rattus norvegicus breakpoint cluster region (Bcr), mRNA [XM_228091]	**25,241**
Acox1	Rattus norvegicus acyl-Coenzyme A oxidase 1, palmitoyl (Acox1), mRNA [NM_017340]	**25,234**
0	Unknown	**25,231**
Ints3	Uncharacterized protein [Source:UniProtKB/TrEMBL;Acc:D3ZAI3] [ENSRNOT00000020575]	**25,208**
Adra2b	Rattus norvegicus adrenergic, alpha-2B-, receptor (Adra2b), mRNA [NM_138505]	**25,194**
Casc3	Rattus norvegicus cancer susceptibility candidate 3 (Casc3), mRNA [NM_147144]	**25,192**
Nhlrc1	Rattus norvegicus NHL repeat containing 1 (Nhlrc1), mRNA [NM_199236]	**25,184**
0	Rattus norvegicus TL0AAA50YL16 mRNA sequence. [FQ212907]	**25,180**

RGD1559508	PREDICTED: Rattus norvegicus similar to hypothetical protein 4930474N05 (RGD1559508), mRNA [XM_577489]	**25,172**
Slc25a30	Rattus norvegicus solute carrier family 25, member 30 (Slc25a30), nuclear gene encoding mitochondrial protein, mRNA [NM_001013187]	**25,155**
Fchsd1	Rattus norvegicus FCH and double SH3 domains 1 (Fchsd1), transcript variant 2, mRNA [NM_001107392]	**25,152**
Dhh	Rattus norvegicus desert hedgehog homolog (Drosophila) (Dhh), mRNA [NM_053367]	**25,144**
Wasl	Rattus norvegicus Wiskott-Aldrich syndrome-like (Wasl), mRNA [NM_001110365]	**25,133**
rnf141	Rattus norvegicus ring finger protein 141 (rnf141), mRNA [NM_001001800]	**25,127**
Stambp	Rattus norvegicus Stam binding protein (Stambp), mRNA [NM_138531]	**25,114**
Tmc1	Rattus norvegicus transmembrane channel-like 1 (Tmc1), mRNA [NM_001108521]	**25,106**
Igsf11	Rattus norvegicus immunoglobulin superfamily, member 11 (Igsf11), mRNA [NM_001013120]	**25,105**
0	transient receptor potential cation channel subfamily M member 3 [Source:RefSeq peptide;Acc:NP_001178491] [ENSRNOT00000017755]	**25,105**
Pacsin2	Rattus norvegicus protein kinase C and casein kinase substrate in neurons 2 (Pacsin2), mRNA [NM_130740]	**25,102**
Fezf2	Rattus norvegicus Fez family zinc finger 2 (Fezf2), mRNA [NM_001107251]	**25,102**
Rab2b	Rattus norvegicus RAB2B, member RAS oncogene family (Rab2b), mRNA [NM_001037645]	**25,090**
Irgq	Rattus norvegicus immunity-related GTPase family, Q (Irgq), mRNA [NM_001135742]	**25,086**
0	Unknown	**25,085**
Furin	Rattus norvegicus furin (paired basic amino acid cleaving enzyme) (Furin), mRNA [NM_019331]	**25,083**
RGD1566107	Rattus norvegicus similar to cAMP responsive element binding protein 5 (RGD1566107), mRNA [NM_001134621]	**25,081**
Ywhae	Rattus norvegicus tyrosine 3-monooxygenase/tryptophan 5-monooxygenase activation protein, epsilon polypeptide (Ywhae), mRNA [NM_031603]	**25,075**
Arhgap1	Rattus norvegicus Rho GTPase activating protein 1 (Arhgap1), mRNA [NM_001107747]	**25,073**
0	Unknown	**25,051**
RGD1562091	Rattus norvegicus similar to expressed sequence C79127 (RGD1562091), mRNA [NM_001106231]	**25,037**
Man2a1	Rattus norvegicus mannosidase, alpha, class 2A, member 1 (Man2a1), mRNA [NM_012979]	**25,034**
RGD1565496	Rattus norvegicus similar to Butyrate-induced transcript 1 (RGD1565496), mRNA [NM_001106831]	**25,033**
Tor1aip2	Rattus norvegicus torsin A interacting protein 2 (Tor1aip2), transcript variant 1, mRNA [NM_199100]	**25,002**
RGD1560884	Rattus norvegicus similar to myosin tail domain-containing protein (RGD1560884), mRNA [NM_001134641]	**24,998**
Ccna2	Rattus norvegicus cyclin A2 (Ccna2), mRNA [NM_053702]	**24,994**
LOC100365745	PREDICTED: Rattus norvegicus hypothetical protein LOC100365745 (LOC100365745), mRNA [XM_002725938]	**24,984**
LOC687796	PREDICTED: Rattus norvegicus hypothetical protein LOC687796, transcript variant 2 (LOC687796), mRNA [XM_001078497]	**24,984**
RGD1306502	Rattus norvegicus similar to hypothetical protein FLJ11193 (RGD1306502), mRNA [NM_001107654]	**24,983**
Timp3	Rattus norvegicus TIMP metallopeptidase inhibitor 3 (Timp3), mRNA [NM_012886]	**24,964**
Rics	PREDICTED: Rattus norvegicus Rho GTPase-activating protein (Rics), mRNA [XM_001056872]	**24,959**
Rarg	Rattus norvegicus retinoic acid receptor, gamma (Rarg), transcript variant 1, mRNA [NM_001135249]	**24,952**
LOC681825	PREDICTED: Rattus norvegicus similar to Prefoldin subunit 3 (Von Hippel-Lindau-binding protein 1) (VHL-binding protein 1) (VBP-1) (LOC681825), mRNA [XM_001058594]	**24,951**

Ctdspl	Rattus norvegicus CTD (carboxy-terminal domain, RNA polymerase II, polypeptide A) small phosphatase-like (Ctdspl), mRNA [NM_001106865]	**24,945**
Cacna1e	Rattus norvegicus calcium channel, voltage-dependent, R type, alpha 1E subunit (Cacna1e), mRNA [NM_019294]	**24,945**
Zfp469	Rattus norvegicus zinc finger protein 469 (Zfp469), mRNA [NM_001107123]	**24,942**
Pde6d	Rattus norvegicus phosphodiesterase 6D, cGMP-specific, rod, delta (Pde6d), mRNA [NM_001108806]	**24,941**
siat7D	PREDICTED: Rattus norvegicus alpha-2,6-sialyltransferase ST6GalNAc IV (siat7D), mRNA [XM_001080040]	**24,930**
Rbks	Rattus norvegicus ribokinase (Rbks), mRNA [NM_001108703]	**24,930**
LOC100192313	Rattus norvegicus hypothetical protein LOC100192313 (LOC100192313), mRNA [NM_001136261]	**24,919**
Psenen	Rattus norvegicus presenilin enhancer 2 homolog (C. elegans) (Psenen), mRNA [NM_001008764]	**24,912**
Il34	Rattus norvegicus interleukin 34 (Il34), mRNA [NM_001025766]	**24,908**
Smtn	Rattus norvegicus smoothelin (Smtn), mRNA [NM_001013049]	**24,904**
0	DRNBTB09 Rat DRG Library Rattus norvegicus cDNA clone DRNBTB09 5', mRNA sequence [BG673348]	**24,904**
Sdc2	Syndecan-2 [Source:UniProtKB/Swiss-Prot;Acc:P34900] [ENSRNOT00000007255]	**24,896**
Hrk	Rattus norvegicus harakiri, BCL2 interacting protein (contains only BH3 domain) (Hrk), mRNA [NM_057130]	**24,891**
0	Unknown	**24,889**
Spg21	Rattus norvegicus spastic paraplegia 21 homolog (human) (Spg21), mRNA [NM_001006987]	**24,876**
RGD1310945	PREDICTED: Rattus norvegicus similar to hypothetical protein FLJ23305 (RGD1310945), mRNA [XM_001065531]	**24,875**
Nlrp1b	PREDICTED: Rattus norvegicus similar to NACHT, leucine rich repeat and PYD containing 1 (LOC691998), mRNA [XM_001080760]	**24,874**
0	Unknown	**24,853**
Pla2g7	Rattus norvegicus phospholipase A2, group VII (platelet-activating factor acetylhydrolase, plasma) (Pla2g7), mRNA [NM_001009353]	**24,849**
0	motile sperm domain-containing protein 2 [Source:RefSeq peptide;Acc:NP_001128060] [ENSRNOT00000004533]	**24,848**
Rell2	Rattus norvegicus RELT-like 2 (Rell2), mRNA [NM_001014149]	**24,846**
0	RVL7467 Wackym-Soares normalized rat vestibular cDNA library Rattus norvegicus cDNA 5', mRNA sequence [DV717012]	**24,840**
Tfb1m	Rattus norvegicus transcription factor B1, mitochondrial (Tfb1m), nuclear gene encoding mitochondrial protein, mRNA [NM_181474]	**24,838**
Brd4	Rattus norvegicus bromodomain containing 4 (Brd4), mRNA [NM_001100903]	**24,827**
Ppp3cb	Rattus norvegicus protein phosphatase 3, catalytic subunit, beta isoform (Ppp3cb), mRNA [NM_017042]	**24,827**
Gp1bb	Rattus norvegicus glycoprotein Ib (platelet), beta polypeptide (Gp1bb), mRNA [NM_053930]	**24,822**
Sos2	Rattus norvegicus son of sevenless homolog 2 (Drosophila) (Sos2), mRNA [NM_001135561]	**24,805**
Ap2b1	Rattus norvegicus adaptor-related protein complex 2, beta 1 subunit (Ap2b1), mRNA [NM_080583]	**24,803**
Zmat5	Uncharacterized protein [Source:UniProtKB/TrEMBL;Acc:D3ZLD2] [ENSRNOT00000010332]	**24,794**
RGD1308448	Rattus norvegicus similar to RIKEN cDNA B130016O10 gene (RGD1308448), mRNA [NM_001107671]	**24,780**
38596	Rattus norvegicus septin 5 (Sept5), mRNA [NM_053931]	**24,780**
Ppy	Rattus norvegicus pancreatic polypeptide (Ppy), mRNA [NM_012626]	**24,771**
Saps1	Rattus norvegicus SAPS domain family, member 1 (Saps1), mRNA [NM_001135849]	**24,769**
Rhd	Rattus norvegicus Rh blood group, D antigen (Rhd), mRNA [NM_022505]	**24,766**

Parm1	Rattus norvegicus prostate androgen-regulated mucin-like protein 1 (Parm1), mRNA [NM_173114]	**24,758**
0	gene regulated by estrogen in breast cancer protein Gene [Source:MGI Symbol;Acc:MGI:2149712] [ENSRNOT00000032417]	**24,747**
Ghr	Rattus norvegicus growth hormone receptor (Ghr), mRNA [NM_017094]	**24,744**
Drg2	Uncharacterized protein [Source:UniProtKB/TrEMBL;Acc:D3ZDC1] [ENSRNOT00000005101]	**24,744**
Dag1	PREDICTED: Rattus norvegicus dystroglycan 1 (dystrophin-associated glycoprotein 1) (Dag1), mRNA [XM_001074892]	**24,735**
Ltbp4	Rattus norvegicus latent transforming growth factor beta binding protein 4 (Ltbp4), mRNA [NM_001170336]	**24,732**
Pdxp	Rattus norvegicus pyridoxal (pyridoxine, vitamin B6) phosphatase (Pdxp), mRNA [NM_001135819]	**24,722**
Numb	Rattus norvegicus numb homolog (Drosophila) (Numb), mRNA [NM_133287]	**24,721**
Tnk2	Rattus norvegicus tyrosine kinase, non-receptor, 2 (Tnk2), mRNA [NM_001008336]	**24,716**
RGD1305415	Uncharacterized protein [Source:UniProtKB/TrEMBL;Acc:D3ZQL6] [ENSRNOT00000036357]	**24,713**
Tbccd1	Rattus norvegicus TBCC domain containing 1 (Tbccd1), mRNA [NM_001012016]	**24,705**
0	Unknown	**24,702**
Pcdhga12	Rattus norvegicus protocadherin gamma subfamily A, 12 (Pcdhga12), mRNA [NM_001037337]	**24,696**
Cdca3	Rattus norvegicus cell division cycle associated 3 (Cdca3), mRNA [NM_001007648]	**24,688**
Fxyd6	Rattus norvegicus FXYD domain-containing ion transport regulator 6 (Fxyd6), mRNA [NM_022005]	**24,682**
0	Unknown	**24,673**
Slc35e3	Rattus norvegicus solute carrier family 35, member E3 (Slc35e3), mRNA [NM_001134687]	**24,673**
0	Rattus norvegicus, 23 clones, strain BN/SsNHsdMCW RNOR03327679, whole genome shotgun sequence [AABR03128084]	**24,668**
Dpf2	Rattus norvegicus D4, zinc and double PHD fingers family 2 (Dpf2), mRNA [NM_001108516]	**24,655**
0	Unknown	**24,653**
Pycr1	Rattus norvegicus pyrroline-5-carboxylate reductase 1 (Pycr1), mRNA [NM_001105857]	**24,649**
Tnk2	Rattus norvegicus tyrosine kinase, non-receptor, 2 (Tnk2), mRNA [NM_001008336]	**24,648**
Zfp317	Rattus norvegicus zinc finger protein 317 (Zfp317), mRNA [NM_001134634]	**24,638**
0	Unknown	**24,635**
Cdc23	Rattus norvegicus CDC23 (cell division cycle 23, yeast, homolog) (Cdc23), mRNA [NM_001100659]	**24,626**
Ndufa3	PREDICTED: Rattus norvegicus NADH dehydrogenase (ubiquinone) 1 alpha subcomplex, 3 (Ndufa3), mRNA [XM_001076462]	**24,623**
Ankmy2	Rattus norvegicus ankyrin repeat and MYND domain containing 2 (Ankmy2), mRNA [NM_001108019]	**24,615**
Rbm38	Rattus norvegicus RNA binding motif protein 38 (Rbm38), mRNA [NM_001108965]	**24,609**
LRRTM1	Rattus norvegicus leucine rich repeat transmembrane neuronal 1 (LRRTM1), mRNA [NM_001109374]	**24,608**
Rerg	RERG_HUMAN (Q96A58) Ras-related and estrogen-regulated growth inhibitor, partial (19%) [TC629838]	**24,603**
Olr575	Rattus norvegicus olfactory receptor 575 (Olr575), mRNA [NM_001000327]	**24,600**
Rfk	Rattus norvegicus riboflavin kinase (Rfk), mRNA [NM_001014106]	**24,587**
Tmcc1	Uncharacterized protein [Source:UniProtKB/TrEMBL;Acc:D3ZH14] [ENSRNOT00000042418]	**24,583**
Lrp3	Rattus norvegicus low density lipoprotein receptor-related protein 3 (Lrp3), mRNA [NM_053541]	**24,582**

Lrp10	Rattus norvegicus low-density lipoprotein receptor-related protein 10 (Lrp10), mRNA [NM_001037777]	**24,580**
Nploc4	Rattus norvegicus nuclear protein localization 4 homolog (S. cerevisiae) (Nploc4), mRNA [NM_080577]	**24,573**
0	Unknown	**24,570**
0	Unknown	**24,551**
Araf	Rattus norvegicus v-raf murine sarcoma 3611 viral oncogene homolog (Araf), transcript variant 1, mRNA [NM_022532]	**24,547**
0	forkhead box protein K2 [Source:RefSeq peptide;Acc:NP_001100545] [ENSRNOT00000068086]	**24,541**
0	Histone H2B type 1 [Source:UniProtKB/Swiss-Prot;Acc:Q00715] [ENSRNOT00000024276]	**24,534**
Gad2	Rattus norvegicus glutamate decarboxylase 2 (Gad2), mRNA [NM_012563]	**24,520**
Olr1105	Rattus norvegicus olfactory receptor 1105 (Olr1105), mRNA [NM_001001078]	**24,516**
Fam101b	Rattus norvegicus family with sequence similarity 101, member B (Fam101b), mRNA [NM_001007611]	**24,511**
Tcf12	Rattus norvegicus transcription factor 12 (Tcf12), mRNA [NM_013176]	**24,500**
Etv1	Rattus norvegicus ets variant 1 (Etv1), transcript variant 1, mRNA [NM_001163156]	**24,472**
Ddx19b	Rattus norvegicus DEAD (Asp-Glu-Ala-As) box polypeptide 19B (Ddx19b), mRNA [NM_001005895]	**24,466**
E2f3	Rattus norvegicus E2F transcription factor 3 (E2f3), mRNA [NM_001137626]	**24,458**
38047	Uncharacterized protein [Source:UniProtKB/TrEMBL;Acc:D3ZNC9] [ENSRNOT00000022076]	**24,452**
Mast2	Rattus norvegicus microtubule associated serine/threonine kinase 2 (Mast2), mRNA [NM_001108005]	**24,451**
Slc18a3	Rattus norvegicus solute carrier family 18 (vesicular acetylcholine), member 3 (Slc18a3), mRNA [NM_031663]	**24,447**
0	Unknown	**24,441**
Ankrd29	Rattus norvegicus ankyrin repeat domain 29 (Ankrd29), mRNA [NM_001190372]	**24,440**
Asb8	Rattus norvegicus ankyrin repeat and SOCS box-containing 8 (Asb8), mRNA [NM_001108109]	**24,433**
Gpr115	Uncharacterized protein [Source:UniProtKB/TrEMBL;Acc:D3ZTY4] [ENSRNOT00000016906]	**24,433**
RGD1564961	PREDICTED: Rattus norvegicus similar to retinoblastoma-binding protein 1 isoform I (RGD1564961), mRNA [XM_226650]	**24,431**
Nr4a3	Rattus norvegicus nuclear receptor subfamily 4, group A, member 3 (Nr4a3), transcript variant 1, mRNA [NM_031628]	**24,424**
Uts2	Rattus norvegicus urotensin 2 (Uts2), mRNA [NM_019160]	**24,416**
0	Unknown	**24,413**
0	Unknown	**24,409**
Iws1	Rattus norvegicus IWS1 homolog (S. cerevisiae) (Iws1), mRNA [NM_001034918]	**24,408**
Ntan1	Rattus norvegicus N-terminal asparagine amidase (Ntan1), mRNA [NM_001025124]	**24,404**
Nckap5l	Uncharacterized protein [Source:UniProtKB/TrEMBL;Acc:D3Z9A7] [ENSRNOT00000022937]	**24,397**
LOC689065	Rattus norvegicus hypothetical protein LOC689065 (LOC689065), mRNA [NM_001109521]	**24,391**
RGD1308106	Rattus norvegicus LOC361719 (RGD1308106), mRNA [NM_001134575]	**24,378**
Stk24	Rattus norvegicus TL0AEA11YD24 mRNA sequence. [FQ227921]	**24,378**
Pogz	Rattus norvegicus pogo transposable element with ZNF domain (Pogz), mRNA [NM_001107693]	**24,375**
Fam110b	Rattus norvegicus family with sequence similarity 110, member B (Fam110b), mRNA [NM_001024341]	**24,367**

Map6d1	Rattus norvegicus MAP6 domain containing 1 (Map6d1), mRNA [NM_001108844]	**24,360**
0	Rattus norvegicus chromosome 10, 11 clones, strain BN/SsNHsdMCW RNOR03214576, whole genome shotgun sequence [AABR03073588]	**24,343**
0	Unknown	**24,328**
Map1lc3a	Rattus norvegicus microtubule-associated protein 1 light chain 3 alpha (Map1lc3a), mRNA [NM_199500]	**24,327**
Casq1	Rattus norvegicus calsequestrin 1 (fast-twitch, skeletal muscle) (Casq1), nuclear gene encoding mitochondrial protein, mRNA [NM_001159594]	**24,326**
Nkiras1	Rattus norvegicus NFKB inhibitor interacting Ras-like 1 (Nkiras1), mRNA [NM_001107252]	**24,321**
Rab10	Rattus norvegicus RAB10, member RAS oncogene family (Rab10), mRNA [NM_017359]	**24,316**
Zbtb4	PREDICTED: Rattus norvegicus zinc finger and BTB domain containing 4 (Zbtb4), mRNA [XM_001079524]	**24,314**
Scamp3	Rattus norvegicus secretory carrier membrane protein 3 (Scamp3), mRNA [NM_031724]	**24,308**
Slc1a3	Rattus norvegicus solute carrier family 1 (glial high affinity glutamate transporter), member 3 (Slc1a3), mRNA [NM_019225]	**24,300**
Ctrl	Rattus norvegicus chymotrypsin-like (Ctrl), mRNA [NM_054009]	**24,298**
Slc17a5	Rattus norvegicus solute carrier family 17 (anion/sugar transporter), member 5 (Slc17a5), mRNA [NM_001009713]	**24,297**
Pak6	Rattus norvegicus p21 protein (Cdc42/Rac)-activated kinase 6 (Pak6), mRNA [NM_001106498]	**24,297**
Ctnnd2	PREDICTED: Rattus norvegicus catenin (cadherin-associated protein), delta 2 (neural plakophilin-related arm-repeat protein) (Ctnnd2), mRNA [XM_001064375]	**24,294**
0	CB544633 AMGNNUC:SRPB2-00179-D4-A srpb2 (10220) Rattus norvegicus cDNA clone srpb2-00179-d4 5', mRNA sequence [CB544633]	**24,291**
Drd5	Rattus norvegicus dopamine receptor D5 (Drd5), mRNA [NM_012768]	**24,290**
Ankib1	Rattus norvegicus ankyrin repeat and IBR domain containing 1 (Ankib1), mRNA [NM_001134781]	**24,286**
Chm	Rattus norvegicus choroideremia (Rab escort protein 1) (Chm), mRNA [NM_017067]	**24,283**
Sirpa	Rattus norvegicus signal-regulatory protein alpha (Sirpa), mRNA [NM_013016]	**24,283**
Mansc1	Rattus norvegicus MANSC domain containing 1 (Mansc1), mRNA [NM_001109603]	**24,278**
Baiap2	Rattus norvegicus BAI1-associated protein 2 (Baiap2), mRNA [NM_057196]	**24,277**
LOC688267	PREDICTED: Rattus norvegicus hypothetical protein LOC688267 (LOC688267), mRNA [XM_002742439]	**24,277**
LOC687399	Rattus norvegicus hypothetical protein LOC687399 (LOC687399), mRNA [NM_001101015]	**24,274**
RGD1309870	Rattus norvegicus hypothetical LOC289778 (RGD1309870), mRNA [NM_001106018]	**24,272**
0	Unknown	**24,267**
0	Transcriptional regulator ATRX [Source:UniProtKB/Swiss-Prot;Acc:P70486] [ENSRNOT00000033355]	**24,265**
0	Rattus norvegicus similar to DAZ-associated protein 1 (LOC309292), mRNA [XM_219766]	**24,257**
Rad54l2	Rattus norvegicus Rad54 like 2 (S. cerevisiae) (Rad54l2), mRNA [NM_001134520]	**24,253**
Dusp19	Rattus norvegicus dual specificity phosphatase 19 (Dusp19), mRNA [NM_001107739]	**24,250**
Nek8	Rattus norvegicus NIMA (never in mitosis gene a)- related kinase 8 (Nek8), mRNA [NM_001105804]	**24,242**
St8sia3	Rattus norvegicus ST8 alpha-N-acetyl-neuraminide alpha-2,8-sialyltransferase 3 (St8sia3), mRNA [NM_013029]	**24,242**
Onecut1	Rattus norvegicus one cut homeobox 1 (Onecut1), mRNA [NM_022671]	**24,236**
Cpne5	Rattus norvegicus copine V (Cpne5), mRNA [NM_001107616]	**24,232**

0	Unknown	**24,223**
Rpl7l1	PREDICTED: Rattus norvegicus ribosomal protein L7-like 1 (Rpl7l1), mRNA [XM_001065509]	**24,220**
Erlin1	Rattus norvegicus ER lipid raft associated 1 (Erlin1), mRNA [NM_001106353]	**24,209**
Xk	Rattus norvegicus X-linked Kx blood group (McLeod syndrome) homolog (Xk), mRNA [NM_001012227]	**24,208**
Ccnd1	Rattus norvegicus cyclin D1 (Ccnd1), mRNA [NM_171992]	**24,208**
Ngf	PREDICTED: Rattus norvegicus nerve growth factor (beta polypeptide) (Ngf), partial mRNA [XM_001067130]	**24,205**
Myo1d	Rattus norvegicus myosin ID (Myo1d), mRNA [NM_012983]	**24,203**
Unc5c	Rattus norvegicus unc-5 homolog C (C. elegans) (Unc5c), mRNA [NM_199407]	**24,202**
Zfp799	Rattus norvegicus zinc finger protein 799 (Zfp799), mRNA [NM_001009537]	**24,190**
Tnfsf13	Rattus norvegicus tumor necrosis factor (ligand) superfamily, member 13 (Tnfsf13), mRNA [NM_001009623]	**24,181**
Il1r2	Rattus norvegicus interleukin 1 receptor, type II (Il1r2), mRNA [NM_053953]	**24,176**
Map2	Rattus norvegicus microtubule-associated protein 2 (Map2), mRNA [NM_013066]	**24,166**
RGD1308601	Rattus norvegicus similar to hypothetical protein (RGD1308601), mRNA [NM_001107374]	**24,165**
Epb41l1	Rattus norvegicus erythrocyte membrane protein band 4.1-like 1 (Epb41l1), transcript variant 2, mRNA [NM_021681]	**24,159**
Shoc2	Rattus norvegicus soc-2 (suppressor of clear) homolog (C. elegans) (Shoc2), mRNA [NM_001013155]	**24,159**
Lmf2	Rattus norvegicus lipase maturation factor 2 (Lmf2), mRNA [NM_001079939]	**24,159**
Lcn2	Rattus norvegicus lipocalin 2 (Lcn2), mRNA [NM_130741]	**24,158**
0	Rattus norvegicus similar to 40S ribosomal protein S8 (LOC302393), mRNA [XM_228533]	**24,151**
Atxn1l	Uncharacterized protein [Source:UniProtKB/TrEMBL;Acc:D3ZBC4] [ENSRNOT00000059225]	**24,148**
0	Angiomotin-like 1 (Predicted)Uncharacterized protein [Source:UniProtKB/TrEMBL;Acc:D3ZK60] [ENSRNOT00000011955]	**24,147**
Myadm	Rattus norvegicus myeloid-associated differentiation marker (Myadm), mRNA [NM_183332]	**24,137**
LOC500392	Rattus norvegicus similar to hypothetical protein FLJ25692 (LOC500392), mRNA [NM_001024340]	**24,134**
Pygo2	Rattus norvegicus pygopus 2 (Pygo2), mRNA [NM_001106447]	**24,129**
0	Unknown	**24,118**
RGD1309762	PREDICTED: Rattus norvegicus similar to KIAA0614 protein (RGD1309762), mRNA [XM_222205]	**24,111**
Aco2	Rattus norvegicus aconitase 2, mitochondrial (Aco2), nuclear gene encoding mitochondrial protein, mRNA [NM_024398]	**24,101**
Nol3	Rattus norvegicus nucleolar protein 3 (apoptosis repressor with CARD domain) (Nol3), mRNA [NM_053516]	**24,100**
Myst3	Rattus norvegicus MYST histone acetyltransferase (monocytic leukemia) 3 (Myst3), mRNA [NM_001100570]	**24,098**
Mapk6	Rattus norvegicus mitogen-activated protein kinase 6 (Mapk6), mRNA [NM_031622]	**24,087**
Tmem38a	Rattus norvegicus transmembrane protein 38a (Tmem38a), mRNA [NM_001100175]	**24,086**
Tspan2	Rattus norvegicus tetraspanin 2 (Tspan2), mRNA [NM_022589]	**24,085**
Sfrs3	Rattus norvegicus splicing factor, arginine/serine-rich 3 (Sfrs3), mRNA [NM_001047907]	**24,083**
Ankrd12	Rattus norvegicus ankyrin repeat domain 12 (Ankrd12), mRNA [NM_001108238]	**24,081**
LOC298139	Rattus norvegicus similar to RIKEN cDNA 2310003M01 (LOC298139), mRNA [NM_001013930]	**24,076**

Alkbh	Rattus norvegicus alkB, alkylation repair homolog (E. coli) (Alkbh), mRNA [NM_001108718]	**24,070**
0	Unknown	**24,057**
Bcas3	Rattus norvegicus breast carcinoma amplified sequence 3 (Bcas3), mRNA [NM_001173430]	**24,055**
Atf2	Rattus norvegicus activating transcription factor 2 (Atf2), mRNA [NM_031018]	**24,050**
Ssr3	Rattus norvegicus signal sequence receptor, gamma (Ssr3), mRNA [NM_031120]	**24,046**
0	Unknown	**24,044**
0	Unknown	**24,038**
Ndufb4l1	Uncharacterized protein [Source:UniProtKB/TrEMBL;Acc:D3ZV29] [ENSRNOT00000044617]	**24,037**
0	Unknown	**24,023**
0	Nol6 protein [Source:UniProtKB/TrEMBL;Acc:B0BNB6] [ENSRNOT00000068046]	**24,003**
Tp53bp2	PREDICTED: Rattus norvegicus tumor protein p53 binding protein, 2, transcript variant 1 (Tp53bp2), mRNA [XM_001063503]	**24,000**
Ankrd55	PREDICTED: Rattus norvegicus ankyrin repeat domain 55 (Ankrd55), mRNA [XM_342195]	**23,996**
Tom1l2	Rattus norvegicus target of myb1-like 2 (chicken) (Tom1l2), mRNA [NM_001108277]	**23,992**
Pusl1	Rattus norvegicus pseudouridylate synthase-like 1 (Pusl1), mRNA [NM_001108699]	**23,991**
Agfg1	Rattus norvegicus ArfGAP with FG repeats 1 (Agfg1), mRNA [NM_001135596]	**23,979**
LOC500213	PREDICTED: Rattus norvegicus similar to T-box transcription factor TBX15 (T-box protein 15) (MmTBx8) (LOC500213), mRNA [XM_575564]	**23,974**
Rab3d	Rattus norvegicus RAB3D, member RAS oncogene family (Rab3d), mRNA [NM_080580]	**23,974**
Nos1	Rattus norvegicus nitric oxide synthase 1, neuronal (Nos1), mRNA [NM_052799]	**23,958**
Cdk2	Rattus norvegicus cyclin dependent kinase 2 (Cdk2), mRNA [NM_199501]	**23,949**
0	Unknown	**23,948**
Srrm2	Uncharacterized protein [Source:UniProtKB/TrEMBL;Acc:D4AAC6] [ENSRNOT00000006370]	**23,948**
Foxj3	Rattus norvegicus forkhead box J3 (Foxj3), mRNA [NM_001107971]	**23,945**
Myoz3	Synaptopodin [Source:UniProtKB/Swiss-Prot;Acc:Q9Z327] [ENSRNOT00000025989]	**23,945**
Ube2ql1	Rattus norvegicus ubiquitin-conjugating enzyme E2Q family-like 1 (Ube2ql1), mRNA [NM_001145163]	**23,942**
Mgll	Rattus norvegicus monoglyceride lipase (Mgll), mRNA [NM_138502]	**23,939**
Per1	Rattus norvegicus period homolog 1 (Drosophila) (Per1), mRNA [NM_001034125]	**23,936**
Arl5b	Rattus norvegicus ADP-ribosylation factor-like 5B (Arl5b), mRNA [NM_001015031]	**23,924**
LOC689939	PREDICTED: Rattus norvegicus similar to KIAA0999 protein (LOC689939), mRNA [XM_001072611]	**23,924**
Mybbp1a	Rattus norvegicus MYB binding protein (P160) 1a (Mybbp1a), mRNA [NM_031668]	**23,913**
0	Q4SRM6_TETNG (Q4SRM6) Chromosome undetermined SCAF14516, whole genome shotgun sequence. (Fragment), partial (5%) [TC616541]	**23,911**
Stradb	Rattus norvegicus STE20-related kinase adaptor beta (Stradb), mRNA [NM_001109307]	**23,893**
Dusp7	Dual specificity protein phosphatase 7 [Source:UniProtKB/Swiss-Prot;Acc:Q63340] [ENSRNOT00000014770]	**23,880**
Znhit1	Uncharacterized protein [Source:UniProtKB/TrEMBL;Acc:D3ZNG9] [ENSRNOT00000001922]	**23,869**
Col27a1	Rattus norvegicus collagen, type XXVII, alpha 1 (Col27a1), mRNA [NM_198747]	**23,856**

Phkg2	Rattus norvegicus phosphorylase kinase, gamma 2 (testis) (Phkg2), mRNA [NM_080584]	**23,846**
Stx16	Rattus norvegicus syntaxin 16 (Stx16), mRNA [NM_001108610]	**23,829**
Rgs7bp	Rattus norvegicus regulator of G-protein signaling 7 binding protein (Rgs7bp), mRNA [NM_001012347]	**23,821**
Mfap3l	Rattus norvegicus microfibrillar-associated protein 3-like (Mfap3l), mRNA [NM_001012049]	**23,818**
Col20a1	Uncharacterized protein [Source:UniProtKB/TrEMBL;Acc:D3ZII5] [ENSRNOT00000040270]	**23,810**
Tpm4	Rattus norvegicus tropomyosin 4 (Tpm4), mRNA [NM_012678]	**23,808**
0	Unknown	**23,793**
Kcnma1	Rattus norvegicus potassium large conductance calcium-activated channel, subfamily M, alpha member 1 (Kcnma1), mRNA [NM_031828]	**23,790**
Tmc4	Rattus norvegicus transmembrane channel-like 4 (Tmc4), mRNA [NM_001034104]	**23,778**
Strn4	Rattus norvegicus striatin, calmodulin binding protein 4 (Strn4), transcript variant 1, mRNA [NM_001107480]	**23,769**
Ocrl	Rattus norvegicus oculocerebrorenal syndrome of Lowe (Ocrl), mRNA [NM_001108256]	**23,765**
Xylt1	Xylosyltransferase 1 [Source:UniProtKB/Swiss-Prot;Acc:Q9EPI1] [ENSRNOT00000025007]	**23,763**
Drap1	Rattus norvegicus Dr1 associated protein 1 (negative cofactor 2 alpha) (Drap1), mRNA [NM_001077668]	**23,762**
Sp1	Rattus norvegicus Sp1 transcription factor (Sp1), mRNA [NM_012655]	**23,760**
LOC500726	Uncharacterized protein [Source:UniProtKB/TrEMBL;Acc:D4A1G8] [ENSRNOT00000030246]	**23,760**
0	Unknown	**23,758**
Arnt2	Rattus norvegicus aryl hydrocarbon receptor nuclear translocator 2 (Arnt2), mRNA [NM_012781]	**23,753**
RGD1311648	Rattus norvegicus similar to hypothetical protein FLJ21820 (RGD1311648), mRNA [NM_001014075]	**23,750**
Pou3f1	Rattus norvegicus POU class 3 homeobox 1 (Pou3f1), mRNA [NM_138838]	**23,747**
Olr1366	Rattus norvegicus olfactory receptor 1366 (Olr1366), mRNA [NM_001000980]	**23,742**
Usp48	Rattus norvegicus ubiquitin specific peptidase 48 (Usp48), mRNA [NM_198785]	**23,731**
Trove2	Rattus norvegicus TROVE domain family, member 2 (Trove2), mRNA [NM_001107183]	**23,720**
Nop2	Rattus norvegicus NOP2 nucleolar protein homolog (yeast) (Nop2), mRNA [NM_001191785]	**23,718**
Ube3b	Rattus norvegicus ubiquitin protein ligase E3B (Ube3b), mRNA [NM_001143894]	**23,711**
LOC680319	Rattus norvegicus hypothetical protein LOC680319 (LOC680319), mRNA [NM_001109401]	**23,704**
Slc8a3	Rattus norvegicus solute carrier family 8 (sodium/calcium exchanger), member 3 (Slc8a3), mRNA [NM_078620]	**23,703**
Freq	Rattus norvegicus frequenin homolog (Drosophila) (Freq), mRNA [NM_024366]	**23,702**
Eri3	Uncharacterized protein [Source:UniProtKB/TrEMBL;Acc:D4AA56] [ENSRNOT00000026222]	**23,701**
Sms	Rattus norvegicus spermine synthase (Sms), mRNA [NM_001033899]	**23,698**
Shisa2	Rattus norvegicus shisa homolog 2 (Xenopus laevis) (Shisa2), mRNA [NM_001191936]	**23,694**
0	MMNPT2S01 Na+-phosphate cotransporter type II {Mus musculus} (exp=-1; wgp=0; cg=0), partial (12%) [TC607345]	**23,682**
Zfp46	Rattus norvegicus zinc finger protein 46 (Zfp46), mRNA [NM_001106691]	**23,662**
Eif1	Rattus norvegicus eukaryotic translation initiation factor 1 (Eif1), mRNA [NM_001105837]	**23,655**
B3gat3	Rattus norvegicus beta-1,3-glucuronyltransferase 3 (glucuronosyltransferase I) (B3gat3), mRNA [NM_001128184]	**23,652**

Sptbn2	Rattus norvegicus spectrin, beta, non-erythrocytic 2 (Sptbn2), mRNA [NM_019167]	**23,647**
Znf598	Rattus norvegicus zinc finger protein 598 (Znf598), mRNA [NM_001105770]	**23,645**
Nr4a1	Rattus norvegicus nuclear receptor subfamily 4, group A, member 1 (Nr4a1), mRNA [NM_024388]	**23,643**
Shisa7	Rattus norvegicus shisa homolog 7 (Xenopus laevis) (Shisa7), mRNA [NM_001145175]	**23,642**
Mmp14	Rattus norvegicus matrix metallopeptidase 14 (membrane-inserted) (Mmp14), mRNA [NM_031056]	**23,634**
0	Unknown	**23,627**
Scrt1	Rattus norvegicus scratch homolog 1, zinc finger protein (Drosophila) (Scrt1), mRNA [NM_001130570]	**23,626**
Grip1	Rattus norvegicus glutamate receptor interacting protein 1 (Grip1), mRNA [NM_032069]	**23,625**
Ppp1r1b	Rattus norvegicus protein phosphatase 1, regulatory (inhibitor) subunit 1B (Ppp1r1b), mRNA [NM_138521]	**23,620**
Nos2	Rattus norvegicus nitric oxide synthase 2, inducible (Nos2), mRNA [NM_012611]	**23,598**
Ampd3	Rattus norvegicus adenosine monophosphate deaminase 3 (Ampd3), mRNA [NM_031544]	**23,591**
Usf2	Rattus norvegicus upstream transcription factor 2, c-fos interacting (Usf2), mRNA [NM_031139]	**23,584**
Smad5	Rattus norvegicus SMAD family member 5 (Smad5), mRNA [NM_021692]	**23,575**
Tmem167b	Rattus norvegicus transmembrane protein 167B (Tmem167b), mRNA [NM_001135260]	**23,536**
0	Unknown	**23,535**
Mcart1	Rattus norvegicus mitochondrial carrier triple repeat 1 (Mcart1), nuclear gene encoding mitochondrial protein, mRNA [NM_001024785]	**23,534**
0	Unknown	**23,533**
Cyp46a1	Rattus norvegicus cytochrome P450, family 46, subfamily a, polypeptide 1 (Cyp46a1), mRNA [NM_001108723]	**23,506**
RGD1565886	PREDICTED: Rattus norvegicus RGD1565886 (RGD1565886), mRNA [XM_237943]	**23,504**
Zbtb16	Rattus norvegicus zinc finger and BTB domain containing 16 (Zbtb16), mRNA [NM_001013181]	**23,501**
Slc25a39	Rattus norvegicus solute carrier family 25, member 39 (Slc25a39), mRNA [NM_001024792]	**23,494**
Ccdc60	Rattus norvegicus coiled-coil domain containing 60 (Ccdc60), mRNA [NM_001034945]	**23,486**
Kctd7	Rattus norvegicus potassium channel tetramerisation domain containing 7 (Kctd7), mRNA [NM_001128194]	**23,484**
Sorbs2	Rattus norvegicus sorbin and SH3 domain containing 2 (Sorbs2), mRNA [NM_053770]	**23,480**
Npas4	Rattus norvegicus neuronal PAS domain protein 4 (Npas4), mRNA [NM_153626]	**23,478**
Letmd1	Rattus norvegicus LETM1 domain containing 1 (Letmd1), mRNA [NM_001122781]	**23,478**
Pptc7	Rattus norvegicus PTC7 protein phosphatase homolog (S. cerevisiae) (Pptc7), mRNA [NM_001107141]	**23,446**
0	Q3U6G1_MOUSE (Q3U6G1) Bone marrow macrophage cDNA, RIKEN full-length enriched library, clone:I830125G18 product:biliverdin reductase B (flavin reductase (NADPH)), full insert sequence, partial (78%) [TC609126]	**23,434**
Srek1ip1	Rattus norvegicus splicing regulatory glutamine/lysine-rich protein 1 interacting protein 1 (Srek1ip1), mRNA [NM_001008373]	**23,428**
RGD1560171	PREDICTED: Rattus norvegicus similar to PRO0149 protein (RGD1560171), mRNA [XM_001054786]	**23,427**
0	GSCR1_HUMAN (Q9NZM4) Glioma tumor suppressor candidate region gene 1 protein, partial (8%) [TC610073]	**23,423**
0	Unknown	**23,422**
Mfap5	Rattus norvegicus microfibrillar associated protein 5 (Mfap5), mRNA [NM_001108644]	**23,407**

Smad4	Rattus norvegicus SMAD family member 4 (Smad4), mRNA [NM_019275]	**23,406**
Dpm2	Rattus norvegicus dolichyl-phosphate mannosyltransferase polypeptide 2, regulatory subunit (Dpm2), mRNA [NM_019252]	**23,405**
Otor	Rattus norvegicus otoraplin (Otor), mRNA [NM_001108960]	**23,398**
Rod1	Rattus norvegicus ROD1 regulator of differentiation 1 (S. pombe) (Rod1), mRNA [NM_031346]	**23,390**
LOC291686	PREDICTED: Rattus norvegicus similar to 60S ribosomal protein L23a (LOC291686), mRNA [XM_002725324]	**23,377**
0	Rattus norvegicus similar to glyceraldehyde-3-phosphate dehydrogenase (LOC297809), mRNA [XM_216324]	**23,373**
Gtf3c2	Rattus norvegicus general transcription factor IIIC, polypeptide 2, beta (Gtf3c2), mRNA [NM_001025120]	**23,369**
Fam167a	Rattus norvegicus family with sequence similarity 167, member A (Fam167a), mRNA [NM_001109102]	**23,352**
LRRTM1	Rattus norvegicus leucine rich repeat transmembrane neuronal 1 (LRRTM1), mRNA [NM_001109374]	**23,352**
Slc12a5	Rattus norvegicus solute carrier family 12 (potassium-chloride transporter), member 5 (Slc12a5), mRNA [NM_134363]	**23,350**
Casc4	PREDICTED: Rattus norvegicus cancer susceptibility candidate 4, transcript variant 3 (Casc4), mRNA [XM_001077018]	**23,350**
Serping1	Rattus norvegicus serine (or cysteine) peptidase inhibitor, clade G, member 1 (Serping1), mRNA [NM_199093]	**23,340**
RGD1307399	Rattus norvegicus similar to chromosome 20 open reading frame 30; HSPC274 protein (RGD1307399), mRNA [NM_001048043]	**23,337**
0	Unknown	**23,330**
Diaph3	PREDICTED: Rattus norvegicus diaphanous homolog 3 (Drosophila) (Diaph3), mRNA [XM_001074393]	**23,325**
Ptprr	Rattus norvegicus protein tyrosine phosphatase, receptor type, R (Ptprr), transcript variant 1, mRNA [NM_053594]	**23,324**
Map3k7	Rattus norvegicus mitogen activated protein kinase kinase kinase 7 (Map3k7), mRNA [NM_001107920]	**23,319**
Dok7	Rattus norvegicus docking protein 7 (Dok7), mRNA [NM_001130062]	**23,314**
Ncln	Rattus norvegicus nicalin homolog (zebrafish) (Ncln), mRNA [NM_001014082]	**23,312**
Cdc2l6	Rattus norvegicus cell division cycle 2-like 6 (CDK8-like) (Cdc2l6), mRNA [NM_001107634]	**23,312**
Fam134b	Rattus norvegicus family with sequence similarity 134, member B (Fam134b), mRNA [NM_001034912]	**23,299**
Arc	Rattus norvegicus activity-regulated cytoskeleton-associated protein (Arc), mRNA [NM_019361]	**23,287**
LOC501389	PREDICTED: Rattus norvegicus similar to chromosome 9 open reading frame 36 (LOC501389), partial mRNA [XM_576802]	**23,283**
Tet1	Rattus norvegicus tet oncogene 1 (Tet1), mRNA [NM_001107643]	**23,278**
Dnm3	Rattus norvegicus dynamin 3 (Dnm3), mRNA [NM_138538]	**23,275**
Tbc1d25	Rattus norvegicus TBC1 domain family, member 25 (Tbc1d25), mRNA [NM_001106955]	**23,273**
Utp6	PREDICTED: Rattus norvegicus UTP6, small subunit (SSU) processome component, homolog (yeast), transcript variant 2 (Utp6), mRNA [XM_002724525]	**23,255**
Sel1l	Rattus norvegicus sel-1 suppressor of lin-12-like (C. elegans) (Sel1l), mRNA [NM_177933]	**23,254**
Pcsk1n	Rattus norvegicus proprotein convertase subtilisin/kexin type 1 inhibitor (Pcsk1n), mRNA [NM_019279]	**23,250**
Grlf1	Uncharacterized protein [Source:UniProtKB/TrEMBL;Acc:D4AD82] [ENSRNOT00000021223]	**23,247**
Abcg1	Rattus norvegicus ATP-binding cassette, subfamily G (WHITE), member 1 (Abcg1), mRNA [NM_053502]	**23,246**
Olr1262	Rattus norvegicus olfactory receptor 1262 (Olr1262), mRNA [NM_001000803]	**23,244**
Snrpa	Rattus norvegicus small nuclear ribonucleoprotein polypeptide A (Snrpa), mRNA [NM_001008303]	**23,241**

Cpne8	Rattus norvegicus copine VIII (Cpne8), mRNA [NM_001108750]	**23,241**
Rap2b	Rattus norvegicus RAP2B, member of RAS oncogene family (Rap2b), mRNA [NM_133410]	**23,234**
Copa	Rattus norvegicus coatomer protein complex subunit alpha (Copa), mRNA [NM_001134540]	**23,233**
Pcp4l1	Rattus norvegicus Purkinje cell protein 4-like 1 (Pcp4l1), mRNA [NM_001126093]	**23,232**
Ncoa5	Rattus norvegicus nuclear receptor coactivator 5 (Ncoa5), mRNA [NM_001106543]	**23,231**
Ston2	Rattus norvegicus stonin 2 (Ston2), mRNA [NM_001135874]	**23,225**
Nfkbib	Rattus norvegicus nuclear factor of kappa light polypeptide gene enhancer in B-cells inhibitor, beta (Nfkbib), mRNA [NM_030867]	**23,225**
0	EST352149 Rat gene index, normalized rat, norvegicus, Bento Soares Rattus norvegicus cDNA clone RGIHD72 5' end, mRNA sequence [AW920845]	**23,221**
0	Q5BK04_RAT (Q5BK04) LOC363306 protein (Fragment), partial (23%) [TC600364]	**23,221**
Nsd1	Rattus norvegicus nuclear receptor binding SET domain protein 1 (Nsd1), mRNA [NM_001107337]	**23,216**
Pcdhb3	Rattus norvegicus protocadherin beta 3 (Pcdhb3), mRNA [NM_001014783]	**23,212**
Slc7a14	Rattus norvegicus solute carrier family 7 (cationic amino acid transporter, y+ system), member 14 (Slc7a14), mRNA [NM_001134615]	**23,203**
Pcnp	Rattus norvegicus PEST proteolytic signal containing nuclear protein (Pcnp), mRNA [NM_001047846]	**23,201**
Stag1	Rattus norvegicus stromal antigen 1 (Stag1), mRNA [NM_001108179]	**23,197**
Prdx6	Rattus norvegicus peroxiredoxin 6 (Prdx6), mRNA [NM_053576]	**23,191**
Alg2	Rattus norvegicus asparagine-linked glycosylation 2, alpha-1,3-mannosyltransferase homolog (S. cerevisiae) (Alg2), mRNA [NM_001100710]	**23,186**
0	Uncharacterized protein [Source:UniProtKB/TrEMBL;Acc:D3ZV40] [ENSRNOT00000009733]	**23,175**
Slc16a1	Rattus norvegicus solute carrier family 16, member 1 (monocarboxylic acid transporter 1) (Slc16a1), mRNA [NM_012716]	**23,168**
0	Unknown	**23,164**
Pon2	Rattus norvegicus paraoxonase 2 (Pon2), mRNA [NM_001013082]	**23,155**
Ephb1	Rattus norvegicus Eph receptor B1 (Ephb1), mRNA [NM_001104528]	**23,150**
Pcdha1	Rattus norvegicus protocadherin alpha 1 (Pcdha1), mRNA [NM_199503]	**23,147**
Acsm5	Rattus norvegicus acyl-CoA synthetase medium-chain family member 5 (Acsm5), mRNA [NM_001014162]	**23,147**
RGD1563106	Rattus norvegicus similar to novel protein (RGD1563106), mRNA [NM_001107010]	**23,141**
Cpne8	Rattus norvegicus copine VIII (Cpne8), mRNA [NM_001108750]	**23,137**
Hist1h2bm	Rattus norvegicus histone cluster 1, H2bm (Hist1h2bm), mRNA [NM_001108414]	**23,123**
Traf3	Rattus norvegicus Tnf receptor-associated factor 3 (Traf3), mRNA [NM_001108724]	**23,122**
Gnas	Rattus norvegicus GNAS complex locus (Gnas), transcript variant 5, mRNA [NM_001159656]	**23,120**
Smoc1	Rattus norvegicus SPARC related modular calcium binding 1 (Smoc1), mRNA [NM_001002835]	**23,106**
Usp6nl	Rattus norvegicus USP6 N-terminal like (Usp6nl), mRNA [NM_001106120]	**23,100**
Pmepa1	Rattus norvegicus prostate transmembrane protein, androgen induced 1 (Pmepa1), mRNA [NM_001107807]	**23,095**
Map2k7	Rattus norvegicus mitogen activated protein kinase kinase 7 (Map2k7), mRNA [NM_001025425]	**23,086**
Mapkapk2	Rattus norvegicus mitogen-activated protein kinase-activated protein kinase 2 (Mapkapk2), mRNA [NM_178102]	**23,080**

0	family with sequence similarity 184, member A Gene [Source:MGI Symbol;Acc:MGI:1923156] [ENSRNOT00000000475]	**23,077**
Olr1395	Rattus norvegicus olfactory receptor 1395 (Olr1395), mRNA [NM_001001092]	**23,074**
Ust	Rattus norvegicus uronyl-2-sulfotransferase (Ust), mRNA [NM_001108458]	**23,063**
Man1c1	Rattus norvegicus mannosidase, alpha, class 1C, member 1 (Man1c1), mRNA [NM_001108687]	**23,053**
0	CR467520 Rat pBluescript Lion Rattus norvegicus cDNA clone LIONp463D12400 3', mRNA sequence [CR467520]	**23,053**
Reep1	Rattus norvegicus receptor accessory protein 1 (Reep1), nuclear gene encoding mitochondrial protein, mRNA [NM_001108633]	**23,050**
RGD1307621	Rattus norvegicus hypothetical LOC314168 (RGD1307621), mRNA [NM_001108025]	**23,049**
Dlgap2	Rattus norvegicus discs, large (Drosophila) homolog-associated protein 2 (Dlgap2), mRNA [NM_053901]	**23,047**
RGD1308782	Rattus norvegicus similar to Zinc finger protein OZF (POZF-1) (RGD1308782), mRNA [NM_001025677]	**23,041**
0	Echinoderm microtubule-associated protein-like 5 [Source:UniProtKB/Swiss-Prot;Acc:Q6ED65] [ENSRNOT00000005978]	**23,036**
0	PREDICTED: Rattus norvegicus RALBP1 associated Eps domain containing 2 (Reps2), miscRNA [XR_085945]	**23,031**
LOC100364162	PREDICTED: Rattus norvegicus ring finger protein 11-like (LOC100364162), mRNA [XM_002726577]	**23,029**
Scmh1	Rattus norvegicus sex comb on midleg homolog 1 (Drosophila) (Scmh1), mRNA [NM_001109669]	**23,026**
Hist3h2a	Rattus norvegicus histone cluster 3, H2a (Hist3h2a), mRNA [NM_021840]	**23,025**
Arl2bp	Rattus norvegicus ADP-ribosylation factor-like 2 binding protein (Arl2bp), mRNA [NM_001024906]	**23,021**
Fam123a	PREDICTED: Rattus norvegicus family with sequence similarity 123A, transcript variant 2 (Fam123a), mRNA [XM_573799]	**23,016**
Ascl5	PREDICTED: Rattus norvegicus achaete-scute complex homolog 5 (Drosophila) (Ascl5), mRNA [XM_344144]	**23,014**
RGD1560891	RCG36219Uncharacterized protein [Source:UniProtKB/TrEMBL;Acc:D4ACQ4] [ENSRNOT00000044006]	**22,996**
Fam12b	Rattus norvegicus family with sequence similarity 12, member B (epididymal) (Fam12b), mRNA [NM_178103]	**22,992**
Cry2	Rattus norvegicus cryptochrome 2 (photolyase-like) (Cry2), mRNA [NM_133405]	**22,991**
RGD1566264	Uncharacterized protein [Source:UniProtKB/TrEMBL;Acc:D3ZFF1] [ENSRNOT00000051929]	**22,989**
Mavs	Rattus norvegicus mitochondrial antiviral signaling protein (Mavs), nuclear gene encoding mitochondrial protein, mRNA [NM_001005556]	**22,980**
Tnni2	Rattus norvegicus troponin I type 2 (skeletal, fast) (Tnni2), mRNA [NM_017185]	**22,978**
Bcl2l1	Rattus norvegicus Bcl2-like 1 (Bcl2l1), nuclear gene encoding mitochondrial protein, transcript variant 3, mRNA [NM_001033670]	**22,977**
Cyp4f6	Rattus norvegicus cytochrome P450, family 4, subfamily f, polypeptide 6 (Cyp4f6), mRNA [NM_153318]	**22,958**
Hsd17b7	Rattus norvegicus hydroxysteroid (17-beta) dehydrogenase 7 (Hsd17b7), mRNA [NM_017235]	**22,941**
Ahcyl1	Rattus norvegicus adenosylhomocysteinase-like 1 (Ahcyl1), mRNA [NM_001108561]	**22,931**
Nras	Rattus norvegicus neuroblastoma ras oncogene (Nras), mRNA [NM_080766]	**22,929**
Prok2	Rattus norvegicus prokineticin 2 (Prok2), transcript variant 1, mRNA [NM_001037541]	**22,921**
Dbndd2	Rattus norvegicus dysbindin (dystrobrevin binding protein 1) domain containing 2 (Dbndd2), mRNA [NM_001047111]	**22,916**
Arhgap26	Rattus norvegicus Rho GTPase activating protein 26 (Arhgap26), mRNA [NM_001107389]	**22,910**
Gatm	Rattus norvegicus glycine amidinotransferase (L-arginine:glycine amidinotransferase) (Gatm), nuclear gene encoding mitochondrial protein, mRNA [NM_031031]	**22,906**
Lrba	Rattus norvegicus LPS-responsive vesicle trafficking, beach and anchor containing (Lrba), mRNA [NM_001108555]	**22,904**

Gabrb2	Rattus norvegicus gamma-aminobutyric acid (GABA) A receptor, beta 2 (Gabrb2), mRNA [NM_012957]	**22,897**
0	Q8R1R5_RAT (Q8R1R5) MIC2L1, partial (9%) [TC628118]	**22,897**
RGD1311249	Rattus norvegicus similar to RIKEN cDNA B230312A22 (RGD1311249), mRNA [NM_001013931]	**22,890**
Dmd	Rattus norvegicus dystrophin (Dmd), transcript variant Dp71c, mRNA [NM_001005246]	**22,890**
Arfgap3	Rattus norvegicus ADP-ribosylation factor GTPase activating protein 3 (Arfgap3), mRNA [NM_001044273]	**22,882**
Hormad2	Rattus norvegicus HORMA domain containing 2 (Hormad2), mRNA [NM_001017501]	**22,880**
0	Unknown	**22,876**
Rhbdl3	Rattus norvegicus rhomboid, veinlet-like 3 (Drosophila) (Rhbdl3), mRNA [NM_001105819]	**22,856**
0	Rattus norvegicus activity and neurotransmitter-induced early gene 2 (ania-2) mRNA, 3'UTR. [AF030087]	**22,855**
Pycrl	Rattus norvegicus pyrroline-5-carboxylate reductase-like (Pycrl), mRNA [NM_001011993]	**22,847**
0	Uncharacterized protein [Source:UniProtKB/TrEMBL;Acc:D4A4B0] [ENSRNOT00000032008]	**22,847**
Wipf2	Rattus norvegicus WAS/WASL interacting protein family, member 2 (Wipf2), mRNA [NM_001191825]	**22,842**
Fam25a	Rattus norvegicus family with sequence similarity 25, member A (Fam25a), mRNA [NM_001134849]	**22,839**
Tmem101	Rattus norvegicus transmembrane protein 101 (Tmem101), mRNA [NM_001191650]	**22,837**
Lhfpl3	Uncharacterized protein [Source:UniProtKB/TrEMBL;Acc:D4AA53] [ENSRNOT00000043281]	**22,833**
Fbxo42	Rattus norvegicus F-box protein 42 (Fbxo42), mRNA [NM_001108691]	**22,825**
0	RCG37698, isoform CRA_aUncharacterized protein [Source:UniProtKB/TrEMBL;Acc:D4AAY1] [ENSRNOT00000037022]	**22,823**
Arhgef11	Rattus norvegicus Rho guanine nucleotide exchange factor (GEF) 11 (Arhgef11), mRNA [NM_023982]	**22,819**
0	Rattus norvegicus similar to 60S RIBOSOMAL PROTEIN L29 (P23) (LOC291941), mRNA [XM_226367]	**22,815**
0	Unknown	**22,811**
Synj1	Rattus norvegicus synaptojanin 1 (Synj1), mRNA [NM_053476]	**22,809**
0	Unknown	**22,807**
Sall3	Rattus norvegicus sal-like 3 (Drosophila) (Sall3), mRNA [NM_001108892]	**22,794**
0	Unknown	**22,793**
0	GLIS family zinc finger 1 (Predicted), isoform CRA_bUncharacterized protein [Source:UniProtKB/TrEMBL;Acc:D4A5Q8] [ENSRNOT00000032433]	**22,790**
Gabrb1	Rattus norvegicus gamma-aminobutyric acid (GABA) A receptor, beta 1 (Gabrb1), mRNA [NM_012956]	**22,786**
LOC313672	Rattus norvegicus kazrin (LOC313672), mRNA [NM_001014070]	**22,782**
Abi2	Rattus norvegicus abl-interactor 2 (Abi2), mRNA [NM_173143]	**22,782**
Spon1	Rattus norvegicus spondin 1, extracellular matrix protein (Spon1), mRNA [NM_172067]	**22,769**
LOC498662	Rattus norvegicus similar to RIKEN cDNA 2610019F03 (LOC498662), mRNA [NM_001100791]	**22,766**
Stox2	Rattus norvegicus storkhead box 2 (Stox2), transcript variant 1, mRNA [NM_001134863]	**22,762**
Rab3b	Rattus norvegicus RAB3B, member RAS oncogene family (Rab3b), mRNA [NM_031091]	**22,757**
RGD1565310	PREDICTED: Rattus norvegicus similar to RIKEN cDNA 1110018J12 (RGD1565310), mRNA [XM_001057346]	**22,748**
RGD1565183	Uncharacterized protein [Source:UniProtKB/TrEMBL;Acc:D3ZG08] [ENSRNOT00000049254]	**22,748**

Msl2	PREDICTED: Rattus norvegicus male-specific lethal 2-like 1 (Drosophila) (Msl2l1), mRNA [XM_001071576]	**22,742**
0	Unknown	**22,741**
Slc25a42	Rattus norvegicus solute carrier family 25, member 42 (Slc25a42), mRNA [NM_001127590]	**22,741**
LOC296300	Rattus norvegicus similar to zinc finger protein 341 (LOC296300), mRNA [NM_001047859]	**22,737**
Plekhg2	Rattus norvegicus pleckstrin homology domain containing, family G (with RhoGef domain) member 2 (Plekhg2), mRNA [NM_001134972]	**22,733**
Dhcr24	Rattus norvegicus 24-dehydrocholesterol reductase (Dhcr24), mRNA [NM_001080148]	**22,730**
Pak3	Rattus norvegicus p21 protein (Cdc42/Rac)-activated kinase 3 (Pak3), mRNA [NM_019210]	**22,730**
Scaf1	Rattus norvegicus SR-related CTD-associated factor 1 (Scaf1), mRNA [NM_019384]	**22,730**
Hlf	PREDICTED: Rattus norvegicus hepatic leukemia factor (Hlf), mRNA [XM_001081149]	**22,728**
Hcrt	Rattus norvegicus hypocretin (Hcrt), mRNA [NM_013179]	**22,723**
0	Unknown	**22,713**
Gria2	Rattus norvegicus glutamate receptor, ionotropic, AMPA 2 (Gria2), transcript variant 2, mRNA [NM_001083811]	**22,707**
Pcsk6	Rattus norvegicus proprotein convertase subtilisin/kexin type 6 (Pcsk6), mRNA [NM_012999]	**22,700**
Scn11a	Rattus norvegicus sodium channel, voltage-gated, type XI, alpha (Scn11a), mRNA [NM_019265]	**22,692**
Tmem8b	Uncharacterized protein [Source:UniProtKB/TrEMBL;Acc:D4AD81] [ENSRNOT00000021227]	**22,692**
Rnf216	Rattus norvegicus ring finger protein 216 (Rnf216), mRNA [NM_001107122]	**22,687**
0	U2af2 protein [Source:UniProtKB/TrEMBL;Acc:Q5EB64] [ENSRNOT00000021391]	**22,686**
Golga7	Rattus norvegicus golgi autoantigen, golgin subfamily a, 7 (Golga7), mRNA [NM_001007731]	**22,685**
Zdhhc3	Rattus norvegicus zinc finger, DHHC-type containing 3 (Zdhhc3), mRNA [NM_001039014]	**22,681**
Cnih2	Rattus norvegicus cornichon homolog 2 (Drosophila) (Cnih2), mRNA [NM_001025132]	**22,680**
Ube2o	PREDICTED: Rattus norvegicus ubiquitin-conjugating enzyme E2O (Ube2o), mRNA [XM_001081723]	**22,679**
Sgsm1	Rattus norvegicus small G protein signaling modulator 1 (Sgsm1), mRNA [NM_001105937]	**22,677**
Ftsjd2	Rattus norvegicus FtsJ methyltransferase domain containing 2 (Ftsjd2), mRNA [NM_001014031]	**22,673**
RGD1562629	PREDICTED: Rattus norvegicus similar to neurobeachin (RGD1562629), miscRNA [XR_085753]	**22,671**
Acss1	Rattus norvegicus acyl-CoA synthetase short-chain family member 1 (Acss1), nuclear gene encoding mitochondrial protein, mRNA [NM_001106524]	**22,668**
0	Uncharacterized protein [Source:UniProtKB/TrEMBL;Acc:D4AE00] [ENSRNOT00000026030]	**22,667**
Tulp4	Rattus norvegicus tubby like protein 4 (Tulp4), mRNA [NM_001109137]	**22,665**
Pde1a	Rattus norvegicus phosphodiesterase 1A, calmodulin-dependent (Pde1a), mRNA [NM_030871]	**22,660**
Gas7	Rattus norvegicus mRNA for GAS-7 protein. [AJ131902]	**22,655**
Elovl1	Rattus norvegicus elongation of very long chain fatty acids (FEN1/Elo2, SUR4/Elo3, yeast)-like 1 (Elovl1), mRNA [NM_001044275]	**22,653**
Olr1535	Rattus norvegicus olfactory receptor 1535 (Olr1535), mRNA [NM_001000945]	**22,645**
Stfa2l1	Rattus norvegicus stefin A2-like 1 (Stfa2l1), mRNA [NM_001004129]	**22,644**
0	Unknown	**22,638**

Capza1	Rattus norvegicus capping protein (actin filament) muscle Z-line, alpha 1 (Capza1), mRNA [NM_001109625]	**22,634**
Rimbp2	Rattus norvegicus RIM binding protein 2 (Rimbp2), mRNA [NM_001100488]	**22,632**
Hspc159	Rattus norvegicus galectin-related protein (Hspc159), mRNA [NM_001134730]	**22,630**
0	Unknown	**22,627**
Taf10	Rattus norvegicus TAF10 RNA polymerase II, TATA box binding protein (TBP)-associated factor (Taf10), mRNA [NM_001134735]	**22,627**
Fchsd2	Rattus norvegicus FCH and double SH3 domains 2 (Fchsd2), mRNA [NM_001107539]	**22,623**
Slc44a2	Rattus norvegicus solute carrier family 44, member 2 (Slc44a2), mRNA [NM_001134715]	**22,613**
Lhfp	Rattus norvegicus lipoma HMGIC fusion partner (Lhfp), mRNA [NM_001109183]	**22,607**
0	Unknown	**22,603**
Cdc42	Rattus norvegicus cell division cycle 42 (GTP binding protein) (Cdc42), mRNA [NM_171994]	**22,601**
RGD1309586	Rattus norvegicus similar to probable ATP-dependent RNA helicase - mouse (RGD1309586), mRNA [NM_001108858]	**22,595**
Galnt1	Polypeptide N-acetylgalactosaminyltransferase 1Polypeptide N-acetylgalactosaminyltransferase 1 soluble form [Source:UniProtKB/Swiss-Prot;Acc:Q10473] [ENSRNOT00000022117]	**22,591**
Slc10a7	Rattus norvegicus solute carrier family 10 (sodium/bile acid cotransporter family), member 7 (Slc10a7), mRNA [NM_001010948]	**22,589**
LOC684998	PREDICTED: Rattus norvegicus hypothetical protein LOC684998 (LOC684998), mRNA [XM_001061848]	**22,582**
RGD1306595	Rattus norvegicus similar to hypothetical protein (RGD1306595), mRNA [NM_001025626]	**22,581**
Kdelr1	Rattus norvegicus KDEL (Lys-Asp-Glu-Leu) endoplasmic reticulum protein retention receptor 1 (Kdelr1), mRNA [NM_001017385]	**22,579**
Zfp384	Rattus norvegicus zinc finger protein 384 (Zfp384), transcript variant 1, mRNA [NM_133429]	**22,579**
A2bp1	Rattus norvegicus ataxin 2 binding protein 1 (A2bp1), mRNA [NM_001106974]	**22,569**
Fam195b	Rattus norvegicus family with sequence similarity 195, member B (Fam195b), mRNA [NM_001108311]	**22,567**
Tspan11	Rattus norvegicus tetraspanin 11 (Tspan11), mRNA [NM_001024262]	**22,557**
Add1	Rattus norvegicus adducin 1 (alpha) (Add1), mRNA [NM_016990]	**22,555**
Slc9a7	Rattus norvegicus solute carrier family 9 (sodium/hydrogen exchanger), member 7 (Slc9a7), mRNA [NM_001108242]	**22,553**
Ppbp	Rattus norvegicus pro-platelet basic protein (chemokine (C-X-C motif) ligand 7) (Ppbp), mRNA [NM_153721]	**22,551**
RGD1311084	Rattus norvegicus similar to 1700113K14Rik protein (RGD1311084), mRNA [NM_001100979]	**22,549**
0	CFP1_MOUSE (Q8BGU5) Cyclin fold protein 1, partial (69%) [TC584108]	**22,544**
Thtpa	Rattus norvegicus thiamine triphosphatase (Thtpa), mRNA [NM_001007682]	**22,537**
Clint1	Rattus norvegicus clathrin interactor 1 (Clint1), mRNA [NM_001002022]	**22,536**
Inpp5a	inositol polyphosphate-5-phosphatase A [Source:RefSeq peptide;Acc:NP_001102393] [ENSRNOT00000054890]	**22,533**
Ugt1a6	Rattus norvegicus UDP glucuronosyltransferase 1 family, polypeptide A6 (Ugt1a6), transcript variant 1, mRNA [NM_001039691]	**22,520**
Pvrl3	Rattus norvegicus poliovirus receptor-related 3 (Pvrl3), mRNA [NM_001105883]	**22,518**
Chic1	Uncharacterized protein [Source:UniProtKB/TrEMBL;Acc:D3ZJX3] [ENSRNOT00000015693]	**22,511**
Bri3bp	Rattus norvegicus Bri3 binding protein (Bri3bp), mRNA [NM_001017487]	**22,506**
Nf2	Rattus norvegicus neurofibromin 2 (merlin) (Nf2), mRNA [NM_013193]	**22,500**

Rnf40	Rattus norvegicus ring finger protein 40 (Rnf40), mRNA [NM_153471]	**22,500**
Cox11	Rattus norvegicus COX11 homolog, cytochrome c oxidase assembly protein (yeast) (Cox11), nuclear gene encoding mitochondrial protein, mRNA [NM_001109575]	**22,498**
Nsd1	Rattus norvegicus nuclear receptor binding SET domain protein 1 (Nsd1), mRNA [NM_001107337]	**22,497**
Cic	Rattus norvegicus capicua homolog (Drosophila) (Cic), mRNA [NM_001107490]	**22,490**
Rybp	Rattus norvegicus RING1 and YY1 binding protein (Rybp), mRNA [NM_001107879]	**22,489**
Zbtb7a	Rattus norvegicus zinc finger and BTB domain containing 7a (Zbtb7a), mRNA [NM_054002]	**22,467**
Synj1	Rattus norvegicus synaptojanin 1 (Synj1), mRNA [NM_053476]	**22,465**
F2r	Rattus norvegicus coagulation factor II (thrombin) receptor (F2r), mRNA [NM_012950]	**22,464**
Trpc4ap	Rattus norvegicus transient receptor potential cation channel, subfamily C, member 4 associated protein (Trpc4ap), mRNA [NM_001100748]	**22,463**
LOC688741	PREDICTED: Rattus norvegicus hypothetical protein LOC688741 (LOC688741), mRNA [XM_002724948]	**22,462**
Tbc1d24	Rattus norvegicus TBC1 domain family, member 24 (Tbc1d24), mRNA [NM_001105769]	**22,461**
Gabbr1	Rattus norvegicus GABA B receptor 1g mRNA, complete cds. [AF312319]	**22,452**
LOC680955	RCG43690, isoform CRA_aUncharacterized protein [Source:UniProtKB/TrEMBL;Acc:D3ZHC6] [ENSRNOT00000061674]	**22,447**
LOC100365064	PREDICTED: Rattus norvegicus zinc finger protein, multitype 1-like (LOC100365064), mRNA [XM_002725423]	**22,445**
LOC681351	PREDICTED: Rattus norvegicus similar to apolipoprotein L, 3 (LOC681351), mRNA [XM_001061370]	**22,442**
Nipsnap3b	Rattus norvegicus nipsnap homolog 3B (C. elegans) (Nipsnap3b), mRNA [NM_001009422]	**22,436**
Eif4e3	Rattus norvegicus eukaryotic translation initiation factor 4E family member 3 (Eif4e3), mRNA [NM_001106612]	**22,431**
0	Unknown	**22,429**
Gga3	Rattus norvegicus golgi associated, gamma adaptin ear containing, ARF binding protein 3 (Gga3), mRNA [NM_001108304]	**22,421**
Pxn	Rattus norvegicus paxillin (Pxn), mRNA [NM_001012147]	**22,420**
Ccdc71	Rattus norvegicus coiled-coil domain containing 71 (Ccdc71), mRNA [NM_001024904]	**22,417**
0	Rattus norvegicus TL0AEA68YG04 mRNA sequence. [FQ231741]	**22,411**
Mageb17	PREDICTED: Rattus norvegicus melanoma antigen family B, 17 (Mageb17), mRNA [XM_001063539]	**22,397**
Npc2	Rattus norvegicus Niemann-Pick disease, type C2 (Npc2), mRNA [NM_173118]	**22,390**
Znf703	Rattus norvegicus zinc finger protein 703 (Znf703), mRNA [NM_001109425]	**22,388**
Hist1h2bc	Rattus norvegicus histone cluster 1, H2bc (Hist1h2bc), mRNA [NM_001109400]	**22,383**
Dscaml1	Rattus norvegicus Down syndrome cell adhesion molecule-like 1 (Dscaml1), mRNA [NM_001108141]	**22,372**
Rorb	Nuclear receptor ROR-beta [Source:UniProtKB/Swiss-Prot;Acc:P45446] [ENSRNOT00000018137]	**22,372**
Zfp563	Rattus norvegicus zinc finger protein 563 (Zfp563), mRNA [NM_001134561]	**22,355**
0	EST231431 Normalized rat ovary, Bento Soares Rattus sp. cDNA clone ROVCI85 3' end, mRNA sequence [AI234869]	**22,355**
Casp7	Rattus norvegicus caspase 7 (Casp7), mRNA [NM_022260]	**22,353**
Kcnip4	Rattus norvegicus Kv channel interacting protein 4 (Kcnip4), mRNA [NM_181365]	**22,351**
Cyp51	Rattus norvegicus cytochrome P450, family 51 (Cyp51), mRNA [NM_012941]	**22,349**

Gria1	Rattus norvegicus glutamate receptor, ionotropic, AMPA 1 (Gria1), mRNA [NM_031608]	**22,337**
Zbtb2	Rattus norvegicus zinc finger and BTB domain containing 2 (Zbtb2), mRNA [NM_001107460]	**22,334**
Slc35e1	Rattus norvegicus solute carrier family 35, member E1 (Slc35e1), mRNA [NM_001109107]	**22,329**
0	Unknown	**22,327**
Srr	Rattus norvegicus serine racemase (Srr), mRNA [NM_198757]	**22,322**
Prkab2	Rattus norvegicus protein kinase, AMP-activated, beta 2 non-catalytic subunit (Prkab2), mRNA [NM_022627]	**22,317**
Mta1	Rattus norvegicus metastasis associated 1 (Mta1), mRNA [NM_022588]	**22,317**
Grik2	Glutamate receptor, ionotropic kainate 2 [Source:UniProtKB/Swiss-Prot;Acc:P42260] [ENSRNOT00000000415]	**22,308**
N4bp1	PREDICTED: Rattus norvegicus Nedd4 binding protein 1 (N4bp1), mRNA [XM_001068071]	**22,308**
Robo1	Rattus norvegicus roundabout homolog 1 (Drosophila) (Robo1), mRNA [NM_022188]	**22,305**
RGD1566107	Rattus norvegicus similar to cAMP responsive element binding protein 5 (RGD1566107), mRNA [NM_001134621]	**22,300**
Nt5dc3	Rattus norvegicus 5'-nucleotidase domain containing 3 (Nt5dc3), mRNA [NM_001134887]	**22,299**
Csde1	Rattus norvegicus cold shock domain containing E1, RNA binding (Csde1), mRNA [NM_054006]	**22,299**
Arpc4	Rattus norvegicus actin related protein 2/3 complex, subunit 4 (Arpc4), mRNA [NM_001106615]	**22,298**
RGD1564887	PREDICTED: Rattus norvegicus similar to 9130011E15Rik protein (RGD1564887), mRNA [XM_574677]	**22,297**
Rela	Rattus norvegicus v-rel reticuloendotheliosis viral oncogene homolog A (avian) (Rela), mRNA [NM_199267]	**22,296**
Erg	Rattus norvegicus v-ets erythroblastosis virus E26 oncogene homolog (avian) (Erg), mRNA [NM_133397]	**22,292**
0	PREDICTED: Rattus norvegicus similar to sidekick 1 (RGD1560686), miscRNA [XR_085987]	**22,284**
Rin2	Rattus norvegicus Ras and Rab interactor 2 (Rin2), mRNA [NM_001107786]	**22,284**
Rasl11a	Rattus norvegicus RAS-like family 11 member A (Rasl11a), mRNA [NM_001002829]	**22,279**
Rsad2	Rattus norvegicus radical S-adenosyl methionine domain containing 2 (Rsad2), mRNA [NM_138881]	**22,278**
Igsf1	Rattus norvegicus immunoglobulin superfamily, member 1 (Igsf1), mRNA [NM_175763]	**22,276**
Lsp1	Rattus norvegicus lymphocyte-specific protein 1 (Lsp1), mRNA [NM_001025420]	**22,275**
Ubqln4	Rattus norvegicus ubiquilin 4 (Ubqln4), mRNA [NM_001107688]	**22,267**
Lasp1	Rattus norvegicus LIM and SH3 protein 1 (Lasp1), mRNA [NM_032613]	**22,265**
Nod2	Rattus norvegicus nucleotide-binding oligomerization domain containing 2 (Nod2), mRNA [NM_001106172]	**22,265**
Fstl4	Rattus norvegicus follistatin-like 4 (Fstl4), mRNA [NM_001107000]	**22,261**
Camk1g	Rattus norvegicus calcium/calmodulin-dependent protein kinase IG (Camk1g), mRNA [NM_182842]	**22,254**
RT1-DMa	Rattus norvegicus RT1 class II, locus DMa (RT1-DMa), mRNA [NM_198741]	**22,251**
0	Unknown	**22,250**
Tspyl5	Uncharacterized protein [Source:UniProtKB/TrEMBL;Acc:D3ZLU5] [ENSRNOT00000008227]	**22,232**
Slc25a39	Rattus norvegicus solute carrier family 25, member 39 (Slc25a39), mRNA [NM_001024792]	**22,206**
Tjap1	Rattus norvegicus tight junction associated protein 1 (Tjap1), mRNA [NM_001108203]	**22,194**
0	Unknown	**22,193**

0	Unknown	**22,187**
0	Unknown	**22,185**
Rnf150	Rattus norvegicus ring finger protein 150 (Rnf150), mRNA [NM_001191093]	**22,183**
0	Unknown	**22,183**
0	Unknown	**22,182**
0	Rattus norvegicus TL0ACA22YL15 mRNA sequence. [FQ217597]	**22,180**
Ptpn12	Rattus norvegicus protein tyrosine phosphatase, non-receptor type 12 (Ptpn12), mRNA [NM_057115]	**22,180**
RGD1562035	PREDICTED: Rattus norvegicus similar to mast cell protease 1-like 3 precursor (RGD1562035), mRNA [XM_001057648]	**22,179**
Ppif	Rattus norvegicus peptidylprolyl isomerase F (Ppif), nuclear gene encoding mitochondrial protein, mRNA [NM_172243]	**22,175**
0	AGENCOURT_109870505 NIH_MGC_420 Rattus norvegicus cDNA clone IMAGE:9033411 5', mRNA sequence [EV768969]	**22,166**
Tomm34	Rattus norvegicus translocase of outer mitochondrial membrane 34 (Tomm34), nuclear gene encoding mitochondrial protein, mRNA [NM_001044244]	**22,161**
0	M-phase phosphoprotein 8 [Source:RefSeq peptide;Acc:NP_001017375] [ENSRNOT00000028112]	**22,158**
Flrt1	Rattus norvegicus fibronectin leucine rich transmembrane protein 1 (Flrt1), mRNA [NM_001109160]	**22,154**
0	Glucose-6-phosphate 1-dehydrogenase [Source:UniProtKB/TrEMBL;Acc:D4A851] [ENSRNOT00000057468]	**22,154**
Grina	Rattus norvegicus glutamate receptor, ionotropic, N-methyl D-aspartate-associated protein 1 (glutamate binding) (Grina), mRNA [NM_153308]	**22,154**
0	U2af2 protein [Source:UniProtKB/TrEMBL;Acc:Q5EB64] [ENSRNOT00000021391]	**22,146**
Fgf2	Rattus norvegicus fibroblast growth factor 2 (Fgf2), mRNA [NM_019305]	**22,144**
Slc7a14	Rattus norvegicus solute carrier family 7 (cationic amino acid transporter, y+ system), member 14 (Slc7a14), mRNA [NM_001134615]	**22,143**
Sos1	Rattus norvegicus Son of sevenless homolog 1 (Drosophila) (Sos1), mRNA [NM_001100716]	**22,143**
Tfg	Rattus norvegicus Trk-fused gene (Tfg), mRNA [NM_001012144]	**22,142**
Syt17	Rattus norvegicus synaptotagmin XVII (Syt17), mRNA [NM_138849]	**22,142**
Fam43a	Rattus norvegicus family with sequence similarity 43, member A (Fam43a), mRNA [NM_001039002]	**22,140**
Fgd1	Rattus norvegicus FYVE, RhoGEF and PH domain containing 1 (Fgd1), mRNA [NM_001037546]	**22,128**
Cygb	Rattus norvegicus cytoglobin (Cygb), mRNA [NM_130744]	**22,125**
Hsd17b11	Rattus norvegicus hydroxysteroid (17-beta) dehydrogenase 11 (Hsd17b11), mRNA [NM_001004209]	**22,121**
Slc43a2	Rattus norvegicus solute carrier family 43, member 2 (Slc43a2), mRNA [NM_001105812]	**22,117**
0	RVL15663 Wackym-Soares normalized rat vestibular cDNA library Rattus norvegicus cDNA 5', mRNA sequence [DV723748]	**22,108**
Rnf216	Rattus norvegicus ring finger protein 216 (Rnf216), mRNA [NM_001107122]	**22,107**
Kcnq1	Rattus norvegicus potassium voltage-gated channel, KQT-like subfamily, member 1 (Kcnq1), mRNA [NM_032073]	**22,104**
Ext1	Rattus norvegicus exostoses (multiple) 1 (Ext1), mRNA [NM_001130540]	**22,094**
Ifnar1	Rattus norvegicus interferon (alpha, beta and omega) receptor 1 (Ifnar1), mRNA [NM_001105893]	**22,093**
Bsn	Rattus norvegicus bassoon (Bsn), mRNA [NM_019146]	**22,093**
0	Unknown	**22,091**

Rap2ip	Rattus norvegicus Rap2 interacting protein (Rap2ip), mRNA [NM_198758]	**22,082**
Gcnt2	Rattus norvegicus glucosaminyl (N-acetyl) transferase 2, I-branching enzyme (Gcnt2), mRNA [NM_001001511]	**22,079**
LOC100360337	PREDICTED: Rattus norvegicus zinc finger protein 128 (LOC100360337), mRNA [XM_002725494]	**22,078**
Lrfn1	Rattus norvegicus leucine rich repeat and fibronectin type III domain containing 1 (Lrfn1), transcript variant 1, mRNA [NM_001127694]	**22,076**
Lce1l	Rattus norvegicus late cornified envelope 1L (Lce1l), mRNA [NM_001109493]	**22,076**
Foxk1	Rattus norvegicus forkhead box K1 (Foxk1), mRNA [NM_001037219]	**22,070**
0	EST350998 Rat gene index, normalized rat, norvegicus, Bento Soares Rattus norvegicus cDNA clone RGIGF73 5' end, mRNA sequence [AW919694]	**22,066**
Nf2	Rattus norvegicus neurofibromin 2 (merlin) (Nf2), mRNA [NM_013193]	**22,066**
Eif4g2	Rattus norvegicus eukaryotic translation initiation factor 4, gamma 2 (Eif4g2), mRNA [NM_001017374]	**22,063**
0	Uncharacterized protein [Source:UniProtKB/TrEMBL;Acc:D3ZR73] [ENSRNOT00000054883]	**22,062**
Sumo2	Rattus norvegicus SMT3 suppressor of mif two 3 homolog 2 (S. cerevisiae) (Sumo2), mRNA [NM_133594]	**22,056**
Sssca1	Rattus norvegicus Sjogren's syndrome/scleroderma autoantigen 1 homolog (human) (Sssca1), mRNA [NM_001109537]	**22,054**
Cldn12	Rattus norvegicus claudin 12 (Cldn12), mRNA [NM_001100813]	**22,050**
Cacna1e	Rattus norvegicus voltage-activated calcium channel alpha-1 subunit (rbe-ii) mRNA, complete cds. [L15453]	**22,047**
Egfr	Rattus norvegicus epidermal growth factor receptor (Egfr), mRNA [NM_031507]	**22,040**
Dlg3	Rattus norvegicus discs, large homolog 3 (Drosophila) (Dlg3), mRNA [NM_031639]	**22,037**
Arhgef2	Rattus norvegicus rho/rac guanine nucleotide exchange factor (GEF) 2 (Arhgef2), mRNA [NM_001012079]	**22,034**
Id2	Rattus norvegicus inhibitor of DNA binding 2 (Id2), mRNA [NM_013060]	**22,026**
Eid1	Rattus norvegicus EP300 interacting inhibitor of differentiation 1 (Eid1), mRNA [NM_001109205]	**22,022**
Scgb1a1	Rattus norvegicus secretoglobin, family 1A, member 1 (uteroglobin) (Scgb1a1), mRNA [NM_013051]	**22,020**
Ncoa1	Rattus norvegicus nuclear receptor coactivator 1 (Ncoa1), mRNA [NM_001108012]	**22,019**
Tmem185b	Rattus norvegicus transmembrane protein 185B (Tmem185b), mRNA [NM_001191668]	**22,013**
Sorbs3	Rattus norvegicus sorbin and SH3 domain containing 3 (Sorbs3), mRNA [NM_001005762]	**22,012**
LOC685890	PREDICTED: Rattus norvegicus hypothetical protein LOC685890 (LOC685890), mRNA [XM_001065654]	**22,008**
Tpm1	Rattus norvegicus tropomyosin 1, alpha (Tpm1), transcript variant 2, mRNA [NM_001034069]	**22,002**
Zfp346	Rattus norvegicus zinc finger protein 346 (Zfp346), mRNA [NM_001107338]	**22,001**
Neto1	Rattus norvegicus neuropilin (NRP) and tolloid (TLL)-like 1 (Neto1), mRNA [NM_001107371]	**21,999**
Trim5	Rattus norvegicus tripartite motif-containing 5 (Trim5), mRNA [NM_001014023]	**21,995**
Gpr137	Rattus norvegicus G protein-coupled receptor 137 (Gpr137), mRNA [NM_001109562]	**21,994**
Slc38a6	Rattus norvegicus solute carrier family 38, member 6 (Slc38a6), mRNA [NM_001013099]	**21,989**
Spg20	Rattus norvegicus spastic paraplegia 20 (Troyer syndrome) homolog (human) (Spg20), mRNA [NM_001106433]	**21,987**
Cops7a	Rattus norvegicus COP9 constitutive photomorphogenic homolog subunit 7A (Arabidopsis) (Cops7a), transcript variant 2, mRNA [NM_001047098]	**21,985**
Zfp39	Rattus norvegicus zinc finger protein 39 (Zfp39), mRNA [NM_001107004]	**21,975**

RGD1311095	Rattus norvegicus similar to hypothetical protein FLJ20259 (RGD1311095), mRNA [NM_001134532]	**21,974**
Il17b	Rattus norvegicus interleukin 17B (Il17b), mRNA [NM_053789]	**21,972**
R3hdm1	Rattus norvegicus R3H domain containing 1 (R3hdm1), mRNA [NM_001134867]	**21,965**
E2f6	Rattus norvegicus E2F transcription factor 6 (E2f6), mRNA [NM_001100717]	**21,965**
0	Cytochrome c oxidase subunit 3 [Source:UniProtKB/Swiss-Prot;Acc:P05505] [ENSRNOT00000049683]	**21,961**
Sdc3	Rattus norvegicus syndecan 3 (Sdc3), mRNA [NM_053893]	**21,957**
Ccdc49	Rattus norvegicus coiled-coil domain containing 49 (Ccdc49), mRNA [NM_001108295]	**21,956**
Sap130	Uncharacterized protein [Source:UniProtKB/TrEMBL;Acc:D3ZLP9] [ENSRNOT00000022761]	**21,947**
Faf2	Rattus norvegicus Fas associated factor family member 2 (Faf2), mRNA [NM_001017445]	**21,947**
Kif3c	Rattus norvegicus kinesin family member 3C (Kif3c), mRNA [NM_053486]	**21,939**
0	Rattus norvegicus similar to cytoplasmic beta-actin (LOC302827), mRNA [XM_229147]	**21,933**
Exd2	Rattus norvegicus exonuclease 3'-5' domain containing 2 (Exd2), mRNA [NM_001108715]	**21,931**
Pou3f2	Rattus norvegicus POU class 3 homeobox 2 (Pou3f2), mRNA [NM_172085]	**21,929**
Arhgdib	Rattus norvegicus Rho, GDP dissociation inhibitor (GDI) beta (Arhgdib), mRNA [NM_001009600]	**21,926**
Stox2	Rattus norvegicus storkhead box 2 (Stox2), transcript variant 2, mRNA [NM_173107]	**21,917**
Gtf3c5	Rattus norvegicus general transcription factor IIIC, polypeptide 5 (Gtf3c5), mRNA [NM_001079941]	**21,917**
0	Uncharacterized protein [Source:UniProtKB/TrEMBL;Acc:D3ZD05] [ENSRNOT00000014135]	**21,917**
Clcn3	Rattus norvegicus chloride channel 3 (Clcn3), mRNA [NM_053363]	**21,915**
0	Unknown	**21,912**
B3gnt2	Rattus norvegicus UDP-GlcNAc:betaGal beta-1,3-N-acetylglucosaminyltransferase 2 (B3gnt2), mRNA [NM_001107240]	**21,904**
Slc18a2	Rattus norvegicus solute carrier family 18 (vesicular monoamine), member 2 (Slc18a2), mRNA [NM_013031]	**21,901**
Apom	Rattus norvegicus apolipoprotein M (Apom), mRNA [NM_019373]	**21,889**
0	Rattus norvegicus similar to lacrimal gland protein (LOC365230), mRNA [XM_344882]	**21,885**
Tcta	Rattus norvegicus T-cell leukemia translocation altered gene (Tcta), mRNA [NM_001014005]	**21,882**
Prcp	Rattus norvegicus prolylcarboxypeptidase (angiotensinase C) (Prcp), mRNA [NM_001106281]	**21,877**
0	FQ099121 Rattus norvegicus brain Sprague-Dawley Rattus norvegicus cDNA 5', mRNA sequence [FQ099121]	**21,871**
0	Uncharacterized protein [Source:UniProtKB/TrEMBL;Acc:D3ZJE3] [ENSRNOT00000057150]	**21,861**
Ahcyl2	Rattus norvegicus adenosylhomocysteinase-like 2 (Ahcyl2), mRNA [NM_001173510]	**21,852**
Zfp192	Rattus norvegicus zinc finger protein 192 (Zfp192), mRNA [NM_001100574]	**21,841**
Vldlr	Rattus norvegicus very low density lipoprotein receptor (Vldlr), mRNA [NM_013155]	**21,840**
Ahrr	Rattus norvegicus aryl-hydrocarbon receptor repressor (Ahrr), mRNA [NM_001024285]	**21,839**
Ep300	PREDICTED: Rattus norvegicus E1A binding protein p300 (Ep300), mRNA [XM_576312]	**21,831**
Fam120a	Rattus norvegicus family with sequence similarity 120A (Fam120a), mRNA [NM_001191816]	**21,829**
Nhp2l1	Rattus norvegicus NHP2 non-histone chromosome protein 2-like 1 (S. cerevisiae) (Nhp2l1), mRNA [NM_212515]	**21,822**

Efr3a	Rattus norvegicus EFR3 homolog A (S. cerevisiae) (Efr3a), mRNA [NM_001130564]	**21,821**
Fam3a	Rattus norvegicus family with sequence similarity 3, member A (Fam3a), mRNA [NM_001109324]	**21,819**
0	Unknown	**21,819**
Rcor3	Rattus norvegicus REST corepressor 3 (Rcor3), mRNA [NM_001134985]	**21,818**
RGD1305733	Rattus norvegicus similar to RIKEN cDNA 2900011O08 (RGD1305733), mRNA [NM_001014114]	**21,816**
0	Rattus norvegicus similar to mKIAA1107 protein (LOC305122), mRNA [XM_223143]	**21,809**
Cks2	Rattus norvegicus CDC28 protein kinase regulatory subunit 2 (Cks2), mRNA [NM_001126083]	**21,802**
0	Unknown	**21,795**
Dclk1	Rattus norvegicus doublecortin-like kinase 1 (Dclk1), transcript variant 1, mRNA [NM_053343]	**21,793**
Mfap3	Rattus norvegicus microfibrillar-associated protein 3 (Mfap3), mRNA [NM_001007609]	**21,792**
0	Unknown	**21,792**
Calb2	Rattus norvegicus calbindin 2 (Calb2), mRNA [NM_053988]	**21,778**
0	Unknown	**21,775**
Rnf13	Rattus norvegicus ring finger protein 13 (Rnf13), mRNA [NM_001109444]	**21,775**
Grb10	Rattus norvegicus growth factor receptor bound protein 10 (Grb10), mRNA [NM_001109093]	**21,771**
Grem2	Rattus norvegicus gremlin 2, cysteine knot superfamily, homolog (Xenopus laevis) (Grem2), mRNA [NM_001105974]	**21,770**
Pcdhb2	Rattus norvegicus protocadherin beta 2 (Pcdhb2), mRNA [NM_001109123]	**21,769**
Fam131b	Rattus norvegicus family with sequence similarity 131, member B (Fam131b), mRNA [NM_001025046]	**21,764**
0	Rattus norvegicus strain F344 hepatocyte malignant transforming factor mRNA, complete sequence. [GU969272]	**21,753**
Lrrc61	Rattus norvegicus leucine rich repeat containing 61 (Lrrc61), mRNA [NM_001109231]	**21,753**
Rere	Rattus norvegicus arginine-glutamic acid dipeptide (RE) repeats (Rere), mRNA [NM_053885]	**21,727**
RGD1310552	Rattus norvegicus similar to hypothetical protein MGC38960 (RGD1310552), mRNA [NM_001106838]	**21,727**
Grb2	Rattus norvegicus growth factor receptor bound protein 2 (Grb2), mRNA [NM_030846]	**21,725**
Per3	Rattus norvegicus period homolog 3 (Drosophila) (Per3), mRNA [NM_023978]	**21,720**
Pvrl2	Rattus norvegicus poliovirus receptor-related 2 (Pvrl2), mRNA [NM_001012064]	**21,718**
Polr3f	Rattus norvegicus polymerase (RNA) III (DNA directed) polypeptide F (Polr3f), mRNA [NM_001107784]	**21,706**
Slc16a14	Rattus norvegicus solute carrier family 16, member 14 (monocarboxylic acid transporter 14) (Slc16a14), mRNA [NM_001108229]	**21,706**
Rrp7a	Rattus norvegicus ribosomal RNA processing 7 homolog A (S. cerevisiae) (Rrp7a), mRNA [NM_001130568]	**21,698**
Abhd12	Rattus norvegicus abhydrolase domain containing 12 (Abhd12), mRNA [NM_001024314]	**21,690**
Rnf126	Rattus norvegicus ring finger protein 126 (Rnf126), mRNA [NM_001033702]	**21,678**
Eif2c2	Rattus norvegicus eukaryotic translation initiation factor 2C, 2 (Eif2c2), mRNA [NM_021597]	**21,670**
0	Unknown	**21,667**
Pear1	Rattus norvegicus platelet endothelial aggregation receptor 1 (Pear1), mRNA [NM_001134959]	**21,665**
Pabpc1	Rattus norvegicus poly(A) binding protein, cytoplasmic 1 (Pabpc1), mRNA [NM_134353]	**21,664**

Eraf	Rattus norvegicus erythroid associated factor (Eraf), mRNA [NM_001106299]	**21,661**
LOC679547	PREDICTED: Rattus norvegicus hypothetical protein LOC679547 (LOC679547), mRNA [XM_002724484]	**21,630**
0	Unknown	**21,619**
0	Unknown	**21,612**
Etnk1	Rattus norvegicus ethanolamine kinase 1 (Etnk1), mRNA [NM_001107894]	**21,604**
Col5a3	Rattus norvegicus collagen, type V, alpha 3 (Col5a3), mRNA [NM_021760]	**21,604**
0	Unknown	**21,602**
Mxd4	Rattus norvegicus Max dimerization protein 4 (Mxd4), mRNA [NM_001108364]	**21,591**
Ncoa5	Rattus norvegicus nuclear receptor coactivator 5 (Ncoa5), mRNA [NM_001106543]	**21,590**
Gapt	Rattus norvegicus Grb2-binding adaptor protein, transmembrane (Gapt), mRNA [NM_001109491]	**21,585**
Ssh3	Rattus norvegicus slingshot homolog 3 (Drosophila) (Ssh3), mRNA [NM_001012217]	**21,581**
0	Unknown	**21,577**
Plbd2	Rattus norvegicus phospholipase B domain containing 2 (Plbd2), mRNA [NM_139255]	**21,574**
Pdgfrb	Rattus norvegicus platelet derived growth factor receptor, beta polypeptide (Pdgfrb), mRNA [NM_031525]	**21,571**
Fbxl17	Rattus norvegicus F-box and leucine-rich repeat protein 17 (Fbxl17), mRNA [NM_001108235]	**21,561**
Alcam	Rattus norvegicus activated leukocyte cell adhesion molecule (Alcam), mRNA [NM_031753]	**21,560**
Cks1b	Rattus norvegicus CDC28 protein kinase regulatory subunit 1B (Cks1b), mRNA [NM_001135749]	**21,557**
RGD1307682	Rattus norvegicus similar to hypothetical protein FLJ21827 (RGD1307682), mRNA [NM_001024760]	**21,554**
Chtf8	Rattus norvegicus CTF8, chromosome transmission fidelity factor 8 homolog (S. cerevisiae) (Chtf8), mRNA [NM_001194951]	**21,553**
0	Unknown	**21,553**
Myot	Rattus norvegicus myotilin (Myot), mRNA [NM_001106148]	**21,552**
Dnah11	PREDICTED: Rattus norvegicus dynein, axonemal, heavy chain 11 (Dnah11), mRNA [XM_001061747]	**21,551**
0	Unknown	**21,551**
Myo16	Rattus norvegicus myosin XVI (Myo16), mRNA [NM_138893]	**21,548**
Tpi1	Rattus norvegicus triosephosphate isomerase 1 (Tpi1), mRNA [NM_022922]	**21,544**
Add3	Rattus norvegicus adducin 3 (gamma) (Add3), transcript variant 1, mRNA [NM_001164103]	**21,523**
Ren	Rattus norvegicus renin (Ren), mRNA [NM_012642]	**21,522**
0	RCG22732Uncharacterized protein [Source:UniProtKB/TrEMBL;Acc:D3ZEH2] [ENSRNOT00000037521]	**21,522**
Zbtb44	Rattus norvegicus zinc finger and BTB domain containing 44 (Zbtb44), mRNA [NM_001034942]	**21,522**
Blcap	Rattus norvegicus bladder cancer associated protein homolog (human) (Blcap), mRNA [NM_133582]	**21,520**
Lmbrd2	Rattus norvegicus LMBR1 domain containing 2 (Lmbrd2), mRNA [NM_001109177]	**21,517**
0	Unknown	**21,512**
Zmiz1	Rattus norvegicus zinc finger, MIZ-type containing 1 (Zmiz1), mRNA [NM_001108393]	**21,512**
Nr4a2	Rattus norvegicus nuclear receptor subfamily 4, group A, member 2 (Nr4a2), mRNA [NM_019328]	**21,510**

Sh3bgrl	Rattus norvegicus SH3 domain binding glutamic acid-rich protein like (Sh3bgrl), mRNA [NM_001173339]	**21,509**
Ube2l6	Rattus norvegicus ubiquitin-conjugating enzyme E2L 6 (Ube2l6), mRNA [NM_001024755]	**21,507**
0	Unknown	**21,505**
Zyx	Rattus norvegicus zyxin (Zyx), mRNA [NM_053761]	**21,502**
0	telomerase-binding protein EST1A [Source:RefSeq peptide;Acc:NP_001099278] [ENSRNOT00000052412]	**21,500**
0	Unknown	**21,494**
Csnk1g1	Rattus norvegicus casein kinase 1, gamma 1 (Csnk1g1), mRNA [NM_022288]	**21,490**
0	Unknown	**21,490**
0	Unknown	**21,488**
0	Unknown	**21,485**
Serf2	Rattus norvegicus small EDRK-rich factor 2 (Serf2), mRNA [NM_001098782]	**21,471**
Fam36a	Rattus norvegicus family with sequence similarity 36, member A (Fam36a), mRNA [NM_001105976]	**21,470**
Th	Rattus norvegicus tyrosine hydroxylase (Th), mRNA [NM_012740]	**21,468**
Tmem231	Transmembrane protein 231 [Source:UniProtKB/Swiss-Prot;Acc:Q5FVM1] [ENSRNOT00000031243]	**21,463**
Pcdhac2	Rattus norvegicus protocadherin alpha subfamily C, 2 (Pcdhac2), mRNA [NM_201422]	**21,459**
Eef2k	Eukaryotic elongation factor 2 kinase [Source:UniProtKB/Swiss-Prot;Acc:P70531] [ENSRNOT00000022726]	**21,458**
39508	Rattus norvegicus membrane-associated ring finger (C3HC4) 8 (March8), mRNA [NM_001107882]	**21,448**
Vwa5b1	Rattus norvegicus von Willebrand factor A domain containing 5B1 (Vwa5b1), mRNA [NM_001107988]	**21,439**
Arl5a	Rattus norvegicus ADP-ribosylation factor-like 5A (Arl5a), mRNA [NM_053979]	**21,436**
0	Unknown	**21,433**
Zfp84	Rattus norvegicus zinc finger protein 84 (Zfp84), mRNA [NM_001107500]	**21,425**
Rasgef1a	PREDICTED: Rattus norvegicus RasGEF domain family, member 1A (Rasgef1a), mRNA [XM_232315]	**21,418**
0	Unknown	**21,413**
Lgals9	Rattus norvegicus lectin, galactoside-binding, soluble, 9 (Lgals9), mRNA [NM_012977]	**21,412**
0	UI-R-C0-hz-h-08-0-UI.s1 UI-R-C0 Rattus norvegicus cDNA clone UI-R-C0-hz-h-08-0-UI 3', mRNA sequence [AA998304]	**21,412**
Rnf26	Rattus norvegicus ring finger protein 26 (Rnf26), mRNA [NM_001113748]	**21,406**
Slc10a3	Rattus norvegicus solute carrier family 10 (sodium/bile acid cotransporter family), member 3 (Slc10a3), mRNA [NM_001024368]	**21,404**
Rundc3b	Rattus norvegicus RUN domain containing 3B (Rundc3b), mRNA [NM_001047116]	**21,403**
0	Unknown	**21,395**
Dusp16	Rattus norvegicus dual specificity phosphatase 16 (Dusp16), mRNA [NM_001106624]	**21,394**
Rab6a	Rattus norvegicus RAB6A, member RAS oncogene family (Rab6a), mRNA [NM_053366]	**21,391**
0	Unknown	**21,379**
Ube2cbp	Rattus norvegicus ubiquitin-conjugating enzyme E2C binding protein (Ube2cbp), mRNA [NM_001039610]	**21,378**
Crat	Rattus norvegicus carnitine acetyltransferase (Crat), nuclear gene encoding mitochondrial protein, mRNA [NM_001004085]	**21,375**

Utrn	Rattus norvegicus utrophin (Utrn), mRNA [NM_013070]	**21,369**
0	Unknown	**21,368**
Plagl2	Rattus norvegicus pleiomorphic adenoma gene-like 2 (Plagl2), mRNA [NM_001106528]	**21,365**
Epm2a	Laforin [Source:UniProtKB/Swiss-Prot;Acc:Q91XQ2] [ENSRNOT00000061930]	**21,365**
0	Rattus norvegicus, 15 clones, strain BN/SsNHsdMCW RNOR03326388, whole genome shotgun sequence [AABR03129123]	**21,355**
Hnf1b	Rattus norvegicus HNF1 homeobox B (Hnf1b), mRNA [NM_013103]	**21,352**
0	Unknown	**21,349**
Accn1	Rattus norvegicus amiloride-sensitive cation channel 1, neuronal (Accn1), transcript variant 1, mRNA [NM_012892]	**21,347**
Irf2	Rattus norvegicus interferon regulatory factor 2 (Irf2), mRNA [NM_001047086]	**21,347**
Epb41l4a	Rattus norvegicus erythrocyte membrane protein band 4.1 like 4A (Epb41l4a), mRNA [NM_001107397]	**21,346**
Tmem176b	Rattus norvegicus transmembrane protein 176B (Tmem176b), mRNA [NM_134390]	**21,340**
LOC684861	PREDICTED: Rattus norvegicus similar to Indoleamine 2,3-dioxygenase (IDO) (Indoleamine-pyrrole 2,3-dioxygenase) (LOC684861), mRNA [XM_001072229]	**21,337**
Bcl6b	Rattus norvegicus B-cell CLL/lymphoma 6, member B (zinc finger protein) (Bcl6b), mRNA [NM_001108279]	**21,336**
Ythdf1	Rattus norvegicus YTH domain family, member 1 (Ythdf1), mRNA [NM_001024756]	**21,332**
Cx3cr1	Rattus norvegicus chemokine (C-X3-C motif) receptor 1 (Cx3cr1), mRNA [NM_133534]	**21,332**
0	UI-R-FJ0-cpz-f-19-0-UI.r1 UI-R-FJ0 Rattus norvegicus cDNA clone UI-R-FJ0-cpz-f-19-0-UI 5', mRNA sequence [CA512374]	**21,331**
0	RVL16467 Wackym-Soares normalized rat vestibular cDNA library Rattus norvegicus cDNA 5', mRNA sequence [DV724416]	**21,328**
0	Unknown	**21,326**
0	Unknown	**21,323**
Apobec1	Rattus norvegicus apolipoprotein B mRNA editing enzyme, catalytic polypeptide 1 (Apobec1), mRNA [NM_012907]	**21,318**
Jph1	Rattus norvegicus junctophilin 1 (Jph1), mRNA [NM_001106630]	**21,294**
Mmgt2	Rattus norvegicus membrane magnesium transporter 2 (Mmgt2), mRNA [NM_001013967]	**21,292**
Ypel5	Rattus norvegicus yippee-like 5 (Drosophila) (Ypel5), mRNA [NM_001035221]	**21,290**
Atxn7l2	Uncharacterized protein [Source:UniProtKB/TrEMBL;Acc:D4A9B8] [ENSRNOT00000037336]	**21,290**
Pygo1	Rattus norvegicus pygopus 1 (Pygo1), mRNA [NM_001191117]	**21,281**
Brunol4	Rattus norvegicus bruno-like 4, RNA binding protein (Drosophila) (Brunol4), mRNA [NM_001107400]	**21,278**
Ccdc6	Uncharacterized protein [Source:UniProtKB/TrEMBL;Acc:D4AEK9] [ENSRNOT00000032159]	**21,275**
Tp53inp2	Tumor protein p53-inducible nuclear protein 2 [Source:UniProtKB/Swiss-Prot;Acc:Q8CHM3] [ENSRNOT00000055310]	**21,273**
Taf12	Rattus norvegicus TAF12 RNA polymerase II, TATA box binding protein (TBP)-associated factor (Taf12), mRNA [NM_001115036]	**21,266**
Rpp25	Rattus norvegicus ribonuclease P 25 subunit (human) (Rpp25), mRNA [NM_001012124]	**21,266**
Hapln4	Rattus norvegicus hyaluronan and proteoglycan link protein 4 (Hapln4), mRNA [NM_001108398]	**21,259**
Znf142	Rattus norvegicus zinc finger protein 142 (Znf142), mRNA [NM_001108225]	**21,258**
Ube2v1	Rattus norvegicus ubiquitin-conjugating enzyme E2 variant 1 (Ube2v1), mRNA [NM_001110345]	**21,258**

Bicd1	Rattus norvegicus bicaudal D homolog 1 (Drosophila) (Bicd1), mRNA [NM_001108653]	**21,247**
0	Unknown	**21,245**
Hn1	Rattus norvegicus hematological and neurological expressed 1 (Hn1), mRNA [NM_001005876]	**21,242**
Pdgfa	Rattus norvegicus platelet-derived growth factor alpha polypeptide (Pdgfa), mRNA [NM_012801]	**21,241**
Zbtb20	Rattus norvegicus zinc finger and BTB domain containing 20 (Zbtb20), mRNA [NM_001105880]	**21,233**
0	Unknown	**21,232**
LOC684800	PREDICTED: Rattus norvegicus similar to stromal membrane-associated protein 1, transcript variant 2 (LOC684800), mRNA [XM_002727177]	**21,230**
S100a5	Rattus norvegicus S100 calcium binding protein A5 (S100a5), mRNA [NM_001106438]	**21,227**
Slc25a18	Rattus norvegicus solute carrier family 25 (mitochondrial carrier), member 18 (Slc25a18), nuclear gene encoding mitochondrial protein, mRNA [NM_001044280]	**21,219**
Ogdh	Rattus norvegicus oxoglutarate (alpha-ketoglutarate) dehydrogenase (lipoamide) (Ogdh), nuclear gene encoding mitochondrial protein, mRNA [NM_001017461]	**21,207**
Spry3	Rattus norvegicus sprouty homolog 3 (Drosophila) (Spry3), mRNA [NM_001109063]	**21,203**
0	Uncharacterized protein [Source:UniProtKB/TrEMBL;Acc:D4A533] [ENSRNOT00000004274]	**21,201**
0	Unknown	**21,189**
Ikzf5	Rattus norvegicus IKAROS family zinc finger 5 (Ikzf5), mRNA [NM_001107555]	**21,187**
Grb10	Rattus norvegicus growth factor receptor bound protein 10 (Grb10), mRNA [NM_001109093]	**21,185**
Hgs	Rattus norvegicus hepatocyte growth factor-regulated tyrosine kinase substrate (Hgs), mRNA [NM_019387]	**21,184**
Acap3	Rattus norvegicus ArfGAP with coiled-coil, ankyrin repeat and PH domains 3 (Acap3), mRNA [NM_001107999]	**21,180**
0	Unknown	**21,176**
Reep1	Rattus norvegicus receptor accessory protein 1 (Reep1), nuclear gene encoding mitochondrial protein, mRNA [NM_001108633]	**21,165**
0	Unknown	**21,156**
Gga1	Rattus norvegicus golgi associated, gamma adaptin ear containing, ARF binding protein 1 (Gga1), mRNA [NM_001011994]	**21,148**
Abl1	Rattus norvegicus c-abl oncogene 1, receptor tyrosine kinase (Abl1), mRNA [NM_001100850]	**21,142**
0	Unknown	**21,140**
Pcdh17	Rattus norvegicus protocadherin 17 (Pcdh17), mRNA [NM_001107279]	**21,138**
0	Unknown	**21,127**
Obfc2b	Rattus norvegicus oligonucleotide/oligosaccharide-binding fold containing 2B (Obfc2b), mRNA [NM_001034939]	**21,125**
Dcaf5	Rattus norvegicus DDB1 and CUL4 associated factor 5 (Dcaf5), nuclear gene encoding mitochondrial protein, mRNA [NM_001100718]	**21,116**
RGD1303130	Rattus norvegicus kidney predominant protein NCU-G1 (RGD1303130), mRNA [NM_001004226]	**21,115**
Syngr2	Rattus norvegicus synaptogyrin 2 (Syngr2), mRNA [NM_053553]	**21,112**
0	Unknown	**21,103**
Nek11	Rattus norvegicus NIMA (never in mitosis gene a)- related kinase 11 (Nek11), mRNA [NM_001108182]	**21,100**
Cacng6	Rattus norvegicus calcium channel, voltage-dependent, gamma subunit 6 (Cacng6), mRNA [NM_080694]	**21,097**

Trib2	Rattus norvegicus TL0AEA74YF17 mRNA sequence. [FQ231260]	**21,084**
Arcn1	Rattus norvegicus archain 1 (Arcn1), mRNA [NM_001007662]	**21,078**
Selm	Rattus norvegicus selenoprotein M (Selm), mRNA [NM_001115013]	**21,078**
RGD1304595	Rattus norvegicus similar to RIKEN cDNA 6330416G13 gene (RGD1304595), mRNA [NM_001106661]	**21,078**
0	Unknown	**21,071**
LOC501979	PREDICTED: Rattus norvegicus similar to ATPase, H+ transporting, V1 subunit F (LOC501979), mRNA [XM_577408]	**21,061**
Mbnl2	Rattus norvegicus muscleblind-like 2 (Mbnl2), mRNA [NM_001111064]	**21,059**
Elovl2	Rattus norvegicus elongation of very long chain fatty acids (FEN1/Elo2, SUR4/Elo3, yeast)-like 2 (Elovl2), mRNA [NM_001109118]	**21,057**
Scd4	Uncharacterized protein [Source:UniProtKB/TrEMBL;Acc:D3ZNA4] [ENSRNOT00000017834]	**21,056**
Snap25	Rattus norvegicus synaptosomal-associated protein 25 (Snap25), mRNA [NM_030991]	**21,054**
0	Unknown	**21,051**
MGC94542	Rattus norvegicus similar to RIKEN cDNA 5430437P03 (MGC94542), mRNA [NM_001006964]	**21,046**
Aanat	Rattus norvegicus arylalkylamine N-acetyltransferase (Aanat), mRNA [NM_012818]	**21,045**
Sema4d	Rattus norvegicus sema domain, immunoglobulin domain (Ig), transmembrane domain (TM) and short cytoplasmic domain, (semaphorin) 4D (Sema4d), mRNA [NM_001170563]	**21,044**
Car7	Rattus norvegicus carbonic anhydrase 7 (Car7), mRNA [NM_001106165]	**21,041**
Caskin2	Rattus norvegicus cask-interacting protein 2 (Caskin2), mRNA [NM_001107065]	**21,040**
RGD1310769	Rattus norvegicus similar to HSPC288 (RGD1310769), mRNA [NM_001106749]	**21,037**
Masp1	Rattus norvegicus mannan-binding lectin serine peptidase 1 (Masp1), mRNA [NM_022257]	**21,035**
Mta2	Rattus norvegicus metastasis associated 1 family, member 2 (Mta2), mRNA [NM_001100740]	**21,032**
Nudt16l1	Rattus norvegicus nudix (nucleoside diphosphate linked moiety X)-type motif 16-like 1 (Nudt16l1), mRNA [NM_001100782]	**21,031**
0	Unknown	**21,031**
0	XM_750817 gamma-tubulin complex component GCP4 {Aspergillus fumigatus Af293} (exp=-1; wgp=0; cg=0), partial (3%) [TC606369]	**21,028**
Pou6f1	Rattus norvegicus POU class 6 homeobox 1 (Pou6f1), mRNA [NM_001105746]	**21,028**
Aig1	Rattus norvegicus androgen-induced 1 (Aig1), mRNA [NM_001134425]	**21,025**
Vom1r30	Rattus norvegicus vomeronasal 1 receptor 30 (Vom1r30), mRNA [NM_001009511]	**21,018**
Fitm2	Rattus norvegicus fat storage-inducing transmembrane protein 2 (Fitm2), mRNA [NM_001107799]	**21,011**
Gatsl3	Rattus norvegicus GATS protein-like 3 (Gatsl3), mRNA [NM_001025128]	**21,002**
Dbt	Rattus norvegicus dihydrolipoamide branched chain transacylase E2 (Dbt), nuclear gene encoding mitochondrial protein, mRNA [NM_053312]	**21,000**
Cdr2	Rattus norvegicus cerebellar degeneration-related 2 (Cdr2), mRNA [NM_001025682]	**20,997**
Ubxn2a	Rattus norvegicus UBX domain protein 2A (Ubxn2a), mRNA [NM_001109482]	**20,992**
Rarres1	Rattus norvegicus retinoic acid receptor responder (tazarotene induced) 1 (Rarres1), mRNA [NM_001014790]	**20,986**
Rimbp2	Rattus norvegicus RIM binding protein 2 (Rimbp2), mRNA [NM_001100488]	**20,985**
Ensa	Rattus norvegicus endosulfine alpha (Ensa), transcript variant 2, mRNA [NM_021842]	**20,981**

Arhgef7	Rattus norvegicus Rho guanine nucleotide exchange factor (GEF7) (Arhgef7), transcript variant 3, mRNA [NM_053740]	**20,979**
Samd14	Rattus norvegicus sterile alpha motif domain containing 14 (Samd14), mRNA [NM_001024966]	**20,978**
0	Unknown	**20,973**
Foxk2	Rattus norvegicus forkhead box K2 (Foxk2), mRNA [NM_001107075]	**20,970**
Gorasp1	Rattus norvegicus golgi reassembly stacking protein 1 (Gorasp1), mRNA [NM_019385]	**20,966**
Art5	Rattus norvegicus ADP-ribosyltransferase 5 (Art5), mRNA [NM_001013039]	**20,965**
Papd4	Rattus norvegicus PAP associated domain containing 4 (Papd4), mRNA [NM_001008372]	**20,963**
Hhipl1	Uncharacterized protein [Source:UniProtKB/TrEMBL;Acc:D3ZJC9] [ENSRNOT00000034883]	**20,963**
Mad2l1bp	Rattus norvegicus MAD2L1 binding protein (Mad2l1bp), mRNA [NM_001009699]	**20,962**
Ulk1	Rattus norvegicus Unc-51 like kinase 1 (C. elegans) (Ulk1), mRNA [NM_001108341]	**20,960**
Nfasc	Rattus norvegicus neurofascin (Nfasc), transcript variant 1, mRNA [NM_001160314]	**20,955**
Rab31	Rattus norvegicus RAB31, member RAS oncogene family (Rab31), mRNA [NM_145094]	**20,949**
Sfrs1	Rattus norvegicus splicing factor, arginine/serine-rich 1 (Sfrs1), mRNA [NM_001109552]	**20,945**
Rhbdf1	Rattus norvegicus rhomboid 5 homolog 1 (Drosophila) (Rhbdf1), mRNA [NM_001030034]	**20,943**
Tesb	Rattus norvegicus testis specific basic protein (Tesb), mRNA [NM_001113183]	**20,941**
Vps4a	Rattus norvegicus vacuolar protein sorting 4 homolog A (S. cerevisiae) (Vps4a), mRNA [NM_145678]	**20,940**
Itgb5	Rattus norvegicus integrin, beta 5 (Itgb5), mRNA [NM_147139]	**20,938**
Nono	Rattus norvegicus non-POU domain containing, octamer-binding (Nono), mRNA [NM_001012356]	**20,929**
LOC679168	PREDICTED: Rattus norvegicus hypothetical protein LOC679168 (LOC679168), mRNA [XM_001055022]	**20,929**
RGD1566399	PREDICTED: Rattus norvegicus similar to MYST histone acetyltransferase monocytic leukemia 4 (RGD1566399), miscRNA [XR_008228]	**20,928**
Calm1	Rattus norvegicus calmodulin 1 (Calm1), mRNA [NM_031969]	**20,926**
Stim2	Rattus norvegicus stromal interaction molecule 2 (Stim2), mRNA [NM_001105750]	**20,918**
Ints7	Rattus norvegicus integrator complex subunit 7 (Ints7), mRNA [NM_001191675]	**20,909**
Slc24a5	Rattus norvegicus solute carrier family 24, member 5 (Slc24a5), mRNA [NM_001107769]	**20,898**
Fam117b	Rattus norvegicus family with sequence similarity 117, member B (Fam117b), mRNA [NM_001108797]	**20,896**
0	Unknown	**20,888**
0	Unknown	**20,886**
RGD1306186	Rattus norvegicus similar to RIKEN cDNA 4930569K13 (RGD1306186), mRNA [NM_001024986]	**20,881**
0	Rattus norvegicus cDNA clone IMAGE:7314743. [BC089965]	**20,871**
Oaz3	Rattus norvegicus ornithine decarboxylase antizyme 3 (Oaz3), mRNA [NM_001101018]	**20,850**
Katnal1	Rattus norvegicus katanin p60 subunit A-like 1 (Katnal1), mRNA [NM_001006956]	**20,849**
0	Unknown	**20,848**
Vasn	Rattus norvegicus vasorin (Vasn), mRNA [NM_001109382]	**20,848**
Olr472	Rattus norvegicus olfactory receptor 472 (Olr472), mRNA [NM_001000301]	**20,847**

Casp2	Rattus norvegicus caspase 2 (Casp2), mRNA [NM_022522]	**20,835**
RGD735029	Rattus norvegicus SEL1 domain containing protein RGD735029 (RGD735029), nuclear gene encoding mitochondrial protein, mRNA [NM_199493]	**20,830**
Mkl1	PREDICTED: Rattus norvegicus megakaryoblastic leukemia (translocation) 1 (Mkl1), mRNA [XM_235497]	**20,830**
Ids	Uncharacterized protein [Source:UniProtKB/TrEMBL;Acc:D3ZPY7] [ENSRNOT00000042925]	**20,830**
0	PREDICTED: Rattus norvegicus similar to high mobility group protein 17 (LOC688424), mRNA [XM_001066874]	**20,825**
Usp21	Rattus norvegicus ubiquitin specific peptidase 21 (Usp21), mRNA [NM_001127638]	**20,804**
Cyp2j4	Rattus norvegicus cytochrome P450, family 2, subfamily j, polypeptide 4 (Cyp2j4), mRNA [NM_023025]	**20,797**
Nsf	Rattus norvegicus N-ethylmaleimide-sensitive factor (Nsf), mRNA [NM_021748]	**20,794**
Kcna1	Rattus norvegicus potassium voltage-gated channel, shaker-related subfamily, member 1 (Kcna1), mRNA [NM_173095]	**20,791**
Olr1381	Rattus norvegicus olfactory receptor 1381 (Olr1381), mRNA [NM_001000523]	**20,788**
Slc9a9	PREDICTED: Rattus norvegicus similar to solute carrier family 9 (sodium/hydrogen exchanger), isoform 9 (RGD1560736), mRNA [XM_001064905]	**20,788**
Bloc1s3	PREDICTED: Rattus norvegicus biogenesis of lysosomal organelles complex-1, subunit 3 (Bloc1s3), mRNA [XM_001058094]	**20,785**
Mid1	Rattus norvegicus midline 1 (Mid1), mRNA [NM_022927]	**20,785**
Cacng8	Rattus norvegicus calcium channel, voltage-dependent, gamma subunit 8 (Cacng8), mRNA [NM_080696]	**20,777**
Comt	Rattus norvegicus catechol-O-methyltransferase (Comt), mRNA [NM_012531]	**20,774**
Pnrc1	Rattus norvegicus proline-rich nuclear receptor coactivator 1 (Pnrc1), mRNA [NM_173322]	**20,770**
Daam1	Rattus norvegicus dishevelled associated activator of morphogenesis 1 (Daam1), mRNA [NM_001108030]	**20,767**
Cnst	PREDICTED: Rattus norvegicus similar to 9630058J23Rik protein (RGD1564833), mRNA [XM_573525]	**20,766**
Clptm1	Rattus norvegicus cleft lip and palate associated transmembrane protein 1 (Clptm1), mRNA [NM_001106232]	**20,765**
Nedd4	Rattus norvegicus neural precursor cell expressed, developmentally down-regulated 4 (Nedd4), mRNA [NM_012986]	**20,765**
0	NID2_HUMAN (Q14112) Nidogen-2 precursor (NID-2) (Osteonidogen), partial (7%) [TC622511]	**20,764**
Ndufa10	Rattus norvegicus NADH dehydrogenase (ubiquinone) 1 alpha subcomplex 10 (Ndufa10), nuclear gene encoding mitochondrial protein, mRNA [NM_199495]	**20,763**
Gpr132	Rattus norvegicus G protein-coupled receptor 132 (Gpr132), mRNA [NM_001170595]	**20,758**
Usp46	Rattus norvegicus ubiquitin specific peptidase 46 (Usp46), mRNA [NM_001191596]	**20,754**
Spryd3	Rattus norvegicus SPRY domain containing 3 (Spryd3), mRNA [NM_001191790]	**20,754**
0	protease, serine, 28 Gene [Source:MGI Symbol;Acc:MGI:2149951] [ENSRNOT00000060388]	**20,749**
Samd9l	PREDICTED: Rattus norvegicus sterile alpha motif domain containing 9-like (Samd9l), mRNA [XM_001069386]	**20,746**
Defb24	Rattus norvegicus defensin beta 24 (Defb24), mRNA [NM_001037508]	**20,745**
Sohlh1	Rattus norvegicus spermatogenesis and oogenesis specific basic helix-loop-helix 1 (Sohlh1), mRNA [NM_001191852]	**20,744**
Hmg20a	Rattus norvegicus high mobility group 20A (Hmg20a), mRNA [NM_001108150]	**20,729**
0	Unknown	**20,723**
Trove2	Rattus norvegicus TROVE domain family, member 2 (Trove2), mRNA [NM_001107183]	**20,722**

Cacna1g	Rattus norvegicus calcium channel, voltage-dependent, T type, alpha 1G subunit (Cacna1g), mRNA [NM_031601]	**20,720**
Cisd3	Rattus norvegicus CDGSH iron sulfur domain 3 (Cisd3), mRNA [NM_001105835]	**20,715**
Samd5	Rattus norvegicus sterile alpha motif domain containing 5 (Samd5), mRNA [NM_001108901]	**20,713**
Lphn1	Rattus norvegicus latrophilin 1 (Lphn1), mRNA [NM_022962]	**20,712**
RGD1310423	Rattus norvegicus similar to hypothetical protein FLJ31737 (RGD1310423), mRNA [NM_001029920]	**20,712**
0	Unknown	**20,707**
Rrm2b	Rattus norvegicus ribonucleotide reductase M2 B (TP53 inducible) (Rrm2b), mRNA [NM_001130543]	**20,705**
0	Q479S2_DECAR (Q479S2) Histidine kinase internal region precursor, partial (7%) [TC592164]	**20,701**
0	Uncharacterized protein [Source:UniProtKB/TrEMBL;Acc:D3ZDX5] [ENSRNOT00000043345]	**20,701**
0	Uncharacterized protein [Source:UniProtKB/TrEMBL;Acc:D3Z932] [ENSRNOT00000066351]	**20,698**
Ctnnbip1	Rattus norvegicus catenin, beta-interacting protein 1 (Ctnnbip1), mRNA [NM_001173388]	**20,696**
Gpr56	Rattus norvegicus G protein-coupled receptor 56 (Gpr56), mRNA [NM_152242]	**20,691**
Slc44a2	Rattus norvegicus solute carrier family 44, member 2 (Slc44a2), mRNA [NM_001134715]	**20,688**
LOC685671	PREDICTED: Rattus norvegicus similar to myocyte enhancer factor 2C (LOC685671), miscRNA [XR_006259]	**20,675**
RGD1565030	PREDICTED: Rattus norvegicus similar to open reading frame A (RGD1565030), mRNA [XM_574008]	**20,673**
Dcx	Rattus norvegicus neuronal migration protein doublecortin mRNA, complete cds. [AF155959]	**20,670**
Rnf187	Rattus norvegicus ring finger protein 187 (Rnf187), mRNA [NM_001164264]	**20,669**
Ubxn7	Rattus norvegicus UBX domain protein 7 (Ubxn7), mRNA [NM_001107086]	**20,668**
Fbxo21	Rattus norvegicus F-box protein 21 (Fbxo21), mRNA [NM_001108338]	**20,665**
Folr2	Rattus norvegicus folate receptor 2 (fetal) (Folr2), mRNA [NM_001106283]	**20,656**
RGD1308127	Rattus norvegicus similar to 2700078E11Rik protein (RGD1308127), mRNA [NM_001014248]	**20,653**
0	Uncharacterized protein [Source:UniProtKB/TrEMBL;Acc:D3ZI48] [ENSRNOT00000063788]	**20,632**
Phactr1	Rattus norvegicus phosphatase and actin regulator 1 (Phactr1), mRNA [NM_214457]	**20,631**
Tesk1	Rattus norvegicus testis-specific kinase 1 (Tesk1), mRNA [NM_031578]	**20,630**
0	Rattus norvegicus similar to Xlr3a protein (LOC367970), mRNA [XM_346375]	**20,629**
Dmwd	PREDICTED: Rattus norvegicus dystrophia myotonica, WD repeat containing (Dmwd), mRNA [XM_001056337]	**20,628**
0	PREDICTED: Rattus norvegicus similar to c114 SLIT-like testicular protein (RGD1561285), mRNA [XM_220927]	**20,623**
0	myeloid/lymphoid or mixed-lineage leukemia 3 Gene [Source:MGI Symbol;Acc:MGI:2444959] [ENSRNOT00000010349]	**20,621**
Vasp	Rattus norvegicus vasodilator-stimulated phosphoprotein (Vasp), mRNA [NM_001108475]	**20,620**
RGD1565693	PREDICTED: Rattus norvegicus similar to GLE1-like, RNA export mediator isoform 1 (RGD1565693), mRNA [XM_001059991]	**20,616**
Prss27	Rattus norvegicus protease, serine 27 (Prss27), mRNA [NM_182949]	**20,616**
C1ql3	Rattus norvegicus complement component 1, q subcomponent-like 3 (C1ql3), mRNA [NM_001109403]	**20,614**
Tnfaip1	Rattus norvegicus tumor necrosis factor, alpha-induced protein 1 (endothelial) (Tnfaip1), mRNA [NM_182950]	**20,610**
Snph	Rattus norvegicus syntaphilin (Snph), mRNA [NM_001106525]	**20,605**

Dr1	Rattus norvegicus down-regulator of transcription 1 (Dr1), mRNA [NM_001011914]	**20,598**
MGC93975	Rattus norvegicus similar to 2310044H10Rik protein (MGC93975), mRNA [NM_001004221]	**20,598**
Fjx1	Rattus norvegicus four jointed box 1 (Drosophila) (Fjx1), mRNA [NM_001108955]	**20,595**
LOC498368	Rattus norvegicus similar to RIKEN cDNA 0610040J01 (LOC498368), mRNA [NM_001017500]	**20,594**
RGD1310444	PREDICTED: Rattus norvegicus LOC363015 (RGD1310444), mRNA [XM_002729871]	**20,587**
RGD1566078	Uncharacterized protein [Source:UniProtKB/TrEMBL;Acc:D3ZWM0] [ENSRNOT00000013187]	**20,585**
Klf16	Rattus norvegicus Kruppel-like factor 16 (Klf16), mRNA [NM_001127604]	**20,583**
Clock	Rattus norvegicus clock homolog (mouse) (Clock), mRNA [NM_021856]	**20,578**
0	Unknown	**20,577**
Synj2	Rattus norvegicus synaptojanin 2 (Synj2), transcript variant 3, mRNA [NM_032071]	**20,574**
Fitm2	Rattus norvegicus fat storage-inducing transmembrane protein 2 (Fitm2), mRNA [NM_001107799]	**20,570**
Zscan10	Rattus norvegicus zinc finger and SCAN domain containing 10 (Zscan10), mRNA [NM_001106981]	**20,561**
Snrpn	Rattus norvegicus small nuclear ribonucleoprotein polypeptide N (Snrpn), mRNA [NM_031117]	**20,560**
Dfnb31	Rattus norvegicus deafness, autosomal recessive 31 (Dfnb31), mRNA [NM_181088]	**20,558**
Baz2a	Rattus norvegicus bromodomain adjacent to zinc finger domain, 2A (Baz2a), mRNA [NM_001107158]	**20,557**
Dcaf5	Rattus norvegicus DDB1 and CUL4 associated factor 5 (Dcaf5), nuclear gene encoding mitochondrial protein, mRNA [NM_001100718]	**20,554**
Man1b1	Endoplasmic reticulum mannosyl-oligosaccharide 1,2-alpha-mannosidase [Source:UniProtKB/Swiss-Prot;Acc:B2GUY0] [ENSRNOT00000016846]	**20,550**
0	Unknown	**20,549**
Arntl2	Rattus norvegicus aryl hydrocarbon receptor nuclear translocator-like 2 (Arntl2), mRNA [NM_133391]	**20,548**
0	Uncharacterized protein [Source:UniProtKB/TrEMBL;Acc:D3ZBI5] [ENSRNOT00000049803]	**20,548**
Gprin1	Uncharacterized protein [Source:UniProtKB/TrEMBL;Acc:D3ZEG5] [ENSRNOT00000024258]	**20,547**
0	Cell division control protein 42 homolog [Source:UniProtKB/Swiss-Prot;Acc:Q8CFN2] [ENSRNOT00000018118]	**20,540**
Ipo7	Rattus norvegicus importin 7 (Ipo7), mRNA [NM_001107545]	**20,537**
Clcn6	Rattus norvegicus chloride channel 6 (Clcn6), mRNA [NM_001106479]	**20,530**
Cbx5	Rattus norvegicus chromobox homolog 5 (HP1 alpha homolog, Drosophila) (Cbx5), mRNA [NM_001106797]	**20,530**
0	Unknown	**20,529**
Cpsf6	Rattus norvegicus cleavage and polyadenylation specific factor 6 (Cpsf6), mRNA [NM_001106785]	**20,527**
Trim26	Rattus norvegicus tripartite motif-containing 26 (Trim26), mRNA [NM_001011665]	**20,524**
0	Unknown	**20,523**
0	Unknown	**20,514**
LOC100363283	PREDICTED: Rattus norvegicus UBX domain protein 2A (LOC100363283), mRNA [XM_002729616]	**20,509**
Tsga14	Rattus norvegicus testis specific, 14 (Tsga14), mRNA [NM_001025770]	**20,508**
0	AMGNNUC:NRHY5-00189-A7-A W Rat hypothalamus (10471) Rattus norvegicus cDNA clone nrhy5-00189-a7 5', mRNA sequence [CB580197]	**20,501**
LOC686412	PREDICTED: Rattus norvegicus similar to sal-like 4 isoform a (LOC686412), mRNA [XM_001074001]	**20,501**

Mmp17	Rattus norvegicus matrix metallopeptidase 17 (Mmp17), mRNA [NM_001105925]	**20,497**
Bcl7b	Rattus norvegicus B-cell CLL/lymphoma 7B (Bcl7b), mRNA [NM_001109021]	**20,493**
Pid1	Rattus norvegicus phosphotyrosine interaction domain containing 1 (Pid1), mRNA [NM_001110493]	**20,492**
Adipor1	Rattus norvegicus adiponectin receptor 1 (Adipor1), mRNA [NM_207587]	**20,491**
Fam48a	Rattus norvegicus family with sequence similarity 48, member A (Fam48a), mRNA [NM_001014170]	**20,489**
0	Unknown	**20,484**
LOC500420	LOC500420 protein [Source:UniProtKB/TrEMBL;Acc:Q3SWT2] [ENSRNOT00000030559]	**20,483**
Med11	Rattus norvegicus mediator complex subunit 11 (Med11), mRNA [NM_001105799]	**20,472**
Mboat4	Rattus norvegicus membrane bound O-acyltransferase domain containing 4 (Mboat4), mRNA [NM_001107317]	**20,470**
Camta1	Rattus norvegicus similar to KIAA0833 protein (Camta1), transcript variant 1, mRNA [NM_001195559]	**20,459**
Rasgef1c	Rattus norvegicus RasGEF domain family, member 1C (Rasgef1c), mRNA [NM_001108273]	**20,453**
Shisa4	Rattus norvegicus shisa homolog 4 (Xenopus laevis) (Shisa4), mRNA [NM_001077826]	**20,452**
0	Unknown	**20,448**
0	Unknown	**20,445**
0	Uncharacterized protein [Source:UniProtKB/TrEMBL;Acc:D4A428] [ENSRNOT00000040254]	**20,444**
Tgoln1	Rattus norvegicus trans-golgi network protein (Tgoln1), mRNA [NM_138840]	**20,442**
Cbx6	Rattus norvegicus chromobox homolog 6 (Cbx6), mRNA [NM_001012119]	**20,433**
Mrps10	Rattus norvegicus mitochondrial ribosomal protein S10 (Mrps10), nuclear gene encoding mitochondrial protein, mRNA [NM_001008859]	**20,429**
Limd2	Rattus norvegicus LIM domain containing 2 (Limd2), mRNA [NM_001025715]	**20,429**
Cox16	Rattus norvegicus COX16 cytochrome c oxidase assembly homolog (Cox16), nuclear gene encoding mitochondrial protein, mRNA [NM_001163153]	**20,428**
Olr233	Rattus norvegicus olfactory receptor 233 (Olr233), mRNA [NM_001000207]	**20,427**
Mapk4	Mitogen-activated protein kinase 4 [Source:UniProtKB/Swiss-Prot;Acc:Q63454] [ENSRNOT00000047271]	**20,425**
Mlst8	Rattus norvegicus MTOR associated protein, LST8 homolog (S. cerevisiae) (Mlst8), mRNA [NM_022404]	**20,419**
Zbtb24	Rattus norvegicus zinc finger and BTB domain containing 24 (Zbtb24), mRNA [NM_001098667]	**20,417**
Znrf2	Rattus norvegicus zinc and ring finger 2 (Znrf2), mRNA [NM_001108628]	**20,412**
H1fx	Uncharacterized protein [Source:UniProtKB/TrEMBL;Acc:D3ZIX4] [ENSRNOT00000033687]	**20,410**
0	Unknown	**20,404**
Fam186b	Rattus norvegicus family with sequence similarity 186, member B (Fam186b), mRNA [NM_001134633]	**20,403**
Olfm2	Rattus norvegicus olfactomedin 2 (Olfm2), mRNA [NM_001015017]	**20,402**
Fkbp8	Rattus norvegicus FK506 binding protein 8 (Fkbp8), mRNA [NM_001037180]	**20,400**
Crlf2	Rattus norvegicus cytokine receptor-like factor 2 (Crlf2), mRNA [NM_134465]	**20,392**
Clvs1	Rattus norvegicus clavesin 1 (Clvs1), mRNA [NM_001108969]	**20,391**
Agap1	Rattus norvegicus ArfGAP with GTPase domain, ankyrin repeat and PH domain 1 (Agap1), mRNA [NM_001108230]	**20,388**

Sbno1	Rattus norvegicus strawberry notch homolog 1 (Drosophila) (Sbno1), mRNA [NM_001107138]	**20,383**
Dcx	Rattus norvegicus doublecortin (Dcx), mRNA [NM_053379]	**20,380**
Pcdhga2	Rattus norvegicus protocadherin gamma subfamily A, 2 (Pcdhga2), mRNA [NM_001037139]	**20,375**
Ddx41	Rattus norvegicus DEAD (Asp-Glu-Ala-Asp) box polypeptide 41 (Ddx41), mRNA [NM_001108046]	**20,372**
Lsm6	Rattus norvegicus LSM6 homolog, U6 small nuclear RNA associated (S. cerevisiae) (Lsm6), mRNA [NM_001126085]	**20,368**
Lphn2	Rattus norvegicus latrophilin 2 (Lphn2), transcript variant 2, mRNA [NM_001190475]	**20,367**
RGD1304952	Rattus norvegicus similar to RIKEN cDNA C530028O21 gene (RGD1304952), mRNA [NM_001014059]	**20,366**
Mapkapk2	Rattus norvegicus mitogen-activated protein kinase-activated protein kinase 2 (Mapkapk2), mRNA [NM_178102]	**20,362**
Negr1	Rattus norvegicus neuronal growth regulator 1 (Negr1), mRNA [NM_021682]	**20,355**
Pcolce	Rattus norvegicus procollagen C-endopeptidase enhancer (Pcolce), mRNA [NM_019237]	**20,353**
Srpk2	Rattus norvegicus SFRS protein kinase 2 (Srpk2), mRNA [NM_001106575]	**20,351**
Phf2	Rattus norvegicus PHD finger protein 2 (Phf2), mRNA [NM_001107342]	**20,349**
Lrrc8b	leucine rich repeat containing 8 family, member B [Source:RefSeq peptide;Acc:NP_001100674] [ENSRNOT00000002897]	**20,348**
Tbx3	Rattus norvegicus T-box 3 (Tbx3), mRNA [NM_181638]	**20,344**
Ankrd40	Rattus norvegicus ankyrin repeat domain 40 (Ankrd40), mRNA [NM_001134699]	**20,343**
Cnpy3	Rattus norvegicus canopy 3 homolog (zebrafish) (Cnpy3), mRNA [NM_001134710]	**20,341**
Trim37	Rattus norvegicus tripartite motif-containing 37 (Trim37), mRNA [NM_001108288]	**20,338**
0	Shultzomica03641 Rat lung airway and parenchyma cDNA libraries Rattus norvegicus cDNA clone Contig3221 5', mRNA sequence [CF110390]	**20,337**
Rexo1	Rattus norvegicus REX1, RNA exonuclease 1 homolog (S. cerevisiae) (Rexo1), mRNA [NM_001012114]	**20,328**
Tusc2	Rattus norvegicus tumor suppressor candidate 2 (Tusc2), mRNA [NM_001109297]	**20,326**
0	Unknown	**20,316**
Zfp318	PREDICTED: Rattus norvegicus zinc finger protein 318 (Zfp318), mRNA [XM_002727181]	**20,316**
Fgf22	Rattus norvegicus fibroblast growth factor 22 (Fgf22), mRNA [NM_130751]	**20,313**
Porcn	Rattus norvegicus porcupine homolog (Drosophila) (Porcn), mRNA [NM_001173355]	**20,313**
Tmem42	Rattus norvegicus transmembrane protein 42 (Tmem42), mRNA [NM_001191886]	**20,303**
Pde4b	Rattus norvegicus phosphodiesterase 4B, cAMP specific (Pde4b), mRNA [NM_017031]	**20,295**
Pttg1ip	Rattus norvegicus pituitary tumor-transforming 1 interacting protein (Pttg1ip), mRNA [NM_001013238]	**20,294**
Mmp16	Rattus norvegicus matrix metallopeptidase 16 (Mmp16), mRNA [NM_080776]	**20,292**
Fam108c1	Rattus norvegicus family with sequence similarity 108, member C1 (Fam108c1), mRNA [NM_001100736]	**20,285**
Zfp346	Rattus norvegicus zinc finger protein 346 (Zfp346), mRNA [NM_001107338]	**20,281**
RGD1309540	Rattus norvegicus similar to hypothetical protein MGC40841; similar to hypothetical protein MGC4707 (RGD1309540), mRNA [NM_001013918]	**20,274**
Nalcn	Rattus norvegicus sodium leak channel, non-selective (Nalcn), mRNA [NM_153630]	**20,273**
0	Rattus norvegicus similar to RIKEN cDNA 1700001F09 (LOC316932), mRNA [XM_229506]	**20,269**
RGD1564177	PREDICTED: Rattus norvegicus RGD1564177 (RGD1564177), mRNA [XM_001054841]	**20,267**

Rnf152	Rattus norvegicus ring finger protein 152 (Rnf152), mRNA [NM_001106305]	**20,264**
Zfyve20	Rattus norvegicus zinc finger, FYVE domain containing 20 (Zfyve20), mRNA [NM_001107875]	**20,263**
Gtf2h3	Rattus norvegicus general transcription factor IIH, polypeptide 3 (Gtf2h3), mRNA [NM_001024236]	**20,262**
0	PREDICTED: Rattus norvegicus similar to dachshund b (LOC680770), mRNA [XM_001058786]	**20,252**
Kcnq2	Rattus norvegicus potassium voltage-gated channel, KQT-like subfamily, member 2 (Kcnq2), mRNA [NM_133322]	**20,251**
Cyp4a8	Rattus norvegicus cytochrome P450, family 4, subfamily a, polypeptide 8 (Cyp4a8), mRNA [NM_031605]	**20,251**
Kcnq2	Rattus norvegicus potassium voltage-gated channel, KQT-like subfamily, member 2 (Kcnq2), mRNA [NM_133322]	**20,248**
0	Unknown	**20,247**
RGD1563070	Rattus norvegicus similar to hypothetical protein (RGD1563070), mRNA [NM_001134541]	**20,238**
Neurl	Rattus norvegicus neuralized homolog (Drosophila) (Neurl), mRNA [NM_001107605]	**20,238**
Kcng2	Rattus norvegicus potassium voltage-gated channel, subfamily G, member 2 (Kcng2), mRNA [NM_001107372]	**20,236**
Ick	Rattus norvegicus intestinal cell kinase (Ick), mRNA [NM_138886]	**20,236**
Igsf8	Rattus norvegicus immunoglobulin superfamily, member 8 (Igsf8), mRNA [NM_001014787]	**20,233**
Atl1	Rattus norvegicus atlastin GTPase 1 (Atl1), mRNA [NM_001009831]	**20,231**
RGD1310444	RCG31867Uncharacterized protein [Source:UniProtKB/TrEMBL;Acc:D3ZIP8] [ENSRNOT00000033969]	**20,223**
Efcab4a	Rattus norvegicus EF-hand calcium binding domain 4A (Efcab4a), mRNA [NM_001127541]	**20,218**
Mxra8	Rattus norvegicus matrix-remodelling associated 8 (Mxra8), mRNA [NM_001007002]	**20,212**
Tcf3	Rattus norvegicus transcription factor 3 (Tcf3), mRNA [NM_001107865]	**20,212**
Gpr83	Rattus norvegicus G protein-coupled receptor 83 (Gpr83), mRNA [NM_080411]	**20,205**
Lhx9	Rattus norvegicus LIM homeobox 9 (Lhx9), mRNA [NM_181367]	**20,205**
0	Unknown	**20,205**
Trps1	Rattus norvegicus trichorhinophalangeal syndrome I homolog (human) (Trps1), mRNA [NM_001134837]	**20,198**
Ppm1d	Rattus norvegicus protein phosphatase 1D magnesium-dependent, delta isoform (Ppm1d), mRNA [NM_001105825]	**20,197**
Smtnl1	Rattus norvegicus smoothelin-like 1 (Smtnl1), mRNA [NM_001191739]	**20,195**
Satb1	Rattus norvegicus SATB homeobox 1 (Satb1), mRNA [NM_001012129]	**20,193**
Snx24	Rattus norvegicus sorting nexin 24 (Snx24), mRNA [NM_001008364]	**20,189**
Kif21b	Rattus norvegicus kinesin family member 21B (Kif21b), mRNA [NM_001105990]	**20,187**
Tcf7	PREDICTED: Rattus norvegicus transcription factor 7, T-cell specific, transcript variant 1 (Tcf7), mRNA [XM_001073458]	**20,186**
Brsk1	Rattus norvegicus BR serine/threonine kinase 1 (Brsk1), mRNA [NM_001127337]	**20,184**
Sgk1	Rattus norvegicus serum/glucocorticoid regulated kinase 1 (Sgk1), transcript variant 3, mRNA [NM_019232]	**20,181**
Dhdds	Rattus norvegicus dehydrodolichyl diphosphate synthase (Dhdds), mRNA [NM_001011978]	**20,180**
Pard3	Rattus norvegicus par-3 (partitioning defective 3) homolog (C. elegans) (Pard3), mRNA [NM_031235]	**20,179**
0	Unknown	**20,173**
Bat2	Rattus norvegicus HLA-B associated transcript 2 (Bat2), mRNA [NM_212462]	**20,173**

Mal2	Rattus norvegicus mal, T-cell differentiation protein 2 (Mal2), mRNA [NM_198786]	**20,166**
Spock1	PREDICTED: Rattus norvegicus sparc/osteonectin, cwcv and kazal-like domains proteoglycan (testican) 1 (Spock1), mRNA [XM_001067546]	**20,164**
Tulp1	Rattus norvegicus tubby like protein 1 (Tulp1), mRNA [NM_001107642]	**20,162**
Supt6h	Rattus norvegicus suppressor of Ty 6 homolog (S. cerevisiae) (Supt6h), mRNA [NM_001191820]	**20,159**
Tns3	Uncharacterized protein [Source:UniProtKB/TrEMBL;Acc:D3ZCL6] [ENSRNOT00000012661]	**20,151**
Nid1	Nidogen-1 [Source:UniProtKB/Swiss-Prot;Acc:P08460] [ENSRNOT00000003349]	**20,150**
Plat	Rattus norvegicus plasminogen activator, tissue (Plat), mRNA [NM_013151]	**20,146**
0	Unknown	**20,138**
RGD1305793	Rattus norvegicus similar to hypothetical protein FLJ20154 (RGD1305793), mRNA [NM_001127484]	**20,138**
LOC687861	PREDICTED: Rattus norvegicus similar to methylmalonic aciduria (cobalamin deficiency) type B homolog (LOC687861), mRNA [XM_001080395]	**20,125**
Olr868	Rattus norvegicus olfactory receptor 868 (Olr868), mRNA [NM_001000821]	**20,116**
Tmem98	Rattus norvegicus transmembrane protein 98 (Tmem98), mRNA [NM_001007672]	**20,114**
Ssr1	Rattus norvegicus signal sequence receptor, alpha (Ssr1), mRNA [NM_001008891]	**20,108**
Srgap1	Rattus norvegicus SLIT-ROBO Rho GTPase activating protein 1 (Srgap1), mRNA [NM_001191784]	**20,096**
Rps6ka2	Rattus norvegicus ribosomal protein S6 kinase polypeptide 2 (Rps6ka2), mRNA [NM_057128]	**20,091**
Zbtb6	Rattus norvegicus zinc finger and BTB domain containing 6 (Zbtb6), mRNA [NM_001108953]	**20,090**
Ubtd2	PREDICTED: Rattus norvegicus ubiquitin domain containing 2 (Ubtd2), mRNA [XM_213249]	**20,086**
LOC302495	Rattus norvegicus hypothetical LOC302495 (LOC302495), mRNA [NM_001106950]	**20,063**
Tmtc3	Rattus norvegicus transmembrane and tetratricopeptide repeat containing 3 (Tmtc3), mRNA [NM_001135858]	**20,053**
Nmnat1	Rattus norvegicus nicotinamide nucleotide adenylyltransferase 1 (Nmnat1), mRNA [NM_001037556]	**20,048**
Amica1	Uncharacterized protein [Source:UniProtKB/TrEMBL;Acc:D4A1C2] [ENSRNOT00000007588]	**20,048**
0	Q4RCF0_TETNG (Q4RCF0) Chromosome undetermined SCAF19066, whole genome shotgun sequence. (Fragment), partial (8%) [TC624062]	**20,047**
Col4a3bp	Rattus norvegicus collagen, type IV, alpha 3 (Goodpasture antigen) binding protein (Col4a3bp), mRNA [NM_001108935]	**20,045**
0	S18L2_MOUSE (Q9D174) SS18-like protein 2, complete [TC585393]	**20,044**
Pdlim7	Rattus norvegicus PDZ and LIM domain 7 (Pdlim7), mRNA [NM_173125]	**20,037**
Slc35a3	Rattus norvegicus solute carrier family 35 (UDP-N-acetylglucosamine (UDP-GlcNAc) transporter), member A3 (Slc35a3), mRNA [NM_001012082]	**20,034**
Traf4af1	Rattus norvegicus TRAF4 associated factor 1 (Traf4af1), mRNA [NM_001004264]	**20,029**
Zhx1	Rattus norvegicus zinc fingers and homeoboxes 1 (Zhx1), mRNA [NM_133620]	**20,029**
Slc12a8	Rattus norvegicus solute carrier family 12 (potassium/chloride transporters), member 8 (Slc12a8), mRNA [NM_153625]	**20,026**
0	Nope protein [Source:UniProtKB/TrEMBL;Acc:B5DFA9] [ENSRNOT00000044706]	**20,024**
0	Unknown	**20,023**
Zfp280b	Rattus norvegicus zinc finger protein 280b (Zfp280b), mRNA [NM_001106384]	**20,022**
Cacng2	Rattus norvegicus calcium channel, voltage-dependent, gamma subunit 2 (Cacng2), mRNA [NM_053351]	**20,021**
0	Unknown	**20,018**

Pskh1	Rattus norvegicus protein serine kinase H1 (Pskh1), mRNA [NM_001108897]	**20,016**
RGD1309594	Rattus norvegicus similar to RIKEN cDNA 1810043G02; DNA segment, Chr 10, Johns Hopkins University 13, expressed (RGD1309594), mRNA [NM_001008351]	**20,016**
Efna2	Rattus norvegicus ephrin A2 (Efna2), mRNA [NM_001168670]	**20,007**
Frzb	Rattus norvegicus frizzled-related protein (Frzb), mRNA [NM_001100527]	**20,006**
Pip4k2c	Rattus norvegicus phosphatidylinositol-5-phosphate 4-kinase, type II, gamma (Pip4k2c), mRNA [NM_080480]	**20,000**
Lamc1	Rattus norvegicus laminin, gamma 1 (Lamc1), mRNA [NM_053966]	**19,998**
Ankrd39	Rattus norvegicus ankyrin repeat domain 39 (Ankrd39), mRNA [NM_001135014]	**19,997**
Kcnj3	Rattus norvegicus potassium inwardly-rectifying channel, subfamily J, member 3 (Kcnj3), mRNA [NM_031610]	**19,997**
0	Unknown	**19,990**
0	BC076587 Apbb2 protein {Mus musculus} (exp=-1; wgp=0; cg=0), partial (4%) [TC625977]	**19,989**
Ankrd40	Rattus norvegicus ankyrin repeat domain 40 (Ankrd40), mRNA [NM_001134699]	**19,985**
Spire1	Rattus norvegicus spire homolog 1 (Drosophila) (Spire1), mRNA [NM_001107381]	**19,981**
Syt9	Rattus norvegicus synaptotagmin IX (Syt9), mRNA [NM_053324]	**19,980**
RGD1563701	PREDICTED: Rattus norvegicus similar to BC068281 protein (RGD1563701), mRNA [XM_001070724]	**19,975**
Fgf13	Rattus norvegicus fibroblast growth factor 13 (Fgf13), mRNA [NM_053428]	**19,971**
Ero1l	Rattus norvegicus ERO1-like (S. cerevisiae) (Ero1l), mRNA [NM_138528]	**19,970**
0	Unknown	**19,963**
Usp31	Rattus norvegicus ubiquitin specific peptidase 31 (Usp31), mRNA [NM_001107548]	**19,961**
Sec31a	Rattus norvegicus SEC31 homolog A (S. cerevisiae) (Sec31a), mRNA [NM_033021]	**19,961**
0	Q5MPV8_PREEN (Q5MPV8) Thap11 (Fragment), partial (52%) [TC586886]	**19,956**
Osr2	Rattus norvegicus odd-skipped related 2 (Drosophila) (Osr2), mRNA [NM_001012118]	**19,955**
Nhlh2	Rattus norvegicus nescient helix loop helix 2 (Nhlh2), mRNA [NM_001106457]	**19,951**
0	Unknown	**19,949**
Rnft2	Rattus norvegicus ring finger protein, transmembrane 2 (Rnft2), mRNA [NM_001107144]	**19,945**
Mlph	Rattus norvegicus melanophilin (Mlph), mRNA [NM_001012135]	**19,945**
0	Uncharacterized protein [Source:UniProtKB/TrEMBL;Acc:D3ZPT5] [ENSRNOT00000067382]	**19,943**
RGD1564755	Rattus norvegicus similar to putative pheromone receptor (Go-VN5) (RGD1564755), mRNA [NM_001099465]	**19,939**
Cetn4	Unknown	**19,936**
Zhx3	Rattus norvegicus zinc fingers and homeoboxes 3 (Zhx3), mRNA [NM_001047097]	**19,935**
Olr875	Rattus norvegicus olfactory receptor 875 (Olr875), mRNA [NM_001000054]	**19,932**
0	Unknown	**19,927**
Zcchc8	Rattus norvegicus zinc finger, CCHC domain containing 8 (Zcchc8), mRNA [NM_001105929]	**19,923**
Ranbp1	Rattus norvegicus RAN binding protein 1 (Ranbp1), mRNA [NM_001108324]	**19,922**

Ecop	Rattus norvegicus EGFR-coamplified and overexpressed protein (Ecop), mRNA [NM_001108630]	**19,921**
Rps6ka1	Rattus norvegicus ribosomal protein S6 kinase polypeptide 1 (Rps6ka1), mRNA [NM_031107]	**19,919**
0	Unknown	**19,916**
Socs2	Rattus norvegicus suppressor of cytokine signaling 2 (Socs2), mRNA [NM_058208]	**19,903**
Tbc1d7	Rattus norvegicus TBC1 domain family, member 7 (Tbc1d7), mRNA [NM_001108411]	**19,899**
Ypel4	Rattus norvegicus yippee-like 4 (Drosophila) (Ypel4), mRNA [NM_001024369]	**19,897**
Ifi30	Rattus norvegicus interferon gamma inducible protein 30 (Ifi30), mRNA [NM_001030026]	**19,892**
Tox3	Rattus norvegicus TOX high mobility group box family member 3 (Tox3), mRNA [NM_001106171]	**19,888**
0	Unknown	**19,885**
Vgf	Rattus norvegicus VGF nerve growth factor inducible (Vgf), mRNA [NM_030997]	**19,880**
0	Rattus norvegicus TL0AEA5YN17 mRNA sequence. [FQ233918]	**19,877**
Satb2	Rattus norvegicus SATB homeobox 2 (Satb2), mRNA [NM_001109306]	**19,870**
Scamp2	Rattus norvegicus secretory carrier membrane protein 2 (Scamp2), mRNA [NM_023955]	**19,865**
Ogfrl1	Rattus norvegicus opioid growth factor receptor-like 1 (Ogfrl1), mRNA [NM_001025708]	**19,862**
Pak7	Rattus norvegicus p21 protein (Cdc42/Rac)-activated kinase 7 (Pak7), mRNA [NM_001107781]	**19,840**
0	RVL8568 Wackym-Soares normalized rat vestibular cDNA library Rattus norvegicus cDNA 5', mRNA sequence [DV717996]	**19,837**
Slc25a22	Rattus norvegicus solute carrier family 25 (mitochondrial carrier, glutamate), member 22 (Slc25a22), nuclear gene encoding mitochondrial protein, mRNA [NM_001014027]	**19,837**
Sstr2	Rattus norvegicus somatostatin receptor 2 (Sstr2), mRNA [NM_019348]	**19,833**
Cdc2l6	Rattus norvegicus cell division cycle 2-like 6 (CDK8-like) (Cdc2l6), mRNA [NM_001107634]	**19,833**
Kcnab2	Rattus norvegicus potassium voltage-gated channel, shaker-related subfamily, beta member 2 (Kcnab2), mRNA [NM_017304]	**19,832**
Rabgap1	Rattus norvegicus RAB GTPase activating protein 1 (Rabgap1), mRNA [NM_001107841]	**19,828**
Nckipsd	Rattus norvegicus NCK interacting protein with SH3 domain (Nckipsd), mRNA [NM_001106857]	**19,826**
Nudt15	Rattus norvegicus nudix (nucleoside diphosphate linked moiety X)-type motif 15 (Nudt15), mRNA [NM_001106049]	**19,824**
0	PREDICTED: Rattus norvegicus similar to Protein C9orf126 homolog (LOC690538), mRNA [XM_001074743]	**19,822**
Pygo2	Rattus norvegicus pygopus 2 (Pygo2), mRNA [NM_001106447]	**19,814**
Pde1b	Rattus norvegicus phosphodiesterase 1B, calmodulin-dependent (Pde1b), mRNA [NM_022710]	**19,812**
Rfx7	Rattus norvegicus regulatory factor X, 7 (Rfx7), mRNA [NM_001127490]	**19,808**
Tpcn1	Rattus norvegicus two pore segment channel 1 (Tpcn1), mRNA [NM_139332]	**19,804**
Prdm4	Rattus norvegicus PR domain containing 4 (Prdm4), mRNA [NM_133312]	**19,802**
RGD1308759	Rattus norvegicus similar to KIAA0892 protein (RGD1308759), mRNA [NM_001106077]	**19,800**
RGD735175	Rattus norvegicus hypothetical protein MGC:72616 (RGD735175), mRNA [NM_199112]	**19,796**
Gtf2ird2	General transcription factor II I repeat domain-containing 2 [Source:UniProtKB/TrEMBL;Acc:Q2V6E6] [ENSRNOT00000002030]	**19,794**
Hdac11	Rattus norvegicus histone deacetylase 11 (Hdac11), mRNA [NM_001106610]	**19,792**

Tceb3	Rattus norvegicus transcription elongation factor B (SIII), polypeptide 3 (Tceb3), mRNA [NM_017103]	**19,788**
Tmem86a	Rattus norvegicus transmembrane protein 86A (Tmem86a), mRNA [NM_001135016]	**19,783**
Wdtc1	Rattus norvegicus WD and tetratricopeptide repeats 1 (Wdtc1), mRNA [NM_001107908]	**19,774**
Elof1	Rattus norvegicus elongation factor 1 homolog (S. cerevisiae) (Elof1), mRNA [NM_001126098]	**19,768**
Cpsf1	Rattus norvegicus cleavage and polyadenylation specific factor 1 (Cpsf1), mRNA [NM_001130571]	**19,759**
0	Q6X2J5_9SAUR (Q6X2J5) NADH dehydrogenase subunit 4 (Fragment), partial (6%) [TC646703]	**19,758**
Atp7a	Rattus norvegicus ATPase, Cu++ transporting, alpha polypeptide (Atp7a), mRNA [NM_052803]	**19,758**
Ptprf	Rattus norvegicus protein tyrosine phosphatase, receptor type, F (Ptprf), mRNA [NM_019249]	**19,755**
0	Unknown	**19,755**
0	Solute carrier family 26, member 8 (Predicted)Uncharacterized protein [Source:UniProtKB/TrEMBL;Acc:D3Z800] [ENSRNOT00000000615]	**19,753**
Myo6	PREDICTED: Rattus norvegicus myosin VI (Myo6), mRNA [XM_236444]	**19,752**
RGD1310352	Rattus norvegicus similar to HTGN29 protein; keratinocytes associated transmembrane protein 2 (RGD1310352), mRNA [NM_001106999]	**19,751**
LOC498201	PREDICTED: Rattus norvegicus hypothetical LOC498201 (LOC498201), mRNA [XM_573419]	**19,747**
0	Unknown	**19,741**
0	methyl-CpG binding domain protein 5 Gene [Source:MGI Symbol;Acc:MGI:2138934] [ENSRNOT00000034632]	**19,738**
Nipsnap1	Rattus norvegicus nipsnap homolog 1 (C. elegans) (Nipsnap1), mRNA [NM_001100730]	**19,738**
Necap2	Rattus norvegicus NECAP endocytosis associated 2 (Necap2), mRNA [NM_199096]	**19,734**
LOC681458	PREDICTED: Rattus norvegicus similar to stearoyl-coenzyme A desaturase 3 (LOC681458), mRNA [XM_001056911]	**19,731**
Pcdhb11	PREDICTED: Rattus norvegicus protocadherin beta 11 (Pcdhb11), mRNA [XM_001055576]	**19,725**
RGD1561065	Rattus norvegicus similar to mKIAA1111 protein (RGD1561065), mRNA [NM_001108253]	**19,723**
0	Protein FAM134A [Source:UniProtKB/Swiss-Prot;Acc:Q3MHU5] [ENSRNOT00000025135]	**19,721**
LOC679038	Rattus norvegicus similar to mammalian retrotransposon derived 8b (LOC679038), mRNA [NM_001100998]	**19,720**
Cmtm5	Rattus norvegicus CKLF-like MARVEL transmembrane domain containing 5 (Cmtm5), mRNA [NM_001106034]	**19,718**
Prelid1	Rattus norvegicus PRELI domain containing 1 (Prelid1), mRNA [NM_001009636]	**19,711**
RGD1565648	PREDICTED: Rattus norvegicus protein S100-A11-like (LOC100362296), mRNA [XM_002729212]	**19,710**
Asap1	Rattus norvegicus ArfGAP with SH3 domain, ankyrin repeat and PH domain 1 (Asap1), mRNA [NM_001044245]	**19,705**
Tpra1	Rattus norvegicus transmembrane protein, adipocyte asscociated 1 (Tpra1), mRNA [NM_053534]	**19,704**
0	Q2FXP0_STAAU (Q2FXP0) Formamidopyrimidine-DNA glycosylase , partial (6%) [TC596639]	**19,691**
Mdga1	Rattus norvegicus MAM domain containing glycosylphosphatidylinositol anchor 1 (Mdga1), mRNA [NM_001107618]	**19,685**
Rabif	Rattus norvegicus RAB interacting factor (Rabif), mRNA [NM_001007678]	**19,682**
Sstr2	Rattus norvegicus somatostatin receptor 2 (Sstr2), mRNA [NM_019348]	**19,681**
Ano6	Rattus norvegicus anoctamin 6 (Ano6), mRNA [NM_001108108]	**19,680**
Prrx1	Rattus norvegicus paired related homeobox 1 (Prrx1), mRNA [NM_153821]	**19,679**
Rab35	Rattus norvegicus RAB35, member RAS oncogene family (Rab35), mRNA [NM_001013046]	**19,674**

Vom2r71	Rattus norvegicus vomeronasal 2 receptor, 71 (Vom2r71), mRNA [NM_001099516]	**19,670**
0	Q7TPN2_MOUSE (Q7TPN2) Ube2o protein, partial (38%) [TC615970]	**19,669**
Crtc3	PREDICTED: Rattus norvegicus CREB regulated transcription coactivator 3 (Crtc3), mRNA [XM_001066604]	**19,667**
Vom1r78	Rattus norvegicus vomeronasal 1 receptor 78 (Vom1r78), mRNA [NM_001008924]	**19,666**
Olr546	Rattus norvegicus olfactory receptor 546 (Olr546), mRNA [NM_001001054]	**19,660**
Olr232	Rattus norvegicus olfactory receptor 232 (Olr232), mRNA [NM_001001035]	**19,660**
Ak3l1	Rattus norvegicus adenylate kinase 3-like 1, mRNA (cDNA clone MGC:93541 IMAGE:7107348), complete cds. [BC087024]	**19,658**
Hdac1	Rattus norvegicus histone deacetylase 1 (Hdac1), mRNA [NM_001025409]	**19,657**
Chst7	Rattus norvegicus carbohydrate (N-acetylglucosamine 6-O) sulfotransferase 7 (Chst7), mRNA [NM_207600]	**19,654**
Ywhah	Rattus norvegicus tyrosine 3-monooxygenase/tryptophan 5-monooxygenase activation protein, eta polypeptide (Ywhah), mRNA [NM_013052]	**19,650**
RGD1560108	Rattus norvegicus similar to RIKEN cDNA 2700081O15 (RGD1560108), mRNA [NM_001109161]	**19,646**
Nucks1	Rattus norvegicus nuclear casein kinase and cyclin-dependent kinase substrate 1 (Nucks1), mRNA [NM_022799]	**19,641**
0	Unknown	**19,636**
Cln6	Rattus norvegicus ceroid-lipofuscinosis, neuronal 6 (Cln6), mRNA [NM_001191794]	**19,633**
Dmrt3	Rattus norvegicus doublesex and mab-3 related transcription factor 3 (Dmrt3), mRNA [NM_001106358]	**19,633**
Cox6a2	Rattus norvegicus cytochrome c oxidase, subunit VIa, polypeptide 2 (Cox6a2), nuclear gene encoding mitochondrial protein, transcript variant 1, mRNA [NM_012812]	**19,631**
RGD1565712	Rattus norvegicus similar to Hypothetical protein MGC59495 (RGD1565712), mRNA [NM_001127579]	**19,631**
Pfn2	Rattus norvegicus profilin 2 (Pfn2), mRNA [NM_030873]	**19,627**
Pigb	Rattus norvegicus phosphatidylinositol glycan anchor biosynthesis, class B (Pigb), mRNA [NM_001108166]	**19,623**
0	Unknown	**19,618**
0	Unknown	**19,617**
Grb2	Rattus norvegicus growth factor receptor bound protein 2 (Grb2), mRNA [NM_030846]	**19,612**
Ids	PREDICTED: Rattus norvegicus iduronate 2-sulfatase (Ids), mRNA [XM_001054447]	**19,605**
Meaf6	Rattus norvegicus MYST/Esa1-associated factor 6 (Meaf6), mRNA [NM_001113784]	**19,596**
Atg5	Rattus norvegicus ATG5 autophagy related 5 homolog (S. cerevisiae) (Atg5), mRNA [NM_001014250]	**19,592**
Susd4	Rattus norvegicus sushi domain containing 4 (Susd4), mRNA [NM_001105982]	**19,591**
Ezh1	Rattus norvegicus enhancer of zeste homolog 1 (Drosophila) (Ezh1), mRNA [NM_001107051]	**19,589**
RGD1311595	PREDICTED: Rattus norvegicus similar to KIAA2026 protein (RGD1311595), mRNA [XM_001079649]	**19,588**
Cap1	Rattus norvegicus CAP, adenylate cyclase-associated protein 1 (yeast) (Cap1), mRNA [NM_022383]	**19,586**
Col12a1	Collagen alpha-1(XII) chain [Source:UniProtKB/Swiss-Prot;Acc:P70560] [ENSRNOT00000043691]	**19,583**
Ythdc1	Rattus norvegicus YTH domain containing 1 (Ythdc1), mRNA [NM_133423]	**19,582**
Scoc	Rattus norvegicus short coiled-coil protein (Scoc), mRNA [NM_001013235]	**19,578**
0	Unknown	**19,568**

Gpd2	Rattus norvegicus glycerol-3-phosphate dehydrogenase 2, mitochondrial (Gpd2), nuclear gene encoding mitochondrial protein, mRNA [NM_012736]	**19,565**
Map1lc3b	Rattus norvegicus microtubule-associated protein 1 light chain 3 beta (Map1lc3b), mRNA [NM_022867]	**19,560**
Zfp57	Rattus norvegicus zinc finger protein 57 (Zfp57), mRNA [NM_213565]	**19,560**
Fam187b	Uncharacterized protein [Source:UniProtKB/TrEMBL;Acc:D3ZT17] [ENSRNOT00000028593]	**19,558**
Jazf1	Uncharacterized protein [Source:UniProtKB/TrEMBL;Acc:D3ZA80] [ENSRNOT00000039580]	**19,555**
Ccdc153	Rattus norvegicus coiled-coil domain containing 153 (Ccdc153), mRNA [NM_001013953]	**19,551**
Tbc1d9b	Rattus norvegicus TBC1 domain family, member 9B (Tbc1d9b), mRNA [NM_001108274]	**19,549**
Ccdc101	Rattus norvegicus coiled-coil domain containing 101 (Ccdc101), mRNA [NM_001114502]	**19,548**
Foxred2	Rattus norvegicus FAD-dependent oxidoreductase domain containing 2 (Foxred2), mRNA [NM_001191787]	**19,543**
Ube2v1	Rattus norvegicus ubiquitin-conjugating enzyme E2 variant 1 (Ube2v1), mRNA [NM_001110345]	**19,538**
Erc2	Rattus norvegicus ELKS/RAB6-interacting/CAST family member 2 (Erc2), mRNA [NM_170787]	**19,533**
Tspyl2	Rattus norvegicus TSPY-like 2 (Tspyl2), mRNA [NM_001191618]	**19,532**
RGD1561149	Rattus norvegicus similar to mKIAA1522 protein (RGD1561149), mRNA [NM_001134629]	**19,530**
Lcp1	Rattus norvegicus lymphocyte cytosolic protein 1 (Lcp1), mRNA [NM_001012044]	**19,524**
Hist2h3c	PREDICTED: Rattus norvegicus similar to CG31613-PA (LOC679950), mRNA [XM_001055051]	**19,520**
LOC686205	PREDICTED: Rattus norvegicus hypothetical protein LOC686205 (LOC686205), mRNA [XM_001072876]	**19,514**
Picalm	Rattus norvegicus phosphatidylinositol binding clathrin assembly protein (Picalm), mRNA [NM_053554]	**19,513**
0	Uncharacterized protein [Source:UniProtKB/TrEMBL;Acc:D3Z8Y2] [ENSRNOT00000034387]	**19,508**
Tas2r113	Rattus norvegicus taste receptor, type 2, member 113 (Tas2r113), mRNA [NM_001166689]	**19,507**
Slc6a18	Rattus norvegicus solute carrier family 6, member 18 (Slc6a18), mRNA [NM_017163]	**19,499**
Pigh	Rattus norvegicus phosphatidylinositol glycan anchor biosynthesis, class H (Pigh), mRNA [NM_001108714]	**19,499**
Ube2m	Rattus norvegicus ubiquitin-conjugating enzyme E2M (UBC12 homolog, yeast) (Ube2m), mRNA [NM_001108471]	**19,496**
Atxn1	Rattus norvegicus ataxin 1 (Atxn1), mRNA [NM_012726]	**19,494**
0	Unknown	**19,493**
RGD1308226	Rattus norvegicus similar to hypothetical protein FLJ32786 (RGD1308226), mRNA [NM_001106589]	**19,493**
0	Protein jagged-2 [Source:UniProtKB/Swiss-Prot;Acc:P97607] [ENSRNOT00000019066]	**19,491**
Pafah1b3	Rattus norvegicus platelet-activating factor acetylhydrolase, isoform 1b, subunit 3 (Pafah1b3), mRNA [NM_053654]	**19,490**
Defb51	Rattus norvegicus defensin beta 51 (Defb51), mRNA [NM_001037547]	**19,488**
0	Histone H2A type 2-A [Source:UniProtKB/Swiss-Prot;Acc:P0CC09] [ENSRNOT00000047696]	**19,480**
Rassf5	Rattus norvegicus Ras association (RalGDS/AF-6) domain family member 5 (Rassf5), mRNA [NM_019365]	**19,479**
Acd	Rattus norvegicus adrenocortical dysplasia homolog (mouse) (Acd), mRNA [NM_001037193]	**19,479**
Ppfia3	Liprin-alpha-3 [Source:UniProtKB/Swiss-Prot;Acc:Q91Z79] [ENSRNOT00000028142]	**19,478**
Mtch2	Rattus norvegicus mitochondrial carrier homolog 2 (C. elegans) (Mtch2), nuclear gene encoding mitochondrial protein, mRNA [NM_001106488]	**19,478**

Psors1c2	Rattus norvegicus psoriasis susceptibility 1 candidate 2 (human) (Psors1c2), mRNA [NM_001166016]	**19,476**
Ptpn1	Rattus norvegicus protein tyrosine phosphatase, non-receptor type 1 (Ptpn1), mRNA [NM_012637]	**19,475**
Plekhh1	Rattus norvegicus pleckstrin homology domain containing, family H (with MyTH4 domain) member 1 (Plekhh1), mRNA [NM_001108036]	**19,471**
0	Unknown	**19,469**
Acox1	Peroxisomal acyl-coenzyme A oxidase 1Peroxisomal acyl-CoA oxidase 1, A chainPeroxisomal acyl-CoA oxidase 1, B chainPeroxisomal acyl-CoA oxidase 1, C chain [Source:UniProtKB/Swiss-Prot;Acc:P07872] [ENSRNOT00000042372]	**19,467**
Lzts2	Rattus norvegicus leucine zipper, putative tumor suppressor 2 (Lzts2), mRNA [NM_001014247]	**19,465**
Abat	Rattus norvegicus 4-aminobutyrate aminotransferase (Abat), nuclear gene encoding mitochondrial protein, mRNA [NM_031003]	**19,462**
0	Rattus norvegicus cDNA clone IMAGE:7377987. [BC158826]	**19,461**
Tmem59	Rattus norvegicus transmembrane protein 59 (Tmem59), mRNA [NM_001139465]	**19,460**
0	Unknown	**19,459**
Sned1	Rattus norvegicus isolate No:6 insulin responsive sequence DNA binding protein-1 mRNA, partial cds. [AF439716]	**19,458**
Defb20	Rattus norvegicus defensin beta 20 (Defb20), mRNA [NM_001037517]	**19,454**
Cd164	Rattus norvegicus CD164 molecule, sialomucin (Cd164), mRNA [NM_031812]	**19,451**
0	Unknown	**19,451**
Ammecr1l	Rattus norvegicus AMME chromosomal region gene 1-like (Ammecr1l), mRNA [NM_001107399]	**19,446**
Tmem188	Rattus norvegicus transmembrane protein 188 (Tmem188), mRNA [NM_001106173]	**19,444**
0	Unknown	**19,444**
Arf3	Rattus norvegicus ADP-ribosylation factor 3 (Arf3), mRNA [NM_080904]	**19,443**
Ppp2r5c	Rattus norvegicus protein phosphatase 2, regulatory subunit B', gamma isoform (Ppp2r5c), mRNA [NM_001191112]	**19,439**
Park7	Rattus norvegicus Parkinson disease (autosomal recessive, early onset) 7, mRNA (cDNA clone MGC:108615 IMAGE:7376914), complete cds. [BC091128]	**19,437**
Pcdhgb2	Uncharacterized protein [Source:UniProtKB/TrEMBL;Acc:D3ZFX5] [ENSRNOT00000065447]	**19,437**
0	Unknown	**19,429**
Cuedc1	Rattus norvegicus CUE domain containing 1 (Cuedc1), mRNA [NM_001013971]	**19,421**
0	Unknown	**19,419**
Acap2	Rattus norvegicus ArfGAP with coiled-coil, ankyrin repeat and PH domains 2 (Acap2), mRNA [NM_001034006]	**19,419**
0	TGF-beta activated kinase 1/MAP3K7 binding protein 3 Gene [Source:MGI Symbol;Acc:MGI:1913974] [ENSRNOT00000004854]	**19,414**
Cxxc1	Rattus norvegicus CXXC finger 1 (PHD domain) (Cxxc1), mRNA [NM_001079698]	**19,394**
Fzr1	Rattus norvegicus fizzy/cell division cycle 20 related 1 (Drosophila) (Fzr1), mRNA [NM_001108074]	**19,392**
LOC100233213	Rattus norvegicus hypothetical protein LOC100233213 (LOC100233213), mRNA [NM_001143803]	**19,390**
Cndp1	Rattus norvegicus carnosine dipeptidase 1 (metallopeptidase M20 family) (Cndp1), mRNA [NM_001007687]	**19,387**
Adcyap1r1	Rattus norvegicus adenylate cyclase activating polypeptide 1 receptor 1 (Adcyap1r1), mRNA [NM_133511]	**19,383**
0	Unknown	**19,378**

Suclg2	Rattus norvegicus succinate-CoA ligase, GDP-forming, beta subunit (Suclg2), nuclear gene encoding mitochondrial protein, mRNA [NM_001100750]	**19,374**
Gpr137b	Rattus norvegicus G protein-coupled receptor 137B (Gpr137b), mRNA [NM_001105978]	**19,373**
Ntrk2	Rattus norvegicus neurotrophic tyrosine kinase, receptor, type 2 (Ntrk2), transcript variant 1, mRNA [NM_012731]	**19,369**
Psma3	Rattus norvegicus proteasome (prosome, macropain) subunit, alpha type 3 (Psma3), mRNA [NM_017280]	**19,369**
Scrg1	Rattus norvegicus stimulator of chondrogenesis 1 (Scrg1), mRNA [NM_033499]	**19,367**
Ganab	Rattus norvegicus glucosidase, alpha; neutral AB (Ganab), mRNA [NM_001106334]	**19,366**
0	Q80UV2_MOUSE (Q80UV2) AU042671 protein, partial (40%) [TC598606]	**19,359**
Map3k7ip2	Rattus norvegicus mitogen-activated protein kinase kinase kinase 7 interacting protein 2 (Map3k7ip2), mRNA [NM_001012062]	**19,350**
0	Rattus norvegicus zinc finger protein 184, mRNA (cDNA clone MGC:156842 IMAGE:7381364), complete cds. [BC128784]	**19,349**
RGD1560088	Uncharacterized protein [Source:UniProtKB/TrEMBL;Acc:D3ZQH7] [ENSRNOT00000047207]	**19,344**
LOC680370	Rattus norvegicus hypothetical protein LOC680370 (LOC680370), mRNA [NM_001109402]	**19,342**
Rgs20	Rattus norvegicus regulator of G-protein signaling 20 (Rgs20), mRNA [NM_001127495]	**19,341**
Fkbp1a	Rattus norvegicus FK506 binding protein 1a (Fkbp1a), mRNA [NM_013102]	**19,341**
Gli3	Rattus norvegicus GLI-Kruppel family member GLI3 (Gli3), mRNA [NM_080405]	**19,340**
Abhd14b	Rattus norvegicus abhydrolase domain containing 14b (Abhd14b), mRNA [NM_001007664]	**19,337**
Elovl4	Rattus norvegicus elongation of very long chain fatty acids (FEN1/Elo2, SUR4/Elo3, yeast)-like 4 (Elovl4), mRNA [NM_001191796]	**19,337**
Pars2	Rattus norvegicus prolyl-tRNA synthetase 2, mitochondrial (putative) (Pars2), nuclear gene encoding mitochondrial protein, mRNA [NM_001014064]	**19,337**
LOC362795	Rattus norvegicus immunoglobulin G heavy chain, mRNA (cDNA clone IMAGE:7385053). [BC098733]	**19,336**
RGD1310495	Uncharacterized protein [Source:UniProtKB/TrEMBL;Acc:D3ZDM4] [ENSRNOT00000000720]	**19,334**
Mn1	Rattus norvegicus meningioma 1 (Mn1), mRNA [NM_001191928]	**19,333**
Grm1	Rattus norvegicus glutamate receptor, metabotropic 1 (Grm1), transcript variant 1, mRNA [NM_017011]	**19,332**
0	Unknown	**19,330**
Arid5b	Rattus norvegicus AT rich interactive domain 5B (Mrf1 like) (Arid5b), mRNA [NM_001107624]	**19,329**
LOC688948	Uncharacterized protein [Source:UniProtKB/TrEMBL;Acc:D4AAJ9] [ENSRNOT00000041748]	**19,325**
Hpcal4	Rattus norvegicus hippocalcin-like 4 (Hpcal4), mRNA [NM_017357]	**19,325**
Acvr1c	Rattus norvegicus activin A receptor, type IC (Acvr1c), mRNA [NM_139090]	**19,324**
0	Uncharacterized protein [Source:UniProtKB/TrEMBL;Acc:D3ZNE4] [ENSRNOT00000056975]	**19,321**
0	Unknown	**19,312**
Slitrk2	Rattus norvegicus SLIT and NTRK-like family, member 2 (Slitrk2), mRNA [NM_001107587]	**19,302**
RGD1564257	Rattus norvegicus similar to hypothetical protein FLJ32825 (RGD1564257), mRNA [NM_001163723]	**19,294**
0	EST346778 Normalized rat embryo, Bento Soares Rattus sp. cDNA clone RGICV78 5' end, mRNA sequence [AW915474]	**19,293**
LOC499718	PREDICTED: Rattus norvegicus hypothetical LOC499718 (LOC499718), mRNA [XM_001078222]	**19,282**

Kpna3	Rattus norvegicus karyopherin alpha 3 (Kpna3), mRNA [NM_001014792]	**19,280**
0	Unknown	**19,277**
Impa2	Rattus norvegicus inositol (myo)-1(or 4)-monophosphatase 2 (Impa2), mRNA [NM_172224]	**19,275**
0	Potassium voltage-gated channel subfamily C member 2 [Source:UniProtKB/Swiss-Prot;Acc:P22462] [ENSRNOT00000049943]	**19,275**
Phlpp1	Rattus norvegicus PH domain and leucine rich repeat protein phosphatase 1 (Phlpp1), mRNA [NM_021657]	**19,268**
Nrn1	Rattus norvegicus neuritin 1 (Nrn1), mRNA [NM_053346]	**19,268**
Ankh	Rattus norvegicus ankylosis, progressive homolog (mouse) (Ankh), mRNA [NM_053714]	**19,261**
RGD1310427	Rattus norvegicus similar to KIAA0090 protein (RGD1310427), mRNA [NM_001108690]	**19,254**
Kcnip1	Rattus norvegicus Kv channel-interacting protein 1 (Kcnip1), mRNA [NM_022929]	**19,252**
0	Unknown	**19,251**
Ap1m1	Rattus norvegicus adaptor-related protein complex 1, mu 1 subunit (Ap1m1), mRNA [NM_001044239]	**19,247**
Pigt	Rattus norvegicus phosphatidylinositol glycan anchor biosynthesis, class T (Pigt), mRNA [NM_001106540]	**19,244**
Ankrd54	Rattus norvegicus ankyrin repeat domain 54 (Ankrd54), mRNA [NM_001025285]	**19,242**
Lass2	Rattus norvegicus LAG1 homolog, ceramide synthase 2 (Lass2), mRNA [NM_001033700]	**19,241**
Phlpp2	Rattus norvegicus PH domain and leucine rich repeat protein phosphatase 2 (Phlpp2), mRNA [NM_001109131]	**19,240**
Aadat	Rattus norvegicus aminoadipate aminotransferase (Aadat), mRNA [NM_017193]	**19,239**
Atp7b	Rattus norvegicus ATPase, Cu++ transporting, beta polypeptide (Atp7b), mRNA [NM_012511]	**19,239**
Prodh2	Rattus norvegicus proline dehydrogenase (oxidase) 2 (Prodh2), mRNA [NM_001038588]	**19,237**
LOC691298	PREDICTED: Rattus norvegicus similar to peptidoglycan recognition protein 2 (LOC691298), mRNA [XM_002729754]	**19,236**
Slc16a11	Rattus norvegicus solute carrier family 16 (monocarboxylic acid transporters), member 11 (Slc16a11), mRNA [NM_001105797]	**19,234**
LOC503202	PREDICTED: Rattus norvegicus similar to seven transmembrane helix receptor (LOC503202), mRNA [XM_002727072]	**19,233**
0	Unknown	**19,232**
Ephb6	Rattus norvegicus Eph receptor B6 (Ephb6), mRNA [NM_001107857]	**19,230**
0	Neuronal PAS domain protein 3 (Predicted), isoform CRA_aUncharacterized protein [Source:UniProtKB/TrEMBL;Acc:D3ZZI2] [ENSRNOT00000067066]	**19,230**
LOC100362981	LRRGT00010 [Source:UniProtKB/TrEMBL;Acc:Q6TXI9] [ENSRNOT00000047211]	**19,227**
Klf15	Rattus norvegicus Kruppel-like factor 15 (Klf15), mRNA [NM_053536]	**19,221**
Arnt2	Rattus norvegicus aryl hydrocarbon receptor nuclear translocator 2 (Arnt2), mRNA [NM_012781]	**19,219**
Ccna1	Rattus norvegicus cyclin A1 (Ccna1), mRNA [NM_001011949]	**19,209**
0	U1 small nuclear ribonucleoprotein C [Source:UniProtKB/TrEMBL;Acc:D3ZCL3] [ENSRNOT00000000586]	**19,208**
Suox	Rattus norvegicus sulfite oxidase (Suox), nuclear gene encoding mitochondrial protein, mRNA [NM_031127]	**19,207**
Smo	Rattus norvegicus smoothened homolog (Drosophila) (Smo), mRNA [NM_012807]	**19,193**
0	Unknown	**19,189**
Igf1	Rattus norvegicus insulin-like growth factor 1 (Igf1), transcript variant 4, mRNA [NM_001082479]	**19,181**

Pdgfa	Rattus norvegicus platelet-derived growth factor alpha polypeptide (Pdgfa), mRNA [NM_012801]	**19,169**
0	Uncharacterized protein [Source:UniProtKB/TrEMBL;Acc:D3ZAP4] [ENSRNOT00000020882]	**19,165**
Srm	Rattus norvegicus spermidine synthase (Srm), mRNA [NM_053464]	**19,163**
0	Unknown	**19,154**
Kcnip3	Rattus norvegicus Kv channel interacting protein 3, calsenilin (Kcnip3), mRNA [NM_032462]	**19,148**
0	Uncharacterized protein [Source:UniProtKB/TrEMBL;Acc:D4A9W8] [ENSRNOT00000014537]	**19,147**
0	Unknown	**19,147**
Hs3st1	Rattus norvegicus heparan sulfate (glucosamine) 3-O-sulfotransferase 1 (Hs3st1), mRNA [NM_053391]	**19,146**
Zfp423	Rattus norvegicus zinc finger protein 423 (Zfp423), mRNA [NM_053583]	**19,138**
Kcnk5	Rattus norvegicus potassium channel, subfamily K, member 5 (Kcnk5), mRNA [NM_001039516]	**19,138**
Acta2	Rattus norvegicus smooth muscle alpha-actin (Acta2), mRNA [NM_031004]	**19,138**
Hs2st1	Rattus norvegicus heparan sulfate 2-O-sulfotransferase 1 (Hs2st1), mRNA [NM_001100518]	**19,117**
Tnpo3	Rattus norvegicus transportin 3 (Tnpo3), mRNA [NM_001106587]	**19,116**
0	SAGA-associated factor 29 homolog [Source:UniProtKB/Swiss-Prot;Acc:P0C606] [ENSRNOT00000026146]	**19,114**
Zfp91	Rattus norvegicus zinc finger protein 91 (Zfp91), mRNA [NM_001169120]	**19,113**
Sfrs15	Rattus norvegicus splicing factor, arginine/serine-rich 15 (Sfrs15), mRNA [NM_001037347]	**19,105**
0	Uncharacterized protein [Source:UniProtKB/TrEMBL;Acc:D3ZAS6] [ENSRNOT00000000895]	**19,102**
Tcn2	Rattus norvegicus transcobalamin 2 (Tcn2), mRNA [NM_022534]	**19,095**
Notch3	Rattus norvegicus Notch homolog 3 (Drosophila) (Notch3), mRNA [NM_020087]	**19,094**
Rai1	Uncharacterized protein [Source:UniProtKB/TrEMBL;Acc:D4A4Z4] [ENSRNOT00000039411]	**19,082**
0	PREDICTED: Rattus norvegicus similar to minichromosome maintenance protein 8 isoform 1 (RGD1560557), miscRNA [XR_007676]	**19,080**
Adprh	Rattus norvegicus ADP-ribosylarginine hydrolase (Adprh), mRNA [NM_183325]	**19,070**
0	Rattus norvegicus similar to px19-like protein (LOC309182), mRNA [XM_219534]	**19,070**
Rrm2	Rattus norvegicus ribonucleotide reductase M2 (Rrm2), mRNA [NM_001025740]	**19,068**
Cd93	Rattus norvegicus CD93 molecule (Cd93), mRNA [NM_053383]	**19,067**
G4	Rattus norvegicus G4 protein (G4), mRNA [NM_001003975]	**19,062**
0	Unknown	**19,060**
0	Unknown	**19,059**
Eno2	Gamma-enolase [Source:UniProtKB/Swiss-Prot;Acc:P07323] [ENSRNOT00000005601]	**19,057**
LOC688421	PREDICTED: Rattus norvegicus similar to Spetex-2C protein (LOC688421), mRNA [XM_001066867]	**19,057**
C1ql1	Rattus norvegicus complement component 1, q subcomponent-like 1 (C1ql1), mRNA [NM_001108838]	**19,052**
0	Protein tyrosine phosphatase, receptor type, B (Predicted), isoform CRA_bUncharacterized protein [Source:UniProtKB/TrEMBL;Acc:D3ZE19] [ENSRNOT00000058384]	**19,045**
Msl3l2	Rattus norvegicus male-specific lethal 3-like 2 (Drosophila) (Msl3l2), mRNA [NM_001014032]	**19,045**

Bat3	Rattus norvegicus HLA-B-associated transcript 3 (Bat3), transcript variant 2, mRNA [NM_053609]	**19,044**
RGD1310686	Rattus norvegicus similar to chromosome 16 open reading frame 5 (RGD1310686), mRNA [NM_001008360]	**19,038**
0	Rattus norvegicus, 25 clones, strain BN/SsNHsdMCW RNOR03324481, whole genome shotgun sequence [AABR03128056]	**19,037**
Stxbp2	Rattus norvegicus syntaxin binding protein 2 (Stxbp2), mRNA [NM_031126]	**19,035**
0	Unknown	**19,033**
Syngap1	Rattus norvegicus synaptic Ras GTPase activating protein 1 homolog (rat) (Syngap1), transcript variant 1, mRNA [NM_181092]	**19,029**
Gdf10	Rattus norvegicus growth differentiation factor 10 (Gdf10), mRNA [NM_024375]	**19,029**
Gpr123	Rattus norvegicus G protein-coupled receptor 123 (Gpr123), mRNA [NM_001107559]	**19,028**
Amph	Rattus norvegicus amphiphysin (Amph), mRNA [NM_022217]	**19,026**
Hif1a	Rattus norvegicus hypoxia-inducible factor 1, alpha subunit (basic helix-loop-helix transcription factor) (Hif1a), mRNA [NM_024359]	**19,021**
LOC689587	PREDICTED: Rattus norvegicus similar to chromosome 9 open reading frame 79 (LOC689587), mRNA [XM_001071271]	**19,013**
Gapdh	Rattus norvegicus glyceraldehyde-3-phosphate dehydrogenase (Gapdh), mRNA [NM_017008]	**19,010**
RGD1305938	Rattus norvegicus TL0AAA77YA11 mRNA sequence. [FQ211901]	**19,009**
Snx10	Rattus norvegicus sorting nexin 10 (Snx10), mRNA [NM_001013085]	**19,008**
0	Unknown	**19,005**
Mlx	Rattus norvegicus MAX-like protein X (Mlx), mRNA [NM_001034112]	**19,004**
Nxph3	Neurexophilin-3 [Source:UniProtKB/Swiss-Prot;Acc:Q9Z2N5] [ENSRNOT00000007133]	**19,004**
Rnf123	Rattus norvegicus ring finger protein 123 (Rnf123), mRNA [NM_001191580]	**18,998**
Lphn2	Rattus norvegicus latrophilin 2 (Lphn2), transcript variant 1, mRNA [NM_134408]	**18,998**
Sec24a	Rattus norvegicus SEC24 family, member A (S. cerevisiae) (Sec24a), mRNA [NM_001105780]	**18,996**
Smpd3	Rattus norvegicus sphingomyelin phosphodiesterase 3, neutral membrane (Smpd3), mRNA [NM_053605]	**18,996**
Ptprn2	Rattus norvegicus protein tyrosine phosphatase, receptor type, N polypeptide 2 (Ptprn2), mRNA [NM_031600]	**18,994**
0	Unknown	**18,989**
Inpp5j	Rattus norvegicus inositol polyphosphate-5-phosphatase J (Inpp5j), mRNA [NM_133562]	**18,984**
Cyp26b1	Rattus norvegicus cytochrome P450, family 26, subfamily b, polypeptide 1 (Cyp26b1), mRNA [NM_181087]	**18,979**
Ptk2b	Rattus norvegicus PTK2B protein tyrosine kinase 2 beta (Ptk2b), mRNA [NM_017318]	**18,977**
0	Unknown	**18,976**
Khdrbs2	Rattus norvegicus KH domain containing, RNA binding, signal transduction associated 2 (Khdrbs2), mRNA [NM_133318]	**18,974**
Tomm22	Rattus norvegicus translocase of outer mitochondrial membrane 22 homolog (yeast) (Tomm22), nuclear gene encoding mitochondrial protein, mRNA [NM_212514]	**18,971**
Kctd11	Rattus norvegicus potassium channel tetramerisation domain containing 11 (Kctd11), mRNA [NM_001108831]	**18,970**
Cyb561d1	Rattus norvegicus cytochrome b-561 domain containing 1 (Cyb561d1), mRNA [NM_001108562]	**18,970**
Gtf3c6	Rattus norvegicus general transcription factor IIIC, polypeptide 6, alpha (Gtf3c6), mRNA [NM_001108537]	**18,969**
Ddah1	Rattus norvegicus dimethylarginine dimethylaminohydrolase 1 (Ddah1), mRNA [NM_022297]	**18,968**

Ttc17	Rattus norvegicus tetratricopeptide repeat domain 17 (Ttc17), mRNA [NM_001107752]	**18,966**
Sptan1	Rattus norvegicus spectrin, alpha, non-erythrocytic 1 (Sptan1), mRNA [NM_171983]	**18,965**
Vps37d	Rattus norvegicus similar to vacuolar protein sorting 37D (LOC687208), mRNA [NM_001128193]	**18,963**
Gba	Rattus norvegicus glucosidase, beta, acid (Gba), mRNA [NM_001127639]	**18,959**
Zc3h7b	Rattus norvegicus zinc finger CCCH-type containing 7B (Zc3h7b), mRNA [NM_001130695]	**18,958**
LOC682968	PREDICTED: Rattus norvegicus similar to Retinal homeobox protein Rx (DRx1) (DRx) (LOC682968), mRNA [XM_001063896]	**18,957**
Snx17	Rattus norvegicus sorting nexin 17 (Snx17), mRNA [NM_001011981]	**18,956**
Gramd2	Uncharacterized protein [Source:UniProtKB/TrEMBL;Acc:D3ZIZ1] [ENSRNOT00000036798]	**18,949**
0	Uncharacterized protein [Source:UniProtKB/TrEMBL;Acc:D3ZFR5] [ENSRNOT00000016784]	**18,949**
Ankrd17	Rattus norvegicus ankyrin repeat domain 17 (Ankrd17), mRNA [NM_001105999]	**18,948**
0	Rattus norvegicus similar to putative pheromone receptor (LOC301894), mRNA [XM_229376]	**18,946**
Cspg4	Rattus norvegicus chondroitin sulfate proteoglycan 4 (Cspg4), mRNA [NM_031022]	**18,944**
Khsrp	Rattus norvegicus KH-type splicing regulatory protein (Khsrp), mRNA [NM_133602]	**18,944**
Ascl1	Rattus norvegicus achaete-scute complex homolog 1 (Drosophila) (Ascl1), mRNA [NM_022384]	**18,943**
0	Uncharacterized protein [Source:UniProtKB/TrEMBL;Acc:D3ZU72] [ENSRNOT00000059829]	**18,943**
Wwc2	Rattus norvegicus WW and C2 domain containing 2 (Wwc2), mRNA [NM_001109111]	**18,943**
39508	Rattus norvegicus membrane-associated ring finger (C3HC4) 8 (March8), mRNA [NM_001107882]	**18,940**
Hcn3	Rattus norvegicus hyperpolarization-activated cyclic nucleotide-gated potassium channel 3 (Hcn3), mRNA [NM_053685]	**18,939**
Ifngr2	Rattus norvegicus interferon gamma receptor 2 (Ifngr2), mRNA [NM_001108313]	**18,933**
0	Unknown	**18,930**
Ergic3	Rattus norvegicus ERGIC and golgi 3 (Ergic3), mRNA [NM_001106533]	**18,922**
Fgf13	Rattus norvegicus fibroblast growth factor 13 (Fgf13), mRNA [NM_053428]	**18,919**
Znf503	Rattus norvegicus zinc finger protein 503 (Znf503), mRNA [NM_001107250]	**18,918**
Nhlh1	Rattus norvegicus nescient helix loop helix 1 (Nhlh1), mRNA [NM_001105970]	**18,916**
LOC687001	Rattus norvegicus similar to histone deacetylase 9 isoform 5 (LOC687001), mRNA [NM_001200045]	**18,913**
Max	Rattus norvegicus MYC associated factor X (Max), mRNA [NM_022210]	**18,910**
Tmem184b	Rattus norvegicus transmembrane protein 184B (Tmem184b), mRNA [NM_001173370]	**18,909**
0	Unknown	**18,909**
Ube2ql1	Rattus norvegicus ubiquitin-conjugating enzyme E2Q family-like 1 (Ube2ql1), mRNA [NM_001145163]	**18,908**
Cenpn	Rattus norvegicus centromere protein N (Cenpn), mRNA [NM_001008366]	**18,902**
Ugt2b5	Rattus norvegicus UDP glucuronosyltransferase 2 family, polypeptide B5 (Ugt2b5), mRNA [NM_153314]	**18,900**
Rasl12	Rattus norvegicus RAS-like, family 12 (Rasl12), mRNA [NM_001108162]	**18,895**
0	Unknown	**18,895**
Ppat	Rattus norvegicus phosphoribosyl pyrophosphate amidotransferase (Ppat), mRNA [NM_057198]	**18,892**

Clstn3	Rattus norvegicus calsyntenin 3 (Clstn3), mRNA [NM_134376]	**18,890**
Stac	PREDICTED: Rattus norvegicus SH3 and cysteine rich domain (Stac), mRNA [XM_001076458]	**18,887**
Zmat3	Rattus norvegicus zinc finger, matrin type 3 (Zmat3), mRNA [NM_022548]	**18,887**
Cd44	Rattus norvegicus Cd44 molecule (Cd44), mRNA [NM_012924]	**18,887**
Jph4	Rattus norvegicus junctophilin 4 (Jph4), mRNA [NM_001003711]	**18,884**
Sox6	Rattus norvegicus SRY (sex determining region Y)-box 6 (Sox6), mRNA [NM_001024751]	**18,882**
Dcun1d4	Rattus norvegicus DCN1, defective in cullin neddylation 1, domain containing 4 (S. cerevisiae) (Dcun1d4), mRNA [NM_001108359]	**18,881**
Xrra1	Rattus norvegicus X-ray radiation resistance associated 1 (Xrra1), mRNA [NM_001113753]	**18,880**
Mt2A	Rattus norvegicus metallothionein 2A (Mt2A), mRNA [NM_001137564]	**18,879**
0	Unknown	**18,877**
LOC303448	Rattus norvegicus similar to glyceraldehyde-3-phosphate dehydrogenase (LOC303448), mRNA [NM_001037190]	**18,867**
Jazf1	PREDICTED: Rattus norvegicus JAZF zinc finger 1 (Jazf1), mRNA [XM_002726367]	**18,860**
0	Unknown	**18,857**
Mamstr	Uncharacterized protein [Source:UniProtKB/TrEMBL;Acc:D3ZHU3] [ENSRNOT00000029167]	**18,857**
0	Unknown	**18,857**
Bag3	Rattus norvegicus Bcl2-associated athanogene 3 (Bag3), mRNA [NM_001011936]	**18,848**
0	Unknown	**18,846**
Pppde2	Rattus norvegicus PPPDE peptidase domain containing 2 (Pppde2), mRNA [NM_001025703]	**18,846**
Nt5dc3	Rattus norvegicus 5'-nucleotidase domain containing 3 (Nt5dc3), mRNA [NM_001134887]	**18,844**
Kcnd1	Rattus norvegicus potassium voltage-gated channel, Shal-related subfamily, member 1 (Kcnd1), mRNA [NM_001105748]	**18,840**
RGD735065	Rattus norvegicus similar to GI:13385412-like protein splice form I (RGD735065), mRNA [NM_199379]	**18,837**
Msx1	Rattus norvegicus msh homeobox 1 (Msx1), mRNA [NM_031059]	**18,835**
Cdh11	Rattus norvegicus cadherin 11 (Cdh11), mRNA [NM_053392]	**18,833**
Clvs1	Rattus norvegicus clavesin 1 (Clvs1), mRNA [NM_001108969]	**18,832**
Kcne1l	Rattus norvegicus potassium voltage-gated channel, Isk-related family, member 1-like (Kcne1l), mRNA [NM_001101003]	**18,827**
Fam117b	Rattus norvegicus family with sequence similarity 117, member B (Fam117b), mRNA [NM_001108797]	**18,826**
Mfap2	Rattus norvegicus microfibrillar-associated protein 2 (Mfap2), mRNA [NM_001107989]	**18,825**
Dnah10	PREDICTED: Rattus norvegicus dynein, axonemal, heavy polypeptide 10 (Dnah10), mRNA [XM_001078937]	**18,824**
Fmo1	Rattus norvegicus flavin containing monooxygenase 1 (Fmo1), mRNA [NM_012792]	**18,822**
Kcna6	Rattus norvegicus potassium voltage gated channel, shaker related subfamily, member 6 (Kcna6), mRNA [NM_023954]	**18,818**
Tmem132b	Rattus norvegicus transmembrane protein 132B (Tmem132b), mRNA [NM_001134536]	**18,815**
Mpped1	Rattus norvegicus metallophosphoesterase domain containing 1 (Mpped1), mRNA [NM_001130569]	**18,814**
Defb27	Rattus norvegicus defensin beta 27 (Defb27), mRNA [NM_001037519]	**18,814**
0	Rattus norvegicus similar to citrate synthase; citrate synthase precursor (LOC299750), mRNA [XM_235086]	**18,813**

Phb	Rattus norvegicus prohibitin (Phb), mRNA [NM_031851]	**18,813**
RGD1563482	Rattus norvegicus similar to hypothetical protein FLJ38663 (RGD1563482), mRNA [NM_001109065]	**18,810**
Reln	Rattus norvegicus reelin (Reln), mRNA [NM_080394]	**18,808**
Zfp280d	Rattus norvegicus zinc finger protein 280D (Zfp280d), mRNA [NM_001108165]	**18,804**
0	kelch repeat and BTB domain-containing protein 4 [Source:RefSeq peptide;Acc:NP_001101216] [ENSRNOT00000012876]	**18,798**
Dpysl2	Rattus norvegicus dihydropyrimidinase-like 2 (Dpysl2), mRNA [NM_001105717]	**18,797**
Prrxl1	Rattus norvegicus paired related homeobox protein-like 1 (Prrxl1), mRNA [NM_145767]	**18,797**
Dcaf4	Rattus norvegicus DDB1 and CUL4 associated factor 4 (Dcaf4), mRNA [NM_001108716]	**18,791**
RGD1309492	Rattus norvegicus similar to mKIAA1737 protein (RGD1309492), mRNA [NM_001108044]	**18,787**
0	Calcipressin-2 [Source:UniProtKB/Swiss-Prot;Acc:Q8CH27] [ENSRNOT00000029593]	**18,779**
Pabpc4	Rattus norvegicus poly(A) binding protein, cytoplasmic 4 (Pabpc4), mRNA [NM_001100538]	**18,778**
LOC689786	PREDICTED: Rattus norvegicus similar to ribosomal protein L35a (LOC689786), mRNA [XM_001072004]	**18,774**
0	Unknown	**18,774**
Pus10	Rattus norvegicus pseudouridylate synthase 10 (Pus10), mRNA [NM_001025278]	**18,771**
Fam81a	Rattus norvegicus family with sequence similarity 81, member A (Fam81a), mRNA [NM_001108163]	**18,768**
Psmf1	Rattus norvegicus proteasome inhibitor subunit 1 (Psmf1), mRNA [NM_001101005]	**18,766**
Nlk	Rattus norvegicus nemo like kinase (Nlk), mRNA [NM_001191924]	**18,764**
Slc19a2	Rattus norvegicus solute carrier family 19 (thiamine transporter), member 2 (Slc19a2), mRNA [NM_001030024]	**18,762**
Fcgr2a	Rattus norvegicus Fc fragment of IgG, low affinity IIa, receptor (CD32) (Fcgr2a), mRNA [NM_053843]	**18,762**
Dclk1	Rattus norvegicus doublecortin-like kinase 1 (Dclk1), transcript variant 1, mRNA [NM_053343]	**18,760**
Thrap3	Rattus norvegicus thyroid hormone receptor associated protein 3 (Thrap3), mRNA [NM_001009693]	**18,749**
Trpv1	Rattus norvegicus transient receptor potential cation channel, subfamily V, member 1 (Trpv1), mRNA [NM_031982]	**18,748**
LOC686076	PREDICTED: Rattus norvegicus similar to suppressor of initiator codon mutations, related sequence 1 (LOC686076), mRNA [XM_001066439]	**18,746**
Ncam2	Rattus norvegicus fasciclin II GPI-linked protein isoform mRNA, complete cds. [AY495696]	**18,744**
Arx	Rattus norvegicus aristaless related homeobox (Arx), mRNA [NM_001100174]	**18,742**
RGD1561149	Rattus norvegicus similar to mKIAA1522 protein (RGD1561149), mRNA [NM_001134629]	**18,739**
0	Unknown	**18,735**
Bag2	Rattus norvegicus Bcl2-associated athanogene 2 (Bag2), mRNA [NM_001128195]	**18,733**
0	PREDICTED: Rattus norvegicus dachshund homolog 2 (Drosophila) (Dach2), mRNA [XM_001055336]	**18,724**
LOC682812	PREDICTED: Rattus norvegicus similar to WD repeat domain 40A, transcript variant 3 (LOC682812), mRNA [XM_001059949]	**18,723**
Tprkb	Rattus norvegicus Tp53rk binding protein (Tprkb), mRNA [NM_001013926]	**18,715**
Thop1	Rattus norvegicus thimet oligopeptidase 1 (Thop1), mRNA [NM_172075]	**18,711**
0	Unknown	**18,708**
Kctd16	Rattus norvegicus potassium channel tetramerisation domain containing 16 (Kctd16), mRNA [NM_001172155]	**18,706**

0	Unknown	**18,706**
0	Uncharacterized protein [Source:UniProtKB/TrEMBL;Acc:D3ZYC5] [ENSRNOT00000043960]	**18,702**
RGD1359127	Rattus norvegicus similar to RIKEN cDNA 2310011J03 (RGD1359127), mRNA [NM_001007657]	**18,702**
Olr184	Rattus norvegicus olfactory receptor 184 (Olr184), mRNA [NM_001000182]	**18,694**
0	BC046289 Sec22l3 protein {Mus musculus} (exp=-1; wgp=0; cg=0), complete [TC638555]	**18,693**
Adra1d	Rattus norvegicus adrenergic, alpha-1D-, receptor (Adra1d), mRNA [NM_024483]	**18,691**
Glrb	Rattus norvegicus glycine receptor, beta (Glrb), mRNA [NM_053296]	**18,686**
0	UI-R-DY1-com-a-12-0-UI.s1 UI-R-DY1 Rattus norvegicus cDNA clone UI-R-DY1-com-a-12-0-UI 3', mRNA sequence [BQ210702]	**18,685**
Lhx4	Rattus norvegicus LIM homeobox 4 (Lhx4), mRNA [NM_001108348]	**18,681**
Sfrs4	Rattus norvegicus splicing factor, arginine/serine-rich 4 (Sfrs4), mRNA [NM_001108685]	**18,678**
Ostm1	Rattus norvegicus osteopetrosis associated transmembrane protein 1 (Ostm1), mRNA [NM_001029925]	**18,676**
Nat13	Rattus norvegicus N-acetyltransferase 13 (Nat13), mRNA [NM_001105881]	**18,675**
Serinc2	Rattus norvegicus serine incorporator 2 (Serinc2), mRNA [NM_001031656]	**18,673**
Pcdhga7	Rattus norvegicus protocadherin gamma subfamily A, 7 (Pcdhga7), mRNA [NM_001014773]	**18,673**
Clec12b	Rattus norvegicus C-type lectin domain family 12, member B (Clec12b), mRNA [NM_001109353]	**18,669**
Cacna1i	Rattus norvegicus calcium channel, voltage-dependent, T type, alpha 1I subunit (Cacna1i), mRNA [NM_020084]	**18,668**
Fgf14	Rattus norvegicus Fgf14b mRNA for fibroblast growth factor 14b, complete cds. [AB008908]	**18,664**
0	Unknown	**18,663**
0	Unknown	**18,658**
Acp2	Rattus norvegicus acid phosphatase 2, lysosomal (Acp2), mRNA [NM_016988]	**18,655**
RGD1565170	Uncharacterized protein [Source:UniProtKB/TrEMBL;Acc:D3ZTX9] [ENSRNOT00000043836]	**18,654**
LOC296235	Rattus norvegicus similar to Cystatin S precursor (LM protein) (LOC296235), mRNA [NM_001037350]	**18,648**
0	Unknown	**18,648**
Fads2	Rattus norvegicus fatty acid desaturase 2 (Fads2), mRNA [NM_031344]	**18,636**
Nudt5	Rattus norvegicus nudix (nucleoside diphosphate linked moiety X)-type motif 5 (Nudt5), mRNA [NM_001007733]	**18,636**
Nr3c2	Rattus norvegicus nuclear receptor subfamily 3, group C, member 2 (Nr3c2), mRNA [NM_013131]	**18,635**
Fgf12	Rattus norvegicus fibroblast growth factor 12 (Fgf12), mRNA [NM_130814]	**18,633**
Ka11	Rattus norvegicus type I keratin KA11 (Ka11), mRNA [NM_001008750]	**18,627**
0	Unknown	**18,622**
Tnnt2	Rattus norvegicus troponin T type 2 (cardiac) (Tnnt2), mRNA [NM_012676]	**18,620**
Ppfibp1	Rattus norvegicus PTPRF interacting protein, binding protein 1 (liprin beta 1) (Ppfibp1), mRNA [NM_001107896]	**18,616**
Bcl2l1	Rattus norvegicus Bcl2-like 1 (Bcl2l1), nuclear gene encoding mitochondrial protein, transcript variant 3, mRNA [NM_001033670]	**18,615**
Ndrg3	Rattus norvegicus N-myc downstream regulated gene 3 (Ndrg3), mRNA [NM_001013923]	**18,611**
0	Unknown	**18,607**

Sufu	Rattus norvegicus suppressor of fused homolog (Drosophila) (Sufu), mRNA [NM_001024899]	**18,607**
Smek2	Rattus norvegicus SMEK homolog 2, suppressor of mek1 (Dictyostelium) (Smek2), mRNA [NM_001108367]	**18,604**
Dll4	Rattus norvegicus delta-like 4 (Drosophila) (Dll4), mRNA [NM_001107760]	**18,603**
Bard1	Rattus norvegicus BRCA1 associated RING domain 1 (Bard1), mRNA [NM_022622]	**18,596**
RGD1562211	Rattus norvegicus similar to ubiquitin specific protease 51 (RGD1562211), mRNA [NM_001108252]	**18,595**
Aldh3a2	Rattus norvegicus aldehyde dehydrogenase 3 family, member A2 (Aldh3a2), mRNA [NM_031731]	**18,594**
MGC114464	Rattus norvegicus similar to expressed sequence AI836003 (MGC114464), mRNA [NM_001024909]	**18,591**
Ramp1	Rattus norvegicus receptor (G protein-coupled) activity modifying protein 1 (Ramp1), mRNA [NM_031645]	**18,583**
Ankrd57	Rattus norvegicus ankyrin repeat domain 57 (Ankrd57), mRNA [NM_001109364]	**18,581**
Prosc	Rattus norvegicus proline synthetase co-transcribed homolog (bacterial) (Prosc), mRNA [NM_001107320]	**18,574**
Calu	Rattus norvegicus calumenin (Calu), transcript variant 2, mRNA [NM_001033898]	**18,573**
0	Unknown	**18,571**
0	Histone deacetylase 7 [Source:UniProtKB/Swiss-Prot;Acc:Q99P96] [ENSRNOT00000011159]	**18,568**
0	Unknown	**18,560**
0	Unknown	**18,557**
Mybl2	Rattus norvegicus myeloblastosis oncogene-like 2 (Mybl2), mRNA [NM_001106536]	**18,555**
Kpna6	Rattus norvegicus karyopherin alpha 6 (importin alpha 7) (Kpna6), mRNA [NM_001015029]	**18,554**
0	Uncharacterized protein [Source:UniProtKB/TrEMBL;Acc:D3ZKX0] [ENSRNOT00000022136]	**18,552**
Siat7F	Rattus norvegicus sialyltransferase 7F (Siat7F), mRNA [NM_001015036]	**18,547**
Wdr70	Rattus norvegicus WD repeat domain 70 (Wdr70), mRNA [NM_001013909]	**18,539**
Gpd2	Rattus norvegicus glycerol-3-phosphate dehydrogenase 2, mitochondrial (Gpd2), nuclear gene encoding mitochondrial protein, mRNA [NM_012736]	**18,536**
Sertad2	Rattus norvegicus SERTA domain containing 2 (Sertad2), mRNA [NM_001024903]	**18,534**
Utp15	Rattus norvegicus UTP15, U3 small nucleolar ribonucleoprotein, homolog (S. cerevisiae) (Utp15), mRNA [NM_001107647]	**18,533**
Hsf1	Rattus norvegicus heat shock transcription factor 1 (Hsf1), mRNA [NM_024393]	**18,526**
Htr5a	Rattus norvegicus 5-hydroxytryptamine (serotonin) receptor 5A (Htr5a), mRNA [NM_013148]	**18,526**
Zbbx	Rattus norvegicus zinc finger, B-box domain containing (Zbbx), mRNA [NM_001029922]	**18,523**
RGD1565725	PREDICTED: Rattus norvegicus similar to hypothetical protein FLJ23834 (RGD1565725), mRNA [XM_001069513]	**18,521**
Tmod1	Rattus norvegicus tropomodulin 1 (Tmod1), mRNA [NM_013044]	**18,520**
Eif4g2	Rattus norvegicus eukaryotic translation initiation factor 4, gamma 2 (Eif4g2), mRNA [NM_001017374]	**18,519**
Cyp4a1	Rattus norvegicus cytochrome P450, family 4, subfamily a, polypeptide 1 (Cyp4a1), mRNA [NM_175837]	**18,519**
Kif11	Rattus norvegicus kinesin family member 11 (Kif11), mRNA [NM_001169112]	**18,518**
Id4	Rattus norvegicus inhibitor of DNA binding 4 (Id4), mRNA [NM_175582]	**18,516**
Parp11	Uncharacterized protein [Source:UniProtKB/TrEMBL;Acc:D3ZPT5] [ENSRNOT00000027092]	**18,515**

0	Unknown	**18,511**
0	Unknown	**18,510**
Tctn1	Rattus norvegicus tectonic family member 1 (Tctn1), mRNA [NM_001177613]	**18,509**
0	Unknown	**18,509**
Mxi1	Rattus norvegicus MAX interactor 1 (Mxi1), mRNA [NM_013160]	**18,506**
Polr2e	Rattus norvegicus polymerase (RNA) II (DNA directed) polypeptide E (Polr2e), mRNA [NM_001109614]	**18,506**
Tbl1xr1	Rattus norvegicus transducin (beta)-like 1 X-linked receptor 1 (Tbl1xr1), mRNA [NM_001108941]	**18,502**
Mcm6	Rattus norvegicus minichromosome maintenance complex component 6 (Mcm6), mRNA [NM_017287]	**18,502**
0	Unknown	**18,492**
Prtfdc1	Rattus norvegicus phosphoribosyl transferase domain containing 1 (Prtfdc1), mRNA [NM_001106127]	**18,485**
0	Rattus norvegicus cdig1U mRNA for hypothetical protein, complete cds. [AB086233]	**18,485**
Atcay	Rattus norvegicus ataxia, cerebellar, Cayman type (Atcay), mRNA [NM_001040190]	**18,481**
Top3b	Rattus norvegicus topoisomerase (DNA) III beta (Top3b), mRNA [NM_001105861]	**18,480**
LOC100360244	Rattus norvegicus LRRGT00053-like (LOC100360244), mRNA [NM_001177863]	**18,476**
0	Unknown	**18,474**
RGD1309534	Rattus norvegicus similar to RIKEN cDNA 4931406C07 (RGD1309534), mRNA [NM_001014206]	**18,467**
Ppp2r5e	Rattus norvegicus protein phosphatase 2, regulatory subunit B', epsilon isoform (Ppp2r5e), mRNA [NM_001106740]	**18,458**
0	Unknown	**18,458**
0	Unknown	**18,457**
Carf	Rattus norvegicus calcium response factor (Carf), mRNA [NM_001106915]	**18,454**
Snap29	Rattus norvegicus synaptosomal-associated protein 29 (Snap29), mRNA [NM_053810]	**18,452**
0	Unknown	**18,452**
Lhfpl2	Rattus norvegicus lipoma HMGIC fusion partner-like 2 (Lhfpl2), mRNA [NM_001106402]	**18,452**
Zim1	Rattus norvegicus zinc finger, imprinted 1 (Zim1), mRNA [NM_001107473]	**18,452**
Fstl1	Rattus norvegicus follistatin-like 1 (Fstl1), mRNA [NM_024369]	**18,450**
Arid5a	Rattus norvegicus AT rich interactive domain 5A (Mrf1 like) (Arid5a), mRNA [NM_001034934]	**18,450**
Adra1a	Rattus norvegicus adrenergic, alpha-1A-, receptor (Adra1a), mRNA [NM_017191]	**18,450**
Pi16	Rattus norvegicus peptidase inhibitor 16 (Pi16), mRNA [NM_001170481]	**18,443**
Iqsec3	Rattus norvegicus IQ motif and Sec7 domain 3 (Iqsec3), mRNA [NM_207617]	**18,442**
RGD1310597	Rattus norvegicus similar to RIKEN cDNA 1200014M14 (RGD1310597), mRNA [NM_001025647]	**18,441**
Inha	Rattus norvegicus inhibin alpha (Inha), mRNA [NM_012590]	**18,440**
Syt2	Rattus norvegicus synaptotagmin II (Syt2), mRNA [NM_012665]	**18,439**
Sdcbp	Rattus norvegicus syndecan binding protein (Sdcbp), mRNA [NM_031986]	**18,439**
0	Unknown	**18,436**

Fbxl5	Rattus norvegicus F-box and leucine-rich repeat protein 5 (Fbxl5), mRNA [NM_001107222]	**18,433**
Adcy9	Rattus norvegicus adenylate cyclase 9 (Adcy9), mRNA [NM_001106980]	**18,433**
Aif1l	Rattus norvegicus allograft inflammatory factor 1-like (Aif1l), mRNA [NM_001108578]	**18,424**
0	TULP3_MOUSE (O88413) Tubby-related protein 3 (Tubby-like protein 3), partial (40%) [TC600284]	**18,422**
Nol4	Rattus norvegicus nucleolar protein 4 (Nol4), mRNA [NM_001107401]	**18,416**
Iqcf1	Rattus norvegicus IQ motif containing F1 (Iqcf1), mRNA [NM_001109361]	**18,415**
Mdm4	Rattus norvegicus Mdm4 p53 binding protein homolog (mouse) (Mdm4), mRNA [NM_001012026]	**18,409**
Lman2l	Rattus norvegicus lectin, mannose-binding 2-like (Lman2l), mRNA [NM_001106900]	**18,406**
Camk2n2	Rattus norvegicus calcium/calmodulin-dependent protein kinase II inhibitor 2 (Camk2n2), mRNA [NM_021678]	**18,404**
Pcgf3	Rattus norvegicus polycomb group ring finger 3 (Pcgf3), mRNA [NM_001107245]	**18,404**
Abi1	Rattus norvegicus abl-interactor 1 (Abi1), mRNA [NM_024397]	**18,403**
0	HUMGLYSYN glycogen synthase kinase 3 {Homo sapiens} (exp=-1; wgp=0; cg=0), partial (4%) [TC645757]	**18,401**
Klhl13	Klhl13 protein [Source:UniProtKB/TrEMBL;Acc:Q3MHT7] [ENSRNOT00000067114]	**18,396**
Trim31	Rattus norvegicus tripartite motif-containing 31 (Trim31), mRNA [NM_001106376]	**18,393**
0	Unknown	**18,385**
0	Unknown	**18,382**
0	Similar to RIKEN cDNA B230206N24 (Predicted), isoform CRA_bUncharacterized protein [Source:UniProtKB/TrEMBL;Acc:D3ZQA9] [ENSRNOT00000056493]	**18,382**
Lrrc59	Rattus norvegicus leucine rich repeat containing 59 (Lrrc59), mRNA [NM_001008280]	**18,377**
Cbln2	Rattus norvegicus cerebellin 2 precursor (Cbln2), mRNA [NM_001012740]	**18,367**
Ttbk2	Rattus norvegicus tau tubulin kinase 2 (Ttbk2), mRNA [NM_001107766]	**18,365**
Ovol2	Rattus norvegicus ovo-like 2 (Drosophila) (Ovol2), mRNA [NM_001106519]	**18,365**
Gys1	Rattus norvegicus glycogen synthase 1, muscle (Gys1), mRNA [NM_001109615]	**18,364**
Shh	Rattus norvegicus sonic hedgehog (Shh), mRNA [NM_017221]	**18,363**
Atp11a	Rattus norvegicus ATPase, class VI, type 11A (Atp11a), mRNA [NM_001107324]	**18,363**
LOC690617	Rattus norvegicus hypothetical protein LOC690617 (LOC690617), mRNA [NM_001109605]	**18,352**
Dlgap2	Rattus norvegicus discs, large (Drosophila) homolog-associated protein 2 (Dlgap2), mRNA [NM_053901]	**18,351**
0	Unknown	**18,348**
Ppp2r5c	Rattus norvegicus protein phosphatase 2, regulatory subunit B', gamma isoform (Ppp2r5c), mRNA [NM_001191112]	**18,341**
Sbk1	Rattus norvegicus SH3-binding domain kinase 1 (Sbk1), mRNA [NM_147135]	**18,331**
0	Unknown	**18,324**
0	Unknown	**18,323**
LOC100366216	PREDICTED: Rattus norvegicus nuclear antigen Sp100-like (LOC100366216), mRNA [XM_002727237]	**18,320**
Gstz1	Rattus norvegicus glutathione transferase zeta 1 (Gstz1), mRNA [NM_001109445]	**18,319**

0	Unknown	**18,315**
Ermap	PREDICTED: Rattus norvegicus erythroblast membrane-associated protein (Ermap), mRNA [XM_001073566]	**18,313**
0	SOCS7_HUMAN (O14512) Suppressor of cytokine signaling 7 (SOCS-7) (Nck, Ash and phospholipase C gamma-binding protein) (Nck-associated protein 4) (NAP-4) (Fragment), partial (5%) [TC628777]	**18,312**
Lpo	Rattus norvegicus lactoperoxidase (Lpo), mRNA [NM_001105829]	**18,312**
Sdhaf2	Rattus norvegicus succinate dehydrogenase complex assembly factor 2 (Sdhaf2), nuclear gene encoding mitochondrial protein, mRNA [NM_001008371]	**18,311**
Rbm25	Rattus norvegicus RNA binding motif protein 25 (Rbm25), mRNA [NM_001108984]	**18,309**
LOC688319	PREDICTED: Rattus norvegicus similar to RAS-related C3 botulinum substrate 3 (LOC688319), mRNA [XM_001081814]	**18,306**
Ulk3	PREDICTED: Rattus norvegicus unc-51-like kinase 3 (C. elegans) (Ulk3), mRNA [XM_001077085]	**18,302**
Pfas	Rattus norvegicus phosphoribosylformylglycinamidine synthase (Pfas), mRNA [NM_001105791]	**18,302**
RGD1309313	Rattus norvegicus similar to RIKEN cDNA 4930538D17 (RGD1309313), mRNA [NM_001014030]	**18,302**
Olr23	Rattus norvegicus olfactory receptor 23 (Olr23), mRNA [NM_001000119]	**18,299**
0	Uncharacterized protein [Source:UniProtKB/TrEMBL;Acc:D3Z839] [ENSRNOT00000043647]	**18,298**
0	Unknown	**18,298**
Gpsm1	Rattus norvegicus G-protein signaling modulator 1 (AGS3-like, C. elegans) (Gpsm1), transcript variant 1, mRNA [NM_144745]	**18,296**
Mgat5	Rattus norvegicus mannosyl (alpha-1,6-)-glycoprotein beta-1,6-N-acetyl-glucosaminyltransferase (Mgat5), mRNA [NM_023095]	**18,295**
RGD1564927	Rattus norvegicus similar to TGFB-induced factor 2 (RGD1564927), mRNA [NM_001134983]	**18,292**
Olr168	Rattus norvegicus olfactory receptor 168 (Olr168), mRNA [NM_001000174]	**18,279**
Kirrel	Rattus norvegicus kin of IRRE like (Drosophila) (Kirrel), mRNA [NM_207606]	**18,278**
Hic2	Rattus norvegicus hypermethylated in cancer 2 (Hic2), mRNA [NM_001105862]	**18,275**
Plcd4	Rattus norvegicus phospholipase C, delta 4 (Plcd4), mRNA [NM_080688]	**18,272**
Nrxn3	Rattus norvegicus neurexin 3 (Nrxn3), mRNA [NM_053817]	**18,270**
LOC689065	Rattus norvegicus hypothetical protein LOC689065 (LOC689065), mRNA [NM_001109521]	**18,269**
Rufy3	Rattus norvegicus RUN and FYVE domain containing 3 (Rufy3), mRNA [NM_001025127]	**18,263**
0	Suppressor of Ty 5 homolog (S. cerevisiae), isoform CRA_aUncharacterized protein [Source:UniProtKB/TrEMBL;Acc:D3ZSS2] [ENSRNOT00000048905]	**18,259**
Zfp143	Rattus norvegicus zinc finger protein 143 (Zfp143), mRNA [NM_001012169]	**18,258**
Cherp	Rattus norvegicus calcium homeostasis endoplasmic reticulum protein (Cherp), mRNA [NM_001106064]	**18,254**
0	Uncharacterized protein [Source:UniProtKB/TrEMBL;Acc:D3ZU97] [ENSRNOT00000059797]	**18,240**
Atf6	Rattus norvegicus activating transcription factor 6 (Atf6), mRNA [NM_001107196]	**18,237**
Sort1	Rattus norvegicus sortilin 1 (Sort1), mRNA [NM_031767]	**18,236**
0	HSN2_RAT (Q6IFS7) Protein HSN2 precursor, complete [TC618038]	**18,234**
0	Uncharacterized protein [Source:UniProtKB/TrEMBL;Acc:D3Z9J4] [ENSRNOT00000013418]	**18,233**

Plekhn1	Rattus norvegicus pleckstrin homology domain containing, family N member 1 (Plekhn1), mRNA [NM_001134523]	**18,225**
Cadm3	Rattus norvegicus cell adhesion molecule 3 (Cadm3), mRNA [NM_001047103]	**18,220**
Dda1	Rattus norvegicus DET1 and DDB1 associated 1 (Dda1), mRNA [NM_001134790]	**18,220**
Zkscan2	Uncharacterized protein [Source:UniProtKB/TrEMBL;Acc:D3ZXU0] [ENSRNOT00000020281]	**18,218**
Rmnd1	Rattus norvegicus required for meiotic nuclear division 1 homolog (S. cerevisiae) (Rmnd1), mRNA [NM_001040128]	**18,210**
Crispld2	Rattus norvegicus cysteine-rich secretory protein LCCL domain containing 2 (Crispld2), mRNA [NM_138518]	**18,205**
0	1PNS_B Chain B, Crystal Structure Of A Streptomycin Dependent Ribosome From E. Coli, 30s Subunit Of 70s Ribosome. This File, 1pns, Contains The 30s Subunit, Two Trnas, And One Mrna Molecule. The 50s Ribosomal Subunit Is In File 1pnu. {Escherichia coli} (e	**18,200**
0	Uncharacterized protein [Source:UniProtKB/TrEMBL;Acc:D4A3Y5] [ENSRNOT00000057785]	**18,198**
0	Uncharacterized protein [Source:UniProtKB/TrEMBL;Acc:D3ZIV5] [ENSRNOT00000049602]	**18,195**
0	Unknown	**18,194**
LOC363326	Rattus norvegicus hypothetical LOC363326 (LOC363326), mRNA [NM_001126289]	**18,194**
Parp14	Rattus norvegicus poly (ADP-ribose) polymerase family, member 14 (Parp14), mRNA [NM_001191659]	**18,192**
Slc4a4	Rattus norvegicus solute carrier family 4, sodium bicarbonate cotransporter, member 4 (Slc4a4), mRNA [NM_053424]	**18,187**
Tmem97	Rattus norvegicus transmembrane protein 97 (Tmem97), mRNA [NM_001008334]	**18,184**
Meaf6	Rattus norvegicus MYST/Esa1-associated factor 6 (Meaf6), mRNA [NM_001113784]	**18,182**
LOC685608	PREDICTED: Rattus norvegicus hypothetical protein LOC685608 (LOC685608), mRNA [XM_001065355]	**18,182**
Fam162a	Rattus norvegicus family with sequence similarity 162, member A (Fam162a), mRNA [NM_001029903]	**18,182**
Fat4	Rattus norvegicus FAT tumor suppressor homolog 4 (Drosophila) (Fat4), mRNA [NM_001191705]	**18,173**
Ephb2	Rattus norvegicus Eph receptor B2 (Ephb2), mRNA [NM_001127319]	**18,173**
Mobkl1b	Rattus norvegicus MOB1, Mps One Binder kinase activator-like 1B (yeast) (Mobkl1b), mRNA [NM_001033891]	**18,165**
Fam122a	Rattus norvegicus family with sequence similarity 122A (Fam122a), mRNA [NM_001014029]	**18,164**
Clip2	Rattus norvegicus CAP-GLY domain containing linker protein 2 (Clip2), mRNA [NM_021997]	**18,163**
Chn2	Rattus norvegicus chimerin (chimaerin) 2 (Chn2), mRNA [NM_032084]	**18,160**
0	Transient receptor potential cation channel subfamily M member 7 [Source:UniProtKB/Swiss-Prot;Acc:Q925B3] [ENSRNOT00000048818]	**18,160**
Eif2s3x	Rattus norvegicus eukaryotic translation initiation factor 2, subunit 3, structural gene X-linked (Eif2s3x), mRNA [NM_001100542]	**18,159**
Ptges3	Rattus norvegicus prostaglandin E synthase 3 (cytosolic) (Ptges3), mRNA [NM_001130989]	**18,152**
0	Uncharacterized protein [Source:UniProtKB/TrEMBL;Acc:D4A7M0] [ENSRNOT00000011178]	**18,146**
Rgr	Rattus norvegicus retinal G protein coupled receptor (Rgr), mRNA [NM_001107299]	**18,144**
Apaf1	Rattus norvegicus apoptotic peptidase activating factor 1 (Apaf1), mRNA [NM_023979]	**18,142**
0	Unknown	**18,141**
Glg1	Rattus norvegicus golgi apparatus protein 1 (Glg1), mRNA [NM_017211]	**18,139**
RGD1308134	Rattus norvegicus similar to RIKEN cDNA 1110020A23 (RGD1308134), mRNA [NM_001127521]	**18,137**
Ndor1	Rattus norvegicus NADPH dependent diflavin oxidoreductase 1 (Ndor1), mRNA [NM_001107818]	**18,127**

Lrrc4b	Leucine-rich repeat-containing protein 4B [Source:UniProtKB/Swiss-Prot;Acc:P0CC10] [ENSRNOT00000026259]	**18,126**
0	Unknown	**18,123**
0	Unknown	**18,123**
RGD1308026	Rattus norvegicus similar to 2310047B19Rik protein (RGD1308026), mRNA [NM_001108755]	**18,122**
Rhbdl1	Rattus norvegicus rhomboid, veinlet-like 1 (Drosophila) (Rhbdl1), mRNA [NM_001191822]	**18,122**
Slc6a2	Rattus norvegicus solute carrier family 6 (neurotransmitter transporter, noradrenalin), member 2 (Slc6a2), mRNA [NM_031343]	**18,121**
Polr2f	DNA-directed RNA polymerases I, II, and III subunit RPABC2 [Source:UniProtKB/Swiss-Prot;Acc:O88828] [ENSRNOT00000015009]	**18,119**
Gse1	PREDICTED: Rattus norvegicus genetic suppressor element 1 (Gse1), mRNA [XM_001078877]	**18,119**
Znf474	Rattus norvegicus zinc finger protein 474 (Znf474), mRNA [NM_001107380]	**18,113**
Pcdhga5	Rattus norvegicus protocadherin gamma subfamily A, 5 (Pcdhga5), mRNA [NM_001037137]	**18,110**
Pygb	Rattus norvegicus phosphorylase, glycogen; brain (Pygb), mRNA [NM_013188]	**18,108**
Tacc1	Rattus norvegicus transforming, acidic coiled-coil containing protein 1 (Tacc1), mRNA [NM_001004107]	**18,108**
Hmg20a	Rattus norvegicus high mobility group 20A (Hmg20a), mRNA [NM_001108150]	**18,108**
0	Unknown	**18,104**
Noxa1	Rattus norvegicus NADPH oxidase activator 1 (Noxa1), mRNA [NM_001100171]	**18,103**
Rab5c	Rattus norvegicus RAB5C, member RAS oncogene family (Rab5c), mRNA [NM_001105840]	**18,102**
Ints9	PREDICTED: Rattus norvegicus integrator complex subunit 9 (Ints9), mRNA [XM_001066109]	**18,101**
Commd10	Rattus norvegicus COMM domain containing 10 (Commd10), mRNA [NM_001004276]	**18,101**
Atp11b	Atp11b protein [Source:UniProtKB/TrEMBL;Acc:Q5RJS7] [ENSRNOT00000016961]	**18,099**
Pdha1	Rattus norvegicus pyruvate dehydrogenase (lipoamide) alpha 1 (Pdha1), nuclear gene encoding mitochondrial protein, mRNA [NM_001004072]	**18,099**
Polr2e	Rattus norvegicus polymerase (RNA) II (DNA directed) polypeptide E (Polr2e), mRNA [NM_001109614]	**18,094**
Kcnk1	Rattus norvegicus potassium channel, subfamily K, member 1 (Kcnk1), mRNA [NM_021688]	**18,093**
Ngb	Rattus norvegicus neuroglobin (Ngb), mRNA [NM_033359]	**18,093**
Efr3a	Rattus norvegicus EFR3 homolog A (S. cerevisiae) (Efr3a), mRNA [NM_001130564]	**18,087**
Apod	Rattus norvegicus apolipoprotein D (Apod), mRNA [NM_012777]	**18,085**
0	Unknown	**18,084**
Dhrs3	Rattus norvegicus dehydrogenase/reductase (SDR family) member 3 (Dhrs3), mRNA [NM_001037199]	**18,084**
Plekhg5	Rattus norvegicus pleckstrin homology domain containing, family G (with RhoGef domain) member 5 (Plekhg5), mRNA [NM_201272]	**18,081**
Syt3	Rattus norvegicus synaptotagmin III (Syt3), mRNA [NM_019122]	**18,080**
Entpd6	Rattus norvegicus ectonucleoside triphosphate diphosphohydrolase 6 (Entpd6), mRNA [NM_053498]	**18,079**
Crbn	Rattus norvegicus cereblon (Crbn), mRNA [NM_001015003]	**18,077**
Znf286a	Rattus norvegicus zinc finger protein 286A (Znf286a), mRNA [NM_001191921]	**18,077**
Ppargc1b	Rattus norvegicus peroxisome proliferator-activated receptor gamma, coactivator 1 beta (Ppargc1b), mRNA [NM_176075]	**18,074**
0	Unknown	**18,074**

B3galt5	Rattus norvegicus UDP-Gal:betaGlcNAc beta 1,3-galactosyltransferase, polypeptide 5 (B3galt5), mRNA [NM_001105887]	**18,074**
0	Unknown	**18,072**
Cln3	Rattus norvegicus ceroid-lipofuscinosis, neuronal 3 (Cln3), mRNA [NM_001006971]	**18,067**
Emilin1	Rattus norvegicus elastin microfibril interfacer 1 (Emilin1), mRNA [NM_001106710]	**18,067**
Map2k4	Rattus norvegicus mitogen activated protein kinase kinase 4 (Map2k4), mRNA [NM_001030023]	**18,066**
Ptpn3	Ptpn3 protein [Source:UniProtKB/TrEMBL;Acc:Q562B7] [ENSRNOT00000059627]	**18,066**
Chaf1b	Rattus norvegicus chromatin assembly factor 1, subunit B (p60) (Chaf1b), mRNA [NM_001024741]	**18,064**
LOC691777	PREDICTED: Rattus norvegicus hypothetical protein LOC691777 (LOC691777), mRNA [XM_002724616]	**18,063**
Pigq	Rattus norvegicus phosphatidylinositol glycan anchor biosynthesis, class Q (Pigq), mRNA [NM_001007607]	**18,061**
Urb2	Rattus norvegicus URB2 ribosome biogenesis 2 homolog (S. cerevisiae) (Urb2), mRNA [NM_001135708]	**18,057**
0	Unknown	**18,055**
Pqlc3	Rattus norvegicus PQ loop repeat containing 3 (Pqlc3), mRNA [NM_001034952]	**18,053**
Sema3c	Rattus norvegicus sema domain, immunoglobulin domain (Ig), short basic domain, secreted, (semaphorin) 3C (Sema3c), mRNA [NM_001106578]	**18,052**
Foxg1	Rattus norvegicus forkhead box G1 (Foxg1), mRNA [NM_012560]	**18,051**
Sox12	Rattus norvegicus SRY (sex determining region Y)-box 12 (Sox12), mRNA [NM_001168650]	**18,045**
Sigmar1	Rattus norvegicus sigma non-opioid intracellular receptor 1 (Sigmar1), mRNA [NM_030996]	**18,038**
Chmp1b	Rattus norvegicus chromatin modifying protein 1B (Chmp1b), mRNA [NM_001109533]	**18,029**
Mef2a	Rattus norvegicus myocyte enhancer factor 2a (Mef2a), mRNA [NM_001014035]	**18,029**
0	AF440762 septin SEPT8_v2 {Homo sapiens} (exp=-1; wgp=0; cg=0), partial (97%) [TC612969]	**18,027**
B4galt6	Rattus norvegicus UDP-Gal:betaGlcNAc beta 1,4-galactosyltransferase, polypeptide 6 (B4galt6), mRNA [NM_031740]	**18,022**
Garnl1	Rattus norvegicus GTPase activating Rap/RanGAP domain-like 1 (Garnl1), mRNA [NM_020083]	**18,017**
Oxa1l	Rattus norvegicus oxidase assembly 1-like (Oxa1l), mRNA [NM_001168583]	**18,014**
Stap2	Rattus norvegicus signal transducing adaptor family member 2 (Stap2), mRNA [NM_001025026]	**18,010**
Tmem119	Rattus norvegicus transmembrane protein 119 (Tmem119), mRNA [NM_001107155]	**18,005**
0	Unknown	**18,005**
LOC100363290	PREDICTED: Rattus norvegicus hypothetical protein LOC100363290 (LOC100363290), mRNA [XM_002728135]	**18,004**
Ano1	Rattus norvegicus anoctamin 1, calcium activated chloride channel (Ano1), mRNA [NM_001107564]	**18,001**
Ntrk2	Rattus norvegicus neurotrophic tyrosine kinase, receptor, type 2 (Ntrk2), transcript variant 3, mRNA [NM_001163169]	**17,998**
0	Rattus norvegicus similar to RIKEN cDNA 1810009A15 (LOC293720), mRNA [XM_215148]	**17,993**
Snx9	Rattus norvegicus sorting nexin 9 (Snx9), mRNA [NM_001127637]	**17,990**
Serpina4	Rattus norvegicus serine (or cysteine) proteinase inhibitor, clade A (alpha-1 antiproteinase, antitrypsin), member 4 (Serpina4), mRNA [NM_145097]	**17,990**
Kcnh1	Rattus norvegicus potassium voltage-gated channel, subfamily H (eag-related), member 1 (Kcnh1), mRNA [NM_031742]	**17,989**
Tmem88b	Rattus norvegicus transmembrane protein 88B (Tmem88b), mRNA [NM_001109426]	**17,989**
RGD1308106	Rattus norvegicus LOC361719 (RGD1308106), mRNA [NM_001134575]	**17,989**

Abhd6	Rattus norvegicus abhydrolase domain containing 6 (Abhd6), mRNA [NM_001007680]	**17,986**
Plekhf2	Rattus norvegicus pleckstrin homology domain containing, family F (with FYVE domain) member 2 (Plekhf2), mRNA [NM_001108655]	**17,972**
Vcam1	Rattus norvegicus vascular cell adhesion molecule 1 (Vcam1), mRNA [NM_012889]	**17,966**
Pbx4	Rattus norvegicus pre-B-cell leukemia homeobox 4 (Pbx4), mRNA [NM_001108399]	**17,965**
RGD1563216	Rattus norvegicus similar to HESB like domain containing 1 (RGD1563216), mRNA [NM_001109278]	**17,964**
Aldoc	Rattus norvegicus aldolase C, fructose-bisphosphate (Aldoc), mRNA [NM_012497]	**17,960**
0	Uncharacterized protein [Source:UniProtKB/TrEMBL;Acc:D3ZN05] [ENSRNOT00000052028]	**17,954**
Gm52	Rattus norvegicus envelope glycoprotein syncytin-A (Gm52), mRNA [NM_001014771]	**17,952**
Cops7b	Rattus norvegicus COP9 constitutive photomorphogenic homolog subunit 7B (Arabidopsis) (Cops7b), mRNA [NM_001108807]	**17,945**
RGD1563108	PREDICTED: Rattus norvegicus similar to chromosome 21 open reading frame 29 (RGD1563108), mRNA [XM_001079415]	**17,938**
0	Uncharacterized protein [Source:UniProtKB/TrEMBL;Acc:D3ZMP1] [ENSRNOT00000044539]	**17,931**
0	Unknown	**17,931**
Ankrd13c	Rattus norvegicus ankyrin repeat domain 13C (Ankrd13c), mRNA [NM_001191570]	**17,928**
Vps37c	Rattus norvegicus vacuolar protein sorting 37 homolog C (S. cerevisiae) (Vps37c), mRNA [NM_001107463]	**17,926**
0	Rattus norvegicus partial mRNA for immunoglobulin alpha heavy chain (partial), complete constant region. [AJ510151]	**17,926**
0	Unknown	**17,921**
0	RCG43605, isoform CRA_aUncharacterized protein [Source:UniProtKB/TrEMBL;Acc:D3ZHC7] [ENSRNOT00000061670]	**17,919**
Zfp455	Rattus norvegicus zinc finger protein 455 (Zfp455), mRNA [NM_173314]	**17,918**
Shc1	Rattus norvegicus SHC (Src homology 2 domain containing) transforming protein 1 (Shc1), nuclear gene encoding mitochondrial protein, transcript variant 1, mRNA [NM_001164060]	**17,917**
Mfge8	Rattus norvegicus milk fat globule-EGF factor 8 protein (Mfge8), transcript variant 2, mRNA [NM_012811]	**17,909**
Vegfa	Rattus norvegicus vascular endothelial growth factor A (Vegfa), transcript variant 1, mRNA [NM_031836]	**17,909**
Pum2	Rattus norvegicus pumilio homolog 2 (Drosophila) (Pum2), mRNA [NM_001106715]	**17,906**
Erbb4	Rattus norvegicus v-erb-a erythroblastic leukemia viral oncogene homolog 4 (avian) (Erbb4), mRNA [NM_021687]	**17,906**
Nek4	Rattus norvegicus NIMA (never in mitosis gene a)-related kinase 4 (Nek4), mRNA [NM_001013134]	**17,899**
Ldb2	Rattus norvegicus LIM domain binding 2 (Ldb2), mRNA [NM_001106009]	**17,898**
Gfra1	Rattus norvegicus GDNF family receptor alpha 1 (Gfra1), mRNA [NM_012959]	**17,894**
Zfp212	Zfp212 protein [Source:UniProtKB/TrEMBL;Acc:Q52KK5] [ENSRNOT00000009054]	**17,885**
Slc25a44	Rattus norvegicus solute carrier family 25, member 44 (Slc25a44), mRNA [NM_001108947]	**17,884**
0	Unknown	**17,884**
Klf3	Rattus norvegicus Kruppel-like factor 3 (basic) (Klf3), mRNA [NM_001105742]	**17,879**
Prpg1	Rattus norvegicus proline-rich proteoglycan 1 (Prpg1), mRNA [NM_172064]	**17,870**
Xiap	Rattus norvegicus X-linked inhibitor of apoptosis (Xiap), mRNA [NM_022231]	**17,866**
Supt16h	Rattus norvegicus suppressor of Ty 16 homolog (S. cerevisiae) (Supt16h), mRNA [NM_001107261]	**17,860**

Rspo2	Rattus norvegicus R-spondin 2 homolog (Xenopus laevis) (Rspo2), mRNA [NM_001130575]	**17,859**
0	RVL11956 Wackym-Soares normalized rat vestibular cDNA library Rattus norvegicus cDNA 5', mRNA sequence [DV720880]	**17,852**
Cpeb4	Rattus norvegicus cytoplasmic polyadenylation element binding protein 4 (Cpeb4), mRNA [NM_001106992]	**17,851**
LOC685953	Platelet glycoprotein 4 [Source:UniProtKB/Swiss-Prot;Acc:Q07969] [ENSRNOT00000066224]	**17,845**
0	HSU97670 eukaryotic translation initiation factor eIF3, p35 subunit {Homo sapiens} (exp=-1; wgp=0; cg=0), partial (26%) [TC586860]	**17,845**
Pom121	Rattus norvegicus nuclear pore membrane protein 121 (Pom121), mRNA [NM_053622]	**17,839**
Sgpl1	Rattus norvegicus sphingosine-1-phosphate lyase 1 (Sgpl1), mRNA [NM_173116]	**17,833**
Tmem143	Rattus norvegicus transmembrane protein 143 (Tmem143), mRNA [NM_001107513]	**17,827**
Ap1s2	Rattus norvegicus adaptor-related protein complex 1, sigma 2 subunit (Ap1s2), mRNA [NM_001127531]	**17,827**
Jak1	Rattus norvegicus Janus kinase 1 (Jak1), mRNA [NM_053466]	**17,827**
0	Unknown	**17,825**
Ahdc1	Rattus norvegicus AT hook, DNA binding motif, containing 1 (Ahdc1), mRNA [NM_001134956]	**17,821**
RGD1307410	RGD1307410 protein [Source:UniProtKB/TrEMBL;Acc:Q5EB78] [ENSRNOT00000054978]	**17,817**
Acvr1	Rattus norvegicus activin A receptor, type I (Acvr1), mRNA [NM_024486]	**17,812**
Znfx1	Rattus norvegicus zinc finger, NFX1-type containing 1 (Znfx1), mRNA [NM_001047860]	**17,805**
Lmna	Rattus norvegicus lamin A (Lmna), transcript variant 2, mRNA [NM_001002016]	**17,805**
Gzf1	Rattus norvegicus GDNF-inducible zinc finger protein 1 (Gzf1), mRNA [NM_001107788]	**17,803**
Ppig	Rattus norvegicus peptidylprolyl isomerase G (Ppig), mRNA [NM_031793]	**17,801**
LOC681186	PREDICTED: Rattus norvegicus hypothetical protein LOC681186 (LOC681186), mRNA [XM_001060674]	**17,800**
RGD1309104	Rattus norvegicus similar to RIKEN cDNA 1700025G04 gene (RGD1309104), mRNA [NM_001105959]	**17,799**
0	Myristoylated alanine-rich C-kinase substrate [Source:UniProtKB/Swiss-Prot;Acc:P30009] [ENSRNOT00000000707]	**17,791**
0	Rattus norvegicus similar to Pyruvate kinase, M2 isozyme (LOC301604), mRNA [XM_237391]	**17,789**
0	Unknown	**17,788**
Hist2h2be	Histone H2B [Source:UniProtKB/TrEMBL;Acc:D3ZZP9] [ENSRNOT00000028770]	**17,778**
Cby1	Rattus norvegicus chibby homolog 1 (Drosophila) (Cby1), mRNA [NM_145676]	**17,773**
Syt11	Rattus norvegicus synaptotagmin XI (Syt11), mRNA [NM_031667]	**17,770**
Tmem25	Rattus norvegicus transmembrane protein 25 (Tmem25), mRNA [NM_001109528]	**17,767**
Ambra1	Rattus norvegicus autophagy/beclin 1 regulator 1 (Ambra1), mRNA [NM_001134341]	**17,765**
Rnf182	Rattus norvegicus ring finger protein 182 (Rnf182), mRNA [NM_001109117]	**17,759**
0	PREDICTED: Rattus norvegicus similar to deleted in malignant brain tumors 1 isoform a precursor (LOC678825), mRNA [XM_001053354]	**17,758**
Leng4	Rattus norvegicus leukocyte receptor cluster (LRC) member 4 (Leng4), mRNA [NM_001134978]	**17,756**
0	Unknown	**17,755**
Jsrp1	Rattus norvegicus junctional sarcoplasmic reticulum protein 1 (Jsrp1), mRNA [NM_001109591]	**17,755**
Suhw3	Uncharacterized protein [Source:UniProtKB/TrEMBL;Acc:D3ZCC6] [ENSRNOT00000044435]	**17,754**

Rnd2	Rattus norvegicus Rho family GTPase 2 (Rnd2), mRNA [NM_001010953]	**17,752**
Klhl23	Rattus norvegicus kelch-like 23 (Drosophila) (Klhl23), mRNA [NM_001134504]	**17,751**
Adora1	Adenosine receptor A1 [Source:UniProtKB/Swiss-Prot;Acc:P25099] [ENSRNOT00000004602]	**17,750**
0	Rattus norvegicus TL0ADA38YF09 mRNA sequence. [FQ221288]	**17,750**
Igsf9b	Uncharacterized protein [Source:UniProtKB/TrEMBL;Acc:D3ZB51] [ENSRNOT00000012391]	**17,750**
0	Uncharacterized protein [Source:UniProtKB/TrEMBL;Acc:D4A1Q6] [ENSRNOT00000056074]	**17,750**
0	Unknown	**17,748**
0	Unknown	**17,736**
N4bp2l1	Rattus norvegicus NEDD4 binding protein 2-like 1 (N4bp2l1), mRNA [NM_001035222]	**17,729**
0	Unknown	**17,725**
Ube2a	Rattus norvegicus ubiquitin-conjugating enzyme E2A (RAD6 homolog) (Ube2a), mRNA [NM_001013933]	**17,723**
Vps25	Rattus norvegicus vacuolar protein sorting 25 homolog (S. cerevisiae) (Vps25), mRNA [NM_001173451]	**17,719**
0	Unknown	**17,718**
0	PREDICTED: Rattus norvegicus similar to TDPOZ2 (RGD1563451), mRNA [XM_001053863]	**17,715**
LOC680787	PREDICTED: Rattus norvegicus rCG56785-like (LOC680787), mRNA [XM_002730096]	**17,714**
Ddx41	Rattus norvegicus DEAD (Asp-Glu-Ala-Asp) box polypeptide 41 (Ddx41), mRNA [NM_001108046]	**17,714**
Usp22	Ubiquitin carboxyl-terminal hydrolase [Source:UniProtKB/TrEMBL;Acc:D3ZTX7] [ENSRNOT00000050061]	**17,708**
Senp5	PREDICTED: Rattus norvegicus Sumo1/sentrin/SMT3 specific peptidase 5 (Senp5), mRNA [XM_221369]	**17,707**
RGD1561410	PREDICTED: Rattus norvegicus similar to sentrin 15 (RGD1559489), mRNA [XM_235207]	**17,703**
Farp1	Rattus norvegicus FERM, RhoGEF (Arhgef) and pleckstrin domain protein 1 (chondrocyte-derived) (Farp1), mRNA [NM_001107287]	**17,697**
Gpc1	Rattus norvegicus glypican 1 (Gpc1), mRNA [NM_030828]	**17,696**
Adhfe1	Rattus norvegicus alcohol dehydrogenase, iron containing, 1 (Adhfe1), nuclear gene encoding mitochondrial protein, mRNA [NM_001025423]	**17,691**
Dcxr	Rattus norvegicus dicarbonyl L-xylulose reductase (Dcxr), mRNA [NM_134387]	**17,690**
Slc2a13	Rattus norvegicus solute carrier family 2 (facilitated glucose transporter), member 13 (Slc2a13), mRNA [NM_133611]	**17,690**
Gad1	Rattus norvegicus glutamate decarboxylase 1 (Gad1), mRNA [NM_017007]	**17,685**
0	Uncharacterized protein [Source:UniProtKB/TrEMBL;Acc:D3ZEB9] [ENSRNOT00000066253]	**17,682**
Add3	Rattus norvegicus adducin 3 (gamma) (Add3), transcript variant 1, mRNA [NM_001164103]	**17,669**
Arhgef9	Rattus norvegicus Cdc42 guanine nucleotide exchange factor (GEF) 9 (Arhgef9), mRNA [NM_023957]	**17,667**
0	Unknown	**17,664**
Slmo1	Rattus norvegicus slowmo homolog 1 (Drosophila) (Slmo1), mRNA [NM_001109570]	**17,661**
Trappc9	Rattus norvegicus trafficking protein particle complex 9 (Trappc9), mRNA [NM_001034156]	**17,660**
Shc3	Rattus norvegicus SHC (Src homology 2 domain containing) transforming protein 3 (Shc3), mRNA [NM_001105743]	**17,660**
0	beta 1,3-galactosyltransferase-like Gene [Source:MGI Symbol;Acc:MGI:2685903] [ENSRNOT00000001199]	**17,655**
Phldb1	Rattus norvegicus pleckstrin homology-like domain, family B, member 1 (Phldb1), mRNA [NM_001191578]	**17,650**

Slc25a24	Rattus norvegicus solute carrier family 25 (mitochondrial carrier, phosphate carrier), member 24 (Slc25a24), nuclear gene encoding mitochondrial protein, mRNA [NM_001127544]	**17,634**
Adcy5	Rattus norvegicus adenylate cyclase 5 (Adcy5), mRNA [NM_022600]	**17,632**
Pcdh11x	Uncharacterized protein [Source:UniProtKB/TrEMBL;Acc:D4A497] [ENSRNOT00000045001]	**17,631**
Fam175b	Rattus norvegicus family with sequence similarity 175, member B (Fam175b), mRNA [NM_001106307]	**17,629**
Chtf8	Rattus norvegicus CTF8, chromosome transmission fidelity factor 8 homolog (S. cerevisiae) (Chtf8), mRNA [NM_001194951]	**17,628**
0	Unknown	**17,628**
Zfp238	Rattus norvegicus zinc finger protein 238 (Zfp238), mRNA [NM_022678]	**17,622**
Ttc4	Rattus norvegicus tetratricopeptide repeat domain 4 (Ttc4), mRNA [NM_001013214]	**17,621**
Tubg2	Rattus norvegicus tubulin, gamma 2 (Tubg2), mRNA [NM_001191075]	**17,621**
0	Cytochrome c oxidase subunit 2 [Source:UniProtKB/Swiss-Prot;Acc:P00406] [ENSRNOT00000043693]	**17,620**
Gpsm3	Rattus norvegicus G-protein signaling modulator 3 (AGS3-like, C. elegans) (Gpsm3), mRNA [NM_001003974]	**17,619**
LOC688507	PREDICTED: Rattus norvegicus similar to Spetex-2F protein (LOC688507), mRNA [XM_001067216]	**17,615**
Dctn4	Dynactin subunit 4 [Source:UniProtKB/Swiss-Prot;Acc:Q9QUR2] [ENSRNOT00000026394]	**17,613**
Hrh3	Rattus norvegicus histamine receptor H3 (Hrh3), mRNA [NM_053506]	**17,613**
0	Q7TS76_MOUSE (Q7TS76) Sip1 protein, partial (13%) [TC617524]	**17,611**
RGD1306520	Rattus norvegicus similar to receptor-interacting factor 1 (RGD1306520), mRNA [NM_001127485]	**17,609**
Lzic	Rattus norvegicus leucine zipper and CTNNBIP1 domain containing (Lzic), mRNA [NM_001013241]	**17,609**
0	Unknown	**17,608**
Htra3	Uncharacterized protein [Source:UniProtKB/TrEMBL;Acc:D3ZA76] [ENSRNOT00000010852]	**17,608**
Baiap2l1	Rattus norvegicus BAI1-associated protein 2-like 1 (Baiap2l1), mRNA [NM_001034140]	**17,605**
C7	PREDICTED: Rattus norvegicus complement component 7 (C7), mRNA [XM_226803]	**17,600**
Ar	Rattus norvegicus androgen receptor (Ar), mRNA [NM_012502]	**17,600**
Hspb9	Rattus norvegicus heat shock protein, alpha-crystallin-related, B9 (Hspb9), mRNA [NM_001108835]	**17,598**
Map3k9	PREDICTED: Rattus norvegicus mitogen-activated protein kinase kinase kinase 9 (Map3k9), mRNA [XM_002726763]	**17,595**
Dmrtc1b	PREDICTED: Rattus norvegicus DMRT-like family C1b (Dmrtc1b), mRNA [XM_001055549]	**17,595**
Dclre1a	Rattus norvegicus DNA cross-link repair 1A, PSO2 homolog (S. cerevisiae) (Dclre1a), mRNA [NM_001106201]	**17,588**
Mtch1	Rattus norvegicus mitochondrial carrier homolog 1 (C. elegans) (Mtch1), nuclear gene encoding mitochondrial protein, mRNA [NM_001100833]	**17,587**
Pldn	Rattus norvegicus pallidin homolog (mouse) (Pldn), mRNA [NM_001025714]	**17,584**
LOC683498	PREDICTED: Rattus norvegicus similar to Actin, aortic smooth muscle (Alpha-actin-2) (LOC683498), mRNA [XM_001066215]	**17,582**
Tpcn1	Rattus norvegicus two pore segment channel 1 (Tpcn1), mRNA [NM_139332]	**17,582**
0	Unknown	**17,579**
Camta2	Rattus norvegicus calmodulin binding transcription activator 2 (Camta2), mRNA [NM_001105801]	**17,576**
Pgf	Rattus norvegicus placental growth factor (Pgf), mRNA [NM_053595]	**17,574**

Lin28	Rattus norvegicus lin-28 homolog (C. elegans) (Lin28), mRNA [NM_001109269]	**17,570**
Lipn	Rattus norvegicus lipase, family member N (Lipn), mRNA [NM_001191955]	**17,568**
Rpl29	Rattus norvegicus ribosomal protein L29 (Rpl29), mRNA [NM_017150]	**17,565**
Grin2b	Rattus norvegicus glutamate receptor, ionotropic, N-methyl D-aspartate 2B (Grin2b), mRNA [NM_012574]	**17,565**
Olr125	Rattus norvegicus olfactory receptor 125 (Olr125), mRNA [NM_001000740]	**17,564**
0	Uncharacterized protein [Source:UniProtKB/TrEMBL;Acc:D3ZPQ3] [ENSRNOT00000056551]	**17,561**
Chi3l1	Rattus norvegicus chitinase 3-like 1 (Chi3l1), mRNA [NM_053560]	**17,558**
Olr260	Rattus norvegicus olfactory receptor 260 (Olr260), mRNA [NM_001000946]	**17,555**
Eif1ay	Rattus norvegicus eukaryotic translation initiation factor 1A, Y-linked (Eif1ay), mRNA [NM_001106963]	**17,555**
0	Unknown	**17,555**
Dnajc17	Rattus norvegicus DnaJ (Hsp40) homolog, subfamily C, member 17 (Dnajc17), mRNA [NM_001191740]	**17,554**
Ptp4a3	Rattus norvegicus protein tyrosine phosphatase type IVA, member 3 (Ptp4a3), mRNA [NM_001114405]	**17,554**
0	SYT1_RAT (P21707) Synaptotagmin-1 (Synaptotagmin I) (SytI) (p65), complete [TC577208]	**17,554**
Zfand3	Rattus norvegicus zinc finger, AN1-type domain 3 (Zfand3), mRNA [NM_001012175]	**17,551**
0	Q4QQW4_RAT (Q4QQW4) Histone deacetylase 1, partial (42%) [TC647523]	**17,550**
Eif3f	Rattus norvegicus eukaryotic translation initiation factor 3, subunit F (Eif3f), mRNA [NM_001106292]	**17,547**
Gpam	Rattus norvegicus glycerol-3-phosphate acyltransferase, mitochondrial (Gpam), nuclear gene encoding mitochondrial protein, mRNA [NM_017274]	**17,544**
Rnps1	Rattus norvegicus ribonucleic acid binding protein S1 (Rnps1), mRNA [NM_001011890]	**17,544**
Dmrta2	Rattus norvegicus DMRT-like family A2 (Dmrta2), mRNA [NM_001107951]	**17,538**
Hp1bp3	Rattus norvegicus heterochromatin protein 1, binding protein 3 (Hp1bp3), mRNA [NM_199108]	**17,537**
Rap2a	RAS related protein 2a [Source:RefSeq peptide;Acc:NP_446193] [ENSRNOT00000032395]	**17,536**
Olr19	Rattus norvegicus olfactory receptor 19 (Olr19), mRNA [NM_001000117]	**17,535**
Tmem229a	Rattus norvegicus transmembrane protein 229A (Tmem229a), mRNA [NM_001109480]	**17,534**
Ttc26	Rattus norvegicus tetratricopeptide repeat domain 26 (Ttc26), mRNA [NM_001025045]	**17,532**
Slc1a2	Rattus norvegicus solute carrier family 1 (glial high affinity glutamate transporter), member 2 (Slc1a2), transcript variant 2, mRNA [NM_001035233]	**17,528**
0	Unknown	**17,527**
Fut8	Rattus norvegicus fucosyltransferase 8 (alpha (1,6) fucosyltransferase) (Fut8), mRNA [NM_001002289]	**17,517**
Ppp2r4	Rattus norvegicus protein phosphatase 2A activator, regulatory subunit 4 (Ppp2r4), mRNA [NM_001108577]	**17,513**
0	PWWP domain-containing protein 2A [Source:RefSeq peptide;Acc:NP_001120768] [ENSRNOT00000005227]	**17,512**
Utp15	Rattus norvegicus UTP15, U3 small nucleolar ribonucleoprotein, homolog (S. cerevisiae) (Utp15), mRNA [NM_001107647]	**17,511**
Man1a1	Rattus norvegicus mannosidase, alpha, class 1A, member 1 (Man1a1), mRNA [NM_001033656]	**17,508**
RGD1308127	Rattus norvegicus similar to 2700078E11Rik protein (RGD1308127), mRNA [NM_001014248]	**17,508**
Rqcd1	Rattus norvegicus rcd1 (required for cell differentiation) homolog 1 (S. pombe) (Rqcd1), mRNA [NM_001009357]	**17,506**
Slc6a17	Rattus norvegicus solute carrier family 6 (neurotransmitter transporter), member 17 (Slc6a17), mRNA [NM_001033079]	**17,500**

0	Unknown	**17,494**
0	Q9UL04_HUMAN (Q9UL04) Membrane protein CH1, partial (9%) [TC585082]	**17,492**
Pkia	Rattus norvegicus protein kinase (cAMP-dependent, catalytic) inhibitor alpha (Pkia), mRNA [NM_053772]	**17,490**
Adrbk1	Rattus norvegicus adrenergic, beta, receptor kinase 1 (Adrbk1), mRNA [NM_012776]	**17,486**
Vcpip1	Deubiquitinating protein VCIP135 [Source:UniProtKB/Swiss-Prot;Acc:Q8CF97] [ENSRNOT00000009136]	**17,484**
0	Unknown	**17,483**
Cenpt	Rattus norvegicus centromere protein T (Cenpt), mRNA [NM_001024257]	**17,477**
Fkbp10	Rattus norvegicus FK506 binding protein 10 (Fkbp10), mRNA [NM_001014120]	**17,477**
LOC691684	PREDICTED: Rattus norvegicus similar to MIC2 like 1 (LOC691684), mRNA [XM_001079259]	**17,476**
Thy1	Rattus norvegicus Thy-1 cell surface antigen (Thy1), mRNA [NM_012673]	**17,475**
Pcbd1	Rattus norvegicus pterin-4 alpha-carbinolamine dehydratase/dimerization cofactor of hepatocyte nuclear factor 1 alpha (Pcbd1), mRNA [NM_001007601]	**17,473**
Tmem175	Rattus norvegicus transmembrane protein 175 (Tmem175), mRNA [NM_001013991]	**17,469**
Dot1l	Rattus norvegicus DOT1-like, histone H3 methyltransferase (S. cerevisiae) (Dot1l), mRNA [NM_001108733]	**17,468**
Osbpl2	Rattus norvegicus oxysterol binding protein-like 2 (Osbpl2), mRNA [NM_001013079]	**17,464**
Cntn4	Rattus norvegicus contactin 4 (Cntn4), mRNA [NM_053879]	**17,459**
Tecta	Rattus norvegicus tectorin alpha (Tecta), mRNA [NM_001106814]	**17,458**
Mynn	Rattus norvegicus myoneurin (Mynn), mRNA [NM_001012178]	**17,454**
Pigh	Rattus norvegicus phosphatidylinositol glycan anchor biosynthesis, class H (Pigh), mRNA [NM_001108714]	**17,452**
RGD1562018	Rattus norvegicus similar to Protein C14orf101 homolog (RGD1562018), mRNA [NM_001170475]	**17,446**
Rcc1	Rattus norvegicus regulator of chromosome condensation 1 (Rcc1), mRNA [NM_001128189]	**17,444**
Rbbp9	Rattus norvegicus retinoblastoma binding protein 9 (Rbbp9), mRNA [NM_019219]	**17,444**
0	Uncharacterized protein [Source:UniProtKB/TrEMBL;Acc:D3ZML2] [ENSRNOT00000054868]	**17,444**
Kb21	Rattus norvegicus type II keratin Kb21 (Kb21), mRNA [NM_001008814]	**17,442**
Sp4	Rattus norvegicus Sp4 transcription factor (Sp4), mRNA [NM_012761]	**17,442**
Stau1	Rattus norvegicus staufen RNA binding protein homolog 1 (Drosophila) (Stau1), transcript variant 1, mRNA [NM_053436]	**17,440**
0	AGENCOURT_118864812 NIH_MGC_248 Rattus norvegicus cDNA clone IMAGE:9113290 5', mRNA sequence [EX491730]	**17,440**
Dcun1d3	Rattus norvegicus DCN1, defective in cullin neddylation 1, domain containing 3 (S. cerevisiae) (Dcun1d3), mRNA [NM_001024886]	**17,437**
Gpr126	PREDICTED: Rattus norvegicus G protein-coupled receptor 126 (Gpr126), mRNA [XM_218313]	**17,433**
0	Unknown	**17,431**
Hmgb1	Rattus norvegicus high mobility group box 1 (Hmgb1), mRNA [NM_012963]	**17,430**
Phlda2	Rattus norvegicus pleckstrin homology-like domain, family A, member 2 (Phlda2), transcript variant 1, mRNA [NM_001100521]	**17,429**
Chd4	PREDICTED: Rattus norvegicus chromodomain helicase DNA binding protein 4 (Chd4), mRNA [XM_232354]	**17,422**
Tsku	Rattus norvegicus tsukushin (Tsku), mRNA [NM_001009965]	**17,420**

0	Unknown	**17,419**
Rap1gds1	Rattus norvegicus RAP1, GTP-GDP dissociation stimulator 1 (Rap1gds1), mRNA [NM_001107728]	**17,413**
MGC125086	Rattus norvegicus similar to RIKEN cDNA 5133401N09 (MGC125086), mRNA [NM_001037362]	**17,410**
Dhrs1	Rattus norvegicus dehydrogenase/reductase (SDR family) member 1 (Dhrs1), mRNA [NM_001007621]	**17,409**
Robo2	Rattus norvegicus roundabout homolog 2 (Drosophila) (Robo2), mRNA [NM_032106]	**17,404**
Lasp1	Rattus norvegicus LIM and SH3 protein 1 (Lasp1), mRNA [NM_032613]	**17,399**
0	Unknown	**17,399**
RGD1306502	Rattus norvegicus similar to hypothetical protein FLJ11193 (RGD1306502), mRNA [NM_001107654]	**17,399**
Gpr17	Rattus norvegicus G protein-coupled receptor 17 (Gpr17), mRNA [NM_001071777]	**17,397**
Ccnt1	Rattus norvegicus cyclin T1 (Ccnt1), mRNA [NM_001108110]	**17,394**
0	Unknown	**17,394**
0	Q6P501_RAT (Q6P501) Lysosomal-associated protein transmembrane 4 alpha, partial (11%) [TC646831]	**17,391**
Fbln7	Uncharacterized protein [Source:UniProtKB/TrEMBL;Acc:D3ZSY7] [ENSRNOT00000023708]	**17,387**
Slco3a1	Rattus norvegicus solute carrier organic anion transporter family, member 3a1 (Slco3a1), mRNA [NM_177481]	**17,382**
0	Unknown	**17,380**
Rgs17	Rattus norvegicus regulator of G-protein signaling 17 (Rgs17), mRNA [NM_001107459]	**17,378**
Exoc4	Rattus norvegicus exocyst complex component 4 (Exoc4), mRNA [NM_053875]	**17,377**
Ctnnd1	Rattus norvegicus catenin (cadherin associated protein), delta 1 (Ctnnd1), mRNA [NM_001107740]	**17,374**
Slc25a29	Rattus norvegicus solute carrier family 25, member 29 (Slc25a29), nuclear gene encoding mitochondrial protein, mRNA [NM_001010958]	**17,366**
Tmem111	Rattus norvegicus transmembrane protein 111 (Tmem111), mRNA [NM_001008355]	**17,360**
Vof16	Rattus norvegicus ischemia related factor vof-16 (Vof16), non-coding RNA [NR_037614]	**17,359**
Rasl10b	Rattus norvegicus RAS-like, family 10, member B (Rasl10b), mRNA [NM_001191648]	**17,357**
Osbp2	Rattus norvegicus oxysterol binding protein 2 (Osbp2), mRNA [NM_001107232]	**17,355**
Kitlg	Rattus norvegicus KIT ligand (Kitlg), transcript variant 1, mRNA [NM_021843]	**17,354**
Ogfod1	Rattus norvegicus 2-oxoglutarate and iron-dependent oxygenase domain containing 1 (Ogfod1), mRNA [NM_001107411]	**17,351**
Grpel2	Rattus norvegicus GrpE-like 2, mitochondrial (Grpel2), nuclear gene encoding mitochondrial protein, mRNA [NM_001109513]	**17,351**
Nox1	Rattus norvegicus NADPH oxidase 1 (Nox1), mRNA [NM_053683]	**17,348**
Qpctl	Rattus norvegicus glutaminyl-peptide cyclotransferase-like (Qpctl), mRNA [NM_001106230]	**17,345**
Igf1	Rattus norvegicus insulin-like growth factor 1 (Igf1), transcript variant 2, mRNA [NM_178866]	**17,337**
RGD1563973	PREDICTED: Rattus norvegicus similar to Gene model 50 (RGD1563973), mRNA [XM_001056614]	**17,336**
0	Unknown	**17,333**
Phf5a	Rattus norvegicus PHD finger protein 5A (Phf5a), mRNA [NM_138888]	**17,327**
Uhmk1	Rattus norvegicus U2AF homology motif (UHM) kinase 1 (Uhmk1), mRNA [NM_017293]	**17,325**
Scrn3	Rattus norvegicus secernin 3 (Scrn3), mRNA [NM_001013162]	**17,325**

Tmem222	Rattus norvegicus transmembrane protein 222 (Tmem222), mRNA [NM_001113780]	**17,323**
Zdhhc8	Rattus norvegicus zinc finger, DHHC-type containing 8 (Zdhhc8), mRNA [NM_001039021]	**17,317**
Alpk3	Rattus norvegicus alpha-kinase 3 (Alpk3), mRNA [NM_001191895]	**17,309**
Fxyd2	Rattus norvegicus FXYD domain-containing ion transport regulator 2 (Fxyd2), transcript variant a, mRNA [NM_145717]	**17,307**
Rhod	Rattus norvegicus ras homolog gene family, member D (Rhod), mRNA [NM_001106323]	**17,306**
Slc7a1	Rattus norvegicus solute carrier family 7 (cationic amino acid transporter, y+ system), member 1 (Slc7a1), mRNA [NM_013111]	**17,304**
0	Unknown	**17,304**
Vsnl1	Rattus norvegicus visinin-like 1 (Vsnl1), mRNA [NM_012686]	**17,303**
Mina	Rattus norvegicus myc induced nuclear antigen (Mina), mRNA [NM_153309]	**17,299**
Mt2A	Rattus norvegicus metallothionein 2A (Mt2A), mRNA [NM_001137564]	**17,298**
Pabpc4	Rattus norvegicus poly(A) binding protein, cytoplasmic 4 (Pabpc4), mRNA [NM_001100538]	**17,296**
LOC687144	PREDICTED: Rattus norvegicus similar to RT1 class I, CE4 (LOC687144), mRNA [XM_001077245]	**17,294**
Heatr2	Rattus norvegicus HEAT repeat containing 2 (Heatr2), mRNA [NM_001134857]	**17,290**
0	Unknown	**17,288**
RGD1565591	Q6NV61_MOUSE (Q6NV61) Ski protein, partial (48%) [TC590169]	**17,287**
0	Rattus norvegicus similar to ubiquitin-conjugating enzyme E2D 1, UBC4/5 homolog; ubiquitin-conjugating enzyme E2D 1 (LOC361831), mRNA [XM_342125]	**17,287**
LOC304558	Uncharacterized protein [Source:UniProtKB/TrEMBL;Acc:D3ZXP1] [ENSRNOT00000046920]	**17,286**
Spag4l	Rattus norvegicus sperm associated antigen 4-like (Spag4l), mRNA [NM_001106530]	**17,284**
RGD1311564	Rattus norvegicus LOC360590 (RGD1311564), mRNA [NM_001108286]	**17,279**
Xiap	Rattus norvegicus X-linked inhibitor of apoptosis (Xiap), mRNA [NM_022231]	**17,279**
Arpc5	Rattus norvegicus actin related protein 2/3 complex, subunit 5 (Arpc5), mRNA [NM_001025717]	**17,277**
Enpp3	Rattus norvegicus ectonucleotide pyrophosphatase/phosphodiesterase 3 (Enpp3), mRNA [NM_019370]	**17,276**
Olr522	Rattus norvegicus olfactory receptor 522 (Olr522), mRNA [NM_001000562]	**17,275**
RGD1309049	Rattus norvegicus similar to RIKEN cDNA 4933415F23 (RGD1309049), mRNA [NM_001013956]	**17,272**
Syngr1	Rattus norvegicus synaptogyrin 1 (Syngr1), mRNA [NM_019166]	**17,271**
Tle3	Rattus norvegicus transducin-like enhancer of split 3 (E(sp1) homolog, Drosophila) (Tle3), mRNA [NM_053400]	**17,267**
Cyp2j4	Rattus norvegicus cytochrome P450, family 2, subfamily j, polypeptide 4 (Cyp2j4), mRNA [NM_023025]	**17,262**
Pde7b	Rattus norvegicus phosphodiesterase 7B (Pde7b), mRNA [NM_080894]	**17,262**
Ndrg3	Rattus norvegicus N-myc downstream regulated gene 3 (Ndrg3), mRNA [NM_001013923]	**17,262**
Gps1	Rattus norvegicus G protein pathway suppressor 1 (Gps1), mRNA [NM_053969]	**17,259**
Fam124a	Fam124a protein [Source:UniProtKB/TrEMBL;Acc:Q6AXP0] [ENSRNOT00000012990]	**17,259**
Pogz	Rattus norvegicus pogo transposable element with ZNF domain (Pogz), mRNA [NM_001107693]	**17,256**
Aaas	Rattus norvegicus achalasia, adrenocortical insufficiency, alacrimia (Allgrove, triple-A) (Aaas), mRNA [NM_001106795]	**17,253**

Kank1	Rattus norvegicus KN motif and ankyrin repeat domains 1 (Kank1), mRNA [NM_001037197]	**17,251**
0	Uncharacterized protein [Source:UniProtKB/TrEMBL;Acc:D4A7X0] [ENSRNOT00000034908]	**17,247**
Cnp	Rattus norvegicus 2',3'-cyclic nucleotide 3' phosphodiesterase (Cnp), mRNA [NM_012809]	**17,245**
Bbs4	Rattus norvegicus Bardet-Biedl syndrome 4 (Bbs4), mRNA [NM_001106826]	**17,245**
Tnfaip8	Rattus norvegicus tumor necrosis factor, alpha-induced protein 8 (Tnfaip8), mRNA [NM_001107387]	**17,243**
Ctf1	Rattus norvegicus cardiotrophin 1 (Ctf1), mRNA [NM_017129]	**17,240**
RGD1566033	PREDICTED: Rattus norvegicus similar to BC003940 protein (RGD1566033), mRNA [XM_001070540]	**17,240**
Kcnj12	Rattus norvegicus potassium inwardly-rectifying channel, subfamily J, member 12 (Kcnj12), mRNA [NM_053981]	**17,238**
Mip	Rattus norvegicus major intrinsic protein of lens fiber (Mip), mRNA [NM_001105719]	**17,237**
Dpf1	Rattus norvegicus D4, zinc and double PHD fingers family 1 (Dpf1), mRNA [NM_001105729]	**17,232**
LOC691317	Uncharacterized protein [Source:UniProtKB/TrEMBL;Acc:D3ZDG4] [ENSRNOT00000012080]	**17,231**
Pcbp4	Rattus norvegicus poly(rC) binding protein 4 (Pcbp4), mRNA [NM_001191883]	**17,231**
RGD1308019	Rattus norvegicus similar to hypothetical protein FLJ20245 (RGD1308019), mRNA [NM_001107816]	**17,229**
Rbm25	Rattus norvegicus RNA binding motif protein 25 (Rbm25), mRNA [NM_001108984]	**17,229**
0	Unknown	**17,225**
Gbas	Rattus norvegicus glioblastoma amplified sequence (Gbas), nuclear gene encoding mitochondrial protein, mRNA [NM_001017486]	**17,225**
Cd38	Rattus norvegicus CD38 molecule (Cd38), mRNA [NM_013127]	**17,224**
Carm1	Rattus norvegicus coactivator-associated arginine methyltransferase 1 (Carm1), transcript variant 1, mRNA [NM_001030041]	**17,222**
Ado	Rattus norvegicus 2-aminoethanethiol (cysteamine) dioxygenase (Ado), mRNA [NM_001107626]	**17,221**
Znf488	Uncharacterized protein [Source:UniProtKB/TrEMBL;Acc:D4AAF0] [ENSRNOT00000031496]	**17,220**
Nup210	Rattus norvegicus nucleoporin 210 (Nup210), mRNA [NM_053322]	**17,219**
Mcm3	Rattus norvegicus minichromosome maintenance complex component 3 (Mcm3), mRNA [NM_001191805]	**17,216**
RGD1559917	Rattus norvegicus similar to SMT3 suppressor of mif two 3 homolog 2 (LOC287993), mRNA [XM_212687]	**17,215**
Dgat2	Rattus norvegicus diacylglycerol O-acyltransferase homolog 2 (mouse) (Dgat2), mRNA [NM_001012345]	**17,211**
Fam13c1	PREDICTED: Rattus norvegicus family with sequence similarity 13, member C1 (Fam13c1), mRNA [XM_001080212]	**17,205**
RGD1559864	PREDICTED: Rattus norvegicus similar to mKIAA1045 protein, transcript variant 1 (RGD1559864), mRNA [XM_002729465]	**17,202**
Cryl1	Rattus norvegicus crystallin, lambda 1 (Cryl1), mRNA [NM_175757]	**17,202**
Chd3	PREDICTED: Rattus norvegicus chromodomain helicase DNA binding protein 3 (Chd3), mRNA [XM_220602]	**17,195**
0	Unknown	**17,194**
Tmem106c	Rattus norvegicus transmembrane protein 106C (Tmem106c), mRNA [NM_001008358]	**17,191**
0	Unknown	**17,189**
RGD1303066	Rattus norvegicus similar to RIKEN cDNA 2610110G12 (RGD1303066), mRNA [NM_212498]	**17,188**
LOC689919	PREDICTED: Rattus norvegicus similar to 40S ribosomal protein S26 (LOC689919), mRNA [XM_001072530]	**17,187**
Amac1	Rattus norvegicus acyl-malonyl condensing enzyme 1 (Amac1), mRNA [NM_001127658]	**17,179**

Igfbp4	Rattus norvegicus insulin-like growth factor binding protein 4 (Igfbp4), mRNA [NM_001004274]	**17,176**
Grin1	Rattus norvegicus glutamate receptor, ionotropic, N-methyl D-aspartate 1 (Grin1), mRNA [NM_017010]	**17,174**
B3galt6	Rattus norvegicus UDP-Gal:betaGal beta 1,3-galactosyltransferase, polypeptide 6 (B3galt6), mRNA [NM_001106699]	**17,173**
0	Unknown	**17,168**
Arhgap42	PREDICTED: Rattus norvegicus similar to novel protein similar to human oligophrenin 1 (OPHN1), transcript variant 1 (RGD1564081), mRNA [XM_001073215]	**17,167**
Slitrk5	Rattus norvegicus SLIT and NTRK-like family, member 5 (Slitrk5), mRNA [NM_001107284]	**17,163**
0	Unknown	**17,161**
Ndufb4	Rattus norvegicus NADH dehydrogenase (ubiquinone) 1 beta subcomplex 4 (Ndufb4), nuclear gene encoding mitochondrial protein, mRNA [NM_001037338]	**17,160**
0	Unknown	**17,154**
Foxi1	Rattus norvegicus forkhead box I1 (Foxi1), mRNA [NM_001105776]	**17,154**
Mak16	Rattus norvegicus MAK16 homolog (S. cerevisiae) (Mak16), mRNA [NM_001014002]	**17,152**
RGD1565149	Rattus norvegicus similar to chromosome 16 open reading frame 7 (RGD1565149), mRNA [NM_001107440]	**17,150**
Ing4	Rattus norvegicus inhibitor of growth family, member 4 (Ing4), mRNA [NM_001079887]	**17,149**
0	Unknown	**17,148**
LOC687746	PREDICTED: Rattus norvegicus similar to Mitochondrial import inner membrane translocase subunit Tim17-B (LOC687746), mRNA [XM_001080001]	**17,146**
Clstn2	Rattus norvegicus calsyntenin 2 (Clstn2), mRNA [NM_134377]	**17,145**
Abcc4	Rattus norvegicus ATP-binding cassette, subfamily C (CFTR/MRP), member 4 (Abcc4), mRNA [NM_133411]	**17,145**
0	Unknown	**17,141**
Tank	Rattus norvegicus TRAF family member-associated NFKB activator (Tank), transcript variant 1, mRNA [NM_145788]	**17,139**
Bcl7b	Rattus norvegicus B-cell CLL/lymphoma 7B (Bcl7b), mRNA [NM_001109021]	**17,132**
Samd10	Rattus norvegicus sterile alpha motif domain containing 10 (Samd10), mRNA [NM_001191967]	**17,128**
Dlg3	Rattus norvegicus discs, large homolog 3 (Drosophila) (Dlg3), mRNA [NM_031639]	**17,127**
Rnf114	Rattus norvegicus ring finger protein 114 (Rnf114), mRNA [NM_001001517]	**17,127**
Fcer1g	Rattus norvegicus Fc fragment of IgE, high affinity I, receptor for; gamma polypeptide (Fcer1g), mRNA [NM_001131001]	**17,127**
Vsig10l	Unknown	**17,127**
Kcnk13	Rattus norvegicus potassium channel, subfamily K, member 13 (Kcnk13), mRNA [NM_022293]	**17,120**
0	Unknown	**17,116**
0	Unknown	**17,114**
Plxdc1	Rattus norvegicus plexin domain containing 1 (Plxdc1), mRNA [NM_001107046]	**17,112**
Sult1a1	Rattus norvegicus sulfotransferase family, cytosolic, 1A, phenol-preferring, member 1 (Sult1a1), mRNA [NM_031834]	**17,109**
Ubxn2a	Rattus norvegicus UBX domain protein 2A (Ubxn2a), mRNA [NM_001109482]	**17,109**

Olr1238	Rattus norvegicus olfactory receptor 1238 (Olr1238), mRNA [NM_001001013]	**17,104**
Dnajb13	Rattus norvegicus DnaJ (Hsp40) related, subfamily B, member 13 (Dnajb13), mRNA [NM_001005885]	**17,102**
0	Unknown	**17,102**
Gpr37	Rattus norvegicus G protein-coupled receptor 37 (Gpr37), mRNA [NM_057201]	**17,093**
0	Uncharacterized protein [Source:UniProtKB/TrEMBL;Acc:D4A319] [ENSRNOT00000007828]	**17,090**
0	Unknown	**17,088**
Mtvr2	Rattus norvegicus mammary tumor virus receptor 2 (Mtvr2), mRNA [NM_001015013]	**17,085**
Sf3b1	Rattus norvegicus splicing factor 3b, subunit 1 (Sf3b1), mRNA [NM_053426]	**17,085**
Foxj2	Rattus norvegicus forkhead box J2 (Foxj2), mRNA [NM_001109352]	**17,084**
0	Unknown	**17,076**
Swap70	Rattus norvegicus SWAP switching B-cell complex 70 (Swap70), mRNA [NM_001106288]	**17,073**
0	EST352678 Rat gene index, normalized rat, norvegicus, Bento Soares Rattus norvegicus cDNA clone RGIHT15 5' end, mRNA sequence [AW921374]	**17,072**
Eif4ebp1	Rattus norvegicus eukaryotic translation initiation factor 4E binding protein 1 (Eif4ebp1), mRNA [NM_053857]	**17,070**
Dbp	Rattus norvegicus D site of albumin promoter (albumin D-box) binding protein (Dbp), mRNA [NM_012543]	**17,069**
Syngr3	Rattus norvegicus synaptogyrin 3 (Syngr3), mRNA [NM_001106985]	**17,064**
LOC685050	PREDICTED: Rattus norvegicus hypothetical protein LOC685050 (LOC685050), mRNA [XM_001062068]	**17,062**
Mettl2	Rattus norvegicus methyltransferase like 2 (Mettl2), mRNA [NM_001108839]	**17,061**
Abtb2	Rattus norvegicus ankyrin repeat and BTB (POZ) domain containing 2 (Abtb2), mRNA [NM_134403]	**17,057**
Hecw2	Rattus norvegicus HECT, C2 and WW domain containing E3 ubiquitin protein ligase 2 (Hecw2), mRNA [NM_001108218]	**17,056**
Arrb2	Rattus norvegicus arrestin, beta 2 (Arrb2), mRNA [NM_012911]	**17,050**
Vgll4	Rattus norvegicus vestigial like 4 (Drosophila) (Vgll4), mRNA [NM_001015004]	**17,049**
Ptk7	Rattus norvegicus PTK7 protein tyrosine kinase 7 (Ptk7), mRNA [NM_001106889]	**17,048**
Mmp27	Rattus norvegicus matrix metallopeptidase 27 (Mmp27), mRNA [NM_001106799]	**17,046**
0	Rattus norvegicus chromosome 1, 5 clones, strain BN/SsNHsdMCW RNOR03207167, whole genome shotgun sequence [AABR03001102]	**17,045**
Slc2a6	Rattus norvegicus solute carrier family 2 (facilitated glucose transporter), member 6 (Slc2a6), mRNA [NM_001106562]	**17,044**
Tmed10	Rattus norvegicus transmembrane emp24-like trafficking protein 10 (yeast) (Tmed10), mRNA [NM_053467]	**17,042**
Trim9	Rattus norvegicus tripartite motif-containing 9 (Trim9), mRNA [NM_130420]	**17,042**
0	Unknown	**17,041**
0	Unknown	**17,037**
Taf15	Rattus norvegicus TAF15 RNA polymerase II, TATA box binding protein (TBP)-associated factor (Taf15), mRNA [NM_001105824]	**17,037**
Gria4	Rattus norvegicus glutamate receptor, ionotrophic, AMPA 4 (Gria4), transcript variant 3, mRNA [NM_001113185]	**17,037**
Tm9sf3	PREDICTED: Rattus norvegicus transmembrane 9 superfamily member 3 (Tm9sf3), mRNA [XM_002725800]	**17,036**
Adora1	Rattus norvegicus adenosine A1 receptor (Adora1), mRNA [NM_017155]	**17,035**

Slc26a11	PREDICTED: Rattus norvegicus solute carrier family 26, member 11 (Slc26a11), partial mRNA [XM_340944]	**17,031**
Gpr114	Rattus norvegicus G protein-coupled receptor 114 (Gpr114), mRNA [NM_001107410]	**17,031**
Fam193a	Uncharacterized protein [Source:UniProtKB/TrEMBL;Acc:D3ZIG8] [ENSRNOT00000018616]	**17,030**
Kcnv2	Rattus norvegicus potassium channel, subfamily V, member 2 (Kcnv2), mRNA [NM_001106370]	**17,030**
Hccs	Rattus norvegicus holocytochrome c synthetase (Hccs), mRNA [NM_001191732]	**17,027**
Rpgr	Rattus norvegicus retinitis pigmentosa GTPase regulator (Rpgr), mRNA [NM_001127601]	**17,022**
Pi4k2a	Rattus norvegicus phosphatidylinositol 4-kinase type 2 alpha (Pi4k2a), mRNA [NM_053735]	**17,020**
0	Rattus norvegicus similar to 60S RIBOSOMAL PROTEIN L29 (P23) (LOC294100), mRNA [XM_220073]	**17,017**
0	Unknown	**17,017**
Dgki	Rattus norvegicus diacylglycerol kinase, iota (Dgki), mRNA [NM_198782]	**17,014**
Aifm2	Rattus norvegicus apoptosis-inducing factor, mitochondrion-associated 2 (Aifm2), nuclear gene encoding mitochondrial protein, mRNA [NM_001139483]	**17,012**
Sfrs6	Rattus norvegicus splicing factor, arginine/serine-rich 6 (Sfrs6), mRNA [NM_001014185]	**17,010**
Rtkn	Rattus norvegicus rhotekin (Rtkn), mRNA [NM_184046]	**17,007**
0	Unknown	**17,004**
Palm	Rattus norvegicus paralemmin (Palm), mRNA [NM_130829]	**17,002**
0	Unknown	**16,998**
Serinc4	Rattus norvegicus serine incorporator 4 (Serinc4), mRNA [NM_001110811]	**16,997**
0	Unknown	**16,996**
Ccbe1	Uncharacterized protein [Source:UniProtKB/TrEMBL;Acc:D4A1T8] [ENSRNOT00000034069]	**16,995**
Cyp4f4	Rattus norvegicus cytochrome P450, family 4, subfamily f, polypeptide 4 (Cyp4f4), mRNA [NM_173123]	**16,993**
0	BF284235 EST448826 Rat Gene Index, normalized rat, Rattus norvegicus cDNA Rattus norvegicus cDNA clone RGIEN17 3' sequence, mRNA sequence [BF284235]	**16,991**
0	Unknown	**16,987**
Rhbdf2	Rattus norvegicus rhomboid 5 homolog 2 (Drosophila) (Rhbdf2), mRNA [NM_001107067]	**16,981**
Slc41a1	Rattus norvegicus solute carrier family 41, member 1 (Slc41a1), mRNA [NM_001108855]	**16,979**
Fam168b	RCG22336, isoform CRA_bUncharacterized protein [Source:UniProtKB/TrEMBL;Acc:D4AEP3] [ENSRNOT00000039480]	**16,978**
Gramd3	Rattus norvegicus GRAM domain containing 3 (Gramd3), mRNA [NM_001014011]	**16,978**
RGD1560792	PREDICTED: Rattus norvegicus RGD1560792 (RGD1560792), mRNA [XM_001081860]	**16,975**
Ablim3	Rattus norvegicus actin binding LIM protein family, member 3 (Ablim3), mRNA [NM_001191698]	**16,974**
Tmcc3	Rattus norvegicus transmembrane and coiled-coil domain family 3 (Tmcc3), mRNA [NM_001108084]	**16,971**
LOC100151767	Rattus norvegicus hypothetical LOC100151767 (LOC100151767), mRNA [NM_001127503]	**16,970**
LOC688708	Uncharacterized protein [Source:UniProtKB/TrEMBL;Acc:D3ZP46] [ENSRNOT00000034463]	**16,965**
RGD1306271	Uncharacterized protein [Source:UniProtKB/TrEMBL;Acc:D3Z9D0] [ENSRNOT00000040391]	**16,964**

LOC682950	PREDICTED: Rattus norvegicus similar to guanine nucleotide binding protein, alpha stimulating, olfactory type (LOC682950), mRNA [XM_001060921]	**16,962**
0	Unknown	**16,954**
Npat	Rattus norvegicus nuclear protein, ataxia-telangiectasia locus (Npat), mRNA [NM_001108147]	**16,948**
Vwa3a	Rattus norvegicus von Willebrand factor A domain containing 3A (Vwa3a), transcript variant 2, mRNA [NM_001079885]	**16,948**
RGD1311634	Rattus norvegicus similar to RIKEN cDNA 3200002M19 (RGD1311634), mRNA [NM_001170435]	**16,947**
Erc1	Rattus norvegicus ELKS/RAB6-interacting/CAST family member 1 (Erc1), mRNA [NM_170788]	**16,944**
Gpm6b	Rattus norvegicus glycoprotein m6b (Gpm6b), mRNA [NM_138846]	**16,944**
Msrb2	Rattus norvegicus methionine sulfoxide reductase B2 (Msrb2), mRNA [NM_001031660]	**16,942**
Defb29	Rattus norvegicus defensin beta 29 (Defb29), mRNA [NM_001037368]	**16,941**
Snx27	Rattus norvegicus sorting nexin family member 27 (Snx27), transcript variant 2, mRNA [NM_152847]	**16,938**
LOC691522	PREDICTED: Rattus norvegicus hypothetical protein LOC691522 (LOC691522), mRNA [XM_001078640]	**16,937**
Lysmd1	Rattus norvegicus LysM, putative peptidoglycan-binding, domain containing 1 (Lysmd1), mRNA [NM_001024302]	**16,936**
Rhoa	Rattus norvegicus ras homolog gene family, member A (Rhoa), mRNA [NM_057132]	**16,933**
Ppt2	Rattus norvegicus palmitoyl-protein thioesterase 2 (Ppt2), mRNA [NM_019367]	**16,932**
Pxmp4	Rattus norvegicus peroxisomal membrane protein 4 (Pxmp4), mRNA [NM_172223]	**16,930**
Mpzl1	Rattus norvegicus myelin protein zero-like 1 (Mpzl1), mRNA [NM_001007728]	**16,929**
Ctns	Rattus norvegicus cystinosis, nephropathic (Ctns), mRNA [NM_001191647]	**16,926**
Abcc2	Rattus norvegicus ATP-binding cassette, subfamily C (CFTR/MRP), member 2 (Abcc2), mRNA [NM_012833]	**16,920**
0	Unknown	**16,917**
Hdac8	Rattus norvegicus histone deacetylase 8 (Hdac8), mRNA [NM_001126373]	**16,914**
Plxna2	Rattus norvegicus plexin A2 (Plxna2), mRNA [NM_001105988]	**16,910**
Tacr1	Rattus norvegicus tachykinin receptor 1 (Tacr1), mRNA [NM_012667]	**16,905**
Vkorc1l1	Rattus norvegicus vitamin K epoxide reductase complex, subunit 1-like 1 (Vkorc1l1), mRNA [NM_203338]	**16,903**
Zinki	Rattus norvegicus Arg3.1/Arc mRNA-binding zinc finger protein (Zinki), mRNA [NM_001142758]	**16,902**
Trib2	Rattus norvegicus tribbles homolog 2 (Drosophila) (Trib2), mRNA [NM_001108015]	**16,900**
Usp15	Rattus norvegicus ubiquitin specific peptidase 15 (Usp15), mRNA [NM_145184]	**16,900**
0	Uncharacterized protein [Source:UniProtKB/TrEMBL;Acc:D3ZGU0] [ENSRNOT00000041039]	**16,899**
RGD1311422	PREDICTED: Rattus norvegicus similar to CG8841-PA (RGD1311422), mRNA [XM_001081678]	**16,893**
0	EPO_MOUSE (P07321) Erythropoietin precursor, partial (7%) [TC593578]	**16,891**
Prdm10	Uncharacterized protein [Source:UniProtKB/TrEMBL;Acc:D3ZQ79] [ENSRNOT00000010423]	**16,891**
LOC688311	PREDICTED: Rattus norvegicus similar to ADP-ribosylation factor-like 1, transcript variant 1 (LOC688311), mRNA [XM_001081794]	**16,890**
0	Unknown	**16,890**
0	Unknown	**16,887**

Bcl2l1	Rattus norvegicus Bcl2-like 1 (Bcl2l1), nuclear gene encoding mitochondrial protein, transcript variant 2, mRNA [NM_031535]	**16,886**
Zcchc3	RCG37285Uncharacterized protein [Source:UniProtKB/TrEMBL;Acc:D3ZZ10] [ENSRNOT00000009859]	**16,885**
Nr2f1	Rattus norvegicus nuclear receptor subfamily 2, group F, member 1 (Nr2f1), mRNA [NM_031130]	**16,881**
Fzd1	Rattus norvegicus frizzled homolog 1 (Drosophila) (Fzd1), mRNA [NM_021266]	**16,879**
Capn5	Rattus norvegicus calpain 5 (Capn5), mRNA [NM_134461]	**16,878**
0	TBC1 domain family member 5 [Source:RefSeq peptide;Acc:NP_001128234] [ENSRNOT00000061800]	**16,876**
Trmt61a	Rattus norvegicus tRNA methyltransferase 61 homolog A (S. cerevisiae) (Trmt61a), mRNA [NM_001007706]	**16,873**
Sestd1	Rattus norvegicus SEC14 and spectrin domains 1 (Sestd1), mRNA [NM_001134514]	**16,871**
0	Unknown	**16,865**
Twsg1	Rattus norvegicus twisted gastrulation homolog 1 (Drosophila) (Twsg1), mRNA [NM_001108811]	**16,863**
RGD1311870	Uncharacterized protein [Source:UniProtKB/TrEMBL;Acc:D3ZKN3] [ENSRNOT00000029077]	**16,863**
Spry3	Rattus norvegicus sprouty homolog 3 (Drosophila) (Spry3), mRNA [NM_001109063]	**16,860**
Sik1	Rattus norvegicus salt-inducible kinase 1 (Sik1), mRNA [NM_021693]	**16,860**
Slit2	Rattus norvegicus slit homolog 2 (Drosophila) (Slit2), mRNA [NM_022632]	**16,860**
Pald	Rattus norvegicus paladin (Pald), mRNA [NM_001034128]	**16,859**
0	Uncharacterized protein [Source:UniProtKB/TrEMBL;Acc:D3ZGC8] [ENSRNOT00000068019]	**16,853**
Brunol4	CUGBP Elav-like family member 4 [Source:RefSeq peptide;Acc:NP_001100870] [ENSRNOT00000061151]	**16,851**
Sh2b3	Rattus norvegicus SH2B adaptor protein 3 (Sh2b3), mRNA [NM_031621]	**16,850**
Ppp1r14c	Rattus norvegicus protein phosphatase 1, regulatory (inhibitor) subunit 14c (Ppp1r14c), mRNA [NM_133425]	**16,849**
Rad23a	Rattus norvegicus RAD23 homolog A (S. cerevisiae) (Rad23a), mRNA [NM_001013190]	**16,835**
Dgkz	Rattus norvegicus diacylglycerol kinase zeta (Dgkz), mRNA [NM_031143]	**16,829**
Rgs16	Rattus norvegicus regulator of G-protein signaling 16 (Rgs16), mRNA [NM_001077589]	**16,826**
LOC690126	PREDICTED: Rattus norvegicus hypothetical protein LOC690126 (LOC690126), miscRNA [XR_006805]	**16,826**
Pax6	Rattus norvegicus paired box 6 (Pax6), mRNA [NM_013001]	**16,823**
Gmppa	Rattus norvegicus GDP-mannose pyrophosphorylase A (Gmppa), mRNA [NM_001025056]	**16,815**
0	Unknown	**16,810**
Eri1	Rattus norvegicus exoribonuclease 1 (Eri1), mRNA [NM_001014143]	**16,807**
Rbpj	Rattus norvegicus recombination signal binding protein for immunoglobulin kappa J region (Rbpj), mRNA [NM_001106631]	**16,806**
Pef1	Rattus norvegicus penta-EF hand domain containing 1 (Pef1), mRNA [NM_001007651]	**16,805**
Slc29a3	Rattus norvegicus solute carrier family 29 (nucleoside transporters), member 3 (Slc29a3), mRNA [NM_181639]	**16,803**
Ascc1	Rattus norvegicus activating signal cointegrator 1 complex subunit 1 (Ascc1), mRNA [NM_001007632]	**16,801**
0	Limkain-b1 [Source:UniProtKB/Swiss-Prot;Acc:Q8VIG2] [ENSRNOT00000003193]	**16,794**
Bmpr1b	Rattus norvegicus bone morphogenetic protein receptor, type IB (Bmpr1b), mRNA [NM_001024259]	**16,789**
Papd5	Rattus norvegicus PAP associated domain containing 5 (Papd5), mRNA [NM_001107416]	**16,787**

Cdc26	Rattus norvegicus cell division cycle 26 (Cdc26), mRNA [NM_001013240]	**16,785**
Rapgef1	PREDICTED: Rattus norvegicus Rap guanine nucleotide exchange factor (GEF) 1 (Rapgef1), mRNA [XM_216018]	**16,779**
Slc39a4	Rattus norvegicus solute carrier family 39 (zinc transporter), member 4 (Slc39a4), mRNA [NM_001077669]	**16,777**
Mepce	Rattus norvegicus methylphosphate capping enzyme (Mepce), mRNA [NM_001100678]	**16,773**
Scly	Rattus norvegicus selenocysteine lyase (Scly), mRNA [NM_001007755]	**16,773**
0	Unknown	**16,771**
0	PREDICTED: Rattus norvegicus similar to RIKEN cDNA 1700001F09 (RGD1562624), mRNA [XM_002742438]	**16,771**
0	Unknown	**16,768**
Trappc5	Rattus norvegicus trafficking protein particle complex 5 (Trappc5), mRNA [NM_001108850]	**16,764**
Gria4	Rattus norvegicus glutamate receptor, ionotrophic, AMPA 4 (Gria4), transcript variant 1, mRNA [NM_017263]	**16,764**
Pbx3	Rattus norvegicus pre-B-cell leukemia homeobox 3 (Pbx3), mRNA [NM_001107834]	**16,763**
Sall3	Rattus norvegicus sal-like 3 (Drosophila) (Sall3), mRNA [NM_001108892]	**16,763**
LOC690479	PREDICTED: Rattus norvegicus hypothetical protein LOC690479 (LOC690479), mRNA [XM_001074561]	**16,759**
Arhgef3	Rattus norvegicus Rho guanine nucleotide exchange factor (GEF) 3 (Arhgef3), mRNA [NM_001106061]	**16,758**
Ptdss1	Rattus norvegicus phosphatidylserine synthase 1 (Ptdss1), mRNA [NM_001012113]	**16,757**
0	PREDICTED: Rattus norvegicus similar to dachshund b (LOC680770), mRNA [XM_001058786]	**16,753**
Uqcrb	Rattus norvegicus ubiquinol-cytochrome c reductase binding protein (Uqcrb), nuclear gene encoding mitochondrial protein, mRNA [NM_001127553]	**16,751**
Lypla2	Rattus norvegicus lysophospholipase 2 (Lypla2), mRNA [NM_031342]	**16,741**
Ms4a10	Rattus norvegicus membrane-spanning 4-domains, subfamily A, member 10 (Ms4a10), mRNA [NM_001106336]	**16,739**
0	Rattus norvegicus TL0AEA74YH08 mRNA sequence. [FQ231255]	**16,738**
0	TBC1 domain family, member 8B Gene [Source:MGI Symbol;Acc:MGI:1918101] [ENSRNOT00000051757]	**16,737**
LOC363306	PREDICTED: Rattus norvegicus hypothetical protein LOC363306 (LOC363306), mRNA [XM_001068086]	**16,736**
Nr2c1	Rattus norvegicus nuclear receptor subfamily 2, group C, member 1 (Nr2c1), mRNA [NM_145780]	**16,733**
Etl4	PREDICTED: Rattus norvegicus similar to KIAA1217 (RGD1563437), mRNA [XM_001069190]	**16,731**
RGD1562608	Rattus norvegicus similar to KIAA1328 protein (RGD1562608), mRNA [NM_001134607]	**16,728**
0	Unknown	**16,726**
Ctdp1	Rattus norvegicus CTD (carboxy-terminal domain, RNA polymerase II, polypeptide A) phosphatase, subunit 1 (Ctdp1), mRNA [NM_001106131]	**16,723**
Xpo4	Rattus norvegicus exportin 4 (Xpo4), mRNA [NM_001106042]	**16,718**
Plcxd2	Rattus norvegicus phosphatidylinositol-specific phospholipase C, X domain containing 2 (Plcxd2), mRNA [NM_001134481]	**16,718**
0	Uncharacterized protein [Source:UniProtKB/TrEMBL;Acc:D3Z994] [ENSRNOT00000022945]	**16,712**
Doxl2	Rattus norvegicus diamine oxidase-like protein 2 (Doxl2), mRNA [NM_199291]	**16,711**
Pnpla5	Rattus norvegicus patatin-like phospholipase domain containing 5 (Pnpla5), mRNA [NM_001130497]	**16,710**
0	Unknown	**16,708**

Slc38a6	Rattus norvegicus solute carrier family 38, member 6 (Slc38a6), mRNA [NM_001013099]	**16,706**
Dnajc14	Rattus norvegicus DnaJ (Hsp40) homolog, subfamily C, member 14 (Dnajc14), mRNA [NM_053690]	**16,704**
Ube2d3	Rattus norvegicus ubiquitin-conjugating enzyme E2D 3 (UBC4/5 homolog, yeast) (Ube2d3), mRNA [NM_031237]	**16,703**
Wars	Rattus norvegicus tryptophanyl-tRNA synthetase (Wars), mRNA [NM_001013170]	**16,702**
LOC685079	PREDICTED: Rattus norvegicus similar to Protein SYS1 homolog (LOC685079), mRNA [XM_001062200]	**16,698**
RGD1305415	Uncharacterized protein [Source:UniProtKB/TrEMBL;Acc:D3ZQL6] [ENSRNOT00000036357]	**16,694**
Nusap1	Rattus norvegicus nucleolar and spindle associated protein 1 (Nusap1), mRNA [NM_001107762]	**16,690**
Add1	Rattus norvegicus adducin 1 (alpha) (Add1), mRNA [NM_016990]	**16,689**
Atp8b2	Rattus norvegicus Atpase, class I, type 8B, member 2 (Atp8b2), mRNA [NM_001024798]	**16,688**
LOC687675	PREDICTED: Rattus norvegicus similar to heparan sulfate D-glucosaminyl 3-O-sulfotransferase 4 (LOC687675), mRNA [XM_001079681]	**16,688**
LOC100364346	Uncharacterized protein [Source:UniProtKB/TrEMBL;Acc:D4ACG5] [ENSRNOT00000048875]	**16,687**
0	Unknown	**16,685**
Ntrk3	Rattus norvegicus neurotrophic tyrosine kinase, receptor, type 3 (Ntrk3), mRNA [NM_019248]	**16,684**
Rhobtb1	Rattus norvegicus Rho-related BTB domain containing 1 (Rhobtb1), mRNA [NM_001107622]	**16,683**
Ftsj2	Rattus norvegicus FtsJ homolog 2 (E. coli) (Ftsj2), mRNA [NM_001107125]	**16,673**
St3gal4	Rattus norvegicus ST3 beta-galactoside alpha-2,3-sialyltransferase 4 (St3gal4), mRNA [NM_203337]	**16,672**
0	Unknown	**16,666**
0	Uncharacterized protein [Source:UniProtKB/TrEMBL;Acc:D4A7T3] [ENSRNOT00000002842]	**16,659**
Wbp11	Rattus norvegicus WW domain binding protein 11 (Wbp11), mRNA [NM_001009661]	**16,657**
Ficd	Rattus norvegicus FIC domain containing (Ficd), mRNA [NM_001010946]	**16,656**
LOC363434	PREDICTED: Rattus norvegicus similar to Discs large homolog 5 (Placenta and prostate DLG) (Discs large protein P-dlg) (LOC363434), partial mRNA [XM_343756]	**16,651**
RGD1562987	Rattus norvegicus similar to cDNA sequence BC031181 (RGD1562987), mRNA [NM_001173472]	**16,649**
Garnl1	Rattus norvegicus GTPase activating Rap/RanGAP domain-like 1 (Garnl1), mRNA [NM_020083]	**16,648**
Nadk	Rattus norvegicus NAD kinase (Nadk), mRNA [NM_001109678]	**16,643**
Znf641	Rattus norvegicus zinc finger protein 641 (Znf641), mRNA [NM_001106792]	**16,641**
Slc6a7	Rattus norvegicus solute carrier family 6 (neurotransmitter transporter, L-proline), member 7 (Slc6a7), mRNA [NM_053996]	**16,639**
Lrrc8b	Rattus norvegicus leucine rich repeat containing 8 family, member B (Lrrc8b), mRNA [NM_001107204]	**16,634**
Plod2	Rattus norvegicus procollagen lysine, 2-oxoglutarate 5-dioxygenase 2 (Plod2), transcript variant 1, mRNA [NM_175869]	**16,633**
Rreb1	Rattus norvegicus ras responsive element binding protein 1 (Rreb1), mRNA [NM_001107348]	**16,632**
Znf668	Rattus norvegicus zinc finger protein 668 (Znf668), mRNA [NM_001107553]	**16,628**
Map6	Rattus norvegicus microtubule-associated protein 6 (Map6), mRNA [NM_017204]	**16,627**
Abcb9	Rattus norvegicus ATP-binding cassette, subfamily B (MDR/TAP), member 9 (Abcb9), mRNA [NM_022238]	**16,625**
0	Unknown	**16,624**

Plp2	Rattus norvegicus proteolipid protein 2 (colonic epithelium-enriched) (Plp2), mRNA [NM_207601]	**16,623**
Lrrc29	Rattus norvegicus leucine rich repeat containing 29 (Lrrc29), mRNA [NM_001195616]	**16,621**
0	PREDICTED: Rattus norvegicus mastermind like 2 (Drosophila) (Maml2), mRNA [XM_001072241]	**16,614**
Stk38	Rattus norvegicus serine/threonine kinase 38 (Stk38), mRNA [NM_001015025]	**16,613**
Col18a1	Rattus norvegicus collagen, type XVIII, alpha 1 (Col18a1), mRNA [NM_053489]	**16,612**
0	Unknown	**16,610**
0	Rattus norvegicus RBSC-skeletrophin/dystrophin-like polypeptide (2210008I1k) mRNA, 2210008I1k-4E2 allele, complete cds. [AY675183]	**16,601**
0	Unknown	**16,597**
LOC100360801	PREDICTED: Rattus norvegicus mCG11385-like (LOC100360801), mRNA [XM_002728402]	**16,594**
Gdf11	PREDICTED: Rattus norvegicus growth differentiation factor 11 (Gdf11), mRNA [XM_343148]	**16,593**
Asf1a	Rattus norvegicus ASF1 anti-silencing function 1 homolog A (S. cerevisiae) (Asf1a), mRNA [NM_001106389]	**16,587**
Kpna1	Rattus norvegicus karyopherin alpha 1/importin alpha 5 (Kpna1) mRNA, complete cds. [AY351984]	**16,584**
0	AGENCOURT_31538552 NIH_MGC_270 Rattus norvegicus cDNA clone IMAGE:7442365 5', mRNA sequence [CV107694]	**16,583**
Tnrc6b	Rattus norvegicus trinucleotide repeat containing 6B (Tnrc6b), mRNA [NM_138845]	**16,583**
Kab	Uncharacterized protein [Source:UniProtKB/TrEMBL;Acc:D3ZET9] [ENSRNOT00000005532]	**16,578**
Sfxn5	Rattus norvegicus sideroflexin 5 (Sfxn5), nuclear gene encoding mitochondrial protein, mRNA [NM_153298]	**16,578**
Phf19	Rattus norvegicus PHD finger protein 19 (Phf19), mRNA [NM_001106570]	**16,578**
Dazap1	Rattus norvegicus DAZ associated protein 1 (Dazap1), mRNA [NM_001025742]	**16,574**
0	Unknown	**16,569**
Orai3	Rattus norvegicus ORAI calcium release-activated calcium modulator 3 (Orai3), mRNA [NM_001014024]	**16,567**
0	Unknown	**16,565**
0	Unknown	**16,564**
Tppp	Rattus norvegicus tubulin polymerization promoting protein (Tppp), mRNA [NM_001108461]	**16,560**
Olr94	Rattus norvegicus olfactory receptor 94 (Olr94), mRNA [NM_001001023]	**16,557**
Atp10d	Uncharacterized protein [Source:UniProtKB/TrEMBL;Acc:D3ZN41] [ENSRNOT00000003146]	**16,557**
RGD1306772	Rattus norvegicus similar to RIKEN cDNA 1110008J03 (RGD1306772), mRNA [NM_001044226]	**16,555**
Zbtb9	Rattus norvegicus zinc finger and BTB domain containing 9 (Zbtb9), mRNA [NM_213564]	**16,553**
Frmd8	Rattus norvegicus FERM domain containing 8 (Frmd8), mRNA [NM_001008348]	**16,551**
Espn	Rattus norvegicus espin (Espn), mRNA [NM_019622]	**16,549**
Anxa2	Rattus norvegicus annexin A2 (Anxa2), mRNA [NM_019905]	**16,548**
Crabp1	Rattus norvegicus cellular retinoic acid binding protein 1 (Crabp1), mRNA [NM_001105716]	**16,548**
B3galt1	Rattus norvegicus UDP-Gal:betaGlcNAc beta 1,3-galactosyltransferase, polypeptide 1 (B3galt1), mRNA [NM_001108954]	**16,547**
Herc3	Rattus norvegicus hect domain and RLD 3 (Herc3), mRNA [NM_001108631]	**16,545**
Syp	Synaptophysin [Source:UniProtKB/Swiss-Prot;Acc:P07825] [ENSRNOT00000013724]	**16,542**

LOC500227	Rattus norvegicus hypothetical gene supported by BC079424 (LOC500227), mRNA [NM_001024330]	**16,540**
0	Productively rearranged V-lambda-2 [Source:UniProtKB/TrEMBL;Acc:A0N4E8] [ENSRNOT00000036565]	**16,539**
Tsr2	Rattus norvegicus TSR2, 20S rRNA accumulation, homolog (S. cerevisiae) (Tsr2), mRNA [NM_001115027]	**16,535**
0	Unknown	**16,533**
0	spectrin beta chain, brain 1 [Source:RefSeq peptide;Acc:NP_001013148] [ENSRNOT00000007441]	**16,531**
Plagl1	Rattus norvegicus pleiomorphic adenoma gene-like 1 (Plagl1), mRNA [NM_012760]	**16,530**
RGD1561931	Uncharacterized protein [Source:UniProtKB/TrEMBL;Acc:D3ZGX1] [ENSRNOT00000035653]	**16,527**
Pla2g3	Rattus norvegicus phospholipase A2, group III (Pla2g3), mRNA [NM_001106015]	**16,524**
LOC681303	PREDICTED: Rattus norvegicus similar to tumor endothelial marker 8 isoform 1 precursor (LOC681303), mRNA [XM_002725159]	**16,519**
0	Uncharacterized protein [Source:UniProtKB/TrEMBL;Acc:D3ZS91] [ENSRNOT00000041424]	**16,518**
Aph1a	Rattus norvegicus anterior pharynx defective 1 homolog A (C. elegans) (Aph1a), mRNA [NM_001014255]	**16,518**
Cacnb2	Rattus norvegicus calcium channel, voltage-dependent, beta 2 subunit (Cacnb2), mRNA [NM_053851]	**16,516**
Pgpep1	Rattus norvegicus pyroglutamyl-peptidase I (Pgpep1), mRNA [NM_201988]	**16,514**
Rtn1	Rattus norvegicus reticulon 1 (Rtn1), mRNA [NM_053865]	**16,512**
Arpc5l	Rattus norvegicus actin related protein 2/3 complex, subunit 5-like, mRNA (cDNA clone IMAGE:7377370), complete cds. [BC098820]	**16,512**
RGD1310862	PREDICTED: Rattus norvegicus similar to adult retina protein (RGD1310862), mRNA [XM_001064739]	**16,507**
Rhob	Rattus norvegicus ras homolog gene family, member B (Rhob), mRNA [NM_022542]	**16,505**
Arfgap2	Rattus norvegicus ADP-ribosylation factor GTPase activating protein 2 (Arfgap2), mRNA [NM_001033707]	**16,503**
0	MUSZFXAA zinc finger protein {Mus musculus} (exp=-1; wgp=0; cg=0), partial (15%) [TC614718]	**16,499**
Pxk	Rattus norvegicus PX domain containing serine/threonine kinase (Pxk), mRNA [NM_182821]	**16,499**
Ache	Rattus norvegicus acetylcholinesterase (Ache), mRNA [NM_172009]	**16,497**
0	Unknown	**16,492**
Tmem192	Rattus norvegicus transmembrane protein 192 (Tmem192), mRNA [NM_001014141]	**16,488**
Tsg101	Rattus norvegicus tumor susceptibility gene 101 (Tsg101), mRNA [NM_181628]	**16,488**
Btbd4	Rattus norvegicus BTB (POZ) domain containing 4 (Btbd4), mRNA [NM_001107808]	**16,488**
Wdr51a	Rattus norvegicus WD repeat domain 51A (Wdr51a), mRNA [NM_001109296]	**16,487**
Calr	Rattus norvegicus calreticulin (Calr), mRNA [NM_022399]	**16,474**
Sgce	Rattus norvegicus sarcoglycan, epsilon (Sgce), mRNA [NM_001002023]	**16,473**
LOC100364372	Uncharacterized protein [Source:UniProtKB/TrEMBL;Acc:D3Z844] [ENSRNOT00000058934]	**16,470**
0	Uncharacterized protein [Source:UniProtKB/TrEMBL;Acc:D3ZFY7] [ENSRNOT00000018667]	**16,468**
Syt4	Rattus norvegicus synaptotagmin IV (Syt4), mRNA [NM_031693]	**16,467**
Txndc12	Rattus norvegicus thioredoxin domain containing 12 (endoplasmic reticulum) (Txndc12), mRNA [NM_001100840]	**16,465**
LOC606294	Rattus norvegicus hypothetical protein LOC606294 (LOC606294), mRNA [NM_001031627]	**16,463**
0	Q2ALT1_9BACI (Q2ALT1) Erythromycin esterase precursor, partial (5%) [TC641238]	**16,462**

0	PREDICTED: Rattus norvegicus similar to SAM and SH3 domain containing protein 1 (Proline-glutamate repeat-containing protein) (RGD1566017), miscRNA [XR_085686]	**16,450**
0	Glyceraldehyde-3-phosphate dehydrogenase [Source:UniProtKB/TrEMBL;Acc:D3ZQ40] [ENSRNOT00000047010]	**16,448**
Dpf3	Rattus norvegicus D4, zinc and double PHD fingers, family 3 (Dpf3), mRNA [NM_001191818]	**16,448**
Scn3a	Rattus norvegicus sodium channel, voltage-gated, type III, alpha (Scn3a), mRNA [NM_013119]	**16,446**
Gabrq	Rattus norvegicus gamma-aminobutyric acid (GABA) receptor, theta (Gabrq), mRNA [NM_031733]	**16,445**
Bace1	Rattus norvegicus beta-site APP cleaving enzyme 1 (Bace1), mRNA [NM_019204]	**16,445**
0	Unknown	**16,436**
Sh3d19	Similar to SH3 domain protein D19 (Predicted)Uncharacterized protein [Source:UniProtKB/TrEMBL;Acc:D3Z8S0] [ENSRNOT00000015802]	**16,434**
0	Unknown	**16,434**
0	Cytochrome c oxidase subunit 1 [Source:UniProtKB/Swiss-Prot;Acc:P05503] [ENSRNOT00000050156]	**16,429**
Cdc42ep2	Rattus norvegicus CDC42 effector protein (Rho GTPase binding) 2 (Cdc42ep2), mRNA [NM_001009689]	**16,425**
Setd7	Rattus norvegicus SET domain containing (lysine methyltransferase) 7 (Setd7), mRNA [NM_001109558]	**16,424**
Zfp275	Rattus norvegicus zinc finger protein 275 (Zfp275), mRNA [NM_001106343]	**16,421**
0	Unknown	**16,421**
Bid	Rattus norvegicus BH3 interacting domain death agonist (Bid), mRNA [NM_022684]	**16,419**
0	expressed sequence C86695 Gene [Source:MGI Symbol;Acc:MGI:2142877] [ENSRNOT00000066415]	**16,413**
Cyb5d1	Rattus norvegicus cytochrome b5 domain containing 1 (Cyb5d1), mRNA [NM_001191890]	**16,409**
Slc17a8	Rattus norvegicus solute carrier family 17 (sodium-dependent inorganic phosphate cotransporter), member 8 (Slc17a8), mRNA [NM_153725]	**16,404**
Pir	Rattus norvegicus pirin (iron-binding nuclear protein) (Pir), mRNA [NM_001009474]	**16,403**
Nap1l1	Rattus norvegicus nucleosome assembly protein 1-like 1 (Nap1l1), mRNA [NM_053561]	**16,402**
0	Q8BUD8_MOUSE (Q8BUD8) 10 days lactation, adult female mammary gland cDNA, RIKEN full-length enriched library, clone:D730020H24 product:ZINC FINGER PROTEIN homolog (Muscleblind-like 2, isoform 1), partial (9%) [TC592330]	16,399
Alkbh6	Rattus norvegicus alkB, alkylation repair homolog 6 (E. coli) (Alkbh6), mRNA [NM_001127450]	**16,397**
Adck4	Rattus norvegicus aarF domain containing kinase 4 (Adck4), mRNA [NM_001012065]	**16,395**
Atp2b3	Rattus norvegicus ATPase, Ca++ transporting, plasma membrane 3 (Atp2b3), mRNA [NM_133288]	**16,393**
0	PREDICTED: Rattus norvegicus similar to glycoprotein, synaptic 2 (RGD1560015), mRNA [XM_001062160]	**16,393**
Fam176a	Rattus norvegicus family with sequence similarity 176, member A (Fam176a), mRNA [NM_001109243]	**16,391**
Tmed5	Rattus norvegicus transmembrane emp24 protein transport domain containing 5 (Tmed5), mRNA [NM_001007619]	**16,391**
Mobkl1b	Rattus norvegicus MOB1, Mps One Binder kinase activator-like 1B (yeast) (Mobkl1b), mRNA [NM_001033891]	**16,386**
Stk32b	Rattus norvegicus serine/threonine kinase 32B (Stk32b), mRNA [NM_001107224]	**16,386**
Olr237	Rattus norvegicus olfactory receptor 237 (Olr237), mRNA [NM_001000210]	**16,382**
Dab1	Rattus norvegicus disabled homolog 1 (Drosophila) (Dab1), mRNA [NM_153621]	**16,380**
Arfip2	Rattus norvegicus ADP-ribosylation factor interacting protein 2 (Arfip2), mRNA [NM_001004222]	**16,378**

RGD1563581	PREDICTED: Rattus norvegicus similar to S100 calcium binding protein A11 (calizzarin) (RGD1563581), mRNA [XM_001064539]	**16,377**
0	Unknown	**16,375**
Hnrnpul2	Uncharacterized protein [Source:UniProtKB/TrEMBL;Acc:D4ABT8] [ENSRNOT00000026416]	**16,375**
0	Unknown	**16,373**
RGD1562846	Rattus norvegicus similar to Docking protein 5 (Downstream of tyrosine kinase 5) (Protein dok-5) (RGD1562846), mRNA [NM_001109344]	**16,370**
Vom1r37	Rattus norvegicus vomeronasal 1 receptor 37 (Vom1r37), mRNA [NM_001008923]	**16,370**
Ppm1l	Rattus norvegicus protein phosphatase 1 (formerly 2C)-like (Ppm1l), mRNA [NM_001107681]	**16,366**
Mtmr4	Rattus norvegicus myotubularin related protein 4 (Mtmr4), mRNA [NM_001105827]	**16,364**
Tollip	Rattus norvegicus toll interacting protein (Tollip), mRNA [NM_001109668]	**16,358**
RGD1561563	PREDICTED: Rattus norvegicus similar to RIKEN cDNA 1700013B16 (RGD1561563), mRNA [XM_001066615]	**16,357**
Rad52	Rattus norvegicus RAD52 homolog (S. cerevisiae) (Rad52), mRNA [NM_001106617]	**16,352**
RGD1564019	PREDICTED: Rattus norvegicus similar to GTPase activating RANGAP domain-like 3 (RGD1564019), mRNA [XM_001078585]	**16,348**
LOC690398	PREDICTED: Rattus norvegicus hypothetical protein LOC690398 (LOC690398), mRNA [XM_001074336]	**16,345**
0	Uncharacterized protein [Source:UniProtKB/TrEMBL;Acc:D3ZME9] [ENSRNOT00000056797]	**16,340**
Timd2	Rattus norvegicus T-cell immunoglobulin and mucin domain containing 2 (Timd2), mRNA [NM_001013855]	**16,337**
Sdc1	Rattus norvegicus syndecan 1 (Sdc1), mRNA [NM_013026]	**16,337**
Rbm4	Rattus norvegicus RNA binding motif protein 4 (Rbm4), mRNA [NM_001170484]	**16,335**
Stxbp5l	Uncharacterized protein [Source:UniProtKB/TrEMBL;Acc:D3ZDG1] [ENSRNOT00000041715]	**16,335**
Terf2	Rattus norvegicus telomeric repeat binding factor 2 (Terf2), mRNA [NM_001108448]	**16,334**
0	AF155065 brain protein {Homo sapiens} (exp=-1; wgp=0; cg=0), partial (12%) [TC626613]	**16,330**
Snx18	Rattus norvegicus sorting nexin 18 (Snx18), mRNA [NM_001107652]	**16,329**
Ergic3	Rattus norvegicus ERGIC and golgi 3 (Ergic3), mRNA [NM_001106533]	**16,328**
0	Unknown	**16,320**
Napepld	Rattus norvegicus N-acyl phosphatidylethanolamine phospholipase D (Napepld), mRNA [NM_199381]	**16,314**
Afap1l2	PREDICTED: Rattus norvegicus actin filament associated protein 1-like 2 (Afap1l2), partial mRNA [XM_001064140]	**16,310**
0	Q3X9T6_METFL (Q3X9T6) CDP-diacylglycerol--serine O-phosphatidyltransferase , partial (6%) [TC628492]	**16,307**
Cux1	Homeobox protein cut-like 1 [Source:UniProtKB/Swiss-Prot;Acc:P53565] [ENSRNOT00000001928]	**16,306**
Larp1	PREDICTED: Rattus norvegicus La ribonucleoprotein domain family, member 1 (Larp1), partial mRNA [XM_001075150]	**16,305**
Itgb3bp	Rattus norvegicus integrin beta 3 binding protein (beta3-endonexin) (Itgb3bp), mRNA [NM_001013213]	**16,301**
Dnajc18	Rattus norvegicus DnaJ (Hsp40) homolog, subfamily C, member 18 (Dnajc18), mRNA [NM_001013887]	**16,300**
Aldh3a1	Rattus norvegicus aldehyde dehydrogenase 3 family, member A1 (Aldh3a1), mRNA [NM_031972]	**16,294**
Lrrc37a	PREDICTED: Rattus norvegicus leucine rich repeat containing 37, member A3 (Lrrc37a3), mRNA [XM_220928]	**16,293**
Plec	Rattus norvegicus plectin (Plec), transcript variant 6, mRNA [NM_022401]	**16,291**
0	Unknown	**16,290**

H1f0	Rattus norvegicus H1 histone family, member 0 (H1f0), mRNA [NM_012578]	**16,286**
Txn2	Rattus norvegicus thioredoxin 2 (Txn2), nuclear gene encoding mitochondrial protein, mRNA [NM_053331]	**16,285**
Nlgn1	Rattus norvegicus neuroligin 1 (Nlgn1), mRNA [NM_053868]	**16,284**
Zfyve20	Rattus norvegicus zinc finger, FYVE domain containing 20 (Zfyve20), mRNA [NM_001107875]	**16,284**
Hipk2	Rattus norvegicus homeodomain interacting protein kinase 2 (Hipk2), mRNA [NM_001108622]	**16,282**
RGD1562342	Rattus norvegicus similar to RIKEN cDNA 1110012D08 (RGD1562342), mRNA [NM_001109281]	**16,276**
Ecop	Rattus norvegicus EGFR-coamplified and overexpressed protein (Ecop), mRNA [NM_001108630]	**16,274**
Snapc5	Rattus norvegicus small nuclear RNA activating complex, polypeptide 5 (Snapc5), mRNA [NM_001109643]	**16,271**
0	Uncharacterized protein [Source:UniProtKB/TrEMBL;Acc:D4A7E5] [ENSRNOT00000023364]	**16,270**
Opn1mw	Rattus norvegicus opsin 1 (cone pigments), medium-wave-sensitive (Opn1mw), mRNA [NM_053548]	**16,267**
0	Unknown	**16,267**
Rnf165	Rattus norvegicus ring finger protein 165 (Rnf165), mRNA [NM_001164505]	**16,266**
Lypd1	Rattus norvegicus Ly6/Plaur domain containing 1 (Lypd1), mRNA [NM_001007727]	**16,263**
St6gal1	Rattus norvegicus ST6 beta-galactosamide alpha-2,6-sialyltranferase 1 (St6gal1), transcript variant 2, mRNA [NM_147205]	**16,262**
Tceb2	Rattus norvegicus transcription elongation factor B (SIII), polypeptide 2 (Tceb2), mRNA [NM_031129]	**16,262**
Prrc1	Rattus norvegicus proline-rich coiled-coil 1 (Prrc1), mRNA [NM_001033887]	**16,260**
Gk	Rattus norvegicus glycerol kinase (Gk), mRNA [NM_024381]	**16,252**
Mapk8	Mitogen-activated protein kinase 8 [Source:UniProtKB/Swiss-Prot;Acc:P49185] [ENSRNOT00000027338]	**16,249**
0	Uncharacterized protein [Source:UniProtKB/TrEMBL;Acc:D3ZMT2] [ENSRNOT00000044934]	**16,248**
Rcc2	PREDICTED: Rattus norvegicus regulator of chromosome condensation 2 (Rcc2), mRNA [XM_216557]	**16,247**
Nat8l	Rattus norvegicus N-acetyltransferase 8-like (Nat8l), mRNA [NM_001191681]	**16,247**
Thoc4	Rattus norvegicus THO complex 4 (Thoc4), mRNA [NM_001109602]	**16,245**
Ankhd1	Rattus norvegicus ankyrin repeat and KH domain containing 1 (Ankhd1), mRNA [NM_001204053]	**16,241**
Med13	Rattus norvegicus mediator complex subunit 13 (Med13), mRNA [NM_001107035]	**16,239**
Slc35e3	Rattus norvegicus solute carrier family 35, member E3 (Slc35e3), mRNA [NM_001134687]	**16,238**
Dapk2	Rattus norvegicus death-associated kinase 2 (Dapk2), mRNA [NM_001013109]	**16,236**
0	AGENCOURT_31541565 NIH_MGC_270 Rattus norvegicus cDNA clone IMAGE:7444018 5', mRNA sequence [CV110972]	**16,235**
0	Unknown	**16,234**
Large	Rattus norvegicus like-glycosyltransferase (Large), mRNA [NM_001108439]	**16,234**
MGC94190	Rattus norvegicus similar to 0610007L01Rik protein (MGC94190), mRNA [NM_001004204]	**16,232**
0	Rattus norvegicus similar to glyceraldehyde-3-phosphate dehydrogenase (phosphorylating) (EC 1.2.1.12) - mouse (LOC302722), mRNA [XM_228999]	**16,231**
Ptpn1	Tyrosine-protein phosphatase non-receptor type 1 [Source:UniProtKB/Swiss-Prot;Acc:P20417] [ENSRNOT00000014309]	**16,229**
Invs	Rattus norvegicus inversin (Invs), mRNA [NM_001107932]	**16,229**

Hras	Rattus norvegicus Harvey rat sarcoma virus oncogene (Hras), transcript variant 2, mRNA [NM_001098241]	**16,227**
Rasgrf2	Rattus norvegicus RAS protein-specific guanine nucleotide-releasing factor 2 (Rasgrf2), mRNA [NM_053721]	**16,226**
Gstt2	Rattus norvegicus glutathione S-transferase, theta 2 (Gstt2), mRNA [NM_012796]	**16,224**
LOC686781	PREDICTED: Rattus norvegicus similar to NFkB interacting protein 1 (LOC686781), mRNA [XM_001075705]	**16,224**
Lrrc4c	Rattus norvegicus leucine rich repeat containing 4C (Lrrc4c), mRNA [NM_001107753]	**16,221**
Fam65b	Rattus norvegicus family with sequence similarity 65, member B (Fam65b), mRNA [NM_001014009]	**16,221**
Prex2	Rattus norvegicus phosphatidylinositol-3,4,5-trisphosphate-dependent Rac exchange factor 2 (Prex2), mRNA [NM_001107899]	**16,219**
Mreg	Rattus norvegicus melanoregulin (Mreg), mRNA [NM_001192002]	**16,218**
LOC690478	Rattus norvegicus similar to keratin associated protein 10-7 (LOC690478), mRNA [NM_001109595]	**16,216**
Lpin2	Rattus norvegicus lipin 2 (Lpin2), mRNA [NM_001108236]	**16,212**
Abi1	Rattus norvegicus abl-interactor 1 (Abi1), mRNA [NM_024397]	**16,210**
Galnt2	Rattus norvegicus UDP-N-acetyl-alpha-D-galactosamine:polypeptide N-acetylgalactosaminyltransferase 2 (GalNAc-T2) (Galnt2), mRNA [NM_001106196]	**16,208**
Rad18	Rattus norvegicus RAD18 homolog (S. cerevisiae) (Rad18), mRNA [NM_001077673]	**16,205**
RGD1306119	Rattus norvegicus similar to transcriptional regulating protein 132 (RGD1306119), mRNA [NM_001169116]	**16,203**
0	Unknown	**16,201**
Suv39h1	Rattus norvegicus suppressor of variegation 3-9 homolog 1 (Drosophila) (Suv39h1), mRNA [NM_001106956]	**16,199**
Zdhhc22	Rattus norvegicus zinc finger, DHHC-type containing 22 (Zdhhc22), mRNA [NM_001039325]	**16,197**
0	Unknown	**16,197**
Gramd1b	Rattus norvegicus GRAM domain containing 1B (Gramd1b), mRNA [NM_001191616]	**16,197**
RGD1307041	Rattus norvegicus similar to hypothetical protein FLJ11305 (RGD1307041), mRNA [NM_001169144]	**16,196**
0	Unknown	**16,190**
Sgcz	Rattus norvegicus sarcoglycan zeta (Sgcz), mRNA [NM_001108875]	**16,188**
0	Ras-related protein Rab-6A [Source:UniProtKB/Swiss-Prot;Acc:Q9WVB1] [ENSRNOT00000025104]	**16,186**
0	Ankyrin repeat and sterile alpha motif domain-containing protein 1B [Source:UniProtKB/Swiss-Prot;Acc:P0C6S7] [ENSRNOT00000038501]	**16,182**
Znf23	Rattus norvegicus zinc finger protein 23 (KOX 16) (Znf23), mRNA [NM_001107428]	**16,179**
Prkcb	Rattus norvegicus protein kinase C, beta (Prkcb), transcript variant 2, mRNA [NM_001172305]	**16,174**
Ubox5	Rattus norvegicus U-box domain containing 5 (Ubox5), mRNA [NM_001033997]	**16,174**
Kcnn3	Rattus norvegicus potassium intermediate/small conductance calcium-activated channel, subfamily N, member 3 (Kcnn3), mRNA [NM_019315]	**16,172**
LOC312502	PREDICTED: Rattus norvegicus similar to RAB11 family interacting protein 5 (class I) isoform 1 (LOC312502), miscRNA [XR_085818]	**16,168**
0	Uncharacterized protein [Source:UniProtKB/TrEMBL;Acc:D3ZP11] [ENSRNOT00000032583]	**16,168**
Irgq	Rattus norvegicus immunity-related GTPase family, Q (Irgq), mRNA [NM_001135742]	**16,164**
0	Q6ZPJ1_MOUSE (Q6ZPJ1) MKIAA1736 protein (Fragment), partial (21%) [TC624205]	**16,164**
Ppm1f	Rattus norvegicus protein phosphatase 1F (PP2C domain containing) (Ppm1f), mRNA [NM_175755]	**16,160**

Stat3	Rattus norvegicus signal transducer and activator of transcription 3 (Stat3), mRNA [NM_012747]	**16,160**
RGD1559513	Uncharacterized protein [Source:UniProtKB/TrEMBL;Acc:D4ADJ9] [ENSRNOT00000033492]	**16,159**
Tcea2	Rattus norvegicus transcription elongation factor A (SII), 2 (Tcea2), mRNA [NM_057098]	**16,157**
Vipr2	Rattus norvegicus vasoactive intestinal peptide receptor 2 (Vipr2), mRNA [NM_017238]	**16,156**
Fnbp4	Rattus norvegicus formin binding protein 4 (Fnbp4), mRNA [NM_001013159]	**16,148**
U2af1l4	Rattus norvegicus U2 small nuclear RNA auxiliary factor 1-like 4 (U2af1l4), mRNA [NM_001008775]	**16,147**
Fam20b	Rattus norvegicus family with sequence similarity 20, member B (Fam20b), mRNA [NM_001107187]	**16,147**
Ezh1	Rattus norvegicus enhancer of zeste homolog 1 (Drosophila) (Ezh1), mRNA [NM_001107051]	**16,145**
Lhb	Rattus norvegicus luteinizing hormone beta (Lhb), transcript variant 1, mRNA [NM_012858]	**16,145**
Ramp2	Rattus norvegicus receptor (G protein-coupled) activity modifying protein 2 (Ramp2), mRNA [NM_031646]	**16,144**
Agpat3	Rattus norvegicus 1-acylglycerol-3-phosphate O-acyltransferase 3 (Agpat3), mRNA [NM_001106378]	**16,142**
Msl1	Rattus norvegicus male-specific lethal 1 homolog (Drosophila) (Msl1), mRNA [NM_001107048]	**16,141**
Hdhd3	Rattus norvegicus haloacid dehalogenase-like hydrolase domain containing 3 (Hdhd3), mRNA [NM_001109511]	**16,139**
0	Uncharacterized protein [Source:UniProtKB/TrEMBL;Acc:D3Z9Y5] [ENSRNOT00000044331]	**16,137**
Rtn2	Rattus norvegicus reticulon 2 (Rtn2), mRNA [NM_201562]	**16,136**
Trim8	Rattus norvegicus tripartite motif-containing 8 (Trim8), mRNA [NM_001128083]	**16,135**
Pde5a	Rattus norvegicus phosphodiesterase 5A, cGMP-specific (Pde5a), mRNA [NM_133584]	**16,135**
Ccnd2	Rattus norvegicus cyclin D2 (Ccnd2), mRNA [NM_022267]	**16,134**
0	Unknown	**16,133**
Wnt7b	Rattus norvegicus wingless-type MMTV integration site family, member 7B (Wnt7b), mRNA [NM_001009695]	**16,132**
Map3k13	Rattus norvegicus mitogen-activated protein kinase kinase kinase 13 (Map3k13), mRNA [NM_001013978]	**16,127**
Pglyrp4	Rattus norvegicus peptidoglycan recognition protein 4 (Pglyrp4), mRNA [NM_001191708]	**16,125**
Grb7	Rattus norvegicus growth factor receptor bound protein 7 (Grb7), mRNA [NM_053403]	**16,121**
0	Uncharacterized protein [Source:UniProtKB/TrEMBL;Acc:D3ZD87] [ENSRNOT00000046906]	**16,121**
0	Unknown	**16,119**
0	Unknown	**16,117**
0	BC052456 Fcho2 {Mus musculus} (exp=-1; wgp=0; cg=0), partial (11%) [TC610872]	**16,114**
Fam155a	PREDICTED: Rattus norvegicus family with sequence similarity 155, member A (Fam155a), mRNA [XM_001076507]	**16,109**
0	Unknown	**16,103**
Cplx3	Rattus norvegicus complexin 3 (Cplx3), mRNA [NM_001109295]	**16,101**
Nfya	Rattus norvegicus nuclear transcription factor-Y alpha (Nfya), mRNA [NM_012865]	**16,100**
Tmem170b	Rattus norvegicus transmembrane protein 170B (Tmem170b), mRNA [NM_001008774]	**16,097**
LOC360479	Rattus norvegicus similar to hypothetical protein (LOC360479), mRNA [NM_001014115]	**16,091**

0	PREDICTED: Rattus norvegicus similar to RNA binding protein gene with multiple splicing, transcript variant 2 (RGD1561067), mRNA [XM_001059526]	**16,090**
0	Myeloid/lymphoid or mixed-lineage leukemia (Mapped)Uncharacterized protein [Source:UniProtKB/TrEMBL;Acc:D3ZLI0] [ENSRNOT00000040881]	**16,090**
Car9	Rattus norvegicus carbonic anhydrase 9 (Car9), mRNA [NM_001107956]	**16,088**
Ctu1	Rattus norvegicus cytosolic thiouridylase subunit 1 homolog (S. pombe) (Ctu1), mRNA [NM_001106251]	**16,084**
Ankrd34b	Rattus norvegicus ankyrin repeat domain 34B (Ankrd34b), mRNA [NM_001109174]	**16,082**
Rgs8	Rattus norvegicus regulator of G-protein signaling 8 (Rgs8), mRNA [NM_019344]	**16,075**
Papolb	Rattus norvegicus poly (A) polymerase beta (testis specific) (Papolb), mRNA [NM_001012020]	**16,074**
Zc3h14	Rattus norvegicus zinc finger CCCH type containing 14 (Zc3h14), transcript variant 1, mRNA [NM_001033951]	**16,073**
Mfhas1	Rattus norvegicus malignant fibrous histiocytoma amplified sequence 1 (Mfhas1), mRNA [NM_001107316]	**16,071**
Ppm1k	Rattus norvegicus protein phosphatase 1K (PP2C domain containing) (Ppm1k), nuclear gene encoding mitochondrial protein, mRNA [NM_001107863]	**16,066**
Sh2d3c	Rattus norvegicus SH2 domain containing 3C (Sh2d3c), mRNA [NM_001108579]	**16,066**
Rbm33	Rattus norvegicus TL0AAA88YH13 mRNA sequence. [FQ211558]	**16,064**
Suv39h1	Rattus norvegicus suppressor of variegation 3-9 homolog 1 (Drosophila) (Suv39h1), mRNA [NM_001106956]	**16,058**
Ttc13	Rattus norvegicus tetratricopeptide repeat domain 13 (Ttc13), mRNA [NM_001136162]	**16,057**
Map2k6	Rattus norvegicus mitogen-activated protein kinase kinase 6 (Map2k6), mRNA [NM_053703]	**16,056**
Thsd1	Rattus norvegicus thrombospondin, type I, domain containing 1 (Thsd1), mRNA [NM_001108878]	**16,055**
Qser1	Rattus norvegicus glutamine and serine rich 1 (Qser1), mRNA [NM_001139493]	**16,055**
0	AGENCOURT_31530923 NIH_MGC_270 Rattus norvegicus cDNA clone IMAGE:7445488 5', mRNA sequence [CV108834]	**16,053**
Pppde1	Rattus norvegicus PPPDE peptidase domain containing 1 (Pppde1), mRNA [NM_001013873]	**16,050**
RGD1305422	PREDICTED: Rattus norvegicus similar to mKIAA0226 protein, transcript variant 2 (RGD1305422), mRNA [XM_002724728]	**16,050**
Adora2a	Rattus norvegicus adenosine A2a receptor (Adora2a), mRNA [NM_053294]	**16,048**
Glt25d2	Uncharacterized protein [Source:UniProtKB/TrEMBL;Acc:D3Z9Z7] [ENSRNOT00000030109]	**16,047**
Olr1400	Rattus norvegicus olfactory receptor 1400 (Olr1400), mRNA [NM_001001096]	**16,047**
Abcc1	Rattus norvegicus ATP-binding cassette, subfamily C (CFTR/MRP), member 1 (Abcc1), mRNA [NM_022281]	**16,045**
0	Unknown	**16,043**
Perp	Rattus norvegicus PERP, TP53 apoptosis effector (Perp), mRNA [NM_001106265]	**16,039**
Parp6	Rattus norvegicus poly (ADP-ribose) polymerase family, member 6 (Parp6), mRNA [NM_001106828]	**16,031**
Rab4b	Rattus norvegicus RAB4B, member RAS oncogene family (Rab4b), mRNA [NM_017355]	**16,030**
0	Unknown	**16,030**
LOC691632	PREDICTED: Rattus norvegicus similar to MIC2 like 1 (LOC691632), mRNA [XM_001079095]	**16,027**
RGD1307439	Rattus norvegicus similar to hypothetical protein MGC35138 (RGD1307439), mRNA [NM_001127540]	**16,025**
Lats2	Rattus norvegicus large tumor suppressor 2 (Lats2), mRNA [NM_001107267]	**16,021**

Setdb1	Uncharacterized protein [Source:UniProtKB/TrEMBL;Acc:D3ZIW7] [ENSRNOT00000028709]	**16,019**
Rap1a	Rattus norvegicus RAP1A, member of RAS oncogene family (Rap1a), mRNA [NM_001005765]	**16,016**
Vom2r18	Rattus norvegicus vomeronasal 2 receptor, 18 (Vom2r18), mRNA [NM_173318]	**16,016**
Pias1	Rattus norvegicus protein inhibitor of activated STAT, 1 (Pias1), mRNA [NM_001106829]	**16,016**
RGD1564018	Uncharacterized protein [Source:UniProtKB/TrEMBL;Acc:D4A328] [ENSRNOT00000011092]	**16,014**
RGD1306151	Rattus norvegicus similar to hypothetical protein DKFZp761D0211 (RGD1306151), mRNA [NM_001108652]	**16,013**
Atp9b	Rattus norvegicus ATPase, class II, type 9B (Atp9b), mRNA [NM_001106130]	**16,013**
Nefh	Rattus norvegicus neurofilament, heavy polypeptide (Nefh), mRNA [NM_012607]	**16,010**
Wnt3a	PREDICTED: Rattus norvegicus wingless-type MMTV integration site family, member 3A (Wnt3a), mRNA [XM_220546]	**16,008**
Impdh1	Rattus norvegicus IMP (inosine monophosphate) dehydrogenase 1 (Impdh1), mRNA [NM_001108619]	**16,006**
Dlg2	Rattus norvegicus discs, large homolog 2 (Drosophila) (Dlg2), mRNA [NM_022282]	**16,001**
Degs1	Rattus norvegicus degenerative spermatocyte homolog 1, lipid desaturase (Drosophila) (Degs1), mRNA [NM_053323]	**15,989**
Smad6	Rattus norvegicus SMAD family member 6 (Smad6), mRNA [NM_001109002]	**15,988**
Kctd15	Rattus norvegicus potassium channel tetramerisation domain containing 15 (Kctd15), mRNA [NM_001109141]	**15,986**
Phf11	Rattus norvegicus PHD finger protein 11 (Phf11), mRNA [NM_001024272]	**15,985**
0	RCG32422, isoform CRA_aUncharacterized protein [Source:UniProtKB/TrEMBL;Acc:D4ABM7] [ENSRNOT00000021070]	**15,984**
Hps5	Rattus norvegicus Hermansky-Pudlak syndrome 5 (Hps5), mRNA [NM_001135612]	**15,981**
Crk	Rattus norvegicus v-crk sarcoma virus CT10 oncogene homolog (avian) (Crk), mRNA [NM_019302]	**15,976**
Kbtbd3	Rattus norvegicus kelch repeat and BTB (POZ) domain containing 3 (Kbtbd3), mRNA [NM_001108121]	**15,975**
Tgs1	Rattus norvegicus trimethylguanosine synthase homolog (S. cerevisiae) (Tgs1), mRNA [NM_001107904]	**15,975**
0	Unknown	**15,972**
RGD1564031	PREDICTED: Rattus norvegicus similar to transcription elongation factor B (SIII), polypeptide 2 (RGD1564031), mRNA [XM_001081217]	**15,970**
Lrrc26	Rattus norvegicus leucine rich repeat containing 26 (Lrrc26), mRNA [NM_001014053]	**15,969**
Adarb2	Rattus norvegicus adenosine deaminase, RNA-specific, B2 (Adarb2), mRNA [NM_133302]	**15,969**
Lpar2	Rattus norvegicus lysophosphatidic acid receptor 2 (Lpar2), mRNA [NM_001109109]	**15,968**
Glud1	Rattus norvegicus glutamate dehydrogenase 1 (Glud1), nuclear gene encoding mitochondrial protein, mRNA [NM_012570]	**15,968**
Cyp4f5	Rattus norvegicus cytochrome P450, family 4, subfamily f, polypeptide 5 (Cyp4f5), mRNA [NM_173124]	**15,968**
0	PREDICTED: Rattus norvegicus similar to KIAA1447 protein (RGD1306500), mRNA [XM_001081783]	**15,966**
Dkkl1	Rattus norvegicus dickkopf-like 1 (Dkkl1), mRNA [NM_001109145]	**15,965**
RGD1559808	Uncharacterized protein [Source:UniProtKB/TrEMBL;Acc:D4AEB9] [ENSRNOT00000045419]	**15,963**
Sec23a	Rattus norvegicus Sec23 homolog A (S. cerevisiae) (Sec23a), mRNA [NM_001105732]	**15,961**
Nxph3	Rattus norvegicus neurexophilin 3 (Nxph3), mRNA [NM_021679]	**15,958**
LOC687090	Rattus norvegicus hypothetical protein LOC687090 (LOC687090), mRNA [NM_001110318]	**15,954**
Slc35b2	Rattus norvegicus solute carrier family 35, member B2 (Slc35b2), mRNA [NM_001037215]	**15,952**

Ptpn9	Rattus norvegicus protein tyrosine phosphatase, non-receptor type 9 (Ptpn9), mRNA [NM_001013040]	**15,950**
Arsg	Rattus norvegicus arylsulfatase G (Arsg), mRNA [NM_001047877]	**15,948**
Usp9x	Rattus norvegicus ubiquitin specific peptidase 9, X-linked (Usp9x), transcript variant 2, mRNA [NM_001135923]	**15,948**
42248	Rattus norvegicus selenoprotein 15 (Sep15), mRNA [NM_133297]	**15,942**
Klhl26	Rattus norvegicus kelch-like 26 (Drosophila) (Klhl26), mRNA [NM_001106075]	**15,941**
Adamts9	Rattus norvegicus a disintegrin-like and metalloprotease (reprolysin type) with thrombospondin type 1 motif, 9 (Adamts9), mRNA [NM_001107877]	**15,927**
Pigy	Rattus norvegicus phosphatidylinositol glycan anchor biosynthesis, class Y (Pigy), nuclear gene encoding mitochondrial protein, mRNA [NM_001024370]	**15,925**
Maoa	Rattus norvegicus monoamine oxidase A (Maoa), nuclear gene encoding mitochondrial protein, mRNA [NM_033653]	**15,924**
Plcb1	Rattus norvegicus phospholipase C, beta 1 (phosphoinositide-specific) (Plcb1), mRNA [NM_001077641]	**15,924**
Cdr2l	Similar to paraneoplastic antigen (Predicted)Uncharacterized protein [Source:UniProtKB/TrEMBL;Acc:D4ABP3] [ENSRNOT00000035865]	**15,924**
Prss32	Rattus norvegicus protease, serine, 32 (Prss32), mRNA [NM_001106983]	**15,921**
Rfk	Rattus norvegicus riboflavin kinase (Rfk), mRNA [NM_001014106]	**15,920**
Olr1341	Rattus norvegicus olfactory receptor 1341 (Olr1341), mRNA [NM_001000482]	**15,912**
Gpr135	Rattus norvegicus G protein-coupled receptor 135 (Gpr135), mRNA [NM_181771]	**15,910**
Zfp422	Rattus norvegicus zinc finger protein 422 (Zfp422), mRNA [NM_001012745]	**15,909**
Map4k1	Rattus norvegicus mitogen activated protein kinase kinase kinase kinase 1 (Map4k1), mRNA [NM_001106243]	**15,908**
0	PREDICTED: Rattus norvegicus similar to olfactory receptor Olr1374 (LOC690140), mRNA [XM_001073432]	**15,904**
Wnt7a	Rattus norvegicus wingless-type MMTV integration site family, member 7A (Wnt7a), mRNA [NM_001100473]	**15,903**
Suv420h2	Rattus norvegicus suppressor of variegation 4-20 homolog 2 (Drosophila) (Suv420h2), mRNA [NM_001107475]	**15,902**
0	Unknown	**15,901**
LOC690311	PREDICTED: Rattus norvegicus similar to Alpha-1-antitrypsin-related protein precursor (LOC690311), mRNA [XM_001074053]	**15,898**
Gimap1	Rattus norvegicus GTPase, IMAP family member 1 (Gimap1), mRNA [NM_001034849]	**15,897**
0	SRY-box containing gene 5 Gene [Source:MGI Symbol;Acc:MGI:98367] [ENSRNOT00000020729]	**15,894**
RGD1562211	Rattus norvegicus similar to ubiquitin specific protease 51 (RGD1562211), mRNA [NM_001108252]	**15,893**
Wdr25l	Rattus norvegicus WD repeat domain 25-like (Wdr25l), mRNA [NM_001135894]	**15,891**
0	Uncharacterized protein [Source:UniProtKB/TrEMBL;Acc:D3ZKG2] [ENSRNOT00000043675]	**15,889**
RGD1562618	Rattus norvegicus similar to RIKEN cDNA 6030419C18 gene (RGD1562618), mRNA [NM_001113791]	**15,886**
Polk	Rattus norvegicus polymerase (DNA directed) kappa (Polk), mRNA [NM_138516]	**15,886**
Zc3h14	Rattus norvegicus zinc finger CCCH type containing 14 (Zc3h14), transcript variant 2, mRNA [NM_138920]	**15,885**
Tmem132e	Rattus norvegicus transmembrane protein 132E (Tmem132e), mRNA [NM_001134410]	**15,884**
0	PREDICTED: Rattus norvegicus similar to DUB-1 (RGD1562061), mRNA [XM_219062]	**15,878**
Rasa3	Rattus norvegicus RAS p21 protein activator 3 (Rasa3), mRNA [NM_031574]	**15,878**
Dtx3	Rattus norvegicus deltex homolog 3 (Drosophila) (Dtx3), mRNA [NM_001191989]	**15,874**

0	Q3QL41_9GAMM (Q3QL41) Phosphoesterase, PA-phosphatase related precursor, partial (13%) [TC587259]	**15,870**
Gna12	Rattus norvegicus guanine nucleotide binding protein (G protein) alpha 12 (Gna12), mRNA [NM_031034]	**15,865**
0	Unknown	**15,865**
Btf3	Rattus norvegicus basic transcription factor 3 (Btf3), mRNA [NM_001008309]	**15,863**
0	Unknown	**15,859**
Tp53inp1	Rattus norvegicus tumor protein p53 inducible nuclear protein 1 (Tp53inp1), mRNA [NM_181084]	**15,854**
Foxl2	Uncharacterized protein [Source:UniProtKB/TrEMBL;Acc:D4A0S1] [ENSRNOT00000023091]	**15,851**
Sox4	PREDICTED: Rattus norvegicus SRY (sex determining region Y)-box 4 (Sox4), mRNA [XM_344594]	**15,847**
Synpo2l	Uncharacterized protein [Source:UniProtKB/TrEMBL;Acc:D3ZZ68] [ENSRNOT00000011956]	**15,846**
0	Unknown	**15,845**
0	Glycerol kinase [Source:UniProtKB/Swiss-Prot;Acc:Q63060] [ENSRNOT00000042729]	**15,836**
Pomt1	Rattus norvegicus protein-O-mannosyltransferase 1 (Pomt1), mRNA [NM_053406]	**15,831**
Cdh2	Rattus norvegicus cadherin 2 (Cdh2), mRNA [NM_031333]	**15,830**
0	PREDICTED: Rattus norvegicus ubiquitination factor E4B (Ube4b), mRNA [XM_233679]	**15,828**
0	Rattus norvegicus similar to gtf2ird2 (LOC360795), mRNA [XM_341065]	**15,825**
Fbxo17	Rattus norvegicus F-box protein 17 (Fbxo17), mRNA [NM_001013064]	**15,825**
RGD1565923	PREDICTED: Rattus norvegicus similar to hypothetical protein FLJ20972 (RGD1565923), mRNA [XM_575900]	**15,825**
Sephs1	Rattus norvegicus selenophosphate synthetase 1 (Sephs1), mRNA [NM_001104630]	**15,824**
S100a11	Rattus norvegicus S100 calcium binding protein A11 (calizzarin) (S100a11), mRNA [NM_001004095]	**15,823**
Olr1335	Rattus norvegicus olfactory receptor 1335 (Olr1335), mRNA [NM_001000479]	**15,820**
0	Unknown	**15,817**
RGD1306526	Rattus norvegicus hypothetical LOC310764 (RGD1306526), mRNA [NM_001107713]	**15,817**
Galnt1	Rattus norvegicus UDP-N-acetyl-alpha-D-galactosamine:polypeptide N-acetylgalactosaminyltransferase 1 (GalNAc-T1) (Galnt1), mRNA [NM_024373]	**15,815**
Prpf18	Rattus norvegicus PRP18 pre-mRNA processing factor 18 homolog (S. cerevisiae) (Prpf18), mRNA [NM_138523]	**15,812**
0	Unknown	**15,812**
Pxk	Rattus norvegicus PX domain containing serine/threonine kinase (Pxk), mRNA [NM_182821]	**15,807**
Zdhhc2	Rattus norvegicus zinc finger, DHHC-type containing 2 (Zdhhc2), mRNA [NM_145096]	**15,803**
Prickle2	Rattus norvegicus prickle homolog 2 (Drosophila) (Prickle2), mRNA [NM_001107876]	**15,801**
Trank1	Rattus norvegicus tetratricopeptide repeat and ankyrin repeat containing 1 (Trank1), mRNA [NM_001191799]	**15,798**
Ank1	Rattus norvegicus ankyrin 1, erythrocytic (Ank1), mRNA [NM_001107322]	**15,795**
Tlx3	PREDICTED: Rattus norvegicus T-cell leukemia, homeobox 3 (Tlx3), mRNA [XM_001064411]	**15,793**
Slc20a2	Rattus norvegicus solute carrier family 20 (phosphate transporter), member 2 (Slc20a2), mRNA [NM_017223]	**15,793**
Srfbp1	Rattus norvegicus serum response factor binding protein 1 (Srfbp1), mRNA [NM_001005536]	**15,792**

Cabp4	Rattus norvegicus calcium binding protein 4 (Cabp4), mRNA [NM_001108926]	**15,791**
Dnal1	Rattus norvegicus dynein, axonemal, light chain 1 (Dnal1), mRNA [NM_001109477]	**15,791**
Olr1513	Rattus norvegicus olfactory receptor 1513 (Olr1513), mRNA [NM_001000035]	**15,790**
Serinc5	Rattus norvegicus serine incorporator 5 (Serinc5), mRNA [NM_133395]	**15,784**
Pcdh19	Rattus norvegicus protocadherin 19 (Pcdh19), mRNA [NM_001169129]	**15,782**
Pitrm1	Rattus norvegicus pitrilysin metallopeptidase 1 (Pitrm1), nuclear gene encoding mitochondrial protein, mRNA [NM_001107363]	**15,777**
Mrps18a	Rattus norvegicus mitochondrial ribosomal protein S18A (Mrps18a), nuclear gene encoding mitochondrial protein, mRNA [NM_198756]	**15,777**
0	PREDICTED: Rattus norvegicus similar to zinc finger protein 617 (LOC684006), mRNA [XM_001068456]	**15,767**
LOC682999	Rattus norvegicus hypothetical protein LOC682999 (LOC682999), mRNA [NM_001127600]	**15,765**
Mrpl37	Rattus norvegicus mitochondrial ribosomal protein L37 (Mrpl37), nuclear gene encoding mitochondrial protein, mRNA [NM_001004235]	**15,762**
Man2b1	Rattus norvegicus mannosidase, alpha, class 2B, member 1 (Man2b1), mRNA [NM_199404]	**15,762**
Optn	Rattus norvegicus optineurin (Optn), mRNA [NM_145081]	**15,762**
Plscr3	Rattus norvegicus phospholipid scramblase 3 (Plscr3), mRNA [NM_001012139]	**15,761**
Mpdu1	Rattus norvegicus mannose-P-dolichol utilization defect 1 (Mpdu1), mRNA [NM_001107011]	**15,760**
0	Q99LS0_MOUSE (Q99LS0) Esophageal cancer related gene 4 protein, partial (13%) [TC630296]	**15,754**
Cd22	Rattus norvegicus CD22 molecule (Cd22), mRNA [NM_001107503]	**15,747**
Txnip	Rattus norvegicus thioredoxin interacting protein (Txnip), mRNA [NM_001008767]	**15,746**
Med14	Rattus norvegicus mediator complex subunit 14 (Med14), mRNA [NM_001191727]	**15,740**
Sec61a2	Rattus norvegicus Sec61 alpha 2 subunit (S. cerevisiae) (Sec61a2), mRNA [NM_001170343]	**15,735**
Ipmk	Rattus norvegicus inositol polyphosphate multikinase (Ipmk), mRNA [NM_134417]	**15,735**
Zfp395	Rattus norvegicus zinc finger protein 395 (Zfp395), mRNA [NM_001107271]	**15,734**
RGD1560175	Putative uncharacterized protein RGD1560175_predictedUncharacterized protein [Source:UniProtKB/TrEMBL;Acc:D4ABR5] [ENSRNOT00000046208]	**15,733**
Dlx3	Rattus norvegicus distal-less homeobox 3 (Dlx3), mRNA [NM_001105832]	**15,729**
Sfrs11	Rattus norvegicus splicing factor, arginine/serine-rich 11 (Sfrs11), mRNA [NM_001035255]	**15,727**
Stk39	Rattus norvegicus serine/threonine kinase 39, STE20/SPS1 homolog (yeast) (Stk39), mRNA [NM_019362]	**15,725**
Ehd4	Rattus norvegicus EH-domain containing 4 (Ehd4), mRNA [NM_139324]	**15,723**
Slc38a1	Rattus norvegicus solute carrier family 38, member 1 (Slc38a1), mRNA [NM_138832]	**15,722**
Nkx6-1	Rattus norvegicus NK6 homeobox 1 (Nkx6-1), mRNA [NM_031737]	**15,722**
RGD1564560	Rattus norvegicus similar to RCK (RGD1564560), mRNA [NM_001109292]	**15,721**
0	Unknown	**15,720**
Rock2	Rattus norvegicus Rho-associated coiled-coil containing protein kinase 2 (Rock2), mRNA [NM_013022]	**15,717**
LOC686809	PREDICTED: Rattus norvegicus similar to protein 7 transactivated by hepatitis B virus X antigen (LOC686809), mRNA [XM_001075804]	**15,715**
0	Unknown	**15,713**

Susd5	Sushi domain containing 5 (Predicted)Uncharacterized protein [Source:UniProtKB/TrEMBL;Acc:D3ZSC1] [ENSRNOT00000028675]	**15,710**
Tmem106b	Rattus norvegicus transmembrane protein 106B (Tmem106b), mRNA [NM_001004267]	**15,709**
LOC681931	PREDICTED: Rattus norvegicus similar to PRP19/PSO4 homolog (LOC681931), mRNA [XM_001059015]	**15,708**
Ifltd1	Rattus norvegicus intermediate filament tail domain containing 1 (Ifltd1), mRNA [NM_001191980]	**15,706**
Arih1	Rattus norvegicus ariadne ubiquitin-conjugating enzyme E2 binding protein homolog 1 (Drosophila) (Arih1), mRNA [NM_001013108]	**15,704**
Rap2ip	Rattus norvegicus Rap2 interacting protein (Rap2ip), mRNA [NM_198758]	**15,703**
Fbxo21	Rattus norvegicus F-box protein 21 (Fbxo21), mRNA [NM_001108338]	**15,697**
Ceacam6	Rattus norvegicus carcinoembryonic antigen-related cell adhesion molecule 6 (Ceacam6), mRNA [NM_001103358]	**15,696**
0	Unknown	**15,690**
Mx1	Rattus norvegicus myxovirus (influenza virus) resistance 1 (Mx1), mRNA [NM_173096]	**15,690**
Ccnd3	Rattus norvegicus cyclin D3 (Ccnd3), mRNA [NM_012766]	**15,689**
Zfp74	PREDICTED: Rattus norvegicus zinc finger protein 569 (Znf569), mRNA [XM_001078778]	**15,687**
0	Unknown	**15,682**
Sry	Rattus norvegicus sex determining region Y (Sry), mRNA [NM_012772]	**15,682**
Ywhag	Rattus norvegicus tyrosine 3-monooxygenase/tryptophan 5-monooxygenase activation protein, gamma polypeptide (Ywhag), mRNA [NM_019376]	**15,681**
Cnnm2	Rattus norvegicus cyclin M2 (Cnnm2), mRNA [NM_001011942]	**15,679**
Nfkb2	Rattus norvegicus nuclear factor of kappa light polypeptide gene enhancer in B-cells 2, p49/p100 (Nfkb2), mRNA [NM_001008349]	**15,679**
Shank2	Rattus norvegicus SH3 and multiple ankyrin repeat domains 2 (Shank2), transcript variant 1, mRNA [NM_201350]	**15,679**
Atoh7	Rattus norvegicus atonal homolog 7 (Drosophila) (Atoh7), mRNA [NM_001170482]	**15,677**
0	Unknown	**15,673**
0	Unknown	**15,669**
Tmem87b	Rattus norvegicus transmembrane protein 87B (Tmem87b), mRNA [NM_001191854]	**15,668**
0	Rattus norvegicus similar to Nedd4 binding protein 1 (LOC365705), mRNA [XM_345172]	**15,668**
0	Unknown	**15,668**
0	Unknown	**15,668**
Spem1	Rattus norvegicus spermatid maturation 1 (Spem1), mRNA [NM_001109653]	**15,667**
Zbtb42	Rattus norvegicus zinc finger and BTB domain containing 42 (Zbtb42), mRNA [NM_001126302]	**15,665**
Spata2	Rattus norvegicus spermatogenesis associated 2 (Spata2), mRNA [NM_053675]	**15,662**
RGD1563866	PREDICTED: Rattus norvegicus RGD1563866, transcript variant 2 (RGD1563866), mRNA [XM_236231]	**15,662**
Znf652	Rattus norvegicus zinc finger protein 652 (Znf652), mRNA [NM_001080207]	**15,660**
Acot7	Rattus norvegicus acyl-CoA thioesterase 7 (Acot7), transcript variant 1, mRNA [NM_001146061]	**15,657**
RGD1306941	Rattus norvegicus similar to CG31122-PA (RGD1306941), mRNA [NM_001108219]	**15,651**
0	Unknown	**15,650**
Slc35c2	Rattus norvegicus solute carrier family 35, member C2 (Slc35c2), mRNA [NM_001107803]	**15,650**

0	Q3FJK6_9BURK (Q3FJK6) Biotin--acetyl-CoA-carboxylase ligase, partial (5%) [TC585832]	**15,650**
LOC680319	Rattus norvegicus hypothetical protein LOC680319 (LOC680319), mRNA [NM_001109401]	**15,646**
RGD1311309	PREDICTED: Rattus norvegicus similar to 2510002A14Rik protein, transcript variant 2 (RGD1311309), mRNA [XM_002724960]	**15,645**
LOC688297	PREDICTED: Rattus norvegicus similar to Retinal rod rhodopsin-sensitive cGMP 3,5-cyclic phosphodiesterase gamma-subunit (GMP-PDE gamma), transcript variant 1 (LOC688297), mRNA [XM_001081804]	**15,644**
Slc5a7	Rattus norvegicus solute carrier family 5 (choline transporter), member 7 (Slc5a7), mRNA [NM_053521]	**15,643**
Phc2	Rattus norvegicus polyhomeotic homolog 2 (Drosophila) (Phc2), mRNA [NM_001013169]	**15,641**
Mvk	Rattus norvegicus mevalonate kinase (Mvk), mRNA [NM_031063]	**15,638**
Fgf5	Rattus norvegicus fibroblast growth factor 5 (Fgf5), mRNA [NM_022211]	**15,637**
Dnajc30	Rattus norvegicus DnaJ (Hsp40) homolog, subfamily C, member 30 (Dnajc30), mRNA [NM_001109024]	**15,636**
Ercc1	Rattus norvegicus excision repair cross-complementing rodent repair deficiency, complementation group 1 (Ercc1), mRNA [NM_001106228]	**15,635**
Acbd7	Rattus norvegicus acyl-Coenzyme A binding domain containing 7 (Acbd7), mRNA [NM_001126079]	**15,632**
Cdh11	Rattus norvegicus cadherin 11 (Cdh11), mRNA [NM_053392]	**15,630**
Cda	Rattus norvegicus cytidine deaminase (Cda), mRNA [NM_001108688]	**15,629**
Gadd45b	Rattus norvegicus growth arrest and DNA-damage-inducible, beta (Gadd45b), mRNA [NM_001008321]	**15,628**
Iqgap1	Rattus norvegicus IQ motif containing GTPase activating protein 1 (Iqgap1), mRNA [NM_001108489]	**15,623**
0	Unknown	**15,622**
0	Similar to HLA-B associated transcript-2 isoform aUncharacterized protein [Source:UniProtKB/TrEMBL;Acc:D3ZUK2] [ENSRNOT00000066368]	**15,620**
Cdk5r1	Rattus norvegicus cyclin-dependent kinase 5, regulatory subunit 1 (Cdk5r1), mRNA [NM_053891]	**15,618**
0	Uncharacterized protein [Source:UniProtKB/TrEMBL;Acc:D4AAV5] [ENSRNOT00000019301]	**15,615**
Pias2	Rattus norvegicus protein inhibitor of activated STAT, 2 (Pias2), mRNA [NM_053337]	**15,614**
Slc1a7	Rattus norvegicus solute carrier family 1 (glutamate transporter), member 7 (Slc1a7), mRNA [NM_001108973]	**15,614**
Cd2ap	Rattus norvegicus CD2-associated protein (Cd2ap), mRNA [NM_181475]	**15,611**
Wnt4	Rattus norvegicus wingless-type MMTV integration site family, member 4 (Wnt4), mRNA [NM_053402]	**15,610**
Armc1	Rattus norvegicus armadillo repeat containing 1 (Armc1), mRNA [NM_001106425]	**15,608**
Rps6ka2	Rattus norvegicus ribosomal protein S6 kinase polypeptide 2 (Rps6ka2), mRNA [NM_057128]	**15,607**
0	Unknown	**15,604**
Hps5	Rattus norvegicus Hermansky-Pudlak syndrome 5 (Hps5), mRNA [NM_001135612]	**15,601**
0	Unknown	**15,597**
0	Rattus norvegicus similar to hypothetical protein (LOC306346), mRNA [XM_224724]	**15,595**
Krt25	Rattus norvegicus keratin 25 (Krt25), mRNA [NM_001008822]	**15,595**
0	Q4RDS8_TETNG (Q4RDS8) Chromosome undetermined SCAF15796, whole genome shotgun sequence. (Fragment), partial (6%) [TC625290]	**15,595**
0	Unknown	**15,594**
RGD1561270	PREDICTED: Rattus norvegicus similar to Zinc finger protein 248 (RGD1561270), mRNA [XM_001057394]	**15,591**

RGD1561672	PREDICTED: Rattus norvegicus similar to novel protein (RGD1561672), mRNA [XM_001065540]	**15,590**
Zdhhc5	Rattus norvegicus zinc finger, DHHC-type containing 5 (Zdhhc5), mRNA [NM_001039338]	**15,587**
Egr2	Rattus norvegicus early growth response 2 (Egr2), mRNA [NM_053633]	**15,586**
Fam171b	Uncharacterized protein [Source:UniProtKB/TrEMBL;Acc:D3ZTG3] [ENSRNOT00000006504]	**15,584**
Khdrbs1	Rattus norvegicus KH domain containing, RNA binding, signal transduction associated 1 (Khdrbs1), mRNA [NM_130405]	**15,577**
Sf3a2	Rattus norvegicus splicing factor 3a, subunit 2 (Sf3a2), mRNA [NM_001011986]	**15,575**
Ap1s1	Rattus norvegicus adaptor-related protein complex 1, sigma 1 subunit, mRNA (cDNA clone MGC:188494 IMAGE:5623505), complete cds. [BC168682]	**15,574**
RGD1562415	Unknown	**15,570**
LOC687707	Rattus norvegicus hypothetical protein LOC687707 (LOC687707), mRNA [NM_001145538]	**15,569**
Rbm33	Rattus norvegicus RNA binding motif protein 33 (Rbm33), mRNA [NM_001191859]	**15,566**
Acbd3	Rattus norvegicus acyl-Coenzyme A binding domain containing 3 (Acbd3), mRNA [NM_182843]	**15,566**
Lancl3	Rattus norvegicus LanC lantibiotic synthetase component C-like 3 (bacterial) (Lancl3), mRNA [NM_001191728]	**15,557**
Dstyk	Rattus norvegicus dual serine/threonine and tyrosine protein kinase (Dstyk), mRNA [NM_199463]	**15,556**
Snx19	Rattus norvegicus sorting nexin 19 (Snx19), mRNA [NM_001108131]	**15,552**
Yipf5	Rattus norvegicus Yip1 domain family, member 5 (Yipf5), mRNA [NM_001014150]	**15,552**
Cc2d1a	Rattus norvegicus coiled-coil and C2 domain containing 1A (Cc2d1a), mRNA [NM_001013869]	**15,544**
Bcas1	Rattus norvegicus breast carcinoma amplified sequence 1 (Bcas1), mRNA [NM_145670]	**15,542**
Vsx1	Rattus norvegicus visual system homeobox 1 (Vsx1), mRNA [NM_001109546]	**15,542**
Chrnb2	Rattus norvegicus cholinergic receptor, nicotinic, beta 2 (neuronal) (Chrnb2), mRNA [NM_019297]	**15,540**
Mbd2	Rattus norvegicus methyl-CpG binding domain protein 2 (Mbd2), mRNA [NM_001115025]	**15,539**
Rgl3	Rattus norvegicus ral guanine nucleotide dissociation stimulator-like 3 (Rgl3), mRNA [NM_001106805]	**15,538**
Arl4c	PREDICTED: Rattus norvegicus ADP-ribosylation factor-like 4C (Arl4c), miscRNA [XR_085918]	**15,538**
0	Unknown	**15,536**
RGD1565096	Rattus norvegicus similar to TSG118.1 (RGD1565096), mRNA [NM_001134611]	**15,534**
Plxnb2	Rattus norvegicus plexin B2 (Plxnb2), mRNA [NM_001108106]	**15,531**
RGD1306148	PREDICTED: Rattus norvegicus similar to KIAA0368 (RGD1306148), miscRNA [XR_085838]	**15,531**
Cdca7l	Rattus norvegicus cell division cycle associated 7 like (Cdca7l), mRNA [NM_001034953]	**15,529**
Pias4	Rattus norvegicus protein inhibitor of activated STAT, 4 (Pias4), mRNA [NM_001100757]	**15,528**
0	Unknown	**15,527**
Clec11a	Rattus norvegicus C-type lectin domain family 11, member a (Clec11a), mRNA [NM_001012459]	**15,526**
Narg2	Rattus norvegicus NMDA receptor regulated 2 (Narg2), mRNA [NM_001191108]	**15,524**
RGD1310358	Rattus norvegicus similar to NNX3 (RGD1310358), mRNA [NM_001107507]	**15,520**
Isoc1	Rattus norvegicus isochorismatase domain containing 1 (Isoc1), mRNA [NM_001014242]	**15,519**

Pax2	Rattus norvegicus paired box 2 (Pax2), mRNA [NM_001106361]	**15,518**
Slc27a4	Rattus norvegicus solute carrier family 27 (fatty acid transporter), member 4 (Slc27a4), mRNA [NM_001100706]	**15,517**
Prss53	Rattus norvegicus protease, serine, 53 (Prss53), mRNA [NM_001109156]	**15,516**
LOC100361585	Uncharacterized protein [Source:UniProtKB/TrEMBL;Acc:D3ZA11] [ENSRNOT00000022866]	**15,515**
0	Unknown	**15,513**
Sncaip	Rattus norvegicus synuclein, alpha interacting protein (Sncaip), mRNA [NM_001107379]	**15,512**
0	Unknown	**15,510**
Arhgap8	Rattus norvegicus Rho GTPase activating protein 8 (Arhgap8), mRNA [NM_001004242]	**15,506**
LOC361128	Rattus norvegicus similar to TR4 orphan receptor associated protein TRA16 (LOC361128), mRNA [NM_001047104]	**15,506**
Chp	Rattus norvegicus calcium binding protein p22 (Chp), mRNA [NM_024139]	**15,504**
0	Unknown	**15,500**
LOC688511	PREDICTED: Rattus norvegicus similar to similar to 60S ribosomal protein L12 (LOC688511), miscRNA [XR_085815]	**15,499**
Dclk3	Rattus norvegicus doublecortin-like kinase 3 (Dclk3), mRNA [NM_001191800]	**15,495**
Cish	Rattus norvegicus cytokine inducible SH2-containing protein (Cish), mRNA [NM_031804]	**15,491**
Rap1gap	PREDICTED: Rattus norvegicus Rap1 GTPase-activating protein (Rap1gap), mRNA [XM_001070178]	**15,490**
Klf11	Rattus norvegicus Kruppel-like factor 11 (Klf11), mRNA [NM_001037354]	**15,482**
Scn8a	Rattus norvegicus sodium channel, voltage gated, type VIII, alpha subunit (Scn8a), mRNA [NM_019266]	**15,482**
0	Unknown	**15,481**
Troap	RCG50654Uncharacterized protein [Source:UniProtKB/TrEMBL;Acc:D3ZLZ9] [ENSRNOT00000043479]	**15,480**
Kalrn	Rattus norvegicus kalirin, RhoGEF kinase (Kalrn), mRNA [NM_032062]	**15,480**
Foxo6	Uncharacterized protein [Source:UniProtKB/TrEMBL;Acc:D3ZV21] [ENSRNOT00000050941]	**15,476**
Ncor2	Rattus norvegicus nuclear receptor co-repressor 2 (Ncor2), mRNA [NM_001108334]	**15,474**
Ccl9	Rattus norvegicus chemokine (C-C motif) ligand 9 (Ccl9), mRNA [NM_001012357]	**15,473**
0	Unknown	**15,471**
0	Unknown	**15,469**
0	Unknown	**15,462**
Camlg	Rattus norvegicus calcium modulating ligand (Camlg), mRNA [NM_053334]	**15,461**
Gabra3	Rattus norvegicus gamma-aminobutyric acid (GABA) A receptor, alpha 3 (Gabra3), mRNA [NM_017069]	**15,460**
0	Unknown	**15,458**
Zc3h18	Rattus norvegicus zinc finger CCCH-type containing 18 (Zc3h18), mRNA [NM_201416]	**15,456**
0	Unknown	**15,456**
0	Unknown	**15,456**
0	Unknown	**15,454**
Dcaf10	Rattus norvegicus DDB1 and CUL4 associated factor 10 (Dcaf10), mRNA [NM_001107935]	**15,450**

Gabbr1	Rattus norvegicus gamma-aminobutyric acid (GABA) B receptor 1 (Gabbr1), mRNA [NM_031028]	**15,449**
Lrfn3	Rattus norvegicus leucine rich repeat and fibronectin type III domain containing 3 (Lrfn3), mRNA [NM_001107502]	**15,449**
LOC498972	PREDICTED: Rattus norvegicus similar to copine II (LOC498972), mRNA [XM_002725371]	**15,446**
Tmed7	Rattus norvegicus transmembrane emp24 protein transport domain containing 7 (Tmed7), mRNA [NM_001105758]	**15,444**
0	Unknown	**15,442**
Ppp1cb	Rattus norvegicus protein phosphatase 1, catalytic subunit, beta isoform (Ppp1cb), mRNA [NM_013065]	**15,441**
Spry1	Rattus norvegicus sprouty homolog 1, antagonist of FGF signaling (Drosophila) (Spry1), mRNA [NM_001106427]	**15,439**
Park2	Rattus norvegicus Parkinson disease (autosomal recessive, juvenile) 2, parkin (Park2), mRNA [NM_020093]	**15,439**
Cd3eap	Rattus norvegicus CD3e molecule, epsilon associated protein (Cd3eap), mRNA [NM_001109416]	**15,437**
Tmem176a	Rattus norvegicus transmembrane protein 176A (Tmem176a), mRNA [NM_001039008]	**15,436**
Dppa3l1	PREDICTED: Rattus norvegicus similar to developmental pluripotency-associated 3 (LOC691014), mRNA [XM_001076514]	**15,434**
0	Unknown	**15,433**
Kbtbd5	Rattus norvegicus kelch repeat and BTB (POZ) domain containing 5 (Kbtbd5), mRNA [NM_001108195]	**15,432**
Dscam	Rattus norvegicus Down syndrome cell adhesion molecule (Dscam), mRNA [NM_133587]	**15,431**
Ap3b1	Rattus norvegicus adaptor-related protein complex 3, beta 1 subunit (Ap3b1), mRNA [NM_001107646]	**15,431**
Ttbk1	Uncharacterized protein [Source:UniProtKB/TrEMBL;Acc:D3ZAU7] [ENSRNOT00000024824]	**15,430**
Kif23	Rattus norvegicus kinesin family member 23 (Kif23), mRNA [NM_001108155]	**15,430**
0	Unknown	**15,429**
Ppcdc	Rattus norvegicus phosphopantothenoylcysteine decarboxylase (Ppcdc), mRNA [NM_001108763]	**15,428**
Xpo5	Rattus norvegicus exportin 5 (Xpo5), mRNA [NM_001108789]	**15,426**
0	Unknown	**15,425**
RGD1311517	Rattus norvegicus similar to RIKEN cDNA 9430015G10 (RGD1311517), mRNA [NM_001014072]	**15,423**
Ptprj	Rattus norvegicus protein tyrosine phosphatase, receptor type, J (Ptprj), mRNA [NM_017269]	**15,423**
Mobkl1a	Rattus norvegicus MOB1, Mps One Binder kinase activator-like 1A (yeast) (Mobkl1a), mRNA [NM_001108357]	**15,422**
LOC688786	Rattus norvegicus similar to CG14483-PA (LOC688786), mRNA [NM_001195245]	**15,420**
Spats2	Rattus norvegicus spermatogenesis associated, serine-rich 2 (Spats2), mRNA [NM_001191614]	**15,419**
0	Uncharacterized protein [Source:UniProtKB/TrEMBL;Acc:D3ZKM8] [ENSRNOT00000030027]	**15,417**
Rassf2	Rattus norvegicus Ras association (RalGDS/AF-6) domain family member 2 (Rassf2), mRNA [NM_001037096]	**15,414**
Agk	Rattus norvegicus acylglycerol kinase (Agk), nuclear gene encoding mitochondrial protein, mRNA [NM_001127497]	**15,412**
Tmem87a	Uncharacterized protein [Source:UniProtKB/TrEMBL;Acc:D3ZWX1] [ENSRNOT00000011280]	**15,411**
Tex261	Rattus norvegicus testis expressed 261 (Tex261), mRNA [NM_001017537]	**15,404**
LOC100363994	PREDICTED: Rattus norvegicus zinc finger protein 455-like (LOC100363994), mRNA [XM_002725924]	**15,403**
Scn4b	Rattus norvegicus sodium channel, voltage-gated, type IV, beta (Scn4b), mRNA [NM_001008880]	**15,401**
Kcnq3	Rattus norvegicus potassium voltage-gated channel, KQT-like subfamily, member 3 (Kcnq3), mRNA [NM_031597]	**15,400**

Rab3a	Rattus norvegicus RAB3A, member RAS oncogene family (Rab3a), mRNA [NM_013018]	**15,399**
RGD1564496	PREDICTED: Rattus norvegicus RGD1564496 (RGD1564496), mRNA [XM_001073145]	**15,397**
Gng11	Rattus norvegicus guanine nucleotide binding protein (G protein), gamma 11 (Gng11), mRNA [NM_022396]	**15,395**
0	Q5XIM8_RAT (Q5XIM8) Lipin 1 (Predicted), partial (4%) [TC586232]	**15,395**
Dnajc4	Rattus norvegicus DnaJ (Hsp40) homolog, subfamily C, member 4 (Dnajc4), mRNA [NM_001013196]	**15,394**
LOC679651	Rattus norvegicus hypothetical protein LOC679651 (LOC679651), mRNA [NM_001195277]	**15,391**
0	Unknown	**15,391**
Adam19	Rattus norvegicus a disintegrin and metallopeptidase domain 19 (meltrin beta) (Adam19), mRNA [NM_001160228]	**15,388**
Srpr	Rattus norvegicus signal recognition particle receptor ('docking protein') (Srpr), mRNA [NM_001034150]	**15,386**
Mlh3	Rattus norvegicus mutL homolog 3 (E. coli) (Mlh3), mRNA [NM_001108043]	**15,385**
Yipf3	Rattus norvegicus Yip1 domain family, member 3 (Yipf3), mRNA [NM_001007801]	**15,385**
LOC684776	PREDICTED: Rattus norvegicus similar to immunoglobin superfamily, member 21 (LOC684776), mRNA [XM_001071901]	**15,380**
Atrnl1	PREDICTED: Rattus norvegicus attractin like 1 (Atrnl1), mRNA [XM_217657]	**15,376**
0	BM387339 UI-R-CN1-cjj-c-03-0-UI.s1 UI-R-CN1 Rattus norvegicus cDNA clone UI-R-CN1-cjj-c-03-0-UI 3', mRNA sequence [BM387339]	**15,376**
Pdzd2	Rattus norvegicus PDZ domain containing 2 (Pdzd2), mRNA [NM_022940]	**15,374**
RGD1564463	Uncharacterized protein [Source:UniProtKB/TrEMBL;Acc:D3ZXB2] [ENSRNOT00000004815]	**15,372**
Pcdh18	Rattus norvegicus protocadherin 18 (Pcdh18), mRNA [NM_001100524]	**15,370**
Tpi1	Rattus norvegicus triosephosphate isomerase 1 (Tpi1), mRNA [NM_022922]	**15,364**
Ptgr2	Rattus norvegicus prostaglandin reductase 2 (Ptgr2), mRNA [NM_001015009]	**15,357**
0	Unknown	**15,352**
0	Unknown	**15,349**
Sftpb	Rattus norvegicus surfactant protein B (Sftpb), mRNA [NM_138842]	**15,349**
Tex11	Uncharacterized protein [Source:UniProtKB/TrEMBL;Acc:D3Z868] [ENSRNOT00000041731]	**15,347**
Prc1	Rattus norvegicus protein regulator of cytokinesis 1 (Prc1), mRNA [NM_001107529]	**15,345**
Duxbl	Uncharacterized protein [Source:UniProtKB/TrEMBL;Acc:D3ZTT0] [ENSRNOT00000052224]	**15,343**
Tmem132b	Rattus norvegicus transmembrane protein 132B (Tmem132b), mRNA [NM_001134536]	**15,342**
LOC678704	Rattus norvegicus hypothetical protein LOC678704 (LOC678704), mRNA [NM_001109369]	**15,340**
Olr3	Rattus norvegicus olfactory receptor 3 (Olr3), mRNA [NM_001000110]	**15,337**
Man2a1	Rattus norvegicus mannosidase, alpha, class 2A, member 1 (Man2a1), mRNA [NM_012979]	**15,332**
0	RCG49325, isoform CRA_bUncharacterized protein [Source:UniProtKB/TrEMBL;Acc:D3ZR95] [ENSRNOT00000025495]	**15,332**
Sytl5	Rattus norvegicus synaptotagmin-like 5 (Sytl5), mRNA [NM_178333]	**15,332**
0	Solute carrier family 39 (Zinc transporter), member 10 (Predicted), isoform CRA_aUncharacterized protein [Source:UniProtKB/TrEMBL;Acc:D4A517] [ENSRNOT00000016262]	**15,331**
Vapa	Rattus norvegicus VAMP (vesicle-associated membrane protein)-associated protein A (Vapa), mRNA [NM_031631]	**15,330**

Foxi3	Rattus norvegicus forkhead box I3 (Foxi3), mRNA [NM_001109349]	**15,329**
Kpna4	Rattus norvegicus karyopherin alpha 4 (importin alpha 3) (Kpna4), mRNA [NM_001014793]	**15,325**
Cmpk1	Rattus norvegicus cytidine monophosphate (UMP-CMP) kinase 1 (Cmpk1), mRNA [NM_001025655]	**15,320**
Mb	Rattus norvegicus myoglobin (Mb), mRNA [NM_021588]	**15,320**
Naaladl1	Rattus norvegicus N-acetylated alpha-linked acidic dipeptidase-like 1 (Naaladl1), mRNA [NM_031759]	**15,315**
Hus1	Rattus norvegicus HUS1 checkpoint homolog (S. pombe) (Hus1), mRNA [NM_001109092]	**15,313**
0	Unknown	**15,313**
Nr1h4	Rattus norvegicus nuclear receptor subfamily 1, group H, member 4 (Nr1h4), mRNA [NM_021745]	**15,312**
Slc28a1	Rattus norvegicus solute carrier family 28 (sodium-coupled nucleoside transporter), member 1 (Slc28a1), mRNA [NM_053863]	**15,310**
Mta1	Rattus norvegicus metastasis associated 1 (Mta1), mRNA [NM_022588]	**15,307**
Luzp1	Rattus norvegicus leucine zipper protein 1 (Luzp1), mRNA [NM_030830]	**15,306**
Tbc1d10a	Rattus norvegicus TBC1 domain family, member 10a (Tbc1d10a), mRNA [NM_001015022]	**15,306**
Clcf1	Rattus norvegicus cardiotrophin-like cytokine factor 1 (Clcf1), mRNA [NM_207615]	**15,296**
Ttll3	Rattus norvegicus tubulin tyrosine ligase-like family, member 3 (Ttll3), mRNA [NM_001108640]	**15,296**
0	Myeloid/lymphoid or mixed-lineage leukemia (Mapped)Uncharacterized protein [Source:UniProtKB/TrEMBL;Acc:D3ZLI0] [ENSRNOT00000020573]	**15,295**
Ska2	Rattus norvegicus spindle and kinetochore associated complex subunit 2 (Ska2), mRNA [NM_001009624]	**15,295**
Rbm16	Rattus norvegicus RNA binding motif protein 16 (Rbm16), mRNA [NM_139094]	**15,293**
Dusp5	Rattus norvegicus dual specificity phosphatase 5 (Dusp5), mRNA [NM_133578]	**15,291**
LOC688289	EGF-containing fibulin-like extracellular matrix protein 2 [Source:RefSeq peptide;Acc:NP_001005907] [ENSRNOT00000027971]	**15,280**
Ube2z	Rattus norvegicus ubiquitin-conjugating enzyme E2Z (Ube2z), mRNA [NM_001037643]	**15,279**
Fam172a	Rattus norvegicus family with sequence similarity 172, member A (Fam172a), mRNA [NM_001106401]	**15,279**
Dynll1	Rattus norvegicus dynein light chain LC8-type 1 (Dynll1), mRNA [NM_053319]	**15,279**
Nek6	Rattus norvegicus NIMA (never in mitosis gene a)-related kinase 6 (Nek6), mRNA [NM_182953]	**15,273**
Sphk2	Rattus norvegicus sphingosine kinase 2 (Sphk2), mRNA [NM_001012066]	**15,271**
Qser1	Rattus norvegicus glutamine and serine rich 1 (Qser1), mRNA [NM_001139493]	**15,271**
0	Unknown	**15,270**
0	Unknown	**15,270**
Vamp8	Rattus norvegicus vesicle-associated membrane protein 8 (Vamp8), mRNA [NM_031827]	**15,268**
Igsf21	Uncharacterized protein [Source:UniProtKB/TrEMBL;Acc:D3ZH04] [ENSRNOT00000051793]	**15,265**
Acy3	Rattus norvegicus aspartoacylase (aminocyclase) 3 (Acy3), mRNA [NM_001009603]	**15,264**
0	Uncharacterized protein [Source:UniProtKB/TrEMBL;Acc:D4ADU3] [ENSRNOT00000014310]	**15,263**
Ncam2	Rattus norvegicus neural cell adhesion molecule 2 (Ncam2), mRNA [NM_203409]	**15,262**
Abca2	Rattus norvegicus ATP-binding cassette, subfamily A (ABC1), member 2 (Abca2), mRNA [NM_024396]	**15,256**
Cdc25b	Rattus norvegicus cell division cycle 25 homolog B (S. pombe) (Cdc25b), mRNA [NM_133572]	**15,255**

0	Unknown	**15,255**
0	Unknown	**15,255**
Gpr56	Rattus norvegicus G protein-coupled receptor 56 (Gpr56), mRNA [NM_152242]	**15,251**
Lin37	Rattus norvegicus lin-37 homolog (C. elegans) (Lin37), mRNA [NM_001106245]	**15,251**
Cep120	Rattus norvegicus centrosomal protein 120 (Cep120), mRNA [NM_001191697]	**15,250**
RGD1305455	Rattus norvegicus similar to hypothetical protein FLJ10925 (RGD1305455), mRNA [NM_001024969]	**15,250**
Hdlbp	Rattus norvegicus high density lipoprotein binding protein (Hdlbp), mRNA [NM_172039]	**15,250**
0	Uncharacterized protein [Source:UniProtKB/TrEMBL;Acc:D3ZA84] [ENSRNOT00000031988]	**15,245**
0	BC062892 brother of CDO {Mus musculus} (exp=-1; wgp=0; cg=0), partial (11%) [TC613432]	**15,243**
Hs6st2	Rattus norvegicus heparan sulfate 6-O-sulfotransferase 2 (Hs6st2), mRNA [NM_001191726]	**15,241**
Aco1	Rattus norvegicus aconitase 1, soluble (Aco1), mRNA [NM_017321]	**15,240**
Znf496	PREDICTED: Rattus norvegicus zinc finger protein 496 (Znf496), mRNA [XM_220512]	**15,239**
Rab3c	Rattus norvegicus RAB3C, member RAS oncogene family (Rab3c), mRNA [NM_133536]	**15,239**
Rit2	Rattus norvegicus Ras-like without CAAX 2 (Rit2), mRNA [NM_001013060]	**15,235**
Grinl1a	Rattus norvegicus glutamate receptor, ionotropic, N-methyl D-aspartate-like 1A (Grinl1a), mRNA [NM_183402]	**15,234**
Dfna5	Rattus norvegicus deafness, autosomal dominant 5 (human) (Dfna5), mRNA [NM_001191749]	**15,234**
Pgap1	Rattus norvegicus post-GPI attachment to proteins 1 (Pgap1), mRNA [NM_201990]	**15,231**
Nedd1	Rattus norvegicus neural precursor cell expressed, developmentally down-regulated 1 (Nedd1), mRNA [NM_001106779]	**15,230**
36951	Rattus norvegicus membrane-associated ring finger (C3HC4) 1 (March1), mRNA [NM_001135838]	**15,229**
Fundc1	Rattus norvegicus FUN14 domain containing 1 (Fundc1), mRNA [NM_001025027]	**15,229**
Anks1a	Rattus norvegicus ankyrin repeat and sterile alpha motif domain containing 1A (Anks1a), mRNA [NM_001107613]	**15,225**
Ap2m1	Rattus norvegicus adaptor-related protein complex 2, mu 1 subunit (Ap2m1), mRNA [NM_053837]	**15,224**
Lrrc28	Rattus norvegicus leucine rich repeat containing 28 (Lrrc28), mRNA [NM_001108486]	**15,222**
0	Unknown	**15,221**
LOC498972	PREDICTED: Rattus norvegicus similar to copine II (LOC498972), partial mRNA [XM_574259]	**15,221**
Sorcs3	Rattus norvegicus sortilin-related VPS10 domain containing receptor 3 (Sorcs3), mRNA [NM_001106367]	**15,217**
0	Unknown	**15,216**
0	Unknown	**15,213**
Mustn1	Rattus norvegicus musculoskeletal, embryonic nuclear protein 1 (Mustn1), mRNA [NM_181368]	**15,213**
Gtpbp1	Rattus norvegicus GTP binding protein 1 (Gtpbp1), mRNA [NM_001199315]	**15,213**
Dzip3	Similar to Ubiquitin ligase protein DZIP3 (DAZ-interacting protein 3 homolog) (Predicted)Uncharacterized protein [Source:UniProtKB/TrEMBL;Acc:D3ZY61] [ENSRNOT00000002678]	**15,213**
Pomc	Rattus norvegicus proopiomelanocortin (Pomc), mRNA [NM_139326]	**15,213**
0	Unknown	**15,212**

Frmd3	Rattus norvegicus FERM domain containing 3 (Frmd3), mRNA [NM_001106662]	**15,210**
Sox2	Rattus norvegicus SRY (sex determining region Y)-box 2 (Sox2), mRNA [NM_001109181]	**15,209**
0	Unknown	**15,207**
Cdx1	PREDICTED: Rattus norvegicus caudal type homeo box 1 (Cdx1), mRNA [XM_344691]	**15,206**
Traf6	Rattus norvegicus Tnf receptor-associated factor 6 (Traf6), mRNA [NM_001107754]	**15,204**
Grinl1a	Rattus norvegicus glutamate receptor, ionotropic, N-methyl D-aspartate-like 1A (Grinl1a), mRNA [NM_183402]	**15,202**
Cox15	Rattus norvegicus COX15 homolog, cytochrome c oxidase assembly protein (yeast) (Cox15), nuclear gene encoding mitochondrial protein, mRNA [NM_001033699]	**15,194**
Morn1	Rattus norvegicus MORN repeat containing 1 (Morn1), mRNA [NM_001005544]	**15,194**
P2rx6	Rattus norvegicus purinergic receptor P2X, ligand-gated ion channel, 6 (P2rx6), mRNA [NM_012721]	**15,194**
0	UI-R-Y0-acg-g-03-0-UI.s1 UI-R-Y0 Rattus norvegicus cDNA clone UI-R-Y0-acg-g-03-0-UI 3', mRNA sequence [AI716836]	**15,194**
Rnf2	Rattus norvegicus ring finger protein 2 (Rnf2), mRNA [NM_001025667]	**15,192**
Qk	Rattus norvegicus quaking (Qk), mRNA [NM_001115021]	**15,191**
Rgl1	Rattus norvegicus ral guanine nucleotide dissociation stimulator,-like 1 (Rgl1), mRNA [NM_001105957]	**15,188**
0	Unknown	**15,186**
Dpp10	Rattus norvegicus dipeptidylpeptidase 10 (Dpp10), mRNA [NM_001012205]	**15,186**
Cds1	Rattus norvegicus CDP-diacylglycerol synthase 1 (Cds1), mRNA [NM_031242]	**15,183**
Agrn	Rattus norvegicus agrin (Agrn), mRNA [NM_175754]	**15,182**
0	Unknown	**15,182**
Tubg2	Rattus norvegicus tubulin, gamma 2 (Tubg2), mRNA [NM_001191075]	**15,181**
RGD1562963	Rattus norvegicus similar to chromosome 6 open reading frame 52 (RGD1562963), mRNA [NM_001145021]	**15,181**
Ankrd46	Rattus norvegicus ankyrin repeat domain 46 (Ankrd46), mRNA [NM_001013948]	**15,180**
Paqr3	Rattus norvegicus progestin and adipoQ receptor family member III (Paqr3), mRNA [NM_001012033]	**15,176**
Prkar2a	Rattus norvegicus protein kinase, cAMP dependent regulatory, type II alpha (Prkar2a), mRNA [NM_019264]	**15,170**
0	Unknown	**15,167**
M6prbp1	PREDICTED: Rattus norvegicus mannose-6-phosphate receptor binding protein 1 (M6prbp1), mRNA [XM_236783]	**15,167**
0	Unknown	**15,164**
Nol9	Nol9 protein [Source:UniProtKB/TrEMBL;Acc:Q4G021] [ENSRNOT00000013535]	**15,164**
LOC680426	PREDICTED: Rattus norvegicus similar to CG7220-PA, isoform A (LOC680426), mRNA [XM_001057119]	**15,163**
Adam23	Rattus norvegicus ADAM metallopeptidase domain 23 (Adam23), mRNA [NM_001029899]	**15,162**
0	Unknown	**15,161**
RGD1562674	PREDICTED: Rattus norvegicus similar to kinase suppressor of ras 2 (RGD1562674), mRNA [XM_222212]	**15,161**
Chd4	PREDICTED: Rattus norvegicus chromodomain helicase DNA binding protein 4 (Chd4), mRNA [XM_001063352]	**15,160**
Alkbh7	Rattus norvegicus alkB, alkylation repair homolog 7 (E. coli) (Alkbh7), mRNA [NM_001109384]	**15,159**

Snd1	Rattus norvegicus staphylococcal nuclease and tudor domain containing 1 (Snd1), mRNA [NM_022694]	**15,158**
Gpr173	Rattus norvegicus G-protein coupled receptor 173 (Gpr173), mRNA [NM_022255]	**15,152**
Ppia	Rattus norvegicus peptidylprolyl isomerase A (cyclophilin A) (Ppia), mRNA [NM_017101]	**15,152**
Slc12a3	Rattus norvegicus solute carrier family 12 (sodium/chloride transporters), member 3 (Slc12a3), mRNA [NM_019345]	**15,151**
0	Unknown	**15,150**
Vwce	Uncharacterized protein [Source:UniProtKB/TrEMBL;Acc:D3ZSS3] [ENSRNOT00000028268]	**15,142**
LOC303566	Rattus norvegicus E2F1-inducible gene (LOC303566), mRNA [NM_001017988]	**15,142**
Tmed2	Rattus norvegicus transmembrane emp24 domain trafficking protein 2 (Tmed2), mRNA [NM_031722]	**15,141**
Sf3b4	Rattus norvegicus splicing factor 3b, subunit 4 (Sf3b4), mRNA [NM_001011951]	**15,140**
RGD1561738	PREDICTED: Rattus norvegicus similar to interferon induced transmembrane protein 2 (1-8D) (RGD1561738), miscRNA [XR_008822]	**15,139**
0	Unknown	**15,138**
Dnajc8	Rattus norvegicus DnaJ (Hsp40) homolog, subfamily C, member 8 (Dnajc8), mRNA [NM_001013168]	**15,138**
Lef1	Lymphoid enhancer-binding factor 1 [Source:UniProtKB/Swiss-Prot;Acc:Q9QXN1] [ENSRNOT00000013694]	**15,138**
Hs1bp3	Uncharacterized protein [Source:UniProtKB/TrEMBL;Acc:D4A6D9] [ENSRNOT00000007991]	**15,134**
Lrrc28	Rattus norvegicus leucine rich repeat containing 28 (Lrrc28), mRNA [NM_001108486]	**15,133**
Cyp2t1	Rattus norvegicus cytochrome P450, family 2, subfamily t, polypeptide 1 (Cyp2t1), mRNA [NM_134369]	**15,131**
Hist1h4b	Rattus norvegicus histone cluster 1, H4b (Hist1h4b), mRNA [NM_022686]	**15,130**
0	Unknown	**15,129**
Mkks	Rattus norvegicus McKusick-Kaufman syndrome (Mkks), mRNA [NM_001008353]	**15,125**
Tcf4	Rattus norvegicus transcription factor 4 (Tcf4), mRNA [NM_053369]	**15,122**
Yif1	Rattus norvegicus Yip1 interacting factor homolog (S. cerevisiae) (Yif1), mRNA [NM_172017]	**15,120**
Taf2	Rattus norvegicus TAF2 RNA polymerase II, TATA box binding protein (TBP)-associated factor (Taf2), mRNA [NM_133319]	**15,119**
0	Uncharacterized protein [Source:UniProtKB/TrEMBL;Acc:D3ZK69] [ENSRNOT00000039927]	**15,119**
Pbld	Rattus norvegicus phenazine biosynthesis-like protein domain containing (Pbld), mRNA [NM_138530]	**15,118**
0	Uncharacterized protein [Source:UniProtKB/TrEMBL;Acc:D3Z8X2] [ENSRNOT00000066542]	**15,113**
Pecam1	Rattus norvegicus platelet/endothelial cell adhesion molecule 1 (Pecam1), mRNA [NM_031591]	**15,112**
Seli	Rattus norvegicus selenoprotein I (Seli), mRNA [NM_001134754]	**15,111**
Ubxn2b	Rattus norvegicus UBX domain protein 2B (Ubxn2b), mRNA [NM_001107905]	**15,108**
Ghrl	Rattus norvegicus ghrelin/obestatin prepropeptide (Ghrl), mRNA [NM_021669]	**15,108**
Cnot6l	Rattus norvegicus CCR4-NOT transcription complex, subunit 6-like (Cnot6l), mRNA [NM_001108355]	**15,102**
RGD1559908	PREDICTED: Rattus norvegicus similar to hypothetical protein (RGD1559908), miscRNA [XR_086058]	**15,099**
Scpep1	Rattus norvegicus serine carboxypeptidase 1 (Scpep1), mRNA [NM_133383]	**15,099**
0	Unknown	**15,090**
0	Unknown	**15,090**

Numa1	Numa1 protein [Source:UniProtKB/TrEMBL;Acc:Q4G051] [ENSRNOT00000000474]	**15,088**
0	RCG33933Uncharacterized protein [Source:UniProtKB/TrEMBL;Acc:D3ZIT0] [ENSRNOT00000013706]	**15,086**
0	PREDICTED: Rattus norvegicus similar to MAP/microtubule affinity-regulating kinase 4 (MAP/microtubule affinity-regulating kinase like 1) (RGD1561231), partial mRNA [XM_001081347]	**15,086**
Tmem18	Rattus norvegicus transmembrane protein 18 (Tmem18), mRNA [NM_001007748]	**15,082**
Emx1	PREDICTED: Rattus norvegicus empty spiracles homeobox 1 (Emx1), mRNA [XM_001073769]	**15,079**
Rnf121	ring finger protein 121 [Source:RefSeq peptide;Acc:NP_001101010] [ENSRNOT00000027321]	**15,078**
Insr	Rattus norvegicus insulin receptor (Insr), mRNA [NM_017071]	**15,078**
0	Unknown	**15,075**
LOC365476	PREDICTED: Rattus norvegicus similar to chromosome 10 open reading frame 79 (LOC365476), mRNA [XM_345041]	**15,072**
Cdkn1b	Rattus norvegicus cyclin-dependent kinase inhibitor 1B (Cdkn1b), mRNA [NM_031762]	**15,065**
0	Unknown	**15,063**
0	Rattus norvegicus similar to 60S ribosomal protein L23a (LOC365309), mRNA [XM_344922]	**15,063**
Lrig2	Rattus norvegicus leucine-rich repeats and immunoglobulin-like domains 2 (Lrig2), mRNA [NM_001107710]	**15,055**
Gnrhr	Rattus norvegicus gonadotropin releasing hormone receptor (Gnrhr), mRNA [NM_031038]	**15,054**
Pvalb	Rattus norvegicus parvalbumin (Pvalb), mRNA [NM_022499]	**15,053**
Gucy1a3	Guanylate cyclase soluble subunit alpha-3 [Source:UniProtKB/Swiss-Prot;Acc:P19686] [ENSRNOT00000017190]	**15,053**
Syt1	Rattus norvegicus synaptotagmin I (Syt1), mRNA [NM_001033680]	**15,052**
Stmn1	Rattus norvegicus stathmin 1 (Stmn1), mRNA [NM_017166]	**15,051**
Agfg2	Rattus norvegicus ArfGAP with FG repeats 2 (Agfg2), mRNA [NM_001107131]	**15,051**
Tmod4	Rattus norvegicus tropomodulin 4 (Tmod4), mRNA [NM_001106449]	**15,050**
Ttc23	Rattus norvegicus tetratricopeptide repeat domain 23 (Ttc23), mRNA [NM_001025681]	**15,043**
Ubp1	Rattus norvegicus upstream binding protein 1 (LBP-1a) (Ubp1), mRNA [NM_001191819]	**15,042**
0	R-spondin-2 [Source:RefSeq peptide;Acc:NP_001124047] [ENSRNOT00000007526]	**15,042**
0	Unknown	**15,037**
Mpp3	PREDICTED: Rattus norvegicus membrane protein, palmitoylated 3 (MAGUK p55 subfamily member 3) (Mpp3), mRNA [XM_340911]	**15,037**
Rlbp1l2	Rattus norvegicus retinaldehyde binding protein 1-like 2 (Rlbp1l2), mRNA [NM_001108459]	**15,034**
0	Unknown	**15,034**
0	Unknown	**15,033**
Mapkap1	Rattus norvegicus mitogen-activated protein kinase associated protein 1 (Mapkap1), mRNA [NM_001011964]	**15,032**
Fam194a	Rattus norvegicus family with sequence similarity 194, member A (Fam194a), mRNA [NM_001024300]	**15,032**
Col3a1	Rattus norvegicus collagen, type III, alpha 1 (Col3a1), mRNA [NM_032085]	**15,029**
Tshz3	Rattus norvegicus teashirt zinc finger homeobox 3 (Tshz3), mRNA [NM_001107506]	**15,027**
Il1rap	Rattus norvegicus interleukin 1 receptor accessory protein (Il1rap), transcript variant 2, mRNA [NM_001167840]	**15,022**

0	Ubiquitin carboxyl-terminal hydrolase [Source:UniProtKB/TrEMBL;Acc:D3ZGB0] [ENSRNOT00000003800]	**15,018**
Fam13b1	Rattus norvegicus family with sequence similarity 13, member B1 (Fam13b1), mRNA [NM_001106158]	**15,018**
0	HECT domain containing 2 Gene [Source:MGI Symbol;Acc:MGI:2442663] [ENSRNOT00000024963]	**15,017**
Ttll13	Rattus norvegicus tubulin tyrosine ligase-like family, member 13 (Ttll13), mRNA [NM_001134962]	**15,006**
LOC688786	Rattus norvegicus similar to CG14483-PA (LOC688786), mRNA [NM_001195245]	**15,006**
0	Unknown	**15,005**
0	Unknown	**15,005**
0	Unknown	**15,004**
Vom1r54	Rattus norvegicus vomeronasal 1 receptor 54 (Vom1r54), mRNA [NM_001008963]	**15,002**
Osbpl7	Rattus norvegicus oxysterol binding protein-like 7 (Osbpl7), mRNA [NM_001107044]	**15,000**
Samd5	Rattus norvegicus sterile alpha motif domain containing 5 (Samd5), mRNA [NM_001108901]	**14,996**
0	Unknown	**14,993**
LOC679199	PREDICTED: Rattus norvegicus similar to LanC lantibiotic synthetase component C-like 3 (LOC679199), mRNA [XM_001055199]	**14,991**
0	Unknown	**14,986**
Glb1l	Rattus norvegicus galactosidase, beta 1-like (Glb1l), mRNA [NM_001127529]	**14,984**
nod3l	Rattus norvegicus NOD3-like protein (nod3l), mRNA [NM_001024360]	**14,982**
Znf467	Rattus norvegicus zinc finger protein 467 (Znf467), mRNA [NM_001024327]	**14,982**
Brd3	Rattus norvegicus bromodomain containing 3 (Brd3), mRNA [NM_001108575]	**14,976**
Epn2	Rattus norvegicus epsin 2 (Epn2), transcript variant 2, mRNA [NM_001033914]	**14,974**
Olr1431	Rattus norvegicus olfactory receptor 1431 (Olr1431), mRNA [NM_001000778]	**14,974**
RGD1305689	Rattus norvegicus similar to DNA segment, Chr 14, ERATO Doi 449, expressed (RGD1305689), mRNA [NM_001008297]	**14,973**
Plxnd1	Rattus norvegicus plexin D1 (Plxnd1), mRNA [NM_001107881]	**14,973**
0	Rattus norvegicus similar to 60S ribosomal protein L10 (QM protein homolog) (LOC289691), mRNA [XM_223490]	**14,973**
0	Uncharacterized protein [Source:UniProtKB/TrEMBL;Acc:D3ZSU2] [ENSRNOT00000023777]	**14,971**
Stard13	Rattus norvegicus StAR-related lipid transfer (START) domain containing 13 (Stard13), mRNA [NM_001109060]	**14,971**
Zwilch	PREDICTED: Rattus norvegicus Zwilch, kinetochore associated, homolog (Drosophila) (Zwilch), mRNA [XM_001078534]	**14,970**
Bpgm	Rattus norvegicus 2,3-bisphosphoglycerate mutase (Bpgm), mRNA [NM_199382]	**14,969**
Kdm5b	Rattus norvegicus lysine (K)-specific demethylase 5B (Kdm5b), mRNA [NM_001107177]	**14,967**
0	Unknown	**14,966**
Sparc	Rattus norvegicus secreted protein, acidic, cysteine-rich (osteonectin) (Sparc), mRNA [NM_012656]	**14,963**
Pcdhb10	Rattus norvegicus protocadherin beta 10 (Pcdhb10), mRNA [NM_001114603]	**14,963**
0	Rattus norvegicus similar to glyceraldehyde-3-phosphate dehydrogenase (phosphorylating) (EC 1.2.1.12) - mouse (LOC288024), mRNA [XM_221353]	**14,962**
0	neurobeachin Gene [Source:MGI Symbol;Acc:MGI:1347075] [ENSRNOT00000020916]	**14,955**

LOC687611	PREDICTED: Rattus norvegicus similar to transmembrane protein induced by tumor necrosis factor alpha (LOC687611), mRNA [XM_001079386]	**14,952**
Crip2	Rattus norvegicus cysteine-rich protein 2 (Crip2), mRNA [NM_022501]	**14,949**
Tsc22d2	Rattus norvegicus TSC22 domain family, member 2 (Tsc22d2), mRNA [NM_001191960]	**14,947**
Acap2	Rattus norvegicus ArfGAP with coiled-coil, ankyrin repeat and PH domains 2 (Acap2), mRNA [NM_001034006]	**14,946**
0	Rattus norvegicus chromosome 8, 32 clones, strain BN/SsNHsdMCW RNOR03312986, whole genome shotgun sequence [AABR03063211]	**14,944**
Zfp148	Rattus norvegicus zinc finger protein 148 (Zfp148), mRNA [NM_031615]	**14,944**
Stard7	Rattus norvegicus StAR-related lipid transfer (START) domain containing 7 (Stard7), mRNA [NM_001106503]	**14,943**
LOC688778	PREDICTED: Rattus norvegicus similar to fatty aldehyde dehydrogenase-like (LOC688778), mRNA [XM_001068253]	**14,940**
Neu3	Rattus norvegicus sialidase 3 (membrane sialidase) (Neu3), mRNA [NM_054010]	**14,937**
0	Rattus norvegicus similar to RIKEN cDNA 4933431D05 (LOC287855), mRNA [XM_221162]	**14,936**
Ubqln4	Rattus norvegicus ubiquilin 4 (Ubqln4), mRNA [NM_001107688]	**14,932**
LOC686143	PREDICTED: Rattus norvegicus similar to keratinocytes proline-rich protein, transcript variant 1 (LOC686143), mRNA [XM_001066677]	**14,932**
Slc1a2	Rattus norvegicus solute carrier family 1 (glial high affinity glutamate transporter), member 2 (Slc1a2), transcript variant 1, mRNA [NM_017215]	**14,930**
0	Unknown	**14,928**
0	Q87XI0_PSESM (Q87XI0) Cobalamin synthesis protein/P47K family protein, partial (7%) [TC596564]	**14,924**
0	Unknown	**14,924**
0	Unknown	**14,919**
Garnl4	Rattus norvegicus GTPase activating Rap/RanGAP domain-like 4 (Garnl4), mRNA [NM_001107019]	**14,916**
Fam63b	PREDICTED: Rattus norvegicus family with sequence similarity 63, member B, transcript variant 1 (Fam63b), mRNA [XM_001054973]	**14,914**
Dlx4	Rattus norvegicus distal-less homeobox 4 (Dlx4), mRNA [NM_001107040]	**14,908**
Cks1b	Rattus norvegicus CDC28 protein kinase regulatory subunit 1B (Cks1b), mRNA [NM_001135749]	**14,907**
Stxbp4	Rattus norvegicus syntaxin binding protein 4 (Stxbp4), mRNA [NM_001107038]	**14,907**
Ep400	Rattus norvegicus E1A binding protein p400 (Ep400), mRNA [NM_001107149]	**14,905**
Golga2	Rattus norvegicus golgi autoantigen, golgin subfamily a, 2 (Golga2), mRNA [NM_022596]	**14,903**
Atf2	Rattus norvegicus activating transcription factor 2 (Atf2), mRNA [NM_031018]	**14,902**
0	Uncharacterized protein [Source:UniProtKB/TrEMBL;Acc:D4A1U8] [ENSRNOT00000007843]	**14,901**
Map6	Rattus norvegicus microtubule-associated protein 6 (Map6), mRNA [NM_017204]	**14,901**
0	Unknown	**14,898**
Rgs4	Rattus norvegicus regulator of G-protein signaling 4 (Rgs4), mRNA [NM_017214]	**14,895**
Prlh	Rattus norvegicus prolactin releasing hormone (Prlh), mRNA [NM_022222]	**14,894**
Efna1	Rattus norvegicus ephrin A1 (Efna1), mRNA [NM_053599]	**14,892**
Olr386	Rattus norvegicus olfactory receptor 386 (Olr386), mRNA [NM_001000856]	**14,890**
Jarid1a	PREDICTED: Rattus norvegicus jumonji, AT rich interactive domain 1A (Rbp2 like) (Jarid1a), mRNA [XM_002729425]	**14,890**
0	Unknown	**14,888**

RGD1309414	Uncharacterized protein [Source:UniProtKB/TrEMBL;Acc:D3ZG24] [ENSRNOT00000012696]	**14,886**
Lrrc58	Rattus norvegicus leucine rich repeat containing 58 (Lrrc58), mRNA [NM_001195558]	**14,882**
0	Unknown	**14,879**
Itgb8	Rattus norvegicus integrin, beta 8 (Itgb8), mRNA [NM_001108726]	**14,877**
Zfp94	Rattus norvegicus zinc finger protein 94 (Zfp94), mRNA [NM_001037212]	**14,871**
Gck	Rattus norvegicus glucokinase (Gck), mRNA [NM_012565]	**14,869**
Slc12a5	Rattus norvegicus solute carrier family 12 (potassium-chloride transporter), member 5 (Slc12a5), mRNA [NM_134363]	**14,868**
Upk1a	Rattus norvegicus uroplakin 1A (Upk1a), mRNA [NM_001108911]	**14,866**
Tceb2	Rattus norvegicus transcription elongation factor B (SIII), polypeptide 2 (Tceb2), mRNA [NM_031129]	**14,865**
0	Unknown	**14,865**
RGD1564379	Rattus norvegicus RGD1564379 (RGD1564379), mRNA [NM_001126295]	**14,855**
Bcl7c	Rattus norvegicus B-cell CLL/lymphoma 7C (Bcl7c), mRNA [NM_001106298]	**14,854**
Nkd2	Rattus norvegicus naked cuticle homolog 2 (Drosophila) (Nkd2), mRNA [NM_001107454]	**14,851**
0	Unknown	**14,850**
0	Q8R4C3_CRIGR (Q8R4C3) Csr1, partial (32%) [TC596558]	**14,845**
Olr1227	Rattus norvegicus olfactory receptor 1227 (Olr1227), mRNA [NM_001000443]	**14,843**
Ybx1	Rattus norvegicus Y box binding protein 1 (Ybx1), mRNA [NM_031563]	**14,840**
0	probable histone-lysine N-methyltransferase NSD2 [Source:RefSeq peptide;Acc:NP_001178481] [ENSRNOT00000050238]	**14,838**
Pacsin1	Rattus norvegicus protein kinase C and casein kinase substrate in neurons 1 (Pacsin1), mRNA [NM_017294]	**14,833**
Kif1b	Rattus norvegicus kinesin family member 1B (Kif1b), mRNA [NM_057200]	**14,833**
Flywch1	PREDICTED: Rattus norvegicus FLYWCH-type zinc finger 1 (Flywch1), mRNA [XM_001056224]	**14,831**
Ghr	Rattus norvegicus growth hormone receptor (Ghr), mRNA [NM_017094]	**14,826**
Spinlw1	Rattus norvegicus serine peptidase inhibitor-like, with Kunitz and WAP domains 1 (eppin) (Spinlw1), mRNA [NM_001109457]	**14,826**
0	Unknown	**14,822**
Trappc1	Rattus norvegicus trafficking protein particle complex 1 (Trappc1), mRNA [NM_001039378]	**14,819**
0	Unknown	**14,818**
0	Unknown	**14,816**
Dmtf1	Rattus norvegicus cyclin D binding myb-like transcription factor 1 (Dmtf1), mRNA [NM_053693]	**14,814**
0	Unknown	**14,812**
Cdig2	Rattus norvegicus Cdig2 protein (Cdig2), mRNA [NM_153624]	**14,809**
Fat4	Rattus norvegicus FAT tumor suppressor homolog 4 (Drosophila) (Fat4), mRNA [NM_001191705]	**14,809**
RGD1311739	Rattus norvegicus similar to RIKEN cDNA 1700037H04 (RGD1311739), mRNA [NM_001025691]	**14,804**
Aqp4	Rattus norvegicus aquaporin 4 (Aqp4), transcript variant 2, mRNA [NM_001142366]	**14,804**
Kcnc4	Rattus norvegicus potassium voltage gated channel, Shaw-related subfamily, member 4 (Kcnc4), mRNA [NM_001122776]	**14,803**

Ubl5	Rattus norvegicus ubiquitin-like 5 (Ubl5), mRNA [NM_001048243]	**14,799**
Gipc1	Rattus norvegicus GIPC PDZ domain containing family, member 1 (Gipc1), mRNA [NM_053341]	**14,798**
App	Rattus norvegicus amyloid beta (A4) precursor protein (App), mRNA [NM_019288]	**14,797**
Best1	Rattus norvegicus bestrophin 1 (Best1), mRNA [NM_001011940]	**14,795**
0	RCG37698, isoform CRA_aUncharacterized protein [Source:UniProtKB/TrEMBL;Acc:D4AAY1] [ENSRNOT00000037022]	**14,795**
Csnk1g3	Rattus norvegicus casein kinase 1, gamma 3 (Csnk1g3), mRNA [NM_022855]	**14,793**
RGD1566001	Rattus norvegicus similar to DNA segment, Chr 4, Brigham & Womens Genetics 0951 expressed (RGD1566001), mRNA [NM_001109263]	**14,787**
Grm1	Rattus norvegicus glutamate receptor, metabotropic 1 (Grm1), transcript variant 1, mRNA [NM_017011]	**14,787**
Xpnpep1	Rattus norvegicus X-prolyl aminopeptidase (aminopeptidase P) 1, soluble (Xpnpep1), mRNA [NM_131913]	**14,785**
Klhl26	Rattus norvegicus kelch-like 26 (Drosophila) (Klhl26), mRNA [NM_001106075]	**14,785**
Csnk2a1	Rattus norvegicus casein kinase 2, alpha 1 polypeptide (Csnk2a1), mRNA [NM_053824]	**14,784**
LOC681380	PREDICTED: Rattus norvegicus similar to spermatogenesis associated glutamate (E)-rich protein 4b (LOC681380), mRNA [XM_002727269]	**14,780**
LOC681647	PREDICTED: Rattus norvegicus similar to F43G9.2 (LOC681647), mRNA [XM_001057182]	**14,778**
Rtn4rl2	Rattus norvegicus reticulon 4 receptor-like 2 (Rtn4rl2), mRNA [NM_181380]	**14,777**
Bmi1	Rattus norvegicus Bmi1 polycomb ring finger oncogene (Bmi1), mRNA [NM_001107368]	**14,777**
Dusp22	Rattus norvegicus dual specificity phosphatase 22 (Dusp22), mRNA [NM_001108412]	**14,772**
Clec2l	Rattus norvegicus C-type lectin domain family 2, member L (Clec2l), mRNA [NM_001044233]	**14,771**
Flrt2	Rattus norvegicus fibronectin leucine rich transmembrane protein 2 (Flrt2), mRNA [NM_001106750]	**14,770**
Lrch1	Rattus norvegicus leucine-rich repeats and calponin homology (CH) domain containing 1 (Lrch1), mRNA [NM_001134727]	**14,765**
Bbx	Rattus norvegicus bobby sox homolog (Drosophila) (Bbx), mRNA [NM_001079938]	**14,763**
Pbxip1	Rattus norvegicus pre-B-cell leukemia homeobox interacting protein 1 (Pbxip1), mRNA [NM_001100976]	**14,763**
Gng12	Guanine nucleotide binding protein gamma 12 subunit [Source:UniProtKB/TrEMBL;Acc:O35359] [ENSRNOT00000007403]	**14,761**
Entpd2	Rattus norvegicus ectonucleoside triphosphate diphosphohydrolase 2 (Entpd2), mRNA [NM_172030]	**14,760**
Pcdhgb7	Rattus norvegicus protocadherin gamma subfamily B, 7 (Pcdhgb7), mRNA [NM_001012215]	**14,757**
Tcea1	Rattus norvegicus transcription elongation factor A (SII) 1 (Tcea1), mRNA [NM_001025735]	**14,756**
Haus1	HAUS augmin-like complex subunit 1 [Source:UniProtKB/Swiss-Prot;Acc:Q9R0A8] [ENSRNOT00000023101]	**14,753**
Lix1	Rattus norvegicus Lix1 homolog (chicken) (Lix1), mRNA [NM_001106214]	**14,750**
0	Unknown	**14,749**
Srd5a2	Rattus norvegicus steroid-5-alpha-reductase, alpha polypeptide 2 (3-oxo-5 alpha-steroid delta 4-dehydrogenase alpha 2) (Srd5a2), mRNA [NM_022711]	**14,749**
Unc5a	Rattus norvegicus unc-5 homolog A (C. elegans) (Unc5a), mRNA [NM_022206]	**14,745**
Fam160b2	Rattus norvegicus family with sequence similarity 160, member B2 (Fam160b2), mRNA [NM_001170474]	**14,744**
Rspo3	Rattus norvegicus R-spondin 3 homolog (Xenopus laevis) (Rspo3), mRNA [NM_001100990]	**14,742**
Hps4	Rattus norvegicus Hermansky-Pudlak syndrome 4 homolog (human) (Hps4), mRNA [NM_001107148]	**14,740**

Gigyf1	Rattus norvegicus GRB10 interacting GYF protein 1 (Gigyf1), mRNA [NM_001107133]	**14,739**
0	Stathmin [Source:UniProtKB/TrEMBL;Acc:D3Z8V0] [ENSRNOT00000050325]	**14,737**
Ak3	Rattus norvegicus adenylate kinase 3 (Ak3), nuclear gene encoding mitochondrial protein, mRNA [NM_013218]	**14,729**
Slc16a2	Rattus norvegicus solute carrier family 16, member 2 (monocarboxylic acid transporter 8) (Slc16a2), mRNA [NM_147216]	**14,726**
0	Unknown	**14,726**
LOC680160	PREDICTED: Rattus norvegicus similar to keratin associated protein 4-7, transcript variant 2 (LOC680160), mRNA [XM_001055726]	**14,726**
Eno1	Rattus norvegicus enolase 1, (alpha) (Eno1), transcript variant 2, mRNA [NM_001109908]	**14,724**
Msl2	PREDICTED: Rattus norvegicus male-specific lethal 2-like 1 (Drosophila) (Msl2l1), mRNA [XM_236567]	**14,721**
Mmd2	Rattus norvegicus monocyte to macrophage differentiation-associated 2 (Mmd2), mRNA [NM_001037217]	**14,716**
Znf213	Rattus norvegicus zinc finger protein 213 (Znf213), mRNA [NM_001105764]	**14,715**
Gnptab	Uncharacterized protein [Source:UniProtKB/TrEMBL;Acc:D3ZJS1] [ENSRNOT00000006946]	**14,715**
Bcdin3d	Rattus norvegicus BCDIN3 domain containing (Bcdin3d), mRNA [NM_001108751]	**14,715**
Pggt1b	Rattus norvegicus protein geranylgeranyltransferase type I, beta subunit (Pggt1b), mRNA [NM_031082]	**14,714**
Lrfn5	Rattus norvegicus leucine rich repeat and fibronectin type III domain containing 5 (Lrfn5), mRNA [NM_001108024]	**14,714**
Znf703	Rattus norvegicus zinc finger protein 703 (Znf703), mRNA [NM_001109425]	**14,713**
Fam118a	Rattus norvegicus family with sequence similarity 118, member A (Fam118a), mRNA [NM_001173336]	**14,713**
0	predicted gene 6924 Gene [Source:MGI Symbol;Acc:MGI:3647600] [ENSRNOT00000043774]	**14,706**
Lhfpl4	Rattus norvegicus lipoma HMGIC fusion partner-like 4 (Lhfpl4), mRNA [NM_181387]	**14,706**
LOC100366258	Uncharacterized protein [Source:UniProtKB/TrEMBL;Acc:D3ZL26] [ENSRNOT00000051182]	**14,704**
Mertk	Rattus norvegicus c-mer proto-oncogene tyrosine kinase (Mertk), mRNA [NM_022943]	**14,703**
Ube2k	Rattus norvegicus ubiquitin-conjugating enzyme E2K (UBC1 homolog, yeast) (Ube2k), mRNA [NM_001106006]	**14,703**
Hspa12a	Rattus norvegicus heat shock protein 12A (Hspa12a), mRNA [NM_001107445]	**14,696**
RGD1304728	Uncharacterized protein [Source:UniProtKB/TrEMBL;Acc:D3ZNS7] [ENSRNOT00000018808]	**14,694**
Chkb	Rattus norvegicus choline kinase beta (Chkb), mRNA [NM_017177]	**14,692**
Gstcd	Rattus norvegicus glutathione S-transferase, C-terminal domain containing (Gstcd), mRNA [NM_001107725]	**14,691**
Prkd3	Rattus norvegicus protein kinase D3 (Prkd3), mRNA [NM_001024263]	**14,689**
Pold4	Rattus norvegicus polymerase (DNA-directed), delta 4 (Pold4), mRNA [NM_001013195]	**14,689**
RGD1565785	Rattus norvegicus similar to chromosome X open reading frame 21 (RGD1565785), mRNA [NM_001109318]	**14,687**
Tnfrsf21	Rattus norvegicus tumor necrosis factor receptor superfamily, member 21 (Tnfrsf21), mRNA [NM_001108207]	**14,684**
Wdr5	Rattus norvegicus WD repeat domain 5 (Wdr5), mRNA [NM_001039034]	**14,684**
0	Nuclear receptor corepressor 1 [Source:UniProtKB/Swiss-Prot;Acc:Q9WUB5] [ENSRNOT00000057679]	**14,680**
Cd320	Rattus norvegicus CD320 molecule (Cd320), mRNA [NM_001014201]	**14,680**
Hgf	Rattus norvegicus hepatocyte growth factor (Hgf), mRNA [NM_017017]	**14,676**
Hcrtr1	Rattus norvegicus hypocretin (orexin) receptor 1 (Hcrtr1), mRNA [NM_013064]	**14,676**

Rhoj	Rattus norvegicus ras homolog gene family, member J (Rhoj), mRNA [NM_001008320]	**14,676**
Dlx5	Rattus norvegicus distal-less homeobox 5 (Dlx5), mRNA [NM_012943]	**14,676**
0	Unknown	**14,672**
Prr14	PREDICTED: Rattus norvegicus proline rich 14 (Prr14), mRNA [XM_001080025]	**14,671**
Unc5c	Rattus norvegicus unc-5 homolog C (C. elegans) (Unc5c), mRNA [NM_199407]	**14,657**
Acot2	Rattus norvegicus acyl-CoA thioesterase 2 (Acot2), nuclear gene encoding mitochondrial protein, mRNA [NM_138907]	**14,657**
0	Unknown	**14,648**
Fam129b	Rattus norvegicus family with sequence similarity 129, member B (Fam129b), mRNA [NM_001109885]	**14,645**
Braf	V-raf murine sarcoma viral oncogene B1-like protein [Source:UniProtKB/TrEMBL;Acc:Q99MC6] [ENSRNOT00000014604]	**14,644**
LOC685125	Rattus norvegicus cDNA clone IMAGE:7132976. [BC166431]	**14,644**
Usp24	Putative uncharacterized protein [Source:UniProtKB/TrEMBL;Acc:Q4G007] [ENSRNOT00000029228]	**14,643**
Tex19	Rattus norvegicus testis expressed 19 (Tex19), mRNA [NM_001109622]	**14,641**
RGD1560784	Uncharacterized protein [Source:UniProtKB/TrEMBL;Acc:D3ZL45] [ENSRNOT00000049864]	**14,637**
Ubap1	Rattus norvegicus ubiquitin-associated protein 1 (Ubap1), mRNA [NM_001012190]	**14,635**
Sumo2	Rattus norvegicus SMT3 suppressor of mif two 3 homolog 2 (S. cerevisiae) (Sumo2), mRNA [NM_133594]	**14,633**
Mtap	Rattus norvegicus methylthioadenosine phosphorylase (Mtap), mRNA [NM_001047867]	**14,631**
Cpeb3	Uncharacterized protein [Source:UniProtKB/TrEMBL;Acc:D3Z9M2] [ENSRNOT00000047387]	**14,627**
Zfp174	Uncharacterized protein [Source:UniProtKB/TrEMBL;Acc:D3ZNW1] [ENSRNOT00000010059]	**14,627**
Jagn1	Rattus norvegicus jagunal homolog 1 (Drosophila) (Jagn1), mRNA [NM_001044272]	**14,627**
Fbxo3	Rattus norvegicus F-box protein 3 (Fbxo3), mRNA [NM_001109606]	**14,626**
0	Uncharacterized protein [Source:UniProtKB/TrEMBL;Acc:D3ZE14] [ENSRNOT00000027981]	**14,623**
Ccl24	Rattus norvegicus chemokine (C-C motif) ligand 24 (Ccl24), mRNA [NM_001013045]	**14,623**
Giyd2	Rattus norvegicus GIY-YIG domain containing 2 (Giyd2), mRNA [NM_001009292]	**14,623**
0	Unknown	**14,622**
Ptchd1	Rattus norvegicus patched domain containing 1 (Ptchd1), mRNA [NM_001191734]	**14,621**
Sdr39u1	Rattus norvegicus short chain dehydrogenase/reductase family 39U, member 1 (Sdr39u1), mRNA [NM_001108378]	**14,617**
Srprb	Rattus norvegicus signal recognition particle receptor, B subunit (Srprb), mRNA [NM_001013252]	**14,617**
0	Unknown	**14,617**
Vgll3	Uncharacterized protein [Source:UniProtKB/TrEMBL;Acc:D3ZZ09] [ENSRNOT00000042316]	**14,615**
Cish	Rattus norvegicus cytokine inducible SH2-containing protein (Cish), mRNA [NM_031804]	**14,613**
0	PREDICTED: Rattus norvegicus similar to novel protein (RGD1560386), partial mRNA [XM_001069736]	**14,611**
0	Unknown	**14,610**
Prepl	Rattus norvegicus prolyl endopeptidase-like (Prepl), mRNA [NM_001010951]	**14,607**
Zdhhc8	Rattus norvegicus zinc finger, DHHC-type containing 8 (Zdhhc8), mRNA [NM_001039021]	**14,605**

Rogdi	Rattus norvegicus rogdi homolog (Drosophila) (Rogdi), mRNA [NM_001024864]	**14,604**
Fxyd6	Rattus norvegicus FXYD domain-containing ion transport regulator 6 (Fxyd6), mRNA [NM_022005]	**14,598**
Selm	Rattus norvegicus selenoprotein M (Selm), mRNA [NM_001115013]	**14,598**
Ctnna2	Rattus norvegicus catenin (cadherin associated protein), alpha 2 (Ctnna2), mRNA [NM_001106598]	**14,597**
Chek1	Rattus norvegicus CHK1 checkpoint homolog (S. pombe) (Chek1), mRNA [NM_080400]	**14,596**
Zmat4	Rattus norvegicus zinc finger, matrin type 4 (Zmat4), mRNA [NM_001134747]	**14,592**
Ifitm1	Rattus norvegicus interferon induced transmembrane protein 1 (Ifitm1), mRNA [NM_001106314]	**14,588**
0	Unknown	**14,587**
Oaf	Rattus norvegicus OAF homolog (Drosophila) (Oaf), mRNA [NM_001014090]	**14,585**
0	Unknown	**14,584**
0	SMR1 proteinSMR1-related undecapeptideSMR1-related hexapeptideSialorphinSubmandibular gland peptide T [Source:UniProtKB/Swiss-Prot;Acc:P13432] [ENSRNOT00000002664]	**14,583**
Klhdc5	Rattus norvegicus kelch domain containing 5 (Klhdc5), mRNA [NM_001109257]	**14,578**
Gal3st3	Rattus norvegicus galactose-3-O-sulfotransferase 3 (Gal3st3), mRNA [NM_001024290]	**14,577**
0	Rattus norvegicus TL0AAA45YD16 mRNA sequence. [FQ213860]	**14,576**
Narg1	Rattus norvegicus NMDA receptor regulated 1 (Narg1), mRNA [NM_001107674]	**14,576**
Ptpn21	Rattus norvegicus protein tyrosine phosphatase, non-receptor type 21 (Ptpn21), mRNA [NM_133545]	**14,575**
Nkain3	Rattus norvegicus Na+/K+ transporting ATPase interacting 3 (Nkain3), mRNA [NM_001109540]	**14,571**
Lrp6	Rattus norvegicus low density lipoprotein receptor-related protein 6 (Lrp6), mRNA [NM_001107892]	**14,570**
RGD1304770	Rattus norvegicus similar to Na+ dependent glucose transporter 1 (RGD1304770), mRNA [NM_001134547]	**14,568**
RGD1307155	Rattus norvegicus similar to CG18661-PA (RGD1307155), mRNA [NM_001037188]	**14,563**
Nop10	Rattus norvegicus NOP10 ribonucleoprotein homolog (yeast) (Nop10), mRNA [NM_001126100]	**14,561**
Ralbp1	Rattus norvegicus ralA binding protein 1 (Ralbp1), mRNA [NM_032067]	**14,561**
Hap1	Rattus norvegicus huntingtin-associated protein 1 (Hap1), transcript variant 1, mRNA [NM_024133]	**14,560**
Pdha1	Rattus norvegicus pyruvate dehydrogenase (lipoamide) alpha 1 (Pdha1), nuclear gene encoding mitochondrial protein, mRNA [NM_001004072]	**14,560**
Fdft1	Rattus norvegicus farnesyl diphosphate farnesyl transferase 1 (Fdft1), mRNA [NM_019238]	**14,548**
Gabarap	Rattus norvegicus GABA(A) receptor-associated protein (Gabarap), mRNA [NM_172036]	**14,547**
Slc6a12	Rattus norvegicus solute carrier family 6 (neurotransmitter transporter, betaine/GABA), member 12 (Slc6a12), mRNA [NM_017335]	**14,546**
Mdm2	Rattus norvegicus Mdm2 p53 binding protein homolog (mouse) (Mdm2), mRNA [NM_001108099]	**14,545**
RGD1564482	Rattus norvegicus RGD1564482 (RGD1564482), non-coding RNA [NR_036617]	**14,545**
Mrps21	Rattus norvegicus mitochondrial ribosomal protein S21 (Mrps21), nuclear gene encoding mitochondrial protein, mRNA [NM_001126094]	**14,539**
Tapt1	PREDICTED: Rattus norvegicus transmembrane anterior posterior transformation 1 (Tapt1), mRNA [XM_001059604]	**14,538**
Lrrc20	Rattus norvegicus leucine rich repeat containing 20 (Lrrc20), mRNA [NM_001109171]	**14,536**
LOC100188936	Rattus norvegicus hypothetical protein LOC100188936 (LOC100188936), mRNA [NM_001134704]	**14,536**

0	Tmem216 protein [Source:UniProtKB/TrEMBL;Acc:B6ID01] [ENSRNOT00000065430]	**14,535**
Sprr3	Rattus norvegicus small proline-rich protein 3 (Sprr3), mRNA [NM_001107686]	**14,534**
Mpped2	Rattus norvegicus metallophosphoesterase domain containing 2 (Mpped2), mRNA [NM_198778]	**14,534**
Nhej1	Rattus norvegicus nonhomologous end-joining factor 1 (Nhej1), mRNA [NM_001014217]	**14,532**
Omg	Rattus norvegicus oligodendrocyte-myelin glycoprotein (Omg), mRNA [NM_001005898]	**14,530**
Mier2	Rattus norvegicus mesoderm induction early response 1, family member 2 (Mier2), mRNA [NM_001108737]	**14,528**
Sypl2	Rattus norvegicus synaptophysin-like 2 (Sypl2), mRNA [NM_001108563]	**14,528**
RGD1561843	Uncharacterized protein [Source:UniProtKB/TrEMBL;Acc:D3ZIJ2] [ENSRNOT00000045791]	**14,527**
Anxa10	Rattus norvegicus annexin A10 (Anxa10), mRNA [NM_001109110]	**14,525**
Anxa9	PREDICTED: Rattus norvegicus annexin A9 (Anxa9), mRNA [XM_002726022]	**14,521**
Hif3a	Rattus norvegicus hypoxia inducible factor 3, alpha subunit (Hif3a), mRNA [NM_022528]	**14,521**
RGD1565059	Rattus norvegicus similar to hypothetical protein E130311K13 (RGD1565059), mRNA [NM_001127562]	**14,517**
Fkrp	Rattus norvegicus fukutin related protein (Fkrp), mRNA [NM_001025678]	**14,516**
0	Unknown	**14,515**
Ermp1	Rattus norvegicus endoplasmic reticulum metallopeptidase 1 (Ermp1), mRNA [NM_184050]	**14,515**
Snrnp70	Rattus norvegicus small nuclear ribonucleoprotein 70 (U1) (Snrnp70), mRNA [NM_001108483]	**14,514**
Wdr45	Rattus norvegicus WD repeat domain 45 (Wdr45), mRNA [NM_001013958]	**14,514**
RGD1309922	Rattus norvegicus similar to 2610301G19Rik protein (RGD1309922), mRNA [NM_001170472]	**14,513**
Necap1	Rattus norvegicus NECAP endocytosis associated 1 (Necap1), mRNA [NM_001029919]	**14,509**
Kcnmb2	Rattus norvegicus potassium large conductance calcium-activated channel, subfamily M, beta member 2 (Kcnmb2), mRNA [NM_176861]	**14,508**
Nanos3	Rattus norvegicus nanos homolog 3 (Drosophila) (Nanos3), mRNA [NM_001105945]	**14,507**
0	Uncharacterized protein [Source:UniProtKB/TrEMBL;Acc:D3Z919] [ENSRNOT00000068178]	**14,505**
RGD1306233	Rattus norvegicus similar to hypothetical protein MGC29761 (RGD1306233), mRNA [NM_001106564]	**14,504**
Cln5	Rattus norvegicus ceroid-lipofuscinosis, neuronal 5 (Cln5), mRNA [NM_001191689]	**14,502**
Atpaf1	Rattus norvegicus ATP synthase mitochondrial F1 complex assembly factor 1 (Atpaf1), nuclear gene encoding mitochondrial protein, mRNA [NM_001107959]	**14,501**
Ccrn4l	Rattus norvegicus CCR4 carbon catabolite repression 4-like (S. cerevisiae) (Ccrn4l), mRNA [NM_138526]	**14,499**
Lpcat4	Rattus norvegicus lysophosphatidylcholine acyltransferase 4 (Lpcat4), mRNA [NM_001106494]	**14,497**
0	Unknown	**14,488**
KIFC2	Rattus norvegicus kinesin family member C2 (KIFC2), mRNA [NM_198752]	**14,487**
Mapk9	Rattus norvegicus mitogen-activated protein kinase 9 (Mapk9), mRNA [NM_017322]	**14,486**
0	Unknown	**14,485**
Epdr1	Rattus norvegicus ependymin related protein 1 (zebrafish) (Epdr1), mRNA [NM_001007625]	**14,484**
Kcna2	Rattus norvegicus potassium voltage-gated channel, shaker-related subfamily, member 2 (Kcna2), mRNA [NM_012970]	**14,477**

Tmem67	Rattus norvegicus transmembrane protein 67 (Tmem67), mRNA [NM_001107916]	**14,477**
Spry2	Rattus norvegicus sprouty homolog 2 (Drosophila) (Spry2), mRNA [NM_001012046]	**14,475**
Cdh23	Rattus norvegicus cadherin 23 (otocadherin) (Cdh23), mRNA [NM_053644]	**14,471**
Tril	Rattus norvegicus TLR4 interactor with leucine-rich repeats (Tril), mRNA [NM_001034010]	**14,464**
0	Uncharacterized protein [Source:UniProtKB/TrEMBL;Acc:D3ZSM9] [ENSRNOT00000001661]	**14,456**
Hoxc5	Rattus norvegicus homeo box C5 (Hoxc5), mRNA [NM_001108116]	**14,454**
0	CB544318 AMGNNUC:NRDG1-00127-B11-A nrdg1 (10855) Rattus norvegicus cDNA clone nrdg1-00127-b11 5', mRNA sequence [CB544318]	**14,453**
Olr1405	Rattus norvegicus olfactory receptor 1405 (Olr1405), mRNA [NM_001001012]	**14,448**
Gapdh	Rattus norvegicus glyceraldehyde-3-phosphate dehydrogenase (Gapdh), mRNA [NM_017008]	**14,447**
Zdhhc18	Rattus norvegicus zinc finger, DHHC-type containing 18 (Zdhhc18), mRNA [NM_001039339]	**14,447**
Dbp	Rattus norvegicus D site of albumin promoter (albumin D-box) binding protein (Dbp), mRNA [NM_012543]	**14,443**
0	Unknown	**14,442**
Zdbf2	PREDICTED: Rattus norvegicus zinc finger, DBF-type containing 2 (Zdbf2), mRNA [XM_576579]	**14,442**
Zfp141	PREDICTED: Rattus norvegicus similar to zinc finger protein 420 (LOC690343), mRNA [XM_001074160]	**14,440**
Fstl3	Follistatin-related protein 3 [Source:UniProtKB/Swiss-Prot;Acc:Q99PW7] [ENSRNOT00000012578]	**14,440**
Fam86a	Rattus norvegicus family with sequence similarity 86, member A (Fam86a), mRNA [NM_001106975]	**14,439**
Ankrd13c	Rattus norvegicus ankyrin repeat domain 13C (Ankrd13c), mRNA [NM_001191570]	**14,436**
0	Q6IE24_RAT (Q6IE24) Ubiquitin specific protease 54, complete [TC592916]	**14,435**
Ubxn8	Rattus norvegicus UBX domain protein 8 (Ubxn8), mRNA [NM_001106086]	**14,434**
0	Uncharacterized protein [Source:UniProtKB/TrEMBL;Acc:D3ZMD5] [ENSRNOT00000056810]	**14,429**
Ppp2r2c	Rattus norvegicus protein phosphatase 2 (formerly 2A), regulatory subunit B, gamma isoform (Ppp2r2c), mRNA [NM_057116]	**14,426**
Bag2	Rattus norvegicus Bcl2-associated athanogene 2 (Bag2), mRNA [NM_001128195]	**14,424**
Scd	Rattus norvegicus stearoyl-CoA desaturase (delta-9-desaturase) (Scd), mRNA [NM_031841]	**14,423**
Ears2	Rattus norvegicus glutamyl-tRNA synthetase 2 mitochondrial (putative) (Ears2), nuclear gene encoding mitochondrial protein, mRNA [NM_001159493]	**14,423**
Rfx3	Rattus norvegicus regulatory factor X, 3 (influences HLA class II expression) (Rfx3), mRNA [NM_001012172]	**14,419**
Tubgcp3	Rattus norvegicus tubulin, gamma complex associated protein 3 (Tubgcp3), mRNA [NM_001107323]	**14,418**
Cntn2	Rattus norvegicus contactin 2 (axonal) (Cntn2), mRNA [NM_012884]	**14,416**
Trim25	Rattus norvegicus tripartite motif-containing 25 (Trim25), mRNA [NM_001009536]	**14,410**
0	Uncharacterized protein [Source:UniProtKB/TrEMBL;Acc:D3ZAT9] [ENSRNOT00000013599]	**14,409**
RGD1307443	Rattus norvegicus similar to mKIAA0319 protein (RGD1307443), mRNA [NM_001197023]	**14,409**
Zbtb25	Rattus norvegicus zinc finger and BTB domain containing 25 (Zbtb25), mRNA [NM_199496]	**14,408**
Thrsp	Rattus norvegicus thyroid hormone responsive (Thrsp), mRNA [NM_012703]	**14,407**
Insr	Rattus norvegicus insulin receptor (Insr), mRNA [NM_017071]	**14,404**

0	PREDICTED: Rattus norvegicus similar to procollagen, type IV, alpha 6 (LOC363458), partial miscRNA [XR_086365]	**14,404**
Eci3	Rattus norvegicus enoyl-Coenzyme A delta isomerase 3 (Eci3), mRNA [NM_001009275]	**14,401**
0	Unknown	**14,400**
Wipi1	Rattus norvegicus WD repeat domain, phosphoinositide interacting 1 (Wipi1), mRNA [NM_001127297]	**14,400**
Cacna1a	Rattus norvegicus calcium channel, voltage-dependent, P/Q type, alpha 1A subunit (Cacna1a), mRNA [NM_012918]	**14,399**
Kcnh7	Rattus norvegicus potassium voltage-gated channel, subfamily H (eag-related), member 7 (Kcnh7), mRNA [NM_131912]	**14,396**
0	LRRG00114LRRGT00001 [Source:UniProtKB/TrEMBL;Acc:Q6QI94] [ENSRNOT00000046112]	**14,395**
0	Unknown	**14,389**
Mmp15	Rattus norvegicus matrix metallopeptidase 15 (Mmp15), mRNA [NM_001106168]	**14,389**
0	Unknown	**14,382**
Rasa3	Rattus norvegicus mRNA for R-ras GTPase activating protein, partial cds. [AB028626]	**14,381**
Atp2b1	Rattus norvegicus ATPase, Ca++ transporting, plasma membrane 1 (Atp2b1), mRNA [NM_053311]	**14,381**
Snai3	Rattus norvegicus snail homolog 3 (Drosophila) (Snai3), mRNA [NM_001107439]	**14,379**
Prom1	Rattus norvegicus prominin 1 (Prom1), transcript variant 1, mRNA [NM_021751]	**14,379**
Atf4	Rattus norvegicus activating transcription factor 4 (tax-responsive enhancer element B67) (Atf4), mRNA [NM_024403]	**14,377**
Mettl7a	Rattus norvegicus methyltransferase like 7A (Mettl7a), mRNA [NM_001037355]	**14,377**
0	ATP synthase subunit a [Source:UniProtKB/Swiss-Prot;Acc:P05504] [ENSRNOT00000046108]	**14,374**
Cpne6	Rattus norvegicus copine VI (Cpne6), mRNA [NM_001191113]	**14,371**
0	Unknown	**14,370**
0	Unknown	**14,369**
Pcdhb4	Rattus norvegicus protocadherin beta 4 (Pcdhb4), mRNA [NM_001114601]	**14,367**
Rtbdn	Rattus norvegicus retbindin (Rtbdn), mRNA [NM_001107165]	**14,366**
Pfkl	Rattus norvegicus phosphofructokinase, liver (Pfkl), mRNA [NM_013190]	**14,366**
Itga10	Rattus norvegicus integrin, alpha 10 (Itga10), mRNA [NM_001107699]	**14,365**
0	Unknown	**14,363**
LOC680549	Rattus norvegicus TL0ADA40YE19 mRNA sequence. [FQ221048]	**14,361**
Leng1	Rattus norvegicus leukocyte receptor cluster (LRC) member 1 (Leng1), mRNA [NM_001106218]	**14,361**
0	Unknown	**14,357**
Rag1ap1	Rattus norvegicus recombination activating gene 1 activating protein 1 (Rag1ap1), mRNA [NM_001106445]	**14,357**
0	AW143110 EST293406 Normalized rat brain, Bento Soares Rattus sp. cDNA clone RGIBD54 5' end, mRNA sequence [AW143110]	**14,357**
Rab34	Rattus norvegicus RAB34, member RAS oncogene family (Rab34), mRNA [NM_001012140]	**14,356**
Lyplal1	Rattus norvegicus lysophospholipase-like 1 (Lyplal1), mRNA [NM_001105986]	**14,354**
Gulp1	Rattus norvegicus GULP, engulfment adaptor PTB domain containing 1 (Gulp1), mRNA [NM_001013171]	**14,351**
LOC681766	Rattus norvegicus hypothetical protein LOC681766 (LOC681766), mRNA [NM_001163562]	**14,351**

Dda1	Rattus norvegicus DET1 and DDB1 associated 1 (Dda1), mRNA [NM_001134790]	**14,348**
Mef2b	Rattus norvegicus myocyte enhancer factor 2B (Mef2b), mRNA [NM_001017507]	**14,346**
LOC302680	Rattus norvegicus similar to CXORF15 (LOC302680), mRNA [NM_001037187]	**14,339**
Vom1r55	Rattus norvegicus vomeronasal 1 receptor 55 (Vom1r55), mRNA [NM_001166763]	**14,338**
Kcnk2	Rattus norvegicus potassium channel, subfamily K, member 2 (Kcnk2), transcript variant 1, mRNA [NM_172041]	**14,336**
0	Unknown	**14,335**
Ube2q2	Uncharacterized protein [Source:UniProtKB/TrEMBL;Acc:D4A1G2] [ENSRNOT00000019539]	**14,331**
0	inverted formin, FH2 and WH2 domain containing Gene [Source:MGI Symbol;Acc:MGI:1917685] [ENSRNOT00000031962]	**14,331**
Slc26a10	solute carrier family 26 member 10 [Source:RefSeq peptide;Acc:NP_001128067] [ENSRNOT00000052288]	**14,327**
Prtg	Rattus norvegicus protogenin homolog (Gallus gallus) (Prtg), mRNA [NM_001037651]	**14,327**
Foxk1	Rattus norvegicus forkhead box K1 (Foxk1), mRNA [NM_001037219]	**14,326**
Tyrobp	Rattus norvegicus Tyro protein tyrosine kinase binding protein (Tyrobp), mRNA [NM_212525]	**14,325**
0	Q53UD0_BRABE (Q53UD0) Paired box protein Pax2/5/8 (Fragment), partial (9%) [TC618096]	**14,323**
Gpd1	Rattus norvegicus glycerol-3-phosphate dehydrogenase 1 (soluble) (Gpd1), mRNA [NM_022215]	**14,321**
Ugt1a9	Rattus norvegicus UDP glucuronosyltransferase 1 family, polypeptide A9 (Ugt1a9), mRNA [NM_201425]	**14,321**
RGD1565693	PREDICTED: Rattus norvegicus similar to GLE1-like, RNA export mediator isoform 1 (RGD1565693), mRNA [XM_001059991]	**14,319**
Cnr1	Rattus norvegicus cannabinoid receptor 1 (brain) (Cnr1), mRNA [NM_012784]	**14,319**
Rab7b	Rattus norvegicus Rab7b, member RAS oncogene family (Rab7b), mRNA [NM_001109328]	**14,318**
Cdh4	PREDICTED: Rattus norvegicus cadherin 4 (Cdh4), mRNA [XM_001061943]	**14,314**
Bpil1	Rattus norvegicus bactericidal/permeability-increasing protein-like 1 (Bpil1), mRNA [NM_001106531]	**14,314**
0	Unknown	**14,312**
Maob	Rattus norvegicus monoamine oxidase B (Maob), nuclear gene encoding mitochondrial protein, mRNA [NM_013198]	**14,311**
RGD1311946	Rattus norvegicus similar to RIKEN cDNA 1810055G02 (RGD1311946), mRNA [NM_001025683]	**14,311**
Krcc1	Rattus norvegicus lysine-rich coiled-coil 1 (Krcc1), mRNA [NM_001009413]	**14,310**
0	Unknown	**14,309**
0	Rattus norvegicus similar to Glyceraldehyde 3-phosphate dehydrogenase (GAPDH) (LOC293022), mRNA [XM_218807]	**14,305**
0	ADAMTS-like 5 (Predicted), isoform CRA_cUncharacterized protein [Source:UniProtKB/TrEMBL;Acc:D3ZG32] [ENSRNOT00000042159]	**14,304**
0	Unknown	**14,300**
Sod2	Rattus norvegicus superoxide dismutase 2, mitochondrial (Sod2), nuclear gene encoding mitochondrial protein, mRNA [NM_017051]	**14,299**
RGD1308154	PREDICTED: Rattus norvegicus similar to CG11388-PA (RGD1308154), mRNA [XM_344027]	**14,295**
Zmynd11	Rattus norvegicus zinc finger, MYND domain containing 11 (Zmynd11), transcript variant 2, mRNA [NM_203367]	**14,294**
Btnl5	Rattus norvegicus butyrophilin-like 5 (Btnl5), mRNA [NM_001166351]	**14,293**
Abhd3	Rattus norvegicus abhydrolase domain containing 3 (Abhd3), mRNA [NM_001106162]	**14,292**
Fmnl1	Rattus norvegicus formin-like 1 (Fmnl1), mRNA [NM_001105846]	**14,290**

Ednrb	Rattus norvegicus endothelin receptor type B (Ednrb), mRNA [NM_017333]	**14,290**
0	Q7TQF0_MOUSE (Q7TQF0) Nid2 protein, partial (17%) [TC601702]	**14,290**
Rnf180	Rattus norvegicus ring finger protein 180 (Rnf180), mRNA [NM_001134986]	**14,284**
0	Unknown	**14,277**
RGD1311703	Rattus norvegicus similar to sid2057p (RGD1311703), mRNA [NM_001013898]	**14,277**
Ripk1	Rattus norvegicus receptor (TNFRSF)-interacting serine-threonine kinase 1 (Ripk1), mRNA [NM_001107350]	**14,276**
Alpi	Rattus norvegicus alkaline phosphatase, intestinal (Alpi), mRNA [NM_022665]	**14,275**
Gtf2e1	Rattus norvegicus general transcription factor IIE, polypeptide 1 (alpha subunit) (Gtf2e1), mRNA [NM_001100556]	**14,274**
0	Unknown	**14,269**
Clasp1	PREDICTED: Rattus norvegicus cytoplasmic linker associated protein 1 (Clasp1), mRNA [XM_001053715]	**14,268**
0	Unknown	**14,266**
Gpr146	Rattus norvegicus G protein-coupled receptor 146 (Gpr146), mRNA [NM_001109062]	**14,265**
RGD1563941	Rattus norvegicus similar to hypothetical protein FLJ20010 (RGD1563941), mRNA [NM_001109293]	**14,264**
Galntl6	Rattus norvegicus UDP-N-acetyl-alpha-D-galactosamine:polypeptide N-acetylgalactosaminyltransferase-like 6 (Galntl6), mRNA [NM_001135756]	**14,263**
0	Unknown	**14,261**
RGD1307222	Uncharacterized protein [Source:UniProtKB/TrEMBL;Acc:D3ZKG9] [ENSRNOT00000003687]	**14,259**
C2cd2l	Rattus norvegicus C2 calcium-dependent domain containing 2-like (C2cd2l), mRNA [NM_001011996]	**14,257**
Dnajc15	Rattus norvegicus DnaJ (Hsp40) homolog, subfamily C, member 15 (Dnajc15), mRNA [NM_001106050]	**14,255**
Ppp2r4	Rattus norvegicus protein phosphatase 2A activator, regulatory subunit 4 (Ppp2r4), mRNA [NM_001108577]	**14,251**
0	Unknown	**14,250**
Aldh5a1	Succinate-semialdehyde dehydrogenase, mitochondrial [Source:UniProtKB/Swiss-Prot;Acc:P51650] [ENSRNOT00000031384]	**14,250**
0	Unknown	**14,250**
LOC365723	PREDICTED: Rattus norvegicus similar to zinc finger protein 458 (LOC365723), miscRNA [XR_086165]	**14,250**
0	Unknown	**14,249**
Zkscan2	PREDICTED: Rattus norvegicus zinc finger with KRAB and SCAN domains 2 (Zkscan2), mRNA [XM_001079250]	**14,243**
0	Unknown	**14,241**
Tox2	Rattus norvegicus TOX high mobility group box family member 2 (Tox2), mRNA [NM_199392]	**14,240**
Cck	Rattus norvegicus cholecystokinin (Cck), mRNA [NM_012829]	**14,232**
Plekhf2	Rattus norvegicus pleckstrin homology domain containing, family F (with FYVE domain) member 2 (Plekhf2), mRNA [NM_001108655]	**14,227**
Cbx6	Rattus norvegicus chromobox homolog 6 (Cbx6), mRNA [NM_001012119]	**14,222**
0	Uncharacterized protein [Source:UniProtKB/TrEMBL;Acc:D3ZP34] [ENSRNOT00000061713]	**14,222**
Pdgfc	Rattus norvegicus platelet derived growth factor C (Pdgfc), mRNA [NM_031317]	**14,220**
Tmem185a	Rattus norvegicus transmembrane protein 185A (Tmem185a), mRNA [NM_001135712]	**14,219**
Dnah17	Uncharacterized protein [Source:UniProtKB/TrEMBL;Acc:D4A2Y8] [ENSRNOT00000004035]	**14,216**

Nsg2	Rattus norvegicus neuron specific gene family member 2 (Nsg2), mRNA [NM_001034152]	**14,213**
Ets2	Rattus norvegicus v-ets erythroblastosis virus E26 oncogene homolog 2 (avian) (Ets2), mRNA [NM_001107107]	**14,213**
0	Unknown	**14,212**
0	Unknown	**14,211**
Mbnl2	Rattus norvegicus muscleblind-like 2 (Mbnl2), mRNA [NM_001111064]	**14,211**
Actl7a	Rattus norvegicus actin-like 7a (Actl7a), mRNA [NM_001011973]	**14,209**
RGD1561678	PREDICTED: Rattus norvegicus similar to Ten-m1 (RGD1561678), mRNA [XM_002730177]	**14,205**
Cpne3	Rattus norvegicus copine III (Cpne3), mRNA [NM_001107917]	**14,205**
Clybl	Rattus norvegicus citrate lyase beta like (Clybl), mRNA [NM_001100685]	**14,201**
Gpr158	Rattus norvegicus G protein-coupled receptor 158 (Gpr158), mRNA [NM_001170326]	**14,197**
Setd5	Rattus norvegicus SET domain containing 5 (Setd5), mRNA [NM_001106614]	**14,196**
Egr4	Rattus norvegicus early growth response 4 (Egr4), mRNA [NM_019137]	**14,195**
Ppm1h	Protein phosphatase 1H [Source:UniProtKB/Swiss-Prot;Acc:Q5M821] [ENSRNOT00000005798]	**14,194**
Mecr	Rattus norvegicus mitochondrial trans-2-enoyl-CoA reductase (Mecr), nuclear gene encoding mitochondrial protein, mRNA [NM_017209]	**14,194**
0	Rattus norvegicus TL0ADA44YP20 mRNA sequence. [FQ219888]	**14,193**
0	Unknown	**14,187**
Lrrtm4	Rattus norvegicus leucine rich repeat transmembrane neuronal 4 (Lrrtm4), mRNA [NM_001134746]	**14,183**
Trim41	Rattus norvegicus tripartite motif-containing 41 (Trim41), mRNA [NM_001134737]	**14,181**
Actr1b	Rattus norvegicus ARP1 actin-related protein 1 homolog B (yeast) (Actr1b), mRNA [NM_001039028]	**14,179**
Wnt5a	Rattus norvegicus wingless-type MMTV integration site family, member 5A (Wnt5a), mRNA [NM_022631]	**14,176**
0	Unknown	**14,175**
Slc2a12	Rattus norvegicus solute carrier family 2 (facilitated glucose transporter), member 12 (Slc2a12), mRNA [NM_001107451]	**14,174**
Alg6	Rattus norvegicus asparagine-linked glycosylation 6, alpha-1,3-glucosyltransferase homolog (S. cerevisiae) (Alg6), mRNA [NM_001033709]	**14,173**
Snx3	Rattus norvegicus sorting nexin 3 (Snx3), mRNA [NM_001044283]	**14,172**
0	Unknown	**14,170**
Chmp4b	PREDICTED: Rattus norvegicus chromatin modifying protein 4B (Chmp4b), mRNA [XM_002726259]	**14,169**
Rtn3	Rattus norvegicus reticulon 3 (Rtn3), transcript variant 2, mRNA [NM_001009953]	**14,169**
Zfp41	Uncharacterized protein [Source:UniProtKB/TrEMBL;Acc:D3ZZK5] [ENSRNOT00000009823]	**14,168**
Pex10	Rattus norvegicus peroxisomal biogenesis factor 10 (Pex10), mRNA [NM_001109405]	**14,166**
Tube1	Rattus norvegicus tubulin, epsilon 1 (Tube1), mRNA [NM_001108536]	**14,164**
Irs1	Rattus norvegicus insulin receptor substrate 1 (Irs1), mRNA [NM_012969]	**14,164**
Reps1	Rattus norvegicus RALBP1 associated Eps domain containing 1 (Reps1), mRNA [NM_001106264]	**14,163**
Angptl6	Rattus norvegicus angiopoietin-like 6 (Angptl6), mRNA [NM_001106702]	**14,159**
Ccdc86	Rattus norvegicus coiled-coil domain containing 86 (Ccdc86), mRNA [NM_001006974]	**14,159**

Arl15	BC026093 ADP-ribosylation factor related protein 2 {Homo sapiens} (exp=-1; wgp=0; cg=0), complete [TC622058]	**14,159**
Zbtb41	Rattus norvegicus zinc finger and BTB domain containing 41 (Zbtb41), mRNA [NM_001191670]	**14,157**
0	AMGNNUC:NRHY6-00002-H8-A W Rat hypothalamus (10470) Rattus norvegicus cDNA clone nrhy6-00002-h8 5', mRNA sequence [CB750240]	**14,156**
Mtcp1	PREDICTED: Rattus norvegicus mature T-cell proliferation 1 (Mtcp1), mRNA [XM_001070795]	**14,156**
0	Uncharacterized protein [Source:UniProtKB/TrEMBL;Acc:D4A3M9] [ENSRNOT00000023028]	**14,155**
Ptprs	Rattus norvegicus protein tyrosine phosphatase, receptor type, S (Ptprs), mRNA [NM_019140]	**14,155**
Zfp318	PREDICTED: Rattus norvegicus zinc finger protein 318 (Zfp318), mRNA [XM_002727181]	**14,154**
Arhgef7	Rattus norvegicus Rho guanine nucleotide exchange factor (GEF7) (Arhgef7), transcript variant 3, mRNA [NM_053740]	**14,153**
0	Rattus norvegicus similar to UBE2I protein (LOC305375), mRNA [XM_223442]	**14,149**
Pdzd7	Rattus norvegicus PDZ domain containing 7 (Pdzd7), mRNA [NM_001106362]	**14,148**
Tmub1	Rattus norvegicus transmembrane and ubiquitin-like domain containing 1 (Tmub1), transcript variant 2, mRNA [NM_001080153]	**14,141**
0	Neuronal acetylcholine receptor subunit alpha-7 [Source:UniProtKB/Swiss-Prot;Acc:Q05941] [ENSRNOT00000020760]	**14,138**
0	Rattus norvegicus similar to Pyruvate kinase, M2 isozyme (LOC290412), mRNA [XM_224416]	**14,138**
Slamf8	Rattus norvegicus SLAM family member 8 (Slamf8), mRNA [NM_001105973]	**14,138**
Arhgap29	Rattus norvegicus Rho GTPase activating protein 29 (Arhgap29), mRNA [NM_001009405]	**14,136**
0	Uncharacterized protein [Source:UniProtKB/TrEMBL;Acc:D4A7E7] [ENSRNOT00000011386]	**14,135**
Olr1343	Rattus norvegicus olfactory receptor 1343 (Olr1343), mRNA [NM_001001088]	**14,135**
Prr3	Rattus norvegicus proline rich 3 (Prr3), mRNA [NM_212544]	**14,134**
LOC497899	Rattus norvegicus similar to hypothetical protein 4930503F14 (LOC497899), mRNA [NM_001017472]	**14,131**
RGD1563996	PREDICTED: Rattus norvegicus similar to Protein UNQ9166/PRO28631 precursor (RGD1563996), mRNA [XM_001077526]	**14,125**
0	Uncharacterized protein [Source:UniProtKB/TrEMBL;Acc:D4AAK9] [ENSRNOT00000038851]	**14,124**
0	Unknown	**14,124**
Kcnq1	Rattus norvegicus potassium voltage-gated channel, KQT-like subfamily, member 1 (Kcnq1), mRNA [NM_032073]	**14,123**
Sgpp1	PREDICTED: Rattus norvegicus sphingosine-1-phosphate phosphatase 1 (Sgpp1), mRNA [XM_001080791]	**14,122**
Zik1	PREDICTED: Rattus norvegicus zinc finger protein interacting with K protein 1 homolog (mouse) (Zik1), mRNA [XM_001076034]	**14,121**
Selplg	Rattus norvegicus selectin P ligand (Selplg), mRNA [NM_001013230]	**14,119**
0	Unknown	**14,118**
Cant1	Rattus norvegicus calcium activated nucleotidase 1 (Cant1), mRNA [NM_144754]	**14,115**
Aqp2	Rattus norvegicus aquaporin 2 (collecting duct) (Aqp2), mRNA [NM_012909]	**14,113**
Pde2a	Rattus norvegicus phosphodiesterase 2A, cGMP-stimulated (Pde2a), transcript variant 2, mRNA [NM_031079]	**14,113**
Tbx2	Rattus norvegicus T-box 2 (Tbx2), mRNA [NM_001107033]	**14,112**
RGD1309710	Rattus norvegicus similar to RIKEN cDNA 0610038D11 (RGD1309710), mRNA [NM_001106330]	**14,109**
Grik5	Rattus norvegicus glutamate receptor, ionotropic, kainate 5 (Grik5), mRNA [NM_031508]	**14,108**
LOC690146	PREDICTED: Rattus norvegicus similar to zinc finger protein 11B (LOC690146), mRNA [XM_001073454]	**14,107**

Rbp4	Rattus norvegicus retinol binding protein 4, plasma (Rbp4), mRNA [NM_013162]	**14,106**
LOC683469	Rattus norvegicus similar to RNA polymerase II transcription factor SIII subunit A2 (Elongin A2) (EloA2) (Transcription elongation factor B polypeptide 3B) (LOC683469), mRNA [NM_001115040]	**14,105**
RGD1564016	PREDICTED: Rattus norvegicus RGD1564016 (RGD1564016), mRNA [XM_579897]	**14,103**
Kcnk13	Rattus norvegicus potassium channel, subfamily K, member 13 (Kcnk13), mRNA [NM_022293]	**14,103**
Ambra1	Rattus norvegicus autophagy/beclin 1 regulator 1 (Ambra1), mRNA [NM_001134341]	**14,100**
0	Rattus norvegicus similar to 60S ribosomal protein L23a (LOC292743), mRNA [XM_218374]	**14,095**
Myo1c	Rattus norvegicus myosin IC (Myo1c), mRNA [NM_023092]	**14,094**
0	Unknown	**14,093**
Rabl5	Rattus norvegicus RAB, member RAS oncogene family-like 5 (Rabl5), mRNA [NM_001011902]	**14,091**
Card6	Rattus norvegicus caspase recruitment domain family, member 6 (Card6), mRNA [NM_001106413]	**14,090**
0	Unknown	**14,089**
Rab22a	Rattus norvegicus RAB22A, member RAS oncogene family (Rab22a), mRNA [NM_001108966]	**14,083**
Calcoco2	PREDICTED: Rattus norvegicus calcium binding and coiled-coil domain 2 (Calcoco2), mRNA [XM_220903]	**14,082**
Bard1	Rattus norvegicus BRCA1 associated RING domain 1 (Bard1), mRNA [NM_022622]	**14,082**
Dusp9	Rattus norvegicus dual specificity phosphatase 9 (Dusp9), mRNA [NM_001037973]	**14,081**
Fusip1	Rattus norvegicus FUS interacting protein (serine-arginine rich) 1 (Fusip1), mRNA [NM_001025738]	**14,081**
Rnase1	Rattus norvegicus ribonuclease, RNase A family, 1 (pancreatic) (Rnase1), mRNA [NM_001029904]	**14,081**
Ccdc109a	Rattus norvegicus coiled-coil domain containing 109A (Ccdc109a), mRNA [NM_001106398]	**14,079**
Rab30	Rattus norvegicus RAB30, member RAS oncogene family (Rab30), mRNA [NM_001015012]	**14,078**
0	Unknown	**14,075**
Gpbp1	Rattus norvegicus GC-rich promoter binding protein 1 (Gpbp1), mRNA [NM_001106410]	**14,072**
Ptov1	Rattus norvegicus prostate tumor overexpressed 1 (Ptov1), mRNA [NM_001008304]	**14,071**
Dd25	Rattus norvegicus hypothetical protein Dd25 (Dd25), mRNA [NM_199403]	**14,070**
Klhdc10	Rattus norvegicus kelch domain containing 10 (Klhdc10), mRNA [NM_001017456]	**14,067**
Gtdc1	Rattus norvegicus glycosyltransferase-like domain containing 1 (Gtdc1), mRNA [NM_001024274]	**14,065**
0	Unknown	**14,064**
Synj2bp	Rattus norvegicus synaptojanin 2 binding protein (Synj2bp), mRNA [NM_022599]	**14,061**
0	Uncharacterized protein [Source:UniProtKB/TrEMBL;Acc:D4A0A1] [ENSRNOT00000016712]	**14,060**
Lipe	Rattus norvegicus lipase, hormone sensitive (Lipe), mRNA [NM_012859]	**14,059**
Apool	Rattus norvegicus apolipoprotein O-like (Apool), mRNA [NM_001014105]	**14,058**
Mest	Rattus norvegicus mesoderm specific transcript homolog (mouse) (Mest), mRNA [NM_001009617]	**14,053**
Slc24a6	Rattus norvegicus solute carrier family 24 (sodium/potassium/calcium exchanger), member 6 (Slc24a6), mRNA [NM_001017488]	**14,053**
0	Unknown	**14,051**

LOC687739	PREDICTED: Rattus norvegicus hypothetical protein LOC687739 (LOC687739), mRNA [XM_001079981]	**14,051**
0	Unknown	**14,051**
Top1	Rattus norvegicus topoisomerase (DNA) I (Top1), mRNA [NM_022615]	**14,047**
Pgam1	Rattus norvegicus phosphoglycerate mutase 1 (brain) (Pgam1), mRNA [NM_053290]	**14,044**
0	Uncharacterized protein [Source:UniProtKB/TrEMBL;Acc:D3ZRA7] [ENSRNOT00000025489]	**14,043**
Xrcc6	Rattus norvegicus X-ray repair complementing defective repair in Chinese hamster cells 6 (Xrcc6), mRNA [NM_139080]	**14,043**
Treml2	PREDICTED: Rattus norvegicus triggering receptor expressed on myeloid cells-like 2 (Treml2), mRNA [XM_001059150]	**14,041**
Agrn	Rattus norvegicus agrin (Agrn), mRNA [NM_175754]	**14,040**
0	Unknown	**14,039**
LOC682469	PREDICTED: Rattus norvegicus similar to jumonji domain containing 1B (LOC682469), mRNA [XM_001061636]	**14,039**
Slc8a1	Rattus norvegicus solute carrier family 8 (sodium/calcium exchanger), member 1 (Slc8a1), mRNA [NM_019268]	**14,039**
Crtc3	PREDICTED: Rattus norvegicus CREB regulated transcription coactivator 3 (Crtc3), mRNA [XM_001066604]	**14,036**
Gpc2	Rattus norvegicus glypican 2 (Gpc2), mRNA [NM_138511]	**14,035**
0	Unknown	**14,033**
LOC302228	PREDICTED: Rattus norvegicus similar to Spindlin-like protein 2 (SPIN-2) (LOC302228), mRNA [XM_229842]	**14,032**
Utp14a	Rattus norvegicus UTP14, U3 small nucleolar ribonucleoprotein, homolog A (yeast) (Utp14a), mRNA [NM_001014113]	**14,021**
Grip2	Rattus norvegicus glutamate receptor interacting protein 2 (Grip2), mRNA [NM_138535]	**14,019**
Aak1	Rattus norvegicus AP2 associated kinase 1 (Aak1), mRNA [NM_001173450]	**14,017**
Cnot7	Rattus norvegicus CCR4-NOT transcription complex, subunit 7 (Cnot7), mRNA [NM_001107313]	**14,015**
0	Unknown	**14,014**
Klf10	Rattus norvegicus Kruppel-like factor 10 (Klf10), mRNA [NM_031135]	**14,012**
Nfatc4	Rattus norvegicus nuclear factor of activated T-cells, cytoplasmic, calcineurin-dependent 4 (Nfatc4), mRNA [NM_001107264]	**14,012**
Hpse	Rattus norvegicus heparanase (Hpse), mRNA [NM_022605]	**14,009**
Lmo4	Rattus norvegicus LIM domain only 4 (Lmo4), mRNA [NM_001009708]	**14,008**
Ncald	Rattus norvegicus neurocalcin delta (Ncald), mRNA [NM_001024371]	**14,007**
Stx16	syntaxin-16 [Source:RefSeq peptide;Acc:NP_001102080] [ENSRNOT00000007054]	**14,007**
0	Rattus norvegicus similar to glyceraldehyde-3-phosphate dehydrogenase (LOC294447), mRNA [XM_228217]	**14,007**
Ehmt1	Rattus norvegicus euchromatic histone-lysine N-methyltransferase 1 (Ehmt1), mRNA [NM_001108572]	**14,006**
Bhlhe23	Rattus norvegicus basic helix-loop-helix family, member e23 (Bhlhe23), mRNA [NM_001109211]	**14,005**
0	Unknown	**14,005**
LOC690347	PREDICTED: Rattus norvegicus hypothetical protein LOC690347 (LOC690347), mRNA [XM_001074177]	**14,003**
Eif4enif1	Rattus norvegicus eukaryotic translation initiation factor 4E nuclear import factor 1 (Eif4enif1), mRNA [NM_001107230]	**14,001**
0	Uncharacterized protein [Source:UniProtKB/TrEMBL;Acc:D3ZSR6] [ENSRNOT00000064626]	**14,000**
0	Uncharacterized protein [Source:UniProtKB/TrEMBL;Acc:D4A5N6] [ENSRNOT00000050854]	**13,999**

Kctd6	Rattus norvegicus potassium channel tetramerisation domain containing 6 (Kctd6), mRNA [NM_001107253]	**13,997**
Thra	Rattus norvegicus thyroid hormone receptor alpha (Thra), transcript variant TRalpha1, mRNA [NM_001017960]	**13,996**
LOC689919	PREDICTED: Rattus norvegicus similar to 40S ribosomal protein S26 (LOC689919), mRNA [XM_001072530]	**13,994**
Slc30a7	Rattus norvegicus solute carrier family 30 (zinc transporter), member 7 (Slc30a7), mRNA [NM_001191715]	**13,994**
Lipa	Rattus norvegicus lipase A, lysosomal acid, cholesterol esterase (Lipa), mRNA [NM_012732]	**13,992**
LOC498154	Rattus norvegicus hypothetical protein LOC498154 (LOC498154), mRNA [NM_001025033]	**13,991**
Slc23a2	Rattus norvegicus solute carrier family 23 (nucleobase transporters), member 2 (Slc23a2), mRNA [NM_017316]	**13,987**
Serpine2	Rattus norvegicus serine (or cysteine) peptidase inhibitor, clade E, member 2 (Serpine2), mRNA [NM_019197]	**13,987**
Larp5	Rattus norvegicus La ribonucleoprotein domain family, member 5 (Larp5), mRNA [NM_001107361]	**13,978**
Brpf3	Rattus norvegicus bromodomain and PHD finger containing, 3 (Brpf3), mRNA [NM_001107615]	**13,975**
Nr3c2	Rattus norvegicus nuclear receptor subfamily 3, group C, member 2 (Nr3c2), mRNA [NM_013131]	**13,973**
Wdr45l	Rattus norvegicus Wdr45 like (Wdr45l), mRNA [NM_001039587]	**13,972**
Hiatl1	Rattus norvegicus hippocampus abundant transcript-like 1 (Hiatl1), mRNA [NM_001107334]	**13,970**
LOC680874	PREDICTED: Rattus norvegicus hypothetical protein LOC680874 (LOC680874), miscRNA [XR_005897]	**13,969**
Nek1	Rattus norvegicus NIMA (never in mitosis gene a)-related kinase 1 (Nek1), mRNA [NM_001106082]	**13,966**
Fuz	Rattus norvegicus fuzzy homolog (Drosophila) (Fuz), mRNA [NM_001037646]	**13,960**
MGC116197	Rattus norvegicus similar to RIKEN cDNA 1700001E04 (MGC116197), mRNA [NM_001025755]	**13,959**
Pla2g12a	Rattus norvegicus phospholipase A2, group XIIA (Pla2g12a), mRNA [NM_001108565]	**13,957**
0	Collagen alpha-1(XI) chain [Source:UniProtKB/Swiss-Prot;Acc:P20909] [ENSRNOT00000024138]	**13,953**
Plekhh2	Rattus norvegicus pleckstrin homology domain containing, family H (with MyTH4 domain) member 2 (Plekhh2), mRNA [NM_001191770]	**13,951**
Homer3	Rattus norvegicus homer homolog 3 (Drosophila) (Homer3), mRNA [NM_053310]	**13,950**
Npff	Rattus norvegicus neuropeptide FF-amide peptide precursor (Npff), mRNA [NM_022586]	**13,949**
Radil	Rattus norvegicus Ras association and DIL domains (Radil), mRNA [NM_001037218]	**13,949**
0	Uncharacterized protein [Source:UniProtKB/TrEMBL;Acc:D3ZHI0] [ENSRNOT00000061428]	**13,944**
Apol3	Rattus norvegicus apolipoprotein L, 3 (Apol3), mRNA [NM_001013175]	**13,943**
LOC619574	Rattus norvegicus hypothetical protein LOC619574 (LOC619574), mRNA [NM_001034959]	**13,942**
Dio2	Rattus norvegicus deiodinase, iodothyronine, type II (Dio2), mRNA [NM_031720]	**13,942**
Klhl30	PREDICTED: Rattus norvegicus kelch-like 30 (Drosophila) (Klhl30), mRNA [XM_237394]	**13,942**
Rdh11	Rattus norvegicus retinol dehydrogenase 11 (all-trans/9-cis/11-cis) (Rdh11), mRNA [NM_001012193]	**13,940**
Iqcf2	PREDICTED: Rattus norvegicus IQ motif containing F2 (Iqcf2), mRNA [XM_001070183]	**13,939**
Olr733	Rattus norvegicus olfactory receptor 733 (Olr733), mRNA [NM_001000618]	**13,939**
Fam149b1	Rattus norvegicus family with sequence similarity 149, member B1 (Fam149b1), mRNA [NM_001013878]	**13,937**
LOC100294508	Rattus norvegicus dyslexia susceptibility 2-like (LOC100294508), mRNA [NM_001159655]	**13,937**
Pigm	Rattus norvegicus phosphatidylinositol glycan anchor biosynthesis, class M (Pigm), mRNA [NM_024144]	**13,935**

Tspan33	Rattus norvegicus tetraspanin 33 (Tspan33), mRNA [NM_001109227]	**13,935**
MAST1	Rattus norvegicus microtubule associated serine/threonine kinase 1 (MAST1), mRNA [NM_181089]	**13,931**
Ghrh	Rattus norvegicus growth hormone releasing hormone (Ghrh), mRNA [NM_031577]	**13,931**
Anapc11	Rattus norvegicus anaphase promoting complex subunit 11 (Anapc11), mRNA [NM_001126082]	**13,929**
Aldh4a1	Rattus norvegicus aldehyde dehydrogenase 4 family, member A1 (Aldh4a1), nuclear gene encoding mitochondrial protein, mRNA [NM_001134698]	**13,928**
Phtf2	Rattus norvegicus putative homeodomain transcription factor 2 (Phtf2), mRNA [NM_001106577]	**13,927**
Cecr6	PREDICTED: Rattus norvegicus cat eye syndrome chromosome region, candidate 6 homolog (human) (Cecr6), mRNA [XM_575656]	**13,924**
Lman2l	VIP36-like protein [Source:RefSeq peptide;Acc:NP_001100370] [ENSRNOT00000021111]	**13,922**
Hiat1	Rattus norvegicus hippocampus abundant gene transcript 1 (Hiat1), mRNA [NM_001106467]	**13,919**
Oxa1l	Rattus norvegicus oxidase assembly 1-like (Oxa1l), mRNA [NM_001168583]	**13,918**
0	Glyceraldehyde-3-phosphate dehydrogenase [Source:UniProtKB/TrEMBL;Acc:D3Z7Z7] [ENSRNOT00000038921]	**13,918**
RGD1561318	PREDICTED: Rattus norvegicus similar to SET domain-containing protein (RGD1561318), mRNA [XM_001066702]	**13,915**
Slc39a14	Rattus norvegicus solute carrier family 39 (zinc transporter), member 14 (Slc39a14), mRNA [NM_001107275]	**13,914**
0	Uncharacterized protein [Source:UniProtKB/TrEMBL;Acc:D3ZH31] [ENSRNOT00000010612]	**13,912**
0	zinc finger protein 866 Gene [Source:MGI Symbol;Acc:MGI:3584369] [ENSRNOT00000034446]	**13,911**
Gpr180	Rattus norvegicus G protein-coupled receptor 180 (Gpr180), mRNA [NM_001006994]	**13,905**
Nolc1	Rattus norvegicus nucleolar and coiled-body phosphoprotein 1 (Nolc1), mRNA [NM_022869]	**13,903**
RGD1559917	Rattus norvegicus similar to SMT3 suppressor of mif two 3 homolog 2 (LOC287993), mRNA [XM_212687]	**13,902**
Ints7	Rattus norvegicus integrator complex subunit 7 (Ints7), mRNA [NM_001191675]	**13,901**
Raver2	Rattus norvegicus ribonucleoprotein, PTB-binding 2 (Raver2), mRNA [NM_001191867]	**13,901**
Vti1a	Rattus norvegicus vesicle transport through interaction with t-SNAREs homolog 1A (yeast) (Vti1a), mRNA [NM_023101]	**13,900**
Gpr158	Rattus norvegicus G protein-coupled receptor 158 (Gpr158), mRNA [NM_001170326]	**13,896**
RGD1563680	Uncharacterized protein [Source:UniProtKB/TrEMBL;Acc:D3ZYC4] [ENSRNOT00000006177]	**13,895**
0	Unknown	**13,895**
Tnrc6a	Rattus norvegicus trinucleotide repeat containing 6a (Tnrc6a), mRNA [NM_001107549]	**13,893**
Nr2c2	Rattus norvegicus nuclear receptor subfamily 2, group C, member 2 (Nr2c2), mRNA [NM_017323]	**13,890**
Acta1	Rattus norvegicus actin, alpha 1, skeletal muscle (Acta1), mRNA [NM_019212]	**13,888**
Ablim2	Rattus norvegicus actin binding LIM protein family, member 2 (Ablim2), transcript variant 1, mRNA [NM_001177695]	**13,887**
0	Unknown	**13,887**
Sox15	Rattus norvegicus SRY (sex determining region Y)-box 15 (Sox15), mRNA [NM_001108830]	**13,885**
Gcc2	Rattus norvegicus GRIP and coiled-coil domain containing 2 (Gcc2), mRNA [NM_001107633]	**13,885**
Lypd5	Rattus norvegicus Ly6/Plaur domain containing 5 (Lypd5), mRNA [NM_001192010]	**13,881**
0	Q4BL67_BURVI (Q4BL67) Phospholipase/Carboxylesterase, partial (5%) [TC592585]	**13,881**
0	PREDICTED: Rattus norvegicus dynein, axonemal, heavy polypeptide 9 (Dnah9), mRNA [XM_002727722]	**13,881**

Znf496	Rattus norvegicus similar to D130067D09 protein (LOC287361), mRNA [XM_220512]	**13,880**
Cd47	Rattus norvegicus Cd47 molecule (Cd47), mRNA [NM_019195]	**13,880**
Olr1714	Rattus norvegicus olfactory receptor 1714 (Olr1714), mRNA [NM_214456]	**13,877**
Fgfr1op2	Rattus norvegicus FGFR1 oncogene partner 2 (Fgfr1op2), mRNA [NM_201421]	**13,876**
Gipr	Rattus norvegicus gastric inhibitory polypeptide receptor (Gipr), mRNA [NM_012714]	**13,875**
0	Uncharacterized protein [Source:UniProtKB/TrEMBL;Acc:D3ZI73] [ENSRNOT00000026047]	**13,875**
0	Unknown	**13,874**
0	PREDICTED: Rattus norvegicus similar to SET domain-containing protein (RGD1561853), mRNA [XM_001064143]	**13,871**
0	Unknown	**13,871**
0	Unknown	**13,870**
Ubr7	Rattus norvegicus ubiquitin protein ligase E3 component n-recognin 7 (putative) (Ubr7), mRNA [NM_001007705]	**13,870**
Nipa2	Rattus norvegicus non imprinted in Prader-Willi/Angelman syndrome 2 homolog (human) (Nipa2), mRNA [NM_001107518]	**13,869**
0	Unknown	**13,867**
Lpin1	Rattus norvegicus lipin 1 (Lpin1), mRNA [NM_001012111]	**13,867**
RGD1312026	Rattus norvegicus similar to RIKEN cDNA C230081A13 (RGD1312026), mRNA [NM_001108149]	**13,865**
Zfp868	Rattus norvegicus zinc finger protein 868 (Zfp868), mRNA [NM_001009538]	**13,864**
Tmem88	Rattus norvegicus transmembrane protein 88 (Tmem88), mRNA [NM_001128155]	**13,864**
Zc3h3	Rattus norvegicus zinc finger CCCH type containing 3 (Zc3h3), mRNA [NM_001134865]	**13,863**
0	Unknown	**13,861**
0	Unknown	**13,857**
0	Unknown	**13,855**
RGD1563982	similar to F-box only protein 27 (RGD1563982), mRNA [Source:RefSeq DNA;Acc:NM_001110491] [ENSRNOT00000026997]	**13,854**
Tmem43	Rattus norvegicus transmembrane protein 43 (Tmem43), mRNA [NM_001007745]	**13,854**
Spa17	Rattus norvegicus sperm autoantigenic protein 17 (Spa17), mRNA [NM_053482]	**13,852**
RGD1563888	Rattus norvegicus similar to DNA segment, Chr 16, ERATO Doi 472, expressed (RGD1563888), mRNA [NM_001108312]	**13,851**
Larp1	BC001460 la related protein {Homo sapiens} (exp=-1; wgp=0; cg=0), partial (3%) [TC629660]	**13,850**
Capn6	Rattus norvegicus calpain 6 (Capn6), mRNA [NM_031808]	**13,850**
Zfp426	Rattus norvegicus zinc finger protein 426 (Zfp426), mRNA [NM_001079943]	**13,849**
Mcf2l	Rattus norvegicus MCF.2 cell line derived transforming sequence-like (Mcf2l), mRNA [NM_053951]	**13,847**
Cyb5d2	Rattus norvegicus cytochrome b5 domain containing 2 (Cyb5d2), mRNA [NM_001007671]	**13,841**
Iapp	Islet amyloid polypeptide [Source:UniProtKB/Swiss-Prot;Acc:P12969] [ENSRNOT00000016614]	**13,838**
Aak1	Rattus norvegicus AP2 associated kinase 1 (Aak1), mRNA [NM_001173450]	**13,836**
Tfip11	Rattus norvegicus tuftelin interacting protein 11 (Tfip11), mRNA [NM_001008291]	**13,835**
0	Ubiquitin carboxyl-terminal hydrolase [Source:UniProtKB/TrEMBL;Acc:D3Z8K5] [ENSRNOT00000035084]	**13,834**

Tbc1d14	Rattus norvegicus TBC1 domain family, member 14 (Tbc1d14), transcript variant 1, mRNA [NM_001012152]	**13,833**
Mtpap	Rattus norvegicus mitochondrial poly(A) polymerase (Mtpap), nuclear gene encoding mitochondrial protein, mRNA [NM_001107359]	**13,833**
0	Unknown	**13,828**
Abhd8	Rattus norvegicus abhydrolase domain containing 8 (Abhd8), mRNA [NM_001107301]	**13,828**
Acvr2b	Rattus norvegicus activin A receptor, type IIB (Acvr2b), mRNA [NM_031554]	**13,826**
Sec63	Rattus norvegicus SEC63 homolog (S. cerevisiae) (Sec63), mRNA [NM_001107637]	**13,823**
Rab32	Rattus norvegicus RAB32, member RAS oncogene family (Rab32), mRNA [NM_001108902]	**13,821**
G7c	Rattus norvegicus G7c protein (G7c), mRNA [NM_212499]	**13,821**
Fam195b	Rattus norvegicus family with sequence similarity 195, member B (Fam195b), mRNA [NM_001108311]	**13,820**
Lipl3	Uncharacterized protein [Source:UniProtKB/TrEMBL;Acc:D4AA61] [ENSRNOT00000026219]	**13,819**
Hsbp1	Rattus norvegicus heat shock factor binding protein 1 (Hsbp1), mRNA [NM_173119]	**13,818**
Fam65a	Protein FAM65A [Source:UniProtKB/Swiss-Prot;Acc:Q4FZU8] [ENSRNOT00000023710]	**13,817**
Elovl6	Rattus norvegicus ELOVL family member 6, elongation of long chain fatty acids (yeast) (Elovl6), mRNA [NM_134383]	**13,815**
0	Unknown	**13,814**
Rnf39	Rattus norvegicus ring finger protein 39 (Rnf39), mRNA [NM_134374]	**13,812**
Nrg1	Rattus norvegicus neuregulin 1 (Nrg1), mRNA [NM_031588]	**13,811**
Ralbp1	Rattus norvegicus ralA binding protein 1 (Ralbp1), mRNA [NM_032067]	**13,809**
Dip2c	Rattus norvegicus DIP2 disco-interacting protein 2 homolog C (Drosophila) (Dip2c), mRNA [NM_001107360]	**13,807**
Golph3	Rattus norvegicus golgi phosphoprotein 3 (coat-protein) (Golph3), mRNA [NM_023977]	**13,804**
Ndel1	Rattus norvegicus nudE nuclear distribution gene E homolog (A. nidulans)-like 1 (Ndel1), mRNA [NM_133320]	**13,804**
Ppm1l	Rattus norvegicus protein phosphatase 1 (formerly 2C)-like (Ppm1l), mRNA [NM_001107681]	**13,798**
Arhgef12	Rattus norvegicus Rho guanine nucleotide exchange factor (GEF) 12 (Arhgef12), mRNA [NM_001013246]	**13,798**
Dedd	Rattus norvegicus death effector domain-containing (Dedd), mRNA [NM_031800]	**13,796**
Ctbp2	Rattus norvegicus C-terminal binding protein 2 (Ctbp2), mRNA [NM_053335]	**13,796**
Arrdc3	Rattus norvegicus arrestin domain containing 3 (Arrdc3), mRNA [NM_001007797]	**13,795**
Dlgap1	Rattus norvegicus discs, large (Drosophila) homolog-associated protein 1 (Dlgap1), mRNA [NM_022946]	**13,791**
Caprin1	Rattus norvegicus cell cycle associated protein 1 (Caprin1), mRNA [NM_001012185]	**13,791**
Son	Rattus norvegicus Son DNA binding protein (Son), transcript variant 2, mRNA [NM_001170328]	**13,791**
Senp7	Rattus norvegicus SUMO1/sentrin specific peptidase 7 (Senp7), mRNA [NM_001105888]	**13,789**
Thap7	Rattus norvegicus THAP domain containing 7 (Thap7), mRNA [NM_001105863]	**13,789**
Atp5g2	Rattus norvegicus ATP synthase, H+ transporting, mitochondrial F0 complex, subunit C2 (subunit 9) (Atp5g2), nuclear gene encoding mitochondrial protein, mRNA [NM_133556]	**13,788**
Stag2	Rattus norvegicus stromal antigen 2 (Stag2), mRNA [NM_001173507]	**13,782**
Ildr2	Uncharacterized protein [Source:UniProtKB/TrEMBL;Acc:D3ZGJ1] [ENSRNOT00000031367]	**13,781**

Stxbp6	Rattus norvegicus syntaxin binding protein 6 (amisyn) (Stxbp6), mRNA [NM_001191872]	**13,781**
LOC497952	PREDICTED: Rattus norvegicus similar to Ubiquitin-associated protein 2-like (LOC497952), miscRNA [XR_005763]	**13,780**
Tmem211	Uncharacterized protein [Source:UniProtKB/TrEMBL;Acc:D3Z827] [ENSRNOT00000043717]	**13,778**
0	Q571D7_MOUSE (Q571D7) MKIAA0268 protein (Fragment), partial (20%) [TC587453]	**13,778**
Vps13b	Rattus norvegicus vacuolar protein sorting 13 homolog B (yeast) (Vps13b), mRNA [NM_001134886]	**13,776**
0	RGD1304924 protein [Source:UniProtKB/TrEMBL;Acc:B0BNI8] [ENSRNOT00000064652]	**13,775**
0	Unknown	**13,773**
0	AA944379 EST199878 Normalized rat embryo, Bento Soares Rattus sp. cDNA clone REMAG24 3' end, mRNA sequence [AA944379]	**13,772**
RGD1310773	Rattus norvegicus similar to hypothetical protein FLJ31810 (RGD1310773), mRNA [NM_001107926]	**13,771**
Fem1a	Rattus norvegicus fem-1 homolog a (C. elegans) (Fem1a), mRNA [NM_001025706]	**13,763**
Tgfbr2	Rattus norvegicus transforming growth factor, beta receptor II (Tgfbr2), mRNA [NM_031132]	**13,759**
Trim16	Rattus norvegicus tripartite motif-containing 16 (Trim16), mRNA [NM_001135033]	**13,755**
Galt	Rattus norvegicus galactose-1-phosphate uridylyltransferase (Galt), mRNA [NM_001013089]	**13,755**
Rxfp1	Rattus norvegicus relaxin/insulin-like family peptide receptor 1 (Rxfp1), mRNA [NM_201417]	**13,753**
0	Unknown	**13,753**
0	Uncharacterized protein [Source:UniProtKB/TrEMBL;Acc:D3ZJB6] [ENSRNOT00000040584]	**13,753**
Dgat2l6	Rattus norvegicus diacylglycerol O-acyltransferase 2-like 6 (Dgat2l6), mRNA [NM_001109370]	**13,753**
Dpp6	Rattus norvegicus dipeptidylpeptidase 6 (Dpp6), mRNA [NM_022850]	**13,752**
Phka2	Rattus norvegicus phosphorylase kinase, alpha 2 (Phka2), mRNA [NM_001190994]	**13,751**
Rhobtb3	Rattus norvegicus Rho-related BTB domain containing 3 (Rhobtb3), mRNA [NM_001107645]	**13,751**
Ict1	Rattus norvegicus immature colon carcinoma transcript 1 (Ict1), mRNA [NM_001191656]	**13,749**
Il17d	PREDICTED: Rattus norvegicus interleukin 17D (Il17d), mRNA [XM_001079675]	**13,748**
0	Unknown	**13,747**
Nptn	Rattus norvegicus neuroplastin (Nptn), mRNA [NM_019380]	**13,747**
Zcchc2	Rattus norvegicus zinc finger, CCHC domain containing 2 (Zcchc2), mRNA [NM_001122677]	**13,744**
0	Catenin delta-2 [Source:UniProtKB/Swiss-Prot;Acc:O35116] [ENSRNOT00000044871]	**13,743**
Ankrd13c	Rattus norvegicus ankyrin repeat domain 13C (Ankrd13c), mRNA [NM_001191570]	**13,736**
0	Unknown	**13,731**
Thsd7b	Rattus norvegicus thrombospondin, type I, domain containing 7B (Thsd7b), mRNA [NM_001191669]	**13,728**
Guca2a	Rattus norvegicus guanylate cyclase activator 2a (guanylin) (Guca2a), mRNA [NM_013118]	**13,727**
Scyl2	Rattus norvegicus SCY1-like 2 (S. cerevisiae) (Scyl2), mRNA [NM_001191780]	**13,727**
Map1s	Rattus norvegicus microtubule-associated protein 1S (Map1s), mRNA [NM_001106070]	**13,726**
Fbxl19	Rattus norvegicus F-box and leucine-rich repeat protein 19 (Fbxl19), mRNA [NM_001107552]	**13,725**
Olr1598	Rattus norvegicus olfactory receptor 1598 (Olr1598), mRNA [NM_001000910]	**13,723**

Porf-2	Rattus norvegicus preoptic regulatory factor-2 (Porf-2), mRNA [NM_173122]	**13,721**
Gls	Rattus norvegicus glutaminase (Gls), nuclear gene encoding mitochondrial protein, transcript variant 1, mRNA [NM_012569]	**13,720**
Kcnh3	Rattus norvegicus potassium voltage-gated channel, subfamily H (eag-related), member 3 (Kcnh3), mRNA [NM_017108]	**13,719**
Fam155a	PREDICTED: Rattus norvegicus family with sequence similarity 155, member A (Fam155a), mRNA [XM_001076507]	**13,718**
Suv420h1	Rattus norvegicus suppressor of variegation 4-20 homolog 1 (Drosophila) (Suv420h1), mRNA [NM_001108512]	**13,713**
LOC684773	PREDICTED: Rattus norvegicus similar to histone 2a (LOC684773), mRNA [XM_001071887]	**13,712**
LOC100362255	PREDICTED: Rattus norvegicus G protein-coupled receptor 98 (LOC100362255), mRNA [XM_002729130]	**13,708**
Pla2g4b	Rattus norvegicus phospholipase A2, group IVB (cytosolic) (Pla2g4b), mRNA [NM_001107764]	**13,708**
Gucy1a2	Rattus norvegicus guanylate cyclase 1, soluble, alpha 2 (Gucy1a2), mRNA [NM_023956]	**13,707**
Clip1	Rattus norvegicus CAP-GLY domain containing linker protein 1 (Clip1), mRNA [NM_031745]	**13,707**
Pex12	Rattus norvegicus peroxisomal biogenesis factor 12 (Pex12), mRNA [NM_053921]	**13,706**
LOC684993	Rattus norvegicus hypothetical protein LOC684993 (LOC684993), mRNA [NM_001109451]	**13,701**
0	Unknown	**13,698**
Casc4	Uncharacterized protein [Source:UniProtKB/TrEMBL;Acc:D3Z812] [ENSRNOT00000038291]	**13,698**
Gp1bb	Rattus norvegicus glycoprotein Ib (platelet), beta polypeptide (Gp1bb), mRNA [NM_053930]	**13,697**
Arrb1	Rattus norvegicus arrestin, beta 1 (Arrb1), mRNA [NM_012910]	**13,696**
Nrsn2	Rattus norvegicus neurensin 2 (Nrsn2), mRNA [NM_001109561]	**13,695**
Rbbp5	Rattus norvegicus retinoblastoma binding protein 5 (Rbbp5), mRNA [NM_001107174]	**13,693**
0	PREDICTED: Rattus norvegicus rCG53928-like (LOC100364438), mRNA [XM_002725571]	**13,692**
Mylk	Rattus norvegicus myosin light chain kinase (Mylk), mRNA [NM_001105874]	**13,691**
Strn	Rattus norvegicus striatin, calmodulin binding protein (Strn), mRNA [NM_019148]	**13,689**
Arl8b	Rattus norvegicus ADP-ribosylation factor-like 8B (Arl8b), mRNA [NM_001024332]	**13,688**
Cox11	Rattus norvegicus COX11 homolog, cytochrome c oxidase assembly protein (yeast) (Cox11), nuclear gene encoding mitochondrial protein, mRNA [NM_001109575]	**13,686**
Rnf144b	Rattus norvegicus ring finger protein 144B (Rnf144b), mRNA [NM_001108881]	**13,684**
Sf3a1	Rattus norvegicus splicing factor 3a, subunit 1 (Sf3a1), mRNA [NM_001107235]	**13,681**
RGD1559682	PREDICTED: Rattus norvegicus similar to peptidylprolyl isomerase A (cyclophilin A)) (RGD1559682), mRNA [XM_341363]	**13,680**
Znf512b	Rattus norvegicus zinc finger protein 512B (Znf512b), mRNA [NM_001107809]	**13,680**
Pex11b	Rattus norvegicus peroxisomal biogenesis factor 11 beta (Pex11b), mRNA [NM_001025684]	**13,679**
0	Unknown	**13,673**
Slitrk1	Rattus norvegicus SLIT and NTRK-like family, member 1 (Slitrk1), mRNA [NM_001107283]	**13,673**
Srek1ip1	Rattus norvegicus splicing regulatory glutamine/lysine-rich protein 1 interacting protein 1 (Srek1ip1), mRNA [NM_001008373]	**13,672**
Nkx3-1	Rattus norvegicus NK3 homeobox 1 (Nkx3-1), mRNA [NM_001034144]	**13,671**
Commd6	Rattus norvegicus COMM domain containing 6 (Commd6), mRNA [NM_001109105]	**13,670**

Mtmr2	Rattus norvegicus myotubularin related protein 2 (Mtmr2), mRNA [NM_001108123]	**13,668**
LOC499465	Rattus norvegicus hypothetical protein LOC499465 (LOC499465), mRNA [NM_001025038]	**13,667**
Gtpbp3	tRNA modification GTPase GTPBP3, mitochondrial [Source:UniProtKB/Swiss-Prot;Acc:Q5PQQ1] [ENSRNOT00000060355]	**13,666**
Fam198a	AMGNNUC:TRBA2-00002-F12-WZE trba2 (10286) Rattus norvegicus cDNA clone trba2-00002-f12, mRNA sequence [CB606389]	**13,665**
Ogn	Rattus norvegicus osteoglycin (Ogn), mRNA [NM_001106103]	**13,663**
Ap1s1	Rattus norvegicus adaptor-related protein complex 1, sigma 1 subunit (Ap1s1), mRNA [NM_001108331]	**13,663**
B3gntl1	Rattus norvegicus UDP-GlcNAc:betaGal beta-1,3-N-acetylglucosaminyltransferase-like 1 (B3gntl1), mRNA [NM_001015035]	**13,659**
Rras2	Rattus norvegicus related RAS viral (r-ras) oncogene homolog 2 (Rras2), mRNA [NM_001013434]	**13,658**
Trappc10	Rattus norvegicus trafficking protein particle complex 10 (Trappc10), mRNA [NM_001173528]	**13,657**
Ddo	Rattus norvegicus D-aspartate oxidase (Ddo), mRNA [NM_001109465]	**13,649**
RGD1562218	Rattus norvegicus similar to RIKEN cDNA 0610039J04 (RGD1562218), mRNA [NM_001034919]	**13,646**
Rinl	Rattus norvegicus Ras and Rab interactor-like (Rinl), mRNA [NM_001106241]	**13,646**
Cln8	Rattus norvegicus ceroid-lipofuscinosis, neuronal 8 (Cln8), mRNA [NM_001007686]	**13,645**
0	Unknown	**13,644**
Pnmt	Rattus norvegicus phenylethanolamine-N-methyltransferase (Pnmt), mRNA [NM_031526]	**13,639**
0	Unknown	**13,638**
LOC680448	Uncharacterized protein [Source:UniProtKB/TrEMBL;Acc:D4ACU2] [ENSRNOT00000060468]	**13,637**
Ifit2	Rattus norvegicus interferon-induced protein with tetratricopeptide repeats 2 (Ifit2), mRNA [NM_001024753]	**13,636**
Yaf2	Rattus norvegicus YY1 associated factor 2 (Yaf2), mRNA [NM_001134871]	**13,636**
0	Unknown	**13,634**
Peli1	Rattus norvegicus pellino 1 (Peli1), mRNA [NM_001100565]	**13,633**
Gabrg1	Rattus norvegicus gamma-aminobutyric acid (GABA) A receptor, gamma 1 (Gabrg1), mRNA [NM_080586]	**13,632**
0	PREDICTED: Rattus norvegicus similar to translocating chain-associating membrane protein 2, transcript variant 1 (LOC684534), mRNA [XM_001070862]	**13,625**
Abl2	Rattus norvegicus v-abl Abelson murine leukemia viral oncogene homolog 2 (arg, Abelson-related gene) (Abl2), mRNA [NM_001107186]	**13,625**
0	PREDICTED: Rattus norvegicus TRAF domain and POZ/BTB containing protein T1-like (LOC100365687), partial mRNA [XM_002725996]	**13,623**
Ghr	Rattus norvegicus growth hormone receptor (Ghr), mRNA [NM_017094]	**13,622**
Mphosph9	Rattus norvegicus M-phase phosphoprotein 9 (Mphosph9), mRNA [NM_001170554]	**13,621**
Nlrx1	Rattus norvegicus NLR family member X1 (Nlrx1), mRNA [NM_001025010]	**13,620**
0	Unknown	**13,618**
Hrh2	Rattus norvegicus histamine receptor H 2 (Hrh2), mRNA [NM_012965]	**13,616**
Wibg	Rattus norvegicus within bgcn homolog (Drosophila) (Wibg), mRNA [NM_001108986]	**13,616**
RGD1566033	PREDICTED: Rattus norvegicus similar to BC003940 protein (RGD1566033), mRNA [XM_001070540]	**13,612**
Odz3	Rattus norvegicus odz, odd Oz/ten-m homolog 3 (Drosophila) (Odz3), mRNA [NM_001169133]	**13,612**

Npr2	Rattus norvegicus natriuretic peptide receptor B/guanylate cyclase B (atrionatriuretic peptide receptor B) (Npr2), mRNA [NM_053838]	**13,612**
0	Unknown	**13,610**
Kitlg	Rattus norvegicus KIT ligand (Kitlg), transcript variant 1, mRNA [NM_021843]	**13,609**
0	Uncharacterized protein [Source:UniProtKB/TrEMBL;Acc:D4A553] [ENSRNOT00000016227]	**13,607**
0	Unknown	**13,606**
Ivd	Rattus norvegicus isovaleryl coenzyme A dehydrogenase (Ivd), nuclear gene encoding mitochondrial protein, mRNA [NM_012592]	**13,602**
0	Glyceraldehyde-3-phosphate dehydrogenase [Source:UniProtKB/TrEMBL;Acc:D3ZEN2] [ENSRNOT00000022122]	**13,602**
Nalcn	Rattus norvegicus sodium leak channel, non-selective (Nalcn), mRNA [NM_153630]	**13,600**
Gria3	Rattus norvegicus glutamate receptor, ionotrophic, AMPA 3 (Gria3), transcript variant 1, mRNA [NM_032990]	**13,599**
0	Unknown	**13,593**
0	Unknown	**13,591**
Tbc1d5	Rattus norvegicus TBC1 domain family, member 5 (Tbc1d5), mRNA [NM_001134762]	**13,591**
Isoc2b	Rattus norvegicus isochorismatase domain containing 2b (Isoc2b), mRNA [NM_001008367]	**13,587**
Flt4	Rattus norvegicus fms-related tyrosine kinase 4 (Flt4), mRNA [NM_053652]	**13,584**
Mt1a	Rattus norvegicus metallothionein 1a (Mt1a), mRNA [NM_138826]	**13,584**
LOC688146	PREDICTED: Rattus norvegicus similar to radical S-adenosyl methionine domain containing 1 (LOC688146), mRNA [XM_001081294]	**13,582**
Cttnbp2nl	Rattus norvegicus CTTNBP2 N-terminal like (Cttnbp2nl), mRNA [NM_001107712]	**13,580**
Nkx2-5	Rattus norvegicus NK2 transcription factor related, locus 5 (Drosophila) (Nkx2-5), mRNA [NM_053651]	**13,580**
Car14	Rattus norvegicus carbonic anhydrase 14 (Car14), mRNA [NM_001109655]	**13,576**
Atp2b1	Rattus norvegicus ATPase, Ca++ transporting, plasma membrane 1 (Atp2b1), mRNA [NM_053311]	**13,575**
Frmpd4	Rattus norvegicus FERM and PDZ domain containing 4 (Frmpd4), mRNA [NM_001106960]	**13,575**
LOC684558	AGENCOURT_113686907 NIH_MGC_431 Rattus norvegicus cDNA clone IMAGE:9088341 5', mRNA sequence [EV775254]	**13,572**
0	Rattus norvegicus similar to CG17293-PA (LOC289641), mRNA [XM_223435]	**13,571**
0	Uncharacterized protein [Source:UniProtKB/TrEMBL;Acc:D3ZP37] [ENSRNOT00000060917]	**13,571**
Znf618	PREDICTED: Rattus norvegicus zinc finger protein 618 (Znf618), mRNA [XM_233016]	**13,567**
Atp11c-ps1	Uncharacterized protein [Source:UniProtKB/TrEMBL;Acc:D3Z9J3] [ENSRNOT00000049120]	**13,566**
0	Q3UXV4_MOUSE (Q3UXV4) 12 days embryo male wolffian duct includes surrounding region cDNA, RIKEN full-length enriched library, clone:6720421K10 product:protein phosphatase 3, catalytic subunit, beta isoform, full insert sequence, partial (47%) [TC597142]	**13,566**
Pik3r5	Rattus norvegicus phosphoinositide-3-kinase, regulatory subunit 5 (Pik3r5), mRNA [NM_001191923]	**13,565**
Ano10	PREDICTED: Rattus norvegicus anoctamin 10 (Ano10), mRNA [XM_236774]	**13,564**
Arpc2	Rattus norvegicus actin related protein 2/3 complex, subunit 2 (Arpc2), mRNA [NM_001106919]	**13,563**
Tmprss7	Rattus norvegicus transmembrane serine protease 7 (Tmprss7), mRNA [NM_001105882]	**13,561**
Grk4	Rattus norvegicus G protein-coupled receptor kinase 4 (Grk4), mRNA [NM_022928]	**13,560**
Eid2b	PREDICTED: Rattus norvegicus similar to hypothetical protein FLJ38944 (RGD1560818), mRNA [XM_002725600]	**13,559**

Alkbh5	Uncharacterized protein [Source:UniProtKB/TrEMBL;Acc:D3ZKD3] [ENSRNOT00000034006]	**13,559**
0	Rattus norvegicus TL0AAA49YL04 mRNA sequence. [FQ213104]	**13,555**
Lmtk2	Rattus norvegicus lemur tyrosine kinase 2 (Lmtk2), mRNA [NM_001137641]	**13,553**
Kcng1	Rattus norvegicus potassium voltage-gated channel, subfamily G, member 1 (Kcng1), mRNA [NM_001106545]	**13,551**
Fam5b	Rattus norvegicus family with sequence similarity 5, member B (Fam5b), mRNA [NM_173115]	**13,550**
Tnpo2	Rattus norvegicus transportin 2 (Tnpo2), mRNA [NM_001107166]	**13,548**
Pcdhg@	Protocadherin-T1 [Source:UniProtKB/TrEMBL;Acc:Q9JIU6] [ENSRNOT00000067185]	**13,547**
Slc39a6	Rattus norvegicus solute carrier family 39 (zinc transporter), member 6 (Slc39a6), mRNA [NM_001024745]	**13,546**
Ppp1r12b	Rattus norvegicus protein phosphatase 1, regulatory (inhibitor) subunit 12B (Ppp1r12b), mRNA [NM_001107178]	**13,545**
Trmt6	Rattus norvegicus tRNA methyltransferase 6 homolog (S. cerevisiae) (Trmt6), mRNA [NM_001107779]	**13,545**
Cenpq	Rattus norvegicus centromere protein Q (Cenpq), mRNA [NM_001014215]	**13,544**
Isg20l2	Rattus norvegicus interferon stimulated exonuclease gene 20-like 2 (Isg20l2), mRNA [NM_001007741]	**13,542**
0	Mitogen-activated protein kinase 4 [Source:UniProtKB/Swiss-Prot;Acc:Q63454] [ENSRNOT00000020851]	**13,542**
Ell	Rattus norvegicus elongation factor RNA polymerase II (Ell), mRNA [NM_001107304]	**13,541**
Serpina11	Rattus norvegicus serine (or cysteine) peptidase inhibitor, clade A (alpha-1 antiproteinase, antitrypsin), member 11 (Serpina11), transcript variant 2, mRNA [NM_001166352]	**13,537**
Paqr9	PREDICTED: Rattus norvegicus progestin and adipoQ receptor family member IX (Paqr9), partial mRNA [XM_236503]	**13,536**
Tmem107	Rattus norvegicus transmembrane protein 107 (Tmem107), mRNA [NM_001109648]	**13,533**
Ndp	Rattus norvegicus Norrie disease (pseudoglioma) (human) (Ndp), mRNA [NM_001108814]	**13,532**
Tgfb2	Rattus norvegicus transforming growth factor, beta 2 (Tgfb2), mRNA [NM_031131]	**13,530**
LOC498675	Rattus norvegicus hypothetical LOC498675 (LOC498675), mRNA [NM_001109113]	**13,527**
0	sprouty homolog 3 [Source:RefSeq peptide;Acc:NP_001102533] [ENSRNOT00000011128]	**13,524**
Kcnc2	Rattus norvegicus potassium voltage gated channel, Shaw-related subfamily, member 2 (Kcnc2), transcript variant a, mRNA [NM_139216]	**13,524**
Nt5m	Rattus norvegicus 5',3'-nucleotidase, mitochondrial (Nt5m), nuclear gene encoding mitochondrial protein, mRNA [NM_001105785]	**13,523**
0	Unknown	**13,522**
Spag8	Rattus norvegicus sperm associated antigen 8 (Spag8), mRNA [NM_001173555]	**13,521**
LOC690079	Rattus norvegicus hypothetical protein LOC690079 (LOC690079), mRNA [NM_001109566]	**13,521**
0	fetal Alzheimer antigen [Source:RefSeq peptide;Acc:NP_001121007] [ENSRNOT00000055100]	**13,520**
Sumf1	Rattus norvegicus sulfatase modifying factor 1 (Sumf1), mRNA [NM_001108639]	**13,519**
Gnb1	Rattus norvegicus guanine nucleotide binding protein (G protein), beta polypeptide 1 (Gnb1), mRNA [NM_030987]	**13,519**
Lsm3	Rattus norvegicus LSM3 homolog, U6 small nuclear RNA associated (S. cerevisiae) (Lsm3), mRNA [NM_001106611]	**13,515**
0	Unknown	**13,513**
PVR	Rattus norvegicus poliovirus receptor (PVR), mRNA [NM_017076]	**13,513**
0	Unknown	**13,508**

Rpap1	Rattus norvegicus RNA polymerase II associated protein 1 (Rpap1), mRNA [NM_001033999]	**13,508**
Megf9	Rattus norvegicus multiple EGF-like-domains 9 (Megf9), mRNA [NM_001107940]	**13,508**
Ddx42	Rattus norvegicus DEAD (Asp-Glu-Ala-Asp) box polypeptide 42 (Ddx42), mRNA [NM_001107059]	**13,508**
Rnf170	PREDICTED: Rattus norvegicus ring finger protein 170 (Rnf170), mRNA [XM_001061874]	**13,507**
Tbc1d22a	PREDICTED: Rattus norvegicus TBC1 domain family, member 22a (Tbc1d22a), mRNA [XM_001078492]	**13,505**
Rabep2	Rattus norvegicus rabaptin, RAB GTPase binding effector protein 2 (Rabep2), mRNA [NM_030585]	**13,500**
0	Uncharacterized protein [Source:UniProtKB/TrEMBL;Acc:D4AAW3] [ENSRNOT00000055945]	**13,500**
LOC679580	PREDICTED: Rattus norvegicus similar to MIC2 like 1 (LOC679580), mRNA [XM_001053578]	**13,500**
0	Uncharacterized protein [Source:UniProtKB/TrEMBL;Acc:D4ACX2] [ENSRNOT00000047604]	**13,500**
Txndc11	Rattus norvegicus thioredoxin domain containing 11 (Txndc11), mRNA [NM_001127532]	**13,495**
0	Unknown	**13,494**
LOC684558	PREDICTED: Rattus norvegicus similar to regulator of nonsense transcripts 1 (LOC684558), mRNA [XM_001070971]	**13,492**
Nav2	Rattus norvegicus neuron navigator 2 (Nav2), mRNA [NM_138529]	**13,487**
Ppp1r16b	Rattus norvegicus protein phosphatase 1, regulatory (inhibitor) subunit 16B (Ppp1r16b), mRNA [NM_001191072]	**13,487**
Map4	Rattus norvegicus microtubule-associated protein 4 (Map4), mRNA [NM_001024278]	**13,486**
Etv5	Rattus norvegicus ets variant 5 (Etv5), mRNA [NM_001107082]	**13,486**
Acsl4	Rattus norvegicus acyl-CoA synthetase long-chain family member 4 (Acsl4), mRNA [NM_053623]	**13,483**
Fkbp5	Rattus norvegicus FK506 binding protein 5 (Fkbp5), mRNA [NM_001012174]	**13,483**
Schip1	Rattus norvegicus schwannomin interacting protein 1 (Schip1), mRNA [NM_001100666]	**13,481**
Grik4	Rattus norvegicus glutamate receptor, ionotropic, kainate 4 (Grik4), mRNA [NM_012572]	**13,480**
Gpr26	Rattus norvegicus G protein-coupled receptor 26 (Gpr26), mRNA [NM_138841]	**13,479**
Zhx2	Zinc fingers and homeoboxes protein 2 [Source:UniProtKB/Swiss-Prot;Acc:Q80VX4] [ENSRNOT00000007326]	**13,479**
RGD1564400	Rattus norvegicus similar to Eukaryotic translation initiation factor 5 (eIF-5) (LOC295660), mRNA [XM_212955]	**13,478**
0	Unknown	**13,478**
Myo6	PREDICTED: Rattus norvegicus myosin VI (Myo6), mRNA [XM_001061392]	**13,474**
Lrp6	Rattus norvegicus low density lipoprotein receptor-related protein 6 (Lrp6), mRNA [NM_001107892]	**13,473**
RGD1308923	Rattus norvegicus LOC362678 (RGD1308923), mRNA [NM_001108698]	**13,473**
Hibch	Rattus norvegicus 3-hydroxyisobutyryl-Coenzyme A hydrolase (Hibch), nuclear gene encoding mitochondrial protein, mRNA [NM_001013112]	**13,472**
Pnma2	Rattus norvegicus paraneoplastic antigen MA2 (Pnma2), mRNA [NM_001107272]	**13,471**
Slc29a4	Rattus norvegicus solute carrier family 29 (nucleoside transporters), member 4 (Slc29a4), mRNA [NM_001105911]	**13,470**
0	Uncharacterized protein [Source:UniProtKB/TrEMBL;Acc:D3ZES7] [ENSRNOT00000017536]	**13,466**
Rnls	Rattus norvegicus renalase, FAD-dependent amine oxidase (Rnls), mRNA [NM_001014167]	**13,463**
Zfp748	Rattus norvegicus TL0AAA77YJ08 mRNA sequence. [FQ211879]	**13,462**
Tnrc6b	Rattus norvegicus trinucleotide repeat containing 6B (Tnrc6b), mRNA [NM_138845]	**13,462**

Sgcb	Rattus norvegicus sarcoglycan, beta (dystrophin-associated glycoprotein) (Sgcb), mRNA [NM_001191068]	**13,461**
Trim65	Rattus norvegicus tripartite motif-containing 65 (Trim65), mRNA [NM_001135714]	**13,458**
Maged1	Rattus norvegicus melanoma antigen, family D, 1 (Maged1), mRNA [NM_053409]	**13,458**
Pld2	Rattus norvegicus phospholipase D2 (Pld2), mRNA [NM_033299]	**13,456**
Sema6b	Rattus norvegicus sema domain, transmembrane domain (TM), and cytoplasmic domain, (semaphorin) 6B (Sema6b), mRNA [NM_053471]	**13,455**
Ddi2	Rattus norvegicus DNA-damage inducible protein 2 (Ddi2), mRNA [NM_001034149]	**13,452**
Hs2st1	Rattus norvegicus heparan sulfate 2-O-sulfotransferase 1 (Hs2st1), mRNA [NM_001100518]	**13,448**
0	Rattus norvegicus similar to hypothetical protein MGC31495 (LOC360655), mRNA [XM_340932]	**13,446**
Dpy30	Rattus norvegicus dpy-30 homolog (C. elegans) (Dpy30), transcript variant 1, mRNA [NM_173117]	**13,446**
Pitpna	Rattus norvegicus phosphatidylinositol transfer protein, alpha (Pitpna), mRNA [NM_017231]	**13,446**
Slc39a14	Rattus norvegicus solute carrier family 39 (zinc transporter), member 14 (Slc39a14), mRNA [NM_001107275]	**13,445**
Mrrf	Rattus norvegicus mitochondrial ribosome recycling factor (Mrrf), nuclear gene encoding mitochondrial protein, mRNA [NM_001008354]	**13,443**
Fam76b	Rattus norvegicus family with sequence similarity 76, member B (Fam76b), mRNA [NM_001108994]	**13,443**
0	Unknown	**13,441**
Fbxo11	Rattus norvegicus F-box protein 11 (Fbxo11), mRNA [NM_181631]	**13,440**
Jakmip3	Rattus norvegicus janus kinase and microtubule interacting protein 3 (Jakmip3), mRNA [NM_001163277]	**13,438**
Amz2	Rattus norvegicus archaelysin family metallopeptidase 2 (Amz2), mRNA [NM_001014121]	**13,434**
Nrip3	Rattus norvegicus nuclear receptor interacting protein 3 (Nrip3), mRNA [NM_001108498]	**13,433**
0	Unknown	**13,431**
Rnf44	Rattus norvegicus ring finger protein 44 (Rnf44), mRNA [NM_001024795]	**13,431**
Mkl1	PREDICTED: Rattus norvegicus megakaryoblastic leukemia (translocation) 1 (Mkl1), mRNA [XM_001077101]	**13,430**
Crygd	Rattus norvegicus crystallin, gamma D (Crygd), mRNA [NM_033095]	**13,424**
LOC688807	PREDICTED: Rattus norvegicus hypothetical protein LOC688807 (LOC688807), mRNA [XM_001068381]	**13,424**
Dpagt1	Rattus norvegicus dolichyl-phosphate (UDP-N-acetylglucosamine) N-acetylglucosaminephosphotransferase 1 (GlcNAc-1-P transferase) (Dpagt1), mRNA [NM_199388]	**13,423**
Fcho2	Rattus norvegicus FCH domain only 2 (Fcho2), mRNA [NM_001191632]	**13,420**
Rras	Rattus norvegicus Harvey rat sarcoma virus oncogene, subgroup R (Rras), mRNA [NM_001108481]	**13,419**
Athl1	Uncharacterized protein [Source:UniProtKB/TrEMBL;Acc:D3ZR78] [ENSRNOT00000020004]	**13,419**
Htr6	Rattus norvegicus 5-hydroxytryptamine (serotonin) receptor 6 (Htr6), mRNA [NM_024365]	**13,419**
Mapk1	Rattus norvegicus mitogen activated protein kinase 1 (Mapk1), mRNA [NM_053842]	**13,419**
RGD1308134	Rattus norvegicus similar to RIKEN cDNA 1110020A23 (RGD1308134), mRNA [NM_001127521]	**13,416**
0	Unknown	**13,414**
0	PREDICTED: Rattus norvegicus similar to glyceraldehyde-3-phosphate dehydrogenase (RGD1560826), mRNA [XM_001075200]	**13,414**
LOC501224	PREDICTED: Rattus norvegicus similar to RIKEN cDNA 2610042L04 (LOC501224), miscRNA [XR_005447]	**13,413**

C1qtnf5	Rattus norvegicus C1q and tumor necrosis factor related protein 5 (C1qtnf5), mRNA [NM_001012123]	**13,411**
Wars2	Rattus norvegicus tryptophanyl tRNA synthetase 2 (mitochondrial) (Wars2), nuclear gene encoding mitochondrial protein, mRNA [NM_001168641]	**13,409**
Hrasls5	Rattus norvegicus HRAS-like suppressor family, member 5 (Hrasls5), mRNA [NM_001039007]	**13,408**
Slc22a6	Rattus norvegicus solute carrier family 22 (organic anion transporter), member 6 (Slc22a6), mRNA [NM_017224]	**13,406**
Mlec	Rattus norvegicus malectin (Mlec), mRNA [NM_001013983]	**13,404**
Kcnk4	Rattus norvegicus potassium channel, subfamily K, member 4 (Kcnk4), mRNA [NM_053804]	**13,396**
Gpc5	Rattus norvegicus glypican 5 (Gpc5), mRNA [NM_001107285]	**13,396**
0	Unknown	**13,395**
Ptger3	Rattus norvegicus prostaglandin E receptor 3 (subtype EP3) (Ptger3), mRNA [NM_012704]	**13,394**
0	Unknown	**13,394**
Cntn6	Rattus norvegicus contactin 6 (Cntn6), mRNA [NM_013225]	**13,390**
Pcp4	Rattus norvegicus Purkinje cell protein 4 (Pcp4), mRNA [NM_013002]	**13,390**
Slc18a1	Rattus norvegicus solute carrier family 18 (vesicular monoamine), member 1 (Slc18a1), mRNA [NM_013152]	**13,388**
Btg2	Rattus norvegicus BTG family, member 2 (Btg2), mRNA [NM_017259]	**13,387**
Gpx1	Rattus norvegicus glutathione peroxidase 1 (Gpx1), mRNA [NM_030826]	**13,385**
Ppp1r13b	Rattus norvegicus protein phosphatase 1, regulatory (inhibitor) subunit 13B (Ppp1r13b), mRNA [NM_001108062]	**13,384**
Pcyt1a	Choline-phosphate cytidylyltransferase A [Source:UniProtKB/Swiss-Prot;Acc:P19836] [ENSRNOT00000002403]	**13,382**
Znf23	Rattus norvegicus zinc finger protein 23 (KOX 16) (Znf23), mRNA [NM_001107428]	**13,382**
Ptprd	PREDICTED: Rattus norvegicus protein tyrosine phosphatase, receptor type, D (Ptprd), mRNA [XM_233065]	**13,380**
Dgcr8	Rattus norvegicus DiGeorge syndrome critical region gene 8 (Dgcr8), mRNA [NM_001105865]	**13,380**
0	Unknown	**13,379**
Mpst	Rattus norvegicus mercaptopyruvate sulfurtransferase (Mpst), nuclear gene encoding mitochondrial protein, mRNA [NM_138843]	**13,379**
RGD1565095	Rattus norvegicus similar to hypothetical protein MGC52110 (RGD1565095), mRNA [NM_001195488]	**13,378**
Apoc4	Rattus norvegicus apolipoprotein C-IV (Apoc4), mRNA [NM_001109419]	**13,377**
Rap2a	Rattus norvegicus RAS related protein 2a (Rap2a), mRNA [NM_053741]	**13,376**
Nppc	Rattus norvegicus natriuretic peptide precursor C (Nppc), mRNA [NM_053750]	**13,376**
Tbc1d5	Rattus norvegicus TBC1 domain family, member 5 (Tbc1d5), mRNA [NM_001134762]	**13,375**
Rnf167	Rattus norvegicus ring finger protein 167 (Rnf167), mRNA [NM_001008361]	**13,371**
Lrig1	Uncharacterized protein [Source:UniProtKB/TrEMBL;Acc:D3ZD84] [ENSRNOT00000017384]	**13,369**
RGD1308154	Uncharacterized protein [Source:UniProtKB/TrEMBL;Acc:D4ADL7] [ENSRNOT00000002356]	**13,367**
LOC361914	Rattus norvegicus similar to solute carrier family 7 (cationic amino acid transporter, y+ system), member 12 (LOC361914), mRNA [NM_001017465]	**13,365**
Sar1b	Rattus norvegicus SAR1 homolog B (S. cerevisiae) (Sar1b), mRNA [NM_001009622]	**13,364**
Ppp1r3a	Rattus norvegicus protein phosphatase 1, regulatory (inhibitor) subunit 3A (Ppp1r3a), mRNA [NM_001109222]	**13,363**

Pigz	Rattus norvegicus phosphatidylinositol glycan anchor biosynthesis, class Z (Pigz), mRNA [NM_001109525]	**13,360**
Megf11	PREDICTED: Rattus norvegicus multiple EGF-like-domains 11 (Megf11), mRNA [XM_001078620]	**13,358**
Map7d2	PREDICTED: Rattus norvegicus MAP7 domain containing 2 (Map7d2), mRNA [XM_001054594]	**13,356**
Arhgef18	Rattus norvegicus rho/rac guanine nucleotide exchange factor (GEF) 18 (Arhgef18), mRNA [NM_001107115]	**13,351**
Nkg7	Rattus norvegicus natural killer cell group 7 sequence (Nkg7), mRNA [NM_133540]	**13,351**
Mapk1ip1l	Rattus norvegicus mitogen-activated protein kinase 1 interacting protein 1-like (Mapk1ip1l), mRNA [NM_001108373]	**13,348**
Slc11a2	Rattus norvegicus solute carrier family 11 (proton-coupled divalent metal ion transporters), member 2 (Slc11a2), mRNA [NM_013173]	**13,346**
0	Unknown	**13,346**
Col1a1	Rattus norvegicus collagen, type I, alpha 1 (Col1a1), mRNA [NM_053304]	**13,345**
Tbc1d2b	Rattus norvegicus TBC1 domain family, member 2B (Tbc1d2b), mRNA [NM_001108175]	**13,343**
Prokr2	Rattus norvegicus prokineticin receptor 2 (Prokr2), mRNA [NM_138978]	**13,343**
Nnt	Rattus norvegicus nicotinamide nucleotide transhydrogenase (Nnt), nuclear gene encoding mitochondrial protein, mRNA [NM_001013157]	**13,343**
LOC499602	Rattus norvegicus hypothetical protein LOC499602 (LOC499602), mRNA [NM_001025039]	**13,342**
Cdipt	Rattus norvegicus CDP-diacylglycerol--inositol 3-phosphatidyltransferase (phosphatidylinositol synthase) (Cdipt), mRNA [NM_138899]	**13,341**
MGC72627	Rattus norvegicus similar to RIKEN cDNA A330021E22, mRNA (cDNA clone MGC:72627 IMAGE:5600085), complete cds. [BC061963]	**13,339**
Etv5	Rattus norvegicus ets variant 5 (Etv5), mRNA [NM_001107082]	**13,336**
Samsn1	Rattus norvegicus SAM domain, SH3 domain and nuclear localization signals, 1 (Samsn1), mRNA [NM_130821]	**13,336**
Fam76a	Rattus norvegicus family with sequence similarity 76, member A (Fam76a), mRNA [NM_001108686]	**13,333**
Znf688	Uncharacterized proteinZinc finger protein 688 (Predicted), isoform CRA_b [Source:UniProtKB/TrEMBL;Acc:D3ZAB7] [ENSRNOT00000024858]	**13,332**
Pdpk1	Rattus norvegicus 3-phosphoinositide dependent protein kinase-1 (Pdpk1), mRNA [NM_031081]	**13,330**
0	Rod-derived cone viability factor (RCG38817) [Source:UniProtKB/TrEMBL;Acc:B5LNR2] [ENSRNOT00000024434]	**13,329**
Mlx	Rattus norvegicus MAX-like protein X (Mlx), mRNA [NM_001034112]	**13,329**
Steap3	Rattus norvegicus STEAP family member 3 (Steap3), mRNA [NM_133314]	**13,329**
Polb	DNA polymerase beta [Source:UniProtKB/Swiss-Prot;Acc:P06766] [ENSRNOT00000026039]	**13,327**
LOC680443	PREDICTED: Rattus norvegicus hypothetical protein LOC680443 (LOC680443), mRNA [XM_002727352]	**13,327**
0	Unknown	**13,326**
0	Unknown	**13,325**
Dos	Rattus norvegicus downstream of Stk11 (Dos), mRNA [NM_001108070]	**13,322**
Birc6	Rattus norvegicus baculoviral IAP repeat-containing 6 (Birc6), mRNA [NM_001170596]	**13,320**
LOC691684	PREDICTED: Rattus norvegicus similar to MIC2 like 1 (LOC691684), mRNA [XM_001079259]	**13,320**
Rgs7	Rattus norvegicus regulator of G-protein signaling 7 (Rgs7), mRNA [NM_019343]	**13,319**
Fam49b	Rattus norvegicus family with sequence similarity 49, member B (Fam49b), mRNA [NM_001126267]	**13,318**
Rprd1a	Uncharacterized protein [Source:UniProtKB/TrEMBL;Acc:D4AAU4] [ENSRNOT00000021624]	**13,318**
Casc5	Rattus norvegicus cancer susceptibility candidate 5 (Casc5), mRNA [NM_001170594]	**13,316**

Reep4	Rattus norvegicus receptor accessory protein 4 (Reep4), mRNA [NM_001025279]	**13,315**
Isl2	Rattus norvegicus ISL LIM homeobox 2 (Isl2), mRNA [NM_020471]	**13,313**
0	Unknown	**13,308**
Cnrip1	Rattus norvegicus cannabinoid receptor interacting protein 1 (Cnrip1), mRNA [NM_001014232]	**13,306**
Icmt	Rattus norvegicus isoprenylcysteine carboxyl methyltransferase (Icmt), mRNA [NM_133310]	**13,303**
Plxdc2	Rattus norvegicus plexin domain containing 2 (Plxdc2), mRNA [NM_001108422]	**13,301**
RGD1559643	Rattus norvegicus similar to hypothetical protein A430031N04 (RGD1559643), mRNA [NM_001109056]	**13,300**
Tfpt	Rattus norvegicus TCF3 (E2A) fusion partner (Tfpt), mRNA [NM_138870]	**13,297**
Gpkow	Rattus norvegicus G patch domain and KOW motifs (Gpkow), mRNA [NM_001109381]	**13,296**
0	Unknown	**13,295**
Mcam	Rattus norvegicus melanoma cell adhesion molecule (Mcam), transcript variant 1, mRNA [NM_023983]	**13,295**
0	Unknown	**13,295**
Panx2	Rattus norvegicus pannexin 2 (Panx2), mRNA [NM_199409]	**13,295**
Lingo4	Rattus norvegicus leucine rich repeat and Ig domain containing 4 (Lingo4), mRNA [NM_001109189]	**13,294**
Zfpm2	Rattus norvegicus zinc finger protein, multitype 2 (Zfpm2), mRNA [NM_001130501]	**13,292**
0	Unknown	**13,291**
0	Uncharacterized protein [Source:UniProtKB/TrEMBL;Acc:D3ZDF2] [ENSRNOT00000042956]	**13,287**
Slc25a16	Rattus norvegicus solute carrier family 25 (mitochondrial carrier, Graves disease autoantigen), member 16 (Slc25a16), nuclear gene encoding mitochondrial protein, mRNA [NM_001100860]	**13,287**
0	AGENCOURT_109870696 NIH_MGC_418 Rattus norvegicus cDNA clone IMAGE:9022683 5', mRNA sequence [EV763695]	**13,286**
Hyal1	Rattus norvegicus hyaluronoglucosaminidase 1 (Hyal1), mRNA [NM_207616]	**13,283**
Limch1	Rattus norvegicus LIM and calponin homology domains 1 (Limch1), mRNA [NM_001191678]	**13,283**
LOC680813	PREDICTED: Rattus norvegicus hypothetical protein LOC680813 (LOC680813), partial mRNA [XM_001058991]	**13,280**
Eif4ebp3	Rattus norvegicus eukaryotic translation initiation factor 4E binding protein 3 (Eif4ebp3), mRNA [NM_001202552]	**13,280**
0	Unknown	**13,277**
Olr1051	Rattus norvegicus olfactory receptor 1051 (Olr1051), mRNA [NM_001001364]	**13,277**
0	Unknown	**13,275**
Np	Rattus norvegicus nucleoside phosphorylase (Np), mRNA [NM_001106031]	**13,274**
Spryd3	Rattus norvegicus SPRY domain containing 3 (Spryd3), mRNA [NM_001191790]	**13,273**
Plcb3	Rattus norvegicus phospholipase C, beta 3 (phosphatidylinositol-specific) (Plcb3), mRNA [NM_033350]	**13,272**
Stau2	Rattus norvegicus staufen, RNA binding protein, homolog 2 (Drosophila) (Stau2), transcript variant LS, mRNA [NM_134466]	**13,269**
Itgbl1	Rattus norvegicus integrin, beta-like 1 (Itgbl1), mRNA [NM_001017505]	**13,268**
Hnrnpab	Rattus norvegicus heterogeneous nuclear ribonucleoprotein A/B (Hnrnpab), mRNA [NM_031330]	**13,264**
LOC257650	Rattus norvegicus hippyragranin (LOC257650), mRNA [NM_147142]	**13,264**

Creb3l3	Rattus norvegicus cAMP responsive element binding protein 3-like 3 (Creb3l3), mRNA [NM_001012115]	**13,263**
Abce1	Rattus norvegicus ATP-binding cassette, subfamily E (OABP), member 1 (Abce1), mRNA [NM_001108446]	**13,263**
Pfkfb2	Rattus norvegicus 6-phosphofructo-2-kinase/fructose-2,6-biphosphatase 2 (Pfkfb2), transcript variant 3, mRNA [NM_080477]	**13,261**
0	AB211061 ES cell associated transcript 8 {Mus musculus} (exp=-1; wgp=0; cg=0), partial (32%) [TC600461]	**13,260**
Armc8	Rattus norvegicus armadillo repeat containing 8 (Armc8), mRNA [NM_001173354]	**13,255**
Kif12	Rattus norvegicus kinesin family member 12 (Kif12), mRNA [NM_001012102]	**13,255**
0	Uncharacterized protein [Source:UniProtKB/TrEMBL;Acc:D3ZKR7] [ENSRNOT00000050021]	**13,255**
LOC690082	PREDICTED: Rattus norvegicus similar to melanoma ubiquitous mutated protein (LOC690082), mRNA [XM_001073181]	**13,249**
Zfc3h1	PREDICTED: Rattus norvegicus proline/serine-rich coiled-coil 2, transcript variant 2 (Psrc2), mRNA [XM_001078700]	**13,248**
Nans	Rattus norvegicus N-acetylneuraminic acid synthase (Nans), mRNA [NM_001106655]	**13,247**
0	Calsyntenin-1Soluble Alc-alphaCTF1-alpha [Source:UniProtKB/Swiss-Prot;Acc:Q6Q0N0] [ENSRNOT00000022100]	**13,245**
Znf865	Rattus norvegicus zinc finger protein 865 (Znf865), mRNA [NM_001134544]	**13,241**
Znf574	Rattus norvegicus zinc finger protein 574 (Znf574), mRNA [NM_001024258]	**13,241**
0	Rattus norvegicus similar to glyceraldehyde-3-phosphate dehydrogenase (phosphorylating) (EC 1.2.1.12) - mouse (LOC363090), mRNA [XM_343421]	**13,238**
Fat1	Rattus norvegicus FAT tumor suppressor homolog 1 (Drosophila) (Fat1), mRNA [NM_031819]	**13,236**
Aptx	Rattus norvegicus aprataxin (Aptx), mRNA [NM_148889]	**13,235**
Pak1	Rattus norvegicus p21 protein (Cdc42/Rac)-activated kinase 1 (Pak1), mRNA [NM_017198]	**13,233**
Ddt	Rattus norvegicus D-dopachrome tautomerase (Ddt), mRNA [NM_024131]	**13,232**
Eif4b	Rattus norvegicus eukaryotic translation initiation factor 4B (Eif4b), mRNA [NM_001008324]	**13,232**
0	Unknown	**13,232**
0	Unknown	**13,219**
Olr297	Rattus norvegicus olfactory receptor 297 (Olr297), mRNA [NM_001000234]	**13,218**
0	Rattus norvegicus TL0ADA43YL03 mRNA sequence. [FQ220235]	**13,211**
0	Unknown	**13,208**
Banp	Rattus norvegicus Btg3 associated nuclear protein (Banp), mRNA [NM_001106191]	**13,207**
Rgs3	Rattus norvegicus regulator of G-protein signaling 3 (Rgs3), mRNA [NM_019340]	**13,207**
0	Uncharacterized protein [Source:UniProtKB/TrEMBL;Acc:D3ZS52] [ENSRNOT00000050090]	**13,204**
0	AF326545 syntaxin binding protein 1 {Mus musculus} (exp=-1; wgp=0; cg=0), partial (5%) [TC627900]	**13,204**
0	Rattus norvegicus perlecan mRNA, partial cds. [U75305]	**13,202**
Slc35a5	Rattus norvegicus solute carrier family 35, member A5 (Slc35a5), mRNA [NM_001191927]	**13,199**
Rexo2	Rattus norvegicus REX2, RNA exonuclease 2 homolog (S. cerevisiae) (Rexo2), mRNA [NM_001008326]	**13,198**
Pcdhga11	Rattus norvegicus protocadherin gamma subfamily A, 11 (Pcdhga11), mRNA [NM_001037153]	**13,197**
Noxo1	Rattus norvegicus NADPH oxidase organizer 1 (Noxo1), mRNA [NM_001106986]	**13,197**

Mfng	Rattus norvegicus MFNG O-fucosylpeptide 3-beta-N-acetylglucosaminyltransferase (Mfng), mRNA [NM_199110]	**13,196**
Nipa1	Rattus norvegicus non imprinted in Prader-Willi/Angelman syndrome 1 homolog (human) (Nipa1), mRNA [NM_001107519]	**13,196**
Prkacb	Rattus norvegicus protein kinase, cAMP dependent, catalytic, beta (Prkacb), mRNA [NM_001077645]	**13,192**
RGD1304694	Uncharacterized protein C22orf9 homolog [Source:UniProtKB/Swiss-Prot;Acc:Q4G008] [ENSRNOT00000042070]	**13,192**
Lpcat1	Rattus norvegicus lysophosphatidylcholine acyltransferase 1 (Lpcat1), mRNA [NM_001100735]	**13,182**
Mfsd9	Rattus norvegicus major facilitator superfamily domain containing 9 (Mfsd9), mRNA [NM_001108215]	**13,181**
Prkcdbp	Rattus norvegicus protein kinase C, delta binding protein (Prkcdbp), mRNA [NM_134449]	**13,181**
Usp9x	Rattus norvegicus ubiquitin specific peptidase 9, X-linked (Usp9x), transcript variant 1, mRNA [NM_001135893]	**13,180**
0	Unknown	**13,179**
Cadm2	Rattus norvegicus cell adhesion molecule 2 (Cadm2), mRNA [NM_001047102]	**13,179**
Extl3	Rattus norvegicus exostoses (multiple)-like 3 (Extl3), mRNA [NM_020097]	**13,178**
0	RVL6921 Wackym-Soares normalized rat vestibular cDNA library Rattus norvegicus cDNA 5', mRNA sequence [DV716564]	**13,176**
Elk4	Rattus norvegicus ELK4, ETS-domain protein (SRF accessory protein 1) (Elk4), mRNA [NM_001107173]	**13,175**
Pdzd4	Rattus norvegicus PDZ domain containing 4 (Pdzd4), mRNA [NM_001135836]	**13,173**
Crmp1	Rattus norvegicus collapsin response mediator protein 1 (Crmp1), mRNA [NM_012932]	**13,170**
0	UI-R-DZ1-cne-j-11-0-UI.s1 UI-R-DZ1 Rattus norvegicus cDNA clone UI-R-DZ1-cne-j-11-0-UI 3', mRNA sequence [CN543219]	**13,169**
LOC682999	Rattus norvegicus hypothetical protein LOC682999 (LOC682999), mRNA [NM_001127600]	**13,169**
0	Thyroid hormone receptor associated protein 2 (Predicted)Uncharacterized protein [Source:UniProtKB/TrEMBL;Acc:D3ZJC6] [ENSRNOT00000042331]	**13,167**
0	Unknown	**13,166**
Pragmin	Rattus norvegicus pragma of Rnd2 (Pragmin), mRNA [NM_001107315]	**13,166**
Stip1	Rattus norvegicus stress-induced phosphoprotein 1 (Stip1), mRNA [NM_138911]	**13,162**
0	PREDICTED: Rattus norvegicus zinc finger protein 407 (Zfp407), mRNA [XM_001061052]	**13,162**
0	Unknown	**13,158**
Zfp365	Rattus norvegicus zinc finger protein 365 (Zfp365), mRNA [NM_001025145]	**13,158**
RGD1563034	PREDICTED: Rattus norvegicus similar to ETS domain transcription factor ERF (Ets2 repressor factor) (RGD1563034), mRNA [XM_218458]	**13,157**
Grin2a	Rattus norvegicus glutamate receptor, ionotropic, N-methyl D-aspartate 2A (Grin2a), mRNA [NM_012573]	**13,156**
0	Glyceraldehyde-3-phosphate dehydrogenase [Source:UniProtKB/TrEMBL;Acc:D3ZME3] [ENSRNOT00000032908]	**13,156**
Akt2	Rattus norvegicus v-akt murine thymoma viral oncogene homolog 2 (Akt2), mRNA [NM_017093]	**13,151**
Zfp800	Rattus norvegicus zinc finger protein 800 (Zfp800), mRNA [NM_001109225]	**13,150**
Fcgbpl1	Rattus norvegicus Fc fragment of IgG binding protein-like 1 (Fcgbpl1), mRNA [NM_001164656]	**13,149**
0	Unknown	**13,145**
Tpm1	Rattus norvegicus tropomyosin 1, alpha (Tpm1), transcript variant 1, mRNA [NM_001034068]	**13,141**
Adnp	Rattus norvegicus activity-dependent neuroprotector homeobox (Adnp), mRNA [NM_022681]	**13,138**

0	BF521738 UI-R-C2-mt-d-06-0-UI.r2 UI-R-C2 Rattus norvegicus cDNA clone UI-R-C2-mt-d-06-0-UI 5', mRNA sequence [BF521738]	**13,136**
C1galt1	Rattus norvegicus core 1 synthase, glycoprotein-N-acetylgalactosamine 3-beta-galactosyltransferase, 1 (C1galt1), mRNA [NM_022950]	**13,135**
Gpr68	Rattus norvegicus G protein-coupled receptor 68 (Gpr68), mRNA [NM_001108049]	**13,134**
LOC685079	RCG32134, isoform CRA_aUncharacterized protein [Source:UniProtKB/TrEMBL;Acc:D3ZY25] [ENSRNOT00000030054]	**13,134**
LOC363306	PREDICTED: Rattus norvegicus hypothetical protein LOC363306 (LOC363306), mRNA [XM_001068086]	**13,131**
Myh10	Rattus norvegicus myosin, heavy chain 10, non-muscle (Myh10), mRNA [NM_031520]	**13,129**
0	Unknown	**13,128**
Ppp2r5b	Rattus norvegicus protein phosphatase 2, regulatory subunit B', beta isoform (Ppp2r5b), mRNA [NM_181379]	**13,127**
Gabarapl1	Rattus norvegicus GABA(A) receptor-associated protein like 1 (Gabarapl1), mRNA [NM_001044294]	**13,126**
0	Dedicator of cyto-kinesis 3 (Predicted)Uncharacterized protein [Source:UniProtKB/TrEMBL;Acc:D4A998] [ENSRNOT00000019867]	**13,126**
0	Unknown	**13,125**
Shbg	Rattus norvegicus sex hormone binding globulin (Shbg), mRNA [NM_012650]	**13,125**
Bin1	Rattus norvegicus bridging integrator 1 (Bin1), mRNA [NM_053959]	**13,123**
Fzd7	Uncharacterized protein [Source:UniProtKB/TrEMBL;Acc:D4ADM3] [ENSRNOT00000033707]	**13,121**
Amigo1	Rattus norvegicus adhesion molecule with Ig like domain 1 (Amigo1), mRNA [NM_206881]	**13,120**
Stra6	Rattus norvegicus stimulated by retinoic acid gene 6 (Stra6), mRNA [NM_001029924]	**13,117**
Met	Rattus norvegicus met proto-oncogene (Met), mRNA [NM_031517]	**13,117**
Spns2	Rattus norvegicus spinster homolog 2 (Spns2), mRNA [NM_001144991]	**13,117**
Ccl27	Rattus norvegicus chemokine (C-C motif) ligand 27 (Ccl27), mRNA [NM_001108660]	**13,116**
Tmsb10	Rattus norvegicus thymosin, beta 10 (Tmsb10), mRNA [NM_021261]	**13,112**
Spc25	Rattus norvegicus SPC25, NDC80 kinetochore complex component, homolog (S. cerevisiae) (Spc25), mRNA [NM_001009654]	**13,111**
Pfkfb3	Rattus norvegicus 6-phosphofructo-2-kinase/fructose-2,6-biphosphatase 3 (Pfkfb3), mRNA [NM_057135]	**13,111**
Kcnk9	Rattus norvegicus potassium channel, subfamily K, member 9 (Kcnk9), mRNA [NM_053405]	**13,110**
0	Unknown	**13,110**
0	Unknown	**13,109**
0	Unknown	**13,104**
Elavl4	Rattus norvegicus ELAV (embryonic lethal, abnormal vision, Drosophila)-like 4 (Hu antigen D) (Elavl4), mRNA [NM_001077651]	**13,103**
Cmtm4	Rattus norvegicus CKLF-like MARVEL transmembrane domain containing 4 (Cmtm4), mRNA [NM_001172151]	**13,103**
Zfp36l2	Rattus norvegicus zinc finger protein 36, C3H type-like 2 (Zfp36l2), mRNA [NM_001036626]	**13,102**
LOC688019	Uncharacterized protein [Source:UniProtKB/TrEMBL;Acc:D3ZAX9] [ENSRNOT00000005443]	**13,102**
Fubp3	Rattus norvegicus far upstream element (FUSE) binding protein 3 (Fubp3), mRNA [NM_001039337]	**13,102**
Ksr1	Rattus norvegicus kinase suppressor of ras 1 (Ksr1), mRNA [NM_001108284]	**13,100**
Olr528	Rattus norvegicus olfactory receptor 528 (Olr528), mRNA [NM_001000319]	**13,099**
Asam	Rattus norvegicus adipocyte-specific adhesion molecule (Asam), mRNA [NM_173154]	**13,098**

Pcdha2	Rattus norvegicus protocadherin alpha 2 (Pcdha2), mRNA [NM_199504]	**13,097**
Inpp4a	Rattus norvegicus inositol polyphosphate-4-phosphatase, type 1 (Inpp4a), mRNA [NM_031002]	**13,097**
Siae	Rattus norvegicus sialic acid acetylesterase (Siae), mRNA [NM_001108759]	**13,096**
0	Rattus norvegicus TL0AEA14YL01 mRNA sequence. [FQ227170]	**13,096**
Pitx3	Rattus norvegicus paired-like homeodomain 3 (Pitx3), mRNA [NM_019247]	**13,094**
Msi1	Rattus norvegicus Musashi homolog 1(Drosophila) (Msi1), mRNA [NM_148890]	**13,093**
Unc119	Rattus norvegicus UNC-119 homolog (C. elegans) (Unc119), mRNA [NM_017188]	**13,092**
Calhm2	Rattus norvegicus calcium homeostasis modulator 2 (Calhm2), mRNA [NM_001008306]	**13,092**
Mrm1	Rattus norvegicus mitochondrial rRNA methyltransferase 1 homolog (S. cerevisiae) (Mrm1), nuclear gene encoding mitochondrial protein, mRNA [NM_001108832]	**13,086**
0	Unknown	**13,084**
0	Histone deacetylase 7 [Source:UniProtKB/Swiss-Prot;Acc:Q99P96] [ENSRNOT00000011159]	**13,084**
0	Rattus norvegicus similar to olfactory receptor MOR111-1 (LOC288810), mRNA [XM_222344]	**13,082**
Rps6ka3	Rattus norvegicus ribosomal protein S6 kinase polypeptide 3 (Rps6ka3), mRNA [NM_001192004]	**13,079**
RGD1564657	PREDICTED: Rattus norvegicus similar to cathepsin 1 precursor (RGD1564657), mRNA [XM_001065135]	**13,076**
Prkab1	Rattus norvegicus protein kinase, AMP-activated, beta 1 non-catalytic subunit (Prkab1), mRNA [NM_031976]	**13,073**
Klf9	Rattus norvegicus Kruppel-like factor 9 (Klf9), mRNA [NM_057211]	**13,073**
Fam96b	Rattus norvegicus family with sequence similarity 96, member B (Fam96b), mRNA [NM_001144854]	**13,072**
RGD1562720	Rattus norvegicus similar to hypothetical protein FLJ25369 (RGD1562720), mRNA [NM_001109240]	**13,071**
Tmem50b	Rattus norvegicus transmembrane protein 50B (Tmem50b), mRNA [NM_001025014]	**13,069**
Gprc5a	Rattus norvegicus G protein-coupled receptor, family C, group 5, member A (Gprc5a), mRNA [NM_001079890]	**13,069**
Adamts7	Rattus norvegicus ADAM metallopeptidase with thrombospondin type 1 motif, 7 (Adamts7), mRNA [NM_001047101]	**13,068**
Plekhb2	Rattus norvegicus pleckstrin homology domain containing, family B (evectins) member 2 (Plekhb2), mRNA [NM_001106899]	**13,065**
0	Unknown	**13,063**
Entpd2	Rattus norvegicus ectonucleoside triphosphate diphosphohydrolase 2 (Entpd2), mRNA [NM_172030]	**13,057**
Med12	Rattus norvegicus mediator complex subunit 12 (Med12), mRNA [NM_001193292]	**13,057**
Fgfr2	Rattus norvegicus fibroblast growth factor receptor 2 (Fgfr2), transcript variant a, mRNA [NM_012712]	**13,056**
Adh4	Rattus norvegicus alcohol dehydrogenase 4 (class II), pi polypeptide (Adh4), mRNA [NM_017270]	**13,056**
LOC361646	Rattus norvegicus similar to K04F10.2 (LOC361646), mRNA [NM_001134574]	**13,055**
Mtss1	Rattus norvegicus metastasis suppressor 1 (Mtss1), mRNA [NM_001130563]	**13,052**
0	predicted gene 101 Pseudogene [Source:MGI Symbol;Acc:MGI:2684947] [ENSRNOT00000039049]	**13,052**
Prps1	Rattus norvegicus phosphoribosyl pyrophosphate synthetase 1 (Prps1), mRNA [NM_017243]	**13,052**
Trim62	PREDICTED: Rattus norvegicus tripartite motif-containing 62 (Trim62), mRNA [XM_001060201]	**13,052**
Psmd11	Rattus norvegicus proteasome (prosome, macropain) 26S subunit, non-ATPase, 11 (Psmd11), mRNA [NM_001107027]	**13,048**

Pltp	Rattus norvegicus phospholipid transfer protein (Pltp), mRNA [NM_001168543]	**13,043**
Acn9	Rattus norvegicus ACN9 homolog (S. cerevisiae) (Acn9), mRNA [NM_001047914]	**13,043**
Narg1	Rattus norvegicus NMDA receptor regulated 1 (Narg1), mRNA [NM_001107674]	**13,040**
Adck2	Rattus norvegicus aarF domain containing kinase 2 (Adck2), mRNA [NM_001107855]	**13,040**
Meis3	Rattus norvegicus Meis homeobox 3 (Meis3), mRNA [NM_001108472]	**13,038**
Pfas	Rattus norvegicus phosphoribosylformylglycinamidine synthase (Pfas), mRNA [NM_001105791]	**13,038**
Itpkc	Rattus norvegicus inositol 1,4,5-trisphosphate 3-kinase C (Itpkc), mRNA [NM_178094]	**13,037**
LOC682329	PREDICTED: Rattus norvegicus similar to Homeobox protein Nkx-3.2 (Bagpipe homeobox protein homolog 1) (LOC682329), mRNA [XM_001061045]	**13,036**
Htra1	Rattus norvegicus HtrA serine peptidase 1 (Htra1), mRNA [NM_031721]	**13,035**
Cbx7	Rattus norvegicus chromobox homolog 7 (Cbx7), mRNA [NM_199117]	**13,033**
Cym	Rattus norvegicus chymosin (Cym), mRNA [NM_020091]	**13,032**
0	PREDICTED: Rattus norvegicus adenomatosis polyposis coli down-regulated 1 (Apcdd1), mRNA [XM_001071384]	**13,028**
0	Unknown	**13,025**
0	Unknown	**13,025**
RGD1308601	Rattus norvegicus similar to hypothetical protein (RGD1308601), mRNA [NM_001107374]	**13,024**
Clip1	Rattus norvegicus CAP-GLY domain containing linker protein 1 (Clip1), mRNA [NM_031745]	**13,024**
Vom1r23	Rattus norvegicus vomeronasal 1 receptor 23 (Vom1r23), mRNA [NM_001008941]	**13,024**
0	Unknown	**13,023**
Deaf1	Rattus norvegicus deformed epidermal autoregulatory factor 1 (Drosophila) (Deaf1), mRNA [NM_031801]	**13,022**
RGD1562755	Uncharacterized protein [Source:UniProtKB/TrEMBL;Acc:D3ZNM1] [ENSRNOT00000051559]	**13,021**
0	Rattus norvegicus zinc finger protein 1 (DZF1) mRNA, partial cds. [U78129]	**13,021**
Galp	Rattus norvegicus galanin-like peptide (Galp), mRNA [NM_022633]	**13,019**
Axin1	Rattus norvegicus axin 1 (Axin1), mRNA [NM_024405]	**13,019**
0	Unknown	**13,018**
0	Unknown	**13,016**
Myo9a	Rattus norvegicus myosin IXA (Myo9a), mRNA [NM_134335]	**13,015**
Cdkn3	Rattus norvegicus cyclin-dependent kinase inhibitor 3 (Cdkn3), mRNA [NM_001106028]	**13,013**
Rec8	Rattus norvegicus REC8 homolog (yeast) (Rec8), mRNA [NM_001011916]	**13,008**
Map2k5	Rattus norvegicus mitogen activated protein kinase kinase 5 (Map2k5), transcript variant 2, mRNA [NM_017246]	**13,007**
LOC683674	PREDICTED: Rattus norvegicus similar to Protein C7orf26 homolog (LOC683674), mRNA [XM_001064018]	**13,007**
0	Condensin complex subunit 2 [Source:UniProtKB/TrEMBL;Acc:D4A8M7] [ENSRNOT00000016157]	**13,003**
Tecpr1	Rattus norvegicus tectonin beta-propeller repeat containing 1 (Tecpr1), mRNA [NM_001037191]	**13,001**
Ubn2	Rattus norvegicus ubinuclein 2 (Ubn2), mRNA [NM_001134553]	**12,998**

Rxrg	Rattus norvegicus retinoid X receptor gamma (Rxrg), mRNA [NM_031765]	**12,995**
LOC680441	Uncharacterized protein [Source:UniProtKB/TrEMBL;Acc:D3ZSR8] [ENSRNOT00000051510]	**12,995**
RGD1306286	Uncharacterized protein [Source:UniProtKB/TrEMBL;Acc:D3ZLP7] [ENSRNOT00000022767]	**12,995**
Uqcc	Rattus norvegicus ubiquinol-cytochrome c reductase complex chaperone (Uqcc), nuclear gene encoding mitochondrial protein, mRNA [NM_001109446]	**12,994**
0	Unknown	**12,992**
Dnm1	Rattus norvegicus dynamin 1 (Dnm1), mRNA [NM_080689]	**12,991**
Chchd5	Rattus norvegicus coiled-coil-helix-coiled-coil-helix domain containing 5 (Chchd5), mRNA [NM_001106509]	**12,991**
0	Uncharacterized protein [Source:UniProtKB/TrEMBL;Acc:D3ZG23] [ENSRNOT00000046737]	**12,991**
LOC682967	PREDICTED: Rattus norvegicus similar to Protein disulfide-isomerase TXNDC10 precursor (Thioredoxin domain-containing protein 10) (LOC682967), mRNA [XM_001063895]	**12,989**
Pprc1	Rattus norvegicus peroxisome proliferator-activated receptor gamma, coactivator-related 1 (Pprc1), mRNA [NM_001106363]	**12,985**
Dclre1c	Rattus norvegicus DNA cross-link repair 1C, PSO2 homolog (S. cerevisiae) (Dclre1c), mRNA [NM_147145]	**12,983**
Magea11	Rattus norvegicus melanoma antigen family A, 11 (Magea11), mRNA [NM_001013962]	**12,982**
Csnk2b	Rattus norvegicus casein kinase 2, beta polypeptide (Csnk2b), transcript variant 1, mRNA [NM_031021]	**12,982**
0	Q80V97_MOUSE (Q80V97) Itga9 protein (Fragment), partial (67%) [TC615359]	**12,978**
Arl6ip1	Rattus norvegicus ADP-ribosylation factor-like 6 interacting protein 1 (Arl6ip1), mRNA [NM_198737]	**12,976**
Ctdspl2	Rattus norvegicus CTD (carboxy-terminal domain, RNA polymerase II, polypeptide A) small phosphatase like 2 (Ctdspl2), mRNA [NM_001014048]	**12,975**
0	Unknown	**12,973**
0	Unknown	**12,972**
Polr3g	Rattus norvegicus polymerase (RNA) III (DNA directed) polypeptide G (Polr3g), mRNA [NM_001109468]	**12,970**
Ring1	Rattus norvegicus ring finger protein 1 (Ring1), mRNA [NM_212549]	**12,969**
Leprot	Rattus norvegicus leptin receptor overlapping transcript (Leprot), mRNA [NM_020099]	**12,969**
Elf2	Rattus norvegicus E74-like factor 2 (Elf2), transcript variant 2, mRNA [NM_001012181]	**12,968**
Pyy	Rattus norvegicus peptide YY (mapped) (Pyy), mRNA [NM_001034080]	**12,967**
LOC100359999	PREDICTED: Rattus norvegicus hypothetical protein LOC100359999 (LOC100359999), partial mRNA [XM_002729469]	**12,967**
Acadm	Rattus norvegicus acyl-Coenzyme A dehydrogenase, C-4 to C-12 straight chain (Acadm), nuclear gene encoding mitochondrial protein, mRNA [NM_016986]	**12,966**
0	Rattus norvegicus cDNA clone IMAGE:7318973. [BC091362]	**12,966**
Zeb1	Rattus norvegicus zinc finger E-box binding homeobox 1 (Zeb1), mRNA [NM_013164]	**12,964**
Dmd	Rattus norvegicus dystrophin (Dmd), transcript variant Dp71a, mRNA [NM_012698]	**12,963**
Pptc7	Rattus norvegicus PTC7 protein phosphatase homolog (S. cerevisiae) (Pptc7), mRNA [NM_001107141]	**12,962**
RGD1304953	Rattus norvegicus similar to SSTK-interacting protein (RGD1304953), mRNA [NM_001108556]	**12,959**
Lrrn4cl	Rattus norvegicus LRRN4 C-terminal like (Lrrn4cl), mRNA [NM_001109579]	**12,958**

Jph3	Rattus norvegicus junctophilin 3 (Jph3), mRNA [NM_001107437]	**12,957**
Ephb1	Rattus norvegicus Eph receptor B1 (Ephb1), mRNA [NM_001104528]	**12,955**
Scd1	Rattus norvegicus stearoyl-Coenzyme A desaturase 1 (Scd1), mRNA [NM_139192]	**12,951**
Napg	Rattus norvegicus N-ethylmaleimide-sensitive factor attachment protein, gamma (Napg), mRNA [NM_001107384]	**12,951**
0	Unknown	**12,950**
Pinx1	Rattus norvegicus PIN2-interacting protein 1 (Pinx1), mRNA [NM_001083337]	**12,942**
0	Q6QAQ5_PIG (Q6QAQ5) 60S ribosomal protein P1 (Fragment), partial (22%) [TC645425]	**12,940**
Grhl2	Rattus norvegicus grainyhead-like 2 (Drosophila) (Grhl2), mRNA [NM_001134527]	**12,940**
Bsdc1	Rattus norvegicus BSD domain containing 1 (Bsdc1), mRNA [NM_001106636]	**12,940**
0	Unknown	**12,935**
Atg14	Rattus norvegicus ATG14 autophagy related 14 homolog (S. cerevisiae) (Atg14), mRNA [NM_001107258]	**12,934**
Plekha7	Rattus norvegicus pleckstrin homology domain containing, family A member 7 (Plekha7), mRNA [NM_001144861]	**12,930**
0	PREDICTED: Rattus norvegicus similar to MIC2 like 1 (LOC501437), partial mRNA [XM_001074315]	**12,930**
Fgd6	Rattus norvegicus FYVE, RhoGEF and PH domain containing 6 (Fgd6), mRNA [NM_001137645]	**12,930**
Ntrk3	Rattus norvegicus neurotrophic tyrosine kinase, receptor, type 3 (Ntrk3), mRNA [NM_019248]	**12,930**
Utp14a	Rattus norvegicus UTP14, U3 small nucleolar ribonucleoprotein, homolog A (yeast) (Utp14a), mRNA [NM_001014113]	**12,927**
Ubiad1	Rattus norvegicus UbiA prenyltransferase domain containing 1 (Ubiad1), mRNA [NM_001107993]	**12,927**
Ebp	Rattus norvegicus emopamil binding protein (sterol isomerase) (Ebp), mRNA [NM_057137]	**12,923**
Kcnj9	Rattus norvegicus potassium inwardly-rectifying channel, subfamily J, member 9 (Kcnj9), mRNA [NM_053834]	**12,921**
0	Uncharacterized protein [Source:UniProtKB/TrEMBL;Acc:D3Z9L4] [ENSRNOT00000057467]	**12,921**
Otud7b	Rattus norvegicus OTU domain containing 7B (Otud7b), mRNA [NM_001107697]	**12,920**
Spred1	Rattus norvegicus sprouty-related, EVH1 domain containing 1 (Spred1), mRNA [NM_001047089]	**12,918**
Car4	Rattus norvegicus carbonic anhydrase 4 (Car4), mRNA [NM_019174]	**12,917**
0	Unknown	**12,916**
Chfr	Rattus norvegicus checkpoint with forkhead and ring finger domains (Chfr), mRNA [NM_001009258]	**12,915**
Dusp3	Rattus norvegicus dual specificity phosphatase 3 (Dusp3), mRNA [NM_001173376]	**12,914**
Tmod2	Rattus norvegicus tropomodulin 2 (Tmod2), mRNA [NM_031613]	**12,914**
Olr1688	Rattus norvegicus olfactory receptor 1688 (Olr1688), mRNA [NM_001000275]	**12,911**
Lyl1	Rattus norvegicus lymphoblastic leukemia derived sequence 1 (Lyl1), mRNA [NM_001007677]	**12,911**
0	Uncharacterized protein [Source:UniProtKB/TrEMBL;Acc:D3ZI73] [ENSRNOT00000026047]	**12,908**
Itga4	Rattus norvegicus integrin, alpha 4 (Itga4), mRNA [NM_001107737]	**12,907**
Syt16	Uncharacterized protein [Source:UniProtKB/TrEMBL;Acc:D3ZB68] [ENSRNOT00000012374]	**12,906**
Krt84	Rattus norvegicus keratin 84 (Krt84), mRNA [NM_001008812]	**12,905**
Bzw1	Rattus norvegicus basic leucine zipper and W2 domains 1 (Bzw1), mRNA [NM_198789]	**12,903**

Pcdhb9	Rattus norvegicus protocadherin beta 9 (Pcdhb9), mRNA [NM_001109390]	**12,903**
0	Uncharacterized protein [Source:UniProtKB/TrEMBL;Acc:D3ZRM8] [ENSRNOT00000018544]	**12,902**
Dip2b	PREDICTED: Rattus norvegicus DIP2 disco-interacting protein 2 homolog B (Drosophila) (Dip2b), mRNA [XM_001063280]	**12,899**
Rcan1	Rattus norvegicus regulator of calcineurin 1 (Rcan1), mRNA [NM_153724]	**12,897**
40603	Rattus norvegicus membrane-associated ring finger (C3HC4) 11 (March11), mRNA [NM_001101828]	**12,897**
RGD1560470	Uncharacterized protein [Source:UniProtKB/TrEMBL;Acc:D3ZTF0] [ENSRNOT00000040995]	**12,895**
RGD1305235	Rattus norvegicus similar to RIKEN cDNA 1700052N19 (RGD1305235), mRNA [NM_001017447]	**12,894**
0	Steroidogenic factor 1 [Source:UniProtKB/Swiss-Prot;Acc:P50569] [ENSRNOT00000042720]	**12,892**
Lxn	Rattus norvegicus latexin (Lxn), mRNA [NM_031655]	**12,889**
Dynll1	Rattus norvegicus dynein light chain LC8-type 1 (Dynll1), mRNA [NM_053319]	**12,887**
Crtc2	Rattus norvegicus CREB regulated transcription coactivator 2 (Crtc2), mRNA [NM_001033895]	**12,885**
Cndp2	Rattus norvegicus CNDP dipeptidase 2 (metallopeptidase M20 family) (Cndp2), mRNA [NM_001010920]	**12,884**
Pcdhga3	Rattus norvegicus protocadherin gamma subfamily A, 3 (Pcdhga3), mRNA [NM_001037154]	**12,882**
0	Q3U6G1_MOUSE (Q3U6G1) Bone marrow macrophage cDNA, RIKEN full-length enriched library, clone:I830125G18 product:biliverdin reductase B (flavin reductase (NADPH)), full insert sequence, partial (78%) [TC609126]	**12,882**
0	PREDICTED: Rattus norvegicus similar to cactin CG1676-PA (LOC303590), miscRNA [XR_006385]	**12,881**
0	Unknown	**12,880**
Lypd2	Rattus norvegicus Ly6/Plaur domain containing 2 (Lypd2), mRNA [NM_001130545]	**12,880**
0	Uncharacterized protein [Source:UniProtKB/TrEMBL;Acc:D3ZE32] [ENSRNOT00000015993]	**12,877**
Tmem47	Rattus norvegicus transmembrane protein 47 (Tmem47), mRNA [NM_001109317]	**12,877**
Lrrc16a	Rattus norvegicus leucine rich repeat containing 16A (Lrrc16a), mRNA [NM_001191692]	**12,876**
0	Q920N8_MOUSE (Q920N8) Protein kinase C thetaII, partial (79%) [TC595074]	**12,876**
Lyst	Rattus norvegicus lysosomal trafficking regulator (Lyst), mRNA [NM_053518]	**12,875**
LOC682033	Rattus norvegicus similar to Protein phosphatase 2A, 59 kDa regulatory subunit B (PP2A PR59) (PP2A B-PR59) (LOC682033), mRNA [NM_001139492]	**12,875**
Lin7c	Rattus norvegicus lin-7 homolog C (C. elegans) (Lin7c), mRNA [NM_021851]	**12,874**
0	Unknown	**12,872**
Mpp3	MAGUK p55 subfamily member 3 [Source:UniProtKB/Swiss-Prot;Acc:O88954] [ENSRNOT00000028264]	**12,872**
Slc22a12	Rattus norvegicus solute carrier family 22 (organic anion/urate transporter), member 12 (Slc22a12), mRNA [NM_001034943]	**12,866**
RGD1562291	PREDICTED: Rattus norvegicus similar to Human T-cell leukemia virus enhancer factor (Forkhead box protein N2) (RGD1562291), mRNA [XM_237502]	**12,866**
Taf1	Rattus norvegicus TAF1 RNA polymerase II, TATA box binding protein (TBP)-associated factor (Taf1), mRNA [NM_001191723]	**12,865**
Pafah1b1	Rattus norvegicus platelet-activating factor acetylhydrolase, isoform 1b, subunit 1 (Pafah1b1), mRNA [NM_031763]	**12,861**
0	Unknown	**12,861**

0	Uncharacterized protein [Source:UniProtKB/TrEMBL;Acc:D3ZDX5] [ENSRNOT00000043345]	**12,859**
Chn1	Rattus norvegicus chimerin (chimaerin) 1 (Chn1), mRNA [NM_032083]	**12,854**
0	Unknown	**12,853**
Mdh2	Rattus norvegicus malate dehydrogenase 2, NAD (mitochondrial) (Mdh2), nuclear gene encoding mitochondrial protein, mRNA [NM_031151]	**12,849**
0	Unknown	**12,844**
0	Unknown	**12,843**
LOC687270	PREDICTED: Rattus norvegicus similar to glyceraldehyde-3-phosphate dehydrogenase (LOC687270), miscRNA [XR_085886]	**12,841**
Ralyl	Rattus norvegicus RALY RNA binding protein-like (Ralyl), mRNA [NM_001024978]	**12,839**
Chd8	Rattus norvegicus chromodomain helicase DNA binding protein 8 (Chd8), mRNA [NM_022933]	**12,839**
Mkrn1	Rattus norvegicus makorin ring finger protein 1 (Mkrn1), mRNA [NM_001004233]	**12,835**
RGD1563986	Rattus norvegicus similar to RIKEN cDNA E330009J07 gene (RGD1563986), mRNA [NM_001044701]	**12,834**
Tpo	Rattus norvegicus thyroid peroxidase (Tpo), mRNA [NM_019353]	**12,834**
Cap2	Rattus norvegicus CAP, adenylate cyclase-associated protein, 2 (yeast) (Cap2), mRNA [NM_053874]	**12,831**
Zfp187	Uncharacterized protein [Source:UniProtKB/TrEMBL;Acc:D3ZFY2] [ENSRNOT00000024693]	**12,830**
0	Unknown	**12,828**
Adamts15	Rattus norvegicus ADAM metallopeptidase with thrombospondin type 1 motif, 15 (Adamts15), mRNA [NM_001106810]	**12,827**
Rad23b	Rattus norvegicus RAD23 homolog B (S. cerevisiae) (Rad23b), mRNA [NM_001025275]	**12,821**
Larp5	Rattus norvegicus La ribonucleoprotein domain family, member 5 (Larp5), mRNA [NM_001107361]	**12,820**
Pdik1l	Rattus norvegicus PDLIM1 interacting kinase 1 like (Pdik1l), mRNA [NM_001107984]	**12,820**
Sgsm1	small G protein signaling modulator 1 [Source:RefSeq peptide;Acc:NP_001099407] [ENSRNOT00000056824]	**12,815**
Grhl1	Uncharacterized protein [Source:UniProtKB/TrEMBL;Acc:D3ZZW4] [ENSRNOT00000038519]	**12,810**
Plcb2	1-phosphatidylinositol-4,5-bisphosphate phosphodiesterase beta-2 [Source:UniProtKB/Swiss-Prot;Acc:O89040] [ENSRNOT00000011911]	**12,808**
Olr687	Rattus norvegicus olfactory receptor 687 (Olr687), mRNA [NM_001000356]	**12,807**
Aer61	Rattus norvegicus glycosyltransferase Aer61 (Aer61), mRNA [NM_001009502]	**12,803**
RGD1561849	Rattus norvegicus similar to RIKEN cDNA 3110035E14 (RGD1561849), mRNA [NM_001109260]	**12,802**
0	Oct 2 Pou-Homeo domain protein [Source:UniProtKB/TrEMBL;Acc:Q63388] [ENSRNOT00000027655]	**12,802**
Pabpn1	Rattus norvegicus poly(A) binding protein, nuclear 1 (Pabpn1), mRNA [NM_001135008]	**12,801**
Muc4	PREDICTED: Rattus norvegicus mucin 4, cell surface associated (Muc4), mRNA [XM_221384]	**12,801**
Ranbp1	Rattus norvegicus RAN binding protein 1 (Ranbp1), mRNA [NM_001108324]	**12,800**
LOC684822	PREDICTED: Rattus norvegicus similar to transcription elongation factor B (SIII), polypeptide 2 (LOC684822), mRNA [XM_002729867]	**12,800**
Intu	Uncharacterized protein [Source:UniProtKB/TrEMBL;Acc:D4ACE5] [ENSRNOT00000014116]	**12,796**
Rpp21	Rattus norvegicus ribonuclease P 21 subunit (human) (Rpp21), mRNA [NM_001002831]	**12,796**
RGD1560527	PREDICTED: Rattus norvegicus similar to serine/threonine kinase (RGD1560527), mRNA [XM_001055752]	**12,794**
Mapk10	Rattus norvegicus mitogen activated protein kinase 10 (Mapk10), mRNA [NM_012806]	**12,791**

Pdk3	Rattus norvegicus pyruvate dehydrogenase kinase, isozyme 3 (Pdk3), nuclear gene encoding mitochondrial protein, mRNA [NM_001106581]	**12,790**
Nat15	Rattus norvegicus N-acetyltransferase 15 (GCN5-related, putative) (Nat15), mRNA [NM_001014226]	**12,785**
Cisd2	Rattus norvegicus CDGSH iron sulfur domain 2 (Cisd2), mRNA [NM_001191608]	**12,785**
Trim7	PREDICTED: Rattus norvegicus tripartite motif-containing 7, transcript variant 2 (Trim7), mRNA [XM_002724489]	**12,780**
Ppp3cc	Rattus norvegicus protein phosphatase 3, catalytic subunit, gamma isoform (Ppp3cc), mRNA [NM_134367]	**12,780**
Lcat	Rattus norvegicus lecithin cholesterol acyltransferase (Lcat), mRNA [NM_017024]	**12,780**
Efs	Rattus norvegicus embryonal Fyn-associated substrate (Efs), mRNA [NM_001106033]	**12,780**
Ykt6	Rattus norvegicus YKT6 v-SNARE homolog (S. cerevisiae) (Ykt6), mRNA [NM_031692]	**12,777**
Unc84b	PREDICTED: Rattus norvegicus unc-84 homolog B (C. elegans) (Unc84b), mRNA [XM_235483]	**12,777**
Rcvrn	Rattus norvegicus recoverin (Rcvrn), mRNA [NM_080901]	**12,776**
Cryga	Rattus norvegicus crystallin, gamma A (Cryga), mRNA [NM_001080936]	**12,775**
RGD1565117	Uncharacterized protein [Source:UniProtKB/TrEMBL;Acc:D3ZJ54] [ENSRNOT00000045687]	**12,774**
Man2c1	Rattus norvegicus mannosidase, alpha, class 2C, member 1 (Man2c1), mRNA [NM_139256]	**12,773**
Sgk196	Rattus norvegicus protein kinase-like protein SgK196 (Sgk196), mRNA [NM_001024883]	**12,773**
LOC688657	PREDICTED: Rattus norvegicus similar to Olfactory receptor 5D13 (LOC688657), mRNA [XM_001067781]	**12,773**
Taok3	Rattus norvegicus TAO kinase 3 (Taok3), mRNA [NM_001024254]	**12,771**
Amigo2	Rattus norvegicus adhesion molecule with Ig like domain 2 (Amigo2), mRNA [NM_182816]	**12,766**
0	Unknown	**12,765**
Psrc1	Rattus norvegicus proline/serine-rich coiled-coil 1 (Psrc1), mRNA [NM_001044302]	**12,762**
0	Unknown	**12,759**
Nr1i2	Rattus norvegicus nuclear receptor subfamily 1, group I, member 2 (Nr1i2), mRNA [NM_052980]	**12,757**
Nedd9	Rattus norvegicus neural precursor cell expressed, developmentally down-regulated 9 (Nedd9), mRNA [NM_001011922]	**12,754**
Eif2s3x	Rattus norvegicus eukaryotic translation initiation factor 2, subunit 3, structural gene X-linked (Eif2s3x), mRNA [NM_001100542]	**12,753**
ST7	Rattus norvegicus suppression of tumorigenicity 7 (ST7), transcript variant 1, mRNA [NM_001004102]	**12,753**
Gnal	Rattus norvegicus guanine nucleotide binding protein, alpha stimulating, olfactory type (Gnal), mRNA [NM_001191836]	**12,751**
Ces2a	Rattus norvegicus carboxylesterase 2A (Ces2a), mRNA [NM_144743]	**12,751**
Fosl1	Rattus norvegicus fos-like antigen 1 (Fosl1), mRNA [NM_012953]	**12,751**
Ppp2r3c	Rattus norvegicus protein phosphatase 2, regulatory subunit B'', gamma (Ppp2r3c), mRNA [NM_001014196]	**12,749**
Chd6	Rattus norvegicus chromodomain helicase DNA binding protein 6 (Chd6), mRNA [NM_001107797]	**12,749**
Adrbk2	Rattus norvegicus adrenergic, beta, receptor kinase 2 (Adrbk2), mRNA [NM_012897]	**12,748**
LOC690096	Uncharacterized protein [Source:UniProtKB/TrEMBL;Acc:D3ZEI0] [ENSRNOT00000012255]	**12,747**
Slmap	Rattus norvegicus sarcolemma associated protein (Slmap), mRNA [NM_001106060]	**12,743**
Tbc1d20	Rattus norvegicus TBC1 domain family, member 20 (Tbc1d20), mRNA [NM_001004281]	**12,743**
Mapt	Rattus norvegicus microtubule-associated protein tau (Mapt), mRNA [NM_017212]	**12,740**

RGD1304982	Rattus norvegicus similar to RIKEN cDNA 2810025M15 (RGD1304982), mRNA [NM_001113775]	**12,739**
Ecel1	Rattus norvegicus endothelin converting enzyme-like 1 (Ecel1), mRNA [NM_021776]	**12,739**
0	Uncharacterized protein [Source:UniProtKB/TrEMBL;Acc:D3ZC05] [ENSRNOT00000046525]	**12,739**
Ns5atp9	Rattus norvegicus NS5A (hepatitis C virus) transactivated protein 9 (Ns5atp9), mRNA [NM_201418]	**12,737**
0	Unknown	**12,736**
Usp32	Rattus norvegicus ubiquitin specific peptidase 32 (Usp32), mRNA [NM_001107032]	**12,735**
Slc4a8	Rattus norvegicus solute carrier family 4, sodium bicarbonate cotransporter, member 8 (Slc4a8), mRNA [NM_199497]	**12,735**
Ret	Rattus norvegicus ret proto-oncogene (Ret), transcript variant 2, mRNA [NM_001110099]	**12,730**
LOC685634	RCG31132Uncharacterized protein [Source:UniProtKB/TrEMBL;Acc:D4ACP2] [ENSRNOT00000019224]	**12,729**
0	Rattus norvegicus similar to glyceraldehyde-3-phosphate dehydrogenase (phosphorylating) (EC 1.2.1.12) - mouse (LOC302809), mRNA [XM_229109]	**12,729**
Ap3m2	Rattus norvegicus adaptor-related protein complex 3, mu 2 subunit (Ap3m2), mRNA [NM_133305]	**12,729**
Epb4.1l5	Rattus norvegicus erythrocyte protein band 4.1-like 5 (Epb4.1l5), mRNA [NM_001012023]	**12,729**
Col6a1	PREDICTED: Rattus norvegicus collagen, type VI, alpha 1 (Col6a1), mRNA [XM_001079629]	**12,729**
Olr1002	Rattus norvegicus olfactory receptor 1002 (Olr1002), mRNA [NM_001001371]	**12,728**
Ankib1	Rattus norvegicus ankyrin repeat and IBR domain containing 1 (Ankib1), mRNA [NM_001134781]	**12,727**
RGD1305713	Rattus norvegicus similar to RIKEN cDNA 3110040N11 (RGD1305713), mRNA [NM_001024749]	**12,727**
Nmt2	Rattus norvegicus N-myristoyltransferase 2 (Nmt2), mRNA [NM_207590]	**12,724**
Got2	Rattus norvegicus glutamic-oxaloacetic transaminase 2, mitochondrial (aspartate aminotransferase 2) (Got2), nuclear gene encoding mitochondrial protein, mRNA [NM_013177]	**12,721**
Basp1	Rattus norvegicus brain abundant, membrane attached signal protein 1 (Basp1), mRNA [NM_022300]	**12,720**
0	Unknown	**12,718**
Il33	Rattus norvegicus interleukin 33 (Il33), mRNA [NM_001014166]	**12,718**
Sema6c	Rattus norvegicus sema domain, transmembrane domain (TM), and cytoplasmic domain, (semaphorin) 6C (Sema6c), mRNA [NM_017308]	**12,716**
Fis1	Rattus norvegicus fission 1 (mitochondrial outer membrane) homolog (S. cerevisiae) (Fis1), nuclear gene encoding mitochondrial protein, mRNA [NM_001105919]	**12,712**
Fam32a	Rattus norvegicus family with sequence similarity 32, member A (Fam32a), mRNA [NM_001128078]	**12,710**
Fgfbp3	Rattus norvegicus fibroblast growth factor binding protein 3 (Fgfbp3), mRNA [NM_001109165]	**12,709**
Gpx2	Rattus norvegicus glutathione peroxidase 2 (Gpx2), mRNA [NM_183403]	**12,708**
RGD1563217	Rattus norvegicus similar to RIKEN cDNA 4930451I11 (RGD1563217), mRNA [NM_001109155]	**12,706**
Usp30	Rattus norvegicus ubiquitin specific peptidase 30 (Usp30), mRNA [NM_001107153]	**12,703**
C1ql2	Rattus norvegicus complement component 1, q subcomponent-like 2 (C1ql2), mRNA [NM_001105949]	**12,703**
0	Gdpd5 protein [Source:UniProtKB/TrEMBL;Acc:B5DF39] [ENSRNOT00000055321]	**12,703**
0	Uncharacterized protein [Source:UniProtKB/TrEMBL;Acc:D3ZFK7] [ENSRNOT00000050217]	**12,703**

Lass1	Rattus norvegicus LAG1 homolog, ceramide synthase 1 (Lass1), mRNA [NM_001044230]	**12,700**
Bat1	Rattus norvegicus HLA-B associated transcript 1 (Bat1), mRNA [NM_133300]	**12,699**
Nfkb1	Nuclear factor NF-kappa-B p105 subunitNuclear factor NF-kappa-B p50 subunit [Source:UniProtKB/Swiss-Prot;Acc:Q63369] [ENSRNOT00000036838]	**12,693**
Snx12	Rattus norvegicus sorting nexin 12 (Snx12), mRNA [NM_001108817]	**12,693**
Znf511	Rattus norvegicus zinc finger protein 511 (Znf511), mRNA [NM_001106309]	**12,692**
Brsk2	PREDICTED: Rattus norvegicus brain serine/threonine kinase 2 (Brsk2), mRNA [XM_001063734]	**12,690**
Foxd4	Forkhead box protein D4 [Source:UniProtKB/Swiss-Prot;Acc:Q63249] [ENSRNOT00000066752]	**12,689**
Gfra2	Rattus norvegicus GDNF family receptor alpha 2 (Gfra2), mRNA [NM_012750]	**12,687**
Hm13	Rattus norvegicus histocompatibility 13 (Hm13), mRNA [NM_001107789]	**12,687**
Rpl23a	Rattus norvegicus ribosomal protein L23a (Rpl23a), mRNA [NM_001108283]	**12,684**
LOC679580	PREDICTED: Rattus norvegicus similar to MIC2 like 1 (LOC679580), mRNA [XM_001053578]	**12,684**
Kcnmb2	Rattus norvegicus potassium large conductance calcium-activated channel, subfamily M, beta member 2 (Kcnmb2), mRNA [NM_176861]	**12,681**
Eya2	Rattus norvegicus eyes absent homolog 2 (Drosophila) (Eya2), mRNA [NM_130427]	**12,679**
Col9a1	Rattus norvegicus collagen, type IX, alpha 1 (Col9a1), mRNA [NM_001100842]	**12,679**
Eif5	Rattus norvegicus eukaryotic translation initiation factor 5 (Eif5), mRNA [NM_020075]	**12,678**
H2afy2	Rattus norvegicus H2A histone family, member Y2 (H2afy2), mRNA [NM_001135807]	**12,678**
Ppp2r2a	Rattus norvegicus protein phosphatase 2 (formerly 2A), regulatory subunit B, alpha isoform (Ppp2r2a), mRNA [NM_053999]	**12,676**
Colq	Acetylcholinesterase collagenic tail peptide [Source:UniProtKB/Swiss-Prot;Acc:O35167] [ENSRNOT00000026550]	**12,674**
Bmpr2	Rattus norvegicus bone morphogenetic protein receptor, type II (serine/threonine kinase) (Bmpr2), mRNA [NM_080407]	**12,673**
Cyb5r1	Rattus norvegicus cytochrome b5 reductase 1 (Cyb5r1), mRNA [NM_001013126]	**12,673**
0	Uncharacterized protein [Source:UniProtKB/TrEMBL;Acc:D3ZDF4] [ENSRNOT00000024704]	**12,672**
LOC684755	PREDICTED: Rattus norvegicus similar to zinc finger like protein 1, transcript variant 3 (LOC684755), mRNA [XM_001075288]	**12,670**
LOC688452	Rattus norvegicus hypothetical protein LOC688452 (LOC688452), mRNA [NM_001135720]	**12,668**
Tmem136	Rattus norvegicus transmembrane protein 136 (Tmem136), mRNA [NM_001108136]	**12,668**
0	Unknown	**12,666**
Tmem134	Rattus norvegicus transmembrane protein 134 (Tmem134), transcript variant 2, mRNA [NM_001078648]	**12,666**
0	Unknown	**12,665**
Mtmr9	Rattus norvegicus myotubularin related protein 9 (Mtmr9), mRNA [NM_001005761]	**12,660**
RGD1563870	PREDICTED: Rattus norvegicus similar to CG3104-PA, isoform A (RGD1563870), miscRNA [XR_086302]	**12,656**
0	Unknown	**12,654**
Aagab	Rattus norvegicus alpha- and gamma-adaptin binding protein (Aagab), mRNA [NM_134398]	**12,651**
0	Unknown	**12,647**
RGD1562174	Uncharacterized protein [Source:UniProtKB/TrEMBL;Acc:D3Z8I3] [ENSRNOT00000038543]	**12,647**

Mrps18b	Rattus norvegicus mitochondrial ribosomal protein S18B (Mrps18b), nuclear gene encoding mitochondrial protein, mRNA [NM_212534]	**12,646**
Fbxo7	Rattus norvegicus F-box protein 7 (Fbxo7), mRNA [NM_001012222]	**12,644**
Bok	Rattus norvegicus BCL2-related ovarian killer (Bok), mRNA [NM_017312]	**12,643**
0	Q6GQU6_MOUSE (Q6GQU6) CDNA sequence BC072620, partial (56%) [TC612613]	**12,642**
Lyg2	PREDICTED: Rattus norvegicus lysozyme G-like 2 (Lyg2), mRNA [XM_578787]	**12,642**
Narf	Rattus norvegicus nuclear prelamin A recognition factor (Narf), mRNA [NM_001039207]	**12,641**
Rsb66	Rattus norvegicus Rsb-66 protein (Rsb66), mRNA [NM_181694]	**12,639**
Wbscr27	Rattus norvegicus Williams Beuren syndrome chromosome region 27 (Wbscr27), mRNA [NM_001109499]	**12,639**
Adamts12	Rattus norvegicus ADAM metallopeptidase with thrombospondin type 1 motif, 12 (Adamts12), mRNA [NM_001106420]	**12,638**
0	Unknown	**12,633**
Homer1	Rattus norvegicus homer homolog 1 (Drosophila) (Homer1), mRNA [NM_031707]	**12,631**
Apoc1	Rattus norvegicus apolipoprotein C-I (Apoc1), transcript variant 1, mRNA [NM_012824]	**12,629**
Nell2	Rattus norvegicus NEL-like 2 (chicken) (Nell2), mRNA [NM_031070]	**12,629**
Eif1ay	Rattus norvegicus eukaryotic translation initiation factor 1A, Y-linked (Eif1ay), mRNA [NM_001106963]	**12,627**
0	Unknown	**12,626**
Slc2a1	Rattus norvegicus solute carrier family 2 (facilitated glucose transporter), member 1 (Slc2a1), mRNA [NM_138827]	**12,625**
Olr1509	Rattus norvegicus olfactory receptor 1509 (Olr1509), mRNA [NM_001000528]	**12,624**
0	AW918633 EST349937 Rat gene index, normalized rat, norvegicus, Bento Soares Rattus norvegicus cDNA clone RGIEV66 5' end, mRNA sequence [AW918633]	**12,623**
0	Unknown	**12,622**
Orai2	Rattus norvegicus ORAI calcium release-activated calcium modulator 2 (Orai2), mRNA [NM_001170403]	**12,620**
Dhtkd1	Rattus norvegicus dehydrogenase E1 and transketolase domain containing 1 (Dhtkd1), nuclear gene encoding mitochondrial protein, mRNA [NM_001025720]	**12,618**
Lrp4	Rattus norvegicus low density lipoprotein receptor-related protein 4 (Lrp4), mRNA [NM_031322]	**12,618**
Zwint	Rattus norvegicus ZW10 interactor (Zwint), mRNA [NM_147138]	**12,615**
Olr1411	Rattus norvegicus olfactory receptor 1411 (Olr1411), mRNA [NM_001000783]	**12,615**
LOC501391	Rattus norvegicus cDNA clone IMAGE:7456737. [BC158634]	**12,610**
Bves	Rattus norvegicus blood vessel epicardial substance (Bves), mRNA [NM_001077590]	**12,610**
Trim26	Rattus norvegicus tripartite motif-containing 26 (Trim26), mRNA [NM_001011665]	**12,608**
Ifi35	Rattus norvegicus interferon-induced protein 35 (Ifi35), mRNA [NM_001009625]	**12,606**
Vars2	Rattus norvegicus valyl-tRNA synthetase 2, mitochondrial (putative) (Vars2), nuclear gene encoding mitochondrial protein, mRNA [NM_213563]	**12,606**
Add2	Rattus norvegicus adducin 2 (beta) (Add2), transcript variant 2, mRNA [NM_012491]	**12,605**
LOC679651	Rattus norvegicus hypothetical protein LOC679651 (LOC679651), mRNA [NM_001195277]	**12,604**
Fam185a	Rattus norvegicus family with sequence similarity 185, member A (Fam185a), mRNA [NM_001128188]	**12,603**

0	Rattus norvegicus similar to Pyruvate kinase, M2 isozyme (LOC315231), mRNA [XM_235589]	**12,602**
0	Rattus norvegicus chromosome 1 strain BN/SsNHsdMCW RNOR03207803, whole genome shotgun sequence [AABR03002941]	**12,601**
Lrp8	Uncharacterized protein [Source:UniProtKB/TrEMBL;Acc:D3ZE47] [ENSRNOT00000017575]	**12,601**
Ndst2	Rattus norvegicus N-deacetylase/N-sulfotransferase (heparan glucosaminyl) 2 (Ndst2), mRNA [NM_001105740]	**12,598**
Arrdc4	Rattus norvegicus arrestin domain containing 4 (Arrdc4), mRNA [NM_001047853]	**12,598**
Akirin1	Rattus norvegicus akirin 1 (Akirin1), mRNA [NM_001030054]	**12,597**
Prm3	Rattus norvegicus protamine 3 (Prm3), mRNA [NM_001002855]	**12,593**
0	Unknown	**12,590**
Syt7	Rattus norvegicus synaptotagmin VII (Syt7), mRNA [NM_021659]	**12,587**
Tcfe2a	Rattus norvegicus transcription factor E2a (Tcfe2a), transcript variant 1, mRNA [NM_133524]	**12,584**
Dlg1	Rattus norvegicus discs, large homolog 1 (Drosophila) (Dlg1), mRNA [NM_012788]	**12,581**
LOC683746	PREDICTED: Rattus norvegicus similar to thyroid autoantigen (LOC683746), mRNA [XM_001067309]	**12,580**
LOC501251	PREDICTED: Rattus norvegicus similar to Discs large homolog 5 (Placenta and prostate DLG) (Discs large protein P-dlg) (LOC501251), mRNA [XM_576670]	**12,580**
Jakmip3	Rattus norvegicus janus kinase and microtubule interacting protein 3 (Jakmip3), mRNA [NM_001163277]	**12,580**
0	Unknown	**12,579**
Cd3d	Rattus norvegicus CD3 molecule, delta (Cd3d), mRNA [NM_013169]	**12,577**
0	Unknown	**12,577**
Dhx37	probable ATP-dependent RNA helicase DHX37 [Source:RefSeq peptide;Acc:NP_001099396] [ENSRNOT00000030823]	**12,570**
Set	Rattus norvegicus SET nuclear oncogene (Set), mRNA [NM_001012504]	**12,569**
LOC685437	PREDICTED: Rattus norvegicus hypothetical protein LOC685437 (LOC685437), mRNA [XM_001063788]	**12,565**
Nebl	Uncharacterized protein [Source:UniProtKB/TrEMBL;Acc:D4A164] [ENSRNOT00000037601]	**12,565**
C2	Rattus norvegicus complement component 2 (C2), mRNA [NM_172222]	**12,564**
Pspn	Rattus norvegicus persephin (Pspn), mRNA [NM_013014]	**12,562**
Vwa1	Rattus norvegicus von Willebrand factor A domain containing 1 (Vwa1), mRNA [NM_001013938]	**12,562**
0	Ras-related protein Rab-28 [Source:UniProtKB/Swiss-Prot;Acc:P51158] [ENSRNOT00000041310]	**12,554**
Nfatc3	Rattus norvegicus nuclear factor of activated T-cells, cytoplasmic, calcineurin-dependent 3 (Nfatc3), mRNA [NM_001108447]	**12,553**
Clock	Rattus norvegicus clock homolog (mouse) (Clock), mRNA [NM_021856]	**12,551**
Atg9a	Rattus norvegicus ATG9 autophagy related 9 homolog A (S. cerevisiae) (Atg9a), mRNA [NM_001014218]	**12,550**
Rasgrp4	Rattus norvegicus RAS guanyl releasing protein 4 (Rasgrp4), mRNA [NM_130824]	**12,549**
Pak1ip1	Rattus norvegicus PAK1 interacting protein 1 (Pak1ip1), mRNA [NM_001037356]	**12,548**
Eps8l2	Rattus norvegicus EPS8-like 2 (Eps8l2), mRNA [NM_001108508]	**12,547**
Mea1	Rattus norvegicus male-enhanced antigen 1 (Mea1), mRNA [NM_001044286]	**12,547**
0	Unknown	**12,547**

Akna	Rattus norvegicus AT-hook transcription factor (Akna), mRNA [NM_001108668]	**12,546**
Pdp2	Rattus norvegicus pyruvate dehyrogenase phosphatase catalytic subunit 2 (Pdp2), mRNA [NM_145091]	**12,545**
0	Unknown	**12,541**
Vom2r35	Rattus norvegicus vomeronasal 2 receptor, 35 (Vom2r35), mRNA [NM_001099473]	**12,541**
0	Q29A56_DROPS (Q29A56) GA18264-PA (Fragment), partial (3%) [TC648872]	**12,540**
RGD1562136	Rattus norvegicus similar to D1Ertd622e protein (RGD1562136), mRNA [NM_001109086]	**12,539**
Col15a1	Col15a1 protein [Source:UniProtKB/TrEMBL;Acc:Q4G024] [ENSRNOT00000017217]	**12,539**
Mex3d	PREDICTED: Rattus norvegicus ring finger (C3HC4 type) and KH domain containing 1 (Rkhd1), mRNA [XM_234921]	**12,539**
0	RIKEN cDNA D430041D05 gene Gene [Source:MGI Symbol;Acc:MGI:2181743] [ENSRNOT00000015073]	**12,534**
Cd6	Rattus norvegicus Cd6 molecule (Cd6), mRNA [NM_175577]	**12,532**
Cnnm4	Metal transporter CNNM4 [Source:UniProtKB/Swiss-Prot;Acc:P0C588] [ENSRNOT00000021434]	**12,529**
Sord	Rattus norvegicus sorbitol dehydrogenase (Sord), mRNA [NM_017052]	**12,528**
G2e3	Rattus norvegicus G2/M-phase specific E3 ubiquitin ligase (G2e3), mRNA [NM_001106726]	**12,528**
Phf1	Rattus norvegicus PHD finger protein 1 (Phf1), mRNA [NM_212538]	**12,526**
LOC246295	Rattus norvegicus glycine-, glutamate-, thienylcyclohexylpiperidine-binding protein (LOC246295), mRNA [NM_145082]	**12,526**
Fibp	Rattus norvegicus fibroblast growth factor (acidic) intracellular binding protein (Fibp), mRNA [NM_172334]	**12,520**
Tbc1d13	Uncharacterized protein [Source:UniProtKB/TrEMBL;Acc:D4AEG7] [ENSRNOT00000021431]	**12,517**
0	Rattus norvegicus similar to glyceraldehyde-3-phosphate dehydrogenase (LOC305750), mRNA [XM_223850]	**12,517**
0	Rattus norvegicus similar to glyceraldehyde-3-phosphate dehydrogenase (LOC291543), mRNA [XM_225871]	**12,515**
Nlgn2	Rattus norvegicus neuroligin 2 (Nlgn2), mRNA [NM_053992]	**12,515**
Adrm1	Rattus norvegicus adhesion regulating molecule 1 (Adrm1), mRNA [NM_031708]	**12,514**
0	Unknown	**12,512**
Slc35e2	Rattus norvegicus solute carrier family 35, member E2 (Slc35e2), mRNA [NM_001107998]	**12,510**
Eif3h	Eukaryotic translation initiation factor 3 subunit H [Source:UniProtKB/Swiss-Prot;Acc:Q6P9U8] [ENSRNOT00000005786]	**12,508**
0	Unknown	**12,506**
Fbxw11	Rattus norvegicus F-box and WD repeat domain containing 11 (Fbxw11), mRNA [NM_001106993]	**12,506**
RGD1560846	Rattus norvegicus similar to hypothetical protein MGC40178 (RGD1560846), mRNA [NM_001134603]	**12,505**
Nfyb	Rattus norvegicus nuclear transcription factor-Y beta (Nfyb), mRNA [NM_031553]	**12,504**
Dstn	Rattus norvegicus destrin (Dstn), mRNA [NM_001033666]	**12,504**
Pdpr	Rattus norvegicus pyruvate dehydrogenase phosphatase regulatory subunit (Pdpr), mRNA [NM_001107430]	**12,504**
Otof	Otoferlin [Source:UniProtKB/Swiss-Prot;Acc:Q9ERC5] [ENSRNOT00000013712]	**12,503**
RGD1311358	Rattus norvegicus similar to RIKEN cDNA 2410017P07 (RGD1311358), mRNA [NM_001017448]	**12,501**
LOC686139	PREDICTED: Rattus norvegicus similar to abhydrolase domain containing 11 (LOC686139), mRNA [XM_001066660]	**12,499**
Vom2r8	Rattus norvegicus vomeronasal 2 receptor, 8 (Vom2r8), mRNA [NM_001099464]	**12,498**

Eno1	Rattus norvegicus enolase 1, (alpha), mRNA (cDNA clone MGC:93770 IMAGE:7108936), complete cds. [BC090069]	**12,496**
Lrrtm2	Rattus norvegicus leucine rich repeat transmembrane neuronal 2 (Lrrtm2), mRNA [NM_001109469]	**12,495**
Atp6v1f	Rattus norvegicus ATPase, H transporting, lysosomal V1 subunit F (Atp6v1f), mRNA [NM_053884]	**12,494**
Kctd17	Rattus norvegicus potassium channel tetramerisation domain containing 17 (Kctd17), mRNA [NM_001134529]	**12,491**
Elmo2	Rattus norvegicus engulfment and cell motility 2 (Elmo2), mRNA [NM_001134955]	**12,491**
Arf5	Rattus norvegicus ADP-ribosylation factor 5 (Arf5), mRNA [NM_024149]	**12,482**
RGD1563263	Rattus norvegicus similar to RIKEN cDNA 1700029I15 (RGD1563263), mRNA [NM_001109201]	**12,482**
0	RNGZAL4 guanine nucleotide-binding protein {Rattus norvegicus} (exp=-1; wgp=0; cg=0), complete [TC578187]	**12,478**
RGD1562551	Rattus norvegicus similar to C20orf82 (RGD1562551), mRNA [NM_001134552]	**12,478**
0	Unknown	**12,477**
0	Unknown	**12,476**
RGD1561238	PREDICTED: Rattus norvegicus similar to ring finger protein 122 homolog (RGD1561238), mRNA [XM_001069254]	**12,475**
RGD1566359	Uncharacterized protein [Source:UniProtKB/TrEMBL;Acc:D4ADM9] [ENSRNOT00000029899]	**12,470**
Mesp1	Rattus norvegicus mesoderm posterior 1 homolog (mouse) (Mesp1), mRNA [NM_001107531]	**12,470**
0	RVL6632 Wackym-Soares normalized rat vestibular cDNA library Rattus norvegicus cDNA 5', mRNA sequence [DV716430]	**12,470**
Agk	Rattus norvegicus acylglycerol kinase (Agk), nuclear gene encoding mitochondrial protein, mRNA [NM_001127497]	**12,469**
0	Stathmin [Source:UniProtKB/Swiss-Prot;Acc:P13668] [ENSRNOT00000022574]	**12,468**
Elovl5	Rattus norvegicus ELOVL family member 5, elongation of long chain fatty acids (yeast) (Elovl5), mRNA [NM_134382]	**12,468**
0	Rattus norvegicus similar to Glyceraldehyde 3-phosphate dehydrogenase (GAPDH) (38 kDa BFA-dependent ADP-ribosylation substrate) (BARS-38) (LOC364400), mRNA [XM_344418]	**12,467**
RGD1565819	Rattus norvegicus similar to C20orf174 (RGD1565819), mRNA [NM_001171096]	**12,464**
LOC500034	Rattus norvegicus similar to CG3570-PA (LOC500034), mRNA [NM_001109221]	**12,464**
Man1b1	Endoplasmic reticulum mannosyl-oligosaccharide 1,2-alpha-mannosidase [Source:UniProtKB/Swiss-Prot;Acc:B2GUY0] [ENSRNOT00000016846]	**12,464**
0	Unknown	**12,461**
Stag2	Rattus norvegicus stromal antigen 2 (Stag2), mRNA [NM_001173507]	**12,461**
Bbs4	Rattus norvegicus Bardet-Biedl syndrome 4 (Bbs4), mRNA [NM_001106826]	**12,459**
Ptp4a1	Rattus norvegicus protein tyrosine phosphatase type IVA, member 1 (Ptp4a1), mRNA [NM_031579]	**12,457**
0	Unknown	**12,456**
Abca8a	PREDICTED: Rattus norvegicus ATP-binding cassette, sub-family A (ABC1), member 8a (Abca8a), mRNA [XM_221100]	**12,452**
Tet2	Uncharacterized protein [Source:UniProtKB/TrEMBL;Acc:D4AC33] [ENSRNOT00000046775]	**12,452**
Stt3b	Rattus norvegicus STT3, subunit of the oligosaccharyltransferase complex, homolog B (S. cerevisiae) (Stt3b), mRNA [NM_001170539]	**12,452**
Gyg1	Rattus norvegicus glycogenin 1 (Gyg1), mRNA [NM_031043]	**12,450**
Olr7	Rattus norvegicus olfactory receptor 7 (Olr7), mRNA [NM_001000768]	**12,448**
0	Rattus norvegicus TL0AEA6YG01 mRNA sequence. [FQ231471]	**12,448**

Rab23	Rattus norvegicus RAB23, member RAS oncogene family (Rab23), mRNA [NM_001109005]	**12,448**
Rpl28	Rattus norvegicus ribosomal protein L28 (Rpl28), mRNA [NM_022697]	**12,447**
Sprr1b	PREDICTED: Rattus norvegicus small proline-rich protein 1B (cornifin) (Sprr1b), mRNA [XM_001065728]	**12,445**
Cacna2d2	Rattus norvegicus calcium channel, voltage-dependent, alpha 2/delta subunit 2 (Cacna2d2), mRNA [NM_175592]	**12,444**
0	Unknown	**12,439**
Usp48	Rattus norvegicus ubiquitin specific peptidase 48 (Usp48), mRNA [NM_198785]	**12,439**
Znf672	Rattus norvegicus zinc finger protein 672 (Znf672), mRNA [NM_001007669]	**12,439**
Pcsk7	Rattus norvegicus proprotein convertase subtilisin/kexin type 7 (Pcsk7), mRNA [NM_019246]	**12,437**
Bad	Rattus norvegicus BCL2-associated agonist of cell death (Bad), mRNA [NM_022698]	**12,436**
RGD1563235	Rattus norvegicus similar to 1700054N08Rik protein (RGD1563235), mRNA [NM_001107441]	**12,436**
Papolg	Rattus norvegicus poly(A) polymerase gamma (Papolg), mRNA [NM_001107244]	**12,436**
Aif1	Rattus norvegicus allograft inflammatory factor 1 (Aif1), mRNA [NM_017196]	**12,436**
Sh3gl1	Rattus norvegicus SH3-domain GRB2-like 1 (Sh3gl1), mRNA [NM_031239]	**12,434**
Olr1091	Rattus norvegicus olfactory receptor 1091 (Olr1091), mRNA [NM_001000593]	**12,433**
0	Unknown	**12,432**
Lrrtm3	Rattus norvegicus leucine rich repeat transmembrane neuronal 3 (Lrrtm3), mRNA [NM_001106387]	**12,431**
Pear1	Rattus norvegicus platelet endothelial aggregation receptor 1 (Pear1), mRNA [NM_001134959]	**12,430**
Efcab4b	PREDICTED: Rattus norvegicus EF-hand calcium binding domain 4B (Efcab4b), mRNA [XM_575675]	**12,428**
Tmem132d	Rattus norvegicus transmembrane protein 132D (Tmem132d), mRNA [NM_198727]	**12,427**
0	Rattus norvegicus TL0AAA52YK09 mRNA sequence. [FQ212560]	**12,424**
0	AGENCOURT_118846637 NIH_MGC_248 Rattus norvegicus cDNA clone IMAGE:9112865 5', mRNA sequence [EX493553]	**12,422**
0	Rattus norvegicus TL0ADA16YE04 mRNA sequence. [FQ223021]	**12,421**
Maf	Rattus norvegicus v-maf musculoaponeurotic fibrosarcoma oncogene homolog (avian) (Maf), mRNA [NM_019318]	**12,421**
0	Unknown	**12,420**
Exph5	PREDICTED: Rattus norvegicus exophilin 5, transcript variant 2 (Exph5), mRNA [XM_002727059]	**12,418**
Crim1	Rattus norvegicus cysteine rich transmembrane BMP regulator 1 (chordin like) (Crim1), mRNA [NM_001169103]	**12,414**
Agpat5	Rattus norvegicus 1-acylglycerol-3-phosphate O-acyltransferase 5 (lysophosphatidic acid acyltransferase, epsilon) (Agpat5), mRNA [NM_001134744]	**12,414**
Ptar1	Rattus norvegicus protein prenyltransferase alpha subunit repeat containing 1 (Ptar1), mRNA [NM_001105760]	**12,412**
Faah	Rattus norvegicus fatty acid amide hydrolase (Faah), mRNA [NM_024132]	**12,411**
0	Unknown	**12,410**
Dot1l	Rattus norvegicus DOT1-like, histone H3 methyltransferase (S. cerevisiae) (Dot1l), mRNA [NM_001108733]	**12,409**
Ddhd1	Rattus norvegicus DDHD domain containing 1 (Ddhd1), mRNA [NM_001033066]	**12,408**
0	Uncharacterized protein [Source:UniProtKB/TrEMBL;Acc:D3ZRC6] [ENSRNOT00000050943]	**12,408**

Bpgm	Rattus norvegicus 2,3-bisphosphoglycerate mutase (Bpgm), mRNA [NM_199382]	**12,407**
Ythdf2	Rattus norvegicus YTH domain family, member 2 (Ythdf2), mRNA [NM_001047099]	**12,407**
Clec4b2	Rattus norvegicus C-type lectin domain family 4, member b2 (Clec4b2), mRNA [NM_001005896]	**12,406**
Slc4a2	Rattus norvegicus solute carrier family 4 (anion exchanger), member 2 (Slc4a2), mRNA [NM_017048]	**12,406**
Igsf1	Rattus norvegicus immunoglobulin superfamily, member 1 (Igsf1), mRNA [NM_175763]	**12,405**
RGD1563296	PREDICTED: Rattus norvegicus similar to component of oligomeric golgi complex 5 isoform 1 (RGD1563296), mRNA [XM_001076141]	**12,405**
Dguok	Rattus norvegicus deoxyguanosine kinase (Dguok), nuclear gene encoding mitochondrial protein, mRNA [NM_001106602]	**12,402**
Slc30a6	Rattus norvegicus solute carrier family 30 (zinc transporter), member 6 (Slc30a6), mRNA [NM_001106708]	**12,400**
Large	Rattus norvegicus like-glycosyltransferase (Large), mRNA [NM_001108439]	**12,400**
0	Unknown	**12,399**
Tmem150c	Rattus norvegicus transmembrane protein 150C (Tmem150c), mRNA [NM_001108354]	**12,398**
Evpl	Rattus norvegicus envoplakin (Evpl), mRNA [NM_001107066]	**12,397**
Bahd1	Similar to mKIAA0945 protein (Predicted)Uncharacterized protein [Source:UniProtKB/TrEMBL;Acc:D3ZHT3] [ENSRNOT00000013878]	**12,396**
Dusp16	Rattus norvegicus dual specificity phosphatase 16 (Dusp16), mRNA [NM_001106624]	**12,395**
Znrf2	Rattus norvegicus zinc and ring finger 2 (Znrf2), mRNA [NM_001108628]	**12,394**
Alg10	Rattus norvegicus asparagine-linked glycosylation 10, alpha-1,2-glucosyltransferase homolog (S. pombe) (Alg10), mRNA [NM_139101]	**12,393**
0	Uncharacterized protein [Source:UniProtKB/TrEMBL;Acc:D3ZWB3] [ENSRNOT00000018647]	**12,391**
0	Unknown	**12,390**
Prkci	Rattus norvegicus protein kinase C, iota (Prkci), mRNA [NM_032059]	**12,390**
0	Rattus norvegicus similar to small GTPase (LOC287708), mRNA [XM_213475]	**12,390**
Otud6b	Rattus norvegicus OTU domain containing 6B (Otud6b), mRNA [NM_001106639]	**12,385**
Gpr45	Rattus norvegicus G protein-coupled receptor 45 (Gpr45), mRNA [NM_001106906]	**12,384**
0	Q8R0G6_MOUSE (Q8R0G6) Ttc19 protein (Fragment), partial (56%) [TC585660]	**12,383**
Fert2	Rattus norvegicus fer (fms/fps related) protein kinase, testis specific 2 (Fert2), mRNA [NM_001106928]	**12,383**
Lpxn	Rattus norvegicus leupaxin (Lpxn), mRNA [NM_001009649]	**12,383**
0	Unknown	**12,383**
Aasdhppt	Rattus norvegicus aminoadipate-semialdehyde dehydrogenase-phosphopantetheinyl transferase (Aasdhppt), mRNA [NM_001106798]	**12,381**
Mboat2	Rattus norvegicus membrane bound O-acyltransferase domain containing 2 (Mboat2), mRNA [NM_001108016]	**12,380**
Btg4	Rattus norvegicus B-cell translocation gene 4 (Btg4), mRNA [NM_001013176]	**12,380**
RGD1306880	RCG50366Uncharacterized protein [Source:UniProtKB/TrEMBL;Acc:D4A7C3] [ENSRNOT00000011586]	**12,378**
0	Unknown	**12,378**
0	Unknown	**12,378**
Rreb1	Rattus norvegicus ras responsive element binding protein 1 (Rreb1), mRNA [NM_001107348]	**12,377**
Rfk	Rattus norvegicus riboflavin kinase (Rfk), mRNA [NM_001014106]	**12,376**

Mtmr7	Rattus norvegicus myotubularin related protein 7 (Mtmr7), mRNA [NM_001107312]	**12,373**
Vpreb3	Rattus norvegicus pre-B lymphocyte 3 (Vpreb3), mRNA [NM_001108930]	**12,364**
Vom2r34	Rattus norvegicus vomeronasal 2 receptor, 34 (Vom2r34), mRNA [NM_001099657]	**12,364**
Rfc5	Rattus norvegicus replication factor C (activator 1) 5 (Rfc5), mRNA [NM_001107146]	**12,363**
Nucb1	Rattus norvegicus nucleobindin 1 (Nucb1), mRNA [NM_053463]	**12,362**
0	Unknown	**12,359**
Terf2ip	Rattus norvegicus telomeric repeat binding factor 2, interacting protein (Terf2ip), mRNA [NM_001013143]	**12,354**
Grid2ip	Rattus norvegicus glutamate receptor, ionotropic, delta 2 (Grid2) interacting protein (Grid2ip), mRNA [NM_001105910]	**12,353**
LOC652955	Rattus norvegicus goliath (LOC652955), mRNA [NM_001037658]	**12,352**
RGD1309188	Rattus norvegicus similar to hypothetical protein BC011833 (RGD1309188), mRNA [NM_001108129]	**12,347**
Slc7a13	Rattus norvegicus solute carrier family 7, (cationic amino acid transporter, y+ system) member 13 (Slc7a13), mRNA [NM_001012100]	**12,346**
Ttc14	Rattus norvegicus tetratricopeptide repeat domain 14 (Ttc14), mRNA [NM_001107666]	**12,345**
0	Unknown	**12,344**
Fastk	Rattus norvegicus Fas-activated serine/threonine kinase (Fastk), mRNA [NM_001011967]	**12,344**
0	Unknown	**12,343**
LOC684996	Rattus norvegicus similar to chromosome 7 open reading frame 11 (LOC684996), mRNA [NM_001109452]	**12,342**
Grin1	Rattus norvegicus glutamate receptor, ionotropic, N-methyl D-aspartate 1 (Grin1), mRNA [NM_017010]	**12,341**
Vps26a	Rattus norvegicus vacuolar protein sorting 26 homolog A (S. pombe) (Vps26a), mRNA [NM_001007740]	**12,338**
Prickle1	Rattus norvegicus prickle homolog 1 (Drosophila) (Prickle1), mRNA [NM_199396]	**12,337**
LOC686921	PREDICTED: Rattus norvegicus hypothetical protein LOC686921 (LOC686921), mRNA [XM_001076338]	**12,334**
Sfxn2	sideroflexin 2 [Source:RefSeq peptide;Acc:NP_001013090] [ENSRNOT00000027112]	**12,334**
Kcnh3	Rattus norvegicus potassium voltage-gated channel, subfamily H (eag-related), member 3 (Kcnh3), mRNA [NM_017108]	**12,333**
0	Q5BK04_RAT (Q5BK04) LOC363306 protein (Fragment), partial (78%) [TC626013]	**12,332**
0	Rattus norvegicus similar to glyceraldehyde-3-phosphate dehydrogenase (phosphorylating) (EC 1.2.1.12) - mouse (LOC289157), mRNA [XM_222813]	**12,330**
RGD1564167	PREDICTED: Rattus norvegicus similar to basic transcription factor 3 (RGD1564167), mRNA [XM_002727687]	**12,327**
Lrrc8d	Rattus norvegicus leucine rich repeat containing 8 family, member D (Lrrc8d), mRNA [NM_001008338]	**12,327**
RGD1564228	PREDICTED: Rattus norvegicus similar to Naglu (RGD1564228), mRNA [XM_001081442]	**12,323**
Agpat3	Rattus norvegicus 1-acylglycerol-3-phosphate O-acyltransferase 3 (Agpat3), mRNA [NM_001106378]	**12,321**
RGD1564676	Rattus norvegicus similar to NF-X1 type zinc finger containing protein (2J511) (LOC364700), mRNA [XM_344589]	**12,321**
0	AW527166 UI-R-BO1-ajm-c-07-0-UI.s1 UI-R-BO1 Rattus norvegicus cDNA clone UI-R-BO1-ajm-c-07-0-UI 3', mRNA sequence [AW527166]	**12,316**
Golt1b	Rattus norvegicus golgi transport 1 homolog B (S. cerevisiae) (Golt1b), mRNA [NM_001113783]	**12,315**
Dpy19l1	Rattus norvegicus dpy-19-like 1 (C. elegans) (Dpy19l1), mRNA [NM_001191791]	**12,313**
Flnb	Rattus norvegicus filamin, beta (Flnb), mRNA [NM_001107288]	**12,311**

Ttc33	Rattus norvegicus tetratricopeptide repeat domain 33 (Ttc33), mRNA [NM_001106414]	**12,309**
Pfdn2	Rattus norvegicus prefoldin subunit 2 (Pfdn2), mRNA [NM_001109476]	**12,308**
0	Rattus norvegicus similar to glyceraldehyde-3-phosphate dehydrogenase (LOC302351), mRNA [XM_228478]	**12,305**
LOC678708	PREDICTED: Rattus norvegicus similar to histone 1, H2ai (LOC678708), mRNA [XM_002724915]	**12,305**
RGD1306941	Rattus norvegicus similar to CG31122-PA (RGD1306941), mRNA [NM_001108219]	**12,304**
Stk24	Rattus norvegicus serine/threonine kinase 24 (STE20 homolog, yeast) (Stk24), mRNA [NM_001127494]	**12,302**
0	Unknown	**12,300**
Nav2	Rattus norvegicus neuron navigator 2 (Nav2), mRNA [NM_138529]	**12,298**
Rab33b	Rattus norvegicus RAB33B, member of RAS oncogene family (Rab33b), mRNA [NM_001108944]	**12,297**
0	Unknown	**12,296**
Rnf32	Rattus norvegicus ring finger protein 32 (Rnf32), mRNA [NM_001012095]	**12,296**
0	Q4IH13_GIBZE (Q4IH13) Predicted protein, partial (6%) [TC640504]	**12,296**
0	Unknown	**12,295**
Ywhaz	Rattus norvegicus tyrosine 3-monooxygenase/tryptophan 5-monooxygenase activation protein, zeta polypeptide (Ywhaz), mRNA [NM_013011]	**12,290**
0	PREDICTED: Rattus norvegicus similar to hypothetical protein 4932411N23 (RGD1561151), mRNA [XM_001066024]	**12,290**
RGD1359634	Rattus norvegicus similar to RIKEN cDNA 1700088E04 (RGD1359634), mRNA [NM_001007708]	**12,289**
0	Unknown	**12,289**
0	Unknown	**12,289**
0	Unknown	**12,289**
0	Unknown	**12,287**
RGD1309374	Uncharacterized protein [Source:UniProtKB/TrEMBL;Acc:D3ZU62] [ENSRNOT00000020492]	**12,285**
0	RIKEN cDNA 2510006D16 gene Gene [Source:MGI Symbol;Acc:MGI:1924049] [ENSRNOT00000000136]	**12,284**
Scamp1	Rattus norvegicus secretory carrier membrane protein 1 (Scamp1), mRNA [NM_001100636]	**12,283**
Htr7	5-hydroxytryptamine receptor 7 [Source:UniProtKB/Swiss-Prot;Acc:P32305] [ENSRNOT00000025493]	**12,283**
Olr1606	Rattus norvegicus olfactory receptor 1606 (Olr1606), mRNA [NM_001000502]	**12,269**
LOC317165	Rattus norvegicus similar to Set alpha isoform (LOC317165), mRNA [NM_001047892]	**12,265**
Kat5	Rattus norvegicus K(lysine) acetyltransferase 5 (Kat5), mRNA [NM_001005872]	**12,262**
0	RIKEN cDNA 9430031J16 gene Gene [Source:MGI Symbol;Acc:MGI:2443135] [ENSRNOT00000041337]	**12,261**
0	Unknown	**12,258**
0	Ankyrin repeat and SOCS box protein 14 [Source:UniProtKB/Swiss-Prot;Acc:P0C927] [ENSRNOT00000017887]	**12,257**
RGD1309759	PREDICTED: Rattus norvegicus similar to cDNA sequence BC013529, transcript variant 2 (RGD1309759), mRNA [XM_001059157]	**12,257**
Tshr	Rattus norvegicus thyroid stimulating hormone receptor (Tshr), mRNA [NM_012888]	**12,257**
0	Uncharacterized protein [Source:UniProtKB/TrEMBL;Acc:D3ZHQ7] [ENSRNOT00000057958]	**12,256**
Csad	Rattus norvegicus cysteine sulfinic acid decarboxylase (Csad), transcript variant 1, mRNA [NM_021750]	**12,251**

LOC691849	Rattus norvegicus hypothetical protein LOC691849 (LOC691849), mRNA [NM_001198796]	**12,247**
Wfikkn1	Rattus norvegicus WAP, follistatin/kazal, immunoglobulin, kunitz and netrin domain containing 1 (Wfikkn1), mRNA [NM_001129776]	**12,241**
Pramef8	Rattus norvegicus PRAME family member 8 (Pramef8), mRNA [NM_001135782]	**12,240**
Tceal1	Rattus norvegicus transcription elongation factor A (SII)-like 1 (Tceal1), mRNA [NM_001009675]	**12,239**
0	Uncharacterized protein [Source:UniProtKB/TrEMBL;Acc:D3ZKN0] [ENSRNOT00000045362]	**12,237**
Rab11fip4	Rattus norvegicus RAB11 family interacting protein 4 (class II) (Rab11fip4), mRNA [NM_001107023]	**12,237**
Cbx3	Rattus norvegicus chromobox homolog 3 (HP1 gamma homolog, Drosophila) (Cbx3), mRNA [NM_001008313]	**12,235**
Pclo	Rattus norvegicus piccolo (presynaptic cytomatrix protein) (Pclo), transcript variant 1, mRNA [NM_020098]	**12,234**
E2f1	Rattus norvegicus E2F transcription factor 1 (E2f1), mRNA [NM_001100778]	**12,233**
0	Uncharacterized protein [Source:UniProtKB/TrEMBL;Acc:D3ZDF4] [ENSRNOT00000024704]	**12,233**
Rcbtb1	Rattus norvegicus regulator of chromosome condensation (RCC1) and BTB (POZ) domain containing protein 1 (Rcbtb1), mRNA [NM_001108380]	**12,233**
Ophn1	Rattus norvegicus oligophrenin 1 (Ophn1), mRNA [NM_001107848]	**12,233**
LOC692032	Uncharacterized protein [Source:UniProtKB/TrEMBL;Acc:D4A4N1] [ENSRNOT00000039400]	**12,233**
LOC680119	PREDICTED: Rattus norvegicus hypothetical protein LOC680119 (LOC680119), mRNA [XM_001055803]	**12,230**
Csnk1e	Rattus norvegicus casein kinase 1, epsilon (Csnk1e), mRNA [NM_031617]	**12,230**
Appl2	Rattus norvegicus adaptor protein, phosphotyrosine interaction, PH domain and leucine zipper containing 2 (Appl2), mRNA [NM_001108741]	**12,228**
Fstl1	Rattus norvegicus follistatin-like 1 (Fstl1), mRNA [NM_024369]	**12,228**
Rala	Rattus norvegicus v-ral simian leukemia viral oncogene homolog A (ras related) (Rala), mRNA [NM_031093]	**12,222**
LOC366449	PREDICTED: Rattus norvegicus hypothetical LOC366449 (LOC366449), miscRNA [XR_006808]	**12,221**
Drd2	Rattus norvegicus dopamine receptor D2 (Drd2), mRNA [NM_012547]	**12,221**
Bola3	Rattus norvegicus bolA homolog 3 (E. coli) (Bola3), mRNA [NM_001106601]	**12,220**
0	Unknown	**12,218**
Grm7	Rattus norvegicus glutamate receptor, metabotropic 7 (Grm7), mRNA [NM_031040]	**12,217**
0	Unknown	**12,216**
Marcks	PREDICTED: Rattus norvegicus myristoylated alanine rich protein kinase C substrate (Marcks), mRNA [XM_002728965]	**12,216**
Pold2	Rattus norvegicus polymerase (DNA directed), delta 2, regulatory subunit (Pold2), mRNA [NM_001013050]	**12,214**
Gpr12	Rattus norvegicus G protein-coupled receptor 12 (Gpr12), transcript variant 2, mRNA [NM_030831]	**12,214**
LOC684529	PREDICTED: Rattus norvegicus similar to cathepsin O (LOC684529), mRNA [XM_001070844]	**12,213**
Plcd4	Rattus norvegicus phospholipase C, delta 4 (Plcd4), mRNA [NM_080688]	**12,213**
Ndufaf4	Rattus norvegicus NADH dehydrogenase (ubiquinone) 1 alpha subcomplex, assembly factor 4 (Ndufaf4), nuclear gene encoding mitochondrial protein, mRNA [NM_198783]	**12,213**
Cyp27b1	Rattus norvegicus cytochrome P450, family 27, subfamily b, polypeptide 1 (Cyp27b1), nuclear gene encoding mitochondrial protein, mRNA [NM_053763]	**12,212**
Tdpoz1	PREDICTED: Rattus norvegicus TD and POZ domain containing 1 (Tdpoz1), mRNA [XM_345239]	**12,211**

Krtap31-1	Rattus norvegicus keratin associated protein 31-1 (Krtap31-1), mRNA [NM_001109409]	**12,210**
Mgat5	Rattus norvegicus mannosyl (alpha-1,6-)-glycoprotein beta-1,6-N-acetyl-glucosaminyltransferase (Mgat5), mRNA [NM_023095]	**12,210**
0	Unknown	**12,207**
0	predicted gene 71 Gene [Source:MGI Symbol;Acc:MGI:2684917] [ENSRNOT00000033412]	**12,207**
Vdac2	Rattus norvegicus voltage-dependent anion channel 2 (Vdac2), nuclear gene encoding mitochondrial protein, mRNA [NM_031354]	**12,207**
Zfp426l2	Rattus norvegicus zinc finger protein 426-like 2 (Zfp426l2), mRNA [NM_001134585]	**12,207**
0	Unknown	**12,207**
0	Unknown	**12,204**
Vps13d	Rattus norvegicus vacuolar protein sorting 13 homolog D (S. cerevisiae) (Vps13d), mRNA [NM_001108006]	**12,204**
0	Unknown	**12,203**
Cabp7	Rattus norvegicus calcium binding protein 7 (Cabp7), mRNA [NM_001007730]	**12,202**
Btd	Rattus norvegicus biotinidase (Btd), mRNA [NM_001012047]	**12,199**
Dtx4	Rattus norvegicus deltex homolog 4 (Drosophila) (Dtx4), mRNA [NM_001047855]	**12,198**
0	Glyceraldehyde-3-phosphate dehydrogenase [Source:UniProtKB/TrEMBL;Acc:D3Z7Z7] [ENSRNOT00000038921]	**12,195**
Gabpa	Rattus norvegicus GA binding protein transcription factor, alpha subunit (Gabpa), mRNA [NM_001108841]	**12,192**
0	Uncharacterized protein [Source:UniProtKB/TrEMBL;Acc:D3ZU53] [ENSRNOT00000001469]	**12,191**
Adamts18	Rattus norvegicus ADAM metallopeptidase with thrombospondin type 1 motif, 18 (Adamts18), mRNA [NM_001191944]	**12,190**
Jub	Rattus norvegicus jub, ajuba homolog (Xenopus laevis) (Jub), mRNA [NM_053503]	**12,190**
Gemin5	Rattus norvegicus gem (nuclear organelle) associated protein 5 (Gemin5), mRNA [NM_001172089]	**12,188**
Heatr5b	Rattus norvegicus HEAT repeat containing 5B (Heatr5b), mRNA [NM_001191064]	**12,187**
0	Unknown	**12,186**
0	Unknown	**12,185**
0	Unknown	**12,185**
0	integrin alpha V Gene [Source:MGI Symbol;Acc:MGI:96608] [ENSRNOT00000006961]	**12,185**
Msx2	Rattus norvegicus msh homeobox 2 (Msx2), mRNA [NM_012982]	**12,184**
Vom1r24	Rattus norvegicus vomeronasal 1 receptor 24 (Vom1r24), mRNA [NM_001008936]	**12,181**
Srxn1	Rattus norvegicus sulfiredoxin 1 homolog (S. cerevisiae) (Srxn1), mRNA [NM_001047858]	**12,179**
Mad1l1	Rattus norvegicus MAD1 mitotic arrest deficient-like 1 (yeast) (Mad1l1), mRNA [NM_001109387]	**12,175**
Olr1615	Rattus norvegicus olfactory receptor 1615 (Olr1615), mRNA [NM_001001003]	**12,173**
Abr	Rattus norvegicus active BCR-related gene (Abr), mRNA [NM_001105814]	**12,173**
0	Uncharacterized protein [Source:UniProtKB/TrEMBL;Acc:D4ACN9] [ENSRNOT00000018433]	**12,172**
Vkorc1	Rattus norvegicus vitamin K epoxide reductase complex, subunit 1 (Vkorc1), mRNA [NM_203335]	**12,171**
39142	Rattus norvegicus membrane-associated ring finger (C3HC4) 7 (March7), mRNA [NM_001012087]	**12,170**
0	Unknown	**12,169**

Stmn2	Rattus norvegicus stathmin-like 2 (Stmn2), mRNA [NM_053440]	**12,164**
Sh3rf1	Rattus norvegicus SH3 domain containing ring finger 1 (Sh3rf1), mRNA [NM_198764]	**12,163**
Rpl18a	Rattus norvegicus ribosomal protein L18A (Rpl18a), mRNA [NM_212510]	**12,161**
Gpr61	Rattus norvegicus G protein-coupled receptor 61 (Gpr61), mRNA [NM_001107715]	**12,160**
Gpr22	Rattus norvegicus G protein-coupled receptor 22 (Gpr22), mRNA [NM_001106722]	**12,159**
RGD1561537	PREDICTED: Rattus norvegicus similar to putative repair and recombination helicase RAD26L, transcript variant 1 (RGD1561537), mRNA [XM_001058999]	**12,157**
LOC100362820	PREDICTED: Rattus norvegicus Protein FAM186A-like (LOC100362820), mRNA [XM_002729856]	**12,156**
Samd8	Rattus norvegicus sterile alpha motif domain containing 8 (Samd8), mRNA [NM_001012040]	**12,154**
LOC683415	PREDICTED: Rattus norvegicus similar to adrenal mitochondrial protease (LOC683415), mRNA [XM_001065830]	**12,153**
Chsy1	Rattus norvegicus chondroitin sulfate synthase 1 (Chsy1), mRNA [NM_001106268]	**12,153**
Ciz1	Rattus norvegicus CDKN1A interacting zinc finger protein 1 (Ciz1), mRNA [NM_001106568]	**12,153**
0	EST450602 Rat Gene Index, normalized rat, Rattus norvegicus cDNA Rattus norvegicus cDNA clone RGIFL60, mRNA sequence [BF286011]	**12,153**
Elf2	Rattus norvegicus E74-like factor 2 (Elf2), transcript variant 2, mRNA [NM_001012181]	**12,152**
LOC678918	PREDICTED: Rattus norvegicus similar to NADH dehydrogenase (ubiquinone) 1 beta subcomplex 3 (LOC678918), mRNA [XM_001053805]	**12,152**
Col12a1	Collagen alpha-1(XII) chain [Source:UniProtKB/Swiss-Prot;Acc:P70560] [ENSRNOT00000051159]	**12,152**
Rgs19	Rattus norvegicus regulator of G-protein signaling 19 (Rgs19), mRNA [NM_021661]	**12,146**
Nqo2	Rattus norvegicus NAD(P)H dehydrogenase, quinone 2 (Nqo2), mRNA [NM_001004214]	**12,145**
0	Unknown	**12,145**
0	Unknown	**12,143**
RGD1309903	Uncharacterized protein [Source:UniProtKB/TrEMBL;Acc:D3ZF86] [ENSRNOT00000015927]	**12,142**
Insig1	Rattus norvegicus insulin induced gene 1 (Insig1), mRNA [NM_022392]	**12,141**
Lipe	Rattus norvegicus lipase, hormone sensitive (Lipe), mRNA [NM_012859]	**12,141**
Myh9	Rattus norvegicus myosin, heavy chain 9, non-muscle (Myh9), mRNA [NM_013194]	**12,137**
Folh1	Rattus norvegicus folate hydrolase 1 (Folh1), mRNA [NM_057185]	**12,137**
Twistnb	Rattus norvegicus TWIST neighbor (Twistnb), mRNA [NM_001108707]	**12,137**
0	Unknown	**12,136**
RGD1307225	Rattus norvegicus similar to MEGF6 (RGD1307225), mRNA [NM_001107663]	**12,136**
0	Unknown	**12,136**
Muc6	Uncharacterized protein [Source:UniProtKB/TrEMBL;Acc:D4A0Z2] [ENSRNOT00000026749]	**12,135**
Lrrc51	Rattus norvegicus leucine rich repeat containing 51 (Lrrc51), mRNA [NM_001106284]	**12,132**
0	Unknown	**12,131**
Klf5	Rattus norvegicus Kruppel-like factor 5 (Klf5), mRNA [NM_053394]	**12,130**
Mtfr1	Rattus norvegicus TL0ACA31YL20 mRNA sequence. [FQ217237]	**12,130**

Ctsd	Rattus norvegicus cathepsin D (Ctsd), mRNA [NM_134334]	**12,129**
0	Unknown	**12,125**
Uprt	Uncharacterized protein [Source:UniProtKB/TrEMBL;Acc:D4ABC7] [ENSRNOT00000033494]	**12,124**
Vom2r16	Rattus norvegicus vomeronasal 2 receptor, 16 (Vom2r16), mRNA [NM_001099655]	**12,119**
Fam19a5	Rattus norvegicus family with sequence similarity 19 (chemokine (C-C motif)-like), member A5 (Fam19a5), mRNA [NM_001191991]	**12,119**
Cdc27	Rattus norvegicus cell division cycle 27 homolog (S. cerevisiae) (Cdc27), mRNA [NM_001024793]	**12,117**
Hist1h1d	Rattus norvegicus histone cluster 1, H1d (Hist1h1d), mRNA [NM_133285]	**12,117**
Prkd3	Rattus norvegicus protein kinase D3 (Prkd3), mRNA [NM_001024263]	**12,116**
Sez6	Rattus norvegicus seizure related 6 homolog (mouse) (Sez6), mRNA [NM_001105754]	**12,116**
Map2k4	Rattus norvegicus mitogen activated protein kinase kinase 4 (Map2k4), mRNA [NM_001030023]	**12,116**
Nfasc	Rattus norvegicus neurofascin (Nfasc), transcript variant 4, mRNA [NM_001160315]	**12,115**
Ttc4	tetratricopeptide repeat domain 4 [Source:RefSeq peptide;Acc:NP_001013232] [ENSRNOT00000010229]	**12,114**
Olr859	Rattus norvegicus olfactory receptor 859 (Olr859), mRNA [NM_001000585]	**12,114**
0	Uncharacterized protein [Source:UniProtKB/TrEMBL;Acc:D3ZH07] [ENSRNOT00000049215]	**12,114**
Rbpj	Rattus norvegicus recombination signal binding protein for immunoglobulin kappa J region (Rbpj), mRNA [NM_001106631]	**12,110**
Olr1415	Rattus norvegicus olfactory receptor 1415 (Olr1415), mRNA [NM_001000781]	**12,108**
Edem3	Rattus norvegicus ER degradation enhancer, mannosidase alpha-like 3 (Edem3), mRNA [NM_001191671]	**12,108**
Smurf2	Rattus norvegicus SMAD specific E3 ubiquitin protein ligase 2 (Smurf2), mRNA [NM_001107061]	**12,107**
Adipor2	Rattus norvegicus adiponectin receptor 2 (Adipor2), mRNA [NM_001037979]	**12,105**
Eif2a	Rattus norvegicus eukaryotic translation initiation factor 2A (Eif2a), mRNA [NM_001109339]	**12,104**
LOC679534	PREDICTED: Rattus norvegicus hypothetical protein LOC679534 (LOC679534), mRNA [XM_002726891]	**12,101**
LOC500532	PREDICTED: Rattus norvegicus hypothetical LOC500532 (LOC500532), miscRNA [XR_008784]	**12,100**
Gpr19	Rattus norvegicus G protein-coupled receptor 19 (Gpr19), mRNA [NM_080579]	**12,098**
Lrrc7	Rattus norvegicus leucine rich repeat containing 7 (Lrrc7), mRNA [NM_057142]	**12,098**
Rpusd3	Rattus norvegicus RNA pseudouridylate synthase domain containing 3 (Rpusd3), mRNA [NM_001108641]	**12,096**
Aurkaip1	Rattus norvegicus aurora kinase A interacting protein 1 (Aurkaip1), mRNA [NM_001004237]	**12,096**
Gfm2	Ribosome-releasing factor 2, mitochondrial [Source:UniProtKB/Swiss-Prot;Acc:Q5BJP6] [ENSRNOT00000052072]	**12,095**
0	Unknown	**12,095**
RGD1564788	PREDICTED: Rattus norvegicus similar to Werner syndrome helicase homolog, transcript variant 1 (RGD1564788), mRNA [XM_001059940]	**12,092**
0	BC075646 Luzp2 protein {Mus musculus} (exp=-1; wgp=0; cg=0), partial (83%) [TC635744]	**12,090**
Prr22	Rattus norvegicus proline rich 22 (Prr22), mRNA [NM_001126375]	**12,089**
Rps6kb2	Rattus norvegicus ribosomal protein S6 kinase, polypeptide 2 (Rps6kb2), mRNA [NM_001010962]	**12,088**
Olr757	Rattus norvegicus olfactory receptor 757 (Olr757), mRNA [NM_001000613]	**12,086**
Rya3	Rattus norvegicus antimicrobial peptide RYA3 (Rya3), mRNA [NM_001109208]	**12,084**

Adnp2	Rattus norvegicus ADNP homeobox 2 (Adnp2), mRNA [NM_001127373]	**12,081**
0	Unknown	**12,080**
LOC365985	Rattus norvegicus similar to adenylate kinase 5 isoform 1 (LOC365985), mRNA [NM_001108951]	**12,077**
Tctn3	PREDICTED: Rattus norvegicus tectonic family member 3 (Tctn3), mRNA [XM_001053561]	**12,076**
Ibtk	Uncharacterized protein [Source:UniProtKB/TrEMBL;Acc:D3ZAK7] [ENSRNOT00000056930]	**12,075**
Smarca2	Rattus norvegicus SWI/SNF related, matrix associated, actin dependent regulator of chromatin, subfamily a, member 2 (Smarca2), mRNA [NM_001004446]	**12,072**
Smndc1	Rattus norvegicus survival motor neuron domain containing 1 (Smndc1), transcript variant 2, mRNA [NM_001025400]	**12,072**
Cabc1	Rattus norvegicus chaperone, ABC1 activity of bc1 complex homolog (S. pombe) (Cabc1), nuclear gene encoding mitochondrial protein, mRNA [NM_001013185]	**12,071**
Cdh7	Rattus norvegicus cadherin 7, type 2 (Cdh7), mRNA [NM_001012737]	**12,071**
Pgm3	Rattus norvegicus phosphoglucomutase 3 (Pgm3), mRNA [NM_001108772]	**12,069**
Ret	Rattus norvegicus ret proto-oncogene (Ret), transcript variant 1, mRNA [NM_012643]	**12,069**
Fbxw5	Rattus norvegicus F-box and WD repeat domain containing 5 (Fbxw5), mRNA [NM_001025730]	**12,069**
0	Uncharacterized protein [Source:UniProtKB/TrEMBL;Acc:D3ZUL0] [ENSRNOT00000013651]	**12,067**
Ugt8	Rattus norvegicus UDP glycosyltransferase 8 (Ugt8), mRNA [NM_019276]	**12,066**
Sprn	Rattus norvegicus shadow of prion protein homolog (zebrafish) (Sprn), mRNA [NM_001031845]	**12,064**
0	Unknown	**12,059**
Rbm24	Rattus norvegicus RNA binding motif protein 24 (Rbm24), mRNA [NM_001191100]	**12,057**
Baat	Rattus norvegicus bile acid Coenzyme A: amino acid N-acyltransferase (glycine N-choloyltransferase) (Baat), mRNA [NM_017300]	**12,057**
Pigu	Rattus norvegicus phosphatidylinositol glycan anchor biosynthesis, class U (Pigu), mRNA [NM_181637]	**12,056**
0	Unknown	**12,055**
0	Unknown	**12,054**
Pum1	Rattus norvegicus pumilio homolog 1 (Drosophila) (Pum1), mRNA [NM_001108684]	**12,049**
Sc5dl	Rattus norvegicus sterol-C5-desaturase (ERG3 delta-5-desaturase homolog, S. cerevisiae)-like (Sc5dl), mRNA [NM_053642]	**12,048**
Umod	Rattus norvegicus uromodulin (Umod), mRNA [NM_017082]	**12,048**
Myt1	Rattus norvegicus myelin transcription factor 1 (Myt1), mRNA [NM_001108615]	**12,043**
Ggt7	Rattus norvegicus gamma-glutamyltransferase 7 (Ggt7), mRNA [NM_130423]	**12,043**
Tuba3a	Rattus norvegicus tubulin, alpha 3A (Tuba3a), mRNA [NM_001040008]	**12,041**
Chmp1a	Rattus norvegicus chromatin modifying protein 1A (Chmp1a), mRNA [NM_001083313]	**12,041**
0	Rattus norvegicus similar to glyceraldehyde-3-phosphate dehydrogenase (LOC296027), mRNA [XM_215798]	**12,040**
Adam15	Rattus norvegicus a disintegrin and metallopeptidase domain 15 (metargidin) (Adam15), mRNA [NM_020308]	**12,040**
0	roundabout 2 [Source:RefSeq peptide;Acc:NP_115289] [ENSRNOT00000044546]	**12,036**
LOC100365115	PREDICTED: Rattus norvegicus LY6/PLAUR domain containing 6B-like (LOC100365115), mRNA [XM_002726150]	**12,035**

Bscl2	Rattus norvegicus Bernardinelli-Seip congenital lipodystrophy 2 homolog (human) (Bscl2), mRNA [NM_001012171]	**12,035**
Cyp2j3	Rattus norvegicus cytochrome P450, family 2, subfamily j, polypeptide 3 (Cyp2j3), mRNA [NM_175766]	**12,032**
Pgbd5	Rattus norvegicus piggyBac transposable element derived 5 (Pgbd5), mRNA [NM_001106198]	**12,032**
Rg9mtd1	Rattus norvegicus RNA (guanine-9-) methyltransferase domain containing 1 (Rg9mtd1), nuclear gene encoding mitochondrial protein, mRNA [NM_001008337]	**12,032**
0	PREDICTED: Rattus norvegicus similar to stathmin 1/oncoprotein 18) (RGD1565395), mRNA [XM_001081318]	**12,031**
Arsk	Rattus norvegicus arylsulfatase family, member K (Arsk), mRNA [NM_001047917]	**12,025**
Far2	Uncharacterized protein [Source:UniProtKB/TrEMBL;Acc:D4A2S1] [ENSRNOT00000002528]	**12,023**
Irs2	Rattus norvegicus insulin receptor substrate 2 (Irs2), mRNA [NM_001168633]	**12,022**
Itpk1	Rattus norvegicus inositol 1,3,4-triphosphate 5/6 kinase (Itpk1), mRNA [NM_001191985]	**12,021**
RGD1563669	Rattus norvegicus similar to Mediator of RNA polymerase II transcription, subunit 9 homolog (RGD1563669), mRNA [NM_001127302]	**12,021**
0	Unknown	**12,018**
0	Unknown	**12,018**
Ccnc	Rattus norvegicus cyclin C (Ccnc), mRNA [NM_001100472]	**12,014**
0	Unknown	**12,014**
0	Unknown	**12,014**
Ccdc39	Rattus norvegicus coiled-coil domain containing 39 (Ccdc39), mRNA [NM_001107667]	**12,011**
Mlxip	PREDICTED: Rattus norvegicus MLX interacting protein (Mlxip), mRNA [XM_001079320]	**12,010**
Zfr	Zinc finger RNA-binding protein [Source:UniProtKB/Swiss-Prot;Acc:Q562A2] [ENSRNOT00000016196]	**12,008**
Olr1516	Rattus norvegicus olfactory receptor 1516 (Olr1516), mRNA [NM_001000038]	**12,006**
Lhx6	Rattus norvegicus LIM homeobox 6 (Lhx6), mRNA [NM_001107837]	**12,004**
LOC302192	PREDICTED: Rattus norvegicus similar to RIKEN cDNA 1700001E04 (LOC302192), mRNA [XM_001065392]	**12,001**
Csda	Rattus norvegicus cold shock domain protein A (Csda), mRNA [NM_031979]	**12,000**
Slc6a9	Rattus norvegicus solute carrier family 6 (neurotransmitter transporter, glycine), member 9 (Slc6a9), mRNA [NM_053818]	**12,000**
0	Uncharacterized protein [Source:UniProtKB/TrEMBL;Acc:D3ZK01] [ENSRNOT00000045607]	**11,998**
Myo9b	Rattus norvegicus myosin IXb (Myo9b), mRNA [NM_012984]	**11,997**
LOC500028	Rattus norvegicus hypothetical protein LOC500028 (LOC500028), mRNA [NM_001047954]	**11,996**
Stk3	Rattus norvegicus serine/threonine kinase 3 (STE20 homolog, yeast) (Stk3), mRNA [NM_031735]	**11,995**
Lmln	Rattus norvegicus leishmanolysin-like (metallopeptidase M8 family) (Lmln), mRNA [NM_001108843]	**11,995**
0	Unknown	**11,994**
0	Rattus norvegicus similar to Pyruvate kinase, M2 isozyme (LOC315231), mRNA [XM_235589]	**11,993**
0	Unknown	**11,993**
Tmem41b	Rattus norvegicus transmembrane protein 41B (Tmem41b), mRNA [NM_001012358]	**11,993**
0	Unknown	**11,988**

RGD1359600	Rattus norvegicus LEA_4 domain containing protein RGD1359600 (RGD1359600), mRNA [NM_001007688]	**11,987**
Actr3	Rattus norvegicus ARP3 actin-related protein 3 homolog (yeast) (Actr3), mRNA [NM_031068]	**11,984**
Nelf	Rattus norvegicus nasal embryonic LHRH factor (Nelf), mRNA [NM_057190]	**11,984**
Camk2d	Rattus norvegicus calcium/calmodulin-dependent protein kinase II delta (Camk2d), mRNA [NM_012519]	**11,984**
Pex5l	Rattus norvegicus peroxisomal biogenesis factor 5-like (Pex5l), mRNA [NM_173152]	**11,982**
Rybp	Rattus norvegicus RING1 and YY1 binding protein (Rybp), mRNA [NM_001107879]	**11,980**
LOC502894	Rattus norvegicus hypothetical protein LOC502894 (LOC502894), mRNA [NM_001025064]	**11,979**
Hdac1	Rattus norvegicus histone deacetylase 1 (Hdac1), mRNA [NM_001025409]	**11,978**
0	PREDICTED: Rattus norvegicus similar to putative nucleic acid binding protein RY-1 (LOC685367), mRNA [XM_001063506]	**11,977**
Idi2	Rattus norvegicus isopentenyl-diphosphate delta isomerase 2 (Idi2), mRNA [NM_001192008]	**11,977**
Ror2	Rattus norvegicus receptor tyrosine kinase-like orphan receptor 2 (Ror2), mRNA [NM_001107339]	**11,977**
Armc7	Rattus norvegicus armadillo repeat containing 7 (Armc7), mRNA [NM_001127522]	**11,974**
Fhl3	Rattus norvegicus four and a half LIM domains 3 (Fhl3), mRNA [NM_001107979]	**11,974**
Crebl2	Rattus norvegicus cAMP responsive element binding protein-like 2 (Crebl2), mRNA [NM_001015027]	**11,974**
0	Unknown	**11,973**
Kirrel3	Rattus norvegicus kin of IRRE like 3 (Drosophila) (Kirrel3), mRNA [NM_001048215]	**11,972**
Clptm1l	Rattus norvegicus CLPTM1-like (Clptm1l), mRNA [NM_001108240]	**11,971**
Smpd3	Rattus norvegicus sphingomyelin phosphodiesterase 3, neutral membrane (Smpd3), mRNA [NM_053605]	**11,970**
Plekho1	Rattus norvegicus pleckstrin homology domain containing, family O member 1 (Plekho1), mRNA [NM_001025119]	**11,970**
Sh3bp5	Rattus norvegicus SH3-domain binding protein 5 (BTK-associated) (Sh3bp5), mRNA [NM_054011]	**11,967**
LOC684122	PREDICTED: Rattus norvegicus similar to development and differentiation enhancing factor-like 1 (LOC684122), mRNA [XM_001069024]	**11,967**
Csrnp2	Rattus norvegicus cysteine-serine-rich nuclear protein 2 (Csrnp2), mRNA [NM_001108113]	**11,966**
0	Unknown	**11,966**
Gja8	Rattus norvegicus gap junction membrane channel protein alpha 8 (Gja8), mRNA [NM_153465]	**11,965**
Dhx33	Rattus norvegicus DEAH (Asp-Glu-Ala-His) box polypeptide 33 (Dhx33), mRNA [NM_001105802]	**11,965**
C8g	Rattus norvegicus complement component 8, gamma polypeptide (C8g), mRNA [NM_001106555]	**11,963**
Ttyh1	Rattus norvegicus tweety homolog 1 (Drosophila) (Ttyh1), mRNA [NM_001106225]	**11,957**
0	Uncharacterized protein [Source:UniProtKB/TrEMBL;Acc:D4A0B5] [ENSRNOT00000064837]	**11,956**
Dusp3	Rattus norvegicus dual specificity phosphatase 3 (Dusp3), mRNA [NM_001173376]	**11,955**
Psme4	Rattus norvegicus proteasome (prosome, macropain) activator subunit 4 (Psme4), mRNA [NM_001025140]	**11,952**
Rptn	Uncharacterized protein [Source:UniProtKB/TrEMBL;Acc:D3ZRF0] [ENSRNOT00000025249]	**11,948**
B3gnt4	Rattus norvegicus UDP-GlcNAc:betaGal beta-1,3-N-acetylglucosaminyltransferase 4 (B3gnt4), mRNA [NM_001105938]	**11,945**
Phactr3	Rattus norvegicus phosphatase and actin regulator 3 (Phactr3), mRNA [NM_214459]	**11,945**
Slc24a1	Rattus norvegicus solute carrier family 24 (sodium/potassium/calcium exchanger), member 1 (Slc24a1), mRNA [NM_020090]	**11,945**

Elmo2	Rattus norvegicus engulfment and cell motility 2 (Elmo2), mRNA [NM_001134955]	**11,945**
RGD1306215	Rattus norvegicus similar to hypothetical protein MGC36831 (RGD1306215), mRNA [NM_001106556]	**11,944**
0	Unknown	**11,943**
Coro7	Rattus norvegicus coronin 7 (Coro7), mRNA [NM_001191639]	**11,940**
0	Uncharacterized protein [Source:UniProtKB/TrEMBL;Acc:D3ZFF7] [ENSRNOT00000020342]	**11,939**
0	Uncharacterized protein [Source:UniProtKB/TrEMBL;Acc:D3ZFV6] [ENSRNOT00000068346]	**11,939**
Rpl10	Rattus norvegicus ribosomal protein L10 (Rpl10), mRNA [NM_031100]	**11,937**
Rab15	Rattus norvegicus RAB15, member RAS oncogene family (Rab15), mRNA [NM_198749]	**11,934**
H1foo	Rattus norvegicus H1 histone family, member O, oocyte-specific (H1foo), mRNA [NM_001109351]	**11,931**
Ccdc49	Rattus norvegicus coiled-coil domain containing 49 (Ccdc49), mRNA [NM_001108295]	**11,931**
Akap7	A-kinase anchoring protein 18 ,isoform delta [Source:RefSeq peptide;Acc:NP_001001801] [ENSRNOT00000017617]	**11,929**
0	Unknown	**11,929**
Trim3	Rattus norvegicus tripartite motif-containing 3 (Trim3), mRNA [NM_031786]	**11,928**
0	Unknown	**11,926**
Top1	Rattus norvegicus topoisomerase (DNA) I (Top1), mRNA [NM_022615]	**11,921**
Ccdc135	Rattus norvegicus coiled-coil domain containing 135 (Ccdc135), mRNA [NM_001106169]	**11,921**
Zfp748	PREDICTED: Rattus norvegicus zinc finger protein 748 (Zfp748), mRNA [XM_001056356]	**11,921**
F2rl1	Rattus norvegicus coagulation factor II (thrombin) receptor-like 1 (F2rl1), mRNA [NM_053897]	**11,918**
Rrbp1	PREDICTED: Rattus norvegicus ribosome binding protein 1, transcript variant 3 (Rrbp1), mRNA [XM_001053669]	**11,916**
Eps8	Uncharacterized protein [Source:UniProtKB/TrEMBL;Acc:D3ZWU6] [ENSRNOT00000009328]	**11,915**
0	Unknown	**11,915**
0	Unknown	**11,915**
Padi1	Rattus norvegicus peptidyl arginine deiminase, type I (Padi1), mRNA [NM_019332]	**11,913**
RGD1306926	Uncharacterized protein [Source:UniProtKB/TrEMBL;Acc:D3ZX03] [ENSRNOT00000054974]	**11,913**
0	Unknown	**11,907**
RGD1306556	PREDICTED: Rattus norvegicus similar to hypothetical protein A530094D01 (RGD1306556), mRNA [XM_001080521]	**11,906**
Pten	Rattus norvegicus phosphatase and tensin homolog (Pten), mRNA [NM_031606]	**11,906**
Bin3	Rattus norvegicus bridging integrator 3 (Bin3), mRNA [NM_001013186]	**11,905**
Rabgap1l	Rattus norvegicus RAB GTPase activating protein 1-like (Rabgap1l), mRNA [NM_001107190]	**11,904**
Mtss1	Rattus norvegicus metastasis suppressor 1 (Mtss1), mRNA [NM_001130563]	**11,904**
Amn1	Rattus norvegicus antagonist of mitotic exit network 1 homolog (S. cerevisiae) (Amn1), mRNA [NM_001008333]	**11,903**
Fam122b	Rattus norvegicus family with sequence similarity 122B (Fam122b), mRNA [NM_001166586]	**11,903**
Pcmtd2	Rattus norvegicus protein-L-isoaspartate (D-aspartate) O-methyltransferase domain containing 2 (Pcmtd2), mRNA [NM_001107810]	**11,903**
Slc20a2	Rattus norvegicus solute carrier family 20 (phosphate transporter), member 2 (Slc20a2), mRNA [NM_017223]	**11,903**

Hist1h1b	Rattus norvegicus histone cluster 1, H1b (Hist1h1b), mRNA [NM_001109417]	**11,899**
0	Rattus norvegicus similar to glyceraldehyde-3-phosphate dehydrogenase (phosphorylating) (EC 1.2.1.12) - mouse (LOC313443), mRNA [XM_233295]	**11,898**
Neu1	Rattus norvegicus sialidase 1 (lysosomal sialidase) (Neu1), mRNA [NM_031522]	**11,897**
Dgkg	Rattus norvegicus diacylglycerol kinase, gamma (Dgkg), mRNA [NM_013126]	**11,894**
Fhdc1	Rattus norvegicus FH2 domain containing 1 (Fhdc1), mRNA [NM_001106437]	**11,892**
Cyp2s1	Rattus norvegicus cytochrome P450, family 2, subfamily s, polypeptide 1 (Cyp2s1), mRNA [NM_001107495]	**11,890**
0	Rattus norvegicus similar to 60S ribosomal protein L23a (LOC299190), mRNA [XM_234397]	**11,888**
Ttc7b	Rattus norvegicus tetratricopeptide repeat domain 7B (Ttc7b), mRNA [NM_001108719]	**11,887**
Atp7b	Rattus norvegicus ATPase, Cu++ transporting, beta polypeptide (Atp7b), mRNA [NM_012511]	**11,887**
Lrfn2	Rattus norvegicus leucine rich repeat and fibronectin type III domain containing 2 (Lrfn2), mRNA [NM_001039699]	**11,885**
M6prbp1	PREDICTED: Rattus norvegicus mannose-6-phosphate receptor binding protein 1 (M6prbp1), mRNA [XM_001061015]	**11,883**
Znf667	Rattus norvegicus zinc finger protein 667 (Znf667), mRNA [NM_001008557]	**11,882**
0	STML1_MOUSE (Q8CI66) Stomatin-like protein 1 (SLP-1), partial (6%) [TC590815]	**11,880**
Pqlc1	Rattus norvegicus PQ loop repeat containing 1 (Pqlc1), mRNA [NM_001013189]	**11,879**
0	Uncharacterized protein [Source:UniProtKB/TrEMBL;Acc:D3ZU50] [ENSRNOT00000001479]	**11,878**
Kcnb1	Rattus norvegicus potassium voltage gated channel, Shab-related subfamily, member 1 (Kcnb1), mRNA [NM_013186]	**11,878**
Tspyl4	Rattus norvegicus TSPY-like 4 (Tspyl4), mRNA [NM_001012075]	**11,876**
0	Unknown	**11,875**
0	Unknown	**11,875**
Spcs2	Rattus norvegicus signal peptidase complex subunit 2 homolog (S. cerevisiae) (Spcs2), mRNA [NM_001191601]	**11,871**
RGD1561678	PREDICTED: Rattus norvegicus similar to Ten-m1 (RGD1561678), mRNA [XM_002730177]	**11,871**
Ift80	Rattus norvegicus intraflagellar transport 80 homolog (Chlamydomonas) (Ift80), mRNA [NM_001013911]	**11,870**
0	Unknown	**11,870**
Pigs	Rattus norvegicus phosphatidylinositol glycan anchor biosynthesis, class S (Pigs), mRNA [NM_001006602]	**11,870**
RGD1307722	Rattus norvegicus similar to hypothetical protein MGC20700 (RGD1307722), mRNA [NM_001108730]	**11,868**
Tox4	Rattus norvegicus TOX high mobility group box family member 4 (Tox4), mRNA [NM_173324]	**11,866**
Cd200	Rattus norvegicus Cd200 molecule (Cd200), mRNA [NM_031518]	**11,865**
0	Unknown	**11,865**
Sorcs2	Rattus norvegicus sortilin-related VPS10 domain containing receptor 2 (Sorcs2), mRNA [NM_001107225]	**11,864**
Ccdc147	Uncharacterized protein [Source:UniProtKB/TrEMBL;Acc:D3ZGP0] [ENSRNOT00000017283]	**11,864**
Casp9	Rattus norvegicus caspase 9, apoptosis-related cysteine peptidase (Casp9), mRNA [NM_031632]	**11,863**
Ng23	Rattus norvegicus Ng23 protein (Ng23), mRNA [NM_001004069]	**11,862**
Atp6v0a2	Rattus norvegicus ATPase, H+ transporting, lysosomal V0 subunit A2 (Atp6v0a2), mRNA [NM_053775]	**11,859**

LOC683713	Rattus norvegicus similar to Galactosylceramide sulfotransferase (GalCer sulfotransferase) (Cerebroside sulfotransferase) (3-phosphoadenylylsulfate:galactosylceramide 3-sulfotransferase) (3-phosphoadenosine-5phosphosulfate:GalCer sulfotransferase) (LOC683	**11,858**
Tram1	Rattus norvegicus translocation associated membrane protein 1 (Tram1), mRNA [NM_001007701]	**11,858**
0	Unknown	**11,855**
Npap60	Rattus norvegicus nuclear pore associated protein (Npap60), mRNA [NM_012991]	**11,854**
Cnr1	Rattus norvegicus cannabinoid receptor 1 (brain) (Cnr1), mRNA [NM_012784]	**11,851**
Rftn2	Uncharacterized protein [Source:UniProtKB/TrEMBL;Acc:D3ZD65] [ENSRNOT00000020910]	**11,850**
RGD1559971	PREDICTED: Rattus norvegicus similar to Na+ dependent glucose transporter 1 (RGD1559971), mRNA [XM_001062835]	**11,848**
Phf20l1	Rattus norvegicus PHD finger protein 20-like 1, mRNA (cDNA clone IMAGE:7389535), complete cds. [BC096896]	**11,847**
Ppm1e	Rattus norvegicus protein phosphatase 1E (PP2C domain containing) (Ppm1e), mRNA [NM_198773]	**11,846**
Siah1a	Rattus norvegicus seven in absentia 1A (Siah1a), mRNA [NM_080905]	**11,846**
Lypd6b	Rattus norvegicus LY6/PLAUR domain containing 6B (Lypd6b), mRNA [NM_001134580]	**11,845**
0	PREDICTED: Rattus norvegicus similar to monoacylglycerol O-acyltransferase 2 (LOC687054), mRNA [XM_001076877]	**11,845**
LOC691797	PREDICTED: Rattus norvegicus similar to developmental pluripotency-associated 3 (LOC691797), mRNA [XM_001079667]	**11,844**
RT1-CE11	Rattus norvegicus RT1 class I, locus CE11 (RT1-CE11), mRNA [NM_001008834]	**11,843**
0	Unknown	**11,843**
RGD1562989	PREDICTED: Rattus norvegicus similar to serine/threonine kinase (RGD1562989), mRNA [XM_229637]	**11,839**
0	Unknown	**11,838**
Sdsl	Rattus norvegicus serine dehydratase-like (Sdsl), mRNA [NM_001108336]	**11,837**
Vdac3	Rattus norvegicus voltage-dependent anion channel 3 (Vdac3), mRNA [NM_031355]	**11,834**
Lmod2	Rattus norvegicus leiomodin 2 (cardiac) (Lmod2), mRNA [NM_001100964]	**11,829**
RGD1306613	Rattus norvegicus similar to RIKEN cDNA 1600012F09 (RGD1306613), mRNA [NM_001107356]	**11,826**
Hnrnpu	Rattus norvegicus heterogeneous nuclear ribonucleoprotein U (Hnrnpu), mRNA [NM_057139]	**11,826**
LOC691365	Uncharacterized protein [Source:UniProtKB/TrEMBL;Acc:D3ZMI8] [ENSRNOT00000048115]	**11,825**
Lgi4	Rattus norvegicus leucine-rich repeat LGI family, member 4 (Lgi4), mRNA [NM_199499]	**11,823**
Scn1b	Rattus norvegicus sodium channel, voltage-gated, type I, beta (Scn1b), mRNA [NM_017288]	**11,819**
Zfp385c	Uncharacterized protein [Source:UniProtKB/TrEMBL;Acc:D3ZE95] [ENSRNOT00000067868]	**11,818**
Usp53	Rattus norvegicus ubiquitin specific peptidase 53 (Usp53), mRNA [NM_001106468]	**11,818**
Tm9sf4	Rattus norvegicus transmembrane 9 superfamily protein member 4 (Tm9sf4), mRNA [NM_001025649]	**11,818**
Mrgprb13	Rattus norvegicus MAS-related GPR, member B13 (Mrgprb13), mRNA [NM_001002283]	**11,817**
Chmp4b	PREDICTED: Rattus norvegicus chromatin modifying protein 4B (Chmp4b), mRNA [XM_001073409]	**11,815**
Dnmt3b	Rattus norvegicus DNA (cytosine-5-)-methyltransferase 3 beta (Dnmt3b), mRNA [NM_001003959]	**11,814**
0	Rattus norvegicus similar to glyceraldehyde-3-phosphate dehydrogenase (phosphorylating) (EC 1.2.1.12) - mouse (LOC365427), mRNA [XM_345008]	**11,814**

Kif3b	Rattus norvegicus kinesin family member 3B (Kif3b), mRNA [NM_001106529]	**11,812**
0	Uncharacterized protein [Source:UniProtKB/TrEMBL;Acc:D3ZKR7] [ENSRNOT00000046075]	**11,809**
Ranbp10	Rattus norvegicus RAN binding protein 10 (Ranbp10), mRNA [NM_001135875]	**11,808**
0	PREDICTED: Rattus norvegicus similar to centromere autoantigen H (LOC684611), mRNA [XM_001071235]	**11,807**
Tmx1	Rattus norvegicus thioredoxin-related transmembrane protein 1 (Tmx1), mRNA [NM_001024800]	**11,805**
Xpnpep3	Rattus norvegicus X-prolyl aminopeptidase (aminopeptidase P) 3, putative (Xpnpep3), mRNA [NM_001130582]	**11,805**
Pank2	Rattus norvegicus pantothenate kinase 2 (Hallervorden-Spatz syndrome) (Pank2), nuclear gene encoding mitochondrial protein, mRNA [NM_001106513]	**11,802**
Pip5k1a	Rattus norvegicus phosphatidylinositol-4-phosphate 5-kinase, type 1, alpha (Pip5k1a), mRNA [NM_001042621]	**11,802**
0	Unknown	**11,801**
Kcnk2	Rattus norvegicus potassium channel, subfamily K, member 2 (Kcnk2), transcript variant 2, mRNA [NM_172042]	**11,801**
Uap1	Rattus norvegicus UDP-N-acteylglucosamine pyrophosphorylase 1 (Uap1), mRNA [NM_001191930]	**11,800**
Aup1	Rattus norvegicus ancient ubiquitous protein 1 (Aup1), mRNA [NM_001079899]	**11,800**
Prkd2	Rattus norvegicus protein kinase D2 (Prkd2), mRNA [NM_001013895]	**11,799**
Tmx2	Rattus norvegicus thioredoxin-related transmembrane protein 2 (Tmx2), mRNA [NM_001007643]	**11,799**
Yipf2	Rattus norvegicus Yip1 domain family, member 2 (Yipf2), mRNA [NM_001014208]	**11,798**
0	Unknown	**11,798**
Spred3	Rattus norvegicus sprouty-related, EVH1 domain containing 3 (Spred3), mRNA [NM_001173344]	**11,797**
0	Unknown	**11,794**
Vom2r41	Rattus norvegicus vomeronasal 2 receptor, 41 (Vom2r41), mRNA [NM_001099500]	**11,793**
Gca	Rattus norvegicus grancalcin (Gca), mRNA [NM_001106483]	**11,791**
Gnas	Rattus norvegicus GNAS complex locus (Gnas), transcript variant 3, mRNA [NM_019132]	**11,791**
0	Unknown	**11,790**
Fam110a	Rattus norvegicus family with sequence similarity 110, member A (Fam110a), mRNA [NM_001014050]	**11,788**
Olr689	Rattus norvegicus olfactory receptor 689 (Olr689), mRNA [NM_001000997]	**11,787**
Cyp4x1	Rattus norvegicus cytochrome P450, family 4, subfamily x, polypeptide 1 (Cyp4x1), mRNA [NM_145675]	**11,787**
Znf76	Rattus norvegicus zinc finger protein 76 (expressed in testis) (Znf76), mRNA [NM_001134755]	**11,785**
Scnm1	Rattus norvegicus sodium channel modifier 1 (Scnm1), mRNA [NM_001107696]	**11,785**
Fzd3	Rattus norvegicus frizzled homolog 3 (Drosophila) (Fzd3), mRNA [NM_153474]	**11,783**
Acad11	Rattus norvegicus acyl-Coenzyme A dehydrogenase family, member 11 (Acad11), mRNA [NM_001108181]	**11,782**
Parp6	Rattus norvegicus poly (ADP-ribose) polymerase family, member 6 (Parp6), mRNA [NM_001106828]	**11,782**
Apaf1	Rattus norvegicus apoptotic peptidase activating factor 1 (Apaf1), mRNA [NM_023979]	**11,778**
Dock9	Rattus norvegicus dedicator of cytokinesis 9 (Dock9), mRNA [NM_001105759]	**11,775**
Pcdhb15	PREDICTED: Rattus norvegicus protocadherin beta 15 (Pcdhb15), mRNA [XM_001055818]	**11,774**

Traf4	Rattus norvegicus Tnf receptor associated factor 4 (Traf4), mRNA [NM_001107017]	**11,774**
Thrap3	Rattus norvegicus thyroid hormone receptor associated protein 3 (Thrap3), mRNA [NM_001009693]	**11,771**
Hyal3	Rattus norvegicus hyaluronoglucosaminidase 3 (Hyal3), mRNA [NM_207599]	**11,771**
Ptma	Rattus norvegicus prothymosin alpha (Ptma), mRNA [NM_021740]	**11,771**
Grifin	Rattus norvegicus galectin-related inter-fiber protein (Grifin), mRNA [NM_057187]	**11,770**
Ncoa6	Nuclear receptor coactivator 6 [Source:UniProtKB/Swiss-Prot;Acc:Q9JLI4] [ENSRNOT00000024714]	**11,768**
Pacrg	Rattus norvegicus Park2 co-regulated (Pacrg), mRNA [NM_001077677]	**11,768**
LOC682215	PREDICTED: Rattus norvegicus hypothetical protein LOC682215 (LOC682215), mRNA [XM_001060486]	**11,767**
Slc22a8	Rattus norvegicus solute carrier family 22 (organic anion transporter), member 8 (Slc22a8), mRNA [NM_031332]	**11,767**
RGD1304878	PREDICTED: Rattus norvegicus similar to 2410024A21Rik protein (RGD1304878), miscRNA [XR_005460]	**11,766**
Pftk1	Rattus norvegicus PFTAIRE protein kinase 1 (Pftk1), mRNA [NM_001108617]	**11,763**
0	protein phosphatase 2, regulatory subunit B (B56), delta isoform Gene [Source:MGI Symbol;Acc:MGI:2388481] [ENSRNOT00000022862]	**11,761**
LOC690000	Uncharacterized protein [Source:UniProtKB/TrEMBL;Acc:D3ZWS2] [ENSRNOT00000020079]	**11,761**
0	Uncharacterized protein [Source:UniProtKB/TrEMBL;Acc:D3ZEZ6] [ENSRNOT00000066536]	**11,758**
Sstr5	Rattus norvegicus somatostatin receptor 5 (Sstr5), mRNA [NM_012882]	**11,757**
Slc36a4	Rattus norvegicus solute carrier family 36 (proton/amino acid symporter), member 4 (Slc36a4), mRNA [NM_001108127]	**11,757**
Nek9	Rattus norvegicus NIMA (never in mitosis gene a)- related kinase 9 (Nek9), mRNA [NM_001106747]	**11,755**
Pofut1	Rattus norvegicus protein O-fucosyltransferase 1 (Pofut1), mRNA [NM_001002278]	**11,755**
Brms1l	Rattus norvegicus breast cancer metastasis-suppressor 1-like (Brms1l), mRNA [NM_001106731]	**11,755**
Vasn	Rattus norvegicus vasorin (Vasn), mRNA [NM_001109382]	**11,755**
Slc43a2	Rattus norvegicus solute carrier family 43, member 2 (Slc43a2), mRNA [NM_001105812]	**11,746**
0	Unknown	**11,745**
0	Rattus norvegicus TL0ABA36YD02 mRNA sequence. [FQ210020]	**11,744**
Pnoc	Rattus norvegicus prepronociceptin (Pnoc), mRNA [NM_013007]	**11,742**
Mast3	Rattus norvegicus microtubule associated serine/threonine kinase 3 (Mast3), mRNA [NM_001134796]	**11,741**
LOC688459	Rattus norvegicus hypothetical protein LOC688459 (LOC688459), mRNA [NM_001109502]	**11,741**
Cbp	Rattus norvegicus Csk binding protein (Cbp), mRNA [NM_022253]	**11,741**
Efha2	Rattus norvegicus EF hand domain family, member A2 (Efha2), mRNA [NM_001191892]	**11,741**
Chst15	Rattus norvegicus carbohydrate (N-acetylgalactosamine 4-sulfate 6-O) sulfotransferase 15 (Chst15), mRNA [NM_173310]	**11,740**
0	Unknown	**11,740**
Fam120b	Rattus norvegicus family with sequence similarity 120B (Fam120b), mRNA [NM_001107466]	**11,740**
Cdk6	Rattus norvegicus cyclin-dependent kinase 6 (Cdk6), mRNA [NM_001191861]	**11,739**
LOC686295	PREDICTED: Rattus norvegicus similar to CG17293-PA (LOC686295), mRNA [XM_001073342]	**11,739**
Ikbkb	Rattus norvegicus inhibitor of kappa light polypeptide gene enhancer in B-cells, kinase beta (Ikbkb), mRNA [NM_053355]	**11,739**

Myf5	Rattus norvegicus myogenic factor 5 (Myf5), mRNA [NM_001106783]	**11,737**
Kras	Rattus norvegicus v-Ki-ras2 Kirsten rat sarcoma viral oncogene homolog (Kras), mRNA [NM_031515]	**11,734**
RGD1559513	PREDICTED: Rattus norvegicus similar to DEAD (Asp-Glu-Ala-Asp) box polypeptide 41 (RGD1559513), mRNA [XM_001059856]	**11,730**
Gpr108	Rattus norvegicus G protein-coupled receptor 108 (Gpr108), mRNA [NM_199399]	**11,730**
RGD1563307	Uncharacterized protein [Source:UniProtKB/TrEMBL;Acc:D4A466] [ENSRNOT00000030832]	**11,726**
Adam10	PREDICTED: Rattus norvegicus ADAM metallopeptidase domain 10 (Adam10), mRNA [XM_217197]	**11,722**
0	Unknown	**11,721**
Ubac2	Rattus norvegicus UBA domain containing 2 (Ubac2), mRNA [NM_001034937]	**11,721**
0	Unknown	**11,721**
Spata6	Rattus norvegicus spermatogenesis associated 6 (Spata6), mRNA [NM_134392]	**11,721**
0	Unknown	**11,720**
Fam84a	Rattus norvegicus family with sequence similarity 84, member A (Fam84a), mRNA [NM_001127299]	**11,719**
0	PREDICTED: Rattus norvegicus similar to Mblk1-related protein-2 (LOC679031), mRNA [XM_001054322]	**11,717**
Arl2	Rattus norvegicus ADP-ribosylation factor-like 2 (Arl2), mRNA [NM_031711]	**11,717**
Rin2	Rattus norvegicus Ras and Rab interactor 2 (Rin2), mRNA [NM_001107786]	**11,716**
Znf593	Rattus norvegicus zinc finger protein 593 (Znf593), mRNA [NM_001106689]	**11,715**
Fads6	Rattus norvegicus fatty acid desaturase domain family, member 6 (Fads6), mRNA [NM_001107064]	**11,714**
Pcdhb13	Protocadherin-T4 [Source:UniProtKB/TrEMBL;Acc:Q9JIU1] [ENSRNOT00000027172]	**11,713**
Abcb7	Rattus norvegicus ATP-binding cassette, subfamily B (MDR/TAP), member 7 (Abcb7), nuclear gene encoding mitochondrial protein, mRNA [NM_212518]	**11,713**
M6pr	Rattus norvegicus mannose-6-phosphate receptor, cation dependent (M6pr), mRNA [NM_001007700]	**11,712**
0	AGENCOURT_26626347 NIH_MGC_253 Rattus norvegicus cDNA clone IMAGE:7301841 5', mRNA sequence [CO388903]	**11,712**
G3bp1	Rattus norvegicus GTPase activating protein (SH3 domain) binding protein 1 (G3bp1), mRNA [NM_133565]	**11,710**
Zdhhc1	Rattus norvegicus zinc finger, DHHC-type containing 1 (Zdhhc1), mRNA [NM_001039099]	**11,710**
Zfp426	Rattus norvegicus zinc finger protein 426 (Zfp426), mRNA [NM_001079943]	**11,707**
0	Uncharacterized protein [Source:UniProtKB/TrEMBL;Acc:D3ZUZ3] [ENSRNOT00000045859]	**11,707**
0	Unknown	**11,706**
Ndufaf1	Rattus norvegicus NADH dehydrogenase (ubiquinone) 1 alpha subcomplex, assembly factor 1 (Ndufaf1), mRNA [NM_001106500]	**11,704**
Cyp2s1	Rattus norvegicus cytochrome P450, family 2, subfamily s, polypeptide 1 (Cyp2s1), mRNA [NM_001107495]	**11,704**
0	Unknown	**11,703**
0	Tetratricopeptide repeat protein GNN [Source:UniProtKB/Swiss-Prot;Acc:Q6P3V7] [ENSRNOT00000059545]	**11,700**
Pdap1	Rattus norvegicus PDGFA associated protein 1 (Pdap1), mRNA [NM_022595]	**11,699**
Def8	Rattus norvegicus differentially expressed in FDCP 8 homolog (mouse) (Def8), mRNA [NM_001024774]	**11,699**
0	Unknown	**11,698**

Zfp53	Rattus norvegicus zinc finger protein 53 (Zfp53), mRNA [NM_001107468]	**11,698**
Zar1l	PREDICTED: Rattus norvegicus similar to Zygote arrest 1 (Oocyte-specific maternal effect factor) (LOC689594), mRNA [XM_001071298]	**11,697**
Chtf8	Rattus norvegicus CTF8, chromosome transmission fidelity factor 8 homolog (S. cerevisiae) (Chtf8), mRNA [NM_001194951]	**11,695**
Kif1b	Rattus norvegicus kinesin family member 1B (Kif1b), mRNA [NM_057200]	**11,694**
Gimap9	Rattus norvegicus GTPase, IMAP family member 9 (Gimap9), mRNA [NM_001008398]	**11,694**
Timp2	Rattus norvegicus TIMP metallopeptidase inhibitor 2 (Timp2), mRNA [NM_021989]	**11,692**
Dcun1d1	Rattus norvegicus DCN1, defective in cullin neddylation 1, domain containing 1 (S. cerevisiae) (Dcun1d1), mRNA [NM_001107668]	**11,690**
Cacnb4	Rattus norvegicus calcium channel, voltage-dependent, beta 4 subunit (Cacnb4), mRNA [NM_001105733]	**11,688**
Zfp322a	Rattus norvegicus zinc finger protein 322a (Zfp322a), mRNA [NM_001135084]	**11,687**
0	Q8CHN9_RAT (Q8CHN9) Transcription factor 1, complete [TC573664]	**11,686**
Oprm1	Rattus norvegicus opioid receptor, mu 1 (Oprm1), transcript variant MOR-1A, mRNA [NM_001038597]	**11,686**
Abo	Rattus norvegicus ABO blood group (transferase A, alpha 1-3-N-acetylgalactosaminyltransferase; transferase B, alpha 1-3-galactosyltransferase) (Abo), mRNA [NM_023094]	**11,685**
Ccdc28a	Rattus norvegicus coiled-coil domain containing 28A (Ccdc28a), mRNA [NM_001037789]	**11,685**
Birc5	Rattus norvegicus baculoviral IAP repeat-containing 5 (Birc5), mRNA [NM_022274]	**11,682**
Rpl28	Rattus norvegicus ribosomal protein L28 (Rpl28), mRNA [NM_022697]	**11,681**
Rgs2	Rattus norvegicus regulator of G-protein signaling 2 (Rgs2), mRNA [NM_053453]	**11,679**
Clcn5	Rattus norvegicus chloride channel 5 (Clcn5), mRNA [NM_017106]	**11,678**
Fabp12	Rattus norvegicus fatty acid binding protein 12 (Fabp12), mRNA [NM_001134614]	**11,677**
Sppl2a	Rattus norvegicus signal peptide peptidase-like 2A (Sppl2a), mRNA [NM_001107770]	**11,675**
Xpnpep3	Rattus norvegicus X-prolyl aminopeptidase (aminopeptidase P) 3, putative (Xpnpep3), mRNA [NM_001130582]	**11,675**
Il20	Rattus norvegicus interleukin 20 (Il20), mRNA [NM_001143881]	**11,674**
Armc5	Rattus norvegicus armadillo repeat containing 5 (Armc5), mRNA [NM_001009455]	**11,674**
Slc4a10	Rattus norvegicus solute carrier family 4, sodium bicarbonate transporter, member 10 (Slc4a10), mRNA [NM_178092]	**11,671**
0	Unknown	**11,670**
B2m	Rattus norvegicus beta-2 microglobulin (B2m), mRNA [NM_012512]	**11,669**
Ccr9	Rattus norvegicus chemokine (C-C motif) receptor 9 (Ccr9), mRNA [NM_172329]	**11,667**
Nacc1	Rattus norvegicus nucleus accumbens associated 1, BEN and BTB (POZ) domain containing (Nacc1), mRNA [NM_134413]	**11,665**
LOC691786	PREDICTED: Rattus norvegicus similar to Stannin (LOC691786), mRNA [XM_001079624]	**11,664**
Kctd5	Rattus norvegicus potassium channel tetramerisation domain containing 5 (Kctd5), mRNA [NM_001105768]	**11,664**
0	Unknown	**11,660**
0	Rattus norvegicus similar to RIKEN cDNA A830059I20 (LOC299265), mRNA [XM_234480]	**11,659**
RT1-DMb	Rattus norvegicus RT1 class II, locus DMb (RT1-DMb), mRNA [NM_198740]	**11,659**
Rap1b	Rattus norvegicus RAP1B, member of RAS oncogene family (Rap1b), mRNA [NM_134346]	**11,658**

Grpr	Rattus norvegicus gastrin releasing peptide receptor (Grpr), mRNA [NM_012706]	**11,658**
LOC100364244	PREDICTED: Rattus norvegicus rCG43589-like (LOC100364244), mRNA [XM_002727401]	**11,657**
Map3k10	Rattus norvegicus mitogen activated protein kinase kinase kinase 10 (Map3k10), mRNA [NM_001191621]	**11,655**
0	Unknown	**11,654**
0	Unknown	**11,653**
Arpp21	Rattus norvegicus cAMP-regulated phosphoprotein 21 (Arpp21), transcript variant 1, mRNA [NM_001135046]	**11,653**
0	Uncharacterized protein [Source:UniProtKB/TrEMBL;Acc:D3ZUQ8] [ENSRNOT00000032636]	**11,653**
Arhgef6	Rattus norvegicus Rac/Cdc42 guanine nucleotide exchange factor (GEF) 6 (Arhgef6), mRNA [NM_001005565]	**11,652**
Camk2b	Rattus norvegicus calcium/calmodulin-dependent protein kinase II beta (Camk2b), transcript variant 1, mRNA [NM_001042354]	**11,651**
Rufy3	Rattus norvegicus RUN and FYVE domain containing 3 (Rufy3), mRNA [NM_001025127]	**11,650**
Abcc10	Rattus norvegicus ATP-binding cassette, subfamily C (CFTR/MRP), member 10 (Abcc10), mRNA [NM_001108201]	**11,649**
Efhc1	Rattus norvegicus EF-hand domain (C-terminal) containing 1 (Efhc1), mRNA [NM_001122947]	**11,648**
LOC500893	Rattus norvegicus similar to GLI-Kruppel family member GLI4 (LOC500893), mRNA [NM_001029926]	**11,646**
Capn2	Rattus norvegicus calpain 2 (Capn2), mRNA [NM_017116]	**11,646**
Dnm2	Rattus norvegicus dynamin 2 (Dnm2), mRNA [NM_013199]	**11,645**
Tef	Rattus norvegicus thyrotrophic embryonic factor (Tef), mRNA [NM_019194]	**11,645**
Ptpro	Rattus norvegicus protein tyrosine phosphatase, receptor type, O (Ptpro), mRNA [NM_017336]	**11,644**
Dnmbp	PREDICTED: Rattus norvegicus dynamin binding protein (Dnmbp), mRNA [XM_219860]	**11,641**
LOC690137	PREDICTED: Rattus norvegicus similar to transmembrane protein induced by tumor necrosis factor alpha (LOC690137), mRNA [XM_001073419]	**11,640**
Chdh	Rattus norvegicus choline dehydrogenase (Chdh), nuclear gene encoding mitochondrial protein, mRNA [NM_198731]	**11,638**
0	Unknown	**11,638**
Parp16	Rattus norvegicus poly (ADP-ribose) polymerase family, member 16 (Parp16), mRNA [NM_001014093]	**11,638**
Rpgr	Rattus norvegicus retinitis pigmentosa GTPase regulator (Rpgr), mRNA [NM_001127601]	**11,636**
Oxr1	Rattus norvegicus oxidation resistance 1 (Oxr1), transcript variant 1, mRNA [NM_001197907]	**11,636**
Slc13a5	Rattus norvegicus solute carrier family 13 (sodium-dependent citrate transporter), member 5 (Slc13a5), mRNA [NM_170668]	**11,636**
Epb49	Rattus norvegicus erythrocyte membrane protein band 4.9 (dematin) (Epb49), mRNA [NM_001108385]	**11,635**
0	Unknown	**11,635**
0	Uncharacterized protein [Source:UniProtKB/TrEMBL;Acc:D3ZV94] [ENSRNOT00000064107]	**11,634**
Nudt16	Rattus norvegicus nudix (nucleoside diphosphate linked moiety X)-type motif 16 (Nudt16), mRNA [NM_001127554]	**11,633**
Pcnxl3	Uncharacterized protein [Source:UniProtKB/TrEMBL;Acc:D3ZSQ1] [ENSRNOT00000064136]	**11,633**
RGD1562339	Uncharacterized protein [Source:UniProtKB/TrEMBL;Acc:D4AC80] [ENSRNOT00000002408]	**11,631**
Cpg1	Rattus norvegicus candidate plasticity gene 1 (Cpg1), mRNA [NM_178104]	**11,630**
0	Uncharacterized protein [Source:UniProtKB/TrEMBL;Acc:D3ZT95] [ENSRNOT00000004563]	**11,629**
LOC691920	PREDICTED: Rattus norvegicus similar to kinesin-like motor protein C20orf23 (LOC691920), mRNA [XM_001080099]	**11,629**

Bre	Rattus norvegicus brain and reproductive organ-expressed protein (Bre), mRNA [NM_199270]	**11,628**
Vezf1	PREDICTED: Rattus norvegicus vascular endothelial zinc finger 1 (Vezf1), partial mRNA [XM_001081191]	**11,626**
Fam120a	Rattus norvegicus family with sequence similarity 120A (Fam120a), mRNA [NM_001191816]	**11,625**
Ing3	Rattus norvegicus inhibitor of growth family, member 3 (Ing3), mRNA [NM_001034107]	**11,623**
0	Unknown	**11,622**
0	Unknown	**11,622**
Dctn5	Rattus norvegicus dynactin 5 (Dctn5), mRNA [NM_001037778]	**11,622**
0	Uncharacterized protein [Source:UniProtKB/TrEMBL;Acc:D4A105] [ENSRNOT00000026728]	**11,620**
Pcp2	Rattus norvegicus Purkinje cell protein 2 (Pcp2), mRNA [NM_001107116]	**11,619**
Gpr68	Rattus norvegicus G protein-coupled receptor 68 (Gpr68), mRNA [NM_001108049]	**11,618**
0	P53 PG-III protein [Source:UniProtKB/TrEMBL;Acc:Q6I9N3] [ENSRNOT00000049514]	**11,616**
Znf521	zinc finger protein 521 [Source:RefSeq peptide;Acc:NP_001100873] [ENSRNOT00000022637]	**11,616**
Ptgdrl	Rattus norvegicus prostaglandin D receptor-like (Ptgdrl), mRNA [NM_022241]	**11,615**
Rhox2	Rattus norvegicus reproductive homeobox on X chromosome 2 (Rhox2), mRNA [NM_001025746]	**11,615**
0	Unknown	**11,614**
Klk11	Rattus norvegicus kallikrein related-peptidase 11 (Klk11), mRNA [NM_001106252]	**11,612**
Ccdc94	Rattus norvegicus coiled-coil domain containing 94 (Ccdc94), mRNA [NM_001109673]	**11,610**
LOC689996	Uncharacterized protein [Source:UniProtKB/TrEMBL;Acc:D3Z801] [ENSRNOT00000058976]	**11,610**
Il6r	Rattus norvegicus interleukin 6 receptor (Il6r), mRNA [NM_017020]	**11,609**
0	Unknown	**11,608**
LOC502876	PREDICTED: Rattus norvegicus similar to protein phosphatase 1, regulatory subunit 15B (LOC502876), miscRNA [XR_086238]	**11,608**
Ttf1	Similar to RNA polymerase I transcription termination factor 1 (Predicted), isoform CRA_bUncharacterized protein [Source:UniProtKB/TrEMBL;Acc:D4A1Z7] [ENSRNOT00000061029]	**11,607**
Pdzrn3	PREDICTED: Rattus norvegicus PDZ domain containing RING finger 3 (Pdzrn3), mRNA [XM_232226]	**11,607**
Zdhhc17	Rattus norvegicus zinc finger, DHHC-type containing 17 (Zdhhc17), mRNA [NM_001039340]	**11,603**
0	Uncharacterized protein [Source:UniProtKB/TrEMBL;Acc:D3ZKV4] [ENSRNOT00000022344]	**11,603**
Olr1720	Rattus norvegicus olfactory receptor 1720 (Olr1720), mRNA [NM_001001116]	**11,602**
RGD1561916	Uncharacterized protein [Source:UniProtKB/TrEMBL;Acc:D3ZGG6] [ENSRNOT00000017903]	**11,597**
Pqlc1	Rattus norvegicus PQ loop repeat containing 1 (Pqlc1), mRNA [NM_001013189]	**11,596**
0	Unknown	**11,593**
Napg	Rattus norvegicus N-ethylmaleimide-sensitive factor attachment protein, gamma (Napg), mRNA [NM_001107384]	**11,592**
Arhgap23	PREDICTED: Rattus norvegicus Rho GTPase activating protein 23 (Arhgap23), mRNA [XM_001081375]	**11,590**
RGD1305254	Uncharacterized protein [Source:UniProtKB/TrEMBL;Acc:D3ZWI0] [ENSRNOT00000036343]	**11,589**
LOC497978	Rattus norvegicus similar to diacylglycerol kinase epsilon (LOC497978), mRNA [NM_001039341]	**11,588**

N4bp2l2	Rattus norvegicus NEDD4 binding protein 2-like 2 (N4bp2l2), mRNA [NM_001005533]	**11,587**
Cltc	Rattus norvegicus clathrin, heavy chain (Hc) (Cltc), mRNA [NM_019299]	**11,587**
0	Uncharacterized protein [Source:UniProtKB/TrEMBL;Acc:D3ZKR7] [ENSRNOT00000046075]	**11,587**
Hdhd2	Rattus norvegicus haloacid dehalogenase-like hydrolase domain containing 2 (Hdhd2), mRNA [NM_001014151]	**11,585**
Psgb1	Rattus norvegicus pregnancy-specific beta 1-glycoprotein (Psgb1), mRNA [NM_021677]	**11,583**
Mzf1	Rattus norvegicus myeloid zinc finger 1 (Mzf1), mRNA [NM_001108470]	**11,582**
Commd9	Rattus norvegicus COMM domain containing 9 (Commd9), mRNA [NM_001033692]	**11,582**
RGD1563070	Rattus norvegicus similar to hypothetical protein (RGD1563070), mRNA [NM_001134541]	**11,582**
0	Rattus norvegicus similar to glyceraldehyde-3-phosphate dehydrogenase (LOC305194), mRNA [XM_223212]	**11,581**
Cdkl3	Rattus norvegicus cyclin-dependent kinase-like 3 (Cdkl3), transcript variant 2, mRNA [NM_021772]	**11,580**
Hrh3	Rattus norvegicus histamine receptor H3 (Hrh3), mRNA [NM_053506]	**11,580**
LOC501089	PREDICTED: Rattus norvegicus similar to Discs large homolog 5 (Placenta and prostate DLG) (Discs large protein P-dlg) (LOC501089), mRNA [XM_576504]	**11,579**
Gnaz	Rattus norvegicus guanine nucleotide binding protein (G protein), alpha z polypeptide (Gnaz), mRNA [NM_013189]	**11,576**
Vamp4	Rattus norvegicus vesicle-associated membrane protein 4 (Vamp4), mRNA [NM_001108856]	**11,575**
Olr741	Rattus norvegicus olfactory receptor 741 (Olr741), mRNA [NM_001000575]	**11,575**
Yes1	Rattus norvegicus Yamaguchi sarcoma viral (v-yes) oncogene homolog 1 (Yes1), mRNA [NM_033298]	**11,574**
0	Uncharacterized protein [Source:UniProtKB/TrEMBL;Acc:D3ZJU2] [ENSRNOT00000004966]	**11,571**
Agt	Rattus norvegicus angiotensinogen (serpin peptidase inhibitor, clade A, member 8) (Agt), mRNA [NM_134432]	**11,570**
RGD1564224	PREDICTED: Rattus norvegicus similar to hypothetical protein MGC18079 (RGD1564224), mRNA [XM_577106]	**11,570**
RGD1306682	Rattus norvegicus similar to RIKEN cDNA 1810046J19 (RGD1306682), mRNA [NM_001108296]	**11,569**
Rnf2	Rattus norvegicus ring finger protein 2 (Rnf2), mRNA [NM_001025667]	**11,567**
0	Unknown	**11,566**
RGD1306282	PREDICTED: Rattus norvegicus similar to RIKEN cDNA 4432406C05 (RGD1306282), mRNA [XM_235171]	**11,565**
Nrip3	Rattus norvegicus nuclear receptor interacting protein 3 (Nrip3), mRNA [NM_001108498]	**11,559**
Tmem218	Rattus norvegicus transmembrane protein 218 (Tmem218), mRNA [NM_001008325]	**11,556**
Numbl	Rattus norvegicus numb homolog (Drosophila)-like (Numbl), mRNA [NM_001033888]	**11,554**
0	Unknown	**11,554**
0	BC012801 gamma tubulin ring complex protein (76p gene) {Homo sapiens} (exp=-1; wgp=0; cg=0), partial (7%) [TC627508]	**11,553**
Vsig8	Rattus norvegicus V-set and immunoglobulin domain containing 8 (Vsig8), mRNA [NM_001105972]	**11,552**
0	Unknown	**11,551**
Usp8	Rattus norvegicus ubiquitin specific peptidase 8 (Usp8), mRNA [NM_001106502]	**11,549**
RGD1310727	Rattus norvegicus LOC363070 (RGD1310727), mRNA [NM_001108764]	**11,549**
Alpi	Rattus norvegicus alkaline phosphatase, intestinal (Alpi), mRNA [NM_022665]	**11,548**

0	Unknown	**11,547**
Rab9b	Rattus norvegicus RAB9B, member RAS oncogene family (Rab9b), mRNA [NM_001109018]	**11,547**
0	Unknown	**11,545**
Phex	Rattus norvegicus phosphate regulating endopeptidase homolog, X-linked (Phex), mRNA [NM_013004]	**11,544**
Brcc3	Rattus norvegicus BRCA1/BRCA2-containing complex, subunit 3 (Brcc3), mRNA [NM_001127300]	**11,543**
Rundc1	PREDICTED: Rattus norvegicus RUN domain containing 1 (Rundc1), mRNA [XM_001081465]	**11,541**
Olr1347	Rattus norvegicus olfactory receptor 1347 (Olr1347), mRNA [NM_001001121]	**11,540**
Rpl10a	Rattus norvegicus ribosomal protein L10A (Rpl10a), mRNA [NM_031065]	**11,540**
Olr1439	Rattus norvegicus olfactory receptor 1439 (Olr1439), mRNA [NM_001000016]	**11,539**
Txndc16	Uncharacterized protein [Source:UniProtKB/TrEMBL;Acc:D3ZQK4] [ENSRNOT00000008372]	**11,538**
RGD1565752	Uncharacterized protein [Source:UniProtKB/TrEMBL;Acc:D3ZE25] [ENSRNOT00000058376]	**11,535**
0	Unknown	**11,532**
0	Unknown	**11,530**
Bnc1	Rattus norvegicus basonuclin 1 (Bnc1), mRNA [NM_001108916]	**11,529**
RGD1308626	Rattus norvegicus similar to 9630044O09Rik protein (RGD1308626), mRNA [NM_001014139]	**11,529**
Zfp365	Rattus norvegicus zinc finger protein 365 (Zfp365), mRNA [NM_001025145]	**11,528**
0	Rattus norvegicus similar to dJ1070B1.1 (similar to KIAA0316 and KIAA0967 proteins) (LOC315916), mRNA [XM_236515]	**11,528**
RGD1307365	PREDICTED: Rattus norvegicus similar to KIAA1009 protein (RGD1307365), mRNA [XM_001054857]	**11,525**
Cacna1b	Rattus norvegicus calcium channel, voltage-dependent, N type, alpha 1B subunit (Cacna1b), transcript variant 1, mRNA [NM_001195199]	**11,523**
Plod3	Rattus norvegicus procollagen-lysine, 2-oxoglutarate 5-dioxygenase 3 (Plod3), mRNA [NM_178101]	**11,520**
Cnih	Rattus norvegicus cornichon homolog (Drosophila) (Cnih), mRNA [NM_001106029]	**11,519**
Wnt9b	Rattus norvegicus wingless-type MMTV integration site family, member 9B (Wnt9b), mRNA [NM_001107055]	**11,517**
Myl9	Rattus norvegicus myosin, light chain 9, regulatory (Myl9), mRNA [NM_001100885]	**11,517**
Oaz2	Rattus norvegicus ornithine decarboxylase antizyme 2 (Oaz2), mRNA [NM_001109899]	**11,517**
Rpl36a	Rattus norvegicus ribosomal protein L36a (Rpl36a), mRNA [NM_001128065]	**11,512**
Ctrb1	Rattus norvegicus chymotrypsinogen B1 (Ctrb1), mRNA [NM_012536]	**11,511**
Dgcr2	Rattus norvegicus DiGeorge syndrome critical region gene 2 (Dgcr2), mRNA [NM_001012146]	**11,510**
Ppm1b	Rattus norvegicus protein phosphatase 1B, magnesium dependent, beta isoform (Ppm1b), mRNA [NM_033096]	**11,509**
Rnase1l1	Rattus norvegicus ribonuclease, RNase A family, 1-like 1 (pancreatic) (Rnase1l1), mRNA [NM_001013232]	**11,508**
Akap10	Rattus norvegicus A kinase (PRKA) anchor protein 10 (Akap10), nuclear gene encoding mitochondrial protein, mRNA [NM_001114606]	**11,508**
Rbmx2	Rattus norvegicus RNA binding motif protein, X-linked 2 (Rbmx2), mRNA [NM_001113786]	**11,507**
Atp11b	Atp11b protein [Source:UniProtKB/TrEMBL;Acc:Q5RJS7] [ENSRNOT00000016961]	**11,506**
Epha4	Rattus norvegicus Eph receptor A4 (Epha4), mRNA [NM_001162411]	**11,504**
0	Unknown	**11,504**

0	A disintegrin-like and metalloprotease (Reprolysin type) with thrombospondin type 1 motif, 6 (Predicted), isoform CRA_aUncharacterized protein [Source:UniProtKB/TrEMBL;Acc:D3ZZF9] [ENSRNOT00000035876]	**11,501**
RGD1309707	Rattus norvegicus similar to RIKEN cDNA 4930431E10 (RGD1309707), mRNA [NM_001109598]	**11,501**
Tmem38b	Rattus norvegicus transmembrane protein 38B (Tmem38b), mRNA [NM_001014191]	**11,500**
Purb	Rattus norvegicus purine rich element binding protein B (Purb), mRNA [NM_001017503]	**11,499**
LOC100125385	Rattus norvegicus hypothetical protein LOC100125385 (LOC100125385), mRNA [NM_001103364]	**11,498**
0	Unknown	**11,496**
Mrpl35	Rattus norvegicus mitochondrial ribosomal protein L35 (Mrpl35), nuclear gene encoding mitochondrial protein, mRNA [NM_001106596]	**11,494**
H6pd	Rattus norvegicus hexose-6-phosphate dehydrogenase (glucose 1-dehydrogenase) (H6pd), mRNA [NM_001106698]	**11,493**
Apc2	Rattus norvegicus adenomatosis polyposis coli 2 (Apc2), mRNA [NM_001106769]	**11,492**
Adra2a	Rattus norvegicus adrenergic, alpha-2A-, receptor (Adra2a), mRNA [NM_012739]	**11,492**
Olr1459	Rattus norvegicus olfactory receptor 1459 (Olr1459), mRNA [NM_001000770]	**11,491**
0	Unknown	**11,489**
Ascc2	Rattus norvegicus activating signal cointegrator 1 complex subunit 2 (Ascc2), mRNA [NM_001109091]	**11,486**
0	Rattus norvegicus similar to mKIAA1511 protein (LOC298457), mRNA [XM_233446]	**11,484**
Suv420h1	Rattus norvegicus suppressor of variegation 4-20 homolog 1 (Drosophila) (Suv420h1), mRNA [NM_001108512]	**11,483**
Rnf151	Rattus norvegicus ring finger protein 151 (Rnf151), mRNA [NM_001106987]	**11,483**
Acads	Rattus norvegicus acyl-Coenzyme A dehydrogenase, C-2 to C-3 short chain (Acads), nuclear gene encoding mitochondrial protein, mRNA [NM_022512]	**11,482**
Dom3z	Rattus norvegicus DOM-3 homolog Z (C. elegans) (Dom3z), mRNA [NM_212497]	**11,481**
Ndufaf4	Rattus norvegicus NADH dehydrogenase (ubiquinone) 1 alpha subcomplex, assembly factor 4 (Ndufaf4), nuclear gene encoding mitochondrial protein, mRNA [NM_198783]	**11,481**
Dlat	Rattus norvegicus dihydrolipoamide S-acetyltransferase (Dlat), nuclear gene encoding mitochondrial protein, mRNA [NM_031025]	**11,478**
Doc2g	Rattus norvegicus double C2, gamma (Doc2g), mRNA [NM_001011937]	**11,477**
0	Unknown	**11,476**
Cyp4v3	Rattus norvegicus cytochrome P450, family 4, subfamily v, polypeptide 3 (Cyp4v3), mRNA [NM_001135600]	**11,476**
0	Rattus norvegicus similar to glyceraldehyde-3-phosphate dehydrogenase (phosphorylating) (EC 1.2.1.12) - mouse (LOC293386), mRNA [XM_219208]	**11,475**
Tlcd1	Rattus norvegicus TLC domain containing 1 (Tlcd1), mRNA [NM_001013858]	**11,474**
Has1	Rattus norvegicus hyaluronan synthase 1 (Has1), mRNA [NM_172323]	**11,470**
ST7	Rattus norvegicus suppression of tumorigenicity 7 (ST7), transcript variant 1, mRNA [NM_001004102]	**11,467**
Tmtc4	Rattus norvegicus transmembrane and tetratricopeptide repeat containing 4 (Tmtc4), mRNA [NM_001134414]	**11,466**
Tstd2	Rattus norvegicus thiosulfate sulfurtransferase (rhodanese)-like domain containing 2 (Tstd2), mRNA [NM_001108663]	**11,462**
Tmie	Rattus norvegicus transmembrane inner ear (Tmie), mRNA [NM_001109299]	**11,462**

Git2	Rattus norvegicus G protein-coupled receptor kinase interacting ArfGAP 2 (Git2), mRNA [NM_001005553]	**11,462**
Olr337	Rattus norvegicus olfactory receptor 337 (Olr337), mRNA [NM_001000507]	**11,462**
Fam55b	Rattus norvegicus family with sequence similarity 55, member B (Fam55b), mRNA [NM_001025055]	**11,460**
0	TOP1_RAT (Q9WUL0) DNA topoisomerase 1 (DNA topoisomerase I) , complete [TC575805]	**11,459**
0	Rattus norvegicus similar to glyceraldehyde-3-phosphate dehydrogenase (phosphorylating) (EC 1.2.1.12) - mouse (LOC315007), mRNA [XM_235346]	**11,459**
0	PREDICTED: Rattus norvegicus similar to glyceraldehyde-3-phosphate dehydrogenase (RGD1565238), partial mRNA [XM_001081651]	**11,455**
LOC687553	PREDICTED: Rattus norvegicus similar to Forkhead box protein L1 (Forkhead-related protein FKHL11) (Transcription factor FKH-6) (LOC687553), mRNA [XM_001079078]	**11,453**
Lrp1	Rattus norvegicus low density lipoprotein-related protein 1 (alpha-2-macroglobulin receptor) (Lrp1), mRNA [NM_001130490]	**11,453**
Rps6ka5	Rattus norvegicus ribosomal protein S6 kinase, polypeptide 5 (Rps6ka5), mRNA [NM_001108048]	**11,452**
Syt6	Rattus norvegicus synaptotagmin VI (Syt6), mRNA [NM_022191]	**11,451**
LOC686921	PREDICTED: Rattus norvegicus hypothetical protein LOC686921 (LOC686921), mRNA [XM_001076338]	**11,451**
Rft1	Rattus norvegicus RFT1 homolog (S. cerevisiae) (Rft1), mRNA [NM_001135866]	**11,451**
Sbsn	Rattus norvegicus suprabasin (Sbsn), transcript variant 2, mRNA [NM_001044231]	**11,450**
Orc5l	Rattus norvegicus origin recognition complex, subunit 5-like (yeast) (Orc5l), mRNA [NM_001014186]	**11,449**
Hk2	Rattus norvegicus hexokinase 2 (Hk2), mRNA [NM_012735]	**11,449**
Tcf20	Rattus norvegicus transcription factor 20 (Tcf20), mRNA [NM_001130574]	**11,447**
Tmem169	Rattus norvegicus transmembrane protein 169 (Tmem169), mRNA [NM_001109574]	**11,445**
Bnip3l	Rattus norvegicus BCL2/adenovirus E1B interacting protein 3-like (Bnip3l), mRNA [NM_080888]	**11,444**
Cdgap	Rattus norvegicus Cdc42 GTPase-activating protein (Cdgap), mRNA [NM_001105879]	**11,444**
0	PREDICTED: Rattus norvegicus polymerase (DNA directed) nu (Poln), miscRNA [XR_085614]	**11,443**
Igfbp1	Rattus norvegicus insulin-like growth factor binding protein 1 (Igfbp1), mRNA [NM_013144]	**11,441**
0	Unknown	**11,440**
Zfp410	Rattus norvegicus zinc finger protein 410 (Zfp410), mRNA [NM_001108042]	**11,440**
Prkrip1	Rattus norvegicus Prkr interacting protein 1 (IL11 inducible) (Prkrip1), mRNA [NM_001098793]	**11,437**
0	Unknown	**11,435**
0	Unknown	**11,434**
Tle4	Rattus norvegicus transducin-like enhancer of split 4 (E(sp1) homolog, Drosophila) (Tle4), mRNA [NM_019141]	**11,434**
Olr1519	Rattus norvegicus olfactory receptor 1519 (Olr1519), mRNA [NM_001000037]	**11,433**
0	Rattus norvegicus unknown protein mRNA, partial cds. [U89745]	**11,433**
Ptms	Rattus norvegicus parathymosin (Ptms), mRNA [NM_031975]	**11,433**
Yap1	Rattus norvegicus yes-associated protein 1 (Yap1), mRNA [NM_001034002]	**11,432**
0	Unknown	**11,432**

0	Unknown	**11,431**
Rragc	Rattus norvegicus Ras-related GTP binding C (Rragc), mRNA [NM_001048184]	**11,430**
0	Unknown	**11,428**
LOC689442	PREDICTED: Rattus norvegicus hypothetical protein LOC689442 (LOC689442), mRNA [XM_001070806]	**11,427**
Gfap	Rattus norvegicus glial fibrillary acidic protein (Gfap), mRNA [NM_017009]	**11,421**
LOC681994	PREDICTED: Rattus norvegicus similar to developmental endothelial locus-1 isoform b, transcript variant 3 (LOC681994), mRNA [XM_001057753]	**11,420**
0	Unknown	**11,419**
Arhgef3	Rattus norvegicus Rho guanine nucleotide exchange factor (GEF) 3 (Arhgef3), mRNA [NM_001106061]	**11,418**
Leng8	Rattus norvegicus leukocyte receptor cluster (LRC) member 8 (Leng8), mRNA [NM_001037790]	**11,415**
Rftn1	Rattus norvegicus raftlin lipid raft linker 1 (Rftn1), mRNA [NM_001135011]	**11,415**
RGD1305704	PREDICTED: Rattus norvegicus similar to c-Mpl binding protein (RGD1305704), miscRNA [XR_006033]	**11,415**
LOC500077	Rattus norvegicus similar to RIKEN cDNA 3110062M04 (LOC500077), mRNA [NM_001024325]	**11,414**
Pfkfb3	Rattus norvegicus 6-phosphofructo-2-kinase/fructose-2,6-biphosphatase 3 (Pfkfb3), mRNA [NM_057135]	**11,411**
Gabra5	Rattus norvegicus gamma-aminobutyric acid (GABA) A receptor, alpha 5 (Gabra5), mRNA [NM_017295]	**11,411**
Apc2	Rattus norvegicus adenomatosis polyposis coli 2 (Apc2), mRNA [NM_001106769]	**11,407**
LOC690402	Rattus norvegicus similar to Short palate, lung and nasal epithelium carcinoma-associated protein 3 homolog precursor (LOC690402), mRNA [NM_001109588]	**11,407**
Sdhc	Rattus norvegicus succinate dehydrogenase complex, subunit C, integral membrane protein (Sdhc), nuclear gene encoding mitochondrial protein, mRNA [NM_001005534]	**11,403**
St3gal5	Rattus norvegicus ST3 beta-galactoside alpha-2,3-sialyltransferase 5 (St3gal5), mRNA [NM_031337]	**11,401**
0	Rattus norvegicus similar to glyceraldehyde-3-phosphate dehydrogenase (LOC311121), mRNA [XM_230014]	**11,400**
0	Rattus norvegicus TL0ADA6YC10 mRNA sequence. [FQ228611]	**11,399**
Prkci	Rattus norvegicus protein kinase C, iota (Prkci), mRNA [NM_032059]	**11,399**
Trerf1	Rattus norvegicus transcriptional regulating factor 1 (Trerf1), mRNA [NM_001108199]	**11,398**
Tmem51	Rattus norvegicus transmembrane protein 51 (Tmem51), mRNA [NM_001109273]	**11,398**
Cytsa	Rattus norvegicus cytospin A (Cytsa), mRNA [NM_001039455]	**11,396**
Epha3	Rattus norvegicus Eph receptor A3 (Epha3), mRNA [NM_031564]	**11,395**
Ppp1r1b	Rattus norvegicus protein phosphatase 1, regulatory (inhibitor) subunit 1B (Ppp1r1b), mRNA [NM_138521]	**11,395**
Olr1248	Rattus norvegicus olfactory receptor 1248 (Olr1248), mRNA [NM_001000452]	**11,395**
LOC690096	PREDICTED: Rattus norvegicus similar to ribosomal protein L28 (LOC690096), mRNA [XM_001073240]	**11,395**
RGD1307830	Uncharacterized protein [Source:UniProtKB/TrEMBL;Acc:D3ZN01] [ENSRNOT00000059984]	**11,393**
Gpr176	Probable G-protein coupled receptor 176 [Source:UniProtKB/Swiss-Prot;Acc:Q64017] [ENSRNOT00000007882]	**11,392**
Syt13	Rattus norvegicus synaptotagmin XIII (Syt13), mRNA [NM_030839]	**11,391**
Mrps36	Rattus norvegicus mitochondrial ribosomal protein S36 (Mrps36), nuclear gene encoding mitochondrial protein, mRNA [NM_001191605]	**11,389**

Phkb	Rattus norvegicus phosphorylase kinase, beta (Phkb), mRNA [NM_001014152]	**11,389**
Pcdhb18	PREDICTED: Rattus norvegicus protocadherin beta 18 (Pcdhb18), mRNA [XM_001055991]	**11,389**
Tcte4	Rattus norvegicus t-complex-associated testis expressed 4 (Tcte4), mRNA [NM_001166303]	**11,388**
Olr132	Rattus norvegicus olfactory receptor 132 (Olr132), mRNA [NM_001001273]	**11,388**
0	DNA-binding protein A [Source:UniProtKB/Swiss-Prot;Acc:Q62764] [ENSRNOT00000007347]	**11,385**
RGD1559979	PREDICTED: Rattus norvegicus similar to APH1B homolog (C.elegans) (RGD1559979), mRNA [XM_001081687]	**11,384**
Ghdc	Rattus norvegicus GH3 domain containing (Ghdc), mRNA [NM_001191651]	**11,384**
0	Unknown	**11,382**
Nhlrc3	Uncharacterized protein [Source:UniProtKB/TrEMBL;Acc:D4A2F6] [ENSRNOT00000014562]	**11,382**
Tmed7	Rattus norvegicus transmembrane emp24 protein transport domain containing 7 (Tmed7), mRNA [NM_001105758]	**11,381**
Akr1a1	Rattus norvegicus aldo-keto reductase family 1, member A1 (aldehyde reductase) (Akr1a1), mRNA [NM_031000]	**11,381**
Nos3	Rattus norvegicus nitric oxide synthase 3, endothelial cell (Nos3), mRNA [NM_021838]	**11,378**
Blzf1	Rattus norvegicus basic leucine zipper nuclear factor 1 (Blzf1), mRNA [NM_001017494]	**11,378**
Bbs1	Rattus norvegicus Bardet-Biedl syndrome 1 (Bbs1), mRNA [NM_001107569]	**11,376**
0	Unknown	**11,375**
Slc19a2	Rattus norvegicus solute carrier family 19 (thiamine transporter), member 2 (Slc19a2), mRNA [NM_001030024]	**11,374**
Zdhhc24	Rattus norvegicus zinc finger, DHHC-type containing 24 (Zdhhc24), mRNA [NM_001039100]	**11,374**
Gk5	Uncharacterized protein [Source:UniProtKB/TrEMBL;Acc:D3ZN47] [ENSRNOT00000067835]	**11,374**
RGD1564419	Rattus norvegicus similar to hypothetical gene supported by BC025338 (RGD1564419), mRNA [NM_001109234]	**11,373**
Pdgfb	Rattus norvegicus platelet-derived growth factor beta polypeptide (simian sarcoma viral (v-sis) oncogene homolog) (Pdgfb), mRNA [NM_031524]	**11,373**
Rbp1	Rattus norvegicus retinol binding protein 1, cellular (Rbp1), mRNA [NM_012733]	**11,367**
0	BC052076 expressed sequence AW050020 {Mus musculus} (exp=-1; wgp=0; cg=0), partial (32%) [TC589792]	**11,366**
Osbpl1a	Rattus norvegicus oxysterol binding protein-like 1A (Osbpl1a), mRNA [NM_172023]	**11,365**
Zg16	Rattus norvegicus zymogen granule protein 16 (Zg16), mRNA [NM_134409]	**11,365**
Rps19bp1	Rattus norvegicus ribosomal protein S19 binding protein 1 (Rps19bp1), mRNA [NM_001130578]	**11,365**
Osbpl6	Rattus norvegicus oxysterol binding protein-like 6 (Osbpl6), mRNA [NM_001107735]	**11,364**
Eif4h	Rattus norvegicus eukaryotic translation initiation factor 4H (Eif4h), mRNA [NM_001006957]	**11,364**
Ptbp2	Rattus norvegicus polypyrimidine tract binding protein 2 (Ptbp2), mRNA [NM_001005555]	**11,361**
Atp4b	Rattus norvegicus ATPase, H+/K+ exchanging, beta polypeptide (Atp4b), mRNA [NM_012510]	**11,361**
Olr1401	Rattus norvegicus olfactory receptor 1401 (Olr1401), mRNA [NM_001000003]	**11,359**
Rexo4	Rattus norvegicus REX4, RNA exonuclease 4 homolog (S. cerevisiae) (Rexo4), mRNA [NM_001033884]	**11,357**
Slc25a28	Rattus norvegicus solute carrier family 25, member 28 (Slc25a28), mRNA [NM_001109515]	**11,352**
RGD621352	Rattus norvegicus similar to RIKEN cDNA 1500031L02 (RGD621352), mRNA [NM_138865]	**11,351**
Eml1	Rattus norvegicus echinoderm microtubule associated protein like 1 (Eml1), mRNA [NM_001025741]	**11,349**

0	Unknown	**11,347**
Adam22	PREDICTED: Rattus norvegicus a disintegrin and metalloprotease domain (ADAM) 22 (Adam22), mRNA [XM_002726324]	**11,346**
Nop56	Rattus norvegicus NOP56 ribonucleoprotein homolog (yeast) (Nop56), mRNA [NM_001025732]	**11,346**
Ager	Rattus norvegicus advanced glycosylation end product-specific receptor (Ager), mRNA [NM_053336]	**11,346**
RGD1309730	Rattus norvegicus similar to RIKEN cDNA B230118H07 (RGD1309730), mRNA [NM_001106491]	**11,345**
Olr425	Rattus norvegicus olfactory receptor 425 (Olr425), mRNA [NM_001000393]	**11,342**
Olr1280	Rattus norvegicus olfactory receptor 1280 (Olr1280), mRNA [NM_001000517]	**11,342**
Cspg5	Rattus norvegicus chondroitin sulfate proteoglycan 5 (Cspg5), transcript variant 1, mRNA [NM_019284]	**11,340**
Prnp	Rattus norvegicus prion protein (Prnp), mRNA [NM_012631]	**11,337**
Neo1	PREDICTED: Rattus norvegicus neogenin homolog 1 (chicken) (Neo1), mRNA [XM_001074913]	**11,336**
Vash1	Uncharacterized protein [Source:UniProtKB/TrEMBL;Acc:D4AE85] [ENSRNOT00000014017]	**11,331**
Scgb1c1	Rattus norvegicus secretoglobin, family 1C, member 1 (Scgb1c1), mRNA [NM_001107561]	**11,325**
Dsel	RCG24512Uncharacterized protein [Source:UniProtKB/TrEMBL;Acc:D3ZYE3] [ENSRNOT00000043890]	**11,325**
0	Unknown	**11,323**
Sptbn1	Rattus norvegicus spectrin, beta, non-erythrocytic 1 (Sptbn1), mRNA [NM_001013130]	**11,322**
0	Rattus norvegicus similar to KIAA0856 protein (LOC315676), mRNA [XM_236249]	**11,322**
Hbg1	Rattus norvegicus hemoglobin, gamma A (Hbg1), mRNA [NM_172093]	**11,322**
Gna11	Rattus norvegicus guanine nucleotide binding protein, alpha 11 (Gna11), mRNA [NM_031033]	**11,321**
0	Q3UKK2_MOUSE (Q3UKK2) 15 days pregnant adult female placenta cDNA, RIKEN full-length enriched library, clone:I530020K06 product:pregnancy-specific glycoprotein 30, full insert sequence, partial (9%) [TC612990]	**11,321**
0	Tudor domain-containing protein PHF20L1 [Source:UniProtKB/Swiss-Prot;Acc:Q4V9H5] [ENSRNOT00000007564]	**11,320**
Xpc	Rattus norvegicus xeroderma pigmentosum, complementation group C (Xpc), mRNA [NM_001107874]	**11,320**
She	PREDICTED: Rattus norvegicus Src homology 2 domain containing E (She), mRNA [XM_001062249]	**11,320**
Scyl3	Rattus norvegicus SCY1-like 3 (S. cerevisiae) (Scyl3), mRNA [NM_001191828]	**11,320**
Dnajb4	Rattus norvegicus DnaJ (Hsp40) homolog, subfamily B, member 4 (Dnajb4), mRNA [NM_001013076]	**11,319**
Rps26	Rattus norvegicus ribosomal protein S26 (Rps26), mRNA [NM_013224]	**11,317**
0	Unknown	**11,317**
Pnrc2	Rattus norvegicus proline-rich nuclear receptor coactivator 2 (Pnrc2), mRNA [NM_001103360]	**11,317**
0	Rattus norvegicus similar to glyceraldehyde-3-phosphate dehydrogenase (phosphorylating) (EC 1.2.1.12) - mouse (LOC301496), mRNA [XM_237274]	**11,315**
Rpl15	Rattus norvegicus ribosomal protein L15 (Rpl15), mRNA [NM_139114]	**11,315**
Diaph2	PREDICTED: Rattus norvegicus diaphanous homolog 2 (Drosophila) (Diaph2), mRNA [XM_001066898]	**11,312**
Heatr6	Rattus norvegicus HEAT repeat containing 6 (Heatr6), mRNA [NM_001079897]	**11,312**
0	Unknown	**11,311**

Kcnh8	Rattus norvegicus potassium voltage-gated channel, subfamily H (eag-related), member 8 (Kcnh8), mRNA [NM_145095]	**11,310**
0	Unknown	**11,309**
0	AI407942 EST236232 Normalized rat ovary, Bento Soares Rattus sp. cDNA clone ROVEE68 3' end, mRNA sequence [AI407942]	**11,308**
0	Unknown	**11,308**
LOC690415	Rattus norvegicus hypothetical protein LOC690415 (LOC690415), mRNA [NM_001109590]	**11,308**
RGD1559731	Uncharacterized protein [Source:UniProtKB/TrEMBL;Acc:D3ZCG1] [ENSRNOT00000005659]	**11,308**
Wdr13	Rattus norvegicus WD repeat domain 13 (Wdr13), mRNA [NM_001108247]	**11,308**
Bet1l	Rattus norvegicus blocked early in transport 1 homolog (S. cerevisiae) like (Bet1l), mRNA [NM_019368]	**11,307**
Cyth2	Rattus norvegicus cytohesin 2 (Cyth2), mRNA [NM_053911]	**11,305**
RGD1310899	Rattus norvegicus similar to CGI-35 protein (RGD1310899), mRNA [NM_001044235]	**11,305**
0	Uncharacterized protein [Source:UniProtKB/TrEMBL;Acc:D3ZL75] [ENSRNOT00000022583]	**11,304**
0	Unknown	**11,303**
0	Uncharacterized protein [Source:UniProtKB/TrEMBL;Acc:D3ZD60] [ENSRNOT00000038336]	**11,303**
0	Uncharacterized protein [Source:UniProtKB/TrEMBL;Acc:D3ZXZ9] [ENSRNOT00000020215]	**11,302**
0	Unknown	**11,302**
Klhl14	Rattus norvegicus kelch-like 14 (Drosophila) (Klhl14), mRNA [NM_001108885]	**11,302**
Pmm2	Rattus norvegicus phosphomannomutase 2 (Pmm2), mRNA [NM_001106973]	**11,301**
Tmem115	Rattus norvegicus transmembrane protein 115 (Tmem115), mRNA [NM_001108779]	**11,301**
Tnfsf8	PREDICTED: Rattus norvegicus tumor necrosis factor (ligand) superfamily, member 8 (Tnfsf8), mRNA [XM_001064723]	**11,301**
Btbd6	Rattus norvegicus BTB (POZ) domain containing 6 (Btbd6), mRNA [NM_001077683]	**11,301**
Me1	Rattus norvegicus malic enzyme 1, NADP(+)-dependent, cytosolic (Me1), mRNA [NM_012600]	**11,301**
Alkbh4	Rattus norvegicus alkB, alkylation repair homolog 4 (E. coli) (Alkbh4), mRNA [NM_001105920]	**11,301**
Rasal2	Rattus norvegicus RAS protein activator like 2 (Rasal2), mRNA [NM_001107188]	**11,298**
0	Uncharacterized protein [Source:UniProtKB/TrEMBL;Acc:D4ACN9] [ENSRNOT00000037120]	**11,298**
Ttyh2	PREDICTED: Rattus norvegicus tweety homolog 2 (Drosophila) (Ttyh2), mRNA [XM_001081641]	**11,297**
Mtmr2	Rattus norvegicus myotubularin related protein 2 (Mtmr2), mRNA [NM_001108123]	**11,297**
RGD1309808	Rattus norvegicus similar to apolipoprotein L2; apolipoprotein L-II (RGD1309808), mRNA [NM_001134801]	**11,296**
Crkrs	Rattus norvegicus Cdc2-related kinase, arginine/serine-rich (Crkrs), transcript variant 2, mRNA [NM_138916]	**11,295**
Fgfr2	Rattus norvegicus fibroblast growth factor receptor 2 (Fgfr2), transcript variant a, mRNA [NM_012712]	**11,294**
Kdsr	Rattus norvegicus 3-ketodihydrosphingosine reductase (Kdsr), mRNA [NM_001108342]	**11,294**
Eif5a2	Rattus norvegicus eukaryotic translation initiation factor 5A2 (Eif5a2), mRNA [NM_001100697]	**11,294**
Egr3	Rattus norvegicus early growth response 3 (Egr3), mRNA [NM_017086]	**11,292**
Bles03	Rattus norvegicus basophilic leukemia expressed protein BLES03 (Bles03), transcript variant 1, mRNA [NM_001024233]	**11,292**
Marveld1	Rattus norvegicus MARVEL domain containing 1 (Marveld1), mRNA [NM_001107590]	**11,291**

Rcor2	Rattus norvegicus REST corepressor 2 (Rcor2), mRNA [NM_001013994]	**11,290**
Mtmr12	Rattus norvegicus myotubularin related protein 12 (Mtmr12), mRNA [NM_001012077]	**11,287**
RGD1309492	Rattus norvegicus similar to mKIAA1737 protein (RGD1309492), mRNA [NM_001108044]	**11,287**
St13	Rattus norvegicus suppression of tumorigenicity 13 (St13), mRNA [NM_031122]	**11,287**
Matn2	PREDICTED: Rattus norvegicus matrilin 2 (Matn2), mRNA [XM_216941]	**11,285**
LOC100360737	PREDICTED: Rattus norvegicus rCG54380-like (LOC100360737), mRNA [XM_002725537]	**11,282**
0	Unknown	**11,282**
0	Unknown	**11,282**
0	Unknown	**11,282**
Stat1	Rattus norvegicus signal transducer and activator of transcription 1 (Stat1), transcript variant alpha, mRNA [NM_032612]	**11,281**
Vamp7	Rattus norvegicus vesicle-associated membrane protein 7 (Vamp7), mRNA [NM_053531]	**11,278**
Gtf3c4	Rattus norvegicus general transcription factor IIIC, polypeptide 4 (Gtf3c4), mRNA [NM_001109473]	**11,277**
Bgn	Rattus norvegicus biglycan (Bgn), mRNA [NM_017087]	**11,277**
Ndufv3	Rattus norvegicus NADH dehydrogenase (ubiquinone) flavoprotein 3 (Ndufv3), nuclear gene encoding mitochondrial protein, transcript variant 1, mRNA [NM_022607]	**11,274**
Strc	Uncharacterized protein [Source:UniProtKB/TrEMBL;Acc:D3ZUE8] [ENSRNOT00000020130]	**11,274**
Tcfap2d	Rattus norvegicus transcription factor AP-2, delta (Tcfap2d), mRNA [NM_001106895]	**11,272**
RGD1309139	Rattus norvegicus similar to CG5435-PA (RGD1309139), mRNA [NM_001134578]	**11,269**
Coq4	Rattus norvegicus coenzyme Q4 homolog (S. cerevisiae) (Coq4), nuclear gene encoding mitochondrial protein, mRNA [NM_001031662]	**11,265**
RGD1311892	Rattus norvegicus similar to hypothetical protein FLJ10901 (RGD1311892), mRNA [NM_001134502]	**11,264**
Dnhd1	PREDICTED: Rattus norvegicus dynein heavy chain domain 1 (Dnhd1), miscRNA [XR_085725]	**11,259**
0	Unknown	**11,259**
Gbp5	Rattus norvegicus guanylate binding protein 5 (Gbp5), mRNA [NM_001108569]	**11,257**
Set	Rattus norvegicus SET nuclear oncogene (Set), mRNA [NM_001012504]	**11,256**
RGD1306000	Rattus norvegicus similar to CG8043-PA (RGD1306000), mRNA [NM_001108827]	**11,255**
0	Unknown	**11,251**
LOC688459	Rattus norvegicus hypothetical protein LOC688459 (LOC688459), mRNA [NM_001109502]	**11,250**
0	Unknown	**11,249**
Itpr2	Rattus norvegicus inositol 1,4,5-triphosphate receptor, type 2 (Itpr2), mRNA [NM_031046]	**11,248**
Ppapdc3	Rattus norvegicus phosphatidic acid phosphatase type 2 domain containing 3 (Ppapdc3), mRNA [NM_001012349]	**11,247**
0	Unknown	**11,247**
Khnyn	PREDICTED: Rattus norvegicus similar to mKIAA0323 protein (RGD1565688), miscRNA [XR_085623]	**11,246**
Olr1309	Rattus norvegicus olfactory receptor 1309 (Olr1309), mRNA [NM_001000466]	**11,245**
Lrrc4	Rattus norvegicus leucine rich repeat containing 4 (Lrrc4), mRNA [NM_001037336]	**11,244**

Etv6	Rattus norvegicus ets variant 6 (Etv6), mRNA [NM_001037353]	**11,244**
0	Q6C5E2_YARLI (Q6C5E2) Similarity (Fragment), partial (11%) [TC594916]	**11,241**
Snrk	Rattus norvegicus SNF related kinase (Snrk), mRNA [NM_138833]	**11,240**
Tpra1	Transmembrane protein adipocyte-associated 1 [Source:UniProtKB/Swiss-Prot;Acc:Q791F6] [ENSRNOT00000022534]	**11,240**
Gzf1	Rattus norvegicus GDNF-inducible zinc finger protein 1 (Gzf1), mRNA [NM_001107788]	**11,238**
0	Unknown	**11,237**
Phldb2	PREDICTED: Rattus norvegicus pleckstrin homology-like domain, family B, member 2 (Phldb2), mRNA [XM_001064525]	**11,237**
Uba6	Rattus norvegicus ubiquitin-like modifier activating enzyme 6 (Uba6), mRNA [NM_001107213]	**11,236**
0	Unknown	**11,236**
Prr19	Rattus norvegicus proline rich 19 (Prr19), mRNA [NM_001173428]	**11,236**
Flot1	Rattus norvegicus flotillin 1 (Flot1), mRNA [NM_022701]	**11,233**
Ppp1cb	Rattus norvegicus protein phosphatase 1, catalytic subunit, beta isoform (Ppp1cb), mRNA [NM_013065]	**11,233**
Gstk1	Rattus norvegicus glutathione S-transferase kappa 1 (Gstk1), nuclear gene encoding mitochondrial protein, mRNA [NM_181371]	**11,231**
Senp1	PREDICTED: Rattus norvegicus Sumo1/sentrin/SMT3 specific peptidase 1 (Senp1), mRNA [XM_001060796]	**11,230**
RGD1307537	Rattus norvegicus similar to RIKEN cDNA 4933417A18 (RGD1307537), mRNA [NM_001013885]	**11,230**
Sema3g	Rattus norvegicus sema domain, immunoglobulin domain (Ig), short basic domain, secreted, (semaphorin) 3G (Sema3g), mRNA [NM_001100882]	**11,228**
0	Q2H348_CHAGB (Q2H348) Predicted protein, partial (5%) [TC596046]	**11,227**
Cdk5rap2	Rattus norvegicus CDK5 regulatory subunit associated protein 2 (Cdk5rap2), mRNA [NM_173134]	**11,227**
Ociad1	Rattus norvegicus OCIA domain containing 1 (Ociad1), mRNA [NM_001013874]	**11,226**
Ankrd13a	Rattus norvegicus ankyrin repeat domain 13a (Ankrd13a), mRNA [NM_001012148]	**11,223**
0	Unknown	**11,222**
LOC688173	PREDICTED: Rattus norvegicus similar to Telethonin (Titin cap protein) (LOC688173), mRNA [XM_001081394]	**11,222**
0	Unknown	**11,221**
LOC298138	PREDICTED: Rattus norvegicus similar to RAS and EF hand domain containing (LOC298138), miscRNA [XR_006670]	**11,219**
Baz2b	Rattus norvegicus bromodomain adjacent to zinc finger domain, 2B (Baz2b), mRNA [NM_001108260]	**11,217**
Fnbp1l	Rattus norvegicus formin binding protein 1-like (Fnbp1l), mRNA [NM_001039609]	**11,211**
Irx1	Rattus norvegicus iroquois homeobox 1 (Irx1), mRNA [NM_001107331]	**11,209**
Tmem39b	Rattus norvegicus transmembrane protein 39b (Tmem39b), mRNA [NM_001014192]	**11,206**
0	Uncharacterized protein [Source:UniProtKB/TrEMBL;Acc:D3ZQI7] [ENSRNOT00000043346]	**11,206**
Eef1e1	Rattus norvegicus eukaryotic translation elongation factor 1 epsilon 1 (Eef1e1), mRNA [NM_001106106]	**11,200**
LOC686428	PREDICTED: Rattus norvegicus similar to Emu2 (LOC686428), mRNA [XM_001069996]	**11,199**
0	Uncharacterized protein [Source:UniProtKB/TrEMBL;Acc:D4A1I9] [ENSRNOT00000066634]	**11,199**
Josd1	Rattus norvegicus Josephin domain containing 1 (Josd1), mRNA [NM_001025009]	**11,195**
Tial1	Rattus norvegicus Tia1 cytotoxic granule-associated RNA binding protein-like 1 (Tial1), mRNA [NM_001013193]	**11,194**

Poldip3	Rattus norvegicus polymerase (DNA-directed), delta interacting protein 3 (Poldip3), mRNA [NM_001130506]	**11,194**
Prg2	Rattus norvegicus proteoglycan 2, bone marrow (Prg2), mRNA [NM_031619]	**11,192**
Slc24a2	Rattus norvegicus solute carrier family 24 (sodium/potassium/calcium exchanger), member 2 (Slc24a2), mRNA [NM_031743]	**11,192**
Wdr19	Rattus norvegicus WD repeat domain 19 (Wdr19), mRNA [NM_001191679]	**11,188**
Cdh10	Rattus norvegicus cadherin 10 (Cdh10), mRNA [NM_001168631]	**11,188**
Coq9	Rattus norvegicus coenzyme Q9 homolog (S. cerevisiae) (Coq9), nuclear gene encoding mitochondrial protein, mRNA [NM_001035257]	**11,188**
Slc9a5	Rattus norvegicus solute carrier family 9 (sodium/hydrogen exchanger), member 5 (Slc9a5), mRNA [NM_138858]	**11,187**
LOC689207	PREDICTED: Rattus norvegicus similar to proteoglycan 4 (LOC689207), mRNA [XM_001069975]	**11,187**
Ip6k1	Rattus norvegicus inositol hexakisphosphate kinase 1 (Ip6k1), mRNA [NM_053316]	**11,185**
Rgs17	Rattus norvegicus regulator of G-protein signaling 17 (Rgs17), mRNA [NM_001107459]	**11,184**
Zfp395	Rattus norvegicus zinc finger protein 395 (Zfp395), mRNA [NM_001107271]	**11,184**
LOC501375	PREDICTED: Rattus norvegicus similar to glutamate receptor, ionotropic, N-methyl D-aspartate-like 1A (LOC501375), mRNA [XM_576788]	**11,183**
Usp10	Rattus norvegicus ubiquitin specific peptidase 10 (Usp10), mRNA [NM_001034146]	**11,183**
LOC100364957	PREDICTED: Rattus norvegicus RGD1560755 protein-like (LOC100364957), mRNA [XM_002725203]	**11,183**
0	Unknown	**11,181**
0	Rattus norvegicus TL0AEA54YG18 mRNA sequence. [FQ225308]	**11,181**
0	Putative uncharacterized proteinUncharacterized protein [Source:UniProtKB/TrEMBL;Acc:D4A5B2] [ENSRNOT00000004034]	**11,181**
0	Unknown	**11,176**
0	Unknown	**11,171**
0	Unknown	**11,167**
Tmem33	Rattus norvegicus transmembrane protein 33 (Tmem33), transcript variant 1, mRNA [NM_021671]	**11,167**
0	Clasp1 protein [Source:UniProtKB/TrEMBL;Acc:Q5M928] [ENSRNOT00000061516]	**11,167**
Ak7	Rattus norvegicus adenylate kinase 7 (Ak7), mRNA [NM_001108055]	**11,167**
Ttc19	Rattus norvegicus tetratricopeptide repeat domain 19 (Ttc19), mRNA [NM_001109644]	**11,163**
Ccdc142	Uncharacterized protein [Source:UniProtKB/TrEMBL;Acc:D3ZV00] [ENSRNOT00000035329]	**11,162**
Mgat4b	Rattus norvegicus mannosyl (alpha-1,3-)-glycoprotein beta-1,4-N-acetylglucosaminyltransferase, isozyme B (Mgat4b), mRNA [NM_001127533]	**11,160**
Ube2i	Rattus norvegicus ubiquitin-conjugating enzyme E2I (UBC9 homolog, yeast) (Ube2i), mRNA [NM_013050]	**11,158**
Prmt6	Rattus norvegicus protein arginine methyltransferase 6 (Prmt6), mRNA [NM_001106466]	**11,157**
LOC678704	Rattus norvegicus similar to Probable cation-transporting ATPase 3 (ATPase family homolog up-regulated in senescence cells 1) (LOC360728), mRNA [XM_341001]	**11,157**
Pax8	Rattus norvegicus paired box 8 (Pax8), mRNA [NM_031141]	**11,157**
Nid67	Rattus norvegicus putative small membrane protein NID67 (Nid67), mRNA [NM_173126]	**11,156**
Abhd13	Rattus norvegicus abhydrolase domain containing 13 (Abhd13), mRNA [NM_001107327]	**11,155**
Dok3	Rattus norvegicus docking protein 3 (Dok3), mRNA [NM_001107336]	**11,154**

Pak2	Rattus norvegicus p21 protein (Cdc42/Rac)-activated kinase 2 (Pak2), mRNA [NM_053306]	**11,152**
Pcdha3	Rattus norvegicus protocadherin alpha 3 (Pcdha3), mRNA [NM_053941]	**11,152**
St5	Rattus norvegicus suppression of tumorigenicity 5 (St5), mRNA [NM_001107547]	**11,152**
Olr1144	Rattus norvegicus olfactory receptor 1144 (Olr1144), mRNA [NM_001000876]	**11,152**
0	Unknown	**11,151**
Mmp1a	Rattus norvegicus matrix metallopeptidase 1a (interstitial collagenase) (Mmp1a), mRNA [NM_001134530]	**11,149**
0	Unknown	**11,146**
Dffb	Rattus norvegicus DNA fragmentation factor, beta polypeptide (caspase-activated DNase) (Dffb), mRNA [NM_053362]	**11,146**
0	Unknown	**11,144**
Ppargc1a	Rattus norvegicus peroxisome proliferator-activated receptor gamma, coactivator 1 alpha (Ppargc1a), mRNA [NM_031347]	**11,144**
Zbtb8os	PREDICTED: Rattus norvegicus zinc finger and BTB domain containing 8 opposite strand (Zbtb8os), miscRNA [XR_086264]	**11,144**
Nat13	Rattus norvegicus N-acetyltransferase 13 (Nat13), mRNA [NM_001105881]	**11,143**
0	Unknown	**11,142**
Ppp6c	Rattus norvegicus protein phosphatase 6, catalytic subunit (Ppp6c), mRNA [NM_133589]	**11,141**
RGD1563302	Rattus norvegicus RGD1563302 (RGD1563302), mRNA [NM_001162899]	**11,137**
Alg8	Rattus norvegicus asparagine-linked glycosylation 8, alpha-1,3-glucosyltransferase homolog (S. cerevisiae) (Alg8), mRNA [NM_001034127]	**11,136**
0	Unknown	**11,134**
Insc	Rattus norvegicus inscuteable homolog (Drosophila) (Insc), mRNA [NM_001106285]	**11,133**
Mllt10	Rattus norvegicus myeloid/lymphoid or mixed-lineage leukemia (trithorax homolog, Drosophila); translocated to, 10 (Mllt10), mRNA [NM_001012162]	**11,133**
Rsbn1	Rattus norvegicus round spermatid basic protein 1 (Rsbn1), mRNA [NM_001191710]	**11,132**
Olr789	Rattus norvegicus olfactory receptor 789 (Olr789), mRNA [NM_001000377]	**11,130**
Chn1	Rattus norvegicus chimerin (chimaerin) 1 (Chn1), mRNA [NM_032083]	**11,129**
Rnase13	Rattus norvegicus ribonuclease, RNase A family, 13 (non-active) (Rnase13), mRNA [NM_001012231]	**11,127**
Fancl	Rattus norvegicus Fanconi anemia, complementation group L (Fancl), mRNA [NM_001191684]	**11,124**
Hps1	Rattus norvegicus Hermansky-Pudlak syndrome 1 homolog (human) (Hps1), mRNA [NM_040669]	**11,122**
Zfp26	Rattus norvegicus zinc finger protein 26 (Zfp26), mRNA [NM_001108995]	**11,122**
Dll3	Rattus norvegicus delta-like 3 (Drosophila) (Dll3), mRNA [NM_053666]	**11,121**
37500	Rattus norvegicus septin 2 (Sept2), mRNA [NM_057148]	**11,121**
Mllt4	Rattus norvegicus myeloid/lymphoid or mixed-lineage leukemia (trithorax homolog, Drosophila); translocated to, 4 (Mllt4), mRNA [NM_013217]	**11,120**
Gtf2h3	Rattus norvegicus general transcription factor IIH, polypeptide 3 (Gtf2h3), mRNA [NM_001024236]	**11,119**
0	Transcription factor E2F5 [Source:UniProtKB/Swiss-Prot;Acc:Q62814] [ENSRNOT00000014361]	**11,117**
0	Exportin 7, isoform CRA_aUncharacterized protein [Source:UniProtKB/TrEMBL;Acc:D3ZBB4] [ENSRNOT00000018440]	**11,114**
Araf	Rattus norvegicus v-raf murine sarcoma 3611 viral oncogene homolog (Araf), transcript variant 1, mRNA [NM_022532]	**11,114**

Calm3	Rattus norvegicus calmodulin 3 (Calm3), mRNA [NM_012518]	**11,113**
0	Uncharacterized protein [Source:UniProtKB/TrEMBL;Acc:D3ZBI5] [ENSRNOT00000042103]	**11,113**
0	Unknown	**11,111**
Olr92	Rattus norvegicus olfactory receptor 92 (Olr92), mRNA [NM_001000140]	**11,111**
Kcnk16	Rattus norvegicus potassium channel, subfamily K, member 16 (Kcnk16), mRNA [NM_001109520]	**11,111**
Cenpv	Uncharacterized protein [Source:UniProtKB/TrEMBL;Acc:D4A9A3] [ENSRNOT00000004127]	**11,109**
Epha4	Rattus norvegicus Eph receptor A4 (Epha4), mRNA [NM_001162411]	**11,108**
St8sia1	Rattus norvegicus ST8 alpha-N-acetyl-neuraminide alpha-2,8-sialyltransferase 1 (St8sia1), mRNA [NM_012813]	**11,107**
LOC100365635	PREDICTED: Rattus norvegicus ankyrin repeat domain 49-like (LOC100365635), miscRNA [XR_085597]	**11,105**
0	Putative uncharacterized protein vp130S [Source:UniProtKB/TrEMBL;Acc:Q810D7] [ENSRNOT00000037528]	**11,104**
Rnf183	Uncharacterized protein [Source:UniProtKB/TrEMBL;Acc:D4A718] [ENSRNOT00000019924]	**11,103**
Arhgap20	Rattus norvegicus Rho GTPase activating protein 20 (Arhgap20), mRNA [NM_213629]	**11,101**
LOC303448	Rattus norvegicus similar to glyceraldehyde-3-phosphate dehydrogenase (LOC303448), mRNA [NM_001037190]	**11,099**
Tmem37	Rattus norvegicus transmembrane protein 37 (Tmem37), mRNA [NM_139095]	**11,098**
0	Unknown	**11,097**
Smad1	Rattus norvegicus SMAD family member 1 (Smad1), mRNA [NM_013130]	**11,096**
Cd44	Rattus norvegicus Cd44 molecule (Cd44), mRNA [NM_012924]	**11,094**
0	Histone H2B-3 [Source:UniProtKB/TrEMBL;Acc:Q9Z2Q9] [ENSRNOT00000042005]	**11,094**
LOC100361049	LRRGT00168 [Source:UniProtKB/TrEMBL;Acc:Q6QI40] [ENSRNOT00000044735]	**11,092**
Med29	Rattus norvegicus mediator complex subunit 29 (Med29), mRNA [NM_001106237]	**11,092**
Sumo1	Rattus norvegicus SMT3 suppressor of mif two 3 homolog 1 (S. cerevisiae) (Sumo1), mRNA [NM_001009672]	**11,091**
0	Uncharacterized protein [Source:UniProtKB/TrEMBL;Acc:D3ZEL6] [ENSRNOT00000045639]	**11,090**
Tollip	Rattus norvegicus toll interacting protein (Tollip), mRNA [NM_001109668]	**11,090**
RGD1307615	Rattus norvegicus similar to hypothetical protein FLJ13045 (RGD1307615), mRNA [NM_001108573]	**11,089**
E2f7	Rattus norvegicus E2F transcription factor 7 (E2f7), mRNA [NM_001108092]	**11,089**
0	Unknown	**11,089**
0	Unknown	**11,087**
0	Unknown	**11,086**
Pitx1	Rattus norvegicus paired-like homeodomain 1 (Pitx1), mRNA [NM_053624]	**11,086**
Pcdh7	Rattus norvegicus protocadherin 7 (Pcdh7), mRNA [NM_001004087]	**11,084**
Kcnd2	Rattus norvegicus potassium voltage-gated channel, Shal-related subfamily, member 2 (Kcnd2), mRNA [NM_031730]	**11,084**
Mansc1	Rattus norvegicus MANSC domain containing 1 (Mansc1), mRNA [NM_001109603]	**11,083**
Nat15	Rattus norvegicus N-acetyltransferase 15 (GCN5-related, putative) (Nat15), mRNA [NM_001014226]	**11,083**
Stx12	Rattus norvegicus syntaxin 12 (Stx12), mRNA [NM_022939]	**11,081**

Acsf2	Rattus norvegicus acyl-CoA synthetase family member 2 (Acsf2), mRNA [NM_001034951]	**11,079**
0	similar to RIKEN cDNA 1700081O22 (LOC363337), mRNA [Source:RefSeq DNA;Acc:NM_001014221] [ENSRNOT00000041051]	**11,079**
RGD1308759	Rattus norvegicus similar to KIAA0892 protein (RGD1308759), mRNA [NM_001106077]	**11,078**
Chodl	Rattus norvegicus chondrolectin (Chodl), mRNA [NM_001105894]	**11,077**
0	Unknown	**11,074**
Rfc1	Rattus norvegicus replication factor C (activator 1) 1 (Rfc1), mRNA [NM_053547]	**11,071**
Ccnyl1	PREDICTED: Rattus norvegicus cyclin Y-like 1 (Ccnyl1), mRNA [XM_237211]	**11,068**
Doxl1	Rattus norvegicus diamine oxidase-like protein 1 (Doxl1), mRNA [NM_199233]	**11,065**
Bmp3	Rattus norvegicus bone morphogenetic protein 3 (Bmp3), mRNA [NM_017105]	**11,064**
0	Unknown	**11,062**
Toag1	Rattus norvegicus tolerance-associated gene 1 (Toag1), mRNA [NM_001110838]	**11,062**
LOC100361389	PREDICTED: Rattus norvegicus hypothetical protein LOC100361389 (LOC100361389), mRNA [XM_002727821]	**11,058**
0	MMU18644 methyltransferase-like protein 1 {Mus musculus} (exp=-1; wgp=0; cg=0), partial (30%) [TC638589]	**11,058**
Mrpl10	Rattus norvegicus mitochondrial ribosomal protein L10 (Mrpl10), nuclear gene encoding mitochondrial protein, mRNA [NM_001109620]	**11,055**
0	Unknown	**11,055**
Ppfia2	Rattus norvegicus protein tyrosine phosphatase, receptor type, f polypeptide (PTPRF), interacting protein (liprin), alpha 2 (Ppfia2), mRNA [NM_001108745]	**11,055**
LOC688090	Rattus norvegicus similar to RT1 class II histocompatibility antigen, B-1 beta chain precursor (RT1.B-beta(1)) (LOC688090), mRNA [NM_001101017]	**11,049**
Gucy1a2	Rattus norvegicus guanylate cyclase 1, soluble, alpha 2 (Gucy1a2), mRNA [NM_023956]	**11,049**
Vegfa	Rattus norvegicus vascular endothelial growth factor A (Vegfa), transcript variant 3, mRNA [NM_001110334]	**11,048**
0	Unknown	**11,047**
0	Unknown	**11,047**
Olr325	Rattus norvegicus olfactory receptor 325 (Olr325), mRNA [NM_001000247]	**11,045**
0	Unknown	**11,044**
Slc7a12	Rattus norvegicus solute carrier family 7 (cationic amino acid transporter, y+ system), member 12 (Slc7a12), mRNA [NM_001011948]	**11,040**
Tmem49	Rattus norvegicus transmembrane protein 49 (Tmem49), mRNA [NM_138839]	**11,039**
0	CCD13_HUMAN (Q8IYE1) Coiled-coil domain-containing protein 13, partial (11%) [TC590118]	**11,038**
Nat12	Rattus norvegicus N-acetyltransferase 12 (GCN5-related, putative) (Nat12), mRNA [NM_001109099]	**11,038**
Olr1605	Rattus norvegicus olfactory receptor 1605 (Olr1605), mRNA [NM_001000088]	**11,035**
0	Glyceraldehyde-3-phosphate dehydrogenase [Source:UniProtKB/TrEMBL;Acc:D3ZKR3] [ENSRNOT00000008094]	**11,034**
0	Unknown	**11,032**
RGD1561766	PREDICTED: Rattus norvegicus similar to basic transcription factor 3 (RGD1561766), miscRNA [XR_009374]	**11,030**
Mgat1	Rattus norvegicus mannosyl (alpha-1,3-)-glycoprotein beta-1,2-N-acetylglucosaminyltransferase (Mgat1), mRNA [NM_030861]	**11,029**

Senp8	Rattus norvegicus SUMO/sentrin specific peptidase family member 8 (Senp8), mRNA [NM_001012355]	**11,027**
P4hb	Rattus norvegicus prolyl 4-hydroxylase, beta polypeptide (P4hb), mRNA [NM_012998]	**11,026**
0	Unknown	**11,025**
Abl1	Rattus norvegicus c-abl oncogene 1, receptor tyrosine kinase (Abl1), mRNA [NM_001100850]	**11,024**
Mier3	Rattus norvegicus mesoderm induction early response 1, family member 3 (Mier3), mRNA [NM_001168000]	**11,023**
Derl3	Rattus norvegicus Der1-like domain family, member 3 (Derl3), mRNA [NM_001109577]	**11,023**
Gpr112l	PREDICTED: Rattus norvegicus G protein-coupled receptor 112 like (Gpr112l), mRNA [XM_001058470]	**11,022**
Ube3a	Rattus norvegicus ubiquitin protein ligase E3A (Ube3a), mRNA [NM_001191837]	**11,022**
Cbfa2t2	Rattus norvegicus core-binding factor, runt domain, alpha subunit 2; translocated to, 2 (Cbfa2t2), mRNA [NM_001168542]	**11,017**
Galr3	Rattus norvegicus galanin receptor 3 (Galr3), mRNA [NM_019173]	**11,016**
Sycn	Rattus norvegicus syncollin (Sycn), mRNA [NM_139086]	**11,015**
Nanp	Rattus norvegicus N-acetylneuraminic acid phosphatase (Nanp), mRNA [NM_001009409]	**11,015**
Tpst1	Rattus norvegicus tyrosylprotein sulfotransferase 1 (Tpst1), mRNA [NM_001011903]	**11,014**
LOC100363969	PREDICTED: Rattus norvegicus zinc finger protein 457-like (LOC100363969), miscRNA [XR_085746]	**11,014**
B4galt5	Rattus norvegicus UDP-Gal:betaGlcNAc beta 1,4-galactosyltransferase, polypeptide 5 (B4galt5), mRNA [NM_001108608]	**11,014**
Xkr4	Rattus norvegicus XK, Kell blood group complex subunit-related family, member 4 (Xkr4), mRNA [NM_001011971]	**11,014**
Rps6ka3	Rattus norvegicus ribosomal protein S6 kinase polypeptide 3 (Rps6ka3), mRNA [NM_001192004]	**11,014**
LOC683923	PREDICTED: Rattus norvegicus hypothetical protein LOC683923 (LOC683923), mRNA [XM_001068075]	**11,011**
Sphkap	Rattus norvegicus SPHK1 interactor, AKAP domain containing (Sphkap), mRNA [NM_001127492]	**11,010**
Pelp1	Rattus norvegicus proline, glutamate and leucine rich protein 1 (Pelp1), mRNA [NM_001024270]	**11,010**
Ttl	Rattus norvegicus tubulin tyrosine ligase (Ttl), mRNA [NM_138536]	**11,010**
Dixdc1	Rattus norvegicus DIX domain containing 1 (Dixdc1), mRNA [NM_001037654]	**11,010**
0	Unknown	**11,009**
0	Uncharacterized protein [Source:UniProtKB/TrEMBL;Acc:D3ZD32] [ENSRNOT00000067364]	**11,009**
LOC687118	PREDICTED: Rattus norvegicus similar to death effector domain-containing DNA binding protein 2 (LOC687118), mRNA [XM_001077155]	**11,002**
0	Unknown	**11,002**
Pcnx	Rattus norvegicus pecanex homolog (Drosophila) (Pcnx), mRNA [NM_001170347]	**10,998**
Zfp828	Rattus norvegicus zinc finger protein 828 (Zfp828), mRNA [NM_001107329]	**10,996**
Artn	Rattus norvegicus artemin (Artn), mRNA [NM_053397]	**10,995**
Cacna1i	Rattus norvegicus calcium channel, voltage-dependent, T type, alpha 1I subunit (Cacna1i), mRNA [NM_020084]	**10,995**
Fance	Rattus norvegicus Fanconi anemia, complementation group E (Fance), mRNA [NM_001191718]	**10,993**
0	Unknown	**10,990**
Dppa3	Rattus norvegicus developmental pluripotency-associated 3 (Dppa3), mRNA [NM_001047864]	**10,990**

Coq5	Rattus norvegicus coenzyme Q5 homolog, methyltransferase (S. cerevisiae) (Coq5), nuclear gene encoding mitochondrial protein, mRNA [NM_001039022]	**10,988**
0	Unknown	**10,987**
Usp32	Rattus norvegicus ubiquitin specific peptidase 32 (Usp32), mRNA [NM_001107032]	**10,985**
Dmrta2	Rattus norvegicus DMRT-like family A2 (Dmrta2), mRNA [NM_001107951]	**10,983**
0	Unknown	**10,983**
Fbxo25	Rattus norvegicus F-box protein 25 (Fbxo25), mRNA [NM_001014239]	**10,982**
0	Unknown	**10,981**
Sfrs2ip	Uncharacterized protein [Source:UniProtKB/TrEMBL;Acc:D4ABH1] [ENSRNOT00000007003]	**10,981**
Mapk8ip3	Rattus norvegicus mitogen-activated protein kinase 8 interacting protein 3 (Mapk8ip3), mRNA [NM_001100673]	**10,980**
Map3k1	Rattus norvegicus mitogen activated protein kinase kinase kinase 1 (Map3k1), mRNA [NM_053887]	**10,979**
0	Rattus norvegicus cDNA clone IMAGE:7323401. [BC088301]	**10,978**
Atp6v1c1	Rattus norvegicus ATPase, H+ transporting, lysosomal V1 subunit C1 (Atp6v1c1), mRNA [NM_001011992]	**10,975**
Cox8a	Rattus norvegicus cytochrome c oxidase subunit VIIIa (Cox8a), mRNA [NM_134345]	**10,975**
Slc16a13	Rattus norvegicus solute carrier family 16, member 13 (monocarboxylic acid transporter 13) (Slc16a13), mRNA [NM_001005530]	**10,974**
Nrbp2	Rattus norvegicus nuclear receptor binding protein 2 (Nrbp2), mRNA [NM_001135007]	**10,974**
0	AT rich interactive domain 1B (SWI-like) Gene [Source:MGI Symbol;Acc:MGI:1926129] [ENSRNOT00000022939]	**10,969**
Dmwd	PREDICTED: Rattus norvegicus dystrophia myotonica, WD repeat containing (Dmwd), mRNA [XM_002725588]	**10,968**
0	ubiquitin protein ligase E3 component n-recognin 2 [Source:RefSeq peptide;Acc:NP_001171542] [ENSRNOT00000061531]	**10,967**
0	Unknown	**10,966**
Ttll12	Rattus norvegicus tubulin tyrosine ligase-like family, member 12 (Ttll12), mRNA [NM_001135922]	**10,965**
0	Unknown	**10,964**
Gpkow	Rattus norvegicus G patch domain and KOW motifs (Gpkow), mRNA [NM_001109381]	**10,962**
Ankk1	Rattus norvegicus ankyrin repeat and kinase domain containing 1 (Ankk1), mRNA [NM_001108999]	**10,961**
Eif2ak2	Rattus norvegicus eukaryotic translation initiation factor 2-alpha kinase 2 (Eif2ak2), mRNA [NM_019335]	**10,961**
0	Unknown	**10,960**
Slc34a1	Rattus norvegicus solute carrier family 34 (sodium phosphate), member 1 (Slc34a1), mRNA [NM_013030]	**10,960**
Gorab	Rattus norvegicus golgin, RAB6-interacting (Gorab), mRNA [NM_001100563]	**10,956**
Sh3bp5	Rattus norvegicus SH3-domain binding protein 5 (BTK-associated) (Sh3bp5), mRNA [NM_054011]	**10,956**
LOC681989	Rattus norvegicus similar to defective SPErmatogenesis family member (spe-39) (LOC681989), mRNA [NM_001101004]	**10,956**
Bok	Rattus norvegicus BCL2-related ovarian killer (Bok), mRNA [NM_017312]	**10,955**
0	Unknown	**10,953**
Cbl	PREDICTED: Rattus norvegicus Cas-Br-M (murine) ecotropic retroviral transforming sequence, transcript variant 1 (Cbl), mRNA [XM_001066453]	**10,952**
Nnat	Rattus norvegicus neuronatin (Nnat), transcript variant 1, mRNA [NM_053601]	**10,951**

0	Unknown	**10,948**
0	Unknown	**10,946**
0	Unknown	**10,945**
0	Unknown	**10,945**
Dtd1	Rattus norvegicus D-tyrosyl-tRNA deacylase 1 homolog (S. cerevisiae) (Dtd1), nuclear gene encoding mitochondrial protein, mRNA [NM_001108594]	**10,944**
Pfdn1	Rattus norvegicus prefoldin subunit 1 (Pfdn1), mRNA [NM_001108427]	**10,944**
0	Unknown	**10,943**
Mtpn	Rattus norvegicus myotrophin (Mtpn), mRNA [NM_024374]	**10,943**
Timm17b	Rattus norvegicus translocase of inner mitochondrial membrane 17 homolog B (yeast) (Timm17b), nuclear gene encoding mitochondrial protein, mRNA [NM_001108249]	**10,940**
Tm4sf4	Rattus norvegicus transmembrane 4 L six family member 4 (Tm4sf4), mRNA [NM_053785]	**10,940**
RGD1309708	Rattus norvegicus similar to RIKEN cDNA 4930455F23 (RGD1309708), mRNA [NM_001014131]	**10,938**
0	Glyceraldehyde-3-phosphate dehydrogenase [Source:UniProtKB/TrEMBL;Acc:D3ZDX2] [ENSRNOT00000051605]	**10,937**
Rab5a	Rattus norvegicus RAB5A, member RAS oncogene family (Rab5a), mRNA [NM_022692]	**10,935**
Coro7	Rattus norvegicus coronin 7 (Coro7), mRNA [NM_001191639]	**10,933**
Prr7	Rattus norvegicus proline rich 7 (synaptic) (Prr7), mRNA [NM_001109116]	**10,930**
Snph	Rattus norvegicus syntaphilin (Snph), mRNA [NM_001106525]	**10,930**
Gsdmd	Rattus norvegicus gasdermin D (Gsdmd), mRNA [NM_001130553]	**10,928**
0	Unknown	**10,925**
Ufl1	Rattus norvegicus E3 UFM1-protein ligase 1 (Ufl1), mRNA [NM_001126279]	**10,924**
Asb3	Rattus norvegicus ankyrin repeat and SOCS box-containing 3 (Asb3), mRNA [NM_001108864]	**10,921**
0	Unknown	**10,919**
Pknox1	Rattus norvegicus PBX/knotted 1 homeobox 1 (Pknox1), mRNA [NM_001013074]	**10,916**
Pthlh	Rattus norvegicus parathyroid hormone-like hormone (Pthlh), mRNA [NM_012636]	**10,915**
0	Unknown	**10,915**
Map7	Rattus norvegicus microtubule-associated protein 7 (Map7), transcript variant 2, mRNA [NM_001198638]	**10,915**
0	Uncharacterized protein [Source:UniProtKB/TrEMBL;Acc:D3ZZI9] [ENSRNOT00000043132]	**10,913**
0	Unknown	**10,912**
Rab8b	Rattus norvegicus RAB8B, member RAS oncogene family (Rab8b), mRNA [NM_153317]	**10,911**
RGD1562127	PREDICTED: Rattus norvegicus similar to chromosome 11 open reading frame 9 (RGD1562127), mRNA [XM_001080934]	**10,911**
0	Unknown	**10,910**
Dctn4	Rattus norvegicus dynactin 4 (Dctn4), mRNA [NM_053404]	**10,908**
RGD1309748	Rattus norvegicus similar to CG4768-PA (RGD1309748), mRNA [NM_001106972]	**10,902**

Spats1	Rattus norvegicus spermatogenesis associated, serine-rich 1 (Spats1), mRNA [NM_181376]	**10,900**
Phyhd1	Rattus norvegicus phytanoyl-CoA dioxygenase domain containing 1 (Phyhd1), mRNA [NM_001013081]	**10,900**
Fbxl12	Rattus norvegicus F-box and leucine-rich repeat protein 12 (Fbxl12), mRNA [NM_001025700]	**10,899**
0	Unknown	**10,897**
0	PREDICTED: Rattus norvegicus hypothetical protein LOC687735 (LOC687735), mRNA [XM_001079963]	**10,896**
0	Uncharacterized protein [Source:UniProtKB/TrEMBL;Acc:D3ZHS3] [ENSRNOT00000013881]	**10,894**
Rspry1	Rattus norvegicus ring finger and SPRY domain containing 1 (Rspry1), mRNA [NM_001100945]	**10,893**
0	Unknown	**10,893**
Foxk2	Rattus norvegicus forkhead box K2 (Foxk2), mRNA [NM_001107075]	**10,892**
Zfp12	PREDICTED: Rattus norvegicus zinc finger protein 12 (Zfp12), mRNA [XM_001072483]	**10,890**
Zp3r	Rattus norvegicus zona pellucida 3 receptor (Zp3r), mRNA [NM_182815]	**10,889**
Kifap3	Rattus norvegicus kinesin-associated protein 3 (Kifap3), mRNA [NM_001105964]	**10,889**
Kcnrg	Rattus norvegicus potassium channel regulator (Kcnrg), mRNA [NM_001191687]	**10,887**
Dnah8	PREDICTED: Rattus norvegicus dynein, axonemal, heavy chain 8 (Dnah8), mRNA [XM_228058]	**10,886**
Zmym3	Rattus norvegicus zinc finger, MYM-type 3 (Zmym3), mRNA [NM_001040155]	**10,885**
Sdccag3	Rattus norvegicus serologically defined colon cancer antigen 3 (Sdccag3), mRNA [NM_001013135]	**10,885**
Yod1	Rattus norvegicus YOD1 OTU deubiquinating enzyme 1 homolog (S. cerevisiae) (Yod1), mRNA [NM_001008889]	**10,885**
Cmtm4	Rattus norvegicus CKLF-like MARVEL transmembrane domain containing 4 (Cmtm4), mRNA [NM_001172151]	**10,885**
Dyrk2	Rattus norvegicus dual-specificity tyrosine-(Y)-phosphorylation regulated kinase 2 (Dyrk2), mRNA [NM_001108100]	**10,883**
Gabarapl1	Rattus norvegicus GABA(A) receptor-associated protein like 1 (Gabarapl1), mRNA [NM_001044294]	**10,883**
Secisbp2l	Rattus norvegicus SECIS binding protein 2-like (Secisbp2l), mRNA [NM_001168527]	**10,881**
Rhbdl3	Rattus norvegicus rhomboid, veinlet-like 3 (Drosophila) (Rhbdl3), mRNA [NM_001105819]	**10,881**
Ywhaq	Rattus norvegicus tyrosine 3-monooxygenase/tryptophan 5-monooxygenase activation protein, theta polypeptide (Ywhaq), mRNA [NM_013053]	**10,881**
Taf1c	Rattus norvegicus TATA box binding protein (Tbp)-associated factor, RNA polymerase I, C (Taf1c), mRNA [NM_001014155]	**10,880**
Epn3	Rattus norvegicus epsin 3 (Epn3), mRNA [NM_001024791]	**10,880**
Rab13	Rattus norvegicus RAB13, member RAS oncogene family (Rab13), mRNA [NM_031092]	**10,879**
Sfrp5	Rattus norvegicus secreted frizzled-related protein 5 (Sfrp5), mRNA [NM_001107591]	**10,877**
Ccno	Rattus norvegicus cyclin O (Ccno), mRNA [NM_001109175]	**10,877**
Dtnb	Rattus norvegicus dystrobrevin, beta (Dtnb), mRNA [NM_001012191]	**10,876**
LOC361990	Rattus norvegicus similar to DKFZP547E1010 protein (LOC361990), mRNA [NM_001014175]	**10,874**
0	PREDICTED: Rattus norvegicus rCG56028-like (LOC100363825), miscRNA [XR_085819]	**10,873**
Slc19a1	Rattus norvegicus solute carrier family 19 (folate transporter), member 1 (Slc19a1), transcript variant 1, mRNA [NM_017299]	**10,871**
Olr1070	Rattus norvegicus olfactory receptor 1070 (Olr1070), mRNA [NM_001000591]	**10,870**
0	Uncharacterized protein [Source:UniProtKB/TrEMBL;Acc:D3Z930] [ENSRNOT00000031558]	**10,866**

Ciapin1	Rattus norvegicus cytokine induced apoptosis inhibitor 1 (Ciapin1), mRNA [NM_001007689]	**10,865**
Tp63	Rattus norvegicus tumor protein p63 (Tp63), transcript variant 3, mRNA [NM_001127341]	**10,864**
Far1	Rattus norvegicus TL0ADA37YA18 mRNA sequence. [FQ221385]	**10,864**
Nfat5	Rattus norvegicus nuclear factor of activated T-cells 5 (Nfat5), mRNA [NM_001107425]	**10,864**
RGD1562143	PREDICTED: Rattus norvegicus similar to Ctps protein (RGD1562143), mRNA [XM_001062088]	**10,863**
Dnajb1	Rattus norvegicus DnaJ (Hsp40) homolog, subfamily B, member 1 (Dnajb1), mRNA [NM_001108441]	**10,858**
0	Unknown	**10,856**
Wdfy1	Rattus norvegicus WD repeat and FYVE domain containing 1 (Wdfy1), mRNA [NM_001008331]	**10,856**
Hoxa9l	RCG52500, isoform CRA_aUncharacterized protein [Source:UniProtKB/TrEMBL;Acc:D3ZSU5] [ENSRNOT00000009487]	**10,854**
Zfp354c	Rattus norvegicus zinc finger protein 354C (Zfp354c), mRNA [NM_023988]	**10,853**
0	Unknown	**10,852**
Dnaja3	Rattus norvegicus DnaJ (Hsp40) homolog, subfamily A, member 3 (Dnaja3), transcript variant 1, mRNA [NM_001038595]	**10,852**
LOC311578	Rattus norvegicus mRNA for CSG142, partial cds. [AB190506]	**10,850**
Sorl1	Rattus norvegicus sortilin-related receptor, LDLR class A repeats-containing (Sorl1), mRNA [NM_053519]	**10,850**
Ppapdc1a	Rattus norvegicus phosphatidic acid phosphatase type 2 domain containing 1A (Ppapdc1a), mRNA [NM_001191631]	**10,848**
Elmod2	Rattus norvegicus ELMO/CED-12 domain containing 2 (Elmod2), mRNA [NM_001109506]	**10,846**
0	Rattus norvegicus similar to glyceraldehyde-3-phosphate dehydrogenase (LOC317168), mRNA [XM_228411]	**10,845**
RGD1561832	PREDICTED: Rattus norvegicus similar to CDNA sequence BC043301 (RGD1561832), mRNA [XM_001080370]	**10,844**
Zfand2a	Rattus norvegicus zinc finger, AN1-type domain 2A (Zfand2a), mRNA [NM_001008363]	**10,843**
Sepx1	Rattus norvegicus selenoprotein X, 1 (Sepx1), mRNA [NM_001044285]	**10,842**
0	Rattus norvegicus similar to glyceraldehyde-3-phosphate dehydrogenase (phosphorylating) (EC 1.2.1.12) - mouse (LOC295452), mRNA [XM_227696]	**10,842**
LOC688776	Uncharacterized protein [Source:UniProtKB/TrEMBL;Acc:D4AAL7] [ENSRNOT00000049703]	**10,840**
0	PREDICTED: Rattus norvegicus zinc finger protein 770 (Znf770), mRNA [XM_001079020]	**10,839**
Smarcc1	Rattus norvegicus SWI/SNF related, matrix associated, actin dependent regulator of chromatin, subfamily c, member 1 (Smarcc1), mRNA [NM_001106861]	**10,837**
Rpl18a	Rattus norvegicus ribosomal protein L18A (Rpl18a), mRNA [NM_212510]	**10,837**
0	Unknown	**10,833**
Cbfb	Rattus norvegicus core-binding factor, beta subunit (Cbfb), mRNA [NM_001013191]	**10,832**
0	Q2U0J3_ASPOR (Q2U0J3) Synaptic vesicle transporter SVOP and related transporters, partial (3%) [TC622715]	**10,829**
0	Uncharacterized protein [Source:UniProtKB/TrEMBL;Acc:D4ADG6] [ENSRNOT00000045033]	**10,828**
0	Unknown	**10,826**
0	Unknown	**10,826**

Cdc2l5	PREDICTED: Rattus norvegicus cell division cycle 2-like 5 (cholinesterase-related cell division controller), transcript variant 2 (Cdc2l5), mRNA [XM_001053609]	**10,824**
LOC681849	Uncharacterized protein [Source:UniProtKB/TrEMBL;Acc:D4A3C4] [ENSRNOT00000007809]	**10,824**
Fam84a	Rattus norvegicus family with sequence similarity 84, member A (Fam84a), mRNA [NM_001127299]	**10,824**
0	Unknown	**10,822**
0	Unknown	**10,821**
0	Unknown	**10,820**
0	voltage-dependent anion channel 1 (Vdac1), nuclear gene encoding mitochondrial protein, mRNA [Source:RefSeq DNA;Acc:NM_031353] [ENSRNOT00000006775]	**10,819**
Erbb2	Rattus norvegicus v-erb-b2 erythroblastic leukemia viral oncogene homolog 2, neuro/glioblastoma derived oncogene homolog (avian) (Erbb2), mRNA [NM_017003]	**10,815**
Lcmt2	Rattus norvegicus leucine carboxyl methyltransferase 2 (Lcmt2), mRNA [NM_001011956]	**10,812**
Cpsf2	Rattus norvegicus cleavage and polyadenylation specific factor 2 (Cpsf2), mRNA [NM_001106753]	**10,811**
Agfg1	Rattus norvegicus ArfGAP with FG repeats 1 (Agfg1), mRNA [NM_001135596]	**10,809**
Evx1	Rattus norvegicus even-skipped homeobox 1 (Evx1), mRNA [NM_001191972]	**10,809**
Col9a1	Rattus norvegicus collagen, type IX, alpha 1 (Col9a1), mRNA [NM_001100842]	**10,808**
Smpd4	Rattus norvegicus sphingomyelin phosphodiesterase 4, neutral membrane (Smpd4), mRNA [NM_001167806]	**10,806**
Hibch	Rattus norvegicus 3-hydroxyisobutyryl-Coenzyme A hydrolase (Hibch), nuclear gene encoding mitochondrial protein, mRNA [NM_001013112]	**10,805**
Ddx19a	Rattus norvegicus DEAD (Asp-Glu-Ala-Asp) box polypeptide 19a (Ddx19a), mRNA [NM_001005381]	**10,805**
Ldlr	Rattus norvegicus low density lipoprotein receptor (Ldlr), mRNA [NM_175762]	**10,804**
0	U1 small nuclear ribonucleoprotein C [Source:UniProtKB/TrEMBL;Acc:D3ZCL3] [ENSRNOT00000000586]	**10,801**
Fgfr3	Rattus norvegicus fibroblast growth factor receptor 3 (Fgfr3), mRNA [NM_053429]	**10,799**
Med24	Rattus norvegicus mediator complex subunit 24 (Med24), mRNA [NM_001034079]	**10,798**
Ucma	Rattus norvegicus upper zone of growth plate and cartilage matrix associated (Ucma), mRNA [NM_001106121]	**10,798**
Olr1750	Rattus norvegicus olfactory receptor 1750 (Olr1750), mRNA [NM_212493]	**10,798**
Smarce1	Rattus norvegicus SWI/SNF related, matrix associated, actin dependent regulator of chromatin, subfamily e, member 1 (Smarce1), mRNA [NM_001024993]	**10,797**
Atp2a2	Rattus norvegicus ATPase, Ca++ transporting, cardiac muscle, slow twitch 2 (Atp2a2), transcript variant 2, mRNA [NM_001110139]	**10,796**
Pkm2	Rattus norvegicus pyruvate kinase, muscle (Pkm2), mRNA [NM_053297]	**10,796**
RGD1306613	Rattus norvegicus similar to RIKEN cDNA 1600012F09 (RGD1306613), mRNA [NM_001107356]	**10,794**
Dync1li2	Rattus norvegicus dynein, cytoplasmic 1 light intermediate chain 2 (Dync1li2), mRNA [NM_031026]	**10,793**
Ng35	Rattus norvegicus Ng35 pseudogene (Ng35), mRNA [NM_001134991]	**10,793**
0	BQ210707 UI-R-DY1-com-a-24-0-UI.s1 NCI_CGAP_DY1 Rattus norvegicus cDNA clone IMAGE:7337090 3', mRNA sequence [BQ210707]	**10,792**
Lrrc36	Rattus norvegicus leucine rich repeat containing 36 (Lrrc36), mRNA [NM_001004088]	**10,790**

Wee1	Rattus norvegicus wee 1 homolog (S. pombe) (Wee1), mRNA [NM_001012742]	**10,789**
Iqgap1	Rattus norvegicus IQ motif containing GTPase activating protein 1 (Iqgap1), mRNA [NM_001108489]	**10,787**
RGD1563194	PREDICTED: Rattus norvegicus similar to ribosomal protein L13 (RGD1563194), miscRNA [XR_009316]	**10,787**
Dnajc14	Rattus norvegicus DnaJ (Hsp40) homolog, subfamily C, member 14 (Dnajc14), mRNA [NM_053690]	**10,785**
0	Uncharacterized protein [Source:UniProtKB/TrEMBL;Acc:D4A268] [ENSRNOT00000007350]	**10,785**
Hes6	Rattus norvegicus hairy and enhancer of split 6 (Drosophila) (Hes6), mRNA [NM_001013179]	**10,785**
Nme4	Rattus norvegicus non-metastatic cells 4, protein expressed in (Nme4), nuclear gene encoding mitochondrial protein, mRNA [NM_001109478]	**10,785**
Nfkbil2	Rattus norvegicus nuclear factor of kappa light polypeptide gene enhancer in B-cells inhibitor-like 2 (Nfkbil2), mRNA [NM_001130572]	**10,779**
RGD1306410	Rattus norvegicus similar to CG14980-PB (RGD1306410), mRNA [NM_001014126]	**10,778**
0	TBC domain-containing protein kinase-like protein [Source:RefSeq peptide;Acc:NP_001127985] [ENSRNOT00000015502]	**10,777**
0	Unknown	**10,777**
Ttc39a	Rattus norvegicus tetratricopeptide repeat domain 39A (Ttc39a), mRNA [NM_001134519]	**10,775**
Slc25a25	Rattus norvegicus solute carrier family 25 (mitochondrial carrier, phosphate carrier), member 25 (Slc25a25), nuclear gene encoding mitochondrial protein, mRNA [NM_145677]	**10,774**
Slc2a4	Rattus norvegicus solute carrier family 2 (facilitated glucose transporter), member 4 (Slc2a4), mRNA [NM_012751]	**10,773**
Snrnp27	Rattus norvegicus small nuclear ribonucleoprotein 27 (U4/U6.U5) (Snrnp27), mRNA [NM_001108636]	**10,770**
Mospd1	Rattus norvegicus motile sperm domain containing 1 (Mospd1), mRNA [NM_001014107]	**10,769**
Cep164	PREDICTED: Rattus norvegicus centrosomal protein 164kDa (Cep164), mRNA [XM_001064241]	**10,768**
0	Unknown	**10,768**
Fgfrl1	Rattus norvegicus fibroblast growth factor receptor-like 1 (Fgfrl1), mRNA [NM_199114]	**10,767**
Atp1b1	Rattus norvegicus ATPase, Na+/K+ transporting, beta 1 polypeptide (Atp1b1), mRNA [NM_013113]	**10,762**
0	Unknown	**10,761**
LOC683753	PREDICTED: Rattus norvegicus hypothetical protein LOC683753 (LOC683753), mRNA [XM_001067334]	**10,761**
Lix1l	Rattus norvegicus Lix1 homolog (mouse)-like (Lix1l), mRNA [NM_001024303]	**10,761**
Myo9b	Rattus norvegicus myosin IXb (Myo9b), mRNA [NM_012984]	**10,760**
Mapk1ip1	Rattus norvegicus mitogen-activated protein kinase 1 interacting protein 1 (Mapk1ip1), mRNA [NM_001122782]	**10,760**
0	Q7TQ74_RAT (Q7TQ74) Ac1573, partial (11%) [TC600249]	**10,758**
Nid2	Rattus norvegicus nidogen 2 (Nid2), mRNA [NM_001012005]	**10,757**
Ccdc80	Rattus norvegicus coiled-coil domain containing 80 (Ccdc80), mRNA [NM_022543]	**10,755**
0	Uncharacterized protein [Source:UniProtKB/TrEMBL;Acc:D3ZIY8] [ENSRNOT00000029667]	**10,755**
Abhd14a	Rattus norvegicus abhydrolase domain containing 14A (Abhd14a), mRNA [NM_001009670]	**10,754**
Bai2	Rattus norvegicus brain-specific angiogenesis inhibitor 2 (Bai2), mRNA [NM_001107914]	**10,753**
42248	Rattus norvegicus selenoprotein 15 (Sep15), mRNA [NM_133297]	**10,753**
Ube2j1	Rattus norvegicus ubiquitin-conjugating enzyme E2, J1 (UBC6 homolog, yeast) (Ube2j1), mRNA [NM_001106642]	**10,752**

Pacsin2	Rattus norvegicus protein kinase C and casein kinase substrate in neurons 2 (Pacsin2), mRNA [NM_130740]	**10,752**
0	Unknown	**10,751**
Rtp4	Rattus norvegicus receptor (chemosensory) transporter protein 4 (Rtp4), mRNA [NM_001108321]	**10,751**
Fpgs	Rattus norvegicus folylpolyglutamate synthase (Fpgs), nuclear gene encoding mitochondrial protein, mRNA [NM_001146125]	**10,750**
0	Unknown	**10,749**
Sgms1	Rattus norvegicus sphingomyelin synthase 1 (Sgms1), mRNA [NM_181386]	**10,749**
Cycs	Rattus norvegicus cytochrome c, somatic (Cycs), nuclear gene encoding mitochondrial protein, mRNA [NM_012839]	**10,748**
Kel	Rattus norvegicus Kell blood group, metallo-endopeptidase (Kel), mRNA [NM_001191611]	**10,747**
RT1-EC2	Rattus norvegicus RT1 class Ib, locus EC2 (RT1-EC2), mRNA [NM_012645]	**10,745**
0	Uncharacterized protein [Source:UniProtKB/TrEMBL;Acc:D4A2X3] [ENSRNOT00000038101]	**10,743**
0	Uncharacterized protein [Source:UniProtKB/TrEMBL;Acc:D3ZKL6] [ENSRNOT00000044160]	**10,741**
Rhoq	Rattus norvegicus ras homolog gene family, member Q (Rhoq), mRNA [NM_053522]	**10,741**
Anks6	Rattus norvegicus ankyrin repeat and sterile alpha motif domain containing 6 (Anks6), mRNA [NM_001015028]	**10,740**
Zfand1	PREDICTED: Rattus norvegicus zinc finger, AN1-type domain 1 (Zfand1), mRNA [XM_342213]	**10,739**
Ptgir	Rattus norvegicus prostaglandin I2 (prostacyclin) receptor (IP) (Ptgir), mRNA [NM_001077644]	**10,739**
Olr1681	Rattus norvegicus olfactory receptor 1681 (Olr1681), mRNA [NM_001001006]	**10,736**
RGD1308319	Uncharacterized protein [Source:UniProtKB/TrEMBL;Acc:D3ZBT5] [ENSRNOT00000050012]	**10,734**
Rngtt	Rattus norvegicus RNA guanylyltransferase and 5'-phosphatase (Rngtt), mRNA [NM_001107923]	**10,734**
Reck	Rattus norvegicus reversion-inducing-cysteine-rich protein with kazal motifs (Reck), mRNA [NM_001107954]	**10,734**
Bbs9	PREDICTED: Rattus norvegicus Bardet-Biedl syndrome 9 (Bbs9), mRNA [XM_235942]	**10,733**
0	Unknown	**10,730**
Havcr1	Rattus norvegicus hepatitis A virus cellular receptor 1 (Havcr1), mRNA [NM_173149]	**10,730**
Tacr2	Rattus norvegicus tachykinin receptor 2 (Tacr2), mRNA [NM_080768]	**10,728**
0	Unknown	**10,726**
0	Unknown	**10,725**
Prpf38b	Rattus norvegicus PRP38 pre-mRNA processing factor 38 (yeast) domain containing B (Prpf38b), mRNA [NM_001024305]	**10,725**
Inpp4a	Rattus norvegicus inositol polyphosphate-4-phosphatase, type 1 (Inpp4a), mRNA [NM_031002]	**10,725**
Ppp1r15a	Rattus norvegicus protein phosphatase 1, regulatory (inhibitor) subunit 15A (Ppp1r15a), mRNA [NM_133546]	**10,723**
Fkbp4	Rattus norvegicus FK506 binding protein 4 (Fkbp4), mRNA [NM_001191863]	**10,720**
Zfand1	Putative uncharacterized protein RGD1309519_predictedUncharacterized protein [Source:UniProtKB/TrEMBL;Acc:D3ZQI4] [ENSRNOT00000013905]	**10,719**
Pitpnc1	Uncharacterized protein [Source:UniProtKB/TrEMBL;Acc:D4AE57] [ENSRNOT00000021498]	**10,719**
Ddx3x	Rattus norvegicus DEAD (Asp-Glu-Ala-Asp) box polypeptide 3, X-linked (Ddx3x), mRNA [NM_001108246]	**10,716**
Prom2	Rattus norvegicus prominin 2 (Prom2), mRNA [NM_138857]	**10,715**

0	Unknown	**10,715**
Etnk2	Rattus norvegicus ethanolamine kinase 2 (Etnk2), mRNA [NM_001108343]	**10,714**
Ptprz1	Rattus norvegicus protein tyrosine phosphatase, receptor-type, Z polypeptide 1 (Ptprz1), transcript variant 1, mRNA [NM_013080]	**10,714**
0	Unknown	**10,713**
Hes1	Rattus norvegicus hairy and enhancer of split 1 (Drosophila) (Hes1), mRNA [NM_024360]	**10,713**
LOC679715	PREDICTED: Rattus norvegicus similar to ribosomal protein L13 (LOC679715), mRNA [XM_001054162]	**10,713**
0	Unknown	**10,713**
Ptprm	Rattus norvegicus protein tyrosine phosphatase, receptor type, M (Ptprm), mRNA [NM_001168632]	**10,711**
0	Unknown	**10,710**
Fam104a	Rattus norvegicus family with sequence similarity 104, member A (Fam104a), mRNA [NM_001034958]	**10,709**
Polr3f	Rattus norvegicus polymerase (RNA) III (DNA directed) polypeptide F (Polr3f), mRNA [NM_001107784]	**10,709**
Tmem104	Rattus norvegicus transmembrane protein 104 (Tmem104), mRNA [NM_001191655]	**10,709**
Camk2b	Rattus norvegicus calcium/calmodulin-dependent protein kinase II beta (Camk2b), transcript variant 2, mRNA [NM_021739]	**10,706**
Tmem106b	Rattus norvegicus transmembrane protein 106B (Tmem106b), mRNA [NM_001004267]	**10,706**
Wnt16	Rattus norvegicus wingless-type MMTV integration site family, member 16 (Wnt16), mRNA [NM_001109223]	**10,704**
Rpl23a	Rattus norvegicus ribosomal protein L23a (Rpl23a), mRNA [NM_001108283]	**10,701**
Fam18a	Rattus norvegicus family with sequence similarity 18, member A (Fam18a), mRNA [NM_001108263]	**10,701**
Ncf1	Rattus norvegicus neutrophil cytosolic factor 1 (Ncf1), mRNA [NM_053734]	**10,697**
Peg10	PREDICTED: Rattus norvegicus paternally expressed 10 (Peg10), mRNA [XM_001053454]	**10,696**
Ttc38	Rattus norvegicus tetratricopeptide repeat domain 38 (Ttc38), mRNA [NM_001130499]	**10,696**
Ccr10	Rattus norvegicus chemokine (C-C motif) receptor 10 (Ccr10), mRNA [NM_001108836]	**10,695**
Cyp4v3	Rattus norvegicus cytochrome P450, family 4, subfamily v, polypeptide 3 (Cyp4v3), mRNA [NM_001135600]	**10,695**
Tmem82	Putative uncharacterized protein RGD1566169_predictedUncharacterized protein [Source:UniProtKB/TrEMBL;Acc:D4A8K4] [ENSRNOT00000016176]	**10,693**
0	Unknown	**10,693**
0	SC23A_MOUSE (Q01405) Protein transport protein Sec23A (SEC23-related protein A), complete [TC596010]	**10,692**
RGD1560151	PREDICTED: Rattus norvegicus similar to predicted CDS, mechanosensory transduction channel NOMPC (1O503) (RGD1560151), mRNA [XM_576520]	**10,692**
Znf629	Rattus norvegicus zinc finger protein-like mRNA, complete sequence. [AY589489]	**10,686**
0	Unknown	**10,684**
Gprc5c	G-protein coupled receptor family C group 5 member C [Source:UniProtKB/Swiss-Prot;Acc:Q3KRC4] [ENSRNOT00000004256]	**10,681**
Slc30a4	Rattus norvegicus solute carrier family 30 (zinc transporter), member 4 (Slc30a4), mRNA [NM_172066]	**10,681**
Dennd5a	Rattus norvegicus DENN/MADD domain containing 5A (Dennd5a), mRNA [NM_001107546]	**10,678**
Mpz	Rattus norvegicus myelin protein zero (Mpz), mRNA [NM_017027]	**10,677**

0	Uncharacterized protein [Source:UniProtKB/TrEMBL;Acc:D4AA66] [ENSRNOT00000056882]	**10,676**
Rfc1	Rattus norvegicus replication factor C (activator 1) 1 (Rfc1), mRNA [NM_053547]	**10,675**
0	Uncharacterized protein [Source:UniProtKB/TrEMBL;Acc:D3ZAF9] [ENSRNOT00000005478]	**10,673**
Cyth1	Rattus norvegicus cytohesin 1 (Cyth1), mRNA [NM_053910]	**10,672**
RGD1304810	Rattus norvegicus similar to 6430573F11Rik protein (RGD1304810), mRNA [NM_001107314]	**10,672**
0	Uncharacterized protein [Source:UniProtKB/TrEMBL;Acc:D3ZHU8] [ENSRNOT00000001891]	**10,670**
Nptx2	Rattus norvegicus neuronal pentraxin 2 (Nptx2), mRNA [NM_001034199]	**10,666**
LOC680200	Rattus norvegicus similar to zinc finger protein 455 (LOC680200), mRNA [NM_001139491]	**10,664**
Tmem35	Rattus norvegicus transmembrane protein 35 (Tmem35), mRNA [NM_001001799]	**10,662**
RGD1565469	Rattus norvegicus RGD1565469 (RGD1565469), mRNA [NM_001109340]	**10,662**
0	Unknown	**10,661**
0	Uncharacterized protein [Source:UniProtKB/TrEMBL;Acc:D3Z969] [ENSRNOT00000036676]	**10,660**
Cgref1	Rattus norvegicus cell growth regulator with EF hand domain 1 (Cgref1), mRNA [NM_139087]	**10,658**
Abcc5	Rattus norvegicus ATP-binding cassette, subfamily C (CFTR/MRP), member 5 (Abcc5), mRNA [NM_053924]	**10,657**
Wars2	Rattus norvegicus tryptophanyl tRNA synthetase 2 (mitochondrial) (Wars2), nuclear gene encoding mitochondrial protein, mRNA [NM_001168641]	**10,657**
Vash2	Rattus norvegicus vasohibin 2 (Vash2), mRNA [NM_001109082]	**10,657**
Mbp	Rattus norvegicus myelin basic protein (Mbp), transcript variant 6, mRNA [NM_001025289]	**10,655**
Aldh18a1	Rattus norvegicus aldehyde dehydrogenase 18 family, member A1 (Aldh18a1), nuclear gene encoding mitochondrial protein, mRNA [NM_001108524]	**10,655**
Col19a1	Uncharacterized protein [Source:UniProtKB/TrEMBL;Acc:D3ZCQ0] [ENSRNOT00000017169]	**10,654**
Sh3bp2	Rattus norvegicus SH3-domain binding protein 2 (Sh3bp2), mRNA [NM_001100684]	**10,653**
Ide	Rattus norvegicus insulin degrading enzyme (Ide), mRNA [NM_013159]	**10,652**
LOC680799	Rattus norvegicus hypothetical protein LOC680799 (LOC680799), transcript variant 2, mRNA [NM_001163519]	**10,650**
0	Rattus norvegicus TL0AEA97YK17 mRNA sequence. [FQ230487]	**10,648**
0	Unknown	**10,646**
Crip	Rattus norvegicus cysteine-rich intestinal protein (Crip), mRNA [NM_001134933]	**10,645**
0	PREDICTED: Rattus norvegicus similar to TDPOZ3 (RGD1564313), mRNA [XM_578051]	**10,642**
0	Unknown	**10,641**
Ythdf3	Rattus norvegicus YTH domain family, member 3 (Ythdf3), mRNA [NM_001108546]	**10,640**
Setl1	Rattus norvegicus SET translocation-like 1 (Setl1), mRNA [NM_194353]	**10,639**
Rpl37a	Rattus norvegicus ribosomal protein L37a (Rpl37a), transcript variant 2, mRNA [NM_001108801]	**10,638**
0	Unknown	**10,637**
Phc1	Rattus norvegicus polyhomeotic homolog 1 (Drosophila) (Phc1), mRNA [NM_001107886]	**10,637**
Mafb	Rattus norvegicus v-maf musculoaponeurotic fibrosarcoma oncogene homolog B (avian) (Mafb), mRNA [NM_019316]	**10,636**

RGD1311458	Rattus norvegicus similar to cDNA sequence BC027231; hypothetical protein MGC27931 (RGD1311458), mRNA [NM_001009678]	**10,632**
Btbd9	Rattus norvegicus BTB (POZ) domain containing 9 (Btbd9), mRNA [NM_001013073]	**10,630**
Fmr1	Rattus norvegicus fragile X mental retardation 1 (Fmr1), mRNA [NM_052804]	**10,630**
0	Rattus norvegicus similar to splicing-related factor RNPS1 (LOC312994), mRNA [XM_232688]	**10,627**
0	Unknown	**10,626**
0	YLP motif-containing protein 1 [Source:UniProtKB/Swiss-Prot;Acc:P0CB49] [ENSRNOT00000047060]	**10,626**
0	Rattus norvegicus similar to glyceraldehyde-3-phosphate dehydrogenase (phosphorylating) (EC 1.2.1.12) - mouse (LOC291545), mRNA [XM_225859]	**10,626**
Snta1	Rattus norvegicus syntrophin, acidic 1 (Snta1), mRNA [NM_001100901]	**10,626**
LOC641520	Rattus norvegicus popeye domain-containing 3 (LOC641520), mRNA [NM_001037369]	**10,625**
Olr702	PREDICTED: Rattus norvegicus olfactory receptor 702 (Olr702), partial mRNA [XM_001077079]	**10,622**
Tmem200b	Uncharacterized protein [Source:UniProtKB/TrEMBL;Acc:D3Z9B6] [ENSRNOT00000047917]	**10,621**
Rgs6	PREDICTED: Rattus norvegicus regulator of G-protein signaling 6 (Rgs6), mRNA [XM_001063200]	**10,620**
Hipk1	Rattus norvegicus homeodomain interacting protein kinase 1 (Hipk1), mRNA [NM_001100986]	**10,618**
0	Uncharacterized protein [Source:UniProtKB/TrEMBL;Acc:D3ZKK0] [ENSRNOT00000062088]	**10,618**
Tmem56	Rattus norvegicus transmembrane protein 56 (Tmem56), mRNA [NM_001135879]	**10,618**
0	Q9NRI8_HUMAN (Q9NRI8) HT014, complete [TC634078]	**10,618**
Kdm4a	Rattus norvegicus lysine (K)-specific demethylase 4A (Kdm4a), mRNA [NM_001107966]	**10,617**
Apobec3f	Rattus norvegicus apolipoprotein B mRNA editing enzyme, catalytic polypeptide-like 3F (Apobec3f), mRNA [NM_001033703]	**10,616**
Gng5	Rattus norvegicus guanine nucleotide binding protein (G protein), gamma 5 (Gng5), mRNA [NM_024377]	**10,614**
Nde1	Rattus norvegicus nudE nuclear distribution gene E homolog 1 (A. nidulans) (Nde1), mRNA [NM_053347]	**10,613**
0	Unknown	**10,611**
0	Unknown	**10,611**
Batf2	Similar to RIKEN cDNA 4933430F08 (Predicted), isoform CRA_bUncharacterized protein [Source:UniProtKB/TrEMBL;Acc:D3ZT85] [ENSRNOT00000028527]	**10,610**
RGD1563556	Uncharacterized protein [Source:UniProtKB/TrEMBL;Acc:D3ZT87] [ENSRNOT00000029887]	**10,610**
Ppp1r1a	Rattus norvegicus protein phosphatase 1, regulatory (inhibitor) subunit 1A (Ppp1r1a), mRNA [NM_022676]	**10,609**
0	Unknown	**10,609**
Glce	RCG57892Uncharacterized protein [Source:UniProtKB/TrEMBL;Acc:D3ZIK0] [ENSRNOT00000038641]	**10,609**
LOC100364959	PREDICTED: Rattus norvegicus basic transcription factor 3-like (LOC100364959), mRNA [XM_002727692]	**10,609**
0	Uncharacterized protein [Source:UniProtKB/TrEMBL;Acc:D3ZVJ6] [ENSRNOT00000037445]	**10,608**
Sdpr	Rattus norvegicus serum deprivation response (Sdpr), mRNA [NM_001007712]	**10,604**
Vcan	Rattus norvegicus versican (Vcan), transcript variant 2, mRNA [NM_053663]	**10,604**
Znf282	Rattus norvegicus zinc finger protein 282 (Znf282), mRNA [NM_001106592]	**10,604**

Tcea1	Rattus norvegicus transcription elongation factor A (SII) 1 (Tcea1), mRNA [NM_001025735]	**10,603**
0	Protein tyrosine phosphatase delta [Source:UniProtKB/TrEMBL;Acc:Q62990] [ENSRNOT00000025426]	**10,602**
Plce1	Rattus norvegicus phospholipase C, epsilon 1 (Plce1), mRNA [NM_053758]	**10,600**
Arl6	Rattus norvegicus ADP-ribosylation factor-like 6 (Arl6), mRNA [NM_001108842]	**10,597**
0	predicted gene 12258 Gene [Source:MGI Symbol;Acc:MGI:3651534] [ENSRNOT00000067580]	**10,597**
0	Uncharacterized protein [Source:UniProtKB/TrEMBL;Acc:D4A0Y6] [ENSRNOT00000038176]	**10,597**
Eif4a1	Rattus norvegicus eukaryotic translation initiation factor 4A, isoform 1 (Eif4a1), mRNA [NM_199372]	**10,597**
Zc3h12c	Rattus norvegicus zinc finger CCCH type containing 12C (Zc3h12c), mRNA [NM_001108146]	**10,594**
LOC690206	Rattus norvegicus hypothetical protein LOC690206 (LOC690206), mRNA [NM_001109567]	**10,593**
Dnajc21	Rattus norvegicus DnaJ (Hsp40) homolog, subfamily C, member 21 (Dnajc21), mRNA [NM_138856]	**10,592**
0	Unknown	**10,592**
Wnk4	Rattus norvegicus WNK lysine deficient protein kinase 4 (Wnk4), mRNA [NM_175579]	**10,587**
0	Unknown	**10,587**
Tmem90b	Rattus norvegicus transmembrane protein 90B (Tmem90b), mRNA [NM_001025020]	**10,586**
Slc16a8	Monocarboxylate transporter 3 [Source:UniProtKB/Swiss-Prot;Acc:O70461] [ENSRNOT00000016247]	**10,586**
Mzf1	Rattus norvegicus myeloid zinc finger 1 (Mzf1), mRNA [NM_001108470]	**10,584**
0	LRRG00123LRRGT00103RCG65927 [Source:UniProtKB/TrEMBL;Acc:Q6QI85] [ENSRNOT00000043973]	**10,583**
Ppard	Rattus norvegicus peroxisome proliferator-activated receptor delta (Ppard), mRNA [NM_013141]	**10,582**
Trub2	Rattus norvegicus TruB pseudouridine (psi) synthase homolog 2 (E. coli) (Trub2), mRNA [NM_001014257]	**10,582**
Cask	Rattus norvegicus calcium/calmodulin-dependent serine protein kinase (MAGUK family) (Cask), mRNA [NM_022184]	**10,579**
0	Unknown	**10,577**
0	Rattus norvegicus similar to glyceraldehyde-3-phosphate dehydrogenase (LOC294417), mRNA [XM_228193]	**10,577**
Mrps21l	PREDICTED: Rattus norvegicus mitochondrial ribosomal protein S21-like (Mrps21l), mRNA [XM_001057655]	**10,575**
0	Unknown	**10,574**
Prdx3	Rattus norvegicus peroxiredoxin 3 (Prdx3), nuclear gene encoding mitochondrial protein, mRNA [NM_022540]	**10,574**
RGD1565705	PREDICTED: Rattus norvegicus similar to chr2 synaptotagmin (RGD1565705), mRNA [XM_002726849]	**10,571**
0	Uncharacterized protein [Source:UniProtKB/TrEMBL;Acc:D3ZKR7] [ENSRNOT00000059076]	**10,571**
0	Uncharacterized protein [Source:UniProtKB/TrEMBL;Acc:D3ZM66] [ENSRNOT00000042277]	**10,569**
0	Unknown	**10,568**
0	Ribosomal protein [Source:UniProtKB/TrEMBL;Acc:D3Z9F6] [ENSRNOT00000045023]	**10,568**
Ptdss2	Rattus norvegicus phosphatidylserine synthase 2 (Ptdss2), mRNA [NM_001106316]	**10,568**
Cacna1a	Rattus norvegicus calcium channel, voltage-dependent, P/Q type, alpha 1A subunit (Cacna1a), mRNA [NM_012918]	**10,566**
0	Uncharacterized protein [Source:UniProtKB/TrEMBL;Acc:D3ZIH7] [ENSRNOT00000033109]	**10,565**
Slc12a2	Rattus norvegicus solute carrier family 12 (sodium/potassium/chloride transporters), member 2 (Slc12a2), mRNA [NM_031798]	**10,564**

Vwf	von Willebrand factor [Source:UniProtKB/Swiss-Prot;Acc:Q62935] [ENSRNOT00000026643]	**10,563**
Olr68	Rattus norvegicus olfactory receptor 68 (Olr68), mRNA [NM_001000133]	**10,562**
Usp53	Rattus norvegicus ubiquitin specific peptidase 53 (Usp53), mRNA [NM_001106468]	**10,562**
Itsn1	Rattus norvegicus intersectin 1 (SH3 domain protein) (Itsn1), transcript variant 1, mRNA [NM_001136096]	**10,561**
Vom2r75	Rattus norvegicus vomeronasal 2 receptor, 75 (Vom2r75), mRNA [NM_173320]	**10,560**
Aqp11	Rattus norvegicus aquaporin 11 (Aqp11), mRNA [NM_173105]	**10,560**
Spata20	Rattus norvegicus spermatogenesis associated 20 (Spata20), mRNA [NM_199402]	**10,554**
0	Unknown	**10,554**
Nt5c3	Rattus norvegicus 5'-nucleotidase, cytosolic III (Nt5c3), mRNA [NM_001107862]	**10,551**
Slc22a3	Rattus norvegicus solute carrier family 22 (extraneuronal monoamine transporter), member 3 (Slc22a3), mRNA [NM_019230]	**10,551**
Tyro3	Rattus norvegicus TYRO3 protein tyrosine kinase (Tyro3), mRNA [NM_017092]	**10,550**
LOC499746	LOC499746 protein [Source:UniProtKB/TrEMBL;Acc:Q498T7] [ENSRNOT00000013278]	**10,544**
Exosc2	Rattus norvegicus exosome component 2 (Exosc2), mRNA [NM_001108952]	**10,543**
Osbpl9	Rattus norvegicus oxysterol binding protein-like 9 (Osbpl9), mRNA [NM_001044234]	**10,543**
Cdc25a	Rattus norvegicus cell division cycle 25 homolog A (S. pombe) (Cdc25a), mRNA [NM_133571]	**10,543**
Ranbp1	Rattus norvegicus RAN binding protein 1 (Ranbp1), mRNA [NM_001108324]	**10,542**
Mdk	Rattus norvegicus midkine (Mdk), mRNA [NM_030859]	**10,539**
Hba-a2	Rattus norvegicus hemoglobin alpha, adult chain 2 (Hba-a2), mRNA [NM_013096]	**10,538**
Dock7	Rattus norvegicus dedicator of cytokinesis 7 (Dock7), mRNA [NM_001191574]	**10,537**
0	RVL6519 Wackym-Soares normalized rat vestibular cDNA library Rattus norvegicus cDNA 5', mRNA sequence [DV716357]	**10,537**
RGD1561557	Rattus norvegicus similar to chromosome 21 open reading frame 29 (RGD1561557), mRNA [NM_001108929]	**10,537**
Clmn	Rattus norvegicus calmin (Clmn), mRNA [NM_001106755]	**10,536**
RGD1310553	Rattus norvegicus similar to expressed sequence AI597479 (RGD1310553), mRNA [NM_001008517]	**10,535**
Otud5	Rattus norvegicus OTU domain containing 5 (Otud5), mRNA [NM_001037496]	**10,534**
Mtx1	Rattus norvegicus Metaxin 1 (Mtx1), nuclear gene encoding mitochondrial protein, mRNA [NM_001100667]	**10,533**
Tm6sf2	Rattus norvegicus transmembrane 6 superfamily member 2 (Tm6sf2), mRNA [NM_001127654]	**10,532**
B4galt2	Rattus norvegicus UDP-Gal:betaGlcNAc beta 1,4- galactosyltransferase, polypeptide 2 (B4galt2), mRNA [NM_001107965]	**10,530**
Eri2	Rattus norvegicus exoribonuclease 2 (Eri2), mRNA [NM_001191114]	**10,529**
RGD1561662	Uncharacterized protein [Source:UniProtKB/TrEMBL;Acc:D3ZVE5] [ENSRNOT00000020516]	**10,529**
Mag	Rattus norvegicus myelin-associated glycoprotein (Mag), mRNA [NM_017190]	**10,527**
Cul4b	Rattus norvegicus cullin 4B (Cul4b), mRNA [NM_001106951]	**10,524**
RT1-CE2	Rattus norvegicus RT1 class I, locus CE2 (RT1-CE2), mRNA [NM_001008840]	**10,522**
A26c2	PREDICTED: Rattus norvegicus ANKRD26-like family C, member 2 (A26c2), mRNA [XM_001055615]	**10,521**
Znf763	Rattus norvegicus zinc finger protein 763 (Znf763), mRNA [NM_001108063]	**10,519**

Hltf	Rattus norvegicus helicase-like transcription factor (Hltf), mRNA [NM_001106478]	**10,518**
Olr1734	Rattus norvegicus olfactory receptor 1734 (Olr1734), mRNA [NM_001001119]	**10,517**
Mll4	Uncharacterized protein [Source:UniProtKB/TrEMBL;Acc:D3ZKG0] [ENSRNOT00000046359]	**10,514**
Kif16b	Rattus norvegicus kinesin family member 16B (Kif16b), mRNA [NM_001107783]	**10,512**
Caprin1	Rattus norvegicus cell cycle associated protein 1 (Caprin1), mRNA [NM_001012185]	**10,511**
RGD1565432	Small VCP/p97-interacting protein [Source:UniProtKB/Swiss-Prot;Acc:P0C0A9] [ENSRNOT00000056024]	**10,510**
Vipr1	Rattus norvegicus vasoactive intestinal peptide receptor 1 (Vipr1), mRNA [NM_012685]	**10,510**
Rasgrp1	Rattus norvegicus RAS guanyl releasing protein 1 (calcium and DAG-regulated) (Rasgrp1), mRNA [NM_019211]	**10,506**
Tmem39a	Rattus norvegicus transmembrane protein 39a (Tmem39a), mRNA [NM_001013865]	**10,504**
0	T-cell receptor beta, variable 5.2 Gene [Source:MGI Symbol;Acc:MGI:98602] [ENSRNOT00000067917]	**10,503**
0	Unknown	**10,503**
Rapgef5	Rattus norvegicus Rap guanine nucleotide exchange factor (GEF) 5 (Rapgef5), mRNA [NM_001047915]	**10,502**
Acin1	Rattus norvegicus apoptotic chromatin condensation inducer 1 (Acin1), mRNA [NM_001170468]	**10,500**
Hopx	Rattus norvegicus HOP homeobox (Hopx), mRNA [NM_133621]	**10,499**
Zfp469	Rattus norvegicus zinc finger protein 469 (Zfp469), mRNA [NM_001107123]	**10,495**
Ssfa2	Rattus norvegicus sperm specific antigen 2 (Ssfa2), mRNA [NM_001107738]	**10,495**
Erh	Rattus norvegicus enhancer of rudimentary homolog (Drosophila) (Erh), mRNA [NM_001109442]	**10,494**
S1pr3	Cardiac sphingosine-1-phosphate specific receptor [Source:UniProtKB/TrEMBL;Acc:Q9QZG4] [ENSRNOT00000019473]	**10,494**
Wfs1	Rattus norvegicus Wolfram syndrome 1 homolog (human) (Wfs1), mRNA [NM_031823]	**10,493**
LOC314600	Rattus norvegicus similar to zinc finger protein 422, related sequence 1 (LOC314600), mRNA [NM_001126281]	**10,489**
Pex14	Rattus norvegicus peroxisomal biogenesis factor 14 (Pex14), mRNA [NM_172063]	**10,488**
Zswim3	Rattus norvegicus zinc finger, SWIM-type containing 3 (Zswim3), mRNA [NM_001107801]	**10,488**
0	Unknown	**10,486**
0	Unknown	**10,486**
RGD1309362	Rattus norvegicus similar to interferon-inducible GTPase (RGD1309362), mRNA [NM_001024884]	**10,480**
Rg9mtd2	Rattus norvegicus RNA (guanine-9-) methyltransferase domain containing 2 (Rg9mtd2), mRNA [NM_001044232]	**10,479**
Fbxw8	Rattus norvegicus F-box and WD repeat domain containing 8 (Fbxw8), mRNA [NM_001107145]	**10,475**
0	Unknown	**10,474**
0	Uncharacterized protein [Source:UniProtKB/TrEMBL;Acc:D3ZD85] [ENSRNOT00000017383]	**10,474**
0	Unknown	**10,473**
Sh2b1	Rattus norvegicus SH2B adaptor protein 1 (Sh2b1), transcript variant 1, mRNA [NM_134456]	**10,473**
0	Unknown	**10,472**
Atg7	Rattus norvegicus ATG7 autophagy related 7 homolog (S. cerevisiae) (Atg7), mRNA [NM_001012097]	**10,471**
Rnf14	Rattus norvegicus ring finger protein 14 (Rnf14), mRNA [NM_001034995]	**10,469**

RGD1561231	PREDICTED: Rattus norvegicus similar to MAP/microtubule affinity-regulating kinase 4 (MAP/microtubule affinity-regulating kinase like 1) (RGD1561231), mRNA [XM_225619]	**10,469**
Pramel7	PREDICTED: Rattus norvegicus similar to PRAMEI7 (RGD1565990), mRNA [XM_001074348]	**10,468**
Rpgrip1l	Rattus norvegicus Rpgrip1-like (Rpgrip1l), mRNA [NM_001107414]	**10,468**
Efna5	Rattus norvegicus ephrin A5 (Efna5), mRNA [NM_053903]	**10,468**
0	Unknown	**10,466**
0	Sirtuin 1 ((Silent mating type information regulation 2, homolog) 1 (S. cerevisiae) (Predicted), isoform CRA_a)Uncharacterized protein [Source:UniProtKB/TrEMBL;Acc:D4A0K3] [ENSRNOT00000067413]	**10,465**
Olr1555	Rattus norvegicus olfactory receptor 1555 (Olr1555), mRNA [NM_001000726]	**10,465**
0	Rattus norvegicus hypothetical gene supported by NM_022920 (LOC360236), mRNA [XM_346394]	**10,464**
0	Unknown	**10,464**
Sfrs2	Rattus norvegicus splicing factor, arginine/serine-rich 2 (Sfrs2), mRNA [NM_001009720]	**10,462**
Wwp1	Rattus norvegicus WW domain containing E3 ubiquitin protein ligase 1 (Wwp1), mRNA [NM_001024757]	**10,462**
Rbm14	RNA binding motif protein 4 [Source:RefSeq peptide;Acc:NP_001163955] [ENSRNOT00000026621]	**10,461**
Icmt	Rattus norvegicus isoprenylcysteine carboxyl methyltransferase (Icmt), mRNA [NM_133310]	**10,460**
RGD1306924	RCG27015, isoform CRA_aUncharacterized protein [Source:UniProtKB/TrEMBL;Acc:D3ZVR0] [ENSRNOT00000018460]	**10,459**
0	similar to RIKEN cDNA 1700001E04 (MGC116197), mRNA [Source:RefSeq DNA;Acc:NM_001025755] [ENSRNOT00000043269]	**10,459**
Mterfd2	Rattus norvegicus MTERF domain containing 2 (Mterfd2), mRNA [NM_001037209]	**10,456**
0	Uncharacterized protein [Source:UniProtKB/TrEMBL;Acc:D4ADA8] [ENSRNOT00000012904]	**10,456**
Rtdr1	Rattus norvegicus rhabdoid tumor deletion region gene 1 (Rtdr1), mRNA [NM_001127557]	**10,456**
Bcl11b	Rattus norvegicus B-cell CLL/lymphoma 11B (zinc finger protein) (Bcl11b), mRNA [NM_001108057]	**10,454**
0	Uncharacterized protein [Source:UniProtKB/TrEMBL;Acc:D4ABL4] [ENSRNOT00000060251]	**10,454**
Ptpn11	Rattus norvegicus protein tyrosine phosphatase, non-receptor type 11 (Ptpn11), transcript variant 1, mRNA [NM_001177593]	**10,454**
Pacsin1	Rattus norvegicus protein kinase C and casein kinase substrate in neurons 1 (Pacsin1), mRNA [NM_017294]	**10,453**
Capza2	Rattus norvegicus capping protein (actin filament) muscle Z-line, alpha 2 (Capza2), mRNA [NM_001009180]	**10,450**
Glb1	Rattus norvegicus galactosidase, beta 1 (Glb1), mRNA [NM_001108192]	**10,449**
Jmjd8	Rattus norvegicus jumonji domain containing 8 (Jmjd8), mRNA [NM_001014116]	**10,448**
Trim44	Rattus norvegicus tripartite motif-containing 44 (Trim44), mRNA [NM_001013203]	**10,448**
LOC685994	PREDICTED: Rattus norvegicus similar to Spetex-2C protein (LOC685994), mRNA [XM_001066107]	**10,447**
Mettl4	Rattus norvegicus methyltransferase like 4 (Mettl4), mRNA [NM_001191814]	**10,442**
0	Unknown	**10,442**
Farp2	Rattus norvegicus FERM, RhoGEF and pleckstrin domain protein 2 (Farp2), mRNA [NM_001108233]	**10,440**
Ogg1	Rattus norvegicus 8-oxoguanine DNA glycosylase (Ogg1), nuclear gene encoding mitochondrial protein, mRNA [NM_030870]	**10,440**
Thsd7a	Rattus norvegicus thrombospondin, type I, domain containing 7A (Thsd7a), mRNA [NM_001191970]	**10,439**

Atf6b	Rattus norvegicus activating transcription factor 6 beta (Atf6b), mRNA [NM_001002809]	**10,436**
Sel1l3	PREDICTED: Rattus norvegicus similar to RIKEN cDNA 2310045A20 (RGD1562860), mRNA [XM_341223]	**10,436**
Me2	Rattus norvegicus malic enzyme 2, NAD(+)-dependent, mitochondrial (Me2), nuclear gene encoding mitochondrial protein, mRNA [NM_001107376]	**10,435**
Fam107b	Rattus norvegicus family with sequence similarity 107, member B (Fam107b), mRNA [NM_001025034]	**10,433**
0	PCTK2_RAT (O35831) Serine/threonine-protein kinase PCTAIRE-2 (PCTAIRE-motif protein kinase 2) , complete [TC581304]	**10,433**
LOC685179	Uncharacterized protein [Source:UniProtKB/TrEMBL;Acc:D3ZPF5] [ENSRNOT00000061933]	**10,432**
0	Unknown	**10,432**
Cstf2	Rattus norvegicus cleavage stimulation factor, 3' pre-RNA subunit 2 (Cstf2), mRNA [NM_001131014]	**10,428**
Robo2	Rattus norvegicus roundabout homolog 2 (Drosophila) (Robo2), mRNA [NM_032106]	**10,428**
Fam116a	Rattus norvegicus family with sequence similarity 116, member A (Fam116a), mRNA [NM_001134467]	**10,425**
Ttc30b	Rattus norvegicus tetratricopeptide repeat domain 30B (Ttc30b), mRNA [NM_001127607]	**10,423**
Slc35a4	Rattus norvegicus solute carrier family 35, member A4 (Slc35a4), mRNA [NM_147140]	**10,420**
Dcun1d1	Rattus norvegicus DCN1, defective in cullin neddylation 1, domain containing 1 (S. cerevisiae) (Dcun1d1), mRNA [NM_001107668]	**10,417**
0	Uncharacterized protein [Source:UniProtKB/TrEMBL;Acc:D3ZBC5] [ENSRNOT00000035888]	**10,416**
Brwd3	Uncharacterized protein [Source:UniProtKB/TrEMBL;Acc:D3Z8C5] [ENSRNOT00000050336]	**10,416**
Olr1454	Rattus norvegicus olfactory receptor 1454 (Olr1454), mRNA [NM_001000020]	**10,415**
0	Unknown	**10,413**
Cbx1	PREDICTED: Rattus norvegicus chromobox homolog 1 (HP1 beta homolog Drosophila) (Cbx1), mRNA [XM_001081346]	**10,412**
Elovl1	Rattus norvegicus elongation of very long chain fatty acids (FEN1/Elo2, SUR4/Elo3, yeast)-like 1 (Elovl1), mRNA [NM_001044275]	**10,409**
Alox12b	Rattus norvegicus arachidonate 12-lipoxygenase, 12R type (Alox12b), mRNA [NM_001039377]	**10,408**
Pnpla6	PREDICTED: Rattus norvegicus patatin-like phospholipase domain containing 6 (Pnpla6), mRNA [XM_001057249]	**10,407**
MGC93975	Rattus norvegicus similar to 2310044H10Rik protein (MGC93975), mRNA [NM_001004221]	**10,406**
0	Unknown	**10,405**
LOC498933	Rattus norvegicus LOC498933 (LOC498933), mRNA [NM_001025764]	**10,404**
RGD1310429	Rattus norvegicus similar to Protein Njmu-R1 (RGD1310429), mRNA [NM_001139506]	**10,404**
Ccdc15	PREDICTED: Rattus norvegicus coiled-coil domain containing 15 (Ccdc15), mRNA [XM_001061074]	**10,402**
Defb25	Rattus norvegicus defensin beta 25 (Defb25), mRNA [NM_001037516]	**10,402**
Kcnk12	Rattus norvegicus potassium channel, subfamily K, member 12 (Kcnk12), mRNA [NM_022292]	**10,401**
Rab27b	Rattus norvegicus RAB27B, member RAS oncogene family (Rab27b), mRNA [NM_053459]	**10,400**
0	Unknown	**10,395**
Murc	Rattus norvegicus muscle-related coiled-coil protein (Murc), mRNA [NM_001107931]	**10,394**
LOC100363412	Uncharacterized protein [Source:UniProtKB/TrEMBL;Acc:D3ZGD4] [ENSRNOT00000064810]	**10,391**
Bcan	Rattus norvegicus brevican (Bcan), transcript variant 1, mRNA [NM_001033665]	**10,390**

0	Rattus norvegicus similar to DNA methyltransferase 3A (LOC289651), mRNA [XM_223446]	**10,390**
Abhd13	Rattus norvegicus abhydrolase domain containing 13 (Abhd13), mRNA [NM_001107327]	**10,389**
Hsp90aa1	Rattus norvegicus heat shock protein 90, alpha (cytosolic), class A member 1 (Hsp90aa1), mRNA [NM_175761]	**10,388**
0	Unknown	**10,386**
Cmtm3	Rattus norvegicus CKLF-like MARVEL transmembrane domain containing 3 (Cmtm3), mRNA [NM_001106164]	**10,386**
Calu	Rattus norvegicus calumenin (Calu), transcript variant 2, mRNA [NM_001033898]	**10,384**
0	Rattus norvegicus similar to NHP2-like protein 1 (High mobility group-like nuclear protein 2 homolog 1) ([U4/U6.U5] tri-snRNP 15.5 kDa protein) (OTK27) (LOC298245), mRNA [XM_233180]	**10,384**
Defa10	Rattus norvegicus defensin alpha 10 (Defa10), mRNA [NM_001033074]	**10,383**
0	TCDD-inducible poly(ADP-ribose) polymerase (Predicted)Uncharacterized protein [Source:UniProtKB/TrEMBL;Acc:D3ZMH5] [ENSRNOT00000015387]	**10,383**
0	FM054771 etcmhea Rattus norvegicus cDNA clone etcmheaP0020N18 5', mRNA sequence [FM054771]	**10,382**
Apbb1	Rattus norvegicus amyloid beta (A4) precursor protein-binding, family B, member 1 (Fe65) (Apbb1), mRNA [NM_080478]	**10,379**
Ap2b1	Rattus norvegicus adaptor-related protein complex 2, beta 1 subunit (Ap2b1), mRNA [NM_080583]	**10,379**
0	Unknown	**10,376**
Kdelr2	Rattus norvegicus KDEL (Lys-Asp-Glu-Leu) endoplasmic reticulum protein retention receptor 2 (Kdelr2), mRNA [NM_001013122]	**10,375**
LOC684035	PREDICTED: Rattus norvegicus similar to arginyl aminopeptidase (aminopeptidase B)-like 1 (LOC684035), miscRNA [XR_085919]	**10,374**
Gtf3c1	Rattus norvegicus general transcription factor IIIC, polypeptide 1, alpha (Gtf3c1), mRNA [NM_133541]	**10,373**
Bmp1	Rattus norvegicus bone morphogenetic protein 1 (Bmp1), mRNA [NM_031323]	**10,371**
0	Unknown	**10,371**
Glis2	Rattus norvegicus GLIS family zinc finger 2 (Glis2), mRNA [NM_001106978]	**10,369**
Vdac1	Rattus norvegicus voltage-dependent anion channel 1 (Vdac1), nuclear gene encoding mitochondrial protein, mRNA [NM_031353]	**10,368**
Tead3	Rattus norvegicus TEA domain family member 3 (Tead3), mRNA [NM_001098216]	**10,363**
Lass1	Rattus norvegicus LAG1 homolog, ceramide synthase 1 (Lass1), mRNA [NM_001044230]	**10,363**
Cntn1	Rattus norvegicus contactin 1 (Cntn1), mRNA [NM_057118]	**10,363**
RGD1565947	Rattus norvegicus similar to netrin 4 (RGD1565947), mRNA [NM_001106780]	**10,362**
Uqcrq	Rattus norvegicus ubiquinol-cytochrome c reductase, complex III subunit VII (Uqcrq), nuclear gene encoding mitochondrial protein, mRNA [NM_001025134]	**10,361**
Fth1	Rattus norvegicus ferritin, heavy polypeptide 1 (Fth1), mRNA [NM_012848]	**10,361**
0	Unknown	**10,361**
Lkap	Rattus norvegicus limkain b1 (Lkap), mRNA [NM_133421]	**10,360**
Dmrtc1b	PREDICTED: Rattus norvegicus DMRT-like family C1b (Dmrtc1b), mRNA [XM_001055549]	**10,360**
Vps35	Rattus norvegicus vacuolar protein sorting 35 homolog (S. cerevisiae) (Vps35), mRNA [NM_001105718]	**10,358**
0	Unknown	**10,352**

Lrrc43	Rattus norvegicus leucine rich repeat containing 43 (Lrrc43), mRNA [NM_001170396]	**10,352**
0	LRRG00131 [Source:UniProtKB/TrEMBL;Acc:Q6QI77] [ENSRNOT00000051980]	**10,352**
Dnajb2	Rattus norvegicus DnaJ (Hsp40) homolog, subfamily B, member 2 (Dnajb2), mRNA [NM_001109541]	**10,351**
RGD1307071	Uncharacterized protein [Source:UniProtKB/TrEMBL;Acc:D4A8C1] [ENSRNOT00000036527]	**10,351**
Smarcal1	Rattus norvegicus Swi/SNF related matrix associated, actin dependent regulator of chromatin, subfamily a-like 1 (Smarcal1), mRNA [NM_001108222]	**10,350**
0	PREDICTED: Rattus norvegicus similar to glyceraldehyde-3-phosphate dehydrogenase (RGD1560797), mRNA [XM_001076325]	**10,350**
Arsa	Rattus norvegicus arylsulfatase A (Arsa), mRNA [NM_001034933]	**10,349**
0	RVL7343 Wackym-Soares normalized rat vestibular cDNA library Rattus norvegicus cDNA 5', mRNA sequence [DV716912]	**10,349**
Cenpa	Rattus norvegicus centromere protein A (Cenpa), mRNA [NM_001106711]	**10,346**
Hbegf	Rattus norvegicus heparin-binding EGF-like growth factor (Hbegf), mRNA [NM_012945]	**10,346**
Creb1	Rattus norvegicus cAMP responsive element binding protein 1 (Creb1), transcript variant A, mRNA [NM_134443]	**10,346**
Dapk1	Rattus norvegicus death associated protein kinase 1 (Dapk1), mRNA [NM_001107335]	**10,344**
N6amt2	Rattus norvegicus N-6 adenine-specific DNA methyltransferase 2 (putative) (N6amt2), mRNA [NM_001134990]	**10,344**
0	Uncharacterized protein [Source:UniProtKB/TrEMBL;Acc:D4A9C5] [ENSRNOT00000011365]	**10,344**
Rhoc	Rattus norvegicus ras homolog gene family, member C (Rhoc), mRNA [NM_001106461]	**10,343**
LOC690340	PREDICTED: Rattus norvegicus similar to spermatogenesis associated glutamate (E)-rich protein 4d (LOC690340), mRNA [XM_001074151]	**10,343**
Cd82	Rattus norvegicus Cd82 molecule (Cd82), mRNA [NM_031797]	**10,343**
Sertad3	Rattus norvegicus SERTA domain containing 3 (Sertad3), mRNA [NM_001017513]	**10,342**
Scgn	Rattus norvegicus secretagogin, EF-hand calcium binding protein (Scgn), mRNA [NM_201561]	**10,342**
Pklr	Rattus norvegicus pyruvate kinase, liver and RBC (Pklr), nuclear gene encoding mitochondrial protein, mRNA [NM_012624]	**10,342**
0	RVL9296 Wackym-Soares normalized rat vestibular cDNA library Rattus norvegicus cDNA 5', mRNA sequence [DV718677]	**10,341**
0	Unknown	**10,340**
Atp5d	Rattus norvegicus ATP synthase, H+ transporting, mitochondrial F1 complex, delta subunit (Atp5d), nuclear gene encoding mitochondrial protein, mRNA [NM_139106]	**10,337**
Gdpd4	PREDICTED: Rattus norvegicus glycerophosphodiester phosphodiesterase domain containing 4 (Gdpd4), mRNA [XM_001059822]	**10,337**
0	Unknown	**10,336**
Cstf1	Rattus norvegicus cleavage stimulation factor, 3' pre-RNA, subunit 1 (Cstf1), mRNA [NM_001013161]	**10,335**
Ril	Rattus norvegicus reversion induced LIM gene (Ril), mRNA [NM_017062]	**10,335**
Zmynd8	Rattus norvegicus zinc finger, MYND-type containing 8 (Zmynd8), mRNA [NM_001100838]	**10,331**
Kif18a	Rattus norvegicus kinesin family member 18A (Kif18a), mRNA [NM_001137642]	**10,331**
0	Unknown	**10,330**
0	Unknown	**10,329**
0	Unknown	**10,328**

Picalm	Rattus norvegicus phosphatidylinositol binding clathrin assembly protein (Picalm), mRNA [NM_053554]	**10,328**
Smchd1	Uncharacterized protein [Source:UniProtKB/TrEMBL;Acc:D4AAG8] [ENSRNOT00000019359]	**10,327**
Ppp1r12a	Rattus norvegicus protein phosphatase 1, regulatory (inhibitor) subunit 12A (Ppp1r12a), mRNA [NM_053890]	**10,327**
Fech	Rattus norvegicus ferrochelatase (Fech), nuclear gene encoding mitochondrial protein, mRNA [NM_001108434]	**10,326**
Slc9a8	Rattus norvegicus solute carrier family 9 (sodium/hydrogen exchanger), member 8 (Slc9a8), mRNA [NM_001025281]	**10,324**
LOC685300	AGENCOURT_17644684 NIH_MGC_237 Rattus norvegicus cDNA clone IMAGE:7115807 5', mRNA sequence [CK471043]	**10,323**
Pou3f3	Rattus norvegicus POU class 3 homeobox 3 (Pou3f3), mRNA [NM_138837]	**10,322**
Kif13a	Rattus norvegicus kinesin family member 13A (Kif13a), mRNA [NM_001107462]	**10,321**
0	Unknown	**10,319**
Bin1	Rattus norvegicus bridging integrator 1 (Bin1), mRNA [NM_053959]	**10,319**
Fbxo33	Rattus norvegicus F-box protein 33 (Fbxo33), mRNA [NM_001108023]	**10,318**
Pet112l	Uncharacterized protein [Source:UniProtKB/TrEMBL;Acc:D3ZJF6] [ENSRNOT00000057128]	**10,318**
Ngfr	Rattus norvegicus nerve growth factor receptor (TNFR superfamily, member 16) (Ngfr), mRNA [NM_012610]	**10,317**
Bpi	Rattus norvegicus bactericidal/permeability-increasing protein (Bpi), mRNA [NM_001004079]	**10,312**
Rpl28	Rattus norvegicus ribosomal protein L28 (Rpl28), mRNA [NM_022697]	**10,311**
Cpne7	Rattus norvegicus copine VII (Cpne7), mRNA [NM_001108454]	**10,311**
Wsb2	Rattus norvegicus WD repeat and SOCS box-containing 2 (Wsb2), mRNA [NM_001007616]	**10,311**
Stk4	Rattus norvegicus serine/threonine kinase 4 (Stk4), mRNA [NM_001107800]	**10,307**
Tbc1d14	Rattus norvegicus TBC1 domain family, member 14 (Tbc1d14), transcript variant 1, mRNA [NM_001012152]	**10,307**
0	Unknown	**10,306**
LOC292543	Rattus norvegicus similar to solute carrier family 7 (cationic amino acid transporter, y+ system), member 3 (LOC292543), mRNA [NM_001025639]	**10,306**
0	Uncharacterized protein [Source:UniProtKB/TrEMBL;Acc:D4A538] [ENSRNOT00000016240]	**10,305**
0	RNA binding motif, single stranded interacting protein Gene [Source:MGI Symbol;Acc:MGI:2444477] [ENSRNOT00000039904]	**10,302**
Tmem183a	Rattus norvegicus transmembrane protein 183A (Tmem183a), mRNA [NM_001013871]	**10,302**
Tmem174	Rattus norvegicus transmembrane protein 174 (Tmem174), mRNA [NM_001024298]	**10,301**
0	Uncharacterized protein [Source:UniProtKB/TrEMBL;Acc:D4A814] [ENSRNOT00000030906]	**10,298**
Vps13d	Rattus norvegicus vacuolar protein sorting 13 homolog D (S. cerevisiae) (Vps13d), mRNA [NM_001108006]	**10,295**
Golga3	Rattus norvegicus golgi autoantigen, golgin subfamily a, 3 (Golga3), mRNA [NM_001107847]	**10,293**
LOC681371	PREDICTED: Rattus norvegicus hypothetical protein LOC681371 (LOC681371), miscRNA [XR_085969]	**10,292**
Olr80	Rattus norvegicus olfactory receptor 80 (Olr80), mRNA [NM_001001270]	**10,292**
Tacc2	Rattus norvegicus transforming, acidic coiled-coil containing protein 2 (Tacc2), transcript variant 1, mRNA [NM_001004415]	**10,291**
0	NADH-ubiquinone oxidoreductase chain 4 [Source:UniProtKB/Swiss-Prot;Acc:P05508] [ENSRNOT00000042928]	**10,291**
0	GTPase KRasGTPase KRas, N-terminally processed [Source:UniProtKB/Swiss-Prot;Acc:P08644] [ENSRNOT00000012588]	**10,290**
Dffa	Rattus norvegicus DNA fragmentation factor, alpha subunit (Dffa), mRNA [NM_053679]	**10,289**

0	RNA binding motif protein 4 [Source:RefSeq peptide;Acc:NP_001163955] [ENSRNOT00000054844]	**10,285**
LOC498122	Rattus norvegicus similar to CG15908-PA (LOC498122), mRNA [NM_001109058]	**10,285**
0	Rattus norvegicus similar to glyceraldehyde-3-phosphate dehydrogenase (LOC293458), mRNA [XM_219295]	**10,285**
0	Unknown	**10,284**
Txlna	Rattus norvegicus taxilin alpha (Txlna), mRNA [NM_001127633]	**10,284**
LOC691169	PREDICTED: Rattus norvegicus hypothetical protein LOC691169 (LOC691169), mRNA [XM_001077081]	**10,283**
RGD1305713	Rattus norvegicus similar to RIKEN cDNA 3110040N11 (RGD1305713), mRNA [NM_001024749]	**10,283**
Ccdc85c	PREDICTED: Rattus norvegicus similar to CG17265-PA (LOC690965), mRNA [XM_001076332]	**10,281**
Cp110	Rattus norvegicus CP110 protein (Cp110), mRNA [NM_001108501]	**10,280**
Pik3ip1	Rattus norvegicus phosphoinositide-3-kinase interacting protein 1 (Pik3ip1), mRNA [NM_001017453]	**10,279**
LOC689766	Rattus norvegicus hypothetical protein LOC689766 (LOC689766), mRNA [NM_001109549]	**10,272**
Polh	Rattus norvegicus polymerase (DNA directed), eta (Polh), mRNA [NM_001108204]	**10,271**
Sema4f	Rattus norvegicus sema domain, immunoglobulin domain (Ig), transmembrane domain (TM) and short cytoplasmic domain, (semaphorin) 4F (Sema4f), mRNA [NM_019272]	**10,271**
Olr1196	Rattus norvegicus olfactory receptor 1196 (Olr1196), mRNA [NM_001000435]	**10,270**
Smc1a	Rattus norvegicus structural maintenance of chromosomes 1A (Smc1a), mRNA [NM_031683]	**10,269**
LOC291863	Rattus norvegicus carboxylesterase-like (LOC291863), mRNA [NM_001013889]	**10,269**
Arhgef4	PREDICTED: Rattus norvegicus Rho guanine nucleotide exchange factor (GEF) 4 (Arhgef4), mRNA [XM_237049]	**10,268**
Ccdc23	Rattus norvegicus coiled-coil domain containing 23 (Ccdc23), transcript variant 1, mRNA [NM_001038994]	**10,267**
Gmeb2	Rattus norvegicus glucocorticoid modulatory element binding protein 2 (Gmeb2), mRNA [NM_031803]	**10,267**
Slc25a28	Rattus norvegicus solute carrier family 25, member 28 (Slc25a28), mRNA [NM_001109515]	**10,263**
Ipo11	PREDICTED: Rattus norvegicus importin 11 (Ipo11), mRNA [XM_002725887]	**10,263**
Cstf2t	Rattus norvegicus cleavage stimulation factor, 3' pre-RNA subunit 2, tau (Cstf2t), mRNA [NM_001107586]	**10,262**
Gas2	Rattus norvegicus growth arrest-specific 2 (Gas2), mRNA [NM_001127504]	**10,261**
0	Unknown	**10,259**
Angptl2	Rattus norvegicus angiopoietin-like 2 (Angptl2), mRNA [NM_133569]	**10,258**
Tacc1	Rattus norvegicus transforming, acidic coiled-coil containing protein 1 (Tacc1), mRNA [NM_001004107]	**10,258**
0	E3 ubiquitin-protein ligase PDZRN3 [Source:UniProtKB/Swiss-Prot;Acc:P68907] [ENSRNOT00000008032]	**10,257**
0	Unknown	**10,257**
Shisa5	Rattus norvegicus shisa homolog 5 (Xenopus laevis) (Shisa5), mRNA [NM_001006989]	**10,257**
RGD1307890	Rattus norvegicus similar to C1orf25 (RGD1307890), mRNA [NM_001037192]	**10,254**
Ang	Rattus norvegicus angiogenin, ribonuclease, RNase A family, 5 (Ang), mRNA [NM_001012359]	**10,252**
Kalrn	Rattus norvegicus kalirin, RhoGEF kinase (Kalrn), mRNA [NM_032062]	**10,251**

0	Rattus norvegicus similar to glyceraldehyde-3-phosphate dehydrogenase (phosphorylating) (EC 1.2.1.12) - mouse (LOC290634), mRNA [XM_214287]	**10,251**
Acss2	Rattus norvegicus acyl-CoA synthetase short-chain family member 2 (Acss2), mRNA [NM_001107793]	**10,251**
0	Q9ERD6_MOUSE (Q9ERD6) Ral-A exchange factor RalGPS2, partial (28%) [TC597248]	**10,250**
Ak2	Rattus norvegicus adenylate kinase 2 (Ak2), nuclear gene encoding mitochondrial protein, transcript variant 2, mRNA [NM_001033967]	**10,250**
RGD1566102	PREDICTED: Rattus norvegicus RGD1566102 (RGD1566102), mRNA [XM_001056364]	**10,249**
0	RCG49325, isoform CRA_bUncharacterized protein [Source:UniProtKB/TrEMBL;Acc:D3ZR95] [ENSRNOT00000025495]	**10,248**
LOC685406	Rattus norvegicus LRRGT00062 (LOC685406), mRNA [NM_001047977]	**10,248**
Sdc4	Rattus norvegicus syndecan 4 (Sdc4), mRNA [NM_012649]	**10,247**
Myh6	Rattus norvegicus myosin, heavy chain 6, cardiac muscle, alpha (Myh6), mRNA [NM_017239]	**10,246**
Slc6a16	PREDICTED: Rattus norvegicus solute carrier family 6, member 16 (Slc6a16), mRNA [XM_001080708]	**10,243**
0	Rattus norvegicus Ac1-283 mRNA, complete cds. [AY325224]	**10,243**
RGD1561246	PREDICTED: Rattus norvegicus similar to put. precursor MuIFN-alpha 5 (RGD1561246), mRNA [XM_001053102]	**10,243**
0	Unknown	**10,243**
Zfat	Rattus norvegicus zinc finger and AT hook domain containing (Zfat), mRNA [NM_001134957]	**10,242**
Cdkn1a	Rattus norvegicus cyclin-dependent kinase inhibitor 1A (Cdkn1a), mRNA [NM_080782]	**10,241**
Dok6	Rattus norvegicus docking protein 6 (Dok6), mRNA [NM_001191943]	**10,240**
Aldh3a2	Rattus norvegicus aldehyde dehydrogenase 3 family, member A2 (Aldh3a2), mRNA [NM_031731]	**10,240**
Ube4a	Rattus norvegicus ubiquitination factor E4A (UFD2 homolog, yeast) (Ube4a), mRNA [NM_207610]	**10,240**
Ptk2	Rattus norvegicus PTK2 protein tyrosine kinase 2 (Ptk2), mRNA [NM_013081]	**10,240**
Timm8a1	Rattus norvegicus translocase of inner mitochondrial membrane 8 homolog a1 (yeast) (Timm8a1), nuclear gene encoding mitochondrial protein, mRNA [NM_053370]	**10,239**
0	Q8TE64_HUMAN (Q8TE64) Pur-gamma B-form, partial (50%) [TC600362]	**10,239**
Galnt13	Rattus norvegicus UDP-N-acetyl-alpha-D-galactosamine:polypeptide N-acetylgalactosaminyltransferase 13 (GalNAc-T13) (Galnt13), mRNA [NM_199106]	**10,238**
Spag6	Rattus norvegicus sperm associated antigen 6 (Spag6), mRNA [NM_001034960]	**10,237**
Lix1	Rattus norvegicus Lix1 homolog (chicken) (Lix1), mRNA [NM_001106214]	**10,236**
Cerk	Rattus norvegicus ceramide kinase (Cerk), mRNA [NM_001134861]	**10,235**
Prss8	Rattus norvegicus protease, serine, 8 (Prss8), mRNA [NM_138836]	**10,234**
0	Unknown	**10,232**
Cidea	Rattus norvegicus cell death-inducing DFFA-like effector a (Cidea), mRNA [NM_001170467]	**10,231**
0	Rattus norvegicus, 23 clones, strain BN/SsNHsdMCW RNOR03327639, whole genome shotgun sequence [AABR03128582]	**10,231**
0	Unknown	**10,230**
Reg3a	Rattus norvegicus regenerating islet-derived 3 alpha (Reg3a), transcript variant 1, mRNA [NM_172077]	**10,227**

LOC679087	PREDICTED: Rattus norvegicus similar to swan (LOC679087), mRNA [XM_001054639]	**10,227**
Slc22a5	Rattus norvegicus solute carrier family 22 (organic cation/carnitine transporter), member 5 (Slc22a5), mRNA [NM_019269]	**10,226**
Igf1r	Rattus norvegicus insulin-like growth factor 1 receptor (Igf1r), mRNA [NM_052807]	**10,225**
Zfyve26	Rattus norvegicus zinc finger, FYVE domain containing 26 (Zfyve26), mRNA [NM_001108038]	**10,225**
0	Unknown	**10,225**
Spop	Rattus norvegicus speckle-type POZ protein (Spop), mRNA [NM_001100496]	**10,223**
Otub2	Rattus norvegicus OTU domain, ubiquitin aldehyde binding 2 (Otub2), mRNA [NM_001108053]	**10,223**
Etf1	Rattus norvegicus eukaryotic translation termination factor 1 (Etf1), mRNA [NM_001008344]	**10,221**
0	Uncharacterized protein [Source:UniProtKB/TrEMBL;Acc:D3ZAC0] [ENSRNOT00000055179]	**10,217**
Jph2	Rattus norvegicus junctophilin 2 (Jph2), mRNA [NM_001037974]	**10,216**
0	KIAA1454-like protein [Source:UniProtKB/TrEMBL;Acc:Q99MF7] [ENSRNOT00000009385]	**10,216**
Arid2	Uncharacterized protein [Source:UniProtKB/TrEMBL;Acc:D3ZJU0] [ENSRNOT00000006970]	**10,215**
Sco1	Rattus norvegicus SCO cytochrome oxidase deficient homolog 1 (yeast) (Sco1), nuclear gene encoding mitochondrial protein, mRNA [NM_001173374]	**10,213**
Zc3h6	Rattus norvegicus zinc finger CCCH type containing 6 (Zc3h6), mRNA [NM_001107772]	**10,213**
Tmem220	Uncharacterized protein [Source:UniProtKB/TrEMBL;Acc:D4A6Z7] [ENSRNOT00000004516]	**10,210**
Pcdhb20	Rattus norvegicus protocadherin beta 20 (Pcdhb20), mRNA [NM_001109395]	**10,210**
Rasal1	Rattus norvegicus RAS protein activator like 1 (GAP1 like) (Rasal1), mRNA [NM_001108335]	**10,208**
Phtf2	Rattus norvegicus putative homeodomain transcription factor 2 (Phtf2), mRNA [NM_001106577]	**10,208**
0	Uncharacterized protein [Source:UniProtKB/TrEMBL;Acc:D3ZYR2] [ENSRNOT00000020691]	**10,207**
Psat1	Rattus norvegicus phosphoserine aminotransferase 1 (Psat1), mRNA [NM_198738]	**10,206**
Prrg1	Rattus norvegicus proline rich Gla (G-carboxyglutamic acid) 1 (Prrg1), mRNA [NM_001191888]	**10,206**
Mgat3	Rattus norvegicus mannosyl (beta-1,4-)-glycoprotein beta-1,4-N-acetylglucosaminyltransferase (Mgat3), mRNA [NM_019239]	**10,206**
Rnf125	Rattus norvegicus ring finger protein 125 (Rnf125), mRNA [NM_001108424]	**10,205**
0	Uncharacterized protein [Source:UniProtKB/TrEMBL;Acc:D3ZZG9] [ENSRNOT00000054682]	**10,205**
Epha7	Rattus norvegicus Eph receptor A7 (Epha7), mRNA [NM_134331]	**10,205**
Defb9	Rattus norvegicus defensin beta 9 (Defb9), mRNA [NM_001037509]	**10,203**
Vom1r59	Rattus norvegicus vomeronasal 1 receptor 59 (Vom1r59), mRNA [NM_001008944]	**10,202**
Eif6	Rattus norvegicus eukaryotic translation initiation factor 6 (Eif6), mRNA [NM_001037352]	**10,202**
Atad5	PREDICTED: Rattus norvegicus ATPase family, AAA domain containing 5, transcript variant 1 (Atad5), mRNA [XM_001080963]	**10,201**
RGD1562079	Rattus norvegicus RGD1562079 (RGD1562079), mRNA [NM_001177687]	**10,200**
Olr217	Rattus norvegicus olfactory receptor 217 (Olr217), mRNA [NM_001000199]	**10,200**
Bhmt	Rattus norvegicus betaine-homocysteine methyltransferase (Bhmt), mRNA [NM_030850]	**10,199**
Ccdc106	Uncharacterized protein [Source:UniProtKB/TrEMBL;Acc:D4AAV8] [ENSRNOT00000021613]	**10,199**

Fstl3	Rattus norvegicus follistatin-like 3 (secreted glycoprotein) (Fstl3), mRNA [NM_053629]	**10,198**
Rbck1	Rattus norvegicus RanBP-type and C3HC4-type zinc finger containing 1 (Rbck1), mRNA [NM_021764]	**10,198**
Pfkl	Rattus norvegicus phosphofructokinase, liver (Pfkl), mRNA [NM_013190]	**10,196**
Drd2	Rattus norvegicus dopamine receptor D2 (Drd2), mRNA [NM_012547]	**10,196**
Flna	Rattus norvegicus filamin A, alpha (Flna), mRNA [NM_001134599]	**10,195**
Dmrt2	Rattus norvegicus doublesex and mab-3 related transcription factor 2 (Dmrt2), mRNA [NM_001107597]	**10,191**
0	Rattus norvegicus similar to glyceraldehyde-3-phosphate dehydrogenase (phosphorylating) (EC 1.2.1.12) - mouse (LOC311475), mRNA [XM_230709]	**10,189**
0	Unknown	**10,187**
Kcnj9	Rattus norvegicus potassium inwardly-rectifying channel, subfamily J, member 9 (Kcnj9), mRNA [NM_053834]	**10,187**
LOC501308	PREDICTED: Rattus norvegicus hypothetical gene supported by BC059164 (LOC501308), mRNA [XM_576721]	**10,187**
Tmem132c	PREDICTED: Rattus norvegicus transmembrane protein 132C (Tmem132c), mRNA [XM_002724836]	**10,183**
Trpm8	Rattus norvegicus transient receptor potential cation channel, subfamily M, member 8 (Trpm8), mRNA [NM_134371]	**10,182**
Peg12	Rattus norvegicus paternally expressed 12 (Peg12), mRNA [NM_001170562]	**10,182**
Oscar	Rattus norvegicus osteoclast associated, immunoglobulin-like receptor (Oscar), mRNA [NM_001184973]	**10,182**
RGD1307749	Rattus norvegicus similar to RIKEN cDNA 1600013K19 (RGD1307749), mRNA [NM_001106759]	**10,181**
RGD1565616	Rattus norvegicus RGD1565616 (RGD1565616), mRNA [NM_001109206]	**10,180**
Glce	PREDICTED: Rattus norvegicus glucuronic acid epimerase (Glce), mRNA [XM_343404]	**10,180**
0	Glutamate receptor subunit GluR1 [Source:UniProtKB/TrEMBL;Acc:Q924I5] [ENSRNOT00000003279]	**10,180**
Fam38b	PREDICTED: Rattus norvegicus similar to CG8486-PA, isoform A (LOC682889), mRNA [XM_001063568]	**10,179**
Ccdc115	Rattus norvegicus coiled-coil domain containing 115 (Ccdc115), mRNA [NM_001108793]	**10,179**
Erlin2	Rattus norvegicus ER lipid raft associated 2 (Erlin2), mRNA [NM_001106088]	**10,178**
Tmem127	Rattus norvegicus transmembrane protein 127 (Tmem127), mRNA [NM_001100978]	**10,178**
Prpf4	Rattus norvegicus PRP4 pre-mRNA processing factor 4 homolog (yeast) (Prpf4), mRNA [NM_001106659]	**10,178**
Bcl2	Rattus norvegicus B-cell CLL/lymphoma 2 (Bcl2), nuclear gene encoding mitochondrial protein, mRNA [NM_016993]	**10,177**
Chuk	Rattus norvegicus conserved helix-loop-helix ubiquitous kinase (Chuk), mRNA [NM_001107588]	**10,177**
L2hgdh	Rattus norvegicus L-2-hydroxyglutarate dehydrogenase (L2hgdh), nuclear gene encoding mitochondrial protein, mRNA [NM_001108028]	**10,177**
0	Q448P6_SOLUS (Q448P6) Protein kinase precursor, partial (3%) [TC606896]	**10,175**
0	Unknown	**10,172**
0	Unknown	**10,171**
0	Unknown	**10,171**
0	Unknown	**10,167**
Fam101a	Rattus norvegicus family with sequence similarity 101, member A (Fam101a), mRNA [NM_001109547]	**10,166**
0	Rattus norvegicus similar to ribosomal protein L23a (LOC297884), mRNA [XM_232762]	**10,166**

Sh3tc2	Sh3tc2 protein [Source:UniProtKB/TrEMBL;Acc:Q5EB84] [ENSRNOT00000026174]	**10,165**
Ndrg3	Rattus norvegicus N-myc downstream regulated gene 3 (Ndrg3), mRNA [NM_001013923]	**10,165**
0	RNCAII8 carbonic anhydrase II {Rattus norvegicus} (exp=-1; wgp=0; cg=0), partial (32%) [TC633165]	**10,164**
Nod1	Rattus norvegicus nucleotide-binding oligomerization domain containing 1 (Nod1), mRNA [NM_001109236]	**10,164**
Hadh	Rattus norvegicus hydroxyacyl-Coenzyme A dehydrogenase (Hadh), nuclear gene encoding mitochondrial protein, mRNA [NM_057186]	**10,163**
Vwa5b2	Rattus norvegicus von Willebrand factor A domain containing 5B2 (Vwa5b2), mRNA [NM_001134535]	**10,163**
LOC367117	Rattus norvegicus similar to RIKEN cDNA 2900055D03 (LOC367117), mRNA [NM_001047919]	**10,163**
0	Glyceraldehyde-3-phosphate dehydrogenase [Source:UniProtKB/TrEMBL;Acc:D4A3W5] [ENSRNOT00000036706]	**10,162**
40787	Rattus norvegicus septin 11 (Sept11), mRNA [NM_001107208]	**10,162**
Rcn1	Rattus norvegicus reticulocalbin 1, EF-hand calcium binding domain (Rcn1), mRNA [NM_001108586]	**10,161**
0	Unknown	**10,161**
Ext2	Rattus norvegicus exostoses (multiple) 2 (Ext2), mRNA [NM_001107751]	**10,161**
0	Unknown	**10,160**
Mllt3	Rattus norvegicus myeloid/lymphoid or mixed-lineage leukemia (trithorax homolog, Drosophila); translocated to, 3 (Mllt3), mRNA [NM_053718]	**10,159**
Nup43	Rattus norvegicus nucleoporin 43 (Nup43), mRNA [NM_001128191]	**10,156**
LOC303448	Rattus norvegicus similar to glyceraldehyde-3-phosphate dehydrogenase (LOC303448), mRNA [NM_001037190]	**10,153**
Srrp	Rattus norvegicus serine-arginine repressor protein (Srrp), mRNA [NM_001135711]	**10,152**
Gyltl1b	Rattus norvegicus glycosyltransferase-like 1B (Gyltl1b), mRNA [NM_199107]	**10,150**
Pdzd8	Rattus norvegicus PDZ domain containing 8 (Pdzd8), mRNA [NM_001107446]	**10,148**
Pphln1	Rattus norvegicus periphilin 1 (Pphln1), mRNA [NM_001108992]	**10,147**
Eif5	Rattus norvegicus eukaryotic translation initiation factor 5 (Eif5), mRNA [NM_020075]	**10,147**
Rxra	Rattus norvegicus retinoid X receptor alpha (Rxra), mRNA [NM_012805]	**10,143**
0	Q43UY0_SOLUS (Q43UY0) Virulence factor MVIN-like, partial (4%) [TC643072]	**10,143**
Nlk	Rattus norvegicus nemo like kinase (Nlk), mRNA [NM_001191924]	**10,142**
Ces5a	Rattus norvegicus carboxylesterase 5A (Ces5a), mRNA [NM_001012056]	**10,142**
Enoph1	Rattus norvegicus enolase-phosphatase 1 (Enoph1), mRNA [NM_001009391]	**10,141**
RGD1560873	PREDICTED: Rattus norvegicus similar to RIKEN cDNA E230015L20 gene (RGD1560873), miscRNA [XR_085636]	**10,140**
Dnajc6	Rattus norvegicus DnaJ (Hsp40) homolog, subfamily C, member 6 (Dnajc6), mRNA [NM_001107949]	**10,139**
0	Unknown	**10,138**
Bst2	Rattus norvegicus bone marrow stromal cell antigen 2 (Bst2), mRNA [NM_198134]	**10,136**
Nrip1	Rattus norvegicus nuclear receptor interacting protein 1 (Nrip1), mRNA [NM_001100560]	**10,135**
0	Unknown	**10,134**
Arid4a	Rattus norvegicus AT rich interactive domain 4A (Rbp1 like) (Arid4a), mRNA [NM_001108029]	**10,133**
0	Unknown	**10,132**

Rad9b	Rattus norvegicus RAD9 homolog B (S. cerevisiae) (Rad9b), mRNA [NM_001030042]	**10,132**
0	Unknown	**10,130**
Tsc22d1	Rattus norvegicus TSC22 domain family, member 1 (Tsc22d1), transcript variant 2, mRNA [NM_013043]	**10,130**
Zbtb5	Rattus norvegicus zinc finger and BTB domain containing 5 (Zbtb5), mRNA [NM_001106657]	**10,129**
0	Unknown	**10,127**
Mocs2	Rattus norvegicus molybdenum cofactor synthesis 2 (Mocs2), transcript variant 2, mRNA [NM_001007633]	**10,127**
Rab18	Rattus norvegicus RAB18, member RAS oncogene family (Rab18), mRNA [NM_001012468]	**10,123**
Erf	Rattus norvegicus Ets2 repressor factor (Erf), mRNA [NM_001170335]	**10,120**
Ttc9b	Rattus norvegicus tetratricopeptide repeat domain 9B (Ttc9b), mRNA [NM_001108478]	**10,120**
0	Rattus norvegicus similar to cell division cycle associated 3; gene rich cluster, C8 gene; trigger of mitotic entry 1 (LOC316373), mRNA [XM_237105]	**10,120**
0	Uncharacterized protein [Source:UniProtKB/TrEMBL;Acc:D3ZRC6] [ENSRNOT00000050943]	**10,117**
Ppp2ca	Rattus norvegicus protein phosphatase 2, catalytic subunit, alpha isoform (Ppp2ca), mRNA [NM_017039]	**10,116**
0	Uncharacterized protein [Source:UniProtKB/TrEMBL;Acc:D3ZQX9] [ENSRNOT00000027019]	**10,116**
0	Q6TXI1_RAT (Q6TXI1) LRRGT00018, partial (5%) [TC597668]	**10,114**
Lce1d	Uncharacterized protein [Source:UniProtKB/TrEMBL;Acc:D3ZFL2] [ENSRNOT00000012464]	**10,110**
Sptlc2	Rattus norvegicus serine palmitoyltransferase, long chain base subunit 2 (Sptlc2), mRNA [NM_001037097]	**10,109**
0	Unknown	**10,109**
0	Exportin 7, isoform CRA_aUncharacterized protein [Source:UniProtKB/TrEMBL;Acc:D3ZBB4] [ENSRNOT00000058431]	**10,108**
RGD1563273	Rattus norvegicus similar to hypothetical protein 9630041N07 (RGD1563273), mRNA [NM_001134600]	**10,108**
Dnah10	PREDICTED: Rattus norvegicus dynein, axonemal, heavy polypeptide 10 (Dnah10), mRNA [XM_001078937]	**10,108**
Slc41a3	Rattus norvegicus solute carrier family 41, member 3 (Slc41a3), mRNA [NM_001037492]	**10,104**
0	Rattus norvegicus similar to olfactory receptor MOR26-1 (LOC293253), mRNA [XM_219179]	**10,104**
Kif3a	Rattus norvegicus kinesin family member 3a (Kif3a), mRNA [NM_053377]	**10,101**
Cdh5	Rattus norvegicus cadherin 5 (Cdh5), mRNA [NM_001107407]	**10,101**
Grem1	Rattus norvegicus gremlin 1, cysteine knot superfamily, homolog (Xenopus laevis) (Grem1), mRNA [NM_019282]	**10,100**
0	Unknown	**10,100**
LOC100360315	PREDICTED: Rattus norvegicus spermatogenesis associated glutamate (E)-rich protein 4e-like (LOC100360315), mRNA [XM_002730116]	**10,100**
LOC685046	Rattus norvegicus hypothetical protein LOC685046 (LOC685046), mRNA [NM_001109453]	**10,098**
0	AW920953 EST352257 Rat gene index, normalized rat, norvegicus, Bento Soares Rattus norvegicus cDNA clone RGIHK22 5' end, mRNA sequence [AW920953]	**10,098**
Syn2	Rattus norvegicus synapsin II (Syn2), transcript variant 2, mRNA [NM_019159]	**10,097**
Gstm2	Rattus norvegicus glutathione S-transferase mu 2 (Gstm2), mRNA [NM_177426]	**10,097**
RGD1561728	Rattus norvegicus similar to high mobility group protein (LOC307239), mRNA [XM_225740]	**10,096**

RGD1311186	Rattus norvegicus similar to RIKEN cDNA 1810014F10 gene (RGD1311186), mRNA [NM_001106310]	**10,096**
0	Uncharacterized protein [Source:UniProtKB/TrEMBL;Acc:D3ZE13] [ENSRNOT00000027984]	**10,095**
RGD1563564	PREDICTED: Rattus norvegicus similar to GTP-binding protein NGB (RGD1563564), mRNA [XM_002728505]	**10,095**
Nat11	Rattus norvegicus N-acetyltransferase 11 (Nat11), mRNA [NM_001108518]	**10,094**
Ephx2	Rattus norvegicus epoxide hydrolase 2, cytoplasmic (Ephx2), mRNA [NM_022936]	**10,093**
LOC100363350	Uncharacterized protein [Source:UniProtKB/TrEMBL;Acc:D4A1D7] [ENSRNOT00000037353]	**10,093**
Pcdhb7	Protocadherin-T3 [Source:UniProtKB/TrEMBL;Acc:Q9JIU3] [ENSRNOT00000027188]	**10,092**
Cables1	Rattus norvegicus Cdk5 and Abl enzyme substrate 1 (Cables1), mRNA [NM_001107404]	**10,091**
Baz1b	Rattus norvegicus bromodomain adjacent to zinc finger domain, 1B (Baz1b), mRNA [NM_001191916]	**10,090**
38777	PREDICTED: Rattus norvegicus membrane-associated ring finger (C3HC4) 6 (March6), mRNA [XM_215517]	**10,089**
St18	Rattus norvegicus suppression of tumorigenicity 18 (St18), mRNA [NM_153310]	**10,089**
Dctn6	Rattus norvegicus dynactin 6 (Dctn6), mRNA [NM_001106085]	**10,086**
0	Ptpn3 protein [Source:UniProtKB/TrEMBL;Acc:Q562B7] [ENSRNOT00000015276]	**10,085**
LOC680813	PREDICTED: Rattus norvegicus hypothetical protein LOC680813 (LOC680813), partial mRNA [XM_001058991]	**10,084**
Otud4	Rattus norvegicus OTU domain containing 4 (Otud4), mRNA [NM_001191700]	**10,083**
0	Uncharacterized protein [Source:UniProtKB/TrEMBL;Acc:D3ZXK4] [ENSRNOT00000025560]	**10,083**
Lgr4	Rattus norvegicus leucine-rich repeat-containing G protein-coupled receptor 4 (Lgr4), mRNA [NM_173328]	**10,083**
Gabrg2	Rattus norvegicus gamma-aminobutyric acid (GABA) A receptor, gamma 2 (Gabrg2), mRNA [NM_183327]	**10,082**
Dpcd	Rattus norvegicus deleted in primary ciliary dyskinesia (Dpcd), mRNA [NM_001013905]	**10,080**
Car11	Rattus norvegicus carbonic anhydrase 11 (Car11), mRNA [NM_175708]	**10,080**
Akap5	Rattus norvegicus A kinase (PRKA) anchor protein 5 (Akap5), mRNA [NM_133515]	**10,080**
Rtn4rl1	Rattus norvegicus reticulon 4 receptor-like 1 (Rtn4rl1), mRNA [NM_181377]	**10,079**
Glt25d1	Rattus norvegicus glycosyltransferase 25 domain containing 1 (Glt25d1), mRNA [NM_001106067]	**10,078**
LOC680377	Uncharacterized protein [Source:UniProtKB/TrEMBL;Acc:D3ZLL4] [ENSRNOT00000047918]	**10,078**
0	Unknown	**10,078**
Gypc	Rattus norvegicus glycophorin C (Gerbich blood group) (Gypc), mRNA [NM_001013233]	**10,076**
Hdac1	Rattus norvegicus histone deacetylase 1 (Hdac1), mRNA [NM_001025409]	**10,076**
Fgfr2	Rattus norvegicus fibroblast growth factor receptor 2 (Fgfr2), transcript variant a, mRNA [NM_012712]	**10,074**
G3bp1	Rattus norvegicus GTPase activating protein (SH3 domain) binding protein 1 (G3bp1), mRNA [NM_133565]	**10,074**
Skp2	Rattus norvegicus S-phase kinase-associated protein 2 (p45) (Skp2), mRNA [NM_001106416]	**10,073**
Ppia	Rattus norvegicus peptidylprolyl isomerase A (cyclophilin A) (Ppia), mRNA [NM_017101]	**10,073**
Grasp	Rattus norvegicus GRP1 (general receptor for phosphoinositides 1)-associated scaffold protein (Grasp), mRNA [NM_138894]	**10,069**
Hisppd2a	Rattus norvegicus histidine acid phosphatase domain containing 2A (Hisppd2a), mRNA [NM_001080783]	**10,067**
0	Unknown	**10,066**

0	Unknown	**10,059**
0	Radial spokehead-like 2 (Predicted)Uncharacterized protein [Source:UniProtKB/TrEMBL;Acc:D3ZP42] [ENSRNOT00000068451]	**10,059**
Ltc4s	Rattus norvegicus leukotriene C4 synthase (Ltc4s), mRNA [NM_053639]	**10,057**
Rnf160	Rattus norvegicus ring finger protein 160 (Rnf160), mRNA [NM_001024235]	**10,057**
LOC686860	PREDICTED: Rattus norvegicus similar to RT1 class I, CE15 (LOC686860), partial mRNA [XM_001076061]	**10,056**
Tnfrsf19	Rattus norvegicus tumor necrosis factor receptor superfamily, member 19 (Tnfrsf19), mRNA [NM_001044229]	**10,054**
Rsrc1	Rattus norvegicus arginine/serine-rich coiled-coil 1 (Rsrc1), mRNA [NM_001014172]	**10,054**
0	Unknown	**10,052**
0	Unknown	**10,052**
Park2	Rattus norvegicus Parkinson disease (autosomal recessive, juvenile) 2, parkin (Park2), mRNA [NM_020093]	**10,051**
RGD1305422	Rattus norvegicus similar to mKIAA0226 protein (LOC303885), mRNA [XM_221382]	**10,051**
Klhdc7b	PREDICTED: Rattus norvegicus kelch domain containing 7B (Klhdc7b), mRNA [XM_001055129]	**10,048**
Mdh1b	Rattus norvegicus malate dehydrogenase 1B, NAD (soluble) (Mdh1b), mRNA [NM_001108221]	**10,045**
Nrg2	Rattus norvegicus neuregulin 2 (Nrg2), mRNA [NM_001136151]	**10,044**
Dnajc17	Rattus norvegicus DnaJ (Hsp40) homolog, subfamily C, member 17 (Dnajc17), mRNA [NM_001191740]	**10,043**
Eraf	Rattus norvegicus erythroid associated factor (Eraf), mRNA [NM_001106299]	**10,040**
0	Uncharacterized protein [Source:UniProtKB/TrEMBL;Acc:D3ZCX4] [ENSRNOT00000008851]	**10,038**
Nsun5	Rattus norvegicus NOL1/NOP2/Sun domain family, member 5 (Nsun5), mRNA [NM_001191593]	**10,037**
Cyp4a2	Rattus norvegicus cytochrome P450, family 4, subfamily a, polypeptide 2 (Cyp4a2), mRNA [NM_001044770]	**10,036**
0	Uncharacterized protein [Source:UniProtKB/TrEMBL;Acc:D3ZW51] [ENSRNOT00000040758]	**10,035**
Cbx2	Rattus norvegicus chromobox homolog 2 (Pc class homolog, Drosophila) (Cbx2), mRNA [NM_001107071]	**10,035**
Col5a1	Rattus norvegicus collagen, type V, alpha 1 (Col5a1), mRNA [NM_134452]	**10,035**
Pou5f1	Rattus norvegicus POU class 5 homeobox 1 (Pou5f1), mRNA [NM_001009178]	**10,035**
Fcho1	Rattus norvegicus FCH domain only 1 (Fcho1), mRNA [NM_001106069]	**10,034**
Fbxl22	Rattus norvegicus F-box and leucine-rich repeat protein 22 (Fbxl22), mRNA [NM_001108769]	**10,032**
Tcte4	Rattus norvegicus t-complex-associated testis expressed 4 (Tcte4), mRNA [NM_001166303]	**10,030**
0	Unknown	**10,029**
Nkrf	PREDICTED: Rattus norvegicus NFKB repressing factor (Nkrf), mRNA [XM_233308]	**10,029**
LOC361990	Rattus norvegicus similar to DKFZP547E1010 protein (LOC361990), mRNA [NM_001014175]	**10,028**
Tbc1d2	Rattus norvegicus TBC1 domain family, member 2 (Tbc1d2), mRNA [NM_001107933]	**10,028**
Garnl4	Rattus norvegicus GTPase activating Rap/RanGAP domain-like 4 (Garnl4), mRNA [NM_001107019]	**10,027**
Eapp	Rattus norvegicus E2F-associated phosphoprotein (Eapp), transcript variant 2, mRNA [NM_001106729]	**10,022**
Kcnd2	Rattus norvegicus potassium voltage-gated channel, Shal-related subfamily, member 2 (Kcnd2), mRNA [NM_031730]	**10,022**
Otof	Otoferlin [Source:UniProtKB/Swiss-Prot;Acc:Q9ERC5] [ENSRNOT00000044278]	**10,022**

Ano7	Rattus norvegicus anoctamin 7 (Ano7), mRNA [NM_001004071]	**10,021**
Pcbp1	Uncharacterized protein [Source:UniProtKB/TrEMBL;Acc:D3ZS68] [ENSRNOT00000023806]	**10,020**
0	Transcriptional regulator ATRX [Source:UniProtKB/Swiss-Prot;Acc:P70486] [ENSRNOT00000033355]	**10,019**
Mtmr11	Rattus norvegicus myotubularin related protein 11 (Mtmr11), mRNA [NM_001191096]	**10,019**
Apex1	Rattus norvegicus APEX nuclease (multifunctional DNA repair enzyme) 1 (Apex1), mRNA [NM_024148]	**10,019**
Palmd	Rattus norvegicus palmdelphin (Palmd), mRNA [NM_001025688]	**10,019**
Cd53	Rattus norvegicus Cd53 molecule (Cd53), mRNA [NM_012523]	**10,018**
Clrn2	Rattus norvegicus clarin 2 (Clrn2), mRNA [NM_001191097]	**10,018**
Nacad	Rattus norvegicus NAC alpha domain containing (Nacad), mRNA [NM_001100655]	**10,014**
Timm23	Rattus norvegicus translocase of inner mitochondrial membrane 23 homolog (yeast) (Timm23), nuclear gene encoding mitochondrial protein, mRNA [NM_019352]	**10,014**
Slc12a6	Rattus norvegicus solute carrier family 12, member 6 (Slc12a6), mRNA [NM_001109630]	**10,013**
LOC494538	UI-R-DA0-byo-b-03-0-UI.s1 UI-R-DA0 Rattus norvegicus cDNA clone UI-R-DA0-byo-b-03-0-UI 3', mRNA sequence [BI279486]	**10,011**
Clec4f	Rattus norvegicus C-type lectin domain family 4, member f (Clec4f), mRNA [NM_053753]	**10,011**
Pgp	Rattus norvegicus phosphoglycolate phosphatase (Pgp), mRNA [NM_001169152]	**10,010**
MGC116197	Rattus norvegicus similar to RIKEN cDNA 1700001E04 (MGC116197), mRNA [NM_001025755]	**10,010**
Ppp2r5a	Rattus norvegicus protein phosphatase 2, regulatory subunit B', alpha isoform (Ppp2r5a), mRNA [NM_001107891]	**10,008**
Egfl7	Rattus norvegicus EGF-like-domain, multiple 7 (Egfl7), mRNA [NM_139104]	**10,007**
Trak2	Rattus norvegicus trafficking protein, kinesin binding 2 (Trak2), mRNA [NM_133560]	**10,005**
0	interferon gamma induced GTPase [Source:RefSeq peptide;Acc:NP_001008765] [ENSRNOT00000039335]	**10,004**
Apbb2	Rattus norvegicus similar to Amyloid beta A4 precursor protein-binding family B member 2 (Fe65-like protein) (LOC305338), mRNA [XM_223399]	**10,002**
0	Unknown	**10,002**
Pde4d	Rattus norvegicus phosphodiesterase 4D, cAMP-specific (phosphodiesterase E3 dunce homolog, Drosophila) (Pde4d), transcript variant 5, mRNA [NM_017032]	**10,000**
Fnbp1l	Rattus norvegicus formin binding protein 1-like (Fnbp1l), mRNA [NM_001039609]	**9,997**
Tapbp	Rattus norvegicus TAP binding protein (Tapbp), mRNA [NM_033098]	**9,997**
0	Unknown	**9,996**
Car2	Rattus norvegicus carbonic anhydrase II (Car2), mRNA [NM_019291]	**9,994**
Cd83	Rattus norvegicus CD83 molecule (Cd83), mRNA [NM_001108410]	**9,994**
Grid2	Rattus norvegicus glutamate receptor, ionotropic, delta 2 (Grid2), mRNA [NM_024379]	**9,994**
Col4a1	Rattus norvegicus collagen, type IV, alpha 1 (Col4a1), mRNA [NM_001135009]	**9,991**
Zcchc3	PREDICTED: Rattus norvegicus zinc finger, CCHC domain containing 3 (Zcchc3), mRNA [XM_001072887]	**9,990**
0	Collagen alpha-1(XI) chain [Source:UniProtKB/Swiss-Prot;Acc:P20909] [ENSRNOT00000023693]	**9,990**

0	PREDICTED: Rattus norvegicus similar to TAF11 RNA polymerase II, TATA box binding protein (TBP)-associated factor (RGD1562272), mRNA [XM_001081219]	**9,989**
Olr1738	Rattus norvegicus olfactory receptor 1738 (Olr1738), mRNA [NM_001006599]	**9,988**
Tgfbr3	Rattus norvegicus transforming growth factor, beta receptor III (Tgfbr3), mRNA [NM_017256]	**9,986**
Nsd1	Rattus norvegicus nuclear receptor binding SET domain protein 1 (Nsd1), mRNA [NM_001107337]	**9,985**
RT1-CE7	Rattus norvegicus RT1 class I, locus CE7 (RT1-CE7), mRNA [NM_001008845]	**9,983**
Esrp1	Rattus norvegicus epithelial splicing regulatory protein 1 (Esrp1), mRNA [NM_001127564]	**9,983**
Stam	Rattus norvegicus signal transducing adaptor molecule (SH3 domain and ITAM motif) 1 (Stam), mRNA [NM_001109121]	**9,981**
LOC690769	RCG51260, isoform CRA_aUncharacterized protein [Source:UniProtKB/TrEMBL;Acc:D3ZIQ9] [ENSRNOT00000025716]	**9,980**
Ktelc1	Rattus norvegicus KTEL (Lys-Tyr-Glu-Leu) containing 1 (Ktelc1), mRNA [NM_001100652]	**9,980**
Nr4a2	Rattus norvegicus nuclear receptor subfamily 4, group A, member 2 (Nr4a2), mRNA [NM_019328]	**9,980**
Stx16	Rattus norvegicus syntaxin 16 (Stx16), mRNA [NM_001108610]	**9,978**
RGD1560166	PREDICTED: Rattus norvegicus similar to Probable G-protein coupled receptor 62 (hGPCR8) (RGD1560166), mRNA [XM_001073569]	**9,977**
Gpi	Rattus norvegicus glucose phosphate isomerase (Gpi), mRNA [NM_207592]	**9,975**
RGD1564535	Uncharacterized protein [Source:UniProtKB/TrEMBL;Acc:D3ZL60] [ENSRNOT00000062001]	**9,975**
Pde3a	Rattus norvegicus phosphodiesterase 3A, cGMP inhibited (Pde3a), mRNA [NM_017337]	**9,974**
Hdac4	PREDICTED: Rattus norvegicus histone deacetylase 4 (Hdac4), mRNA [XM_001067733]	**9,974**
0	PREDICTED: Rattus norvegicus similar to transglutaminase 7 (LOC691932), mRNA [XM_001080162]	**9,972**
Ercc8	Rattus norvegicus excision repair cross-complementing rodent repair deficiency, complementation group 8 (Ercc8), mRNA [NM_001107650]	**9,972**
0	Unknown	**9,970**
Cdh20	Rattus norvegicus cadherin 20 (Cdh20), mRNA [NM_001012748]	**9,970**
LOC500035	Rattus norvegicus hypothetical protein LOC500035 (LOC500035), mRNA [NM_001047955]	**9,967**
Ddah2	Rattus norvegicus dimethylarginine dimethylaminohydrolase 2 (Ddah2), transcript variant 1, mRNA [NM_212532]	**9,967**
0	AHR_RAT (P41738) Aryl hydrocarbon receptor precursor (Ah receptor) (AhR), complete [TC573854]	**9,964**
Dcaf8	Rattus norvegicus DDB1 and CUL4 associated factor 8 (Dcaf8), mRNA [NM_001014231]	**9,963**
Ccdc43	Rattus norvegicus coiled-coil domain containing 43 (Ccdc43), mRNA [NM_001100728]	**9,962**
RT1-N3	Rattus norvegicus RT1 class Ib, locus N3 (RT1-N3), mRNA [NM_001008855]	**9,961**
Sntg1	Rattus norvegicus syntrophin, gamma 1 (Sntg1), mRNA [NM_001191981]	**9,957**
0	RCG46688, isoform CRA_aUncharacterized protein [Source:UniProtKB/TrEMBL;Acc:D3ZRP0] [ENSRNOT00000039466]	**9,957**
Grik2	Rattus norvegicus glutamate receptor, ionotropic, kainate 2 (Grik2), mRNA [NM_019309]	**9,956**
0	Unknown	**9,954**
0	Rattus norvegicus similar to 14-3-3 PROTEIN TAU (14-3-3 PROTEIN THETA) (LOC316483), mRNA [XM_237259]	**9,952**
0	Uncharacterized protein [Source:UniProtKB/TrEMBL;Acc:D3ZKL6] [ENSRNOT00000044160]	**9,951**
LOC691561	PREDICTED: Rattus norvegicus hypothetical protein LOC691561 (LOC691561), mRNA [XM_001078835]	**9,950**

0	Rattus norvegicus similar to putative homeobox protein (LOC294473), mRNA [XM_228242]	**9,949**
Bhmt2	Rattus norvegicus betaine-homocysteine methyltransferase 2 (Bhmt2), mRNA [NM_001014256]	**9,943**
Chrm1	Rattus norvegicus cholinergic receptor, muscarinic 1 (Chrm1), mRNA [NM_080773]	**9,943**
Ano10	Uncharacterized protein [Source:UniProtKB/TrEMBL;Acc:D3ZBU6] [ENSRNOT00000035371]	**9,941**
Ap3d1	Rattus norvegicus adaptor-related protein complex 3, delta 1 subunit (Ap3d1), mRNA [NM_001100719]	**9,939**
Ric8b	Rattus norvegicus resistance to inhibitors of cholinesterase 8 homolog B (C. elegans) (Ric8b), mRNA [NM_175598]	**9,939**
Enpp1	Rattus norvegicus ectonucleotide pyrophosphatase/phosphodiesterase 1 (Enpp1), mRNA [NM_053535]	**9,937**
0	Uncharacterized protein [Source:UniProtKB/TrEMBL;Acc:D3ZVJ1] [ENSRNOT00000068427]	**9,935**
Ankle2	Rattus norvegicus ankyrin repeat and LEM domain containing 2 (Ankle2), mRNA [NM_001047901]	**9,935**
0	Metallothionein 1 [Source:UniProtKB/TrEMBL;Acc:Q7M082] [ENSRNOT00000058957]	**9,934**
LOC684993	Rattus norvegicus hypothetical protein LOC684993 (LOC684993), mRNA [NM_001109451]	**9,934**
Trpc5	Rattus norvegicus transient receptor potential cation channel, subfamily C, member 5 (Trpc5), mRNA [NM_080898]	**9,932**
Fam118b	Rattus norvegicus family with sequence similarity 118, member B (Fam118b), mRNA [NM_001025283]	**9,932**
Zfp46	Rattus norvegicus zinc finger protein 46 (Zfp46), mRNA [NM_001106691]	**9,931**
0	Uncharacterized protein [Source:UniProtKB/TrEMBL;Acc:D3ZV89] [ENSRNOT00000059958]	**9,930**
Gab2	Rattus norvegicus GRB2-associated binding protein 2 (Gab2), mRNA [NM_053417]	**9,928**
Prcc	Rattus norvegicus papillary renal cell carcinoma (translocation-associated) (Prcc), mRNA [NM_001107700]	**9,926**
Atp13a1	Rattus norvegicus ATPase type 13A1 (Atp13a1), mRNA [NM_001106079]	**9,925**
0	Uncharacterized protein [Source:UniProtKB/TrEMBL;Acc:D3ZBT9] [ENSRNOT00000020886]	**9,924**
Tmem135	Rattus norvegicus transmembrane protein 135 (Tmem135), mRNA [NM_001013896]	**9,923**
LOC498145	Rattus norvegicus similar to RIKEN cDNA 2810453I06 (LOC498145), mRNA [NM_001017485]	**9,922**
0	Uncharacterized protein [Source:UniProtKB/TrEMBL;Acc:D3ZKR7] [ENSRNOT00000050021]	**9,921**
0	Rattus norvegicus similar to glyceraldehyde-3-phosphate dehydrogenase (LOC315534), mRNA [XM_235981]	**9,919**
Mrps17	Rattus norvegicus mitochondrial ribosomal protein S17 (Mrps17), nuclear gene encoding mitochondrial protein, mRNA [NM_001105923]	**9,919**
0	Unknown	**9,917**
Trim69	Rattus norvegicus tripartite motif-containing 69 (Trim69), mRNA [NM_001013160]	**9,915**
Car1	Rattus norvegicus carbonic anhydrase 1 (Car1), mRNA [NM_001107660]	**9,914**
Tsc22d1	Rattus norvegicus TSC22 domain family, member 1 (Tsc22d1), transcript variant 1, mRNA [NM_001109912]	**9,912**
Wfdc10	Rattus norvegicus WAP four-disulfide core domain 10 (Wfdc10), mRNA [NM_001109461]	**9,911**
Tns3	PREDICTED: Rattus norvegicus similar to novel protein similar to Tensin Tns (RGD1564174), mRNA [XM_002725003]	**9,911**
LOC683516	PREDICTED: Rattus norvegicus similar to Zinc finger X-linked protein ZXDB (LOC683516), partial mRNA [XM_001066304]	**9,910**
0	BC034115 Creg2 protein {Mus musculus} (exp=-1; wgp=0; cg=0), partial (34%) [TC605666]	**9,910**
Rab27a	Rattus norvegicus RAB27A, member RAS oncogene family (Rab27a), mRNA [NM_017317]	**9,907**
LOC680039	Rattus norvegicus hypothetical protein LOC680039 (LOC680039), mRNA [NM_001109388]	**9,907**

Pyroxd2	Rattus norvegicus pyridine nucleotide-disulphide oxidoreductase domain 2 (Pyroxd2), mRNA [NM_001004261]	**9,906**
Atp2a1	Rattus norvegicus ATPase, Ca++ transporting, cardiac muscle, fast twitch 1 (Atp2a1), mRNA [NM_058213]	**9,906**
Krt5	Rattus norvegicus keratin 5 (Krt5), mRNA [NM_183333]	**9,904**
Lpar3	Rattus norvegicus lysophosphatidic acid receptor 3 (Lpar3), mRNA [NM_023969]	**9,902**
Alms1	Rattus norvegicus Alstrom syndrome 1 homolog (human) (Alms1), mRNA [NM_001106604]	**9,902**
Lzts1	Rattus norvegicus leucine zipper, putative tumor suppressor 1 (Lzts1), mRNA [NM_153470]	**9,901**
H2afy	Rattus norvegicus H2A histone family, member Y (H2afy), mRNA [NM_017182]	**9,901**
0	Unknown	**9,900**
Snip	Rattus norvegicus SNAP25-interacting protein (Snip), mRNA [NM_019378]	**9,900**
0	Unknown	**9,897**
Atad1	Rattus norvegicus ATPase family, AAA domain containing 1 (Atad1), mRNA [NM_001035002]	**9,897**
Krba1	Uncharacterized protein [Source:UniProtKB/TrEMBL;Acc:D3ZKZ3] [ENSRNOT00000043856]	**9,896**
RGD1311501	Rattus norvegicus similar to chromosome 9 open reading frame 7 (RGD1311501), mRNA [NM_001106561]	**9,895**
0	Rattus norvegicus similar to glyceraldehyde-3-phosphate dehydrogenase (LOC290902), mRNA [XM_225042]	**9,895**
Aldh18a1	Rattus norvegicus aldehyde dehydrogenase 18 family, member A1 (Aldh18a1), nuclear gene encoding mitochondrial protein, mRNA [NM_001108524]	**9,893**
Tgif1	Rattus norvegicus TGFB-induced factor homeobox 1 (Tgif1), mRNA [NM_001015020]	**9,891**
Ly6h	Rattus norvegicus lymphocyte antigen 6 complex, locus H (Ly6h), mRNA [NM_001134839]	**9,887**
RGD1560978	Rattus norvegicus similar to hypothetical protein (RGD1560978), mRNA [NM_001134631]	**9,885**
Olr310	Rattus norvegicus olfactory receptor 310 (Olr310), mRNA [NM_001000765]	**9,885**
Ecsit	Rattus norvegicus ECSIT homolog (Drosophila) (Ecsit), nuclear gene encoding mitochondrial protein, mRNA [NM_001006986]	**9,883**
LOC688778	PREDICTED: Rattus norvegicus similar to fatty aldehyde dehydrogenase-like (LOC688778), mRNA [XM_001068253]	**9,882**
RGD1560137	PREDICTED: Rattus norvegicus similar to expressed sequence AU021034 (RGD1560137), miscRNA [XR_008828]	**9,880**
Slc9a6	PREDICTED: Rattus norvegicus solute carrier family 9 (sodium/hydrogen exchanger), member 6 (Slc9a6), mRNA [XM_001053956]	**9,879**
Try10	Rattus norvegicus trypsin 10 (Try10), mRNA [NM_001004097]	**9,879**
Tmem110	Rattus norvegicus transmembrane protein 110 (Tmem110), mRNA [NM_198774]	**9,879**
Slc29a2	Rattus norvegicus solute carrier family 29 (nucleoside transporters), member 2 (Slc29a2), mRNA [NM_031738]	**9,878**
Trappc6a	Rattus norvegicus trafficking protein particle complex 6A (Trappc6a), mRNA [NM_001109410]	**9,877**
Scn1a	Rattus norvegicus sodium channel, voltage-gated, type I, alpha (Scn1a), mRNA [NM_030875]	**9,877**
Rcbtb1	Rattus norvegicus regulator of chromosome condensation (RCC1) and BTB (POZ) domain containing protein 1 (Rcbtb1), mRNA [NM_001108380]	**9,876**
0	LRRGT00061 [Source:UniProtKB/TrEMBL;Acc:Q6TUI3] [ENSRNOT00000050740]	**9,876**
Nr1h3	Rattus norvegicus nuclear receptor subfamily 1, group H, member 3 (Nr1h3), mRNA [NM_031627]	**9,874**
LOC689727	PREDICTED: Rattus norvegicus hypothetical protein LOC689727 (LOC689727), mRNA [XM_001071797]	**9,870**
LOC686591	PREDICTED: Rattus norvegicus similar to paraneoplastic antigen MA2 (LOC686591), mRNA [XM_001074878]	**9,867**

Znf644	Rattus norvegicus zinc finger protein 644 (Znf644), mRNA [NM_001139484]	**9,866**
0	Unknown	**9,865**
Grk4	Rattus norvegicus G protein-coupled receptor kinase 4 (Grk4), mRNA [NM_022928]	**9,865**
Elac1	Rattus norvegicus elaC homolog 1 (E. coli) (Elac1), mRNA [NM_001107406]	**9,864**
0	Unknown	**9,859**
Olr769	Rattus norvegicus olfactory receptor 769 (Olr769), mRNA [NM_001000371]	**9,858**
Akt1	Rattus norvegicus v-akt murine thymoma viral oncogene homolog 1 (Akt1), mRNA [NM_033230]	**9,858**
0	Rattus norvegicus similar to RIKEN cDNA 0610012D17 (LOC294368), mRNA [XM_228129]	**9,857**
Sectm1b	Rattus norvegicus secreted and transmembrane 1B (Sectm1b), mRNA [NM_199082]	**9,857**
RGD1561656	Rattus norvegicus similar to loricrin - mouse (LOC364810), mRNA [XM_344647]	**9,856**
Fam169b	PREDICTED: Rattus norvegicus hypothetical protein LOC680906 (LOC680906), mRNA [XM_001059420]	**9,853**
Pcdhb17	PREDICTED: Rattus norvegicus protocadherin beta 17 (Pcdhb17), mRNA [XM_001065253]	**9,853**
Sdccag3	Rattus norvegicus serologically defined colon cancer antigen 3 (Sdccag3), mRNA [NM_001013135]	**9,852**
0	dual specificity phosphatase 14 [Source:RefSeq peptide;Acc:NP_001073362] [ENSRNOT00000043148]	**9,851**
0	Unknown	**9,850**
Tusc1	PREDICTED: Rattus norvegicus tumor suppressor candidate 1 (Tusc1), mRNA [XM_001060960]	**9,849**
0	Ral GTPase-activating protein subunit alpha-2 [Source:UniProtKB/Swiss-Prot;Acc:P86411] [ENSRNOT00000015414]	**9,847**
0	Unknown	**9,847**
0	Uncharacterized protein [Source:UniProtKB/TrEMBL;Acc:D3ZY99] [ENSRNOT00000033247]	**9,847**
Usp36	Rattus norvegicus ubiquitin specific peptidase 36 (Usp36), mRNA [NM_001107069]	**9,847**
Itga11	Rattus norvegicus integrin, alpha 11 (Itga11), mRNA [NM_001108156]	**9,846**
0	Unknown	**9,845**
Rras2	Rattus norvegicus related RAS viral (r-ras) oncogene homolog 2 (Rras2), mRNA [NM_001013434]	**9,845**
Stam	Rattus norvegicus signal transducing adaptor molecule (SH3 domain and ITAM motif) 1 (Stam), mRNA [NM_001109121]	**9,844**
Hsdl2	Rattus norvegicus hydroxysteroid dehydrogenase like 2 (Hsdl2), mRNA [NM_001025697]	**9,840**
Ctbp2	Rattus norvegicus C-terminal binding protein 2 (Ctbp2), mRNA [NM_053335]	**9,838**
0	LIM domain only protein 3 [Source:UniProtKB/Swiss-Prot;Acc:Q99MB5] [ENSRNOT00000010623]	**9,836**
Laptm4b	Rattus norvegicus lysosomal protein transmembrane 4 beta (Laptm4b), mRNA [NM_001013174]	**9,833**
0	PREDICTED: Rattus norvegicus similar to TDPOZ2 (RGD1562545), mRNA [XM_001061198]	**9,832**
0	Rattus norvegicus similar to glyceraldehyde-3-phosphate dehydrogenase (LOC290492), mRNA [XM_224528]	**9,832**
Taok1	Rattus norvegicus TAO kinase 1 (Taok1), mRNA [NM_173327]	**9,831**
Vwc2	Rattus norvegicus von Willebrand factor C domain containing 2 (Vwc2), mRNA [NM_001109312]	**9,831**
Cox4i2	Rattus norvegicus cytochrome c oxidase subunit IV isoform 2 (Cox4i2), nuclear gene encoding mitochondrial protein, mRNA [NM_053472]	**9,830**
0	Unknown	**9,829**

Hcrtr2	Rattus norvegicus hypocretin (orexin) receptor 2 (Hcrtr2), mRNA [NM_013074]	**9,827**
Polr2i	Rattus norvegicus polymerase (RNA) II (DNA directed) polypeptide I (Polr2i), mRNA [NM_001106244]	**9,827**
Sv2c	Rattus norvegicus synaptic vesicle glycoprotein 2c (Sv2c), mRNA [NM_031593]	**9,827**
RGD1311378	Rattus norvegicus similar to RIKEN cDNA 2010011I20 (RGD1311378), mRNA [NM_001106547]	**9,825**
Dynlt1	Rattus norvegicus dynein light chain Tctex-type 1 (Dynlt1), mRNA [NM_031318]	**9,824**
0	Uncharacterized protein [Source:UniProtKB/TrEMBL;Acc:D3Z9J4] [ENSRNOT00000048141]	**9,823**
Dhx57	Rattus norvegicus DEAH (Asp-Glu-Ala-Asp/His) box polypeptide 57 (Dhx57), mRNA [NM_001191907]	**9,822**
Tspyl1	Rattus norvegicus TSPY-like 1 (Tspyl1), mRNA [NM_001013033]	**9,821**
0	Unknown	**9,821**
Errfi1	Rattus norvegicus ERBB receptor feedback inhibitor 1 (Errfi1), mRNA [NM_001014071]	**9,820**
0	cathepsin Q-like 2 [Source:RefSeq peptide;Acc:NP_001002813] [ENSRNOT00000045847]	**9,820**
0	Uncharacterized protein [Source:UniProtKB/TrEMBL;Acc:D4A3B7] [ENSRNOT00000009262]	**9,820**
Scn2b	Rattus norvegicus sodium channel, voltage-gated, type II, beta (Scn2b), mRNA [NM_012877]	**9,820**
RGD1564036	Rattus norvegicus similar to RIKEN cDNA 3010026O09 (RGD1564036), mRNA [NM_001109030]	**9,820**
Cachd1	Rattus norvegicus cache domain containing 1 (Cachd1), mRNA [NM_001191758]	**9,820**
Ppia	Rattus norvegicus peptidylprolyl isomerase A (cyclophilin A) (Ppia), mRNA [NM_017101]	**9,820**
Slc16a10	Rattus norvegicus solute carrier family 16 (monocarboxylic acid transporters), member 10 (Slc16a10), mRNA [NM_138831]	**9,819**
0	Q9D2U9_MOUSE (Q9D2U9) Adult male cerebellum cDNA, RIKEN full-length enriched library, clone:1500011O09 product:HISTONE H2B, full insert sequence (Histone 3, H2ba) (Histone protein Hist3h2ba), complete [TC601505]	**9,817**
Slc7a6os	Rattus norvegicus solute carrier family 7, member 6 opposite strand (Slc7a6os), mRNA [NM_139328]	**9,816**
Hnrnpa3	Rattus norvegicus heterogeneous nuclear ribonucleoprotein A3 (Hnrnpa3), transcript variant 1, mRNA [NM_001111294]	**9,816**
Dnajc5	Rattus norvegicus DnaJ (Hsp40) homolog, subfamily C, member 5 (Dnajc5), mRNA [NM_024161]	**9,811**
0	Unknown	**9,810**
MGC94915	Rattus norvegicus similar to hypothetical protein (MGC94915), mRNA [NM_001007009]	**9,810**
0	Unknown	**9,808**
Atic	Rattus norvegicus 5-aminoimidazole-4-carboxamide ribonucleotide formyltransferase/IMP cyclohydrolase (Atic), mRNA [NM_031014]	**9,808**
0	Unknown	**9,806**
0	Putative uncharacterized protein RGD1560252_predictedUncharacterized protein [Source:UniProtKB/TrEMBL;Acc:D4A3Y2] [ENSRNOT00000023411]	**9,805**
Prrt4	Rattus norvegicus proline-rich transmembrane protein 4 (Prrt4), mRNA [NM_001109226]	**9,804**
Map3k12	Rattus norvegicus mitogen activated protein kinase kinase kinase 12 (Map3k12), mRNA [NM_013055]	**9,803**
0	Bax inhibitor 1 [Source:UniProtKB/Swiss-Prot;Acc:P55062] [ENSRNOT00000045927]	**9,802**
0	Unknown	**9,799**
Yif1	Rattus norvegicus Yip1 interacting factor homolog (S. cerevisiae) (Yif1), mRNA [NM_172017]	**9,799**

Pink1	Rattus norvegicus PTEN induced putative kinase 1 (Pink1), nuclear gene encoding mitochondrial protein, mRNA [NM_001106694]	**9,797**
Znf608	Rattus norvegicus zinc finger protein 608 (Znf608), mRNA [NM_001107378]	**9,796**
RGD1561039	Rattus norvegicus similar to RIKEN cDNA 1700065I17 (RGD1561039), mRNA [NM_001109126]	**9,795**
Olr1468	Rattus norvegicus olfactory receptor 1468 (Olr1468), mRNA [NM_001000724]	**9,795**
0	Unknown	**9,794**
Magi2	Rattus norvegicus membrane associated guanylate kinase, WW and PDZ domain containing 2 (Magi2), mRNA [NM_053621]	**9,794**
RGD1308093	PREDICTED: Rattus norvegicus similar to FLJ00128 protein (RGD1308093), mRNA [XM_001075554]	**9,792**
Heatr3	Uncharacterized protein [Source:UniProtKB/TrEMBL;Acc:D3ZV81] [ENSRNOT00000020793]	**9,790**
Kchip2	Rattus norvegicus A-type potassium channel modulatory protein 2 (Kchip2), mRNA [NM_001034005]	**9,790**
Pdcd6ip	Rattus norvegicus programmed cell death 6 interacting protein (Pdcd6ip), mRNA [NM_001029910]	**9,790**
RGD1309730	Rattus norvegicus similar to RIKEN cDNA B230118H07 (RGD1309730), mRNA [NM_001106491]	**9,790**
Tmem209	Rattus norvegicus transmembrane protein 209 (Tmem209), mRNA [NM_001014055]	**9,789**
Gabrb3	Rattus norvegicus gamma-aminobutyric acid (GABA) A receptor, beta 3 (Gabrb3), mRNA [NM_017065]	**9,789**
Akirin2	Rattus norvegicus akirin 2 (Akirin2), mRNA [NM_001039914]	**9,788**
St3gal3	Rattus norvegicus ST3 beta-galactoside alpha-2,3-sialyltransferase 3 (St3gal3), mRNA [NM_031697]	**9,787**
LOC685636	PREDICTED: Rattus norvegicus hypothetical protein LOC685636 (LOC685636), mRNA [XM_001064613]	**9,785**
Impa1	Rattus norvegicus Inositol (myo)-1(or 4)-monophosphatase 1 (Impa1), mRNA [NM_032057]	**9,785**
Setd2	Rattus norvegicus SET domain containing 2 (Setd2), mRNA [NM_001108189]	**9,782**
RGD1311783	Rattus norvegicus similar to RIKEN cDNA 2010012O05 (RGD1311783), mRNA [NM_001134509]	**9,781**
Fam83e	PREDICTED: Rattus norvegicus family with sequence similarity 83, member E (Fam83e), mRNA [XM_001079852]	**9,779**
RGD1562552	Rattus norvegicus similar to hypothetical protein LOC340061 (RGD1562552), mRNA [NM_001109122]	**9,779**
Fanca	Rattus norvegicus Fanconi anemia, complementation group A (Fanca), mRNA [NM_001108455]	**9,779**
Cdc37	Rattus norvegicus cell division cycle 37 homolog (S. cerevisiae) (Cdc37), mRNA [NM_053743]	**9,777**
Lrrc7	Rattus norvegicus leucine rich repeat containing 7 (Lrrc7), mRNA [NM_057142]	**9,776**
0	Unknown	**9,774**
Iars	Rattus norvegicus isoleucyl-tRNA synthetase (Iars), mRNA [NM_001100572]	**9,773**
Atg5	Rattus norvegicus ATG5 autophagy related 5 homolog (S. cerevisiae) (Atg5), mRNA [NM_001014250]	**9,769**
Crygf	Rattus norvegicus crystallin, gamma F (Crygf), mRNA [NM_001109557]	**9,769**
Shc4	Rattus norvegicus SHC (Src homology 2 domain containing) family, member 4 (Shc4), mRNA [NM_001191065]	**9,766**
Chac2	Rattus norvegicus ChaC, cation transport regulator homolog 2 (E. coli) (Chac2), mRNA [NM_001025016]	**9,764**
Asns	Rattus norvegicus asparagine synthetase (Asns), mRNA [NM_013079]	**9,763**
Stat5a	Rattus norvegicus signal transducer and activator of transcription 5A (Stat5a), mRNA [NM_017064]	**9,761**
0	Rattus norvegicus TL0AEA9YC09 mRNA sequence. [FQ230424]	**9,758**
Fgf17	Rattus norvegicus fibroblast growth factor 17 (Fgf17), mRNA [NM_019198]	**9,758**

Olr875	Rattus norvegicus olfactory receptor 875 (Olr875), mRNA [NM_001000054]	**9,757**
Cog3	Rattus norvegicus component of oligomeric golgi complex 3 (Cog3), mRNA [NM_001012157]	**9,756**
0	transcription factor Spi-C [Source:RefSeq peptide;Acc:NP_001101550] [ENSRNOT00000007542]	**9,755**
RGD1562024	Rattus norvegicus RGD1562024 (RGD1562024), mRNA [NM_001113788]	**9,753**
Etfdh	Rattus norvegicus electron-transferring-flavoprotein dehydrogenase (Etfdh), nuclear gene encoding mitochondrial protein, mRNA [NM_198742]	**9,753**
LOC100125362	Rattus norvegicus hypothetical protein LOC100125362 (LOC100125362), mRNA [NM_001103354]	**9,752**
Crcp	Rattus norvegicus CGRP receptor component (Crcp), mRNA [NM_053670]	**9,751**
Pars2	Rattus norvegicus prolyl-tRNA synthetase 2, mitochondrial (putative) (Pars2), nuclear gene encoding mitochondrial protein, mRNA [NM_001014064]	**9,751**
Ptpro	Rattus norvegicus protein tyrosine phosphatase, receptor type, O (Ptpro), mRNA [NM_017336]	**9,751**
LOC502371	PREDICTED: Rattus norvegicus similar to 40S ribosomal protein S21 (LOC502371), mRNA [XM_001053958]	**9,751**
Shq1	Rattus norvegicus SHQ1 homolog (S. cerevisiae) (Shq1), mRNA [NM_001134713]	**9,749**
Ampd1	Rattus norvegicus adenosine monophosphate deaminase 1 (isoform M) (Ampd1), mRNA [NM_138876]	**9,749**
Cdc34	Rattus norvegicus cell division cycle 34 homolog (S. cerevisiae) (Cdc34), mRNA [NM_001013103]	**9,749**
Fndc3a	Rattus norvegicus fibronectin type III domain containing 3a (Fndc3a), mRNA [NM_001107278]	**9,749**
Rnf41	Rattus norvegicus ring finger protein 41 (Rnf41), mRNA [NM_001012195]	**9,747**
Impad1	Rattus norvegicus inositol monophosphatase domain containing 1 (Impad1), mRNA [NM_001008772]	**9,745**
0	Unknown	**9,744**
Slc25a16	Rattus norvegicus solute carrier family 25 (mitochondrial carrier, Graves disease autoantigen), member 16 (Slc25a16), nuclear gene encoding mitochondrial protein, mRNA [NM_001100860]	**9,743**
Fgr	Rattus norvegicus Gardner-Rasheed feline sarcoma viral (v-fgr) oncogene homolog (Fgr), mRNA [NM_024145]	**9,743**
Ensa	Rattus norvegicus endosulfine alpha (Ensa), transcript variant 1, mRNA [NM_001033974]	**9,742**
Golga7b	PREDICTED: Rattus norvegicus golgi autoantigen, golgin subfamily a, 7B (Golga7b), mRNA [XM_001055463]	**9,742**
0	Q7VBZ4_PROMA (Q7VBZ4) Diaminopimelate epimerase, partial (5%) [TC594147]	**9,741**
0	Unknown	**9,739**
LOC314942	PREDICTED: Rattus norvegicus similar to CUB and Sushi multiple domains 3 isoform 1, transcript variant 4 (LOC314942), mRNA [XM_001063221]	**9,738**
Hddc2	Rattus norvegicus HD domain containing 2 (Hddc2), mRNA [NM_001108460]	**9,738**
Tex19	Rattus norvegicus testis expressed 19 (Tex19), mRNA [NM_001109622]	**9,736**
Xrn1	Uncharacterized protein [Source:UniProtKB/TrEMBL;Acc:D4ABN8] [ENSRNOT00000014481]	**9,735**
LOC687057	PREDICTED: Rattus norvegicus similar to Calponin-2 (Calponin H2, smooth muscle) (Neutral calponin) (LOC687057), mRNA [XM_001076906]	**9,730**
Ankrd6	Rattus norvegicus ankyrin repeat domain 6 (Ankrd6), mRNA [NM_001134969]	**9,730**
Inppl1	Rattus norvegicus inositol polyphosphate phosphatase-like 1 (Inppl1), mRNA [NM_022944]	**9,727**
Mmp28	Rattus norvegicus matrix metallopeptidase 28 (Mmp28), mRNA [NM_001079888]	**9,725**

Prkrir	Rattus norvegicus protein-kinase, interferon-inducible double stranded RNA dependent inhibitor, repressor of (P58 repressor) (Prkrir), mRNA [NM_001191630]	**9,724**
0	Rattus norvegicus similar to necdin (LOC292493), mRNA [XM_218132]	**9,724**
Dgkb	Rattus norvegicus diacylglycerol kinase, beta (Dgkb), mRNA [NM_019304]	**9,723**
LOC298018	RCG31985Uncharacterized protein [Source:UniProtKB/TrEMBL;Acc:D4AE08] [ENSRNOT00000014053]	**9,723**
0	Unknown	**9,723**
Gjb4	Rattus norvegicus gap junction protein, beta 4 (Gjb4), mRNA [NM_053984]	**9,721**
Zfp827	PREDICTED: Rattus norvegicus similar to hypothetical protein (RGD1560620), mRNA [XM_001074445]	**9,720**
Zmat3	Rattus norvegicus zinc finger, matrin type 3 (Zmat3), mRNA [NM_022548]	**9,718**
Tmem184c	Rattus norvegicus transmembrane protein 184C (Tmem184c), mRNA [NM_178330]	**9,717**
0	RCG43751Uncharacterized protein [Source:UniProtKB/TrEMBL;Acc:D3ZKG1] [ENSRNOT00000044594]	**9,715**
0	Rattus norvegicus similar to cytoplasmic beta-actin (LOC298169), mRNA [XM_233107]	**9,714**
Dyrk2	Rattus norvegicus dual-specificity tyrosine-(Y)-phosphorylation regulated kinase 2 (Dyrk2), mRNA [NM_001108100]	**9,713**
Ikbke	Rattus norvegicus inhibitor of kappa light polypeptide gene enhancer in B-cells, kinase epsilon (Ikbke), mRNA [NM_001108854]	**9,713**
Spdya	Rattus norvegicus speedy homolog A (Xenopus laevis) (Spdya), mRNA [NM_138855]	**9,712**
RGD1562877	AGENCOURT_31521904 NIH_MGC_238 Rattus norvegicus cDNA clone IMAGE:7456091 5', mRNA sequence [CV103892]	**9,711**
Nrbp	Rattus norvegicus nuclear receptor binding protein (Nrbp), mRNA [NM_001034997]	**9,711**
Mcpt8	Rattus norvegicus mast cell protease 8 (Mcpt8), mRNA [NM_021598]	**9,711**
Tmem163	Rattus norvegicus transmembrane protein 163 (Tmem163), mRNA [NM_001110763]	**9,710**
Agxt2l2	Rattus norvegicus alanine-glyoxylate aminotransferase 2-like 2 (Agxt2l2), mRNA [NM_001128196]	**9,709**
Hs6st3	PREDICTED: Rattus norvegicus heparan sulfate 6-O-sulfotransferase 3 (Hs6st3), mRNA [XM_344461]	**9,708**
Nxph1	Rattus norvegicus neurexophilin 1 (Nxph1), mRNA [NM_012994]	**9,700**
Retnlg	Rattus norvegicus resistin-like gamma (Retnlg), mRNA [NM_181625]	**9,697**
Elavl3	Rattus norvegicus ELAV (embryonic lethal, abnormal vision, Drosophila)-like 3 (Hu antigen C) (Elavl3), mRNA [NM_172324]	**9,697**
Sntb1	Rattus norvegicus syntrophin, beta 1 (Sntb1), mRNA [NM_001130542]	**9,697**
Atg10	Rattus norvegicus autophagy-related 10 (S. cerevisiae) (Atg10), mRNA [NM_001109505]	**9,696**
Cog1	Rattus norvegicus component of oligomeric golgi complex 1 (Cog1), mRNA [NM_001107062]	**9,695**
Stk3	Rattus norvegicus serine/threonine kinase 3 (STE20 homolog, yeast) (Stk3), mRNA [NM_031735]	**9,693**
Gmds	Rattus norvegicus GDP-mannose 4, 6-dehydratase (Gmds), mRNA [NM_001039606]	**9,692**
Selt	Rattus norvegicus selenoprotein T (Selt), mRNA [NM_001014253]	**9,691**
0	IQ domain-containing protein D [Source:UniProtKB/Swiss-Prot;Acc:Q5XIR6] [ENSRNOT00000063923]	**9,690**
Cacna1b	Rattus norvegicus calcium channel, voltage-dependent, N type, alpha 1B subunit (Cacna1b), transcript variant 2, mRNA [NM_147141]	**9,690**
Ptges	Rattus norvegicus prostaglandin E synthase (Ptges), mRNA [NM_021583]	**9,687**
0	Uncharacterized protein [Source:UniProtKB/TrEMBL;Acc:D4ACF9] [ENSRNOT00000036989]	**9,686**

RGD1562657	Uncharacterized protein [Source:UniProtKB/TrEMBL;Acc:D3ZVB0] [ENSRNOT00000036185]	**9,686**
Adam10	PREDICTED: Rattus norvegicus ADAM metallopeptidase domain 10 (Adam10), mRNA [XM_001054737]	**9,684**
RGD1311344	PREDICTED: Rattus norvegicus similar to RIKEN cDNA 2810039F03 (RGD1311344), miscRNA [XR_007345]	**9,684**
Mis12	Rattus norvegicus MIS12, MIND kinetochore complex component, homolog (S. pombe) (Mis12), mRNA [NM_001047972]	**9,681**
0	Unknown	**9,681**
Ifitm2	Rattus norvegicus interferon induced transmembrane protein 2 (Ifitm2), mRNA [NM_030833]	**9,679**
LOC307974	Rattus norvegicus similar to dJ1016N21.1 (novel protein (ortholog of Drosophila Pecanex (PCX), similar to KIAA0805)) (LOC307974), mRNA [XM_226603]	**9,679**
Cfp	Rattus norvegicus complement factor properdin (Cfp), mRNA [NM_001106757]	**9,678**
Smarcd3	Rattus norvegicus SWI/SNF related, matrix associated, actin dependent regulator of chromatin, subfamily d, member 3 (Smarcd3), mRNA [NM_001011966]	**9,677**
Dock1	Rattus norvegicus dedicator of cyto-kinesis 1 (Dock1), mRNA [NM_001143858]	**9,677**
Csnk1d	Rattus norvegicus casein kinase 1, delta (Csnk1d), mRNA [NM_139060]	**9,677**
Edem2	Rattus norvegicus ER degradation enhancer, mannosidase alpha-like 2 (Edem2), mRNA [NM_001004230]	**9,676**
Plk5	Rattus norvegicus polo-like kinase 5 (Plk5), mRNA [NM_001170557]	**9,675**
0	Unknown	**9,673**
0	Unknown	**9,672**
Abp10	PREDICTED: Rattus norvegicus annexin V-binding protein ABP-10 (Abp10), mRNA [XM_001060944]	**9,671**
Fam81a	Rattus norvegicus family with sequence similarity 81, member A (Fam81a), mRNA [NM_001108163]	**9,671**
Krtap13-2	Rattus norvegicus keratin associated protein 13-2 (Krtap13-2), nuclear gene encoding mitochondrial protein, mRNA [NM_001109325]	**9,671**
LOC499749	Rattus norvegicus similar to RIKEN cDNA C430004E15 (LOC499749), mRNA [NM_001024309]	**9,670**
RGD1306008	Rattus norvegicus similar to RIKEN cDNA 4930511I11 (RGD1306008), mRNA [NM_001113777]	**9,668**
Ublcp1	Rattus norvegicus ubiquitin-like domain containing CTD phosphatase 1 (Ublcp1), mRNA [NM_001014117]	**9,666**
P4ha1	Rattus norvegicus prolyl 4-hydroxylase, alpha polypeptide I (P4ha1), mRNA [NM_172062]	**9,666**
Vegfa	Rattus norvegicus vascular endothelial growth factor A (Vegfa), transcript variant 3, mRNA [NM_001110334]	**9,665**
Nxt2	Rattus norvegicus nuclear transport factor 2-like export factor 2 (Nxt2), mRNA [NM_001108120]	**9,665**
0	DNA-directed RNA polymerase I subunit RPA1 [Source:UniProtKB/Swiss-Prot;Acc:O54889] [ENSRNOT00000013417]	**9,663**
Emd	Rattus norvegicus emerin (Emd), mRNA [NM_012948]	**9,662**
Tmem43	Rattus norvegicus transmembrane protein 43 (Tmem43), mRNA [NM_001007745]	**9,662**
Inpp5b	Rattus norvegicus inositol polyphosphate-5-phosphatase B (Inpp5b), nuclear gene encoding mitochondrial protein, mRNA [NM_001100755]	**9,659**
Usp11	Rattus norvegicus ubiquitin specific peptidase 11 (Usp11), mRNA [NM_001008861]	**9,658**
Eif2ak2	Rattus norvegicus eukaryotic translation initiation factor 2-alpha kinase 2 (Eif2ak2), mRNA [NM_019335]	**9,655**
Fez2	Rattus norvegicus fasciculation and elongation protein zeta 2 (zygin II) (Fez2), mRNA [NM_053600]	**9,653**
Lgals4	Rattus norvegicus lectin, galactoside-binding, soluble, 4 (Lgals4), mRNA [NM_012975]	**9,651**

RGD1564386	PREDICTED: Rattus norvegicus similar to TDPOZ3 (RGD1564386), mRNA [XM_001074961]	**9,651**
0	Q99MR2_MOUSE (Q99MR2) EPHB4, partial (63%) [TC591623]	**9,650**
0	Uncharacterized protein [Source:UniProtKB/TrEMBL;Acc:D4A319] [ENSRNOT00000007828]	**9,648**
0	Unknown	**9,648**
Rin1	Rattus norvegicus Ras and Rab interactor 1 (Rin1), mRNA [NM_139038]	**9,645**
0	SUMO-conjugating enzyme UBC9 [Source:UniProtKB/Swiss-Prot;Acc:P63281] [ENSRNOT00000024406]	**9,645**
Slc14a1	Rattus norvegicus solute carrier family 14 (urea transporter), member 1 (Slc14a1), mRNA [NM_019346]	**9,642**
Tekt4	Rattus norvegicus tektin 4 (Tekt4), mRNA [NM_001013965]	**9,641**
Nrip1	Rattus norvegicus nuclear receptor interacting protein 1 (Nrip1), mRNA [NM_001100560]	**9,641**
Tcof1	Rattus norvegicus Treacher Collins-Franceschetti syndrome 1 homolog (human) (Tcof1), mRNA [NM_001106143]	**9,639**
RGD1305014	Rattus norvegicus similar to RIKEN cDNA 2310057M21 (RGD1305014), mRNA [NM_001014025]	**9,638**
Pmp2	Rattus norvegicus peripheral myelin protein 2 (Pmp2), mRNA [NM_001109514]	**9,636**
Serpina11	Rattus norvegicus serine (or cysteine) peptidase inhibitor, clade A (alpha-1 antiproteinase, antitrypsin), member 11 (Serpina11), transcript variant 1, mRNA [NM_001008776]	**9,635**
LOC288913	Rattus norvegicus similar to LEYDIG CELL TUMOR 10 KD PROTEIN (LOC288913), mRNA [NM_198728]	**9,635**
0	Unknown	**9,633**
Relt	Rattus norvegicus RELT tumor necrosis factor receptor (Relt), mRNA [NM_001108495]	**9,633**
Vom1r40	Rattus norvegicus vomeronasal 1 receptor 40 (Vom1r40), mRNA [NM_001008904]	**9,632**
0	Unknown	**9,629**
0	Unknown	**9,628**
Urb2	Rattus norvegicus URB2 ribosome biogenesis 2 homolog (S. cerevisiae) (Urb2), mRNA [NM_001135708]	**9,628**
Chchd8	Rattus norvegicus coiled-coil-helix-coiled-coil-helix domain containing 8 (Chchd8), mRNA [NM_001127655]	**9,627**
0	Unknown	**9,625**
Foxi2	Forkhead box protein I2 [Source:UniProtKB/Swiss-Prot;Acc:Q63248] [ENSRNOT00000025569]	**9,625**
Tubb3	Rattus norvegicus tubulin, beta 3 (Tubb3), mRNA [NM_139254]	**9,623**
Rxrb	Rattus norvegicus retinoid X receptor beta (Rxrb), mRNA [NM_206849]	**9,622**
Arsa	arylsulfatase A [Source:RefSeq peptide;Acc:NP_001030105] [ENSRNOT00000017783]	**9,620**
0	Unknown	**9,620**
Wdr37	Rattus norvegicus WD repeat domain 37 (Wdr37), mRNA [NM_001107362]	**9,619**
0	Uncharacterized protein [Source:UniProtKB/TrEMBL;Acc:D3ZZX4] [ENSRNOT00000066953]	**9,619**
P2ry4	Rattus norvegicus pyrimidinergic receptor P2Y, G-protein coupled, 4 (P2ry4), mRNA [NM_031680]	**9,618**
Lrch4	Rattus norvegicus leucine-rich repeats and calponin homology (CH) domain containing 4 (Lrch4), mRNA [NM_001127551]	**9,617**
0	Unknown	**9,617**
0	Unknown	**9,615**

0	Unknown	**9,614**
Kcmf1	Rattus norvegicus potassium channel modulatory factor 1 (Kcmf1), mRNA [NM_001128192]	**9,612**
0	Suppressor of Ty 5 homolog (S. cerevisiae), isoform CRA_aUncharacterized protein [Source:UniProtKB/TrEMBL;Acc:D3ZSS2] [ENSRNOT00000068115]	**9,612**
Pdlim5	Rattus norvegicus PDZ and LIM domain 5 (Pdlim5), mRNA [NM_053326]	**9,611**
Wbp1	Rattus norvegicus WW domain binding protein 1 (Wbp1), mRNA [NM_001106600]	**9,610**
Ppapdc1b	Rattus norvegicus phosphatidic acid phosphatase type 2 domain containing 1B (Ppapdc1b), mRNA [NM_001109411]	**9,610**
Ube2e2	Rattus norvegicus ubiquitin-conjugating enzyme E2E 2 (UBC4/5 homolog, yeast) (Ube2e2), mRNA [NM_001108371]	**9,607**
0	Unknown	**9,606**
Adra1b	Rattus norvegicus adrenergic, alpha-1B-, receptor (Adra1b), mRNA [NM_016991]	**9,606**
Pdzk1ip1	Rattus norvegicus PDZK1 interacting protein 1 (Pdzk1ip1), mRNA [NM_130401]	**9,606**
Mfap1a	Rattus norvegicus microfibrillar-associated protein 1A (Mfap1a), mRNA [NM_001191964]	**9,605**
0	Uncharacterized protein [Source:UniProtKB/TrEMBL;Acc:D4A2W5] [ENSRNOT00000065969]	**9,604**
Efha2	Rattus norvegicus EF hand domain family, member A2 (Efha2), mRNA [NM_001191892]	**9,604**
Scx	Rattus norvegicus scleraxis (Scx), mRNA [NM_001130508]	**9,599**
Slc44a1	Choline transporter-like protein 1 [Source:UniProtKB/Swiss-Prot;Acc:Q8VII6] [ENSRNOT00000024229]	**9,598**
Mapk8ip2	Mapk8ip2 protein [Source:UniProtKB/TrEMBL;Acc:Q3B8P9] [ENSRNOT00000055792]	**9,594**
Pddc1	Rattus norvegicus Parkinson disease 7 domain containing 1 (Pddc1), mRNA [NM_001107563]	**9,594**
Fam54b	Rattus norvegicus family with sequence similarity 54, member B (Fam54b), mRNA [NM_001013935]	**9,594**
Ppp1r2	Rattus norvegicus protein phosphatase 1, regulatory (inhibitor) subunit 2 (Ppp1r2), mRNA [NM_138823]	**9,592**
Lrrc47	Rattus norvegicus leucine rich repeat containing 47 (Lrrc47), mRNA [NM_001135666]	**9,591**
Mare	Rattus norvegicus alpha globin regulatory element containing gene (Mare), mRNA [NM_001034936]	**9,591**
Zfp748	Rattus norvegicus TL0AAA77YJ08 mRNA sequence. [FQ211879]	**9,590**
Actrt2	Rattus norvegicus actin-related protein T2 (Actrt2), mRNA [NM_001013937]	**9,589**
Plekha1	Rattus norvegicus pleckstrin homology domain containing, family A (phosphoinositide binding specific) member 1 (Plekha1), mRNA [NM_001079894]	**9,588**
Sipa1	Rattus norvegicus signal-induced proliferation-associated 1 (Sipa1), mRNA [NM_001004089]	**9,588**
Enc1	Rattus norvegicus ectodermal-neural cortex 1 (Enc1), mRNA [NM_001003401]	**9,587**
RGD1309102	Rattus norvegicus similar to TRS85 homolog (RGD1309102), mRNA [NM_001106160]	**9,585**
0	Unknown	**9,583**
Cnot2	Rattus norvegicus CCR4-NOT transcription complex, subunit 2 (Cnot2), mRNA [NM_001011988]	**9,582**
Uggt1	Rattus norvegicus UDP-glucose glycoprotein glucosyltransferase 1 (Uggt1), mRNA [NM_133596]	**9,582**
Pla2g5	Rattus norvegicus phospholipase A2, group V (Pla2g5), mRNA [NM_017174]	**9,581**
Agtr1b	Rattus norvegicus angiotensin II receptor, type 1b (Agtr1b), mRNA [NM_031009]	**9,580**

Diaph3	PREDICTED: Rattus norvegicus diaphanous homolog 3 (Drosophila), transcript variant 2 (Diaph3), mRNA [XM_224392]	**9,579**
Myh3	Rattus norvegicus myosin, heavy chain 3, skeletal muscle, embryonic (Myh3), mRNA [NM_012604]	**9,579**
Arsj	Rattus norvegicus arylsulfatase family, member J (Arsj), mRNA [NM_001047887]	**9,579**
Ppara	Rattus norvegicus peroxisome proliferator activated receptor alpha (Ppara), mRNA [NM_013196]	**9,577**
Camk2a	Rattus norvegicus calcium/calmodulin-dependent protein kinase II alpha (Camk2a), mRNA [NM_012920]	**9,577**
0	Unknown	**9,574**
Olr777	Rattus norvegicus olfactory receptor 777 (Olr777), mRNA [NM_001000579]	**9,572**
Dcaf7	Rattus norvegicus DDB1 and CUL4 associated factor 7 (Dcaf7), mRNA [NM_001107057]	**9,572**
Klhl22	Rattus norvegicus kelch-like 22 (Drosophila) (Klhl22), mRNA [NM_001107079]	**9,571**
Ppp1r3d	Rattus norvegicus protein phosphatase 1, regulatory subunit 3D (Ppp1r3d), mRNA [NM_001109564]	**9,571**
Yeats4	Rattus norvegicus YEATS domain containing 4 (Yeats4), mRNA [NM_001127527]	**9,570**
Nek7	Rattus norvegicus NIMA (never in mitosis gene a)-related kinase 7 (Nek7), mRNA [NM_001108346]	**9,570**
Kcne2	Rattus norvegicus potassium voltage-gated channel, Isk-related family, member 2 (Kcne2), mRNA [NM_133603]	**9,569**
Map1d	Rattus norvegicus methionine aminopeptidase 1D (Map1d), mRNA [NM_001107812]	**9,566**
Ttc1	Rattus norvegicus tetratricopeptide repeat domain 1 (Ttc1), mRNA [NM_001005529]	**9,566**
Ctdspl	Rattus norvegicus CTD (carboxy-terminal domain, RNA polymerase II, polypeptide A) small phosphatase-like (Ctdspl), mRNA [NM_001106865]	**9,566**
0	RCG29400Uncharacterized protein [Source:UniProtKB/TrEMBL;Acc:D3ZPS3] [ENSRNOT00000060296]	**9,565**
Ch25h	Rattus norvegicus cholesterol 25-hydroxylase (Ch25h), mRNA [NM_001025415]	**9,564**
Hsd11b2	Rattus norvegicus hydroxysteroid 11-beta dehydrogenase 2 (Hsd11b2), mRNA [NM_017081]	**9,564**
0	Rattus norvegicus similar to RIKEN cDNA 1700001E04 (LOC316188), mRNA [XM_236878]	**9,562**
Fam13a1	Rattus norvegicus family with sequence similarity 13, member A1 (Fam13a1), mRNA [NM_001100862]	**9,562**
Gatad1	Uncharacterized protein [Source:UniProtKB/TrEMBL;Acc:D3ZHD5] [ENSRNOT00000011666]	**9,561**
LOC310177	Uncharacterized protein [Source:UniProtKB/TrEMBL;Acc:D3ZS22] [ENSRNOT00000030328]	**9,561**
Cisd3	Rattus norvegicus CDGSH iron sulfur domain 3 (Cisd3), mRNA [NM_001105835]	**9,560**
Mpp6	Rattus norvegicus membrane protein, palmitoylated 6 (MAGUK p55 subfamily member 6) (Mpp6), mRNA [NM_001134982]	**9,560**
0	Unknown	**9,559**
0	Unknown	**9,557**
Dnal4	Rattus norvegicus dynein, axonemal, light chain 4 (Dnal4), mRNA [NM_001009666]	**9,557**
0	Unknown	**9,556**
Nr2c2	Rattus norvegicus nuclear receptor subfamily 2, group C, member 2 (Nr2c2), mRNA [NM_017323]	**9,552**
Zfp61	Rattus norvegicus zinc finger protein 61 (Zfp61), mRNA [NM_001017512]	**9,551**
Rnf25	Rattus norvegicus ring finger protein 25 (Rnf25), mRNA [NM_001012004]	**9,550**
Cib1	Rattus norvegicus calcium and integrin binding 1 (calmyrin) (Cib1), mRNA [NM_031145]	**9,550**
Cenpb	Uncharacterized protein [Source:UniProtKB/TrEMBL;Acc:D4A5E3] [ENSRNOT00000002929]	**9,549**

Ppp1r3d	Rattus norvegicus protein phosphatase 1, regulatory subunit 3D (Ppp1r3d), mRNA [NM_001109564]	**9,549**
Chac1	Rattus norvegicus ChaC, cation transport regulator homolog 1 (E. coli) (Chac1), mRNA [NM_001173437]	**9,548**
RGD1564845	Rattus norvegicus similar to RIKEN cDNA 3830403N18 (LOC363507), mRNA [XM_343831]	**9,548**
0	FQ131320 Rattus norvegicus 11-12 days foetus Sprague-Dawley Rattus norvegicus cDNA clone TL0ADA22YJ10 5', mRNA sequence [FQ131320]	**9,547**
Kctd4	Rattus norvegicus potassium channel tetramerisation domain containing 4 (Kctd4), mRNA [NM_001109650]	**9,547**
Adrb2	Rattus norvegicus adrenergic, beta-2-, receptor, surface (Adrb2), mRNA [NM_012492]	**9,546**
Pmaip1	Rattus norvegicus phorbol-12-myristate-13-acetate-induced protein 1 (Pmaip1), mRNA [NM_001008385]	**9,545**
Atmin	Rattus norvegicus ATM interactor (Atmin), mRNA [NM_001191786]	**9,545**
Plekha1	Rattus norvegicus pleckstrin homology domain containing, family A (phosphoinositide binding specific) member 1 (Plekha1), mRNA [NM_001079894]	**9,543**
Tfrc	Rattus norvegicus transferrin receptor (Tfrc), mRNA [NM_022712]	**9,543**
Tlk2	Rattus norvegicus tousled-like kinase 2 (Tlk2), mRNA [NM_001191652]	**9,542**
Ubr4	Rattus norvegicus ubiquitin protein ligase E3 component n-recognin 4 (Ubr4), mRNA [NM_001039026]	**9,542**
Whsc2	Rattus norvegicus Wolf-Hirschhorn syndrome candidate 2 (human) (Whsc2), mRNA [NM_001008339]	**9,541**
Cdyl2	Rattus norvegicus chromodomain protein, Y chromosome-like 2 (Cdyl2), mRNA [NM_001106189]	**9,541**
RGD1311066	Rattus norvegicus similar to RIKEN cDNA 0610011L14 gene (RGD1311066), mRNA [NM_001106535]	**9,541**
Lrtm2	Rattus norvegicus leucine-rich repeats and transmembrane domains 2 (Lrtm2), mRNA [NM_001109430]	**9,538**
LOC500877	Rattus norvegicus Ab1-152 (LOC500877), mRNA [NM_001047963]	**9,538**
Polr3g	Rattus norvegicus polymerase (RNA) III (DNA directed) polypeptide G (Polr3g), mRNA [NM_001109468]	**9,538**
Fgfr1	Rattus norvegicus Fibroblast growth factor receptor 1 (Fgfr1), mRNA [NM_024146]	**9,535**
Rpp30	Rattus norvegicus ribonuclease P/MRP 30 subunit (human) (Rpp30), mRNA [NM_001191083]	**9,534**
0	Unknown	**9,534**
Fat3	Rattus norvegicus FAT tumor suppressor homolog 3 (Drosophila) (Fat3), mRNA [NM_138544]	**9,533**
Ltbr	Rattus norvegicus lymphotoxin beta receptor (TNFR superfamily, member 3) (Ltbr), mRNA [NM_001008315]	**9,532**
Ly6g5c	Rattus norvegicus lymphocyte antigen 6 complex, locus G5C (Ly6g5c), mRNA [NM_198739]	**9,531**
0	Uncharacterized protein [Source:UniProtKB/TrEMBL;Acc:D3ZU94] [ENSRNOT00000059801]	**9,531**
Gpr85	Rattus norvegicus G protein-coupled receptor 85 (Gpr85), mRNA [NM_022254]	**9,530**
Mrpl50	Rattus norvegicus mitochondrial ribosomal protein L50 (Mrpl50), nuclear gene encoding mitochondrial protein, mRNA [NM_001108665]	**9,529**
0	Rattus norvegicus similar to glyceraldehyde-3-phosphate dehydrogenase (LOC297785), mRNA [XM_216321]	**9,527**
Brwd3	PREDICTED: Rattus norvegicus bromodomain and WD repeat domain containing 3 (Brwd3), mRNA [XM_001054667]	**9,525**
Cox11	Rattus norvegicus COX11 homolog, cytochrome c oxidase assembly protein (yeast) (Cox11), nuclear gene encoding mitochondrial protein, mRNA [NM_001109575]	**9,525**
0	Unknown	**9,524**
Thap4	Rattus norvegicus THAP domain containing 4 (Thap4), mRNA [NM_001005564]	**9,523**

Fam167a	Rattus norvegicus family with sequence similarity 167, member A (Fam167a), mRNA [NM_001109102]	**9,522**
Stx8	Rattus norvegicus syntaxin 8 (Stx8), mRNA [NM_031656]	**9,521**
Clns1a	Rattus norvegicus chloride channel, nucleotide-sensitive, 1A (Clns1a), mRNA [NM_031719]	**9,521**
0	Unknown	**9,520**
Epb41l1	Rattus norvegicus erythrocyte membrane protein band 4.1-like 1 (Epb41l1), transcript variant 2, mRNA [NM_021681]	**9,520**
Ccdc130	Rattus norvegicus coiled-coil domain containing 130 (Ccdc130), mRNA [NM_001037644]	**9,520**
Cdc42ep5	Rattus norvegicus CDC42 effector protein (Rho GTPase binding) 5 (Cdc42ep5), mRNA [NM_001108469]	**9,518**
Tmem130	Rattus norvegicus transmembrane protein 130 (Tmem130), mRNA [NM_001170399]	**9,518**
0	Unknown	**9,517**
Htr1a	Rattus norvegicus 5-hydroxytryptamine (serotonin) receptor 1A (Htr1a), mRNA [NM_012585]	**9,516**
Fam160b1	LOC361774 (Predicted)Uncharacterized protein [Source:UniProtKB/TrEMBL;Acc:D4A3I5] [ENSRNOT00000023247]	**9,516**
Chst10	Rattus norvegicus carbohydrate sulfotransferase 10 (Chst10), mRNA [NM_080397]	**9,514**
RGD1563351	PREDICTED: Rattus norvegicus RGD1563351 (RGD1563351), mRNA [XM_238200]	**9,514**
RGD1565598	PREDICTED: Rattus norvegicus similar to serine/threonine kinase (RGD1565598), mRNA [XM_001055923]	**9,512**
Olr206	Rattus norvegicus olfactory receptor 206 (Olr206), mRNA [NM_001000193]	**9,511**
0	Unknown	**9,509**
0	Unknown	**9,509**
0	Uncharacterized protein [Source:UniProtKB/TrEMBL;Acc:D3ZIS8] [ENSRNOT00000060124]	**9,509**
Cd163l1	Rattus norvegicus CD163 molecule-like 1 (Cd163l1), mRNA [NM_001106312]	**9,509**
Cldn10	Rattus norvegicus claudin 10 (Cldn10), mRNA [NM_001106058]	**9,509**
Slc22a15	Rattus norvegicus solute carrier family 22, member 15 (Slc22a15), mRNA [NM_001107707]	**9,507**
Slc4a10	Rattus norvegicus solute carrier family 4, sodium bicarbonate transporter, member 10 (Slc4a10), mRNA [NM_178092]	**9,506**
Fubp1	Rattus norvegicus far upstream element (FUSE) binding protein 1 (Fubp1), mRNA [NM_001037653]	**9,506**
0	Unknown	**9,506**
0	PREDICTED: Rattus norvegicus multiple EGF-like-domains 11 (Megf11), mRNA [XM_001078620]	**9,504**
Pcdha13	Rattus norvegicus protocadherin alpha 13 (Pcdha13), mRNA [NM_053934]	**9,503**
Kdr	Rattus norvegicus kinase insert domain receptor (Kdr), mRNA [NM_013062]	**9,503**
0	Unknown	**9,502**
Wfdc10	Rattus norvegicus WAP four-disulfide core domain 10 (Wfdc10), mRNA [NM_001109461]	**9,501**
RGD1559442	Rattus norvegicus similar to SET binding factor 2 (LOC691036), mRNA [NM_001134970]	**9,500**
Txnrd1	Rattus norvegicus thioredoxin reductase 1 (Txnrd1), mRNA [NM_031614]	**9,497**
Camkv	Rattus norvegicus CaM kinase-like vesicle-associated (Camkv), mRNA [NM_024000]	**9,496**
0	Unknown	**9,496**
Rpp14	Rattus norvegicus ribonuclease P 14 subunit (human) (Rpp14), transcript variant 1, mRNA [NM_001108372]	**9,495**

0	Uncharacterized protein [Source:UniProtKB/TrEMBL;Acc:D4A2F4] [ENSRNOT00000014567]	**9,494**
Hipk4	Rattus norvegicus homeodomain interacting protein kinase 4 (Hipk4), mRNA [NM_001024776]	**9,493**
Pom121l2	Rattus norvegicus POM121 membrane glycoprotein-like 2 (Pom121l2), transcript variant 1, mRNA [NM_001162930]	**9,492**
Dph1	Rattus norvegicus DPH1 homolog (S. cerevisiae) (Dph1), mRNA [NM_001105809]	**9,491**
Prss36	Rattus norvegicus protease, serine, 36 (Prss36), mRNA [NM_001011560]	**9,491**
Evc	Rattus norvegicus Ellis van Creveld syndrome homolog (human) (Evc), mRNA [NM_001170439]	**9,490**
Tes	Rattus norvegicus testis derived transcript (Tes), mRNA [NM_001039344]	**9,490**
Polr3a	PREDICTED: Rattus norvegicus polymerase (RNA) III (DNA directed) polypeptide A (Polr3a), partial mRNA [XM_341388]	**9,489**
0	Rattus norvegicus similar to glyceraldehyde-3-phosphate-dehydrogenase (EC 1.2.1.12) (LOC314605), mRNA [XM_234875]	**9,488**
Atf1	Rattus norvegicus activating transcription factor 1 (Atf1), mRNA [NM_001100895]	**9,488**
0	Unknown	**9,486**
Olr752	Rattus norvegicus olfactory receptor 752 (Olr752), mRNA [NM_001000367]	**9,484**
Znf704	PREDICTED: Rattus norvegicus zinc finger protein 704 (Znf704), mRNA [XM_226953]	**9,482**
0	Unknown	**9,480**
Gfpt1	Rattus norvegicus glutamine fructose-6-phosphate transaminase 1 (Gfpt1), mRNA [NM_001005879]	**9,480**
Lrat	Rattus norvegicus lecithin-retinol acyltransferase (phosphatidylcholine-retinol-O-acyltransferase) (Lrat), mRNA [NM_022280]	**9,479**
Bach1	Rattus norvegicus BTB and CNC homology 1, basic leucine zipper transcription factor 1 (Bach1), mRNA [NM_001107113]	**9,478**
Ly6h	Rattus norvegicus lymphocyte antigen 6 complex, locus H (Ly6h), mRNA [NM_001134839]	**9,478**
Kif26a	Rattus norvegicus kinesin family member 26A (Kif26a), mRNA [NM_001170348]	**9,477**
Klhl29	Rattus norvegicus kelch-like 29 (Drosophila) (Klhl29), mRNA [NM_001106713]	**9,476**
RGD1303144	Rattus norvegicus HypB, AroK, ADK and rho factor domain containing protein RGD1303144 (RGD1303144), mRNA [NM_001004266]	**9,474**
Vps13b	Rattus norvegicus vacuolar protein sorting 13 homolog B (yeast) (Vps13b), mRNA [NM_001134886]	**9,472**
MGC116197	Rattus norvegicus similar to RIKEN cDNA 1700001E04 (MGC116197), mRNA [NM_001025755]	**9,472**
0	Uncharacterized protein [Source:UniProtKB/TrEMBL;Acc:D4AE17] [ENSRNOT00000002068]	**9,470**
0	Rattus norvegicus similar to glyceraldehyde-3-phosphate dehydrogenase (phosphorylating) (EC 1.2.1.12) - mouse (LOC293463), mRNA [XM_219301]	**9,467**
Maf1	Rattus norvegicus MAF1 homolog (S. cerevisiae) (Maf1), mRNA [NM_001014085]	**9,464**
LOC689926	Rattus norvegicus hypothetical protein LOC689926 (LOC689926), mRNA [NM_001163002]	**9,463**
Pdp1	Rattus norvegicus pyruvate dehyrogenase phosphatase catalytic subunit 1 (Pdp1), nuclear gene encoding mitochondrial protein, mRNA [NM_019372]	**9,461**
RGD1311345	Rattus norvegicus similar to CG9752-PA (RGD1311345), mRNA [NM_001173436]	**9,461**
Rtn4r	Rattus norvegicus reticulon 4 receptor (Rtn4r), mRNA [NM_053613]	**9,459**
Bphl	Rattus norvegicus biphenyl hydrolase-like (serine hydrolase) (Bphl), mRNA [NM_001037206]	**9,459**
Foxp3	Rattus norvegicus forkhead box P3 (Foxp3), mRNA [NM_001108250]	**9,456**

0	Q52289_PSEPU (Q52289) UxpA protein, partial (5%) [TC624946]	**9,455**
Tal1	Rattus norvegicus T-cell acute lymphocytic leukemia 1 (Tal1), mRNA [NM_001107958]	**9,455**
Mln	Rattus norvegicus motilin (Mln), mRNA [NM_001110056]	**9,455**
Dph2	Rattus norvegicus DPH2 homolog (S. cerevisiae) (Dph2), mRNA [NM_001015007]	**9,454**
Pmf1	Rattus norvegicus polyamine-modulated factor 1 (Pmf1), mRNA [NM_001191568]	**9,453**
Apip	Rattus norvegicus APAF1 interacting protein (Apip), mRNA [NM_001106492]	**9,452**
Cenpk	Rattus norvegicus centromere protein K (Cenpk), mRNA [NM_001106407]	**9,449**
RGD1560028	Rattus norvegicus similar to RIKEN cDNA C130060K24 gene (RGD1560028), mRNA [NM_001109239]	**9,449**
Zbtb34	PREDICTED: Rattus norvegicus zinc finger and BTB domain containing 34 (Zbtb34), mRNA [XM_001069858]	**9,448**
Wdr20a	Rattus norvegicus WD repeat domain 20a (Wdr20a), mRNA [NM_001100894]	**9,447**
Slc4a11	Rattus norvegicus solute carrier family 4, sodium borate transporter, member 11 (Slc4a11), mRNA [NM_001107775]	**9,445**
0	RVL797 Wackym-Soares normalized rat vestibular cDNA library Rattus norvegicus cDNA 5', mRNA sequence [CX569749]	**9,445**
RGD1563748	PREDICTED: Rattus norvegicus similar to ankyrin-like protein (RGD1563748), mRNA [XM_214327]	**9,445**
Trim35	Rattus norvegicus tripartite motif-containing 35 (Trim35), mRNA [NM_001025142]	**9,442**
0	Unknown	**9,441**
Gtf2h5	Rattus norvegicus general transcription factor IIH, polypeptide 5 (Gtf2h5), mRNA [NM_001126088]	**9,440**
0	Uncharacterized protein [Source:UniProtKB/TrEMBL;Acc:D4ACT2] [ENSRNOT00000008002]	**9,438**
RGD1564803	PREDICTED: Rattus norvegicus RGD1564803 (RGD1564803), mRNA [XM_579791]	**9,437**
Aldoc	Rattus norvegicus aldolase C, fructose-bisphosphate (Aldoc), mRNA [NM_012497]	**9,436**
RGD1311357	PREDICTED: Rattus norvegicus similar to A530083I02Rik protein (RGD1311357), mRNA [XM_001070093]	**9,434**
Pcbp3	Rattus norvegicus poly(rC) binding protein 3 (Pcbp3), mRNA [NM_001011945]	**9,434**
Eif4g3	Rattus norvegicus eukaryotic translation initiation factor 4 gamma, 3 (Eif4g3), mRNA [NM_001106693]	**9,433**
Antxr1	Rattus norvegicus anthrax toxin receptor 1 (Antxr1), mRNA [NM_001044249]	**9,433**
Pan3	PREDICTED: Rattus norvegicus PAN3 polyA specific ribonuclease subunit homolog (S. cerevisiae), transcript variant 2 (Pan3), mRNA [XM_346914]	**9,432**
Mitd1	Rattus norvegicus MIT, microtubule interacting and transport, domain containing 1 (Mitd1), mRNA [NM_001009714]	**9,431**
Serhl2	Rattus norvegicus serine hydrolase-like 2 (Serhl2), mRNA [NM_001130579]	**9,431**
Znf553	PREDICTED: Rattus norvegicus zinc finger protein 553 (Znf553), mRNA [XM_219345]	**9,430**
Dcaf11	Rattus norvegicus DDB1 and CUL4 associated factor 11 (Dcaf11), mRNA [NM_001009686]	**9,430**
Trmt6	Rattus norvegicus tRNA methyltransferase 6 homolog (S. cerevisiae) (Trmt6), mRNA [NM_001107779]	**9,430**
Rabggta	Rattus norvegicus Rab geranylgeranyltransferase, alpha subunit (Rabggta), mRNA [NM_031654]	**9,430**
0	Unknown	**9,429**
RGD1308470	Rattus norvegicus similar to RIKEN cDNA 4933433P14 gene (RGD1308470), mRNA [NM_001014198]	**9,428**
Usp47	Rattus norvegicus ubiquitin specific peptidase 47 (Usp47), mRNA [NM_001107542]	**9,427**

0	Rattus norvegicus TL0ACA43YD07 mRNA sequence. [FQ216390]	**9,426**
Rufy4	PREDICTED: Rattus norvegicus RUN and FYVE domain containing 4 (Rufy4), mRNA [XM_001056259]	**9,425**
0	Unknown	**9,424**
Mdp1	Rattus norvegicus magnesium-dependent phosphatase 1 (Mdp1), mRNA [NM_001106039]	**9,422**
Cyp2u1	Rattus norvegicus cytochrome P450, family 2, subfamily u, polypeptide 1 (Cyp2u1), mRNA [NM_001024779]	**9,420**
Oas1b	Rattus norvegicus 2-5 oligoadenylate synthetase 1B (Oas1b), mRNA [NM_144752]	**9,418**
0	Uncharacterized protein [Source:UniProtKB/TrEMBL;Acc:D3ZAP7] [ENSRNOT00000008901]	**9,417**
Kdelc2	Rattus norvegicus KDEL (Lys-Asp-Glu-Leu) containing 2 (Kdelc2), mRNA [NM_001025123]	**9,417**
Slc4a1	Rattus norvegicus solute carrier family 4 (anion exchanger), member 1 (Slc4a1), mRNA [NM_012651]	**9,416**
0	Unknown	**9,412**
Twist1	Rattus norvegicus twist homolog 1 (Drosophila) (Twist1), mRNA [NM_053530]	**9,412**
Ss18l1	Rattus norvegicus synovial sarcoma translocation gene on chromosome 18-like 1 (Ss18l1), mRNA [NM_138918]	**9,412**
Col23a1	Rattus norvegicus collagen, type XXIII, alpha 1 (Col23a1), mRNA [NM_181636]	**9,410**
LOC500974	PREDICTED: Rattus norvegicus similar to CDNA sequence BC024479 (LOC500974), miscRNA [XR_005433]	**9,409**
Dusp6	Rattus norvegicus dual specificity phosphatase 6 (Dusp6), mRNA [NM_053883]	**9,408**
0	PREDICTED: Rattus norvegicus similar to Protein KIAA0586 (LOC690035), miscRNA [XR_086273]	**9,404**
0	PREDICTED: Rattus norvegicus anillin, actin binding protein (Anln), mRNA [XM_001067026]	**9,404**
0	Unknown	**9,403**
0	transcription factor CP2 [Source:RefSeq peptide;Acc:NP_001128186] [ENSRNOT00000055374]	**9,403**
Pign	Rattus norvegicus phosphatidylinositol glycan, class N (Pign), mRNA [NM_001100584]	**9,399**
Pdcd7	Rattus norvegicus programmed cell death 7 (Pdcd7), mRNA [NM_001108768]	**9,399**
0	Unknown	**9,398**
Anp32e	Rattus norvegicus acidic (leucine-rich) nuclear phosphoprotein 32 family, member E (Anp32e), mRNA [NM_001013200]	**9,397**
0	Unknown	**9,397**
Cbx1	RCG35120, isoform CRA_aUncharacterized protein [Source:UniProtKB/TrEMBL;Acc:D4A3T3] [ENSRNOT00000011665]	**9,397**
Steap4	Rattus norvegicus STEAP family member 4 (Steap4), mRNA [NM_001044265]	**9,396**
LOC501222	Rattus norvegicus TL0AAA47YE23 mRNA sequence. [FQ213532]	**9,393**
0	Q412C4_KINRA (Q412C4) Amine oxidase, partial (3%) [TC617018]	**9,390**
Eif2c4	Rattus norvegicus eukaryotic translation initiation factor 2C, 4 (Eif2c4), mRNA [NM_001106686]	**9,388**
0	Unknown	**9,388**
Rshl1	Rattus norvegicus radial spokehead-like 1 (Rshl1), mRNA [NM_001024748]	**9,387**
0	Q6DC77_BRARE (Q6DC77) Zgc:101065, partial (7%) [TC593872]	**9,387**
0	Unknown	**9,387**
0	Unknown	**9,385**

0	Q2JMC8_SYNJB (Q2JMC8) TonB family protein, partial (7%) [TC596892]	**9,385**
0	Rattus norvegicus cDNA clone IMAGE:7300863. [BC084702]	**9,384**
RGD1563375	PREDICTED: Rattus norvegicus similar to small nuclear ribonucleoparticle-associated protein (RGD1563375), mRNA [XM_345546]	**9,384**
0	Unknown	**9,384**
Geft	Rattus norvegicus RhoA/RAC/CDC42 exchange factor (Geft), mRNA [NM_199395]	**9,383**
0	Q9VXG3_DROME (Q9VXG3) CG9968-PB, isoform B, partial (4%) [TC635273]	**9,382**
LOC680643	PREDICTED: Rattus norvegicus similar to MIC2 like 1 (LOC680643), mRNA [XM_001058120]	**9,382**
RGD1564541	Rattus norvegicus similar to hypothetical protein FLJ22965 (RGD1564541), mRNA [NM_001107950]	**9,381**
Cybasc3	Rattus norvegicus cytochrome b, ascorbate dependent 3 (Cybasc3), mRNA [NM_001014164]	**9,380**
RGD1561878	PREDICTED: Rattus norvegicus similar to mKIAA0978 protein (RGD1561878), mRNA [XM_002726270]	**9,380**
Doc2b	Rattus norvegicus double C2-like domains, beta (Doc2b), mRNA [NM_031142]	**9,380**
Stmn2	Rattus norvegicus stathmin-like 2 (Stmn2), mRNA [NM_053440]	**9,379**
Ubtd1	Rattus norvegicus ubiquitin domain containing 1 (Ubtd1), mRNA [NM_001013153]	**9,379**
Dppa3	Rattus norvegicus developmental pluripotency-associated 3 (Dppa3), mRNA [NM_001047864]	**9,378**
Anp32b	Rattus norvegicus acidic (leucine-rich) nuclear phosphoprotein 32 family, member B (Anp32b), mRNA [NM_131911]	**9,378**
Ftsjd2	Rattus norvegicus FtsJ methyltransferase domain containing 2 (Ftsjd2), mRNA [NM_001014031]	**9,377**
Cyp2b15	Rattus norvegicus cytochrome P450, family 2, subfamily b, polypeptide 15 (Cyp2b15), mRNA [NM_001135668]	**9,372**
0	Unknown	**9,371**
Parp16	Rattus norvegicus poly (ADP-ribose) polymerase family, member 16 (Parp16), mRNA [NM_001014093]	**9,368**
Gpr162	Rattus norvegicus G protein-coupled receptor 162 (Gpr162), mRNA [NM_001108646]	**9,368**
Alkbh8	Rattus norvegicus alkB, alkylation repair homolog 8 (E. coli) (Alkbh8), mRNA [NM_001191909]	**9,367**
Chst14	Rattus norvegicus carbohydrate (N-acetylgalactosamine 4-0) sulfotransferase 14 (Chst14), mRNA [NM_001109639]	**9,365**
0	Unknown	**9,363**
Slc17a6	Rattus norvegicus solute carrier family 17 (sodium-dependent inorganic phosphate cotransporter), member 6 (Slc17a6), mRNA [NM_053427]	**9,363**
0	Unknown	**9,362**
Gja10	Rattus norvegicus gap junction protein, alpha 10 (Gja10), mRNA [NM_001173508]	**9,360**
0	Rattus norvegicus TL0ADA32YM01 mRNA sequence. [FQ221714]	**9,360**
0	PREDICTED: Rattus norvegicus similar to taube nuss (RGD1562299), mRNA [XM_234348]	**9,358**
Cyhr1	Rattus norvegicus cysteine and histidine rich 1 (Cyhr1), mRNA [NM_001025122]	**9,358**
0	Rattus norvegicus similar to T cell receptor V-alpha J-alpha (LOC290165), mRNA [XM_224125]	**9,358**
Mthfr	Methylenetetrahydrofolate reductase [Source:UniProtKB/TrEMBL;Acc:D4A7E8] [ENSRNOT00000011384]	**9,358**
LOC681224	PREDICTED: Rattus norvegicus similar to cyclin D binding myb-like transcription factor 1 (LOC681224), partial mRNA [XM_002725335]	**9,356**
0	Uncharacterized protein [Source:UniProtKB/TrEMBL;Acc:D3ZX94] [ENSRNOT00000013367]	**9,355**
Ascl2	Rattus norvegicus achaete-scute complex homolog 2 (Drosophila) (Ascl2), mRNA [NM_031503]	**9,355**

0	Unknown	**9,355**
Relt	Rattus norvegicus RELT tumor necrosis factor receptor (Relt), mRNA [NM_001108495]	**9,354**
LOC685796	PREDICTED: Rattus norvegicus similar to similar to RIKEN cDNA 1700001E04 (LOC685796), mRNA [XM_001065304]	**9,353**
Fam193b	Rattus norvegicus family with sequence similarity 193, member B (Fam193b), mRNA [NM_001170408]	**9,353**
0	Glyceraldehyde-3-phosphate dehydrogenase [Source:UniProtKB/TrEMBL;Acc:D4A6J7] [ENSRNOT00000002985]	**9,351**
Arfgef1	ADP-ribosylation factor guanine nucleotide-exchange factor 1(Brefeldin A-inhibited) (Predicted), isoform CRA_aUncharacterized protein [Source:UniProtKB/TrEMBL;Acc:D4A631] [ENSRNOT00000007766]	**9,350**
Slc25a36	PREDICTED: Rattus norvegicus solute carrier family 25, member 36 (Slc25a36), mRNA [XM_001065705]	**9,349**
Wars	Rattus norvegicus tryptophanyl-tRNA synthetase (Wars), mRNA [NM_001013170]	**9,349**
0	Uncharacterized protein [Source:UniProtKB/TrEMBL;Acc:D3Z884] [ENSRNOT00000049147]	**9,346**
Rufy2	Rattus norvegicus RUN and FYVE domain containing 2 (Rufy2), mRNA [NM_001168586]	**9,346**
RGD1305704	Rattus norvegicus cDNA clone IMAGE:5598328, **** WARNING: chimeric clone ****. [BC168677]	**9,346**
Dcun1d4	Rattus norvegicus DCN1, defective in cullin neddylation 1, domain containing 4 (S. cerevisiae) (Dcun1d4), mRNA [NM_001108359]	**9,345**
Smcr8	Rattus norvegicus Smith-Magenis syndrome chromosome region, candidate 8 homolog (human) (Smcr8), mRNA [NM_001191919]	**9,345**
0	Unknown	**9,345**
Nrg4	Rattus norvegicus neuregulin 4 (Nrg4), mRNA [NM_001191109]	**9,343**
Itgb4	Rattus norvegicus integrin, beta 4 (Itgb4), mRNA [NM_013180]	**9,342**
Trim47	Rattus norvegicus tripartite motif-containing 47 (Trim47), mRNA [NM_001109585]	**9,341**
Chat	Rattus norvegicus choline acetyltransferase (Chat), mRNA [NM_001170593]	**9,341**
Amigo2	Rattus norvegicus adhesion molecule with Ig like domain 2 (Amigo2), mRNA [NM_182816]	**9,341**
Rassf9	Rattus norvegicus Ras association (RalGDS/AF-6) domain family (N-terminal) member 9 (Rassf9), mRNA [NM_022959]	**9,340**
Tmcc2	PREDICTED: Rattus norvegicus transmembrane and coiled-coil domain family 2 (Tmcc2), mRNA [XM_223107]	**9,339**
Olr714	Rattus norvegicus olfactory receptor 714 (Olr714), mRNA [NM_001000923]	**9,339**
Tada3l	Rattus norvegicus transcriptional adaptor 3 (NGG1 homolog, yeast)-like (Tada3l), mRNA [NM_001025734]	**9,339**
Phospho1	Rattus norvegicus phosphatase, orphan 1 (Phospho1), mRNA [NM_001105833]	**9,338**
0	Unknown	**9,338**
0	Unknown	**9,338**
Serpinb8	Rattus norvegicus serpin peptidase inhibitor, clade B (ovalbumin), member 8 (Serpinb8), mRNA [NM_001105948]	**9,337**
Nelf	Rattus norvegicus nasal embryonic LHRH factor (Nelf), mRNA [NM_057190]	**9,336**
Lce1f	Rattus norvegicus late cornified envelope 1F (Lce1f), mRNA [NM_001109188]	**9,335**
0	CB545809 AMGNNUC:MRPE3-00116-C9-A placenta embryo D17 (10379) Rattus norvegicus cDNA clone mrpe3-00116-c9 5', mRNA sequence [CB545809]	**9,334**
Slc35e1	Rattus norvegicus solute carrier family 35, member E1 (Slc35e1), mRNA [NM_001109107]	**9,334**
0	Unknown	**9,334**

Tnfsf15	Rattus norvegicus tumor necrosis factor (ligand) superfamily, member 15 (Tnfsf15), mRNA [NM_145765]	**9,333**
0	Unknown	**9,333**
Dgcr2	Rattus norvegicus TL0AAA82YH19 mRNA sequence. [FQ211720]	**9,332**
Cast	Rattus norvegicus calpastatin (Cast), transcript variant 1, mRNA [NM_053295]	**9,328**
Fndc1	Rattus norvegicus fibronectin type III domain containing 1 (Fndc1), mRNA [NM_001038615]	**9,328**
Erbb2	Rattus norvegicus v-erb-b2 erythroblastic leukemia viral oncogene homolog 2, neuro/glioblastoma derived oncogene homolog (avian) (Erbb2), mRNA [NM_017003]	**9,324**
Mtmr3	Rattus norvegicus myotubularin related protein 3 (Mtmr3), mRNA [NM_001012038]	**9,324**
Wrap53	Rattus norvegicus WD repeat containing, antisense to TP53 (Wrap53), mRNA [NM_001007610]	**9,324**
Acoxl	Rattus norvegicus acyl-Coenzyme A oxidase-like (Acoxl), mRNA [NM_001106508]	**9,322**
Uroc1	Uncharacterized protein [Source:UniProtKB/TrEMBL;Acc:D3ZDW1] [ENSRNOT00000038251]	**9,321**
Mobkl3	Rattus norvegicus MOB1, Mps One Binder kinase activator-like 3 (yeast) (Mobkl3), mRNA [NM_133528]	**9,319**
Dlx1	Rattus norvegicus distal-less homeobox 1 (Dlx1), mRNA [NM_001100531]	**9,319**
Actc1	Rattus norvegicus actin, alpha, cardiac muscle 1 (Actc1), mRNA [NM_019183]	**9,318**
Inpp5f	Rattus norvegicus inositol polyphosphate-5-phosphatase F (Inpp5f), mRNA [NM_001107554]	**9,318**
Sorcs3	Rattus norvegicus sortilin-related VPS10 domain containing receptor 3 (Sorcs3), mRNA [NM_001106367]	**9,317**
Als2	Rattus norvegicus amyotrophic lateral sclerosis 2 (juvenile) homolog (human) (Als2), mRNA [NM_001013413]	**9,316**
0	Unknown	**9,316**
Cds2	Rattus norvegicus CDP-diacylglycerol synthase (phosphatidate cytidylyltransferase) 2 (Cds2), mRNA [NM_053643]	**9,315**
St8sia5	Rattus norvegicus ST8 alpha-N-acetyl-neuraminide alpha-2,8-sialyltransferase 5 (St8sia5), mRNA [NM_213628]	**9,314**
Nop10	Rattus norvegicus NOP10 ribonucleoprotein homolog (yeast) (Nop10), mRNA [NM_001126100]	**9,312**
Mrpl42	Rattus norvegicus mitochondrial ribosomal protein L42 (Mrpl42), nuclear gene encoding mitochondrial protein, mRNA [NM_001106782]	**9,311**
Cirh1a	Rattus norvegicus cirrhosis, autosomal recessive 1A (cirhin) (Cirh1a), mRNA [NM_001009640]	**9,311**
0	Uncharacterized protein [Source:UniProtKB/TrEMBL;Acc:D3ZIW7] [ENSRNOT00000068671]	**9,311**
0	RIKEN cDNA 5330417C22 gene Gene [Source:MGI Symbol;Acc:MGI:1923930] [ENSRNOT00000027493]	**9,311**
LOC689316	PREDICTED: Rattus norvegicus hypothetical protein LOC689316 (LOC689316), miscRNA [XR_085654]	**9,311**
Pcdhb22	Protocadherin-T5 [Source:UniProtKB/TrEMBL;Acc:Q9JIU0] [ENSRNOT00000027133]	**9,309**
Tada2b	Rattus norvegicus transcriptional adaptor 2 (ADA2 homolog, yeast)-beta (Tada2b), mRNA [NM_001170455]	**9,307**
Eed	Rattus norvegicus embryonic ectoderm development (Eed), mRNA [NM_001106278]	**9,306**
0	Unknown	**9,304**
Mpp5	Rattus norvegicus membrane protein, palmitoylated 5 (MAGUK p55 subfamily member 5) (Mpp5), mRNA [NM_001108034]	**9,304**
Flvcr2	Rattus norvegicus feline leukemia virus subgroup C cellular receptor family, member 2 (Flvcr2), mRNA [NM_199109]	**9,302**
RGD1307983	PREDICTED: Rattus norvegicus similar to HSPC043 protein (RGD1307983), mRNA [XM_001061972]	**9,299**
Plekhm2	Rattus norvegicus pleckstrin homology domain containing, family M (with RUN domain) member 2 (Plekhm2), mRNA [NM_001191767]	**9,297**

Fut11	Rattus norvegicus fucosyltransferase 11 (alpha (1,3) fucosyltransferase) (Fut11), mRNA [NM_173308]	**9,297**
0	Rattus norvegicus similar to Peptidyl-prolyl cis-trans isomerase A (PPIase) (Rotamase) (Cyclophilin A) (Cyclosporin A-binding protein) (SP18) (LOC314776), mRNA [XM_235075]	**9,296**
LOC690319	PREDICTED: Rattus norvegicus similar to Alpha-1-antitrypsin-related protein precursor (LOC690319), mRNA [XM_001074075]	**9,296**
Ggn	Rattus norvegicus gametogenetin (Ggn), mRNA [NM_001013065]	**9,296**
Polr1a	Rattus norvegicus polymerase (RNA) I polypeptide A (Polr1a), mRNA [NM_031772]	**9,294**
Snx13	Rattus norvegicus sorting nexin 13 (Snx13), mRNA [NM_001108708]	**9,293**
Slc16a5	Rattus norvegicus solute carrier family 16, member 5 (monocarboxylic acid transporter 6) (Slc16a5), mRNA [NM_001109568]	**9,292**
Hcn2	Rattus norvegicus hyperpolarization activated cyclic nucleotide-gated potassium channel 2 (Hcn2), mRNA [NM_053684]	**9,290**
Rasl11b	Rattus norvegicus RAS-like family 11 member B (Rasl11b), mRNA [NM_001002830]	**9,289**
0	FQ076898 Rattus norvegicus brain Sprague-Dawley Rattus norvegicus cDNA 5', mRNA sequence [FQ076898]	**9,288**
Qpctl	Rattus norvegicus glutaminyl-peptide cyclotransferase-like (Qpctl), mRNA [NM_001106230]	**9,288**
0	Uncharacterized protein [Source:UniProtKB/TrEMBL;Acc:D3ZAC0] [ENSRNOT00000036112]	**9,288**
0	Ryanodine receptor 1 [Source:UniProtKB/TrEMBL;Acc:O35208] [ENSRNOT00000027893]	**9,288**
Cdk4	Rattus norvegicus cyclin-dependent kinase 4 (Cdk4), mRNA [NM_053593]	**9,286**
0	Unknown	**9,286**
0	Unknown	**9,285**
Pcdhga9	Rattus norvegicus protocadherin gamma subfamily A, 9 (Pcdhga9), mRNA [NM_001037158]	**9,285**
0	Rattus norvegicus TL0AAA57YH24 mRNA sequence. [FQ212370]	**9,284**
LOC501296	PREDICTED: Rattus norvegicus hypothetical gene supported by BC059164 (LOC501296), partial mRNA [XM_001081221]	**9,284**
LOC679383	Rattus norvegicus similar to DNA segment, Chr 5, ERATO Doi 135, expressed (LOC679383), mRNA [NM_001128287]	**9,283**
Kcnt2	Rattus norvegicus potassium channel, subfamily T, member 2 (Kcnt2), mRNA [NM_198762]	**9,283**
Zbtb43	Rattus norvegicus zinc finger and BTB domain containing 43 (Zbtb43), mRNA [NM_001012094]	**9,283**
Cops8	Rattus norvegicus COP9 constitutive photomorphogenic homolog subunit 8 (Arabidopsis) (Cops8), mRNA [NM_001013227]	**9,283**
0	Rattus norvegicus similar to glyceraldehyde-3-phosphate dehydrogenase (phosphorylating) (EC 1.2.1.12) - mouse (LOC298960), mRNA [XM_234091]	**9,282**
Ammecr1l	Rattus norvegicus AMME chromosomal region gene 1-like (Ammecr1l), mRNA [NM_001107399]	**9,282**
Extl2	Rattus norvegicus exostoses (multiple)-like 2 (Extl2), mRNA [NM_001100704]	**9,281**
Unc45b	Rattus norvegicus unc-45 homolog B (C. elegans) (Unc45b), mRNA [NM_001107028]	**9,280**
Cdkn2a	Rattus norvegicus cyclin-dependent kinase inhibitor 2A (Cdkn2a), mRNA [NM_031550]	**9,279**
Nus1	Rattus norvegicus nuclear undecaprenyl pyrophosphate synthase 1 homolog (S. cerevisiae) (Nus1), mRNA [NM_001164157]	**9,278**
Olr1385	Rattus norvegicus olfactory receptor 1385 (Olr1385), mRNA [NM_214833]	**9,276**
Zbtb48	Rattus norvegicus zinc finger and BTB domain containing 48 (Zbtb48), mRNA [NM_001013216]	**9,276**
Klhl15	Rattus norvegicus kelch-like 15 (Drosophila) (Klhl15), mRNA [NM_001108021]	**9,275**

Egln3	Rattus norvegicus EGL nine homolog 3 (C. elegans) (Egln3), mRNA [NM_019371]	**9,274**
Ift52	Rattus norvegicus intraflagellar transport 52 homolog (Chlamydomonas) (Ift52), mRNA [NM_001177685]	**9,273**
LOC100363421	PREDICTED: Rattus norvegicus hypothetical protein LOC100363421 (LOC100363421), mRNA [XM_002726502]	**9,272**
Rnf10	Rattus norvegicus ring finger protein 10 (Rnf10), mRNA [NM_001011904]	**9,272**
Cldn1	Rattus norvegicus claudin 1 (Cldn1), mRNA [NM_031699]	**9,271**
Nxph4	Rattus norvegicus neurexophilin 4 (Nxph4), mRNA [NM_021680]	**9,270**
Lemd2	Rattus norvegicus LEM domain containing 2 (Lemd2), mRNA [NM_001039032]	**9,270**
Ap3s1	Rattus norvegicus adaptor-related protein complex 3, sigma 1 subunit (Ap3s1), mRNA [NM_001106933]	**9,269**
Tmem223	Rattus norvegicus transmembrane protein 223 (Tmem223), mRNA [NM_001191104]	**9,269**
RT1-Bb	Rattus norvegicus RT1 class II, locus Bb (RT1-Bb), mRNA [NM_001004084]	**9,268**
Mgat5b	Rattus norvegicus mannosyl (alpha-1,6-)-glycoprotein beta-1,6-N-acetyl-glucosaminyltransferase, isozyme B (Mgat5b), mRNA [NM_001107068]	**9,267**
Lman1l	Rattus norvegicus lectin, mannose-binding, 1 like (Lman1l), mRNA [NM_001012465]	**9,266**
Erh	Rattus norvegicus enhancer of rudimentary homolog (Drosophila) (Erh), mRNA [NM_001109442]	**9,265**
Depdc6	PREDICTED: Rattus norvegicus DEP domain containing 6 (Depdc6), mRNA [XM_001066889]	**9,264**
RGD1305939	Rattus norvegicus hypothetical LOC300074 (RGD1305939), mRNA [NM_001130581]	**9,263**
Cass4	Rattus norvegicus Cas scaffolding protein family member 4 (Cass4), mRNA [NM_001191744]	**9,263**
Emp1	Rattus norvegicus epithelial membrane protein 1 (Emp1), mRNA [NM_012843]	**9,263**
Dpysl3	Rattus norvegicus dihydropyrimidinase-like 3 (Dpysl3), mRNA [NM_012934]	**9,261**
0	Unknown	**9,261**
RGD1565512	Rattus norvegicus similar to hypothetical protein 4932418E24 (RGD1565512), mRNA [NM_001134617]	**9,261**
Klhdc3	Rattus norvegicus kelch domain containing 3 (Klhdc3), mRNA [NM_001012203]	**9,259**
0	Unknown	**9,258**
MGC112715	Rattus norvegicus hypothetical protein LOC690899 (MGC112715), mRNA [NM_001044300]	**9,258**
0	ATG2 autophagy related 2 homolog B (S. cerevisiae) Gene [Source:MGI Symbol;Acc:MGI:1923809] [ENSRNOT00000006131]	**9,257**
Gphb5	Rattus norvegicus glycoprotein hormone beta 5 (Gphb5), mRNA [NM_001007013]	**9,257**
Trrap	Rattus norvegicus transformation/transcription domain-associated protein (Trrap), mRNA [NM_001105907]	**9,256**
Itgb5	Rattus norvegicus integrin, beta 5 (Itgb5), mRNA [NM_147139]	**9,256**
LOC304239	Uncharacterized protein [Source:UniProtKB/TrEMBL;Acc:D3ZH07] [ENSRNOT00000001187]	**9,255**
0	Rattus norvegicus similar to spindlin (LOC294635), mRNA [XM_226656]	**9,253**
Vps33a	Rattus norvegicus vacuolar protein sorting 33 homolog A (S. cerevisiae) (Vps33a), mRNA [NM_022961]	**9,253**
Myof	Rattus norvegicus myoferlin (Myof), mRNA [NM_001191636]	**9,253**
LOC685385	Uncharacterized protein [Source:UniProtKB/TrEMBL;Acc:D3ZPS5] [ENSRNOT00000056523]	**9,252**
Srgap2	Rattus norvegicus SLIT-ROBO Rho GTPase activating protein 2 (Srgap2), mRNA [NM_001134958]	**9,252**
0	Unknown	**9,252**

Oxsr1	Rattus norvegicus oxidative-stress responsive 1 (Oxsr1), mRNA [NM_001108194]	**9,252**
Sec24c	Rattus norvegicus SEC24 family, member C (S. cerevisiae) (Sec24c), mRNA [NM_001109456]	**9,251**
0	Unknown	**9,250**
Gpr156	Rattus norvegicus G protein-coupled receptor 156 (Gpr156), mRNA [NM_153295]	**9,250**
0	Unknown	**9,249**
0	Unknown	**9,248**
0	Q84NG9_VITVI (Q84NG9) 2S albumin, partial (6%) [TC640666]	**9,247**
RGD1560859	PREDICTED: Rattus norvegicus similar to 2300003P22Rik protein (RGD1560859), miscRNA [XR_008965]	**9,246**
Riok3	Rattus norvegicus RIO kinase 3 (yeast) (Riok3), mRNA [NM_001108423]	**9,246**
Cops2	Rattus norvegicus COP9 constitutive photomorphogenic homolog subunit 2 (Arabidopsis) (Cops2), mRNA [NM_153297]	**9,244**
Paqr6	Rattus norvegicus progestin and adipoQ receptor family member VI (Paqr6), mRNA [NM_001191077]	**9,243**
Ormdl2	Rattus norvegicus ORM1-like 2 (S. cerevisiae) (Ormdl2), mRNA [NM_001105940]	**9,241**
0	Uncharacterized protein [Source:UniProtKB/TrEMBL;Acc:D4A3V3] [ENSRNOT00000011481]	**9,241**
0	Unknown	**9,241**
LOC690130	PREDICTED: Rattus norvegicus similar to ras homolog gene family, member f (LOC690130), mRNA [XM_001073389]	**9,240**
0	Unknown	**9,240**
0	Unknown	**9,240**
Lrrc40	Rattus norvegicus leucine rich repeat containing 40 (Lrrc40), mRNA [NM_001034926]	**9,240**
Snap23	Rattus norvegicus synaptosomal-associated protein 23 (Snap23), mRNA [NM_022689]	**9,236**
RGD1561777	PREDICTED: Rattus norvegicus similar to Na+ dependent glucose transporter 1 (RGD1561777), mRNA [XM_001063079]	**9,235**
Stk11	Rattus norvegicus serine/threonine kinase 11 (Stk11), mRNA [NM_001108069]	**9,233**
Erp44	Rattus norvegicus endoplasmic reticulum protein 44 (Erp44), mRNA [NM_001008317]	**9,233**
Atrn	Rattus norvegicus attractin (Atrn), mRNA [NM_031351]	**9,232**
Med23	Mediator of RNA polymerase II transcription subunit 23 [Source:UniProtKB/Swiss-Prot;Acc:Q5EB59] [ENSRNOT00000018404]	**9,231**
RGD1562874	PREDICTED: Rattus norvegicus similar to hypothetical protein DKFZp434E1818.1 - human (fragment) (RGD1562874), mRNA [XM_001077456]	**9,230**
Api5	Rattus norvegicus apoptosis inhibitor 5 (Api5), mRNA [NM_001127379]	**9,226**
Atad3a	Rattus norvegicus ATPase family, AAA domain containing 3A (Atad3a), nuclear gene encoding mitochondrial protein, mRNA [NM_001034922]	**9,226**
Nos1	Rattus norvegicus nitric oxide synthase 1, neuronal (Nos1), mRNA [NM_052799]	**9,223**
Dirc2	Rattus norvegicus disrupted in renal carcinoma 2 (human) (Dirc2), mRNA [NM_001012017]	**9,222**
0	Uncharacterized protein [Source:UniProtKB/TrEMBL;Acc:D3ZHU8] [ENSRNOT00000001891]	**9,220**
Fam76a	Rattus norvegicus family with sequence similarity 76, member A (Fam76a), mRNA [NM_001108686]	**9,218**
LOC684441	PREDICTED: Rattus norvegicus similar to Peptidyl-prolyl cis-trans isomerase NIMA-interacting 4 (Rotamase Pin4) (PPIase Pin4) (LOC684441), miscRNA [XR_086666]	**9,218**
0	Uncharacterized protein [Source:UniProtKB/TrEMBL;Acc:D3ZK13] [ENSRNOT00000061879]	**9,218**

Nudcd3	Rattus norvegicus NudC domain containing 3 (Nudcd3), mRNA [NM_001103362]	**9,217**
Tmem184a	Rattus norvegicus transmembrane protein 184A (Tmem184a), mRNA [NM_001025413]	**9,216**
RGD1563917	PREDICTED: Rattus norvegicus similar to Nuclear autoantigen Sp-100 (Speckled 100 kDa) (RGD1563917), mRNA [XM_001063206]	**9,216**
Luc7l2	Rattus norvegicus LUC7-like 2 (S. cerevisiae) (Luc7l2), mRNA [NM_001107853]	**9,215**
Entpd4	Rattus norvegicus ectonucleoside triphosphate diphosphohydrolase 4 (Entpd4), mRNA [NM_001108384]	**9,215**
0	Rattus norvegicus similar to glyceraldehyde-3-phosphate dehydrogenase (phosphorylating) (EC 1.2.1.12) - mouse (LOC305630), mRNA [XM_223741]	**9,215**
Cntf	Rattus norvegicus ciliary neurotrophic factor (Cntf), mRNA [NM_013166]	**9,211**
0	Unknown	**9,210**
Fry	Rattus norvegicus furry homolog (Drosophila) (Fry), mRNA [NM_001170398]	**9,208**
Ccdc44	Rattus norvegicus coiled-coil domain containing 44 (Ccdc44), mRNA [NM_001108302]	**9,207**
Lancl2	Rattus norvegicus LanC lantibiotic synthetase component C-like 2 (bacterial) (Lancl2), mRNA [NM_001014187]	**9,206**
Gsg1l	PREDICTED: Rattus norvegicus GSG1-like (Gsg1l), mRNA [XM_574558]	**9,205**
Aof1	Rattus norvegicus amine oxidase (flavin containing) domain 1 (Aof1), mRNA [NM_001107343]	**9,203**
0	Uncharacterized protein [Source:UniProtKB/TrEMBL;Acc:D3ZQW1] [ENSRNOT00000015065]	**9,202**
Chrm2	Rattus norvegicus cholinergic receptor, muscarinic 2 (Chrm2), mRNA [NM_031016]	**9,202**
Slc24a4	Rattus norvegicus solute carrier family 24 (sodium/potassium/calcium exchanger), member 4 (Slc24a4), mRNA [NM_001108051]	**9,202**
Zdhhc13	Rattus norvegicus zinc finger, DHHC-type containing 13 (Zdhhc13), mRNA [NM_001039037]	**9,201**
Dbndd1	Rattus norvegicus dysbindin (dystrobrevin binding protein 1) domain containing 1 (Dbndd1), mRNA [NM_001014156]	**9,200**
Rab32	Rattus norvegicus RAB32, member RAS oncogene family (Rab32), mRNA [NM_001108902]	**9,199**
Gde1	Rattus norvegicus glycerophosphodiester phosphodiesterase 1 (Gde1), mRNA [NM_032615]	**9,199**
Tmem178	Rattus norvegicus transmembrane protein 178 (Tmem178), mRNA [NM_001004282]	**9,198**
0	Rattus norvegicus similar to caspase 3, apoptosis related cysteine protease (LOC292581), mRNA [XM_218253]	**9,198**
Map3k2	Rattus norvegicus mitogen activated protein kinase kinase kinase 2 (Map3k2), mRNA [NM_138503]	**9,197**
Cyp2b3	Rattus norvegicus cytochrome P450, family 2, subfamily b, polypeptide 3 (Cyp2b3), mRNA [NM_173294]	**9,197**
0	Unknown	**9,195**
Strn3	Rattus norvegicus striatin, calmodulin binding protein 3 (Strn3), mRNA [NM_001029897]	**9,195**
Pcdhb8	Rattus norvegicus protocadherin beta 8 (Pcdhb8), mRNA [NM_001014779]	**9,195**
Zc3h6	Rattus norvegicus zinc finger CCCH type containing 6 (Zc3h6), mRNA [NM_001107772]	**9,194**
Fam102b	Rattus norvegicus family with sequence similarity 102, member B (Fam102b), mRNA [NM_001163568]	**9,191**
0	Unknown	**9,191**
Gstt1	Rattus norvegicus glutathione S-transferase theta 1 (Gstt1), mRNA [NM_053293]	**9,190**
Sec62	Rattus norvegicus SEC62 homolog (S. cerevisiae) (Sec62), mRNA [NM_001034129]	**9,190**
Lrp11	Rattus norvegicus low density lipoprotein receptor-related protein 11 (Lrp11), mRNA [NM_001106217]	**9,188**

0	Unknown	**9,186**
LOC100188984	Rattus norvegicus hypothetical protein LOC100188984 (LOC100188984), mRNA [NM_001134998]	**9,185**
Atg4b	Rattus norvegicus ATG4 autophagy related 4 homolog B (S. cerevisiae) (Atg4b), mRNA [NM_001025711]	**9,185**
Calml4	Rattus norvegicus calmodulin-like 4 (Calml4), mRNA [NM_001127575]	**9,185**
Itga1	Rattus norvegicus integrin, alpha 1 (Itga1), mRNA [NM_030994]	**9,181**
LOC100361845	PREDICTED: Rattus norvegicus hypothetical protein LOC100361845 (LOC100361845), mRNA [XM_002729636]	**9,179**
Mbd3	Rattus norvegicus methyl-CpG binding domain protein 3 (Mbd3), mRNA [NM_001108735]	**9,179**
Mical3	Rattus norvegicus microtubule associated monoxygenase, calponin and LIM domain containing 3 (Mical3), mRNA [NM_001191085]	**9,177**
RGD1565023	PREDICTED: Rattus norvegicus similar to Suppressor of S. cerevisiae gcr2 (RGD1565023), mRNA [XM_001055900]	**9,174**
LOC678966	PREDICTED: Rattus norvegicus hypothetical protein LOC678966 (LOC678966), partial mRNA [XM_001054033]	**9,173**
0	Unknown	**9,169**
Srf	Rattus norvegicus serum response factor (c-fos serum response element-binding transcription factor) (Srf), mRNA [NM_001109302]	**9,169**
LOC691632	PREDICTED: Rattus norvegicus similar to MIC2 like 1 (LOC691632), mRNA [XM_001079095]	**9,167**
Tor2a	Rattus norvegicus torsin family 2, member A (Tor2a), mRNA [NM_001007744]	**9,165**
Pln	Rattus norvegicus phospholamban (Pln), mRNA [NM_022707]	**9,165**
0	Rattus norvegicus chromosome 7, 20 clones, strain BN/SsNHsdMCW RNOR03304384, whole genome shotgun sequence [AABR03055433]	**9,164**
Vps13a	Rattus norvegicus vacuolar protein sorting 13 homolog A (S. cerevisiae) (Vps13a), mRNA [NM_001100975]	**9,164**
Gla	Rattus norvegicus galactosidase, alpha (Gla), mRNA [NM_001108820]	**9,163**
Ndrg2	Rattus norvegicus N-myc downstream regulated gene 2 (Ndrg2), mRNA [NM_133583]	**9,163**
0	Uncharacterized protein [Source:UniProtKB/TrEMBL;Acc:D3ZX00] [ENSRNOT00000046987]	**9,163**
Gucy1b2	Rattus norvegicus guanylate cyclase 1, soluble, beta 2 (Gucy1b2), mRNA [NM_012770]	**9,162**
LOC682097	PREDICTED: Rattus norvegicus similar to TBP-associated factor 4 (LOC682097), miscRNA [XR_086218]	**9,159**
0	Unknown	**9,159**
Hsd3b5	Rattus norvegicus hydroxy-delta-5-steroid dehydrogenase, 3 beta- and steroid delta-isomerase 5 (Hsd3b5), mRNA [NM_012584]	**9,158**
LOC680531	Rattus norvegicus similar to CG3880-PA (LOC680531), mRNA [NM_001109418]	**9,158**
Hs3st6	Rattus norvegicus heparan sulfate (glucosamine) 3-O-sulfotransferase 6 (Hs3st6), mRNA [NM_001109450]	**9,156**
Dcps	Rattus norvegicus decapping enzyme, scavenger (Dcps), mRNA [NM_153302]	**9,156**
Rab14	Rattus norvegicus RAB14, member RAS oncogene family (Rab14), mRNA [NM_053589]	**9,155**
U2surp	PREDICTED: Rattus norvegicus similar to CG9346-PA, transcript variant 1 (RGD1307882), mRNA [XM_002729954]	**9,155**
Tmc5	Rattus norvegicus transmembrane channel-like 5 (Tmc5), mRNA [NM_001012216]	**9,153**
Rassf8	Rattus norvegicus Ras association (RalGDS/AF-6) domain family (N-terminal) member 8 (Rassf8), mRNA [NM_001191753]	**9,152**
MGC95208	Rattus norvegicus similar to 4930453N24Rik protein (MGC95208), mRNA [NM_001005552]	**9,150**
Dhx8	Rattus norvegicus DEAH (Asp-Glu-Ala-His) box polypeptide 8 (Dhx8), mRNA [NM_001047844]	**9,149**
Rab7l1	Rattus norvegicus RAB7, member RAS oncogene family-like 1 (Rab7l1), mRNA [NM_133590]	**9,149**

Trim27	Rattus norvegicus tripartite motif-containing 27 (Trim27), transcript variant 1, mRNA [NM_001134974]	**9,149**
0	Unknown	**9,148**
RGD1560737	PREDICTED: Rattus norvegicus similar to elicitor-like mating protein M81 (RGD1560737), mRNA [XM_225445]	**9,147**
Hbp1	Rattus norvegicus HMG-box transcription factor 1 (Hbp1), mRNA [NM_013221]	**9,144**
0	Unknown	**9,143**
Rnf144b	Rattus norvegicus ring finger protein 144B (Rnf144b), mRNA [NM_001108881]	**9,143**
Odc1	Rattus norvegicus ornithine decarboxylase 1 (Odc1), mRNA [NM_012615]	**9,142**
0	Unknown	**9,140**
RGD1308644	RCG50901Uncharacterized protein [Source:UniProtKB/TrEMBL;Acc:D3ZAP3] [ENSRNOT00000031717]	**9,140**
Olr50	Rattus norvegicus olfactory receptor 50 (Olr50), mRNA [NM_001000131]	**9,139**
0	Unknown	**9,139**
Pcdh9	Rattus norvegicus protocadherin 9 (Pcdh9), mRNA [NM_001191688]	**9,139**
LOC680430	PREDICTED: Rattus norvegicus similar to germinal histone H4 gene (LOC680430), mRNA [XM_001057149]	**9,138**
Krt80	Rattus norvegicus keratin 80 (Krt80), mRNA [NM_001008815]	**9,138**
0	PREDICTED: Rattus norvegicus similar to TDPOZ2 (RGD1562545), mRNA [XM_001061198]	**9,138**
Acin1	Rattus norvegicus apoptotic chromatin condensation inducer 1 (Acin1), mRNA [NM_001170468]	**9,137**
Rhoh	Rattus norvegicus ras homolog gene family, member H (Rhoh), mRNA [NM_001013430]	**9,135**
Armcx3	Rattus norvegicus armadillo repeat containing, X-linked 3 (Armcx3), mRNA [NM_001014273]	**9,134**
0	ERG6_MAGGR (Q5EN22) Sterol 24-C-methyltransferase (Delta(24)-sterol C-methyltransferase) , partial (5%) [TC583516]	**9,134**
Ift52	Rattus norvegicus intraflagellar transport 52 homolog (Chlamydomonas) (Ift52), mRNA [NM_001177685]	**9,133**
LOC289378	Rattus norvegicus similar to B0432.8 (LOC289378), mRNA [NM_001105987]	**9,133**
RGD1565819	Rattus norvegicus similar to C20orf174 (RGD1565819), mRNA [NM_001171096]	**9,133**
0	Unknown	**9,133**
RGD1565767	Ribosomal protein L15 [Source:UniProtKB/TrEMBL;Acc:D3ZF52] [ENSRNOT00000032528]	**9,132**
Ccdc64	Rattus norvegicus coiled-coil domain containing 64 (Ccdc64), mRNA [NM_001191667]	**9,132**
0	Unknown	**9,131**
Fam126b	Rattus norvegicus family with sequence similarity 126, member B (Fam126b), mRNA [NM_001025710]	**9,131**
Tmem40	Rattus norvegicus transmembrane protein 40 (Tmem40), mRNA [NM_001191573]	**9,130**
Hsd17b1	Rattus norvegicus hydroxysteroid (17-beta) dehydrogenase 1 (Hsd17b1), mRNA [NM_012851]	**9,128**
Ccdc89	Rattus norvegicus coiled-coil domain containing 89 (Ccdc89), mRNA [NM_001134833]	**9,127**
Tmcc2	Similar to RIKEN cDNA 1110063G11 (Predicted)Uncharacterized protein [Source:UniProtKB/TrEMBL;Acc:D3ZE26] [ENSRNOT00000000036]	**9,125**
Rdh13	Rattus norvegicus retinol dehydrogenase 13 (all-trans/9-cis) (Rdh13), nuclear gene encoding mitochondrial protein, mRNA [NM_001108468]	**9,124**
0	Unknown	**9,123**
Inhbb	Rattus norvegicus inhibin beta-B (Inhbb), mRNA [NM_080771]	**9,121**

Pank3	Rattus norvegicus pantothenate kinase 3 (Pank3), mRNA [NM_001108272]	**9,120**
0	Unknown	**9,120**
Vav3	Rattus norvegicus vav 3 guanine nucleotide exchange factor (Vav3), mRNA [NM_001191714]	**9,118**
Mobkl3	Rattus norvegicus MOB1, Mps One Binder kinase activator-like 3 (yeast) (Mobkl3), mRNA [NM_133528]	**9,118**
Pqlc2	Rattus norvegicus PQ loop repeat containing 2 (Pqlc2), mRNA [NM_001108689]	**9,115**
Gldn	Rattus norvegicus gliomedin (Gldn), mRNA [NM_181382]	**9,115**
Dnaja4	Rattus norvegicus DnaJ (Hsp40) homolog, subfamily A, member 4 (Dnaja4), mRNA [NM_001025411]	**9,115**
Pih1d2	Rattus norvegicus PIH1 domain containing 2 (Pih1d2), mRNA [NM_001191793]	**9,112**
Paqr8	Rattus norvegicus progestin and adipoQ receptor family member VIII (Paqr8), mRNA [NM_001014099]	**9,111**
LOC679140	PREDICTED: Rattus norvegicus similar to exosome component 1, transcript variant 1 (LOC679140), mRNA [XM_001054866]	**9,110**
0	Unknown	**9,108**
Trub2	Rattus norvegicus TruB pseudouridine (psi) synthase homolog 2 (E. coli) (Trub2), mRNA [NM_001014257]	**9,108**
0	Histone H3 [Source:UniProtKB/TrEMBL;Acc:D3ZXC3] [ENSRNOT00000046278]	**9,107**
Mex3c	Rattus norvegicus mex-3 homolog C (C. elegans) (Mex3c), mRNA [NM_001107377]	**9,104**
Fam46a	Rattus norvegicus family with sequence similarity 46, member A (Fam46a), mRNA [NM_001106844]	**9,100**
0	Unknown	**9,100**
0	Uncharacterized protein [Source:UniProtKB/TrEMBL;Acc:D3ZJ63] [ENSRNOT00000042360]	**9,099**
LOC682968	PREDICTED: Rattus norvegicus similar to Retinal homeobox protein Rx (DRx1) (DRx) (LOC682968), mRNA [XM_001063896]	**9,099**
Itsn1	Rattus norvegicus intersectin 1 (SH3 domain protein) (Itsn1), transcript variant 2, mRNA [NM_019227]	**9,098**
0	Unknown	**9,097**
0	PREDICTED: Rattus norvegicus up-regulator of carnitine transporter, OCTN2 (LOC303140), mRNA [XM_001073573]	**9,096**
Zcchc2	Rattus norvegicus zinc finger, CCHC domain containing 2 (Zcchc2), mRNA [NM_001122677]	**9,095**
0	probable tRNA pseudouridine synthase 2 [Source:RefSeq peptide;Acc:NP_001014279] [ENSRNOT00000040550]	**9,095**
Mtf1	Rattus norvegicus metal-regulatory transcription factor 1 (Mtf1), mRNA [NM_001108677]	**9,095**
Nrbp2	Rattus norvegicus nuclear receptor binding protein 2 (Nrbp2), mRNA [NM_001135007]	**9,092**
LOC691572	PREDICTED: Rattus norvegicus hypothetical protein LOC691572 (LOC691572), mRNA [XM_001078882]	**9,092**
0	Unknown	**9,089**
Nrxn1	Rattus norvegicus neurexin 1 (Nrxn1), mRNA [NM_021767]	**9,087**
0	Q6MG19_RAT (Q6MG19) Discoidin domain receptor family, member 1, partial (18%) [TC622836]	**9,086**
Prss35	Rattus norvegicus protease, serine, 35 (Prss35), mRNA [NM_001008560]	**9,085**
Ipo11	PREDICTED: Rattus norvegicus importin 11 (Ipo11), mRNA [XM_002725887]	**9,084**
0	Unknown	**9,084**
G6pc3	Rattus norvegicus glucose 6 phosphatase, catalytic, 3 (G6pc3), mRNA [NM_176077]	**9,084**
Pdxdc1	Rattus norvegicus pyridoxal-dependent decarboxylase domain containing 1 (Pdxdc1), mRNA [NM_001134961]	**9,084**

RGD1564308	Uncharacterized protein [Source:UniProtKB/TrEMBL;Acc:D3ZVL4] [ENSRNOT00000059317]	**9,080**
LOC100363193	Rattus norvegicus LRRGT00076-like (LOC100363193), mRNA [NM_001177824]	**9,076**
0	Unknown	**9,075**
Osbp	Rattus norvegicus oxysterol binding protein (Osbp), mRNA [NM_001108927]	**9,075**
Mark1	Rattus norvegicus MAP/microtubule affinity-regulating kinase 1 (Mark1), mRNA [NM_053947]	**9,074**
Cat	Rattus norvegicus catalase (Cat), mRNA [NM_012520]	**9,074**
Sgef	Uncharacterized protein [Source:UniProtKB/TrEMBL;Acc:D4A1D2] [ENSRNOT00000019553]	**9,073**
Ssr1	Rattus norvegicus signal sequence receptor, alpha (Ssr1), mRNA [NM_001008891]	**9,073**
Pde4d	Rattus norvegicus phosphodiesterase 4D, cAMP-specific (phosphodiesterase E3 dunce homolog, Drosophila) (Pde4d), transcript variant 1, mRNA [NM_001113328]	**9,072**
0	Rattus norvegicus TL0ADA51YN11 mRNA sequence. [FQ228778]	**9,070**
RGD1560695	PREDICTED: Rattus norvegicus similar to hypothetical protein 4930474N05 (RGD1560695), mRNA [XM_001074018]	**9,069**
RGD1306410	Rattus norvegicus similar to CG14980-PB (RGD1306410), mRNA [NM_001014126]	**9,068**
0	Unknown	**9,067**
LOC683516	PREDICTED: Rattus norvegicus similar to Zinc finger X-linked protein ZXDB (LOC683516), partial mRNA [XM_001066304]	**9,066**
LOC689257	Uncharacterized protein [Source:UniProtKB/TrEMBL;Acc:D3ZF17] [ENSRNOT00000058586]	**9,065**
Tnfrsf1b	Tumor necrosis factor receptor superfamily member 1B [Source:UniProtKB/Swiss-Prot;Acc:Q80WY6] [ENSRNOT00000022478]	**9,065**
RGD1559864	PREDICTED: Rattus norvegicus similar to mKIAA1045 protein, transcript variant 2 (RGD1559864), mRNA [XM_575808]	**9,064**
Osbpl11	Rattus norvegicus oxysterol binding protein-like 11 (Osbpl11), mRNA [NM_001107090]	**9,062**
Bdh1	Rattus norvegicus 3-hydroxybutyrate dehydrogenase, type 1 (Bdh1), nuclear gene encoding mitochondrial protein, mRNA [NM_053995]	**9,062**
Dis3l2	Rattus norvegicus DIS3 mitotic control homolog (S. cerevisiae)-like 2 (Dis3l2), transcript variant 1, mRNA [NM_001109007]	**9,062**
Lppr1	Rattus norvegicus lipid phosphate phosphatase-related protein type 1 (Lppr1), mRNA [NM_201271]	**9,059**
0	Uncharacterized protein [Source:UniProtKB/TrEMBL;Acc:D3ZBK3] [ENSRNOT00000060360]	**9,059**
RGD1310311	Rattus norvegicus similar to chromosome 14 open reading frame 104 (RGD1310311), mRNA [NM_001014197]	**9,059**
Kcnk2	Rattus norvegicus potassium channel, subfamily K, member 2 (Kcnk2), transcript variant 1, mRNA [NM_172041]	**9,058**
Vom2r55	Rattus norvegicus vomeronasal 2 receptor, 55 (Vom2r55), mRNA [NM_001099495]	**9,054**
0	Unknown	**9,054**
Sesn1	Rattus norvegicus sestrin 1 (Sesn1), mRNA [NM_001106396]	**9,053**
Olr1286	Rattus norvegicus olfactory receptor 1286 (Olr1286), mRNA [NM_001000799]	**9,049**
N-pac	Putative oxidoreductase GLYR1 [Source:UniProtKB/Swiss-Prot;Acc:Q5RKH0] [ENSRNOT00000004159]	**9,049**
0	Uncharacterized protein [Source:UniProtKB/TrEMBL;Acc:D3ZQ92] [ENSRNOT00000026116]	**9,048**
Adcy1	Rattus norvegicus adenylate cyclase 1 (brain) (Adcy1), mRNA [NM_001107239]	**9,047**
LOC691543	Rattus norvegicus hypothetical protein LOC691543 (LOC691543), mRNA [NM_001109645]	**9,047**
Slc27a2	Rattus norvegicus solute carrier family 27 (fatty acid transporter), member 2 (Slc27a2), mRNA [NM_031736]	**9,046**

Dars2	Rattus norvegicus aspartyl-tRNA synthetase 2 (mitochondrial) (Dars2), nuclear gene encoding mitochondrial protein, mRNA [NM_001034143]	**9,045**
Sec24c	Rattus norvegicus SEC24 family, member C (S. cerevisiae) (Sec24c), mRNA [NM_001109456]	**9,045**
Elovl6	Rattus norvegicus ELOVL family member 6, elongation of long chain fatty acids (yeast) (Elovl6), mRNA [NM_134383]	**9,041**
0	BC042626 Chmp2b protein {Mus musculus} (exp=-1; wgp=0; cg=0), partial (49%) [TC588602]	**9,040**
Gpr89	Rattus norvegicus G protein-coupled receptor 89 (Gpr89), mRNA [NM_001139486]	**9,039**
0	Williams Beuren syndrome chromosome region 27 [Source:RefSeq peptide;Acc:NP_001102969] [ENSRNOT00000002002]	**9,037**
0	Unknown	**9,036**
Sypl1	Rattus norvegicus synaptophysin-like 1 (Sypl1), mRNA [NM_001014263]	**9,036**
Kdelr3	Rattus norvegicus KDEL (Lys-Asp-Glu-Leu) endoplasmic reticulum protein retention receptor 3 (Kdelr3), mRNA [NM_001127546]	**9,035**
Hes5	Rattus norvegicus hairy and enhancer of split 5 (Drosophila) (Hes5), mRNA [NM_024383]	**9,035**
Nkr-p1c	Rattus norvegicus killer cell lectin-like receptor subfamily B member (Nkr-p1c), mRNA [NM_001040189]	**9,033**
Sh2d5	PREDICTED: Rattus norvegicus SH2 domain containing 5 (Sh2d5), mRNA [XM_001070433]	**9,033**
0	Unknown	**9,032**
Zfp263	Rattus norvegicus zinc finger protein 263 (Zfp263), mRNA [NM_001105763]	**9,029**
0	Unknown	**9,029**
LOC100364812	PREDICTED: Rattus norvegicus hypothetical protein LOC100364812 (LOC100364812), mRNA [XM_002725256]	**9,027**
Lypla1	Rattus norvegicus lysophospholipase 1 (Lypla1), mRNA [NM_013006]	**9,026**
Steap2	Rattus norvegicus six transmembrane epithelial antigen of the prostate 2 (Steap2), mRNA [NM_001107846]	**9,026**
0	Uncharacterized protein [Source:UniProtKB/TrEMBL;Acc:D3ZTC4] [ENSRNOT00000034159]	**9,025**
Mesp2	Rattus norvegicus mesoderm posterior 2 homolog (mouse) (Mesp2), mRNA [NM_001106273]	**9,024**
0	Rattus norvegicus similar to 40S ribosomal protein S7 (S8) (LOC367108), mRNA [XM_345950]	**9,024**
LOC679835	FM119812 etnohea Rattus norvegicus cDNA clone etnoheaP0047K24 3', mRNA sequence [FM119812]	**9,023**
Sdhd	Rattus norvegicus succinate dehydrogenase complex, subunit D, integral membrane protein (Sdhd), nuclear gene encoding mitochondrial protein, mRNA [NM_198788]	**9,022**
RGD1560883	PREDICTED: Rattus norvegicus similar to KIAA0825 protein (RGD1560883), mRNA [XM_226616]	**9,021**
0	Unknown	**9,019**
0	Unknown	**9,018**
C1qtnf2	Rattus norvegicus C1q and tumor necrosis factor related protein 2 (C1qtnf2), mRNA [NM_001191918]	**9,018**
Abcb10	Rattus norvegicus ATP-binding cassette, subfamily B (MDR/TAP), member 10 (Abcb10), nuclear gene encoding mitochondrial protein, mRNA [NM_001012166]	**9,016**
Klf6	Rattus norvegicus Kruppel-like factor 6 (Klf6), mRNA [NM_031642]	**9,016**
0	Unknown	**9,015**
0	Rattus norvegicus TL0AAA53YP17 mRNA sequence. [FQ212466]	**9,015**
Gria3	Rattus norvegicus glutamate receptor, ionotrophic, AMPA 3 (Gria3), transcript variant 2, mRNA [NM_001112742]	**9,015**

RGD1564859	PREDICTED: Rattus norvegicus RGD1564859 (RGD1564859), mRNA [XM_347061]	**9,015**
Rfxank	Rattus norvegicus regulatory factor X-associated ankyrin-containing protein (Rfxank), mRNA [NM_001013136]	**9,014**
Bcar1	Rattus norvegicus breast cancer anti-estrogen resistance 1 (Bcar1), mRNA [NM_012931]	**9,013**
RGD1310794	Rattus norvegicus similar to RIKEN cDNA C030048B08 (RGD1310794), mRNA [NM_001024246]	**9,012**
Sin3a	Rattus norvegicus SIN3 homolog A, transcription regulator (yeast) (Sin3a), mRNA [NM_001108761]	**9,012**
Vps53	Rattus norvegicus vacuolar protein sorting 53 homolog (S. cerevisiae) (Vps53), mRNA [NM_001105813]	**9,011**
RGD1562136	Rattus norvegicus similar to D1Ertd622e protein (RGD1562136), mRNA [NM_001109086]	**9,010**
Klhl18	Uncharacterized protein [Source:UniProtKB/TrEMBL;Acc:D4A902] [ENSRNOT00000056141]	**9,009**
Pnmal2	Rattus norvegicus PNMA-like 2 (Pnmal2), mRNA [NM_001107481]	**9,009**
Map4k4	Rattus norvegicus mitogen-activated protein kinase kinase kinase kinase 4 (Map4k4), mRNA [NM_001106904]	**9,007**
0	Rattus norvegicus similar to glyceraldehyde-3-phosphate dehydrogenase (LOC295420), mRNA [XM_227654]	**9,006**
Hibadh	Rattus norvegicus 3-hydroxyisobutyrate dehydrogenase (Hibadh), mRNA [NM_022243]	**9,006**
Sh3glb1	Rattus norvegicus SH3-domain GRB2-like endophilin B1 (Sh3glb1), mRNA [NM_001011929]	**9,006**
0	Unknown	**9,005**
Kdm4dl	PREDICTED: Rattus norvegicus similar to jumonji domain containing 2D (RGD1563367), mRNA [XM_576355]	**9,005**
0	Unknown	**9,005**
0	Unknown	**9,004**
Ythdf2	Rattus norvegicus YTH domain family, member 2 (Ythdf2), mRNA [NM_001047099]	**9,001**
0	Unknown	**9,001**
0	Unknown	**9,000**
Gtrgeo22	Rattus norvegicus gene trap ROSA b-geo 22 (Gtrgeo22), mRNA [NM_001109621]	**8,998**
0	Unknown	**8,998**
Lrfn4	Rattus norvegicus leucine rich repeat and fibronectin type III domain containing 4 (Lrfn4), mRNA [NM_001109508]	**8,998**
Uhmk1	Rattus norvegicus U2AF homology motif (UHM) kinase 1 (Uhmk1), mRNA [NM_017293]	**8,995**
Esf1	Rattus norvegicus ESF1, nucleolar pre-rRNA processing protein, homolog (S. cerevisiae) (Esf1), mRNA [NM_001100771]	**8,994**
Pik3ca	Rattus norvegicus phosphoinositide-3-kinase, catalytic, alpha polypeptide (Pik3ca), mRNA [NM_133399]	**8,992**
Pepd	Rattus norvegicus peptidase D (Pepd), mRNA [NM_001009641]	**8,991**
Alg14	Rattus norvegicus asparagine-linked glycosylation 14 homolog (S. cerevisiae) (Alg14), mRNA [NM_001014176]	**8,987**
Pou3f3	Rattus norvegicus POU class 3 homeobox 3 (Pou3f3), mRNA [NM_138837]	**8,985**
Ccl12	Rattus norvegicus chemokine (C-C motif) ligand 12 (Ccl12), mRNA [NM_001105822]	**8,985**
Bean1	Uncharacterized protein [Source:UniProtKB/TrEMBL;Acc:D3ZFF6] [ENSRNOT00000017774]	**8,982**
Ccnj	Rattus norvegicus cyclin J (Ccnj), mRNA [NM_001106369]	**8,981**
Optn	Rattus norvegicus optineurin (Optn), mRNA [NM_145081]	**8,980**
Surf2	Rattus norvegicus surfeit 2 (Surf2), mRNA [NM_001033866]	**8,980**

0	Rattus norvegicus TL0ADA36YM24 mRNA sequence. [FQ221400]	**8,979**
Chac2	Rattus norvegicus ChaC, cation transport regulator homolog 2 (E. coli) (Chac2), mRNA [NM_001025016]	**8,979**
Tmem68	Rattus norvegicus transmembrane protein 68 (Tmem68), mRNA [NM_001107903]	**8,977**
RGD1565158	PREDICTED: Rattus norvegicus similar to RIKEN cDNA 4921537P18 (RGD1565158), mRNA [XM_001077736]	**8,974**
LOC685030	PREDICTED: Rattus norvegicus similar to paired immunoglobin-like type 2 receptor beta (LOC685030), mRNA [XM_002724831]	**8,973**
Oscp1	Rattus norvegicus organic solute carrier partner 1 (Oscp1), mRNA [NM_001029923]	**8,972**
Pikfyve	Similar to phosphatidylinositol-3-phosphate/phosphatidylinositol 5-kinase, type III isoform 2Uncharacterized protein [Source:UniProtKB/TrEMBL;Acc:D3ZT14] [ENSRNOT00000020447]	**8,972**
0	Unknown	**8,966**
Gphn	Rattus norvegicus gephyrin (Gphn), mRNA [NM_022865]	**8,965**
Mex3c	Rattus norvegicus mex-3 homolog C (C. elegans) (Mex3c), mRNA [NM_001107377]	**8,965**
Strbp	Rattus norvegicus spermatid perinuclear RNA binding protein (Strbp), mRNA [NM_053416]	**8,964**
Ebpl	Rattus norvegicus emopamil binding protein-like (Ebpl), mRNA [NM_001108381]	**8,962**
0	PREDICTED: Rattus norvegicus similar to H3 histone, family 3B (LOC685984), mRNA [XM_001066068]	**8,962**
Polr3b	Rattus norvegicus polymerase (RNA) III (DNA directed) polypeptide B (Polr3b), mRNA [NM_001191878]	**8,961**
0	AGENCOURT_109866019 NIH_MGC_420 Rattus norvegicus cDNA clone IMAGE:9032542 5', mRNA sequence [EV763082]	**8,961**
Lrriq1	PREDICTED: Rattus norvegicus leucine-rich repeats and IQ motif containing 1 (Lrriq1), mRNA [XM_001080693]	**8,961**
LOC690349	Rattus norvegicus hypothetical protein LOC690349 (LOC690349), mRNA [NM_001109581]	**8,959**
Pgrmc1	Rattus norvegicus progesterone receptor membrane component 1 (Pgrmc1), mRNA [NM_021766]	**8,957**
Brms1	Rattus norvegicus breast cancer metastasis-suppressor 1 (Brms1), mRNA [NM_001009605]	**8,956**
Pgm2	Rattus norvegicus phosphoglucomutase 2 (Pgm2), mRNA [NM_001106007]	**8,955**
Tnks2	Rattus norvegicus tankyrase, TRF1-interacting ankyrin-related ADP-ribose polymerase 2 (Tnks2), mRNA [NM_001107607]	**8,955**
0	Unknown	**8,954**
Aktip	Rattus norvegicus AKT interacting protein (Aktip), mRNA [NM_001011926]	**8,952**
0	Rattus norvegicus similar to glyceraldehyde-3-phosphate dehydrogenase (LOC307263), mRNA [XM_225724]	**8,952**
0	Uncharacterized protein [Source:UniProtKB/TrEMBL;Acc:D4ACD7] [ENSRNOT00000014120]	**8,951**
Slc9a2	Rattus norvegicus solute carrier family 9 (sodium/hydrogen exchanger), member 2 (Slc9a2), transcript variant 1, mRNA [NM_001113335]	**8,949**
0	Uncharacterized protein [Source:UniProtKB/TrEMBL;Acc:D3Z9J9] [ENSRNOT00000015767]	**8,948**
Calm3	Rattus norvegicus calmodulin 3 (Calm3), mRNA [NM_012518]	**8,948**
Mapk11	Rattus norvegicus mitogen-activated protein kinase 11 (Mapk11), mRNA [NM_001109532]	**8,948**
Mat2b	Rattus norvegicus methionine adenosyltransferase II, beta (Mat2b), mRNA [NM_001044282]	**8,948**
Hrk	Rattus norvegicus harakiri, BCL2 interacting protein (contains only BH3 domain) (Hrk), mRNA [NM_057130]	**8,947**
Drg1	Rattus norvegicus developmentally regulated GTP binding protein 1 (Drg1), mRNA [NM_001009685]	**8,947**
Cab39l	Rattus norvegicus calcium binding protein 39-like (Cab39l), mRNA [NM_001011917]	**8,947**

Aff4	Rattus norvegicus AF4/FMR2 family, member 4 (Aff4), mRNA [NM_001107001]	**8,946**
Pfdn6	Rattus norvegicus prefoldin subunit 6 (Pfdn6), mRNA [NM_212506]	**8,944**
Hmgn1	Rattus norvegicus high-mobility group nucleosome binding domain 1 (Hmgn1), mRNA [NM_001013184]	**8,943**
0	PREDICTED: Rattus norvegicus similar to Discs large homolog 5 (Placenta and prostate DLG) (Discs large protein P-dlg) (LOC691410), mRNA [XM_001078125]	**8,943**
0	Unknown	**8,943**
Maff	Rattus norvegicus v-maf musculoaponeurotic fibrosarcoma oncogene homolog F (avian) (Maff), mRNA [NM_001130573]	**8,941**
Ulk2	Rattus norvegicus Unc-51 like kinase 2 (C. elegans) (Ulk2), mRNA [NM_001191645]	**8,939**
RGD1565119	PREDICTED: Rattus norvegicus similar to Mitochondrial carrier triple repeat 1 (RGD1565119), mRNA [XM_001078261]	**8,939**
Rab39	Rattus norvegicus RAB39, member RAS oncogene family (Rab39), mRNA [NM_001108148]	**8,939**
Vax2	Rattus norvegicus ventral anterior homeobox 2 (Vax2), mRNA [NM_022637]	**8,939**
Lhfpl2	Rattus norvegicus lipoma HMGIC fusion partner-like 2 (Lhfpl2), mRNA [NM_001106402]	**8,938**
Gspt2	Rattus norvegicus G1 to S phase transition 2 (Gspt2), mRNA [NM_001109319]	**8,938**
Cdc45l	Rattus norvegicus CDC45 cell division cycle 45-like (S. cerevisiae) (Cdc45l), mRNA [NM_001105866]	**8,938**
Mad2l1	Rattus norvegicus MAD2 mitotic arrest deficient-like 1 (yeast) (Mad2l1), mRNA [NM_001106594]	**8,937**
Brdt	Rattus norvegicus bromodomain, testis-specific (Brdt), mRNA [NM_001012031]	**8,937**
Susd2	Rattus norvegicus sushi domain containing 2 (Susd2), mRNA [NM_001106381]	**8,936**
Nipal4	Rattus norvegicus NIPA-like domain containing 4 (Nipal4), mRNA [NM_001106995]	**8,935**
0	Unknown	**8,934**
Hist2h2ab	Rattus norvegicus histone cluster 2, H2ab (Hist2h2ab), mRNA [NM_001111341]	**8,932**
Rspry1	Rattus norvegicus ring finger and SPRY domain containing 1 (Rspry1), mRNA [NM_001100945]	**8,932**
Slc22a15	Rattus norvegicus solute carrier family 22, member 15 (Slc22a15), mRNA [NM_001107707]	**8,931**
Racgap1	Rattus norvegicus Rac GTPase-activating protein 1 (Racgap1), mRNA [NM_001108112]	**8,931**
Spg20	Rattus norvegicus spastic paraplegia 20 (Troyer syndrome) homolog (human) (Spg20), mRNA [NM_001106433]	**8,929**
0	AI060108 UI-R-C1-li-g-08-0-UI.s1 UI-R-C1 Rattus norvegicus cDNA clone UI-R-C1-li-g-08-0-UI 3', mRNA sequence [AI060108]	**8,927**
Dnhd1	PREDICTED: Rattus norvegicus dynein heavy chain domain 1 (Dnhd1), miscRNA [XR_085725]	**8,927**
Zfp637	Rattus norvegicus zinc finger protein 637 (Zfp637), mRNA [NM_001134908]	**8,926**
Htt	Rattus norvegicus huntingtin (Htt), mRNA [NM_024357]	**8,926**
Utf1	Rattus norvegicus undifferentiated embryonic cell transcription factor 1 (Utf1), mRNA [NM_001131032]	**8,925**
0	PREDICTED: Rattus norvegicus similar to Zgc:56193 (RGD1564447), mRNA [XM_001058987]	**8,924**
0	Rattus norvegicus similar to Glyceraldehyde 3-phosphate dehydrogenase (GAPDH) (38 kDa BFA-dependent ADP-ribosylation substrate) (BARS-38) (LOC299213), mRNA [XM_234433]	**8,923**
0	Unknown	**8,923**
0	Unknown	**8,921**

Wdr20a	Rattus norvegicus WD repeat domain 20a (Wdr20a), mRNA [NM_001100894]	**8,917**
Plunc	Rattus norvegicus palate, lung, and nasal epithelium associated (Plunc), mRNA [NM_172031]	**8,916**
0	Unknown	**8,916**
0	Unknown	**8,915**
Csnk2a2	Rattus norvegicus casein kinase 2, alpha prime polypeptide (Csnk2a2), mRNA [NM_001107409]	**8,915**
Pdrg1	Rattus norvegicus p53 and DNA damage regulated 1 (Pdrg1), mRNA [NM_001014762]	**8,912**
Nfx1	Rattus norvegicus nuclear transcription factor, X-box binding 1 (Nfx1), mRNA [NM_001024784]	**8,912**
Mdga2	Rattus norvegicus MAM domain containing glycosylphosphatidylinositol anchor 2 (Mdga2), mRNA [NM_199269]	**8,912**
0	Unknown	**8,911**
RGD1304580	Rattus norvegicus similar to Hypothetical protein MGC38513 (RGD1304580), mRNA [NM_001009283]	**8,911**
Apoe	Rattus norvegicus apolipoprotein E (Apoe), mRNA [NM_138828]	**8,911**
Ogfod1	Rattus norvegicus 2-oxoglutarate and iron-dependent oxygenase domain containing 1 (Ogfod1), mRNA [NM_001107411]	**8,910**
RGD1564809	Uncharacterized protein [Source:UniProtKB/TrEMBL;Acc:D3ZC90] [ENSRNOT00000038184]	**8,909**
Sumo1	Rattus norvegicus SMT3 suppressor of mif two 3 homolog 1 (S. cerevisiae) (Sumo1), mRNA [NM_001009672]	**8,908**
Ctsk	Rattus norvegicus cathepsin K (Ctsk), mRNA [NM_031560]	**8,907**
Myh7	Rattus norvegicus myosin, heavy chain 7, cardiac muscle, beta (Myh7), mRNA [NM_017240]	**8,905**
Os9	Rattus norvegicus osteosarcoma amplified 9 (Os9), mRNA [NM_001007265]	**8,904**
0	Unknown	**8,902**
Mbnl1	Rattus norvegicus muscleblind-like 1 (Drosophila) (Mbnl1), mRNA [NM_001191566]	**8,902**
Nit2	Rattus norvegicus nitrilase family, member 2 (Nit2), mRNA [NM_001034126]	**8,901**
Alkbh4	Rattus norvegicus alkB, alkylation repair homolog 4 (E. coli) (Alkbh4), mRNA [NM_001105920]	**8,901**
0	Unknown	**8,901**
Abcf2	Rattus norvegicus ATP-binding cassette, subfamily F (GCN20), member 2 (Abcf2), nuclear gene encoding mitochondrial protein, mRNA [NM_001109666]	**8,899**
Clic6	Rattus norvegicus chloride intracellular channel 6 (Clic6), nuclear gene encoding mitochondrial protein, mRNA [NM_176078]	**8,899**
Nme3	Rattus norvegicus non-metastatic cells 3, protein expressed in (Nme3), mRNA [NM_053507]	**8,899**
0	Rattus norvegicus similar to MAP/microtubule affinity-regulating kinase 2 isoform a; ELKL motif kinase 1; ELKL motif kinase (LOC290818), mRNA [XM_224940]	**8,896**
RGD1560383	RCG34143Uncharacterized protein [Source:UniProtKB/TrEMBL;Acc:D4AC62] [ENSRNOT00000014414]	**8,896**
Cacna1d	Rattus norvegicus calcium channel, voltage-dependent, L type, alpha 1D subunit (Cacna1d), mRNA [NM_017298]	**8,895**
Podxl	Rattus norvegicus podocalyxin-like (Podxl), mRNA [NM_138848]	**8,893**
LOC100364057	PREDICTED: Rattus norvegicus Btf3l4 protein-like, transcript variant 1 (LOC100364057), mRNA [XM_002726573]	**8,893**
LOC688695	PREDICTED: Rattus norvegicus similar to SON protein (LOC688695), mRNA [XM_001067956]	**8,892**
Ccdc134	Rattus norvegicus coiled-coil domain containing 134 (Ccdc134), mRNA [NM_001024355]	**8,889**

Thap6	THAP domain containing 6 [Source:RefSeq peptide;Acc:NP_001100679] [ENSRNOT00000003431]	**8,888**
Pik3c2b	Rattus norvegicus phosphoinositide-3-kinase, class 2, beta polypeptide (Pik3c2b), mRNA [NM_001105951]	**8,886**
0	Rattus norvegicus similar to glyceraldehyde-3-phosphate dehydrogenase (LOC289434), mRNA [XM_213991]	**8,885**
0	Rattus norvegicus similar to RIKEN cDNA 1700001E04 (LOC363321), mRNA [XM_343661]	**8,885**
0	PREDICTED: Rattus norvegicus 2-deoxyribose-5-phosphate aldolase homolog (C. elegans) (Dera), miscRNA [XR_085824]	**8,884**
Btf3	Rattus norvegicus basic transcription factor 3 (Btf3), mRNA [NM_001008309]	**8,884**
Fam116b	Rattus norvegicus family with sequence similarity 116, member B (Fam116b), mRNA [NM_001108749]	**8,883**
Chic2	Rattus norvegicus cysteine-rich hydrophobic domain 2 (Chic2), mRNA [NM_001105736]	**8,882**
0	Uncharacterized protein [Source:UniProtKB/TrEMBL;Acc:D3ZJK8] [ENSRNOT00000065214]	**8,879**
Ptgds	Rattus norvegicus prostaglandin D2 synthase (brain) (Ptgds), mRNA [NM_013015]	**8,879**
Bbs10	Rattus norvegicus Bardet-Biedl syndrome 10 (Bbs10), mRNA [NM_001109286]	**8,879**
0	Uncharacterized protein [Source:UniProtKB/TrEMBL;Acc:D3ZKR7] [ENSRNOT00000040456]	**8,879**
Lancl1	Rattus norvegicus LanC lantibiotic synthetase component C-like 1 (bacterial) (Lancl1), mRNA [NM_053723]	**8,879**
Gtf2ird1	Rattus norvegicus GTF2I repeat domain containing 1 (Gtf2ird1), mRNA [NM_001001504]	**8,877**
Mcf2l	Rattus norvegicus MCF.2 cell line derived transforming sequence-like (Mcf2l), mRNA [NM_053951]	**8,877**
Tmprss3	Rattus norvegicus transmembrane protease, serine 3 (Tmprss3), mRNA [NM_001107619]	**8,876**
0	AN1-type zinc finger and ubiquitin domain-containing protein 1 [Source:RefSeq peptide;Acc:NP_775454] [ENSRNOT00000043556]	**8,874**
LOC680236	PREDICTED: Rattus norvegicus hypothetical protein LOC680236 (LOC680236), mRNA [XM_001056256]	**8,874**
0	Uncharacterized protein [Source:UniProtKB/TrEMBL;Acc:D3Z944] [ENSRNOT00000052233]	**8,873**
RGD1311910	Rattus norvegicus similar to hypothetical p38 protein (RGD1311910), mRNA [NM_001107373]	**8,872**
Minpp1	Rattus norvegicus multiple inositol polyphosphate histidine phosphatase 1 (Minpp1), mRNA [NM_019263]	**8,872**
Dip2a	Rattus norvegicus DIP2 disco-interacting protein 2 homolog A (Drosophila) (Dip2a), mRNA [NM_001191564]	**8,871**
0	Rattus norvegicus similar to glyceraldehyde-3-phosphate dehydrogenase (phosphorylating) (EC 1.2.1.12) - mouse (LOC288024), mRNA [XM_221353]	**8,870**
Tbce	Rattus norvegicus tubulin folding cofactor E (Tbce), mRNA [NM_001012161]	**8,869**
Sh3rf3	PREDICTED: Rattus norvegicus SH3 multiple domains 4, transcript variant 2 (Sh3md4), mRNA [XM_001054117]	**8,868**
Rbm20	Rattus norvegicus RNA binding motif protein 20 (Rbm20), mRNA [NM_001107611]	**8,868**
Vcpip1	Rattus norvegicus valosin containing protein (p97)/p47 complex interacting protein 1 (Vcpip1), mRNA [NM_176857]	**8,865**
0	Rattus sp. membrane protein-73 mRNA, partial cds; mitochondrial gene for mitochondrial product. [S63519]	**8,864**
0	Rattus norvegicus TL0AAA50YN10 mRNA sequence. [FQ212883]	**8,863**
Ebi3	Rattus norvegicus Epstein-Barr virus induced 3 (Ebi3), mRNA [NM_001109421]	**8,862**
B4galt7	Rattus norvegicus xylosylprotein beta1,4-galactosyltransferase, polypeptide 7 (galactosyltransferase I) (B4galt7), mRNA [NM_001031661]	**8,862**
Gtpbp2	Rattus norvegicus GTP binding protein 2 (Gtpbp2), mRNA [NM_001013225]	**8,860**
Zfp36l3	PREDICTED: Rattus norvegicus zinc finger protein 36, C3H type-like 3 (Zfp36l3), mRNA [XM_001053657]	**8,860**

Znf532	Rattus norvegicus zinc finger protein 532 (Znf532), mRNA [NM_001107382]	**8,859**
Spetex-2F	Rattus norvegicus Spetex-2F protein (Spetex-2F), mRNA [NM_001009968]	**8,859**
Acr	Rattus norvegicus acrosin (Acr), mRNA [NM_012490]	**8,855**
Slc5a1	Rattus norvegicus solute carrier family 5 (sodium/glucose cotransporter), member 1 (Slc5a1), mRNA [NM_013033]	**8,853**
Pttg1	Rattus norvegicus pituitary tumor-transforming 1 (Pttg1), mRNA [NM_022391]	**8,852**
Slc1a1	Rattus norvegicus solute carrier family 1 (neuronal/epithelial high affinity glutamate transporter, system Xag), member 1 (Slc1a1), mRNA [NM_013032]	**8,848**
0	Uncharacterized protein [Source:UniProtKB/TrEMBL;Acc:D3ZKR7] [ENSRNOT00000059076]	**8,846**
RGD1307799	Rattus norvegicus similar to RIKEN cDNA 2400003C14 (RGD1307799), mRNA [NM_001017454]	**8,845**
0	Unknown	**8,843**
Ddx24	Rattus norvegicus DEAD (Asp-Glu-Ala-Asp) box polypeptide 24 (Ddx24), mRNA [NM_199119]	**8,842**
LOC100360849	PREDICTED: Rattus norvegicus hypothetical protein LOC100360849 (LOC100360849), mRNA [XM_002729568]	**8,841**
Fam188a	Rattus norvegicus family with sequence similarity 188, member A (Fam188a), mRNA [NM_001106122]	**8,838**
Olr311	Rattus norvegicus olfactory receptor 311 (Olr311), mRNA [NM_001000555]	**8,837**
39326	Rattus norvegicus septin 7 (Sept7), transcript variant 2, mRNA [NM_001113740]	**8,836**
0	Unknown	**8,836**
Acer3	PREDICTED: Rattus norvegicus phytoceramidase, alkaline (Phca), mRNA [XM_001065019]	**8,836**
RGD1310587	Rattus norvegicus similar to hypothetical protein FLJ14146 (RGD1310587), mRNA [NM_001100857]	**8,833**
0	Rattus norvegicus similar to putative pheromone receptor (LOC302028), mRNA [XM_229568]	**8,831**
0	Unknown	**8,831**
Atox1	Rattus norvegicus ATX1 antioxidant protein 1 homolog (yeast) (Atox1), mRNA [NM_053359]	**8,830**
Nupl1	Rattus norvegicus nucleoporin like 1 (Nupl1), mRNA [NM_139091]	**8,827**
Calca	Rattus norvegicus calcitonin-related polypeptide alpha (Calca), transcript variant 2, mRNA [NM_001033955]	**8,825**
0	Unknown	**8,823**
Pctk2	Rattus norvegicus PCTAIRE protein kinase 2 (Pctk2), mRNA [NM_001108082]	**8,821**
RGD1561102	Rattus norvegicus similar to ribosomal protein S12 (RGD1561102), mRNA [NM_001106777]	**8,821**
Cited1	Rattus norvegicus Cbp/p300-interacting transactivator with Glu/Asp-rich carboxy-terminal domain 1 (Cited1), mRNA [NM_172055]	**8,820**
Uchl1	Rattus norvegicus ubiquitin carboxyl-terminal esterase L1 (ubiquitin thiolesterase) (Uchl1), mRNA [NM_017237]	**8,818**
0	Unknown	**8,818**
RGD1565806	Uncharacterized protein [Source:UniProtKB/TrEMBL;Acc:D4A9L3] [ENSRNOT00000035742]	**8,817**
Ins1	Rattus norvegicus insulin 1 (Ins1), mRNA [NM_019129]	**8,816**
Gucy2d	Rattus norvegicus guanylate cyclase 2d (Gucy2d), mRNA [NM_130737]	**8,815**
Olr1253	Rattus norvegicus olfactory receptor 1253 (Olr1253), mRNA [NM_001000805]	**8,815**
Il3ra	Rattus norvegicus interleukin 3 receptor, alpha (Il3ra), mRNA [NM_139260]	**8,813**

0	PREDICTED: Rattus norvegicus similar to monoacylglycerol O-acyltransferase 2 (LOC685560), mRNA [XM_001064308]	**8,813**
Cyth3	Rattus norvegicus cytohesin 3 (Cyth3), mRNA [NM_053912]	**8,810**
Traf3	Rattus norvegicus Tnf receptor-associated factor 3 (Traf3), mRNA [NM_001108724]	**8,810**
Iqub	Rattus norvegicus IQ motif and ubiquitin domain containing (Iqub), mRNA [NM_001034130]	**8,809**
Cyr61	Rattus norvegicus cysteine-rich, angiogenic inducer, 61 (Cyr61), mRNA [NM_031327]	**8,809**
Zrsr2	Uncharacterized protein [Source:UniProtKB/TrEMBL;Acc:D3ZHQ8] [ENSRNOT00000045300]	**8,808**
0	Unknown	**8,807**
Tpd52l1	Rattus norvegicus tumor protein D52-like 1 (Tpd52l1), mRNA [NM_001044295]	**8,804**
Slc25a46	Rattus norvegicus solute carrier family 25, member 46 (Slc25a46), mRNA [NM_001100515]	**8,802**
Leprotl1	Rattus norvegicus leptin receptor overlapping transcript-like 1 (Leprotl1), mRNA [NM_001013188]	**8,802**
Lgals8	Rattus norvegicus lectin, galactoside-binding, soluble, 8 (Lgals8), mRNA [NM_053862]	**8,801**
0	LRRGT00181 [Source:UniProtKB/TrEMBL;Acc:Q6QI27] [ENSRNOT00000041132]	**8,800**
LOC681820	PREDICTED: Rattus norvegicus hypothetical protein LOC681820 (LOC681820), mRNA [XM_001058575]	**8,800**
LOC691153	Rattus norvegicus hypothetical protein LOC691153 (LOC691153), mRNA [NM_001109627]	**8,800**
Fam134b	Rattus norvegicus family with sequence similarity 134, member B (Fam134b), mRNA [NM_001034912]	**8,799**
Zfp161	Rattus norvegicus zinc finger protein 161 (Zfp161), mRNA [NM_172325]	**8,799**
Kcna5	Rattus norvegicus potassium voltage-gated channel, shaker-related subfamily, member 5 (Kcna5), mRNA [NM_012972]	**8,799**
0	Unknown	**8,798**
Rab11fip2	Rattus norvegicus RAB11 family interacting protein 2 (class I) (Rab11fip2), mRNA [NM_001107447]	**8,798**
Npm3	Uncharacterized protein [Source:UniProtKB/TrEMBL;Acc:D3ZYK9] [ENSRNOT00000023963]	**8,797**
0	immunoglobulin joining chain Gene [Source:MGI Symbol;Acc:MGI:96493] [ENSRNOT00000004866]	**8,797**
Hsd17b8	Rattus norvegicus hydroxysteroid (17-beta) dehydrogenase 8 (Hsd17b8), mRNA [NM_212529]	**8,796**
Atmin	Rattus norvegicus ATM interactor (Atmin), mRNA [NM_001191786]	**8,796**
Casp6	Rattus norvegicus caspase 6 (Casp6), mRNA [NM_031775]	**8,795**
Cacna2d1	Rattus norvegicus calcium channel, voltage-dependent, alpha2/delta subunit 1 (Cacna2d1), transcript variant 2, mRNA [NM_001110848]	**8,794**
LOC684270	PREDICTED: Rattus norvegicus similar to isochorismatase domain containing 2, transcript variant 2 (LOC684270), mRNA [XM_001059344]	**8,794**
0	Unknown	**8,793**
LOC683720	PREDICTED: Rattus norvegicus similar to keratin 6L (LOC683720), mRNA [XM_002726981]	**8,788**
Ripk1	Rattus norvegicus receptor (TNFRSF)-interacting serine-threonine kinase 1 (Ripk1), mRNA [NM_001107350]	**8,788**
0	Unknown	**8,787**
Alg9	Rattus norvegicus asparagine-linked glycosylation 9, alpha-1,2-mannosyltransferase homolog (S. cerevisiae) (Alg9), mRNA [NM_001109000]	**8,786**
Rpl10l	Uncharacterized protein [Source:UniProtKB/TrEMBL;Acc:D4A1P2] [ENSRNOT00000047597]	**8,784**
RGD1566010	PREDICTED: Rattus norvegicus RGD1566010 (RGD1566010), mRNA [XM_001067260]	**8,784**
0	Unknown	**8,783**

Fam96b	Rattus norvegicus family with sequence similarity 96, member B (Fam96b), mRNA [NM_001144854]	**8,782**
Plcb1	Rattus norvegicus phospholipase C, beta 1 (phosphoinositide-specific) (Plcb1), mRNA [NM_001077641]	**8,781**
0	Unknown	**8,780**
Tox	Rattus norvegicus thymocyte selection-associated high mobility group box (Tox), mRNA [NM_001108654]	**8,779**
RGD1305508	PREDICTED: Rattus norvegicus similar to hypothetical protein MGC23280 (RGD1305508), miscRNA [XR_009409]	**8,778**
0	PREDICTED: Rattus norvegicus similar to keratinocytes proline-rich protein, transcript variant 1 (LOC686143), mRNA [XM_001066677]	**8,777**
Dhrsx	Rattus norvegicus dehydrogenase/reductase (SDR family) X-linked (Dhrsx), mRNA [NM_001105914]	**8,777**
Raly	Rattus norvegicus RNA binding protein, autoantigenic (hnRNP-associated with lethal yellow homolog (mouse)) (Raly), mRNA [NM_001011958]	**8,777**
0	Unknown	**8,775**
Gmfb	Rattus norvegicus glia maturation factor, beta (Gmfb), mRNA [NM_031032]	**8,775**
Nr1d2	Rattus norvegicus nuclear receptor subfamily 1, group D, member 2 (Nr1d2), mRNA [NM_147210]	**8,775**
Zfp191	Rattus norvegicus zinc finger protein 191 (Zfp191), mRNA [NM_182955]	**8,773**
0	Unknown	**8,772**
Pgm1	Rattus norvegicus phosphoglucomutase 1 (Pgm1), mRNA [NM_017033]	**8,772**
0	Rattus norvegicus similar to putative protein kinase (LOC308211), mRNA [XM_218001]	**8,771**
Ptcra	Pre T-cell antigen receptor alpha [Source:UniProtKB/Swiss-Prot;Acc:P0C6B3] [ENSRNOT00000067894]	**8,771**
Qsox2	Rattus norvegicus quiescin Q6 sulfhydryl oxidase 2 (Qsox2), mRNA [NM_001109434]	**8,771**
Vcan	Rattus norvegicus versican (Vcan), transcript variant 2, mRNA [NM_053663]	**8,770**
Tnfrsf12a	Rattus norvegicus tumor necrosis factor receptor superfamily, member 12a (Tnfrsf12a), mRNA [NM_181086]	**8,768**
RGD1562310	Rattus norvegicus similar to hypothetical protein FLJ21415 (RGD1562310), mRNA [NM_001109066]	**8,767**
Zbtb33	Rattus norvegicus zinc finger and BTB domain containing 33 (Zbtb33), mRNA [NM_001109314]	**8,765**
Anapc11	Rattus norvegicus anaphase promoting complex subunit 11 (Anapc11), mRNA [NM_001126082]	**8,764**
0	Uncharacterized protein [Source:UniProtKB/TrEMBL;Acc:D3ZKR7] [ENSRNOT00000050021]	**8,764**
Mcfd2	Rattus norvegicus multiple coagulation factor deficiency 2 (Mcfd2), mRNA [NM_139253]	**8,763**
Pipox	Rattus norvegicus pipecolic acid oxidase (Pipox), mRNA [NM_001012009]	**8,761**
Tgm2	Rattus norvegicus transglutaminase 2, C polypeptide (Tgm2), mRNA [NM_019386]	**8,760**
0	Rattus norvegicus similar to glyceraldehyde-3-phosphate dehydrogenase (LOC311121), mRNA [XM_230014]	**8,760**
Akt3	Rattus norvegicus v-akt murine thymoma viral oncogene homolog 3 (protein kinase B, gamma) (Akt3), mRNA [NM_031575]	**8,758**
Tbc1d22b	Rattus norvegicus TBC1 domain family, member 22B (Tbc1d22b), mRNA [NM_001025059]	**8,757**
Asb6	Rattus norvegicus ankyrin repeat and SOCS box-containing 6 (Asb6), mRNA [NM_001011963]	**8,757**
RGD1561890	PREDICTED: Rattus norvegicus similar to craniofacial development protein 1 (RGD1561890), mRNA [XM_233989]	**8,754**
Crtap	Rattus norvegicus cartilage associated protein (Crtap), mRNA [NM_001108785]	**8,754**
Calcb	Rattus norvegicus calcitonin-related polypeptide, beta (Calcb), mRNA [NM_138513]	**8,752**

LOC301193	PREDICTED: Rattus norvegicus similar to Discs large homolog 5 (Placenta and prostate DLG) (Discs large protein P-dlg) (LOC301193), mRNA [XM_002727321]	**8,750**
Pik3r1	Rattus norvegicus phosphoinositide-3-kinase, regulatory subunit 1 (alpha) (Pik3r1), mRNA [NM_013005]	**8,749**
Hs3st6	Rattus norvegicus heparan sulfate (glucosamine) 3-O-sulfotransferase 6 (Hs3st6), mRNA [NM_001109450]	**8,747**
Orc3l	Rattus norvegicus origin recognition complex, subunit 3-like (yeast) (Orc3l), mRNA [NM_001025282]	**8,747**
Peo1	Rattus norvegicus progressive external ophthalmoplegia 1 (Peo1), mRNA [NM_001107599]	**8,747**
Cdc40	Rattus norvegicus cell division cycle 40 homolog (S. cerevisiae) (Cdc40), mRNA [NM_001108538]	**8,746**
Krtap3-1	Uncharacterized protein [Source:UniProtKB/TrEMBL;Acc:D3ZZR8] [ENSRNOT00000016752]	**8,746**
Dbi	Rattus norvegicus LRRGT00046 mRNA, complete cds. [AY383701]	**8,744**
Aqp4	Rattus norvegicus aquaporin 4 (Aqp4), transcript variant 2, mRNA [NM_001142366]	**8,744**
0	Unknown	**8,743**
Neurl1B	Rattus norvegicus neuralized homolog 1B (Drosophila) (Neurl1B), mRNA [NM_001142652]	**8,743**
Lmbr1	Rattus norvegicus limb region 1 homolog (mouse) (Lmbr1), mRNA [NM_001191858]	**8,742**
Tmbim1	Rattus norvegicus transmembrane BAX inhibitor motif containing 1 (Tmbim1), mRNA [NM_001007713]	**8,741**
Arhgap4	Rattus norvegicus Rho GTPase activating protein 4 (Arhgap4), mRNA [NM_144740]	**8,741**
Spint2	Rattus norvegicus serine peptidase inhibitor, Kunitz type, 2 (Spint2), transcript variant 1, mRNA [NM_001082549]	**8,741**
Plekhf1	Rattus norvegicus pleckstrin homology domain containing, family F (with FYVE domain) member 1 (Plekhf1), mRNA [NM_001013148]	**8,740**
0	Unknown	**8,740**
Hist2h3c2	Rattus norvegicus histone cluster 2, H3c2 (Hist2h3c2), mRNA [NM_001107698]	**8,740**
Gm672	Rattus norvegicus gene model 672, (NCBI) (Gm672), mRNA [NM_001108891]	**8,737**
0	Unknown	**8,737**
0	Q2DQC2_9DELT (Q2DQC2) HipA protein, DNA binding regulator, partial (5%) [TC592031]	**8,737**
Pla2g1b	Rattus norvegicus phospholipase A2, group IB, pancreas (Pla2g1b), mRNA [NM_031585]	**8,736**
Rab5a	Rattus norvegicus RAB5A, member RAS oncogene family (Rab5a), mRNA [NM_022692]	**8,735**
Pds5b	Rattus norvegicus PDS5, regulator of cohesion maintenance, homolog B (S. cerevisiae) (Pds5b), transcript variant 1, mRNA [NM_001102383]	**8,735**
Calml3	Rattus norvegicus calmodulin-like 3 (Calml3), mRNA [NM_001012054]	**8,735**
Unc13b	Rattus norvegicus unc-13 homolog B (C. elegans) (Unc13b), transcript variant 2, mRNA [NM_001042579]	**8,734**
Zcchc7	Rattus norvegicus zinc finger, CCHC domain containing 7 (Zcchc7), mRNA [NM_001106658]	**8,734**
0	Nuclear receptor coactivator 3 [Source:UniProtKB/Swiss-Prot;Acc:Q9EPU2] [ENSRNOT00000044234]	**8,730**
Coch	cochlin [Source:RefSeq peptide;Acc:NP_001102180] [ENSRNOT00000007365]	**8,728**
0	Unknown	**8,728**
Tshz1	BC017636 Sdccag33 protein {Mus musculus} (exp=-1; wgp=0; cg=0), partial (27%) [TC593766]	**8,728**
Krt14	Rattus norvegicus keratin 14 (Krt14), mRNA [NM_001008751]	**8,728**
0	Uncharacterized protein [Source:UniProtKB/TrEMBL;Acc:D3ZXM8] [ENSRNOT00000059741]	**8,727**

Cst3	Rattus norvegicus cystatin C (Cst3), mRNA [NM_012837]	**8,727**
Il1rapl1	Rattus norvegicus interleukin 1 receptor accessory protein-like 1 (Il1rapl1), mRNA [NM_177935]	**8,726**
Lyn	Rattus norvegicus v-yes-1 Yamaguchi sarcoma viral related oncogene homolog (Lyn), transcript variant 1, mRNA [NM_030857]	**8,726**
LOC497848	PREDICTED: Rattus norvegicus hypothetical LOC497848 (LOC497848), miscRNA [XR_006411]	**8,725**
0	Cytoplasmic polyadenylation element binding protein 2 (Predicted)Uncharacterized protein [Source:UniProtKB/TrEMBL;Acc:D3ZHK8] [ENSRNOT00000006727]	**8,725**
Gulo	Rattus norvegicus gulonolactone (L-) oxidase (Gulo), mRNA [NM_022220]	**8,724**
0	Unknown	**8,724**
Nsfl1c	Rattus norvegicus NSFL1 (p97) cofactor (p47) (Nsfl1c), mRNA [NM_031981]	**8,723**
Acly	Rattus norvegicus ATP citrate lyase (Acly), transcript variant 1, mRNA [NM_016987]	**8,723**
Znf394	Rattus norvegicus zinc finger protein 394 (Znf394), mRNA [NM_145724]	**8,721**
Adi1	Rattus norvegicus acireductone dioxygenase 1 (Adi1), mRNA [NM_199097]	**8,721**
Ctbp1	Rattus norvegicus C-terminal binding protein 1 (Ctbp1), mRNA [NM_019201]	**8,720**
Aof1	Rattus norvegicus amine oxidase (flavin containing) domain 1 (Aof1), mRNA [NM_001107343]	**8,719**
Fzd9	Rattus norvegicus frizzled homolog 9 (Drosophila) (Fzd9), mRNA [NM_153305]	**8,718**
Myc	Rattus norvegicus myelocytomatosis oncogene (Myc), mRNA [NM_012603]	**8,717**
Stk11ip	Rattus norvegicus serine/threonine kinase 11 interacting protein (Stk11ip), mRNA [NM_001106922]	**8,716**
Derl1	Rattus norvegicus Der1-like domain family, member 1 (Derl1), mRNA [NM_001014202]	**8,713**
Dusp14	Rattus norvegicus dual specificity phosphatase 14 (Dusp14), mRNA [NM_001079893]	**8,713**
Echdc3	Rattus norvegicus enoyl Coenzyme A hydratase domain containing 3 (Echdc3), nuclear gene encoding mitochondrial protein, mRNA [NM_001101010]	**8,712**
LOC688562	PREDICTED: Rattus norvegicus similar to U1 small nuclear ribonucleoprotein C (U1 snRNP protein C) (U1C protein) (U1-C) (LOC688562), mRNA [XM_001067425]	**8,711**
Slc1a5	Rattus norvegicus solute carrier family 1 (neutral amino acid transporter), member 5 (Slc1a5), mRNA [NM_175758]	**8,711**
Tmem44	Uncharacterized protein [Source:UniProtKB/TrEMBL;Acc:D4ADT4] [ENSRNOT00000002353]	**8,711**
0	Unknown	**8,709**
Nmnat3	Rattus norvegicus nicotinamide nucleotide adenylyltransferase 3 (Nmnat3), nuclear gene encoding mitochondrial protein, mRNA [NM_001013224]	**8,709**
Prtn3	Rattus norvegicus proteinase 3 (Prtn3), mRNA [NM_001024264]	**8,706**
Mamdc4	Rattus norvegicus MAM domain containing 4 (Mamdc4), mRNA [NM_145768]	**8,706**
Gap43	Rattus norvegicus growth associated protein 43 (Gap43), mRNA [NM_017195]	**8,705**
LOC296884	Rattus norvegicus cDNA clone IMAGE:7132615. [BC083591]	**8,703**
Zfp426l	Rattus norvegicus zinc finger protein 426-like (Zfp426l), mRNA [NM_001135018]	**8,703**
0	Unknown	**8,702**
0	Rattus norvegicus similar to 60S ribosomal protein L10 (QM protein homolog) (LOC301174), mRNA [XM_236837]	**8,701**

Grid1	Rattus norvegicus glutamate receptor, ionotropic, delta 1 (Grid1), mRNA [NM_024378]	**8,701**
LOC499843	Rattus norvegicus LRRGT00091 (LOC499843), mRNA [NM_001047953]	**8,701**
Impact	Rattus norvegicus imprinted and ancient (Impact), mRNA [NM_001012235]	**8,700**
Sae1	Rattus norvegicus SUMO1 activating enzyme subunit 1 (Sae1), mRNA [NM_001012063]	**8,700**
Gnpda2	Rattus norvegicus glucosamine-6-phosphate deaminase 2 (Gnpda2), mRNA [NM_001106005]	**8,700**
LOC500118	Rattus norvegicus similar to RIKEN cDNA D330028D13 (LOC500118), mRNA [NM_001025771]	**8,700**
0	Unknown	**8,696**
RGD1559985	PREDICTED: Rattus norvegicus similar to chromosome 10 open reading frame 92 (RGD1559985), mRNA [XM_574582]	**8,695**
0	Unknown	**8,694**
0	Uncharacterized protein [Source:UniProtKB/TrEMBL;Acc:D4A283] [ENSRNOT00000007407]	**8,693**
Pdcl	Rattus norvegicus phosducin-like (Pdcl), mRNA [NM_022247]	**8,690**
Tspan12	Rattus norvegicus tetraspanin 12 (Tspan12), mRNA [NM_001015026]	**8,688**
Elmod2	Rattus norvegicus ELMO/CED-12 domain containing 2 (Elmod2), mRNA [NM_001109506]	**8,686**
Adh1	Rattus norvegicus alcohol dehydrogenase 1 (class I) (Adh1), mRNA [NM_019286]	**8,684**
Zkscan3	Rattus norvegicus zinc finger with KRAB and SCAN domains 3 (Zkscan3), mRNA [NM_001012053]	**8,683**
RGD1309374	Uncharacterized protein [Source:UniProtKB/TrEMBL;Acc:D3ZU62] [ENSRNOT00000020492]	**8,683**
Rasa2	Rattus norvegicus RAS p21 protein activator 2 (Rasa2), mRNA [NM_001105724]	**8,682**
Polb	Rattus norvegicus polymerase (DNA directed), beta (Polb), mRNA [NM_017141]	**8,681**
0	Unknown	**8,678**
Clu	Rattus norvegicus clusterin (Clu), mRNA [NM_053021]	**8,678**
Tmem170b	Rattus norvegicus transmembrane protein 170B (Tmem170b), mRNA [NM_001008774]	**8,677**
Trappc3	Rattus norvegicus trafficking protein particle complex 3 (Trappc3), mRNA [NM_001008376]	**8,677**
Ypel3	Rattus norvegicus yippee-like 3 (Drosophila) (Ypel3), mRNA [NM_001135698]	**8,677**
Glipr2	PREDICTED: Rattus norvegicus GLI pathogenesis-related 2 (Glipr2), mRNA [XM_001054584]	**8,677**
Usp20	Rattus norvegicus ubiquitin specific peptidase 20 (Usp20), mRNA [NM_001107827]	**8,674**
RGD1311080	PREDICTED: Rattus norvegicus similar to RIKEN cDNA A930038C07 (RGD1311080), miscRNA [XR_008378]	**8,673**
Immt	Rattus norvegicus inner membrane protein, mitochondrial (Immt), nuclear gene encoding mitochondrial protein, mRNA [NM_001034928]	**8,673**
RGD1305793	Rattus norvegicus similar to hypothetical protein FLJ20154 (RGD1305793), mRNA [NM_001127484]	**8,672**
Ddr2	Rattus norvegicus discoidin domain receptor tyrosine kinase 2 (Ddr2), mRNA [NM_031764]	**8,672**
Camk4	Rattus norvegicus calcium/calmodulin-dependent protein kinase IV (Camk4), mRNA [NM_012727]	**8,670**
0	Unknown	**8,668**
Zfp853	PREDICTED: Rattus norvegicus zinc finger protein 853 (Zfp853), mRNA [XM_002724787]	**8,666**
Cdc37l1	Rattus norvegicus cell division cycle 37 homolog (S. cerevisiae)-like 1 (Cdc37l1), mRNA [NM_001011941]	**8,666**
0	Unknown	**8,663**

Lce1m	Rattus norvegicus late cornified envelope 1M (Lce1m), mRNA [NM_001109500]	**8,663**
LOC363746	PREDICTED: Rattus norvegicus similar to 40S ribosomal protein S7 (S8) (LOC363746), miscRNA [XR_006358]	**8,663**
0	Unknown	**8,661**
Ttc13	Rattus norvegicus tetratricopeptide repeat domain 13 (Ttc13), mRNA [NM_001136162]	**8,656**
Srpk1	Rattus norvegicus SFRS protein kinase 1 (Srpk1), mRNA [NM_001025726]	**8,656**
Adpgk	Rattus norvegicus ADP-dependent glucokinase (Adpgk), mRNA [NM_001100723]	**8,655**
0	Unknown	**8,655**
LOC680262	PREDICTED: Rattus norvegicus hypothetical protein LOC680262, transcript variant 1 (LOC680262), mRNA [XM_001056376]	**8,653**
LOC691543	Rattus norvegicus hypothetical protein LOC691543 (LOC691543), mRNA [NM_001109645]	**8,653**
0	AA996428 UI-R-C0-he-g-09-0-UI.s1 UI-R-C0 Rattus norvegicus cDNA clone UI-R-C0-he-g-09-0-UI 3', mRNA sequence [AA996428]	**8,650**
LOC683402	Rattus norvegicus similar to AP-3 complex subunit sigma-2 (Adapter-related protein complex 3 sigma-2 subunit) (Sigma-adaptin 3b) (AP-3 complex sigma-3B subunit) (Sigma-3B-adaptin) (LOC683402), mRNA [NM_001115039]	**8,649**
Nlk	Rattus norvegicus nemo like kinase (Nlk), mRNA [NM_001191924]	**8,649**
Spata18	Rattus norvegicus spermatogenesis associated 18 (Spata18), mRNA [NM_199374]	**8,648**
Cr1l	Rattus norvegicus complement component (3b/4b) receptor 1-like (Cr1l), transcript variant 3, mRNA [NM_001005265]	**8,647**
0	Unknown	**8,646**
Fam155b	Uncharacterized protein [Source:UniProtKB/TrEMBL;Acc:D4A4E6] [ENSRNOT00000057920]	**8,646**
Lman1	Rattus norvegicus lectin, mannose-binding, 1 (Lman1), mRNA [NM_053886]	**8,645**
0	Unknown	**8,644**
0	Unknown	**8,643**
Tarsl2	Rattus norvegicus threonyl-tRNA synthetase-like 2 (Tarsl2), mRNA [NM_001014020]	**8,643**
0	Unknown	**8,643**
Ldb1	Rattus norvegicus LIM domain binding 1 (Ldb1), mRNA [NM_001107601]	**8,643**
0	Unknown	**8,642**
Tmed9	Rattus norvegicus transmembrane emp24 protein transport domain containing 9 (Tmed9), mRNA [NM_001009703]	**8,641**
Rnf152	Rattus norvegicus ring finger protein 152 (Rnf152), mRNA [NM_001106305]	**8,641**
Kif5a	Rattus norvegicus kinesin family member 5A (Kif5a), mRNA [NM_212523]	**8,640**
Trappc2	Rattus norvegicus trafficking protein particle complex 2 (Trappc2), mRNA [NM_001024965]	**8,639**
RGD1559679	PREDICTED: Rattus norvegicus similar to Interferon alpha-1 precursor (RGD1559679), mRNA [XM_578466]	**8,638**
0	Unknown	**8,638**
Prrg4	Rattus norvegicus proline rich Gla (G-carboxyglutamic acid) 4 (transmembrane) (Prrg4), mRNA [NM_001109203]	**8,636**
Zfp709	Rattus norvegicus zinc finger protein 709 (Zfp709), mRNA [NM_153731]	**8,635**
Bend5	Rattus norvegicus BEN domain containing 5 (Bend5), mRNA [NM_001108672]	**8,635**
Brca2	Rattus norvegicus breast cancer 2 (Brca2), mRNA [NM_031542]	**8,635**

Arl4c	PREDICTED: Rattus norvegicus ADP-ribosylation factor-like 4C (Arl4c), miscRNA [XR_086347]	**8,634**
Btg3	Rattus norvegicus BTG family, member 3 (Btg3), mRNA [NM_019290]	**8,633**
Ccdc56	Rattus norvegicus coiled-coil domain containing 56 (Ccdc56), mRNA [NM_001109047]	**8,631**
0	Unknown	**8,631**
Letm2	Rattus norvegicus leucine zipper-EF-hand containing transmembrane protein 2 (Letm2), nuclear gene encoding mitochondrial protein, mRNA [NM_001012158]	**8,631**
St3gal2	Rattus norvegicus ST3 beta-galactoside alpha-2,3-sialyltransferase 2 (St3gal2), mRNA [NM_031695]	**8,630**
Psmb11	Rattus norvegicus proteasome (prosome, macropain) subunit, beta type, 11 (Psmb11), mRNA [NM_001106032]	**8,629**
Zdhhc15	Rattus norvegicus zinc finger, DHHC-type containing 15 (Zdhhc15), mRNA [NM_001039101]	**8,629**
0	Unknown	**8,629**
Akap2	Rattus norvegicus A kinase (PRKA) anchor protein 2 (Akap2), mRNA [NM_001011974]	**8,629**
Zbtb10	Rattus norvegicus zinc finger and BTB domain containing 10 (Zbtb10), mRNA [NM_024489]	**8,629**
0	Uncharacterized protein [Source:UniProtKB/TrEMBL;Acc:D4A391] [ENSRNOT00000066991]	**8,628**
Ftsjd1	Rattus norvegicus FtsJ methyltransferase domain containing 1 (Ftsjd1), mRNA [NM_001106186]	**8,627**
Duox1	Rattus norvegicus dual oxidase 1 (Duox1), mRNA [NM_153739]	**8,627**
Wdr60	Rattus norvegicus WD repeat domain 60 (Wdr60), mRNA [NM_001191773]	**8,627**
0	Unknown	**8,626**
Cdh29	Uncharacterized protein [Source:UniProtKB/TrEMBL;Acc:D4AAX3] [ENSRNOT00000046259]	**8,626**
Blvrb	Rattus norvegicus biliverdin reductase B (flavin reductase (NADPH)) (Blvrb), mRNA [NM_001106236]	**8,626**
Siah2	Rattus norvegicus seven in absentia 2 (Siah2), mRNA [NM_134457]	**8,625**
0	Unknown	**8,625**
RT1-S3	Rattus norvegicus RT1 class Ib, locus S3 (RT1-S3), mRNA [NM_001008886]	**8,625**
Prr5l	Rattus norvegicus proline rich 5 like (Prr5l), mRNA [NM_001080150]	**8,625**
Ormdl3	Rattus norvegicus ORM1-like 3 (S. cerevisiae) (Ormdl3), mRNA [NM_001047897]	**8,624**
RGD1561582	Uncharacterized protein [Source:UniProtKB/TrEMBL;Acc:D3ZHH6] [ENSRNOT00000006999]	**8,623**
Pcdhgb8	Rattus norvegicus protocadherin gamma subfamily B, 8 (Pcdhgb8), mRNA [NM_001037159]	**8,623**
Rwdd4	Rattus norvegicus RWD domain containing 4A (Rwdd4), mRNA [NM_001034994]	**8,622**
Tmem2	Rattus norvegicus transmembrane protein 2 (Tmem2), mRNA [NM_001107596]	**8,618**
0	Unknown	**8,615**
Cdyl	Rattus norvegicus chromodomain protein, Y-like (Cdyl), mRNA [NM_001014145]	**8,614**
Man1c1	Rattus norvegicus mannosidase, alpha, class 1C, member 1 (Man1c1), mRNA [NM_001108687]	**8,613**
Spry4	Rattus norvegicus sprouty homolog 4 (Drosophila) (Spry4), mRNA [NM_001106150]	**8,613**
Arhgap5	Rattus norvegicus Rho GTPase activating protein 5 (Arhgap5), mRNA [NM_001047869]	**8,611**
Sardh	Rattus norvegicus sarcosine dehydrogenase (Sardh), nuclear gene encoding mitochondrial protein, mRNA [NM_053664]	**8,610**

0	FM095807 etnofat Rattus norvegicus cDNA clone etnofatP0038D05 5', mRNA sequence [FM095807]	**8,610**
Itsn1	Rattus norvegicus intersectin 1 (SH3 domain protein) (Itsn1), transcript variant 2, mRNA [NM_019227]	**8,610**
RT1-T24-2	Rattus norvegicus RT1 class I, locus T24, gene 2 (RT1-T24-2), non-coding RNA [NR_002151]	**8,609**
Recql5	Rattus norvegicus RecQ protein-like 5 (Recql5), mRNA [NM_001105853]	**8,609**
0	Unknown	**8,608**
Rhbdd3	Rattus norvegicus rhomboid domain containing 3 (Rhbdd3), mRNA [NM_001013875]	**8,607**
Ndufs7	Rattus norvegicus NADH dehydrogenase (ubiquinone) Fe-S protein 7 (Ndufs7), nuclear gene encoding mitochondrial protein, mRNA [NM_001008525]	**8,607**
Fkbp3	Rattus norvegicus FK506 binding protein 3 (Fkbp3), mRNA [NM_001106736]	**8,607**
Agps	Rattus norvegicus alkylglycerone phosphate synthase (Agps), mRNA [NM_053350]	**8,607**
Lipogenin	Rattus norvegicus Lipogenin (Lipogenin), mRNA [NM_145790]	**8,606**
Gorasp2	Rattus norvegicus golgi reassembly stacking protein 2 (Gorasp2), mRNA [NM_001007720]	**8,603**
0	Unknown	**8,600**
Tssk4	Rattus norvegicus testis-specific serine kinase 4 (Tssk4), mRNA [NM_001110487]	**8,600**
0	Unknown	**8,600**
Fam115a	Similar to mKIAA0738 protein (Predicted), isoform CRA_aUncharacterized protein [Source:UniProtKB/TrEMBL;Acc:D3Z8A4] [ENSRNOT00000024428]	**8,600**
0	Uncharacterized protein [Source:UniProtKB/TrEMBL;Acc:D3ZE74] [ENSRNOT00000050658]	**8,600**
Foxq1	Rattus norvegicus forkhead box Q1 (Foxq1), mRNA [NM_022858]	**8,598**
Wdr1	Rattus norvegicus WD repeat domain 1 (Wdr1), mRNA [NM_001014135]	**8,598**
Gpc3	Rattus norvegicus glypican 3 (Gpc3), mRNA [NM_012774]	**8,598**
Hiatl1	Rattus norvegicus hippocampus abundant transcript-like 1 (Hiatl1), mRNA [NM_001107334]	**8,597**
0	Suppressor of G2 allele of SKP1 homolog [Source:UniProtKB/Swiss-Prot;Acc:B0BN85] [ENSRNOT00000042606]	**8,597**
0	Uncharacterized protein [Source:UniProtKB/TrEMBL;Acc:D3ZKR7] [ENSRNOT00000059076]	**8,596**
Slc6a6	Rattus norvegicus solute carrier family 6 (neurotransmitter transporter, taurine), member 6 (Slc6a6), mRNA [NM_017206]	**8,596**
0	Kinesin-like protein kif1a [Source:UniProtKB/TrEMBL;Acc:Q2P9S1] [ENSRNOT00000050557]	**8,595**
Cyb561	Rattus norvegicus cytochrome b-561 (Cyb561), mRNA [NM_001107056]	**8,595**
Ly96	Rattus norvegicus lymphocyte antigen 96 (Ly96), mRNA [NM_001024279]	**8,594**
RGD1310348	Rattus norvegicus similar to Ser/Thr-rich protein T10 in DGCR region (RGD1310348), mRNA [NM_001108323]	**8,593**
0	Rattus norvegicus similar to putative homeobox protein (LOC294473), mRNA [XM_228242]	**8,593**
0	Unknown	**8,593**
Rasa4	PREDICTED: Rattus norvegicus similar to Rasa4 protein, transcript variant 1 (RGD1565457), mRNA [XM_002724808]	**8,592**
LOC680716	PREDICTED: Rattus norvegicus hypothetical protein LOC680716 (LOC680716), mRNA [XM_001058554]	**8,592**
Ahnak	Rattus norvegicus AHNAK nucleoprotein (Ahnak), mRNA [NM_001191951]	**8,591**

Nln	Rattus norvegicus neurolysin (metallopeptidase M3 family) (Nln), nuclear gene encoding mitochondrial protein, mRNA [NM_053970]	**8,591**
Ly86	Rattus norvegicus lymphocyte antigen 86 (Ly86), mRNA [NM_001106128]	**8,591**
Mlxipl	Rattus norvegicus MLX interacting protein-like (Mlxipl), mRNA [NM_133552]	**8,591**
Fam55c	Rattus norvegicus family with sequence similarity 55, member C (Fam55c), mRNA [NM_001109435]	**8,590**
Ndufa10l1	Rattus norvegicus NADH dehydrogenase (ubiquinone) 1 alpha subcomplex 10-like 1 (Ndufa10l1), mRNA [NM_182671]	**8,589**
Aqp5	Rattus norvegicus aquaporin 5 (Aqp5), mRNA [NM_012779]	**8,587**
Pcdh17	Rattus norvegicus protocadherin 17 (Pcdh17), mRNA [NM_001107279]	**8,587**
0	Rattus norvegicus similar to RIKEN cDNA 4933431D05 (LOC364949), mRNA [XM_344727]	**8,585**
Prkd3	Rattus norvegicus protein kinase D3 (Prkd3), mRNA [NM_001024263]	**8,584**
Nlrp1a	Rattus norvegicus NLR family, pyrin domain containing 1A (Nlrp1a), mRNA [NM_001145755]	**8,584**
N-pac	Rattus norvegicus cytokine-like nuclear factor n-pac (N-pac), mRNA [NM_001007800]	**8,583**
Kpna1	Rattus norvegicus karyopherin alpha 1 (Kpna1), mRNA [NM_198726]	**8,583**
0	Unknown	**8,583**
Nrp2	Rattus norvegicus neuropilin 2 (Nrp2), mRNA [NM_030869]	**8,582**
Cst11	Rattus norvegicus cystatin 11 (Cst11), mRNA [NM_139085]	**8,582**
Map3k7ip1	Rattus norvegicus mitogen-activated protein kinase kinase kinase 7 interacting protein 1 (Map3k7ip1), mRNA [NM_001109976]	**8,582**
Sfrs11	Rattus norvegicus splicing factor, arginine/serine-rich 11 (Sfrs11), mRNA [NM_001035255]	**8,582**
Ucp2	Rattus norvegicus uncoupling protein 2 (mitochondrial, proton carrier) (Ucp2), nuclear gene encoding mitochondrial protein, mRNA [NM_019354]	**8,582**
LOC100361404	PREDICTED: Rattus norvegicus hypothetical protein LOC100361404 (LOC100361404), mRNA [XM_002729057]	**8,581**
0	Unknown	**8,579**
0	Q3TS78_MOUSE (Q3TS78) In vitro fertilized eggs cDNA, RIKEN full-length enriched library, clone:7420459N23 product:transportin 1, full insert sequence. (Fragment), partial (11%) [TC640129]	**8,578**
RGD1559578	Rattus norvegicus RGD1559578 (RGD1559578), mRNA [NM_001134497]	**8,578**
Pgam5	Rattus norvegicus phosphoglycerate mutase family member 5 (Pgam5), nuclear gene encoding mitochondrial protein, mRNA [NM_001025272]	**8,578**
LOC680555	PREDICTED: Rattus norvegicus similar to EMI domain containing 1 (LOC680555), partial mRNA [XM_001057708]	**8,577**
0	Unknown	**8,576**
Dnajb6	Rattus norvegicus DnaJ (Hsp40) homolog, subfamily B, member 6 (Dnajb6), mRNA [NM_001013209]	**8,575**
Rabac1	Rattus norvegicus Rab acceptor 1 (prenylated) (Rabac1), mRNA [NM_031774]	**8,574**
Mkrn2	Rattus norvegicus makorin, ring finger protein, 2 (Mkrn2), mRNA [NM_001008314]	**8,574**
0	Ig epsilon chain C region [Source:UniProtKB/Swiss-Prot;Acc:P01855] [ENSRNOT00000006912]	**8,573**
Hist1h1b	Rattus norvegicus histone cluster 1, H1b (Hist1h1b), mRNA [NM_001109417]	**8,571**
Ccdc58	Rattus norvegicus coiled-coil domain containing 58 (Ccdc58), mRNA [NM_001105875]	**8,570**
RGD1307235	Rattus norvegicus similar to RIKEN cDNA 2310035C23 (RGD1307235), mRNA [NM_001134546]	**8,569**
Dstn	Rattus norvegicus destrin (Dstn), mRNA [NM_001033666]	**8,569**

Glycam1	Rattus norvegicus glycosylation dependent cell adhesion molecule 1 (Glycam1), mRNA [NM_012794]	**8,567**
0	PREDICTED: Rattus norvegicus SRY-box containing gene 9 (LOC100361122), mRNA [XM_002727836]	**8,564**
LOC501282	PREDICTED: Rattus norvegicus similar to lymphocyte antigen 6 complex, locus E ligand (LOC501282), mRNA [XM_576697]	**8,564**
Sstr3	Rattus norvegicus somatostatin receptor 3 (Sstr3), mRNA [NM_133522]	**8,562**
Ttc3	Rattus norvegicus tetratricopeptide repeat domain 3 (Ttc3), mRNA [NM_001108315]	**8,561**
Dmtf1	Rattus norvegicus cyclin D binding myb-like transcription factor 1 (Dmtf1), mRNA [NM_053693]	**8,560**
0	Unknown	**8,560**
Bmp2k	Uncharacterized protein [Source:UniProtKB/TrEMBL;Acc:D4A0Z6] [ENSRNOT00000060868]	**8,559**
Snrpb	Rattus norvegicus small nuclear ribonucleoprotein polypeptides B and B1 (Snrpb), mRNA [NM_134358]	**8,559**
Unc84b	Uncharacterized protein [Source:UniProtKB/TrEMBL;Acc:D3ZJ67] [ENSRNOT00000046399]	**8,559**
Ppp1r15b	Rattus norvegicus protein phosphatase 1, regulatory (inhibitor) subunit 15b (Ppp1r15b), mRNA [NM_001107175]	**8,558**
RGD1311066	RGD1311066 proteinSimilar to RIKEN cDNA 0610011L14 gene [Source:UniProtKB/TrEMBL;Acc:B1WC03] [ENSRNOT00000027369]	**8,558**
Rpl24	Rattus norvegicus ribosomal protein L24 (Rpl24), mRNA [NM_022515]	**8,556**
RGD1561530	PREDICTED: Rattus norvegicus similar to Tle6 protein (RGD1561530), miscRNA [XR_009018]	**8,555**
0	Similar to intracellular membrane-associated calcium-independent phospholipase A2 gamma (Predicted), isoform CRA_bUncharacterized protein [Source:UniProtKB/TrEMBL;Acc:D3ZRC4] [ENSRNOT00000047296]	**8,555**
MGC94199	Rattus norvegicus similar to RIKEN cDNA 2610301B20; EST AI428449 (MGC94199), mRNA [NM_001007746]	**8,555**
Il8ra	Rattus norvegicus interleukin 8 receptor, alpha (Il8ra), mRNA [NM_019310]	**8,554**
Ribc1	Rattus norvegicus RIB43A domain with coiled-coils 1 (Ribc1), mRNA [NM_001007715]	**8,551**
Mmd	Rattus norvegicus monocyte to macrophage differentiation-associated (Mmd), mRNA [NM_001007673]	**8,551**
Fst	Rattus norvegicus follistatin (Fst), mRNA [NM_012561]	**8,551**
Pla2g2c	Rattus norvegicus phospholipase A2, group IIC (Pla2g2c), mRNA [NM_019202]	**8,550**
Sgtb	Rattus norvegicus small glutamine-rich tetratricopeptide repeat (TPR)-containing, beta (Sgtb), mRNA [NM_181629]	**8,549**
Cyp2c11	Rattus norvegicus cytochrome P450, subfamily 2, polypeptide 11 (Cyp2c11), mRNA [NM_019184]	**8,548**
Ndn	Rattus norvegicus necdin homolog (mouse) (Ndn), mRNA [NM_001008558]	**8,548**
Wdr13	Rattus norvegicus WD repeat domain 13 (Wdr13), mRNA [NM_001108247]	**8,548**
Lrrc38	Rattus norvegicus leucine rich repeat containing 38 (Lrrc38), mRNA [NM_001107991]	**8,547**
Mybphl	Rattus norvegicus myosin binding protein H-like (Mybphl), mRNA [NM_001014042]	**8,547**
Lppr4	Rattus norvegicus lipid phosphate phosphatase-related protein type 4 (Lppr4), mRNA [NM_001001508]	**8,546**
Fam21c	Rattus norvegicus family with sequence similarity 21, member C (Fam21c), mRNA [NM_199207]	**8,545**
Mettl9	Rattus norvegicus methyltransferase like 9 (Mettl9), mRNA [NM_001163164]	**8,544**
Tmie	Rattus norvegicus transmembrane inner ear (Tmie), mRNA [NM_001109299]	**8,541**
Nat9	Rattus norvegicus N-acetyltransferase 9 (GCN5-related, putative) (Nat9), mRNA [NM_001134835]	**8,541**
Ccdc115	coiled-coil domain-containing protein 115 [Source:RefSeq peptide;Acc:NP_001102263] [ENSRNOT00000017722]	**8,541**

Xpo5	Rattus norvegicus exportin 5 (Xpo5), mRNA [NM_001108789]	**8,540**
Cntnap4	Rattus norvegicus contactin associated protein-like 4 (Cntnap4), mRNA [NM_001107432]	**8,540**
LOC690333	Rattus norvegicus hypothetical protein LOC690333 (LOC690333), mRNA [NM_001109580]	**8,538**
Irf9	Rattus norvegicus interferon regulatory factor 9 (Irf9), mRNA [NM_001012041]	**8,538**
Maml1	Rattus norvegicus mastermind like 1 (Drosophila) (Maml1), mRNA [NM_001106997]	**8,538**
LOC500700	PREDICTED: Rattus norvegicus similar to chromosome 14 open reading frame 145 (LOC500700), miscRNA [XR_085864]	**8,537**
0	Uncharacterized protein [Source:UniProtKB/TrEMBL;Acc:D3ZL00] [ENSRNOT00000056427]	**8,536**
Hdgfrp3	Rattus norvegicus hepatoma-derived growth factor, related protein 3 (Hdgfrp3), mRNA [NM_145785]	**8,536**
Gapdh	Rattus norvegicus glyceraldehyde-3-phosphate dehydrogenase (Gapdh), mRNA [NM_017008]	**8,536**
Syne1	Rattus norvegicus candidate plasticity protein 2b (cPG2B) mRNA, complete cds. [AY597251]	**8,535**
Chd1	Rattus norvegicus chromodomain helicase DNA binding protein 1 (Chd1), mRNA [NM_001107465]	**8,534**
Ckap5	Ckap5 protein [Source:UniProtKB/TrEMBL;Acc:Q32Q01] [ENSRNOT00000021837]	**8,533**
Vom2r12	Rattus norvegicus vomeronasal 2 receptor, 12 (Vom2r12), mRNA [NM_001099488]	**8,532**
Jmjd1c	Rattus norvegicus jumonji domain containing 1C (Jmjd1c), mRNA [NM_001191719]	**8,530**
Clcn3	Rattus norvegicus chloride channel 3 (Clcn3), mRNA [NM_053363]	**8,529**
Cant1	Rattus norvegicus calcium activated nucleotidase 1 (Cant1), mRNA [NM_144754]	**8,529**
Ankrd34c	Rattus norvegicus ankyrin repeat domain 34C (Ankrd34c), mRNA [NM_001106845]	**8,528**
Tmco1	Rattus norvegicus transmembrane and coiled-coil domains 1 (Tmco1), mRNA [NM_001009631]	**8,526**
MGC112883	Rattus norvegicus LOC500651 (MGC112883), mRNA [NM_001047743]	**8,526**
Cyp2w1	PREDICTED: Rattus norvegicus cytochrome P450, family 2, subfamily W, polypeptide 1 (Cyp2w1), mRNA [XM_001074391]	**8,525**
0	Unknown	**8,525**
0	Unknown	**8,523**
Eaf1	Rattus norvegicus ELL associated factor 1 (Eaf1), mRNA [NM_001107293]	**8,522**
LOC687056	Rattus norvegicus hypothetical protein LOC687056 (LOC687056), mRNA [NM_001134996]	**8,522**
0	Unknown	**8,518**
Vangl1	Rattus norvegicus vang-like 1 (van gogh, Drosophila) (Vangl1), mRNA [NM_001109584]	**8,518**
Fxyd3	Rattus norvegicus FXYD domain-containing ion transport regulator 3 (Fxyd3), mRNA [NM_172317]	**8,518**
Csmd1	Rattus norvegicus CUB and Sushi multiple domains 1 (Csmd1), mRNA [NM_001037327]	**8,518**
Olr1201	Rattus norvegicus olfactory receptor 1201 (Olr1201), mRNA [NM_001000818]	**8,517**
Jph3	Rattus norvegicus junctophilin 3 (Jph3), mRNA [NM_001107437]	**8,517**
Trappc6a	Rattus norvegicus trafficking protein particle complex 6A (Trappc6a), mRNA [NM_001109410]	**8,517**
0	Unknown	**8,513**
Aldh1l1	Rattus norvegicus aldehyde dehydrogenase 1 family, member L1 (Aldh1l1), mRNA [NM_022547]	**8,512**
Sost	Rattus norvegicus sclerosteosis (Sost), mRNA [NM_030584]	**8,511**

Acadsb	Rattus norvegicus acyl-Coenzyme A dehydrogenase, short/branched chain (Acadsb), nuclear gene encoding mitochondrial protein, mRNA [NM_013084]	**8,511**
Igfbp2	Rattus norvegicus insulin-like growth factor binding protein 2 (Igfbp2), mRNA [NM_013122]	**8,510**
Whamm	Rattus norvegicus WAS protein homolog associated with actin, golgi membranes and microtubules (Whamm), mRNA [NM_001130728]	**8,510**
0	PREDICTED: Rattus norvegicus mCG114897-like (LOC100363987), miscRNA [XR_085743]	**8,508**
LOC685513	Uncharacterized protein [Source:UniProtKB/TrEMBL;Acc:D3ZVY9] [ENSRNOT00000036953]	**8,507**
Prpsap2	Rattus norvegicus phosphoribosyl pyrophosphate synthetase-associated protein 2 (Prpsap2), mRNA [NM_057131]	**8,506**
LOC691692	PREDICTED: Rattus norvegicus hypothetical protein LOC691692 (LOC691692), mRNA [XM_001079285]	**8,505**
Snrpn	Rattus norvegicus small nuclear ribonucleoprotein polypeptide N (Snrpn), mRNA [NM_031117]	**8,501**
Pdcd6ip	Rattus norvegicus programmed cell death 6 interacting protein (Pdcd6ip), mRNA [NM_001029910]	**8,500**
Tll1	Rattus norvegicus tolloid-like 1 (Tll1), mRNA [NM_001106081]	**8,499**
Fgd4	Rattus norvegicus FYVE, RhoGEF and PH domain containing 4 (Fgd4), mRNA [NM_139263]	**8,499**
Rsph1	Rattus norvegicus radial spoke head 1 homolog (Chlamydomonas) (Rsph1), mRNA [NM_001012176]	**8,497**
0	PREDICTED: Rattus norvegicus CG13585-like (LOC100359425), mRNA [XM_002728168]	**8,497**
0	Uncharacterized protein [Source:UniProtKB/TrEMBL;Acc:D4A2S8] [ENSRNOT00000034757]	**8,496**
Snx29	Rattus norvegicus sorting nexin 29 (Snx29), mRNA [NM_001109526]	**8,495**
Magix	Rattus norvegicus MAGI family member, X-linked (Magix), mRNA [NM_001014109]	**8,495**
0	Unknown	**8,494**
Qprt	Rattus norvegicus quinolinate phosphoribosyltransferase (Qprt), mRNA [NM_001009646]	**8,492**
0	Unknown	**8,492**
Ccdc65	Rattus norvegicus coiled-coil domain containing 65 (Ccdc65), mRNA [NM_001014203]	**8,490**
Cltb	Rattus norvegicus clathrin, light chain (Lcb) (Cltb), mRNA [NM_053835]	**8,487**
Fam161a	Rattus norvegicus family with sequence similarity 161, member A (Fam161a), mRNA [NM_001013876]	**8,487**
Plcg1	Rattus norvegicus phospholipase C, gamma 1 (Plcg1), mRNA [NM_013187]	**8,486**
Loxl1	Rattus norvegicus lysyl oxidase-like 1 (Loxl1), mRNA [NM_001012125]	**8,485**
LOC680207	PREDICTED: Rattus norvegicus hypothetical protein LOC680207 (LOC680207), mRNA [XM_001056119]	**8,484**
Zdhhc14	Rattus norvegicus zinc finger, DHHC-type containing 14 (Zdhhc14), mRNA [NM_001039343]	**8,484**
0	Uncharacterized protein [Source:UniProtKB/TrEMBL;Acc:D3ZCX4] [ENSRNOT00000008851]	**8,484**
Mfsd9	Rattus norvegicus major facilitator superfamily domain containing 9 (Mfsd9), mRNA [NM_001108215]	**8,482**
Traf3ip2	Rattus norvegicus Traf3 interacting protein 2 (Traf3ip2), mRNA [NM_001044248]	**8,481**
Tpmt	Rattus norvegicus thiopurine S-methyltransferase (Tpmt), mRNA [NM_001079531]	**8,481**
0	PREDICTED: Rattus norvegicus dynein, axonemal, heavy polypeptide 9, transcript variant 2 (Dnah9), mRNA [XM_002724507]	**8,480**
Zfp189	Rattus norvegicus zinc finger protein 189 (Zfp189), mRNA [NM_001107930]	**8,478**
Dnm2	Rattus norvegicus dynamin 2 (Dnm2), mRNA [NM_013199]	**8,477**

Ap1ar	Rattus norvegicus adaptor-related protein complex 1 associated regulatory protein (Ap1ar), mRNA [NM_001191850]	**8,477**
Unc13a	Rattus norvegicus unc-13 homolog A (C. elegans) (Unc13a), mRNA [NM_022861]	**8,476**
Ahsa2	Rattus norvegicus AHA1, activator of heat shock protein ATPase homolog 2 (yeast) (Ahsa2), mRNA [NM_001107241]	**8,475**
Wnt1	Rattus norvegicus wingless-type MMTV integration site family, member 1 (Wnt1), mRNA [NM_001105714]	**8,474**
Asb2	Rattus norvegicus ankyrin repeat and SOCS box-containing 2 (Asb2), mRNA [NM_001011984]	**8,474**
Fhod1	Rattus norvegicus formin homology 2 domain containing 1 (Fhod1), mRNA [NM_001191600]	**8,474**
Znf518a	Rattus norvegicus zinc finger protein 518A (Znf518a), mRNA [NM_001030038]	**8,472**
Ilk	Rattus norvegicus integrin-linked kinase (Ilk), mRNA [NM_133409]	**8,470**
LOC100174910	Rattus norvegicus glutaredoxin-like protein (LOC100174910), mRNA [NM_001131003]	**8,470**
Ccdc50	Rattus norvegicus coiled-coil domain containing 50 (Ccdc50), mRNA [NM_182736]	**8,469**
Atl2	Rattus norvegicus atlastin GTPase 2 (Atl2), mRNA [NM_001100671]	**8,468**
Plxna3	Rattus norvegicus plexin A3 (Plxna3), mRNA [NM_001107581]	**8,468**
0	Rattus norvegicus similar to mammary tumor virus receptor 2 isoform 2 (LOC361441), mRNA [XM_341720]	**8,468**
Taar8c	Rattus norvegicus trace amine-associated receptor 8c (Taar8c), mRNA [NM_175600]	**8,467**
Xpo6	Rattus norvegicus exportin 6 (Xpo6), mRNA [NM_001011935]	**8,465**
Kptn	kaptin [Source:RefSeq peptide;Acc:NP_001100927] [ENSRNOT00000002043]	**8,465**
Slc7a4	Rattus norvegicus solute carrier family 7 (cationic amino acid transporter, y+ system), member 4 (Slc7a4), mRNA [NM_001107078]	**8,465**
Mapk14	Rattus norvegicus mitogen activated protein kinase 14 (Mapk14), mRNA [NM_031020]	**8,464**
Pycr1	pyrroline-5-carboxylate reductase 1 [Source:RefSeq peptide;Acc:NP_001099327] [ENSRNOT00000054949]	**8,462**
Ndufa10l1	Rattus norvegicus NADH dehydrogenase (ubiquinone) 1 alpha subcomplex 10-like 1 (Ndufa10l1), mRNA [NM_182671]	**8,462**
Sema3a	Rattus norvegicus sema domain, immunoglobulin domain (Ig), short basic domain, secreted, (semaphorin) 3A (Sema3a), mRNA [NM_017310]	**8,461**
Casq2	Rattus norvegicus calsequestrin 2 (cardiac muscle) (Casq2), nuclear gene encoding mitochondrial protein, mRNA [NM_017131]	**8,460**
Phf15	Rattus norvegicus PHD finger protein 15 (Phf15), mRNA [NM_001106998]	**8,460**
RGD1311952	Rattus norvegicus similar to Protein C20orf177 (RGD1311952), mRNA [NM_001134551]	**8,459**
LOC300024	Lymphocyte antigen 6B [Source:UniProtKB/Swiss-Prot;Acc:Q63317] [ENSRNOT00000009395]	**8,459**
Serinc3	Rattus norvegicus serine incorporator 3 (Serinc3), mRNA [NM_001008312]	**8,457**
Srp68	Rattus norvegicus signal recognition particle 68 (Srp68), mRNA [NM_001108840]	**8,457**
Adcy3	Rattus norvegicus adenylate cyclase 3 (Adcy3), mRNA [NM_130779]	**8,456**
Zcchc4	Rattus norvegicus zinc finger, CCHC domain containing 4 (Zcchc4), mRNA [NM_001108360]	**8,456**
Ctsr	Rattus norvegicus cathepsin R (Ctsr), mRNA [NM_175581]	**8,456**
Fam35a	Rattus norvegicus family with sequence similarity 35, member A (Fam35a), mRNA [NM_001025028]	**8,455**
Mcm5	Rattus norvegicus minichromosome maintenance complex component 5 (Mcm5), mRNA [NM_001106170]	**8,454**
Bspry	Rattus norvegicus B-box and SPRY domain containing (Bspry), mRNA [NM_022261]	**8,453**
Tubgcp6	Rattus norvegicus tubulin, gamma complex associated protein 6 (Tubgcp6), mRNA [NM_001108748]	**8,453**

Dnase2a	Rattus norvegicus deoxyribonuclease II alpha (Dnase2a), mRNA [NM_138539]	**8,453**
Rwdd2b	Rattus norvegicus RWD domain containing 2B (Rwdd2b), mRNA [NM_001100559]	**8,453**
RGD1565192	Rattus norvegicus similar to 1810013D10Rik protein (RGD1565192), mRNA [NM_001134639]	**8,453**
0	Unknown	**8,452**
Rbak	Rattus norvegicus RB-associated KRAB zinc finger (Rbak), mRNA [NM_001191664]	**8,452**
Olr1557	Rattus norvegicus olfactory receptor 1557 (Olr1557), mRNA [NM_001000050]	**8,450**
LOC691889	PREDICTED: Rattus norvegicus similar to ATPase, aminophospholipid transporter-like, class I, type 8A, member 2 (LOC691889), miscRNA [XR_086029]	**8,449**
RGD1306520	Rattus norvegicus similar to receptor-interacting factor 1 (RGD1306520), mRNA [NM_001127485]	**8,448**
0	cadherin, EGF LAG seven-pass G-type receptor 1 (flamingo homolog, Drosophila) Gene [Source:MGI Symbol;Acc:MGI:1100883] [ENSRNOT00000036025]	**8,447**
RGD1308049	Uncharacterized protein [Source:UniProtKB/TrEMBL;Acc:D3ZT97] [ENSRNOT00000028509]	**8,446**
0	Unknown	**8,443**
LOC308198	PREDICTED: Rattus norvegicus similar to serine/threonine kinase (LOC308198), partial mRNA [XM_217963]	**8,441**
0	Rattus norvegicus similar to glyceraldehyde-3-phosphate dehydrogenase (phosphorylating) (EC 1.2.1.12) - mouse (LOC300277), mRNA [XM_217067]	**8,441**
Anapc1	Rattus norvegicus anaphase promoting complex subunit 1 (Anapc1), mRNA [NM_001107771]	**8,440**
0	Uncharacterized protein [Source:UniProtKB/TrEMBL;Acc:D3ZES0] [ENSRNOT00000031521]	**8,440**
LOC680155	Uncharacterized protein [Source:UniProtKB/TrEMBL;Acc:D3ZXU7] [ENSRNOT00000020271]	**8,439**
Calcrl	Rattus norvegicus calcitonin receptor-like (Calcrl), mRNA [NM_012717]	**8,439**
Znrd1	Rattus norvegicus zinc ribbon domain containing, 1 (Znrd1), transcript variant 2, mRNA [NM_213567]	**8,438**
Dck	Rattus norvegicus deoxycytidine kinase (Dck), mRNA [NM_024158]	**8,437**
0	Unknown	**8,436**
Plekha4	Rattus norvegicus pleckstrin homology domain containing, family A (phosphoinositide binding specific) member 4 (Plekha4), mRNA [NM_199101]	**8,436**
Brd2	Rattus norvegicus bromodomain containing 2 (Brd2), mRNA [NM_212495]	**8,435**
Fcrla	Rattus norvegicus Fc receptor-like A (Fcrla), mRNA [NM_001100682]	**8,434**
LOC691113	RCG56346Uncharacterized protein [Source:UniProtKB/TrEMBL;Acc:D3Z8R8] [ENSRNOT00000015807]	**8,434**
Hcn4	Rattus norvegicus hyperpolarization activated cyclic nucleotide-gated potassium channel 4 (Hcn4), mRNA [NM_021658]	**8,433**
0	Unknown	**8,433**
0	Unknown	**8,432**
Sertad4	Rattus norvegicus SERTA domain containing 4 (Sertad4), mRNA [NM_001108351]	**8,432**
Nuak1	Rattus norvegicus NUAK family, SNF1-like kinase, 1 (Nuak1), mRNA [NM_001106774]	**8,432**
LOC683538	Uncharacterized protein [Source:UniProtKB/TrEMBL;Acc:D3ZNK1] [ENSRNOT00000061761]	**8,431**

Svep1	Sushi, von Willebrand factor type A, EGF and pentraxin domain-containing protein 1 [Source:UniProtKB/Swiss-Prot;Acc:P0C6B8] [ENSRNOT00000047200]	**8,429**
Fktn	Rattus norvegicus fukutin (Fktn), mRNA [NM_001108667]	**8,429**
Agl	Rattus norvegicus amylo-1,6-glucosidase, 4-alpha-glucanotransferase (Agl), mRNA [NM_001108564]	**8,429**
LOC690576	PREDICTED: Rattus norvegicus similar to RIKEN cDNA 4930555G01 (RGD1564665), partial mRNA [XM_002727373]	**8,428**
Ggcx	Rattus norvegicus gamma-glutamyl carboxylase (Ggcx), mRNA [NM_031756]	**8,426**
Mmp25	PREDICTED: Rattus norvegicus matrix metalloproteinase 25 (Mmp25), mRNA [XM_002742434]	**8,424**
Echdc2	Rattus norvegicus enoyl Coenzyme A hydratase domain containing 2 (Echdc2), mRNA [NM_001106675]	**8,424**
0	Unknown	**8,423**
Zfp36	Rattus norvegicus zinc finger protein 36 (Zfp36), mRNA [NM_133290]	**8,422**
0	Unknown	**8,421**
Snw1	Rattus norvegicus SNW domain containing 1 (Snw1), mRNA [NM_001109279]	**8,420**
RGD1565283	Rattus norvegicus similar to novel protein (RGD1565283), mRNA [NM_001109074]	**8,416**
Dynll2	Rattus norvegicus dynein light chain LC8-type 2 (Dynll2), mRNA [NM_080697]	**8,415**
0	AB017635 ERCC4 {Cricetulus griseus} (exp=-1; wgp=0; cg=0), partial (26%) [TC614683]	**8,414**
0	Uncharacterized protein [Source:UniProtKB/TrEMBL;Acc:D3ZAG8] [ENSRNOT00000037316]	**8,414**
Crlf1	Rattus norvegicus cytokine receptor-like factor 1 (Crlf1), mRNA [NM_001106074]	**8,414**
Me1	Rattus norvegicus malic enzyme 1, NADP(+)-dependent, cytosolic (Me1), mRNA [NM_012600]	**8,412**
Foxo4	Rattus norvegicus forkhead box O4 (Foxo4), mRNA [NM_001106943]	**8,412**
Cpd	Rattus norvegicus carboxypeptidase D (Cpd), mRNA [NM_012836]	**8,410**
RGD1563982	Rattus norvegicus similar to F-box only protein 27 (RGD1563982), mRNA [NM_001110491]	**8,409**
Timm22	PREDICTED: Rattus norvegicus translocase of inner mitochondrial membrane 22 homolog (yeast) (Timm22), mRNA [XM_001080619]	**8,408**
Mcf2	PREDICTED: Rattus norvegicus MCF.2 cell line derived transforming sequence (Mcf2), mRNA [XM_002727688]	**8,408**
LOC100361198	PREDICTED: Rattus norvegicus rCG43589-like (LOC100361198), mRNA [XM_002730083]	**8,407**
Ptprh	Rattus norvegicus protein tyrosine phosphatase, receptor type, H (Ptprh), mRNA [NM_001191945]	**8,407**
Lpgat1	Rattus norvegicus lysophosphatidylglycerol acyltransferase 1 (Lpgat1), mRNA [NM_001109376]	**8,406**
Cabp7	Rattus norvegicus calcium binding protein 7 (Cabp7), mRNA [NM_001007730]	**8,403**
Lrp11	Rattus norvegicus low density lipoprotein receptor-related protein 11 (Lrp11), mRNA [NM_001106217]	**8,403**
Ptprt	Rattus norvegicus protein tyrosine phosphatase, receptor type, T (Ptprt), mRNA [NM_001108603]	**8,401**
RGD1310852	Rattus norvegicus similar to RIKEN cDNA 9130401M01 (RGD1310852), mRNA [NM_001025007]	**8,400**
Gpr20	Rattus norvegicus G protein-coupled receptor 20 (Gpr20), mRNA [NM_022216]	**8,400**
Xpa	Rattus norvegicus xeroderma pigmentosum, complementation group A (Xpa), mRNA [NM_001106656]	**8,398**
0	Uncharacterized protein [Source:UniProtKB/TrEMBL;Acc:D3ZA96] [ENSRNOT00000034797]	**8,398**
Znf592	Rattus norvegicus zinc finger protein 592 (Znf592), mRNA [NM_001106272]	**8,397**

Rnf187	Rattus norvegicus ring finger protein 187 (Rnf187), mRNA [NM_001164264]	**8,395**
Qsox1	Rattus norvegicus quiescin Q6 sulfhydryl oxidase 1 (Qsox1), transcript variant 2, mRNA [NM_053431]	**8,393**
Slfnl1	Rattus norvegicus schlafen-like 1 (Slfnl1), mRNA [NM_001024347]	**8,392**
Kat5	Rattus norvegicus K(lysine) acetyltransferase 5 (Kat5), mRNA [NM_001005872]	**8,391**
RGD1565983	Rattus norvegicus similar to apurinic/apyrimidinic endonuclease 2 (RGD1565983), mRNA [NM_001079892]	**8,387**
Tbc1d30	Uncharacterized protein [Source:UniProtKB/TrEMBL;Acc:D3ZG06] [ENSRNOT00000024679]	**8,387**
Dnajc17	Rattus norvegicus DnaJ (Hsp40) homolog, subfamily C, member 17 (Dnajc17), mRNA [NM_001191740]	**8,387**
LOC497848	PREDICTED: Rattus norvegicus hypothetical LOC497848 (LOC497848), miscRNA [XR_007272]	**8,386**
Rapgef2	Rattus norvegicus Rap guanine nucleotide exchange factor (GEF) 2 (Rapgef2), mRNA [NM_001107684]	**8,386**
RGD1560672	Rattus norvegicus similar to novel protein (RGD1560672), mRNA [NM_001109073]	**8,386**
0	Uncharacterized protein [Source:UniProtKB/TrEMBL;Acc:D3Z995] [ENSRNOT00000022941]	**8,385**
Gpr182	Rattus norvegicus G protein-coupled receptor 182 (Gpr182), mRNA [NM_053302]	**8,384**
Wdr44	WD repeat-containing protein 44 [Source:UniProtKB/Swiss-Prot;Acc:Q9R037] [ENSRNOT00000040742]	**8,384**
0	Unknown	**8,383**
Unc119b	Rattus norvegicus unc-119 homolog B (C. elegans) (Unc119b), mRNA [NM_001105934]	**8,382**
Trpm3	Rattus norvegicus transient receptor potential cation channel, subfamily M, member 3 (Trpm3), mRNA [NM_001191562]	**8,381**
Zfp40	Rattus norvegicus zinc finger protein 40 (Zfp40), mRNA [NM_001168642]	**8,380**
Rbl2	Rattus norvegicus retinoblastoma-like 2 (Rbl2), mRNA [NM_031094]	**8,378**
Serpina5	Rattus norvegicus serine (or cysteine) peptidase inhibitor, clade A, member 5 (Serpina5), mRNA [NM_022957]	**8,377**
Gfer	Rattus norvegicus growth factor, augmenter of liver regeneration (Gfer), mRNA [NM_013222]	**8,377**
Hspa4	Rattus norvegicus heat shock protein 4 (Hspa4), mRNA [NM_153629]	**8,377**
Psd2	Rattus norvegicus pleckstrin and Sec7 domain containing 2 (Psd2), mRNA [NM_001107395]	**8,377**
0	Unknown	**8,377**
Gpcpd1	Rattus norvegicus glycerophosphocholine phosphodiesterase GDE1 homolog (S. cerevisiae) (Gpcpd1), mRNA [NM_198779]	**8,377**
Tdrkh	Rattus norvegicus tudor and KH domain containing (Tdrkh), mRNA [NM_001014038]	**8,375**
Ubtf	Rattus norvegicus upstream binding transcription factor, RNA polymerase I (Ubtf), transcript variant 2, mRNA [NM_001127690]	**8,375**
Olr160	Rattus norvegicus olfactory receptor 160 (Olr160), mRNA [NM_001000738]	**8,375**
0	Rattus norvegicus similar to heterogeneous nuclear ribonucleoprotein K (LOC294952), mRNA [XM_227009]	**8,374**
Cadps	Rattus norvegicus Ca++-dependent secretion activator (Cadps), mRNA [NM_013219]	**8,374**
Mrpl45	Rattus norvegicus mitochondrial ribosomal protein L45 (Mrpl45), nuclear gene encoding mitochondrial protein, mRNA [NM_001105834]	**8,373**
Slc8a1	Rattus norvegicus solute carrier family 8 (sodium/calcium exchanger), member 1 (Slc8a1), mRNA [NM_019268]	**8,373**
0	Unknown	**8,372**
0	Unknown	**8,370**
Pi4kb	Rattus norvegicus phosphatidylinositol 4-kinase, catalytic, beta (Pi4kb), mRNA [NM_031083]	**8,370**

Cttn	Rattus norvegicus cortactin (Cttn), mRNA [NM_021868]	**8,370**
Homer2	Rattus norvegicus homer homolog 2 (Drosophila) (Homer2), mRNA [NM_053309]	**8,369**
0	Unknown	**8,369**
Fam124b	RCG64106Uncharacterized protein [Source:UniProtKB/TrEMBL;Acc:D4ACJ9] [ENSRNOT00000030626]	**8,368**
0	Unknown	**8,366**
RGD1564599	Rattus norvegicus hypothetical protein LOC689600 (LOC689600), mRNA [NM_001106351]	**8,366**
0	Rattus norvegicus similar to glutamine repeat protein 1 (LOC365129), mRNA [XM_344830]	**8,366**
Ddit4l	Rattus norvegicus DNA-damage-inducible transcript 4-like (Ddit4l), mRNA [NM_080399]	**8,366**
Rcan3	Rattus norvegicus RCAN family member 3 (Rcan3), mRNA [NM_001012746]	**8,365**
Msh5	Rattus norvegicus mutS homolog 5 (E. coli) (Msh5), mRNA [NM_212536]	**8,365**
0	Unknown	**8,364**
Actn1	Rattus norvegicus actinin, alpha 1 (Actn1), mRNA [NM_031005]	**8,364**
0	Uncharacterized protein [Source:UniProtKB/TrEMBL;Acc:D3ZKL6] [ENSRNOT00000044160]	**8,362**
Mpzl2	Rattus norvegicus myelin protein zero-like 2 (Mpzl2), mRNA [NM_001106818]	**8,362**
Snurf	Rattus norvegicus SNRPN upstream reading frame (Snurf), mRNA [NM_130738]	**8,362**
Tbx1	Rattus norvegicus T-box 1 (Tbx1), mRNA [NM_001108322]	**8,361**
0	Unknown	**8,361**
RGD1566265	Rattus norvegicus similar to RIKEN cDNA 2610002M06 (RGD1566265), mRNA [NM_001134589]	**8,361**
Kdm2b	Rattus norvegicus lysine (K)-specific demethylase 2B (Kdm2b), mRNA [NM_001100679]	**8,360**
Alg12	Rattus norvegicus asparagine-linked glycosylation 12 homolog (yeast, alpha-1,6-mannosyltransferase) (Alg12), mRNA [NM_001108104]	**8,359**
Txnrd1	Rattus norvegicus thioredoxin reductase 1 (Txnrd1), mRNA [NM_031614]	**8,358**
Dmtf1	Rattus norvegicus cyclin D binding myb-like transcription factor 1 (Dmtf1), mRNA [NM_053693]	**8,357**
Ubtf	Rattus norvegicus upstream binding transcription factor, RNA polymerase I (Ubtf), transcript variant 1, mRNA [NM_001105723]	**8,357**
Palld	Rattus norvegicus similar to palladin; CGI-151 protein, mRNA (cDNA clone MGC:94544 IMAGE:7189104), complete cds. [BC088409]	**8,356**
Slc45a1	Rattus norvegicus solute carrier family 45, member 1 (Slc45a1), mRNA [NM_144747]	**8,356**
Tmem144	Rattus norvegicus transmembrane protein 144 (Tmem144), mRNA [NM_001108551]	**8,356**
0	Nuclease-sensitive element-binding protein 1 [Source:UniProtKB/Swiss-Prot;Acc:P62961] [ENSRNOT00000058700]	**8,356**
0	Uncharacterized protein [Source:UniProtKB/TrEMBL;Acc:D4ADJ6] [ENSRNOT00000026340]	**8,354**
Nsmce2	Rattus norvegicus non-SMC element 2, MMS21 homolog (S. cerevisiae) (Nsmce2), mRNA [NM_001024876]	**8,353**
Trpc1	Rattus norvegicus transient receptor potential cation channel, subfamily C, member 1 (Trpc1), mRNA [NM_053558]	**8,353**
Eci2	Rattus norvegicus enoyl-Coenzyme A delta isomerase 2 (Eci2), mRNA [NM_001006966]	**8,352**
Piga	Rattus norvegicus phosphatidylinositol glycan anchor biosynthesis, class A (Piga), mRNA [NM_001108816]	**8,352**
LOC498705	Rattus norvegicus hypothetical protein LOC498705 (LOC498705), mRNA [NM_001047934]	**8,351**
Col11a2	Rattus norvegicus collagen, type XI, alpha 2 (Col11a2), mRNA [NM_212528]	**8,350**

Oxt	Rat oxytocin mRNA, complete cds. [M25649]	**8,350**
0	Uncharacterized protein [Source:UniProtKB/TrEMBL;Acc:D3ZW22] [ENSRNOT00000037429]	**8,349**
Lclat1	Similar to lysocardiolipin acyltransferase isoform 1 (Predicted)Uncharacterized protein [Source:UniProtKB/TrEMBL;Acc:D3ZFF4] [ENSRNOT00000043641]	**8,348**
0	Rattus norvegicus similar to glyceraldehyde-3-phosphate dehydrogenase (LOC289349), mRNA [XM_223044]	**8,347**
0	Unknown	**8,347**
Zfp9	Rattus norvegicus zinc finger protein 9 (Zfp9), mRNA [NM_001127635]	**8,345**
Tinf2	Rattus norvegicus TERF1 (TRF1)-interacting nuclear factor 2 (Tinf2), mRNA [NM_001006962]	**8,342**
0	Uncharacterized protein [Source:UniProtKB/TrEMBL;Acc:D3ZZD9] [ENSRNOT00000068382]	**8,341**
Etaa1	Rattus norvegicus Ewing tumor-associated antigen 1 (Etaa1), mRNA [NM_001109094]	**8,341**
Taf5l	Rattus norvegicus TAF5-like RNA polymerase II, p300/CBP-associated factor (PCAF)-associated factor (Taf5l), mRNA [NM_001107442]	**8,341**
RGD1563440	Rattus norvegicus similar to hypothetical protein (RGD1563440), mRNA [NM_001100965]	**8,341**
Olr775	Rattus norvegicus olfactory receptor 775 (Olr775), mRNA [NM_001000374]	**8,340**
Prep	Rattus norvegicus prolyl endopeptidase (Prep), mRNA [NM_031324]	**8,340**
Olr1303	Rattus norvegicus olfactory receptor 1303 (Olr1303), mRNA [NM_001000796]	**8,339**
Ccnt2	Rattus norvegicus cyclin T2 (Ccnt2), mRNA [NM_001107171]	**8,338**
Fosl2	Rattus norvegicus fos-like antigen 2 (Fosl2), mRNA [NM_012954]	**8,338**
Itfg1	Rattus norvegicus integrin alpha FG-GAP repeat containing 1 (Itfg1), mRNA [NM_133557]	**8,338**
Farp1	Rattus norvegicus FERM, RhoGEF (Arhgef) and pleckstrin domain protein 1 (chondrocyte-derived) (Farp1), mRNA [NM_001107287]	**8,336**
Tubg1	Rattus norvegicus tubulin, gamma 1 (Tubg1), mRNA [NM_145778]	**8,336**
Prl8a3	Rattus norvegicus prolactin family 8, subfamily a, member 3 (Prl8a3), mRNA [NM_020079]	**8,336**
Ogt	Rattus norvegicus O-linked N-acetylglucosamine (GlcNAc) transferase (UDP-N-acetylglucosamine:polypeptide-N-acetylglucosaminyl transferase) (Ogt), mRNA [NM_017107]	**8,335**
Yars	Rattus norvegicus tyrosyl-tRNA synthetase (Yars), mRNA [NM_001025696]	**8,335**
Gss	Rattus norvegicus glutathione synthetase (Gss), mRNA [NM_012962]	**8,334**
LOC679989	PREDICTED: Rattus norvegicus similar to t-complex 11 protein (LOC679989), mRNA [XM_001055198]	**8,331**
Ccdc126	Rattus norvegicus coiled-coil domain containing 126 (Ccdc126), mRNA [NM_001109232]	**8,330**
0	Uncharacterized protein [Source:UniProtKB/TrEMBL;Acc:D3ZER7] [ENSRNOT00000043011]	**8,330**
Wwp2	Rattus norvegicus WW domain containing E3 ubiquitin protein ligase 2 (Wwp2), mRNA [NM_001106184]	**8,329**
C1d	Rattus norvegicus C1D nuclear receptor co-repressor (C1d), mRNA [NM_001106021]	**8,327**
Podxl2	Rattus norvegicus podocalyxin-like 2 (Podxl2), mRNA [NM_001106607]	**8,327**
0	Uncharacterized protein [Source:UniProtKB/TrEMBL;Acc:D3ZJS8] [ENSRNOT00000037641]	**8,327**
Rwdd1	Rattus norvegicus RWD domain containing 1 (Rwdd1), mRNA [NM_147146]	**8,327**
Fam164a	Rattus norvegicus family with sequence similarity 164, member A (Fam164a), mRNA [NM_001107661]	**8,327**

Crlf1	Rattus norvegicus cytokine receptor-like factor 1 (Crlf1), mRNA [NM_001106074]	**8,323**
Tmem81	Rattus norvegicus transmembrane protein 81 (Tmem81), mRNA [NM_001017490]	**8,322**
Spast	Rattus norvegicus spastin (Spast), mRNA [NM_001108702]	**8,321**
0	Unknown	**8,321**
Tpmt	thiopurine S-methyltransferase [Source:RefSeq peptide;Acc:NP_001072999] [ENSRNOT00000022085]	**8,321**
Ccdc102a	Rattus norvegicus coiled-coil domain containing 102A (Ccdc102a), mRNA [NM_001108437]	**8,319**
0	Unknown	**8,318**
Adamts15	Rattus norvegicus ADAM metallopeptidase with thrombospondin type 1 motif, 15 (Adamts15), mRNA [NM_001106810]	**8,317**
0	Unknown	**8,317**
0	Neuronal cell adhesion molecule [Source:UniProtKB/Swiss-Prot;Acc:P97686] [ENSRNOT00000059737]	**8,316**
Gns	Rattus norvegicus glucosamine (N-acetyl)-6-sulfatase (Gns), mRNA [NM_001011989]	**8,314**
Pde3b	Rattus norvegicus phosphodiesterase 3B, cGMP-inhibited (Pde3b), mRNA [NM_017229]	**8,311**
Pard6b	Rattus norvegicus par-6 (partitioning defective 6) homolog beta (C. elegans) (Pard6b), mRNA [NM_001108609]	**8,309**
0	Band 4.1-like protein 4B [Source:UniProtKB/Swiss-Prot;Acc:B2RYE5] [ENSRNOT00000014982]	**8,306**
Anxa6	Rattus norvegicus annexin A6 (Anxa6), mRNA [NM_024156]	**8,306**
0	RVL9223 Wackym-Soares normalized rat vestibular cDNA library Rattus norvegicus cDNA 5', mRNA sequence [DV718613]	**8,306**
LOC689412	Rattus norvegicus similar to CG4025-PA (LOC689412), mRNA [NM_001109538]	**8,302**
Isyna1	Rattus norvegicus inositol-3-phosphate synthase 1 (Isyna1), mRNA [NM_001013880]	**8,302**
Ube2b	Rattus norvegicus ubiquitin-conjugating enzyme E2B (RAD6 homolog, S. cerevisiae) (Ube2b), mRNA [NM_031138]	**8,301**
Resp18	Rattus norvegicus regulated endocrine-specific protein 18 (Resp18), mRNA [NM_019278]	**8,301**
Nr3c1	Rattus norvegicus nuclear receptor subfamily 3, group C, member 1 (Nr3c1), mRNA [NM_012576]	**8,300**
Asah1	Rattus norvegicus N-acylsphingosine amidohydrolase (acid ceramidase) 1 (Asah1), mRNA [NM_053407]	**8,299**
0	Unknown	**8,298**
Gtpbp4	Rattus norvegicus GTP binding protein 4 (Gtpbp4), mRNA [NM_053689]	**8,297**
Pomt2	Rattus norvegicus protein-O-mannosyltransferase 2 (Pomt2), mRNA [NM_001047114]	**8,293**
MGC94891	Rattus norvegicus hypothetical protein LOC681210 (MGC94891), mRNA [NM_001044277]	**8,292**
RGD1562351	Rattus norvegicus similar to chromosome 7 open reading frame 23 (RGD1562351), mRNA [NM_001109219]	**8,292**
Olr1338	Rattus norvegicus olfactory receptor 1338 (Olr1338), mRNA [NM_001000789]	**8,290**
Cep68	Uncharacterized protein [Source:UniProtKB/TrEMBL;Acc:D3ZZ61] [ENSRNOT00000039523]	**8,290**
Irf1	Rattus norvegicus interferon regulatory factor 1 (Irf1), mRNA [NM_012591]	**8,290**
Capns1	Rattus norvegicus calpain, small subunit 1 (Capns1), mRNA [NM_017118]	**8,289**
Ccar1	Rattus norvegicus cell division cycle and apoptosis regulator 1 (Ccar1), mRNA [NM_001108535]	**8,289**
LOC690479	Uncharacterized protein [Source:UniProtKB/TrEMBL;Acc:D3ZTZ5] [ENSRNOT00000034798]	**8,287**
Hcfc1	Rattus norvegicus host cell factor C1 (Hcfc1), mRNA [NM_001139507]	**8,287**

Dcp1b	Similar to decapping enzyme Dcp1b (Predicted), isoform CRA_aUncharacterized protein [Source:UniProtKB/TrEMBL;Acc:D3ZZJ7] [ENSRNOT00000009832]	**8,285**
Dus4l	Rattus norvegicus dihydrouridine synthase 4-like (S. cerevisiae) (Dus4l), mRNA [NM_001135803]	**8,285**
0	Unknown	**8,284**
0	Unknown	**8,284**
Stk36	Rattus norvegicus serine/threonine kinase 36 (fused homolog, Drosophila) (Stk36), mRNA [NM_001173986]	**8,282**
Slc7a10	Rattus norvegicus solute carrier family 7, (neutral amino acid transporter, y+ system) member 10 (Slc7a10), mRNA [NM_053726]	**8,282**
0	Unknown	**8,282**
Pomt2	Rattus norvegicus protein-O-mannosyltransferase 2 (Pomt2), mRNA [NM_001047114]	**8,282**
Gjc2	Rattus norvegicus gap junction protein, gamma 2 (Gjc2), mRNA [NM_001100784]	**8,282**
Vps37a	Rattus norvegicus vacuolar protein sorting 37 homolog A (S. cerevisiae) (Vps37a), mRNA [NM_001024867]	**8,282**
Lrig2	Rattus norvegicus leucine-rich repeats and immunoglobulin-like domains 2 (Lrig2), mRNA [NM_001107710]	**8,280**
RGD1565257	Rattus norvegicus similar to zinc finger protein 650 (RGD1565257), mRNA [NM_001134550]	**8,279**
Ppp4c	Rattus norvegicus protein phosphatase 4, catalytic subunit (Ppp4c), mRNA [NM_134359]	**8,278**
Ppp2r5e	Rattus norvegicus protein phosphatase 2, regulatory subunit B', epsilon isoform (Ppp2r5e), mRNA [NM_001106740]	**8,278**
Igsf10	Rattus norvegicus immunoglobulin superfamily, member 10 (Igsf10), mRNA [NM_198768]	**8,277**
0	Unknown	**8,277**
Olr1375	Rattus norvegicus olfactory receptor 1375 (Olr1375), mRNA [NM_214829]	**8,277**
0	Unknown	**8,277**
0	Uncharacterized protein [Source:UniProtKB/TrEMBL;Acc:D4A3B3] [ENSRNOT00000065050]	**8,277**
Adss	Rattus norvegicus adenylosuccinate synthase (Adss), mRNA [NM_001105975]	**8,277**
Plekhh3	Rattus norvegicus pleckstrin homology domain containing, family H (with MyTH4 domain) member 3 (Plekhh3), mRNA [NM_001037202]	**8,277**
RGD1311078	Rattus norvegicus LOC360664 (RGD1311078), mRNA [NM_001108307]	**8,277**
Dok4	Rattus norvegicus docking protein 4 (Dok4), mRNA [NM_001108438]	**8,276**
Rae1	Rattus norvegicus RAE1 RNA export 1 homolog (S. pombe) (Rae1), mRNA [NM_001033708]	**8,275**
Cnfn	Rattus norvegicus cornifelin (Cnfn), mRNA [NM_001108909]	**8,275**
Cd300lg	PREDICTED: Rattus norvegicus Cd300 molecule-like family member G, transcript variant 1 (Cd300lg), mRNA [XM_001061779]	**8,274**
0	Unknown	**8,272**
0	Unknown	**8,272**
Mcm3	Rattus norvegicus minichromosome maintenance complex component 3 (Mcm3), mRNA [NM_001191805]	**8,271**
Lsm1	Rattus norvegicus LSM1 homolog, U6 small nuclear RNA associated (S. cerevisiae) (Lsm1), mRNA [NM_001108876]	**8,271**
0	Unknown	**8,270**
0	Unknown	**8,270**
Mif4gd	Rattus norvegicus MIF4G domain containing (Mif4gd), mRNA [NM_001014122]	**8,269**

RGD1309888	Rattus norvegicus similar to RIKEN cDNA 1500002O20 (RGD1309888), mRNA [NM_001014243]	**8,268**
Cd2bp2	Rattus norvegicus Cd2 (cytoplasmic tail) binding protein 2 (Cd2bp2), mRNA [NM_001106297]	**8,267**
Sft2d1	Rattus norvegicus SFT2 domain containing 1 (Sft2d1), mRNA [NM_001008302]	**8,266**
0	Solute carrier family 25 member 40 [Source:UniProtKB/Swiss-Prot;Acc:Q498U3] [ENSRNOT00000030352]	**8,266**
Psme1	Rattus norvegicus proteasome (prosome, macropain) activator subunit 1 (Psme1), mRNA [NM_017264]	**8,264**
Il17re	Rattus norvegicus interleukin 17 receptor E (Il17re), mRNA [NM_001004091]	**8,263**
0	Unknown	**8,262**
0	Rattus norvegicus similar to RIKEN cDNA 1700001F09 (LOC364257), mRNA [XM_344300]	**8,260**
Pex5	Rattus norvegicus peroxisomal biogenesis factor 5 (Pex5), mRNA [NM_001170584]	**8,259**
Olr286	Rattus norvegicus olfactory receptor 286 (Olr286), mRNA [NM_001000554]	**8,258**
Olr115	Rattus norvegicus olfactory receptor 115 (Olr115), mRNA [NM_001000152]	**8,258**
Tex101	Rattus norvegicus testis expressed 101 (Tex101), mRNA [NM_139037]	**8,258**
Trnp1	Uncharacterized protein [Source:UniProtKB/TrEMBL;Acc:D3ZTW4] [ENSRNOT00000032841]	**8,257**
Gstm3	Rattus norvegicus glutathione S-transferase mu 3 (Gstm3), mRNA [NM_020540]	**8,256**
Tmem90b	Rattus norvegicus transmembrane protein 90B (Tmem90b), mRNA [NM_001025020]	**8,254**
0	Uncharacterized protein [Source:UniProtKB/TrEMBL;Acc:D3ZC82] [ENSRNOT00000030501]	**8,252**
Crip3	Rattus norvegicus cysteine-rich protein 3 (Crip3), mRNA [NM_001109303]	**8,252**
Ptpn14	Rattus norvegicus protein tyrosine phosphatase, non-receptor type 14 (Ptpn14), mRNA [NM_001107200]	**8,252**
0	Unknown	**8,250**
Dnajb2	Rattus norvegicus DnaJ (Hsp40) homolog, subfamily B, member 2 (Dnajb2), mRNA [NM_001109541]	**8,249**
LOC679800	PREDICTED: Rattus norvegicus similar to MIC2 like 1 (LOC679800), mRNA [XM_001054523]	**8,249**
0	Unknown	**8,248**
LOC301521	PREDICTED: Rattus norvegicus Coiled-coil domain containing 108-like (LOC301521), mRNA [XM_002730048]	**8,247**
Ifitm3	Rattus norvegicus interferon induced transmembrane protein 3 (Ifitm3), mRNA [NM_001136124]	**8,245**
0	Uncharacterized protein [Source:UniProtKB/TrEMBL;Acc:D3ZVS0] [ENSRNOT00000035275]	**8,245**
0	Uncharacterized protein [Source:UniProtKB/TrEMBL;Acc:D3ZKR9] [ENSRNOT00000022373]	**8,242**
Nfkbie	Rattus norvegicus nuclear factor of kappa light polypeptide gene enhancer in B-cells inhibitor, epsilon (Nfkbie), mRNA [NM_199111]	**8,241**
0	Unknown	**8,236**
Znf746	Rattus norvegicus zinc finger protein 746 (Znf746), mRNA [NM_001100852]	**8,235**
MGC95152	Rattus norvegicus similar to B230212L03Rik protein (MGC95152), mRNA [NM_001007645]	**8,235**
Eif4e	Rattus norvegicus eukaryotic translation initiation factor 4E (Eif4e), mRNA [NM_053974]	**8,232**
Cnot6	Rattus norvegicus CCR4-NOT transcription complex, subunit 6 (Cnot6), mRNA [NM_001013856]	**8,229**
Snurf	Rattus norvegicus SNRPN upstream reading frame (Snurf), mRNA [NM_130738]	**8,228**
Olr1462	Rattus norvegicus olfactory receptor 1462 (Olr1462), mRNA [NM_001001097]	**8,227**

Prss34	Rattus norvegicus protease, serine, 34 (Prss34), mRNA [NM_001105772]	**8,227**
Iqgap3	Rattus norvegicus IQ motif containing GTPase activating protein 3 (Iqgap3), mRNA [NM_001191709]	**8,227**
RGD1310271	RCG31097, isoform CRA_aUncharacterized protein [Source:UniProtKB/TrEMBL;Acc:D3ZMX5] [ENSRNOT00000027564]	**8,226**
Pik3cd	Rattus norvegicus phosphoinositide-3-kinase, catalytic, delta polypeptide (Pik3cd), mRNA [NM_001108978]	**8,225**
0	Unknown	**8,224**
0	RCG26214Uncharacterized protein [Source:UniProtKB/TrEMBL;Acc:D3ZVK4] [ENSRNOT00000028891]	**8,224**
Cp110	Rattus norvegicus CP110 protein (Cp110), mRNA [NM_001108501]	**8,224**
Ccdc48	Rattus norvegicus coiled-coil domain containing 48 (Ccdc48), mRNA [NM_001163921]	**8,224**
Ncstn	Rattus norvegicus nicastrin (Ncstn), mRNA [NM_174864]	**8,222**
Prp2l1	Rat proline-rich protein (PRP-1) [Source:UniProtKB/TrEMBL;Acc:Q63455] [ENSRNOT00000004642]	**8,222**
Acer2	Rattus norvegicus alkaline ceramidase 2 (Acer2), mRNA [NM_001107943]	**8,222**
LOC691807	Uncharacterized protein C4orf3 homolog [Source:UniProtKB/Swiss-Prot;Acc:Q498U0] [ENSRNOT00000019647]	**8,221**
0	Unknown	**8,220**
0	Uncharacterized protein [Source:UniProtKB/TrEMBL;Acc:D3ZQH1] [ENSRNOT00000001957]	**8,218**
Tmem63c	Rattus norvegicus transmembrane protein 63c (Tmem63c), mRNA [NM_001108045]	**8,218**
Nqo1	Rattus norvegicus NAD(P)H dehydrogenase, quinone 1 (Nqo1), mRNA [NM_017000]	**8,218**
Apoo	Rattus norvegicus apolipoprotein O (Apoo), mRNA [NM_001199178]	**8,218**
LOC680758	PREDICTED: Rattus norvegicus similar to spermatogenesis associated glutamate (E)-rich protein 4d (LOC680758), mRNA [XM_001058748]	**8,216**
Nup98	Rattus norvegicus nucleoporin 98 (Nup98), mRNA [NM_031074]	**8,216**
Them4	Rattus norvegicus thioesterase superfamily member 4 (Them4), mRNA [NM_001025017]	**8,216**
0	Unknown	**8,215**
Rims4	Rattus norvegicus regulating synaptic membrane exocytosis 4 (Rims4), mRNA [NM_170666]	**8,215**
0	Unknown	**8,214**
Fam98a	Rattus norvegicus family with sequence similarity 98, member A (Fam98a), mRNA [NM_001014073]	**8,214**
Gpr177	Rattus norvegicus G protein-coupled receptor 177 (Gpr177), transcript variant 1, mRNA [NM_199408]	**8,213**
Zfp386	Rattus norvegicus zinc finger protein 386 (Kruppel-like) (Zfp386), mRNA [NM_019620]	**8,212**
0	Uncharacterized protein [Source:UniProtKB/TrEMBL;Acc:D3ZRC6] [ENSRNOT00000050943]	**8,212**
Mgea5	Rattus norvegicus meningioma expressed antigen 5 (hyaluronidase) (Mgea5), mRNA [NM_131904]	**8,211**
Akr1cl2	Rattus norvegicus aldo-keto reductase family 1, member C-like 2 (Akr1cl2), mRNA [NM_001008342]	**8,210**
Gabra2	Rattus norvegicus gamma-aminobutyric acid (GABA-A) receptor, subunit alpha 2 (Gabra2), mRNA [NM_001135779]	**8,209**
0	Unknown	**8,208**
0	Unknown	**8,207**
RGD1564804	Rattus norvegicus similar to chromosome 1 open reading frame 50 (RGD1564804), mRNA [NM_001107969]	**8,207**
LOC683684	PREDICTED: Rattus norvegicus hypothetical protein LOC683684 (LOC683684), mRNA [XM_001067050]	**8,206**

Fip1l1	Rattus norvegicus FIP1 like 1 (S. cerevisiae) (Fip1l1), mRNA [NM_001008295]	**8,206**
Mylk2	Rattus norvegicus myosin light chain kinase 2 (Mylk2), mRNA [NM_057209]	**8,205**
Gpr153	Rattus norvegicus G protein-coupled receptor 153 (Gpr153), mRNA [NM_001034855]	**8,204**
Ttc5	Rattus norvegicus tetratricopeptide repeat domain 5 (Ttc5), mRNA [NM_001013131]	**8,203**
Tmem123	Rattus norvegicus transmembrane protein 123 (Tmem123), mRNA [NM_001014205]	**8,203**
Wwtr1	Rattus norvegicus WW domain containing transcription regulator 1 (Wwtr1), mRNA [NM_001024869]	**8,201**
Zfp354a	Rattus norvegicus zinc finger protein 354A (Zfp354a), mRNA [NM_052798]	**8,201**
LOC100360599	PREDICTED: Rattus norvegicus rCG33743-like (LOC100360599), mRNA [XM_002724606]	**8,200**
Prnd	Rattus norvegicus prion protein 2 (dublet) (Prnd), mRNA [NM_001102431]	**8,199**
Mrps21	Rattus norvegicus mitochondrial ribosomal protein S21 (Mrps21), nuclear gene encoding mitochondrial protein, mRNA [NM_001126094]	**8,196**
Lin9	PREDICTED: Rattus norvegicus lin-9 homolog (C. elegans) (Lin9), mRNA [XM_001067807]	**8,192**
Pglyrp1	Rattus norvegicus peptidoglycan recognition protein 1 (Pglyrp1), mRNA [NM_053373]	**8,191**
RGD1310964	Rattus norvegicus similar to RIKEN cDNA 9430031J16 (RGD1310964), mRNA [NM_001107132]	**8,190**
0	Uncharacterized protein [Source:UniProtKB/TrEMBL;Acc:D3ZJC7] [ENSRNOT00000068355]	**8,190**
Poldip3	Rattus norvegicus polymerase (DNA-directed), delta interacting protein 3 (Poldip3), mRNA [NM_001130506]	**8,190**
Ipo13	Rattus norvegicus importin 13 (Ipo13), mRNA [NM_053778]	**8,189**
0	Histone H3 [Source:UniProtKB/TrEMBL;Acc:D3ZCY4] [ENSRNOT00000043897]	**8,189**
Enc1	Rattus norvegicus ectodermal-neural cortex 1 (Enc1), mRNA [NM_001003401]	**8,187**
Ube2f	Rattus norvegicus ubiquitin-conjugating enzyme E2F (putative) (Ube2f), mRNA [NM_001008381]	**8,186**
RT1-CE2	Rattus norvegicus RT1 class I, locus CE2 (RT1-CE2), mRNA [NM_001008840]	**8,183**
Prpf19	Rattus norvegicus PRP19/PSO4 pre-mRNA processing factor 19 homolog (S. cerevisiae) (Prpf19), mRNA [NM_139333]	**8,183**
Tspan6	Rattus norvegicus tetraspanin 6 (Tspan6), mRNA [NM_001100672]	**8,182**
LOC680489	Rattus norvegicus similar to claudin 1 (LOC680489), mRNA [NM_001109415]	**8,181**
Cd14	Rattus norvegicus CD14 molecule (Cd14), mRNA [NM_021744]	**8,181**
LOC501810	PREDICTED: Rattus norvegicus similar to Leukosialin precursor (Leucocyte sialoglycoprotein) (Sialophorin) (Ly-48) (B cell differentiation antigen LP-3) (CD43 antigen) (LOC501810), miscRNA [XR_005724]	**8,181**
Cyp7b1	Rattus norvegicus cytochrome P450, family 7, subfamily b, polypeptide 1 (Cyp7b1), mRNA [NM_019138]	**8,179**
C1qtnf6	Rattus norvegicus C1q and tumor necrosis factor related protein 6 (C1qtnf6), mRNA [NM_001034932]	**8,178**
Dclk2	Rattus norvegicus doublecortin-like kinase 2 (Dclk2), transcript variant 2, mRNA [NM_001009691]	**8,177**
LOC682404	PREDICTED: Rattus norvegicus similar to CG12929-PA (LOC682404), mRNA [XM_001061367]	**8,177**
0	Unknown	**8,176**
0	Uncharacterized protein [Source:UniProtKB/TrEMBL;Acc:D3ZDE7] [ENSRNOT00000049155]	**8,175**
Ank3	Rattus norvegicus ankyrin 3, node of Ranvier (Ank3), transcript variant 2, mRNA [NM_001033984]	**8,173**
LOC100360189	PREDICTED: Rattus norvegicus rCG56785-like (LOC100360189), mRNA [XM_002730167]	**8,172**

Klhl23	Rattus norvegicus kelch-like 23 (Drosophila) (Klhl23), mRNA [NM_001134504]	**8,171**
Nap1l5	Rattus norvegicus nucleosome assembly protein 1-like 5 (Nap1l5), mRNA [NM_001044293]	**8,171**
Tmem50a	Rattus norvegicus transmembrane protein 50A (Tmem50a), mRNA [NM_001127525]	**8,170**
Ube3c	PREDICTED: Rattus norvegicus ubiquitin protein ligase E3C (Ube3c), mRNA [XM_001055148]	**8,170**
Pja2	Rattus norvegicus praja 2, RING-H2 motif containing (Pja2), mRNA [NM_138896]	**8,169**
0	predicted gene 10022 Gene [Source:MGI Symbol;Acc:MGI:3809058] [ENSRNOT00000037001]	**8,168**
Sephs1	Rattus norvegicus selenophosphate synthetase 1 (Sephs1), mRNA [NM_001104630]	**8,168**
Zc3h15	Rattus norvegicus zinc finger CCCH-type containing 15 (Zc3h15), mRNA [NM_001010963]	**8,167**
Nr2f6	Rattus norvegicus nuclear receptor subfamily 2, group F, member 6 (Nr2f6), mRNA [NM_139113]	**8,167**
0	Unknown	**8,167**
Aldh1b1	Rattus norvegicus aldehyde dehydrogenase 1 family, member B1 (Aldh1b1), nuclear gene encoding mitochondrial protein, mRNA [NM_001011975]	**8,165**
Ropn1l	Rattus norvegicus ropporin 1-like (Ropn1l), mRNA [NM_001191086]	**8,164**
Cd46	Rattus norvegicus CD46 molecule, complement regulatory protein (Cd46), mRNA [NM_019190]	**8,163**
Rbm9	Rattus norvegicus RNA binding motif protein 9 (Rbm9), mRNA [NM_001079895]	**8,163**
Dab2ip	Rattus norvegicus DAB2 interacting protein (Dab2ip), mRNA [NM_138710]	**8,162**
0	Proprotein convertase subtilisin/kexin type 5 [Source:UniProtKB/Swiss-Prot;Acc:P41413] [ENSRNOT00000016164]	**8,162**
Zfp444	Rattus norvegicus zinc finger protein 444 (Zfp444), mRNA [NM_001169143]	**8,162**
Pola1	Rattus norvegicus polymerase (DNA directed), alpha 1 (Pola1), mRNA [NM_053479]	**8,161**
0	Unknown	**8,159**
Ap2a1	adaptor-related protein complex 2, alpha 1 subunit [Source:RefSeq peptide;Acc:NP_001100981] [ENSRNOT00000020304]	**8,159**
Pdlim1	Rattus norvegicus PDZ and LIM domain 1 (Pdlim1), mRNA [NM_017365]	**8,157**
Sit1	Rattus norvegicus signaling threshold regulating transmembrane adaptor 1 (Sit1), mRNA [NM_001024344]	**8,157**
RGD1561426	PREDICTED: Rattus norvegicus RGD1561426 (RGD1561426), mRNA [XM_001069564]	**8,156**
0	Uncharacterized protein [Source:UniProtKB/TrEMBL;Acc:D3Z9M1] [ENSRNOT00000056416]	**8,156**
0	Unknown	**8,155**
Rbm7	Rattus norvegicus RNA binding motif protein 7 (Rbm7), mRNA [NM_001108143]	**8,154**
0	Unknown	**8,154**
Ptpn5	Rattus norvegicus protein tyrosine phosphatase, non-receptor type 5 (Ptpn5), mRNA [NM_019253]	**8,154**
0	Unknown	**8,154**
Wdr78	Rattus norvegicus WD repeat domain 78 (Wdr78), mRNA [NM_001024786]	**8,153**
LOC305806	Rattus norvegicus similar to glutaredoxin 1 (thioltransferase); glutaredoxin (LOC305806), mRNA [NM_001013993]	**8,153**
Sgsm2	Rattus norvegicus small G protein signaling modulator 2 (Sgsm2), mRNA [NM_001107020]	**8,153**
Ing1	Rattus norvegicus inhibitor of growth family, member 1 (Ing1), mRNA [NM_001038591]	**8,152**
0	PREDICTED: Rattus norvegicus rCG58102-like (LOC100363350), mRNA [XM_002729901]	**8,152**

Mtnr1a	Rattus norvegicus melatonin receptor 1A (Mtnr1a), mRNA [NM_053676]	**8,151**
0	Uncharacterized protein [Source:UniProtKB/TrEMBL;Acc:D3ZHQ9] [ENSRNOT00000045241]	**8,150**
Ptprn	Rattus norvegicus protein tyrosine phosphatase, receptor type, N (Ptprn), mRNA [NM_053881]	**8,149**
RGD1563904	Rattus norvegicus similar to melanoma antigen family B, 4 (RGD1563904), mRNA [NM_001109367]	**8,149**
0	Uncharacterized protein [Source:UniProtKB/TrEMBL;Acc:D3ZQT0] [ENSRNOT00000011090]	**8,147**
Fbxo15	Rattus norvegicus F-box protein 15 (Fbxo15), mRNA [NM_001108436]	**8,146**
RGD1308470	Rattus norvegicus similar to RIKEN cDNA 4933433P14 gene (RGD1308470), mRNA [NM_001014198]	**8,146**
Lins1	Uncharacterized protein [Source:UniProtKB/TrEMBL;Acc:D3ZVQ9] [ENSRNOT00000018463]	**8,145**
Mas1	Rattus norvegicus MAS1 oncogene (Mas1), mRNA [NM_012757]	**8,145**
RGD1563956	Uncharacterized protein [Source:UniProtKB/TrEMBL;Acc:D4A8N3] [ENSRNOT00000030686]	**8,143**
Krt4	Rattus norvegicus keratin 4 (Krt4), mRNA [NM_001008806]	**8,142**
RGD1311122	Rattus norvegicus similar to RIKEN cDNA 1110003E01 (RGD1311122), mRNA [NM_001037792]	**8,142**
0	Unknown	**8,141**
0	Kinesin-like protein KIF1B [Source:UniProtKB/Swiss-Prot;Acc:O88658] [ENSRNOT00000029605]	**8,141**
38231	Rattus norvegicus septin 4 (Sept4), nuclear gene encoding mitochondrial protein, mRNA [NM_001011893]	**8,140**
Bpnt1	Rattus norvegicus 3'(2'), 5'-bisphosphate nucleotidase 1 (Bpnt1), mRNA [NM_171990]	**8,140**
Cast	Rattus norvegicus calpastatin (Cast), transcript variant 1, mRNA [NM_053295]	**8,138**
Wdr77	Rattus norvegicus WD repeat domain 77 (Wdr77), mRNA [NM_001008771]	**8,138**
Prim1	Rattus norvegicus DNA primase, p49 subunit (Prim1), mRNA [NM_001008768]	**8,137**
0	Unknown	**8,137**
Eml4	Rattus norvegicus echinoderm microtubule associated protein like 4 (Eml4), mRNA [NM_001108008]	**8,135**
LOC688420	PREDICTED: Rattus norvegicus similar to claudin 4 (LOC688420), mRNA [XM_001066865]	**8,133**
Afaf	Rattus norvegicus Acrosome formation associated factor (Afaf), mRNA [NM_001039345]	**8,132**
Atp4a	Rattus norvegicus ATPase, H+/K+ exchanging, alpha polypeptide (Atp4a), mRNA [NM_012509]	**8,131**
RGD1359108	Rattus norvegicus similar to RIKEN cDNA 3110043O21 (RGD1359108), mRNA [NM_001007702]	**8,130**
Btbd10	Rattus norvegicus BTB (POZ) domain containing 10 (Btbd10), mRNA [NM_001014022]	**8,126**
MGC116121	Rattus norvegicus similar to RIKEN cDNA 2700062C07 (MGC116121), mRNA [NM_001024905]	**8,124**
Fscn3	Rattus norvegicus fascin homolog 3, actin-bundling protein, testicular (Strongylocentrotus purpuratus) (Fscn3), mRNA [NM_001004232]	**8,124**
Npl	Rattus norvegicus N-acetylneuraminate pyruvate lyase (Npl), mRNA [NM_001013984]	**8,123**
Ears2	Rattus norvegicus glutamyl-tRNA synthetase 2 mitochondrial (putative) (Ears2), nuclear gene encoding mitochondrial protein, mRNA [NM_001159493]	**8,123**
0	Unknown	**8,123**
0	Unknown	**8,121**
Prmt2	Rattus norvegicus protein arginine methyltransferase 2 (Prmt2), mRNA [NM_001025144]	**8,121**

Olr63	Rattus norvegicus olfactory receptor 63 (Olr63), mRNA [NM_001000542]	**8,119**
Znf655	Rattus norvegicus zinc finger protein 655 (Znf655), mRNA [NM_001008362]	**8,119**
0	Unknown	**8,118**
Olr1319	Rattus norvegicus olfactory receptor 1319 (Olr1319), mRNA [NM_001000470]	**8,118**
Uhrf2	Rattus norvegicus ubiquitin-like with PHD and ring finger domains 2 (Uhrf2), mRNA [NM_001107585]	**8,118**
Nap1l4	Rattus norvegicus nucleosome assembly protein 1-like 4 (Nap1l4), mRNA [NM_001012170]	**8,118**
Camsap1l1	Rattus norvegicus calmodulin regulated spectrin-associated protein 1-like 1 (Camsap1l1), mRNA [NM_001134503]	**8,115**
Btbd3	Rattus norvegicus BTB (POZ) domain containing 3 (Btbd3), mRNA [NM_001107782]	**8,114**
Slc22a13	Rattus norvegicus solute carrier family 22 (organic anion transporter), member 13 (Slc22a13), mRNA [NM_001126285]	**8,113**
Ndrg4	Rattus norvegicus N-myc downstream regulated gene 4 (Ndrg4), mRNA [NM_031967]	**8,113**
Ahr	Rattus norvegicus aryl hydrocarbon receptor (Ahr), mRNA [NM_013149]	**8,113**
RGD1564964	Rattus norvegicus similar to WD repeat domain 11 protein (RGD1564964), mRNA [NM_001134564]	**8,112**
0	Unknown	**8,111**
Fmr1	Rattus norvegicus fragile X mental retardation 1 (Fmr1), mRNA [NM_052804]	**8,107**
Fam59b	Rattus norvegicus family with sequence similarity 59, member B (Fam59b), mRNA [NM_001191874]	**8,106**
Ncoa7	Uncharacterized protein [Source:UniProtKB/TrEMBL;Acc:D3ZM65] [ENSRNOT00000018849]	**8,104**
Ptprr	Rattus norvegicus protein tyrosine phosphatase, receptor type, R (Ptprr), transcript variant 1, mRNA [NM_053594]	**8,102**
Bcat1	Rattus norvegicus branched chain aminotransferase 1, cytosolic (Bcat1), mRNA [NM_017253]	**8,101**
Pxmp3	Rattus norvegicus peroxisomal membrane protein 3 (Pxmp3), mRNA [NM_017234]	**8,101**
LOC679566	Rattus norvegicus hypothetical protein LOC679566 (LOC679566), mRNA [NM_001109372]	**8,101**
Galns	Rattus norvegicus galactosamine (N-acetyl)-6-sulfate sulfatase (Galns), mRNA [NM_001047851]	**8,100**
Ptges3l1	Rattus norvegicus prostaglandin E synthase 3-like 1 (Ptges3l1), mRNA [NM_001014272]	**8,100**
Adora2b	Rattus norvegicus adenosine A2B receptor (Adora2b), mRNA [NM_017161]	**8,099**
Poll	Rattus norvegicus polymerase (DNA directed), lambda (Poll), mRNA [NM_001014168]	**8,098**
Slc5a3	Rattus norvegicus solute carrier family 5 (sodium/myo-inositol cotransporter), member 3 (Slc5a3), mRNA [NM_053715]	**8,098**
Myh13	Myosin heavy chain [Source:UniProtKB/TrEMBL;Acc:Q9Z2B7] [ENSRNOT00000055257]	**8,098**
Mfsd6	Rattus norvegicus major facilitator superfamily domain containing 6 (Mfsd6), mRNA [NM_001106911]	**8,097**
Znf839	PREDICTED: Rattus norvegicus zinc finger protein 839 (Znf839), mRNA [XM_001071353]	**8,097**
Prex1	Rattus norvegicus phosphatidylinositol-3,4,5-trisphosphate-dependent Rac exchange factor 1 (Prex1), mRNA [NM_001135718]	**8,096**
Mtm1	Rattus norvegicus myotubularin 1 (Mtm1), mRNA [NM_001013047]	**8,096**
0	Glyceraldehyde-3-phosphate dehydrogenase [Source:UniProtKB/TrEMBL;Acc:D3ZT40] [ENSRNOT00000041811]	**8,096**
0	Unknown	**8,096**
LOC302680	similar to CXORF15 (LOC302680), mRNA [Source:RefSeq DNA;Acc:NM_001037187] [ENSRNOT00000006843]	**8,095**
Tbc1d19	Rattus norvegicus TBC1 domain family, member 19 (Tbc1d19), mRNA [NM_001106008]	**8,095**

0	Q5SZT6_MOUSE (Q5SZT6) OTTMUSP00000000447, partial (4%) [TC640179]	**8,093**
Pigl	Rattus norvegicus phosphatidylinositol glycan anchor biosynthesis, class L (Pigl), mRNA [NM_138901]	**8,090**
Rnf123	Rattus norvegicus ring finger protein 123 (Rnf123), mRNA [NM_001191580]	**8,090**
Xrcc1	Rattus norvegicus X-ray repair complementing defective repair in Chinese hamster cells 1 (Xrcc1), mRNA [NM_053435]	**8,090**
Stx5	Rattus norvegicus syntaxin 5 (Stx5), mRNA [NM_031704]	**8,090**
Olr806	Rattus norvegicus olfactory receptor 806 (Olr806), mRNA [NM_001000976]	**8,089**
Camsap1	Rattus norvegicus calmodulin regulated spectrin-associated protein 1 (Camsap1), mRNA [NM_001168549]	**8,089**
Vps36	Rattus norvegicus vacuolar protein sorting 36 homolog (S. cerevisiae) (Vps36), mRNA [NM_001106092]	**8,086**
Tst	Rattus norvegicus thiosulfate sulfurtransferase (Tst), nuclear gene encoding mitochondrial protein, mRNA [NM_012808]	**8,086**
Cpeb1	Rattus norvegicus cytoplasmic polyadenylation element binding protein 1 (Cpeb1), mRNA [NM_001106276]	**8,086**
LOC498829	PREDICTED: Rattus norvegicus Ab2-143 (LOC498829), miscRNA [XR_005439]	**8,085**
Grin2c	Rattus norvegicus glutamate receptor, ionotropic, N-methyl D-aspartate 2C (Grin2c), mRNA [NM_012575]	**8,084**
0	Unknown	**8,082**
Tdrd12	PREDICTED: Rattus norvegicus similar to CG11133-PA (LOC689639), mRNA [XM_001071461]	**8,081**
Tk2	Rattus norvegicus thymidine kinase 2, mitochondrial (Tk2), nuclear gene encoding mitochondrial protein, mRNA [NM_001106166]	**8,080**
0	Unknown	**8,080**
Srp72	Rattus norvegicus signal recognition particle 72 (Srp72), mRNA [NM_001170601]	**8,078**
Zcchc17	Rattus norvegicus zinc finger, CCHC domain containing 17 (Zcchc17), mRNA [NM_001109267]	**8,078**
Olr834	Rattus norvegicus olfactory receptor 834 (Olr834), mRNA [NM_001000407]	**8,077**
0	Dedicator of cytokinesis 5 (Predicted)Uncharacterized protein [Source:UniProtKB/TrEMBL;Acc:D3ZYS9] [ENSRNOT00000058887]	**8,077**
Dppa2	PREDICTED: Rattus norvegicus similar to developmental pluripotency-associated 2 (LOC685364), mRNA [XM_001063497]	**8,076**
RT1-A1	Rattus norvegicus RT1 class Ia, locus A1 (RT1-A1), mRNA [NM_001008827]	**8,076**
0	Unknown	**8,074**
0	Uncharacterized protein [Source:UniProtKB/TrEMBL;Acc:D3ZRC6] [ENSRNOT00000050943]	**8,073**
RGD1564915	PREDICTED: Rattus norvegicus similar to TDPOZ2 (RGD1564915), mRNA [XM_578033]	**8,072**
Lsm11	Rattus norvegicus LSM11, U7 small nuclear RNA associated (Lsm11), mRNA [NM_001173384]	**8,070**
Sox2	Rattus norvegicus SRY (sex determining region Y)-box 2 (Sox2), mRNA [NM_001109181]	**8,066**
0	Unknown	**8,066**
0	AGENCOURT_27827201 NIH_MGC_252 Rattus norvegicus cDNA clone IMAGE:7307928 5', mRNA sequence [CO396750]	**8,063**
Sel1l	Rattus norvegicus sel-1 suppressor of lin-12-like (C. elegans) (Sel1l), mRNA [NM_177933]	**8,062**
Mtf2	Rattus norvegicus metal response element binding transcription factor 2 (Mtf2), mRNA [NM_001100898]	**8,061**
Sned1	Rattus norvegicus sushi, nidogen and EGF-like domains 1 (Sned1), mRNA [NM_001167842]	**8,061**
Tpd52l2	Rattus norvegicus tumor protein D52-like 2 (Tpd52l2), mRNA [NM_198744]	**8,060**
Klhl21	Rattus norvegicus kelch-like 21 (Drosophila) (Klhl21), mRNA [NM_001107996]	**8,059**

Pigw	Rattus norvegicus phosphatidylinositol glycan anchor biosynthesis, class W (Pigw), mRNA [NM_194461]	**8,058**
0	Unknown	**8,057**
Unc79	Uncharacterized protein [Source:UniProtKB/TrEMBL;Acc:D3ZSV8] [ENSRNOT00000066306]	**8,057**
Fen1	Rattus norvegicus flap structure-specific endonuclease 1 (Fen1), mRNA [NM_053430]	**8,057**
0	Unknown	**8,057**
Zfp64	Rattus norvegicus zinc finger protein 64 (Zfp64), mRNA [NM_001012093]	**8,055**
0	PREDICTED: Rattus norvegicus similar to eyes absent 4 isoform a (LOC360684), partial mRNA [XM_340956]	**8,055**
Rapgef6	Rattus norvegicus Rap guanine nucleotide exchange factor (GEF) 6 (Rapgef6), mRNA [NM_001107003]	**8,054**
Fam122b	Rattus norvegicus family with sequence similarity 122B (Fam122b), mRNA [NM_001166586]	**8,053**
Zfp1	PREDICTED: Rattus norvegicus zinc finger protein 1 (Zfp1), mRNA [XM_001077676]	**8,053**
Top2a	Rattus norvegicus topoisomerase (DNA) II alpha (Top2a), mRNA [NM_022183]	**8,052**
Rfng	Rattus norvegicus RFNG O-fucosylpeptide 3-beta-N-acetylglucosaminyltransferase (Rfng), mRNA [NM_021849]	**8,051**
0	Unknown	**8,051**
0	Unknown	**8,051**
0	Ig gamma-2A chain C region [Source:UniProtKB/Swiss-Prot;Acc:P20760] [ENSRNOT00000066209]	**8,050**
Metrn	Rattus norvegicus meteorin, glial cell differentiation regulator (Metrn), mRNA [NM_001009962]	**8,048**
Rbbp4	Rattus norvegicus retinoblastoma binding protein 4 (Rbbp4), mRNA [NM_001107912]	**8,047**
Zfyve19	Rattus norvegicus zinc finger, FYVE domain containing 19 (Zfyve19), mRNA [NM_001034948]	**8,046**
Vegfc	Rattus norvegicus vascular endothelial growth factor C (Vegfc), mRNA [NM_053653]	**8,046**
Kpna5	Rattus norvegicus karyopherin alpha 5 (importin alpha 6) (Kpna5), mRNA [NM_001025113]	**8,045**
Flrt3	Rattus norvegicus fibronectin leucine rich transmembrane protein 3 (Flrt3), mRNA [NM_001126291]	**8,044**
Zdhhc21	Rattus norvegicus zinc finger, DHHC-type containing 21 (Zdhhc21), mRNA [NM_001039009]	**8,042**
Bcat2	Branched-chain-amino-acid aminotransferase, mitochondrial [Source:UniProtKB/Swiss-Prot;Acc:O35854] [ENSRNOT00000028474]	**8,040**
0	Unknown	**8,040**
0	Unknown	**8,040**
Adcy2	Rattus norvegicus adenylate cyclase 2 (brain) (Adcy2), mRNA [NM_031007]	**8,039**
Uso1	Rattus norvegicus USO1 homolog, vesicle docking protein (yeast) (Uso1), mRNA [NM_019379]	**8,038**
0	Unknown	**8,037**
0	Unknown	**8,035**
Gcsh	Rattus norvegicus glycine cleavage system protein H (aminomethyl carrier) (Gcsh), nuclear gene encoding mitochondrial protein, mRNA [NM_133598]	**8,034**
0	Unknown	**8,033**
0	Unknown	**8,030**
0	Unknown	**8,030**

Prps1	Rattus norvegicus phosphoribosyl pyrophosphate synthetase 1 (Prps1), mRNA [NM_017243]	**8,029**
LOC687565	Rattus norvegicus similar to density-regulated protein (LOC687565), mRNA [NM_001115046]	**8,029**
Disp1	Rattus norvegicus dispatched homolog 1 (Drosophila) (Disp1), mRNA [NM_001105983]	**8,028**
Fgfr3	Rattus norvegicus fibroblast growth factor receptor 3 (Fgfr3), mRNA [NM_053429]	**8,025**
LOC363337	Rattus norvegicus similar to RIKEN cDNA 1700081O22 (LOC363337), mRNA [NM_001014221]	**8,024**
Zfp786	Rattus norvegicus zinc finger protein 786 (Zfp786), mRNA [NM_001127594]	**8,024**
0	Uncharacterized protein [Source:UniProtKB/TrEMBL;Acc:D3ZVK0] [ENSRNOT00000009701]	**8,023**
Kcnk6	Rattus norvegicus potassium channel, subfamily K, member 6 (Kcnk6), mRNA [NM_053806]	**8,022**
Wdr25l	Rattus norvegicus WD repeat domain 25-like (Wdr25l), mRNA [NM_001135894]	**8,021**
0	PREDICTED: Rattus norvegicus similar to glyceraldehyde-3-phosphate dehydrogenase (phosphorylating) (EC 1.2.1.12) (RGD1564958), mRNA [XM_001066279]	**8,020**
Rpl10a	Rattus norvegicus ribosomal protein L10A (Rpl10a), mRNA [NM_031065]	**8,020**
Zfp93	Rattus norvegicus zinc finger protein 93 (Zfp93), mRNA [NM_001106546]	**8,019**
0	Unknown	**8,019**
Trpm5	Rattus norvegicus transient receptor potential cation channel, subfamily M, member 5 (Trpm5), mRNA [NM_001191896]	**8,018**
Nrp1	Rattus norvegicus neuropilin 1 (Nrp1), mRNA [NM_145098]	**8,018**
Tmem74	Uncharacterized protein [Source:UniProtKB/TrEMBL;Acc:D3ZR33] [ENSRNOT00000006791]	**8,018**
0	Rattus norvegicus TL0ACA48YA23 mRNA sequence. [FQ215489]	**8,013**
Gcn1l1	Rattus norvegicus GCN1 general control of amino-acid synthesis 1-like 1 (yeast) (Gcn1l1), mRNA [NM_001168664]	**8,013**
Ncapd3	Rattus norvegicus non-SMC condensin II complex, subunit D3 (Ncapd3), mRNA [NM_001034000]	**8,013**
Rpap2	Rattus norvegicus RNA polymerase II associated protein 2 (Rpap2), mRNA [NM_001013987]	**8,010**
0	PREDICTED: Rattus norvegicus hypothetical gene supported by BC082068 (LOC501476), mRNA [XM_002730162]	**8,010**
LOC683034	PREDICTED: Rattus norvegicus hypothetical protein LOC683034, transcript variant 2 (LOC683034), mRNA [XM_001061393]	**8,010**
0	Mannan-binding lectin serine protease 1Mannan-binding lectin serine protease 1 heavy chainMannan-binding lectin serine protease 1 light chain [Source:UniProtKB/Swiss-Prot;Acc:Q8CHN8] [ENSRNOT00000002512]	**8,010**
Rapgef4	Rap guanine nucleotide exchange factor 4 [Source:UniProtKB/Swiss-Prot;Acc:Q9Z1C7] [ENSRNOT00000010630]	**8,009**
Mfn2	Rattus norvegicus mitofusin 2 (Mfn2), nuclear gene encoding mitochondrial protein, mRNA [NM_130894]	**8,009**
Cbr1	Rattus norvegicus carbonyl reductase 1 (Cbr1), mRNA [NM_019170]	**8,009**
Parp1	Rattus norvegicus poly (ADP-ribose) polymerase 1 (Parp1), mRNA [NM_013063]	**8,006**
Phf20	Rattus norvegicus PHD finger protein 20 (Phf20), mRNA [NM_001107795]	**8,006**
Tubd1	Rattus norvegicus tubulin, delta 1 (Tubd1), mRNA [NM_001105826]	**8,003**
0	Unknown	**8,003**
Hs3st3a1	Rattus norvegicus heparan sulfate (glucosamine) 3-O-sulfotransferase 3A1 (Hs3st3a1), mRNA [NM_001172067]	**8,003**
Lrrn4cl	Rattus norvegicus LRRN4 C-terminal like (Lrrn4cl), mRNA [NM_001109579]	**8,002**

Gpt2	Rattus norvegicus glutamic pyruvate transaminase (alanine aminotransferase) 2 (Gpt2), mRNA [NM_001012057]	**8,001**
Myo5b	Rattus norvegicus myosin Vb (Myo5b), mRNA [NM_017083]	**8,001**
Sumf2	Rattus norvegicus sulfatase modifying factor 2 (Sumf2), mRNA [NM_001025125]	**8,000**
Scara5	Rattus norvegicus scavenger receptor class A, member 5 (putative) (Scara5), mRNA [NM_001135855]	**8,000**
Habp4	Rattus norvegicus hyaluronan binding protein 4 (Habp4), mRNA [NM_001079940]	**7,998**
0	similar to RIKEN cDNA 1700001E04 (MGC116197), mRNA [Source:RefSeq DNA;Acc:NM_001025755] [ENSRNOT00000043269]	**7,998**
Usp13	Rattus norvegicus ubiquitin specific protease 13 (isopeptidase T-3) (Usp13), mRNA [NM_001107665]	**7,997**
0	P90550_LEIDO (P90550) 39 kDa antigen, partial (5%) [TC612167]	**7,997**
LOC688387	PREDICTED: Rattus norvegicus hypothetical protein LOC688387 (LOC688387), mRNA [XM_001066721]	**7,995**
LOC363337	Rattus norvegicus similar to RIKEN cDNA 1700081O22 (LOC363337), mRNA [NM_001014221]	**7,995**
LOC688463	PREDICTED: Rattus norvegicus hypothetical protein LOC688463 (LOC688463), mRNA [XM_001064240]	**7,994**
Pamr1	Rattus norvegicus peptidase domain containing associated with muscle regeneration 1 (Pamr1), mRNA [NM_001107755]	**7,994**
B9d1	Rattus norvegicus B9 protein domain 1 (B9d1), mRNA [NM_001105786]	**7,993**
Jak2	Rattus norvegicus Janus kinase 2 (Jak2), mRNA [NM_031514]	**7,993**
Myt1l	Rattus norvegicus myelin transcription factor 1-like (Myt1l), mRNA [NM_053888]	**7,991**
0	Rattus norvegicus similar to zinc finger protein 91 (HPF7, HTF10) (LOC308338), mRNA [XM_218198]	**7,991**
Ccdc85a	Rattus norvegicus coiled-coil domain containing 85A (Ccdc85a), mRNA [NM_001191553]	**7,991**
0	Unknown	**7,991**
Baiap2l2	Uncharacterized protein [Source:UniProtKB/TrEMBL;Acc:D4A901] [ENSRNOT00000016362]	**7,988**
Sptbn4	Sptbn4 protein [Source:UniProtKB/TrEMBL;Acc:Q5BJU8] [ENSRNOT00000028393]	**7,986**
Acyp2	Rattus norvegicus acylphosphatase 2, muscle type (Acyp2), mRNA [NM_001169145]	**7,985**
Spg7	Rattus norvegicus spastic paraplegia 7 homolog (human) (Spg7), nuclear gene encoding mitochondrial protein, mRNA [NM_181388]	**7,985**
0	Unknown	**7,983**
Pkn2	Rattus norvegicus protein kinase N2 (Pkn2), mRNA [NM_001105755]	**7,983**
Ipo5	Uncharacterized protein [Source:UniProtKB/TrEMBL;Acc:D4A781] [ENSRNOT00000014859]	**7,983**
Zbtb8a	Rattus norvegicus zinc finger and BTB domain containing 8a (Zbtb8a), mRNA [NM_001107913]	**7,979**
Harbi1	Rattus norvegicus harbinger transposase derived 1 (Harbi1), mRNA [NM_001113793]	**7,979**
Camkk2	Rattus norvegicus calcium/calmodulin-dependent protein kinase kinase 2, beta (Camkk2), mRNA [NM_031338]	**7,977**
Nptn	Rattus norvegicus neuroplastin (Nptn), mRNA [NM_019380]	**7,977**
Lrrc25	Rattus norvegicus TL0ADA9YE12 mRNA sequence. [FQ228377]	**7,977**
Nudcd2	Rattus norvegicus NudC domain containing 2 (Nudcd2), mRNA [NM_001009621]	**7,974**
Rnf215	Rattus norvegicus ring finger protein 215 (Rnf215), mRNA [NM_001107234]	**7,969**
0	Rattus norvegicus similar to glyceraldehyde-3-phosphate dehydrogenase (phosphorylating) (EC 1.2.1.12) - mouse (LOC303889), mRNA [XM_221419]	**7,969**

LOC100360662	PREDICTED: Rattus norvegicus thioredoxin-like 4-like (LOC100360662), mRNA [XM_002729753]	**7,969**
Lace1	Rattus norvegicus lactation elevated 1 (Lace1), mRNA [NM_001037656]	**7,968**
Pigg	PREDICTED: Rattus norvegicus phosphatidylinositol glycan anchor biosynthesis, class G, transcript variant 3 (Pigg), mRNA [XM_223731]	**7,967**
Fv1	Rattus norvegicus Friend virus susceptibility 1 (Fv1), mRNA [NM_001014018]	**7,967**
Ldoc1l	Uncharacterized protein [Source:UniProtKB/TrEMBL;Acc:D3ZI44] [ENSRNOT00000039588]	**7,964**
Atoh8	Rattus norvegicus atonal homolog 8 (Drosophila) (Atoh8), mRNA [NM_001109241]	**7,963**
Hist1h3f	PREDICTED: Rattus norvegicus histone cluster 1, H3f (Hist1h3f), mRNA [XM_001071516]	**7,962**
Cyb5r2	Rattus norvegicus cytochrome b5 reductase 2 (Cyb5r2), mRNA [NM_001014244]	**7,962**
Dync1li2	Rattus norvegicus dynein, cytoplasmic 1 light intermediate chain 2 (Dync1li2), mRNA [NM_031026]	**7,962**
0	Unknown	**7,961**
0	Unknown	**7,960**
Lias	Rattus norvegicus lipoic acid synthetase (Lias), nuclear gene encoding mitochondrial protein, mRNA [NM_001012037]	**7,959**
0	Unknown	**7,959**
0	Unknown	**7,958**
0	ORML3_RAT (Q6QI25) ORM1-like protein 3 (Liver regeneration-related protein LRRGT00183), complete [TC597420]	**7,956**
Srbd1	Uncharacterized protein [Source:UniProtKB/TrEMBL;Acc:D4A9B0] [ENSRNOT00000019894]	**7,955**
Olr259	Rattus norvegicus olfactory receptor 259 (Olr259), mRNA [NM_001000222]	**7,955**
0	Unknown	**7,954**
0	Unknown	**7,952**
Hsf2	Rattus norvegicus heat shock transcription factor 2 (Hsf2), mRNA [NM_031694]	**7,951**
Atp6v0e1	Rattus norvegicus ATPase, H+ transporting, lysosomal, V0 subunit e1 (Atp6v0e1), mRNA [NM_053578]	**7,951**
Hlx	Rattus norvegicus H2.0-like homeobox (Hlx), mRNA [NM_001077674]	**7,951**
Gys1	Rattus norvegicus glycogen synthase 1, muscle (Gys1), mRNA [NM_001109615]	**7,950**
Ccny	Rattus norvegicus cyclin Y (Ccny), mRNA [NM_001191833]	**7,946**
R3hcc1	PREDICTED: Rattus norvegicus R3H domain and coiled-coil containing 1 (R3hcc1), mRNA [XM_001069252]	**7,942**
Fgf1	Rattus norvegicus fibroblast growth factor 1 (Fgf1), mRNA [NM_012846]	**7,942**
LOC100365858	PREDICTED: Rattus norvegicus zinc finger CCCH type, antiviral 1-like (LOC100365858), miscRNA [XR_085680]	**7,941**
0	Uncharacterized protein [Source:UniProtKB/TrEMBL;Acc:D3ZLM0] [ENSRNOT00000044997]	**7,940**
RGD1563547	Rattus norvegicus RGD1563547 (RGD1563547), mRNA [NM_001108266]	**7,940**
Hnrnpk	Rattus norvegicus heterogeneous nuclear ribonucleoprotein K (Hnrnpk), mRNA [NM_057141]	**7,940**
Bicd2	Rattus norvegicus bicaudal D homolog 2 (Drosophila) (Bicd2), transcript variant 1, mRNA [NM_001033674]	**7,939**
Olr1531	Rattus norvegicus olfactory receptor 1531 (Olr1531), mRNA [NM_001001102]	**7,937**
0	Unknown	**7,937**
0	Unknown	**7,936**

Nav3	Rattus norvegicus neuron navigator 3 (Nav3), mRNA [NM_001191782]	**7,935**
Mllt4	Rattus norvegicus myeloid/lymphoid or mixed-lineage leukemia (trithorax homolog, Drosophila); translocated to, 4 (Mllt4), mRNA [NM_013217]	**7,935**
Rrn3	Rattus norvegicus RRN3 RNA polymerase I transcription factor homolog (S. cerevisiae) (Rrn3), mRNA [NM_001135846]	**7,935**
Cenpc1	Rattus norvegicus centromere protein C 1 (Cenpc1), mRNA [NM_001004098]	**7,934**
0	Unknown	**7,934**
Xcl1	Rattus norvegicus chemokine (C motif) ligand 1 (Xcl1), mRNA [NM_134361]	**7,933**
Spin1	Rattus norvegicus spindlin 1 (Spin1), mRNA [NM_001024796]	**7,933**
Hmg1l1	Rattus norvegicus high-mobility group (nonhistone chromosomal) protein 1-like 1 (Hmg1l1), mRNA [NM_001109373]	**7,931**
Bbc3	Rattus norvegicus Bcl-2 binding component 3 (Bbc3), mRNA [NM_173837]	**7,931**
Ezr	Rattus norvegicus ezrin (Ezr), mRNA [NM_019357]	**7,931**
Fitm1	Rattus norvegicus fat storage-inducing transmembrane protein 1 (Fitm1), mRNA [NM_001106037]	**7,931**
0	Rattus norvegicus similar to glyceraldehyde-3-phosphate dehydrogenase (phosphorylating) (EC 1.2.1.12) - mouse (LOC294844), mRNA [XM_226873]	**7,930**
0	Rattus norvegicus clone PLRR-14 polymorphic leucine-rich repeat protein mRNA, partial cds. [AF406815]	**7,930**
Nr1h2	Rattus norvegicus nuclear receptor subfamily 1, group H, member 2 (Nr1h2), mRNA [NM_031626]	**7,930**
Uck2	Rattus norvegicus uridine-cytidine kinase 2 (Uck2), mRNA [NM_001102408]	**7,928**
Epha6	Ephrin type-A receptor 6 [Source:UniProtKB/Swiss-Prot;Acc:P54758] [ENSRNOT00000047310]	**7,927**
Adcyap1	Rattus norvegicus adenylate cyclase activating polypeptide 1 (Adcyap1), mRNA [NM_016989]	**7,927**
Olr1366	Rattus norvegicus olfactory receptor 1366 (Olr1366), mRNA [NM_001000980]	**7,926**
0	Q63ZX6_MOUSE (Q63ZX6) Phf20l1 protein (Fragment), partial (4%) [TC630334]	**7,926**
Fxr2	Rattus norvegicus fragile X mental retardation, autosomal homolog 2 (Fxr2), mRNA [NM_001100647]	**7,926**
RGD1307461	Rattus norvegicus similar to RIKEN cDNA 6430571L13 gene; similar to g20 protein (RGD1307461), mRNA [NM_001106854]	**7,924**
Pnma1	Rattus norvegicus paraneoplastic antigen MA1 (Pnma1), mRNA [NM_130820]	**7,923**
Zfyve9	Rattus norvegicus zinc finger, FYVE domain containing 9 (Zfyve9), mRNA [NM_001107952]	**7,923**
0	Uncharacterized protein [Source:UniProtKB/TrEMBL;Acc:D3ZFR8] [ENSRNOT00000036063]	**7,923**
Tcrb	AGENCOURT_28622268 NIH_MGC_250 Rattus norvegicus cDNA clone IMAGE:7384586 5', mRNA sequence [CO562512]	**7,920**
0	Rattus norvegicus similar to Dcra (LOC360703), mRNA [XM_340974]	**7,919**
Efcab1	Rattus norvegicus EF hand calcium binding domain 1 (Efcab1), mRNA [NM_001106930]	**7,919**
Sms	Rattus norvegicus spermine synthase (Sms), mRNA [NM_001033899]	**7,919**
Pik3ca	Rattus norvegicus phosphoinositide-3-kinase, catalytic, alpha polypeptide (Pik3ca), mRNA [NM_133399]	**7,918**
Acrbp	Rattus norvegicus acrosin binding protein (Acrbp), mRNA [NM_001025049]	**7,918**
Arhgef19	Rattus norvegicus Rho guanine nucleotide exchange factor (GEF) 19 (Arhgef19), mRNA [NM_001108692]	**7,917**
Pcgf1	Rattus norvegicus polycomb group ring finger 1 (Pcgf1), mRNA [NM_001007000]	**7,913**
Plag1	Rattus norvegicus pleiomorphic adenoma gene 1 (Plag1), mRNA [NM_001008316]	**7,913**

Tmeff1	Rattus norvegicus transmembrane protein with EGF-like and two follistatin-like domains 1 (Tmeff1), mRNA [NM_023020]	**7,913**
0	PREDICTED: Rattus norvegicus similar to glyceraldehyde-3-phosphate dehydrogenase (phosphorylating) (EC 1.2.1.12) (RGD1561881), mRNA [XM_001078200]	**7,909**
0	Unknown	**7,909**
Gdap1l1	Rattus norvegicus ganglioside-induced differentiation-associated protein 1-like 1 (Gdap1l1), mRNA [NM_001107798]	**7,908**
Brwd3	Uncharacterized protein [Source:UniProtKB/TrEMBL;Acc:D3Z8C5] [ENSRNOT00000050336]	**7,908**
Hrh1	Rattus norvegicus histamine receptor H 1 (Hrh1), mRNA [NM_017018]	**7,907**
Zkscan3	Rattus norvegicus zinc finger with KRAB and SCAN domains 3 (Zkscan3), mRNA [NM_001012053]	**7,907**
Got1	Rattus norvegicus glutamic-oxaloacetic transaminase 1, soluble (aspartate aminotransferase 1) (Got1), mRNA [NM_012571]	**7,906**
Armc7	Rattus norvegicus armadillo repeat containing 7 (Armc7), mRNA [NM_001127522]	**7,906**
0	Unknown	**7,906**
Mum1l1	Rattus norvegicus melanoma associated antigen (mutated) 1-like 1 (Mum1l1), mRNA [NM_001109321]	**7,906**
Bag4	Rattus norvegicus BCL2-associated athanogene 4 (Bag4), mRNA [NM_001025130]	**7,903**
0	Unknown	**7,903**
RGD1311357	PREDICTED: Rattus norvegicus similar to A530083I02Rik protein (RGD1311357), mRNA [XM_001070093]	**7,903**
RGD1564722	Uncharacterized protein [Source:UniProtKB/TrEMBL;Acc:D4ABT2] [ENSRNOT00000049766]	**7,902**
Slc30a3	Rattus norvegicus solute carrier family 30 (zinc transporter), member 3 (Slc30a3), mRNA [NM_001013243]	**7,899**
Sync	Rattus norvegicus syncoilin (Sync), mRNA [NM_001108683]	**7,899**
Gle1	Rattus norvegicus GLE1 RNA export mediator homolog (yeast) (Gle1), mRNA [NM_001025731]	**7,899**
Calm1	Rattus norvegicus calmodulin 1 (Calm1), mRNA [NM_031969]	**7,898**
Jdp2	Rattus norvegicus Jun dimerization protein 2 (Jdp2), mRNA [NM_053894]	**7,895**
Eif3j	Rattus norvegicus eukaryotic translation initiation factor 3, subunit J (Eif3j), mRNA [NM_001077670]	**7,894**
Plcd3	Rattus norvegicus phospholipase C, delta 3 (Plcd3), mRNA [NM_001105845]	**7,894**
Wbp5	Rattus norvegicus WW domain binding protein 5 (Wbp5), mRNA [NM_001127502]	**7,894**
Hps3	Rattus norvegicus Hermansky-Pudlak syndrome 3 homolog (human) (Hps3), mRNA [NM_001107664]	**7,893**
RGD1359449	Rattus norvegicus gasdermin domain containing protein RGD1359449 (RGD1359449), mRNA [NM_001014084]	**7,892**
0	Unknown	**7,892**
Sbds	Rattus norvegicus Shwachman-Bodian-Diamond syndrome homolog (human) (Sbds), mRNA [NM_001008289]	**7,891**
0	Unknown	**7,890**
LOC378467	Rattus norvegicus promethin (LOC378467), mRNA [NM_194354]	**7,890**
Triobp	Uncharacterized protein [Source:UniProtKB/TrEMBL;Acc:D3ZHT2] [ENSRNOT00000067341]	**7,889**
Herpud2	Rattus norvegicus HERPUD family member 2 (Herpud2), mRNA [NM_001024988]	**7,886**
Mrgprb4	Rattus norvegicus MAS-related GPR, member B4 (Mrgprb4), mRNA [NM_001002287]	**7,886**
Tpd52l1	Rattus norvegicus tumor protein D52-like 1 (Tpd52l1), mRNA [NM_001044295]	**7,885**

Polr3d	Rattus norvegicus polymerase (RNA) III (DNA directed) polypeptide D (Polr3d), mRNA [NM_001031653]	**7,884**
Pcolce	Rattus norvegicus procollagen C-endopeptidase enhancer (Pcolce), mRNA [NM_019237]	**7,882**
Chrnb4	Rattus norvegicus cholinergic receptor, nicotinic, beta 4 (Chrnb4), mRNA [NM_052806]	**7,881**
0	Unknown	**7,878**
Calm2	Rattus norvegicus calmodulin 2 (Calm2), mRNA [NM_017326]	**7,878**
Wdr89	Rattus norvegicus WD repeat domain 89 (Wdr89), mRNA [NM_001014078]	**7,877**
Tead1	Rattus norvegicus TEA domain family member 1 (Tead1), mRNA [NM_001198589]	**7,877**
Glyatl3	Rattus norvegicus glycine-N-acyltransferase-like 3 (Glyatl3), mRNA [NM_001145062]	**7,876**
Stxbp5	Rattus norvegicus syntaxin binding protein 5 (tomosyn) (Stxbp5), transcript variant b, mRNA [NM_178345]	**7,876**
Cinp	Rattus norvegicus cyclin-dependent kinase 2-interacting protein (Cinp), mRNA [NM_001106758]	**7,875**
Abhd5	Rattus norvegicus abhydrolase domain containing 5 (Abhd5), mRNA [NM_212524]	**7,874**
0	predicted gene 884 Gene [Source:MGI Symbol;Acc:MGI:2685730] [ENSRNOT00000043156]	**7,873**
0	Unknown	**7,872**
Ptcd3	Rattus norvegicus Pentatricopeptide repeat domain 3 (Ptcd3), nuclear gene encoding mitochondrial protein, mRNA [NM_001134718]	**7,872**
0	RCG22822Uncharacterized protein [Source:UniProtKB/TrEMBL;Acc:D3ZYH3] [ENSRNOT00000048236]	**7,872**
Ngrn	Rattus norvegicus neugrin, neurite outgrowth associated (Ngrn), transcript variant 2, mRNA [NM_001033900]	**7,871**
Pex11g	Rattus norvegicus peroxisomal biogenesis factor 11 gamma (Pex11g), mRNA [NM_001105902]	**7,871**
Rpp14	Rattus norvegicus ribonuclease P 14 subunit (human) (Rpp14), transcript variant 1, mRNA [NM_001108372]	**7,871**
RT1-CE4	Rattus norvegicus RT1 class I, locus CE4 (RT1-CE4), mRNA [NM_001008842]	**7,871**
Capn5	Rattus norvegicus calpain 5 (Capn5), mRNA [NM_134461]	**7,871**
Cxadrl1	Rattus norvegicus coxsackie virus and adenovirus receptor-like 1 (Cxadrl1), mRNA [NM_199406]	**7,871**
LOC311026	PREDICTED: Rattus norvegicus similar to mKIAA1461 protein (LOC311026), miscRNA [XR_007266]	**7,870**
Gpx3	Rattus norvegicus glutathione peroxidase 3 (Gpx3), mRNA [NM_022525]	**7,868**
Slc25a20	Rattus norvegicus solute carrier family 25 (carnitine/acylcarnitine translocase), member 20 (Slc25a20), nuclear gene encoding mitochondrial protein, mRNA [NM_053965]	**7,867**
Parp2	Rattus norvegicus poly (ADP-ribose) polymerase 2 (Parp2), mRNA [NM_001106030]	**7,866**
Olr53	Rattus norvegicus olfactory receptor 53 (Olr53), mRNA [NM_001001008]	**7,865**
Ryk	Rattus norvegicus receptor-like tyrosine kinase (Ryk), mRNA [NM_080402]	**7,864**
Ndst3	Rattus norvegicus N-deacetylase/N-sulfotransferase (heparan glucosaminyl) 3 (Ndst3), mRNA [NM_001191716]	**7,862**
0	Unknown	**7,862**
0	Unknown	**7,860**
0	Unknown	**7,860**
Aatk	Rattus norvegicus apoptosis-associated tyrosine kinase (Aatk), mRNA [NM_001168703]	**7,860**
RGD1311072	Rattus norvegicus similar to 2410001C21Rik protein (RGD1311072), mRNA [NM_001033890]	**7,860**

Bahd1	PREDICTED: Rattus norvegicus bromo adjacent homology domain containing 1 (Bahd1), mRNA [XM_347035]	**7,859**
Agbl1	PREDICTED: Rattus norvegicus similar to hypothetical protein D430020F16 (RGD1560280), mRNA [XM_001063833]	**7,859**
Tmem149	Rattus norvegicus transmembrane protein 149 (Tmem149), mRNA [NM_001134610]	**7,859**
Fgf11	Rattus norvegicus fibroblast growth factor 11 (Fgf11), mRNA [NM_130816]	**7,858**
Ehd3	Rattus norvegicus EH-domain containing 3 (Ehd3), mRNA [NM_138890]	**7,855**
Ubqln1	Rattus norvegicus ubiquilin 1 (Ubqln1), mRNA [NM_053747]	**7,854**
Rbm42	Rattus norvegicus RNA binding motif protein 42 (Rbm42), mRNA [NM_001014159]	**7,852**
LOC499124	Rattus norvegicus mouse zinc finger protein 14-like (LOC499124), mRNA [NM_001100991]	**7,851**
Ddhd2	Uncharacterized protein [Source:UniProtKB/TrEMBL;Acc:D3ZJ91] [ENSRNOT00000020832]	**7,851**
Glt25d1	Rattus norvegicus glycosyltransferase 25 domain containing 1 (Glt25d1), mRNA [NM_001106067]	**7,849**
Sc4mol	Rattus norvegicus sterol-C4-methyl oxidase-like (Sc4mol), mRNA [NM_080886]	**7,849**
Wdr43	Rattus norvegicus WD repeat domain 43 (Wdr43), mRNA [NM_001037791]	**7,848**
Spr	Rattus norvegicus sepiapterin reductase (7,8-dihydrobiopterin:NADP+ oxidoreductase) (Spr), mRNA [NM_019181]	**7,847**
Pbrm1	PREDICTED: Rattus norvegicus polybromo 1, transcript variant 2 (Pbrm1), mRNA [XM_001059164]	**7,847**
Thg1l	Rattus norvegicus tRNA-histidine guanylyltransferase 1-like (S. cerevisiae) (Thg1l), mRNA [NM_001013966]	**7,847**
Gabrd	Rattus norvegicus gamma-aminobutyric acid (GABA) A receptor, delta (Gabrd), mRNA [NM_017289]	**7,846**
LOC299558	PREDICTED: Rattus norvegicus similar to Zinc finger protein 44 (Zinc finger protein KOX7) (Gonadotropin-inducible transcription repressor 2) (GIOT-2) (LOC299558), miscRNA [XR_009217]	**7,845**
RGD1559750	PREDICTED: Rattus norvegicus similar to 5-aminoimidazole-4-carboxamide ribonucleotide formyltransferase/IMP cyclohydrolase (RGD1559750), mRNA [XM_227632]	**7,845**
Trim66	Rattus norvegicus tripartite motif-containing 66 (Trim66), mRNA [NM_001191839]	**7,844**
Hist3h2bb	Rattus norvegicus histone cluster 3, H2bb (Hist3h2bb), mRNA [NM_001109641]	**7,844**
Fibcd1	Rattus norvegicus fibrinogen C domain containing 1 (Fibcd1), mRNA [NM_001107829]	**7,842**
Olr1744	Rattus norvegicus olfactory receptor 1744 (Olr1744), mRNA [NM_001001426]	**7,842**
Ppil1	Rattus norvegicus peptidylprolyl isomerase (cyclophilin)-like 1 (Ppil1), mRNA [NM_001034188]	**7,842**
LOC686537	PREDICTED: Rattus norvegicus similar to Carcinoembryonic antigen-related cell adhesion molecule 3 precursor (Carcinoembryonic antigen CGM1) (LOC686537), mRNA [XM_001074635]	**7,842**
0	Uncharacterized protein [Source:UniProtKB/TrEMBL;Acc:D3Z9A9] [ENSRNOT00000015727]	**7,840**
0	Uncharacterized protein [Source:UniProtKB/TrEMBL;Acc:D3Z881] [ENSRNOT00000012600]	**7,838**
Nudcd2	Rattus norvegicus NudC domain containing 2 (Nudcd2), mRNA [NM_001009621]	**7,837**
Gpx8	Rattus norvegicus glutathione peroxidase 8 (Gpx8), mRNA [NM_001106411]	**7,837**
Hey1	Rattus norvegicus hairy/enhancer-of-split related with YRPW motif 1 (Hey1), mRNA [NM_001191845]	**7,835**
Scg3	Rattus norvegicus secretogranin III (Scg3), mRNA [NM_053856]	**7,835**
Ash1l	Rattus norvegicus ash1 (absent, small, or homeotic)-like (Drosophila) (Ash1l), mRNA [NM_001107689]	**7,834**

0	Unknown	**7,833**
Ints2	Rattus norvegicus integrator complex subunit 2, mRNA (cDNA clone MGC:156742 IMAGE:7107619), complete cds. [BC128757]	**7,833**
Rassf5	Rattus norvegicus Ras association (RalGDS/AF-6) domain family member 5 (Rassf5), mRNA [NM_019365]	**7,832**
0	ribonuclease III, nuclear Gene [Source:MGI Symbol;Acc:MGI:1261425] [ENSRNOT00000048026]	**7,832**
0	AMGNNUC:SRPG2-00063-G11-A srpg2 (10238) Rattus norvegicus cDNA clone srpg2-00063-g11 5', mRNA sequence [CB547488]	**7,831**
Trmt1	Rattus norvegicus TRM1 tRNA methyltransferase 1 homolog (S. cerevisiae) (Trmt1), mRNA [NM_001013870]	**7,829**
Mrpl49	Rattus norvegicus mitochondrial ribosomal protein L49 (Mrpl49), nuclear gene encoding mitochondrial protein, mRNA [NM_001047883]	**7,829**
Pign	Rattus norvegicus phosphatidylinositol glycan, class N (Pign), mRNA [NM_001100584]	**7,826**
Olr1625	Rattus norvegicus olfactory receptor 1625 (Olr1625), mRNA [NM_001000971]	**7,826**
0	RGD1308143 protein [Source:UniProtKB/TrEMBL;Acc:Q5XI45] [ENSRNOT00000056439]	**7,826**
Dip2a	Rattus norvegicus DIP2 disco-interacting protein 2 homolog A (Drosophila) (Dip2a), mRNA [NM_001191564]	**7,825**
0	Unknown	**7,825**
Nmral1	Rattus norvegicus NmrA-like family domain containing 1 (Nmral1), mRNA [NM_001191588]	**7,824**
Hdgfrp3	Rattus norvegicus hepatoma-derived growth factor, related protein 3 (Hdgfrp3), mRNA [NM_145785]	**7,822**
0	Unknown	**7,822**
Dicer1	PREDICTED: Rattus norvegicus dicer 1, ribonuclease type III (Dicer1), mRNA [XM_001068155]	**7,821**
Olr1077	Rattus norvegicus olfactory receptor 1077 (Olr1077), mRNA [NM_001000941]	**7,821**
Scp2	Rattus norvegicus sterol carrier protein 2 (Scp2), mRNA [NM_138508]	**7,821**
0	Uncharacterized protein [Source:UniProtKB/TrEMBL;Acc:D4A391] [ENSRNOT00000066991]	**7,821**
Col11a2	Rattus norvegicus collagen, type XI, alpha 2 (Col11a2), mRNA [NM_212528]	**7,820**
Cd200	Rattus norvegicus Cd200 molecule (Cd200), mRNA [NM_031518]	**7,819**
Kank1	Rattus norvegicus KN motif and ankyrin repeat domains 1 (Kank1), mRNA [NM_001037197]	**7,818**
Sfrs18	Rattus norvegicus splicing factor, arginine/serine-rich 18 (Sfrs18), mRNA [NM_001025274]	**7,816**
Camp	Rattus norvegicus cathelicidin antimicrobial peptide (Camp), mRNA [NM_001100724]	**7,816**
0	Unknown	**7,816**
Adat3	Rattus norvegicus adenosine deaminase, tRNA-specific 3, TAD3 homolog (S. cerevisiae) (Adat3), mRNA [NM_001103361]	**7,816**
Ctxn2	Rattus norvegicus cortexin 2 (Ctxn2), mRNA [NM_001162935]	**7,816**
Plcd1	Rattus norvegicus phospholipase C, delta 1 (Plcd1), mRNA [NM_017035]	**7,813**
Rcor1	Rattus norvegicus REST corepressor 1 (Rcor1), mRNA [NM_001108060]	**7,813**
0	Unknown	**7,812**
Fgfr1	Rattus norvegicus Fibroblast growth factor receptor 1 (Fgfr1), mRNA [NM_024146]	**7,812**
Ccrl2	Rattus norvegicus chemokine (C-C motif) receptor-like 2 (Ccrl2), mRNA [NM_001108191]	**7,810**
Pigo	Uncharacterized protein [Source:UniProtKB/TrEMBL;Acc:D3ZTP8] [ENSRNOT00000013213]	**7,810**
Kctd2	PREDICTED: Rattus norvegicus potassium channel tetramerisation domain containing 2 (Kctd2), mRNA [XM_001081684]	**7,810**

Ces2i	Uncharacterized protein [Source:UniProtKB/TrEMBL;Acc:D3ZE31] [ENSRNOT00000015997]	**7,810**
Gpr98	PREDICTED: Rattus norvegicus G protein-coupled receptor 98 (Gpr98), mRNA [XM_002725894]	**7,810**
Aen	Rattus norvegicus apoptosis enhancing nuclease (Aen), mRNA [NM_001108487]	**7,809**
0	Unknown	**7,808**
0	O16480_CAEEL (O16480) Serpentine receptor, class t protein 8, partial (5%) [TC627292]	**7,807**
Shmt2	Rattus norvegicus serine hydroxymethyltransferase 2 (mitochondrial) (Shmt2), nuclear gene encoding mitochondrial protein, mRNA [NM_001008322]	**7,806**
Rmi1	Uncharacterized protein [Source:UniProtKB/TrEMBL;Acc:D3ZHX4] [ENSRNOT00000025812]	**7,805**
Taf8	Rattus norvegicus TAF8 RNA polymerase II, TATA box binding protein (TBP)-associated factor (Taf8), mRNA [NM_001108197]	**7,803**
Gabpb1l	Rattus norvegicus GA binding protein transcription factor, beta subunit 1-like (Gabpb1l), mRNA [NM_001135015]	**7,802**
Ctsa	Rattus norvegicus cathepsin A (Ctsa), mRNA [NM_001011959]	**7,801**
Notch4	Rattus norvegicus Notch homolog 4 (Drosophila) (Notch4), mRNA [NM_001002827]	**7,801**
LOC500475	Rattus norvegicus similar to hypothetical protein 4933430I17 (LOC500475), mRNA [NM_001024346]	**7,800**
Slc7a1	Rattus norvegicus solute carrier family 7 (cationic amino acid transporter, y+ system), member 1 (Slc7a1), mRNA [NM_013111]	**7,799**
Kat2a	Rattus norvegicus K(lysine) acetyltransferase 2A (Kat2a), mRNA [NM_001107050]	**7,799**
Myt1	Rattus norvegicus myelin transcription factor 1 (Myt1), mRNA [NM_001108615]	**7,797**
Map3k7ip1	Rattus norvegicus mitogen-activated protein kinase kinase kinase 7 interacting protein 1 (Map3k7ip1), mRNA [NM_001109976]	**7,797**
LOC682861	PREDICTED: Rattus norvegicus similar to adenomatosis polyposis coli down-regulated 1 (LOC682861), mRNA [XM_001063452]	**7,796**
Rom1	Rattus norvegicus retinal outer segment membrane protein 1 (Rom1), mRNA [NM_001009690]	**7,796**
LOC684293	PREDICTED: Rattus norvegicus similar to synaptotagmin XIV (LOC684293), mRNA [XM_001069763]	**7,795**
LOC689074	Rattus norvegicus similar to Protein KIAA1543 (LOC689074), mRNA [NM_001144840]	**7,795**
0	Unknown	**7,794**
0	Rattus norvegicus similar to RIKEN cDNA A230102I05 (LOC308316), mRNA [XM_218209]	**7,793**
RGD1562997	Unknown	**7,793**
Per3	Rattus norvegicus period homolog 3 (Drosophila) (Per3), mRNA [NM_023978]	**7,791**
Tbccd1	Rattus norvegicus TBCC domain containing 1 (Tbccd1), mRNA [NM_001012016]	**7,790**
Ppfia4	Rattus norvegicus protein tyrosine phosphatase, receptor type, f polypeptide (PTPRF), interacting protein (liprin), alpha 4 (Ppfia4), mRNA [NM_080409]	**7,790**
Akna	Rattus norvegicus AT-hook transcription factor (Akna), mRNA [NM_001108668]	**7,789**
Znf511	Rattus norvegicus zinc finger protein 511 (Znf511), mRNA [NM_001106309]	**7,789**
RGD1307357	Rattus norvegicus similar to hypothetical protein DKFZp434A1319 (RGD1307357), mRNA [NM_001106180]	**7,788**
0	Uncharacterized protein [Source:UniProtKB/TrEMBL;Acc:D3ZLJ4] [ENSRNOT00000041935]	**7,788**
Zdhhc6	Rattus norvegicus zinc finger, DHHC-type containing 6 (Zdhhc6), mRNA [NM_001037652]	**7,787**
0	Unknown	**7,786**

Shroom3	Rattus norvegicus shroom family member 3 (Shroom3), mRNA [NM_001100889]	**7,786**
0	Unknown	**7,786**
0	Glyceraldehyde-3-phosphate dehydrogenase [Source:UniProtKB/TrEMBL;Acc:D3Z8U8] [ENSRNOT00000026712]	**7,784**
LOC690490	PREDICTED: Rattus norvegicus similar to similar to RIKEN cDNA 1700001E04 (LOC690490), mRNA [XM_001074598]	**7,783**
Wdr8	Rattus norvegicus WD repeat domain 8 (Wdr8), mRNA [NM_001014262]	**7,781**
Ireb2	Rattus norvegicus iron responsive element binding protein 2 (Ireb2), mRNA [NM_022863]	**7,781**
Nupr1	Rattus norvegicus nuclear protein, transcriptional regulator, 1 (Nupr1), mRNA [NM_053611]	**7,780**
LOC494539	PREDICTED: Rattus norvegicus spectrin beta-like (LOC494539), miscRNA [XR_085708]	**7,779**
Slc38a4	Rattus norvegicus solute carrier family 38, member 4 (Slc38a4), mRNA [NM_130748]	**7,779**
Ms4a15	Rattus norvegicus membrane-spanning 4-domains, subfamily A, member 15 (Ms4a15), mRNA [NM_001191897]	**7,779**
Gmeb1	Rattus norvegicus glucocorticoid modulatory element binding protein 1 (Gmeb1), mRNA [NM_001109268]	**7,779**
Igf2r	Rattus norvegicus insulin-like growth factor 2 receptor (Igf2r), mRNA [NM_012756]	**7,778**
0	Unknown	**7,778**
Cd69	Rattus norvegicus Cd69 molecule (Cd69), mRNA [NM_134327]	**7,777**
0	Hydin protein [Source:UniProtKB/TrEMBL;Acc:Q5BJQ9] [ENSRNOT00000059128]	**7,775**
Il1rn	Rattus norvegicus interleukin 1 receptor antagonist (Il1rn), mRNA [NM_022194]	**7,773**
Srebf1	Sterol regulatory element-binding protein 1Processed sterol regulatory element-binding protein 1 [Source:UniProtKB/Swiss-Prot;Acc:P56720] [ENSRNOT00000047053]	**7,772**
Med16	Rattus norvegicus mediator complex subunit 16 (Med16), mRNA [NM_001106768]	**7,770**
0	Uncharacterized protein [Source:UniProtKB/TrEMBL;Acc:D3ZXP2] [ENSRNOT00000041819]	**7,769**
Fkbp1b	Rattus norvegicus FK506 binding protein 1b (Fkbp1b), mRNA [NM_022675]	**7,768**
Pip5k1a	Rattus norvegicus phosphatidylinositol-4-phosphate 5-kinase, type 1, alpha (Pip5k1a), mRNA [NM_001042621]	**7,768**
Znf516	Rattus norvegicus zinc finger protein 516 (Znf516), mRNA [NM_001191695]	**7,768**
Fem1b	Rattus norvegicus fem-1 homolog b (C. elegans) (Fem1b), mRNA [NM_001108157]	**7,767**
Fah	Rattus norvegicus fumarylacetoacetate hydrolase (Fah), mRNA [NM_017181]	**7,765**
0	Rattus norvegicus TL0AEA7YD23 mRNA sequence. [FQ231120]	**7,764**
LOC301748	PREDICTED: Rattus norvegicus similar to RIKEN cDNA 1700001E04 (LOC301748), mRNA [XM_002730085]	**7,764**
Anks1a	Rattus norvegicus ankyrin repeat and sterile alpha motif domain containing 1A (Anks1a), mRNA [NM_001107613]	**7,763**
Msgn1	Rattus norvegicus mesogenin 1 (Msgn1), mRNA [NM_001109551]	**7,762**
Usp2	Rattus norvegicus ubiquitin specific peptidase 2 (Usp2), mRNA [NM_053774]	**7,762**
Zfp212	PREDICTED: Rattus norvegicus Zinc finger protein 212 (Zfp212), mRNA [XM_001053455]	**7,762**
Fam179a	Uncharacterized protein [Source:UniProtKB/TrEMBL;Acc:D4A1L0] [ENSRNOT00000036374]	**7,761**
Dbc1	Rattus norvegicus deleted in bladder cancer 1 (human) (Dbc1), mRNA [NM_080482]	**7,760**
Cab39	Rattus norvegicus calcium binding protein 39 (Cab39), mRNA [NM_001106924]	**7,758**

Mcm7	Rattus norvegicus minichromosome maintenance complex component 7 (Mcm7), mRNA [NM_001004203]	**7,758**
Gpr21	Rattus norvegicus G protein-coupled receptor 21 (Gpr21), mRNA [NM_001109554]	**7,757**
LOC500251	Rattus norvegicus hypothetical protein LOC500251 (LOC500251), mRNA [NM_001025047]	**7,757**
Kcnn2	Rattus norvegicus potassium intermediate/small conductance calcium-activated channel, subfamily N, member 2 (Kcnn2), mRNA [NM_019314]	**7,756**
RGD1307569	PREDICTED: Rattus norvegicus similar to Protein C21orf63 homolog precursor, transcript variant 1 (RGD1307569), mRNA [XM_001073261]	**7,754**
Nol10	Rattus norvegicus nucleolar protein 10 (Nol10), mRNA [NM_001014076]	**7,753**
Kcnh2	Rattus norvegicus potassium voltage-gated channel, subfamily H (eag-related), member 2 (Kcnh2), mRNA [NM_053949]	**7,753**
Psx1	PREDICTED: Rattus norvegicus placenta specific homeobox 1 (Psx1), mRNA [XM_231064]	**7,753**
Usp7	Rattus norvegicus ubiquitin specific peptidase 7 (herpes virus-associated) (Usp7), mRNA [NM_001024790]	**7,752**
LOC687295	PREDICTED: Rattus norvegicus similar to translocase of inner mitochondrial membrane 50 homolog, transcript variant 2 (LOC687295), mRNA [XM_001073346]	**7,752**
Kctd3	Rattus norvegicus potassium channel tetramerisation domain containing 3 (Kctd3), mRNA [NM_001107199]	**7,749**
Cox19	Rattus norvegicus COX19 cytochrome c oxidase assembly homolog (S. cerevisiae) (Cox19), mRNA [NM_001107126]	**7,748**
Slit1	Rattus norvegicus slit homolog 1 (Drosophila) (Slit1), mRNA [NM_022953]	**7,748**
Hip1r	Rattus norvegicus huntingtin interacting protein 1 related (Hip1r), transcript variant 1, mRNA [NM_001134763]	**7,748**
Nup214	Rattus norvegicus nucleoporin 214 (Nup214), mRNA [NM_001168559]	**7,747**
0	Rattus norvegicus similar to hypothetical protein DKFZp761B107 (LOC305415), mRNA [XM_223492]	**7,746**
Fam8a1	Uncharacterized protein [Source:UniProtKB/TrEMBL;Acc:D3ZBT7] [ENSRNOT00000060500]	**7,744**
Igfbp7	Rattus norvegicus insulin-like growth factor binding protein 7 (Igfbp7), mRNA [NM_001013048]	**7,744**
LOC366300	PREDICTED: Rattus norvegicus hypothetical LOC366300 (LOC366300), mRNA [XM_002726499]	**7,744**
0	RGD1311624 protein [Source:UniProtKB/TrEMBL;Acc:Q4G026] [ENSRNOT00000054990]	**7,743**
0	Unknown	**7,742**
0	Unknown	**7,742**
Prune	Rattus norvegicus prune homolog (Drosophila) (Prune), mRNA [NM_001007697]	**7,742**
Tppp3	Rattus norvegicus tubulin polymerization-promoting protein family member 3 (Tppp3), mRNA [NM_001009639]	**7,741**
Dimt1l	Rattus norvegicus DIM1 dimethyladenosine transferase 1-like (S. cerevisiae) (Dimt1l), mRNA [NM_001106408]	**7,741**
Akap1	A-kinase anchor protein 1, mitochondrial [Source:UniProtKB/Swiss-Prot;Acc:O88884] [ENSRNOT00000003251]	**7,740**
Ankrd50	Rattus norvegicus ankyrin repeat domain 50 (Ankrd50), mRNA [NM_001191606]	**7,740**
Akr1b10	Rattus norvegicus aldo-keto reductase family 1, member B10 (aldose reductase) (Akr1b10), mRNA [NM_001013084]	**7,739**
Farp2	Rattus norvegicus FERM, RhoGEF and pleckstrin domain protein 2 (Farp2), mRNA [NM_001108233]	**7,736**
Gnai3	Rattus norvegicus guanine nucleotide binding protein (G protein), alpha inhibiting 3 (Gnai3), mRNA [NM_013106]	**7,736**
Klhl35	Uncharacterized protein [Source:UniProtKB/TrEMBL;Acc:D4A6V5] [ENSRNOT00000023386]	**7,734**
Ska2	Rattus norvegicus spindle and kinetochore associated complex subunit 2 (Ska2), mRNA [NM_001009624]	**7,733**
0	mannosyl-oligosaccharide 1,2-alpha-mannosidase IA [Source:RefSeq peptide;Acc:NP_001028828] [ENSRNOT00000041738]	**7,733**

Spata13	Rattus norvegicus spermatogenesis associated 13 (Spata13), mRNA [NM_001191686]	**7,733**
Myocd	Rattus norvegicus myocardin (Myocd), mRNA [NM_182667]	**7,732**
0	Uncharacterized protein [Source:UniProtKB/TrEMBL;Acc:D3ZPI3] [ENSRNOT00000038791]	**7,732**
Ppfia2	Rattus norvegicus protein tyrosine phosphatase, receptor type, f polypeptide (PTPRF), interacting protein (liprin), alpha 2 (Ppfia2), mRNA [NM_001108745]	**7,731**
Arap2	Rattus norvegicus ArfGAP with RhoGAP domain, ankyrin repeat and PH domain 2 (Arap2), mRNA [NM_001107216]	**7,731**
Cyp2b3	Rattus norvegicus cytochrome P450, family 2, subfamily b, polypeptide 3 (Cyp2b3), mRNA [NM_173294]	**7,729**
Igfbp3	Rattus norvegicus insulin-like growth factor binding protein 3 (Igfbp3), mRNA [NM_012588]	**7,727**
Mtdh	Rattus norvegicus metadherin (Mtdh), mRNA [NM_133398]	**7,727**
Secisbp2l	Rattus norvegicus SECIS binding protein 2-like (Secisbp2l), mRNA [NM_001168527]	**7,726**
LOC687465	PREDICTED: Rattus norvegicus hypothetical protein LOC687465 (LOC687465), mRNA [XM_001078693]	**7,726**
Psmd1	Rattus norvegicus proteasome (prosome, macropain) 26S subunit, non-ATPase, 1 (Psmd1), mRNA [NM_031978]	**7,726**
RGD1307603	Rattus norvegicus similar to hypothetical protein MGC37914 (RGD1307603), mRNA [NM_001134508]	**7,725**
LOC308954	Rattus norvegicus similar to hypothetical protein MGC50721 (LOC308954), mRNA [NM_001024777]	**7,725**
0	Unknown	**7,724**
Adck1	Rattus norvegicus aarF domain containing kinase 1 (Adck1), mRNA [NM_001108985]	**7,722**
Cdc73	Rattus norvegicus cell division cycle 73, Paf1/RNA polymerase II complex component, homolog (S. cerevisiae) (Cdc73), mRNA [NM_001024769]	**7,722**
0	Q80XK5_MOUSE (Q80XK5) Frmpd1 protein (Fragment), partial (23%) [TC584718]	**7,721**
Rnasel	Rattus norvegicus ribonuclease L (2',5'-oligoisoadenylate synthetase-dependent) (Rnasel), mRNA [NM_182673]	**7,720**
Pomgnt1	Rattus norvegicus protein O-linked mannose beta1,2-N-acetylglucosaminyltransferase (Pomgnt1), mRNA [NM_001007747]	**7,720**
Arhgap10	Rattus norvegicus Rho GTPase activating protein 10 (Arhgap10), mRNA [NM_001109501]	**7,720**
Apbb1ip	Rattus norvegicus amyloid beta (A4) precursor protein-binding, family B, member 1 interacting protein (Apbb1ip), mRNA [NM_001100577]	**7,719**
0	PREDICTED: Rattus norvegicus SPT2, Suppressor of Ty, domain containing 1 (S. cerevisiae) (Spty2d1), mRNA [XM_002726380]	**7,718**
Crebzf	Rattus norvegicus CREB/ATF bZIP transcription factor (Crebzf), mRNA [NM_001106279]	**7,718**
Dennd3	PREDICTED: Rattus norvegicus DENN/MADD domain containing 3 (Dennd3), mRNA [XM_001072997]	**7,718**
Hars2l	Rattus norvegicus histidyl-tRNA synthetase 2-like (Hars2l), mRNA [NM_001014012]	**7,717**
RGD1305481	Rattus norvegicus hypothetical LOC294030 (RGD1305481), mRNA [NM_001047856]	**7,717**
Scgb3a1	Rattus norvegicus secretoglobin, family 3A, member 1 (Scgb3a1), mRNA [NM_001013180]	**7,716**
Dcaf6	Similar to IQ motif and WD repeats 1 (Predicted)Uncharacterized protein [Source:UniProtKB/TrEMBL;Acc:D4A8L4] [ENSRNOT00000004199]	**7,715**
Olr1701	Rattus norvegicus olfactory receptor 1701 (Olr1701), mRNA [NM_001001114]	**7,713**
0	Unknown	**7,713**
RGD1559859	PREDICTED: Rattus norvegicus RGD1559859 (RGD1559859), mRNA [XM_001072031]	**7,713**
Twistnb	Rattus norvegicus TWIST neighbor (Twistnb), mRNA [NM_001108707]	**7,713**
0	Unknown	**7,713**

Snapc5	Rattus norvegicus small nuclear RNA activating complex, polypeptide 5 (Snapc5), mRNA [NM_001109643]	**7,711**
Coch	Rattus norvegicus coagulation factor C homolog, cochlin (Limulus polyphemus) (Coch), mRNA [NM_001108710]	**7,710**
S100a10	Rattus norvegicus S100 calcium binding protein A10 (S100a10), mRNA [NM_031114]	**7,710**
Gpld1	Rattus norvegicus glycosylphosphatidylinositol specific phospholipase D1 (Gpld1), mRNA [NM_001100512]	**7,710**
0	Unknown	**7,709**
Dstn	Rattus norvegicus destrin (Dstn), mRNA [NM_001033666]	**7,708**
Evl	Rattus norvegicus Enah/Vasp-like (Evl), mRNA [NM_024147]	**7,707**
Klhl24	Rattus norvegicus kelch-like 24 (Drosophila) (Klhl24), mRNA [NM_181473]	**7,707**
RGD1560046	PREDICTED: Rattus norvegicus similar to 3110052M02Rik protein (RGD1560046), mRNA [XM_574335]	**7,706**
0	membrane-bound transcription factor site-2 protease [Source:RefSeq peptide;Acc:NP_001030179] [ENSRNOT00000010089]	**7,706**
Fbxo28	Rattus norvegicus F-box protein 28 (Fbxo28), mRNA [NM_001107203]	**7,705**
Uqcrh	Rattus norvegicus ubiquinol-cytochrome c reductase hinge protein (Uqcrh), nuclear gene encoding mitochondrial protein, mRNA [NM_001009480]	**7,704**
0	2',5'-phosphodiesterase 12 [Source:UniProtKB/Swiss-Prot;Acc:Q6AXQ5] [ENSRNOT00000017800]	**7,704**
LOC680722	PREDICTED: Rattus norvegicus hypothetical protein LOC680722 (LOC680722), mRNA [XM_001058566]	**7,703**
Vom1r22	Rattus norvegicus vomeronasal 1 receptor 22 (Vom1r22), mRNA [NM_001008900]	**7,703**
Prpg1	Rattus norvegicus proline-rich proteoglycan 1 (Prpg1), mRNA [NM_172064]	**7,701**
0	Unknown	**7,700**
Spcs3	Rattus norvegicus signal peptidase complex subunit 3 homolog (S. cerevisiae) (Spcs3), mRNA [NM_001191073]	**7,700**
Zxdc	Uncharacterized protein [Source:UniProtKB/TrEMBL;Acc:D3ZYP0] [ENSRNOT00000038244]	**7,700**
Rbm15b	RCG25263Uncharacterized protein [Source:UniProtKB/TrEMBL;Acc:D3ZHD6] [ENSRNOT00000018995]	**7,699**
Cdc25b	Rattus norvegicus cell division cycle 25 homolog B (S. pombe) (Cdc25b), mRNA [NM_133572]	**7,699**
Tnfrsf4	Rattus norvegicus tumor necrosis factor receptor superfamily, member 4 (Tnfrsf4), mRNA [NM_013049]	**7,697**
Grsf1	Rattus norvegicus G-rich RNA sequence binding factor 1 (Grsf1), mRNA [NM_001100890]	**7,696**
Supv3l1	Rattus norvegicus suppressor of var1, 3-like 1 (S. cerevisiae) (Supv3l1), mRNA [NM_001012462]	**7,695**
Znf597	Rattus norvegicus zinc finger protein 597 (Znf597), mRNA [NM_153732]	**7,694**
Galnt3	Rattus norvegicus UDP-N-acetyl-alpha-D-galactosamine:polypeptide N-acetylgalactosaminyltransferase 3 (GalNAc-T3) (Galnt3), mRNA [NM_001015032]	**7,694**
Arfgap2	Rattus norvegicus ADP-ribosylation factor GTPase activating protein 2 (Arfgap2), mRNA [NM_001033707]	**7,694**
Arrdc2	Rattus norvegicus arrestin domain containing 2 (Arrdc2), mRNA [NM_001107303]	**7,693**
Nup160	Rattus norvegicus nucleoporin 160 (Nup160), mRNA [NM_001107744]	**7,693**
Lgr5	Rattus norvegicus leucine rich repeat containing G protein coupled receptor 5 (Lgr5), mRNA [NM_001106784]	**7,691**
0	Unknown	**7,690**
Med10	Rattus norvegicus mediator complex subunit 10 (Med10), mRNA [NM_001106097]	**7,690**
Apex2	Uncharacterized protein [Source:UniProtKB/TrEMBL;Acc:D3ZHV4] [ENSRNOT00000038157]	**7,689**

0	Golgi integral membrane protein 4 [Source:UniProtKB/Swiss-Prot;Acc:Q5BJK8] [ENSRNOT00000064588]	**7,686**
0	Unknown	**7,685**
Mtmr6	Rattus norvegicus myotubularin related protein 6 (Mtmr6), mRNA [NM_001107268]	**7,685**
Rab11fip3	Peroxisomal 2,4-dienoyl-CoA reductase [Source:UniProtKB/Swiss-Prot;Acc:Q9Z2M4] [ENSRNOT00000027467]	**7,683**
Zfp90	Rattus norvegicus zinc finger protein 90 (Zfp90), mRNA [NM_001025765]	**7,683**
Vstm2b	Rattus norvegicus V-set and transmembrane domain containing 2B (Vstm2b), mRNA [NM_001108479]	**7,681**
Tll2	Rattus norvegicus tolloid-like 2 (Tll2), mRNA [NM_001191898]	**7,680**
0	Rattus norvegicus similar to ets variant gene 5 (LOC294776), mRNA [XM_226808]	**7,680**
Rhpn2	Rattus norvegicus rhophilin, Rho GTPase binding protein 2 (Rhpn2), mRNA [NM_001107505]	**7,680**
0	Unknown	**7,679**
Lypd3	Rattus norvegicus Ly6/Plaur domain containing 3 (Lypd3), mRNA [NM_021759]	**7,678**
LOC298795	Rattus norvegicus similar to 14-3-3 protein sigma (LOC298795), mRNA [NM_001013941]	**7,677**
Mrpl40	Rattus norvegicus mitochondrial ribosomal protein L40 (Mrpl40), nuclear gene encoding mitochondrial protein, mRNA [NM_001024865]	**7,671**
Ppp3r1	Rattus norvegicus protein phosphatase 3, regulatory subunit B, alpha isoform (Ppp3r1), mRNA [NM_017309]	**7,670**
Lass5	Rattus norvegicus LAG1 homolog, ceramide synthase 5 (Lass5), mRNA [NM_001108993]	**7,669**
Haus8	Rattus norvegicus HAUS augmin-like complex, subunit 8 (Haus8), mRNA [NM_001024971]	**7,669**
0	Palladin [Source:UniProtKB/Swiss-Prot;Acc:P0C5E3] [ENSRNOT00000013636]	**7,669**
Tmem223	Rattus norvegicus transmembrane protein 223 (Tmem223), mRNA [NM_001191104]	**7,668**
LOC691254	PREDICTED: Rattus norvegicus hypothetical protein LOC691254 (LOC691254), mRNA [XM_001077402]	**7,668**
0	Unknown	**7,667**
0	Unknown	**7,666**
0	Uncharacterized protein [Source:UniProtKB/TrEMBL;Acc:D3Z9J6] [ENSRNOT00000043839]	**7,666**
RGD1563888	Rattus norvegicus similar to DNA segment, Chr 16, ERATO Doi 472, expressed (RGD1563888), mRNA [NM_001108312]	**7,666**
Snx16	Rattus norvegicus sorting nexin 16 (Snx16), mRNA [NM_022289]	**7,665**
RGD1565390	PREDICTED: Rattus norvegicus similar to putative protein kinase (RGD1565390), mRNA [XM_344843]	**7,664**
0	Unknown	**7,661**
Nfkbid	PREDICTED: Rattus norvegicus nuclear factor of kappa light polypeptide gene enhancer in B-cells inhibitor, delta (Nfkbid), mRNA [XM_001079086]	**7,660**
Actbl2	Rattus norvegicus actin, beta-like 2 (Actbl2), mRNA [NM_001106409]	**7,660**
Gpr27	Rattus norvegicus G protein-coupled receptor 27 (Gpr27), mRNA [NM_023099]	**7,657**
Zfp426l	Rattus norvegicus zinc finger protein 426-like (Zfp426l), mRNA [NM_001135018]	**7,655**
Ino80b	Rattus norvegicus INO80 complex subunit B (Ino80b), mRNA [NM_001048233]	**7,654**
Sfrs9	Rattus norvegicus splicing factor, arginine/serine-rich 9 (Sfrs9), mRNA [NM_001009255]	**7,654**
Unc84a	Rattus norvegicus unc-84 homolog A (C. elegans) (Unc84a), mRNA [NM_001007147]	**7,653**
LOC679934	Rattus norvegicus similar to chromosome 3 open reading frame 10 (LOC679934), mRNA [NM_001195476]	**7,651**

Klf14	Rattus norvegicus Kruppel-like factor 14 (Klf14), mRNA [NM_001135094]	**7,650**
Rif1	Uncharacterized protein [Source:UniProtKB/TrEMBL;Acc:D3ZLT9] [ENSRNOT00000008201]	**7,649**
Zc3h13	Rattus norvegicus zinc finger CCCH type containing 13 (Zc3h13), mRNA [NM_001170471]	**7,649**
0	TF7L2_HUMAN (Q9NQB0) Transcription factor 7-like 2 (HMG box transcription factor 4) (T-cell-specific transcription factor 4) (TCF-4) (hTCF-4), partial (58%) [TC603124]	**7,649**
0	Unknown	**7,647**
RGD1563667	Rattus norvegicus similar to TDPOZ3 (RGD1563667), mRNA [NM_001100985]	**7,647**
Fam123c	Uncharacterized protein [Source:UniProtKB/TrEMBL;Acc:D3ZHS8] [ENSRNOT00000033700]	**7,646**
S100a6	Rattus norvegicus S100 calcium binding protein A6 (S100a6), mRNA [NM_053485]	**7,645**
Vezt	Rattus norvegicus vezatin, adherens junctions transmembrane protein (Vezt), mRNA [NM_001006984]	**7,644**
Cdkl2	Rattus norvegicus cyclin-dependent kinase-like 2 (CDC2-related kinase) (Cdkl2), mRNA [NM_001012035]	**7,643**
Eif2s1	Rattus norvegicus eukaryotic translation initiation factor 2, subunit 1 alpha (Eif2s1), mRNA [NM_019356]	**7,643**
LOC300308	Rattus norvegicus similar to hypothetical protein 4930509O22 (LOC300308), mRNA [NM_001013952]	**7,642**
Hexim2	Rattus norvegicus hexamthylene bis-acetamide inducible 2 (Hexim2), mRNA [NM_001107054]	**7,642**
Csrnp1	Rattus norvegicus cysteine-serine-rich nuclear protein 1 (Csrnp1), mRNA [NM_001108786]	**7,642**
0	Unknown	**7,641**
Rwdd2b	Rattus norvegicus RWD domain containing 2B (Rwdd2b), mRNA [NM_001100559]	**7,641**
Mgat2	Rattus norvegicus mannosyl (alpha-1,6-)-glycoprotein beta-1,2-N-acetylglucosaminyltransferase (Mgat2), mRNA [NM_053604]	**7,640**
Macrod1	Rattus norvegicus MACRO domain containing 1 (Macrod1), mRNA [NM_139337]	**7,638**
Mccc2	Rattus norvegicus methylcrotonoyl-Coenzyme A carboxylase 2 (beta) (Mccc2), nuclear gene encoding mitochondrial protein, mRNA [NM_001012177]	**7,637**
Zfp84	Rattus norvegicus zinc finger protein 84 (Zfp84), mRNA [NM_001107500]	**7,637**
0	Rattus norvegicus Ac1-290 mRNA, complete cds. [AY321322]	**7,636**
Mettl6	Rattus norvegicus methyltransferase like 6 (Mettl6), mRNA [NM_001007623]	**7,636**
Stk17b	Rattus norvegicus serine/threonine kinase 17b (Stk17b), mRNA [NM_133392]	**7,635**
Rbm3	Rattus norvegicus RNA binding motif (RNP1, RRM) protein 3 (Rbm3), mRNA [NM_053696]	**7,634**
RGD1311745	Rattus norvegicus similar to RIKEN cDNA 1110059G10 (RGD1311745), mRNA [NM_001008329]	**7,634**
0	Uncharacterized protein [Source:UniProtKB/TrEMBL;Acc:D3ZNC7] [ENSRNOT00000057981]	**7,634**
0	Unknown	**7,633**
RGD1565859	PREDICTED: Rattus norvegicus RGD1565859 (RGD1565859), mRNA [XM_001063482]	**7,632**
Slco4c1	Rattus norvegicus solute carrier organic anion transporter family, member 4C1 (Slco4c1), mRNA [NM_001002024]	**7,632**
Gcat	Rattus norvegicus glycine C-acetyltransferase (2-amino-3-ketobutyrate-coenzyme A ligase) (Gcat), nuclear gene encoding mitochondrial protein, mRNA [NM_001024277]	**7,631**
RGD1306746	Rattus norvegicus similar to Hypothetical protein MGC25529 (RGD1306746), mRNA [NM_001107872]	**7,631**

Trim11	Rattus norvegicus tripartite motif-containing 11 (Trim11), mRNA [NM_001108276]	**7,629**
Zp3	Rattus norvegicus zona pellucida glycoprotein 3 (sperm receptor) (Zp3), mRNA [NM_053762]	**7,627**
0	Unknown	**7,627**
RGD1308430	Rattus norvegicus similar to 1700123O20Rik protein (RGD1308430), mRNA [NM_001108376]	**7,626**
0	Unknown	**7,625**
Phyh	Rattus norvegicus phytanoyl-CoA 2-hydroxylase (Phyh), mRNA [NM_053674]	**7,624**
0	Unknown	**7,623**
0	Unknown	**7,623**
0	Unknown	**7,623**
0	Unknown	**7,623**
Pde10a	Rattus norvegicus phosphodiesterase 10A (Pde10a), mRNA [NM_022236]	**7,622**
LOC688083	PREDICTED: Rattus norvegicus similar to LRP16 protein, transcript variant 2 (LOC688083), mRNA [XM_001080245]	**7,621**
Gpr165	Rattus norvegicus G protein-coupled receptor 165 (Gpr165), mRNA [NM_001106582]	**7,621**
Usp25	Rattus norvegicus ubiquitin specific peptidase 25 (Usp25), mRNA [NM_001107114]	**7,621**
Rtn4rl1	Rattus norvegicus reticulon 4 receptor-like 1 (Rtn4rl1), mRNA [NM_181377]	**7,620**
Gabra4	Rattus norvegicus gamma-aminobutyric acid (GABA) A receptor, alpha 4 (Gabra4), mRNA [NM_080587]	**7,619**
Cdk105	Rattus norvegicus CDK105 protein (Cdk105), mRNA [NM_134415]	**7,618**
Ephx1	Rattus norvegicus epoxide hydrolase 1, microsomal (Ephx1), transcript variant 2, mRNA [NM_012844]	**7,617**
0	Unknown	**7,614**
0	Uncharacterized protein [Source:UniProtKB/TrEMBL;Acc:D4A391] [ENSRNOT00000066991]	**7,614**
Serpinb6a	Rattus norvegicus serine (or cysteine) peptidase inhibitor, clade B, member 6a (Serpinb6a), mRNA [NM_199085]	**7,614**
Lenep	Rattus norvegicus lens epithelial protein (Lenep), mRNA [NM_053614]	**7,613**
0	Unknown	**7,612**
LOC684274	Rattus norvegicus similar to RIKEN cDNA 2810012L14 (LOC684274), mRNA [NM_001115041]	**7,611**
Bcat2	Rattus norvegicus branched chain aminotransferase 2, mitochondrial (Bcat2), nuclear gene encoding mitochondrial protein, mRNA [NM_022400]	**7,610**
Coq2	Rattus norvegicus coenzyme Q2 homolog, prenyltransferase (yeast) (Coq2), nuclear gene encoding mitochondrial protein, mRNA [NM_001044255]	**7,610**
Gpha2	Rattus norvegicus glycoprotein hormone alpha 2 (Gpha2), mRNA [NM_133619]	**7,609**
Usp1	Rattus norvegicus ubiquitin specific peptidase 1 (Usp1), mRNA [NM_001015015]	**7,605**
0	Unknown	**7,605**
LOC687708	Rattus norvegicus hypothetical protein LOC687708 (LOC687708), mRNA [NM_001101016]	**7,605**
Eif5b	Rattus norvegicus eukaryotic translation initiation factor 5B (Eif5b), mRNA [NM_001110141]	**7,604**
Zbtb38	Rattus norvegicus zinc finger and BTB domain containing 38 (Zbtb38), mRNA [NM_001012471]	**7,602**
Ckmt1	Rattus norvegicus creatine kinase, mitochondrial 1 (Ckmt1), nuclear gene encoding mitochondrial protein, mRNA [NM_001012738]	**7,602**

Sstr4	Rattus norvegicus somatostatin receptor 4 (Sstr4), mRNA [NM_013036]	**7,601**
Osgepl1	Rattus norvegicus O-sialoglycoprotein endopeptidase-like 1 (Osgepl1), mRNA [NM_001024787]	**7,600**
B3galt2	Rattus norvegicus UDP-Gal:betaGlcNAc beta 1,3-galactosyltransferase, polypeptide 2 (B3galt2), mRNA [NM_001109492]	**7,600**
0	Rattus norvegicus similar to glyceraldehyde-3-phosphate dehydrogenase (phosphorylating) (EC 1.2.1.12) - mouse (LOC302809), mRNA [XM_229109]	**7,600**
Npy5r	Rattus norvegicus neuropeptide Y receptor Y5 (Npy5r), mRNA [NM_012869]	**7,599**
Gpr149	Rattus norvegicus G protein-coupled receptor 149 (Gpr149), mRNA [NM_138891]	**7,599**
LOC685909	Rattus norvegicus similar to H2A histone family, member V isoform 1 (LOC685909), mRNA [NM_001106019]	**7,599**
Rnf168	Rattus norvegicus ring finger protein 168 (Rnf168), mRNA [NM_001127597]	**7,599**
Zfp653	Rattus norvegicus zinc finger protein 653 (Zfp653), mRNA [NM_001106807]	**7,598**
RGD1306917	Rattus norvegicus similar to RIKEN cDNA 2900010M23 (RGD1306917), mRNA [NM_001108528]	**7,595**
Prkag3	Rattus norvegicus protein kinase, AMP-activated, gamma 3 non-catalytic subunit (Prkag3), mRNA [NM_001106921]	**7,595**
Armc9	Rattus norvegicus armadillo repeat containing 9 (Armc9), mRNA [NM_001109663]	**7,593**
Tnks2	Rattus norvegicus tankyrase, TRF1-interacting ankyrin-related ADP-ribose polymerase 2 (Tnks2), mRNA [NM_001107607]	**7,593**
Zeb2	Rattus norvegicus zinc finger E-box binding homeobox 2 (Zeb2), mRNA [NM_001033701]	**7,593**
Slc35b4	Rattus norvegicus solute carrier family 35, member B4 (Slc35b4), mRNA [NM_001106590]	**7,592**
RGD1310262	Rattus norvegicus hypothetical LOC304650 (RGD1310262), mRNA [NM_001107161]	**7,591**
Cabyr	Rattus norvegicus calcium binding tyrosine-(Y)-phosphorylation regulated (Cabyr), mRNA [NM_001143893]	**7,590**
Mon1a	Rattus norvegicus MON1 homolog A (yeast) (Mon1a), mRNA [NM_001126284]	**7,588**
0	Unknown	**7,588**
RGD1309821	Rattus norvegicus similar to KIAA1161 protein (RGD1309821), mRNA [NM_001108971]	**7,588**
RGD1563634	Rattus norvegicus similar to R31449_3 (RGD1563634), mRNA [NM_001081447]	**7,587**
0	Uncharacterized protein [Source:UniProtKB/TrEMBL;Acc:D3ZJN7] [ENSRNOT00000003207]	**7,587**
LOC498601	PREDICTED: Rattus norvegicus similar to cyclin B2 (LOC498601), miscRNA [XR_006448]	**7,586**
C1ql4	Complement component 1, q subcomponent-like 4 (Predicted)Uncharacterized protein [Source:UniProtKB/TrEMBL;Acc:D3ZMN4] [ENSRNOT00000003152]	**7,586**
Plscr1	Rattus norvegicus phospholipid scramblase 1 (Plscr1), mRNA [NM_057194]	**7,586**
Zfp709l2	Rattus norvegicus zinc finger protein 709-like 2 (Zfp709l2), mRNA [NM_001163062]	**7,586**
Rgs11	Rattus norvegicus regulator of G-protein signaling 11 (Rgs11), mRNA [NM_019338]	**7,583**
Vav2	Rattus norvegicus vav 2 guanine nucleotide exchange factor (Vav2), mRNA [NM_001106563]	**7,583**
Slc16a6	Rattus norvegicus solute carrier family 16, member 6 (monocarboxylic acid transporter 7) (Slc16a6), mRNA [NM_198760]	**7,582**
Cisd2	Rattus norvegicus CDGSH iron sulfur domain 2 (Cisd2), mRNA [NM_001191608]	**7,582**
0	Uncharacterized protein [Source:UniProtKB/TrEMBL;Acc:D3ZAK7] [ENSRNOT00000038415]	**7,581**
Mrpl35	Rattus norvegicus mitochondrial ribosomal protein L35 (Mrpl35), nuclear gene encoding mitochondrial protein, mRNA [NM_001106596]	**7,579**

Ttll11	Ttll11 protein [Source:UniProtKB/TrEMBL;Acc:B5DFG3] [ENSRNOT00000032204]	**7,579**
RGD1559662	PREDICTED: Rattus norvegicus similar to implantation serine proteinase 2 (RGD1559662), mRNA [XM_001059769]	**7,577**
Micb	Rattus norvegicus MHC class I polypeptide-related sequence B (Micb), mRNA [NM_001017468]	**7,577**
RGD1563365	PREDICTED: Rattus norvegicus similar to DNA segment, Chr 10, Wayne State University 102, expressed (RGD1563365), mRNA [XM_002729760]	**7,577**
0	PREDICTED: Rattus norvegicus similar to High mobility group protein 1 (HMG-1) (High mobility group protein B1) (Amphoterin) (Heparin-binding protein p30) (LOC685091), mRNA [XM_001062256]	**7,574**
0	Uncharacterized protein [Source:UniProtKB/TrEMBL;Acc:D3Z8J8] [ENSRNOT00000058230]	**7,572**
0	Unknown	**7,570**
LOC100302465	Rattus norvegicus hypothetical LOC100302465 (LOC100302465), mRNA [NM_001163214]	**7,570**
LOC688909	PREDICTED: Rattus norvegicus hypothetical protein LOC688909 (LOC688909), mRNA [XM_001068783]	**7,568**
LOC680039	Rattus norvegicus hypothetical protein LOC680039 (LOC680039), mRNA [NM_001109388]	**7,568**
0	Unknown	**7,567**
Pdcl2	PREDICTED: Rattus norvegicus phosducin-like 2 (Pdcl2), mRNA [XM_001076368]	**7,566**
LOC685685	PREDICTED: Rattus norvegicus similar to S100 calcium-binding protein, ventral prostate (LOC685685), mRNA [XM_001064819]	**7,565**
LOC689207	PREDICTED: Rattus norvegicus similar to proteoglycan 4 (LOC689207), mRNA [XM_001069975]	**7,565**
Emilin1	Rattus norvegicus elastin microfibril interfacer 1 (Emilin1), mRNA [NM_001106710]	**7,565**
0	Unknown	**7,565**
Angel2	Rattus norvegicus angel homolog 2 (Drosophila) (Angel2), mRNA [NM_001135119]	**7,564**
0	Unknown	**7,564**
Odf3b	Rattus norvegicus outer dense fiber of sperm tails 3B (Odf3b), mRNA [NM_001191992]	**7,563**
Pot1a	Rattus norvegicus protection of telomeres 1A (Pot1a), mRNA [NM_001024322]	**7,563**
Nradd	Rattus norvegicus neurotrophin receptor associated death domain (Nradd), mRNA [NM_139259]	**7,563**
Slain2	Rattus norvegicus SLAIN motif family, member 2 (Slain2), mRNA [NM_001107214]	**7,563**
Grm6	Rattus norvegicus glutamate receptor, metabotropic 6 (Grm6), mRNA [NM_022920]	**7,563**
Paip2	Rattus norvegicus poly(A) binding protein interacting protein 2 (Paip2), mRNA [NM_001014148]	**7,562**
C1qb	Rattus norvegicus complement component 1, q subcomponent, B chain (C1qb), mRNA [NM_019262]	**7,562**
Ctps2	Rattus norvegicus CTP synthase II (Ctps2), mRNA [NM_001034998]	**7,561**
Cpt2	Rattus norvegicus carnitine palmitoyltransferase 2 (Cpt2), nuclear gene encoding mitochondrial protein, mRNA [NM_012930]	**7,561**
0	Unknown	**7,561**
0	Bromodomain and WD repeat domain containing 1 (Predicted), isoform CRA_bUncharacterized protein [Source:UniProtKB/TrEMBL;Acc:D3ZFG0] [ENSRNOT00000002231]	**7,559**
0	BC043133 Cnot2 protein {Mus musculus} (exp=-1; wgp=0; cg=0), partial (14%) [TC583782]	**7,558**
0	Unknown	**7,558**

Il23a	Rattus norvegicus Interleukin 23, alpha subunit p19 (Il23a), mRNA [NM_130410]	**7,554**
Arap2	Rattus norvegicus ArfGAP with RhoGAP domain, ankyrin repeat and PH domain 2 (Arap2), mRNA [NM_001107216]	**7,553**
Foxa1	Rattus norvegicus forkhead box A1 (Foxa1), mRNA [NM_012742]	**7,551**
LOC316717	PREDICTED: Rattus norvegicus protein serine-threonine phosphatase catalytic subunit PP-1c (LOC316717), miscRNA [XR_009632]	**7,551**
Rwdd4	Rattus norvegicus RWD domain containing 4A (Rwdd4), mRNA [NM_001034994]	**7,548**
0	Unknown	**7,546**
0	Unknown	**7,546**
Smarca4	Rattus norvegicus SWI/SNF related, matrix associated, actin dependent regulator of chromatin, subfamily a, member 4 (Smarca4), mRNA [NM_134368]	**7,546**
Ube2a	Rattus norvegicus ubiquitin-conjugating enzyme E2A (RAD6 homolog) (Ube2a), mRNA [NM_001013933]	**7,545**
Fam71e1	Uncharacterized protein [Source:UniProtKB/TrEMBL;Acc:D4A176] [ENSRNOT00000043724]	**7,545**
Olr750	Rattus norvegicus olfactory receptor 750 (Olr750), mRNA [NM_001000366]	**7,544**
Mff	Rattus norvegicus mitochondrial fission factor (Mff), nuclear gene encoding mitochondrial protein, mRNA [NM_001039015]	**7,543**
Ccng1	Rattus norvegicus cyclin G1 (Ccng1), mRNA [NM_012923]	**7,543**
Gria2	Rattus norvegicus glutamate receptor, ionotropic, AMPA 2 (Gria2), transcript variant 2, mRNA [NM_001083811]	**7,542**
LOC686141	PREDICTED: Rattus norvegicus similar to Spetex-2C protein (LOC686141), mRNA [XM_573722]	**7,542**
Tcfap4	Rattus norvegicus transcription factor AP4 (Tcfap4), mRNA [NM_001108267]	**7,541**
0	Rattus norvegicus similar to RIKEN cDNA 1700081O22 (LOC364963), mRNA [XM_344734]	**7,541**
Rhot1	Rattus norvegicus ras homolog gene family, member T1 (Rhot1), mRNA [NM_001107026]	**7,541**
Crcp	Rattus norvegicus CGRP receptor component (Crcp), mRNA [NM_053670]	**7,540**
0	Uncharacterized protein [Source:UniProtKB/TrEMBL;Acc:D3ZY88] [ENSRNOT00000018184]	**7,539**
Nfatc2	Rattus norvegicus nuclear factor of activated T-cells, cytoplasmic, calcineurin-dependent 2 (Nfatc2), mRNA [NM_001107805]	**7,538**
Acsl6	Rattus norvegicus acyl-CoA synthetase long-chain family member 6 (Acsl6), mRNA [NM_130739]	**7,538**
Tspan13	Rattus norvegicus tetraspanin 13 (Tspan13), mRNA [NM_001013244]	**7,537**
RGD1560972	PREDICTED: Rattus norvegicus similar to putative protein kinase (RGD1560972), mRNA [XM_577704]	**7,537**
Vom2r72	Rattus norvegicus vomeronasal 2 receptor, 72 (Vom2r72), mRNA [NM_001099517]	**7,536**
Duxbl	PREDICTED: Rattus norvegicus double homeobox B-like (Duxbl), mRNA [XM_001056985]	**7,536**
Tomm6	PREDICTED: Rattus norvegicus translocase of outer mitochondrial membrane 6 homolog (yeast) (Tomm6), mRNA [XM_001060392]	**7,536**
LOC499234	PREDICTED: Rattus norvegicus similar to NACHT, leucine rich repeat and PYD containing 14-like (LOC499234), partial mRNA [XM_002728785]	**7,535**
Ufd1l	Rattus norvegicus ubiquitin fusion degradation 1 like (yeast) (Ufd1l), mRNA [NM_053418]	**7,534**
LOC691781	PREDICTED: Rattus norvegicus similar to Tubulin alpha-2 chain (Alpha-tubulin 2) (LOC691781), partial miscRNA [XR_086096]	**7,533**
Tjp2	Rattus norvegicus tight junction protein 2 (Tjp2), mRNA [NM_053773]	**7,532**
Ang1	Rattus norvegicus angiogenin, ribonuclease A family, member 1 (Ang1), mRNA [NM_001006992]	**7,532**
Mif	MIF=macrophage migration inhibitory factor [rats, liver, mRNA, 525 nt]. [S73424]	**7,531**

Slc25a27	Rattus norvegicus solute carrier family 25, member 27 (Slc25a27), nuclear gene encoding mitochondrial protein, mRNA [NM_053500]	**7,531**
Wnk3	Rattus norvegicus WNK lysine deficient protein kinase 3 (Wnk3), mRNA [NM_001163607]	**7,530**
Olr128	Rattus norvegicus olfactory receptor 128 (Olr128), mRNA [NM_001000159]	**7,530**
0	Uncharacterized protein [Source:UniProtKB/TrEMBL;Acc:D3ZQ22] [ENSRNOT00000048946]	**7,530**
Rad51	Rattus norvegicus RAD51 homolog (RecA homolog, E. coli) (S. cerevisiae) (Rad51), mRNA [NM_001109204]	**7,530**
Gopc	Rattus norvegicus golgi associated PDZ and coiled-coil motif containing (Gopc), mRNA [NM_001107631]	**7,529**
Wtap	Rattus norvegicus Wilms tumor 1 associated protein (Wtap), transcript variant 2, mRNA [NM_001113543]	**7,529**
Casp8ap2	Rattus norvegicus caspase 8 associated protein 2 (Casp8ap2), mRNA [NM_001107921]	**7,527**
Slc25a16	Rat mitochondrial solute carrier protein mRNA, 5' end. [M32973]	**7,524**
Zmynd17	Rattus norvegicus zinc finger, MYND-type containing 17 (Zmynd17), mRNA [NM_001106025]	**7,524**
Asb1	Rattus norvegicus ankyrin repeat and SOCS box-containing 1 (Asb1), mRNA [NM_001108232]	**7,524**
Calml4	Rattus norvegicus calmodulin-like 4 (Calml4), mRNA [NM_001127575]	**7,524**
0	Unknown	**7,523**
Sgcb	Rattus norvegicus sarcoglycan, beta (dystrophin-associated glycoprotein) (Sgcb), mRNA [NM_001191068]	**7,523**
Cxcl12	Rattus norvegicus chemokine (C-X-C motif) ligand 12 (stromal cell-derived factor 1) (Cxcl12), transcript variant 3, mRNA [NM_001033883]	**7,522**
Slc46a1	Rattus norvegicus solute carrier family 46 (folate transporter), member 1 (Slc46a1), mRNA [NM_001013969]	**7,521**
Oat	Rattus norvegicus ornithine aminotransferase (gyrate atrophy) (Oat), nuclear gene encoding mitochondrial protein, mRNA [NM_022521]	**7,519**
Lpin3	Rattus norvegicus lipin 3 (Lpin3), mRNA [NM_001014184]	**7,519**
Ptpru	Rattus norvegicus protein tyrosine phosphatase, receptor type, U (Ptpru), mRNA [NM_001191575]	**7,519**
Tmlhe	Rattus norvegicus trimethyllysine hydroxylase, epsilon (Tmlhe), nuclear gene encoding mitochondrial protein, mRNA [NM_133387]	**7,519**
Fxyd3	Rattus norvegicus FXYD domain-containing ion transport regulator 3 (Fxyd3), mRNA [NM_172317]	**7,516**
Fam82a2	Rattus norvegicus family with sequence similarity 82, member A2 (Fam82a2), mRNA [NM_001014046]	**7,516**
0	PREDICTED: Rattus norvegicus similar to spermatogenesis associated glutamate (E)-rich protein 4d (LOC363324), mRNA [XM_343663]	**7,516**
Lhx3	LIM homeodomain protein 3b [Source:UniProtKB/TrEMBL;Acc:Q91ZX3] [ENSRNOT00000025047]	**7,515**
Olr162	Rattus norvegicus olfactory receptor 162 (Olr162), mRNA [NM_001000950]	**7,514**
Egr3	Rattus norvegicus early growth response 3 (Egr3), mRNA [NM_017086]	**7,513**
Fam176a	Rattus norvegicus family with sequence similarity 176, member A (Fam176a), mRNA [NM_001109243]	**7,512**
Zpbp2	Rattus norvegicus zona pellucida binding protein 2 (Zpbp2), mRNA [NM_001007011]	**7,509**
Pxmp2	Rattus norvegicus peroxisomal membrane protein 2 (Pxmp2), mRNA [NM_031587]	**7,508**
Actl6b	Rattus norvegicus actin-like 6B (Actl6b), mRNA [NM_001105917]	**7,507**
Prkra	Rattus norvegicus protein kinase, interferon inducible double stranded RNA dependent activator (Prkra), mRNA [NM_001024780]	**7,506**
Fzd8	Rattus norvegicus frizzled homolog 8 (Drosophila) (Fzd8), mRNA [NM_001044251]	**7,506**
RGD1564456	PREDICTED: Rattus norvegicus similar to chromosome 10 open reading frame 18 (RGD1564456), mRNA [XM_001068906]	**7,504**
Gmcl1	Rattus norvegicus germ cell-less homolog 1 (Drosophila) (Gmcl1), transcript variant 1, mRNA [NM_001033931]	**7,504**

0	Unknown	**7,504**
LOC682660	PREDICTED: Rattus norvegicus similar to LanC lantibiotic synthetase component C-like 3 (LOC682660), mRNA [XM_001062515]	**7,503**
0	Q86YD5_HUMAN (Q86YD5) LOC143458 protein (Fragment), partial (31%) [TC642266]	**7,503**
Tmem219	PREDICTED: Rattus norvegicus transmembrane protein 219 (Tmem219), mRNA [XM_001080074]	**7,501**
Thap1	Rattus norvegicus THAP domain containing, apoptosis associated protein 1 (Thap1), mRNA [NM_001008340]	**7,500**
Ppp3ca	Rattus norvegicus protein phosphatase 3, catalytic subunit, alpha isoform (Ppp3ca), mRNA [NM_017041]	**7,500**
Asna1	Rattus norvegicus arsA arsenite transporter, ATP-binding, homolog 1 (bacterial) (Asna1), mRNA [NM_001100505]	**7,499**
Urg4	Rattus norvegicus up-regulated gene 4 (Urg4), nuclear gene encoding mitochondrial protein, mRNA [NM_001077660]	**7,498**
Mapkapk3	Rattus norvegicus mitogen-activated protein kinase-activated protein kinase 3 (Mapkapk3), mRNA [NM_001012127]	**7,497**
Cirbp	Rattus norvegicus cold inducible RNA binding protein (Cirbp), mRNA [NM_031147]	**7,497**
Znf512b	Rattus norvegicus zinc finger protein 512B (Znf512b), mRNA [NM_001107809]	**7,496**
Nkap	Rattus norvegicus NFKB activating protein (Nkap), mRNA [NM_001024872]	**7,495**
RGD1566386	Rattus norvegicus similar to Hypothetical protein A430033K04 (RGD1566386), mRNA [NM_001107127]	**7,495**
Nkd2	Rattus norvegicus naked cuticle homolog 2 (Drosophila) (Nkd2), mRNA [NM_001107454]	**7,492**
Dynlt3	Rattus norvegicus dynein light chain Tctex-type 3 (Dynlt3), mRNA [NM_001013228]	**7,491**
LOC300314	PREDICTED: Rattus norvegicus similar to Protein KIAA0196 (LOC300314), mRNA [XM_238532]	**7,489**
Atp5s	Rattus norvegicus ATP synthase, H+ transporting, mitochondrial F0 complex, subunit s (factor B) (Atp5s), nuclear gene encoding mitochondrial protein, mRNA [NM_001007749]	**7,489**
Rgs12	Rattus norvegicus regulator of G-protein signaling 12 (Rgs12), mRNA [NM_019339]	**7,489**
0	Unknown	**7,488**
Insm1	PREDICTED: Rattus norvegicus insulinoma-associated 1 (Insm1), mRNA [XM_001055419]	**7,488**
0	Uncharacterized protein [Source:UniProtKB/TrEMBL;Acc:D3ZQC4] [ENSRNOT00000050888]	**7,487**
Poldip2	Rattus norvegicus polymerase (DNA-directed), delta interacting protein 2 (Poldip2), mRNA [NM_001105816]	**7,486**
Hsp90ab1	Heat shock protein HSP 90-beta [Source:UniProtKB/Swiss-Prot;Acc:P34058] [ENSRNOT00000026920]	**7,485**
Trub2	Rattus norvegicus TruB pseudouridine (psi) synthase homolog 2 (E. coli) (Trub2), mRNA [NM_001014257]	**7,484**
Unc5b	Rattus norvegicus unc-5 homolog B (C. elegans) (Unc5b), mRNA [NM_022207]	**7,484**
Gpr172a	Rattus norvegicus G protein-coupled receptor 172A (Gpr172a), mRNA [NM_001109670]	**7,484**
Hamp	Rattus norvegicus hepcidin antimicrobial peptide (Hamp), mRNA [NM_053469]	**7,484**
Parg	Rattus norvegicus poly (ADP-ribose) glycohydrolase (Parg), mRNA [NM_031339]	**7,483**
LOC685792	PREDICTED: Rattus norvegicus similar to Discs large homolog 5 (Placenta and prostate DLG) (Discs large protein P-dlg) (LOC685792), mRNA [XM_217497]	**7,482**
0	Unknown	**7,480**
0	Uncharacterized protein [Source:UniProtKB/TrEMBL;Acc:D3ZJM8] [ENSRNOT00000061644]	**7,480**
Wnt3	Rattus norvegicus wingless-type MMTV integration site family, member 3 (Wnt3), mRNA [NM_001105715]	**7,479**

Acaca	Rattus norvegicus acetyl-coenzyme A carboxylase alpha (Acaca), mRNA [NM_022193]	**7,479**
RGD1562608	Rattus norvegicus similar to KIAA1328 protein (RGD1562608), mRNA [NM_001134607]	**7,478**
Smcr7	PREDICTED: Rattus norvegicus Smith-Magenis syndrome chromosome region, candidate 7 homolog (human) (Smcr7), mRNA [XM_001077609]	**7,478**
0	Unknown	**7,477**
RGD1566047	PREDICTED: Rattus norvegicus similar to MAP/microtubule affinity-regulating kinase 4 (MAP/microtubule affinity-regulating kinase like 1) (RGD1566047), mRNA [XM_574062]	**7,475**
Pla2g2f	Rattus norvegicus phospholipase A2, group IIF (Pla2g2f), mRNA [NM_001109587]	**7,475**
Ubac1	Rattus norvegicus UBA domain containing 1 (Ubac1), mRNA [NM_001007742]	**7,475**
Tmtc2	Rattus norvegicus transmembrane and tetratricopeptide repeat containing 2 (Tmtc2), mRNA [NM_001171177]	**7,474**
Tyms	Rattus norvegicus thymidylate synthetase (Tyms), mRNA [NM_019179]	**7,473**
Chad	Rattus norvegicus chondroadherin (Chad), mRNA [NM_019164]	**7,473**
Pts	Rattus norvegicus 6-pyruvoyl-tetrahydropterin synthase (Pts), mRNA [NM_017220]	**7,472**
Rgc32	Rattus norvegicus response gene to complement 32 (Rgc32), mRNA [NM_054008]	**7,470**
Wdr86	Rattus norvegicus WD repeat domain 86 (Wdr86), mRNA [NM_001110489]	**7,470**
Msh4	Rattus norvegicus mutS homolog 4 (E. coli) (Msh4), mRNA [NM_001106477]	**7,469**
Kcna7	Rattus norvegicus potassium voltage-gated channel, shaker-related subfamily, member 7 (Kcna7), mRNA [NM_001108914]	**7,469**
0	TBC1 domain family, member 23 (Predicted), isoform CRA_cUncharacterized protein [Source:UniProtKB/TrEMBL;Acc:D4A0K5] [ENSRNOT00000002240]	**7,468**
Tmbim6	Rattus norvegicus transmembrane BAX inhibitor motif containing 6 (Tmbim6), mRNA [NM_019381]	**7,466**
Atp5g2	Rattus norvegicus ATP synthase, H+ transporting, mitochondrial F0 complex, subunit C2 (subunit 9) (Atp5g2), nuclear gene encoding mitochondrial protein, mRNA [NM_133556]	**7,466**
Styk1	Similar to NOK kinase (Predicted)Uncharacterized protein [Source:UniProtKB/TrEMBL;Acc:D3ZHY0] [ENSRNOT00000013833]	**7,466**
Fam65c	Uncharacterized protein [Source:UniProtKB/TrEMBL;Acc:D3ZX40] [ENSRNOT00000035463]	**7,464**
0	Uncharacterized protein [Source:UniProtKB/TrEMBL;Acc:D3ZE96] [ENSRNOT00000005587]	**7,464**
Col22a1	PREDICTED: Rattus norvegicus collagen, type XXII, alpha 1 (Col22a1), mRNA [XM_243609]	**7,463**
RGD1308544	PREDICTED: Rattus norvegicus LOC361192 (RGD1308544), mRNA [XM_001063171]	**7,462**
Tmem17	Rattus norvegicus transmembrane protein 17 (Tmem17), mRNA [NM_001010961]	**7,460**
Slc2a3	Rattus norvegicus solute carrier family 2 (facilitated glucose transporter), member 3 (Slc2a3), mRNA [NM_017102]	**7,459**
Lcn12	Rattus norvegicus lipocalin 12 (Lcn12), mRNA [NM_001128138]	**7,459**
Rpl6	Rattus norvegicus ribosomal protein L6 (Rpl6), mRNA [NM_053971]	**7,458**
RGD1304693	Rattus norvegicus similar to CG14803-PA (RGD1304693), mRNA [NM_001135780]	**7,455**
0	Uncharacterized protein [Source:UniProtKB/TrEMBL;Acc:D3ZQ32] [ENSRNOT00000039210]	**7,453**
LOC100366030	Rattus norvegicus rCG37858-like (LOC100366030), mRNA [NM_001177909]	**7,452**
Foxj1	Rattus norvegicus forkhead box J1 (Foxj1), mRNA [NM_053832]	**7,452**

Clip4	Rattus norvegicus CAP-GLY domain containing linker protein family, member 4 (Clip4), mRNA [NM_001013942]	**7,452**
RGD1303142	Rattus norvegicus oxidative stress responsive gene (RGD1303142), mRNA [NM_201560]	**7,452**
0	Unknown	**7,451**
Ern1	Rattus norvegicus endoplasmic reticulum to nucleus signaling 1 (Ern1), mRNA [NM_001191926]	**7,450**
0	Unknown	**7,450**
LOC687346	PREDICTED: Rattus norvegicus similar to BRCA1-associated protein (BRAP2) (Impedes mitogenic signal propagation) (IMP) (LOC687346), mRNA [XM_001075972]	**7,448**
Myo18a	Rattus norvegicus myosin XVIIIa (Myo18a), mRNA [NM_001172137]	**7,447**
Cuta	Rattus norvegicus cutA divalent cation tolerance homolog (E. coli) (Cuta), transcript variant 1, mRNA [NM_212494]	**7,447**
Nme6	Rattus norvegicus non-metastatic cells 6, protein expressed in (nucleoside-diphosphate kinase) (Nme6), mRNA [NM_001191884]	**7,446**
0	Rattus norvegicus similar to NOL1R protein; Williams-Beuren syndrome critical region protein 20 (LOC305339), mRNA [XM_223400]	**7,445**
Mre11a	Rattus norvegicus MRE11 meiotic recombination 11 homolog A (S. cerevisiae) (Mre11a), mRNA [NM_022279]	**7,445**
0	Unknown	**7,445**
0	Uncharacterized protein [Source:UniProtKB/TrEMBL;Acc:D3Z932] [ENSRNOT00000066351]	**7,444**
Baz1b	Rattus norvegicus bromodomain adjacent to zinc finger domain, 1B (Baz1b), mRNA [NM_001191916]	**7,443**
Cdon	Rattus norvegicus Cdon homolog (mouse) (Cdon), mRNA [NM_017358]	**7,442**
Mrpl48	Rattus norvegicus mitochondrial ribosomal protein L48 (Mrpl48), nuclear gene encoding mitochondrial protein, mRNA [NM_001106282]	**7,442**
Ccdc104	Rattus norvegicus coiled-coil domain containing 104 (Ccdc104), mRNA [NM_001024866]	**7,441**
Lyz2	Rattus norvegicus lysozyme 2 (Lyz2), mRNA [NM_012771]	**7,441**
Hsd17b12	Rattus norvegicus hydroxysteroid (17-beta) dehydrogenase 12 (Hsd17b12), mRNA [NM_032066]	**7,441**
Bex2	Rattus norvegicus brain expressed X-linked 2 (Bex2), mRNA [NM_001077435]	**7,440**
Grk5	Rattus norvegicus G protein-coupled receptor kinase 5 (Grk5), mRNA [NM_030829]	**7,438**
RGD1565566	PREDICTED: Rattus norvegicus similar to 60S ribosomal protein L18a (RGD1565566), mRNA [XM_574336]	**7,435**
0	Uncharacterized protein [Source:UniProtKB/TrEMBL;Acc:D4A9I0] [ENSRNOT00000035986]	**7,434**
Aspdh	Rattus norvegicus aspartate dehydrogenase domain containing (Aspdh), mRNA [NM_001009643]	**7,434**
Tmem48	Rattus norvegicus transmembrane protein 48 (Tmem48), mRNA [NM_001025023]	**7,434**
Bmp2k	Uncharacterized protein [Source:UniProtKB/TrEMBL;Acc:D4A0Z6] [ENSRNOT00000060868]	**7,433**
Pgrmc1	Rattus norvegicus progesterone receptor membrane component 1 (Pgrmc1), mRNA [NM_021766]	**7,432**
Stim2	Rattus norvegicus stromal interaction molecule 2 (Stim2), mRNA [NM_001105750]	**7,431**
Acvr2b	Rattus norvegicus activin A receptor, type IIB (Acvr2b), mRNA [NM_031554]	**7,430**
0	Unknown	**7,429**
Stard8	Rattus norvegicus StAR-related lipid transfer (START) domain containing 8 (Stard8), mRNA [NM_001107849]	**7,428**
Rfx2	Rattus norvegicus regulatory factor X, 2 (influences HLA class II expression) (Rfx2), mRNA [NM_001106877]	**7,427**
Ckap2l	Uncharacterized protein [Source:UniProtKB/TrEMBL;Acc:D3Z8Z9] [ENSRNOT00000039794]	**7,427**

Pop1	Rattus norvegicus processing of precursor 1, ribonuclease P/MRP subunit (S. cerevisiae) (Pop1), mRNA [NM_001130550]	**7,427**
Cct8	Rattus norvegicus chaperonin containing Tcp1, subunit 8 (theta) (Cct8), mRNA [NM_001105897]	**7,424**
RGD1562747	Rattus norvegicus similar to RIKEN cDNA 1110012L19 (RGD1562747), mRNA [NM_001106212]	**7,423**
0	Unknown	**7,421**
0	Unknown	**7,420**
0	Unknown	**7,420**
Cox6a1	Rattus norvegicus cytochrome c oxidase, subunit VIa, polypeptide 1 (Cox6a1), nuclear gene encoding mitochondrial protein, mRNA [NM_012814]	**7,419**
RGD1308695	Rattus norvegicus similar to RIKEN cDNA 1200016B10 (RGD1308695), mRNA [NM_001105960]	**7,417**
0	PREDICTED: Rattus norvegicus rCG41835-like (LOC100359996), mRNA [XM_002728584]	**7,416**
Tmco4	Rattus norvegicus transmembrane and coiled-coil domains 4 (Tmco4), mRNA [NM_001034949]	**7,416**
Gdap1	Rattus norvegicus ganglioside-induced differentiation-associated-protein 1 (Gdap1), mRNA [NM_001107897]	**7,416**
Abcd3	Rattus norvegicus ATP-binding cassette, subfamily D (ALD), member 3 (Abcd3), mRNA [NM_012804]	**7,415**
Nefl	Rattus norvegicus neurofilament, light polypeptide (Nefl), mRNA [NM_031783]	**7,414**
Lpar6	Rattus norvegicus lysophosphatidic acid receptor 6 (Lpar6), mRNA [NM_001045843]	**7,414**
Vom1r38	Rattus norvegicus vomeronasal 1 receptor 38 (Vom1r38), mRNA [NM_001008964]	**7,413**
Bet1	Rattus norvegicus blocked early in transport 1 homolog (S. cerevisiae) (Bet1), mRNA [NM_019251]	**7,413**
Fam151a	Rattus norvegicus family with sequence similarity 151, member A (Fam151a), mRNA [NM_001005558]	**7,412**
Vegfa	Rattus norvegicus vascular endothelial growth factor A (Vegfa), transcript variant 1, mRNA [NM_031836]	**7,411**
Ppp1cb	Rattus norvegicus protein phosphatase 1, catalytic subunit, beta isoform (Ppp1cb), mRNA [NM_013065]	**7,411**
LOC689410	PREDICTED: Rattus norvegicus hypothetical protein LOC689410 (LOC689410), mRNA [XM_001070689]	**7,411**
Ifitm7	Uncharacterized protein [Source:UniProtKB/TrEMBL;Acc:D3ZY98] [ENSRNOT00000039434]	**7,409**
LOC685203	Uncharacterized protein [Source:UniProtKB/TrEMBL;Acc:D3ZBW5] [ENSRNOT00000059159]	**7,409**
Lass6	PREDICTED: Rattus norvegicus LAG1 homolog, ceramide synthase 6 (Lass6), mRNA [XM_001058317]	**7,408**
Zcchc10	Zinc finger CCHC domain-containing protein 10 [Source:UniProtKB/Swiss-Prot;Acc:Q5EB97] [ENSRNOT00000009098]	**7,406**
0	Unknown	**7,406**
Nxf1	Rattus norvegicus nuclear RNA export factor 1 (Nxf1), mRNA [NM_021579]	**7,405**
0	Unknown	**7,403**
RGD1308722	Rattus norvegicus similar to RIKEN cDNA A130042E20; open reading frame 57 (RGD1308722), mRNA [NM_001108529]	**7,402**
Nr1h5p	Uncharacterized protein [Source:UniProtKB/TrEMBL;Acc:D3ZFA7] [ENSRNOT00000036229]	**7,402**
Gp5	Rattus norvegicus glycoprotein V (platelet) (Gp5), mRNA [NM_012795]	**7,400**
Rps8	Rattus norvegicus ribosomal protein S8 (Rps8), mRNA [NM_031706]	**7,399**
Ndc80	Rattus norvegicus NDC80 homolog, kinetochore complex component (S. cerevisiae) (Ndc80), mRNA [NM_001126270]	**7,397**
Pif1	Rattus norvegicus Pif1/Rrm3 DNA-helicase-like protein (Pif1), mRNA [NM_001044253]	**7,396**
0	Unknown	**7,396**

0	Unknown	**7,396**
Tspo	Rattus norvegicus translocator protein (Tspo), mRNA [NM_012515]	**7,396**
Mtmr4	Rattus norvegicus myotubularin related protein 4 (Mtmr4), mRNA [NM_001105827]	**7,395**
Pnma3	Rattus norvegicus paraneoplastic antigen MA3 (Pnma3), mRNA [NM_001106342]	**7,394**
Clcn1	Rattus norvegicus chloride channel 1 (Clcn1), mRNA [NM_013147]	**7,394**
Yipf4	Rattus norvegicus Yip1 domain family, member 4 (Yipf4), mRNA [NM_001009712]	**7,393**
Nthl1	Rattus norvegicus nth (endonuclease III)-like 1 (E.coli) (Nthl1), mRNA [NM_001105728]	**7,392**
Thumpd1	Rattus norvegicus THUMP domain containing 1 (Thumpd1), mRNA [NM_001009688]	**7,391**
Gtpbp6	Rattus norvegicus GTP binding protein 6 (putative) (Gtpbp6), mRNA [NM_001135840]	**7,390**
Cpsf6	Rattus norvegicus cleavage and polyadenylation specific factor 6 (Cpsf6), mRNA [NM_001106785]	**7,390**
Itm2a	Rattus norvegicus integral membrane protein 2A (Itm2a), mRNA [NM_001025712]	**7,389**
Tmem168	Rattus norvegicus transmembrane protein 168 (Tmem168), mRNA [NM_001014054]	**7,388**
0	Uncharacterized protein [Source:UniProtKB/TrEMBL;Acc:D4ACK9] [ENSRNOT00000019278]	**7,388**
Gh1	Rattus norvegicus growth hormone 1 (Gh1), mRNA [NM_001034848]	**7,388**
Dbnl	Drebrin-like protein [Source:UniProtKB/Swiss-Prot;Acc:Q9JHL4] [ENSRNOT00000017375]	**7,386**
Rps6kb1	Rattus norvegicus ribosomal protein S6 kinase, polypeptide 1 (Rps6kb1), mRNA [NM_031985]	**7,386**
0	Rattus norvegicus TL0AEA9YM10 mRNA sequence. [FQ230244]	**7,385**
Myo9b	Rattus norvegicus myosin IXb (Myo9b), mRNA [NM_012984]	**7,384**
0	Histone H3.1 [Source:UniProtKB/Swiss-Prot;Acc:Q6LED0] [ENSRNOT00000059330]	**7,382**
Mrps35	Rattus norvegicus mitochondrial ribosomal protein S35 (Mrps35), nuclear gene encoding mitochondrial protein, mRNA [NM_001106628]	**7,382**
Ucn2	Rattus norvegicus urocortin 2 (Ucn2), mRNA [NM_133385]	**7,381**
0	Uncharacterized protein [Source:UniProtKB/TrEMBL;Acc:D4A8N4] [ENSRNOT00000004178]	**7,381**
0	Uncharacterized protein [Source:UniProtKB/TrEMBL;Acc:D4A1J6] [ENSRNOT00000021816]	**7,380**
Msl1	Rattus norvegicus male-specific lethal 1 homolog (Drosophila) (Msl1), mRNA [NM_001107048]	**7,380**
0	Solute carrier family 37 (Glycerol-3-phosphate transporter), member 3 (Predicted), isoform CRA_cUncharacterized protein [Source:UniProtKB/TrEMBL;Acc:D3ZZN8] [ENSRNOT00000011915]	**7,379**
0	microtubule-actin crosslinking factor 1 [Source:RefSeq peptide;Acc:NP_001129230] [ENSRNOT00000021951]	**7,377**
Olr321	Rattus norvegicus olfactory receptor 321 (Olr321), mRNA [NM_001000244]	**7,377**
Gsr	Rattus norvegicus glutathione reductase (Gsr), mRNA [NM_053906]	**7,375**
0	UI-R-EB1-clg-p-22-0-UI.s1 UI-R-EB1 Rattus norvegicus cDNA clone UI-R-EB1-clg-p-22-0-UI 3', mRNA sequence [BQ202508]	**7,375**
0	Unknown	**7,375**
0	AMGNNUC:NRHY7-00027-F7-A nrhy7 (10850) Rattus norvegicus cDNA clone nrhy7-00027-f7 5', mRNA sequence [CB546704]	**7,374**
0	Unknown	**7,374**
Ttc8	Rattus norvegicus tetratricopeptide repeat domain 8 (Ttc8), mRNA [NM_001106752]	**7,372**

Uqcr11	Rattus norvegicus ubiquinol-cytochrome c reductase, complex III subunit XI (Uqcr11), nuclear gene encoding mitochondrial protein, mRNA [NM_001126097]	**7,370**
0	Unknown	**7,367**
Il18bp	Rattus norvegicus interleukin 18 binding protein (Il18bp), mRNA [NM_053374]	**7,367**
Necab2	Rattus norvegicus N-terminal EF-hand calcium binding protein 2 (Necab2), mRNA [NM_133415]	**7,367**
Tbc1d8	Rattus norvegicus TBC1 domain family, member 8 (Tbc1d8), mRNA [NM_001191067]	**7,367**
Znf691	Rattus norvegicus zinc finger protein 691 (Znf691), mRNA [NM_001107968]	**7,366**
LOC682058	PREDICTED: Rattus norvegicus similar to nucleolar protein with MIF4G domain 1 (LOC682058), mRNA [XM_001059617]	**7,363**
0	Unknown	**7,363**
LOC499770	Rattus norvegicus similar to LOC495800 protein (LOC499770), mRNA [NM_001047110]	**7,363**
Crabp2	Rattus norvegicus cellular retinoic acid binding protein 2 (Crabp2), mRNA [NM_017244]	**7,362**
0	Unknown	**7,360**
Exoc2	Rattus norvegicus exocyst complex component 2 (Exoc2), mRNA [NM_134414]	**7,360**
0	Uncharacterized protein [Source:UniProtKB/TrEMBL;Acc:D3ZZF2] [ENSRNOT00000005894]	**7,359**
RGD1305645	Uncharacterized protein [Source:UniProtKB/TrEMBL;Acc:D4A540] [ENSRNOT00000036990]	**7,359**
Mgat4a	Rattus norvegicus mannosyl (alpha-1,3-)-glycoprotein beta-1,4-N-acetylglucosaminyltransferase, isozyme A (Mgat4a), transcript variant 1, mRNA [NM_001160155]	**7,359**
0	Uncharacterized protein [Source:UniProtKB/TrEMBL;Acc:D4ACU3] [ENSRNOT00000060466]	**7,358**
Spryd4	Rattus norvegicus SPRY domain containing 4 (Spryd4), mRNA [NM_001037765]	**7,357**
Ap4s1	Rattus norvegicus adaptor-related protein complex AP-4, sigma 1 (Ap4s1), mRNA [NM_001130999]	**7,357**
Nab1	Rattus norvegicus Ngfi-A binding protein 1 (Nab1), mRNA [NM_022856]	**7,357**
Grinl1a	Rattus norvegicus glutamate receptor, ionotropic, N-methyl D-aspartate-like 1A (Grinl1a), mRNA [NM_183402]	**7,357**
LOC683077	Rattus norvegicus similar to praja1, RING-H2 motif containing (LOC683077), mRNA [NM_001101006]	**7,355**
Rps15	Rattus norvegicus ribosomal protein S15 (Rps15), mRNA [NM_017151]	**7,354**
Aarsd1	Rattus norvegicus alanyl-tRNA synthetase domain containing 1 (Aarsd1), mRNA [NM_001034109]	**7,354**
Ndufaf4	Rattus norvegicus NADH dehydrogenase (ubiquinone) 1 alpha subcomplex, assembly factor 4 (Ndufaf4), nuclear gene encoding mitochondrial protein, mRNA [NM_198783]	**7,353**
RGD1309058	RCG20883Uncharacterized protein [Source:UniProtKB/TrEMBL;Acc:D4A7T7] [ENSRNOT00000014814]	**7,352**
L3mbtl2	Rattus norvegicus l(3)mbt-like 2 (Drosophila) (L3mbtl2), mRNA [NM_001033695]	**7,350**
Mizf	PREDICTED: Rattus norvegicus MBD2-interacting zinc finger (Mizf), mRNA [XM_002727050]	**7,349**
0	Uncharacterized protein [Source:UniProtKB/TrEMBL;Acc:D3ZV19] [ENSRNOT00000009759]	**7,349**
0	Unknown	**7,347**
0	Unknown	**7,347**
LOC60591	Rattus norvegicus calcium-activated potassium channel beta subunit (LOC60591), mRNA [NM_022203]	**7,345**

Cep152	PREDICTED: Rattus norvegicus centrosomal protein 152 (Cep152), mRNA [XM_002726171]	**7,345**
Micalcl	Rattus norvegicus MICAL C-terminal like (Micalcl), mRNA [NM_182669]	**7,344**
Slc2a8	Rattus norvegicus solute carrier family 2, (facilitated glucose transporter) member 8 (Slc2a8), mRNA [NM_053494]	**7,343**
RGD1311558	Rattus norvegicus similar to 4930506M07Rik protein (RGD1311558), mRNA [NM_001079705]	**7,342**
RGD1566099	Rattus norvegicus similar to novel protein (RGD1566099), mRNA [NM_001108347]	**7,342**
LOC680155	Uncharacterized protein [Source:UniProtKB/TrEMBL;Acc:D3ZXU7] [ENSRNOT00000020271]	**7,340**
RGD1564571	PREDICTED: Rattus norvegicus similar to DC-SIGN (RGD1564571), mRNA [XM_221808]	**7,338**
Grinl1a	Rattus norvegicus glutamate receptor, ionotropic, N-methyl D-aspartate-like 1A (Grinl1a), mRNA [NM_183402]	**7,338**
RGD1563046	Rattus norvegicus similar to cerberus-like (RGD1563046), mRNA [NM_001115031]	**7,337**
Gnb5	Rattus norvegicus guanine nucleotide binding protein (G protein), beta 5 (Gnb5), mRNA [NM_031770]	**7,337**
Rnf217	Rattus norvegicus ring finger protein 217 (Rnf217), mRNA [NM_001106204]	**7,337**
LOC501312	PREDICTED: Rattus norvegicus hypothetical gene supported by BC082068 (LOC501312), mRNA [XM_576725]	**7,336**
Ctbp1	Rattus norvegicus C-terminal binding protein 1 (Ctbp1), mRNA [NM_019201]	**7,336**
RGD1563833	PREDICTED: Rattus norvegicus similar to putative homeobox protein (RGD1563833), mRNA [XM_577929]	**7,336**
0	PREDICTED: Rattus norvegicus ubiquitination factor E4B (Ube4b), mRNA [XM_233679]	**7,335**
Prkcd	Rattus norvegicus protein kinase C, delta (Prkcd), mRNA [NM_133307]	**7,334**
Olr323	Rattus norvegicus olfactory receptor 323 (Olr323), mRNA [NM_001000245]	**7,334**
0	Uncharacterized protein [Source:UniProtKB/TrEMBL;Acc:D3ZTN0] [ENSRNOT00000035247]	**7,334**
LOC100360108	PREDICTED: Rattus norvegicus cell surface receptor FDFACT-like (LOC100360108), mRNA [XM_002727949]	**7,332**
Klk7	Rattus norvegicus kallikrein-related peptidase 7 (Klk7), mRNA [NM_001106254]	**7,331**
Tmem86a	Rattus norvegicus transmembrane protein 86A (Tmem86a), mRNA [NM_001135016]	**7,330**
Zbtb45	Rattus norvegicus zinc finger and BTB domain containing 45 (Zbtb45), mRNA [NM_001107478]	**7,330**
RGD1559921	PREDICTED: Rattus norvegicus similar to Macrophage migration inhibitory factor (MIF) (Delayed early response protein 6) (RGD1559921), mRNA [XM_001080942]	**7,330**
Prpf38b	Rattus norvegicus PRP38 pre-mRNA processing factor 38 (yeast) domain containing B (Prpf38b), mRNA [NM_001024305]	**7,329**
0	Rattus norvegicus similar to actin-related protein 2 (LOC301861), mRNA [XM_217505]	**7,329**
0	Unknown	**7,328**
Rundc1	Q96C34_HUMAN (Q96C34) RUNDC1 protein (Fragment), partial (40%) [TC601870]	**7,328**
Rab40c	Rattus norvegicus Rab40c, member RAS oncogene family (Rab40c), mRNA [NM_182675]	**7,325**
Zfp568	Uncharacterized protein [Source:UniProtKB/TrEMBL;Acc:D4A3P6] [ENSRNOT00000039872]	**7,325**
Diras1	Rattus norvegicus DIRAS family, GTP-binding RAS-like 1 (Diras1), mRNA [NM_001108987]	**7,325**
Bub3	Rattus norvegicus budding uninhibited by benzimidazoles 3 homolog (S. cerevisiae) (Bub3), mRNA [NM_001047906]	**7,322**
Vegp2	Rattus norvegicus von Ebners gland protein 2 (Vegp2), mRNA [NM_053574]	**7,321**
Cnot1	Rattus norvegicus CCR4-NOT transcription complex, subunit 1 (Cnot1), mRNA [NM_001134840]	**7,321**

0	Histone H3 [Source:UniProtKB/TrEMBL;Acc:D4A602] [ENSRNOT00000030033]	**7,320**
0	Unknown	**7,320**
Dnah2	Axonemal dynein heavy chain [Source:UniProtKB/TrEMBL;Acc:Q62820] [ENSRNOT00000045220]	**7,320**
Sp9	Rattus norvegicus trans-acting transcription factor 9 (Sp9), mRNA [NM_001191902]	**7,319**
RGD1560880	Rattus norvegicus similar to RIKEN cDNA 2310002J15 (RGD1560880), mRNA [NM_001109194]	**7,319**
RGD1559505	Rattus norvegicus similar to RIKEN cDNA 9030409E16 (RGD1559505), mRNA [NM_001134608]	**7,319**
RGD1560978	Rattus norvegicus similar to hypothetical protein (RGD1560978), mRNA [NM_001134631]	**7,316**
Sox11	Rattus norvegicus SRY (sex determining region Y)-box 11 (Sox11), mRNA [NM_053349]	**7,316**
Dgcr14	Rattus norvegicus DiGeorge syndrome critical region gene 14 (Dgcr14), mRNA [NM_001012472]	**7,316**
Nek7	NIMA-related kinase 7 [Source:RefSeq peptide;Acc:NP_001101816] [ENSRNOT00000000817]	**7,315**
Prc1	Rattus norvegicus protein regulator of cytokinesis 1 (Prc1), mRNA [NM_001107529]	**7,313**
0	Unknown	**7,313**
Cercam	Rattus norvegicus cerebral endothelial cell adhesion molecule (Cercam), mRNA [NM_001011962]	**7,313**
Asb18	Rattus norvegicus ankyrin repeat and SOCS box-containing 18 (Asb18), mRNA [NM_001108231]	**7,313**
0	Rattus norvegicus similar to cell division cycle associated 3; gene rich cluster, C8 gene; trigger of mitotic entry 1 (LOC316373), mRNA [XM_237105]	**7,312**
Stard13	Rattus norvegicus StAR-related lipid transfer (START) domain containing 13 (Stard13), mRNA [NM_001109060]	**7,312**
0	Uncharacterized protein [Source:UniProtKB/TrEMBL;Acc:D3ZIF9] [ENSRNOT00000067842]	**7,311**
Tsc22d3	Rattus norvegicus TSC22 domain family, member 3 (Tsc22d3), mRNA [NM_031345]	**7,310**
Olr481	Rattus norvegicus olfactory receptor 481 (Olr481), mRNA [NM_001000684]	**7,310**
0	Uncharacterized protein [Source:UniProtKB/TrEMBL;Acc:D3Z840] [ENSRNOT00000050759]	**7,309**
F8	Rattus norvegicus coagulation factor VIII, procoagulant component (F8), mRNA [NM_183331]	**7,309**
0	Rattus norvegicus chromosome 20, major histocompatibility complex, assembled from 40 BACs, strain Brown Norway (BN/ssNHsd), RT1n haplotype; segment 5/11 [BX883046]	**7,308**
Tdp1	Rattus norvegicus tyrosyl-DNA phosphodiesterase 1 (Tdp1), mRNA [NM_001031657]	**7,308**
Fn1	Rattus norvegicus fibronectin 1 (Fn1), mRNA [NM_019143]	**7,308**
Arnt	Rattus norvegicus aryl hydrocarbon receptor nuclear translocator (Arnt), mRNA [NM_012780]	**7,307**
0	Uncharacterized protein [Source:UniProtKB/TrEMBL;Acc:D4A9K1] [ENSRNOT00000031814]	**7,306**
Cct6a	Rattus norvegicus chaperonin containing Tcp1, subunit 6A (zeta 1) (Cct6a), mRNA [NM_001033684]	**7,304**
LOC683722	Rattus norvegicus similar to Fgfr1 oncogene partner (LOC683722), mRNA [NM_001101008]	**7,304**
0	Unknown	**7,303**
Rnmt	mRNA cap guanine-N7 methyltransferase [Source:UniProtKB/Swiss-Prot;Acc:Q5U2U7] [ENSRNOT00000022410]	**7,303**
Med30	Rattus norvegicus mediator complex subunit 30 (Med30), mRNA [NM_001130539]	**7,301**
Krt15	Rattus norvegicus keratin 15 (Krt15), mRNA [NM_001004022]	**7,300**

0	Unknown	**7,298**
Olr221	Rattus norvegicus olfactory receptor 221 (Olr221), mRNA [NM_001000200]	**7,298**
Fam181b	Rattus norvegicus family with sequence similarity 181, member B (Fam181b), mRNA [NM_001109150]	**7,297**
Ccdc124	Rattus norvegicus coiled-coil domain containing 124 (Ccdc124), mRNA [NM_001106071]	**7,294**
Trip4	Rattus norvegicus thyroid hormone receptor interactor 4 (Trip4), mRNA [NM_001134981]	**7,293**
Dpy19l4	Putative uncharacterized protein RGD1305828_predictedUncharacterized protein [Source:UniProtKB/TrEMBL;Acc:D3Z939] [ENSRNOT00000037869]	**7,293**
Atp11b	Atp11b protein [Source:UniProtKB/TrEMBL;Acc:Q5RJS7] [ENSRNOT00000016961]	**7,290**
Tmed7	Rattus norvegicus transmembrane emp24 protein transport domain containing 7 (Tmed7), mRNA [NM_001105758]	**7,290**
Klhl21	Rattus norvegicus kelch-like 21 (Drosophila) (Klhl21), mRNA [NM_001107996]	**7,290**
0	Rattus norvegicus similar to glyceraldehyde-3-phosphate dehydrogenase (LOC313970), mRNA [XM_234037]	**7,289**
G2e3	Rattus norvegicus G2/M-phase specific E3 ubiquitin ligase (G2e3), mRNA [NM_001106726]	**7,289**
Apoa1bp	Rattus norvegicus apolipoprotein A-I binding protein (Apoa1bp), mRNA [NM_001106440]	**7,287**
0	Similar to oxysterol-binding protein-like protein 8 isoform a (Similar to oxysterol-binding protein-like protein 8 isoform a (Predicted), isoform CRA_a) [Source:UniProtKB/TrEMBL;Acc:B5DF26] [ENSRNOT00000029993]	**7,287**
0	Uncharacterized protein [Source:UniProtKB/TrEMBL;Acc:D4A4F9] [ENSRNOT00000057735]	**7,287**
Idi1	Rattus norvegicus isopentenyl-diphosphate delta isomerase 1 (Idi1), mRNA [NM_053539]	**7,286**
Ssr2	Rattus norvegicus signal sequence receptor, beta (Ssr2), mRNA [NM_001106442]	**7,283**
0	Unknown	**7,282**
Vegfb	Rattus norvegicus vascular endothelial growth factor B (Vegfb), mRNA [NM_053549]	**7,282**
Serf1	PREDICTED: Rattus norvegicus small EDRK-rich factor 1 (Serf1), mRNA [XM_001070716]	**7,281**
Grk6	Rattus norvegicus G protein-coupled receptor kinase 6 (Grk6), transcript variant 1, mRNA [NM_031657]	**7,281**
LOC365238	Rattus norvegicus similar to CG15432-PA (LOC365238), mRNA [NM_001108913]	**7,281**
Rdx	Rattus norvegicus radixin (Rdx), mRNA [NM_001005889]	**7,279**
Lfng	Rattus norvegicus LFNG O-fucosylpeptide 3-beta-N-acetylglucosaminyltransferase (Lfng), mRNA [NM_133393]	**7,278**
Spata24	Rattus norvegicus spermatogenesis associated 24 (Spata24), mRNA [NM_001025636]	**7,277**
0	BQ195967 UI-R-CN1-cmn-g-04-0-UI.s1 UI-R-CN1 Rattus norvegicus cDNA clone UI-R-CN1-cmn-g-04-0-UI 3', mRNA sequence [BQ195967]	**7,277**
Cd79a	PREDICTED: Rattus norvegicus Cd79a molecule, immunoglobulin-associated alpha (Cd79a), mRNA [XM_002725547]	**7,276**
RGD1562502	Rattus norvegicus similar to RIKEN cDNA 2610029G23 (RGD1562502), mRNA [NM_001108818]	**7,275**
0	Unknown	**7,275**
0	Unknown	**7,275**
Olr862	Rattus norvegicus olfactory receptor 862 (Olr862), mRNA [NM_001001071]	**7,273**
Spg20	Rattus norvegicus spastic paraplegia 20 (Troyer syndrome) homolog (human) (Spg20), mRNA [NM_001106433]	**7,273**
Rsl1d1	Rattus norvegicus ribosomal L1 domain containing 1 (Rsl1d1), mRNA [NM_001008876]	**7,272**

0	Unknown	**7,270**
LOC497940	Rattus norvegicus similar to RIKEN cDNA 2810408A11 (LOC497940), mRNA [NM_001017476]	**7,270**
LOC690937	PREDICTED: Rattus norvegicus similar to Discs large homolog 5 (Placenta and prostate DLG) (Discs large protein P-dlg) (LOC690937), mRNA [XM_001076213]	**7,269**
Hectd1	Uncharacterized protein [Source:UniProtKB/TrEMBL;Acc:D3ZLS5] [ENSRNOT00000008459]	**7,267**
Stk32c	Rattus norvegicus serine/threonine kinase 32C (Stk32c), mRNA [NM_001108922]	**7,267**
Rnd1	Rattus norvegicus Rho family GTPase 1 (Rnd1), mRNA [NM_001013222]	**7,267**
Azin1	Rattus norvegicus antizyme inhibitor 1 (Azin1), mRNA [NM_022585]	**7,266**
0	Rattus norvegicus similar to protein-tyrosine-phosphatase (EC 3.1.3.48) isoenzyme AcP2 - rat (LOC289297), mRNA [XM_222985]	**7,266**
Nars2	Rattus norvegicus asparaginyl-tRNA synthetase 2 (mitochondrial)(putative) (Nars2), nuclear gene encoding mitochondrial protein, mRNA [NM_001034921]	**7,265**
Zfp458	Rattus norvegicus zinc finger protein 458 (Zfp458), transcript variant 1, mRNA [NM_001142962]	**7,264**
Lonrf2	Uncharacterized protein [Source:UniProtKB/TrEMBL;Acc:D3ZX48] [ENSRNOT00000033964]	**7,263**
Csrnp3	Similar to TGF-beta induced apotosis protein 2 (Predicted)Uncharacterized protein [Source:UniProtKB/TrEMBL;Acc:D4AE74] [ENSRNOT00000007197]	**7,262**
G3bp2	Rattus norvegicus GTPase activating protein (SH3 domain) binding protein 2 (G3bp2), mRNA [NM_001013989]	**7,262**
Cse1l	Rattus norvegicus CSE1 chromosome segregation 1-like (yeast) (Cse1l), mRNA [NM_001108607]	**7,261**
Plekha3	Rattus norvegicus pleckstrin homology domain-containing, family A (phosphoinositide binding specific) member 3 (Plekha3), mRNA [NM_001013077]	**7,260**
Slco1a4	Rattus norvegicus solute carrier organic anion transporter family, member 1a4 (Slco1a4), mRNA [NM_131906]	**7,260**
RGD1306783	Rattus norvegicus similar to 2810422O20Rik protein (RGD1306783), mRNA [NM_001025668]	**7,260**
Synrg	Rattus norvegicus synergin, gamma (Synrg), mRNA [NM_053419]	**7,259**
Vamp4	Rattus norvegicus vesicle-associated membrane protein 4 (Vamp4), mRNA [NM_001108856]	**7,259**
Mfsd7	Rattus norvegicus major facilitator superfamily domain containing 7 (Mfsd7), mRNA [NM_001107246]	**7,257**
0	Unknown	**7,257**
Gls	Rattus norvegicus glutaminase (Gls), nuclear gene encoding mitochondrial protein, transcript variant 2, mRNA [NM_001109968]	**7,256**
Aqp9	Rattus norvegicus aquaporin 9 (Aqp9), mRNA [NM_022960]	**7,255**
Ano4	Rattus norvegicus anoctamin 4 (Ano4), mRNA [NM_001106778]	**7,254**
Wfdc1	Rattus norvegicus WAP four-disulfide core domain 1 (Wfdc1), mRNA [NM_133581]	**7,253**
Gmcl1l	Uncharacterized protein [Source:UniProtKB/TrEMBL;Acc:D3ZV83] [ENSRNOT00000048887]	**7,252**
0	Q2PC21_BOVIN (Q2PC21) HCAP-G protein (Fragment), partial (88%) [TC601153]	**7,252**
Zfp52	Rattus norvegicus zinc finger protein 52 (Zfp52), mRNA [NM_001014158]	**7,248**
Zfp706	Rattus norvegicus zinc finger protein 706 (Zfp706), mRNA [NM_001126087]	**7,247**
0	Unknown	**7,247**

Hnrnpc	Rattus norvegicus heterogeneous nuclear ribonucleoprotein C (C1/C2) (Hnrnpc), mRNA [NM_001025633]	**7,246**
Tmem229b	Rattus norvegicus transmembrane protein 229B (Tmem229b), mRNA [NM_001109359]	**7,245**
0	Uncharacterized protein [Source:UniProtKB/TrEMBL;Acc:D3ZXV2] [ENSRNOT00000043588]	**7,245**
Sbf1	PREDICTED: Rattus norvegicus SET binding factor 1 (Sbf1), mRNA [XM_001054780]	**7,245**
0	Unknown	**7,242**
LOC684024	PREDICTED: Rattus norvegicus similar to dual specificity phosphatase 5 (LOC684024), mRNA [XM_001068576]	**7,242**
Tmed3	Rattus norvegicus transmembrane emp24 protein transport domain containing 3 (Tmed3), mRNA [NM_001004249]	**7,241**
Prp2	Rattus norvegicus proline rich protein 2 (Prp2), mRNA [NM_001013211]	**7,240**
0	Unknown	**7,238**
Cenpl	Rattus norvegicus centromere protein L (Cenpl), mRNA [NM_001033061]	**7,237**
0	PREDICTED: Rattus norvegicus ADAMTS-like 1 (Adamtsl1), mRNA [XM_342858]	**7,237**
Lrtm2	Rattus norvegicus leucine-rich repeats and transmembrane domains 2 (Lrtm2), mRNA [NM_001109430]	**7,237**
0	Uncharacterized protein [Source:UniProtKB/TrEMBL;Acc:D3ZQC4] [ENSRNOT00000050888]	**7,236**
Arid3b	Rattus norvegicus AT rich interactive domain 3B (Bright like) (Arid3b), mRNA [NM_001109001]	**7,236**
Slc15a1	Rattus norvegicus solute carrier family 15 (oligopeptide transporter), member 1 (Slc15a1), transcript variant 1, mRNA [NM_057121]	**7,236**
0	Unknown	**7,236**
Slc30a9	Rattus norvegicus solute carrier family 30 (zinc transporter), member 9 (Slc30a9), mRNA [NM_001109088]	**7,236**
Slc10a5	Rattus norvegicus solute carrier family 10 (sodium/bile acid cotransporter family), member 5 (Slc10a5), mRNA [NM_001025280]	**7,236**
0	Uncharacterized protein [Source:UniProtKB/TrEMBL;Acc:D4A6W7] [ENSRNOT00000009090]	**7,235**
Slc25a38	Rattus norvegicus solute carrier family 25, member 38 (Slc25a38), nuclear gene encoding mitochondrial protein, mRNA [NM_001030032]	**7,235**
MGC95208	Rattus norvegicus similar to 4930453N24Rik protein (MGC95208), mRNA [NM_001005552]	**7,234**
Mterfd3	Rattus norvegicus MTERF domain containing 3 (Mterfd3), mRNA [NM_001014265]	**7,234**
Serp1	Rattus norvegicus stress-associated endoplasmic reticulum protein 1 (Serp1), mRNA [NM_030835]	**7,233**
Znf644	Rattus norvegicus zinc finger protein 644 (Znf644), mRNA [NM_001139484]	**7,233**
Rela	Rattus norvegicus v-rel reticuloendotheliosis viral oncogene homolog A (avian) (Rela), mRNA [NM_199267]	**7,232**
Vom2r76	Rattus norvegicus vomeronasal 2 receptor, 76 (Vom2r76), mRNA [NM_001099505]	**7,232**
Sptb	Rattus norvegicus spectrin, beta, erythrocytic (Sptb), mRNA [NM_212522]	**7,231**
Ppp1r9a	Rattus norvegicus protein phosphatase 1, regulatory (inhibitor) subunit 9A (Ppp1r9a), mRNA [NM_053473]	**7,231**
LOC361016	Rattus norvegicus similar to RIKEN cDNA 4933406L09, mRNA (cDNA clone IMAGE:7301570), complete cds. [BC086559]	**7,229**
Trim28	Rattus norvegicus tripartite motif-containing 28 (Trim28), mRNA [NM_053916]	**7,229**
Chml	Rattus norvegicus choroideremia-like (Rab escort protein 2) (Chml), mRNA [NM_001109524]	**7,229**
Mrpl17	Rattus norvegicus mitochondrial ribosomal protein L17 (Mrpl17), nuclear gene encoding mitochondrial protein, mRNA [NM_133539]	**7,229**
Siglec15	Rattus norvegicus sialic acid binding Ig-like lectin 15 (Siglec15), mRNA [NM_001191942]	**7,226**
Gpd1l	Rattus norvegicus glycerol-3-phosphate dehydrogenase 1-like (Gpd1l), mRNA [NM_001191885]	**7,226**

LOC679800	PREDICTED: Rattus norvegicus similar to MIC2 like 1 (LOC679800), mRNA [XM_001054523]	**7,225**
Crls1	Rattus norvegicus cardiolipin synthase 1 (Crls1), mRNA [NM_001014258]	**7,225**
Tjp1	Rattus norvegicus tight junction protein 1 (Tjp1), mRNA [NM_001106266]	**7,222**
0	Unknown	**7,221**
0	Unknown	**7,220**
LOC688390	Rattus norvegicus hypothetical protein LOC688390 (LOC688390), mRNA [NM_001109496]	**7,220**
Hoxc6	PREDICTED: Rattus norvegicus homeo box C6, transcript variant 1 (Hoxc6), mRNA [XM_001069410]	**7,219**
RGD1310016	Uncharacterized protein [Source:UniProtKB/TrEMBL;Acc:D3ZNB8] [ENSRNOT00000021783]	**7,219**
0	Unknown	**7,218**
Rab33a	Rattus norvegicus RAB33A, member RAS oncogene family (Rab33a), mRNA [NM_001108257]	**7,217**
Fam178a	Rattus norvegicus family with sequence similarity 178, member A (Fam178a), mRNA [NM_001134612]	**7,217**
Pim3	Rattus norvegicus pim-3 oncogene (Pim3), mRNA [NM_022602]	**7,215**
0	PREDICTED: Rattus norvegicus similar to H3 histone, family 3B (RGD1564548), mRNA [XM_001072064]	**7,212**
Tm6sf1	Rattus norvegicus transmembrane 6 superfamily member 1 (Tm6sf1), mRNA [NM_001108490]	**7,211**
Paqr7	Rattus norvegicus progestin and adipoQ receptor family member VII (Paqr7), mRNA [NM_001034081]	**7,210**
Pex14	Rattus norvegicus peroxisomal biogenesis factor 14 (Pex14), mRNA [NM_172063]	**7,209**
0	Uncharacterized protein [Source:UniProtKB/TrEMBL;Acc:D3ZTT2] [ENSRNOT00000059588]	**7,208**
Dync1i2	Rattus norvegicus dynein cytoplasmic 1 intermediate chain 2 (Dync1i2), mRNA [NM_053880]	**7,208**
Bfar	Rattus norvegicus bifunctional apoptosis regulator (Bfar), mRNA [NM_001013125]	**7,207**
Snrnp27	Rattus norvegicus small nuclear ribonucleoprotein 27 (U4/U6.U5) (Snrnp27), mRNA [NM_001108636]	**7,206**
Fbxo34	Rattus norvegicus F-box protein 34 (Fbxo34), mRNA [NM_001107257]	**7,206**
Znf513	Rattus norvegicus zinc finger protein 513 (Znf513), mRNA [NM_001012110]	**7,206**
Lmx1a	Rattus norvegicus LIM homeobox transcription factor 1 alpha (Lmx1a), mRNA [NM_001105967]	**7,204**
Yrdc	Rattus norvegicus yrdC domain containing (E.coli) (Yrdc), nuclear gene encoding mitochondrial protein, mRNA [NM_175604]	**7,203**
Gpr161	G protein-coupled receptor 161 [Source:RefSeq peptide;Acc:NP_001178601] [ENSRNOT00000067745]	**7,203**
RGD1306928	PREDICTED: Rattus norvegicus similar to hypothetical protein MGC13138, transcript variant 1 (RGD1306928), mRNA [XM_001080215]	**7,203**
Mfap4	Rattus norvegicus microfibrillar-associated protein 4 (Mfap4), mRNA [NM_001034124]	**7,203**
Acap3	Rattus norvegicus ArfGAP with coiled-coil, ankyrin repeat and PH domains 3 (Acap3), mRNA [NM_001107999]	**7,203**
0	Unknown	**7,202**
Wdfy2	Rattus norvegicus WD repeat and FYVE domain containing 2 (Wdfy2), mRNA [NM_001107269]	**7,202**
0	Glyceraldehyde-3-phosphate dehydrogenase [Source:UniProtKB/TrEMBL;Acc:D3ZWV2] [ENSRNOT00000030148]	**7,201**
LOC684652	PREDICTED: Rattus norvegicus hypothetical protein LOC684652 (LOC684652), partial mRNA [XM_001055278]	**7,201**
LOC502894	Rattus norvegicus hypothetical protein LOC502894 (LOC502894), mRNA [NM_001025064]	**7,200**
Zmynd15	Rattus norvegicus zinc finger, MYND-type containing 15 (Zmynd15), mRNA [NM_001105800]	**7,199**

Rnd3	Rattus norvegicus Rho family GTPase 3 (Rnd3), mRNA [NM_001007641]	**7,198**
0	Unknown	**7,198**
0	Rattus norvegicus strain BN/SsNHsdMCW RNOR03327191, whole genome shotgun sequence [AABR03127998]	**7,197**
0	Unknown	**7,197**
Adar	Rattus norvegicus adenosine deaminase, RNA-specific (Adar), mRNA [NM_031006]	**7,196**
Josd2	Rattus norvegicus Josephin domain containing 2 (Josd2), mRNA [NM_001106256]	**7,196**
Ralbp1	Rattus norvegicus ralA binding protein 1 (Ralbp1), mRNA [NM_032067]	**7,196**
Tmem55b	Rattus norvegicus transmembrane protein 55B (Tmem55b), mRNA [NM_001014233]	**7,195**
0	Unknown	**7,195**
Nt5c3l	Rattus norvegicus 5'-nucleotidase, cytosolic III-like (Nt5c3l), mRNA [NM_001007723]	**7,195**
Zbtb11	Rattus norvegicus zinc finger and BTB domain containing 11 (Zbtb11), mRNA [NM_001107097]	**7,195**
0	Unknown	**7,194**
Cpt1c	Rattus norvegicus carnitine palmitoyltransferase 1c (Cpt1c), mRNA [NM_001034925]	**7,191**
Camk2g	Rattus norvegicus calcium/calmodulin-dependent protein kinase II gamma (Camk2g), mRNA [NM_133605]	**7,190**
Fyttd1	Rattus norvegicus forty-two-three domain containing 1 (Fyttd1), mRNA [NM_001047899]	**7,190**
Ing2	Rattus norvegicus inhibitor of growth family, member 2 (Ing2), mRNA [NM_001106083]	**7,190**
Tex264	Rattus norvegicus testis expressed 264 (Tex264), mRNA [NM_001007665]	**7,188**
0	Unknown	**7,188**
0	Rattus norvegicus strain Sprague-Dawley morphine related protein-1 mRNA, complete cds. [AF145796]	**7,187**
Masp2	Rattus Norvegicus mRNA for MAp19 protein, partial clone prl-5. [Y18568]	**7,187**
0	Unknown	**7,187**
0	Uncharacterized protein [Source:UniProtKB/TrEMBL;Acc:D3ZER7] [ENSRNOT00000043011]	**7,186**
0	Unknown	**7,185**
Tmprss6	Rattus norvegicus transmembrane protease, serine 6 (Tmprss6), mRNA [NM_001130556]	**7,185**
Serpinb2	Rattus norvegicus serpin peptidase inhibitor, clade B (ovalbumin), member 2 (Serpinb2), mRNA [NM_021696]	**7,185**
0	Rattus norvegicus similar to Elongation factor 1-gamma (EF-1-gamma) (eEF-1B gamma) (LOC364451), mRNA [XM_344447]	**7,185**
Gkap1	Rattus norvegicus G kinase anchoring protein 1 (Gkap1), mRNA [NM_001012160]	**7,184**
Fgd4	Rattus norvegicus FYVE, RhoGEF and PH domain containing 4 (Fgd4), mRNA [NM_139263]	**7,184**
Inpp1	Rattus norvegicus inositol polyphosphate-1-phosphatase (Inpp1), mRNA [NM_001012131]	**7,184**
Tmem181	Rattus norvegicus transmembrane protein 181 (Tmem181), mRNA [NM_001130939]	**7,184**
Fau	Rattus norvegicus Finkel-Biskis-Reilly murine sarcoma virus (FBR-MuSV) ubiquitously expressed (Fau), transcript variant 1, mRNA [NM_001012739]	**7,184**
Me3	Rattus norvegicus malic enzyme 3, NADP(+)-dependent, mitochondrial (Me3), nuclear gene encoding mitochondrial protein, mRNA [NM_001108491]	**7,183**

Tcea1	Rattus norvegicus transcription elongation factor A (SII) 1 (Tcea1), mRNA [NM_001025735]	**7,183**
Rnf19a	Rattus norvegicus ring finger protein 19A (Rnf19a), mRNA [NM_001130560]	**7,183**
Lcmt1	Rattus norvegicus leucine carboxyl methyltransferase 1 (Lcmt1), mRNA [NM_199405]	**7,183**
Serac1	Similar to serine active site containing 1 (Predicted)Uncharacterized protein [Source:UniProtKB/TrEMBL;Acc:D3ZEJ3] [ENSRNOT00000060527]	**7,182**
Csf2ra	Rattus norvegicus granulocyte-macrophage colony stimulating receptor alpha (Csf2ra), mRNA [NM_001037660]	**7,181**
Tor1aip1	Rattus norvegicus torsin A interacting protein 1 (Tor1aip1), mRNA [NM_145092]	**7,179**
0	Unknown	**7,179**
Ddx3x	Rattus norvegicus DEAD (Asp-Glu-Ala-Asp) box polypeptide 3, X-linked (Ddx3x), mRNA [NM_001108246]	**7,177**
0	Rattus norvegicus similar to Aspartate aminotransferase, mitochondrial precursor (Transaminase A) (Glutamate oxaloacetate transaminase-2) (LOC296593), mRNA [XM_231092]	**7,177**
Pgm2l1	Rattus norvegicus phosphoglucomutase 2-like 1 (Pgm2l1), mRNA [NM_001109454]	**7,177**
Neurl2	Rattus norvegicus neuralized homolog 2 (Drosophila) (Neurl2), mRNA [NM_001107802]	**7,176**
Aoc3	Rattus norvegicus amine oxidase, copper containing 3 (vascular adhesion protein 1) (Aoc3), mRNA [NM_031582]	**7,176**
Ccnf	Rattus norvegicus cyclin F (Ccnf), mRNA [NM_001100474]	**7,175**
Tlk1	Rattus norvegicus tousled-like kinase 1 (Tlk1), mRNA [NM_001107734]	**7,175**
0	Unknown	**7,174**
Nudt13	Rattus norvegicus nudix (nucleoside diphosphate linked moiety X)-type motif 13 (Nudt13), mRNA [NM_001127636]	**7,173**
Dsc2	Rattus norvegicus desmocollin 2 (Dsc2), mRNA [NM_001033688]	**7,171**
Bzrap1	Peripheral-type benzodiazepine receptor-associated protein 1 [Source:UniProtKB/Swiss-Prot;Acc:Q9JIR0] [ENSRNOT00000045158]	**7,171**
0	PREDICTED: Rattus norvegicus similar to KIAA0953 protein (RGD1310845), mRNA [XM_233942]	**7,171**
Scrn1	Rattus norvegicus secernin 1 (Scrn1), mRNA [NM_001025063]	**7,170**
RGD1559442	Rattus norvegicus similar to SET binding factor 2 (LOC691036), mRNA [NM_001134970]	**7,169**
Atmin	Rattus norvegicus ATM interactor (Atmin), mRNA [NM_001191786]	**7,169**
Pde9a	Rattus norvegicus phosphodiesterase 9A (Pde9a), mRNA [NM_138543]	**7,167**
0	Unknown	**7,167**
Abhd10	Rattus norvegicus abhydrolase domain containing 10 (Abhd10), mRNA [NM_001123352]	**7,166**
RGD1564515	PREDICTED: Rattus norvegicus similar to alpha 1B-glycoprotein (RGD1564515), mRNA [XM_001069779]	**7,166**
Lca5	Rattus norvegicus Leber congenital amaurosis 5 (human) (Lca5), mRNA [NM_001013954]	**7,165**
0	PREDICTED: Rattus norvegicus single-stranded DNA binding protein 2 (Ssbp2), mRNA [XM_001065491]	**7,164**
Zfand6	Rattus norvegicus zinc finger, AN1-type domain 6 (Zfand6), mRNA [NM_001007630]	**7,162**
Mtmr10	Rattus norvegicus myotubularin related protein 10 (Mtmr10), mRNA [NM_001100846]	**7,162**
Copg	Rattus norvegicus coatomer protein complex, subunit gamma (Copg), mRNA [NM_001031822]	**7,161**
Stx2	Rattus norvegicus syntaxin 2 (Stx2), mRNA [NM_012748]	**7,160**
0	Unknown	**7,160**

Znf865	Rattus norvegicus zinc finger protein 865 (Znf865), mRNA [NM_001134544]	**7,159**
RGD1311267	Rattus norvegicus similar to RIKEN cDNA 4931426K16 gene (RGD1311267), mRNA [NM_001039024]	**7,158**
0	Rattus norvegicus similar to glyceraldehyde-3-phosphate dehydrogenase (phosphorylating) (EC 1.2.1.12) - mouse (LOC294405), mRNA [XM_215391]	**7,157**
Gnb2	Rattus norvegicus guanine nucleotide binding protein (G protein), beta polypeptide 2 (Gnb2), mRNA [NM_031037]	**7,157**
Tas2r109	Rattus norvegicus taste receptor, type 2, member 109 (Tas2r109), mRNA [NM_001080939]	**7,157**
Lnp	Rattus norvegicus limb and neural patterns (Lnp), mRNA [NM_001077429]	**7,156**
0	Unknown	**7,154**
Ptpn6	Rattus norvegicus protein tyrosine phosphatase, non-receptor type 6 (Ptpn6), mRNA [NM_053908]	**7,153**
Ppap2c	Rattus norvegicus phosphatidic acid phosphatase type 2c (Ppap2c), mRNA [NM_139252]	**7,151**
Ap1b1	Rattus norvegicus adaptor-related protein complex 1, beta 1 subunit (Ap1b1), mRNA [NM_017277]	**7,150**
Fam53a	Rattus norvegicus family with sequence similarity 53, member A (Fam53a), mRNA [NM_001107228]	**7,150**
Gpr172a	Rattus norvegicus G protein-coupled receptor 172A (Gpr172a), mRNA [NM_001109670]	**7,149**
Pnldc1	Rattus norvegicus poly(A)-specific ribonuclease (PARN)-like domain containing 1 (Pnldc1), mRNA [NM_001025724]	**7,149**
RGD1311273	Rattus norvegicus similar to RIKEN cDNA 9530058B02 (RGD1311273), mRNA [NM_001109475]	**7,148**
Pak4	Rattus norvegicus p21 protein (Cdc42/Rac)-activated kinase 4 (Pak4), mRNA [NM_001106238]	**7,148**
0	Q4KM71_RAT (Q4KM71) NonO/p54nrb homolog, complete [TC583734]	**7,147**
0	PREDICTED: Rattus norvegicus similar to RIKEN cDNA 1500001A10 (RGD1560213), miscRNA [XR_085996]	**7,147**
Gsn	Rattus norvegicus gelsolin (Gsn), mRNA [NM_001004080]	**7,146**
Inpp5k	Rattus norvegicus inositol polyphosphate-5-phosphatase K (Inpp5k), mRNA [NM_001013859]	**7,146**
0	Lysophosphatidylcholine acyltransferase 2B [Source:UniProtKB/Swiss-Prot;Acc:Q4V8A1] [ENSRNOT00000037141]	**7,145**
Spsb1	Rattus norvegicus splA/ryanodine receptor domain and SOCS box containing 1 (Spsb1), mRNA [NM_001107994]	**7,145**
Bag1	Rattus norvegicus BCL2-associated athanogene (Bag1), mRNA [NM_001106647]	**7,145**
Fam35a	Rattus norvegicus family with sequence similarity 35, member A (Fam35a), mRNA [NM_001025028]	**7,145**
Fgfbp3	Rattus norvegicus fibroblast growth factor binding protein 3 (Fgfbp3), mRNA [NM_001109165]	**7,145**
RGD1311433	Uncharacterized protein [Source:UniProtKB/TrEMBL;Acc:D3Z9R2] [ENSRNOT00000003700]	**7,144**
Higd2a	Rattus norvegicus HIG1 hypoxia inducible domain family, member 2A (Higd2a), mRNA [NM_001106102]	**7,144**
Rad54l	Rattus norvegicus RAD54 like (S. cerevisiae) (Rad54l), mRNA [NM_001134960]	**7,144**
0	Unknown	**7,143**
Olr1614	Rattus norvegicus olfactory receptor 1614 (Olr1614), mRNA [NM_001000841]	**7,142**
Ephb3	Rattus norvegicus Eph receptor B3 (Ephb3), mRNA [NM_001105868]	**7,141**
Cpeb1	Rattus norvegicus cytoplasmic polyadenylation element binding protein 1 (Cpeb1), mRNA [NM_001106276]	**7,141**
Taf5l	Rattus norvegicus TAF5-like RNA polymerase II, p300/CBP-associated factor (PCAF)-associated factor (Taf5l), mRNA [NM_001107442]	**7,141**
Prdm5	Uncharacterized protein [Source:UniProtKB/TrEMBL;Acc:D3ZRZ9] [ENSRNOT00000009569]	**7,140**

Isg20	Rattus norvegicus interferon stimulated exonuclease gene 20 (Isg20), mRNA [NM_001008510]	**7,138**
Mrc2	Rattus norvegicus mannose receptor, C type 2 (Mrc2), mRNA [NM_001024687]	**7,138**
0	PREDICTED: Rattus norvegicus similar to Emu2 (LOC685612), mRNA [XM_001064529]	**7,138**
Flcn	Rattus norvegicus folliculin (Flcn), mRNA [NM_199390]	**7,137**
RGD1562165	PREDICTED: Rattus norvegicus similar to GABA(A) receptor-associated protein like 2 (RGD1562165), partial mRNA [XM_001058517]	**7,136**
0	Unknown	**7,135**
Acp5	Rattus norvegicus acid phosphatase 5, tartrate resistant (Acp5), mRNA [NM_019144]	**7,135**
0	Unknown	**7,134**
Kiss1r	Rattus norvegicus KISS1 receptor (Kiss1r), mRNA [NM_023992]	**7,134**
0	Pyruvate kinase [Source:UniProtKB/TrEMBL;Acc:D3ZH85] [ENSRNOT00000015275]	**7,133**
Herc6	PREDICTED: Rattus norvegicus potential ubiquitin ligase (Herc6), mRNA [XM_001064216]	**7,133**
Pet112l	PREDICTED: Rattus norvegicus PET112-like (yeast) (Pet112l), mRNA [XM_001066526]	**7,133**
Tmem141	Rattus norvegicus transmembrane protein 141 (Tmem141), mRNA [NM_001109197]	**7,132**
Rnf20	Rattus norvegicus ring finger protein 20 (Rnf20), mRNA [NM_001107929]	**7,132**
0	Unknown	**7,131**
Rrh	Rattus norvegicus retinal pigment epithelium derived rhodopsin homolog (Rrh), mRNA [NM_001107726]	**7,130**
0	Unknown	**7,129**
LOC301816	PREDICTED: Rattus norvegicus similar to Spindlin-like protein 2 (SPIN-2) (LOC301816), mRNA [XM_229292]	**7,128**
RGD1305110	Rattus norvegicus similar to KIAA1841 protein (RGD1305110), mRNA [NM_001100971]	**7,126**
Bcl2l2	Rattus norvegicus Bcl2-like 2 (Bcl2l2), mRNA [NM_021850]	**7,126**
Nos1ap	Rattus norvegicus nitric oxide synthase 1 (neuronal) adaptor protein (Nos1ap), mRNA [NM_138922]	**7,126**
0	Homeobox protein cut-like 1 [Source:UniProtKB/Swiss-Prot;Acc:P53565] [ENSRNOT00000059486]	**7,125**
Stx6	Rattus norvegicus syntaxin 6 (Stx6), mRNA [NM_031665]	**7,125**
Rnf168	Rattus norvegicus ring finger protein 168 (Rnf168), mRNA [NM_001127597]	**7,122**
Fam105a	Rattus norvegicus family with sequence similarity 105, member A (Fam105a), mRNA [NM_001037648]	**7,122**
LOC690478	Rattus norvegicus similar to keratin associated protein 10-7 (LOC690478), mRNA [NM_001109595]	**7,122**
Snx4	Rattus norvegicus sorting nexin 4 (Snx4), mRNA [NM_001127550]	**7,121**
0	Uncharacterized protein [Source:UniProtKB/TrEMBL;Acc:D3ZRK3] [ENSRNOT00000059370]	**7,121**
Agpat5	Rattus norvegicus 1-acylglycerol-3-phosphate O-acyltransferase 5 (lysophosphatidic acid acyltransferase, epsilon) (Agpat5), mRNA [NM_001134744]	**7,121**
Ptch1	Rattus norvegicus patched homolog 1 (Drosophila) (Ptch1), mRNA [NM_053566]	**7,120**
Alb	Rattus norvegicus albumin (Alb), mRNA [NM_134326]	**7,119**
Jun	Rattus norvegicus Jun oncogene (Jun), mRNA [NM_021835]	**7,119**
Il6st	Rattus norvegicus interleukin 6 signal transducer (Il6st), mRNA [NM_001008725]	**7,119**

Pnpla7	Rattus norvegicus patatin-like phospholipase domain containing 7 (Pnpla7), mRNA [NM_144738]	**7,119**
0	Uncharacterized protein [Source:UniProtKB/TrEMBL;Acc:D3ZCG9] [ENSRNOT00000005644]	**7,118**
Gpx7	Rattus norvegicus glutathione peroxidase 7 (Gpx7), mRNA [NM_001106673]	**7,116**
Lamc1	Rattus norvegicus laminin, gamma 1 (Lamc1), mRNA [NM_053966]	**7,116**
Mpv17l2	Rattus norvegicus MPV17 mitochondrial membrane protein-like 2 (Mpv17l2), nuclear gene encoding mitochondrial protein, mRNA [NM_001106072]	**7,116**
Isy1	Rattus norvegicus ISY1 splicing factor homolog (S. cerevisiae) (Isy1), mRNA [NM_001014188]	**7,115**
Ppp2r1b	Rattus norvegicus protein phosphatase 2 (formerly 2A), regulatory subunit A, beta isoform (Ppp2r1b), mRNA [NM_001025418]	**7,115**
Fgf21	Rattus norvegicus fibroblast growth factor 21 (Fgf21), mRNA [NM_130752]	**7,111**
Lrrc47	Rattus norvegicus leucine rich repeat containing 47 (Lrrc47), mRNA [NM_001135666]	**7,111**
Arid4a	Rattus norvegicus AT rich interactive domain 4A (Rbp1 like) (Arid4a), mRNA [NM_001108029]	**7,109**
Scand1	Rattus norvegicus SCAN domain-containing 1 (Scand1), mRNA [NM_001108599]	**7,108**
0	Uncharacterized protein [Source:UniProtKB/TrEMBL;Acc:D3ZQF0] [ENSRNOT00000047238]	**7,108**
Il17ra	Rattus norvegicus interleukin 17 receptor A (Il17ra), mRNA [NM_001107883]	**7,106**
0	Unknown	**7,106**
Cdkn2b	Rattus norvegicus cyclin-dependent kinase inhibitor 2B (p15, inhibits CDK4) (Cdkn2b), mRNA [NM_130812]	**7,106**
RGD1561520	PREDICTED: Rattus norvegicus similar to Ab2-162 (RGD1561520), mRNA [XM_345159]	**7,105**
RGD1305572	Rattus norvegicus similar to hypothetical protein MGC30618 (RGD1305572), mRNA [NM_001024970]	**7,105**
Tmem186	Rattus norvegicus transmembrane protein 186 (Tmem186), mRNA [NM_001025756]	**7,104**
Kcnq4	Potassium voltage-gated channel subfamily KQT member 4 [Source:UniProtKB/Swiss-Prot;Acc:Q9JK96] [ENSRNOT00000014231]	**7,103**
Grk6	Rattus norvegicus G protein-coupled receptor kinase 6 (Grk6), transcript variant 2, mRNA [NM_001112713]	**7,103**
0	Unknown	**7,103**
0	Uncharacterized protein [Source:UniProtKB/TrEMBL;Acc:D4A553] [ENSRNOT00000016227]	**7,102**
Nedd8	Rattus norvegicus neural precursor cell expressed, developmentally down-regulated 8 (Nedd8), mRNA [NM_138878]	**7,102**
Alg13	Rattus norvegicus asparagine-linked glycosylation 13 homolog (S. cerevisiae) (Alg13), mRNA [NM_001013951]	**7,100**
Xylt2	Rattus norvegicus xylosyltransferase II (Xylt2), mRNA [NM_022296]	**7,100**
Nkiras1	Rattus norvegicus NFKB inhibitor interacting Ras-like 1 (Nkiras1), mRNA [NM_001107252]	**7,099**
Vps33b	Rattus norvegicus vacuolar protein sorting 33 homolog B (yeast) (Vps33b), mRNA [NM_022286]	**7,098**
Znf804a	Rattus norvegicus similar to hypothetical protein (LOC295695), mRNA [XM_230042]	**7,098**
Extl1	Rattus norvegicus exostoses (multiple)-like 1 (Extl1), mRNA [NM_001107985]	**7,097**
0	Unknown	**7,097**
Olr196	Rattus norvegicus olfactory receptor 196 (Olr196), mRNA [NM_001000187]	**7,095**
Crygf	Rattus norvegicus crystallin, gamma F (Crygf), mRNA [NM_001109557]	**7,095**
0	Unknown	**7,094**
0	Coiled-coil domain-containing-like [Source:UniProtKB/TrEMBL;Acc:Q5M816] [ENSRNOT00000015782]	**7,093**

0	Unknown	**7,093**
Senp6	Rattus norvegicus SUMO1/sentrin specific peptidase 6 (Senp6), mRNA [NM_001106842]	**7,092**
Taz	Rattus norvegicus tafazzin (Taz), nuclear gene encoding mitochondrial protein, mRNA [NM_001025748]	**7,091**
0	Unknown	**7,091**
Arl13b	Rattus norvegicus ADP-ribosylation factor-like 13B (Arl13b), mRNA [NM_001107101]	**7,091**
Zfp758	PREDICTED: Rattus norvegicus similar to reduced expression 2 (LOC690559), mRNA [XM_001074805]	**7,091**
Bri3	Rattus norvegicus brain protein I3 (Bri3), mRNA [NM_001009604]	**7,090**
Olr606	Rattus norvegicus olfactory receptor 606 (Olr606), mRNA [NM_001000927]	**7,090**
Cluap1	Rattus norvegicus clusterin associated protein 1 (Cluap1), mRNA [NM_001014225]	**7,088**
0	Rattus norvegicus similar to Alpha enolase (2-phospho-D-glycerate hydro-lyase) (Non-neural enolase) (NNE) (Enolase 1) (LOC287338), mRNA [XM_220490]	**7,088**
Kif13a	Rattus norvegicus kinesin family member 13A (Kif13a), mRNA [NM_001107462]	**7,088**
RGD1311756	Rattus norvegicus similar to hypothetical protein FLJ20950 (RGD1311756), mRNA [NM_001173557]	**7,087**
Rfwd3	Rattus norvegicus similar to hypothetical protein FLJ34389 (LOC361409), mRNA [XM_341690]	**7,086**
RGD1311273	Rattus norvegicus similar to RIKEN cDNA 9530058B02 (RGD1311273), mRNA [NM_001109475]	**7,084**
Kif5c	Rattus norvegicus kinesin family member 5C (Kif5c), mRNA [NM_001107730]	**7,083**
Eef2k	Rattus norvegicus eukaryotic elongation factor-2 kinase (Eef2k), mRNA [NM_012947]	**7,082**
Bdnf	Rattus norvegicus brain-derived neurotrophic factor (Bdnf), mRNA [NM_012513]	**7,082**
0	Rattus norvegicus similar to Proteasome activator complex subunit 2 (Proteasome activator 28-beta subunit) (PA28beta) (PA28b) (Activator of multicatalytic protease subunit 2) (11S regulator complex beta subunit) (REG-beta) (LOC304754), mRNA [XM_222590]	**7,081**
Lat	Rattus norvegicus linker for activation of T cells (Lat), mRNA [NM_030853]	**7,081**
Heca	Rattus norvegicus headcase homolog (Drosophila) (Heca), mRNA [NM_001107514]	**7,079**
Socs4	Rattus norvegicus suppressor of cytokine signaling 4 (Socs4), mRNA [NM_001107256]	**7,078**
Gjc3	Gap junction protein [Source:UniProtKB/TrEMBL;Acc:Q80XF8] [ENSRNOT00000001797]	**7,077**
0	Rattus norvegicus similar to glyceraldehyde-3-phosphate dehydrogenase (LOC310648), mRNA [XM_227423]	**7,076**
LOC100366273	Uncharacterized protein [Source:UniProtKB/TrEMBL;Acc:D4A1X2] [ENSRNOT00000014629]	**7,076**
Bicc1	Rattus norvegicus bicaudal C homolog 1 (Drosophila) (Bicc1), mRNA [NM_001108531]	**7,076**
0	Unknown	**7,075**
Ubr5	E3 ubiquitin-protein ligase UBR5 [Source:UniProtKB/Swiss-Prot;Acc:Q62671] [ENSRNOT00000009115]	**7,074**
0	Unknown	**7,074**
Sec61a1	Rattus norvegicus Sec61 alpha 1 subunit (S. cerevisiae) (Sec61a1), mRNA [NM_199256]	**7,072**
LOC691141	Rattus norvegicus hypothetical protein LOC691141 (LOC691141), mRNA [NM_001109624]	**7,072**
0	Uncharacterized protein [Source:UniProtKB/TrEMBL;Acc:D4A6G8] [ENSRNOT00000048964]	**7,071**
Egfl8	Rattus norvegicus EGF-like-domain, multiple 8 (Egfl8), transcript variant 1, mRNA [NM_001004070]	**7,071**

Olr1	Rattus norvegicus oxidized low density lipoprotein (lectin-like) receptor 1 (Olr1), mRNA [NM_133306]	**7,071**
LOC678769	Rattus norvegicus similar to Cysteine protease ATG4A (Autophagy-related protein 4 homolog A) (Autophagin-2) (Autophagy-related cysteine endopeptidase 2) (AUT-like 2 cysteine endopeptidase) (LOC678769), mRNA [NM_001126298]	**7,070**
Gnptg	Rattus norvegicus N-acetylglucosamine-1-phosphate transferase, gamma subunit (Gnptg), mRNA [NM_001100493]	**7,070**
Zmynd19	Rattus norvegicus zinc finger, MYND-type containing 19 (Zmynd19), mRNA [NM_198770]	**7,070**
0	Rattus norvegicus LOC361297 (LOC361297), mRNA [XM_341582]	**7,069**
Psme2	Rattus norvegicus proteasome (prosome, macropain) activator subunit 2 (Psme2), mRNA [NM_017257]	**7,068**
Adam8	Uncharacterized protein [Source:UniProtKB/TrEMBL;Acc:D3ZB52] [ENSRNOT00000024369]	**7,067**
Ccng2	Rattus norvegicus cyclin G2 (Ccng2), mRNA [NM_001105725]	**7,066**
0	Unknown	**7,065**
RGD1566085	Uncharacterized protein [Source:UniProtKB/TrEMBL;Acc:D3Z8P5] [ENSRNOT00000051678]	**7,064**
0	Unknown	**7,064**
Dcn	Rattus norvegicus decorin (Dcn), mRNA [NM_024129]	**7,064**
Guf1	Rattus norvegicus GUF1 GTPase homolog (S. cerevisiae) (Guf1), mRNA [NM_001107215]	**7,063**
Magel2	RCG63550Uncharacterized protein [Source:UniProtKB/TrEMBL;Acc:D3ZTL5] [ENSRNOT00000013483]	**7,062**
Kif2a	Kinesin-like protein KIF2A [Source:UniProtKB/Swiss-Prot;Acc:Q9WV63] [ENSRNOT00000019225]	**7,062**
Recql4	Rattus norvegicus RecQ protein-like 4 (Recql4), mRNA [NM_001130494]	**7,061**
Acvr2a	Rattus norvegicus activin A receptor, type IIA (Acvr2a), mRNA [NM_031571]	**7,061**
Slc5a5	Rattus norvegicus solute carrier family 5 (sodium iodide symporter), member 5 (Slc5a5), mRNA [NM_052983]	**7,061**
RGD1560666	PREDICTED: Rattus norvegicus similar to KIAA1280 protein (RGD1560666), mRNA [XM_001069576]	**7,060**
Slc38a2	Rattus norvegicus solute carrier family 38, member 2 (Slc38a2), mRNA [NM_181090]	**7,059**
Bhlha9	Uncharacterized protein [Source:UniProtKB/TrEMBL;Acc:D3ZY30] [ENSRNOT00000032934]	**7,058**
LOC293103	Rattus norvegicus similar to RIKEN cDNA 0610007P06 (LOC293103), mRNA [NM_001013897]	**7,058**
Asf1b	Rattus norvegicus ASF1 anti-silencing function 1 homolog B (S. cerevisiae) (Asf1b), mRNA [NM_001107160]	**7,058**
Cadm4	Rattus norvegicus cell adhesion molecule 4 (Cadm4), mRNA [NM_001047107]	**7,056**
0	Unknown	**7,056**
0	Unknown	**7,056**
Cdc42ep1	Rattus norvegicus CDC42 effector protein (Rho GTPase binding) 1 (Cdc42ep1), mRNA [NM_001079700]	**7,054**
0	Unknown	**7,054**
Mapk8ip1	Rattus norvegicus mitogen-activated protein kinase 8 interacting protein 1 (Mapk8ip1), mRNA [NM_053777]	**7,054**
Mcl1	Rattus norvegicus myeloid cell leukemia sequence 1 (Mcl1), nuclear gene encoding mitochondrial protein, mRNA [NM_021846]	**7,053**
Clcc1	Rattus norvegicus chloride channel CLIC-like 1 (Clcc1), mRNA [NM_133414]	**7,052**
0	Unknown	**7,050**
Rpa1	Rattus norvegicus replication protein A1 (Rpa1), mRNA [NM_001047843]	**7,050**

Zfp169	Uncharacterized protein [Source:UniProtKB/TrEMBL;Acc:D3Z9A8] [ENSRNOT00000022932]	**7,049**
0	BC030489 Anln protein {Mus musculus} (exp=-1; wgp=0; cg=0), partial (14%) [TC595131]	**7,048**
Zbtb1	Rattus norvegicus zinc finger and BTB domain containing 1 (Zbtb1), mRNA [NM_001004444]	**7,047**
0	Unknown	**7,047**
LOC679983	AMGNNUC:URRG1-00065-A12-A urrg1 (14046) Rattus norvegicus cDNA clone urrg1-00065-a12 5', mRNA sequence [CB547742]	**7,047**
0	Unknown	**7,046**
Crebl2	Rattus norvegicus cAMP responsive element binding protein-like 2 (Crebl2), mRNA [NM_001015027]	**7,046**
0	Unknown	**7,046**
0	Unknown	**7,046**
Itga10	Rattus norvegicus integrin, alpha 10 (Itga10), mRNA [NM_001107699]	**7,045**
Pou2af1	Rattus norvegicus POU class 2 associating factor 1 (Pou2af1), mRNA [NM_001109599]	**7,044**
0	Unknown	**7,044**
Dbnl	Rattus norvegicus drebrin-like (Dbnl), mRNA [NM_031352]	**7,044**
Ndufaf3	Rattus norvegicus NADH dehydrogenase (ubiquinone) 1 alpha subcomplex, assembly factor 3 (Ndufaf3), nuclear gene encoding mitochondrial protein, transcript variant 1, mRNA [NM_020080]	**7,042**
0	Unknown	**7,039**
0	Unknown	**7,037**
Fam5c	Rattus norvegicus family with sequence similarity 5, member C (Fam5c), mRNA [NM_173121]	**7,036**
Ap2a2	Rattus norvegicus adaptor-related protein complex 2, alpha 2 subunit (Ap2a2), mRNA [NM_031008]	**7,035**
Ppp1r1c	Rattus norvegicus protein phosphatase 1, regulatory (inhibitor) subunit 1C (Ppp1r1c), mRNA [NM_001109200]	**7,035**
Krtap9-5	PREDICTED: Rattus norvegicus hypothetical protein LOC680468 (LOC680468), mRNA [XM_001057319]	**7,035**
Ddit4	Rattus norvegicus DNA-damage-inducible transcript 4 (Ddit4), mRNA [NM_080906]	**7,034**
LOC687536	PREDICTED: Rattus norvegicus similar to Forkhead box protein F1 (Forkhead-related protein FKHL5) (Forkhead-related transcription factor 1) (FREAC-1) (Hepatocyte nuclear factor 3 forkhead homolog 8) (HFH-8) (LOC687536), mRNA [XM_001079002]	**7,033**
RGD1560936	PREDICTED: Rattus norvegicus similar to 60S ribosomal protein L13 (RGD1560936), mRNA [XM_219309]	**7,033**
Cdo1	Rattus norvegicus cysteine dioxygenase, type I (Cdo1), mRNA [NM_052809]	**7,033**
0	Unknown	**7,033**
Stard3	Rattus norvegicus StAR-related lipid transfer (START) domain containing 3 (Stard3), mRNA [NM_001014229]	**7,033**
Sms	Rattus norvegicus spermine synthase (Sms), mRNA [NM_001033899]	**7,032**
RGD1307325	Rattus norvegicus similar to RIKEN cDNA 4933411K20 (RGD1307325), mRNA [NM_001014001]	**7,032**
Pno1	Rattus norvegicus partner of NOB1 homolog (S. cerevisiae) (Pno1), mRNA [NM_199083]	**7,032**
RGD1564469	Uncharacterized protein [Source:UniProtKB/TrEMBL;Acc:D3ZJT4] [ENSRNOT00000040909]	**7,031**
0	Unknown	**7,030**
0	Unknown	**7,030**

Hnrpd	Rattus norvegicus heterogeneous nuclear ribonucleoprotein D (Hnrpd), transcript variant 1, mRNA [NM_024404]	**7,030**
0	CTGE5_MOUSE (Q8R311) Cutaneous T-cell lymphoma-associated antigen 5 homolog (cTAGE-5 protein) (Meningioma-expressed antigen 6), partial (9%) [TC630795]	**7,028**
0	Unknown	**7,028**
0	Unknown	**7,027**
Dcun1d2	Rattus norvegicus DCN1, defective in cullin neddylation 1, domain containing 2 (S. cerevisiae) (Dcun1d2), mRNA [NM_001134798]	**7,025**
Fam160a2	Rattus norvegicus family with sequence similarity 160, member A2 (Fam160a2), mRNA [NM_001005538]	**7,025**
Gnb3	Rattus norvegicus guanine nucleotide binding protein (G protein), beta polypeptide 3 (Gnb3), mRNA [NM_021858]	**7,025**
Fam125a	Rattus norvegicus family with sequence similarity 125, member A (Fam125a), mRNA [NM_199375]	**7,023**
Lrrc23	Rattus norvegicus leucine rich repeat containing 23 (Lrrc23), mRNA [NM_001013165]	**7,023**
Nudt4	Rattus norvegicus nudix (nucleoside diphosphate linked moiety X)-type motif 4 (Nudt4), mRNA [NM_053598]	**7,022**
Olr1088	PREDICTED: Rattus norvegicus olfactory receptor 1088 (Olr1088), mRNA [XM_001077880]	**7,020**
Olr399	Rattus norvegicus olfactory receptor 399 (Olr399), mRNA [NM_001000560]	**7,019**
0	Unknown	**7,019**
0	Unknown	**7,016**
Cyp2a2	Rattus norvegicus cytochrome P450, family 2, subfamily a, polypeptide 2 (Cyp2a2), mRNA [NM_012693]	**7,015**
Amacr	Rattus norvegicus alpha-methylacyl-CoA racemase (Amacr), nuclear gene encoding mitochondrial protein, mRNA [NM_012816]	**7,013**
Slc38a10	Putative uncharacterized protein RGD1306356Uncharacterized protein [Source:UniProtKB/TrEMBL;Acc:D3ZH43] [ENSRNOT00000006225]	**7,012**
Hspb8	Rattus norvegicus heat shock protein B8 (Hspb8), mRNA [NM_053612]	**7,012**
Kcnk10	Rattus norvegicus potassium channel, subfamily K, member 10 (Kcnk10), mRNA [NM_023096]	**7,011**
Rhobtb2	Rattus norvegicus Rho-related BTB domain containing 2 (Rhobtb2), mRNA [NM_001013133]	**7,009**
LOC685398	PREDICTED: Rattus norvegicus similar to 1A6/DRIM (down-regulated in metastasis) interacting protein (LOC685398), mRNA [XM_001063635]	**7,009**
0	AGENCOURT_26629706 NIH_MGC_253 Rattus norvegicus cDNA clone IMAGE:7305279 5', mRNA sequence [CO385661]	**7,009**
Trim33	Uncharacterized protein [Source:UniProtKB/TrEMBL;Acc:D3ZUK4] [ENSRNOT00000025600]	**7,007**
Lig4	Rattus norvegicus ligase IV, DNA, ATP-dependent (Lig4), mRNA [NM_001106095]	**7,007**
Fam136a	Rattus norvegicus family with sequence similarity 136, member A (Fam136a), mRNA [NM_001106605]	**7,007**
Sesn3	Rattus norvegicus sestrin 3 (Sesn3), mRNA [NM_001108125]	**7,006**
Ccdc12	Rattus norvegicus coiled-coil domain containing 12 (Ccdc12), mRNA [NM_001108783]	**7,006**
LOC498685	Rattus norvegicus similar to UPF0308 protein C9orf21 (LOC498685), mRNA [NM_001109114]	**7,005**
Zfp639	Rattus norvegicus zinc finger protein 639 (Zfp639), mRNA [NM_001080907]	**7,004**
Gpc4	Rattus norvegicus glypican 4 (Gpc4), mRNA [NM_001014108]	**7,004**
Tfdp2	Rattus norvegicus transcription factor Dp-2 (E2F dimerization partner 2) (Tfdp2), mRNA [NM_001106847]	**7,004**
Cadps2	PREDICTED: Rattus norvegicus Ca++-dependent secretion activator 2, transcript variant 1 (Cadps2), mRNA [XM_001060172]	**7,003**
Xkr7	Rattus norvegicus XK, Kell blood group complex subunit-related family, member 7 (Xkr7), mRNA [NM_001012092]	**7,002**

Mrpl16	Rattus norvegicus mitochondrial ribosomal protein L16 (Mrpl16), nuclear gene encoding mitochondrial protein, mRNA [NM_001009647]	**7,002**
Slc25a33	Rattus norvegicus solute carrier family 25, member 33 (Slc25a33), nuclear gene encoding mitochondrial protein, mRNA [NM_001127574]	**7,002**
0	SEC24 related gene family, member D (S. cerevisiae) (Predicted), isoform CRA_bUncharacterized protein [Source:UniProtKB/TrEMBL;Acc:D3ZTC8] [ENSRNOT00000020433]	**7,001**
RGD1564342	Uncharacterized protein [Source:UniProtKB/TrEMBL;Acc:D3ZFA2] [ENSRNOT00000030991]	**7,001**
Pcdhga8	Rattus norvegicus protocadherin gamma subfamily A, 8 (Pcdhga8), mRNA [NM_001037156]	**7,001**
Rpp30	Rattus norvegicus ribonuclease P/MRP 30 subunit (human) (Rpp30), mRNA [NM_001191083]	**7,000**
Dennd2a	Uncharacterized protein [Source:UniProtKB/TrEMBL;Acc:D3ZLQ1] [ENSRNOT00000034549]	**7,000**
Abhd1	Rattus norvegicus abhydrolase domain containing 1 (Abhd1), mRNA [NM_001008520]	**7,000**
0	Unknown	**6,999**
0	FM101283 etnofat Rattus norvegicus cDNA clone etnofatP0059F08 5', mRNA sequence [FM101283]	**6,998**
0	set=Set beta isoform {alternatively spliced} [rats, neonatal kidney, mRNA, 2026 nt]. [S68987]	**6,998**
Ankrd34b	Rattus norvegicus ankyrin repeat domain 34B (Ankrd34b), mRNA [NM_001109174]	**6,997**
Lrrc4	Rattus norvegicus leucine rich repeat containing 4 (Lrrc4), mRNA [NM_001037336]	**6,997**
Gpr101	Rattus norvegicus G protein-coupled receptor 101 (Gpr101), mRNA [NM_001108258]	**6,996**
Ddx21	Rattus norvegicus DEAD (Asp-Glu-Ala-Asp) box polypeptide 21 (Ddx21), mRNA [NM_001037201]	**6,995**
Gna13	Rattus norvegicus guanine nucleotide binding protein (G protein), alpha 13 (Gna13), mRNA [NM_001013119]	**6,995**
Defb36	Rattus norvegicus defensin beta 36 (Defb36), mRNA [NM_001037515]	**6,995**
LOC688869	Rattus norvegicus similar to cytochrome c oxidase, subunit VIb polypeptide 1 (LOC688869), nuclear gene encoding mitochondrial protein, mRNA [NM_001145273]	**6,994**
Lsm6	Rattus norvegicus LSM6 homolog, U6 small nuclear RNA associated (S. cerevisiae) (Lsm6), mRNA [NM_001126085]	**6,992**
Ctsh	Rattus norvegicus cathepsin H (Ctsh), mRNA [NM_012939]	**6,992**
Phf3	Rattus norvegicus PHD finger protein 3 (Phf3), mRNA [NM_001108791]	**6,992**
0	Unknown	**6,991**
Btbd17	Rattus norvegicus BTB (POZ) domain containing 17 (Btbd17), mRNA [NM_001134534]	**6,991**
Znf498	Rattus norvegicus zinc finger protein 498 (Znf498), mRNA [NM_001108851]	**6,991**
Atp13a2	Rattus norvegicus ATPase type 13A2 (Atp13a2), mRNA [NM_001173432]	**6,991**
Pdlim2	Rattus norvegicus PDZ and LIM domain 2 (Pdlim2), mRNA [NM_001007622]	**6,990**
Rad51l3	Rattus norvegicus RAD51-like 3 (S. cerevisiae) (Rad51l3), mRNA [NM_001107029]	**6,988**
Nat14	Rattus norvegicus N-acetyltransferase 14 (Nat14), mRNA [NM_001108466]	**6,988**
Mterf	Rattus norvegicus mitochondrial transcription termination factor (Mterf), nuclear gene encoding mitochondrial protein, mRNA [NM_053499]	**6,987**
Prpsap1	Rattus norvegicus phosphoribosyl pyrophosphate synthetase-associated protein 1 (Prpsap1), mRNA [NM_022545]	**6,987**
0	Rattus norvegicus similar to glyceraldehyde-3-phosphate dehydrogenase (phosphorylating) (EC 1.2.1.12) - mouse (LOC301496), mRNA [XM_237274]	**6,987**

Msra	Rattus norvegicus methionine sulfoxide reductase A (Msra), mRNA [NM_053307]	**6,986**
Mylip	Rattus norvegicus myosin regulatory light chain interacting protein (Mylip), mRNA [NM_001107344]	**6,986**
0	Beta-crystallin A3Beta-crystallin A3, isoform A1, Delta4 formBeta-crystallin A3, isoform A1, Delta7 formBeta-crystallin A3, isoform A1, Delta8 form [Source:UniProtKB/Swiss-Prot;Acc:P14881] [ENSRNOT00000067029]	**6,985**
0	Uncharacterized protein [Source:UniProtKB/TrEMBL;Acc:D3ZKT9] [ENSRNOT00000066171]	**6,985**
Gpatch4	Rattus norvegicus G patch domain containing 4 (Gpatch4), mRNA [NM_001024979]	**6,985**
Lhfpl5	Rattus norvegicus lipoma HMGIC fusion partner-like 5 (Lhfpl5), mRNA [NM_001013906]	**6,985**
Znf608	Rattus norvegicus zinc finger protein 608 (Znf608), mRNA [NM_001107378]	**6,983**
Arid3b	Rattus norvegicus AT rich interactive domain 3B (Bright like) (Arid3b), mRNA [NM_001109001]	**6,981**
Ctsll3	RCG24317, isoform CRA_aUncharacterized protein [Source:UniProtKB/TrEMBL;Acc:D3ZJV2] [ENSRNOT00000061398]	**6,980**
0	Unknown	**6,980**
Arpp19	Rattus norvegicus cAMP-regulated phosphoprotein 19 (Arpp19), mRNA [NM_031660]	**6,980**
Syt12	Rattus norvegicus synaptotagmin XII (Syt12), mRNA [NM_138835]	**6,980**
Grik1	Rattus norvegicus glutamate receptor, ionotropic, kainate 1 (Grik1), transcript variant 2, mRNA [NM_017241]	**6,978**
Loh12cr1	Rattus norvegicus loss of heterozygosity, 12, chromosomal region 1 homolog (human) (Loh12cr1), mRNA [NM_001108650]	**6,978**
Snx20	Rattus norvegicus sorting nexin 20 (Snx20), mRNA [NM_001024999]	**6,975**
0	Unknown	**6,974**
LOC294497	Rattus norvegicus similar to double homeobox, 4; double homeobox protein 4 (LOC294497), mRNA [XM_228268]	**6,973**
Eif4b	Rattus norvegicus eukaryotic translation initiation factor 4B (Eif4b), mRNA [NM_001008324]	**6,973**
RGD1307554	Rattus norvegicus similar to CG16812-PA (RGD1307554), mRNA [NM_001109664]	**6,972**
RGD1565641	Rattus norvegicus RGD1565641 (RGD1565641), mRNA [NM_001109179]	**6,970**
Mfsd5	Rattus norvegicus major facilitator superfamily domain containing 5 (Mfsd5), mRNA [NM_001126282]	**6,969**
Pnmal1	Rattus norvegicus PNMA-like 1 (Pnmal1), mRNA [NM_001108473]	**6,968**
Ppp1r16a	Rattus norvegicus protein phosphatase 1, regulatory (inhibitor) subunit 16A (Ppp1r16a), mRNA [NM_001130566]	**6,968**
RGD1559904	Rattus norvegicus similar to mKIAA1429 protein (RGD1559904), mRNA [NM_001107915]	**6,968**
Tpd52l1	Rattus norvegicus tumor protein D52-like 1 (Tpd52l1), mRNA [NM_001044295]	**6,968**
0	Rattus norvegicus TL0ABA19YF11 mRNA sequence. [FQ210991]	**6,967**
Cacng6	Rattus norvegicus calcium channel, voltage-dependent, gamma subunit 6 (Cacng6), mRNA [NM_080694]	**6,967**
Cacna1c	Rattus norvegicus calcium channel, voltage-dependent, L type, alpha 1C subunit (Cacna1c), mRNA [NM_012517]	**6,966**
LOC680155	Uncharacterized protein [Source:UniProtKB/TrEMBL;Acc:D3ZXU7] [ENSRNOT00000020271]	**6,966**
Anapc13	Rattus norvegicus anaphase promoting complex subunit 13 (Anapc13), mRNA [NM_001173983]	**6,966**
LOC686108	PREDICTED: Rattus norvegicus hypothetical protein LOC686108 (LOC686108), mRNA [XM_002729139]	**6,964**
RGD1564420	Uncharacterized protein [Source:UniProtKB/TrEMBL;Acc:D4AEB6] [ENSRNOT00000013315]	**6,962**
Trim39	Rattus norvegicus tripartite motif-containing 39 (Trim39), mRNA [NM_213562]	**6,961**

0	FM135825 etnokid Rattus norvegicus cDNA clone etnokidP0036F18 5', mRNA sequence [FM135825]	**6,958**
Mmaa	Rattus norvegicus methylmalonic aciduria (cobalamin deficiency) cblA type (Mmaa), nuclear gene encoding mitochondrial protein, mRNA [NM_001106174]	**6,957**
Ubfd1	Rattus norvegicus ubiquitin family domain containing 1 (Ubfd1), mRNA [NM_001034911]	**6,956**
0	Unknown	**6,954**
RGD1565972	Rattus norvegicus similar to hypothetical protein 4932411G14 (RGD1565972), mRNA [NM_001134606]	**6,954**
LOC500354	Rattus norvegicus similar to C030030A07Rik protein (LOC500354), mRNA [NM_001037797]	**6,953**
Fastkd5	PREDICTED: Rattus norvegicus similar to hypothetical protein FLJ13149 (RGD1563839), miscRNA [XR_085786]	**6,953**
Mettl14	Rattus norvegicus methyltransferase like 14 (Mettl14), mRNA [NM_001106470]	**6,951**
Oaz2	Rattus norvegicus ornithine decarboxylase antizyme 2 (Oaz2), mRNA [NM_001109899]	**6,950**
Vom2r24	Rattus norvegicus vomeronasal 2 receptor, 24 (Vom2r24), mRNA [NM_001099493]	**6,950**
Rictor	PREDICTED: Rattus norvegicus RPTOR independent companion of MTOR, complex 2 (Rictor), mRNA [XM_226812]	**6,950**
LOC684996	Rattus norvegicus similar to chromosome 7 open reading frame 11 (LOC684996), mRNA [NM_001109452]	**6,949**
Hnrnpl	Rattus norvegicus heterogeneous nuclear ribonucleoprotein L (Hnrnpl), transcript variant 1, mRNA [NM_001134760]	**6,946**
Prosapip1	Rattus norvegicus ProSAPiP1 protein (Prosapip1), mRNA [NM_172022]	**6,946**
Magi1	Rattus norvegicus membrane associated guanylate kinase, WW and PDZ domain containing 1 (Magi1), mRNA [NM_001030045]	**6,946**
Tmem161a	Rattus norvegicus transmembrane protein 161A (Tmem161a), mRNA [NM_001108874]	**6,946**
LOC362473	PREDICTED: Rattus norvegicus similar to Elongation factor 1-gamma (EF-1-gamma) (eEF-1B gamma) (LOC362473), miscRNA [XR_008347]	**6,944**
Rtp3	Rattus norvegicus receptor (chemosensory) transporter protein 3 (Rtp3), mRNA [NM_001108190]	**6,943**
Atp6v1a	Rattus norvegicus ATPase, H+ transporting, lysosomal V1 subunit A (Atp6v1a), mRNA [NM_001108318]	**6,943**
0	pleiomorphic adenoma gene-like 2 [Source:RefSeq peptide;Acc:NP_001099998] [ENSRNOT00000013383]	**6,942**
Olr1414	Rattus norvegicus olfactory receptor 1414 (Olr1414), mRNA [NM_001000782]	**6,941**
0	Unknown	**6,940**
Gin1	Rattus norvegicus gypsy retrotransposon integrase 1 (Gin1), mRNA [NM_001025012]	**6,939**
Mcm4	Rattus norvegicus minichromosome maintenance complex component 4 (Mcm4), mRNA [NM_033651]	**6,939**
RGD1306730	Rattus norvegicus similar to cDNA sequence BC025641 (RGD1306730), mRNA [NM_001039608]	**6,939**
Zfp414	Rattus norvegicus zinc finger protein 414 (Zfp414), mRNA [NM_001009664]	**6,939**
Zdhhc12	Rattus norvegicus zinc finger, DHHC-type containing 12 (Zdhhc12), mRNA [NM_001013239]	**6,938**
Itpka	Rattus norvegicus inositol 1,4,5-trisphosphate 3-kinase A (Itpka), mRNA [NM_031045]	**6,937**
Grem2	Rattus norvegicus gremlin 2, cysteine knot superfamily, homolog (Xenopus laevis) (Grem2), mRNA [NM_001105974]	**6,936**
0	Unknown	**6,936**
0	Unknown	**6,935**
RGD1310495	PREDICTED: Rattus norvegicus similar to KIAA1919 protein (RGD1310495), mRNA [XM_001063192]	**6,935**
R3hdml	Rattus norvegicus R3H domain (binds single-stranded nucleic acids) containing-like (R3hdml), mRNA [NM_001108962]	**6,932**

Cox6b2	Rattus norvegicus cytochrome c oxidase subunit VIb polypeptide 2 (Cox6b2), mRNA [NM_001039085]	**6,931**
RGD1304595	Rattus norvegicus similar to RIKEN cDNA 6330416G13 gene (RGD1304595), mRNA [NM_001106661]	**6,931**
LOC686567	PREDICTED: Rattus norvegicus similar to Epiplakin (LOC686567), mRNA [XM_001074770]	**6,931**
Cyp2d4	Rattus norvegicus cytochrome P450, family 2, subfamily d, polypeptide 4 (Cyp2d4), mRNA [NM_138515]	**6,930**
Chmp4bl1	PREDICTED: Rattus norvegicus similar to Charged multivesicular body protein 4b (Chromatin-modifying protein 4b) (CHMP4b) (LOC679886), mRNA [XM_002726346]	**6,930**
Tg	Rattus norvegicus thyroglobulin (Tg), mRNA [NM_030988]	**6,929**
Mapk3	Rattus norvegicus mitogen activated protein kinase 3 (Mapk3), mRNA [NM_017347]	**6,929**
Ehd2	Rattus norvegicus EH-domain containing 2 (Ehd2), mRNA [NM_001024897]	**6,928**
0	Unknown	**6,928**
RGD1561241	PREDICTED: Rattus norvegicus similar to Mblk1-related protein-1 (RGD1561241), mRNA [XM_001058603]	**6,928**
Pappa	Rattus norvegicus pregnancy-associated plasma protein A (Pappa), mRNA [NM_001107939]	**6,927**
Gprc5d	Rattus norvegicus G protein-coupled receptor, family C, group 5, member D (Gprc5d), mRNA [NM_001109254]	**6,926**
LOC690977	PREDICTED: Rattus norvegicus similar to RIKEN cDNA 5031410I06 (LOC690977), mRNA [XM_002727316]	**6,924**
0	Uncharacterized protein [Source:UniProtKB/TrEMBL;Acc:D3ZET0] [ENSRNOT00000035939]	**6,924**
0	Unknown	**6,924**
Lrrn1	Rattus norvegicus leucine rich repeat neuronal 1 (Lrrn1), mRNA [NM_001037363]	**6,924**
Or7e24	Putative gustatory receptor clone PTE01 [Source:UniProtKB/Swiss-Prot;Acc:P35894] [ENSRNOT00000067321]	**6,922**
Slc36a4	Rattus norvegicus solute carrier family 36 (proton/amino acid symporter), member 4 (Slc36a4), mRNA [NM_001108127]	**6,920**
Hexb	Rattus norvegicus hexosaminidase B (Hexb), mRNA [NM_001011946]	**6,920**
LOC681754	PREDICTED: Rattus norvegicus similar to cytochrome c oxidase, subunit VIb polypeptide 1 (LOC681754), mRNA [XM_001058263]	**6,920**
Wfdc2	Rattus norvegicus WAP four-disulfide core domain 2 (Wfdc2), mRNA [NM_173109]	**6,919**
Rps6kc1	Rattus norvegicus ribosomal protein S6 kinase, polypeptide 1 (Rps6kc1), mRNA [NM_001105984]	**6,919**
0	Uncharacterized protein [Source:UniProtKB/TrEMBL;Acc:D3ZR79] [ENSRNOT00000032299]	**6,918**
0	Uncharacterized protein [Source:UniProtKB/TrEMBL;Acc:D3ZRP8] [ENSRNOT00000031672]	**6,918**
Olr120	Rattus norvegicus olfactory receptor 120 (Olr120), mRNA [NM_001000154]	**6,917**
Gata1	Rattus norvegicus GATA binding protein 1 (Gata1), mRNA [NM_012764]	**6,916**
Clk2	Rattus norvegicus CDC-like kinase 2 (Clk2), mRNA [NM_001014254]	**6,916**
Wdr47	Rattus norvegicus WD repeat domain 47 (Wdr47), mRNA [NM_001100702]	**6,916**
Snca	synuclein SYN2 {alternatively spliced} [rats, mRNA, 695 nt]. [S73008]	**6,914**
Cp	Rattus norvegicus GPI-anchored ceruloplasmin mRNA, complete cds. [AF202115]	**6,912**
Polr2e	Rattus norvegicus polymerase (RNA) II (DNA directed) polypeptide E (Polr2e), mRNA [NM_001109614]	**6,912**
0	Unknown	**6,912**
Zbtb2	Rattus norvegicus zinc finger and BTB domain containing 2 (Zbtb2), mRNA [NM_001107460]	**6,911**

0	Uncharacterized protein [Source:UniProtKB/TrEMBL;Acc:D3ZPH4] [ENSRNOT00000061920]	**6,911**
Cpsf4l	PREDICTED: Rattus norvegicus cleavage and polyadenylation specific factor 4-like (Cpsf4l), mRNA [XM_001081632]	**6,910**
Pdgfd	Rattus norvegicus platelet-derived growth factor, D polypeptide (Pdgfd), mRNA [NM_023962]	**6,910**
0	Unknown	**6,909**
LOC686388	PREDICTED: Rattus norvegicus similar to Prostatic steroid-binding protein C1 chain precursor (Prostatein peptide C1) (LOC686388), mRNA [XM_001073908]	**6,906**
LOC500413	Rattus norvegicus hypothetical protein LOC500413 (LOC500413), mRNA [NM_001171802]	**6,906**
0	Unknown	**6,906**
0	Rattus norvegicus similar to Alpha enolase (2-phospho-D-glycerate hydro-lyase) (Non-neural enolase) (NNE) (Enolase 1) (LOC310585), mRNA [XM_227366]	**6,906**
RGD1560248	PREDICTED: Rattus norvegicus similar to formin-like 2 isoform B, transcript variant 2 (RGD1560248), mRNA [XM_002726151]	**6,905**
Rnase4	Rattus norvegicus ribonuclease, RNase A family 4 (Rnase4), mRNA [NM_020082]	**6,905**
Jam3	Rattus norvegicus junctional adhesion molecule 3 (Jam3), mRNA [NM_001004269]	**6,905**
Gak	Rattus norvegicus cyclin G associated kinase (Gak), mRNA [NM_031030]	**6,903**
0	Unknown	**6,903**
0	Unknown	**6,901**
Olr209	Rattus norvegicus olfactory receptor 209 (Olr209), mRNA [NM_001000736]	**6,900**
Rgnef	Rattus norvegicus Rho-guanine nucleotide exchange factor (Rgnef), mRNA [NM_001108542]	**6,900**
Numa1	PREDICTED: Rattus norvegicus nuclear mitotic apparatus protein 1 (Numa1), mRNA [XM_218972]	**6,900**
Psmd7	Rattus norvegicus proteasome (prosome, macropain) 26S subunit, non-ATPase, 7 (Psmd7), mRNA [NM_001107426]	**6,899**
Trex1	Rattus norvegicus three prime repair exonuclease 1 (Trex1), mRNA [NM_001024989]	**6,898**
0	Unknown	**6,898**
Irf6	Rattus norvegicus interferon regulatory factor 6 (Irf6), mRNA [NM_001108859]	**6,898**
Gdpd1	Rattus norvegicus glycerophosphodiester phosphodiesterase domain containing 1 (Gdpd1), mRNA [NM_001044238]	**6,897**
0	Unknown	**6,895**
Alg11	Rattus norvegicus asparagine-linked glycosylation 11, alpha-1,2-mannosyltransferase homolog (yeast) (Alg11), mRNA [NM_001108401]	**6,895**
Dnlz	Rattus norvegicus DNL-type zinc finger (Dnlz), mRNA [NM_001130990]	**6,895**
0	Uncharacterized protein [Source:UniProtKB/TrEMBL;Acc:D3ZWP2] [ENSRNOT00000025138]	**6,893**
LOC682377	PREDICTED: Rattus norvegicus similar to olfactory receptor 697 (LOC682377), partial mRNA [XM_001061252]	**6,893**
Mpv17l	Rattus norvegicus MPV17 mitochondrial membrane protein-like (Mpv17l), nuclear gene encoding mitochondrial protein, mRNA [NM_001098240]	**6,891**
Whsc1l1	Rattus norvegicus Wolf-Hirschhorn syndrome candidate 1-like 1 (human) (Whsc1l1), mRNA [NM_001106090]	**6,889**
Tnks	Rattus norvegicus tankyrase, TRF1-interacting ankyrin-related ADP-ribose polymerase (Tnks), mRNA [NM_001106084]	**6,886**
LOC680039	Rattus norvegicus hypothetical protein LOC680039 (LOC680039), mRNA [NM_001109388]	**6,886**
LOC686031	Rattus norvegicus hypothetical protein LOC686031 (LOC686031), mRNA [NM_001109490]	**6,885**

Cbln3	Rattus norvegicus cerebellin 3 precursor (Cbln3), mRNA [NM_001109330]	**6,884**
Actg1	Rattus norvegicus actin, gamma 1 (Actg1), mRNA [NM_001127449]	**6,884**
Mgat4a	Rattus norvegicus mannosyl (alpha-1,3-)-glycoprotein beta-1,4-N-acetylglucosaminyltransferase, isozyme A (Mgat4a), transcript variant 1, mRNA [NM_001160155]	**6,882**
Mrgpre	Rattus norvegicus MAS-related GPR, member E (Mrgpre), mRNA [NM_001002288]	**6,881**
Sall2	Rattus norvegicus sal-like 2 (Drosophila) (Sall2), mRNA [NM_001107262]	**6,880**
LOC680227	Rattus norvegicus LRRGT00193 (LOC680227), mRNA [NM_001047974]	**6,880**
0	Unknown	**6,879**
Cpne9	Rattus norvegicus copine family member IX (Cpne9), mRNA [NM_001024982]	**6,878**
Alpk1	Uncharacterized protein [Source:UniProtKB/TrEMBL;Acc:D3ZTA7] [ENSRNOT00000030798]	**6,876**
0	Unknown	**6,874**
LOC686041	Rattus norvegicus cDNA clone IMAGE:6889638, partial cds. [BC059164]	**6,873**
Tpst2	Rattus norvegicus tyrosylprotein sulfotransferase 2 (Tpst2), mRNA [NM_001008508]	**6,873**
Daxx	Rattus norvegicus death-domain associated protein (Daxx), mRNA [NM_080891]	**6,872**
LOC686980	PREDICTED: Rattus norvegicus similar to arsenate resistance protein 2 (LOC686980), mRNA [XM_001076594]	**6,872**
0	Rattus norvegicus similar to 60S ribosomal protein L13a (Transplantation antigen P198) (Tum-P198 antigen) (LOC365089), mRNA [XM_344809]	**6,871**
0	Unknown	**6,869**
Smg5	Rattus norvegicus Smg-5 homolog, nonsense mediated mRNA decay factor (C. elegans), mRNA (cDNA clone IMAGE:9034114), partial cds. [BC169031]	**6,868**
0	Uncharacterized protein [Source:UniProtKB/TrEMBL;Acc:D3Z9L4] [ENSRNOT00000015730]	**6,867**
Vapb	Vesicle-associated membrane protein-associated protein B [Source:UniProtKB/Swiss-Prot;Acc:Q9Z269] [ENSRNOT00000007554]	**6,867**
Uba6	Rattus norvegicus ubiquitin-like modifier activating enzyme 6 (Uba6), mRNA [NM_001107213]	**6,866**
Trip12	Rattus norvegicus thyroid hormone receptor interactor 12 (Trip12), mRNA [NM_001031659]	**6,864**
LOC501080	PREDICTED: Rattus norvegicus similar to Discs large homolog 5 (Placenta and prostate DLG) (Discs large protein P-dlg) (LOC501080), mRNA [XM_576492]	**6,864**
Ccnk	Rattus norvegicus cyclin K (Ccnk), mRNA [NM_001109672]	**6,864**
0	Unknown	**6,862**
Birc7	PREDICTED: Rattus norvegicus baculoviral IAP repeat-containing 7 (Birc7), mRNA [XM_001058257]	**6,861**
Fcgr2a	Low affinity immunoglobulin gamma Fc region receptor III [Source:UniProtKB/Swiss-Prot;Acc:P27645] [ENSRNOT00000045427]	**6,861**
Mtrr	Rattus norvegicus 5-methyltetrahydrofolate-homocysteine methyltransferase reductase (Mtrr), mRNA [NM_001039003]	**6,859**
Krtcap2	Rattus norvegicus keratinocyte associated protein 2 (Krtcap2), mRNA [NM_001106444]	**6,859**
Spsb3	Rattus norvegicus splA/ryanodine receptor domain and SOCS box containing 3 (Spsb3), mRNA [NM_001106988]	**6,859**
Dcbld2	Rattus norvegicus discoidin, CUB and LCCL domain containing 2 (Dcbld2), mRNA [NM_130419]	**6,857**
Akr1cl2	Rattus norvegicus aldo-keto reductase family 1, member C-like 2 (Akr1cl2), mRNA [NM_001008342]	**6,856**

Pnkp	Rattus norvegicus polynucleotide kinase 3'-phosphatase (Pnkp), mRNA [NM_001004259]	**6,856**
Dbr1	Rattus norvegicus debranching enzyme homolog 1 (S. cerevisiae) (Dbr1), mRNA [NM_001109437]	**6,856**
Ccdc23	Rattus norvegicus coiled-coil domain containing 23 (Ccdc23), transcript variant 1, mRNA [NM_001038994]	**6,855**
0	Uncharacterized protein [Source:UniProtKB/TrEMBL;Acc:D3ZFM9] [ENSRNOT00000064974]	**6,853**
Aldh2	Rattus norvegicus aldehyde dehydrogenase 2 family (mitochondrial) (Aldh2), nuclear gene encoding mitochondrial protein, mRNA [NM_032416]	**6,853**
Rbm12	Rattus norvegicus RNA binding motif protein 12 (Rbm12), mRNA [NM_001037657]	**6,852**
Dll3	Rattus norvegicus delta-like 3 (Drosophila) (Dll3), mRNA [NM_053666]	**6,852**
0	PREDICTED: Rattus norvegicus similar to double homeobox 4c (LOC680963), mRNA [XM_002725876]	**6,852**
Slc7a11	Rattus norvegicus solute carrier family 7 (cationic amino acid transporter, y+ system), member 11 (Slc7a11), mRNA [NM_001107673]	**6,851**
0	Unknown	**6,851**
0	PRD11_HUMAN (Q9NQV5) PR domain-containing protein 11, partial (36%) [TC604616]	**6,850**
Fundc2	Rattus norvegicus FUN14 domain containing 2 (Fundc2), mRNA [NM_001135757]	**6,850**
Abcb9	Rattus norvegicus ATP-binding cassette, subfamily B (MDR/TAP), member 9 (Abcb9), mRNA [NM_022238]	**6,850**
Rgc32	Rattus norvegicus response gene to complement 32 (Rgc32), mRNA [NM_054008]	**6,849**
Anp32a	Rattus norvegicus acidic (leucine-rich) nuclear phosphoprotein 32 family, member A (Anp32a), mRNA [NM_012903]	**6,849**
Npdc1	Rattus norvegicus neural proliferation, differentiation and control, 1 (Npdc1), mRNA [NM_001004231]	**6,849**
0	Unknown	**6,848**
Olr1186	Rattus norvegicus olfactory receptor 1186 (Olr1186), mRNA [NM_001000862]	**6,848**
Olr1226	Rattus norvegicus olfactory receptor 1226 (Olr1226), mRNA [NM_001000442]	**6,848**
Tbc1d2b	Rattus norvegicus TBC1 domain family, member 2B (Tbc1d2b), mRNA [NM_001108175]	**6,847**
0	Unknown	**6,846**
0	Unknown	**6,846**
0	Unknown	**6,846**
N4bp3	Rattus norvegicus Nedd4 binding protein 3 (N4bp3), mRNA [NM_001033893]	**6,845**
Syt4	Rattus norvegicus synaptotagmin IV (Syt4), mRNA [NM_031693]	**6,845**
0	Unknown	**6,845**
Rptor	Rattus norvegicus regulatory associated protein of MTOR, complex 1 (Rptor), mRNA [NM_001134499]	**6,845**
Fbxw12	Uncharacterized protein [Source:UniProtKB/TrEMBL;Acc:D4A956] [ENSRNOT00000028113]	**6,841**
Adam9	Rattus norvegicus ADAM metallopeptidase domain 9 (meltrin gamma) (Adam9), mRNA [NM_001014772]	**6,840**
Stard10	Rattus norvegicus StAR-related lipid transfer (START) domain containing 10 (Stard10), mRNA [NM_001013069]	**6,840**
Slc25a19	Rattus norvegicus solute carrier family 25 (mitochondrial thiamine pyrophosphate carrier), member 19 (Slc25a19), nuclear gene encoding mitochondrial protein, mRNA [NM_001007674]	**6,840**
RGD1308541	Uncharacterized protein [Source:UniProtKB/TrEMBL;Acc:D3ZFP6] [ENSRNOT00000031783]	**6,839**
Rit1	Rattus norvegicus Ras-like without CAAX 1 (Rit1), mRNA [NM_001109185]	**6,838**

Exoc5	Rattus norvegicus exocyst complex component 5 (Exoc5), mRNA [NM_022204]	**6,837**
Abca5	Rattus norvegicus ATP-binding cassette, subfamily A (ABC1), member 5 (Abca5), mRNA [NM_173307]	**6,836**
Rpl6	Rattus norvegicus ribosomal protein L6 (Rpl6), mRNA [NM_053971]	**6,836**
Il12rb2	Rattus norvegicus interleukin 12 receptor, beta 2 (Il12rb2), mRNA [NM_001191750]	**6,835**
Olr143	Rattus norvegicus olfactory receptor 143 (Olr143), mRNA [NM_001000163]	**6,834**
Nup153	Rattus norvegicus nucleoporin 153 (Nup153), mRNA [NM_001100470]	**6,834**
Sppl2b	Rattus norvegicus signal peptide peptidase-like 2B (Sppl2b), mRNA [NM_001014200]	**6,833**
Arpc1b	Rattus norvegicus actin related protein 2/3 complex, subunit 1B (Arpc1b), mRNA [NM_019289]	**6,832**
Mrpl50	Rattus norvegicus mitochondrial ribosomal protein L50 (Mrpl50), nuclear gene encoding mitochondrial protein, mRNA [NM_001108665]	**6,831**
0	DP13A_MOUSE (Q8K3H0) DCC-interacting protein 13 alpha (Dip13 alpha) (Adapter protein containing PH domain, PTB domain and leucine zipper motif 1), partial (54%) [TC594026]	**6,831**
Bnipl	Rattus norvegicus BCL2/adenovirus E1B 19kD interacting protein like (Bnipl), mRNA [NM_001128187]	**6,830**
0	Unknown	**6,830**
0	Protein FAM162A [Source:UniProtKB/Swiss-Prot;Acc:Q4QQV3] [ENSRNOT00000003069]	**6,830**
0	PREDICTED: Rattus norvegicus similar to Tle6 protein (RGD1561530), miscRNA [XR_009018]	**6,830**
Nosip	Rattus norvegicus nitric oxide synthase interacting protein (Nosip), mRNA [NM_001106260]	**6,829**
LOC500265	PREDICTED: Rattus norvegicus nucleoporin 50-like (LOC500265), miscRNA [XR_007267]	**6,827**
Neurl	Rattus norvegicus neuralized homolog (Drosophila) (Neurl), mRNA [NM_001107605]	**6,827**
0	Unknown	**6,826**
Amot	Uncharacterized protein [Source:UniProtKB/TrEMBL;Acc:D3ZZ27] [ENSRNOT00000049482]	**6,826**
LOC500959	Rattus norvegicus triosephosphate isomerase (LOC500959), mRNA [NM_001033072]	**6,825**
0	Unknown	**6,824**
LOC690350	PREDICTED: Rattus norvegicus similar to LSM7 homolog, U6 small nuclear RNA associated (LOC690350), mRNA [XM_001074288]	**6,822**
Polr3g	Rattus norvegicus polymerase (RNA) III (DNA directed) polypeptide G (Polr3g), mRNA [NM_001109468]	**6,821**
Vwa3a	Rattus norvegicus von Willebrand factor A domain containing 3A (Vwa3a), transcript variant 1, mRNA [NM_001198653]	**6,821**
Cldn4	Rattus norvegicus claudin 4 (Cldn4), mRNA [NM_001012022]	**6,821**
0	Unknown	**6,820**
Fkbp15	Uncharacterized protein [Source:UniProtKB/TrEMBL;Acc:D3ZR68] [ENSRNOT00000002083]	**6,820**
Rsrc1	Rattus norvegicus arginine/serine-rich coiled-coil 1 (Rsrc1), mRNA [NM_001014172]	**6,818**
Gdpd2	Rattus norvegicus glycerophosphodiester phosphodiesterase domain containing 2 (Gdpd2), mRNA [NM_001106944]	**6,818**
Lgmn	Rattus norvegicus legumain (Lgmn), mRNA [NM_022226]	**6,818**
0	Unknown	**6,818**
0	Uncharacterized protein [Source:UniProtKB/TrEMBL;Acc:D3ZQ32] [ENSRNOT00000039210]	**6,817**

RGD1308930	PREDICTED: Rattus norvegicus similar to Protein phosphatase 1, regulatory subunit 3D (Protein phosphatase 1, regulatory subunit 6) (Protein phosphatase 1 binding subunit R6) (RGD1308930), mRNA [XM_002725247]	**6,817**
0	Unknown	**6,817**
Ruvbl2	Rattus norvegicus RuvB-like 2 (E. coli) (Ruvbl2), mRNA [NM_001025405]	**6,816**
Spata3	Rattus norvegicus spermatogenesis associated 3 (Spata3), mRNA [NM_001108805]	**6,816**
0	Uncharacterized protein [Source:UniProtKB/TrEMBL;Acc:D3ZBD2] [ENSRNOT00000040453]	**6,813**
Luc7l2	Rattus norvegicus LUC7-like 2 (S. cerevisiae) (Luc7l2), mRNA [NM_001107853]	**6,811**
Obfc1	Rattus norvegicus oligonucleotide/oligosaccharide-binding fold containing 1 (Obfc1), mRNA [NM_001011943]	**6,811**
0	Unknown	**6,810**
Prr16	Rattus norvegicus proline rich 16 (Prr16), mRNA [NM_001108432]	**6,810**
Megf10	Rattus norvegicus multiple EGF-like domains 10 (Megf10), mRNA [NM_001100657]	**6,808**
0	Unknown	**6,807**
Ep400	Rattus norvegicus E1A binding protein p400 (Ep400), mRNA [NM_001107149]	**6,807**
Acsm3	Rattus norvegicus acyl-CoA synthetase medium-chain family member 3 (Acsm3), mRNA [NM_033231]	**6,806**
0	Unknown	**6,806**
Dscr3	Rattus norvegicus Down syndrome critical region gene 3 (Dscr3), mRNA [NM_001108316]	**6,806**
RGD1560398	PREDICTED: Rattus norvegicus RGD1560398 (RGD1560398), mRNA [XM_001080358]	**6,805**
Ccl5	Rattus norvegicus chemokine (C-C motif) ligand 5 (Ccl5), mRNA [NM_031116]	**6,804**
Olr390	Rattus norvegicus olfactory receptor 390 (Olr390), mRNA [NM_001000558]	**6,803**
0	Unknown	**6,802**
Rilpl1	Rattus norvegicus Rab interacting lysosomal protein-like 1 (Rilpl1), mRNA [NM_001191665]	**6,802**
Mybl1	Rattus norvegicus myeloblastosis oncogene-like 1 (Mybl1), mRNA [NM_001106632]	**6,801**
0	PREDICTED: Rattus norvegicus similar to polymerase (RNA) II (DNA directed) polypeptide H (RGD1565904), mRNA [XM_001071711]	**6,800**
Mrgprg	Rattus norvegicus MAS-related GPR, member G (Mrgprg), mRNA [NM_203470]	**6,800**
Prodh	Rattus norvegicus proline dehydrogenase (Prodh), nuclear gene encoding mitochondrial protein, mRNA [NM_001135778]	**6,800**
Lgi1	Rattus norvegicus leucine-rich, glioma inactivated 1 (Lgi1), mRNA [NM_145769]	**6,799**
Igsf3	Rattus norvegicus immunoglobulin superfamily, member 3 (Igsf3), mRNA [NM_001106455]	**6,799**
Usp42	Rattus norvegicus ubiquitin specific peptidase 42 (Usp42), mRNA [NM_001105909]	**6,798**
0	Unknown	**6,798**
Trpc3	Rattus norvegicus transient receptor potential cation channel, subfamily C, member 3 (Trpc3), mRNA [NM_021771]	**6,798**
0	Rattus norvegicus TL0ADA18YA08 mRNA sequence. [FQ222908]	**6,797**
Dock6	Rattus norvegicus dedicator of cytokinesis 6 (Dock6), mRNA [NM_001108997]	**6,796**
0	Unknown	**6,796**
0	Unknown	**6,795**

0	Unknown	**6,794**
0	Unknown	**6,793**
0	Uncharacterized protein [Source:UniProtKB/TrEMBL;Acc:D3ZRC6] [ENSRNOT00000050943]	**6,792**
Lsm7	Rattus norvegicus LSM7 homolog, U6 small nuclear RNA associated (S. cerevisiae) (Lsm7), mRNA [NM_001108732]	**6,792**
Tchp	Rattus norvegicus trichoplein, keratin filament binding (Tchp), mRNA [NM_001191666]	**6,792**
0	immunoglobulin superfamily member 3 [Source:RefSeq peptide;Acc:NP_001099925] [ENSRNOT00000035518]	**6,791**
Acbd5	Rattus norvegicus acyl-Coenzyme A binding domain containing 5 (Acbd5), mRNA [NM_001077635]	**6,791**
Col9a2	Rattus norvegicus collagen, type IX, alpha 2 (Col9a2), mRNA [NM_001108675]	**6,790**
Cyc1	Rattus norvegicus cytochrome c-1 (Cyc1), nuclear gene encoding mitochondrial protein, mRNA [NM_001130491]	**6,790**
RGD1563047	Rattus norvegicus similar to von Ebner minor salivary gland protein (RGD1563047), mRNA [NM_001077680]	**6,789**
Ppp1r1a	Rattus norvegicus protein phosphatase 1, regulatory (inhibitor) subunit 1A (Ppp1r1a), mRNA [NM_022676]	**6,788**
Olr1252	Rattus norvegicus olfactory receptor 1252 (Olr1252), mRNA [NM_001000454]	**6,788**
Nedd9	Rattus norvegicus neural precursor cell expressed, developmentally down-regulated 9 (Nedd9), mRNA [NM_001011922]	**6,788**
Eea1	Rattus norvegicus early endosome antigen 1 (Eea1), mRNA [NM_001108086]	**6,787**
0	Unknown	**6,786**
RGD1311899	Rattus norvegicus similar to RIKEN cDNA 2210016L21 gene (RGD1311899), mRNA [NM_001009630]	**6,786**
0	Unknown	**6,786**
Phgdh	Rattus norvegicus phosphoglycerate dehydrogenase (Phgdh), mRNA [NM_031620]	**6,785**
Klhl20	Rattus norvegicus kelch-like 20 (Drosophila) (Klhl20), mRNA [NM_001107192]	**6,784**
Fastkd3	Rattus norvegicus FAST kinase domains 3 (Fastkd3), mRNA [NM_001082574]	**6,782**
0	Unknown	**6,781**
Ltk	Rattus norvegicus leukocyte receptor tyrosine kinase (Ltk), mRNA [NM_001107763]	**6,780**
Znf641	Rattus norvegicus zinc finger protein 641 (Znf641), mRNA [NM_001106792]	**6,779**
0	Unknown	**6,778**
LOC690344	Rattus norvegicus similar to Protein UNQ655/PRO1286 homolog precursor (LOC690344), mRNA [NM_001168661]	**6,777**
LOC317456	Rattus norvegicus hypothetical LOC317456 (LOC317456), mRNA [NM_001047894]	**6,777**
0	Rattus norvegicus similar to glyceraldehyde-3-phosphate dehydrogenase (LOC299727), mRNA [XM_235039]	**6,775**
Slc35b1	Rattus norvegicus solute carrier family 35, member B1 (Slc35b1), mRNA [NM_199081]	**6,775**
0	Unknown	**6,775**
Zdhhc4	Rattus norvegicus zinc finger, DHHC-type containing 4 (Zdhhc4), mRNA [NM_001013123]	**6,775**
Tpte	Rattus norvegicus transmembrane phosphatase with tensin homology (Tpte), mRNA [NM_001108877]	**6,774**
RGD1359378	Rattus norvegicus mesenchymal stem cell protein DSCD75 (RGD1359378), mRNA [NM_001007658]	**6,773**
0	Unknown	**6,773**
Pik3ca	Rattus norvegicus phosphoinositide-3-kinase, catalytic, alpha polypeptide (Pik3ca), mRNA [NM_133399]	**6,772**

Vom1r6	Rattus norvegicus vomeronasal 1 receptor 6 (Vom1r6), mRNA [NM_001009512]	**6,771**
Aldh1a1	Rattus norvegicus aldehyde dehydrogenase 1 family, member A1 (Aldh1a1), mRNA [NM_022407]	**6,771**
Zfp329	Rattus norvegicus zinc finger protein 329 (Zfp329), mRNA [NM_001107477]	**6,770**
Tmem57	Rattus norvegicus transmembrane protein 57 (Tmem57), mRNA [NM_001025699]	**6,770**
St7l	Rattus norvegicus suppression of tumorigenicity 7-like (St7l), mRNA [NM_001007639]	**6,768**
Sec3l1	Rattus norvegicus SEC3-like 1 (S. cerevisiae) (Sec3l1), mRNA [NM_001024770]	**6,768**
Trabd	Rattus norvegicus TraB domain containing (Trabd), mRNA [NM_001106788]	**6,767**
Scx	Rattus norvegicus scleraxis (Scx), mRNA [NM_001130508]	**6,767**
Sytl4	Rattus norvegicus synaptotagmin-like 4 (Sytl4), mRNA [NM_080410]	**6,765**
Fam76b	Rattus norvegicus family with sequence similarity 76, member B (Fam76b), mRNA [NM_001108994]	**6,765**
Grinl1a	Rattus norvegicus glutamate receptor, ionotropic, N-methyl D-aspartate-like 1A (Grinl1a), mRNA [NM_183402]	**6,765**
Rdh11	Rattus norvegicus retinol dehydrogenase 11 (all-trans/9-cis/11-cis) (Rdh11), mRNA [NM_001012193]	**6,764**
Lgi2	Rattus norvegicus leucine-rich repeat LGI family, member 2 (Lgi2), mRNA [NM_001107219]	**6,763**
Klhl11	Rattus norvegicus kelch-like 11 (Drosophila) (Klhl11), mRNA [NM_001105838]	**6,762**
0	Unknown	**6,760**
Krt83	Rattus norvegicus keratin 83 (Krt83), mRNA [NM_001101675]	**6,759**
Pex26	Rattus norvegicus peroxisomal biogenesis factor 26 (Pex26), mRNA [NM_001106618]	**6,759**
0	Unknown	**6,759**
Mrpl53	Rattus norvegicus mitochondrial ribosomal protein L53 (Mrpl53), nuclear gene encoding mitochondrial protein, mRNA [NM_001108635]	**6,757**
Pqbp1	Rattus norvegicus polyglutamine binding protein 1 (Pqbp1), mRNA [NM_001013957]	**6,753**
RGD1560065	Rattus norvegicus similar to RIKEN cDNA 2410004B18 (RGD1560065), mRNA [NM_001134616]	**6,753**
Wfdc2	Rattus norvegicus WAP four-disulfide core domain 2 (Wfdc2), mRNA [NM_173109]	**6,753**
Cyb5r4	Rattus norvegicus cytochrome b5 reductase 4 (Cyb5r4), mRNA [NM_133427]	**6,753**
0	Uncharacterized protein [Source:UniProtKB/TrEMBL;Acc:D4ABX1] [ENSRNOT00000032406]	**6,751**
Klhl8	Rattus norvegicus kelch-like 8 (Drosophila) (Klhl8), mRNA [NM_001105995]	**6,749**
0	Unknown	**6,749**
Acsf3	Similar to C50H11.1, isoform CRA_aUncharacterized protein [Source:UniProtKB/TrEMBL;Acc:D3ZUX7] [ENSRNOT00000020313]	**6,748**
Igtp	Rattus norvegicus interferon gamma induced GTPase (Igtp), mRNA [NM_001008765]	**6,748**
LOC317416	PREDICTED: Rattus norvegicus similar to inter alpha-trypsin inhibitor, heavy chain 4 (LOC317416), miscRNA [XR_006139]	**6,748**
Fgd4	Rattus norvegicus FYVE, RhoGEF and PH domain containing 4 (Fgd4), mRNA [NM_139263]	**6,748**
Actg2	Rattus norvegicus actin, gamma 2, smooth muscle, enteric (Actg2), mRNA [NM_012893]	**6,747**
0	Unknown	**6,747**
Pxdn	PREDICTED: Rattus norvegicus peroxidasin homolog (Drosophila) (Pxdn), partial mRNA [XM_001053103]	**6,746**
Vom2r56	Rattus norvegicus vomeronasal 2 receptor, 56 (Vom2r56), mRNA [NM_001099484]	**6,745**

Rragd	Rattus norvegicus Ras-related GTP binding D (Rragd), mRNA [NM_001106641]	**6,744**
0	Unknown	**6,744**
Ppfibp2	Rattus norvegicus PTPRF interacting protein, binding protein 2 (liprin beta 2) (Ppfibp2), mRNA [NM_001100582]	**6,744**
Orc6l	Rattus norvegicus origin recognition complex, subunit 6 like (yeast) (Orc6l), mRNA [NM_001033690]	**6,743**
Txndc15	Rattus norvegicus thioredoxin domain containing 15 (Txndc15), mRNA [NM_001024998]	**6,743**
Sipa1l2	Rattus norvegicus signal-induced proliferation-associated 1 like 2 (Sipa1l2), mRNA [NM_001009704]	**6,743**
Il2rb	Rattus norvegicus interleukin 2 receptor, beta (Il2rb), mRNA [NM_013195]	**6,743**
Map2k1ip1	Rattus norvegicus mitogen-activated protein kinase kinase 1 interacting protein 1 (Map2k1ip1), mRNA [NM_001008375]	**6,743**
RGD1564664	Rattus norvegicus similar to LOC387763 protein (RGD1564664), mRNA [NM_001110055]	**6,742**
Purb	Rattus norvegicus purine rich element binding protein B (Purb), mRNA [NM_001017503]	**6,741**
Pmpca	Rattus norvegicus peptidase (mitochondrial processing) alpha (Pmpca), nuclear gene encoding mitochondrial protein, mRNA [NM_001003673]	**6,741**
0	Unknown	**6,739**
Pkd1	PREDICTED: Rattus norvegicus polycystic kidney disease 1 homolog (human), transcript variant 2 (Pkd1), mRNA [XM_002727754]	**6,739**
Insig2	Insulin-induced gene 2 protein [Source:UniProtKB/Swiss-Prot;Acc:Q80UA9] [ENSRNOT00000003391]	**6,738**
Gldc	Rattus norvegicus glycine dehydrogenase (decarboxylating) (Gldc), nuclear gene encoding mitochondrial protein, mRNA [NM_001107583]	**6,737**
RGD1305592	Rattus norvegicus similar to RIKEN cDNA 2900092E17 (RGD1305592), mRNA [NM_001013901]	**6,733**
Gtf2f1	Rattus norvegicus general transcription factor IIF, polypeptide 1 (Gtf2f1), mRNA [NM_001007711]	**6,733**
Mov10	Rattus norvegicus Moloney leukemia virus 10 (Mov10), mRNA [NM_001107711]	**6,732**
Kcnma1	Rattus norvegicus potassium large conductance calcium-activated channel, subfamily M, alpha member 1 (Kcnma1), mRNA [NM_031828]	**6,732**
Setbp1	PREDICTED: Rattus norvegicus SET binding protein 1 (Setbp1), mRNA [XM_225744]	**6,731**
Sox14	Rattus norvegicus SRY (sex determining region Y)-box 14 (Sox14), mRNA [NM_001106850]	**6,731**
Ostbeta	Uncharacterized protein [Source:UniProtKB/TrEMBL;Acc:D3ZYJ2] [ENSRNOT00000042353]	**6,731**
0	Rattus norvegicus TL0ADA12YH10 mRNA sequence. [FQ223342]	**6,730**
Fam53b	Rattus norvegicus family with sequence similarity 53, member B (Fam53b), mRNA [NM_001107556]	**6,729**
LOC362901	Rattus norvegicus Ac1254 (LOC362901), mRNA [NM_001008877]	**6,729**
Rpgr	Rattus norvegicus retinitis pigmentosa GTPase regulator (Rpgr), mRNA [NM_001127601]	**6,729**
0	Unknown	**6,729**
0	Unknown	**6,728**
LOC685226	Uncharacterized protein [Source:UniProtKB/TrEMBL;Acc:D3Z9V3] [ENSRNOT00000045972]	**6,727**
RGD1563091	PREDICTED: Rattus norvegicus similar to OEF2 (RGD1563091), mRNA [XM_575365]	**6,726**
0	Unknown	**6,725**
RT1-Cl	Rattus norvegicus RT1 class Ib, locus Cl (RT1-Cl), mRNA [NM_206848]	**6,725**
Ttc19	Rattus norvegicus tetratricopeptide repeat domain 19 (Ttc19), mRNA [NM_001109644]	**6,724**

Atp5j	Rattus norvegicus ATP synthase, H+ transporting, mitochondrial F0 complex, subunit F6 (Atp5j), nuclear gene encoding mitochondrial protein, mRNA [NM_053602]	**6,723**
Znf606	PREDICTED: Rattus norvegicus zinc finger protein 606 (Znf606), mRNA [XM_001063437]	**6,722**
Spag4	Rattus norvegicus sperm associated antigen 4 (Spag4), mRNA [NM_031792]	**6,722**
Nrcam	Rattus norvegicus neuronal cell adhesion molecule (Nrcam), mRNA [NM_013150]	**6,720**
Idh3g	Rattus norvegicus isocitrate dehydrogenase 3 (NAD), gamma (Idh3g), nuclear gene encoding mitochondrial protein, mRNA [NM_031551]	**6,720**
0	Unknown	**6,720**
Lrrc46	Rattus norvegicus leucine rich repeat containing 46 (Lrrc46), mRNA [NM_001004201]	**6,719**
0	Q264C8_MYCVN (Q264C8) Response regulator receiver:LytTr DNA-binding region, partial (9%) [TC583267]	**6,719**
Ndufa7	Rattus norvegicus NADH dehydrogenase (ubiquinone) 1 alpha subcomplex, 7 (B14.5a) (Ndufa7), nuclear gene encoding mitochondrial protein, mRNA [NM_001106772]	**6,719**
0	Uncharacterized protein [Source:UniProtKB/TrEMBL;Acc:D3ZYM6] [ENSRNOT00000057216]	**6,718**
Smad2	Rattus norvegicus SMAD family member 2 (Smad2), mRNA [NM_019191]	**6,718**
Slit3	Rattus norvegicus slit homolog 3 (Drosophila) (Slit3), mRNA [NM_031321]	**6,718**
Ncr1	Rattus norvegicus natural cytotoxicity triggering receptor 1 (Ncr1), mRNA [NM_057199]	**6,717**
Vps29	Rattus norvegicus vacuolar protein sorting 29 homolog (S. cerevisiae) (Vps29), mRNA [NM_001105932]	**6,716**
Ddx49	Rattus norvegicus DEAD (Asp-Glu-Ala-Asp) box polypeptide 49 (Ddx49), mRNA [NM_001115023]	**6,716**
Hspa2	Rattus norvegicus heat shock protein 2 (Hspa2), mRNA [NM_021863]	**6,715**
Itm2c	Rattus norvegicus integral membrane protein 2C (Itm2c), mRNA [NM_001009674]	**6,715**
Wdr75	Rattus norvegicus WD repeat domain 75 (Wdr75), mRNA [NM_001047889]	**6,714**
Kank4	Rattus norvegicus KN motif and ankyrin repeat domains 4 (Kank4), mRNA [NM_001107947]	**6,714**
Slc30a10	Rattus norvegicus solute carrier family 30, member 10 (Slc30a10), mRNA [NM_001105985]	**6,714**
Nes	Rattus norvegicus nestin (Nes), mRNA [NM_012987]	**6,713**
LOC307974	Rattus norvegicus similar to dJ1016N21.1 (novel protein (ortholog of Drosophila Pecanex (PCX), similar to KIAA0805)) (LOC307974), mRNA [XM_226603]	**6,713**
0	Uncharacterized protein [Source:UniProtKB/TrEMBL;Acc:D4A2D0] [ENSRNOT00000043578]	**6,713**
Frmd4a	Rattus norvegicus FERM domain containing 4A (Frmd4a), mRNA [NM_001191821]	**6,713**
Slc35d1	Rattus norvegicus solute carrier family 35 (UDP-glucuronic acid/UDP-N-acetylgalactosamine dual transporter), member D1 (Slc35d1), mRNA [NM_001106668]	**6,712**
Ccnjl	Rattus norvegicus cyclin J-like (Ccnjl), mRNA [NM_001037773]	**6,711**
Itgb1	Rattus norvegicus integrin, beta 1 (Itgb1), mRNA [NM_017022]	**6,710**
Gucy1b3	Rattus norvegicus guanylate cyclase 1, soluble, beta 3 (Gucy1b3), mRNA [NM_012769]	**6,710**
Hao1	Rattus norvegicus hydroxyacid oxidase (glycolate oxidase) 1 (Hao1), mRNA [NM_001107780]	**6,709**
Olr825	Rattus norvegicus olfactory receptor 825 (Olr825), mRNA [NM_001000904]	**6,708**

Fam114a2	Uncharacterized protein [Source:UniProtKB/TrEMBL;Acc:D3ZC89] [ENSRNOT00000058619]	**6,708**
RGD1565972	Rattus norvegicus similar to hypothetical protein 4932411G14 (RGD1565972), mRNA [NM_001134606]	**6,707**
0	Unknown	**6,707**
Fdxacb1	Rattus norvegicus ferredoxin-fold anticodon binding domain containing 1 (Fdxacb1), mRNA [NM_001108145]	**6,706**
0	Uncharacterized protein [Source:UniProtKB/TrEMBL;Acc:D3ZDA4] [ENSRNOT00000065619]	**6,705**
Rnaset2	Rattus norvegicus ribonuclease T2 (Rnaset2), mRNA [NM_001106210]	**6,705**
Ss18	Rattus norvegicus synovial sarcoma translocation, Chromosome 18 (Ss18), mRNA [NM_001100900]	**6,704**
Kti12	Rattus norvegicus KTI12 homolog, chromatin associated (S. cerevisiae) (Kti12), mRNA [NM_001044288]	**6,703**
Ahcy	Rattus norvegicus adenosylhomocysteinase (Ahcy), mRNA [NM_017201]	**6,702**
Amd1	Rattus norvegicus adenosylmethionine decarboxylase 1 (Amd1), mRNA [NM_031011]	**6,702**
Pcsk1	Rattus norvegicus proprotein convertase subtilisin/kexin type 1 (Pcsk1), mRNA [NM_017091]	**6,702**
Psip1	Rattus norvegicus PC4 and SFRS1 interacting protein 1 (Psip1), mRNA [NM_175765]	**6,701**
Zfp2	Rattus norvegicus zinc finger protein 2 (Zfp2), mRNA [NM_001127558]	**6,701**
Pirt	Rattus norvegicus phosphoinositide-interacting regulator of transient receptor potential channels (Pirt), mRNA [NM_001109032]	**6,700**
Aoc2	Rattus norvegicus amine oxidase, copper containing 2 (retina-specific) (Aoc2), non-coding RNA [NR_033180]	**6,700**
Serp2	Rattus norvegicus stress-associated endoplasmic reticulum protein family member 2 (Serp2), mRNA [NM_001109104]	**6,700**
Irx5	Rattus norvegicus iroquois homeobox 5 (Irx5), mRNA [NM_001030044]	**6,700**
Fam71d	Rattus norvegicus family with sequence similarity 71, member D (Fam71d), mRNA [NM_001017469]	**6,699**
RGD1311847	Rattus norvegicus similar to 1700030K09Rik protein (RGD1311847), mRNA [NM_001013879]	**6,699**
Cstf3	Rattus norvegicus cleavage stimulation factor, 3' pre-RNA, subunit 3 (Cstf3), mRNA [NM_001077672]	**6,698**
Trappc10	Rattus norvegicus trafficking protein particle complex 10 (Trappc10), mRNA [NM_001173528]	**6,698**
Chmp7	Rattus norvegicus CHMP family, member 7 (Chmp7), mRNA [NM_001108872]	**6,698**
Cdc37	Rattus norvegicus cell division cycle 37 homolog (S. cerevisiae) (Cdc37), mRNA [NM_053743]	**6,697**
Xpo6	Rattus norvegicus exportin 6 (Xpo6), mRNA [NM_001011935]	**6,697**
LOC686785	PREDICTED: Rattus norvegicus similar to N-acetyltransferase, homolog of S. cerevisiae ARD1 (LOC686785), miscRNA [XR_009033]	**6,697**
Nup98	Rattus norvegicus nucleoporin (Nup98) mRNA, complete cds. [L39991]	**6,696**
Mog	Rattus norvegicus myelin oligodendrocyte glycoprotein (Mog), mRNA [NM_022668]	**6,695**
Pou3f4	Rattus norvegicus POU class 3 homeobox 4 (Pou3f4), mRNA [NM_017252]	**6,694**
Senp3	Rattus norvegicus Sumo1/sentrin/SMT3 specific peptidase 3 (Senp3), mRNA [NM_001013116]	**6,693**
Snapap	Rattus norvegicus SNAP-associated protein (Snapap), transcript variant 2, mRNA [NM_001025648]	**6,693**
LOC684395	PREDICTED: Rattus norvegicus hypothetical protein LOC684395 (LOC684395), mRNA [XM_001070185]	**6,692**
RGD1305500	Rattus norvegicus similar to hypothetical protein FLJ13188 (RGD1305500), mRNA [NM_001107448]	**6,692**
Aipl1	Rattus norvegicus aryl hydrocarbon receptor-interacting protein-like 1 (Aipl1), mRNA [NM_021590]	**6,690**
St6gal2	AMGNNUC:NRHY4-00050-E9-AV W Rat hypothalamus (10464) Rattus norvegicus cDNA clone nrhy4-00050-e9, mRNA sequence [CB605923]	**6,690**

0	Unknown	**6,689**
Opalin	Rattus norvegicus oligodendrocytic myelin paranodal and inner loop protein (Opalin), mRNA [NM_001017386]	**6,689**
F2rl2	Rattus norvegicus coagulation factor II (thrombin) receptor-like 2 (F2rl2), mRNA [NM_053313]	**6,688**
Hadha	Rattus norvegicus hydroxyacyl-Coenzyme A dehydrogenase/3-ketoacyl-Coenzyme A thiolase/enoyl-Coenzyme A hydratase (trifunctional protein), alpha subunit (Hadha), nuclear gene encoding mitochondrial protein, mRNA [NM_130826]	**6,688**
0	Unknown	**6,688**
Casr	Rattus norvegicus calcium-sensing receptor (Casr), mRNA [NM_016996]	**6,686**
Rev3l	Rattus norvegicus REV3-like, catalytic subunit of DNA polymerase zeta (yeast) (Rev3l), mRNA [NM_001083966]	**6,685**
Gpr98	PREDICTED: Rattus norvegicus G protein-coupled receptor 98 (Gpr98), mRNA [XM_002725894]	**6,685**
Arhgef15	Rattus norvegicus Rho guanine nucleotide exchange factor (GEF) 15 (Arhgef15), mRNA [NM_001105789]	**6,684**
LOC688778	PREDICTED: Rattus norvegicus similar to fatty aldehyde dehydrogenase-like (LOC688778), mRNA [XM_001068253]	**6,684**
Sult5a1	Rattus norvegicus sulfotransferase family 5A, member 1 (Sult5a1), transcript variant 2, mRNA [NM_001106194]	**6,683**
Mdfic	Rattus norvegicus MyoD family inhibitor domain containing (Mdfic), mRNA [NM_001105668]	**6,683**
Abcb8	Rattus norvegicus ATP-binding cassette, subfamily B (MDR/TAP), member 8 (Abcb8), nuclear gene encoding mitochondrial protein, mRNA [NM_001007796]	**6,682**
0	Unknown	**6,682**
Cd81	Rattus norvegicus Cd81 molecule (Cd81), mRNA [NM_013087]	**6,680**
LOC500684	Rattus norvegicus hypothetical protein LOC500684 (LOC500684), mRNA [NM_001047959]	**6,680**
Grid2	Rattus norvegicus glutamate receptor, ionotropic, delta 2 (Grid2), mRNA [NM_024379]	**6,679**
0	Unknown	**6,679**
0	Core histone macro-H2A.1 [Source:UniProtKB/Swiss-Prot;Acc:Q02874] [ENSRNOT00000015632]	**6,678**
0	Unknown	**6,677**
LOC686041	Rattus norvegicus cDNA clone IMAGE:6889638, partial cds. [BC059164]	**6,677**
Cldnd2	Rattus norvegicus claudin domain containing 2 (Cldnd2), mRNA [NM_001109143]	**6,676**
Plekho2	Uncharacterized protein [Source:UniProtKB/TrEMBL;Acc:D3Z9K4] [ENSRNOT00000042633]	**6,675**
Acpp	Rattus norvegicus acid phosphatase, prostate (Acpp), transcript variant 1, mRNA [NM_001134901]	**6,673**
Abcc5	Rattus norvegicus ATP-binding cassette, subfamily C (CFTR/MRP), member 5 (Abcc5), mRNA [NM_053924]	**6,673**
0	Z20D2_MOUSE (O88878) Zinc finger A20 domain-containing protein 2 (Zinc finger protein 216), complete [TC586066]	**6,671**
RGD1563120	PREDICTED: Rattus norvegicus similar to RIKEN cDNA 2210009G21 (RGD1563120), mRNA [XM_001081289]	**6,671**
Znf286a	Rattus norvegicus zinc finger protein 286A (Znf286a), mRNA [NM_001191921]	**6,670**
Amz2	Rattus norvegicus archaelysin family metallopeptidase 2 (Amz2), mRNA [NM_001014121]	**6,669**
Btaf1	Rattus norvegicus BTAF1 RNA polymerase II, B-TFIID transcription factor-associated, (Mot1 homolog, S. cerevisiae) (Btaf1), mRNA [NM_001191917]	**6,669**
0	Unknown	**6,666**

Nxph1	Rattus norvegicus neurexophilin 1 (Nxph1), mRNA [NM_012994]	**6,665**
Fars2	Rattus norvegicus phenylalanyl-tRNA synthetase 2, mitochondrial (Fars2), nuclear gene encoding mitochondrial protein, mRNA [NM_001013139]	**6,665**
Cd99	Rattus norvegicus CD99 antigen (Cd99), mRNA [NM_001100804]	**6,664**
Ehmt2	Rattus norvegicus euchromatic histone lysine N-methyltransferase 2 (Ehmt2), mRNA [NM_212463]	**6,664**
Gins1	Rattus norvegicus GINS complex subunit 1 (Psf1 homolog) (Gins1), mRNA [NM_001109207]	**6,663**
Ebf2	Rattus norvegicus early B-cell factor 2 (Ebf2), mRNA [NM_001108383]	**6,663**
Fam19a2	Unknown	**6,661**
0	Unknown	**6,661**
0	Unknown	**6,659**
0	Unknown	**6,658**
Zbed4	Rattus norvegicus zinc finger, BED-type containing 4 (Zbed4), mRNA [NM_001134800]	**6,657**
B3galt4	Rattus norvegicus UDP-Gal:betaGlcNAc beta 1,3-galactosyltransferase, polypeptide 4 (B3galt4), mRNA [NM_133553]	**6,657**
Spopl	Rattus norvegicus speckle-type POZ protein-like (Spopl), mRNA [NM_001134447]	**6,657**
RGD1309077	Rattus norvegicus similar to putative RNA methyltransferase (RGD1309077), mRNA [NM_001108282]	**6,655**
Trim23	Rattus norvegicus tripartite motif-containing 23 (Trim23), mRNA [NM_001100637]	**6,655**
Sp7	Rattus norvegicus Sp7 transcription factor (Sp7), transcript variant 1, mRNA [NM_001037632]	**6,654**
Man2a2	Rattus norvegicus mannosidase 2, alpha 2 (Man2a2), mRNA [NM_001107527]	**6,654**
Mrpl34	Rattus norvegicus mitochondrial ribosomal protein L34 (Mrpl34), nuclear gene encoding mitochondrial protein, mRNA [NM_001006965]	**6,654**
0	Uncharacterized protein [Source:UniProtKB/TrEMBL;Acc:D3ZF29] [ENSRNOT00000012203]	**6,653**
0	AGENCOURT_28718588 NIH_MGC_249 Rattus norvegicus cDNA clone IMAGE:7378234 5', mRNA sequence [CO802318]	**6,653**
0	Unknown	**6,653**
LOC685240	PREDICTED: Rattus norvegicus similar to High mobility group protein 1 (HMG-1) (High mobility group protein B1) (Amphoterin) (Heparin-binding protein p30) (LOC685240), miscRNA [XR_085878]	**6,652**
Zbtb7b	Rattus norvegicus zinc finger and BTB domain containing 7B (Zbtb7b), mRNA [NM_001106446]	**6,652**
0	Unknown	**6,651**
Slc9a3	Rattus norvegicus solute carrier family 9 (sodium/hydrogen exchanger), member 3 (Slc9a3), mRNA [NM_012654]	**6,649**
Apba3	Rattus norvegicus amyloid beta (A4) precursor protein-binding, family A, member 3 (Apba3), mRNA [NM_031781]	**6,649**
Nr0b1	Rattus norvegicus nuclear receptor subfamily 0, group B, member 1 (Nr0b1), mRNA [NM_053317]	**6,649**
Fam71a	Rattus norvegicus family with sequence similarity 71, member A (Fam71a), mRNA [NM_001109084]	**6,649**
Znf521	Rattus norvegicus zinc finger protein 521 (Znf521), mRNA [NM_001107403]	**6,647**
Epha5	Rattus norvegicus EphA5 (Epha5), transcript variant 2, mRNA [NM_024367]	**6,647**
Lpcat3	Rattus norvegicus lysophosphatidylcholine acyltransferase 3 (Lpcat3), mRNA [NM_001012189]	**6,647**
Shd	Rattus norvegicus src homology 2 domain-containing transforming protein D (Shd), mRNA [NM_001108223]	**6,646**
0	Unknown	**6,646**

LOC361963	PREDICTED: Rattus norvegicus similar to phosphoglycerate mutase (EC 5.4.2.1) B chain - rat (LOC361963), miscRNA [XR_086660]	**6,644**
Cited4	Rattus norvegicus Cbp/p300-interacting transactivator, with Glu/Asp-rich carboxy-terminal domain, 4 (Cited4), mRNA [NM_053699]	**6,644**
0	Unknown	**6,644**
Agrp	Rattus norvegicus agouti related protein homolog (mouse) (Agrp), mRNA [NM_033650]	**6,643**
Timm23	Rattus norvegicus translocase of inner mitochondrial membrane 23 homolog (yeast) (Timm23), nuclear gene encoding mitochondrial protein, mRNA [NM_019352]	**6,643**
Zbtb37	Rattus norvegicus zinc finger and BTB domain containing 37 (Zbtb37), mRNA [NM_001107191]	**6,641**
Gripap1	Rattus norvegicus GRIP1 associated protein 1 (Gripap1), mRNA [NM_053807]	**6,640**
0	Uncharacterized protein [Source:UniProtKB/TrEMBL;Acc:D4A0B5] [ENSRNOT00000064837]	**6,640**
Mrpl23	Rattus norvegicus mitochondrial ribosomal protein L23 (Mrpl23), nuclear gene encoding mitochondrial protein, mRNA [NM_022529]	**6,640**
Bace2	Rattus norvegicus beta-site APP-cleaving enzyme 2 (Bace2), mRNA [NM_001002802]	**6,640**
LOC100362727	PREDICTED: Rattus norvegicus ubiquitin carboxyl-terminal hydrolase CYLD (LOC100362727), mRNA [XM_002728578]	**6,639**
Acaa2	Rattus norvegicus acetyl-Coenzyme A acyltransferase 2 (Acaa2), nuclear gene encoding mitochondrial protein, mRNA [NM_130433]	**6,638**
Zgpat	Rattus norvegicus zinc finger, CCCH-type with G patch domain (Zgpat), mRNA [NM_001009656]	**6,638**
Ric3	Rattus norvegicus resistance to inhibitors of cholinesterase 3 homolog (C. elegans) (Ric3), mRNA [NM_001115045]	**6,638**
Ftsj1	Uncharacterized protein [Source:UniProtKB/TrEMBL;Acc:D3ZZA1] [ENSRNOT00000006365]	**6,638**
0	Thyroid hormone receptor associated protein 2 (Predicted)Uncharacterized protein [Source:UniProtKB/TrEMBL;Acc:D3ZJC6] [ENSRNOT00000042331]	**6,637**
Dnajc11	Rattus norvegicus DnaJ (Hsp40) homolog, subfamily C, member 11 (Dnajc11), mRNA [NM_001108694]	**6,636**
Lrrc16b	Leucine-rich repeat-containing protein 16B [Source:UniProtKB/Swiss-Prot;Acc:Q5XHY1] [ENSRNOT00000032252]	**6,636**
Usp24	Putative uncharacterized protein [Source:UniProtKB/TrEMBL;Acc:Q4G007] [ENSRNOT00000029228]	**6,635**
Phf7	Rattus norvegicus PHD finger protein 7 (Phf7), mRNA [NM_001012211]	**6,635**
Oas2	Rattus norvegicus 2'-5' oligoadenylate synthetase 2 (Oas2), mRNA [NM_001009715]	**6,635**
Adam11	Rattus norvegicus ADAM metallopeptidase domain 11 (Adam11), mRNA [NM_001108300]	**6,635**
Dap3	Rattus norvegicus death associated protein 3 (Dap3), nuclear gene encoding mitochondrial protein, mRNA [NM_001011950]	**6,635**
LOC681367	PREDICTED: Rattus norvegicus hypothetical protein LOC681367 (LOC681367), mRNA [XM_001061427]	**6,634**
0	Unknown	**6,633**
S100a1	Rattus norvegicus S100 calcium binding protein A1 (S100a1), mRNA [NM_001007636]	**6,633**
Anubl1	Rattus norvegicus AN1, ubiquitin-like, homolog (Xenopus laevis) (Anubl1), mRNA [NM_173332]	**6,633**
Usp28	Rattus norvegicus ubiquitin specific peptidase 28 (Usp28), mRNA [NM_001108144]	**6,633**
Upp1	Rattus norvegicus uridine phosphorylase 1 (Upp1), mRNA [NM_001030025]	**6,633**
Fhl4	Rattus norvegicus four and a half LIM domains 4 (Fhl4), mRNA [NM_001013172]	**6,632**
Zdhhc7	Rattus norvegicus zinc finger, DHHC-type containing 7 (Zdhhc7), mRNA [NM_133394]	**6,632**
0	Unknown	**6,632**

Epn3	Rattus norvegicus epsin 3 (Epn3), mRNA [NM_001024791]	**6,632**
Giot1	Rattus norvegicus gonadotropin inducible ovarian transcription factor 1 (Giot1), mRNA [NM_133563]	**6,631**
RGD1305178	Rattus norvegicus similar to Hypothetical protein MGC11690 (RGD1305178), mRNA [NM_001025003]	**6,630**
Heatr5a	Uncharacterized protein [Source:UniProtKB/TrEMBL;Acc:D3ZKL2] [ENSRNOT00000035309]	**6,629**
0	Unknown	**6,629**
Zadh2	Rattus norvegicus zinc binding alcohol dehydrogenase, domain containing 2 (Zadh2), mRNA [NM_001106129]	**6,629**
Casp3	Rattus norvegicus caspase 3 (Casp3), mRNA [NM_012922]	**6,628**
Ns5atp4	Rattus norvegicus NS5A (hepatitis C virus) transactivated protein 4 (Ns5atp4), mRNA [NM_207607]	**6,625**
RGD1564927	Rattus norvegicus similar to TGFB-induced factor 2 (RGD1564927), mRNA [NM_001134983]	**6,624**
0	DDX27_MOUSE (Q921N6) Probable ATP-dependent RNA helicase DDX27 (DEAD box protein 27) , partial (74%) [TC616929]	**6,624**
Pcdhga10	Rattus norvegicus protocadherin gamma subfamily A, 10 (Pcdhga10), mRNA [NM_001037135]	**6,624**
0	Unknown	**6,623**
Ccdc76	Rattus norvegicus coiled-coil domain containing 76 (Ccdc76), mRNA [NM_001033902]	**6,622**
Cd6	Rattus norvegicus Cd6 molecule (Cd6), mRNA [NM_175577]	**6,622**
Atp8b3	Uncharacterized protein [Source:UniProtKB/TrEMBL;Acc:D3ZE62] [ENSRNOT00000039813]	**6,621**
L3mbtl	Uncharacterized protein [Source:UniProtKB/TrEMBL;Acc:D4A6H6] [ENSRNOT00000045677]	**6,620**
Uba52	Rattus norvegicus ubiquitin A-52 residue ribosomal protein fusion product 1 (Uba52), mRNA [NM_031687]	**6,619**
RGD1305627	Rattus norvegicus hypothetical LOC314467 (RGD1305627), mRNA [NM_001134560]	**6,618**
Exnef	Rattus norvegicus exonuclease NEF-sp (Exnef), mRNA [NM_001079706]	**6,618**
LOC687426	PREDICTED: Rattus norvegicus similar to G protein-coupled receptor 133 (LOC687426), miscRNA [XR_085591]	**6,617**
0	Unknown	**6,617**
RGD1311501	Rattus norvegicus similar to chromosome 9 open reading frame 7 (RGD1311501), mRNA [NM_001106561]	**6,617**
Iqcb1	Rattus norvegicus IQ motif containing B1 (Iqcb1), mRNA [NM_001107092]	**6,616**
Odz2	Rattus norvegicus odz, odd Oz/ten-m homolog 2 (Drosophila) (Odz2), mRNA [NM_020088]	**6,615**
0	Unknown	**6,613**
Slc1a6	Rattus norvegicus solute carrier family 1 (high affinity aspartate/glutamate transporter), member 6 (Slc1a6), mRNA [NM_032065]	**6,612**
Mcpt1l4	Mast cell protease 1-like 4 [Source:UniProtKB/TrEMBL;Acc:Q6IE57] [ENSRNOT00000043182]	**6,612**
Evx2	PREDICTED: Rattus norvegicus even skipped homeotic gene 2 (Evx2), mRNA [XM_001063297]	**6,612**
Btbd12	PREDICTED: Rattus norvegicus BTB (POZ) domain containing 12 (Btbd12), mRNA [XM_001079342]	**6,612**
Pogk	Rattus norvegicus pogo transposable element with KRAB domain (Pogk), mRNA [NM_001107194]	**6,611**
Pebp1	Rattus norvegicus phosphatidylethanolamine binding protein 1 (Pebp1), mRNA [NM_017236]	**6,611**
Gdi2	Rattus norvegicus GDP dissociation inhibitor 2 (Gdi2), mRNA [NM_017276]	**6,610**
Magmas	Rattus norvegicus mitochondria-associated protein involved in granulocyte-macrophage colony-stimulating factor signal transduction (Magmas), nuclear gene encoding mitochondrial protein, mRNA [NM_001100136]	**6,608**

0	WSP1_SCHPO (O36027) Wiskott-Aldrich syndrome homolog protein 1, partial (5%) [TC645988]	**6,607**
Scarf1	Rattus norvegicus scavenger receptor class F, member 1 (Scarf1), mRNA [NM_001107022]	**6,606**
Cyfip1	Rattus norvegicus cytoplasmic FMR1 interacting protein 1 (Cyfip1), mRNA [NM_001107517]	**6,606**
Smpd2	Rattus norvegicus sphingomyelin phosphodiesterase 2, neutral membrane (Smpd2), mRNA [NM_031360]	**6,605**
LOC100362172	LRRGT00112-like (LOC100362172), mRNA [Source:RefSeq DNA;Acc:NM_001177820] [ENSRNOT00000043267]	**6,605**
Smox	Rattus norvegicus spermine oxidase (Smox), mRNA [NM_001134854]	**6,603**
Senp2	Rattus norvegicus Sumo1/sentrin/SMT3 specific peptidase 2 (Senp2), mRNA [NM_023989]	**6,601**
Srpx2	Rattus norvegicus sushi-repeat-containing protein, X-linked 2 (Srpx2), mRNA [NM_001108243]	**6,601**
Mpn2	PREDICTED: Rattus norvegicus marapsin 2 (Mpn2), mRNA [XM_001077024]	**6,601**
Hbb-b1	Rattus norvegicus hemoglobin, beta adult major chain (Hbb-b1), mRNA [NM_198776]	**6,600**
0	Rattus norvegicus similar to 25kDa nuclear protein (LOC301683), mRNA [XM_237488]	**6,598**
LOC502684	Rattus norvegicus hypothetical protein LOC502684 (LOC502684), mRNA [NM_001025060]	**6,597**
Mut	PREDICTED: Rattus norvegicus methylmalonyl-Coenzyme A mutase (Mut), mRNA [XM_001067239]	**6,596**
Kcnq5l	Potassium voltage-gated channel KQT-like subfamily member 5 [Source:UniProtKB/TrEMBL;Acc:Q8K3W7] [ENSRNOT00000040034]	**6,595**
Cys1	Rattus norvegicus cystin 1 (Cys1), mRNA [NM_001109597]	**6,594**
0	Unknown	**6,593**
Krtap16-5	Rattus norvegicus keratin associated protein 16-5 (Krtap16-5), mRNA [NM_001109424]	**6,593**
Olr801	Rattus norvegicus olfactory receptor 801 (Olr801), mRNA [NM_001001004]	**6,591**
Spag9	Rattus norvegicus sperm associated antigen 9 (Spag9), mRNA [NM_001108290]	**6,591**
Styxl1	Rattus norvegicus serine/threonine/tyrosine interacting-like 1 (Styxl1), mRNA [NM_001037788]	**6,588**
Treml1	Rattus norvegicus triggering receptor expressed on myeloid cells-like 1 (Treml1), mRNA [NM_001192001]	**6,587**
Odf3	Rattus norvegicus outer dense fiber of sperm tails 3 (Odf3), mRNA [NM_001108924]	**6,587**
RGD1564327	Rattus norvegicus similar to integrin alpha 8 (RGD1564327), mRNA [NM_001173972]	**6,587**
Tcerg1	Rattus norvegicus transcription elongation regulator 1 (Tcerg1), mRNA [NM_001107390]	**6,586**
0	Unknown	**6,586**
Miip	Rattus norvegicus migration and invasion inhibitory protein (Miip), mRNA [NM_001017450]	**6,583**
Itpkb	Rattus norvegicus inositol 1,4,5-trisphosphate 3-kinase B (Itpkb), mRNA [NM_019312]	**6,583**
RGD1565225	PREDICTED: Rattus norvegicus similar to Interferon alpha-1 precursor (RGD1565225), mRNA [XM_575856]	**6,580**
Ccpg1	Rattus norvegicus cell cycle progression 1 (Ccpg1), mRNA [NM_001108770]	**6,579**
Ptpn4	Rattus norvegicus protein tyrosine phosphatase, non-receptor type 4 (Ptpn4), mRNA [NM_001100479]	**6,579**
H1f0	Rattus norvegicus H1 histone family, member 0 (H1f0), mRNA [NM_012578]	**6,577**
Trpc1	Rattus norvegicus transient receptor potential cation channel, subfamily C, member 1 (Trpc1), mRNA [NM_053558]	**6,576**
Tmprss5	Rattus norvegicus transmembrane protease, serine 5 (Tmprss5), mRNA [NM_153311]	**6,576**
Kcnh2	Rattus norvegicus potassium voltage-gated channel, subfamily H (eag-related), member 2 (Kcnh2), mRNA [NM_053949]	**6,575**

LOC685152	Uncharacterized protein [Source:UniProtKB/TrEMBL;Acc:D3ZLY4] [ENSRNOT00000028246]	**6,575**
Pcsk6	Rattus norvegicus proprotein convertase subtilisin/kexin type 6 (Pcsk6), mRNA [NM_012999]	**6,574**
Hspb7	Rattus norvegicus heat shock protein family, member 7 (cardiovascular) (Hspb7), mRNA [NM_031607]	**6,573**
0	serine/threonine-protein kinase PINK1, mitochondrial [Source:RefSeq peptide;Acc:NP_001100164] [ENSRNOT00000020820]	**6,572**
Ptdss1	Rattus norvegicus phosphatidylserine synthase 1 (Ptdss1), mRNA [NM_001012113]	**6,571**
Thumpd2	Rattus norvegicus THUMP domain containing 2 (Thumpd2), transcript variant 2, mRNA [NM_001012108]	**6,571**
0	Unknown	**6,569**
0	Uncharacterized protein [Source:UniProtKB/TrEMBL;Acc:D3ZN76] [ENSRNOT00000026111]	**6,568**
0	Succinate-semialdehyde dehydrogenase, mitochondrial [Source:UniProtKB/Swiss-Prot;Acc:P51650] [ENSRNOT00000050919]	**6,568**
0	PREDICTED: Rattus norvegicus similar to hypothetical protein 4930509O22 (RGD1566007), mRNA [XM_346086]	**6,564**
Ube2v2	Rattus norvegicus ubiquitin-conjugating enzyme E2 variant 2 (Ube2v2), mRNA [NM_183052]	**6,563**
Rnaseh2a	Rattus norvegicus ribonuclease H2, subunit A (Rnaseh2a), mRNA [NM_001013234]	**6,563**
Bnip3	Rattus norvegicus BCL2/adenovirus E1B interacting protein 3 (Bnip3), nuclear gene encoding mitochondrial protein, mRNA [NM_053420]	**6,562**
Mrpl36	Rattus norvegicus mitochondrial ribosomal protein L36 (Mrpl36), nuclear gene encoding mitochondrial protein, mRNA [NM_001108879]	**6,562**
Rlim	Rattus norvegicus ring finger protein, LIM domain interacting (Rlim), mRNA [NM_001024892]	**6,562**
Usp3	Rattus norvegicus ubiquitin specific peptidase 3 (Usp3), mRNA [NM_001025424]	**6,562**
Limk2	Rattus norvegicus LIM domain kinase 2 (Limk2), mRNA [NM_024135]	**6,559**
0	Unknown	**6,559**
Lcn10	Rattus norvegicus lipocalin 10 (Lcn10), mRNA [NM_001128137]	**6,559**
Pold1	Rattus norvegicus polymerase (DNA directed), delta 1, catalytic subunit (Pold1), mRNA [NM_021662]	**6,558**
Vom1r12	Rattus norvegicus vomeronasal 1 receptor 12 (Vom1r12), mRNA [NM_001008962]	**6,558**
0	Unknown	**6,557**
Aloxe3	Rattus norvegicus arachidonate lipoxygenase 3 (Aloxe3), mRNA [NM_001105793]	**6,555**
0	Unknown	**6,555**
Tardbp	Rattus norvegicus TAR DNA binding protein (Tardbp), mRNA [NM_001011979]	**6,555**
0	Unknown	**6,555**
Fig4	Rattus norvegicus FIG4 homolog (S. cerevisiae) (Fig4), mRNA [NM_001047096]	**6,554**
RGD1562161	Rattus norvegicus similar to chromosome X open reading frame 23 (RGD1562161), mRNA [NM_001127568]	**6,554**
Rfwd2	Rattus norvegicus ring finger and WD repeat domain 2 (Rfwd2), mRNA [NM_001025126]	**6,552**
Cr1l	Rattus norvegicus complement component (3b/4b) receptor 1-like (Cr1l), transcript variant 1, mRNA [NM_001005330]	**6,552**
Sec23ip	Rattus norvegicus SEC23 interacting protein (Sec23ip), mRNA [NM_001134859]	**6,552**
Ltb	Rattus norvegicus lymphotoxin beta (TNF superfamily, member 3) (Ltb), mRNA [NM_212507]	**6,551**
Zfp277	PREDICTED: Rattus norvegicus similar to zinc finger protein 277 isoform 1 (LOC298977), mRNA [XM_216701]	**6,550**
0	Unknown	**6,550**

Kif27	Rattus norvegicus kinesin family member 27 (Kif27), mRNA [NM_198050]	**6,549**
Kcnf1	Rattus norvegicus potassium voltage-gated channel, subfamily F, member 1 (Kcnf1), mRNA [NM_001169104]	**6,549**
Rpl39	Rattus norvegicus ribosomal protein L39 (Rpl39), mRNA [NM_012875]	**6,548**
0	Uncharacterized protein [Source:UniProtKB/TrEMBL;Acc:D3ZJ52] [ENSRNOT00000006574]	**6,547**
Dusp23	dual specificity protein phosphatase 23 [Source:RefSeq peptide;Acc:NP_001178759] [ENSRNOT00000030449]	**6,546**
0	Uncharacterized protein [Source:UniProtKB/TrEMBL;Acc:D3ZE35] [ENSRNOT00000027967]	**6,546**
0	Unknown	**6,545**
Gsto1	Rattus norvegicus glutathione S-transferase omega 1 (Gsto1), mRNA [NM_001007602]	**6,544**
0	Rattus norvegicus similar to 40S ribosomal protein S7 (S8) (LOC289932), mRNA [XM_223834]	**6,544**
Zfp324	RCG27445Uncharacterized protein [Source:UniProtKB/TrEMBL;Acc:D3ZB44] [ENSRNOT00000036874]	**6,543**
Cit	Rattus norvegicus citron (Cit), mRNA [NM_001029911]	**6,543**
LOC684903	PREDICTED: Rattus norvegicus similar to Probable ATP-dependent RNA helicase DHX34 (DEAH box protein 34), transcript variant 2 (LOC684903), mRNA [XM_001062397]	**6,543**
Glt8d3	Rattus norvegicus glycosyltransferase 8 domain containing 3 (Glt8d3), mRNA [NM_001100887]	**6,542**
Cpsf4	Rattus norvegicus cleavage and polyadenylation specific factor 4 (Cpsf4), mRNA [NM_001012351]	**6,541**
0	Unknown	**6,540**
Kcnn1	Rattus norvegicus potassium intermediate/small conductance calcium-activated channel, subfamily N, member 1 (Kcnn1), mRNA [NM_019313]	**6,538**
RGD1559841	PREDICTED: Rattus norvegicus similar to expressed sequence AW413431 (RGD1559841), mRNA [XM_002725411]	**6,536**
Lmo7	Rattus norvegicus LIM domain 7 (Lmo7), mRNA [NM_001001515]	**6,536**
Copb1	Rattus norvegicus coatomer protein complex, subunit beta 1 (Copb1), mRNA [NM_080781]	**6,536**
Acot1	Rattus norvegicus acyl-CoA thioesterase 1 (Acot1), mRNA [NM_031315]	**6,536**
0	Uncharacterized protein [Source:UniProtKB/TrEMBL;Acc:D3ZA88] [ENSRNOT00000055210]	**6,536**
0	AGENCOURT_31532477 NIH_MGC_238 Rattus norvegicus cDNA clone IMAGE:7456410 5', mRNA sequence [CV119824]	**6,535**
Car8	Rattus norvegicus carbonic anhydrase 8 (Car8), mRNA [NM_001009662]	**6,532**
Cc2d1a	Rattus norvegicus coiled-coil and C2 domain containing 1A (Cc2d1a), mRNA [NM_001013869]	**6,532**
0	Unknown	**6,532**
Igfbp2	Rattus norvegicus insulin-like growth factor binding protein 2 (Igfbp2), mRNA [NM_013122]	**6,531**
Dnajc14	Rattus norvegicus DnaJ (Hsp40) homolog, subfamily C, member 14 (Dnajc14), mRNA [NM_053690]	**6,531**
0	Uncharacterized protein [Source:UniProtKB/TrEMBL;Acc:D4A9Q0] [ENSRNOT00000029274]	**6,531**
LOC691414	Uncharacterized protein [Source:UniProtKB/TrEMBL;Acc:D3ZAB4] [ENSRNOT00000045621]	**6,530**
Tmprss11e	PREDICTED: Rattus norvegicus transmembrane protease, serine 11e (Tmprss11e), mRNA [XM_001074738]	**6,530**
0	Uncharacterized protein [Source:UniProtKB/TrEMBL;Acc:D3ZCM1] [ENSRNOT00000024516]	**6,529**
Schip1	Rattus norvegicus schwannomin interacting protein 1 (Schip1), mRNA [NM_001100666]	**6,529**
Dhx36	Rattus norvegicus DEAH (Asp-Glu-Ala-His) box polypeptide 36 (Dhx36), mRNA [NM_001107678]	**6,528**

Ttll1	Rattus norvegicus tubulin tyrosine ligase-like family, member 1 (Ttll1), mRNA [NM_001012200]	**6,527**
0	Q9QXT9_MOUSE (Q9QXT9) KID2 (Zinc finger protein 354B), partial (12%) [TC642135]	**6,527**
Arfgef2	Rattus norvegicus ADP-ribosylation factor guanine nucleotide-exchange factor 2 (brefeldin A-inhibited) (Arfgef2), mRNA [NM_181083]	**6,526**
Avpr2	Rattus norvegicus arginine vasopressin receptor 2 (Avpr2), mRNA [NM_019136]	**6,526**
Mier1	Rattus norvegicus mesoderm induction early response 1 homolog (Xenopus laevis) (Mier1), mRNA [NM_001131012]	**6,526**
RGD1563349	Rattus norvegicus similar to RIKEN cDNA 9330182L06 (RGD1563349), mRNA [NM_001109345]	**6,525**
0	Unknown	**6,524**
Lefty2	Rattus norvegicus Left-right determination factor 2 (Lefty2), mRNA [NM_001007556]	**6,523**
Ube2g2	Rattus norvegicus ubiquitin-conjugating enzyme E2G 2 (UBC7 homolog, yeast) (Ube2g2), mRNA [NM_001106380]	**6,523**
Loxhd1	Rattus norvegicus lipoxygenase homology domains 1 (Loxhd1), mRNA [NM_001106132]	**6,522**
Pard6g	Rattus norvegicus par-6 partitioning defective 6 homolog gamma (C. elegans) (Pard6g), mRNA [NM_001100973]	**6,522**
Atg16l1	Rattus norvegicus ATG16 autophagy related 16-like 1 (S. cerevisiae) (Atg16l1), mRNA [NM_001108809]	**6,522**
Rpl10	Rattus norvegicus ribosomal protein L10 (Rpl10), mRNA [NM_031100]	**6,521**
Tex28	Rattus norvegicus testis expressed 28 (Tex28), mRNA [NM_001191103]	**6,521**
Elp2	Rattus norvegicus elongation protein 2 homolog (S. cerevisiae) (Elp2), mRNA [NM_001034145]	**6,521**
0	Uncharacterized protein [Source:UniProtKB/TrEMBL;Acc:D3ZHR9] [ENSRNOT00000056217]	**6,520**
0	PREDICTED: Rattus norvegicus similar to Dedicator of cytokinesis protein 10 (Protein zizimin 3) (RGD1561963), miscRNA [XR_085916]	**6,520**
Nfe2l2	Rattus norvegicus nuclear factor, erythroid derived 2, like 2 (Nfe2l2), mRNA [NM_031789]	**6,519**
RGD1308049	Uncharacterized protein [Source:UniProtKB/TrEMBL;Acc:D3ZT97] [ENSRNOT00000028509]	**6,519**
0	Unknown	**6,518**
0	Q8R4G5_MOUSE (Q8R4G5) Truncated N-acetylglucosaminyltransferase V, partial (35%) [TC588091]	**6,518**
0	Rattus norvegicus similar to endo B cytokeratin (LOC315493), mRNA [XM_235966]	**6,517**
Hddc3	Rattus norvegicus HD domain containing 3 (Hddc3), mRNA [NM_001107528]	**6,516**
0	Unknown	**6,515**
0	Uncharacterized protein [Source:UniProtKB/TrEMBL;Acc:D3ZW60] [ENSRNOT00000005861]	**6,514**
0	Q3VJI6_9CHLB (Q3VJI6) Helix-turn-helix, Fis-type, partial (5%) [TC623116]	**6,513**
Nagk	Rattus norvegicus N-acetylglucosamine kinase (Nagk), mRNA [NM_001037768]	**6,513**
Tmem103	Rattus norvegicus transmembrane protein 103 (Tmem103), mRNA [NM_001108782]	**6,512**
Ryr2	Rattus norvegicus ryanodine receptor 2, cardiac (Ryr2), transcript variant 2, mRNA [NM_001191043]	**6,512**
Mustn1	Rattus norvegicus musculoskeletal, embryonic nuclear protein 1 (Mustn1), mRNA [NM_181368]	**6,511**
Slc6a15	Rattus norvegicus solute carrier family 6 (neutral amino acid transporter), member 15 (Slc6a15), mRNA [NM_172321]	**6,510**
Gucy1a3	Rattus norvegicus guanylate cyclase 1, soluble, alpha 3 (Gucy1a3), mRNA [NM_017090]	**6,510**
Egln1	Egl nine homolog 1 [Source:UniProtKB/Swiss-Prot;Acc:P59722] [ENSRNOT00000026767]	**6,510**
Sri	Rattus norvegicus sorcin (Sri), mRNA [NM_001128190]	**6,509**

Dqx1	Rattus norvegicus DEAQ box RNA-dependent ATPase 1 (Dqx1), mRNA [NM_001109407]	**6,509**
LOC682419	PREDICTED: Rattus norvegicus similar to putative pheromone receptor (Go-VN4) (LOC682419), partial mRNA [XM_001061453]	**6,509**
0	Unknown	**6,508**
Pafah2	Rattus norvegicus platelet-activating factor acetylhydrolase 2 (Pafah2), mRNA [NM_177932]	**6,507**
Lmo7	Rattus norvegicus LIM domain 7 (Lmo7), mRNA [NM_001001515]	**6,507**
Ccdc8	Rattus norvegicus coiled-coil domain containing 8 (Ccdc8), mRNA [NM_001009533]	**6,506**
Slc27a5	Rattus norvegicus solute carrier family 27 (fatty acid transporter), member 5 (Slc27a5), mRNA [NM_024143]	**6,505**
Vps41	Rattus norvegicus vacuolar protein sorting 41 homolog (S. cerevisiae) (Vps41), mRNA [NM_001107355]	**6,503**
0	Unknown	**6,503**
Cpne2	PREDICTED: Rattus norvegicus copine II (Cpne2), mRNA [XM_001062196]	**6,503**
RGD1306192	Rattus norvegicus similar to RIKEN cDNA 2310079N02 (RGD1306192), mRNA [NM_001134573]	**6,502**
Ncrna00117	Rattus norvegicus non-protein coding RNA 117 (Ncrna00117), mRNA [NM_001106022]	**6,501**
0	Unknown	**6,501**
Dab2	Rattus norvegicus disabled homolog 2 (Drosophila) (Dab2), mRNA [NM_024159]	**6,500**
0	Unknown	**6,498**
T2	Rattus norvegicus brachyury 2 (T2), mRNA [NM_001161835]	**6,497**
Ppp1cc	Rattus norvegicus protein phosphatase 1, catalytic subunit, gamma isoform (Ppp1cc), mRNA [NM_022498]	**6,497**
Fxr1	Rattus norvegicus fragile X mental retardation, autosomal homolog 1 (Fxr1), mRNA [NM_001012179]	**6,497**
LOC689927	Rattus norvegicus similar to keratin associated protein 10-10 (LOC689927), mRNA [NM_001109555]	**6,495**
LOC100365943	PREDICTED: Rattus norvegicus proteasome subunit alpha type-3-like (LOC100365943), mRNA [XM_002724523]	**6,494**
LOC688765	Rattus norvegicus hypothetical protein LOC688765 (LOC688765), mRNA [NM_001109512]	**6,494**
Sspo	Rattus norvegicus SCO-spondin (Sspo), mRNA [NM_001007016]	**6,494**
0	AW254563 UI-R-BJ0-aej-h-01-0-UI.s1 UI-R-BJ0 Rattus norvegicus cDNA clone UI-R-BJ0-aej-h-01-0-UI 3', mRNA sequence [AW254563]	**6,494**
Olr1349	Rattus norvegicus olfactory receptor 1349 (Olr1349), mRNA [NM_001000484]	**6,494**
Gstm3	glutathione S-transferase Mu 3 [Source:RefSeq peptide;Acc:NP_065415] [ENSRNOT00000025800]	**6,493**
Cytsb	Rattus norvegicus cytospin B (Cytsb), mRNA [NM_001039017]	**6,492**
0	Uncharacterized protein [Source:UniProtKB/TrEMBL;Acc:D3Z8B7] [ENSRNOT00000057522]	**6,492**
Gng5	Rattus norvegicus guanine nucleotide binding protein (G protein), gamma 5 (Gng5), mRNA [NM_024377]	**6,491**
0	Uncharacterized protein [Source:UniProtKB/TrEMBL;Acc:D4AD94] [ENSRNOT00000036604]	**6,491**
Ift122	PREDICTED: Rattus norvegicus intraflagellar transport 122 homolog (Chlamydomonas) (Ift122), mRNA [XM_001054604]	**6,490**
LOC679576	PREDICTED: Rattus norvegicus hypothetical protein LOC679576 (LOC679576), mRNA [XM_001053562]	**6,490**
Lsm6	Rattus norvegicus LSM6 homolog, U6 small nuclear RNA associated (S. cerevisiae) (Lsm6), mRNA [NM_001126085]	**6,490**
Mpi	Rattus norvegicus mannose phosphate isomerase (mapped) (Mpi), mRNA [NM_001004081]	**6,490**
Cdk5rap2	Rattus norvegicus CDK5 regulatory subunit associated protein 2 (Cdk5rap2), mRNA [NM_173134]	**6,490**

Tmem165	Rattus norvegicus transmembrane protein 165 (Tmem165), mRNA [NM_001024802]	**6,487**
Tube1	Rattus norvegicus tubulin, epsilon 1 (Tube1), mRNA [NM_001108536]	**6,486**
Ahnak2	PREDICTED: Rattus norvegicus similar to KIAA2019 protein (RGD1309696), mRNA [XM_234572]	**6,486**
0	Unknown	**6,486**
Vps45	Rattus norvegicus vacuolar protein sorting 45 homolog (S. cerevisiae) (Vps45), mRNA [NM_172072]	**6,486**
0	Unknown	**6,485**
Ccdc21	Rattus norvegicus coiled-coil domain containing 21 (Ccdc21), mRNA [NM_001080151]	**6,484**
0	Uncharacterized protein [Source:UniProtKB/TrEMBL;Acc:D3ZHU8] [ENSRNOT00000001891]	**6,483**
Fastkd1	Rattus norvegicus FAST kinase domains 1 (Fastkd1), mRNA [NM_001191738]	**6,483**
Hhat	PREDICTED: Rattus norvegicus hedgehog acyltransferase (Hhat), mRNA [XM_002728056]	**6,481**
Enpp4	Rattus norvegicus ectonucleotide pyrophosphatase/phosphodiesterase 4 (Enpp4), mRNA [NM_001106892]	**6,481**
Mysm1	Uncharacterized protein [Source:UniProtKB/TrEMBL;Acc:D4A7T9] [ENSRNOT00000039554]	**6,480**
Tmem120a	Rattus norvegicus transmembrane protein 120A (Tmem120a), mRNA [NM_001010945]	**6,480**
Rora	Rattus norvegicus RAR-related orphan receptor A (Rora), mRNA [NM_001106834]	**6,480**
Otub1	Rattus norvegicus OTU domain, ubiquitin aldehyde binding 1 (Otub1), mRNA [NM_001106332]	**6,480**
Id3	Rattus norvegicus inhibitor of DNA binding 3 (Id3), mRNA [NM_013058]	**6,477**
0	Uncharacterized protein [Source:UniProtKB/TrEMBL;Acc:D3ZRA6] [ENSRNOT00000040629]	**6,475**
0	Rattus norvegicus similar to 60S ribosomal protein L10 (QM protein homolog) (LOC366036), mRNA [XM_345350]	**6,475**
LOC685003	PREDICTED: Rattus norvegicus similar to Leydig cell tumor 10 kDa protein (LOC685003), mRNA [XM_001061861]	**6,474**
Ttc9	Rattus norvegicus tetratricopeptide repeat domain 9 (Ttc9), mRNA [NM_001134731]	**6,474**
Zswim2	Rattus norvegicus zinc finger, SWIM-type containing 2 (Zswim2), mRNA [NM_001011960]	**6,473**
Ptrh2	Rattus norvegicus peptidyl-tRNA hydrolase 2 (Ptrh2), nuclear gene encoding mitochondrial protein, mRNA [NM_001013860]	**6,473**
C5	PREDICTED: Rattus norvegicus complement component 5 (C5), mRNA [XM_345342]	**6,473**
Lta4h	Rattus norvegicus leukotriene A4 hydrolase (Lta4h), mRNA [NM_001030031]	**6,471**
Znf692	Rattus norvegicus zinc finger protein 692 (Znf692), mRNA [NM_001126272]	**6,470**
RGD1359616	Rattus norvegicus similar to 2010321M09Rik protein (RGD1359616), mRNA [NM_001007663]	**6,469**
Cpne8	Rattus norvegicus copine VIII (Cpne8), mRNA [NM_001108750]	**6,468**
Tm9sf2	Rattus norvegicus transmembrane 9 superfamily member 2 (Tm9sf2), mRNA [NM_001005554]	**6,467**
Epm2aip1	PREDICTED: Rattus norvegicus EPM2A (laforin) interacting protein 1 (Epm2aip1), mRNA [XM_236659]	**6,465**
LOC680531	Rattus norvegicus similar to CG3880-PA (LOC680531), mRNA [NM_001109418]	**6,465**
Cdk10	Rattus norvegicus cyclin-dependent kinase 10 (Cdk10), transcript variant 3, mRNA [NM_001025722]	**6,464**
Zic5	Rattus norvegicus Zic family member 5 (odd-paired homolog, Drosophila) (Zic5), mRNA [NM_001108391]	**6,464**
Rps27	Rattus norvegicus ribosomal protein S27 (Rps27), mRNA [NM_053597]	**6,464**
Psma4	Rattus norvegicus proteasome (prosome, macropain) subunit, alpha type 4 (Psma4), mRNA [NM_017281]	**6,463**

Tmem30a	Rattus norvegicus transmembrane protein 30A (Tmem30a), mRNA [NM_001004248]	**6,462**
LOC100233177	Rattus norvegicus hypothetical protein LOC100233177 (LOC100233177), mRNA [NM_001134531]	**6,462**
Msh2	Rattus norvegicus mutS homolog 2 (E. coli) (Msh2), mRNA [NM_031058]	**6,459**
Rbm18	Rattus norvegicus RNA binding motif protein 18 (Rbm18), mRNA [NM_001107838]	**6,459**
Clcnkb	Rattus norvegicus chloride channel Kb (Clcnkb), mRNA [NM_173103]	**6,458**
Prp15	Rattus norvegicus proline-rich protein 15 (Prp15), mRNA [NM_012632]	**6,458**
Sh2d4a	Rattus norvegicus SH2 domain containing 4A (Sh2d4a), mRNA [NM_001012048]	**6,458**
0	Unknown	**6,457**
LOC689963	Rattus norvegicus hypothetical protein LOC689963 (LOC689963), mRNA [NM_001109560]	**6,457**
Mrap	Rattus norvegicus melanocortin 2 receptor accessory protein (Mrap), mRNA [NM_001135834]	**6,455**
Olr1536	Rattus norvegicus olfactory receptor 1536 (Olr1536), mRNA [NM_001001353]	**6,455**
0	Uncharacterized protein [Source:UniProtKB/TrEMBL;Acc:D3ZZG2] [ENSRNOT00000065209]	**6,455**
0	Uncharacterized protein [Source:UniProtKB/TrEMBL;Acc:D3ZLK5] [ENSRNOT00000061377]	**6,454**
0	Unknown	**6,454**
MGC116197	Rattus norvegicus similar to RIKEN cDNA 1700001E04 (MGC116197), mRNA [NM_001025755]	**6,454**
Klhdc8a	Rattus norvegicus kelch domain containing 8A (Klhdc8a), mRNA [NM_001100683]	**6,453**
Uxs1	Rattus norvegicus UDP-glucuronate decarboxylase 1 (Uxs1), mRNA [NM_139336]	**6,453**
0	Unknown	**6,453**
0	Unknown	**6,453**
Adam4	Rattus norvegicus a disintegrin and metalloprotease domain 4 (Adam4), mRNA [NM_020305]	**6,451**
0	NADH-ubiquinone oxidoreductase chain 1 [Source:UniProtKB/Swiss-Prot;Acc:P03889] [ENSRNOT00000047550]	**6,450**
Pfkfb1	Rattus norvegicus 6-phosphofructo-2-kinase/fructose-2,6-biphosphatase 1 (Pfkfb1), mRNA [NM_012621]	**6,450**
Mybl2	Rattus norvegicus myeloblastosis oncogene-like 2 (Mybl2), mRNA [NM_001106536]	**6,450**
Etv4	Rattus norvegicus ets variant 4 (Etv4), mRNA [NM_001108299]	**6,449**
Aadacl1	Rattus norvegicus arylacetamide deacetylase-like 1 (Aadacl1), mRNA [NM_001127524]	**6,449**
0	Rattus norvegicus similar to RIKEN cDNA 4933431D05 (LOC301906), mRNA [XM_217507]	**6,449**
0	Unknown	**6,449**
Bcar1	Rattus norvegicus breast cancer anti-estrogen resistance 1 (Bcar1), mRNA [NM_012931]	**6,449**
Unc119b	Rattus norvegicus unc-119 homolog B (C. elegans) (Unc119b), mRNA [NM_001105934]	**6,448**
Hcca2	Rattus norvegicus HCCA2 protein (Hcca2), mRNA [NM_001109159]	**6,448**
Cry1	Rattus norvegicus cryptochrome 1 (photolyase-like) (Cry1), mRNA [NM_198750]	**6,447**
Pstpip2	PREDICTED: Rattus norvegicus proline-serine-threonine phosphatase-interacting protein 2 (Pstpip2), mRNA [XM_225698]	**6,447**
0	Unknown	**6,446**
Nob1	Rattus norvegicus NIN1/RPN12 binding protein 1 homolog (S. cerevisiae) (Nob1), mRNA [NM_199086]	**6,446**

Itfg3	Rattus norvegicus integrin alpha FG-GAP repeat containing 3 (Itfg3), mRNA [NM_001009701]	**6,446**
Pik3ca	Rattus norvegicus phosphoinositide-3-kinase, catalytic, alpha polypeptide (Pik3ca), mRNA [NM_133399]	**6,446**
Fbxo6	Rattus norvegicus F-box protein 6 (Fbxo6), mRNA [NM_138917]	**6,446**
Rbm5	Rattus norvegicus RNA binding motif protein 5 (Rbm5), mRNA [NM_001100548]	**6,445**
0	Unknown	**6,445**
0	Unknown	**6,445**
Olr230	Rattus norvegicus olfactory receptor 230 (Olr230), mRNA [NM_001000205]	**6,444**
Morn5	Rattus norvegicus MORN repeat containing 5 (Morn5), mRNA [NM_001047912]	**6,444**
Hisppd2a	Rattus norvegicus histidine acid phosphatase domain containing 2A (Hisppd2a), mRNA [NM_001080783]	**6,443**
Mrs2	Rattus norvegicus MRS2 magnesium homeostasis factor homolog (S. cerevisiae) (Mrs2), mRNA [NM_024001]	**6,442**
0	Unknown	**6,442**
Vps24	Rattus norvegicus vacuolar protein sorting 24 homolog (S. cerevisiae) (Vps24), mRNA [NM_172331]	**6,441**
RGD1563065	PREDICTED: Rattus norvegicus similar to 3110047P20Rik protein (RGD1563065), mRNA [XM_001078520]	**6,439**
Flad1	Rattus norvegicus flavin adenine dinucleotide synthetase (Flad1), mRNA [NM_001110138]	**6,439**
Jakmip2	Rattus norvegicus janus kinase and microtubule interacting protein 2 (Jakmip2), mRNA [NM_001107391]	**6,438**
0	Unknown	**6,438**
0	Unknown	**6,437**
Clec4a3	Rattus norvegicus C-type lectin domain family 4, member a3 (Clec4a3), mRNA [NM_001005891]	**6,436**
Zfp382	Rattus norvegicus zinc finger protein 382 (Zfp382), mRNA [NM_144749]	**6,434**
Lrrc8c	Rattus norvegicus leucine rich repeat containing 8 family, member C (Lrrc8c), mRNA [NM_001037179]	**6,433**
0	set=Set beta isoform {alternatively spliced} [rats, neonatal kidney, mRNA, 2026 nt]. [S68987]	**6,433**
B4galnt4	Rattus norvegicus beta-1,4-N-acetyl-galactosaminyl transferase 4 (B4galnt4), mRNA [NM_001107562]	**6,431**
Pigv	Rattus norvegicus phosphatidylinositol glycan anchor biosynthesis, class V (Pigv), mRNA [NM_001010966]	**6,430**
Lphn3	Rattus norvegicus latrophilin 3 (Lphn3), mRNA [NM_130822]	**6,430**
Prkag2	Rattus norvegicus protein kinase, AMP-activated, gamma 2 non-catalytic subunit (Prkag2), mRNA [NM_184051]	**6,430**
Nmbr	Rattus norvegicus neuromedin B receptor (Nmbr), mRNA [NM_012799]	**6,430**
Olr892	Rattus norvegicus olfactory receptor 892 (Olr892), mRNA [NM_001000060]	**6,429**
Shprh	Rattus norvegicus SNF2 histone linker PHD RING helicase (Shprh), mRNA [NM_001107470]	**6,429**
Trpc4	Rattus norvegicus transient receptor potential cation channel, subfamily C, member 4 (Trpc4), transcript variant alpha, mRNA [NM_080396]	**6,429**
0	DRABAD11 Rat DRG Library Rattus norvegicus cDNA clone DRABAD11 5', mRNA sequence [BG664185]	**6,429**
Copb2	Rattus norvegicus coatomer protein complex, subunit beta 2 (beta prime) (Copb2), mRNA [NM_021765]	**6,428**
0	Uncharacterized protein [Source:UniProtKB/TrEMBL;Acc:D3ZY49] [ENSRNOT00000001562]	**6,428**
Kcne4	Rattus norvegicus potassium voltage-gated channel, Isk-related family, member 4 (Kcne4), mRNA [NM_212526]	**6,425**
Slc20a2	Rattus norvegicus solute carrier family 20 (phosphate transporter), member 2 (Slc20a2), mRNA [NM_017223]	**6,424**

Trim2	Rattus norvegicus tripartite motif-containing 2 (Trim2), mRNA [NM_001108552]	**6,424**
Elovl7	Rattus norvegicus ELOVL family member 7, elongation of long chain fatty acids (yeast) (Elovl7), mRNA [NM_001191844]	**6,423**
Tmem177	Rattus norvegicus transmembrane protein 177 (Tmem177), mRNA [NM_001037776]	**6,422**
Gas2l1	Rattus norvegicus growth arrest-specific 2 like 1 (Gas2l1), mRNA [NM_001108365]	**6,422**
Sptlc1	Rattus norvegicus serine palmitoyltransferase, long chain base subunit 1 (Sptlc1), mRNA [NM_001108406]	**6,422**
0	Unknown	**6,419**
Hn1l	Rattus norvegicus hematological and neurological expressed 1-like (Hn1l), mRNA [NM_001013182]	**6,418**
LOC681086	RCG61899Uncharacterized protein [Source:UniProtKB/TrEMBL;Acc:D3ZZU7] [ENSRNOT00000020600]	**6,417**
Fbxw4	Rattus norvegicus F-box and WD repeat domain containing 4 (Fbxw4), mRNA [NM_001107600]	**6,417**
0	Unknown	**6,414**
Kctd10	Rattus norvegicus potassium channel tetramerization domain containing 10 (Kctd10), mRNA [NM_001009973]	**6,414**
LOC691241	PREDICTED: Rattus norvegicus similar to MIC2 like 1 (LOC691241), mRNA [XM_001077347]	**6,414**
LOC100125367	Rattus norvegicus hypothetical protein LOC100125367 (LOC100125367), mRNA [NM_001103357]	**6,414**
0	Unknown	**6,414**
Gabpa	Rattus norvegicus GA binding protein transcription factor, alpha subunit (Gabpa), mRNA [NM_001108841]	**6,413**
0	Unknown	**6,410**
Stx2	Rattus norvegicus syntaxin 2 (Stx2), mRNA [NM_012748]	**6,410**
Serpina3n	Rattus norvegicus serine (or cysteine) peptidase inhibitor, clade A, member 3N (Serpina3n), mRNA [NM_031531]	**6,409**
0	Unknown	**6,408**
0	Unknown	**6,408**
0	Unknown	**6,407**
Ttpal	Rattus norvegicus tocopherol (alpha) transfer protein-like (Ttpal), mRNA [NM_001106537]	**6,407**
Xpot	Rattus norvegicus exportin, tRNA (nuclear export receptor for tRNAs) (Xpot), mRNA [NM_001108102]	**6,406**
Ube2k	Rattus norvegicus ubiquitin-conjugating enzyme E2K (UBC1 homolog, yeast) (Ube2k), mRNA [NM_001106006]	**6,406**
Uros	Rattus norvegicus uroporphyrinogen III synthase (Uros), mRNA [NM_001012068]	**6,406**
Eml5	PREDICTED: Rattus norvegicus echinoderm microtubule associated protein like 5, transcript variant 2 (Eml5), mRNA [XM_001064078]	**6,405**
Hmox1	Rattus norvegicus heme oxygenase (decycling) 1 (Hmox1), mRNA [NM_012580]	**6,405**
Plaur	Rattus norvegicus plasminogen activator, urokinase receptor (Plaur), transcript variant 1, mRNA [NM_134352]	**6,404**
Limd1	Rattus norvegicus LIM domains containing 1 (Limd1), mRNA [NM_001112737]	**6,404**
Gtpbp8	Rattus norvegicus GTP-binding protein 8 (putative) (Gtpbp8), mRNA [NM_001025015]	**6,403**
RGD1560888	Rattus norvegicus similar to Cell division protein kinase 8 (Protein kinase K35) (RGD1560888), mRNA [NM_001109061]	**6,403**
LOC688285	PREDICTED: Rattus norvegicus hypothetical protein LOC688285 (LOC688285), mRNA [XM_001081744]	**6,401**
Zdhhc20	Rattus norvegicus zinc finger, DHHC-type containing 20 (Zdhhc20), mRNA [NM_001039336]	**6,401**
0	Unknown	**6,399**

Gphn	Rattus norvegicus gephyrin (Gphn), mRNA [NM_022865]	**6,399**
Gas2l1	Rattus norvegicus growth arrest-specific 2 like 1 (Gas2l1), mRNA [NM_001108365]	**6,399**
Polr3k	Rattus norvegicus polymerase (RNA) III (DNA directed) polypeptide K (Polr3k), mRNA [NM_001014259]	**6,397**
Ccdc37	Coiled-coil domain containing 37 (Predicted), isoform CRA_cUncharacterized protein [Source:UniProtKB/TrEMBL;Acc:D3ZEI2] [ENSRNOT00000024222]	**6,397**
0	Rattus norvegicus similar to RIKEN cDNA 4432405B04 (LOC360802), mRNA [XM_341074]	**6,397**
LOC691995	Rattus norvegicus hypothetical protein LOC691995 (LOC691995), mRNA [NM_001103353]	**6,396**
0	Rattus norvegicus similar to RIKEN cDNA 4933431D05 (LOC363395), mRNA [XM_343725]	**6,396**
0	Unknown	**6,395**
0	Unknown	**6,395**
Arid4b	Rattus norvegicus AT rich interactive domain 4B (Rbp1 like) (Arid4b), mRNA [NM_053421]	**6,395**
Ftl	Rattus norvegicus ferritin, light polypeptide (Ftl), mRNA [NM_022500]	**6,395**
RGD1562378	PREDICTED: Rattus norvegicus histone H4 variant H4-v.1 (RGD1562378), mRNA [XM_575704]	**6,394**
Shmt1	Rattus norvegicus serine hydroxymethyltransferase 1 (soluble) (Shmt1), nuclear gene encoding mitochondrial protein, mRNA [NM_001047842]	**6,394**
Zfp202	Rattus norvegicus zinc finger protein 202 (Zfp202), mRNA [NM_001109290]	**6,393**
Gns	Rattus norvegicus glucosamine (N-acetyl)-6-sulfatase (Gns), mRNA [NM_001011989]	**6,393**
0	Unknown	**6,393**
Scnn1b	Rattus norvegicus sodium channel, nonvoltage-gated 1, beta (Scnn1b), mRNA [NM_012648]	**6,393**
LOC499806	Rattus norvegicus similar to RIKEN cDNA 4933404M02 (LOC499806), mRNA [NM_001024311]	**6,392**
0	Unknown	**6,392**
Fcer1g	Rattus norvegicus Fc fragment of IgE, high affinity I, receptor for; gamma polypeptide (Fcer1g), mRNA [NM_001131001]	**6,388**
Slc26a9	Rattus norvegicus solute carrier family 26, member 9 (Slc26a9), mRNA [NM_001107172]	**6,388**
0	Unknown	**6,388**
Rnaseh2b	Rattus norvegicus ribonuclease H2, subunit B (Rnaseh2b), mRNA [NM_001007007]	**6,388**
0	Unknown	**6,387**
Rrp1	Rattus norvegicus ribosomal RNA processing 1 homolog (S. cerevisiae) (Rrp1), mRNA [NM_001012073]	**6,387**
Kank3	Rattus norvegicus KN motif and ankyrin repeat domains 3 (Kank3), mRNA [NM_001108989]	**6,386**
Opn4	Rattus norvegicus opsin 4 (Opn4), mRNA [NM_138860]	**6,384**
0	PREDICTED: Rattus norvegicus similar to high mobility group nucleosomal binding domain 1 (LOC691642), mRNA [XM_001079135]	**6,384**
Niacr1	Rattus norvegicus niacin receptor 1 (Niacr1), mRNA [NM_181476]	**6,384**
Agl	Rattus norvegicus amylo-1,6-glucosidase, 4-alpha-glucanotransferase (Agl), mRNA [NM_001108564]	**6,382**
RGD1307254	Rattus norvegicus similar to RIKEN cDNA 1200011I18 (RGD1307254), mRNA [NM_001024875]	**6,381**
Pdss2	Rattus norvegicus prenyl (decaprenyl) diphosphate synthase, subunit 2 (Pdss2), mRNA [NM_001014249]	**6,380**
0	Unknown	**6,379**

Ccnc	Rattus norvegicus cyclin C (Ccnc), mRNA [NM_001100472]	**6,378**
0	Unknown	**6,376**
Osmr	Rattus norvegicus oncostatin M receptor (Osmr), mRNA [NM_001005384]	**6,375**
Coro2a	Rattus norvegicus coronin, actin binding protein 2A (Coro2a), mRNA [NM_001012101]	**6,375**
RGD1308775	Rattus norvegicus similar to RIKEN cDNA 4921536K21 (RGD1308775), mRNA [NM_001107233]	**6,375**
Znf512	Uncharacterized protein [Source:UniProtKB/TrEMBL;Acc:D3ZIF0] [ENSRNOT00000006658]	**6,371**
Ptn	Rattus norvegicus pleiotrophin (Ptn), mRNA [NM_017066]	**6,371**
Ppwd1	Rattus norvegicus peptidylprolyl isomerase domain and WD repeat containing 1 (Ppwd1), mRNA [NM_001106406]	**6,370**
Zbtb43	Rattus norvegicus zinc finger and BTB domain containing 43 (Zbtb43), mRNA [NM_001012094]	**6,370**
Tns1	Rattus norvegicus tensin 1 (Tns1), mRNA [NM_001191810]	**6,370**
0	Unknown	**6,369**
0	Unknown	**6,369**
Zswim5	Rattus norvegicus zinc finger, SWIM domain containing 5 (Zswim5), mRNA [NM_001107963]	**6,369**
RGD1565012	PREDICTED: Rattus norvegicus similar to beta-1,3-N-acetylglucosaminyltransferase bGnT-5 (RGD1565012), mRNA [XM_231163]	**6,368**
Hbs1l	Rattus norvegicus Hbs1-like (S. cerevisiae) (Hbs1l), mRNA [NM_001011934]	**6,368**
0	Unknown	**6,367**
Orai1	Rattus norvegicus ORAI calcium release-activated calcium modulator 1 (Orai1), mRNA [NM_001013982]	**6,367**
Phlda3	Rattus norvegicus pleckstrin homology-like domain, family A, member 3 (Phlda3), mRNA [NM_001012206]	**6,364**
Ddx51	Rattus norvegicus DEAD (Asp-Glu-Ala-Asp) box polypeptide 51 (Ddx51), mRNA [NM_001107150]	**6,363**
Nub1	Rattus norvegicus negative regulator of ubiquitin-like proteins 1 (Nub1), mRNA [NM_001013925]	**6,363**
N4bp2	Uncharacterized protein [Source:UniProtKB/TrEMBL;Acc:D3ZLS6] [ENSRNOT00000031792]	**6,363**
0	B double prime 1, subunit of RNA polymerase III transcription initiation factor IIIB (Predicted), isoform CRA_aUncharacterized protein [Source:UniProtKB/TrEMBL;Acc:D3ZDI4] [ENSRNOT00000024023]	**6,361**
Zfp280d	Rattus norvegicus zinc finger protein 280D (Zfp280d), mRNA [NM_001108165]	**6,361**
Upf2	Rattus norvegicus UPF2 regulator of nonsense transcripts homolog (yeast) (Upf2), mRNA [NM_001108421]	**6,361**
RGD1566149	Rattus norvegicus similar to CDNA sequence BC017647 (RGD1566149), mRNA [NM_001109038]	**6,360**
0	Rattus norvegicus, 30 clones, strain BN/SsNHsdMCW RNOR03322613, whole genome shotgun sequence [AABR03128500]	**6,360**
Map4k5	Rattus norvegicus mitogen-activated protein kinase kinase kinase kinase 5 (Map4k5), mRNA [NM_001192016]	**6,360**
Adarb1	Rattus norvegicus adenosine deaminase, RNA-specific, B1 (Adarb1), transcript variant 1, mRNA [NM_012894]	**6,359**
Stard3nl	Rattus norvegicus STARD3 N-terminal like (Stard3nl), mRNA [NM_001008298]	**6,358**
0	Q6P9S9_MOUSE (Q6P9S9) Zinc finger, ZZ domain containing 3, partial (20%) [TC642834]	**6,357**
0	Rattus norvegicus similar to glyceraldehyde-3-phosphate dehydrogenase (LOC313741), mRNA [XM_233699]	**6,357**
LOC499124	Rattus norvegicus mouse zinc finger protein 14-like (LOC499124), mRNA [NM_001100991]	**6,357**
0	Unknown	**6,355**

Slc35a2	Rattus norvegicus solute carrier family 35 (UDP-galactose transporter), member A2 (Slc35a2), mRNA [NM_001127642]	**6,354**
Engase	Rattus norvegicus endo-beta-N-acetylglucosaminidase (Engase), mRNA [NM_001107070]	**6,353**
Abi3	Rattus norvegicus ABI family, member 3 (Abi3), mRNA [NM_001013118]	**6,352**
Traf3ip3	Rattus norvegicus TRAF3 interacting protein 3 (Traf3ip3), mRNA [NM_001014132]	**6,352**
Hmgb1	Rattus norvegicus high mobility group box 1 (Hmgb1), mRNA [NM_012963]	**6,352**
Wbscr28	Uncharacterized protein [Source:UniProtKB/TrEMBL;Acc:D3ZKV0] [ENSRNOT00000033499]	**6,352**
Arfip1	Rattus norvegicus ADP-ribosylation factor interacting protein 1 (Arfip1), mRNA [NM_021763]	**6,351**
Tuba1b	Rattus norvegicus tubulin, alpha 1B (Tuba1b), mRNA [NM_001044270]	**6,349**
LOC317456	Rattus norvegicus hypothetical LOC317456 (LOC317456), mRNA [NM_001047894]	**6,349**
Sln	Rattus norvegicus sarcolipin (Sln), mRNA [NM_001013247]	**6,346**
Tigd3	Rattus norvegicus tigger transposable element derived 3 (Tigd3), mRNA [NM_001107573]	**6,346**
Slc6a2	Rattus norvegicus solute carrier family 6 (neurotransmitter transporter, noradrenalin), member 2 (Slc6a2), mRNA [NM_031343]	**6,346**
Snrk	Rattus norvegicus SNF related kinase (Snrk), mRNA [NM_138833]	**6,345**
0	Unknown	**6,345**
Hnrnpab	Rattus norvegicus heterogeneous nuclear ribonucleoprotein A/B (Hnrnpab), mRNA [NM_031330]	**6,345**
Pxmp2	Rattus norvegicus peroxisomal membrane protein 2 (Pxmp2), mRNA [NM_031587]	**6,344**
Olr1547	Rattus norvegicus olfactory receptor 1547 (Olr1547), mRNA [NM_001001107]	**6,343**
Rpl30	Rattus norvegicus ribosomal protein L30 (Rpl30), mRNA [NM_022699]	**6,343**
LOC501110	Rattus norvegicus similar to Glutathione S-transferase A1 (GTH1) (HA subunit 1) (GST-epsilon) (GSTA1-1) (GST class-alpha) (LOC501110), mRNA [NM_001024361]	**6,343**
Mks1	Rattus norvegicus Meckel syndrome, type 1 (Mks1), mRNA [NM_001034917]	**6,343**
Gtf2h2	Rattus norvegicus general transcription factor IIH, polypeptide 2 (Gtf2h2), mRNA [NM_001077428]	**6,341**
Rpe	Rattus norvegicus ribulose-5-phosphate-3-epimerase (Rpe), mRNA [NM_001033903]	**6,341**
Klhl36	Rattus norvegicus kelch-like 36 (Drosophila) (Klhl36), mRNA [NM_001017511]	**6,340**
Wdr6	Rattus norvegicus WD repeat domain 6 (Wdr6), mRNA [NM_001006988]	**6,340**
Fam89a	Rattus norvegicus family with sequence similarity 89, member A (Fam89a), mRNA [NM_001011711]	**6,339**
Lyzl6	Rattus norvegicus lysozyme-like 6 (Lyzl6), mRNA [NM_001135833]	**6,339**
Gda	Rattus norvegicus guanine deaminase (Gda), mRNA [NM_031776]	**6,339**
Brwd2	PREDICTED: Rattus norvegicus bromodomain and WD repeat domain containing 2 (Brwd2), mRNA [XM_219377]	**6,339**
Fbxo3	Rattus norvegicus F-box protein 3 (Fbxo3), mRNA [NM_001109606]	**6,339**
Grm3	Rattus norvegicus glutamate receptor, metabotropic 3 (Grm3), mRNA [NM_001105712]	**6,338**
Best2	Rattus norvegicus bestrophin 2 (Best2), mRNA [NM_001108895]	**6,338**
LOC685634	PREDICTED: Rattus norvegicus hypothetical protein LOC685634 (LOC685634), mRNA [XM_001064606]	**6,337**
RGD1559884	Rattus norvegicus similar to RIKEN cDNA 9930023K05 (RGD1559884), mRNA [NM_001134613]	**6,335**

Tf	Rattus norvegicus transferrin (Tf), mRNA [NM_001013110]	**6,334**
Ttc39b	Rattus norvegicus tetratricopeptide repeat domain 39B (Ttc39b), mRNA [NM_001106665]	**6,333**
0	Histone H4Osteogenic growth peptide [Source:UniProtKB/Swiss-Prot;Acc:P62804] [ENSRNOT00000068510]	**6,332**
Ccbl1	Rattus norvegicus cysteine conjugate-beta lyase, cytoplasmic (Ccbl1), mRNA [NM_001013164]	**6,332**
0	Rattus norvegicus similar to ribosomal protein S27a (LOC291599), mRNA [XM_225950]	**6,330**
Camsap1l1	Rattus norvegicus calmodulin regulated spectrin-associated protein 1-like 1 (Camsap1l1), mRNA [NM_001134503]	**6,330**
Iqcg	Rattus norvegicus IQ motif containing G (Iqcg), mRNA [NM_001014230]	**6,330**
Hmgb2	Rattus norvegicus high mobility group box 2 (Hmgb2), mRNA [NM_017187]	**6,329**
0	Unknown	**6,328**
Plxdc1	Rattus norvegicus plexin domain containing 1 (Plxdc1), mRNA [NM_001107046]	**6,328**
Slc3a2	Rattus norvegicus solute carrier family 3 (activators of dibasic and neutral amino acid transport), member 2 (Slc3a2), mRNA [NM_019283]	**6,327**
Olr361	Rattus norvegicus olfactory receptor 361 (Olr361), mRNA [NM_001000556]	**6,327**
Tcf25	Uncharacterized protein [Source:UniProtKB/TrEMBL;Acc:D3ZC46] [ENSRNOT00000046155]	**6,326**
LOC100361840	PREDICTED: Rattus norvegicus LIM homeobox transcription factor 1, beta-like (LOC100361840), mRNA [XM_002742444]	**6,326**
0	Unknown	**6,326**
Car12	Rattus norvegicus carbonic anyhydrase 12 (Car12), mRNA [NM_001080756]	**6,326**
RGD1563073	PREDICTED: Rattus norvegicus similar to SIGLEC-like 1 (RGD1563073), mRNA [XM_002725572]	**6,326**
LOC100363621	PREDICTED: Rattus norvegicus ecto-NOX disulfide-thiol exchanger 1 (LOC100363621), mRNA [XM_002725125]	**6,325**
Ets2	Rattus norvegicus v-ets erythroblastosis virus E26 oncogene homolog 2 (avian) (Ets2), mRNA [NM_001107107]	**6,325**
RGD1311186	Rattus norvegicus similar to RIKEN cDNA 1810014F10 gene (RGD1311186), mRNA [NM_001106310]	**6,324**
0	Unknown	**6,324**
Srcrb4d	Uncharacterized protein [Source:UniProtKB/TrEMBL;Acc:D3ZQH3] [ENSRNOT00000001953]	**6,323**
Slc7a6	Rattus norvegicus solute carrier family 7 (cationic amino acid transporter, y+ system), member 6 (Slc7a6), mRNA [NM_001107424]	**6,323**
0	Nebulin (Predicted)Uncharacterized protein [Source:UniProtKB/TrEMBL;Acc:D3ZM87] [ENSRNOT00000038926]	**6,322**
Pus10	pseudouridylate synthase 10 [Source:RefSeq peptide;Acc:NP_001020449] [ENSRNOT00000008115]	**6,322**
Pml	PREDICTED: Rattus norvegicus promyelocytic leukemia (Pml), mRNA [XM_236296]	**6,322**
LOC684139	PREDICTED: Rattus norvegicus similar to zinc finger protein 582 (LOC684139), mRNA [XM_001069100]	**6,320**
LOC686298	PREDICTED: Rattus norvegicus similar to protein phosphatase 2C eta isoform 2 (LOC686298), mRNA [XM_001073372]	**6,320**
0	Unknown	**6,319**
RGD1304624	Rattus norvegicus similar to RIKEN cDNA 2700097O09 (RGD1304624), mRNA [NM_001108022]	**6,319**
Ctsc	Rattus norvegicus cathepsin C (Ctsc), mRNA [NM_017097]	**6,319**
Kbtbd2	Rattus norvegicus kelch repeat and BTB (POZ) domain containing 2 (Kbtbd2), mRNA [NM_001107861]	**6,319**
Xrcc5	Rattus norvegicus X-ray repair complementing defective repair in Chinese hamster cells 5 (Xrcc5), mRNA [NM_177419]	**6,318**
Tnnc2	Rattus norvegicus troponin C type 2 (fast) (Tnnc2), mRNA [NM_001037351]	**6,317**

Psd	Rattus norvegicus pleckstrin and Sec7 domain containing (Psd), mRNA [NM_134370]	**6,316**
Asphd2	Rattus norvegicus aspartate beta-hydroxylase domain containing 2 (Asphd2), mRNA [NM_001009716]	**6,316**
Atcay	Rattus norvegicus ataxia, cerebellar, Cayman type (Atcay), mRNA [NM_001040190]	**6,316**
0	Uncharacterized protein [Source:UniProtKB/TrEMBL;Acc:D3ZW42] [ENSRNOT00000065829]	**6,316**
B3galnt1	Rattus norvegicus beta-1,3-N-acetylgalactosaminyltransferase 1 (B3galnt1), mRNA [NM_001013158]	**6,315**
0	Unknown	**6,315**
Pkm2	Rattus norvegicus pyruvate kinase, muscle (Pkm2), mRNA [NM_053297]	**6,314**
Uckl1	Rattus norvegicus uridine-cytidine kinase 1-like 1 (Uckl1), mRNA [NM_001109212]	**6,313**
0	Uncharacterized protein [Source:UniProtKB/TrEMBL;Acc:D3Z8C0] [ENSRNOT00000042031]	**6,313**
Dusp10	Rattus norvegicus dual specificity phosphatase 10 (Dusp10), mRNA [NM_001105734]	**6,311**
Parp3	Rattus norvegicus poly (ADP-ribose) polymerase family, member 3 (Parp3), mRNA [NM_001008328]	**6,311**
Psmd3	Rattus norvegicus proteasome (prosome, macropain) 26S subunit, non-ATPase, 3 (Psmd3), mRNA [NM_001008281]	**6,311**
Taf8	Rattus norvegicus TAF8 RNA polymerase II, TATA box binding protein (TBP)-associated factor (Taf8), mRNA [NM_001108197]	**6,309**
0	Unknown	**6,308**
Tbx18	Rattus norvegicus T-box18 (Tbx18), mRNA [NM_001108173]	**6,308**
Ppm1a	Rattus norvegicus protein phosphatase 1A, magnesium dependent, alpha isoform (Ppm1a), mRNA [NM_017038]	**6,307**
Arrdc5	Rattus norvegicus arrestin domain containing 5 (Arrdc5), mRNA [NM_001109408]	**6,307**
Cdk7	Cell division protein kinase 7 [Source:UniProtKB/Swiss-Prot;Acc:P51952] [ENSRNOT00000025026]	**6,307**
Bcl2l1	Rattus norvegicus Bcl2-like 1 (Bcl2l1), nuclear gene encoding mitochondrial protein, transcript variant 1, mRNA [NM_001033672]	**6,306**
Pik3cb	Rattus norvegicus phosphoinositide-3-kinase, catalytic, beta polypeptide (Pik3cb), mRNA [NM_053481]	**6,306**
Dnm1l	Rattus norvegicus dynamin 1-like (Dnm1l), mRNA [NM_053655]	**6,302**
Wdr25l	Rattus norvegicus WD repeat domain 25-like (Wdr25l), mRNA [NM_001135894]	**6,301**
Sarnp	Rattus norvegicus SAP domain containing ribonucleoprotein (Sarnp), mRNA [NM_001033070]	**6,301**
Tbc1d5	Rattus norvegicus TBC1 domain family, member 5 (Tbc1d5), mRNA [NM_001134762]	**6,301**
Acap1	Rattus norvegicus ArfGAP with coiled-coil, ankyrin repeat and PH domains 1 (Acap1), mRNA [NM_001105796]	**6,301**
Nin	Rattus norvegicus ninein (GSK3B interacting protein) (Nin), mRNA [NM_001106737]	**6,298**
0	Rattus norvegicus similar to Hypothetical protein LOC216150 (LOC365006), mRNA [XM_344770]	**6,297**
0	Unknown	**6,297**
Sfrs14	Rattus norvegicus splicing factor, arginine/serine-rich 14 (Sfrs14), mRNA [NM_001108397]	**6,296**
Prima1	Rattus norvegicus proline rich membrane anchor 1 (Prima1), mRNA [NM_001108721]	**6,295**
RGD1304879	Rattus norvegicus similar to Zinc finger protein 398 (Zinc finger DNA binding protein p52/p71) (RGD1304879), mRNA [NM_001014056]	**6,295**
0	Unknown	**6,295**
Rpia	Rattus norvegicus ribose 5-phosphate isomerase A (Rpia), mRNA [NM_001108632]	**6,294**
Ttk	Rattus norvegicus Ttk protein kinase (Ttk), mRNA [NM_001108172]	**6,294**

Zbtb9	Rattus norvegicus zinc finger and BTB domain containing 9 (Zbtb9), mRNA [NM_213564]	**6,293**
0	Unknown	**6,293**
RGD1311406	PREDICTED: Rattus norvegicus similar to RIKEN cDNA B430306N03 gene (RGD1311406), mRNA [XM_001063911]	**6,293**
0	Unknown	**6,293**
Ddr1	Rattus norvegicus discoidin domain receptor tyrosine kinase 1 (Ddr1), transcript variant 1, mRNA [NM_013137]	**6,293**
Rassf7	Rattus norvegicus Ras association (RalGDS/AF-6) domain family (N-terminal) member 7 (Rassf7), mRNA [NM_001106317]	**6,292**
0	Q3U348_MOUSE (Q3U348) NOD-derived CD11c +ve dendritic cells cDNA, RIKEN full-length enriched library, clone:F630112C03 product:Rap2 interacting protein x homolog, partial (31%) [TC632655]	**6,292**
Il17rd	Rattus norvegicus interleukin 17 receptor D (Il17rd), mRNA [NM_001191937]	**6,292**
Duox2	Rattus norvegicus dual oxidase 2 (Duox2), mRNA [NM_024141]	**6,291**
Smarce1	Rattus norvegicus SWI/SNF related, matrix associated, actin dependent regulator of chromatin, subfamily e, member 1 (Smarce1), mRNA [NM_001024993]	**6,291**
LOC500625	Rattus norvegicus hypothetical protein LOC500625 (LOC500625), mRNA [NM_001025151]	**6,291**
Rnf34	Rattus norvegicus ring finger protein 34 (Rnf34), mRNA [NM_001004075]	**6,291**
Tril	Rattus norvegicus TLR4 interactor with leucine-rich repeats (Tril), mRNA [NM_001034010]	**6,290**
Nsmce1	Rattus norvegicus non-SMC element 1 homolog (S. cerevisiae) (Nsmce1), mRNA [NM_001039611]	**6,289**
Tshr	Rattus norvegicus thyroid stimulating hormone receptor (Tshr), mRNA [NM_012888]	**6,288**
LOC679229	PREDICTED: Rattus norvegicus similar to zinc finger protein 397, transcript variant 2 (LOC679229), mRNA [XM_001055142]	**6,287**
Klhl9	Rattus norvegicus kelch-like 9 (Drosophila) (Klhl9), mRNA [NM_001107944]	**6,287**
Frem2	Uncharacterized protein [Source:UniProtKB/TrEMBL;Acc:D3ZG74] [ENSRNOT00000031487]	**6,287**
Atg10	Rattus norvegicus autophagy-related 10 (S. cerevisiae) (Atg10), mRNA [NM_001109505]	**6,286**
Itgad	Rattus norvegicus integrin, alpha D (Itgad), mRNA [NM_031691]	**6,286**
Rsph10b	Rattus norvegicus radial spoke head 10 homolog B (Chlamydomonas) (Rsph10b), mRNA [NM_001013867]	**6,286**
Fas	Rattus norvegicus Fas (TNF receptor superfamily, member 6) (Fas), mRNA [NM_139194]	**6,285**
0	PREDICTED: Rattus norvegicus maltase-glucoamylase (Mgam), mRNA [XM_231714]	**6,285**
LOC679861	AGENCOURT_114819228 NIH_MGC_433 Rattus norvegicus cDNA clone IMAGE:9098661 5', mRNA sequence [EV777635]	**6,285**
0	GAPR1_MOUSE (Q9CYL5) Golgi-associated plant pathogenesis-related protein 1 (Golgi-associated PR-1 protein) (GAPR-1) (Glioma pathogenesis-related protein 2) (GliPR 2), complete [TC625589]	**6,284**
Gfra2	Rattus norvegicus GDNF family receptor alpha 2 (Gfra2), mRNA [NM_012750]	**6,284**
LOC685106	PREDICTED: Rattus norvegicus similar to ribosomal protein L6 (LOC685106), mRNA [XM_001062312]	**6,282**
Mrpl51	Rattus norvegicus mitochondrial ribosomal protein L51 (Mrpl51), nuclear gene encoding mitochondrial protein, mRNA [NM_001106621]	**6,280**
Prmt8	PREDICTED: Rattus norvegicus similar to Protein arginine N-methyltransferase 4 (Heterogeneous nuclear ribonucleoprotein methyltransferase-like protein 4) (LOC688502), mRNA [XM_002726433]	**6,280**
Asb11	Rattus norvegicus ankyrin repeat and SOCS box-containing 11 (Asb11), mRNA [NM_001106962]	**6,280**

0	Ryanodine receptor type 3 [Source:UniProtKB/TrEMBL;Acc:Q9R273] [ENSRNOT00000049582]	**6,279**
Thap3	Rattus norvegicus THAP domain containing, apoptosis associated protein 3 (Thap3), mRNA [NM_001108695]	**6,279**
Lage3	Rattus norvegicus L antigen family, member 3 (Lage3), mRNA [NM_001106345]	**6,277**
RGD1564325	40S ribosomal protein S24 [Source:UniProtKB/TrEMBL;Acc:D3ZFZ8] [ENSRNOT00000047252]	**6,277**
Slco2b1	Rattus norvegicus solute carrier organic anion transporter family, member 2b1 (Slco2b1), mRNA [NM_080786]	**6,276**
0	Unknown	**6,276**
0	Unknown	**6,275**
Spdef	Rattus norvegicus SAM pointed domain containing ets transcription factor (Spdef), mRNA [NM_001109530]	**6,275**
St13	Rattus norvegicus suppression of tumorigenicity 13 (St13), mRNA [NM_031122]	**6,275**
Ecg2	Rattus norvegicus esophagus cancer-related protein 2 (Ecg2), mRNA [NM_001002816]	**6,274**
Btla	Rattus norvegicus B and T lymphocyte associated (Btla), mRNA [NM_213630]	**6,274**
RGD1309808	Rattus norvegicus similar to apolipoprotein L2; apolipoprotein L-II (RGD1309808), mRNA [NM_001134801]	**6,274**
Smarcb1	Rattus norvegicus SWI/SNF related, matrix associated, actin dependent regulator of chromatin, subfamily b, member 1 (Smarcb1), mRNA [NM_001025728]	**6,274**
Fam198b	Rattus norvegicus family with sequence similarity 198, member B (Fam198b), mRNA [NM_199105]	**6,273**
0	Q4FAC5_VIBAL (Q4FAC5) Collagenase, partial (3%) [TC596950]	**6,272**
Olr1083	Rattus norvegicus olfactory receptor 1083 (Olr1083), mRNA [NM_001000710]	**6,272**
RGD1561795	Rattus norvegicus similar to RIKEN cDNA 1700012B09 (RGD1561795), mRNA [NM_001109289]	**6,272**
Gja3	Rattus norvegicus gap junction protein, alpha 3 (Gja3), mRNA [NM_024376]	**6,272**
Pigp	Rattus norvegicus phosphatidylinositol glycan anchor biosynthesis, class P (Pigp), mRNA [NM_001099758]	**6,272**
Cnga1	Rattus norvegicus cyclic nucleotide gated channel alpha 1 (Cnga1), mRNA [NM_053497]	**6,271**
Ddx47	Rattus norvegicus DEAD (Asp-Glu-Ala-Asp) box polypeptide 47 (Ddx47), mRNA [NM_001015005]	**6,271**
0	Rattus norvegicus TL0AEA64YE20 mRNA sequence. [FQ232826]	**6,270**
Obox5	Uncharacterized protein [Source:UniProtKB/TrEMBL;Acc:D3ZYG1] [ENSRNOT00000045660]	**6,269**
Mtmr15	Rattus norvegicus myotubularin related protein 15 (Mtmr15), mRNA [NM_001191633]	**6,269**
0	Rattus norvegicus, 23 clones, strain BN/SsNHsdMCW RNOR03327657, whole genome shotgun sequence [AABR03128419]	**6,269**
0	Unknown	**6,267**
Saa4	Rattus norvegicus serum amyloid A4, constitutive (Saa4), mRNA [NM_001009478]	**6,267**
Parn	PREDICTED: Rattus norvegicus poly(A)-specific ribonuclease (deadenylation nuclease) (Parn), mRNA [XM_001075629]	**6,265**
Ccdc32	Rattus norvegicus coiled-coil domain containing 32 (Ccdc32), mRNA [NM_001024245]	**6,265**
Uck1	Rattus norvegicus uridine-cytidine kinase 1 (Uck1), mRNA [NM_001107831]	**6,264**
0	Unknown	**6,263**
Vapb	Rattus norvegicus VAMP (vesicle-associated membrane protein)-associated protein B and C (Vapb), mRNA [NM_021847]	**6,262**
0	Unknown	**6,262**

Trim2	Rattus norvegicus tripartite motif-containing 2 (Trim2), mRNA [NM_001108552]	**6,262**
Rtn4	Rattus norvegicus reticulon 4 (Rtn4), mRNA [NM_031831]	**6,262**
0	Unknown	**6,262**
0	Uncharacterized protein [Source:UniProtKB/TrEMBL;Acc:D4AD38] [ENSRNOT00000036164]	**6,261**
LOC681740	PREDICTED: Rattus norvegicus similar to jumonji protein, transcript variant 4 (LOC681740), mRNA [XM_001056897]	**6,259**
0	Unknown	**6,258**
Rnf111	Rattus norvegicus ring finger protein 111 (Rnf111), mRNA [NM_001106836]	**6,258**
0	PREDICTED: Rattus norvegicus similar to high mobility group nucleosomal binding domain 1 (LOC680964), mRNA [XM_001059675]	**6,258**
Shc2	Rattus norvegicus SHC (Src homology 2 domain containing) transforming protein 2 (Shc2), mRNA [NM_001108065]	**6,257**
Isca1	Rattus norvegicus iron-sulfur cluster assembly 1 homolog (S. cerevisiae) (Isca1), mRNA [NM_181626]	**6,257**
LOC681152	PREDICTED: Rattus norvegicus similar to cytoplasmic beta-actin (LOC681152), mRNA [XM_001060515]	**6,256**
Galr2	Rattus norvegicus galanin receptor 2 (Galr2), mRNA [NM_019172]	**6,256**
Mrps11	Rattus norvegicus mitochondrial ribosomal protein S11 (Mrps11), nuclear gene encoding mitochondrial protein, mRNA [NM_001109148]	**6,256**
RGD1561796	Uncharacterized protein [Source:UniProtKB/TrEMBL;Acc:D3ZS66] [ENSRNOT00000068577]	**6,256**
Tbcd	PREDICTED: Rattus norvegicus tubulin-specific chaperone d (Tbcd), partial mRNA [XM_340955]	**6,256**
0	Unknown	**6,255**
Magi3	Rattus norvegicus membrane associated guanylate kinase, WW and PDZ domain containing 3 (Magi3), mRNA [NM_139084]	**6,255**
Olr1435	Rattus norvegicus olfactory receptor 1435 (Olr1435), mRNA [NM_001000777]	**6,255**
Lonrf3	Rattus norvegicus LON peptidase N-terminal domain and ring finger 3 (Lonrf3), mRNA [NM_001191585]	**6,254**
Krtap19-3	PREDICTED: Rattus norvegicus keratin associated protein 19-3 (Krtap19-3), mRNA [XM_001063382]	**6,253**
Mettl9	Rattus norvegicus methyltransferase like 9 (Mettl9), mRNA [NM_001163164]	**6,252**
Echdc1	Rattus norvegicus enoyl Coenzyme A hydratase domain containing 1 (Echdc1), mRNA [NM_001007734]	**6,250**
0	Unknown	**6,250**
0	Unknown	**6,249**
0	Uncharacterized protein [Source:UniProtKB/TrEMBL;Acc:D3ZDB2] [ENSRNOT00000068478]	**6,248**
0	Unknown	**6,248**
Capn2	Rattus norvegicus calpain 2 (Capn2), mRNA [NM_017116]	**6,248**
Spats2l	Rattus norvegicus spermatogenesis associated, serine-rich 2-like (Spats2l), mRNA [NM_001014102]	**6,248**
Bat4	Rattus norvegicus HLA-B associated transcript 4 (Bat4), mRNA [NM_001034157]	**6,247**
Ccdc33	Rattus norvegicus coiled-coil domain containing 33 (Ccdc33), mRNA [NM_001014091]	**6,247**
Rbms1	Rattus norvegicus RNA binding motif, single stranded interacting protein 1 (Rbms1), mRNA [NM_001012184]	**6,247**
Mfsd6	Rattus norvegicus major facilitator superfamily domain containing 6 (Mfsd6), mRNA [NM_001106911]	**6,246**
Inpp5a	Rattus norvegicus inositol polyphosphate-5-phosphatase A (Inpp5a), mRNA [NM_001108923]	**6,246**
0	Unknown	**6,246**

Prok1	Rattus norvegicus prokineticin 1 (Prok1), mRNA [NM_138851]	**6,243**
Cflar	Rattus norvegicus CASP8 and FADD-like apoptosis regulator, mRNA (cDNA clone MGC:108616 IMAGE:7376876), complete cds. [BC089781]	**6,243**
Ror1	Rattus norvegicus receptor tyrosine kinase-like orphan receptor 1 (Ror1), mRNA [NM_001108671]	**6,242**
0	Uncharacterized protein [Source:UniProtKB/TrEMBL;Acc:D3ZG39] [ENSRNOT00000058806]	**6,241**
Ppp1cc	Rattus norvegicus protein phosphatase 1, catalytic subunit, gamma isoform (Ppp1cc), mRNA [NM_022498]	**6,241**
0	Unknown	**6,240**
0	Unknown	**6,240**
RGD1306058	Rattus norvegicus similar to RIKEN cDNA 1110007C09 (RGD1306058), mRNA [NM_001134572]	**6,240**
LOC684745	PREDICTED: Rattus norvegicus similar to germinal histone H4 gene (LOC684745), mRNA [XM_001071818]	**6,239**
Ndufaf4	Rattus norvegicus NADH dehydrogenase (ubiquinone) 1 alpha subcomplex, assembly factor 4 (Ndufaf4), nuclear gene encoding mitochondrial protein, mRNA [NM_198783]	**6,239**
Pcnxl2	Uncharacterized protein [Source:UniProtKB/TrEMBL;Acc:D4AB99] [ENSRNOT00000030077]	**6,238**
Dip2c	Rattus norvegicus DIP2 disco-interacting protein 2 homolog C (Drosophila) (Dip2c), mRNA [NM_001107360]	**6,238**
0	RGD1565425 protein [Source:UniProtKB/TrEMBL;Acc:B0BMZ8] [ENSRNOT00000065777]	**6,238**
Hs3st3a1	Rattus norvegicus heparan sulfate (glucosamine) 3-O-sulfotransferase 3A1 (Hs3st3a1), mRNA [NM_001172067]	**6,238**
Plac1l	PREDICTED: Rattus norvegicus placenta-specific 1-like (Plac1l), mRNA [XM_001076035]	**6,237**
Rbm24	Rattus norvegicus RNA binding motif protein 24 (Rbm24), mRNA [NM_001191100]	**6,237**
RGD1304931	Rattus norvegicus similar to RIKEN cDNA 2310042D19 (RGD1304931), mRNA [NM_001108001]	**6,236**
0	Unknown	**6,235**
Rtp1	Rattus norvegicus receptor (chemosensory) transporter protein 1 (Rtp1), mRNA [NM_001105870]	**6,233**
0	Uncharacterized protein [Source:UniProtKB/TrEMBL;Acc:D3ZSP3] [ENSRNOT00000068004]	**6,232**
Hspb1	Rattus norvegicus heat shock protein 1 (Hspb1), mRNA [NM_031970]	**6,231**
RGD1308261	Rattus norvegicus similar to RIKEN cDNA 1190005P17 (RGD1308261), mRNA [NM_001134464]	**6,229**
RGD1563863	Rattus norvegicus similar to expressed sequence AI553587 (RGD1563863), mRNA [NM_001127559]	**6,228**
0	Unknown	**6,227**
Scg2	Rattus norvegicus secretogranin II (chromogranin C) (Scg2), mRNA [NM_022669]	**6,227**
Cabp1	Rattus norvegicus calcium binding protein 1 (Cabp1), transcript variant 3, mRNA [NM_001033676]	**6,225**
LOC292449	PREDICTED: Rattus norvegicus similar to hypothetical protein (LOC292449), miscRNA [XR_085688]	**6,225**
0	Unknown	**6,225**
Fbn2	Rattus norvegicus fibrillin 2 (Fbn2), mRNA [NM_031826]	**6,224**
Rhebl1	Rattus norvegicus Ras homolog enriched in brain like 1 (Rhebl1), mRNA [NM_182825]	**6,224**
Gpatc2	Rattus norvegicus G patch domain containing 2 (Gpatc2), mRNA [NM_001011909]	**6,224**
Gmfg	Rattus norvegicus glia maturation factor, gamma (Gmfg), mRNA [NM_181091]	**6,224**
RGD1563979	PREDICTED: Rattus norvegicus similar to over-expressed breast tumor protein (RGD1563979), mRNA [XM_001064610]	**6,223**

Dll1	Rattus norvegicus delta-like 1 (Drosophila) (Dll1), mRNA [NM_032063]	**6,223**
Ankrd12	Rattus norvegicus ankyrin repeat domain 12 (Ankrd12), mRNA [NM_001108238]	**6,223**
Rp1	Rattus norvegicus retinitis pigmentosa 1 (human) (Rp1), mRNA [NM_001195676]	**6,223**
Gramd1a	Rattus norvegicus GRAM domain containing 1A (Gramd1a), mRNA [NM_001014160]	**6,222**
0	Homeobox protein cut-like 1 [Source:UniProtKB/Swiss-Prot;Acc:P53565] [ENSRNOT00000044841]	**6,222**
Znf483	Rattus norvegicus zinc finger protein 483 (Znf483), mRNA [NM_133422]	**6,222**
Rnf138	Rattus norvegicus ring finger protein 138 (Rnf138), mRNA [NM_053588]	**6,221**
Dusp4	Rattus norvegicus dual specificity phosphatase 4 (Dusp4), mRNA [NM_022199]	**6,218**
0	Unknown	**6,218**
LOC100366132	PREDICTED: Rattus norvegicus Glyceraldehyde-3-phosphate dehydrogenase-like (LOC100366132), mRNA [XM_002726560]	**6,217**
RGD1563667	Rattus norvegicus similar to TDPOZ3 (RGD1563667), mRNA [NM_001100985]	**6,216**
Zscan18	Uncharacterized protein [Source:UniProtKB/TrEMBL;Acc:D3ZCL4] [ENSRNOT00000036880]	**6,216**
0	Unknown	**6,216**
LOC681140	PREDICTED: Rattus norvegicus similar to 14-3-3 protein theta (14-3-3 protein tau) (14-3-3 protein T-cell) (HS1 protein) (LOC681140), mRNA [XM_001060456]	**6,216**
Arpp21	Rattus norvegicus cAMP-regulated phosphoprotein 21 (Arpp21), transcript variant 2, mRNA [NM_001135045]	**6,215**
0	Unknown	**6,215**
LOC684681	PREDICTED: Rattus norvegicus similar to Histone H1.2 (H1 VAR.1) (H1c) (LOC684681), mRNA [XM_001071565]	**6,214**
0	Rattus norvegicus TL0ADA12YN23 mRNA sequence. [FQ223309]	**6,214**
Bicd2	Rattus norvegicus bicaudal D homolog 2 (Drosophila) (Bicd2), transcript variant 1, mRNA [NM_001033674]	**6,214**
0	Unknown	**6,213**
mrpl11	Rattus norvegicus mitochondrial ribosomal protein L11 (mrpl11), nuclear gene encoding mitochondrial protein, mRNA [NM_001006973]	**6,213**
Cxadr	Rattus norvegicus coxsackie virus and adenovirus receptor (Cxadr), mRNA [NM_053570]	**6,212**
Ndufab1	Rattus norvegicus NADH dehydrogenase (ubiquinone) 1, alpha/beta subcomplex, 1 (Ndufab1), nuclear gene encoding mitochondrial protein, mRNA [NM_001106294]	**6,210**
0	Vesicle transport through interaction with t-SNAREs homolog 1B [Source:UniProtKB/Swiss-Prot;Acc:P58200] [ENSRNOT00000015814]	**6,210**
Pnn	Rattus norvegicus pinin, desmosome associated protein (Pnn), mRNA [NM_001109023]	**6,209**
Mcc	Rattus norvegicus mutated in colorectal cancers (Mcc), mRNA [NM_001170534]	**6,208**
Chrdl1	Rattus norvegicus kohjirin (Chrdl1), mRNA [NM_199502]	**6,207**
Lrmp	PREDICTED: Rattus norvegicus lymphoid-restricted membrane protein (Lrmp), mRNA [XM_001074768]	**6,206**
0	Uncharacterized protein [Source:UniProtKB/TrEMBL;Acc:D4A273] [ENSRNOT00000007422]	**6,205**
RGD1566369	40S ribosomal protein S8 [Source:UniProtKB/TrEMBL;Acc:D3ZIE1] [ENSRNOT00000006662]	**6,205**
Plcg1	Rattus norvegicus phospholipase C, gamma 1 (Plcg1), mRNA [NM_013187]	**6,205**
0	LOC499746 protein [Source:UniProtKB/TrEMBL;Acc:Q498T7] [ENSRNOT00000062007]	**6,205**

0	Unknown	**6,204**
Dmbx1	Rattus norvegicus diencephalon/mesencephalon homeobox 1 (Dmbx1), mRNA [NM_001107961]	**6,204**
Ccdc152	Rattus norvegicus coiled-coil domain containing 152 (Ccdc152), mRNA [NM_001191959]	**6,204**
RGD1559999	PREDICTED: Rattus norvegicus similar to DNA segment, Chr 5, ERATO Doi 577, expressed (RGD1559999), mRNA [XM_001069829]	**6,204**
Exosc9	Rattus norvegicus exosome component 9 (Exosc9), mRNA [NM_001025406]	**6,203**
Akap13	Rattus norvegicus A kinase (PRKA) anchor protein 13 (Akap13), mRNA [NM_001106271]	**6,203**
Arse	Rattus norvegicus arylsulfatase E (chondrodysplasia punctata 1) (Arse), mRNA [NM_001047885]	**6,202**
Cript	Rattus norvegicus cysteine-rich PDZ-binding protein (Cript), mRNA [NM_019907]	**6,202**
LOC691725	PREDICTED: Rattus norvegicus similar to Nucleolar RNA helicase 2 (Nucleolar RNA helicase II) (Nucleolar RNA helicase Gu) (RH II/Gu) (Gu-alpha) (DEAD box protein 21) (LOC691725), miscRNA [XR_086290]	**6,202**
0	Unknown	**6,202**
Supt7l	Rattus norvegicus suppressor of Ty 7 (S. cerevisiae)-like (Supt7l), mRNA [NM_001108010]	**6,201**
RGD1304827	Rattus norvegicus hypothetical LOC316091 (RGD1304827), mRNA [NM_001009437]	**6,201**
LOC688916	PREDICTED: Rattus norvegicus hypothetical protein LOC688916 (LOC688916), mRNA [XM_001068819]	**6,200**
LOC689725	Uncharacterized protein [Source:UniProtKB/TrEMBL;Acc:D3ZCW3] [ENSRNOT00000037765]	**6,198**
LOC680097	PREDICTED: Rattus norvegicus similar to germinal histone H4 gene (LOC680097), mRNA [XM_001055696]	**6,198**
0	Uncharacterized protein [Source:UniProtKB/TrEMBL;Acc:D3ZQ99] [ENSRNOT00000046318]	**6,197**
Fzd2	Rattus norvegicus frizzled homolog 2 (Drosophila) (Fzd2), mRNA [NM_172035]	**6,196**
Ddrgk1	Rattus norvegicus DDRGK domain containing 1 (Ddrgk1), mRNA [NM_001106512]	**6,196**
Lactb2	Rattus norvegicus lactamase, beta 2 (Lactb2), mRNA [NM_001024247]	**6,196**
Cul5	Rattus norvegicus cullin 5 (Cul5), mRNA [NM_022683]	**6,194**
Slu7	Rattus norvegicus SLU7 splicing factor homolog (S. cerevisiae) (Slu7), mRNA [NM_001100550]	**6,194**
Myeov2	Rattus norvegicus myeloma overexpressed 2 (Myeov2), mRNA [NM_001109044]	**6,194**
Ddr2	Rattus norvegicus discoidin domain receptor tyrosine kinase 2 (Ddr2), mRNA [NM_031764]	**6,193**
Zfp3	Rattus norvegicus zinc finger protein 3 (Zfp3), mRNA [NM_001109034]	**6,192**
Fadd	Rattus norvegicus Fas (TNFRSF6)-associated via death domain (Fadd), mRNA [NM_152937]	**6,192**
0	Uncharacterized protein [Source:UniProtKB/TrEMBL;Acc:D3ZRI2] [ENSRNOT00000012282]	**6,192**
Pitpnm2	Rattus norvegicus phosphatidylinositol transfer protein, membrane-associated 2 (Pitpnm2), mRNA [NM_001107139]	**6,191**
Tgm4	Rattus norvegicus transglutaminase 4 (prostate) (Tgm4), mRNA [NM_022713]	**6,191**
Actg1	Rattus norvegicus actin, gamma 1 (Actg1), mRNA [NM_001127449]	**6,190**
Myo10	Rattus norvegicus myosin X (Myo10), mRNA [NM_001107657]	**6,189**
Usp35	PREDICTED: Rattus norvegicus ubiquitin specific peptidase 35 (Usp35), mRNA [XM_001064027]	**6,189**
0	Uncharacterized protein [Source:UniProtKB/TrEMBL;Acc:D3ZHI0] [ENSRNOT00000061428]	**6,186**
RGD1309350	Rattus norvegicus similar to transthyretin (4L369) (RGD1309350), mRNA [NM_001134507]	**6,184**

Tmem27	Rattus norvegicus transmembrane protein 27 (Tmem27), mRNA [NM_020976]	**6,184**
Fam108a1	Rattus norvegicus family with sequence similarity 108, member A1 (Fam108a1), mRNA [NM_001006983]	**6,183**
Pold3	Rattus norvegicus polymerase (DNA-directed), delta 3, accessory subunit (Pold3), mRNA [NM_001024750]	**6,182**
0	Unknown	**6,181**
RGD1563613	Uncharacterized protein [Source:UniProtKB/TrEMBL;Acc:D4AE10] [ENSRNOT00000044779]	**6,181**
Zfp335	Uncharacterized protein [Source:UniProtKB/TrEMBL;Acc:D3ZXD5] [ENSRNOT00000023521]	**6,180**
Unc13c	Rattus norvegicus unc-13 homolog C (C. elegans) (Unc13c), mRNA [NM_173146]	**6,180**
0	Unknown	**6,178**
Casp2	Rattus norvegicus caspase 2 (Casp2), mRNA [NM_022522]	**6,178**
LOC682138	PREDICTED: Rattus norvegicus similar to DEAD (Asp-Glu-Ala-Asp) box polypeptide 43 (LOC682138), mRNA [XM_001060057]	**6,177**
Rem2	Rattus norvegicus RAS (RAD and GEM) like GTP binding 2 (Rem2), mRNA [NM_022685]	**6,177**
RGD1562612	PREDICTED: Rattus norvegicus similar to RIKEN cDNA 1110003E01 (RGD1562612), mRNA [XM_577381]	**6,176**
Rabepk	Rattus norvegicus Rab9 effector protein with kelch motifs (Rabepk), mRNA [NM_001024871]	**6,175**
Ppm1b	Rattus norvegicus protein phosphatase 1B, magnesium dependent, beta isoform (Ppm1b), mRNA [NM_033096]	**6,175**
0	Unknown	**6,175**
Abcb4	Rattus norvegicus ATP-binding cassette, subfamily B (MDR/TAP), member 4 (Abcb4), mRNA [NM_012690]	**6,175**
Wwc1	PREDICTED: Rattus norvegicus WW and C2 domain containing 1, transcript variant 2 (Wwc1), mRNA [XM_002724669]	**6,173**
Fam49a	Rattus norvegicus family with sequence similarity 49, member A (Fam49a), mRNA [NM_001106718]	**6,173**
0	Uncharacterized protein [Source:UniProtKB/TrEMBL;Acc:D3ZUC5] [ENSRNOT00000046312]	**6,173**
Alas1	Rattus norvegicus aminolevulinate, delta-, synthase 1 (Alas1), mRNA [NM_024484]	**6,171**
Gon4l	Rattus norvegicus gon-4-like (C. elegans) (Gon4l), mRNA [NM_001024797]	**6,171**
Zbtb20	Rattus norvegicus zinc finger and BTB domain containing 20 (Zbtb20), mRNA [NM_001105880]	**6,170**
Traf7	Rattus norvegicus Tnf receptor-associated factor 7 (Traf7), mRNA [NM_001127548]	**6,170**
RGD1566036	Rattus norvegicus similar to RIKEN cDNA 2310008H04 (RGD1566036), mRNA [NM_001100988]	**6,168**
Mtor	Rattus norvegicus mechanistic target of rapamycin (serine/threonine kinase) (Mtor), mRNA [NM_019906]	**6,168**
Dbx2	Homeodomain protein Dbx2 [Source:UniProtKB/TrEMBL;Acc:Q5DWV0] [ENSRNOT00000009143]	**6,167**
Prmt5	Rattus norvegicus protein arginine methyltransferase 5 (Prmt5), mRNA [NM_001108867]	**6,167**
N6amt1	Rattus norvegicus N-6 adenine-specific DNA methyltransferase 1 (putative) (N6amt1), mRNA [NM_001191592]	**6,166**
Vip	Rattus norvegicus vasoactive intestinal peptide (Vip), mRNA [NM_053991]	**6,165**
Zbtb39	Rattus norvegicus zinc finger and BTB domain containing 39 (Zbtb39), mRNA [NM_001130537]	**6,165**
0	Unknown	**6,164**
Rab9a	Rattus norvegicus RAB9A, member RAS oncogene family (Rab9a), mRNA [NM_053458]	**6,163**
0	Unknown	**6,163**
Haus3	Rattus norvegicus HAUS augmin-like complex, subunit 3 (Haus3), mRNA [NM_001109412]	**6,160**

Slc40a1	Rattus norvegicus solute carrier family 39 (iron-regulated transporter), member 1 (Slc40a1), mRNA [NM_133315]	**6,160**
Abcb1b	Rattus norvegicus ATP-binding cassette, subfamily B (MDR/TAP), member 1B (Abcb1b), mRNA [NM_012623]	**6,159**
Atp5i	Rattus norvegicus ATP synthase, H+ transporting, mitochondrial F0 complex, subunit E (Atp5i), nuclear gene encoding mitochondrial protein, mRNA [NM_080481]	**6,159**
0	PREDICTED: Rattus norvegicus 40S ribosomal protein SA-like (LOC100365517), mRNA [XM_002726683]	**6,158**
Bcap29	Rattus norvegicus B-cell receptor-associated protein 29 (Bcap29), mRNA [NM_001006980]	**6,158**
Aacs	Rattus norvegicus acetoacetyl-CoA synthetase (Aacs), mRNA [NM_023104]	**6,158**
Kcnab1	Rattus norvegicus potassium voltage-gated channel, shaker-related subfamily, beta member 1 (Kcnab1), mRNA [NM_017303]	**6,158**
Glra2	Rattus norvegicus glycine receptor, alpha 2 (Glra2), mRNA [NM_012568]	**6,155**
Map7d1	Rattus norvegicus MAP7 domain containing 1 (Map7d1), mRNA [NM_001109438]	**6,155**
Slc12a7	Rattus norvegicus solute carrier family 12 (potassium/chloride transporters), member 7 (Slc12a7), mRNA [NM_001013144]	**6,154**
LOC684623	Uncharacterized protein [Source:UniProtKB/TrEMBL;Acc:D3ZKJ1] [ENSRNOT00000003659]	**6,154**
Slc35e4	Rattus norvegicus solute carrier family 35, member E4 (Slc35e4), mRNA [NM_153316]	**6,154**
Nkx6-3	Rattus norvegicus NK6 homeobox 3 (Nkx6-3), mRNA [NM_001109455]	**6,154**
0	Unknown	**6,153**
Tmem194b	Rattus norvegicus transmembrane protein 194B (Tmem194b), mRNA [NM_001134642]	**6,153**
Mapk13	Rattus norvegicus mitogen activated protein kinase 13 (Mapk13), mRNA [NM_019231]	**6,153**
RGD1310861	Rattus norvegicus similar to RIKEN cDNA 1500011H22 (RGD1310861), mRNA [NM_001008290]	**6,153**
Cd97	Rattus norvegicus CD97 molecule (Cd97), mRNA [NM_001012164]	**6,152**
Rgnef	Rattus norvegicus Rho-guanine nucleotide exchange factor (Rgnef), mRNA [NM_001108542]	**6,151**
Crlf3	Rattus norvegicus unknown mRNA sequence. [AF072835]	**6,151**
Elavl2	Rattus norvegicus ELAV (embryonic lethal, abnormal vision, Drosophila)-like 2 (Hu antigen B) (Elavl2), mRNA [NM_173309]	**6,149**
0	Unknown	**6,148**
Cyp2a1	Rattus norvegicus cytochrome P450, family 2, subfamily a, polypeptide 1 (Cyp2a1), mRNA [NM_012692]	**6,148**
Dner	PREDICTED: Rattus norvegicus delta/notch-like EGF repeat containing (Dner), mRNA [XM_001064351]	**6,147**
Ngfrap1	Rattus norvegicus nerve growth factor receptor (TNFRSF16) associated protein 1 (Ngfrap1), mRNA [NM_053401]	**6,147**
0	Unknown	**6,146**
Nif3l1	Rattus norvegicus NIF3 NGG1 interacting factor 3-like 1 (S. pombe) (Nif3l1), mRNA [NM_001024763]	**6,146**
Olr13	Rattus norvegicus olfactory receptor 13 (Olr13), mRNA [NM_001000116]	**6,146**
Rpl24	Rattus norvegicus ribosomal protein L24 (Rpl24), mRNA [NM_022515]	**6,144**
LOC679934	Rattus norvegicus similar to chromosome 3 open reading frame 10 (LOC679934), mRNA [NM_001195476]	**6,143**
0	Unknown	**6,143**
Kcnj14	Rattus norvegicus potassium inwardly-rectifying channel, subfamily J, member 14 (Kcnj14), mRNA [NM_170718]	**6,143**
0	Unknown	**6,142**

Skp2	Rattus norvegicus S-phase kinase-associated protein 2 (p45) (Skp2), mRNA [NM_001106416]	**6,142**
Atp6v0d1	Rattus norvegicus ATPase, H+ transporting, lysosomal V0 subunit D1 (Atp6v0d1), mRNA [NM_001011927]	**6,141**
LOC679824	PREDICTED: Rattus norvegicus hypothetical protein LOC679824 (LOC679824), miscRNA [XR_085739]	**6,141**
LOC683410	PREDICTED: Rattus norvegicus similar to ankyrin repeat domain 33, transcript variant 2 (LOC683410), mRNA [XM_002726976]	**6,140**
Irf1	Rattus norvegicus interferon regulatory factor 1 (Irf1), mRNA [NM_012591]	**6,140**
Tle1	Rattus norvegicus transducin-like enhancer of split 1 (E(sp1) homolog, Drosophila) (Tle1), mRNA [NM_001173433]	**6,139**
Ccdc88b	Rattus norvegicus coiled-coil domain containing 88B (Ccdc88b), mRNA [NM_001108517]	**6,139**
0	Unknown	**6,138**
0	Unknown	**6,137**
Ercc5	Rattus norvegicus excision repair cross-complementing rodent repair deficiency, complementation group 5 (Ercc5), mRNA [NM_001106910]	**6,137**
Ube2q1	Rattus norvegicus ubiquitin-conjugating enzyme E2Q family member 1 (Ube2q1), mRNA [NM_001106448]	**6,136**
LOC501307	Rattus norvegicus similar to spermatogenesis associated glutamate (E)-rich protein 4d, mRNA (cDNA clone IMAGE:7310837). [BC088860]	**6,136**
0	large tumor suppressor Gene [Source:MGI Symbol;Acc:MGI:1333883] [ENSRNOT00000020098]	**6,136**
Pip5k1b	Rattus norvegicus phosphatidylinositol-4-phosphate 5-kinase, type I, beta (Pip5k1b), mRNA [NM_001012743]	**6,136**
Tfeb	Rattus norvegicus transcription factor EB (Tfeb), mRNA [NM_001025707]	**6,135**
Gnb1l	Uncharacterized protein [Source:UniProtKB/TrEMBL;Acc:D3ZSP4] [ENSRNOT00000002596]	**6,134**
0	CD209 antigen-like protein C [Source:RefSeq peptide;Acc:NP_001178024] [ENSRNOT00000061750]	**6,133**
Spetex-2A	Rattus norvegicus Spetex-2A protein (Spetex-2A), mRNA [NM_001009976]	**6,131**
Tnfsf13b	Rattus norvegicus tumor necrosis factor (ligand) superfamily, member 13b (Tnfsf13b), mRNA [NM_001109112]	**6,131**
0	PREDICTED: Rattus norvegicus pro-histogranin (LOC502940), miscRNA [XR_009615]	**6,131**
0	Rattus norvegicus similar to putative protein kinase (LOC292372), mRNA [XM_217965]	**6,130**
Cdc20	Rattus norvegicus cell division cycle 20 homolog (S. cerevisiae) (Cdc20), mRNA [NM_171993]	**6,129**
0	Unknown	**6,128**
Vps54	Rattus norvegicus vacuolar protein sorting 54 homolog (S. cerevisiae) (Vps54), mRNA [NM_173147]	**6,127**
Bub1	Rattus norvegicus budding uninhibited by benzimidazoles 1 homolog (S. cerevisiae) (Bub1), mRNA [NM_001106507]	**6,127**
Slc25a26	Rattus norvegicus solute carrier family 25, member 26 (Slc25a26), nuclear gene encoding mitochondrial protein, mRNA [NM_001108638]	**6,126**
LOC500959	Rattus norvegicus triosephosphate isomerase (LOC500959), mRNA [NM_001033072]	**6,126**
LOC100363289	Rattus norvegicus LRRGT00022-like (LOC100363289), mRNA [NM_001177825]	**6,125**
Lrrc1	Rattus norvegicus leucine rich repeat containing 1 (Lrrc1), mRNA [NM_001014268]	**6,124**
Tctex1d2	Rattus norvegicus Tctex1 domain containing 2 (Tctex1d2), mRNA [NM_001109054]	**6,124**
Bnip1	Rattus norvegicus BCL2/adenovirus E1B interacting protein 1 (Bnip1), mRNA [NM_080897]	**6,122**
Fbxo8	Rattus norvegicus F-box protein 8 (Fbxo8), mRNA [NM_001012050]	**6,122**
0	Unknown	**6,121**
LOC303590	PREDICTED: Rattus norvegicus similar to cactin CG1676-PA (LOC303590), miscRNA [XR_006385]	**6,121**

Hspa4l	Rattus norvegicus heat shock protein 4-like (Hspa4l), mRNA [NM_001106428]	**6,120**
Hmgcl	Rattus norvegicus 3-hydroxymethyl-3-methylglutaryl-Coenzyme A lyase (Hmgcl), nuclear gene encoding mitochondrial protein, mRNA [NM_024386]	**6,120**
Lipt2	Rattus norvegicus lipoyl(octanoyl) transferase 2 (putative) (Lipt2), nuclear gene encoding mitochondrial protein, mRNA [NM_001108917]	**6,117**
0	Uncharacterized protein [Source:UniProtKB/TrEMBL;Acc:D3ZHM7] [ENSRNOT00000036931]	**6,116**
Hist1h2ail	Rattus norvegicus histone cluster 1, H2ai-like (Hist1h2ail), mRNA [NM_001013056]	**6,115**
Brp16	Rattus norvegicus brain protein 16 (Brp16), mRNA [NM_001007707]	**6,113**
Zzz3	Rattus norvegicus zinc finger, ZZ-type containing 3 (Zzz3), mRNA [NM_001134549]	**6,112**
Pus3	Rattus norvegicus pseudouridylate synthase 3 (Pus3), mRNA [NM_001108134]	**6,112**
Chrd	Rattus norvegicus chordin (Chrd), mRNA [NM_057134]	**6,111**
Ralgds	Rattus norvegicus ral guanine nucleotide dissociation stimulator (Ralgds), mRNA [NM_019250]	**6,111**
LOC363337	Rattus norvegicus similar to RIKEN cDNA 1700081O22 (LOC363337), mRNA [NM_001014221]	**6,111**
Nkain4	Rattus norvegicus Na+/K+ transporting ATPase interacting 4 (Nkain4), mRNA [NM_001106550]	**6,111**
0	Unknown	**6,110**
Doc2a	Rattus norvegicus double C2-like domains, alpha (Doc2a), mRNA [NM_022937]	**6,110**
Wdr24	Rattus norvegicus WD repeat domain 24 (Wdr24), mRNA [NM_001191084]	**6,109**
0	Unknown	**6,109**
RGD1566215	Rattus norvegicus similar to Coatomer gamma-2 subunit (Gamma-2 coat protein) (Gamma-2 COP) (RGD1566215), mRNA [NM_001106929]	**6,108**
Dusp1	Rattus norvegicus dual specificity phosphatase 1 (Dusp1), mRNA [NM_053769]	**6,108**
St8sia4	Rattus norvegicus ST8 alpha-N-acetyl-neuraminide alpha-2,8-sialyltransferase 4 (St8sia4), mRNA [NM_053914]	**6,107**
0	YTH domain family protein 3 [Source:RefSeq peptide;Acc:NP_001102016] [ENSRNOT00000052093]	**6,106**
Adcy7	Rattus norvegicus adenylate cyclase 7 (Adcy7), mRNA [NM_053396]	**6,105**
Katnal2	PREDICTED: Rattus norvegicus katanin p60 subunit A-like 2 (Katnal2), mRNA [XM_001055413]	**6,105**
0	Unknown	**6,104**
LOC686041	Rattus norvegicus cDNA clone IMAGE:6889638, partial cds. [BC059164]	**6,104**
LOC689425	Rattus norvegicus hypothetical protein LOC689425 (LOC689425), mRNA [NM_001135002]	**6,102**
Anapc10	Rattus norvegicus anaphase promoting complex subunit 10 (Anapc10), mRNA [NM_001108445]	**6,102**
Sstr1	Rattus norvegicus somatostatin receptor 1 (Sstr1), mRNA [NM_012719]	**6,100**
Mlycd	Rattus norvegicus malonyl-CoA decarboxylase (Mlycd), nuclear gene encoding mitochondrial protein, mRNA [NM_053477]	**6,100**
Olr1162	Rattus norvegicus olfactory receptor 1162 (Olr1162), mRNA [NM_001000870]	**6,098**
Ndufv3	Rattus norvegicus NADH dehydrogenase (ubiquinone) flavoprotein 3 (Ndufv3), nuclear gene encoding mitochondrial protein, transcript variant 1, mRNA [NM_022607]	**6,098**
0	Uncharacterized protein [Source:UniProtKB/TrEMBL;Acc:D3ZGN8] [ENSRNOT00000049028]	**6,097**
0	Unknown	**6,096**

0	Unknown	**6,096**
Mia	Rattus norvegicus melanoma inhibitory activity (Mia), mRNA [NM_030852]	**6,094**
Mtdh	Rattus norvegicus metadherin (Mtdh), mRNA [NM_133398]	**6,094**
Nsun6	Rattus norvegicus NOL1/NOP2/Sun domain family, member 6 (Nsun6), mRNA [NM_001107367]	**6,094**
Jag2	PREDICTED: Rattus norvegicus jagged 2 (Jag2), mRNA [XM_001073124]	**6,094**
Col6a3	Rattus norvegicus procollagen, type VI, alpha 3 (Col6a3), mRNA [NM_001109008]	**6,092**
RGD1559748	Rattus norvegicus similar to Palate lung and nasal carcinoma-like protein precursor (Tongue plunc-like protein) (RGD1559748), mRNA [NM_001107792]	**6,092**
Trim45	Rattus norvegicus tripartite motif-containing 45 (Trim45), mRNA [NM_001106453]	**6,091**
Cby3	PREDICTED: Rattus norvegicus chibby homolog 3 (Drosophila) (Cby3), mRNA [XM_001074756]	**6,091**
Olr1475	Rattus norvegicus olfactory receptor 1475 (Olr1475), mRNA [NM_001000027]	**6,090**
Iqce	Uncharacterized protein [Source:UniProtKB/TrEMBL;Acc:D3ZUL9] [ENSRNOT00000051565]	**6,090**
Neto2	Neuropilin and tolloid-like protein 2 [Source:UniProtKB/Swiss-Prot;Acc:C6K2K4] [ENSRNOT00000021739]	**6,089**
0	Uncharacterized protein [Source:UniProtKB/TrEMBL;Acc:D3ZJP9] [ENSRNOT00000033716]	**6,089**
Triml2	Rattus norvegicus tripartite motif family-like 2 (Triml2), mRNA [NM_001191690]	**6,089**
Itga6	Integrin alpha 6 subchain [Source:UniProtKB/TrEMBL;Acc:Q924W3] [ENSRNOT00000002075]	**6,087**
Bcl6	Rattus norvegicus B-cell CLL/lymphoma 6 (Bcl6), mRNA [NM_001107084]	**6,087**
Gnb4	Rattus norvegicus guanine nucleotide binding protein (G protein), beta polypeptide 4 (Gnb4), mRNA [NM_001013910]	**6,085**
Utx	Uncharacterized protein [Source:UniProtKB/TrEMBL;Acc:D4ADF1] [ENSRNOT00000005742]	**6,084**
Yy1	Rattus norvegicus YY1 transcription factor (Yy1), mRNA [NM_173290]	**6,084**
LOC685909	Rattus norvegicus similar to H2A histone family, member V isoform 1 (LOC685909), mRNA [NM_001106019]	**6,083**
LOC686240	PREDICTED: Rattus norvegicus similar to NMDA receptor regulated 1-like, transcript variant 2 (LOC686240), mRNA [XM_001073064]	**6,082**
Dusp26	Rattus norvegicus dual specificity phosphatase 26 (putative) (Dusp26), mRNA [NM_001012352]	**6,082**
Ccdc90b	Rattus norvegicus coiled-coil domain containing 90B (Ccdc90b), mRNA [NM_001024885]	**6,082**
RGD1309765	Rattus norvegicus similar to hypothetical protein (RGD1309765), mRNA [NM_001169115]	**6,082**
Fam161a	Rattus norvegicus family with sequence similarity 161, member A (Fam161a), mRNA [NM_001013876]	**6,081**
Yme1l1	Rattus norvegicus YME1-like 1 (S. cerevisiae) (Yme1l1), nuclear gene encoding mitochondrial protein, mRNA [NM_053682]	**6,081**
Tph1	Rattus norvegicus tryptophan hydroxylase 1 (Tph1), mRNA [NM_001100634]	**6,081**
Thumpd1	Rattus norvegicus THUMP domain containing 1 (Thumpd1), mRNA [NM_001009688]	**6,080**
0	Unknown	**6,080**
fut10	Rattus norvegicus alpha3-fucosyltransferase (fut10), mRNA [NM_001012360]	**6,080**
Ints12	Rattus norvegicus integrator complex subunit 12 (Ints12), mRNA [NM_001007640]	**6,079**
Commd3	Rattus norvegicus COMM domain containing 3 (Commd3), mRNA [NM_198732]	**6,079**
0	Uncharacterized protein [Source:UniProtKB/TrEMBL;Acc:D4A3V7] [ENSRNOT00000023451]	**6,079**

Oxsm	Rattus norvegicus 3-oxoacyl-ACP synthase, mitochondrial (Oxsm), nuclear gene encoding mitochondrial protein, mRNA [NM_001100508]	**6,079**
0	Rattus norvegicus similar to SEPIAPTERIN REDUCTASE (SPR) (LOC297402), mRNA [XM_216190]	**6,077**
Kcns3	Rattus norvegicus potassium voltage-gated channel, delayed-rectifier, subfamily S, member 3 (Kcns3), mRNA [NM_031778]	**6,077**
Lrrc68	Rattus norvegicus leucine rich repeat containing 68 (Lrrc68), mRNA [NM_001107482]	**6,077**
Pih1d1	Rattus norvegicus PIH1 domain containing 1 (Pih1d1), mRNA [NM_001024868]	**6,076**
LOC686456	PREDICTED: Rattus norvegicus similar to zinc finger protein 218 (LOC686456), mRNA [XM_001074194]	**6,075**
0	Unknown	**6,075**
Klk1l	Rattus norvegicus kallikrein 1-like peptidase (Klk1l), mRNA [NM_012593]	**6,074**
Amz1	Rattus norvegicus archaelysin family metallopeptidase 1 (Amz1), mRNA [NM_001047092]	**6,074**
0	Unknown	**6,073**
Arih2	Rattus norvegicus ariadne homolog 2 (Drosophila) (Arih2), mRNA [NM_001012275]	**6,073**
Ankrd1	Rattus norvegicus ankyrin repeat domain 1 (cardiac muscle) (Ankrd1), mRNA [NM_013220]	**6,073**
0	Uncharacterized protein [Source:UniProtKB/TrEMBL;Acc:D3ZD16] [ENSRNOT00000012794]	**6,072**
0	Uncharacterized protein [Source:UniProtKB/TrEMBL;Acc:D3ZKK0] [ENSRNOT00000062086]	**6,071**
Tars2	Rattus norvegicus threonyl-tRNA synthetase 2, mitochondrial (putative) (Tars2), nuclear gene encoding mitochondrial protein, mRNA [NM_001014040]	**6,070**
Gmpr	Rattus norvegicus guanosine monophosphate reductase (Gmpr), mRNA [NM_057188]	**6,070**
RGD1311307	Rattus norvegicus similar to 1300014I06Rik protein (RGD1311307), mRNA [NM_001025719]	**6,067**
Fyco1	Rattus norvegicus FYVE and coiled-coil domain containing 1 (Fyco1), mRNA [NM_001106870]	**6,066**
Maea	Rattus norvegicus macrophage erythroblast attacher (Maea), mRNA [NM_001008319]	**6,065**
0	Rattus norvegicus TL0ADA9YM19 mRNA sequence. [FQ228322]	**6,065**
Prdx2	Rattus norvegicus peroxiredoxin 2 (Prdx2), nuclear gene encoding mitochondrial protein, mRNA [NM_017169]	**6,065**
Mpdz	Rattus norvegicus multiple PDZ domain protein (Mpdz), mRNA [NM_019196]	**6,063**
Cd8b	Rattus norvegicus CD8b molecule (Cd8b), mRNA [NM_031539]	**6,063**
Tssc4	Rattus norvegicus tumor suppressing subtransferable candidate 4 (Tssc4), mRNA [NM_001013194]	**6,062**
Tchh	PREDICTED: Rattus norvegicus trichohyalin (Tchh), mRNA [XM_001054694]	**6,062**
Far1	Rattus norvegicus fatty acyl CoA reductase 1 (Far1), mRNA [NM_001011933]	**6,062**
RGD1564964	Rattus norvegicus similar to WD repeat domain 11 protein (RGD1564964), mRNA [NM_001134564]	**6,062**
Ifna1	Rattus norvegicus interferon-alpha 1 (Ifna1), mRNA [NM_001014786]	**6,061**
Pan2	Rattus norvegicus PAN2 polyA specific ribonuclease subunit homolog (S. cerevisiae) (Pan2), mRNA [NM_001008862]	**6,060**
Smchd1	PREDICTED: Rattus norvegicus similar to RIKEN cDNA 4931400A14 (RGD1307234), mRNA [XM_001056555]	**6,060**
Xpo1	Rattus norvegicus exportin 1, CRM1 homolog (yeast) (Xpo1), mRNA [NM_053490]	**6,058**
Tmem212	Rattus norvegicus transmembrane protein 212 (Tmem212), transcript variant 2, mRNA [NM_001164439]	**6,058**
LOC681908	Rattus norvegicus hypothetical protein LOC681908 (LOC681908), mRNA [NM_001127609]	**6,055**

0	Unknown	**6,054**
0	Uncharacterized protein [Source:UniProtKB/TrEMBL;Acc:D3Z8H1] [ENSRNOT00000027841]	**6,054**
0	Unknown	**6,053**
0	Unknown	**6,052**
Zfp207	Rattus norvegicus zinc finger protein 207 (Zfp207), mRNA [NM_001039020]	**6,051**
Mfap1a	Rattus norvegicus microfibrillar-associated protein 1A (Mfap1a), mRNA [NM_001191964]	**6,051**
RGD1565775	LOC361980 protein [Source:UniProtKB/TrEMBL;Acc:Q56A23] [ENSRNOT00000027385]	**6,051**
Creld1	Rattus norvegicus cysteine-rich with EGF-like domains 1 (Creld1), mRNA [NM_001024783]	**6,050**
0	Unknown	**6,049**
0	Unknown	**6,049**
Mcf2l	Rattus norvegicus MCF.2 cell line derived transforming sequence-like (Mcf2l), mRNA [NM_053951]	**6,049**
Cytip	Rattus norvegicus cytohesin 1 interacting protein (Cytip), mRNA [NM_001012086]	**6,049**
Spock3	Rattus norvegicus sparc/osteonectin, cwcv and kazal-like domains proteoglycan (testican) 3 (Spock3), mRNA [NM_001107310]	**6,048**
Tmcc3	Rattus norvegicus transmembrane and coiled-coil domain family 3 (Tmcc3), mRNA [NM_001108084]	**6,047**
E2f1	Rattus norvegicus E2F transcription factor 1 (E2f1), mRNA [NM_001100778]	**6,046**
Aurkc	Rattus norvegicus aurora kinase C (Aurkc), mRNA [NM_001106221]	**6,046**
0	UI-R-BJ1-awi-h-01-0-UI.s1 UI-R-BJ1 Rattus norvegicus cDNA clone UI-R-BJ1-awi-h-01-0-UI 3', mRNA sequence [BE113996]	**6,046**
Enpp4	Rattus norvegicus ectonucleotide pyrophosphatase/phosphodiesterase 4 (Enpp4), mRNA [NM_001106892]	**6,045**
0	Unknown	**6,043**
Myo19	Rattus norvegicus myosin XIX (Myo19), mRNA [NM_001163736]	**6,041**
Olr1583	Rattus norvegicus olfactory receptor 1583 (Olr1583), mRNA [NM_001000080]	**6,038**
Nampt	Rattus norvegicus nicotinamide phosphoribosyltransferase (Nampt), mRNA [NM_177928]	**6,037**
Bpil3	Rattus norvegicus bactericidal/permeability-increasing protein-like 3 (Bpil3), mRNA [NM_001107791]	**6,037**
Mif	Rattus norvegicus macrophage migration inhibitory factor (Mif), mRNA [NM_031051]	**6,037**
Akap11	Rattus norvegicus A kinase (PRKA) anchor protein 11 (Akap11), mRNA [NM_012773]	**6,037**
Irak3	Rattus norvegicus interleukin-1 receptor-associated kinase 3 (Irak3), mRNA [NM_001108101]	**6,036**
Pik3c2a	Rattus norvegicus phosphoinositide-3-kinase, class 2, alpha polypeptide (Pik3c2a), mRNA [NM_001108500]	**6,035**
Sbno1	Rattus norvegicus strawberry notch homolog 1 (Drosophila) (Sbno1), mRNA [NM_001107138]	**6,034**
Usp40	Rattus norvegicus ubiquitin specific peptidase 40 (Usp40), mRNA [NM_001134885]	**6,033**
Dmtf1	Rattus norvegicus cyclin D binding myb-like transcription factor 1 (Dmtf1), mRNA [NM_053693]	**6,031**
Cyp2d3	Rattus norvegicus cytochrome P450, family 2, subfamily d, polypeptide 3 (Cyp2d3), mRNA [NM_173093]	**6,030**
Scfd2	Rattus norvegicus sec1 family domain containing 2 (Scfd2), mRNA [NM_001017499]	**6,030**
Mettl10	Rattus norvegicus methyltransferase like 10 (Mettl10), mRNA [NM_001108504]	**6,030**
Grinl1a	Rattus norvegicus glutamate receptor, ionotropic, N-methyl D-aspartate-like 1A (Grinl1a), mRNA [NM_183402]	**6,028**

Pgm2l1	Rattus norvegicus phosphoglucomutase 2-like 1 (Pgm2l1), mRNA [NM_001109454]	**6,028**
0	Unknown	**6,026**
Rnf43	Rattus norvegicus ring finger protein 43 (Rnf43), mRNA [NM_001135921]	**6,026**
Itih3	Rattus norvegicus inter-alpha trypsin inhibitor, heavy chain 3 (Itih3), mRNA [NM_017351]	**6,025**
0	Uncharacterized protein [Source:UniProtKB/TrEMBL;Acc:D3Z9X6] [ENSRNOT00000064120]	**6,024**
0	Unknown	**6,023**
Atpif1	Rattus norvegicus ATPase inhibitory factor 1 (Atpif1), nuclear gene encoding mitochondrial protein, mRNA [NM_012915]	**6,023**
Pnck	Rattus norvegicus pregnancy upregulated non-ubiquitously expressed CaM kinase (Pnck), mRNA [NM_017275]	**6,022**
Gtf2ird2	Rattus norvegicus similar to gtf2ird2 (LOC288606), mRNA [XM_213757]	**6,022**
RGD1308380	Rattus norvegicus similar to RIKEN cDNA 2310079F23 (RGD1308380), mRNA [NM_001130061]	**6,022**
Dact2	Rattus norvegicus dapper, antagonist of beta-catenin, homolog 2 (Xenopus laevis) (Dact2), mRNA [NM_001107464]	**6,022**
0	Unknown	**6,021**
Ofd1	Rattus norvegicus oral-facial-digital syndrome 1 gene homolog (human) (Ofd1), mRNA [NM_001106961]	**6,021**
0	Unknown	**6,020**
LOC685563	PREDICTED: Rattus norvegicus similar to Discs large homolog 5 (Placenta and prostate DLG) (Discs large protein P-dlg) (LOC685563), mRNA [XM_001064312]	**6,019**
0	Uncharacterized protein [Source:UniProtKB/TrEMBL;Acc:D3ZS15] [ENSRNOT00000050823]	**6,018**
Cul4a	Rattus norvegicus cullin 4A (Cul4a), mRNA [NM_001127301]	**6,018**
0	Unknown	**6,017**
0	Unknown	**6,017**
0	Unknown	**6,017**
Myadml2	Rattus norvegicus myeloid-associated differentiation marker-like 2 (Myadml2), mRNA [NM_001128185]	**6,017**
Ubr2	Rattus norvegicus ubiquitin protein ligase E3 component n-recognin 2 (Ubr2), mRNA [NM_001178071]	**6,016**
Sh3glb2	Rattus norvegicus SH3-domain GRB2-like endophilin B2 (Sh3glb2), mRNA [NM_001009692]	**6,016**
Cdkn2aip	Rattus norvegicus CDKN2A interacting protein (Cdkn2aip), mRNA [NM_001014000]	**6,016**
Elf1	Rattus norvegicus E74-like factor 1 (Elf1), mRNA [NM_053520]	**6,016**
Ostf1	Rattus norvegicus osteoclast stimulating factor 1 (Ostf1), mRNA [NM_148892]	**6,016**
Acot4	Rattus norvegicus acyl-CoA thioesterase 4 (Acot4), mRNA [NM_001109440]	**6,015**
Itm2b	Rattus norvegicus integral membrane protein 2B (Itm2b), mRNA [NM_001006963]	**6,015**
Esrp2	Rattus norvegicus epithelial splicing regulatory protein 2 (Esrp2), mRNA [NM_001107423]	**6,014**
0	Rattus norvegicus obscurin (Obscurin) mRNA, Obscurin-S allele, partial cds. [AY167411]	**6,014**
Syk	Rattus norvegicus spleen tyrosine kinase (Syk), mRNA [NM_012758]	**6,014**
Usp40	Rattus norvegicus ubiquitin specific peptidase 40 (Usp40), mRNA [NM_001134885]	**6,014**
0	Unknown	**6,013**

Cpamd8	PREDICTED: Rattus norvegicus similar to Murinoglobulin 1 homolog (RGD1566313), mRNA [XM_213006]	**6,013**
0	Unknown	**6,009**
RT1-CE3	Rattus norvegicus RT1 class I, locus CE3 (RT1-CE3), mRNA [NM_001008841]	**6,009**
Nop14	Rattus norvegicus NOP14 nucleolar protein homolog (yeast) (Nop14), mRNA [NM_001106013]	**6,008**
Uspl1	Rattus norvegicus ubiquitin specific peptidase like 1 (Uspl1), transcript variant 1, mRNA [NM_001198555]	**6,007**
Cryz	Rattus norvegicus crystallin, zeta (Cryz), mRNA [NM_001012183]	**6,006**
0	Uncharacterized protein [Source:UniProtKB/TrEMBL;Acc:D4A6W5] [ENSRNOT00000009094]	**6,006**
Kpnb1	Rattus norvegicus karyopherin (importin) beta 1 (Kpnb1), mRNA [NM_017063]	**6,006**
Map4k3	Rattus norvegicus mitogen-activated protein kinase kinase kinase kinase 3 (Map4k3), mRNA [NM_133407]	**6,006**
Chmp7	Rattus norvegicus CHMP family, member 7 (Chmp7), mRNA [NM_001108872]	**6,005**
Grpel2	Rattus norvegicus GrpE-like 2, mitochondrial (Grpel2), nuclear gene encoding mitochondrial protein, mRNA [NM_001109513]	**6,005**
0	Unknown	**6,004**
Sfrs12	Rattus norvegicus splicing factor, arginine/serine-rich 12 (Sfrs12), mRNA [NM_020092]	**6,002**
Lamp2	Rattus norvegicus lysosomal-associated membrane protein 2 (Lamp2), mRNA [NM_017068]	**6,002**
Pex16	Rattus norvegicus peroxisomal biogenesis factor 16 (Pex16), mRNA [NM_001012088]	**6,001**
Usp29	Rattus norvegicus ubiquitin specific peptidase 29 (Usp29), mRNA [NM_001108465]	**6,000**
LOC100361882	PREDICTED: Rattus norvegicus rCG56785-like (LOC100361882), mRNA [XM_002730111]	**6,000**
Tm4sf20	Rattus norvegicus transmembrane 4 L six family member 20 (Tm4sf20), mRNA [NM_001109618]	**5,998**
0	Unknown	**5,998**
Adcy6	Rattus norvegicus adenylate cyclase 6 (Adcy6), mRNA [NM_012821]	**5,997**
0	Unknown	**5,997**
Dtnbp1	Rattus norvegicus distrobrevin binding protein 1 (Dtnbp1), mRNA [NM_001037664]	**5,996**
0	Unknown	**5,996**
Chpt1	Rattus norvegicus choline phosphotransferase 1 (Chpt1), mRNA [NM_001007750]	**5,996**
Rnaset2	Rattus norvegicus ribonuclease T2 (Rnaset2), mRNA [NM_001106210]	**5,995**
0	Unknown	**5,994**
0	Unknown	**5,994**
Gls2	Rattus norvegicus glutaminase 2 (liver, mitochondrial) (Gls2), nuclear gene encoding mitochondrial protein, mRNA [NM_138904]	**5,994**
0	Uncharacterized protein [Source:UniProtKB/TrEMBL;Acc:D3Z9Y0] [ENSRNOT00000022886]	**5,993**
Mrfap1	MORF4 family-associated protein 1 [Source:UniProtKB/Swiss-Prot;Acc:Q5M820] [ENSRNOT00000043744]	**5,993**
0	Unknown	**5,993**
0	Unknown	**5,991**
Timm23	Rattus norvegicus translocase of inner mitochondrial membrane 23 homolog (yeast) (Timm23), nuclear gene encoding mitochondrial protein, mRNA [NM_019352]	**5,990**

0	Vitamin K-dependent gamma-carboxylase [Source:UniProtKB/Swiss-Prot;Acc:O88496] [ENSRNOT00000058024]	**5,990**
Thap11	Rattus norvegicus THAP domain containing 11 (Thap11), mRNA [NM_001107422]	**5,989**
0	Unknown	**5,989**
Exoc6	Rattus norvegicus exocyst complex component 6 (Exoc6), mRNA [NM_019277]	**5,989**
0	Q3X4I5_9ACTN (Q3X4I5) Queuine/other tRNA-ribosyltransferase:Queuine tRNA-ribosyltransferase , partial (5%) [TC646930]	**5,988**
B3gat2	Rattus norvegicus beta-1,3-glucuronyltransferase 2 (glucuronosyltransferase S) (B3gat2), mRNA [NM_022609]	**5,985**
0	Unknown	**5,985**
Lrp1b	Rattus norvegicus low density lipoprotein-related protein 1B (deleted in tumors) (Lrp1b), mRNA [NM_001107843]	**5,985**
0	Unknown	**5,984**
0	plakophilin 4 Gene [Source:MGI Symbol;Acc:MGI:109281] [ENSRNOT00000059246]	**5,984**
0	RVL12088 Wackym-Soares normalized rat vestibular cDNA library Rattus norvegicus cDNA 5', mRNA sequence [DV720985]	**5,984**
Nrcam	Rattus norvegicus neuronal cell adhesion molecule (Nrcam), mRNA [NM_013150]	**5,983**
Cttn	Rattus norvegicus cortactin (Cttn), mRNA [NM_021868]	**5,983**
Adk	Rattus norvegicus adenosine kinase (Adk), mRNA [NM_012895]	**5,982**
Hprt1	Rattus norvegicus hypoxanthine phosphoribosyltransferase 1 (Hprt1), mRNA [NM_012583]	**5,981**
Ddx18	Rattus norvegicus DEAD (Asp-Glu-Ala-Asp) box polypeptide 18 (Ddx18), mRNA [NM_001006996]	**5,981**
LOC290577	PREDICTED: Rattus norvegicus hypothetical LOC290577 (LOC290577), mRNA [XM_002728376]	**5,981**
RGD1563714	Rattus norvegicus RGD1563714 (RGD1563714), mRNA [NM_001126297]	**5,980**
Akr7a2	Rattus norvegicus aldo-keto reductase family 7, member A2 (aflatoxin aldehyde reductase) (Akr7a2), mRNA [NM_134407]	**5,978**
Lamb3	Rattus norvegicus laminin, beta 3 (Lamb3), mRNA [NM_001100841]	**5,977**
Pdcd2	Rattus norvegicus programmed cell death 2 (Pdcd2), mRNA [NM_031638]	**5,977**
LOC498276	Rattus norvegicus Fc gamma receptor II beta (LOC498276), mRNA [NM_001135992]	**5,977**
Dusp27	Rattus norvegicus dual specificity phosphatase 27 (putative) (Dusp27), mRNA [NM_001191929]	**5,977**
0	Copper-transporting ATPase 1 [Source:UniProtKB/Swiss-Prot;Acc:P70705] [ENSRNOT00000003395]	**5,976**
LOC100364138	PREDICTED: Rattus norvegicus ferritin light chain 1-like (LOC100364138), mRNA [XM_002726633]	**5,976**
0	Uncharacterized protein [Source:UniProtKB/TrEMBL;Acc:D4ADX4] [ENSRNOT00000060718]	**5,976**
0	Hepatic leukemia factor [Source:UniProtKB/Swiss-Prot;Acc:Q64709] [ENSRNOT00000055664]	**5,975**
Atp6v1a	Rattus norvegicus ATPase, H+ transporting, lysosomal V1 subunit A (Atp6v1a), mRNA [NM_001108318]	**5,974**
Megf8	PREDICTED: Rattus norvegicus multiple EGF-like-domains 8 (Megf8), mRNA [XM_341803]	**5,973**
0	RGD1565316 protein [Source:UniProtKB/TrEMBL;Acc:B2RYK6] [ENSRNOT00000056421]	**5,972**
Myeov2	Rattus norvegicus myeloma overexpressed 2 (Myeov2), mRNA [NM_001109044]	**5,972**
Sf4	Rattus norvegicus splicing factor 4 (Sf4), mRNA [NM_001011920]	**5,972**
Bloc1s2	Rattus norvegicus biogenesis of lysosomal organelles complex-1, subunit 2 (Bloc1s2), mRNA [NM_001037349]	**5,972**
RGD1306000	Rattus norvegicus similar to CG8043-PA (RGD1306000), mRNA [NM_001108827]	**5,972**

Tm7sf3	Rattus norvegicus transmembrane 7 superfamily member 3 (Tm7sf3), mRNA [NM_001011970]	**5,971**
Tmem53	Rattus norvegicus transmembrane protein 53 (Tmem53), mRNA [NM_001107964]	**5,971**
Stau2	Rattus norvegicus staufen, RNA binding protein, homolog 2 (Drosophila) (Stau2), transcript variant LL, mRNA [NM_001007149]	**5,970**
Ambp	Rattus norvegicus alpha-1-microglobulin/bikunin precursor (Ambp), mRNA [NM_012901]	**5,970**
0	Unknown	**5,969**
Zfyve1	Rattus norvegicus zinc finger, FYVE domain containing 1 (Zfyve1), mRNA [NM_001106743]	**5,968**
Rps27a	Rattus norvegicus ribosomal protein S27a (Rps27a), mRNA [NM_031113]	**5,967**
F11r	Rattus norvegicus F11 receptor (F11r), mRNA [NM_053796]	**5,967**
Ube2s	Rattus norvegicus ubiquitin-conjugating enzyme E2S (Ube2s), mRNA [NM_001106224]	**5,967**
Lysmd3	Rattus norvegicus LysM, putative peptidoglycan-binding, domain containing 3 (Lysmd3), mRNA [NM_001009698]	**5,966**
Znf292	Rattus norvegicus zinc finger protein 292 (Znf292), mRNA [NM_001008879]	**5,966**
Gtf2i	Rattus norvegicus general transcription factor II I (Gtf2i), mRNA [NM_001001512]	**5,965**
RGD1307597	Rattus norvegicus similar to mKIAA0317 protein (RGD1307597), mRNA [NM_001106744]	**5,964**
Rtf1	Rattus norvegicus Rtf1, Paf1/RNA polymerase II complex component, homolog (S. cerevisiae) (Rtf1), mRNA [NM_001108958]	**5,963**
Gusb	Rattus norvegicus glucuronidase, beta (Gusb), mRNA [NM_017015]	**5,962**
Tacc3	Rattus norvegicus transforming, acidic coiled-coil containing protein 3 (Tacc3), mRNA [NM_001004424]	**5,960**
Slmo2	Rattus norvegicus slowmo homolog 2 (Drosophila) (Slmo2), mRNA [NM_001009543]	**5,960**
0	Unknown	**5,960**
Ccl2	Rattus norvegicus chemokine (C-C motif) ligand 2 (Ccl2), mRNA [NM_031530]	**5,960**
0	Unknown	**5,959**
RGD1309138	Rattus norvegicus similar to hypothetical protein MGC9912 (RGD1309138), mRNA [NM_001108981]	**5,959**
0	Rattus norvegicus TL0AAA45YD16 mRNA sequence. [FQ213860]	**5,959**
Mapk12	Rattus norvegicus mitogen-activated protein kinase 12 (Mapk12), mRNA [NM_021746]	**5,958**
Sdf2	Rattus norvegicus stromal cell derived factor 2 (Sdf2), mRNA [NM_001105803]	**5,958**
Chek2	Rattus norvegicus CHK2 checkpoint homolog (S. pombe) (Chek2), mRNA [NM_053677]	**5,958**
LOC679630	PREDICTED: Rattus norvegicus similar to eukaryotic translation initiation factor 4, gamma 1 isoform a (LOC679630), mRNA [XM_002727916]	**5,958**
Aatk	Rattus norvegicus apoptosis-associated tyrosine kinase (Aatk), mRNA [NM_001168703]	**5,957**
Mul1	Rattus norvegicus mitochondrial ubiquitin ligase activator of NFKB 1 (Mul1), nuclear gene encoding mitochondrial protein, mRNA [NM_001106695]	**5,954**
Baz1a	Rattus norvegicus bromodomain adjacent to zinc finger domain, 1A (Baz1a), mRNA [NM_001170568]	**5,954**
St6galnac3	Rattus norvegicus ST6 (alpha-N-acetyl-neuraminyl-2,3-beta-galactosyl-1,3)-N-acetylgalactosaminide alpha-2,6-sialyltransferase 3 (St6galnac3), mRNA [NM_019123]	**5,954**
Olr1135	Rattus norvegicus olfactory receptor 1135 (Olr1135), mRNA [NM_001000878]	**5,953**
Msmb	Rattus norvegicus microseminoprotein, beta (Msmb), mRNA [NM_019188]	**5,953**
LOC302022	Rattus norvegicus similar to nidogen 2 protein (LOC302022), mRNA [NM_001127530]	**5,953**

Syt8	Rattus norvegicus synaptotagmin VIII (Syt8), mRNA [NM_053325]	**5,952**
0	PREDICTED: Rattus norvegicus similar to RIKEN cDNA D230037D09 gene (RGD1566396), mRNA [XM_001067433]	**5,951**
0	Unknown	**5,951**
RGD1310324	Rattus norvegicus hypothetical LOC287388 (RGD1310324), mRNA [NM_001163492]	**5,950**
Neurl4	Rattus norvegicus neuralized homolog 4 (Drosophila) (Neurl4), mRNA [NM_001107013]	**5,950**
LOC501234	PREDICTED: Rattus norvegicus hypothetical gene supported by BC082068 (LOC501234), mRNA [XM_576655]	**5,950**
0	Rattus norvegicus similar to ribosomal protein S15 (LOC290226), mRNA [XM_224191]	**5,949**
Tmem200a	Rattus norvegicus transmembrane protein 200A (Tmem200a), mRNA [NM_001109135]	**5,949**
Gtf3a	Rattus norvegicus general transcription factor III A (Gtf3a), mRNA [NM_001113570]	**5,948**
Tcea3	Rattus norvegicus transcription elongation factor A (SII), 3 (Tcea3), mRNA [NM_001015008]	**5,947**
0	PREDICTED: Rattus norvegicus similar to ribosomal protein L30 (RGD1562394), mRNA [XM_001081708]	**5,946**
LOC499219	Rattus norvegicus hypothetical protein LOC499219 (LOC499219), mRNA [NM_001047938]	**5,945**
Fam135a	Rattus norvegicus family with sequence similarity 135, member A (Fam135a), mRNA [NM_001134597]	**5,945**
RGD1562080	PREDICTED: Rattus norvegicus similar to Hypothetical protein CBG10141 (RGD1562080), mRNA [XM_574108]	**5,945**
Hist1h2bh	Rattus norvegicus histone cluster 1, H2bh (Hist1h2bh), mRNA [NM_001107352]	**5,944**
Slc17a9	Rattus norvegicus solute carrier family 17, member 9 (Slc17a9), mRNA [NM_001108613]	**5,944**
Gcc2	Rattus norvegicus GRIP and coiled-coil domain containing 2 (Gcc2), mRNA [NM_001107633]	**5,944**
Pcyox1l	Rattus norvegicus prenylcysteine oxidase 1 like (Pcyox1l), mRNA [NM_001134542]	**5,944**
0	Uncharacterized protein [Source:UniProtKB/TrEMBL;Acc:D3ZEZ9] [ENSRNOT00000064331]	**5,943**
Mgst1	Rattus norvegicus microsomal glutathione S-transferase 1 (Mgst1), mRNA [NM_134349]	**5,943**
0	Unknown	**5,942**
Tmprss9	Rattus norvegicus transmembrane protease, serine 9 (Tmprss9), mRNA [NM_001047100]	**5,942**
Rhov	Rattus norvegicus ras homolog gene family, member V (Rhov), mRNA [NM_138542]	**5,942**
Nphp3	Rattus norvegicus nephronophthisis 3 (adolescent) (Nphp3), mRNA [NM_001191882]	**5,940**
Fau	Rattus norvegicus Finkel-Biskis-Reilly murine sarcoma virus (FBR-MuSV) ubiquitously expressed (Fau), transcript variant 1, mRNA [NM_001012739]	**5,939**
Wdr54	Rattus norvegicus WD repeat domain 54 (Wdr54), mRNA [NM_001109245]	**5,938**
Elmod3	Rattus norvegicus ELMO/CED-12 domain containing 3 (Elmod3), mRNA [NM_001013087]	**5,938**
0	Unknown	**5,935**
Etaa1	Rattus norvegicus Ewing tumor-associated antigen 1 (Etaa1), mRNA [NM_001109094]	**5,934**
0	Uncharacterized protein [Source:UniProtKB/TrEMBL;Acc:D3ZR70] [ENSRNOT00000030150]	**5,933**
LOC683761	PREDICTED: Rattus norvegicus similar to RT1 class I, CE11 (LOC683761), partial mRNA [XM_001053482]	**5,932**
Cyp20a1	Rattus norvegicus cytochrome P450, family 20, subfamily a, polypeptide 1 (Cyp20a1), mRNA [NM_199401]	**5,932**
Cbx3	Rattus norvegicus chromobox homolog 3 (HP1 gamma homolog, Drosophila) (Cbx3), mRNA [NM_001008313]	**5,931**

Hist2h4	Rattus norvegicus histone cluster 2, H4 (Hist2h4), mRNA [NM_001123469]	**5,931**
0	Unknown	**5,931**
Anxa7	Rattus norvegicus annexin A7 (Anxa7), mRNA [NM_130416]	**5,930**
0	Unknown	**5,930**
LOC686999	Rattus norvegicus similar to M phase phosphoprotein 6 (LOC686999), mRNA [NM_001129881]	**5,930**
Yipf4	Rattus norvegicus Yip1 domain family, member 4 (Yipf4), mRNA [NM_001009712]	**5,930**
Aldoa	Rattus norvegicus aldolase A, fructose-bisphosphate (Aldoa), transcript variant 1, mRNA [NM_012495]	**5,930**
Tmem14a	Rattus norvegicus transmembrane protein 14A (Tmem14a), mRNA [NM_001108790]	**5,929**
0	Unknown	**5,928**
Exosc5	Rattus norvegicus exosome component 5 (Exosc5), mRNA [NM_001107493]	**5,928**
RGD1561792	Rattus norvegicus similar to CG14903-PA (RGD1561792), mRNA [NM_001134524]	**5,927**
Pnck	Rattus norvegicus pregnancy upregulated non-ubiquitously expressed CaM kinase (Pnck), mRNA [NM_017275]	**5,925**
Pdcd7	Rattus norvegicus programmed cell death 7 (Pdcd7), mRNA [NM_001108768]	**5,924**
RGD1306063	Rattus norvegicus similar to HT021 (RGD1306063), mRNA [NM_001106026]	**5,923**
RGD1566052	Rattus norvegicus similar to elongation protein 4 homolog (RGD1566052), mRNA [NM_001115047]	**5,922**
Aurka	Rattus norvegicus aurora kinase A (Aurka), mRNA [NM_153296]	**5,921**
Pisd	Uncharacterized protein [Source:UniProtKB/TrEMBL;Acc:D3ZAW2] [ENSRNOT00000024813]	**5,920**
Rpa2	Rattus norvegicus replication protein A2 (Rpa2), mRNA [NM_021582]	**5,920**
0	Unknown	**5,919**
0	Unknown	**5,918**
Zc3h11a	Rattus norvegicus zinc finger CCCH-type containing 11A (Zc3h11a), mRNA [NM_001047902]	**5,918**
0	Unknown	**5,918**
0	Rattus norvegicus similar to 60S RIBOSOMAL PROTEIN L5 (LOC298243), mRNA [XM_233179]	**5,916**
Fgfr1op2	Rattus norvegicus FGFR1 oncogene partner 2 (Fgfr1op2), mRNA [NM_201421]	**5,916**
0	Unknown	**5,916**
Spaca4	Rattus norvegicus sperm acrosome associated 4 (Spaca4), mRNA [NM_001109623]	**5,915**
Znf296	PREDICTED: Rattus norvegicus zinc finger protein 296 (Znf296), mRNA [XM_001075949]	**5,915**
0	Unknown	**5,914**
Olr420	Rattus norvegicus olfactory receptor 420 (Olr420), mRNA [NM_001000389]	**5,914**
Fancf	PREDICTED: Rattus norvegicus Fanconi anemia, complementation group F (Fancf), mRNA [XM_001080926]	**5,913**
Lpgat1	Rattus norvegicus lysophosphatidylglycerol acyltransferase 1 (Lpgat1), mRNA [NM_001109376]	**5,913**
Hus1	Rattus norvegicus HUS1 checkpoint homolog (S. pombe) (Hus1), mRNA [NM_001109092]	**5,912**
0	Unknown	**5,912**
Coro6	Rattus norvegicus coronin 6 (Coro6), mRNA [NM_139115]	**5,912**

Cxcr4	Rattus norvegicus chemokine (C-X-C motif) receptor 4 (Cxcr4), mRNA [NM_022205]	**5,912**
Hnrnph2	Rattus norvegicus heterogeneous nuclear ribonucleoprotein H2 (H') (Hnrnph2), mRNA [NM_001014019]	**5,912**
0	Unknown	**5,911**
Wrb	Rattus norvegicus tryptophan rich basic protein (Wrb), mRNA [NM_199373]	**5,911**
Tp73	Rattus norvegicus tumor protein p73 (Tp73), mRNA [NM_001108696]	**5,910**
Vps52	Rattus norvegicus vacuolar protein sorting 52 homolog (S. cerevisiae) (Vps52), mRNA [NM_033097]	**5,910**
0	Rattus norvegicus similar to c-Mpl binding protein (LOC366985), mRNA [XM_345871]	**5,909**
RT1-CE13	Rattus norvegicus RT1 class I, locus CE13 (RT1-CE13), mRNA [NM_001008836]	**5,909**
Kcnab1	Rattus norvegicus potassium voltage-gated channel, shaker-related subfamily, beta member 1 (Kcnab1), mRNA [NM_017303]	**5,908**
Dnase1l1	Rattus norvegicus deoxyribonuclease 1-like 1 (Dnase1l1), mRNA [NM_001014223]	**5,908**
0	Unknown	**5,907**
RGD1307365	PREDICTED: Rattus norvegicus similar to KIAA1009 protein (RGD1307365), mRNA [XM_001054857]	**5,907**
LOC690728	Rattus norvegicus similar to Protein C12orf11 (Sarcoma antigen NY-SAR-95) (LOC690728), mRNA [NM_001109608]	**5,906**
Luc7l	Rattus norvegicus LUC7-like (S. cerevisiae) (Luc7l), mRNA [NM_001024269]	**5,905**
Cdc42se2	Rattus norvegicus CDC42 small effector 2 (Cdc42se2), mRNA [NM_001126089]	**5,904**
0	Unknown	**5,902**
Klri2	Rattus norvegicus killer cell lectin-like receptor family I member 2 (Klri2), mRNA [NM_001012648]	**5,902**
RGD1565690	Uncharacterized protein [Source:UniProtKB/TrEMBL;Acc:D3ZF21] [ENSRNOT00000039316]	**5,901**
0	Unknown	**5,900**
Hsp90ab1	Rattus norvegicus heat shock protein 90 alpha (cytosolic), class B member 1 (Hsp90ab1), mRNA [NM_001004082]	**5,900**
Dok1	Rattus norvegicus docking protein 1 (Dok1), mRNA [NM_001025416]	**5,900**
Cryaa	Rattus norvegicus crystallin, alpha A (Cryaa), mRNA [NM_012534]	**5,900**
RGD1564839	Uncharacterized protein [Source:UniProtKB/TrEMBL;Acc:D3ZKU5] [ENSRNOT00000043590]	**5,899**
0	Unknown	**5,898**
Ppp1r14b	Rattus norvegicus protein phosphatase 1, regulatory (inhibitor) subunit 14B (Ppp1r14b), mRNA [NM_172045]	**5,898**
0	Unknown	**5,897**
0	Unknown	**5,896**
Sp110	Rattus norvegicus SP110 nuclear body protein (Sp110), mRNA [NM_001034137]	**5,895**
RGD1309108	PREDICTED: Rattus norvegicus similar to hypothetical protein FLJ23554 (RGD1309108), mRNA [XM_236102]	**5,894**
Kbtbd8	Rattus norvegicus kelch repeat and BTB (POZ) domain containing 8 (Kbtbd8), mRNA [NM_001109250]	**5,894**
Sp100	Rattus norvegicus SP100 nuclear antigen (Sp100), mRNA [NM_001014220]	**5,894**
Actr3b	Rattus norvegicus ARP3 actin-related protein 3 homolog B (yeast) (Actr3b), mRNA [NM_001191860]	**5,894**
Slc25a1	Rattus norvegicus solute carrier family 25 (mitochondrial carrier, citrate transporter), member 1 (Slc25a1), nuclear gene encoding mitochondrial protein, mRNA [NM_017307]	**5,893**

Dio1	Rattus norvegicus deiodinase, iodothyronine, type I (Dio1), mRNA [NM_021653]	**5,892**
Bysl	Rattus norvegicus bystin-like (Bysl), mRNA [NM_182674]	**5,892**
Pacsin3	Rattus norvegicus protein kinase C and casein kinase substrate in neurons 3 (Pacsin3), mRNA [NM_001009966]	**5,891**
Clgn	Rattus norvegicus calmegin (Clgn), mRNA [NM_001109472]	**5,890**
0	Unknown	**5,888**
Traip	Rattus norvegicus TRAF-interacting protein (Traip), mRNA [NM_001109004]	**5,888**
0	Uncharacterized protein [Source:UniProtKB/TrEMBL;Acc:D4A5F0] [ENSRNOT00000002917]	**5,887**
Map4k2	Rattus norvegicus mitogen activated protein kinase kinase kinase kinase 2 (Map4k2), mRNA [NM_001106329]	**5,886**
Fth1	Rattus norvegicus ferritin, heavy polypeptide 1 (Fth1), mRNA [NM_012848]	**5,885**
Casp9	Rattus norvegicus caspase 9, apoptosis-related cysteine peptidase (Casp9), mRNA [NM_031632]	**5,885**
Ptprk	Rattus norvegicus protein tyrosine phosphatase, receptor type, K, extracellular region (Ptprk), mRNA [NM_001029902]	**5,885**
Mdm1	Rattus norvegicus Mdm1 nuclear protein homolog (mouse) (Mdm1), mRNA [NM_001017459]	**5,884**
LOC688818	PREDICTED: Rattus norvegicus similar to NEDD4-binding protein 1 (N4BP1) (LOC688818), mRNA [XM_001068419]	**5,884**
Crem	Rattus norvegicus cAMP responsive element modulator (Crem), transcript variant 3, mRNA [NM_001110860]	**5,884**
0	AE001111 A. fulgidus predicted coding region AF2391 {Archaeoglobus fulgidus DSM 4304} (exp=-1; wgp=0; cg=1), partial (26%) [TC624087]	**5,883**
Smcr7l	Rattus norvegicus Smith-Magenis syndrome chromosome region, candidate 7-like (human) (Smcr7l), mRNA [NM_001007709]	**5,883**
Eef1g	Rattus norvegicus eukaryotic translation elongation factor 1 gamma (Eef1g), mRNA [NM_001004223]	**5,882**
0	Unknown	**5,881**
Sephs2	Rattus norvegicus selenophosphate synthetase 2 (Sephs2), mRNA [NM_001079889]	**5,881**
Zkscan5	Rattus norvegicus zinc finger with KRAB and SCAN domains 5 (Zkscan5), mRNA [NM_001107119]	**5,880**
0	Unknown	**5,879**
0	Rattus norvegicus similar to ribosomal protein S24 (LOC315035), mRNA [XM_235376]	**5,878**
0	Rattus norvegicus similar to melanocyte-specific gene 1 protein (LOC309188), mRNA [XM_219543]	**5,877**
Taf2	Rattus norvegicus TAF2 RNA polymerase II, TATA box binding protein (TBP)-associated factor (Taf2), mRNA [NM_133319]	**5,877**
0	Uncharacterized protein [Source:UniProtKB/TrEMBL;Acc:D4A6Z9] [ENSRNOT00000057567]	**5,877**
Tmem2	Rattus norvegicus transmembrane protein 2 (Tmem2), mRNA [NM_001107596]	**5,876**
Megf6	Rattus norvegicus multiple EGF-like-domains 6 (Megf6), mRNA [NM_022955]	**5,876**
Rfc1	Rattus norvegicus replication factor C (activator 1) 1 (Rfc1), mRNA [NM_053547]	**5,875**
Aga	Rattus norvegicus aspartylglucosaminidase (Aga), mRNA [NM_001031641]	**5,875**
Pik3r3	Rattus norvegicus phosphoinositide-3-kinase, regulatory subunit 3 (gamma) (Pik3r3), mRNA [NM_022213]	**5,874**
0	Unknown	**5,874**
Qtrt1	Rattus norvegicus queuine tRNA-ribosyltransferase 1 (Qtrt1), mRNA [NM_022250]	**5,874**
Fau	Rattus norvegicus Finkel-Biskis-Reilly murine sarcoma virus (FBR-MuSV) ubiquitously expressed (Fau), transcript variant 1, mRNA [NM_001012739]	**5,873**

Znf777	Rattus norvegicus zinc finger protein 777 (Znf777), mRNA [NM_001109348]	**5,870**
Rfwd3	Rattus norvegicus TL0AEA87YO10 mRNA sequence. [FQ230829]	**5,870**
Lysmd2	Rattus norvegicus LysM, putative peptidoglycan-binding, domain containing 2 (Lysmd2), mRNA [NM_001106839]	**5,869**
RT1-CE14	Rattus norvegicus RT1 class I, locus CE14 (RT1-CE14), transcript variant 1, mRNA [NM_001008837]	**5,869**
RGD1309829	Rattus norvegicus similar to dJ842G6.1.1 (novel protein) (RGD1309829), mRNA [NM_001126371]	**5,869**
0	Rattus norvegicus TL0ACA41YI14 mRNA sequence. [FQ216706]	**5,868**
Phospho2	Rattus norvegicus phosphatase, orphan 2 (Phospho2), mRNA [NM_001007642]	**5,868**
Dcaf17	Rattus norvegicus DDB1 and CUL4 associated factor 17 (Dcaf17), mRNA [NM_001177688]	**5,866**
0	UI-R-BS1-azr-f-06-0-UI.s1 UI-R-BS1 Rattus norvegicus cDNA clone UI-R-BS1-azr-f-06-0-UI 3', mRNA sequence [BE118692]	**5,866**
Wwc2	Rattus norvegicus WW and C2 domain containing 2 (Wwc2), mRNA [NM_001109111]	**5,866**
Olr916	Rattus norvegicus olfactory receptor 916 (Olr916), mRNA [NM_001000587]	**5,865**
Memo1	Rattus norvegicus mediator of cell motility 1 (Memo1), mRNA [NM_001029917]	**5,864**
Zfp364	Rattus norvegicus zinc finger protein 364 (Zfp364), mRNA [NM_001108560]	**5,864**
Rdbp	Rattus norvegicus RD RNA-binding protein (Rdbp), transcript variant 1, mRNA [NM_212548]	**5,861**
RGD1562012	Rattus norvegicus RGD1562012 (RGD1562012), mRNA [NM_001173471]	**5,860**
Chmp5	Rattus norvegicus chromatin modifying protein 5 (Chmp5), mRNA [NM_001025410]	**5,860**
Rpl12	Uncharacterized protein [Source:UniProtKB/TrEMBL;Acc:D3ZJE2] [ENSRNOT00000003002]	**5,859**
Hcfc2	Rattus norvegicus host cell factor C2 (Hcfc2), mRNA [NM_001008357]	**5,858**
Itgb1	Rattus norvegicus integrin, beta 1 (Itgb1), mRNA [NM_017022]	**5,858**
Gpr139	Rattus norvegicus G protein-coupled receptor 139 (Gpr139), mRNA [NM_001024241]	**5,858**
Psen2	Rattus norvegicus presenilin 2 (Psen2), mRNA [NM_031087]	**5,856**
Efcab2	Rattus norvegicus EF-hand calcium binding domain 2 (Efcab2), mRNA [NM_001105977]	**5,856**
Gabarapl2	Rattus norvegicus GABA(A) receptor-associated protein like 2 (Gabarapl2), mRNA [NM_022706]	**5,856**
Elk4	Rattus norvegicus ELK4, ETS-domain protein (SRF accessory protein 1) (Elk4), mRNA [NM_001107173]	**5,856**
Cog8	Rattus norvegicus component of oligomeric golgi complex 8 (Cog8), mRNA [NM_001106182]	**5,855**
Aebp1	Rattus norvegicus AE binding protein 1 (Aebp1), mRNA [NM_001100970]	**5,855**
Rngtt	Rattus norvegicus RNA guanylyltransferase and 5'-phosphatase (Rngtt), mRNA [NM_001107923]	**5,855**
0	Uncharacterized protein [Source:UniProtKB/TrEMBL;Acc:D4A3B9] [ENSRNOT00000042234]	**5,854**
LOC688393	Rattus norvegicus similar to XPA binding protein 1 (LOC688393), mRNA [NM_001127572]	**5,854**
Ubxn11	Rattus norvegicus UBX domain protein 11 (Ubxn11), mRNA [NM_138853]	**5,853**
Klf4	Rattus norvegicus Kruppel-like factor 4 (gut) (Klf4), mRNA [NM_053713]	**5,852**
Tbx15	Rattus norvegicus T-box 15 (Tbx15), mRNA [NM_001106451]	**5,851**
0	Rattus norvegicus similar to High mobility group protein 1 (HMG-1) (Amphoterin) (Heparin-binding protein p30) (LOC364873), mRNA [XM_344684]	**5,851**
RGD1560271	Rattus norvegicus similar to inhibitor of MyoD family-a (RGD1560271), mRNA [NM_001109301]	**5,849**

0	Unknown	**5,849**
Prpf40a	Rattus norvegicus PRP40 pre-mRNA processing factor 40 homolog A (S. cerevisiae) (Prpf40a), mRNA [NM_001106480]	**5,848**
RGD1563528	Rattus norvegicus similar to Brain protein 44-like (RGD1563528), mRNA [NM_001109365]	**5,847**
Zdhhc23	Rattus norvegicus zinc finger, DHHC-type containing 23 (Zdhhc23), mRNA [NM_213627]	**5,847**
0	AA957328 UI-R-E1-fu-g-08-0-UI.s1 UI-R-E1 Rattus norvegicus cDNA clone UI-R-E1-fu-g-08-0-UI 3', mRNA sequence [AA957328]	**5,845**
0	Rattus norvegicus similar to RIKEN cDNA 1700081O22 (LOC363336), mRNA [XM_343674]	**5,845**
0	Mast cell protease 1-like 2 [Source:UniProtKB/TrEMBL;Acc:Q6IE56] [ENSRNOT00000038253]	**5,845**
0	Unknown	**5,844**
Trib3	Rattus norvegicus tribbles homolog 3 (Drosophila) (Trib3), mRNA [NM_144755]	**5,844**
Glmn	Rattus norvegicus glomulin, FKBP associated protein (Glmn), mRNA [NM_001105993]	**5,843**
LOC689842	PREDICTED: Rattus norvegicus similar to Nucleolar GTP-binding protein 1 (Chronic renal failure gene protein) (GTP-binding protein NGB) (LOC689842), miscRNA [XR_006738]	**5,843**
Clta	Rattus norvegicus clathrin, light chain (Lca) (Clta), mRNA [NM_031974]	**5,842**
LOC685020	PREDICTED: Rattus norvegicus paired immunoglobin-like type 2 receptor alpha (Pilra), mRNA [XM_213732]	**5,842**
Snx33	Rattus norvegicus sorting nexin 33 (Snx33), mRNA [NM_001127488]	**5,841**
LOC679774	PREDICTED: Rattus norvegicus hypothetical protein LOC679774 (LOC679774), mRNA [XM_001054422]	**5,841**
Chchd3	Rattus norvegicus coiled-coil-helix-coiled-coil-helix domain containing 3 (Chchd3), mRNA [NM_001106588]	**5,841**
0	Unknown	**5,840**
Med8	Rattus norvegicus mediator complex subunit 8 (Med8), mRNA [NM_001108673]	**5,839**
Ppa2	Rattus norvegicus pyrophosphatase (inorganic) 2 (Ppa2), nuclear gene encoding mitochondrial protein, mRNA [NM_001135871]	**5,837**
Eef1g	Rattus norvegicus eukaryotic translation elongation factor 1 gamma (Eef1g), mRNA [NM_001004223]	**5,837**
0	LRRGT00198 [Source:UniProtKB/TrEMBL;Acc:Q6QI10] [ENSRNOT00000040322]	**5,836**
Ret	Rattus norvegicus ret proto-oncogene (Ret), transcript variant 1, mRNA [NM_012643]	**5,836**
Dut	Rattus norvegicus deoxyuridine triphosphatase (Dut), nuclear gene encoding mitochondrial protein, transcript variant 1, mRNA [NM_053592]	**5,836**
0	Unknown	**5,835**
0	Unknown	**5,835**
Osm	Rattus norvegicus oncostatin M (Osm), mRNA [NM_001006961]	**5,835**
Hspa12b	Rattus norvegicus heat shock protein 12B (Hspa12b), mRNA [NM_001107778]	**5,835**
Zfand2b	Rattus norvegicus zinc finger, AN1 type domain 2B (Zfand2b), mRNA [NM_001025745]	**5,835**
Fhit	Rattus norvegicus fragile histidine triad gene (Fhit), mRNA [NM_021774]	**5,835**
Srp19	Rattus norvegicus signal recognition particle 19 (Srp19), mRNA [NM_001106157]	**5,834**
0	Rattus norvegicus TL0AEA65YG02 mRNA sequence. [FQ232533]	**5,833**
Gstcd	Rattus norvegicus glutathione S-transferase, C-terminal domain containing (Gstcd), mRNA [NM_001107725]	**5,833**
Cbx3	Rattus norvegicus chromobox homolog 3 (HP1 gamma homolog, Drosophila) (Cbx3), mRNA [NM_001008313]	**5,832**

Cldn12	Rattus norvegicus claudin 12 (Cldn12), mRNA [NM_001100813]	**5,832**
Klhl32	Uncharacterized protein [Source:UniProtKB/TrEMBL;Acc:D3ZR28] [ENSRNOT00000050653]	**5,831**
RGD1560212	Rattus norvegicus similar to DNA segment, Chr 18, Wayne State University 98, expressed (RGD1560212), mRNA [NM_001077676]	**5,829**
Tyw3	Similar to RIKEN cDNA 5230400J09 (Predicted), isoform CRA_aUncharacterized protein [Source:UniProtKB/TrEMBL;Acc:D3ZHR8] [ENSRNOT00000032874]	**5,829**
Otud7a	PREDICTED: Rattus norvegicus OTU domain containing 7A (Otud7a), mRNA [XM_219703]	**5,828**
Erap1	Rattus norvegicus endoplasmic reticulum aminopeptidase 1 (Erap1), mRNA [NM_030836]	**5,828**
Cops3	Rattus norvegicus COP9 constitutive photomorphogenic homolog subunit 3 (Arabidopsis) (Cops3), mRNA [NM_001004200]	**5,828**
0	PREDICTED: Rattus norvegicus hypothetical protein LOC680789 (LOC680789), partial mRNA [XM_001058892]	**5,828**
Mid1ip1	Rattus norvegicus MID1 interacting protein 1 (gastrulation specific G12 homolog (zebrafish)) (Mid1ip1), mRNA [NM_206950]	**5,827**
0	Unknown	**5,827**
Mprip	Rattus norvegicus myosin phosphatase Rho interacting protein (Mprip), transcript variant 1, mRNA [NM_053814]	**5,825**
Leprel2	Rattus norvegicus leprecan-like 2 (Leprel2), mRNA [NM_001106620]	**5,824**
Mcm10	Rattus norvegicus minichromosome maintenance complex component 10 (Mcm10), mRNA [NM_001107366]	**5,824**
0	Unknown	**5,824**
0	PREDICTED: Rattus norvegicus similar to RIKEN cDNA 9030221M09 gene (RGD1305469), mRNA [XM_001066163]	**5,821**
RGD1565309	PREDICTED: Rattus norvegicus similar to hypothetical protein MGC33370 (RGD1565309), mRNA [XM_573520]	**5,821**
LOC690276	Rattus norvegicus hypothetical protein LOC690276 (LOC690276), mRNA [NM_001109573]	**5,821**
Olr56	Rattus norvegicus olfactory receptor 56 (Olr56), mRNA [NM_001000130]	**5,821**
0	Unknown	**5,820**
Otud3	Rattus norvegicus OTU domain containing 3 (Otud3), mRNA [NM_001191983]	**5,820**
Spag5	Rattus norvegicus sperm associated antigen 5 (Spag5), mRNA [NM_001044224]	**5,819**
Smc6	Rattus norvegicus structural maintenance of chromosomes 6 (Smc6), mRNA [NM_001108014]	**5,819**
0	Uncharacterized protein [Source:UniProtKB/TrEMBL;Acc:D3ZFD6] [ENSRNOT00000065986]	**5,816**
Wdr18	Rattus norvegicus WD repeat domain 18 (Wdr18), mRNA [NM_001039027]	**5,814**
Gcsh	Rattus norvegicus glycine cleavage system protein H (aminomethyl carrier) (Gcsh), nuclear gene encoding mitochondrial protein, mRNA [NM_133598]	**5,814**
0	Q3QF41_9GAMM (Q3QF41) Recombination protein O, RecO, partial (5%) [TC612601]	**5,814**
0	PREDICTED: Rattus norvegicus similar to hypothetical protein FLJ32859 (RGD1306995), mRNA [XM_001069247]	**5,812**
LOC685580	RCG48030Uncharacterized protein [Source:UniProtKB/TrEMBL;Acc:D3ZML1] [ENSRNOT00000027137]	**5,812**
Bhlha15	Rattus norvegicus basic helix-loop-helix family, member a15 (Bhlha15), mRNA [NM_012863]	**5,811**
0	Unknown	**5,809**
Htr2b	Rattus norvegicus 5-hydroxytryptamine (serotonin) receptor 2B (Htr2b), mRNA [NM_017250]	**5,808**
Cep78	Rattus norvegicus centrosomal protein 78 (Cep78), mRNA [NM_021741]	**5,807**

0	Unknown	**5,807**
Kcns2	Rattus norvegicus potassium voltage-gated channel, delayed-rectifier, subfamily S, member 2 (Kcns2), mRNA [NM_023966]	**5,806**
RT1-A2	Rattus norvegicus RT1 class Ia, locus A2 (RT1-A2), mRNA [NM_001008829]	**5,804**
Edc4	Rattus norvegicus enhancer of mRNA decapping 4 (Edc4), mRNA [NM_001033068]	**5,804**
Pgrmc1	Rattus norvegicus progesterone receptor membrane component 1 (Pgrmc1), mRNA [NM_021766]	**5,803**
Invs	Rattus norvegicus inversin (Invs), mRNA [NM_001107932]	**5,803**
0	Uncharacterized protein [Source:UniProtKB/TrEMBL;Acc:D4A4N9] [ENSRNOT00000016068]	**5,802**
0	Uncharacterized protein [Source:UniProtKB/TrEMBL;Acc:D3ZHQ7] [ENSRNOT00000032430]	**5,802**
Cgm4	Rattus norvegicus carcinoembryonic antigen gene family 4 (Cgm4), mRNA [NM_012525]	**5,802**
Fert2	Rattus norvegicus fer (fms/fps related) protein kinase, testis specific 2 (Fert2), mRNA [NM_001106928]	**5,801**
Osbpl7	Rattus norvegicus oxysterol binding protein-like 7 (Osbpl7), mRNA [NM_001107044]	**5,801**
Ints4	Rattus norvegicus integrator complex subunit 4 (Ints4), mRNA [NM_001191629]	**5,800**
Ttll1	Rattus norvegicus tubulin tyrosine ligase-like family, member 1 (Ttll1), mRNA [NM_001012200]	**5,799**
Ugt2b37	Rat UDP glucuronosyltransferase-21 (UDPGTr-21) mRNA, 3' end. [M33747]	**5,799**
Sds	Rattus norvegicus serine dehydratase (Sds), mRNA [NM_053962]	**5,799**
Tlr6	Rattus norvegicus toll-like receptor 6 (Tlr6), mRNA [NM_207604]	**5,798**
Brf2	Rattus norvegicus BRF2, subunit of RNA polymerase III transcription initiation factor, BRF1-like (Brf2), mRNA [NM_001024773]	**5,797**
0	Unknown	**5,797**
Kdm4d	Rattus norvegicus lysine (K)-specific demethylase 4D (Kdm4d), mRNA [NM_001079712]	**5,797**
Pam	Rattus norvegicus peptidylglycine alpha-amidating monooxygenase (Pam), mRNA [NM_013000]	**5,796**
Lrrc33	Rattus norvegicus leucine rich repeat containing 33 (Lrrc33), mRNA [NM_001024995]	**5,796**
Zfp251	Rattus norvegicus zinc finger protein 251 (Zfp251), mRNA [NM_001191911]	**5,794**
Tert	Rattus norvegicus telomerase reverse transcriptase (Tert), mRNA [NM_053423]	**5,794**
0	Uncharacterized protein [Source:UniProtKB/TrEMBL;Acc:D4A3Z9] [ENSRNOT00000064084]	**5,794**
0	Unknown	**5,794**
Ccdc52	Rattus norvegicus coiled-coil domain containing 52 (Ccdc52), mRNA [NM_001008285]	**5,794**
RGD1308461	PREDICTED: Rattus norvegicus similar to RIKEN cDNA 4632415K11 (RGD1308461), mRNA [XM_002725396]	**5,794**
0	Unknown	**5,793**
Tmem90a	Rattus norvegicus transmembrane protein 90A (Tmem90a), mRNA [NM_001109485]	**5,792**
Hoxd4	Rattus norvegicus homeo box D4 (Hoxd4), mRNA [NM_001105885]	**5,792**
0	BF412119 UI-R-BT1-bmy-h-08-0-UI.s1 UI-R-BT1 Rattus norvegicus cDNA clone UI-R-BT1-bmy-h-08-0-UI 3', mRNA sequence [BF412119]	**5,791**
RGD1304587	Rattus norvegicus similar to RIKEN cDNA 2310033P09 (RGD1304587), mRNA [NM_001100551]	**5,790**
Itgam	integrin alpha-M [Source:RefSeq peptide;Acc:NP_036843] [ENSRNOT00000026748]	**5,790**
Sar1a	Rattus norvegicus SAR1 homolog A (S. cerevisiae) (Sar1a), mRNA [NM_001007739]	**5,788**

RGD1310081	RGD1310081 protein [Source:UniProtKB/TrEMBL;Acc:Q5XHZ1] [ENSRNOT00000020170]	**5,787**
Cpz	Rattus norvegicus carboxypeptidase Z (Cpz), mRNA [NM_031766]	**5,787**
Arpc5l	Rattus norvegicus actin related protein 2/3 complex, subunit 5-like (Arpc5l), mRNA [NM_001037767]	**5,787**
Emb	Rattus norvegicus embigin homolog (mouse) (Emb), mRNA [NM_053719]	**5,786**
RGD1561277	PREDICTED: Rattus norvegicus RGD1561277 (RGD1561277), mRNA [XM_579757]	**5,786**
Pde7a	Rattus norvegicus phosphodiesterase 7A (Pde7a), mRNA [NM_031080]	**5,785**
Skap2	Rattus norvegicus src kinase associated phosphoprotein 2 (Skap2), mRNA [NM_130413]	**5,785**
Lppr3	Rattus norvegicus lipid phosphate phosphatase-related protein type 3 (Lppr3), mRNA [NM_181634]	**5,784**
Ikzf2	Rattus norvegicus IKAROS family zinc finger 2 (Ikzf2), mRNA [NM_001106916]	**5,784**
0	Dystonin (Predicted)Uncharacterized protein [Source:UniProtKB/TrEMBL;Acc:D3ZC56] [ENSRNOT00000044452]	**5,783**
Mrpl46	Rattus norvegicus mitochondrial ribosomal protein L46 (Mrpl46), nuclear gene encoding mitochondrial protein, mRNA [NM_001013068]	**5,782**
Hapln3	Rattus norvegicus hyaluronan and proteoglycan link protein 3 (Hapln3), mRNA [NM_001008559]	**5,782**
0	Unknown	**5,781**
Ccm2	Rattus norvegicus cerebral cavernous malformation 2 (Ccm2), mRNA [NM_001126275]	**5,779**
Ddx20	Rattus norvegicus DEAD (Asp-Glu-Ala-Asp) box polypeptide 20 (Ddx20), mRNA [NM_001191711]	**5,779**
0	Unknown	**5,779**
Kcnj11	Rattus norvegicus potassium inwardly rectifying channel, subfamily J, member 11 (Kcnj11), mRNA [NM_031358]	**5,779**
Gar1	Rattus norvegicus GAR1 ribonucleoprotein homolog (yeast) (Gar1), mRNA [NM_001024306]	**5,778**
0	Rattus norvegicus similar to protocadherin alpha 12 (LOC317356), mRNA [XM_228736]	**5,777**
Olr485	Rattus norvegicus olfactory receptor 485 (Olr485), mRNA [NM_001000307]	**5,777**
Cyld	Rattus norvegicus cylindromatosis (turban tumor syndrome) (Cyld), mRNA [NM_001017380]	**5,776**
0	PREDICTED: Rattus norvegicus similar to fatty aldehyde dehydrogenase-like (LOC683865), mRNA [XM_001067816]	**5,775**
Ppp1r3e	Rattus norvegicus protein phosphatase 1, regulatory (inhibitor) subunit 3E (Ppp1r3e), mRNA [NM_001169575]	**5,775**
Chga	Rattus norvegicus chromogranin A (Chga), mRNA [NM_021655]	**5,775**
0	Unknown	**5,773**
Fahd1	Rattus norvegicus fumarylacetoacetate hydrolase domain containing 1 (Fahd1), nuclear gene encoding mitochondrial protein, mRNA [NM_001024991]	**5,773**
Ipcef1	Rattus norvegicus interactor protein for cytohesin exchange factors 1 (Ipcef1), mRNA [NM_001170799]	**5,773**
0	Unknown	**5,771**
Otx1	Rattus norvegicus orthodenticle homeobox 1 (Otx1), mRNA [NM_013109]	**5,771**
Tmsb4x	Rattus norvegicus thymosin beta 4, X-linked (Tmsb4x), mRNA [NM_031136]	**5,771**
Olr545	Rattus norvegicus olfactory receptor 545 (Olr545), mRNA [NM_001001053]	**5,771**
Fam148b	Uncharacterized protein [Source:UniProtKB/TrEMBL;Acc:D3ZDJ2] [ENSRNOT00000012048]	**5,770**

0	SAM68_RAT (Q91V33) KH domain-containing, RNA-binding, signal transduction-associated protein 1 (p21 Ras GTPase-activating protein-associated p62) (GAP-associated tyrosine phosphoprotein p62) (Src-associated in mitosis 68 kDa protein) (Sam68) (p68), partia	**5,770**
Mettl9	Rattus norvegicus methyltransferase like 9 (Mettl9), mRNA [NM_001163164]	**5,769**
RGD1307218	Rattus norvegicus similar to RIKEN cDNA 2810432L12 (RGD1307218), mRNA [NM_001014190]	**5,769**
Tnfrsf13b	PREDICTED: Rattus norvegicus tumor necrosis factor receptor superfamily, member 13B (Tnfrsf13b), mRNA [XM_001079369]	**5,768**
RGD1306809	Rattus norvegicus similar to hypothetical protein FLJ30596 (RGD1306809), mRNA [NM_001044252]	**5,768**
RGD1565883	Rattus norvegicus similar to hypothetical protein FLJ14721 (RGD1565883), mRNA [NM_001109067]	**5,768**
Ptcd1	Rattus norvegicus pentatricopeptide repeat domain 1 (Ptcd1), nuclear gene encoding mitochondrial protein, mRNA [NM_001109665]	**5,767**
Stat1	Rattus norvegicus signal transducer and activator of transcription 1 (Stat1), transcript variant beta, mRNA [NM_001034164]	**5,767**
Kctd3	Rattus norvegicus potassium channel tetramerisation domain containing 3 (Kctd3), mRNA [NM_001107199]	**5,767**
Pcm1	Rattus norvegicus pericentriolar material 1 (Pcm1), mRNA [NM_031076]	**5,766**
Rad1	Rattus norvegicus RAD1 homolog (S. pombe) (Rad1), mRNA [NM_001106419]	**5,765**
Pcdh11x	PREDICTED: Rattus norvegicus protocadherin 11 X-linked (Pcdh11x), mRNA [XM_001055860]	**5,764**
Cyp2b15	Cytochrome P450 2B15 [Source:UniProtKB/Swiss-Prot;Acc:Q64583] [ENSRNOT00000002054]	**5,764**
Atg16l1	Rattus norvegicus ATG16 autophagy related 16-like 1 (S. cerevisiae) (Atg16l1), mRNA [NM_001108809]	**5,763**
Esyt1	Rattus norvegicus extended synaptotagmin-like protein 1 (Esyt1), mRNA [NM_017249]	**5,763**
Trim47	Rattus norvegicus tripartite motif-containing 47 (Trim47), mRNA [NM_001109585]	**5,763**
0	Uncharacterized protein [Source:UniProtKB/TrEMBL;Acc:D3ZHQ7] [ENSRNOT00000057958]	**5,763**
Hck	Rattus norvegicus hemopoietic cell kinase (Hck), mRNA [NM_013185]	**5,761**
Nfrkb	Rattus norvegicus nuclear factor related to kappa B binding protein (Nfrkb), mRNA [NM_001108133]	**5,761**
RGD1305420	Rattus norvegicus similar to Nef associated protein 1 (RGD1305420), mRNA [NM_001025652]	**5,760**
Synm	Rattus norvegicus synemin, intermediate filament protein (Synm), mRNA [NM_001134858]	**5,759**
Ccdc72	coiled-coil domain-containing protein 72 [Source:RefSeq peptide;Acc:NP_001119520] [ENSRNOT00000068775]	**5,759**
Dlec1	PREDICTED: Rattus norvegicus deleted in lung and esophageal cancer 1 isoform DLEC1-N1 (Dlec1), partial mRNA [XM_001062041]	**5,758**
Bat5	Rattus norvegicus HLA-B associated transcript 5 (Bat5), mRNA [NM_212531]	**5,758**
Trit1	Rattus norvegicus tRNA isopentenyltransferase 1 (Trit1), mRNA [NM_001108676]	**5,756**
Yipf1	Rattus norvegicus Yip1 domain family, member 1 (Yipf1), mRNA [NM_199383]	**5,756**
Cbx8	Rattus norvegicus chromobox homolog 8 (Pc class homolog, Drosophila) (Cbx8), mRNA [NM_001034078]	**5,755**
Tax1bp1	Rattus norvegicus Tax1 (human T-cell leukemia virus type I) binding protein 1 (Tax1bp1), mRNA [NM_001004199]	**5,755**
Usp12	Rattus norvegicus ubiquitin specific peptidase 12 (Usp12), mRNA [NM_001166576]	**5,755**
0	UI-R-C2p-rx-c-04-0-UI.r1 UI-R-C2p Rattus norvegicus cDNA clone UI-R-C2p-rx-c-04-0-UI 5', mRNA sequence [BF545174]	**5,755**
RGD1306576	Rattus norvegicus similar to hypothetical protein (RGD1306576), mRNA [NM_001106648]	**5,755**
Opa1	Rattus norvegicus optic atrophy 1 homolog (human) (Opa1), nuclear gene encoding mitochondrial protein, mRNA [NM_133585]	**5,754**
0	Unknown	**5,754**

Mocs3	Rattus norvegicus molybdenum cofactor synthesis 3 (Mocs3), mRNA [NM_001107804]	**5,754**
Ppp2r2d	Rattus norvegicus protein phosphatase 2, regulatory subunit B, delta isoform (Ppp2r2d), mRNA [NM_144746]	**5,754**
Alad	Rattus norvegicus aminolevulinate, delta-, dehydratase (Alad), mRNA [NM_012899]	**5,753**
Gcom1	Rattus norvegicus GRINL1A complex locus (Gcom1), mRNA [NM_001014211]	**5,752**
0	Unknown	**5,751**
Mtrr	Rattus norvegicus 5-methyltetrahydrofolate-homocysteine methyltransferase reductase (Mtrr), mRNA [NM_001039003]	**5,751**
Lrrc49	Rattus norvegicus leucine rich repeat containing 49 (Lrrc49), mRNA [NM_001134469]	**5,750**
0	grp75=75 kda glucose regulated protein [rats, Sprague-Dawley, brain, mRNA, 3001 nt]. [S78556]	**5,750**
Ndfip2	Rattus norvegicus Nedd4 family interacting protein 2 (Ndfip2), mRNA [NM_001108390]	**5,748**
Tnfaip2	Rattus norvegicus tumor necrosis factor, alpha-induced protein 2 (Tnfaip2), mRNA [NM_001137633]	**5,748**
Papss2	Rattus norvegicus 3'-phosphoadenosine 5'-phosphosulfate synthase 2 (Papss2), mRNA [NM_001106375]	**5,748**
Gemin4	Rattus norvegicus gem (nuclear organelle) associated protein 4 (Gemin4), mRNA [NM_001109037]	**5,748**
0	Rattus norvegicus similar to dJ132F21.2 (Contains a novel protein similar to the L82E from Drosophila) (LOC366238), mRNA [XM_345461]	**5,747**
0	Rattus norvegicus similar to RIKEN cDNA 1110011K10 (LOC315466), mRNA [XM_235929]	**5,747**
LOC687071	PREDICTED: Rattus norvegicus similar to alanine-glyoxylate aminotransferase 2-like 1 (LOC687071), mRNA [XM_001076965]	**5,746**
Ednra	Rattus norvegicus endothelin receptor type A (Ednra), mRNA [NM_012550]	**5,746**
Stk25	Rattus norvegicus serine/threonine kinase 25 (STE20 homolog, yeast) (Stk25), mRNA [NM_184049]	**5,746**
Erf	Rattus norvegicus Ets2 repressor factor (Erf), mRNA [NM_001170335]	**5,746**
0	Unknown	**5,744**
Tmem213	Uncharacterized protein [Source:UniProtKB/TrEMBL;Acc:D3ZZA5] [ENSRNOT00000018331]	**5,744**
Zfx	Rattus norvegicus zinc finger protein X-linked (Zfx), mRNA [NM_001109017]	**5,743**
Ipo5	Uncharacterized protein [Source:UniProtKB/TrEMBL;Acc:D4A781] [ENSRNOT00000014859]	**5,743**
Arih2	Rattus norvegicus ariadne homolog 2 (Drosophila) (Arih2), mRNA [NM_001012275]	**5,743**
0	Unknown	**5,743**
0	DNJB6_MOUSE (O54946) DnaJ homolog subfamily B member 6 (Heat shock protein J2) (HSJ-2) (MRJ) (mDj4), partial (29%) [TC587510]	**5,742**
Exoc7	Rattus norvegicus exocyst complex component 7 (Exoc7), mRNA [NM_022691]	**5,742**
0	Unknown	**5,742**
F2r	Rattus norvegicus coagulation factor II (thrombin) receptor (F2r), mRNA [NM_012950]	**5,742**
0	Rattus norvegicus similar to ribosomal protein S27a (LOC364669), mRNA [XM_344565]	**5,741**
0	Uncharacterized protein [Source:UniProtKB/TrEMBL;Acc:D4A9I1] [ENSRNOT00000037171]	**5,741**
Bag4	Rattus norvegicus BCL2-associated athanogene 4 (Bag4), mRNA [NM_001025130]	**5,739**
Morc2	Rattus norvegicus MORC family CW-type zinc finger 2 (Morc2), mRNA [NM_001106016]	**5,739**
0	Cytochrome b [Source:UniProtKB/Swiss-Prot;Acc:P00159] [ENSRNOT00000042098]	**5,739**
Zfyve28	Rattus norvegicus zinc finger, FYVE domain containing 28 (Zfyve28), mRNA [NM_001107227]	**5,738**

Dhx57	Rattus norvegicus DEAH (Asp-Glu-Ala-Asp/His) box polypeptide 57 (Dhx57), mRNA [NM_001191907]	**5,737**
LOC679976	PREDICTED: Rattus norvegicus similar to ribosomal protein S27a (LOC679976), mRNA [XM_001057026]	**5,736**
Cdc42se2	Rattus norvegicus CDC42 small effector 2 (Cdc42se2), mRNA [NM_001126089]	**5,735**
Iffo1	Rattus norvegicus intermediate filament family orphan 1 (Iffo1), mRNA [NM_001108647]	**5,734**
Cee	Rattus norvegicus conserved edge expressed protein (Cee), mRNA [NM_001163320]	**5,734**
RGD1563222	Rattus norvegicus similar to RIKEN cDNA A930018P22 (RGD1563222), mRNA [NM_001108585]	**5,734**
Rffl	Rattus norvegicus ring finger and FYVE like domain containing protein (Rffl), mRNA [NM_001004068]	**5,732**
RGD1306208	Rattus norvegicus hypothetical LOC296483 (RGD1306208), mRNA [NM_001106551]	**5,732**
0	Uncharacterized protein [Source:UniProtKB/TrEMBL;Acc:D3ZKL6] [ENSRNOT00000044160]	**5,732**
0	Unknown	**5,730**
Tmem107	Rattus norvegicus transmembrane protein 107 (Tmem107), mRNA [NM_001109648]	**5,729**
Rnpep	Rattus norvegicus arginyl aminopeptidase (aminopeptidase B) (Rnpep), mRNA [NM_031097]	**5,729**
Peli3	Rattus norvegicus pellino 3 (Peli3), mRNA [NM_001127542]	**5,727**
LOC299282	Rattus norvegicus Serine protease inhibitor (LOC299282), mRNA [NM_182474]	**5,727**
MGC125239	Rattus norvegicus hypothetical protein LOC686179 (MGC125239), mRNA [NM_001077231]	**5,723**
Grb14	Rattus norvegicus growth factor receptor bound protein 14 (Grb14), mRNA [NM_031623]	**5,723**
Cttnbp2	Rattus norvegicus cortactin binding protein 2 (Cttnbp2), mRNA [NM_001114401]	**5,723**
0	Unknown	**5,722**
Park7	Rattus norvegicus Parkinson disease (autosomal recessive, early onset) 7 (Park7), mRNA [NM_057143]	**5,722**
Akap17b	Uncharacterized protein [Source:UniProtKB/TrEMBL;Acc:D3ZDR8] [ENSRNOT00000039809]	**5,722**
0	Uncharacterized protein [Source:UniProtKB/TrEMBL;Acc:D3ZGV8] [ENSRNOT00000004513]	**5,722**
0	Unknown	**5,721**
Ybx1	Rattus norvegicus Y box binding protein 1 (Ybx1), mRNA [NM_031563]	**5,720**
Sbno2	Rattus norvegicus strawberry notch homolog 2 (Drosophila) (Sbno2), mRNA [NM_001108068]	**5,720**
Zfp37	Rattus norvegicus zinc finger protein 37 (Zfp37), mRNA [NM_058209]	**5,719**
0	Rattus norvegicus similar to Zfp-29 (LOC365296), mRNA [XM_344914]	**5,718**
0	Unknown	**5,718**
Fam20a	Rattus norvegicus family with sequence similarity 20, member A (Fam20a), mRNA [NM_001012237]	**5,718**
Csnk1d	Rattus norvegicus casein kinase 1, delta (Csnk1d), mRNA [NM_139060]	**5,717**
Oxct1	Rattus norvegicus 3-oxoacid CoA transferase 1 (Oxct1), nuclear gene encoding mitochondrial protein, mRNA [NM_001127580]	**5,717**
Mdm2	Rattus norvegicus Mdm2 p53 binding protein homolog (mouse) (Mdm2), mRNA [NM_001108099]	**5,716**
0	similar to RIKEN cDNA 1700081O22 (LOC363337), mRNA [Source:RefSeq DNA;Acc:NM_001014221] [ENSRNOT00000041051]	**5,715**
Nup205	Rattus norvegicus nucleoporin 205 (Nup205), mRNA [NM_001108620]	**5,714**
Epo	Rattus norvegicus erythropoietin (Epo), mRNA [NM_017001]	**5,714**

Dph1	Rattus norvegicus DPH1 homolog (S. cerevisiae) (Dph1), mRNA [NM_001105809]	**5,714**
Ddx60	PREDICTED: Rattus norvegicus DEAD (Asp-Glu-Ala-Asp) box polypeptide 60 (Ddx60), mRNA [XM_224795]	**5,714**
Defb22	Rattus norvegicus defensin beta 22 (Defb22), mRNA [NM_134391]	**5,714**
Nfyb	Rattus norvegicus nuclear transcription factor-Y beta (Nfyb), mRNA [NM_031553]	**5,714**
Fam163b	Rattus norvegicus family with sequence similarity 163, member B (Fam163b), mRNA [NM_001109458]	**5,713**
Wnt5b	Rattus norvegicus wingless-type MMTV integration site family, member 5B (Wnt5b), mRNA [NM_001100489]	**5,713**
Nr2f6	Rattus norvegicus nuclear receptor subfamily 2, group F, member 6 (Nr2f6), mRNA [NM_139113]	**5,713**
Prima1	Rattus norvegicus proline rich membrane anchor 1 (Prima1), mRNA [NM_001108721]	**5,713**
LOC100359750	PREDICTED: Rattus norvegicus hypothetical protein LOC100359750 (LOC100359750), mRNA [XM_002728371]	**5,710**
Hspa13	Rattus norvegicus heat shock protein 13 (Hspa13), mRNA [NM_019271]	**5,708**
Mto1	Rattus norvegicus mitochondrial translation optimization 1 homolog (S. cerevisiae) (Mto1), nuclear gene encoding mitochondrial protein, mRNA [NM_001106841]	**5,707**
Smarcad1	Rattus norvegicus SWI/SNF-related, matrix-associated actin-dependent regulator of chromatin, subfamily a, containing DEAD/H box 1 (Smarcad1), mRNA [NM_001107864]	**5,706**
Gjb1	Rattus norvegicus gap junction protein, beta 1 (Gjb1), mRNA [NM_017251]	**5,704**
Usp45	Rattus norvegicus ubiquitin specific peptidase 45 (Usp45), mRNA [NM_001107918]	**5,703**
0	aldolase 1 A retrogene 1 Gene [Source:MGI Symbol;Acc:MGI:2447811] [ENSRNOT00000029141]	**5,702**
RGD1561459	Rattus norvegicus similar to RIKEN cDNA 1810020D17 (RGD1561459), mRNA [NM_001108493]	**5,701**
Prkcz	Rattus norvegicus protein kinase C, zeta (Prkcz), mRNA [NM_022507]	**5,701**
Mtnr1b	Rattus norvegicus melatonin receptor 1B (Mtnr1b), mRNA [NM_001100641]	**5,700**
Neu4	Rattus norvegicus sialidase 4 (Neu4), mRNA [NM_001108234]	**5,700**
Olr838	Rattus norvegicus olfactory receptor 838 (Olr838), mRNA [NM_001000581]	**5,699**
Rsbn1l	Rattus norvegicus round spermatid basic protein 1-like (Rsbn1l), mRNA [NM_001135872]	**5,698**
0	Unknown	**5,698**
Atl3	Rattus norvegicus atlastin GTPase 3 (Atl3), mRNA [NM_001044241]	**5,698**
Map9	Rattus norvegicus microtubule-associated protein 9 (Map9), mRNA [NM_001135716]	**5,697**
Pdcl3	Rattus norvegicus phosducin-like 3 (Pdcl3), mRNA [NM_001025709]	**5,693**
0	Unknown	**5,693**
Mrpl1	Rattus norvegicus mitochondrial ribosomal protein L1 (Mrpl1), nuclear gene encoding mitochondrial protein, mRNA [NM_001105997]	**5,691**
Renbp	Rattus norvegicus renin binding protein (Renbp), mRNA [NM_031095]	**5,690**
Tmem116	Rattus norvegicus transmembrane protein 116 (Tmem116), mRNA [NM_001159625]	**5,689**
LOC501302	PREDICTED: Rattus norvegicus hypothetical gene supported by BC082068 (LOC501302), mRNA [XM_576715]	**5,689**
Slc25a17	Rattus norvegicus solute carrier family 25 (mitochondrial carrier, peroxisomal membrane protein), member 17 (Slc25a17), nuclear gene encoding mitochondrial protein, mRNA [NM_001126269]	**5,687**

Csnk1a1	Rattus norvegicus casein kinase 1, alpha 1 (Csnk1a1), mRNA [NM_053615]	**5,686**
0	Histone H4Osteogenic growth peptide [Source:UniProtKB/Swiss-Prot;Acc:P62804] [ENSRNOT00000046363]	**5,685**
0	Unknown	**5,685**
RGD1565210	PREDICTED: Rattus norvegicus RGD1565210 (RGD1565210), mRNA [XM_001071740]	**5,683**
LOC366709	PREDICTED: Rattus norvegicus similar to prohibitin (LOC366709), miscRNA [XR_006662]	**5,683**
RGD1563666	Rattus norvegicus similar to chromosome X open reading frame 43 (RGD1563666), mRNA [NM_001134568]	**5,682**
Slc8a1	Rattus norvegicus solute carrier family 8 (sodium/calcium exchanger), member 1 (Slc8a1), mRNA [NM_019268]	**5,682**
0	Myelin-associated oligodendrocyte basic protein [Source:UniProtKB/Swiss-Prot;Acc:Q63327] [ENSRNOT00000064831]	**5,681**
Wfdc3	Rattus norvegicus WAP four-disulfide core domain 3 (Wfdc3), mRNA [NM_001106541]	**5,681**
0	RCG29594Uncharacterized protein [Source:UniProtKB/TrEMBL;Acc:D3ZE42] [ENSRNOT00000000022]	**5,680**
Drd4	Rattus norvegicus dopamine receptor D4 (Drd4), mRNA [NM_012944]	**5,679**
LOC685908	PREDICTED: Rattus norvegicus similar to Discs large homolog 5 (Placenta and prostate DLG) (Discs large protein P-dlg) (LOC685908), mRNA [XM_001065719]	**5,679**
0	Uncharacterized protein [Source:UniProtKB/TrEMBL;Acc:D3ZHQ7] [ENSRNOT00000032430]	**5,679**
0	Uncharacterized protein [Source:UniProtKB/TrEMBL;Acc:D3ZJV0] [ENSRNOT00000006968]	**5,679**
Ccdc54	Uncharacterized protein [Source:UniProtKB/TrEMBL;Acc:D4A958] [ENSRNOT00000035340]	**5,679**
0	LRRGT00153 [Source:UniProtKB/TrEMBL;Acc:Q6QI55] [ENSRNOT00000051766]	**5,678**
0	Unknown	**5,677**
Tmem60	Rattus norvegicus transmembrane protein 60 (Tmem60), mRNA [NM_001191610]	**5,676**
Zbtb5	Rattus norvegicus zinc finger and BTB domain containing 5 (Zbtb5), mRNA [NM_001106657]	**5,676**
Rt1.aa	Rattus norvegicus MHC class I RT1.Aa alpha-chain (Rt1.aa), mRNA [NM_001134701]	**5,676**
Ttr	Rattus norvegicus transthyretin (Ttr), mRNA [NM_012681]	**5,675**
0	Uncharacterized protein [Source:UniProtKB/TrEMBL;Acc:D3ZTL3] [ENSRNOT00000034097]	**5,675**
LOC690948	PREDICTED: Rattus norvegicus similar to paired-Ig-like receptor A11 (LOC690948), mRNA [XM_002725502]	**5,675**
0	WD repeat domain 34 [Source:UniProtKB/TrEMBL;Acc:Q66H05] [ENSRNOT00000021132]	**5,674**
Tdrd3	Rattus norvegicus tudor domain containing 3 (Tdrd3), mRNA [NM_001012043]	**5,674**
Pcgf3	Rattus norvegicus polycomb group ring finger 3 (Pcgf3), mRNA [NM_001107245]	**5,674**
Flot2	Rattus norvegicus flotillin 2 (Flot2), mRNA [NM_031830]	**5,674**
Dspp	Rattus norvegicus dentin sialophosphoprotein (Dspp), mRNA [NM_012790]	**5,674**
Bsg	Rattus norvegicus basigin (Bsg), transcript variant 1, mRNA [NM_001109882]	**5,674**
LOC499643	Rattus norvegicus similar to hypothetical protein FLJ25371, mRNA (cDNA clone MGC:105887 IMAGE:7132360), complete cds. [BC085354]	**5,674**
0	Unknown	**5,674**
Dnah1	Rattus norvegicus dynein, axonemal, heavy chain 1 (Dnah1), mRNA [NM_001033655]	**5,672**
Glb1l2	Rattus norvegicus galactosidase, beta 1-like 2 (Glb1l2), mRNA [NM_001192018]	**5,672**

Gng10	Rattus norvegicus guanine nucleotide binding protein (G protein), gamma 10 (Gng10), mRNA [NM_053660]	**5,672**
RGD1308195	Rattus norvegicus similar to secreted Ly6/uPAR related protein 2 (RGD1308195), mRNA [NM_001130551]	**5,671**
Arf1	Rattus norvegicus ADP-ribosylation factor 1 (Arf1), mRNA [NM_022518]	**5,671**
0	Uncharacterized protein [Source:UniProtKB/TrEMBL;Acc:D3ZI04] [ENSRNOT00000060225]	**5,669**
Rab43	Rattus norvegicus RAB43, member RAS oncogene family (Rab43), mRNA [NM_001024331]	**5,669**
Tcfap2a	Rattus norvegicus transcription factor AP-2, alpha (Tcfap2a), mRNA [NM_001107345]	**5,669**
Ptcd2	Rattus norvegicus pentatricopeptide repeat domain 2 (Ptcd2), mRNA [NM_001107648]	**5,669**
Aamp	Rattus norvegicus angio-associated, migratory cell protein (Aamp), mRNA [NM_001106920]	**5,669**
0	Unknown	**5,667**
Olr1635	Rattus norvegicus olfactory receptor 1635 (Olr1635), mRNA [NM_001000970]	**5,666**
Srp72	Rattus norvegicus signal recognition particle 72 (Srp72), mRNA [NM_001170601]	**5,666**
Rpn2	Rattus norvegicus ribophorin II (Rpn2), mRNA [NM_031698]	**5,665**
LOC100360472	PREDICTED: Rattus norvegicus Trf-proximal protein homolog (LOC100360472), mRNA [XM_002730018]	**5,665**
0	Uncharacterized protein [Source:UniProtKB/TrEMBL;Acc:D3ZBR3] [ENSRNOT00000056667]	**5,664**
Nrf1	Rattus norvegicus nuclear respiratory factor 1 (Nrf1), mRNA [NM_001100708]	**5,663**
LOC683626	Rattus norvegicus similar to limb-bud and heart (LOC683626), mRNA [NM_001129880]	**5,663**
Stmn3	Rattus norvegicus stathmin-like 3 (Stmn3), mRNA [NM_024346]	**5,662**
Emid1	Rattus norvegicus EMI domain containing 1 (Emid1), mRNA [NM_001109467]	**5,662**
LOC685304	Rattus norvegicus hypothetical protein LOC685304 (LOC685304), mRNA [NM_001195612]	**5,661**
0	Unknown	**5,661**
0	Uncharacterized protein [Source:UniProtKB/TrEMBL;Acc:D3ZRX9] [ENSRNOT00000065093]	**5,661**
Ttll7	Uncharacterized protein [Source:UniProtKB/TrEMBL;Acc:D4ACG4] [ENSRNOT00000055318]	**5,661**
0	Unknown	**5,659**
0	Unknown	**5,659**
Cdc42bpg	Rattus norvegicus CDC42 binding protein kinase gamma (DMPK-like) (Cdc42bpg), mRNA [NM_001130013]	**5,658**
0	Unknown	**5,658**
Mcts1	Rattus norvegicus malignant T cell amplified sequence 1 (Mcts1), mRNA [NM_001044237]	**5,657**
Clpb	Rattus norvegicus ClpB caseinolytic peptidase B homolog (E. coli) (Clpb), mRNA [NM_022947]	**5,657**
0	Unknown	**5,657**
0	Unknown	**5,657**
0	Unknown	**5,657**
Nufip1	Rattus norvegicus nuclear fragile X mental retardation protein interacting protein 1 (Nufip1), mRNA [NM_001007758]	**5,656**
Ppp1ca	Rattus norvegicus protein phosphatase 1, catalytic subunit, alpha isoform (Ppp1ca), mRNA [NM_031527]	**5,656**
0	Unknown	**5,656**

Ptprn2	Rattus norvegicus protein tyrosine phosphatase, receptor type, N polypeptide 2 (Ptprn2), mRNA [NM_031600]	**5,655**
LOC100125361	Rattus norvegicus zinc finger protein LOC100125361 (LOC100125361), mRNA [NM_001109677]	**5,654**
Rims2	Rattus norvegicus regulating synaptic membrane exocytosis 2 (Rims2), transcript variant 1, mRNA [NM_053945]	**5,654**
Rtn4	Rattus norvegicus reticulon 4 (Rtn4), mRNA [NM_031831]	**5,654**
0	Uncharacterized protein [Source:UniProtKB/TrEMBL;Acc:D3Z8R9] [ENSRNOT00000015806]	**5,653**
Wdr26	Rattus norvegicus WD repeat domain 26 (Wdr26), mRNA [NM_001109081]	**5,653**
0	Uncharacterized protein [Source:UniProtKB/TrEMBL;Acc:D3ZCX9] [ENSRNOT00000041504]	**5,653**
MGC116202	Rattus norvegicus hypothetical protein LOC688736 (MGC116202), mRNA [NM_001044292]	**5,653**
Nip30	Rattus norvegicus NEFA-interacting nuclear protein NIP30 (Nip30), mRNA [NM_001014014]	**5,653**
0	Unknown	**5,651**
0	Unknown	**5,650**
Nat1	Rattus norvegicus N-acetyltransferase 1 (Nat1), transcript variant 1, mRNA [NM_053853]	**5,649**
0	Uncharacterized protein [Source:UniProtKB/TrEMBL;Acc:D3ZEA5] [ENSRNOT00000047885]	**5,649**
Mtfr1	Rattus norvegicus mitochondrial fission regulator 1 (Mtfr1), nuclear gene encoding mitochondrial protein, mRNA [NM_001100977]	**5,648**
Acan	Rattus norvegicus aggrecan (Acan), mRNA [NM_022190]	**5,648**
Larp6	Rattus norvegicus La ribonucleoprotein domain family, member 6 (Larp6), mRNA [NM_001108154]	**5,648**
0	Unknown	**5,647**
Gjd3	PREDICTED: Rattus norvegicus gap junction protein, delta 3 (Gjd3), mRNA [XM_001081399]	**5,646**
Vwa5a	Rattus norvegicus von Willebrand factor A domain containing 5A (Vwa5a), mRNA [NM_198755]	**5,646**
Catsperg1	Rattus norvegicus cation channel, sperm-associated, gamma 1 (Catsperg1), mRNA [NM_001170340]	**5,646**
0	Unknown	**5,645**
Spata9	Rattus norvegicus spermatogenesis associated 9 (Spata9), mRNA [NM_001106399]	**5,645**
MGC109340	Rattus norvegicus similar to Microsomal signal peptidase 23 kDa subunit (SPase 22 kDa subunit) (SPC22/23) (MGC109340), mRNA [NM_001024267]	**5,645**
Nit1	Rattus norvegicus nitrilase 1 (Nit1), transcript variant 1, mRNA [NM_182668]	**5,644**
Sh3bp4	Rattus norvegicus SH3-domain binding protein 4 (Sh3bp4), mRNA [NM_022693]	**5,644**
Galnt6	Rattus norvegicus UDP-N-acetyl-alpha-D-galactosamine:polypeptide N-acetylgalactosaminyltransferase 6 (Galnt6), mRNA [NM_001172063]	**5,643**
0	Unknown	**5,641**
0	Unknown	**5,640**
Gadd45gip1	Rattus norvegicus growth arrest and DNA-damage-inducible, gamma interacting protein 1 (Gadd45gip1), mRNA [NM_001100504]	**5,640**
Golsyn	PREDICTED: Rattus norvegicus Golgi-localized protein (Golsyn), mRNA [XM_001064007]	**5,640**
0	Unknown	**5,639**
Map2k3	Rattus norvegicus mitogen activated protein kinase kinase 3 (Map2k3), mRNA [NM_001100674]	**5,639**
Nfkbia	Rattus norvegicus nuclear factor of kappa light polypeptide gene enhancer in B-cells inhibitor, alpha (Nfkbia), mRNA [NM_001105720]	**5,638**

Nkrf	Uncharacterized protein [Source:UniProtKB/TrEMBL;Acc:D3ZSG0] [ENSRNOT00000016660]	**5,638**
RGD1564290	PREDICTED: Rattus norvegicus similar to ribosomal protein S27a (RGD1564290), mRNA [XM_001060136]	**5,638**
Pdxk	Rattus norvegicus pyridoxal (pyridoxine, vitamin B6) kinase (Pdxk), mRNA [NM_031769]	**5,635**
Tagln	Rattus norvegicus transgelin (Tagln), mRNA [NM_031549]	**5,634**
Dync1li1	Rattus norvegicus dynein cytoplasmic 1 light intermediate chain 1 (Dync1li1), mRNA [NM_145772]	**5,633**
0	Uncharacterized protein [Source:UniProtKB/TrEMBL;Acc:D4A0Y6] [ENSRNOT00000038176]	**5,633**
Manea	Rattus norvegicus mannosidase, endo-alpha (Manea), mRNA [NM_080785]	**5,632**
0	Uncharacterized protein [Source:UniProtKB/TrEMBL;Acc:D3ZFL8] [ENSRNOT00000035387]	**5,631**
Ccdc25	Rattus norvegicus coiled-coil domain containing 25 (Ccdc25), mRNA [NM_001108382]	**5,631**
Ard1a	Rattus norvegicus ARD1 homolog A, N-acetyltransferase (S. cerevisiae) (Ard1a), mRNA [NM_001135839]	**5,631**
0	Unknown	**5,630**
Dnajc28	Rattus norvegicus DnaJ (Hsp40) homolog, subfamily C, member 28 (Dnajc28), mRNA [NM_001014124]	**5,630**
0	Unknown	**5,629**
Chd1	Rattus norvegicus chromodomain helicase DNA binding protein 1 (Chd1), mRNA [NM_001107465]	**5,627**
Acot4	Rattus norvegicus acyl-CoA thioesterase 4 (Acot4), mRNA [NM_001109440]	**5,627**
Rrp15	Rattus norvegicus ribosomal RNA processing 15 homolog (S. cerevisiae) (Rrp15), mRNA [NM_001009702]	**5,626**
Map3k14	Rattus norvegicus mitogen-activated protein kinase kinase kinase 14 (Map3k14), mRNA [NM_001108301]	**5,626**
Gtf2h1	Rattus norvegicus general transcription factor IIH, polypeptide 1 (Gtf2h1), mRNA [NM_001108485]	**5,626**
Aadacl1	Rattus norvegicus arylacetamide deacetylase-like 1 (Aadacl1), mRNA [NM_001127524]	**5,625**
Sdhb	Rattus norvegicus succinate dehydrogenase complex, subunit B, iron sulfur (Ip) (Sdhb), nuclear gene encoding mitochondrial protein, mRNA [NM_001100539]	**5,625**
Sdf4	Rattus norvegicus stromal cell derived factor 4 (Sdf4), mRNA [NM_130412]	**5,625**
Dus3l	Rattus norvegicus dihydrouridine synthase 3-like (S. cerevisiae) (Dus3l), mRNA [NM_001034923]	**5,625**
Nbr1	Rattus norvegicus neighbor of Brca1 gene 1 (Nbr1), mRNA [NM_001024765]	**5,624**
LOC680498	PREDICTED: Rattus norvegicus similar to CG31613-PA (LOC680498), mRNA [XM_001057443]	**5,624**
0	Uncharacterized protein [Source:UniProtKB/TrEMBL;Acc:D4A3Y9] [ENSRNOT00000011560]	**5,623**
0	Unknown	**5,621**
Tmx4	Rattus norvegicus thioredoxin-related transmembrane protein 4 (Tmx4), mRNA [NM_001100529]	**5,620**
0	Unknown	**5,620**
Slc39a11	Rattus norvegicus solute carrier family 39 (metal ion transporter), member 11 (Slc39a11), mRNA [NM_001013042]	**5,620**
Tmem194b	Rattus norvegicus transmembrane protein 194B (Tmem194b), mRNA [NM_001134642]	**5,618**
Gmps	Rattus norvegicus guanine monphosphate synthetase (Gmps), mRNA [NM_001024754]	**5,618**
Slc32a1	Rattus norvegicus solute carrier family 32 (GABA vesicular transporter), member 1 (Slc32a1), mRNA [NM_031782]	**5,618**
Vom1r53	Rattus norvegicus vomeronasal 1 receptor 53 (Vom1r53), mRNA [NM_001008934]	**5,617**

Aip	Rattus norvegicus aryl-hydrocarbon receptor-interacting protein (Aip), mRNA [NM_172327]	**5,616**
Cflar	Rattus norvegicus CASP8 and FADD-like apoptosis regulator (Cflar), transcript variant 1, mRNA [NM_001033864]	**5,616**
Abo2	Rattus norvegicus histo-blood group ABO system transferase 2 (Abo2), mRNA [NM_001160263]	**5,616**
Donson	Rattus norvegicus downstream neighbor of SON (Donson), mRNA [NM_001008287]	**5,615**
Anxa1	Rattus norvegicus annexin A1 (Anxa1), mRNA [NM_012904]	**5,615**
0	Unknown	**5,615**
Ppil2	Rattus norvegicus peptidylprolyl isomerase (cyclophilin)-like 2 (Ppil2), mRNA [NM_001017383]	**5,613**
Myoc	Rattus norvegicus myocilin (Myoc), mRNA [NM_030865]	**5,612**
Yeats2	Rattus norvegicus YEATS domain containing 2 (Yeats2), mRNA [NM_001109057]	**5,612**
Cdkn2d	Rattus norvegicus similar to cyclin-dependent kinase inhibitor 2D (Cdkn2d), mRNA [NM_001009719]	**5,612**
LOC100360604	Uncharacterized protein [Source:UniProtKB/TrEMBL;Acc:D3ZPN7] [ENSRNOT00000049571]	**5,611**
LOC681309	PREDICTED: Rattus norvegicus similar to Thrombospondin-3 precursor (LOC681309), miscRNA [XR_006034]	**5,611**
0	Unknown	**5,611**
Mageb3	Rattus norvegicus melanoma antigen family B, 3 (Mageb3), mRNA [NM_001106965]	**5,610**
Eno2	Rattus norvegicus enolase 2, gamma, neuronal (Eno2), mRNA [NM_139325]	**5,609**
0	RVL23074 Wackym-Soares normalized rat vestibular cDNA library Rattus norvegicus cDNA 5', mRNA sequence [DY471857]	**5,608**
0	Rattus norvegicus similar to 60S ribosomal protein L21 (LOC364769), mRNA [XM_344624]	**5,607**
C2	Rattus norvegicus complement component 2 (C2), mRNA [NM_172222]	**5,607**
Necab3	Rattus norvegicus N-terminal EF-hand calcium binding protein 3 (Necab3), mRNA [NM_001098724]	**5,607**
Prrx2	Rattus norvegicus paired related homeobox 2 (Prrx2), mRNA [NM_001105739]	**5,607**
RGD1560046	PREDICTED: Rattus norvegicus similar to 3110052M02Rik protein (RGD1560046), mRNA [XM_002725490]	**5,607**
0	RCG63555RCG63717Uncharacterized protein [Source:UniProtKB/TrEMBL;Acc:D3Z9R8] [ENSRNOT00000051411]	**5,606**
RGD1566052	Rattus norvegicus similar to elongation protein 4 homolog (RGD1566052), mRNA [NM_001115047]	**5,605**
0	Unknown	**5,605**
Gpr152	PREDICTED: Rattus norvegicus G protein-coupled receptor 152 (Gpr152), mRNA [XM_002725764]	**5,605**
Wdr4	Rattus norvegicus WD repeat domain 4 (Wdr4), mRNA [NM_001135667]	**5,605**
0	Unknown	**5,603**
0	Unknown	**5,603**
Haus8	Rattus norvegicus HAUS augmin-like complex, subunit 8 (Haus8), mRNA [NM_001024971]	**5,602**
0	Uncharacterized protein [Source:UniProtKB/TrEMBL;Acc:D4ACT3] [ENSRNOT00000042604]	**5,601**
Lipc	Rattus norvegicus lipase, hepatic (Lipc), mRNA [NM_012597]	**5,600**
0	Unknown	**5,600**
Gad1	Rattus norvegicus glutamate decarboxylase 1 (Gad1), mRNA [NM_017007]	**5,599**
Wdr53	Rattus norvegicus WD repeat domain 53 (Wdr53), mRNA [NM_001109055]	**5,599**

Tm4sf19	Rattus norvegicus transmembrane 4 L six family member 19 (Tm4sf19), mRNA [NM_001105873]	**5,599**
RGD1306001	Rattus norvegicus similar to 2210021J22Rik protein (RGD1306001), mRNA [NM_001130696]	**5,598**
LOC286987	Rattus norvegicus hemiferrin, transferrin-like protein (LOC286987), mRNA [NM_173321]	**5,598**
Vps18	Rattus norvegicus vacuolar protein sorting 18 homolog (S. cerevisiae) (Vps18), mRNA [NM_001106499]	**5,597**
Dtx2	Rattus norvegicus deltex homolog 2 (Drosophila) (Dtx2), mRNA [NM_001107157]	**5,597**
0	Unknown	**5,596**
Dgcr6	Rattus norvegicus DiGeorge syndrome critical region gene 6 (Dgcr6), mRNA [NM_001107080]	**5,595**
Nell2	Rattus norvegicus NEL-like 2 (chicken) (Nell2), mRNA [NM_031070]	**5,595**
Clp1	Rattus norvegicus CLP1, cleavage and polyadenylation factor I subunit, homolog (S. cerevisiae) (Clp1), mRNA [NM_001009599]	**5,594**
Angptl4	Rattus norvegicus angiopoietin-like 4 (Angptl4), mRNA [NM_199115]	**5,594**
0	RCG40058Uncharacterized protein [Source:UniProtKB/TrEMBL;Acc:D4A9P7] [ENSRNOT00000026267]	**5,594**
0	Uncharacterized protein [Source:UniProtKB/TrEMBL;Acc:D3ZVM3] [ENSRNOT00000059301]	**5,593**
0	zinc finger X-chromosomal protein [Source:RefSeq peptide;Acc:NP_001102487] [ENSRNOT00000007501]	**5,590**
Sox18	Rattus norvegicus SRY (sex determining region Y)-box 18 (Sox18), mRNA [NM_001024781]	**5,590**
Slc25a12	Rattus norvegicus similar to solute carrier family 25 (mitochondrial carrier, Aralar), member 12 (LOC362145), mRNA [XM_342445]	**5,590**
Jrkl	Rattus norvegicus jerky homolog-like (mouse) (Jrkl), mRNA [NM_001108122]	**5,589**
0	Unknown	**5,589**
Grb14	Rattus norvegicus growth factor receptor bound protein 14 (Grb14), mRNA [NM_031623]	**5,588**
Dlst	Rattus norvegicus dihydrolipoamide S-succinyltransferase (E2 component of 2-oxo-glutarate complex) (Dlst), mRNA [NM_001006981]	**5,588**
Nfil3	Rattus norvegicus nuclear factor, interleukin 3 regulated (Nfil3), mRNA [NM_053727]	**5,588**
0	Q6XUM4_9PSED (Q6XUM4) Integrase-like protein, partial (6%) [TC644955]	**5,588**
Eif2b1	Rattus norvegicus eukaryotic translation initiation factor 2B, subunit 1 alpha (Eif2b1), mRNA [NM_172029]	**5,587**
0	Unknown	**5,585**
Ergic2	Rattus norvegicus ERGIC and golgi 2 (Ergic2), mRNA [NM_001024984]	**5,584**
Utp3	Rattus norvegicus UTP3, small subunit (SSU) processome component, homolog (S. cerevisiae) (Utp3), mRNA [NM_001012036]	**5,584**
0	Uncharacterized protein [Source:UniProtKB/TrEMBL;Acc:D3ZEA8] [ENSRNOT00000044296]	**5,583**
Idh1	Rattus norvegicus isocitrate dehydrogenase 1 (NADP+), soluble (Idh1), mRNA [NM_031510]	**5,581**
Nov	Rattus norvegicus nephroblastoma overexpressed gene (Nov), mRNA [NM_030868]	**5,581**
LOC100363170	PREDICTED: Rattus norvegicus rCG53134-like (LOC100363170), mRNA [XM_002730165]	**5,581**
Acat1	Rattus norvegicus acetyl-coenzyme A acetyltransferase 1 (Acat1), nuclear gene encoding mitochondrial protein, mRNA [NM_017075]	**5,580**
Smurf2	Rattus norvegicus SMAD specific E3 ubiquitin protein ligase 2 (Smurf2), mRNA [NM_001107061]	**5,580**
Mthfd2	Rattus norvegicus methylenetetrahydrofolate dehydrogenase (NADP+ dependent) 2, methenyltetrahydrofolate cyclohydrolase (Mthfd2), nuclear gene encoding mitochondrial protein, mRNA [NM_001109398]	**5,579**
RGD1304592	Rattus norvegicus similar to KIAA0528 protein (RGD1304592), mRNA [NM_001134581]	**5,578**

0	Unknown	**5,578**
0	CB547247 AMGNNUC:SRPB2-00217-C8-A srpb2 (10220) Rattus norvegicus cDNA clone srpb2-00217-c8 5', mRNA sequence [CB547247]	**5,576**
Pin1	Rattus norvegicus peptidylprolyl cis/trans isomerase, NIMA-interacting 1 (Pin1), mRNA [NM_001106701]	**5,576**
Lims1	Rattus norvegicus LIM and senescent cell antigen-like domains 1 (Lims1), mRNA [NM_001145456]	**5,576**
Angpt1	Rattus norvegicus angiopoietin 1 (Angpt1), mRNA [NM_053546]	**5,576**
Lhx6	Rattus norvegicus LIM homeobox 6 (Lhx6), mRNA [NM_001107837]	**5,576**
0	RCG23367Uncharacterized protein [Source:UniProtKB/TrEMBL;Acc:D3ZPX0] [ENSRNOT00000047731]	**5,575**
Vav2	Rattus norvegicus vav 2 guanine nucleotide exchange factor (Vav2), mRNA [NM_001106563]	**5,575**
Crisp1	Rattus norvegicus cysteine-rich secretory protein 1 (Crisp1), mRNA [NM_022859]	**5,574**
RGD1310269	Rattus norvegicus hypothetical LOC314472 (RGD1310269), mRNA [NM_001126280]	**5,573**
Pgls	Rattus norvegicus 6-phosphogluconolactonase (Pgls), mRNA [NM_001106066]	**5,573**
Capsl	Rattus norvegicus calcyphosine-like (Capsl), mRNA [NM_001106417]	**5,573**
0	Rattus norvegicus similar to Ran-interacting protein MOG1 (LOC290273), mRNA [XM_224238]	**5,573**
Rrm1-ps1	PREDICTED: Rattus norvegicus similar to Ribonucleotide reductase M1 (LOC364182), miscRNA [XR_086014]	**5,572**
0	Uncharacterized protein [Source:UniProtKB/TrEMBL;Acc:D4ADL2] [ENSRNOT00000049830]	**5,571**
0	Unknown	**5,571**
Cox8b	Rattus norvegicus cytochrome c oxidase, subunit VIIIb (Cox8b), nuclear gene encoding mitochondrial protein, mRNA [NM_012786]	**5,570**
Atp2c1	Rattus norvegicus ATPase, Ca++ transporting, type 2C, member 1 (Atp2c1), mRNA [NM_131907]	**5,570**
RGD1309621	PREDICTED: Rattus norvegicus similar to hypothetical protein FLJ10652 (RGD1309621), mRNA [XM_001075984]	**5,570**
LOC288913	Rattus norvegicus similar to LEYDIG CELL TUMOR 10 KD PROTEIN (LOC288913), mRNA [NM_198728]	**5,569**
Ptcd1	Rattus norvegicus pentatricopeptide repeat domain 1 (Ptcd1), nuclear gene encoding mitochondrial protein, mRNA [NM_001109665]	**5,568**
Lingo3	Rattus norvegicus leucine rich repeat and Ig domain containing 3 (Lingo3), mRNA [NM_001109609]	**5,568**
Ppp1r10	Rattus norvegicus protein phosphatase 1, regulatory subunit 10 (Ppp1r10), mRNA [NM_022951]	**5,568**
Twf1	Rattus norvegicus twinfilin, actin-binding protein, homolog 1 (Drosophila) (Twf1), mRNA [NM_001008521]	**5,568**
0	Unknown	**5,567**
RGD1307934	PREDICTED: Rattus norvegicus similar to DNA segment, Chr 19, ERATO Doi 386, expressed (RGD1307934), mRNA [XM_001055620]	**5,566**
LOC687516	Unknown	**5,566**
Kcnj4	Rattus norvegicus potassium inwardly-rectifying channel, subfamily J, member 4 (Kcnj4), mRNA [NM_053870]	**5,565**
Notch1	Rattus norvegicus Notch homolog 1, translocation-associated (Drosophila) (Notch1), mRNA [NM_001105721]	**5,562**
Rapgef5	Rattus norvegicus Rap guanine nucleotide exchange factor (GEF) 5 (Rapgef5), mRNA [NM_001047915]	**5,562**
Khnyn	PREDICTED: Rattus norvegicus similar to mKIAA0323 protein (RGD1565688), miscRNA [XR_086021]	**5,561**
Slc31a2	Rattus norvegicus solute carrier family 31 (copper transporters), member 2 (Slc31a2), mRNA [NM_001033693]	**5,561**
Pgm3	Rattus norvegicus phosphoglucomutase 3, mRNA (cDNA clone MGC:188729 IMAGE:9027278), complete cds. [BC166838]	**5,560**
Slc15a4	Rattus norvegicus solute carrier family 15, member 4 (Slc15a4), mRNA [NM_144758]	**5,557**

0	RVL17833 Wackym-Soares normalized rat vestibular cDNA library Rattus norvegicus cDNA 5', mRNA sequence [DV725625]	**5,557**
0	Uncharacterized protein [Source:UniProtKB/TrEMBL;Acc:D3ZLA5] [ENSRNOT00000047517]	**5,556**
Magt1	Rattus norvegicus magnesium transporter 1 (Magt1), mRNA [NM_053946]	**5,556**
Ndufs4	Rattus norvegicus NADH dehydrogenase (ubiquinone) Fe-S protein 4 (Ndufs4), nuclear gene encoding mitochondrial protein, mRNA [NM_001025146]	**5,556**
RGD1565796	Rattus norvegicus similar to RIKEN cDNA A930005I04 gene (RGD1565796), mRNA [NM_001109089]	**5,554**
Gstm5	Rattus norvegicus glutathione S-transferase, mu 5 (Gstm5), mRNA [NM_172038]	**5,554**
Nagpa	Rattus norvegicus N-acetylglucosamine-1-phosphodiester alpha-N-acetylglucosaminidase (Nagpa), mRNA [NM_001108265]	**5,554**
0	Rattus norvegicus similar to Heterogeneous nuclear ribonucleoprotein A1 (Helix-destabilizing protein) (Single-strand binding protein) (hnRNP core protein A1) (HDP) (LOC312921), mRNA [XM_232629]	**5,553**
0	Unknown	**5,553**
0	Rattus norvegicus similar to 4930572L20Rik protein (LOC363857), mRNA [XM_344065]	**5,552**
Prmt7	Rattus norvegicus protein arginine methyltransferase 7 (Prmt7), mRNA [NM_001014153]	**5,552**
B4galt3	Rattus norvegicus UDP-Gal:betaGlcNAc beta 1,4-galactosyltransferase, polypeptide 3 (B4galt3), mRNA [NM_001009539]	**5,552**
Tmem59l	Transmembrane protein 59-like [Source:UniProtKB/Swiss-Prot;Acc:Q5HZE8] [ENSRNOT00000038139]	**5,551**
Rap1a	Rattus norvegicus RAP1A, member of RAS oncogene family (Rap1a), mRNA [NM_001005765]	**5,551**
Serpine3	Uncharacterized protein [Source:UniProtKB/TrEMBL;Acc:D4A9F7] [ENSRNOT00000051307]	**5,550**
Nepn	Rattus norvegicus nephrocan (Nepn), mRNA [NM_001107632]	**5,549**
Spata1	Rattus norvegicus spermatogenesis associated 1 (Spata1), mRNA [NM_001014177]	**5,548**
Cd300lg	PREDICTED: Rattus norvegicus Cd300 molecule-like family member G, transcript variant 1 (Cd300lg), mRNA [XM_001061779]	**5,548**
Rsl1d1	Rattus norvegicus ribosomal L1 domain containing 1 (Rsl1d1), mRNA [NM_001008876]	**5,546**
Rest	Rattus norvegicus RE1-silencing transcription factor (Rest), mRNA [NM_031788]	**5,546**
Veph1	Rattus norvegicus ventricular zone expressed PH domain homolog 1 (zebrafish) (Veph1), mRNA [NM_001014171]	**5,546**
0	Unknown	**5,545**
0	RGD1565232 (Predicted)Uncharacterized protein [Source:UniProtKB/TrEMBL;Acc:D3ZUG5] [ENSRNOT00000066977]	**5,544**
0	Unknown	**5,544**
Ints5	Rattus norvegicus integrator complex subunit 5 (Ints5), mRNA [NM_001107576]	**5,543**
Tmem129	Rattus norvegicus transmembrane protein 129 (Tmem129), mRNA [NM_001126274]	**5,543**
Tdo2	Rattus norvegicus tryptophan 2,3-dioxygenase (Tdo2), mRNA [NM_022403]	**5,543**
Hsp90aa1	Rattus norvegicus heat shock protein 90, alpha (cytosolic), class A member 1 (Hsp90aa1), mRNA [NM_175761]	**5,542**
0	Unknown	**5,542**
0	Unknown	**5,542**
Grn	Rattus norvegicus granulin (Grn), transcript variant 1, mRNA [NM_017113]	**5,541**
Pde4dl1	PREDICTED: Rattus norvegicus phosphodiesterase 4D, cAMP-specific-like 1 (Pde4dl1), mRNA [XM_574187]	**5,540**

Fam132a	Rattus norvegicus family with sequence similarity 132, member A (Fam132a), mRNA [NM_001108000]	**5,540**
Esr2	Rattus norvegicus estrogen receptor 2 (ER beta) (Esr2), mRNA [NM_012754]	**5,536**
Glt6d1	Rattus norvegicus glycosyltransferase 6 domain containing 1 (Glt6d1), mRNA [NM_001106559]	**5,536**
Hmgcs1	Rattus norvegicus 3-hydroxy-3-methylglutaryl-Coenzyme A synthase 1 (soluble) (Hmgcs1), mRNA [NM_017268]	**5,535**
Slc12a9	Rattus norvegicus solute carrier family 12 (potassium/chloride transporters), member 9 (Slc12a9), mRNA [NM_134405]	**5,534**
LOC685964	Rattus norvegicus hypothetical protein LOC685964 (LOC685964), mRNA [NM_001109489]	**5,533**
Depdc5	Rattus norvegicus DEP domain containing 5 (Depdc5), mRNA [NM_001107229]	**5,532**
LOC682206	PREDICTED: Rattus norvegicus similar to Zinc finger protein 208 (LOC682206), mRNA [XM_001060419]	**5,532**
0	Rattus norvegicus similar to 3-phosphoglycerate dehydrogenase (LOC294985), mRNA [XM_227056]	**5,531**
Adm	Rattus norvegicus adrenomedullin (Adm), mRNA [NM_012715]	**5,530**
Stard5	Rattus norvegicus StAR-related lipid transfer (START) domain containing 5 (Stard5), mRNA [NM_001192011]	**5,530**
Vta1	Rattus norvegicus Vps20-associated 1 homolog (S. cerevisiae) (Vta1), mRNA [NM_001025640]	**5,529**
Slco1c1	Rattus norvegicus solute carrier organic anion transporter family, member 1c1 (Slco1c1), mRNA [NM_053441]	**5,529**
Dync1i1	Rattus norvegicus dynein cytoplasmic 1 intermediate chain 1 (Dync1i1), mRNA [NM_019234]	**5,528**
Hapln2	Rattus norvegicus hyaluronan and proteoglycan link protein 2 (Hapln2), mRNA [NM_022285]	**5,528**
Smpd1	Rattus norvegicus sphingomyelin phosphodiesterase 1, acid lysosomal (Smpd1), mRNA [NM_001006997]	**5,527**
Mrpl32	Rattus norvegicus mitochondrial ribosomal protein L32 (Mrpl32), nuclear gene encoding mitochondrial protein, mRNA [NM_001106116]	**5,527**
Nol12	Rattus norvegicus nucleolar protein 12 (Nol12), mRNA [NM_001012747]	**5,527**
Cdk9	Rattus norvegicus cyclin-dependent kinase 9 (Cdk9), mRNA [NM_001007743]	**5,527**
Mpnd	Rattus norvegicus MPN domain containing (Mpnd), mRNA [NM_001085406]	**5,527**
Ola1	Rattus norvegicus Obg-like ATPase 1 (Ola1), mRNA [NM_001033927]	**5,525**
0	Unknown	**5,525**
0	Unknown	**5,524**
RGD1560187	Rattus norvegicus similar to Hypothetical UPF0327 protein (RGD1560187), mRNA [NM_001173556]	**5,524**
LOC690918	Rattus norvegicus hypothetical protein LOC690918 (LOC690918), mRNA [NM_001109613]	**5,523**
Akap11	Rattus norvegicus A kinase (PRKA) anchor protein 11 (Akap11), mRNA [NM_012773]	**5,523**
Zp4	Rattus norvegicus zona pellucida glycoprotein 4 (Zp4), mRNA [NM_172330]	**5,523**
0	Unknown	**5,522**
RGD1560997	PREDICTED: Rattus norvegicus similar to ribosomal protein S27a (RGD1560997), mRNA [XM_001053937]	**5,522**
Frmd6	FERM domain-containing protein 6 [Source:UniProtKB/Swiss-Prot;Acc:Q8VII0] [ENSRNOT00000009610]	**5,521**
RGD1562601	Uncharacterized protein [Source:UniProtKB/TrEMBL;Acc:D3ZLH3] [ENSRNOT00000048420]	**5,521**
0	MST4_MOUSE (Q99JT2) Serine/threonine-protein kinase MST4 (STE20-like kinase MST4) (MST-4) (Mammalian STE20-like protein kinase 4) , partial (32%) [TC630882]	**5,520**
Znf764	PREDICTED: Rattus norvegicus zinc finger protein 764, transcript variant 1 (Znf764), mRNA [XM_001079970]	**5,519**

Slc25a15	Rattus norvegicus solute carrier family 25 (mitochondrial carrier; ornithine transporter) member 15 (Slc25a15), nuclear gene encoding mitochondrial protein, mRNA [NM_001047880]	**5,518**
Whsc1	Rattus norvegicus Wolf-Hirschhorn syndrome candidate 1 (human) (Whsc1), mRNA [NM_001191552]	**5,517**
Nme7	Rattus norvegicus non-metastatic cells 7, protein expressed in (nucleoside-diphosphate kinase) (Nme7), mRNA [NM_138532]	**5,517**
Abca3	PREDICTED: Rattus norvegicus ATP-binding cassette, sub-family A (ABC1), member 3 (Abca3), mRNA [XM_220219]	**5,517**
Cyp2c79	Uncharacterized protein [Source:UniProtKB/TrEMBL;Acc:D3ZDQ1] [ENSRNOT00000017367]	**5,516**
0	zinc finger protein 62 Gene [Source:MGI Symbol;Acc:MGI:99662] [ENSRNOT00000003373]	**5,516**
Jakmip3	Rattus norvegicus janus kinase and microtubule interacting protein 3 (Jakmip3), mRNA [NM_001163277]	**5,516**
Tiprl	Rattus norvegicus TIP41, TOR signaling pathway regulator-like (S. cerevisiae) (Tiprl), mRNA [NM_001109667]	**5,515**
Ech1	Rattus norvegicus enoyl coenzyme A hydratase 1, peroxisomal (Ech1), mRNA [NM_022594]	**5,515**
Mrfap1	Rattus norvegicus Mof4 family associated protein 1 (Mrfap1), mRNA [NM_001009264]	**5,514**
Znf711	Uncharacterized protein [Source:UniProtKB/TrEMBL;Acc:D3ZHB2] [ENSRNOT00000006237]	**5,513**
Bcl2l13	Rattus norvegicus BCL2-like 13 (apoptosis facilitator) (Bcl2l13), nuclear gene encoding mitochondrial protein, mRNA [NM_001107885]	**5,513**
Cox7a2l	Rattus norvegicus cytochrome c oxidase subunit VIIa polypeptide 2 like (Cox7a2l), nuclear gene encoding mitochondrial protein, mRNA [NM_001106704]	**5,512**
Spns1	Rattus norvegicus spinster homolog 1 (Drosophila) (Spns1), mRNA [NM_001039208]	**5,511**
Tmem171	Rattus norvegicus transmembrane protein 171 (Tmem171), mRNA [NM_001013903]	**5,511**
Fxc1	Rattus norvegicus fractured callus expressed transcript 1 (Fxc1), mRNA [NM_053371]	**5,511**
Rc3h2	Rattus norvegicus ring finger and CCCH-type zinc finger domains 2 (Rc3h2), mRNA [NM_001107839]	**5,511**
0	Rattus norvegicus similar to beta-actin (LOC302340), mRNA [XM_228468]	**5,510**
0	Unknown	**5,510**
LOC100364597	PREDICTED: Rattus norvegicus 5-nucleotidase, cytosolic II-like (LOC100364597), mRNA [XM_002725789]	**5,510**
0	Unknown	**5,510**
RGD1560581	PREDICTED: Rattus norvegicus similar to glyceraldehyde-3-phosphate dehydrogenase (RGD1560581), miscRNA [XR_009063]	**5,510**
Tal2	Rattus norvegicus T-cell acute lymphocytic leukemia 2 (Tal2), mRNA [NM_001109462]	**5,509**
Mcoln1	Rattus norvegicus mucolipin 1 (Mcoln1), mRNA [NM_001105903]	**5,509**
Ifi44	Rattus norvegicus interferon-induced protein 44 (Ifi44), mRNA [NM_001107729]	**5,509**
Pms2	Rattus norvegicus PMS2 postmeiotic segregation increased 2 (S. cerevisiae) (Pms2), mRNA [NM_001105908]	**5,508**
0	Unknown	**5,508**
Ccdc27	Rattus norvegicus coiled-coil domain containing 27 (Ccdc27), mRNA [NM_001191063]	**5,507**
Kat3	Rattus norvegicus kynurenine aminotransferase III (Kat3), mRNA [NM_001015037]	**5,507**
Cdc14a	Rattus norvegicus CDC14 cell division cycle 14 homolog A (S. cerevisiae) (Cdc14a), transcript variant 2, mRNA [NM_001107718]	**5,507**
0	Unknown	**5,506**
LOC691975	PREDICTED: Rattus norvegicus similar to 40S ribosomal protein S17 (LOC691975), mRNA [XM_001080507]	**5,506**

0	Unknown	**5,505**
Kif5b	Rattus norvegicus kinesin family member 5B (Kif5b), mRNA [NM_057202]	**5,505**
RGD1561667	PREDICTED: Rattus norvegicus similar to putative protein kinase (RGD1561667), mRNA [XM_001054195]	**5,505**
Abo2	Rattus norvegicus histo-blood group ABO system transferase 2 (Abo2), mRNA [NM_001160263]	**5,502**
Taf1b	Rattus norvegicus TATA box binding protein (Tbp)-associated factor, RNA polymerase I, B (Taf1b), mRNA [NM_001191107]	**5,502**
Rage	Rattus norvegicus renal tumor antigen (Rage), mRNA [NM_001010965]	**5,501**
Kif26b	Rattus norvegicus kinesin family member 26B (Kif26b), mRNA [NM_001109079]	**5,501**
Rpl39	Rattus norvegicus ribosomal protein L39 (Rpl39), mRNA [NM_012875]	**5,499**
Nars	Rattus norvegicus asparaginyl-tRNA synthetase (Nars), transcript variant 1, mRNA [NM_001025635]	**5,497**
Slc25a37	Rattus norvegicus solute carrier family 25, member 37 (Slc25a37), nuclear gene encoding mitochondrial protein, mRNA [NM_001013996]	**5,497**
0	Unknown	**5,496**
Olr1867	Rattus norvegicus olfactory receptor 1867 (Olr1867), mRNA [NM_001101022]	**5,495**
LOC681153	PREDICTED: Rattus norvegicus hypothetical protein LOC681153 (LOC681153), mRNA [XM_001060542]	**5,494**
LOC687121	PREDICTED: Rattus norvegicus similar to Shc SH2-domain binding protein 1 (LOC687121), mRNA [XM_001077162]	**5,493**
Suds3	Uncharacterized protein [Source:UniProtKB/TrEMBL;Acc:D3ZYN5] [ENSRNOT00000001504]	**5,490**
Kctd19	PREDICTED: Rattus norvegicus potassium channel tetramerisation domain containing 19 (Kctd19), mRNA [XM_001075666]	**5,489**
Nop56	Rattus norvegicus NOP56 ribonucleoprotein homolog (yeast) (Nop56), mRNA [NM_001025732]	**5,489**
0	Rattus norvegicus TL0ABA3YL04 mRNA sequence. [FQ209772]	**5,489**
Ndufb7	Rattus norvegicus NADH dehydrogenase (ubiquinone) 1 beta subcomplex, 7 (Ndufb7), nuclear gene encoding mitochondrial protein, mRNA [NM_001108442]	**5,488**
Aph1b	Rattus norvegicus anterior pharynx defective 1 homolog B (C. elegans) (Aph1b), mRNA [NM_001047090]	**5,487**
0	PREDICTED: Rattus norvegicus similar to Multisynthetase complex auxiliary component p43 (LOC365837), miscRNA [XR_005797]	**5,487**
Dhx35	Rattus norvegicus DEAH (Asp-Glu-Ala-His) box polypeptide 35 (Dhx35), mRNA [NM_001108601]	**5,486**
0	Rattus norvegicus TL0ADA52YC03 mRNA sequence. [FQ228753]	**5,486**
Eml4	Rattus norvegicus echinoderm microtubule associated protein like 4 (Eml4), mRNA [NM_001108008]	**5,486**
Nolc1	Rattus norvegicus nucleolar and coiled-body phosphoprotein 1 (Nolc1), mRNA [NM_022869]	**5,485**
LOC100233176	Rattus norvegicus hypothetical protein LOC100233176 (LOC100233176), transcript variant 1, mRNA [NM_001142941]	**5,484**
RGD1308299	Rattus norvegicus similar to chromosome 6 open reading frame 130 (RGD1308299), mRNA [NM_001134596]	**5,483**
Accn4	Rattus norvegicus amiloride-sensitive cation channel 4, pituitary (Accn4), mRNA [NM_022234]	**5,483**
Ppp5c	Rattus norvegicus protein phosphatase 5, catalytic subunit (Ppp5c), mRNA [NM_031729]	**5,482**
Dse	Rattus norvegicus dermatan sulfate epimerase (Dse), mRNA [NM_001108933]	**5,482**
Mslnl	Uncharacterized protein [Source:UniProtKB/TrEMBL;Acc:D4AB62] [ENSRNOT00000060286]	**5,482**
Psmc3ip	Rattus norvegicus PSMC3 interacting protein (Psmc3ip), mRNA [NM_134458]	**5,481**
Erc2	Rattus norvegicus ELKS/RAB6-interacting/CAST family member 2 (Erc2), mRNA [NM_170787]	**5,480**

Amt	Rattus norvegicus aminomethyltransferase (Amt), nuclear gene encoding mitochondrial protein, mRNA [NM_001014004]	**5,479**
Hip1r	Rattus norvegicus huntingtin interacting protein 1 related (Hip1r), transcript variant 1, mRNA [NM_001134763]	**5,478**
Cog6	Rattus norvegicus component of oligomeric golgi complex 6 (Cog6), mRNA [NM_001004262]	**5,478**
Qrich2	Uncharacterized protein [Source:UniProtKB/TrEMBL;Acc:D3ZG97] [ENSRNOT00000034817]	**5,477**
LOC685544	Rattus norvegicus hypothetical protein LOC685544 (LOC685544), mRNA [NM_001109474]	**5,477**
Krtap16-5	Rattus norvegicus keratin associated protein 16-5 (Krtap16-5), mRNA [NM_001109424]	**5,477**
RGD1305679	Rattus norvegicus similar to 9530008L14Rik protein (RGD1305679), mRNA [NM_001014144]	**5,476**
Olr1387	Rattus norvegicus olfactory receptor 1387 (Olr1387), mRNA [NM_001000001]	**5,475**
Pld3	Rattus norvegicus phospholipase D family, member 3 (Pld3), mRNA [NM_001012167]	**5,474**
Slc22a25	Rattus norvegicus solute carrier family 22, member 25 (Slc22a25), mRNA [NM_138908]	**5,474**
Cd99l2	Rattus norvegicus CD99 molecule-like 2 (Cd99l2), mRNA [NM_134459]	**5,474**
Evc2	Rattus norvegicus Ellis van Creveld syndrome 2 homolog (human) (Evc2), mRNA [NM_001106012]	**5,473**
0	Uncharacterized protein [Source:UniProtKB/TrEMBL;Acc:D3ZH87] [ENSRNOT00000047160]	**5,473**
Cnst	Uncharacterized protein [Source:UniProtKB/TrEMBL;Acc:D3ZKK3] [ENSRNOT00000003641]	**5,472**
Ccdc99	Rattus norvegicus coiled-coil domain containing 99 (Ccdc99), mRNA [NM_001034138]	**5,472**
Fshb	Rattus norvegicus follicle stimulating hormone, beta polypeptide (Fshb), mRNA [NM_001007597]	**5,472**
Atg2a	Rattus norvegicus ATG2 autophagy related 2 homolog A (S. cerevisiae) (Atg2a), mRNA [NM_001109545]	**5,471**
LOC100364718	PREDICTED: Rattus norvegicus myeloid/lymphoid or mixed-lineage leukemia 3-like (LOC100364718), mRNA [XM_002726310]	**5,471**
Xab2	Rattus norvegicus XPA binding protein 2 (Xab2), mRNA [NM_139109]	**5,471**
Ero1lb	Rattus norvegicus ERO1-like beta (S. cerevisiae) (Ero1lb), mRNA [NM_001191893]	**5,470**
0	Unknown	**5,469**
0	Unknown	**5,469**
0	Unknown	**5,468**
Hsp90ab1	Rattus norvegicus heat shock protein 90 alpha (cytosolic), class B member 1 (Hsp90ab1), mRNA [NM_001004082]	**5,468**
RGD1309906	Rattus norvegicus similar to RIKEN cDNA 2310004I24 gene (RGD1309906), mRNA [NM_001009246]	**5,468**
0	Uncharacterized protein [Source:UniProtKB/TrEMBL;Acc:D3ZVD4] [ENSRNOT00000020523]	**5,467**
0	Rattus norvegicus TL0ABA20YK18 mRNA sequence. [FQ210882]	**5,467**
Fahd2a	Rattus norvegicus fumarylacetoacetate hydrolase domain containing 2A (Fahd2a), mRNA [NM_001134834]	**5,466**
LOC314328	Rattus norvegicus similar to chromosome 14 open reading frame 166B (LOC314328), mRNA [NM_001037783]	**5,466**
Rsl24d1	Rattus norvegicus ribosomal L24 domain containing 1 (Rsl24d1), mRNA [NM_001014212]	**5,466**
Aadacl3	Rattus norvegicus arylacetamide deacetylase-like 3 (Aadacl3), mRNA [NM_001191768]	**5,465**
Olr567	Rattus norvegicus olfactory receptor 567 (Olr567), mRNA [NM_001000326]	**5,465**
Slpil2	Rattus norvegicus antileukoproteinase-like 2 (Slpil2), mRNA [NM_001008872]	**5,465**
0	LRRGT00148 [Source:UniProtKB/TrEMBL;Acc:Q6QI60] [ENSRNOT00000058693]	**5,464**

Cox7b	Rattus norvegicus cytochrome c oxidase subunit VIIb (Cox7b), nuclear gene encoding mitochondrial protein, mRNA [NM_182819]	**5,463**
Cdc6	Rattus norvegicus cell division cycle 6 homolog (S. cerevisiae) (Cdc6), mRNA [NM_001108298]	**5,462**
Fbp2	Rattus norvegicus fructose-1,6-bisphosphatase 2 (Fbp2), mRNA [NM_053716]	**5,462**
0	Unknown	**5,459**
RGD1563996	PREDICTED: Rattus norvegicus similar to Protein UNQ9166/PRO28631 precursor (RGD1563996), mRNA [XM_001077526]	**5,458**
Mrps14	Rattus norvegicus mitochondrial ribosomal protein S14 (Mrps14), nuclear gene encoding mitochondrial protein, mRNA [NM_001105963]	**5,458**
Lnx2	Rattus norvegicus ligand of numb-protein X 2 (Lnx2), mRNA [NM_001108329]	**5,458**
Tbrg4	Rattus norvegicus transforming growth factor beta regulator 4 (Tbrg4), mRNA [NM_001012154]	**5,457**
0	Unknown	**5,455**
Tmem199	Rattus norvegicus transmembrane protein 199 (Tmem199), mRNA [NM_001024992]	**5,454**
Egf	Rattus norvegicus epidermal growth factor (Egf), mRNA [NM_012842]	**5,454**
Pcdhb9	Rattus norvegicus protocadherin beta 9 (Pcdhb9), mRNA [NM_001109390]	**5,453**
Brp44l	Rattus norvegicus brain protein 44-like (Brp44l), mRNA [NM_133561]	**5,453**
0	Unknown	**5,452**
Rab21	Rattus norvegicus RAB21, member RAS oncogene family (Rab21), mRNA [NM_001004238]	**5,451**
Tmod3	Rattus norvegicus tropomodulin 3 (Tmod3), mRNA [NM_001011997]	**5,449**
Btg1	Rattus norvegicus B-cell translocation gene 1, anti-proliferative (Btg1), mRNA [NM_017258]	**5,448**
Vav3	Rattus norvegicus vav 3 guanine nucleotide exchange factor (Vav3), mRNA [NM_001191714]	**5,447**
0	Unknown	**5,446**
Spsb4	Rattus norvegicus splA/ryanodine receptor domain and SOCS box containing 4 (Spsb4), mRNA [NM_001106849]	**5,446**
Actr1a	Rattus norvegicus ARP1 actin-related protein 1 homolog A, centractin alpha (yeast) (Actr1a), mRNA [NM_001106364]	**5,446**
Slc6a20	Rattus norvegicus solute carrier family 6 (proline IMINO transporter), member 20 (Slc6a20), mRNA [NM_133296]	**5,445**
Nog	Rattus norvegicus noggin (Nog), mRNA [NM_012990]	**5,445**
LOC690422	PREDICTED: Rattus norvegicus hypothetical protein LOC690422, transcript variant 2 (LOC690422), mRNA [XM_001072932]	**5,444**
0	Unknown	**5,442**
LOC690386	Rattus norvegicus hypothetical protein LOC690386 (LOC690386), mRNA [NM_001109586]	**5,442**
0	Histone H4Osteogenic growth peptide [Source:UniProtKB/Swiss-Prot;Acc:P62804] [ENSRNOT00000049464]	**5,441**
0	Unknown	**5,441**
LOC682666	PREDICTED: Rattus norvegicus hypothetical protein LOC682666 (LOC682666), mRNA [XM_002728534]	**5,441**
Aqp1	Rattus norvegicus aquaporin 1 (Aqp1), mRNA [NM_012778]	**5,441**
0	Serine/threonine-protein kinase TAO2 [Source:UniProtKB/Swiss-Prot;Acc:Q9JLS3] [ENSRNOT00000055018]	**5,441**
LOC363337	Rattus norvegicus similar to RIKEN cDNA 1700081O22 (LOC363337), mRNA [NM_001014221]	**5,440**
Serf1	Similar to small EDRK-rich factor 1 (Predicted), isoform CRA_aUncharacterized protein [Source:UniProtKB/TrEMBL;Acc:D3ZDW2] [ENSRNOT00000024291]	**5,440**

RGD1306917	Rattus norvegicus similar to RIKEN cDNA 2900010M23 (RGD1306917), mRNA [NM_001108528]	**5,440**
Smarcd2	Rattus norvegicus SWI/SNF related, matrix associated, actin dependent regulator of chromatin, subfamily d, member 2 (Smarcd2), mRNA [NM_031983]	**5,440**
Igfbp6	Rattus norvegicus insulin-like growth factor binding protein 6 (Igfbp6), mRNA [NM_013104]	**5,438**
Abcb1b	Rattus norvegicus ATP-binding cassette, subfamily B (MDR/TAP), member 1B (Abcb1b), mRNA [NM_012623]	**5,438**
Lrrc55	Rattus norvegicus leucine rich repeat containing 55 (Lrrc55), mRNA [NM_001122975]	**5,437**
Olr1169	Rattus norvegicus olfactory receptor 1169 (Olr1169), mRNA [NM_001000434]	**5,437**
0	Unknown	**5,437**
0	Unknown	**5,437**
0	Unknown	**5,436**
RGD1304719	Rattus norvegicus similar to RIKEN cDNA 2810002N01 (RGD1304719), mRNA [NM_001037769]	**5,436**
Bclaf1	Rattus norvegicus BCL2-associated transcription factor 1 (Bclaf1), mRNA [NM_001047852]	**5,436**
Tgs1	Rattus norvegicus trimethylguanosine synthase homolog (S. cerevisiae) (Tgs1), mRNA [NM_001107904]	**5,436**
Olr153	Rattus norvegicus olfactory receptor 153 (Olr153), mRNA [NM_001000167]	**5,436**
0	Unknown	**5,435**
0	Nipped-B homolog (Drosophila)Uncharacterized protein [Source:UniProtKB/TrEMBL;Acc:D3ZG77] [ENSRNOT00000021181]	**5,433**
Mosc2	Rattus norvegicus MOCO sulphurase C-terminal domain containing 2 (Mosc2), mRNA [NM_134410]	**5,433**
Mafk	Rattus norvegicus v-maf musculoaponeurotic fibrosarcoma oncogene homolog K (avian) (Mafk), mRNA [NM_145673]	**5,433**
RGD1563975	Rattus norvegicus similar to testis expressed sequence 13B (RGD1563975), mRNA [NM_001195270]	**5,432**
0	Unknown	**5,431**
Cage1	Rattus norvegicus cancer antigen 1 (Cage1), mRNA [NM_001012052]	**5,431**
0	Rattus norvegicus similar to GLE1-like, RNA export mediator; GLE1 (yeast homolog)-like, RNA export mediator; hGLE1 (LOC299220), mRNA [XM_234444]	**5,431**
Hsph1	Rattus norvegicus heat shock 105/110 protein 1 (Hsph1), mRNA [NM_001011901]	**5,429**
0	PREDICTED: Rattus norvegicus hypothetical protein LOC100363159 (LOC100363159), mRNA [XM_002728167]	**5,428**
0	Unknown	**5,428**
Prpf8	Rattus norvegicus PRP8 pre-mRNA processing factor 8 homolog (S. cerevisiae) (Prpf8), mRNA [NM_001191590]	**5,427**
Apbb3	Rattus norvegicus amyloid beta (A4) precursor protein-binding, family B, member 3 (Apbb3), mRNA [NM_053957]	**5,426**
0	USE1_MOUSE (Q9CQ56) USE1-like protein (D12 protein), partial (69%) [TC603663]	**5,426**
Thnsl1	Rattus norvegicus threonine synthase-like 1 (S. cerevisiae) (Thnsl1), mRNA [NM_001025035]	**5,426**
Zrsr1	Rattus norvegicus zinc finger (CCCH type), RNA binding motif and serine/arginine rich 1 (Zrsr1), mRNA [NM_001017504]	**5,425**
0	Uncharacterized protein [Source:UniProtKB/TrEMBL;Acc:D3ZG84] [ENSRNOT00000005064]	**5,422**
0	Unknown	**5,422**
Mblac1	Rattus norvegicus metallo-beta-lactamase domain containing 1 (Mblac1), mRNA [NM_001024996]	**5,421**

Sfrs1	Rattus norvegicus splicing factor, arginine/serine-rich 1 (Sfrs1), mRNA [NM_001109552]	**5,421**
LOC361985	Rattus norvegicus similar to NICE-3 (LOC361985), mRNA [NM_001014174]	**5,421**
Zfyve27	Rattus norvegicus zinc finger, FYVE domain containing 27 (Zfyve27), mRNA [NM_199104]	**5,420**
0	Uncharacterized protein [Source:UniProtKB/TrEMBL;Acc:D3ZH07] [ENSRNOT00000049215]	**5,420**
Ftsj1	Uncharacterized protein [Source:UniProtKB/TrEMBL;Acc:D3ZZA1] [ENSRNOT00000006365]	**5,420**
Sult2b1	Rattus norvegicus sulfotransferase family, cytosolic, 2B, member 1 (Sult2b1), mRNA [NM_001039665]	**5,420**
0	40S ribosomal protein S8 [Source:UniProtKB/TrEMBL;Acc:D3ZT31] [ENSRNOT00000001785]	**5,420**
0	Unknown	**5,420**
0	similar to CDNA sequence BC017647 (RGD1566149), mRNA [Source:RefSeq DNA;Acc:NM_001109038] [ENSRNOT00000045107]	**5,419**
0	Unknown	**5,419**
LOC100360919	Rattus norvegicus Immunoglobulin lambda-like polypeptide 1-like (LOC100360919), mRNA [NM_001190341]	**5,419**
Csgalnact2	Rattus norvegicus chondroitin sulfate N-acetylgalactosaminyltransferase 2 (Csgalnact2), mRNA [NM_001106616]	**5,418**
Afg3l2	Rattus norvegicus AFG3(ATPase family gene 3)-like 2 (yeast) (Afg3l2), mRNA [NM_001134864]	**5,418**
Rpl27	Rattus norvegicus ribosomal protein L27 (Rpl27), mRNA [NM_022514]	**5,417**
Pds5a	Rattus norvegicus PDS5, regulator of cohesion maintenance, homolog A (S. cerevisiae) (Pds5a), mRNA [NM_001083624]	**5,417**
Tlk2	Rattus norvegicus tousled-like kinase 2 (Tlk2), mRNA [NM_001191652]	**5,416**
0	Unknown	**5,415**
0	Unknown	**5,414**
0	ZN364_MOUSE (Q9D0C1) Zinc finger protein 364 (Rabring 7), partial (82%) [TC589182]	**5,414**
Ccpg1	Rattus norvegicus cell cycle progression 1 (Ccpg1), mRNA [NM_001108770]	**5,413**
RGD1309779	Rattus norvegicus similar to ENSANGP00000021391 (RGD1309779), mRNA [NM_001108766]	**5,413**
0	Unknown	**5,413**
0	PREDICTED: Rattus norvegicus similar to peptide deformylase-like protein (LOC690214), mRNA [XM_001073696]	**5,412**
0	Unknown	**5,411**
Tnfrsf25	Rattus norvegicus tumor necrosis factor receptor superfamily, member 25 (Tnfrsf25), mRNA [NM_001137644]	**5,410**
Cdk105	Rattus norvegicus CDK105 protein (Cdk105), mRNA [NM_134415]	**5,410**
Tmem147	Rattus norvegicus transmembrane protein 147 (Tmem147), mRNA [NM_001038494]	**5,408**
0	Unknown	**5,408**
Eapp	Rattus norvegicus E2F-associated phosphoprotein (Eapp), transcript variant 1, mRNA [NM_001134987]	**5,407**
RGD1564338	PREDICTED: Rattus norvegicus similar to aminoacylase 1 (RGD1564338), mRNA [XM_346293]	**5,407**
Rpl12	Uncharacterized protein [Source:UniProtKB/TrEMBL;Acc:D3ZJE2] [ENSRNOT00000003002]	**5,407**
Ccdc82	Rattus norvegicus coiled-coil domain containing 82 (Ccdc82), mRNA [NM_001007660]	**5,406**
0	Uncharacterized protein [Source:UniProtKB/TrEMBL;Acc:D3ZG02] [ENSRNOT00000029178]	**5,406**
Zfp280b	Rattus norvegicus zinc finger protein 280b (Zfp280b), mRNA [NM_001106384]	**5,405**

Cecr5	Rattus norvegicus cat eye syndrome chromosome region, candidate 5 homolog (human) (Cecr5), mRNA [NM_001107884]	**5,404**
0	Rattus norvegicus similar to ribosomal protein S15 (LOC295574), mRNA [XM_227850]	**5,403**
Clpp	PREDICTED: Rattus norvegicus ClpP caseinolytic peptidase, ATP-dependent, proteolytic subunit homolog (E. coli) (Clpp), mRNA [XM_217313]	**5,403**
Fbxl2	PREDICTED: Rattus norvegicus F-box and leucine-rich repeat protein 2 (Fbxl2), mRNA [XM_001076670]	**5,403**
0	Rattus norvegicus similar to RIKEN cDNA 1700039A02 (LOC363397), mRNA [XM_343727]	**5,403**
Akap10	Rattus norvegicus A kinase (PRKA) anchor protein 10 (Akap10), nuclear gene encoding mitochondrial protein, mRNA [NM_001114606]	**5,403**
0	Uncharacterized protein [Source:UniProtKB/TrEMBL;Acc:D3ZY83] [ENSRNOT00000033315]	**5,403**
Prr11	Rattus norvegicus proline rich 11 (Prr11), mRNA [NM_001108287]	**5,403**
0	Natural resistance-associated macrophage protein 2 [Source:UniProtKB/Swiss-Prot;Acc:O54902] [ENSRNOT00000026494]	**5,402**
Trmt5	Rattus norvegicus TRM5 tRNA methyltransferase 5 homolog (S. cerevisiae) (Trmt5), mRNA [NM_001108713]	**5,402**
0	Uncharacterized protein [Source:UniProtKB/TrEMBL;Acc:D3ZNV0] [ENSRNOT00000064946]	**5,402**
LOC497978	Rattus norvegicus similar to diacylglycerol kinase epsilon (LOC497978), mRNA [NM_001039341]	**5,402**
Mospd1	Rattus norvegicus motile sperm domain containing 1 (Mospd1), mRNA [NM_001014107]	**5,401**
RGD1563835	60S ribosomal protein L27 [Source:UniProtKB/TrEMBL;Acc:D3ZTK5] [ENSRNOT00000042756]	**5,401**
Cald1	Rattus norvegicus caldesmon 1 (Cald1), mRNA [NM_013146]	**5,401**
Gba2	Rattus norvegicus glucosidase beta 2 (Gba2), mRNA [NM_001013091]	**5,400**
Crygs	Rattus norvegicus crystallin, gamma S (Crygs), mRNA [NM_001109553]	**5,399**
Car6	Rattus norvegicus carbonic anhydrase 6 (Car6), mRNA [NM_001134841]	**5,399**
0	Unknown	**5,399**
0	RVL17674 Wackym-Soares normalized rat vestibular cDNA library Rattus norvegicus cDNA 5', mRNA sequence [DV725494]	**5,399**
0	Unknown	**5,398**
0	Unknown	**5,397**
0	Unknown	**5,397**
Rnf103	Rattus norvegicus ring finger protein 103 (Rnf103), mRNA [NM_053438]	**5,395**
0	Unknown	**5,394**
Tysnd1	Rattus norvegicus trypsin domain containing 1 (Tysnd1), mRNA [NM_001108932]	**5,394**
Hist1h2af	Rattus norvegicus histone cluster 1, H2af (Hist1h2af), mRNA [NM_001024282]	**5,391**
0	Unknown	**5,391**
Mrps18c	Rattus norvegicus mitochondrial ribosomal protein S18C (Mrps18c), nuclear gene encoding mitochondrial protein, mRNA [NM_001105996]	**5,390**
Mesdc2	Rattus norvegicus mesoderm development candidate 2 (Mesdc2), mRNA [NM_001008345]	**5,389**
Tradd	Rattus norvegicus TNFRSF1A-associated via death domain (Tradd), mRNA [NM_001100480]	**5,388**
Alg6	Rattus norvegicus asparagine-linked glycosylation 6, alpha-1,3-glucosyltransferase homolog (S. cerevisiae) (Alg6), mRNA [NM_001033709]	**5,388**
LOC100361629	Uncharacterized protein [Source:UniProtKB/TrEMBL;Acc:D4A202] [ENSRNOT00000061017]	**5,387**
Mkx	Iroquois homeobox protein-like 1 (Predicted)Uncharacterized protein [Source:UniProtKB/TrEMBL;Acc:D3ZUL2] [ENSRNOT00000025623]	**5,386**

Psap	Rattus norvegicus prosaposin (Psap), transcript variant 1, mRNA [NM_013013]	**5,385**
RGD1311269	Rattus norvegicus similar to hypothetical protein FLJ37953 (RGD1311269), transcript variant 1, mRNA [NM_001170473]	**5,385**
Sc65	Rattus norvegicus synaptonemal complex protein SC65 (Sc65), mRNA [NM_021581]	**5,385**
Klhl2	PREDICTED: Rattus norvegicus kelch-like 2, Mayven (Drosophila) (Klhl2), mRNA [XM_001073589]	**5,384**
Dynlrb2	Rattus norvegicus dynein light chain roadblock-type 2 (Dynlrb2), mRNA [NM_001108451]	**5,384**
Orc4l	Rattus norvegicus origin recognition complex, subunit 4-like (yeast) (Orc4l), mRNA [NM_199092]	**5,384**
Ttf2	Rattus norvegicus transcription termination factor, RNA polymerase II (Ttf2), mRNA [NM_001106454]	**5,383**
Spon2	Rattus norvegicus spondin 2, extracellular matrix protein (Spon2), mRNA [NM_138533]	**5,382**
Lrrcc1	Rattus norvegicus leucine rich repeat and coiled-coil domain containing 1 (Lrrcc1), mRNA [NM_001100645]	**5,382**
Canx	Rattus norvegicus calnexin (Canx), mRNA [NM_172008]	**5,382**
Acot3	Rattus norvegicus acyl-CoA thioesterase 3 (Acot3), mRNA [NM_001108041]	**5,382**
0	Q69Z61_MOUSE (Q69Z61) MKIAA1935 protein (Fragment), partial (13%) [TC582589]	**5,381**
Pck2	Rattus norvegicus phosphoenolpyruvate carboxykinase 2 (mitochondrial) (Pck2), nuclear gene encoding mitochondrial protein, mRNA [NM_001108377]	**5,380**
Htr2a	Rattus norvegicus 5-hydroxytryptamine (serotonin) receptor 2A (Htr2a), mRNA [NM_017254]	**5,380**
Serinc1	Rattus norvegicus serine incorporator 1 (Serinc1), mRNA [NM_182951]	**5,379**
Rab3il1	Rattus norvegicus RAB3A interacting protein (rabin3)-like 1 (Rab3il1), mRNA [NM_134411]	**5,378**
0	Unknown	**5,378**
Asmt	Rattus norvegicus acetylserotonin O-methyltransferase (Asmt), mRNA [NM_144759]	**5,377**
RGD1565459	Uncharacterized protein [Source:UniProtKB/TrEMBL;Acc:D3ZXK6] [ENSRNOT00000042056]	**5,377**
Usf1	Rattus norvegicus upstream transcription factor 1 (Usf1), mRNA [NM_031777]	**5,376**
Mypn	Rattus norvegicus myopalladin (Mypn), mRNA [NM_001107628]	**5,376**
Epha10	Rattus norvegicus EPH receptor A10 (Epha10), mRNA [NM_001135709]	**5,375**
0	Unknown	**5,375**
Pcgf6	Rattus norvegicus polycomb group ring finger 6 (Pcgf6), mRNA [NM_001013154]	**5,375**
Tcfe2a	Rattus norvegicus transcription factor E2a (Tcfe2a), transcript variant 1, mRNA [NM_133524]	**5,374**
Cd3eap	Rattus norvegicus CD3e molecule, epsilon associated protein (Cd3eap), mRNA [NM_001109416]	**5,374**
0	Unknown	**5,374**
Stk38l	Rattus norvegicus serine/threonine kinase 38 like (Stk38l), mRNA [NM_001083336]	**5,374**
Elfn1	Rattus norvegicus extracellular leucine-rich repeat and fibronectin type III domain containing 1 (Elfn1), mRNA [NM_001105913]	**5,372**
Cep152	PREDICTED: Rattus norvegicus centrosomal protein 152 (Cep152), mRNA [XM_002726171]	**5,371**
Timeless	Rattus norvegicus timeless homolog (Drosophila) (Timeless), mRNA [NM_031340]	**5,370**
Mrpl3	Rattus norvegicus mitochondrial ribosomal protein L3 (Mrpl3), nuclear gene encoding mitochondrial protein, mRNA [NM_001106852]	**5,370**
Padi3	Rattus norvegicus peptidyl arginine deiminase, type III (Padi3), mRNA [NM_017230]	**5,370**

Vom2r13	Rattus norvegicus vomeronasal 2 receptor, 13 (Vom2r13), mRNA [NM_001099489]	**5,370**
Gabarapl2	Rattus norvegicus GABA(A) receptor-associated protein like 2 (Gabarapl2), mRNA [NM_022706]	**5,369**
0	Unknown	**5,369**
Cox18	Rattus norvegicus COX18 cytochrome c oxidase assembly homolog (S. cerevisiae) (Cox18), nuclear gene encoding mitochondrial protein, mRNA [NM_001106000]	**5,368**
Fam65c	Uncharacterized protein [Source:UniProtKB/TrEMBL;Acc:D3ZX40] [ENSRNOT00000035463]	**5,366**
Csf1r	Rattus norvegicus colony stimulating factor 1 receptor (Csf1r), mRNA [NM_001029901]	**5,366**
Polr3h	Rattus norvegicus polymerase (RNA) III (DNA directed) polypeptide H (Polr3h), mRNA [NM_001127295]	**5,366**
RGD1307161	Rattus norvegicus similar to 0610010K06Rik protein (RGD1307161), mRNA [NM_001025669]	**5,365**
Strap	Rattus norvegicus serine/threonine kinase receptor associated protein (Strap), mRNA [NM_001011969]	**5,365**
0	Unknown	**5,365**
Mall	Rattus norvegicus mal, T-cell differentiation protein-like (Mall), mRNA [NM_001014182]	**5,364**
Centd3	PREDICTED: Rattus norvegicus centaurin, delta 3 (Centd3), miscRNA [XR_085668]	**5,364**
Mark4	Rattus norvegicus MAP/microtubule affinity-regulating kinase 4 (Mark4), mRNA [NM_001191071]	**5,364**
Igfbpl1	Rattus norvegicus insulin-like growth factor binding protein-like 1 (Igfbpl1), mRNA [NM_001108972]	**5,364**
Itch	Rattus norvegicus itchy E3 ubiquitin protein ligase homolog (mouse) (Itch), mRNA [NM_001005887]	**5,364**
0	Unknown	**5,361**
Pdzrn4	Rattus norvegicus PDZ domain containing RING finger 4 (Pdzrn4), mRNA [NM_001108107]	**5,361**
Sdccag10	Rattus norvegicus serologically defined colon cancer antigen 10 (Sdccag10), mRNA [NM_001013199]	**5,361**
0	Unknown	**5,360**
Aftph	PREDICTED: Rattus norvegicus aftiphilin (Aftph), mRNA [XM_001055784]	**5,359**
0	Unknown	**5,357**
Socs7	Rattus norvegicus similar to SH2 domain containing SOCS box protein SOCS7 (LOC287659), mRNA [XM_213443]	**5,357**
Npepo	Rattus norvegicus aminopeptidase O (Npepo), mRNA [NM_001012346]	**5,357**
Hif1an	Rattus norvegicus hypoxia-inducible factor 1, alpha subunit inhibitor (Hif1an), mRNA [NM_001113749]	**5,357**
Lsg1	Rattus norvegicus large subunit GTPase 1 homolog (S. cerevisiae) (Lsg1), mRNA [NM_001013421]	**5,356**
Fam151b	Rattus norvegicus family with sequence similarity 151, member B (Fam151b), mRNA [NM_001134748]	**5,356**
Myh3	Rattus norvegicus myosin, heavy chain 3, skeletal muscle, embryonic (Myh3), mRNA [NM_012604]	**5,355**
0	Uncharacterized protein [Source:UniProtKB/TrEMBL;Acc:D3ZEB6] [ENSRNOT00000010789]	**5,355**
0	phosphatidylinositol glycan anchor biosynthesis, class A [Source:RefSeq peptide;Acc:NP_001102286] [ENSRNOT00000004733]	**5,354**
RGD1305007	Rattus norvegicus similar to RIKEN cDNA 1110031I02 (RGD1305007), mRNA [NM_001013976]	**5,353**
RGD1563259	Uncharacterized protein [Source:UniProtKB/TrEMBL;Acc:D3ZXP2] [ENSRNOT00000041819]	**5,352**
Olr1229	Rattus norvegicus olfactory receptor 1229 (Olr1229), mRNA [NM_001000444]	**5,350**
LOC499779	Uncharacterized protein C9orf119 homolog [Source:UniProtKB/Swiss-Prot;Acc:Q63ZV7] [ENSRNOT00000030558]	**5,349**

Fam114a2	PREDICTED: Rattus norvegicus family with sequence similarity 114, member A2 (Fam114a2), mRNA [XM_220442]	**5,349**
Thoc6	Rattus norvegicus THO complex 6 homolog (Drosophila) (Thoc6), mRNA [NM_024384]	**5,348**
Lgals4	Rattus norvegicus lectin, galactoside-binding, soluble, 4 (Lgals4), mRNA [NM_012975]	**5,347**
0	Unknown	**5,346**
0	Unknown	**5,346**
0	Rattus norvegicus cDNA clone IMAGE:7388791. [BC158842]	**5,346**
Mepe	Rattus norvegicus matrix extracellular phosphoglycoprotein (Mepe), mRNA [NM_024142]	**5,346**
RGD1566314	Uncharacterized protein [Source:UniProtKB/TrEMBL;Acc:D3ZPU0] [ENSRNOT00000027087]	**5,345**
Raf1	Rattus norvegicus v-raf-leukemia viral oncogene 1 (Raf1), mRNA [NM_012639]	**5,345**
Ceacam20	Rattus norvegicus carcinoembryonic antigen-related cell adhesion molecule 20 (Ceacam20), mRNA [NM_001170324]	**5,345**
Zfp438	Rattus norvegicus zinc finger protein 438 (Zfp438), mRNA [NM_001107358]	**5,345**
37681	Rattus norvegicus membrane-associated ring finger (C3HC4) 3 (March3), mRNA [NM_001007759]	**5,344**
0	Unknown	**5,344**
Rabep1	Rattus norvegicus rabaptin, RAB GTPase binding effector protein 1 (Rabep1), mRNA [NM_019124]	**5,344**
Folr1	Rattus norvegicus folate receptor 1 (adult) (Folr1), mRNA [NM_133527]	**5,344**
0	Unknown	**5,344**
RT1-M5	Rattus norvegicus RT1 class Ib, locus M5 (RT1-M5), transcript variant 2, mRNA [NM_001048045]	**5,343**
0	Oxysterol-binding protein [Source:UniProtKB/TrEMBL;Acc:D3ZHZ3] [ENSRNOT00000042964]	**5,342**
Als2cr4	Rattus norvegicus amyotrophic lateral sclerosis 2 (juvenile) chromosome region, candidate 4 (Als2cr4), mRNA [NM_001108220]	**5,342**
0	Unknown	**5,342**
Acot8	Rattus norvegicus acyl-CoA thioesterase 8 (Acot8), mRNA [NM_130756]	**5,342**
Glt8d1	Rattus norvegicus glycosyltransferase 8 domain containing 1 (Glt8d1), mRNA [NM_001007683]	**5,342**
Tmem45a	Rattus norvegicus transmembrane protein 45A (Tmem45a), mRNA [NM_001191074]	**5,341**
0	Unknown	**5,341**
Atp6v1d	Rattus norvegicus ATPase, H+ transporting, lysosomal V1 subunit D (Atp6v1d), mRNA [NM_199386]	**5,340**
0	Unknown	**5,339**
0	Unknown	**5,339**
LOC688972	PREDICTED: Rattus norvegicus similar to Glycophorin (LOC688972), mRNA [XM_001069014]	**5,339**
Nmb	Rattus norvegicus neuromedin B (Nmb), mRNA [NM_001109149]	**5,338**
Epc2	Rattus norvegicus enhancer of polycomb homolog 2 (Drosophila) (Epc2), mRNA [NM_001108581]	**5,338**
0	Unknown	**5,337**
Manba	Rattus norvegicus mannosidase, beta A, lysosomal (Manba), mRNA [NM_001031655]	**5,334**
0	Uncharacterized protein [Source:UniProtKB/TrEMBL;Acc:D3ZIK8] [ENSRNOT00000025790]	**5,334**
Tsen15	Rattus norvegicus tRNA splicing endonuclease 15 homolog (S. cerevisiae) (Tsen15), mRNA [NM_001105958]	**5,333**

Med22	Rattus norvegicus mediator complex subunit 22 (Med22), mRNA [NM_001077679]	**5,333**
Olr889	Rattus norvegicus olfactory receptor 889 (Olr889), mRNA [NM_001000061]	**5,331**
0	Unknown	**5,331**
Irf7	Rattus norvegicus interferon regulatory factor 7 (Irf7), mRNA [NM_001033691]	**5,330**
0	Unknown	**5,330**
Sfmbt1	Rattus norvegicus Scm-like with four mbt domains 1 (Sfmbt1), mRNA [NM_031647]	**5,329**
0	Unknown	**5,329**
Cggbp1	Rattus norvegicus CGG triplet repeat binding protein 1 (Cggbp1), mRNA [NM_001105900]	**5,328**
Osbpl11	Rattus norvegicus oxysterol binding protein-like 11 (Osbpl11), mRNA [NM_001107090]	**5,328**
0	Unknown	**5,328**
Fosl2	Rattus norvegicus fos-like antigen 2 (Fosl2), mRNA [NM_012954]	**5,328**
Pgam2	Rattus norvegicus phosphoglycerate mutase 2 (muscle) (Pgam2), mRNA [NM_017328]	**5,327**
Piwil2	Rattus norvegicus piwi-like 2 (Drosophila) (Piwil2), mRNA [NM_001107276]	**5,327**
Tmem11	Rattus norvegicus transmembrane protein 11 (Tmem11), transcript variant 2, mRNA [NM_001166165]	**5,326**
Mgat4c	Rattus norvegicus mannosyl (alpha-1,3-)-glycoprotein beta-1,4-N-acetylglucosaminyltransferase, isozyme C (putative) (Mgat4c), mRNA [NM_001135814]	**5,326**
Nphs2	Rattus norvegicus nephrosis 2 homolog (human) (Nphs2), mRNA [NM_130828]	**5,326**
0	Uncharacterized protein [Source:UniProtKB/TrEMBL;Acc:D3ZK88] [ENSRNOT00000061600]	**5,325**
Dyt1	Rattus norvegicus dystonia 1 (Dyt1), mRNA [NM_153303]	**5,324**
Gas8	Rattus norvegicus growth arrest specific 8 (Gas8), mRNA [NM_001039030]	**5,324**
Smok2a	Rattus norvegicus sperm motility kinase 2A (Smok2a), mRNA [NM_001191619]	**5,324**
0	Unknown	**5,323**
Ptpn22	Rattus norvegicus protein tyrosine phosphatase, non-receptor type 22 (lymphoid) (Ptpn22), mRNA [NM_001106460]	**5,323**
Dhodh	Rattus norvegicus dihydroorotate dehydrogenase (Dhodh), nuclear gene encoding mitochondrial protein, mRNA [NM_001008553]	**5,321**
Hdac3	Rattus norvegicus histone deacetylase 3 (Hdac3), mRNA [NM_053448]	**5,320**
Cdyl2	Rattus norvegicus chromodomain protein, Y chromosome-like 2 (Cdyl2), mRNA [NM_001106189]	**5,319**
LOC682202	Uncharacterized protein [Source:UniProtKB/TrEMBL;Acc:D3ZFR6] [ENSRNOT00000043066]	**5,319**
Amh	Rattus norvegicus anti-Mullerian hormone (Amh), mRNA [NM_012902]	**5,319**
Cog7	Rattus norvegicus component of oligomeric golgi complex 7 (Cog7), mRNA [NM_001033889]	**5,319**
Rpusd2	Rattus norvegicus RNA pseudouridylate synthase domain containing 2 (Rpusd2), mRNA [NM_001135845]	**5,319**
Klf13	Rattus norvegicus Kruppel-like factor 13 (Klf13), mRNA [NM_001109147]	**5,316**
Agpat6	Rattus norvegicus 1-acylglycerol-3-phosphate O-acyltransferase 6 (lysophosphatidic acid acyltransferase, zeta) (Agpat6), mRNA [NM_001047849]	**5,316**
Nupl1	Nucleoporin p58/p45 [Source:UniProtKB/Swiss-Prot;Acc:P70581] [ENSRNOT00000017204]	**5,315**
0	Unknown	**5,315**

Rab11fip4	Rattus norvegicus RAB11 family interacting protein 4 (class II) (Rab11fip4), mRNA [NM_001107023]	**5,315**
LOC499782	Rattus norvegicus similar to 60S ribosomal protein L12 (LOC499782), mRNA [NM_001109198]	**5,314**
LOC363060	Rattus norvegicus similar to RIKEN cDNA 1600029D21 (LOC363060), mRNA [NM_001014209]	**5,314**
Tbx21	Rattus norvegicus T-box 21 (Tbx21), mRNA [NM_001107043]	**5,314**
0	Unknown	**5,314**
Ctr9	Rattus norvegicus Ctr9, Paf1/RNA polymerase II complex component, homolog (S. cerevisiae) (Ctr9), mRNA [NM_001100661]	**5,314**
Chm	Rattus norvegicus choroideremia (Rab escort protein 1) (Chm), mRNA [NM_017067]	**5,313**
0	Unknown	**5,312**
Pfkm	Rattus norvegicus phosphofructokinase, muscle (Pfkm), mRNA [NM_031715]	**5,312**
Sgol2	Uncharacterized protein [Source:UniProtKB/TrEMBL;Acc:D3ZWI4] [ENSRNOT00000029526]	**5,312**
0	Unknown	**5,312**
Pbp2	Rattus norvegicus phosphatidylethanolamine binding protein 2 (Pbp2), mRNA [NM_001105756]	**5,311**
0	Rattus norvegicus TL0AEA5YK19 mRNA sequence. [FQ233969]	**5,309**
Skil	Uncharacterized protein [Source:UniProtKB/TrEMBL;Acc:D3ZWL1] [ENSRNOT00000013191]	**5,308**
LOC690460	Rattus norvegicus similar to keratin associated protein 10-7 (LOC690460), mRNA [NM_001109593]	**5,308**
Gpr25	RCG45981Uncharacterized protein [Source:UniProtKB/TrEMBL;Acc:D4A7G6] [ENSRNOT00000011368]	**5,307**
0	Uncharacterized protein [Source:UniProtKB/TrEMBL;Acc:D3ZKC7] [ENSRNOT00000064975]	**5,307**
LOC681336	PREDICTED: Rattus norvegicus similar to spermatogenesis associated glutamate (E)-rich protein 4d (LOC681336), mRNA [XM_001061313]	**5,306**
RGD1560010	Rattus norvegicus RGD1560010 (RGD1560010), mRNA [NM_001107685]	**5,306**
Fam91a1	Rattus norvegicus family with sequence similarity 91, member A1 (Fam91a1), mRNA [NM_001127578]	**5,305**
LOC682131	PREDICTED: Rattus norvegicus similar to KRAB-zinc finger protein (LOC682131), mRNA [XM_001060021]	**5,304**
Tect2	Rattus norvegicus tectonic 2 (Tect2), mRNA [NM_001080782]	**5,304**
Ccnt2	Rattus norvegicus cyclin T2 (Ccnt2), mRNA [NM_001107171]	**5,304**
RGD1310185	Uncharacterized protein [Source:UniProtKB/TrEMBL;Acc:D3ZCD2] [ENSRNOT00000032301]	**5,304**
0	Unknown	**5,304**
0	Uncharacterized protein [Source:UniProtKB/TrEMBL;Acc:D3ZAW7] [ENSRNOT00000041850]	**5,304**
0	Unknown	**5,303**
0	Unknown	**5,302**
Tob1	Rattus norvegicus transducer of ErbB-2.1 (Tob1), mRNA [NM_133317]	**5,300**
Yaf2	Rattus norvegicus YY1 associated factor 2 (Yaf2), mRNA [NM_001134871]	**5,299**
Rpe	Rattus norvegicus ribulose-5-phosphate-3-epimerase (Rpe), mRNA [NM_001033903]	**5,299**
Sympk	Rattus norvegicus symplekin (Sympk), mRNA [NM_001100830]	**5,299**
Plaa	Rattus norvegicus phospholipase A2, activating protein (Plaa), mRNA [NM_053866]	**5,298**
Dnai2	Rattus norvegicus dynein, axonemal, intermediate chain 2 (Dnai2), mRNA [NM_001007726]	**5,298**

Nisch	Nischarin [Source:UniProtKB/Swiss-Prot;Acc:Q4G017] [ENSRNOT00000066465]	**5,298**
0	Unknown	**5,297**
Hsp90aa1	Rattus norvegicus heat shock protein 90, alpha (cytosolic), class A member 1 (Hsp90aa1), mRNA [NM_175761]	**5,296**
Cldn11	Rattus norvegicus claudin 11 (Cldn11), mRNA [NM_053457]	**5,296**
Snrnp35	Rattus norvegicus small nuclear ribonucleoprotein 35 (U11/U12) (Snrnp35), mRNA [NM_001014127]	**5,295**
LOC688632	PREDICTED: Rattus norvegicus similar to 60S ribosomal protein L17 (L23) (Amino acid starvation-induced protein) (ASI) (LOC688632), mRNA [XM_001067694]	**5,295**
Pdia4	Rattus norvegicus protein disulfide isomerase family A, member 4 (Pdia4), mRNA [NM_053849]	**5,294**
Nanos1	Uncharacterized protein [Source:UniProtKB/TrEMBL;Acc:D4A1F8] [ENSRNOT00000036203]	**5,294**
LOC501033	Uncharacterized protein [Source:UniProtKB/TrEMBL;Acc:D3ZGF2] [ENSRNOT00000018553]	**5,294**
Socs3	Rattus norvegicus suppressor of cytokine signaling 3 (Socs3), mRNA [NM_053565]	**5,293**
Mdfic	Rattus norvegicus MyoD family inhibitor domain containing (Mdfic), mRNA [NM_001105668]	**5,292**
Rnf149	Uncharacterized protein [Source:UniProtKB/TrEMBL;Acc:D3ZI66] [ENSRNOT00000018684]	**5,292**
Cntnap5a	Rattus norvegicus contactin associated protein-like 5A (Cntnap5a), mRNA [NM_001047865]	**5,291**
Herc4	Rattus norvegicus hect domain and RLD 4 (Herc4), mRNA [NM_001012074]	**5,291**
Dnd1	Rattus norvegicus dead end homolog 1 (zebrafish) (Dnd1), mRNA [NM_001109379]	**5,290**
0	Unknown	**5,288**
Blvra	Rattus norvegicus biliverdin reductase A (Blvra), mRNA [NM_053850]	**5,288**
Phpt1	Rattus norvegicus phosphohistidine phosphatase 1 (Phpt1), mRNA [NM_001106558]	**5,288**
0	Unknown	**5,288**
0	Unknown	**5,288**
0	Rattus norvegicus similar to 60S ribosomal protein L12 (LOC296165), mRNA [XM_215850]	**5,287**
Dynlrb2	Rattus norvegicus dynein light chain roadblock-type 2 (Dynlrb2), mRNA [NM_001108451]	**5,287**
0	Unknown	**5,286**
Ica1l	Rattus norvegicus islet cell autoantigen 1-like (Ica1l), mRNA [NM_199400]	**5,286**
RGD1311463	Rattus norvegicus similar to RIKEN cDNA 2700007P21 (RGD1311463), mRNA [NM_001014045]	**5,286**
Mmadhc	Rattus norvegicus methylmalonic aciduria (cobalamin deficiency) cblD type, with homocystinuria (Mmadhc), nuclear gene encoding mitochondrial protein, mRNA [NM_001004280]	**5,286**
Hint3	Histidine triad nucleotide-binding protein 3 [Source:UniProtKB/Swiss-Prot;Acc:Q8K3P7] [ENSRNOT00000019047]	**5,285**
LOC100366173	PREDICTED: Rattus norvegicus Zinc finger protein 383-like, transcript variant 3 (LOC100366173), mRNA [XM_002725562]	**5,284**
0	Q6NW38_HUMAN (Q6NW38) IVNS1ABP protein, partial (5%) [TC590756]	**5,280**
Tpd52l3	Rattus norvegicus tumor protein D52-like 3 (Tpd52l3), mRNA [NM_001106349]	**5,278**
Tmem117	PREDICTED: Rattus norvegicus transmembrane protein 117 (Tmem117), mRNA [XM_001058576]	**5,278**
Pigz	Rattus norvegicus phosphatidylinositol glycan anchor biosynthesis, class Z (Pigz), mRNA [NM_001109525]	**5,278**

Ica1	Rattus norvegicus islet cell autoantigen 1 (Ica1), mRNA [NM_030844]	**5,277**
0	Uncharacterized protein [Source:UniProtKB/TrEMBL;Acc:D3ZBR0] [ENSRNOT00000045189]	**5,277**
Nmur1	Rattus norvegicus neuromedin U receptor 1 (Nmur1), mRNA [NM_023100]	**5,276**
Adat1	Rattus norvegicus adenosine deaminase, tRNA-specific 1 (Adat1), mRNA [NM_001109611]	**5,276**
0	Unknown	**5,276**
Ifitm6	Uncharacterized protein [Source:UniProtKB/TrEMBL;Acc:D3ZR91] [ENSRNOT00000054875]	**5,275**
Olr202	Rattus norvegicus olfactory receptor 202 (Olr202), mRNA [NM_001000550]	**5,275**
Psg19	Rattus norvegicus pregnancy specific glycoprotein 19 (Psg19), mRNA [NM_019126]	**5,274**
0	Unknown	**5,274**
0	Probable O-sialoglycoprotein endopeptidase 2 [Source:UniProtKB/Swiss-Prot;Acc:Q4V7F3] [ENSRNOT00000039880]	**5,273**
0	Fam40a protein [Source:UniProtKB/TrEMBL;Acc:Q4FZS7] [ENSRNOT00000033776]	**5,272**
0	Unknown	**5,272**
0	Rattus norvegicus similar to heat shock protein 84 - mouse (LOC291871), mRNA [XM_226259]	**5,271**
Wdr35l	Rattus norvegicus WD repeat domain 35-like (Wdr35l), mRNA [NM_001099340]	**5,270**
LOC688966	Rattus norvegicus similar to K11B4.2 (LOC688966), mRNA [NM_001145726]	**5,270**
RGD1559982	PREDICTED: Rattus norvegicus similar to paraneoplastic antigen like 6A (RGD1559982), mRNA [XM_001058237]	**5,270**
Ankrd60	RCG40922Uncharacterized protein [Source:UniProtKB/TrEMBL;Acc:D3ZTI6] [ENSRNOT00000055082]	**5,270**
0	Unknown	**5,269**
RGD1563378	RCG36341Uncharacterized protein [Source:UniProtKB/TrEMBL;Acc:D3ZC26] [ENSRNOT00000050544]	**5,269**
Med31	Rattus norvegicus mediator complex subunit 31 (Med31), mRNA [NM_001135813]	**5,269**
LOC100363987	PREDICTED: Rattus norvegicus mCG114897-like (LOC100363987), miscRNA [XR_085743]	**5,268**
0	Adenosylhomocysteinase [Source:UniProtKB/TrEMBL;Acc:D4AB27] [ENSRNOT00000017748]	**5,268**
0	Unknown	**5,267**
Slc27a3	Rattus norvegicus solute carrier family 27 (fatty acid transporter), member 3 (Slc27a3), mRNA [NM_001106439]	**5,266**
Kifc3	Rattus norvegicus kinesin family member C3 (Kifc3), mRNA [NM_001103352]	**5,266**
Cttn	Rattus norvegicus cortactin (Cttn), mRNA [NM_021868]	**5,264**
LOC301839	PREDICTED: Rattus norvegicus similar to Spindlin-like protein 2 (SPIN-2) (LOC301839), mRNA [XM_001061753]	**5,263**
Sdccag3	Rattus norvegicus serologically defined colon cancer antigen 3 (Sdccag3), mRNA [NM_001013135]	**5,262**
0	Uncharacterized protein [Source:UniProtKB/TrEMBL;Acc:D3ZMU6] [ENSRNOT00000057016]	**5,262**
Tmco3	Rattus norvegicus transmembrane and coiled-coil domains 3 (Tmco3), mRNA [NM_001135857]	**5,261**
Med6	Rattus norvegicus mediator complex subunit 6 (Med6), mRNA [NM_001106742]	**5,261**
Hebp2	Rattus norvegicus heme binding protein 2 (Hebp2), mRNA [NM_001107515]	**5,261**
Rab26	Rattus norvegicus RAB26, member RAS oncogene family (Rab26), mRNA [NM_133580]	**5,260**
0	Unknown	**5,259**

LOC691130	PREDICTED: Rattus norvegicus hypothetical protein LOC691130 (LOC691130), mRNA [XM_001076936]	**5,259**
Flt4	Rattus norvegicus fms-related tyrosine kinase 4 (Flt4), mRNA [NM_053652]	**5,259**
Slc4a3	Rattus norvegicus solute carrier family 4 (anion exchanger), member 3 (Slc4a3), mRNA [NM_017049]	**5,259**
Psip1	Rattus norvegicus PC4 and SFRS1 interacting protein 1 (Psip1), mRNA [NM_175765]	**5,258**
Fads3	Rattus norvegicus fatty acid desaturase 3 (Fads3), mRNA [NM_173137]	**5,258**
0	Rattus norvegicus similar to hypothetical protein (LOC301869), mRNA [XM_229350]	**5,257**
Pdia3	Rattus norvegicus protein disulfide isomerase family A, member 3 (Pdia3), mRNA [NM_017319]	**5,257**
Cdc42ep3	Rattus norvegicus CDC42 effector protein (Rho GTPase binding) 3 (Cdc42ep3), mRNA [NM_001048044]	**5,256**
Pnkd	Rattus norvegicus paroxysmal nonkinesiogenic dyskinesia (Pnkd), nuclear gene encoding mitochondrial protein, transcript variant 3, mRNA [NM_001134753]	**5,255**
Tmlhe	Rattus norvegicus trimethyllysine hydroxylase, epsilon (Tmlhe), nuclear gene encoding mitochondrial protein, mRNA [NM_133387]	**5,255**
Cep135	Uncharacterized protein [Source:UniProtKB/TrEMBL;Acc:D3ZI35] [ENSRNOT00000032156]	**5,255**
Ccdc87	Rattus norvegicus coiled-coil domain containing 87 (Ccdc87), mRNA [NM_001109527]	**5,255**
Rrs1	Rattus norvegicus RRS1 ribosome biogenesis regulator homolog (S. cerevisiae) (Rrs1), mRNA [NM_001079699]	**5,255**
Prkar1a	Rattus norvegicus protein kinase, cAMP-dependent, regulatory, type I, alpha (Prkar1a), mRNA [NM_013181]	**5,254**
0	Unknown	**5,254**
Pik3ca	Rattus norvegicus phosphoinositide-3-kinase, catalytic, alpha polypeptide (Pik3ca), mRNA [NM_133399]	**5,253**
Hapln1	Rattus norvegicus hyaluronan and proteoglycan link protein 1 (Hapln1), mRNA [NM_019189]	**5,253**
Zfp347	Rattus norvegicus zinc finger protein 347 (Zfp347), mRNA [NM_133390]	**5,252**
Efemp1	Rattus norvegicus EGF-containing fibulin-like extracellular matrix protein 1 (Efemp1), mRNA [NM_001012039]	**5,251**
Anks3	Rattus norvegicus ankyrin repeat and sterile alpha motif domain containing 3 (Anks3), mRNA [NM_001009676]	**5,251**
Fgf9	Rattus norvegicus fibroblast growth factor 9 (Fgf9), mRNA [NM_012952]	**5,251**
Ly96	Rattus norvegicus lymphocyte antigen 96 (Ly96), mRNA [NM_001024279]	**5,251**
0	Rattus norvegicus similar to hippocampal cholinergic neurostimulating peptide precursor protein (LOC293883), mRNA [XM_219774]	**5,251**
Atp6v0b	Rattus norvegicus ATPase, H+ transporting, lysosomal V0 subunit B (Atp6v0b), mRNA [NM_001106681]	**5,250**
0	Unknown	**5,250**
0	Histone H2A type 1-F [Source:UniProtKB/Swiss-Prot;Acc:Q64598] [ENSRNOT00000041929]	**5,250**
0	Glyceraldehyde-3-phosphate dehydrogenase [Source:UniProtKB/TrEMBL;Acc:D3ZKR3] [ENSRNOT00000008094]	**5,249**
Lrfn1	Rattus norvegicus leucine rich repeat and fibronectin type III domain containing 1 (Lrfn1), transcript variant 2, mRNA [NM_001108910]	**5,248**
Smpdl3a	Rattus norvegicus sphingomyelin phosphodiesterase, acid-like 3A (Smpdl3a), mRNA [NM_001005539]	**5,248**
Mib1	Rattus norvegicus mindbomb homolog 1 (Drosophila) (Mib1), mRNA [NM_001107405]	**5,248**
Ndufb8	Rattus norvegicus NADH dehydrogenase (ubiquinone) 1 beta subcomplex 8 (Ndufb8), nuclear gene encoding mitochondrial protein, mRNA [NM_001106360]	**5,246**
Gabrg3	Rattus norvegicus gamma-aminobutyric acid (GABA) A receptor, gamma 3 (Gabrg3), mRNA [NM_024370]	**5,246**

Olr227	Rattus norvegicus olfactory receptor 227 (Olr227), mRNA [NM_001000203]	**5,245**
Gadd45a	Rattus norvegicus growth arrest and DNA-damage-inducible, alpha (Gadd45a), mRNA [NM_024127]	**5,245**
Entpd7	Rattus norvegicus ectonucleoside triphosphate diphosphohydrolase 7 (Entpd7), mRNA [NM_001107595]	**5,244**
Zfp281	Rattus norvegicus zinc finger protein 281 (Zfp281), mRNA [NM_001012030]	**5,244**
Scarb2	Rattus norvegicus scavenger receptor class B, member 2 (Scarb2), mRNA [NM_054001]	**5,243**
0	Unknown	**5,243**
Znf287	Rattus norvegicus zinc finger protein 287 (Znf287), mRNA [NM_001107008]	**5,243**
Noc3l	Rattus norvegicus nucleolar complex associated 3 homolog (S. cerevisiae) (Noc3l), mRNA [NM_001108523]	**5,243**
0	Unknown	**5,242**
Gtf2a1	Rattus norvegicus general transcription factor IIA, 1 (Gtf2a1), mRNA [NM_022208]	**5,241**
0	Unknown	**5,240**
Zfp219	Rattus norvegicus zinc finger protein 219 (Zfp219), mRNA [NM_001007681]	**5,240**
Znf646	Uncharacterized protein [Source:UniProtKB/TrEMBL;Acc:D3ZZ84] [ENSRNOT00000036187]	**5,239**
Ptpn18	Rattus norvegicus protein tyrosine phosphatase, non-receptor type 18 (Ptpn18), mRNA [NM_001013111]	**5,239**
0	Uncharacterized protein [Source:UniProtKB/TrEMBL;Acc:D3ZCQ8] [ENSRNOT00000047654]	**5,239**
Wipi2	Rattus norvegicus WD repeat domain, phosphoinositide interacting 2 (Wipi2), mRNA [NM_001007615]	**5,238**
Slc12a4	Rattus norvegicus solute carrier family 12 (potassium/chloride transporters), member 4 (Slc12a4), mRNA [NM_019229]	**5,237**
LOC100158225	Rattus norvegicus hypothetical protein LOC100158225 (LOC100158225), mRNA [NM_001127606]	**5,237**
Wdr31	Rattus norvegicus WD repeat domain 31 (Wdr31), mRNA [NM_001011976]	**5,237**
RGD1561481	PREDICTED: Rattus norvegicus similar to ubiquitin specific protease 12 (RGD1561481), mRNA [XM_577055]	**5,236**
Rhbg	Rattus norvegicus Rh family, B glycoprotein (Rhbg), mRNA [NM_183054]	**5,236**
0	Rattus norvegicus similar to KIAA1895 protein (LOC293775), mRNA [XM_219621]	**5,235**
0	Peptidyl-prolyl cis-trans isomerase [Source:UniProtKB/TrEMBL;Acc:D3ZUZ9] [ENSRNOT00000011302]	**5,235**
0	Uncharacterized protein [Source:UniProtKB/TrEMBL;Acc:D4A0Y6] [ENSRNOT00000038176]	**5,235**
Rab11a	Rattus norvegicus RAB11a, member RAS oncogene family (Rab11a), mRNA [NM_031152]	**5,235**
Med27	Rattus norvegicus mediator complex subunit 27 (Med27), mRNA [NM_001106565]	**5,235**
0	Unknown	**5,235**
0	Uncharacterized protein [Source:UniProtKB/TrEMBL;Acc:D3ZG82] [ENSRNOT00000063806]	**5,234**
0	Uncharacterized protein [Source:UniProtKB/TrEMBL;Acc:D3Z934] [ENSRNOT00000019780]	**5,232**
Lmo1	Rattus norvegicus LIM domain only 1 (Lmo1), mRNA [NM_139112]	**5,232**
Zfp276	Uncharacterized protein [Source:UniProtKB/TrEMBL;Acc:D4A710] [ENSRNOT00000022324]	**5,232**
0	Small nuclear ribonucleoprotein-associated protein B [Source:UniProtKB/Swiss-Prot;Acc:P17136] [ENSRNOT00000043260]	**5,232**
Nfrkb	Rattus norvegicus nuclear factor related to kappa B binding protein (Nfrkb), mRNA [NM_001108133]	**5,232**
Slco4a1	Rattus norvegicus solute carrier organic anion transporter family, member 4a1 (Slco4a1), mRNA [NM_133608]	**5,231**

Rpl31	Rattus norvegicus ribosomal protein L31 (Rpl31), mRNA [NM_022506]	**5,230**
Kif3b	Rattus norvegicus kinesin family member 3B (Kif3b), mRNA [NM_001106529]	**5,229**
Lrrc14	Rattus norvegicus leucine rich repeat containing 14 (Lrrc14), mRNA [NM_001024354]	**5,229**
Ptpla	Rattus norvegicus protein tyrosine phosphatase-like (proline instead of catalytic arginine), member a (Ptpla), mRNA [NM_001101001]	**5,229**
Dopey2	Rattus norvegicus dopey family member 2 (Dopey2), mRNA [NM_001191660]	**5,228**
LOC499781	Uncharacterized protein C9orf117 homolog [Source:UniProtKB/Swiss-Prot;Acc:Q4V7B0] [ENSRNOT00000020660]	**5,227**
0	Rattus norvegicus similar to Peptidylprolyl isomerase-like 2 (LOC302108), mRNA [XM_217532]	**5,227**
Fbxo2	Rattus norvegicus F-box protein 2 (Fbxo2), mRNA [NM_053511]	**5,226**
Alppl2	PREDICTED: Rattus norvegicus similar to alkaline phosphatase (EC 3.1.3.1) precursor, placental-like - human (RGD1565100), mRNA [XM_001064732]	**5,226**
Exoc6b	Rattus norvegicus exocyst complex component 6B (Exoc6b), mRNA [NM_001109246]	**5,226**
RGD1561940	PREDICTED: Rattus norvegicus similar to KB07 protein (RGD1561940), partial mRNA [XM_002725261]	**5,225**
Ece2	Rattus norvegicus endothelin-converting enzyme 2 (Ece2), mRNA [NM_001002815]	**5,225**
RGD1559531	Uncharacterized protein [Source:UniProtKB/TrEMBL;Acc:D3ZQ96] [ENSRNOT00000048673]	**5,225**
Ftsjd1	Rattus norvegicus FtsJ methyltransferase domain containing 1 (Ftsjd1), mRNA [NM_001106186]	**5,225**
Tnrc6b	Rattus norvegicus trinucleotide repeat containing 6B (Tnrc6b), mRNA [NM_138845]	**5,224**
Ppil4	Rattus norvegicus peptidylprolyl isomerase (cyclophilin)-like 4 (Ppil4), mRNA [NM_001108457]	**5,223**
RGD1565025	RCG48116, isoform CRA_aUncharacterized protein [Source:UniProtKB/TrEMBL;Acc:D4A806] [ENSRNOT00000028197]	**5,221**
Ptpn2	Rattus norvegicus protein tyrosine phosphatase, non-receptor type 2 (Ptpn2), mRNA [NM_053990]	**5,219**
Golt1a	Rattus norvegicus golgi transport 1 homolog A (S. cerevisiae) (Golt1a), mRNA [NM_001109070]	**5,219**
Fam69a	Rattus norvegicus family with sequence similarity 69, member A (Fam69a), mRNA [NM_001170456]	**5,219**
0	Unknown	**5,219**
0	Unknown	**5,218**
S100z	Rattus norvegicus S100 calcium binding protein Z (S100z), mRNA [NM_001191701]	**5,218**
Ces1a	Rattus norvegicus carboxylesterase 1A (Ces1a), mRNA [NM_001190375]	**5,218**
Glyctk	Rattus norvegicus glycerate kinase (Glyctk), mRNA [NM_001109449]	**5,217**
0	Unknown	**5,217**
Nap5	PREDICTED: Rattus norvegicus Nck-associated protein 5 (Nap5), mRNA [XM_344134]	**5,217**
Nvl	Rattus norvegicus nuclear VCP-like (Nvl), mRNA [NM_001105980]	**5,216**
Pikfyve	Similar to phosphatidylinositol-3-phosphate/phosphatidylinositol 5-kinase, type III isoform 2Uncharacterized protein [Source:UniProtKB/TrEMBL;Acc:D3ZT14] [ENSRNOT00000020447]	**5,215**
Osgin1	Rattus norvegicus oxidative stress induced growth inhibitor 1 (Osgin1), mRNA [NM_138504]	**5,213**
Rufy2	Rattus norvegicus RUN and FYVE domain containing 2 (Rufy2), mRNA [NM_001168586]	**5,212**
Kctd21	Rattus norvegicus potassium channel tetramerisation domain containing 21 (Kctd21), mRNA [NM_001109151]	**5,212**

Ctu1	Rattus norvegicus cytosolic thiouridylase subunit 1 homolog (S. pombe) (Ctu1), mRNA [NM_001106251]	**5,212**
0	Unknown	**5,210**
Morc3	Rattus norvegicus MORC family CW-type zinc finger 3 (Morc3), mRNA [NM_001107109]	**5,210**
0	Unknown	**5,209**
Kcnmb3	Rattus norvegicus potassium large conductance calcium-activated channel, subfamily M beta member 3 (Kcnmb3), mRNA [NM_001104560]	**5,209**
Avil	Rattus norvegicus advillin (Avil), mRNA [NM_024401]	**5,209**
Snx8	Rattus norvegicus sorting nexin 8 (Snx8), mRNA [NM_001105912]	**5,209**
0	Unknown	**5,209**
RGD1308117	Rattus norvegicus similar to 9930012K11Rik protein (RGD1308117), mRNA [NM_001134571]	**5,209**
Lmbr1l	Rattus norvegicus limb region 1-like homolog (mouse) (Lmbr1l), mRNA [NM_001013950]	**5,209**
Ostc	Rattus norvegicus oligosaccharyltransferase complex subunit (Ostc), mRNA [NM_001108566]	**5,208**
Galnt14	Rattus norvegicus UDP-N-acetyl-alpha-D-galactosamine:polypeptide N-acetylgalactosaminyltransferase 14 (GalNAc-T14) (Galnt14), mRNA [NM_001012109]	**5,208**
Timm13	Rattus norvegicus translocase of inner mitochondrial membrane 13 homolog (yeast) (Timm13), nuclear gene encoding mitochondrial protein, mRNA [NM_145781]	**5,207**
Oprm1	Rattus norvegicus opioid receptor, mu 1 (Oprm1), transcript variant MOR-1B2, mRNA [NM_001038599]	**5,207**
Serp2	Rattus norvegicus stress-associated endoplasmic reticulum protein family member 2 (Serp2), mRNA [NM_001109104]	**5,207**
0	Unknown	**5,206**
0	Rattus norvegicus similar to Glyceraldehyde 3-phosphate dehydrogenase (GAPDH) (38 kDa BFA-dependent ADP-ribosylation substrate) (BARS-38) (LOC362444), mRNA [XM_342766]	**5,206**
0	Leucine-rich repeat-containing protein KIAA1731 homolog [Source:UniProtKB/Swiss-Prot;Acc:A4L9P8] [ENSRNOT00000029634]	**5,206**
Eif2b5	Rattus norvegicus eukaryotic translation initiation factor 2B, subunit 5 epsilon (Eif2b5), mRNA [NM_138866]	**5,206**
0	Uncharacterized protein [Source:UniProtKB/TrEMBL;Acc:D3ZAQ6] [ENSRNOT00000024122]	**5,205**
LOC684280	PREDICTED: Rattus norvegicus similar to Class I histocompatibility antigen, Non-RT1.A alpha-1 chain precursor (LOC684280), partial mRNA [XM_001069706]	**5,205**
0	Putative uncharacterized protein RGD1559942_predictedUncharacterized protein [Source:UniProtKB/TrEMBL;Acc:D3ZXR2] [ENSRNOT00000059680]	**5,205**
Ccng2	Rattus norvegicus cyclin G2 (Ccng2), mRNA [NM_001105725]	**5,204**
0	Unknown	**5,202**
0	Unknown	**5,202**
Camsap1	Rattus norvegicus calmodulin regulated spectrin-associated protein 1 (Camsap1), mRNA [NM_001168549]	**5,201**
Nt5dc2	Rattus norvegicus 5'-nucleotidase domain containing 2 (Nt5dc2), mRNA [NM_001009271]	**5,200**
0	Unknown	**5,200**
Tut1	Rattus norvegicus terminal uridylyl transferase 1, U6 snRNA-specific (Tut1), mRNA [NM_001033901]	**5,200**

Camkk1	Rattus norvegicus calcium/calmodulin-dependent protein kinase kinase 1, alpha (Camkk1), mRNA [NM_031662]	**5,199**
Phrf1	Rattus norvegicus PHD and ring finger domains 1 (Phrf1), mRNA [NM_139093]	**5,199**
Tmem158	Rattus norvegicus transmembrane protein 158 (Tmem158), mRNA [NM_057212]	**5,198**
RGD1560187	Rattus norvegicus similar to protein of fungal metazoan origin like (11.1 kD) (2C514) (LOC362641), mRNA [XM_342957]	**5,198**
Chrm3	Rattus norvegicus cholinergic receptor, muscarinic 3 (Chrm3), mRNA [NM_012527]	**5,196**
0	Unknown	**5,195**
Tmem79	Rattus norvegicus transmembrane protein 79 (Tmem79), mRNA [NM_001033896]	**5,194**
0	AGENCOURT_31522257 NIH_MGC_269 Rattus norvegicus cDNA clone IMAGE:7460337 5', mRNA sequence [CV117922]	**5,194**
Csrp2	Rattus norvegicus cysteine and glycine-rich protein 2 (Csrp2), mRNA [NM_177425]	**5,194**
Faim	Rattus norvegicus Fas apoptotic inhibitory molecule (Faim), mRNA [NM_080895]	**5,193**
Zfp51	Rattus norvegicus zinc finger protein 51 (Zfp51), mRNA [NM_001107467]	**5,193**
Apoh	Rattus norvegicus apolipoprotein H (beta-2-glycoprotein I) (Apoh), mRNA [NM_001009626]	**5,192**
LOC688298	Rattus norvegicus hypothetical protein LOC688298 (LOC688298), mRNA [NM_001143896]	**5,192**
0	Unknown	**5,192**
Kcnb2	Rattus norvegicus potassium voltage gated channel, Shab-related subfamily, member 2 (Kcnb2), mRNA [NM_054000]	**5,191**
0	Unknown	**5,190**
Zfp105	Rattus norvegicus zinc finger protein 105 (Zfp105), mRNA [NM_001012128]	**5,190**
0	Uncharacterized protein [Source:UniProtKB/TrEMBL;Acc:D4ABH4] [ENSRNOT00000014695]	**5,189**
Mtmr1	Rattus norvegicus myotubularin related protein 1 (Mtmr1), mRNA [NM_001191725]	**5,189**
Ednra	Rattus norvegicus endothelin receptor type A (Ednra), mRNA [NM_012550]	**5,189**
Slc39a7	Rattus norvegicus solute carrier family 39 (zinc transporter), member 7 (Slc39a7), transcript variant 2, mRNA [NM_001008885]	**5,188**
LOC688310	PREDICTED: Rattus norvegicus similar to CG5500-PA (LOC688310), mRNA [XM_001081793]	**5,188**
0	Unknown	**5,187**
Havcr2	Rattus norvegicus hepatitis A virus cellular receptor 2 (Havcr2), mRNA [NM_001100762]	**5,187**
Mkln1	Rattus norvegicus muskelin 1, intracellular mediator containing kelch motifs (Mkln1), mRNA [NM_031359]	**5,186**
Nes	Rattus norvegicus nestin (Nes), mRNA [NM_012987]	**5,186**
Smug1	Rattus norvegicus single-strand-selective monofunctional uracil-DNA glycosylase 1 (Smug1), mRNA [NM_177934]	**5,186**
RGD1311578	Rattus norvegicus similar to PRO1853 homolog (RGD1311578), mRNA [NM_001008318]	**5,185**
Zfp131	Rattus norvegicus zinc finger protein 131 (Zfp131), mRNA [NM_001100698]	**5,184**
Smarcc1	Rattus norvegicus SWI/SNF related, matrix associated, actin dependent regulator of chromatin, subfamily c, member 1 (Smarcc1), mRNA [NM_001106861]	**5,184**
0	Uncharacterized protein [Source:UniProtKB/TrEMBL;Acc:D4ADK1] [ENSRNOT00000046038]	**5,183**
Fam92a1	Uncharacterized protein [Source:UniProtKB/TrEMBL;Acc:D3ZP87] [ENSRNOT00000022249]	**5,183**
0	Unknown	**5,182**

0	Unknown	**5,181**
Fkbp7	Rattus norvegicus FK506 binding protein 7 (Fkbp7), mRNA [NM_001106485]	**5,181**
Cggbp1	Rattus norvegicus CGG triplet repeat binding protein 1 (Cggbp1), mRNA [NM_001105900]	**5,180**
0	Unknown	**5,180**
RGD1307465	Rattus norvegicus similar to RIKEN cDNA 8430406I07 (RGD1307465), mRNA [NM_001009655]	**5,178**
Tagln3	Rattus norvegicus transgelin 3 (Tagln3), transcript variant 2, mRNA [NM_031676]	**5,178**
Rps2-ps7	PREDICTED: Rattus norvegicus similar to 40S ribosomal protein S2 (LOC688435), mRNA [XM_002725434]	**5,177**
Apobec2	Rattus norvegicus apolipoprotein B mRNA editing enzyme, catalytic polypeptide-like 2 (Apobec2), mRNA [NM_001106883]	**5,177**
Rbak	Rattus norvegicus RB-associated KRAB zinc finger (Rbak), mRNA [NM_001191664]	**5,175**
Vom1r48	Rattus norvegicus vomeronasal 1 receptor 48 (Vom1r48), mRNA [NM_001008931]	**5,175**
Tnnc1	Rattus norvegicus troponin C type 1 (slow) (Tnnc1), mRNA [NM_001034105]	**5,175**
Slc43a3	Rattus norvegicus solute carrier family 43, member 3 (Slc43a3), mRNA [NM_001107743]	**5,175**
Slc44a4	Rattus norvegicus solute carrier family 44, member 4 (Slc44a4), mRNA [NM_212541]	**5,172**
0	Unknown	**5,172**
0	PREDICTED: Rattus norvegicus RGD1564086 (RGD1564086), mRNA [XM_001078704]	**5,171**
0	Q6UX82_HUMAN (Q6UX82) Phospholipase inhibitor, partial (16%) [TC595358]	**5,171**
0	Rab3 GTPase-activating protein catalytic subunit [Source:UniProtKB/Swiss-Prot;Acc:P69735] [ENSRNOT00000005289]	**5,170**
0	BF543486 UI-R-C3-sz-e-01-0-UI.r1 UI-R-C3 Rattus norvegicus cDNA clone UI-R-C3-sz-e-01-0-UI 5', mRNA sequence [BF543486]	**5,169**
Slc26a10	solute carrier family 26 member 10 [Source:RefSeq peptide;Acc:NP_001128067] [ENSRNOT00000052288]	**5,169**
Olr165	Rattus norvegicus olfactory receptor 165 (Olr165), mRNA [NM_001000172]	**5,169**
Ppil4	peptidyl-prolyl cis-trans isomerase-like 4 [Source:RefSeq peptide;Acc:NP_001101927] [ENSRNOT00000021437]	**5,169**
Wif1	Rattus norvegicus Wnt inhibitory factor 1 (Wif1), mRNA [NM_053738]	**5,168**
Fzd6	Rattus norvegicus frizzled homolog 6 (Drosophila) (Fzd6), mRNA [NM_001130536]	**5,167**
Anapc7	Rattus norvegicus anaphase promoting complex subunit 7 (Anapc7), mRNA [NM_001107142]	**5,167**
Odz3	Rattus norvegicus odz, odd Oz/ten-m homolog 3 (Drosophila) (Odz3), mRNA [NM_001169133]	**5,166**
Btbd1	Rattus norvegicus BTB (POZ) domain containing 1 (Btbd1), mRNA [NM_001011932]	**5,166**
Parp8	PREDICTED: Rattus norvegicus poly (ADP-ribose) polymerase family, member 8 (Parp8), mRNA [XM_001068789]	**5,166**
Agtr1a	Rattus norvegicus angiotensin II receptor, type 1a (Agtr1a), mRNA [NM_030985]	**5,165**
0	Unknown	**5,165**
Fshr	Rattus norvegicus follicle stimulating hormone receptor (Fshr), mRNA [NM_199237]	**5,164**
0	Unknown	**5,162**
Hapln4	Rattus norvegicus hyaluronan and proteoglycan link protein 4 (Hapln4), mRNA [NM_001108398]	**5,161**
LOC684919	PREDICTED: Rattus norvegicus similar to zinc finger protein 449 (LOC684919), mRNA [XM_001072469]	**5,161**
RGD1562381	Uncharacterized protein [Source:UniProtKB/TrEMBL;Acc:D3ZM01] [ENSRNOT00000040565]	**5,161**

Lin54	Rattus norvegicus lin-54 homolog (C. elegans) (Lin54), mRNA [NM_001100564]	**5,161**
Leng8	Rattus norvegicus leukocyte receptor cluster (LRC) member 8 (Leng8), mRNA [NM_001037790]	**5,161**
Scand3	Rattus norvegicus SCAN domain containing 3 (Scand3), mRNA [NM_001105924]	**5,160**
0	Unknown	**5,160**
Dak	Rattus norvegicus dihydroxyacetone kinase 2 homolog (S. cerevisiae) (Dak), mRNA [NM_001039031]	**5,160**
Ints10	Rattus norvegicus integrator complex subunit 10 (Ints10), mRNA [NM_001134416]	**5,159**
Aen	Rattus norvegicus apoptosis enhancing nuclease (Aen), mRNA [NM_001108487]	**5,158**
Otub2	Rattus norvegicus OTU domain, ubiquitin aldehyde binding 2 (Otub2), mRNA [NM_001108053]	**5,157**
Zfp319	Rattus norvegicus zinc finger protein 319 (Zfp319), mRNA [NM_001170478]	**5,157**
Top2a	DNA topoisomerase 2-alpha [Source:UniProtKB/Swiss-Prot;Acc:P41516] [ENSRNOT00000050725]	**5,157**
Epc2	Rattus norvegicus enhancer of polycomb homolog 2 (Drosophila) (Epc2), mRNA [NM_001108581]	**5,157**
0	Rattus norvegicus chromosome 10, 7 clones, strain BN/SsNHsdMCW RNOR03214123, whole genome shotgun sequence [AABR03073170]	**5,157**
Tcp11l1	Rattus norvegicus t-complex 11 like 1 (Tcp11l1), mRNA [NM_001109202]	**5,156**
RGD1564952	PREDICTED: Rattus norvegicus similar to zinc finger, RAN-binding domain containing 3 (RGD1564952), mRNA [XM_002728000]	**5,155**
Surf6	Rattus norvegicus surfeit 6 (Surf6), mRNA [NM_001015014]	**5,154**
0	Rattus norvegicus chromosome 20, 4 clones, strain BN/SsNHsdMCW RNOR03269132, whole genome shotgun sequence [AABR03116353]	**5,154**
Mthfd2	Rattus norvegicus methylenetetrahydrofolate dehydrogenase (NADP+ dependent) 2, methenyltetrahydrofolate cyclohydrolase (Mthfd2), nuclear gene encoding mitochondrial protein, mRNA [NM_001109398]	**5,154**
LOC679682	Rattus norvegicus similar to ES cell-expressed Ras (LOC679682), mRNA [NM_001109375]	**5,153**
0	Uncharacterized protein [Source:UniProtKB/TrEMBL;Acc:D3ZSV9] [ENSRNOT00000011770]	**5,153**
Pc	Rattus norvegicus pyruvate carboxylase (Pc), nuclear gene encoding mitochondrial protein, mRNA [NM_012744]	**5,153**
Phgr1	PREDICTED: Rattus norvegicus similar to RIKEN cDNA 1810007E14 (RGD1566202), mRNA [XM_001080765]	**5,153**
Naprt1	Rattus norvegicus nicotinate phosphoribosyltransferase domain containing 1 (Naprt1), mRNA [NM_207609]	**5,152**
LOC690000	Uncharacterized protein [Source:UniProtKB/TrEMBL;Acc:D3ZWS2] [ENSRNOT00000020079]	**5,152**
Farsb	Rattus norvegicus phenylalanyl-tRNA synthetase, beta subunit (Farsb), mRNA [NM_001004252]	**5,152**
0	Unknown	**5,152**
RGD1306954	Rattus norvegicus similar to RIKEN cDNA 1110004E09 (RGD1306954), mRNA [NM_001008288]	**5,152**
0	Unknown	**5,152**
Clec1a	Rattus norvegicus C-type lectin domain family 1, member a (Clec1a), mRNA [NM_001109253]	**5,150**
Xrcc6bp1	Rattus norvegicus XRCC6 binding protein 1 (Xrcc6bp1), mRNA [NM_001134466]	**5,150**
Rps24	Rattus norvegicus ribosomal protein S24 (Rps24), mRNA [NM_031112]	**5,149**
Sts	Rattus norvegicus steroid sulfatase (Sts), mRNA [NM_012661]	**5,149**
Svop	Rattus norvegicus SV2 related protein (Svop), mRNA [NM_134404]	**5,148**
Mrpl19	Rattus norvegicus mitochondrial ribosomal protein L19 (Mrpl19), nuclear gene encoding mitochondrial protein, mRNA [NM_001029898]	**5,148**

Fcgr1a	Rattus norvegicus Fc fragment of IgG, high affinity Ia, receptor (CD64) (Fcgr1a), mRNA [NM_001100836]	**5,148**
0	Unknown	**5,148**
Kif4b	Uncharacterized protein [Source:UniProtKB/TrEMBL;Acc:D3ZYT4] [ENSRNOT00000006165]	**5,147**
Abcf2	Rattus norvegicus ATP-binding cassette, subfamily F (GCN20), member 2 (Abcf2), nuclear gene encoding mitochondrial protein, mRNA [NM_001109666]	**5,147**
0	Unknown	**5,147**
Lrrc48	Rattus norvegicus leucine rich repeat containing 48 (Lrrc48), mRNA [NM_001013857]	**5,145**
Lpar2	Rattus norvegicus lysophosphatidic acid receptor 2 (Lpar2), mRNA [NM_001109109]	**5,145**
0	Unknown	**5,144**
Obsl1	Uncharacterized protein [Source:UniProtKB/TrEMBL;Acc:D3ZZ80] [ENSRNOT00000020673]	**5,144**
0	Unknown	**5,142**
Rbm41	Rattus norvegicus RNA binding motif protein 41 (Rbm41), mRNA [NM_001109420]	**5,142**
0	AI555711 UI-R-C2p-qr-b-08-0-UI.s1 UI-R-C2p Rattus norvegicus cDNA clone UI-R-C2p-qr-b-08-0-UI 3', mRNA sequence [AI555711]	**5,142**
Optc	Rattus norvegicus opticin (Optc), mRNA [NM_001107176]	**5,142**
Sik1	Serine/threonine-protein kinase SIK1 [Source:UniProtKB/Swiss-Prot;Acc:Q9R1U5] [ENSRNOT00000001579]	**5,140**
Pcyox1	Rattus norvegicus prenylcysteine oxidase 1 (Pcyox1), mRNA [NM_145085]	**5,140**
Zfp445	Rattus norvegicus zinc finger protein 445 (Zfp445), mRNA [NM_001191802]	**5,140**
Fam71f1	Rattus norvegicus family with sequence similarity 71, member F1 (Fam71f1), mRNA [NM_001024324]	**5,140**
Olr921	Rattus norvegicus olfactory receptor 921 (Olr921), mRNA [NM_001001385]	**5,139**
Gpn2	GPN-loop GTPase 2 [Source:UniProtKB/Swiss-Prot;Acc:D4A7C0] [ENSRNOT00000009275]	**5,138**
Stbd1	Rattus norvegicus starch binding domain 1 (Stbd1), mRNA [NM_001013988]	**5,138**
Mixl1	Rattus norvegicus Mix1 homeobox-like 1 (Xenopus laevis) (Mixl1), mRNA [NM_001105979]	**5,137**
Kcng3	Rattus norvegicus potassium voltage-gated channel, subfamily G, member 3 (Kcng3), transcript variant 1, mRNA [NM_133426]	**5,137**
LOC691661	Rattus norvegicus similar to hypothetical protein FLJ35105 (LOC297949), mRNA [XM_232841]	**5,136**
0	Uncharacterized protein [Source:UniProtKB/TrEMBL;Acc:D3ZRD1] [ENSRNOT00000013290]	**5,136**
Flt3	Rattus norvegicus fms-related tyrosine kinase 3 (Flt3), mRNA [NM_001100822]	**5,136**
Wdr55	Rattus norvegicus WD repeat domain 55 (Wdr55), mRNA [NM_001017932]	**5,136**
Lztfl1	Rattus norvegicus leucine zipper transcription factor-like 1 (Lztfl1), mRNA [NM_001024266]	**5,135**
Trim36	Rattus norvegicus tripartite motif-containing 36 (Trim36), mRNA [NM_001106147]	**5,135**
RGD1561955	Uncharacterized protein [Source:UniProtKB/TrEMBL;Acc:D3ZSZ6] [ENSRNOT00000058079]	**5,133**
Lamp1	Rattus norvegicus lysosomal-associated membrane protein 1 (Lamp1), mRNA [NM_012857]	**5,132**
Oprd1	Rattus norvegicus opioid receptor, delta 1 (Oprd1), mRNA [NM_012617]	**5,131**
Neil1	Rattus norvegicus nei endonuclease VIII-like 1 (E. coli) (Neil1), mRNA [NM_001025754]	**5,131**
Smyd3	Rattus norvegicus SET and MYND domain containing 3 (Smyd3), mRNA [NM_001025762]	**5,130**

Slc26a2	Rattus norvegicus solute carrier family 26 (sulfate transporter), member 2 (Slc26a2), mRNA [NM_057127]	**5,130**
Trim39	Rattus norvegicus tripartite motif-containing 39 (Trim39), mRNA [NM_213562]	**5,129**
0	Rattus norvegicus similar to hypothetical protein MGC18873 (LOC302398), mRNA [XM_228559]	**5,129**
Haus1	Rattus norvegicus HAUS augmin-like complex, subunit 1 (Haus1), mRNA [NM_138864]	**5,129**
Trim67	Rattus norvegicus tripartite motif-containing 67 (Trim67), mRNA [NM_001135715]	**5,129**
Vamp5	Rattus norvegicus vesicle-associated membrane protein 5 (Vamp5), mRNA [NM_053555]	**5,128**
0	Unknown	**5,128**
LOC100359653	PREDICTED: Rattus norvegicus rCG43168-like (LOC100359653), mRNA [XM_002728431]	**5,128**
LOC690105	PREDICTED: Rattus norvegicus hypothetical protein LOC690105 (LOC690105), mRNA [XM_002726579]	**5,127**
Cpa2	Rattus norvegicus carboxypeptidase A2 (pancreatic) (Cpa2), mRNA [NM_001013083]	**5,127**
Adat1	Rattus norvegicus adenosine deaminase, tRNA-specific 1 (Adat1), mRNA [NM_001109611]	**5,126**
Zcchc9	Rattus norvegicus zinc finger, CCHC domain containing 9 (Zcchc9), mRNA [NM_001013156]	**5,126**
LOC687696	Rattus norvegicus similar to AMSH-family protein (LOC687696), mRNA [NM_001115048]	**5,125**
Hist1h2aa	Rattus norvegicus histone cluster 1, H2aa (Hist1h2aa), mRNA [NM_021839]	**5,125**
LOC691259	Rattus norvegicus hypothetical protein LOC691259 (LOC691259), mRNA [NM_001109632]	**5,125**
Map3k6	Rattus norvegicus mitogen-activated protein kinase kinase kinase 6 (Map3k6), mRNA [NM_001107909]	**5,124**
Sars	Rattus norvegicus seryl-tRNA synthetase (Sars), mRNA [NM_001007606]	**5,124**
Tle2	Rattus norvegicus transducin-like enhancer of split 2 (E(sp1) homolog, Drosophila) (Tle2), mRNA [NM_001039013]	**5,123**
Cacng5	Rattus norvegicus calcium channel, voltage-dependent, gamma subunit 5 (Cacng5), mRNA [NM_080693]	**5,122**
Ndfip1	Rattus norvegicus Nedd4 family interacting protein 1 (Ndfip1), mRNA [NM_001013059]	**5,122**
Rhobtb1	Rattus norvegicus Rho-related BTB domain containing 1 (Rhobtb1), mRNA [NM_001107622]	**5,122**
Hook3	Rattus norvegicus hook homolog 3 (Drosophila) (Hook3), mRNA [NM_001136098]	**5,121**
Clca4	Rattus norvegicus chloride channel accessory 4 (Clca4), mRNA [NM_201419]	**5,121**
RGD1312005	Rattus norvegicus similar to DD1 (RGD1312005), mRNA [NM_001106144]	**5,121**
LOC362710	Rattus norvegicus hypothetical LOC362710 (LOC362710), mRNA [NM_001126372]	**5,120**
Ankrd28	Q505D1_MOUSE (Q505D1) Ankyrin repeat domain 28, partial (8%) [TC599514]	**5,119**
Hpx	Rattus norvegicus hemopexin (Hpx), mRNA [NM_053318]	**5,118**
0	Unknown	**5,116**
0	Unknown	**5,115**
0	Rattus norvegicus similar to KIAA1771 protein (LOC313388), mRNA [XM_242940]	**5,115**
0	Unknown	**5,114**
Dlk2	Rattus norvegicus delta-like 2 homolog (Drosophila) (Dlk2), mRNA [NM_001108202]	**5,112**
0	tRNA-splicing endonuclease subunit Sen15 [Source:RefSeq peptide;Acc:NP_001099428] [ENSRNOT00000003226]	**5,112**
Slc39a12	Rattus norvegicus solute carrier family 39 (zinc transporter), member 12 (Slc39a12), mRNA [NM_001106124]	**5,111**

0	Unknown	**5,111**
Ccdc34	Rattus norvegicus coiled-coil domain containing 34 (Ccdc34), mRNA [NM_001108587]	**5,110**
Dnajc25	Rattus norvegicus DnaJ (Hsp40) homolog, subfamily C, member 25 (Dnajc25), mRNA [NM_001025021]	**5,110**
Epha2	Rattus norvegicus Eph receptor A2 (Epha2), mRNA [NM_001108977]	**5,109**
0	Uncharacterized protein [Source:UniProtKB/TrEMBL;Acc:D4A886] [ENSRNOT00000052276]	**5,109**
LOC678766	PREDICTED: Rattus norvegicus similar to ras homolog gene family, member U (LOC678766), mRNA [XM_001053087]	**5,108**
0	Rattus norvegicus chromosome 7, 38 clones, strain BN/SsNHsdMCW RNOR03304491, whole genome shotgun sequence [AABR03059028]	**5,107**
Ly9	Rattus norvegicus lymphocyte antigen 9 (Ly9), mRNA [NM_001191673]	**5,107**
0	Unknown	**5,107**
Gamt	Rattus norvegicus guanidinoacetate N-methyltransferase (Gamt), mRNA [NM_012793]	**5,106**
0	Unknown	**5,106**
Rnf213	PREDICTED: Rattus norvegicus ring finger protein 213 (Rnf213), mRNA [XM_001081768]	**5,106**
Tubgcp2	Rattus norvegicus tubulin, gamma complex associated protein 2 (Tubgcp2), mRNA [NM_001107560]	**5,106**
0	RCG47285, isoform CRA_bUncharacterized protein [Source:UniProtKB/TrEMBL;Acc:D3ZEH6] [ENSRNOT00000024237]	**5,105**
Kcnmb4	Rattus norvegicus potassium large conductance calcium-activated channel, subfamily M, beta member 4 (Kcnmb4), mRNA [NM_023960]	**5,104**
0	Unknown	**5,104**
Morn2	Rattus norvegicus MORN repeat containing 2 (Morn2), transcript variant 1, mRNA [NM_001126086]	**5,104**
Alg5	Rattus norvegicus asparagine-linked glycosylation 5, dolichyl-phosphate beta-glucosyltransferase homolog (S. cerevisiae) (Alg5), mRNA [NM_001025407]	**5,104**
RGD1566215	Rattus norvegicus similar to Coatomer gamma-2 subunit (Gamma-2 coat protein) (Gamma-2 COP) (RGD1566215), mRNA [NM_001106929]	**5,103**
Efha1	Rattus norvegicus EF-hand domain family, member A1 (Efha1), mRNA [NM_134396]	**5,103**
Rps17	Rattus norvegicus ribosomal protein S17 (Rps17), mRNA [NM_017152]	**5,103**
Cbx3	Rattus norvegicus chromobox homolog 3 (HP1 gamma homolog, Drosophila) (Cbx3), mRNA [NM_001008313]	**5,103**
Rdh8	Rattus norvegicus retinol dehydrogenase 8 (all-trans) (Rdh8), mRNA [NM_001168593]	**5,102**
RGD1563441	Rattus norvegicus similar to RIKEN cDNA A030009H04 (RGD1563441), mRNA [NM_001126292]	**5,102**
Vps4b	Rattus norvegicus vacuolar protein sorting 4 homolog B (S. cerevisiae) (Vps4b), mRNA [NM_001025716]	**5,102**
Egln2	Rattus norvegicus EGL nine homolog 2 (C. elegans) (Egln2), mRNA [NM_001004083]	**5,099**
Usp43	Rattus norvegicus ubiquitin specific peptidase 43 (Usp43), mRNA [NM_001100968]	**5,099**
Dcaf12l1	Rattus norvegicus DDB1 and CUL4 associated factor 12-like 1 (Dcaf12l1), mRNA [NM_001107941]	**5,099**
0	Unknown	**5,098**
0	Unknown	**5,098**
Rpusd1	Rattus norvegicus RNA pseudouridylate synthase domain containing 1 (Rpusd1), mRNA [NM_001105774]	**5,098**
Cntnap5	Rattus norvegicus contactin associated protein-like 5 (Cntnap5), mRNA [NM_001047866]	**5,097**
0	Mast cell protease 1-like 2 [Source:UniProtKB/TrEMBL;Acc:Q6IE56] [ENSRNOT00000038253]	**5,097**

Vwa2	PREDICTED: Rattus norvegicus von Willebrand factor A domain containing 2 (Vwa2), miscRNA [XR_008134]	**5,097**
LOC680711	PREDICTED: Rattus norvegicus hypothetical protein LOC680711 (LOC680711), mRNA [XM_001056892]	**5,095**
0	Nipped-B homolog (Drosophila)Uncharacterized protein [Source:UniProtKB/TrEMBL;Acc:D3ZG77] [ENSRNOT00000021181]	**5,093**
Hexdc	Rattus norvegicus hexosaminidase (glycosyl hydrolase family 20, catalytic domain) containing (Hexdc), mRNA [NM_001142562]	**5,093**
Paip1	Rattus norvegicus poly(A) binding protein interacting protein 1 (Paip1), mRNA [NM_001108937]	**5,093**
0	Uncharacterized protein [Source:UniProtKB/TrEMBL;Acc:D4A1S8] [ENSRNOT00000046898]	**5,092**
Ube2c	Rattus norvegicus ubiquitin-conjugating enzyme E2C (Ube2c), mRNA [NM_001106542]	**5,090**
Fbxl4	Rattus norvegicus F-box and leucine-rich repeat protein 4 (Fbxl4), mRNA [NM_001107919]	**5,090**
0	Unknown	**5,090**
RGD1563170	PREDICTED: Rattus norvegicus RGD1563170 (RGD1563170), mRNA [XM_001054677]	**5,089**
LOC100233176	Rattus norvegicus hypothetical protein LOC100233176 (LOC100233176), transcript variant 2, mRNA [NM_001142942]	**5,089**
Retsat	Rattus norvegicus retinol saturase (all trans retinol 13,14 reductase) (Retsat), mRNA [NM_145084]	**5,089**
Ncoa2	Rattus norvegicus nuclear receptor coactivator 2 (Ncoa2), mRNA [NM_031822]	**5,088**
Klk12	Rattus norvegicus kallikrein related-peptidase 12 (Klk12), mRNA [NM_001107508]	**5,087**
RGD1563015	Rattus norvegicus RGD1563015 (RGD1563015), mRNA [NM_001134627]	**5,087**
Fez2	Rattus norvegicus fasciculation and elongation protein zeta 2 (zygin II), mRNA (cDNA clone MGC:112579 IMAGE:7128839), complete cds. [BC100060]	**5,087**
Nudcd1	Rattus norvegicus NudC domain containing 1 (Nudcd1), mRNA [NM_001130561]	**5,087**
Tomm40	Rattus norvegicus translocase of outer mitochondrial membrane 40 homolog (yeast) (Tomm40), nuclear gene encoding mitochondrial protein, mRNA [NM_212520]	**5,087**
RGD1564906	Glutathione S-transferase [Source:UniProtKB/TrEMBL;Acc:D3ZBD6] [ENSRNOT00000000233]	**5,086**
0	Unknown	**5,086**
Pcyt2	Rattus norvegicus phosphate cytidylyltransferase 2, ethanolamine (Pcyt2), mRNA [NM_053568]	**5,086**
0	Unknown	**5,085**
Ttc3	Rattus norvegicus tetratricopeptide repeat domain 3 (Ttc3), mRNA [NM_001108315]	**5,084**
Gpr113	Rattus norvegicus G protein-coupled receptor 113 (Gpr113), mRNA [NM_001106712]	**5,083**
RGD1307225	Rattus norvegicus similar to MEGF6 (RGD1307225), mRNA [NM_001107663]	**5,083**
0	Unknown	**5,083**
0	Q8K0W6_MOUSE (Q8K0W6) LOC545728 protein (Fragment), partial (29%) [TC590048]	**5,083**
Ctxn3	Rattus norvegicus cortexin 3 (Ctxn3), mRNA [NM_001134696]	**5,082**
Snx32	PREDICTED: Rattus norvegicus sorting nexin 32 (Snx32), mRNA [XM_001073782]	**5,082**
Gal	Rattus norvegicus galanin prepropeptide (Gal), mRNA [NM_033237]	**5,081**
RGD1565657	similar to germ cell-less (RGD1565657), mRNA [Source:RefSeq DNA;Acc:NM_001106728] [ENSRNOT00000041559]	**5,081**
0	Unknown	**5,080**

Mmp17	Rattus norvegicus matrix metallopeptidase 17 (Mmp17), mRNA [NM_001105925]	**5,080**
RGD1564171	Rattus norvegicus RGD1564171 (RGD1564171), mRNA [NM_001109186]	**5,080**
0	Uncharacterized protein [Source:UniProtKB/TrEMBL;Acc:D3ZSU9] [ENSRNOT00000050431]	**5,080**
Spink2	Rattus norvegicus serine peptidase inhibitor, Kazal type 2 (acrosin-trypsin inhibitor) (Spink2), mRNA [NM_001008870]	**5,080**
0	Uncharacterized protein [Source:UniProtKB/TrEMBL;Acc:D3ZJ17] [ENSRNOT00000006594]	**5,080**
Fam176b	Rattus norvegicus family with sequence similarity 176, member B (Fam176b), mRNA [NM_001108679]	**5,080**
Fam183b	Uncharacterized protein [Source:UniProtKB/TrEMBL;Acc:D3Z8P9] [ENSRNOT00000003841]	**5,079**
Stard4	Rattus norvegicus StAR-related lipid transfer (START) domain containing 4 (Stard4), mRNA [NM_001106159]	**5,079**
RGD1564325	40S ribosomal protein S24 [Source:UniProtKB/TrEMBL;Acc:D3ZFZ8] [ENSRNOT00000047252]	**5,079**
Dohh	Rattus norvegicus deoxyhypusine hydroxylase/monooxygenase (Dohh), mRNA [NM_001025006]	**5,078**
0	Unknown	**5,077**
Lrit1	Leucine-rich repeat, immunoglobulin-like domain and transmembrane domain-containing protein 1 [Source:UniProtKB/Swiss-Prot;Acc:Q9JMH2] [ENSRNOT00000017773]	**5,076**
0	Uncharacterized protein [Source:UniProtKB/TrEMBL;Acc:D4A3R8] [ENSRNOT00000049208]	**5,076**
N5	Rattus norvegicus DNA binding protein N5 (N5), mRNA [NM_022857]	**5,076**
Axl	Rattus norvegicus Axl receptor tyrosine kinase (Axl), transcript variant 1, mRNA [NM_031794]	**5,076**
Tmem57	Rattus norvegicus transmembrane protein 57 (Tmem57), mRNA [NM_001025699]	**5,076**
Cdk105	Rattus norvegicus CDK105 protein (Cdk105), mRNA [NM_134415]	**5,076**
RGD1563425	Rattus norvegicus similar to FLJ35784 protein (RGD1563425), mRNA [NM_001109059]	**5,075**
Prkcsh	Rattus norvegicus protein kinase C substrate 80K-H (Prkcsh), mRNA [NM_001106806]	**5,075**
Pdgfb	Rattus norvegicus platelet-derived growth factor beta polypeptide (simian sarcoma viral (v-sis) oncogene homolog) (Pdgfb), mRNA [NM_031524]	**5,075**
0	Unknown	**5,072**
0	Rattus norvegicus similar to expressed sequence AI850305 (LOC314660), mRNA [XM_234947]	**5,072**
LOC690725	PREDICTED: Rattus norvegicus hypothetical protein LOC690725 (LOC690725), mRNA [XM_001075387]	**5,072**
Tmem107	Rattus norvegicus transmembrane protein 107 (Tmem107), mRNA [NM_001109648]	**5,072**
0	Rattus norvegicus similar to glyceraldehyde-3-phosphate dehydrogenase (phosphorylating) (EC 1.2.1.12) - mouse (LOC308706), mRNA [XM_218761]	**5,072**
Brd1	Rattus norvegicus bromodomain containing 1 (Brd1), mRNA [NM_001108103]	**5,071**
0	Uncharacterized protein [Source:UniProtKB/TrEMBL;Acc:D3ZDE9] [ENSRNOT00000041674]	**5,070**
Fxc1	Rattus norvegicus fractured callus expressed transcript 1 (Fxc1), mRNA [NM_053371]	**5,070**
LOC296778	Rattus norvegicus similar to RIKEN cDNA 4933431D05 (LOC296778), mRNA [XM_216072]	**5,070**
Kidins220	Rattus norvegicus kinase D-interacting substrate 220 (Kidins220), mRNA [NM_053795]	**5,069**
Tspan11	Rattus norvegicus tetraspanin 11 (Tspan11), mRNA [NM_001024262]	**5,068**
Nup62	Rattus norvegicus nucleoporin 62 (Nup62), mRNA [NM_023098]	**5,068**

Stk35	Rattus norvegicus serine/threonine kinase 35 (Stk35), mRNA [NM_001107773]	**5,067**
Eral1	Rattus norvegicus Era (G-protein)-like 1 (E. coli) (Eral1), mRNA [NM_001013229]	**5,067**
Saps2	Rattus norvegicus SAPS domain family, member 2 (Saps2), mRNA [NM_001106789]	**5,067**
Trub1	Rattus norvegicus TruB pseudouridine (psi) synthase homolog 1 (E. coli) (Trub1), mRNA [NM_001012173]	**5,067**
Anp32b	Rattus norvegicus acidic (leucine-rich) nuclear phosphoprotein 32 family, member B (Anp32b), mRNA [NM_131911]	**5,065**
RGD1564048	PREDICTED: Rattus norvegicus similar to Protein C20orf46 (RGD1564048), mRNA [XM_001060786]	**5,062**
Afap1l1	Rattus norvegicus actin filament associated protein 1-like 1 (Afap1l1), mRNA [NM_001106142]	**5,061**
Pramef12	PREDICTED: Rattus norvegicus PRAME family member 12 (Pramef12), mRNA [XM_001077034]	**5,061**
Ngef	Rattus norvegicus neuronal guanine nucleotide exchange factor (Ngef), mRNA [NM_001136241]	**5,060**
Secisbp2	Rattus norvegicus SECIS binding protein 2 (Secisbp2), mRNA [NM_024002]	**5,057**
Slc7a15	Rattus norvegicus solute carrier family 7 (cationic amino acid transporter, y+ system), member 15 (Slc7a15), mRNA [NM_001106714]	**5,057**
Vom2r72	Rattus norvegicus vomeronasal 2 receptor, 72 (Vom2r72), mRNA [NM_001099517]	**5,056**
Pi4k2b	Rattus norvegicus phosphatidylinositol 4-kinase type 2 beta (Pi4k2b), mRNA [NM_001005883]	**5,055**
Gpsm2	Rattus norvegicus G-protein signaling modulator 2 (AGS3-like, C. elegans) (Gpsm2), mRNA [NM_001191962]	**5,055**
RGD1563159	Rattus norvegicus RGD1563159 (RGD1563159), mRNA [NM_001108428]	**5,054**
LOC689741	PREDICTED: Rattus norvegicus similar to Der1-like domain family, member 1 (LOC689741), mRNA [XM_001071836]	**5,054**
Nrbf2	Rattus norvegicus nuclear receptor binding factor 2 (Nrbf2), mRNA [NM_022186]	**5,054**
Scube1	Rattus norvegicus signal peptide, CUB domain, EGF-like 1 (Scube1), mRNA [NM_001134884]	**5,052**
RGD1307947	Rattus norvegicus similar to RIKEN cDNA C430008C19 (RGD1307947), mRNA [NM_001014083]	**5,051**
Myh11	Rattus norvegicus myosin, heavy chain 11, smooth muscle (Myh11), mRNA [NM_001170600]	**5,051**
Cog4	Rattus norvegicus component of oligomeric golgi complex 4 (Cog4), mRNA [NM_001108449]	**5,051**
0	Unknown	**5,049**
LOC685007	PREDICTED: Rattus norvegicus similar to unc-93 homolog A (LOC685007), mRNA [XM_001061886]	**5,049**
Ankrd27	Uncharacterized protein [Source:UniProtKB/TrEMBL;Acc:D3ZBZ8] [ENSRNOT00000017669]	**5,047**
Rnf219	Rattus norvegicus ring finger protein 219 (Rnf219), mRNA [NM_001108389]	**5,045**
Mt3	Rattus norvegicus metallothionein 3 (Mt3), mRNA [NM_053968]	**5,045**
Bdh2	Rattus norvegicus 3-hydroxybutyrate dehydrogenase, type 2 (Bdh2), mRNA [NM_001106473]	**5,045**
0	Unknown	**5,044**
0	Unknown	**5,043**
Rab44	Uncharacterized protein [Source:UniProtKB/TrEMBL;Acc:D4A226] [ENSRNOT00000036899]	**5,042**
LOC679404	PREDICTED: Rattus norvegicus similar to spermatogenesis associated glutamate (E)-rich protein 4d (LOC679404), mRNA [XM_001056196]	**5,042**
Mthfd2	Rattus norvegicus methylenetetrahydrofolate dehydrogenase (NADP+ dependent) 2, methenyltetrahydrofolate cyclohydrolase (Mthfd2), nuclear gene encoding mitochondrial protein, mRNA [NM_001109398]	**5,041**
Gtf2ird1	Rattus norvegicus GTF2I repeat domain containing 1 (Gtf2ird1), mRNA [NM_001001504]	**5,041**

Csf3r	Rattus norvegicus colony stimulating factor 3 receptor (granulocyte) (Csf3r), mRNA [NM_001106685]	**5,041**
Sult4a1	Rattus norvegicus sulfotransferase family 4A, member 1 (Sult4a1), mRNA [NM_031641]	**5,040**
Zfp7	Rattus norvegicus zinc finger protein 7 (Zfp7), mRNA [NM_001142757]	**5,040**
Galnt10	Rattus norvegicus UDP-N-acetyl-alpha-D-galactosamine:polypeptide N-acetylgalactosaminyltransferase 10 (GalNAc-T10) (Galnt10), mRNA [NM_130742]	**5,039**
LOC293589	Rattus norvegicus TL0AAA51YB05 mRNA sequence. [FQ212842]	**5,038**
0	Unknown	**5,038**
RGD1560073	Uncharacterized protein [Source:UniProtKB/TrEMBL;Acc:D3ZCN9] [ENSRNOT00000043250]	**5,034**
0	Uncharacterized protein [Source:UniProtKB/TrEMBL;Acc:D3ZAL9] [ENSRNOT00000068454]	**5,034**
Mrps26	Rattus norvegicus mitochondrial ribosomal protein S26 (Mrps26), nuclear gene encoding mitochondrial protein, mRNA [NM_001013206]	**5,033**
Papss1	Rattus norvegicus 3'-phosphoadenosine 5'-phosphosulfate synthase 1 (Papss1), mRNA [NM_001106471]	**5,033**
Bwk1	Rattus norvegicus Bwk1 leukemia-related gene (Bwk1), mRNA [NM_198743]	**5,032**
Fn1	Rattus norvegicus fibronectin 1 (Fn1), mRNA [NM_019143]	**5,032**
Gpt	Rattus norvegicus glutamic-pyruvate transaminase (alanine aminotransferase) (Gpt), mRNA [NM_031039]	**5,030**
Ifrd2	Rattus norvegicus interferon-related developmental regulator 2 (Ifrd2), mRNA [NM_001047871]	**5,029**
Slc9a4	Rattus norvegicus solute carrier family 9 (sodium/hydrogen exchanger), member 4 (Slc9a4), mRNA [NM_173098]	**5,029**
Cybb	Rattus norvegicus cytochrome b-245, beta polypeptide (Cybb), mRNA [NM_023965]	**5,028**
0	myoneurin [Source:RefSeq peptide;Acc:NP_001012178] [ENSRNOT00000058909]	**5,028**
Smptb	Rattus norvegicus polypyrimidine tract-binding protein (Smptb), mRNA [NM_182818]	**5,027**
Rad51ap1	Rattus norvegicus RAD51 associated protein 1 (Rad51ap1), mRNA [NM_001079711]	**5,026**
Wdr44	PREDICTED: Rattus norvegicus WD repeat domain 44 (Wdr44), mRNA [XM_001061437]	**5,025**
Puf60	Rattus norvegicus poly-U binding splicing factor 60 (Puf60), mRNA [NM_001191880]	**5,024**
LOC690269	PREDICTED: Rattus norvegicus hypothetical protein LOC690269 (LOC690269), mRNA [XM_002727149]	**5,024**
LOC503192	Zinc finger protein 6 [Source:UniProtKB/TrEMBL;Acc:P97662] [ENSRNOT00000019204]	**5,024**
Vps72	Rattus norvegicus vacuolar protein sorting 72 homolog (S. cerevisiae) (Vps72), mRNA [NM_001107695]	**5,022**
RGD1561635	PREDICTED: Rattus norvegicus similar to 40S ribosomal protein S17 (RGD1561635), mRNA [XM_002724878]	**5,021**
Scrib	Rattus norvegicus scribbled homolog (Drosophila) (Scrib), mRNA [NM_001191879]	**5,020**
Slc30a1	Rattus norvegicus solute carrier family 30 (zinc transporter), member 1 (Slc30a1), mRNA [NM_022853]	**5,020**
Ehd1	Rattus norvegicus EH-domain containing 1 (Ehd1), mRNA [NM_001011939]	**5,019**
Magee1	Rattus norvegicus melanoma antigen, family E, 1 (Magee1), mRNA [NM_001079891]	**5,019**
RGD1306001	Rattus norvegicus similar to 2210021J22Rik protein (RGD1306001), mRNA [NM_001130696]	**5,018**
Bspry	Rattus norvegicus B-box and SPRY domain containing (Bspry), mRNA [NM_022261]	**5,018**
0	Unknown	**5,017**
Nrtn	Rattus norvegicus neurturin (Nrtn), mRNA [NM_053399]	**5,017**

0	Unknown	**5,016**
Siat7E	PREDICTED: Rattus norvegicus sialyltransferase 7E (Siat7E), mRNA [XM_001078153]	**5,016**
0	Uncharacterized protein [Source:UniProtKB/TrEMBL;Acc:D3Z8Y2] [ENSRNOT00000064614]	**5,015**
0	Uncharacterized protein [Source:UniProtKB/TrEMBL;Acc:D4AEI9] [ENSRNOT00000034494]	**5,014**
Bex1	Rattus norvegicus brain expressed, X-linked 1 (Bex1), mRNA [NM_001037365]	**5,014**
0	Ankyrin repeat and SOCS box protein 14 [Source:UniProtKB/Swiss-Prot;Acc:P0C927] [ENSRNOT00000017847]	**5,014**
Rock1	Rattus norvegicus Rho-associated coiled-coil containing protein kinase 1 (Rock1), mRNA [NM_031098]	**5,013**
0	Unknown	**5,013**
Tmem121	Uncharacterized protein [Source:UniProtKB/TrEMBL;Acc:D3ZMR5] [ENSRNOT00000006872]	**5,012**
Sec16b	Rattus norvegicus SEC16 homolog B (S. cerevisiae) (Sec16b), mRNA [NM_053571]	**5,012**
Cacybp	Rattus norvegicus calcyclin binding protein (Cacybp), mRNA [NM_001004208]	**5,012**
Itpripl1	Rattus norvegicus inositol 1,4,5-triphosphate receptor interacting protein-like 1 (Itpripl1), mRNA [NM_001025043]	**5,011**
0	Unknown	**5,010**
Mat2a	Rattus norvegicus methionine adenosyltransferase II, alpha (Mat2a), mRNA [NM_134351]	**5,010**
Rpl23	Rattus norvegicus ribosomal protein L23 (Rpl23), mRNA [NM_001007599]	**5,009**
Chrne	Rattus norvegicus cholinergic receptor, nicotinic, epsilon (Chrne), mRNA [NM_017194]	**5,009**
Dync1i2	Rattus norvegicus dynein cytoplasmic 1 intermediate chain 2 (Dync1i2), mRNA [NM_053880]	**5,009**
0	Unknown	**5,009**
Tomm6	RCG43475Uncharacterized protein [Source:UniProtKB/TrEMBL;Acc:D3ZJS3] [ENSRNOT00000018959]	**5,009**
Snx7	Rattus norvegicus sorting nexin 7 (Snx7), mRNA [NM_001012083]	**5,008**
Sec24b	Rattus norvegicus SEC24 family, member B (S. cerevisiae) (Sec24b), mRNA [NM_001106474]	**5,008**
LOC688302	PREDICTED: Rattus norvegicus similar to spermatogenesis associated glutamate (E)-rich protein 4d (LOC688302), partial mRNA [XM_002730119]	**5,008**
0	Uncharacterized protein [Source:UniProtKB/TrEMBL;Acc:D4A621] [ENSRNOT00000064915]	**5,007**
Myo1b	Rattus norvegicus myosin Ib (Myo1b), mRNA [NM_053986]	**5,007**
Pla2g15	Rattus norvegicus phospholipase A2, group XV (Pla2g15), mRNA [NM_001004277]	**5,007**
Nasp	Rattus norvegicus nuclear autoantigenic sperm protein (histone-binding) (Nasp), mRNA [NM_001005543]	**5,006**
Klc4	Rattus norvegicus kinesin light chain 4 (Klc4), mRNA [NM_001009601]	**5,006**
0	Unknown	**5,004**
Rplp0	Rattus norvegicus ribosomal protein, large, P0 (Rplp0), mRNA [NM_022402]	**5,004**
0	Uncharacterized protein [Source:UniProtKB/TrEMBL;Acc:D3ZPD3] [ENSRNOT00000056348]	**5,003**
Kifap3	Rattus norvegicus kinesin-associated protein 3 (Kifap3), mRNA [NM_001105964]	**5,003**
Prg4	Rattus norvegicus proteoglycan 4, (megakaryocyte stimulating factor, articular superficial zone protein, camptodactyly, arthropathy, coxa vara, pericarditis syndrome) (Prg4), mRNA [NM_001105962]	**5,003**

0	Unknown	**5,003**
Tceal8	Rattus norvegicus transcription elongation factor A (SII)-like 8 (Tceal8), mRNA [NM_001014275]	**5,001**
Hs3st5	Rattus norvegicus heparan sulfate (glucosamine) 3-O-sulfotransferase 5 (Hs3st5), mRNA [NM_001106392]	**5,000**
