# Supplementary material for: The RNA-Binding Protein hnRNP K Mediates the Effect of BDNF on Dendritic mRNA Metabolism and Regulates Synaptic NMDA Receptors in Hippocampal Neurons
Source: eNeuro. 2017 Dec 12;4(6):ENEURO.0268-17.2017. doi: 10.1523/ENEURO.0268-17.2017 (PMC5732018; doi:10.1523/ENEURO.0268-17.2017)
Supplement: Table 1-2 — Download Table 1-2, PDF file. [file sup_enu-eN-NWR-0268-17-s05.pdf]

Table 1-2 - Biological processes associated with mRNAs co-immunoprecipitated with hnRNP K as assessed by Gene Ontology

GO biological process	Rattus norvegicus (reference list)	uploaded list	(expected)	(over/under)	(fold enrichment)	(P-value)
glutamate receptor signaling pathway (GO:0007215)	44	39	17.26	+	2.26	3.81E-02
amino acid transport (GO:0006865)	90	76	35.31	+	2.15	1.46E-05
synapse assembly (GO:0007416)	51	43	20.01	+	2.15	4.35E-02
dendrite morphogenesis (GO:0048813)	57	48	22.36	+	2.15	1.34E-02
dendrite development (GO:0016358)	106	89	41.58	+	2.14	8.56E-07
regulation of neuronal synaptic plasticity (GO:0048168)	71	59	27.85	+	2.12	1.46E-03
regulation of synaptic plasticity (GO:0048167)	181	148	71.00	+	2.08	6.28E-12
forebrain cell migration (GO:0021885)	70	57	27.46	+	2.08	4.35E-03
regulation of dendrite morphogenesis (GO:0048814)	94	76	36.88	+	2.06	8.66E-05
telencephalon cell migration (GO:0022029)	67	54	26.28	+	2.05	1.14E-02
positive regulation of dendrite development (GO:1900006)	91	73	35.70	+	2.04	2.24E-04
negative regulation of synaptic transmission (GO:0050805)	64	51	25.11	+	2.03	2.96E-02
learning (GO:0007612)	159	126	62.37	+	2.02	6.53E-09
regulation of synapse structure or activity (GO:0050803)	280	221	109.84	+	2.01	3.29E-17
positive regulation of synapse assembly (GO:0051965)	66	52	25.89	+	2.01	3.27E-02
synaptic vesicle cycle (GO:0099504)	94	74	36.88	+	2.01	3.75E-04
regulation of postsynaptic membrane potential (GO:0060078)	65	51	25.50	+	2.00	4.48E-02
neurotransmitter secretion (GO:0007269)	102	80	40.01	+	2.00	1.29E-04
signal release from synapse (GO:0099643)	102	80	40.01	+	2.00	1.29E-04
regulation of synapse assembly (GO:0051963)	83	65	32.56	+	2.00	2.79E-03
presynaptic process involved in chemical synaptic transmission (GO:0099531)	106	83	41.58	+	2.00	7.57E-05
regulation of dendritic spine development (GO:0060998)	68	53	26.68	+	1.99	3.58E-02
vesicle-mediated transport in synapse (GO:0099003)	86	67	33.74	+	1.99	2.23E-03
synapse organization (GO:0050808)	139	108	54.53	+	1.98	7.85E-07
central nervous system neuron development (GO:0021954)	85	66	33.34	+	1.98	3.04E-03
neurotransmitter transport (GO:0006836)	147	114	57.67	+	1.98	2.71E-07
regulation of calcium ion transmembrane transporter activity (GO:1901019)	71	55	27.85	+	1.97	2.85E-02
cerebral cortex development (GO:0021987)	128	99	50.21	+	1.97	5.75E-06
regulation of neurotransmitter transport (GO:0051588)	70	54	27.46	+	1.97	3.90E-02
regulation of potassium ion transport (GO:0043266)	86	66	33.74	+	1.96	4.51E-03
regulation of synapse organization (GO:0050807)	120	92	47.07	+	1.95	3.35E-05
protein lipidation (GO:0006497)	77	59	30.21	+	1.95	1.80E-02
calcium ion regulated exocytosis (GO:0017156)	81	62	31.78	+	1.95	1.05E-02

learning or memory (GO:0007611)	272	208	106.70	+	1.95	1.31E-14
regulation of dendrite development (GO:0050773)	157	120	61.59	+	1.95	2.02E-07
regulation of neurotransmitter levels (GO:0001505)	199	152	78.07	+	1.95	5.29E-10
synaptic vesicle localization (GO:0097479)	110	84	43.15	+	1.95	1.80E-04
regulation of cation channel activity (GO:2001257)	96	73	37.66	+	1.94	1.66E-03
synaptic vesicle transport (GO:0048489)	108	82	42.37	+	1.94	3.32E-04
establishment of synaptic vesicle localization (GO:0097480)	108	82	42.37	+	1.94	3.32E-04
modulation of synaptic transmission (GO:0050804)	340	258	133.38	+	1.93	2.81E-18
regulation of calcium ion-dependent exocytosis (GO:0017158)	74	56	29.03	+	1.93	4.54E-02
lipoprotein biosynthetic process (GO:0042158)	82	62	32.17	+	1.93	1.54E-02
negative regulation of neuron projection development (GO:0010977)	147	111	57.67	+	1.92	2.13E-06
hippocampus development (GO:0021766)	106	80	41.58	+	1.92	6.09E-04
regulation of sodium ion transport (GO:0002028)	80	60	31.38	+	1.91	2.83E-02
establishment of vesicle localization (GO:0051650)	160	120	62.77	+	1.91	6.20E-07
regulation of ion transmembrane transporter activity (GO:0032412)	191	143	74.93	+	1.91	1.17E-08
neuron projection morphogenesis (GO:0048812)	425	318	166.72	+	1.91	3.61E-22
memory (GO:0007613)	131	98	51.39	+	1.91	3.53E-05
negative regulation of neuron differentiation (GO:0045665)	222	166	87.09	+	1.91	2.19E-10
establishment of cell polarity (GO:0030010)	99	74	38.84	+	1.91	2.58E-03
response to osmotic stress (GO:0006970)	87	65	34.13	+	1.90	1.30E-02
positive regulation of exocytosis (GO:0045921)	83	62	32.56	+	1.90	2.24E-02
regulation of transmembrane transporter activity (GO:0022898)	197	147	77.28	+	1.90	7.28E-09
developmental growth involved in morphogenesis (GO:0060560)	122	91	47.86	+	1.90	1.39E-04
regulation of axonogenesis (GO:0050770)	181	135	71.00	+	1.90	6.17E-08
vesicle localization (GO:0051648)	169	126	66.30	+	1.90	3.06E-07
cell morphogenesis involved in neuron differentiation (GO:0048667)	389	290	152.60	+	1.90	9.16E-20
Ras protein signal transduction (GO:0007265)	129	96	50.61	+	1.90	6.44E-05
negative regulation of neurogenesis (GO:0050768)	285	212	111.80	+	1.90	1.09E-13
pallium development (GO:0021543)	203	151	79.63	+	1.90	4.54E-09
neuron projection development (GO:0031175)	592	439	232.23	+	1.89	7.68E-31
adult locomotory behavior (GO:0008344)	100	74	39.23	+	1.89	3.72E-03
negative regulation of nervous system development (GO:0051961)	307	227	120.43	+	1.88	1.52E-14
axonogenesis (GO:0007409)	325	240	127.49	+	1.88	1.89E-15
regulation of transporter activity (GO:0032409)	210	155	82.38	+	1.88	4.04E-09
associative learning (GO:0008306)	95	70	37.27	+	1.88	8.59E-03
negative regulation of cell development (GO:0010721)	340	250	133.38	+	1.87	5.66E-16
regulation of neuron projection development (GO:0010975)	498	366	195.36	+	1.87	1.52E-24

positive regulation of neuron projection development (GO:0010976)	293	215	114.94	+	1.87	2.63E-13
chemical synaptic transmission (GO:0007268)	334	245	131.02	+	1.87	1.75E-15
trans-synaptic signaling (GO:0099537)	334	245	131.02	+	1.87	1.75E-15
synaptic signaling (GO:0099536)	334	245	131.02	+	1.87	1.75E-15
anterograde trans-synaptic signaling (GO:0098916)	334	245	131.02	+	1.87	1.75E-15
positive regulation of synaptic transmission (GO:0050806)	131	96	51.39	+	1.87	1.31E-04
cognition (GO:0050890)	303	222	118.86	+	1.87	9.52E-14
regulation of exocytosis (GO:0017157)	183	134	71.79	+	1.87	2.41E-07
regulation of cell morphogenesis involved in differentiation (GO:0010769)	366	268	143.58	+	1.87	4.53E-17
negative regulation of cell morphogenesis involved in differentiation (GO:0010771)	115	84	45.11	+	1.86	1.12E-03
negative regulation of canonical Wnt signaling pathway (GO:0090090)	100	73	39.23	+	1.86	7.13E-03
axon development (GO:0061564)	359	262	140.83	+	1.86	1.88E-16
action potential (GO:0001508)	92	67	36.09	+	1.86	2.10E-02
endothelium development (GO:0003158)	95	69	37.27	+	1.85	1.64E-02
regulation of protein complex disassembly (GO:0043244)	87	63	34.13	+	1.85	4.83E-02
neuron projection guidance (GO:0097485)	195	141	76.50	+	1.84	1.74E-07
circadian rhythm (GO:0007623)	166	120	65.12	+	1.84	5.15E-06
telencephalon development (GO:0021537)	292	211	114.55	+	1.84	2.38E-12
Golgi organization (GO:0007030)	90	65	35.31	+	1.84	3.78E-02
cerebellum development (GO:0021549)	126	91	49.43	+	1.84	5.69E-04
negative regulation of protein serine/threonine kinase activity (GO:0071901)	122	88	47.86	+	1.84	9.72E-04
axon guidance (GO:0007411)	193	139	75.71	+	1.84	3.10E-07
metencephalon development (GO:0022037)	138	99	54.14	+	1.83	2.15E-04
single-organism behavior (GO:0044708)	470	337	184.38	+	1.83	1.12E-20
negative regulation of cell projection organization (GO:0031345)	173	124	67.87	+	1.83	4.44E-06
negative regulation of neuron apoptotic process (GO:0043524)	160	114	62.77	+	1.82	2.92E-05
adult behavior (GO:0030534)	170	121	66.69	+	1.81	1.05E-05
signal transduction by protein phosphorylation (GO:0023014)	214	152	83.95	+	1.81	1.05E-07
ensheathment of neurons (GO:0007272)	96	68	37.66	+	1.81	4.32E-02
axon ensheathment (GO:0008366)	96	68	37.66	+	1.81	4.32E-02
positive regulation of cell morphogenesis involved in differentiation (GO:0010770)	185	131	72.57	+	1.81	3.09E-06
regulation of circadian rhythm (GO:0042752)	109	77	42.76	+	1.80	1.20E-02
regulation of neuron differentiation (GO:0045664)	662	467	259.69	+	1.80	3.21E-28
locomotory behavior (GO:0007626)	224	158	87.87	+	1.80	6.92E-08
negative regulation of protein complex assembly (GO:0031333)	105	74	41.19	+	1.80	2.05E-02
regulation of extent of cell growth (GO:0061387)	115	81	45.11	+	1.80	7.33E-03
establishment or maintenance of cell polarity (GO:0007163)	152	107	59.63	+	1.79	1.55E-04

regulation of neuron apoptotic process (GO:0043523)	243	171	95.33	+	1.79	1.19E-08
neuron development (GO:0048666)	760	534	298.14	+	1.79	2.62E-32
gliogenesis (GO:0042063)	218	153	85.52	+	1.79	2.07E-07
positive regulation of cell projection organization (GO:0031346)	372	261	145.93	+	1.79	2.60E-14
positive regulation of neuron differentiation (GO:0045666)	384	269	150.64	+	1.79	9.60E-15
negative regulation of ion transport (GO:0043271)	130	91	51.00	+	1.78	2.13E-03
MAPK cascade (GO:0000165)	163	114	63.94	+	1.78	7.69E-05
cellular response to insulin stimulus (GO:0032869)	159	111	62.37	+	1.78	1.30E-04
neural tube formation (GO:0001841)	122	85	47.86	+	1.78	6.15E-03
regulation of receptor activity (GO:0010469)	122	85	47.86	+	1.78	6.15E-03
carboxylic acid transport (GO:0046942)	211	147	82.77	+	1.78	8.15E-07
potassium ion transmembrane transport (GO:0071805)	135	94	52.96	+	1.77	1.72E-03
cellular potassium ion transport (GO:0071804)	135	94	52.96	+	1.77	1.72E-03
regulation of membrane potential (GO:0042391)	375	261	147.11	+	1.77	6.68E-14
glial cell differentiation (GO:0010001)	171	119	67.08	+	1.77	4.85E-05
exocytosis (GO:0006887)	207	144	81.20	+	1.77	1.38E-06
regulation of proteasomal ubiquitin-dependent protein catabolic process (GO:0032434)	105	73	41.19	+	1.77	3.76E-02
potassium ion transport (GO:0006813)	150	104	58.84	+	1.77	4.96E-04
protein localization to plasma membrane (GO:0072659)	150	104	58.84	+	1.77	4.96E-04
positive regulation of nervous system development (GO:0051962)	540	374	211.84	+	1.77	1.13E-20
regulation of cell morphogenesis (GO:0022604)	582	403	228.31	+	1.77	1.79E-22
negative regulation of cytoskeleton organization (GO:0051494)	117	81	45.90	+	1.76	1.38E-02
regulation of neuron death (GO:1901214)	318	220	124.75	+	1.76	4.43E-11
limbic system development (GO:0021761)	136	94	53.35	+	1.76	2.35E-03
regulation of smooth muscle cell proliferation (GO:0048660)	136	94	53.35	+	1.76	2.35E-03
regulation of cell projection organization (GO:0031344)	657	454	257.73	+	1.76	1.67E-25
regulated exocytosis (GO:0045055)	126	87	49.43	+	1.76	6.55E-03
regulation of ion transmembrane transport (GO:0034765)	396	273	155.35	+	1.76	3.76E-14
behavior (GO:0007610)	633	436	248.32	+	1.76	4.21E-24
primary neural tube formation (GO:0014020)	109	75	42.76	+	1.75	4.00E-02
cell morphogenesis involved in differentiation (GO:0000904)	554	381	217.33	+	1.75	1.36E-20
regulation of cell size (GO:0008361)	192	132	75.32	+	1.75	1.54E-05
epithelial tube formation (GO:0072175)	144	99	56.49	+	1.75	1.47E-03
protein dephosphorylation (GO:0006470)	176	121	69.04	+	1.75	7.02E-05
peptidyl-serine phosphorylation (GO:0018105)	144	99	56.49	+	1.75	1.47E-03
regulation of cation transmembrane transport (GO:1904062)	227	156	89.05	+	1.75	5.77E-07
regulation of neurogenesis (GO:0050767)	802	551	314.61	+	1.75	5.14E-31

positive regulation of neurogenesis (GO:0050769)	476	327	186.73	+	1.75	3.14E-17
regulation of nervous system development (GO:0051960)	897	616	351.88	+	1.75	4.81E-35
regulation of protein polymerization (GO:0032271)	172	118	67.47	+	1.75	1.19E-04
embryonic epithelial tube formation (GO:0001838)	143	98	56.10	+	1.75	1.94E-03
forebrain development (GO:0030900)	443	303	173.78	+	1.74	1.60E-15
regulation of transmembrane transport (GO:0034762)	411	281	161.23	+	1.74	3.42E-14
regulation of actin filament length (GO:0030832)	158	108	61.98	+	1.74	5.57E-04
regulation of actin polymerization or depolymerization (GO:0008064)	158	108	61.98	+	1.74	5.57E-04
neural tube development (GO:0021915)	180	123	70.61	+	1.74	7.40E-05
cellular response to hypoxia (GO:0071456)	142	97	55.70	+	1.74	2.55E-03
regulation of cellular component size (GO:0032535)	359	245	140.83	+	1.74	5.65E-12
regulation of small GTPase mediated signal transduction (GO:0051056)	223	152	87.48	+	1.74	1.73E-06
negative regulation of protein kinase activity (GO:0006469)	229	156	89.83	+	1.74	1.05E-06
rhythmic process (GO:0048511)	341	232	133.77	+	1.73	4.49E-11
regulation of regulated secretory pathway (GO:1903305)	122	83	47.86	+	1.73	1.99E-02
cellular response to decreased oxygen levels (GO:0036294)	150	102	58.84	+	1.73	1.59E-03
regulation of Ras protein signal transduction (GO:0046578)	200	136	78.46	+	1.73	1.71E-05
hindbrain development (GO:0030902)	178	121	69.83	+	1.73	1.28E-04
brain development (GO:0007420)	821	558	322.07	+	1.73	2.71E-30
negative regulation of neuron death (GO:1901215)	209	142	81.99	+	1.73	8.08E-06
regulation of heart contraction (GO:0008016)	165	112	64.73	+	1.73	4.56E-04
head development (GO:0060322)	861	584	337.76	+	1.73	1.22E-31
dephosphorylation (GO:0016311)	270	183	105.92	+	1.73	4.23E-08
regulation of MAP kinase activity (GO:0043405)	301	204	118.08	+	1.73	2.67E-09
organic anion transport (GO:0015711)	242	164	94.93	+	1.73	5.23E-07
negative regulation of MAPK cascade (GO:0043409)	155	105	60.80	+	1.73	1.27E-03
cell growth (GO:0016049)	145	98	56.88	+	1.72	3.52E-03
central nervous system development (GO:0007417)	1018	688	399.35	+	1.72	1.77E-37
small GTPase mediated signal transduction (GO:0007264)	336	227	131.81	+	1.72	1.75E-10
regulation of cell development (GO:0060284)	971	656	380.91	+	1.72	1.67E-35
peptidyl-serine modification (GO:0018209)	160	108	62.77	+	1.72	1.01E-03
negative regulation of kinase activity (GO:0033673)	246	166	96.50	+	1.72	5.46E-07
plasma membrane organization (GO:0007009)	209	141	81.99	+	1.72	1.42E-05
gland morphogenesis (GO:0022612)	129	87	50.61	+	1.72	1.62E-02
protein localization to cell periphery (GO:1990778)	162	109	63.55	+	1.72	1.03E-03
positive regulation of cell growth (GO:0030307)	162	109	63.55	+	1.72	1.03E-03
neuron differentiation (GO:0030182)	968	651	379.73	+	1.71	1.23E-34

negative regulation of Wnt signaling pathway (GO:0030178)	137	92	53.74	+	1.71	9.99E-03
positive regulation of cell development (GO:0010720)	575	386	225.57	+	1.71	3.94E-19
signal release (GO:0023061)	176	118	69.04	+	1.71	3.86E-04
nephron development (GO:0072006)	130	87	51.00	+	1.71	2.16E-02
response to insulin (GO:0032868)	246	164	96.50	+	1.70	1.66E-06
cellular response to oxygen levels (GO:0071453)	162	108	63.55	+	1.70	1.80E-03
positive regulation of cellular catabolic process (GO:0031331)	195	130	76.50	+	1.70	1.15E-04
phospholipid biosynthetic process (GO:0008654)	123	82	48.25	+	1.70	4.68E-02
regulation of metal ion transport (GO:0010959)	360	240	141.22	+	1.70	1.25E-10
second-messenger-mediated signaling (GO:0019932)	147	98	57.67	+	1.70	6.28E-03
tube formation (GO:0035148)	159	106	62.37	+	1.70	2.31E-03
transmembrane receptor protein tyrosine kinase signaling pathway (GO:0007169)	339	226	132.99	+	1.70	7.19E-10
vesicle organization (GO:0016050)	206	137	80.81	+	1.70	5.53E-05
endosomal transport (GO:0016197)	191	127	74.93	+	1.69	1.93E-04
morphogenesis of embryonic epithelium (GO:0016331)	167	111	65.51	+	1.69	1.42E-03
regulation of actin filament polymerization (GO:0030833)	137	91	53.74	+	1.69	1.74E-02
secretion by cell (GO:0032940)	383	254	150.25	+	1.69	3.78E-11
regulation of vesicle-mediated transport (GO:0060627)	454	301	178.10	+	1.69	1.18E-13
regulation of protein serine/threonine kinase activity (GO:0071900)	433	287	169.86	+	1.69	6.89E-13
regulation of ion transport (GO:0043269)	596	395	233.80	+	1.69	1.05E-18
regulation of proteolysis involved in cellular protein catabolic process (GO:1903050)	163	108	63.94	+	1.69	2.38E-03
regulation of catabolic process (GO:0009894)	455	301	178.49	+	1.69	1.56E-13
protein localization to membrane (GO:0072657)	297	196	116.51	+	1.68	7.06E-08
generation of neurons (GO:0048699)	1493	985	585.69	+	1.68	2.61E-51
kidney epithelium development (GO:0072073)	141	93	55.31	+	1.68	1.79E-02
regulation of cellular catabolic process (GO:0031329)	311	205	122.00	+	1.68	2.63E-08
establishment of protein localization to membrane (GO:0090150)	173	114	67.87	+	1.68	1.48E-03
response to hypoxia (GO:0001666)	375	247	147.11	+	1.68	1.82E-10
phospholipid metabolic process (GO:0006644)	249	164	97.68	+	1.68	3.82E-06
cell surface receptor signaling pathway involved in cell-cell signaling (GO:1905114)	249	164	97.68	+	1.68	3.82E-06
cell projection organization (GO:0030030)	952	627	373.46	+	1.68	8.92E-31
cell projection morphogenesis (GO:0048858)	636	418	249.50	+	1.68	2.57E-19
regulation of proteasomal protein catabolic process (GO:0061136)	140	92	54.92	+	1.68	2.34E-02
regulation of insulin secretion (GO:0050796)	172	113	67.47	+	1.67	1.93E-03
endomembrane system organization (GO:0010256)	419	275	164.37	+	1.67	9.37E-12
regulation of protein catabolic process (GO:0042176)	311	204	122.00	+	1.67	4.49E-08
regulation of cellular protein catabolic process (GO:1903362)	183	120	71.79	+	1.67	9.23E-04

Golgi vesicle transport (GO:0048193)	180	118	70.61	+	1.67	1.19E-03
nervous system development (GO:0007399)	2238	1467	877.94	+	1.67	8.42E-79
positive regulation of catabolic process (GO:0009896)	255	167	100.03	+	1.67	3.96E-06
neurogenesis (GO:0022008)	1608	1053	630.80	+	1.67	7.90E-54
glycerophospholipid metabolic process (GO:0006650)	168	110	65.90	+	1.67	3.22E-03
branching morphogenesis of an epithelial tube (GO:0048754)	165	108	64.73	+	1.67	4.14E-03
negative regulation of cell migration (GO:0030336)	217	142	85.13	+	1.67	7.79E-05
negative regulation of protein phosphorylation (GO:0001933)	399	261	156.52	+	1.67	7.11E-11
positive regulation of protein serine/threonine kinase activity (GO:0071902)	263	172	103.17	+	1.67	2.43E-06
response to light stimulus (GO:0009416)	300	196	117.69	+	1.67	1.60E-07
negative regulation of transferase activity (GO:0051348)	274	179	107.49	+	1.67	1.16E-06
negative regulation of cell growth (GO:0030308)	173	113	67.87	+	1.67	2.54E-03
glycoprotein biosynthetic process (GO:0009101)	245	160	96.11	+	1.66	1.08E-05
response to decreased oxygen levels (GO:0036293)	386	252	151.42	+	1.66	2.51E-10
regulation of developmental growth (GO:0048638)	337	220	132.20	+	1.66	1.05E-08
anion transport (GO:0006820)	360	235	141.22	+	1.66	1.84E-09
muscle cell development (GO:0055001)	141	92	55.31	+	1.66	3.07E-02
cell part morphogenesis (GO:0032990)	660	430	258.91	+	1.66	2.83E-19
intracellular signal transduction (GO:0035556)	1302	848	510.76	+	1.66	2.27E-41
central nervous system neuron differentiation (GO:0021953)	192	125	75.32	+	1.66	7.42E-04
regulation of cell growth (GO:0001558)	395	257	154.95	+	1.66	2.01E-10
morphogenesis of a branching epithelium (GO:0061138)	197	128	77.28	+	1.66	5.84E-04
secretion (GO:0046903)	511	332	200.46	+	1.66	4.14E-14
epithelial tube morphogenesis (GO:0060562)	348	226	136.52	+	1.66	8.44E-09
vacuolar transport (GO:0007034)	228	148	89.44	+	1.65	6.31E-05
regulation of nucleotide metabolic process (GO:0006140)	208	135	81.60	+	1.65	2.79E-04
glycoprotein metabolic process (GO:0009100)	282	183	110.63	+	1.65	1.20E-06
developmental growth (GO:0048589)	410	266	160.84	+	1.65	9.69E-11
positive regulation of protein catabolic process (GO:0045732)	182	118	71.40	+	1.65	2.03E-03
regulation of endocytosis (GO:0030100)	213	138	83.56	+	1.65	2.19E-04
negative regulation of cellular component organization (GO:0051129)	633	410	248.32	+	1.65	8.06E-18
cellular response to peptide (GO:1901653)	286	185	112.19	+	1.65	1.22E-06
negative regulation of phosphorylation (GO:0042326)	436	282	171.04	+	1.65	2.21E-11
regulation of purine nucleotide metabolic process (GO:1900542)	201	130	78.85	+	1.65	5.93E-04
negative regulation of locomotion (GO:0040013)	263	170	103.17	+	1.65	6.93E-06
connective tissue development (GO:0061448)	212	137	83.17	+	1.65	2.83E-04
actin filament-based process (GO:0030029)	424	274	166.33	+	1.65	6.03E-11

cell division (GO:0051301)	277	179	108.66	+	1.65	2.58E-06
regulation of protein kinase activity (GO:0045859)	659	425	258.52	+	1.64	3.01E-18
cell-cell signaling (GO:0007267)	704	454	276.17	+	1.64	1.11E-19
regulation of kinase activity (GO:0043549)	718	463	281.66	+	1.64	4.00E-20
negative regulation of cellular component movement (GO:0051271)	259	167	101.60	+	1.64	1.15E-05
striated muscle cell differentiation (GO:0051146)	197	127	77.28	+	1.64	9.83E-04
negative regulation of intracellular signal transduction (GO:1902532)	466	300	182.81	+	1.64	5.09E-12
angiogenesis (GO:0001525)	258	166	101.21	+	1.64	1.48E-05
negative regulation of protein modification process (GO:0031400)	527	339	206.74	+	1.64	7.28E-14
actin filament organization (GO:0007015)	185	119	72.57	+	1.64	2.66E-03
morphogenesis of a branching structure (GO:0001763)	207	133	81.20	+	1.64	6.05E-04
positive regulation of developmental growth (GO:0048639)	179	115	70.22	+	1.64	4.38E-03
positive regulation of MAP kinase activity (GO:0043406)	179	115	70.22	+	1.64	4.38E-03
protein polyubiquitination (GO:0000209)	162	104	63.55	+	1.64	1.52E-02
regulation of anatomical structure size (GO:0090066)	519	333	203.60	+	1.64	2.00E-13
organelle localization (GO:0051640)	385	247	151.03	+	1.64	2.59E-09
regulation of protein binding (GO:0043393)	184	118	72.18	+	1.63	3.43E-03
tube morphogenesis (GO:0035239)	393	252	154.17	+	1.63	1.58E-09
response to oxygen levels (GO:0070482)	418	268	163.98	+	1.63	2.78E-10
cellular response to peptide hormone stimulus (GO:0071375)	259	166	101.60	+	1.63	1.91E-05
positive regulation of kinase activity (GO:0033674)	437	280	171.43	+	1.63	8.03E-11
positive regulation of protein kinase activity (GO:0045860)	403	258	158.09	+	1.63	9.67E-10
establishment of organelle localization (GO:0051656)	314	201	123.18	+	1.63	4.72E-07
actin cytoskeleton organization (GO:0030036)	383	245	150.25	+	1.63	4.29E-09
eye morphogenesis (GO:0048592)	158	101	61.98	+	1.63	2.51E-02
positive regulation of growth (GO:0045927)	263	168	103.17	+	1.63	1.93E-05
morphogenesis of an epithelium (GO:0002009)	489	312	191.83	+	1.63	4.09E-12
negative regulation of cell differentiation (GO:0045596)	665	424	260.87	+	1.63	2.37E-17
protein transport (GO:0015031)	946	603	371.10	+	1.62	6.36E-26
cellular response to growth factor stimulus (GO:0071363)	463	295	181.63	+	1.62	3.01E-11
activation of protein kinase activity (GO:0032147)	234	149	91.80	+	1.62	1.79E-04
regulation of actin filament-based process (GO:0032970)	322	205	126.32	+	1.62	4.78E-07
muscle cell differentiation (GO:0042692)	269	171	105.53	+	1.62	1.95E-05
response to growth factor (GO:0070848)	498	316	195.36	+	1.62	5.29E-12
epithelial cell development (GO:0002064)	227	144	89.05	+	1.62	3.77E-04
establishment of protein localization (GO:0045184)	1072	679	420.53	+	1.61	6.89E-29
single-organism membrane organization (GO:0044802)	605	383	237.33	+	1.61	6.10E-15

urogenital system development (GO:0001655)	354	224	138.87	+	1.61	1.09E-07
blood vessel development (GO:0001568)	452	286	177.31	+	1.61	1.73E-10
cellular response to organonitrogen compound (GO:0071417)	498	315	195.36	+	1.61	8.71E-12
negative regulation of phosphorus metabolic process (GO:0010563)	552	349	216.54	+	1.61	2.63E-13
negative regulation of phosphate metabolic process (GO:0045936)	552	349	216.54	+	1.61	2.63E-13
positive regulation of transferase activity (GO:0051347)	503	318	197.32	+	1.61	6.79E-12
regulation of binding (GO:0051098)	315	199	123.57	+	1.61	1.66E-06
regulation of transferase activity (GO:0051338)	809	511	317.36	+	1.61	1.13E-20
regulation of autophagy (GO:0010506)	198	125	77.67	+	1.61	3.50E-03
vasculature development (GO:0001944)	480	303	188.30	+	1.61	3.88E-11
regulation of blood circulation (GO:1903522)	252	159	98.86	+	1.61	1.10E-04
aging (GO:0007568)	398	251	156.13	+	1.61	9.18E-09
negative regulation of cell motility (GO:2000146)	230	145	90.23	+	1.61	4.84E-04
ameboidal-type cell migration (GO:0001667)	165	104	64.73	+	1.61	3.25E-02
regulation of actin cytoskeleton organization (GO:0032956)	284	179	111.41	+	1.61	1.53E-05
regulation of canonical Wnt signaling pathway (GO:0060828)	173	109	67.87	+	1.61	1.98E-02
response to peptide hormone (GO:0043434)	454	286	178.10	+	1.61	2.84E-10
glycosylation (GO:0070085)	213	134	83.56	+	1.60	1.67E-03
regulation of muscle system process (GO:0090257)	202	127	79.24	+	1.60	3.50E-03
response to carbohydrate (GO:0009743)	237	149	92.97	+	1.60	3.78E-04
growth (GO:0040007)	484	304	189.87	+	1.60	6.33E-11
cell morphogenesis (GO:0000902)	880	552	345.21	+	1.60	6.33E-22
renal system development (GO:0072001)	311	195	122.00	+	1.60	4.43E-06
response to peptide (GO:1901652)	504	316	197.71	+	1.60	2.32E-11
regulation of cytoskeleton organization (GO:0051493)	418	262	163.98	+	1.60	5.54E-09
negative regulation of transport (GO:0051051)	490	307	192.22	+	1.60	6.29E-11
metal ion transport (GO:0030001)	543	340	213.01	+	1.60	2.44E-12
developmental maturation (GO:0021700)	211	132	82.77	+	1.59	2.72E-03
regulation of protein modification by small protein conjugation or removal (GO:1903320)	211	132	82.77	+	1.59	2.72E-03
protein homooligomerization (GO:0051260)	283	177	111.02	+	1.59	3.18E-05
enzyme linked receptor protein signaling pathway (GO:0007167)	520	325	203.99	+	1.59	1.39E-11
regulation of epithelial cell migration (GO:0010632)	168	105	65.90	+	1.59	4.16E-02
protein phosphorylation (GO:0006468)	781	488	306.38	+	1.59	1.03E-18
regulation of transport (GO:0051049)	1813	1132	711.22	+	1.59	1.35E-48
tissue morphogenesis (GO:0048729)	596	372	233.80	+	1.59	1.52E-13
cellular response to hormone stimulus (GO:0032870)	561	350	220.07	+	1.59	1.45E-12
cell-cell signaling by wnt (GO:0198738)	202	126	79.24	+	1.59	5.70E-03

Wnt signaling pathway (GO:0016055)	202	126	79.24	+	1.59	5.70E-03
regulation of protein ubiquitination (GO:0031396)	186	116	72.97	+	1.59	1.54E-02
single-organism carbohydrate metabolic process (GO:0044723)	470	293	184.38	+	1.59	4.52E-10
regulation of cellular localization (GO:0060341)	839	523	329.13	+	1.59	4.66E-20
regulation of ossification (GO:0030278)	199	124	78.07	+	1.59	7.29E-03
positive regulation of ion transport (GO:0043270)	249	155	97.68	+	1.59	3.74E-04
negative regulation of protein transport (GO:0051224)	209	130	81.99	+	1.59	4.43E-03
nitrogen compound transport (GO:0071705)	402	250	157.70	+	1.59	3.93E-08
response to glucose (GO:0009749)	185	115	72.57	+	1.58	1.96E-02
negative regulation of establishment of protein localization (GO:1904950)	222	138	87.09	+	1.58	2.11E-03
cellular response to steroid hormone stimulus (GO:0071383)	206	128	80.81	+	1.58	5.67E-03
protein glycosylation (GO:0006486)	203	126	79.63	+	1.58	7.25E-03
macromolecule glycosylation (GO:0043413)	203	126	79.63	+	1.58	7.25E-03
glycerolipid metabolic process (GO:0046486)	245	152	96.11	+	1.58	6.08E-04
regulation of cellular component biogenesis (GO:0044087)	774	480	303.63	+	1.58	9.42E-18
single-organism cellular localization (GO:1902580)	695	431	272.64	+	1.58	1.19E-15
kidney development (GO:0001822)	292	181	114.55	+	1.58	3.99E-05
regulation of calcium ion transport (GO:0051924)	234	145	91.80	+	1.58	1.27E-03
tube development (GO:0035295)	681	421	267.15	+	1.58	5.22E-15
cellular response to extracellular stimulus (GO:0031668)	212	131	83.17	+	1.58	5.59E-03
negative regulation of developmental process (GO:0051093)	884	546	346.78	+	1.57	3.11E-20
response to hormone (GO:0009725)	965	596	378.56	+	1.57	2.26E-22
protein localization (GO:0008104)	1523	940	597.45	+	1.57	1.79E-37
cellular component morphogenesis (GO:0032989)	966	596	378.95	+	1.57	2.87E-22
protein autophosphorylation (GO:0046777)	198	122	77.67	+	1.57	1.50E-02
response to alkaloid (GO:0043279)	211	130	82.77	+	1.57	7.11E-03
regulation of anatomical structure morphogenesis (GO:0022603)	1017	626	398.96	+	1.57	2.58E-23
cellular response to nutrient levels (GO:0031669)	182	112	71.40	+	1.57	4.04E-02
regulation of growth (GO:0040008)	639	393	250.67	+	1.57	1.69E-13
cellular response to nitrogen compound (GO:1901699)	574	353	225.17	+	1.57	7.41E-12
cellular response to external stimulus (GO:0071496)	309	190	121.22	+	1.57	3.01E-05
response to steroid hormone (GO:0048545)	457	281	179.28	+	1.57	6.41E-09
response to ketone (GO:1901654)	296	182	116.12	+	1.57	6.33E-05
regulation of cellular component organization (GO:0051128)	2292	1409	899.12	+	1.57	1.22E-58
ossification (GO:0001503)	262	161	102.78	+	1.57	4.59E-04
response to organonitrogen compound (GO:0010243)	993	610	389.54	+	1.57	1.98E-22
muscle structure development (GO:0061061)	458	281	179.67	+	1.56	8.06E-09

regulation of GTPase activity (GO:0043087)	495	303	194.18	+	1.56	1.37E-09
response to mechanical stimulus (GO:0009612)	278	170	109.06	+	1.56	2.72E-04
blood vessel morphogenesis (GO:0048514)	350	214	137.30	+	1.56	5.10E-06
mesenchyme development (GO:0060485)	193	118	75.71	+	1.56	3.08E-02
heart development (GO:0007507)	553	338	216.94	+	1.56	6.60E-11
positive regulation of cellular component organization (GO:0051130)	1203	735	471.92	+	1.56	4.29E-27
negative regulation of cell communication (GO:0010648)	1159	708	454.66	+	1.56	6.10E-26
response to drug (GO:0042493)	614	375	240.86	+	1.56	2.46E-12
phosphorus metabolic process (GO:0006793)	1725	1053	676.70	+	1.56	1.12E-40
negative regulation of organelle organization (GO:0010639)	300	183	117.69	+	1.55	9.92E-05
phosphate-containing compound metabolic process (GO:0006796)	1723	1051	675.91	+	1.55	1.85E-40
negative regulation of growth (GO:0045926)	246	150	96.50	+	1.55	1.96E-03
organic substance transport (GO:0071702)	1547	943	606.87	+	1.55	1.23E-35
negative regulation of signaling (GO:0023057)	1162	708	455.84	+	1.55	1.22E-25
regulation of localization (GO:0032879)	2487	1515	975.62	+	1.55	2.07E-61
ion transport (GO:0006811)	1064	648	417.39	+	1.55	4.04E-23
cellular response to endogenous stimulus (GO:0071495)	1038	632	407.19	+	1.55	1.94E-22
response to abiotic stimulus (GO:0009628)	1245	758	488.40	+	1.55	1.26E-27
regulation of cellular response to growth factor stimulus (GO:0090287)	230	140	90.23	+	1.55	5.26E-03
autophagy (GO:0006914)	301	183	118.08	+	1.55	1.24E-04
circulatory system development (GO:0072359)	854	519	335.01	+	1.55	1.04E-17
cardiovascular system development (GO:0072358)	854	519	335.01	+	1.55	1.04E-17
establishment of localization in cell (GO:0051649)	1170	711	458.98	+	1.55	1.85E-25
regulation of cell differentiation (GO:0045595)	1651	1003	647.67	+	1.55	1.13E-37
modification-dependent protein catabolic process (GO:0019941)	400	243	156.92	+	1.55	6.54E-07
vesicle-mediated transport (GO:0016192)	960	583	376.60	+	1.55	3.34E-20
ubiquitin-dependent protein catabolic process (GO:0006511)	392	238	153.78	+	1.55	1.07E-06
regulation of system process (GO:0044057)	496	301	194.57	+	1.55	4.30E-09
muscle system process (GO:0003012)	234	142	91.80	+	1.55	5.17E-03
proteasomal protein catabolic process (GO:0010498)	211	128	82.77	+	1.55	1.80E-02
regulation of nucleocytoplasmic transport (GO:0046822)	216	131	84.73	+	1.55	1.40E-02
regulation of MAPK cascade (GO:0043408)	681	413	267.15	+	1.55	2.19E-13
regulation of protein complex assembly (GO:0043254)	343	208	134.55	+	1.55	1.65E-05
response to glucocorticoid (GO:0051384)	269	163	105.53	+	1.54	8.91E-04
cellular response to abiotic stimulus (GO:0071214)	307	186	120.43	+	1.54	1.21E-04
inner ear development (GO:0048839)	213	129	83.56	+	1.54	1.79E-02
carbohydrate derivative biosynthetic process (GO:1901137)	469	284	183.98	+	1.54	2.42E-08

lung development (GO:0030324)	233	141	91.40	+	1.54	6.52E-03
divalent inorganic cation transport (GO:0072511)	258	156	101.21	+	1.54	1.86E-03
cellular localization (GO:0051641)	1645	994	645.31	+	1.54	2.10E-36
phosphorylation (GO:0016310)	1063	642	417.00	+	1.54	5.25E-22
divalent metal ion transport (GO:0070838)	255	154	100.03	+	1.54	2.36E-03
regulation of intracellular transport (GO:0032386)	467	282	183.20	+	1.54	3.84E-08
cell development (GO:0048468)	1575	951	617.85	+	1.54	1.72E-34
regulation of cellular protein localization (GO:1903827)	545	329	213.80	+	1.54	6.75E-10
positive regulation of MAPK cascade (GO:0043410)	474	286	185.94	+	1.54	2.95E-08
regulation of lipid metabolic process (GO:0019216)	300	181	117.69	+	1.54	2.46E-04
modification-dependent macromolecule catabolic process (GO:0043632)	408	246	160.05	+	1.54	9.87E-07
response to nitrogen compound (GO:1901698)	1092	658	428.38	+	1.54	2.14E-22
negative regulation of transcription from RNA polymerase II promoter (GO:0000122)	757	456	296.96	+	1.54	1.43E-14
negative regulation of signal transduction (GO:0009968)	1041	627	408.37	+	1.54	3.85E-21
response to endogenous stimulus (GO:0009719)	1620	975	635.51	+	1.53	5.68E-35
respiratory tube development (GO:0030323)	236	142	92.58	+	1.53	8.05E-03
protein oligomerization (GO:0051259)	482	290	189.08	+	1.53	2.82E-08
regulation of Wnt signaling pathway (GO:0030111)	246	148	96.50	+	1.53	4.87E-03
apoptotic process (GO:0006915)	675	406	264.79	+	1.53	1.39E-12
intracellular protein transport (GO:0006886)	587	353	230.27	+	1.53	1.37E-10
regulation of phosphorylation (GO:0042325)	1399	841	548.81	+	1.53	2.31E-29
carbohydrate metabolic process (GO:0005975)	559	336	219.29	+	1.53	6.21E-10
carbohydrate homeostasis (GO:0033500)	213	128	83.56	+	1.53	2.81E-02
glucose homeostasis (GO:0042593)	213	128	83.56	+	1.53	2.81E-02
regulation of secretion by cell (GO:1903530)	694	417	272.25	+	1.53	6.29E-13
regulation of epithelial cell proliferation (GO:0050678)	323	194	126.71	+	1.53	1.11E-04
response to acid chemical (GO:0001101)	403	242	158.09	+	1.53	1.98E-06
regulation of intracellular protein transport (GO:0033157)	370	222	145.15	+	1.53	1.14E-05
response to estradiol (GO:0032355)	230	138	90.23	+	1.53	1.30E-02
cell death (GO:0008219)	745	447	292.25	+	1.53	5.96E-14
regulation of cell communication (GO:0010646)	2764	1658	1084.28	+	1.53	6.80E-64
regulation of signaling (GO:0023051)	2786	1671	1092.91	+	1.53	1.86E-64
regulation of multicellular organismal development (GO:2000026)	1784	1070	699.84	+	1.53	2.63E-38
positive regulation of apoptotic process (GO:0043065)	584	350	229.10	+	1.53	2.72E-10
cellular macromolecule localization (GO:0070727)	1093	655	428.77	+	1.53	1.05E-21
sulfur compound metabolic process (GO:0006790)	272	163	106.70	+	1.53	1.72E-03
cellular protein localization (GO:0034613)	1085	650	425.63	+	1.53	1.75E-21

in utero embryonic development (GO:0001701)	429	257	168.29	+	1.53	6.98E-07
response to monosaccharide (GO:0034284)	207	124	81.20	+	1.53	4.54E-02
positive regulation of cell differentiation (GO:0045597)	940	563	368.75	+	1.53	3.69E-18
intracellular transport (GO:0046907)	904	541	354.63	+	1.53	2.91E-17
macromolecule localization (GO:0033036)	1795	1074	704.16	+	1.53	4.75E-38
ion transmembrane transport (GO:0034220)	709	424	278.13	+	1.52	7.09E-13
apoptotic signaling pathway (GO:0097190)	271	162	106.31	+	1.52	2.16E-03
positive regulation of cell death (GO:0010942)	629	376	246.75	+	1.52	4.27E-11
positive regulation of cellular component biogenesis (GO:0044089)	405	242	158.88	+	1.52	3.05E-06
protein targeting (GO:0006605)	318	190	124.75	+	1.52	2.22E-04
response to purine-containing compound (GO:0014074)	211	126	82.77	+	1.52	4.43E-02
regulation of peptide hormone secretion (GO:0090276)	211	126	82.77	+	1.52	4.43E-02
positive regulation of transport (GO:0051050)	990	591	388.37	+	1.52	6.54E-19
positive regulation of programmed cell death (GO:0043068)	588	351	230.67	+	1.52	4.12E-10
programmed cell death (GO:0012501)	702	419	275.39	+	1.52	1.45E-12
organic hydroxy compound metabolic process (GO:1901615)	300	179	117.69	+	1.52	5.99E-04
regulation of secretion (GO:0051046)	743	443	291.47	+	1.52	2.29E-13
protein ubiquitination (GO:0016567)	515	307	202.03	+	1.52	1.83E-08
ear development (GO:0043583)	240	143	94.15	+	1.52	1.22E-02
cellular response to organic cyclic compound (GO:0071407)	549	327	215.37	+	1.52	3.89E-09
response to wounding (GO:0009611)	455	271	178.49	+	1.52	3.80E-07
regulation of protein modification process (GO:0031399)	1612	960	632.37	+	1.52	1.01E-32
regulation of phosphate metabolic process (GO:0019220)	1622	965	636.29	+	1.52	9.15E-33
muscle organ development (GO:0007517)	264	157	103.56	+	1.52	4.36E-03
taxis (GO:0042330)	412	245	161.62	+	1.52	3.62E-06
regulation of phosphorus metabolic process (GO:0051174)	1623	965	636.68	+	1.52	1.14E-32
regulation of protein stability (GO:0031647)	217	129	85.13	+	1.52	4.26E-02
regulation of protein localization (GO:0032880)	971	577	380.91	+	1.51	5.63E-18
heart morphogenesis (GO:0003007)	229	136	89.83	+	1.51	2.54E-02
response to corticosteroid (GO:0031960)	293	174	114.94	+	1.51	1.21E-03
regulation of peptidyl-tyrosine phosphorylation (GO:0050730)	224	133	87.87	+	1.51	3.27E-02
camera-type eye development (GO:0043010)	337	200	132.20	+	1.51	1.57E-04
positive regulation of catalytic activity (GO:0043085)	1252	743	491.14	+	1.51	5.88E-24
regulation of hormone secretion (GO:0046883)	273	162	107.09	+	1.51	3.30E-03
regulation of protein phosphorylation (GO:0001932)	1297	769	508.80	+	1.51	8.12E-25
protein modification by small protein conjugation (GO:0032446)	555	329	217.72	+	1.51	5.75E-09
response to reactive oxygen species (GO:0000302)	221	131	86.70	+	1.51	4.15E-02

eye development (GO:0001654)	383	227	150.25	+	1.51	2.02E-05
chemotaxis (GO:0006935)	410	243	160.84	+	1.51	5.66E-06
cytoskeleton organization (GO:0007010)	859	509	336.98	+	1.51	2.76E-15
regulation of developmental process (GO:0050793)	2362	1399	926.58	+	1.51	2.30E-49
cellular response to lipid (GO:0071396)	522	309	204.77	+	1.51	3.33E-08
positive regulation of developmental process (GO:0051094)	1300	769	509.97	+	1.51	1.54E-24
positive regulation of GTPase activity (GO:0043547)	443	262	173.78	+	1.51	1.51E-06
negative regulation of cell proliferation (GO:0008285)	629	372	246.75	+	1.51	2.44E-10
cellular response to oxygen-containing compound (GO:1901701)	937	554	367.57	+	1.51	1.04E-16
inorganic cation transmembrane transport (GO:0098662)	467	276	183.20	+	1.51	5.36E-07
cellular protein modification process (GO:0006464)	2383	1408	934.82	+	1.51	3.54E-49
protein modification process (GO:0036211)	2383	1408	934.82	+	1.51	3.54E-49
regulation of cellular response to stress (GO:0080135)	530	313	207.91	+	1.51	3.13E-08
positive regulation of molecular function (GO:0044093)	1557	919	610.79	+	1.50	8.03E-30
negative regulation of multicellular organismal process (GO:0051241)	1064	628	417.39	+	1.50	3.47E-19
response to extracellular stimulus (GO:0009991)	593	350	232.63	+	1.50	1.81E-09
positive regulation of protein modification process (GO:0031401)	1068	630	418.96	+	1.50	3.35E-19
response to metal ion (GO:0010038)	434	256	170.25	+	1.50	3.08E-06
small molecule biosynthetic process (GO:0044283)	334	197	131.02	+	1.50	3.06E-04
positive regulation of phosphorylation (GO:0042327)	919	542	360.51	+	1.50	4.49E-16
positive regulation of secretion by cell (GO:1903532)	390	230	152.99	+	1.50	2.38E-05
regulation of organelle organization (GO:0033043)	1099	648	431.12	+	1.50	8.46E-20
regulation of hormone levels (GO:0010817)	489	288	191.83	+	1.50	2.95E-07
regulation of intracellular signal transduction (GO:1902531)	1513	891	593.53	+	1.50	1.85E-28
cellular component disassembly (GO:0022411)	282	166	110.63	+	1.50	3.83E-03
establishment of protein localization to organelle (GO:0072594)	267	157	104.74	+	1.50	8.14E-03
regulation of cell migration (GO:0030334)	711	418	278.92	+	1.50	1.46E-11
macromolecule modification (GO:0043412)	2576	1514	1010.53	+	1.50	2.93E-52
epithelium development (GO:0060429)	1009	593	395.82	+	1.50	1.49E-17
system development (GO:0048731)	4152	2439	1628.78	+	1.50	1.90E-93
protein modification by small protein conjugation or removal (GO:0070647)	676	397	265.19	+	1.50	8.74E-11
response to organic cyclic compound (GO:0014070)	1209	710	474.28	+	1.50	1.40E-21
regulation of biological quality (GO:0065008)	3322	1949	1303.18	+	1.50	3.23E-70
gland development (GO:0048732)	537	315	210.66	+	1.50	5.60E-08
chordate embryonic development (GO:0043009)	699	410	274.21	+	1.50	3.79E-11
positive regulation of signaling (GO:0023056)	1468	861	575.88	+	1.50	8.16E-27
monovalent inorganic cation transport (GO:0015672)	370	217	145.15	+	1.50	9.91E-05

respiratory system development (GO:0060541)	266	156	104.35	+	1.49	1.01E-02
membrane organization (GO:0061024)	747	438	293.04	+	1.49	4.57E-12
positive regulation of phosphorus metabolic process (GO:0010562)	1037	608	406.80	+	1.49	7.44E-18
positive regulation of phosphate metabolic process (GO:0045937)	1037	608	406.80	+	1.49	7.44E-18
positive regulation of intracellular signal transduction (GO:1902533)	884	518	346.78	+	1.49	1.00E-14
positive regulation of cell communication (GO:0010647)	1461	856	573.13	+	1.49	1.70E-26
anatomical structure formation involved in morphogenesis (GO:0048646)	990	580	388.37	+	1.49	8.00E-17
sensory organ development (GO:0007423)	589	345	231.06	+	1.49	6.69E-09
negative regulation of response to stimulus (GO:0048585)	1346	788	528.02	+	1.49	5.59E-24
response to inorganic substance (GO:0010035)	629	368	246.75	+	1.49	1.34E-09
positive regulation of protein phosphorylation (GO:0001934)	877	513	344.04	+	1.49	2.02E-14
response to radiation (GO:0009314)	477	279	187.12	+	1.49	1.15E-06
embryo development ending in birth or egg hatching (GO:0009792)	708	414	277.74	+	1.49	4.32E-11
inorganic ion transmembrane transport (GO:0098660)	551	322	216.15	+	1.49	4.97E-08
regulation of cellular component movement (GO:0051270)	813	475	318.93	+	1.49	4.63E-13
regulation of signal transduction (GO:0009966)	2441	1426	957.57	+	1.49	2.62E-47
cation transmembrane transport (GO:0098655)	529	309	207.52	+	1.49	1.39E-07
single-organism localization (GO:1902578)	2021	1180	792.81	+	1.49	8.55E-38
blood circulation (GO:0008015)	370	216	145.15	+	1.49	1.51E-04
response to nutrient levels (GO:0031667)	567	331	222.43	+	1.49	2.85E-08
sensory organ morphogenesis (GO:0090596)	281	164	110.23	+	1.49	7.27E-03
regulation of homeostatic process (GO:0032844)	473	276	185.55	+	1.49	1.81E-06
regulation of protein transport (GO:0051223)	763	445	299.32	+	1.49	6.02E-12
response to oxygen-containing compound (GO:1901700)	1710	996	670.81	+	1.48	1.15E-30
positive regulation of secretion (GO:0051047)	419	244	164.37	+	1.48	2.31E-05
positive regulation of hydrolase activity (GO:0051345)	732	426	287.15	+	1.48	3.47E-11
cation transport (GO:0006812)	720	419	282.45	+	1.48	5.88E-11
regulation of protein targeting (GO:1903533)	299	174	117.29	+	1.48	4.11E-03
regulation of molecular function (GO:0065009)	2574	1497	1009.75	+	1.48	4.89E-49
response to oxidative stress (GO:0006979)	411	239	161.23	+	1.48	3.75E-05
multicellular organism development (GO:0007275)	4547	2644	1783.73	+	1.48	6.00E-99
circulatory system process (GO:0003013)	375	218	147.11	+	1.48	1.77E-04
small molecule catabolic process (GO:0044282)	246	143	96.50	+	1.48	4.21E-02
cellular divalent inorganic cation homeostasis (GO:0072503)	346	201	135.73	+	1.48	6.42E-04
cellular protein catabolic process (GO:0044257)	482	280	189.08	+	1.48	2.06E-06
divalent inorganic cation homeostasis (GO:0072507)	372	216	145.93	+	1.48	2.24E-04
cellular calcium ion homeostasis (GO:0006874)	329	191	129.06	+	1.48	1.38E-03

transport (GO:0006810)	3305	1918	1296.51	+	1.48	2.16E-65
muscle tissue development (GO:0060537)	324	188	127.10	+	1.48	1.78E-03
cation homeostasis (GO:0055080)	555	322	217.72	+	1.48	1.10E-07
cell migration (GO:0016477)	712	413	279.31	+	1.48	1.45E-10
inorganic ion homeostasis (GO:0098771)	571	331	224.00	+	1.48	6.27E-08
regulation of locomotion (GO:0040012)	787	456	308.73	+	1.48	7.23E-12
positive regulation of cellular protein metabolic process (GO:0032270)	1348	781	528.80	+	1.48	1.68E-22
establishment of localization (GO:0051234)	3456	2001	1355.75	+	1.48	4.01E-68
anatomical structure morphogenesis (GO:0009653)	2139	1238	839.10	+	1.48	2.79E-38
regulation of establishment of protein localization (GO:0070201)	819	474	321.28	+	1.48	2.31E-12
cellular chemical homeostasis (GO:0055082)	617	357	242.04	+	1.47	1.19E-08
RNA biosynthetic process (GO:0032774)	1215	703	476.63	+	1.47	9.29E-20
positive regulation of cell cycle (GO:0045787)	299	173	117.29	+	1.47	6.21E-03
single-organism transport (GO:0044765)	1801	1042	706.51	+	1.47	3.01E-31
positive regulation of proteolysis (GO:0045862)	294	170	115.33	+	1.47	7.97E-03
chemical homeostasis (GO:0048878)	927	536	363.65	+	1.47	2.84E-14
nucleic acid-templated transcription (GO:0097659)	1202	695	471.53	+	1.47	2.01E-19
proteolysis involved in cellular protein catabolic process (GO:0051603)	467	270	183.20	+	1.47	6.57E-06
negative regulation of nucleic acid-templated transcription (GO:1903507)	1095	633	429.56	+	1.47	2.22E-17
negative regulation of cell death (GO:0060548)	967	559	379.34	+	1.47	5.43E-15
negative regulation of transcription, DNA-templated (GO:0045892)	1076	622	422.10	+	1.47	5.08E-17
regulation of mitotic cell cycle (GO:0007346)	391	226	153.38	+	1.47	1.53E-04
transcription, DNA-templated (GO:0006351)	1201	694	471.14	+	1.47	2.50E-19
cell proliferation (GO:0008283)	566	327	222.03	+	1.47	1.21E-07
reproductive structure development (GO:0048608)	502	290	196.93	+	1.47	1.71E-06
positive regulation of organelle organization (GO:0010638)	580	335	227.53	+	1.47	7.06E-08
single-multicellular organism process (GO:0044707)	5153	2976	2021.46	+	1.47	3.88E-112
positive regulation of signal transduction (GO:0009967)	1306	754	512.33	+	1.47	3.36E-21
ion homeostasis (GO:0050801)	577	333	226.35	+	1.47	8.90E-08
single-organism intracellular transport (GO:1902582)	371	214	145.54	+	1.47	4.16E-04
negative regulation of RNA biosynthetic process (GO:1902679)	1110	640	435.44	+	1.47	2.32E-17
protein localization to organelle (GO:0033365)	477	275	187.12	+	1.47	5.97E-06
mitotic cell cycle (GO:0000278)	484	279	189.87	+	1.47	4.56E-06
regulation of apoptotic signaling pathway (GO:2001233)	380	219	149.07	+	1.47	3.12E-04
peptidyl-amino acid modification (GO:0018193)	703	405	275.78	+	1.47	6.63E-10
striated muscle tissue development (GO:0014706)	309	178	121.22	+	1.47	5.65E-03
regulation of cell motility (GO:2000145)	750	432	294.22	+	1.47	9.94E-11

response to lipid (GO:0033993)	1099	633	431.12	+	1.47	4.85E-17
reproductive system development (GO:0061458)	507	292	198.89	+	1.47	1.99E-06
metal ion homeostasis (GO:0055065)	488	281	191.44	+	1.47	4.39E-06
wound healing (GO:0042060)	337	194	132.20	+	1.47	1.93E-03
regulation of multicellular organismal process (GO:0051239)	2658	1530	1042.70	+	1.47	7.83E-48
tissue development (GO:0009888)	1608	925	630.80	+	1.47	2.09E-26
positive regulation of transcription from RNA polymerase II promoter (GO:0045944)	1050	604	411.90	+	1.47	5.28E-16
negative regulation of apoptotic process (GO:0043066)	864	497	338.94	+	1.47	1.18E-12
negative regulation of cellular macromolecule biosynthetic process (GO:2000113)	1250	719	490.36	+	1.47	1.17E-19
locomotion (GO:0040011)	980	563	384.44	+	1.46	1.31E-14
positive regulation of cellular protein localization (GO:1903829)	343	197	134.55	+	1.46	1.82E-03
single-organism biosynthetic process (GO:0044711)	841	483	329.91	+	1.46	4.15E-12
cell differentiation (GO:0030154)	3267	1875	1281.60	+	1.46	3.06E-60
localization (GO:0051179)	4363	2504	1711.55	+	1.46	1.32E-86
single-organism organelle organization (GO:1902589)	1369	785	537.04	+	1.46	1.97E-21
protein catabolic process (GO:0030163)	513	294	201.24	+	1.46	2.80E-06
negative regulation of RNA metabolic process (GO:0051253)	1157	663	453.88	+	1.46	1.67E-17
positive regulation of protein metabolic process (GO:0051247)	1438	824	564.11	+	1.46	1.33E-22
transmembrane transport (GO:0055085)	1042	597	408.76	+	1.46	1.95E-15
regulation of cell death (GO:0010941)	1550	888	608.05	+	1.46	1.22E-24
negative regulation of programmed cell death (GO:0043069)	878	503	344.43	+	1.46	1.52E-12
regulation of catalytic activity (GO:0050790)	2047	1172	803.01	+	1.46	5.64E-34
positive regulation of intracellular transport (GO:0032388)	290	166	113.76	+	1.46	1.85E-02
positive regulation of nucleic acid-templated transcription (GO:1903508)	1328	760	520.96	+	1.46	2.04E-20
positive regulation of transcription, DNA-templated (GO:0045893)	1328	760	520.96	+	1.46	2.04E-20
epithelial cell differentiation (GO:0030855)	519	297	203.60	+	1.46	2.63E-06
embryo development (GO:0009790)	1049	600	411.51	+	1.46	2.21E-15
positive regulation of RNA biosynthetic process (GO:1902680)	1331	761	522.14	+	1.46	2.42E-20
mitotic cell cycle process (GO:1903047)	453	259	177.71	+	1.46	3.75E-05
negative regulation of macromolecule biosynthetic process (GO:0010558)	1315	751	515.86	+	1.46	6.62E-20
calcium ion homeostasis (GO:0055074)	345	197	135.34	+	1.46	2.66E-03
organophosphate metabolic process (GO:0019637)	703	401	275.78	+	1.45	3.28E-09
animal organ development (GO:0048513)	3159	1801	1239.24	+	1.45	1.83E-55
cellular response to chemical stimulus (GO:0070887)	2174	1239	852.83	+	1.45	2.07E-35
negative regulation of cellular biosynthetic process (GO:0031327)	1374	782	539.00	+	1.45	1.74E-20
positive regulation of RNA metabolic process (GO:0051254)	1383	787	542.53	+	1.45	1.27E-20
regulation of apoptotic process (GO:0042981)	1412	803	553.91	+	1.45	4.82E-21

negative regulation of biosynthetic process (GO:0009890)	1405	799	551.16	+	1.45	6.44E-21
cellular response to organic substance (GO:0071310)	1771	1007	694.74	+	1.45	2.02E-27
cellular cation homeostasis (GO:0030003)	482	274	189.08	+	1.45	2.27E-05
positive regulation of multicellular organismal process (GO:0051240)	1543	877	605.30	+	1.45	2.88E-23
regulation of programmed cell death (GO:0043067)	1427	811	559.79	+	1.45	3.25E-21
negative regulation of nitrogen compound metabolic process (GO:0051172)	1422	808	557.83	+	1.45	4.24E-21
cellular metal ion homeostasis (GO:0006875)	426	242	167.11	+	1.45	1.96E-04
single-organism developmental process (GO:0044767)	5217	2962	2046.57	+	1.45	1.40E-102
carbohydrate derivative metabolic process (GO:1901135)	828	470	324.81	+	1.45	6.34E-11
negative regulation of gene expression (GO:0010629)	1390	789	545.28	+	1.45	2.13E-20
embryonic morphogenesis (GO:0048598)	615	349	241.26	+	1.45	1.97E-07
cellular lipid metabolic process (GO:0044255)	749	425	293.82	+	1.45	1.33E-09
anatomical structure development (GO:0048856)	4940	2803	1937.90	+	1.45	6.13E-95
negative regulation of nucleobase-containing compound metabolic process (GO:0045934)	1299	737	509.58	+	1.45	9.02E-19
negative regulation of cellular process (GO:0048523)	4088	2319	1603.67	+	1.45	4.64E-74
regulation of cell cycle (GO:0051726)	811	460	318.15	+	1.45	1.38E-10
developmental process (GO:0032502)	5248	2976	2058.73	+	1.45	1.28E-102
response to organic substance (GO:0010033)	2713	1538	1064.28	+	1.45	1.62E-44
movement of cell or subcellular component (GO:0006928)	1122	636	440.15	+	1.44	1.14E-15
organophosphate biosynthetic process (GO:0090407)	337	191	132.20	+	1.44	6.32E-03
protein complex subunit organization (GO:0071822)	1034	586	405.63	+	1.44	3.49E-14
lipid biosynthetic process (GO:0008610)	383	217	150.25	+	1.44	1.21E-03
cellular ion homeostasis (GO:0006873)	496	281	194.57	+	1.44	1.96E-05
positive regulation of nucleobase-containing compound metabolic process (GO:0045935)	1617	916	634.33	+	1.44	4.82E-24
regulation of transcription from RNA polymerase II promoter (GO:0006357)	1771	1003	694.74	+	1.44	1.03E-26
cellular developmental process (GO:0048869)	3473	1965	1362.42	+	1.44	2.32E-59
cofactor metabolic process (GO:0051186)	327	185	128.28	+	1.44	1.04E-02
positive regulation of nitrogen compound metabolic process (GO:0051173)	1727	977	677.48	+	1.44	9.58E-26
negative regulation of protein metabolic process (GO:0051248)	998	564	391.50	+	1.44	2.57E-13
cellular response to stress (GO:0033554)	1412	796	553.91	+	1.44	7.85E-20
skeletal system development (GO:0001501)	456	257	178.88	+	1.44	1.41E-04
protein complex assembly (GO:0006461)	921	519	361.30	+	1.44	8.06E-12
protein complex biogenesis (GO:0070271)	921	519	361.30	+	1.44	8.06E-12
nucleobase-containing compound biosynthetic process (GO:0034654)	1475	831	578.62	+	1.44	8.57E-21
heterocycle biosynthetic process (GO:0018130)	1532	863	600.99	+	1.44	1.00E-21
positive regulation of macromolecule metabolic process (GO:0010604)	2718	1531	1066.24	+	1.44	8.49E-43
regulation of cell cycle process (GO:0010564)	476	268	186.73	+	1.44	7.71E-05

positive regulation of cellular metabolic process (GO:0031325)	2727	1535	1069.77	+	1.43	9.00E-43
organ morphogenesis (GO:0009887)	933	525	366.00	+	1.43	6.97E-12
negative regulation of biological process (GO:0048519)	4402	2476	1726.85	+	1.43	3.95E-77
aromatic compound biosynthetic process (GO:0019438)	1546	868	606.48	+	1.43	1.82E-21
positive regulation of cellular biosynthetic process (GO:0031328)	1689	947	662.57	+	1.43	1.31E-23
organic cyclic compound biosynthetic process (GO:1901362)	1636	917	641.78	+	1.43	1.09E-22
positive regulation of cellular process (GO:0048522)	4627	2593	1815.12	+	1.43	2.30E-80
negative regulation of cellular protein metabolic process (GO:0032269)	939	526	368.36	+	1.43	1.39E-11
regulation of cellular protein metabolic process (GO:0032268)	2291	1283	898.73	+	1.43	1.40E-33
negative regulation of molecular function (GO:0044092)	1020	571	400.13	+	1.43	9.12E-13
positive regulation of biological process (GO:0048518)	5076	2840	1991.25	+	1.43	6.24E-90
cellular catabolic process (GO:0044248)	1095	612	429.56	+	1.42	9.29E-14
positive regulation of metabolic process (GO:0009893)	2913	1628	1142.73	+	1.42	3.57E-44
positive regulation of biosynthetic process (GO:0009891)	1726	964	677.09	+	1.42	1.39E-23
positive regulation of gene expression (GO:0010628)	1650	921	647.28	+	1.42	2.88E-22
positive regulation of cell proliferation (GO:0008284)	887	495	347.96	+	1.42	1.75E-10
microtubule cytoskeleton organization (GO:0000226)	362	202	142.01	+	1.42	8.44E-03
cellular homeostasis (GO:0019725)	733	409	287.55	+	1.42	3.40E-08
regulation of protein metabolic process (GO:0051246)	2436	1359	955.61	+	1.42	3.29E-35
positive regulation of cell migration (GO:0030335)	427	238	167.51	+	1.42	1.07E-03
positive regulation of macromolecule biosynthetic process (GO:0010557)	1564	871	613.54	+	1.42	1.45E-20
cellular macromolecule catabolic process (GO:0044265)	627	349	245.96	+	1.42	1.66E-06
negative regulation of macromolecule metabolic process (GO:0010605)	2186	1215	857.54	+	1.42	3.58E-30
cell adhesion (GO:0007155)	871	484	341.68	+	1.42	6.54E-10
negative regulation of metabolic process (GO:0009892)	2389	1326	937.18	+	1.41	3.18E-33
regulation of sequence-specific DNA binding transcription factor activity (GO:0051090)	346	192	135.73	+	1.41	2.14E-02
biological adhesion (GO:0022610)	882	489	346.00	+	1.41	6.83E-10
negative regulation of cellular metabolic process (GO:0031324)	2237	1240	877.55	+	1.41	1.93E-30
localization of cell (GO:0051674)	793	439	311.08	+	1.41	1.60E-08
cell motility (GO:0048870)	793	439	311.08	+	1.41	1.60E-08
negative regulation of cell cycle (GO:0045786)	327	181	128.28	+	1.41	4.66E-02
regulation of response to stimulus (GO:0048583)	3270	1810	1282.78	+	1.41	1.14E-47
regulation of protein secretion (GO:0050708)	409	226	160.45	+	1.41	3.99E-03
positive regulation of locomotion (GO:0040017)	455	251	178.49	+	1.41	1.10E-03
lipid metabolic process (GO:0006629)	959	529	376.20	+	1.41	1.44E-10
cellular component organization (GO:0016043)	4702	2593	1844.54	+	1.41	1.01E-73
regulation of cell proliferation (GO:0042127)	1562	859	612.75	+	1.40	9.41E-19

regulation of body fluid levels (GO:0050878)	353	194	138.48	+	1.40	3.37E-02
single-organism catabolic process (GO:0044712)	608	334	238.51	+	1.40	1.55E-05
positive regulation of establishment of protein localization (GO:1904951)	521	286	204.38	+	1.40	2.34E-04
positive regulation of cell motility (GO:2000147)	441	242	173.00	+	1.40	2.67E-03
positive regulation of cellular component movement (GO:0051272)	452	248	177.31	+	1.40	1.95E-03
positive regulation of protein transport (GO:0051222)	485	266	190.26	+	1.40	7.51E-04
cellular component assembly (GO:0022607)	1865	1019	731.62	+	1.39	4.17E-22
homeostatic process (GO:0042592)	1407	768	551.95	+	1.39	1.25E-15
cell-cell adhesion (GO:0098609)	596	325	233.80	+	1.39	5.21E-05
embryonic organ development (GO:0048568)	477	260	187.12	+	1.39	1.68E-03
negative regulation of catalytic activity (GO:0043086)	751	409	294.61	+	1.39	6.89E-07
catabolic process (GO:0009056)	1326	722	520.17	+	1.39	2.89E-14
macromolecule catabolic process (GO:0009057)	713	388	279.70	+	1.39	2.30E-06
covalent chromatin modification (GO:0016569)	379	206	148.68	+	1.39	3.43E-02
single-organism metabolic process (GO:0044710)	2872	1559	1126.65	+	1.38	1.89E-35
organic substance catabolic process (GO:1901575)	1264	686	495.85	+	1.38	4.15E-13
regulation of hydrolase activity (GO:0051336)	1184	642	464.47	+	1.38	5.34E-12
cellular component organization or biogenesis (GO:0071840)	4917	2661	1928.88	+	1.38	1.47E-68
mitochondrion organization (GO:0007005)	480	259	188.30	+	1.38	3.81E-03
pattern specification process (GO:0007389)	456	246	178.88	+	1.38	7.37E-03
endocytosis (GO:0006897)	445	240	174.57	+	1.37	1.01E-02
response to external stimulus (GO:0009605)	1979	1067	776.34	+	1.37	1.76E-21
organelle organization (GO:0006996)	2821	1520	1106.64	+	1.37	7.72E-33
small molecule metabolic process (GO:0044281)	1428	767	560.19	+	1.37	5.14E-14
positive regulation of response to stimulus (GO:0048584)	1778	954	697.49	+	1.37	3.59E-18
cell surface receptor signaling pathway (GO:0007166)	1656	887	649.63	+	1.37	1.95E-16
regulation of response to stress (GO:0080134)	1142	611	447.99	+	1.36	2.99E-10
single organism cell adhesion (GO:0098602)	473	253	185.55	+	1.36	9.64E-03
single organismal cell-cell adhesion (GO:0016337)	448	239	175.75	+	1.36	2.23E-02
regulation of cell adhesion (GO:0030155)	619	330	242.83	+	1.36	3.40E-04
hemopoiesis (GO:0030097)	580	309	227.53	+	1.36	9.58E-04
regulation of primary metabolic process (GO:0080090)	5149	2738	2019.89	+	1.36	4.59E-64
nucleobase-containing small molecule metabolic process (GO:0055086)	471	250	184.77	+	1.35	1.90E-02
microtubule-based process (GO:0007017)	528	280	207.13	+	1.35	5.25E-03
regulation of macromolecule metabolic process (GO:0060255)	5150	2725	2020.28	+	1.35	1.06E-61
regulation of metabolic process (GO:0019222)	5499	2908	2157.19	+	1.35	3.36E-67
regulation of cellular metabolic process (GO:0031323)	5218	2759	2046.96	+	1.35	1.83E-62

cell cycle (GO:0007049)	760	401	298.14	+	1.35	3.85E-05
macromolecular complex assembly (GO:0065003)	1206	635	473.10	+	1.34	1.55E-09
regulation of nucleobase-containing compound metabolic process (GO:0019219)	3481	1829	1365.55	+	1.34	4.41E-35
macromolecular complex subunit organization (GO:0043933)	1743	915	683.76	+	1.34	8.36E-15
regulation of nitrogen compound metabolic process (GO:0051171)	3740	1963	1467.16	+	1.34	4.31E-38
developmental process involved in reproduction (GO:0003006)	733	384	287.55	+	1.34	1.70E-04
hematopoietic or lymphoid organ development (GO:0048534)	638	334	250.28	+	1.33	1.48E-03
regulation of nucleic acid-templated transcription (GO:1903506)	3003	1572	1178.04	+	1.33	2.67E-28
regulation of transcription, DNA-templated (GO:0006355)	2977	1558	1167.84	+	1.33	6.15E-28
regulation of RNA biosynthetic process (GO:2001141)	3010	1575	1180.79	+	1.33	2.77E-28
regulation of RNA metabolic process (GO:0051252)	3133	1637	1229.04	+	1.33	2.22E-29
regulation of gene expression (GO:0010468)	3647	1904	1430.67	+	1.33	2.48E-35
regulation of cellular macromolecule biosynthetic process (GO:2000112)	3355	1748	1316.13	+	1.33	2.87E-31
response to cytokine (GO:0034097)	668	348	262.05	+	1.33	1.29E-03
regulation of macromolecule biosynthetic process (GO:0010556)	3463	1803	1358.49	+	1.33	2.36E-32
cellular protein metabolic process (GO:0044267)	3251	1692	1275.33	+	1.33	8.68E-30
cellular component biogenesis (GO:0044085)	2108	1097	826.94	+	1.33	2.24E-17
cell cycle process (GO:0022402)	723	376	283.62	+	1.33	4.92E-04
response to stress (GO:0006950)	2989	1554	1172.55	+	1.33	1.40E-26
regulation of cellular biosynthetic process (GO:0031326)	3632	1887	1424.79	+	1.32	9.04E-34
regulation of biosynthetic process (GO:0009889)	3696	1919	1449.90	+	1.32	2.42E-34
RNA metabolic process (GO:0016070)	2014	1044	790.07	+	1.32	7.32E-16
cellular metabolic process (GO:0044237)	7028	3639	2757.00	+	1.32	5.61E-81
chromatin organization (GO:0006325)	564	292	221.25	+	1.32	1.98E-02
cellular response to DNA damage stimulus (GO:0006974)	582	300	228.31	+	1.31	2.04E-02
cellular macromolecule metabolic process (GO:0044260)	5345	2752	2096.78	+	1.31	4.39E-52
cellular aromatic compound metabolic process (GO:0006725)	3120	1601	1223.94	+	1.31	4.73E-25
carboxylic acid metabolic process (GO:0019752)	689	353	270.29	+	1.31	4.76E-03
organic acid metabolic process (GO:0006082)	771	395	302.45	+	1.31	1.04E-03
oxoacid metabolic process (GO:0043436)	693	355	271.86	+	1.31	4.48E-03
organic cyclic compound metabolic process (GO:1901360)	3285	1682	1288.67	+	1.31	3.15E-26
immune system development (GO:0002520)	672	344	263.62	+	1.30	7.09E-03
heterocycle metabolic process (GO:0046483)	3061	1565	1200.79	+	1.30	1.13E-23
biosynthetic process (GO:0009058)	3255	1664	1276.90	+	1.30	1.46E-25
nucleobase-containing compound metabolic process (GO:0006139)	2943	1502	1154.50	+	1.30	3.63E-22
organic substance biosynthetic process (GO:1901576)	3177	1620	1246.30	+	1.30	3.17E-24
cellular biosynthetic process (GO:0044249)	3109	1583	1219.62	+	1.30	2.94E-23

regulation of proteolysis (GO:0030162)	656	332	257.34	+	1.29	2.76E-02
reproductive process (GO:0022414)	1402	709	549.99	+	1.29	8.93E-08
reproduction (GO:0000003)	1405	710	551.16	+	1.29	9.78E-08
nucleic acid metabolic process (GO:0090304)	2470	1248	968.95	+	1.29	3.70E-16
regulation of response to external stimulus (GO:0032101)	735	371	288.33	+	1.29	9.56E-03
organic substance metabolic process (GO:0071704)	7647	3859	2999.83	+	1.29	6.37E-74
primary metabolic process (GO:0044238)	7212	3629	2829.18	+	1.28	1.00E-65
metabolic process (GO:0008152)	8072	4019	3166.55	+	1.27	3.72E-71
macromolecule metabolic process (GO:0043170)	6052	3006	2374.13	+	1.27	7.84E-45
macromolecule biosynthetic process (GO:0009059)	2384	1181	935.21	+	1.26	1.32E-12
cellular macromolecule biosynthetic process (GO:0034645)	2357	1167	924.62	+	1.26	2.40E-12
protein metabolic process (GO:0019538)	3867	1903	1516.98	+	1.25	4.00E-22
single organism reproductive process (GO:0044702)	1261	620	494.68	+	1.25	1.06E-04
nitrogen compound metabolic process (GO:0006807)	4129	2018	1619.76	+	1.25	1.91E-22
single-organism cellular process (GO:0044763)	10837	5219	4251.22	+	1.23	7.15E-86
single-organism process (GO:0044699)	12411	5950	4868.69	+	1.22	2.64E-109
biological regulation (GO:0065007)	11690	5601	4585.85	+	1.22	5.99E-95
multi-organism process (GO:0051704)	1944	930	762.61	+	1.22	3.83E-06
cellular nitrogen compound metabolic process (GO:0034641)	3794	1814	1488.34	+	1.22	1.46E-15
cellular nitrogen compound biosynthetic process (GO:0044271)	2277	1083	893.24	+	1.21	3.99E-07
regulation of biological process (GO:0050789)	11093	5274	4351.65	+	1.21	8.62E-78
regulation of cellular process (GO:0050794)	10637	5047	4172.77	+	1.21	7.99E-70
cellular process (GO:0009987)	14095	6673	5529.30	+	1.21	2.86E-130
multicellular organismal process (GO:0032501)	7149	3384	2804.47	+	1.21	3.57E-34
gene expression (GO:0010467)	2601	1210	1020.34	+	1.19	3.79E-06
biological_process (GO:0008150)	17404	8026	6827.38	+	1.18	3.68E-193
response to stimulus (GO:0050896)	8233	3757	3229.71	+	1.16	3.58E-26
cellular response to stimulus (GO:0051716)	6618	2966	2596.16	+	1.14	1.55E-13
cell communication (GO:0007154)	5491	2438	2154.05	+	1.13	2.70E-08
signaling (GO:0023052)	5365	2362	2104.62	+	1.12	1.40E-06
single organism signaling (GO:0044700)	5362	2360	2103.45	+	1.12	1.58E-06
response to chemical (GO:0042221)	4894	2154	1919.86	+	1.12	1.48E-05
signal transduction (GO:0007165)	5098	2183	1999.88	+	1.09	2.09E-02
system process (GO:0003008)	2700	920	1059.18	-	.87	1.62E-02
neurological system process (GO:0050877)	2229	661	874.41	-	.76	1.49E-11
peptide biosynthetic process (GO:0043043)	693	198	271.86	-	.73	9.26E-03
translation (GO:0006412)	675	190	264.79	-	.72	4.77E-03

G-protein coupled receptor signaling pathway (GO:0007186)	2278	535	893.63	-	.60	5.19E-38
sensory perception (GO:0007600)	1865	403	731.62	-	.55	3.21E-39
Unclassified (UNCLASSIFIED)	6377	1303	2501.62	-	.52	0.00E00
production of molecular mediator of immune response (GO:0002440)	144	25	56.49	-	.44	1.62E-02
immunoglobulin production (GO:0002377)	127	21	49.82	-	.42	2.64E-02
detection of stimulus (GO:0051606)	1605	264	629.62	-	.42	1.28E-60
detection of stimulus involved in sensory perception (GO:0050906)	1509	216	591.96	-	.36	2.34E-70
sensory perception of chemical stimulus (GO:0007606)	1510	209	592.35	-	.35	1.02E-73
detection of chemical stimulus (GO:0009593)	1477	197	579.41	-	.34	1.53E-75
sensory perception of smell (GO:0007608)	1427	188	559.79	-	.34	6.21E-74
protein activation cascade (GO:0072376)	104	13	40.80	-	.32	3.02E-03
detection of chemical stimulus involved in sensory perception (GO:0050907)	1445	177	566.86	-	.31	9.92E-82
detection of chemical stimulus involved in sensory perception of smell (GO:0050911)	1389	167	544.89	-	.31	5.38E-80
complement activation (GO:0006956)	93	10	36.48	-	.27	1.76E-03
humoral immune response mediated by circulating immunoglobulin (GO:0002455)	84	7	32.95	-	.21	4.06E-04
complement activation, classical pathway (GO:0006958)	79	5	30.99	-	< 0.2	7.73E-05
