# Supplementary material for: The RNA-Binding Protein hnRNP K Mediates the Effect of BDNF on Dendritic mRNA Metabolism and Regulates Synaptic NMDA Receptors in Hippocampal Neurons
Source: eNeuro. 2017 Dec 12;4(6):ENEURO.0268-17.2017. doi: 10.1523/ENEURO.0268-17.2017 (PMC5732018; doi:10.1523/ENEURO.0268-17.2017)
Supplement: Table 1-3 — Download Table 1-3, PDF file. [file sup_enu-eN-NWR-0268-17-s06.pdf]

Table 1-3 - Cellular components associated with mRNAs co-immunoprecipitated with hnRNP K as assessed by Gene Ontology

GO Cellular component	Rattus norvegicus (reference list)	uploaded list	(expected)	(over/under)	(fold enrichment)	(P-value)
presynaptic active zone (GO:0048786)	28	27	10.98	+	2.46	3.90E-02
neuron projection membrane (GO:0032589)	49	44	19.22	+	2.29	1.07E-03
dendritic shaft (GO:0043198)	58	52	22.75	+	2.29	1.23E-04
exocytic vesicle membrane (GO:0099501)	62	55	24.32	+	2.26	7.55E-05
synaptic vesicle membrane (GO:0030672)	62	55	24.32	+	2.26	7.55E-05
neurotransmitter receptor complex (GO:0098878)	50	43	19.61	+	2.19	4.10E-03
ionotropic glutamate receptor complex (GO:0008328)	50	43	19.61	+	2.19	4.10E-03
T-tubule (GO:0030315)	60	51	23.54	+	2.17	7.57E-04
presynaptic membrane (GO:0042734)	74	62	29.03	+	2.14	8.37E-05
neuron spine (GO:0044309)	155	129	60.80	+	2.12	1.90E-11
dendritic spine (GO:0043197)	153	127	60.02	+	2.12	3.62E-11
excitatory synapse (GO:0060076)	255	211	100.03	+	2.11	2.07E-19
postsynaptic density (GO:0014069)	235	194	92.19	+	2.10	1.29E-17
postsynaptic specialization (GO:0099572)	235	194	92.19	+	2.10	1.29E-17
main axon (GO:0044304)	74	61	29.03	+	2.10	1.82E-04
terminal bouton (GO:0043195)	111	90	43.54	+	2.07	5.53E-07
synaptic vesicle (GO:0008021)	154	124	60.41	+	2.05	5.34E-10
voltage-gated potassium channel complex (GO:0008076)	76	61	29.81	+	2.05	4.33E-04
presynapse (GO:0098793)	330	263	129.46	+	2.03	2.45E-22
clathrin-coated pit (GO:0005905)	54	43	21.18	+	2.03	2.49E-02
potassium channel complex (GO:0034705)	78	62	30.60	+	2.03	4.79E-04
postsynapse (GO:0098794)	428	340	167.90	+	2.03	3.73E-29
postsynaptic membrane (GO:0045211)	197	156	77.28	+	2.02	2.20E-12
exocytic vesicle (GO:0070382)	170	134	66.69	+	2.01	2.76E-10
neuromuscular junction (GO:0031594)	61	48	23.93	+	2.01	1.17E-02
synaptic membrane (GO:0097060)	267	210	104.74	+	2.00	6.90E-17
synapse part (GO:0044456)	677	532	265.58	+	2.00	1.27E-45
dendrite (GO:0030425)	581	452	227.92	+	1.98	2.36E-37
synapse (GO:0045202)	842	653	330.31	+	1.98	8.07E-55
transport vesicle membrane (GO:0030658)	117	90	45.90	+	1.96	6.37E-06
axon part (GO:0033267)	307	236	120.43	+	1.96	5.45E-18
sarcolemma (GO:0042383)	150	115	58.84	+	1.95	6.56E-08
clathrin-coated vesicle (GO:0030136)	97	74	38.05	+	1.94	1.85E-04

neuron projection terminus (GO:0044306)	198	151	77.67	+	1.94	1.09E-10
axon terminus (GO:0043679)	185	141	72.57	+	1.94	7.31E-10
leading edge membrane (GO:0031256)	133	100	52.17	+	1.92	2.93E-06
axon (GO:0030424)	550	412	215.76	+	1.91	1.84E-30
transport vesicle (GO:0030133)	292	217	114.55	+	1.89	7.62E-15
plasma membrane raft (GO:0044853)	101	75	39.62	+	1.89	4.17E-04
caveola (GO:0005901)	92	68	36.09	+	1.88	1.65E-03
Golgi-associated vesicle (GO:0005798)	69	51	27.07	+	1.88	3.16E-02
neuron projection (GO:0043005)	1165	861	457.02	+	1.88	1.28E-64
growth cone (GO:0030426)	171	126	67.08	+	1.88	9.50E-08
filopodium (GO:0030175)	91	67	35.70	+	1.88	2.23E-03
somatodendritic compartment (GO:0036477)	865	635	339.33	+	1.87	1.20E-45
site of polarized growth (GO:0030427)	176	129	69.04	+	1.87	7.91E-08
membrane region (GO:0098589)	396	290	155.35	+	1.87	1.67E-19
cytoplasmic vesicle membrane (GO:0030659)	283	206	111.02	+	1.86	3.97E-13
cell-cell contact zone (GO:0044291)	73	53	28.64	+	1.85	3.53E-02
Golgi membrane (GO:0000139)	390	283	152.99	+	1.85	1.85E-18
membrane microdomain (GO:0098857)	337	243	132.20	+	1.84	2.65E-15
membrane raft (GO:0045121)	337	243	132.20	+	1.84	2.65E-15
endosome membrane (GO:0010008)	240	173	94.15	+	1.84	1.98E-10
cytoplasmic vesicle part (GO:0044433)	305	219	119.65	+	1.83	1.91E-13
coated vesicle (GO:0030135)	152	109	59.63	+	1.83	6.89E-06
neuron part (GO:0097458)	1518	1088	595.49	+	1.83	3.00E-76
vesicle membrane (GO:0012506)	311	222	122.00	+	1.82	2.17E-13
cell leading edge (GO:0031252)	349	249	136.91	+	1.82	3.45E-15
neuronal cell body (GO:0043025)	623	441	244.40	+	1.80	1.33E-27
endocytic vesicle (GO:0030139)	128	90	50.21	+	1.79	3.11E-04
Golgi apparatus part (GO:0044431)	577	405	226.35	+	1.79	1.70E-24
endosomal part (GO:0044440)	258	181	101.21	+	1.79	5.27E-10
cell body (GO:0044297)	700	491	274.60	+	1.79	4.28E-30
ruffle (GO:0001726)	145	101	56.88	+	1.78	9.34E-05
trans-Golgi network (GO:0005802)	158	110	61.98	+	1.77	2.62E-05
vacuolar membrane (GO:0005774)	421	293	165.15	+	1.77	9.97E-17
ion channel complex (GO:0034702)	262	182	102.78	+	1.77	1.02E-09
whole membrane (GO:0098805)	1122	776	440.15	+	1.76	3.77E-47
lamellipodium (GO:0030027)	162	112	63.55	+	1.76	2.79E-05
early endosome (GO:0005769)	246	170	96.50	+	1.76	8.37E-09

cation channel complex (GO:0034703)	155	107	60.80	+	1.76	6.05E-05
late endosome (GO:0005770)	177	122	69.43	+	1.76	8.06E-06
vacuolar part (GO:0044437)	441	301	173.00	+	1.74	4.20E-16
bounding membrane of organelle (GO:0098588)	1191	805	467.21	+	1.72	1.89E-45
transmembrane transporter complex (GO:1902495)	289	194	113.37	+	1.71	3.36E-09
transporter complex (GO:1990351)	293	196	114.94	+	1.71	3.49E-09
endosome (GO:0005768)	642	429	251.85	+	1.70	5.04E-22
lytic vacuole membrane (GO:0098852)	213	142	83.56	+	1.70	3.90E-06
lysosomal membrane (GO:0005765)	213	142	83.56	+	1.70	3.90E-06
organelle subcompartment (GO:0031984)	258	172	101.21	+	1.70	9.25E-08
perinuclear region of cytoplasm (GO:0048471)	637	424	249.89	+	1.70	1.95E-21
cell projection membrane (GO:0031253)	263	175	103.17	+	1.70	7.33E-08
Golgi subcompartment (GO:0098791)	245	163	96.11	+	1.70	3.27E-07
focal adhesion (GO:0005925)	390	259	152.99	+	1.69	2.66E-12
Golgi apparatus (GO:0005794)	1165	771	457.02	+	1.69	2.78E-40
cell-substrate adherens junction (GO:0005924)	395	261	154.95	+	1.68	3.61E-12
plasma membrane region (GO:0098590)	920	606	360.90	+	1.68	1.85E-30
cell projection part (GO:0044463)	1015	668	398.17	+	1.68	9.20E-34
cell-substrate junction (GO:0030055)	400	263	156.92	+	1.68	4.87E-12
adherens junction (GO:0005912)	470	308	184.38	+	1.67	3.37E-14
vacuole (GO:0005773)	979	641	384.05	+	1.67	1.13E-31
myelin sheath (GO:0043209)	208	136	81.60	+	1.67	2.49E-05
cell junction (GO:0030054)	1122	732	440.15	+	1.66	3.92E-36
cell projection (GO:0042995)	1921	1247	753.59	+	1.65	6.05E-64
anchoring junction (GO:0070161)	485	313	190.26	+	1.65	1.33E-13
mitochondrial outer membrane (GO:0005741)	138	88	54.14	+	1.63	1.70E-02
plasma membrane protein complex (GO:0098797)	476	302	186.73	+	1.62	3.58E-12
organelle outer membrane (GO:0031968)	158	100	61.98	+	1.61	6.32E-03
cytoplasmic, membrane-bounded vesicle (GO:0016023)	925	585	362.87	+	1.61	4.54E-25
microbody (GO:0042579)	136	86	53.35	+	1.61	2.82E-02
peroxisome (GO:0005777)	136	86	53.35	+	1.61	2.82E-02
extrinsic component of plasma membrane (GO:0019897)	146	92	57.27	+	1.61	1.71E-02
intrinsic component of organelle membrane (GO:0031300)	143	90	56.10	+	1.60	2.20E-02
extrinsic component of membrane (GO:0019898)	260	163	101.99	+	1.60	1.66E-05
outer membrane (GO:0019867)	163	102	63.94	+	1.60	8.09E-03
basolateral plasma membrane (GO:0016323)	232	145	91.01	+	1.59	1.20E-04
cytoplasmic region (GO:0099568)	298	186	116.90	+	1.59	2.29E-06

perikaryon (GO:0043204)	141	88	55.31	+	1.59	3.60E-02
cytoplasmic side of plasma membrane (GO:0009898)	164	102	64.34	+	1.59	1.03E-02
integral component of organelle membrane (GO:0031301)	140	87	54.92	+	1.58	4.61E-02
cytoplasmic vesicle (GO:0031410)	1035	643	406.02	+	1.58	7.46E-26
intracellular vesicle (GO:0097708)	1037	643	406.80	+	1.58	1.21E-25
lytic vacuole (GO:0000323)	429	266	168.29	+	1.58	1.70E-09
lysosome (GO:0005764)	429	266	168.29	+	1.58	1.70E-09
cytoplasmic side of membrane (GO:0098562)	176	108	69.04	+	1.56	1.00E-02
endoplasmic reticulum membrane (GO:0005789)	686	420	269.11	+	1.56	4.05E-15
endomembrane system (GO:0012505)	3241	1982	1271.41	+	1.56	7.78E-87
nuclear outer membrane-endoplasmic reticulum membrane network (GO:0042175)	703	429	275.78	+	1.56	2.96E-15
endoplasmic reticulum part (GO:0044432)	772	471	302.85	+	1.56	6.37E-17
plasma membrane receptor complex (GO:0098802)	164	100	64.34	+	1.55	2.67E-02
endoplasmic reticulum (GO:0005783)	1355	824	531.55	+	1.55	4.76E-31
actin-based cell projection (GO:0098858)	191	116	74.93	+	1.55	7.48E-03
organelle membrane (GO:0031090)	1949	1183	764.57	+	1.55	3.55E-46
secretory vesicle (GO:0099503)	449	269	176.14	+	1.53	3.86E-08
actin cytoskeleton (GO:0015629)	430	257	168.68	+	1.52	1.31E-07
contractile fiber part (GO:0044449)	191	114	74.93	+	1.52	1.85E-02
microtubule (GO:0005874)	322	192	126.32	+	1.52	3.28E-05
cell cortex (GO:0005938)	239	142	93.76	+	1.51	2.33E-03
contractile fiber (GO:0043292)	214	127	83.95	+	1.51	8.20E-03
sarcomere (GO:0030017)	177	105	69.43	+	1.51	4.89E-02
myofibril (GO:0030016)	204	121	80.03	+	1.51	1.36E-02
receptor complex (GO:0043235)	320	188	125.53	+	1.50	1.21E-04
transferase complex, transferring phosphorus-containing groups (GO:0061695)	239	139	93.76	+	1.48	8.35E-03
ubiquitin ligase complex (GO:0000151)	273	158	107.09	+	1.48	2.70E-03
transcription factor complex (GO:0005667)	313	181	122.79	+	1.47	5.50E-04
nucleoplasm (GO:0005654)	2113	1216	828.90	+	1.47	3.06E-37
nuclear body (GO:0016604)	287	164	112.59	+	1.46	3.53E-03
membrane protein complex (GO:0098796)	1031	586	404.45	+	1.45	3.04E-15
transferase complex (GO:1990234)	701	398	274.99	+	1.45	1.11E-09
intrinsic component of plasma membrane (GO:0031226)	1162	655	455.84	+	1.44	1.56E-16
plasma membrane part (GO:0044459)	2252	1269	883.43	+	1.44	4.13E-35
cell-cell junction (GO:0005911)	407	229	159.66	+	1.43	1.41E-04
integral component of plasma membrane (GO:0005887)	1094	615	429.16	+	1.43	3.74E-15
vesicle (GO:0031982)	3401	1904	1334.17	+	1.43	8.19E-55

membrane-bounded vesicle (GO:0031988)	3254	1820	1276.51	+	1.43	1.22E-51
nuclear envelope (GO:0005635)	384	214	150.64	+	1.42	6.81E-04
apical plasma membrane (GO:0016324)	309	170	121.22	+	1.40	1.82E-02
cytoplasmic part (GO:0044444)	6819	3737	2675.01	+	1.40	1.24E-119
nucleoplasm part (GO:0044451)	668	366	262.05	+	1.40	5.31E-07
extracellular vesicle (GO:1903561)	2610	1430	1023.87	+	1.40	1.45E-34
extracellular organelle (GO:0043230)	2617	1433	1026.62	+	1.40	1.56E-34
extracellular exosome (GO:0070062)	2596	1419	1018.38	+	1.39	8.64E-34
cytoplasm (GO:0005737)	9562	5217	3751.06	+	1.39	2.70E-202
nuclear chromatin (GO:0000790)	310	169	121.61	+	1.39	3.06E-02
apical part of cell (GO:0045177)	408	222	160.05	+	1.39	2.12E-03
cytosol (GO:0005829)	1984	1079	778.30	+	1.39	8.02E-24
catalytic complex (GO:1902494)	1058	575	415.04	+	1.39	1.96E-11
nuclear part (GO:0044428)	3385	1837	1327.89	+	1.38	3.94E-44
nuclear lumen (GO:0031981)	2921	1584	1145.87	+	1.38	9.62E-37
intracellular membrane-bounded organelle (GO:0043231)	9568	5184	3753.41	+	1.38	1.22E-192
intracellular organelle lumen (GO:0070013)	3296	1782	1292.98	+	1.38	1.35E-41
organelle lumen (GO:0043233)	3301	1784	1294.94	+	1.38	1.48E-41
membrane-enclosed lumen (GO:0031974)	3301	1784	1294.94	+	1.38	1.48E-41
polymeric cytoskeletal fiber (GO:0099513)	560	301	219.68	+	1.37	9.92E-05
supramolecular fiber (GO:0099512)	560	301	219.68	+	1.37	9.92E-05
protein complex (GO:0043234)	4040	2170	1584.84	+	1.37	5.02E-51
membrane-bounded organelle (GO:0043227)	10720	5757	4205.33	+	1.37	5.27E-225
cytoskeleton (GO:0005856)	1760	944	690.43	+	1.37	1.02E-18
intracellular organelle part (GO:0044446)	6833	3647	2680.50	+	1.36	2.59E-99
organelle part (GO:0044422)	7014	3737	2751.51	+	1.36	6.32E-102
organelle envelope (GO:0031967)	974	515	382.09	+	1.35	2.74E-08
envelope (GO:0031975)	981	518	384.83	+	1.35	2.90E-08
microtubule cytoskeleton (GO:0015630)	1010	533	396.21	+	1.35	1.61E-08
mitochondrion (GO:0005739)	1740	915	682.58	+	1.34	8.19E-16
nucleus (GO:0005634)	6007	3155	2356.47	+	1.34	6.05E-73
intracellular part (GO:0044424)	12356	6477	4847.11	+	1.34	6.48E-255
chromatin (GO:0000785)	479	251	187.91	+	1.34	6.62E-03
intracellular organelle (GO:0043229)	10800	5656	4236.71	+	1.33	6.44E-188
nuclear chromosome part (GO:0044454)	488	254	191.44	+	1.33	9.19E-03
intracellular (GO:0005622)	12864	6689	5046.39	+	1.33	2.52E-263
organelle (GO:0043226)	11850	6159	4648.61	+	1.32	1.70E-215

nucleolus (GO:0005730)	815	423	319.71	+	1.32	1.40E-05
nuclear chromosome (GO:0000228)	521	268	204.38	+	1.31	1.18E-02
cytoskeletal part (GO:0044430)	1358	698	532.73	+	1.31	1.17E-09
mitochondrial membrane (GO:0031966)	558	285	218.90	+	1.30	1.05E-02
mitochondrial envelope (GO:0005740)	603	307	236.55	+	1.30	6.14E-03
chromosomal part (GO:0044427)	790	402	309.91	+	1.30	2.40E-04
mitochondrial part (GO:0044429)	786	396	308.34	+	1.28	7.64E-04
cell surface (GO:0009986)	838	421	328.74	+	1.28	4.52E-04
macromolecular complex (GO:0032991)	5064	2539	1986.55	+	1.28	5.22E-39
chromosome (GO:0005694)	862	432	338.15	+	1.28	4.08E-04
intracellular non-membrane-bounded organelle (GO:0043232)	3946	1971	1547.97	+	1.27	3.25E-27
non-membrane-bounded organelle (GO:0043228)	3946	1971	1547.97	+	1.27	3.25E-27
extracellular region part (GO:0044421)	3745	1858	1469.12	+	1.26	7.15E-24
extracellular region (GO:0005576)	4135	2005	1622.11	+	1.24	1.93E-21
cell part (GO:0044464)	15800	7555	6198.15	+	1.22	3.36E-211
cell (GO:0005623)	15869	7569	6225.22	+	1.22	3.38E-208
membrane (GO:0016020)	9765	4560	3830.69	+	1.19	1.49E-49
cell periphery (GO:0071944)	5396	2464	2116.79	+	1.16	2.33E-14
cellular_component (GO:0005575)	18430	8402	7229.87	+	1.16	1.73E-218
plasma membrane (GO:0005886)	5275	2400	2069.32	+	1.16	3.85E-13
membrane part (GO:0044425)	7677	3378	3011.59	+	1.12	6.32E-13
cytosolic part (GO:0044445)	455	117	178.49	-	.66	5.95E-04
ribosome (GO:0005840)	537	129	210.66	-	.61	7.82E-07
large ribosomal subunit (GO:0015934)	260	61	101.99	-	.60	9.05E-03
ribosomal subunit (GO:0044391)	402	89	157.70	-	.56	1.72E-06
cytosolic large ribosomal subunit (GO:0022625)	224	44	87.87	-	.50	1.92E-04
small ribosomal subunit (GO:0015935)	143	28	56.10	-	.50	3.05E-02
cytosolic ribosome (GO:0022626)	341	66	133.77	-	.49	6.05E-08
Unclassified (UNCLASSIFIED)	5351	927	2099.13	-	.44	0.00E00
cytosolic small ribosomal subunit (GO:0022627)	109	17	42.76	-	.40	7.84E-03
immunoglobulin complex (GO:0019814)	64	3	25.11	-	< 0.2	4.49E-05
