# Supplementary material for: The RNA-Binding Protein hnRNP K Mediates the Effect of BDNF on Dendritic mRNA Metabolism and Regulates Synaptic NMDA Receptors in Hippocampal Neurons
Source: eNeuro. 2017 Dec 12;4(6):ENEURO.0268-17.2017. doi: 10.1523/ENEURO.0268-17.2017 (PMC5732018; doi:10.1523/ENEURO.0268-17.2017)
Supplement: Table 1-4 — Download Table 1-4, PDF file. [file sup_enu-eN-NWR-0268-17-s07.pdf]

Table 1-4 - Molecular functions associated with mRNAs co-immunoprecipitated with hnRNP K as assessed by Gene Ontology

GO Molecular function	Rattus norvegicus (reference list)	uploaded list	(expected)	(over/under)	(fold enrichment)	(P-value)
transmembrane receptor protein tyrosine kinase activity (GO:0004714)	57	45	22.36	+	2.01	4.02E-02
SH3 domain binding (GO:0017124)	94	74	36.88	+	2.01	1.14E-04
PDZ domain binding (GO:0030165)	117	90	45.90	+	1.96	1.28E-05
amino acid transmembrane transporter activity (GO:0015171)	81	62	31.78	+	1.95	3.20E-03
calmodulin binding (GO:0005516)	152	116	59.63	+	1.95	1.42E-07
calcium-dependent protein binding (GO:0048306)	69	52	27.07	+	1.92	3.24E-02
transmembrane receptor protein kinase activity (GO:0019199)	73	55	28.64	+	1.92	1.88E-02
ion channel binding (GO:0044325)	124	93	48.64	+	1.91	2.32E-05
channel regulator activity (GO:0016247)	127	95	49.82	+	1.91	1.84E-05
receptor signaling protein serine/threonine kinase activity (GO:0004702)	89	65	34.91	+	1.86	8.13E-03
beta-catenin binding (GO:0008013)	84	61	32.95	+	1.85	1.88E-02
potassium ion transmembrane transporter activity (GO:0015079)	142	102	55.70	+	1.83	3.84E-05
protein domain specific binding (GO:0019904)	690	484	270.68	+	1.79	2.48E-29
potassium channel activity (GO:0005267)	117	82	45.90	+	1.79	2.32E-03
syntaxin binding (GO:0019905)	95	66	37.27	+	1.77	3.22E-02
ubiquitin protein ligase binding (GO:0031625)	275	190	107.88	+	1.76	1.04E-09
receptor signaling protein activity (GO:0005057)	122	84	47.86	+	1.76	3.38E-03
ubiquitin-like protein ligase binding (GO:0044389)	279	192	109.45	+	1.75	1.10E-09
carboxylic acid transmembrane transporter activity (GO:0046943)	109	75	42.76	+	1.75	1.22E-02
protein C-terminus binding (GO:0008022)	210	144	82.38	+	1.75	1.06E-06
voltage-gated channel activity (GO:0022832)	182	124	71.40	+	1.74	2.31E-05
voltage-gated ion channel activity (GO:0005244)	182	124	71.40	+	1.74	2.31E-05
organic acid transmembrane transporter activity (GO:0005342)	112	76	43.94	+	1.73	1.69E-02
protein N-terminus binding (GO:0047485)	118	80	46.29	+	1.73	1.02E-02
phosphoprotein phosphatase activity (GO:0004721)	163	110	63.94	+	1.72	2.39E-04
transcription factor activity, RNA polymerase II transcription factor binding (GO:0001076)	129	87	50.61	+	1.72	4.92E-03
phosphatase activity (GO:0016791)	261	175	102.39	+	1.71	8.34E-08
histone deacetylase binding (GO:0042826)	109	73	42.76	+	1.71	3.84E-02
SNARE binding (GO:0000149)	129	86	50.61	+	1.70	8.62E-03
phosphatase binding (GO:0019902)	185	123	72.57	+	1.69	9.66E-05
voltage-gated cation channel activity (GO:0022843)	134	89	52.57	+	1.69	6.82E-03
kinase binding (GO:0019900)	661	439	259.30	+	1.69	8.74E-22
transcription corepressor activity (GO:0003714)	154	102	60.41	+	1.69	1.54E-03
protein phosphatase binding (GO:0019903)	139	92	54.53	+	1.69	5.38E-03
metal ion transmembrane transporter activity (GO:0046873)	397	262	155.74	+	1.68	7.35E-12
protein kinase binding (GO:0019901)	594	392	233.02	+	1.68	9.42E-19
small GTPase binding (GO:0031267)	276	182	108.27	+	1.68	1.23E-07
GTPase binding (GO:0051020)	305	201	119.65	+	1.68	1.32E-08

protein kinase regulator activity (GO:0019887)	161	106	63.16	+	1.68	1.23E-03
kinase regulator activity (GO:0019207)	181	119	71.00	+	1.68	2.78E-04
phosphoric ester hydrolase activity (GO:0042578)	347	228	136.12	+	1.67	6.83E-10
protein tyrosine kinase activity (GO:0004713)	128	84	50.21	+	1.67	1.94E-02
nucleoside-triphosphatase regulator activity (GO:0060589)	247	160	96.90	+	1.65	5.62E-06
magnesium ion binding (GO:0000287)	204	132	80.03	+	1.65	1.41E-04
Ras GTPase binding (GO:0017016)	261	168	102.39	+	1.64	3.51E-06
enzyme activator activity (GO:0008047)	378	243	148.28	+	1.64	1.00E-09
gated channel activity (GO:0022836)	302	194	118.47	+	1.64	2.33E-07
divalent inorganic cation transmembrane transporter activity (GO:0072509)	161	103	63.16	+	1.63	5.96E-03
GTPase regulator activity (GO:0030695)	225	142	88.26	+	1.61	1.89E-04
GTPase activator activity (GO:0005096)	205	129	80.42	+	1.60	8.32E-04
enzyme binding (GO:0019899)	1866	1172	732.01	+	1.60	2.64E-52
phosphotransferase activity, alcohol group as acceptor (GO:0016773)	707	444	277.35	+	1.60	1.16E-17
ubiquitin-like protein ligase activity (GO:0061659)	201	126	78.85	+	1.60	1.36E-03
protein kinase activity (GO:0004672)	604	378	236.94	+	1.60	1.72E-14
cation channel activity (GO:0005261)	291	182	114.16	+	1.59	5.91E-06
protein serine/threonine kinase activity (GO:0004674)	426	266	167.11	+	1.59	1.67E-09
ubiquitin protein ligase activity (GO:0061630)	197	123	77.28	+	1.59	2.23E-03
core promoter binding (GO:0001047)	154	96	60.41	+	1.59	3.42E-02
substrate-specific channel activity (GO:0022838)	411	256	161.23	+	1.59	5.72E-09
kinase activity (GO:0016301)	806	501	316.18	+	1.58	2.30E-19
ion channel activity (GO:0005216)	399	248	156.52	+	1.58	1.53E-08
transcription factor activity, transcription factor binding (GO:0000989)	451	280	176.92	+	1.58	7.71E-10
transcription factor activity, protein binding (GO:0000988)	452	280	177.31	+	1.58	9.76E-10
protein homodimerization activity (GO:0042803)	779	482	305.59	+	1.58	3.58E-18
identical protein binding (GO:0042802)	1285	795	504.09	+	1.58	8.20E-32
transcriptional repressor activity, RNA polymerase II transcription regulatory region sequence-specific binding (GO:0001227)	177	109	69.43	+	1.57	1.58E-02
transferase activity, transferring phosphorus-containing groups (GO:0016772)	952	581	373.46	+	1.56	4.20E-21
secondary active transmembrane transporter activity (GO:0015291)	225	137	88.26	+	1.55	2.06E-03
passive transmembrane transporter activity (GO:0022803)	446	271	174.96	+	1.55	1.64E-08
channel activity (GO:0015267)	446	271	174.96	+	1.55	1.64E-08
transcription cofactor activity (GO:0003712)	401	243	157.31	+	1.54	2.49E-07
inorganic cation transmembrane transporter activity (GO:0022890)	494	299	193.79	+	1.54	2.08E-09
substrate-specific transmembrane transporter activity (GO:0022891)	885	535	347.17	+	1.54	2.07E-18
protein heterodimerization activity (GO:0046982)	541	327	212.23	+	1.54	2.10E-10
ion transmembrane transporter activity (GO:0015075)	756	455	296.57	+	1.53	5.49E-15
transcription coactivator activity (GO:0003713)	228	137	89.44	+	1.53	4.01E-03
cytoskeletal protein binding (GO:0008092)	822	493	322.46	+	1.53	3.70E-16
ubiquitin-like protein transferase activity (GO:0019787)	412	247	161.62	+	1.53	4.58E-07
ubiquitin-protein transferase activity (GO:0004842)	397	237	155.74	+	1.52	1.52E-06
protein dimerization activity (GO:0046983)	1243	742	487.61	+	1.52	4.03E-25

cation transmembrane transporter activity (GO:0008324)	578	345	226.74	+	1.52	2.08E-10
anion transmembrane transporter activity (GO:0008509)	186	111	72.97	+	1.52	4.78E-02
phosphatidylinositol binding (GO:0035091)	208	124	81.60	+	1.52	1.71E-02
substrate-specific transporter activity (GO:0022892)	1050	625	411.90	+	1.52	2.05E-20
G-protein coupled receptor binding (GO:0001664)	261	155	102.39	+	1.51	1.68E-03
transmembrane transporter activity (GO:0022857)	977	577	383.27	+	1.51	6.07E-18
transcription factor binding (GO:0008134)	559	330	219.29	+	1.50	2.61E-09
transcription factor activity, RNA polymerase II core promoter proximal region sequence-specific binding (GO:0000982)	353	208	138.48	+	1.50	4.26E-05
RNA polymerase II core promoter proximal region sequence-specific DNA binding (GO:0000978)	331	195	129.85	+	1.50	1.18E-04
monovalent inorganic cation transmembrane transporter activity (GO:0015077)	338	199	132.59	+	1.50	9.05E-05
phospholipid binding (GO:0005543)	338	198	132.59	+	1.49	1.38E-04
transporter activity (GO:0005215)	1231	716	482.91	+	1.48	2.90E-21
molecular function regulator (GO:0098772)	1156	671	453.48	+	1.48	1.53E-19
core promoter proximal region sequence-specific DNA binding (GO:0000987)	350	203	137.30	+	1.48	1.89E-04
core promoter proximal region DNA binding (GO:0001159)	352	204	138.09	+	1.48	1.85E-04
RNA polymerase II transcription factor activity, sequence-specific DNA binding (GO:0000981)	631	365	247.53	+	1.47	2.10E-09
chromatin binding (GO:0003682)	511	295	200.46	+	1.47	3.89E-07
GTP binding (GO:0005525)	376	216	147.50	+	1.46	1.48E-04
protein complex binding (GO:0032403)	857	492	336.19	+	1.46	7.11E-13
protein binding (GO:0005515)	6478	3702	2541.24	+	1.46	1.36E-145
regulatory region nucleic acid binding (GO:0001067)	787	449	308.73	+	1.45	3.72E-11
regulatory region DNA binding (GO:0000975)	784	447	307.55	+	1.45	4.69E-11
tubulin binding (GO:0015631)	279	159	109.45	+	1.45	1.14E-02
transcription regulatory region DNA binding (GO:0044212)	781	445	306.38	+	1.45	5.91E-11
macromolecular complex binding (GO:0044877)	1408	802	552.34	+	1.45	1.03E-21
guanyl ribonucleotide binding (GO:0032561)	397	226	155.74	+	1.45	1.46E-04
transferase activity (GO:0016740)	2243	1275	879.90	+	1.45	1.21E-36
guanyl nucleotide binding (GO:0019001)	398	226	156.13	+	1.45	1.75E-04
enzyme regulator activity (GO:0030234)	854	484	335.01	+	1.44	9.69E-12
RNA polymerase II regulatory region DNA binding (GO:0001012)	563	319	220.86	+	1.44	5.01E-07
sequence-specific double-stranded DNA binding (GO:1990837)	655	371	256.95	+	1.44	1.77E-08
RNA polymerase II regulatory region sequence-specific DNA binding (GO:0000977)	557	314	218.50	+	1.44	1.16E-06
transcription regulatory region sequence-specific DNA binding (GO:0000976)	629	354	246.75	+	1.43	1.10E-07
amide binding (GO:0033218)	292	164	114.55	+	1.43	1.78E-02
actin binding (GO:0003779)	367	206	143.97	+	1.43	1.38E-03
transcriptional activator activity, RNA polymerase II transcription regulatory region sequence-specific binding (GO:0001228)	337	189	132.20	+	1.43	4.14E-03
double-stranded DNA binding (GO:0003690)	739	414	289.90	+	1.43	4.55E-09
anion binding (GO:0043168)	275	154	107.88	+	1.43	3.81E-02
active transmembrane transporter activity (GO:0022804)	356	198	139.65	+	1.42	3.99E-03
purine nucleotide binding (GO:0017076)	1786	978	700.63	+	1.40	7.50E-22
ribonucleotide binding (GO:0032553)	1792	979	702.98	+	1.39	1.43E-21
purine ribonucleotide binding (GO:0032555)	1776	970	696.70	+	1.39	2.63E-21

nucleoside binding (GO:0001882)	1750	955	686.50	+	1.39	8.16E-21
hydrolase activity, acting on ester bonds (GO:0016788)	726	396	284.80	+	1.39	3.49E-07
ribonucleoside binding (GO:0032549)	1743	950	683.76	+	1.39	1.57E-20
purine ribonucleoside binding (GO:0032550)	1740	948	682.58	+	1.39	1.97E-20
purine nucleoside binding (GO:0001883)	1743	949	683.76	+	1.39	2.26E-20
adenyl nucleotide binding (GO:0030554)	1428	777	560.19	+	1.39	4.77E-16
purine ribonucleoside triphosphate binding (GO:0035639)	1732	942	679.44	+	1.39	4.60E-20
carbohydrate derivative binding (GO:0097367)	2125	1155	833.61	+	1.39	2.19E-25
adenyl ribonucleotide binding (GO:0032559)	1420	770	557.05	+	1.38	1.55E-15
zinc ion binding (GO:0008270)	1072	580	420.53	+	1.38	6.48E-11
ATP binding (GO:0005524)	1385	745	543.32	+	1.37	3.55E-14
sequence-specific DNA binding (GO:0043565)	981	524	384.83	+	1.36	8.32E-09
lipid binding (GO:0008289)	625	333	245.18	+	1.36	9.51E-05
nucleic acid binding transcription factor activity (GO:0001071)	997	530	391.11	+	1.36	1.27E-08
transcription factor activity, sequence-specific DNA binding (GO:0003700)	997	530	391.11	+	1.36	1.27E-08
ion binding (GO:0043167)	3686	1949	1445.97	+	1.35	3.66E-40
nucleotide binding (GO:0000166)	2285	1208	896.38	+	1.35	2.01E-22
nucleoside phosphate binding (GO:1901265)	2285	1208	896.38	+	1.35	2.01E-22
small molecule binding (GO:0036094)	2465	1302	966.99	+	1.35	2.22E-24
metal ion binding (GO:0046872)	3472	1831	1362.02	+	1.34	1.59E-36
cation binding (GO:0043169)	3515	1853	1378.89	+	1.34	5.56E-37
calcium ion binding (GO:0005509)	679	357	266.36	+	1.34	1.14E-04
receptor binding (GO:0005102)	1545	804	606.08	+	1.33	2.75E-12
transition metal ion binding (GO:0046914)	1375	709	539.40	+	1.31	8.60E-10
catalytic activity (GO:0003824)	5653	2898	2217.60	+	1.31	8.92E-55
pyrophosphatase activity (GO:0016462)	724	366	284.02	+	1.29	3.00E-03
hydrolase activity, acting on acid anhydrides, in phosphorus-containing anhydrides (GO:0016818)	726	367	284.80	+	1.29	2.91E-03
binding (GO:0005488)	12007	6014	4710.20	+	1.28	9.38E-160
hydrolase activity, acting on acid anhydrides (GO:0016817)	733	367	287.55	+	1.28	6.68E-03
poly(A) RNA binding (GO:0044822)	1181	591	463.29	+	1.28	6.79E-06
DNA binding (GO:0003677)	1817	908	712.79	+	1.27	3.34E-10
nucleoside-triphosphatase activity (GO:0017111)	678	337	265.97	+	1.27	2.95E-02
heterocyclic compound binding (GO:1901363)	5569	2727	2184.65	+	1.25	4.82E-35
organic cyclic compound binding (GO:0097159)	5646	2762	2214.86	+	1.25	2.30E-35
hydrolase activity (GO:0016787)	2384	1155	935.21	+	1.24	2.98E-10
RNA binding (GO:0003723)	1651	769	647.67	+	1.19	2.01E-03
nucleic acid binding (GO:0003676)	3721	1713	1459.70	+	1.17	2.23E-09
molecular_function (GO:0003674)	16806	7710	6592.79	+	1.17	3.54E-154
signal transducer activity (GO:0004871)	2687	803	1054.08	-	.76	2.21E-14
receptor activity (GO:0004872)	2626	718	1030.15	-	.70	3.55E-24
molecular transducer activity (GO:0060089)	2626	718	1030.15	-	.70	3.55E-24
signaling receptor activity (GO:0038023)	2424	621	950.91	-	.65	7.87E-30

transmembrane receptor activity (GO:0099600)	2369	582	929.33	-	.63	2.49E-34
transmembrane signaling receptor activity (GO:0004888)	2326	557	912.46	-	.61	6.71E-37
Unclassified (UNCLASSIFIED)	6975	1619	2736.21	-	.59	0.00E00
structural constituent of ribosome (GO:0003735)	497	113	194.97	-	.58	2.23E-07
G-protein coupled receptor activity (GO:0004930)	1955	368	766.92	-	.48	1.32E-58
olfactory receptor activity (GO:0004984)	1389	167	544.89	-	.31	1.64E-80
odorant binding (GO:0005549)	369	40	144.75	-	.28	8.39E-22
taste receptor activity (GO:0008527)	42	2	16.48	-	< 0.2	2.64E-02
immunoglobulin receptor binding (GO:0034987)	64	2	25.11	-	< 0.2	1.03E-05
