# Supplementary material for: The RNA-Binding Protein hnRNP K Mediates the Effect of BDNF on Dendritic mRNA Metabolism and Regulates Synaptic NMDA Receptors in Hippocampal Neurons
Source: eNeuro. 2017 Dec 12;4(6):ENEURO.0268-17.2017. doi: 10.1523/ENEURO.0268-17.2017 (PMC5732018; doi:10.1523/ENEURO.0268-17.2017)
Supplement: Table 1-5 — Download Table 1-5, PDF file. [file sup_enu-eN-NWR-0268-17-s08.pdf]

Table 1-5 - hnRNP K co-immunoprecipitated transcripts regulated by BDNF

Gene Symbol	Description	Fold change
LOC302228	PREDICTED: Rattus norvegicus similar to Spindlin-like protein 2 (SPIN-2) (LOC302228), mRNA [XM_229842]	**0,085**
Tuba3a	Rattus norvegicus tubulin, alpha 3A (Tuba3a), mRNA [NM_001040008]	**0,204**
Fabp12	Rattus norvegicus fatty acid binding protein 12 (Fabp12), mRNA [NM_001134614]	**0,215**
Slc12a8	Rattus norvegicus solute carrier family 12 (potassium/chloride transporters), member 8 (Slc12a8), mRNA [NM_153625]	**0,222**
Nog	Rattus norvegicus noggin (Nog), mRNA [NM_012990]	**0,225**
0	Unknown	**0,233**
Gpx2	Rattus norvegicus glutathione peroxidase 2 (Gpx2), mRNA [NM_183403]	**0,251**
Ltb	Rattus norvegicus lymphotoxin beta (TNF superfamily, member 3) (Ltb), mRNA [NM_212507]	**0,255**
Ahrr	Rattus norvegicus aryl-hydrocarbon receptor repressor (Ahrr), mRNA [NM_001024285]	**0,271**
0	Uncharacterized protein [Source:UniProtKB/TrEMBL;Acc:D3ZUC5] [ENSRNOT00000046312]	**0,273**
Megf8	PREDICTED: Rattus norvegicus multiple EGF-like-domains 8 (Megf8), mRNA [XM_341803]	**0,274**
Pdcl2	PREDICTED: Rattus norvegicus phosducin-like 2 (Pdcl2), mRNA [XM_001076368]	**0,284**
Mepe	Rattus norvegicus matrix extracellular phosphoglycoprotein (Mepe), mRNA [NM_024142]	**0,289**
Galr3	Rattus norvegicus galanin receptor 3 (Galr3), mRNA [NM_019173]	**0,290**
LOC685685	PREDICTED: Rattus norvegicus similar to S100 calcium-binding protein, ventral prostate (LOC685685), mRNA [XM_001064819]	**0,306**
Sec16b	Rattus norvegicus SEC16 homolog B (S. cerevisiae) (Sec16b), mRNA [NM_053571]	**0,314**
Fam20a	Rattus norvegicus family with sequence similarity 20, member A (Fam20a), mRNA [NM_001012237]	**0,314**
Wnt3	Rattus norvegicus wingless-type MMTV integration site family, member 3 (Wnt3), mRNA [NM_001105715]	**0,314**
Obsl1	Uncharacterized protein [Source:UniProtKB/TrEMBL;Acc:D3ZZ80] [ENSRNOT00000020673]	**0,315**
Ppil4	Rattus norvegicus peptidylprolyl isomerase (cyclophilin)-like 4 (Ppil4), mRNA [NM_001108457]	**0,316**
0	Unknown	**0,320**
Rbm41	Rattus norvegicus RNA binding motif protein 41 (Rbm41), mRNA [NM_001109420]	**0,321**
Pqlc2	Rattus norvegicus PQ loop repeat containing 2 (Pqlc2), mRNA [NM_001108689]	**0,325**
Rassf9	Rattus norvegicus Ras association (RalGDS/AF-6) domain family (N-terminal) member 9 (Rassf9), mRNA [NM_022959]	**0,328**
Cpa2	Rattus norvegicus carboxypeptidase A2 (pancreatic) (Cpa2), mRNA [NM_001013083]	**0,331**
0	PREDICTED: Rattus norvegicus hypothetical protein LOC687735 (LOC687735), mRNA [XM_001079963]	**0,332**
Ano1	Rattus norvegicus anoctamin 1, calcium activated chloride channel (Ano1), mRNA [NM_001107564]	**0,332**
Twistnb	Rattus norvegicus TWIST neighbor (Twistnb), mRNA [NM_001108707]	**0,333**
Lgi2	Rattus norvegicus leucine-rich repeat LGI family, member 2 (Lgi2), mRNA [NM_001107219]	**0,338**
Olr1395	Rattus norvegicus olfactory receptor 1395 (Olr1395), mRNA [NM_001001092]	**0,340**

0	PREDICTED: Rattus norvegicus dynein, axonemal, heavy polypeptide 9 (Dnah9), mRNA [XM_002727722]	**0,342**
Tmem79	Rattus norvegicus transmembrane protein 79 (Tmem79), mRNA [NM_001033896]	**0,343**
LOC682131	PREDICTED: Rattus norvegicus similar to KRAB-zinc finger protein (LOC682131), mRNA [XM_001060021]	**0,344**
Gusb	Rattus norvegicus glucuronidase, beta (Gusb), mRNA [NM_017015]	**0,345**
Pola1	Rattus norvegicus polymerase (DNA directed), alpha 1 (Pola1), mRNA [NM_053479]	**0,346**
Tdrkh	Rattus norvegicus tudor and KH domain containing (Tdrkh), mRNA [NM_001014038]	**0,349**
Top2a	Rattus norvegicus topoisomerase (DNA) II alpha (Top2a), mRNA [NM_022183]	**0,351**
Lrrc55	Rattus norvegicus leucine rich repeat containing 55 (Lrrc55), mRNA [NM_001122975]	**0,352**
Alpk3	Rattus norvegicus alpha-kinase 3 (Alpk3), mRNA [NM_001191895]	**0,353**
0	Rab3 GTPase-activating protein catalytic subunit [Source:UniProtKB/Swiss-Prot;Acc:P69735] [ENSRNOT00000005289]	**0,353**
0	Unknown	**0,353**
Nob1	Rattus norvegicus NIN1/RPN12 binding protein 1 homolog (S. cerevisiae) (Nob1), mRNA [NM_199086]	**0,353**
Gnb1l	Uncharacterized protein [Source:UniProtKB/TrEMBL;Acc:D3ZSP4] [ENSRNOT00000002596]	**0,356**
Cyb5r4	Rattus norvegicus cytochrome b5 reductase 4 (Cyb5r4), mRNA [NM_133427]	**0,358**
Gpatch4	Rattus norvegicus G patch domain containing 4 (Gpatch4), mRNA [NM_001024979]	**0,360**
Plac1l	PREDICTED: Rattus norvegicus placenta-specific 1-like (Plac1l), mRNA [XM_001076035]	**0,360**
Kif1c	Rattus norvegicus kinesin family member 1C (Kif1c), mRNA [NM_145877]	**0,361**
Chmp4bl1	PREDICTED: Rattus norvegicus similar to Charged multivesicular body protein 4b (Chromatin-modifying protein 4b) (CHMP4b) (LOC679886), mRNA [XM_002726346]	**0,363**
Hexim2	Rattus norvegicus hexamthylene bis-acetamide inducible 2 (Hexim2), mRNA [NM_001107054]	**0,363**
Atl3	Rattus norvegicus atlastin GTPase 3 (Atl3), mRNA [NM_001044241]	**0,363**
Slc25a37	Rattus norvegicus solute carrier family 25, member 37 (Slc25a37), nuclear gene encoding mitochondrial protein, mRNA [NM_001013996]	**0,363**
Ctxn3	Rattus norvegicus cortexin 3 (Ctxn3), mRNA [NM_001134696]	**0,365**
RGD1565690	Uncharacterized protein [Source:UniProtKB/TrEMBL;Acc:D3ZF21] [ENSRNOT00000039316]	**0,365**
0	PREDICTED: Rattus norvegicus similar to RIKEN cDNA D230037D09 gene (RGD1566396), mRNA [XM_001067433]	**0,365**
Alb	Rattus norvegicus albumin (Alb), mRNA [NM_134326]	**0,366**
0	Uncharacterized protein [Source:UniProtKB/TrEMBL;Acc:D3ZKC7] [ENSRNOT00000064975]	**0,366**
Mapk12	Rattus norvegicus mitogen-activated protein kinase 12 (Mapk12), mRNA [NM_021746]	**0,367**
Stfa2l1	Rattus norvegicus stefin A2-like 1 (Stfa2l1), mRNA [NM_001004129]	**0,369**
Abcf2	Rattus norvegicus ATP-binding cassette, subfamily F (GCN20), member 2 (Abcf2), nuclear gene encoding mitochondrial protein, mRNA [NM_001109666]	**0,369**
0	Unknown	**0,373**
Mcm6	Rattus norvegicus minichromosome maintenance complex component 6 (Mcm6), mRNA [NM_017287]	**0,374**
LOC685953	Platelet glycoprotein 4 [Source:UniProtKB/Swiss-Prot;Acc:Q07969] [ENSRNOT00000066224]	**0,375**

Hspa2	Rattus norvegicus heat shock protein 2 (Hspa2), mRNA [NM_021863]	**0,376**
Lrrc48	Rattus norvegicus leucine rich repeat containing 48 (Lrrc48), mRNA [NM_001013857]	**0,376**
Btg3	Rattus norvegicus BTG family, member 3 (Btg3), mRNA [NM_019290]	**0,377**
Mcpt1l4	Mast cell protease 1-like 4 [Source:UniProtKB/TrEMBL;Acc:Q6IE57] [ENSRNOT00000043182]	**0,379**
Fam53a	Rattus norvegicus family with sequence similarity 53, member A (Fam53a), mRNA [NM_001107228]	**0,379**
0	Unknown	**0,380**
Manba	Rattus norvegicus mannosidase, beta A, lysosomal (Manba), mRNA [NM_001031655]	**0,381**
LOC691692	PREDICTED: Rattus norvegicus hypothetical protein LOC691692 (LOC691692), mRNA [XM_001079285]	**0,381**
Piwil2	Rattus norvegicus piwi-like 2 (Drosophila) (Piwil2), mRNA [NM_001107276]	**0,382**
0	Unknown	**0,383**
Med6	Rattus norvegicus mediator complex subunit 6 (Med6), mRNA [NM_001106742]	**0,386**
0	Unknown	**0,387**
Bclaf1	Rattus norvegicus BCL2-associated transcription factor 1 (Bclaf1), mRNA [NM_001047852]	**0,387**
Itm2a	Rattus norvegicus integral membrane protein 2A (Itm2a), mRNA [NM_001025712]	**0,389**
Pcdha1	Rattus norvegicus protocadherin alpha 1 (Pcdha1), mRNA [NM_199503]	**0,390**
RGD1305627	Rattus norvegicus hypothetical LOC314467 (RGD1305627), mRNA [NM_001134560]	**0,391**
LOC502876	PREDICTED: Rattus norvegicus similar to protein phosphatase 1, regulatory subunit 15B (LOC502876), miscRNA [XR_086238]	**0,391**
Cenpl	Rattus norvegicus centromere protein L (Cenpl), mRNA [NM_001033061]	**0,392**
Rnf168	Rattus norvegicus ring finger protein 168 (Rnf168), mRNA [NM_001127597]	**0,393**
Glt8d3	Rattus norvegicus glycosyltransferase 8 domain containing 3 (Glt8d3), mRNA [NM_001100887]	**0,393**
N4bp2	Uncharacterized protein [Source:UniProtKB/TrEMBL;Acc:D3ZLS6] [ENSRNOT00000031792]	**0,393**
Eml4	Rattus norvegicus echinoderm microtubule associated protein like 4 (Eml4), mRNA [NM_001108008]	**0,394**
Slco4a1	Rattus norvegicus solute carrier organic anion transporter family, member 4a1 (Slco4a1), mRNA [NM_133608]	**0,394**
Stim2	Rattus norvegicus stromal interaction molecule 2 (Stim2), mRNA [NM_001105750]	**0,395**
Acan	Rattus norvegicus aggrecan (Acan), mRNA [NM_022190]	**0,396**
RGD1565309	PREDICTED: Rattus norvegicus similar to hypothetical protein MGC33370 (RGD1565309), mRNA [XM_573520]	**0,396**
0	predicted gene 884 Gene [Source:MGI Symbol;Acc:MGI:2685730] [ENSRNOT00000043156]	**0,396**
Clgn	Rattus norvegicus calmegin (Clgn), mRNA [NM_001109472]	**0,397**
0	Uncharacterized protein [Source:UniProtKB/TrEMBL;Acc:D4A3R8] [ENSRNOT00000049208]	**0,399**
C1ql4	Complement component 1, q subcomponent-like 4 (Predicted)Uncharacterized protein [Source:UniProtKB/TrEMBL;Acc:D3ZMN4] [ENSRNOT00000003152]	**0,399**
Gas2l1	Rattus norvegicus growth arrest-specific 2 like 1 (Gas2l1), mRNA [NM_001108365]	**0,400**
0	Unknown	**0,400**
Ttk	Rattus norvegicus Ttk protein kinase (Ttk), mRNA [NM_001108172]	**0,400**

Crebzf	Rattus norvegicus CREB/ATF bZIP transcription factor (Crebzf), mRNA [NM_001106279]	**0,401**
Fanca	Rattus norvegicus Fanconi anemia, complementation group A (Fanca), mRNA [NM_001108455]	**0,403**
Osbpl11	Rattus norvegicus oxysterol binding protein-like 11 (Osbpl11), mRNA [NM_001107090]	**0,403**
Tfeb	Rattus norvegicus transcription factor EB (Tfeb), mRNA [NM_001025707]	**0,403**
Ifi44	Rattus norvegicus interferon-induced protein 44 (Ifi44), mRNA [NM_001107729]	**0,403**
0	Unknown	**0,404**
LOC679824	PREDICTED: Rattus norvegicus hypothetical protein LOC679824 (LOC679824), miscRNA [XR_085739]	**0,405**
0	Unknown	**0,405**
Ndufb7	Rattus norvegicus NADH dehydrogenase (ubiquinone) 1 beta subcomplex, 7 (Ndufb7), nuclear gene encoding mitochondrial protein, mRNA [NM_001108442]	**0,405**
0	Unknown	**0,406**
Rrs1	Rattus norvegicus RRS1 ribosome biogenesis regulator homolog (S. cerevisiae) (Rrs1), mRNA [NM_001079699]	**0,406**
LOC100362255	PREDICTED: Rattus norvegicus G protein-coupled receptor 98 (LOC100362255), mRNA [XM_002729130]	**0,406**
Acot4	Rattus norvegicus acyl-CoA thioesterase 4 (Acot4), mRNA [NM_001109440]	**0,406**
Sec23ip	Rattus norvegicus SEC23 interacting protein (Sec23ip), mRNA [NM_001134859]	**0,407**
Timeless	Rattus norvegicus timeless homolog (Drosophila) (Timeless), mRNA [NM_031340]	**0,408**
Thnsl1	Rattus norvegicus threonine synthase-like 1 (S. cerevisiae) (Thnsl1), mRNA [NM_001025035]	**0,409**
Tchp	Rattus norvegicus trichoplein, keratin filament binding (Tchp), mRNA [NM_001191666]	**0,410**
Zfp398	Rattus norvegicus zinc finger protein 398 (Zfp398), mRNA [NM_001109230]	**0,411**
Renbp	Rattus norvegicus renin binding protein (Renbp), mRNA [NM_031095]	**0,411**
0	Unknown	**0,412**
Aen	Rattus norvegicus apoptosis enhancing nuclease (Aen), mRNA [NM_001108487]	**0,413**
0	Unknown	**0,414**
RGD1566052	Rattus norvegicus similar to elongation protein 4 homolog (RGD1566052), mRNA [NM_001115047]	**0,414**
0	Uncharacterized protein [Source:UniProtKB/TrEMBL;Acc:D4A0Y6] [ENSRNOT00000038176]	**0,414**
Rad51	Rattus norvegicus RAD51 homolog (RecA homolog, E. coli) (S. cerevisiae) (Rad51), mRNA [NM_001109204]	**0,414**
Nrf1	Rattus norvegicus nuclear respiratory factor 1 (Nrf1), mRNA [NM_001100708]	**0,414**
Atp9b	Rattus norvegicus ATPase, class II, type 9B (Atp9b), mRNA [NM_001106130]	**0,415**
0	Unknown	**0,415**
Rrm1-ps1	PREDICTED: Rattus norvegicus similar to Ribonucleotide reductase M1 (LOC364182), miscRNA [XR_086014]	**0,415**
Aco2	Rattus norvegicus aconitase 2, mitochondrial (Aco2), nuclear gene encoding mitochondrial protein, mRNA [NM_024398]	**0,415**
Dhodh	Rattus norvegicus dihydroorotate dehydrogenase (Dhodh), nuclear gene encoding mitochondrial protein, mRNA [NM_001008553]	**0,415**
Mrs2	Rattus norvegicus MRS2 magnesium homeostasis factor homolog (S. cerevisiae) (Mrs2), mRNA [NM_024001]	**0,415**
Nme6	Rattus norvegicus non-metastatic cells 6, protein expressed in (nucleoside-diphosphate kinase) (Nme6), mRNA [NM_001191884]	**0,415**

Cnga1	Rattus norvegicus cyclic nucleotide gated channel alpha 1 (Cnga1), mRNA [NM_053497]	**0,416**
0	PREDICTED: Rattus norvegicus similar to Multisynthetase complex auxiliary component p43 (LOC365837), miscRNA [XR_005797]	**0,416**
RGD1306001	Rattus norvegicus similar to 2210021J22Rik protein (RGD1306001), mRNA [NM_001130696]	**0,416**
Galr2	Rattus norvegicus galanin receptor 2 (Galr2), mRNA [NM_019172]	**0,417**
Slu7	Rattus norvegicus SLU7 splicing factor homolog (S. cerevisiae) (Slu7), mRNA [NM_001100550]	**0,417**
Taf8	Rattus norvegicus TAF8 RNA polymerase II, TATA box binding protein (TBP)-associated factor (Taf8), mRNA [NM_001108197]	**0,417**
Tmsb10	Rattus norvegicus thymosin, beta 10 (Tmsb10), mRNA [NM_021261]	**0,417**
LOC678766	PREDICTED: Rattus norvegicus similar to ras homolog gene family, member U (LOC678766), mRNA [XM_001053087]	**0,418**
0	Unknown	**0,418**
0	Uncharacterized protein [Source:UniProtKB/TrEMBL;Acc:D4A0Y6] [ENSRNOT00000038176]	**0,419**
Smarca4	Rattus norvegicus SWI/SNF related, matrix associated, actin dependent regulator of chromatin, subfamily a, member 4 (Smarca4), mRNA [NM_134368]	**0,419**
Itfg3	Rattus norvegicus integrin alpha FG-GAP repeat containing 3 (Itfg3), mRNA [NM_001009701]	**0,419**
RGD1307465	Rattus norvegicus similar to RIKEN cDNA 8430406I07 (RGD1307465), mRNA [NM_001009655]	**0,420**
0	Unknown	**0,420**
0	Protein FAM162A [Source:UniProtKB/Swiss-Prot;Acc:Q4QQV3] [ENSRNOT00000003069]	**0,421**
0	RVL23074 Wackym-Soares normalized rat vestibular cDNA library Rattus norvegicus cDNA 5', mRNA sequence [DY471857]	**0,421**
F2rl2	Rattus norvegicus coagulation factor II (thrombin) receptor-like 2 (F2rl2), mRNA [NM_053313]	**0,421**
Echdc2	Rattus norvegicus enoyl Coenzyme A hydratase domain containing 2 (Echdc2), mRNA [NM_001106675]	**0,421**
Mkx	Iroquois homeobox protein-like 1 (Predicted)Uncharacterized protein [Source:UniProtKB/TrEMBL;Acc:D3ZUL2] [ENSRNOT00000025623]	**0,422**
0	Unknown	**0,422**
Prl6a1	Rattus norvegicus prolactin family 6, subfamily a, member 1 (Prl6a1), mRNA [NM_022176]	**0,422**
Iqub	Rattus norvegicus IQ motif and ubiquitin domain containing (Iqub), mRNA [NM_001034130]	**0,423**
LOC690326	Rattus norvegicus hypothetical protein LOC690326 (LOC690326), mRNA [NM_001109578]	**0,424**
Ofd1	Rattus norvegicus oral-facial-digital syndrome 1 gene homolog (human) (Ofd1), mRNA [NM_001106961]	**0,424**
Prpf19	Rattus norvegicus PRP19/PSO4 pre-mRNA processing factor 19 homolog (S. cerevisiae) (Prpf19), mRNA [NM_139333]	**0,424**
Zfp786	Rattus norvegicus zinc finger protein 786 (Zfp786), mRNA [NM_001127594]	**0,424**
Kbtbd2	Rattus norvegicus kelch repeat and BTB (POZ) domain containing 2 (Kbtbd2), mRNA [NM_001107861]	**0,424**
RGD1304931	Rattus norvegicus similar to RIKEN cDNA 2310042D19 (RGD1304931), mRNA [NM_001108001]	**0,424**
Tmem121	Uncharacterized protein [Source:UniProtKB/TrEMBL;Acc:D3ZMR5] [ENSRNOT00000006872]	**0,425**
Mrc2	Rattus norvegicus mannose receptor, C type 2 (Mrc2), mRNA [NM_001024687]	**0,425**
Ifltd1	Rattus norvegicus intermediate filament tail domain containing 1 (Ifltd1), mRNA [NM_001191980]	**0,425**
Polr3g	Rattus norvegicus polymerase (RNA) III (DNA directed) polypeptide G (Polr3g), mRNA [NM_001109468]	**0,425**
Yif1	Rattus norvegicus Yip1 interacting factor homolog (S. cerevisiae) (Yif1), mRNA [NM_172017]	**0,425**

Crem	Rattus norvegicus cAMP responsive element modulator (Crem), transcript variant 3, mRNA [NM_001110860]	**0,426**
RGD1560672	Rattus norvegicus similar to novel protein (RGD1560672), mRNA [NM_001109073]	**0,426**
Tlr6	Rattus norvegicus toll-like receptor 6 (Tlr6), mRNA [NM_207604]	**0,426**
0	Unknown	**0,426**
LOC100158225	Rattus norvegicus hypothetical protein LOC100158225 (LOC100158225), mRNA [NM_001127606]	**0,426**
Sdccag10	Rattus norvegicus serologically defined colon cancer antigen 10 (Sdccag10), mRNA [NM_001013199]	**0,428**
Lrrc14	Rattus norvegicus leucine rich repeat containing 14 (Lrrc14), mRNA [NM_001024354]	**0,428**
Clp1	Rattus norvegicus CLP1, cleavage and polyadenylation factor I subunit, homolog (S. cerevisiae) (Clp1), mRNA [NM_001009599]	**0,428**
Faim	Rattus norvegicus Fas apoptotic inhibitory molecule (Faim), mRNA [NM_080895]	**0,429**
Dync1i1	Rattus norvegicus dynein cytoplasmic 1 intermediate chain 1 (Dync1i1), mRNA [NM_019234]	**0,429**
Vip	Rattus norvegicus vasoactive intestinal peptide (Vip), mRNA [NM_053991]	**0,430**
RGD1559859	PREDICTED: Rattus norvegicus RGD1559859 (RGD1559859), mRNA [XM_001072031]	**0,430**
Slc31a2	Rattus norvegicus solute carrier family 31 (copper transporters), member 2 (Slc31a2), mRNA [NM_001033693]	**0,430**
Hs3st5	Rattus norvegicus heparan sulfate (glucosamine) 3-O-sulfotransferase 5 (Hs3st5), mRNA [NM_001106392]	**0,430**
LOC683410	PREDICTED: Rattus norvegicus similar to ankyrin repeat domain 33, transcript variant 2 (LOC683410), mRNA [XM_002726976]	**0,430**
0	Unknown	**0,431**
Cadps2	PREDICTED: Rattus norvegicus Ca++-dependent secretion activator 2, transcript variant 1 (Cadps2), mRNA [XM_001060172]	**0,431**
Adra1b	Rattus norvegicus adrenergic, alpha-1B-, receptor (Adra1b), mRNA [NM_016991]	**0,431**
Anp32b	Rattus norvegicus acidic (leucine-rich) nuclear phosphoprotein 32 family, member B (Anp32b), mRNA [NM_131911]	**0,432**
RGD1312005	Rattus norvegicus similar to DD1 (RGD1312005), mRNA [NM_001106144]	**0,432**
RGD1304879	Rattus norvegicus similar to Zinc finger protein 398 (Zinc finger DNA binding protein p52/p71) (RGD1304879), mRNA [NM_001014056]	**0,432**
Btg1	Rattus norvegicus B-cell translocation gene 1, anti-proliferative (Btg1), mRNA [NM_017258]	**0,432**
Slc39a7	Rattus norvegicus solute carrier family 39 (zinc transporter), member 7 (Slc39a7), transcript variant 2, mRNA [NM_001008885]	**0,433**
0	Unknown	**0,433**
Tut1	Rattus norvegicus terminal uridylyl transferase 1, U6 snRNA-specific (Tut1), mRNA [NM_001033901]	**0,433**
Adcy9	Rattus norvegicus adenylate cyclase 9 (Adcy9), mRNA [NM_001106980]	**0,433**
Cage1	Rattus norvegicus cancer antigen 1 (Cage1), mRNA [NM_001012052]	**0,433**
Mettl6	Rattus norvegicus methyltransferase like 6 (Mettl6), mRNA [NM_001007623]	**0,433**
Spag5	Rattus norvegicus sperm associated antigen 5 (Spag5), mRNA [NM_001044224]	**0,434**
Zmynd19	Rattus norvegicus zinc finger, MYND-type containing 19 (Zmynd19), mRNA [NM_198770]	**0,434**
Rrp15	Rattus norvegicus ribosomal RNA processing 15 homolog (S. cerevisiae) (Rrp15), mRNA [NM_001009702]	**0,434**
Mkrn1	Rattus norvegicus makorin ring finger protein 1 (Mkrn1), mRNA [NM_001004233]	**0,434**
Zic5	Rattus norvegicus Zic family member 5 (odd-paired homolog, Drosophila) (Zic5), mRNA [NM_001108391]	**0,434**
LOC690422	PREDICTED: Rattus norvegicus hypothetical protein LOC690422, transcript variant 2 (LOC690422), mRNA [XM_001072932]	**0,435**

Slc22a25	Rattus norvegicus solute carrier family 22, member 25 (Slc22a25), mRNA [NM_138908]	**0,435**
Crisp1	Rattus norvegicus cysteine-rich secretory protein 1 (Crisp1), mRNA [NM_022859]	**0,436**
0	Uncharacterized protein [Source:UniProtKB/TrEMBL;Acc:D3ZWP2] [ENSRNOT00000025138]	**0,436**
0	Q9QXT9_MOUSE (Q9QXT9) KID2 (Zinc finger protein 354B), partial (12%) [TC642135]	**0,437**
Gda	Rattus norvegicus guanine deaminase (Gda), mRNA [NM_031776]	**0,437**
Cyp4v3	Rattus norvegicus cytochrome P450, family 4, subfamily v, polypeptide 3 (Cyp4v3), mRNA [NM_001135600]	**0,437**
Zfp105	Rattus norvegicus zinc finger protein 105 (Zfp105), mRNA [NM_001012128]	**0,437**
Olr868	Rattus norvegicus olfactory receptor 868 (Olr868), mRNA [NM_001000821]	**0,437**
Znf711	Uncharacterized protein [Source:UniProtKB/TrEMBL;Acc:D3ZHB2] [ENSRNOT00000006237]	**0,437**
Tnfaip2	Rattus norvegicus tumor necrosis factor, alpha-induced protein 2 (Tnfaip2), mRNA [NM_001137633]	**0,437**
Ambp	Rattus norvegicus alpha-1-microglobulin/bikunin precursor (Ambp), mRNA [NM_012901]	**0,438**
Gnrhr	Rattus norvegicus gonadotropin releasing hormone receptor (Gnrhr), mRNA [NM_031038]	**0,438**
Zfp276	Uncharacterized protein [Source:UniProtKB/TrEMBL;Acc:D4A710] [ENSRNOT00000022324]	**0,438**
Hsp90ab1	Rattus norvegicus heat shock protein 90 alpha (cytosolic), class B member 1 (Hsp90ab1), mRNA [NM_001004082]	**0,439**
Cops3	Rattus norvegicus COP9 constitutive photomorphogenic homolog subunit 3 (Arabidopsis) (Cops3), mRNA [NM_001004200]	**0,439**
Ppil4	peptidyl-prolyl cis-trans isomerase-like 4 [Source:RefSeq peptide;Acc:NP_001101927] [ENSRNOT00000021437]	**0,439**
Hsp90aa1	Rattus norvegicus heat shock protein 90, alpha (cytosolic), class A member 1 (Hsp90aa1), mRNA [NM_175761]	**0,439**
0	Unknown	**0,439**
Commd3	Rattus norvegicus COMM domain containing 3 (Commd3), mRNA [NM_198732]	**0,439**
0	Ryanodine receptor 1 [Source:UniProtKB/TrEMBL;Acc:O35208] [ENSRNOT00000027893]	**0,440**
Snrnp35	Rattus norvegicus small nuclear ribonucleoprotein 35 (U11/U12) (Snrnp35), mRNA [NM_001014127]	**0,440**
Gnai3	Rattus norvegicus guanine nucleotide binding protein (G protein), alpha inhibiting 3 (Gnai3), mRNA [NM_013106]	**0,440**
Pcdha3	Rattus norvegicus protocadherin alpha 3 (Pcdha3), mRNA [NM_053941]	**0,441**
Abca3	PREDICTED: Rattus norvegicus ATP-binding cassette, sub-family A (ABC1), member 3 (Abca3), mRNA [XM_220219]	**0,441**
Smurf2	Rattus norvegicus SMAD specific E3 ubiquitin protein ligase 2 (Smurf2), mRNA [NM_001107061]	**0,441**
Smc6	Rattus norvegicus structural maintenance of chromosomes 6 (Smc6), mRNA [NM_001108014]	**0,442**
Chga	Rattus norvegicus chromogranin A (Chga), mRNA [NM_021655]	**0,442**
0	Q69Z61_MOUSE (Q69Z61) MKIAA1935 protein (Fragment), partial (13%) [TC582589]	**0,442**
Bspry	Rattus norvegicus B-box and SPRY domain containing (Bspry), mRNA [NM_022261]	**0,443**
Rsl1d1	Rattus norvegicus ribosomal L1 domain containing 1 (Rsl1d1), mRNA [NM_001008876]	**0,443**
LOC100233176	Rattus norvegicus hypothetical protein LOC100233176 (LOC100233176), transcript variant 2, mRNA [NM_001142942]	**0,443**
Cisd2	Rattus norvegicus CDGSH iron sulfur domain 2 (Cisd2), mRNA [NM_001191608]	**0,443**
Grem1	Rattus norvegicus gremlin 1, cysteine knot superfamily, homolog (Xenopus laevis) (Grem1), mRNA [NM_019282]	**0,443**
Anks3	Rattus norvegicus ankyrin repeat and sterile alpha motif domain containing 3 (Anks3), mRNA [NM_001009676]	**0,443**

Surf6	Rattus norvegicus surfeit 6 (Surf6), mRNA [NM_001015014]	**0,443**
0	Unknown	**0,443**
Slc5a7	Rattus norvegicus solute carrier family 5 (choline transporter), member 7 (Slc5a7), mRNA [NM_053521]	**0,443**
Dhx35	Rattus norvegicus DEAH (Asp-Glu-Ala-His) box polypeptide 35 (Dhx35), mRNA [NM_001108601]	**0,443**
Zbed4	Rattus norvegicus zinc finger, BED-type containing 4 (Zbed4), mRNA [NM_001134800]	**0,444**
Fbp2	Rattus norvegicus fructose-1,6-bisphosphatase 2 (Fbp2), mRNA [NM_053716]	**0,444**
0	Histone H3 [Source:UniProtKB/TrEMBL;Acc:D3ZCY4] [ENSRNOT00000043897]	**0,444**
Rtn4	Rattus norvegicus reticulon 4 (Rtn4), mRNA [NM_031831]	**0,444**
Apba3	Rattus norvegicus amyloid beta (A4) precursor protein-binding, family A, member 3 (Apba3), mRNA [NM_031781]	**0,444**
Plbd2	Rattus norvegicus phospholipase B domain containing 2 (Plbd2), mRNA [NM_139255]	**0,444**
Med27	Rattus norvegicus mediator complex subunit 27 (Med27), mRNA [NM_001106565]	**0,444**
RGD1564048	PREDICTED: Rattus norvegicus similar to Protein C20orf46 (RGD1564048), mRNA [XM_001060786]	**0,444**
Afap1l1	Rattus norvegicus actin filament associated protein 1-like 1 (Afap1l1), mRNA [NM_001106142]	**0,445**
Gemin4	Rattus norvegicus gem (nuclear organelle) associated protein 4 (Gemin4), mRNA [NM_001109037]	**0,445**
Mdfic	Rattus norvegicus MyoD family inhibitor domain containing (Mdfic), mRNA [NM_001105668]	**0,445**
Slc35c2	Rattus norvegicus solute carrier family 35, member C2 (Slc35c2), mRNA [NM_001107803]	**0,445**
RGD1563091	PREDICTED: Rattus norvegicus similar to OEF2 (RGD1563091), mRNA [XM_575365]	**0,446**
Polr3b	Rattus norvegicus polymerase (RNA) III (DNA directed) polypeptide B (Polr3b), mRNA [NM_001191878]	**0,446**
Grpel2	Rattus norvegicus GrpE-like 2, mitochondrial (Grpel2), nuclear gene encoding mitochondrial protein, mRNA [NM_001109513]	**0,446**
Loxl1	Rattus norvegicus lysyl oxidase-like 1 (Loxl1), mRNA [NM_001012125]	**0,446**
Styk1	Similar to NOK kinase (Predicted)Uncharacterized protein [Source:UniProtKB/TrEMBL;Acc:D3ZHY0] [ENSRNOT00000013833]	**0,447**
Rnf219	Rattus norvegicus ring finger protein 219 (Rnf219), mRNA [NM_001108389]	**0,447**
0	PREDICTED: Rattus norvegicus mCG114897-like (LOC100363987), miscRNA [XR_085743]	**0,447**
Il17ra	Rattus norvegicus interleukin 17 receptor A (Il17ra), mRNA [NM_001107883]	**0,447**
Prkar1a	Rattus norvegicus protein kinase, cAMP-dependent, regulatory, type I, alpha (Prkar1a), mRNA [NM_013181]	**0,447**
Trim36	Rattus norvegicus tripartite motif-containing 36 (Trim36), mRNA [NM_001106147]	**0,447**
Tbc1d2b	Rattus norvegicus TBC1 domain family, member 2B (Tbc1d2b), mRNA [NM_001108175]	**0,447**
0	Unknown	**0,447**
Ddx20	Rattus norvegicus DEAD (Asp-Glu-Ala-Asp) box polypeptide 20 (Ddx20), mRNA [NM_001191711]	**0,447**
Kcnmb4	Rattus norvegicus potassium large conductance calcium-activated channel, subfamily M, beta member 4 (Kcnmb4), mRNA [NM_023960]	**0,448**
Gpsm2	Rattus norvegicus G-protein signaling modulator 2 (AGS3-like, C. elegans) (Gpsm2), mRNA [NM_001191962]	**0,448**
Ccpg1	Rattus norvegicus cell cycle progression 1 (Ccpg1), mRNA [NM_001108770]	**0,448**
Mrpl32	Rattus norvegicus mitochondrial ribosomal protein L32 (Mrpl32), nuclear gene encoding mitochondrial protein, mRNA [NM_001106116]	**0,448**
Fbxo2	Rattus norvegicus F-box protein 2 (Fbxo2), mRNA [NM_053511]	**0,449**

Sar1a	Rattus norvegicus SAR1 homolog A (S. cerevisiae) (Sar1a), mRNA [NM_001007739]	**0,449**
Stard4	Rattus norvegicus StAR-related lipid transfer (START) domain containing 4 (Stard4), mRNA [NM_001106159]	**0,449**
0	LRRGT00148 [Source:UniProtKB/TrEMBL;Acc:Q6QI60] [ENSRNOT00000058693]	**0,449**
Kcnh2	Rattus norvegicus potassium voltage-gated channel, subfamily H (eag-related), member 2 (Kcnh2), mRNA [NM_053949]	**0,450**
Larp6	Rattus norvegicus La ribonucleoprotein domain family, member 6 (Larp6), mRNA [NM_001108154]	**0,450**
Chd1	Rattus norvegicus chromodomain helicase DNA binding protein 1 (Chd1), mRNA [NM_001107465]	**0,450**
Rbm20	Rattus norvegicus RNA binding motif protein 20 (Rbm20), mRNA [NM_001107611]	**0,450**
0	Unknown	**0,450**
LOC681309	PREDICTED: Rattus norvegicus similar to Thrombospondin-3 precursor (LOC681309), miscRNA [XR_006034]	**0,450**
Stk35	Rattus norvegicus serine/threonine kinase 35 (Stk35), mRNA [NM_001107773]	**0,450**
Fam135a	Rattus norvegicus family with sequence similarity 135, member A (Fam135a), mRNA [NM_001134597]	**0,451**
Dohh	Rattus norvegicus deoxyhypusine hydroxylase/monooxygenase (Dohh), mRNA [NM_001025006]	**0,451**
0	Unknown	**0,451**
Fcer1g	Rattus norvegicus Fc fragment of IgE, high affinity I, receptor for; gamma polypeptide (Fcer1g), mRNA [NM_001131001]	**0,451**
Fibcd1	Rattus norvegicus fibrinogen C domain containing 1 (Fibcd1), mRNA [NM_001107829]	**0,451**
Bex1	Rattus norvegicus brain expressed, X-linked 1 (Bex1), mRNA [NM_001037365]	**0,451**
Tmx4	Rattus norvegicus thioredoxin-related transmembrane protein 4 (Tmx4), mRNA [NM_001100529]	**0,451**
Ppap2c	Rattus norvegicus phosphatidic acid phosphatase type 2c (Ppap2c), mRNA [NM_139252]	**0,451**
Ccdc34	Rattus norvegicus coiled-coil domain containing 34 (Ccdc34), mRNA [NM_001108587]	**0,451**
0	Unknown	**0,451**
Zbtb5	Rattus norvegicus zinc finger and BTB domain containing 5 (Zbtb5), mRNA [NM_001106657]	**0,452**
Ropn1l	Rattus norvegicus ropporin 1-like (Ropn1l), mRNA [NM_001191086]	**0,452**
0	Unknown	**0,452**
Fam175b	Rattus norvegicus family with sequence similarity 175, member B (Fam175b), mRNA [NM_001106307]	**0,452**
Znf804a	Rattus norvegicus similar to hypothetical protein (LOC295695), mRNA [XM_230042]	**0,452**
Kidins220	Rattus norvegicus kinase D-interacting substrate 220 (Kidins220), mRNA [NM_053795]	**0,452**
Myh7	Rattus norvegicus myosin, heavy chain 7, cardiac muscle, beta (Myh7), mRNA [NM_017240]	**0,452**
Grinl1a	Rattus norvegicus glutamate receptor, ionotropic, N-methyl D-aspartate-like 1A (Grinl1a), mRNA [NM_183402]	**0,452**
0	Unknown	**0,452**
Stard3	Rattus norvegicus StAR-related lipid transfer (START) domain containing 3 (Stard3), mRNA [NM_001014229]	**0,452**
Olr1519	Rattus norvegicus olfactory receptor 1519 (Olr1519), mRNA [NM_001000037]	**0,452**
Slc32a1	Rattus norvegicus solute carrier family 32 (GABA vesicular transporter), member 1 (Slc32a1), mRNA [NM_031782]	**0,453**
0	Unknown	**0,453**
0	Unknown	**0,453**

0	PREDICTED: Rattus norvegicus similar to peptide deformylase-like protein (LOC690214), mRNA [XM_001073696]	**0,453**
Dync1i2	Rattus norvegicus dynein cytoplasmic 1 intermediate chain 2 (Dync1i2), mRNA [NM_053880]	**0,453**
Ndfip2	Rattus norvegicus Nedd4 family interacting protein 2 (Ndfip2), mRNA [NM_001108390]	**0,453**
Svop	Rattus norvegicus SV2 related protein (Svop), mRNA [NM_134404]	**0,453**
RGD1559841	PREDICTED: Rattus norvegicus similar to expressed sequence AW413431 (RGD1559841), mRNA [XM_002725411]	**0,454**
0	Rattus norvegicus similar to KIAA1895 protein (LOC293775), mRNA [XM_219621]	**0,454**
0	IQ domain-containing protein D [Source:UniProtKB/Swiss-Prot;Acc:Q5XIR6] [ENSRNOT00000063923]	**0,454**
Mrps14	Rattus norvegicus mitochondrial ribosomal protein S14 (Mrps14), nuclear gene encoding mitochondrial protein, mRNA [NM_001105963]	**0,454**
Exosc2	Rattus norvegicus exosome component 2 (Exosc2), mRNA [NM_001108952]	**0,454**
Tmem57	Rattus norvegicus transmembrane protein 57 (Tmem57), mRNA [NM_001025699]	**0,454**
0	Leucine-rich repeat-containing protein KIAA1731 homolog [Source:UniProtKB/Swiss-Prot;Acc:A4L9P8] [ENSRNOT00000029634]	**0,455**
Thap3	Rattus norvegicus THAP domain containing, apoptosis associated protein 3 (Thap3), mRNA [NM_001108695]	**0,455**
Zfp364	Rattus norvegicus zinc finger protein 364 (Zfp364), mRNA [NM_001108560]	**0,455**
0	Uncharacterized protein [Source:UniProtKB/TrEMBL;Acc:D3ZYM6] [ENSRNOT00000057216]	**0,455**
Cyc1	Rattus norvegicus cytochrome c-1 (Cyc1), nuclear gene encoding mitochondrial protein, mRNA [NM_001130491]	**0,455**
Rtf1	Rattus norvegicus Rtf1, Paf1/RNA polymerase II complex component, homolog (S. cerevisiae) (Rtf1), mRNA [NM_001108958]	**0,456**
LOC499779	Uncharacterized protein C9orf119 homolog [Source:UniProtKB/Swiss-Prot;Acc:Q63ZV7] [ENSRNOT00000030558]	**0,456**
Cdc42se2	Rattus norvegicus CDC42 small effector 2 (Cdc42se2), mRNA [NM_001126089]	**0,456**
0	Unknown	**0,456**
Tmem107	Rattus norvegicus transmembrane protein 107 (Tmem107), mRNA [NM_001109648]	**0,456**
Slc5a1	Rattus norvegicus solute carrier family 5 (sodium/glucose cotransporter), member 1 (Slc5a1), mRNA [NM_013033]	**0,457**
Rest	Rattus norvegicus RE1-silencing transcription factor (Rest), mRNA [NM_031788]	**0,457**
Hexdc	Rattus norvegicus hexosaminidase (glycosyl hydrolase family 20, catalytic domain) containing (Hexdc), mRNA [NM_001142562]	**0,457**
Hspa13	Rattus norvegicus heat shock protein 13 (Hspa13), mRNA [NM_019271]	**0,457**
Dusp26	Rattus norvegicus dual specificity phosphatase 26 (putative) (Dusp26), mRNA [NM_001012352]	**0,457**
RGD1308299	Rattus norvegicus similar to chromosome 6 open reading frame 130 (RGD1308299), mRNA [NM_001134596]	**0,457**
RGD1309906	Rattus norvegicus similar to RIKEN cDNA 2310004I24 gene (RGD1309906), mRNA [NM_001009246]	**0,457**
Klhl8	Rattus norvegicus kelch-like 8 (Drosophila) (Klhl8), mRNA [NM_001105995]	**0,457**
Clcc1	Rattus norvegicus chloride channel CLIC-like 1 (Clcc1), mRNA [NM_133414]	**0,457**
0	Unknown	**0,457**
LOC688310	PREDICTED: Rattus norvegicus similar to CG5500-PA (LOC688310), mRNA [XM_001081793]	**0,457**
0	Unknown	**0,457**
Kctd11	Rattus norvegicus potassium channel tetramerisation domain containing 11 (Kctd11), mRNA [NM_001108831]	**0,457**
Bmp3	Rattus norvegicus bone morphogenetic protein 3 (Bmp3), mRNA [NM_017105]	**0,458**

Pus10	pseudouridylate synthase 10 [Source:RefSeq peptide;Acc:NP_001020449] [ENSRNOT00000008115]	**0,458**
Gpr98	PREDICTED: Rattus norvegicus G protein-coupled receptor 98 (Gpr98), mRNA [XM_002725894]	**0,458**
Ica1	Rattus norvegicus islet cell autoantigen 1 (Ica1), mRNA [NM_030844]	**0,458**
Eef1g	Rattus norvegicus eukaryotic translation elongation factor 1 gamma (Eef1g), mRNA [NM_001004223]	**0,459**
Man2a2	Rattus norvegicus mannosidase 2, alpha 2 (Man2a2), mRNA [NM_001107527]	**0,459**
Ercc5	Rattus norvegicus excision repair cross-complementing rodent repair deficiency, complementation group 5 (Ercc5), mRNA [NM_001106910]	**0,459**
0	Uncharacterized protein [Source:UniProtKB/TrEMBL;Acc:D3ZHI0] [ENSRNOT00000061428]	**0,459**
Eif2a	Rattus norvegicus eukaryotic translation initiation factor 2A (Eif2a), mRNA [NM_001109339]	**0,459**
Dmtf1	Rattus norvegicus cyclin D binding myb-like transcription factor 1 (Dmtf1), mRNA [NM_053693]	**0,459**
Lrmp	PREDICTED: Rattus norvegicus lymphoid-restricted membrane protein (Lrmp), mRNA [XM_001074768]	**0,459**
Mslnl	Uncharacterized protein [Source:UniProtKB/TrEMBL;Acc:D4AB62] [ENSRNOT00000060286]	**0,459**
Ngrn	Rattus norvegicus neugrin, neurite outgrowth associated (Ngrn), transcript variant 2, mRNA [NM_001033900]	**0,460**
Sars	Rattus norvegicus seryl-tRNA synthetase (Sars), mRNA [NM_001007606]	**0,460**
Tgm2	Rattus norvegicus transglutaminase 2, C polypeptide (Tgm2), mRNA [NM_019386]	**0,460**
Mitd1	Rattus norvegicus MIT, microtubule interacting and transport, domain containing 1 (Mitd1), mRNA [NM_001009714]	**0,460**
0	Rattus norvegicus similar to NOL1R protein; Williams-Beuren syndrome critical region protein 20 (LOC305339), mRNA [XM_223400]	**0,460**
Camsap1	Rattus norvegicus calmodulin regulated spectrin-associated protein 1 (Camsap1), mRNA [NM_001168549]	**0,460**
Twf1	Rattus norvegicus twinfilin, actin-binding protein, homolog 1 (Drosophila) (Twf1), mRNA [NM_001008521]	**0,460**
Sympk	Rattus norvegicus symplekin (Sympk), mRNA [NM_001100830]	**0,460**
Snx20	Rattus norvegicus sorting nexin 20 (Snx20), mRNA [NM_001024999]	**0,461**
Rpl23	Rattus norvegicus ribosomal protein L23 (Rpl23), mRNA [NM_001007599]	**0,461**
RGD1306001	Rattus norvegicus similar to 2210021J22Rik protein (RGD1306001), mRNA [NM_001130696]	**0,461**
Pigg	Uncharacterized protein [Source:UniProtKB/TrEMBL;Acc:D3ZDH0] [ENSRNOT00000033973]	**0,461**
Dlst	Rattus norvegicus dihydrolipoamide S-succinyltransferase (E2 component of 2-oxo-glutarate complex) (Dlst), mRNA [NM_001006981]	**0,461**
Wdr4	Rattus norvegicus WD repeat domain 4 (Wdr4), mRNA [NM_001135667]	**0,461**
Ddrgk1	Rattus norvegicus DDRGK domain containing 1 (Ddrgk1), mRNA [NM_001106512]	**0,461**
Fez2	Rattus norvegicus fasciculation and elongation protein zeta 2 (zygin II), mRNA (cDNA clone MGC:112579 IMAGE:7128839), complete cds. [BC100060]	**0,461**
Kcnj4	Rattus norvegicus potassium inwardly-rectifying channel, subfamily J, member 4 (Kcnj4), mRNA [NM_053870]	**0,461**
Scrib	Rattus norvegicus scribbled homolog (Drosophila) (Scrib), mRNA [NM_001191879]	**0,462**
Pfkm	Rattus norvegicus phosphofructokinase, muscle (Pfkm), mRNA [NM_031715]	**0,462**
Slc27a3	Rattus norvegicus solute carrier family 27 (fatty acid transporter), member 3 (Slc27a3), mRNA [NM_001106439]	**0,462**
0	Uncharacterized protein [Source:UniProtKB/TrEMBL;Acc:D3ZJS8] [ENSRNOT00000037641]	**0,462**
Cdkn2d	Rattus norvegicus similar to cyclin-dependent kinase inhibitor 2D (Cdkn2d), mRNA [NM_001009719]	**0,463**

Dhx36	Rattus norvegicus DEAH (Asp-Glu-Ala-His) box polypeptide 36 (Dhx36), mRNA [NM_001107678]	**0,463**
Mpp6	Rattus norvegicus membrane protein, palmitoylated 6 (MAGUK p55 subfamily member 6) (Mpp6), mRNA [NM_001134982]	**0,463**
0	Uncharacterized protein [Source:UniProtKB/TrEMBL;Acc:D3ZAL9] [ENSRNOT00000068454]	**0,463**
RGD1310324	Rattus norvegicus hypothetical LOC287388 (RGD1310324), mRNA [NM_001163492]	**0,463**
0	Unknown	**0,463**
Atp8b3	Uncharacterized protein [Source:UniProtKB/TrEMBL;Acc:D3ZE62] [ENSRNOT00000039813]	**0,463**
Kif3b	Rattus norvegicus kinesin family member 3B (Kif3b), mRNA [NM_001106529]	**0,464**
Hadha	Rattus norvegicus hydroxyacyl-Coenzyme A dehydrogenase/3-ketoacyl-Coenzyme A thiolase/enoyl-Coenzyme A hydratase (trifunctional protein), alpha subunit (Hadha), nuclear gene encoding mitochondrial protein, mRNA [NM_130826]	**0,464**
Lrrn4cl	Rattus norvegicus LRRN4 C-terminal like (Lrrn4cl), mRNA [NM_001109579]	**0,464**
RGD1561796	Uncharacterized protein [Source:UniProtKB/TrEMBL;Acc:D3ZS66] [ENSRNOT00000068577]	**0,464**
Jag2	PREDICTED: Rattus norvegicus jagged 2 (Jag2), mRNA [XM_001073124]	**0,464**
Pip5k1b	Rattus norvegicus phosphatidylinositol-4-phosphate 5-kinase, type I, beta (Pip5k1b), mRNA [NM_001012743]	**0,464**
Dnd1	Rattus norvegicus dead end homolog 1 (zebrafish) (Dnd1), mRNA [NM_001109379]	**0,464**
Alas1	Rattus norvegicus aminolevulinate, delta-, synthase 1 (Alas1), mRNA [NM_024484]	**0,464**
Vom2r15	Rattus norvegicus vomeronasal 2 receptor, 15 (Vom2r15), mRNA [NM_001099490]	**0,464**
Evc2	Rattus norvegicus Ellis van Creveld syndrome 2 homolog (human) (Evc2), mRNA [NM_001106012]	**0,464**
Uck1	Rattus norvegicus uridine-cytidine kinase 1 (Uck1), mRNA [NM_001107831]	**0,464**
0	Unknown	**0,464**
Fam21c	Rattus norvegicus family with sequence similarity 21, member C (Fam21c), mRNA [NM_199207]	**0,464**
Uspl1	Rattus norvegicus ubiquitin specific peptidase like 1 (Uspl1), transcript variant 1, mRNA [NM_001198555]	**0,464**
Ing2	Rattus norvegicus inhibitor of growth family, member 2 (Ing2), mRNA [NM_001106083]	**0,464**
Glycam1	Rattus norvegicus glycosylation dependent cell adhesion molecule 1 (Glycam1), mRNA [NM_012794]	**0,465**
LOC100366173	PREDICTED: Rattus norvegicus Zinc finger protein 383-like, transcript variant 3 (LOC100366173), mRNA [XM_002725562]	**0,465**
Rapgef6	Rattus norvegicus Rap guanine nucleotide exchange factor (GEF) 6 (Rapgef6), mRNA [NM_001107003]	**0,465**
0	Unknown	**0,465**
Rptor	Rattus norvegicus regulatory associated protein of MTOR, complex 1 (Rptor), mRNA [NM_001134499]	**0,465**
Rage	Rattus norvegicus renal tumor antigen (Rage), mRNA [NM_001010965]	**0,465**
Fxc1	Rattus norvegicus fractured callus expressed transcript 1 (Fxc1), mRNA [NM_053371]	**0,465**
Mog	Rattus norvegicus myelin oligodendrocyte glycoprotein (Mog), mRNA [NM_022668]	**0,465**
RGD1560137	PREDICTED: Rattus norvegicus similar to expressed sequence AU021034 (RGD1560137), miscRNA [XR_008828]	**0,466**
Exoc7	Rattus norvegicus exocyst complex component 7 (Exoc7), mRNA [NM_022691]	**0,466**
Ppp1r10	Rattus norvegicus protein phosphatase 1, regulatory subunit 10 (Ppp1r10), mRNA [NM_022951]	**0,466**
Chrm3	Rattus norvegicus cholinergic receptor, muscarinic 3 (Chrm3), mRNA [NM_012527]	**0,466**

Xrcc5	Rattus norvegicus X-ray repair complementing defective repair in Chinese hamster cells 5 (Xrcc5), mRNA [NM_177419]	**0,466**
Nkap	Rattus norvegicus NFKB activating protein (Nkap), mRNA [NM_001024872]	**0,466**
Pde9a	Rattus norvegicus phosphodiesterase 9A (Pde9a), mRNA [NM_138543]	**0,467**
Itpka	Rattus norvegicus inositol 1,4,5-trisphosphate 3-kinase A (Itpka), mRNA [NM_031045]	**0,467**
Cacna1h	Rattus norvegicus calcium channel, voltage-dependent, T type, alpha 1H subunit (Cacna1h), mRNA [NM_153814]	**0,467**
Rgnef	Rattus norvegicus Rho-guanine nucleotide exchange factor (Rgnef), mRNA [NM_001108542]	**0,467**
Rab11fip3	Peroxisomal 2,4-dienoyl-CoA reductase [Source:UniProtKB/Swiss-Prot;Acc:Q9Z2M4] [ENSRNOT00000027467]	**0,467**
Tmem141	Rattus norvegicus transmembrane protein 141 (Tmem141), mRNA [NM_001109197]	**0,467**
Pam	Rattus norvegicus peptidylglycine alpha-amidating monooxygenase (Pam), mRNA [NM_013000]	**0,467**
Ndufaf4	Rattus norvegicus NADH dehydrogenase (ubiquinone) 1 alpha subcomplex, assembly factor 4 (Ndufaf4), nuclear gene encoding mitochondrial protein, mRNA [NM_198783]	**0,468**
0	Unknown	**0,468**
Fahd2a	Rattus norvegicus fumarylacetoacetate hydrolase domain containing 2A (Fahd2a), mRNA [NM_001134834]	**0,468**
Rfc1	Rattus norvegicus replication factor C (activator 1) 1 (Rfc1), mRNA [NM_053547]	**0,468**
Sfrs14	Rattus norvegicus splicing factor, arginine/serine-rich 14 (Sfrs14), mRNA [NM_001108397]	**0,468**
0	Unknown	**0,468**
Fxc1	Rattus norvegicus fractured callus expressed transcript 1 (Fxc1), mRNA [NM_053371]	**0,468**
Kcnab1	Rattus norvegicus potassium voltage-gated channel, shaker-related subfamily, beta member 1 (Kcnab1), mRNA [NM_017303]	**0,469**
Tmod3	Rattus norvegicus tropomodulin 3 (Tmod3), mRNA [NM_001011997]	**0,469**
Alad	Rattus norvegicus aminolevulinate, delta-, dehydratase (Alad), mRNA [NM_012899]	**0,469**
Pcdhb9	Rattus norvegicus protocadherin beta 9 (Pcdhb9), mRNA [NM_001109390]	**0,469**
0	BC062892 brother of CDO {Mus musculus} (exp=-1; wgp=0; cg=0), partial (11%) [TC613432]	**0,469**
Pih1d1	Rattus norvegicus PIH1 domain containing 1 (Pih1d1), mRNA [NM_001024868]	**0,469**
Zrsr2	Uncharacterized protein [Source:UniProtKB/TrEMBL;Acc:D3ZHQ8] [ENSRNOT00000045300]	**0,469**
LOC687565	Rattus norvegicus similar to density-regulated protein (LOC687565), mRNA [NM_001115046]	**0,469**
Zbtb8a	Rattus norvegicus zinc finger and BTB domain containing 8a (Zbtb8a), mRNA [NM_001107913]	**0,469**
Klhl21	Rattus norvegicus kelch-like 21 (Drosophila) (Klhl21), mRNA [NM_001107996]	**0,469**
Farsb	Rattus norvegicus phenylalanyl-tRNA synthetase, beta subunit (Farsb), mRNA [NM_001004252]	**0,469**
Cdk105	Rattus norvegicus CDK105 protein (Cdk105), mRNA [NM_134415]	**0,470**
Pdia4	Rattus norvegicus protein disulfide isomerase family A, member 4 (Pdia4), mRNA [NM_053849]	**0,470**
Chmp5	Rattus norvegicus chromatin modifying protein 5 (Chmp5), mRNA [NM_001025410]	**0,470**
0	Unknown	**0,470**
Gpr146	Rattus norvegicus G protein-coupled receptor 146 (Gpr146), mRNA [NM_001109062]	**0,470**
Grb7	Rattus norvegicus growth factor receptor bound protein 7 (Grb7), mRNA [NM_053403]	**0,470**

Nolc1	Rattus norvegicus nucleolar and coiled-body phosphoprotein 1 (Nolc1), mRNA [NM_022869]	**0,470**
Pxmp2	Rattus norvegicus peroxisomal membrane protein 2 (Pxmp2), mRNA [NM_031587]	**0,470**
Rpl6	Rattus norvegicus ribosomal protein L6 (Rpl6), mRNA [NM_053971]	**0,470**
Tdrd3	Rattus norvegicus tudor domain containing 3 (Tdrd3), mRNA [NM_001012043]	**0,470**
Obfc1	Rattus norvegicus oligonucleotide/oligosaccharide-binding fold containing 1 (Obfc1), mRNA [NM_001011943]	**0,471**
Igfbpl1	Rattus norvegicus insulin-like growth factor binding protein-like 1 (Igfbpl1), mRNA [NM_001108972]	**0,471**
0	BC075646 Luzp2 protein {Mus musculus} (exp=-1; wgp=0; cg=0), partial (83%) [TC635744]	**0,471**
Nufip1	Rattus norvegicus nuclear fragile X mental retardation protein interacting protein 1 (Nufip1), mRNA [NM_001007758]	**0,471**
Uqcrh	Rattus norvegicus ubiquinol-cytochrome c reductase hinge protein (Uqcrh), nuclear gene encoding mitochondrial protein, mRNA [NM_001009480]	**0,471**
Gtf2f1	Rattus norvegicus general transcription factor IIF, polypeptide 1 (Gtf2f1), mRNA [NM_001007711]	**0,471**
Brp44l	Rattus norvegicus brain protein 44-like (Brp44l), mRNA [NM_133561]	**0,471**
Tmigd1	Rattus norvegicus transmembrane and immunoglobulin domain containing 1 (Tmigd1), mRNA [NM_001135029]	**0,471**
Cd99l2	Rattus norvegicus CD99 molecule-like 2 (Cd99l2), mRNA [NM_134459]	**0,471**
RGD1561777	PREDICTED: Rattus norvegicus similar to Na+ dependent glucose transporter 1 (RGD1561777), mRNA [XM_001063079]	**0,472**
Oxct1	Rattus norvegicus 3-oxoacid CoA transferase 1 (Oxct1), nuclear gene encoding mitochondrial protein, mRNA [NM_001127580]	**0,472**
Pacsin3	Rattus norvegicus protein kinase C and casein kinase substrate in neurons 3 (Pacsin3), mRNA [NM_001009966]	**0,472**
RGD1309058	RCG20883Uncharacterized protein [Source:UniProtKB/TrEMBL;Acc:D4A7T7] [ENSRNOT00000014814]	**0,472**
0	plakophilin 4 Gene [Source:MGI Symbol;Acc:MGI:109281] [ENSRNOT00000059246]	**0,472**
Slc7a6	Rattus norvegicus solute carrier family 7 (cationic amino acid transporter, y+ system), member 6 (Slc7a6), mRNA [NM_001107424]	**0,472**
0	Uncharacterized protein [Source:UniProtKB/TrEMBL;Acc:D3Z8B7] [ENSRNOT00000057522]	**0,472**
Vps29	Rattus norvegicus vacuolar protein sorting 29 homolog (S. cerevisiae) (Vps29), mRNA [NM_001105932]	**0,472**
0	Uncharacterized protein [Source:UniProtKB/TrEMBL;Acc:D3ZAC0] [ENSRNOT00000055179]	**0,472**
Nt5dc2	Rattus norvegicus 5'-nucleotidase domain containing 2 (Nt5dc2), mRNA [NM_001009271]	**0,472**
Cd3eap	Rattus norvegicus CD3e molecule, epsilon associated protein (Cd3eap), mRNA [NM_001109416]	**0,472**
Ss18	Rattus norvegicus synovial sarcoma translocation, Chromosome 18 (Ss18), mRNA [NM_001100900]	**0,472**
RGD1310269	Rattus norvegicus hypothetical LOC314472 (RGD1310269), mRNA [NM_001126280]	**0,472**
Aoc3	Rattus norvegicus amine oxidase, copper containing 3 (vascular adhesion protein 1) (Aoc3), mRNA [NM_031582]	**0,472**
Pgm3	Rattus norvegicus phosphoglucomutase 3, mRNA (cDNA clone MGC:188729 IMAGE:9027278), complete cds. [BC166838]	**0,472**
0	Unknown	**0,472**
Sdhb	Rattus norvegicus succinate dehydrogenase complex, subunit B, iron sulfur (Ip) (Sdhb), nuclear gene encoding mitochondrial protein, mRNA [NM_001100539]	**0,472**
Cys1	Rattus norvegicus cystin 1 (Cys1), mRNA [NM_001109597]	**0,472**
Trim2	Rattus norvegicus tripartite motif-containing 2 (Trim2), mRNA [NM_001108552]	**0,473**
Lrp5	Rattus norvegicus low density lipoprotein receptor-related protein 5 (Lrp5), mRNA [NM_001106321]	**0,473**

Tyw3	Similar to RIKEN cDNA 5230400J09 (Predicted), isoform CRA_aUncharacterized protein [Source:UniProtKB/TrEMBL;Acc:D3ZHR8] [ENSRNOT00000032874]	**0,473**
Casp3	Rattus norvegicus caspase 3 (Casp3), mRNA [NM_012922]	**0,473**
Nsmce1	Rattus norvegicus non-SMC element 1 homolog (S. cerevisiae) (Nsmce1), mRNA [NM_001039611]	**0,473**
Ogn	Rattus norvegicus osteoglycin (Ogn), mRNA [NM_001106103]	**0,473**
Casp6	Rattus norvegicus caspase 6 (Casp6), mRNA [NM_031775]	**0,473**
Phgdh	Rattus norvegicus phosphoglycerate dehydrogenase (Phgdh), mRNA [NM_031620]	**0,473**
RGD1563273	Rattus norvegicus similar to hypothetical protein 9630041N07 (RGD1563273), mRNA [NM_001134600]	**0,473**
Ndufaf3	Rattus norvegicus NADH dehydrogenase (ubiquinone) 1 alpha subcomplex, assembly factor 3 (Ndufaf3), nuclear gene encoding mitochondrial protein, transcript variant 1, mRNA [NM_020080]	**0,473**
Cinp	Rattus norvegicus cyclin-dependent kinase 2-interacting protein (Cinp), mRNA [NM_001106758]	**0,473**
0	Uncharacterized protein [Source:UniProtKB/TrEMBL;Acc:D3ZHM7] [ENSRNOT00000036931]	**0,473**
Slc4a11	Rattus norvegicus solute carrier family 4, sodium borate transporter, member 11 (Slc4a11), mRNA [NM_001107775]	**0,474**
Cdc37	Rattus norvegicus cell division cycle 37 homolog (S. cerevisiae) (Cdc37), mRNA [NM_053743]	**0,474**
Hddc3	Rattus norvegicus HD domain containing 3 (Hddc3), mRNA [NM_001107528]	**0,474**
Mapkapk3	Rattus norvegicus mitogen-activated protein kinase-activated protein kinase 3 (Mapkapk3), mRNA [NM_001012127]	**0,474**
0	Nipped-B homolog (Drosophila)Uncharacterized protein [Source:UniProtKB/TrEMBL;Acc:D3ZG77] [ENSRNOT00000021181]	**0,474**
Oat	Rattus norvegicus ornithine aminotransferase (gyrate atrophy) (Oat), nuclear gene encoding mitochondrial protein, mRNA [NM_022521]	**0,474**
Trim45	Rattus norvegicus tripartite motif-containing 45 (Trim45), mRNA [NM_001106453]	**0,474**
0	Unknown	**0,474**
Mfap1a	Rattus norvegicus microfibrillar-associated protein 1A (Mfap1a), mRNA [NM_001191964]	**0,474**
0	Uncharacterized protein [Source:UniProtKB/TrEMBL;Acc:D4A3Y9] [ENSRNOT00000011560]	**0,475**
Rgc32	Rattus norvegicus response gene to complement 32 (Rgc32), mRNA [NM_054008]	**0,475**
Impad1	Rattus norvegicus inositol monophosphatase domain containing 1 (Impad1), mRNA [NM_001008772]	**0,475**
0	Unknown	**0,475**
Btaf1	Rattus norvegicus BTAF1 RNA polymerase II, B-TFIID transcription factor-associated, (Mot1 homolog, S. cerevisiae) (Btaf1), mRNA [NM_001191917]	**0,475**
Mblac1	Rattus norvegicus metallo-beta-lactamase domain containing 1 (Mblac1), mRNA [NM_001024996]	**0,475**
Tp73	Rattus norvegicus tumor protein p73 (Tp73), mRNA [NM_001108696]	**0,475**
Chchd3	Rattus norvegicus coiled-coil-helix-coiled-coil-helix domain containing 3 (Chchd3), mRNA [NM_001106588]	**0,475**
Vta1	Rattus norvegicus Vps20-associated 1 homolog (S. cerevisiae) (Vta1), mRNA [NM_001025640]	**0,475**
Rpa2	Rattus norvegicus replication protein A2 (Rpa2), mRNA [NM_021582]	**0,475**
Ormdl2	Rattus norvegicus ORM1-like 2 (S. cerevisiae) (Ormdl2), mRNA [NM_001105940]	**0,475**
LOC684776	PREDICTED: Rattus norvegicus similar to immunoglobin superfamily, member 21 (LOC684776), mRNA [XM_001071901]	**0,475**

Gcc2	Rattus norvegicus GRIP and coiled-coil domain containing 2 (Gcc2), mRNA [NM_001107633]	**0,475**
RGD1566052	Rattus norvegicus similar to elongation protein 4 homolog (RGD1566052), mRNA [NM_001115047]	**0,475**
Rnaset2	Rattus norvegicus ribonuclease T2 (Rnaset2), mRNA [NM_001106210]	**0,475**
Prodh	Rattus norvegicus proline dehydrogenase (Prodh), nuclear gene encoding mitochondrial protein, mRNA [NM_001135778]	**0,476**
RGD1310587	Rattus norvegicus similar to hypothetical protein FLJ14146 (RGD1310587), mRNA [NM_001100857]	**0,476**
LOC679229	PREDICTED: Rattus norvegicus similar to zinc finger protein 397, transcript variant 2 (LOC679229), mRNA [XM_001055142]	**0,476**
RGD1559808	Uncharacterized protein [Source:UniProtKB/TrEMBL;Acc:D4AEB9] [ENSRNOT00000045419]	**0,476**
RGD1309077	Rattus norvegicus similar to putative RNA methyltransferase (RGD1309077), mRNA [NM_001108282]	**0,476**
0	ZN364_MOUSE (Q9D0C1) Zinc finger protein 364 (Rabring 7), partial (82%) [TC589182]	**0,476**
LOC687696	Rattus norvegicus similar to AMSH-family protein (LOC687696), mRNA [NM_001115048]	**0,476**
Dlk2	Rattus norvegicus delta-like 2 homolog (Drosophila) (Dlk2), mRNA [NM_001108202]	**0,476**
0	Unknown	**0,476**
0	Centrosomal protein 1 (Predicted)Uncharacterized protein [Source:UniProtKB/TrEMBL;Acc:D3ZRZ3] [ENSRNOT00000007906]	**0,476**
Casq2	Rattus norvegicus calsequestrin 2 (cardiac muscle) (Casq2), nuclear gene encoding mitochondrial protein, mRNA [NM_017131]	**0,476**
Hspa4l	Rattus norvegicus heat shock protein 4-like (Hspa4l), mRNA [NM_001106428]	**0,477**
0	Rattus norvegicus similar to heat shock protein 84 - mouse (LOC291871), mRNA [XM_226259]	**0,477**
Ndufb8	Rattus norvegicus NADH dehydrogenase (ubiquinone) 1 beta subcomplex 8 (Ndufb8), nuclear gene encoding mitochondrial protein, mRNA [NM_001106360]	**0,477**
Cyb561	Rattus norvegicus cytochrome b-561 (Cyb561), mRNA [NM_001107056]	**0,477**
Prep	Rattus norvegicus prolyl endopeptidase (Prep), mRNA [NM_031324]	**0,477**
Wdr53	Rattus norvegicus WD repeat domain 53 (Wdr53), mRNA [NM_001109055]	**0,477**
0	SEC24 related gene family, member D (S. cerevisiae) (Predicted), isoform CRA_bUncharacterized protein [Source:UniProtKB/TrEMBL;Acc:D3ZTC8] [ENSRNOT00000020433]	**0,477**
Zfand2b	Rattus norvegicus zinc finger, AN1 type domain 2B (Zfand2b), mRNA [NM_001025745]	**0,477**
RGD1309730	Rattus norvegicus similar to RIKEN cDNA B230118H07 (RGD1309730), mRNA [NM_001106491]	**0,477**
Scg2	Rattus norvegicus secretogranin II (chromogranin C) (Scg2), mRNA [NM_022669]	**0,477**
LOC298795	Rattus norvegicus similar to 14-3-3 protein sigma (LOC298795), mRNA [NM_001013941]	**0,477**
Map3k6	Rattus norvegicus mitogen-activated protein kinase kinase kinase 6 (Map3k6), mRNA [NM_001107909]	**0,477**
0	Unknown	**0,478**
RGD1306928	PREDICTED: Rattus norvegicus similar to hypothetical protein MGC13138, transcript variant 1 (RGD1306928), mRNA [XM_001080215]	**0,478**
Etaa1	Rattus norvegicus Ewing tumor-associated antigen 1 (Etaa1), mRNA [NM_001109094]	**0,478**
0	Unknown	**0,478**
RGD1311847	Rattus norvegicus similar to 1700030K09Rik protein (RGD1311847), mRNA [NM_001013879]	**0,478**
Mlycd	Rattus norvegicus malonyl-CoA decarboxylase (Mlycd), nuclear gene encoding mitochondrial protein, mRNA [NM_053477]	**0,478**

Nqo1	Rattus norvegicus NAD(P)H dehydrogenase, quinone 1 (Nqo1), mRNA [NM_017000]	**0,478**
RGD1309779	Rattus norvegicus similar to ENSANGP00000021391 (RGD1309779), mRNA [NM_001108766]	**0,478**
Lrrc49	Rattus norvegicus leucine rich repeat containing 49 (Lrrc49), mRNA [NM_001134469]	**0,478**
0	Unknown	**0,479**
Aurka	Rattus norvegicus aurora kinase A (Aurka), mRNA [NM_153296]	**0,479**
0	Uncharacterized protein [Source:UniProtKB/TrEMBL;Acc:D3ZDB2] [ENSRNOT00000068478]	**0,479**
RGD1561890	PREDICTED: Rattus norvegicus similar to craniofacial development protein 1 (RGD1561890), mRNA [XM_233989]	**0,479**
Lyz2	Rattus norvegicus lysozyme 2 (Lyz2), mRNA [NM_012771]	**0,479**
0	Rattus norvegicus strain BN/SsNHsdMCW RNOR03327191, whole genome shotgun sequence [AABR03127998]	**0,479**
Dgcr14	Rattus norvegicus DiGeorge syndrome critical region gene 14 (Dgcr14), mRNA [NM_001012472]	**0,479**
Lysmd2	Rattus norvegicus LysM, putative peptidoglycan-binding, domain containing 2 (Lysmd2), mRNA [NM_001106839]	**0,479**
Tnfsf13b	Rattus norvegicus tumor necrosis factor (ligand) superfamily, member 13b (Tnfsf13b), mRNA [NM_001109112]	**0,479**
Tyro3	Rattus norvegicus TYRO3 protein tyrosine kinase (Tyro3), mRNA [NM_017092]	**0,479**
0	SAM68_RAT (Q91V33) KH domain-containing, RNA-binding, signal transduction-associated protein 1 (p21 Ras GTPase-activating protein-associated p62) (GAP-associated tyrosine phosphoprotein p62) (Src-associated in mitosis 68 kDa protein) (Sam68) (p68), partia	**0,479**
Baz1a	Rattus norvegicus bromodomain adjacent to zinc finger domain, 1A (Baz1a), mRNA [NM_001170568]	**0,479**
Ccdc72	coiled-coil domain-containing protein 72 [Source:RefSeq peptide;Acc:NP_001119520] [ENSRNOT00000068775]	**0,479**
0	Rattus sp. membrane protein-73 mRNA, partial cds; mitochondrial gene for mitochondrial product. [S63519]	**0,479**
Whamm	Rattus norvegicus WAS protein homolog associated with actin, golgi membranes and microtubules (Whamm), mRNA [NM_001130728]	**0,479**
Rgs11	Rattus norvegicus regulator of G-protein signaling 11 (Rgs11), mRNA [NM_019338]	**0,480**
Enpp4	Rattus norvegicus ectonucleotide pyrophosphatase/phosphodiesterase 4 (Enpp4), mRNA [NM_001106892]	**0,480**
RGD1309765	Rattus norvegicus similar to hypothetical protein (RGD1309765), mRNA [NM_001169115]	**0,480**
Pkd1	PREDICTED: Rattus norvegicus polycystic kidney disease 1 homolog (human), transcript variant 2 (Pkd1), mRNA [XM_002727754]	**0,480**
Upp1	Rattus norvegicus uridine phosphorylase 1 (Upp1), mRNA [NM_001030025]	**0,480**
LOC361985	Rattus norvegicus similar to NICE-3 (LOC361985), mRNA [NM_001014174]	**0,480**
0	Unknown	**0,480**
Sdccag3	Rattus norvegicus serologically defined colon cancer antigen 3 (Sdccag3), mRNA [NM_001013135]	**0,480**
Tpmt	thiopurine S-methyltransferase [Source:RefSeq peptide;Acc:NP_001072999] [ENSRNOT00000022085]	**0,480**
Fgd4	Rattus norvegicus FYVE, RhoGEF and PH domain containing 4 (Fgd4), mRNA [NM_139263]	**0,480**
Rufy2	Rattus norvegicus RUN and FYVE domain containing 2 (Rufy2), mRNA [NM_001168586]	**0,480**
LOC303590	PREDICTED: Rattus norvegicus similar to cactin CG1676-PA (LOC303590), miscRNA [XR_006385]	**0,480**
Zgpat	Rattus norvegicus zinc finger, CCCH-type with G patch domain (Zgpat), mRNA [NM_001009656]	**0,480**
Strap	Rattus norvegicus serine/threonine kinase receptor associated protein (Strap), mRNA [NM_001011969]	**0,480**
Gpn2	GPN-loop GTPase 2 [Source:UniProtKB/Swiss-Prot;Acc:D4A7C0] [ENSRNOT00000009275]	**0,481**

0	Uncharacterized protein [Source:UniProtKB/TrEMBL;Acc:D3ZVD4] [ENSRNOT00000020523]	**0,481**
0	Rattus norvegicus chromosome 10, 7 clones, strain BN/SsNHsdMCW RNOR03214123, whole genome shotgun sequence [AABR03073170]	**0,481**
Ccdc25	Rattus norvegicus coiled-coil domain containing 25 (Ccdc25), mRNA [NM_001108382]	**0,481**
0	Unknown	**0,481**
Grinl1a	Rattus norvegicus glutamate receptor, ionotropic, N-methyl D-aspartate-like 1A (Grinl1a), mRNA [NM_183402]	**0,481**
0	Unknown	**0,481**
Pigp	Rattus norvegicus phosphatidylinositol glycan anchor biosynthesis, class P (Pigp), mRNA [NM_001099758]	**0,481**
RGD1564906	Glutathione S-transferase [Source:UniProtKB/TrEMBL;Acc:D3ZBD6] [ENSRNOT00000000233]	**0,481**
Arid4b	Rattus norvegicus AT rich interactive domain 4B (Rbp1 like) (Arid4b), mRNA [NM_053421]	**0,481**
Nudt13	Rattus norvegicus nudix (nucleoside diphosphate linked moiety X)-type motif 13 (Nudt13), mRNA [NM_001127636]	**0,481**
Cggbp1	Rattus norvegicus CGG triplet repeat binding protein 1 (Cggbp1), mRNA [NM_001105900]	**0,481**
LOC684274	Rattus norvegicus similar to RIKEN cDNA 2810012L14 (LOC684274), mRNA [NM_001115041]	**0,481**
Rplp0	Rattus norvegicus ribosomal protein, large, P0 (Rplp0), mRNA [NM_022402]	**0,481**
RGD1305178	Rattus norvegicus similar to Hypothetical protein MGC11690 (RGD1305178), mRNA [NM_001025003]	**0,481**
Csnk1d	Rattus norvegicus casein kinase 1, delta (Csnk1d), mRNA [NM_139060]	**0,481**
Whsc1	Rattus norvegicus Wolf-Hirschhorn syndrome candidate 1 (human) (Whsc1), mRNA [NM_001191552]	**0,481**
Mcc	Rattus norvegicus mutated in colorectal cancers (Mcc), mRNA [NM_001170534]	**0,481**
Cox7a2l	Rattus norvegicus cytochrome c oxidase subunit VIIa polypeptide 2 like (Cox7a2l), nuclear gene encoding mitochondrial protein, mRNA [NM_001106704]	**0,481**
0	Unknown	**0,481**
Fhod1	Rattus norvegicus formin homology 2 domain containing 1 (Fhod1), mRNA [NM_001191600]	**0,482**
Hsp90ab1	Heat shock protein HSP 90-beta [Source:UniProtKB/Swiss-Prot;Acc:P34058] [ENSRNOT00000026920]	**0,482**
Sdf4	Rattus norvegicus stromal cell derived factor 4 (Sdf4), mRNA [NM_130412]	**0,482**
Ubxn7	Rattus norvegicus UBX domain protein 7 (Ubxn7), mRNA [NM_001107086]	**0,482**
Nmb	Rattus norvegicus neuromedin B (Nmb), mRNA [NM_001109149]	**0,482**
Rab9a	Rattus norvegicus RAB9A, member RAS oncogene family (Rab9a), mRNA [NM_053458]	**0,482**
Ddr2	Rattus norvegicus discoidin domain receptor tyrosine kinase 2 (Ddr2), mRNA [NM_031764]	**0,482**
Rad9b	Rattus norvegicus RAD9 homolog B (S. cerevisiae) (Rad9b), mRNA [NM_001030042]	**0,482**
LOC502894	Rattus norvegicus hypothetical protein LOC502894 (LOC502894), mRNA [NM_001025064]	**0,482**
0	Uncharacterized protein [Source:UniProtKB/TrEMBL;Acc:D3ZGV8] [ENSRNOT00000004513]	**0,482**
0	Unknown	**0,482**
Gpr162	Rattus norvegicus G protein-coupled receptor 162 (Gpr162), mRNA [NM_001108646]	**0,482**
0	Unknown	**0,482**
Dmbx1	Rattus norvegicus diencephalon/mesencephalon homeobox 1 (Dmbx1), mRNA [NM_001107961]	**0,482**

0	Unknown	**0,482**
RGD1560065	Rattus norvegicus similar to RIKEN cDNA 2410004B18 (RGD1560065), mRNA [NM_001134616]	**0,482**
Znf518a	Rattus norvegicus zinc finger protein 518A (Znf518a), mRNA [NM_001030038]	**0,482**
Clta	Rattus norvegicus clathrin, light chain (Lca) (Clta), mRNA [NM_031974]	**0,483**
LOC100125367	Rattus norvegicus hypothetical protein LOC100125367 (LOC100125367), mRNA [NM_001103357]	**0,483**
Kcnk13	Rattus norvegicus potassium channel, subfamily K, member 13 (Kcnk13), mRNA [NM_022293]	**0,483**
Engase	Rattus norvegicus endo-beta-N-acetylglucosaminidase (Engase), mRNA [NM_001107070]	**0,483**
Ccdc104	Rattus norvegicus coiled-coil domain containing 104 (Ccdc104), mRNA [NM_001024866]	**0,483**
C1d	Rattus norvegicus C1D nuclear receptor co-repressor (C1d), mRNA [NM_001106021]	**0,483**
Nasp	Rattus norvegicus nuclear autoantigenic sperm protein (histone-binding) (Nasp), mRNA [NM_001005543]	**0,483**
Inpp5f	Rattus norvegicus inositol polyphosphate-5-phosphatase F (Inpp5f), mRNA [NM_001107554]	**0,483**
Zfp354a	Rattus norvegicus zinc finger protein 354A (Zfp354a), mRNA [NM_052798]	**0,483**
0	Unknown	**0,483**
Rdbp	Rattus norvegicus RD RNA-binding protein (Rdbp), transcript variant 1, mRNA [NM_212548]	**0,483**
Mrfap1	Rattus norvegicus Mof4 family associated protein 1 (Mrfap1), mRNA [NM_001009264]	**0,483**
0	Kinesin-like protein kif1a [Source:UniProtKB/TrEMBL;Acc:Q2P9S1] [ENSRNOT00000050557]	**0,483**
LOC679682	Rattus norvegicus similar to ES cell-expressed Ras (LOC679682), mRNA [NM_001109375]	**0,483**
Nup62	Rattus norvegicus nucleoporin 62 (Nup62), mRNA [NM_023098]	**0,483**
0	Unknown	**0,483**
Fen1	Rattus norvegicus flap structure-specific endonuclease 1 (Fen1), mRNA [NM_053430]	**0,483**
0	Unknown	**0,483**
Kcnj14	Rattus norvegicus potassium inwardly-rectifying channel, subfamily J, member 14 (Kcnj14), mRNA [NM_170718]	**0,484**
RGD1306063	Rattus norvegicus similar to HT021 (RGD1306063), mRNA [NM_001106026]	**0,484**
Ccdc12	Rattus norvegicus coiled-coil domain containing 12 (Ccdc12), mRNA [NM_001108783]	**0,484**
Rnf168	Rattus norvegicus ring finger protein 168 (Rnf168), mRNA [NM_001127597]	**0,484**
0	RCG63555RCG63717Uncharacterized protein [Source:UniProtKB/TrEMBL;Acc:D3Z9R8] [ENSRNOT00000051411]	**0,484**
Cacybp	Rattus norvegicus calcyclin binding protein (Cacybp), mRNA [NM_001004208]	**0,484**
Rsph10b	Rattus norvegicus radial spoke head 10 homolog B (Chlamydomonas) (Rsph10b), mRNA [NM_001013867]	**0,484**
Pipox	Rattus norvegicus pipecolic acid oxidase (Pipox), mRNA [NM_001012009]	**0,484**
0	Unknown	**0,484**
Prr7	Rattus norvegicus proline rich 7 (synaptic) (Prr7), mRNA [NM_001109116]	**0,484**
Vasn	Rattus norvegicus vasorin (Vasn), mRNA [NM_001109382]	**0,484**
Gcat	Rattus norvegicus glycine C-acetyltransferase (2-amino-3-ketobutyrate-coenzyme A ligase) (Gcat), nuclear gene encoding mitochondrial protein, mRNA [NM_001024277]	**0,484**

Med10	Rattus norvegicus mediator complex subunit 10 (Med10), mRNA [NM_001106097]	**0,484**
Mrps26	Rattus norvegicus mitochondrial ribosomal protein S26 (Mrps26), nuclear gene encoding mitochondrial protein, mRNA [NM_001013206]	**0,484**
Tax1bp1	Rattus norvegicus Tax1 (human T-cell leukemia virus type I) binding protein 1 (Tax1bp1), mRNA [NM_001004199]	**0,484**
Eif2s1	Rattus norvegicus eukaryotic translation initiation factor 2, subunit 1 alpha (Eif2s1), mRNA [NM_019356]	**0,485**
Kctd3	Rattus norvegicus potassium channel tetramerisation domain containing 3 (Kctd3), mRNA [NM_001107199]	**0,485**
Efcab2	Rattus norvegicus EF-hand calcium binding domain 2 (Efcab2), mRNA [NM_001105977]	**0,485**
Akap1	A-kinase anchor protein 1, mitochondrial [Source:UniProtKB/Swiss-Prot;Acc:O88884] [ENSRNOT00000003251]	**0,485**
Erc2	Rattus norvegicus ELKS/RAB6-interacting/CAST family member 2 (Erc2), mRNA [NM_170787]	**0,485**
Wdr24	Rattus norvegicus WD repeat domain 24 (Wdr24), mRNA [NM_001191084]	**0,485**
Ttr	Rattus norvegicus transthyretin (Ttr), mRNA [NM_012681]	**0,485**
Dnhd1	PREDICTED: Rattus norvegicus dynein heavy chain domain 1 (Dnhd1), miscRNA [XR_085725]	**0,485**
RGD1561792	Rattus norvegicus similar to CG14903-PA (RGD1561792), mRNA [NM_001134524]	**0,485**
RGD1564964	Rattus norvegicus similar to WD repeat domain 11 protein (RGD1564964), mRNA [NM_001134564]	**0,485**
Mfsd6	Rattus norvegicus major facilitator superfamily domain containing 6 (Mfsd6), mRNA [NM_001106911]	**0,485**
Galnt14	Rattus norvegicus UDP-N-acetyl-alpha-D-galactosamine:polypeptide N-acetylgalactosaminyltransferase 14 (GalNAc-T14) (Galnt14), mRNA [NM_001012109]	**0,485**
Ep400	Rattus norvegicus E1A binding protein p400 (Ep400), mRNA [NM_001107149]	**0,485**
Clns1a	Rattus norvegicus chloride channel, nucleotide-sensitive, 1A (Clns1a), mRNA [NM_031719]	**0,485**
Lgi4	Rattus norvegicus leucine-rich repeat LGI family, member 4 (Lgi4), mRNA [NM_199499]	**0,485**
Sp110	Rattus norvegicus SP110 nuclear body protein (Sp110), mRNA [NM_001034137]	**0,485**
LOC688459	Rattus norvegicus hypothetical protein LOC688459 (LOC688459), mRNA [NM_001109502]	**0,485**
0	Rattus norvegicus TL0ABA19YF11 mRNA sequence. [FQ210991]	**0,485**
0	Unknown	**0,485**
Mtrr	Rattus norvegicus 5-methyltetrahydrofolate-homocysteine methyltransferase reductase (Mtrr), mRNA [NM_001039003]	**0,486**
Sipa1l2	Rattus norvegicus signal-induced proliferation-associated 1 like 2 (Sipa1l2), mRNA [NM_001009704]	**0,486**
Aph1b	Rattus norvegicus anterior pharynx defective 1 homolog B (C. elegans) (Aph1b), mRNA [NM_001047090]	**0,486**
0	Unknown	**0,486**
RGD1304719	Rattus norvegicus similar to RIKEN cDNA 2810002N01 (RGD1304719), mRNA [NM_001037769]	**0,486**
RGD1560846	Rattus norvegicus similar to hypothetical protein MGC40178 (RGD1560846), mRNA [NM_001134603]	**0,486**
RGD1304587	Rattus norvegicus similar to RIKEN cDNA 2310033P09 (RGD1304587), mRNA [NM_001100551]	**0,486**
Zfp281	Rattus norvegicus zinc finger protein 281 (Zfp281), mRNA [NM_001012030]	**0,486**
Hist1h2bh	Rattus norvegicus histone cluster 1, H2bh (Hist1h2bh), mRNA [NM_001107352]	**0,486**
Idi2	Rattus norvegicus isopentenyl-diphosphate delta isomerase 2 (Idi2), mRNA [NM_001192008]	**0,486**
Pik3ca	Rattus norvegicus phosphoinositide-3-kinase, catalytic, alpha polypeptide (Pik3ca), mRNA [NM_133399]	**0,486**

0	Uncharacterized protein [Source:UniProtKB/TrEMBL;Acc:D4ABH4] [ENSRNOT00000014695]	**0,486**
Anxa10	Rattus norvegicus annexin A10 (Anxa10), mRNA [NM_001109110]	**0,486**
Ybx1	Rattus norvegicus Y box binding protein 1 (Ybx1), mRNA [NM_031563]	**0,486**
Irf1	Rattus norvegicus interferon regulatory factor 1 (Irf1), mRNA [NM_012591]	**0,487**
Mthfd2	Rattus norvegicus methylenetetrahydrofolate dehydrogenase (NADP+ dependent) 2, methenyltetrahydrofolate cyclohydrolase (Mthfd2), nuclear gene encoding mitochondrial protein, mRNA [NM_001109398]	**0,487**
Tuba1b	Rattus norvegicus tubulin, alpha 1B (Tuba1b), mRNA [NM_001044270]	**0,487**
LOC641520	Rattus norvegicus popeye domain-containing 3 (LOC641520), mRNA [NM_001037369]	**0,487**
Bicd2	Rattus norvegicus bicaudal D homolog 2 (Drosophila) (Bicd2), transcript variant 1, mRNA [NM_001033674]	**0,487**
Psmd1	Rattus norvegicus proteasome (prosome, macropain) 26S subunit, non-ATPase, 1 (Psmd1), mRNA [NM_031978]	**0,487**
Rabep1	Rattus norvegicus rabaptin, RAB GTPase binding effector protein 1 (Rabep1), mRNA [NM_019124]	**0,487**
Zcchc17	Rattus norvegicus zinc finger, CCHC domain containing 17 (Zcchc17), mRNA [NM_001109267]	**0,487**
Pikfyve	Similar to phosphatidylinositol-3-phosphate/phosphatidylinositol 5-kinase, type III isoform 2Uncharacterized protein [Source:UniProtKB/TrEMBL;Acc:D3ZT14] [ENSRNOT00000020447]	**0,487**
Znf286a	Rattus norvegicus zinc finger protein 286A (Znf286a), mRNA [NM_001191921]	**0,487**
N4bp3	Rattus norvegicus Nedd4 binding protein 3 (N4bp3), mRNA [NM_001033893]	**0,487**
RGD1305500	Rattus norvegicus similar to hypothetical protein FLJ13188 (RGD1305500), mRNA [NM_001107448]	**0,487**
RGD1564599	Rattus norvegicus hypothetical protein LOC689600 (LOC689600), mRNA [NM_001106351]	**0,487**
Hmgcs1	Rattus norvegicus 3-hydroxy-3-methylglutaryl-Coenzyme A synthase 1 (soluble) (Hmgcs1), mRNA [NM_017268]	**0,487**
Fam193b	Rattus norvegicus family with sequence similarity 193, member B (Fam193b), mRNA [NM_001170408]	**0,487**
LOC296778	Rattus norvegicus similar to RIKEN cDNA 4933431D05 (LOC296778), mRNA [XM_216072]	**0,487**
Sh3bp4	Rattus norvegicus SH3-domain binding protein 4 (Sh3bp4), mRNA [NM_022693]	**0,487**
Phospho2	Rattus norvegicus phosphatase, orphan 2 (Phospho2), mRNA [NM_001007642]	**0,487**
Pxdn	PREDICTED: Rattus norvegicus peroxidasin homolog (Drosophila) (Pxdn), partial mRNA [XM_001053103]	**0,487**
Slc25a46	Rattus norvegicus solute carrier family 25, member 46 (Slc25a46), mRNA [NM_001100515]	**0,488**
RGD1309730	Rattus norvegicus similar to RIKEN cDNA B230118H07 (RGD1309730), mRNA [NM_001106491]	**0,488**
Tect2	Rattus norvegicus tectonic 2 (Tect2), mRNA [NM_001080782]	**0,488**
Pitrm1	Rattus norvegicus pitrilysin metallopeptidase 1 (Pitrm1), nuclear gene encoding mitochondrial protein, mRNA [NM_001107363]	**0,488**
RT1-A2	Rattus norvegicus RT1 class Ia, locus A2 (RT1-A2), mRNA [NM_001008829]	**0,488**
Mgat4a	Rattus norvegicus mannosyl (alpha-1,3-)-glycoprotein beta-1,4-N-acetylglucosaminyltransferase, isozyme A (Mgat4a), transcript variant 1, mRNA [NM_001160155]	**0,488**
0	Copper-transporting ATPase 1 [Source:UniProtKB/Swiss-Prot;Acc:P70705] [ENSRNOT00000003395]	**0,488**
Sf4	Rattus norvegicus splicing factor 4 (Sf4), mRNA [NM_001011920]	**0,488**
Mcf2	PREDICTED: Rattus norvegicus MCF.2 cell line derived transforming sequence (Mcf2), mRNA [XM_002727688]	**0,488**

Gng10	Rattus norvegicus guanine nucleotide binding protein (G protein), gamma 10 (Gng10), mRNA [NM_053660]	**0,488**
Znf629	Rattus norvegicus zinc finger protein-like mRNA, complete sequence. [AY589489]	**0,488**
Cdk105	Rattus norvegicus CDK105 protein (Cdk105), mRNA [NM_134415]	**0,488**
Hip1r	Rattus norvegicus huntingtin interacting protein 1 related (Hip1r), transcript variant 1, mRNA [NM_001134763]	**0,488**
RGD1359616	Rattus norvegicus similar to 2010321M09Rik protein (RGD1359616), mRNA [NM_001007663]	**0,488**
Suds3	Uncharacterized protein [Source:UniProtKB/TrEMBL;Acc:D3ZYN5] [ENSRNOT00000001504]	**0,489**
Ppp1cc	Rattus norvegicus protein phosphatase 1, catalytic subunit, gamma isoform (Ppp1cc), mRNA [NM_022498]	**0,489**
Ptprm	Rattus norvegicus protein tyrosine phosphatase, receptor type, M (Ptprm), mRNA [NM_001168632]	**0,489**
Serf1	PREDICTED: Rattus norvegicus small EDRK-rich factor 1 (Serf1), mRNA [XM_001070716]	**0,489**
Rpl39	Rattus norvegicus ribosomal protein L39 (Rpl39), mRNA [NM_012875]	**0,489**
0	tRNA-splicing endonuclease subunit Sen15 [Source:RefSeq peptide;Acc:NP_001099428] [ENSRNOT00000003226]	**0,489**
Haus3	Rattus norvegicus HAUS augmin-like complex, subunit 3 (Haus3), mRNA [NM_001109412]	**0,489**
Slco1c1	Rattus norvegicus solute carrier organic anion transporter family, member 1c1 (Slco1c1), mRNA [NM_053441]	**0,489**
Rt1.aa	Rattus norvegicus MHC class I RT1.Aa alpha-chain (Rt1.aa), mRNA [NM_001134701]	**0,489**
Fam5c	Rattus norvegicus family with sequence similarity 5, member C (Fam5c), mRNA [NM_173121]	**0,489**
Aldh2	Rattus norvegicus aldehyde dehydrogenase 2 family (mitochondrial) (Aldh2), nuclear gene encoding mitochondrial protein, mRNA [NM_032416]	**0,489**
Tmem17	Rattus norvegicus transmembrane protein 17 (Tmem17), mRNA [NM_001010961]	**0,489**
Map3k7ip1	Rattus norvegicus mitogen-activated protein kinase kinase kinase 7 interacting protein 1 (Map3k7ip1), mRNA [NM_001109976]	**0,489**
0	Rattus norvegicus similar to expressed sequence AI850305 (LOC314660), mRNA [XM_234947]	**0,489**
Ctr9	Rattus norvegicus Ctr9, Paf1/RNA polymerase II complex component, homolog (S. cerevisiae) (Ctr9), mRNA [NM_001100661]	**0,489**
Acyp2	Rattus norvegicus acylphosphatase 2, muscle type (Acyp2), mRNA [NM_001169145]	**0,489**
Rock1	Rattus norvegicus Rho-associated coiled-coil containing protein kinase 1 (Rock1), mRNA [NM_031098]	**0,489**
Secisbp2	Rattus norvegicus SECIS binding protein 2 (Secisbp2), mRNA [NM_024002]	**0,489**
RGD1561635	PREDICTED: Rattus norvegicus similar to 40S ribosomal protein S17 (RGD1561635), mRNA [XM_002724878]	**0,490**
Atg16l1	Rattus norvegicus ATG16 autophagy related 16-like 1 (S. cerevisiae) (Atg16l1), mRNA [NM_001108809]	**0,490**
Atp5s	Rattus norvegicus ATP synthase, H+ transporting, mitochondrial F0 complex, subunit s (factor B) (Atp5s), nuclear gene encoding mitochondrial protein, mRNA [NM_001007749]	**0,490**
Erbb2	Rattus norvegicus v-erb-b2 erythroblastic leukemia viral oncogene homolog 2, neuro/glioblastoma derived oncogene homolog (avian) (Erbb2), mRNA [NM_017003]	**0,490**
0	Uncharacterized protein [Source:UniProtKB/TrEMBL;Acc:D4A886] [ENSRNOT00000052276]	**0,490**
Pnmal2	Rattus norvegicus PNMA-like 2 (Pnmal2), mRNA [NM_001107481]	**0,490**
Cdh7	Rattus norvegicus cadherin 7, type 2 (Cdh7), mRNA [NM_001012737]	**0,490**
Traf3ip3	Rattus norvegicus TRAF3 interacting protein 3 (Traf3ip3), mRNA [NM_001014132]	**0,490**
Cog8	Rattus norvegicus component of oligomeric golgi complex 8 (Cog8), mRNA [NM_001106182]	**0,490**

Slc12a6	Rattus norvegicus solute carrier family 12, member 6 (Slc12a6), mRNA [NM_001109630]	**0,490**
RGD1564803	PREDICTED: Rattus norvegicus RGD1564803 (RGD1564803), mRNA [XM_579791]	**0,490**
Eef1g	Rattus norvegicus eukaryotic translation elongation factor 1 gamma (Eef1g), mRNA [NM_001004223]	**0,490**
0	Q80XK5_MOUSE (Q80XK5) Frmpd1 protein (Fragment), partial (23%) [TC584718]	**0,490**
0	Unknown	**0,490**
LOC688302	PREDICTED: Rattus norvegicus similar to spermatogenesis associated glutamate (E)-rich protein 4d (LOC688302), partial mRNA [XM_002730119]	**0,490**
Snca	synuclein SYN2 {alternatively spliced} [rats, mRNA, 695 nt]. [S73008]	**0,490**
Cdk105	Rattus norvegicus CDK105 protein (Cdk105), mRNA [NM_134415]	**0,490**
Nat1	Rattus norvegicus N-acetyltransferase 1 (Nat1), transcript variant 1, mRNA [NM_053853]	**0,490**
Pin1	Rattus norvegicus peptidylprolyl cis/trans isomerase, NIMA-interacting 1 (Pin1), mRNA [NM_001106701]	**0,490**
Vegfc	Rattus norvegicus vascular endothelial growth factor C (Vegfc), mRNA [NM_053653]	**0,491**
Slc25a17	Rattus norvegicus solute carrier family 25 (mitochondrial carrier, peroxisomal membrane protein), member 17 (Slc25a17), nuclear gene encoding mitochondrial protein, mRNA [NM_001126269]	**0,491**
Grik1	Rattus norvegicus glutamate receptor, ionotropic, kainate 1 (Grik1), transcript variant 2, mRNA [NM_017241]	**0,491**
Tmem168	Rattus norvegicus transmembrane protein 168 (Tmem168), mRNA [NM_001014054]	**0,491**
Gpr98	PREDICTED: Rattus norvegicus G protein-coupled receptor 98 (Gpr98), mRNA [XM_002725894]	**0,491**
Xpo6	Rattus norvegicus exportin 6 (Xpo6), mRNA [NM_001011935]	**0,491**
0	Rattus norvegicus TL0ADA12YN23 mRNA sequence. [FQ223309]	**0,491**
Neurl4	Rattus norvegicus neuralized homolog 4 (Drosophila) (Neurl4), mRNA [NM_001107013]	**0,491**
Ubac1	Rattus norvegicus UBA domain containing 1 (Ubac1), mRNA [NM_001007742]	**0,491**
Rmi1	Uncharacterized protein [Source:UniProtKB/TrEMBL;Acc:D3ZHX4] [ENSRNOT00000025812]	**0,491**
Btbd1	Rattus norvegicus BTB (POZ) domain containing 1 (Btbd1), mRNA [NM_001011932]	**0,491**
Xpot	Rattus norvegicus exportin, tRNA (nuclear export receptor for tRNAs) (Xpot), mRNA [NM_001108102]	**0,491**
LOC682206	PREDICTED: Rattus norvegicus similar to Zinc finger protein 208 (LOC682206), mRNA [XM_001060419]	**0,492**
Mgat4a	Rattus norvegicus mannosyl (alpha-1,3-)-glycoprotein beta-1,4-N-acetylglucosaminyltransferase, isozyme A (Mgat4a), transcript variant 1, mRNA [NM_001160155]	**0,492**
Erh	Rattus norvegicus enhancer of rudimentary homolog (Drosophila) (Erh), mRNA [NM_001109442]	**0,492**
Slc15a4	Rattus norvegicus solute carrier family 15, member 4 (Slc15a4), mRNA [NM_144758]	**0,492**
Nr2f6	Rattus norvegicus nuclear receptor subfamily 2, group F, member 6 (Nr2f6), mRNA [NM_139113]	**0,492**
Mrps18c	Rattus norvegicus mitochondrial ribosomal protein S18C (Mrps18c), nuclear gene encoding mitochondrial protein, mRNA [NM_001105996]	**0,492**
0	Unknown	**0,492**
0	Succinate-semialdehyde dehydrogenase, mitochondrial [Source:UniProtKB/Swiss-Prot;Acc:P51650] [ENSRNOT00000050919]	**0,492**
0	Kinesin-like protein KIF1B [Source:UniProtKB/Swiss-Prot;Acc:O88658] [ENSRNOT00000029605]	**0,492**

LOC311026	PREDICTED: Rattus norvegicus similar to mKIAA1461 protein (LOC311026), miscRNA [XR_007266]	**0,492**
RGD1311910	Rattus norvegicus similar to hypothetical p38 protein (RGD1311910), mRNA [NM_001107373]	**0,492**
Metrn	Rattus norvegicus meteorin, glial cell differentiation regulator (Metrn), mRNA [NM_001009962]	**0,492**
0	Unknown	**0,492**
Inppl1	Rattus norvegicus inositol polyphosphate phosphatase-like 1 (Inppl1), mRNA [NM_022944]	**0,492**
0	Unknown	**0,492**
Efha1	Rattus norvegicus EF-hand domain family, member A1 (Efha1), mRNA [NM_134396]	**0,492**
RGD1565192	Rattus norvegicus similar to 1810013D10Rik protein (RGD1565192), mRNA [NM_001134639]	**0,492**
Pdss2	Rattus norvegicus prenyl (decaprenyl) diphosphate synthase, subunit 2 (Pdss2), mRNA [NM_001014249]	**0,492**
0	Unknown	**0,492**
Zfp131	Rattus norvegicus zinc finger protein 131 (Zfp131), mRNA [NM_001100698]	**0,492**
Rsrc1	Rattus norvegicus arginine/serine-rich coiled-coil 1 (Rsrc1), mRNA [NM_001014172]	**0,492**
Rpp14	Rattus norvegicus ribonuclease P 14 subunit (human) (Rpp14), transcript variant 1, mRNA [NM_001108372]	**0,492**
Snrnp27	Rattus norvegicus small nuclear ribonucleoprotein 27 (U4/U6.U5) (Snrnp27), mRNA [NM_001108636]	**0,492**
Fam71e1	Uncharacterized protein [Source:UniProtKB/TrEMBL;Acc:D4A176] [ENSRNOT00000043724]	**0,492**
Jph3	Rattus norvegicus junctophilin 3 (Jph3), mRNA [NM_001107437]	**0,492**
0	Uncharacterized protein [Source:UniProtKB/TrEMBL;Acc:D3ZQW1] [ENSRNOT00000015065]	**0,493**
Mex3d	PREDICTED: Rattus norvegicus ring finger (C3HC4 type) and KH domain containing 1 (Rkhd1), mRNA [XM_234921]	**0,493**
Chd1	Rattus norvegicus chromodomain helicase DNA binding protein 1 (Chd1), mRNA [NM_001107465]	**0,493**
0	Fam40a protein [Source:UniProtKB/TrEMBL;Acc:Q4FZS7] [ENSRNOT00000033776]	**0,493**
RGD1308261	Rattus norvegicus similar to RIKEN cDNA 1190005P17 (RGD1308261), mRNA [NM_001134464]	**0,493**
Gpt	Rattus norvegicus glutamic-pyruvate transaminase (alanine aminotransferase) (Gpt), mRNA [NM_031039]	**0,493**
Acot8	Rattus norvegicus acyl-CoA thioesterase 8 (Acot8), mRNA [NM_130756]	**0,493**
Zbtb43	Rattus norvegicus zinc finger and BTB domain containing 43 (Zbtb43), mRNA [NM_001012094]	**0,493**
0	Uncharacterized protein [Source:UniProtKB/TrEMBL;Acc:D4A3V3] [ENSRNOT00000011481]	**0,493**
Pik3r3	Rattus norvegicus phosphoinositide-3-kinase, regulatory subunit 3 (gamma) (Pik3r3), mRNA [NM_022213]	**0,493**
0	ATG2 autophagy related 2 homolog B (S. cerevisiae) Gene [Source:MGI Symbol;Acc:MGI:1923809] [ENSRNOT00000006131]	**0,493**
Bcl6	Rattus norvegicus B-cell CLL/lymphoma 6 (Bcl6), mRNA [NM_001107084]	**0,493**
Parn	PREDICTED: Rattus norvegicus poly(A)-specific ribonuclease (deadenylation nuclease) (Parn), mRNA [XM_001075629]	**0,493**
0	Unknown	**0,493**
Bat4	Rattus norvegicus HLA-B associated transcript 4 (Bat4), mRNA [NM_001034157]	**0,493**
0	Unknown	**0,493**
Bnip3	Rattus norvegicus BCL2/adenovirus E1B interacting protein 3 (Bnip3), nuclear gene encoding mitochondrial protein, mRNA [NM_053420]	**0,493**
Phf3	Rattus norvegicus PHD finger protein 3 (Phf3), mRNA [NM_001108791]	**0,494**

Pqbp1	Rattus norvegicus polyglutamine binding protein 1 (Pqbp1), mRNA [NM_001013957]	**0,494**
Prom1	Rattus norvegicus prominin 1 (Prom1), transcript variant 1, mRNA [NM_021751]	**0,494**
RGD1561481	PREDICTED: Rattus norvegicus similar to ubiquitin specific protease 12 (RGD1561481), mRNA [XM_577055]	**0,494**
Zfp386	Rattus norvegicus zinc finger protein 386 (Kruppel-like) (Zfp386), mRNA [NM_019620]	**0,494**
Mrpl34	Rattus norvegicus mitochondrial ribosomal protein L34 (Mrpl34), nuclear gene encoding mitochondrial protein, mRNA [NM_001006965]	**0,494**
LOC687346	PREDICTED: Rattus norvegicus similar to BRCA1-associated protein (BRAP2) (Impedes mitogenic signal propagation) (IMP) (LOC687346), mRNA [XM_001075972]	**0,494**
E2f1	Rattus norvegicus E2F transcription factor 1 (E2f1), mRNA [NM_001100778]	**0,494**
Phf7	Rattus norvegicus PHD finger protein 7 (Phf7), mRNA [NM_001012211]	**0,494**
Pno1	Rattus norvegicus partner of NOB1 homolog (S. cerevisiae) (Pno1), mRNA [NM_199083]	**0,494**
Rnf152	Rattus norvegicus ring finger protein 152 (Rnf152), mRNA [NM_001106305]	**0,494**
Nov	Rattus norvegicus nephroblastoma overexpressed gene (Nov), mRNA [NM_030868]	**0,494**
Ephb1	Rattus norvegicus Eph receptor B1 (Ephb1), mRNA [NM_001104528]	**0,494**
RGD1564839	Uncharacterized protein [Source:UniProtKB/TrEMBL;Acc:D3ZKU5] [ENSRNOT00000043590]	**0,494**
0	Unknown	**0,494**
Sh3rf3	PREDICTED: Rattus norvegicus SH3 multiple domains 4, transcript variant 2 (Sh3md4), mRNA [XM_001054117]	**0,494**
0	Unknown	**0,494**
0	Unknown	**0,495**
Fau	Rattus norvegicus Finkel-Biskis-Reilly murine sarcoma virus (FBR-MuSV) ubiquitously expressed (Fau), transcript variant 1, mRNA [NM_001012739]	**0,495**
Ppp1r1a	Rattus norvegicus protein phosphatase 1, regulatory (inhibitor) subunit 1A (Ppp1r1a), mRNA [NM_022676]	**0,495**
Klhdc8a	Rattus norvegicus kelch domain containing 8A (Klhdc8a), mRNA [NM_001100683]	**0,495**
Mlx	Rattus norvegicus MAX-like protein X (Mlx), mRNA [NM_001034112]	**0,495**
Map2k3	Rattus norvegicus mitogen activated protein kinase kinase 3 (Map2k3), mRNA [NM_001100674]	**0,495**
Smchd1	PREDICTED: Rattus norvegicus similar to RIKEN cDNA 4931400A14 (RGD1307234), mRNA [XM_001056555]	**0,495**
Mrpl19	Rattus norvegicus mitochondrial ribosomal protein L19 (Mrpl19), nuclear gene encoding mitochondrial protein, mRNA [NM_001029898]	**0,495**
Hsf2	Rattus norvegicus heat shock transcription factor 2 (Hsf2), mRNA [NM_031694]	**0,495**
0	Unknown	**0,495**
Grk6	Rattus norvegicus G protein-coupled receptor kinase 6 (Grk6), transcript variant 1, mRNA [NM_031657]	**0,495**
Accn4	Rattus norvegicus amiloride-sensitive cation channel 4, pituitary (Accn4), mRNA [NM_022234]	**0,495**
Ifrd2	Rattus norvegicus interferon-related developmental regulator 2 (Ifrd2), mRNA [NM_001047871]	**0,495**
0	Unknown	**0,495**
0	Unknown	**0,495**
0	Rattus norvegicus chromosome 1, 5 clones, strain BN/SsNHsdMCW RNOR03207167, whole genome shotgun sequence [AABR03001102]	**0,495**

Tubgcp2	Rattus norvegicus tubulin, gamma complex associated protein 2 (Tubgcp2), mRNA [NM_001107560]	**0,495**
Srp68	Rattus norvegicus signal recognition particle 68 (Srp68), mRNA [NM_001108840]	**0,495**
Pold3	Rattus norvegicus polymerase (DNA-directed), delta 3, accessory subunit (Pold3), mRNA [NM_001024750]	**0,495**
Rapgef5	Rattus norvegicus Rap guanine nucleotide exchange factor (GEF) 5 (Rapgef5), mRNA [NM_001047915]	**0,495**
Cep135	Uncharacterized protein [Source:UniProtKB/TrEMBL;Acc:D3ZI35] [ENSRNOT00000032156]	**0,495**
Dok1	Rattus norvegicus docking protein 1 (Dok1), mRNA [NM_001025416]	**0,495**
Ppp1ca	Rattus norvegicus protein phosphatase 1, catalytic subunit, alpha isoform (Ppp1ca), mRNA [NM_031527]	**0,495**
Tm7sf3	Rattus norvegicus transmembrane 7 superfamily member 3 (Tm7sf3), mRNA [NM_001011970]	**0,496**
Prkcsh	Rattus norvegicus protein kinase C substrate 80K-H (Prkcsh), mRNA [NM_001106806]	**0,496**
Puf60	Rattus norvegicus poly-U binding splicing factor 60 (Puf60), mRNA [NM_001191880]	**0,496**
Rab40c	Rattus norvegicus Rab40c, member RAS oncogene family (Rab40c), mRNA [NM_182675]	**0,496**
LOC690126	PREDICTED: Rattus norvegicus hypothetical protein LOC690126 (LOC690126), miscRNA [XR_006805]	**0,496**
Agtr1a	Rattus norvegicus angiotensin II receptor, type 1a (Agtr1a), mRNA [NM_030985]	**0,496**
Pigo	Uncharacterized protein [Source:UniProtKB/TrEMBL;Acc:D3ZTP8] [ENSRNOT00000013213]	**0,496**
Usp42	Rattus norvegicus ubiquitin specific peptidase 42 (Usp42), mRNA [NM_001105909]	**0,496**
0	Uncharacterized protein [Source:UniProtKB/TrEMBL;Acc:D3ZG02] [ENSRNOT00000029178]	**0,496**
RGD1307597	Rattus norvegicus similar to mKIAA0317 protein (RGD1307597), mRNA [NM_001106744]	**0,496**
LOC679934	Rattus norvegicus similar to chromosome 3 open reading frame 10 (LOC679934), mRNA [NM_001195476]	**0,496**
Pdcl3	Rattus norvegicus phosducin-like 3 (Pdcl3), mRNA [NM_001025709]	**0,496**
Vav3	Rattus norvegicus vav 3 guanine nucleotide exchange factor (Vav3), mRNA [NM_001191714]	**0,496**
Dnase2a	Rattus norvegicus deoxyribonuclease II alpha (Dnase2a), mRNA [NM_138539]	**0,496**
Wwc1	PREDICTED: Rattus norvegicus WW and C2 domain containing 1, transcript variant 2 (Wwc1), mRNA [XM_002724669]	**0,496**
Lyl1	Rattus norvegicus lymphoblastic leukemia derived sequence 1 (Lyl1), mRNA [NM_001007677]	**0,496**
Rpl24	Rattus norvegicus ribosomal protein L24 (Rpl24), mRNA [NM_022515]	**0,496**
Mcts1	Rattus norvegicus malignant T cell amplified sequence 1 (Mcts1), mRNA [NM_001044237]	**0,496**
0	Unknown	**0,496**
Bub1	Rattus norvegicus budding uninhibited by benzimidazoles 1 homolog (S. cerevisiae) (Bub1), mRNA [NM_001106507]	**0,496**
Gmfg	Rattus norvegicus glia maturation factor, gamma (Gmfg), mRNA [NM_181091]	**0,496**
Lrtm2	Rattus norvegicus leucine-rich repeats and transmembrane domains 2 (Lrtm2), mRNA [NM_001109430]	**0,496**
LOC499124	Rattus norvegicus mouse zinc finger protein 14-like (LOC499124), mRNA [NM_001100991]	**0,496**
Rnasel	Rattus norvegicus ribonuclease L (2',5'-oligoisoadenylate synthetase-dependent) (Rnasel), mRNA [NM_182673]	**0,496**
Dgcr2	Rattus norvegicus TL0AAA82YH19 mRNA sequence. [FQ211720]	**0,496**
Ddit4	Rattus norvegicus DNA-damage-inducible transcript 4 (Ddit4), mRNA [NM_080906]	**0,496**
Dnase1l1	Rattus norvegicus deoxyribonuclease 1-like 1 (Dnase1l1), mRNA [NM_001014223]	**0,496**

Myo19	Rattus norvegicus myosin XIX (Myo19), mRNA [NM_001163736]	**0,496**
Hdac1	Rattus norvegicus histone deacetylase 1 (Hdac1), mRNA [NM_001025409]	**0,496**
LOC684903	PREDICTED: Rattus norvegicus similar to Probable ATP-dependent RNA helicase DHX34 (DEAH box protein 34), transcript variant 2 (LOC684903), mRNA [XM_001062397]	**0,497**
Smpd1	Rattus norvegicus sphingomyelin phosphodiesterase 1, acid lysosomal (Smpd1), mRNA [NM_001006997]	**0,497**
Herc4	Rattus norvegicus hect domain and RLD 4 (Herc4), mRNA [NM_001012074]	**0,497**
Mboat4	Rattus norvegicus membrane bound O-acyltransferase domain containing 4 (Mboat4), mRNA [NM_001107317]	**0,497**
Gsg1l	PREDICTED: Rattus norvegicus GSG1-like (Gsg1l), mRNA [XM_574558]	**0,497**
Fam134c	Rattus norvegicus family with sequence similarity 134, member C (Fam134c), mRNA [NM_001135804]	**0,497**
Eef2k	Eukaryotic elongation factor 2 kinase [Source:UniProtKB/Swiss-Prot;Acc:P70531] [ENSRNOT00000022726]	**0,497**
Elmod2	Rattus norvegicus ELMO/CED-12 domain containing 2 (Elmod2), mRNA [NM_001109506]	**0,497**
Cerk	Rattus norvegicus ceramide kinase (Cerk), mRNA [NM_001134861]	**0,497**
Dtx2	Rattus norvegicus deltex homolog 2 (Drosophila) (Dtx2), mRNA [NM_001107157]	**0,497**
Hpx	Rattus norvegicus hemopexin (Hpx), mRNA [NM_053318]	**0,497**
0	Unknown	**0,497**
Ezr	Rattus norvegicus ezrin (Ezr), mRNA [NM_019357]	**0,498**
Taz	Rattus norvegicus tafazzin (Taz), nuclear gene encoding mitochondrial protein, mRNA [NM_001025748]	**0,498**
Alms1	Rattus norvegicus Alstrom syndrome 1 homolog (human) (Alms1), mRNA [NM_001106604]	**0,498**
0	Unknown	**0,498**
Fam98a	Rattus norvegicus family with sequence similarity 98, member A (Fam98a), mRNA [NM_001014073]	**0,498**
Idh1	Rattus norvegicus isocitrate dehydrogenase 1 (NADP+), soluble (Idh1), mRNA [NM_031510]	**0,498**
RGD1563365	PREDICTED: Rattus norvegicus similar to DNA segment, Chr 10, Wayne State University 102, expressed (RGD1563365), mRNA [XM_002729760]	**0,498**
Etaa1	Rattus norvegicus Ewing tumor-associated antigen 1 (Etaa1), mRNA [NM_001109094]	**0,498**
Kcnk6	Rattus norvegicus potassium channel, subfamily K, member 6 (Kcnk6), mRNA [NM_053806]	**0,498**
Nop56	Rattus norvegicus NOP56 ribonucleoprotein homolog (yeast) (Nop56), mRNA [NM_001025732]	**0,498**
LOC688869	Rattus norvegicus similar to cytochrome c oxidase, subunit VIb polypeptide 1 (LOC688869), nuclear gene encoding mitochondrial protein, mRNA [NM_001145273]	**0,498**
RGD1311899	Rattus norvegicus similar to RIKEN cDNA 2210016L21 gene (RGD1311899), mRNA [NM_001009630]	**0,498**
Flcn	Rattus norvegicus folliculin (Flcn), mRNA [NM_199390]	**0,498**
Tnrc6b	Rattus norvegicus trinucleotide repeat containing 6B (Tnrc6b), mRNA [NM_138845]	**0,498**
Ftsj1	Uncharacterized protein [Source:UniProtKB/TrEMBL;Acc:D3ZZA1] [ENSRNOT00000006365]	**0,498**
Atg16l1	Rattus norvegicus ATG16 autophagy related 16-like 1 (S. cerevisiae) (Atg16l1), mRNA [NM_001108809]	**0,498**
0	Unknown	**0,498**

0	Unknown	**0,498**
Pnmal1	Rattus norvegicus PNMA-like 1 (Pnmal1), mRNA [NM_001108473]	**0,498**
RGD1563996	PREDICTED: Rattus norvegicus similar to Protein UNQ9166/PRO28631 precursor (RGD1563996), mRNA [XM_001077526]	**0,498**
B3gat2	Rattus norvegicus beta-1,3-glucuronyltransferase 2 (glucuronosyltransferase S) (B3gat2), mRNA [NM_022609]	**0,498**
Yipf1	Rattus norvegicus Yip1 domain family, member 1 (Yipf1), mRNA [NM_199383]	**0,499**
Sc4mol	Rattus norvegicus sterol-C4-methyl oxidase-like (Sc4mol), mRNA [NM_080886]	**0,499**
Cdc42se2	Rattus norvegicus CDC42 small effector 2 (Cdc42se2), mRNA [NM_001126089]	**0,499**
Usp53	Rattus norvegicus ubiquitin specific peptidase 53 (Usp53), mRNA [NM_001106468]	**0,499**
Ptpn2	Rattus norvegicus protein tyrosine phosphatase, non-receptor type 2 (Ptpn2), mRNA [NM_053990]	**0,499**
MGC94891	Rattus norvegicus hypothetical protein LOC681210 (MGC94891), mRNA [NM_001044277]	**0,499**
Aldh1b1	Rattus norvegicus aldehyde dehydrogenase 1 family, member B1 (Aldh1b1), nuclear gene encoding mitochondrial protein, mRNA [NM_001011975]	**0,499**
Cct6a	Rattus norvegicus chaperonin containing Tcp1, subunit 6A (zeta 1) (Cct6a), mRNA [NM_001033684]	**0,499**
Igfbp2	Rattus norvegicus insulin-like growth factor binding protein 2 (Igfbp2), mRNA [NM_013122]	**0,499**
Lrrcc1	Rattus norvegicus leucine rich repeat and coiled-coil domain containing 1 (Lrrcc1), mRNA [NM_001100645]	**0,499**
0	Uncharacterized protein [Source:UniProtKB/TrEMBL;Acc:D4A6W5] [ENSRNOT00000009094]	**0,499**
Syne1	Rattus norvegicus candidate plasticity protein 2b (cPG2B) mRNA, complete cds. [AY597251]	**0,499**
Sec24b	Rattus norvegicus SEC24 family, member B (S. cerevisiae) (Sec24b), mRNA [NM_001106474]	**0,499**
0	roundabout 2 [Source:RefSeq peptide;Acc:NP_115289] [ENSRNOT00000044546]	**0,499**
Mdm1	Rattus norvegicus Mdm1 nuclear protein homolog (mouse) (Mdm1), mRNA [NM_001017459]	**0,499**
Gfm2	Ribosome-releasing factor 2, mitochondrial [Source:UniProtKB/Swiss-Prot;Acc:Q5BJP6] [ENSRNOT00000052072]	**0,499**
Mrpl45	Rattus norvegicus mitochondrial ribosomal protein L45 (Mrpl45), nuclear gene encoding mitochondrial protein, mRNA [NM_001105834]	**0,499**
0	Q9NRI8_HUMAN (Q9NRI8) HT014, complete [TC634078]	**0,499**
Hnrnpa3	Rattus norvegicus heterogeneous nuclear ribonucleoprotein A3 (Hnrnpa3), transcript variant 1, mRNA [NM_001111294]	**0,499**
Nop14	Rattus norvegicus NOP14 nucleolar protein homolog (yeast) (Nop14), mRNA [NM_001106013]	**0,499**
RGD1564676	Rattus norvegicus similar to NF-X1 type zinc finger containing protein (2J511) (LOC364700), mRNA [XM_344589]	**0,499**
Gstcd	Rattus norvegicus glutathione S-transferase, C-terminal domain containing (Gstcd), mRNA [NM_001107725]	**0,499**
0	Unknown	**0,500**
Ppp2r5e	Rattus norvegicus protein phosphatase 2, regulatory subunit B', epsilon isoform (Ppp2r5e), mRNA [NM_001106740]	**0,500**
Adarb1	Rattus norvegicus adenosine deaminase, RNA-specific, B1 (Adarb1), transcript variant 1, mRNA [NM_012894]	**0,500**
Aph1a	Rattus norvegicus anterior pharynx defective 1 homolog A (C. elegans) (Aph1a), mRNA [NM_001014255]	**0,500**
Rps27a	Rattus norvegicus ribosomal protein S27a (Rps27a), mRNA [NM_031113]	**0,500**
Vps4a	Rattus norvegicus vacuolar protein sorting 4 homolog A (S. cerevisiae) (Vps4a), mRNA [NM_145678]	**0,500**
Rsl1d1	Rattus norvegicus ribosomal L1 domain containing 1 (Rsl1d1), mRNA [NM_001008876]	**0,500**
0	Unknown	**0,500**

Kcnn2	Rattus norvegicus potassium intermediate/small conductance calcium-activated channel, subfamily N, member 2 (Kcnn2), mRNA [NM_019314]	**0,500**
Tmem149	Rattus norvegicus transmembrane protein 149 (Tmem149), mRNA [NM_001134610]	**0,500**
LOC691995	Rattus norvegicus hypothetical protein LOC691995 (LOC691995), mRNA [NM_001103353]	**0,500**
Ccdc32	Rattus norvegicus coiled-coil domain containing 32 (Ccdc32), mRNA [NM_001024245]	**0,500**
Atpif1	Rattus norvegicus ATPase inhibitory factor 1 (Atpif1), nuclear gene encoding mitochondrial protein, mRNA [NM_012915]	**0,500**
0	Uncharacterized protein [Source:UniProtKB/TrEMBL;Acc:D3ZMU6] [ENSRNOT00000057016]	**0,500**
Tmem14a	Rattus norvegicus transmembrane protein 14A (Tmem14a), mRNA [NM_001108790]	**0,500**
Nol12	Rattus norvegicus nucleolar protein 12 (Nol12), mRNA [NM_001012747]	**0,500**
Ppp1r9a	Rattus norvegicus protein phosphatase 1, regulatory (inhibitor) subunit 9A (Ppp1r9a), mRNA [NM_053473]	**0,500**
Ttc23	Rattus norvegicus tetratricopeptide repeat domain 23 (Ttc23), mRNA [NM_001025681]	**0,500**
Rgs12	Rattus norvegicus regulator of G-protein signaling 12 (Rgs12), mRNA [NM_019339]	**0,500**
Brca2	Rattus norvegicus breast cancer 2 (Brca2), mRNA [NM_031542]	**0,500**
Dnajc21	Rattus norvegicus DnaJ (Hsp40) homolog, subfamily C, member 21 (Dnajc21), mRNA [NM_138856]	**0,500**
Rpia	Rattus norvegicus ribose 5-phosphate isomerase A (Rpia), mRNA [NM_001108632]	**0,500**
Sec24c	Rattus norvegicus SEC24 family, member C (S. cerevisiae) (Sec24c), mRNA [NM_001109456]	**0,501**
Kif13a	Rattus norvegicus kinesin family member 13A (Kif13a), mRNA [NM_001107462]	**0,501**
0	Rattus norvegicus similar to ribosomal protein S27a (LOC291599), mRNA [XM_225950]	**0,501**
Me1	Rattus norvegicus malic enzyme 1, NADP(+)-dependent, cytosolic (Me1), mRNA [NM_012600]	**0,501**
Arpc5l	Rattus norvegicus actin related protein 2/3 complex, subunit 5-like (Arpc5l), mRNA [NM_001037767]	**0,501**
Invs	Rattus norvegicus inversin (Invs), mRNA [NM_001107932]	**0,501**
B4galt3	Rattus norvegicus UDP-Gal:betaGlcNAc beta 1,4-galactosyltransferase, polypeptide 3 (B4galt3), mRNA [NM_001009539]	**0,501**
Bag1	Rattus norvegicus BCL2-associated athanogene (Bag1), mRNA [NM_001106647]	**0,501**
Rwdd1	Rattus norvegicus RWD domain containing 1 (Rwdd1), mRNA [NM_147146]	**0,501**
Prc1	Rattus norvegicus protein regulator of cytokinesis 1 (Prc1), mRNA [NM_001107529]	**0,501**
Mrpl46	Rattus norvegicus mitochondrial ribosomal protein L46 (Mrpl46), nuclear gene encoding mitochondrial protein, mRNA [NM_001013068]	**0,501**
RGD1309829	Rattus norvegicus similar to dJ842G6.1.1 (novel protein) (RGD1309829), mRNA [NM_001126371]	**0,501**
Zswim5	Rattus norvegicus zinc finger, SWIM domain containing 5 (Zswim5), mRNA [NM_001107963]	**0,501**
Dnajb6	Rattus norvegicus DnaJ (Hsp40) homolog, subfamily B, member 6 (Dnajb6), mRNA [NM_001013209]	**0,501**
Ptgr2	Rattus norvegicus prostaglandin reductase 2 (Ptgr2), mRNA [NM_001015009]	**0,501**
Atmin	Rattus norvegicus ATM interactor (Atmin), mRNA [NM_001191786]	**0,501**
Slc25a18	Rattus norvegicus solute carrier family 25 (mitochondrial carrier), member 18 (Slc25a18), nuclear gene encoding mitochondrial protein, mRNA [NM_001044280]	**0,502**
Xylt2	Rattus norvegicus xylosyltransferase II (Xylt2), mRNA [NM_022296]	**0,502**
Copb2	Rattus norvegicus coatomer protein complex, subunit beta 2 (beta prime) (Copb2), mRNA [NM_021765]	**0,502**

Tmem147	Rattus norvegicus transmembrane protein 147 (Tmem147), mRNA [NM_001038494]	**0,502**
Mrpl51	Rattus norvegicus mitochondrial ribosomal protein L51 (Mrpl51), nuclear gene encoding mitochondrial protein, mRNA [NM_001106621]	**0,502**
Slc4a3	Rattus norvegicus solute carrier family 4 (anion exchanger), member 3 (Slc4a3), mRNA [NM_017049]	**0,502**
Zfyve20	Rattus norvegicus zinc finger, FYVE domain containing 20 (Zfyve20), mRNA [NM_001107875]	**0,502**
Ruvbl2	Rattus norvegicus RuvB-like 2 (E. coli) (Ruvbl2), mRNA [NM_001025405]	**0,502**
Fam183b	Uncharacterized protein [Source:UniProtKB/TrEMBL;Acc:D3Z8P9] [ENSRNOT00000003841]	**0,502**
0	ORML3_RAT (Q6QI25) ORM1-like protein 3 (Liver regeneration-related protein LRRGT00183), complete [TC597420]	**0,502**
0	RVL9223 Wackym-Soares normalized rat vestibular cDNA library Rattus norvegicus cDNA 5', mRNA sequence [DV718613]	**0,502**
0	Unknown	**0,502**
RGD1564952	PREDICTED: Rattus norvegicus similar to zinc finger, RAN-binding domain containing 3 (RGD1564952), mRNA [XM_002728000]	**0,502**
Slc12a9	Rattus norvegicus solute carrier family 12 (potassium/chloride transporters), member 9 (Slc12a9), mRNA [NM_134405]	**0,502**
Rasa3	Rattus norvegicus mRNA for R-ras GTPase activating protein, partial cds. [AB028626]	**0,502**
Tril	Rattus norvegicus TLR4 interactor with leucine-rich repeats (Tril), mRNA [NM_001034010]	**0,502**
Coq2	Rattus norvegicus coenzyme Q2 homolog, prenyltransferase (yeast) (Coq2), nuclear gene encoding mitochondrial protein, mRNA [NM_001044255]	**0,502**
0	Peptidyl-prolyl cis-trans isomerase [Source:UniProtKB/TrEMBL;Acc:D3ZUZ9] [ENSRNOT00000011302]	**0,503**
Mcoln1	Rattus norvegicus mucolipin 1 (Mcoln1), mRNA [NM_001105903]	**0,503**
Rps6kc1	Rattus norvegicus ribosomal protein S6 kinase, polypeptide 1 (Rps6kc1), mRNA [NM_001105984]	**0,503**
RGD1304592	Rattus norvegicus similar to KIAA0528 protein (RGD1304592), mRNA [NM_001134581]	**0,503**
Hsp90aa1	Rattus norvegicus heat shock protein 90, alpha (cytosolic), class A member 1 (Hsp90aa1), mRNA [NM_175761]	**0,503**
RGD1306233	Rattus norvegicus similar to hypothetical protein MGC29761 (RGD1306233), mRNA [NM_001106564]	**0,503**
LOC362473	PREDICTED: Rattus norvegicus similar to Elongation factor 1-gamma (EF-1-gamma) (eEF-1B gamma) (LOC362473), miscRNA [XR_008347]	**0,503**
Sae1	Rattus norvegicus SUMO1 activating enzyme subunit 1 (Sae1), mRNA [NM_001012063]	**0,503**
Ddhd2	Uncharacterized protein [Source:UniProtKB/TrEMBL;Acc:D3ZJ91] [ENSRNOT00000020832]	**0,503**
Luc7l	Rattus norvegicus LUC7-like (S. cerevisiae) (Luc7l), mRNA [NM_001024269]	**0,503**
Pck2	Rattus norvegicus phosphoenolpyruvate carboxykinase 2 (mitochondrial) (Pck2), nuclear gene encoding mitochondrial protein, mRNA [NM_001108377]	**0,503**
38777	PREDICTED: Rattus norvegicus membrane-associated ring finger (C3HC4) 6 (March6), mRNA [XM_215517]	**0,503**
0	Unknown	**0,503**
RGD1304728	Uncharacterized protein [Source:UniProtKB/TrEMBL;Acc:D3ZNS7] [ENSRNOT00000018808]	**0,503**
0	Unknown	**0,503**
0	Uncharacterized protein [Source:UniProtKB/TrEMBL;Acc:D3ZQ99] [ENSRNOT00000046318]	**0,503**
Pcdhb20	Rattus norvegicus protocadherin beta 20 (Pcdhb20), mRNA [NM_001109395]	**0,503**
Agpat6	Rattus norvegicus 1-acylglycerol-3-phosphate O-acyltransferase 6 (lysophosphatidic acid acyltransferase, zeta) (Agpat6), mRNA [NM_001047849]	**0,503**

0	Uncharacterized protein [Source:UniProtKB/TrEMBL;Acc:D3ZEA8] [ENSRNOT00000044296]	**0,503**
Usp40	Rattus norvegicus ubiquitin specific peptidase 40 (Usp40), mRNA [NM_001134885]	**0,503**
0	PREDICTED: Rattus norvegicus 40S ribosomal protein SA-like (LOC100365517), mRNA [XM_002726683]	**0,503**
Itgb1	Rattus norvegicus integrin, beta 1 (Itgb1), mRNA [NM_017022]	**0,503**
0	Uncharacterized protein [Source:UniProtKB/TrEMBL;Acc:D3ZF29] [ENSRNOT00000012203]	**0,503**
Pgm2l1	Rattus norvegicus phosphoglucomutase 2-like 1 (Pgm2l1), mRNA [NM_001109454]	**0,503**
Bicd2	Rattus norvegicus bicaudal D homolog 2 (Drosophila) (Bicd2), transcript variant 1, mRNA [NM_001033674]	**0,503**
0	Uncharacterized protein [Source:UniProtKB/TrEMBL;Acc:D3ZFL8] [ENSRNOT00000035387]	**0,504**
0	Unknown	**0,504**
LOC690146	PREDICTED: Rattus norvegicus similar to zinc finger protein 11B (LOC690146), mRNA [XM_001073454]	**0,504**
Rspo3	Rattus norvegicus R-spondin 3 homolog (Xenopus laevis) (Rspo3), mRNA [NM_001100990]	**0,504**
0	Unknown	**0,504**
LOC317456	Rattus norvegicus hypothetical LOC317456 (LOC317456), mRNA [NM_001047894]	**0,504**
Prrx2	Rattus norvegicus paired related homeobox 2 (Prrx2), mRNA [NM_001105739]	**0,504**
Pcm1	Rattus norvegicus pericentriolar material 1 (Pcm1), mRNA [NM_031076]	**0,504**
Grinl1a	Rattus norvegicus glutamate receptor, ionotropic, N-methyl D-aspartate-like 1A (Grinl1a), mRNA [NM_183402]	**0,504**
Fbxw4	Rattus norvegicus F-box and WD repeat domain containing 4 (Fbxw4), mRNA [NM_001107600]	**0,504**
0	Unknown	**0,504**
0	Unknown	**0,504**
Stat1	Rattus norvegicus signal transducer and activator of transcription 1 (Stat1), transcript variant beta, mRNA [NM_001034164]	**0,504**
Bcat1	Rattus norvegicus branched chain aminotransferase 1, cytosolic (Bcat1), mRNA [NM_017253]	**0,504**
Slc27a5	Rattus norvegicus solute carrier family 27 (fatty acid transporter), member 5 (Slc27a5), mRNA [NM_024143]	**0,504**
Odz2	Rattus norvegicus odz, odd Oz/ten-m homolog 2 (Drosophila) (Odz2), mRNA [NM_020088]	**0,504**
Alg5	Rattus norvegicus asparagine-linked glycosylation 5, dolichyl-phosphate beta-glucosyltransferase homolog (S. cerevisiae) (Alg5), mRNA [NM_001025407]	**0,504**
Akt1	Rattus norvegicus v-akt murine thymoma viral oncogene homolog 1 (Akt1), mRNA [NM_033230]	**0,504**
Slc25a38	Rattus norvegicus solute carrier family 25, member 38 (Slc25a38), nuclear gene encoding mitochondrial protein, mRNA [NM_001030032]	**0,504**
Plekhh3	Rattus norvegicus pleckstrin homology domain containing, family H (with MyTH4 domain) member 3 (Plekhh3), mRNA [NM_001037202]	**0,504**
0	Unknown	**0,504**
Usp12	Rattus norvegicus ubiquitin specific peptidase 12 (Usp12), mRNA [NM_001166576]	**0,504**
Dynlt3	Rattus norvegicus dynein light chain Tctex-type 3 (Dynlt3), mRNA [NM_001013228]	**0,504**
Vps24	Rattus norvegicus vacuolar protein sorting 24 homolog (S. cerevisiae) (Vps24), mRNA [NM_172331]	**0,504**
LOC681754	PREDICTED: Rattus norvegicus similar to cytochrome c oxidase, subunit VIb polypeptide 1 (LOC681754), mRNA [XM_001058263]	**0,505**
Mettl9	Rattus norvegicus methyltransferase like 9 (Mettl9), mRNA [NM_001163164]	**0,505**

Taf5l	Rattus norvegicus TAF5-like RNA polymerase II, p300/CBP-associated factor (PCAF)-associated factor (Taf5l), mRNA [NM_001107442]	**0,505**
Nop56	Rattus norvegicus NOP56 ribonucleoprotein homolog (yeast) (Nop56), mRNA [NM_001025732]	**0,505**
Exosc9	Rattus norvegicus exosome component 9 (Exosc9), mRNA [NM_001025406]	**0,505**
Rae1	Rattus norvegicus RAE1 RNA export 1 homolog (S. pombe) (Rae1), mRNA [NM_001033708]	**0,505**
LOC681367	PREDICTED: Rattus norvegicus hypothetical protein LOC681367 (LOC681367), mRNA [XM_001061427]	**0,505**
0	Unknown	**0,505**
Ndufb4	Rattus norvegicus NADH dehydrogenase (ubiquinone) 1 beta subcomplex 4 (Ndufb4), nuclear gene encoding mitochondrial protein, mRNA [NM_001037338]	**0,505**
Fam160a2	Rattus norvegicus family with sequence similarity 160, member A2 (Fam160a2), mRNA [NM_001005538]	**0,505**
RGD1307934	PREDICTED: Rattus norvegicus similar to DNA segment, Chr 19, ERATO Doi 386, expressed (RGD1307934), mRNA [XM_001055620]	**0,505**
Ptcd3	Rattus norvegicus Pentatricopeptide repeat domain 3 (Ptcd3), nuclear gene encoding mitochondrial protein, mRNA [NM_001134718]	**0,505**
Nxph3	Neurexophilin-3 [Source:UniProtKB/Swiss-Prot;Acc:Q9Z2N5] [ENSRNOT00000007133]	**0,505**
LOC688909	PREDICTED: Rattus norvegicus hypothetical protein LOC688909 (LOC688909), mRNA [XM_001068783]	**0,506**
0	Unknown	**0,506**
Pgls	Rattus norvegicus 6-phosphogluconolactonase (Pgls), mRNA [NM_001106066]	**0,506**
Ccdc50	Rattus norvegicus coiled-coil domain containing 50 (Ccdc50), mRNA [NM_182736]	**0,506**
RGD1311501	Rattus norvegicus similar to chromosome 9 open reading frame 7 (RGD1311501), mRNA [NM_001106561]	**0,506**
Olr1335	Rattus norvegicus olfactory receptor 1335 (Olr1335), mRNA [NM_001000479]	**0,506**
Tssc4	Rattus norvegicus tumor suppressing subtransferable candidate 4 (Tssc4), mRNA [NM_001013194]	**0,506**
Ap3d1	Rattus norvegicus adaptor-related protein complex 3, delta 1 subunit (Ap3d1), mRNA [NM_001100719]	**0,506**
Ascl2	Rattus norvegicus achaete-scute complex homolog 2 (Drosophila) (Ascl2), mRNA [NM_031503]	**0,506**
Zfp7	Rattus norvegicus zinc finger protein 7 (Zfp7), mRNA [NM_001142757]	**0,506**
Rabepk	Rattus norvegicus Rab9 effector protein with kelch motifs (Rabepk), mRNA [NM_001024871]	**0,506**
Elp2	Rattus norvegicus elongation protein 2 homolog (S. cerevisiae) (Elp2), mRNA [NM_001034145]	**0,506**
Efemp1	Rattus norvegicus EGF-containing fibulin-like extracellular matrix protein 1 (Efemp1), mRNA [NM_001012039]	**0,506**
Manea	Rattus norvegicus mannosidase, endo-alpha (Manea), mRNA [NM_080785]	**0,506**
LOC689316	PREDICTED: Rattus norvegicus hypothetical protein LOC689316 (LOC689316), miscRNA [XR_085654]	**0,506**
RGD1310861	Rattus norvegicus similar to RIKEN cDNA 1500011H22 (RGD1310861), mRNA [NM_001008290]	**0,506**
RGD1309708	Rattus norvegicus similar to RIKEN cDNA 4930455F23 (RGD1309708), mRNA [NM_001014131]	**0,506**
Prmt7	Rattus norvegicus protein arginine methyltransferase 7 (Prmt7), mRNA [NM_001014153]	**0,506**
0	Uncharacterized protein [Source:UniProtKB/TrEMBL;Acc:D3ZRD1] [ENSRNOT00000013290]	**0,506**
Rbm15b	RCG25263Uncharacterized protein [Source:UniProtKB/TrEMBL;Acc:D3ZHD6] [ENSRNOT00000018995]	**0,506**
Car4	Rattus norvegicus carbonic anhydrase 4 (Car4), mRNA [NM_019174]	**0,506**
Cirbp	Rattus norvegicus cold inducible RNA binding protein (Cirbp), mRNA [NM_031147]	**0,506**

0	Uncharacterized protein [Source:UniProtKB/TrEMBL;Acc:D4A3Z9] [ENSRNOT00000064084]	**0,506**
Gtf2h3	Rattus norvegicus general transcription factor IIH, polypeptide 3 (Gtf2h3), mRNA [NM_001024236]	**0,506**
0	Uncharacterized protein [Source:UniProtKB/TrEMBL;Acc:D3ZDE9] [ENSRNOT00000041674]	**0,506**
Ebpl	Rattus norvegicus emopamil binding protein-like (Ebpl), mRNA [NM_001108381]	**0,506**
Dync1li2	Rattus norvegicus dynein, cytoplasmic 1 light intermediate chain 2 (Dync1li2), mRNA [NM_031026]	**0,506**
LOC100233176	Rattus norvegicus hypothetical protein LOC100233176 (LOC100233176), transcript variant 1, mRNA [NM_001142941]	**0,506**
Cry1	Rattus norvegicus cryptochrome 1 (photolyase-like) (Cry1), mRNA [NM_198750]	**0,506**
Rpl31	Rattus norvegicus ribosomal protein L31 (Rpl31), mRNA [NM_022506]	**0,507**
Ahcy	Rattus norvegicus adenosylhomocysteinase (Ahcy), mRNA [NM_017201]	**0,507**
Aldh1a1	Rattus norvegicus aldehyde dehydrogenase 1 family, member A1 (Aldh1a1), mRNA [NM_022407]	**0,507**
MGC125239	Rattus norvegicus hypothetical protein LOC686179 (MGC125239), mRNA [NM_001077231]	**0,507**
0	Unknown	**0,507**
Ptchd1	Rattus norvegicus patched domain containing 1 (Ptchd1), mRNA [NM_001191734]	**0,507**
MGC116202	Rattus norvegicus hypothetical protein LOC688736 (MGC116202), mRNA [NM_001044292]	**0,507**
Thumpd1	Rattus norvegicus THUMP domain containing 1 (Thumpd1), mRNA [NM_001009688]	**0,507**
Ripk1	Rattus norvegicus receptor (TNFRSF)-interacting serine-threonine kinase 1 (Ripk1), mRNA [NM_001107350]	**0,507**
Ufd1l	Rattus norvegicus ubiquitin fusion degradation 1 like (yeast) (Ufd1l), mRNA [NM_053418]	**0,507**
0	Unknown	**0,507**
Hmgn1	Rattus norvegicus high-mobility group nucleosome binding domain 1 (Hmgn1), mRNA [NM_001013184]	**0,507**
0	ERG6_MAGGR (Q5EN22) Sterol 24-C-methyltransferase (Delta(24)-sterol C-methyltransferase) , partial (5%) [TC583516]	**0,507**
mrpl11	Rattus norvegicus mitochondrial ribosomal protein L11 (mrpl11), nuclear gene encoding mitochondrial protein, mRNA [NM_001006973]	**0,507**
Dscr3	Rattus norvegicus Down syndrome critical region gene 3 (Dscr3), mRNA [NM_001108316]	**0,508**
Col23a1	Rattus norvegicus collagen, type XXIII, alpha 1 (Col23a1), mRNA [NM_181636]	**0,508**
Rnaset2	Rattus norvegicus ribonuclease T2 (Rnaset2), mRNA [NM_001106210]	**0,508**
Rpl30	Rattus norvegicus ribosomal protein L30 (Rpl30), mRNA [NM_022699]	**0,508**
0	Unknown	**0,508**
0	Unknown	**0,508**
0	DNJB6_MOUSE (O54946) DnaJ homolog subfamily B member 6 (Heat shock protein J2) (HSJ-2) (MRJ) (mDj4), partial (29%) [TC587510]	**0,508**
0	Uncharacterized protein [Source:UniProtKB/TrEMBL;Acc:D3ZR79] [ENSRNOT00000032299]	**0,508**
Man1a1	Rattus norvegicus mannosidase, alpha, class 1A, member 1 (Man1a1), mRNA [NM_001033656]	**0,508**
Nsfl1c	Rattus norvegicus NSFL1 (p97) cofactor (p47) (Nsfl1c), mRNA [NM_031981]	**0,508**
0	Unknown	**0,508**
0	AT rich interactive domain 1B (SWI-like) Gene [Source:MGI Symbol;Acc:MGI:1926129] [ENSRNOT00000022939]	**0,508**
LOC100364597	PREDICTED: Rattus norvegicus 5-nucleotidase, cytosolic II-like (LOC100364597), mRNA [XM_002725789]	**0,508**

Coq9	Rattus norvegicus coenzyme Q9 homolog (S. cerevisiae) (Coq9), nuclear gene encoding mitochondrial protein, mRNA [NM_001035257]	**0,508**
Sppl2b	Rattus norvegicus signal peptide peptidase-like 2B (Sppl2b), mRNA [NM_001014200]	**0,508**
Tcte4	Rattus norvegicus t-complex-associated testis expressed 4 (Tcte4), mRNA [NM_001166303]	**0,508**
Rps27	Rattus norvegicus ribosomal protein S27 (Rps27), mRNA [NM_053597]	**0,508**
Mrpl40	Rattus norvegicus mitochondrial ribosomal protein L40 (Mrpl40), nuclear gene encoding mitochondrial protein, mRNA [NM_001024865]	**0,508**
Vangl1	Rattus norvegicus vang-like 1 (van gogh, Drosophila) (Vangl1), mRNA [NM_001109584]	**0,508**
Sptlc1	Rattus norvegicus serine palmitoyltransferase, long chain base subunit 1 (Sptlc1), mRNA [NM_001108406]	**0,508**
Pim3	Rattus norvegicus pim-3 oncogene (Pim3), mRNA [NM_022602]	**0,508**
Usp40	Rattus norvegicus ubiquitin specific peptidase 40 (Usp40), mRNA [NM_001134885]	**0,508**
Mrpl35	Rattus norvegicus mitochondrial ribosomal protein L35 (Mrpl35), nuclear gene encoding mitochondrial protein, mRNA [NM_001106596]	**0,509**
0	Unknown	**0,509**
Lin54	Rattus norvegicus lin-54 homolog (C. elegans) (Lin54), mRNA [NM_001100564]	**0,509**
0	Unknown	**0,509**
Hapln3	Rattus norvegicus hyaluronan and proteoglycan link protein 3 (Hapln3), mRNA [NM_001008559]	**0,509**
RGD1311578	Rattus norvegicus similar to PRO1853 homolog (RGD1311578), mRNA [NM_001008318]	**0,509**
RGD1562351	Rattus norvegicus similar to chromosome 7 open reading frame 23 (RGD1562351), mRNA [NM_001109219]	**0,509**
Anxa7	Rattus norvegicus annexin A7 (Anxa7), mRNA [NM_130416]	**0,509**
Smarcal1	Rattus norvegicus Swi/SNF related matrix associated, actin dependent regulator of chromatin, subfamily a-like 1 (Smarcal1), mRNA [NM_001108222]	**0,509**
Fau	Rattus norvegicus Finkel-Biskis-Reilly murine sarcoma virus (FBR-MuSV) ubiquitously expressed (Fau), transcript variant 1, mRNA [NM_001012739]	**0,509**
Epc2	Rattus norvegicus enhancer of polycomb homolog 2 (Drosophila) (Epc2), mRNA [NM_001108581]	**0,509**
Abhd14a	Rattus norvegicus abhydrolase domain containing 14A (Abhd14a), mRNA [NM_001009670]	**0,509**
Oaz2	Rattus norvegicus ornithine decarboxylase antizyme 2 (Oaz2), mRNA [NM_001109899]	**0,509**
Layn	Rattus norvegicus layilin (Layn), mRNA [NM_001191997]	**0,509**
LOC679976	PREDICTED: Rattus norvegicus similar to ribosomal protein S27a (LOC679976), mRNA [XM_001057026]	**0,509**
0	Q8R4G5_MOUSE (Q8R4G5) Truncated N-acetylglucosaminyltransferase V, partial (35%) [TC588091]	**0,509**
Rpl24	Rattus norvegicus ribosomal protein L24 (Rpl24), mRNA [NM_022515]	**0,509**
0	Unknown	**0,509**
RGD1311378	Rattus norvegicus similar to RIKEN cDNA 2010011I20 (RGD1311378), mRNA [NM_001106547]	**0,509**
Aadat	Rattus norvegicus aminoadipate aminotransferase (Aadat), mRNA [NM_017193]	**0,509**
Ppp1r3d	Rattus norvegicus protein phosphatase 1, regulatory subunit 3D (Ppp1r3d), mRNA [NM_001109564]	**0,509**
Ap2a2	Rattus norvegicus adaptor-related protein complex 2, alpha 2 subunit (Ap2a2), mRNA [NM_031008]	**0,509**

Abcb10	Rattus norvegicus ATP-binding cassette, subfamily B (MDR/TAP), member 10 (Abcb10), nuclear gene encoding mitochondrial protein, mRNA [NM_001012166]	**0,510**
LOC686809	PREDICTED: Rattus norvegicus similar to protein 7 transactivated by hepatitis B virus X antigen (LOC686809), mRNA [XM_001075804]	**0,510**
0	Unknown	**0,510**
Cacng5	Rattus norvegicus calcium channel, voltage-dependent, gamma subunit 5 (Cacng5), mRNA [NM_080693]	**0,510**
Echdc1	Rattus norvegicus enoyl Coenzyme A hydratase domain containing 1 (Echdc1), mRNA [NM_001007734]	**0,510**
Senp6	Rattus norvegicus SUMO1/sentrin specific peptidase 6 (Senp6), mRNA [NM_001106842]	**0,510**
Igf2r	Rattus norvegicus insulin-like growth factor 2 receptor (Igf2r), mRNA [NM_012756]	**0,510**
Rnf160	Rattus norvegicus ring finger protein 160 (Rnf160), mRNA [NM_001024235]	**0,510**
Rbm42	Rattus norvegicus RNA binding motif protein 42 (Rbm42), mRNA [NM_001014159]	**0,510**
Dner	PREDICTED: Rattus norvegicus delta/notch-like EGF repeat containing (Dner), mRNA [XM_001064351]	**0,510**
Wdr75	Rattus norvegicus WD repeat domain 75 (Wdr75), mRNA [NM_001047889]	**0,510**
0	Unknown	**0,510**
Mbp	Rattus norvegicus myelin basic protein (Mbp), transcript variant 1, mRNA [NM_001025291]	**0,510**
Psmc3ip	Rattus norvegicus PSMC3 interacting protein (Psmc3ip), mRNA [NM_134458]	**0,510**
Phrf1	Rattus norvegicus PHD and ring finger domains 1 (Phrf1), mRNA [NM_139093]	**0,510**
Ifit2	Rattus norvegicus interferon-induced protein with tetratricopeptide repeats 2 (Ifit2), mRNA [NM_001024753]	**0,510**
Tlk2	Rattus norvegicus tousled-like kinase 2 (Tlk2), mRNA [NM_001191652]	**0,510**
Itgb5	Rattus norvegicus integrin, beta 5 (Itgb5), mRNA [NM_147139]	**0,510**
Srebf1	Sterol regulatory element-binding protein 1Processed sterol regulatory element-binding protein 1 [Source:UniProtKB/Swiss-Prot;Acc:P56720] [ENSRNOT00000047053]	**0,510**
Spdya	Rattus norvegicus speedy homolog A (Xenopus laevis) (Spdya), mRNA [NM_138855]	**0,510**
Cdkl2	Rattus norvegicus cyclin-dependent kinase-like 2 (CDC2-related kinase) (Cdkl2), mRNA [NM_001012035]	**0,510**
Ppp1cc	Rattus norvegicus protein phosphatase 1, catalytic subunit, gamma isoform (Ppp1cc), mRNA [NM_022498]	**0,510**
Nit2	Rattus norvegicus nitrilase family, member 2 (Nit2), mRNA [NM_001034126]	**0,510**
Prom1	Rattus norvegicus prominin 1 (Prom1), transcript variant 1, mRNA [NM_021751]	**0,511**
Dnhd1	PREDICTED: Rattus norvegicus dynein heavy chain domain 1 (Dnhd1), miscRNA [XR_085725]	**0,511**
Gabarapl2	Rattus norvegicus GABA(A) receptor-associated protein like 2 (Gabarapl2), mRNA [NM_022706]	**0,511**
Cycs	Rattus norvegicus cytochrome c, somatic (Cycs), nuclear gene encoding mitochondrial protein, mRNA [NM_012839]	**0,511**
0	Unknown	**0,511**
Ctbp1	Rattus norvegicus C-terminal binding protein 1 (Ctbp1), mRNA [NM_019201]	**0,511**
Myh13	Myosin heavy chain [Source:UniProtKB/TrEMBL;Acc:Q9Z2B7] [ENSRNOT00000055257]	**0,511**
Rundc1	PREDICTED: Rattus norvegicus RUN domain containing 1 (Rundc1), mRNA [XM_001081465]	**0,511**
Rnf19a	Rattus norvegicus ring finger protein 19A (Rnf19a), mRNA [NM_001130560]	**0,511**

Lipt2	Rattus norvegicus lipoyl(octanoyl) transferase 2 (putative) (Lipt2), nuclear gene encoding mitochondrial protein, mRNA [NM_001108917]	**0,511**
Lzts1	Rattus norvegicus leucine zipper, putative tumor suppressor 1 (Lzts1), mRNA [NM_153470]	**0,511**
Gpr149	Rattus norvegicus G protein-coupled receptor 149 (Gpr149), mRNA [NM_138891]	**0,511**
Chuk	Rattus norvegicus conserved helix-loop-helix ubiquitous kinase (Chuk), mRNA [NM_001107588]	**0,511**
Pgf	Rattus norvegicus placental growth factor (Pgf), mRNA [NM_053595]	**0,511**
0	Unknown	**0,511**
0	Unknown	**0,511**
Harbi1	Rattus norvegicus harbinger transposase derived 1 (Harbi1), mRNA [NM_001113793]	**0,511**
0	Uncharacterized protein [Source:UniProtKB/TrEMBL;Acc:D3ZCM1] [ENSRNOT00000024516]	**0,511**
Gins1	Rattus norvegicus GINS complex subunit 1 (Psf1 homolog) (Gins1), mRNA [NM_001109207]	**0,511**
Ddx3x	Rattus norvegicus DEAD (Asp-Glu-Ala-Asp) box polypeptide 3, X-linked (Ddx3x), mRNA [NM_001108246]	**0,511**
Znf777	Rattus norvegicus zinc finger protein 777 (Znf777), mRNA [NM_001109348]	**0,511**
Hsph1	Rattus norvegicus heat shock 105/110 protein 1 (Hsph1), mRNA [NM_001011901]	**0,511**
Tsen15	Rattus norvegicus tRNA splicing endonuclease 15 homolog (S. cerevisiae) (Tsen15), mRNA [NM_001105958]	**0,512**
Chmp7	Rattus norvegicus CHMP family, member 7 (Chmp7), mRNA [NM_001108872]	**0,512**
Hnrnpab	Rattus norvegicus heterogeneous nuclear ribonucleoprotein A/B (Hnrnpab), mRNA [NM_031330]	**0,512**
RGD1560936	PREDICTED: Rattus norvegicus similar to 60S ribosomal protein L13 (RGD1560936), mRNA [XM_219309]	**0,512**
Aip	Rattus norvegicus aryl-hydrocarbon receptor-interacting protein (Aip), mRNA [NM_172327]	**0,512**
Gpc4	Rattus norvegicus glypican 4 (Gpc4), mRNA [NM_001014108]	**0,512**
Ola1	Rattus norvegicus Obg-like ATPase 1 (Ola1), mRNA [NM_001033927]	**0,512**
Rnf20	Rattus norvegicus ring finger protein 20 (Rnf20), mRNA [NM_001107929]	**0,512**
Ctsc	Rattus norvegicus cathepsin C (Ctsc), mRNA [NM_017097]	**0,512**
Smyd3	Rattus norvegicus SET and MYND domain containing 3 (Smyd3), mRNA [NM_001025762]	**0,512**
Ube2s	Rattus norvegicus ubiquitin-conjugating enzyme E2S (Ube2s), mRNA [NM_001106224]	**0,512**
Zdhhc14	Rattus norvegicus zinc finger, DHHC-type containing 14 (Zdhhc14), mRNA [NM_001039343]	**0,512**
Prg4	Rattus norvegicus proteoglycan 4, (megakaryocyte stimulating factor, articular superficial zone protein, camptodactyly, arthropathy, coxa vara, pericarditis syndrome) (Prg4), mRNA [NM_001105962]	**0,512**
Ipo5	Uncharacterized protein [Source:UniProtKB/TrEMBL;Acc:D4A781] [ENSRNOT00000014859]	**0,512**
Mrpl36	Rattus norvegicus mitochondrial ribosomal protein L36 (Mrpl36), nuclear gene encoding mitochondrial protein, mRNA [NM_001108879]	**0,512**
Ccng1	Rattus norvegicus cyclin G1 (Ccng1), mRNA [NM_012923]	**0,512**
Anapc13	Rattus norvegicus anaphase promoting complex subunit 13 (Anapc13), mRNA [NM_001173983]	**0,512**
Cee	Rattus norvegicus conserved edge expressed protein (Cee), mRNA [NM_001163320]	**0,512**
Sts	Rattus norvegicus steroid sulfatase (Sts), mRNA [NM_012661]	**0,512**
Ube2k	Rattus norvegicus ubiquitin-conjugating enzyme E2K (UBC1 homolog, yeast) (Ube2k), mRNA [NM_001106006]	**0,512**

0	Unknown	**0,512**
Angptl4	Rattus norvegicus angiopoietin-like 4 (Angptl4), mRNA [NM_199115]	**0,512**
Grb14	Rattus norvegicus growth factor receptor bound protein 14 (Grb14), mRNA [NM_031623]	**0,513**
LOC100361629	Uncharacterized protein [Source:UniProtKB/TrEMBL;Acc:D4A202] [ENSRNOT00000061017]	**0,513**
0	Unknown	**0,513**
Pinx1	Rattus norvegicus PIN2-interacting protein 1 (Pinx1), mRNA [NM_001083337]	**0,513**
0	Uncharacterized protein [Source:UniProtKB/TrEMBL;Acc:D4A2X3] [ENSRNOT00000038101]	**0,513**
0	Unknown	**0,513**
Tcerg1	Rattus norvegicus transcription elongation regulator 1 (Tcerg1), mRNA [NM_001107390]	**0,513**
Ubxn2a	Rattus norvegicus UBX domain protein 2A (Ubxn2a), mRNA [NM_001109482]	**0,513**
P2rx6	Rattus norvegicus purinergic receptor P2X, ligand-gated ion channel, 6 (P2rx6), mRNA [NM_012721]	**0,513**
Shroom3	Rattus norvegicus shroom family member 3 (Shroom3), mRNA [NM_001100889]	**0,513**
Mapk14	Rattus norvegicus mitogen activated protein kinase 14 (Mapk14), mRNA [NM_031020]	**0,513**
Tars2	Rattus norvegicus threonyl-tRNA synthetase 2, mitochondrial (putative) (Tars2), nuclear gene encoding mitochondrial protein, mRNA [NM_001014040]	**0,513**
Trub1	Rattus norvegicus TruB pseudouridine (psi) synthase homolog 1 (E. coli) (Trub1), mRNA [NM_001012173]	**0,513**
Mfn2	Rattus norvegicus mitofusin 2 (Mfn2), nuclear gene encoding mitochondrial protein, mRNA [NM_130894]	**0,513**
N-pac	Putative oxidoreductase GLYR1 [Source:UniProtKB/Swiss-Prot;Acc:Q5RKH0] [ENSRNOT00000004159]	**0,513**
0	Unknown	**0,513**
RGD1561832	PREDICTED: Rattus norvegicus similar to CDNA sequence BC043301 (RGD1561832), mRNA [XM_001080370]	**0,513**
Arl4c	PREDICTED: Rattus norvegicus ADP-ribosylation factor-like 4C (Arl4c), miscRNA [XR_086347]	**0,513**
LOC290577	PREDICTED: Rattus norvegicus hypothetical LOC290577 (LOC290577), mRNA [XM_002728376]	**0,513**
Rit1	Rattus norvegicus Ras-like without CAAX 1 (Rit1), mRNA [NM_001109185]	**0,513**
Timm23	Rattus norvegicus translocase of inner mitochondrial membrane 23 homolog (yeast) (Timm23), nuclear gene encoding mitochondrial protein, mRNA [NM_019352]	**0,513**
Lsg1	Rattus norvegicus large subunit GTPase 1 homolog (S. cerevisiae) (Lsg1), mRNA [NM_001013421]	**0,514**
0	Unknown	**0,514**
LOC685634	PREDICTED: Rattus norvegicus hypothetical protein LOC685634 (LOC685634), mRNA [XM_001064606]	**0,514**
0	Unknown	**0,514**
Atg10	Rattus norvegicus autophagy-related 10 (S. cerevisiae) (Atg10), mRNA [NM_001109505]	**0,514**
Phpt1	Rattus norvegicus phosphohistidine phosphatase 1 (Phpt1), mRNA [NM_001106558]	**0,514**
Ppp2r2d	Rattus norvegicus protein phosphatase 2, regulatory subunit B, delta isoform (Ppp2r2d), mRNA [NM_144746]	**0,514**
Znf511	Rattus norvegicus zinc finger protein 511 (Znf511), mRNA [NM_001106309]	**0,514**
Usp10	Rattus norvegicus ubiquitin specific peptidase 10 (Usp10), mRNA [NM_001034146]	**0,514**

Mtdh	Rattus norvegicus metadherin (Mtdh), mRNA [NM_133398]	**0,514**
Slc22a5	Rattus norvegicus solute carrier family 22 (organic cation/carnitine transporter), member 5 (Slc22a5), mRNA [NM_019269]	**0,514**
Osbpl7	Rattus norvegicus oxysterol binding protein-like 7 (Osbpl7), mRNA [NM_001107044]	**0,514**
Gpr172a	Rattus norvegicus G protein-coupled receptor 172A (Gpr172a), mRNA [NM_001109670]	**0,514**
Zfp202	Rattus norvegicus zinc finger protein 202 (Zfp202), mRNA [NM_001109290]	**0,514**
Klhl23	Rattus norvegicus kelch-like 23 (Drosophila) (Klhl23), mRNA [NM_001134504]	**0,514**
Mapk8ip1	Rattus norvegicus mitogen-activated protein kinase 8 interacting protein 1 (Mapk8ip1), mRNA [NM_053777]	**0,514**
Pnkd	Rattus norvegicus paroxysmal nonkinesiogenic dyskinesia (Pnkd), nuclear gene encoding mitochondrial protein, transcript variant 3, mRNA [NM_001134753]	**0,514**
0	AW918633 EST349937 Rat gene index, normalized rat, norvegicus, Bento Soares Rattus norvegicus cDNA clone RGIEV66 5' end, mRNA sequence [AW918633]	**0,514**
0	Unknown	**0,514**
Cluap1	Rattus norvegicus clusterin associated protein 1 (Cluap1), mRNA [NM_001014225]	**0,514**
Lias	Rattus norvegicus lipoic acid synthetase (Lias), nuclear gene encoding mitochondrial protein, mRNA [NM_001012037]	**0,514**
Hspa4	Rattus norvegicus heat shock protein 4 (Hspa4), mRNA [NM_153629]	**0,514**
LOC679934	Rattus norvegicus similar to chromosome 3 open reading frame 10 (LOC679934), mRNA [NM_001195476]	**0,514**
0	Unknown	**0,514**
0	predicted gene 71 Gene [Source:MGI Symbol;Acc:MGI:2684917] [ENSRNOT00000033412]	**0,515**
RGD1310794	Rattus norvegicus similar to RIKEN cDNA C030048B08 (RGD1310794), mRNA [NM_001024246]	**0,515**
MGC95208	Rattus norvegicus similar to 4930453N24Rik protein (MGC95208), mRNA [NM_001005552]	**0,515**
Timm13	Rattus norvegicus translocase of inner mitochondrial membrane 13 homolog (yeast) (Timm13), nuclear gene encoding mitochondrial protein, mRNA [NM_145781]	**0,515**
Rtn4rl1	Rattus norvegicus reticulon 4 receptor-like 1 (Rtn4rl1), mRNA [NM_181377]	**0,515**
B3galnt1	Rattus norvegicus beta-1,3-N-acetylgalactosaminyltransferase 1 (B3galnt1), mRNA [NM_001013158]	**0,515**
Akna	Rattus norvegicus AT-hook transcription factor (Akna), mRNA [NM_001108668]	**0,515**
Rfwd2	Rattus norvegicus ring finger and WD repeat domain 2 (Rfwd2), mRNA [NM_001025126]	**0,515**
Klhl36	Rattus norvegicus kelch-like 36 (Drosophila) (Klhl36), mRNA [NM_001017511]	**0,515**
0	Uncharacterized protein [Source:UniProtKB/TrEMBL;Acc:D3ZI04] [ENSRNOT00000060225]	**0,515**
Ech1	Rattus norvegicus enoyl coenzyme A hydratase 1, peroxisomal (Ech1), mRNA [NM_022594]	**0,515**
LOC681336	PREDICTED: Rattus norvegicus similar to spermatogenesis associated glutamate (E)-rich protein 4d (LOC681336), mRNA [XM_001061313]	**0,515**
0	Unknown	**0,515**
Cog7	Rattus norvegicus component of oligomeric golgi complex 7 (Cog7), mRNA [NM_001033889]	**0,515**
Cbx1	RCG35120, isoform CRA_aUncharacterized protein [Source:UniProtKB/TrEMBL;Acc:D4A3T3] [ENSRNOT00000011665]	**0,515**
0	Nipped-B homolog (Drosophila)Uncharacterized protein [Source:UniProtKB/TrEMBL;Acc:D3ZG77] [ENSRNOT00000021181]	**0,515**

Spink2	Rattus norvegicus serine peptidase inhibitor, Kazal type 2 (acrosin-trypsin inhibitor) (Spink2), mRNA [NM_001008870]	**0,515**
Taf5l	Rattus norvegicus TAF5-like RNA polymerase II, p300/CBP-associated factor (PCAF)-associated factor (Taf5l), mRNA [NM_001107442]	**0,515**
LOC680489	Rattus norvegicus similar to claudin 1 (LOC680489), mRNA [NM_001109415]	**0,515**
Myh6	Rattus norvegicus myosin, heavy chain 6, cardiac muscle, alpha (Myh6), mRNA [NM_017239]	**0,515**
Tmlhe	Rattus norvegicus trimethyllysine hydroxylase, epsilon (Tmlhe), nuclear gene encoding mitochondrial protein, mRNA [NM_133387]	**0,515**
0	WD repeat domain 34 [Source:UniProtKB/TrEMBL;Acc:Q66H05] [ENSRNOT00000021132]	**0,515**
Park7	Rattus norvegicus Parkinson disease (autosomal recessive, early onset) 7 (Park7), mRNA [NM_057143]	**0,515**
Ptpla	Rattus norvegicus protein tyrosine phosphatase-like (proline instead of catalytic arginine), member a (Ptpla), mRNA [NM_001101001]	**0,515**
Atp6v1c1	Rattus norvegicus ATPase, H+ transporting, lysosomal V1 subunit C1 (Atp6v1c1), mRNA [NM_001011992]	**0,515**
Syt4	Rattus norvegicus synaptotagmin IV (Syt4), mRNA [NM_031693]	**0,515**
Pcgf6	Rattus norvegicus polycomb group ring finger 6 (Pcgf6), mRNA [NM_001013154]	**0,515**
Tcfe2a	Rattus norvegicus transcription factor E2a (Tcfe2a), transcript variant 1, mRNA [NM_133524]	**0,515**
Gspt2	Rattus norvegicus G1 to S phase transition 2 (Gspt2), mRNA [NM_001109319]	**0,515**
Vps36	Rattus norvegicus vacuolar protein sorting 36 homolog (S. cerevisiae) (Vps36), mRNA [NM_001106092]	**0,515**
B4galt7	Rattus norvegicus xylosylprotein beta1,4-galactosyltransferase, polypeptide 7 (galactosyltransferase I) (B4galt7), mRNA [NM_001031661]	**0,515**
Bex2	Rattus norvegicus brain expressed X-linked 2 (Bex2), mRNA [NM_001077435]	**0,515**
Clpb	Rattus norvegicus ClpB caseinolytic peptidase B homolog (E. coli) (Clpb), mRNA [NM_022947]	**0,515**
Mthfd2	Rattus norvegicus methylenetetrahydrofolate dehydrogenase (NADP+ dependent) 2, methenyltetrahydrofolate cyclohydrolase (Mthfd2), nuclear gene encoding mitochondrial protein, mRNA [NM_001109398]	**0,516**
0	Unknown	**0,516**
Dhx57	Rattus norvegicus DEAH (Asp-Glu-Ala-Asp/His) box polypeptide 57 (Dhx57), mRNA [NM_001191907]	**0,516**
Upf2	Rattus norvegicus UPF2 regulator of nonsense transcripts homolog (yeast) (Upf2), mRNA [NM_001108421]	**0,516**
Tpd52l1	Rattus norvegicus tumor protein D52-like 1 (Tpd52l1), mRNA [NM_001044295]	**0,516**
Jub	Rattus norvegicus jub, ajuba homolog (Xenopus laevis) (Jub), mRNA [NM_053503]	**0,516**
LOC499806	Rattus norvegicus similar to RIKEN cDNA 4933404M02 (LOC499806), mRNA [NM_001024311]	**0,516**
RGD1564325	40S ribosomal protein S24 [Source:UniProtKB/TrEMBL;Acc:D3ZFZ8] [ENSRNOT00000047252]	**0,516**
Rnmt	mRNA cap guanine-N7 methyltransferase [Source:UniProtKB/Swiss-Prot;Acc:Q5U2U7] [ENSRNOT00000022410]	**0,516**
Anks1a	Rattus norvegicus ankyrin repeat and sterile alpha motif domain containing 1A (Anks1a), mRNA [NM_001107613]	**0,516**
0	RCG47285, isoform CRA_bUncharacterized protein [Source:UniProtKB/TrEMBL;Acc:D3ZEH6] [ENSRNOT00000024237]	**0,516**
Sumf1	Rattus norvegicus sulfatase modifying factor 1 (Sumf1), mRNA [NM_001108639]	**0,516**
Brwd2	PREDICTED: Rattus norvegicus bromodomain and WD repeat domain containing 2 (Brwd2), mRNA [XM_219377]	**0,516**
Tmlhe	Rattus norvegicus trimethyllysine hydroxylase, epsilon (Tmlhe), nuclear gene encoding mitochondrial protein, mRNA [NM_133387]	**0,516**
C8g	Rattus norvegicus complement component 8, gamma polypeptide (C8g), mRNA [NM_001106555]	**0,516**

LOC691725	PREDICTED: Rattus norvegicus similar to Nucleolar RNA helicase 2 (Nucleolar RNA helicase II) (Nucleolar RNA helicase Gu) (RH II/Gu) (Gu-alpha) (DEAD box protein 21) (LOC691725), miscRNA [XR_086290]	**0,516**
0	Uncharacterized protein [Source:UniProtKB/TrEMBL;Acc:D4ADK1] [ENSRNOT00000046038]	**0,516**
Fxr2	Rattus norvegicus fragile X mental retardation, autosomal homolog 2 (Fxr2), mRNA [NM_001100647]	**0,516**
LOC686980	PREDICTED: Rattus norvegicus similar to arsenate resistance protein 2 (LOC686980), mRNA [XM_001076594]	**0,516**
Usf1	Rattus norvegicus upstream transcription factor 1 (Usf1), mRNA [NM_031777]	**0,516**
Clptm1l	Rattus norvegicus CLPTM1-like (Clptm1l), mRNA [NM_001108240]	**0,516**
RGD1306576	Rattus norvegicus similar to hypothetical protein (RGD1306576), mRNA [NM_001106648]	**0,516**
Ccdc90b	Rattus norvegicus coiled-coil domain containing 90B (Ccdc90b), mRNA [NM_001024885]	**0,516**
Guf1	Rattus norvegicus GUF1 GTPase homolog (S. cerevisiae) (Guf1), mRNA [NM_001107215]	**0,516**
Pde7a	Rattus norvegicus phosphodiesterase 7A (Pde7a), mRNA [NM_031080]	**0,516**
Alg14	Rattus norvegicus asparagine-linked glycosylation 14 homolog (S. cerevisiae) (Alg14), mRNA [NM_001014176]	**0,516**
Purb	Rattus norvegicus purine rich element binding protein B (Purb), mRNA [NM_001017503]	**0,516**
0	Unknown	**0,516**
LOC499124	Rattus norvegicus mouse zinc finger protein 14-like (LOC499124), mRNA [NM_001100991]	**0,516**
Rpl39	Rattus norvegicus ribosomal protein L39 (Rpl39), mRNA [NM_012875]	**0,516**
Cog4	Rattus norvegicus component of oligomeric golgi complex 4 (Cog4), mRNA [NM_001108449]	**0,516**
0	RVL17674 Wackym-Soares normalized rat vestibular cDNA library Rattus norvegicus cDNA 5', mRNA sequence [DV725494]	**0,516**
Pgam5	Rattus norvegicus phosphoglycerate mutase family member 5 (Pgam5), nuclear gene encoding mitochondrial protein, mRNA [NM_001025272]	**0,517**
Amd1	Rattus norvegicus adenosylmethionine decarboxylase 1 (Amd1), mRNA [NM_031011]	**0,517**
Ppp5c	Rattus norvegicus protein phosphatase 5, catalytic subunit (Ppp5c), mRNA [NM_031729]	**0,517**
0	Unknown	**0,517**
Btbd17	Rattus norvegicus BTB (POZ) domain containing 17 (Btbd17), mRNA [NM_001134534]	**0,517**
Mdp1	Rattus norvegicus magnesium-dependent phosphatase 1 (Mdp1), mRNA [NM_001106039]	**0,517**
Pink1	Rattus norvegicus PTEN induced putative kinase 1 (Pink1), nuclear gene encoding mitochondrial protein, mRNA [NM_001106694]	**0,517**
RGD1309492	Rattus norvegicus similar to mKIAA1737 protein (RGD1309492), mRNA [NM_001108044]	**0,517**
Gls	Rattus norvegicus glutaminase (Gls), nuclear gene encoding mitochondrial protein, transcript variant 2, mRNA [NM_001109968]	**0,517**
0	Rattus norvegicus similar to heterogeneous nuclear ribonucleoprotein K (LOC294952), mRNA [XM_227009]	**0,517**
Rasgef1c	Rattus norvegicus RasGEF domain family, member 1C (Rasgef1c), mRNA [NM_001108273]	**0,517**
Wfdc2	Rattus norvegicus WAP four-disulfide core domain 2 (Wfdc2), mRNA [NM_173109]	**0,517**
0	zinc finger X-chromosomal protein [Source:RefSeq peptide;Acc:NP_001102487] [ENSRNOT00000007501]	**0,517**
Rwdd4	Rattus norvegicus RWD domain containing 4A (Rwdd4), mRNA [NM_001034994]	**0,517**
Pacsin2	Rattus norvegicus protein kinase C and casein kinase substrate in neurons 2 (Pacsin2), mRNA [NM_130740]	**0,517**
Tmem74	Uncharacterized protein [Source:UniProtKB/TrEMBL;Acc:D3ZR33] [ENSRNOT00000006791]	**0,517**

Trpc4	Rattus norvegicus transient receptor potential cation channel, subfamily C, member 4 (Trpc4), transcript variant alpha, mRNA [NM_080396]	**0,517**
Nr1h3	Rattus norvegicus nuclear receptor subfamily 1, group H, member 3 (Nr1h3), mRNA [NM_031627]	**0,517**
Nsmce2	Rattus norvegicus non-SMC element 2, MMS21 homolog (S. cerevisiae) (Nsmce2), mRNA [NM_001024876]	**0,517**
Prmt8	PREDICTED: Rattus norvegicus similar to Protein arginine N-methyltransferase 4 (Heterogeneous nuclear ribonucleoprotein methyltransferase-like protein 4) (LOC688502), mRNA [XM_002726433]	**0,517**
Hadh	Rattus norvegicus hydroxyacyl-Coenzyme A dehydrogenase (Hadh), nuclear gene encoding mitochondrial protein, mRNA [NM_057186]	**0,517**
Eml4	Rattus norvegicus echinoderm microtubule associated protein like 4 (Eml4), mRNA [NM_001108008]	**0,517**
0	Unknown	**0,517**
Pdap1	Rattus norvegicus PDGFA associated protein 1 (Pdap1), mRNA [NM_022595]	**0,517**
Sfrs9	Rattus norvegicus splicing factor, arginine/serine-rich 9 (Sfrs9), mRNA [NM_001009255]	**0,517**
Usp36	Rattus norvegicus ubiquitin specific peptidase 36 (Usp36), mRNA [NM_001107069]	**0,517**
LOC687536	PREDICTED: Rattus norvegicus similar to Forkhead box protein F1 (Forkhead-related protein FKHL5) (Forkhead-related transcription factor 1) (FREAC-1) (Hepatocyte nuclear factor 3 forkhead homolog 8) (HFH-8) (LOC687536), mRNA [XM_001079002]	**0,517**
Cecr5	Rattus norvegicus cat eye syndrome chromosome region, candidate 5 homolog (human) (Cecr5), mRNA [NM_001107884]	**0,517**
Trip4	Rattus norvegicus thyroid hormone receptor interactor 4 (Trip4), mRNA [NM_001134981]	**0,517**
RGD1565641	Rattus norvegicus RGD1565641 (RGD1565641), mRNA [NM_001109179]	**0,518**
Appl2	Rattus norvegicus adaptor protein, phosphotyrosine interaction, PH domain and leucine zipper containing 2 (Appl2), mRNA [NM_001108741]	**0,518**
Ppm1a	Rattus norvegicus protein phosphatase 1A, magnesium dependent, alpha isoform (Ppm1a), mRNA [NM_017038]	**0,518**
Nfrkb	Rattus norvegicus nuclear factor related to kappa B binding protein (Nfrkb), mRNA [NM_001108133]	**0,518**
Aamp	Rattus norvegicus angio-associated, migratory cell protein (Aamp), mRNA [NM_001106920]	**0,518**
Ska2	Rattus norvegicus spindle and kinetochore associated complex subunit 2 (Ska2), mRNA [NM_001009624]	**0,518**
Cdkl3	Rattus norvegicus cyclin-dependent kinase-like 3 (Cdkl3), transcript variant 2, mRNA [NM_021772]	**0,518**
Tmem60	Rattus norvegicus transmembrane protein 60 (Tmem60), mRNA [NM_001191610]	**0,518**
Larp5	Rattus norvegicus La ribonucleoprotein domain family, member 5 (Larp5), mRNA [NM_001107361]	**0,518**
Tmem50a	Rattus norvegicus transmembrane protein 50A (Tmem50a), mRNA [NM_001127525]	**0,518**
LOC100364957	PREDICTED: Rattus norvegicus RGD1560755 protein-like (LOC100364957), mRNA [XM_002725203]	**0,518**
Naprt1	Rattus norvegicus nicotinate phosphoribosyltransferase domain containing 1 (Naprt1), mRNA [NM_207609]	**0,518**
0	PREDICTED: Rattus norvegicus hypothetical protein LOC680789 (LOC680789), partial mRNA [XM_001058892]	**0,518**
0	Unknown	**0,518**
Hprt1	Rattus norvegicus hypoxanthine phosphoribosyltransferase 1 (Hprt1), mRNA [NM_012583]	**0,518**
Gpld1	Rattus norvegicus glycosylphosphatidylinositol specific phospholipase D1 (Gpld1), mRNA [NM_001100512]	**0,518**
Vps33b	Rattus norvegicus vacuolar protein sorting 33 homolog B (yeast) (Vps33b), mRNA [NM_022286]	**0,518**
LOC682058	PREDICTED: Rattus norvegicus similar to nucleolar protein with MIF4G domain 1 (LOC682058), mRNA [XM_001059617]	**0,518**
Cltb	Rattus norvegicus clathrin, light chain (Lcb) (Cltb), mRNA [NM_053835]	**0,518**

Krcc1	Rattus norvegicus lysine-rich coiled-coil 1 (Krcc1), mRNA [NM_001009413]	**0,518**
Ankrd34b	Rattus norvegicus ankyrin repeat domain 34B (Ankrd34b), mRNA [NM_001109174]	**0,518**
Mfap1a	Rattus norvegicus microfibrillar-associated protein 1A (Mfap1a), mRNA [NM_001191964]	**0,518**
Gdap1	Rattus norvegicus ganglioside-induced differentiation-associated-protein 1 (Gdap1), mRNA [NM_001107897]	**0,519**
0	Unknown	**0,519**
Abcd3	Rattus norvegicus ATP-binding cassette, subfamily D (ALD), member 3 (Abcd3), mRNA [NM_012804]	**0,519**
RGD1560978	Rattus norvegicus similar to hypothetical protein (RGD1560978), mRNA [NM_001134631]	**0,519**
Prom2	Rattus norvegicus prominin 2 (Prom2), mRNA [NM_138857]	**0,519**
Ndufaf1	Rattus norvegicus NADH dehydrogenase (ubiquinone) 1 alpha subcomplex, assembly factor 1 (Ndufaf1), mRNA [NM_001106500]	**0,519**
Large	Rattus norvegicus like-glycosyltransferase (Large), mRNA [NM_001108439]	**0,519**
Krt80	Rattus norvegicus keratin 80 (Krt80), mRNA [NM_001008815]	**0,519**
MGC112715	Rattus norvegicus hypothetical protein LOC690899 (MGC112715), mRNA [NM_001044300]	**0,519**
0	Q6NW38_HUMAN (Q6NW38) IVNS1ABP protein, partial (5%) [TC590756]	**0,519**
Fam104a	Rattus norvegicus family with sequence similarity 104, member A (Fam104a), mRNA [NM_001034958]	**0,519**
Pus3	Rattus norvegicus pseudouridylate synthase 3 (Pus3), mRNA [NM_001108134]	**0,519**
Trim39	Rattus norvegicus tripartite motif-containing 39 (Trim39), mRNA [NM_213562]	**0,519**
Actr1b	Rattus norvegicus ARP1 actin-related protein 1 homolog B (yeast) (Actr1b), mRNA [NM_001039028]	**0,519**
0	Unknown	**0,519**
Msl1	Rattus norvegicus male-specific lethal 1 homolog (Drosophila) (Msl1), mRNA [NM_001107048]	**0,519**
Qsox1	Rattus norvegicus quiescin Q6 sulfhydryl oxidase 1 (Qsox1), transcript variant 2, mRNA [NM_053431]	**0,519**
Cd3eap	Rattus norvegicus CD3e molecule, epsilon associated protein (Cd3eap), mRNA [NM_001109416]	**0,519**
RGD1308544	PREDICTED: Rattus norvegicus LOC361192 (RGD1308544), mRNA [XM_001063171]	**0,519**
Ubqln1	Rattus norvegicus ubiquilin 1 (Ubqln1), mRNA [NM_053747]	**0,519**
0	Rattus norvegicus similar to RIKEN cDNA 4933431D05 (LOC363395), mRNA [XM_343725]	**0,519**
Dnmt3b	Rattus norvegicus DNA (cytosine-5-)-methyltransferase 3 beta (Dnmt3b), mRNA [NM_001003959]	**0,519**
Mfsd5	Rattus norvegicus major facilitator superfamily domain containing 5 (Mfsd5), mRNA [NM_001126282]	**0,519**
Scp2	Rattus norvegicus sterol carrier protein 2 (Scp2), mRNA [NM_138508]	**0,519**
RGD1305645	Uncharacterized protein [Source:UniProtKB/TrEMBL;Acc:D4A540] [ENSRNOT00000036990]	**0,519**
Tmem2	Rattus norvegicus transmembrane protein 2 (Tmem2), mRNA [NM_001107596]	**0,519**
LOC100361389	PREDICTED: Rattus norvegicus hypothetical protein LOC100361389 (LOC100361389), mRNA [XM_002727821]	**0,519**
Usp3	Rattus norvegicus ubiquitin specific peptidase 3 (Usp3), mRNA [NM_001025424]	**0,519**
Tmbim1	Rattus norvegicus transmembrane BAX inhibitor motif containing 1 (Tmbim1), mRNA [NM_001007713]	**0,520**
Serp2	Rattus norvegicus stress-associated endoplasmic reticulum protein family member 2 (Serp2), mRNA [NM_001109104]	**0,520**
Serp2	Rattus norvegicus stress-associated endoplasmic reticulum protein family member 2 (Serp2), mRNA [NM_001109104]	**0,520**

Gtf2h2	Rattus norvegicus general transcription factor IIH, polypeptide 2 (Gtf2h2), mRNA [NM_001077428]	**0,520**
Cyp7b1	Rattus norvegicus cytochrome P450, family 7, subfamily b, polypeptide 1 (Cyp7b1), mRNA [NM_019138]	**0,520**
Emid1	Rattus norvegicus EMI domain containing 1 (Emid1), mRNA [NM_001109467]	**0,520**
Slc25a20	Rattus norvegicus solute carrier family 25 (carnitine/acylcarnitine translocase), member 20 (Slc25a20), nuclear gene encoding mitochondrial protein, mRNA [NM_053965]	**0,520**
0	protease, serine, 28 Gene [Source:MGI Symbol;Acc:MGI:2149951] [ENSRNOT00000060388]	**0,520**
Itgb5	Rattus norvegicus integrin, beta 5 (Itgb5), mRNA [NM_147139]	**0,520**
Bcl2l2	Rattus norvegicus Bcl2-like 2 (Bcl2l2), mRNA [NM_021850]	**0,520**
Krtcap2	Rattus norvegicus keratinocyte associated protein 2 (Krtcap2), mRNA [NM_001106444]	**0,520**
Adamts9	Rattus norvegicus a disintegrin-like and metalloprotease (reprolysin type) with thrombospondin type 1 motif, 9 (Adamts9), mRNA [NM_001107877]	**0,520**
Bin1	Rattus norvegicus bridging integrator 1 (Bin1), mRNA [NM_053959]	**0,520**
0	Unknown	**0,520**
Wnt16	Rattus norvegicus wingless-type MMTV integration site family, member 16 (Wnt16), mRNA [NM_001109223]	**0,520**
Orc4l	Rattus norvegicus origin recognition complex, subunit 4-like (yeast) (Orc4l), mRNA [NM_199092]	**0,520**
0	Unknown	**0,520**
Pnoc	Rattus norvegicus prepronociceptin (Pnoc), mRNA [NM_013007]	**0,520**
Rbm12	Rattus norvegicus RNA binding motif protein 12 (Rbm12), mRNA [NM_001037657]	**0,520**
Tmem223	Rattus norvegicus transmembrane protein 223 (Tmem223), mRNA [NM_001191104]	**0,520**
Ptn	Rattus norvegicus pleiotrophin (Ptn), mRNA [NM_017066]	**0,520**
Dip2a	Rattus norvegicus DIP2 disco-interacting protein 2 homolog A (Drosophila) (Dip2a), mRNA [NM_001191564]	**0,520**
Trub2	Rattus norvegicus TruB pseudouridine (psi) synthase homolog 2 (E. coli) (Trub2), mRNA [NM_001014257]	**0,520**
Ppa2	Rattus norvegicus pyrophosphatase (inorganic) 2 (Ppa2), nuclear gene encoding mitochondrial protein, mRNA [NM_001135871]	**0,520**
0	PREDICTED: Rattus norvegicus maltase-glucoamylase (Mgam), mRNA [XM_231714]	**0,520**
Skap2	Rattus norvegicus src kinase associated phosphoprotein 2 (Skap2), mRNA [NM_130413]	**0,520**
0	Unknown	**0,520**
Vps4b	Rattus norvegicus vacuolar protein sorting 4 homolog B (S. cerevisiae) (Vps4b), mRNA [NM_001025716]	**0,520**
0	Unknown	**0,520**
0	Unknown	**0,521**
0	Unknown	**0,521**
Ube2b	Rattus norvegicus ubiquitin-conjugating enzyme E2B (RAD6 homolog, S. cerevisiae) (Ube2b), mRNA [NM_031138]	**0,521**
0	Q3QF41_9GAMM (Q3QF41) Recombination protein O, RecO, partial (5%) [TC612601]	**0,521**
LOC293103	Rattus norvegicus similar to RIKEN cDNA 0610007P06 (LOC293103), mRNA [NM_001013897]	**0,521**
Doc2a	Rattus norvegicus double C2-like domains, alpha (Doc2a), mRNA [NM_022937]	**0,521**
Ankmy2	Rattus norvegicus ankyrin repeat and MYND domain containing 2 (Ankmy2), mRNA [NM_001108019]	**0,521**

Dcaf6	Similar to IQ motif and WD repeats 1 (Predicted)Uncharacterized protein [Source:UniProtKB/TrEMBL;Acc:D4A8L4] [ENSRNOT00000004199]	**0,521**
Frmd3	Rattus norvegicus FERM domain containing 3 (Frmd3), mRNA [NM_001106662]	**0,521**
Lrrc43	Rattus norvegicus leucine rich repeat containing 43 (Lrrc43), mRNA [NM_001170396]	**0,521**
Cadps	Rattus norvegicus Ca++-dependent secretion activator (Cadps), mRNA [NM_013219]	**0,521**
LOC683753	PREDICTED: Rattus norvegicus hypothetical protein LOC683753 (LOC683753), mRNA [XM_001067334]	**0,521**
Tlk1	Rattus norvegicus tousled-like kinase 1 (Tlk1), mRNA [NM_001107734]	**0,521**
RGD1566036	Rattus norvegicus similar to RIKEN cDNA 2310008H04 (RGD1566036), mRNA [NM_001100988]	**0,521**
Klhl35	Uncharacterized protein [Source:UniProtKB/TrEMBL;Acc:D4A6V5] [ENSRNOT00000023386]	**0,521**
Dbnl	Drebrin-like protein [Source:UniProtKB/Swiss-Prot;Acc:Q9JHL4] [ENSRNOT00000017375]	**0,521**
0	Rattus norvegicus similar to Hypothetical protein LOC216150 (LOC365006), mRNA [XM_344770]	**0,521**
Osgin1	Rattus norvegicus oxidative stress induced growth inhibitor 1 (Osgin1), mRNA [NM_138504]	**0,521**
Ppp2r5a	Rattus norvegicus protein phosphatase 2, regulatory subunit B', alpha isoform (Ppp2r5a), mRNA [NM_001107891]	**0,521**
Psma4	Rattus norvegicus proteasome (prosome, macropain) subunit, alpha type 4 (Psma4), mRNA [NM_017281]	**0,521**
0	Unknown	**0,521**
Gpr61	Rattus norvegicus G protein-coupled receptor 61 (Gpr61), mRNA [NM_001107715]	**0,521**
0	CCD13_HUMAN (Q8IYE1) Coiled-coil domain-containing protein 13, partial (11%) [TC590118]	**0,521**
Ccrn4l	Rattus norvegicus CCR4 carbon catabolite repression 4-like (S. cerevisiae) (Ccrn4l), mRNA [NM_138526]	**0,521**
0	PREDICTED: Rattus norvegicus similar to ribosomal protein L30 (RGD1562394), mRNA [XM_001081708]	**0,521**
Zbtb43	Rattus norvegicus zinc finger and BTB domain containing 43 (Zbtb43), mRNA [NM_001012094]	**0,521**
Znf296	PREDICTED: Rattus norvegicus zinc finger protein 296 (Znf296), mRNA [XM_001075949]	**0,521**
Pds5b	Rattus norvegicus PDS5, regulator of cohesion maintenance, homolog B (S. cerevisiae) (Pds5b), transcript variant 1, mRNA [NM_001102383]	**0,521**
Dctn5	Rattus norvegicus dynactin 5 (Dctn5), mRNA [NM_001037778]	**0,522**
Ptdss2	Rattus norvegicus phosphatidylserine synthase 2 (Ptdss2), mRNA [NM_001106316]	**0,522**
Pdcd2	Rattus norvegicus programmed cell death 2 (Pdcd2), mRNA [NM_031638]	**0,522**
0	Rattus norvegicus TL0AEA65YG02 mRNA sequence. [FQ232533]	**0,522**
RGD1307569	PREDICTED: Rattus norvegicus similar to Protein C21orf63 homolog precursor, transcript variant 1 (RGD1307569), mRNA [XM_001073261]	**0,522**
Armc7	Rattus norvegicus armadillo repeat containing 7 (Armc7), mRNA [NM_001127522]	**0,522**
Rnaseh2a	Rattus norvegicus ribonuclease H2, subunit A (Rnaseh2a), mRNA [NM_001013234]	**0,522**
Mpnd	Rattus norvegicus MPN domain containing (Mpnd), mRNA [NM_001085406]	**0,522**
Idh3g	Rattus norvegicus isocitrate dehydrogenase 3 (NAD), gamma (Idh3g), nuclear gene encoding mitochondrial protein, mRNA [NM_031551]	**0,522**
Cab39	Rattus norvegicus calcium binding protein 39 (Cab39), mRNA [NM_001106924]	**0,522**
Klhl11	Rattus norvegicus kelch-like 11 (Drosophila) (Klhl11), mRNA [NM_001105838]	**0,522**
Prpf40a	Rattus norvegicus PRP40 pre-mRNA processing factor 40 homolog A (S. cerevisiae) (Prpf40a), mRNA [NM_001106480]	**0,522**
Rab33a	Rattus norvegicus RAB33A, member RAS oncogene family (Rab33a), mRNA [NM_001108257]	**0,522**

Arpp21	Rattus norvegicus cAMP-regulated phosphoprotein 21 (Arpp21), transcript variant 2, mRNA [NM_001135045]	**0,522**
0	Rattus norvegicus similar to Peptidylprolyl isomerase-like 2 (LOC302108), mRNA [XM_217532]	**0,522**
RGD1308049	Uncharacterized protein [Source:UniProtKB/TrEMBL;Acc:D3ZT97] [ENSRNOT00000028509]	**0,522**
Mrpl3	Rattus norvegicus mitochondrial ribosomal protein L3 (Mrpl3), nuclear gene encoding mitochondrial protein, mRNA [NM_001106852]	**0,522**
Mapt	Rattus norvegicus microtubule-associated protein tau (Mapt), mRNA [NM_017212]	**0,522**
Hmgb2	Rattus norvegicus high mobility group box 2 (Hmgb2), mRNA [NM_017187]	**0,522**
0	Unknown	**0,522**
RGD1304624	Rattus norvegicus similar to RIKEN cDNA 2700097O09 (RGD1304624), mRNA [NM_001108022]	**0,522**
LOC686041	Rattus norvegicus cDNA clone IMAGE:6889638, partial cds. [BC059164]	**0,522**
Tmed9	Rattus norvegicus transmembrane emp24 protein transport domain containing 9 (Tmed9), mRNA [NM_001009703]	**0,522**
0	Unknown	**0,522**
Fgf9	Rattus norvegicus fibroblast growth factor 9 (Fgf9), mRNA [NM_012952]	**0,522**
Trim35	Rattus norvegicus tripartite motif-containing 35 (Trim35), mRNA [NM_001025142]	**0,522**
0	Uncharacterized protein [Source:UniProtKB/TrEMBL;Acc:D3ZHU8] [ENSRNOT00000001891]	**0,523**
Dennd3	PREDICTED: Rattus norvegicus DENN/MADD domain containing 3 (Dennd3), mRNA [XM_001072997]	**0,523**
Aftph	PREDICTED: Rattus norvegicus aftiphilin (Aftph), mRNA [XM_001055784]	**0,523**
Cbr1	Rattus norvegicus carbonyl reductase 1 (Cbr1), mRNA [NM_019170]	**0,523**
Ppapdc3	Rattus norvegicus phosphatidic acid phosphatase type 2 domain containing 3 (Ppapdc3), mRNA [NM_001012349]	**0,523**
Wtap	Rattus norvegicus Wilms tumor 1 associated protein (Wtap), transcript variant 2, mRNA [NM_001113543]	**0,523**
0	Uncharacterized protein [Source:UniProtKB/TrEMBL;Acc:D3ZRI2] [ENSRNOT00000012282]	**0,523**
Sfmbt1	Rattus norvegicus Scm-like with four mbt domains 1 (Sfmbt1), mRNA [NM_031647]	**0,523**
Ecsit	Rattus norvegicus ECSIT homolog (Drosophila) (Ecsit), nuclear gene encoding mitochondrial protein, mRNA [NM_001006986]	**0,523**
Apex1	Rattus norvegicus APEX nuclease (multifunctional DNA repair enzyme) 1 (Apex1), mRNA [NM_024148]	**0,523**
Rwdd2b	Rattus norvegicus RWD domain containing 2B (Rwdd2b), mRNA [NM_001100559]	**0,523**
Ndufa7	Rattus norvegicus NADH dehydrogenase (ubiquinone) 1 alpha subcomplex, 7 (B14.5a) (Ndufa7), nuclear gene encoding mitochondrial protein, mRNA [NM_001106772]	**0,523**
Bhmt	Rattus norvegicus betaine-homocysteine methyltransferase (Bhmt), mRNA [NM_030850]	**0,523**
RGD1565767	Ribosomal protein L15 [Source:UniProtKB/TrEMBL;Acc:D3ZF52] [ENSRNOT00000032528]	**0,523**
Arhgef19	Rattus norvegicus Rho guanine nucleotide exchange factor (GEF) 19 (Arhgef19), mRNA [NM_001108692]	**0,523**
Prmt2	Rattus norvegicus protein arginine methyltransferase 2 (Prmt2), mRNA [NM_001025144]	**0,523**
Ng35	Rattus norvegicus Ng35 pseudogene (Ng35), mRNA [NM_001134991]	**0,523**
0	Unknown	**0,523**
RGD1305508	PREDICTED: Rattus norvegicus similar to hypothetical protein MGC23280 (RGD1305508), miscRNA [XR_009409]	**0,523**
0	Unknown	**0,523**

Pum2	Rattus norvegicus pumilio homolog 2 (Drosophila) (Pum2), mRNA [NM_001106715]	**0,523**
Dnajc17	Rattus norvegicus DnaJ (Hsp40) homolog, subfamily C, member 17 (Dnajc17), mRNA [NM_001191740]	**0,523**
Dtnb	Rattus norvegicus dystrobrevin, beta (Dtnb), mRNA [NM_001012191]	**0,523**
0	Unknown	**0,523**
LOC679566	Rattus norvegicus hypothetical protein LOC679566 (LOC679566), mRNA [NM_001109372]	**0,523**
RGD1565469	Rattus norvegicus RGD1565469 (RGD1565469), mRNA [NM_001109340]	**0,523**
Akap11	Rattus norvegicus A kinase (PRKA) anchor protein 11 (Akap11), mRNA [NM_012773]	**0,523**
Gnb4	Rattus norvegicus guanine nucleotide binding protein (G protein), beta polypeptide 4 (Gnb4), mRNA [NM_001013910]	**0,524**
0	Unknown	**0,524**
Wrb	Rattus norvegicus tryptophan rich basic protein (Wrb), mRNA [NM_199373]	**0,524**
Fam82a2	Rattus norvegicus family with sequence similarity 82, member A2 (Fam82a2), mRNA [NM_001014046]	**0,524**
LOC361990	Rattus norvegicus similar to DKFZP547E1010 protein (LOC361990), mRNA [NM_001014175]	**0,524**
Xrn1	Uncharacterized protein [Source:UniProtKB/TrEMBL;Acc:D4ABN8] [ENSRNOT00000014481]	**0,524**
LOC498154	Rattus norvegicus hypothetical protein LOC498154 (LOC498154), mRNA [NM_001025033]	**0,524**
Mdm2	Rattus norvegicus Mdm2 p53 binding protein homolog (mouse) (Mdm2), mRNA [NM_001108099]	**0,524**
Klf5	Rattus norvegicus Kruppel-like factor 5 (Klf5), mRNA [NM_053394]	**0,524**
Akr1cl2	Rattus norvegicus aldo-keto reductase family 1, member C-like 2 (Akr1cl2), mRNA [NM_001008342]	**0,524**
0	ubiquitin protein ligase E3 component n-recognin 2 [Source:RefSeq peptide;Acc:NP_001171542] [ENSRNOT00000061531]	**0,524**
0	Rattus norvegicus TL0ADA18YA08 mRNA sequence. [FQ222908]	**0,524**
Rhebl1	Rattus norvegicus Ras homolog enriched in brain like 1 (Rhebl1), mRNA [NM_182825]	**0,524**
Npat	Rattus norvegicus nuclear protein, ataxia-telangiectasia locus (Npat), mRNA [NM_001108147]	**0,524**
Cox7b	Rattus norvegicus cytochrome c oxidase subunit VIIb (Cox7b), nuclear gene encoding mitochondrial protein, mRNA [NM_182819]	**0,524**
Sft2d1	Rattus norvegicus SFT2 domain containing 1 (Sft2d1), mRNA [NM_001008302]	**0,524**
Ttc9	Rattus norvegicus tetratricopeptide repeat domain 9 (Ttc9), mRNA [NM_001134731]	**0,524**
LOC690344	Rattus norvegicus similar to Protein UNQ655/PRO1286 homolog precursor (LOC690344), mRNA [NM_001168661]	**0,524**
Enpp1	Rattus norvegicus ectonucleotide pyrophosphatase/phosphodiesterase 1 (Enpp1), mRNA [NM_053535]	**0,524**
Phf20	Rattus norvegicus PHD finger protein 20 (Phf20), mRNA [NM_001107795]	**0,524**
Cyb5d2	Rattus norvegicus cytochrome b5 domain containing 2 (Cyb5d2), mRNA [NM_001007671]	**0,524**
0	Transcription factor E2F5 [Source:UniProtKB/Swiss-Prot;Acc:Q62814] [ENSRNOT00000014361]	**0,524**
0	Bromodomain and WD repeat domain containing 1 (Predicted), isoform CRA_bUncharacterized protein [Source:UniProtKB/TrEMBL;Acc:D3ZFG0] [ENSRNOT00000002231]	**0,524**
0	Uncharacterized protein [Source:UniProtKB/TrEMBL;Acc:D4A268] [ENSRNOT00000007350]	**0,524**
0	Unknown	**0,524**
0	Uncharacterized protein [Source:UniProtKB/TrEMBL;Acc:D3ZBI5] [ENSRNOT00000042103]	**0,524**

Neu4	Rattus norvegicus sialidase 4 (Neu4), mRNA [NM_001108234]	**0,524**
Apbb3	Rattus norvegicus amyloid beta (A4) precursor protein-binding, family B, member 3 (Apbb3), mRNA [NM_053957]	**0,524**
Lsm6	Rattus norvegicus LSM6 homolog, U6 small nuclear RNA associated (S. cerevisiae) (Lsm6), mRNA [NM_001126085]	**0,524**
Usp28	Rattus norvegicus ubiquitin specific peptidase 28 (Usp28), mRNA [NM_001108144]	**0,524**
Slc9a2	Rattus norvegicus solute carrier family 9 (sodium/hydrogen exchanger), member 2 (Slc9a2), transcript variant 1, mRNA [NM_001113335]	**0,524**
LOC302022	Rattus norvegicus similar to nidogen 2 protein (LOC302022), mRNA [NM_001127530]	**0,524**
Fkbp5	Rattus norvegicus FK506 binding protein 5 (Fkbp5), mRNA [NM_001012174]	**0,524**
Arhgef4	PREDICTED: Rattus norvegicus Rho guanine nucleotide exchange factor (GEF) 4 (Arhgef4), mRNA [XM_237049]	**0,524**
Tef	Rattus norvegicus thyrotrophic embryonic factor (Tef), mRNA [NM_019194]	**0,524**
Btbd10	Rattus norvegicus BTB (POZ) domain containing 10 (Btbd10), mRNA [NM_001014022]	**0,524**
Cdk10	Rattus norvegicus cyclin-dependent kinase 10 (Cdk10), transcript variant 3, mRNA [NM_001025722]	**0,524**
Chrd	Rattus norvegicus chordin (Chrd), mRNA [NM_057134]	**0,525**
0	Unknown	**0,525**
Slc36a4	Rattus norvegicus solute carrier family 36 (proton/amino acid symporter), member 4 (Slc36a4), mRNA [NM_001108127]	**0,525**
RGD1308380	Rattus norvegicus similar to RIKEN cDNA 2310079F23 (RGD1308380), mRNA [NM_001130061]	**0,525**
Sc65	Rattus norvegicus synaptonemal complex protein SC65 (Sc65), mRNA [NM_021581]	**0,525**
Qsox2	Rattus norvegicus quiescin Q6 sulfhydryl oxidase 2 (Qsox2), mRNA [NM_001109434]	**0,525**
Itm2b	Rattus norvegicus integral membrane protein 2B (Itm2b), mRNA [NM_001006963]	**0,525**
Esf1	Rattus norvegicus ESF1, nucleolar pre-rRNA processing protein, homolog (S. cerevisiae) (Esf1), mRNA [NM_001100771]	**0,525**
0	Unknown	**0,525**
Tmem219	PREDICTED: Rattus norvegicus transmembrane protein 219 (Tmem219), mRNA [XM_001080074]	**0,525**
0	AB017635 ERCC4 {Cricetulus griseus} (exp=-1; wgp=0; cg=0), partial (26%) [TC614683]	**0,525**
Dusp1	Rattus norvegicus dual specificity phosphatase 1 (Dusp1), mRNA [NM_053769]	**0,525**
Ndn	Rattus norvegicus necdin homolog (mouse) (Ndn), mRNA [NM_001008558]	**0,525**
Rbmx2	Rattus norvegicus RNA binding motif protein, X-linked 2 (Rbmx2), mRNA [NM_001113786]	**0,525**
Spg7	Rattus norvegicus spastic paraplegia 7 homolog (human) (Spg7), nuclear gene encoding mitochondrial protein, mRNA [NM_181388]	**0,525**
0	Unknown	**0,525**
Ptges	Rattus norvegicus prostaglandin E synthase (Ptges), mRNA [NM_021583]	**0,525**
Slc30a9	Rattus norvegicus solute carrier family 30 (zinc transporter), member 9 (Slc30a9), mRNA [NM_001109088]	**0,525**
0	SMR1 proteinSMR1-related undecapeptideSMR1-related hexapeptideSialorphinSubmandibular gland peptide T [Source:UniProtKB/Swiss-Prot;Acc:P13432] [ENSRNOT00000002664]	**0,525**
LOC293589	Rattus norvegicus TL0AAA51YB05 mRNA sequence. [FQ212842]	**0,525**
Fgfr1op2	Rattus norvegicus FGFR1 oncogene partner 2 (Fgfr1op2), mRNA [NM_201421]	**0,525**
Tmem57	Rattus norvegicus transmembrane protein 57 (Tmem57), mRNA [NM_001025699]	**0,525**

Ttc13	Rattus norvegicus tetratricopeptide repeat domain 13 (Ttc13), mRNA [NM_001136162]	**0,525**
Pramef12	PREDICTED: Rattus norvegicus PRAME family member 12 (Pramef12), mRNA [XM_001077034]	**0,525**
Hcfc2	Rattus norvegicus host cell factor C2 (Hcfc2), mRNA [NM_001008357]	**0,525**
LOC684270	PREDICTED: Rattus norvegicus similar to isochorismatase domain containing 2, transcript variant 2 (LOC684270), mRNA [XM_001059344]	**0,525**
Znf644	Rattus norvegicus zinc finger protein 644 (Znf644), mRNA [NM_001139484]	**0,525**
Ammecr1l	Rattus norvegicus AMME chromosomal region gene 1-like (Ammecr1l), mRNA [NM_001107399]	**0,525**
0	Unknown	**0,525**
Lins1	Uncharacterized protein [Source:UniProtKB/TrEMBL;Acc:D3ZVQ9] [ENSRNOT00000018463]	**0,525**
Pdlim5	Rattus norvegicus PDZ and LIM domain 5 (Pdlim5), mRNA [NM_053326]	**0,525**
RGD1564290	PREDICTED: Rattus norvegicus similar to ribosomal protein S27a (RGD1564290), mRNA [XM_001060136]	**0,525**
Ergic2	Rattus norvegicus ERGIC and golgi 2 (Ergic2), mRNA [NM_001024984]	**0,525**
Paip2	Rattus norvegicus poly(A) binding protein interacting protein 2 (Paip2), mRNA [NM_001014148]	**0,526**
Cops8	Rattus norvegicus COP9 constitutive photomorphogenic homolog subunit 8 (Arabidopsis) (Cops8), mRNA [NM_001013227]	**0,526**
Txnip	Rattus norvegicus thioredoxin interacting protein (Txnip), mRNA [NM_001008767]	**0,526**
Kcnb2	Rattus norvegicus potassium voltage gated channel, Shab-related subfamily, member 2 (Kcnb2), mRNA [NM_054000]	**0,526**
Eea1	Rattus norvegicus early endosome antigen 1 (Eea1), mRNA [NM_001108086]	**0,526**
0	Uncharacterized protein [Source:UniProtKB/TrEMBL;Acc:D4ADX4] [ENSRNOT00000060718]	**0,526**
Rpl6	Rattus norvegicus ribosomal protein L6 (Rpl6), mRNA [NM_053971]	**0,526**
Ptpn6	Rattus norvegicus protein tyrosine phosphatase, non-receptor type 6 (Ptpn6), mRNA [NM_053908]	**0,526**
0	Unknown	**0,526**
Ntrk2	Rattus norvegicus neurotrophic tyrosine kinase, receptor, type 2 (Ntrk2), transcript variant 3, mRNA [NM_001163169]	**0,526**
RGD1566099	Rattus norvegicus similar to novel protein (RGD1566099), mRNA [NM_001108347]	**0,526**
Rab7l1	Rattus norvegicus RAB7, member RAS oncogene family-like 1 (Rab7l1), mRNA [NM_133590]	**0,526**
RGD1562079	Rattus norvegicus RGD1562079 (RGD1562079), mRNA [NM_001177687]	**0,526**
Inpp1	Rattus norvegicus inositol polyphosphate-1-phosphatase (Inpp1), mRNA [NM_001012131]	**0,526**
Pigy	Rattus norvegicus phosphatidylinositol glycan anchor biosynthesis, class Y (Pigy), nuclear gene encoding mitochondrial protein, mRNA [NM_001024370]	**0,526**
Lsm6	Rattus norvegicus LSM6 homolog, U6 small nuclear RNA associated (S. cerevisiae) (Lsm6), mRNA [NM_001126085]	**0,526**
Ccdc21	Rattus norvegicus coiled-coil domain containing 21 (Ccdc21), mRNA [NM_001080151]	**0,526**
LOC682861	PREDICTED: Rattus norvegicus similar to adenomatosis polyposis coli down-regulated 1 (LOC682861), mRNA [XM_001063452]	**0,526**
0	Unknown	**0,526**
Cenpc1	Rattus norvegicus centromere protein C 1 (Cenpc1), mRNA [NM_001004098]	**0,526**
Pear1	Rattus norvegicus platelet endothelial aggregation receptor 1 (Pear1), mRNA [NM_001134959]	**0,526**
Tmem44	Uncharacterized protein [Source:UniProtKB/TrEMBL;Acc:D4ADT4] [ENSRNOT00000002353]	**0,526**

Zfp64	Rattus norvegicus zinc finger protein 64 (Zfp64), mRNA [NM_001012093]	**0,526**
Lrrc47	Rattus norvegicus leucine rich repeat containing 47 (Lrrc47), mRNA [NM_001135666]	**0,526**
Acbd5	Rattus norvegicus acyl-Coenzyme A binding domain containing 5 (Acbd5), mRNA [NM_001077635]	**0,526**
Etf1	Rattus norvegicus eukaryotic translation termination factor 1 (Etf1), mRNA [NM_001008344]	**0,526**
Sephs1	Rattus norvegicus selenophosphate synthetase 1 (Sephs1), mRNA [NM_001104630]	**0,526**
Uros	Rattus norvegicus uroporphyrinogen III synthase (Uros), mRNA [NM_001012068]	**0,526**
Pgm2	Rattus norvegicus phosphoglucomutase 2 (Pgm2), mRNA [NM_001106007]	**0,526**
Csrnp3	Similar to TGF-beta induced apotosis protein 2 (Predicted)Uncharacterized protein [Source:UniProtKB/TrEMBL;Acc:D4AE74] [ENSRNOT00000007197]	**0,526**
Mllt4	Rattus norvegicus myeloid/lymphoid or mixed-lineage leukemia (trithorax homolog, Drosophila); translocated to, 4 (Mllt4), mRNA [NM_013217]	**0,526**
Pgap1	Rattus norvegicus post-GPI attachment to proteins 1 (Pgap1), mRNA [NM_201990]	**0,526**
Mid1ip1	Rattus norvegicus MID1 interacting protein 1 (gastrulation specific G12 homolog (zebrafish)) (Mid1ip1), mRNA [NM_206950]	**0,526**
Srp19	Rattus norvegicus signal recognition particle 19 (Srp19), mRNA [NM_001106157]	**0,526**
Rpusd2	Rattus norvegicus RNA pseudouridylate synthase domain containing 2 (Rpusd2), mRNA [NM_001135845]	**0,526**
Nrtn	Rattus norvegicus neurturin (Nrtn), mRNA [NM_053399]	**0,526**
Tspyl1	Rattus norvegicus TSPY-like 1 (Tspyl1), mRNA [NM_001013033]	**0,527**
RGD1311269	Rattus norvegicus similar to hypothetical protein FLJ37953 (RGD1311269), transcript variant 1, mRNA [NM_001170473]	**0,527**
RGD1561878	PREDICTED: Rattus norvegicus similar to mKIAA0978 protein (RGD1561878), mRNA [XM_002726270]	**0,527**
Cyp4f1	Rattus norvegicus cytochrome P450, family 4, subfamily f, polypeptide 1 (Cyp4f1), mRNA [NM_019623]	**0,527**
Acot2	Rattus norvegicus acyl-CoA thioesterase 2 (Acot2), nuclear gene encoding mitochondrial protein, mRNA [NM_138907]	**0,527**
Ngfrap1	Rattus norvegicus nerve growth factor receptor (TNFRSF16) associated protein 1 (Ngfrap1), mRNA [NM_053401]	**0,527**
Spsb3	Rattus norvegicus splA/ryanodine receptor domain and SOCS box containing 3 (Spsb3), mRNA [NM_001106988]	**0,527**
Slc7a6os	Rattus norvegicus solute carrier family 7, member 6 opposite strand (Slc7a6os), mRNA [NM_139328]	**0,527**
Fkbp3	Rattus norvegicus FK506 binding protein 3 (Fkbp3), mRNA [NM_001106736]	**0,527**
Gkap1	Rattus norvegicus G kinase anchoring protein 1 (Gkap1), mRNA [NM_001012160]	**0,527**
Sgms1	Rattus norvegicus sphingomyelin synthase 1 (Sgms1), mRNA [NM_181386]	**0,527**
Hars2l	Rattus norvegicus histidyl-tRNA synthetase 2-like (Hars2l), mRNA [NM_001014012]	**0,527**
St5	Rattus norvegicus suppression of tumorigenicity 5 (St5), mRNA [NM_001107547]	**0,527**
Csad	Rattus norvegicus cysteine sulfinic acid decarboxylase (Csad), transcript variant 1, mRNA [NM_021750]	**0,527**
Txndc15	Rattus norvegicus thioredoxin domain containing 15 (Txndc15), mRNA [NM_001024998]	**0,527**
0	Unknown	**0,527**
Setd5	Rattus norvegicus SET domain containing 5 (Setd5), mRNA [NM_001106614]	**0,527**
Igsf21	Uncharacterized protein [Source:UniProtKB/TrEMBL;Acc:D3ZH04] [ENSRNOT00000051793]	**0,527**
Mrpl48	Rattus norvegicus mitochondrial ribosomal protein L48 (Mrpl48), nuclear gene encoding mitochondrial protein, mRNA [NM_001106282]	**0,527**

Otub2	Rattus norvegicus OTU domain, ubiquitin aldehyde binding 2 (Otub2), mRNA [NM_001108053]	**0,527**
Lpgat1	Rattus norvegicus lysophosphatidylglycerol acyltransferase 1 (Lpgat1), mRNA [NM_001109376]	**0,527**
Lix1	Rattus norvegicus Lix1 homolog (chicken) (Lix1), mRNA [NM_001106214]	**0,527**
Ano10	PREDICTED: Rattus norvegicus anoctamin 10 (Ano10), mRNA [XM_236774]	**0,527**
Ttc3	Rattus norvegicus tetratricopeptide repeat domain 3 (Ttc3), mRNA [NM_001108315]	**0,527**
Apoe	Rattus norvegicus apolipoprotein E (Apoe), mRNA [NM_138828]	**0,527**
RGD1311186	Rattus norvegicus similar to RIKEN cDNA 1810014F10 gene (RGD1311186), mRNA [NM_001106310]	**0,527**
Cnr1	Rattus norvegicus cannabinoid receptor 1 (brain) (Cnr1), mRNA [NM_012784]	**0,527**
MGC95208	Rattus norvegicus similar to 4930453N24Rik protein (MGC95208), mRNA [NM_001005552]	**0,527**
Peo1	Rattus norvegicus progressive external ophthalmoplegia 1 (Peo1), mRNA [NM_001107599]	**0,527**
Higd2a	Rattus norvegicus HIG1 hypoxia inducible domain family, member 2A (Higd2a), mRNA [NM_001106102]	**0,527**
Etfdh	Rattus norvegicus electron-transferring-flavoprotein dehydrogenase (Etfdh), nuclear gene encoding mitochondrial protein, mRNA [NM_198742]	**0,527**
LOC685909	Rattus norvegicus similar to H2A histone family, member V isoform 1 (LOC685909), mRNA [NM_001106019]	**0,527**
Neo1	PREDICTED: Rattus norvegicus neogenin homolog 1 (chicken) (Neo1), mRNA [XM_001074913]	**0,527**
Grid1	Rattus norvegicus glutamate receptor, ionotropic, delta 1 (Grid1), mRNA [NM_024378]	**0,527**
Pxmp3	Rattus norvegicus peroxisomal membrane protein 3 (Pxmp3), mRNA [NM_017234]	**0,527**
Pacsin2	Rattus norvegicus protein kinase C and casein kinase substrate in neurons 2 (Pacsin2), mRNA [NM_130740]	**0,527**
0	PREDICTED: Rattus norvegicus similar to eyes absent 4 isoform a (LOC360684), partial mRNA [XM_340956]	**0,528**
Zc3h6	Rattus norvegicus zinc finger CCCH type containing 6 (Zc3h6), mRNA [NM_001107772]	**0,528**
0	Unknown	**0,528**
Aof1	Rattus norvegicus amine oxidase (flavin containing) domain 1 (Aof1), mRNA [NM_001107343]	**0,528**
Aadacl1	Rattus norvegicus arylacetamide deacetylase-like 1 (Aadacl1), mRNA [NM_001127524]	**0,528**
Eapp	Rattus norvegicus E2F-associated phosphoprotein (Eapp), transcript variant 2, mRNA [NM_001106729]	**0,528**
LOC100360604	Uncharacterized protein [Source:UniProtKB/TrEMBL;Acc:D3ZPN7] [ENSRNOT00000049571]	**0,528**
Ctxn2	Rattus norvegicus cortexin 2 (Ctxn2), mRNA [NM_001162935]	**0,528**
Adck1	Rattus norvegicus aarF domain containing kinase 1 (Adck1), mRNA [NM_001108985]	**0,528**
MGC125086	Rattus norvegicus similar to RIKEN cDNA 5133401N09 (MGC125086), mRNA [NM_001037362]	**0,528**
0	LAMBV_CHICK (Q01636) Laminin beta-1 chain variant (Laminin beta-1-2 chain) (Fragment), partial (11%) [TC599571]	**0,528**
Abcf2	Rattus norvegicus ATP-binding cassette, subfamily F (GCN20), member 2 (Abcf2), nuclear gene encoding mitochondrial protein, mRNA [NM_001109666]	**0,528**
Kdr	Rattus norvegicus kinase insert domain receptor (Kdr), mRNA [NM_013062]	**0,528**
0	Unknown	**0,528**
Nfil3	Rattus norvegicus nuclear factor, interleukin 3 regulated (Nfil3), mRNA [NM_053727]	**0,528**
Gdap1l1	Rattus norvegicus ganglioside-induced differentiation-associated protein 1-like 1 (Gdap1l1), mRNA [NM_001107798]	**0,528**

Ngef	Rattus norvegicus neuronal guanine nucleotide exchange factor (Ngef), mRNA [NM_001136241]	**0,528**
Slc30a3	Rattus norvegicus solute carrier family 30 (zinc transporter), member 3 (Slc30a3), mRNA [NM_001013243]	**0,528**
Fgfbp3	Rattus norvegicus fibroblast growth factor binding protein 3 (Fgfbp3), mRNA [NM_001109165]	**0,528**
Rnf10	Rattus norvegicus ring finger protein 10 (Rnf10), mRNA [NM_001011904]	**0,528**
Poll	Rattus norvegicus polymerase (DNA directed), lambda (Poll), mRNA [NM_001014168]	**0,528**
Cryga	Rattus norvegicus crystallin, gamma A (Cryga), mRNA [NM_001080936]	**0,528**
RGD1560978	Rattus norvegicus similar to hypothetical protein (RGD1560978), mRNA [NM_001134631]	**0,528**
Lrp11	Rattus norvegicus low density lipoprotein receptor-related protein 11 (Lrp11), mRNA [NM_001106217]	**0,528**
0	Unknown	**0,528**
Arfgef2	Rattus norvegicus ADP-ribosylation factor guanine nucleotide-exchange factor 2 (brefeldin A-inhibited) (Arfgef2), mRNA [NM_181083]	**0,528**
Taf1c	Rattus norvegicus TATA box binding protein (Tbp)-associated factor, RNA polymerase I, C (Taf1c), mRNA [NM_001014155]	**0,528**
Acp5	Rattus norvegicus acid phosphatase 5, tartrate resistant (Acp5), mRNA [NM_019144]	**0,528**
Vegfb	Rattus norvegicus vascular endothelial growth factor B (Vegfb), mRNA [NM_053549]	**0,528**
Map4	Rattus norvegicus microtubule-associated protein 4 (Map4), mRNA [NM_001024278]	**0,528**
Tomm6	PREDICTED: Rattus norvegicus translocase of outer mitochondrial membrane 6 homolog (yeast) (Tomm6), mRNA [XM_001060392]	**0,528**
Geft	Rattus norvegicus RhoA/RAC/CDC42 exchange factor (Geft), mRNA [NM_199395]	**0,528**
Plaa	Rattus norvegicus phospholipase A2, activating protein (Plaa), mRNA [NM_053866]	**0,528**
Pc	Rattus norvegicus pyruvate carboxylase (Pc), nuclear gene encoding mitochondrial protein, mRNA [NM_012744]	**0,528**
Copb1	Rattus norvegicus coatomer protein complex, subunit beta 1 (Copb1), mRNA [NM_080781]	**0,528**
Znf655	Rattus norvegicus zinc finger protein 655 (Znf655), mRNA [NM_001008362]	**0,528**
Hisppd2a	Rattus norvegicus histidine acid phosphatase domain containing 2A (Hisppd2a), mRNA [NM_001080783]	**0,528**
Ptprk	Rattus norvegicus protein tyrosine phosphatase, receptor type, K, extracellular region (Ptprk), mRNA [NM_001029902]	**0,528**
0	Unknown	**0,529**
0	Uncharacterized protein [Source:UniProtKB/TrEMBL;Acc:D3ZHU8] [ENSRNOT00000001891]	**0,529**
Vps54	Rattus norvegicus vacuolar protein sorting 54 homolog (S. cerevisiae) (Vps54), mRNA [NM_173147]	**0,529**
LOC100363170	PREDICTED: Rattus norvegicus rCG53134-like (LOC100363170), mRNA [XM_002730165]	**0,529**
Atg2a	Rattus norvegicus ATG2 autophagy related 2 homolog A (S. cerevisiae) (Atg2a), mRNA [NM_001109545]	**0,529**
Flt3	Rattus norvegicus fms-related tyrosine kinase 3 (Flt3), mRNA [NM_001100822]	**0,529**
RGD1562551	Rattus norvegicus similar to C20orf82 (RGD1562551), mRNA [NM_001134552]	**0,529**
0	Rattus norvegicus TL0ABA20YK18 mRNA sequence. [FQ210882]	**0,529**
Ly6h	Rattus norvegicus lymphocyte antigen 6 complex, locus H (Ly6h), mRNA [NM_001134839]	**0,529**
Crat	Rattus norvegicus carnitine acetyltransferase (Crat), nuclear gene encoding mitochondrial protein, mRNA [NM_001004085]	**0,529**
0	Unknown	**0,529**
0	Uncharacterized protein [Source:UniProtKB/TrEMBL;Acc:D4A1J6] [ENSRNOT00000021816]	**0,529**

0	Unknown	**0,529**
RGD1564327	Rattus norvegicus similar to integrin alpha 8 (RGD1564327), mRNA [NM_001173972]	**0,529**
Anxa2	Rattus norvegicus annexin A2 (Anxa2), mRNA [NM_019905]	**0,529**
Pebp1	Rattus norvegicus phosphatidylethanolamine binding protein 1 (Pebp1), mRNA [NM_017236]	**0,529**
0	Unknown	**0,529**
Zfp212	PREDICTED: Rattus norvegicus Zinc finger protein 212 (Zfp212), mRNA [XM_001053455]	**0,529**
Vdac3	Rattus norvegicus voltage-dependent anion channel 3 (Vdac3), mRNA [NM_031355]	**0,529**
Rab34	Rattus norvegicus RAB34, member RAS oncogene family (Rab34), mRNA [NM_001012140]	**0,529**
Xpo5	Rattus norvegicus exportin 5 (Xpo5), mRNA [NM_001108789]	**0,530**
Yme1l1	Rattus norvegicus YME1-like 1 (S. cerevisiae) (Yme1l1), nuclear gene encoding mitochondrial protein, mRNA [NM_053682]	**0,530**
Eif2ak2	Rattus norvegicus eukaryotic translation initiation factor 2-alpha kinase 2 (Eif2ak2), mRNA [NM_019335]	**0,530**
0	Rattus norvegicus similar to mammary tumor virus receptor 2 isoform 2 (LOC361441), mRNA [XM_341720]	**0,530**
Tnfaip8	Rattus norvegicus tumor necrosis factor, alpha-induced protein 8 (Tnfaip8), mRNA [NM_001107387]	**0,530**
Ndel1	Rattus norvegicus nudE nuclear distribution gene E homolog (A. nidulans)-like 1 (Ndel1), mRNA [NM_133320]	**0,530**
Apoo	Rattus norvegicus apolipoprotein O (Apoo), mRNA [NM_001199178]	**0,530**
0	Unknown	**0,530**
Sbno2	Rattus norvegicus strawberry notch homolog 2 (Drosophila) (Sbno2), mRNA [NM_001108068]	**0,530**
0	Unknown	**0,530**
Pknox1	Rattus norvegicus PBX/knotted 1 homeobox 1 (Pknox1), mRNA [NM_001013074]	**0,530**
Ppp2r1b	Rattus norvegicus protein phosphatase 2 (formerly 2A), regulatory subunit A, beta isoform (Ppp2r1b), mRNA [NM_001025418]	**0,530**
Tbrg4	Rattus norvegicus transforming growth factor beta regulator 4 (Tbrg4), mRNA [NM_001012154]	**0,530**
Camsap1	Rattus norvegicus calmodulin regulated spectrin-associated protein 1 (Camsap1), mRNA [NM_001168549]	**0,530**
RGD1566314	Uncharacterized protein [Source:UniProtKB/TrEMBL;Acc:D3ZPU0] [ENSRNOT00000027087]	**0,530**
Npap60	Rattus norvegicus nuclear pore associated protein (Npap60), mRNA [NM_012991]	**0,530**
0	Uncharacterized protein [Source:UniProtKB/TrEMBL;Acc:D3ZRP8] [ENSRNOT00000031672]	**0,530**
LOC681989	Rattus norvegicus similar to defective SPErmatogenesis family member (spe-39) (LOC681989), mRNA [NM_001101004]	**0,530**
0	40S ribosomal protein S8 [Source:UniProtKB/TrEMBL;Acc:D3ZT31] [ENSRNOT00000001785]	**0,530**
Poldip2	Rattus norvegicus polymerase (DNA-directed), delta interacting protein 2 (Poldip2), mRNA [NM_001105816]	**0,530**
RGD1308049	Uncharacterized protein [Source:UniProtKB/TrEMBL;Acc:D3ZT97] [ENSRNOT00000028509]	**0,530**
Ddx21	Rattus norvegicus DEAD (Asp-Glu-Ala-Asp) box polypeptide 21 (Ddx21), mRNA [NM_001037201]	**0,530**
Tubg1	Rattus norvegicus tubulin, gamma 1 (Tubg1), mRNA [NM_145778]	**0,530**
Coq5	Rattus norvegicus coenzyme Q5 homolog, methyltransferase (S. cerevisiae) (Coq5), nuclear gene encoding mitochondrial protein, mRNA [NM_001039022]	**0,530**
Fn1	Rattus norvegicus fibronectin 1 (Fn1), mRNA [NM_019143]	**0,530**

Tmem11	Rattus norvegicus transmembrane protein 11 (Tmem11), transcript variant 2, mRNA [NM_001166165]	**0,530**
Ppp4c	Rattus norvegicus protein phosphatase 4, catalytic subunit (Ppp4c), mRNA [NM_134359]	**0,530**
Cln5	Rattus norvegicus ceroid-lipofuscinosis, neuronal 5 (Cln5), mRNA [NM_001191689]	**0,530**
Flot1	Rattus norvegicus flotillin 1 (Flot1), mRNA [NM_022701]	**0,530**
Sbno1	Rattus norvegicus strawberry notch homolog 1 (Drosophila) (Sbno1), mRNA [NM_001107138]	**0,530**
0	Unknown	**0,530**
Ftl	Rattus norvegicus ferritin, light polypeptide (Ftl), mRNA [NM_022500]	**0,530**
Lrrc28	Rattus norvegicus leucine rich repeat containing 28 (Lrrc28), mRNA [NM_001108486]	**0,530**
Yars	Rattus norvegicus tyrosyl-tRNA synthetase (Yars), mRNA [NM_001025696]	**0,530**
LOC100364138	PREDICTED: Rattus norvegicus ferritin light chain 1-like (LOC100364138), mRNA [XM_002726633]	**0,530**
Mosc2	Rattus norvegicus MOCO sulphurase C-terminal domain containing 2 (Mosc2), mRNA [NM_134410]	**0,530**
Gss	Rattus norvegicus glutathione synthetase (Gss), mRNA [NM_012962]	**0,530**
RGD1306917	Rattus norvegicus similar to RIKEN cDNA 2900010M23 (RGD1306917), mRNA [NM_001108528]	**0,530**
Ermp1	Rattus norvegicus endoplasmic reticulum metallopeptidase 1 (Ermp1), mRNA [NM_184050]	**0,530**
Trak2	Rattus norvegicus trafficking protein, kinesin binding 2 (Trak2), mRNA [NM_133560]	**0,530**
Pbrm1	PREDICTED: Rattus norvegicus polybromo 1, transcript variant 2 (Pbrm1), mRNA [XM_001059164]	**0,530**
Vps53	Rattus norvegicus vacuolar protein sorting 53 homolog (S. cerevisiae) (Vps53), mRNA [NM_001105813]	**0,530**
0	RCG40058Uncharacterized protein [Source:UniProtKB/TrEMBL;Acc:D4A9P7] [ENSRNOT00000026267]	**0,531**
Ptk2	Rattus norvegicus PTK2 protein tyrosine kinase 2 (Ptk2), mRNA [NM_013081]	**0,531**
Gramd1a	Rattus norvegicus GRAM domain containing 1A (Gramd1a), mRNA [NM_001014160]	**0,531**
Zfp653	Rattus norvegicus zinc finger protein 653 (Zfp653), mRNA [NM_001106807]	**0,531**
LOC686240	PREDICTED: Rattus norvegicus similar to NMDA receptor regulated 1-like, transcript variant 2 (LOC686240), mRNA [XM_001073064]	**0,531**
Phlda2	Rattus norvegicus pleckstrin homology-like domain, family A, member 2 (Phlda2), transcript variant 1, mRNA [NM_001100521]	**0,531**
0	Unknown	**0,531**
Mff	Rattus norvegicus mitochondrial fission factor (Mff), nuclear gene encoding mitochondrial protein, mRNA [NM_001039015]	**0,531**
Acsf3	Similar to C50H11.1, isoform CRA_aUncharacterized protein [Source:UniProtKB/TrEMBL;Acc:D3ZUX7] [ENSRNOT00000020313]	**0,531**
Senp3	Rattus norvegicus Sumo1/sentrin/SMT3 specific peptidase 3 (Senp3), mRNA [NM_001013116]	**0,531**
Sptbn4	Sptbn4 protein [Source:UniProtKB/TrEMBL;Acc:Q5BJU8] [ENSRNOT00000028393]	**0,531**
0	Uncharacterized protein [Source:UniProtKB/TrEMBL;Acc:D3Z9Y0] [ENSRNOT00000022886]	**0,531**
RGD1311433	Uncharacterized protein [Source:UniProtKB/TrEMBL;Acc:D3Z9R2] [ENSRNOT00000003700]	**0,531**
Bcan	Rattus norvegicus brevican (Bcan), transcript variant 1, mRNA [NM_001033665]	**0,531**
RGD1562502	Rattus norvegicus similar to RIKEN cDNA 2610029G23 (RGD1562502), mRNA [NM_001108818]	**0,531**
Slc46a1	Rattus norvegicus solute carrier family 46 (folate transporter), member 1 (Slc46a1), mRNA [NM_001013969]	**0,531**

LOC314942	PREDICTED: Rattus norvegicus similar to CUB and Sushi multiple domains 3 isoform 1, transcript variant 4 (LOC314942), mRNA [XM_001063221]	**0,531**
Mtmr3	Rattus norvegicus myotubularin related protein 3 (Mtmr3), mRNA [NM_001012038]	**0,531**
Bri3	Rattus norvegicus brain protein I3 (Bri3), mRNA [NM_001009604]	**0,531**
Cpsf4	Rattus norvegicus cleavage and polyadenylation specific factor 4 (Cpsf4), mRNA [NM_001012351]	**0,531**
Abcc5	Rattus norvegicus ATP-binding cassette, subfamily C (CFTR/MRP), member 5 (Abcc5), mRNA [NM_053924]	**0,531**
0	Unknown	**0,531**
Epdr1	Rattus norvegicus ependymin related protein 1 (zebrafish) (Epdr1), mRNA [NM_001007625]	**0,531**
Tm9sf2	Rattus norvegicus transmembrane 9 superfamily member 2 (Tm9sf2), mRNA [NM_001005554]	**0,531**
Lrriq1	PREDICTED: Rattus norvegicus leucine-rich repeats and IQ motif containing 1 (Lrriq1), mRNA [XM_001080693]	**0,531**
LOC686041	Rattus norvegicus cDNA clone IMAGE:6889638, partial cds. [BC059164]	**0,531**
0	AW920953 EST352257 Rat gene index, normalized rat, norvegicus, Bento Soares Rattus norvegicus cDNA clone RGIHK22 5' end, mRNA sequence [AW920953]	**0,531**
0	BC043133 Cnot2 protein {Mus musculus} (exp=-1; wgp=0; cg=0), partial (14%) [TC583782]	**0,531**
Tbc1d2b	Rattus norvegicus TBC1 domain family, member 2B (Tbc1d2b), mRNA [NM_001108175]	**0,532**
Srgap2	Rattus norvegicus SLIT-ROBO Rho GTPase activating protein 2 (Srgap2), mRNA [NM_001134958]	**0,532**
Phf19	Rattus norvegicus PHD finger protein 19 (Phf19), mRNA [NM_001106570]	**0,532**
Lxn	Rattus norvegicus latexin (Lxn), mRNA [NM_031655]	**0,532**
Dcaf11	Rattus norvegicus DDB1 and CUL4 associated factor 11 (Dcaf11), mRNA [NM_001009686]	**0,532**
Trmt61a	Rattus norvegicus tRNA methyltransferase 61 homolog A (S. cerevisiae) (Trmt61a), mRNA [NM_001007706]	**0,532**
Zfyve28	Rattus norvegicus zinc finger, FYVE domain containing 28 (Zfyve28), mRNA [NM_001107227]	**0,532**
0	Unknown	**0,532**
Prkrir	Rattus norvegicus protein-kinase, interferon-inducible double stranded RNA dependent inhibitor, repressor of (P58 repressor) (Prkrir), mRNA [NM_001191630]	**0,532**
Rragc	Rattus norvegicus Ras-related GTP binding C (Rragc), mRNA [NM_001048184]	**0,532**
MGC94190	Rattus norvegicus similar to 0610007L01Rik protein (MGC94190), mRNA [NM_001004204]	**0,532**
Garnl4	Rattus norvegicus GTPase activating Rap/RanGAP domain-like 4 (Garnl4), mRNA [NM_001107019]	**0,532**
Atp6v1a	Rattus norvegicus ATPase, H+ transporting, lysosomal V1 subunit A (Atp6v1a), mRNA [NM_001108318]	**0,532**
Atp6v0e1	Rattus norvegicus ATPase, H+ transporting, lysosomal, V0 subunit e1 (Atp6v0e1), mRNA [NM_053578]	**0,532**
Tfip11	Rattus norvegicus tuftelin interacting protein 11 (Tfip11), mRNA [NM_001008291]	**0,532**
LOC691849	Rattus norvegicus hypothetical protein LOC691849 (LOC691849), mRNA [NM_001198796]	**0,532**
0	Uncharacterized protein [Source:UniProtKB/TrEMBL;Acc:D4A1S8] [ENSRNOT00000046898]	**0,532**
Ccpg1	Rattus norvegicus cell cycle progression 1 (Ccpg1), mRNA [NM_001108770]	**0,532**
RGD1566369	40S ribosomal protein S8 [Source:UniProtKB/TrEMBL;Acc:D3ZIE1] [ENSRNOT00000006662]	**0,532**

Smarcc1	Rattus norvegicus SWI/SNF related, matrix associated, actin dependent regulator of chromatin, subfamily c, member 1 (Smarcc1), mRNA [NM_001106861]	**0,532**
RGD1307235	Rattus norvegicus similar to RIKEN cDNA 2310035C23 (RGD1307235), mRNA [NM_001134546]	**0,532**
Rnf103	Rattus norvegicus ring finger protein 103 (Rnf103), mRNA [NM_053438]	**0,532**
RGD1562987	Rattus norvegicus similar to cDNA sequence BC031181 (RGD1562987), mRNA [NM_001173472]	**0,532**
Bpgm	Rattus norvegicus 2,3-bisphosphoglycerate mutase (Bpgm), mRNA [NM_199382]	**0,532**
Pcdhb15	PREDICTED: Rattus norvegicus protocadherin beta 15 (Pcdhb15), mRNA [XM_001055818]	**0,532**
Adss	Rattus norvegicus adenylosuccinate synthase (Adss), mRNA [NM_001105975]	**0,532**
Panx2	Rattus norvegicus pannexin 2 (Panx2), mRNA [NM_199409]	**0,533**
Nxph1	Rattus norvegicus neurexophilin 1 (Nxph1), mRNA [NM_012994]	**0,533**
Ocel1	Rattus norvegicus occludin/ELL domain containing 1 (Ocel1), mRNA [NM_001106065]	**0,533**
Nat8l	Rattus norvegicus N-acetyltransferase 8-like (Nat8l), mRNA [NM_001191681]	**0,533**
0	Unknown	**0,533**
0	AGENCOURT_28718588 NIH_MGC_249 Rattus norvegicus cDNA clone IMAGE:7378234 5', mRNA sequence [CO802318]	**0,533**
0	Rattus norvegicus similar to 60S ribosomal protein L21 (LOC364769), mRNA [XM_344624]	**0,533**
Nt5c3l	Rattus norvegicus 5'-nucleotidase, cytosolic III-like (Nt5c3l), mRNA [NM_001007723]	**0,533**
Trappc3	Rattus norvegicus trafficking protein particle complex 3 (Trappc3), mRNA [NM_001008376]	**0,533**
0	RVL12088 Wackym-Soares normalized rat vestibular cDNA library Rattus norvegicus cDNA 5', mRNA sequence [DV720985]	**0,533**
Pon2	Rattus norvegicus paraoxonase 2 (Pon2), mRNA [NM_001013082]	**0,533**
0	Unknown	**0,533**
Ndufab1	Rattus norvegicus NADH dehydrogenase (ubiquinone) 1, alpha/beta subcomplex, 1 (Ndufab1), nuclear gene encoding mitochondrial protein, mRNA [NM_001106294]	**0,533**
Serpinb6a	Rattus norvegicus serine (or cysteine) peptidase inhibitor, clade B, member 6a (Serpinb6a), mRNA [NM_199085]	**0,533**
0	Rattus norvegicus TL0AEA5YK19 mRNA sequence. [FQ233969]	**0,533**
Crlf3	Rattus norvegicus unknown mRNA sequence. [AF072835]	**0,533**
0	Rattus norvegicus similar to SEPIAPTERIN REDUCTASE (SPR) (LOC297402), mRNA [XM_216190]	**0,533**
RGD1565775	LOC361980 protein [Source:UniProtKB/TrEMBL;Acc:Q56A23] [ENSRNOT00000027385]	**0,533**
0	Uncharacterized protein [Source:UniProtKB/TrEMBL;Acc:D3ZZD9] [ENSRNOT00000068382]	**0,533**
Fbxo8	Rattus norvegicus F-box protein 8 (Fbxo8), mRNA [NM_001012050]	**0,533**
Gpr172a	Rattus norvegicus G protein-coupled receptor 172A (Gpr172a), mRNA [NM_001109670]	**0,533**
Lrrc37a	PREDICTED: Rattus norvegicus leucine rich repeat containing 37, member A3 (Lrrc37a3), mRNA [XM_220928]	**0,533**
Veph1	Rattus norvegicus ventricular zone expressed PH domain homolog 1 (zebrafish) (Veph1), mRNA [NM_001014171]	**0,533**
Id3	Rattus norvegicus inhibitor of DNA binding 3 (Id3), mRNA [NM_013058]	**0,533**
Pou3f2	Rattus norvegicus POU class 3 homeobox 2 (Pou3f2), mRNA [NM_172085]	**0,533**

Lztfl1	Rattus norvegicus leucine zipper transcription factor-like 1 (Lztfl1), mRNA [NM_001024266]	**0,533**
0	grp75=75 kda glucose regulated protein [rats, Sprague-Dawley, brain, mRNA, 3001 nt]. [S78556]	**0,533**
0	Uncharacterized protein [Source:UniProtKB/TrEMBL;Acc:D4A0Y6] [ENSRNOT00000038176]	**0,533**
RGD1559643	Rattus norvegicus similar to hypothetical protein A430031N04 (RGD1559643), mRNA [NM_001109056]	**0,533**
0	Uncharacterized protein [Source:UniProtKB/TrEMBL;Acc:D3ZAQ6] [ENSRNOT00000024122]	**0,533**
0	Unknown	**0,533**
Ak2	Rattus norvegicus adenylate kinase 2 (Ak2), nuclear gene encoding mitochondrial protein, transcript variant 2, mRNA [NM_001033967]	**0,533**
0	Unknown	**0,533**
Nmt2	Rattus norvegicus N-myristoyltransferase 2 (Nmt2), mRNA [NM_207590]	**0,533**
RGD1306000	Rattus norvegicus similar to CG8043-PA (RGD1306000), mRNA [NM_001108827]	**0,533**
Gnpda2	Rattus norvegicus glucosamine-6-phosphate deaminase 2 (Gnpda2), mRNA [NM_001106005]	**0,533**
LOC690728	Rattus norvegicus similar to Protein C12orf11 (Sarcoma antigen NY-SAR-95) (LOC690728), mRNA [NM_001109608]	**0,533**
Mtx1	Rattus norvegicus Metaxin 1 (Mtx1), nuclear gene encoding mitochondrial protein, mRNA [NM_001100667]	**0,533**
RGD1562161	Rattus norvegicus similar to chromosome X open reading frame 23 (RGD1562161), mRNA [NM_001127568]	**0,533**
0	RCG23367Uncharacterized protein [Source:UniProtKB/TrEMBL;Acc:D3ZPX0] [ENSRNOT00000047731]	**0,533**
RGD1305110	Rattus norvegicus similar to KIAA1841 protein (RGD1305110), mRNA [NM_001100971]	**0,534**
Edc4	Rattus norvegicus enhancer of mRNA decapping 4 (Edc4), mRNA [NM_001033068]	**0,534**
Dirc2	Rattus norvegicus disrupted in renal carcinoma 2 (human) (Dirc2), mRNA [NM_001012017]	**0,534**
Uqcrb	Rattus norvegicus ubiquinol-cytochrome c reductase binding protein (Uqcrb), nuclear gene encoding mitochondrial protein, mRNA [NM_001127553]	**0,534**
0	Unknown	**0,534**
0	Rattus norvegicus similar to 40S ribosomal protein S7 (S8) (LOC289932), mRNA [XM_223834]	**0,534**
Igfbp2	Rattus norvegicus insulin-like growth factor binding protein 2 (Igfbp2), mRNA [NM_013122]	**0,534**
Ppp1r12b	Rattus norvegicus protein phosphatase 1, regulatory (inhibitor) subunit 12B (Ppp1r12b), mRNA [NM_001107178]	**0,534**
Vom2r13	Rattus norvegicus vomeronasal 2 receptor, 13 (Vom2r13), mRNA [NM_001099489]	**0,534**
Jakmip2	Rattus norvegicus janus kinase and microtubule interacting protein 2 (Jakmip2), mRNA [NM_001107391]	**0,534**
RGD1560187	Rattus norvegicus similar to protein of fungal metazoan origin like (11.1 kD) (2C514) (LOC362641), mRNA [XM_342957]	**0,534**
Ugt1a6	Rattus norvegicus UDP glucuronosyltransferase 1 family, polypeptide A6 (Ugt1a6), transcript variant 1, mRNA [NM_001039691]	**0,534**
Slc19a1	Rattus norvegicus solute carrier family 19 (folate transporter), member 1 (Slc19a1), transcript variant 1, mRNA [NM_017299]	**0,534**
RGD1305420	Rattus norvegicus similar to Nef associated protein 1 (RGD1305420), mRNA [NM_001025652]	**0,534**
Josd1	Rattus norvegicus Josephin domain containing 1 (Josd1), mRNA [NM_001025009]	**0,534**
Crcp	Rattus norvegicus CGRP receptor component (Crcp), mRNA [NM_053670]	**0,534**
Psat1	Rattus norvegicus phosphoserine aminotransferase 1 (Psat1), mRNA [NM_198738]	**0,534**
Tmem81	Rattus norvegicus transmembrane protein 81 (Tmem81), mRNA [NM_001017490]	**0,534**

Enc1	Rattus norvegicus ectodermal-neural cortex 1 (Enc1), mRNA [NM_001003401]	**0,534**
0	Unknown	**0,535**
Fgfrl1	Rattus norvegicus fibroblast growth factor receptor-like 1 (Fgfrl1), mRNA [NM_199114]	**0,535**
0	Unknown	**0,535**
Usp24	Putative uncharacterized protein [Source:UniProtKB/TrEMBL;Acc:Q4G007] [ENSRNOT00000029228]	**0,535**
0	Unknown	**0,535**
Cyp4f5	Rattus norvegicus cytochrome P450, family 4, subfamily f, polypeptide 5 (Cyp4f5), mRNA [NM_173124]	**0,535**
Elmo2	Rattus norvegicus engulfment and cell motility 2 (Elmo2), mRNA [NM_001134955]	**0,535**
Sdhc	Rattus norvegicus succinate dehydrogenase complex, subunit C, integral membrane protein (Sdhc), nuclear gene encoding mitochondrial protein, mRNA [NM_001005534]	**0,535**
Ppm1l	Rattus norvegicus protein phosphatase 1 (formerly 2C)-like (Ppm1l), mRNA [NM_001107681]	**0,535**
Smchd1	Uncharacterized protein [Source:UniProtKB/TrEMBL;Acc:D4AAG8] [ENSRNOT00000019359]	**0,535**
Mgat1	Rattus norvegicus mannosyl (alpha-1,3-)-glycoprotein beta-1,2-N-acetylglucosaminyltransferase (Mgat1), mRNA [NM_030861]	**0,535**
Tmem192	Rattus norvegicus transmembrane protein 192 (Tmem192), mRNA [NM_001014141]	**0,535**
Drg1	Rattus norvegicus developmentally regulated GTP binding protein 1 (Drg1), mRNA [NM_001009685]	**0,535**
Brd2	Rattus norvegicus bromodomain containing 2 (Brd2), mRNA [NM_212495]	**0,535**
Fry	Rattus norvegicus furry homolog (Drosophila) (Fry), mRNA [NM_001170398]	**0,535**
0	STML1_MOUSE (Q8CI66) Stomatin-like protein 1 (SLP-1), partial (6%) [TC590815]	**0,535**
Whsc2	Rattus norvegicus Wolf-Hirschhorn syndrome candidate 2 (human) (Whsc2), mRNA [NM_001008339]	**0,535**
Sult1a1	Rattus norvegicus sulfotransferase family, cytosolic, 1A, phenol-preferring, member 1 (Sult1a1), mRNA [NM_031834]	**0,535**
0	Golgi integral membrane protein 4 [Source:UniProtKB/Swiss-Prot;Acc:Q5BJK8] [ENSRNOT00000064588]	**0,535**
Wdr77	Rattus norvegicus WD repeat domain 77 (Wdr77), mRNA [NM_001008771]	**0,535**
Nrcam	Rattus norvegicus neuronal cell adhesion molecule (Nrcam), mRNA [NM_013150]	**0,535**
Vamp4	Rattus norvegicus vesicle-associated membrane protein 4 (Vamp4), mRNA [NM_001108856]	**0,535**
LOC100174910	Rattus norvegicus glutaredoxin-like protein (LOC100174910), mRNA [NM_001131003]	**0,535**
Gak	Rattus norvegicus cyclin G associated kinase (Gak), mRNA [NM_031030]	**0,535**
Anp32b	Rattus norvegicus acidic (leucine-rich) nuclear phosphoprotein 32 family, member B (Anp32b), mRNA [NM_131911]	**0,535**
0	Unknown	**0,535**
Pcdh17	Rattus norvegicus protocadherin 17 (Pcdh17), mRNA [NM_001107279]	**0,535**
Lppr4	Rattus norvegicus lipid phosphate phosphatase-related protein type 4 (Lppr4), mRNA [NM_001001508]	**0,535**
Msrb2	Rattus norvegicus methionine sulfoxide reductase B2 (Msrb2), mRNA [NM_001031660]	**0,536**
Bloc1s3	PREDICTED: Rattus norvegicus biogenesis of lysosomal organelles complex-1, subunit 3 (Bloc1s3), mRNA [XM_001058094]	**0,536**
0	EST350998 Rat gene index, normalized rat, norvegicus, Bento Soares Rattus norvegicus cDNA clone RGIGF73 5' end, mRNA sequence [AW919694]	**0,536**

Cyp2j4	Rattus norvegicus cytochrome P450, family 2, subfamily j, polypeptide 4 (Cyp2j4), mRNA [NM_023025]	**0,536**
Traf3ip2	Rattus norvegicus Traf3 interacting protein 2 (Traf3ip2), mRNA [NM_001044248]	**0,536**
Smarce1	Rattus norvegicus SWI/SNF related, matrix associated, actin dependent regulator of chromatin, subfamily e, member 1 (Smarce1), mRNA [NM_001024993]	**0,536**
0	PREDICTED: Rattus norvegicus zinc finger protein 770 (Znf770), mRNA [XM_001079020]	**0,536**
LOC298018	RCG31985Uncharacterized protein [Source:UniProtKB/TrEMBL;Acc:D4AE08] [ENSRNOT00000014053]	**0,536**
Nkain4	Rattus norvegicus Na+/K+ transporting ATPase interacting 4 (Nkain4), mRNA [NM_001106550]	**0,536**
Ncoa7	Uncharacterized protein [Source:UniProtKB/TrEMBL;Acc:D3ZM65] [ENSRNOT00000018849]	**0,536**
Rpl12	Uncharacterized protein [Source:UniProtKB/TrEMBL;Acc:D3ZJE2] [ENSRNOT00000003002]	**0,536**
Gorab	Rattus norvegicus golgin, RAB6-interacting (Gorab), mRNA [NM_001100563]	**0,536**
Cyhr1	Rattus norvegicus cysteine and histidine rich 1 (Cyhr1), mRNA [NM_001025122]	**0,536**
Chmp4b	PREDICTED: Rattus norvegicus chromatin modifying protein 4B (Chmp4b), mRNA [XM_001073409]	**0,536**
Alkbh7	Rattus norvegicus alkB, alkylation repair homolog 7 (E. coli) (Alkbh7), mRNA [NM_001109384]	**0,536**
Atp5j	Rattus norvegicus ATP synthase, H+ transporting, mitochondrial F0 complex, subunit F6 (Atp5j), nuclear gene encoding mitochondrial protein, mRNA [NM_053602]	**0,536**
0	Q571D7_MOUSE (Q571D7) MKIAA0268 protein (Fragment), partial (20%) [TC587453]	**0,536**
Pcdha2	Rattus norvegicus protocadherin alpha 2 (Pcdha2), mRNA [NM_199504]	**0,536**
RGD1559864	PREDICTED: Rattus norvegicus similar to mKIAA1045 protein, transcript variant 2 (RGD1559864), mRNA [XM_575808]	**0,536**
0	Unknown	**0,536**
Magi3	Rattus norvegicus membrane associated guanylate kinase, WW and PDZ domain containing 3 (Magi3), mRNA [NM_139084]	**0,536**
Ptrh2	Rattus norvegicus peptidyl-tRNA hydrolase 2 (Ptrh2), nuclear gene encoding mitochondrial protein, mRNA [NM_001013860]	**0,536**
0	Unknown	**0,537**
Lmx1a	Rattus norvegicus LIM homeobox transcription factor 1 alpha (Lmx1a), mRNA [NM_001105967]	**0,537**
LOC685908	PREDICTED: Rattus norvegicus similar to Discs large homolog 5 (Placenta and prostate DLG) (Discs large protein P-dlg) (LOC685908), mRNA [XM_001065719]	**0,537**
LOC499219	Rattus norvegicus hypothetical protein LOC499219 (LOC499219), mRNA [NM_001047938]	**0,537**
Rpl15	Rattus norvegicus ribosomal protein L15 (Rpl15), mRNA [NM_139114]	**0,537**
Uso1	Rattus norvegicus USO1 homolog, vesicle docking protein (yeast) (Uso1), mRNA [NM_019379]	**0,537**
0	Uncharacterized protein [Source:UniProtKB/TrEMBL;Acc:D3ZDX5] [ENSRNOT00000043345]	**0,537**
Pex5	Rattus norvegicus peroxisomal biogenesis factor 5 (Pex5), mRNA [NM_001170584]	**0,537**
0	Unknown	**0,537**
LOC100359750	PREDICTED: Rattus norvegicus hypothetical protein LOC100359750 (LOC100359750), mRNA [XM_002728371]	**0,537**
Synj2bp	Rattus norvegicus synaptojanin 2 binding protein (Synj2bp), mRNA [NM_022599]	**0,537**
Exosc5	Rattus norvegicus exosome component 5 (Exosc5), mRNA [NM_001107493]	**0,537**

Clpp	PREDICTED: Rattus norvegicus ClpP caseinolytic peptidase, ATP-dependent, proteolytic subunit homolog (E. coli) (Clpp), mRNA [XM_217313]	**0,537**
Apc2	Rattus norvegicus adenomatosis polyposis coli 2 (Apc2), mRNA [NM_001106769]	**0,537**
0	Unknown	**0,537**
St7l	Rattus norvegicus suppression of tumorigenicity 7-like (St7l), mRNA [NM_001007639]	**0,537**
Lmo7	Rattus norvegicus LIM domain 7 (Lmo7), mRNA [NM_001001515]	**0,537**
Utp3	Rattus norvegicus UTP3, small subunit (SSU) processome component, homolog (S. cerevisiae) (Utp3), mRNA [NM_001012036]	**0,537**
0	Rattus norvegicus TL0AEA54YG18 mRNA sequence. [FQ225308]	**0,537**
LOC100364718	PREDICTED: Rattus norvegicus myeloid/lymphoid or mixed-lineage leukemia 3-like (LOC100364718), mRNA [XM_002726310]	**0,537**
RGD1309138	Rattus norvegicus similar to hypothetical protein MGC9912 (RGD1309138), mRNA [NM_001108981]	**0,537**
Itfg1	Rattus norvegicus integrin alpha FG-GAP repeat containing 1 (Itfg1), mRNA [NM_133557]	**0,537**
Cd200	Rattus norvegicus Cd200 molecule (Cd200), mRNA [NM_031518]	**0,537**
Ctps2	Rattus norvegicus CTP synthase II (Ctps2), mRNA [NM_001034998]	**0,537**
Spats2l	Rattus norvegicus spermatogenesis associated, serine-rich 2-like (Spats2l), mRNA [NM_001014102]	**0,537**
Serf1	Similar to small EDRK-rich factor 1 (Predicted), isoform CRA_aUncharacterized protein [Source:UniProtKB/TrEMBL;Acc:D3ZDW2] [ENSRNOT00000024291]	**0,537**
0	Unknown	**0,537**
0	Unknown	**0,537**
Rspry1	Rattus norvegicus ring finger and SPRY domain containing 1 (Rspry1), mRNA [NM_001100945]	**0,537**
0	Unknown	**0,537**
Slc30a1	Rattus norvegicus solute carrier family 30 (zinc transporter), member 1 (Slc30a1), mRNA [NM_022853]	**0,537**
Nbr1	Rattus norvegicus neighbor of Brca1 gene 1 (Nbr1), mRNA [NM_001024765]	**0,537**
Acat1	Rattus norvegicus acetyl-coenzyme A acetyltransferase 1 (Acat1), nuclear gene encoding mitochondrial protein, mRNA [NM_017075]	**0,537**
LOC680200	Rattus norvegicus similar to zinc finger protein 455 (LOC680200), mRNA [NM_001139491]	**0,537**
Fam155a	PREDICTED: Rattus norvegicus family with sequence similarity 155, member A (Fam155a), mRNA [XM_001076507]	**0,537**
Fbxw11	Rattus norvegicus F-box and WD repeat domain containing 11 (Fbxw11), mRNA [NM_001106993]	**0,537**
Slc35b2	Rattus norvegicus solute carrier family 35, member B2 (Slc35b2), mRNA [NM_001037215]	**0,537**
Kcnk2	Rattus norvegicus potassium channel, subfamily K, member 2 (Kcnk2), transcript variant 2, mRNA [NM_172042]	**0,538**
0	Q6C5E2_YARLI (Q6C5E2) Similarity (Fragment), partial (11%) [TC594916]	**0,538**
RGD1306954	Rattus norvegicus similar to RIKEN cDNA 1110004E09 (RGD1306954), mRNA [NM_001008288]	**0,538**
0	Unknown	**0,538**
Bdnf	Rattus norvegicus brain-derived neurotrophic factor (Bdnf), mRNA [NM_012513]	**0,538**
Dab2ip	Rattus norvegicus DAB2 interacting protein (Dab2ip), mRNA [NM_138710]	**0,538**
Cdk5rap2	Rattus norvegicus CDK5 regulatory subunit associated protein 2 (Cdk5rap2), mRNA [NM_173134]	**0,538**
Ptpn5	Rattus norvegicus protein tyrosine phosphatase, non-receptor type 5 (Ptpn5), mRNA [NM_019253]	**0,538**

Ccdc65	Rattus norvegicus coiled-coil domain containing 65 (Ccdc65), mRNA [NM_001014203]	**0,538**
Fgfr3	Rattus norvegicus fibroblast growth factor receptor 3 (Fgfr3), mRNA [NM_053429]	**0,538**
Podxl	Rattus norvegicus podocalyxin-like (Podxl), mRNA [NM_138848]	**0,538**
Bcar1	Rattus norvegicus breast cancer anti-estrogen resistance 1 (Bcar1), mRNA [NM_012931]	**0,538**
Man2a1	Rattus norvegicus mannosidase, alpha, class 2A, member 1 (Man2a1), mRNA [NM_012979]	**0,538**
Thap4	Rattus norvegicus THAP domain containing 4 (Thap4), mRNA [NM_001005564]	**0,538**
Lpgat1	Rattus norvegicus lysophosphatidylglycerol acyltransferase 1 (Lpgat1), mRNA [NM_001109376]	**0,538**
Necab3	Rattus norvegicus N-terminal EF-hand calcium binding protein 3 (Necab3), mRNA [NM_001098724]	**0,538**
Pot1a	Rattus norvegicus protection of telomeres 1A (Pot1a), mRNA [NM_001024322]	**0,538**
Ccbl1	Rattus norvegicus cysteine conjugate-beta lyase, cytoplasmic (Ccbl1), mRNA [NM_001013164]	**0,538**
LOC685106	PREDICTED: Rattus norvegicus similar to ribosomal protein L6 (LOC685106), mRNA [XM_001062312]	**0,538**
Ostc	Rattus norvegicus oligosaccharyltransferase complex subunit (Ostc), mRNA [NM_001108566]	**0,538**
Stat1	Rattus norvegicus signal transducer and activator of transcription 1 (Stat1), transcript variant alpha, mRNA [NM_032612]	**0,538**
0	microtubule-actin crosslinking factor 1 [Source:RefSeq peptide;Acc:NP_001129230] [ENSRNOT00000021951]	**0,538**
Eif2b5	Rattus norvegicus eukaryotic translation initiation factor 2B, subunit 5 epsilon (Eif2b5), mRNA [NM_138866]	**0,538**
Lenep	Rattus norvegicus lens epithelial protein (Lenep), mRNA [NM_053614]	**0,538**
0	dual specificity phosphatase 14 [Source:RefSeq peptide;Acc:NP_001073362] [ENSRNOT00000043148]	**0,538**
Qrich2	Uncharacterized protein [Source:UniProtKB/TrEMBL;Acc:D3ZG97] [ENSRNOT00000034817]	**0,538**
Actg1	Rattus norvegicus actin, gamma 1 (Actg1), mRNA [NM_001127449]	**0,538**
0	Unknown	**0,538**
0	Rattus norvegicus TL0ACA41YI14 mRNA sequence. [FQ216706]	**0,539**
Capn2	Rattus norvegicus calpain 2 (Capn2), mRNA [NM_017116]	**0,539**
0	Unknown	**0,539**
LOC683034	PREDICTED: Rattus norvegicus hypothetical protein LOC683034, transcript variant 2 (LOC683034), mRNA [XM_001061393]	**0,539**
0	Unknown	**0,539**
Stau2	Rattus norvegicus staufen, RNA binding protein, homolog 2 (Drosophila) (Stau2), transcript variant LL, mRNA [NM_001007149]	**0,539**
RGD1307615	Rattus norvegicus similar to hypothetical protein FLJ13045 (RGD1307615), mRNA [NM_001108573]	**0,539**
Epha4	Rattus norvegicus Eph receptor A4 (Epha4), mRNA [NM_001162411]	**0,539**
RGD1305014	Rattus norvegicus similar to RIKEN cDNA 2310057M21 (RGD1305014), mRNA [NM_001014025]	**0,539**
Prps1	Rattus norvegicus phosphoribosyl pyrophosphate synthetase 1 (Prps1), mRNA [NM_017243]	**0,539**
Duxbl	PREDICTED: Rattus norvegicus double homeobox B-like (Duxbl), mRNA [XM_001056985]	**0,539**
Mvk	Rattus norvegicus mevalonate kinase (Mvk), mRNA [NM_031063]	**0,539**
RGD1563296	PREDICTED: Rattus norvegicus similar to component of oligomeric golgi complex 5 isoform 1 (RGD1563296), mRNA [XM_001076141]	**0,539**
RGD1565432	Small VCP/p97-interacting protein [Source:UniProtKB/Swiss-Prot;Acc:P0C0A9] [ENSRNOT00000056024]	**0,539**

LOC499782	Rattus norvegicus similar to 60S ribosomal protein L12 (LOC499782), mRNA [NM_001109198]	**0,539**
Rgc32	Rattus norvegicus response gene to complement 32 (Rgc32), mRNA [NM_054008]	**0,539**
0	Unknown	**0,539**
0	Unknown	**0,539**
LOC680799	Rattus norvegicus hypothetical protein LOC680799 (LOC680799), transcript variant 2, mRNA [NM_001163519]	**0,539**
Gmcl1	Rattus norvegicus germ cell-less homolog 1 (Drosophila) (Gmcl1), transcript variant 1, mRNA [NM_001033931]	**0,539**
Chst10	Rattus norvegicus carbohydrate sulfotransferase 10 (Chst10), mRNA [NM_080397]	**0,539**
0	EPO_MOUSE (P07321) Erythropoietin precursor, partial (7%) [TC593578]	**0,539**
Lrrc8c	Rattus norvegicus leucine rich repeat containing 8 family, member C (Lrrc8c), mRNA [NM_001037179]	**0,539**
Thsd7b	Rattus norvegicus thrombospondin, type I, domain containing 7B (Thsd7b), mRNA [NM_001191669]	**0,539**
Plekhb2	Rattus norvegicus pleckstrin homology domain containing, family B (evectins) member 2 (Plekhb2), mRNA [NM_001106899]	**0,539**
Erp44	Rattus norvegicus endoplasmic reticulum protein 44 (Erp44), mRNA [NM_001008317]	**0,539**
Disp1	Rattus norvegicus dispatched homolog 1 (Drosophila) (Disp1), mRNA [NM_001105983]	**0,539**
Dbnl	Rattus norvegicus drebrin-like (Dbnl), mRNA [NM_031352]	**0,539**
0	Unknown	**0,539**
H1f0	Rattus norvegicus H1 histone family, member 0 (H1f0), mRNA [NM_012578]	**0,539**
Pgrmc1	Rattus norvegicus progesterone receptor membrane component 1 (Pgrmc1), mRNA [NM_021766]	**0,539**
Nvl	Rattus norvegicus nuclear VCP-like (Nvl), mRNA [NM_001105980]	**0,539**
Pcyt1a	Choline-phosphate cytidylyltransferase A [Source:UniProtKB/Swiss-Prot;Acc:P19836] [ENSRNOT00000002403]	**0,539**
Asb11	Rattus norvegicus ankyrin repeat and SOCS box-containing 11 (Asb11), mRNA [NM_001106962]	**0,539**
Gal	Rattus norvegicus galanin prepropeptide (Gal), mRNA [NM_033237]	**0,539**
0	Uncharacterized protein [Source:UniProtKB/TrEMBL;Acc:D3ZHU8] [ENSRNOT00000001891]	**0,539**
Nthl1	Rattus norvegicus nth (endonuclease III)-like 1 (E.coli) (Nthl1), mRNA [NM_001105728]	**0,539**
Ccdc134	Rattus norvegicus coiled-coil domain containing 134 (Ccdc134), mRNA [NM_001024355]	**0,540**
Sumo2	Rattus norvegicus SMT3 suppressor of mif two 3 homolog 2 (S. cerevisiae) (Sumo2), mRNA [NM_133594]	**0,540**
LOC500413	Rattus norvegicus hypothetical protein LOC500413 (LOC500413), mRNA [NM_001171802]	**0,540**
Gldc	Rattus norvegicus glycine dehydrogenase (decarboxylating) (Gldc), nuclear gene encoding mitochondrial protein, mRNA [NM_001107583]	**0,540**
Mterf	Rattus norvegicus mitochondrial transcription termination factor (Mterf), nuclear gene encoding mitochondrial protein, mRNA [NM_053499]	**0,540**
Snx27	Rattus norvegicus sorting nexin family member 27 (Snx27), transcript variant 2, mRNA [NM_152847]	**0,540**
0	Unknown	**0,540**
Snapap	Rattus norvegicus SNAP-associated protein (Snapap), transcript variant 2, mRNA [NM_001025648]	**0,540**
Spg20	Rattus norvegicus spastic paraplegia 20 (Troyer syndrome) homolog (human) (Spg20), mRNA [NM_001106433]	**0,540**
Aof1	Rattus norvegicus amine oxidase (flavin containing) domain 1 (Aof1), mRNA [NM_001107343]	**0,540**
Ift52	Rattus norvegicus intraflagellar transport 52 homolog (Chlamydomonas) (Ift52), mRNA [NM_001177685]	**0,540**

Tmem107	Rattus norvegicus transmembrane protein 107 (Tmem107), mRNA [NM_001109648]	**0,540**
Prkag2	Rattus norvegicus protein kinase, AMP-activated, gamma 2 non-catalytic subunit (Prkag2), mRNA [NM_184051]	**0,540**
LOC691975	PREDICTED: Rattus norvegicus similar to 40S ribosomal protein S17 (LOC691975), mRNA [XM_001080507]	**0,540**
RGD1304595	Rattus norvegicus similar to RIKEN cDNA 6330416G13 gene (RGD1304595), mRNA [NM_001106661]	**0,540**
0	Unknown	**0,540**
0	Unknown	**0,540**
0	Uncharacterized protein [Source:UniProtKB/TrEMBL;Acc:D4AEI9] [ENSRNOT00000034494]	**0,540**
Eif4b	Rattus norvegicus eukaryotic translation initiation factor 4B (Eif4b), mRNA [NM_001008324]	**0,540**
Ndrg4	Rattus norvegicus N-myc downstream regulated gene 4 (Ndrg4), mRNA [NM_031967]	**0,540**
Arhgap20	Rattus norvegicus Rho GTPase activating protein 20 (Arhgap20), mRNA [NM_213629]	**0,540**
RGD1560073	Uncharacterized protein [Source:UniProtKB/TrEMBL;Acc:D3ZCN9] [ENSRNOT00000043250]	**0,540**
Gng5	Rattus norvegicus guanine nucleotide binding protein (G protein), gamma 5 (Gng5), mRNA [NM_024377]	**0,540**
0	Unknown	**0,540**
Zdhhc4	Rattus norvegicus zinc finger, DHHC-type containing 4 (Zdhhc4), mRNA [NM_001013123]	**0,540**
Cyth3	Rattus norvegicus cytohesin 3 (Cyth3), mRNA [NM_053912]	**0,540**
Tmtc3	Rattus norvegicus transmembrane and tetratricopeptide repeat containing 3 (Tmtc3), mRNA [NM_001135858]	**0,540**
0	Uncharacterized protein [Source:UniProtKB/TrEMBL;Acc:D3ZJV0] [ENSRNOT00000006968]	**0,540**
Pqlc1	Rattus norvegicus PQ loop repeat containing 1 (Pqlc1), mRNA [NM_001013189]	**0,540**
Nars2	Rattus norvegicus asparaginyl-tRNA synthetase 2 (mitochondrial)(putative) (Nars2), nuclear gene encoding mitochondrial protein, mRNA [NM_001034921]	**0,540**
RGD1564809	Uncharacterized protein [Source:UniProtKB/TrEMBL;Acc:D3ZC90] [ENSRNOT00000038184]	**0,540**
0	PREDICTED: Rattus norvegicus similar to Mblk1-related protein-2 (LOC679031), mRNA [XM_001054322]	**0,540**
RT1-A1	Rattus norvegicus RT1 class Ia, locus A1 (RT1-A1), mRNA [NM_001008827]	**0,540**
RGD1566386	Rattus norvegicus similar to Hypothetical protein A430033K04 (RGD1566386), mRNA [NM_001107127]	**0,540**
Sfrs11	Rattus norvegicus splicing factor, arginine/serine-rich 11 (Sfrs11), mRNA [NM_001035255]	**0,540**
Tesk1	Rattus norvegicus testis-specific kinase 1 (Tesk1), mRNA [NM_031578]	**0,540**
Dph2	Rattus norvegicus DPH2 homolog (S. cerevisiae) (Dph2), mRNA [NM_001015007]	**0,540**
Zmym3	Rattus norvegicus zinc finger, MYM-type 3 (Zmym3), mRNA [NM_001040155]	**0,540**
0	Uncharacterized protein [Source:UniProtKB/TrEMBL;Acc:D4A553] [ENSRNOT00000016227]	**0,540**
H2afy	Rattus norvegicus H2A histone family, member Y (H2afy), mRNA [NM_017182]	**0,540**
Hnrnpl	Rattus norvegicus heterogeneous nuclear ribonucleoprotein L (Hnrnpl), transcript variant 1, mRNA [NM_001134760]	**0,540**
0	Uncharacterized protein [Source:UniProtKB/TrEMBL;Acc:D4AAV5] [ENSRNOT00000019301]	**0,540**
Sh3bp5	Rattus norvegicus SH3-domain binding protein 5 (BTK-associated) (Sh3bp5), mRNA [NM_054011]	**0,541**

Ndufv3	Rattus norvegicus NADH dehydrogenase (ubiquinone) flavoprotein 3 (Ndufv3), nuclear gene encoding mitochondrial protein, transcript variant 1, mRNA [NM_022607]	**0,541**
Cpt2	Rattus norvegicus carnitine palmitoyltransferase 2 (Cpt2), nuclear gene encoding mitochondrial protein, mRNA [NM_012930]	**0,541**
Derl1	Rattus norvegicus Der1-like domain family, member 1 (Derl1), mRNA [NM_001014202]	**0,541**
Capsl	Rattus norvegicus calcyphosine-like (Capsl), mRNA [NM_001106417]	**0,541**
Pthlh	Rattus norvegicus parathyroid hormone-like hormone (Pthlh), mRNA [NM_012636]	**0,541**
Impact	Rattus norvegicus imprinted and ancient (Impact), mRNA [NM_001012235]	**0,541**
Scarb2	Rattus norvegicus scavenger receptor class B, member 2 (Scarb2), mRNA [NM_054001]	**0,541**
Dbx2	Homeodomain protein Dbx2 [Source:UniProtKB/TrEMBL;Acc:Q5DWV0] [ENSRNOT00000009143]	**0,541**
Lancl1	Rattus norvegicus LanC lantibiotic synthetase component C-like 1 (bacterial) (Lancl1), mRNA [NM_053723]	**0,541**
Atrn	Rattus norvegicus attractin (Atrn), mRNA [NM_031351]	**0,541**
Btbd6	Rattus norvegicus BTB (POZ) domain containing 6 (Btbd6), mRNA [NM_001077683]	**0,541**
Acly	Rattus norvegicus ATP citrate lyase (Acly), transcript variant 1, mRNA [NM_016987]	**0,541**
Zcchc2	Rattus norvegicus zinc finger, CCHC domain containing 2 (Zcchc2), mRNA [NM_001122677]	**0,541**
Sdc1	Rattus norvegicus syndecan 1 (Sdc1), mRNA [NM_013026]	**0,541**
Itgb1	Rattus norvegicus integrin, beta 1 (Itgb1), mRNA [NM_017022]	**0,541**
Cdk5rap2	Rattus norvegicus CDK5 regulatory subunit associated protein 2 (Cdk5rap2), mRNA [NM_173134]	**0,541**
Cntn2	Rattus norvegicus contactin 2 (axonal) (Cntn2), mRNA [NM_012884]	**0,541**
Ppp2r3c	Rattus norvegicus protein phosphatase 2, regulatory subunit B'', gamma (Ppp2r3c), mRNA [NM_001014196]	**0,541**
Phf1	Rattus norvegicus PHD finger protein 1 (Phf1), mRNA [NM_212538]	**0,541**
Prpsap2	Rattus norvegicus phosphoribosyl pyrophosphate synthetase-associated protein 2 (Prpsap2), mRNA [NM_057131]	**0,541**
Gtpbp8	Rattus norvegicus GTP-binding protein 8 (putative) (Gtpbp8), mRNA [NM_001025015]	**0,541**
Rwdd2b	Rattus norvegicus RWD domain containing 2B (Rwdd2b), mRNA [NM_001100559]	**0,541**
Atp1b1	Rattus norvegicus ATPase, Na+/K+ transporting, beta 1 polypeptide (Atp1b1), mRNA [NM_013113]	**0,541**
Ccdc124	Rattus norvegicus coiled-coil domain containing 124 (Ccdc124), mRNA [NM_001106071]	**0,541**
0	UI-R-C2p-rx-c-04-0-UI.r1 UI-R-C2p Rattus norvegicus cDNA clone UI-R-C2p-rx-c-04-0-UI 5', mRNA sequence [BF545174]	**0,541**
Cxxc4	Rattus norvegicus CXXC finger 4 (Cxxc4), mRNA [NM_053342]	**0,541**
Eraf	Rattus norvegicus erythroid associated factor (Eraf), mRNA [NM_001106299]	**0,541**
Fam86a	Rattus norvegicus family with sequence similarity 86, member A (Fam86a), mRNA [NM_001106975]	**0,541**
0	Unknown	**0,541**
Cpt1c	Rattus norvegicus carnitine palmitoyltransferase 1c (Cpt1c), mRNA [NM_001034925]	**0,541**
Ube3a	Rattus norvegicus ubiquitin protein ligase E3A (Ube3a), mRNA [NM_001191837]	**0,541**
Cisd3	Rattus norvegicus CDGSH iron sulfur domain 3 (Cisd3), mRNA [NM_001105835]	**0,541**
Gpc2	Rattus norvegicus glypican 2 (Gpc2), mRNA [NM_138511]	**0,541**

Nap1l5	Rattus norvegicus nucleosome assembly protein 1-like 5 (Nap1l5), mRNA [NM_001044293]	**0,541**
0	Uncharacterized protein [Source:UniProtKB/TrEMBL;Acc:D4ACD7] [ENSRNOT00000014120]	**0,542**
Fam89a	Rattus norvegicus family with sequence similarity 89, member A (Fam89a), mRNA [NM_001011711]	**0,542**
Itpripl1	Rattus norvegicus inositol 1,4,5-triphosphate receptor interacting protein-like 1 (Itpripl1), mRNA [NM_001025043]	**0,542**
Ttc5	Rattus norvegicus tetratricopeptide repeat domain 5 (Ttc5), mRNA [NM_001013131]	**0,542**
Slc35b1	Rattus norvegicus solute carrier family 35, member B1 (Slc35b1), mRNA [NM_199081]	**0,542**
Aldh3a2	Rattus norvegicus aldehyde dehydrogenase 3 family, member A2 (Aldh3a2), mRNA [NM_031731]	**0,542**
Vipr1	Rattus norvegicus vasoactive intestinal peptide receptor 1 (Vipr1), mRNA [NM_012685]	**0,542**
0	Unknown	**0,542**
Eif4h	Rattus norvegicus eukaryotic translation initiation factor 4H (Eif4h), mRNA [NM_001006957]	**0,542**
Hnrnpc	Rattus norvegicus heterogeneous nuclear ribonucleoprotein C (C1/C2) (Hnrnpc), mRNA [NM_001025633]	**0,542**
Mrpl53	Rattus norvegicus mitochondrial ribosomal protein L53 (Mrpl53), nuclear gene encoding mitochondrial protein, mRNA [NM_001108635]	**0,542**
Gas2	Rattus norvegicus growth arrest-specific 2 (Gas2), mRNA [NM_001127504]	**0,542**
RGD1562012	Rattus norvegicus RGD1562012 (RGD1562012), mRNA [NM_001173471]	**0,542**
Pisd	Uncharacterized protein [Source:UniProtKB/TrEMBL;Acc:D3ZAW2] [ENSRNOT00000024813]	**0,542**
0	RNGZAL4 guanine nucleotide-binding protein {Rattus norvegicus} (exp=-1; wgp=0; cg=0), complete [TC578187]	**0,542**
Bpnt1	Rattus norvegicus 3'(2'), 5'-bisphosphate nucleotidase 1 (Bpnt1), mRNA [NM_171990]	**0,542**
Sms	Rattus norvegicus spermine synthase (Sms), mRNA [NM_001033899]	**0,542**
RGD1311458	Rattus norvegicus similar to cDNA sequence BC027231; hypothetical protein MGC27931 (RGD1311458), mRNA [NM_001009678]	**0,542**
Aatk	Rattus norvegicus apoptosis-associated tyrosine kinase (Aatk), mRNA [NM_001168703]	**0,542**
Zfp706	Rattus norvegicus zinc finger protein 706 (Zfp706), mRNA [NM_001126087]	**0,542**
Pik3ca	Rattus norvegicus phosphoinositide-3-kinase, catalytic, alpha polypeptide (Pik3ca), mRNA [NM_133399]	**0,542**
Hps5	Rattus norvegicus Hermansky-Pudlak syndrome 5 (Hps5), mRNA [NM_001135612]	**0,542**
Rtn2	Rattus norvegicus reticulon 2 (Rtn2), mRNA [NM_201562]	**0,542**
Odc1	Rattus norvegicus ornithine decarboxylase 1 (Odc1), mRNA [NM_012615]	**0,542**
Pcdh9	Rattus norvegicus protocadherin 9 (Pcdh9), mRNA [NM_001191688]	**0,542**
Tspan13	Rattus norvegicus tetraspanin 13 (Tspan13), mRNA [NM_001013244]	**0,542**
Prkrip1	Rattus norvegicus Prkr interacting protein 1 (IL11 inducible) (Prkrip1), mRNA [NM_001098793]	**0,542**
Ostf1	Rattus norvegicus osteoclast stimulating factor 1 (Ostf1), mRNA [NM_148892]	**0,542**
LOC688966	Rattus norvegicus similar to K11B4.2 (LOC688966), mRNA [NM_001145726]	**0,542**
0	TCDD-inducible poly(ADP-ribose) polymerase (Predicted)Uncharacterized protein [Source:UniProtKB/TrEMBL;Acc:D3ZMH5] [ENSRNOT00000015387]	**0,542**
Lrrc23	Rattus norvegicus leucine rich repeat containing 23 (Lrrc23), mRNA [NM_001013165]	**0,542**
Uck2	Rattus norvegicus uridine-cytidine kinase 2 (Uck2), mRNA [NM_001102408]	**0,542**

Zfyve19	Rattus norvegicus zinc finger, FYVE domain containing 19 (Zfyve19), mRNA [NM_001034948]	**0,542**
0	Unknown	**0,542**
Rpn2	Rattus norvegicus ribophorin II (Rpn2), mRNA [NM_031698]	**0,542**
Nat9	Rattus norvegicus N-acetyltransferase 9 (GCN5-related, putative) (Nat9), mRNA [NM_001134835]	**0,542**
Pde3b	Rattus norvegicus phosphodiesterase 3B, cGMP-inhibited (Pde3b), mRNA [NM_017229]	**0,542**
Nfkbie	Rattus norvegicus nuclear factor of kappa light polypeptide gene enhancer in B-cells inhibitor, epsilon (Nfkbie), mRNA [NM_199111]	**0,543**
Lpcat1	Rattus norvegicus lysophosphatidylcholine acyltransferase 1 (Lpcat1), mRNA [NM_001100735]	**0,543**
Gatad1	Uncharacterized protein [Source:UniProtKB/TrEMBL;Acc:D3ZHD5] [ENSRNOT00000011666]	**0,543**
Slc39a12	Rattus norvegicus solute carrier family 39 (zinc transporter), member 12 (Slc39a12), mRNA [NM_001106124]	**0,543**
0	Unknown	**0,543**
Zfp709	Rattus norvegicus zinc finger protein 709 (Zfp709), mRNA [NM_153731]	**0,543**
Rnaseh2b	Rattus norvegicus ribonuclease H2, subunit B (Rnaseh2b), mRNA [NM_001007007]	**0,543**
Ccdc27	Rattus norvegicus coiled-coil domain containing 27 (Ccdc27), mRNA [NM_001191063]	**0,543**
Mpv17l2	Rattus norvegicus MPV17 mitochondrial membrane protein-like 2 (Mpv17l2), nuclear gene encoding mitochondrial protein, mRNA [NM_001106072]	**0,543**
Smarcd2	Rattus norvegicus SWI/SNF related, matrix associated, actin dependent regulator of chromatin, subfamily d, member 2 (Smarcd2), mRNA [NM_031983]	**0,543**
Bok	Rattus norvegicus BCL2-related ovarian killer (Bok), mRNA [NM_017312]	**0,543**
0	Uncharacterized protein [Source:UniProtKB/TrEMBL;Acc:D3ZRC6] [ENSRNOT00000050943]	**0,543**
0	Rattus norvegicus TL0AAA57YH24 mRNA sequence. [FQ212370]	**0,543**
Stk3	Rattus norvegicus serine/threonine kinase 3 (STE20 homolog, yeast) (Stk3), mRNA [NM_031735]	**0,543**
Map9	Rattus norvegicus microtubule-associated protein 9 (Map9), mRNA [NM_001135716]	**0,543**
Ciapin1	Rattus norvegicus cytokine induced apoptosis inhibitor 1 (Ciapin1), mRNA [NM_001007689]	**0,543**
Cyp2d4	Rattus norvegicus cytochrome P450, family 2, subfamily d, polypeptide 4 (Cyp2d4), mRNA [NM_138515]	**0,543**
Cyp20a1	Rattus norvegicus cytochrome P450, family 20, subfamily a, polypeptide 1 (Cyp20a1), mRNA [NM_199401]	**0,543**
Rtn4	Rattus norvegicus reticulon 4 (Rtn4), mRNA [NM_031831]	**0,543**
Snx4	Rattus norvegicus sorting nexin 4 (Snx4), mRNA [NM_001127550]	**0,543**
LOC366449	PREDICTED: Rattus norvegicus hypothetical LOC366449 (LOC366449), miscRNA [XR_006808]	**0,543**
Vav2	Rattus norvegicus vav 2 guanine nucleotide exchange factor (Vav2), mRNA [NM_001106563]	**0,543**
Fitm2	Rattus norvegicus fat storage-inducing transmembrane protein 2 (Fitm2), mRNA [NM_001107799]	**0,543**
Dynlt1	Rattus norvegicus dynein light chain Tctex-type 1 (Dynlt1), mRNA [NM_031318]	**0,543**
Zfp191	Rattus norvegicus zinc finger protein 191 (Zfp191), mRNA [NM_182955]	**0,543**
0	R-spondin-2 [Source:RefSeq peptide;Acc:NP_001124047] [ENSRNOT00000007526]	**0,544**
0	Unknown	**0,544**
Ahnak	Rattus norvegicus AHNAK nucleoprotein (Ahnak), mRNA [NM_001191951]	**0,544**

Rps15	Rattus norvegicus ribosomal protein S15 (Rps15), mRNA [NM_017151]	**0,544**
Hcca2	Rattus norvegicus HCCA2 protein (Hcca2), mRNA [NM_001109159]	**0,544**
Ubtf	Rattus norvegicus upstream binding transcription factor, RNA polymerase I (Ubtf), transcript variant 2, mRNA [NM_001127690]	**0,544**
Eif4g2	Rattus norvegicus eukaryotic translation initiation factor 4, gamma 2 (Eif4g2), mRNA [NM_001017374]	**0,544**
Lhfp	Rattus norvegicus lipoma HMGIC fusion partner (Lhfp), mRNA [NM_001109183]	**0,544**
Cdk7	Cell division protein kinase 7 [Source:UniProtKB/Swiss-Prot;Acc:P51952] [ENSRNOT00000025026]	**0,544**
Tmem110	Rattus norvegicus transmembrane protein 110 (Tmem110), mRNA [NM_198774]	**0,544**
Prss8	Rattus norvegicus protease, serine, 8 (Prss8), mRNA [NM_138836]	**0,544**
Fubp1	Rattus norvegicus far upstream element (FUSE) binding protein 1 (Fubp1), mRNA [NM_001037653]	**0,544**
0	YTH domain family protein 3 [Source:RefSeq peptide;Acc:NP_001102016] [ENSRNOT00000052093]	**0,544**
Wdr26	Rattus norvegicus WD repeat domain 26 (Wdr26), mRNA [NM_001109081]	**0,544**
Ctsa	Rattus norvegicus cathepsin A (Ctsa), mRNA [NM_001011959]	**0,544**
RGD1560187	Rattus norvegicus similar to Hypothetical UPF0327 protein (RGD1560187), mRNA [NM_001173556]	**0,544**
Thrap3	Rattus norvegicus thyroid hormone receptor associated protein 3 (Thrap3), mRNA [NM_001009693]	**0,544**
Ccdc76	Rattus norvegicus coiled-coil domain containing 76 (Ccdc76), mRNA [NM_001033902]	**0,544**
Acadm	Rattus norvegicus acyl-Coenzyme A dehydrogenase, C-4 to C-12 straight chain (Acadm), nuclear gene encoding mitochondrial protein, mRNA [NM_016986]	**0,544**
Mrps35	Rattus norvegicus mitochondrial ribosomal protein S35 (Mrps35), nuclear gene encoding mitochondrial protein, mRNA [NM_001106628]	**0,544**
Fam19a2	Unknown	**0,544**
Bai2	Rattus norvegicus brain-specific angiogenesis inhibitor 2 (Bai2), mRNA [NM_001107914]	**0,544**
Pcsk7	Rattus norvegicus proprotein convertase subtilisin/kexin type 7 (Pcsk7), mRNA [NM_019246]	**0,544**
Usp30	Rattus norvegicus ubiquitin specific peptidase 30 (Usp30), mRNA [NM_001107153]	**0,544**
Tmem127	Rattus norvegicus transmembrane protein 127 (Tmem127), mRNA [NM_001100978]	**0,544**
Cyp26b1	Rattus norvegicus cytochrome P450, family 26, subfamily b, polypeptide 1 (Cyp26b1), mRNA [NM_181087]	**0,544**
Tmem150c	Rattus norvegicus transmembrane protein 150C (Tmem150c), mRNA [NM_001108354]	**0,544**
Stap2	Rattus norvegicus signal transducing adaptor family member 2 (Stap2), mRNA [NM_001025026]	**0,544**
Znf532	Rattus norvegicus zinc finger protein 532 (Znf532), mRNA [NM_001107382]	**0,544**
Ppp1r16a	Rattus norvegicus protein phosphatase 1, regulatory (inhibitor) subunit 16A (Ppp1r16a), mRNA [NM_001130566]	**0,544**
Ubr7	Rattus norvegicus ubiquitin protein ligase E3 component n-recognin 7 (putative) (Ubr7), mRNA [NM_001007705]	**0,544**
Tbc1d2	Rattus norvegicus TBC1 domain family, member 2 (Tbc1d2), mRNA [NM_001107933]	**0,544**
Mrpl1	Rattus norvegicus mitochondrial ribosomal protein L1 (Mrpl1), nuclear gene encoding mitochondrial protein, mRNA [NM_001105997]	**0,544**
Trub2	Rattus norvegicus TruB pseudouridine (psi) synthase homolog 2 (E. coli) (Trub2), mRNA [NM_001014257]	**0,544**
0	E3 ubiquitin-protein ligase PDZRN3 [Source:UniProtKB/Swiss-Prot;Acc:P68907] [ENSRNOT00000008032]	**0,544**
RGD1311072	Rattus norvegicus similar to 2410001C21Rik protein (RGD1311072), mRNA [NM_001033890]	**0,544**

Cryaa	Rattus norvegicus crystallin, alpha A (Cryaa), mRNA [NM_012534]	**0,544**
Tes	Rattus norvegicus testis derived transcript (Tes), mRNA [NM_001039344]	**0,544**
Pcnxl2	Uncharacterized protein [Source:UniProtKB/TrEMBL;Acc:D4AB99] [ENSRNOT00000030077]	**0,544**
L2hgdh	Rattus norvegicus L-2-hydroxyglutarate dehydrogenase (L2hgdh), nuclear gene encoding mitochondrial protein, mRNA [NM_001108028]	**0,545**
0	Unknown	**0,545**
Cbln2	Rattus norvegicus cerebellin 2 precursor (Cbln2), mRNA [NM_001012740]	**0,545**
Amz2	Rattus norvegicus archaelysin family metallopeptidase 2 (Amz2), mRNA [NM_001014121]	**0,545**
Acox1	Peroxisomal acyl-coenzyme A oxidase 1Peroxisomal acyl-CoA oxidase 1, A chainPeroxisomal acyl-CoA oxidase 1, B chainPeroxisomal acyl-CoA oxidase 1, C chain [Source:UniProtKB/Swiss-Prot;Acc:P07872] [ENSRNOT00000042372]	**0,545**
Zc3h11a	Rattus norvegicus zinc finger CCCH-type containing 11A (Zc3h11a), mRNA [NM_001047902]	**0,545**
Pacsin1	Rattus norvegicus protein kinase C and casein kinase substrate in neurons 1 (Pacsin1), mRNA [NM_017294]	**0,545**
0	BC042626 Chmp2b protein {Mus musculus} (exp=-1; wgp=0; cg=0), partial (49%) [TC588602]	**0,545**
LOC681647	PREDICTED: Rattus norvegicus similar to F43G9.2 (LOC681647), mRNA [XM_001057182]	**0,545**
0	Rattus norvegicus similar to RIKEN cDNA 4933431D05 (LOC301906), mRNA [XM_217507]	**0,545**
0	Unknown	**0,545**
LOC100233177	Rattus norvegicus hypothetical protein LOC100233177 (LOC100233177), mRNA [NM_001134531]	**0,545**
Crim1	Rattus norvegicus cysteine rich transmembrane BMP regulator 1 (chordin like) (Crim1), mRNA [NM_001169103]	**0,545**
0	Uncharacterized protein [Source:UniProtKB/TrEMBL;Acc:D3ZQ22] [ENSRNOT00000048946]	**0,545**
Mpp5	Rattus norvegicus membrane protein, palmitoylated 5 (MAGUK p55 subfamily member 5) (Mpp5), mRNA [NM_001108034]	**0,545**
RGD1562378	PREDICTED: Rattus norvegicus histone H4 variant H4-v.1 (RGD1562378), mRNA [XM_575704]	**0,545**
0	Uncharacterized protein [Source:UniProtKB/TrEMBL;Acc:D3ZBT9] [ENSRNOT00000020886]	**0,545**
Ccdc56	Rattus norvegicus coiled-coil domain containing 56 (Ccdc56), mRNA [NM_001109047]	**0,545**
0	DP13A_MOUSE (Q8K3H0) DCC-interacting protein 13 alpha (Dip13 alpha) (Adapter protein containing PH domain, PTB domain and leucine zipper motif 1), partial (54%) [TC594026]	**0,545**
Iars	Rattus norvegicus isoleucyl-tRNA synthetase (Iars), mRNA [NM_001100572]	**0,545**
Slc5a3	Rattus norvegicus solute carrier family 5 (sodium/myo-inositol cotransporter), member 3 (Slc5a3), mRNA [NM_053715]	**0,545**
Rapgef2	Rattus norvegicus Rap guanine nucleotide exchange factor (GEF) 2 (Rapgef2), mRNA [NM_001107684]	**0,545**
Smad1	Rattus norvegicus SMAD family member 1 (Smad1), mRNA [NM_013130]	**0,545**
RGD1563106	Rattus norvegicus similar to novel protein (RGD1563106), mRNA [NM_001107010]	**0,545**
Pik3ip1	Rattus norvegicus phosphoinositide-3-kinase interacting protein 1 (Pik3ip1), mRNA [NM_001017453]	**0,545**
0	Unknown	**0,545**
Enc1	Rattus norvegicus ectodermal-neural cortex 1 (Enc1), mRNA [NM_001003401]	**0,545**
ST7	Rattus norvegicus suppression of tumorigenicity 7 (ST7), transcript variant 1, mRNA [NM_001004102]	**0,545**
LOC690977	PREDICTED: Rattus norvegicus similar to RIKEN cDNA 5031410I06 (LOC690977), mRNA [XM_002727316]	**0,545**

Znf512b	Rattus norvegicus zinc finger protein 512B (Znf512b), mRNA [NM_001107809]	**0,545**
0	Unknown	**0,545**
Ccnyl1	PREDICTED: Rattus norvegicus cyclin Y-like 1 (Ccnyl1), mRNA [XM_237211]	**0,545**
Leprot	Rattus norvegicus leptin receptor overlapping transcript (Leprot), mRNA [NM_020099]	**0,545**
Mmd2	Rattus norvegicus monocyte to macrophage differentiation-associated 2 (Mmd2), mRNA [NM_001037217]	**0,545**
Ctbp2	Rattus norvegicus C-terminal binding protein 2 (Ctbp2), mRNA [NM_053335]	**0,545**
Cltc	Rattus norvegicus clathrin, heavy chain (Hc) (Cltc), mRNA [NM_019299]	**0,545**
0	Unknown	**0,545**
LOC308954	Rattus norvegicus similar to hypothetical protein MGC50721 (LOC308954), mRNA [NM_001024777]	**0,546**
RGD1303142	Rattus norvegicus oxidative stress responsive gene (RGD1303142), mRNA [NM_201560]	**0,546**
Nup98	Rattus norvegicus nucleoporin (Nup98) mRNA, complete cds. [L39991]	**0,546**
Klf14	Rattus norvegicus Kruppel-like factor 14 (Klf14), mRNA [NM_001135094]	**0,546**
Tns3	PREDICTED: Rattus norvegicus similar to novel protein similar to Tensin Tns (RGD1564174), mRNA [XM_002725003]	**0,546**
Gnptg	Rattus norvegicus N-acetylglucosamine-1-phosphate transferase, gamma subunit (Gnptg), mRNA [NM_001100493]	**0,546**
Slc9a6	PREDICTED: Rattus norvegicus solute carrier family 9 (sodium/hydrogen exchanger), member 6 (Slc9a6), mRNA [XM_001053956]	**0,546**
Wipi2	Rattus norvegicus WD repeat domain, phosphoinositide interacting 2 (Wipi2), mRNA [NM_001007615]	**0,546**
0	Unknown	**0,546**
0	Unknown	**0,546**
Wdr1	Rattus norvegicus WD repeat domain 1 (Wdr1), mRNA [NM_001014135]	**0,546**
MGC94199	Rattus norvegicus similar to RIKEN cDNA 2610301B20; EST AI428449 (MGC94199), mRNA [NM_001007746]	**0,546**
Hras	Rattus norvegicus Harvey rat sarcoma virus oncogene (Hras), transcript variant 2, mRNA [NM_001098241]	**0,546**
Pdp2	Rattus norvegicus pyruvate dehyrogenase phosphatase catalytic subunit 2 (Pdp2), mRNA [NM_145091]	**0,546**
Znf496	PREDICTED: Rattus norvegicus zinc finger protein 496 (Znf496), mRNA [XM_220512]	**0,546**
0	Unknown	**0,546**
Vamp7	Rattus norvegicus vesicle-associated membrane protein 7 (Vamp7), mRNA [NM_053531]	**0,546**
Inhbb	Rattus norvegicus inhibin beta-B (Inhbb), mRNA [NM_080771]	**0,546**
Mybl2	Rattus norvegicus myeloblastosis oncogene-like 2 (Mybl2), mRNA [NM_001106536]	**0,546**
Kcnk1	Rattus norvegicus potassium channel, subfamily K, member 1 (Kcnk1), mRNA [NM_021688]	**0,546**
Cyp2c79	Uncharacterized protein [Source:UniProtKB/TrEMBL;Acc:D3ZDQ1] [ENSRNOT00000017367]	**0,546**
Rxrb	Rattus norvegicus retinoid X receptor beta (Rxrb), mRNA [NM_206849]	**0,546**
Xkr8	Rattus norvegicus XK, Kell blood group complex subunit-related family, member 8 (Xkr8), mRNA [NM_001012099]	**0,546**
Gsdmd	Rattus norvegicus gasdermin D (Gsdmd), mRNA [NM_001130553]	**0,546**
Atic	Rattus norvegicus 5-aminoimidazole-4-carboxamide ribonucleotide formyltransferase/IMP cyclohydrolase (Atic), mRNA [NM_031014]	**0,546**
Mical3	Rattus norvegicus microtubule associated monoxygenase, calponin and LIM domain containing 3 (Mical3), mRNA [NM_001191085]	**0,546**

0	Rattus norvegicus similar to RIKEN cDNA 4933431D05 (LOC287855), mRNA [XM_221162]	**0,546**
0	Rattus norvegicus TL0ADA32YM01 mRNA sequence. [FQ221714]	**0,546**
Rras	Rattus norvegicus Harvey rat sarcoma virus oncogene, subgroup R (Rras), mRNA [NM_001108481]	**0,546**
Glud1	Rattus norvegicus glutamate dehydrogenase 1 (Glud1), nuclear gene encoding mitochondrial protein, mRNA [NM_012570]	**0,546**
Rpl10	Rattus norvegicus ribosomal protein L10 (Rpl10), mRNA [NM_031100]	**0,546**
0	Rattus norvegicus TL0ADA51YN11 mRNA sequence. [FQ228778]	**0,546**
Bend5	Rattus norvegicus BEN domain containing 5 (Bend5), mRNA [NM_001108672]	**0,547**
RGD1562601	Uncharacterized protein [Source:UniProtKB/TrEMBL;Acc:D3ZLH3] [ENSRNOT00000048420]	**0,547**
RGD1306613	Rattus norvegicus similar to RIKEN cDNA 1600012F09 (RGD1306613), mRNA [NM_001107356]	**0,547**
Ccdc23	Rattus norvegicus coiled-coil domain containing 23 (Ccdc23), transcript variant 1, mRNA [NM_001038994]	**0,547**
Hook3	Rattus norvegicus hook homolog 3 (Drosophila) (Hook3), mRNA [NM_001136098]	**0,547**
Zadh2	Rattus norvegicus zinc binding alcohol dehydrogenase, domain containing 2 (Zadh2), mRNA [NM_001106129]	**0,547**
Ube2g2	Rattus norvegicus ubiquitin-conjugating enzyme E2G 2 (UBC7 homolog, yeast) (Ube2g2), mRNA [NM_001106380]	**0,547**
Gabrg2	Rattus norvegicus gamma-aminobutyric acid (GABA) A receptor, gamma 2 (Gabrg2), mRNA [NM_183327]	**0,547**
LOC501234	PREDICTED: Rattus norvegicus hypothetical gene supported by BC082068 (LOC501234), mRNA [XM_576655]	**0,547**
0	Uncharacterized protein [Source:UniProtKB/TrEMBL;Acc:D4A9K1] [ENSRNOT00000031814]	**0,547**
Cacna2d1	Rattus norvegicus calcium channel, voltage-dependent, alpha2/delta subunit 1 (Cacna2d1), transcript variant 2, mRNA [NM_001110848]	**0,547**
Pex14	Rattus norvegicus peroxisomal biogenesis factor 14 (Pex14), mRNA [NM_172063]	**0,547**
Myeov2	Rattus norvegicus myeloma overexpressed 2 (Myeov2), mRNA [NM_001109044]	**0,547**
LOC681086	RCG61899Uncharacterized protein [Source:UniProtKB/TrEMBL;Acc:D3ZZU7] [ENSRNOT00000020600]	**0,547**
Anxa1	Rattus norvegicus annexin A1 (Anxa1), mRNA [NM_012904]	**0,547**
Slc11a2	Rattus norvegicus solute carrier family 11 (proton-coupled divalent metal ion transporters), member 2 (Slc11a2), mRNA [NM_013173]	**0,547**
Fam105a	Rattus norvegicus family with sequence similarity 105, member A (Fam105a), mRNA [NM_001037648]	**0,547**
Hsf2	Rattus norvegicus heat shock transcription factor 2 (Hsf2), mRNA [NM_031694]	**0,547**
Flrt3	Rattus norvegicus fibronectin leucine rich transmembrane protein 3 (Flrt3), mRNA [NM_001126291]	**0,547**
Enoph1	Rattus norvegicus enolase-phosphatase 1 (Enoph1), mRNA [NM_001009391]	**0,547**
0	Unknown	**0,547**
Acads	Rattus norvegicus acyl-Coenzyme A dehydrogenase, C-2 to C-3 short chain (Acads), nuclear gene encoding mitochondrial protein, mRNA [NM_022512]	**0,547**
Slc41a3	Rattus norvegicus solute carrier family 41, member 3 (Slc41a3), mRNA [NM_001037492]	**0,547**
Tspan6	Rattus norvegicus tetraspanin 6 (Tspan6), mRNA [NM_001100672]	**0,547**
LOC679383	Rattus norvegicus similar to DNA segment, Chr 5, ERATO Doi 135, expressed (LOC679383), mRNA [NM_001128287]	**0,547**
Hnrnpab	Rattus norvegicus heterogeneous nuclear ribonucleoprotein A/B (Hnrnpab), mRNA [NM_031330]	**0,547**
Mizf	PREDICTED: Rattus norvegicus MBD2-interacting zinc finger (Mizf), mRNA [XM_002727050]	**0,547**

Anapc11	Rattus norvegicus anaphase promoting complex subunit 11 (Anapc11), mRNA [NM_001126082]	**0,547**
Ppp1r1a	Rattus norvegicus protein phosphatase 1, regulatory (inhibitor) subunit 1A (Ppp1r1a), mRNA [NM_022676]	**0,547**
Minpp1	Rattus norvegicus multiple inositol polyphosphate histidine phosphatase 1 (Minpp1), mRNA [NM_019263]	**0,547**
Ttc30b	Rattus norvegicus tetratricopeptide repeat domain 30B (Ttc30b), mRNA [NM_001127607]	**0,547**
Bnip3l	Rattus norvegicus BCL2/adenovirus E1B interacting protein 3-like (Bnip3l), mRNA [NM_080888]	**0,547**
Csrnp2	Rattus norvegicus cysteine-serine-rich nuclear protein 2 (Csrnp2), mRNA [NM_001108113]	**0,547**
Tceal8	Rattus norvegicus transcription elongation factor A (SII)-like 8 (Tceal8), mRNA [NM_001014275]	**0,547**
Alg9	Rattus norvegicus asparagine-linked glycosylation 9, alpha-1,2-mannosyltransferase homolog (S. cerevisiae) (Alg9), mRNA [NM_001109000]	**0,548**
Kcnd2	Rattus norvegicus potassium voltage-gated channel, Shal-related subfamily, member 2 (Kcnd2), mRNA [NM_031730]	**0,548**
Rrbp1	PREDICTED: Rattus norvegicus ribosome binding protein 1, transcript variant 3 (Rrbp1), mRNA [XM_001053669]	**0,548**
Glul	Rattus norvegicus glutamate-ammonia ligase (glutamine synthetase) (Glul), mRNA [NM_017073]	**0,548**
LOC501282	PREDICTED: Rattus norvegicus similar to lymphocyte antigen 6 complex, locus E ligand (LOC501282), mRNA [XM_576697]	**0,548**
RGD1560398	PREDICTED: Rattus norvegicus RGD1560398 (RGD1560398), mRNA [XM_001080358]	**0,548**
Zfp382	Rattus norvegicus zinc finger protein 382 (Zfp382), mRNA [NM_144749]	**0,548**
LOC312502	PREDICTED: Rattus norvegicus similar to RAB11 family interacting protein 5 (class I) isoform 1 (LOC312502), miscRNA [XR_085818]	**0,548**
Prkd3	Rattus norvegicus protein kinase D3 (Prkd3), mRNA [NM_001024263]	**0,548**
0	Uncharacterized protein [Source:UniProtKB/TrEMBL;Acc:D3ZIY8] [ENSRNOT00000029667]	**0,548**
0	PREDICTED: Rattus norvegicus similar to Zgc:56193 (RGD1564447), mRNA [XM_001058987]	**0,548**
0	Unknown	**0,548**
Amn1	Rattus norvegicus antagonist of mitotic exit network 1 homolog (S. cerevisiae) (Amn1), mRNA [NM_001008333]	**0,548**
Nos1ap	Rattus norvegicus nitric oxide synthase 1 (neuronal) adaptor protein (Nos1ap), mRNA [NM_138922]	**0,548**
Serping1	Rattus norvegicus serine (or cysteine) peptidase inhibitor, clade G, member 1 (Serping1), mRNA [NM_199093]	**0,548**
Agpat3	Rattus norvegicus 1-acylglycerol-3-phosphate O-acyltransferase 3 (Agpat3), mRNA [NM_001106378]	**0,548**
Fau	Rattus norvegicus Finkel-Biskis-Reilly murine sarcoma virus (FBR-MuSV) ubiquitously expressed (Fau), transcript variant 1, mRNA [NM_001012739]	**0,548**
B4galt2	Rattus norvegicus UDP-Gal:betaGlcNAc beta 1,4- galactosyltransferase, polypeptide 2 (B4galt2), mRNA [NM_001107965]	**0,548**
Ribc1	Rattus norvegicus RIB43A domain with coiled-coils 1 (Ribc1), mRNA [NM_001007715]	**0,548**
Errfi1	Rattus norvegicus ERBB receptor feedback inhibitor 1 (Errfi1), mRNA [NM_001014071]	**0,548**
Pnkp	Rattus norvegicus polynucleotide kinase 3'-phosphatase (Pnkp), mRNA [NM_001004259]	**0,548**
Slc17a6	Rattus norvegicus solute carrier family 17 (sodium-dependent inorganic phosphate cotransporter), member 6 (Slc17a6), mRNA [NM_053427]	**0,548**
Hdgfrp3	Rattus norvegicus hepatoma-derived growth factor, related protein 3 (Hdgfrp3), mRNA [NM_145785]	**0,548**
RGD1309651	Uncharacterized protein [Source:UniProtKB/TrEMBL;Acc:D3ZSX5] [ENSRNOT00000023721]	**0,548**
Unc119b	Rattus norvegicus unc-119 homolog B (C. elegans) (Unc119b), mRNA [NM_001105934]	**0,548**
RGD1311273	Rattus norvegicus similar to RIKEN cDNA 9530058B02 (RGD1311273), mRNA [NM_001109475]	**0,548**

Smndc1	Rattus norvegicus survival motor neuron domain containing 1 (Smndc1), transcript variant 2, mRNA [NM_001025400]	**0,548**
Vkorc1	Rattus norvegicus vitamin K epoxide reductase complex, subunit 1 (Vkorc1), mRNA [NM_203335]	**0,548**
Calm2	Rattus norvegicus calmodulin 2 (Calm2), mRNA [NM_017326]	**0,548**
Aqp11	Rattus norvegicus aquaporin 11 (Aqp11), mRNA [NM_173105]	**0,548**
Ccdc28a	Rattus norvegicus coiled-coil domain containing 28A (Ccdc28a), mRNA [NM_001037789]	**0,548**
Slc35f2	Rattus norvegicus solute carrier family 35, member F2 (Slc35f2), mRNA [NM_001106822]	**0,548**
Kctd3	Rattus norvegicus potassium channel tetramerisation domain containing 3 (Kctd3), mRNA [NM_001107199]	**0,548**
Angpt1	Rattus norvegicus angiopoietin 1 (Angpt1), mRNA [NM_053546]	**0,548**
RGD1311267	Rattus norvegicus similar to RIKEN cDNA 4931426K16 gene (RGD1311267), mRNA [NM_001039024]	**0,548**
Cacna1b	Rattus norvegicus calcium channel, voltage-dependent, N type, alpha 1B subunit (Cacna1b), transcript variant 2, mRNA [NM_147141]	**0,548**
Pdzrn4	Rattus norvegicus PDZ domain containing RING finger 4 (Pdzrn4), mRNA [NM_001108107]	**0,548**
Tardbp	Rattus norvegicus TAR DNA binding protein (Tardbp), mRNA [NM_001011979]	**0,548**
Mapk1ip1	Rattus norvegicus mitogen-activated protein kinase 1 interacting protein 1 (Mapk1ip1), mRNA [NM_001122782]	**0,549**
Trappc6a	Rattus norvegicus trafficking protein particle complex 6A (Trappc6a), mRNA [NM_001109410]	**0,549**
Pgm2l1	Rattus norvegicus phosphoglucomutase 2-like 1 (Pgm2l1), mRNA [NM_001109454]	**0,549**
Ilk	Rattus norvegicus integrin-linked kinase (Ilk), mRNA [NM_133409]	**0,549**
0	BC030489 Anln protein {Mus musculus} (exp=-1; wgp=0; cg=0), partial (14%) [TC595131]	**0,549**
Nans	Rattus norvegicus N-acetylneuraminic acid synthase (Nans), mRNA [NM_001106655]	**0,549**
Kcnk10	Rattus norvegicus potassium channel, subfamily K, member 10 (Kcnk10), mRNA [NM_023096]	**0,549**
Cr1l	Rattus norvegicus complement component (3b/4b) receptor 1-like (Cr1l), transcript variant 3, mRNA [NM_001005265]	**0,549**
0	Unknown	**0,549**
0	Transcriptional regulator ATRX [Source:UniProtKB/Swiss-Prot;Acc:P70486] [ENSRNOT00000033355]	**0,549**
Rras2	Rattus norvegicus related RAS viral (r-ras) oncogene homolog 2 (Rras2), mRNA [NM_001013434]	**0,549**
0	Unknown	**0,549**
Bbs9	PREDICTED: Rattus norvegicus Bardet-Biedl syndrome 9 (Bbs9), mRNA [XM_235942]	**0,549**
Ints7	Rattus norvegicus integrator complex subunit 7 (Ints7), mRNA [NM_001191675]	**0,549**
Cops2	Rattus norvegicus COP9 constitutive photomorphogenic homolog subunit 2 (Arabidopsis) (Cops2), mRNA [NM_153297]	**0,549**
0	Uncharacterized protein [Source:UniProtKB/TrEMBL;Acc:D3Z9M1] [ENSRNOT00000056416]	**0,549**
Gmps	Rattus norvegicus guanine monphosphate synthetase (Gmps), mRNA [NM_001024754]	**0,549**
0	Unknown	**0,549**
Ddah2	Rattus norvegicus dimethylarginine dimethylaminohydrolase 2 (Ddah2), transcript variant 1, mRNA [NM_212532]	**0,549**
G3bp1	Rattus norvegicus GTPase activating protein (SH3 domain) binding protein 1 (G3bp1), mRNA [NM_133565]	**0,549**
Cript	Rattus norvegicus cysteine-rich PDZ-binding protein (Cript), mRNA [NM_019907]	**0,549**
Akap10	Rattus norvegicus A kinase (PRKA) anchor protein 10 (Akap10), nuclear gene encoding mitochondrial protein, mRNA [NM_001114606]	**0,549**

0	Uncharacterized protein [Source:UniProtKB/TrEMBL;Acc:D4ACK9] [ENSRNOT00000019278]	**0,549**
Pde2a	Rattus norvegicus phosphodiesterase 2A, cGMP-stimulated (Pde2a), transcript variant 2, mRNA [NM_031079]	**0,549**
Fam69a	Rattus norvegicus family with sequence similarity 69, member A (Fam69a), mRNA [NM_001170456]	**0,549**
Nus1	Rattus norvegicus nuclear undecaprenyl pyrophosphate synthase 1 homolog (S. cerevisiae) (Nus1), mRNA [NM_001164157]	**0,549**
Fam114a2	Uncharacterized protein [Source:UniProtKB/TrEMBL;Acc:D3ZC89] [ENSRNOT00000058619]	**0,549**
LOC678918	PREDICTED: Rattus norvegicus similar to NADH dehydrogenase (ubiquinone) 1 beta subcomplex 3 (LOC678918), mRNA [XM_001053805]	**0,549**
Mlec	Rattus norvegicus malectin (Mlec), mRNA [NM_001013983]	**0,549**
0	Unknown	**0,549**
Stard13	Rattus norvegicus StAR-related lipid transfer (START) domain containing 13 (Stard13), mRNA [NM_001109060]	**0,549**
Eif2s3x	Rattus norvegicus eukaryotic translation initiation factor 2, subunit 3, structural gene X-linked (Eif2s3x), mRNA [NM_001100542]	**0,549**
Pold2	Rattus norvegicus polymerase (DNA directed), delta 2, regulatory subunit (Pold2), mRNA [NM_001013050]	**0,549**
Tmco3	Rattus norvegicus transmembrane and coiled-coil domains 3 (Tmco3), mRNA [NM_001135857]	**0,550**
Parp8	PREDICTED: Rattus norvegicus poly (ADP-ribose) polymerase family, member 8 (Parp8), mRNA [XM_001068789]	**0,550**
0	Unknown	**0,550**
Zfand2a	Rattus norvegicus zinc finger, AN1-type domain 2A (Zfand2a), mRNA [NM_001008363]	**0,550**
Wfikkn1	Rattus norvegicus WAP, follistatin/kazal, immunoglobulin, kunitz and netrin domain containing 1 (Wfikkn1), mRNA [NM_001129776]	**0,550**
Atp6v1a	Rattus norvegicus ATPase, H+ transporting, lysosomal V1 subunit A (Atp6v1a), mRNA [NM_001108318]	**0,550**
Fkbp7	Rattus norvegicus FK506 binding protein 7 (Fkbp7), mRNA [NM_001106485]	**0,550**
0	Cytoplasmic polyadenylation element binding protein 2 (Predicted)Uncharacterized protein [Source:UniProtKB/TrEMBL;Acc:D3ZHK8] [ENSRNOT00000006727]	**0,550**
Lmo7	Rattus norvegicus LIM domain 7 (Lmo7), mRNA [NM_001001515]	**0,550**
Ppwd1	Rattus norvegicus peptidylprolyl isomerase domain and WD repeat containing 1 (Ppwd1), mRNA [NM_001106406]	**0,550**
Cnp	Rattus norvegicus 2',3'-cyclic nucleotide 3' phosphodiesterase (Cnp), mRNA [NM_012809]	**0,550**
LOC684035	PREDICTED: Rattus norvegicus similar to arginyl aminopeptidase (aminopeptidase B)-like 1 (LOC684035), miscRNA [XR_085919]	**0,550**
Plp2	Rattus norvegicus proteolipid protein 2 (colonic epithelium-enriched) (Plp2), mRNA [NM_207601]	**0,550**
Fgd4	Rattus norvegicus FYVE, RhoGEF and PH domain containing 4 (Fgd4), mRNA [NM_139263]	**0,550**
Plekhn1	Rattus norvegicus pleckstrin homology domain containing, family N member 1 (Plekhn1), mRNA [NM_001134523]	**0,550**
LOC679800	PREDICTED: Rattus norvegicus similar to MIC2 like 1 (LOC679800), mRNA [XM_001054523]	**0,550**
Rnf217	Rattus norvegicus ring finger protein 217 (Rnf217), mRNA [NM_001106204]	**0,550**
Spa17	Rattus norvegicus sperm autoantigenic protein 17 (Spa17), mRNA [NM_053482]	**0,550**
LOC684558	AGENCOURT_113686907 NIH_MGC_431 Rattus norvegicus cDNA clone IMAGE:9088341 5', mRNA sequence [EV775254]	**0,550**
Hnrnpk	Rattus norvegicus heterogeneous nuclear ribonucleoprotein K (Hnrnpk), mRNA [NM_057141]	**0,550**
Sarnp	Rattus norvegicus SAP domain containing ribonucleoprotein (Sarnp), mRNA [NM_001033070]	**0,550**
Prnp	Rattus norvegicus prion protein (Prnp), mRNA [NM_012631]	**0,550**

RGD1311358	Rattus norvegicus similar to RIKEN cDNA 2410017P07 (RGD1311358), mRNA [NM_001017448]	**0,550**
Ppp1r14c	Rattus norvegicus protein phosphatase 1, regulatory (inhibitor) subunit 14c (Ppp1r14c), mRNA [NM_133425]	**0,550**
LOC501110	Rattus norvegicus similar to Glutathione S-transferase A1 (GTH1) (HA subunit 1) (GST-epsilon) (GSTA1-1) (GST class-alpha) (LOC501110), mRNA [NM_001024361]	**0,550**
Slc35a4	Rattus norvegicus solute carrier family 35, member A4 (Slc35a4), mRNA [NM_147140]	**0,550**
Snw1	Rattus norvegicus SNW domain containing 1 (Snw1), mRNA [NM_001109279]	**0,550**
Ufl1	Rattus norvegicus E3 UFM1-protein ligase 1 (Ufl1), mRNA [NM_001126279]	**0,550**
Dlx1	Rattus norvegicus distal-less homeobox 1 (Dlx1), mRNA [NM_001100531]	**0,550**
Pmpca	Rattus norvegicus peptidase (mitochondrial processing) alpha (Pmpca), nuclear gene encoding mitochondrial protein, mRNA [NM_001003673]	**0,550**
Tmem120a	Rattus norvegicus transmembrane protein 120A (Tmem120a), mRNA [NM_001010945]	**0,550**
Ythdf2	Rattus norvegicus YTH domain family, member 2 (Ythdf2), mRNA [NM_001047099]	**0,550**
0	Unknown	**0,550**
0	Unknown	**0,551**
LOC500118	Rattus norvegicus similar to RIKEN cDNA D330028D13 (LOC500118), mRNA [NM_001025771]	**0,551**
Mcm4	Rattus norvegicus minichromosome maintenance complex component 4 (Mcm4), mRNA [NM_033651]	**0,551**
Sephs1	Rattus norvegicus selenophosphate synthetase 1 (Sephs1), mRNA [NM_001104630]	**0,551**
Tmem169	Rattus norvegicus transmembrane protein 169 (Tmem169), mRNA [NM_001109574]	**0,551**
Icmt	Rattus norvegicus isoprenylcysteine carboxyl methyltransferase (Icmt), mRNA [NM_133310]	**0,551**
Nupr1	Rattus norvegicus nuclear protein, transcriptional regulator, 1 (Nupr1), mRNA [NM_053611]	**0,551**
0	Unknown	**0,551**
LOC684993	Rattus norvegicus hypothetical protein LOC684993 (LOC684993), mRNA [NM_001109451]	**0,551**
0	Unknown	**0,551**
RGD1311122	Rattus norvegicus similar to RIKEN cDNA 1110003E01 (RGD1311122), mRNA [NM_001037792]	**0,551**
Atp13a1	Rattus norvegicus ATPase type 13A1 (Atp13a1), mRNA [NM_001106079]	**0,551**
Usp47	Rattus norvegicus ubiquitin specific peptidase 47 (Usp47), mRNA [NM_001107542]	**0,551**
Dda1	Rattus norvegicus DET1 and DDB1 associated 1 (Dda1), mRNA [NM_001134790]	**0,551**
Tollip	Rattus norvegicus toll interacting protein (Tollip), mRNA [NM_001109668]	**0,551**
Slc39a14	Rattus norvegicus solute carrier family 39 (zinc transporter), member 14 (Slc39a14), mRNA [NM_001107275]	**0,551**
Ftsjd2	Rattus norvegicus FtsJ methyltransferase domain containing 2 (Ftsjd2), mRNA [NM_001014031]	**0,551**
Dmrt3	Rattus norvegicus doublesex and mab-3 related transcription factor 3 (Dmrt3), mRNA [NM_001106358]	**0,551**
0	Rattus norvegicus TL0AEA7YD23 mRNA sequence. [FQ231120]	**0,551**
0	Rattus norvegicus TL0AEA9YC09 mRNA sequence. [FQ230424]	**0,551**
0	Unknown	**0,551**
Rnf111	Rattus norvegicus ring finger protein 111 (Rnf111), mRNA [NM_001106836]	**0,551**

RGD1563888	Rattus norvegicus similar to DNA segment, Chr 16, ERATO Doi 472, expressed (RGD1563888), mRNA [NM_001108312]	**0,551**
Nod1	Rattus norvegicus nucleotide-binding oligomerization domain containing 1 (Nod1), mRNA [NM_001109236]	**0,551**
Ubtd1	Rattus norvegicus ubiquitin domain containing 1 (Ubtd1), mRNA [NM_001013153]	**0,551**
0	Unknown	**0,551**
Armc5	Rattus norvegicus armadillo repeat containing 5 (Armc5), mRNA [NM_001009455]	**0,551**
0	Unknown	**0,551**
Folr1	Rattus norvegicus folate receptor 1 (adult) (Folr1), mRNA [NM_133527]	**0,551**
Ccdc8	Rattus norvegicus coiled-coil domain containing 8 (Ccdc8), mRNA [NM_001009533]	**0,551**
Elk4	Rattus norvegicus ELK4, ETS-domain protein (SRF accessory protein 1) (Elk4), mRNA [NM_001107173]	**0,551**
0	Unknown	**0,551**
0	AA996428 UI-R-C0-he-g-09-0-UI.s1 UI-R-C0 Rattus norvegicus cDNA clone UI-R-C0-he-g-09-0-UI 3', mRNA sequence [AA996428]	**0,551**
Rpl10l	Uncharacterized protein [Source:UniProtKB/TrEMBL;Acc:D4A1P2] [ENSRNOT00000047597]	**0,551**
Lrrc40	Rattus norvegicus leucine rich repeat containing 40 (Lrrc40), mRNA [NM_001034926]	**0,551**
0	set=Set beta isoform {alternatively spliced} [rats, neonatal kidney, mRNA, 2026 nt]. [S68987]	**0,551**
0	Rattus norvegicus TL0ADA52YC03 mRNA sequence. [FQ228753]	**0,551**
Eif2c4	Rattus norvegicus eukaryotic translation initiation factor 2C, 4 (Eif2c4), mRNA [NM_001106686]	**0,551**
0	Unknown	**0,551**
Slc24a4	Rattus norvegicus solute carrier family 24 (sodium/potassium/calcium exchanger), member 4 (Slc24a4), mRNA [NM_001108051]	**0,551**
Ard1a	Rattus norvegicus ARD1 homolog A, N-acetyltransferase (S. cerevisiae) (Ard1a), mRNA [NM_001135839]	**0,551**
Anks6	Rattus norvegicus ankyrin repeat and sterile alpha motif domain containing 6 (Anks6), mRNA [NM_001015028]	**0,551**
Napg	Rattus norvegicus N-ethylmaleimide-sensitive factor attachment protein, gamma (Napg), mRNA [NM_001107384]	**0,551**
0	Unknown	**0,551**
Ptp4a2	Rattus norvegicus protein tyrosine phosphatase 4a2 (Ptp4a2), mRNA [NM_053475]	**0,551**
Nnt	Rattus norvegicus nicotinamide nucleotide transhydrogenase (Nnt), nuclear gene encoding mitochondrial protein, mRNA [NM_001013157]	**0,552**
LOC689919	PREDICTED: Rattus norvegicus similar to 40S ribosomal protein S26 (LOC689919), mRNA [XM_001072530]	**0,552**
Spc25	Rattus norvegicus SPC25, NDC80 kinetochore complex component, homolog (S. cerevisiae) (Spc25), mRNA [NM_001009654]	**0,552**
0	Unknown	**0,552**
Slc22a15	Rattus norvegicus solute carrier family 22, member 15 (Slc22a15), mRNA [NM_001107707]	**0,552**
Rcn1	Rattus norvegicus reticulocalbin 1, EF-hand calcium binding domain (Rcn1), mRNA [NM_001108586]	**0,552**
Tppp3	Rattus norvegicus tubulin polymerization-promoting protein family member 3 (Tppp3), mRNA [NM_001009639]	**0,552**
0	Uncharacterized protein [Source:UniProtKB/TrEMBL;Acc:D3ZE35] [ENSRNOT00000027967]	**0,552**
Dennd5a	Rattus norvegicus DENN/MADD domain containing 5A (Dennd5a), mRNA [NM_001107546]	**0,552**
Gmds	Rattus norvegicus GDP-mannose 4, 6-dehydratase (Gmds), mRNA [NM_001039606]	**0,552**
Lrrc4c	Rattus norvegicus leucine rich repeat containing 4C (Lrrc4c), mRNA [NM_001107753]	**0,552**

Scgb1c1	Rattus norvegicus secretoglobin, family 1C, member 1 (Scgb1c1), mRNA [NM_001107561]	**0,552**
Frzb	Rattus norvegicus frizzled-related protein (Frzb), mRNA [NM_001100527]	**0,552**
Kcnf1	Rattus norvegicus potassium voltage-gated channel, subfamily F, member 1 (Kcnf1), mRNA [NM_001169104]	**0,552**
0	Unknown	**0,552**
Tmem43	Rattus norvegicus transmembrane protein 43 (Tmem43), mRNA [NM_001007745]	**0,552**
LOC288913	Rattus norvegicus similar to LEYDIG CELL TUMOR 10 KD PROTEIN (LOC288913), mRNA [NM_198728]	**0,552**
Plekha1	Rattus norvegicus pleckstrin homology domain containing, family A (phosphoinositide binding specific) member 1 (Plekha1), mRNA [NM_001079894]	**0,552**
Klhl22	Rattus norvegicus kelch-like 22 (Drosophila) (Klhl22), mRNA [NM_001107079]	**0,552**
Gtpbp4	Rattus norvegicus GTP binding protein 4 (Gtpbp4), mRNA [NM_053689]	**0,552**
Gna14	Rattus norvegicus guanine nucleotide binding protein, alpha 14 (Gna14), mRNA [NM_001013151]	**0,552**
Tlcd1	Rattus norvegicus TLC domain containing 1 (Tlcd1), mRNA [NM_001013858]	**0,552**
0	Uncharacterized protein [Source:UniProtKB/TrEMBL;Acc:D4A533] [ENSRNOT00000004274]	**0,552**
Siat7E	PREDICTED: Rattus norvegicus sialyltransferase 7E (Siat7E), mRNA [XM_001078153]	**0,552**
Hmgb1	Rattus norvegicus high mobility group box 1 (Hmgb1), mRNA [NM_012963]	**0,552**
Stx6	Rattus norvegicus syntaxin 6 (Stx6), mRNA [NM_031665]	**0,552**
Rrm2	Rattus norvegicus ribonucleotide reductase M2 (Rrm2), mRNA [NM_001025740]	**0,552**
Acvr1c	Rattus norvegicus activin A receptor, type IC (Acvr1c), mRNA [NM_139090]	**0,552**
Nav3	Rattus norvegicus neuron navigator 3 (Nav3), mRNA [NM_001191782]	**0,552**
Ap1ar	Rattus norvegicus adaptor-related protein complex 1 associated regulatory protein (Ap1ar), mRNA [NM_001191850]	**0,552**
Atp11c-ps1	Uncharacterized protein [Source:UniProtKB/TrEMBL;Acc:D3Z9J3] [ENSRNOT00000049120]	**0,552**
Sfrs11	Rattus norvegicus splicing factor, arginine/serine-rich 11 (Sfrs11), mRNA [NM_001035255]	**0,552**
0	AA944379 EST199878 Normalized rat embryo, Bento Soares Rattus sp. cDNA clone REMAG24 3' end, mRNA sequence [AA944379]	**0,552**
0	Unknown	**0,552**
0	Unknown	**0,552**
Gadd45gip1	Rattus norvegicus growth arrest and DNA-damage-inducible, gamma interacting protein 1 (Gadd45gip1), mRNA [NM_001100504]	**0,552**
Nhlrc1	Rattus norvegicus NHL repeat containing 1 (Nhlrc1), mRNA [NM_199236]	**0,552**
Yipf4	Rattus norvegicus Yip1 domain family, member 4 (Yipf4), mRNA [NM_001009712]	**0,552**
Slc9a9	PREDICTED: Rattus norvegicus similar to solute carrier family 9 (sodium/hydrogen exchanger), isoform 9 (RGD1560736), mRNA [XM_001064905]	**0,552**
Agpat5	Rattus norvegicus 1-acylglycerol-3-phosphate O-acyltransferase 5 (lysophosphatidic acid acyltransferase, epsilon) (Agpat5), mRNA [NM_001134744]	**0,552**
0	Unknown	**0,552**
Uhrf2	Rattus norvegicus ubiquitin-like with PHD and ring finger domains 2 (Uhrf2), mRNA [NM_001107585]	**0,552**

Rpusd3	Rattus norvegicus RNA pseudouridylate synthase domain containing 3 (Rpusd3), mRNA [NM_001108641]	**0,552**
Igsf10	Rattus norvegicus immunoglobulin superfamily, member 10 (Igsf10), mRNA [NM_198768]	**0,553**
0	Unknown	**0,553**
0	Unknown	**0,553**
Gpx1	Rattus norvegicus glutathione peroxidase 1 (Gpx1), mRNA [NM_030826]	**0,553**
Senp2	Rattus norvegicus Sumo1/sentrin/SMT3 specific peptidase 2 (Senp2), mRNA [NM_023989]	**0,553**
N4bp2l1	Rattus norvegicus NEDD4 binding protein 2-like 1 (N4bp2l1), mRNA [NM_001035222]	**0,553**
LOC688632	PREDICTED: Rattus norvegicus similar to 60S ribosomal protein L17 (L23) (Amino acid starvation-induced protein) (ASI) (LOC688632), mRNA [XM_001067694]	**0,553**
Lysmd3	Rattus norvegicus LysM, putative peptidoglycan-binding, domain containing 3 (Lysmd3), mRNA [NM_001009698]	**0,553**
Npdc1	Rattus norvegicus neural proliferation, differentiation and control, 1 (Npdc1), mRNA [NM_001004231]	**0,553**
RGD1564482	Rattus norvegicus RGD1564482 (RGD1564482), non-coding RNA [NR_036617]	**0,553**
Ube3c	PREDICTED: Rattus norvegicus ubiquitin protein ligase E3C (Ube3c), mRNA [XM_001055148]	**0,553**
0	CR467520 Rat pBluescript Lion Rattus norvegicus cDNA clone LIONp463D12400 3', mRNA sequence [CR467520]	**0,553**
0	Rattus norvegicus TL0ADA38YF09 mRNA sequence. [FQ221288]	**0,553**
40787	Rattus norvegicus septin 11 (Sept11), mRNA [NM_001107208]	**0,553**
Fat1	Rattus norvegicus FAT tumor suppressor homolog 1 (Drosophila) (Fat1), mRNA [NM_031819]	**0,553**
Cdig2	Rattus norvegicus Cdig2 protein (Cdig2), mRNA [NM_153624]	**0,553**
Tmsb4x	Rattus norvegicus thymosin beta 4, X-linked (Tmsb4x), mRNA [NM_031136]	**0,553**
RGD1309808	Rattus norvegicus similar to apolipoprotein L2; apolipoprotein L-II (RGD1309808), mRNA [NM_001134801]	**0,553**
LOC310177	Uncharacterized protein [Source:UniProtKB/TrEMBL;Acc:D3ZS22] [ENSRNOT00000030328]	**0,553**
Rdx	Rattus norvegicus radixin (Rdx), mRNA [NM_001005889]	**0,553**
Klf10	Rattus norvegicus Kruppel-like factor 10 (Klf10), mRNA [NM_031135]	**0,553**
RGD1563956	Uncharacterized protein [Source:UniProtKB/TrEMBL;Acc:D4A8N3] [ENSRNOT00000030686]	**0,553**
LOC378467	Rattus norvegicus promethin (LOC378467), mRNA [NM_194354]	**0,553**
Oprm1	Rattus norvegicus opioid receptor, mu 1 (Oprm1), transcript variant MOR-1A, mRNA [NM_001038597]	**0,553**
Nmral1	Rattus norvegicus NmrA-like family domain containing 1 (Nmral1), mRNA [NM_001191588]	**0,553**
Fitm2	Rattus norvegicus fat storage-inducing transmembrane protein 2 (Fitm2), mRNA [NM_001107799]	**0,553**
Adpgk	Rattus norvegicus ADP-dependent glucokinase (Adpgk), mRNA [NM_001100723]	**0,553**
0	Uncharacterized protein [Source:UniProtKB/TrEMBL;Acc:D3ZKL6] [ENSRNOT00000044160]	**0,553**
Peg12	Rattus norvegicus paternally expressed 12 (Peg12), mRNA [NM_001170562]	**0,553**
Sgcb	Rattus norvegicus sarcoglycan, beta (dystrophin-associated glycoprotein) (Sgcb), mRNA [NM_001191068]	**0,553**
Tmem50b	Rattus norvegicus transmembrane protein 50B (Tmem50b), mRNA [NM_001025014]	**0,553**
Nid67	Rattus norvegicus putative small membrane protein NID67 (Nid67), mRNA [NM_173126]	**0,553**

Trappc9	Rattus norvegicus trafficking protein particle complex 9 (Trappc9), mRNA [NM_001034156]	**0,553**
Plod1	Rattus norvegicus procollagen-lysine 1, 2-oxoglutarate 5-dioxygenase 1 (Plod1), mRNA [NM_053827]	**0,553**
Vdac1	Rattus norvegicus voltage-dependent anion channel 1 (Vdac1), nuclear gene encoding mitochondrial protein, mRNA [NM_031353]	**0,553**
0	Unknown	**0,553**
RGD1562846	Rattus norvegicus similar to Docking protein 5 (Downstream of tyrosine kinase 5) (Protein dok-5) (RGD1562846), mRNA [NM_001109344]	**0,553**
Necap2	Rattus norvegicus NECAP endocytosis associated 2 (Necap2), mRNA [NM_199096]	**0,553**
0	Unknown	**0,553**
Slc35a2	Rattus norvegicus solute carrier family 35 (UDP-galactose transporter), member A2 (Slc35a2), mRNA [NM_001127642]	**0,553**
Numa1	PREDICTED: Rattus norvegicus nuclear mitotic apparatus protein 1 (Numa1), mRNA [XM_218972]	**0,553**
0	Unknown	**0,554**
Klhl13	Klhl13 protein [Source:UniProtKB/TrEMBL;Acc:Q3MHT7] [ENSRNOT00000067114]	**0,554**
Kcnc1	Rattus norvegicus potassium voltage gated channel, Shaw-related subfamily, member 1 (Kcnc1), mRNA [NM_012856]	**0,554**
0	Uncharacterized protein [Source:UniProtKB/TrEMBL;Acc:D3Z9A9] [ENSRNOT00000015727]	**0,554**
LOC497978	Rattus norvegicus similar to diacylglycerol kinase epsilon (LOC497978), mRNA [NM_001039341]	**0,554**
Fundc2	Rattus norvegicus FUN14 domain containing 2 (Fundc2), mRNA [NM_001135757]	**0,554**
Pcyox1	Rattus norvegicus prenylcysteine oxidase 1 (Pcyox1), mRNA [NM_145085]	**0,554**
Mcfd2	Rattus norvegicus multiple coagulation factor deficiency 2 (Mcfd2), mRNA [NM_139253]	**0,554**
Paip1	Rattus norvegicus poly(A) binding protein interacting protein 1 (Paip1), mRNA [NM_001108937]	**0,554**
Tmed7	Rattus norvegicus transmembrane emp24 protein transport domain containing 7 (Tmed7), mRNA [NM_001105758]	**0,554**
Pdha1	Rattus norvegicus pyruvate dehydrogenase (lipoamide) alpha 1 (Pdha1), nuclear gene encoding mitochondrial protein, mRNA [NM_001004072]	**0,554**
B2m	Rattus norvegicus beta-2 microglobulin (B2m), mRNA [NM_012512]	**0,554**
RGD1564325	40S ribosomal protein S24 [Source:UniProtKB/TrEMBL;Acc:D3ZFZ8] [ENSRNOT00000047252]	**0,554**
0	Unknown	**0,554**
Tnks2	Rattus norvegicus tankyrase, TRF1-interacting ankyrin-related ADP-ribose polymerase 2 (Tnks2), mRNA [NM_001107607]	**0,554**
Psmd7	Rattus norvegicus proteasome (prosome, macropain) 26S subunit, non-ATPase, 7 (Psmd7), mRNA [NM_001107426]	**0,554**
Smurf2	Rattus norvegicus SMAD specific E3 ubiquitin protein ligase 2 (Smurf2), mRNA [NM_001107061]	**0,554**
RGD621352	Rattus norvegicus similar to RIKEN cDNA 1500031L02 (RGD621352), mRNA [NM_138865]	**0,554**
Trim11	Rattus norvegicus tripartite motif-containing 11 (Trim11), mRNA [NM_001108276]	**0,554**
Trabd	Rattus norvegicus TraB domain containing (Trabd), mRNA [NM_001106788]	**0,554**
Bola3	Rattus norvegicus bolA homolog 3 (E. coli) (Bola3), mRNA [NM_001106601]	**0,554**
Unc13c	Rattus norvegicus unc-13 homolog C (C. elegans) (Unc13c), mRNA [NM_173146]	**0,554**
Utx	Uncharacterized protein [Source:UniProtKB/TrEMBL;Acc:D4ADF1] [ENSRNOT00000005742]	**0,554**
Srf	Rattus norvegicus serum response factor (c-fos serum response element-binding transcription factor) (Srf), mRNA [NM_001109302]	**0,554**
0	Unknown	**0,554**

Fth1	Rattus norvegicus ferritin, heavy polypeptide 1 (Fth1), mRNA [NM_012848]	**0,554**
Acer3	PREDICTED: Rattus norvegicus phytoceramidase, alkaline (Phca), mRNA [XM_001065019]	**0,554**
Reep4	Rattus norvegicus receptor accessory protein 4 (Reep4), mRNA [NM_001025279]	**0,554**
Hsp90aa1	Rattus norvegicus heat shock protein 90, alpha (cytosolic), class A member 1 (Hsp90aa1), mRNA [NM_175761]	**0,554**
0	Collagen alpha-1(XI) chain [Source:UniProtKB/Swiss-Prot;Acc:P20909] [ENSRNOT00000024138]	**0,554**
Pdha1	Rattus norvegicus pyruvate dehydrogenase (lipoamide) alpha 1 (Pdha1), nuclear gene encoding mitochondrial protein, mRNA [NM_001004072]	**0,554**
0	Unknown	**0,554**
Nrip1	Rattus norvegicus nuclear receptor interacting protein 1 (Nrip1), mRNA [NM_001100560]	**0,554**
RGD1309534	Rattus norvegicus similar to RIKEN cDNA 4931406C07 (RGD1309534), mRNA [NM_001014206]	**0,554**
Slc26a2	Rattus norvegicus solute carrier family 26 (sulfate transporter), member 2 (Slc26a2), mRNA [NM_057127]	**0,554**
RGD1309139	Rattus norvegicus similar to CG5435-PA (RGD1309139), mRNA [NM_001134578]	**0,554**
Ndufv3	Rattus norvegicus NADH dehydrogenase (ubiquinone) flavoprotein 3 (Ndufv3), nuclear gene encoding mitochondrial protein, transcript variant 1, mRNA [NM_022607]	**0,554**
Stk38	Rattus norvegicus serine/threonine kinase 38 (Stk38), mRNA [NM_001015025]	**0,554**
RGD1564964	Rattus norvegicus similar to WD repeat domain 11 protein (RGD1564964), mRNA [NM_001134564]	**0,554**
Shoc2	Rattus norvegicus soc-2 (suppressor of clear) homolog (C. elegans) (Shoc2), mRNA [NM_001013155]	**0,554**
Strada	Rattus norvegicus STE20-related kinase adaptor alpha (Strada), mRNA [NM_182820]	**0,554**
Trappc6a	Rattus norvegicus trafficking protein particle complex 6A (Trappc6a), mRNA [NM_001109410]	**0,554**
Cggbp1	Rattus norvegicus CGG triplet repeat binding protein 1 (Cggbp1), mRNA [NM_001105900]	**0,554**
Hdac1	Rattus norvegicus histone deacetylase 1 (Hdac1), mRNA [NM_001025409]	**0,555**
Man1b1	Endoplasmic reticulum mannosyl-oligosaccharide 1,2-alpha-mannosidase [Source:UniProtKB/Swiss-Prot;Acc:B2GUY0] [ENSRNOT00000016846]	**0,555**
Sstr3	Rattus norvegicus somatostatin receptor 3 (Sstr3), mRNA [NM_133522]	**0,555**
Rab15	Rattus norvegicus RAB15, member RAS oncogene family (Rab15), mRNA [NM_198749]	**0,555**
Wdr44	PREDICTED: Rattus norvegicus WD repeat domain 44 (Wdr44), mRNA [XM_001061437]	**0,555**
Asb6	Rattus norvegicus ankyrin repeat and SOCS box-containing 6 (Asb6), mRNA [NM_001011963]	**0,555**
0	Unknown	**0,555**
Pgp	Rattus norvegicus phosphoglycolate phosphatase (Pgp), mRNA [NM_001169152]	**0,555**
RGD1307365	PREDICTED: Rattus norvegicus similar to KIAA1009 protein (RGD1307365), mRNA [XM_001054857]	**0,555**
Zfp61	Rattus norvegicus zinc finger protein 61 (Zfp61), mRNA [NM_001017512]	**0,555**
Rpl27	Rattus norvegicus ribosomal protein L27 (Rpl27), mRNA [NM_022514]	**0,555**
0	Unknown	**0,555**
Amigo1	Rattus norvegicus adhesion molecule with Ig like domain 1 (Amigo1), mRNA [NM_206881]	**0,555**
Ltbr	Rattus norvegicus lymphotoxin beta receptor (TNFR superfamily, member 3) (Ltbr), mRNA [NM_001008315]	**0,555**
Tcof1	Rattus norvegicus Treacher Collins-Franceschetti syndrome 1 homolog (human) (Tcof1), mRNA [NM_001106143]	**0,555**

0	Tmem216 protein [Source:UniProtKB/TrEMBL;Acc:B6ID01] [ENSRNOT00000065430]	**0,555**
Mkks	Rattus norvegicus McKusick-Kaufman syndrome (Mkks), mRNA [NM_001008353]	**0,555**
Fbxo25	Rattus norvegicus F-box protein 25 (Fbxo25), mRNA [NM_001014239]	**0,555**
RGD1306809	Rattus norvegicus similar to hypothetical protein FLJ30596 (RGD1306809), mRNA [NM_001044252]	**0,555**
Cct8	Rattus norvegicus chaperonin containing Tcp1, subunit 8 (theta) (Cct8), mRNA [NM_001105897]	**0,555**
Mphosph9	Rattus norvegicus M-phase phosphoprotein 9 (Mphosph9), mRNA [NM_001170554]	**0,555**
Rufy3	Rattus norvegicus RUN and FYVE domain containing 3 (Rufy3), mRNA [NM_001025127]	**0,555**
Ppia	Rattus norvegicus peptidylprolyl isomerase A (cyclophilin A) (Ppia), mRNA [NM_017101]	**0,555**
0	Histone H4Osteogenic growth peptide [Source:UniProtKB/Swiss-Prot;Acc:P62804] [ENSRNOT00000049464]	**0,555**
Artn	Rattus norvegicus artemin (Artn), mRNA [NM_053397]	**0,555**
Dera	PREDICTED: Rattus norvegicus 2-deoxyribose-5-phosphate aldolase homolog (C. elegans) (Dera), miscRNA [XR_085824]	**0,555**
Plcd4	Rattus norvegicus phospholipase C, delta 4 (Plcd4), mRNA [NM_080688]	**0,555**
Apoc1	Rattus norvegicus apolipoprotein C-I (Apoc1), transcript variant 1, mRNA [NM_012824]	**0,555**
0	Rattus norvegicus similar to RIKEN cDNA A230102I05 (LOC308316), mRNA [XM_218209]	**0,555**
Eif4ebp1	Rattus norvegicus eukaryotic translation initiation factor 4E binding protein 1 (Eif4ebp1), mRNA [NM_053857]	**0,555**
0	YLP motif-containing protein 1 [Source:UniProtKB/Swiss-Prot;Acc:P0CB49] [ENSRNOT00000047060]	**0,555**
Pdzd8	Rattus norvegicus PDZ domain containing 8 (Pdzd8), mRNA [NM_001107446]	**0,555**
Scyl3	Rattus norvegicus SCY1-like 3 (S. cerevisiae) (Scyl3), mRNA [NM_001191828]	**0,555**
LOC689842	PREDICTED: Rattus norvegicus similar to Nucleolar GTP-binding protein 1 (Chronic renal failure gene protein) (GTP-binding protein NGB) (LOC689842), miscRNA [XR_006738]	**0,556**
0	Rattus norvegicus TL0ADA36YM24 mRNA sequence. [FQ221400]	**0,556**
Timm8a1	Rattus norvegicus translocase of inner mitochondrial membrane 8 homolog a1 (yeast) (Timm8a1), nuclear gene encoding mitochondrial protein, mRNA [NM_053370]	**0,556**
Aldh18a1	Rattus norvegicus aldehyde dehydrogenase 18 family, member A1 (Aldh18a1), nuclear gene encoding mitochondrial protein, mRNA [NM_001108524]	**0,556**
Ggct	Rattus norvegicus gamma-glutamyl cyclotransferase (Ggct), mRNA [NM_001108629]	**0,556**
Zfp329	Rattus norvegicus zinc finger protein 329 (Zfp329), mRNA [NM_001107477]	**0,556**
0	Rattus norvegicus TL0AAA52YK09 mRNA sequence. [FQ212560]	**0,556**
Gpr89	Rattus norvegicus G protein-coupled receptor 89 (Gpr89), mRNA [NM_001139486]	**0,556**
Aldh1l1	Rattus norvegicus aldehyde dehydrogenase 1 family, member L1 (Aldh1l1), mRNA [NM_022547]	**0,556**
Dffa	Rattus norvegicus DNA fragmentation factor, alpha subunit (Dffa), mRNA [NM_053679]	**0,556**
Ndrg2	Rattus norvegicus N-myc downstream regulated gene 2 (Ndrg2), mRNA [NM_133583]	**0,556**
Kif18a	Rattus norvegicus kinesin family member 18A (Kif18a), mRNA [NM_001137642]	**0,556**
Hsd11b2	Rattus norvegicus hydroxysteroid 11-beta dehydrogenase 2 (Hsd11b2), mRNA [NM_017081]	**0,556**

0	Rattus norvegicus similar to ribosomal protein S24 (LOC315035), mRNA [XM_235376]	**0,556**
RGD1307218	Rattus norvegicus similar to RIKEN cDNA 2810432L12 (RGD1307218), mRNA [NM_001014190]	**0,556**
Dcun1d1	Rattus norvegicus DCN1, defective in cullin neddylation 1, domain containing 1 (S. cerevisiae) (Dcun1d1), mRNA [NM_001107668]	**0,556**
Flywch1	PREDICTED: Rattus norvegicus FLYWCH-type zinc finger 1 (Flywch1), mRNA [XM_001056224]	**0,556**
Thoc6	Rattus norvegicus THO complex 6 homolog (Drosophila) (Thoc6), mRNA [NM_024384]	**0,556**
Fbxo3	Rattus norvegicus F-box protein 3 (Fbxo3), mRNA [NM_001109606]	**0,556**
Rap1gap	PREDICTED: Rattus norvegicus Rap1 GTPase-activating protein (Rap1gap), mRNA [XM_001070178]	**0,556**
Spop	Rattus norvegicus speckle-type POZ protein (Spop), mRNA [NM_001100496]	**0,556**
0	Coiled-coil domain-containing-like [Source:UniProtKB/TrEMBL;Acc:Q5M816] [ENSRNOT00000015782]	**0,556**
0	SC23A_MOUSE (Q01405) Protein transport protein Sec23A (SEC23-related protein A), complete [TC596010]	**0,556**
Vps26b	Rattus norvegicus vacuolar protein sorting 26 homolog B (S. pombe) (Vps26b), mRNA [NM_001106809]	**0,556**
Ftsj2	Rattus norvegicus FtsJ homolog 2 (E. coli) (Ftsj2), mRNA [NM_001107125]	**0,556**
Pts	Rattus norvegicus 6-pyruvoyl-tetrahydropterin synthase (Pts), mRNA [NM_017220]	**0,556**
Rhoc	Rattus norvegicus ras homolog gene family, member C (Rhoc), mRNA [NM_001106461]	**0,556**
Slc35d3	Rattus norvegicus solute carrier family 35, member D3 (Slc35d3), mRNA [NM_001107522]	**0,556**
Shmt2	Rattus norvegicus serine hydroxymethyltransferase 2 (mitochondrial) (Shmt2), nuclear gene encoding mitochondrial protein, mRNA [NM_001008322]	**0,556**
Rpp21	Rattus norvegicus ribonuclease P 21 subunit (human) (Rpp21), mRNA [NM_001002831]	**0,556**
Ndufaf4	Rattus norvegicus NADH dehydrogenase (ubiquinone) 1 alpha subcomplex, assembly factor 4 (Ndufaf4), nuclear gene encoding mitochondrial protein, mRNA [NM_198783]	**0,556**
Cox6a1	Rattus norvegicus cytochrome c oxidase, subunit VIa, polypeptide 1 (Cox6a1), nuclear gene encoding mitochondrial protein, mRNA [NM_012814]	**0,556**
Gpr68	Rattus norvegicus G protein-coupled receptor 68 (Gpr68), mRNA [NM_001108049]	**0,556**
Csgalnact2	Rattus norvegicus chondroitin sulfate N-acetylgalactosaminyltransferase 2 (Csgalnact2), mRNA [NM_001106616]	**0,556**
0	Unknown	**0,556**
Npr2	Rattus norvegicus natriuretic peptide receptor B/guanylate cyclase B (atrionatriuretic peptide receptor B) (Npr2), mRNA [NM_053838]	**0,556**
Rasgef1a	PREDICTED: Rattus norvegicus RasGEF domain family, member 1A (Rasgef1a), mRNA [XM_232315]	**0,556**
Abce1	Rattus norvegicus ATP-binding cassette, subfamily E (OABP), member 1 (Abce1), mRNA [NM_001108446]	**0,556**
RGD1311463	Rattus norvegicus similar to RIKEN cDNA 2700007P21 (RGD1311463), mRNA [NM_001014045]	**0,556**
RGD1559442	Rattus norvegicus similar to SET binding factor 2 (LOC691036), mRNA [NM_001134970]	**0,556**
RGD1307225	Rattus norvegicus similar to MEGF6 (RGD1307225), mRNA [NM_001107663]	**0,556**
0	Unknown	**0,557**
Hes6	Rattus norvegicus hairy and enhancer of split 6 (Drosophila) (Hes6), mRNA [NM_001013179]	**0,557**
Sall3	Rattus norvegicus sal-like 3 (Drosophila) (Sall3), mRNA [NM_001108892]	**0,557**
Ccdc115	coiled-coil domain-containing protein 115 [Source:RefSeq peptide;Acc:NP_001102263] [ENSRNOT00000017722]	**0,557**

Cpsf2	Rattus norvegicus cleavage and polyadenylation specific factor 2 (Cpsf2), mRNA [NM_001106753]	**0,557**
Parp1	Rattus norvegicus poly (ADP-ribose) polymerase 1 (Parp1), mRNA [NM_013063]	**0,557**
Cdc2l6	Rattus norvegicus cell division cycle 2-like 6 (CDK8-like) (Cdc2l6), mRNA [NM_001107634]	**0,557**
Asah1	Rattus norvegicus N-acylsphingosine amidohydrolase (acid ceramidase) 1 (Asah1), mRNA [NM_053407]	**0,557**
Np	Rattus norvegicus nucleoside phosphorylase (Np), mRNA [NM_001106031]	**0,557**
Mrpl37	Rattus norvegicus mitochondrial ribosomal protein L37 (Mrpl37), nuclear gene encoding mitochondrial protein, mRNA [NM_001004235]	**0,557**
Lancl3	Rattus norvegicus LanC lantibiotic synthetase component C-like 3 (bacterial) (Lancl3), mRNA [NM_001191728]	**0,557**
0	Unknown	**0,557**
Nkd2	Rattus norvegicus naked cuticle homolog 2 (Drosophila) (Nkd2), mRNA [NM_001107454]	**0,557**
Gba	Rattus norvegicus glucosidase, beta, acid (Gba), mRNA [NM_001127639]	**0,557**
Ssh3	Rattus norvegicus slingshot homolog 3 (Drosophila) (Ssh3), mRNA [NM_001012217]	**0,557**
LOC686041	Rattus norvegicus cDNA clone IMAGE:6889638, partial cds. [BC059164]	**0,557**
Deaf1	Rattus norvegicus deformed epidermal autoregulatory factor 1 (Drosophila) (Deaf1), mRNA [NM_031801]	**0,557**
Camkv	Rattus norvegicus CaM kinase-like vesicle-associated (Camkv), mRNA [NM_024000]	**0,557**
Zwilch	PREDICTED: Rattus norvegicus Zwilch, kinetochore associated, homolog (Drosophila) (Zwilch), mRNA [XM_001078534]	**0,557**
Bcl11b	Rattus norvegicus B-cell CLL/lymphoma 11B (zinc finger protein) (Bcl11b), mRNA [NM_001108057]	**0,557**
Kptn	kaptin [Source:RefSeq peptide;Acc:NP_001100927] [ENSRNOT00000002043]	**0,557**
Rfc1	Rattus norvegicus replication factor C (activator 1) 1 (Rfc1), mRNA [NM_053547]	**0,557**
Wdr78	Rattus norvegicus WD repeat domain 78 (Wdr78), mRNA [NM_001024786]	**0,557**
Cnot1	Rattus norvegicus CCR4-NOT transcription complex, subunit 1 (Cnot1), mRNA [NM_001134840]	**0,557**
Fzd9	Rattus norvegicus frizzled homolog 9 (Drosophila) (Fzd9), mRNA [NM_153305]	**0,557**
Gmeb1	Rattus norvegicus glucocorticoid modulatory element binding protein 1 (Gmeb1), mRNA [NM_001109268]	**0,557**
Nudt4	Rattus norvegicus nudix (nucleoside diphosphate linked moiety X)-type motif 4 (Nudt4), mRNA [NM_053598]	**0,557**
Ndufb4l1	Uncharacterized protein [Source:UniProtKB/TrEMBL;Acc:D3ZV29] [ENSRNOT00000044617]	**0,557**
Mmd	Rattus norvegicus monocyte to macrophage differentiation-associated (Mmd), mRNA [NM_001007673]	**0,557**
Dnajc12	Rattus norvegicus DnaJ (Hsp40) homolog, subfamily C, member 12 (Dnajc12), mRNA [NM_001034032]	**0,557**
Arnt2	Rattus norvegicus aryl hydrocarbon receptor nuclear translocator 2 (Arnt2), mRNA [NM_012781]	**0,557**
Ankrd13a	Rattus norvegicus ankyrin repeat domain 13a (Ankrd13a), mRNA [NM_001012148]	**0,557**
0	Unknown	**0,557**
RGD1562211	Rattus norvegicus similar to ubiquitin specific protease 51 (RGD1562211), mRNA [NM_001108252]	**0,557**
Plk5	Rattus norvegicus polo-like kinase 5 (Plk5), mRNA [NM_001170557]	**0,557**
0	Dystonin (Predicted)Uncharacterized protein [Source:UniProtKB/TrEMBL;Acc:D3ZC56] [ENSRNOT00000044452]	**0,557**
Ywhaq	Rattus norvegicus tyrosine 3-monooxygenase/tryptophan 5-monooxygenase activation protein, theta polypeptide (Ywhaq), mRNA [NM_013053]	**0,557**
0	Unknown	**0,557**

Trim17	Rattus norvegicus tripartite motif-containing 17 (Trim17), mRNA [NM_022798]	**0,558**
Ank1	Rattus norvegicus ankyrin 1, erythrocytic (Ank1), mRNA [NM_001107322]	**0,558**
0	Uncharacterized protein [Source:UniProtKB/TrEMBL;Acc:D3ZQC4] [ENSRNOT00000050888]	**0,558**
Zfp445	Rattus norvegicus zinc finger protein 445 (Zfp445), mRNA [NM_001191802]	**0,558**
Gpr56	Rattus norvegicus G protein-coupled receptor 56 (Gpr56), mRNA [NM_152242]	**0,558**
0	Unknown	**0,558**
Smarca2	Rattus norvegicus SWI/SNF related, matrix associated, actin dependent regulator of chromatin, subfamily a, member 2 (Smarca2), mRNA [NM_001004446]	**0,558**
Matn2	PREDICTED: Rattus norvegicus matrilin 2 (Matn2), mRNA [XM_216941]	**0,558**
LOC100366273	Uncharacterized protein [Source:UniProtKB/TrEMBL;Acc:D4A1X2] [ENSRNOT00000014629]	**0,558**
Tsc22d1	Rattus norvegicus TSC22 domain family, member 1 (Tsc22d1), transcript variant 1, mRNA [NM_001109912]	**0,558**
Osbpl9	Rattus norvegicus oxysterol binding protein-like 9 (Osbpl9), mRNA [NM_001044234]	**0,558**
Dag1	PREDICTED: Rattus norvegicus dystroglycan 1 (dystrophin-associated glycoprotein 1) (Dag1), mRNA [XM_001074892]	**0,558**
Surf2	Rattus norvegicus surfeit 2 (Surf2), mRNA [NM_001033866]	**0,558**
Centd3	PREDICTED: Rattus norvegicus centaurin, delta 3 (Centd3), miscRNA [XR_085668]	**0,558**
Anp32e	Rattus norvegicus acidic (leucine-rich) nuclear phosphoprotein 32 family, member E (Anp32e), mRNA [NM_001013200]	**0,558**
Slain2	Rattus norvegicus SLAIN motif family, member 2 (Slain2), mRNA [NM_001107214]	**0,558**
Zrsr1	Rattus norvegicus zinc finger (CCCH type), RNA binding motif and serine/arginine rich 1 (Zrsr1), mRNA [NM_001017504]	**0,558**
RGD1307983	PREDICTED: Rattus norvegicus similar to HSPC043 protein (RGD1307983), mRNA [XM_001061972]	**0,558**
0	Rattus norvegicus TL0ADA12YH10 mRNA sequence. [FQ223342]	**0,558**
Grm1	Rattus norvegicus glutamate receptor, metabotropic 1 (Grm1), transcript variant 1, mRNA [NM_017011]	**0,558**
LOC498829	PREDICTED: Rattus norvegicus Ab2-143 (LOC498829), miscRNA [XR_005439]	**0,558**
Znf286a	Rattus norvegicus zinc finger protein 286A (Znf286a), mRNA [NM_001191921]	**0,558**
Dhx57	Rattus norvegicus DEAH (Asp-Glu-Ala-Asp/His) box polypeptide 57 (Dhx57), mRNA [NM_001191907]	**0,558**
Jak2	Rattus norvegicus Janus kinase 2 (Jak2), mRNA [NM_031514]	**0,558**
Ube2l6	Rattus norvegicus ubiquitin-conjugating enzyme E2L 6 (Ube2l6), mRNA [NM_001024755]	**0,558**
Mto1	Rattus norvegicus mitochondrial translation optimization 1 homolog (S. cerevisiae) (Mto1), nuclear gene encoding mitochondrial protein, mRNA [NM_001106841]	**0,558**
Dip2a	Rattus norvegicus DIP2 disco-interacting protein 2 homolog A (Drosophila) (Dip2a), mRNA [NM_001191564]	**0,558**
Bcap29	Rattus norvegicus B-cell receptor-associated protein 29 (Bcap29), mRNA [NM_001006980]	**0,558**
Timm22	PREDICTED: Rattus norvegicus translocase of inner mitochondrial membrane 22 homolog (yeast) (Timm22), mRNA [XM_001080619]	**0,558**
Rpl10a	Rattus norvegicus ribosomal protein L10A (Rpl10a), mRNA [NM_031065]	**0,558**
Bscl2	Rattus norvegicus Bernardinelli-Seip congenital lipodystrophy 2 homolog (human) (Bscl2), mRNA [NM_001012171]	**0,558**
Ythdc1	Rattus norvegicus YTH domain containing 1 (Ythdc1), mRNA [NM_133423]	**0,558**

Akap7	A-kinase anchoring protein 18 ,isoform delta [Source:RefSeq peptide;Acc:NP_001001801] [ENSRNOT00000017617]	**0,558**
LOC685909	Rattus norvegicus similar to H2A histone family, member V isoform 1 (LOC685909), mRNA [NM_001106019]	**0,558**
Znf292	Rattus norvegicus zinc finger protein 292 (Znf292), mRNA [NM_001008879]	**0,558**
RGD1561849	Rattus norvegicus similar to RIKEN cDNA 3110035E14 (RGD1561849), mRNA [NM_001109260]	**0,558**
0	Unknown	**0,558**
Cabyr	Rattus norvegicus calcium binding tyrosine-(Y)-phosphorylation regulated (Cabyr), mRNA [NM_001143893]	**0,558**
Kcnc2	Rattus norvegicus potassium voltage gated channel, Shaw-related subfamily, member 2 (Kcnc2), transcript variant a, mRNA [NM_139216]	**0,558**
0	Unknown	**0,558**
Ppm1b	Rattus norvegicus protein phosphatase 1B, magnesium dependent, beta isoform (Ppm1b), mRNA [NM_033096]	**0,558**
Tmem90b	Rattus norvegicus transmembrane protein 90B (Tmem90b), mRNA [NM_001025020]	**0,558**
Foxj1	Rattus norvegicus forkhead box J1 (Foxj1), mRNA [NM_053832]	**0,558**
Aco1	Rattus norvegicus aconitase 1, soluble (Aco1), mRNA [NM_017321]	**0,558**
RGD1306613	Rattus norvegicus similar to RIKEN cDNA 1600012F09 (RGD1306613), mRNA [NM_001107356]	**0,558**
Hibch	Rattus norvegicus 3-hydroxyisobutyryl-Coenzyme A hydrolase (Hibch), nuclear gene encoding mitochondrial protein, mRNA [NM_001013112]	**0,558**
Exoc6	Rattus norvegicus exocyst complex component 6 (Exoc6), mRNA [NM_019277]	**0,558**
0	Uncharacterized protein [Source:UniProtKB/TrEMBL;Acc:D3ZAF9] [ENSRNOT00000005478]	**0,559**
0	Unknown	**0,559**
Sertad4	Rattus norvegicus SERTA domain containing 4 (Sertad4), mRNA [NM_001108351]	**0,559**
Cdc37l1	Rattus norvegicus cell division cycle 37 homolog (S. cerevisiae)-like 1 (Cdc37l1), mRNA [NM_001011941]	**0,559**
C1qtnf5	Rattus norvegicus C1q and tumor necrosis factor related protein 5 (C1qtnf5), mRNA [NM_001012123]	**0,559**
Dok3	Rattus norvegicus docking protein 3 (Dok3), mRNA [NM_001107336]	**0,559**
Sumo1	Rattus norvegicus SMT3 suppressor of mif two 3 homolog 1 (S. cerevisiae) (Sumo1), mRNA [NM_001009672]	**0,559**
Syt12	Rattus norvegicus synaptotagmin XII (Syt12), mRNA [NM_138835]	**0,559**
Pacrg	Rattus norvegicus Park2 co-regulated (Pacrg), mRNA [NM_001077677]	**0,559**
0	U1 small nuclear ribonucleoprotein C [Source:UniProtKB/TrEMBL;Acc:D3ZCL3] [ENSRNOT00000000586]	**0,559**
Zdhhc7	Rattus norvegicus zinc finger, DHHC-type containing 7 (Zdhhc7), mRNA [NM_133394]	**0,559**
Bag4	Rattus norvegicus BCL2-associated athanogene 4 (Bag4), mRNA [NM_001025130]	**0,559**
Armcx3	Rattus norvegicus armadillo repeat containing, X-linked 3 (Armcx3), mRNA [NM_001014273]	**0,559**
Cul5	Rattus norvegicus cullin 5 (Cul5), mRNA [NM_022683]	**0,559**
Cenpv	Uncharacterized protein [Source:UniProtKB/TrEMBL;Acc:D4A9A3] [ENSRNOT00000004127]	**0,559**
Uba52	Rattus norvegicus ubiquitin A-52 residue ribosomal protein fusion product 1 (Uba52), mRNA [NM_031687]	**0,559**
Hlx	Rattus norvegicus H2.0-like homeobox (Hlx), mRNA [NM_001077674]	**0,559**
Dfna5	Rattus norvegicus deafness, autosomal dominant 5 (human) (Dfna5), mRNA [NM_001191749]	**0,559**
0	Unknown	**0,559**

Vps13d	Rattus norvegicus vacuolar protein sorting 13 homolog D (S. cerevisiae) (Vps13d), mRNA [NM_001108006]	**0,559**
0	Unknown	**0,559**
Bphl	Rattus norvegicus biphenyl hydrolase-like (serine hydrolase) (Bphl), mRNA [NM_001037206]	**0,559**
Aadacl1	Rattus norvegicus arylacetamide deacetylase-like 1 (Aadacl1), mRNA [NM_001127524]	**0,559**
Kpna1	Rattus norvegicus karyopherin alpha 1/importin alpha 5 (Kpna1) mRNA, complete cds. [AY351984]	**0,559**
0	Unknown	**0,559**
Tnnc2	Rattus norvegicus troponin C type 2 (fast) (Tnnc2), mRNA [NM_001037351]	**0,559**
Pard3	Rattus norvegicus par-3 (partitioning defective 3) homolog (C. elegans) (Pard3), mRNA [NM_031235]	**0,559**
LOC502684	Rattus norvegicus hypothetical protein LOC502684 (LOC502684), mRNA [NM_001025060]	**0,559**
Strbp	Rattus norvegicus spermatid perinuclear RNA binding protein (Strbp), mRNA [NM_053416]	**0,559**
Anapc1	Rattus norvegicus anaphase promoting complex subunit 1 (Anapc1), mRNA [NM_001107771]	**0,560**
Rnf25	Rattus norvegicus ring finger protein 25 (Rnf25), mRNA [NM_001012004]	**0,560**
0	Unknown	**0,560**
Sema3f	Rattus norvegicus sema domain, immunoglobulin domain (Ig), short basic domain, secreted, (semaphorin) 3 F (Sema3f), mRNA [NM_001108185]	**0,560**
Folh1	Rattus norvegicus folate hydrolase 1 (Folh1), mRNA [NM_057185]	**0,560**
LOC684280	PREDICTED: Rattus norvegicus similar to Class I histocompatibility antigen, Non-RT1.A alpha-1 chain precursor (LOC684280), partial mRNA [XM_001069706]	**0,560**
Zbtb48	Rattus norvegicus zinc finger and BTB domain containing 48 (Zbtb48), mRNA [NM_001013216]	**0,560**
Hs2st1	Rattus norvegicus heparan sulfate 2-O-sulfotransferase 1 (Hs2st1), mRNA [NM_001100518]	**0,560**
Cspg4	Rattus norvegicus chondroitin sulfate proteoglycan 4 (Cspg4), mRNA [NM_031022]	**0,560**
Myo18a	Rattus norvegicus myosin XVIIIa (Myo18a), mRNA [NM_001172137]	**0,560**
Oxa1l	Rattus norvegicus oxidase assembly 1-like (Oxa1l), mRNA [NM_001168583]	**0,560**
Prkci	Rattus norvegicus protein kinase C, iota (Prkci), mRNA [NM_032059]	**0,560**
0	Uncharacterized protein [Source:UniProtKB/TrEMBL;Acc:D3ZQ92] [ENSRNOT00000026116]	**0,560**
Pnn	Rattus norvegicus pinin, desmosome associated protein (Pnn), mRNA [NM_001109023]	**0,560**
Kcnt2	Rattus norvegicus potassium channel, subfamily T, member 2 (Kcnt2), mRNA [NM_198762]	**0,560**
Psme2	Rattus norvegicus proteasome (prosome, macropain) activator subunit 2 (Psme2), mRNA [NM_017257]	**0,560**
Sco1	Rattus norvegicus SCO cytochrome oxidase deficient homolog 1 (yeast) (Sco1), nuclear gene encoding mitochondrial protein, mRNA [NM_001173374]	**0,560**
LOC686428	PREDICTED: Rattus norvegicus similar to Emu2 (LOC686428), mRNA [XM_001069996]	**0,560**
Akap2	Rattus norvegicus A kinase (PRKA) anchor protein 2 (Akap2), mRNA [NM_001011974]	**0,560**
Ccny	Rattus norvegicus cyclin Y (Ccny), mRNA [NM_001191833]	**0,560**
Tmcc2	PREDICTED: Rattus norvegicus transmembrane and coiled-coil domain family 2 (Tmcc2), mRNA [XM_223107]	**0,560**
0	Q2DQC2_9DELT (Q2DQC2) HipA protein, DNA binding regulator, partial (5%) [TC592031]	**0,560**

0	Rattus norvegicus TL0AAA50YN10 mRNA sequence. [FQ212883]	**0,560**
0	Uncharacterized protein [Source:UniProtKB/TrEMBL;Acc:D3ZXZ9] [ENSRNOT00000020215]	**0,560**
Pxmp2	Rattus norvegicus peroxisomal membrane protein 2 (Pxmp2), mRNA [NM_031587]	**0,560**
Gtpbp6	Rattus norvegicus GTP binding protein 6 (putative) (Gtpbp6), mRNA [NM_001135840]	**0,560**
Dcaf12l1	Rattus norvegicus DDB1 and CUL4 associated factor 12-like 1 (Dcaf12l1), mRNA [NM_001107941]	**0,560**
Camlg	Rattus norvegicus calcium modulating ligand (Camlg), mRNA [NM_053334]	**0,560**
0	Unknown	**0,560**
0	Uncharacterized protein [Source:UniProtKB/TrEMBL;Acc:D3ZLA5] [ENSRNOT00000047517]	**0,560**
RGD1562747	Rattus norvegicus similar to RIKEN cDNA 1110012L19 (RGD1562747), mRNA [NM_001106212]	**0,561**
Abhd8	Rattus norvegicus abhydrolase domain containing 8 (Abhd8), mRNA [NM_001107301]	**0,561**
Zkscan3	Rattus norvegicus zinc finger with KRAB and SCAN domains 3 (Zkscan3), mRNA [NM_001012053]	**0,561**
0	Uncharacterized protein [Source:UniProtKB/TrEMBL;Acc:D3Z8J8] [ENSRNOT00000058230]	**0,561**
Odz3	Rattus norvegicus odz, odd Oz/ten-m homolog 3 (Drosophila) (Odz3), mRNA [NM_001169133]	**0,561**
Gucy1b3	Rattus norvegicus guanylate cyclase 1, soluble, beta 3 (Gucy1b3), mRNA [NM_012769]	**0,561**
0	Stathmin [Source:UniProtKB/Swiss-Prot;Acc:P13668] [ENSRNOT00000022574]	**0,561**
Gabra4	Rattus norvegicus gamma-aminobutyric acid (GABA) A receptor, alpha 4 (Gabra4), mRNA [NM_080587]	**0,561**
Scarb1	Rattus norvegicus scavenger receptor class B, member 1 (Scarb1), mRNA [NM_031541]	**0,561**
Tgs1	Rattus norvegicus trimethylguanosine synthase homolog (S. cerevisiae) (Tgs1), mRNA [NM_001107904]	**0,561**
0	Unknown	**0,561**
Tmeff1	Rattus norvegicus transmembrane protein with EGF-like and two follistatin-like domains 1 (Tmeff1), mRNA [NM_023020]	**0,561**
Rbl2	Rattus norvegicus retinoblastoma-like 2 (Rbl2), mRNA [NM_031094]	**0,561**
Acss1	Rattus norvegicus acyl-CoA synthetase short-chain family member 1 (Acss1), nuclear gene encoding mitochondrial protein, mRNA [NM_001106524]	**0,561**
Sec24c	Rattus norvegicus SEC24 family, member C (S. cerevisiae) (Sec24c), mRNA [NM_001109456]	**0,561**
Vcan	Rattus norvegicus versican (Vcan), transcript variant 2, mRNA [NM_053663]	**0,561**
Cbx6	Rattus norvegicus chromobox homolog 6 (Cbx6), mRNA [NM_001012119]	**0,561**
Aldoc	Rattus norvegicus aldolase C, fructose-bisphosphate (Aldoc), mRNA [NM_012497]	**0,561**
0	Unknown	**0,561**
LOC685398	PREDICTED: Rattus norvegicus similar to 1A6/DRIM (down-regulated in metastasis) interacting protein (LOC685398), mRNA [XM_001063635]	**0,561**
Rps24	Rattus norvegicus ribosomal protein S24 (Rps24), mRNA [NM_031112]	**0,561**
0	Rattus norvegicus TL0AEA64YE20 mRNA sequence. [FQ232826]	**0,561**
Mynn	Rattus norvegicus myoneurin (Mynn), mRNA [NM_001012178]	**0,561**
Smpd4	Rattus norvegicus sphingomyelin phosphodiesterase 4, neutral membrane (Smpd4), mRNA [NM_001167806]	**0,561**
Mpdz	Rattus norvegicus multiple PDZ domain protein (Mpdz), mRNA [NM_019196]	**0,561**

Rgr	Rattus norvegicus retinal G protein coupled receptor (Rgr), mRNA [NM_001107299]	**0,561**
Ap1s2	Rattus norvegicus adaptor-related protein complex 1, sigma 2 subunit (Ap1s2), mRNA [NM_001127531]	**0,561**
LOC365985	Rattus norvegicus similar to adenylate kinase 5 isoform 1 (LOC365985), mRNA [NM_001108951]	**0,561**
Tmem223	Rattus norvegicus transmembrane protein 223 (Tmem223), mRNA [NM_001191104]	**0,561**
Crls1	Rattus norvegicus cardiolipin synthase 1 (Crls1), mRNA [NM_001014258]	**0,561**
Drd2	Rattus norvegicus dopamine receptor D2 (Drd2), mRNA [NM_012547]	**0,561**
0	Rattus norvegicus similar to High mobility group protein 1 (HMG-1) (Amphoterin) (Heparin-binding protein p30) (LOC364873), mRNA [XM_344684]	**0,561**
RGD1563863	Rattus norvegicus similar to expressed sequence AI553587 (RGD1563863), mRNA [NM_001127559]	**0,561**
Brms1l	Rattus norvegicus breast cancer metastasis-suppressor 1-like (Brms1l), mRNA [NM_001106731]	**0,561**
Ankrd12	Rattus norvegicus ankyrin repeat domain 12 (Ankrd12), mRNA [NM_001108238]	**0,561**
0	Unknown	**0,561**
LOC500974	PREDICTED: Rattus norvegicus similar to CDNA sequence BC024479 (LOC500974), miscRNA [XR_005433]	**0,561**
Mettl9	Rattus norvegicus methyltransferase like 9 (Mettl9), mRNA [NM_001163164]	**0,561**
Cln8	Rattus norvegicus ceroid-lipofuscinosis, neuronal 8 (Cln8), mRNA [NM_001007686]	**0,561**
M6pr	Rattus norvegicus mannose-6-phosphate receptor, cation dependent (M6pr), mRNA [NM_001007700]	**0,561**
Klhl9	Rattus norvegicus kelch-like 9 (Drosophila) (Klhl9), mRNA [NM_001107944]	**0,562**
Kiss1r	Rattus norvegicus KISS1 receptor (Kiss1r), mRNA [NM_023992]	**0,562**
Wwox	Rattus norvegicus WW domain-containing oxidoreductase (Wwox), mRNA [NM_001106188]	**0,562**
Cspg5	Rattus norvegicus chondroitin sulfate proteoglycan 5 (Cspg5), transcript variant 1, mRNA [NM_019284]	**0,562**
Ogfod1	Rattus norvegicus 2-oxoglutarate and iron-dependent oxygenase domain containing 1 (Ogfod1), mRNA [NM_001107411]	**0,562**
Fam13a1	Rattus norvegicus family with sequence similarity 13, member A1 (Fam13a1), mRNA [NM_001100862]	**0,562**
Zfp407	PREDICTED: Rattus norvegicus zinc finger protein 407 (Zfp407), mRNA [XM_225679]	**0,562**
Slc35e1	Rattus norvegicus solute carrier family 35, member E1 (Slc35e1), mRNA [NM_001109107]	**0,562**
Ampd1	Rattus norvegicus adenosine monophosphate deaminase 1 (isoform M) (Ampd1), mRNA [NM_138876]	**0,562**
Plch1	Rattus norvegicus TL0AAA86YN08 mRNA sequence. [FQ211591]	**0,562**
Siat7F	Rattus norvegicus sialyltransferase 7F (Siat7F), mRNA [NM_001015036]	**0,562**
Gba2	Rattus norvegicus glucosidase beta 2 (Gba2), mRNA [NM_001013091]	**0,562**
Lrrc33	Rattus norvegicus leucine rich repeat containing 33 (Lrrc33), mRNA [NM_001024995]	**0,562**
Wdr60	Rattus norvegicus WD repeat domain 60 (Wdr60), mRNA [NM_001191773]	**0,562**
Cnr1	Rattus norvegicus cannabinoid receptor 1 (brain) (Cnr1), mRNA [NM_012784]	**0,562**
0	Rattus norvegicus similar to 60S ribosomal protein L10 (QM protein homolog) (LOC366036), mRNA [XM_345350]	**0,562**
Mrpl10	Rattus norvegicus mitochondrial ribosomal protein L10 (Mrpl10), nuclear gene encoding mitochondrial protein, mRNA [NM_001109620]	**0,562**
G4	Rattus norvegicus G4 protein (G4), mRNA [NM_001003975]	**0,562**
RGD1311745	Rattus norvegicus similar to RIKEN cDNA 1110059G10 (RGD1311745), mRNA [NM_001008329]	**0,562**

Kif5b	Rattus norvegicus kinesin family member 5B (Kif5b), mRNA [NM_057202]	**0,562**
Vcam1	Rattus norvegicus vascular cell adhesion molecule 1 (Vcam1), mRNA [NM_012889]	**0,562**
Mier2	Rattus norvegicus mesoderm induction early response 1, family member 2 (Mier2), mRNA [NM_001108737]	**0,562**
Slc12a4	Rattus norvegicus solute carrier family 12 (potassium/chloride transporters), member 4 (Slc12a4), mRNA [NM_019229]	**0,562**
Lipa	Rattus norvegicus lipase A, lysosomal acid, cholesterol esterase (Lipa), mRNA [NM_012732]	**0,562**
Rasl11b	Rattus norvegicus RAS-like family 11 member B (Rasl11b), mRNA [NM_001002830]	**0,562**
0	Unknown	**0,562**
0	Protein tyrosine phosphatase delta [Source:UniProtKB/TrEMBL;Acc:Q62990] [ENSRNOT00000025426]	**0,562**
0	Uncharacterized protein [Source:UniProtKB/TrEMBL;Acc:D3ZET0] [ENSRNOT00000035939]	**0,562**
Kifap3	Rattus norvegicus kinesin-associated protein 3 (Kifap3), mRNA [NM_001105964]	**0,562**
Mrpl49	Rattus norvegicus mitochondrial ribosomal protein L49 (Mrpl49), nuclear gene encoding mitochondrial protein, mRNA [NM_001047883]	**0,562**
0	Unknown	**0,562**
Nelf	Rattus norvegicus nasal embryonic LHRH factor (Nelf), mRNA [NM_057190]	**0,562**
LOC100361404	PREDICTED: Rattus norvegicus hypothetical protein LOC100361404 (LOC100361404), mRNA [XM_002729057]	**0,562**
Tex264	Rattus norvegicus testis expressed 264 (Tex264), mRNA [NM_001007665]	**0,562**
Syt8	Rattus norvegicus synaptotagmin VIII (Syt8), mRNA [NM_053325]	**0,562**
Pold1	Rattus norvegicus polymerase (DNA directed), delta 1, catalytic subunit (Pold1), mRNA [NM_021662]	**0,562**
Pan3	PREDICTED: Rattus norvegicus PAN3 polyA specific ribonuclease subunit homolog (S. cerevisiae), transcript variant 2 (Pan3), mRNA [XM_346914]	**0,563**
R3hcc1	PREDICTED: Rattus norvegicus R3H domain and coiled-coil containing 1 (R3hcc1), mRNA [XM_001069252]	**0,563**
Pyroxd2	Rattus norvegicus pyridine nucleotide-disulphide oxidoreductase domain 2 (Pyroxd2), mRNA [NM_001004261]	**0,563**
Pard6g	Rattus norvegicus par-6 partitioning defective 6 homolog gamma (C. elegans) (Pard6g), mRNA [NM_001100973]	**0,563**
Nxf1	Rattus norvegicus nuclear RNA export factor 1 (Nxf1), mRNA [NM_021579]	**0,563**
Arse	Rattus norvegicus arylsulfatase E (chondrodysplasia punctata 1) (Arse), mRNA [NM_001047885]	**0,563**
Ncoa1	Rattus norvegicus nuclear receptor coactivator 1 (Ncoa1), mRNA [NM_001108012]	**0,563**
0	RCG49325, isoform CRA_bUncharacterized protein [Source:UniProtKB/TrEMBL;Acc:D3ZR95] [ENSRNOT00000025495]	**0,563**
Galnt1	Rattus norvegicus UDP-N-acetyl-alpha-D-galactosamine:polypeptide N-acetylgalactosaminyltransferase 1 (GalNAc-T1) (Galnt1), mRNA [NM_024373]	**0,563**
Cyld	Rattus norvegicus cylindromatosis (turban tumor syndrome) (Cyld), mRNA [NM_001017380]	**0,563**
Pfdn1	Rattus norvegicus prefoldin subunit 1 (Pfdn1), mRNA [NM_001108427]	**0,563**
Nlrx1	Rattus norvegicus NLR family member X1 (Nlrx1), mRNA [NM_001025010]	**0,563**
Yrdc	Rattus norvegicus yrdC domain containing (E.coli) (Yrdc), nuclear gene encoding mitochondrial protein, mRNA [NM_175604]	**0,563**
Zmat3	Rattus norvegicus zinc finger, matrin type 3 (Zmat3), mRNA [NM_022548]	**0,563**
Lmln	Rattus norvegicus leishmanolysin-like (metallopeptidase M8 family) (Lmln), mRNA [NM_001108843]	**0,563**

Phf5a	Rattus norvegicus PHD finger protein 5A (Phf5a), mRNA [NM_138888]	**0,563**
Pdcd7	Rattus norvegicus programmed cell death 7 (Pdcd7), mRNA [NM_001108768]	**0,563**
Glt25d1	Rattus norvegicus glycosyltransferase 25 domain containing 1 (Glt25d1), mRNA [NM_001106067]	**0,563**
RGD1311066	RGD1311066 proteinSimilar to RIKEN cDNA 0610011L14 gene [Source:UniProtKB/TrEMBL;Acc:B1WC03] [ENSRNOT00000027369]	**0,563**
0	Unknown	**0,563**
Mat2b	Rattus norvegicus methionine adenosyltransferase II, beta (Mat2b), mRNA [NM_001044282]	**0,563**
Msra	Rattus norvegicus methionine sulfoxide reductase A (Msra), mRNA [NM_053307]	**0,563**
Rbp4	Rattus norvegicus retinol binding protein 4, plasma (Rbp4), mRNA [NM_013162]	**0,563**
Spcs2	Rattus norvegicus signal peptidase complex subunit 2 homolog (S. cerevisiae) (Spcs2), mRNA [NM_001191601]	**0,563**
Cryl1	Rattus norvegicus crystallin, lambda 1 (Cryl1), mRNA [NM_175757]	**0,563**
0	Uncharacterized protein [Source:UniProtKB/TrEMBL;Acc:D3ZZF2] [ENSRNOT00000005894]	**0,563**
0	Unknown	**0,563**
0	Uncharacterized protein [Source:UniProtKB/TrEMBL;Acc:D3ZN76] [ENSRNOT00000026111]	**0,563**
Srpr	Rattus norvegicus signal recognition particle receptor ('docking protein') (Srpr), mRNA [NM_001034150]	**0,563**
C1H6orf35	Rattus norvegicus chromosome 1 open reading frame, human C6orf35 (C1H6orf35), mRNA [NM_001144860]	**0,563**
Dnajc6	Rattus norvegicus DnaJ (Hsp40) homolog, subfamily C, member 6 (Dnajc6), mRNA [NM_001107949]	**0,563**
LOC494539	PREDICTED: Rattus norvegicus spectrin beta-like (LOC494539), miscRNA [XR_085708]	**0,563**
Rab39	Rattus norvegicus RAB39, member RAS oncogene family (Rab39), mRNA [NM_001108148]	**0,563**
Usp32	Rattus norvegicus ubiquitin specific peptidase 32 (Usp32), mRNA [NM_001107032]	**0,563**
Efr3a	Rattus norvegicus EFR3 homolog A (S. cerevisiae) (Efr3a), mRNA [NM_001130564]	**0,563**
Tle1	Rattus norvegicus transducin-like enhancer of split 1 (E(sp1) homolog, Drosophila) (Tle1), mRNA [NM_001173433]	**0,563**
0	TULP3_MOUSE (O88413) Tubby-related protein 3 (Tubby-like protein 3), partial (40%) [TC600284]	**0,563**
0	Unknown	**0,563**
Narg1	Rattus norvegicus NMDA receptor regulated 1 (Narg1), mRNA [NM_001107674]	**0,563**
Ints12	Rattus norvegicus integrator complex subunit 12 (Ints12), mRNA [NM_001007640]	**0,563**
Lypd1	Rattus norvegicus Ly6/Plaur domain containing 1 (Lypd1), mRNA [NM_001007727]	**0,564**
0	Rattus norvegicus similar to Proteasome activator complex subunit 2 (Proteasome activator 28-beta subunit) (PA28beta) (PA28b) (Activator of multicatalytic protease subunit 2) (11S regulator complex beta subunit) (REG-beta) (LOC304754), mRNA [XM_222590]	**0,564**
Zdhhc1	Rattus norvegicus zinc finger, DHHC-type containing 1 (Zdhhc1), mRNA [NM_001039099]	**0,564**
St3gal5	Rattus norvegicus ST3 beta-galactoside alpha-2,3-sialyltransferase 5 (St3gal5), mRNA [NM_031337]	**0,564**
Klhl25	Rattus norvegicus kelch-like 25 (Drosophila) (Klhl25), mRNA [NM_001039006]	**0,564**
Srek1ip1	Rattus norvegicus splicing regulatory glutamine/lysine-rich protein 1 interacting protein 1 (Srek1ip1), mRNA [NM_001008373]	**0,564**
MGC94542	Rattus norvegicus similar to RIKEN cDNA 5430437P03 (MGC94542), mRNA [NM_001006964]	**0,564**
0	Unknown	**0,564**

LOC100362820	PREDICTED: Rattus norvegicus Protein FAM186A-like (LOC100362820), mRNA [XM_002729856]	**0,564**
Cd93	Rattus norvegicus CD93 molecule (Cd93), mRNA [NM_053383]	**0,564**
Ptpn21	Rattus norvegicus protein tyrosine phosphatase, non-receptor type 21 (Ptpn21), mRNA [NM_133545]	**0,564**
Fbxo6	Rattus norvegicus F-box protein 6 (Fbxo6), mRNA [NM_138917]	**0,564**
Gpx8	Rattus norvegicus glutathione peroxidase 8 (Gpx8), mRNA [NM_001106411]	**0,564**
Dmtf1	Rattus norvegicus cyclin D binding myb-like transcription factor 1 (Dmtf1), mRNA [NM_053693]	**0,564**
Nxph1	Rattus norvegicus neurexophilin 1 (Nxph1), mRNA [NM_012994]	**0,564**
Zfp189	Rattus norvegicus zinc finger protein 189 (Zfp189), mRNA [NM_001107930]	**0,564**
Tnfrsf12a	Rattus norvegicus tumor necrosis factor receptor superfamily, member 12a (Tnfrsf12a), mRNA [NM_181086]	**0,564**
KIFC2	Rattus norvegicus kinesin family member C2 (KIFC2), mRNA [NM_198752]	**0,564**
Apln	Rattus norvegicus apelin (Apln), mRNA [NM_031612]	**0,564**
Mrpl23	Rattus norvegicus mitochondrial ribosomal protein L23 (Mrpl23), nuclear gene encoding mitochondrial protein, mRNA [NM_022529]	**0,564**
Rpl10	Rattus norvegicus ribosomal protein L10 (Rpl10), mRNA [NM_031100]	**0,564**
Pcdhga1	Rattus norvegicus protocadherin gamma subfamily A, 1 (Pcdhga1), mRNA [NM_001037140]	**0,564**
Rpap1	Rattus norvegicus RNA polymerase II associated protein 1 (Rpap1), mRNA [NM_001033999]	**0,564**
Zcchc2	Rattus norvegicus zinc finger, CCHC domain containing 2 (Zcchc2), mRNA [NM_001122677]	**0,564**
Hps5	Rattus norvegicus Hermansky-Pudlak syndrome 5 (Hps5), mRNA [NM_001135612]	**0,564**
Tead2	Rattus norvegicus TEA domain family member 2 (Tead2), mRNA [NM_001107512]	**0,564**
Coro7	Rattus norvegicus coronin 7 (Coro7), mRNA [NM_001191639]	**0,564**
Igsf1	Rattus norvegicus immunoglobulin superfamily, member 1 (Igsf1), mRNA [NM_175763]	**0,564**
Myeov2	Rattus norvegicus myeloma overexpressed 2 (Myeov2), mRNA [NM_001109044]	**0,564**
Farp1	Rattus norvegicus FERM, RhoGEF (Arhgef) and pleckstrin domain protein 1 (chondrocyte-derived) (Farp1), mRNA [NM_001107287]	**0,564**
Arfgap2	Rattus norvegicus ADP-ribosylation factor GTPase activating protein 2 (Arfgap2), mRNA [NM_001033707]	**0,564**
Arhgef3	Rattus norvegicus Rho guanine nucleotide exchange factor (GEF) 3 (Arhgef3), mRNA [NM_001106061]	**0,564**
0	Uncharacterized protein [Source:UniProtKB/TrEMBL;Acc:D4AA66] [ENSRNOT00000056882]	**0,564**
Klhl2	PREDICTED: Rattus norvegicus kelch-like 2, Mayven (Drosophila) (Klhl2), mRNA [XM_001073589]	**0,564**
Gadd45a	Rattus norvegicus growth arrest and DNA-damage-inducible, alpha (Gadd45a), mRNA [NM_024127]	**0,564**
Tcea1	Rattus norvegicus transcription elongation factor A (SII) 1 (Tcea1), mRNA [NM_001025735]	**0,564**
Abhd13	Rattus norvegicus abhydrolase domain containing 13 (Abhd13), mRNA [NM_001107327]	**0,564**
Mon1a	Rattus norvegicus MON1 homolog A (yeast) (Mon1a), mRNA [NM_001126284]	**0,564**
Gpc1	Rattus norvegicus glypican 1 (Gpc1), mRNA [NM_030828]	**0,564**
Dis3l2	Rattus norvegicus DIS3 mitotic control homolog (S. cerevisiae)-like 2 (Dis3l2), transcript variant 1, mRNA [NM_001109007]	**0,564**
Cse1l	Rattus norvegicus CSE1 chromosome segregation 1-like (yeast) (Cse1l), mRNA [NM_001108607]	**0,564**
0	Unknown	**0,564**

0	Similar to oxysterol-binding protein-like protein 8 isoform a (Similar to oxysterol-binding protein-like protein 8 isoform a (Predicted), isoform CRA_a) [Source:UniProtKB/TrEMBL;Acc:B5DF26] [ENSRNOT00000029993]	**0,564**
Fam32a	Rattus norvegicus family with sequence similarity 32, member A (Fam32a), mRNA [NM_001128078]	**0,564**
Sgtb	Rattus norvegicus small glutamine-rich tetratricopeptide repeat (TPR)-containing, beta (Sgtb), mRNA [NM_181629]	**0,564**
0	Unknown	**0,564**
Atp5i	Rattus norvegicus ATP synthase, H+ transporting, mitochondrial F0 complex, subunit E (Atp5i), nuclear gene encoding mitochondrial protein, mRNA [NM_080481]	**0,565**
Grk5	Rattus norvegicus G protein-coupled receptor kinase 5 (Grk5), mRNA [NM_030829]	**0,565**
Tmem87b	Rattus norvegicus transmembrane protein 87B (Tmem87b), mRNA [NM_001191854]	**0,565**
Gtf3c2	Rattus norvegicus general transcription factor IIIC, polypeptide 2, beta (Gtf3c2), mRNA [NM_001025120]	**0,565**
Ipo13	Rattus norvegicus importin 13 (Ipo13), mRNA [NM_053778]	**0,565**
Acad11	Rattus norvegicus acyl-Coenzyme A dehydrogenase family, member 11 (Acad11), mRNA [NM_001108181]	**0,565**
Heatr5b	Rattus norvegicus HEAT repeat containing 5B (Heatr5b), mRNA [NM_001191064]	**0,565**
Mt3	Rattus norvegicus metallothionein 3 (Mt3), mRNA [NM_053968]	**0,565**
Adcy7	Rattus norvegicus adenylate cyclase 7 (Adcy7), mRNA [NM_053396]	**0,565**
Ccnt2	Rattus norvegicus cyclin T2 (Ccnt2), mRNA [NM_001107171]	**0,565**
Srp72	Rattus norvegicus signal recognition particle 72 (Srp72), mRNA [NM_001170601]	**0,565**
0	RVL16467 Wackym-Soares normalized rat vestibular cDNA library Rattus norvegicus cDNA 5', mRNA sequence [DV724416]	**0,565**
0	Q84NG9_VITVI (Q84NG9) 2S albumin, partial (6%) [TC640666]	**0,565**
Rnf167	Rattus norvegicus ring finger protein 167 (Rnf167), mRNA [NM_001008361]	**0,565**
Grinl1a	Rattus norvegicus glutamate receptor, ionotropic, N-methyl D-aspartate-like 1A (Grinl1a), mRNA [NM_183402]	**0,565**
Eml1	Rattus norvegicus echinoderm microtubule associated protein like 1 (Eml1), mRNA [NM_001025741]	**0,565**
Dll3	Rattus norvegicus delta-like 3 (Drosophila) (Dll3), mRNA [NM_053666]	**0,565**
Zfp423	Rattus norvegicus zinc finger protein 423 (Zfp423), mRNA [NM_053583]	**0,565**
Bat5	Rattus norvegicus HLA-B associated transcript 5 (Bat5), mRNA [NM_212531]	**0,565**
0	Unknown	**0,565**
Pnck	Rattus norvegicus pregnancy upregulated non-ubiquitously expressed CaM kinase (Pnck), mRNA [NM_017275]	**0,565**
LOC684755	PREDICTED: Rattus norvegicus similar to zinc finger like protein 1, transcript variant 3 (LOC684755), mRNA [XM_001075288]	**0,565**
B4galt5	Rattus norvegicus UDP-Gal:betaGlcNAc beta 1,4-galactosyltransferase, polypeptide 5 (B4galt5), mRNA [NM_001108608]	**0,565**
Mkrn2	Rattus norvegicus makorin, ring finger protein, 2 (Mkrn2), mRNA [NM_001008314]	**0,565**
Csrp2	Rattus norvegicus cysteine and glycine-rich protein 2 (Csrp2), mRNA [NM_177425]	**0,565**
Mtdh	Rattus norvegicus metadherin (Mtdh), mRNA [NM_133398]	**0,565**
Psme4	Rattus norvegicus proteasome (prosome, macropain) activator subunit 4 (Psme4), mRNA [NM_001025140]	**0,565**
Alkbh6	Rattus norvegicus alkB, alkylation repair homolog 6 (E. coli) (Alkbh6), mRNA [NM_001127450]	**0,565**

Stk38l	Rattus norvegicus serine/threonine kinase 38 like (Stk38l), mRNA [NM_001083336]	**0,565**
RGD1308626	Rattus norvegicus similar to 9630044O09Rik protein (RGD1308626), mRNA [NM_001014139]	**0,565**
Cul4a	Rattus norvegicus cullin 4A (Cul4a), mRNA [NM_001127301]	**0,565**
Pcnxl3	Uncharacterized protein [Source:UniProtKB/TrEMBL;Acc:D3ZSQ1] [ENSRNOT00000064136]	**0,565**
Piga	Rattus norvegicus phosphatidylinositol glycan anchor biosynthesis, class A (Piga), mRNA [NM_001108816]	**0,565**
LOC685563	PREDICTED: Rattus norvegicus similar to Discs large homolog 5 (Placenta and prostate DLG) (Discs large protein P-dlg) (LOC685563), mRNA [XM_001064312]	**0,565**
Sdhd	Rattus norvegicus succinate dehydrogenase complex, subunit D, integral membrane protein (Sdhd), nuclear gene encoding mitochondrial protein, mRNA [NM_198788]	**0,565**
Pomc	Rattus norvegicus proopiomelanocortin (Pomc), mRNA [NM_139326]	**0,565**
Fam131b	Rattus norvegicus family with sequence similarity 131, member B (Fam131b), mRNA [NM_001025046]	**0,565**
Znf521	Rattus norvegicus zinc finger protein 521 (Znf521), mRNA [NM_001107403]	**0,565**
Cited1	Rattus norvegicus Cbp/p300-interacting transactivator with Glu/Asp-rich carboxy-terminal domain 1 (Cited1), mRNA [NM_172055]	**0,565**
Hnrnph2	Rattus norvegicus heterogeneous nuclear ribonucleoprotein H2 (H') (Hnrnph2), mRNA [NM_001014019]	**0,565**
Slc12a2	Rattus norvegicus solute carrier family 12 (sodium/potassium/chloride transporters), member 2 (Slc12a2), mRNA [NM_031798]	**0,566**
Reps1	Rattus norvegicus RALBP1 associated Eps domain containing 1 (Reps1), mRNA [NM_001106264]	**0,566**
Aifm2	Rattus norvegicus apoptosis-inducing factor, mitochondrion-associated 2 (Aifm2), nuclear gene encoding mitochondrial protein, mRNA [NM_001139483]	**0,566**
Ebi3	Rattus norvegicus Epstein-Barr virus induced 3 (Ebi3), mRNA [NM_001109421]	**0,566**
Prkd2	Rattus norvegicus protein kinase D2 (Prkd2), mRNA [NM_001013895]	**0,566**
Dnlz	Rattus norvegicus DNL-type zinc finger (Dnlz), mRNA [NM_001130990]	**0,566**
Tmbim6	Rattus norvegicus transmembrane BAX inhibitor motif containing 6 (Tmbim6), mRNA [NM_019381]	**0,566**
Tle2	Rattus norvegicus transducin-like enhancer of split 2 (E(sp1) homolog, Drosophila) (Tle2), mRNA [NM_001039013]	**0,566**
Sgpp1	PREDICTED: Rattus norvegicus sphingosine-1-phosphate phosphatase 1 (Sgpp1), mRNA [XM_001080791]	**0,566**
Fbxo34	Rattus norvegicus F-box protein 34 (Fbxo34), mRNA [NM_001107257]	**0,566**
Skp2	Rattus norvegicus S-phase kinase-associated protein 2 (p45) (Skp2), mRNA [NM_001106416]	**0,566**
Cndp2	Rattus norvegicus CNDP dipeptidase 2 (metallopeptidase M20 family) (Cndp2), mRNA [NM_001010920]	**0,566**
Eapp	Rattus norvegicus E2F-associated phosphoprotein (Eapp), transcript variant 1, mRNA [NM_001134987]	**0,566**
Map1s	Rattus norvegicus microtubule-associated protein 1S (Map1s), mRNA [NM_001106070]	**0,566**
Vapa	Rattus norvegicus VAMP (vesicle-associated membrane protein)-associated protein A (Vapa), mRNA [NM_031631]	**0,566**
Letmd1	Rattus norvegicus LETM1 domain containing 1 (Letmd1), mRNA [NM_001122781]	**0,566**
Csrnp1	Rattus norvegicus cysteine-serine-rich nuclear protein 1 (Csrnp1), mRNA [NM_001108786]	**0,566**
0	Uncharacterized protein [Source:UniProtKB/TrEMBL;Acc:D3ZUQ8] [ENSRNOT00000032636]	**0,566**
Alg11	Rattus norvegicus asparagine-linked glycosylation 11, alpha-1,2-mannosyltransferase homolog (yeast) (Alg11), mRNA [NM_001108401]	**0,566**

Rdh11	Rattus norvegicus retinol dehydrogenase 11 (all-trans/9-cis/11-cis) (Rdh11), mRNA [NM_001012193]	**0,566**
RGD1560108	Rattus norvegicus similar to RIKEN cDNA 2700081O15 (RGD1560108), mRNA [NM_001109161]	**0,566**
37500	Rattus norvegicus septin 2 (Sept2), mRNA [NM_057148]	**0,566**
Fstl1	Rattus norvegicus follistatin-like 1 (Fstl1), mRNA [NM_024369]	**0,566**
Cyp4f4	Rattus norvegicus cytochrome P450, family 4, subfamily f, polypeptide 4 (Cyp4f4), mRNA [NM_173123]	**0,566**
LOC619574	Rattus norvegicus hypothetical protein LOC619574 (LOC619574), mRNA [NM_001034959]	**0,566**
Lass1	Rattus norvegicus LAG1 homolog, ceramide synthase 1 (Lass1), mRNA [NM_001044230]	**0,566**
0	Uncharacterized protein [Source:UniProtKB/TrEMBL;Acc:D3Z8H1] [ENSRNOT00000027841]	**0,566**
Gpr180	Rattus norvegicus G protein-coupled receptor 180 (Gpr180), mRNA [NM_001006994]	**0,566**
Ctsd	Rattus norvegicus cathepsin D (Ctsd), mRNA [NM_134334]	**0,566**
Grk4	Rattus norvegicus G protein-coupled receptor kinase 4 (Grk4), mRNA [NM_022928]	**0,566**
Pigl	Rattus norvegicus phosphatidylinositol glycan anchor biosynthesis, class L (Pigl), mRNA [NM_138901]	**0,566**
Fam160b1	LOC361774 (Predicted)Uncharacterized protein [Source:UniProtKB/TrEMBL;Acc:D4A3I5] [ENSRNOT00000023247]	**0,566**
0	Q63ZX6_MOUSE (Q63ZX6) Phf20l1 protein (Fragment), partial (4%) [TC630334]	**0,566**
Rpl18a	Rattus norvegicus ribosomal protein L18A (Rpl18a), mRNA [NM_212510]	**0,566**
Eml5	PREDICTED: Rattus norvegicus echinoderm microtubule associated protein like 5, transcript variant 2 (Eml5), mRNA [XM_001064078]	**0,566**
Yaf2	Rattus norvegicus YY1 associated factor 2 (Yaf2), mRNA [NM_001134871]	**0,566**
Fbxo7	Rattus norvegicus F-box protein 7 (Fbxo7), mRNA [NM_001012222]	**0,566**
0	Unknown	**0,566**
Phyhd1	Rattus norvegicus phytanoyl-CoA dioxygenase domain containing 1 (Phyhd1), mRNA [NM_001013081]	**0,566**
LOC257650	Rattus norvegicus hippyragranin (LOC257650), mRNA [NM_147142]	**0,566**
RGD1359108	Rattus norvegicus similar to RIKEN cDNA 3110043O21 (RGD1359108), mRNA [NM_001007702]	**0,566**
Prkab1	Rattus norvegicus protein kinase, AMP-activated, beta 1 non-catalytic subunit (Prkab1), mRNA [NM_031976]	**0,566**
Ppp1r14b	Rattus norvegicus protein phosphatase 1, regulatory (inhibitor) subunit 14B (Ppp1r14b), mRNA [NM_172045]	**0,566**
Kctd19	PREDICTED: Rattus norvegicus potassium channel tetramerisation domain containing 19 (Kctd19), mRNA [XM_001075666]	**0,566**
LOC100363421	PREDICTED: Rattus norvegicus hypothetical protein LOC100363421 (LOC100363421), mRNA [XM_002726502]	**0,567**
0	Unknown	**0,567**
Ptges3	Rattus norvegicus prostaglandin E synthase 3 (cytosolic) (Ptges3), mRNA [NM_001130989]	**0,567**
Eno1	Rattus norvegicus enolase 1, (alpha), mRNA (cDNA clone MGC:93770 IMAGE:7108936), complete cds. [BC090069]	**0,567**
Amz2	Rattus norvegicus archaelysin family metallopeptidase 2 (Amz2), mRNA [NM_001014121]	**0,567**
Elovl6	Rattus norvegicus ELOVL family member 6, elongation of long chain fatty acids (yeast) (Elovl6), mRNA [NM_134383]	**0,567**
Tex261	Rattus norvegicus testis expressed 261 (Tex261), mRNA [NM_001017537]	**0,567**
RGD1564228	PREDICTED: Rattus norvegicus similar to Naglu (RGD1564228), mRNA [XM_001081442]	**0,567**
Rsrc1	Rattus norvegicus arginine/serine-rich coiled-coil 1 (Rsrc1), mRNA [NM_001014172]	**0,567**

0	Uncharacterized protein [Source:UniProtKB/TrEMBL;Acc:D3ZFY7] [ENSRNOT00000018667]	**0,567**
Isoc2b	Rattus norvegicus isochorismatase domain containing 2b (Isoc2b), mRNA [NM_001008367]	**0,567**
Taf12	Rattus norvegicus TAF12 RNA polymerase II, TATA box binding protein (TBP)-associated factor (Taf12), mRNA [NM_001115036]	**0,567**
0	Unknown	**0,567**
Adcyap1	Rattus norvegicus adenylate cyclase activating polypeptide 1 (Adcyap1), mRNA [NM_016989]	**0,567**
Tubd1	Rattus norvegicus tubulin, delta 1 (Tubd1), mRNA [NM_001105826]	**0,567**
Sin3a	Rattus norvegicus SIN3 homolog A, transcription regulator (yeast) (Sin3a), mRNA [NM_001108761]	**0,567**
Stau2	Rattus norvegicus staufen, RNA binding protein, homolog 2 (Drosophila) (Stau2), transcript variant LS, mRNA [NM_134466]	**0,567**
Glyctk	Rattus norvegicus glycerate kinase (Glyctk), mRNA [NM_001109449]	**0,567**
Fbxl4	Rattus norvegicus F-box and leucine-rich repeat protein 4 (Fbxl4), mRNA [NM_001107919]	**0,567**
Slc7a10	Rattus norvegicus solute carrier family 7, (neutral amino acid transporter, y+ system) member 10 (Slc7a10), mRNA [NM_053726]	**0,567**
Arid5a	Rattus norvegicus AT rich interactive domain 5A (Mrf1 like) (Arid5a), mRNA [NM_001034934]	**0,567**
Nedd8	Rattus norvegicus neural precursor cell expressed, developmentally down-regulated 8 (Nedd8), mRNA [NM_138878]	**0,567**
Slc30a6	Rattus norvegicus solute carrier family 30 (zinc transporter), member 6 (Slc30a6), mRNA [NM_001106708]	**0,567**
0	Unknown	**0,567**
Chchd8	Rattus norvegicus coiled-coil-helix-coiled-coil-helix domain containing 8 (Chchd8), mRNA [NM_001127655]	**0,567**
Tshr	Rattus norvegicus thyroid stimulating hormone receptor (Tshr), mRNA [NM_012888]	**0,567**
Vstm2b	Rattus norvegicus V-set and transmembrane domain containing 2B (Vstm2b), mRNA [NM_001108479]	**0,567**
Wbp11	Rattus norvegicus WW domain binding protein 11 (Wbp11), mRNA [NM_001009661]	**0,567**
LOC680498	PREDICTED: Rattus norvegicus similar to CG31613-PA (LOC680498), mRNA [XM_001057443]	**0,567**
Nupl1	Rattus norvegicus nucleoporin like 1 (Nupl1), mRNA [NM_139091]	**0,567**
Ipo5	Uncharacterized protein [Source:UniProtKB/TrEMBL;Acc:D4A781] [ENSRNOT00000014859]	**0,567**
Lrrc8d	Rattus norvegicus leucine rich repeat containing 8 family, member D (Lrrc8d), mRNA [NM_001008338]	**0,567**
Ube2e2	Rattus norvegicus ubiquitin-conjugating enzyme E2E 2 (UBC4/5 homolog, yeast) (Ube2e2), mRNA [NM_001108371]	**0,567**
Pigs	Rattus norvegicus phosphatidylinositol glycan anchor biosynthesis, class S (Pigs), mRNA [NM_001006602]	**0,567**
Hbs1l	Rattus norvegicus Hbs1-like (S. cerevisiae) (Hbs1l), mRNA [NM_001011934]	**0,567**
Atad1	Rattus norvegicus ATPase family, AAA domain containing 1 (Atad1), mRNA [NM_001035002]	**0,567**
Ndufa10l1	Rattus norvegicus NADH dehydrogenase (ubiquinone) 1 alpha subcomplex 10-like 1 (Ndufa10l1), mRNA [NM_182671]	**0,567**
Nrbf2	Rattus norvegicus nuclear receptor binding factor 2 (Nrbf2), mRNA [NM_022186]	**0,567**
Ttc19	Rattus norvegicus tetratricopeptide repeat domain 19 (Ttc19), mRNA [NM_001109644]	**0,567**
Itgb3bp	Rattus norvegicus integrin beta 3 binding protein (beta3-endonexin) (Itgb3bp), mRNA [NM_001013213]	**0,567**
RGD1560891	RCG36219Uncharacterized protein [Source:UniProtKB/TrEMBL;Acc:D4ACQ4] [ENSRNOT00000044006]	**0,567**
Stat5a	Rattus norvegicus signal transducer and activator of transcription 5A (Stat5a), mRNA [NM_017064]	**0,567**
Mtf2	Rattus norvegicus metal response element binding transcription factor 2 (Mtf2), mRNA [NM_001100898]	**0,567**

0	Q3FJK6_9BURK (Q3FJK6) Biotin--acetyl-CoA-carboxylase ligase, partial (5%) [TC585832]	**0,567**
Ube4a	Rattus norvegicus ubiquitination factor E4A (UFD2 homolog, yeast) (Ube4a), mRNA [NM_207610]	**0,568**
Dusp9	Rattus norvegicus dual specificity phosphatase 9 (Dusp9), mRNA [NM_001037973]	**0,568**
Nfkbid	PREDICTED: Rattus norvegicus nuclear factor of kappa light polypeptide gene enhancer in B-cells inhibitor, delta (Nfkbid), mRNA [XM_001079086]	**0,568**
Mut	PREDICTED: Rattus norvegicus methylmalonyl-Coenzyme A mutase (Mut), mRNA [XM_001067239]	**0,568**
Nudcd3	Rattus norvegicus NudC domain containing 3 (Nudcd3), mRNA [NM_001103362]	**0,568**
Cul4b	Rattus norvegicus cullin 4B (Cul4b), mRNA [NM_001106951]	**0,568**
0	Unknown	**0,568**
Pphln1	Rattus norvegicus periphilin 1 (Pphln1), mRNA [NM_001108992]	**0,568**
0	Rattus norvegicus similar to 60S ribosomal protein L12 (LOC296165), mRNA [XM_215850]	**0,568**
0	Rattus norvegicus TL0AEA74YH08 mRNA sequence. [FQ231255]	**0,568**
Abca5	Rattus norvegicus ATP-binding cassette, subfamily A (ABC1), member 5 (Abca5), mRNA [NM_173307]	**0,568**
Rfc1	Rattus norvegicus replication factor C (activator 1) 1 (Rfc1), mRNA [NM_053547]	**0,568**
Mettl14	Rattus norvegicus methyltransferase like 14 (Mettl14), mRNA [NM_001106470]	**0,568**
Cables1	Rattus norvegicus Cdk5 and Abl enzyme substrate 1 (Cables1), mRNA [NM_001107404]	**0,568**
Copg	Rattus norvegicus coatomer protein complex, subunit gamma (Copg), mRNA [NM_001031822]	**0,568**
Rin2	Rattus norvegicus Ras and Rab interactor 2 (Rin2), mRNA [NM_001107786]	**0,568**
Nrp1	Rattus norvegicus neuropilin 1 (Nrp1), mRNA [NM_145098]	**0,568**
Mcm7	Rattus norvegicus minichromosome maintenance complex component 7 (Mcm7), mRNA [NM_001004203]	**0,568**
Entpd5	Rattus norvegicus ectonucleoside triphosphate diphosphohydrolase 5 (Entpd5), mRNA [NM_199394]	**0,568**
Pgrmc2	Rattus norvegicus progesterone receptor membrane component 2 (Pgrmc2), mRNA [NM_001008374]	**0,568**
Chek2	Rattus norvegicus CHK2 checkpoint homolog (S. pombe) (Chek2), mRNA [NM_053677]	**0,568**
Znf608	Rattus norvegicus zinc finger protein 608 (Znf608), mRNA [NM_001107378]	**0,568**
Secisbp2l	Rattus norvegicus SECIS binding protein 2-like (Secisbp2l), mRNA [NM_001168527]	**0,568**
0	Unknown	**0,568**
0	KIAA1454-like protein [Source:UniProtKB/TrEMBL;Acc:Q99MF7] [ENSRNOT00000009385]	**0,568**
LOC361016	Rattus norvegicus similar to RIKEN cDNA 4933406L09, mRNA (cDNA clone IMAGE:7301570), complete cds. [BC086559]	**0,568**
Znf213	Rattus norvegicus zinc finger protein 213 (Znf213), mRNA [NM_001105764]	**0,568**
Mpdu1	Rattus norvegicus mannose-P-dolichol utilization defect 1 (Mpdu1), mRNA [NM_001107011]	**0,568**
RGD1565033	Rattus norvegicus similar to hypothetical protein LOC284018 isoform b (RGD1565033), mRNA [NM_001109050]	**0,568**
Ssfa2	Rattus norvegicus sperm specific antigen 2 (Ssfa2), mRNA [NM_001107738]	**0,568**
Fam96b	Rattus norvegicus family with sequence similarity 96, member B (Fam96b), mRNA [NM_001144854]	**0,568**
Nradd	Rattus norvegicus neurotrophin receptor associated death domain (Nradd), mRNA [NM_139259]	**0,568**

Slc25a22	Rattus norvegicus solute carrier family 25 (mitochondrial carrier, glutamate), member 22 (Slc25a22), nuclear gene encoding mitochondrial protein, mRNA [NM_001014027]	**0,568**
Gng5	Rattus norvegicus guanine nucleotide binding protein (G protein), gamma 5 (Gng5), mRNA [NM_024377]	**0,568**
Stx12	Rattus norvegicus syntaxin 12 (Stx12), mRNA [NM_022939]	**0,568**
Rsbn1l	Rattus norvegicus round spermatid basic protein 1-like (Rsbn1l), mRNA [NM_001135872]	**0,568**
Serp1	Rattus norvegicus stress-associated endoplasmic reticulum protein 1 (Serp1), mRNA [NM_030835]	**0,568**
Ccdc23	Rattus norvegicus coiled-coil domain containing 23 (Ccdc23), transcript variant 1, mRNA [NM_001038994]	**0,568**
Mlh3	Rattus norvegicus mutL homolog 3 (E. coli) (Mlh3), mRNA [NM_001108043]	**0,568**
Gpbp1	Rattus norvegicus GC-rich promoter binding protein 1 (Gpbp1), mRNA [NM_001106410]	**0,568**
Eif5	Rattus norvegicus eukaryotic translation initiation factor 5 (Eif5), mRNA [NM_020075]	**0,568**
Rpl10a	Rattus norvegicus ribosomal protein L10A (Rpl10a), mRNA [NM_031065]	**0,569**
Ankrd54	Rattus norvegicus ankyrin repeat domain 54 (Ankrd54), mRNA [NM_001025285]	**0,569**
LOC682469	PREDICTED: Rattus norvegicus similar to jumonji domain containing 1B (LOC682469), mRNA [XM_001061636]	**0,569**
Tbc1d14	Rattus norvegicus TBC1 domain family, member 14 (Tbc1d14), transcript variant 1, mRNA [NM_001012152]	**0,569**
Wwc2	Rattus norvegicus WW and C2 domain containing 2 (Wwc2), mRNA [NM_001109111]	**0,569**
Neu1	Rattus norvegicus sialidase 1 (lysosomal sialidase) (Neu1), mRNA [NM_031522]	**0,569**
0	Unknown	**0,569**
Pdzrn3	PREDICTED: Rattus norvegicus PDZ domain containing RING finger 3 (Pdzrn3), mRNA [XM_232226]	**0,569**
Mea1	Rattus norvegicus male-enhanced antigen 1 (Mea1), mRNA [NM_001044286]	**0,569**
Psen2	Rattus norvegicus presenilin 2 (Psen2), mRNA [NM_031087]	**0,569**
LOC687090	Rattus norvegicus hypothetical protein LOC687090 (LOC687090), mRNA [NM_001110318]	**0,569**
Jrkl	Rattus norvegicus jerky homolog-like (mouse) (Jrkl), mRNA [NM_001108122]	**0,569**
LOC499749	Rattus norvegicus similar to RIKEN cDNA C430004E15 (LOC499749), mRNA [NM_001024309]	**0,569**
Lrp10	Rattus norvegicus low-density lipoprotein receptor-related protein 10 (Lrp10), mRNA [NM_001037777]	**0,569**
Lage3	Rattus norvegicus L antigen family, member 3 (Lage3), mRNA [NM_001106345]	**0,569**
Wdfy1	Rattus norvegicus WD repeat and FYVE domain containing 1 (Wdfy1), mRNA [NM_001008331]	**0,569**
Set	Rattus norvegicus SET nuclear oncogene (Set), mRNA [NM_001012504]	**0,569**
Hist2h4	Rattus norvegicus histone cluster 2, H4 (Hist2h4), mRNA [NM_001123469]	**0,569**
Gls2	Rattus norvegicus glutaminase 2 (liver, mitochondrial) (Gls2), nuclear gene encoding mitochondrial protein, mRNA [NM_138904]	**0,569**
0	Unknown	**0,569**
Fam43a	Rattus norvegicus family with sequence similarity 43, member A (Fam43a), mRNA [NM_001039002]	**0,569**
Haus8	Rattus norvegicus HAUS augmin-like complex, subunit 8 (Haus8), mRNA [NM_001024971]	**0,569**
Dok4	Rattus norvegicus docking protein 4 (Dok4), mRNA [NM_001108438]	**0,569**
Ndst2	Rattus norvegicus N-deacetylase/N-sulfotransferase (heparan glucosaminyl) 2 (Ndst2), mRNA [NM_001105740]	**0,569**

Fkbp9	Rattus norvegicus FK506 binding protein 9 (Fkbp9), mRNA [NM_001007646]	**0,569**
LOC688420	PREDICTED: Rattus norvegicus similar to claudin 4 (LOC688420), mRNA [XM_001066865]	**0,569**
Ndufaf4	Rattus norvegicus NADH dehydrogenase (ubiquinone) 1 alpha subcomplex, assembly factor 4 (Ndufaf4), nuclear gene encoding mitochondrial protein, mRNA [NM_198783]	**0,569**
Wnt7a	Rattus norvegicus wingless-type MMTV integration site family, member 7A (Wnt7a), mRNA [NM_001100473]	**0,569**
0	Unknown	**0,569**
0	PREDICTED: Rattus norvegicus rCG41835-like (LOC100359996), mRNA [XM_002728584]	**0,569**
Glt8d1	Rattus norvegicus glycosyltransferase 8 domain containing 1 (Glt8d1), mRNA [NM_001007683]	**0,569**
Fam116a	Rattus norvegicus family with sequence similarity 116, member A (Fam116a), mRNA [NM_001134467]	**0,569**
Dnajc8	Rattus norvegicus DnaJ (Hsp40) homolog, subfamily C, member 8 (Dnajc8), mRNA [NM_001013168]	**0,569**
Tcea1	Rattus norvegicus transcription elongation factor A (SII) 1 (Tcea1), mRNA [NM_001025735]	**0,569**
38231	Rattus norvegicus septin 4 (Sept4), nuclear gene encoding mitochondrial protein, mRNA [NM_001011893]	**0,569**
Cox19	Rattus norvegicus COX19 cytochrome c oxidase assembly homolog (S. cerevisiae) (Cox19), mRNA [NM_001107126]	**0,569**
Wdr43	Rattus norvegicus WD repeat domain 43 (Wdr43), mRNA [NM_001037791]	**0,569**
0	P90550_LEIDO (P90550) 39 kDa antigen, partial (5%) [TC612167]	**0,569**
Diablo	Rattus norvegicus diablo homolog (Drosophila) (Diablo), nuclear gene encoding mitochondrial protein, mRNA [NM_001008292]	**0,569**
Ntrk3	Rattus norvegicus neurotrophic tyrosine kinase, receptor, type 3 (Ntrk3), mRNA [NM_019248]	**0,569**
Wdr37	Rattus norvegicus WD repeat domain 37 (Wdr37), mRNA [NM_001107362]	**0,569**
Nudcd1	Rattus norvegicus NudC domain containing 1 (Nudcd1), mRNA [NM_001130561]	**0,570**
0	probable tRNA pseudouridine synthase 2 [Source:RefSeq peptide;Acc:NP_001014279] [ENSRNOT00000040550]	**0,570**
Leprotl1	Rattus norvegicus leptin receptor overlapping transcript-like 1 (Leprotl1), mRNA [NM_001013188]	**0,570**
Pdrg1	Rattus norvegicus p53 and DNA damage regulated 1 (Pdrg1), mRNA [NM_001014762]	**0,570**
Pqlc1	Rattus norvegicus PQ loop repeat containing 1 (Pqlc1), mRNA [NM_001013189]	**0,570**
Npff	Rattus norvegicus neuropeptide FF-amide peptide precursor (Npff), mRNA [NM_022586]	**0,570**
LOC691889	PREDICTED: Rattus norvegicus similar to ATPase, aminophospholipid transporter-like, class I, type 8A, member 2 (LOC691889), miscRNA [XR_086029]	**0,570**
0	Ribosomal protein [Source:UniProtKB/TrEMBL;Acc:D3Z9F6] [ENSRNOT00000045023]	**0,570**
Haus1	HAUS augmin-like complex subunit 1 [Source:UniProtKB/Swiss-Prot;Acc:Q9R0A8] [ENSRNOT00000023101]	**0,570**
Fam176a	Rattus norvegicus family with sequence similarity 176, member A (Fam176a), mRNA [NM_001109243]	**0,570**
MGC112883	Rattus norvegicus LOC500651 (MGC112883), mRNA [NM_001047743]	**0,570**
Osbpl1a	Rattus norvegicus oxysterol binding protein-like 1A (Osbpl1a), mRNA [NM_172023]	**0,570**
Tmem144	Rattus norvegicus transmembrane protein 144 (Tmem144), mRNA [NM_001108551]	**0,570**
Ccdc58	Rattus norvegicus coiled-coil domain containing 58 (Ccdc58), mRNA [NM_001105875]	**0,570**
0	Rattus norvegicus similar to hypothetical protein DKFZp761B107 (LOC305415), mRNA [XM_223492]	**0,570**

Mtvr2	Rattus norvegicus mammary tumor virus receptor 2 (Mtvr2), mRNA [NM_001015013]	**0,570**
Lypla2	Rattus norvegicus lysophospholipase 2 (Lypla2), mRNA [NM_031342]	**0,570**
Cbx3	Rattus norvegicus chromobox homolog 3 (HP1 gamma homolog, Drosophila) (Cbx3), mRNA [NM_001008313]	**0,570**
0	Uncharacterized protein [Source:UniProtKB/TrEMBL;Acc:D4ACU3] [ENSRNOT00000060466]	**0,570**
0	Unknown	**0,570**
Tmem115	Rattus norvegicus transmembrane protein 115 (Tmem115), mRNA [NM_001108779]	**0,570**
Sf3b1	Rattus norvegicus splicing factor 3b, subunit 1 (Sf3b1), mRNA [NM_053426]	**0,570**
Skil	Uncharacterized protein [Source:UniProtKB/TrEMBL;Acc:D3ZWL1] [ENSRNOT00000013191]	**0,570**
Isoc1	Rattus norvegicus isochorismatase domain containing 1 (Isoc1), mRNA [NM_001014242]	**0,570**
Pigt	Rattus norvegicus phosphatidylinositol glycan anchor biosynthesis, class T (Pigt), mRNA [NM_001106540]	**0,570**
Rps17	Rattus norvegicus ribosomal protein S17 (Rps17), mRNA [NM_017152]	**0,570**
Fhit	Rattus norvegicus fragile histidine triad gene (Fhit), mRNA [NM_021774]	**0,570**
Sdf2	Rattus norvegicus stromal cell derived factor 2 (Sdf2), mRNA [NM_001105803]	**0,570**
Abca2	Rattus norvegicus ATP-binding cassette, subfamily A (ABC1), member 2 (Abca2), mRNA [NM_024396]	**0,570**
0	Unknown	**0,570**
Meis1	Rattus norvegicus Meis homeobox 1 (Meis1), mRNA [NM_001134702]	**0,570**
0	Unknown	**0,570**
Pex16	Rattus norvegicus peroxisomal biogenesis factor 16 (Pex16), mRNA [NM_001012088]	**0,570**
LOC684800	PREDICTED: Rattus norvegicus similar to stromal membrane-associated protein 1, transcript variant 2 (LOC684800), mRNA [XM_002727177]	**0,570**
Cbx3	Rattus norvegicus chromobox homolog 3 (HP1 gamma homolog, Drosophila) (Cbx3), mRNA [NM_001008313]	**0,570**
Chd4	PREDICTED: Rattus norvegicus chromodomain helicase DNA binding protein 4 (Chd4), mRNA [XM_001063352]	**0,570**
Nat12	Rattus norvegicus N-acetyltransferase 12 (GCN5-related, putative) (Nat12), mRNA [NM_001109099]	**0,570**
Trpc1	Rattus norvegicus transient receptor potential cation channel, subfamily C, member 1 (Trpc1), mRNA [NM_053558]	**0,570**
Cpne2	PREDICTED: Rattus norvegicus copine II (Cpne2), mRNA [XM_001062196]	**0,570**
Paqr3	Rattus norvegicus progestin and adipoQ receptor family member III (Paqr3), mRNA [NM_001012033]	**0,570**
Srxn1	Rattus norvegicus sulfiredoxin 1 homolog (S. cerevisiae) (Srxn1), mRNA [NM_001047858]	**0,570**
Cpne3	Rattus norvegicus copine III (Cpne3), mRNA [NM_001107917]	**0,570**
0	Unknown	**0,570**
Smarcd3	Rattus norvegicus SWI/SNF related, matrix associated, actin dependent regulator of chromatin, subfamily d, member 3 (Smarcd3), mRNA [NM_001011966]	**0,570**
Neu3	Rattus norvegicus sialidase 3 (membrane sialidase) (Neu3), mRNA [NM_054010]	**0,570**
Tmem184c	Rattus norvegicus transmembrane protein 184C (Tmem184c), mRNA [NM_178330]	**0,570**
RGD1561520	PREDICTED: Rattus norvegicus similar to Ab2-162 (RGD1561520), mRNA [XM_345159]	**0,570**
RGD1559531	Uncharacterized protein [Source:UniProtKB/TrEMBL;Acc:D3ZQ96] [ENSRNOT00000048673]	**0,570**

Nox1	Rattus norvegicus NADPH oxidase 1 (Nox1), mRNA [NM_053683]	**0,570**
0	Unknown	**0,570**
Ccar1	Rattus norvegicus cell division cycle and apoptosis regulator 1 (Ccar1), mRNA [NM_001108535]	**0,570**
LOC298138	PREDICTED: Rattus norvegicus similar to RAS and EF hand domain containing (LOC298138), miscRNA [XR_006670]	**0,570**
0	Unknown	**0,570**
RGD1310597	Rattus norvegicus similar to RIKEN cDNA 1200014M14 (RGD1310597), mRNA [NM_001025647]	**0,570**
0	Unknown	**0,570**
0	PREDICTED: Rattus norvegicus ADAMTS-like 1 (Adamtsl1), mRNA [XM_342858]	**0,570**
0	Rattus norvegicus similar to ribosomal protein S27a (LOC364669), mRNA [XM_344565]	**0,570**
0	Unknown	**0,570**
Hap1	Rattus norvegicus huntingtin-associated protein 1 (Hap1), transcript variant 1, mRNA [NM_024133]	**0,571**
Srp72	Rattus norvegicus signal recognition particle 72 (Srp72), mRNA [NM_001170601]	**0,571**
0	Unknown	**0,571**
Mzf1	Rattus norvegicus myeloid zinc finger 1 (Mzf1), mRNA [NM_001108470]	**0,571**
Galnt3	Rattus norvegicus UDP-N-acetyl-alpha-D-galactosamine:polypeptide N-acetylgalactosaminyltransferase 3 (GalNAc-T3) (Galnt3), mRNA [NM_001015032]	**0,571**
Cnst	PREDICTED: Rattus norvegicus similar to 9630058J23Rik protein (RGD1564833), mRNA [XM_573525]	**0,571**
Vwa5b2	Rattus norvegicus von Willebrand factor A domain containing 5B2 (Vwa5b2), mRNA [NM_001134535]	**0,571**
LOC501296	PREDICTED: Rattus norvegicus hypothetical gene supported by BC059164 (LOC501296), partial mRNA [XM_001081221]	**0,571**
Ncln	Rattus norvegicus nicalin homolog (zebrafish) (Ncln), mRNA [NM_001014082]	**0,571**
Tmed10	Rattus norvegicus transmembrane emp24-like trafficking protein 10 (yeast) (Tmed10), mRNA [NM_053467]	**0,571**
Ogg1	Rattus norvegicus 8-oxoguanine DNA glycosylase (Ogg1), nuclear gene encoding mitochondrial protein, mRNA [NM_030870]	**0,571**
Zfp828	Rattus norvegicus zinc finger protein 828 (Zfp828), mRNA [NM_001107329]	**0,571**
Atad3a	Rattus norvegicus ATPase family, AAA domain containing 3A (Atad3a), nuclear gene encoding mitochondrial protein, mRNA [NM_001034922]	**0,571**
Zkscan3	Rattus norvegicus zinc finger with KRAB and SCAN domains 3 (Zkscan3), mRNA [NM_001012053]	**0,571**
Erap1	Rattus norvegicus endoplasmic reticulum aminopeptidase 1 (Erap1), mRNA [NM_030836]	**0,571**
Znf689	Rattus norvegicus zinc finger protein 689 (Znf689), mRNA [NM_173330]	**0,571**
Klhl24	Rattus norvegicus kelch-like 24 (Drosophila) (Klhl24), mRNA [NM_181473]	**0,571**
Perp	Rattus norvegicus PERP, TP53 apoptosis effector (Perp), mRNA [NM_001106265]	**0,571**
Tacr2	Rattus norvegicus tachykinin receptor 2 (Tacr2), mRNA [NM_080768]	**0,571**
RGD1307071	Uncharacterized protein [Source:UniProtKB/TrEMBL;Acc:D4A8C1] [ENSRNOT00000036527]	**0,571**
Elavl2	Rattus norvegicus ELAV (embryonic lethal, abnormal vision, Drosophila)-like 2 (Hu antigen B) (Elavl2), mRNA [NM_173309]	**0,571**
Mrpl42	Rattus norvegicus mitochondrial ribosomal protein L42 (Mrpl42), nuclear gene encoding mitochondrial protein, mRNA [NM_001106782]	**0,571**
Slc39a13	Rattus norvegicus solute carrier family 39 (zinc transporter), member 13 (Slc39a13), mRNA [NM_001039196]	**0,571**

Cflar	Rattus norvegicus CASP8 and FADD-like apoptosis regulator, mRNA (cDNA clone MGC:108616 IMAGE:7376876), complete cds. [BC089781]	**0,571**
Dnajc14	Rattus norvegicus DnaJ (Hsp40) homolog, subfamily C, member 14 (Dnajc14), mRNA [NM_053690]	**0,571**
Cbx7	Rattus norvegicus chromobox homolog 7 (Cbx7), mRNA [NM_199117]	**0,571**
0	Unknown	**0,571**
RGD1565210	PREDICTED: Rattus norvegicus RGD1565210 (RGD1565210), mRNA [XM_001071740]	**0,571**
Timm23	Rattus norvegicus translocase of inner mitochondrial membrane 23 homolog (yeast) (Timm23), nuclear gene encoding mitochondrial protein, mRNA [NM_019352]	**0,571**
Hexb	Rattus norvegicus hexosaminidase B (Hexb), mRNA [NM_001011946]	**0,571**
Alkbh4	Rattus norvegicus alkB, alkylation repair homolog 4 (E. coli) (Alkbh4), mRNA [NM_001105920]	**0,571**
LOC679651	Rattus norvegicus hypothetical protein LOC679651 (LOC679651), mRNA [NM_001195277]	**0,571**
Birc5	Rattus norvegicus baculoviral IAP repeat-containing 5 (Birc5), mRNA [NM_022274]	**0,571**
Tcf4	Rattus norvegicus transcription factor 4 (Tcf4), mRNA [NM_053369]	**0,572**
Trim27	Rattus norvegicus tripartite motif-containing 27 (Trim27), transcript variant 1, mRNA [NM_001134974]	**0,572**
Psme1	Rattus norvegicus proteasome (prosome, macropain) activator subunit 1 (Psme1), mRNA [NM_017264]	**0,572**
Yif1	Rattus norvegicus Yip1 interacting factor homolog (S. cerevisiae) (Yif1), mRNA [NM_172017]	**0,572**
LOC691920	PREDICTED: Rattus norvegicus similar to kinesin-like motor protein C20orf23 (LOC691920), mRNA [XM_001080099]	**0,572**
Spats1	Rattus norvegicus spermatogenesis associated, serine-rich 1 (Spats1), mRNA [NM_181376]	**0,572**
Rpl28	Rattus norvegicus ribosomal protein L28 (Rpl28), mRNA [NM_022697]	**0,572**
Nup153	Rattus norvegicus nucleoporin 153 (Nup153), mRNA [NM_001100470]	**0,572**
Hltf	Rattus norvegicus helicase-like transcription factor (Hltf), mRNA [NM_001106478]	**0,572**
RGD1305713	Rattus norvegicus similar to RIKEN cDNA 3110040N11 (RGD1305713), mRNA [NM_001024749]	**0,572**
0	Unknown	**0,572**
Cenpk	Rattus norvegicus centromere protein K (Cenpk), mRNA [NM_001106407]	**0,572**
Ddx24	Rattus norvegicus DEAD (Asp-Glu-Ala-Asp) box polypeptide 24 (Ddx24), mRNA [NM_199119]	**0,572**
0	Unknown	**0,572**
Smc1a	Rattus norvegicus structural maintenance of chromosomes 1A (Smc1a), mRNA [NM_031683]	**0,572**
RGD1308319	PREDICTED: Rattus norvegicus similar to KIAA0802 protein (RGD1308319), partial mRNA [XM_002727262]	**0,572**
Nupl1	Nucleoporin p58/p45 [Source:UniProtKB/Swiss-Prot;Acc:P70581] [ENSRNOT00000017204]	**0,572**
Add1	Rattus norvegicus adducin 1 (alpha) (Add1), mRNA [NM_016990]	**0,572**
Ddit4l	Rattus norvegicus DNA-damage-inducible transcript 4-like (Ddit4l), mRNA [NM_080399]	**0,572**
Dnajc5	Rattus norvegicus DnaJ (Hsp40) homolog, subfamily C, member 5 (Dnajc5), mRNA [NM_024161]	**0,572**
Tctex1d2	Rattus norvegicus Tctex1 domain containing 2 (Tctex1d2), mRNA [NM_001109054]	**0,572**
Ctu1	Rattus norvegicus cytosolic thiouridylase subunit 1 homolog (S. pombe) (Ctu1), mRNA [NM_001106251]	**0,572**
Gtf2ird1	Rattus norvegicus GTF2I repeat domain containing 1 (Gtf2ird1), mRNA [NM_001001504]	**0,572**

Dph1	Rattus norvegicus DPH1 homolog (S. cerevisiae) (Dph1), mRNA [NM_001105809]	**0,572**
0	Q6DC77_BRARE (Q6DC77) Zgc:101065, partial (7%) [TC593872]	**0,572**
Olfm3	Rattus norvegicus olfactomedin 3 (Olfm3), mRNA [NM_145777]	**0,572**
Gpsm1	Rattus norvegicus G-protein signaling modulator 1 (AGS3-like, C. elegans) (Gpsm1), transcript variant 1, mRNA [NM_144745]	**0,572**
Zfp148	Rattus norvegicus zinc finger protein 148 (Zfp148), mRNA [NM_031615]	**0,572**
Spast	Rattus norvegicus spastin (Spast), mRNA [NM_001108702]	**0,572**
RGD1560171	PREDICTED: Rattus norvegicus similar to PRO0149 protein (RGD1560171), mRNA [XM_001054786]	**0,572**
Bcl2l1	Rattus norvegicus Bcl2-like 1 (Bcl2l1), nuclear gene encoding mitochondrial protein, transcript variant 3, mRNA [NM_001033670]	**0,572**
Plekha4	Rattus norvegicus pleckstrin homology domain containing, family A (phosphoinositide binding specific) member 4 (Plekha4), mRNA [NM_199101]	**0,572**
Spire1	Rattus norvegicus spire homolog 1 (Drosophila) (Spire1), mRNA [NM_001107381]	**0,572**
Cst3	Rattus norvegicus cystatin C (Cst3), mRNA [NM_012837]	**0,572**
RGD1564788	PREDICTED: Rattus norvegicus similar to Werner syndrome helicase homolog, transcript variant 1 (RGD1564788), mRNA [XM_001059940]	**0,572**
Clip4	Rattus norvegicus CAP-GLY domain containing linker protein family, member 4 (Clip4), mRNA [NM_001013942]	**0,572**
Mettl9	Rattus norvegicus methyltransferase like 9 (Mettl9), mRNA [NM_001163164]	**0,572**
Dstyk	Rattus norvegicus dual serine/threonine and tyrosine protein kinase (Dstyk), mRNA [NM_199463]	**0,572**
LOC289378	Rattus norvegicus similar to B0432.8 (LOC289378), mRNA [NM_001105987]	**0,572**
Gpr135	Rattus norvegicus G protein-coupled receptor 135 (Gpr135), mRNA [NM_181771]	**0,572**
Arpc5	Rattus norvegicus actin related protein 2/3 complex, subunit 5 (Arpc5), mRNA [NM_001025717]	**0,572**
Ing1	Rattus norvegicus inhibitor of growth family, member 1 (Ing1), mRNA [NM_001038591]	**0,572**
0	Unknown	**0,572**
Ptdss1	Rattus norvegicus phosphatidylserine synthase 1 (Ptdss1), mRNA [NM_001012113]	**0,572**
Akap13	Rattus norvegicus A kinase (PRKA) anchor protein 13 (Akap13), mRNA [NM_001106271]	**0,572**
0	Myelin-associated oligodendrocyte basic protein [Source:UniProtKB/Swiss-Prot;Acc:Q63327] [ENSRNOT00000064831]	**0,572**
Def8	Rattus norvegicus differentially expressed in FDCP 8 homolog (mouse) (Def8), mRNA [NM_001024774]	**0,572**
0	Unknown	**0,572**
Nfyb	Rattus norvegicus nuclear transcription factor-Y beta (Nfyb), mRNA [NM_031553]	**0,573**
0	Rattus norvegicus cDNA clone IMAGE:7367251. [BC167759]	**0,573**
Asns	Rattus norvegicus asparagine synthetase (Asns), mRNA [NM_013079]	**0,573**
0	Q6TXI1_RAT (Q6TXI1) LRRGT00018, partial (5%) [TC597668]	**0,573**
0	Uncharacterized protein [Source:UniProtKB/TrEMBL;Acc:D3ZZG2] [ENSRNOT00000065209]	**0,573**
Uchl1	Rattus norvegicus ubiquitin carboxyl-terminal esterase L1 (ubiquitin thiolesterase) (Uchl1), mRNA [NM_017237]	**0,573**
Xpo1	Rattus norvegicus exportin 1, CRM1 homolog (yeast) (Xpo1), mRNA [NM_053490]	**0,573**
Riok3	Rattus norvegicus RIO kinase 3 (yeast) (Riok3), mRNA [NM_001108423]	**0,573**
Calb2	Rattus norvegicus calbindin 2 (Calb2), mRNA [NM_053988]	**0,573**

Plcd4	Rattus norvegicus phospholipase C, delta 4 (Plcd4), mRNA [NM_080688]	**0,573**
Tomm22	Rattus norvegicus translocase of outer mitochondrial membrane 22 homolog (yeast) (Tomm22), nuclear gene encoding mitochondrial protein, mRNA [NM_212514]	**0,573**
Acss2	Rattus norvegicus acyl-CoA synthetase short-chain family member 2 (Acss2), mRNA [NM_001107793]	**0,573**
Pigu	Rattus norvegicus phosphatidylinositol glycan anchor biosynthesis, class U (Pigu), mRNA [NM_181637]	**0,573**
Tshr	Rattus norvegicus thyroid stimulating hormone receptor (Tshr), mRNA [NM_012888]	**0,573**
Serpine2	Rattus norvegicus serine (or cysteine) peptidase inhibitor, clade E, member 2 (Serpine2), mRNA [NM_019197]	**0,573**
Rab18	Rattus norvegicus RAB18, member RAS oncogene family (Rab18), mRNA [NM_001012468]	**0,573**
Trpm8	Rattus norvegicus transient receptor potential cation channel, subfamily M, member 8 (Trpm8), mRNA [NM_134371]	**0,573**
Gpkow	Rattus norvegicus G patch domain and KOW motifs (Gpkow), mRNA [NM_001109381]	**0,573**
Aqp4	Rattus norvegicus aquaporin 4 (Aqp4), transcript variant 2, mRNA [NM_001142366]	**0,573**
Ubxn2a	Rattus norvegicus UBX domain protein 2A (Ubxn2a), mRNA [NM_001109482]	**0,573**
Eif4e	Rattus norvegicus eukaryotic translation initiation factor 4E (Eif4e), mRNA [NM_053974]	**0,573**
Rab9b	Rattus norvegicus RAB9B, member RAS oncogene family (Rab9b), mRNA [NM_001109018]	**0,573**
Isy1	Rattus norvegicus ISY1 splicing factor homolog (S. cerevisiae) (Isy1), mRNA [NM_001014188]	**0,573**
Cdca7l	Rattus norvegicus cell division cycle associated 7 like (Cdca7l), mRNA [NM_001034953]	**0,573**
0	Unknown	**0,573**
F2rl1	Rattus norvegicus coagulation factor II (thrombin) receptor-like 1 (F2rl1), mRNA [NM_053897]	**0,573**
Scpep1	Rattus norvegicus serine carboxypeptidase 1 (Scpep1), mRNA [NM_133383]	**0,573**
Ebp	Rattus norvegicus emopamil binding protein (sterol isomerase) (Ebp), mRNA [NM_057137]	**0,573**
Eef1e1	Rattus norvegicus eukaryotic translation elongation factor 1 epsilon 1 (Eef1e1), mRNA [NM_001106106]	**0,573**
Pak1ip1	Rattus norvegicus PAK1 interacting protein 1 (Pak1ip1), mRNA [NM_001037356]	**0,573**
Saps2	Rattus norvegicus SAPS domain family, member 2 (Saps2), mRNA [NM_001106789]	**0,573**
N4bp2l2	Rattus norvegicus NEDD4 binding protein 2-like 2 (N4bp2l2), mRNA [NM_001005533]	**0,573**
LOC689074	Rattus norvegicus similar to Protein KIAA1543 (LOC689074), mRNA [NM_001144840]	**0,573**
Grem2	Rattus norvegicus gremlin 2, cysteine knot superfamily, homolog (Xenopus laevis) (Grem2), mRNA [NM_001105974]	**0,573**
Erh	Rattus norvegicus enhancer of rudimentary homolog (Drosophila) (Erh), mRNA [NM_001109442]	**0,573**
Pik3ca	Rattus norvegicus phosphoinositide-3-kinase, catalytic, alpha polypeptide (Pik3ca), mRNA [NM_133399]	**0,573**
Tmem163	Rattus norvegicus transmembrane protein 163 (Tmem163), mRNA [NM_001110763]	**0,573**
Mif4gd	Rattus norvegicus MIF4G domain containing (Mif4gd), mRNA [NM_001014122]	**0,573**
Scamp2	Rattus norvegicus secretory carrier membrane protein 2 (Scamp2), mRNA [NM_023955]	**0,573**
Meis3	Rattus norvegicus Meis homeobox 3 (Meis3), mRNA [NM_001108472]	**0,573**
Map4k3	Rattus norvegicus mitogen-activated protein kinase kinase kinase kinase 3 (Map4k3), mRNA [NM_133407]	**0,573**

Slc25a25	Rattus norvegicus solute carrier family 25 (mitochondrial carrier, phosphate carrier), member 25 (Slc25a25), nuclear gene encoding mitochondrial protein, mRNA [NM_145677]	**0,573**
LOC499770	Rattus norvegicus similar to LOC495800 protein (LOC499770), mRNA [NM_001047110]	**0,573**
Ipmk	Rattus norvegicus inositol polyphosphate multikinase (Ipmk), mRNA [NM_134417]	**0,573**
RGD1563194	PREDICTED: Rattus norvegicus similar to ribosomal protein L13 (RGD1563194), miscRNA [XR_009316]	**0,573**
RGD1310358	Rattus norvegicus similar to NNX3 (RGD1310358), mRNA [NM_001107507]	**0,573**
N6amt1	Rattus norvegicus N-6 adenine-specific DNA methyltransferase 1 (putative) (N6amt1), mRNA [NM_001191592]	**0,573**
RGD1310773	Rattus norvegicus similar to hypothetical protein FLJ31810 (RGD1310773), mRNA [NM_001107926]	**0,573**
Giyd2	Rattus norvegicus GIY-YIG domain containing 2 (Giyd2), mRNA [NM_001009292]	**0,573**
Nup205	Rattus norvegicus nucleoporin 205 (Nup205), mRNA [NM_001108620]	**0,573**
Pcsk6	Rattus norvegicus proprotein convertase subtilisin/kexin type 6 (Pcsk6), mRNA [NM_012999]	**0,573**
LOC682404	PREDICTED: Rattus norvegicus similar to CG12929-PA (LOC682404), mRNA [XM_001061367]	**0,573**
Kcnb1	Rattus norvegicus potassium voltage gated channel, Shab-related subfamily, member 1 (Kcnb1), mRNA [NM_013186]	**0,573**
LOC680207	PREDICTED: Rattus norvegicus hypothetical protein LOC680207 (LOC680207), mRNA [XM_001056119]	**0,573**
Nfkbil2	Rattus norvegicus nuclear factor of kappa light polypeptide gene enhancer in B-cells inhibitor-like 2 (Nfkbil2), mRNA [NM_001130572]	**0,573**
Vamp8	Rattus norvegicus vesicle-associated membrane protein 8 (Vamp8), mRNA [NM_031827]	**0,573**
Tmem123	Rattus norvegicus transmembrane protein 123 (Tmem123), mRNA [NM_001014205]	**0,573**
Cd2bp2	Rattus norvegicus Cd2 (cytoplasmic tail) binding protein 2 (Cd2bp2), mRNA [NM_001106297]	**0,573**
Zfp458	Rattus norvegicus zinc finger protein 458 (Zfp458), transcript variant 1, mRNA [NM_001142962]	**0,574**
RGD1309748	Rattus norvegicus similar to CG4768-PA (RGD1309748), mRNA [NM_001106972]	**0,574**
Slit2	Rattus norvegicus slit homolog 2 (Drosophila) (Slit2), mRNA [NM_022632]	**0,574**
Larp5	Rattus norvegicus La ribonucleoprotein domain family, member 5 (Larp5), mRNA [NM_001107361]	**0,574**
Ces5a	Rattus norvegicus carboxylesterase 5A (Ces5a), mRNA [NM_001012056]	**0,574**
Lactb2	Rattus norvegicus lactamase, beta 2 (Lactb2), mRNA [NM_001024247]	**0,574**
Ccdc101	Rattus norvegicus coiled-coil domain containing 101 (Ccdc101), mRNA [NM_001114502]	**0,574**
LOC684558	PREDICTED: Rattus norvegicus similar to regulator of nonsense transcripts 1 (LOC684558), mRNA [XM_001070971]	**0,574**
0	Uncharacterized protein [Source:UniProtKB/TrEMBL;Acc:D3ZXM8] [ENSRNOT00000059741]	**0,574**
Rps6ka1	Rattus norvegicus ribosomal protein S6 kinase polypeptide 1 (Rps6ka1), mRNA [NM_031107]	**0,574**
Mtmr11	Rattus norvegicus myotubularin related protein 11 (Mtmr11), mRNA [NM_001191096]	**0,574**
0	Unknown	**0,574**
Sfrs4	Rattus norvegicus splicing factor, arginine/serine-rich 4 (Sfrs4), mRNA [NM_001108685]	**0,574**
Slc20a2	Rattus norvegicus solute carrier family 20 (phosphate transporter), member 2 (Slc20a2), mRNA [NM_017223]	**0,574**
Znf597	Rattus norvegicus zinc finger protein 597 (Znf597), mRNA [NM_153732]	**0,574**

0	B double prime 1, subunit of RNA polymerase III transcription initiation factor IIIB (Predicted), isoform CRA_aUncharacterized protein [Source:UniProtKB/TrEMBL;Acc:D3ZDI4] [ENSRNOT00000024023]	**0,574**
Trmt6	Rattus norvegicus tRNA methyltransferase 6 homolog (S. cerevisiae) (Trmt6), mRNA [NM_001107779]	**0,574**
Arl8b	Rattus norvegicus ADP-ribosylation factor-like 8B (Arl8b), mRNA [NM_001024332]	**0,574**
Tmem53	Rattus norvegicus transmembrane protein 53 (Tmem53), mRNA [NM_001107964]	**0,574**
0	Unknown	**0,574**
Ythdf1	Rattus norvegicus YTH domain family, member 1 (Ythdf1), mRNA [NM_001024756]	**0,574**
Scg3	Rattus norvegicus secretogranin III (Scg3), mRNA [NM_053856]	**0,574**
0	Unknown	**0,574**
Sh2b3	Rattus norvegicus SH2B adaptor protein 3 (Sh2b3), mRNA [NM_031621]	**0,574**
Pex19	Rattus norvegicus peroxisomal biogenesis factor 19 (Pex19), transcript variant 2, mRNA [NM_001134777]	**0,574**
Rbpj	Rattus norvegicus recombination signal binding protein for immunoglobulin kappa J region (Rbpj), mRNA [NM_001106631]	**0,574**
Sertad2	Rattus norvegicus SERTA domain containing 2 (Sertad2), mRNA [NM_001024903]	**0,574**
Eif3f	Rattus norvegicus eukaryotic translation initiation factor 3, subunit F (Eif3f), mRNA [NM_001106292]	**0,574**
Stx8	Rattus norvegicus syntaxin 8 (Stx8), mRNA [NM_031656]	**0,574**
0	Unknown	**0,574**
Plod3	Rattus norvegicus procollagen-lysine, 2-oxoglutarate 5-dioxygenase 3 (Plod3), mRNA [NM_178101]	**0,574**
P2ry4	Rattus norvegicus pyrimidinergic receptor P2Y, G-protein coupled, 4 (P2ry4), mRNA [NM_031680]	**0,574**
Lrfn2	Rattus norvegicus leucine rich repeat and fibronectin type III domain containing 2 (Lrfn2), mRNA [NM_001039699]	**0,574**
Mmp14	Rattus norvegicus matrix metallopeptidase 14 (membrane-inserted) (Mmp14), mRNA [NM_031056]	**0,574**
Lysmd1	Rattus norvegicus LysM, putative peptidoglycan-binding, domain containing 1 (Lysmd1), mRNA [NM_001024302]	**0,574**
0	Unknown	**0,574**
LOC682999	Rattus norvegicus hypothetical protein LOC682999 (LOC682999), mRNA [NM_001127600]	**0,574**
Hsd17b8	Rattus norvegicus hydroxysteroid (17-beta) dehydrogenase 8 (Hsd17b8), mRNA [NM_212529]	**0,574**
Dnajb1	Rattus norvegicus DnaJ (Hsp40) homolog, subfamily B, member 1 (Dnajb1), mRNA [NM_001108441]	**0,574**
Rora	Rattus norvegicus RAR-related orphan receptor A (Rora), mRNA [NM_001106834]	**0,574**
Fzd2	Rattus norvegicus frizzled homolog 2 (Drosophila) (Fzd2), mRNA [NM_172035]	**0,574**
0	Unknown	**0,575**
0	Unknown	**0,575**
Gtf3c5	Rattus norvegicus general transcription factor IIIC, polypeptide 5 (Gtf3c5), mRNA [NM_001079941]	**0,575**
Unc119	Rattus norvegicus UNC-119 homolog (C. elegans) (Unc119), mRNA [NM_017188]	**0,575**
Vdac2	Rattus norvegicus voltage-dependent anion channel 2 (Vdac2), nuclear gene encoding mitochondrial protein, mRNA [NM_031354]	**0,575**
Necap1	Rattus norvegicus NECAP endocytosis associated 1 (Necap1), mRNA [NM_001029919]	**0,575**
LOC361128	Rattus norvegicus similar to TR4 orphan receptor associated protein TRA16 (LOC361128), mRNA [NM_001047104]	**0,575**

Scd	Rattus norvegicus stearoyl-CoA desaturase (delta-9-desaturase) (Scd), mRNA [NM_031841]	**0,575**
Als2cr4	Rattus norvegicus amyotrophic lateral sclerosis 2 (juvenile) chromosome region, candidate 4 (Als2cr4), mRNA [NM_001108220]	**0,575**
Mustn1	Rattus norvegicus musculoskeletal, embryonic nuclear protein 1 (Mustn1), mRNA [NM_181368]	**0,575**
0	Rattus norvegicus TL0AEA68YG04 mRNA sequence. [FQ231741]	**0,575**
LOC680319	Rattus norvegicus hypothetical protein LOC680319 (LOC680319), mRNA [NM_001109401]	**0,575**
Eif5b	Rattus norvegicus eukaryotic translation initiation factor 5B (Eif5b), mRNA [NM_001110141]	**0,575**
Ecop	Rattus norvegicus EGFR-coamplified and overexpressed protein (Ecop), mRNA [NM_001108630]	**0,575**
Ppm1k	Rattus norvegicus protein phosphatase 1K (PP2C domain containing) (Ppm1k), nuclear gene encoding mitochondrial protein, mRNA [NM_001107863]	**0,575**
Zcchc9	Rattus norvegicus zinc finger, CCHC domain containing 9 (Zcchc9), mRNA [NM_001013156]	**0,575**
0	Solute carrier family 37 (Glycerol-3-phosphate transporter), member 3 (Predicted), isoform CRA_cUncharacterized protein [Source:UniProtKB/TrEMBL;Acc:D3ZZN8] [ENSRNOT00000011915]	**0,575**
Camkk2	Rattus norvegicus calcium/calmodulin-dependent protein kinase kinase 2, beta (Camkk2), mRNA [NM_031338]	**0,575**
Shprh	Rattus norvegicus SNF2 histone linker PHD RING helicase (Shprh), mRNA [NM_001107470]	**0,575**
Klf9	Rattus norvegicus Kruppel-like factor 9 (Klf9), mRNA [NM_057211]	**0,575**
RGD1306917	Rattus norvegicus similar to RIKEN cDNA 2900010M23 (RGD1306917), mRNA [NM_001108528]	**0,575**
Sfrp1	PREDICTED: Rattus norvegicus secreted frizzled-related protein 1 (Sfrp1), mRNA [XM_001072532]	**0,575**
Zfp318	PREDICTED: Rattus norvegicus zinc finger protein 318 (Zfp318), mRNA [XM_002727181]	**0,575**
S100a3	Rattus norvegicus S100 calcium binding protein A3 (S100a3), mRNA [NM_053681]	**0,575**
Ammecr1l	Rattus norvegicus AMME chromosomal region gene 1-like (Ammecr1l), mRNA [NM_001107399]	**0,575**
0	Rattus norvegicus similar to Ran-interacting protein MOG1 (LOC290273), mRNA [XM_224238]	**0,575**
Magmas	Rattus norvegicus mitochondria-associated protein involved in granulocyte-macrophage colony-stimulating factor signal transduction (Magmas), nuclear gene encoding mitochondrial protein, mRNA [NM_001100136]	**0,575**
Srd5a1	Rattus norvegicus steroid-5-alpha-reductase, alpha polypeptide 1 (3-oxo-5 alpha-steroid delta 4-dehydrogenase alpha 1) (Srd5a1), mRNA [NM_017070]	**0,575**
Slc20a2	Rattus norvegicus solute carrier family 20 (phosphate transporter), member 2 (Slc20a2), mRNA [NM_017223]	**0,575**
Fbxl19	Rattus norvegicus F-box and leucine-rich repeat protein 19 (Fbxl19), mRNA [NM_001107552]	**0,575**
Dgat2	Rattus norvegicus diacylglycerol O-acyltransferase homolog 2 (mouse) (Dgat2), mRNA [NM_001012345]	**0,575**
Timm23	Rattus norvegicus translocase of inner mitochondrial membrane 23 homolog (yeast) (Timm23), nuclear gene encoding mitochondrial protein, mRNA [NM_019352]	**0,575**
Gys1	Rattus norvegicus glycogen synthase 1, muscle (Gys1), mRNA [NM_001109615]	**0,575**
0	phosphatidylinositol glycan anchor biosynthesis, class A [Source:RefSeq peptide;Acc:NP_001102286] [ENSRNOT00000004733]	**0,575**
Rab31	Rattus norvegicus RAB31, member RAS oncogene family (Rab31), mRNA [NM_145094]	**0,575**
LOC687675	PREDICTED: Rattus norvegicus similar to heparan sulfate D-glucosaminyl 3-O-sulfotransferase 4 (LOC687675), mRNA [XM_001079681]	**0,575**

Thsd7a	Rattus norvegicus thrombospondin, type I, domain containing 7A (Thsd7a), mRNA [NM_001191970]	**0,575**
Rps8	Rattus norvegicus ribosomal protein S8 (Rps8), mRNA [NM_031706]	**0,575**
RGD735175	Rattus norvegicus hypothetical protein MGC:72616 (RGD735175), mRNA [NM_199112]	**0,575**
RGD1561537	PREDICTED: Rattus norvegicus similar to putative repair and recombination helicase RAD26L, transcript variant 1 (RGD1561537), mRNA [XM_001058999]	**0,575**
Ska2	Rattus norvegicus spindle and kinetochore associated complex subunit 2 (Ska2), mRNA [NM_001009624]	**0,575**
Ap4s1	Rattus norvegicus adaptor-related protein complex AP-4, sigma 1 (Ap4s1), mRNA [NM_001130999]	**0,575**
0	Q7TQM0_MOUSE (Q7TQM0) Fbxo10 protein (Fragment), partial (7%) [TC646406]	**0,575**
Cep78	Rattus norvegicus centrosomal protein 78 (Cep78), mRNA [NM_021741]	**0,575**
Nubp2	Rattus norvegicus nucleotide binding protein 2 (Nubp2), mRNA [NM_001011891]	**0,575**
Mbp	Rattus norvegicus myelin basic protein (Mbp), transcript variant 6, mRNA [NM_001025289]	**0,575**
Nup214	Rattus norvegicus nucleoporin 214 (Nup214), mRNA [NM_001168559]	**0,575**
Cxcl12	Rattus norvegicus chemokine (C-X-C motif) ligand 12 (stromal cell-derived factor 1) (Cxcl12), transcript variant 3, mRNA [NM_001033883]	**0,575**
Dnajc25	Rattus norvegicus DnaJ (Hsp40) homolog, subfamily C, member 25 (Dnajc25), mRNA [NM_001025021]	**0,575**
RGD1310553	Rattus norvegicus similar to expressed sequence AI597479 (RGD1310553), mRNA [NM_001008517]	**0,575**
Ppm1d	Rattus norvegicus protein phosphatase 1D magnesium-dependent, delta isoform (Ppm1d), mRNA [NM_001105825]	**0,575**
RGD1563701	PREDICTED: Rattus norvegicus similar to BC068281 protein (RGD1563701), mRNA [XM_001070724]	**0,576**
0	Unknown	**0,576**
P4hb	Rattus norvegicus prolyl 4-hydroxylase, beta polypeptide (P4hb), mRNA [NM_012998]	**0,576**
Nap1l4	Rattus norvegicus nucleosome assembly protein 1-like 4 (Nap1l4), mRNA [NM_001012170]	**0,576**
0	Unknown	**0,576**
0	Palladin [Source:UniProtKB/Swiss-Prot;Acc:P0C5E3] [ENSRNOT00000013636]	**0,576**
Ptdss1	Rattus norvegicus phosphatidylserine synthase 1 (Ptdss1), mRNA [NM_001012113]	**0,576**
0	Unknown	**0,576**
Samd4a	Rattus norvegicus sterile alpha motif domain containing 4A (Samd4a), mRNA [NM_001107254]	**0,576**
Filip1	Rattus norvegicus filamin A interacting protein 1 (Filip1), mRNA [NM_145682]	**0,576**
Tnfrsf25	Rattus norvegicus tumor necrosis factor receptor superfamily, member 25 (Tnfrsf25), mRNA [NM_001137644]	**0,576**
Zfp35	Rattus norvegicus zinc finger protein 35 (Zfp35), mRNA [NM_001013141]	**0,576**
Sppl2a	Rattus norvegicus signal peptide peptidase-like 2A (Sppl2a), mRNA [NM_001107770]	**0,576**
Suox	Rattus norvegicus sulfite oxidase (Suox), nuclear gene encoding mitochondrial protein, mRNA [NM_031127]	**0,576**
Ccdc126	Rattus norvegicus coiled-coil domain containing 126 (Ccdc126), mRNA [NM_001109232]	**0,576**
Dot1l	Rattus norvegicus DOT1-like, histone H3 methyltransferase (S. cerevisiae) (Dot1l), mRNA [NM_001108733]	**0,576**
RGD1560088	Uncharacterized protein [Source:UniProtKB/TrEMBL;Acc:D3ZQH7] [ENSRNOT00000047207]	**0,576**
Ccdc43	Rattus norvegicus coiled-coil domain containing 43 (Ccdc43), mRNA [NM_001100728]	**0,576**

RGD1311946	Rattus norvegicus similar to RIKEN cDNA 1810055G02 (RGD1311946), mRNA [NM_001025683]	**0,576**
Pygb	Rattus norvegicus phosphorylase, glycogen; brain (Pygb), mRNA [NM_013188]	**0,576**
Arl6ip1	Rattus norvegicus ADP-ribosylation factor-like 6 interacting protein 1 (Arl6ip1), mRNA [NM_198737]	**0,576**
Mrpl16	Rattus norvegicus mitochondrial ribosomal protein L16 (Mrpl16), nuclear gene encoding mitochondrial protein, mRNA [NM_001009647]	**0,576**
Raf1	Rattus norvegicus v-raf-leukemia viral oncogene 1 (Raf1), mRNA [NM_012639]	**0,576**
Znf839	PREDICTED: Rattus norvegicus zinc finger protein 839 (Znf839), mRNA [XM_001071353]	**0,576**
LOC100363621	PREDICTED: Rattus norvegicus ecto-NOX disulfide-thiol exchanger 1 (LOC100363621), mRNA [XM_002725125]	**0,576**
Nkrf	Uncharacterized protein [Source:UniProtKB/TrEMBL;Acc:D3ZSG0] [ENSRNOT00000016660]	**0,576**
Traf3	Rattus norvegicus Tnf receptor-associated factor 3 (Traf3), mRNA [NM_001108724]	**0,576**
Dom3z	Rattus norvegicus DOM-3 homolog Z (C. elegans) (Dom3z), mRNA [NM_212497]	**0,576**
Scn3a	Rattus norvegicus sodium channel, voltage-gated, type III, alpha (Scn3a), mRNA [NM_013119]	**0,576**
Smarcb1	Rattus norvegicus SWI/SNF related, matrix associated, actin dependent regulator of chromatin, subfamily b, member 1 (Smarcb1), mRNA [NM_001025728]	**0,576**
RGD1309188	Rattus norvegicus similar to hypothetical protein BC011833 (RGD1309188), mRNA [NM_001108129]	**0,576**
0	Unknown	**0,576**
Pdcd7	Rattus norvegicus programmed cell death 7 (Pdcd7), mRNA [NM_001108768]	**0,576**
LOC100360849	PREDICTED: Rattus norvegicus hypothetical protein LOC100360849 (LOC100360849), mRNA [XM_002729568]	**0,576**
RGD1564167	PREDICTED: Rattus norvegicus similar to basic transcription factor 3 (RGD1564167), mRNA [XM_002727687]	**0,576**
Cnih	Rattus norvegicus cornichon homolog (Drosophila) (Cnih), mRNA [NM_001106029]	**0,576**
Sec14l2	Rattus norvegicus SEC14-like 2 (S. cerevisiae) (Sec14l2), mRNA [NM_053801]	**0,576**
Tomm34	Rattus norvegicus translocase of outer mitochondrial membrane 34 (Tomm34), nuclear gene encoding mitochondrial protein, mRNA [NM_001044244]	**0,576**
Tmem164	Rattus norvegicus transmembrane protein 164 (Tmem164), mRNA [NM_001109014]	**0,576**
Hibch	Rattus norvegicus 3-hydroxyisobutyryl-Coenzyme A hydrolase (Hibch), nuclear gene encoding mitochondrial protein, mRNA [NM_001013112]	**0,576**
RGD1308759	Rattus norvegicus similar to KIAA0892 protein (RGD1308759), mRNA [NM_001106077]	**0,576**
Synj2	Rattus norvegicus synaptojanin 2 (Synj2), transcript variant 3, mRNA [NM_032071]	**0,576**
Tfg	Rattus norvegicus Trk-fused gene (Tfg), mRNA [NM_001012144]	**0,576**
Fam129b	Rattus norvegicus family with sequence similarity 129, member B (Fam129b), mRNA [NM_001109885]	**0,576**
Suv420h2	Rattus norvegicus suppressor of variegation 4-20 homolog 2 (Drosophila) (Suv420h2), mRNA [NM_001107475]	**0,577**
Apobec1	Rattus norvegicus apolipoprotein B mRNA editing enzyme, catalytic polypeptide 1 (Apobec1), mRNA [NM_012907]	**0,577**
Slc25a24	Rattus norvegicus solute carrier family 25 (mitochondrial carrier, phosphate carrier), member 24 (Slc25a24), nuclear gene encoding mitochondrial protein, mRNA [NM_001127544]	**0,577**
0	Thyroid hormone receptor associated protein 2 (Predicted)Uncharacterized protein [Source:UniProtKB/TrEMBL;Acc:D3ZJC6] [ENSRNOT00000042331]	**0,577**

Agl	Rattus norvegicus amylo-1,6-glucosidase, 4-alpha-glucanotransferase (Agl), mRNA [NM_001108564]	**0,577**
LOC678769	Rattus norvegicus similar to Cysteine protease ATG4A (Autophagy-related protein 4 homolog A) (Autophagin-2) (Autophagy-related cysteine endopeptidase 2) (AUT-like 2 cysteine endopeptidase) (LOC678769), mRNA [NM_001126298]	**0,577**
Msh5	Rattus norvegicus mutS homolog 5 (E. coli) (Msh5), mRNA [NM_212536]	**0,577**
Znf553	PREDICTED: Rattus norvegicus zinc finger protein 553 (Znf553), mRNA [XM_219345]	**0,577**
Zkscan2	Uncharacterized protein [Source:UniProtKB/TrEMBL;Acc:D3ZXU0] [ENSRNOT00000020281]	**0,577**
Slc9a7	Rattus norvegicus solute carrier family 9 (sodium/hydrogen exchanger), member 7 (Slc9a7), mRNA [NM_001108242]	**0,577**
Tsc22d3	Rattus norvegicus TSC22 domain family, member 3 (Tsc22d3), mRNA [NM_031345]	**0,577**
0	Unknown	**0,577**
RGD1560997	PREDICTED: Rattus norvegicus similar to ribosomal protein S27a (RGD1560997), mRNA [XM_001053937]	**0,577**
Zdhhc17	Rattus norvegicus zinc finger, DHHC-type containing 17 (Zdhhc17), mRNA [NM_001039340]	**0,577**
Mif	MIF=macrophage migration inhibitory factor [rats, liver, mRNA, 525 nt]. [S73424]	**0,577**
0	Unknown	**0,577**
Rtdr1	Rattus norvegicus rhabdoid tumor deletion region gene 1 (Rtdr1), mRNA [NM_001127557]	**0,577**
Aard	Rattus norvegicus alanine and arginine rich domain containing protein (Aard), mRNA [NM_145093]	**0,577**
Shc2	Rattus norvegicus SHC (Src homology 2 domain containing) transforming protein 2 (Shc2), mRNA [NM_001108065]	**0,577**
Uckl1	Rattus norvegicus uridine-cytidine kinase 1-like 1 (Uckl1), mRNA [NM_001109212]	**0,577**
Sez6	Rattus norvegicus seizure related 6 homolog (mouse) (Sez6), mRNA [NM_001105754]	**0,577**
0	Uncharacterized protein [Source:UniProtKB/TrEMBL;Acc:D4A319] [ENSRNOT00000007828]	**0,577**
LOC691781	PREDICTED: Rattus norvegicus similar to Tubulin alpha-2 chain (Alpha-tubulin 2) (LOC691781), partial miscRNA [XR_086096]	**0,577**
RGD1563378	RCG36341Uncharacterized protein [Source:UniProtKB/TrEMBL;Acc:D3ZC26] [ENSRNOT00000050544]	**0,577**
0	PREDICTED: Rattus norvegicus similar to spermatogenesis associated glutamate (E)-rich protein 4d (LOC363324), mRNA [XM_343663]	**0,577**
Rpl28	Rattus norvegicus ribosomal protein L28 (Rpl28), mRNA [NM_022697]	**0,577**
Tor1aip2	Rattus norvegicus torsin A interacting protein 2 (Tor1aip2), transcript variant 1, mRNA [NM_199100]	**0,577**
Nlk	Rattus norvegicus nemo like kinase (Nlk), mRNA [NM_001191924]	**0,577**
Agfg2	Rattus norvegicus ArfGAP with FG repeats 2 (Agfg2), mRNA [NM_001107131]	**0,577**
Tmem218	Rattus norvegicus transmembrane protein 218 (Tmem218), mRNA [NM_001008325]	**0,577**
Lrrc8a	Rattus norvegicus leucine rich repeat containing 8 family, member A (Lrrc8a), mRNA [NM_001024782]	**0,577**
Ehd4	Rattus norvegicus EH-domain containing 4 (Ehd4), mRNA [NM_139324]	**0,577**
Banp	Rattus norvegicus Btg3 associated nuclear protein (Banp), mRNA [NM_001106191]	**0,577**
Ogt	Rattus norvegicus O-linked N-acetylglucosamine (GlcNAc) transferase (UDP-N-acetylglucosamine:polypeptide-N-acetylglucosaminyl transferase) (Ogt), mRNA [NM_017107]	**0,577**
LOC100360472	PREDICTED: Rattus norvegicus Trf-proximal protein homolog (LOC100360472), mRNA [XM_002730018]	**0,577**
0	Uncharacterized protein [Source:UniProtKB/TrEMBL;Acc:D3ZRK3] [ENSRNOT00000059370]	**0,577**

0	Unknown	**0,577**
Lgals8	Rattus norvegicus lectin, galactoside-binding, soluble, 8 (Lgals8), mRNA [NM_053862]	**0,577**
Ikbkb	Rattus norvegicus inhibitor of kappa light polypeptide gene enhancer in B-cells, kinase beta (Ikbkb), mRNA [NM_053355]	**0,578**
LOC100151767	Rattus norvegicus hypothetical LOC100151767 (LOC100151767), mRNA [NM_001127503]	**0,578**
Ndufa10	Rattus norvegicus NADH dehydrogenase (ubiquinone) 1 alpha subcomplex 10 (Ndufa10), nuclear gene encoding mitochondrial protein, mRNA [NM_199495]	**0,578**
0	Unknown	**0,578**
Crmp1	Rattus norvegicus collapsin response mediator protein 1 (Crmp1), mRNA [NM_012932]	**0,578**
Car11	Rattus norvegicus carbonic anhydrase 11 (Car11), mRNA [NM_175708]	**0,578**
Ppp1r2	Rattus norvegicus protein phosphatase 1, regulatory (inhibitor) subunit 2 (Ppp1r2), mRNA [NM_138823]	**0,578**
0	Histone H2A type 1-F [Source:UniProtKB/Swiss-Prot;Acc:Q64598] [ENSRNOT00000041929]	**0,578**
Arid2	Uncharacterized protein [Source:UniProtKB/TrEMBL;Acc:D3ZJU0] [ENSRNOT00000006970]	**0,578**
Bsdc1	Rattus norvegicus BSD domain containing 1 (Bsdc1), mRNA [NM_001106636]	**0,578**
Efna1	Rattus norvegicus ephrin A1 (Efna1), mRNA [NM_053599]	**0,578**
Hic2	Rattus norvegicus hypermethylated in cancer 2 (Hic2), mRNA [NM_001105862]	**0,578**
0	Unknown	**0,578**
Atf4	Rattus norvegicus activating transcription factor 4 (tax-responsive enhancer element B67) (Atf4), mRNA [NM_024403]	**0,578**
LOC100188984	Rattus norvegicus hypothetical protein LOC100188984 (LOC100188984), mRNA [NM_001134998]	**0,578**
0	Uncharacterized protein [Source:UniProtKB/TrEMBL;Acc:D3ZHQ7] [ENSRNOT00000032430]	**0,578**
Fam76a	Rattus norvegicus family with sequence similarity 76, member A (Fam76a), mRNA [NM_001108686]	**0,578**
Tspan2	Rattus norvegicus tetraspanin 2 (Tspan2), mRNA [NM_022589]	**0,578**
Rabep2	Rattus norvegicus rabaptin, RAB GTPase binding effector protein 2 (Rabep2), mRNA [NM_030585]	**0,578**
Nup160	Rattus norvegicus nucleoporin 160 (Nup160), mRNA [NM_001107744]	**0,578**
Hn1	Rattus norvegicus hematological and neurological expressed 1 (Hn1), mRNA [NM_001005876]	**0,578**
0	Q87XI0_PSESM (Q87XI0) Cobalamin synthesis protein/P47K family protein, partial (7%) [TC596564]	**0,578**
Tfcp2l1	Rattus norvegicus transcription factor CP2-like 1 (Tfcp2l1), mRNA [NM_001107170]	**0,578**
0	Uncharacterized protein [Source:UniProtKB/TrEMBL;Acc:D3ZFK7] [ENSRNOT00000050217]	**0,578**
Mta1	Rattus norvegicus metastasis associated 1 (Mta1), mRNA [NM_022588]	**0,578**
0	TBC1 domain family, member 23 (Predicted), isoform CRA_cUncharacterized protein [Source:UniProtKB/TrEMBL;Acc:D4A0K5] [ENSRNOT00000002240]	**0,578**
Snrnp27	Rattus norvegicus small nuclear ribonucleoprotein 27 (U4/U6.U5) (Snrnp27), mRNA [NM_001108636]	**0,578**
Ptprr	Rattus norvegicus protein tyrosine phosphatase, receptor type, R (Ptprr), transcript variant 1, mRNA [NM_053594]	**0,578**
0	Unknown	**0,578**
RGD1304694	Uncharacterized protein C22orf9 homolog [Source:UniProtKB/Swiss-Prot;Acc:Q4G008] [ENSRNOT00000042070]	**0,578**

0	set=Set beta isoform {alternatively spliced} [rats, neonatal kidney, mRNA, 2026 nt]. [S68987]	**0,578**
LOC500392	Rattus norvegicus similar to hypothetical protein FLJ25692 (LOC500392), mRNA [NM_001024340]	**0,578**
Phf2	Rattus norvegicus PHD finger protein 2 (Phf2), mRNA [NM_001107342]	**0,578**
0	Unknown	**0,578**
Zfand1	PREDICTED: Rattus norvegicus zinc finger, AN1-type domain 1 (Zfand1), mRNA [XM_342213]	**0,578**
Zfp318	PREDICTED: Rattus norvegicus zinc finger protein 318 (Zfp318), mRNA [XM_002727181]	**0,578**
Pom121	Rattus norvegicus nuclear pore membrane protein 121 (Pom121), mRNA [NM_053622]	**0,578**
Znf598	Rattus norvegicus zinc finger protein 598 (Znf598), mRNA [NM_001105770]	**0,578**
Mobkl3	Rattus norvegicus MOB1, Mps One Binder kinase activator-like 3 (yeast) (Mobkl3), mRNA [NM_133528]	**0,579**
Galm	Rattus norvegicus galactose mutarotase (aldose 1-epimerase) (Galm), mRNA [NM_001007704]	**0,579**
Zfp280d	Rattus norvegicus zinc finger protein 280D (Zfp280d), mRNA [NM_001108165]	**0,579**
Tmem132c	PREDICTED: Rattus norvegicus transmembrane protein 132C (Tmem132c), mRNA [XM_002724836]	**0,579**
RGD1566264	Uncharacterized protein [Source:UniProtKB/TrEMBL;Acc:D3ZFF1] [ENSRNOT00000051929]	**0,579**
Klf15	Rattus norvegicus Kruppel-like factor 15 (Klf15), mRNA [NM_053536]	**0,579**
0	Neuronal cell adhesion molecule [Source:UniProtKB/Swiss-Prot;Acc:P97686] [ENSRNOT00000059737]	**0,579**
Apom	Rattus norvegicus apolipoprotein M (Apom), mRNA [NM_019373]	**0,579**
Trit1	Rattus norvegicus tRNA isopentenyltransferase 1 (Trit1), mRNA [NM_001108676]	**0,579**
Cbx5	Rattus norvegicus chromobox homolog 5 (HP1 alpha homolog, Drosophila) (Cbx5), mRNA [NM_001106797]	**0,579**
Polr3g	Rattus norvegicus polymerase (RNA) III (DNA directed) polypeptide G (Polr3g), mRNA [NM_001109468]	**0,579**
Cr1l	Rattus norvegicus complement component (3b/4b) receptor 1-like (Cr1l), transcript variant 1, mRNA [NM_001005330]	**0,579**
0	GTPase KRasGTPase KRas, N-terminally processed [Source:UniProtKB/Swiss-Prot;Acc:P08644] [ENSRNOT00000012588]	**0,579**
Ypel5	Rattus norvegicus yippee-like 5 (Drosophila) (Ypel5), mRNA [NM_001035221]	**0,579**
Fyttd1	Rattus norvegicus forty-two-three domain containing 1 (Fyttd1), mRNA [NM_001047899]	**0,579**
Add3	Rattus norvegicus adducin 3 (gamma) (Add3), transcript variant 1, mRNA [NM_001164103]	**0,579**
Bad	Rattus norvegicus BCL2-associated agonist of cell death (Bad), mRNA [NM_022698]	**0,579**
Cbfb	Rattus norvegicus core-binding factor, beta subunit (Cbfb), mRNA [NM_001013191]	**0,579**
Strn3	Rattus norvegicus striatin, calmodulin binding protein 3 (Strn3), mRNA [NM_001029897]	**0,579**
RGD1562608	Rattus norvegicus similar to KIAA1328 protein (RGD1562608), mRNA [NM_001134607]	**0,579**
Dpm2	Rattus norvegicus dolichyl-phosphate mannosyltransferase polypeptide 2, regulatory subunit (Dpm2), mRNA [NM_019252]	**0,579**
0	Unknown	**0,579**
Rngtt	Rattus norvegicus RNA guanylyltransferase and 5'-phosphatase (Rngtt), mRNA [NM_001107923]	**0,579**
Agtrap	Rattus norvegicus angiotensin II receptor-associated protein (Agtrap), mRNA [NM_001007654]	**0,579**
Aak1	Rattus norvegicus AP2 associated kinase 1 (Aak1), mRNA [NM_001173450]	**0,579**
Shc4	Rattus norvegicus SHC (Src homology 2 domain containing) family, member 4 (Shc4), mRNA [NM_001191065]	**0,579**

Pbx4	Rattus norvegicus pre-B-cell leukemia homeobox 4 (Pbx4), mRNA [NM_001108399]	**0,579**
0	Unknown	**0,579**
0	Unknown	**0,579**
Pex12	Rattus norvegicus peroxisomal biogenesis factor 12 (Pex12), mRNA [NM_053921]	**0,579**
Gpr27	Rattus norvegicus G protein-coupled receptor 27 (Gpr27), mRNA [NM_023099]	**0,580**
Arhgef3	Rattus norvegicus Rho guanine nucleotide exchange factor (GEF) 3 (Arhgef3), mRNA [NM_001106061]	**0,580**
0	Uncharacterized protein [Source:UniProtKB/TrEMBL;Acc:D3Z930] [ENSRNOT00000031558]	**0,580**
Fam49a	Rattus norvegicus family with sequence similarity 49, member A (Fam49a), mRNA [NM_001106718]	**0,580**
Etnk2	Rattus norvegicus ethanolamine kinase 2 (Etnk2), mRNA [NM_001108343]	**0,580**
Ttc14	Rattus norvegicus tetratricopeptide repeat domain 14 (Ttc14), mRNA [NM_001107666]	**0,580**
0	Unknown	**0,580**
Fis1	Rattus norvegicus fission 1 (mitochondrial outer membrane) homolog (S. cerevisiae) (Fis1), nuclear gene encoding mitochondrial protein, mRNA [NM_001105919]	**0,580**
Zfc3h1	PREDICTED: Rattus norvegicus proline/serine-rich coiled-coil 2, transcript variant 2 (Psrc2), mRNA [XM_001078700]	**0,580**
Dcaf5	Rattus norvegicus DDB1 and CUL4 associated factor 5 (Dcaf5), nuclear gene encoding mitochondrial protein, mRNA [NM_001100718]	**0,580**
Zfp187	Uncharacterized protein [Source:UniProtKB/TrEMBL;Acc:D3ZFY2] [ENSRNOT00000024693]	**0,580**
Znf142	Rattus norvegicus zinc finger protein 142 (Znf142), mRNA [NM_001108225]	**0,580**
Sfrs6	Rattus norvegicus splicing factor, arginine/serine-rich 6 (Sfrs6), mRNA [NM_001014185]	**0,580**
Pdcd6ip	Rattus norvegicus programmed cell death 6 interacting protein (Pdcd6ip), mRNA [NM_001029910]	**0,580**
0	Unknown	**0,580**
Me1	Rattus norvegicus malic enzyme 1, NADP(+)-dependent, cytosolic (Me1), mRNA [NM_012600]	**0,580**
Dgkb	Rattus norvegicus diacylglycerol kinase, beta (Dgkb), mRNA [NM_019304]	**0,580**
Bbs4	Rattus norvegicus Bardet-Biedl syndrome 4 (Bbs4), mRNA [NM_001106826]	**0,580**
LOC690000	PREDICTED: Rattus norvegicus similar to CG3740-PA, transcript variant 2 (LOC690000), mRNA [XM_001073988]	**0,580**
Mtch1	Rattus norvegicus mitochondrial carrier homolog 1 (C. elegans) (Mtch1), nuclear gene encoding mitochondrial protein, mRNA [NM_001100833]	**0,580**
0	Unknown	**0,580**
Acsm5	Rattus norvegicus acyl-CoA synthetase medium-chain family member 5 (Acsm5), mRNA [NM_001014162]	**0,580**
Eif2ak2	Rattus norvegicus eukaryotic translation initiation factor 2-alpha kinase 2 (Eif2ak2), mRNA [NM_019335]	**0,580**
Zscan18	Uncharacterized protein [Source:UniProtKB/TrEMBL;Acc:D3ZCL4] [ENSRNOT00000036880]	**0,580**
Hmg20a	Rattus norvegicus high mobility group 20A (Hmg20a), mRNA [NM_001108150]	**0,580**
Ube3b	Rattus norvegicus ubiquitin protein ligase E3B (Ube3b), mRNA [NM_001143894]	**0,580**
Entpd2	Rattus norvegicus ectonucleoside triphosphate diphosphohydrolase 2 (Entpd2), mRNA [NM_172030]	**0,580**
Slc25a29	Rattus norvegicus solute carrier family 25, member 29 (Slc25a29), nuclear gene encoding mitochondrial protein, mRNA [NM_001010958]	**0,580**
Dennd2a	Uncharacterized protein [Source:UniProtKB/TrEMBL;Acc:D3ZLQ1] [ENSRNOT00000034549]	**0,580**

Ankib1	Rattus norvegicus ankyrin repeat and IBR domain containing 1 (Ankib1), mRNA [NM_001134781]	**0,580**
Pik3r1	Rattus norvegicus phosphoinositide-3-kinase, regulatory subunit 1 (alpha) (Pik3r1), mRNA [NM_013005]	**0,580**
U2surp	PREDICTED: Rattus norvegicus similar to CG9346-PA, transcript variant 1 (RGD1307882), mRNA [XM_002729954]	**0,580**
Wdr31	Rattus norvegicus WD repeat domain 31 (Wdr31), mRNA [NM_001011976]	**0,580**
Atp2a2	Rattus norvegicus ATPase, Ca++ transporting, cardiac muscle, slow twitch 2 (Atp2a2), transcript variant 2, mRNA [NM_001110139]	**0,580**
Hsd17b11	Rattus norvegicus hydroxysteroid (17-beta) dehydrogenase 11 (Hsd17b11), mRNA [NM_001004209]	**0,580**
Katnal1	Rattus norvegicus katanin p60 subunit A-like 1 (Katnal1), mRNA [NM_001006956]	**0,580**
Slc8a1	Rattus norvegicus solute carrier family 8 (sodium/calcium exchanger), member 1 (Slc8a1), mRNA [NM_019268]	**0,580**
Arhgap10	Rattus norvegicus Rho GTPase activating protein 10 (Arhgap10), mRNA [NM_001109501]	**0,580**
Tmem49	Rattus norvegicus transmembrane protein 49 (Tmem49), mRNA [NM_138839]	**0,580**
Ambra1	Rattus norvegicus autophagy/beclin 1 regulator 1 (Ambra1), mRNA [NM_001134341]	**0,580**
Ankrd57	Rattus norvegicus ankyrin repeat domain 57 (Ankrd57), mRNA [NM_001109364]	**0,580**
Polr2e	Rattus norvegicus polymerase (RNA) II (DNA directed) polypeptide E (Polr2e), mRNA [NM_001109614]	**0,580**
Anubl1	Rattus norvegicus AN1, ubiquitin-like, homolog (Xenopus laevis) (Anubl1), mRNA [NM_173332]	**0,580**
Ahsa2	Rattus norvegicus AHA1, activator of heat shock protein ATPase homolog 2 (yeast) (Ahsa2), mRNA [NM_001107241]	**0,580**
Agt	Rattus norvegicus angiotensinogen (serpin peptidase inhibitor, clade A, member 8) (Agt), mRNA [NM_134432]	**0,580**
LOC684996	Rattus norvegicus similar to chromosome 7 open reading frame 11 (LOC684996), mRNA [NM_001109452]	**0,581**
Prim1	Rattus norvegicus DNA primase, p49 subunit (Prim1), mRNA [NM_001008768]	**0,581**
0	Unknown	**0,581**
Rhoq	Rattus norvegicus ras homolog gene family, member Q (Rhoq), mRNA [NM_053522]	**0,581**
0	Unknown	**0,581**
Fas	Rattus norvegicus Fas (TNF receptor superfamily, member 6) (Fas), mRNA [NM_139194]	**0,581**
Slc1a6	Rattus norvegicus solute carrier family 1 (high affinity aspartate/glutamate transporter), member 6 (Slc1a6), mRNA [NM_032065]	**0,581**
Gria4	Rattus norvegicus glutamate receptor, ionotrophic, AMPA 4 (Gria4), transcript variant 3, mRNA [NM_001113185]	**0,581**
Gad1	Rattus norvegicus glutamate decarboxylase 1 (Gad1), mRNA [NM_017007]	**0,581**
Cks2	Rattus norvegicus CDC28 protein kinase regulatory subunit 2 (Cks2), mRNA [NM_001126083]	**0,581**
Dusp3	Rattus norvegicus dual specificity phosphatase 3 (Dusp3), mRNA [NM_001173376]	**0,581**
LOC680692	PREDICTED: Rattus norvegicus similar to Golgi phosphoprotein 2 (Golgi membrane protein GP73) (LOC680692), mRNA [XM_002725221]	**0,581**
Zmynd8	Rattus norvegicus zinc finger, MYND-type containing 8 (Zmynd8), mRNA [NM_001100838]	**0,581**
Syt16	Uncharacterized protein [Source:UniProtKB/TrEMBL;Acc:D3ZB68] [ENSRNOT00000012374]	**0,581**
Bbc3	Rattus norvegicus Bcl-2 binding component 3 (Bbc3), mRNA [NM_173837]	**0,581**
Znf467	Rattus norvegicus zinc finger protein 467 (Znf467), mRNA [NM_001024327]	**0,581**
Plekha3	Rattus norvegicus pleckstrin homology domain-containing, family A (phosphoinositide binding specific) member 3 (Plekha3), mRNA [NM_001013077]	**0,581**

Chd4	PREDICTED: Rattus norvegicus chromodomain helicase DNA binding protein 4 (Chd4), mRNA [XM_232354]	**0,581**
Tcf12	Rattus norvegicus transcription factor 12 (Tcf12), mRNA [NM_013176]	**0,581**
RGD1308093	PREDICTED: Rattus norvegicus similar to FLJ00128 protein (RGD1308093), mRNA [XM_001075554]	**0,581**
Ptprd	PREDICTED: Rattus norvegicus protein tyrosine phosphatase, receptor type, D (Ptprd), mRNA [XM_233065]	**0,581**
Strada	Rattus norvegicus STE20-related kinase adaptor alpha (Strada), mRNA [NM_182820]	**0,581**
Kcne2	Rattus norvegicus potassium voltage-gated channel, Isk-related family, member 2 (Kcne2), mRNA [NM_133603]	**0,581**
Spock3	Rattus norvegicus sparc/osteonectin, cwcv and kazal-like domains proteoglycan (testican) 3 (Spock3), mRNA [NM_001107310]	**0,581**
RGD1304878	PREDICTED: Rattus norvegicus similar to 2410024A21Rik protein (RGD1304878), miscRNA [XR_005460]	**0,581**
Slc26a10	solute carrier family 26 member 10 [Source:RefSeq peptide;Acc:NP_001128067] [ENSRNOT00000052288]	**0,581**
Yy1	Rattus norvegicus YY1 transcription factor (Yy1), mRNA [NM_173290]	**0,581**
0	UI-R-BS1-azr-f-06-0-UI.s1 UI-R-BS1 Rattus norvegicus cDNA clone UI-R-BS1-azr-f-06-0-UI 3', mRNA sequence [BE118692]	**0,581**
Npm3	Uncharacterized protein [Source:UniProtKB/TrEMBL;Acc:D3ZYK9] [ENSRNOT00000023963]	**0,581**
Adra2a	Rattus norvegicus adrenergic, alpha-2A-, receptor (Adra2a), mRNA [NM_012739]	**0,581**
Mif	Rattus norvegicus macrophage migration inhibitory factor (Mif), mRNA [NM_031051]	**0,581**
Fam96b	Rattus norvegicus family with sequence similarity 96, member B (Fam96b), mRNA [NM_001144854]	**0,581**
0	Unknown	**0,581**
Calca	Rattus norvegicus calcitonin-related polypeptide alpha (Calca), transcript variant 2, mRNA [NM_001033955]	**0,581**
Pus10	Rattus norvegicus pseudouridylate synthase 10 (Pus10), mRNA [NM_001025278]	**0,581**
Wdfy2	Rattus norvegicus WD repeat and FYVE domain containing 2 (Wdfy2), mRNA [NM_001107269]	**0,581**
0	Proprotein convertase subtilisin/kexin type 5 [Source:UniProtKB/Swiss-Prot;Acc:P41413] [ENSRNOT00000016164]	**0,581**
Nfe2l2	Rattus norvegicus nuclear factor, erythroid derived 2, like 2 (Nfe2l2), mRNA [NM_031789]	**0,581**
0	Uncharacterized protein [Source:UniProtKB/TrEMBL;Acc:D3ZRC6] [ENSRNOT00000050943]	**0,581**
Pet112l	PREDICTED: Rattus norvegicus PET112-like (yeast) (Pet112l), mRNA [XM_001066526]	**0,581**
Ifi30	Rattus norvegicus interferon gamma inducible protein 30 (Ifi30), mRNA [NM_001030026]	**0,581**
Stbd1	Rattus norvegicus starch binding domain 1 (Stbd1), mRNA [NM_001013988]	**0,581**
Gzf1	Rattus norvegicus GDNF-inducible zinc finger protein 1 (Gzf1), mRNA [NM_001107788]	**0,581**
Rffl	Rattus norvegicus ring finger and FYVE like domain containing protein (Rffl), mRNA [NM_001004068]	**0,582**
Gripap1	Rattus norvegicus GRIP1 associated protein 1 (Gripap1), mRNA [NM_053807]	**0,582**
Eif3j	Rattus norvegicus eukaryotic translation initiation factor 3, subunit J (Eif3j), mRNA [NM_001077670]	**0,582**
Klhl15	Rattus norvegicus kelch-like 15 (Drosophila) (Klhl15), mRNA [NM_001108021]	**0,582**
Rab11fip4	Rattus norvegicus RAB11 family interacting protein 4 (class II) (Rab11fip4), mRNA [NM_001107023]	**0,582**
0	Unknown	**0,582**
Dcun1d4	Rattus norvegicus DCN1, defective in cullin neddylation 1, domain containing 4 (S. cerevisiae) (Dcun1d4), mRNA [NM_001108359]	**0,582**
Hus1	Rattus norvegicus HUS1 checkpoint homolog (S. pombe) (Hus1), mRNA [NM_001109092]	**0,582**

Fam195b	Rattus norvegicus family with sequence similarity 195, member B (Fam195b), mRNA [NM_001108311]	**0,582**
Olr297	Rattus norvegicus olfactory receptor 297 (Olr297), mRNA [NM_001000234]	**0,582**
0	Unknown	**0,582**
0	Unknown	**0,582**
LOC498606	Rattus norvegicus hypothetical protein LOC498606 (LOC498606), mRNA [NM_001025143]	**0,582**
Zmynd11	Rattus norvegicus zinc finger, MYND domain containing 11 (Zmynd11), transcript variant 3, mRNA [NM_203369]	**0,582**
LOC686298	PREDICTED: Rattus norvegicus similar to protein phosphatase 2C eta isoform 2 (LOC686298), mRNA [XM_001073372]	**0,582**
Rab22a	Rattus norvegicus RAB22A, member RAS oncogene family (Rab22a), mRNA [NM_001108966]	**0,582**
0	Unknown	**0,582**
Cpamd8	PREDICTED: Rattus norvegicus similar to Murinoglobulin 1 homolog (RGD1566313), mRNA [XM_213006]	**0,582**
Ranbp1	Rattus norvegicus RAN binding protein 1 (Ranbp1), mRNA [NM_001108324]	**0,582**
Pldn	Rattus norvegicus pallidin homolog (mouse) (Pldn), mRNA [NM_001025714]	**0,582**
0	Unknown	**0,582**
Cbx2	Rattus norvegicus chromobox homolog 2 (Pc class homolog, Drosophila) (Cbx2), mRNA [NM_001107071]	**0,582**
Pias3	Rattus norvegicus protein inhibitor of activated STAT, 3 (Pias3), mRNA [NM_031784]	**0,582**
Kctd5	Rattus norvegicus potassium channel tetramerisation domain containing 5 (Kctd5), mRNA [NM_001105768]	**0,582**
Rnf32	Rattus norvegicus ring finger protein 32 (Rnf32), mRNA [NM_001012095]	**0,582**
Ddt	Rattus norvegicus D-dopachrome tautomerase (Ddt), mRNA [NM_024131]	**0,582**
Nrg1	Rattus norvegicus neuregulin 1 (Nrg1), mRNA [NM_031588]	**0,582**
0	Unknown	**0,582**
Lzts2	Rattus norvegicus leucine zipper, putative tumor suppressor 2 (Lzts2), mRNA [NM_001014247]	**0,582**
Ssr1	Rattus norvegicus signal sequence receptor, alpha (Ssr1), mRNA [NM_001008891]	**0,582**
Idi1	Rattus norvegicus isopentenyl-diphosphate delta isomerase 1 (Idi1), mRNA [NM_053539]	**0,582**
0	Unknown	**0,582**
Kcnd1	Rattus norvegicus potassium voltage-gated channel, Shal-related subfamily, member 1 (Kcnd1), mRNA [NM_001105748]	**0,582**
Irf2bp1	Rattus norvegicus interferon regulatory factor 2 binding protein 1 (Irf2bp1), mRNA [NM_001107483]	**0,582**
0	Unknown	**0,582**
Dtx4	Rattus norvegicus deltex homolog 4 (Drosophila) (Dtx4), mRNA [NM_001047855]	**0,582**
Cog1	Rattus norvegicus component of oligomeric golgi complex 1 (Cog1), mRNA [NM_001107062]	**0,582**
Gabpa	Rattus norvegicus GA binding protein transcription factor, alpha subunit (Gabpa), mRNA [NM_001108841]	**0,582**
Eri1	Rattus norvegicus exoribonuclease 1 (Eri1), mRNA [NM_001014143]	**0,582**
Stmn2	Rattus norvegicus stathmin-like 2 (Stmn2), mRNA [NM_053440]	**0,582**
Tob1	Rattus norvegicus transducer of ErbB-2.1 (Tob1), mRNA [NM_133317]	**0,582**
Foxk2	Rattus norvegicus forkhead box K2 (Foxk2), mRNA [NM_001107075]	**0,582**

Ppapdc2	Rattus norvegicus phosphatidic acid phosphatase type 2 domain containing 2 (Ppapdc2), mRNA [NM_001034854]	**0,582**
0	Uncharacterized protein [Source:UniProtKB/TrEMBL;Acc:D3ZR70] [ENSRNOT00000030150]	**0,582**
Zcchc7	Rattus norvegicus zinc finger, CCHC domain containing 7 (Zcchc7), mRNA [NM_001106658]	**0,582**
Mfng	Rattus norvegicus MFNG O-fucosylpeptide 3-beta-N-acetylglucosaminyltransferase (Mfng), mRNA [NM_199110]	**0,582**
Nrip3	Rattus norvegicus nuclear receptor interacting protein 3 (Nrip3), mRNA [NM_001108498]	**0,582**
Cbx3	Rattus norvegicus chromobox homolog 3 (HP1 gamma homolog, Drosophila) (Cbx3), mRNA [NM_001008313]	**0,582**
Znf667	Rattus norvegicus zinc finger protein 667 (Znf667), mRNA [NM_001008557]	**0,582**
0	LIM domain only protein 3 [Source:UniProtKB/Swiss-Prot;Acc:Q99MB5] [ENSRNOT00000010623]	**0,582**
LOC100362172	LRRGT00112-like (LOC100362172), mRNA [Source:RefSeq DNA;Acc:NM_001177820] [ENSRNOT00000043267]	**0,582**
Lrrn1	Rattus norvegicus leucine rich repeat neuronal 1 (Lrrn1), mRNA [NM_001037363]	**0,582**
Pcp4l1	Rattus norvegicus Purkinje cell protein 4-like 1 (Pcp4l1), mRNA [NM_001126093]	**0,583**
Ctnna2	Rattus norvegicus catenin (cadherin associated protein), alpha 2 (Ctnna2), mRNA [NM_001106598]	**0,583**
Il6st	Rattus norvegicus interleukin 6 signal transducer (Il6st), mRNA [NM_001008725]	**0,583**
0	Q80V97_MOUSE (Q80V97) Itga9 protein (Fragment), partial (67%) [TC615359]	**0,583**
Wwp1	Rattus norvegicus WW domain containing E3 ubiquitin protein ligase 1 (Wwp1), mRNA [NM_001024757]	**0,583**
Mtmr7	Rattus norvegicus myotubularin related protein 7 (Mtmr7), mRNA [NM_001107312]	**0,583**
Parg	Rattus norvegicus poly (ADP-ribose) glycohydrolase (Parg), mRNA [NM_031339]	**0,583**
RGD1359634	Rattus norvegicus similar to RIKEN cDNA 1700088E04 (RGD1359634), mRNA [NM_001007708]	**0,583**
Thoc4	Rattus norvegicus THO complex 4 (Thoc4), mRNA [NM_001109602]	**0,583**
Ptp4a1	Rattus norvegicus protein tyrosine phosphatase type IVA, member 1 (Ptp4a1), mRNA [NM_031579]	**0,583**
0	Rattus norvegicus TL0ABA36YD02 mRNA sequence. [FQ210020]	**0,583**
Lrrc4	Rattus norvegicus leucine rich repeat containing 4 (Lrrc4), mRNA [NM_001037336]	**0,583**
0	Unknown	**0,583**
Med23	Mediator of RNA polymerase II transcription subunit 23 [Source:UniProtKB/Swiss-Prot;Acc:Q5EB59] [ENSRNOT00000018404]	**0,583**
Spryd3	Rattus norvegicus SPRY domain containing 3 (Spryd3), mRNA [NM_001191790]	**0,583**
Pex5l	Rattus norvegicus peroxisomal biogenesis factor 5-like (Pex5l), mRNA [NM_173152]	**0,583**
Tram1	Rattus norvegicus translocation associated membrane protein 1 (Tram1), mRNA [NM_001007701]	**0,583**
Polr2i	Rattus norvegicus polymerase (RNA) II (DNA directed) polypeptide I (Polr2i), mRNA [NM_001106244]	**0,583**
Pcdh19	Rattus norvegicus protocadherin 19 (Pcdh19), mRNA [NM_001169129]	**0,583**
Bcap31	Rattus norvegicus B-cell receptor-associated protein 31 (Bcap31), mRNA [NM_001004224]	**0,583**
Sstr2	Rattus norvegicus somatostatin receptor 2 (Sstr2), mRNA [NM_019348]	**0,583**
Sik1	Serine/threonine-protein kinase SIK1 [Source:UniProtKB/Swiss-Prot;Acc:Q9R1U5] [ENSRNOT00000001579]	**0,583**
Tmem111	Rattus norvegicus transmembrane protein 111 (Tmem111), mRNA [NM_001008355]	**0,583**
Capn1	Rattus norvegicus calpain 1 (Capn1), mRNA [NM_019152]	**0,583**

Zc3h18	Rattus norvegicus zinc finger CCCH-type containing 18 (Zc3h18), mRNA [NM_201416]	**0,583**
0	Unknown	**0,583**
Dusp4	Rattus norvegicus dual specificity phosphatase 4 (Dusp4), mRNA [NM_022199]	**0,583**
Zc3h15	Rattus norvegicus zinc finger CCCH-type containing 15 (Zc3h15), mRNA [NM_001010963]	**0,583**
Irgq	Rattus norvegicus immunity-related GTPase family, Q (Irgq), mRNA [NM_001135742]	**0,583**
Mmgt1	Rattus norvegicus membrane magnesium transporter 1 (Mmgt1), mRNA [NM_001106970]	**0,583**
Znf511	Rattus norvegicus zinc finger protein 511 (Znf511), mRNA [NM_001106309]	**0,583**
LOC688459	Rattus norvegicus hypothetical protein LOC688459 (LOC688459), mRNA [NM_001109502]	**0,583**
Tgoln1	Rattus norvegicus trans-golgi network protein (Tgoln1), mRNA [NM_138840]	**0,583**
0	Unknown	**0,583**
Ybx1	Rattus norvegicus Y box binding protein 1 (Ybx1), mRNA [NM_031563]	**0,583**
RT1-CE3	Rattus norvegicus RT1 class I, locus CE3 (RT1-CE3), mRNA [NM_001008841]	**0,583**
Ptprf	Rattus norvegicus protein tyrosine phosphatase, receptor type, F (Ptprf), mRNA [NM_019249]	**0,583**
0	EST346778 Normalized rat embryo, Bento Soares Rattus sp. cDNA clone RGICV78 5' end, mRNA sequence [AW915474]	**0,583**
Dpp8	Rattus norvegicus dipeptidylpeptidase 8 (Dpp8), mRNA [NM_001108159]	**0,584**
0	Unknown	**0,584**
0	AF440762 septin SEPT8_v2 {Homo sapiens} (exp=-1; wgp=0; cg=0), partial (97%) [TC612969]	**0,584**
Ttpal	Rattus norvegicus tocopherol (alpha) transfer protein-like (Ttpal), mRNA [NM_001106537]	**0,584**
Rab35	Rattus norvegicus RAB35, member RAS oncogene family (Rab35), mRNA [NM_001013046]	**0,584**
0	Unknown	**0,584**
RGD1563300	Uncharacterized protein [Source:UniProtKB/TrEMBL;Acc:D3Z8Q4] [ENSRNOT00000032995]	**0,584**
Epha5	Rattus norvegicus EphA5 (Epha5), transcript variant 2, mRNA [NM_024367]	**0,584**
Rabgap1	Rattus norvegicus RAB GTPase activating protein 1 (Rabgap1), mRNA [NM_001107841]	**0,584**
Adra1d	Rattus norvegicus adrenergic, alpha-1D-, receptor (Adra1d), mRNA [NM_024483]	**0,584**
C2	Rattus norvegicus complement component 2 (C2), mRNA [NM_172222]	**0,584**
Gprc5d	Rattus norvegicus G protein-coupled receptor, family C, group 5, member D (Gprc5d), mRNA [NM_001109254]	**0,584**
Zfp346	Rattus norvegicus zinc finger protein 346 (Zfp346), mRNA [NM_001107338]	**0,584**
Lpar3	Rattus norvegicus lysophosphatidic acid receptor 3 (Lpar3), mRNA [NM_023969]	**0,584**
Ptprr	Rattus norvegicus protein tyrosine phosphatase, receptor type, R (Ptprr), transcript variant 1, mRNA [NM_053594]	**0,584**
Zfp319	Rattus norvegicus zinc finger protein 319 (Zfp319), mRNA [NM_001170478]	**0,584**
Phkb	Rattus norvegicus phosphorylase kinase, beta (Phkb), mRNA [NM_001014152]	**0,584**
RGD1561766	PREDICTED: Rattus norvegicus similar to basic transcription factor 3 (RGD1561766), miscRNA [XR_009374]	**0,584**
Aurkc	Rattus norvegicus aurora kinase C (Aurkc), mRNA [NM_001106221]	**0,584**
Cant1	Rattus norvegicus calcium activated nucleotidase 1 (Cant1), mRNA [NM_144754]	**0,584**

Ptpro	Rattus norvegicus protein tyrosine phosphatase, receptor type, O (Ptpro), mRNA [NM_017336]	**0,584**
Camk4	Rattus norvegicus calcium/calmodulin-dependent protein kinase IV (Camk4), mRNA [NM_012727]	**0,584**
Tjap1	Rattus norvegicus tight junction associated protein 1 (Tjap1), mRNA [NM_001108203]	**0,584**
LOC680319	Rattus norvegicus hypothetical protein LOC680319 (LOC680319), mRNA [NM_001109401]	**0,584**
Ube2c	Rattus norvegicus ubiquitin-conjugating enzyme E2C (Ube2c), mRNA [NM_001106542]	**0,584**
Atp5g2	Rattus norvegicus ATP synthase, H+ transporting, mitochondrial F0 complex, subunit C2 (subunit 9) (Atp5g2), nuclear gene encoding mitochondrial protein, mRNA [NM_133556]	**0,584**
RGD1561530	PREDICTED: Rattus norvegicus similar to Tle6 protein (RGD1561530), miscRNA [XR_009018]	**0,584**
0	Unknown	**0,584**
Rnf138	Rattus norvegicus ring finger protein 138 (Rnf138), mRNA [NM_053588]	**0,584**
Sdhaf2	Rattus norvegicus succinate dehydrogenase complex assembly factor 2 (Sdhaf2), nuclear gene encoding mitochondrial protein, mRNA [NM_001008371]	**0,584**
Polr2e	Rattus norvegicus polymerase (RNA) II (DNA directed) polypeptide E (Polr2e), mRNA [NM_001109614]	**0,584**
Atp11a	Rattus norvegicus ATPase, class VI, type 11A (Atp11a), mRNA [NM_001107324]	**0,584**
Zfat	Rattus norvegicus zinc finger and AT hook domain containing (Zfat), mRNA [NM_001134957]	**0,584**
Tnpo3	Rattus norvegicus transportin 3 (Tnpo3), mRNA [NM_001106587]	**0,584**
Nrcam	Rattus norvegicus neuronal cell adhesion molecule (Nrcam), mRNA [NM_013150]	**0,584**
RGD1560873	PREDICTED: Rattus norvegicus similar to RIKEN cDNA E230015L20 gene (RGD1560873), miscRNA [XR_085636]	**0,584**
Smpd3	Rattus norvegicus sphingomyelin phosphodiesterase 3, neutral membrane (Smpd3), mRNA [NM_053605]	**0,584**
Kcnq3	Rattus norvegicus potassium voltage-gated channel, KQT-like subfamily, member 3 (Kcnq3), mRNA [NM_031597]	**0,584**
Spry4	Rattus norvegicus sprouty homolog 4 (Drosophila) (Spry4), mRNA [NM_001106150]	**0,584**
Ubap2	Rattus norvegicus ubiquitin-associated protein 2 (Ubap2), mRNA [NM_001107928]	**0,584**
Apip	Rattus norvegicus APAF1 interacting protein (Apip), mRNA [NM_001106492]	**0,584**
Ext1	Rattus norvegicus exostoses (multiple) 1 (Ext1), mRNA [NM_001130540]	**0,584**
Ssr3	Rattus norvegicus signal sequence receptor, gamma (Ssr3), mRNA [NM_031120]	**0,584**
Notch4	Rattus norvegicus Notch homolog 4 (Drosophila) (Notch4), mRNA [NM_001002827]	**0,584**
0	Uncharacterized protein [Source:UniProtKB/TrEMBL;Acc:D3ZJC7] [ENSRNOT00000068355]	**0,584**
Snn	Rattus norvegicus stannin (Snn), mRNA [NM_001034083]	**0,584**
Slc25a42	Rattus norvegicus solute carrier family 25, member 42 (Slc25a42), mRNA [NM_001127590]	**0,584**
Ppp1r15a	Rattus norvegicus protein phosphatase 1, regulatory (inhibitor) subunit 15A (Ppp1r15a), mRNA [NM_133546]	**0,584**
LOC498662	Rattus norvegicus similar to RIKEN cDNA 2610019F03 (LOC498662), mRNA [NM_001100791]	**0,584**
LOC680155	Uncharacterized protein [Source:UniProtKB/TrEMBL;Acc:D3ZXU7] [ENSRNOT00000020271]	**0,584**
0	Histone H3 [Source:UniProtKB/TrEMBL;Acc:D3ZXC3] [ENSRNOT00000046278]	**0,585**
Dnajb2	Rattus norvegicus DnaJ (Hsp40) homolog, subfamily B, member 2 (Dnajb2), mRNA [NM_001109541]	**0,585**

Fam149b1	Rattus norvegicus family with sequence similarity 149, member B1 (Fam149b1), mRNA [NM_001013878]	**0,585**
Zfp365	Rattus norvegicus zinc finger protein 365 (Zfp365), mRNA [NM_001025145]	**0,585**
F8	Rattus norvegicus coagulation factor VIII, procoagulant component (F8), mRNA [NM_183331]	**0,585**
0	Unknown	**0,585**
0	Unknown	**0,585**
LOC689158	PREDICTED: Rattus norvegicus similar to 60S ribosomal protein L29 (LOC689158), mRNA [XM_002727076]	**0,585**
Rcbtb1	Rattus norvegicus regulator of chromosome condensation (RCC1) and BTB (POZ) domain containing protein 1 (Rcbtb1), mRNA [NM_001108380]	**0,585**
Ctnnd2	PREDICTED: Rattus norvegicus catenin (cadherin-associated protein), delta 2 (neural plakophilin-related arm-repeat protein) (Ctnnd2), mRNA [XM_001064375]	**0,585**
Slc35e3	Rattus norvegicus solute carrier family 35, member E3 (Slc35e3), mRNA [NM_001134687]	**0,585**
Gga3	Rattus norvegicus golgi associated, gamma adaptin ear containing, ARF binding protein 3 (Gga3), mRNA [NM_001108304]	**0,585**
Casc4	PREDICTED: Rattus norvegicus cancer susceptibility candidate 4, transcript variant 3 (Casc4), mRNA [XM_001077018]	**0,585**
Gstt2	Rattus norvegicus glutathione S-transferase, theta 2 (Gstt2), mRNA [NM_012796]	**0,585**
0	Unknown	**0,585**
Lipe	Rattus norvegicus lipase, hormone sensitive (Lipe), mRNA [NM_012859]	**0,585**
Apoa1bp	Rattus norvegicus apolipoprotein A-I binding protein (Apoa1bp), mRNA [NM_001106440]	**0,585**
RGD1306941	Rattus norvegicus similar to CG31122-PA (RGD1306941), mRNA [NM_001108219]	**0,585**
Plekhf2	Rattus norvegicus pleckstrin homology domain containing, family F (with FYVE domain) member 2 (Plekhf2), mRNA [NM_001108655]	**0,585**
Abcc4	Rattus norvegicus ATP-binding cassette, subfamily C (CFTR/MRP), member 4 (Abcc4), mRNA [NM_133411]	**0,585**
Slc17a8	Rattus norvegicus solute carrier family 17 (sodium-dependent inorganic phosphate cotransporter), member 8 (Slc17a8), mRNA [NM_153725]	**0,585**
Cdr2	Rattus norvegicus cerebellar degeneration-related 2 (Cdr2), mRNA [NM_001025682]	**0,585**
Orai3	Rattus norvegicus ORAI calcium release-activated calcium modulator 3 (Orai3), mRNA [NM_001014024]	**0,585**
Snd1	Rattus norvegicus staphylococcal nuclease and tudor domain containing 1 (Snd1), mRNA [NM_022694]	**0,585**
Tpbg	Rattus norvegicus trophoblast glycoprotein (Tpbg), mRNA [NM_031807]	**0,585**
0	Collagen alpha-1(XI) chain [Source:UniProtKB/Swiss-Prot;Acc:P20909] [ENSRNOT00000023693]	**0,585**
Asb8	Rattus norvegicus ankyrin repeat and SOCS box-containing 8 (Asb8), mRNA [NM_001108109]	**0,585**
Pld1	Rattus norvegicus phospholipase D1 (Pld1), mRNA [NM_030992]	**0,585**
RGD1565117	Uncharacterized protein [Source:UniProtKB/TrEMBL;Acc:D3ZJ54] [ENSRNOT00000045687]	**0,585**
Serinc3	Rattus norvegicus serine incorporator 3 (Serinc3), mRNA [NM_001008312]	**0,585**
Rexo4	Rattus norvegicus REX4, RNA exonuclease 4 homolog (S. cerevisiae) (Rexo4), mRNA [NM_001033884]	**0,585**
Cflar	Rattus norvegicus CASP8 and FADD-like apoptosis regulator (Cflar), transcript variant 2, mRNA [NM_057138]	**0,585**
Gorasp1	Rattus norvegicus golgi reassembly stacking protein 1 (Gorasp1), mRNA [NM_019385]	**0,585**
0	Histone H3 [Source:UniProtKB/TrEMBL;Acc:D4A602] [ENSRNOT00000030033]	**0,585**
0	Uncharacterized protein [Source:UniProtKB/TrEMBL;Acc:D3ZKX0] [ENSRNOT00000022136]	**0,585**

Ccl2	Rattus norvegicus chemokine (C-C motif) ligand 2 (Ccl2), mRNA [NM_031530]	**0,585**
Slc7a1	Rattus norvegicus solute carrier family 7 (cationic amino acid transporter, y+ system), member 1 (Slc7a1), mRNA [NM_013111]	**0,585**
Gnb3	Rattus norvegicus guanine nucleotide binding protein (G protein), beta polypeptide 3 (Gnb3), mRNA [NM_021858]	**0,585**
Aagab	Rattus norvegicus alpha- and gamma-adaptin binding protein (Aagab), mRNA [NM_134398]	**0,585**
RGD1566112	Rattus norvegicus similar to pleckstrin homology domain protein (5V327) (RGD1566112), mRNA [NM_001127566]	**0,586**
Pomgnt1	Rattus norvegicus protein O-linked mannose beta1,2-N-acetylglucosaminyltransferase (Pomgnt1), mRNA [NM_001007747]	**0,586**
Cyth2	Rattus norvegicus cytohesin 2 (Cyth2), mRNA [NM_053911]	**0,586**
Prdx3	Rattus norvegicus peroxiredoxin 3 (Prdx3), nuclear gene encoding mitochondrial protein, mRNA [NM_022540]	**0,586**
Dhrs1	Rattus norvegicus dehydrogenase/reductase (SDR family) member 1 (Dhrs1), mRNA [NM_001007621]	**0,586**
Tubg2	Rattus norvegicus tubulin, gamma 2 (Tubg2), mRNA [NM_001191075]	**0,586**
Rnf114	Rattus norvegicus ring finger protein 114 (Rnf114), mRNA [NM_001001517]	**0,586**
Dagla	Rattus norvegicus diacylglycerol lipase, alpha (Dagla), mRNA [NM_001005886]	**0,586**
Qpctl	Rattus norvegicus glutaminyl-peptide cyclotransferase-like (Qpctl), mRNA [NM_001106230]	**0,586**
Arhgef18	Rattus norvegicus rho/rac guanine nucleotide exchange factor (GEF) 18 (Arhgef18), mRNA [NM_001107115]	**0,586**
Tmco1	Rattus norvegicus transmembrane and coiled-coil domains 1 (Tmco1), mRNA [NM_001009631]	**0,586**
Pqlc3	Rattus norvegicus PQ loop repeat containing 3 (Pqlc3), mRNA [NM_001034952]	**0,586**
Ube2f	Rattus norvegicus ubiquitin-conjugating enzyme E2F (putative) (Ube2f), mRNA [NM_001008381]	**0,586**
Nrbp2	Rattus norvegicus nuclear receptor binding protein 2 (Nrbp2), mRNA [NM_001135007]	**0,586**
0	Unknown	**0,586**
RGD1306682	Rattus norvegicus similar to RIKEN cDNA 1810046J19 (RGD1306682), mRNA [NM_001108296]	**0,586**
Lrrc59	Rattus norvegicus leucine rich repeat containing 59 (Lrrc59), mRNA [NM_001008280]	**0,586**
Igsf3	Rattus norvegicus immunoglobulin superfamily, member 3 (Igsf3), mRNA [NM_001106455]	**0,586**
Pmepa1	prostate transmembrane protein, androgen induced 1 [Source:RefSeq peptide;Acc:NP_001101277] [ENSRNOT00000008261]	**0,586**
Plce1	Rattus norvegicus phospholipase C, epsilon 1 (Plce1), mRNA [NM_053758]	**0,586**
Txnrd1	Rattus norvegicus thioredoxin reductase 1 (Txnrd1), mRNA [NM_031614]	**0,586**
Suv420h1	Rattus norvegicus suppressor of variegation 4-20 homolog 1 (Drosophila) (Suv420h1), mRNA [NM_001108512]	**0,586**
Golga3	Rattus norvegicus golgi autoantigen, golgin subfamily a, 3 (Golga3), mRNA [NM_001107847]	**0,586**
RGD1305899	PREDICTED: Rattus norvegicus similar to Protein C20orf158 (RGD1305899), mRNA [XM_001058977]	**0,586**
Sidt2	Rattus norvegicus SID1 transmembrane family, member 2 (Sidt2), mRNA [NM_001108142]	**0,586**
Sap30bp	Rattus norvegicus SAP30 binding protein (Sap30bp), mRNA [NM_001108305]	**0,586**
Pxk	Rattus norvegicus PX domain containing serine/threonine kinase (Pxk), mRNA [NM_182821]	**0,586**
Fdft1	Rattus norvegicus farnesyl diphosphate farnesyl transferase 1 (Fdft1), mRNA [NM_019238]	**0,586**
Hif1an	Rattus norvegicus hypoxia-inducible factor 1, alpha subunit inhibitor (Hif1an), mRNA [NM_001113749]	**0,586**
Fam193a	Uncharacterized protein [Source:UniProtKB/TrEMBL;Acc:D3ZIG8] [ENSRNOT00000018616]	**0,586**

Gpr19	Rattus norvegicus G protein-coupled receptor 19 (Gpr19), mRNA [NM_080579]	**0,586**
0	Unknown	**0,586**
0	FM101283 etnofat Rattus norvegicus cDNA clone etnofatP0059F08 5', mRNA sequence [FM101283]	**0,586**
Cdc34	Rattus norvegicus cell division cycle 34 homolog (S. cerevisiae) (Cdc34), mRNA [NM_001013103]	**0,586**
0	Unknown	**0,586**
Rlim	Rattus norvegicus ring finger protein, LIM domain interacting (Rlim), mRNA [NM_001024892]	**0,586**
Cpeb1	Rattus norvegicus cytoplasmic polyadenylation element binding protein 1 (Cpeb1), mRNA [NM_001106276]	**0,586**
LOC290595	Rattus norvegicus hypothetical gene supported by AF152002 (LOC290595), mRNA [NM_001106063]	**0,586**
Gtf2ird2	General transcription factor II I repeat domain-containing 2 [Source:UniProtKB/TrEMBL;Acc:Q2V6E6] [ENSRNOT00000002030]	**0,586**
Gpi	Rattus norvegicus glucose phosphate isomerase (Gpi), mRNA [NM_207592]	**0,586**
Syngr3	Rattus norvegicus synaptogyrin 3 (Syngr3), mRNA [NM_001106985]	**0,586**
Nipsnap1	Rattus norvegicus nipsnap homolog 1 (C. elegans) (Nipsnap1), mRNA [NM_001100730]	**0,586**
Robo2	Rattus norvegicus roundabout homolog 2 (Drosophila) (Robo2), mRNA [NM_032106]	**0,586**
Qser1	Rattus norvegicus glutamine and serine rich 1 (Qser1), mRNA [NM_001139493]	**0,586**
Ccdc91	Rattus norvegicus coiled-coil domain containing 91 (Ccdc91), mRNA [NM_001014061]	**0,586**
Adipor1	Rattus norvegicus adiponectin receptor 1 (Adipor1), mRNA [NM_207587]	**0,586**
Oaf	Rattus norvegicus OAF homolog (Drosophila) (Oaf), mRNA [NM_001014090]	**0,586**
0	Unknown	**0,586**
LOC688948	Uncharacterized protein [Source:UniProtKB/TrEMBL;Acc:D4AAJ9] [ENSRNOT00000041748]	**0,586**
RGD1310348	Rattus norvegicus similar to Ser/Thr-rich protein T10 in DGCR region (RGD1310348), mRNA [NM_001108323]	**0,586**
Irak3	Rattus norvegicus interleukin-1 receptor-associated kinase 3 (Irak3), mRNA [NM_001108101]	**0,587**
Slc43a2	Rattus norvegicus solute carrier family 43, member 2 (Slc43a2), mRNA [NM_001105812]	**0,587**
LOC501302	PREDICTED: Rattus norvegicus hypothetical gene supported by BC082068 (LOC501302), mRNA [XM_576715]	**0,587**
Ralb	Rattus norvegicus v-ral simian leukemia viral oncogene homolog B (ras related; GTP binding protein) (Ralb), mRNA [NM_053821]	**0,587**
Mapre1	Rattus norvegicus microtubule-associated protein, RP/EB family, member 1 (Mapre1), mRNA [NM_138509]	**0,587**
0	Unknown	**0,587**
Slit1	Rattus norvegicus slit homolog 1 (Drosophila) (Slit1), mRNA [NM_022953]	**0,587**
Hibadh	Rattus norvegicus 3-hydroxyisobutyrate dehydrogenase (Hibadh), mRNA [NM_022243]	**0,587**
Rap2ip	Rattus norvegicus Rap2 interacting protein (Rap2ip), mRNA [NM_198758]	**0,587**
Xbp1	Rattus norvegicus X-box binding protein 1 (Xbp1), mRNA [NM_001004210]	**0,587**
Bcat2	Branched-chain-amino-acid aminotransferase, mitochondrial [Source:UniProtKB/Swiss-Prot;Acc:O35854] [ENSRNOT00000028474]	**0,587**
Csnk1a1	Rattus norvegicus casein kinase 1, alpha 1 (Csnk1a1), mRNA [NM_053615]	**0,587**
Zbtb38	Rattus norvegicus zinc finger and BTB domain containing 38 (Zbtb38), mRNA [NM_001012471]	**0,587**
Sec63	Rattus norvegicus SEC63 homolog (S. cerevisiae) (Sec63), mRNA [NM_001107637]	**0,587**

Pelp1	Rattus norvegicus proline, glutamate and leucine rich protein 1 (Pelp1), mRNA [NM_001024270]	**0,587**
Gamt	Rattus norvegicus guanidinoacetate N-methyltransferase (Gamt), mRNA [NM_012793]	**0,587**
Dact2	Rattus norvegicus dapper, antagonist of beta-catenin, homolog 2 (Xenopus laevis) (Dact2), mRNA [NM_001107464]	**0,587**
MGC95152	Rattus norvegicus similar to B230212L03Rik protein (MGC95152), mRNA [NM_001007645]	**0,587**
0	Unknown	**0,587**
RGD1303130	Rattus norvegicus kidney predominant protein NCU-G1 (RGD1303130), mRNA [NM_001004226]	**0,587**
Dgkg	Rattus norvegicus diacylglycerol kinase, gamma (Dgkg), mRNA [NM_013126]	**0,587**
Chp	Rattus norvegicus calcium binding protein p22 (Chp), mRNA [NM_024139]	**0,587**
0	Rattus norvegicus similar to 60S RIBOSOMAL PROTEIN L29 (P23) (LOC290219), mRNA [XM_224186]	**0,587**
Ubr4	Rattus norvegicus ubiquitin protein ligase E3 component n-recognin 4 (Ubr4), mRNA [NM_001039026]	**0,587**
0	Q2H348_CHAGB (Q2H348) Predicted protein, partial (5%) [TC596046]	**0,587**
0	Unknown	**0,587**
0	PREDICTED: Rattus norvegicus similar to Emu2 (LOC685612), mRNA [XM_001064529]	**0,587**
Nop10	Rattus norvegicus NOP10 ribonucleoprotein homolog (yeast) (Nop10), mRNA [NM_001126100]	**0,587**
Sspn	Rattus norvegicus sarcospan (Sspn), mRNA [NM_001109255]	**0,587**
Arsa	arylsulfatase A [Source:RefSeq peptide;Acc:NP_001030105] [ENSRNOT00000017783]	**0,587**
Rxrg	Rattus norvegicus retinoid X receptor gamma (Rxrg), mRNA [NM_031765]	**0,587**
MGC116197	Rattus norvegicus similar to RIKEN cDNA 1700001E04 (MGC116197), mRNA [NM_001025755]	**0,587**
0	Unknown	**0,587**
0	Unknown	**0,587**
Znf593	Rattus norvegicus zinc finger protein 593 (Znf593), mRNA [NM_001106689]	**0,587**
Tjp1	Rattus norvegicus tight junction protein 1 (Tjp1), mRNA [NM_001106266]	**0,587**
Ostm1	Rattus norvegicus osteopetrosis associated transmembrane protein 1 (Ostm1), mRNA [NM_001029925]	**0,587**
Utp14a	Rattus norvegicus UTP14, U3 small nucleolar ribonucleoprotein, homolog A (yeast) (Utp14a), mRNA [NM_001014113]	**0,587**
Ell	Rattus norvegicus elongation factor RNA polymerase II (Ell), mRNA [NM_001107304]	**0,587**
Rhot1	Rattus norvegicus ras homolog gene family, member T1 (Rhot1), mRNA [NM_001107026]	**0,587**
Cdh10	Rattus norvegicus cadherin 10 (Cdh10), mRNA [NM_001168631]	**0,587**
RGD1560286	Rattus norvegicus similar to DNA segment, Chr 4, ERATO Doi 22, expressed (RGD1560286), transcript variant 1, mRNA [NM_001114599]	**0,587**
Tmem132e	Rattus norvegicus transmembrane protein 132E (Tmem132e), mRNA [NM_001134410]	**0,588**
Mmp17	Rattus norvegicus matrix metallopeptidase 17 (Mmp17), mRNA [NM_001105925]	**0,588**
Acn9	Rattus norvegicus ACN9 homolog (S. cerevisiae) (Acn9), mRNA [NM_001047914]	**0,588**
RGD1563070	Rattus norvegicus similar to hypothetical protein (RGD1563070), mRNA [NM_001134541]	**0,588**
Phf20l1	Rattus norvegicus PHD finger protein 20-like 1, mRNA (cDNA clone IMAGE:7389535), complete cds. [BC096896]	**0,588**
Slc1a2	Rattus norvegicus solute carrier family 1 (glial high affinity glutamate transporter), member 2 (Slc1a2), transcript variant 1, mRNA [NM_017215]	**0,588**

Tmem143	Rattus norvegicus transmembrane protein 143 (Tmem143), mRNA [NM_001107513]	**0,588**
RGD1562415	Unknown	**0,588**
Ppia	Rattus norvegicus peptidylprolyl isomerase A (cyclophilin A) (Ppia), mRNA [NM_017101]	**0,588**
Zfp46	Rattus norvegicus zinc finger protein 46 (Zfp46), mRNA [NM_001106691]	**0,588**
Frmd8	Rattus norvegicus FERM domain containing 8 (Frmd8), mRNA [NM_001008348]	**0,588**
Emd	Rattus norvegicus emerin (Emd), mRNA [NM_012948]	**0,588**
0	A disintegrin-like and metalloprotease (Reprolysin type) with thrombospondin type 1 motif, 6 (Predicted), isoform CRA_aUncharacterized protein [Source:UniProtKB/TrEMBL;Acc:D3ZZF9] [ENSRNOT00000035876]	**0,588**
RGD1307325	Rattus norvegicus similar to RIKEN cDNA 4933411K20 (RGD1307325), mRNA [NM_001014001]	**0,588**
0	Unknown	**0,588**
LOC100365943	PREDICTED: Rattus norvegicus proteasome subunit alpha type-3-like (LOC100365943), mRNA [XM_002724523]	**0,588**
Bin3	Rattus norvegicus bridging integrator 3 (Bin3), mRNA [NM_001013186]	**0,588**
Slc22a23	Rattus norvegicus solute carrier family 22, member 23 (Slc22a23), mRNA [NM_022624]	**0,588**
Mlst8	Rattus norvegicus MTOR associated protein, LST8 homolog (S. cerevisiae) (Mlst8), mRNA [NM_022404]	**0,588**
0	Uncharacterized protein [Source:UniProtKB/TrEMBL;Acc:D3ZAK7] [ENSRNOT00000038415]	**0,588**
Spns2	Rattus norvegicus spinster homolog 2 (Spns2), mRNA [NM_001144991]	**0,588**
Adora1	Adenosine receptor A1 [Source:UniProtKB/Swiss-Prot;Acc:P25099] [ENSRNOT00000004602]	**0,588**
Gabrb1	Rattus norvegicus gamma-aminobutyric acid (GABA) A receptor, beta 1 (Gabrb1), mRNA [NM_012956]	**0,588**
Mmp2	Rattus norvegicus matrix metallopeptidase 2 (Mmp2), mRNA [NM_031054]	**0,588**
Tm9sf4	Rattus norvegicus transmembrane 9 superfamily protein member 4 (Tm9sf4), mRNA [NM_001025649]	**0,588**
Ddhd1	Rattus norvegicus DDHD domain containing 1 (Ddhd1), mRNA [NM_001033066]	**0,588**
Lypd6b	Rattus norvegicus LY6/PLAUR domain containing 6B (Lypd6b), mRNA [NM_001134580]	**0,588**
Farp2	Rattus norvegicus FERM, RhoGEF and pleckstrin domain protein 2 (Farp2), mRNA [NM_001108233]	**0,588**
Ptch1	Rattus norvegicus patched homolog 1 (Drosophila) (Ptch1), mRNA [NM_053566]	**0,588**
0	Unknown	**0,588**
Gimap9	Rattus norvegicus GTPase, IMAP family member 9 (Gimap9), mRNA [NM_001008398]	**0,588**
MGC93975	Rattus norvegicus similar to 2310044H10Rik protein (MGC93975), mRNA [NM_001004221]	**0,588**
Nptn	Rattus norvegicus neuroplastin (Nptn), mRNA [NM_019380]	**0,588**
0	Uncharacterized protein [Source:UniProtKB/TrEMBL;Acc:D3Z7Z0] [ENSRNOT00000033201]	**0,588**
Usp1	Rattus norvegicus ubiquitin specific peptidase 1 (Usp1), mRNA [NM_001015015]	**0,588**
Agrp	Rattus norvegicus agouti related protein homolog (mouse) (Agrp), mRNA [NM_033650]	**0,588**
Cntf	Rattus norvegicus ciliary neurotrophic factor (Cntf), mRNA [NM_013166]	**0,588**
0	Unknown	**0,588**
Galntl1	Rattus norvegicus UDP-N-acetyl-alpha-D-galactosamine:polypeptide N-acetylgalactosaminyltransferase-like 1 (Galntl1), mRNA [NM_001100863]	**0,588**

Plxnd1	Rattus norvegicus plexin D1 (Plxnd1), mRNA [NM_001107881]	**0,588**
0	Unknown	**0,589**
Dus3l	Rattus norvegicus dihydrouridine synthase 3-like (S. cerevisiae) (Dus3l), mRNA [NM_001034923]	**0,589**
Tbkbp1	Rattus norvegicus TBK1 binding protein 1 (Tbkbp1), mRNA [NM_172021]	**0,589**
Agrn	Rattus norvegicus agrin (Agrn), mRNA [NM_175754]	**0,589**
Ube2i	Rattus norvegicus ubiquitin-conjugating enzyme E2I (UBC9 homolog, yeast) (Ube2i), mRNA [NM_013050]	**0,589**
Eif4a1	Rattus norvegicus eukaryotic translation initiation factor 4A, isoform 1 (Eif4a1), mRNA [NM_199372]	**0,589**
Tspan5	Rattus norvegicus tetraspanin 5 (Tspan5), mRNA [NM_001004090]	**0,589**
Odz3	Rattus norvegicus odz, odd Oz/ten-m homolog 3 (Drosophila) (Odz3), mRNA [NM_001169133]	**0,589**
Tusc2	Rattus norvegicus tumor suppressor candidate 2 (Tusc2), mRNA [NM_001109297]	**0,589**
Rcan1	Rattus norvegicus regulator of calcineurin 1 (Rcan1), mRNA [NM_153724]	**0,589**
Rnf2	Rattus norvegicus ring finger protein 2 (Rnf2), mRNA [NM_001025667]	**0,589**
Taok1	Rattus norvegicus TAO kinase 1 (Taok1), mRNA [NM_173327]	**0,589**
Ccdc116	Rattus norvegicus coiled-coil domain containing 116 (Ccdc116), mRNA [NM_001024740]	**0,589**
Bach1	Rattus norvegicus BTB and CNC homology 1, basic leucine zipper transcription factor 1 (Bach1), mRNA [NM_001107113]	**0,589**
Rab11a	Rattus norvegicus RAB11a, member RAS oncogene family (Rab11a), mRNA [NM_031152]	**0,589**
RGD1307254	Rattus norvegicus similar to RIKEN cDNA 1200011I18 (RGD1307254), mRNA [NM_001024875]	**0,589**
Ppfibp2	Rattus norvegicus PTPRF interacting protein, binding protein 2 (liprin beta 2) (Ppfibp2), mRNA [NM_001100582]	**0,589**
Rbbp5	Rattus norvegicus retinoblastoma binding protein 5 (Rbbp5), mRNA [NM_001107174]	**0,589**
LOC302680	Rattus norvegicus similar to CXORF15 (LOC302680), mRNA [NM_001037187]	**0,589**
RGD1310899	Rattus norvegicus similar to CGI-35 protein (RGD1310899), mRNA [NM_001044235]	**0,589**
Pvalb	Rattus norvegicus parvalbumin (Pvalb), mRNA [NM_022499]	**0,589**
LOC688708	Uncharacterized protein [Source:UniProtKB/TrEMBL;Acc:D3ZP46] [ENSRNOT00000034463]	**0,589**
Tmem165	Rattus norvegicus transmembrane protein 165 (Tmem165), mRNA [NM_001024802]	**0,589**
Abp10	PREDICTED: Rattus norvegicus annexin V-binding protein ABP-10 (Abp10), mRNA [XM_001060944]	**0,589**
Snap29	Rattus norvegicus synaptosomal-associated protein 29 (Snap29), mRNA [NM_053810]	**0,589**
Gk	Rattus norvegicus glycerol kinase (Gk), mRNA [NM_024381]	**0,589**
0	Unknown	**0,589**
LOC500227	Rattus norvegicus hypothetical gene supported by BC079424 (LOC500227), mRNA [NM_001024330]	**0,589**
Glmn	Rattus norvegicus glomulin, FKBP associated protein (Glmn), mRNA [NM_001105993]	**0,589**
Fam110b	Rattus norvegicus family with sequence similarity 110, member B (Fam110b), mRNA [NM_001024341]	**0,589**
Sp4	Rattus norvegicus Sp4 transcription factor (Sp4), mRNA [NM_012761]	**0,589**
Sema3c	Rattus norvegicus sema domain, immunoglobulin domain (Ig), short basic domain, secreted, (semaphorin) 3C (Sema3c), mRNA [NM_001106578]	**0,589**
Rad23b	Rattus norvegicus RAD23 homolog B (S. cerevisiae) (Rad23b), mRNA [NM_001025275]	**0,589**

Adi1	Rattus norvegicus acireductone dioxygenase 1 (Adi1), mRNA [NM_199097]	**0,589**
Zfp90	Rattus norvegicus zinc finger protein 90 (Zfp90), mRNA [NM_001025765]	**0,589**
Dnah10	PREDICTED: Rattus norvegicus dynein, axonemal, heavy polypeptide 10 (Dnah10), mRNA [XM_001078937]	**0,589**
Degs1	Rattus norvegicus degenerative spermatocyte homolog 1, lipid desaturase (Drosophila) (Degs1), mRNA [NM_053323]	**0,589**
0	Unknown	**0,589**
Fam55c	Rattus norvegicus family with sequence similarity 55, member C (Fam55c), mRNA [NM_001109435]	**0,589**
Immt	Rattus norvegicus inner membrane protein, mitochondrial (Immt), nuclear gene encoding mitochondrial protein, mRNA [NM_001034928]	**0,589**
Rpl18a	Rattus norvegicus ribosomal protein L18A (Rpl18a), mRNA [NM_212510]	**0,589**
Pdgfrb	Rattus norvegicus platelet derived growth factor receptor, beta polypeptide (Pdgfrb), mRNA [NM_031525]	**0,589**
Pms2	Rattus norvegicus PMS2 postmeiotic segregation increased 2 (S. cerevisiae) (Pms2), mRNA [NM_001105908]	**0,589**
Tmod1	Rattus norvegicus tropomodulin 1 (Tmod1), mRNA [NM_013044]	**0,589**
Nol3	Rattus norvegicus nucleolar protein 3 (apoptosis repressor with CARD domain) (Nol3), mRNA [NM_053516]	**0,589**
Zbtb39	Rattus norvegicus zinc finger and BTB domain containing 39 (Zbtb39), mRNA [NM_001130537]	**0,589**
Fahd1	Rattus norvegicus fumarylacetoacetate hydrolase domain containing 1 (Fahd1), nuclear gene encoding mitochondrial protein, mRNA [NM_001024991]	**0,589**
Exoc5	Rattus norvegicus exocyst complex component 5 (Exoc5), mRNA [NM_022204]	**0,589**
Trim47	Rattus norvegicus tripartite motif-containing 47 (Trim47), mRNA [NM_001109585]	**0,589**
0	Unknown	**0,589**
Dhdds	Rattus norvegicus dehydrodolichyl diphosphate synthase (Dhdds), mRNA [NM_001011978]	**0,589**
Ankrd40	Rattus norvegicus ankyrin repeat domain 40 (Ankrd40), mRNA [NM_001134699]	**0,589**
Phlpp1	Rattus norvegicus PH domain and leucine rich repeat protein phosphatase 1 (Phlpp1), mRNA [NM_021657]	**0,589**
Gjb2	Rattus norvegicus gap junction protein, beta 2 (Gjb2), mRNA [NM_001004099]	**0,589**
Bmp7	Rattus norvegicus bone morphogenetic protein 7 (Bmp7), mRNA [NM_001191856]	**0,589**
Cab39l	Rattus norvegicus calcium binding protein 39-like (Cab39l), mRNA [NM_001011917]	**0,589**
Dnal4	Rattus norvegicus dynein, axonemal, light chain 4 (Dnal4), mRNA [NM_001009666]	**0,589**
Tex15	Rattus norvegicus testis expressed 15 (Tex15), mRNA [NM_001106087]	**0,589**
Stag2	Rattus norvegicus stromal antigen 2 (Stag2), mRNA [NM_001173507]	**0,589**
Ercc8	Rattus norvegicus excision repair cross-complementing rodent repair deficiency, complementation group 8 (Ercc8), mRNA [NM_001107650]	**0,589**
Csda	Rattus norvegicus cold shock domain protein A (Csda), mRNA [NM_031979]	**0,589**
RGD1310444	PREDICTED: Rattus norvegicus LOC363015 (RGD1310444), mRNA [XM_002729871]	**0,589**
Homer1	Rattus norvegicus homer homolog 1 (Drosophila) (Homer1), mRNA [NM_031707]	**0,589**
Cbln1	Rattus norvegicus cerebellin 1 precursor (Cbln1), mRNA [NM_001109127]	**0,590**
0	Rattus norvegicus similar to 60S RIBOSOMAL PROTEIN L29 (P23) (LOC291920), mRNA [XM_226340]	**0,590**
Pfdn6	Rattus norvegicus prefoldin subunit 6 (Pfdn6), mRNA [NM_212506]	**0,590**

Dppa3	Rattus norvegicus developmental pluripotency-associated 3 (Dppa3), mRNA [NM_001047864]	**0,590**
Mllt1	Rattus norvegicus myeloid/lymphoid or mixed-lineage leukemia (trithorax homolog, Drosophila); translocated to, 1 (Mllt1), mRNA [NM_001106876]	**0,590**
LOC680758	PREDICTED: Rattus norvegicus similar to spermatogenesis associated glutamate (E)-rich protein 4d (LOC680758), mRNA [XM_001058748]	**0,590**
Jun	Rattus norvegicus Jun oncogene (Jun), mRNA [NM_021835]	**0,590**
Eef2k	Rattus norvegicus eukaryotic elongation factor-2 kinase (Eef2k), mRNA [NM_012947]	**0,590**
Ttf2	Rattus norvegicus transcription termination factor, RNA polymerase II (Ttf2), mRNA [NM_001106454]	**0,590**
Slc12a5	Rattus norvegicus solute carrier family 12 (potassium-chloride transporter), member 5 (Slc12a5), mRNA [NM_134363]	**0,590**
C2cd2	Rattus norvegicus C2 calcium-dependent domain containing 2 (C2cd2), mRNA [NM_199391]	**0,590**
Prps1	Rattus norvegicus phosphoribosyl pyrophosphate synthetase 1 (Prps1), mRNA [NM_017243]	**0,590**
Pnck	Rattus norvegicus pregnancy upregulated non-ubiquitously expressed CaM kinase (Pnck), mRNA [NM_017275]	**0,590**
Speg	Rattus norvegicus SPEG complex locus (Speg), transcript variant 1, mRNA [NM_001108802]	**0,590**
Tmem220	Uncharacterized protein [Source:UniProtKB/TrEMBL;Acc:D4A6Z7] [ENSRNOT00000004516]	**0,590**
RGD1309707	Rattus norvegicus similar to RIKEN cDNA 4930431E10 (RGD1309707), mRNA [NM_001109598]	**0,590**
Tstd2	Rattus norvegicus thiosulfate sulfurtransferase (rhodanese)-like domain containing 2 (Tstd2), mRNA [NM_001108663]	**0,590**
Sgsm1	Rattus norvegicus small G protein signaling modulator 1 (Sgsm1), mRNA [NM_001105937]	**0,590**
Gcsh	Rattus norvegicus glycine cleavage system protein H (aminomethyl carrier) (Gcsh), nuclear gene encoding mitochondrial protein, mRNA [NM_133598]	**0,590**
Ube2z	Rattus norvegicus ubiquitin-conjugating enzyme E2Z (Ube2z), mRNA [NM_001037643]	**0,590**
0	PREDICTED: Rattus norvegicus similar to Dedicator of cytokinesis protein 10 (Protein zizimin 3) (RGD1561963), miscRNA [XR_085916]	**0,590**
Sft2d3	Rattus norvegicus SFT2 domain containing 3 (Sft2d3), mRNA [NM_001108887]	**0,590**
Lrp11	Rattus norvegicus low density lipoprotein receptor-related protein 11 (Lrp11), mRNA [NM_001106217]	**0,590**
Sdcbp	Rattus norvegicus syndecan binding protein (Sdcbp), mRNA [NM_031986]	**0,590**
Msl2	PREDICTED: Rattus norvegicus male-specific lethal 2-like 1 (Drosophila) (Msl2l1), mRNA [XM_001071576]	**0,590**
Trim32	Rattus norvegicus tripartite motif-containing 32 (Trim32), mRNA [NM_001012103]	**0,590**
0	CFP1_MOUSE (Q8BGU5) Cyclin fold protein 1, partial (69%) [TC584108]	**0,590**
Ralbp1	Rattus norvegicus ralA binding protein 1 (Ralbp1), mRNA [NM_032067]	**0,590**
0	Unknown	**0,590**
Rab2b	Rattus norvegicus RAB2B, member RAS oncogene family (Rab2b), mRNA [NM_001037645]	**0,590**
Syn2	Rattus norvegicus synapsin II (Syn2), transcript variant 2, mRNA [NM_019159]	**0,590**
Vamp3	Rattus norvegicus vesicle-associated membrane protein 3 (Vamp3), mRNA [NM_057097]	**0,590**
Gls	Rattus norvegicus glutaminase (Gls), nuclear gene encoding mitochondrial protein, transcript variant 1, mRNA [NM_012569]	**0,590**
Oxa1l	Rattus norvegicus oxidase assembly 1-like (Oxa1l), mRNA [NM_001168583]	**0,590**
Zfp40	Rattus norvegicus zinc finger protein 40 (Zfp40), mRNA [NM_001168642]	**0,590**
Cks1b	Rattus norvegicus CDC28 protein kinase regulatory subunit 1B (Cks1b), mRNA [NM_001135749]	**0,590**

Parp6	Rattus norvegicus poly (ADP-ribose) polymerase family, member 6 (Parp6), mRNA [NM_001106828]	**0,590**
Yipf5	Rattus norvegicus Yip1 domain family, member 5 (Yipf5), mRNA [NM_001014150]	**0,590**
LOC680039	Rattus norvegicus hypothetical protein LOC680039 (LOC680039), mRNA [NM_001109388]	**0,590**
RGD1305704	Rattus norvegicus cDNA clone IMAGE:5598328, **** WARNING: chimeric clone ****. [BC168677]	**0,590**
0	Unknown	**0,590**
0	Unknown	**0,590**
0	EST352149 Rat gene index, normalized rat, norvegicus, Bento Soares Rattus norvegicus cDNA clone RGIHD72 5' end, mRNA sequence [AW920845]	**0,590**
0	Uncharacterized protein [Source:UniProtKB/TrEMBL;Acc:D3Z932] [ENSRNOT00000066351]	**0,590**
Sdc4	Rattus norvegicus syndecan 4 (Sdc4), mRNA [NM_012649]	**0,590**
Tmem41b	Rattus norvegicus transmembrane protein 41B (Tmem41b), mRNA [NM_001012358]	**0,590**
Fam46a	Rattus norvegicus family with sequence similarity 46, member A (Fam46a), mRNA [NM_001106844]	**0,591**
Stat3	Rattus norvegicus signal transducer and activator of transcription 3 (Stat3), mRNA [NM_012747]	**0,591**
Kdelc2	Rattus norvegicus KDEL (Lys-Asp-Glu-Leu) containing 2 (Kdelc2), mRNA [NM_001025123]	**0,591**
Ttc1	Rattus norvegicus tetratricopeptide repeat domain 1 (Ttc1), mRNA [NM_001005529]	**0,591**
Prex2	Rattus norvegicus phosphatidylinositol-3,4,5-trisphosphate-dependent Rac exchange factor 2 (Prex2), mRNA [NM_001107899]	**0,591**
0	Unknown	**0,591**
Hgf	Rattus norvegicus hepatocyte growth factor (Hgf), mRNA [NM_017017]	**0,591**
Gabra2	Rattus norvegicus gamma-aminobutyric acid (GABA-A) receptor, subunit alpha 2 (Gabra2), mRNA [NM_001135779]	**0,591**
RGD1307493	PREDICTED: Rattus norvegicus similar to membrane protein expressed in epithelial-like lung adenocarcinoma (RGD1307493), mRNA [XM_001080742]	**0,591**
RGD1311558	Rattus norvegicus similar to 4930506M07Rik protein (RGD1311558), mRNA [NM_001079705]	**0,591**
Tmem39a	Rattus norvegicus transmembrane protein 39a (Tmem39a), mRNA [NM_001013865]	**0,591**
Dcbld2	Rattus norvegicus discoidin, CUB and LCCL domain containing 2 (Dcbld2), mRNA [NM_130419]	**0,591**
Kat5	Rattus norvegicus K(lysine) acetyltransferase 5 (Kat5), mRNA [NM_001005872]	**0,591**
0	Unknown	**0,591**
Gatsl3	Rattus norvegicus GATS protein-like 3 (Gatsl3), mRNA [NM_001025128]	**0,591**
0	Unknown	**0,591**
Trim41	Rattus norvegicus tripartite motif-containing 41 (Trim41), mRNA [NM_001134737]	**0,591**
Commd9	Rattus norvegicus COMM domain containing 9 (Commd9), mRNA [NM_001033692]	**0,591**
Cdc42	Rattus norvegicus cell division cycle 42 (GTP binding protein) (Cdc42), mRNA [NM_171994]	**0,591**
Wdr5	Rattus norvegicus WD repeat domain 5 (Wdr5), mRNA [NM_001039034]	**0,591**
RGD1305587	Rattus norvegicus similar to RIKEN cDNA 2010107G23 (RGD1305587), mRNA [NM_001127452]	**0,591**
Cyr61	Rattus norvegicus cysteine-rich, angiogenic inducer, 61 (Cyr61), mRNA [NM_031327]	**0,591**

0	Vesicle transport through interaction with t-SNAREs homolog 1B [Source:UniProtKB/Swiss-Prot;Acc:P58200] [ENSRNOT00000015814]	**0,591**
Pla2g12a	Rattus norvegicus phospholipase A2, group XIIA (Pla2g12a), mRNA [NM_001108565]	**0,591**
Smok2a	Rattus norvegicus sperm motility kinase 2A (Smok2a), mRNA [NM_001191619]	**0,591**
Pabpc1	Rattus norvegicus poly(A) binding protein, cytoplasmic 1 (Pabpc1), mRNA [NM_134353]	**0,591**
0	Unknown	**0,591**
0	Uncharacterized protein [Source:UniProtKB/TrEMBL;Acc:D3ZAP4] [ENSRNOT00000020882]	**0,591**
Mettl2	Rattus norvegicus methyltransferase like 2 (Mettl2), mRNA [NM_001108839]	**0,591**
Intu	Uncharacterized protein [Source:UniProtKB/TrEMBL;Acc:D4ACE5] [ENSRNOT00000014116]	**0,591**
Rabl4	Rattus norvegicus RAB, member of RAS oncogene family-like 4 (Rabl4), mRNA [NM_001130495]	**0,591**
Fip1l1	Rattus norvegicus FIP1 like 1 (S. cerevisiae) (Fip1l1), mRNA [NM_001008295]	**0,591**
Crkrs	Rattus norvegicus Cdc2-related kinase, arginine/serine-rich (Crkrs), transcript variant 2, mRNA [NM_138916]	**0,591**
0	Rattus norvegicus similar to 60S ribosomal protein L10 (QM protein homolog) (LOC301174), mRNA [XM_236837]	**0,591**
Zfr	Zinc finger RNA-binding protein [Source:UniProtKB/Swiss-Prot;Acc:Q562A2] [ENSRNOT00000016196]	**0,591**
Galnt2	Rattus norvegicus UDP-N-acetyl-alpha-D-galactosamine:polypeptide N-acetylgalactosaminyltransferase 2 (GalNAc-T2) (Galnt2), mRNA [NM_001106196]	**0,591**
Pgam1	Rattus norvegicus phosphoglycerate mutase 1 (brain) (Pgam1), mRNA [NM_053290]	**0,591**
Bin1	Rattus norvegicus bridging integrator 1 (Bin1), mRNA [NM_053959]	**0,591**
Zfp277	PREDICTED: Rattus norvegicus similar to zinc finger protein 277 isoform 1 (LOC298977), mRNA [XM_216701]	**0,591**
0	Q99LS0_MOUSE (Q99LS0) Esophageal cancer related gene 4 protein, partial (13%) [TC630296]	**0,591**
Galnt1	Polypeptide N-acetylgalactosaminyltransferase 1Polypeptide N-acetylgalactosaminyltransferase 1 soluble form [Source:UniProtKB/Swiss-Prot;Acc:Q10473] [ENSRNOT00000022117]	**0,591**
Acot7	Rattus norvegicus acyl-CoA thioesterase 7 (Acot7), transcript variant 1, mRNA [NM_001146061]	**0,591**
Itsn1	Rattus norvegicus intersectin 1 (SH3 domain protein) (Itsn1), transcript variant 1, mRNA [NM_001136096]	**0,591**
Spats2	Rattus norvegicus spermatogenesis associated, serine-rich 2 (Spats2), mRNA [NM_001191614]	**0,591**
LOC684652	PREDICTED: Rattus norvegicus hypothetical protein LOC684652 (LOC684652), partial mRNA [XM_001055278]	**0,591**
Mpi	Rattus norvegicus mannose phosphate isomerase (mapped) (Mpi), mRNA [NM_001004081]	**0,592**
Rnps1	Rattus norvegicus ribonucleic acid binding protein S1 (Rnps1), mRNA [NM_001011890]	**0,592**
RGD1564469	Uncharacterized protein [Source:UniProtKB/TrEMBL;Acc:D3ZJT4] [ENSRNOT00000040909]	**0,592**
Slc16a13	Rattus norvegicus solute carrier family 16, member 13 (monocarboxylic acid transporter 13) (Slc16a13), mRNA [NM_001005530]	**0,592**
0	SYT1_RAT (P21707) Synaptotagmin-1 (Synaptotagmin I) (SytI) (p65), complete [TC577208]	**0,592**
Phactr3	Rattus norvegicus phosphatase and actin regulator 3 (Phactr3), mRNA [NM_214459]	**0,592**
Nrxn2	Rattus norvegicus neurexin 2 (Nrxn2), mRNA [NM_053846]	**0,592**
Thumpd1	Rattus norvegicus THUMP domain containing 1 (Thumpd1), mRNA [NM_001009688]	**0,592**
Rragd	Rattus norvegicus Ras-related GTP binding D (Rragd), mRNA [NM_001106641]	**0,592**

Fasn	Rattus norvegicus fatty acid synthase (Fasn), mRNA [NM_017332]	**0,592**
0	fetal Alzheimer antigen [Source:RefSeq peptide;Acc:NP_001121007] [ENSRNOT00000055100]	**0,592**
Mobkl3	Rattus norvegicus MOB1, Mps One Binder kinase activator-like 3 (yeast) (Mobkl3), mRNA [NM_133528]	**0,592**
RGD1562618	Rattus norvegicus similar to RIKEN cDNA 6030419C18 gene (RGD1562618), mRNA [NM_001113791]	**0,592**
Gtf2ird1	Rattus norvegicus GTF2I repeat domain containing 1 (Gtf2ird1), mRNA [NM_001001504]	**0,592**
Sorcs3	Rattus norvegicus sortilin-related VPS10 domain containing receptor 3 (Sorcs3), mRNA [NM_001106367]	**0,592**
0	UI-R-Y0-acg-g-03-0-UI.s1 UI-R-Y0 Rattus norvegicus cDNA clone UI-R-Y0-acg-g-03-0-UI 3', mRNA sequence [AI716836]	**0,592**
Fosl1	Rattus norvegicus fos-like antigen 1 (Fosl1), mRNA [NM_012953]	**0,592**
RGD1560888	Rattus norvegicus similar to Cell division protein kinase 8 (Protein kinase K35) (RGD1560888), mRNA [NM_001109061]	**0,592**
Cttnbp2nl	Rattus norvegicus CTTNBP2 N-terminal like (Cttnbp2nl), mRNA [NM_001107712]	**0,592**
Agap1	Rattus norvegicus ArfGAP with GTPase domain, ankyrin repeat and PH domain 1 (Agap1), mRNA [NM_001108230]	**0,592**
Galnt13	Rattus norvegicus UDP-N-acetyl-alpha-D-galactosamine:polypeptide N-acetylgalactosaminyltransferase 13 (GalNAc-T13) (Galnt13), mRNA [NM_199106]	**0,592**
Dclk2	Rattus norvegicus doublecortin-like kinase 2 (Dclk2), transcript variant 2, mRNA [NM_001009691]	**0,592**
Ppp2ca	Rattus norvegicus protein phosphatase 2, catalytic subunit, alpha isoform (Ppp2ca), mRNA [NM_017039]	**0,592**
0	Rattus norvegicus similar to 60S RIBOSOMAL PROTEIN L29 (P23) (LOC292938), mRNA [XM_218668]	**0,592**
Lamc1	Rattus norvegicus laminin, gamma 1 (Lamc1), mRNA [NM_053966]	**0,592**
LOC691365	Uncharacterized protein [Source:UniProtKB/TrEMBL;Acc:D3ZMI8] [ENSRNOT00000048115]	**0,592**
Calm1	Rattus norvegicus calmodulin 1 (Calm1), mRNA [NM_031969]	**0,592**
Spr	Rattus norvegicus sepiapterin reductase (7,8-dihydrobiopterin:NADP+ oxidoreductase) (Spr), mRNA [NM_019181]	**0,592**
RT1-CE2	Rattus norvegicus RT1 class I, locus CE2 (RT1-CE2), mRNA [NM_001008840]	**0,592**
Ndufb4	Rattus norvegicus NADH dehydrogenase (ubiquinone) 1 beta subcomplex 4 (Ndufb4), nuclear gene encoding mitochondrial protein, mRNA [NM_001037338]	**0,592**
0	UI-R-DY1-com-a-12-0-UI.s1 UI-R-DY1 Rattus norvegicus cDNA clone UI-R-DY1-com-a-12-0-UI 3', mRNA sequence [BQ210702]	**0,592**
0	Unknown	**0,592**
Carf	Rattus norvegicus calcium response factor (Carf), mRNA [NM_001106915]	**0,592**
Cbx3	Rattus norvegicus chromobox homolog 3 (HP1 gamma homolog, Drosophila) (Cbx3), mRNA [NM_001008313]	**0,592**
Hist1h4b	Rattus norvegicus histone cluster 1, H4b (Hist1h4b), mRNA [NM_022686]	**0,592**
Zfp709l2	Rattus norvegicus zinc finger protein 709-like 2 (Zfp709l2), mRNA [NM_001163062]	**0,592**
Hps4	Rattus norvegicus Hermansky-Pudlak syndrome 4 homolog (human) (Hps4), mRNA [NM_001107148]	**0,592**
Twistnb	Rattus norvegicus TWIST neighbor (Twistnb), mRNA [NM_001108707]	**0,592**
Rexo2	Rattus norvegicus REX2, RNA exonuclease 2 homolog (S. cerevisiae) (Rexo2), mRNA [NM_001008326]	**0,592**
LOC687516	Unknown	**0,592**
Trappc10	Rattus norvegicus trafficking protein particle complex 10 (Trappc10), mRNA [NM_001173528]	**0,592**

Iqsec3	Rattus norvegicus IQ motif and Sec7 domain 3 (Iqsec3), mRNA [NM_207617]	**0,593**
Chsy1	Rattus norvegicus chondroitin sulfate synthase 1 (Chsy1), mRNA [NM_001106268]	**0,593**
Alg10	Rattus norvegicus asparagine-linked glycosylation 10, alpha-1,2-glucosyltransferase homolog (S. pombe) (Alg10), mRNA [NM_139101]	**0,593**
Saps1	Rattus norvegicus SAPS domain family, member 1 (Saps1), mRNA [NM_001135849]	**0,593**
Txndc16	Uncharacterized protein [Source:UniProtKB/TrEMBL;Acc:D3ZQK4] [ENSRNOT00000008372]	**0,593**
Pld2	Rattus norvegicus phospholipase D2 (Pld2), mRNA [NM_033299]	**0,593**
Kras	Rattus norvegicus v-Ki-ras2 Kirsten rat sarcoma viral oncogene homolog (Kras), mRNA [NM_031515]	**0,593**
Nln	Rattus norvegicus neurolysin (metallopeptidase M3 family) (Nln), nuclear gene encoding mitochondrial protein, mRNA [NM_053970]	**0,593**
Arhgap5	Rattus norvegicus Rho GTPase activating protein 5 (Arhgap5), mRNA [NM_001047869]	**0,593**
Igf1	Rattus norvegicus insulin-like growth factor 1 (Igf1), transcript variant 2, mRNA [NM_178866]	**0,593**
Arpp19	Rattus norvegicus cAMP-regulated phosphoprotein 19 (Arpp19), mRNA [NM_031660]	**0,593**
Zbtb7c	Rattus norvegicus zinc finger and BTB domain containing 7C (Zbtb7c), mRNA [NM_001127375]	**0,593**
Cygb	Rattus norvegicus cytoglobin (Cygb), mRNA [NM_130744]	**0,593**
0	XM_750817 gamma-tubulin complex component GCP4 {Aspergillus fumigatus Af293} (exp=-1; wgp=0; cg=0), partial (3%) [TC606369]	**0,593**
Cstf2t	Rattus norvegicus cleavage stimulation factor, 3' pre-RNA subunit 2, tau (Cstf2t), mRNA [NM_001107586]	**0,593**
Mbd2	Rattus norvegicus methyl-CpG binding domain protein 2 (Mbd2), mRNA [NM_001115025]	**0,593**
G3bp2	Rattus norvegicus GTPase activating protein (SH3 domain) binding protein 2 (G3bp2), mRNA [NM_001013989]	**0,593**
0	Uncharacterized protein [Source:UniProtKB/TrEMBL;Acc:D3ZA84] [ENSRNOT00000031988]	**0,593**
Gcap14	PREDICTED: Rattus norvegicus granule cell antiserum positive 14 (Gcap14), mRNA [XM_224672]	**0,593**
Nckipsd	Rattus norvegicus NCK interacting protein with SH3 domain (Nckipsd), mRNA [NM_001106857]	**0,593**
RGD1563441	Rattus norvegicus similar to RIKEN cDNA A030009H04 (RGD1563441), mRNA [NM_001126292]	**0,593**
Lrrc56	Rattus norvegicus leucine rich repeat containing 56 (Lrrc56), mRNA [NM_001024902]	**0,593**
Aldh4a1	Rattus norvegicus aldehyde dehydrogenase 4 family, member A1 (Aldh4a1), nuclear gene encoding mitochondrial protein, mRNA [NM_001134698]	**0,593**
Senp5	PREDICTED: Rattus norvegicus Sumo1/sentrin/SMT3 specific peptidase 5 (Senp5), mRNA [XM_221369]	**0,593**
Cdc45l	Rattus norvegicus CDC45 cell division cycle 45-like (S. cerevisiae) (Cdc45l), mRNA [NM_001105866]	**0,593**
Chn1	Rattus norvegicus chimerin (chimaerin) 1 (Chn1), mRNA [NM_032083]	**0,593**
Spcs3	Rattus norvegicus signal peptidase complex subunit 3 homolog (S. cerevisiae) (Spcs3), mRNA [NM_001191073]	**0,593**
0	BF521738 UI-R-C2-mt-d-06-0-UI.r2 UI-R-C2 Rattus norvegicus cDNA clone UI-R-C2-mt-d-06-0-UI 5', mRNA sequence [BF521738]	**0,593**
Serac1	Similar to serine active site containing 1 (Predicted)Uncharacterized protein [Source:UniProtKB/TrEMBL;Acc:D3ZEJ3] [ENSRNOT00000060527]	**0,593**
Casq1	Rattus norvegicus calsequestrin 1 (fast-twitch, skeletal muscle) (Casq1), nuclear gene encoding mitochondrial protein, mRNA [NM_001159594]	**0,593**
Copa	Rattus norvegicus coatomer protein complex subunit alpha (Copa), mRNA [NM_001134540]	**0,593**
Tbc1d5	Rattus norvegicus TBC1 domain family, member 5 (Tbc1d5), mRNA [NM_001134762]	**0,593**
Etv5	Rattus norvegicus ets variant 5 (Etv5), mRNA [NM_001107082]	**0,593**
Lppr3	Rattus norvegicus lipid phosphate phosphatase-related protein type 3 (Lppr3), mRNA [NM_181634]	**0,593**

Jazf1	PREDICTED: Rattus norvegicus JAZF zinc finger 1 (Jazf1), mRNA [XM_002726367]	**0,593**
RGD1559747	Rattus norvegicus similar to Zinc finger and SCAN domain containing protein 2 (Zinc finger protein 29) (RGD1559747), non-coding RNA [NR_027235]	**0,593**
Rala	Rattus norvegicus v-ral simian leukemia viral oncogene homolog A (ras related) (Rala), mRNA [NM_031093]	**0,593**
Fam83e	PREDICTED: Rattus norvegicus family with sequence similarity 83, member E (Fam83e), mRNA [XM_001079852]	**0,593**
RGD1305235	Rattus norvegicus similar to RIKEN cDNA 1700052N19 (RGD1305235), mRNA [NM_001017447]	**0,593**
Dpf1	Rattus norvegicus D4, zinc and double PHD fingers family 1 (Dpf1), mRNA [NM_001105729]	**0,593**
0	Unknown	**0,593**
0	Uncharacterized protein [Source:UniProtKB/TrEMBL;Acc:D3Z8C0] [ENSRNOT00000042031]	**0,593**
0	Unknown	**0,593**
Fzr1	Rattus norvegicus fizzy/cell division cycle 20 related 1 (Drosophila) (Fzr1), mRNA [NM_001108074]	**0,593**
0	Unknown	**0,593**
Znrf2	Rattus norvegicus zinc and ring finger 2 (Znrf2), mRNA [NM_001108628]	**0,593**
Ankle2	Rattus norvegicus ankyrin repeat and LEM domain containing 2 (Ankle2), mRNA [NM_001047901]	**0,593**
0	Rattus norvegicus similar to putative protein kinase (LOC292372), mRNA [XM_217965]	**0,593**
RGD1310495	Uncharacterized protein [Source:UniProtKB/TrEMBL;Acc:D3ZDM4] [ENSRNOT00000000720]	**0,593**
Slc38a2	Rattus norvegicus solute carrier family 38, member 2 (Slc38a2), mRNA [NM_181090]	**0,593**
Slc1a3	Rattus norvegicus solute carrier family 1 (glial high affinity glutamate transporter), member 3 (Slc1a3), mRNA [NM_019225]	**0,593**
0	RVL15663 Wackym-Soares normalized rat vestibular cDNA library Rattus norvegicus cDNA 5', mRNA sequence [DV723748]	**0,593**
Tgif1	Rattus norvegicus TGFB-induced factor homeobox 1 (Tgif1), mRNA [NM_001015020]	**0,593**
LOC691572	PREDICTED: Rattus norvegicus hypothetical protein LOC691572 (LOC691572), mRNA [XM_001078882]	**0,593**
Ttc9b	Rattus norvegicus tetratricopeptide repeat domain 9B (Ttc9b), mRNA [NM_001108478]	**0,593**
Ccl27	Rattus norvegicus chemokine (C-C motif) ligand 27 (Ccl27), mRNA [NM_001108660]	**0,593**
Herv-frd	Rattus norvegicus HERV-FRD provirus ancestral Env polyprotein (Herv-frd), mRNA [NM_001024239]	**0,593**
Nell2	Rattus norvegicus NEL-like 2 (chicken) (Nell2), mRNA [NM_031070]	**0,593**
Sgcb	Rattus norvegicus sarcoglycan, beta (dystrophin-associated glycoprotein) (Sgcb), mRNA [NM_001191068]	**0,593**
0	U2af2 protein [Source:UniProtKB/TrEMBL;Acc:Q5EB64] [ENSRNOT00000021391]	**0,593**
Vsnl1	Rattus norvegicus visinin-like 1 (Vsnl1), mRNA [NM_012686]	**0,593**
0	Rattus norvegicus chromosome 1, 5 clones, strain BN/SsNHsdMCW RNOR03207166, whole genome shotgun sequence [AABR03000122]	**0,593**
0	Unknown	**0,594**
Tacr3	Rattus norvegicus tachykinin receptor 3 (Tacr3), mRNA [NM_017053]	**0,594**
Grk4	Rattus norvegicus G protein-coupled receptor kinase 4 (Grk4), mRNA [NM_022928]	**0,594**
0	Calcium/calmodulin-dependent protein kinase type 1G [Source:UniProtKB/Swiss-Prot;Acc:Q7TNJ7] [ENSRNOT00000044161]	**0,594**
Sned1	Rattus norvegicus sushi, nidogen and EGF-like domains 1 (Sned1), mRNA [NM_001167842]	**0,594**

Kctd15	Rattus norvegicus potassium channel tetramerisation domain containing 15 (Kctd15), mRNA [NM_001109141]	**0,594**
Fbxo11	Rattus norvegicus F-box protein 11 (Fbxo11), mRNA [NM_181631]	**0,594**
Slc27a4	Rattus norvegicus solute carrier family 27 (fatty acid transporter), member 4 (Slc27a4), mRNA [NM_001100706]	**0,594**
Arfgap3	Rattus norvegicus ADP-ribosylation factor GTPase activating protein 3 (Arfgap3), mRNA [NM_001044273]	**0,594**
Rbak	Rattus norvegicus RB-associated KRAB zinc finger (Rbak), mRNA [NM_001191664]	**0,594**
Dhtkd1	Rattus norvegicus dehydrogenase E1 and transketolase domain containing 1 (Dhtkd1), nuclear gene encoding mitochondrial protein, mRNA [NM_001025720]	**0,594**
Gphn	Rattus norvegicus gephyrin (Gphn), mRNA [NM_022865]	**0,594**
Dusp6	Rattus norvegicus dual specificity phosphatase 6 (Dusp6), mRNA [NM_053883]	**0,594**
0	Unknown	**0,594**
RGD1564019	PREDICTED: Rattus norvegicus similar to GTPase activating RANGAP domain-like 3 (RGD1564019), mRNA [XM_001078585]	**0,594**
Cds1	Rattus norvegicus CDP-diacylglycerol synthase 1 (Cds1), mRNA [NM_031242]	**0,594**
Ncoa6	Nuclear receptor coactivator 6 [Source:UniProtKB/Swiss-Prot;Acc:Q9JLI4] [ENSRNOT00000024714]	**0,594**
Fnbp4	Rattus norvegicus formin binding protein 4 (Fnbp4), mRNA [NM_001013159]	**0,594**
Rab4a	Rattus norvegicus RAB4A, member RAS oncogene family (Rab4a), mRNA [NM_013019]	**0,594**
Tsr2	Rattus norvegicus TSR2, 20S rRNA accumulation, homolog (S. cerevisiae) (Tsr2), mRNA [NM_001115027]	**0,594**
Nos3	Rattus norvegicus nitric oxide synthase 3, endothelial cell (Nos3), mRNA [NM_021838]	**0,594**
Efnb1	Rattus norvegicus ephrin B1 (Efnb1), mRNA [NM_017089]	**0,594**
Cd99	Rattus norvegicus CD99 antigen (Cd99), mRNA [NM_001100804]	**0,594**
0	Unknown	**0,594**
Git2	Rattus norvegicus G protein-coupled receptor kinase interacting ArfGAP 2 (Git2), mRNA [NM_001005553]	**0,594**
0	SUMO-conjugating enzyme UBC9 [Source:UniProtKB/Swiss-Prot;Acc:P63281] [ENSRNOT00000024406]	**0,594**
RGD1307799	Rattus norvegicus similar to RIKEN cDNA 2400003C14 (RGD1307799), mRNA [NM_001017454]	**0,594**
Txndc11	Rattus norvegicus thioredoxin domain containing 11 (Txndc11), mRNA [NM_001127532]	**0,594**
Fgfr1	Rattus norvegicus Fibroblast growth factor receptor 1 (Fgfr1), mRNA [NM_024146]	**0,594**
Wdr48	Rattus norvegicus WD repeat domain 48 (Wdr48), mRNA [NM_001135895]	**0,594**
0	Unknown	**0,594**
Rbks	Rattus norvegicus ribokinase (Rbks), mRNA [NM_001108703]	**0,594**
Ngb	Rattus norvegicus neuroglobin (Ngb), mRNA [NM_033359]	**0,594**
0	Rattus norvegicus similar to RIKEN cDNA 1700081O22 (LOC363336), mRNA [XM_343674]	**0,594**
Slc35c1	Rattus norvegicus solute carrier family 35, member C1 (Slc35c1), mRNA [NM_001107748]	**0,594**
Tbce	Rattus norvegicus tubulin folding cofactor E (Tbce), mRNA [NM_001012161]	**0,594**
0	Unknown	**0,594**
RGD1311703	Rattus norvegicus similar to sid2057p (RGD1311703), mRNA [NM_001013898]	**0,594**

Scly	Rattus norvegicus selenocysteine lyase (Scly), mRNA [NM_001007755]	**0,594**
Ccdc39	Rattus norvegicus coiled-coil domain containing 39 (Ccdc39), mRNA [NM_001107667]	**0,594**
Igf1	Rattus norvegicus insulin-like growth factor 1 (Igf1), transcript variant 4, mRNA [NM_001082479]	**0,594**
RGD1310571	Rattus norvegicus similar to hypothetical protein (RGD1310571), mRNA [NM_001014147]	**0,594**
FAM120C	PREDICTED: Rattus norvegicus family with sequence similarity 120C (FAM120C), mRNA [XM_001068090]	**0,594**
Scara3	Rattus norvegicus scavenger receptor class A, member 3 (Scara3), mRNA [NM_001108870]	**0,594**
Cdkn2aip	Rattus norvegicus CDKN2A interacting protein (Cdkn2aip), mRNA [NM_001014000]	**0,594**
Arrdc4	Rattus norvegicus arrestin domain containing 4 (Arrdc4), mRNA [NM_001047853]	**0,594**
0	PREDICTED: Rattus norvegicus similar to Protein KIAA0586 (LOC690035), miscRNA [XR_086273]	**0,594**
Abhd10	Rattus norvegicus abhydrolase domain containing 10 (Abhd10), mRNA [NM_001123352]	**0,594**
Zfp637	Rattus norvegicus zinc finger protein 637 (Zfp637), mRNA [NM_001134908]	**0,594**
Agfg1	Rattus norvegicus ArfGAP with FG repeats 1 (Agfg1), mRNA [NM_001135596]	**0,594**
Tspan31	Rattus norvegicus tetraspanin 31 (Tspan31), mRNA [NM_001008378]	**0,595**
Leprel2	Rattus norvegicus leprecan-like 2 (Leprel2), mRNA [NM_001106620]	**0,595**
Zmym4	Rattus norvegicus zinc finger, MYM-type 4 (Zmym4), mRNA [NM_001107982]	**0,595**
Tmed2	Rattus norvegicus transmembrane emp24 domain trafficking protein 2 (Tmed2), mRNA [NM_031722]	**0,595**
Abcg4	Rattus norvegicus ATP-binding cassette, subfamily G (WHITE), member 4 (Abcg4), mRNA [NM_001106816]	**0,595**
Rbm4	Rattus norvegicus RNA binding motif protein 4 (Rbm4), mRNA [NM_001170484]	**0,595**
Rps6kb2	Rattus norvegicus ribosomal protein S6 kinase, polypeptide 2 (Rps6kb2), mRNA [NM_001010962]	**0,595**
Apod	Rattus norvegicus apolipoprotein D (Apod), mRNA [NM_012777]	**0,595**
Fkbp1a	Rattus norvegicus FK506 binding protein 1a (Fkbp1a), mRNA [NM_013102]	**0,595**
Arf5	Rattus norvegicus ADP-ribosylation factor 5 (Arf5), mRNA [NM_024149]	**0,595**
Cdc25a	Rattus norvegicus cell division cycle 25 homolog A (S. pombe) (Cdc25a), mRNA [NM_133571]	**0,595**
Slc12a7	Rattus norvegicus solute carrier family 12 (potassium/chloride transporters), member 7 (Slc12a7), mRNA [NM_001013144]	**0,595**
Serinc1	Rattus norvegicus serine incorporator 1 (Serinc1), mRNA [NM_182951]	**0,595**
0	Unknown	**0,595**
RGD1307461	Rattus norvegicus similar to RIKEN cDNA 6430571L13 gene; similar to g20 protein (RGD1307461), mRNA [NM_001106854]	**0,595**
Bcl2l1	Rattus norvegicus Bcl2-like 1 (Bcl2l1), nuclear gene encoding mitochondrial protein, transcript variant 3, mRNA [NM_001033670]	**0,595**
Clock	Rattus norvegicus clock homolog (mouse) (Clock), mRNA [NM_021856]	**0,595**
0	Uncharacterized protein [Source:UniProtKB/TrEMBL;Acc:D4AE00] [ENSRNOT00000026030]	**0,595**
Parp14	Rattus norvegicus poly (ADP-ribose) polymerase family, member 14 (Parp14), mRNA [NM_001191659]	**0,595**
0	Unknown	**0,595**
0	SRY-box containing gene 5 Gene [Source:MGI Symbol;Acc:MGI:98367] [ENSRNOT00000020729]	**0,595**
0	Rattus norvegicus TL0ADA6YC10 mRNA sequence. [FQ228611]	**0,595**

Tmem186	Rattus norvegicus transmembrane protein 186 (Tmem186), mRNA [NM_001025756]	**0,595**
MGC114464	Rattus norvegicus similar to expressed sequence AI836003 (MGC114464), mRNA [NM_001024909]	**0,595**
Myst2	Rattus norvegicus MYST histone acetyltransferase 2 (Myst2), mRNA [NM_181081]	**0,595**
Itga6	Integrin alpha 6 subchain [Source:UniProtKB/TrEMBL;Acc:Q924W3] [ENSRNOT00000002075]	**0,595**
Ubfd1	Rattus norvegicus ubiquitin family domain containing 1 (Ubfd1), mRNA [NM_001034911]	**0,595**
Ppp1cb	Rattus norvegicus protein phosphatase 1, catalytic subunit, beta isoform (Ppp1cb), mRNA [NM_013065]	**0,595**
0	Unknown	**0,595**
Ccnd3	Rattus norvegicus cyclin D3 (Ccnd3), mRNA [NM_012766]	**0,595**
Gga1	Rattus norvegicus golgi associated, gamma adaptin ear containing, ARF binding protein 1 (Gga1), mRNA [NM_001011994]	**0,595**
Capza2	Rattus norvegicus capping protein (actin filament) muscle Z-line, alpha 2 (Capza2), mRNA [NM_001009180]	**0,595**
Fem1a	Rattus norvegicus fem-1 homolog a (C. elegans) (Fem1a), mRNA [NM_001025706]	**0,595**
Tet1	Rattus norvegicus tet oncogene 1 (Tet1), mRNA [NM_001107643]	**0,595**
Tpd52l1	Rattus norvegicus tumor protein D52-like 1 (Tpd52l1), mRNA [NM_001044295]	**0,595**
LOC684681	PREDICTED: Rattus norvegicus similar to Histone H1.2 (H1 VAR.1) (H1c) (LOC684681), mRNA [XM_001071565]	**0,595**
Gstk1	Rattus norvegicus glutathione S-transferase kappa 1 (Gstk1), nuclear gene encoding mitochondrial protein, mRNA [NM_181371]	**0,595**
RGD1560248	PREDICTED: Rattus norvegicus similar to formin-like 2 isoform B, transcript variant 2 (RGD1560248), mRNA [XM_002726151]	**0,595**
Gap43	Rattus norvegicus growth associated protein 43 (Gap43), mRNA [NM_017195]	**0,595**
Pycr1	Rattus norvegicus pyrroline-5-carboxylate reductase 1 (Pycr1), mRNA [NM_001105857]	**0,595**
0	Rattus norvegicus TL0ACA22YL15 mRNA sequence. [FQ217597]	**0,595**
Cbara1	Rattus norvegicus calcium binding atopy-related autoantigen 1 (Cbara1), nuclear gene encoding mitochondrial protein, mRNA [NM_199412]	**0,595**
Pkig	Rattus norvegicus protein kinase inhibitor, gamma (Pkig), mRNA [NM_153469]	**0,596**
0	Unknown	**0,596**
Commd10	Rattus norvegicus COMM domain containing 10 (Commd10), mRNA [NM_001004276]	**0,596**
Gpr108	Rattus norvegicus G protein-coupled receptor 108 (Gpr108), mRNA [NM_199399]	**0,596**
Pde5a	Rattus norvegicus phosphodiesterase 5A, cGMP-specific (Pde5a), mRNA [NM_133584]	**0,596**
Commd2	Rattus norvegicus COMM domain containing 2 (Commd2), mRNA [NM_001109503]	**0,596**
Myh10	Rattus norvegicus myosin, heavy chain 10, non-muscle (Myh10), mRNA [NM_031520]	**0,596**
Api5	Rattus norvegicus apoptosis inhibitor 5 (Api5), mRNA [NM_001127379]	**0,596**
E2f3	Rattus norvegicus E2F transcription factor 3 (E2f3), mRNA [NM_001137626]	**0,596**
Caprin1	Rattus norvegicus cell cycle associated protein 1 (Caprin1), mRNA [NM_001012185]	**0,596**
Nono	Rattus norvegicus non-POU domain containing, octamer-binding (Nono), mRNA [NM_001012356]	**0,596**
Unc84b	Uncharacterized protein [Source:UniProtKB/TrEMBL;Acc:D3ZJ67] [ENSRNOT00000046399]	**0,596**
0	PREDICTED: Rattus norvegicus similar to H3 histone, family 3B (RGD1564548), mRNA [XM_001072064]	**0,596**
RGD1311739	Rattus norvegicus similar to RIKEN cDNA 1700037H04 (RGD1311739), mRNA [NM_001025691]	**0,596**

Dclre1c	Rattus norvegicus DNA cross-link repair 1C, PSO2 homolog (S. cerevisiae) (Dclre1c), mRNA [NM_147145]	**0,596**
0	Unknown	**0,596**
0	Uncharacterized protein [Source:UniProtKB/TrEMBL;Acc:D4A2S8] [ENSRNOT00000034757]	**0,596**
0	Unknown	**0,596**
RGD1311952	Rattus norvegicus similar to Protein C20orf177 (RGD1311952), mRNA [NM_001134551]	**0,596**
Dkk3	Rattus norvegicus dickkopf homolog 3 (Xenopus laevis) (Dkk3), mRNA [NM_138519]	**0,596**
0	Q9UH66_HUMAN (Q9UH66) 7h3 protein (Fragment), partial (4%) [TC581403]	**0,596**
Fam178a	Rattus norvegicus family with sequence similarity 178, member A (Fam178a), mRNA [NM_001134612]	**0,596**
LOC678741	PREDICTED: Rattus norvegicus similar to Zinc finger CCCH-type domain containing protein 6 (LOC678741), mRNA [XM_001053214]	**0,596**
Igf2	Rattus norvegicus insulin-like growth factor 2 (Igf2), transcript variant 1, mRNA [NM_031511]	**0,596**
Nde1	Rattus norvegicus nudE nuclear distribution gene E homolog 1 (A. nidulans) (Nde1), mRNA [NM_053347]	**0,596**
Cic	Rattus norvegicus capicua homolog (Drosophila) (Cic), mRNA [NM_001107490]	**0,596**
Creb1	Rattus norvegicus cAMP responsive element binding protein 1 (Creb1), transcript variant A, mRNA [NM_134443]	**0,596**
Stim1	Rattus norvegicus stromal interaction molecule 1 (Stim1), mRNA [NM_001108496]	**0,596**
Zfand6	Rattus norvegicus zinc finger, AN1-type domain 6 (Zfand6), mRNA [NM_001007630]	**0,596**
Lphn2	Rattus norvegicus latrophilin 2 (Lphn2), transcript variant 2, mRNA [NM_001190475]	**0,596**
Tle3	Rattus norvegicus transducin-like enhancer of split 3 (E(sp1) homolog, Drosophila) (Tle3), mRNA [NM_053400]	**0,596**
0	Unknown	**0,596**
0	Unknown	**0,596**
Prosc	Rattus norvegicus TL0ACA41YL07 mRNA sequence. [FQ216663]	**0,596**
Prkcd	Rattus norvegicus protein kinase C, delta (Prkcd), mRNA [NM_133307]	**0,596**
Rrp1	Rattus norvegicus ribosomal RNA processing 1 homolog (S. cerevisiae) (Rrp1), mRNA [NM_001012073]	**0,596**
Prkcb	Rattus norvegicus protein kinase C, beta (Prkcb), transcript variant 2, mRNA [NM_001172305]	**0,596**
Sema3c	Rattus norvegicus sema domain, immunoglobulin domain (Ig), short basic domain, secreted, (semaphorin) 3C (Sema3c), mRNA [NM_001106578]	**0,596**
0	Uncharacterized protein [Source:UniProtKB/TrEMBL;Acc:D3ZV40] [ENSRNOT00000009733]	**0,596**
Cdk2ap1	Rattus norvegicus CDK2-associated protein 1 (Cdk2ap1), mRNA [NM_001113751]	**0,596**
Ppt1	Rattus norvegicus palmitoyl-protein thioesterase 1 (Ppt1), mRNA [NM_022502]	**0,596**
Phtf2	Rattus norvegicus putative homeodomain transcription factor 2 (Phtf2), mRNA [NM_001106577]	**0,596**
LOC363337	Rattus norvegicus similar to RIKEN cDNA 1700081O22 (LOC363337), mRNA [NM_001014221]	**0,596**
Rhbdl3	Rattus norvegicus rhomboid, veinlet-like 3 (Drosophila) (Rhbdl3), mRNA [NM_001105819]	**0,596**
Trim5	Rattus norvegicus tripartite motif-containing 5 (Trim5), mRNA [NM_001014023]	**0,596**
Ogfrl1	Rattus norvegicus opioid growth factor receptor-like 1 (Ogfrl1), mRNA [NM_001025708]	**0,596**
Gbas	Rattus norvegicus glioblastoma amplified sequence (Gbas), nuclear gene encoding mitochondrial protein, mRNA [NM_001017486]	**0,596**
RGD1561270	PREDICTED: Rattus norvegicus similar to Zinc finger protein 248 (RGD1561270), mRNA [XM_001057394]	**0,596**

Cacnb4	Rattus norvegicus calcium channel, voltage-dependent, beta 4 subunit (Cacnb4), mRNA [NM_001105733]	**0,596**
Rabgef1	Rattus norvegicus RAB guanine nucleotide exchange factor (GEF) 1 (Rabgef1), mRNA [NM_001108333]	**0,596**
0	Unknown	**0,596**
RGD1560046	PREDICTED: Rattus norvegicus similar to 3110052M02Rik protein (RGD1560046), mRNA [XM_002725490]	**0,596**
Plscr1	Rattus norvegicus phospholipid scramblase 1 (Plscr1), mRNA [NM_057194]	**0,596**
Hiatl1	Rattus norvegicus hippocampus abundant transcript-like 1 (Hiatl1), mRNA [NM_001107334]	**0,596**
0	Q4SRM6_TETNG (Q4SRM6) Chromosome undetermined SCAF14516, whole genome shotgun sequence. (Fragment), partial (5%) [TC616541]	**0,596**
0	Unknown	**0,596**
Mks1	Rattus norvegicus Meckel syndrome, type 1 (Mks1), mRNA [NM_001034917]	**0,596**
Elof1	Rattus norvegicus elongation factor 1 homolog (S. cerevisiae) (Elof1), mRNA [NM_001126098]	**0,596**
Gps1	Rattus norvegicus G protein pathway suppressor 1 (Gps1), mRNA [NM_053969]	**0,596**
0	UI-R-BJ1-atd-d-01-0-UI.s1 UI-R-BJ1 Rattus norvegicus cDNA clone UI-R-BJ1-atd-d-01-0-UI 3', mRNA sequence [BE098737]	**0,596**
Crabp2	Rattus norvegicus cellular retinoic acid binding protein 2 (Crabp2), mRNA [NM_017244]	**0,596**
Rgs3	Rattus norvegicus regulator of G-protein signaling 3 (Rgs3), mRNA [NM_019340]	**0,596**
Vgll4	Rattus norvegicus vestigial like 4 (Drosophila) (Vgll4), mRNA [NM_001015004]	**0,597**
Rpl23a	Rattus norvegicus ribosomal protein L23a (Rpl23a), mRNA [NM_001108283]	**0,597**
LOC686295	PREDICTED: Rattus norvegicus similar to CG17293-PA (LOC686295), mRNA [XM_001073342]	**0,597**
Chaf1b	Rattus norvegicus chromatin assembly factor 1, subunit B (p60) (Chaf1b), mRNA [NM_001024741]	**0,597**
Gabbr2	Rattus norvegicus gamma-aminobutyric acid (GABA) B receptor 2 (Gabbr2), mRNA [NM_031802]	**0,597**
LOC685300	AGENCOURT_17644684 NIH_MGC_237 Rattus norvegicus cDNA clone IMAGE:7115807 5', mRNA sequence [CK471043]	**0,597**
Zer1	Rattus norvegicus zer-1 homolog (C. elegans) (Zer1), mRNA [NM_001100707]	**0,597**
Dbt	Rattus norvegicus dihydrolipoamide branched chain transacylase E2 (Dbt), nuclear gene encoding mitochondrial protein, mRNA [NM_053312]	**0,597**
Myo6	PREDICTED: Rattus norvegicus myosin VI (Myo6), mRNA [XM_001061392]	**0,597**
0	Q96FH0_HUMAN (Q96FH0) MEF2B protein, complete [TC593056]	**0,597**
0	Echinoderm microtubule-associated protein-like 5 [Source:UniProtKB/Swiss-Prot;Acc:Q6ED65] [ENSRNOT00000005978]	**0,597**
Hccs	Rattus norvegicus holocytochrome c synthetase (Hccs), mRNA [NM_001191732]	**0,597**
Npr1	Rattus norvegicus natriuretic peptide receptor A/guanylate cyclase A (atrionatriuretic peptide receptor A) (Npr1), mRNA [NM_012613]	**0,597**
LOC363306	PREDICTED: Rattus norvegicus hypothetical protein LOC363306 (LOC363306), mRNA [XM_001068086]	**0,597**
Grpel2	Rattus norvegicus GrpE-like 2, mitochondrial (Grpel2), nuclear gene encoding mitochondrial protein, mRNA [NM_001109513]	**0,597**
0	Unknown	**0,597**
Dars2	Rattus norvegicus aspartyl-tRNA synthetase 2 (mitochondrial) (Dars2), nuclear gene encoding mitochondrial protein, mRNA [NM_001034143]	**0,597**
Scamp4	Rattus norvegicus secretory carrier membrane protein 4 (Scamp4), mRNA [NM_031725]	**0,597**
Prpf38b	Rattus norvegicus PRP38 pre-mRNA processing factor 38 (yeast) domain containing B (Prpf38b), mRNA [NM_001024305]	**0,597**
Fam12b	Rattus norvegicus family with sequence similarity 12, member B (epididymal) (Fam12b), mRNA [NM_178103]	**0,597**

Usp48	Rattus norvegicus ubiquitin specific peptidase 48 (Usp48), mRNA [NM_198785]	**0,597**
Sumo3	Rattus norvegicus SMT3 suppressor of mif two 3 homolog 3 (S. cerevisiae) (Sumo3), mRNA [NM_001024295]	**0,597**
St18	Rattus norvegicus suppression of tumorigenicity 18 (St18), mRNA [NM_153310]	**0,597**
R3hdm1	Rattus norvegicus R3H domain containing 1 (R3hdm1), mRNA [NM_001134867]	**0,597**
Miip	Rattus norvegicus migration and invasion inhibitory protein (Miip), mRNA [NM_001017450]	**0,597**
Taf2	Rattus norvegicus TAF2 RNA polymerase II, TATA box binding protein (TBP)-associated factor (Taf2), mRNA [NM_133319]	**0,597**
Cldn12	Rattus norvegicus claudin 12 (Cldn12), mRNA [NM_001100813]	**0,597**
Nrxn1	Rattus norvegicus neurexin 1 (Nrxn1), mRNA [NM_021767]	**0,597**
Litaf	Rattus norvegicus lipopolysaccharide-induced TNF factor (Litaf), mRNA [NM_001105735]	**0,597**
0	UI-R-FS0-crv-o-03-0-UI.s1 UI-R-FS0 Rattus norvegicus cDNA clone UI-R-FS0-crv-o-03-0-UI 3', mRNA sequence [CB328432]	**0,597**
Nacad	Rattus norvegicus NAC alpha domain containing (Nacad), mRNA [NM_001100655]	**0,597**
MGC94207	Rattus norvegicus similar to RIKEN cDNA C030006K11 (MGC94207), mRNA [NM_001007751]	**0,597**
Nr1d2	Rattus norvegicus nuclear receptor subfamily 1, group D, member 2 (Nr1d2), mRNA [NM_147210]	**0,597**
Ggps1	Rattus norvegicus geranylgeranyl diphosphate synthase 1 (Ggps1), mRNA [NM_001007626]	**0,597**
Prr5l	Rattus norvegicus proline rich 5 like (Prr5l), mRNA [NM_001080150]	**0,597**
Trove2	Rattus norvegicus TROVE domain family, member 2 (Trove2), mRNA [NM_001107183]	**0,597**
Eps8l2	Rattus norvegicus EPS8-like 2 (Eps8l2), mRNA [NM_001108508]	**0,597**
0	Unknown	**0,597**
Frmpd4	Rattus norvegicus FERM and PDZ domain containing 4 (Frmpd4), mRNA [NM_001106960]	**0,597**
Apaf1	Rattus norvegicus apoptotic peptidase activating factor 1 (Apaf1), mRNA [NM_023979]	**0,597**
Cmbl	Rattus norvegicus carboxymethylenebutenolidase homolog (Pseudomonas) (Cmbl), mRNA [NM_001008770]	**0,597**
Srpk1	Rattus norvegicus SFRS protein kinase 1 (Srpk1), mRNA [NM_001025726]	**0,597**
0	Unknown	**0,598**
Pik3c2b	Rattus norvegicus phosphoinositide-3-kinase, class 2, beta polypeptide (Pik3c2b), mRNA [NM_001105951]	**0,598**
Cplx1	Rattus norvegicus complexin 1 (Cplx1), mRNA [NM_022864]	**0,598**
Tor1aip1	Rattus norvegicus torsin A interacting protein 1 (Tor1aip1), mRNA [NM_145092]	**0,598**
Pmepa1	Rattus norvegicus prostate transmembrane protein, androgen induced 1 (Pmepa1), mRNA [NM_001107807]	**0,598**
0	Unknown	**0,598**
Cryab	Rattus norvegicus crystallin, alpha B (Cryab), mRNA [NM_012935]	**0,598**
Rspry1	Rattus norvegicus ring finger and SPRY domain containing 1 (Rspry1), mRNA [NM_001100945]	**0,598**
Mapkapk2	Rattus norvegicus mitogen-activated protein kinase-activated protein kinase 2 (Mapkapk2), mRNA [NM_178102]	**0,598**
siat7D	PREDICTED: Rattus norvegicus alpha-2,6-sialyltransferase ST6GalNAc IV (siat7D), mRNA [XM_001080040]	**0,598**
RGD1564969	PREDICTED: Rattus norvegicus similar to 60S ribosomal protein L29 (P23) (RGD1564969), mRNA [XM_002726901]	**0,598**
Syt17	Rattus norvegicus synaptotagmin XVII (Syt17), mRNA [NM_138849]	**0,598**

Hist3h2a	Rattus norvegicus histone cluster 3, H2a (Hist3h2a), mRNA [NM_021840]	**0,598**
Zc3h14	Rattus norvegicus zinc finger CCCH type containing 14 (Zc3h14), transcript variant 2, mRNA [NM_138920]	**0,598**
Dpp10	Rattus norvegicus dipeptidylpeptidase 10 (Dpp10), mRNA [NM_001012205]	**0,598**
0	Uncharacterized protein [Source:UniProtKB/TrEMBL;Acc:D3ZKR7] [ENSRNOT00000059076]	**0,598**
Epm2a	Laforin [Source:UniProtKB/Swiss-Prot;Acc:Q91XQ2] [ENSRNOT00000061930]	**0,598**
Opn4	Rattus norvegicus opsin 4 (Opn4), mRNA [NM_138860]	**0,598**
Tcfcp2	Rattus norvegicus transcription factor CP2 (Tcfcp2), mRNA [NM_001134714]	**0,598**
Acadsb	Rattus norvegicus acyl-Coenzyme A dehydrogenase, short/branched chain (Acadsb), nuclear gene encoding mitochondrial protein, mRNA [NM_013084]	**0,598**
Med24	Rattus norvegicus mediator complex subunit 24 (Med24), mRNA [NM_001034079]	**0,598**
Trip12	Rattus norvegicus thyroid hormone receptor interactor 12 (Trip12), mRNA [NM_001031659]	**0,598**
Acot1	Rattus norvegicus acyl-CoA thioesterase 1 (Acot1), mRNA [NM_031315]	**0,598**
Ppil1	Rattus norvegicus peptidylprolyl isomerase (cyclophilin)-like 1 (Ppil1), mRNA [NM_001034188]	**0,598**
Fmod	Rattus norvegicus fibromodulin (Fmod), mRNA [NM_080698]	**0,598**
Gle1	Rattus norvegicus GLE1 RNA export mediator homolog (yeast) (Gle1), mRNA [NM_001025731]	**0,598**
LOC681140	PREDICTED: Rattus norvegicus similar to 14-3-3 protein theta (14-3-3 protein tau) (14-3-3 protein T-cell) (HS1 protein) (LOC681140), mRNA [XM_001060456]	**0,598**
Reep1	Rattus norvegicus receptor accessory protein 1 (Reep1), nuclear gene encoding mitochondrial protein, mRNA [NM_001108633]	**0,598**
Usp7	Rattus norvegicus ubiquitin specific peptidase 7 (herpes virus-associated) (Usp7), mRNA [NM_001024790]	**0,598**
Dapk1	Rattus norvegicus death associated protein kinase 1 (Dapk1), mRNA [NM_001107335]	**0,598**
Myo9a	Rattus norvegicus myosin IXA (Myo9a), mRNA [NM_134335]	**0,598**
Car7	Rattus norvegicus carbonic anhydrase 7 (Car7), mRNA [NM_001106165]	**0,598**
Khdrbs1	Rattus norvegicus KH domain containing, RNA binding, signal transduction associated 1 (Khdrbs1), mRNA [NM_130405]	**0,598**
Slc15a2	Rattus norvegicus solute carrier family 15 (H+/peptide transporter), member 2 (Slc15a2), mRNA [NM_031672]	**0,598**
0	Q53UD0_BRABE (Q53UD0) Paired box protein Pax2/5/8 (Fragment), partial (9%) [TC618096]	**0,598**
Mettl7a	Rattus norvegicus methyltransferase like 7A (Mettl7a), mRNA [NM_001037355]	**0,598**
Plxdc2	Rattus norvegicus plexin domain containing 2 (Plxdc2), mRNA [NM_001108422]	**0,598**
LOC688776	Uncharacterized protein [Source:UniProtKB/TrEMBL;Acc:D4AAL7] [ENSRNOT00000049703]	**0,598**
Cbx1	PREDICTED: Rattus norvegicus chromobox homolog 1 (HP1 beta homolog Drosophila) (Cbx1), mRNA [XM_001081346]	**0,598**
Phb	Rattus norvegicus prohibitin (Phb), mRNA [NM_031851]	**0,598**
0	Rattus norvegicus TL0AEA97YK17 mRNA sequence. [FQ230487]	**0,598**
Cdc42se1	Rattus norvegicus CDC42 small effector 1 (Cdc42se1), mRNA [NM_001039044]	**0,598**
Lancl2	Rattus norvegicus LanC lantibiotic synthetase component C-like 2 (bacterial) (Lancl2), mRNA [NM_001014187]	**0,598**

Pdp1	Rattus norvegicus pyruvate dehyrogenase phosphatase catalytic subunit 1 (Pdp1), nuclear gene encoding mitochondrial protein, mRNA [NM_019372]	**0,598**
0	Uncharacterized protein [Source:UniProtKB/TrEMBL;Acc:D3ZKR9] [ENSRNOT00000022373]	**0,598**
0	Rattus norvegicus similar to 60S RIBOSOMAL PROTEIN L29 (P23) (LOC302428), mRNA [XM_228586]	**0,598**
LOC100362727	PREDICTED: Rattus norvegicus ubiquitin carboxyl-terminal hydrolase CYLD (LOC100362727), mRNA [XM_002728578]	**0,598**
Zfp758	PREDICTED: Rattus norvegicus similar to reduced expression 2 (LOC690559), mRNA [XM_001074805]	**0,599**
Tcta	Rattus norvegicus T-cell leukemia translocation altered gene (Tcta), mRNA [NM_001014005]	**0,599**
0	Rattus norvegicus similar to 60S RIBOSOMAL PROTEIN L29 (P23) (LOC294697), mRNA [XM_226719]	**0,599**
Clec16a	PREDICTED: Rattus norvegicus C-type lectin domain family 16, member A (Clec16a), mRNA [XM_213209]	**0,599**
Satb1	Rattus norvegicus SATB homeobox 1 (Satb1), mRNA [NM_001012129]	**0,599**
Gria1	Rattus norvegicus glutamate receptor, ionotropic, AMPA 1 (Gria1), mRNA [NM_031608]	**0,599**
Bloc1s2	Rattus norvegicus biogenesis of lysosomal organelles complex-1, subunit 2 (Bloc1s2), mRNA [NM_001037349]	**0,599**
Srbd1	Uncharacterized protein [Source:UniProtKB/TrEMBL;Acc:D4A9B0] [ENSRNOT00000019894]	**0,599**
Bysl	Rattus norvegicus bystin-like (Bysl), mRNA [NM_182674]	**0,599**
Ly6h	Rattus norvegicus lymphocyte antigen 6 complex, locus H (Ly6h), mRNA [NM_001134839]	**0,599**
Giot1	Rattus norvegicus gonadotropin inducible ovarian transcription factor 1 (Giot1), mRNA [NM_133563]	**0,599**
Nrxn3	Rattus norvegicus neurexin 3 (Nrxn3), mRNA [NM_053817]	**0,599**
Jarid1a	PREDICTED: Rattus norvegicus jumonji, AT rich interactive domain 1A (Rbp2 like) (Jarid1a), mRNA [XM_002729425]	**0,599**
Epha3	Rattus norvegicus Eph receptor A3 (Epha3), mRNA [NM_031564]	**0,599**
Gpd2	Rattus norvegicus glycerol-3-phosphate dehydrogenase 2, mitochondrial (Gpd2), nuclear gene encoding mitochondrial protein, mRNA [NM_012736]	**0,599**
Pcbp4	Rattus norvegicus poly(rC) binding protein 4 (Pcbp4), mRNA [NM_001191883]	**0,599**
Eif4ebp3	Rattus norvegicus eukaryotic translation initiation factor 4E binding protein 3 (Eif4ebp3), mRNA [NM_001202552]	**0,599**
LOC499781	Uncharacterized protein C9orf117 homolog [Source:UniProtKB/Swiss-Prot;Acc:Q4V7B0] [ENSRNOT00000020660]	**0,599**
LOC688311	PREDICTED: Rattus norvegicus similar to ADP-ribosylation factor-like 1, transcript variant 1 (LOC688311), mRNA [XM_001081794]	**0,599**
Arid4a	Rattus norvegicus AT rich interactive domain 4A (Rbp1 like) (Arid4a), mRNA [NM_001108029]	**0,599**
Ppp1r15b	Rattus norvegicus protein phosphatase 1, regulatory (inhibitor) subunit 15b (Ppp1r15b), mRNA [NM_001107175]	**0,599**
Gdi2	Rattus norvegicus GDP dissociation inhibitor 2 (Gdi2), mRNA [NM_017276]	**0,599**
Trim33	Uncharacterized protein [Source:UniProtKB/TrEMBL;Acc:D3ZUK4] [ENSRNOT00000025600]	**0,599**
Rasa2	Rattus norvegicus RAS p21 protein activator 2 (Rasa2), mRNA [NM_001105724]	**0,599**
LOC691477	PREDICTED: Rattus norvegicus similar to 60S ribosomal protein L29 (P23) (LOC691477), mRNA [XM_001078461]	**0,599**
Trpc2	Rattus norvegicus transient receptor potential cation channel, subfamily C, member 2, mRNA (cDNA clone MGC:189397 IMAGE:9091675), complete cds. [BC169022]	**0,599**
Evx2	PREDICTED: Rattus norvegicus even skipped homeotic gene 2 (Evx2), mRNA [XM_001063297]	**0,599**

Rpgr	Rattus norvegicus retinitis pigmentosa GTPase regulator (Rpgr), mRNA [NM_001127601]	**0,599**
0	Unknown	**0,599**
Ccdc85a	Rattus norvegicus coiled-coil domain containing 85A (Ccdc85a), mRNA [NM_001191553]	**0,599**
0	Uncharacterized protein [Source:UniProtKB/TrEMBL;Acc:D3ZGV8] [ENSRNOT00000004410]	**0,599**
Dstn	Rattus norvegicus destrin (Dstn), mRNA [NM_001033666]	**0,599**
Fam35a	Rattus norvegicus family with sequence similarity 35, member A (Fam35a), mRNA [NM_001025028]	**0,599**
0	DRNBTB09 Rat DRG Library Rattus norvegicus cDNA clone DRNBTB09 5', mRNA sequence [BG673348]	**0,599**
Pdgfa	Rattus norvegicus platelet-derived growth factor alpha polypeptide (Pdgfa), mRNA [NM_012801]	**0,599**
Dpf1	Rattus norvegicus D4, zinc and double PHD fingers family 1 (Dpf1), mRNA [NM_001105729]	**0,599**
Cacng2	Rattus norvegicus calcium channel, voltage-dependent, gamma subunit 2 (Cacng2), mRNA [NM_053351]	**0,599**
Dnajc15	Rattus norvegicus DnaJ (Hsp40) homolog, subfamily C, member 15 (Dnajc15), mRNA [NM_001106050]	**0,599**
Slc17a5	Rattus norvegicus solute carrier family 17 (anion/sugar transporter), member 5 (Slc17a5), mRNA [NM_001009713]	**0,599**
Vkorc1l1	Rattus norvegicus vitamin K epoxide reductase complex, subunit 1-like 1 (Vkorc1l1), mRNA [NM_203338]	**0,599**
Uba6	Rattus norvegicus ubiquitin-like modifier activating enzyme 6 (Uba6), mRNA [NM_001107213]	**0,600**
Ntrk2	Rattus norvegicus neurotrophic tyrosine kinase, receptor, type 2 (Ntrk2), transcript variant 1, mRNA [NM_012731]	**0,600**
Rftn2	Uncharacterized protein [Source:UniProtKB/TrEMBL;Acc:D3ZD65] [ENSRNOT00000020910]	**0,600**
0	Unknown	**0,600**
Lphn1	Rattus norvegicus latrophilin 1 (Lphn1), mRNA [NM_022962]	**0,600**
Nacc2	Rattus norvegicus nucleus accumbens associated 2, BEN and BTB (POZ) domain containing (Nacc2), mRNA [NM_001100533]	**0,600**
Hs2st1	Rattus norvegicus heparan sulfate 2-O-sulfotransferase 1 (Hs2st1), mRNA [NM_001100518]	**0,600**
Chm	Rattus norvegicus choroideremia (Rab escort protein 1) (Chm), mRNA [NM_017067]	**0,600**
Mxd4	Rattus norvegicus Max dimerization protein 4 (Mxd4), mRNA [NM_001108364]	**0,600**
0	Uncharacterized protein [Source:UniProtKB/TrEMBL;Acc:D3ZBC5] [ENSRNOT00000035888]	**0,600**
Donson	Rattus norvegicus downstream neighbor of SON (Donson), mRNA [NM_001008287]	**0,600**
Carhsp1	Rattus norvegicus calcium regulated heat stable protein 1 (Carhsp1), mRNA [NM_152790]	**0,600**
Pcdh17	Rattus norvegicus protocadherin 17 (Pcdh17), mRNA [NM_001107279]	**0,600**
C1ql3	Rattus norvegicus complement component 1, q subcomponent-like 3 (C1ql3), mRNA [NM_001109403]	**0,600**
Anxa11	Rattus norvegicus annexin A11 (Anxa11), mRNA [NM_001011918]	**0,600**
Dnm1l	Rattus norvegicus dynamin 1-like (Dnm1l), mRNA [NM_053655]	**0,600**
0	Unknown	**0,600**
Golga2	Rattus norvegicus golgi autoantigen, golgin subfamily a, 2 (Golga2), mRNA [NM_022596]	**0,600**
Elovl2	Rattus norvegicus elongation of very long chain fatty acids (FEN1/Elo2, SUR4/Elo3, yeast)-like 2 (Elovl2), mRNA [NM_001109118]	**0,600**
Atcay	Rattus norvegicus ataxia, cerebellar, Cayman type (Atcay), mRNA [NM_001040190]	**0,600**
0	Rattus norvegicus TL0ABA3YL04 mRNA sequence. [FQ209772]	**0,600**

Sh3glb1	Rattus norvegicus SH3-domain GRB2-like endophilin B1 (Sh3glb1), mRNA [NM_001011929]	**0,600**
Txnl4b	Rattus norvegicus thioredoxin-like 4B (Txnl4b), mRNA [NM_001013891]	**0,600**
Acaca	Rattus norvegicus acetyl-coenzyme A carboxylase alpha (Acaca), mRNA [NM_022193]	**0,600**
Axl	Rattus norvegicus Axl receptor tyrosine kinase (Axl), transcript variant 1, mRNA [NM_031794]	**0,600**
RGD1310311	Rattus norvegicus similar to chromosome 14 open reading frame 104 (RGD1310311), mRNA [NM_001014197]	**0,600**
Slc24a3	Rattus norvegicus solute carrier family 24 (sodium/potassium/calcium exchanger), member 3 (Slc24a3), mRNA [NM_053505]	**0,600**
0	Unknown	**0,600**
Sox2	Rattus norvegicus SRY (sex determining region Y)-box 2 (Sox2), mRNA [NM_001109181]	**0,600**
Ap3m2	Rattus norvegicus adaptor-related protein complex 3, mu 2 subunit (Ap3m2), mRNA [NM_133305]	**0,600**
Cdyl2	Rattus norvegicus chromodomain protein, Y chromosome-like 2 (Cdyl2), mRNA [NM_001106189]	**0,600**
RGD1564887	PREDICTED: Rattus norvegicus similar to 9130011E15Rik protein (RGD1564887), mRNA [XM_574677]	**0,600**
Pptc7	Rattus norvegicus PTC7 protein phosphatase homolog (S. cerevisiae) (Pptc7), mRNA [NM_001107141]	**0,600**
Marcks	PREDICTED: Rattus norvegicus myristoylated alanine rich protein kinase C substrate (Marcks), mRNA [XM_002728965]	**0,600**
0	Rattus norvegicus similar to cell division cycle associated 3; gene rich cluster, C8 gene; trigger of mitotic entry 1 (LOC316373), mRNA [XM_237105]	**0,600**
Limk1	Rattus norvegicus LIM domain kinase 1 (Limk1), mRNA [NM_031727]	**0,600**
Ncdn	Rattus norvegicus neurochondrin (Ncdn), mRNA [NM_053543]	**0,600**
0	Rattus norvegicus cDNA clone IMAGE:7388791. [BC158842]	**0,600**
Ripk1	Rattus norvegicus receptor (TNFRSF)-interacting serine-threonine kinase 1 (Ripk1), mRNA [NM_001107350]	**0,600**
Gpr123	Rattus norvegicus G protein-coupled receptor 123 (Gpr123), mRNA [NM_001107559]	**0,600**
Scn4b	Rattus norvegicus sodium channel, voltage-gated, type IV, beta (Scn4b), mRNA [NM_001008880]	**0,600**
Cck	Rattus norvegicus cholecystokinin (Cck), mRNA [NM_012829]	**0,600**
Rab13	Rattus norvegicus RAB13, member RAS oncogene family (Rab13), mRNA [NM_031092]	**0,600**
Tmem135	Rattus norvegicus transmembrane protein 135 (Tmem135), mRNA [NM_001013896]	**0,600**
Epha6	Ephrin type-A receptor 6 [Source:UniProtKB/Swiss-Prot;Acc:P54758] [ENSRNOT00000047310]	**0,600**
Zik1	PREDICTED: Rattus norvegicus zinc finger protein interacting with K protein 1 homolog (mouse) (Zik1), mRNA [XM_001076034]	**0,600**
Lgr4	Rattus norvegicus leucine-rich repeat-containing G protein-coupled receptor 4 (Lgr4), mRNA [NM_173328]	**0,600**
Abhd12	Rattus norvegicus abhydrolase domain containing 12 (Abhd12), mRNA [NM_001024314]	**0,601**
Sfxn5	Rattus norvegicus sideroflexin 5 (Sfxn5), nuclear gene encoding mitochondrial protein, mRNA [NM_153298]	**0,601**
Pias4	Rattus norvegicus protein inhibitor of activated STAT, 4 (Pias4), mRNA [NM_001100757]	**0,601**
Mrps17	Rattus norvegicus mitochondrial ribosomal protein S17 (Mrps17), nuclear gene encoding mitochondrial protein, mRNA [NM_001105923]	**0,601**
Sfrs12	Rattus norvegicus splicing factor, arginine/serine-rich 12 (Sfrs12), mRNA [NM_020092]	**0,601**
Xpnpep1	Rattus norvegicus X-prolyl aminopeptidase (aminopeptidase P) 1, soluble (Xpnpep1), mRNA [NM_131913]	**0,601**
Psenen	Rattus norvegicus presenilin enhancer 2 homolog (C. elegans) (Psenen), mRNA [NM_001008764]	**0,601**

Map2	Rattus norvegicus microtubule-associated protein 2 (Map2), mRNA [NM_013066]	**0,601**
Osbpl7	Rattus norvegicus oxysterol binding protein-like 7 (Osbpl7), mRNA [NM_001107044]	**0,601**
Otud7a	PREDICTED: Rattus norvegicus OTU domain containing 7A (Otud7a), mRNA [XM_219703]	**0,601**
Aen	Rattus norvegicus apoptosis enhancing nuclease (Aen), mRNA [NM_001108487]	**0,601**
0	Unknown	**0,601**
Amigo2	Rattus norvegicus adhesion molecule with Ig like domain 2 (Amigo2), mRNA [NM_182816]	**0,601**
LOC292199	Rattus norvegicus hypothetical LOC292199 (LOC292199), mRNA [NM_001144859]	**0,601**
LOC100364959	PREDICTED: Rattus norvegicus basic transcription factor 3-like (LOC100364959), mRNA [XM_002727692]	**0,601**
Ranbp10	Rattus norvegicus RAN binding protein 10 (Ranbp10), mRNA [NM_001135875]	**0,601**
RGD1308695	Rattus norvegicus similar to RIKEN cDNA 1200016B10 (RGD1308695), mRNA [NM_001105960]	**0,601**
Ptpn1	Tyrosine-protein phosphatase non-receptor type 1 [Source:UniProtKB/Swiss-Prot;Acc:P20417] [ENSRNOT00000014309]	**0,601**
Aqp5	Rattus norvegicus aquaporin 5 (Aqp5), mRNA [NM_012779]	**0,601**
0	Unknown	**0,601**
Sec14l1	Rattus norvegicus SEC14-like 1 (S. cerevisiae) (Sec14l1), mRNA [NM_001108309]	**0,601**
Gfra2	Rattus norvegicus GDNF family receptor alpha 2 (Gfra2), mRNA [NM_012750]	**0,601**
Elovl1	Rattus norvegicus elongation of very long chain fatty acids (FEN1/Elo2, SUR4/Elo3, yeast)-like 1 (Elovl1), mRNA [NM_001044275]	**0,601**
P2rx3	Rattus norvegicus purinergic receptor P2X, ligand-gated ion channel, 3 (P2rx3), mRNA [NM_031075]	**0,601**
LOC680097	PREDICTED: Rattus norvegicus similar to germinal histone H4 gene (LOC680097), mRNA [XM_001055696]	**0,601**
0	Similar to HLA-B associated transcript-2 isoform aUncharacterized protein [Source:UniProtKB/TrEMBL;Acc:D3ZUK2] [ENSRNOT00000066368]	**0,601**
Rai1	Uncharacterized protein [Source:UniProtKB/TrEMBL;Acc:D4A4Z4] [ENSRNOT00000039411]	**0,601**
Insig2	Insulin-induced gene 2 protein [Source:UniProtKB/Swiss-Prot;Acc:Q80UA9] [ENSRNOT00000003391]	**0,601**
0	Unknown	**0,601**
Zfp799	Rattus norvegicus zinc finger protein 799 (Zfp799), mRNA [NM_001009537]	**0,601**
Rreb1	Rattus norvegicus ras responsive element binding protein 1 (Rreb1), mRNA [NM_001107348]	**0,601**
Gtf2e1	Rattus norvegicus general transcription factor IIE, polypeptide 1 (alpha subunit) (Gtf2e1), mRNA [NM_001100556]	**0,601**
Nrbp2	Rattus norvegicus nuclear receptor binding protein 2 (Nrbp2), mRNA [NM_001135007]	**0,601**
Mnt	Rattus norvegicus max binding protein (Mnt), mRNA [NM_001105807]	**0,601**
Cant1	Rattus norvegicus calcium activated nucleotidase 1 (Cant1), mRNA [NM_144754]	**0,601**
Pnpla2	Rattus norvegicus patatin-like phospholipase domain containing 2 (Pnpla2), mRNA [NM_001108509]	**0,601**
Aff4	Rattus norvegicus AF4/FMR2 family, member 4 (Aff4), mRNA [NM_001107001]	**0,601**
Scn3b	Rattus norvegicus sodium channel, voltage-gated, type III, beta (Scn3b), mRNA [NM_139097]	**0,601**
Jph3	Rattus norvegicus junctophilin 3 (Jph3), mRNA [NM_001107437]	**0,601**
RGD1565310	PREDICTED: Rattus norvegicus similar to RIKEN cDNA 1110018J12 (RGD1565310), mRNA [XM_001057346]	**0,601**
Tsku	Rattus norvegicus tsukushin (Tsku), mRNA [NM_001009965]	**0,601**

Lman1	Rattus norvegicus lectin, mannose-binding, 1 (Lman1), mRNA [NM_053886]	**0,601**
Sdr39u1	Rattus norvegicus short chain dehydrogenase/reductase family 39U, member 1 (Sdr39u1), mRNA [NM_001108378]	**0,601**
0	Unknown	**0,601**
Fam76b	Rattus norvegicus family with sequence similarity 76, member B (Fam76b), mRNA [NM_001108994]	**0,601**
Add2	Rattus norvegicus adducin 2 (beta) (Add2), transcript variant 2, mRNA [NM_012491]	**0,601**
Dynll2	Rattus norvegicus dynein light chain LC8-type 2 (Dynll2), mRNA [NM_080697]	**0,601**
MGC109340	Rattus norvegicus similar to Microsomal signal peptidase 23 kDa subunit (SPase 22 kDa subunit) (SPC22/23) (MGC109340), mRNA [NM_001024267]	**0,601**
Sigmar1	Rattus norvegicus sigma non-opioid intracellular receptor 1 (Sigmar1), mRNA [NM_030996]	**0,601**
Cd68	Rattus norvegicus Cd68 molecule (Cd68), mRNA [NM_001031638]	**0,601**
Slitrk5	Rattus norvegicus SLIT and NTRK-like family, member 5 (Slitrk5), mRNA [NM_001107284]	**0,601**
LOC317165	Rattus norvegicus similar to Set alpha isoform (LOC317165), mRNA [NM_001047892]	**0,601**
Ezh1	Rattus norvegicus enhancer of zeste homolog 1 (Drosophila) (Ezh1), mRNA [NM_001107051]	**0,602**
Polb	Rattus norvegicus polymerase (DNA directed), beta (Polb), mRNA [NM_017141]	**0,602**
0	Uncharacterized protein [Source:UniProtKB/TrEMBL;Acc:D3ZDV6] [ENSRNOT00000011793]	**0,602**
0	Unknown	**0,602**
Entpd3	Rattus norvegicus ectonucleoside triphosphate diphosphohydrolase 3 (Entpd3), mRNA [NM_178106]	**0,602**
0	Rattus norvegicus similar to 60S RIBOSOMAL PROTEIN L29 (P23) (LOC294697), mRNA [XM_226719]	**0,602**
Mrps21	Rattus norvegicus mitochondrial ribosomal protein S21 (Mrps21), nuclear gene encoding mitochondrial protein, mRNA [NM_001126094]	**0,602**
LOC684399	PREDICTED: Rattus norvegicus similar to 60S ribosomal protein L29 (P23) (LOC684399), mRNA [XM_001070213]	**0,602**
LOC497978	Rattus norvegicus similar to diacylglycerol kinase epsilon (LOC497978), mRNA [NM_001039341]	**0,602**
0	Unknown	**0,602**
Cpsf6	Rattus norvegicus cleavage and polyadenylation specific factor 6 (Cpsf6), mRNA [NM_001106785]	**0,602**
Agxt2l2	Rattus norvegicus alanine-glyoxylate aminotransferase 2-like 2 (Agxt2l2), mRNA [NM_001128196]	**0,602**
Slc13a3	Rattus norvegicus solute carrier family 13 (sodium-dependent dicarboxylate transporter), member 3 (Slc13a3), mRNA [NM_022866]	**0,602**
LOC302680	similar to CXORF15 (LOC302680), mRNA [Source:RefSeq DNA;Acc:NM_001037187] [ENSRNOT00000006843]	**0,602**
Snx17	Rattus norvegicus sorting nexin 17 (Snx17), mRNA [NM_001011981]	**0,602**
0	AF155065 brain protein {Homo sapiens} (exp=-1; wgp=0; cg=0), partial (12%) [TC626613]	**0,602**
Taf2	Rattus norvegicus TAF2 RNA polymerase II, TATA box binding protein (TBP)-associated factor (Taf2), mRNA [NM_133319]	**0,602**
Nalcn	Rattus norvegicus sodium leak channel, non-selective (Nalcn), mRNA [NM_153630]	**0,602**
Dnah11	PREDICTED: Rattus norvegicus dynein, axonemal, heavy chain 11 (Dnah11), mRNA [XM_001061747]	**0,602**
Sec31a	Rattus norvegicus SEC31 homolog A (S. cerevisiae) (Sec31a), mRNA [NM_033021]	**0,602**
Trib3	Rattus norvegicus tribbles homolog 3 (Drosophila) (Trib3), mRNA [NM_144755]	**0,602**
0	Unknown	**0,602**

Prrt1	Rattus norvegicus proline-rich transmembrane protein 1 (Prrt1), mRNA [NM_001032285]	**0,602**
Tmem183a	Rattus norvegicus transmembrane protein 183A (Tmem183a), mRNA [NM_001013871]	**0,602**
Gchfr	Rattus norvegicus GTP cyclohydrolase I feedback regulator (Gchfr), mRNA [NM_133595]	**0,602**
Resp18	Rattus norvegicus regulated endocrine-specific protein 18 (Resp18), mRNA [NM_019278]	**0,602**
0	Unknown	**0,602**
Clk2	Rattus norvegicus CDC-like kinase 2 (Clk2), mRNA [NM_001014254]	**0,602**
LOC314600	Rattus norvegicus similar to zinc finger protein 422, related sequence 1 (LOC314600), mRNA [NM_001126281]	**0,602**
Haus8	Rattus norvegicus HAUS augmin-like complex, subunit 8 (Haus8), mRNA [NM_001024971]	**0,602**
Fam164a	Rattus norvegicus family with sequence similarity 164, member A (Fam164a), mRNA [NM_001107661]	**0,602**
Kcna1	Rattus norvegicus potassium voltage-gated channel, shaker-related subfamily, member 1 (Kcna1), mRNA [NM_173095]	**0,602**
Zfp251	Rattus norvegicus zinc finger protein 251 (Zfp251), mRNA [NM_001191911]	**0,602**
Bsn	Rattus norvegicus bassoon (Bsn), mRNA [NM_019146]	**0,602**
Rab5a	Rattus norvegicus RAB5A, member RAS oncogene family (Rab5a), mRNA [NM_022692]	**0,602**
0	RGD1311624 protein [Source:UniProtKB/TrEMBL;Acc:Q4G026] [ENSRNOT00000054990]	**0,602**
Glce	PREDICTED: Rattus norvegicus glucuronic acid epimerase (Glce), mRNA [XM_343404]	**0,602**
0	Unknown	**0,602**
Zbtb4	PREDICTED: Rattus norvegicus zinc finger and BTB domain containing 4 (Zbtb4), mRNA [XM_001079524]	**0,602**
F2r	Rattus norvegicus coagulation factor II (thrombin) receptor (F2r), mRNA [NM_012950]	**0,602**
Unc5c	Rattus norvegicus unc-5 homolog C (C. elegans) (Unc5c), mRNA [NM_199407]	**0,602**
Fam53b	Rattus norvegicus family with sequence similarity 53, member B (Fam53b), mRNA [NM_001107556]	**0,602**
RGD1311422	PREDICTED: Rattus norvegicus similar to CG8841-PA (RGD1311422), mRNA [XM_001081678]	**0,602**
Vps37a	Rattus norvegicus vacuolar protein sorting 37 homolog A (S. cerevisiae) (Vps37a), mRNA [NM_001024867]	**0,603**
Hnrnpu	Rattus norvegicus heterogeneous nuclear ribonucleoprotein U (Hnrnpu), mRNA [NM_057139]	**0,603**
Bat2	Rattus norvegicus HLA-B associated transcript 2 (Bat2), mRNA [NM_212462]	**0,603**
Itih3	Rattus norvegicus inter-alpha trypsin inhibitor, heavy chain 3 (Itih3), mRNA [NM_017351]	**0,603**
Ccdc80	Rattus norvegicus coiled-coil domain containing 80 (Ccdc80), mRNA [NM_022543]	**0,603**
Stxbp5	Rattus norvegicus syntaxin binding protein 5 (tomosyn) (Stxbp5), transcript variant b, mRNA [NM_178345]	**0,603**
RGD1309540	Rattus norvegicus similar to hypothetical protein MGC40841; similar to hypothetical protein MGC4707 (RGD1309540), mRNA [NM_001013918]	**0,603**
Polb	DNA polymerase beta [Source:UniProtKB/Swiss-Prot;Acc:P06766] [ENSRNOT00000026039]	**0,603**
Add2	Rattus norvegicus adducin 2 (beta) (Add2), transcript variant 1, mRNA [NM_001109880]	**0,603**
LOC296884	Rattus norvegicus cDNA clone IMAGE:7132615. [BC083591]	**0,603**
Cap2	Rattus norvegicus CAP, adenylate cyclase-associated protein, 2 (yeast) (Cap2), mRNA [NM_053874]	**0,603**
RGD1306215	Rattus norvegicus similar to hypothetical protein MGC36831 (RGD1306215), mRNA [NM_001106556]	**0,603**
0	Q62262_MOUSE (Q62262) Spermatid perinuclear RNA binding protein, partial (12%) [TC595714]	**0,603**

Lmtk2	Rattus norvegicus lemur tyrosine kinase 2 (Lmtk2), mRNA [NM_001137641]	**0,603**
Alkbh8	Rattus norvegicus alkB, alkylation repair homolog 8 (E. coli) (Alkbh8), mRNA [NM_001191909]	**0,603**
Ets2	Rattus norvegicus v-ets erythroblastosis virus E26 oncogene homolog 2 (avian) (Ets2), mRNA [NM_001107107]	**0,603**
Ythdf3	Rattus norvegicus YTH domain family, member 3 (Ythdf3), mRNA [NM_001108546]	**0,603**
Otx2	Rattus norvegicus orthodenticle homeobox 2 (Otx2), mRNA [NM_001100566]	**0,603**
Iqgap1	Rattus norvegicus IQ motif containing GTPase activating protein 1 (Iqgap1), mRNA [NM_001108489]	**0,603**
Rgs4	Rattus norvegicus regulator of G-protein signaling 4 (Rgs4), mRNA [NM_017214]	**0,603**
RGD1561065	Rattus norvegicus similar to mKIAA1111 protein (RGD1561065), mRNA [NM_001108253]	**0,603**
Alg8	Rattus norvegicus asparagine-linked glycosylation 8, alpha-1,3-glucosyltransferase homolog (S. cerevisiae) (Alg8), mRNA [NM_001034127]	**0,603**
Cdipt	Rattus norvegicus CDP-diacylglycerol--inositol 3-phosphatidyltransferase (phosphatidylinositol synthase) (Cdipt), mRNA [NM_138899]	**0,603**
Fbxw5	Rattus norvegicus F-box and WD repeat domain containing 5 (Fbxw5), mRNA [NM_001025730]	**0,603**
0	Histone H3.1 [Source:UniProtKB/Swiss-Prot;Acc:Q6LED0] [ENSRNOT00000059330]	**0,603**
Ahcyl1	Rattus norvegicus adenosylhomocysteinase-like 1 (Ahcyl1), mRNA [NM_001108561]	**0,603**
Tmtc4	Rattus norvegicus transmembrane and tetratricopeptide repeat containing 4 (Tmtc4), mRNA [NM_001134414]	**0,603**
Fam149a	PREDICTED: Rattus norvegicus family with sequence similarity 149, member A (Fam149a), mRNA [XM_001061521]	**0,603**
Daam1	Rattus norvegicus dishevelled associated activator of morphogenesis 1 (Daam1), mRNA [NM_001108030]	**0,603**
Zfp426	Rattus norvegicus zinc finger protein 426 (Zfp426), mRNA [NM_001079943]	**0,603**
0	Unknown	**0,603**
Stard6	Rattus norvegicus StAR-related lipid transfer (START) domain containing 6 (Stard6), mRNA [NM_001007627]	**0,603**
0	Unknown	**0,603**
Lrrc7	Rattus norvegicus leucine rich repeat containing 7 (Lrrc7), mRNA [NM_057142]	**0,603**
Galntl6	Rattus norvegicus UDP-N-acetyl-alpha-D-galactosamine:polypeptide N-acetylgalactosaminyltransferase-like 6 (Galntl6), mRNA [NM_001135756]	**0,603**
Pou3f3	Rattus norvegicus POU class 3 homeobox 3 (Pou3f3), mRNA [NM_138837]	**0,603**
Clcn3	Rattus norvegicus chloride channel 3 (Clcn3), mRNA [NM_053363]	**0,603**
Golph3	Rattus norvegicus golgi phosphoprotein 3 (coat-protein) (Golph3), mRNA [NM_023977]	**0,603**
B4galnt4	Rattus norvegicus beta-1,4-N-acetyl-galactosaminyl transferase 4 (B4galnt4), mRNA [NM_001107562]	**0,603**
Golga7	Rattus norvegicus golgi autoantigen, golgin subfamily a, 7 (Golga7), mRNA [NM_001007731]	**0,603**
Adnp	Rattus norvegicus activity-dependent neuroprotector homeobox (Adnp), mRNA [NM_022681]	**0,603**
Ubr5	E3 ubiquitin-protein ligase UBR5 [Source:UniProtKB/Swiss-Prot;Acc:Q62671] [ENSRNOT00000009115]	**0,603**
Tecr	Rattus norvegicus trans-2,3-enoyl-CoA reductase (Tecr), mRNA [NM_138549]	**0,603**
Myo9b	Rattus norvegicus myosin IXb (Myo9b), mRNA [NM_012984]	**0,603**
LOC100294508	Rattus norvegicus dyslexia susceptibility 2-like (LOC100294508), mRNA [NM_001159655]	**0,603**
Ankrd46	Rattus norvegicus ankyrin repeat domain 46 (Ankrd46), mRNA [NM_001013948]	**0,603**
Mrpl50	Rattus norvegicus mitochondrial ribosomal protein L50 (Mrpl50), nuclear gene encoding mitochondrial protein, mRNA [NM_001108665]	**0,603**

Synj1	Rattus norvegicus synaptojanin 1 (Synj1), mRNA [NM_053476]	**0,603**
0	AW917568 EST348872 Rat gene index, normalized rat, norvegicus, Bento Soares Rattus norvegicus cDNA clone RGIEF42 5' end, mRNA sequence [AW917568]	**0,603**
Chac2	Rattus norvegicus ChaC, cation transport regulator homolog 2 (E. coli) (Chac2), mRNA [NM_001025016]	**0,603**
Bcl7b	Rattus norvegicus B-cell CLL/lymphoma 7B (Bcl7b), mRNA [NM_001109021]	**0,603**
39508	Rattus norvegicus membrane-associated ring finger (C3HC4) 8 (March8), mRNA [NM_001107882]	**0,603**
Zfp322a	Rattus norvegicus zinc finger protein 322a (Zfp322a), mRNA [NM_001135084]	**0,603**
Pigh	Rattus norvegicus phosphatidylinositol glycan anchor biosynthesis, class H (Pigh), mRNA [NM_001108714]	**0,603**
Ctdsp1	Rattus norvegicus CTD (carboxy-terminal domain, RNA polymerase II, polypeptide A) small phosphatase 1 (Ctdsp1), mRNA [NM_001128079]	**0,603**
Homer2	Rattus norvegicus homer homolog 2 (Drosophila) (Homer2), mRNA [NM_053309]	**0,604**
Bcdin3d	Rattus norvegicus BCDIN3 domain containing (Bcdin3d), mRNA [NM_001108751]	**0,604**
Rufy3	Rattus norvegicus RUN and FYVE domain containing 3 (Rufy3), mRNA [NM_001025127]	**0,604**
RGD1560880	Rattus norvegicus similar to RIKEN cDNA 2310002J15 (RGD1560880), mRNA [NM_001109194]	**0,604**
S100a16	Rattus norvegicus S100 calcium binding protein A16 (S100a16), mRNA [NM_001108557]	**0,604**
0	Putative uncharacterized protein RGD1560252_predictedUncharacterized protein [Source:UniProtKB/TrEMBL;Acc:D4A3Y2] [ENSRNOT00000023411]	**0,604**
Mavs	Rattus norvegicus mitochondrial antiviral signaling protein (Mavs), nuclear gene encoding mitochondrial protein, mRNA [NM_001005556]	**0,604**
Chst1	Rattus norvegicus carbohydrate (keratan sulfate Gal-6) sulfotransferase 1 (Chst1), mRNA [NM_001011955]	**0,604**
Cops7a	Rattus norvegicus COP9 constitutive photomorphogenic homolog subunit 7A (Arabidopsis) (Cops7a), transcript variant 2, mRNA [NM_001047098]	**0,604**
Ptpn4	Rattus norvegicus protein tyrosine phosphatase, non-receptor type 4 (Ptpn4), mRNA [NM_001100479]	**0,604**
Mknk2	Rattus norvegicus MAP kinase-interacting serine/threonine kinase 2 (Mknk2), mRNA [NM_001011985]	**0,604**
Gtpbp3	Rattus norvegicus GTP binding protein 3 (Gtpbp3), nuclear gene encoding mitochondrial protein, mRNA [NM_001011919]	**0,604**
Mllt3	Rattus norvegicus myeloid/lymphoid or mixed-lineage leukemia (trithorax homolog, Drosophila); translocated to, 3 (Mllt3), mRNA [NM_053718]	**0,604**
0	Unknown	**0,604**
0	Unknown	**0,604**
Ap2b1	Rattus norvegicus adaptor-related protein complex 2, beta 1 subunit (Ap2b1), mRNA [NM_080583]	**0,604**
Atg14	Rattus norvegicus ATG14 autophagy related 14 homolog (S. cerevisiae) (Atg14), mRNA [NM_001107258]	**0,604**
Steap2	Rattus norvegicus six transmembrane epithelial antigen of the prostate 2 (Steap2), mRNA [NM_001107846]	**0,604**
Dixdc1	Rattus norvegicus DIX domain containing 1 (Dixdc1), mRNA [NM_001037654]	**0,604**
Znf579	Rattus norvegicus zinc finger protein 579 (Znf579), mRNA [NM_001126276]	**0,604**
0	Rattus norvegicus similar to RIKEN cDNA 1110011K10 (LOC315466), mRNA [XM_235929]	**0,604**
Eif4a1	Rattus norvegicus eukaryotic translation initiation factor 4A, isoform 1 (Eif4a1), mRNA [NM_199372]	**0,604**
0	Uncharacterized protein [Source:UniProtKB/TrEMBL;Acc:D3ZXK4] [ENSRNOT00000025560]	**0,604**
Itpkc	Rattus norvegicus inositol 1,4,5-trisphosphate 3-kinase C (Itpkc), mRNA [NM_178094]	**0,604**

Rnf166	Rattus norvegicus ring finger protein 166 (Rnf166), mRNA [NM_001002279]	**0,604**
Calu	Rattus norvegicus calumenin (Calu), transcript variant 2, mRNA [NM_001033898]	**0,604**
LOC302495	Rattus norvegicus hypothetical LOC302495 (LOC302495), mRNA [NM_001106950]	**0,604**
0	Uncharacterized protein [Source:UniProtKB/TrEMBL;Acc:D4A9I1] [ENSRNOT00000037171]	**0,604**
Vhl	Rattus norvegicus von Hippel-Lindau tumor suppressor (Vhl), mRNA [NM_052801]	**0,604**
Kcnk12	Rattus norvegicus potassium channel, subfamily K, member 12 (Kcnk12), mRNA [NM_022292]	**0,604**
Brcc3	Rattus norvegicus BRCA1/BRCA2-containing complex, subunit 3 (Brcc3), mRNA [NM_001127300]	**0,604**
Clptm1	Rattus norvegicus cleft lip and palate associated transmembrane protein 1 (Clptm1), mRNA [NM_001106232]	**0,604**
Lrrtm4	Rattus norvegicus leucine rich repeat transmembrane neuronal 4 (Lrrtm4), mRNA [NM_001134746]	**0,604**
0	Unknown	**0,604**
Acy3	Rattus norvegicus aspartoacylase (aminocyclase) 3 (Acy3), mRNA [NM_001009603]	**0,604**
Neto2	Neuropilin and tolloid-like protein 2 [Source:UniProtKB/Swiss-Prot;Acc:C6K2K4] [ENSRNOT00000021739]	**0,604**
Agps	Rattus norvegicus alkylglycerone phosphate synthase (Agps), mRNA [NM_053350]	**0,604**
Ttc9c	Rattus norvegicus tetratricopeptide repeat domain 9C (Ttc9c), mRNA [NM_001007693]	**0,604**
Xrra1	Rattus norvegicus X-ray radiation resistance associated 1 (Xrra1), mRNA [NM_001113753]	**0,604**
0	Uncharacterized protein [Source:UniProtKB/TrEMBL;Acc:D3ZKR7] [ENSRNOT00000046075]	**0,604**
Nudt16l1	Rattus norvegicus nudix (nucleoside diphosphate linked moiety X)-type motif 16-like 1 (Nudt16l1), mRNA [NM_001100782]	**0,604**
Usp45	Rattus norvegicus ubiquitin specific peptidase 45 (Usp45), mRNA [NM_001107918]	**0,604**
Lhfpl2	Rattus norvegicus lipoma HMGIC fusion partner-like 2 (Lhfpl2), mRNA [NM_001106402]	**0,604**
RGD1310352	Rattus norvegicus similar to HTGN29 protein; keratinocytes associated transmembrane protein 2 (RGD1310352), mRNA [NM_001106999]	**0,605**
Scarb1	Rattus norvegicus scavenger receptor class B, member 1 (Scarb1), mRNA [NM_031541]	**0,605**
0	Uncharacterized protein [Source:UniProtKB/TrEMBL;Acc:D4ADL2] [ENSRNOT00000049830]	**0,605**
Uprt	Uncharacterized protein [Source:UniProtKB/TrEMBL;Acc:D4ABC7] [ENSRNOT00000033494]	**0,605**
Znf606	PREDICTED: Rattus norvegicus zinc finger protein 606 (Znf606), mRNA [XM_001063437]	**0,605**
0	PREDICTED: Rattus norvegicus similar to DUB-1 (RGD1562061), mRNA [XM_219062]	**0,605**
Cybrd1	Rattus norvegicus cytochrome b reductase 1 (Cybrd1), mRNA [NM_001011954]	**0,605**
LOC688019	Uncharacterized protein [Source:UniProtKB/TrEMBL;Acc:D3ZAX9] [ENSRNOT00000005443]	**0,605**
0	Unknown	**0,605**
Pnma3	Rattus norvegicus paraneoplastic antigen MA3 (Pnma3), mRNA [NM_001106342]	**0,605**
Polh	Rattus norvegicus polymerase (DNA directed), eta (Polh), mRNA [NM_001108204]	**0,605**
Hspa12a	Rattus norvegicus heat shock protein 12A (Hspa12a), mRNA [NM_001107445]	**0,605**
Mtss1	Rattus norvegicus metastasis suppressor 1 (Mtss1), mRNA [NM_001130563]	**0,605**
Plcb1	Rattus norvegicus phospholipase C, beta 1 (phosphoinositide-specific) (Plcb1), mRNA [NM_001077641]	**0,605**
Rap2a	RAS related protein 2a [Source:RefSeq peptide;Acc:NP_446193] [ENSRNOT00000032395]	**0,605**

Acrbp	Rattus norvegicus acrosin binding protein (Acrbp), mRNA [NM_001025049]	**0,605**
0	Unknown	**0,605**
Znf618	PREDICTED: Rattus norvegicus zinc finger protein 618 (Znf618), mRNA [XM_233016]	**0,605**
Mtfr1	Rattus norvegicus TL0ACA31YL20 mRNA sequence. [FQ217237]	**0,605**
Epb41l4a	Rattus norvegicus erythrocyte membrane protein band 4.1 like 4A (Epb41l4a), mRNA [NM_001107397]	**0,605**
Narf	Rattus norvegicus nuclear prelamin A recognition factor (Narf), mRNA [NM_001039207]	**0,605**
Nup210	Rattus norvegicus nucleoporin 210 (Nup210), mRNA [NM_053322]	**0,605**
Zfp84	Rattus norvegicus zinc finger protein 84 (Zfp84), mRNA [NM_001107500]	**0,605**
Ggcx	Rattus norvegicus gamma-glutamyl carboxylase (Ggcx), mRNA [NM_031756]	**0,605**
Cdk2ap2	Rattus norvegicus CDK2-associated protein 2 (Cdk2ap2), mRNA [NM_001109498]	**0,605**
RGD1562291	PREDICTED: Rattus norvegicus similar to Human T-cell leukemia virus enhancer factor (Forkhead box protein N2) (RGD1562291), mRNA [XM_237502]	**0,605**
RGD1559682	PREDICTED: Rattus norvegicus similar to peptidylprolyl isomerase A (cyclophilin A)) (RGD1559682), mRNA [XM_341363]	**0,605**
Chrne	Rattus norvegicus cholinergic receptor, nicotinic, epsilon (Chrne), mRNA [NM_017194]	**0,605**
Gulp1	Rattus norvegicus GULP, engulfment adaptor PTB domain containing 1 (Gulp1), mRNA [NM_001013171]	**0,605**
Srpr	Rattus norvegicus signal recognition particle receptor ('docking protein') (Srpr), mRNA [NM_001034150]	**0,605**
Klk1l	Rattus norvegicus kallikrein 1-like peptidase (Klk1l), mRNA [NM_012593]	**0,605**
Sgsm1	small G protein signaling modulator 1 [Source:RefSeq peptide;Acc:NP_001099407] [ENSRNOT00000056824]	**0,605**
RGD1564036	Rattus norvegicus similar to RIKEN cDNA 3010026O09 (RGD1564036), mRNA [NM_001109030]	**0,605**
Fam71d	Rattus norvegicus family with sequence similarity 71, member D (Fam71d), mRNA [NM_001017469]	**0,605**
Gfra1	Rattus norvegicus GDNF family receptor alpha 1 (Gfra1), mRNA [NM_012959]	**0,605**
Olr1252	Rattus norvegicus olfactory receptor 1252 (Olr1252), mRNA [NM_001000454]	**0,605**
LOC500013	PREDICTED: Rattus norvegicus similar to sterile alpha motif domain containing 9-like (LOC500013), miscRNA [XR_005677]	**0,605**
Pdcd6ip	Rattus norvegicus programmed cell death 6 interacting protein (Pdcd6ip), mRNA [NM_001029910]	**0,605**
Hp1bp3	Rattus norvegicus heterochromatin protein 1, binding protein 3 (Hp1bp3), mRNA [NM_199108]	**0,605**
Hmg20b	Rattus norvegicus high mobility group 20 B (Hmg20b), mRNA [NM_001108731]	**0,605**
Pcdhac2	Rattus norvegicus protocadherin alpha subfamily C, 2 (Pcdhac2), mRNA [NM_201422]	**0,605**
0	Uncharacterized protein [Source:UniProtKB/TrEMBL;Acc:D3ZXP2] [ENSRNOT00000041819]	**0,605**
Polr2f	DNA-directed RNA polymerases I, II, and III subunit RPABC2 [Source:UniProtKB/Swiss-Prot;Acc:O88828] [ENSRNOT00000015009]	**0,605**
Rnf216	Rattus norvegicus ring finger protein 216 (Rnf216), mRNA [NM_001107122]	**0,605**
LOC690350	PREDICTED: Rattus norvegicus similar to LSM7 homolog, U6 small nuclear RNA associated (LOC690350), mRNA [XM_001074288]	**0,605**
Ckmt1	Rattus norvegicus creatine kinase, mitochondrial 1 (Ckmt1), nuclear gene encoding mitochondrial protein, mRNA [NM_001012738]	**0,605**
0	Unknown	**0,606**
LOC689410	PREDICTED: Rattus norvegicus hypothetical protein LOC689410 (LOC689410), mRNA [XM_001070689]	**0,606**

Tbc1d22b	Rattus norvegicus TBC1 domain family, member 22B (Tbc1d22b), mRNA [NM_001025059]	**0,606**
Nop10	Rattus norvegicus NOP10 ribonucleoprotein homolog (yeast) (Nop10), mRNA [NM_001126100]	**0,606**
Parp6	Rattus norvegicus poly (ADP-ribose) polymerase family, member 6 (Parp6), mRNA [NM_001106828]	**0,606**
Sos1	Rattus norvegicus Son of sevenless homolog 1 (Drosophila) (Sos1), mRNA [NM_001100716]	**0,606**
Dbp	Rattus norvegicus D site of albumin promoter (albumin D-box) binding protein (Dbp), mRNA [NM_012543]	**0,606**
Masp2	Rattus Norvegicus mRNA for MAp19 protein, partial clone prl-5. [Y18568]	**0,606**
0	Unknown	**0,606**
RGD1306502	Rattus norvegicus similar to hypothetical protein FLJ11193 (RGD1306502), mRNA [NM_001107654]	**0,606**
0	Unknown	**0,606**
Lsm1	Rattus norvegicus LSM1 homolog, U6 small nuclear RNA associated (S. cerevisiae) (Lsm1), mRNA [NM_001108876]	**0,606**
Btd	Rattus norvegicus biotinidase (Btd), mRNA [NM_001012047]	**0,606**
Cd164	Rattus norvegicus CD164 molecule, sialomucin (Cd164), mRNA [NM_031812]	**0,606**
Itgbl1	Rattus norvegicus integrin, beta-like 1 (Itgbl1), mRNA [NM_001017505]	**0,606**
Lsm2	Rattus norvegicus LSM2 homolog, U6 small nuclear RNA associated (S. cerevisiae) (Lsm2), transcript variant 2, mRNA [NM_001165922]	**0,606**
Fat3	Rattus norvegicus FAT tumor suppressor homolog 3 (Drosophila) (Fat3), mRNA [NM_138544]	**0,606**
0	PREDICTED: Rattus norvegicus similar to 60S ribosomal protein L29 (P23) (RGD1563134), mRNA [XM_001080572]	**0,606**
Bcl2	Rattus norvegicus B-cell CLL/lymphoma 2 (Bcl2), nuclear gene encoding mitochondrial protein, mRNA [NM_016993]	**0,606**
Mt2A	Rattus norvegicus metallothionein 2A (Mt2A), mRNA [NM_001137564]	**0,606**
Ceacam19	Rattus norvegicus carcinoembryonic antigen-related cell adhesion molecule 19 (Ceacam19), mRNA [NM_001198970]	**0,606**
Gpr26	Rattus norvegicus G protein-coupled receptor 26 (Gpr26), mRNA [NM_138841]	**0,606**
Fam120b	Rattus norvegicus family with sequence similarity 120B (Fam120b), mRNA [NM_001107466]	**0,606**
Mcart1l	Rattus norvegicus mitochondrial carrier triple repeat 1-like (Mcart1l), nuclear gene encoding mitochondrial protein, mRNA [NM_001127602]	**0,606**
Ttll1	Rattus norvegicus tubulin tyrosine ligase-like family, member 1 (Ttll1), mRNA [NM_001012200]	**0,606**
Zc3h10	Rattus norvegicus zinc finger CCCH type containing 10 (Zc3h10), mRNA [NM_001191090]	**0,606**
Slc18a2	Rattus norvegicus solute carrier family 18 (vesicular monoamine), member 2 (Slc18a2), mRNA [NM_013031]	**0,606**
Hmg1l1	Rattus norvegicus high-mobility group (nonhistone chromosomal) protein 1-like 1 (Hmg1l1), mRNA [NM_001109373]	**0,606**
LOC497848	PREDICTED: Rattus norvegicus hypothetical LOC497848 (LOC497848), miscRNA [XR_006411]	**0,606**
Ctrl	Rattus norvegicus chymotrypsin-like (Ctrl), mRNA [NM_054009]	**0,606**
Krtap16-5	Rattus norvegicus keratin associated protein 16-5 (Krtap16-5), mRNA [NM_001109424]	**0,606**
Dhx33	Rattus norvegicus DEAH (Asp-Glu-Ala-His) box polypeptide 33 (Dhx33), mRNA [NM_001105802]	**0,606**
Flrt2	Rattus norvegicus fibronectin leucine rich transmembrane protein 2 (Flrt2), mRNA [NM_001106750]	**0,606**
Ywhah	Rattus norvegicus tyrosine 3-monooxygenase/tryptophan 5-monooxygenase activation protein, eta polypeptide (Ywhah), mRNA [NM_013052]	**0,606**
Ptov1	Rattus norvegicus prostate tumor overexpressed 1 (Ptov1), mRNA [NM_001008304]	**0,606**
LOC500893	Rattus norvegicus similar to GLI-Kruppel family member GLI4 (LOC500893), mRNA [NM_001029926]	**0,606**

Ifna1	Rattus norvegicus interferon-alpha 1 (Ifna1), mRNA [NM_001014786]	**0,606**
Neurl2	Rattus norvegicus neuralized homolog 2 (Drosophila) (Neurl2), mRNA [NM_001107802]	**0,606**
Abr	Rattus norvegicus active BCR-related gene (Abr), mRNA [NM_001105814]	**0,606**
RGD1310727	Rattus norvegicus LOC363070 (RGD1310727), mRNA [NM_001108764]	**0,606**
0	Q2U0J3_ASPOR (Q2U0J3) Synaptic vesicle transporter SVOP and related transporters, partial (3%) [TC622715]	**0,606**
RGD1565149	Rattus norvegicus similar to chromosome 16 open reading frame 7 (RGD1565149), mRNA [NM_001107440]	**0,606**
Agpat5	Rattus norvegicus 1-acylglycerol-3-phosphate O-acyltransferase 5 (lysophosphatidic acid acyltransferase, epsilon) (Agpat5), mRNA [NM_001134744]	**0,606**
Ak3l1	Rattus norvegicus adenylate kinase 3-like 1, mRNA (cDNA clone MGC:93541 IMAGE:7107348), complete cds. [BC087024]	**0,606**
Bgn	Rattus norvegicus biglycan (Bgn), mRNA [NM_017087]	**0,606**
Extl1	Rattus norvegicus exostoses (multiple)-like 1 (Extl1), mRNA [NM_001107985]	**0,606**
RGD1308127	Rattus norvegicus similar to 2700078E11Rik protein (RGD1308127), mRNA [NM_001014248]	**0,606**
Atp11b	Atp11b protein [Source:UniProtKB/TrEMBL;Acc:Q5RJS7] [ENSRNOT00000016961]	**0,606**
Sfrs3	Rattus norvegicus splicing factor, arginine/serine-rich 3 (Sfrs3), mRNA [NM_001047907]	**0,607**
0	BC052456 Fcho2 {Mus musculus} (exp=-1; wgp=0; cg=0), partial (11%) [TC610872]	**0,607**
Slmo2	Rattus norvegicus slowmo homolog 2 (Drosophila) (Slmo2), mRNA [NM_001009543]	**0,607**
0	Uncharacterized protein [Source:UniProtKB/TrEMBL;Acc:D3ZG39] [ENSRNOT00000058806]	**0,607**
Fgf13	Rattus norvegicus fibroblast growth factor 13 (Fgf13), mRNA [NM_053428]	**0,607**
Cast	Rattus norvegicus calpastatin (Cast), transcript variant 1, mRNA [NM_053295]	**0,607**
Gclc	Rattus norvegicus glutamate-cysteine ligase, catalytic subunit (Gclc), mRNA [NM_012815]	**0,607**
0	Uncharacterized protein [Source:UniProtKB/TrEMBL;Acc:D3ZGN8] [ENSRNOT00000049028]	**0,607**
RGD1310686	Rattus norvegicus similar to chromosome 16 open reading frame 5 (RGD1310686), mRNA [NM_001008360]	**0,607**
Snx18	Rattus norvegicus sorting nexin 18 (Snx18), mRNA [NM_001107652]	**0,607**
Igfbp6	Rattus norvegicus insulin-like growth factor binding protein 6 (Igfbp6), mRNA [NM_013104]	**0,607**
Chrnb1	Rattus norvegicus cholinergic receptor, nicotinic, beta 1 (muscle) (Chrnb1), mRNA [NM_012528]	**0,607**
Pcbd1	Rattus norvegicus pterin-4 alpha-carbinolamine dehydratase/dimerization cofactor of hepatocyte nuclear factor 1 alpha (Pcbd1), mRNA [NM_001007601]	**0,607**
RGD1565183	Uncharacterized protein [Source:UniProtKB/TrEMBL;Acc:D3ZG08] [ENSRNOT00000049254]	**0,607**
Prss53	Rattus norvegicus protease, serine, 53 (Prss53), mRNA [NM_001109156]	**0,607**
Zdhhc18	Rattus norvegicus zinc finger, DHHC-type containing 18 (Zdhhc18), mRNA [NM_001039339]	**0,607**
Stt3b	Rattus norvegicus STT3, subunit of the oligosaccharyltransferase complex, homolog B (S. cerevisiae) (Stt3b), mRNA [NM_001170539]	**0,607**
Txndc12	Rattus norvegicus thioredoxin domain containing 12 (endoplasmic reticulum) (Txndc12), mRNA [NM_001100840]	**0,607**
0	Dedicator of cyto-kinesis 3 (Predicted)Uncharacterized protein [Source:UniProtKB/TrEMBL;Acc:D4A998] [ENSRNOT00000019867]	**0,607**
0	Unknown	**0,607**

Pyy	Rattus norvegicus peptide YY (mapped) (Pyy), mRNA [NM_001034080]	**0,607**
Top2a	DNA topoisomerase 2-alpha [Source:UniProtKB/Swiss-Prot;Acc:P41516] [ENSRNOT00000050725]	**0,607**
Actr3	Rattus norvegicus ARP3 actin-related protein 3 homolog (yeast) (Actr3), mRNA [NM_031068]	**0,607**
Isca1	Rattus norvegicus iron-sulfur cluster assembly 1 homolog (S. cerevisiae) (Isca1), mRNA [NM_181626]	**0,607**
Rell2	Rattus norvegicus RELT-like 2 (Rell2), mRNA [NM_001014149]	**0,607**
Zfp94	Rattus norvegicus zinc finger protein 94 (Zfp94), mRNA [NM_001037212]	**0,607**
0	Q4RDS8_TETNG (Q4RDS8) Chromosome undetermined SCAF15796, whole genome shotgun sequence. (Fragment), partial (6%) [TC625290]	**0,607**
Slc2a13	Rattus norvegicus solute carrier family 2 (facilitated glucose transporter), member 13 (Slc2a13), mRNA [NM_133611]	**0,607**
Fastk	Rattus norvegicus Fas-activated serine/threonine kinase (Fastk), mRNA [NM_001011967]	**0,607**
Top3b	Rattus norvegicus topoisomerase (DNA) III beta (Top3b), mRNA [NM_001105861]	**0,607**
Cd82	Rattus norvegicus Cd82 molecule (Cd82), mRNA [NM_031797]	**0,607**
Atp2b1	Rattus norvegicus ATPase, Ca++ transporting, plasma membrane 1 (Atp2b1), mRNA [NM_053311]	**0,607**
Epha4	Rattus norvegicus Eph receptor A4 (Epha4), mRNA [NM_001162411]	**0,607**
LOC691807	Uncharacterized protein C4orf3 homolog [Source:UniProtKB/Swiss-Prot;Acc:Q498U0] [ENSRNOT00000019647]	**0,607**
Abcc1	Rattus norvegicus ATP-binding cassette, subfamily C (CFTR/MRP), member 1 (Abcc1), mRNA [NM_022281]	**0,607**
Gucy1a3	Guanylate cyclase soluble subunit alpha-3 [Source:UniProtKB/Swiss-Prot;Acc:P19686] [ENSRNOT00000017190]	**0,607**
Sstr4	Rattus norvegicus somatostatin receptor 4 (Sstr4), mRNA [NM_013036]	**0,607**
RGD1563888	Rattus norvegicus similar to DNA segment, Chr 16, ERATO Doi 472, expressed (RGD1563888), mRNA [NM_001108312]	**0,607**
Cbs	Rattus norvegicus cystathionine beta synthase (Cbs), mRNA [NM_012522]	**0,607**
0	Rattus norvegicus similar to Heterogeneous nuclear ribonucleoprotein A1 (Helix-destabilizing protein) (Single-strand binding protein) (hnRNP core protein A1) (HDP) (LOC312921), mRNA [XM_232629]	**0,607**
LOC690096	Uncharacterized protein [Source:UniProtKB/TrEMBL;Acc:D3ZEI0] [ENSRNOT00000012255]	**0,607**
Wdtc1	Rattus norvegicus WD and tetratricopeptide repeats 1 (Wdtc1), mRNA [NM_001107908]	**0,607**
Insig1	Rattus norvegicus insulin induced gene 1 (Insig1), mRNA [NM_022392]	**0,607**
RGD1308195	Rattus norvegicus similar to secreted Ly6/uPAR related protein 2 (RGD1308195), mRNA [NM_001130551]	**0,607**
Larp1	PREDICTED: Rattus norvegicus La ribonucleoprotein domain family, member 1 (Larp1), partial mRNA [XM_001075150]	**0,607**
Rerg	RERG_HUMAN (Q96A58) Ras-related and estrogen-regulated growth inhibitor, partial (19%) [TC629838]	**0,608**
Nat13	Rattus norvegicus N-acetyltransferase 13 (Nat13), mRNA [NM_001105881]	**0,608**
Spg20	Rattus norvegicus spastic paraplegia 20 (Troyer syndrome) homolog (human) (Spg20), mRNA [NM_001106433]	**0,608**
0	Uncharacterized protein [Source:UniProtKB/TrEMBL;Acc:D3ZW02] [ENSRNOT00000036647]	**0,608**
Camk1g	Rattus norvegicus calcium/calmodulin-dependent protein kinase IG (Camk1g), mRNA [NM_182842]	**0,608**
Nxt2	Rattus norvegicus nuclear transport factor 2-like export factor 2 (Nxt2), mRNA [NM_001108120]	**0,608**
0	RVL7343 Wackym-Soares normalized rat vestibular cDNA library Rattus norvegicus cDNA 5', mRNA sequence [DV716912]	**0,608**
Slc4a8	Rattus norvegicus solute carrier family 4, sodium bicarbonate cotransporter, member 8 (Slc4a8), mRNA [NM_199497]	**0,608**

Cyp4x1	Rattus norvegicus cytochrome P450, family 4, subfamily x, polypeptide 1 (Cyp4x1), mRNA [NM_145675]	**0,608**
Angel2	Rattus norvegicus angel homolog 2 (Drosophila) (Angel2), mRNA [NM_001135119]	**0,608**
Cdca3	Rattus norvegicus cell division cycle associated 3 (Cdca3), mRNA [NM_001007648]	**0,608**
0	Uncharacterized protein [Source:UniProtKB/TrEMBL;Acc:D4A7M0] [ENSRNOT00000011178]	**0,608**
Lsm3	Rattus norvegicus LSM3 homolog, U6 small nuclear RNA associated (S. cerevisiae) (Lsm3), mRNA [NM_001106611]	**0,608**
Fth1	Rattus norvegicus ferritin, heavy polypeptide 1 (Fth1), mRNA [NM_012848]	**0,608**
0	Unknown	**0,608**
Ick	Rattus norvegicus intestinal cell kinase (Ick), mRNA [NM_138886]	**0,608**
Impdh1	Rattus norvegicus IMP (inosine monophosphate) dehydrogenase 1 (Impdh1), mRNA [NM_001108619]	**0,608**
0	Unknown	**0,608**
Dcx	Rattus norvegicus neuronal migration protein doublecortin mRNA, complete cds. [AF155959]	**0,608**
Bcan	Rattus norvegicus brevican (Bcan), transcript variant 2, mRNA [NM_012916]	**0,608**
Ppfibp1	Rattus norvegicus PTPRF interacting protein, binding protein 1 (liprin beta 1) (Ppfibp1), mRNA [NM_001107896]	**0,608**
Slc22a17	Rattus norvegicus solute carrier family 22, member 17 (Slc22a17), mRNA [NM_177421]	**0,608**
Maf	Rattus norvegicus v-maf musculoaponeurotic fibrosarcoma oncogene homolog (avian) (Maf), mRNA [NM_019318]	**0,608**
Dbp	Rattus norvegicus D site of albumin promoter (albumin D-box) binding protein (Dbp), mRNA [NM_012543]	**0,608**
0	Rattus norvegicus similar to 60S RIBOSOMAL PROTEIN L29 (P23) (LOC293697), mRNA [XM_219533]	**0,608**
0	Unknown	**0,608**
0	Solute carrier family 39 (Zinc transporter), member 10 (Predicted), isoform CRA_aUncharacterized protein [Source:UniProtKB/TrEMBL;Acc:D4A517] [ENSRNOT00000016262]	**0,608**
Nat15	Rattus norvegicus N-acetyltransferase 15 (GCN5-related, putative) (Nat15), mRNA [NM_001014226]	**0,608**
Gpr83	Rattus norvegicus G protein-coupled receptor 83 (Gpr83), mRNA [NM_080411]	**0,608**
Zfp422	Rattus norvegicus zinc finger protein 422 (Zfp422), mRNA [NM_001012745]	**0,608**
Ap3m1	Rattus norvegicus adaptor-related protein complex 3, mu 1 subunit (Ap3m1), mRNA [NM_133593]	**0,608**
Reep6	Rattus norvegicus receptor accessory protein 6 (Reep6), mRNA [NM_001013218]	**0,608**
Atf6	Rattus norvegicus activating transcription factor 6 (Atf6), mRNA [NM_001107196]	**0,608**
Ciz1	Rattus norvegicus CDKN1A interacting zinc finger protein 1 (Ciz1), mRNA [NM_001106568]	**0,608**
Gcsh	Rattus norvegicus glycine cleavage system protein H (aminomethyl carrier) (Gcsh), nuclear gene encoding mitochondrial protein, mRNA [NM_133598]	**0,608**
Pomt2	Rattus norvegicus protein-O-mannosyltransferase 2 (Pomt2), mRNA [NM_001047114]	**0,608**
0	Unknown	**0,608**
Rfxank	Rattus norvegicus regulatory factor X-associated ankyrin-containing protein (Rfxank), mRNA [NM_001013136]	**0,608**
0	Unknown	**0,608**
Hdgf	Rattus norvegicus hepatoma-derived growth factor (Hdgf), mRNA [NM_053707]	**0,608**

Msl2	PREDICTED: Rattus norvegicus male-specific lethal 2-like 1 (Drosophila) (Msl2l1), mRNA [XM_236567]	**0,608**
Sertad3	Rattus norvegicus SERTA domain containing 3 (Sertad3), mRNA [NM_001017513]	**0,608**
0	Uncharacterized protein [Source:UniProtKB/TrEMBL;Acc:D4A273] [ENSRNOT00000007422]	**0,608**
Samd8	Rattus norvegicus sterile alpha motif domain containing 8 (Samd8), mRNA [NM_001012040]	**0,608**
Pabpc4	Rattus norvegicus poly(A) binding protein, cytoplasmic 4 (Pabpc4), mRNA [NM_001100538]	**0,608**
Zic4	Rattus norvegicus Zic family member 4 (Zic4), mRNA [NM_001108176]	**0,608**
Faf2	Rattus norvegicus Fas associated factor family member 2 (Faf2), mRNA [NM_001017445]	**0,608**
0	AGENCOURT_118846637 NIH_MGC_248 Rattus norvegicus cDNA clone IMAGE:9112865 5', mRNA sequence [EX493553]	**0,608**
Rasd2	Rattus norvegicus RASD family, member 2 (Rasd2), mRNA [NM_133568]	**0,608**
Papss2	Rattus norvegicus 3'-phosphoadenosine 5'-phosphosulfate synthase 2 (Papss2), mRNA [NM_001106375]	**0,608**
0	Unknown	**0,608**
Dynll1	Rattus norvegicus dynein light chain LC8-type 1 (Dynll1), mRNA [NM_053319]	**0,608**
Nipa1	Rattus norvegicus non imprinted in Prader-Willi/Angelman syndrome 1 homolog (human) (Nipa1), mRNA [NM_001107519]	**0,608**
Trpc1	Rattus norvegicus transient receptor potential cation channel, subfamily C, member 1 (Trpc1), mRNA [NM_053558]	**0,608**
Cacna1g	Rattus norvegicus calcium channel, voltage-dependent, T type, alpha 1G subunit (Cacna1g), mRNA [NM_031601]	**0,609**
Slc1a1	Rattus norvegicus solute carrier family 1 (neuronal/epithelial high affinity glutamate transporter, system Xag), member 1 (Slc1a1), mRNA [NM_013032]	**0,609**
Rgma	Rattus norvegicus RGM domain family, member A (Rgma), mRNA [NM_001107524]	**0,609**
Otud7b	Rattus norvegicus OTU domain containing 7B (Otud7b), mRNA [NM_001107697]	**0,609**
Tnrc6a	Rattus norvegicus trinucleotide repeat containing 6a (Tnrc6a), mRNA [NM_001107549]	**0,609**
Synrg	Rattus norvegicus synergin, gamma (Synrg), mRNA [NM_053419]	**0,609**
Dcaf10	Rattus norvegicus DDB1 and CUL4 associated factor 10 (Dcaf10), mRNA [NM_001107935]	**0,609**
Zfp278	Rattus norvegicus zinc finger protein 278, mRNA (cDNA clone IMAGE:7376163), partial cds. [BC098801]	**0,609**
Cyp4a8	Rattus norvegicus cytochrome P450, family 4, subfamily a, polypeptide 8 (Cyp4a8), mRNA [NM_031605]	**0,609**
0	Unknown	**0,609**
LOC685240	PREDICTED: Rattus norvegicus similar to High mobility group protein 1 (HMG-1) (High mobility group protein B1) (Amphoterin) (Heparin-binding protein p30) (LOC685240), miscRNA [XR_085878]	**0,609**
RGD1308134	Rattus norvegicus similar to RIKEN cDNA 1110020A23 (RGD1308134), mRNA [NM_001127521]	**0,609**
Kirrel3	Rattus norvegicus kin of IRRE like 3 (Drosophila) (Kirrel3), mRNA [NM_001048215]	**0,609**
0	PREDICTED: Rattus norvegicus similar to High mobility group protein 1 (HMG-1) (High mobility group protein B1) (Amphoterin) (Heparin-binding protein p30) (LOC685091), mRNA [XM_001062256]	**0,609**
Ubn2	Rattus norvegicus ubinuclein 2 (Ubn2), mRNA [NM_001134553]	**0,609**
Ddah1	Rattus norvegicus dimethylarginine dimethylaminohydrolase 1 (Ddah1), mRNA [NM_022297]	**0,609**
Zfp563	Rattus norvegicus zinc finger protein 563 (Zfp563), mRNA [NM_001134561]	**0,609**

Hnrpd	Rattus norvegicus heterogeneous nuclear ribonucleoprotein D (Hnrpd), transcript variant 1, mRNA [NM_024404]	**0,609**
0	PREDICTED: Rattus norvegicus dynein, axonemal, heavy polypeptide 9, transcript variant 2 (Dnah9), mRNA [XM_002724507]	**0,609**
Rfk	Rattus norvegicus riboflavin kinase (Rfk), mRNA [NM_001014106]	**0,609**
Il6r	Rattus norvegicus interleukin 6 receptor (Il6r), mRNA [NM_017020]	**0,609**
Khdrbs2	Rattus norvegicus KH domain containing, RNA binding, signal transduction associated 2 (Khdrbs2), mRNA [NM_133318]	**0,609**
0	Rattus norvegicus cDNA clone IMAGE:7377987. [BC158826]	**0,609**
RGD1308923	Rattus norvegicus LOC362678 (RGD1308923), mRNA [NM_001108698]	**0,609**
Lphn3	Rattus norvegicus latrophilin 3 (Lphn3), mRNA [NM_130822]	**0,609**
Eno2	Gamma-enolase [Source:UniProtKB/Swiss-Prot;Acc:P07323] [ENSRNOT00000005601]	**0,609**
Arpc2	Rattus norvegicus actin related protein 2/3 complex, subunit 2 (Arpc2), mRNA [NM_001106919]	**0,609**
Nusap1	Rattus norvegicus nucleolar and spindle associated protein 1 (Nusap1), mRNA [NM_001107762]	**0,609**
Fusip1	Rattus norvegicus FUS interacting protein (serine-arginine rich) 1 (Fusip1), mRNA [NM_001025738]	**0,609**
Timm17b	Rattus norvegicus translocase of inner mitochondrial membrane 17 homolog B (yeast) (Timm17b), nuclear gene encoding mitochondrial protein, mRNA [NM_001108249]	**0,609**
0	Unknown	**0,609**
0	Ral GTPase-activating protein subunit alpha-2 [Source:UniProtKB/Swiss-Prot;Acc:P86411] [ENSRNOT00000015414]	**0,609**
0	Oxysterol-binding protein [Source:UniProtKB/TrEMBL;Acc:D3ZHZ3] [ENSRNOT00000042964]	**0,609**
Dnajb13	Rattus norvegicus DnaJ (Hsp40) related, subfamily B, member 13 (Dnajb13), mRNA [NM_001005885]	**0,609**
RGD1564338	PREDICTED: Rattus norvegicus similar to aminoacylase 1 (RGD1564338), mRNA [XM_346293]	**0,609**
Taf15	Rattus norvegicus TAF15 RNA polymerase II, TATA box binding protein (TBP)-associated factor (Taf15), mRNA [NM_001105824]	**0,609**
Vwa1	Rattus norvegicus von Willebrand factor A domain containing 1 (Vwa1), mRNA [NM_001013938]	**0,609**
Fam73b	Rattus norvegicus family with sequence similarity 73, member B (Fam73b), mRNA [NM_001106566]	**0,609**
Col9a2	Rattus norvegicus collagen, type IX, alpha 2 (Col9a2), mRNA [NM_001108675]	**0,609**
RGD1304693	Rattus norvegicus similar to CG14803-PA (RGD1304693), mRNA [NM_001135780]	**0,609**
Serinc5	Rattus norvegicus serine incorporator 5 (Serinc5), mRNA [NM_133395]	**0,609**
Cyp51	Rattus norvegicus cytochrome P450, family 51 (Cyp51), mRNA [NM_012941]	**0,609**
0	Uncharacterized protein [Source:UniProtKB/TrEMBL;Acc:D3ZIS8] [ENSRNOT00000060124]	**0,609**
RGD1307890	Rattus norvegicus similar to C1orf25 (RGD1307890), mRNA [NM_001037192]	**0,609**
Clock	Rattus norvegicus clock homolog (mouse) (Clock), mRNA [NM_021856]	**0,609**
Amacr	Rattus norvegicus alpha-methylacyl-CoA racemase (Amacr), nuclear gene encoding mitochondrial protein, mRNA [NM_012816]	**0,609**
Actb	Rattus norvegicus actin, beta (Actb), mRNA [NM_031144]	**0,609**
Cnot2	Rattus norvegicus CCR4-NOT transcription complex, subunit 2 (Cnot2), mRNA [NM_001011988]	**0,610**
Maf1	Rattus norvegicus MAF1 homolog (S. cerevisiae) (Maf1), mRNA [NM_001014085]	**0,610**
Hrk	Rattus norvegicus harakiri, BCL2 interacting protein (contains only BH3 domain) (Hrk), mRNA [NM_057130]	**0,610**

LOC685079	PREDICTED: Rattus norvegicus similar to Protein SYS1 homolog (LOC685079), mRNA [XM_001062200]	**0,610**
Acat3	Rattus norvegicus acetyl-Coenzyme A acetyltransferase 3 (Acat3), mRNA [NM_001006995]	**0,610**
Trappc10	Rattus norvegicus trafficking protein particle complex 10 (Trappc10), mRNA [NM_001173528]	**0,610**
Pip5k1a	Rattus norvegicus phosphatidylinositol-4-phosphate 5-kinase, type 1, alpha (Pip5k1a), mRNA [NM_001042621]	**0,610**
0	Rattus norvegicus TL0AAA49YL04 mRNA sequence. [FQ213104]	**0,610**
Tead3	Rattus norvegicus TEA domain family member 3 (Tead3), mRNA [NM_001098216]	**0,610**
Papd5	Rattus norvegicus PAP associated domain containing 5 (Papd5), mRNA [NM_001107416]	**0,610**
Trpc4ap	Rattus norvegicus transient receptor potential cation channel, subfamily C, member 4 associated protein (Trpc4ap), mRNA [NM_001100748]	**0,610**
0	Rattus norvegicus activity and neurotransmitter-induced early gene 2 (ania-2) mRNA, 3'UTR. [AF030087]	**0,610**
Nek1	Rattus norvegicus NIMA (never in mitosis gene a)-related kinase 1 (Nek1), mRNA [NM_001106082]	**0,610**
Nrxn2	Rattus norvegicus neurexin 2 (Nrxn2), mRNA [NM_053846]	**0,610**
Fbn2	Rattus norvegicus fibrillin 2 (Fbn2), mRNA [NM_031826]	**0,610**
Ube2o	PREDICTED: Rattus norvegicus ubiquitin-conjugating enzyme E2O (Ube2o), mRNA [XM_001081723]	**0,610**
RGD1309313	Rattus norvegicus similar to RIKEN cDNA 4930538D17 (RGD1309313), mRNA [NM_001014030]	**0,610**
LOC690349	Rattus norvegicus hypothetical protein LOC690349 (LOC690349), mRNA [NM_001109581]	**0,610**
Flnb	Rattus norvegicus filamin, beta (Flnb), mRNA [NM_001107288]	**0,610**
Olfml1	Rattus norvegicus olfactomedin-like 1 (Olfml1), mRNA [NM_001013192]	**0,610**
0	Uncharacterized protein [Source:UniProtKB/TrEMBL;Acc:D3Z9L4] [ENSRNOT00000057467]	**0,610**
Mfsd9	Rattus norvegicus major facilitator superfamily domain containing 9 (Mfsd9), mRNA [NM_001108215]	**0,610**
Rab27a	Rattus norvegicus RAB27A, member RAS oncogene family (Rab27a), mRNA [NM_017317]	**0,610**
Cdc42bpb	Rattus norvegicus CDC42 binding protein kinase beta (DMPK-like) (Cdc42bpb), mRNA [NM_053620]	**0,610**
Sesn3	Rattus norvegicus sestrin 3 (Sesn3), mRNA [NM_001108125]	**0,610**
B3gnt2	Rattus norvegicus UDP-GlcNAc:betaGal beta-1,3-N-acetylglucosaminyltransferase 2 (B3gnt2), mRNA [NM_001107240]	**0,610**
Znf668	Rattus norvegicus zinc finger protein 668 (Znf668), mRNA [NM_001107553]	**0,610**
Pbx3	Rattus norvegicus pre-B-cell leukemia homeobox 3 (Pbx3), mRNA [NM_001107834]	**0,610**
0	Unknown	**0,610**
Sphkap	Rattus norvegicus SPHK1 interactor, AKAP domain containing (Sphkap), mRNA [NM_001127492]	**0,610**
Tspyl2	Rattus norvegicus TSPY-like 2 (Tspyl2), mRNA [NM_001191618]	**0,610**
LOC683788	Rattus norvegicus similar to Fascin (Singed-like protein) (LOC683788), mRNA [NM_001100806]	**0,610**
Sord	Rattus norvegicus sorbitol dehydrogenase (Sord), mRNA [NM_017052]	**0,610**
Acp2	Rattus norvegicus acid phosphatase 2, lysosomal (Acp2), mRNA [NM_016988]	**0,610**
Tmem178	Rattus norvegicus transmembrane protein 178 (Tmem178), mRNA [NM_001004282]	**0,610**
Pja2	Rattus norvegicus praja 2, RING-H2 motif containing (Pja2), mRNA [NM_138896]	**0,610**
Rnf32	Rattus norvegicus ring finger protein 32 (Rnf32), mRNA [NM_001012095]	**0,610**

Fbrs	PREDICTED: Rattus norvegicus fibrosin (Fbrs), mRNA [XM_002725689]	**0,610**
Scarf1	Rattus norvegicus scavenger receptor class F, member 1 (Scarf1), mRNA [NM_001107022]	**0,610**
Stk4	Rattus norvegicus serine/threonine kinase 4 (Stk4), mRNA [NM_001107800]	**0,610**
LOC691221	PREDICTED: Rattus norvegicus similar to CG1998-PA (LOC691221), mRNA [XM_001077260]	**0,610**
Elac1	Rattus norvegicus elaC homolog 1 (E. coli) (Elac1), mRNA [NM_001107406]	**0,610**
0	Unknown	**0,610**
0	Uncharacterized protein [Source:UniProtKB/TrEMBL;Acc:D3ZIK8] [ENSRNOT00000025790]	**0,610**
Ube2v1	Rattus norvegicus ubiquitin-conjugating enzyme E2 variant 1 (Ube2v1), mRNA [NM_001110345]	**0,610**
Prr5	Rattus norvegicus proline rich 5 (renal) (Prr5), mRNA [NM_001012121]	**0,610**
Ulk2	Rattus norvegicus Unc-51 like kinase 2 (C. elegans) (Ulk2), mRNA [NM_001191645]	**0,610**
Pabpc4	Rattus norvegicus poly(A) binding protein, cytoplasmic 4 (Pabpc4), mRNA [NM_001100538]	**0,610**
RGD735029	Rattus norvegicus SEL1 domain containing protein RGD735029 (RGD735029), nuclear gene encoding mitochondrial protein, mRNA [NM_199493]	**0,610**
RGD1564456	PREDICTED: Rattus norvegicus similar to chromosome 10 open reading frame 18 (RGD1564456), mRNA [XM_001068906]	**0,610**
Dpysl2	Rattus norvegicus dihydropyrimidinase-like 2 (Dpysl2), mRNA [NM_001105717]	**0,610**
RGD1306520	Rattus norvegicus similar to receptor-interacting factor 1 (RGD1306520), mRNA [NM_001127485]	**0,611**
0	FQ131320 Rattus norvegicus 11-12 days foetus Sprague-Dawley Rattus norvegicus cDNA clone TL0ADA22YJ10 5', mRNA sequence [FQ131320]	**0,611**
Tspan9	Rattus norvegicus tetraspanin 9 (Tspan9), mRNA [NM_001107890]	**0,611**
Dusp16	Rattus norvegicus dual specificity phosphatase 16 (Dusp16), mRNA [NM_001106624]	**0,611**
Eno1	Rattus norvegicus enolase 1, (alpha) (Eno1), transcript variant 2, mRNA [NM_001109908]	**0,611**
Arfip2	Rattus norvegicus ADP-ribosylation factor interacting protein 2 (Arfip2), mRNA [NM_001004222]	**0,611**
Slc16a1	Rattus norvegicus solute carrier family 16, member 1 (monocarboxylic acid transporter 1) (Slc16a1), mRNA [NM_012716]	**0,611**
Rpl12	Uncharacterized protein [Source:UniProtKB/TrEMBL;Acc:D3ZJE2] [ENSRNOT00000003002]	**0,611**
LOC500028	Rattus norvegicus hypothetical protein LOC500028 (LOC500028), mRNA [NM_001047954]	**0,611**
Il10rb	Rattus norvegicus interleukin 10 receptor, beta (Il10rb), mRNA [NM_001107111]	**0,611**
FAM120C	Uncharacterized protein [Source:UniProtKB/TrEMBL;Acc:D3ZNI4] [ENSRNOT00000003565]	**0,611**
Sytl5	Rattus norvegicus synaptotagmin-like 5 (Sytl5), mRNA [NM_178333]	**0,611**
Ncam2	Rattus norvegicus fasciclin II GPI-linked protein isoform mRNA, complete cds. [AY495696]	**0,611**
Klhdc5	Rattus norvegicus kelch domain containing 5 (Klhdc5), mRNA [NM_001109257]	**0,611**
Osbpl2	Rattus norvegicus oxysterol binding protein-like 2 (Osbpl2), mRNA [NM_001013079]	**0,611**
Sdc2	Syndecan-2 [Source:UniProtKB/Swiss-Prot;Acc:P34900] [ENSRNOT00000007255]	**0,611**
Dnal1	Rattus norvegicus dynein, axonemal, light chain 1 (Dnal1), mRNA [NM_001109477]	**0,611**
Fam65c	Uncharacterized protein [Source:UniProtKB/TrEMBL;Acc:D3ZX40] [ENSRNOT00000035463]	**0,611**
Sfxn2	sideroflexin 2 [Source:RefSeq peptide;Acc:NP_001013090] [ENSRNOT00000027112]	**0,611**
0	Unknown	**0,611**

Crip2	Rattus norvegicus cysteine-rich protein 2 (Crip2), mRNA [NM_022501]	**0,611**
Nol10	Rattus norvegicus nucleolar protein 10 (Nol10), mRNA [NM_001014076]	**0,611**
Dnajb12	Rattus norvegicus DnaJ (Hsp40) homolog, subfamily B, member 12 (Dnajb12), mRNA [NM_001013907]	**0,611**
Dlg3	Rattus norvegicus discs, large homolog 3 (Drosophila) (Dlg3), mRNA [NM_031639]	**0,611**
0	Unknown	**0,611**
42248	Rattus norvegicus selenoprotein 15 (Sep15), mRNA [NM_133297]	**0,611**
Ralyl	Rattus norvegicus RALY RNA binding protein-like (Ralyl), mRNA [NM_001024978]	**0,611**
Ptcd2	Rattus norvegicus pentatricopeptide repeat domain 2 (Ptcd2), mRNA [NM_001107648]	**0,611**
Hgs	Rattus norvegicus hepatocyte growth factor-regulated tyrosine kinase substrate (Hgs), mRNA [NM_019387]	**0,611**
Tlk2	Rattus norvegicus tousled-like kinase 2 (Tlk2), mRNA [NM_001191652]	**0,611**
Snap23	Rattus norvegicus synaptosomal-associated protein 23 (Snap23), mRNA [NM_022689]	**0,611**
Nudt3	Rattus norvegicus nudix (nucleoside diphosphate linked moiety X)-type motif 3 (Nudt3), mRNA [NM_001024243]	**0,611**
Dyrk2	Rattus norvegicus dual-specificity tyrosine-(Y)-phosphorylation regulated kinase 2 (Dyrk2), mRNA [NM_001108100]	**0,611**
Alg6	Rattus norvegicus asparagine-linked glycosylation 6, alpha-1,3-glucosyltransferase homolog (S. cerevisiae) (Alg6), mRNA [NM_001033709]	**0,611**
0	Uncharacterized protein [Source:UniProtKB/TrEMBL;Acc:D4A7X0] [ENSRNOT00000034908]	**0,611**
Znf503	Rattus norvegicus zinc finger protein 503 (Znf503), mRNA [NM_001107250]	**0,612**
Fdxacb1	Rattus norvegicus ferredoxin-fold anticodon binding domain containing 1 (Fdxacb1), mRNA [NM_001108145]	**0,612**
Sgce	Rattus norvegicus sarcoglycan, epsilon (Sgce), mRNA [NM_001002023]	**0,612**
Znf536	Uncharacterized protein [Source:UniProtKB/TrEMBL;Acc:D3ZJS6] [ENSRNOT00000018956]	**0,612**
Trpc5	Rattus norvegicus transient receptor potential cation channel, subfamily C, member 5 (Trpc5), mRNA [NM_080898]	**0,612**
0	Unknown	**0,612**
0	Unknown	**0,612**
Fndc3a	Rattus norvegicus fibronectin type III domain containing 3a (Fndc3a), mRNA [NM_001107278]	**0,612**
Hdac11	Rattus norvegicus histone deacetylase 11 (Hdac11), mRNA [NM_001106610]	**0,612**
Plat	Rattus norvegicus plasminogen activator, tissue (Plat), mRNA [NM_013151]	**0,612**
Rtp1	Rattus norvegicus receptor (chemosensory) transporter protein 1 (Rtp1), mRNA [NM_001105870]	**0,612**
Ank3	Rattus norvegicus ankyrin 3, node of Ranvier (Ank3), transcript variant 2, mRNA [NM_001033984]	**0,612**
Trim9	Rattus norvegicus tripartite motif-containing 9 (Trim9), mRNA [NM_130420]	**0,612**
Mrps21l	PREDICTED: Rattus norvegicus mitochondrial ribosomal protein S21-like (Mrps21l), mRNA [XM_001057655]	**0,612**
Rpl29	Rattus norvegicus ribosomal protein L29 (Rpl29), mRNA [NM_017150]	**0,612**
Osgepl1	Rattus norvegicus O-sialoglycoprotein endopeptidase-like 1 (Osgepl1), mRNA [NM_001024787]	**0,612**
Slco3a1	Rattus norvegicus solute carrier organic anion transporter family, member 3a1 (Slco3a1), mRNA [NM_177481]	**0,612**
RGD1307155	Rattus norvegicus similar to CG18661-PA (RGD1307155), mRNA [NM_001037188]	**0,612**
Fam120a	Rattus norvegicus family with sequence similarity 120A (Fam120a), mRNA [NM_001191816]	**0,612**

0	Unknown	**0,612**
0	AW143110 EST293406 Normalized rat brain, Bento Soares Rattus sp. cDNA clone RGIBD54 5' end, mRNA sequence [AW143110]	**0,612**
Zdhhc3	Rattus norvegicus zinc finger, DHHC-type containing 3 (Zdhhc3), mRNA [NM_001039014]	**0,612**
Bmp1	Rattus norvegicus bone morphogenetic protein 1 (Bmp1), mRNA [NM_031323]	**0,612**
Gnas	Rattus norvegicus GNAS complex locus (Gnas), transcript variant 5, mRNA [NM_001159656]	**0,612**
RGD1308448	Rattus norvegicus similar to RIKEN cDNA B130016O10 gene (RGD1308448), mRNA [NM_001107671]	**0,612**
Erbb2	Rattus norvegicus v-erb-b2 erythroblastic leukemia viral oncogene homolog 2, neuro/glioblastoma derived oncogene homolog (avian) (Erbb2), mRNA [NM_017003]	**0,612**
Prcc	Rattus norvegicus papillary renal cell carcinoma (translocation-associated) (Prcc), mRNA [NM_001107700]	**0,612**
Ube2a	Rattus norvegicus ubiquitin-conjugating enzyme E2A (RAD6 homolog) (Ube2a), mRNA [NM_001013933]	**0,612**
Prr13	Rattus norvegicus proline rich 13 (Prr13), mRNA [NM_001008379]	**0,612**
0	Unknown	**0,612**
0	Uncharacterized protein [Source:UniProtKB/TrEMBL;Acc:D4A0A1] [ENSRNOT00000016712]	**0,612**
Sar1b	Rattus norvegicus SAR1 homolog B (S. cerevisiae) (Sar1b), mRNA [NM_001009622]	**0,612**
Map4k4	Rattus norvegicus mitogen-activated protein kinase kinase kinase kinase 4 (Map4k4), mRNA [NM_001106904]	**0,612**
Mlx	Rattus norvegicus MAX-like protein X (Mlx), mRNA [NM_001034112]	**0,612**
Yipf2	Rattus norvegicus Yip1 domain family, member 2 (Yipf2), mRNA [NM_001014208]	**0,612**
Nedd1	Rattus norvegicus neural precursor cell expressed, developmentally down-regulated 1 (Nedd1), mRNA [NM_001106779]	**0,612**
Sprr1al	PREDICTED: Rattus norvegicus small proline-rich protein 1A-like (Sprr1al), mRNA [XM_001056859]	**0,612**
Ttc33	Rattus norvegicus tetratricopeptide repeat domain 33 (Ttc33), mRNA [NM_001106414]	**0,612**
0	Q52289_PSEPU (Q52289) UxpA protein, partial (5%) [TC624946]	**0,612**
Irs2	Rattus norvegicus insulin receptor substrate 2 (Irs2), mRNA [NM_001168633]	**0,612**
Lace1	Rattus norvegicus lactation elevated 1 (Lace1), mRNA [NM_001037656]	**0,612**
0	Bax inhibitor 1 [Source:UniProtKB/Swiss-Prot;Acc:P55062] [ENSRNOT00000045927]	**0,612**
Snph	Rattus norvegicus syntaphilin (Snph), mRNA [NM_001106525]	**0,612**
0	RVL20318 Wackym-Soares normalized rat vestibular cDNA library Rattus norvegicus cDNA 5', mRNA sequence [DV727767]	**0,612**
Rhod	Rattus norvegicus ras homolog gene family, member D (Rhod), mRNA [NM_001106323]	**0,612**
0	Uncharacterized protein [Source:UniProtKB/TrEMBL;Acc:D3ZTW2] [ENSRNOT00000036540]	**0,612**
LOC499843	Rattus norvegicus LRRGT00091 (LOC499843), mRNA [NM_001047953]	**0,612**
0	Rattus norvegicus strain F344 hepatocyte malignant transforming factor mRNA, complete sequence. [GU969272]	**0,612**
Crbn	Rattus norvegicus cereblon (Crbn), mRNA [NM_001015003]	**0,612**
Tomm6	RCG43475Uncharacterized protein [Source:UniProtKB/TrEMBL;Acc:D3ZJS3] [ENSRNOT00000018959]	**0,612**
Tnk2	Rattus norvegicus tyrosine kinase, non-receptor, 2 (Tnk2), mRNA [NM_001008336]	**0,612**
Wars	Rattus norvegicus tryptophanyl-tRNA synthetase (Wars), mRNA [NM_001013170]	**0,612**

Kcnh1	Rattus norvegicus potassium voltage-gated channel, subfamily H (eag-related), member 1 (Kcnh1), mRNA [NM_031742]	**0,613**
A2bp1	Rattus norvegicus ataxin 2 binding protein 1 (A2bp1), mRNA [NM_001106974]	**0,613**
Rit2	Rattus norvegicus Ras-like without CAAX 2 (Rit2), mRNA [NM_001013060]	**0,613**
0	voltage-dependent anion channel 1 (Vdac1), nuclear gene encoding mitochondrial protein, mRNA [Source:RefSeq DNA;Acc:NM_031353] [ENSRNOT00000006775]	**0,613**
Rbck1	Rattus norvegicus RanBP-type and C3HC4-type zinc finger containing 1 (Rbck1), mRNA [NM_021764]	**0,613**
Ndufs4	Rattus norvegicus NADH dehydrogenase (ubiquinone) Fe-S protein 4 (Ndufs4), nuclear gene encoding mitochondrial protein, mRNA [NM_001025146]	**0,613**
Sec62	Rattus norvegicus SEC62 homolog (S. cerevisiae) (Sec62), mRNA [NM_001034129]	**0,613**
Bri3bp	Rattus norvegicus Bri3 binding protein (Bri3bp), mRNA [NM_001017487]	**0,613**
LOC686860	PREDICTED: Rattus norvegicus similar to RT1 class I, CE15 (LOC686860), partial mRNA [XM_001076061]	**0,613**
0	PREDICTED: Rattus norvegicus multiple EGF-like-domains 11 (Megf11), mRNA [XM_001078620]	**0,613**
Arap2	Rattus norvegicus ArfGAP with RhoGAP domain, ankyrin repeat and PH domain 2 (Arap2), mRNA [NM_001107216]	**0,613**
Ergic3	Rattus norvegicus ERGIC and golgi 3 (Ergic3), mRNA [NM_001106533]	**0,613**
Tmem103	Rattus norvegicus transmembrane protein 103 (Tmem103), mRNA [NM_001108782]	**0,613**
RGD1562582	Rattus norvegicus similar to KIAA0406-like protein (RGD1562582), mRNA [NM_001134619]	**0,613**
0	Unknown	**0,613**
0	Unknown	**0,613**
0	Uncharacterized protein [Source:UniProtKB/TrEMBL;Acc:D4A1I9] [ENSRNOT00000066634]	**0,613**
Pop1	Rattus norvegicus processing of precursor 1, ribonuclease P/MRP subunit (S. cerevisiae) (Pop1), mRNA [NM_001130550]	**0,613**
Scd1	Rattus norvegicus stearoyl-Coenzyme A desaturase 1 (Scd1), mRNA [NM_139192]	**0,613**
0	Unknown	**0,613**
Tpi1	Rattus norvegicus triosephosphate isomerase 1 (Tpi1), mRNA [NM_022922]	**0,613**
0	Unknown	**0,613**
Opalin	Rattus norvegicus oligodendrocytic myelin paranodal and inner loop protein (Opalin), mRNA [NM_001017386]	**0,613**
Npas2	Rattus norvegicus neuronal PAS domain protein 2 (Npas2), mRNA [NM_001108214]	**0,613**
Vps37d	Rattus norvegicus similar to vacuolar protein sorting 37D (LOC687208), mRNA [NM_001128193]	**0,613**
Gdpd2	Rattus norvegicus glycerophosphodiester phosphodiesterase domain containing 2 (Gdpd2), mRNA [NM_001106944]	**0,613**
Gria3	Rattus norvegicus glutamate receptor, ionotrophic, AMPA 3 (Gria3), transcript variant 2, mRNA [NM_001112742]	**0,613**
Mro	PREDICTED: Rattus norvegicus maestro (Mro), mRNA [XM_001053368]	**0,613**
RGD1310427	Rattus norvegicus similar to KIAA0090 protein (RGD1310427), mRNA [NM_001108690]	**0,613**
Nutf2	Rattus norvegicus nuclear transport factor 2 (Nutf2), mRNA [NM_001007629]	**0,613**
Vgf	Rattus norvegicus VGF nerve growth factor inducible (Vgf), mRNA [NM_030997]	**0,613**
Cdc73	Rattus norvegicus cell division cycle 73, Paf1/RNA polymerase II complex component, homolog (S. cerevisiae) (Cdc73), mRNA [NM_001024769]	**0,613**

Spdef	Rattus norvegicus SAM pointed domain containing ets transcription factor (Spdef), mRNA [NM_001109530]	**0,613**
Sult2b1	Rattus norvegicus sulfotransferase family, cytosolic, 2B, member 1 (Sult2b1), mRNA [NM_001039665]	**0,613**
LOC305633	Rattus norvegicus similar to Antxr2 protein, mRNA (cDNA clone IMAGE:7322598). [BC088294]	**0,613**
Prkcb	Rattus norvegicus protein kinase C, beta (Prkcb), transcript variant 1, mRNA [NM_012713]	**0,613**
Arsb	Rattus norvegicus arylsulfatase B (Arsb), mRNA [NM_033443]	**0,613**
Zdhhc6	Rattus norvegicus zinc finger, DHHC-type containing 6 (Zdhhc6), mRNA [NM_001037652]	**0,613**
Dchs1	Rattus norvegicus dachsous 1 (Drosophila) (Dchs1), mRNA [NM_001107544]	**0,613**
Wbp5	Rattus norvegicus WW domain binding protein 5 (Wbp5), mRNA [NM_001127502]	**0,613**
RGD1563235	Rattus norvegicus similar to 1700054N08Rik protein (RGD1563235), mRNA [NM_001107441]	**0,613**
LOC689927	Rattus norvegicus similar to keratin associated protein 10-10 (LOC689927), mRNA [NM_001109555]	**0,613**
Derl3	Rattus norvegicus Der1-like domain family, member 3 (Derl3), mRNA [NM_001109577]	**0,613**
Cabp7	Rattus norvegicus calcium binding protein 7 (Cabp7), mRNA [NM_001007730]	**0,613**
Capza1	Rattus norvegicus capping protein (actin filament) muscle Z-line, alpha 1 (Capza1), mRNA [NM_001109625]	**0,613**
Spata24	Rattus norvegicus spermatogenesis associated 24 (Spata24), mRNA [NM_001025636]	**0,613**
Gria2	Rattus norvegicus glutamate receptor, ionotropic, AMPA 2 (Gria2), transcript variant 2, mRNA [NM_001083811]	**0,613**
0	similar to RIKEN cDNA 1700001E04 (MGC116197), mRNA [Source:RefSeq DNA;Acc:NM_001025755] [ENSRNOT00000043269]	**0,613**
Nfat5	Rattus norvegicus nuclear factor of activated T-cells 5 (Nfat5), mRNA [NM_001107425]	**0,614**
Ubxn4	Rattus norvegicus UBX domain protein 4 (Ubxn4), mRNA [NM_001012025]	**0,614**
Nab2	Rattus norvegicus Ngfi-A binding protein 2 (Nab2), mRNA [NM_001134874]	**0,614**
Tspyl4	Rattus norvegicus TSPY-like 4 (Tspyl4), mRNA [NM_001012075]	**0,614**
Sall3	Rattus norvegicus sal-like 3 (Drosophila) (Sall3), mRNA [NM_001108892]	**0,614**
Gpr37l1	Rattus norvegicus G protein-coupled receptor 37-like 1 (Gpr37l1), mRNA [NM_145784]	**0,614**
Fam172a	Rattus norvegicus family with sequence similarity 172, member A (Fam172a), mRNA [NM_001106401]	**0,614**
Nalcn	Rattus norvegicus sodium leak channel, non-selective (Nalcn), mRNA [NM_153630]	**0,614**
Akap11	Rattus norvegicus A kinase (PRKA) anchor protein 11 (Akap11), mRNA [NM_012773]	**0,614**
Gprc5b	Rattus norvegicus G protein-coupled receptor, family C, group 5, member B (Gprc5b), mRNA [NM_001106304]	**0,614**
Hcn3	Rattus norvegicus hyperpolarization-activated cyclic nucleotide-gated potassium channel 3 (Hcn3), mRNA [NM_053685]	**0,614**
Gpm6b	Rattus norvegicus glycoprotein m6b (Gpm6b), mRNA [NM_138846]	**0,614**
Nfkb1	Nuclear factor NF-kappa-B p105 subunitNuclear factor NF-kappa-B p50 subunit [Source:UniProtKB/Swiss-Prot;Acc:Q63369] [ENSRNOT00000036838]	**0,614**
Snx13	Rattus norvegicus sorting nexin 13 (Snx13), mRNA [NM_001108708]	**0,614**
Cnnm1	Rattus norvegicus cyclin M1 (Cnnm1), mRNA [NM_001107593]	**0,614**
Ahcyl2	Rattus norvegicus adenosylhomocysteinase-like 2 (Ahcyl2), mRNA [NM_001173510]	**0,614**
Josd2	Rattus norvegicus Josephin domain containing 2 (Josd2), mRNA [NM_001106256]	**0,614**

Rnf26	Rattus norvegicus ring finger protein 26 (Rnf26), mRNA [NM_001113748]	**0,614**
Rgs7	Rattus norvegicus regulator of G-protein signaling 7 (Rgs7), mRNA [NM_019343]	**0,614**
0	Unknown	**0,614**
LOC683077	Rattus norvegicus similar to praja1, RING-H2 motif containing (LOC683077), mRNA [NM_001101006]	**0,614**
Tmem39b	Rattus norvegicus transmembrane protein 39b (Tmem39b), mRNA [NM_001014192]	**0,614**
Mdga2	Rattus norvegicus MAM domain containing glycosylphosphatidylinositol anchor 2 (Mdga2), mRNA [NM_199269]	**0,614**
Scand1	Rattus norvegicus SCAN domain-containing 1 (Scand1), mRNA [NM_001108599]	**0,614**
0	Q7VBZ4_PROMA (Q7VBZ4) Diaminopimelate epimerase, partial (5%) [TC594147]	**0,614**
Pygo2	Rattus norvegicus pygopus 2 (Pygo2), mRNA [NM_001106447]	**0,614**
Nucks1	Rattus norvegicus nuclear casein kinase and cyclin-dependent kinase substrate 1 (Nucks1), mRNA [NM_022799]	**0,614**
Coro1c	Rattus norvegicus coronin, actin binding protein 1C (Coro1c), mRNA [NM_001109327]	**0,614**
Mccc2	Rattus norvegicus methylcrotonoyl-Coenzyme A carboxylase 2 (beta) (Mccc2), nuclear gene encoding mitochondrial protein, mRNA [NM_001012177]	**0,614**
RGD1563070	Rattus norvegicus similar to hypothetical protein (RGD1563070), mRNA [NM_001134541]	**0,614**
Tmem150a	Rattus norvegicus transmembrane protein 150A (Tmem150a), mRNA [NM_139107]	**0,614**
RGD1311309	PREDICTED: Rattus norvegicus similar to 2510002A14Rik protein, transcript variant 2 (RGD1311309), mRNA [XM_002724960]	**0,614**
Lman2l	Rattus norvegicus lectin, mannose-binding 2-like (Lman2l), mRNA [NM_001106900]	**0,614**
RGD1310127	Rattus norvegicus similar to cDNA sequence BC017158 (RGD1310127), mRNA [NM_001035517]	**0,614**
0	Rattus norvegicus cDNA clone IMAGE:7318973. [BC091362]	**0,614**
0	Unknown	**0,614**
Znrd1	Rattus norvegicus zinc ribbon domain containing, 1 (Znrd1), transcript variant 2, mRNA [NM_213567]	**0,614**
RGD1564560	Rattus norvegicus similar to RCK (RGD1564560), mRNA [NM_001109292]	**0,614**
Zim1	Rattus norvegicus zinc finger, imprinted 1 (Zim1), mRNA [NM_001107473]	**0,614**
Grb14	Rattus norvegicus growth factor receptor bound protein 14 (Grb14), mRNA [NM_031623]	**0,614**
0	Unknown	**0,614**
0	Transcription initiation factor TFIID subunit 6 [Source:UniProtKB/Swiss-Prot;Acc:Q63801] [ENSRNOT00000001829]	**0,614**
Kcnq5l	Potassium voltage-gated channel KQT-like subfamily member 5 [Source:UniProtKB/TrEMBL;Acc:Q8K3W7] [ENSRNOT00000040034]	**0,614**
Rps6kb1	Rattus norvegicus ribosomal protein S6 kinase, polypeptide 1 (Rps6kb1), mRNA [NM_031985]	**0,614**
Bet1	Rattus norvegicus blocked early in transport 1 homolog (S. cerevisiae) (Bet1), mRNA [NM_019251]	**0,614**
Znf282	Rattus norvegicus zinc finger protein 282 (Znf282), mRNA [NM_001106592]	**0,614**
Pppde1	Rattus norvegicus PPPDE peptidase domain containing 1 (Pppde1), mRNA [NM_001013873]	**0,614**
Dqx1	Rattus norvegicus DEAQ box RNA-dependent ATPase 1 (Dqx1), mRNA [NM_001109407]	**0,614**
0	PREDICTED: Rattus norvegicus similar to c114 SLIT-like testicular protein (RGD1561285), mRNA [XM_220927]	**0,614**

Pars2	Rattus norvegicus prolyl-tRNA synthetase 2, mitochondrial (putative) (Pars2), nuclear gene encoding mitochondrial protein, mRNA [NM_001014064]	**0,614**
Nr4a2	Rattus norvegicus nuclear receptor subfamily 4, group A, member 2 (Nr4a2), mRNA [NM_019328]	**0,614**
Rai14	Rattus norvegicus retinoic acid induced 14 (Rai14), mRNA [NM_001011947]	**0,614**
Msx1	Rattus norvegicus msh homeobox 1 (Msx1), mRNA [NM_031059]	**0,614**
Tjp2	Rattus norvegicus tight junction protein 2 (Tjp2), mRNA [NM_053773]	**0,614**
0	Q4QQW4_RAT (Q4QQW4) Histone deacetylase 1, partial (42%) [TC647523]	**0,614**
Seli	Rattus norvegicus selenoprotein I (Seli), mRNA [NM_001134754]	**0,614**
Fcrla	Rattus norvegicus Fc receptor-like A (Fcrla), mRNA [NM_001100682]	**0,614**
RGD1565920	PREDICTED: Rattus norvegicus similar to OTTMUSP00000000438 (RGD1565920), mRNA [XM_344713]	**0,614**
Ap3b1	Rattus norvegicus adaptor-related protein complex 3, beta 1 subunit (Ap3b1), mRNA [NM_001107646]	**0,614**
Fam101b	Rattus norvegicus family with sequence similarity 101, member B (Fam101b), mRNA [NM_001007611]	**0,614**
Nsd1	Rattus norvegicus nuclear receptor binding SET domain protein 1 (Nsd1), mRNA [NM_001107337]	**0,614**
Adcy2	Rattus norvegicus adenylate cyclase 2 (brain) (Adcy2), mRNA [NM_031007]	**0,614**
0	Uncharacterized protein [Source:UniProtKB/TrEMBL;Acc:D3ZEB6] [ENSRNOT00000010789]	**0,615**
0	PREDICTED: Rattus norvegicus similar to 60S ribosomal protein L29 (P23) (RGD1559865), mRNA [XM_346093]	**0,615**
Tmem229b	Rattus norvegicus transmembrane protein 229B (Tmem229b), mRNA [NM_001109359]	**0,615**
Ndc80	Rattus norvegicus NDC80 homolog, kinetochore complex component (S. cerevisiae) (Ndc80), mRNA [NM_001126270]	**0,615**
0	Unknown	**0,615**
LOC690096	PREDICTED: Rattus norvegicus similar to ribosomal protein L28 (LOC690096), mRNA [XM_001073240]	**0,615**
Rrp7a	Rattus norvegicus ribosomal RNA processing 7 homolog A (S. cerevisiae) (Rrp7a), mRNA [NM_001130568]	**0,615**
Adhfe1	Rattus norvegicus alcohol dehydrogenase, iron containing, 1 (Adhfe1), nuclear gene encoding mitochondrial protein, mRNA [NM_001025423]	**0,615**
Nadk	Rattus norvegicus NAD kinase (Nadk), mRNA [NM_001109678]	**0,615**
LOC100125364	Rattus norvegicus hypothetical protein LOC100125364 (LOC100125364), mRNA [NM_001103356]	**0,615**
Rnf123	Rattus norvegicus ring finger protein 123 (Rnf123), mRNA [NM_001191580]	**0,615**
Micalcl	Rattus norvegicus MICAL C-terminal like (Micalcl), mRNA [NM_182669]	**0,615**
Itgb8	Rattus norvegicus integrin, beta 8 (Itgb8), mRNA [NM_001108726]	**0,615**
Sptbn2	Rattus norvegicus spectrin, beta, non-erythrocytic 2 (Sptbn2), mRNA [NM_019167]	**0,615**
Rtn1	Rattus norvegicus reticulon 1 (Rtn1), mRNA [NM_053865]	**0,615**
Slc25a44	Rattus norvegicus solute carrier family 25, member 44 (Slc25a44), mRNA [NM_001108947]	**0,615**
Tox3	Rattus norvegicus TOX high mobility group box family member 3 (Tox3), mRNA [NM_001106171]	**0,615**
Ranbp1	Rattus norvegicus RAN binding protein 1 (Ranbp1), mRNA [NM_001108324]	**0,615**
Rhbdf1	Rattus norvegicus rhomboid 5 homolog 1 (Drosophila) (Rhbdf1), mRNA [NM_001030034]	**0,615**
Fam102b	Rattus norvegicus family with sequence similarity 102, member B (Fam102b), mRNA [NM_001163568]	**0,615**

LOC100125362	Rattus norvegicus hypothetical protein LOC100125362 (LOC100125362), mRNA [NM_001103354]	**0,615**
Lhfpl2	Rattus norvegicus lipoma HMGIC fusion partner-like 2 (Lhfpl2), mRNA [NM_001106402]	**0,615**
Atp5g2	Rattus norvegicus ATP synthase, H+ transporting, mitochondrial F0 complex, subunit C2 (subunit 9) (Atp5g2), nuclear gene encoding mitochondrial protein, mRNA [NM_133556]	**0,615**
0	Unknown	**0,615**
0	Unknown	**0,615**
RGD1306595	Rattus norvegicus similar to hypothetical protein (RGD1306595), mRNA [NM_001025626]	**0,615**
0	Rattus norvegicus TL0AEA6YG01 mRNA sequence. [FQ231471]	**0,615**
0	Uncharacterized protein [Source:UniProtKB/TrEMBL;Acc:D3ZXV2] [ENSRNOT00000043588]	**0,615**
Rfc5	Rattus norvegicus replication factor C (activator 1) 5 (Rfc5), mRNA [NM_001107146]	**0,615**
Spock2	Rattus norvegicus sparc/osteonectin, cwcv and kazal-like domains proteoglycan 2 (Spock2), mRNA [NM_001108533]	**0,615**
Cd320	Rattus norvegicus CD320 molecule (Cd320), mRNA [NM_001014201]	**0,615**
Atp2b1	Rattus norvegicus ATPase, Ca++ transporting, plasma membrane 1 (Atp2b1), mRNA [NM_053311]	**0,615**
Mmgt2	Rattus norvegicus membrane magnesium transporter 2 (Mmgt2), mRNA [NM_001013967]	**0,615**
0	Unknown	**0,615**
0	Q8VDS2_MOUSE (Q8VDS2) Ttll3 protein, partial (38%) [TC587171]	**0,615**
Prcp	Rattus norvegicus prolylcarboxypeptidase (angiotensinase C) (Prcp), mRNA [NM_001106281]	**0,615**
Lpin1	Rattus norvegicus lipin 1 (Lpin1), mRNA [NM_001012111]	**0,615**
Atp2b2	Rattus norvegicus ATPase, Ca++ transporting, plasma membrane 2 (Atp2b2), mRNA [NM_012508]	**0,615**
Pigz	Rattus norvegicus phosphatidylinositol glycan anchor biosynthesis, class Z (Pigz), mRNA [NM_001109525]	**0,615**
Rab10	Rattus norvegicus RAB10, member RAS oncogene family (Rab10), mRNA [NM_017359]	**0,615**
Pmp22	Rattus norvegicus peripheral myelin protein 22 (Pmp22), mRNA [NM_017037]	**0,615**
LOC100359980	PREDICTED: Rattus norvegicus smooth muscle and non-muscle myosin alkali light chain 6B-like (LOC100359980), mRNA [XM_002726853]	**0,615**
Mak16	Rattus norvegicus MAK16 homolog (S. cerevisiae) (Mak16), mRNA [NM_001014002]	**0,615**
Sc5dl	Rattus norvegicus sterol-C5-desaturase (ERG3 delta-5-desaturase homolog, S. cerevisiae)-like (Sc5dl), mRNA [NM_053642]	**0,615**
LOC679861	AGENCOURT_114819228 NIH_MGC_433 Rattus norvegicus cDNA clone IMAGE:9098661 5', mRNA sequence [EV777635]	**0,616**
Usp9x	Rattus norvegicus ubiquitin specific peptidase 9, X-linked (Usp9x), transcript variant 2, mRNA [NM_001135923]	**0,616**
Fst	Rattus norvegicus follistatin (Fst), mRNA [NM_012561]	**0,616**
Mcpt8	Rattus norvegicus mast cell protease 8 (Mcpt8), mRNA [NM_021598]	**0,616**
Mare	Rattus norvegicus alpha globin regulatory element containing gene (Mare), mRNA [NM_001034936]	**0,616**
Lrrc7	Rattus norvegicus leucine rich repeat containing 7 (Lrrc7), mRNA [NM_057142]	**0,616**
Iffo1	Rattus norvegicus intermediate filament family orphan 1 (Iffo1), mRNA [NM_001108647]	**0,616**
0	Unknown	**0,616**
Jup	Rattus norvegicus junction plakoglobin (Jup), mRNA [NM_031047]	**0,616**

RGD1563982	Rattus norvegicus similar to F-box only protein 27 (RGD1563982), mRNA [NM_001110491]	**0,616**
Entpd6	Rattus norvegicus ectonucleoside triphosphate diphosphohydrolase 6 (Entpd6), mRNA [NM_053498]	**0,616**
Slc1a2	Rattus norvegicus solute carrier family 1 (glial high affinity glutamate transporter), member 2 (Slc1a2), transcript variant 2, mRNA [NM_001035233]	**0,616**
0	Unknown	**0,616**
0	Rattus norvegicus similar to actin-related protein 2 (LOC301861), mRNA [XM_217505]	**0,616**
0	Uncharacterized protein [Source:UniProtKB/TrEMBL;Acc:D4ADA8] [ENSRNOT00000012904]	**0,616**
Hk1	Rattus norvegicus hexokinase 1 (Hk1), nuclear gene encoding mitochondrial protein, mRNA [NM_012734]	**0,616**
Helz	Rattus norvegicus helicase with zinc finger (Helz), mRNA [NM_001105848]	**0,616**
Cxcl12	Rattus norvegicus chemokine (C-X-C motif) ligand 12 (stromal cell-derived factor 1) (Cxcl12), transcript variant 1, mRNA [NM_022177]	**0,616**
LOC684773	PREDICTED: Rattus norvegicus similar to histone 2a (LOC684773), mRNA [XM_001071887]	**0,616**
Alcam	Rattus norvegicus activated leukocyte cell adhesion molecule (Alcam), mRNA [NM_031753]	**0,616**
RGD1564058	Rattus norvegicus similar to cDNA sequence BC056474 (RGD1564058), mRNA [NM_001105947]	**0,616**
Megf9	Rattus norvegicus multiple EGF-like-domains 9 (Megf9), mRNA [NM_001107940]	**0,616**
Zeb2	Rattus norvegicus zinc finger E-box binding homeobox 2 (Zeb2), mRNA [NM_001033701]	**0,616**
Trak1	Rattus norvegicus trafficking protein, kinesin binding 1 (Trak1), mRNA [NM_001134565]	**0,616**
Rnf41	Rattus norvegicus ring finger protein 41 (Rnf41), mRNA [NM_001012195]	**0,616**
Tspan11	Rattus norvegicus tetraspanin 11 (Tspan11), mRNA [NM_001024262]	**0,616**
0	Unknown	**0,616**
Cast	Rattus norvegicus calpastatin (Cast), transcript variant 1, mRNA [NM_053295]	**0,616**
RGD1561962	Rattus norvegicus TL0ABA17YG18 mRNA sequence. [FQ211110]	**0,616**
LOC300308	Rattus norvegicus similar to hypothetical protein 4930509O22 (LOC300308), mRNA [NM_001013952]	**0,616**
Grik2	Rattus norvegicus glutamate receptor, ionotropic, kainate 2 (Grik2), mRNA [NM_019309]	**0,616**
Pnrc2	Rattus norvegicus proline-rich nuclear receptor coactivator 2 (Pnrc2), mRNA [NM_001103360]	**0,616**
Sh3bp2	Rattus norvegicus SH3-domain binding protein 2 (Sh3bp2), mRNA [NM_001100684]	**0,616**
Kctd17	Rattus norvegicus potassium channel tetramerisation domain containing 17 (Kctd17), mRNA [NM_001134529]	**0,616**
Epm2aip1	PREDICTED: Rattus norvegicus EPM2A (laforin) interacting protein 1 (Epm2aip1), mRNA [XM_236659]	**0,616**
Rad54l2	Rattus norvegicus Rad54 like 2 (S. cerevisiae) (Rad54l2), mRNA [NM_001134520]	**0,616**
RGD1305733	Rattus norvegicus similar to RIKEN cDNA 2900011O08 (RGD1305733), mRNA [NM_001014114]	**0,616**
0	Unknown	**0,616**
Pdgfd	Rattus norvegicus platelet-derived growth factor, D polypeptide (Pdgfd), mRNA [NM_023962]	**0,616**
RGD1309808	Rattus norvegicus similar to apolipoprotein L2; apolipoprotein L-II (RGD1309808), mRNA [NM_001134801]	**0,616**
Ndufa10l1	Rattus norvegicus NADH dehydrogenase (ubiquinone) 1 alpha subcomplex 10-like 1 (Ndufa10l1), mRNA [NM_182671]	**0,616**
Znf483	Rattus norvegicus zinc finger protein 483 (Znf483), mRNA [NM_133422]	**0,616**
Ube2ql1	Rattus norvegicus ubiquitin-conjugating enzyme E2Q family-like 1 (Ube2ql1), mRNA [NM_001145163]	**0,616**

Homer3	Rattus norvegicus homer homolog 3 (Drosophila) (Homer3), mRNA [NM_053310]	**0,616**
LOC688966	Rattus norvegicus similar to K11B4.2 (LOC688966), mRNA [NM_001145726]	**0,616**
MGC116121	Rattus norvegicus similar to RIKEN cDNA 2700062C07 (MGC116121), mRNA [NM_001024905]	**0,617**
LOC100363969	PREDICTED: Rattus norvegicus zinc finger protein 457-like (LOC100363969), miscRNA [XR_085746]	**0,617**
RGD1309228	Rattus norvegicus similar to putative protein, with at least 9 transmembrane domains, of eukaryotic origin (43.9 kD) (2G415) (RGD1309228), mRNA [NM_001017451]	**0,617**
Dhdpsl	Rattus norvegicus dihydrodipicolinate synthase-like, mitochondrial (Dhdpsl), nuclear gene encoding mitochondrial protein, mRNA [NM_001106355]	**0,617**
Brwd3	PREDICTED: Rattus norvegicus bromodomain and WD repeat domain containing 3 (Brwd3), mRNA [XM_001054667]	**0,617**
Cops7b	Rattus norvegicus COP9 constitutive photomorphogenic homolog subunit 7B (Arabidopsis) (Cops7b), mRNA [NM_001108807]	**0,617**
Traf4af1	Rattus norvegicus TRAF4 associated factor 1 (Traf4af1), mRNA [NM_001004264]	**0,617**
Pnpo	Rattus norvegicus pyridoxine 5'-phosphate oxidase (Pnpo), mRNA [NM_022601]	**0,617**
Casc5	Rattus norvegicus cancer susceptibility candidate 5 (Casc5), mRNA [NM_001170594]	**0,617**
LOC366709	PREDICTED: Rattus norvegicus similar to prohibitin (LOC366709), miscRNA [XR_006662]	**0,617**
0	Unknown	**0,617**
Trim16	Rattus norvegicus tripartite motif-containing 16 (Trim16), mRNA [NM_001135033]	**0,617**
Dusp22	Rattus norvegicus dual specificity phosphatase 22 (Dusp22), mRNA [NM_001108412]	**0,617**
Abcc1	Rattus norvegicus ATP-binding cassette, subfamily C (CFTR/MRP), member 1 (Abcc1), mRNA [NM_022281]	**0,617**
0	Unknown	**0,617**
Fam35a	Rattus norvegicus family with sequence similarity 35, member A (Fam35a), mRNA [NM_001025028]	**0,617**
Mum1l1	Rattus norvegicus melanoma associated antigen (mutated) 1-like 1 (Mum1l1), mRNA [NM_001109321]	**0,617**
Ccr10	Rattus norvegicus chemokine (C-C motif) receptor 10 (Ccr10), mRNA [NM_001108836]	**0,617**
0	Rattus norvegicus TL0ADA16YE04 mRNA sequence. [FQ223021]	**0,617**
St3gal4	Rattus norvegicus ST3 beta-galactoside alpha-2,3-sialyltransferase 4 (St3gal4), mRNA [NM_203337]	**0,617**
Furin	Rattus norvegicus furin (paired basic amino acid cleaving enzyme) (Furin), mRNA [NM_019331]	**0,617**
Fam136a	Rattus norvegicus family with sequence similarity 136, member A (Fam136a), mRNA [NM_001106605]	**0,617**
Caskin1	Rattus norvegicus CASK interacting protein 1 (Caskin1), mRNA [NM_080690]	**0,617**
Pomt2	Rattus norvegicus protein-O-mannosyltransferase 2 (Pomt2), mRNA [NM_001047114]	**0,617**
RGD1560166	PREDICTED: Rattus norvegicus similar to Probable G-protein coupled receptor 62 (hGPCR8) (RGD1560166), mRNA [XM_001073569]	**0,617**
Tapt1	PREDICTED: Rattus norvegicus transmembrane anterior posterior transformation 1 (Tapt1), mRNA [XM_001059604]	**0,617**
0	Q99MR2_MOUSE (Q99MR2) EPHB4, partial (63%) [TC591623]	**0,617**
Cdh2	Rattus norvegicus cadherin 2 (Cdh2), mRNA [NM_031333]	**0,617**
0	Unknown	**0,617**
Slc7a11	Rattus norvegicus solute carrier family 7 (cationic amino acid transporter, y+ system), member 11 (Slc7a11), mRNA [NM_001107673]	**0,617**
Igfbp4	Rattus norvegicus insulin-like growth factor binding protein 4 (Igfbp4), mRNA [NM_001004274]	**0,617**

Rab6a	Rattus norvegicus RAB6A, member RAS oncogene family (Rab6a), mRNA [NM_053366]	**0,617**
Srek1ip1	Rattus norvegicus splicing regulatory glutamine/lysine-rich protein 1 interacting protein 1 (Srek1ip1), mRNA [NM_001008373]	**0,617**
Dtnbp1	Rattus norvegicus distrobrevin binding protein 1 (Dtnbp1), mRNA [NM_001037664]	**0,617**
LOC683684	PREDICTED: Rattus norvegicus hypothetical protein LOC683684 (LOC683684), mRNA [XM_001067050]	**0,617**
Sorcs2	Rattus norvegicus sortilin-related VPS10 domain containing receptor 2 (Sorcs2), mRNA [NM_001107225]	**0,617**
Ncstn	Rattus norvegicus nicastrin (Ncstn), mRNA [NM_174864]	**0,617**
Rere	Rattus norvegicus arginine-glutamic acid dipeptide (RE) repeats (Rere), mRNA [NM_053885]	**0,617**
Dmd	Rattus norvegicus dystrophin (Dmd), transcript variant Dp71a, mRNA [NM_012698]	**0,617**
0	MMNPT2S01 Na+-phosphate cotransporter type II {Mus musculus} (exp=-1; wgp=0; cg=0), partial (12%) [TC607345]	**0,617**
0	Unknown	**0,617**
Prr3	Rattus norvegicus proline rich 3 (Prr3), mRNA [NM_212544]	**0,617**
Mobkl1b	Rattus norvegicus MOB1, Mps One Binder kinase activator-like 1B (yeast) (Mobkl1b), mRNA [NM_001033891]	**0,617**
Tnks	Rattus norvegicus tankyrase, TRF1-interacting ankyrin-related ADP-ribose polymerase (Tnks), mRNA [NM_001106084]	**0,617**
RGD1560792	PREDICTED: Rattus norvegicus RGD1560792 (RGD1560792), mRNA [XM_001081860]	**0,617**
Klk7	Rattus norvegicus kallikrein-related peptidase 7 (Klk7), mRNA [NM_001106254]	**0,617**
0	BT006320 At1g66470 {Arabidopsis thaliana} (exp=-1; wgp=0; cg=0), partial (5%) [TC589845]	**0,617**
Vcpip1	Deubiquitinating protein VCIP135 [Source:UniProtKB/Swiss-Prot;Acc:Q8CF97] [ENSRNOT00000009136]	**0,617**
Znf865	Rattus norvegicus zinc finger protein 865 (Znf865), mRNA [NM_001134544]	**0,618**
Pars2	Rattus norvegicus prolyl-tRNA synthetase 2, mitochondrial (putative) (Pars2), nuclear gene encoding mitochondrial protein, mRNA [NM_001014064]	**0,618**
0	Unknown	**0,618**
Trps1	Rattus norvegicus trichorhinophalangeal syndrome I homolog (human) (Trps1), mRNA [NM_001134837]	**0,618**
Srm	Rattus norvegicus spermidine synthase (Srm), mRNA [NM_053464]	**0,618**
Acap2	Rattus norvegicus ArfGAP with coiled-coil, ankyrin repeat and PH domains 2 (Acap2), mRNA [NM_001034006]	**0,618**
Hist1h3f	PREDICTED: Rattus norvegicus histone cluster 1, H3f (Hist1h3f), mRNA [XM_001071516]	**0,618**
LOC100361198	PREDICTED: Rattus norvegicus rCG43589-like (LOC100361198), mRNA [XM_002730083]	**0,618**
39692	Rattus norvegicus septin 8 (Sept8), mRNA [NM_001107002]	**0,618**
0	AW143179 EST293475 Normalized rat brain, Bento Soares Rattus sp. cDNA clone RGIBF04 5' end, mRNA sequence [AW143179]	**0,618**
Pias2	Rattus norvegicus protein inhibitor of activated STAT, 2 (Pias2), mRNA [NM_053337]	**0,618**
Mecr	Rattus norvegicus mitochondrial trans-2-enoyl-CoA reductase (Mecr), nuclear gene encoding mitochondrial protein, mRNA [NM_017209]	**0,618**
Mrgprg	Rattus norvegicus MAS-related GPR, member G (Mrgprg), mRNA [NM_203470]	**0,618**
Mknk1	Rattus norvegicus MAP kinase-interacting serine/threonine kinase 1 (Mknk1), mRNA [NM_001044267]	**0,618**
0	Unknown	**0,618**
Cmpk1	Rattus norvegicus cytidine monophosphate (UMP-CMP) kinase 1 (Cmpk1), mRNA [NM_001025655]	**0,618**

Slc16a11	Rattus norvegicus solute carrier family 16 (monocarboxylic acid transporters), member 11 (Slc16a11), mRNA [NM_001105797]	**0,618**
Mepce	Rattus norvegicus methylphosphate capping enzyme (Mepce), mRNA [NM_001100678]	**0,618**
Ccdc33	Rattus norvegicus coiled-coil domain containing 33 (Ccdc33), mRNA [NM_001014091]	**0,618**
Mxra8	Rattus norvegicus matrix-remodelling associated 8 (Mxra8), mRNA [NM_001007002]	**0,618**
Timd2	Rattus norvegicus T-cell immunoglobulin and mucin domain containing 2 (Timd2), mRNA [NM_001013855]	**0,618**
Vps37b	Rattus norvegicus vacuolar protein sorting 37 homolog B (S. cerevisiae) (Vps37b), mRNA [NM_001105928]	**0,618**
Pik3ca	Rattus norvegicus phosphoinositide-3-kinase, catalytic, alpha polypeptide (Pik3ca), mRNA [NM_133399]	**0,618**
Cntn3	Rattus norvegicus contactin 3 (plasmacytoma associated) (Cntn3), mRNA [NM_019329]	**0,618**
Fbxl12	Rattus norvegicus F-box and leucine-rich repeat protein 12 (Fbxl12), mRNA [NM_001025700]	**0,618**
0	Unknown	**0,618**
Ttyh3	Rattus norvegicus tweety homolog 3 (Drosophila) (Ttyh3), mRNA [NM_001107124]	**0,618**
Bag2	Rattus norvegicus Bcl2-associated athanogene 2 (Bag2), mRNA [NM_001128195]	**0,618**
LOC306766	Rattus norvegicus hypothetical LOC306766 (LOC306766), mRNA [NM_001014007]	**0,618**
Dppa3	Rattus norvegicus developmental pluripotency-associated 3 (Dppa3), mRNA [NM_001047864]	**0,618**
Ubap1	Rattus norvegicus ubiquitin-associated protein 1 (Ubap1), mRNA [NM_001012190]	**0,618**
Fam189b	Rattus norvegicus family with sequence similarity 189, member B (Fam189b), mRNA [NM_001107690]	**0,618**
RGD1309759	PREDICTED: Rattus norvegicus similar to cDNA sequence BC013529, transcript variant 2 (RGD1309759), mRNA [XM_001059157]	**0,618**
LOC685067	PREDICTED: Rattus norvegicus similar to guanylate binding protein family, member 6 (LOC685067), miscRNA [XR_006095]	**0,618**
Atp7a	Rattus norvegicus ATPase, Cu++ transporting, alpha polypeptide (Atp7a), mRNA [NM_052803]	**0,618**
Stk39	Rattus norvegicus serine/threonine kinase 39, STE20/SPS1 homolog (yeast) (Stk39), mRNA [NM_019362]	**0,618**
Agpat3	Rattus norvegicus 1-acylglycerol-3-phosphate O-acyltransferase 3 (Agpat3), mRNA [NM_001106378]	**0,618**
LOC686139	PREDICTED: Rattus norvegicus similar to abhydrolase domain containing 11 (LOC686139), mRNA [XM_001066660]	**0,618**
Surf4	Rattus norvegicus surfeit 4 (Surf4), mRNA [NM_001033868]	**0,618**
0	Q4KM71_RAT (Q4KM71) NonO/p54nrb homolog, complete [TC583734]	**0,618**
Pi16	Rattus norvegicus peptidase inhibitor 16 (Pi16), mRNA [NM_001170481]	**0,618**
Hmgn2	Rattus norvegicus high mobility group nucleosomal binding domain 2 (Hmgn2), mRNA [NM_001025624]	**0,618**
Elovl6	Rattus norvegicus ELOVL family member 6, elongation of long chain fatty acids (yeast) (Elovl6), mRNA [NM_134383]	**0,618**
0	Unknown	**0,618**
Impa1	Rattus norvegicus Inositol (myo)-1(or 4)-monophosphatase 1 (Impa1), mRNA [NM_032057]	**0,618**
Dpcd	Rattus norvegicus deleted in primary ciliary dyskinesia (Dpcd), mRNA [NM_001013905]	**0,618**
0	Unknown	**0,618**
Htr3a	Rattus norvegicus 5-hydroxytryptamine (serotonin) receptor 3a (Htr3a), mRNA [NM_024394]	**0,618**
0	Unknown	**0,618**
Aurkaip1	Rattus norvegicus aurora kinase A interacting protein 1 (Aurkaip1), mRNA [NM_001004237]	**0,618**

Gpr85	Rattus norvegicus G protein-coupled receptor 85 (Gpr85), mRNA [NM_022254]	**0,618**
Snai1	Rattus norvegicus snail homolog 1 (Drosophila) (Snai1), mRNA [NM_053805]	**0,618**
LOC684841	PREDICTED: Rattus norvegicus similar to CG31613-PA (LOC684841), mRNA [XM_001072155]	**0,618**
Srm	Rattus norvegicus spermidine synthase (Srm), mRNA [NM_053464]	**0,618**
LOC292722	Uncharacterized protein [Source:UniProtKB/TrEMBL;Acc:D3Z9I0] [ENSRNOT00000027783]	**0,618**
Dpysl4	Rattus norvegicus dihydropyrimidinase-like 4 (Dpysl4), mRNA [NM_012933]	**0,618**
0	Unknown	**0,618**
Tmem33	Rattus norvegicus transmembrane protein 33 (Tmem33), transcript variant 1, mRNA [NM_021671]	**0,618**
Bet1l	Rattus norvegicus blocked early in transport 1 homolog (S. cerevisiae) like (Bet1l), mRNA [NM_019368]	**0,618**
Dnali1	Rattus norvegicus dynein, axonemal, light intermediate chain 1 (Dnali1), mRNA [NM_001031647]	**0,618**
Ns5atp4	Rattus norvegicus NS5A (hepatitis C virus) transactivated protein 4 (Ns5atp4), mRNA [NM_207607]	**0,618**
Sypl2	Rattus norvegicus synaptophysin-like 2 (Sypl2), mRNA [NM_001108563]	**0,618**
Dcaf5	Rattus norvegicus DDB1 and CUL4 associated factor 5 (Dcaf5), nuclear gene encoding mitochondrial protein, mRNA [NM_001100718]	**0,618**
Ccdc102a	Rattus norvegicus coiled-coil domain containing 102A (Ccdc102a), mRNA [NM_001108437]	**0,618**
0	BF285957 EST450548 Rat Gene Index, normalized rat, Rattus norvegicus cDNA Rattus norvegicus cDNA clone RGIFL30, mRNA sequence [BF285957]	**0,619**
Rfx1	Rattus norvegicus regulatory factor X, 1 (influences HLA class II expression) (Rfx1), mRNA [NM_001105944]	**0,619**
Sms	Rattus norvegicus spermine synthase (Sms), mRNA [NM_001033899]	**0,619**
0	Unknown	**0,619**
S100b	Rattus norvegicus S100 calcium binding protein B (S100b), mRNA [NM_013191]	**0,619**
Zbtb9	Rattus norvegicus zinc finger and BTB domain containing 9 (Zbtb9), mRNA [NM_213564]	**0,619**
Ap2m1	Rattus norvegicus adaptor-related protein complex 2, mu 1 subunit (Ap2m1), mRNA [NM_053837]	**0,619**
Ophn1	Rattus norvegicus oligophrenin 1 (Ophn1), mRNA [NM_001107848]	**0,619**
Grin3a	Rattus norvegicus glutamate receptor, ionotropic, N-methyl-D-aspartate 3A (Grin3a), transcript variant 1, mRNA [NM_138546]	**0,619**
Gstz1	Rattus norvegicus glutathione transferase zeta 1 (Gstz1), mRNA [NM_001109445]	**0,619**
Spry1	Rattus norvegicus sprouty homolog 1, antagonist of FGF signaling (Drosophila) (Spry1), mRNA [NM_001106427]	**0,619**
Apobec2	Rattus norvegicus apolipoprotein B mRNA editing enzyme, catalytic polypeptide-like 2 (Apobec2), mRNA [NM_001106883]	**0,619**
Pcbp3	Rattus norvegicus poly(rC) binding protein 3 (Pcbp3), mRNA [NM_001011945]	**0,619**
Ppapdc1b	Rattus norvegicus phosphatidic acid phosphatase type 2 domain containing 1B (Ppapdc1b), mRNA [NM_001109411]	**0,619**
LOC684112	PREDICTED: Rattus norvegicus similar to KIAA0999 protein (LOC684112), mRNA [XM_001068984]	**0,619**
Tmem189	Rattus norvegicus transmembrane protein 189 (Tmem189), mRNA [NM_001113752]	**0,619**
Ehd1	Rattus norvegicus EH-domain containing 1 (Ehd1), mRNA [NM_001011939]	**0,619**
0	Q8TE64_HUMAN (Q8TE64) Pur-gamma B-form, partial (50%) [TC600362]	**0,619**
Ralgds	Rattus norvegicus ral guanine nucleotide dissociation stimulator (Ralgds), mRNA [NM_019250]	**0,619**

Samd5	Rattus norvegicus sterile alpha motif domain containing 5 (Samd5), mRNA [NM_001108901]	**0,619**
0	Unknown	**0,619**
Ythdf2	Rattus norvegicus YTH domain family, member 2 (Ythdf2), mRNA [NM_001047099]	**0,619**
Ids	PREDICTED: Rattus norvegicus iduronate 2-sulfatase (Ids), mRNA [XM_001054447]	**0,619**
Dazap2	Rattus norvegicus DAZ associated protein 2 (Dazap2), mRNA [NM_001013107]	**0,619**
Ptbp1	Rattus norvegicus polypyrimidine tract binding protein 1 (Ptbp1), transcript variant 2, mRNA [NM_022516]	**0,619**
Znf23	Rattus norvegicus zinc finger protein 23 (KOX 16) (Znf23), mRNA [NM_001107428]	**0,619**
Fnbp1l	Rattus norvegicus formin binding protein 1-like (Fnbp1l), mRNA [NM_001039609]	**0,619**
LOC690340	PREDICTED: Rattus norvegicus similar to spermatogenesis associated glutamate (E)-rich protein 4d (LOC690340), mRNA [XM_001074151]	**0,619**
Iqcb1	Rattus norvegicus IQ motif containing B1 (Iqcb1), mRNA [NM_001107092]	**0,619**
Yeats4	Rattus norvegicus YEATS domain containing 4 (Yeats4), mRNA [NM_001127527]	**0,619**
Cstf2	Rattus norvegicus cleavage stimulation factor, 3' pre-RNA subunit 2 (Cstf2), mRNA [NM_001131014]	**0,619**
Ano6	Rattus norvegicus anoctamin 6 (Ano6), mRNA [NM_001108108]	**0,619**
RGD1311648	Rattus norvegicus similar to hypothetical protein FLJ21820 (RGD1311648), mRNA [NM_001014075]	**0,619**
Sirpa	Rattus norvegicus signal-regulatory protein alpha (Sirpa), mRNA [NM_013016]	**0,619**
Usp13	Rattus norvegicus ubiquitin specific protease 13 (isopeptidase T-3) (Usp13), mRNA [NM_001107665]	**0,619**
Myo16	Rattus norvegicus myosin XVI (Myo16), mRNA [NM_138893]	**0,619**
LOC501251	PREDICTED: Rattus norvegicus similar to Discs large homolog 5 (Placenta and prostate DLG) (Discs large protein P-dlg) (LOC501251), mRNA [XM_576670]	**0,619**
Men1	Rattus norvegicus multiple endocrine neoplasia 1 (Men1), mRNA [NM_019208]	**0,619**
Myd88	Rattus norvegicus myeloid differentiation primary response gene 88 (Myd88), mRNA [NM_198130]	**0,619**
Psip1	Rattus norvegicus PC4 and SFRS1 interacting protein 1 (Psip1), mRNA [NM_175765]	**0,619**
Bag3	Rattus norvegicus Bcl2-associated athanogene 3 (Bag3), mRNA [NM_001011936]	**0,619**
Egfr	Rattus norvegicus epidermal growth factor receptor (Egfr), mRNA [NM_031507]	**0,619**
Zhx3	Rattus norvegicus zinc fingers and homeoboxes 3 (Zhx3), mRNA [NM_001047097]	**0,619**
Bmi1	Rattus norvegicus Bmi1 polycomb ring finger oncogene (Bmi1), mRNA [NM_001107368]	**0,619**
Fam134b	Rattus norvegicus family with sequence similarity 134, member B (Fam134b), mRNA [NM_001034912]	**0,619**
Msh2	Rattus norvegicus mutS homolog 2 (E. coli) (Msh2), mRNA [NM_031058]	**0,619**
G2e3	Rattus norvegicus G2/M-phase specific E3 ubiquitin ligase (G2e3), mRNA [NM_001106726]	**0,619**
Map3k3	Rattus norvegicus mitogen activated protein kinase kinase kinase 3 (Map3k3), mRNA [NM_001107058]	**0,620**
Slc25a16	Rattus norvegicus solute carrier family 25 (mitochondrial carrier, Graves disease autoantigen), member 16 (Slc25a16), nuclear gene encoding mitochondrial protein, mRNA [NM_001100860]	**0,620**
0	Unknown	**0,620**
Rcan2	Rattus norvegicus regulator of calcineurin 2 (Rcan2), mRNA [NM_175578]	**0,620**

Unc79	Uncharacterized protein [Source:UniProtKB/TrEMBL;Acc:D3ZSV8] [ENSRNOT00000066306]	**0,620**
Ppp1r7	Rattus norvegicus protein phosphatase 1, regulatory (inhibitor) subunit 7 (Ppp1r7), mRNA [NM_001009825]	**0,620**
Igsf8	Rattus norvegicus immunoglobulin superfamily, member 8 (Igsf8), mRNA [NM_001014787]	**0,620**
0	protein phosphatase 2, regulatory subunit B (B56), delta isoform Gene [Source:MGI Symbol;Acc:MGI:2388481] [ENSRNOT00000022862]	**0,620**
Caly	Rattus norvegicus calcyon neuron-specific vesicular protein (Caly), transcript variant 1, mRNA [NM_138915]	**0,620**
Ptprn2	Rattus norvegicus protein tyrosine phosphatase, receptor type, N polypeptide 2 (Ptprn2), mRNA [NM_031600]	**0,620**
Spem1	Rattus norvegicus spermatid maturation 1 (Spem1), mRNA [NM_001109653]	**0,620**
Camsap1l1	Rattus norvegicus calmodulin regulated spectrin-associated protein 1-like 1 (Camsap1l1), mRNA [NM_001134503]	**0,620**
Rnf170	PREDICTED: Rattus norvegicus ring finger protein 170 (Rnf170), mRNA [XM_001061874]	**0,620**
Trappc1	Rattus norvegicus trafficking protein particle complex 1 (Trappc1), mRNA [NM_001039378]	**0,620**
0	Q6P501_RAT (Q6P501) Lysosomal-associated protein transmembrane 4 alpha, partial (11%) [TC646831]	**0,620**
Pofut2	Rattus norvegicus protein O-fucosyltransferase 2 (Pofut2), mRNA [NM_001107621]	**0,620**
Ezh1	Rattus norvegicus enhancer of zeste homolog 1 (Drosophila) (Ezh1), mRNA [NM_001107051]	**0,620**
Bmpr1b	Rattus norvegicus bone morphogenetic protein receptor, type IB (Bmpr1b), mRNA [NM_001024259]	**0,620**
Cox6a2	Rattus norvegicus cytochrome c oxidase, subunit VIa, polypeptide 2 (Cox6a2), nuclear gene encoding mitochondrial protein, transcript variant 1, mRNA [NM_012812]	**0,620**
Ppdpf	Rattus norvegicus pancreatic progenitor cell differentiation and proliferation factor homolog (zebrafish) (Ppdpf), mRNA [NM_001009316]	**0,620**
Grin3a	Rattus norvegicus glutamate receptor, ionotropic, N-methyl-D-aspartate 3A (Grin3a), transcript variant 1, mRNA [NM_138546]	**0,620**
Dimt1l	Rattus norvegicus DIM1 dimethyladenosine transferase 1-like (S. cerevisiae) (Dimt1l), mRNA [NM_001106408]	**0,620**
Cmtm5	Rattus norvegicus CKLF-like MARVEL transmembrane domain containing 5 (Cmtm5), mRNA [NM_001106034]	**0,620**
Ap2a1	adaptor-related protein complex 2, alpha 1 subunit [Source:RefSeq peptide;Acc:NP_001100981] [ENSRNOT00000020304]	**0,620**
Psors1c2	Rattus norvegicus psoriasis susceptibility 1 candidate 2 (human) (Psors1c2), mRNA [NM_001166016]	**0,620**
RGD1564379	Rattus norvegicus RGD1564379 (RGD1564379), mRNA [NM_001126295]	**0,620**
Vps25	Rattus norvegicus vacuolar protein sorting 25 homolog (S. cerevisiae) (Vps25), mRNA [NM_001173451]	**0,620**
Ttll7	Uncharacterized protein [Source:UniProtKB/TrEMBL;Acc:D4ACG4] [ENSRNOT00000055318]	**0,620**
Shroom2	Rattus norvegicus shroom family member 2 (Shroom2), mRNA [NM_001047893]	**0,620**
Nudt12	Rattus norvegicus nudix (nucleoside diphosphate linked moiety X)-type motif 12 (Nudt12), mRNA [NM_001109010]	**0,620**
Oxsr1	Rattus norvegicus oxidative-stress responsive 1 (Oxsr1), mRNA [NM_001108194]	**0,620**
0	Unknown	**0,620**
Acap2	Rattus norvegicus ArfGAP with coiled-coil, ankyrin repeat and PH domains 2 (Acap2), mRNA [NM_001034006]	**0,620**
RGD1560010	Rattus norvegicus RGD1560010 (RGD1560010), mRNA [NM_001107685]	**0,620**
LOC687295	PREDICTED: Rattus norvegicus similar to translocase of inner mitochondrial membrane 50 homolog, transcript variant 2 (LOC687295), mRNA [XM_001073346]	**0,620**
Adck4	Rattus norvegicus aarF domain containing kinase 4 (Adck4), mRNA [NM_001012065]	**0,620**

Map3k14	Rattus norvegicus mitogen-activated protein kinase kinase kinase 14 (Map3k14), mRNA [NM_001108301]	**0,620**
Wdr89	Rattus norvegicus WD repeat domain 89 (Wdr89), mRNA [NM_001014078]	**0,620**
Cox8a	Rattus norvegicus cytochrome c oxidase subunit VIIIa (Cox8a), mRNA [NM_134345]	**0,620**
Mt1a	Rattus norvegicus metallothionein 1a (Mt1a), mRNA [NM_138826]	**0,620**
Ankrd13c	Rattus norvegicus ankyrin repeat domain 13C (Ankrd13c), mRNA [NM_001191570]	**0,620**
Fastkd3	Rattus norvegicus FAST kinase domains 3 (Fastkd3), mRNA [NM_001082574]	**0,620**
Tprkb	Rattus norvegicus Tp53rk binding protein (Tprkb), mRNA [NM_001013926]	**0,620**
Foxm1	Rattus norvegicus forkhead box M1 (Foxm1), mRNA [NM_031633]	**0,621**
Mrps11	Rattus norvegicus mitochondrial ribosomal protein S11 (Mrps11), nuclear gene encoding mitochondrial protein, mRNA [NM_001109148]	**0,621**
0	Uncharacterized protein [Source:UniProtKB/TrEMBL;Acc:D3ZPG8] [ENSRNOT00000068110]	**0,621**
N4bp1	PREDICTED: Rattus norvegicus Nedd4 binding protein 1 (N4bp1), mRNA [XM_001068071]	**0,621**
Gpr155	Rattus norvegicus G protein-coupled receptor 155 (Gpr155), mRNA [NM_001107811]	**0,621**
Arf3	Rattus norvegicus ADP-ribosylation factor 3 (Arf3), mRNA [NM_080904]	**0,621**
0	Gdpd5 protein [Source:UniProtKB/TrEMBL;Acc:B5DF39] [ENSRNOT00000055321]	**0,621**
Chd6	Rattus norvegicus chromodomain helicase DNA binding protein 6 (Chd6), mRNA [NM_001107797]	**0,621**
Slc9a5	Rattus norvegicus solute carrier family 9 (sodium/hydrogen exchanger), member 5 (Slc9a5), mRNA [NM_138858]	**0,621**
RGD1308019	Rattus norvegicus similar to hypothetical protein FLJ20245 (RGD1308019), mRNA [NM_001107816]	**0,621**
Gpr4	Rattus norvegicus G protein-coupled receptor 4 (Gpr4), mRNA [NM_001025680]	**0,621**
Heatr2	Rattus norvegicus HEAT repeat containing 2 (Heatr2), mRNA [NM_001134857]	**0,621**
Tnfsf13	Rattus norvegicus tumor necrosis factor (ligand) superfamily, member 13 (Tnfsf13), mRNA [NM_001009623]	**0,621**
Papss2	Rattus norvegicus 3'-phosphoadenosine 5'-phosphosulfate synthase 2 (Papss2), mRNA [NM_001106375]	**0,621**
Gria4	Rattus norvegicus glutamate receptor, ionotrophic, AMPA 4 (Gria4), transcript variant 1, mRNA [NM_017263]	**0,621**
Ctbp1	Rattus norvegicus C-terminal binding protein 1 (Ctbp1), mRNA [NM_019201]	**0,621**
Pdcd6ip	Rattus norvegicus programmed cell death 6 interacting protein (Pdcd6ip), mRNA [NM_001029910]	**0,621**
Mterfd3	Rattus norvegicus MTERF domain containing 3 (Mterfd3), mRNA [NM_001014265]	**0,621**
Ptprz1	Rattus norvegicus protein tyrosine phosphatase, receptor-type, Z polypeptide 1 (Ptprz1), transcript variant 1, mRNA [NM_013080]	**0,621**
Stx1a	Rattus norvegicus syntaxin 1A (brain) (Stx1a), mRNA [NM_053788]	**0,621**
Mybl2	Rattus norvegicus myeloblastosis oncogene-like 2 (Mybl2), mRNA [NM_001106536]	**0,621**
Exoc6b	Rattus norvegicus exocyst complex component 6B (Exoc6b), mRNA [NM_001109246]	**0,621**
Prkacb	Rattus norvegicus protein kinase, cAMP dependent, catalytic, beta (Prkacb), mRNA [NM_001077645]	**0,621**
Ttc17	Rattus norvegicus tetratricopeptide repeat domain 17 (Ttc17), mRNA [NM_001107752]	**0,621**
Stmn2	Rattus norvegicus stathmin-like 2 (Stmn2), mRNA [NM_053440]	**0,621**
Ube2a	Rattus norvegicus ubiquitin-conjugating enzyme E2A (RAD6 homolog) (Ube2a), mRNA [NM_001013933]	**0,621**
Pgap2	Rattus norvegicus post-GPI attachment to proteins 2 (Pgap2), mRNA [NM_053895]	**0,621**

0	Unknown	**0,621**
LOC690105	PREDICTED: Rattus norvegicus hypothetical protein LOC690105 (LOC690105), mRNA [XM_002726579]	**0,621**
Pdcl	Rattus norvegicus phosducin-like (Pdcl), mRNA [NM_022247]	**0,621**
Gabra1	Rattus norvegicus gamma-aminobutyric acid (GABA) A receptor, alpha 1 (Gabra1), mRNA [NM_183326]	**0,621**
Dcun1d1	Rattus norvegicus DCN1, defective in cullin neddylation 1, domain containing 1 (S. cerevisiae) (Dcun1d1), mRNA [NM_001107668]	**0,621**
Npy	Rattus norvegicus neuropeptide Y (Npy), mRNA [NM_012614]	**0,621**
0	Rattus norvegicus similar to RIKEN cDNA 0610012D17 (LOC294368), mRNA [XM_228129]	**0,621**
0	PREDICTED: Rattus norvegicus pro-histogranin (LOC502940), miscRNA [XR_009615]	**0,621**
0	PREDICTED: Rattus norvegicus similar to RIKEN cDNA 9030221M09 gene (RGD1305469), mRNA [XM_001066163]	**0,621**
Mib1	Rattus norvegicus mindbomb homolog 1 (Drosophila) (Mib1), mRNA [NM_001107405]	**0,621**
Aig1	Rattus norvegicus androgen-induced 1 (Aig1), mRNA [NM_001134425]	**0,621**
Rngtt	Rattus norvegicus RNA guanylyltransferase and 5'-phosphatase (Rngtt), mRNA [NM_001107923]	**0,621**
Fancl	Rattus norvegicus Fanconi anemia, complementation group L (Fancl), mRNA [NM_001191684]	**0,621**
0	FM054771 etcmhea Rattus norvegicus cDNA clone etcmheaP0020N18 5', mRNA sequence [FM054771]	**0,621**
Pgpep1	Rattus norvegicus pyroglutamyl-peptidase I (Pgpep1), mRNA [NM_201988]	**0,621**
Ccnjl	Rattus norvegicus cyclin J-like (Ccnjl), mRNA [NM_001037773]	**0,622**
Fgfr2	Rattus norvegicus fibroblast growth factor receptor 2 (Fgfr2), transcript variant a, mRNA [NM_012712]	**0,622**
Fndc5	PREDICTED: Rattus norvegicus fibronectin type III domain containing 5 (Fndc5), mRNA [XM_002729542]	**0,622**
0	Unknown	**0,622**
0	CITE4_MOUSE (Q9WUL8) Cbp/p300-interacting transactivator 4 (MSG1-related protein 2) (MRG-2), partial (8%) [TC620332]	**0,622**
0	Unknown	**0,622**
LOC679715	PREDICTED: Rattus norvegicus similar to ribosomal protein L13 (LOC679715), mRNA [XM_001054162]	**0,622**
Dpy19l4	Putative uncharacterized protein RGD1305828_predictedUncharacterized protein [Source:UniProtKB/TrEMBL;Acc:D3Z939] [ENSRNOT00000037869]	**0,622**
Pcp4	Rattus norvegicus Purkinje cell protein 4 (Pcp4), mRNA [NM_013002]	**0,622**
Srpk2	Rattus norvegicus SFRS protein kinase 2 (Srpk2), mRNA [NM_001106575]	**0,622**
Gltpd1	Rattus norvegicus glycolipid transfer protein domain containing 1 (Gltpd1), mRNA [NM_001007703]	**0,622**
0	Unknown	**0,622**
Eif2c2	Rattus norvegicus eukaryotic translation initiation factor 2C, 2 (Eif2c2), mRNA [NM_021597]	**0,622**
Vamp1	Rattus norvegicus vesicle-associated membrane protein 1 (Vamp1), mRNA [NM_013090]	**0,622**
Grm4	Rattus norvegicus glutamate receptor, metabotropic 4 (Grm4), mRNA [NM_022666]	**0,622**
Cacnb1	Rattus norvegicus calcium channel, voltage-dependent, beta 1 subunit (Cacnb1), mRNA [NM_017346]	**0,622**
Papola	Rattus norvegicus poly (A) polymerase alpha (Papola), mRNA [NM_001108056]	**0,622**
Mrpl40	Rattus norvegicus mitochondrial ribosomal protein L40 (Mrpl40), nuclear gene encoding mitochondrial protein, mRNA [NM_001024865]	**0,622**

nod3l	Rattus norvegicus NOD3-like protein (nod3l), mRNA [NM_001024360]	**0,622**
0	Unknown	**0,622**
0	similar to RIKEN cDNA 1700001E04 (MGC116197), mRNA [Source:RefSeq DNA;Acc:NM_001025755] [ENSRNOT00000043269]	**0,622**
Itga1	Rattus norvegicus integrin, alpha 1 (Itga1), mRNA [NM_030994]	**0,622**
0	Unknown	**0,622**
Fam113a	Rattus norvegicus family with sequence similarity 113, member A (Fam113a), mRNA [NM_001012348]	**0,622**
Rassf3	Rattus norvegicus Ras association (RalGDS/AF-6) domain family member 3 (Rassf3), mRNA [NM_001108747]	**0,622**
Tshz3	Rattus norvegicus teashirt zinc finger homeobox 3 (Tshz3), mRNA [NM_001107506]	**0,622**
Rufy2	Rattus norvegicus RUN and FYVE domain containing 2 (Rufy2), mRNA [NM_001168586]	**0,622**
Lmbr1l	Rattus norvegicus limb region 1-like homolog (mouse) (Lmbr1l), mRNA [NM_001013950]	**0,622**
RGD1562218	Rattus norvegicus similar to RIKEN cDNA 0610039J04 (RGD1562218), mRNA [NM_001034919]	**0,622**
LOC686590	PREDICTED: Rattus norvegicus similar to IQ motif and Sec7 domain 1, transcript variant 2 (LOC686590), mRNA [XM_001073009]	**0,622**
0	Unknown	**0,622**
Tmem82	Putative uncharacterized protein RGD1566169_predictedUncharacterized protein [Source:UniProtKB/TrEMBL;Acc:D4A8K4] [ENSRNOT00000016176]	**0,622**
Chac2	Rattus norvegicus ChaC, cation transport regulator homolog 2 (E. coli) (Chac2), mRNA [NM_001025016]	**0,622**
Agpat1	Rattus norvegicus 1-acylglycerol-3-phosphate O-acyltransferase 1 (lysophosphatidic acid acyltransferase, alpha) (Agpat1), mRNA [NM_212458]	**0,622**
Med12	Rattus norvegicus mediator complex subunit 12 (Med12), mRNA [NM_001193292]	**0,622**
LOC100365064	PREDICTED: Rattus norvegicus zinc finger protein, multitype 1-like (LOC100365064), mRNA [XM_002725423]	**0,622**
Ppp1r13b	Rattus norvegicus protein phosphatase 1, regulatory (inhibitor) subunit 13B (Ppp1r13b), mRNA [NM_001108062]	**0,622**
Atp6ap1	Rattus norvegicus ATPase, H+ transporting, lysosomal accessory protein 1 (Atp6ap1), mRNA [NM_031785]	**0,622**
Tmed4	Rattus norvegicus transmembrane emp24 protein transport domain containing 4 (Tmed4), mRNA [NM_001107238]	**0,622**
Scn2a1	Rattus norvegicus sodium channel, voltage-gated, type II, alpha 1 (Scn2a1), mRNA [NM_012647]	**0,622**
Suclg2	Rattus norvegicus succinate-CoA ligase, GDP-forming, beta subunit (Suclg2), nuclear gene encoding mitochondrial protein, mRNA [NM_001100750]	**0,622**
Tcf7l2	Rattus norvegicus transcription factor 7-like 2 (T-cell specific, HMG-box) (Tcf7l2), mRNA [NM_001191052]	**0,622**
0	Unknown	**0,622**
Taf9b	Rattus norvegicus TAF9B RNA polymerase II, TATA box binding protein (TBP)-associated factor (Taf9b), mRNA [NM_133615]	**0,622**
Glis2	Rattus norvegicus GLIS family zinc finger 2 (Glis2), mRNA [NM_001106978]	**0,622**
Jph1	Rattus norvegicus junctophilin 1 (Jph1), mRNA [NM_001106630]	**0,622**
Btbd2	PREDICTED: Rattus norvegicus BTB (POZ) domain containing 2 (Btbd2), mRNA [XM_576181]	**0,622**
0	Unknown	**0,622**
Akap5	Rattus norvegicus A kinase (PRKA) anchor protein 5 (Akap5), mRNA [NM_133515]	**0,622**
Ano10	Uncharacterized protein [Source:UniProtKB/TrEMBL;Acc:D3ZBU6] [ENSRNOT00000035371]	**0,623**

Arhgef2	Rattus norvegicus rho/rac guanine nucleotide exchange factor (GEF) 2 (Arhgef2), mRNA [NM_001012079]	**0,623**
Abcb1b	Rattus norvegicus ATP-binding cassette, subfamily B (MDR/TAP), member 1B (Abcb1b), mRNA [NM_012623]	**0,623**
Arpp21	Rattus norvegicus cAMP-regulated phosphoprotein 21 (Arpp21), transcript variant 1, mRNA [NM_001135046]	**0,623**
Bcat2	Rattus norvegicus branched chain aminotransferase 2, mitochondrial (Bcat2), nuclear gene encoding mitochondrial protein, mRNA [NM_022400]	**0,623**
Ednrb	Rattus norvegicus endothelin receptor type B (Ednrb), mRNA [NM_017333]	**0,623**
Wbp1	Rattus norvegicus WW domain binding protein 1 (Wbp1), mRNA [NM_001106600]	**0,623**
Cntnap4	Rattus norvegicus contactin associated protein-like 4 (Cntnap4), mRNA [NM_001107432]	**0,623**
Tmem55b	Rattus norvegicus transmembrane protein 55B (Tmem55b), mRNA [NM_001014233]	**0,623**
Cd44	Rattus norvegicus Cd44 molecule (Cd44), mRNA [NM_012924]	**0,623**
Cdh6	Rattus norvegicus cadherin 6 (Cdh6), mRNA [NM_012927]	**0,623**
Selt	Rattus norvegicus selenoprotein T (Selt), mRNA [NM_001014253]	**0,623**
0	Uncharacterized protein [Source:UniProtKB/TrEMBL;Acc:D4ABX1] [ENSRNOT00000032406]	**0,623**
0	Unknown	**0,623**
Stam	Rattus norvegicus signal transducing adaptor molecule (SH3 domain and ITAM motif) 1 (Stam), mRNA [NM_001109121]	**0,623**
Clcn5	Rattus norvegicus chloride channel 5 (Clcn5), mRNA [NM_017106]	**0,623**
Hdgfrp3	Rattus norvegicus hepatoma-derived growth factor, related protein 3 (Hdgfrp3), mRNA [NM_145785]	**0,623**
0	Unknown	**0,623**
Dok6	Rattus norvegicus docking protein 6 (Dok6), mRNA [NM_001191943]	**0,623**
Arf1	Rattus norvegicus ADP-ribosylation factor 1 (Arf1), mRNA [NM_022518]	**0,623**
Raver2	Rattus norvegicus ribonucleoprotein, PTB-binding 2 (Raver2), mRNA [NM_001191867]	**0,623**
Creg1	Rattus norvegicus cellular repressor of E1A-stimulated genes 1 (Creg1), mRNA [NM_001105966]	**0,623**
0	Unknown	**0,623**
Mysm1	Uncharacterized protein [Source:UniProtKB/TrEMBL;Acc:D4A7T9] [ENSRNOT00000039554]	**0,623**
Slc17a9	Rattus norvegicus solute carrier family 17, member 9 (Slc17a9), mRNA [NM_001108613]	**0,623**
Park2	Rattus norvegicus Parkinson disease (autosomal recessive, juvenile) 2, parkin (Park2), mRNA [NM_020093]	**0,623**
Snurf	Rattus norvegicus SNRPN upstream reading frame (Snurf), mRNA [NM_130738]	**0,623**
Kcnd2	Rattus norvegicus potassium voltage-gated channel, Shal-related subfamily, member 2 (Kcnd2), mRNA [NM_031730]	**0,623**
Tpi1	Rattus norvegicus triosephosphate isomerase 1 (Tpi1), mRNA [NM_022922]	**0,623**
Il34	Rattus norvegicus interleukin 34 (Il34), mRNA [NM_001025766]	**0,623**
Kif11	Rattus norvegicus kinesin family member 11 (Kif11), mRNA [NM_001169112]	**0,623**
Hspc159	Rattus norvegicus galectin-related protein (Hspc159), mRNA [NM_001134730]	**0,623**
Nfix	Rattus norvegicus nuclear factor I/X (CCAAT-binding transcription factor) (Nfix), mRNA [NM_030866]	**0,623**
Gorasp2	Rattus norvegicus golgi reassembly stacking protein 2 (Gorasp2), mRNA [NM_001007720]	**0,623**
Fnbp1l	Rattus norvegicus formin binding protein 1-like (Fnbp1l), mRNA [NM_001039609]	**0,623**

Kcng2	Rattus norvegicus potassium voltage-gated channel, subfamily G, member 2 (Kcng2), mRNA [NM_001107372]	**0,623**
Adipor2	Rattus norvegicus adiponectin receptor 2 (Adipor2), mRNA [NM_001037979]	**0,623**
0	Q7TS76_MOUSE (Q7TS76) Sip1 protein, partial (13%) [TC617524]	**0,623**
Nme3	Rattus norvegicus non-metastatic cells 3, protein expressed in (Nme3), mRNA [NM_053507]	**0,623**
Slc4a10	Rattus norvegicus solute carrier family 4, sodium bicarbonate transporter, member 10 (Slc4a10), mRNA [NM_178092]	**0,623**
Isg20	Rattus norvegicus interferon stimulated exonuclease gene 20 (Isg20), mRNA [NM_001008510]	**0,623**
Cntnap1	Rattus norvegicus contactin associated protein 1 (Cntnap1), mRNA [NM_032061]	**0,623**
Casp2	Rattus norvegicus caspase 2 (Casp2), mRNA [NM_022522]	**0,623**
0	PREDICTED: Rattus norvegicus WAP four-disulfide core domain 6A (Wfdc6a), mRNA [XM_001062561]	**0,623**
Fgf2	Rattus norvegicus fibroblast growth factor 2 (Fgf2), mRNA [NM_019305]	**0,623**
0	Unknown	**0,623**
Ephb2	Rattus norvegicus Eph receptor B2 (Ephb2), mRNA [NM_001127319]	**0,623**
Htr6	Rattus norvegicus 5-hydroxytryptamine (serotonin) receptor 6 (Htr6), mRNA [NM_024365]	**0,623**
Ccna2	Rattus norvegicus cyclin A2 (Ccna2), mRNA [NM_053702]	**0,623**
Suv39h1	Rattus norvegicus suppressor of variegation 3-9 homolog 1 (Drosophila) (Suv39h1), mRNA [NM_001106956]	**0,623**
Sos2	Rattus norvegicus son of sevenless homolog 2 (Drosophila) (Sos2), mRNA [NM_001135561]	**0,623**
Lgi3	Rattus norvegicus leucine-rich repeat LGI family, member 3 (Lgi3), mRNA [NM_001107277]	**0,623**
Vom2r56	Rattus norvegicus vomeronasal 2 receptor, 56 (Vom2r56), mRNA [NM_001099484]	**0,623**
Arrb2	Rattus norvegicus arrestin, beta 2 (Arrb2), mRNA [NM_012911]	**0,623**
Id4	Rattus norvegicus inhibitor of DNA binding 4 (Id4), mRNA [NM_175582]	**0,623**
Dnm2	Rattus norvegicus dynamin 2 (Dnm2), mRNA [NM_013199]	**0,624**
Pkp2	Rattus norvegicus plakophilin 2 (Pkp2), mRNA [NM_001100499]	**0,624**
Vps37c	Rattus norvegicus vacuolar protein sorting 37 homolog C (S. cerevisiae) (Vps37c), mRNA [NM_001107463]	**0,624**
0	Unknown	**0,624**
Fgr	Rattus norvegicus Gardner-Rasheed feline sarcoma viral (v-fgr) oncogene homolog (Fgr), mRNA [NM_024145]	**0,624**
Lnx2	Rattus norvegicus ligand of numb-protein X 2 (Lnx2), mRNA [NM_001108329]	**0,624**
Sntg1	Rattus norvegicus syntrophin, gamma 1 (Sntg1), mRNA [NM_001191981]	**0,624**
RGD1562890	Rattus norvegicus RGD1562890 (RGD1562890), non-coding RNA [NR_037704]	**0,624**
Tubb3	Rattus norvegicus tubulin, beta 3 (Tubb3), mRNA [NM_139254]	**0,624**
App	Rattus norvegicus amyloid beta (A4) precursor protein (App), mRNA [NM_019288]	**0,624**
Got2	Rattus norvegicus glutamic-oxaloacetic transaminase 2, mitochondrial (aspartate aminotransferase 2) (Got2), nuclear gene encoding mitochondrial protein, mRNA [NM_013177]	**0,624**
0	Unknown	**0,624**
Jam2	Rattus norvegicus junctional adhesion molecule 2 (Jam2), mRNA [NM_001034004]	**0,624**

Pfn2	Rattus norvegicus profilin 2 (Pfn2), mRNA [NM_030873]	**0,624**
Cyp2j3	Rattus norvegicus cytochrome P450, family 2, subfamily j, polypeptide 3 (Cyp2j3), mRNA [NM_175766]	**0,624**
Fam20b	Rattus norvegicus family with sequence similarity 20, member B (Fam20b), mRNA [NM_001107187]	**0,624**
Tril	Rattus norvegicus TLR4 interactor with leucine-rich repeats (Tril), mRNA [NM_001034010]	**0,624**
Elavl4	Rattus norvegicus ELAV (embryonic lethal, abnormal vision, Drosophila)-like 4 (Hu antigen D) (Elavl4), mRNA [NM_001077651]	**0,624**
Calml4	Rattus norvegicus calmodulin-like 4 (Calml4), mRNA [NM_001127575]	**0,624**
Mospd3	Rattus norvegicus motile sperm domain containing 3 (Mospd3), mRNA [NM_001025629]	**0,624**
LOC500475	Rattus norvegicus similar to hypothetical protein 4933430I17 (LOC500475), mRNA [NM_001024346]	**0,624**
RGD1309621	PREDICTED: Rattus norvegicus similar to hypothetical protein FLJ10652 (RGD1309621), mRNA [XM_001075984]	**0,624**
Tmem59	Rattus norvegicus transmembrane protein 59 (Tmem59), mRNA [NM_001139465]	**0,624**
Pde10a	Rattus norvegicus phosphodiesterase 10A (Pde10a), mRNA [NM_022236]	**0,624**
0	Unknown	**0,624**
Cdh11	Rattus norvegicus cadherin 11 (Cdh11), mRNA [NM_053392]	**0,624**
Pttg1ip	Rattus norvegicus pituitary tumor-transforming 1 interacting protein (Pttg1ip), mRNA [NM_001013238]	**0,624**
Gabrd	Rattus norvegicus gamma-aminobutyric acid (GABA) A receptor, delta (Gabrd), mRNA [NM_017289]	**0,624**
Serinc2	Rattus norvegicus serine incorporator 2 (Serinc2), mRNA [NM_001031656]	**0,624**
RGD1565496	Rattus norvegicus similar to Butyrate-induced transcript 1 (RGD1565496), mRNA [NM_001106831]	**0,624**
Ublcp1	Rattus norvegicus ubiquitin-like domain containing CTD phosphatase 1 (Ublcp1), mRNA [NM_001014117]	**0,624**
Srrm1	Rattus norvegicus serine/arginine repetitive matrix 1 (Srrm1), mRNA [NM_001107986]	**0,624**
Emx2	Rattus norvegicus empty spiracles homeobox 2 (Emx2), mRNA [NM_001109169]	**0,624**
LOC367084	PREDICTED: Rattus norvegicus similar to 60S ribosomal protein L29 (P23) (LOC367084), mRNA [XM_002729903]	**0,624**
Son	Rattus norvegicus Son DNA binding protein (Son), transcript variant 2, mRNA [NM_001170328]	**0,624**
Fam132a	Rattus norvegicus family with sequence similarity 132, member A (Fam132a), mRNA [NM_001108000]	**0,624**
Thumpd2	Rattus norvegicus THUMP domain containing 2 (Thumpd2), transcript variant 2, mRNA [NM_001012108]	**0,624**
Bre	Rattus norvegicus brain and reproductive organ-expressed protein (Bre), mRNA [NM_199270]	**0,624**
Prkcdbp	Rattus norvegicus protein kinase C, delta binding protein (Prkcdbp), mRNA [NM_134449]	**0,624**
LOC100364559	PREDICTED: Rattus norvegicus gene model 691, (NCBI)-like (LOC100364559), mRNA [XM_002726287]	**0,624**
Tacr1	Rattus norvegicus tachykinin receptor 1 (Tacr1), mRNA [NM_012667]	**0,624**
Pprc1	Rattus norvegicus peroxisome proliferator-activated receptor gamma, coactivator-related 1 (Pprc1), mRNA [NM_001106363]	**0,624**
Lrrc4	Rattus norvegicus leucine rich repeat containing 4 (Lrrc4), mRNA [NM_001037336]	**0,624**
RGD1306520	Rattus norvegicus similar to receptor-interacting factor 1 (RGD1306520), mRNA [NM_001127485]	**0,624**
Cxcl16	Rattus norvegicus chemokine (C-X-C motif) ligand 16 (Cxcl16), mRNA [NM_001017478]	**0,624**
Cacnb3	Rattus norvegicus calcium channel, voltage-dependent, beta 3 subunit (Cacnb3), mRNA [NM_012828]	**0,624**
Rarg	Rattus norvegicus retinoic acid receptor, gamma (Rarg), transcript variant 1, mRNA [NM_001135249]	**0,624**

Zfp748	Rattus norvegicus TL0AAA77YJ08 mRNA sequence. [FQ211879]	**0,624**
Nlrp1a	Rattus norvegicus NLR family, pyrin domain containing 1A (Nlrp1a), mRNA [NM_001145755]	**0,624**
Hmox2	Rattus norvegicus heme oxygenase (decycling) 2 (Hmox2), mRNA [NM_024387]	**0,624**
Rab11fip2	Rattus norvegicus RAB11 family interacting protein 2 (class I) (Rab11fip2), mRNA [NM_001107447]	**0,624**
Krt83	Rattus norvegicus keratin 83 (Krt83), mRNA [NM_001101675]	**0,624**
Dbn1	Rattus norvegicus drebrin 1 (Dbn1), mRNA [NM_031024]	**0,624**
Lgi1	Rattus norvegicus leucine-rich, glioma inactivated 1 (Lgi1), mRNA [NM_145769]	**0,624**
Dnajc10	Rattus norvegicus DnaJ (Hsp40) homolog, subfamily C, member 10 (Dnajc10), mRNA [NM_001106486]	**0,625**
RGD1559904	Rattus norvegicus similar to mKIAA1429 protein (RGD1559904), mRNA [NM_001107915]	**0,625**
Fcgr2a	Low affinity immunoglobulin gamma Fc region receptor III [Source:UniProtKB/Swiss-Prot;Acc:P27645] [ENSRNOT00000045427]	**0,625**
Xylb	Rattus norvegicus xylulokinase homolog (H. influenzae) (Xylb), mRNA [NM_001033704]	**0,625**
0	Unknown	**0,625**
LOC678880	PREDICTED: Rattus norvegicus similar to mammalian retrotransposon derived 8b (LOC678880), mRNA [XM_001053139]	**0,625**
Fam13c1	PREDICTED: Rattus norvegicus family with sequence similarity 13, member C1 (Fam13c1), mRNA [XM_001080212]	**0,625**
Ide	Rattus norvegicus insulin degrading enzyme (Ide), mRNA [NM_013159]	**0,625**
Cstf1	Rattus norvegicus cleavage stimulation factor, 3' pre-RNA, subunit 1 (Cstf1), mRNA [NM_001013161]	**0,625**
Slc24a5	Rattus norvegicus solute carrier family 24, member 5 (Slc24a5), mRNA [NM_001107769]	**0,625**
LOC686412	PREDICTED: Rattus norvegicus similar to sal-like 4 isoform a (LOC686412), mRNA [XM_001074001]	**0,625**
Hcrtr1	Rattus norvegicus hypocretin (orexin) receptor 1 (Hcrtr1), mRNA [NM_013064]	**0,625**
0	Uncharacterized protein [Source:UniProtKB/TrEMBL;Acc:D4A2D0] [ENSRNOT00000043578]	**0,625**
Mtus1	Rattus norvegicus mitochondrial tumor suppressor 1 (Mtus1), nuclear gene encoding mitochondrial protein, mRNA [NM_178093]	**0,625**
Impa2	Rattus norvegicus inositol (myo)-1(or 4)-monophosphatase 2 (Impa2), mRNA [NM_172224]	**0,625**
Prpf4	Rattus norvegicus PRP4 pre-mRNA processing factor 4 homolog (yeast) (Prpf4), mRNA [NM_001106659]	**0,625**
Mrps18a	Rattus norvegicus mitochondrial ribosomal protein S18A (Mrps18a), nuclear gene encoding mitochondrial protein, mRNA [NM_198756]	**0,625**
LOC288913	Rattus norvegicus similar to LEYDIG CELL TUMOR 10 KD PROTEIN (LOC288913), mRNA [NM_198728]	**0,625**
0	Rattus norvegicus similar to 60S RIBOSOMAL PROTEIN L29 (P23) (LOC364407), mRNA [XM_344423]	**0,625**
Rprd1b	Rattus norvegicus regulation of nuclear pre-mRNA domain containing 1B (Rprd1b), mRNA [NM_001098727]	**0,625**
Ghr	Rattus norvegicus growth hormone receptor (Ghr), mRNA [NM_017094]	**0,625**
Mink1	PREDICTED: Rattus norvegicus similar to Map4k6-pending protein (LOC303259), mRNA [XM_001079459]	**0,625**
0	Unknown	**0,625**
Ift122	PREDICTED: Rattus norvegicus intraflagellar transport 122 homolog (Chlamydomonas) (Ift122), mRNA [XM_001054604]	**0,625**
0	Unknown	**0,625**
Dlx5	Rattus norvegicus distal-less homeobox 5 (Dlx5), mRNA [NM_012943]	**0,625**
Rc3h2	Rattus norvegicus ring finger and CCCH-type zinc finger domains 2 (Rc3h2), mRNA [NM_001107839]	**0,625**

Fam65a	Protein FAM65A [Source:UniProtKB/Swiss-Prot;Acc:Q4FZU8] [ENSRNOT00000023710]	**0,625**
Rnf181	Rattus norvegicus ring finger protein 181 (Rnf181), mRNA [NM_001007647]	**0,625**
Lrp3	Rattus norvegicus low density lipoprotein receptor-related protein 3 (Lrp3), mRNA [NM_053541]	**0,625**
Taok3	Rattus norvegicus TAO kinase 3 (Taok3), mRNA [NM_001024254]	**0,625**
Invs	Rattus norvegicus inversin (Invs), mRNA [NM_001107932]	**0,625**
Samd4b	Rattus norvegicus sterile alpha motif domain containing 4B (Samd4b), mRNA [NM_001107498]	**0,625**
Ppp2r5e	Rattus norvegicus protein phosphatase 2, regulatory subunit B', epsilon isoform (Ppp2r5e), mRNA [NM_001106740]	**0,625**
LOC687381	PREDICTED: Rattus norvegicus similar to COX10 homolog, cytochrome c oxidase assembly protein, heme A: farnesyltransferase (LOC687381), mRNA [XM_001077717]	**0,625**
Arhgap1	Rattus norvegicus Rho GTPase activating protein 1 (Arhgap1), mRNA [NM_001107747]	**0,625**
Lphn3	Rattus norvegicus latrophilin 3 (Lphn3), mRNA [NM_130822]	**0,625**
0	Unknown	**0,625**
Il20	Rattus norvegicus interleukin 20 (Il20), mRNA [NM_001143881]	**0,625**
Papolg	Rattus norvegicus poly(A) polymerase gamma (Papolg), mRNA [NM_001107244]	**0,625**
Abca8a	PREDICTED: Rattus norvegicus ATP-binding cassette, sub-family A (ABC1), member 8a (Abca8a), mRNA [XM_221100]	**0,625**
Rhov	Rattus norvegicus ras homolog gene family, member V (Rhov), mRNA [NM_138542]	**0,625**
Agfg1	Rattus norvegicus ArfGAP with FG repeats 1 (Agfg1), mRNA [NM_001135596]	**0,625**
0	Rattus norvegicus similar to 60S RIBOSOMAL PROTEIN L29 (P23) (LOC291290), mRNA [XM_225531]	**0,626**
Mcm3	Rattus norvegicus minichromosome maintenance complex component 3 (Mcm3), mRNA [NM_001191805]	**0,626**
Slc29a3	Rattus norvegicus solute carrier family 29 (nucleoside transporters), member 3 (Slc29a3), mRNA [NM_181639]	**0,626**
Ppp6c	Rattus norvegicus protein phosphatase 6, catalytic subunit (Ppp6c), mRNA [NM_133589]	**0,626**
Fam134b	Rattus norvegicus family with sequence similarity 134, member B (Fam134b), mRNA [NM_001034912]	**0,626**
Cnot4	Rattus norvegicus CCR4-NOT transcription complex, subunit 4 (Cnot4), mRNA [NM_001037782]	**0,626**
0	Q3X9T6_METFL (Q3X9T6) CDP-diacylglycerol--serine O-phosphatidyltransferase , partial (6%) [TC628492]	**0,626**
Arhgap4	Rattus norvegicus Rho GTPase activating protein 4 (Arhgap4), mRNA [NM_144740]	**0,626**
Tor1aip1	Rattus norvegicus torsin A interacting protein 1 (Tor1aip1), mRNA [NM_145092]	**0,626**
Ptpdc1	Rattus norvegicus protein tyrosine phosphatase domain containing 1 (Ptpdc1), mRNA [NM_001106104]	**0,626**
Cdh13	Rattus norvegicus cadherin 13 (Cdh13), mRNA [NM_138889]	**0,626**
RGD1566001	Rattus norvegicus similar to DNA segment, Chr 4, Brigham & Womens Genetics 0951 expressed (RGD1566001), mRNA [NM_001109263]	**0,626**
Gdi1	Rattus norvegicus GDP dissociation inhibitor 1 (Gdi1), mRNA [NM_017088]	**0,626**
RGD1563516	PREDICTED: Rattus norvegicus similar to histone protein Hist2h3c1 (RGD1563516), miscRNA [XR_006811]	**0,626**
0	ADAMTS-like 5 (Predicted), isoform CRA_cUncharacterized protein [Source:UniProtKB/TrEMBL;Acc:D3ZG32] [ENSRNOT00000042159]	**0,626**
Dpf2	Rattus norvegicus D4, zinc and double PHD fingers family 2 (Dpf2), mRNA [NM_001108516]	**0,626**
Efha2	Rattus norvegicus EF hand domain family, member A2 (Efha2), mRNA [NM_001191892]	**0,626**

Naf1	Rattus norvegicus nuclear assembly factor 1 homolog (S. cerevisiae) (Naf1), mRNA [NM_001024772]	**0,626**
Scoc	Rattus norvegicus short coiled-coil protein (Scoc), mRNA [NM_001013235]	**0,626**
Cnnm2	Rattus norvegicus cyclin M2 (Cnnm2), mRNA [NM_001011942]	**0,626**
LOC100363283	PREDICTED: Rattus norvegicus UBX domain protein 2A (LOC100363283), mRNA [XM_002729616]	**0,626**
0	Q2ALT1_9BACI (Q2ALT1) Erythromycin esterase precursor, partial (5%) [TC641238]	**0,626**
Rabggta	Rattus norvegicus Rab geranylgeranyltransferase, alpha subunit (Rabggta), mRNA [NM_031654]	**0,626**
LOC680236	PREDICTED: Rattus norvegicus hypothetical protein LOC680236 (LOC680236), mRNA [XM_001056256]	**0,626**
Mll5	PREDICTED: Rattus norvegicus myeloid/lymphoid or mixed-lineage leukemia 5 (trithorax homolog, Drosophila) (Mll5), mRNA [XM_231287]	**0,626**
0	Unknown	**0,626**
Fam5b	Rattus norvegicus family with sequence similarity 5, member B (Fam5b), mRNA [NM_173115]	**0,626**
Clip2	Rattus norvegicus CAP-GLY domain containing linker protein 2 (Clip2), mRNA [NM_021997]	**0,626**
Ndp	Rattus norvegicus Norrie disease (pseudoglioma) (human) (Ndp), mRNA [NM_001108814]	**0,626**
Mylk	Rattus norvegicus myosin light chain kinase (Mylk), mRNA [NM_001105874]	**0,626**
RGD1562091	Rattus norvegicus similar to expressed sequence C79127 (RGD1562091), mRNA [NM_001106231]	**0,626**
Slitrk1	Rattus norvegicus SLIT and NTRK-like family, member 1 (Slitrk1), mRNA [NM_001107283]	**0,626**
Gabpb1l	Rattus norvegicus GA binding protein transcription factor, beta subunit 1-like (Gabpb1l), mRNA [NM_001135015]	**0,626**
Depdc5	Rattus norvegicus DEP domain containing 5 (Depdc5), mRNA [NM_001107229]	**0,626**
Mmp24	Rattus norvegicus matrix metallopeptidase 24 (Mmp24), mRNA [NM_031757]	**0,626**
Nr2f1	Rattus norvegicus nuclear receptor subfamily 2, group F, member 1 (Nr2f1), mRNA [NM_031130]	**0,626**
Cttn	Rattus norvegicus cortactin (Cttn), mRNA [NM_021868]	**0,626**
0	Unknown	**0,626**
Med8	Rattus norvegicus mediator complex subunit 8 (Med8), mRNA [NM_001108673]	**0,626**
0	Unknown	**0,626**
Epb41l1	Rattus norvegicus erythrocyte membrane protein band 4.1-like 1 (Epb41l1), transcript variant 2, mRNA [NM_021681]	**0,626**
Trim25	Rattus norvegicus tripartite motif-containing 25 (Trim25), mRNA [NM_001009536]	**0,626**
Ppt2	Rattus norvegicus palmitoyl-protein thioesterase 2 (Ppt2), mRNA [NM_019367]	**0,626**
0	Unknown	**0,626**
Syvn1	Rattus norvegicus synovial apoptosis inhibitor 1, synoviolin (Syvn1), mRNA [NM_001100739]	**0,626**
Gnl3l	Rattus norvegicus guanine nucleotide binding protein-like 3 (nucleolar)-like (Gnl3l), mRNA [NM_001081958]	**0,626**
Kifap3	Rattus norvegicus kinesin-associated protein 3 (Kifap3), mRNA [NM_001105964]	**0,626**
Luzp1	Rattus norvegicus leucine zipper protein 1 (Luzp1), mRNA [NM_030830]	**0,626**
Mgat5b	Rattus norvegicus mannosyl (alpha-1,6-)-glycoprotein beta-1,6-N-acetyl-glucosaminyltransferase, isozyme B (Mgat5b), mRNA [NM_001107068]	**0,626**
Dclk1	Rattus norvegicus doublecortin-like kinase 1 (Dclk1), transcript variant 1, mRNA [NM_053343]	**0,626**
Sphk2	Rattus norvegicus sphingosine kinase 2 (Sphk2), mRNA [NM_001012066]	**0,626**

RGD735065	Rattus norvegicus similar to GI:13385412-like protein splice form I (RGD735065), mRNA [NM_199379]	**0,626**
0	PREDICTED: Rattus norvegicus similar to 60S ribosomal protein L29 (P23) (LOC690662), mRNA [XM_001075132]	**0,626**
Fat4	Rattus norvegicus FAT tumor suppressor homolog 4 (Drosophila) (Fat4), mRNA [NM_001191705]	**0,626**
0	Unknown	**0,626**
Nepn	Rattus norvegicus nephrocan (Nepn), mRNA [NM_001107632]	**0,626**
0	Rattus norvegicus similar to hypothetical protein (LOC301869), mRNA [XM_229350]	**0,626**
LOC498601	PREDICTED: Rattus norvegicus similar to cyclin B2 (LOC498601), miscRNA [XR_006448]	**0,627**
Pdpn	Rattus norvegicus podoplanin (Pdpn), mRNA [NM_019358]	**0,627**
Ccdc97	Rattus norvegicus coiled-coil domain containing 97 (Ccdc97), mRNA [NM_001106235]	**0,627**
Pcbp1	Uncharacterized protein [Source:UniProtKB/TrEMBL;Acc:D3ZS68] [ENSRNOT00000023806]	**0,627**
Mbd1	Rattus norvegicus methyl-CpG binding domain protein 1 (Mbd1), mRNA [NM_001011924]	**0,627**
Rab8a	Rattus norvegicus RAB8A, member RAS oncogene family (Rab8a), mRNA [NM_053998]	**0,627**
Zfp143	Rattus norvegicus zinc finger protein 143 (Zfp143), mRNA [NM_001012169]	**0,627**
Znf641	Rattus norvegicus zinc finger protein 641 (Znf641), mRNA [NM_001106792]	**0,627**
LOC317456	Rattus norvegicus hypothetical LOC317456 (LOC317456), mRNA [NM_001047894]	**0,627**
Rxfp1	Rattus norvegicus relaxin/insulin-like family peptide receptor 1 (Rxfp1), mRNA [NM_201417]	**0,627**
Mast3	Rattus norvegicus microtubule associated serine/threonine kinase 3 (Mast3), mRNA [NM_001134796]	**0,627**
RGD1307554	Rattus norvegicus similar to CG16812-PA (RGD1307554), mRNA [NM_001109664]	**0,627**
Mthfs	Rattus norvegicus 5,10-methenyltetrahydrofolate synthetase (5-formyltetrahydrofolate cyclo-ligase) (Mthfs), mRNA [NM_001009349]	**0,627**
Snx24	Rattus norvegicus sorting nexin 24 (Snx24), mRNA [NM_001008364]	**0,627**
Faah	Rattus norvegicus fatty acid amide hydrolase (Faah), mRNA [NM_024132]	**0,627**
Arhgap8	Rattus norvegicus Rho GTPase activating protein 8 (Arhgap8), mRNA [NM_001004242]	**0,627**
Efnb3	Rattus norvegicus ephrin B3 (Efnb3), mRNA [NM_001100980]	**0,627**
Meaf6	Rattus norvegicus MYST/Esa1-associated factor 6 (Meaf6), mRNA [NM_001113784]	**0,627**
Stat2	Rattus norvegicus signal transducer and activator of transcription 2 (Stat2), mRNA [NM_001011905]	**0,627**
0	RVL6519 Wackym-Soares normalized rat vestibular cDNA library Rattus norvegicus cDNA 5', mRNA sequence [DV716357]	**0,627**
Slc9a1	Rattus norvegicus solute carrier family 9 (sodium/hydrogen exchanger), member 1 (Slc9a1), mRNA [NM_012652]	**0,627**
Cyp2d4	Cytochrome P450 2D18 [Source:UniProtKB/Swiss-Prot;Acc:Q64680] [ENSRNOT00000011880]	**0,627**
Sulf2	Rattus norvegicus sulfatase 2 (Sulf2), mRNA [NM_001034927]	**0,627**
Zbtb37	Rattus norvegicus zinc finger and BTB domain containing 37 (Zbtb37), mRNA [NM_001107191]	**0,627**
0	Rattus norvegicus similar to glutamine repeat protein 1 (LOC365129), mRNA [XM_344830]	**0,627**
Atp6v1f	Rattus norvegicus ATPase, H transporting, lysosomal V1 subunit F (Atp6v1f), mRNA [NM_053884]	**0,627**
Tmem138	Rattus norvegicus transmembrane protein 138 (Tmem138), mRNA [NM_198777]	**0,627**
St13	Rattus norvegicus suppression of tumorigenicity 13 (St13), mRNA [NM_031122]	**0,627**

Zc3h14	Rattus norvegicus zinc finger CCCH type containing 14 (Zc3h14), transcript variant 1, mRNA [NM_001033951]	**0,627**
Tp53i11	Rattus norvegicus tumor protein p53 inducible protein 11 (Tp53i11), mRNA [NM_001107749]	**0,627**
Hapln4	Rattus norvegicus hyaluronan and proteoglycan link protein 4 (Hapln4), mRNA [NM_001108398]	**0,627**
Ncoa5	Rattus norvegicus nuclear receptor coactivator 5 (Ncoa5), mRNA [NM_001106543]	**0,627**
Ttc7b	Rattus norvegicus tetratricopeptide repeat domain 7B (Ttc7b), mRNA [NM_001108719]	**0,627**
Mobkl1b	Rattus norvegicus MOB1, Mps One Binder kinase activator-like 1B (yeast) (Mobkl1b), mRNA [NM_001033891]	**0,627**
RGD1307443	Rattus norvegicus similar to mKIAA0319 protein (RGD1307443), mRNA [NM_001197023]	**0,627**
Zfp91	Rattus norvegicus zinc finger protein 91 (Zfp91), mRNA [NM_001169120]	**0,627**
RGD1563547	Rattus norvegicus RGD1563547 (RGD1563547), mRNA [NM_001108266]	**0,627**
Amigo2	Rattus norvegicus adhesion molecule with Ig like domain 2 (Amigo2), mRNA [NM_182816]	**0,627**
0	Unknown	**0,627**
Fam125a	Rattus norvegicus family with sequence similarity 125, member A (Fam125a), mRNA [NM_199375]	**0,627**
Dscam	Rattus norvegicus Down syndrome cell adhesion molecule (Dscam), mRNA [NM_133587]	**0,627**
Rgs17	Rattus norvegicus regulator of G-protein signaling 17 (Rgs17), mRNA [NM_001107459]	**0,627**
Toag1	Rattus norvegicus tolerance-associated gene 1 (Toag1), mRNA [NM_001110838]	**0,627**
Tmem167b	Rattus norvegicus transmembrane protein 167B (Tmem167b), mRNA [NM_001135260]	**0,628**
0	Uncharacterized protein [Source:UniProtKB/TrEMBL;Acc:D4A2W5] [ENSRNOT00000065969]	**0,628**
RGD1310185	Uncharacterized protein [Source:UniProtKB/TrEMBL;Acc:D3ZCD2] [ENSRNOT00000032301]	**0,628**
39142	Rattus norvegicus membrane-associated ring finger (C3HC4) 7 (March7), mRNA [NM_001012087]	**0,628**
RGD1304952	Rattus norvegicus similar to RIKEN cDNA C530028O21 gene (RGD1304952), mRNA [NM_001014059]	**0,628**
Eif4g3	Rattus norvegicus eukaryotic translation initiation factor 4 gamma, 3 (Eif4g3), mRNA [NM_001106693]	**0,628**
Coro2b	Coro2b protein [Source:UniProtKB/TrEMBL;Acc:Q5EB67] [ENSRNOT00000020951]	**0,628**
0	Uncharacterized protein [Source:UniProtKB/TrEMBL;Acc:D4ADU3] [ENSRNOT00000014310]	**0,628**
Hmg20a	Rattus norvegicus high mobility group 20A (Hmg20a), mRNA [NM_001108150]	**0,628**
Slmo1	Rattus norvegicus slowmo homolog 1 (Drosophila) (Slmo1), mRNA [NM_001109570]	**0,628**
Cpsf7	Rattus norvegicus cleavage and polyadenylation specific factor 7 (Cpsf7), mRNA [NM_001014245]	**0,628**
LOC679462	Rattus norvegicus similar to Tetraspanin-15 (Tspan-15) (Transmembrane 4 superfamily member 15) (Tetraspan NET-7) (LOC679462), mRNA [NM_001115032]	**0,628**
Wdfy3	Rattus norvegicus WD repeat and FYVE domain containing 3 (Wdfy3), mRNA [NM_001170551]	**0,628**
Nrip3	Rattus norvegicus nuclear receptor interacting protein 3 (Nrip3), mRNA [NM_001108498]	**0,628**
Nploc4	Rattus norvegicus nuclear protein localization 4 homolog (S. cerevisiae) (Nploc4), mRNA [NM_080577]	**0,628**
Tceal1	Rattus norvegicus transcription elongation factor A (SII)-like 1 (Tceal1), mRNA [NM_001009675]	**0,628**
MGC93975	Rattus norvegicus similar to 2310044H10Rik protein (MGC93975), mRNA [NM_001004221]	**0,628**
0	Unknown	**0,628**

0	Unknown	**0,628**
Neurod6	Rattus norvegicus neurogenic differentiation 6 (Neurod6), mRNA [NM_001109237]	**0,628**
Nelf	Rattus norvegicus nasal embryonic LHRH factor (Nelf), mRNA [NM_057190]	**0,628**
0	Unknown	**0,628**
0	Unknown	**0,628**
Col11a2	Rattus norvegicus collagen, type XI, alpha 2 (Col11a2), mRNA [NM_212528]	**0,628**
LOC311352	Rattus norvegicus similar to Adenosine deaminase CG11994-PA (LOC311352), mRNA [NM_001014047]	**0,628**
Sema5a	Rattus norvegicus sema domain, seven thrombospondin repeats (type 1 and type 1-like), transmembrane domain (TM) and short cytoplasmic domain, (semaphorin) 5A (Sema5a), mRNA [NM_001107659]	**0,628**
Zfp426l	Rattus norvegicus zinc finger protein 426-like (Zfp426l), mRNA [NM_001135018]	**0,628**
C5	PREDICTED: Rattus norvegicus complement component 5 (C5), mRNA [XM_345342]	**0,628**
0	Unknown	**0,628**
0	PREDICTED: Rattus norvegicus similar to dachshund b (LOC680770), mRNA [XM_001058786]	**0,628**
RGD1564114	Rattus norvegicus similar to FLJ46082 protein (RGD1564114), mRNA [NM_001127563]	**0,628**
Sbk1	Rattus norvegicus SH3-binding domain kinase 1 (Sbk1), mRNA [NM_147135]	**0,628**
Cep120	Rattus norvegicus centrosomal protein 120 (Cep120), mRNA [NM_001191697]	**0,628**
Gabarapl1	Rattus norvegicus GABA(A) receptor-associated protein like 1 (Gabarapl1), mRNA [NM_001044294]	**0,628**
Cdc42ep3	Rattus norvegicus CDC42 effector protein (Rho GTPase binding) 3 (Cdc42ep3), mRNA [NM_001048044]	**0,628**
Atp2b3	Rattus norvegicus ATPase, Ca++ transporting, plasma membrane 3 (Atp2b3), mRNA [NM_133288]	**0,628**
Serpina5	Rattus norvegicus serine (or cysteine) peptidase inhibitor, clade A, member 5 (Serpina5), mRNA [NM_022957]	**0,628**
0	telomerase-binding protein EST1A [Source:RefSeq peptide;Acc:NP_001099278] [ENSRNOT00000052412]	**0,628**
Col27a1	Rattus norvegicus collagen, type XXVII, alpha 1 (Col27a1), mRNA [NM_198747]	**0,628**
Zdhhc13	Rattus norvegicus zinc finger, DHHC-type containing 13 (Zdhhc13), mRNA [NM_001039037]	**0,628**
Fam100b	PREDICTED: Rattus norvegicus family with sequence similarity 100, member B (Fam100b), mRNA [XM_213532]	**0,628**
Lzic	Rattus norvegicus leucine zipper and CTNNBIP1 domain containing (Lzic), mRNA [NM_001013241]	**0,628**
Zfp141	PREDICTED: Rattus norvegicus similar to zinc finger protein 420 (LOC690343), mRNA [XM_001074160]	**0,628**
Map7	Rattus norvegicus microtubule-associated protein 7 (Map7), transcript variant 2, mRNA [NM_001198638]	**0,628**
Tom1	Rattus norvegicus target of myb1 homolog (chicken) (Tom1), mRNA [NM_001008365]	**0,628**
Usp32	Rattus norvegicus ubiquitin specific peptidase 32 (Usp32), mRNA [NM_001107032]	**0,628**
LOC684139	PREDICTED: Rattus norvegicus similar to zinc finger protein 582 (LOC684139), mRNA [XM_001069100]	**0,628**
Nkain1	PREDICTED: Rattus norvegicus similar to RIKEN cDNA 2610200G18 (RGD1561205), mRNA [XM_575920]	**0,628**
RGD1560383	RCG34143Uncharacterized protein [Source:UniProtKB/TrEMBL;Acc:D4AC62] [ENSRNOT00000014414]	**0,628**
0	Uncharacterized protein [Source:UniProtKB/TrEMBL;Acc:D3ZW60] [ENSRNOT00000005861]	**0,628**
Pcdhb2	Rattus norvegicus protocadherin beta 2 (Pcdhb2), mRNA [NM_001109123]	**0,628**

Api5	Rattus norvegicus apoptosis inhibitor 5 (Api5), mRNA [NM_001127379]	**0,628**
Epb41l3	Rattus norvegicus erythrocyte membrane protein band 4.1-like 3 (Epb41l3), mRNA [NM_053927]	**0,629**
Gosr2	Rattus norvegicus golgi SNAP receptor complex member 2 (Gosr2), mRNA [NM_031685]	**0,629**
Slc7a14	Rattus norvegicus solute carrier family 7 (cationic amino acid transporter, y+ system), member 14 (Slc7a14), mRNA [NM_001134615]	**0,629**
Clip1	Rattus norvegicus CAP-GLY domain containing linker protein 1 (Clip1), mRNA [NM_031745]	**0,629**
0	Unknown	**0,629**
RGD1310081	RGD1310081 protein [Source:UniProtKB/TrEMBL;Acc:Q5XHZ1] [ENSRNOT00000020170]	**0,629**
Shbg	Rattus norvegicus sex hormone binding globulin (Shbg), mRNA [NM_012650]	**0,629**
Zmynd11	Rattus norvegicus zinc finger, MYND domain containing 11 (Zmynd11), transcript variant 2, mRNA [NM_203367]	**0,629**
Qk	Rattus norvegicus quaking (Qk), mRNA [NM_001115021]	**0,629**
0	Unknown	**0,629**
0	Unknown	**0,629**
Atpbd4	Rattus norvegicus ATP binding domain 4 (Atpbd4), mRNA [NM_001014181]	**0,629**
0	Unknown	**0,629**
Kdm5b	Rattus norvegicus lysine (K)-specific demethylase 5B (Kdm5b), mRNA [NM_001107177]	**0,629**
Abhd4	Rattus norvegicus abhydrolase domain containing 4 (Abhd4), mRNA [NM_001108866]	**0,629**
Ptar1	Rattus norvegicus protein prenyltransferase alpha subunit repeat containing 1 (Ptar1), mRNA [NM_001105760]	**0,629**
Lsm11	Rattus norvegicus LSM11, U7 small nuclear RNA associated (Lsm11), mRNA [NM_001173384]	**0,629**
RGD1310429	Rattus norvegicus similar to Protein Njmu-R1 (RGD1310429), mRNA [NM_001139506]	**0,629**
0	EST352678 Rat gene index, normalized rat, norvegicus, Bento Soares Rattus norvegicus cDNA clone RGIHT15 5' end, mRNA sequence [AW921374]	**0,629**
S100a10	Rattus norvegicus S100 calcium binding protein A10 (S100a10), mRNA [NM_031114]	**0,629**
Slc6a8	Rattus norvegicus solute carrier family 6 (neurotransmitter transporter, creatine), member 8 (Slc6a8), mRNA [NM_017348]	**0,629**
Grik4	Rattus norvegicus glutamate receptor, ionotropic, kainate 4 (Grik4), mRNA [NM_012572]	**0,629**
Gnas	Rattus norvegicus GNAS complex locus (Gnas), transcript variant 3, mRNA [NM_019132]	**0,629**
Apool	Rattus norvegicus apolipoprotein O-like (Apool), mRNA [NM_001014105]	**0,629**
Cdo1	Rattus norvegicus cysteine dioxygenase, type I (Cdo1), mRNA [NM_052809]	**0,629**
RGD1311084	Rattus norvegicus similar to 1700113K14Rik protein (RGD1311084), mRNA [NM_001100979]	**0,629**
Gnptab	Uncharacterized protein [Source:UniProtKB/TrEMBL;Acc:D3ZJS1] [ENSRNOT00000006946]	**0,629**
LOC680227	Rattus norvegicus LRRGT00193 (LOC680227), mRNA [NM_001047974]	**0,629**
Fxr1	Rattus norvegicus fragile X mental retardation, autosomal homolog 1 (Fxr1), mRNA [NM_001012179]	**0,629**
Ankrd13b	PREDICTED: Rattus norvegicus similar to novel protein (RGD1564005), mRNA [XM_001080794]	**0,629**
0	Rattus norvegicus similar to 60S ribosomal protein L23a (LOC365309), mRNA [XM_344922]	**0,629**
Tmem86a	Rattus norvegicus transmembrane protein 86A (Tmem86a), mRNA [NM_001135016]	**0,629**

Scara5	Rattus norvegicus scavenger receptor class A, member 5 (putative) (Scara5), mRNA [NM_001135855]	**0,629**
0	RNA binding motif, single stranded interacting protein Gene [Source:MGI Symbol;Acc:MGI:2444477] [ENSRNOT00000039904]	**0,629**
Rabif	Rattus norvegicus RAB interacting factor (Rabif), mRNA [NM_001007678]	**0,629**
LOC100361585	Uncharacterized protein [Source:UniProtKB/TrEMBL;Acc:D3ZA11] [ENSRNOT00000022866]	**0,629**
Car2	Rattus norvegicus carbonic anhydrase II (Car2), mRNA [NM_019291]	**0,629**
Eif2ak1	Rattus norvegicus eukaryotic translation initiation factor 2 alpha kinase 1 (Eif2ak1), mRNA [NM_013223]	**0,629**
Glipr2	PREDICTED: Rattus norvegicus GLI pathogenesis-related 2 (Glipr2), mRNA [XM_001054584]	**0,629**
LOC683538	Uncharacterized protein [Source:UniProtKB/TrEMBL;Acc:D3ZNK1] [ENSRNOT00000061761]	**0,629**
Suv420h1	Rattus norvegicus suppressor of variegation 4-20 homolog 1 (Drosophila) (Suv420h1), mRNA [NM_001108512]	**0,629**
Gfap	Rattus norvegicus glial fibrillary acidic protein (Gfap), mRNA [NM_017009]	**0,629**
Cul2	Rattus norvegicus cullin 2 (Cul2), mRNA [NM_001108417]	**0,629**
Nipsnap3b	Rattus norvegicus nipsnap homolog 3B (C. elegans) (Nipsnap3b), mRNA [NM_001009422]	**0,629**
Ccdc153	Rattus norvegicus coiled-coil domain containing 153 (Ccdc153), mRNA [NM_001013953]	**0,629**
Susd4	Rattus norvegicus sushi domain containing 4 (Susd4), mRNA [NM_001105982]	**0,630**
Tdpoz1	PREDICTED: Rattus norvegicus TD and POZ domain containing 1 (Tdpoz1), mRNA [XM_345239]	**0,630**
Slc6a15	Rattus norvegicus solute carrier family 6 (neutral amino acid transporter), member 15 (Slc6a15), mRNA [NM_172321]	**0,630**
Il17d	PREDICTED: Rattus norvegicus interleukin 17D (Il17d), mRNA [XM_001079675]	**0,630**
Fastkd1	Rattus norvegicus FAST kinase domains 1 (Fastkd1), mRNA [NM_001191738]	**0,630**
Atp1a2	Rattus norvegicus ATPase, Na+/K+ transporting, alpha 2 polypeptide (Atp1a2), mRNA [NM_012505]	**0,630**
Etnk1	Rattus norvegicus ethanolamine kinase 1 (Etnk1), mRNA [NM_001107894]	**0,630**
Arl6	Rattus norvegicus ADP-ribosylation factor-like 6 (Arl6), mRNA [NM_001108842]	**0,630**
Tox4	Rattus norvegicus TOX high mobility group box family member 4 (Tox4), mRNA [NM_173324]	**0,630**
Fam188a	Rattus norvegicus family with sequence similarity 188, member A (Fam188a), mRNA [NM_001106122]	**0,630**
Bcl9	Rattus norvegicus B-cell CLL/lymphoma 9 (Bcl9), mRNA [NM_001107703]	**0,630**
0	Unknown	**0,630**
Lcat	Rattus norvegicus lecithin cholesterol acyltransferase (Lcat), mRNA [NM_017024]	**0,630**
Acot4	Rattus norvegicus acyl-CoA thioesterase 4 (Acot4), mRNA [NM_001109440]	**0,630**
Stag1	Rattus norvegicus stromal antigen 1 (Stag1), mRNA [NM_001108179]	**0,630**
Nr4a2	Rattus norvegicus nuclear receptor subfamily 4, group A, member 2 (Nr4a2), mRNA [NM_019328]	**0,630**
Usp43	Rattus norvegicus ubiquitin specific peptidase 43 (Usp43), mRNA [NM_001100968]	**0,630**
Fam117a	Rattus norvegicus family with sequence similarity 117, member A (Fam117a), mRNA [NM_001109039]	**0,630**
Rerg	PREDICTED: Rattus norvegicus RAS-like, estrogen-regulated, growth-inhibitor (Rerg), mRNA [XM_578417]	**0,630**
Zbtb1	Rattus norvegicus zinc finger and BTB domain containing 1 (Zbtb1), mRNA [NM_001004444]	**0,630**
Eid1	Rattus norvegicus EP300 interacting inhibitor of differentiation 1 (Eid1), mRNA [NM_001109205]	**0,630**

Itsn1	Rattus norvegicus intersectin 1 (SH3 domain protein) (Itsn1), transcript variant 2, mRNA [NM_019227]	**0,630**
Cnnm3	Rattus norvegicus cyclin M3 (Cnnm3), mRNA [NM_001106901]	**0,630**
Ppif	Rattus norvegicus peptidylprolyl isomerase F (Ppif), nuclear gene encoding mitochondrial protein, mRNA [NM_172243]	**0,630**
Rab33b	Rattus norvegicus RAB33B, member of RAS oncogene family (Rab33b), mRNA [NM_001108944]	**0,630**
Chchd5	Rattus norvegicus coiled-coil-helix-coiled-coil-helix domain containing 5 (Chchd5), mRNA [NM_001106509]	**0,630**
Ust	Rattus norvegicus uronyl-2-sulfotransferase (Ust), mRNA [NM_001108458]	**0,630**
Mpped1	Rattus norvegicus metallophosphoesterase domain containing 1 (Mpped1), mRNA [NM_001130569]	**0,630**
0	Rattus norvegicus similar to putative pheromone receptor (LOC302028), mRNA [XM_229568]	**0,630**
Ergic1	PREDICTED: Rattus norvegicus endoplasmic reticulum-golgi intermediate compartment (ERGIC) 1 (Ergic1), mRNA [XM_001066818]	**0,630**
Runx1	Rattus norvegicus runt-related transcription factor 1 (Runx1), mRNA [NM_017325]	**0,630**
RGD1310351	Rattus norvegicus similar to RIKEN cDNA 4732418C07 (RGD1310351), mRNA [NM_001106677]	**0,630**
Sfrs1	Rattus norvegicus splicing factor, arginine/serine-rich 1 (Sfrs1), mRNA [NM_001109552]	**0,630**
Abcg1	Rattus norvegicus ATP-binding cassette, subfamily G (WHITE), member 1 (Abcg1), mRNA [NM_053502]	**0,630**
Bmp2k	Uncharacterized protein [Source:UniProtKB/TrEMBL;Acc:D4A0Z6] [ENSRNOT00000060868]	**0,630**
Atp2b3	Rattus norvegicus ATPase, Ca++ transporting, plasma membrane 3 (Atp2b3), mRNA [NM_133288]	**0,630**
0	Uncharacterized protein [Source:UniProtKB/TrEMBL;Acc:D3ZDF2] [ENSRNOT00000042956]	**0,630**
Macrod1	Rattus norvegicus MACRO domain containing 1 (Macrod1), mRNA [NM_139337]	**0,630**
Rad52	Rattus norvegicus RAD52 homolog (S. cerevisiae) (Rad52), mRNA [NM_001106617]	**0,630**
Abl1	Rattus norvegicus c-abl oncogene 1, receptor tyrosine kinase (Abl1), mRNA [NM_001100850]	**0,630**
Fkrp	Rattus norvegicus fukutin related protein (Fkrp), mRNA [NM_001025678]	**0,630**
Wdr70	Rattus norvegicus WD repeat domain 70 (Wdr70), mRNA [NM_001013909]	**0,630**
Far1	Rattus norvegicus TL0ADA37YA18 mRNA sequence. [FQ221385]	**0,630**
Clasp2	Rattus norvegicus cytoplasmic linker associated protein 2 (Clasp2), mRNA [NM_053722]	**0,630**
0	PREDICTED: Rattus norvegicus similar to Tle6 protein (RGD1561530), miscRNA [XR_009018]	**0,630**
Caprin1	Rattus norvegicus cell cycle associated protein 1 (Caprin1), mRNA [NM_001012185]	**0,630**
Pctk1	Rattus norvegicus PCTAIRE protein kinase 1 (Pctk1), transcript variant 1, mRNA [NM_001004132]	**0,630**
0	Q7TQ74_RAT (Q7TQ74) Ac1573, partial (11%) [TC600249]	**0,631**
Xiap	Rattus norvegicus X-linked inhibitor of apoptosis (Xiap), mRNA [NM_022231]	**0,631**
Abi1	Rattus norvegicus abl-interactor 1 (Abi1), mRNA [NM_024397]	**0,631**
RGD1305823	Rattus norvegicus similar to RIKEN cDNA 0610037P05 (RGD1305823), mRNA [NM_001108261]	**0,631**
Dmrta2	Rattus norvegicus DMRT-like family A2 (Dmrta2), mRNA [NM_001107951]	**0,631**
Znf524	Rattus norvegicus zinc finger protein 524 (Znf524), mRNA [NM_001108905]	**0,631**
Pnma2	Rattus norvegicus paraneoplastic antigen MA2 (Pnma2), mRNA [NM_001107272]	**0,631**
Ppp1r11	Rattus norvegicus protein phosphatase 1, regulatory (inhibitor) subunit 11 (Ppp1r11), mRNA [NM_212542]	**0,631**

Xpo4	Rattus norvegicus exportin 4 (Xpo4), mRNA [NM_001106042]	**0,631**
Kdsr	Rattus norvegicus 3-ketodihydrosphingosine reductase (Kdsr), mRNA [NM_001108342]	**0,631**
0	Q4SB82_TETNG (Q4SB82) Chromosome undetermined SCAF14677, whole genome shotgun sequence, partial (5%) [TC590476]	**0,631**
0	Unknown	**0,631**
Sh3bp5	Rattus norvegicus SH3-domain binding protein 5 (BTK-associated) (Sh3bp5), mRNA [NM_054011]	**0,631**
Map1lc3a	Rattus norvegicus microtubule-associated protein 1 light chain 3 alpha (Map1lc3a), mRNA [NM_199500]	**0,631**
0	Rattus norvegicus similar to DNA methyltransferase 3A (LOC289651), mRNA [XM_223446]	**0,631**
Shisa7	Rattus norvegicus shisa homolog 7 (Xenopus laevis) (Shisa7), mRNA [NM_001145175]	**0,631**
RGD1561678	PREDICTED: Rattus norvegicus similar to Ten-m1 (RGD1561678), mRNA [XM_002730177]	**0,631**
Fam63b	PREDICTED: Rattus norvegicus family with sequence similarity 63, member B, transcript variant 1 (Fam63b), mRNA [XM_001054973]	**0,631**
Nlk	Rattus norvegicus nemo like kinase (Nlk), mRNA [NM_001191924]	**0,631**
U2af1l4	Rattus norvegicus U2 small nuclear RNA auxiliary factor 1-like 4 (U2af1l4), mRNA [NM_001008775]	**0,631**
Tubgcp3	Rattus norvegicus tubulin, gamma complex associated protein 3 (Tubgcp3), mRNA [NM_001107323]	**0,631**
Cdc42ep4	Rattus norvegicus CDC42 effector protein (Rho GTPase binding) 4 (Cdc42ep4), mRNA [NM_001107063]	**0,631**
Med15	Rattus norvegicus mediator complex subunit 15 (Med15), mRNA [NM_001108325]	**0,631**
Tbc1d19	Rattus norvegicus TBC1 domain family, member 19 (Tbc1d19), mRNA [NM_001106008]	**0,631**
Megf9	Rattus norvegicus multiple EGF-like-domains 9 (Megf9), mRNA [NM_001107940]	**0,631**
Stag2	Rattus norvegicus stromal antigen 2 (Stag2), mRNA [NM_001173507]	**0,631**
Smg6	Rattus norvegicus Smg-6 homolog, nonsense mediated mRNA decay factor (C. elegans) (Smg6), mRNA [NM_001105808]	**0,631**
Atp1b2	Rattus norvegicus ATPase, Na+/K+ transporting, beta 2 polypeptide (Atp1b2), mRNA [NM_012507]	**0,631**
Hist3h2bb	Rattus norvegicus histone cluster 3, H2bb (Hist3h2bb), mRNA [NM_001109641]	**0,631**
Ehmt1	Rattus norvegicus euchromatic histone-lysine N-methyltransferase 1 (Ehmt1), mRNA [NM_001108572]	**0,631**
Maoa	Rattus norvegicus monoamine oxidase A (Maoa), nuclear gene encoding mitochondrial protein, mRNA [NM_033653]	**0,631**
Gucy1a2	Rattus norvegicus guanylate cyclase 1, soluble, alpha 2 (Gucy1a2), mRNA [NM_023956]	**0,631**
Fam49b	Rattus norvegicus family with sequence similarity 49, member B (Fam49b), mRNA [NM_001126267]	**0,631**
0	AHR_RAT (P41738) Aryl hydrocarbon receptor precursor (Ah receptor) (AhR), complete [TC573854]	**0,631**
Slc24a2	Rattus norvegicus solute carrier family 24 (sodium/potassium/calcium exchanger), member 2 (Slc24a2), mRNA [NM_031743]	**0,631**
Fam161a	Rattus norvegicus family with sequence similarity 161, member A (Fam161a), mRNA [NM_001013876]	**0,631**
Zdhhc9	Rattus norvegicus zinc finger, DHHC-type containing 9 (Zdhhc9), mRNA [NM_001039016]	**0,631**
0	Uncharacterized protein [Source:UniProtKB/TrEMBL;Acc:D3ZGU0] [ENSRNOT00000041039]	**0,631**
Med1	Rattus norvegicus mediator complex subunit 1 (Med1), mRNA [NM_001134361]	**0,631**
Ercc1	Rattus norvegicus excision repair cross-complementing rodent repair deficiency, complementation group 1 (Ercc1), mRNA [NM_001106228]	**0,631**
Guca2a	Rattus norvegicus guanylate cyclase activator 2a (guanylin) (Guca2a), mRNA [NM_013118]	**0,631**
Fam13b1	Rattus norvegicus family with sequence similarity 13, member B1 (Fam13b1), mRNA [NM_001106158]	**0,632**

Slc12a6	Rattus norvegicus solute carrier family 12, member 6 (Slc12a6), mRNA [NM_001109630]	**0,632**
Senp7	Rattus norvegicus SUMO1/sentrin specific peptidase 7 (Senp7), mRNA [NM_001105888]	**0,632**
RGD1565972	Rattus norvegicus similar to hypothetical protein 4932411G14 (RGD1565972), mRNA [NM_001134606]	**0,632**
Ogdh	Rattus norvegicus TL0ADA19YE17 mRNA sequence. [FQ222842]	**0,632**
Ubox5	Rattus norvegicus U-box domain containing 5 (Ubox5), mRNA [NM_001033997]	**0,632**
Vof16	Rattus norvegicus ischemia related factor vof-16 (Vof16), non-coding RNA [NR_037614]	**0,632**
Atp11b	Atp11b protein [Source:UniProtKB/TrEMBL;Acc:Q5RJS7] [ENSRNOT00000016961]	**0,632**
Shc3	Rattus norvegicus SHC (Src homology 2 domain containing) transforming protein 3 (Shc3), mRNA [NM_001105743]	**0,632**
Cbll1	Rattus norvegicus Cas-Br-M (murine) ecotropic retroviral transforming sequence-like 1 (Cbll1), mRNA [NM_001108018]	**0,632**
Slc35b4	Rattus norvegicus solute carrier family 35, member B4 (Slc35b4), mRNA [NM_001106590]	**0,632**
Depdc6	PREDICTED: Rattus norvegicus DEP domain containing 6 (Depdc6), mRNA [XM_001066889]	**0,632**
Mlf2	Rattus norvegicus myeloid leukemia factor 2 (Mlf2), mRNA [NM_001107889]	**0,632**
Bche	Rattus norvegicus butyrylcholinesterase (Bche), mRNA [NM_022942]	**0,632**
Rpp30	Rattus norvegicus ribonuclease P/MRP 30 subunit (human) (Rpp30), mRNA [NM_001191083]	**0,632**
Kbtbd8	Rattus norvegicus kelch repeat and BTB (POZ) domain containing 8 (Kbtbd8), mRNA [NM_001109250]	**0,632**
Tbc1d9	Rattus norvegicus TBC1 domain family, member 9 (Tbc1d9), mRNA [NM_001134539]	**0,632**
Aup1	Rattus norvegicus ancient ubiquitous protein 1 (Aup1), mRNA [NM_001079899]	**0,632**
Slc25a27	Rattus norvegicus solute carrier family 25, member 27 (Slc25a27), nuclear gene encoding mitochondrial protein, mRNA [NM_053500]	**0,632**
Nat13	Rattus norvegicus N-acetyltransferase 13 (Nat13), mRNA [NM_001105881]	**0,632**
Fam176a	Rattus norvegicus family with sequence similarity 176, member A (Fam176a), mRNA [NM_001109243]	**0,632**
Fcer1g	Rattus norvegicus Fc fragment of IgE, high affinity I, receptor for; gamma polypeptide (Fcer1g), mRNA [NM_001131001]	**0,632**
Wdfy3	Rattus norvegicus WD repeat and FYVE domain containing 3 (Wdfy3), mRNA [NM_001170551]	**0,632**
0	Uncharacterized protein [Source:UniProtKB/TrEMBL;Acc:D3ZH31] [ENSRNOT00000010612]	**0,632**
Paqr4	Rattus norvegicus progestin and adipoQ receptor family member IV (Paqr4), mRNA [NM_001017377]	**0,632**
0	Q8R4C3_CRIGR (Q8R4C3) Csr1, partial (32%) [TC596558]	**0,632**
Pten	Rattus norvegicus phosphatase and tensin homolog (Pten), mRNA [NM_031606]	**0,632**
Gramd3	Rattus norvegicus GRAM domain containing 3 (Gramd3), mRNA [NM_001014011]	**0,632**
Zfand3	Rattus norvegicus zinc finger, AN1-type domain 3 (Zfand3), mRNA [NM_001012175]	**0,632**
0	PREDICTED: Rattus norvegicus similar to TDPOZ2 (RGD1562545), mRNA [XM_001061198]	**0,632**
0	Unknown	**0,632**
Rgs17	Rattus norvegicus regulator of G-protein signaling 17 (Rgs17), mRNA [NM_001107459]	**0,632**
Znf691	Rattus norvegicus zinc finger protein 691 (Znf691), mRNA [NM_001107968]	**0,632**
Myo1d	Rattus norvegicus myosin ID (Myo1d), mRNA [NM_012983]	**0,632**
0	Uncharacterized protein [Source:UniProtKB/TrEMBL;Acc:D3ZC05] [ENSRNOT00000046525]	**0,632**

0	HSU97670 eukaryotic translation initiation factor eIF3, p35 subunit {Homo sapiens} (exp=-1; wgp=0; cg=0), partial (26%) [TC586860]	**0,632**
Rnf165	Rattus norvegicus ring finger protein 165 (Rnf165), mRNA [NM_001164505]	**0,632**
Cux1	Homeobox protein cut-like 1 [Source:UniProtKB/Swiss-Prot;Acc:P53565] [ENSRNOT00000001928]	**0,632**
Rnf14	Rattus norvegicus ring finger protein 14 (Rnf14), mRNA [NM_001034995]	**0,632**
0	Unknown	**0,632**
Avil	Rattus norvegicus advillin (Avil), mRNA [NM_024401]	**0,632**
Dmtf1	Rattus norvegicus cyclin D binding myb-like transcription factor 1 (Dmtf1), mRNA [NM_053693]	**0,632**
Ptprj	Rattus norvegicus protein tyrosine phosphatase, receptor type, J (Ptprj), mRNA [NM_017269]	**0,632**
Fbxo21	Rattus norvegicus F-box protein 21 (Fbxo21), mRNA [NM_001108338]	**0,632**
Lphn2	Rattus norvegicus latrophilin 2 (Lphn2), transcript variant 1, mRNA [NM_134408]	**0,632**
Ankrd13c	Rattus norvegicus ankyrin repeat domain 13C (Ankrd13c), mRNA [NM_001191570]	**0,632**
Bcl7c	Rattus norvegicus B-cell CLL/lymphoma 7C (Bcl7c), mRNA [NM_001106298]	**0,633**
Cdh4	PREDICTED: Rattus norvegicus cadherin 4 (Cdh4), mRNA [XM_001061943]	**0,633**
Mrpl4	Rattus norvegicus mitochondrial ribosomal protein L4 (Mrpl4), nuclear gene encoding mitochondrial protein, mRNA [NM_001108754]	**0,633**
Pcyt1a	Rattus norvegicus phosphate cytidylyltransferase 1, choline, alpha (Pcyt1a), mRNA [NM_078622]	**0,633**
Gpd2	Rattus norvegicus glycerol-3-phosphate dehydrogenase 2, mitochondrial (Gpd2), nuclear gene encoding mitochondrial protein, mRNA [NM_012736]	**0,633**
Smap2	Rattus norvegicus small ArfGAP2 (Smap2), mRNA [NM_001100669]	**0,633**
Dio3	Rattus norvegicus deiodinase, iodothyronine, type III (Dio3), mRNA [NM_017210]	**0,633**
LOC301193	PREDICTED: Rattus norvegicus similar to Discs large homolog 5 (Placenta and prostate DLG) (Discs large protein P-dlg) (LOC301193), mRNA [XM_002727321]	**0,633**
LOC686076	PREDICTED: Rattus norvegicus similar to suppressor of initiator codon mutations, related sequence 1 (LOC686076), mRNA [XM_001066439]	**0,633**
0	Unknown	**0,633**
RGD1561472	PREDICTED: Rattus norvegicus similar to mKIAA2005 protein (RGD1561472), miscRNA [XR_008610]	**0,633**
Narg2	Rattus norvegicus NMDA receptor regulated 2 (Narg2), mRNA [NM_001191108]	**0,633**
0	Unknown	**0,633**
Cpsf2	Rattus norvegicus cleavage and polyadenylation specific factor 2 (Cpsf2), mRNA [NM_001106753]	**0,633**
Stk24	Rattus norvegicus TL0AEA11YD24 mRNA sequence. [FQ227921]	**0,633**
40057	Rattus norvegicus septin 9 (Sept9), transcript variant 2, mRNA [NM_176856]	**0,633**
Ror2	Rattus norvegicus receptor tyrosine kinase-like orphan receptor 2 (Ror2), mRNA [NM_001107339]	**0,633**
Pla2g2d	Rattus norvegicus phospholipase A2, group IID (Pla2g2d), mRNA [NM_001013428]	**0,633**
Hsd17b1	Rattus norvegicus hydroxysteroid (17-beta) dehydrogenase 1 (Hsd17b1), mRNA [NM_012851]	**0,633**
Phf17	Rattus norvegicus PHD finger protein 17 (Phf17), mRNA [NM_001107670]	**0,633**
Rbak	Rattus norvegicus RB-associated KRAB zinc finger (Rbak), mRNA [NM_001191664]	**0,633**

Gabpb1	Rattus norvegicus GA binding protein transcription factor, beta subunit 1 (Gabpb1), mRNA [NM_001039036]	**0,633**
Jund	Rattus norvegicus jun D proto-oncogene (Jund), mRNA [NM_138875]	**0,633**
0	Nuclear receptor corepressor 1 [Source:UniProtKB/Swiss-Prot;Acc:Q9WUB5] [ENSRNOT00000057679]	**0,633**
Zdhhc3	Rattus norvegicus zinc finger, DHHC-type containing 3 (Zdhhc3), mRNA [NM_001039014]	**0,633**
Nedd4l	Rattus norvegicus neural precursor cell expressed, developmentally down-regulated 4-like (Nedd4l), mRNA [NM_001008300]	**0,633**
0	Unknown	**0,633**
Mmp16	Rattus norvegicus matrix metallopeptidase 16 (Mmp16), mRNA [NM_080776]	**0,633**
Spag1	Rattus norvegicus sperm associated antigen 1 (Spag1), mRNA [NM_001012116]	**0,633**
Tmem130	Rattus norvegicus transmembrane protein 130 (Tmem130), mRNA [NM_001170399]	**0,633**
Tbccd1	Rattus norvegicus TBCC domain containing 1 (Tbccd1), mRNA [NM_001012016]	**0,633**
Mtch2	Rattus norvegicus mitochondrial carrier homolog 2 (C. elegans) (Mtch2), nuclear gene encoding mitochondrial protein, mRNA [NM_001106488]	**0,633**
Olig1	Rattus norvegicus oligodendrocyte transcription factor 1 (Olig1), mRNA [NM_021770]	**0,633**
Casp7	Rattus norvegicus caspase 7 (Casp7), mRNA [NM_022260]	**0,633**
Hs3st2	Rattus norvegicus heparan sulfate (glucosamine) 3-O-sulfotransferase 2 (Hs3st2), mRNA [NM_181370]	**0,633**
Ap1s2	Rattus norvegicus adaptor-related protein complex 1, sigma 2 subunit (Ap1s2), mRNA [NM_001127531]	**0,633**
Tmem9	Rattus norvegicus transmembrane protein 9 (Tmem9), mRNA [NM_001105953]	**0,634**
Sh3d19	Similar to SH3 domain protein D19 (Predicted)Uncharacterized protein [Source:UniProtKB/TrEMBL;Acc:D3Z8S0] [ENSRNOT00000015802]	**0,634**
Trappc5	Rattus norvegicus trafficking protein particle complex 5 (Trappc5), mRNA [NM_001108850]	**0,634**
Myoc	Rattus norvegicus myocilin (Myoc), mRNA [NM_030865]	**0,634**
Trpc3	Rattus norvegicus transient receptor potential cation channel, subfamily C, member 3 (Trpc3), mRNA [NM_021771]	**0,634**
0	Q448P6_SOLUS (Q448P6) Protein kinase precursor, partial (3%) [TC606896]	**0,634**
LOC501312	PREDICTED: Rattus norvegicus hypothetical gene supported by BC082068 (LOC501312), mRNA [XM_576725]	**0,634**
Tnfrsf21	Rattus norvegicus tumor necrosis factor receptor superfamily, member 21 (Tnfrsf21), mRNA [NM_001108207]	**0,634**
RGD1566010	PREDICTED: Rattus norvegicus RGD1566010 (RGD1566010), mRNA [XM_001067260]	**0,634**
Tmem185a	Rattus norvegicus transmembrane protein 185A (Tmem185a), mRNA [NM_001135712]	**0,634**
Kpna3	Rattus norvegicus karyopherin alpha 3 (Kpna3), mRNA [NM_001014792]	**0,634**
RGD1565170	Uncharacterized protein [Source:UniProtKB/TrEMBL;Acc:D3ZTX9] [ENSRNOT00000043836]	**0,634**
ST7	Rattus norvegicus suppression of tumorigenicity 7 (ST7), transcript variant 1, mRNA [NM_001004102]	**0,634**
Pttg1	Rattus norvegicus pituitary tumor-transforming 1 (Pttg1), mRNA [NM_022391]	**0,634**
Heatr5a	Uncharacterized protein [Source:UniProtKB/TrEMBL;Acc:D3ZKL2] [ENSRNOT00000035309]	**0,634**
0	Unknown	**0,634**
0	Putative bHLH transcription factor [Source:UniProtKB/TrEMBL;Acc:Q8VD56] [ENSRNOT00000018309]	**0,634**
Gria2	Rattus norvegicus glutamate receptor, ionotropic, AMPA 2 (Gria2), transcript variant 2, mRNA [NM_001083811]	**0,634**
Prrc1	Rattus norvegicus proline-rich coiled-coil 1 (Prrc1), mRNA [NM_001033887]	**0,634**

Rps6ka5	Rattus norvegicus ribosomal protein S6 kinase, polypeptide 5 (Rps6ka5), mRNA [NM_001108048]	**0,634**
Inpp4a	Rattus norvegicus inositol polyphosphate-4-phosphatase, type 1 (Inpp4a), mRNA [NM_031002]	**0,634**
Dazap1	Rattus norvegicus DAZ associated protein 1 (Dazap1), mRNA [NM_001025742]	**0,634**
LOC501375	PREDICTED: Rattus norvegicus similar to glutamate receptor, ionotropic, N-methyl D-aspartate-like 1A (LOC501375), mRNA [XM_576788]	**0,634**
Plag1	Rattus norvegicus pleiomorphic adenoma gene 1 (Plag1), mRNA [NM_001008316]	**0,634**
Arhgef7	Rattus norvegicus Rho guanine nucleotide exchange factor (GEF7) (Arhgef7), transcript variant 3, mRNA [NM_053740]	**0,634**
Ccnk	Rattus norvegicus cyclin K (Ccnk), mRNA [NM_001109672]	**0,634**
0	Q6UX82_HUMAN (Q6UX82) Phospholipase inhibitor, partial (16%) [TC595358]	**0,634**
0	Unknown	**0,634**
Inpp5b	Rattus norvegicus inositol polyphosphate-5-phosphatase B (Inpp5b), nuclear gene encoding mitochondrial protein, mRNA [NM_001100755]	**0,634**
Rhd	Rattus norvegicus Rh blood group, D antigen (Rhd), mRNA [NM_022505]	**0,634**
Dnajb4	Rattus norvegicus DnaJ (Hsp40) homolog, subfamily B, member 4 (Dnajb4), mRNA [NM_001013076]	**0,634**
Zmiz2	Rattus norvegicus zinc finger, MIZ-type containing 2 (Zmiz2), mRNA [NM_001100507]	**0,634**
Prex1	Rattus norvegicus phosphatidylinositol-3,4,5-trisphosphate-dependent Rac exchange factor 1 (Prex1), mRNA [NM_001135718]	**0,634**
Bicd1	Rattus norvegicus bicaudal D homolog 1 (Drosophila) (Bicd1), mRNA [NM_001108653]	**0,634**
Slc12a3	Rattus norvegicus solute carrier family 12 (sodium/chloride transporters), member 3 (Slc12a3), mRNA [NM_019345]	**0,634**
Foxj3	Rattus norvegicus forkhead box J3 (Foxj3), mRNA [NM_001107971]	**0,634**
Mis12	Rattus norvegicus MIS12, MIND kinetochore complex component, homolog (S. pombe) (Mis12), mRNA [NM_001047972]	**0,634**
Ppapdc1a	Rattus norvegicus phosphatidic acid phosphatase type 2 domain containing 1A (Ppapdc1a), mRNA [NM_001191631]	**0,634**
Atg9a	Rattus norvegicus ATG9 autophagy related 9 homolog A (S. cerevisiae) (Atg9a), mRNA [NM_001014218]	**0,634**
Myo6	PREDICTED: Rattus norvegicus myosin VI (Myo6), mRNA [XM_236444]	**0,635**
LOC680802	Rattus norvegicus TL0AAA71YE16 mRNA sequence. [FQ212058]	**0,635**
Ncor1	PREDICTED: Rattus norvegicus nuclear receptor co-repressor 1 (Ncor1), mRNA [XM_001077495]	**0,635**
0	Uncharacterized protein [Source:UniProtKB/TrEMBL;Acc:D3ZVS0] [ENSRNOT00000035275]	**0,635**
Eif4e3	Rattus norvegicus eukaryotic translation initiation factor 4E family member 3 (Eif4e3), mRNA [NM_001106612]	**0,635**
Lgals4	Rattus norvegicus lectin, galactoside-binding, soluble, 4 (Lgals4), mRNA [NM_012975]	**0,635**
Lsm12	Rattus norvegicus LSM12 homolog (S. cerevisiae) (Lsm12), mRNA [NM_001105843]	**0,635**
Add3	Rattus norvegicus adducin 3 (gamma) (Add3), transcript variant 1, mRNA [NM_001164103]	**0,635**
Rab6b	Rattus norvegicus RAB6B, member RAS oncogene family (Rab6b), mRNA [NM_001108775]	**0,635**
Pcdh21	Rattus norvegicus protocadherin 21 (Pcdh21), mRNA [NM_053572]	**0,635**
Cisd3	Rattus norvegicus CDGSH iron sulfur domain 3 (Cisd3), mRNA [NM_001105835]	**0,635**
Rbm38	Rattus norvegicus RNA binding motif protein 38 (Rbm38), mRNA [NM_001108965]	**0,635**
RGD1563049	Rattus norvegicus RGD1563049 (RGD1563049), mRNA [NM_001134521]	**0,635**
Cpne4	Rattus norvegicus copine IV (Cpne4), mRNA [NM_001109003]	**0,635**

Lrrc8b	leucine rich repeat containing 8 family, member B [Source:RefSeq peptide;Acc:NP_001100674] [ENSRNOT00000002897]	**0,635**
Dusp5	Rattus norvegicus dual specificity phosphatase 5 (Dusp5), mRNA [NM_133578]	**0,635**
0	Uncharacterized protein [Source:UniProtKB/TrEMBL;Acc:D4A553] [ENSRNOT00000016227]	**0,635**
RGD1565257	Rattus norvegicus similar to zinc finger protein 650 (RGD1565257), mRNA [NM_001134550]	**0,635**
0	Uncharacterized protein [Source:UniProtKB/TrEMBL;Acc:D3Z944] [ENSRNOT00000052233]	**0,635**
Sorl1	Rattus norvegicus sortilin-related receptor, LDLR class A repeats-containing (Sorl1), mRNA [NM_053519]	**0,635**
LOC100365546	PREDICTED: Rattus norvegicus rCG30673-like (LOC100365546), mRNA [XM_002726646]	**0,635**
RGD1559442	Rattus norvegicus similar to SET binding factor 2 (LOC691036), mRNA [NM_001134970]	**0,635**
0	Uncharacterized protein [Source:UniProtKB/TrEMBL;Acc:D3ZTL3] [ENSRNOT00000034097]	**0,635**
0	Uncharacterized protein [Source:UniProtKB/TrEMBL;Acc:D3ZDF4] [ENSRNOT00000024704]	**0,635**
Mgat5b	Rattus norvegicus mannosyl (alpha-1,6-)-glycoprotein beta-1,6-N-acetyl-glucosaminyltransferase, isozyme B (Mgat5b), mRNA [NM_001107068]	**0,635**
Negr1	Rattus norvegicus neuronal growth regulator 1 (Negr1), mRNA [NM_021682]	**0,635**
Apaf1	Rattus norvegicus apoptotic peptidase activating factor 1 (Apaf1), mRNA [NM_023979]	**0,635**
Map3k7	Rattus norvegicus mitogen activated protein kinase kinase kinase 7 (Map3k7), mRNA [NM_001107920]	**0,635**
Padi2	Rattus norvegicus peptidyl arginine deiminase, type II (Padi2), mRNA [NM_017226]	**0,635**
Tmem35	Rattus norvegicus transmembrane protein 35 (Tmem35), mRNA [NM_001001799]	**0,635**
LOC685179	PREDICTED: Rattus norvegicus similar to SWI/SNF-related matrix-associated actin-dependent regulator of chromatin c2, transcript variant 4 (LOC685179), mRNA [XM_001055795]	**0,635**
0	Rattus norvegicus similar to ribosomal protein S15 (LOC290226), mRNA [XM_224191]	**0,635**
Prkar2b	Rattus norvegicus protein kinase, cAMP dependent regulatory, type II beta (Prkar2b), mRNA [NM_001030020]	**0,635**
Traf3	Rattus norvegicus Tnf receptor-associated factor 3 (Traf3), mRNA [NM_001108724]	**0,635**
Scamp1	Rattus norvegicus secretory carrier membrane protein 1 (Scamp1), mRNA [NM_001100636]	**0,635**
Cd276	Rattus norvegicus Cd276 molecule (Cd276), mRNA [NM_182824]	**0,635**
Perld1	Rattus norvegicus per1-like domain containing 1 (Perld1), mRNA [NM_001143895]	**0,635**
Zbtb7a	Rattus norvegicus zinc finger and BTB domain containing 7a (Zbtb7a), mRNA [NM_054002]	**0,635**
0	Uncharacterized protein [Source:UniProtKB/TrEMBL;Acc:D3ZA88] [ENSRNOT00000055210]	**0,635**
Smarcc1	Rattus norvegicus SWI/SNF related, matrix associated, actin dependent regulator of chromatin, subfamily c, member 1 (Smarcc1), mRNA [NM_001106861]	**0,635**
Cln3	Rattus norvegicus ceroid-lipofuscinosis, neuronal 3 (Cln3), mRNA [NM_001006971]	**0,635**
Map2k6	Rattus norvegicus mitogen-activated protein kinase kinase 6 (Map2k6), mRNA [NM_053703]	**0,635**
Sap25	PREDICTED: Rattus norvegicus similar to FLJ00248 protein (RGD1562406), mRNA [XM_001076431]	**0,635**
Sema4b	Rattus norvegicus sema domain, immunoglobulin domain (Ig), transmembrane domain (TM) and short cytoplasmic domain, (semaphorin) 4B (Sema4b), mRNA [NM_001170462]	**0,635**
Tsc22d2	Rattus norvegicus TSC22 domain family, member 2 (Tsc22d2), mRNA [NM_001191960]	**0,635**

Mrpl52	Rattus norvegicus mitochondrial ribosomal protein L52 (Mrpl52), nuclear gene encoding mitochondrial protein, mRNA [NM_001108375]	**0,635**
Gtdc1	Rattus norvegicus glycosyltransferase-like domain containing 1 (Gtdc1), mRNA [NM_001024274]	**0,635**
Eaf1	Rattus norvegicus ELL associated factor 1 (Eaf1), mRNA [NM_001107293]	**0,635**
0	Rattus norvegicus TL0ADA49YG04 mRNA sequence. [FQ229104]	**0,635**
0	Uncharacterized protein [Source:UniProtKB/TrEMBL;Acc:D3ZF37] [ENSRNOT00000047805]	**0,635**
Dyrk2	Rattus norvegicus dual-specificity tyrosine-(Y)-phosphorylation regulated kinase 2 (Dyrk2), mRNA [NM_001108100]	**0,635**
Nt5c3	Rattus norvegicus 5'-nucleotidase, cytosolic III (Nt5c3), mRNA [NM_001107862]	**0,635**
Csgalnact1	Rattus norvegicus chondroitin sulfate N-acetylgalactosaminyltransferase 1 (Csgalnact1), mRNA [NM_001107309]	**0,635**
Papola	Rattus norvegicus poly (A) polymerase alpha (Papola), mRNA [NM_001108056]	**0,635**
Hnrnpul1	Rattus norvegicus heterogeneous nuclear ribonucleoprotein U-like 1 (Hnrnpul1), mRNA [NM_001108477]	**0,635**
C1galt1	Rattus norvegicus core 1 synthase, glycoprotein-N-acetylgalactosamine 3-beta-galactosyltransferase, 1 (C1galt1), mRNA [NM_022950]	**0,635**
0	Unknown	**0,635**
RGD1563015	Rattus norvegicus RGD1563015 (RGD1563015), mRNA [NM_001134627]	**0,636**
Evl	Rattus norvegicus Enah/Vasp-like (Evl), mRNA [NM_024147]	**0,636**
Me2	Rattus norvegicus malic enzyme 2, NAD(+)-dependent, mitochondrial (Me2), nuclear gene encoding mitochondrial protein, mRNA [NM_001107376]	**0,636**
Ntrk3	Rattus norvegicus neurotrophic tyrosine kinase, receptor, type 3 (Ntrk3), mRNA [NM_019248]	**0,636**
Senp8	Rattus norvegicus SUMO/sentrin specific peptidase family member 8 (Senp8), mRNA [NM_001012355]	**0,636**
0	Unknown	**0,636**
0	Unknown	**0,636**
Cttn	Rattus norvegicus cortactin (Cttn), mRNA [NM_021868]	**0,636**
Map4k5	Rattus norvegicus mitogen-activated protein kinase kinase kinase kinase 5 (Map4k5), mRNA [NM_001192016]	**0,636**
Adap1	Rattus norvegicus ArfGAP with dual PH domains 1 (Adap1), mRNA [NM_133567]	**0,636**
0	PCTK2_RAT (O35831) Serine/threonine-protein kinase PCTAIRE-2 (PCTAIRE-motif protein kinase 2) , complete [TC581304]	**0,636**
Wdr55	Rattus norvegicus WD repeat domain 55 (Wdr55), mRNA [NM_001017932]	**0,636**
Nr2c2	Rattus norvegicus nuclear receptor subfamily 2, group C, member 2 (Nr2c2), mRNA [NM_017323]	**0,636**
Nr3c2	Rattus norvegicus nuclear receptor subfamily 3, group C, member 2 (Nr3c2), mRNA [NM_013131]	**0,636**
Tysnd1	Rattus norvegicus trypsin domain containing 1 (Tysnd1), mRNA [NM_001108932]	**0,636**
Arid4a	Rattus norvegicus AT rich interactive domain 4A (Rbp1 like) (Arid4a), mRNA [NM_001108029]	**0,636**
Blvrb	Rattus norvegicus biliverdin reductase B (flavin reductase (NADPH)) (Blvrb), mRNA [NM_001106236]	**0,636**
Gng8	Rattus norvegicus guanine nucleotide binding protein (G protein), gamma 8 (Gng8), mRNA [NM_139185]	**0,636**
0	Unknown	**0,636**
Commd6	Rattus norvegicus COMM domain containing 6 (Commd6), mRNA [NM_001109105]	**0,636**
Ephb3	Rattus norvegicus Eph receptor B3 (Ephb3), mRNA [NM_001105868]	**0,636**

Spg20	Rattus norvegicus spastic paraplegia 20 (Troyer syndrome) homolog (human) (Spg20), mRNA [NM_001106433]	**0,636**
Frs2	Rattus norvegicus fibroblast growth factor receptor substrate 2 (Frs2), mRNA [NM_001108097]	**0,636**
RGD1303066	Rattus norvegicus similar to RIKEN cDNA 2610110G12 (RGD1303066), mRNA [NM_212498]	**0,636**
0	Neuronal PAS domain protein 3 (Predicted), isoform CRA_aUncharacterized protein [Source:UniProtKB/TrEMBL;Acc:D3ZZI2] [ENSRNOT00000067066]	**0,636**
0	Uncharacterized protein [Source:UniProtKB/TrEMBL;Acc:D4A1N7] [ENSRNOT00000056097]	**0,636**
Tmem179b	Rattus norvegicus transmembrane protein 179B (Tmem179b), mRNA [NM_001109572]	**0,636**
0	Unknown	**0,636**
Nr2c2	Rattus norvegicus nuclear receptor subfamily 2, group C, member 2 (Nr2c2), mRNA [NM_017323]	**0,636**
0	Unknown	**0,636**
Igsf11	Rattus norvegicus immunoglobulin superfamily, member 11 (Igsf11), mRNA [NM_001013120]	**0,636**
RGD1564177	PREDICTED: Rattus norvegicus RGD1564177 (RGD1564177), mRNA [XM_001054841]	**0,636**
0	Unknown	**0,636**
Lrrc16a	Rattus norvegicus leucine rich repeat containing 16A (Lrrc16a), mRNA [NM_001191692]	**0,636**
LOC689412	Rattus norvegicus similar to CG4025-PA (LOC689412), mRNA [NM_001109538]	**0,636**
Pabpn1	Rattus norvegicus poly(A) binding protein, nuclear 1 (Pabpn1), mRNA [NM_001135008]	**0,636**
Zfp426l	Rattus norvegicus zinc finger protein 426-like (Zfp426l), mRNA [NM_001135018]	**0,636**
LOC361914	Rattus norvegicus similar to solute carrier family 7 (cationic amino acid transporter, y+ system), member 12 (LOC361914), mRNA [NM_001017465]	**0,636**
0	BTB/POZ domain-containing protein KCTD1 [Source:UniProtKB/Swiss-Prot;Acc:Q8R4G8] [ENSRNOT00000061504]	**0,636**
Cpne9	Rattus norvegicus copine family member IX (Cpne9), mRNA [NM_001024982]	**0,636**
0	RVL8997 Wackym-Soares normalized rat vestibular cDNA library Rattus norvegicus cDNA 5', mRNA sequence [DV718414]	**0,636**
Pcdhb8	Rattus norvegicus protocadherin beta 8 (Pcdhb8), mRNA [NM_001014779]	**0,636**
0	Uncharacterized protein [Source:UniProtKB/TrEMBL;Acc:D3Z881] [ENSRNOT00000012600]	**0,636**
0	PREDICTED: Rattus norvegicus hypothetical gene supported by BC082068 (LOC501476), mRNA [XM_002730162]	**0,636**
Porf-2	Rattus norvegicus preoptic regulatory factor-2 (Porf-2), mRNA [NM_173122]	**0,636**
0	Rattus norvegicus TL0AEA10YI10 mRNA sequence. [FQ228098]	**0,637**
Pnldc1	Rattus norvegicus poly(A)-specific ribonuclease (PARN)-like domain containing 1 (Pnldc1), mRNA [NM_001025724]	**0,637**
Scrg1	Rattus norvegicus stimulator of chondrogenesis 1 (Scrg1), mRNA [NM_033499]	**0,637**
Ano4	Rattus norvegicus anoctamin 4 (Ano4), mRNA [NM_001106778]	**0,637**
Snrpa	Rattus norvegicus small nuclear ribonucleoprotein polypeptide A (Snrpa), mRNA [NM_001008303]	**0,637**
0	Unknown	**0,637**
Myt1	Rattus norvegicus myelin transcription factor 1 (Myt1), mRNA [NM_001108615]	**0,637**
Tmod4	Rattus norvegicus tropomodulin 4 (Tmod4), mRNA [NM_001106449]	**0,637**

Nat14	Rattus norvegicus N-acetyltransferase 14 (Nat14), mRNA [NM_001108466]	**0,637**
Ncoa2	Rattus norvegicus nuclear receptor coactivator 2 (Ncoa2), mRNA [NM_031822]	**0,637**
Samd9l	PREDICTED: Rattus norvegicus sterile alpha motif domain containing 9-like (Samd9l), mRNA [XM_001069386]	**0,637**
LOC683415	PREDICTED: Rattus norvegicus similar to adrenal mitochondrial protease (LOC683415), mRNA [XM_001065830]	**0,637**
Myh11	Rattus norvegicus myosin, heavy chain 11, smooth muscle (Myh11), mRNA [NM_001170600]	**0,637**
Tmem42	Rattus norvegicus transmembrane protein 42 (Tmem42), mRNA [NM_001191886]	**0,637**
0	Uncharacterized protein [Source:UniProtKB/TrEMBL;Acc:D4A4F9] [ENSRNOT00000057735]	**0,637**
Slc18a1	Rattus norvegicus solute carrier family 18 (vesicular monoamine), member 1 (Slc18a1), mRNA [NM_013152]	**0,637**
RGD1561843	Uncharacterized protein [Source:UniProtKB/TrEMBL;Acc:D3ZIJ2] [ENSRNOT00000045791]	**0,637**
Tnfrsf1a	Rattus norvegicus tumor necrosis factor receptor superfamily, member 1a (Tnfrsf1a), mRNA [NM_013091]	**0,637**
Ptpn9	Rattus norvegicus protein tyrosine phosphatase, non-receptor type 9 (Ptpn9), mRNA [NM_001013040]	**0,637**
Med19	Rattus norvegicus mediator complex subunit 19 (Med19), mRNA [NM_001107741]	**0,637**
0	PREDICTED: Rattus norvegicus ubiquitination factor E4B (Ube4b), mRNA [XM_233679]	**0,637**
0	Uncharacterized protein [Source:UniProtKB/TrEMBL;Acc:D3ZLJ4] [ENSRNOT00000041935]	**0,637**
Pole3	Rattus norvegicus polymerase (DNA directed), epsilon 3 (p17 subunit) (Pole3), mRNA [NM_001007652]	**0,637**
Mtmr2	Rattus norvegicus myotubularin related protein 2 (Mtmr2), mRNA [NM_001108123]	**0,637**
Ap3s1	Rattus norvegicus adaptor-related protein complex 3, sigma 1 subunit (Ap3s1), mRNA [NM_001106933]	**0,637**
LOC685792	PREDICTED: Rattus norvegicus similar to Discs large homolog 5 (Placenta and prostate DLG) (Discs large protein P-dlg) (LOC685792), mRNA [XM_217497]	**0,637**
Rimbp2	Rattus norvegicus RIM binding protein 2 (Rimbp2), mRNA [NM_001100488]	**0,637**
Lef1	Rattus norvegicus lymphoid enhancer binding factor 1 (Lef1), mRNA [NM_130429]	**0,637**
Crtap	cartilage-associated protein [Source:RefSeq peptide;Acc:NP_001102255] [ENSRNOT00000013519]	**0,637**
Lphn1	Rattus norvegicus latrophilin 1 (Lphn1), mRNA [NM_022962]	**0,637**
Mdc1	Rattus norvegicus mediator of DNA damage checkpoint 1 (Mdc1), mRNA [NM_001166275]	**0,637**
Kctd16	Rattus norvegicus potassium channel tetramerisation domain containing 16 (Kctd16), mRNA [NM_001172155]	**0,637**
Lrrc1	Rattus norvegicus leucine rich repeat containing 1 (Lrrc1), mRNA [NM_001014268]	**0,637**
Ube2d3	Rattus norvegicus ubiquitin-conjugating enzyme E2D 3 (UBC4/5 homolog, yeast) (Ube2d3), mRNA [NM_031237]	**0,637**
Diaph3	PREDICTED: Rattus norvegicus diaphanous homolog 3 (Drosophila) (Diaph3), mRNA [XM_001074393]	**0,637**
Slc6a1	Rattus norvegicus solute carrier family 6 (neurotransmitter transporter, GABA), member 1 (Slc6a1), mRNA [NM_024371]	**0,637**
Zfp317	Rattus norvegicus zinc finger protein 317 (Zfp317), mRNA [NM_001134634]	**0,637**
Cpne7	Rattus norvegicus copine VII (Cpne7), mRNA [NM_001108454]	**0,637**
Mbnl2	Rattus norvegicus muscleblind-like 2 (Mbnl2), mRNA [NM_001111064]	**0,638**
Prkra	Rattus norvegicus protein kinase, interferon inducible double stranded RNA dependent activator (Prkra), mRNA [NM_001024780]	**0,638**
LOC100364057	PREDICTED: Rattus norvegicus Btf3l4 protein-like, transcript variant 1 (LOC100364057), mRNA [XM_002726573]	**0,638**

Nkr-p1c	Rattus norvegicus killer cell lectin-like receptor subfamily B member (Nkr-p1c), mRNA [NM_001040189]	**0,638**
Tmem222	Rattus norvegicus transmembrane protein 222 (Tmem222), mRNA [NM_001113780]	**0,638**
Crtap	Rattus norvegicus cartilage associated protein (Crtap), mRNA [NM_001108785]	**0,638**
0	Unknown	**0,638**
Ets2	Rattus norvegicus v-ets erythroblastosis virus E26 oncogene homolog 2 (avian) (Ets2), mRNA [NM_001107107]	**0,638**
0	PREDICTED: Rattus norvegicus similar to KIAA1447 protein (RGD1306500), mRNA [XM_001081783]	**0,638**
Lhx6	Rattus norvegicus LIM homeobox 6 (Lhx6), mRNA [NM_001107837]	**0,638**
Lin9	PREDICTED: Rattus norvegicus lin-9 homolog (C. elegans) (Lin9), mRNA [XM_001067807]	**0,638**
0	Rattus norvegicus TL0AEA68YE13 mRNA sequence. [FQ231763]	**0,638**
Lrrc20	Rattus norvegicus leucine rich repeat containing 20 (Lrrc20), mRNA [NM_001109171]	**0,638**
Lonrf3	Rattus norvegicus LON peptidase N-terminal domain and ring finger 3 (Lonrf3), mRNA [NM_001191585]	**0,638**
Shpk	Rattus norvegicus sedoheptulokinase (Shpk), mRNA [NM_001033682]	**0,638**
Col4a1	Rattus norvegicus collagen, type IV, alpha 1 (Col4a1), mRNA [NM_001135009]	**0,638**
0	Rattus norvegicus similar to 60S RIBOSOMAL PROTEIN L29 (P23) (LOC290219), mRNA [XM_224186]	**0,638**
Fcrla	Rattus norvegicus Fc receptor-like A (Fcrla), mRNA [NM_001100682]	**0,638**
Klf3	Rattus norvegicus Kruppel-like factor 3 (basic) (Klf3), mRNA [NM_001105742]	**0,638**
0	Unknown	**0,638**
Nudt15	Rattus norvegicus nudix (nucleoside diphosphate linked moiety X)-type motif 15 (Nudt15), mRNA [NM_001106049]	**0,638**
0	Unknown	**0,638**
Prpf38b	Rattus norvegicus PRP38 pre-mRNA processing factor 38 (yeast) domain containing B (Prpf38b), mRNA [NM_001024305]	**0,638**
0	Q4RCF0_TETNG (Q4RCF0) Chromosome undetermined SCAF19066, whole genome shotgun sequence. (Fragment), partial (8%) [TC624062]	**0,638**
0	Rattus norvegicus chromosome 7, 38 clones, strain BN/SsNHsdMCW RNOR03304491, whole genome shotgun sequence [AABR03059028]	**0,638**
Gpr12	Rattus norvegicus G protein-coupled receptor 12 (Gpr12), transcript variant 2, mRNA [NM_030831]	**0,638**
Zfp426	Rattus norvegicus zinc finger protein 426 (Zfp426), mRNA [NM_001079943]	**0,638**
Tbc1d10b	Rattus norvegicus TBC1 domain family, member 10b (Tbc1d10b), mRNA [NM_001108921]	**0,638**
Abcb1b	Rattus norvegicus ATP-binding cassette, subfamily B (MDR/TAP), member 1B (Abcb1b), mRNA [NM_012623]	**0,638**
Gpc5	Rattus norvegicus glypican 5 (Gpc5), mRNA [NM_001107285]	**0,638**
Mmachc	Rattus norvegicus methylmalonic aciduria (cobalamin deficiency) cblC type, with homocystinuria (Mmachc), mRNA [NM_001107962]	**0,638**
0	Uncharacterized protein [Source:UniProtKB/TrEMBL;Acc:D3Z9J9] [ENSRNOT00000015767]	**0,638**
Lss	Rattus norvegicus lanosterol synthase (2,3-oxidosqualene-lanosterol cyclase) (Lss), mRNA [NM_031049]	**0,638**
Tnks2	Rattus norvegicus tankyrase, TRF1-interacting ankyrin-related ADP-ribose polymerase 2 (Tnks2), mRNA [NM_001107607]	**0,639**
Plekha7	Rattus norvegicus pleckstrin homology domain containing, family A member 7 (Plekha7), mRNA [NM_001144861]	**0,639**
Ppp3ca	Rattus norvegicus protein phosphatase 3, catalytic subunit, alpha isoform (Ppp3ca), mRNA [NM_017041]	**0,639**
Spon2	Rattus norvegicus spondin 2, extracellular matrix protein (Spon2), mRNA [NM_138533]	**0,639**

Unc5b	Rattus norvegicus unc-5 homolog B (C. elegans) (Unc5b), mRNA [NM_022207]	**0,639**
Med11	Rattus norvegicus mediator complex subunit 11 (Med11), mRNA [NM_001105799]	**0,639**
Kcnh3	Rattus norvegicus potassium voltage-gated channel, subfamily H (eag-related), member 3 (Kcnh3), mRNA [NM_017108]	**0,639**
Chm	Rattus norvegicus choroideremia (Rab escort protein 1) (Chm), mRNA [NM_017067]	**0,639**
Nfx1	Rattus norvegicus nuclear transcription factor, X-box binding 1 (Nfx1), mRNA [NM_001024784]	**0,639**
0	Potassium voltage-gated channel subfamily C member 3 [Source:UniProtKB/Swiss-Prot;Acc:Q01956] [ENSRNOT00000027043]	**0,639**
0	Uncharacterized protein [Source:UniProtKB/TrEMBL;Acc:D3ZC82] [ENSRNOT00000030501]	**0,639**
Gucy1a2	Rattus norvegicus guanylate cyclase 1, soluble, alpha 2 (Gucy1a2), mRNA [NM_023956]	**0,639**
Rgs2	Rattus norvegicus regulator of G-protein signaling 2 (Rgs2), mRNA [NM_053453]	**0,639**
Dfnb31	Rattus norvegicus deafness, autosomal recessive 31 (Dfnb31), mRNA [NM_181088]	**0,639**
Arap2	Rattus norvegicus ArfGAP with RhoGAP domain, ankyrin repeat and PH domain 2 (Arap2), mRNA [NM_001107216]	**0,639**
Tspan12	Rattus norvegicus tetraspanin 12 (Tspan12), mRNA [NM_001015026]	**0,639**
Acvr2b	Rattus norvegicus activin A receptor, type IIB (Acvr2b), mRNA [NM_031554]	**0,639**
Tmem161a	Rattus norvegicus transmembrane protein 161A (Tmem161a), mRNA [NM_001108874]	**0,639**
Slc6a9	Rattus norvegicus solute carrier family 6 (neurotransmitter transporter, glycine), member 9 (Slc6a9), mRNA [NM_053818]	**0,639**
Rara	Rattus norvegicus retinoic acid receptor, alpha (Rara), mRNA [NM_031528]	**0,639**
Onecut1	Rattus norvegicus one cut homeobox 1 (Onecut1), mRNA [NM_022671]	**0,639**
Sfrs2ip	Uncharacterized protein [Source:UniProtKB/TrEMBL;Acc:D4ABH1] [ENSRNOT00000007003]	**0,639**
Gabarap	Rattus norvegicus GABA(A) receptor-associated protein (Gabarap), mRNA [NM_172036]	**0,639**
Actn1	Rattus norvegicus actinin, alpha 1 (Actn1), mRNA [NM_031005]	**0,639**
Supt6h	Rattus norvegicus suppressor of Ty 6 homolog (S. cerevisiae) (Supt6h), mRNA [NM_001191820]	**0,639**
0	Unknown	**0,639**
Hist1h2af	Rattus norvegicus histone cluster 1, H2af (Hist1h2af), mRNA [NM_001024282]	**0,639**
Ywhab	Rattus norvegicus tyrosine 3-monooxygenase/tryptophan 5-monooxygenase activation protein, beta polypeptide (Ywhab), mRNA [NM_019377]	**0,639**
Csrp1	Rattus norvegicus cysteine and glycine-rich protein 1 (Csrp1), mRNA [NM_017148]	**0,639**
Fam126b	Rattus norvegicus family with sequence similarity 126, member B (Fam126b), mRNA [NM_001025710]	**0,639**
0	Unknown	**0,639**
Bhlhe41	PREDICTED: Rattus norvegicus basic helix-loop-helix family, member e41 (Bhlhe41), mRNA [XM_002729454]	**0,639**
Lrig2	Rattus norvegicus leucine-rich repeats and immunoglobulin-like domains 2 (Lrig2), mRNA [NM_001107710]	**0,639**
Glg1	Rattus norvegicus golgi apparatus protein 1 (Glg1), mRNA [NM_017211]	**0,639**
Foxg1	Rattus norvegicus forkhead box G1 (Foxg1), mRNA [NM_012560]	**0,639**
0	Unknown	**0,639**
Nfkbib	Rattus norvegicus nuclear factor of kappa light polypeptide gene enhancer in B-cells inhibitor, beta (Nfkbib), mRNA [NM_030867]	**0,639**
Slc38a6	Rattus norvegicus solute carrier family 38, member 6 (Slc38a6), mRNA [NM_001013099]	**0,639**

Tmem214	Rattus norvegicus transmembrane protein 214 (Tmem214), mRNA [NM_001014195]	**0,639**
Dcaf4	Rattus norvegicus DDB1 and CUL4 associated factor 4 (Dcaf4), mRNA [NM_001108716]	**0,639**
0	Unknown	**0,639**
Sema6b	Rattus norvegicus sema domain, transmembrane domain (TM), and cytoplasmic domain, (semaphorin) 6B (Sema6b), mRNA [NM_053471]	**0,639**
Gtrgeo22	Rattus norvegicus gene trap ROSA b-geo 22 (Gtrgeo22), mRNA [NM_001109621]	**0,639**
Clvs1	Rattus norvegicus clavesin 1 (Clvs1), mRNA [NM_001108969]	**0,640**
Elf2	Rattus norvegicus E74-like factor 2 (Elf2), transcript variant 2, mRNA [NM_001012181]	**0,640**
Popdc2	Rattus norvegicus popeye domain containing 2 (Popdc2), mRNA [NM_199113]	**0,640**
Slc41a1	Rattus norvegicus solute carrier family 41, member 1 (Slc41a1), mRNA [NM_001108855]	**0,640**
Tor2a	Rattus norvegicus torsin family 2, member A (Tor2a), mRNA [NM_001007744]	**0,640**
Shkbp1	SH3KBP1-binding protein 1 [Source:UniProtKB/Swiss-Prot;Acc:P0C5J9] [ENSRNOT00000028348]	**0,640**
Anxa6	Rattus norvegicus annexin A6 (Anxa6), mRNA [NM_024156]	**0,640**
Mfhas1	Rattus norvegicus malignant fibrous histiocytoma amplified sequence 1 (Mfhas1), mRNA [NM_001107316]	**0,640**
0	Unknown	**0,640**
Ptpru	Rattus norvegicus protein tyrosine phosphatase, receptor type, U (Ptpru), mRNA [NM_001191575]	**0,640**
Arx	Rattus norvegicus aristaless related homeobox (Arx), mRNA [NM_001100174]	**0,640**
Prrx1	Rattus norvegicus paired related homeobox 1 (Prrx1), mRNA [NM_153821]	**0,640**
Uhmk1	Rattus norvegicus U2AF homology motif (UHM) kinase 1 (Uhmk1), mRNA [NM_017293]	**0,640**
0	Rattus norvegicus TL0AEA14YL01 mRNA sequence. [FQ227170]	**0,640**
Crtc3	PREDICTED: Rattus norvegicus CREB regulated transcription coactivator 3 (Crtc3), mRNA [XM_001066604]	**0,640**
0	Unknown	**0,640**
Gdf10	Rattus norvegicus growth differentiation factor 10 (Gdf10), mRNA [NM_024375]	**0,640**
Ptges3l1	Rattus norvegicus prostaglandin E synthase 3-like 1 (Ptges3l1), mRNA [NM_001014272]	**0,640**
Thtpa	Rattus norvegicus thiamine triphosphatase (Thtpa), mRNA [NM_001007682]	**0,640**
Os9	Rattus norvegicus osteosarcoma amplified 9 (Os9), mRNA [NM_001007265]	**0,640**
0	Glutamate receptor subunit GluR1 [Source:UniProtKB/TrEMBL;Acc:Q924I5] [ENSRNOT00000003279]	**0,640**
0	Rattus norvegicus cDNA clone IMAGE:7374474. [BC091254]	**0,640**
Cav1	Rattus norvegicus caveolin 1, caveolae protein (Cav1), transcript variant 2, mRNA [NM_133651]	**0,640**
Sharpin	Rattus norvegicus SHANK-associated RH domain interactor (Sharpin), mRNA [NM_031153]	**0,640**
Slc35a3	Rattus norvegicus solute carrier family 35 (UDP-N-acetylglucosamine (UDP-GlcNAc) transporter), member A3 (Slc35a3), mRNA [NM_001012082]	**0,640**
0	Unknown	**0,640**
Isg20l2	Rattus norvegicus interferon stimulated exonuclease gene 20-like 2 (Isg20l2), mRNA [NM_001007741]	**0,640**
Xpnpep3	Rattus norvegicus X-prolyl aminopeptidase (aminopeptidase P) 3, putative (Xpnpep3), mRNA [NM_001130582]	**0,640**
Zfpm2	Rattus norvegicus zinc finger protein, multitype 2 (Zfpm2), mRNA [NM_001130501]	**0,640**

0	PREDICTED: Rattus norvegicus similar to glyceraldehyde-3-phosphate dehydrogenase (RGD1565238), partial mRNA [XM_001081651]	**0,640**
Dr1	Rattus norvegicus down-regulator of transcription 1 (Dr1), mRNA [NM_001011914]	**0,640**
Mina	Rattus norvegicus myc induced nuclear antigen (Mina), mRNA [NM_153309]	**0,640**
Gca	Rattus norvegicus grancalcin (Gca), mRNA [NM_001106483]	**0,640**
Pclo	Rattus norvegicus piccolo (presynaptic cytomatrix protein) (Pclo), transcript variant 1, mRNA [NM_020098]	**0,640**
Asf1a	Rattus norvegicus ASF1 anti-silencing function 1 homolog A (S. cerevisiae) (Asf1a), mRNA [NM_001106389]	**0,640**
Rbp1	Rattus norvegicus retinol binding protein 1, cellular (Rbp1), mRNA [NM_012733]	**0,640**
Cetn4	Unknown	**0,640**
Zfp414	Rattus norvegicus zinc finger protein 414 (Zfp414), mRNA [NM_001009664]	**0,640**
Sarm1	Rattus norvegicus sterile alpha and TIR motif containing 1 (Sarm1), mRNA [NM_001105817]	**0,641**
Prelid1	Rattus norvegicus PRELI domain containing 1 (Prelid1), mRNA [NM_001009636]	**0,641**
Tmub1	Rattus norvegicus transmembrane and ubiquitin-like domain containing 1 (Tmub1), transcript variant 2, mRNA [NM_001080153]	**0,641**
0	Uncharacterized protein [Source:UniProtKB/TrEMBL;Acc:D3ZE74] [ENSRNOT00000050658]	**0,641**
Cmpk2	Rattus norvegicus cytidine monophosphate (UMP-CMP) kinase 2, mitochondrial (Cmpk2), nuclear gene encoding mitochondrial protein, mRNA [NM_001108017]	**0,641**
Brd4	Rattus norvegicus bromodomain containing 4 (Brd4), mRNA [NM_001100903]	**0,641**
Rpgrip1l	Rattus norvegicus Rpgrip1-like (Rpgrip1l), mRNA [NM_001107414]	**0,641**
C1qtnf6	Rattus norvegicus C1q and tumor necrosis factor related protein 6 (C1qtnf6), mRNA [NM_001034932]	**0,641**
Dph3	Rattus norvegicus DPH3, KTI11 homolog (S. cerevisiae) (Dph3), mRNA [NM_001134850]	**0,641**
0	Rattus norvegicus similar to peroxiredoxin 6 (LOC287416), mRNA [XM_220591]	**0,641**
Lrrn2	Rattus norvegicus leucine rich repeat neuronal 2 (Lrrn2), mRNA [NM_001177368]	**0,641**
Cdkn3	Rattus norvegicus cyclin-dependent kinase inhibitor 3 (Cdkn3), mRNA [NM_001106028]	**0,641**
Zcchc4	Rattus norvegicus zinc finger, CCHC domain containing 4 (Zcchc4), mRNA [NM_001108360]	**0,641**
Zfp263	Rattus norvegicus zinc finger protein 263 (Zfp263), mRNA [NM_001105763]	**0,641**
Hyou1	Rattus norvegicus hypoxia up-regulated 1 (Hyou1), transcript variant 1, mRNA [NM_138867]	**0,641**
Cyfip2	Rattus norvegicus cytoplasmic FMR1 interacting protein 2 (Cyfip2), mRNA [NM_001106996]	**0,641**
Csf1r	Rattus norvegicus colony stimulating factor 1 receptor (Csf1r), mRNA [NM_001029901]	**0,641**
Npl	Rattus norvegicus N-acetylneuraminate pyruvate lyase (Npl), mRNA [NM_001013984]	**0,641**
Mgll	Rattus norvegicus monoglyceride lipase (Mgll), mRNA [NM_138502]	**0,641**
Fam155b	Uncharacterized protein [Source:UniProtKB/TrEMBL;Acc:D4A4E6] [ENSRNOT00000057920]	**0,641**
Sfrs15	Rattus norvegicus splicing factor, arginine/serine-rich 15 (Sfrs15), mRNA [NM_001037347]	**0,641**
Fam76b	Rattus norvegicus family with sequence similarity 76, member B (Fam76b), mRNA [NM_001108994]	**0,641**
Ppard	Rattus norvegicus peroxisome proliferator-activated receptor delta (Ppard), mRNA [NM_013141]	**0,641**
Spock2	Rattus norvegicus sparc/osteonectin, cwcv and kazal-like domains proteoglycan 2 (Spock2), mRNA [NM_001108533]	**0,641**

Trim46	Rattus norvegicus tripartite motif-containing 46 (Trim46), mRNA [NM_001107691]	**0,641**
Glb1	Rattus norvegicus galactosidase, beta 1 (Glb1), mRNA [NM_001108192]	**0,641**
Ptprs	Rattus norvegicus protein tyrosine phosphatase, receptor type, S (Ptprs), mRNA [NM_019140]	**0,641**
Tnfrsf4	Rattus norvegicus tumor necrosis factor receptor superfamily, member 4 (Tnfrsf4), mRNA [NM_013049]	**0,641**
Dact3	Rattus norvegicus dapper, antagonist of beta-catenin, homolog 3 (Xenopus laevis) (Dact3), mRNA [NM_001191947]	**0,641**
M6pr	Rattus norvegicus mannose-6-phosphate receptor, cation dependent (M6pr), mRNA [NM_001007700]	**0,641**
0	Cobl-like 1 Gene [Source:MGI Symbol;Acc:MGI:2442894] [ENSRNOT00000001606]	**0,641**
Mad2l1	Rattus norvegicus MAD2 mitotic arrest deficient-like 1 (yeast) (Mad2l1), mRNA [NM_001106594]	**0,641**
Reck	Rattus norvegicus reversion-inducing-cysteine-rich protein with kazal motifs (Reck), mRNA [NM_001107954]	**0,641**
Eif5a2	Rattus norvegicus eukaryotic translation initiation factor 5A2 (Eif5a2), mRNA [NM_001100697]	**0,641**
Nf2	Rattus norvegicus neurofibromin 2 (merlin) (Nf2), mRNA [NM_013193]	**0,641**
Zdhhc20	Rattus norvegicus zinc finger, DHHC-type containing 20 (Zdhhc20), mRNA [NM_001039336]	**0,641**
0	RVL6632 Wackym-Soares normalized rat vestibular cDNA library Rattus norvegicus cDNA 5', mRNA sequence [DV716430]	**0,641**
LOC363746	PREDICTED: Rattus norvegicus similar to 40S ribosomal protein S7 (S8) (LOC363746), miscRNA [XR_006358]	**0,641**
Zdhhc2	Rattus norvegicus zinc finger, DHHC-type containing 2 (Zdhhc2), mRNA [NM_145096]	**0,642**
Hpca	Rattus norvegicus hippocalcin (Hpca), mRNA [NM_017122]	**0,642**
Runx1t1	Rattus norvegicus runt-related transcription factor 1; translocated to, 1 (cyclin D-related) (Runx1t1), mRNA [NM_001108657]	**0,642**
Fance	Rattus norvegicus Fanconi anemia, complementation group E (Fance), mRNA [NM_001191718]	**0,642**
0	Q8R3U9_MOUSE (Q8R3U9) Dlgap4 protein, partial (34%) [TC609401]	**0,642**
0	Potassium voltage-gated channel subfamily D member 3 [Source:UniProtKB/Swiss-Prot;Acc:Q62897] [ENSRNOT00000051835]	**0,642**
Zhx1	Rattus norvegicus zinc fingers and homeoboxes 1 (Zhx1), mRNA [NM_133620]	**0,642**
Slc3a1	Rattus norvegicus solute carrier family 3, member 1 (Slc3a1), mRNA [NM_017216]	**0,642**
Fmr1	Rattus norvegicus fragile X mental retardation 1 (Fmr1), mRNA [NM_052804]	**0,642**
0	Unknown	**0,642**
Fam118b	Rattus norvegicus family with sequence similarity 118, member B (Fam118b), mRNA [NM_001025283]	**0,642**
RGD1563216	Rattus norvegicus similar to HESB like domain containing 1 (RGD1563216), mRNA [NM_001109278]	**0,642**
Jak3	Rattus norvegicus Janus kinase 3 (Jak3), mRNA [NM_012855]	**0,642**
Kcng3	Rattus norvegicus potassium voltage-gated channel, subfamily G, member 3 (Kcng3), transcript variant 1, mRNA [NM_133426]	**0,642**
Mre11a	Rattus norvegicus MRE11 meiotic recombination 11 homolog A (S. cerevisiae) (Mre11a), mRNA [NM_022279]	**0,642**
Mtmr4	Rattus norvegicus myotubularin related protein 4 (Mtmr4), mRNA [NM_001105827]	**0,642**
0	zinc finger protein 62 Gene [Source:MGI Symbol;Acc:MGI:99662] [ENSRNOT00000003373]	**0,642**
Gtpbp1	Rattus norvegicus GTP binding protein 1 (Gtpbp1), mRNA [NM_001199315]	**0,642**
Ampd3	Rattus norvegicus adenosine monophosphate deaminase 3 (Ampd3), mRNA [NM_031544]	**0,642**
0	Unknown	**0,642**

Nicn1	Rattus norvegicus nicolin 1 (Nicn1), mRNA [NM_001034999]	**0,642**
0	Unknown	**0,642**
Fktn	Rattus norvegicus fukutin (Fktn), mRNA [NM_001108667]	**0,642**
Tceb2	Rattus norvegicus transcription elongation factor B (SIII), polypeptide 2 (Tceb2), mRNA [NM_031129]	**0,642**
Atrnl1	PREDICTED: Rattus norvegicus attractin like 1 (Atrnl1), mRNA [XM_217657]	**0,642**
Ppm1e	Rattus norvegicus protein phosphatase 1E (PP2C domain containing) (Ppm1e), mRNA [NM_198773]	**0,642**
LOC689574	Rattus norvegicus hypothetical protein LOC689574 (LOC689574), nuclear gene encoding mitochondrial protein, transcript variant 1, mRNA [NM_001195503]	**0,642**
Pctk2	Rattus norvegicus PCTAIRE protein kinase 2 (Pctk2), mRNA [NM_001108082]	**0,642**
Catsperg1	Rattus norvegicus cation channel, sperm-associated, gamma 1 (Catsperg1), mRNA [NM_001170340]	**0,642**
Ikbke	Rattus norvegicus inhibitor of kappa light polypeptide gene enhancer in B-cells, kinase epsilon (Ikbke), mRNA [NM_001108854]	**0,642**
Snurf	Rattus norvegicus SNRPN upstream reading frame (Snurf), mRNA [NM_130738]	**0,642**
0	Unknown	**0,642**
RGD1565591	PREDICTED: Rattus norvegicus similar to Ski protein (RGD1565591), partial mRNA [XM_001077382]	**0,642**
Dstn	Rattus norvegicus destrin (Dstn), mRNA [NM_001033666]	**0,642**
Rab1b	Rattus norvegicus RAB1B, member RAS oncogene family (Rab1b), mRNA [NM_001109979]	**0,642**
0	Uncharacterized protein [Source:UniProtKB/TrEMBL;Acc:D3ZPT5] [ENSRNOT00000067382]	**0,642**
Pla2g6	Rattus norvegicus phospholipase A2, group VI (cytosolic, calcium-independent) (Pla2g6), mRNA [NM_001005560]	**0,642**
RT1-DMb	Rattus norvegicus RT1 class II, locus DMb (RT1-DMb), mRNA [NM_198740]	**0,642**
Ttc3	Rattus norvegicus tetratricopeptide repeat domain 3 (Ttc3), mRNA [NM_001108315]	**0,642**
0	Unknown	**0,642**
Hif1a	Rattus norvegicus hypoxia-inducible factor 1, alpha subunit (basic helix-loop-helix transcription factor) (Hif1a), mRNA [NM_024359]	**0,642**
Cckbr	Rattus norvegicus cholecystokinin B receptor (Cckbr), mRNA [NM_013165]	**0,643**
Itga10	Rattus norvegicus integrin, alpha 10 (Itga10), mRNA [NM_001107699]	**0,643**
0	Unknown	**0,643**
0	Unknown	**0,643**
Cdc2l5	PREDICTED: Rattus norvegicus cell division cycle 2-like 5 (cholinesterase-related cell division controller), transcript variant 2 (Cdc2l5), mRNA [XM_001053609]	**0,643**
Bbs10	Rattus norvegicus Bardet-Biedl syndrome 10 (Bbs10), mRNA [NM_001109286]	**0,643**
Gpam	Rattus norvegicus glycerol-3-phosphate acyltransferase, mitochondrial (Gpam), nuclear gene encoding mitochondrial protein, mRNA [NM_017274]	**0,643**
Mdfic	Rattus norvegicus MyoD family inhibitor domain containing (Mdfic), mRNA [NM_001105668]	**0,643**
0	Rattus norvegicus similar to melanocyte-specific gene 1 protein (LOC309188), mRNA [XM_219543]	**0,643**
Asb2	Rattus norvegicus ankyrin repeat and SOCS box-containing 2 (Asb2), mRNA [NM_001011984]	**0,643**
Nras	Rattus norvegicus neuroblastoma ras oncogene (Nras), mRNA [NM_080766]	**0,643**

0	Unknown	**0,643**
Irs4	PREDICTED: Rattus norvegicus insulin receptor substrate 4 (Irs4), mRNA [XM_001056753]	**0,643**
0	Q6GQU6_MOUSE (Q6GQU6) CDNA sequence BC072620, partial (56%) [TC612613]	**0,643**
Snrk	Rattus norvegicus SNF related kinase (Snrk), mRNA [NM_138833]	**0,643**
Nfu1	Rattus norvegicus NFU1 iron-sulfur cluster scaffold homolog (S. cerevisiae) (Nfu1), nuclear gene encoding mitochondrial protein, mRNA [NM_001106606]	**0,643**
Rab2b	Rattus norvegicus RAB2B, member RAS oncogene family, mRNA (cDNA clone IMAGE:7316063), partial cds. [BC092636]	**0,643**
Slc7a14	Rattus norvegicus solute carrier family 7 (cationic amino acid transporter, y+ system), member 14 (Slc7a14), mRNA [NM_001134615]	**0,643**
RGD1309762	PREDICTED: Rattus norvegicus similar to KIAA0614 protein (RGD1309762), mRNA [XM_222205]	**0,643**
Camta1	Rattus norvegicus similar to KIAA0833 protein (Camta1), transcript variant 1, mRNA [NM_001195559]	**0,643**
RGD1559896	Rattus norvegicus similar to RIKEN cDNA 2310022B05 (RGD1559896), mRNA [NM_001109134]	**0,643**
Kcnc2	Rattus norvegicus potassium voltage gated channel, Shaw-related subfamily, member 2 (Kcnc2), transcript variant b, mRNA [NM_139217]	**0,643**
B3galt6	Rattus norvegicus UDP-Gal:betaGal beta 1,3-galactosyltransferase, polypeptide 6 (B3galt6), mRNA [NM_001106699]	**0,643**
LOC498368	Rattus norvegicus similar to RIKEN cDNA 0610040J01 (LOC498368), mRNA [NM_001017500]	**0,643**
St8sia3	Rattus norvegicus ST8 alpha-N-acetyl-neuraminide alpha-2,8-sialyltransferase 3 (St8sia3), mRNA [NM_013029]	**0,643**
Glrx1	Rattus norvegicus glutaredoxin 1 (Glrx1), mRNA [NM_022278]	**0,643**
0	Unknown	**0,643**
Csf1	Rattus norvegicus colony stimulating factor 1 (macrophage) (Csf1), mRNA [NM_023981]	**0,643**
Stk32b	Rattus norvegicus serine/threonine kinase 32B (Stk32b), mRNA [NM_001107224]	**0,643**
Sepx1	Rattus norvegicus selenoprotein X, 1 (Sepx1), mRNA [NM_001044285]	**0,643**
Srebf2	Rattus norvegicus sterol regulatory element binding transcription factor 2 (Srebf2), mRNA [NM_001033694]	**0,643**
Hey2	Rattus norvegicus hairy/enhancer-of-split related with YRPW motif 2 (Hey2), mRNA [NM_130417]	**0,643**
Ttc8	Rattus norvegicus tetratricopeptide repeat domain 8 (Ttc8), mRNA [NM_001106752]	**0,643**
Jmjd8	Rattus norvegicus jumonji domain containing 8 (Jmjd8), mRNA [NM_001014116]	**0,644**
Foxp1	Rattus norvegicus forkhead box P1 (Foxp1), mRNA [NM_001034131]	**0,644**
Supt4h1	Rattus norvegicus suppressor of Ty 4 homolog 1 (S. cerevisiae) (Supt4h1), mRNA [NM_001105828]	**0,644**
Zbtb8os	PREDICTED: Rattus norvegicus zinc finger and BTB domain containing 8 opposite strand (Zbtb8os), miscRNA [XR_086264]	**0,644**
Cpeb4	Rattus norvegicus cytoplasmic polyadenylation element binding protein 4 (Cpeb4), mRNA [NM_001106992]	**0,644**
0	Unknown	**0,644**
Scnm1	Rattus norvegicus sodium channel modifier 1 (Scnm1), mRNA [NM_001107696]	**0,644**
LOC300314	PREDICTED: Rattus norvegicus similar to Protein KIAA0196 (LOC300314), mRNA [XM_238532]	**0,644**
Rpe	Rattus norvegicus ribulose-5-phosphate-3-epimerase (Rpe), mRNA [NM_001033903]	**0,644**
Slit3	Rattus norvegicus slit homolog 3 (Drosophila) (Slit3), mRNA [NM_031321]	**0,644**
Cd46	Rattus norvegicus CD46 molecule, complement regulatory protein (Cd46), mRNA [NM_019190]	**0,644**

Gpr137b	Rattus norvegicus G protein-coupled receptor 137B (Gpr137b), mRNA [NM_001105978]	**0,644**
0	Unknown	**0,644**
Pcdhb7	Protocadherin-T3 [Source:UniProtKB/TrEMBL;Acc:Q9JIU3] [ENSRNOT00000027188]	**0,644**
Rap1a	Rattus norvegicus RAP1A, member of RAS oncogene family (Rap1a), mRNA [NM_001005765]	**0,644**
0	Hepatic leukemia factor [Source:UniProtKB/Swiss-Prot;Acc:Q64709] [ENSRNOT00000055664]	**0,644**
Zbtb7b	Rattus norvegicus zinc finger and BTB domain containing 7B (Zbtb7b), mRNA [NM_001106446]	**0,644**
Plekhh1	Rattus norvegicus pleckstrin homology domain containing, family H (with MyTH4 domain) member 1 (Plekhh1), mRNA [NM_001108036]	**0,644**
Tmem71	PREDICTED: Rattus norvegicus transmembrane protein 71 (Tmem71), mRNA [XM_001075116]	**0,644**
Rtn3	Rattus norvegicus reticulon 3 (Rtn3), transcript variant 1, mRNA [NM_080909]	**0,644**
Ptgfrn	Rattus norvegicus prostaglandin F2 receptor negative regulator (Ptgfrn), mRNA [NM_019243]	**0,644**
Srfbp1	Rattus norvegicus serum response factor binding protein 1 (Srfbp1), mRNA [NM_001005536]	**0,644**
Sf1	Rattus norvegicus splicing factor 1 (Sf1), transcript variant 1, mRNA [NM_001110793]	**0,644**
0	Unknown	**0,644**
0	Unknown	**0,644**
38961	Rattus norvegicus septin 6 (Sept6), mRNA [NM_001173429]	**0,644**
Tmed5	Rattus norvegicus transmembrane emp24 protein transport domain containing 5 (Tmed5), mRNA [NM_001007619]	**0,644**
Porcn	Rattus norvegicus porcupine homolog (Drosophila) (Porcn), mRNA [NM_001173355]	**0,644**
Foxk1	Rattus norvegicus forkhead box K1 (Foxk1), mRNA [NM_001037219]	**0,644**
Ibtk	Uncharacterized protein [Source:UniProtKB/TrEMBL;Acc:D3ZAK7] [ENSRNOT00000056930]	**0,644**
Fam3a	Rattus norvegicus family with sequence similarity 3, member A (Fam3a), mRNA [NM_001109324]	**0,644**
Mfap3	Rattus norvegicus microfibrillar-associated protein 3 (Mfap3), mRNA [NM_001007609]	**0,644**
Fzd1	Rattus norvegicus frizzled homolog 1 (Drosophila) (Fzd1), mRNA [NM_021266]	**0,644**
Cnot7	Rattus norvegicus CCR4-NOT transcription complex, subunit 7 (Cnot7), mRNA [NM_001107313]	**0,644**
Slc30a4	Rattus norvegicus solute carrier family 30 (zinc transporter), member 4 (Slc30a4), mRNA [NM_172066]	**0,644**
0	Unknown	**0,644**
0	UI-R-EB1-clg-p-22-0-UI.s1 UI-R-EB1 Rattus norvegicus cDNA clone UI-R-EB1-clg-p-22-0-UI 3', mRNA sequence [BQ202508]	**0,644**
LOC365238	Rattus norvegicus similar to CG15432-PA (LOC365238), mRNA [NM_001108913]	**0,644**
Kcnd3	Rattus norvegicus potassium voltage-gated channel, Shal-related subfamily, member 3 (Kcnd3), mRNA [NM_031739]	**0,644**
0	Uncharacterized protein [Source:UniProtKB/TrEMBL;Acc:D3ZP37] [ENSRNOT00000060917]	**0,645**
Flrt1	Rattus norvegicus fibronectin leucine rich transmembrane protein 1 (Flrt1), mRNA [NM_001109160]	**0,645**
Ppp2r5c	Rattus norvegicus protein phosphatase 2, regulatory subunit B', gamma isoform (Ppp2r5c), mRNA [NM_001191112]	**0,645**
Abcd2	Rattus norvegicus ATP-binding cassette, subfamily D (ALD), member 2 (Abcd2), mRNA [NM_033352]	**0,645**
Sh2b2	Rattus norvegicus SH2B adaptor protein 2 (Sh2b2), mRNA [NM_053669]	**0,645**
0	Tudor domain-containing protein PHF20L1 [Source:UniProtKB/Swiss-Prot;Acc:Q4V9H5] [ENSRNOT00000007564]	**0,645**

Akap6	Rattus norvegicus A kinase (PRKA) anchor protein 6 (Akap6), mRNA [NM_022618]	**0,645**
Tbc1d14	Rattus norvegicus TBC1 domain family, member 14 (Tbc1d14), transcript variant 1, mRNA [NM_001012152]	**0,645**
0	Unknown	**0,645**
Scmh1	Rattus norvegicus sex comb on midleg homolog 1 (Drosophila) (Scmh1), mRNA [NM_001109669]	**0,645**
Utp15	Rattus norvegicus UTP15, U3 small nucleolar ribonucleoprotein, homolog (S. cerevisiae) (Utp15), mRNA [NM_001107647]	**0,645**
Shroom2	Rattus norvegicus shroom family member 2 (Shroom2), mRNA [NM_001047893]	**0,645**
Slc39a1	Rattus norvegicus solute carrier family 39 (zinc transporter), member 1 (Slc39a1), mRNA [NM_001134577]	**0,645**
Tpp1	Rattus norvegicus tripeptidyl peptidase I (Tpp1), mRNA [NM_031357]	**0,645**
Rnf125	Rattus norvegicus ring finger protein 125 (Rnf125), mRNA [NM_001108424]	**0,645**
Zmynd17	Rattus norvegicus zinc finger, MYND-type containing 17 (Zmynd17), mRNA [NM_001106025]	**0,645**
Kcnc3	Rattus norvegicus mRNA sequence. [M84210]	**0,645**
0	Unknown	**0,645**
0	Unknown	**0,645**
Smek2	Rattus norvegicus SMEK homolog 2, suppressor of mek1 (Dictyostelium) (Smek2), mRNA [NM_001108367]	**0,645**
Tk1	Rattus norvegicus thymidine kinase 1, soluble (Tk1), mRNA [NM_052800]	**0,645**
Tcf19	Rattus norvegicus transcription factor 19 (Tcf19), mRNA [NM_213561]	**0,645**
Rph3al	Rattus norvegicus rabphilin 3A-like (without C2 domains) (Rph3al), mRNA [NM_133591]	**0,645**
Scn1a	Rattus norvegicus sodium channel, voltage-gated, type I, alpha (Scn1a), mRNA [NM_030875]	**0,645**
0	Unknown	**0,645**
Ccdc94	Rattus norvegicus coiled-coil domain containing 94 (Ccdc94), mRNA [NM_001109673]	**0,645**
Rab14	Rattus norvegicus RAB14, member RAS oncogene family (Rab14), mRNA [NM_053589]	**0,645**
Cdh11	Rattus norvegicus cadherin 11 (Cdh11), mRNA [NM_053392]	**0,645**
0	Uncharacterized protein [Source:UniProtKB/TrEMBL;Acc:D4AE17] [ENSRNOT00000002068]	**0,645**
Rnft2	Rattus norvegicus ring finger protein, transmembrane 2 (Rnft2), mRNA [NM_001107144]	**0,645**
RGD1564677	PREDICTED: Rattus norvegicus similar to transcription factor ONECUT2 (RGD1564677), partial mRNA [XM_002725360]	**0,645**
0	Unknown	**0,645**
0	Unknown	**0,645**
Fam194a	Rattus norvegicus family with sequence similarity 194, member A (Fam194a), mRNA [NM_001024300]	**0,645**
Tnrc6b	Rattus norvegicus trinucleotide repeat containing 6B (Tnrc6b), mRNA [NM_138845]	**0,645**
0	Unknown	**0,645**
0	Unknown	**0,645**
Pa2g4	Rattus norvegicus proliferation-associated 2G4 (Pa2g4), mRNA [NM_001004206]	**0,645**
Lims1	Rattus norvegicus LIM and senescent cell antigen-like domains 1 (Lims1), mRNA [NM_001145456]	**0,645**
Aif1l	Rattus norvegicus allograft inflammatory factor 1-like (Aif1l), mRNA [NM_001108578]	**0,645**

Trim3	Rattus norvegicus tripartite motif-containing 3 (Trim3), mRNA [NM_031786]	**0,645**
0	Unknown	**0,646**
LOC688319	PREDICTED: Rattus norvegicus similar to RAS-related C3 botulinum substrate 3 (LOC688319), mRNA [XM_001081814]	**0,646**
0	Uncharacterized protein [Source:UniProtKB/TrEMBL;Acc:D3ZS88] [ENSRNOT00000011803]	**0,646**
Rad18	Rattus norvegicus RAD18 homolog (S. cerevisiae) (Rad18), mRNA [NM_001077673]	**0,646**
Acsl1	Rattus norvegicus acyl-CoA synthetase long-chain family member 1 (Acsl1), mRNA [NM_012820]	**0,646**
Nkiras1	Rattus norvegicus NFKB inhibitor interacting Ras-like 1 (Nkiras1), mRNA [NM_001107252]	**0,646**
0	family with sequence similarity 184, member A Gene [Source:MGI Symbol;Acc:MGI:1923156] [ENSRNOT00000000475]	**0,646**
Radil	Rattus norvegicus Ras association and DIL domains (Radil), mRNA [NM_001037218]	**0,646**
Wiz	Rattus norvegicus widely-interspaced zinc finger motifs (Wiz), mRNA [NM_001108064]	**0,646**
Eefsec	Rattus norvegicus eukaryotic elongation factor, selenocysteine-tRNA-specific (Eefsec), mRNA [NM_001109249]	**0,646**
Tmco4	Rattus norvegicus transmembrane and coiled-coil domains 4 (Tmco4), mRNA [NM_001034949]	**0,646**
Rbm24	Rattus norvegicus RNA binding motif protein 24 (Rbm24), mRNA [NM_001191100]	**0,646**
0	Unknown	**0,646**
Camta2	Rattus norvegicus calmodulin binding transcription activator 2 (Camta2), mRNA [NM_001105801]	**0,646**
Smn1	Rattus norvegicus survival motor neuron 1 (Smn1), mRNA [NM_022509]	**0,646**
0	Q29A56_DROPS (Q29A56) GA18264-PA (Fragment), partial (3%) [TC648872]	**0,646**
Fhdc1	Rattus norvegicus FH2 domain containing 1 (Fhdc1), mRNA [NM_001106437]	**0,646**
Fbxl5	Rattus norvegicus F-box and leucine-rich repeat protein 5 (Fbxl5), mRNA [NM_001107222]	**0,646**
Serpina11	Rattus norvegicus serine (or cysteine) peptidase inhibitor, clade A (alpha-1 antiproteinase, antitrypsin), member 11 (Serpina11), transcript variant 1, mRNA [NM_001008776]	**0,646**
LOC684327	PREDICTED: Rattus norvegicus similar to inter-alpha (globulin) inhibitor H5 (LOC684327), mRNA [XM_001069890]	**0,646**
Fam84a	Rattus norvegicus family with sequence similarity 84, member A (Fam84a), mRNA [NM_001127299]	**0,646**
0	Shultzomica03080 Rat lung airway and parenchyma cDNA libraries Rattus norvegicus cDNA clone Contig2706 5', mRNA sequence [CF109829]	**0,646**
0	Unknown	**0,646**
Ddx19b	Rattus norvegicus DEAD (Asp-Glu-Ala-As) box polypeptide 19B (Ddx19b), mRNA [NM_001005895]	**0,646**
Sepp1	Rattus norvegicus selenoprotein P, plasma, 1 (Sepp1), transcript variant 2, mRNA [NM_001083911]	**0,646**
Haus2	PREDICTED: Rattus norvegicus HAUS augmin-like complex, subunit 2, transcript variant 1 (Haus2), mRNA [XM_001053441]	**0,646**
Tspan9	Rattus norvegicus tetraspanin 9 (Tspan9), mRNA [NM_001107890]	**0,646**
RGD1566265	Rattus norvegicus similar to RIKEN cDNA 2610002M06 (RGD1566265), mRNA [NM_001134589]	**0,646**
Nfix	Rattus norvegicus nuclear factor I/X (CCAAT-binding transcription factor) (Nfix), mRNA [NM_030866]	**0,646**
Trim44	Rattus norvegicus tripartite motif-containing 44 (Trim44), mRNA [NM_001013203]	**0,646**
Eif4enif1	Rattus norvegicus eukaryotic translation initiation factor 4E nuclear import factor 1 (Eif4enif1), mRNA [NM_001107230]	**0,646**
Strn	Rattus norvegicus striatin, calmodulin binding protein (Strn), mRNA [NM_019148]	**0,646**

RGD1563613	Uncharacterized protein [Source:UniProtKB/TrEMBL;Acc:D4AE10] [ENSRNOT00000044779]	**0,646**
Rims1	Rattus norvegicus regulating synaptic membrane exocytosis 1 (Rims1), mRNA [NM_052829]	**0,646**
Atf5	Rattus norvegicus activating transcription factor 5 (Atf5), mRNA [NM_172336]	**0,646**
0	Uncharacterized protein [Source:UniProtKB/TrEMBL;Acc:D4A5F0] [ENSRNOT00000002917]	**0,646**
Man1c1	Rattus norvegicus mannosidase, alpha, class 1C, member 1 (Man1c1), mRNA [NM_001108687]	**0,646**
Vps13b	Rattus norvegicus vacuolar protein sorting 13 homolog B (yeast) (Vps13b), mRNA [NM_001134886]	**0,646**
Atxn1	Rattus norvegicus ataxin 1 (Atxn1), mRNA [NM_012726]	**0,646**
0	Rattus norvegicus TL0ACA70YE02 mRNA sequence. [FQ224293]	**0,646**
Rab3c	Rattus norvegicus RAB3C, member RAS oncogene family (Rab3c), mRNA [NM_133536]	**0,646**
Xkr4	Rattus norvegicus XK, Kell blood group complex subunit-related family, member 4 (Xkr4), mRNA [NM_001011971]	**0,646**
Ap2b1	Rattus norvegicus adaptor-related protein complex 2, beta 1 subunit (Ap2b1), mRNA [NM_080583]	**0,646**
Atf6	Rattus norvegicus activating transcription factor 6 (Atf6), mRNA [NM_001107196]	**0,646**
Lyrm2	Rattus norvegicus LYR motif containing 2 (Lyrm2), mRNA [NM_001126096]	**0,646**
LOC365723	PREDICTED: Rattus norvegicus similar to zinc finger protein 458 (LOC365723), miscRNA [XR_086165]	**0,646**
0	Unknown	**0,647**
Tax1bp3	Rattus norvegicus Tax1 (human T-cell leukemia virus type I) binding protein 3 (Tax1bp3), mRNA [NM_001025419]	**0,647**
0	Myeloid/lymphoid or mixed-lineage leukemia (Mapped)Uncharacterized protein [Source:UniProtKB/TrEMBL;Acc:D3ZLI0] [ENSRNOT00000040881]	**0,647**
0	Unknown	**0,647**
Tmem188	Rattus norvegicus transmembrane protein 188 (Tmem188), mRNA [NM_001106173]	**0,647**
B3galt1	Rattus norvegicus UDP-Gal:betaGlcNAc beta 1,3-galactosyltransferase, polypeptide 1 (B3galt1), mRNA [NM_001108954]	**0,647**
Cdc23	Rattus norvegicus CDC23 (cell division cycle 23, yeast, homolog) (Cdc23), mRNA [NM_001100659]	**0,647**
Fam163a	Rattus norvegicus family with sequence similarity 163, member A (Fam163a), mRNA [NM_001109072]	**0,647**
Dbp	Rattus norvegicus D site of albumin promoter (albumin D-box) binding protein (Dbp), mRNA [NM_012543]	**0,647**
Fam81a	Rattus norvegicus family with sequence similarity 81, member A (Fam81a), mRNA [NM_001108163]	**0,647**
Rnf13	Rattus norvegicus ring finger protein 13 (Rnf13), mRNA [NM_001109444]	**0,647**
Zfp426l2	Rattus norvegicus zinc finger protein 426-like 2 (Zfp426l2), mRNA [NM_001134585]	**0,647**
RGD1563216	Rattus norvegicus similar to HESB like domain containing 1 (RGD1563216), mRNA [NM_001109278]	**0,647**
Rcbtb1	Rattus norvegicus regulator of chromosome condensation (RCC1) and BTB (POZ) domain containing protein 1 (Rcbtb1), mRNA [NM_001108380]	**0,647**
Slc25a34	Rattus norvegicus solute carrier family 25, member 34 (Slc25a34), mRNA [NM_001013936]	**0,647**
LOC686326	PREDICTED: Rattus norvegicus similar to Interferon-alpha/beta receptor beta chain precursor (IFN-alpha-REC) (Type I interferon receptor) (IFN-R) (Interferon alpha/beta receptor-2) (LOC686326), mRNA [XM_001073550]	**0,647**
0	MMU64033 Tera {Mus musculus} (exp=-1; wgp=0; cg=0), partial (13%) [TC591393]	**0,647**
RGD1311783	Rattus norvegicus similar to RIKEN cDNA 2010012O05 (RGD1311783), mRNA [NM_001134509]	**0,647**
Ddx47	Rattus norvegicus DEAD (Asp-Glu-Ala-Asp) box polypeptide 47 (Ddx47), mRNA [NM_001015005]	**0,647**

Phactr1	Rattus norvegicus phosphatase and actin regulator 1 (Phactr1), mRNA [NM_214457]	**0,647**
Napg	Rattus norvegicus N-ethylmaleimide-sensitive factor attachment protein, gamma (Napg), mRNA [NM_001107384]	**0,647**
Hint3	Histidine triad nucleotide-binding protein 3 [Source:UniProtKB/Swiss-Prot;Acc:Q8K3P7] [ENSRNOT00000019047]	**0,647**
Nbl1	Rattus norvegicus neuroblastoma, suppression of tumorigenicity 1 (Nbl1), mRNA [NM_031609]	**0,647**
Fktn	Rattus norvegicus fukutin (Fktn), mRNA [NM_001108667]	**0,647**
Hs1bp3	PREDICTED: Rattus norvegicus HCLS1 binding protein 3, transcript variant 2 (Hs1bp3), mRNA [XM_233975]	**0,647**
Plekha1	Rattus norvegicus pleckstrin homology domain containing, family A (phosphoinositide binding specific) member 1 (Plekha1), mRNA [NM_001079894]	**0,647**
Prc1	Rattus norvegicus protein regulator of cytokinesis 1 (Prc1), mRNA [NM_001107529]	**0,647**
Rab27b	Rattus norvegicus RAB27B, member RAS oncogene family (Rab27b), mRNA [NM_053459]	**0,647**
Mll5	Mll5 protein [Source:UniProtKB/TrEMBL;Acc:B1WBR7] [ENSRNOT00000038978]	**0,647**
Oas1b	Rattus norvegicus 2-5 oligoadenylate synthetase 1B (Oas1b), mRNA [NM_144752]	**0,647**
Nfatc3	Rattus norvegicus nuclear factor of activated T-cells, cytoplasmic, calcineurin-dependent 3 (Nfatc3), mRNA [NM_001108447]	**0,647**
Adam10	PREDICTED: Rattus norvegicus ADAM metallopeptidase domain 10 (Adam10), mRNA [XM_001054737]	**0,647**
Usp4	Rattus norvegicus ubiquitin specific peptidase 4 (proto-oncogene) (Usp4), transcript variant 1, mRNA [NM_001135012]	**0,647**
Sox13	Rattus norvegicus SRY (sex determining region Y)-box 13 (Sox13), mRNA [NM_001105952]	**0,647**
Tns3	Uncharacterized protein [Source:UniProtKB/TrEMBL;Acc:D3ZCL6] [ENSRNOT00000012661]	**0,647**
0	PREDICTED: Rattus norvegicus similar to zinc finger protein 617 (LOC684006), mRNA [XM_001068456]	**0,648**
Psma3	Rattus norvegicus proteasome (prosome, macropain) subunit, alpha type 3 (Psma3), mRNA [NM_017280]	**0,648**
Hcfc1r1	Rattus norvegicus host cell factor C1 regulator 1 (XPO1-dependent) (Hcfc1r1), transcript variant 1, mRNA [NM_001185047]	**0,648**
Kcnj6	Rattus norvegicus potassium inwardly-rectifying channel, subfamily J, member 6 (Kcnj6), mRNA [NM_013192]	**0,648**
Pex11g	Rattus norvegicus peroxisomal biogenesis factor 11 gamma (Pex11g), mRNA [NM_001105902]	**0,648**
Gpr177	Rattus norvegicus G protein-coupled receptor 177 (Gpr177), transcript variant 1, mRNA [NM_199408]	**0,648**
RGD1561931	Uncharacterized protein [Source:UniProtKB/TrEMBL;Acc:D3ZGX1] [ENSRNOT00000035653]	**0,648**
RGD1562865	Rattus norvegicus similar to BTB and CNC homology 1, basic leucine zipper transcription factor 2 (RGD1562865), mRNA [NM_001135754]	**0,648**
Ryk	Rattus norvegicus receptor-like tyrosine kinase (Ryk), mRNA [NM_080402]	**0,648**
Gpr20	Rattus norvegicus G protein-coupled receptor 20 (Gpr20), mRNA [NM_022216]	**0,648**
Hyal1	Rattus norvegicus hyaluronoglucosaminidase 1 (Hyal1), mRNA [NM_207616]	**0,648**
Pcdha13	Rattus norvegicus protocadherin alpha 13 (Pcdha13), mRNA [NM_053934]	**0,648**
RGD1306151	Rattus norvegicus similar to hypothetical protein DKFZp761D0211 (RGD1306151), mRNA [NM_001108652]	**0,648**
Banf1	Rattus norvegicus barrier to autointegration factor 1 (Banf1), mRNA [NM_053631]	**0,648**
Dap	Rattus norvegicus death-associated protein (Dap), mRNA [NM_022526]	**0,648**
Man1c1	Rattus norvegicus mannosidase, alpha, class 1C, member 1 (Man1c1), mRNA [NM_001108687]	**0,648**
Chmp4b	PREDICTED: Rattus norvegicus chromatin modifying protein 4B (Chmp4b), mRNA [XM_002726259]	**0,648**

Stau1	Rattus norvegicus staufen RNA binding protein homolog 1 (Drosophila) (Stau1), transcript variant 1, mRNA [NM_053436]	**0,648**
Ptbp2	Rattus norvegicus polypyrimidine tract binding protein 2 (Ptbp2), mRNA [NM_001005555]	**0,648**
0	Potassium voltage-gated channel subfamily D member 3 [Source:UniProtKB/Swiss-Prot;Acc:Q62897] [ENSRNOT00000019997]	**0,648**
Dock9	Rattus norvegicus dedicator of cytokinesis 9 (Dock9), mRNA [NM_001105759]	**0,648**
Mcart1	Rattus norvegicus mitochondrial carrier triple repeat 1 (Mcart1), nuclear gene encoding mitochondrial protein, mRNA [NM_001024785]	**0,648**
Flad1	Rattus norvegicus flavin adenine dinucleotide synthetase (Flad1), mRNA [NM_001110138]	**0,648**
Slc38a6	Rattus norvegicus solute carrier family 38, member 6 (Slc38a6), mRNA [NM_001013099]	**0,648**
Cx3cl1	Rattus norvegicus chemokine (C-X3-C motif) ligand 1 (Cx3cl1), mRNA [NM_134455]	**0,648**
Arpc1b	Rattus norvegicus actin related protein 2/3 complex, subunit 1B (Arpc1b), mRNA [NM_019289]	**0,648**
Gbp2	Rattus norvegicus guanylate binding protein 2 (Gbp2), mRNA [NM_133624]	**0,648**
Prdx6	Rattus norvegicus peroxiredoxin 6 (Prdx6), mRNA [NM_053576]	**0,648**
Nfasc	Rattus norvegicus neurofascin (Nfasc), transcript variant 4, mRNA [NM_001160315]	**0,648**
LOC679651	Rattus norvegicus hypothetical protein LOC679651 (LOC679651), mRNA [NM_001195277]	**0,648**
Lasp1	Rattus norvegicus LIM and SH3 protein 1 (Lasp1), mRNA [NM_032613]	**0,648**
Fchsd2	Rattus norvegicus FCH and double SH3 domains 2 (Fchsd2), mRNA [NM_001107539]	**0,648**
Mras	Rattus norvegicus muscle RAS oncogene homolog (Mras), mRNA [NM_012981]	**0,648**
Adat1	Rattus norvegicus adenosine deaminase, tRNA-specific 1 (Adat1), mRNA [NM_001109611]	**0,648**
Neto1	Rattus norvegicus neuropilin (NRP) and tolloid (TLL)-like 1 (Neto1), mRNA [NM_001107371]	**0,648**
0	BC052076 expressed sequence AW050020 {Mus musculus} (exp=-1; wgp=0; cg=0), partial (32%) [TC589792]	**0,649**
RGD1309710	Rattus norvegicus similar to RIKEN cDNA 0610038D11 (RGD1309710), mRNA [NM_001106330]	**0,649**
Tspan7	Rattus norvegicus tetraspanin 7 (Tspan7), mRNA [NM_001108815]	**0,649**
Scn8a	Rattus norvegicus sodium channel, voltage gated, type VIII, alpha subunit (Scn8a), mRNA [NM_019266]	**0,649**
Sall2	Rattus norvegicus sal-like 2 (Drosophila) (Sall2), mRNA [NM_001107262]	**0,649**
Pdzd11	Rattus norvegicus PDZ domain containing 11 (Pdzd11), mRNA [NM_001106945]	**0,649**
Adora2a	Rattus norvegicus adenosine A2a receptor (Adora2a), mRNA [NM_053294]	**0,649**
RGD1565119	PREDICTED: Rattus norvegicus similar to Mitochondrial carrier triple repeat 1 (RGD1565119), mRNA [XM_001078261]	**0,649**
Ppp1r12a	Rattus norvegicus protein phosphatase 1, regulatory (inhibitor) subunit 12A (Ppp1r12a), mRNA [NM_053890]	**0,649**
Usp6nl	Rattus norvegicus USP6 N-terminal like (Usp6nl), mRNA [NM_001106120]	**0,649**
Pepd	Rattus norvegicus peptidase D (Pepd), mRNA [NM_001009641]	**0,649**
Slc25a16	Rattus norvegicus solute carrier family 25 (mitochondrial carrier, Graves disease autoantigen), member 16 (Slc25a16), nuclear gene encoding mitochondrial protein, mRNA [NM_001100860]	**0,649**
Pnkd	Rattus norvegicus paroxysmal nonkinesiogenic dyskinesia (Pnkd), nuclear gene encoding mitochondrial protein, transcript variant 2, mRNA [NM_001134751]	**0,649**
Sv2b	Rattus norvegicus synaptic vesicle glycoprotein 2b (Sv2b), mRNA [NM_057207]	**0,649**

0	Solute carrier family 39 (Zinc transporter), member 10 (Predicted), isoform CRA_aUncharacterized protein [Source:UniProtKB/TrEMBL;Acc:D4A517] [ENSRNOT00000016262]	**0,649**
Sema6d	Rattus norvegicus sema domain, transmembrane domain (TM), and cytoplasmic domain, (semaphorin) 6D (Sema6d), mRNA [NM_001107768]	**0,649**
LOC501222	Rattus norvegicus TL0AAA47YE23 mRNA sequence. [FQ213532]	**0,649**
B3gnt7	Rattus norvegicus UDP-GlcNAc:betaGal beta-1,3-N-acetylglucosaminyltransferase 7 (B3gnt7), mRNA [NM_001012134]	**0,649**
Ddx19a	Rattus norvegicus DEAD (Asp-Glu-Ala-Asp) box polypeptide 19a (Ddx19a), mRNA [NM_001005381]	**0,649**
Pcsk1	Rattus norvegicus proprotein convertase subtilisin/kexin type 1 (Pcsk1), mRNA [NM_017091]	**0,649**
Zwint	Rattus norvegicus ZW10 interactor (Zwint), mRNA [NM_147138]	**0,649**
Atp2a2	Rattus norvegicus ATPase, Ca++ transporting, cardiac muscle, slow twitch 2 (Atp2a2), transcript variant 3, mRNA [NM_001110823]	**0,649**
Sf3a1	Rattus norvegicus splicing factor 3a, subunit 1 (Sf3a1), mRNA [NM_001107235]	**0,649**
Cdh13	Rattus norvegicus cadherin 13 (Cdh13), mRNA [NM_138889]	**0,649**
0	Unknown	**0,649**
Lhpp	Rattus norvegicus phospholysine phosphohistidine inorganic pyrophosphate phosphatase (Lhpp), mRNA [NM_001009706]	**0,649**
Csk	Rattus norvegicus c-src tyrosine kinase (Csk), mRNA [NM_001030039]	**0,649**
0	T-cell receptor beta, variable 5.2 Gene [Source:MGI Symbol;Acc:MGI:98602] [ENSRNOT00000067917]	**0,649**
Iws1	Rattus norvegicus IWS1 homolog (S. cerevisiae) (Iws1), mRNA [NM_001034918]	**0,649**
LOC499602	Rattus norvegicus hypothetical protein LOC499602 (LOC499602), mRNA [NM_001025039]	**0,649**
Pclo	Rattus norvegicus piccolo (presynaptic cytomatrix protein) (Pclo), transcript variant 2, mRNA [NM_001110797]	**0,649**
Slc35e4	Rattus norvegicus solute carrier family 35, member E4 (Slc35e4), mRNA [NM_153316]	**0,649**
Lrch1	Rattus norvegicus leucine-rich repeats and calponin homology (CH) domain containing 1 (Lrch1), mRNA [NM_001134727]	**0,649**
Pacs1	Rattus norvegicus phosphofurin acidic cluster sorting protein 1 (Pacs1), mRNA [NM_134406]	**0,649**
Fbxo15	Rattus norvegicus F-box protein 15 (Fbxo15), mRNA [NM_001108436]	**0,649**
Tmem30a	Rattus norvegicus transmembrane protein 30A (Tmem30a), mRNA [NM_001004248]	**0,649**
Echdc3	Rattus norvegicus enoyl Coenzyme A hydratase domain containing 3 (Echdc3), nuclear gene encoding mitochondrial protein, mRNA [NM_001101010]	**0,649**
Stt3a	Rattus norvegicus STT3, subunit of the oligosaccharyltransferase complex, homolog A (S. cerevisiae) (Stt3a), mRNA [NM_001134749]	**0,649**
Cnih2	Rattus norvegicus cornichon homolog 2 (Drosophila) (Cnih2), mRNA [NM_001025132]	**0,649**
39326	Rattus norvegicus septin 7 (Sept7), transcript variant 2, mRNA [NM_001113740]	**0,649**
Cmip	Rattus norvegicus c-Maf-inducing protein (Cmip), mRNA [NM_001163273]	**0,650**
0	AMGNNUC:NRHY5-00189-A7-A W Rat hypothalamus (10471) Rattus norvegicus cDNA clone nrhy5-00189-a7 5', mRNA sequence [CB580197]	**0,650**
Baiap2	Rattus norvegicus BAI1-associated protein 2 (Baiap2), mRNA [NM_057196]	**0,650**
Xpo6	Rattus norvegicus exportin 6 (Xpo6), mRNA [NM_001011935]	**0,650**
Pde10a	Rattus norvegicus phosphodiesterase 10A (Pde10a), mRNA [NM_022236]	**0,650**
RGD1564420	Uncharacterized protein [Source:UniProtKB/TrEMBL;Acc:D4AEB6] [ENSRNOT00000013315]	**0,650**

Cpne6	Rattus norvegicus copine VI (Cpne6), mRNA [NM_001191113]	**0,650**
Sptan1	Rattus norvegicus spectrin, alpha, non-erythrocytic 1 (Sptan1), mRNA [NM_171983]	**0,650**
Ctdspl2	Rattus norvegicus CTD (carboxy-terminal domain, RNA polymerase II, polypeptide A) small phosphatase like 2 (Ctdspl2), mRNA [NM_001014048]	**0,650**
Plcxd2	Rattus norvegicus phosphatidylinositol-specific phospholipase C, X domain containing 2 (Plcxd2), mRNA [NM_001134481]	**0,650**
Chn1	Rattus norvegicus chimerin (chimaerin) 1 (Chn1), mRNA [NM_032083]	**0,650**
Mrps36	Rattus norvegicus mitochondrial ribosomal protein S36 (Mrps36), nuclear gene encoding mitochondrial protein, mRNA [NM_001191605]	**0,650**
RGD1561728	Rattus norvegicus similar to high mobility group protein (LOC307239), mRNA [XM_225740]	**0,650**
Fosl2	Rattus norvegicus fos-like antigen 2 (Fosl2), mRNA [NM_012954]	**0,650**
Atp2c1	Rattus norvegicus ATPase, Ca++ transporting, type 2C, member 1 (Atp2c1), mRNA [NM_131907]	**0,650**
Uxt	Rattus norvegicus ubiquitously expressed transcript (Uxt), mRNA [NM_001006982]	**0,650**
LOC497899	Rattus norvegicus similar to hypothetical protein 4930503F14 (LOC497899), mRNA [NM_001017472]	**0,650**
Cpsf6	Rattus norvegicus cleavage and polyadenylation specific factor 6 (Cpsf6), mRNA [NM_001106785]	**0,650**
RT1-CE7	Rattus norvegicus RT1 class I, locus CE7 (RT1-CE7), mRNA [NM_001008845]	**0,650**
Slc2a1	Rattus norvegicus solute carrier family 2 (facilitated glucose transporter), member 1 (Slc2a1), mRNA [NM_138827]	**0,650**
Bahd1	PREDICTED: Rattus norvegicus bromo adjacent homology domain containing 1 (Bahd1), mRNA [XM_347035]	**0,650**
Atf2	Rattus norvegicus activating transcription factor 2 (Atf2), mRNA [NM_031018]	**0,650**
RGD1559908	PREDICTED: Rattus norvegicus similar to hypothetical protein (RGD1559908), miscRNA [XR_086058]	**0,650**
Adprh	Rattus norvegicus ADP-ribosylarginine hydrolase (Adprh), mRNA [NM_183325]	**0,650**
Znfx1	Rattus norvegicus zinc finger, NFX1-type containing 1 (Znfx1), mRNA [NM_001047860]	**0,650**
0	Unknown	**0,650**
Ralbp1	Rattus norvegicus ralA binding protein 1 (Ralbp1), mRNA [NM_032067]	**0,650**
Accn2	Rattus norvegicus amiloride-sensitive cation channel 2, neuronal (Accn2), mRNA [NM_024154]	**0,650**
Pou6f1	Rattus norvegicus POU class 6 homeobox 1 (Pou6f1), mRNA [NM_001105746]	**0,650**
0	Rattus norvegicus TL0AEA61YM21 mRNA sequence. [FQ233449]	**0,650**
Fxyd6	Rattus norvegicus FXYD domain-containing ion transport regulator 6 (Fxyd6), mRNA [NM_022005]	**0,650**
Pdpk1	Rattus norvegicus 3-phosphoinositide dependent protein kinase-1 (Pdpk1), mRNA [NM_031081]	**0,650**
Mxi1	Rattus norvegicus MAX interactor 1 (Mxi1), mRNA [NM_013160]	**0,650**
RGD1307749	Rattus norvegicus similar to RIKEN cDNA 1600013K19 (RGD1307749), mRNA [NM_001106759]	**0,650**
Cntn6	Rattus norvegicus contactin 6 (Cntn6), mRNA [NM_013225]	**0,650**
Klf16	Rattus norvegicus Kruppel-like factor 16 (Klf16), mRNA [NM_001127604]	**0,650**
Gucy1a3	Rattus norvegicus guanylate cyclase 1, soluble, alpha 3 (Gucy1a3), mRNA [NM_017090]	**0,650**
Rgl1	Rattus norvegicus ral guanine nucleotide dissociation stimulator,-like 1 (Rgl1), mRNA [NM_001105957]	**0,650**
Hsbp1	Rattus norvegicus heat shock factor binding protein 1 (Hsbp1), mRNA [NM_173119]	**0,650**
Ing3	Rattus norvegicus inhibitor of growth family, member 3 (Ing3), mRNA [NM_001034107]	**0,651**

Samd14	Rattus norvegicus sterile alpha motif domain containing 14 (Samd14), mRNA [NM_001024966]	**0,651**
Slc25a30	Rattus norvegicus solute carrier family 25, member 30 (Slc25a30), nuclear gene encoding mitochondrial protein, mRNA [NM_001013187]	**0,651**
0	Unknown	**0,651**
Erlin1	Rattus norvegicus ER lipid raft associated 1 (Erlin1), mRNA [NM_001106353]	**0,651**
Reln	Rattus norvegicus reelin (Reln), mRNA [NM_080394]	**0,651**
Olfml3	Rattus norvegicus olfactomedin-like 3 (Olfml3), mRNA [NM_001107708]	**0,651**
Dpy30	Rattus norvegicus dpy-30 homolog (C. elegans) (Dpy30), transcript variant 1, mRNA [NM_173117]	**0,651**
0	PREDICTED: Rattus norvegicus RALBP1 associated Eps domain containing 2 (Reps2), miscRNA [XR_085945]	**0,651**
0	Uncharacterized protein [Source:UniProtKB/TrEMBL;Acc:D3ZNV0] [ENSRNOT00000064946]	**0,651**
Rics	PREDICTED: Rattus norvegicus Rho GTPase-activating protein (Rics), mRNA [XM_001056872]	**0,651**
Vps39	Rattus norvegicus vacuolar protein sorting 39 homolog (S. cerevisiae) (Vps39), mRNA [NM_001012186]	**0,651**
Arpp21	Rattus norvegicus cAMP-regulated phosphoprotein 21 (Arpp21), transcript variant 1, mRNA [NM_001135046]	**0,651**
Mark1	Rattus norvegicus MAP/microtubule affinity-regulating kinase 1 (Mark1), mRNA [NM_053947]	**0,651**
Trim26	Rattus norvegicus tripartite motif-containing 26 (Trim26), mRNA [NM_001011665]	**0,651**
Abi2	Rattus norvegicus abl-interactor 2 (Abi2), mRNA [NM_173143]	**0,651**
Pum1	Rattus norvegicus pumilio homolog 1 (Drosophila) (Pum1), mRNA [NM_001108684]	**0,651**
RGD1311595	PREDICTED: Rattus norvegicus similar to KIAA2026 protein (RGD1311595), mRNA [XM_001079649]	**0,651**
Zfp469	Rattus norvegicus zinc finger protein 469 (Zfp469), mRNA [NM_001107123]	**0,651**
Cetn4	Unknown	**0,651**
Mkl1	PREDICTED: Rattus norvegicus megakaryoblastic leukemia (translocation) 1 (Mkl1), mRNA [XM_235497]	**0,651**
Stradb	Rattus norvegicus STE20-related kinase adaptor beta (Stradb), mRNA [NM_001109307]	**0,651**
Mapt	Rattus norvegicus microtubule-associated protein tau (Mapt), mRNA [NM_017212]	**0,651**
0	Uncharacterized protein [Source:UniProtKB/TrEMBL;Acc:D3ZQC4] [ENSRNOT00000050888]	**0,651**
Marcksl1	Rattus norvegicus MARCKS-like 1 (Marcksl1), mRNA [NM_030862]	**0,651**
Gpd1	Rattus norvegicus glycerol-3-phosphate dehydrogenase 1 (soluble) (Gpd1), mRNA [NM_022215]	**0,651**
Plxna2	Rattus norvegicus plexin A2 (Plxna2), mRNA [NM_001105988]	**0,651**
Sod3	Rattus norvegicus superoxide dismutase 3, extracellular (Sod3), mRNA [NM_012880]	**0,651**
Ak1	Rattus norvegicus adenylate kinase 1 (Ak1), mRNA [NM_024349]	**0,652**
Gabbr1	Rattus norvegicus gamma-aminobutyric acid (GABA) B receptor 1 (Gabbr1), mRNA [NM_031028]	**0,652**
Sema5b	Rattus norvegicus sema domain, seven thrombospondin repeats (type 1 and type 1-like), transmembrane domain (TM) and short cytoplasmic domain, (semaphorin) 5B (Sema5b), mRNA [NM_001107091]	**0,652**
Ascc1	Rattus norvegicus activating signal cointegrator 1 complex subunit 1 (Ascc1), mRNA [NM_001007632]	**0,652**
Dppa3l1	PREDICTED: Rattus norvegicus similar to developmental pluripotency-associated 3 (LOC691014), mRNA [XM_001076514]	**0,652**
0	Histone H2A type 2-A [Source:UniProtKB/Swiss-Prot;Acc:P0CC09] [ENSRNOT00000047696]	**0,652**

Tubg2	Rattus norvegicus tubulin, gamma 2 (Tubg2), mRNA [NM_001191075]	**0,652**
Omg	Rattus norvegicus oligodendrocyte-myelin glycoprotein (Omg), mRNA [NM_001005898]	**0,652**
Rnf208	Rattus norvegicus ring finger protein 208 (Rnf208), mRNA [NM_001109195]	**0,652**
0	Catenin delta-2 [Source:UniProtKB/Swiss-Prot;Acc:O35116] [ENSRNOT00000044871]	**0,652**
Acd	Rattus norvegicus adrenocortical dysplasia homolog (mouse) (Acd), mRNA [NM_001037193]	**0,652**
0	PREDICTED: Rattus norvegicus extracellular leucine-rich repeat and fibronectin type III domain containing 2 (Elfn2), miscRNA [XR_086301]	**0,652**
Lsm7	Rattus norvegicus LSM7 homolog, U6 small nuclear RNA associated (S. cerevisiae) (Lsm7), mRNA [NM_001108732]	**0,652**
Esyt1	Rattus norvegicus extended synaptotagmin-like protein 1 (Esyt1), mRNA [NM_017249]	**0,652**
Jakmip3	Rattus norvegicus janus kinase and microtubule interacting protein 3 (Jakmip3), mRNA [NM_001163277]	**0,652**
Katnal2	PREDICTED: Rattus norvegicus katanin p60 subunit A-like 2 (Katnal2), mRNA [XM_001055413]	**0,652**
Npepps	Rattus norvegicus aminopeptidase puromycin sensitive (Npepps), mRNA [NM_080395]	**0,652**
Csnk1g3	Rattus norvegicus casein kinase 1, gamma 3 (Csnk1g3), mRNA [NM_022855]	**0,652**
Mtpap	Rattus norvegicus mitochondrial poly(A) polymerase (Mtpap), nuclear gene encoding mitochondrial protein, mRNA [NM_001107359]	**0,652**
Fam81a	Rattus norvegicus family with sequence similarity 81, member A (Fam81a), mRNA [NM_001108163]	**0,652**
0	predicted gene 12258 Gene [Source:MGI Symbol;Acc:MGI:3651534] [ENSRNOT00000067580]	**0,652**
Tmub2	Rattus norvegicus transmembrane and ubiquitin-like domain containing 2 (Tmub2), mRNA [NM_001031651]	**0,652**
Asb1	Rattus norvegicus ankyrin repeat and SOCS box-containing 1 (Asb1), mRNA [NM_001108232]	**0,652**
Cotl1	Rattus norvegicus coactosin-like 1 (Dictyostelium) (Cotl1), mRNA [NM_001108452]	**0,652**
Zfp568	Uncharacterized protein [Source:UniProtKB/TrEMBL;Acc:D4A3P6] [ENSRNOT00000039872]	**0,652**
Cyp2j3	Rattus norvegicus cytochrome P450, family 2, subfamily j, polypeptide 3 (Cyp2j3), mRNA [NM_175766]	**0,652**
Tnpo2	Rattus norvegicus transportin 2 (Tnpo2), mRNA [NM_001107166]	**0,652**
Sptbn1	Rattus norvegicus spectrin, beta, non-erythrocytic 1 (Sptbn1), mRNA [NM_001013130]	**0,652**
Cpne6	Rattus norvegicus copine VI (Cpne6), mRNA [NM_001191113]	**0,652**
0	Transforming growth factor beta-2 [Source:UniProtKB/Swiss-Prot;Acc:Q07257] [ENSRNOT00000057338]	**0,652**
0	Uncharacterized protein [Source:UniProtKB/TrEMBL;Acc:D3ZFR5] [ENSRNOT00000016784]	**0,652**
Pcdh18	Rattus norvegicus protocadherin 18 (Pcdh18), mRNA [NM_001100524]	**0,652**
Lpar2	Rattus norvegicus lysophosphatidic acid receptor 2 (Lpar2), mRNA [NM_001109109]	**0,653**
RGD1565772	Rattus norvegicus similar to hypothetical protein A430110N23 (RGD1565772), mRNA [NM_001134545]	**0,653**
LOC100366259	PREDICTED: Rattus norvegicus rCG52099-like (LOC100366259), mRNA [XM_002725118]	**0,653**
RGD1309903	Uncharacterized protein [Source:UniProtKB/TrEMBL;Acc:D3ZF86] [ENSRNOT00000015927]	**0,653**
Plekhb1	Rattus norvegicus pleckstrin homology domain containing, family B (evectins) member 1 (Plekhb1), mRNA [NM_172033]	**0,653**
Acap3	Rattus norvegicus ArfGAP with coiled-coil, ankyrin repeat and PH domains 3 (Acap3), mRNA [NM_001107999]	**0,653**
Prkx	Rattus norvegicus protein kinase, X-linked (Prkx), mRNA [NM_001033963]	**0,653**
Lmf2	Rattus norvegicus lipase maturation factor 2 (Lmf2), mRNA [NM_001079939]	**0,653**

RGD1305464	Rattus norvegicus similar to human chromosome 15 open reading frame 39 (RGD1305464), mRNA [NM_001025011]	**0,653**
Cdk4	Rattus norvegicus cyclin-dependent kinase 4 (Cdk4), mRNA [NM_053593]	**0,653**
RGD1563579	Uncharacterized protein [Source:UniProtKB/TrEMBL;Acc:D4A888] [ENSRNOT00000057412]	**0,653**
0	Q4RQ24_TETNG (Q4RQ24) Chromosome 17 SCAF15006, whole genome shotgun sequence, partial (4%) [TC582327]	**0,653**
Dyrk1b	Rattus norvegicus dual-specificity tyrosine-(Y)-phosphorylation regulated kinase 1b (Dyrk1b), mRNA [NM_001107496]	**0,653**
Slc25a28	Rattus norvegicus solute carrier family 25, member 28 (Slc25a28), mRNA [NM_001109515]	**0,653**
LOC679580	PREDICTED: Rattus norvegicus similar to MIC2 like 1 (LOC679580), mRNA [XM_001053578]	**0,653**
Amac1	Rattus norvegicus acyl-malonyl condensing enzyme 1 (Amac1), mRNA [NM_001127658]	**0,653**
Adck2	Rattus norvegicus aarF domain containing kinase 2 (Adck2), mRNA [NM_001107855]	**0,653**
Sh3kbp1	Rattus norvegicus SH3-domain kinase binding protein 1 (Sh3kbp1), mRNA [NM_053360]	**0,653**
Dmrta2	Rattus norvegicus DMRT-like family A2 (Dmrta2), mRNA [NM_001107951]	**0,653**
Dynll1	Rattus norvegicus dynein light chain LC8-type 1 (Dynll1), mRNA [NM_053319]	**0,653**
Abl2	Rattus norvegicus v-abl Abelson murine leukemia viral oncogene homolog 2 (arg, Abelson-related gene) (Abl2), mRNA [NM_001107186]	**0,653**
Gm52	Rattus norvegicus envelope glycoprotein syncytin-A (Gm52), mRNA [NM_001014771]	**0,653**
0	Unknown	**0,653**
Ints9	PREDICTED: Rattus norvegicus integrator complex subunit 9 (Ints9), mRNA [XM_001066109]	**0,653**
Aaas	Rattus norvegicus achalasia, adrenocortical insufficiency, alacrimia (Allgrove, triple-A) (Aaas), mRNA [NM_001106795]	**0,653**
LOC680441	Uncharacterized protein [Source:UniProtKB/TrEMBL;Acc:D3ZSR8] [ENSRNOT00000051510]	**0,653**
0	Rattus norvegicus TL0ADA44YP20 mRNA sequence. [FQ219888]	**0,653**
Ptpn1	Rattus norvegicus protein tyrosine phosphatase, non-receptor type 1 (Ptpn1), mRNA [NM_012637]	**0,653**
Rag1ap1	Rattus norvegicus recombination activating gene 1 activating protein 1 (Rag1ap1), mRNA [NM_001106445]	**0,653**
Acsl4	Rattus norvegicus acyl-CoA synthetase long-chain family member 4 (Acsl4), mRNA [NM_053623]	**0,653**
Ocrl	Rattus norvegicus oculocerebrorenal syndrome of Lowe (Ocrl), mRNA [NM_001108256]	**0,653**
Basp1	Rattus norvegicus brain abundant, membrane attached signal protein 1 (Basp1), mRNA [NM_022300]	**0,653**
Ganab	Rattus norvegicus glucosidase, alpha; neutral AB (Ganab), mRNA [NM_001106334]	**0,653**
Atg5	Rattus norvegicus ATG5 autophagy related 5 homolog (S. cerevisiae) (Atg5), mRNA [NM_001014250]	**0,653**
Taf6	Rattus norvegicus TAF6 RNA polymerase II, TATA box binding protein (TBP)-associated factor (Taf6), mRNA [NM_001044225]	**0,653**
Pip4k2a	Rattus norvegicus phosphatidylinositol-5-phosphate 4-kinase, type II, alpha (Pip4k2a), mRNA [NM_053926]	**0,653**
0	Unknown	**0,653**
Peg10	PREDICTED: Rattus norvegicus paternally expressed 10 (Peg10), mRNA [XM_001053454]	**0,653**
Mapk6	Rattus norvegicus mitogen-activated protein kinase 6 (Mapk6), mRNA [NM_031622]	**0,653**
Gnpda1	Rattus norvegicus glucosamine-6-phosphate deaminase 1 (Gnpda1), mRNA [NM_001134995]	**0,654**
Pcdhb3	Rattus norvegicus protocadherin beta 3 (Pcdhb3), mRNA [NM_001014783]	**0,654**
LOC680711	PREDICTED: Rattus norvegicus hypothetical protein LOC680711 (LOC680711), mRNA [XM_001056892]	**0,654**

Tmem209	Rattus norvegicus transmembrane protein 209 (Tmem209), mRNA [NM_001014055]	**0,654**
Suhw3	Uncharacterized protein [Source:UniProtKB/TrEMBL;Acc:D3ZCC6] [ENSRNOT00000044435]	**0,654**
Rhbg	Rattus norvegicus Rh family, B glycoprotein (Rhbg), mRNA [NM_183054]	**0,654**
Stx16	Rattus norvegicus syntaxin 16 (Stx16), mRNA [NM_001108610]	**0,654**
Lin7c	Rattus norvegicus lin-7 homolog C (C. elegans) (Lin7c), mRNA [NM_021851]	**0,654**
0	Rattus norvegicus similar to glyceraldehyde-3-phosphate dehydrogenase (phosphorylating) (EC 1.2.1.12) - mouse (LOC365427), mRNA [XM_345008]	**0,654**
LOC683516	PREDICTED: Rattus norvegicus similar to Zinc finger X-linked protein ZXDB (LOC683516), partial mRNA [XM_001066304]	**0,654**
0	1PNS_B Chain B, Crystal Structure Of A Streptomycin Dependent Ribosome From E. Coli, 30s Subunit Of 70s Ribosome. This File, 1pns, Contains The 30s Subunit, Two Trnas, And One Mrna Molecule. The 50s Ribosomal Subunit Is In File 1pnu. {Escherichia coli} (e	**0,654**
LOC688083	PREDICTED: Rattus norvegicus similar to LRP16 protein, transcript variant 2 (LOC688083), mRNA [XM_001080245]	**0,654**
0	slingshot homolog 1 (Drosophila) Gene [Source:MGI Symbol;Acc:MGI:2686240] [ENSRNOT00000044751]	**0,654**
Il33	Rattus norvegicus interleukin 33 (Il33), mRNA [NM_001014166]	**0,654**
Psmb11	Rattus norvegicus proteasome (prosome, macropain) subunit, beta type, 11 (Psmb11), mRNA [NM_001106032]	**0,654**
Ykt6	Rattus norvegicus YKT6 v-SNARE homolog (S. cerevisiae) (Ykt6), mRNA [NM_031692]	**0,654**
Kif16b	Rattus norvegicus kinesin family member 16B (Kif16b), mRNA [NM_001107783]	**0,654**
Prickle1	Rattus norvegicus prickle homolog 1 (Drosophila) (Prickle1), mRNA [NM_199396]	**0,654**
Slc26a1	Rattus norvegicus solute carrier family 26 (sulfate transporter), member 1 (Slc26a1), mRNA [NM_022287]	**0,654**
LOC690079	Rattus norvegicus hypothetical protein LOC690079 (LOC690079), mRNA [NM_001109566]	**0,654**
Chst7	Rattus norvegicus carbohydrate (N-acetylglucosamine 6-O) sulfotransferase 7 (Chst7), mRNA [NM_207600]	**0,654**
Leng1	Rattus norvegicus leukocyte receptor cluster (LRC) member 1 (Leng1), mRNA [NM_001106218]	**0,654**
Stac	PREDICTED: Rattus norvegicus SH3 and cysteine rich domain (Stac), mRNA [XM_001076458]	**0,654**
Mid1	Rattus norvegicus midline 1 (Mid1), mRNA [NM_022927]	**0,654**
Vpreb3	Rattus norvegicus pre-B lymphocyte 3 (Vpreb3), mRNA [NM_001108930]	**0,654**
Pcolce	Rattus norvegicus procollagen C-endopeptidase enhancer (Pcolce), mRNA [NM_019237]	**0,654**
Jak1	Rattus norvegicus Janus kinase 1 (Jak1), mRNA [NM_053466]	**0,654**
0	Unknown	**0,654**
Pggt1b	Rattus norvegicus protein geranylgeranyltransferase type I, beta subunit (Pggt1b), mRNA [NM_031082]	**0,654**
Rnf180	Rattus norvegicus ring finger protein 180 (Rnf180), mRNA [NM_001134986]	**0,654**
Gbp5	Rattus norvegicus guanylate binding protein 5 (Gbp5), mRNA [NM_001108569]	**0,654**
Ncam2	Rattus norvegicus neural cell adhesion molecule 2 (Ncam2), mRNA [NM_203409]	**0,654**
Sumo2	Rattus norvegicus SMT3 suppressor of mif two 3 homolog 2 (S. cerevisiae) (Sumo2), mRNA [NM_133594]	**0,654**
Tmed7	Rattus norvegicus transmembrane emp24 protein transport domain containing 7 (Tmed7), mRNA [NM_001105758]	**0,654**
Ubxn8	Rattus norvegicus UBX domain protein 8 (Ubxn8), mRNA [NM_001106086]	**0,654**

Adnp2	Rattus norvegicus ADNP homeobox 2 (Adnp2), mRNA [NM_001127373]	**0,654**
Ntrk2	Rattus norvegicus neurotrophic tyrosine kinase, receptor, type 2 (Ntrk2), transcript variant 2, mRNA [NM_001163168]	**0,654**
0	LOC100125377 proteinRCG58522 [Source:UniProtKB/TrEMBL;Acc:Q5HZD9] [ENSRNOT00000039741]	**0,655**
Eif1ay	Rattus norvegicus eukaryotic translation initiation factor 1A, Y-linked (Eif1ay), mRNA [NM_001106963]	**0,655**
0	Unknown	**0,655**
0	host cell factor C1 regulator 1 [Source:RefSeq peptide;Acc:NP_001093962] [ENSRNOT00000004702]	**0,655**
0	Similar to intracellular membrane-associated calcium-independent phospholipase A2 gamma (Predicted), isoform CRA_bUncharacterized protein [Source:UniProtKB/TrEMBL;Acc:D3ZRC4] [ENSRNOT00000047296]	**0,655**
Gsk3b	Rattus norvegicus glycogen synthase kinase 3 beta (Gsk3b), mRNA [NM_032080]	**0,655**
RGD1562533	Rattus norvegicus similar to mKIAA0774 protein (RGD1562533), mRNA [NM_001100989]	**0,655**
0	Unknown	**0,655**
LOC301128	Rattus norvegicus similar to jumonji domain containing 2B (LOC301128), mRNA [NM_001044236]	**0,655**
0	Unknown	**0,655**
Notch1	Rattus norvegicus Notch homolog 1, translocation-associated (Drosophila) (Notch1), mRNA [NM_001105721]	**0,655**
0	Uncharacterized protein [Source:UniProtKB/TrEMBL;Acc:D3ZXW1] [ENSRNOT00000030613]	**0,655**
0	AGENCOURT_26626347 NIH_MGC_253 Rattus norvegicus cDNA clone IMAGE:7301841 5', mRNA sequence [CO388903]	**0,655**
Ece1	Endothelin-converting enzyme 1 [Source:UniProtKB/Swiss-Prot;Acc:P42893] [ENSRNOT00000067616]	**0,655**
Zfp169	Uncharacterized protein [Source:UniProtKB/TrEMBL;Acc:D3Z9A8] [ENSRNOT00000022932]	**0,655**
Vps52	Rattus norvegicus vacuolar protein sorting 52 homolog (S. cerevisiae) (Vps52), mRNA [NM_033097]	**0,655**
Vegfa	Rattus norvegicus vascular endothelial growth factor A (Vegfa), transcript variant 3, mRNA [NM_001110334]	**0,655**
Rftn1	Rattus norvegicus raftlin lipid raft linker 1 (Rftn1), mRNA [NM_001135011]	**0,655**
0	Uncharacterized protein [Source:UniProtKB/TrEMBL;Acc:D3ZKR7] [ENSRNOT00000046075]	**0,655**
Ube2m	Rattus norvegicus ubiquitin-conjugating enzyme E2M (UBC12 homolog, yeast) (Ube2m), mRNA [NM_001108471]	**0,655**
LOC686841	Rattus norvegicus similar to Protein EAN57 (LOC686841), mRNA [NM_001044289]	**0,655**
LOC307974	Rattus norvegicus similar to dJ1016N21.1 (novel protein (ortholog of Drosophila Pecanex (PCX), similar to KIAA0805)) (LOC307974), mRNA [XM_226603]	**0,655**
Trove2	Rattus norvegicus TROVE domain family, member 2 (Trove2), mRNA [NM_001107183]	**0,655**
0	Unknown	**0,655**
Zbtb34	PREDICTED: Rattus norvegicus zinc finger and BTB domain containing 34 (Zbtb34), mRNA [XM_001069858]	**0,655**
0	Unknown	**0,655**
Cachd1	Rattus norvegicus cache domain containing 1 (Cachd1), mRNA [NM_001191758]	**0,655**
Met	Rattus norvegicus met proto-oncogene (Met), mRNA [NM_031517]	**0,655**
RGD1305572	Rattus norvegicus similar to hypothetical protein MGC30618 (RGD1305572), mRNA [NM_001024970]	**0,655**
Gpr137	Rattus norvegicus G protein-coupled receptor 137 (Gpr137), mRNA [NM_001109562]	**0,655**

Psmf1	Rattus norvegicus proteasome inhibitor subunit 1 (Psmf1), mRNA [NM_001101005]	**0,655**
Pcyox1l	Rattus norvegicus prenylcysteine oxidase 1 like (Pcyox1l), mRNA [NM_001134542]	**0,655**
Ntrk2	Rattus norvegicus neurotrophic tyrosine kinase, receptor, type 2 (Ntrk2), transcript variant 1, mRNA [NM_012731]	**0,655**
Lsamp	Rattus norvegicus limbic system-associated membrane protein (Lsamp), mRNA [NM_017242]	**0,655**
Zfp853	PREDICTED: Rattus norvegicus zinc finger protein 853 (Zfp853), mRNA [XM_002724787]	**0,655**
LOC684122	PREDICTED: Rattus norvegicus similar to development and differentiation enhancing factor-like 1 (LOC684122), mRNA [XM_001069024]	**0,655**
Fam117b	Rattus norvegicus family with sequence similarity 117, member B (Fam117b), mRNA [NM_001108797]	**0,655**
Chkb	Rattus norvegicus choline kinase beta (Chkb), mRNA [NM_017177]	**0,655**
Prkar2a	Rattus norvegicus protein kinase, cAMP dependent regulatory, type II alpha (Prkar2a), mRNA [NM_019264]	**0,655**
Fam57b	Rattus norvegicus family with sequence similarity 57, member B (Fam57b), mRNA [NM_001106296]	**0,655**
Sh3bgrl3	Rattus norvegicus SH3 domain binding glutamic acid-rich protein-like 3 (Sh3bgrl3), mRNA [NM_001106688]	**0,655**
Pank1	Rattus norvegicus pantothenate kinase 1 (Pank1), mRNA [NM_001106373]	**0,655**
RGD1563556	Uncharacterized protein [Source:UniProtKB/TrEMBL;Acc:D3ZT87] [ENSRNOT00000029887]	**0,655**
Slc10a3	Rattus norvegicus solute carrier family 10 (sodium/bile acid cotransporter family), member 3 (Slc10a3), mRNA [NM_001024368]	**0,655**
Gpr156	Rattus norvegicus G protein-coupled receptor 156 (Gpr156), mRNA [NM_153295]	**0,655**
Zeb1	Rattus norvegicus zinc finger E-box binding homeobox 1 (Zeb1), mRNA [NM_013164]	**0,655**
Tbl1xr1	Rattus norvegicus transducin (beta)-like 1 X-linked receptor 1 (Tbl1xr1), mRNA [NM_001108941]	**0,655**
Eif4b	Rattus norvegicus eukaryotic translation initiation factor 4B (Eif4b), mRNA [NM_001008324]	**0,655**
Traf6	Rattus norvegicus Tnf receptor-associated factor 6 (Traf6), mRNA [NM_001107754]	**0,656**
LOC683516	PREDICTED: Rattus norvegicus similar to Zinc finger X-linked protein ZXDB (LOC683516), partial mRNA [XM_001066304]	**0,656**
Tanc2	Rattus norvegicus tetratricopeptide repeat, ankyrin repeat and coiled-coil containing 2 (Tanc2), mRNA [NM_001191653]	**0,656**
Tbc1d9	Rattus norvegicus TBC1 domain family, member 9 (Tbc1d9), mRNA [NM_001134539]	**0,656**
Rundc1	Q96C34_HUMAN (Q96C34) RUNDC1 protein (Fragment), partial (40%) [TC601870]	**0,656**
RGD1562310	Rattus norvegicus similar to hypothetical protein FLJ21415 (RGD1562310), mRNA [NM_001109066]	**0,656**
Sod2	Rattus norvegicus superoxide dismutase 2, mitochondrial (Sod2), nuclear gene encoding mitochondrial protein, mRNA [NM_017051]	**0,656**
Exoc4	Rattus norvegicus exocyst complex component 4 (Exoc4), mRNA [NM_053875]	**0,656**
Tln1	Rattus norvegicus talin 1 (Tln1), mRNA [NM_001039025]	**0,656**
Pctk1	Rattus norvegicus PCTAIRE protein kinase 1 (Pctk1), transcript variant 2, mRNA [NM_031077]	**0,656**
Uroc1	Uncharacterized protein [Source:UniProtKB/TrEMBL;Acc:D3ZDW1] [ENSRNOT00000038251]	**0,656**
Snapc5	Rattus norvegicus small nuclear RNA activating complex, polypeptide 5 (Snapc5), mRNA [NM_001109643]	**0,656**
RGD1566084	Uncharacterized protein [Source:UniProtKB/TrEMBL;Acc:D3ZPT0] [ENSRNOT00000043197]	**0,656**
Nmur1	Rattus norvegicus neuromedin U receptor 1 (Nmur1), mRNA [NM_023100]	**0,656**
Brwd3	Uncharacterized protein [Source:UniProtKB/TrEMBL;Acc:D3Z8C5] [ENSRNOT00000050336]	**0,656**
Rnf39	Rattus norvegicus ring finger protein 39 (Rnf39), mRNA [NM_134374]	**0,656**

Srd5a3	Rattus norvegicus steroid 5 alpha-reductase 3 (Srd5a3), mRNA [NM_001013990]	**0,656**
Bak1	Rattus norvegicus BCL2-antagonist/killer 1 (Bak1), mRNA [NM_053812]	**0,656**
Slc22a12	Rattus norvegicus solute carrier family 22 (organic anion/urate transporter), member 12 (Slc22a12), mRNA [NM_001034943]	**0,656**
Rnf150	Rattus norvegicus ring finger protein 150 (Rnf150), mRNA [NM_001191093]	**0,656**
Pcif1	Rattus norvegicus PDX1 C-terminal inhibiting factor 1 (Pcif1), mRNA [NM_001108605]	**0,656**
Prmt6	Rattus norvegicus protein arginine methyltransferase 6 (Prmt6), mRNA [NM_001106466]	**0,656**
Dcaf7	Rattus norvegicus DDB1 and CUL4 associated factor 7 (Dcaf7), mRNA [NM_001107057]	**0,656**
Dmwd	PREDICTED: Rattus norvegicus dystrophia myotonica, WD repeat containing (Dmwd), mRNA [XM_002725588]	**0,656**
Cc2d1a	Rattus norvegicus coiled-coil and C2 domain containing 1A (Cc2d1a), mRNA [NM_001013869]	**0,656**
Optn	Rattus norvegicus optineurin (Optn), mRNA [NM_145081]	**0,656**
Dgat2l6	Rattus norvegicus diacylglycerol O-acyltransferase 2-like 6 (Dgat2l6), mRNA [NM_001109370]	**0,656**
0	Kinesin light chain 2 (Predicted), isoform CRA_bKlc2 protein [Source:UniProtKB/TrEMBL;Acc:B2GV74] [ENSRNOT00000041238]	**0,656**
Sobpl	Rattus norvegicus sine oculis-binding protein homolog-like (Drosophila) (Sobpl), mRNA [NM_001104640]	**0,656**
Cald1	Rattus norvegicus caldesmon 1 (Cald1), mRNA [NM_013146]	**0,656**
Ppfia2	Rattus norvegicus protein tyrosine phosphatase, receptor type, f polypeptide (PTPRF), interacting protein (liprin), alpha 2 (Ppfia2), mRNA [NM_001108745]	**0,656**
Hip1	Rattus norvegicus huntingtin interacting protein 1 (Hip1), mRNA [NM_001100475]	**0,656**
G3bp1	Rattus norvegicus GTPase activating protein (SH3 domain) binding protein 1 (G3bp1), mRNA [NM_133565]	**0,656**
Fam5b	Rattus norvegicus family with sequence similarity 5, member B (Fam5b), mRNA [NM_173115]	**0,656**
Plp1	Rattus norvegicus proteolipid protein 1 (Plp1), mRNA [NM_030990]	**0,656**
Fam122b	Rattus norvegicus family with sequence similarity 122B (Fam122b), mRNA [NM_001166586]	**0,657**
Lmbrd2	Rattus norvegicus LMBR1 domain containing 2 (Lmbrd2), mRNA [NM_001109177]	**0,657**
Slc29a4	Rattus norvegicus solute carrier family 29 (nucleoside transporters), member 4 (Slc29a4), mRNA [NM_001105911]	**0,657**
Lrit1	Leucine-rich repeat, immunoglobulin-like domain and transmembrane domain-containing protein 1 [Source:UniProtKB/Swiss-Prot;Acc:Q9JMH2] [ENSRNOT00000017773]	**0,657**
RGD1309079	Rattus norvegicus similar to Ab2-095 (RGD1309079), mRNA [NM_001134472]	**0,657**
Pcdhb9	Rattus norvegicus protocadherin beta 9 (Pcdhb9), mRNA [NM_001109390]	**0,657**
0	Unknown	**0,657**
Akirin1	Rattus norvegicus akirin 1 (Akirin1), mRNA [NM_001030054]	**0,657**
Ostm1	Rattus norvegicus osteopetrosis associated transmembrane protein 1 (Ostm1), mRNA [NM_001029925]	**0,657**
RGD1565591	PREDICTED: Rattus norvegicus similar to Ski protein (RGD1565591), partial mRNA [XM_001077382]	**0,657**
Ppig	Rattus norvegicus peptidylprolyl isomerase G (Ppig), mRNA [NM_031793]	**0,657**
RGD1311345	Rattus norvegicus similar to CG9752-PA (RGD1311345), mRNA [NM_001173436]	**0,657**
Ril	Rattus norvegicus reversion induced LIM gene (Ril), mRNA [NM_017062]	**0,657**

Narg1	Rattus norvegicus NMDA receptor regulated 1 (Narg1), mRNA [NM_001107674]	**0,657**
Fcho2	Rattus norvegicus FCH domain only 2 (Fcho2), mRNA [NM_001191632]	**0,657**
Nlk	Rattus norvegicus nemo like kinase (Nlk), mRNA [NM_001191924]	**0,657**
Vom2r72	Rattus norvegicus vomeronasal 2 receptor, 72 (Vom2r72), mRNA [NM_001099517]	**0,657**
0	Unknown	**0,657**
Ehd1	Rattus norvegicus EH-domain containing 1 (Ehd1), mRNA [NM_001011939]	**0,657**
Slc27a1	Rattus norvegicus solute carrier family 27 (fatty acid transporter), member 1 (Slc27a1), mRNA [NM_053580]	**0,657**
Nphp3	Rattus norvegicus nephronophthisis 3 (adolescent) (Nphp3), mRNA [NM_001191882]	**0,657**
Btrc	Rattus norvegicus beta-transducin repeat containing (Btrc), mRNA [NM_001007148]	**0,657**
Arl2bp	Rattus norvegicus ADP-ribosylation factor-like 2 binding protein (Arl2bp), mRNA [NM_001024906]	**0,657**
0	Q40555_TOBAC (Q40555) Peroxidase, partial (5%) [TC599198]	**0,657**
Olr390	Rattus norvegicus olfactory receptor 390 (Olr390), mRNA [NM_001000558]	**0,657**
Tfrc	Rattus norvegicus transferrin receptor (Tfrc), mRNA [NM_022712]	**0,657**
Pitpnm2	Rattus norvegicus phosphatidylinositol transfer protein, membrane-associated 2 (Pitpnm2), mRNA [NM_001107139]	**0,657**
Kctd13	Rattus norvegicus potassium channel tetramerisation domain containing 13 (Kctd13), mRNA [NM_198736]	**0,657**
Fam176b	Rattus norvegicus family with sequence similarity 176, member B (Fam176b), mRNA [NM_001108679]	**0,657**
Opcml	Rattus norvegicus opioid binding protein/cell adhesion molecule-like (Opcml), mRNA [NM_053848]	**0,657**
Rgs20	Rattus norvegicus regulator of G-protein signaling 20 (Rgs20), mRNA [NM_001127495]	**0,657**
Map2k1ip1	Rattus norvegicus mitogen-activated protein kinase kinase 1 interacting protein 1 (Map2k1ip1), mRNA [NM_001008375]	**0,657**
Smpd3	Rattus norvegicus sphingomyelin phosphodiesterase 3, neutral membrane (Smpd3), mRNA [NM_053605]	**0,657**
RGD1562342	Rattus norvegicus similar to RIKEN cDNA 1110012D08 (RGD1562342), mRNA [NM_001109281]	**0,657**
Fcho1	Rattus norvegicus FCH domain only 1 (Fcho1), mRNA [NM_001106069]	**0,657**
Ado	Rattus norvegicus 2-aminoethanethiol (cysteamine) dioxygenase (Ado), mRNA [NM_001107626]	**0,657**
Mgat5	Rattus norvegicus mannosyl (alpha-1,6-)-glycoprotein beta-1,6-N-acetyl-glucosaminyltransferase (Mgat5), mRNA [NM_023095]	**0,657**
Ret	Rattus norvegicus ret proto-oncogene (Ret), transcript variant 1, mRNA [NM_012643]	**0,657**
Pppde1	Rattus norvegicus PPPDE peptidase domain containing 1 (Pppde1), mRNA [NM_001013873]	**0,657**
Znrf2	Rattus norvegicus zinc and ring finger 2 (Znrf2), mRNA [NM_001108628]	**0,658**
Acsl6	Rattus norvegicus acyl-CoA synthetase long-chain family member 6 (Acsl6), mRNA [NM_130739]	**0,658**
0	Unknown	**0,658**
Alkbh5	Uncharacterized protein [Source:UniProtKB/TrEMBL;Acc:D3ZKD3] [ENSRNOT00000034006]	**0,658**
0	Unknown	**0,658**
0	Q9ERD6_MOUSE (Q9ERD6) Ral-A exchange factor RalGPS2, partial (28%) [TC597248]	**0,658**
Bag4	Rattus norvegicus BCL2-associated athanogene 4 (Bag4), mRNA [NM_001025130]	**0,658**
Nkain3	Rattus norvegicus Na+/K+ transporting ATPase interacting 3 (Nkain3), mRNA [NM_001109540]	**0,658**

Lrfn4	Rattus norvegicus leucine rich repeat and fibronectin type III domain containing 4 (Lrfn4), mRNA [NM_001109508]	**0,658**
Epha7	Rattus norvegicus Eph receptor A7 (Epha7), mRNA [NM_134331]	**0,658**
Shank3	Rattus norvegicus SH3 and multiple ankyrin repeat domains 3 (Shank3), mRNA [NM_021676]	**0,658**
Snapc2	Rattus norvegicus small nuclear RNA activating complex, polypeptide 2 (Snapc2), mRNA [NM_001013121]	**0,658**
0	Unknown	**0,658**
Cib1	Rattus norvegicus calcium and integrin binding 1 (calmyrin) (Cib1), mRNA [NM_031145]	**0,658**
Sfrs18	Rattus norvegicus splicing factor, arginine/serine-rich 18 (Sfrs18), mRNA [NM_001025274]	**0,658**
Kalrn	Rattus norvegicus kalirin, RhoGEF kinase (Kalrn), mRNA [NM_032062]	**0,658**
Tmem109	Rattus norvegicus transmembrane protein 109 (Tmem109), mRNA [NM_001007736]	**0,658**
Rab36	Rattus norvegicus RAB36, member RAS oncogene family (Rab36), mRNA [NM_001109589]	**0,658**
Yipf3	Rattus norvegicus Yip1 domain family, member 3 (Yipf3), mRNA [NM_001007801]	**0,658**
Wnk3	Rattus norvegicus WNK lysine deficient protein kinase 3 (Wnk3), mRNA [NM_001163607]	**0,658**
Tmx1	Rattus norvegicus thioredoxin-related transmembrane protein 1 (Tmx1), mRNA [NM_001024800]	**0,658**
Ino80e	Rattus norvegicus INO80 complex subunit E (Ino80e), mRNA [NM_001013900]	**0,658**
Dus4l	Rattus norvegicus dihydrouridine synthase 4-like (S. cerevisiae) (Dus4l), mRNA [NM_001135803]	**0,658**
0	Unknown	**0,658**
0	2',5'-phosphodiesterase 12 [Source:UniProtKB/Swiss-Prot;Acc:Q6AXQ5] [ENSRNOT00000017800]	**0,658**
Spint2	Rattus norvegicus serine peptidase inhibitor, Kunitz type, 2 (Spint2), transcript variant 1, mRNA [NM_001082549]	**0,658**
PVR	Rattus norvegicus poliovirus receptor (PVR), mRNA [NM_017076]	**0,658**
Rogdi	Rattus norvegicus rogdi homolog (Drosophila) (Rogdi), mRNA [NM_001024864]	**0,658**
Wibg	Rattus norvegicus within bgcn homolog (Drosophila) (Wibg), mRNA [NM_001108986]	**0,658**
0	Unknown	**0,658**
Calm1	Rattus norvegicus calmodulin 1 (Calm1), mRNA [NM_031969]	**0,658**
LOC499330	Rattus norvegicus similar to Nicotinamide riboside kinase 1 (LOC499330), mRNA [NM_001024292]	**0,658**
Camk2b	Rattus norvegicus calcium/calmodulin-dependent protein kinase II beta (Camk2b), transcript variant 2, mRNA [NM_021739]	**0,658**
Vac14	Rattus norvegicus Vac14 homolog (S. cerevisiae) (Vac14), mRNA [NM_177930]	**0,659**
0	Q8R1R5_RAT (Q8R1R5) MIC2L1, partial (9%) [TC628118]	**0,659**
0	Q9UL04_HUMAN (Q9UL04) Membrane protein CH1, partial (9%) [TC585082]	**0,659**
Ccdc86	Rattus norvegicus coiled-coil domain containing 86 (Ccdc86), mRNA [NM_001006974]	**0,659**
Lrrc46	Rattus norvegicus leucine rich repeat containing 46 (Lrrc46), mRNA [NM_001004201]	**0,659**
0	Unknown	**0,659**
Fam108c1	Rattus norvegicus family with sequence similarity 108, member C1 (Fam108c1), mRNA [NM_001100736]	**0,659**
Tgs1	Rattus norvegicus trimethylguanosine synthase homolog (S. cerevisiae) (Tgs1), mRNA [NM_001107904]	**0,659**
0	Unknown	**0,659**

Nat15	Rattus norvegicus N-acetyltransferase 15 (GCN5-related, putative) (Nat15), mRNA [NM_001014226]	**0,659**
Prps2	Rattus norvegicus phosphoribosyl pyrophosphate synthetase 2 (Prps2), mRNA [NM_012634]	**0,659**
Ankrd34a	Rattus norvegicus ankyrin repeat domain 34A (Ankrd34a), mRNA [NM_001024980]	**0,659**
Sema4f	Rattus norvegicus sema domain, immunoglobulin domain (Ig), transmembrane domain (TM) and short cytoplasmic domain, (semaphorin) 4F (Sema4f), mRNA [NM_019272]	**0,659**
Arl5b	Rattus norvegicus ADP-ribosylation factor-like 5B (Arl5b), mRNA [NM_001015031]	**0,659**
RGD1562755	Uncharacterized protein [Source:UniProtKB/TrEMBL;Acc:D3ZNM1] [ENSRNOT00000051559]	**0,659**
Rtn3	Rattus norvegicus reticulon 3 (Rtn3), transcript variant 1, mRNA [NM_080909]	**0,659**
Man1a2	Rattus norvegicus mannosidase, alpha, class 1A, member 2 (Man1a2), mRNA [NM_001106452]	**0,659**
Phyhip	Rattus norvegicus phytanoyl-CoA 2-hydroxylase interacting protein (Phyhip), mRNA [NM_001017376]	**0,659**
0	Uncharacterized protein [Source:UniProtKB/TrEMBL;Acc:D3ZE13] [ENSRNOT00000027984]	**0,659**
Map3k7ip2	Rattus norvegicus mitogen-activated protein kinase kinase kinase 7 interacting protein 2 (Map3k7ip2), mRNA [NM_001012062]	**0,659**
C1qtnf6	Rattus norvegicus C1q and tumor necrosis factor related protein 6 (C1qtnf6), mRNA [NM_001034932]	**0,659**
Gpt2	Rattus norvegicus glutamic pyruvate transaminase (alanine aminotransferase) 2 (Gpt2), mRNA [NM_001012057]	**0,659**
Nfx1	Rattus norvegicus nuclear transcription factor, X-box binding 1 (Nfx1), mRNA [NM_001024784]	**0,659**
Araf	Rattus norvegicus v-raf murine sarcoma 3611 viral oncogene homolog (Araf), transcript variant 1, mRNA [NM_022532]	**0,659**
Sh3bgrl	Rattus norvegicus SH3 domain binding glutamic acid-rich protein like (Sh3bgrl), mRNA [NM_001173339]	**0,659**
Ina	Rattus norvegicus internexin neuronal intermediate filament protein, alpha (Ina), mRNA [NM_019128]	**0,659**
Nfib	Rattus norvegicus nuclear factor I/B (Nfib), mRNA [NM_031566]	**0,659**
Ret	Rattus norvegicus ret proto-oncogene (Ret), transcript variant 2, mRNA [NM_001110099]	**0,660**
Kctd4	Rattus norvegicus potassium channel tetramerisation domain containing 4 (Kctd4), mRNA [NM_001109650]	**0,660**
Aspdh	Rattus norvegicus aspartate dehydrogenase domain containing (Aspdh), mRNA [NM_001009643]	**0,660**
RGD1561849	Rattus norvegicus similar to RIKEN cDNA 3110035E14 (RGD1561849), mRNA [NM_001109260]	**0,660**
Eif1ad	Rattus norvegicus eukaryotic translation initiation factor 1A domain containing (Eif1ad), mRNA [NM_001008305]	**0,660**
Cacna1c	Rattus norvegicus calcium channel, voltage-dependent, L type, alpha 1C subunit (Cacna1c), mRNA [NM_012517]	**0,660**
Fam84a	Rattus norvegicus family with sequence similarity 84, member A (Fam84a), mRNA [NM_001127299]	**0,660**
Cnpy3	Rattus norvegicus canopy 3 homolog (zebrafish) (Cnpy3), mRNA [NM_001134710]	**0,660**
Stk40	Rattus norvegicus serine/threonine kinase 40 (Stk40), mRNA [NM_183056]	**0,660**
Hs6st3	PREDICTED: Rattus norvegicus heparan sulfate 6-O-sulfotransferase 3 (Hs6st3), mRNA [XM_344461]	**0,660**
0	Unknown	**0,660**
Capn2	Rattus norvegicus calpain 2 (Capn2), mRNA [NM_017116]	**0,660**
0	Uncharacterized protein [Source:UniProtKB/TrEMBL;Acc:D3ZHQ7] [ENSRNOT00000057958]	**0,660**
Swap70	Rattus norvegicus SWAP switching B-cell complex 70 (Swap70), mRNA [NM_001106288]	**0,660**
Klf4	Rattus norvegicus Kruppel-like factor 4 (gut) (Klf4), mRNA [NM_053713]	**0,660**

Mocs3	Rattus norvegicus molybdenum cofactor synthesis 3 (Mocs3), mRNA [NM_001107804]	**0,660**
Nptxr	Rattus norvegicus neuronal pentraxin receptor (Nptxr), mRNA [NM_030841]	**0,660**
Cdk9	Rattus norvegicus cyclin-dependent kinase 9 (Cdk9), mRNA [NM_001007743]	**0,660**
Eif2s3x	Rattus norvegicus eukaryotic translation initiation factor 2, subunit 3, structural gene X-linked (Eif2s3x), mRNA [NM_001100542]	**0,660**
Doc2b	Rattus norvegicus double C2-like domains, beta (Doc2b), mRNA [NM_031142]	**0,660**
0	Q479S2_DECAR (Q479S2) Histidine kinase internal region precursor, partial (7%) [TC592164]	**0,660**
Wasf1	Rattus norvegicus WAS protein family, member 1 (Wasf1), mRNA [NM_001025114]	**0,660**
Zfp410	Rattus norvegicus zinc finger protein 410 (Zfp410), mRNA [NM_001108042]	**0,660**
LOC689226	Rattus norvegicus similar to ubiquitin-conjugating enzyme E2R 2 (LOC689226), mRNA [NM_001127573]	**0,660**
0	Rattus norvegicus similar to glyceraldehyde-3-phosphate dehydrogenase (LOC305750), mRNA [XM_223850]	**0,660**
Prkar1b	Rattus norvegicus protein kinase, cAMP dependent regulatory, type I, beta (Prkar1b), mRNA [NM_001033679]	**0,660**
Epb41l3	Rattus norvegicus erythrocyte membrane protein band 4.1-like 3 (Epb41l3), mRNA [NM_053927]	**0,660**
Tmem106b	Rattus norvegicus transmembrane protein 106B (Tmem106b), mRNA [NM_001004267]	**0,660**
Cntnap5a	Rattus norvegicus contactin associated protein-like 5A (Cntnap5a), mRNA [NM_001047865]	**0,660**
Ash1l	Rattus norvegicus ash1 (absent, small, or homeotic)-like (Drosophila) (Ash1l), mRNA [NM_001107689]	**0,660**
0	Unknown	**0,660**
0	Nope protein [Source:UniProtKB/TrEMBL;Acc:B5DFA9] [ENSRNOT00000040854]	**0,660**
Tnrc6b	Rattus norvegicus trinucleotide repeat containing 6B (Tnrc6b), mRNA [NM_138845]	**0,660**
0	Unknown	**0,660**
Rap1a	Rattus norvegicus RAP1A, member of RAS oncogene family (Rap1a), mRNA [NM_001005765]	**0,660**
Defb22	Rattus norvegicus defensin beta 22 (Defb22), mRNA [NM_134391]	**0,661**
Calu	Rattus norvegicus calumenin (Calu), transcript variant 2, mRNA [NM_001033898]	**0,661**
0	Unknown	**0,661**
Gp1bb	Rattus norvegicus glycoprotein Ib (platelet), beta polypeptide (Gp1bb), mRNA [NM_053930]	**0,661**
Nosip	Rattus norvegicus nitric oxide synthase interacting protein (Nosip), mRNA [NM_001106260]	**0,661**
Ipo11	PREDICTED: Rattus norvegicus importin 11 (Ipo11), mRNA [XM_002725887]	**0,661**
0	RGS6_HUMAN (P49758) Regulator of G-protein signaling 6 (RGS6) (S914), partial (11%) [TC592366]	**0,661**
Tecpr1	Rattus norvegicus tectonin beta-propeller repeat containing 1 (Tecpr1), mRNA [NM_001037191]	**0,661**
Atp6v0b	Rattus norvegicus ATPase, H+ transporting, lysosomal V0 subunit B (Atp6v0b), mRNA [NM_001106681]	**0,661**
Cxxc5	Rattus norvegicus CXXC finger 5 (Cxxc5), mRNA [NM_001007628]	**0,661**
Sorcs3	Rattus norvegicus sortilin-related VPS10 domain containing receptor 3 (Sorcs3), mRNA [NM_001106367]	**0,661**
Fez2	Rattus norvegicus fasciculation and elongation protein zeta 2 (zygin II) (Fez2), mRNA [NM_053600]	**0,661**
LOC681994	PREDICTED: Rattus norvegicus similar to developmental endothelial locus-1 isoform b, transcript variant 3 (LOC681994), mRNA [XM_001057753]	**0,661**
Sst	Rattus norvegicus somatostatin (Sst), mRNA [NM_012659]	**0,661**

Erlin1	Rattus norvegicus ER lipid raft associated 1 (Erlin1), mRNA [NM_001106353]	**0,661**
0	Unknown	**0,661**
0	Ataxin 2 (Predicted)Uncharacterized protein [Source:UniProtKB/TrEMBL;Acc:D4A2N8] [ENSRNOT00000001691]	**0,661**
Baalc	Rattus norvegicus brain and acute leukemia, cytoplasmic (Baalc), mRNA [NM_144762]	**0,661**
Grpr	Rattus norvegicus gastrin releasing peptide receptor (Grpr), mRNA [NM_012706]	**0,661**
Grik5	Rattus norvegicus glutamate receptor, ionotropic, kainate 5 (Grik5), mRNA [NM_031508]	**0,661**
0	Rattus norvegicus similar to 60S ribosomal protein L23a (LOC292743), mRNA [XM_218374]	**0,661**
0	Unknown	**0,661**
LOC688314	PREDICTED: Rattus norvegicus similar to stimulated by retinoic acid 13 (LOC688314), mRNA [XM_001081840]	**0,661**
Calml3	Rattus norvegicus calmodulin-like 3 (Calml3), mRNA [NM_001012054]	**0,661**
Jakmip3	Rattus norvegicus janus kinase and microtubule interacting protein 3 (Jakmip3), mRNA [NM_001163277]	**0,661**
Lppr1	Rattus norvegicus lipid phosphate phosphatase-related protein type 1 (Lppr1), mRNA [NM_201271]	**0,661**
Sumo2	Rattus norvegicus SMT3 suppressor of mif two 3 homolog 2 (S. cerevisiae) (Sumo2), mRNA [NM_133594]	**0,661**
Zfp800	Rattus norvegicus zinc finger protein 800 (Zfp800), mRNA [NM_001109225]	**0,661**
0	Unknown	**0,662**
0	Unknown	**0,662**
Fbxo42	Rattus norvegicus F-box protein 42 (Fbxo42), mRNA [NM_001108691]	**0,662**
Hsd17b7	Rattus norvegicus hydroxysteroid (17-beta) dehydrogenase 7 (Hsd17b7), mRNA [NM_017235]	**0,662**
Fundc1	Rattus norvegicus FUN14 domain containing 1 (Fundc1), mRNA [NM_001025027]	**0,662**
Gbf1	Rattus norvegicus golgi-specific brefeldin A resistant guanine nucleotide exchange factor 1 (Gbf1), mRNA [NM_001191634]	**0,662**
LOC294154	similar to chromosome 6 open reading frame 106 isoform a (LOC294154), mRNA [Source:RefSeq DNA;Acc:NM_001039607] [ENSRNOT00000059426]	**0,662**
Parp2	Rattus norvegicus poly (ADP-ribose) polymerase 2 (Parp2), mRNA [NM_001106030]	**0,662**
0	Rattus norvegicus TL0AAA53YP17 mRNA sequence. [FQ212466]	**0,662**
Nr2f2	Rattus norvegicus nuclear receptor subfamily 2, group F, member 2 (Nr2f2), mRNA [NM_080778]	**0,662**
Tob2	Rattus norvegicus transducer of ERBB2, 2 (Tob2), mRNA [NM_001007146]	**0,662**
Pank3	Rattus norvegicus pantothenate kinase 3 (Pank3), mRNA [NM_001108272]	**0,662**
Farp1	Rattus norvegicus FERM, RhoGEF (Arhgef) and pleckstrin domain protein 1 (chondrocyte-derived) (Farp1), mRNA [NM_001107287]	**0,662**
Muc1	Rattus norvegicus mucin 1, cell surface associated (Muc1), mRNA [NM_012602]	**0,662**
Asb3	Rattus norvegicus ankyrin repeat and SOCS box-containing 3 (Asb3), mRNA [NM_001108864]	**0,662**
Clec4a3	Rattus norvegicus C-type lectin domain family 4, member a3 (Clec4a3), mRNA [NM_001005891]	**0,662**
Icam1	Rattus norvegicus intercellular adhesion molecule 1 (Icam1), mRNA [NM_012967]	**0,662**
Prkaa2	Rattus norvegicus protein kinase, AMP-activated, alpha 2 catalytic subunit (Prkaa2), mRNA [NM_023991]	**0,662**
Pip5k1c	Rattus norvegicus phosphatidylinositol-4-phosphate 5-kinase, type I, gamma (Pip5k1c), transcript variant c, mRNA [NM_001009967]	**0,662**

Hsf1	Rattus norvegicus heat shock transcription factor 1 (Hsf1), mRNA [NM_024393]	**0,662**
Cdkn2b	Rattus norvegicus cyclin-dependent kinase inhibitor 2B (p15, inhibits CDK4) (Cdkn2b), mRNA [NM_130812]	**0,662**
0	Unknown	**0,662**
LOC680813	PREDICTED: Rattus norvegicus hypothetical protein LOC680813 (LOC680813), partial mRNA [XM_001058991]	**0,662**
Yipf4	Rattus norvegicus Yip1 domain family, member 4 (Yipf4), mRNA [NM_001009712]	**0,662**
Exoc6b	Rattus norvegicus exocyst complex component 6B (Exoc6b), mRNA [NM_001109246]	**0,662**
Rcor1	Rattus norvegicus REST corepressor 1 (Rcor1), mRNA [NM_001108060]	**0,662**
Srr	Rattus norvegicus serine racemase (Srr), mRNA [NM_198757]	**0,662**
0	Q6IE24_RAT (Q6IE24) Ubiquitin specific protease 54, complete [TC592916]	**0,663**
Adam11	Rattus norvegicus ADAM metallopeptidase domain 11 (Adam11), mRNA [NM_001108300]	**0,663**
0	Unknown	**0,663**
Chrm4	Rattus norvegicus cholinergic receptor, muscarinic 4 (Chrm4), mRNA [NM_031547]	**0,663**
Arl6ip5	Rattus norvegicus ADP-ribosylation-like factor 6 interacting protein 5 (Arl6ip5), mRNA [NM_023972]	**0,663**
MAST1	Rattus norvegicus microtubule associated serine/threonine kinase 1 (MAST1), mRNA [NM_181089]	**0,663**
Fam117b	Rattus norvegicus family with sequence similarity 117, member B (Fam117b), mRNA [NM_001108797]	**0,663**
Gdf11	PREDICTED: Rattus norvegicus growth differentiation factor 11 (Gdf11), mRNA [XM_343148]	**0,663**
0	Unknown	**0,663**
Spon1	Rattus norvegicus spondin 1, extracellular matrix protein (Spon1), mRNA [NM_172067]	**0,663**
Map2k4	Rattus norvegicus mitogen activated protein kinase kinase 4 (Map2k4), mRNA [NM_001030023]	**0,663**
0	Q3U6G1_MOUSE (Q3U6G1) Bone marrow macrophage cDNA, RIKEN full-length enriched library, clone:I830125G18 product:biliverdin reductase B (flavin reductase (NADPH)), full insert sequence, partial (78%) [TC609126]	**0,663**
RGD1559917	Rattus norvegicus similar to SMT3 suppressor of mif two 3 homolog 2 (LOC287993), mRNA [XM_212687]	**0,663**
0	Unknown	**0,663**
0	MTA3_HUMAN (Q9BTC8) Metastasis-associated protein MTA3, partial (5%) [TC620956]	**0,663**
Dlg1	Rattus norvegicus discs, large homolog 1 (Drosophila) (Dlg1), mRNA [NM_012788]	**0,663**
Pbld	Rattus norvegicus phenazine biosynthesis-like protein domain containing (Pbld), mRNA [NM_138530]	**0,663**
Nek4	Rattus norvegicus NIMA (never in mitosis gene a)-related kinase 4 (Nek4), mRNA [NM_001013134]	**0,663**
Lima1	Rattus norvegicus LIM domain and actin binding 1 (Lima1), mRNA [NM_001191615]	**0,663**
0	Unknown	**0,663**
Rap2ip	Rattus norvegicus Rap2 interacting protein (Rap2ip), mRNA [NM_198758]	**0,663**
Bicc1	Rattus norvegicus bicaudal C homolog 1 (Drosophila) (Bicc1), mRNA [NM_001108531]	**0,663**
Dll1	Rattus norvegicus delta-like 1 (Drosophila) (Dll1), mRNA [NM_032063]	**0,663**
Scrt1	Rattus norvegicus scratch homolog 1, zinc finger protein (Drosophila) (Scrt1), mRNA [NM_001130570]	**0,663**
Ikzf5	Rattus norvegicus IKAROS family zinc finger 5 (Ikzf5), mRNA [NM_001107555]	**0,663**

0	Unknown	**0,663**
Pcdhb17	PREDICTED: Rattus norvegicus protocadherin beta 17 (Pcdhb17), mRNA [XM_001065253]	**0,663**
Rpap2	Rattus norvegicus RNA polymerase II associated protein 2 (Rpap2), mRNA [NM_001013987]	**0,663**
RGD1359600	Rattus norvegicus LEA_4 domain containing protein RGD1359600 (RGD1359600), mRNA [NM_001007688]	**0,664**
Nop2	Rattus norvegicus NOP2 nucleolar protein homolog (yeast) (Nop2), mRNA [NM_001191785]	**0,664**
Diras1	Rattus norvegicus DIRAS family, GTP-binding RAS-like 1 (Diras1), mRNA [NM_001108987]	**0,664**
Akt3	Rattus norvegicus v-akt murine thymoma viral oncogene homolog 3 (protein kinase B, gamma) (Akt3), mRNA [NM_031575]	**0,664**
Prrx1	Rattus norvegicus paired related homeobox 1 (Prrx1), mRNA [NM_153821]	**0,664**
Ywhag	Rattus norvegicus tyrosine 3-monooxygenase/tryptophan 5-monooxygenase activation protein, gamma polypeptide (Ywhag), mRNA [NM_019376]	**0,664**
Myo10	Rattus norvegicus myosin X (Myo10), mRNA [NM_001107657]	**0,664**
Spata2L	Rattus norvegicus spermatogenesis associated 2-like (Spata2L), mRNA [NM_001109133]	**0,664**
Pde1a	Rattus norvegicus phosphodiesterase 1A, calmodulin-dependent (Pde1a), mRNA [NM_030871]	**0,664**
Slc25a28	Rattus norvegicus solute carrier family 25, member 28 (Slc25a28), mRNA [NM_001109515]	**0,664**
Aqp4	Rattus norvegicus aquaporin 4 (Aqp4), transcript variant 2, mRNA [NM_001142366]	**0,664**
Kctd4	Rattus norvegicus potassium channel tetramerisation domain containing 4 (Kctd4), mRNA [NM_001109650]	**0,664**
0	CB544318 AMGNNUC:NRDG1-00127-B11-A nrdg1 (10855) Rattus norvegicus cDNA clone nrdg1-00127-b11 5', mRNA sequence [CB544318]	**0,664**
Arid1a	Rattus norvegicus AT rich interactive domain 1A (SWI-like) (Arid1a), mRNA [NM_001106635]	**0,664**
Ints10	Rattus norvegicus integrator complex subunit 10 (Ints10), mRNA [NM_001134416]	**0,664**
Rfesd	Rattus norvegicus Rieske (Fe-S) domain containing (Rfesd), mRNA [NM_001108540]	**0,664**
Gpr75	Rattus norvegicus G protein-coupled receptor 75 (Gpr75), mRNA [NM_001109096]	**0,664**
Dlg2	Rattus norvegicus discs, large homolog 2 (Drosophila) (Dlg2), mRNA [NM_022282]	**0,664**
Ppp3cb	Rattus norvegicus protein phosphatase 3, catalytic subunit, beta isoform (Ppp3cb), mRNA [NM_017042]	**0,664**
Fstl4	Rattus norvegicus follistatin-like 4 (Fstl4), mRNA [NM_001107000]	**0,664**
Zic1	Rattus norvegicus Zic family member 1 (odd-paired homolog, Drosophila) (Zic1), mRNA [NM_022677]	**0,664**
Bzw1	Rattus norvegicus basic leucine zipper and W2 domains 1 (Bzw1), mRNA [NM_198789]	**0,664**
Thrap3	Rattus norvegicus thyroid hormone receptor associated protein 3 (Thrap3), mRNA [NM_001009693]	**0,664**
Brunol4	Rattus norvegicus bruno-like 4, RNA binding protein (Drosophila) (Brunol4), mRNA [NM_001107400]	**0,664**
0	Q5DTV9_MOUSE (Q5DTV9) MKIAA1671 protein (Fragment), partial (75%) [TC598280]	**0,664**
Clasp1	PREDICTED: Rattus norvegicus cytoplasmic linker associated protein 1 (Clasp1), mRNA [XM_001053715]	**0,664**
Cbln3	Rattus norvegicus cerebellin 3 precursor (Cbln3), mRNA [NM_001109330]	**0,664**
Rarres1	Rattus norvegicus retinoic acid receptor responder (tazarotene induced) 1 (Rarres1), mRNA [NM_001014790]	**0,664**
Tmem170b	Rattus norvegicus transmembrane protein 170B (Tmem170b), mRNA [NM_001008774]	**0,665**
0	Unknown	**0,665**
Cacna1c	Rattus norvegicus calcium channel, voltage-dependent, L type, alpha 1C subunit (Cacna1c), mRNA [NM_012517]	**0,665**

0	Unknown	**0,665**
Olr1557	Rattus norvegicus olfactory receptor 1557 (Olr1557), mRNA [NM_001000050]	**0,665**
Wee1	Rattus norvegicus wee 1 homolog (S. pombe) (Wee1), mRNA [NM_001012742]	**0,665**
Peli1	Rattus norvegicus pellino 1 (Peli1), mRNA [NM_001100565]	**0,665**
0	Unknown	**0,665**
0	Unknown	**0,665**
Bcl9l	Rattus norvegicus B-cell CLL/lymphoma 9-like (Bcl9l), mRNA [NM_001106817]	**0,665**
Ncoa3	PREDICTED: Rattus norvegicus nuclear receptor coactivator 3 (Ncoa3), mRNA [XM_215947]	**0,665**
Mt2A	Rattus norvegicus metallothionein 2A (Mt2A), mRNA [NM_001137564]	**0,665**
Gpr37	Rattus norvegicus G protein-coupled receptor 37 (Gpr37), mRNA [NM_057201]	**0,665**
0	Unknown	**0,665**
Top1	Rattus norvegicus topoisomerase (DNA) I (Top1), mRNA [NM_022615]	**0,665**
Pla2g7	Rattus norvegicus phospholipase A2, group VII (platelet-activating factor acetylhydrolase, plasma) (Pla2g7), mRNA [NM_001009353]	**0,665**
Sos2	Rattus norvegicus son of sevenless homolog 2 (Drosophila) (Sos2), mRNA [NM_001135561]	**0,665**
Gria3	Rattus norvegicus glutamate receptor, ionotrophic, AMPA 3 (Gria3), transcript variant 1, mRNA [NM_032990]	**0,665**
Brunol5	Rattus norvegicus bruno-like 5, RNA binding protein (Drosophila) (Brunol5), mRNA [NM_001135603]	**0,665**
0	Unknown	**0,665**
Terc	Rattus norvegicus telomerase RNA component (Terc), telomerase RNA [NR_001567]	**0,665**
Rap1b	Rattus norvegicus RAP1B, member of RAS oncogene family (Rap1b), mRNA [NM_134346]	**0,665**
Kcnj12	Rattus norvegicus potassium inwardly-rectifying channel, subfamily J, member 12 (Kcnj12), mRNA [NM_053981]	**0,665**
Gmfb	Rattus norvegicus glia maturation factor, beta (Gmfb), mRNA [NM_031032]	**0,665**
St3gal2	Rattus norvegicus ST3 beta-galactoside alpha-2,3-sialyltransferase 2 (St3gal2), mRNA [NM_031695]	**0,665**
0	Uncharacterized protein [Source:UniProtKB/TrEMBL;Acc:D3ZBI5] [ENSRNOT00000049803]	**0,665**
Gypc	Rattus norvegicus glycophorin C (Gerbich blood group) (Gypc), mRNA [NM_001013233]	**0,665**
Ptma	Rattus norvegicus prothymosin alpha (Ptma), mRNA [NM_021740]	**0,665**
Senp1	PREDICTED: Rattus norvegicus Sumo1/sentrin/SMT3 specific peptidase 1 (Senp1), mRNA [XM_001060796]	**0,665**
Tacc1	Rattus norvegicus transforming, acidic coiled-coil containing protein 1 (Tacc1), mRNA [NM_001004107]	**0,665**
LOC503175	PREDICTED: Rattus norvegicus similar to Protein KIAA0280 (LOC503175), mRNA [XM_578699]	**0,665**
RGD1561238	PREDICTED: Rattus norvegicus similar to ring finger protein 122 homolog (RGD1561238), mRNA [XM_001069254]	**0,665**
Phc2	Rattus norvegicus polyhomeotic homolog 2 (Drosophila) (Phc2), mRNA [NM_001013169]	**0,665**
0	Shultzomica03641 Rat lung airway and parenchyma cDNA libraries Rattus norvegicus cDNA clone Contig3221 5', mRNA sequence [CF110390]	**0,665**
Fam171b	Uncharacterized protein [Source:UniProtKB/TrEMBL;Acc:D3ZTG3] [ENSRNOT00000006504]	**0,665**
0	Rattus norvegicus similar to cytoplasmic beta-actin (LOC302827), mRNA [XM_229147]	**0,665**
Pllp	Rattus norvegicus plasma membrane proteolipid (plasmolipin) (Pllp), mRNA [NM_022533]	**0,665**

Dock7	Rattus norvegicus dedicator of cytokinesis 7 (Dock7), mRNA [NM_001191574]	**0,665**
0	Unknown	**0,665**
Htr5a	Rattus norvegicus 5-hydroxytryptamine (serotonin) receptor 5A (Htr5a), mRNA [NM_013148]	**0,665**
Ptpn23	Rattus norvegicus protein tyrosine phosphatase, non-receptor type 23 (Ptpn23), mRNA [NM_057204]	**0,666**
Meis2	Rattus norvegicus Meis homeobox 2 (Meis2), mRNA [NM_001107758]	**0,666**
LOC100233213	Rattus norvegicus hypothetical protein LOC100233213 (LOC100233213), mRNA [NM_001143803]	**0,666**
Dclk2	Rattus norvegicus doublecortin-like kinase 2 (Dclk2), transcript variant 2, mRNA [NM_001009691]	**0,666**
Gnao1	Rattus norvegicus guanine nucleotide binding protein (G protein), alpha activating activity polypeptide O (Gnao1), mRNA [NM_017327]	**0,666**
Igtp	Rattus norvegicus interferon gamma induced GTPase (Igtp), mRNA [NM_001008765]	**0,666**
Zfyve20	Rattus norvegicus zinc finger, FYVE domain containing 20 (Zfyve20), mRNA [NM_001107875]	**0,666**
Opa3	Rattus norvegicus optic atrophy 3 (human) (Opa3), nuclear gene encoding mitochondrial protein, mRNA [NM_001107486]	**0,666**
Atl2	Rattus norvegicus atlastin GTPase 2 (Atl2), mRNA [NM_001100671]	**0,666**
LOC682812	PREDICTED: Rattus norvegicus similar to WD repeat domain 40A, transcript variant 3 (LOC682812), mRNA [XM_001059949]	**0,666**
Ulk1	Rattus norvegicus Unc-51 like kinase 1 (C. elegans) (Ulk1), mRNA [NM_001108341]	**0,666**
Rundc3b	Rattus norvegicus RUN domain containing 3B (Rundc3b), mRNA [NM_001047116]	**0,666**
Zmat2	Rattus norvegicus zinc finger, matrin type 2 (Zmat2), mRNA [NM_001135582]	**0,666**
Thap11	Rattus norvegicus THAP domain containing 11 (Thap11), mRNA [NM_001107422]	**0,666**
Rqcd1	Rattus norvegicus rcd1 (required for cell differentiation) homolog 1 (S. pombe) (Rqcd1), mRNA [NM_001009357]	**0,666**
Synj1	Rattus norvegicus synaptojanin 1 (Synj1), mRNA [NM_053476]	**0,666**
0	Unknown	**0,666**
Freq	Rattus norvegicus frequenin homolog (Drosophila) (Freq), mRNA [NM_024366]	**0,666**
Yipf6	Rattus norvegicus Yip1 domain family, member 6 (Yipf6), mRNA [NM_001025747]	**0,666**
0	PREDICTED: Rattus norvegicus similar to novel protein (RGD1560386), partial mRNA [XM_001069736]	**0,666**
Atl1	Rattus norvegicus atlastin GTPase 1 (Atl1), mRNA [NM_001009831]	**0,666**
Cxcl14	Rattus norvegicus chemokine (C-X-C motif) ligand 14 (Cxcl14), mRNA [NM_001013137]	**0,666**
0	Rattus norvegicus similar to glyceraldehyde-3-phosphate dehydrogenase (LOC296027), mRNA [XM_215798]	**0,666**
Adcy5	Rattus norvegicus adenylate cyclase 5 (Adcy5), mRNA [NM_022600]	**0,666**
Ncor2	Rattus norvegicus nuclear receptor co-repressor 2 (Ncor2), mRNA [NM_001108334]	**0,666**
Ube2q2	Uncharacterized protein [Source:UniProtKB/TrEMBL;Acc:D4A1G2] [ENSRNOT00000019539]	**0,666**
0	Unknown	**0,666**
Brsk2	PREDICTED: Rattus norvegicus brain serine/threonine kinase 2 (Brsk2), mRNA [XM_001063734]	**0,666**
Gtlf3b	Rattus norvegicus gene trap locus F3b (Gtlf3b), mRNA [NM_001170541]	**0,666**
Spock1	PREDICTED: Rattus norvegicus sparc/osteonectin, cwcv and kazal-like domains proteoglycan (testican) 1 (Spock1), mRNA [XM_001067546]	**0,666**
Pftk1	Rattus norvegicus PFTAIRE protein kinase 1 (Pftk1), mRNA [NM_001108617]	**0,666**

Ccl24	Rattus norvegicus chemokine (C-C motif) ligand 24 (Ccl24), mRNA [NM_001013045]	**0,666**
0	Unknown	**0,666**
Leng4	Rattus norvegicus leukocyte receptor cluster (LRC) member 4 (Leng4), mRNA [NM_001134978]	**0,666**
RGD1309922	Rattus norvegicus similar to 2610301G19Rik protein (RGD1309922), mRNA [NM_001170472]	**0,666**
Gspt1	Rattus norvegicus G1 to S phase transition 1 (Gspt1), mRNA [NM_001003978]	**0,666**
Bmpr1a	Rattus norvegicus bone morphogenetic protein receptor, type IA (Bmpr1a), mRNA [NM_030849]	**0,666**
RGD1562211	Rattus norvegicus similar to ubiquitin specific protease 51 (RGD1562211), mRNA [NM_001108252]	**0,666**
0	Unknown	**0,666**
Tg	Rattus norvegicus thyroglobulin (Tg), mRNA [NM_030988]	**0,666**
0	RGD1304924 protein [Source:UniProtKB/TrEMBL;Acc:B0BNI8] [ENSRNOT00000064652]	**0,666**
Zfp278	Rattus norvegicus zinc finger protein 278 (Zfp278), mRNA [NM_001107231]	**0,666**
Cd24	Rattus norvegicus CD24 molecule (Cd24), mRNA [NM_012752]	**0,666**
0	Unknown	**0,666**
Rimbp2	Rattus norvegicus RIM binding protein 2 (Rimbp2), mRNA [NM_001100488]	**0,666**
Pygo1	Rattus norvegicus pygopus 1 (Pygo1), mRNA [NM_001191117]	**0,666**
Arhgef9	Rattus norvegicus Cdc42 guanine nucleotide exchange factor (GEF) 9 (Arhgef9), mRNA [NM_023957]	**0,666**
Lta4h	Rattus norvegicus leukotriene A4 hydrolase (Lta4h), mRNA [NM_001030031]	**0,667**
Map3k12	Rattus norvegicus mitogen activated protein kinase kinase kinase 12 (Map3k12), mRNA [NM_013055]	**0,667**
0	Rattus norvegicus similar to 60S ribosomal protein L13a (Transplantation antigen P198) (Tum-P198 antigen) (LOC365089), mRNA [XM_344809]	**0,667**
Exd2	Rattus norvegicus exonuclease 3'-5' domain containing 2 (Exd2), mRNA [NM_001108715]	**0,667**
Pskh1	Rattus norvegicus protein serine kinase H1 (Pskh1), mRNA [NM_001108897]	**0,667**
Pcdhb19	PREDICTED: Rattus norvegicus protocadherin beta 19 (Pcdhb19), mRNA [XM_001056051]	**0,667**
Papolb	Rattus norvegicus poly (A) polymerase beta (testis specific) (Papolb), mRNA [NM_001012020]	**0,667**
0	Unknown	**0,667**
RGD1564463	Uncharacterized protein [Source:UniProtKB/TrEMBL;Acc:D3ZXB2] [ENSRNOT00000004815]	**0,667**
Syt11	Rattus norvegicus synaptotagmin XI (Syt11), mRNA [NM_031667]	**0,667**
0	Uncharacterized protein [Source:UniProtKB/TrEMBL;Acc:D3ZNC7] [ENSRNOT00000057981]	**0,667**
Mtpn	Rattus norvegicus myotrophin (Mtpn), mRNA [NM_024374]	**0,667**
Cbx8	Rattus norvegicus chromobox homolog 8 (Pc class homolog, Drosophila) (Cbx8), mRNA [NM_001034078]	**0,667**
Zfp180	Rattus norvegicus zinc finger protein 180 (Zfp180), mRNA [NM_144757]	**0,667**
Klhl23	Rattus norvegicus kelch-like 23 (Drosophila) (Klhl23), mRNA [NM_001134504]	**0,667**
Rab3d	Rattus norvegicus RAB3D, member RAS oncogene family (Rab3d), mRNA [NM_080580]	**0,667**
Hrnbp3	similar to ataxin 2-binding protein 1 isoform 2 (RGD1560070), mRNA [Source:RefSeq DNA;Acc:NM_001134498] [ENSRNOT00000004524]	**0,667**
LOC305806	Rattus norvegicus similar to glutaredoxin 1 (thioltransferase); glutaredoxin (LOC305806), mRNA [NM_001013993]	**0,667**

0	Unknown	**0,667**
Fzd3	Rattus norvegicus frizzled homolog 3 (Drosophila) (Fzd3), mRNA [NM_153474]	**0,667**
H6pd	Rattus norvegicus hexose-6-phosphate dehydrogenase (glucose 1-dehydrogenase) (H6pd), mRNA [NM_001106698]	**0,667**
Gatc	Rattus norvegicus glutamyl-tRNA(Gln) amidotransferase, subunit C homolog (bacterial) (Gatc), mRNA [NM_001108339]	**0,667**
0	Unknown	**0,667**
0	Uncharacterized protein [Source:UniProtKB/TrEMBL;Acc:D3ZTN0] [ENSRNOT00000035247]	**0,667**
Uhrf1bp1l	Rattus norvegicus UHRF1 binding protein 1-like (Uhrf1bp1l), mRNA [NM_001108753]	**0,667**
0	Unknown	**0,667**
0	Q3QL41_9GAMM (Q3QL41) Phosphoesterase, PA-phosphatase related precursor, partial (13%) [TC587259]	**0,667**
Ccdc40	Rattus norvegicus coiled-coil domain containing 40 (Ccdc40), mRNA [NM_001134688]	**0,667**
Urm1	Rattus norvegicus ubiquitin related modifier 1 homolog (S. cerevisiae) (Urm1), mRNA [NM_001137562]	**0,667**
Mbd6	Rattus norvegicus methyl-CpG binding domain protein 6 (Mbd6), mRNA [NM_001170566]	**0,667**
0	Unknown	**0,667**
Atg7	Rattus norvegicus ATG7 autophagy related 7 homolog (S. cerevisiae) (Atg7), mRNA [NM_001012097]	**0,667**
RGD1311575	Uncharacterized protein [Source:UniProtKB/TrEMBL;Acc:D4A5F4] [ENSRNOT00000002908]	**0,667**
Uvrag	Rattus norvegicus UV radiation resistance associated gene (Uvrag), mRNA [NM_001107536]	**0,667**
Tdrd12	PREDICTED: Rattus norvegicus similar to CG11133-PA (LOC689639), mRNA [XM_001071461]	**0,667**
Nr2e1	Rattus norvegicus nuclear receptor subfamily 2, group E, member 1 (Nr2e1), mRNA [NM_001113197]	**0,667**
B4galnt1	Rattus norvegicus beta-1,4-N-acetyl-galactosaminyl transferase 1 (B4galnt1), mRNA [NM_022860]	**0,668**
Dclre1a	Rattus norvegicus DNA cross-link repair 1A, PSO2 homolog (S. cerevisiae) (Dclre1a), mRNA [NM_001106201]	**0,668**
Zcchc2	Rattus norvegicus zinc finger, CCHC domain containing 2 (Zcchc2), mRNA [NM_001122677]	**0,668**
Mbnl1	Rattus norvegicus muscleblind-like 1 (Drosophila) (Mbnl1), mRNA [NM_001191566]	**0,668**
Cadm2	Rattus norvegicus cell adhesion molecule 2 (Cadm2), mRNA [NM_001047102]	**0,668**
Efha2	Rattus norvegicus EF hand domain family, member A2 (Efha2), mRNA [NM_001191892]	**0,668**
Actn4	Rattus norvegicus actinin alpha 4 (Actn4), mRNA [NM_031675]	**0,668**
RGD1306502	Rattus norvegicus similar to hypothetical protein FLJ11193 (RGD1306502), mRNA [NM_001107654]	**0,668**
Adam8	Uncharacterized protein [Source:UniProtKB/TrEMBL;Acc:D3ZB52] [ENSRNOT00000024369]	**0,668**
Utp14a	Rattus norvegicus UTP14, U3 small nucleolar ribonucleoprotein, homolog A (yeast) (Utp14a), mRNA [NM_001014113]	**0,668**
Fam122a	Rattus norvegicus family with sequence similarity 122A (Fam122a), mRNA [NM_001014029]	**0,668**
Gpr158	Rattus norvegicus G protein-coupled receptor 158 (Gpr158), mRNA [NM_001170326]	**0,668**
Arl15	PREDICTED: Rattus norvegicus ADP-ribosylation factor-like 15 (Arl15), mRNA [XM_001069431]	**0,668**
Ppfia2	Rattus norvegicus protein tyrosine phosphatase, receptor type, f polypeptide (PTPRF), interacting protein (liprin), alpha 2 (Ppfia2), mRNA [NM_001108745]	**0,668**
Dusp8	Rattus norvegicus dual specificity phosphatase 8 (Dusp8), mRNA [NM_001108510]	**0,668**

Pcyt1b	Rattus norvegicus phosphate cytidylyltransferase 1, choline, beta (Pcyt1b), mRNA [NM_173151]	**0,668**
0	RVL9282 Wackym-Soares normalized rat vestibular cDNA library Rattus norvegicus cDNA 5', mRNA sequence [DV718665]	**0,668**
Dgki	Rattus norvegicus diacylglycerol kinase, iota (Dgki), mRNA [NM_198782]	**0,668**
0	Unknown	**0,668**
Camk2b	Rattus norvegicus calcium/calmodulin-dependent protein kinase II beta (Camk2b), transcript variant 1, mRNA [NM_001042354]	**0,668**
Pvrl3	Rattus norvegicus poliovirus receptor-related 3 (Pvrl3), mRNA [NM_001105883]	**0,668**
0	Rattus norvegicus similar to ribosomal protein L23a (LOC297884), mRNA [XM_232762]	**0,668**
0	O16480_CAEEL (O16480) Serpentine receptor, class t protein 8, partial (5%) [TC627292]	**0,668**
Pou3f3	Rattus norvegicus POU class 3 homeobox 3 (Pou3f3), mRNA [NM_138837]	**0,668**
Rbm33	Rattus norvegicus RNA binding motif protein 33 (Rbm33), mRNA [NM_001191859]	**0,668**
0	Uncharacterized protein [Source:UniProtKB/TrEMBL;Acc:D3ZJK8] [ENSRNOT00000065214]	**0,668**
0	Unknown	**0,668**
Zdhhc21	Rattus norvegicus zinc finger, DHHC-type containing 21 (Zdhhc21), mRNA [NM_001039009]	**0,668**
RGD1307041	Rattus norvegicus similar to hypothetical protein FLJ11305 (RGD1307041), mRNA [NM_001169144]	**0,668**
Grik2	Glutamate receptor, ionotropic kainate 2 [Source:UniProtKB/Swiss-Prot;Acc:P42260] [ENSRNOT00000000415]	**0,668**
RGD1563564	PREDICTED: Rattus norvegicus similar to GTP-binding protein NGB (RGD1563564), mRNA [XM_002728505]	**0,669**
Kpna6	Rattus norvegicus karyopherin alpha 6 (importin alpha 7) (Kpna6), mRNA [NM_001015029]	**0,669**
LOC682988	Rattus norvegicus similar to mediator of RNA polymerase II transcription, subunit 18 homolog (LOC682988), mRNA [NM_001115037]	**0,669**
Mrpl47	Rattus norvegicus mitochondrial ribosomal protein L47 (Mrpl47), nuclear gene encoding mitochondrial protein, mRNA [NM_001037183]	**0,669**
Prkaca	Rattus norvegicus protein kinase, cAMP-dependent, catalytic, alpha (Prkaca), mRNA [NM_001100922]	**0,669**
Sorbs2	Rattus norvegicus sorbin and SH3 domain containing 2 (Sorbs2), mRNA [NM_053770]	**0,669**
Syt1	Rattus norvegicus synaptotagmin I (Syt1), mRNA [NM_001033680]	**0,669**
Prdm2	Rattus norvegicus PR domain containing 2, with ZNF domain (Prdm2), mRNA [NM_001077648]	**0,669**
Neurod2	Rattus norvegicus neurogenic differentiation 2 (Neurod2), mRNA [NM_019326]	**0,669**
Lsm16	PREDICTED: Rattus norvegicus LSM16 homolog (EDC3, S. cerevisiae), transcript variant 1 (Lsm16), mRNA [XM_001072079]	**0,669**
LOC684822	PREDICTED: Rattus norvegicus similar to transcription elongation factor B (SIII), polypeptide 2 (LOC684822), mRNA [XM_002729867]	**0,669**
Tsc2	Rattus norvegicus tuberous sclerosis 2 (Tsc2), mRNA [NM_012680]	**0,669**
LOC652956	Rattus norvegicus p55 protein (LOC652956), mRNA [NM_001037659]	**0,669**
0	Unknown	**0,669**
Mgat5	Rattus norvegicus mannosyl (alpha-1,6-)-glycoprotein beta-1,6-N-acetyl-glucosaminyltransferase (Mgat5), mRNA [NM_023095]	**0,669**
LOC305806	Rattus norvegicus similar to glutaredoxin 1 (thioltransferase); glutaredoxin (LOC305806), mRNA [NM_001013993]	**0,669**
0	Uncharacterized protein [Source:UniProtKB/TrEMBL;Acc:D4A5N6] [ENSRNOT00000050854]	**0,669**
Rpl7l1	PREDICTED: Rattus norvegicus ribosomal protein L7-like 1 (Rpl7l1), mRNA [XM_001065509]	**0,669**
0	PREDICTED: Rattus norvegicus similar to MIC2 like 1 (LOC501437), partial mRNA [XM_001074315]	**0,669**

RGD1311575	PREDICTED: Rattus norvegicus hypothetical LOC289568 (RGD1311575), mRNA [XM_002724955]	**0,669**
0	Unknown	**0,669**
Aldh3a2	Rattus norvegicus aldehyde dehydrogenase 3 family, member A2 (Aldh3a2), mRNA [NM_031731]	**0,669**
RGD1562608	Rattus norvegicus similar to KIAA1328 protein (RGD1562608), mRNA [NM_001134607]	**0,669**
Oxr1	Rattus norvegicus oxidation resistance 1 (Oxr1), transcript variant 1, mRNA [NM_001197907]	**0,669**
Slc7a12	Rattus norvegicus solute carrier family 7 (cationic amino acid transporter, y+ system), member 12 (Slc7a12), mRNA [NM_001011948]	**0,669**
Mdga1	Rattus norvegicus MAM domain containing glycosylphosphatidylinositol anchor 1 (Mdga1), mRNA [NM_001107618]	**0,669**
Rab1b	Rattus norvegicus RAB1B, member RAS oncogene family (Rab1b), mRNA [NM_001109979]	**0,669**
Fhl1	Rattus norvegicus four and a half LIM domains 1 (Fhl1), transcript variant 1, mRNA [NM_001033926]	**0,669**
Wfdc3	Rattus norvegicus WAP four-disulfide core domain 3 (Wfdc3), mRNA [NM_001106541]	**0,669**
0	Unknown	**0,669**
Nhlrc3	Uncharacterized protein [Source:UniProtKB/TrEMBL;Acc:D4A2F6] [ENSRNOT00000014562]	**0,670**
Zbtb20	Rattus norvegicus zinc finger and BTB domain containing 20 (Zbtb20), mRNA [NM_001105880]	**0,670**
Rimklb	Uncharacterized protein [Source:UniProtKB/TrEMBL;Acc:D3ZJ79] [ENSRNOT00000032690]	**0,670**
Dguok	Rattus norvegicus deoxyguanosine kinase (Dguok), nuclear gene encoding mitochondrial protein, mRNA [NM_001106602]	**0,670**
Ei24	Rattus norvegicus etoposide induced 2.4 mRNA (Ei24), mRNA [NM_001025660]	**0,670**
0	Unknown	**0,670**
LOC366300	PREDICTED: Rattus norvegicus hypothetical LOC366300 (LOC366300), mRNA [XM_002726499]	**0,670**
LOC682999	Rattus norvegicus hypothetical protein LOC682999 (LOC682999), mRNA [NM_001127600]	**0,670**
Elmod1	Rattus norvegicus ELMO/CED-12 domain containing 1 (Elmod1), mRNA [NM_001191579]	**0,670**
Ppp3cc	Rattus norvegicus protein phosphatase 3, catalytic subunit, gamma isoform (Ppp3cc), mRNA [NM_134367]	**0,670**
0	Glyceraldehyde-3-phosphate dehydrogenase [Source:UniProtKB/TrEMBL;Acc:D3ZEN2] [ENSRNOT00000022122]	**0,670**
0	Unknown	**0,670**
Fubp3	Rattus norvegicus far upstream element (FUSE) binding protein 3 (Fubp3), mRNA [NM_001039337]	**0,670**
Inha	Rattus norvegicus inhibin alpha (Inha), mRNA [NM_012590]	**0,670**
Hist2h2ab	Rattus norvegicus histone cluster 2, H2ab (Hist2h2ab), mRNA [NM_001111341]	**0,670**
Fam8a1	Uncharacterized protein [Source:UniProtKB/TrEMBL;Acc:D3ZBT7] [ENSRNOT00000060500]	**0,670**
Cox11	Rattus norvegicus COX11 homolog, cytochrome c oxidase assembly protein (yeast) (Cox11), nuclear gene encoding mitochondrial protein, mRNA [NM_001109575]	**0,670**
Tnfaip1	Rattus norvegicus tumor necrosis factor, alpha-induced protein 1 (endothelial) (Tnfaip1), mRNA [NM_182950]	**0,670**
0	Unknown	**0,670**
Spcs2	Rattus norvegicus signal peptidase complex subunit 2 homolog (S. cerevisiae) (Spcs2), mRNA [NM_001191601]	**0,670**
0	Rattus norvegicus TL0AAA50YL16 mRNA sequence. [FQ212907]	**0,670**
Lrrc68	Rattus norvegicus leucine rich repeat containing 68 (Lrrc68), mRNA [NM_001107482]	**0,670**

Nudt5	Rattus norvegicus nudix (nucleoside diphosphate linked moiety X)-type motif 5 (Nudt5), mRNA [NM_001007733]	**0,670**
RGD1566399	PREDICTED: Rattus norvegicus similar to MYST histone acetyltransferase monocytic leukemia 4 (RGD1566399), miscRNA [XR_008228]	**0,670**
Shc1	Rattus norvegicus SHC (Src homology 2 domain containing) transforming protein 1 (Shc1), nuclear gene encoding mitochondrial protein, transcript variant 1, mRNA [NM_001164060]	**0,670**
Mef2a	Rattus norvegicus myocyte enhancer factor 2a (Mef2a), mRNA [NM_001014035]	**0,670**
Fat4	Rattus norvegicus FAT tumor suppressor homolog 4 (Drosophila) (Fat4), mRNA [NM_001191705]	**0,670**
LOC688511	PREDICTED: Rattus norvegicus similar to similar to 60S ribosomal protein L12 (LOC688511), miscRNA [XR_085815]	**0,670**
Pigg	PREDICTED: Rattus norvegicus phosphatidylinositol glycan anchor biosynthesis, class G, transcript variant 3 (Pigg), mRNA [XM_223731]	**0,670**
Irf9	Rattus norvegicus interferon regulatory factor 9 (Irf9), mRNA [NM_001012041]	**0,670**
Tbc1d25	Rattus norvegicus TBC1 domain family, member 25 (Tbc1d25), mRNA [NM_001106955]	**0,670**
RGD1562629	PREDICTED: Rattus norvegicus similar to neurobeachin (RGD1562629), miscRNA [XR_085753]	**0,670**
Bat3	Rattus norvegicus HLA-B-associated transcript 3 (Bat3), transcript variant 2, mRNA [NM_053609]	**0,670**
Serinc4	Rattus norvegicus serine incorporator 4 (Serinc4), mRNA [NM_001110811]	**0,670**
Atn1	Rattus norvegicus atrophin 1 (Atn1), mRNA [NM_017228]	**0,670**
Slc1a3	Rattus norvegicus solute carrier family 1 (glial high affinity glutamate transporter), member 3 (Slc1a3), mRNA [NM_019225]	**0,671**
Slc7a8	Rattus norvegicus solute carrier family 7 (cationic amino acid transporter, y+ system), member 8 (Slc7a8), mRNA [NM_053442]	**0,671**
0	Rattus norvegicus similar to RIKEN cDNA 4930415K17 (LOC302803), mRNA [XM_217625]	**0,671**
Abi1	Rattus norvegicus abl-interactor 1 (Abi1), mRNA [NM_024397]	**0,671**
Tpd52l1	Rattus norvegicus tumor protein D52-like 1 (Tpd52l1), mRNA [NM_001044295]	**0,671**
LOC682033	Rattus norvegicus similar to Protein phosphatase 2A, 59 kDa regulatory subunit B (PP2A PR59) (PP2A B-PR59) (LOC682033), mRNA [NM_001139492]	**0,671**
LOC680262	PREDICTED: Rattus norvegicus hypothetical protein LOC680262, transcript variant 1 (LOC680262), mRNA [XM_001056376]	**0,671**
Mcf2l	Rattus norvegicus MCF.2 cell line derived transforming sequence-like (Mcf2l), mRNA [NM_053951]	**0,671**
Ahr	Rattus norvegicus aryl hydrocarbon receptor (Ahr), mRNA [NM_013149]	**0,671**
Ric8b	Rattus norvegicus resistance to inhibitors of cholinesterase 8 homolog B (C. elegans) (Ric8b), mRNA [NM_175598]	**0,671**
Reep5	Rattus norvegicus receptor accessory protein 5 (Reep5), mRNA [NM_001108888]	**0,671**
Plagl2	Rattus norvegicus pleiomorphic adenoma gene-like 2 (Plagl2), mRNA [NM_001106528]	**0,671**
0	Palmitoyl-protein thioesterase 1 [Source:UniProtKB/Swiss-Prot;Acc:P45479] [ENSRNOT00000017998]	**0,671**
Zfhx4	Rattus norvegicus zinc finger homeobox 4 (Zfhx4), mRNA [NM_001191702]	**0,671**
Arhgef12	Rattus norvegicus Rho guanine nucleotide exchange factor (GEF) 12 (Arhgef12), mRNA [NM_001013246]	**0,671**
Cdkn2aipnl	Rattus norvegicus CDKN2A interacting protein N-terminal like (Cdkn2aipnl), mRNA [NM_001008278]	**0,671**
Rab5a	Rattus norvegicus RAB5A, member RAS oncogene family (Rab5a), mRNA [NM_022692]	**0,671**
Alkbh	Rattus norvegicus alkB, alkylation repair homolog (E. coli) (Alkbh), mRNA [NM_001108718]	**0,671**
0	Unknown	**0,671**

Ctsb	Rattus norvegicus cathepsin B (Ctsb), mRNA [NM_022597]	**0,671**
S1pr1	Rattus norvegicus sphingosine-1-phosphate receptor 1 (S1pr1), mRNA [NM_017301]	**0,671**
0	Unknown	**0,671**
Pip4k2c	Rattus norvegicus phosphatidylinositol-5-phosphate 4-kinase, type II, gamma (Pip4k2c), mRNA [NM_080480]	**0,671**
Rbm4b	Rattus norvegicus RNA binding motif protein 4B (Rbm4b), mRNA [NM_001007014]	**0,671**
Hpse	Rattus norvegicus heparanase (Hpse), mRNA [NM_022605]	**0,671**
0	Uncharacterized protein [Source:UniProtKB/TrEMBL;Acc:D3ZH07] [ENSRNOT00000049215]	**0,671**
Stx7	Rattus norvegicus syntaxin 7 (Stx7), mRNA [NM_021869]	**0,671**
0	PREDICTED: Rattus norvegicus dachshund homolog 2 (Drosophila) (Dach2), mRNA [XM_001055336]	**0,671**
Akr1cl2	Rattus norvegicus aldo-keto reductase family 1, member C-like 2 (Akr1cl2), mRNA [NM_001008342]	**0,671**
Erc1	Rattus norvegicus ELKS/RAB6-interacting/CAST family member 1 (Erc1), mRNA [NM_170788]	**0,671**
Cldnd1	Rattus norvegicus claudin domain containing 1 (Cldnd1), mRNA [NM_001006955]	**0,671**
RGD1311357	PREDICTED: Rattus norvegicus similar to A530083I02Rik protein (RGD1311357), mRNA [XM_001070093]	**0,672**
0	Unknown	**0,672**
Atp2a1	Rattus norvegicus ATPase, Ca++ transporting, cardiac muscle, fast twitch 1 (Atp2a1), mRNA [NM_058213]	**0,672**
Tbc1d10a	Rattus norvegicus TBC1 domain family, member 10a (Tbc1d10a), mRNA [NM_001015022]	**0,672**
Ndufa3	PREDICTED: Rattus norvegicus NADH dehydrogenase (ubiquinone) 1 alpha subcomplex, 3 (Ndufa3), mRNA [XM_001076462]	**0,672**
Pnpla6	PREDICTED: Rattus norvegicus patatin-like phospholipase domain containing 6 (Pnpla6), mRNA [XM_001057249]	**0,672**
Gas7	Rattus norvegicus growth arrest specific 7 (Gas7), mRNA [NM_053484]	**0,672**
0	Unknown	**0,672**
Samd5	Rattus norvegicus sterile alpha motif domain containing 5 (Samd5), mRNA [NM_001108901]	**0,672**
Rnf144b	Rattus norvegicus ring finger protein 144B (Rnf144b), mRNA [NM_001108881]	**0,672**
0	Unknown	**0,672**
Klf11	Rattus norvegicus Kruppel-like factor 11 (Klf11), mRNA [NM_001037354]	**0,672**
0	Rattus norvegicus similar to glyceraldehyde-3-phosphate dehydrogenase (LOC297809), mRNA [XM_216324]	**0,672**
Rnf121	ring finger protein 121 [Source:RefSeq peptide;Acc:NP_001101010] [ENSRNOT00000027321]	**0,672**
Sez6l2	Rattus norvegicus seizure related 6 homolog (mouse)-like 2 (Sez6l2), mRNA [NM_001107550]	**0,672**
Chrna5	Rattus norvegicus cholinergic receptor, nicotinic, alpha 5 (Chrna5), mRNA [NM_017078]	**0,672**
RGD1563224	Rattus norvegicus similar to 4930438D12Rik protein (RGD1563224), mRNA [NM_001100995]	**0,672**
0	Unknown	**0,672**
Ugt1a6	Rattus norvegicus UDP glucuronosyltransferase 1 family, polypeptide A6 (Ugt1a6), transcript variant 1, mRNA [NM_001039691]	**0,672**
Trerf1	Rattus norvegicus transcriptional regulating factor 1 (Trerf1), mRNA [NM_001108199]	**0,672**
0	Unknown	**0,672**
Uba1	Rattus norvegicus ubiquitin-like modifier activating enzyme 1 (Uba1), mRNA [NM_001014080]	**0,672**

Ppme1	Rattus norvegicus protein phosphatase methylesterase 1 (Ppme1), mRNA [NM_001191838]	**0,672**
0	Uncharacterized protein [Source:UniProtKB/TrEMBL;Acc:D3Z8X2] [ENSRNOT00000066542]	**0,672**
Ppp2r3a	Rattus norvegicus protein phosphatase 2, regulatory subunit B'', alpha (Ppp2r3a), mRNA [NM_001012202]	**0,672**
0	Q14610_HUMAN (Q14610) Interferon-gamma variant, partial (12%) [TC620342]	**0,672**
0	NID2_HUMAN (Q14112) Nidogen-2 precursor (NID-2) (Osteonidogen), partial (7%) [TC622511]	**0,672**
Hic1	Rattus norvegicus hypermethylated in cancer 1 (Hic1), mRNA [NM_001107021]	**0,672**
Zcchc11	Rattus norvegicus zinc finger, CCHC domain containing 11 (Zcchc11), mRNA [NM_001107953]	**0,672**
LOC691522	PREDICTED: Rattus norvegicus hypothetical protein LOC691522 (LOC691522), mRNA [XM_001078640]	**0,672**
0	Sema domain, transmembrane domain (TM), and cytoplasmic domain, (Semaphorin) 6A (Predicted), isoform CRA_aUncharacterized protein [Source:UniProtKB/TrEMBL;Acc:D3ZAG0] [ENSRNOT00000005476]	**0,672**
Sptan1	Rattus norvegicus spectrin, alpha, non-erythrocytic 1 (Sptan1), mRNA [NM_171983]	**0,673**
0	Rattus norvegicus similar to glyceraldehyde-3-phosphate dehydrogenase (phosphorylating) (EC 1.2.1.12) - mouse (LOC288024), mRNA [XM_221353]	**0,673**
RGD1309104	Rattus norvegicus similar to RIKEN cDNA 1700025G04 gene (RGD1309104), mRNA [NM_001105959]	**0,673**
Sds	Rattus norvegicus serine dehydratase (Sds), mRNA [NM_053962]	**0,673**
Sestd1	Rattus norvegicus SEC14 and spectrin domains 1 (Sestd1), mRNA [NM_001134514]	**0,673**
Cpne8	Rattus norvegicus copine VIII (Cpne8), mRNA [NM_001108750]	**0,673**
Ly9	Rattus norvegicus lymphocyte antigen 9 (Ly9), mRNA [NM_001191673]	**0,673**
LOC360479	Rattus norvegicus similar to hypothetical protein (LOC360479), mRNA [NM_001014115]	**0,673**
Lrpap1	Alpha-2-macroglobulin receptor-associated protein [Source:UniProtKB/Swiss-Prot;Acc:Q99068] [ENSRNOT00000012665]	**0,673**
Sp1	Rattus norvegicus Sp1 transcription factor (Sp1), mRNA [NM_012655]	**0,673**
Fads1	Rattus norvegicus fatty acid desaturase 1 (Fads1), mRNA [NM_053445]	**0,673**
RGD1559513	Uncharacterized protein [Source:UniProtKB/TrEMBL;Acc:D4ADJ9] [ENSRNOT00000033492]	**0,673**
Tspo	Rattus norvegicus translocator protein (Tspo), mRNA [NM_012515]	**0,673**
0	PREDICTED: Rattus norvegicus similar to 60S ribosomal protein L29 (P23) (RGD1566186), mRNA [XM_001070155]	**0,673**
0	Unknown	**0,673**
Elavl1	Rattus norvegicus ELAV (embryonic lethal, abnormal vision, Drosophila)-like 1 (Hu antigen R) (Elavl1), mRNA [NM_001108848]	**0,673**
Naf1	Rattus norvegicus nuclear assembly factor 1 homolog (S. cerevisiae) (Naf1), mRNA [NM_001024772]	**0,673**
Ppm1f	Rattus norvegicus protein phosphatase 1F (PP2C domain containing) (Ppm1f), mRNA [NM_175755]	**0,673**
Mobkl1a	Rattus norvegicus MOB1, Mps One Binder kinase activator-like 1A (yeast) (Mobkl1a), mRNA [NM_001108357]	**0,673**
Dmwd	PREDICTED: Rattus norvegicus dystrophia myotonica, WD repeat containing (Dmwd), mRNA [XM_001056337]	**0,673**
Rgs7bp	Rattus norvegicus regulator of G-protein signaling 7 binding protein (Rgs7bp), mRNA [NM_001012347]	**0,673**
Foxk1	Rattus norvegicus forkhead box K1 (Foxk1), mRNA [NM_001037219]	**0,673**
Dse	Rattus norvegicus dermatan sulfate epimerase (Dse), mRNA [NM_001108933]	**0,673**

LOC100365635	PREDICTED: Rattus norvegicus ankyrin repeat domain 49-like (LOC100365635), miscRNA [XR_085597]	**0,673**
Bbs1	Rattus norvegicus Bardet-Biedl syndrome 1 (Bbs1), mRNA [NM_001107569]	**0,673**
Reep3	Rattus norvegicus receptor accessory protein 3 (Reep3), mRNA [NM_001106386]	**0,673**
0	Unknown	**0,673**
0	E3 ubiquitin-protein ligase HUWE1 [Source:UniProtKB/Swiss-Prot;Acc:P51593] [ENSRNOT00000068719]	**0,673**
0	large tumor suppressor Gene [Source:MGI Symbol;Acc:MGI:1333883] [ENSRNOT00000020098]	**0,673**
0	Unknown	**0,673**
Dad1	Rattus norvegicus defender against cell death 1 (Dad1), mRNA [NM_138910]	**0,673**
LOC690490	PREDICTED: Rattus norvegicus similar to similar to RIKEN cDNA 1700001E04 (LOC690490), mRNA [XM_001074598]	**0,674**
Rcan3	Rattus norvegicus RCAN family member 3 (Rcan3), mRNA [NM_001012746]	**0,674**
Ccdc6	Uncharacterized protein [Source:UniProtKB/TrEMBL;Acc:D4AEK9] [ENSRNOT00000032159]	**0,674**
0	Rattus norvegicus cDNA clone IMAGE:7323401. [BC088301]	**0,674**
Dbn1	Rattus norvegicus drebrin 1 (Dbn1), mRNA [NM_031024]	**0,674**
Mark2	Rattus norvegicus MAP/microtubule affinity-regulating kinase 2 (Mark2), mRNA [NM_021699]	**0,674**
Dnajc17	Rattus norvegicus DnaJ (Hsp40) homolog, subfamily C, member 17 (Dnajc17), mRNA [NM_001191740]	**0,674**
Pkm2	Rattus norvegicus clone UI-R-FJ0-cpu-c-03-0-UI unknown mRNA. [AY724474]	**0,674**
Cercam	Rattus norvegicus cerebral endothelial cell adhesion molecule (Cercam), mRNA [NM_001011962]	**0,674**
Supt16h	Rattus norvegicus suppressor of Ty 16 homolog (S. cerevisiae) (Supt16h), mRNA [NM_001107261]	**0,674**
0	Unknown	**0,674**
Samd12	Rattus norvegicus sterile alpha motif domain containing 12 (Samd12), mRNA [NM_001130562]	**0,674**
Ints2	Rattus norvegicus integrator complex subunit 2, mRNA (cDNA clone MGC:156742 IMAGE:7107619), complete cds. [BC128757]	**0,674**
Cdkn1b	Rattus norvegicus cyclin-dependent kinase inhibitor 1B (Cdkn1b), mRNA [NM_031762]	**0,674**
Col1a1	Rattus norvegicus collagen, type I, alpha 1 (Col1a1), mRNA [NM_053304]	**0,674**
Apex2	Uncharacterized protein [Source:UniProtKB/TrEMBL;Acc:D3ZHV4] [ENSRNOT00000038157]	**0,674**
Gas7	Rattus norvegicus mRNA for GAS-7 protein. [AJ131902]	**0,674**
Otub1	Rattus norvegicus OTU domain, ubiquitin aldehyde binding 1 (Otub1), mRNA [NM_001106332]	**0,674**
0	Unknown	**0,674**
Nif3l1	Rattus norvegicus NIF3 NGG1 interacting factor 3-like 1 (S. pombe) (Nif3l1), mRNA [NM_001024763]	**0,674**
Gna12	Rattus norvegicus guanine nucleotide binding protein (G protein) alpha 12 (Gna12), mRNA [NM_031034]	**0,674**
Ablim3	Rattus norvegicus actin binding LIM protein family, member 3 (Ablim3), mRNA [NM_001191698]	**0,674**
Ccnc	Rattus norvegicus cyclin C (Ccnc), mRNA [NM_001100472]	**0,674**
Zfp53	Rattus norvegicus zinc finger protein 53 (Zfp53), mRNA [NM_001107468]	**0,674**
Lypd5	Rattus norvegicus Ly6/Plaur domain containing 5 (Lypd5), mRNA [NM_001192010]	**0,674**
Usp6nl	Rattus norvegicus USP6 N-terminal like (Usp6nl), mRNA [NM_001106120]	**0,674**

Slc22a23	Rattus norvegicus solute carrier family 22, member 23 (Slc22a23), mRNA [NM_022624]	**0,674**
Pbx2	Rattus norvegicus pre-B-cell leukemia homeobox 2 (Pbx2), mRNA [NM_001002828]	**0,674**
RGD1562699	Rattus norvegicus RGD1562699 (RGD1562699), mRNA [NM_001106141]	**0,674**
Hdac4	PREDICTED: Rattus norvegicus histone deacetylase 4 (Hdac4), mRNA [XM_001067733]	**0,674**
Zfp354c	Rattus norvegicus zinc finger protein 354C (Zfp354c), mRNA [NM_023988]	**0,674**
Pcdh7	Rattus norvegicus protocadherin 7 (Pcdh7), mRNA [NM_001004087]	**0,674**
Mettl10	Rattus norvegicus methyltransferase like 10 (Mettl10), mRNA [NM_001108504]	**0,675**
Nudcd2	Rattus norvegicus NudC domain containing 2 (Nudcd2), mRNA [NM_001009621]	**0,675**
Smad7	Rattus norvegicus SMAD family member 7 (Smad7), mRNA [NM_030858]	**0,675**
Gabarapl1	Rattus norvegicus GABA(A) receptor-associated protein like 1 (Gabarapl1), mRNA [NM_001044294]	**0,675**
0	LRRGT00107 [Source:UniProtKB/TrEMBL;Acc:Q6TUD7] [ENSRNOT00000035353]	**0,675**
Ddx41	Rattus norvegicus DEAD (Asp-Glu-Ala-Asp) box polypeptide 41 (Ddx41), mRNA [NM_001108046]	**0,675**
RGD1561149	Rattus norvegicus similar to mKIAA1522 protein (RGD1561149), mRNA [NM_001134629]	**0,675**
RGD1560612	Uncharacterized protein [Source:UniProtKB/TrEMBL;Acc:D3ZKV2] [ENSRNOT00000041680]	**0,675**
Myc	Rattus norvegicus myelocytomatosis oncogene (Myc), mRNA [NM_012603]	**0,675**
Rft1	Rattus norvegicus RFT1 homolog (S. cerevisiae) (Rft1), mRNA [NM_001135866]	**0,675**
Cyp2s1	Rattus norvegicus cytochrome P450, family 2, subfamily s, polypeptide 1 (Cyp2s1), mRNA [NM_001107495]	**0,675**
Purb	Rattus norvegicus purine rich element binding protein B (Purb), mRNA [NM_001017503]	**0,675**
Fam134a	PREDICTED: Rattus norvegicus family with sequence similarity 134, member A (Fam134a), mRNA [XM_001057799]	**0,675**
Zfp418	Rattus norvegicus zinc finger protein 418 (Zfp418), mRNA [NM_001191620]	**0,675**
Insc	Rattus norvegicus inscuteable homolog (Drosophila) (Insc), mRNA [NM_001106285]	**0,675**
RGD1563680	Uncharacterized protein [Source:UniProtKB/TrEMBL;Acc:D3ZYC4] [ENSRNOT00000006177]	**0,675**
0	Unknown	**0,675**
Rybp	Rattus norvegicus RING1 and YY1 binding protein (Rybp), mRNA [NM_001107879]	**0,675**
L3mbtl	Uncharacterized protein [Source:UniProtKB/TrEMBL;Acc:D4A6H6] [ENSRNOT00000045677]	**0,675**
0	Histone H4Osteogenic growth peptide [Source:UniProtKB/Swiss-Prot;Acc:P62804] [ENSRNOT00000068510]	**0,675**
Rab22a	Rattus norvegicus RAB22A, member RAS oncogene family (Rab22a), mRNA [NM_001108966]	**0,675**
Gja3	Rattus norvegicus gap junction protein, alpha 3 (Gja3), mRNA [NM_024376]	**0,675**
0	Q52LS7_HUMAN (Q52LS7) SCRT1 protein, partial (45%) [TC630587]	**0,675**
0	RVL7467 Wackym-Soares normalized rat vestibular cDNA library Rattus norvegicus cDNA 5', mRNA sequence [DV717012]	**0,675**
Olr1387	Rattus norvegicus olfactory receptor 1387 (Olr1387), mRNA [NM_001000001]	**0,675**
Mgrn1	Rattus norvegicus mahogunin, ring finger 1 (Mgrn1), mRNA [NM_001013964]	**0,675**
Usp54	Rattus norvegicus ubiquitin specific peptidase 54 (Usp54), mRNA [NM_001008863]	**0,675**
Ttll3	Rattus norvegicus tubulin tyrosine ligase-like family, member 3 (Ttll3), mRNA [NM_001108640]	**0,675**

Vcl	Rattus norvegicus vinculin (Vcl), mRNA [NM_001107248]	**0,675**
Foxn3	Rattus norvegicus forkhead box N3 (Foxn3), mRNA [NM_001108047]	**0,675**
Slc25a35	Rattus norvegicus solute carrier family 25, member 35 (Slc25a35), mRNA [NM_001109033]	**0,676**
Prpf18	Rattus norvegicus PRP18 pre-mRNA processing factor 18 homolog (S. cerevisiae) (Prpf18), mRNA [NM_138523]	**0,676**
Bfar	Rattus norvegicus bifunctional apoptosis regulator (Bfar), mRNA [NM_001013125]	**0,676**
Rhobtb3	Rattus norvegicus Rho-related BTB domain containing 3 (Rhobtb3), mRNA [NM_001107645]	**0,676**
0	AGENCOURT_31541565 NIH_MGC_270 Rattus norvegicus cDNA clone IMAGE:7444018 5', mRNA sequence [CV110972]	**0,676**
Xpc	Rattus norvegicus xeroderma pigmentosum, complementation group C (Xpc), mRNA [NM_001107874]	**0,676**
Grm4	Rattus norvegicus glutamate receptor, metabotropic 4 (Grm4), mRNA [NM_022666]	**0,676**
Kcnmb2	Rattus norvegicus potassium large conductance calcium-activated channel, subfamily M, beta member 2 (Kcnmb2), mRNA [NM_176861]	**0,676**
Gramd1b	Rattus norvegicus GRAM domain containing 1B (Gramd1b), mRNA [NM_001191616]	**0,676**
LOC500956	Rattus norvegicus hypothetical protein LOC500956 (LOC500956), mRNA [NM_001025054]	**0,676**
Zmat4	Rattus norvegicus zinc finger, matrin type 4 (Zmat4), mRNA [NM_001134747]	**0,676**
Alg3	Rattus norvegicus asparagine-linked glycosylation 3, alpha-1,3- mannosyltransferase homolog (S. cerevisiae) (Alg3), transcript variant 1, mRNA [NM_001142363]	**0,676**
0	SAGA-associated factor 29 homolog [Source:UniProtKB/Swiss-Prot;Acc:P0C606] [ENSRNOT00000026146]	**0,676**
0	Unknown	**0,676**
Psrc1	Rattus norvegicus proline/serine-rich coiled-coil 1 (Psrc1), mRNA [NM_001044302]	**0,676**
Man2c1	Rattus norvegicus mannosidase, alpha, class 2C, member 1 (Man2c1), mRNA [NM_139256]	**0,676**
LOC686506	Rattus norvegicus hypothetical protein LOC686506 (LOC686506), mRNA [NM_001134729]	**0,676**
Vps26a	Rattus norvegicus vacuolar protein sorting 26 homolog A (S. pombe) (Vps26a), mRNA [NM_001007740]	**0,676**
0	Unknown	**0,676**
Ddx41	Rattus norvegicus DEAD (Asp-Glu-Ala-Asp) box polypeptide 41 (Ddx41), mRNA [NM_001108046]	**0,676**
0	Unknown	**0,676**
Megf11	PREDICTED: Rattus norvegicus multiple EGF-like-domains 11 (Megf11), mRNA [XM_001078620]	**0,676**
LOC690769	RCG51260, isoform CRA_aUncharacterized protein [Source:UniProtKB/TrEMBL;Acc:D3ZIQ9] [ENSRNOT00000025716]	**0,676**
Slc35f1	Rattus norvegicus solute carrier family 35, member F1 (Slc35f1), mRNA [NM_001109338]	**0,676**
Pkmyt1	Rattus norvegicus protein kinase, membrane associated tyrosine/threonine 1 (Pkmyt1), mRNA [NM_001105766]	**0,676**
0	Q87VI1_PSESM (Q87VI1) Rhodanese domain protein/phosphatidylserine decarboxylase, partial (3%) [TC610837]	**0,676**
0	Unknown	**0,677**
Ube2g1	Rattus norvegicus ubiquitin-conjugating enzyme E2G 1 (UBC7 homolog, yeast) (Ube2g1), mRNA [NM_022690]	**0,677**
Slc22a15	Rattus norvegicus solute carrier family 22, member 15 (Slc22a15), mRNA [NM_001107707]	**0,677**
0	PREDICTED: Rattus norvegicus similar to double homeobox 4c (LOC680963), mRNA [XM_002725876]	**0,677**
Nedd4	Rattus norvegicus neural precursor cell expressed, developmentally down-regulated 4 (Nedd4), mRNA [NM_012986]	**0,677**

Prrg1	Rattus norvegicus proline rich Gla (G-carboxyglutamic acid) 1 (Prrg1), mRNA [NM_001191888]	**0,677**
Plekhg5	Rattus norvegicus pleckstrin homology domain containing, family G (with RhoGef domain) member 5 (Plekhg5), mRNA [NM_201272]	**0,677**
Neurod1	Rattus norvegicus neurogenic differentiation 1 (Neurod1), mRNA [NM_019218]	**0,677**
LOC100192313	Rattus norvegicus hypothetical protein LOC100192313 (LOC100192313), mRNA [NM_001136261]	**0,677**
RGD1309821	Rattus norvegicus similar to KIAA1161 protein (RGD1309821), mRNA [NM_001108971]	**0,677**
Camk2n1	Rattus norvegicus calcium/calmodulin-dependent protein kinase II inhibitor 1 (Camk2n1), mRNA [NM_173337]	**0,677**
LOC304239	Uncharacterized protein [Source:UniProtKB/TrEMBL;Acc:D3ZH07] [ENSRNOT00000001187]	**0,677**
0	TGF-beta activated kinase 1/MAP3K7 binding protein 3 Gene [Source:MGI Symbol;Acc:MGI:1913974] [ENSRNOT00000004854]	**0,677**
Ankrd45	Uncharacterized protein [Source:UniProtKB/TrEMBL;Acc:D3Z8M5] [ENSRNOT00000003857]	**0,677**
0	Unknown	**0,677**
Stx1b	Syntaxin-1B [Source:UniProtKB/Swiss-Prot;Acc:P61265] [ENSRNOT00000026063]	**0,677**
Nr1h2	Rattus norvegicus nuclear receptor subfamily 1, group H, member 2 (Nr1h2), mRNA [NM_031626]	**0,677**
Sp4	Rattus norvegicus Sp4 transcription factor (Sp4), mRNA [NM_012761]	**0,677**
Ergic3	Rattus norvegicus ERGIC and golgi 3 (Ergic3), mRNA [NM_001106533]	**0,677**
0	RCG37698, isoform CRA_aUncharacterized protein [Source:UniProtKB/TrEMBL;Acc:D4AAY1] [ENSRNOT00000037022]	**0,677**
0	Unknown	**0,677**
St6galnac3	Rattus norvegicus ST6 (alpha-N-acetyl-neuraminyl-2,3-beta-galactosyl-1,3)-N-acetylgalactosaminide alpha-2,6-sialyltransferase 3 (St6galnac3), mRNA [NM_019123]	**0,677**
Nmnat2	Rattus norvegicus nicotinamide nucleotide adenylyltransferase 2 (Nmnat2), mRNA [NM_001048042]	**0,677**
0	Rattus norvegicus similar to glyceraldehyde-3-phosphate dehydrogenase (phosphorylating) (EC 1.2.1.12) - mouse (LOC301496), mRNA [XM_237274]	**0,677**
Col4a5	PREDICTED: Rattus norvegicus collagen, type IV, alpha 5 (Col4a5), mRNA [XM_001055156]	**0,677**
Tceb2	Rattus norvegicus transcription elongation factor B (SIII), polypeptide 2 (Tceb2), mRNA [NM_031129]	**0,677**
Mef2c	PREDICTED: Rattus norvegicus myocyte enhancer factor 2C (Mef2c), mRNA [XM_001056692]	**0,677**
Eny2	Rattus norvegicus enhancer of yellow 2 homolog (Drosophila) (Eny2), mRNA [NM_001130580]	**0,677**
Sdc3	Rattus norvegicus syndecan 3 (Sdc3), mRNA [NM_053893]	**0,677**
Phldb1	Rattus norvegicus pleckstrin homology-like domain, family B, member 1 (Phldb1), mRNA [NM_001191578]	**0,678**
Rasal2	Rattus norvegicus RAS protein activator like 2 (Rasal2), mRNA [NM_001107188]	**0,678**
Ccdc127	Rattus norvegicus coiled-coil domain containing 127 (Ccdc127), mRNA [NM_198766]	**0,678**
Znf703	Rattus norvegicus zinc finger protein 703 (Znf703), mRNA [NM_001109425]	**0,678**
Fntb	Rattus norvegicus farnesyltransferase, CAAX box, beta (Fntb), mRNA [NM_172034]	**0,678**
Tnrc4	Rattus norvegicus trinucleotide repeat containing 4 (Tnrc4), mRNA [NM_001109190]	**0,678**
Hisppd2a	Rattus norvegicus histidine acid phosphatase domain containing 2A (Hisppd2a), mRNA [NM_001080783]	**0,678**
Shisa7	Rattus norvegicus shisa homolog 7 (Xenopus laevis) (Shisa7), mRNA [NM_001145175]	**0,678**

0	Unknown	**0,678**
Dusp18	Rattus norvegicus dual specificity phosphatase 18 (Dusp18), mRNA [NM_001013128]	**0,678**
Nr4a1	Rattus norvegicus nuclear receptor subfamily 4, group A, member 1 (Nr4a1), mRNA [NM_024388]	**0,678**
Slc4a4	Rattus norvegicus solute carrier family 4, sodium bicarbonate cotransporter, member 4 (Slc4a4), mRNA [NM_053424]	**0,678**
LOC304558	PREDICTED: Rattus norvegicus similar to TPR repeat-containing protein KIAA1043 (LOC304558), mRNA [XM_222260]	**0,678**
Stac2	Rattus norvegicus SH3 and cysteine rich domain 2 (Stac2), mRNA [NM_001108834]	**0,678**
Fbxo46	Rattus norvegicus F-box protein 46 (Fbxo46), mRNA [NM_001025642]	**0,678**
Adam22	PREDICTED: Rattus norvegicus a disintegrin and metalloprotease domain (ADAM) 22 (Adam22), mRNA [XM_002726324]	**0,678**
Mrrf	Rattus norvegicus mitochondrial ribosome recycling factor (Mrrf), nuclear gene encoding mitochondrial protein, mRNA [NM_001008354]	**0,678**
Fbxl22	Rattus norvegicus F-box and leucine-rich repeat protein 22 (Fbxl22), mRNA [NM_001108769]	**0,678**
0	Uncharacterized protein [Source:UniProtKB/TrEMBL;Acc:D4A1B0] [ENSRNOT00000045263]	**0,678**
Mect1	Rattus norvegicus mucoepidermoid carcinoma translocated 1 (Mect1), mRNA [NM_001047115]	**0,678**
Atg12	Rattus norvegicus ATG12 autophagy related 12 homolog (S. cerevisiae) (Atg12), mRNA [NM_001038495]	**0,678**
Ccdc135	Rattus norvegicus coiled-coil domain containing 135 (Ccdc135), mRNA [NM_001106169]	**0,678**
LOC682102	Rattus norvegicus hypothetical protein LOC682102 (LOC682102), mRNA [NM_001134732]	**0,678**
Cacna1b	Rattus norvegicus calcium channel, voltage-dependent, N type, alpha 1B subunit (Cacna1b), transcript variant 1, mRNA [NM_001195199]	**0,678**
0	Uncharacterized protein [Source:UniProtKB/TrEMBL;Acc:D3ZV82] [ENSRNOT00000037181]	**0,678**
Caskin2	Rattus norvegicus cask-interacting protein 2 (Caskin2), mRNA [NM_001107065]	**0,678**
0	Rattus norvegicus similar to 60S RIBOSOMAL PROTEIN L29 (P23) (LOC295470), mRNA [XM_227709]	**0,678**
Gp1bb	Rattus norvegicus glycoprotein Ib (platelet), beta polypeptide (Gp1bb), mRNA [NM_053930]	**0,678**
Hdac7	PREDICTED: Rattus norvegicus histone deacetylase 7 (Hdac7), mRNA [XM_001059057]	**0,678**
RGD1563904	Rattus norvegicus similar to melanoma antigen family B, 4 (RGD1563904), mRNA [NM_001109367]	**0,678**
0	KCNA3_RAT (P15384) Potassium voltage-gated channel subfamily A member 3 (Voltage-gated potassium channel subunit Kv1.3) (RGK5) (RCK3) (KV3), complete [TC574813]	**0,679**
0	Unknown	**0,679**
0	Uncharacterized protein [Source:UniProtKB/TrEMBL;Acc:D3ZT95] [ENSRNOT00000004563]	**0,679**
Chchd10	Rattus norvegicus coiled-coil-helix-coiled-coil-helix domain containing 10 (Chchd10), mRNA [NM_001007008]	**0,679**
Mudeng	Rattus norvegicus MU-2/AP1M2 domain containing, death-inducing (Mudeng), mRNA [NM_001030036]	**0,679**
Pdk2	Rattus norvegicus pyruvate dehydrogenase kinase, isozyme 2 (Pdk2), nuclear gene encoding mitochondrial protein, mRNA [NM_030872]	**0,679**
Mx1	Rattus norvegicus myxovirus (influenza virus) resistance 1 (Mx1), mRNA [NM_173096]	**0,679**
LOC680874	PREDICTED: Rattus norvegicus hypothetical protein LOC680874 (LOC680874), miscRNA [XR_005897]	**0,679**
Mcam	Rattus norvegicus melanoma cell adhesion molecule (Mcam), transcript variant 1, mRNA [NM_023983]	**0,679**
Snx19	Rattus norvegicus sorting nexin 19 (Snx19), mRNA [NM_001108131]	**0,679**
Rhobtb1	Rattus norvegicus Rho-related BTB domain containing 1 (Rhobtb1), mRNA [NM_001107622]	**0,679**

0	DRNBZC09 Rat DRG Library Rattus norvegicus cDNA clone DRNBZC09 5', mRNA sequence [BG671896]	**0,679**
Tp63	Rattus norvegicus tumor protein p63 (Tp63), transcript variant 3, mRNA [NM_001127341]	**0,679**
Hcfc1	Rattus norvegicus host cell factor C1 (Hcfc1), mRNA [NM_001139507]	**0,679**
Agrn	Rattus norvegicus agrin (Agrn), mRNA [NM_175754]	**0,679**
0	Transcriptional regulator ATRX [Source:UniProtKB/Swiss-Prot;Acc:P70486] [ENSRNOT00000033355]	**0,679**
Elmod2	Rattus norvegicus ELMO/CED-12 domain containing 2 (Elmod2), mRNA [NM_001109506]	**0,679**
LOC689226	Rattus norvegicus similar to ubiquitin-conjugating enzyme E2R 2 (LOC689226), mRNA [NM_001127573]	**0,679**
0	Unknown	**0,679**
Oaz3	Rattus norvegicus ornithine decarboxylase antizyme 3 (Oaz3), mRNA [NM_001101018]	**0,679**
Scamp3	Rattus norvegicus secretory carrier membrane protein 3 (Scamp3), mRNA [NM_031724]	**0,679**
RGD1305215	Rattus norvegicus similar to expressed sequence AA960436 (RGD1305215), mRNA [NM_001014013]	**0,679**
0	Uncharacterized protein [Source:UniProtKB/TrEMBL;Acc:D3ZRC6] [ENSRNOT00000050943]	**0,679**
Lig4	Rattus norvegicus ligase IV, DNA, ATP-dependent (Lig4), mRNA [NM_001106095]	**0,679**
Deadc1	Rattus norvegicus deaminase domain containing 1 (Deadc1), mRNA [NM_001115028]	**0,679**
LOC680531	Rattus norvegicus similar to CG3880-PA (LOC680531), mRNA [NM_001109418]	**0,679**
LOC691169	PREDICTED: Rattus norvegicus hypothetical protein LOC691169 (LOC691169), mRNA [XM_001077081]	**0,679**
Sort1	Rattus norvegicus sortilin 1 (Sort1), mRNA [NM_031767]	**0,679**
RGD1307365	PREDICTED: Rattus norvegicus similar to KIAA1009 protein (RGD1307365), mRNA [XM_001054857]	**0,680**
0	Unknown	**0,680**
Glis2	Rattus norvegicus GLIS family zinc finger 2 (Glis2), mRNA [NM_001106978]	**0,680**
Mmgt2	Rattus norvegicus membrane magnesium transporter 2 (Mmgt2), mRNA [NM_001013967]	**0,680**
Arhgdia	Rattus norvegicus Rho GDP dissociation inhibitor (GDI) alpha (Arhgdia), mRNA [NM_001007005]	**0,680**
Kcnma1	Rattus norvegicus potassium large conductance calcium-activated channel, subfamily M, alpha member 1 (Kcnma1), mRNA [NM_031828]	**0,680**
Pdxp	Rattus norvegicus pyridoxal (pyridoxine, vitamin B6) phosphatase (Pdxp), mRNA [NM_001135819]	**0,680**
Kcnk2	Rattus norvegicus potassium channel, subfamily K, member 2 (Kcnk2), transcript variant 1, mRNA [NM_172041]	**0,680**
Zyx	Rattus norvegicus zyxin (Zyx), mRNA [NM_053761]	**0,680**
0	Unknown	**0,680**
Lingo1	Rattus norvegicus leucine rich repeat and Ig domain containing 1 (Lingo1), mRNA [NM_001100722]	**0,680**
0	Uncharacterized protein [Source:UniProtKB/TrEMBL;Acc:D3ZJU2] [ENSRNOT00000004966]	**0,680**
Lhx4	Rattus norvegicus LIM homeobox 4 (Lhx4), mRNA [NM_001108348]	**0,680**
0	Unknown	**0,680**
0	AMGNNUC:NRHY6-00002-H8-A W Rat hypothalamus (10470) Rattus norvegicus cDNA clone nrhy6-00002-h8 5', mRNA sequence [CB750240]	**0,680**
0	Homeobox protein cut-like 1 [Source:UniProtKB/Swiss-Prot;Acc:P53565] [ENSRNOT00000059486]	**0,680**
Vma21	PREDICTED: Rattus norvegicus similar to 2610030H06Rik protein (RGD1566155), mRNA [XM_001067193]	**0,680**

Dgki	Rattus norvegicus diacylglycerol kinase, iota (Dgki), mRNA [NM_198782]	**0,680**
0	Uncharacterized protein [Source:UniProtKB/TrEMBL;Acc:D3Z995] [ENSRNOT00000022941]	**0,680**
RGD1308772	Uncharacterized protein [Source:UniProtKB/TrEMBL;Acc:D3ZIN5] [ENSRNOT00000013780]	**0,680**
LOC691317	Uncharacterized protein [Source:UniProtKB/TrEMBL;Acc:D3ZDG4] [ENSRNOT00000012080]	**0,680**
0	Unknown	**0,680**
Ank2	Uncharacterized protein [Source:UniProtKB/TrEMBL;Acc:D4A4Q9] [ENSRNOT00000055615]	**0,680**
Slc16a3	Rattus norvegicus solute carrier family 16, member 3 (monocarboxylic acid transporter 4) (Slc16a3), mRNA [NM_030834]	**0,680**
0	Unknown	**0,681**
Rabl2b	Rattus norvegicus RAB, member of RAS oncogene family-like 2B (Rabl2b), mRNA [NM_001013221]	**0,681**
Lin7a	Rattus norvegicus lin-7 homolog a (C. elegans) (Lin7a), mRNA [NM_053514]	**0,681**
0	Q5BK04_RAT (Q5BK04) LOC363306 protein (Fragment), partial (78%) [TC626013]	**0,681**
Mark4	Rattus norvegicus MAP/microtubule affinity-regulating kinase 4 (Mark4), mRNA [NM_001191071]	**0,681**
LOC680222	LOC680222 protein [Source:UniProtKB/TrEMBL;Acc:B2GNF2] [ENSRNOT00000047256]	**0,681**
0	Rattus norvegicus similar to zinc finger protein 91 (HPF7, HTF10) (LOC308338), mRNA [XM_218198]	**0,681**
Slc41a2	Rattus norvegicus solute carrier family 41, member 2 (Slc41a2), mRNA [NM_001108742]	**0,681**
Foxo3	Rattus norvegicus forkhead box O3 (Foxo3), mRNA [NM_001106395]	**0,681**
Zcchc3	PREDICTED: Rattus norvegicus zinc finger, CCHC domain containing 3 (Zcchc3), mRNA [XM_001072887]	**0,681**
Tmem132b	Rattus norvegicus transmembrane protein 132B (Tmem132b), mRNA [NM_001134536]	**0,681**
Rsu1	Rattus norvegicus Ras suppressor protein 1 (Rsu1), mRNA [NM_001109404]	**0,681**
Stx17	Rattus norvegicus syntaxin 17 (Stx17), mRNA [NM_145723]	**0,681**
Msl3l2	Rattus norvegicus male-specific lethal 3-like 2 (Drosophila) (Msl3l2), mRNA [NM_001014032]	**0,681**
RGD1563482	Rattus norvegicus similar to hypothetical protein FLJ38663 (RGD1563482), mRNA [NM_001109065]	**0,681**
0	salt inducible kinase 2 Gene [Source:MGI Symbol;Acc:MGI:2445031] [ENSRNOT00000066893]	**0,681**
Mafb	Rattus norvegicus v-maf musculoaponeurotic fibrosarcoma oncogene homolog B (avian) (Mafb), mRNA [NM_019316]	**0,681**
Polr3f	Rattus norvegicus polymerase (RNA) III (DNA directed) polypeptide F (Polr3f), mRNA [NM_001107784]	**0,681**
Cry2	Rattus norvegicus cryptochrome 2 (photolyase-like) (Cry2), mRNA [NM_133405]	**0,681**
Zbtb2	Rattus norvegicus zinc finger and BTB domain containing 2 (Zbtb2), mRNA [NM_001107460]	**0,681**
0	Unknown	**0,681**
Dusp16	Rattus norvegicus dual specificity phosphatase 16 (Dusp16), mRNA [NM_001106624]	**0,681**
Pip4k2b	Rattus norvegicus phosphatidylinositol-5-phosphate 4-kinase, type II, beta (Pip4k2b), mRNA [NM_053550]	**0,681**
0	Unknown	**0,681**
0	Cell division control protein 42 homolog [Source:UniProtKB/Swiss-Prot;Acc:Q8CFN2] [ENSRNOT00000018118]	**0,681**
Dll3	Rattus norvegicus delta-like 3 (Drosophila) (Dll3), mRNA [NM_053666]	**0,681**
Arsk	Rattus norvegicus arylsulfatase family, member K (Arsk), mRNA [NM_001047917]	**0,681**

Kndc1	PREDICTED: Rattus norvegicus kinase non-catalytic C-lobe domain (KIND) containing 1 (Kndc1), mRNA [XM_002725727]	**0,681**
Stard7	Rattus norvegicus StAR-related lipid transfer (START) domain containing 7 (Stard7), mRNA [NM_001106503]	**0,682**
Atp2b4	Rattus norvegicus ATPase, Ca++ transporting, plasma membrane 4 (Atp2b4), mRNA [NM_001005871]	**0,682**
0	Unknown	**0,682**
Gpatc2	Rattus norvegicus G patch domain containing 2 (Gpatc2), mRNA [NM_001011909]	**0,682**
Sorbs2	Rattus norvegicus sorbin and SH3 domain containing 2 (Sorbs2), mRNA [NM_053770]	**0,682**
Wwp2	Rattus norvegicus WW domain containing E3 ubiquitin protein ligase 2 (Wwp2), mRNA [NM_001106184]	**0,682**
Dpp6	Rattus norvegicus dipeptidylpeptidase 6 (Dpp6), mRNA [NM_022850]	**0,682**
0	Unknown	**0,682**
Zbtb41	Rattus norvegicus zinc finger and BTB domain containing 41 (Zbtb41), mRNA [NM_001191670]	**0,682**
Zfp36l3	PREDICTED: Rattus norvegicus zinc finger protein 36, C3H type-like 3 (Zfp36l3), mRNA [XM_001053657]	**0,682**
Mertk	Rattus norvegicus c-mer proto-oncogene tyrosine kinase (Mertk), mRNA [NM_022943]	**0,682**
Gpr45	Rattus norvegicus G protein-coupled receptor 45 (Gpr45), mRNA [NM_001106906]	**0,682**
Pax1	Rattus norvegicus paired box 1 (Pax1), mRNA [NM_001107787]	**0,682**
Wdr25l	Rattus norvegicus WD repeat domain 25-like (Wdr25l), mRNA [NM_001135894]	**0,682**
Slc31a1	Rattus norvegicus solute carrier family 31 (copper transporters), member 1 (Slc31a1), mRNA [NM_133600]	**0,682**
Nfyc	Rattus norvegicus nuclear transcription factor-Y gamma (Nfyc), mRNA [NM_012866]	**0,682**
Stxbp6	Rattus norvegicus syntaxin binding protein 6 (amisyn) (Stxbp6), mRNA [NM_001191872]	**0,682**
Rab8b	Rattus norvegicus RAB8B, member RAS oncogene family (Rab8b), mRNA [NM_153317]	**0,682**
0	AW254563 UI-R-BJ0-aej-h-01-0-UI.s1 UI-R-BJ0 Rattus norvegicus cDNA clone UI-R-BJ0-aej-h-01-0-UI 3', mRNA sequence [AW254563]	**0,682**
Ipo7	Rattus norvegicus importin 7 (Ipo7), mRNA [NM_001107545]	**0,682**
Clcn3	Rattus norvegicus chloride channel 3 (Clcn3), mRNA [NM_053363]	**0,682**
0	Rattus norvegicus similar to Nedd4 binding protein 1 (LOC365705), mRNA [XM_345172]	**0,682**
0	Unknown	**0,682**
Olig2	Rattus norvegicus oligodendrocyte lineage transcription factor 2 (Olig2), mRNA [NM_001100557]	**0,682**
LOC682105	Rattus norvegicus similar to receptor expression enhancing protein 2 (LOC682105), mRNA [NM_001048047]	**0,682**
Zswim4	Rattus norvegicus zinc finger, SWIM-type containing 4 (Zswim4), mRNA [NM_001107163]	**0,682**
0	Rattus norvegicus TL0ADA43YL03 mRNA sequence. [FQ220235]	**0,682**
Prp2l1	Rat proline-rich protein (PRP-1) [Source:UniProtKB/TrEMBL;Acc:Q63455] [ENSRNOT00000004642]	**0,682**
Parm1	Rattus norvegicus prostate androgen-regulated mucin-like protein 1 (Parm1), mRNA [NM_173114]	**0,682**
0	Rattus norvegicus TL0AEA59YG01 mRNA sequence. [FQ234328]	**0,682**
LOC681292	PREDICTED: Rattus norvegicus hypothetical protein LOC681292 (LOC681292), mRNA [XM_001061110]	**0,682**
Lrrtm2	Rattus norvegicus leucine rich repeat transmembrane neuronal 2 (Lrrtm2), mRNA [NM_001109469]	**0,682**
LOC314140	Rattus norvegicus ribose-phosphate pyrophosphokinase I -like (LOC314140), mRNA [NM_001009694]	**0,682**

Rnf123	Rattus norvegicus ring finger protein 123 (Rnf123), mRNA [NM_001191580]	**0,682**
0	Uncharacterized protein [Source:UniProtKB/TrEMBL;Acc:D3ZW51] [ENSRNOT00000040758]	**0,682**
Ednra	Rattus norvegicus endothelin receptor type A (Ednra), mRNA [NM_012550]	**0,683**
Rod1	Rattus norvegicus ROD1 regulator of differentiation 1 (S. pombe) (Rod1), mRNA [NM_031346]	**0,683**
Zmat5	Uncharacterized protein [Source:UniProtKB/TrEMBL;Acc:D3ZLD2] [ENSRNOT00000010332]	**0,683**
Plekhm1	Rattus norvegicus pleckstrin homology domain containing, family M (with RUN domain) member 1 (Plekhm1), mRNA [NM_001009677]	**0,683**
Cacna1g	Rattus norvegicus calcium channel, voltage-dependent, T type, alpha 1G subunit (Cacna1g), mRNA [NM_031601]	**0,683**
Cit	Rattus norvegicus citron (Cit), mRNA [NM_001029911]	**0,683**
Hecw1	Rattus norvegicus HECT, C2 and WW domain containing E3 ubiquitin protein ligase 1 (Hecw1), mRNA [NM_001106117]	**0,683**
0	Unknown	**0,683**
Sema5a	Rattus norvegicus sema domain, seven thrombospondin repeats (type 1 and type 1-like), transmembrane domain (TM) and short cytoplasmic domain, (semaphorin) 5A (Sema5a), mRNA [NM_001107659]	**0,683**
Armc1	Rattus norvegicus armadillo repeat containing 1 (Armc1), mRNA [NM_001106425]	**0,683**
Zfp41	Uncharacterized protein [Source:UniProtKB/TrEMBL;Acc:D3ZZK5] [ENSRNOT00000009823]	**0,683**
Zfp583	Rattus norvegicus zinc finger protein 583 (Zfp583), mRNA [NM_001134609]	**0,683**
Ang1	Rattus norvegicus angiogenin, ribonuclease A family, member 1 (Ang1), mRNA [NM_001006992]	**0,683**
Pi4k2b	Rattus norvegicus phosphatidylinositol 4-kinase type 2 beta (Pi4k2b), mRNA [NM_001005883]	**0,683**
Eif4ebp2	Rattus norvegicus eukaryotic translation initiation factor 4E binding protein 2 (Eif4ebp2), mRNA [NM_001033069]	**0,683**
Agk	Rattus norvegicus acylglycerol kinase (Agk), nuclear gene encoding mitochondrial protein, mRNA [NM_001127497]	**0,683**
LOC303448	Rattus norvegicus similar to glyceraldehyde-3-phosphate dehydrogenase (LOC303448), mRNA [NM_001037190]	**0,683**
Hcn1	Rattus norvegicus hyperpolarization-activated cyclic nucleotide-gated potassium channel 1 (Hcn1), mRNA [NM_053375]	**0,683**
0	Unknown	**0,683**
Sbf1	PREDICTED: Rattus norvegicus SET binding factor 1 (Sbf1), mRNA [XM_001054780]	**0,683**
Gatad2a	Rattus norvegicus GATA zinc finger domain containing 2A (Gatad2a), mRNA [NM_001013881]	**0,683**
0	Unknown	**0,683**
Nhlh1	Rattus norvegicus nescient helix loop helix 1 (Nhlh1), mRNA [NM_001105970]	**0,683**
Fut9	Rattus norvegicus fucosyltransferase 9 (alpha (1,3) fucosyltransferase) (Fut9), mRNA [NM_053465]	**0,683**
M6prbp1	PREDICTED: Rattus norvegicus mannose-6-phosphate receptor binding protein 1 (M6prbp1), mRNA [XM_236783]	**0,683**
Kitlg	Rattus norvegicus KIT ligand (Kitlg), transcript variant 1, mRNA [NM_021843]	**0,683**
Mfap3l	Rattus norvegicus microfibrillar-associated protein 3-like (Mfap3l), mRNA [NM_001012049]	**0,683**
Sgta	Rattus norvegicus small glutamine-rich tetratricopeptide repeat (TPR)-containing, alpha (Sgta), mRNA [NM_022703]	**0,683**
Ctrb1	Rattus norvegicus chymotrypsinogen B1 (Ctrb1), mRNA [NM_012536]	**0,684**
0	Rattus norvegicus similar to RIKEN cDNA 1700001F09 (LOC316932), mRNA [XM_229506]	**0,684**
Fam100a	Rattus norvegicus family with sequence similarity 100, member A (Fam100a), mRNA [NM_001007668]	**0,684**

Tcf3	Rattus norvegicus transcription factor 3 (Tcf3), mRNA [NM_001107865]	**0,684**
RGD1566380	Rattus norvegicus hypothetical gene supported by NM_017187 (RGD1566380), mRNA [NM_001134593]	**0,684**
Fam198b	Rattus norvegicus family with sequence similarity 198, member B (Fam198b), mRNA [NM_199105]	**0,684**
0	Uncharacterized protein [Source:UniProtKB/TrEMBL;Acc:D3Z884] [ENSRNOT00000049147]	**0,684**
Mul1	Rattus norvegicus mitochondrial ubiquitin ligase activator of NFKB 1 (Mul1), nuclear gene encoding mitochondrial protein, mRNA [NM_001106695]	**0,684**
Rtkn	Rattus norvegicus rhotekin (Rtkn), mRNA [NM_184046]	**0,684**
Lats2	Rattus norvegicus large tumor suppressor 2 (Lats2), mRNA [NM_001107267]	**0,684**
Epas1	Rattus norvegicus endothelial PAS domain protein 1 (Epas1), mRNA [NM_023090]	**0,684**
Dedd	Rattus norvegicus death effector domain-containing (Dedd), mRNA [NM_031800]	**0,684**
0	Unknown	**0,684**
0	Rattus norvegicus similar to glyceraldehyde-3-phosphate dehydrogenase (phosphorylating) (EC 1.2.1.12) - mouse (LOC363486), mRNA [XM_343809]	**0,684**
Scai	PREDICTED: Rattus norvegicus similar to Protein C9orf126 homolog (LOC690538), mRNA [XM_001074743]	**0,684**
0	RCG49325, isoform CRA_bUncharacterized protein [Source:UniProtKB/TrEMBL;Acc:D3ZR95] [ENSRNOT00000025495]	**0,684**
Kcnh7	Rattus norvegicus potassium voltage-gated channel, subfamily H (eag-related), member 7 (Kcnh7), mRNA [NM_131912]	**0,684**
Chmp1b	Rattus norvegicus chromatin modifying protein 1B (Chmp1b), mRNA [NM_001109533]	**0,684**
Tm9sf1	Rattus norvegicus transmembrane 9 superfamily member 1 (Tm9sf1), mRNA [NM_001012155]	**0,684**
Rfwd3	Rattus norvegicus TL0AEA87YO10 mRNA sequence. [FQ230829]	**0,684**
Lmna	Rattus norvegicus lamin A (Lmna), transcript variant 2, mRNA [NM_001002016]	**0,684**
0	Rattus norvegicus similar to 60S ribosomal protein L23a (LOC299190), mRNA [XM_234397]	**0,684**
Nfasc	Rattus norvegicus neurofascin (Nfasc), transcript variant 1, mRNA [NM_001160314]	**0,684**
Sema4g	Rattus norvegicus sema domain, immunoglobulin domain (Ig), transmembrane domain (TM) and short cytoplasmic domain, (semaphorin) 4G (Sema4g), mRNA [NM_001108526]	**0,684**
Slc8a1	Rattus norvegicus solute carrier family 8 (sodium/calcium exchanger), member 1 (Slc8a1), mRNA [NM_019268]	**0,685**
Scamp5	Rattus norvegicus secretory carrier membrane protein 5 (Scamp5), mRNA [NM_031726]	**0,685**
Tp53inp2	PREDICTED: Rattus norvegicus tumor protein p53 inducible nuclear protein 2 (Trp53inp2), mRNA [XM_001074013]	**0,685**
0	PREDICTED: Rattus norvegicus similar to H3 histone, family 3B (LOC685984), mRNA [XM_001066068]	**0,685**
Ucp2	Rattus norvegicus uncoupling protein 2 (mitochondrial, proton carrier) (Ucp2), nuclear gene encoding mitochondrial protein, mRNA [NM_019354]	**0,685**
Zfp280d	Rattus norvegicus zinc finger protein 280D (Zfp280d), mRNA [NM_001108165]	**0,685**
Wipf1	Rattus norvegicus WAS/WASL interacting protein family, member 1 (Wipf1), mRNA [NM_057192]	**0,685**
0	FM095807 etnofat Rattus norvegicus cDNA clone etnofatP0038D05 5', mRNA sequence [FM095807]	**0,685**
Btf3	Rattus norvegicus basic transcription factor 3 (Btf3), mRNA [NM_001008309]	**0,685**
0	Uncharacterized protein [Source:UniProtKB/TrEMBL;Acc:D4ACD7] [ENSRNOT00000014120]	**0,685**
Tgfb2	Rattus norvegicus transforming growth factor, beta 2 (Tgfb2), mRNA [NM_031131]	**0,685**

Serpinh1	Rattus norvegicus serine (or cysteine) peptidase inhibitor, clade H, member 1 (Serpinh1), mRNA [NM_017173]	**0,685**
0	Rattus norvegicus similar to glyceraldehyde-3-phosphate dehydrogenase (LOC317168), mRNA [XM_228411]	**0,685**
0	Uncharacterized protein [Source:UniProtKB/TrEMBL;Acc:D3ZJX9] [ENSRNOT00000057090]	**0,685**
0	Unknown	**0,685**
0	Unknown	**0,685**
Ccna2	Rattus norvegicus cyclin A2 (Ccna2), mRNA [NM_053702]	**0,685**
Efr3a	Rattus norvegicus EFR3 homolog A (S. cerevisiae) (Efr3a), mRNA [NM_001130564]	**0,685**
0	AGENCOURT_27851764 NIH_MGC_252 Rattus norvegicus cDNA clone IMAGE:7309973 5', mRNA sequence [CO400749]	**0,685**
Suv39h1	Rattus norvegicus suppressor of variegation 3-9 homolog 1 (Drosophila) (Suv39h1), mRNA [NM_001106956]	**0,685**
Nmnat1	Rattus norvegicus nicotinamide nucleotide adenylyltransferase 1 (Nmnat1), mRNA [NM_001037556]	**0,685**
0	Unknown	**0,685**
Shank1	Rattus norvegicus SH3 and multiple ankyrin repeat domains 1 (Shank1), mRNA [NM_031751]	**0,685**
RGD1307357	Rattus norvegicus similar to hypothetical protein DKFZp434A1319 (RGD1307357), mRNA [NM_001106180]	**0,685**
Ubxn2b	Rattus norvegicus UBX domain protein 2B (Ubxn2b), mRNA [NM_001107905]	**0,686**
Zfp358	Rattus norvegicus zinc finger protein 358 (Zfp358), mRNA [NM_001108328]	**0,686**
Cecr6	PREDICTED: Rattus norvegicus cat eye syndrome chromosome region, candidate 6 homolog (human) (Cecr6), mRNA [XM_575656]	**0,686**
Vwa3a	Rattus norvegicus von Willebrand factor A domain containing 3A (Vwa3a), transcript variant 2, mRNA [NM_001079885]	**0,686**
RGD1562018	Rattus norvegicus similar to Protein C14orf101 homolog (RGD1562018), mRNA [NM_001170475]	**0,686**
0	Unknown	**0,686**
Rabgap1l	Rattus norvegicus RAB GTPase activating protein 1-like (Rabgap1l), mRNA [NM_001107190]	**0,686**
Grid2	Rattus norvegicus glutamate receptor, ionotropic, delta 2 (Grid2), mRNA [NM_024379]	**0,686**
RGD1306783	Rattus norvegicus similar to 2810422O20Rik protein (RGD1306783), mRNA [NM_001025668]	**0,686**
Pogz	Rattus norvegicus pogo transposable element with ZNF domain (Pogz), mRNA [NM_001107693]	**0,686**
Cp	Rattus norvegicus GPI-anchored ceruloplasmin mRNA, complete cds. [AF202115]	**0,686**
Vps37c	Rattus norvegicus vacuolar protein sorting 37 homolog C (S. cerevisiae) (Vps37c), mRNA [NM_001107463]	**0,686**
Csnk2a1	Rattus norvegicus casein kinase 2, alpha 1 polypeptide (Csnk2a1), mRNA [NM_053824]	**0,686**
0	Unknown	**0,686**
Gdf11	Growth/differentiation factor 11 [Source:UniProtKB/Swiss-Prot;Acc:Q9Z217] [ENSRNOT00000010035]	**0,686**
Lgals3bp	Rattus norvegicus lectin, galactoside-binding, soluble, 3 binding protein (Lgals3bp), mRNA [NM_139096]	**0,686**
Aplp1	Rattus norvegicus amyloid beta (A4) precursor-like protein 1 (Aplp1), mRNA [NM_001100802]	**0,686**
0	Unknown	**0,687**
RGD1308759	Rattus norvegicus similar to KIAA0892 protein (RGD1308759), mRNA [NM_001106077]	**0,687**
0	Q4BL67_BURVI (Q4BL67) Phospholipase/Carboxylesterase, partial (5%) [TC592585]	**0,687**
Rdh13	Rattus norvegicus retinol dehydrogenase 13 (all-trans/9-cis) (Rdh13), nuclear gene encoding mitochondrial protein, mRNA [NM_001108468]	**0,687**

0	slingshot homolog 1 (Drosophila) Gene [Source:MGI Symbol;Acc:MGI:2686240] [ENSRNOT00000044751]	**0,687**
Ugcg	Rattus norvegicus UDP-glucose ceramide glucosyltransferase (Ugcg), mRNA [NM_031795]	**0,687**
Cdkn1a	Rattus norvegicus cyclin-dependent kinase inhibitor 1A (Cdkn1a), mRNA [NM_080782]	**0,687**
Ddx42	Rattus norvegicus DEAD (Asp-Glu-Ala-Asp) box polypeptide 42 (Ddx42), mRNA [NM_001107059]	**0,687**
0	Uncharacterized protein [Source:UniProtKB/TrEMBL;Acc:D3ZKR7] [ENSRNOT00000050021]	**0,687**
Fam161a	Rattus norvegicus family with sequence similarity 161, member A (Fam161a), mRNA [NM_001013876]	**0,687**
Sln	Rattus norvegicus sarcolipin (Sln), mRNA [NM_001013247]	**0,687**
0	Uncharacterized protein [Source:UniProtKB/TrEMBL;Acc:D4A8N4] [ENSRNOT00000004178]	**0,687**
LOC100360919	Rattus norvegicus Immunoglobulin lambda-like polypeptide 1-like (LOC100360919), mRNA [NM_001190341]	**0,687**
Dgkz	Rattus norvegicus diacylglycerol kinase zeta (Dgkz), mRNA [NM_031143]	**0,687**
LOC500420	LOC500420 protein [Source:UniProtKB/TrEMBL;Acc:Q3SWT2] [ENSRNOT00000030559]	**0,687**
Kcna7	Rattus norvegicus potassium voltage-gated channel, shaker-related subfamily, member 7 (Kcna7), mRNA [NM_001108914]	**0,687**
0	Unknown	**0,687**
LOC689442	PREDICTED: Rattus norvegicus hypothetical protein LOC689442 (LOC689442), mRNA [XM_001070806]	**0,687**
0	UI-R-BO1-asq-d-09-0-UI.s1 UI-R-BO1 Rattus norvegicus cDNA clone UI-R-BO1-asq-d-09-0-UI 3', mRNA sequence [BE106644]	**0,687**
Tbx6	Rattus norvegicus T-box 6 (Tbx6), mRNA [NM_001108920]	**0,687**
0	Unknown	**0,687**
Yes1	Rattus norvegicus Yamaguchi sarcoma viral (v-yes) oncogene homolog 1 (Yes1), mRNA [NM_033298]	**0,687**
Syt5	Rattus norvegicus synaptotagmin V (Syt5), mRNA [NM_019350]	**0,687**
Msn	Rattus norvegicus moesin (Msn), mRNA [NM_030863]	**0,687**
LOC685244	PREDICTED: Rattus norvegicus similar to IQ motif and Sec7 domain 2, transcript variant 2 (LOC685244), mRNA [XM_002727538]	**0,687**
Acpl2	Rattus norvegicus acid phosphatase-like 2 (Acpl2), mRNA [NM_001007710]	**0,687**
LOC685796	PREDICTED: Rattus norvegicus similar to similar to RIKEN cDNA 1700001E04 (LOC685796), mRNA [XM_001065304]	**0,687**
LOC678704	Rattus norvegicus similar to Probable cation-transporting ATPase 3 (ATPase family homolog up-regulated in senescence cells 1) (LOC360728), mRNA [XM_341001]	**0,687**
0	Unknown	**0,687**
LOC679983	AMGNNUC:URRG1-00065-A12-A urrg1 (14046) Rattus norvegicus cDNA clone urrg1-00065-a12 5', mRNA sequence [CB547742]	**0,687**
Akap10	Rattus norvegicus A kinase (PRKA) anchor protein 10 (Akap10), nuclear gene encoding mitochondrial protein, mRNA [NM_001114606]	**0,687**
Lhx2	Rattus norvegicus LIM homeobox 2 (Lhx2), mRNA [NM_001106571]	**0,687**
Cacna1a	Rattus norvegicus calcium channel, voltage-dependent, P/Q type, alpha 1A subunit (Cacna1a), mRNA [NM_012918]	**0,687**
Thg1l	Rattus norvegicus tRNA-histidine guanylyltransferase 1-like (S. cerevisiae) (Thg1l), mRNA [NM_001013966]	**0,687**
Srgap2	Rattus norvegicus TL0AAA55YK04 mRNA sequence. [FQ212420]	**0,687**
Rfk	Rattus norvegicus riboflavin kinase (Rfk), mRNA [NM_001014106]	**0,688**
0	Uncharacterized protein [Source:UniProtKB/TrEMBL;Acc:D3ZMT2] [ENSRNOT00000044934]	**0,688**

Dnajb9	Rattus norvegicus DnaJ (Hsp40) homolog, subfamily B, member 9 (Dnajb9), mRNA [NM_012699]	**0,688**
Zfp316	Rattus norvegicus zinc finger protein 316 (Zfp316), mRNA [NM_001107121]	**0,688**
0	Unknown	**0,688**
Zfhx2	Rattus norvegicus zinc finger homeobox 2 (Zfhx2), mRNA [NM_001098803]	**0,688**
Cldn19	Rattus norvegicus claudin 19 (Cldn19), mRNA [NM_001008514]	**0,688**
Nop2	Rattus norvegicus NOP2 nucleolar protein homolog (yeast) (Nop2), mRNA [NM_001191785]	**0,688**
Pptc7	Rattus norvegicus PTC7 protein phosphatase homolog (S. cerevisiae) (Pptc7), mRNA [NM_001107141]	**0,688**
Scrn3	Rattus norvegicus secernin 3 (Scrn3), mRNA [NM_001013162]	**0,688**
0	Unknown	**0,688**
Sfxn1	Rattus norvegicus sideroflexin 1 (Sfxn1), mRNA [NM_001012213]	**0,688**
Tle4	Rattus norvegicus transducin-like enhancer of split 4 (E(sp1) homolog, Drosophila) (Tle4), mRNA [NM_019141]	**0,688**
Cpt1a	Rattus norvegicus carnitine palmitoyltransferase 1a, liver (Cpt1a), nuclear gene encoding mitochondrial protein, mRNA [NM_031559]	**0,688**
RGD1306271	Uncharacterized protein [Source:UniProtKB/TrEMBL;Acc:D3Z9D0] [ENSRNOT00000040391]	**0,688**
RGD1564915	PREDICTED: Rattus norvegicus similar to TDPOZ2 (RGD1564915), mRNA [XM_578033]	**0,688**
Lhx9	Rattus norvegicus LIM homeobox 9 (Lhx9), mRNA [NM_181367]	**0,688**
G6pd	Rattus norvegicus glucose-6-phosphate dehydrogenase (G6pd), mRNA [NM_017006]	**0,688**
Slc25a10	Rattus norvegicus solute carrier family 25 (mitochondrial carrier; dicarboxylate transporter), member 10 (Slc25a10), nuclear gene encoding mitochondrial protein, mRNA [NM_133418]	**0,688**
Rad21	Rattus norvegicus RAD21 homolog (S. pombe) (Rad21), mRNA [NM_001025701]	**0,688**
Zkscan1	Rattus norvegicus zinc finger with KRAB and SCAN domains 1 (Zkscan1), mRNA [NM_001025760]	**0,688**
RGD1306926	Uncharacterized protein [Source:UniProtKB/TrEMBL;Acc:D3ZX03] [ENSRNOT00000054974]	**0,688**
0	Unknown	**0,688**
Map6d1	Rattus norvegicus MAP6 domain containing 1 (Map6d1), mRNA [NM_001108844]	**0,688**
Rock2	Rattus norvegicus Rho-associated coiled-coil containing protein kinase 2 (Rock2), mRNA [NM_013022]	**0,688**
0	Unknown	**0,688**
Adamts3	Rattus norvegicus ADAM metallopeptidase with thrombospondin type 1, motif 3 (Adamts3), mRNA [NM_001107212]	**0,688**
Rgma	Rattus norvegicus RGM domain family, member A (Rgma), mRNA [NM_001107524]	**0,688**
0	Rattus norvegicus similar to UBE2I protein (LOC305375), mRNA [XM_223442]	**0,688**
Ankrd55	PREDICTED: Rattus norvegicus ankyrin repeat domain 55 (Ankrd55), mRNA [XM_342195]	**0,688**
Rab11b	Rattus norvegicus RAB11B, member RAS oncogene family (Rab11b), mRNA [NM_032617]	**0,688**
B4galt6	Rattus norvegicus UDP-Gal:betaGlcNAc beta 1,4-galactosyltransferase, polypeptide 6 (B4galt6), mRNA [NM_031740]	**0,689**
Sipa1l1	Rattus norvegicus signal-induced proliferation-associated 1 like 1 (Sipa1l1), mRNA [NM_139330]	**0,689**
Mier1	Rattus norvegicus mesoderm induction early response 1 homolog (Xenopus laevis) (Mier1), mRNA [NM_001131012]	**0,689**
Pak1	Rattus norvegicus p21 protein (Cdc42/Rac)-activated kinase 1 (Pak1), mRNA [NM_017198]	**0,689**

Olr1162	Rattus norvegicus olfactory receptor 1162 (Olr1162), mRNA [NM_001000870]	**0,689**
Nrarp	Rattus norvegicus Notch-regulated ankyrin repeat protein (Nrarp), mRNA [NM_001143750]	**0,689**
Utp6	PREDICTED: Rattus norvegicus UTP6, small subunit (SSU) processome component, homolog (yeast), transcript variant 2 (Utp6), mRNA [XM_002724525]	**0,689**
0	Unknown	**0,689**
Luc7l2	Rattus norvegicus LUC7-like 2 (S. cerevisiae) (Luc7l2), mRNA [NM_001107853]	**0,689**
Krtap16-5	Rattus norvegicus keratin associated protein 16-5 (Krtap16-5), mRNA [NM_001109424]	**0,689**
Sfxn3	Rattus norvegicus sideroflexin 3 (Sfxn3), mRNA [NM_022948]	**0,689**
0	Unknown	**0,689**
0	Uncharacterized protein [Source:UniProtKB/TrEMBL;Acc:D3ZKV4] [ENSRNOT00000022344]	**0,689**
Nucb1	Rattus norvegicus nucleobindin 1 (Nucb1), mRNA [NM_053463]	**0,689**
0	Unknown	**0,689**
0	Unknown	**0,689**
Igf2bp2	PREDICTED: Rattus norvegicus insulin-like growth factor 2 mRNA binding protein 2, transcript variant 2 (Igf2bp2), mRNA [XM_221343]	**0,689**
Ntsr2	Rattus norvegicus neurotensin receptor 2 (Ntsr2), mRNA [NM_022695]	**0,689**
Slitrk3	Rattus norvegicus SLIT and NTRK-like family, member 3 (Slitrk3), mRNA [NM_001107683]	**0,689**
LOC684024	PREDICTED: Rattus norvegicus similar to dual specificity phosphatase 5 (LOC684024), mRNA [XM_001068576]	**0,689**
Cdc2l6	Rattus norvegicus cell division cycle 2-like 6 (CDK8-like) (Cdc2l6), mRNA [NM_001107634]	**0,689**
0	Unknown	**0,689**
0	Unknown	**0,689**
0	UI-R-FS1-cqi-l-02-0-UI.s1 UI-R-FS1 Rattus norvegicus cDNA clone UI-R-FS1-cqi-l-02-0-UI 3', mRNA sequence [BU760441]	**0,689**
Smad4	Rattus norvegicus SMAD family member 4 (Smad4), mRNA [NM_019275]	**0,689**
Cdc26	Rattus norvegicus cell division cycle 26 (Cdc26), mRNA [NM_001013240]	**0,689**
0	PREDICTED: Rattus norvegicus similar to Protein C9orf126 homolog (LOC690538), mRNA [XM_001074743]	**0,689**
Pde4d	Rattus norvegicus phosphodiesterase 4D, cAMP-specific (phosphodiesterase E3 dunce homolog, Drosophila) (Pde4d), transcript variant 5, mRNA [NM_017032]	**0,689**
Tmem101	Rattus norvegicus transmembrane protein 101 (Tmem101), mRNA [NM_001191650]	**0,689**
0	Unknown	**0,689**
Hip1	Rattus norvegicus huntingtin interacting protein 1 (Hip1), mRNA [NM_001100475]	**0,689**
0	AGENCOURT_118864812 NIH_MGC_248 Rattus norvegicus cDNA clone IMAGE:9113290 5', mRNA sequence [EX491730]	**0,689**
Cpeb3	Uncharacterized protein [Source:UniProtKB/TrEMBL;Acc:D3Z9M2] [ENSRNOT00000047387]	**0,689**
0	Unknown	**0,689**
Fam19a5	Rattus norvegicus family with sequence similarity 19 (chemokine (C-C motif)-like), member A5 (Fam19a5), mRNA [NM_001191991]	**0,690**
Rpl28	Rattus norvegicus ribosomal protein L28 (Rpl28), mRNA [NM_022697]	**0,690**

Ltk	Rattus norvegicus leukocyte receptor tyrosine kinase (Ltk), mRNA [NM_001107763]	**0,690**
0	Unknown	**0,690**
LOC683460	PREDICTED: Rattus norvegicus hypothetical protein LOC683460, transcript variant 2 (LOC683460), mRNA [XM_001065403]	**0,690**
Ero1l	Rattus norvegicus ERO1-like (S. cerevisiae) (Ero1l), mRNA [NM_138528]	**0,690**
0	Uncharacterized protein [Source:UniProtKB/TrEMBL;Acc:D3ZKR7] [ENSRNOT00000059076]	**0,690**
LOC686921	PREDICTED: Rattus norvegicus hypothetical protein LOC686921 (LOC686921), mRNA [XM_001076338]	**0,690**
0	Rattus norvegicus hypothetical LOC100125371, mRNA (cDNA clone MGC:105701 IMAGE:7309421), complete cds. [BC089106]	**0,690**
Sdk2	Rattus norvegicus sidekick homolog 2 (chicken) (Sdk2), mRNA [NM_001108303]	**0,690**
Dullard	Rattus norvegicus Dullard homolog (Xenopus laevis) (Dullard), mRNA [NM_001100494]	**0,690**
Wnt7b	Rattus norvegicus wingless-type MMTV integration site family, member 7B (Wnt7b), mRNA [NM_001009695]	**0,690**
0	Rattus norvegicus TL0ADA41YI21 mRNA sequence. [FQ220826]	**0,690**
0	Unknown	**0,690**
Mllt10	Rattus norvegicus myeloid/lymphoid or mixed-lineage leukemia (trithorax homolog, Drosophila); translocated to, 10 (Mllt10), mRNA [NM_001012162]	**0,690**
Cldn19	Rattus norvegicus claudin 19 (Cldn19), mRNA [NM_001008514]	**0,690**
Tpst1	Rattus norvegicus tyrosylprotein sulfotransferase 1 (Tpst1), mRNA [NM_001011903]	**0,690**
RGD1309362	Rattus norvegicus similar to interferon-inducible GTPase (RGD1309362), mRNA [NM_001024884]	**0,690**
Zdbf2	PREDICTED: Rattus norvegicus zinc finger, DBF-type containing 2 (Zdbf2), mRNA [XM_576579]	**0,690**
LOC302192	PREDICTED: Rattus norvegicus similar to RIKEN cDNA 1700001E04 (LOC302192), mRNA [XM_001065392]	**0,690**
Kcnip1	Rattus norvegicus Kv channel-interacting protein 1 (Kcnip1), mRNA [NM_022929]	**0,690**
Arhgdib	Rattus norvegicus Rho, GDP dissociation inhibitor (GDI) beta (Arhgdib), mRNA [NM_001009600]	**0,690**
Lppr5	Rattus norvegicus lipid phosphate phosphatase-related protein type 5 (Lppr5), mRNA [NM_001107720]	**0,690**
Pcdhga9	Rattus norvegicus protocadherin gamma subfamily A, 9 (Pcdhga9), mRNA [NM_001037158]	**0,690**
0	Rattus norvegicus TL0ADA47YE11 mRNA sequence. [FQ229630]	**0,690**
Pou2f1	Rattus norvegicus POU class 2 homeobox 1 (Pou2f1), mRNA [NM_001100639]	**0,690**
Nap1l1	Rattus norvegicus nucleosome assembly protein 1-like 1 (Nap1l1), mRNA [NM_053561]	**0,690**
Nr3c1	Rattus norvegicus nuclear receptor subfamily 3, group C, member 1 (Nr3c1), mRNA [NM_012576]	**0,691**
Apc	Rattus norvegicus adenomatous polyposis coli (Apc), mRNA [NM_012499]	**0,691**
0	Unknown	**0,691**
Olfm2	Rattus norvegicus olfactomedin 2 (Olfm2), mRNA [NM_001015017]	**0,691**
Nppc	Rattus norvegicus natriuretic peptide precursor C (Nppc), mRNA [NM_053750]	**0,691**
Cdh20	Rattus norvegicus cadherin 20 (Cdh20), mRNA [NM_001012748]	**0,691**
Srd5a2	Rattus norvegicus steroid-5-alpha-reductase, alpha polypeptide 2 (3-oxo-5 alpha-steroid delta 4-dehydrogenase alpha 2) (Srd5a2), mRNA [NM_022711]	**0,691**

0	Unknown	**0,691**
Araf	Rattus norvegicus v-raf murine sarcoma 3611 viral oncogene homolog (Araf), transcript variant 1, mRNA [NM_022532]	**0,691**
Rnf14	Rattus norvegicus ring finger protein 14 (Rnf14), mRNA [NM_001034995]	**0,691**
Rhbdl1	Rattus norvegicus rhomboid, veinlet-like 1 (Drosophila) (Rhbdl1), mRNA [NM_001191822]	**0,691**
Stx2	Rattus norvegicus syntaxin 2 (Stx2), mRNA [NM_012748]	**0,691**
0	Unknown	**0,691**
0	Uncharacterized protein [Source:UniProtKB/TrEMBL;Acc:D4AD38] [ENSRNOT00000036164]	**0,691**
Jazf1	PREDICTED: Rattus norvegicus JAZF zinc finger 1 (Jazf1), mRNA [XM_002726367]	**0,691**
Rnasek	Rattus norvegicus ribonuclease, RNase K (Rnasek), mRNA [NM_001137561]	**0,691**
Rybp	Rattus norvegicus RING1 and YY1 binding protein (Rybp), mRNA [NM_001107879]	**0,691**
Ppp1r1c	Rattus norvegicus protein phosphatase 1, regulatory (inhibitor) subunit 1C (Ppp1r1c), mRNA [NM_001109200]	**0,691**
Slc4a4	Rattus norvegicus solute carrier family 4, sodium bicarbonate cotransporter, member 4 (Slc4a4), mRNA [NM_053424]	**0,691**
RGD1561916	Uncharacterized protein [Source:UniProtKB/TrEMBL;Acc:D3ZGG6] [ENSRNOT00000017903]	**0,691**
Pcsk6	Rattus norvegicus proprotein convertase subtilisin/kexin type 6 (Pcsk6), mRNA [NM_012999]	**0,691**
P4ha1	Rattus norvegicus prolyl 4-hydroxylase, alpha polypeptide I (P4ha1), mRNA [NM_172062]	**0,691**
Eif3h	Eukaryotic translation initiation factor 3 subunit H [Source:UniProtKB/Swiss-Prot;Acc:Q6P9U8] [ENSRNOT00000005786]	**0,691**
LOC682920	PREDICTED: Rattus norvegicus similar to phosphatase and actin regulator 4 (LOC682920), mRNA [XM_001063709]	**0,691**
Ly6g6c	Rattus norvegicus lymphocyte antigen 6 complex, locus G6C (Ly6g6c), mRNA [NM_001001969]	**0,691**
0	Uncharacterized protein [Source:UniProtKB/TrEMBL;Acc:D4A391] [ENSRNOT00000066991]	**0,691**
Adcyap1r1	Rattus norvegicus adenylate cyclase activating polypeptide 1 receptor 1 (Adcyap1r1), mRNA [NM_133511]	**0,691**
LOC688430	PREDICTED: Rattus norvegicus similar to Cofilin-1 (Cofilin, non-muscle isoform), transcript variant 1 (LOC688430), mRNA [XM_001067293]	**0,692**
Eln	Rattus norvegicus elastin (Eln), mRNA [NM_012722]	**0,692**
Gnai2	Rattus norvegicus guanine nucleotide binding protein (G protein), alpha inhibiting 2 (Gnai2), mRNA [NM_031035]	**0,692**
Mrpl10	Rattus norvegicus mitochondrial ribosomal protein L10 (Mrpl10), nuclear gene encoding mitochondrial protein, mRNA [NM_001109620]	**0,692**
Kcnq3	Rattus norvegicus potassium voltage-gated channel, KQT-like subfamily, member 3 (Kcnq3), mRNA [NM_031597]	**0,692**
0	Unknown	**0,692**
0	Unknown	**0,692**
Ggn	Rattus norvegicus gametogenetin (Ggn), mRNA [NM_001013065]	**0,692**
Cnot3	Rattus norvegicus CCR4-NOT transcription complex, subunit 3 (Cnot3), mRNA [NM_001107471]	**0,692**
0	Unknown	**0,692**
Prkab2	Rattus norvegicus protein kinase, AMP-activated, beta 2 non-catalytic subunit (Prkab2), mRNA [NM_022627]	**0,692**
Slc10a7	Rattus norvegicus solute carrier family 10 (sodium/bile acid cotransporter family), member 7 (Slc10a7), mRNA [NM_001010948]	**0,692**
Nr4a3	Rattus norvegicus nuclear receptor subfamily 4, group A, member 3 (Nr4a3), transcript variant 1, mRNA [NM_031628]	**0,692**
0	Unknown	**0,692**

Sec61a2	Rattus norvegicus Sec61 alpha 2 subunit (S. cerevisiae) (Sec61a2), mRNA [NM_001170343]	**0,692**
Alpl	Rattus norvegicus alkaline phosphatase, liver/bone/kidney (Alpl), mRNA [NM_013059]	**0,692**
Mrm1	Rattus norvegicus mitochondrial rRNA methyltransferase 1 homolog (S. cerevisiae) (Mrm1), nuclear gene encoding mitochondrial protein, mRNA [NM_001108832]	**0,692**
Klhl14	Rattus norvegicus kelch-like 14 (Drosophila) (Klhl14), mRNA [NM_001108885]	**0,692**
Egln3	Rattus norvegicus EGL nine homolog 3 (C. elegans) (Egln3), mRNA [NM_019371]	**0,692**
Olr1435	Rattus norvegicus olfactory receptor 1435 (Olr1435), mRNA [NM_001000777]	**0,692**
0	Uncharacterized protein [Source:UniProtKB/TrEMBL;Acc:D3ZM70] [ENSRNOT00000006897]	**0,692**
LOC683470	PREDICTED: Rattus norvegicus similar to growth arrest specific 1 (LOC683470), mRNA [XM_001066058]	**0,692**
Raver1	Rattus norvegicus ribonucleoprotein, PTB-binding 1 (Raver1), mRNA [NM_001013939]	**0,693**
Med13	Rattus norvegicus mediator complex subunit 13 (Med13), mRNA [NM_001107035]	**0,693**
Ksr1	Rattus norvegicus kinase suppressor of ras 1 (Ksr1), mRNA [NM_001108284]	**0,693**
Otub1	Rattus norvegicus OTU domain, ubiquitin aldehyde binding 1 (Otub1), mRNA [NM_001106332]	**0,693**
Tmem132b	Rattus norvegicus transmembrane protein 132B (Tmem132b), mRNA [NM_001134536]	**0,693**
Astn1	Rattus norvegicus astrotactin 1 (Astn1), mRNA [NM_001170603]	**0,693**
0	Unknown	**0,693**
Dnajc14	Rattus norvegicus DnaJ (Hsp40) homolog, subfamily C, member 14 (Dnajc14), mRNA [NM_053690]	**0,693**
Pramef8	Rattus norvegicus PRAME family member 8 (Pramef8), mRNA [NM_001135782]	**0,693**
LOC652955	Rattus norvegicus goliath (LOC652955), mRNA [NM_001037658]	**0,693**
0	Unknown	**0,693**
Morn1	Rattus norvegicus MORN repeat containing 1 (Morn1), mRNA [NM_001005544]	**0,693**
Pak4	Rattus norvegicus p21 protein (Cdc42/Rac)-activated kinase 4 (Pak4), mRNA [NM_001106238]	**0,693**
Adamts7	Rattus norvegicus ADAM metallopeptidase with thrombospondin type 1 motif, 7 (Adamts7), mRNA [NM_001047101]	**0,693**
Col11a2	Rattus norvegicus collagen, type XI, alpha 2 (Col11a2), mRNA [NM_212528]	**0,693**
0	Unknown	**0,693**
Calcb	Rattus norvegicus calcitonin-related polypeptide, beta (Calcb), mRNA [NM_138513]	**0,693**
Smcr7	PREDICTED: Rattus norvegicus Smith-Magenis syndrome chromosome region, candidate 7 homolog (human) (Smcr7), mRNA [XM_001077609]	**0,693**
Tbp	Rattus norvegicus TATA box binding protein (Tbp), mRNA [NM_001004198]	**0,693**
LOC500625	Rattus norvegicus hypothetical protein LOC500625 (LOC500625), mRNA [NM_001025151]	**0,693**
0	Uncharacterized protein [Source:UniProtKB/TrEMBL;Acc:D3ZD04] [ENSRNOT00000045918]	**0,693**
Cbln4	Rattus norvegicus cerebellin 4 precursor (Cbln4), mRNA [NM_001109210]	**0,693**
Klf7	Rattus norvegicus Kruppel-like factor 7 (ubiquitous) (Klf7), mRNA [NM_001108800]	**0,693**
Abhd2	Rattus norvegicus abhydrolase domain containing 2 (Abhd2), mRNA [NM_001106275]	**0,693**
0	Unknown	**0,693**

Chtf8	Rattus norvegicus CTF8, chromosome transmission fidelity factor 8 homolog (S. cerevisiae) (Chtf8), mRNA [NM_001194951]	**0,693**
Med25	Rattus norvegicus mediator complex subunit 25 (Med25), mRNA [NM_001170426]	**0,693**
Stxbp5l	Uncharacterized protein [Source:UniProtKB/TrEMBL;Acc:D3ZDG1] [ENSRNOT00000041715]	**0,693**
Kcnh4	Rattus norvegicus potassium voltage-gated channel, subfamily H (eag-related), member 4 (Kcnh4), mRNA [NM_053630]	**0,693**
Elovl7	Rattus norvegicus ELOVL family member 7, elongation of long chain fatty acids (yeast) (Elovl7), mRNA [NM_001191844]	**0,694**
Ifngr2	Rattus norvegicus interferon gamma receptor 2 (Ifngr2), mRNA [NM_001108313]	**0,694**
Mad1l1	Rattus norvegicus MAD1 mitotic arrest deficient-like 1 (yeast) (Mad1l1), mRNA [NM_001109387]	**0,694**
RGD1306772	Rattus norvegicus similar to RIKEN cDNA 1110008J03 (RGD1306772), mRNA [NM_001044226]	**0,694**
Pak4	Rattus norvegicus p21 protein (Cdc42/Rac)-activated kinase 4 (Pak4), mRNA [NM_001106238]	**0,694**
Golga1	Rattus norvegicus golgi autoantigen, golgin subfamily a, 1 (Golga1), mRNA [NM_001107842]	**0,694**
En1	PREDICTED: Rattus norvegicus engrailed homeobox 1, transcript variant 2 (En1), mRNA [XM_001056772]	**0,694**
0	Unknown	**0,694**
RGD1563351	PREDICTED: Rattus norvegicus RGD1563351 (RGD1563351), mRNA [XM_238200]	**0,694**
Clybl	Rattus norvegicus citrate lyase beta like (Clybl), mRNA [NM_001100685]	**0,694**
RGD1566359	Uncharacterized protein [Source:UniProtKB/TrEMBL;Acc:D4ADM9] [ENSRNOT00000029899]	**0,694**
Cyp2w1	PREDICTED: Rattus norvegicus cytochrome P450, family 2, subfamily W, polypeptide 1 (Cyp2w1), mRNA [XM_001074391]	**0,694**
Cnot6l	Rattus norvegicus CCR4-NOT transcription complex, subunit 6-like (Cnot6l), mRNA [NM_001108355]	**0,694**
0	Unknown	**0,694**
Il13ra1	Rattus norvegicus interleukin 13 receptor, alpha 1 (Il13ra1), mRNA [NM_145789]	**0,694**
0	Rattus norvegicus similar to cell division cycle associated 3; gene rich cluster, C8 gene; trigger of mitotic entry 1 (LOC316373), mRNA [XM_237105]	**0,694**
Fam48a	Rattus norvegicus family with sequence similarity 48, member A (Fam48a), mRNA [NM_001014170]	**0,694**
Akap12	Rattus norvegicus A kinase (PRKA) anchor protein 12 (Akap12), transcript variant 1, mRNA [NM_057103]	**0,694**
Tmem43	Rattus norvegicus transmembrane protein 43 (Tmem43), mRNA [NM_001007745]	**0,694**
0	Unknown	**0,694**
0	ROBO1_RAT (O55005) Roundabout homolog 1 precursor, complete [TC573812]	**0,694**
Pdlim7	Rattus norvegicus PDZ and LIM domain 7 (Pdlim7), mRNA [NM_173125]	**0,694**
Kank1	Rattus norvegicus KN motif and ankyrin repeat domains 1 (Kank1), mRNA [NM_001037197]	**0,694**
Slc29a4	Rattus norvegicus solute carrier family 29 (nucleoside transporters), member 4 (Slc29a4), mRNA [NM_001105911]	**0,694**
0	Unknown	**0,694**
Cand2	Rattus norvegicus cullin-associated and neddylation-dissociated 2 (putative) (Cand2), mRNA [NM_181362]	**0,694**
Zinki	Rattus norvegicus Arg3.1/Arc mRNA-binding zinc finger protein (Zinki), mRNA [NM_001142758]	**0,694**
Pir	Rattus norvegicus pirin (iron-binding nuclear protein) (Pir), mRNA [NM_001009474]	**0,695**
Thpo	Rattus norvegicus thrombopoietin (Thpo), mRNA [NM_031133]	**0,695**

Cyb5r1	Rattus norvegicus cytochrome b5 reductase 1 (Cyb5r1), mRNA [NM_001013126]	**0,695**
Hdac8	Rattus norvegicus histone deacetylase 8 (Hdac8), mRNA [NM_001126373]	**0,695**
Atp6v0a2	Rattus norvegicus ATPase, H+ transporting, lysosomal V0 subunit A2 (Atp6v0a2), mRNA [NM_053775]	**0,695**
Akt1s1	Rattus norvegicus AKT1 substrate 1 (proline-rich) (Akt1s1), mRNA [NM_001106259]	**0,695**
Wipf3	Rattus norvegicus WAS/WASL interacting protein family, member 3 (Wipf3), mRNA [NM_147211]	**0,695**
Solh	Rattus norvegicus small optic lobes homolog (Drosophila) (Solh), mRNA [NM_001106990]	**0,695**
Pafah1b3	Rattus norvegicus platelet-activating factor acetylhydrolase, isoform 1b, subunit 3 (Pafah1b3), mRNA [NM_053654]	**0,695**
0	Unknown	**0,695**
0	Unknown	**0,695**
St6gal1	Rattus norvegicus ST6 beta-galactosamide alpha-2,6-sialyltranferase 1 (St6gal1), transcript variant 2, mRNA [NM_147205]	**0,695**
Khnyn	PREDICTED: Rattus norvegicus similar to mKIAA0323 protein (RGD1565688), miscRNA [XR_086021]	**0,695**
0	Unknown	**0,695**
Tfdp2	Rattus norvegicus transcription factor Dp-2 (E2F dimerization partner 2) (Tfdp2), mRNA [NM_001106847]	**0,695**
0	Unknown	**0,695**
Baat	Rattus norvegicus bile acid Coenzyme A: amino acid N-acyltransferase (glycine N-choloyltransferase) (Baat), mRNA [NM_017300]	**0,695**
Rbm25	Rattus norvegicus RNA binding motif protein 25 (Rbm25), mRNA [NM_001108984]	**0,695**
Sirt6	Rattus norvegicus sirtuin (silent mating type information regulation 2 homolog) 6 (S. cerevisiae) (Sirt6), mRNA [NM_001031649]	**0,695**
Col20a1	Uncharacterized protein [Source:UniProtKB/TrEMBL;Acc:D3ZII5] [ENSRNOT00000040270]	**0,695**
Pcdhb18	PREDICTED: Rattus norvegicus protocadherin beta 18 (Pcdhb18), mRNA [XM_001055991]	**0,695**
0	Calcipressin-2 [Source:UniProtKB/Swiss-Prot;Acc:Q8CH27] [ENSRNOT00000029593]	**0,695**
Mtf1	Rattus norvegicus metal-regulatory transcription factor 1 (Mtf1), mRNA [NM_001108677]	**0,695**
Eri2	Rattus norvegicus exoribonuclease 2 (Eri2), mRNA [NM_001191114]	**0,696**
0	Rattus norvegicus similar to Pyruvate kinase, M2 isozyme (LOC290412), mRNA [XM_224416]	**0,696**
Myo5a	Rattus norvegicus myosin VA (Myo5a), mRNA [NM_022178]	**0,696**
Ogdh	Rattus norvegicus oxoglutarate (alpha-ketoglutarate) dehydrogenase (lipoamide) (Ogdh), nuclear gene encoding mitochondrial protein, mRNA [NM_001017461]	**0,696**
RGD1565705	PREDICTED: Rattus norvegicus similar to chr2 synaptotagmin (RGD1565705), mRNA [XM_002726849]	**0,696**
Selm	Rattus norvegicus selenoprotein M (Selm), mRNA [NM_001115013]	**0,696**
Fezf2	Rattus norvegicus Fez family zinc finger 2 (Fezf2), mRNA [NM_001107251]	**0,696**
Tmem25	Rattus norvegicus transmembrane protein 25 (Tmem25), mRNA [NM_001109528]	**0,696**
LOC679087	PREDICTED: Rattus norvegicus similar to swan (LOC679087), mRNA [XM_001054639]	**0,696**
Olr727	Rattus norvegicus olfactory receptor 727 (Olr727), mRNA [NM_001000619]	**0,696**
0	PREDICTED: Rattus norvegicus thymic stromal lymphopoietin (Tslp), mRNA [XM_001067649]	**0,696**
Arf2	Rattus norvegicus ADP-ribosylation factor 2 (Arf2), mRNA [NM_024150]	**0,696**

Ube2k	Rattus norvegicus ubiquitin-conjugating enzyme E2K (UBC1 homolog, yeast) (Ube2k), mRNA [NM_001106006]	**0,696**
Scarf2	Rattus norvegicus scavenger receptor class F, member 2 (Scarf2), mRNA [NM_001105864]	**0,696**
Trhde	Rattus norvegicus thyrotropin-releasing hormone degrading enzyme (Trhde), mRNA [NM_001108991]	**0,696**
Rasal1	Rattus norvegicus RAS protein activator like 1 (GAP1 like) (Rasal1), mRNA [NM_001108335]	**0,696**
Rfx2	Rattus norvegicus regulatory factor X, 2 (influences HLA class II expression) (Rfx2), mRNA [NM_001106877]	**0,696**
Shisa7	Rattus norvegicus shisa homolog 7 (Xenopus laevis) (Shisa7), mRNA [NM_001145175]	**0,696**
0	Unknown	**0,696**
0	Unknown	**0,696**
Pxn	Rattus norvegicus paxillin (Pxn), mRNA [NM_001012147]	**0,696**
RGD1309403	Rattus norvegicus similar to hypothetical protein FLJ12661 (RGD1309403), mRNA [NM_001107676]	**0,696**
Csde1	Rattus norvegicus cold shock domain containing E1, RNA binding (Csde1), mRNA [NM_054006]	**0,696**
Nkiras1	Rattus norvegicus NFKB inhibitor interacting Ras-like 1 (Nkiras1), mRNA [NM_001107252]	**0,696**
0	Unknown	**0,696**
Klhl21	Rattus norvegicus kelch-like 21 (Drosophila) (Klhl21), mRNA [NM_001107996]	**0,696**
Cdc7	Rattus norvegicus cell division cycle 7 homolog (S. cerevisiae) (Cdc7), mRNA [NM_001108352]	**0,696**
Kdm2a	Rattus norvegicus lysine (K)-specific demethylase 2A (Kdm2a), mRNA [NM_001108515]	**0,696**
Znf23	Rattus norvegicus zinc finger protein 23 (KOX 16) (Znf23), mRNA [NM_001107428]	**0,697**
Wnk1	Rattus norvegicus WNK lysine deficient protein kinase 1 (Wnk1), transcript variant 3, mRNA [NM_053794]	**0,697**
Cyp2c11	Rattus norvegicus cytochrome P450, subfamily 2, polypeptide 11 (Cyp2c11), mRNA [NM_019184]	**0,697**
Pfkfb2	Rattus norvegicus 6-phosphofructo-2-kinase/fructose-2,6-biphosphatase 2 (Pfkfb2), transcript variant 3, mRNA [NM_080477]	**0,697**
Kcnma1	Rattus norvegicus potassium large conductance calcium-activated channel, subfamily M, alpha member 1 (Kcnma1), mRNA [NM_031828]	**0,697**
Cox18	Rattus norvegicus COX18 cytochrome c oxidase assembly homolog (S. cerevisiae) (Cox18), nuclear gene encoding mitochondrial protein, mRNA [NM_001106000]	**0,697**
Ankrd12	Rattus norvegicus ankyrin repeat domain 12 (Ankrd12), mRNA [NM_001108238]	**0,697**
Mln	Rattus norvegicus motilin (Mln), mRNA [NM_001110056]	**0,697**
Pmm2	Rattus norvegicus phosphomannomutase 2 (Pmm2), mRNA [NM_001106973]	**0,697**
Plxnb2	Rattus norvegicus plexin B2 (Plxnb2), mRNA [NM_001108106]	**0,697**
Me3	Rattus norvegicus malic enzyme 3, NADP(+)-dependent, mitochondrial (Me3), nuclear gene encoding mitochondrial protein, mRNA [NM_001108491]	**0,697**
RGD1307722	Rattus norvegicus similar to hypothetical protein MGC20700 (RGD1307722), mRNA [NM_001108730]	**0,697**
Tsc2	Rattus norvegicus tuberous sclerosis 2 (Tsc2), mRNA [NM_012680]	**0,697**
Ttc4	Rattus norvegicus tetratricopeptide repeat domain 4 (Ttc4), mRNA [NM_001013214]	**0,697**
H2afy2	Rattus norvegicus H2A histone family, member Y2 (H2afy2), mRNA [NM_001135807]	**0,697**
Col4a1	Rattus norvegicus collagen, type IV, alpha 1 (Col4a1), mRNA [NM_001135009]	**0,697**

Htr5b	Rattus norvegicus 5-hydroxytryptamine (serotonin) receptor 5B (Htr5b), mRNA [NM_024395]	**0,697**
Trim16	Rattus norvegicus tripartite motif-containing 16 (Trim16), mRNA [NM_001135033]	**0,697**
RGD1560883	PREDICTED: Rattus norvegicus similar to KIAA0825 protein (RGD1560883), mRNA [XM_226616]	**0,698**
Polr3f	Rattus norvegicus polymerase (RNA) III (DNA directed) polypeptide F (Polr3f), mRNA [NM_001107784]	**0,698**
Adamts15	Rattus norvegicus ADAM metallopeptidase with thrombospondin type 1 motif, 15 (Adamts15), mRNA [NM_001106810]	**0,698**
Ppp1r9b	Rattus norvegicus protein phosphatase 1, regulatory subunit 9B (Ppp1r9b), mRNA [NM_053474]	**0,698**
Mobkl1a	Rattus norvegicus MOB1, Mps One Binder kinase activator-like 1A (yeast) (Mobkl1a), mRNA [NM_001108357]	**0,698**
Oprm1	Rattus norvegicus opioid receptor, mu 1 (Oprm1), transcript variant 1, mRNA [NM_013071]	**0,698**
Zfp192	Rattus norvegicus zinc finger protein 192 (Zfp192), mRNA [NM_001100574]	**0,698**
Gapdh-ps1	Rattus norvegicus glyceraldehyde-3-phosphate dehydrogenase, pseudogene 1 (Gapdh-ps1), non-coding RNA [NR_003722]	**0,698**
C1ql2	Rattus norvegicus complement component 1, q subcomponent-like 2 (C1ql2), mRNA [NM_001105949]	**0,698**
Tcerg1l	Rattus norvegicus transcription elongation regulator 1-like (Tcerg1l), mRNA [NM_001130077]	**0,698**
Stxbp4	Rattus norvegicus syntaxin binding protein 4 (Stxbp4), mRNA [NM_001107038]	**0,698**
0	Unknown	**0,698**
Kcnk3	Rattus norvegicus potassium channel, subfamily K, member 3 (Kcnk3), mRNA [NM_033376]	**0,698**
Glce	RCG57892Uncharacterized protein [Source:UniProtKB/TrEMBL;Acc:D3ZIK0] [ENSRNOT00000038641]	**0,698**
Cul3	Rattus norvegicus cullin 3 (Cul3), mRNA [NM_001106923]	**0,698**
Lcp1	Rattus norvegicus lymphocyte cytosolic protein 1 (Lcp1), mRNA [NM_001012044]	**0,698**
Ctdsp1	Rattus norvegicus CTD (carboxy-terminal domain, RNA polymerase II, polypeptide A) small phosphatase 1 (Ctdsp1), mRNA [NM_001128079]	**0,698**
0	Rattus norvegicus similar to KIAA1771 protein (LOC313388), mRNA [XM_242940]	**0,698**
0	Unknown	**0,698**
LOC313641	PREDICTED: Rattus norvegicus perlecan (LOC313641), miscRNA [XR_009630]	**0,698**
Ctdspl	Rattus norvegicus CTD (carboxy-terminal domain, RNA polymerase II, polypeptide A) small phosphatase-like (Ctdspl), mRNA [NM_001106865]	**0,698**
0	Unknown	**0,698**
Otud6b	Rattus norvegicus OTU domain containing 6B (Otud6b), mRNA [NM_001106639]	**0,698**
Snrpb	Rattus norvegicus small nuclear ribonucleoprotein polypeptides B and B1 (Snrpb), mRNA [NM_134358]	**0,698**
Ins1	Rattus norvegicus insulin 1 (Ins1), mRNA [NM_019129]	**0,698**
0	Rattus norvegicus similar to ubiquitin-conjugating enzyme E2N (homologous to yeast UBC13); bendless protein (LOC301282), mRNA [XM_236973]	**0,699**
0	Uncharacterized protein [Source:UniProtKB/TrEMBL;Acc:D3Z8T0] [ENSRNOT00000041537]	**0,699**
Epn1	Rattus norvegicus Epsin 1 (Epn1), mRNA [NM_057136]	**0,699**
Col12a1	Collagen alpha-1(XII) chain [Source:UniProtKB/Swiss-Prot;Acc:P70560] [ENSRNOT00000051159]	**0,699**
RGD1309823	PREDICTED: Rattus norvegicus similar to hypothetical protein FLJ21156 (RGD1309823), miscRNA [XR_085840]	**0,699**
0	Unknown	**0,699**

Paqr6	Rattus norvegicus progestin and adipoQ receptor family member VI (Paqr6), mRNA [NM_001191077]	**0,699**
Pcdhb20	Rattus norvegicus protocadherin beta 20 (Pcdhb20), mRNA [NM_001109395]	**0,699**
Copz1	Rattus norvegicus coatomer protein complex, subunit zeta 1 (Copz1), mRNA [NM_001108117]	**0,699**
Lpcat4	Rattus norvegicus lysophosphatidylcholine acyltransferase 4 (Lpcat4), mRNA [NM_001106494]	**0,699**
Magi2	Rattus norvegicus membrane associated guanylate kinase, WW and PDZ domain containing 2 (Magi2), mRNA [NM_053621]	**0,699**
Cd2ap	Rattus norvegicus CD2-associated protein (Cd2ap), mRNA [NM_181475]	**0,699**
Ddx58	Rattus norvegicus DEAD (Asp-Glu-Ala-Asp) box polypeptide 58 (Ddx58), mRNA [NM_001106645]	**0,699**
Dlgap2	Rattus norvegicus discs, large (Drosophila) homolog-associated protein 2 (Dlgap2), mRNA [NM_053901]	**0,699**
Chic2	Rattus norvegicus cysteine-rich hydrophobic domain 2 (Chic2), mRNA [NM_001105736]	**0,699**
Mfap4	Rattus norvegicus microfibrillar-associated protein 4 (Mfap4), mRNA [NM_001034124]	**0,699**
Creb3l1	Rattus norvegicus cAMP responsive element binding protein 3-like 1 (Creb3l1), mRNA [NM_001005562]	**0,699**
Aldh18a1	Rattus norvegicus aldehyde dehydrogenase 18 family, member A1 (Aldh18a1), nuclear gene encoding mitochondrial protein, mRNA [NM_001108524]	**0,700**
Znf295	Rattus norvegicus zinc finger protein 295 (Znf295), mRNA [NM_001107105]	**0,700**
Acbd3	Rattus norvegicus acyl-Coenzyme A binding domain containing 3 (Acbd3), mRNA [NM_182843]	**0,700**
Nek8	Rattus norvegicus NIMA (never in mitosis gene a)- related kinase 8 (Nek8), mRNA [NM_001105804]	**0,700**
Lnp	Rattus norvegicus limb and neural patterns (Lnp), mRNA [NM_001077429]	**0,700**
Gca	Rattus norvegicus grancalcin (Gca), mRNA [NM_001106483]	**0,700**
Usp46	Rattus norvegicus ubiquitin specific peptidase 46 (Usp46), mRNA [NM_001191596]	**0,700**
0	Unknown	**0,700**
0	Unknown	**0,700**
0	HSN2_RAT (Q6IFS7) Protein HSN2 precursor, complete [TC618038]	**0,700**
Nr2c1	Rattus norvegicus nuclear receptor subfamily 2, group C, member 1 (Nr2c1), mRNA [NM_145780]	**0,700**
C1ql3	Rattus norvegicus complement component 1, q subcomponent-like 3 (C1ql3), mRNA [NM_001109403]	**0,700**
Cyfip2	Rattus norvegicus cytoplasmic FMR1 interacting protein 2 (Cyfip2), mRNA [NM_001106996]	**0,700**
Rps6ka3	Rattus norvegicus ribosomal protein S6 kinase polypeptide 3 (Rps6ka3), mRNA [NM_001192004]	**0,700**
0	Uncharacterized protein [Source:UniProtKB/TrEMBL;Acc:D4A391] [ENSRNOT00000066991]	**0,700**
Plekhh1	Rattus norvegicus pleckstrin homology domain containing, family H (with MyTH4 domain) member 1 (Plekhh1), mRNA [NM_001108036]	**0,700**
Eif4e3	Rattus norvegicus eukaryotic translation initiation factor 4E family member 3 (Eif4e3), mRNA [NM_001106612]	**0,700**
Isl2	Rattus norvegicus ISL LIM homeobox 2 (Isl2), mRNA [NM_020471]	**0,701**
Cdx1	PREDICTED: Rattus norvegicus caudal type homeo box 1 (Cdx1), mRNA [XM_344691]	**0,701**
Prkcg	Rattus norvegicus protein kinase C, gamma (Prkcg), mRNA [NM_012628]	**0,701**
Ncam1	Rattus norvegicus neural cell adhesion molecule 1 (Ncam1), mRNA [NM_031521]	**0,701**
0	Unknown	**0,701**

Sec24a	Rattus norvegicus SEC24 family, member A (S. cerevisiae) (Sec24a), mRNA [NM_001105780]	**0,701**
Tbl1x	Rattus norvegicus transducin (beta)-like 1 X-linked (Tbl1x), mRNA [NM_001106964]	**0,701**
Golt1b	Rattus norvegicus golgi transport 1 homolog B (S. cerevisiae) (Golt1b), mRNA [NM_001113783]	**0,701**
Spag4l	Rattus norvegicus sperm associated antigen 4-like (Spag4l), mRNA [NM_001106530]	**0,701**
Meis1	Rattus norvegicus Meis homeobox 1 (Meis1), mRNA [NM_001134702]	**0,701**
Dzip1	Dzip1 protein [Source:UniProtKB/TrEMBL;Acc:Q5EB75] [ENSRNOT00000038596]	**0,701**
RGD1559513	PREDICTED: Rattus norvegicus similar to DEAD (Asp-Glu-Ala-Asp) box polypeptide 41 (RGD1559513), mRNA [XM_001059856]	**0,701**
LOC679580	PREDICTED: Rattus norvegicus similar to MIC2 like 1 (LOC679580), mRNA [XM_001053578]	**0,701**
0	Unknown	**0,701**
LOC690286	PREDICTED: Rattus norvegicus similar to hepatic leukemia factor (LOC690286), mRNA [XM_001073964]	**0,701**
0	Unknown	**0,701**
0	Unknown	**0,701**
0	Unknown	**0,701**
0	Unknown	**0,701**
Slc30a7	Rattus norvegicus solute carrier family 30 (zinc transporter), member 7 (Slc30a7), mRNA [NM_001191715]	**0,701**
0	Putative uncharacterized proteinUncharacterized protein [Source:UniProtKB/TrEMBL;Acc:D4A5B2] [ENSRNOT00000004034]	**0,701**
Crlf2	Rattus norvegicus cytokine receptor-like factor 2 (Crlf2), mRNA [NM_134465]	**0,701**
Zfp174	Uncharacterized protein [Source:UniProtKB/TrEMBL;Acc:D3ZNW1] [ENSRNOT00000010059]	**0,702**
0	Unknown	**0,702**
Cited2	Rattus norvegicus Cbp/p300-interacting transactivator, with Glu/Asp-rich carboxy-terminal domain, 2 (Cited2), mRNA [NM_053698]	**0,702**
Fam123a	PREDICTED: Rattus norvegicus family with sequence similarity 123A, transcript variant 2 (Fam123a), mRNA [XM_573799]	**0,702**
Lppr5	Rattus norvegicus lipid phosphate phosphatase-related protein type 5 (Lppr5), mRNA [NM_001107720]	**0,702**
0	Unknown	**0,702**
Rab6b	Rattus norvegicus RAB6B, member RAS oncogene family (Rab6b), mRNA [NM_001108775]	**0,702**
Arl13b	Rattus norvegicus ADP-ribosylation factor-like 13B (Arl13b), mRNA [NM_001107101]	**0,702**
Arhgef9	Rattus norvegicus Cdc42 guanine nucleotide exchange factor (GEF) 9 (Arhgef9), mRNA [NM_023957]	**0,702**
0	Q8R111_MOUSE (Q8R111) Plekha6 protein (Fragment), partial (70%) [TC597504]	**0,702**
0	LOC100125365 protein [Source:UniProtKB/TrEMBL;Acc:Q5U2N5] [ENSRNOT00000051777]	**0,702**
Sp2	Rattus norvegicus Sp2 transcription factor (Sp2), mRNA [NM_001107045]	**0,702**
0	Unknown	**0,702**
Drg2	Uncharacterized protein [Source:UniProtKB/TrEMBL;Acc:D3ZDC1] [ENSRNOT00000005101]	**0,702**
Aldh4a1	Rattus norvegicus aldehyde dehydrogenase 4 family, member A1 (Aldh4a1), nuclear gene encoding mitochondrial protein, mRNA [NM_001134698]	**0,702**
Rcor3	Rattus norvegicus REST corepressor 3 (Rcor3), mRNA [NM_001134985]	**0,702**
0	Uncharacterized protein [Source:UniProtKB/TrEMBL;Acc:D3ZEI4] [ENSRNOT00000012248]	**0,702**

Vezf1	PREDICTED: Rattus norvegicus vascular endothelial zinc finger 1 (Vezf1), partial mRNA [XM_001081191]	**0,702**
Gli2	Rattus norvegicus GLI family zinc finger 2 (Gli2), mRNA [NM_001107169]	**0,702**
Ganc	Rattus norvegicus glucosidase, alpha; neutral C (Ganc), mRNA [NM_001145840]	**0,702**
Glyatl3	Rattus norvegicus glycine-N-acyltransferase-like 3 (Glyatl3), mRNA [NM_001145062]	**0,702**
Mbtps2	Rattus norvegicus membrane-bound transcription factor peptidase, site 2 (Mbtps2), mRNA [NM_001035007]	**0,702**
Nptxr	Rattus norvegicus neuronal pentraxin receptor (Nptxr), mRNA [NM_030841]	**0,702**
Pde4d	Rattus norvegicus phosphodiesterase 4D, cAMP-specific (phosphodiesterase E3 dunce homolog, Drosophila) (Pde4d), transcript variant 1, mRNA [NM_001113328]	**0,702**
Tbc1d30	Uncharacterized protein [Source:UniProtKB/TrEMBL;Acc:D3ZG06] [ENSRNOT00000024679]	**0,702**
Eid2b	PREDICTED: Rattus norvegicus similar to hypothetical protein FLJ38944 (RGD1560818), mRNA [XM_002725600]	**0,702**
Snx9	Rattus norvegicus sorting nexin 9 (Snx9), mRNA [NM_001127637]	**0,702**
Susd4	Rattus norvegicus sushi domain containing 4 (Susd4), mRNA [NM_001105982]	**0,702**
Sike	Rattus norvegicus suppressor of IKK epsilon (Sike), mRNA [NM_001012182]	**0,703**
E2f1	Rattus norvegicus E2F transcription factor 1 (E2f1), mRNA [NM_001100778]	**0,703**
0	Unknown	**0,703**
Pcdhga7	Rattus norvegicus protocadherin gamma subfamily A, 7 (Pcdhga7), mRNA [NM_001014773]	**0,703**
0	PREDICTED: Rattus norvegicus similar to translocating chain-associating membrane protein 2, transcript variant 1 (LOC684534), mRNA [XM_001070862]	**0,703**
0	AGENCOURT_109870703 NIH_MGC_418 Rattus norvegicus cDNA clone IMAGE:9022754 5', mRNA sequence [EV768930]	**0,703**
RGD1563047	Rattus norvegicus similar to von Ebner minor salivary gland protein (RGD1563047), mRNA [NM_001077680]	**0,703**
0	Sirtuin 1 ((Silent mating type information regulation 2, homolog) 1 (S. cerevisiae) (Predicted), isoform CRA_a)Uncharacterized protein [Source:UniProtKB/TrEMBL;Acc:D4A0K3] [ENSRNOT00000067413]	**0,703**
Pcsk4	Rattus norvegicus proprotein convertase subtilisin/kexin type 4 (Pcsk4), mRNA [NM_133559]	**0,703**
Rorb	Nuclear receptor ROR-beta [Source:UniProtKB/Swiss-Prot;Acc:P45446] [ENSRNOT00000018137]	**0,703**
0	Unknown	**0,703**
Blzf1	Rattus norvegicus basic leucine zipper nuclear factor 1 (Blzf1), mRNA [NM_001017494]	**0,703**
0	Myristoylated alanine-rich C-kinase substrate [Source:UniProtKB/Swiss-Prot;Acc:P30009] [ENSRNOT00000000707]	**0,703**
Angel1	Rattus norvegicus angel homolog 1 (Drosophila) (Angel1), mRNA [NM_001108717]	**0,703**
Urb2	Rattus norvegicus URB2 ribosome biogenesis 2 homolog (S. cerevisiae) (Urb2), mRNA [NM_001135708]	**0,703**
0	Unknown	**0,703**
Ssbp3	Rattus norvegicus single stranded DNA binding protein 3 (Ssbp3), mRNA [NM_053358]	**0,703**
Kcnk5	Rattus norvegicus potassium channel, subfamily K, member 5 (Kcnk5), mRNA [NM_001039516]	**0,703**
0	Unknown	**0,703**
LOC683761	PREDICTED: Rattus norvegicus similar to RT1 class I, CE11 (LOC683761), partial mRNA [XM_001053482]	**0,704**

Zfp39	Rattus norvegicus zinc finger protein 39 (Zfp39), mRNA [NM_001107004]	**0,704**
Igsf11	Rattus norvegicus immunoglobulin superfamily, member 11 (Igsf11), mRNA [NM_001013120]	**0,704**
Sf3a2	Rattus norvegicus splicing factor 3a, subunit 2 (Sf3a2), mRNA [NM_001011986]	**0,704**
Rshl1	Rattus norvegicus radial spokehead-like 1 (Rshl1), mRNA [NM_001024748]	**0,704**
Sema6c	Rattus norvegicus sema domain, transmembrane domain (TM), and cytoplasmic domain, (semaphorin) 6C (Sema6c), mRNA [NM_017308]	**0,704**
0	Glutamate receptor subunit GluR1 [Source:UniProtKB/TrEMBL;Acc:Q924I5] [ENSRNOT00000003279]	**0,704**
LOC100188936	Rattus norvegicus hypothetical protein LOC100188936 (LOC100188936), mRNA [NM_001134704]	**0,704**
Ddx41	Rattus norvegicus DEAD (Asp-Glu-Ala-Asp) box polypeptide 41 (Ddx41), mRNA [NM_001108046]	**0,704**
0	Unknown	**0,704**
Sel1l3	PREDICTED: Rattus norvegicus similar to RIKEN cDNA 2310045A20 (RGD1562860), mRNA [XM_341223]	**0,704**
Cpne8	Rattus norvegicus copine VIII (Cpne8), mRNA [NM_001108750]	**0,704**
0	Unknown	**0,704**
0	Rattus norvegicus similar to endo B cytokeratin (LOC315493), mRNA [XM_235966]	**0,704**
Epn3	Rattus norvegicus epsin 3 (Epn3), mRNA [NM_001024791]	**0,704**
0	Unknown	**0,704**
0	Unknown	**0,704**
Tmem184b	Rattus norvegicus transmembrane protein 184B (Tmem184b), mRNA [NM_001173370]	**0,704**
Prkca	Rattus norvegicus protein kinase C, alpha (Prkca), mRNA [NM_001105713]	**0,704**
0	Unknown	**0,705**
Coro2b	Coro2b protein [Source:UniProtKB/TrEMBL;Acc:Q5EB67] [ENSRNOT00000020951]	**0,705**
Adrm1	Rattus norvegicus adhesion regulating molecule 1 (Adrm1), mRNA [NM_031708]	**0,705**
0	Q8BUD8_MOUSE (Q8BUD8) 10 days lactation, adult female mammary gland cDNA, RIKEN full-length enriched library, clone:D730020H24 product:ZINC FINGER PROTEIN homolog (Muscleblind-like 2, isoform 1), partial (9%) [TC592330]	**0,705**
Rps6ka3	Rattus norvegicus ribosomal protein S6 kinase polypeptide 3 (Rps6ka3), mRNA [NM_001192004]	**0,705**
Gabrb3	Rattus norvegicus gamma-aminobutyric acid (GABA) A receptor, beta 3 (Gabrb3), mRNA [NM_017065]	**0,705**
0	Unknown	**0,705**
Gps2	Rattus norvegicus G protein pathway suppressor 2 (Gps2), mRNA [NM_001017477]	**0,705**
Rnf38	Rattus norvegicus ring finger protein 38 (Rnf38), mRNA [NM_134467]	**0,705**
0	Unknown	**0,705**
Cdr2l	Similar to paraneoplastic antigen (Predicted)Uncharacterized protein [Source:UniProtKB/TrEMBL;Acc:D4ABP3] [ENSRNOT00000035865]	**0,705**
Pank3	Rattus norvegicus pantothenate kinase 3 (Pank3), mRNA [NM_001108272]	**0,705**
Hecw2	Rattus norvegicus HECT, C2 and WW domain containing E3 ubiquitin protein ligase 2 (Hecw2), mRNA [NM_001108218]	**0,705**
Slc24a2	Rattus norvegicus solute carrier family 24 (sodium/potassium/calcium exchanger), member 2 (Slc24a2), mRNA [NM_031743]	**0,705**
Rab7a	Rattus norvegicus RAB7A, member RAS oncogene family (Rab7a), mRNA [NM_023950]	**0,705**

Aqp4	Rattus norvegicus aquaporin 4 (Aqp4), transcript variant 2, mRNA [NM_001142366]	**0,705**
0	Q6QAQ5_PIG (Q6QAQ5) 60S ribosomal protein P1 (Fragment), partial (22%) [TC645425]	**0,705**
RGD1311429	PREDICTED: Rattus norvegicus similar to KIAA1267 protein (RGD1311429), miscRNA [XR_006336]	**0,705**
0	Unknown	**0,705**
Lce1f	Rattus norvegicus late cornified envelope 1F (Lce1f), mRNA [NM_001109188]	**0,705**
Pak7	Rattus norvegicus p21 protein (Cdc42/Rac)-activated kinase 7 (Pak7), mRNA [NM_001107781]	**0,705**
0	Rattus norvegicus zinc finger protein 184, mRNA (cDNA clone MGC:156842 IMAGE:7381364), complete cds. [BC128784]	**0,705**
Mrfap1	MORF4 family-associated protein 1 [Source:UniProtKB/Swiss-Prot;Acc:Q5M820] [ENSRNOT00000043744]	**0,705**
RGD1565030	PREDICTED: Rattus norvegicus similar to open reading frame A (RGD1565030), mRNA [XM_574008]	**0,705**
Brpf3	Rattus norvegicus bromodomain and PHD finger containing, 3 (Brpf3), mRNA [NM_001107615]	**0,705**
0	Unknown	**0,706**
Hbegf	Rattus norvegicus heparin-binding EGF-like growth factor (Hbegf), mRNA [NM_012945]	**0,706**
0	Q3QX63_9RHOB (Q3QX63) Phage baseplate assembly protein V, partial (10%) [TC626448]	**0,706**
Slc7a5	Rattus norvegicus solute carrier family 7 (cationic amino acid transporter, y+ system), member 5 (Slc7a5), mRNA [NM_017353]	**0,706**
Kif3c	Rattus norvegicus kinesin family member 3C (Kif3c), mRNA [NM_053486]	**0,706**
0	Unknown	**0,706**
Ntsr1	Rattus norvegicus neurotensin receptor 1 (Ntsr1), mRNA [NM_001108967]	**0,706**
Ap1s1	Rattus norvegicus adaptor-related protein complex 1, sigma 1 subunit, mRNA (cDNA clone MGC:188494 IMAGE:5623505), complete cds. [BC168682]	**0,706**
Arpc5l	Rattus norvegicus actin related protein 2/3 complex, subunit 5-like, mRNA (cDNA clone IMAGE:7377370), complete cds. [BC098820]	**0,706**
Prkci	Rattus norvegicus protein kinase C, iota (Prkci), mRNA [NM_032059]	**0,706**
Cyfip1	Rattus norvegicus cytoplasmic FMR1 interacting protein 1 (Cyfip1), mRNA [NM_001107517]	**0,706**
0	Unknown	**0,706**
Hapln1	Rattus norvegicus hyaluronan and proteoglycan link protein 1 (Hapln1), mRNA [NM_019189]	**0,706**
Prickle2	Rattus norvegicus prickle homolog 2 (Drosophila) (Prickle2), mRNA [NM_001107876]	**0,706**
0	Unknown	**0,707**
Grm6	Rattus norvegicus glutamate receptor, metabotropic 6 (Grm6), mRNA [NM_022920]	**0,707**
Gjc2	Rattus norvegicus gap junction protein, gamma 2 (Gjc2), mRNA [NM_001100784]	**0,707**
LOC688390	Rattus norvegicus hypothetical protein LOC688390 (LOC688390), mRNA [NM_001109496]	**0,707**
0	Q2I375_HUMAN (Q2I375) QKI, partial (64%) [TC627221]	**0,707**
Wdr8	Rattus norvegicus WD repeat domain 8 (Wdr8), mRNA [NM_001014262]	**0,707**
LOC691684	PREDICTED: Rattus norvegicus similar to MIC2 like 1 (LOC691684), mRNA [XM_001079259]	**0,707**
Hmgb1	Rattus norvegicus high mobility group box 1 (Hmgb1), mRNA [NM_012963]	**0,707**
RGD1559613	Rattus norvegicus RGD1559613 (RGD1559613), mRNA [NM_001109138]	**0,707**

Snx12	Rattus norvegicus sorting nexin 12 (Snx12), mRNA [NM_001108817]	**0,707**
Pgrmc1	Rattus norvegicus progesterone receptor membrane component 1 (Pgrmc1), mRNA [NM_021766]	**0,707**
Satb2	Rattus norvegicus SATB homeobox 2 (Satb2), mRNA [NM_001109306]	**0,707**
Alkbh4	Rattus norvegicus alkB, alkylation repair homolog 4 (E. coli) (Alkbh4), mRNA [NM_001105920]	**0,707**
0	Rattus norvegicus TL0AEA67YN06 mRNA sequence. [FQ231880]	**0,707**
Tet2	Uncharacterized protein [Source:UniProtKB/TrEMBL;Acc:D4AC33] [ENSRNOT00000046775]	**0,707**
0	Suppressor of Ty 5 homolog (S. cerevisiae), isoform CRA_aUncharacterized protein [Source:UniProtKB/TrEMBL;Acc:D3ZSS2] [ENSRNOT00000068115]	**0,707**
Zbtb44	Rattus norvegicus zinc finger and BTB domain containing 44 (Zbtb44), mRNA [NM_001034942]	**0,707**
0	Unknown	**0,708**
LOC680207	PREDICTED: Rattus norvegicus hypothetical protein LOC680207 (LOC680207), mRNA [XM_001056119]	**0,708**
Adam19	Rattus norvegicus a disintegrin and metallopeptidase domain 19 (meltrin beta) (Adam19), mRNA [NM_001160228]	**0,708**
Cdk2	Rattus norvegicus cyclin dependent kinase 2 (Cdk2), mRNA [NM_199501]	**0,708**
Steap3	Rattus norvegicus STEAP family member 3 (Steap3), mRNA [NM_133314]	**0,708**
Hspb6	Rattus norvegicus heat shock protein, alpha-crystallin-related, B6 (Hspb6), mRNA [NM_138887]	**0,708**
Coch	Rattus norvegicus coagulation factor C homolog, cochlin (Limulus polyphemus) (Coch), mRNA [NM_001108710]	**0,708**
Cyb5b	Rattus norvegicus cytochrome b5 type B (outer mitochondrial membrane) (Cyb5b), nuclear gene encoding mitochondrial protein, mRNA [NM_030586]	**0,708**
Kcmf1	Rattus norvegicus potassium channel modulatory factor 1 (Kcmf1), mRNA [NM_001128192]	**0,708**
Hdac1	Rattus norvegicus histone deacetylase 1 (Hdac1), mRNA [NM_001025409]	**0,708**
Nr1d1	Rattus norvegicus nuclear receptor subfamily 1, group D, member 1 (Nr1d1), transcript variant 2, mRNA [NM_145775]	**0,708**
Fyco1	Rattus norvegicus FYVE and coiled-coil domain containing 1 (Fyco1), mRNA [NM_001106870]	**0,708**
Slc38a7	Rattus norvegicus solute carrier family 38, member 7 (Slc38a7), mRNA [NM_001003705]	**0,708**
Eif6	Rattus norvegicus eukaryotic translation initiation factor 6 (Eif6), mRNA [NM_001037352]	**0,708**
40057	Rattus norvegicus septin 9 (Sept9), transcript variant 2, mRNA [NM_176856]	**0,708**
Znf703	Rattus norvegicus zinc finger protein 703 (Znf703), mRNA [NM_001109425]	**0,708**
Aff3	Rattus norvegicus AF4/FMR2 family, member 3 (Aff3), mRNA [NM_001191887]	**0,708**
Zap70	Rattus norvegicus zeta-chain (TCR) associated protein kinase (Zap70), mRNA [NM_001012002]	**0,708**
Smtnl2	Rattus norvegicus smoothelin-like 2 (Smtnl2), mRNA [NM_001190998]	**0,708**
Olr386	Rattus norvegicus olfactory receptor 386 (Olr386), mRNA [NM_001000856]	**0,709**
Ttf1	Similar to RNA polymerase I transcription termination factor 1 (Predicted), isoform CRA_bUncharacterized protein [Source:UniProtKB/TrEMBL;Acc:D4A1Z7] [ENSRNOT00000061029]	**0,709**
0	RCG22822Uncharacterized protein [Source:UniProtKB/TrEMBL;Acc:D3ZYH3] [ENSRNOT00000048236]	**0,709**
Phlpp2	Rattus norvegicus PH domain and leucine rich repeat protein phosphatase 2 (Phlpp2), mRNA [NM_001109131]	**0,709**

Fmo1	Rattus norvegicus flavin containing monooxygenase 1 (Fmo1), mRNA [NM_012792]	**0,709**
0	Rattus norvegicus chromosome 20, major histocompatibility complex, assembled from 40 BACs, strain Brown Norway (BN/ssNHsd), RT1n haplotype; segment 5/11 [BX883046]	**0,709**
0	spectrin beta chain, brain 1 [Source:RefSeq peptide;Acc:NP_001013148] [ENSRNOT00000007441]	**0,709**
Dennd1a	Rattus norvegicus DENN/MADD domain containing 1A (Dennd1a), mRNA [NM_001191747]	**0,709**
LOC363060	Rattus norvegicus similar to RIKEN cDNA 1600029D21 (LOC363060), mRNA [NM_001014209]	**0,709**
LOC498122	Rattus norvegicus similar to CG15908-PA (LOC498122), mRNA [NM_001109058]	**0,709**
0	UI-R-DZ1-cne-j-11-0-UI.s1 UI-R-DZ1 Rattus norvegicus cDNA clone UI-R-DZ1-cne-j-11-0-UI 3', mRNA sequence [CN543219]	**0,709**
Faim2	Rattus norvegicus Fas apoptotic inhibitory molecule 2 (Faim2), mRNA [NM_144756]	**0,709**
Csmd1	Rattus norvegicus CUB and Sushi multiple domains 1 (Csmd1), mRNA [NM_001037327]	**0,709**
Olr1414	Rattus norvegicus olfactory receptor 1414 (Olr1414), mRNA [NM_001000782]	**0,709**
Garnl1	Rattus norvegicus GTPase activating Rap/RanGAP domain-like 1 (Garnl1), mRNA [NM_020083]	**0,709**
0	Unknown	**0,709**
Snx19	Rattus norvegicus sorting nexin 19 (Snx19), mRNA [NM_001108131]	**0,709**
0	Unknown	**0,709**
Nudt7	Rattus norvegicus nudix (nucleoside diphosphate linked moiety X)-type motif 7 (Nudt7), mRNA [NM_001108450]	**0,709**
0	Unknown	**0,709**
Ppp1cb	Rattus norvegicus protein phosphatase 1, catalytic subunit, beta isoform (Ppp1cb), mRNA [NM_013065]	**0,710**
0	PREDICTED: Rattus norvegicus rCG53928-like (LOC100364438), mRNA [XM_002725571]	**0,710**
Nr1i2	Rattus norvegicus nuclear receptor subfamily 1, group I, member 2 (Nr1i2), mRNA [NM_052980]	**0,710**
Exoc2	Rattus norvegicus exocyst complex component 2 (Exoc2), mRNA [NM_134414]	**0,710**
RGD1359158	Rattus norvegicus similar to RIKEN cDNA 1110059E24 (RGD1359158), mRNA [NM_001007737]	**0,710**
Dffb	Rattus norvegicus DNA fragmentation factor, beta polypeptide (caspase-activated DNase) (Dffb), mRNA [NM_053362]	**0,710**
Il2rb	Rattus norvegicus interleukin 2 receptor, beta (Il2rb), mRNA [NM_013195]	**0,710**
Plec	Rattus norvegicus plectin (Plec), transcript variant 6, mRNA [NM_022401]	**0,710**
RGD1305938	Rattus norvegicus TL0AAA77YA11 mRNA sequence. [FQ211901]	**0,710**
Tmem38b	Rattus norvegicus transmembrane protein 38B (Tmem38b), mRNA [NM_001014191]	**0,710**
0	Uncharacterized protein [Source:UniProtKB/TrEMBL;Acc:D4A7E5] [ENSRNOT00000023364]	**0,710**
Xiap	Rattus norvegicus X-linked inhibitor of apoptosis (Xiap), mRNA [NM_022231]	**0,710**
Pln	Rattus norvegicus phospholamban (Pln), mRNA [NM_022707]	**0,710**
Uhmk1	Rattus norvegicus U2AF homology motif (UHM) kinase 1 (Uhmk1), mRNA [NM_017293]	**0,710**
Smg7	Rattus norvegicus Smg-7 homolog, nonsense mediated mRNA decay factor (C. elegans) (Smg7), mRNA [NM_001191549]	**0,710**
Arsg	Rattus norvegicus arylsulfatase G (Arsg), mRNA [NM_001047877]	**0,710**
RGD1311406	PREDICTED: Rattus norvegicus similar to RIKEN cDNA B430306N03 gene (RGD1311406), mRNA [XM_001063911]	**0,711**

Taok2	Rattus norvegicus TAO kinase 2 (Taok2), mRNA [NM_022702]	**0,711**
Chfr	Rattus norvegicus checkpoint with forkhead and ring finger domains (Chfr), mRNA [NM_001009258]	**0,711**
Mapre2	Rattus norvegicus microtubule-associated protein, RP/EB family, member 2 (Mapre2), mRNA [NM_001101000]	**0,711**
Tet3	Uncharacterized protein [Source:UniProtKB/TrEMBL;Acc:D3ZES0] [ENSRNOT00000031312]	**0,711**
0	Unknown	**0,711**
Rnf144a	Rattus norvegicus ring finger protein 144A (Rnf144a), mRNA [NM_001082410]	**0,711**
0	Unknown	**0,711**
Asap1	Rattus norvegicus ArfGAP with SH3 domain, ankyrin repeat and PH domain 1 (Asap1), mRNA [NM_001044245]	**0,711**
Ppp2r4	Rattus norvegicus protein phosphatase 2A activator, regulatory subunit 4 (Ppp2r4), mRNA [NM_001108577]	**0,711**
Grlf1	Uncharacterized protein [Source:UniProtKB/TrEMBL;Acc:D4AD82] [ENSRNOT00000021223]	**0,711**
0	Q8QQ04_CAMPS (Q8QQ04) CMP190R, partial (13%) [TC605838]	**0,711**
Dmrtc1b	PREDICTED: Rattus norvegicus DMRT-like family C1b (Dmrtc1b), mRNA [XM_001055549]	**0,711**
Zswim2	Rattus norvegicus zinc finger, SWIM-type containing 2 (Zswim2), mRNA [NM_001011960]	**0,711**
0	Uncharacterized protein [Source:UniProtKB/TrEMBL;Acc:D4AAW3] [ENSRNOT00000055945]	**0,711**
Aqp1	Rattus norvegicus aquaporin 1 (Aqp1), mRNA [NM_012778]	**0,711**
Usp20	Rattus norvegicus ubiquitin specific peptidase 20 (Usp20), mRNA [NM_001107827]	**0,711**
Araf	Rattus norvegicus v-raf murine sarcoma 3611 viral oncogene homolog (Araf), transcript variant 1, mRNA [NM_022532]	**0,712**
Styxl1	Rattus norvegicus serine/threonine/tyrosine interacting-like 1 (Styxl1), mRNA [NM_001037788]	**0,712**
0	Unknown	**0,712**
RGD1560784	Uncharacterized protein [Source:UniProtKB/TrEMBL;Acc:D3ZL45] [ENSRNOT00000049864]	**0,712**
Pex19	Rattus norvegicus peroxisomal biogenesis factor 19 (Pex19), transcript variant 1, mRNA [NM_001107375]	**0,712**
Rasa4	PREDICTED: Rattus norvegicus similar to Rasa4 protein, transcript variant 1 (RGD1565457), mRNA [XM_002724808]	**0,712**
Foxa1	Rattus norvegicus forkhead box A1 (Foxa1), mRNA [NM_012742]	**0,712**
0	Q6MG19_RAT (Q6MG19) Discoidin domain receptor family, member 1, partial (18%) [TC622836]	**0,712**
0	Uncharacterized protein [Source:UniProtKB/TrEMBL;Acc:D3ZJB6] [ENSRNOT00000040584]	**0,712**
RGD1563349	Rattus norvegicus similar to RIKEN cDNA 9330182L06 (RGD1563349), mRNA [NM_001109345]	**0,712**
Cbll1	Rattus norvegicus Cas-Br-M (murine) ecotropic retroviral transforming sequence-like 1 (Cbll1), mRNA [NM_001108018]	**0,712**
Nhlh2	Rattus norvegicus nescient helix loop helix 2 (Nhlh2), mRNA [NM_001106457]	**0,712**
Prr16	Rattus norvegicus proline rich 16 (Prr16), mRNA [NM_001108432]	**0,712**
Arl5a	Rattus norvegicus ADP-ribosylation factor-like 5A (Arl5a), mRNA [NM_053979]	**0,712**
RGD1563866	PREDICTED: Rattus norvegicus RGD1563866, transcript variant 2 (RGD1563866), mRNA [XM_236231]	**0,712**
Fbxl17	Rattus norvegicus F-box and leucine-rich repeat protein 17 (Fbxl17), mRNA [NM_001108235]	**0,712**
Fam184b	Uncharacterized protein [Source:UniProtKB/TrEMBL;Acc:D3ZTZ0] [ENSRNOT00000004923]	**0,712**
0	Q2FXP0_STAAU (Q2FXP0) Formamidopyrimidine-DNA glycosylase , partial (6%) [TC596639]	**0,712**

LOC687121	PREDICTED: Rattus norvegicus similar to Shc SH2-domain binding protein 1 (LOC687121), mRNA [XM_001077162]	**0,712**
Tnfsf12	Rattus norvegicus tumor necrosis factor ligand superfamily member 12 (Tnfsf12), mRNA [NM_001001513]	**0,712**
LOC688297	PREDICTED: Rattus norvegicus similar to Retinal rod rhodopsin-sensitive cGMP 3,5-cyclic phosphodiesterase gamma-subunit (GMP-PDE gamma), transcript variant 1 (LOC688297), mRNA [XM_001081804]	**0,712**
Xpr1	Rattus norvegicus xenotropic and polytropic retrovirus receptor 1 (Xpr1), mRNA [NM_001105992]	**0,712**
Rfx7	Rattus norvegicus regulatory factor X, 7 (Rfx7), mRNA [NM_001127490]	**0,713**
Baz1b	Rattus norvegicus bromodomain adjacent to zinc finger domain, 1B (Baz1b), mRNA [NM_001191916]	**0,713**
RT1-CE2	Rattus norvegicus RT1 class I, locus CE2 (RT1-CE2), mRNA [NM_001008840]	**0,713**
Timp4	Rattus norvegicus tissue inhibitor of metalloproteinase 4 (Timp4), mRNA [NM_001109393]	**0,713**
Lrrc3b	PREDICTED: Rattus norvegicus leucine rich repeat containing 3B (Lrrc3b), mRNA [XM_002725036]	**0,713**
Gse1	PREDICTED: Rattus norvegicus genetic suppressor element 1 (Gse1), mRNA [XM_001078877]	**0,713**
Tmed7	Rattus norvegicus transmembrane emp24 protein transport domain containing 7 (Tmed7), mRNA [NM_001105758]	**0,713**
Col5a3	Rattus norvegicus collagen, type V, alpha 3 (Col5a3), mRNA [NM_021760]	**0,713**
Ankhd1	Rattus norvegicus ankyrin repeat and KH domain containing 1 (Ankhd1), mRNA [NM_001204053]	**0,713**
Brunol4	CUGBP Elav-like family member 4 [Source:RefSeq peptide;Acc:NP_001100870] [ENSRNOT00000061151]	**0,713**
Dpp8	Rattus norvegicus dipeptidylpeptidase 8 (Dpp8), mRNA [NM_001108159]	**0,713**
Pafah1b1	Rattus norvegicus platelet-activating factor acetylhydrolase, isoform 1b, subunit 1 (Pafah1b1), mRNA [NM_031763]	**0,713**
0	BQ195967 UI-R-CN1-cmn-g-04-0-UI.s1 UI-R-CN1 Rattus norvegicus cDNA clone UI-R-CN1-cmn-g-04-0-UI 3', mRNA sequence [BQ195967]	**0,713**
Lynx1	Rattus norvegicus Ly6/neurotoxin 1 (Lynx1), mRNA [NM_001130546]	**0,713**
0	Q5DBE9_SCHJA (Q5DBE9) SJCHGC01974 protein, partial (22%) [TC633657]	**0,713**
Ptprh	Rattus norvegicus protein tyrosine phosphatase, receptor type, H (Ptprh), mRNA [NM_001191945]	**0,713**
Isyna1	Rattus norvegicus inositol-3-phosphate synthase 1 (Isyna1), mRNA [NM_001013880]	**0,713**
0	Glycerol kinase [Source:UniProtKB/Swiss-Prot;Acc:Q63060] [ENSRNOT00000042729]	**0,713**
Stx2	Rattus norvegicus syntaxin 2 (Stx2), mRNA [NM_012748]	**0,713**
RGD1308782	Rattus norvegicus similar to Zinc finger protein OZF (POZF-1) (RGD1308782), mRNA [NM_001025677]	**0,714**
0	Rattus norvegicus Ac1-283 mRNA, complete cds. [AY325224]	**0,714**
Slc2a3	Rattus norvegicus solute carrier family 2 (facilitated glucose transporter), member 3 (Slc2a3), mRNA [NM_017102]	**0,714**
Gyltl1b	Rattus norvegicus glycosyltransferase-like 1B (Gyltl1b), mRNA [NM_199107]	**0,714**
Pcdh1	PREDICTED: Rattus norvegicus protocadherin 1 (Pcdh1), partial mRNA [XM_225997]	**0,714**
Defb20	Rattus norvegicus defensin beta 20 (Defb20), mRNA [NM_001037517]	**0,714**
0	Rattus norvegicus similar to Pyruvate kinase, M2 isozyme (LOC315231), mRNA [XM_235589]	**0,714**
RGD1559892	PREDICTED: Rattus norvegicus similar to 60S ribosomal protein L29 (P23) (RGD1559892), mRNA [XM_001071693]	**0,714**
0	Unknown	**0,714**
Tst	Rattus norvegicus thiosulfate sulfurtransferase (Tst), nuclear gene encoding mitochondrial protein, mRNA [NM_012808]	**0,714**

Olr1635	Rattus norvegicus olfactory receptor 1635 (Olr1635), mRNA [NM_001000970]	**0,714**
0	Unknown	**0,714**
Pcdhb11	PREDICTED: Rattus norvegicus protocadherin beta 11 (Pcdhb11), mRNA [XM_001055576]	**0,714**
Ppat	Rattus norvegicus phosphoribosyl pyrophosphate amidotransferase (Ppat), mRNA [NM_057198]	**0,714**
Cplx2	Rattus norvegicus complexin 2 (Cplx2), mRNA [NM_053878]	**0,714**
Acap3	Rattus norvegicus ArfGAP with coiled-coil, ankyrin repeat and PH domains 3 (Acap3), mRNA [NM_001107999]	**0,714**
Garnl1	Rattus norvegicus GTPase activating Rap/RanGAP domain-like 1 (Garnl1), mRNA [NM_020083]	**0,714**
Idh3a	Rattus norvegicus isocitrate dehydrogenase 3 (NAD+) alpha (Idh3a), nuclear gene encoding mitochondrial protein, mRNA [NM_053638]	**0,714**
Hist1h2bc	Rattus norvegicus histone cluster 1, H2bc (Hist1h2bc), mRNA [NM_001109400]	**0,714**
0	Unknown	**0,714**
Frem2	Uncharacterized protein [Source:UniProtKB/TrEMBL;Acc:D3ZG74] [ENSRNOT00000031487]	**0,714**
RGD1311249	Rattus norvegicus similar to RIKEN cDNA B230312A22 (RGD1311249), mRNA [NM_001013931]	**0,714**
Sptlc2	Rattus norvegicus serine palmitoyltransferase, long chain base subunit 2 (Sptlc2), mRNA [NM_001037097]	**0,714**
Ddx3x	Rattus norvegicus DEAD (Asp-Glu-Ala-Asp) box polypeptide 3, X-linked (Ddx3x), mRNA [NM_001108246]	**0,714**
Spata6	Rattus norvegicus spermatogenesis associated 6 (Spata6), mRNA [NM_134392]	**0,714**
Mmaa	Rattus norvegicus methylmalonic aciduria (cobalamin deficiency) cblA type (Mmaa), nuclear gene encoding mitochondrial protein, mRNA [NM_001106174]	**0,714**
Prtg	Rattus norvegicus protogenin homolog (Gallus gallus) (Prtg), mRNA [NM_001037651]	**0,714**
Schip1	Rattus norvegicus schwannomin interacting protein 1 (Schip1), mRNA [NM_001100666]	**0,714**
0	PREDICTED: Rattus norvegicus similar to high mobility group protein 17 (LOC688424), mRNA [XM_001066874]	**0,715**
Sncb	Rattus norvegicus synuclein, beta (Sncb), mRNA [NM_080777]	**0,715**
Wipf2	Rattus norvegicus WAS/WASL interacting protein family, member 2 (Wipf2), mRNA [NM_001191825]	**0,715**
Gad2	Rattus norvegicus glutamate decarboxylase 2 (Gad2), mRNA [NM_012563]	**0,715**
Syt2	Rattus norvegicus synaptotagmin II (Syt2), mRNA [NM_012665]	**0,715**
Cblb	Rattus norvegicus Cas-Br-M (murine) ecotropic retroviral transforming sequence b (Cblb), mRNA [NM_133601]	**0,715**
Bhlhe23	Rattus norvegicus basic helix-loop-helix family, member e23 (Bhlhe23), mRNA [NM_001109211]	**0,715**
0	PREDICTED: Rattus norvegicus similar to Tubby-related protein 3 (Tubby-like protein 3) (LOC688749), mRNA [XM_001071995]	**0,715**
Kcnc4	Rattus norvegicus potassium voltage gated channel, Shaw-related subfamily, member 4 (Kcnc4), mRNA [NM_001122776]	**0,715**
0	Unknown	**0,715**
Ppp2r5c	Rattus norvegicus protein phosphatase 2, regulatory subunit B', gamma isoform (Ppp2r5c), mRNA [NM_001191112]	**0,715**
Crip	Rattus norvegicus cysteine-rich intestinal protein (Crip), mRNA [NM_001134933]	**0,715**
0	Unknown	**0,715**
Gpr176	Probable G-protein coupled receptor 176 [Source:UniProtKB/Swiss-Prot;Acc:Q64017] [ENSRNOT00000007882]	**0,715**
Tmem18	Rattus norvegicus transmembrane protein 18 (Tmem18), mRNA [NM_001007748]	**0,715**

Arhgap17	Rattus norvegicus Rho GTPase activating protein 17 (Arhgap17), mRNA [NM_022244]	**0,716**
Trak1	Rattus norvegicus trafficking protein, kinesin binding 1 (Trak1), mRNA [NM_001134565]	**0,716**
Nes	Rattus norvegicus nestin (Nes), mRNA [NM_012987]	**0,716**
Med14	Rattus norvegicus mediator complex subunit 14 (Med14), mRNA [NM_001191727]	**0,716**
Wdr45	Rattus norvegicus WD repeat domain 45 (Wdr45), mRNA [NM_001013958]	**0,716**
Ephb6	Rattus norvegicus Eph receptor B6 (Ephb6), mRNA [NM_001107857]	**0,716**
Zpbp2	Rattus norvegicus zona pellucida binding protein 2 (Zpbp2), mRNA [NM_001007011]	**0,716**
Pglyrp1	Rattus norvegicus peptidoglycan recognition protein 1 (Pglyrp1), mRNA [NM_053373]	**0,716**
Mcl1	Rattus norvegicus myeloid cell leukemia sequence 1 (Mcl1), nuclear gene encoding mitochondrial protein, mRNA [NM_021846]	**0,716**
Egf	Rattus norvegicus epidermal growth factor (Egf), mRNA [NM_012842]	**0,716**
Ccnc	Rattus norvegicus cyclin C (Ccnc), mRNA [NM_001100472]	**0,716**
0	Q3UXV4_MOUSE (Q3UXV4) 12 days embryo male wolffian duct includes surrounding region cDNA, RIKEN full-length enriched library, clone:6720421K10 product:protein phosphatase 3, catalytic subunit, beta isoform, full insert sequence, partial (47%) [TC597142]	**0,716**
Ttll12	Rattus norvegicus tubulin tyrosine ligase-like family, member 12 (Ttll12), mRNA [NM_001135922]	**0,716**
Sssca1	Rattus norvegicus Sjogren's syndrome/scleroderma autoantigen 1 homolog (human) (Sssca1), mRNA [NM_001109537]	**0,716**
Fhl1	Rattus norvegicus four and a half LIM domains 1 (Fhl1), transcript variant 2, mRNA [NM_145669]	**0,716**
Ube2d1	Rattus norvegicus ubiquitin-conjugating enzyme E2D 1, UBC4/5 homolog (yeast) (Ube2d1), mRNA [NM_001108530]	**0,717**
Pde4a	Rattus norvegicus phosphodiesterase 4A, cAMP-specific (phosphodiesterase E2 dunce homolog, Drosophila) (Pde4a), mRNA [NM_013101]	**0,717**
Prrt4	Rattus norvegicus proline-rich transmembrane protein 4 (Prrt4), mRNA [NM_001109226]	**0,717**
Ccnc	Rattus norvegicus cyclin C (Ccnc), mRNA [NM_001100472]	**0,717**
0	Unknown	**0,717**
Olr1366	Rattus norvegicus olfactory receptor 1366 (Olr1366), mRNA [NM_001000980]	**0,717**
0	Q3TS78_MOUSE (Q3TS78) In vitro fertilized eggs cDNA, RIKEN full-length enriched library, clone:7420459N23 product:transportin 1, full insert sequence. (Fragment), partial (11%) [TC640129]	**0,717**
Kif1b	Rattus norvegicus kinesin family member 1B (Kif1b), mRNA [NM_057200]	**0,717**
Olfm1	Rattus norvegicus olfactomedin 1 (Olfm1), mRNA [NM_053573]	**0,717**
Tanc2	Rattus norvegicus tetratricopeptide repeat, ankyrin repeat and coiled-coil containing 2 (Tanc2), mRNA [NM_001191653]	**0,717**
Fam168a	Rattus norvegicus family with sequence similarity 168, member A (Fam168a), mRNA [NM_001108494]	**0,717**
0	Unknown	**0,717**
0	Unknown	**0,717**
Ears2	Rattus norvegicus glutamyl-tRNA synthetase 2 mitochondrial (putative) (Ears2), nuclear gene encoding mitochondrial protein, mRNA [NM_001159493]	**0,717**
Cc2d1a	Rattus norvegicus coiled-coil and C2 domain containing 1A (Cc2d1a), mRNA [NM_001013869]	**0,717**

0	Rattus norvegicus similar to glyceraldehyde-3-phosphate dehydrogenase (phosphorylating) (EC 1.2.1.12) - mouse (LOC302722), mRNA [XM_228999]	**0,717**
H1f0	Rattus norvegicus H1 histone family, member 0 (H1f0), mRNA [NM_012578]	**0,717**
Cox16	Rattus norvegicus COX16 cytochrome c oxidase assembly homolog (Cox16), nuclear gene encoding mitochondrial protein, mRNA [NM_001163153]	**0,718**
0	Q4IH13_GIBZE (Q4IH13) Predicted protein, partial (6%) [TC640504]	**0,718**
RGD1561672	PREDICTED: Rattus norvegicus similar to novel protein (RGD1561672), mRNA [XM_001065540]	**0,718**
Pcbp2	Rattus norvegicus poly(rC) binding protein 2 (Pcbp2), mRNA [NM_001013223]	**0,718**
Fam168b	RCG22336, isoform CRA_bUncharacterized protein [Source:UniProtKB/TrEMBL;Acc:D4AEP3] [ENSRNOT00000039480]	**0,718**
RGD1560175	Putative uncharacterized protein RGD1560175_predictedUncharacterized protein [Source:UniProtKB/TrEMBL;Acc:D4ABR5] [ENSRNOT00000046208]	**0,718**
Plagl1	Rattus norvegicus pleiomorphic adenoma gene-like 1 (Plagl1), mRNA [NM_012760]	**0,718**
Emp3	Rattus norvegicus epithelial membrane protein 3 (Emp3), mRNA [NM_030847]	**0,718**
0	Unknown	**0,718**
Jph4	Rattus norvegicus junctophilin 4 (Jph4), mRNA [NM_001003711]	**0,718**
Znf580	PREDICTED: Rattus norvegicus zinc finger protein 580 (Znf580), mRNA [XM_001072400]	**0,718**
Ubqln2	Rattus norvegicus ubiquilin 2 (Ubqln2), mRNA [NM_001108251]	**0,718**
Angptl6	Rattus norvegicus angiopoietin-like 6 (Angptl6), mRNA [NM_001106702]	**0,718**
0	Unknown	**0,718**
0	Unknown	**0,718**
Fbxo44	Rattus norvegicus TL0AAA48YG12 mRNA sequence. [FQ213337]	**0,718**
Tcea2	Rattus norvegicus transcription elongation factor A (SII), 2 (Tcea2), mRNA [NM_057098]	**0,718**
0	Rattus norvegicus similar to Glyceraldehyde 3-phosphate dehydrogenase (GAPDH) (38 kDa BFA-dependent ADP-ribosylation substrate) (BARS-38) (LOC364400), mRNA [XM_344418]	**0,718**
0	Unknown	**0,718**
RGD1310423	Rattus norvegicus similar to hypothetical protein FLJ31737 (RGD1310423), mRNA [NM_001029920]	**0,718**
Ireb2	Rattus norvegicus iron responsive element binding protein 2 (Ireb2), mRNA [NM_022863]	**0,718**
0	Unknown	**0,718**
Sms	Rattus norvegicus spermine synthase (Sms), mRNA [NM_001033899]	**0,718**
Stox2	Rattus norvegicus storkhead box 2 (Stox2), transcript variant 1, mRNA [NM_001134863]	**0,718**
LOC501618	Rattus norvegicus LRRGT00179 (LOC501618), mRNA [NM_001047970]	**0,718**
Grip1	Rattus norvegicus glutamate receptor interacting protein 1 (Grip1), mRNA [NM_032069]	**0,718**
Rhog	Rattus norvegicus ras homolog gene family, member G (rho G) (Rhog), mRNA [NM_001037195]	**0,718**
Ang	Rattus norvegicus angiogenin, ribonuclease, RNase A family, 5 (Ang), mRNA [NM_001012359]	**0,719**

Vash1	Uncharacterized protein [Source:UniProtKB/TrEMBL;Acc:D4AE85] [ENSRNOT00000014017]	**0,719**
Kab	Uncharacterized protein [Source:UniProtKB/TrEMBL;Acc:D3ZET9] [ENSRNOT00000005532]	**0,719**
Tspan18	Rattus norvegicus tetraspanin 18 (Tspan18), mRNA [NM_001107750]	**0,719**
Col3a1	Rattus norvegicus collagen, type III, alpha 1 (Col3a1), mRNA [NM_032085]	**0,719**
Cryz	Rattus norvegicus crystallin, zeta (Cryz), mRNA [NM_001012183]	**0,719**
RGD1312026	Rattus norvegicus similar to RIKEN cDNA C230081A13 (RGD1312026), mRNA [NM_001108149]	**0,719**
Ywhae	Rattus norvegicus tyrosine 3-monooxygenase/tryptophan 5-monooxygenase activation protein, epsilon polypeptide (Ywhae), mRNA [NM_031603]	**0,719**
0	HUMGLYSYN glycogen synthase kinase 3 {Homo sapiens} (exp=-1; wgp=0; cg=0), partial (4%) [TC645757]	**0,719**
0	Uncharacterized protein [Source:UniProtKB/TrEMBL;Acc:D4A9C5] [ENSRNOT00000011365]	**0,719**
0	Unknown	**0,719**
Wasl	Rattus norvegicus Wiskott-Aldrich syndrome-like (Wasl), mRNA [NM_001110365]	**0,719**
Map3k9	PREDICTED: Rattus norvegicus mitogen-activated protein kinase kinase kinase 9 (Map3k9), mRNA [XM_002726763]	**0,719**
Anapc11	Rattus norvegicus anaphase promoting complex subunit 11 (Anapc11), mRNA [NM_001126082]	**0,719**
Hm13	Rattus norvegicus histocompatibility 13 (Hm13), mRNA [NM_001107789]	**0,720**
LOC100364190	Similar to contactin associated protein-like 2 isoform a, isoform CRA_bUncharacterized protein [Source:UniProtKB/TrEMBL;Acc:D3ZA73] [ENSRNOT00000008688]	**0,720**
LOC688613	Rattus norvegicus hypothetical protein LOC688613 (LOC688613), mRNA [NM_001134845]	**0,720**
LOC363326	Rattus norvegicus hypothetical LOC363326 (LOC363326), mRNA [NM_001126289]	**0,720**
Prr22	Rattus norvegicus proline rich 22 (Prr22), mRNA [NM_001126375]	**0,720**
0	Unknown	**0,720**
Kcnj3	G protein-activated inward rectifier potassium channel 1 [Source:UniProtKB/Swiss-Prot;Acc:P63251] [ENSRNOT00000007335]	**0,720**
Atp6v1g2	Rattus norvegicus ATPase, H+ transporting, lysosomal V1 subunit G2 (Atp6v1g2), mRNA [NM_212490]	**0,720**
Plekhg1	Rattus norvegicus pleckstrin homology domain containing, family G (with RhoGef domain) member 1 (Plekhg1), mRNA [NM_001190999]	**0,720**
0	Unknown	**0,720**
Brd4	Rattus norvegicus bromodomain containing 4 (Brd4), mRNA [NM_001100903]	**0,720**
Pcdh7	Rattus norvegicus protocadherin 7 (Pcdh7), mRNA [NM_001004087]	**0,720**
Gsn	Rattus norvegicus gelsolin (Gsn), mRNA [NM_001004080]	**0,720**
Jazf1	Uncharacterized protein [Source:UniProtKB/TrEMBL;Acc:D3ZA80] [ENSRNOT00000039580]	**0,720**
Fxyd7	Rattus norvegicus FXYD domain-containing ion transport regulator 7 (Fxyd7), mRNA [NM_022008]	**0,720**
Zfp275	Rattus norvegicus zinc finger protein 275 (Zfp275), mRNA [NM_001106343]	**0,720**
Nrn1	Rattus norvegicus neuritin 1 (Nrn1), mRNA [NM_053346]	**0,720**
0	Unknown	**0,720**
Pcdhb13	Protocadherin-T4 [Source:UniProtKB/TrEMBL;Acc:Q9JIU1] [ENSRNOT00000027172]	**0,720**
Golph3l	Rattus norvegicus golgi phosphoprotein 3-like (Golph3l), mRNA [NM_001007698]	**0,720**

Smad5	Rattus norvegicus SMAD family member 5 (Smad5), mRNA [NM_021692]	**0,721**
LOC498972	PREDICTED: Rattus norvegicus similar to copine II (LOC498972), partial mRNA [XM_574259]	**0,721**
0	Unknown	**0,721**
LOC100360337	PREDICTED: Rattus norvegicus zinc finger protein 128 (LOC100360337), mRNA [XM_002725494]	**0,721**
Map3k13	Rattus norvegicus similar to mitogen-activated protein kinase kinase kinase 13; leucine zipper-bearing kinase, mRNA (cDNA clone MGC:94134 IMAGE:7127060), complete cds. [BC081976]	**0,721**
Rasgrf2	Rattus norvegicus RAS protein-specific guanine nucleotide-releasing factor 2 (Rasgrf2), mRNA [NM_053721]	**0,721**
RGD1563634	Rattus norvegicus similar to R31449_3 (RGD1563634), mRNA [NM_001081447]	**0,721**
Olr227	Rattus norvegicus olfactory receptor 227 (Olr227), mRNA [NM_001000203]	**0,721**
Pak3	Rattus norvegicus p21 protein (Cdc42/Rac)-activated kinase 3 (Pak3), mRNA [NM_019210]	**0,721**
G6pc3	Rattus norvegicus glucose 6 phosphatase, catalytic, 3 (G6pc3), mRNA [NM_176077]	**0,721**
Rhox2	Rattus norvegicus reproductive homeobox on X chromosome 2 (Rhox2), mRNA [NM_001025746]	**0,721**
Slc6a9	Rattus norvegicus solute carrier family 6 (neurotransmitter transporter, glycine), member 9 (Slc6a9), mRNA [NM_053818]	**0,721**
RGD1563263	Rattus norvegicus similar to RIKEN cDNA 1700029I15 (RGD1563263), mRNA [NM_001109201]	**0,721**
Cacng3	Rattus norvegicus calcium channel, voltage-dependent, gamma subunit 3 (Cacng3), mRNA [NM_080691]	**0,721**
Zfyve26	Rattus norvegicus zinc finger, FYVE domain containing 26 (Zfyve26), mRNA [NM_001108038]	**0,721**
Bcas3	Rattus norvegicus breast carcinoma amplified sequence 3 (Bcas3), mRNA [NM_001173430]	**0,721**
Ttc7b	Rattus norvegicus tetratricopeptide repeat domain 7B (Ttc7b), mRNA [NM_001108719]	**0,721**
Bspry	Rattus norvegicus B-box and SPRY domain containing (Bspry), mRNA [NM_022261]	**0,722**
RGD1302996	Rattus norvegicus hypothetical protein MGC:15854 (RGD1302996), mRNA [NM_213610]	**0,722**
LOC302495	Rattus norvegicus hypothetical LOC302495 (LOC302495), mRNA [NM_001106950]	**0,722**
Ipo11	PREDICTED: Rattus norvegicus importin 11 (Ipo11), mRNA [XM_002725887]	**0,722**
Slco4c1	Rattus norvegicus solute carrier organic anion transporter family, member 4C1 (Slco4c1), mRNA [NM_001002024]	**0,722**
RGD1308106	Rattus norvegicus LOC361719 (RGD1308106), mRNA [NM_001134575]	**0,722**
Enah	Rattus norvegicus enabled homolog (Drosophila) (Enah), mRNA [NM_001012150]	**0,722**
Calcoco1	Rattus norvegicus calcium binding and coiled coil domain 1 (Calcoco1), mRNA [NM_139190]	**0,722**
RGD1564387	PREDICTED: Rattus norvegicus similar to RIKEN cDNA C030014K22 gene (RGD1564387), mRNA [XM_002728030]	**0,722**
Dcc	Rattus norvegicus deleted in colorectal carcinoma (Dcc), mRNA [NM_012841]	**0,722**
Scnn1b	Rattus norvegicus sodium channel, nonvoltage-gated 1, beta (Scnn1b), mRNA [NM_012648]	**0,722**
Aqp9	Rattus norvegicus aquaporin 9 (Aqp9), mRNA [NM_022960]	**0,722**
Cpne5	Rattus norvegicus copine V (Cpne5), mRNA [NM_001107616]	**0,722**
Cyb5r3	Rattus norvegicus cytochrome b5 reductase 3 (Cyb5r3), mRNA [NM_138877]	**0,722**
Rpp14	Rattus norvegicus ribonuclease P 14 subunit (human) (Rpp14), transcript variant 1, mRNA [NM_001108372]	**0,722**
Spag9	Rattus norvegicus sperm associated antigen 9 (Spag9), mRNA [NM_001108290]	**0,722**

0	Unknown	**0,722**
RGD1307067	Rattus norvegicus LOC362840 (RGD1307067), mRNA [NM_001173974]	**0,723**
Crygf	Rattus norvegicus crystallin, gamma F (Crygf), mRNA [NM_001109557]	**0,723**
0	Unknown	**0,723**
0	Uncharacterized protein [Source:UniProtKB/TrEMBL;Acc:D4A1U8] [ENSRNOT00000007843]	**0,723**
Paip2b	Putative uncharacterized protein RGD1307930_predictedUncharacterized protein [Source:UniProtKB/TrEMBL;Acc:D4AAB9] [ENSRNOT00000019399]	**0,723**
0	C5AR_RABIT (Q9TUE1) C5a anaphylatoxin chemotactic receptor (C5a-R) (C5aR) (CD88 antigen) (Fragment), partial (7%) [TC623897]	**0,723**
0	Unknown	**0,723**
Ccnf	Rattus norvegicus cyclin F (Ccnf), mRNA [NM_001100474]	**0,723**
Myst3	Rattus norvegicus MYST histone acetyltransferase (monocytic leukemia) 3 (Myst3), mRNA [NM_001100570]	**0,723**
Whsc1	Rattus norvegicus Wolf-Hirschhorn syndrome candidate 1 (human) (Whsc1), mRNA [NM_001191552]	**0,723**
0	Unknown	**0,723**
0	Unknown	**0,723**
Tmcc1	Uncharacterized protein [Source:UniProtKB/TrEMBL;Acc:D3ZH14] [ENSRNOT00000042418]	**0,723**
RGD1311564	Rattus norvegicus LOC360590 (RGD1311564), mRNA [NM_001108286]	**0,723**
0	Unknown	**0,723**
LOC685636	PREDICTED: Rattus norvegicus hypothetical protein LOC685636 (LOC685636), mRNA [XM_001064613]	**0,723**
Hspa12a	Rattus norvegicus heat shock protein 12A (Hspa12a), mRNA [NM_001107445]	**0,723**
RT1-CE4	Rattus norvegicus RT1 class I, locus CE4 (RT1-CE4), mRNA [NM_001008842]	**0,724**
Rg9mtd1	Rattus norvegicus RNA (guanine-9-) methyltransferase domain containing 1 (Rg9mtd1), nuclear gene encoding mitochondrial protein, mRNA [NM_001008337]	**0,724**
Dazap2	Rattus norvegicus DAZ associated protein 2 (Dazap2), mRNA [NM_001013107]	**0,724**
Mtmr1	Rattus norvegicus myotubularin related protein 1 (Mtmr1), mRNA [NM_001191725]	**0,724**
Ccrk	Rattus norvegicus cell cycle related kinase (Ccrk), mRNA [NM_001025752]	**0,724**
0	LRRG00114LRRGT00001 [Source:UniProtKB/TrEMBL;Acc:Q6QI94] [ENSRNOT00000046112]	**0,724**
Epb4.1l2	PREDICTED: Rattus norvegicus erythrocyte membrane protein band 4.1-like 2 (Epb4.1l2), mRNA [XM_001053351]	**0,724**
Lsm6	Rattus norvegicus LSM6 homolog, U6 small nuclear RNA associated (S. cerevisiae) (Lsm6), mRNA [NM_001126085]	**0,724**
0	Histone deacetylase 7 [Source:UniProtKB/Swiss-Prot;Acc:Q99P96] [ENSRNOT00000011159]	**0,724**
0	Protein tyrosine phosphatase, receptor type, B (Predicted), isoform CRA_bUncharacterized protein [Source:UniProtKB/TrEMBL;Acc:D3ZE19] [ENSRNOT00000058384]	**0,724**
Sgk1	Rattus norvegicus serum/glucocorticoid regulated kinase 1 (Sgk1), transcript variant 3, mRNA [NM_019232]	**0,724**
Rnf152	Rattus norvegicus ring finger protein 152 (Rnf152), mRNA [NM_001106305]	**0,725**
Zfp26	Rattus norvegicus zinc finger protein 26 (Zfp26), mRNA [NM_001108995]	**0,725**

Kdm2b	Rattus norvegicus lysine (K)-specific demethylase 2B (Kdm2b), mRNA [NM_001100679]	**0,725**
Fgf13	Rattus norvegicus fibroblast growth factor 13 (Fgf13), mRNA [NM_053428]	**0,725**
Hpcal1	Rattus norvegicus hippocalcin-like 1 (Hpcal1), mRNA [NM_017356]	**0,725**
RGD1309492	Rattus norvegicus similar to mKIAA1737 protein (RGD1309492), mRNA [NM_001108044]	**0,725**
0	TOP1_RAT (Q9WUL0) DNA topoisomerase 1 (DNA topoisomerase I) , complete [TC575805]	**0,725**
0	Uncharacterized protein [Source:UniProtKB/TrEMBL;Acc:D3ZQF0] [ENSRNOT00000047238]	**0,725**
Apob48r	Rattus norvegicus apolipoprotein B48 receptor (Apob48r), mRNA [NM_001109154]	**0,725**
Eral1	Rattus norvegicus Era (G-protein)-like 1 (E. coli) (Eral1), mRNA [NM_001013229]	**0,725**
0	Unknown	**0,725**
Pdzd2	Rattus norvegicus PDZ domain containing 2 (Pdzd2), mRNA [NM_022940]	**0,725**
Spry2	Rattus norvegicus sprouty homolog 2 (Drosophila) (Spry2), mRNA [NM_001012046]	**0,725**
0	Uncharacterized protein [Source:UniProtKB/TrEMBL;Acc:D3ZCX9] [ENSRNOT00000041504]	**0,725**
Bcorl1	Rattus norvegicus BCL6 co-repressor-like 1 (Bcorl1), mRNA [NM_001191587]	**0,726**
Mxd1	Rattus norvegicus max dimerization protein 1 (Mxd1), mRNA [NM_001100749]	**0,726**
0	Unknown	**0,726**
Antxr1	Rattus norvegicus anthrax toxin receptor 1 (Antxr1), mRNA [NM_001044249]	**0,726**
Bcl2l12	Rattus norvegicus BCL2-like 12 (proline rich) (Bcl2l12), mRNA [NM_001108480]	**0,726**
Atp8b2	Rattus norvegicus Atpase, class I, type 8B, member 2 (Atp8b2), mRNA [NM_001024798]	**0,726**
Osmr	Rattus norvegicus oncostatin M receptor (Osmr), mRNA [NM_001005384]	**0,726**
0	Unknown	**0,726**
Tmcc2	Similar to RIKEN cDNA 1110063G11 (Predicted)Uncharacterized protein [Source:UniProtKB/TrEMBL;Acc:D3ZE26] [ENSRNOT00000000036]	**0,726**
Slc2a6	Rattus norvegicus solute carrier family 2 (facilitated glucose transporter), member 6 (Slc2a6), mRNA [NM_001106562]	**0,726**
Itga4	Rattus norvegicus integrin, alpha 4 (Itga4), mRNA [NM_001107737]	**0,726**
Sh3tc2	Sh3tc2 protein [Source:UniProtKB/TrEMBL;Acc:Q5EB84] [ENSRNOT00000026174]	**0,726**
Cpsf1	Rattus norvegicus cleavage and polyadenylation specific factor 1 (Cpsf1), mRNA [NM_001130571]	**0,726**
RGD1560859	PREDICTED: Rattus norvegicus similar to 2300003P22Rik protein (RGD1560859), miscRNA [XR_008965]	**0,726**
0	UI-R-FJ0-cpz-f-19-0-UI.r1 UI-R-FJ0 Rattus norvegicus cDNA clone UI-R-FJ0-cpz-f-19-0-UI 5', mRNA sequence [CA512374]	**0,726**
Serpina11	Rattus norvegicus serine (or cysteine) peptidase inhibitor, clade A (alpha-1 antiproteinase, antitrypsin), member 11 (Serpina11), transcript variant 2, mRNA [NM_001166352]	**0,727**
Hist1h2bc	Rattus norvegicus histone cluster 1, H2bc (Hist1h2bc), mRNA [NM_001109400]	**0,727**
Usp21	Rattus norvegicus ubiquitin specific peptidase 21 (Usp21), mRNA [NM_001127638]	**0,727**
Chst14	Rattus norvegicus carbohydrate (N-acetylgalactosamine 4-0) sulfotransferase 14 (Chst14), mRNA [NM_001109639]	**0,727**
Olr857	Rattus norvegicus olfactory receptor 857 (Olr857), mRNA [NM_001000583]	**0,727**
0	Unknown	**0,727**

Wnt5b	Rattus norvegicus wingless-type MMTV integration site family, member 5B (Wnt5b), mRNA [NM_001100489]	**0,727**
Fmnl1	Rattus norvegicus formin-like 1 (Fmnl1), mRNA [NM_001105846]	**0,727**
Myo9b	Rattus norvegicus myosin IXb (Myo9b), mRNA [NM_012984]	**0,727**
0	Rattus norvegicus cDNA clone IMAGE:7314743. [BC089965]	**0,727**
Ly6e	Rattus norvegicus lymphocyte antigen 6 complex, locus E (Ly6e), mRNA [NM_001017467]	**0,727**
Morc3	Rattus norvegicus MORC family CW-type zinc finger 3 (Morc3), mRNA [NM_001107109]	**0,727**
0	Rattus norvegicus clone UI-R-FJ0-cpy-l-05-0-UI unknown mRNA. [AY724520]	**0,727**
Fam172a	Rattus norvegicus family with sequence similarity 172, member A (Fam172a), mRNA [NM_001106401]	**0,727**
Cald1	Rattus norvegicus caldesmon 1 (Cald1), mRNA [NM_013146]	**0,728**
Pla2g2f	Rattus norvegicus phospholipase A2, group IIF (Pla2g2f), mRNA [NM_001109587]	**0,728**
Dctn4	Dynactin subunit 4 [Source:UniProtKB/Swiss-Prot;Acc:Q9QUR2] [ENSRNOT00000026394]	**0,728**
Cap1	Rattus norvegicus CAP, adenylate cyclase-associated protein 1 (yeast) (Cap1), mRNA [NM_022383]	**0,728**
Stk4	Rattus norvegicus serine/threonine kinase 4 (Stk4), mRNA [NM_001107800]	**0,728**
0	Histone H2B-3 [Source:UniProtKB/TrEMBL;Acc:Q9Z2Q9] [ENSRNOT00000042005]	**0,728**
0	Unknown	**0,728**
LOC685385	PREDICTED: Rattus norvegicus similar to S100 calcium binding protein A14 (LOC685385), mRNA [XM_002725995]	**0,728**
0	Unknown	**0,728**
0	Unknown	**0,728**
Cdc14b	Rattus norvegicus CDC14 cell division cycle 14 homolog B (S. cerevisiae) (Cdc14b), mRNA [NM_001108404]	**0,728**
LOC100364162	PREDICTED: Rattus norvegicus ring finger protein 11-like (LOC100364162), mRNA [XM_002726577]	**0,728**
0	Unknown	**0,728**
0	Unknown	**0,728**
Ptk2b	Rattus norvegicus PTK2B protein tyrosine kinase 2 beta (Ptk2b), mRNA [NM_017318]	**0,729**
Mink1	Uncharacterized protein [Source:UniProtKB/TrEMBL;Acc:D3ZMP1] [ENSRNOT00000043732]	**0,729**
Adam23	Rattus norvegicus ADAM metallopeptidase domain 23 (Adam23), mRNA [NM_001029899]	**0,729**
LOC691918	PREDICTED: Rattus norvegicus similar to Centrosomal protein of 27 kDa (Cep27 protein), transcript variant 2 (LOC691918), mRNA [XM_002726165]	**0,729**
Itch	Rattus norvegicus itchy E3 ubiquitin protein ligase homolog (mouse) (Itch), mRNA [NM_001005887]	**0,729**
0	Unknown	**0,729**
Slc39a9	Rattus norvegicus solute carrier family 39 (zinc transporter), member 9 (Slc39a9), mRNA [NM_001034929]	**0,729**
0	Uncharacterized protein [Source:UniProtKB/TrEMBL;Acc:D3ZHQ7] [ENSRNOT00000057958]	**0,729**
0	Unknown	**0,729**
0	Unknown	**0,729**
0	inverted formin, FH2 and WH2 domain containing Gene [Source:MGI Symbol;Acc:MGI:1917685] [ENSRNOT00000031962]	**0,729**

Pde7b	Rattus norvegicus phosphodiesterase 7B (Pde7b), mRNA [NM_080894]	**0,729**
0	Glyceraldehyde-3-phosphate dehydrogenase [Source:UniProtKB/TrEMBL;Acc:D3ZQ40] [ENSRNOT00000047010]	**0,729**
Vom2r16	Rattus norvegicus vomeronasal 2 receptor, 16 (Vom2r16), mRNA [NM_001099655]	**0,729**
Qpctl	Rattus norvegicus glutaminyl-peptide cyclotransferase-like (Qpctl), mRNA [NM_001106230]	**0,729**
Fnbp1	Rattus norvegicus formin binding protein 1 (Fnbp1), mRNA [NM_138914]	**0,730**
0	Unknown	**0,730**
0	Uncharacterized protein [Source:UniProtKB/TrEMBL;Acc:D3ZNE4] [ENSRNOT00000056975]	**0,730**
Kdelr2	Rattus norvegicus KDEL (Lys-Asp-Glu-Leu) endoplasmic reticulum protein retention receptor 2 (Kdelr2), mRNA [NM_001013122]	**0,730**
Nf1	Rattus norvegicus neurofibromin 1 (Nf1), mRNA [NM_012609]	**0,730**
Pdxdc1	Rattus norvegicus pyridoxal-dependent decarboxylase domain containing 1 (Pdxdc1), mRNA [NM_001134961]	**0,730**
Efna2	Rattus norvegicus ephrin A2 (Efna2), mRNA [NM_001168670]	**0,730**
Aldh5a1	Succinate-semialdehyde dehydrogenase, mitochondrial [Source:UniProtKB/Swiss-Prot;Acc:P51650] [ENSRNOT00000031384]	**0,730**
Irf5	Rattus norvegicus interferon regulatory factor 5 (Irf5), mRNA [NM_001106586]	**0,730**
Eps15	Rattus norvegicus epidermal growth factor receptor pathway substrate 15 (Eps15), mRNA [NM_001009424]	**0,730**
0	Unknown	**0,730**
Tpm1	Rattus norvegicus tropomyosin 1, alpha (Tpm1), transcript variant 1, mRNA [NM_001034068]	**0,731**
Sned1	Rattus norvegicus isolate No:6 insulin responsive sequence DNA binding protein-1 mRNA, partial cds. [AF439716]	**0,731**
0	Rattus norvegicus chromosome 10, 11 clones, strain BN/SsNHsdMCW RNOR03214576, whole genome shotgun sequence [AABR03073588]	**0,731**
RGD1565886	PREDICTED: Rattus norvegicus RGD1565886 (RGD1565886), mRNA [XM_237943]	**0,731**
Kdm6b	Rattus norvegicus lysine (K)-specific demethylase 6B (Kdm6b), mRNA [NM_001108829]	**0,731**
Pald	Rattus norvegicus paladin (Pald), mRNA [NM_001034128]	**0,731**
Vsx1	Rattus norvegicus visual system homeobox 1 (Vsx1), mRNA [NM_001109546]	**0,731**
Olr714	Rattus norvegicus olfactory receptor 714 (Olr714), mRNA [NM_001000923]	**0,731**
Gstcd	Rattus norvegicus glutathione S-transferase, C-terminal domain containing (Gstcd), mRNA [NM_001107725]	**0,731**
Cntfr	Rattus norvegicus ciliary neurotrophic factor receptor (Cntfr), mRNA [NM_001003929]	**0,731**
Pus7l	PREDICTED: Rattus norvegicus pseudouridylate synthase 7 homolog (S. cerevisiae)-like (Pus7l), mRNA [XM_001058268]	**0,731**
Casp8ap2	Rattus norvegicus caspase 8 associated protein 2 (Casp8ap2), mRNA [NM_001107921]	**0,731**
Optn	Rattus norvegicus optineurin (Optn), mRNA [NM_145081]	**0,731**
Supt7l	Rattus norvegicus suppressor of Ty 7 (S. cerevisiae)-like (Supt7l), mRNA [NM_001108010]	**0,732**
Steap2	Rattus norvegicus six transmembrane epithelial antigen of the prostate 2 (Steap2), mRNA [NM_001107846]	**0,732**
0	Unknown	**0,732**
0	Unknown	**0,732**
Pde1b	Rattus norvegicus phosphodiesterase 1B, calmodulin-dependent (Pde1b), mRNA [NM_022710]	**0,732**
Pcbp3	Rattus norvegicus poly(rC) binding protein 3 (Pcbp3), mRNA [NM_001011945]	**0,732**

Acvr1b	Rattus norvegicus activin A receptor, type IB (Acvr1b), mRNA [NM_199230]	**0,732**
LOC688741	PREDICTED: Rattus norvegicus hypothetical protein LOC688741 (LOC688741), mRNA [XM_002724948]	**0,732**
Timp3	Rattus norvegicus TIMP metallopeptidase inhibitor 3 (Timp3), mRNA [NM_012886]	**0,732**
Hist1h2bl	Rattus norvegicus histone cluster 1, H2bl (Hist1h2bl), mRNA [NM_022647]	**0,732**
Adra1a	Rattus norvegicus adrenergic, alpha-1A-, receptor (Adra1a), mRNA [NM_017191]	**0,732**
Odf3b	Rattus norvegicus outer dense fiber of sperm tails 3B (Odf3b), mRNA [NM_001191992]	**0,732**
Ccdc15	PREDICTED: Rattus norvegicus coiled-coil domain containing 15 (Ccdc15), mRNA [XM_001061074]	**0,732**
0	Rattus norvegicus similar to putative homeobox protein (LOC294473), mRNA [XM_228242]	**0,732**
Zcchc24	Rattus norvegicus zinc finger, CCHC domain containing 24 (Zcchc24), mRNA [NM_001108394]	**0,732**
Pcgf1	Rattus norvegicus polycomb group ring finger 1 (Pcgf1), mRNA [NM_001007000]	**0,732**
Olr1734	Rattus norvegicus olfactory receptor 1734 (Olr1734), mRNA [NM_001001119]	**0,732**
Atp10d	Uncharacterized protein [Source:UniProtKB/TrEMBL;Acc:D3ZN41] [ENSRNOT00000003146]	**0,732**
RGD1562339	Uncharacterized protein [Source:UniProtKB/TrEMBL;Acc:D4AC80] [ENSRNOT00000002408]	**0,732**
Fam70b	Rattus norvegicus family with sequence similarity 70, member B (Fam70b), mRNA [NM_001106094]	**0,733**
Slc4a10	Rattus norvegicus solute carrier family 4, sodium bicarbonate transporter, member 10 (Slc4a10), mRNA [NM_178092]	**0,733**
0	Unknown	**0,733**
0	Uncharacterized protein [Source:UniProtKB/TrEMBL;Acc:D4A5I4] [ENSRNOT00000037100]	**0,733**
0	DNA-binding protein A [Source:UniProtKB/Swiss-Prot;Acc:Q62764] [ENSRNOT00000007347]	**0,733**
Slc25a19	Rattus norvegicus solute carrier family 25 (mitochondrial thiamine pyrophosphate carrier), member 19 (Slc25a19), nuclear gene encoding mitochondrial protein, mRNA [NM_001007674]	**0,733**
Zdhhc5	Rattus norvegicus zinc finger, DHHC-type containing 5 (Zdhhc5), mRNA [NM_001039338]	**0,733**
Ankrd40	Rattus norvegicus ankyrin repeat domain 40 (Ankrd40), mRNA [NM_001134699]	**0,734**
Hs6st1	Rattus norvegicus heparan sulfate 6-O-sulfotransferase 1 (Hs6st1), mRNA [NM_001108210]	**0,734**
Vom2r18	Rattus norvegicus vomeronasal 2 receptor, 18 (Vom2r18), mRNA [NM_173318]	**0,734**
0	BC076587 Apbb2 protein {Mus musculus} (exp=-1; wgp=0; cg=0), partial (4%) [TC625977]	**0,734**
Pcgf3	Rattus norvegicus polycomb group ring finger 3 (Pcgf3), mRNA [NM_001107245]	**0,734**
Olr1415	Rattus norvegicus olfactory receptor 1415 (Olr1415), mRNA [NM_001000781]	**0,734**
Grip1	Rattus norvegicus glutamate receptor interacting protein 1 (Grip1), mRNA [NM_032069]	**0,734**
Msgn1	Rattus norvegicus mesogenin 1 (Msgn1), mRNA [NM_001109551]	**0,734**
Phyhipl	Rattus norvegicus phytanoyl-CoA 2-hydroxylase interacting protein-like (Phyhipl), mRNA [NM_001012076]	**0,734**
0	Unknown	**0,734**
Dclk1	Rattus norvegicus doublecortin-like kinase 1 (Dclk1), transcript variant 1, mRNA [NM_053343]	**0,734**
0	Unknown	**0,734**
Ube2n	Rattus norvegicus ubiquitin-conjugating enzyme E2N (UBC13 homolog, yeast) (Ube2n), mRNA [NM_053928]	**0,734**

Lsp1	Rattus norvegicus lymphocyte-specific protein 1 (Lsp1), mRNA [NM_001025420]	**0,734**
0	Unknown	**0,734**
Olr1229	Rattus norvegicus olfactory receptor 1229 (Olr1229), mRNA [NM_001000444]	**0,734**
Reep3	Rattus norvegicus receptor accessory protein 3 (Reep3), mRNA [NM_001106386]	**0,735**
Vom1r-ps39	Rattus norvegicus vomeronasal 1 receptor pseudogene 39 (Vom1r-ps39), mRNA [NM_001009509]	**0,735**
Pcmtd2	Rattus norvegicus protein-L-isoaspartate (D-aspartate) O-methyltransferase domain containing 2 (Pcmtd2), mRNA [NM_001107810]	**0,735**
0	Unknown	**0,735**
Noxa1	Rattus norvegicus NADPH oxidase activator 1 (Noxa1), mRNA [NM_001100171]	**0,735**
Fnbp1	Rattus norvegicus formin binding protein 1 (Fnbp1), mRNA [NM_138914]	**0,735**
Rbm25	Rattus norvegicus RNA binding motif protein 25 (Rbm25), mRNA [NM_001108984]	**0,735**
0	Q6NUI3_HUMAN (Q6NUI3) Beta 3-glycosyltransferase-like, partial (23%) [TC629324]	**0,735**
Fam124b	RCG64106Uncharacterized protein [Source:UniProtKB/TrEMBL;Acc:D4ACJ9] [ENSRNOT00000030626]	**0,735**
Capn5	Rattus norvegicus calpain 5 (Capn5), mRNA [NM_134461]	**0,735**
0	Unknown	**0,735**
Camk2g	Rattus norvegicus calcium/calmodulin-dependent protein kinase II gamma (Camk2g), mRNA [NM_133605]	**0,735**
Ulk3	PREDICTED: Rattus norvegicus unc-51-like kinase 3 (C. elegans) (Ulk3), mRNA [XM_001077085]	**0,735**
Hcrtr2	Rattus norvegicus hypocretin (orexin) receptor 2 (Hcrtr2), mRNA [NM_013074]	**0,735**
Orc5l	Rattus norvegicus origin recognition complex, subunit 5-like (yeast) (Orc5l), mRNA [NM_001014186]	**0,735**
Dhh	Rattus norvegicus desert hedgehog homolog (Drosophila) (Dhh), mRNA [NM_053367]	**0,736**
Bub3	Rattus norvegicus budding uninhibited by benzimidazoles 3 homolog (S. cerevisiae) (Bub3), mRNA [NM_001047906]	**0,736**
Sema6d	Rattus norvegicus sema domain, transmembrane domain (TM), and cytoplasmic domain, (semaphorin) 6D (Sema6d), mRNA [NM_001107768]	**0,736**
Pank2	Rattus norvegicus pantothenate kinase 2 (Hallervorden-Spatz syndrome) (Pank2), nuclear gene encoding mitochondrial protein, mRNA [NM_001106513]	**0,736**
Xpo5	Rattus norvegicus exportin 5 (Xpo5), mRNA [NM_001108789]	**0,736**
Col15a1	Col15a1 protein [Source:UniProtKB/TrEMBL;Acc:Q4G024] [ENSRNOT00000017217]	**0,736**
Ppp1r1b	Rattus norvegicus protein phosphatase 1, regulatory (inhibitor) subunit 1B (Ppp1r1b), mRNA [NM_138521]	**0,736**
RGD1566078	Uncharacterized protein [Source:UniProtKB/TrEMBL;Acc:D3ZWM0] [ENSRNOT00000013187]	**0,736**
Nebl	Uncharacterized protein [Source:UniProtKB/TrEMBL;Acc:D4A164] [ENSRNOT00000037601]	**0,736**
Eef1a2	Rattus norvegicus eukaryotic translation elongation factor 1 alpha 2 (Eef1a2), mRNA [NM_012660]	**0,736**
Sos1	Rattus norvegicus Son of sevenless homolog 1 (Drosophila) (Sos1), mRNA [NM_001100716]	**0,736**
Gpr173	Rattus norvegicus G-protein coupled receptor 173 (Gpr173), mRNA [NM_022255]	**0,736**
Prkd3	Rattus norvegicus protein kinase D3 (Prkd3), mRNA [NM_001024263]	**0,736**
Ptms	Rattus norvegicus parathymosin (Ptms), mRNA [NM_031975]	**0,736**
Sh2d3c	Rattus norvegicus SH2 domain containing 3C (Sh2d3c), mRNA [NM_001108579]	**0,737**

Pde4a	Rattus norvegicus phosphodiesterase 4A, cAMP-specific (phosphodiesterase E2 dunce homolog, Drosophila) (Pde4a), mRNA [NM_013101]	**0,737**
Vwc2	Rattus norvegicus von Willebrand factor C domain containing 2 (Vwc2), mRNA [NM_001109312]	**0,737**
Amz1	Rattus norvegicus archaelysin family metallopeptidase 1 (Amz1), mRNA [NM_001047092]	**0,737**
Fem1c	Rattus norvegicus fem-1 homolog c (C. elegans) (Fem1c), mRNA [NM_001106932]	**0,737**
Kif18b	Rattus norvegicus kinesin family member 18B (Kif18b), mRNA [NM_001039019]	**0,737**
Plod2	Rattus norvegicus procollagen lysine, 2-oxoglutarate 5-dioxygenase 2 (Plod2), transcript variant 1, mRNA [NM_175869]	**0,737**
Tctn3	PREDICTED: Rattus norvegicus tectonic family member 3 (Tctn3), mRNA [XM_001053561]	**0,737**
Calcoco2	PREDICTED: Rattus norvegicus calcium binding and coiled-coil domain 2 (Calcoco2), mRNA [XM_220903]	**0,737**
Cd47	Rattus norvegicus Cd47 molecule (Cd47), mRNA [NM_019195]	**0,737**
Vps13d	Rattus norvegicus vacuolar protein sorting 13 homolog D (S. cerevisiae) (Vps13d), mRNA [NM_001108006]	**0,737**
0	AGENCOURT_31543064 NIH_MGC_269 Rattus norvegicus cDNA clone IMAGE:7462807 5', mRNA sequence [CV121387]	**0,738**
Arrdc5	Rattus norvegicus arrestin domain containing 5 (Arrdc5), mRNA [NM_001109408]	**0,738**
LOC685179	Uncharacterized protein [Source:UniProtKB/TrEMBL;Acc:D3ZPF5] [ENSRNOT00000028244]	**0,738**
LOC680443	PREDICTED: Rattus norvegicus hypothetical protein LOC680443 (LOC680443), mRNA [XM_002727352]	**0,738**
Ssh2	Rattus norvegicus slingshot homolog 2 (Drosophila) (Ssh2), mRNA [NM_001107024]	**0,738**
Znf575	Rattus norvegicus zinc finger protein 575 (Znf575), mRNA [NM_001107489]	**0,738**
Appbp2	Rattus norvegicus amyloid beta precursor protein (cytoplasmic tail) binding protein 2 (Appbp2), mRNA [NM_001100969]	**0,738**
Edem2	Rattus norvegicus ER degradation enhancer, mannosidase alpha-like 2 (Edem2), mRNA [NM_001004230]	**0,738**
Utrn	Rattus norvegicus utrophin (Utrn), mRNA [NM_013070]	**0,738**
0	Uncharacterized protein [Source:UniProtKB/TrEMBL;Acc:D3ZJB6] [ENSRNOT00000040584]	**0,739**
Tmcc3	Rattus norvegicus transmembrane and coiled-coil domain family 3 (Tmcc3), mRNA [NM_001108084]	**0,739**
0	Unknown	**0,739**
0	Cytochrome c oxidase subunit 3 [Source:UniProtKB/Swiss-Prot;Acc:P05505] [ENSRNOT00000049683]	**0,739**
Mn1	Rattus norvegicus meningioma 1 (Mn1), mRNA [NM_001191928]	**0,739**
0	Unknown	**0,739**
Tank	Rattus norvegicus TRAF family member-associated NFKB activator (Tank), transcript variant 1, mRNA [NM_145788]	**0,739**
Ring1	Rattus norvegicus ring finger protein 1 (Ring1), mRNA [NM_212549]	**0,739**
Dcxr	Rattus norvegicus dicarbonyl L-xylulose reductase (Dcxr), mRNA [NM_134387]	**0,739**
0	EST231431 Normalized rat ovary, Bento Soares Rattus sp. cDNA clone ROVCI85 3' end, mRNA sequence [AI234869]	**0,739**
Gabrg3	Rattus norvegicus gamma-aminobutyric acid (GABA) A receptor, gamma 3 (Gabrg3), mRNA [NM_024370]	**0,739**
Wrap53	Rattus norvegicus WD repeat containing, antisense to TP53 (Wrap53), mRNA [NM_001007610]	**0,739**
Zbtb5	Rattus norvegicus zinc finger and BTB domain containing 5 (Zbtb5), mRNA [NM_001106657]	**0,739**
Plekhf2	Rattus norvegicus pleckstrin homology domain containing, family F (with FYVE domain) member 2 (Plekhf2), mRNA [NM_001108655]	**0,739**
Klhl26	Rattus norvegicus kelch-like 26 (Drosophila) (Klhl26), mRNA [NM_001106075]	**0,740**

Zfp385d	Rattus norvegicus zinc finger protein 385D (Zfp385d), mRNA [NM_001013992]	**0,740**
Gpkow	Rattus norvegicus G patch domain and KOW motifs (Gpkow), mRNA [NM_001109381]	**0,740**
Adora1	Rattus norvegicus adenosine A1 receptor (Adora1), mRNA [NM_017155]	**0,740**
Tial1	Rattus norvegicus Tia1 cytotoxic granule-associated RNA binding protein-like 1 (Tial1), mRNA [NM_001013193]	**0,740**
Gstm7	Rattus norvegicus glutathione S-transferase, mu 7 (Gstm7), mRNA [NM_031154]	**0,740**
0	Unknown	**0,740**
0	Unknown	**0,740**
RGD1560902	PREDICTED: Rattus norvegicus similar to Cofilin, non-muscle isoform (Cofilin-1) (RGD1560902), mRNA [XM_002724509]	**0,740**
LOC688463	PREDICTED: Rattus norvegicus hypothetical protein LOC688463 (LOC688463), mRNA [XM_001064240]	**0,740**
LOC688778	PREDICTED: Rattus norvegicus similar to fatty aldehyde dehydrogenase-like (LOC688778), mRNA [XM_001068253]	**0,740**
Arhgef7	Rattus norvegicus Rho guanine nucleotide exchange factor (GEF7) (Arhgef7), transcript variant 1, mRNA [NM_001113521]	**0,741**
0	Ubiquitin carboxyl-terminal hydrolase [Source:UniProtKB/TrEMBL;Acc:D3Z8K5] [ENSRNOT00000035084]	**0,741**
Fgf14	Rattus norvegicus fibroblast growth factor-like factor 4D mRNA, partial cds. [AF348523]	**0,741**
Ablim2	Rattus norvegicus actin binding LIM protein family, member 2 (Ablim2), transcript variant 1, mRNA [NM_001177695]	**0,741**
RGD1306410	Rattus norvegicus similar to CG14980-PB (RGD1306410), mRNA [NM_001014126]	**0,741**
Rnf215	Rattus norvegicus ring finger protein 215 (Rnf215), mRNA [NM_001107234]	**0,741**
Slc26a10	solute carrier family 26 member 10 [Source:RefSeq peptide;Acc:NP_001128067] [ENSRNOT00000052288]	**0,741**
Nxph4	Rattus norvegicus neurexophilin 4 (Nxph4), mRNA [NM_021680]	**0,741**
0	Unknown	**0,741**
Fam186b	Rattus norvegicus family with sequence similarity 186, member B (Fam186b), mRNA [NM_001134633]	**0,741**
Fgfbp3	Rattus norvegicus fibroblast growth factor binding protein 3 (Fgfbp3), mRNA [NM_001109165]	**0,741**
0	Unknown	**0,741**
Efhd2	Rattus norvegicus EF-hand domain family, member D2 (Efhd2), mRNA [NM_001031648]	**0,741**
0	Uncharacterized protein [Source:UniProtKB/TrEMBL;Acc:D4A9I0] [ENSRNOT00000035986]	**0,741**
Stk3	Rattus norvegicus serine/threonine kinase 3 (STE20 homolog, yeast) (Stk3), mRNA [NM_031735]	**0,741**
Pbx1	Rattus norvegicus pre-B-cell leukemia homeobox 1 (Pbx1), transcript variant 2, mRNA [NM_001100681]	**0,741**
Ncstn	Rattus norvegicus nicastrin (Ncstn), mRNA [NM_174864]	**0,742**
Fcgr2a	Rattus norvegicus Fc fragment of IgG, low affinity IIa, receptor (CD32) (Fcgr2a), mRNA [NM_053843]	**0,742**
Ptprt	Rattus norvegicus protein tyrosine phosphatase, receptor type, T (Ptprt), mRNA [NM_001108603]	**0,742**
0	CB545809 AMGNNUC:MRPE3-00116-C9-A placenta embryo D17 (10379) Rattus norvegicus cDNA clone mrpe3-00116-c9 5', mRNA sequence [CB545809]	**0,742**
0	Unknown	**0,742**
Zp3r	Rattus norvegicus zona pellucida 3 receptor (Zp3r), mRNA [NM_182815]	**0,742**
0	Uncharacterized protein [Source:UniProtKB/TrEMBL;Acc:D3ZD16] [ENSRNOT00000012794]	**0,742**

0	Histone H2B type 1 [Source:UniProtKB/Swiss-Prot;Acc:Q00715] [ENSRNOT00000024276]	**0,742**
Atp7b	Rattus norvegicus ATPase, Cu++ transporting, beta polypeptide (Atp7b), mRNA [NM_012511]	**0,743**
0	Unknown	**0,743**
0	Unknown	**0,743**
LOC691543	Rattus norvegicus hypothetical protein LOC691543 (LOC691543), mRNA [NM_001109645]	**0,743**
Fam70a	Rattus norvegicus family with sequence similarity 70, member A (Fam70a), mRNA [NM_182822]	**0,743**
Gulo	Rattus norvegicus gulonolactone (L-) oxidase (Gulo), mRNA [NM_022220]	**0,743**
Khnyn	PREDICTED: Rattus norvegicus similar to mKIAA0323 protein (RGD1565688), miscRNA [XR_085623]	**0,743**
Cpeb4	Rattus norvegicus cytoplasmic polyadenylation element binding protein 4 (Cpeb4), mRNA [NM_001106992]	**0,743**
0	PREDICTED: Rattus norvegicus similar to glycoprotein, synaptic 2 (RGD1560015), mRNA [XM_001062160]	**0,743**
Cd8b	Rattus norvegicus CD8b molecule (Cd8b), mRNA [NM_031539]	**0,744**
0	Uncharacterized protein [Source:UniProtKB/TrEMBL;Acc:D3ZU50] [ENSRNOT00000001479]	**0,744**
Kcnk13	Rattus norvegicus potassium channel, subfamily K, member 13 (Kcnk13), mRNA [NM_022293]	**0,744**
Abcd1	Rattus norvegicus ATP-binding cassette, subfamily D (ALD), member 1 (Abcd1), mRNA [NM_001108821]	**0,744**
Capn6	Rattus norvegicus calpain 6 (Capn6), mRNA [NM_031808]	**0,744**
0	Unknown	**0,744**
Bmyc	Rattus norvegicus brain expressed myelocytomatosis oncogene (Bmyc), mRNA [NM_001013163]	**0,744**
Ascl5	PREDICTED: Rattus norvegicus achaete-scute complex homolog 5 (Drosophila) (Ascl5), mRNA [XM_344144]	**0,744**
Znf652	Rattus norvegicus zinc finger protein 652 (Znf652), mRNA [NM_001080207]	**0,745**
Nid2	Rattus norvegicus nidogen 2 (Nid2), mRNA [NM_001012005]	**0,745**
Gatm	Rattus norvegicus glycine amidinotransferase (L-arginine:glycine amidinotransferase) (Gatm), nuclear gene encoding mitochondrial protein, mRNA [NM_031031]	**0,745**
Pusl1	Rattus norvegicus pseudouridylate synthase-like 1 (Pusl1), mRNA [NM_001108699]	**0,745**
Pbx1	Rattus norvegicus pre-B-cell leukemia homeobox 1 (Pbx1), transcript variant 2, mRNA [NM_001100681]	**0,745**
Birc6	Rattus norvegicus baculoviral IAP repeat-containing 6 (Birc6), mRNA [NM_001170596]	**0,745**
Fbxl7	Rattus norvegicus F-box and leucine-rich repeat protein 7 (Fbxl7), mRNA [NM_001108545]	**0,745**
Kcng1	Rattus norvegicus potassium voltage-gated channel, subfamily G, member 1 (Kcng1), mRNA [NM_001106545]	**0,745**
Kcnip3	Rattus norvegicus Kv channel interacting protein 3, calsenilin (Kcnip3), mRNA [NM_032462]	**0,745**
Mblac2	Rattus norvegicus metallo-beta-lactamase domain containing 2 (Mblac2), mRNA [NM_001108934]	**0,746**
Itpk1	Rattus norvegicus inositol 1,3,4-triphosphate 5/6 kinase (Itpk1), mRNA [NM_001191985]	**0,746**
LOC687796	PREDICTED: Rattus norvegicus hypothetical protein LOC687796, transcript variant 2 (LOC687796), mRNA [XM_001078497]	**0,746**
Trim26	Rattus norvegicus tripartite motif-containing 26 (Trim26), mRNA [NM_001011665]	**0,746**
0	Uncharacterized protein [Source:UniProtKB/TrEMBL;Acc:D3ZV19] [ENSRNOT00000009759]	**0,746**
LOC291686	PREDICTED: Rattus norvegicus similar to 60S ribosomal protein L23a (LOC291686), mRNA [XM_002725324]	**0,746**

Tbc1d5	Rattus norvegicus TBC1 domain family, member 5 (Tbc1d5), mRNA [NM_001134762]	**0,746**
LOC690082	PREDICTED: Rattus norvegicus similar to melanoma ubiquitous mutated protein (LOC690082), mRNA [XM_001073181]	**0,746**
LOC678704	Rattus norvegicus hypothetical protein LOC678704 (LOC678704), mRNA [NM_001109369]	**0,747**
RGD1565095	Rattus norvegicus similar to hypothetical protein MGC52110 (RGD1565095), mRNA [NM_001195488]	**0,747**
Hoxd4	Rattus norvegicus homeo box D4 (Hoxd4), mRNA [NM_001105885]	**0,747**
0	Unknown	**0,747**
Megf11	PREDICTED: Rattus norvegicus multiple EGF-like-domains 11 (Megf11), mRNA [XM_001078620]	**0,747**
0	RVL11956 Wackym-Soares normalized rat vestibular cDNA library Rattus norvegicus cDNA 5', mRNA sequence [DV720880]	**0,747**
Cbfa2t3	Rattus norvegicus core-binding factor, runt domain, alpha subunit 2; translocated to, 3 (Cbfa2t3), mRNA [NM_001108453]	**0,747**
0	PREDICTED: Rattus norvegicus similar to Myosin light chain 1 slow a (RGD1560334), mRNA [XM_228900]	**0,747**
0	probable histone-lysine N-methyltransferase NSD2 [Source:RefSeq peptide;Acc:NP_001178481] [ENSRNOT00000050238]	**0,747**
0	Unknown	**0,747**
Arhgap21	Rattus norvegicus Rho GTPase activating protein 21 (Arhgap21), mRNA [NM_001191693]	**0,747**
Cntn4	Rattus norvegicus contactin 4 (Cntn4), mRNA [NM_053879]	**0,747**
Socs4	Rattus norvegicus suppressor of cytokine signaling 4 (Socs4), mRNA [NM_001107256]	**0,747**
Vom1r12	Rattus norvegicus vomeronasal 1 receptor 12 (Vom1r12), mRNA [NM_001008962]	**0,747**
Prkaa1	Rattus norvegicus protein kinase, AMP-activated, alpha 1 catalytic subunit (Prkaa1), mRNA [NM_019142]	**0,748**
Gal3st3	Rattus norvegicus galactose-3-O-sulfotransferase 3 (Gal3st3), mRNA [NM_001024290]	**0,748**
Nsun5	Rattus norvegicus NOL1/NOP2/Sun domain family, member 5 (Nsun5), mRNA [NM_001191593]	**0,748**
LOC690478	Rattus norvegicus similar to keratin associated protein 10-7 (LOC690478), mRNA [NM_001109595]	**0,748**
Dpf2	Rattus norvegicus D4, zinc and double PHD fingers family 2 (Dpf2), mRNA [NM_001108516]	**0,748**
Znf498	Rattus norvegicus zinc finger protein 498 (Znf498), mRNA [NM_001108851]	**0,748**
RGD1309374	Uncharacterized protein [Source:UniProtKB/TrEMBL;Acc:D3ZU62] [ENSRNOT00000020492]	**0,748**
Ermap	PREDICTED: Rattus norvegicus erythroblast membrane-associated protein (Ermap), mRNA [XM_001073566]	**0,748**
0	Unknown	**0,748**
0	HECT domain containing 2 Gene [Source:MGI Symbol;Acc:MGI:2442663] [ENSRNOT00000024963]	**0,748**
0	GLIS family zinc finger 1 (Predicted), isoform CRA_bUncharacterized protein [Source:UniProtKB/TrEMBL;Acc:D4A5Q8] [ENSRNOT00000032433]	**0,748**
Zfp93	Rattus norvegicus zinc finger protein 93 (Zfp93), mRNA [NM_001106546]	**0,748**
0	Unknown	**0,748**
Emx1	PREDICTED: Rattus norvegicus empty spiracles homeobox 1 (Emx1), mRNA [XM_001073769]	**0,749**
Crygf	Rattus norvegicus crystallin, gamma F (Crygf), mRNA [NM_001109557]	**0,749**
Prp2l1	Rat proline-rich protein (PRP-1) [Source:UniProtKB/TrEMBL;Acc:Q63455] [ENSRNOT00000004642]	**0,749**
Nrsn2	Rattus norvegicus neurensin 2 (Nrsn2), mRNA [NM_001109561]	**0,749**
Crebbp	Rattus norvegicus CREB binding protein (Crebbp), mRNA [NM_133381]	**0,749**

Ube2l3	Rattus norvegicus ubiquitin-conjugating enzyme E2L 3 (Ube2l3), mRNA [NM_001108847]	**0,749**
Amica1	Uncharacterized protein [Source:UniProtKB/TrEMBL;Acc:D4A1C2] [ENSRNOT00000007588]	**0,749**
Egr4	Rattus norvegicus early growth response 4 (Egr4), mRNA [NM_019137]	**0,749**
RGD1308319	Uncharacterized protein [Source:UniProtKB/TrEMBL;Acc:D3ZBT5] [ENSRNOT00000050012]	**0,750**
Pax8	Rattus norvegicus paired box 8 (Pax8), mRNA [NM_031141]	**0,750**
Spon1	Rattus norvegicus spondin 1, extracellular matrix protein (Spon1), mRNA [NM_172067]	**0,750**
Clcn6	Rattus norvegicus chloride channel 6 (Clcn6), mRNA [NM_001106479]	**0,750**
0	Unknown	**0,750**
Ftsj1	Uncharacterized protein [Source:UniProtKB/TrEMBL;Acc:D3ZZA1] [ENSRNOT00000006365]	**0,750**
Setd7	Rattus norvegicus SET domain containing (lysine methyltransferase) 7 (Setd7), mRNA [NM_001109558]	**0,750**
Wnk4	Rattus norvegicus WNK lysine deficient protein kinase 4 (Wnk4), mRNA [NM_175579]	**0,750**
Gnat1	Rattus norvegicus guanine nucleotide binding protein (G protein), alpha transducing 1 (Gnat1), mRNA [NM_001108780]	**0,751**
Rpp25	Rattus norvegicus ribonuclease P 25 subunit (human) (Rpp25), mRNA [NM_001012124]	**0,751**
Ubl5	Rattus norvegicus ubiquitin-like 5 (Ubl5), mRNA [NM_001048243]	**0,751**
Klf12	Rattus norvegicus Kruppel-like factor 12 (Klf12), mRNA [NM_001107281]	**0,751**
RGD1309586	Rattus norvegicus similar to probable ATP-dependent RNA helicase - mouse (RGD1309586), mRNA [NM_001108858]	**0,751**
Gng3	Rattus norvegicus guanine nucleotide binding protein (G protein), gamma 3 (Gng3), mRNA [NM_053658]	**0,751**
Tmem175	Rattus norvegicus transmembrane protein 175 (Tmem175), mRNA [NM_001013991]	**0,751**
Athl1	Uncharacterized protein [Source:UniProtKB/TrEMBL;Acc:D3ZR78] [ENSRNOT00000020004]	**0,751**
Kcnc3	Rattus norvegicus potassium voltage gated channel, Shaw-related subfamily, member 3 (Kcnc3), mRNA [NM_053997]	**0,751**
LOC691684	PREDICTED: Rattus norvegicus similar to MIC2 like 1 (LOC691684), mRNA [XM_001079259]	**0,752**
Gapdh	Rattus norvegicus glyceraldehyde-3-phosphate dehydrogenase (Gapdh), mRNA [NM_017008]	**0,752**
S1pr2	Rattus norvegicus sphingosine-1-phosphate receptor 2 (S1pr2), mRNA [NM_017192]	**0,752**
Nrep	Rattus norvegicus neuronal regeneration related protein (Nrep), mRNA [NM_178096]	**0,752**
Numa1	Numa1 protein [Source:UniProtKB/TrEMBL;Acc:Q4G051] [ENSRNOT00000000474]	**0,752**
0	S18L2_MOUSE (Q9D174) SS18-like protein 2, complete [TC585393]	**0,752**
0	Unknown	**0,752**
LOC689065	Rattus norvegicus hypothetical protein LOC689065 (LOC689065), mRNA [NM_001109521]	**0,752**
0	Rattus norvegicus similar to Pyruvate kinase, M2 isozyme (LOC315231), mRNA [XM_235589]	**0,752**
Ecel1	Rattus norvegicus endothelin converting enzyme-like 1 (Ecel1), mRNA [NM_021776]	**0,752**
Recql4	Rattus norvegicus RecQ protein-like 4 (Recql4), mRNA [NM_001130494]	**0,752**
0	Rattus norvegicus similar to 60S RIBOSOMAL PROTEIN L29 (P23) (LOC294100), mRNA [XM_220073]	**0,752**
0	Unknown	**0,753**
0	Unknown	**0,753**

Lcn10	Rattus norvegicus lipocalin 10 (Lcn10), mRNA [NM_001128137]	**0,753**
Rin2	Rattus norvegicus Ras and Rab interactor 2 (Rin2), mRNA [NM_001107786]	**0,753**
Dcakd	Rattus norvegicus dephospho-CoA kinase domain containing (Dcakd), mRNA [NM_001007724]	**0,753**
Kptn	Rattus norvegicus kaptin (actin binding protein) (Kptn), mRNA [NM_001107457]	**0,753**
0	Unknown	**0,753**
Ece1	Rattus norvegicus endothelin converting enzyme 1 (Ece1), mRNA [NM_053596]	**0,753**
Rph3a	Rattus norvegicus rabphilin 3A (Rph3a), mRNA [NM_133518]	**0,753**
Cugbp2	Rattus norvegicus CUG triplet repeat, RNA binding protein 2 (Cugbp2), transcript variant 2, mRNA [NM_001083586]	**0,754**
0	Unknown	**0,754**
Cntn4	Rattus norvegicus contactin 4 (Cntn4), mRNA [NM_053879]	**0,754**
Pex19	Rattus norvegicus peroxisomal biogenesis factor 19 (Pex19), transcript variant 1, mRNA [NM_001107375]	**0,754**
0	AGENCOURT_109870505 NIH_MGC_420 Rattus norvegicus cDNA clone IMAGE:9033411 5', mRNA sequence [EV768969]	**0,754**
Snap91	Rattus norvegicus synaptosomal-associated protein 91 (Snap91), mRNA [NM_031728]	**0,754**
Rbpj	Rattus norvegicus recombination signal binding protein for immunoglobulin kappa J region (Rbpj), mRNA [NM_001106631]	**0,754**
RGD1560286	Rattus norvegicus similar to DNA segment, Chr 4, ERATO Doi 22, expressed (RGD1560286), transcript variant 1, mRNA [NM_001114599]	**0,754**
Serpinb8	Rattus norvegicus serpin peptidase inhibitor, clade B (ovalbumin), member 8 (Serpinb8), mRNA [NM_001105948]	**0,754**
0	Unknown	**0,754**
Nhp2l1	Rattus norvegicus NHP2 non-histone chromosome protein 2-like 1 (S. cerevisiae) (Nhp2l1), mRNA [NM_212515]	**0,755**
Ube2d4	Rattus norvegicus ubiquitin-conjugating enzyme E2D 4 (Ube2d4), mRNA [NM_031001]	**0,755**
Snap25	Rattus norvegicus synaptosomal-associated protein 25 (Snap25), mRNA [NM_030991]	**0,755**
Ppif	Rattus norvegicus peptidylprolyl isomerase F (Ppif), nuclear gene encoding mitochondrial protein, mRNA [NM_172243]	**0,755**
Slc1a4	Rattus norvegicus solute carrier family 1 (glutamate/neutral amino acid transporter), member 4 (Slc1a4), mRNA [NM_198763]	**0,755**
C1qb	Rattus norvegicus complement component 1, q subcomponent, B chain (C1qb), mRNA [NM_019262]	**0,755**
Lyplal1	Rattus norvegicus lysophospholipase-like 1 (Lyplal1), mRNA [NM_001105986]	**0,756**
Mfsd6	Rattus norvegicus major facilitator superfamily domain containing 6 (Mfsd6), mRNA [NM_001106911]	**0,756**
0	Unknown	**0,756**
0	Unknown	**0,756**
Dab2	Rattus norvegicus disabled homolog 2 (Drosophila) (Dab2), mRNA [NM_024159]	**0,757**
Casc3	Rattus norvegicus cancer susceptibility candidate 3 (Casc3), mRNA [NM_147144]	**0,757**
Defb27	Rattus norvegicus defensin beta 27 (Defb27), mRNA [NM_001037519]	**0,757**
0	additional sex combs like 3 (Drosophila) Gene [Source:MGI Symbol;Acc:MGI:2685175] [ENSRNOT00000020661]	**0,757**
Plekhg1	Rattus norvegicus pleckstrin homology domain containing, family G (with RhoGef domain) member 1 (Plekhg1), mRNA [NM_001190999]	**0,757**
Fn3krp	Rattus norvegicus fructosamine-3-kinase-related protein (Fn3krp), mRNA [NM_001107077]	**0,757**
RGD1309870	Rattus norvegicus hypothetical LOC289778 (RGD1309870), mRNA [NM_001106018]	**0,757**

T2	Rattus norvegicus brachyury 2 (T2), mRNA [NM_001161835]	**0,757**
Atp6v0a1	Rattus norvegicus ATPase, H+ transporting, lysosomal V0 subunit A1 (Atp6v0a1), mRNA [NM_031604]	**0,757**
Mxi1	Rattus norvegicus MAX interactor 1 (Mxi1), mRNA [NM_013160]	**0,758**
Ccr9	Rattus norvegicus chemokine (C-C motif) receptor 9 (Ccr9), mRNA [NM_172329]	**0,758**
0	Unknown	**0,758**
Pglyrp4	Rattus norvegicus peptidoglycan recognition protein 4 (Pglyrp4), mRNA [NM_001191708]	**0,758**
Ict1	Rattus norvegicus immature colon carcinoma transcript 1 (Ict1), mRNA [NM_001191656]	**0,758**
Creb3l2	Rattus norvegicus cAMP responsive element binding protein 3-like 2 (Creb3l2), mRNA [NM_001012188]	**0,759**
Ak7	Rattus norvegicus adenylate kinase 7 (Ak7), mRNA [NM_001108055]	**0,759**
Mlph	Rattus norvegicus melanophilin (Mlph), mRNA [NM_001012135]	**0,759**
Map1b	Rattus norvegicus microtubule-associated protein 1B (Map1b), mRNA [NM_019217]	**0,759**
0	Uncharacterized protein [Source:UniProtKB/TrEMBL;Acc:D4A9W8] [ENSRNOT00000014537]	**0,759**
Nol4	Rattus norvegicus nucleolar protein 4 (Nol4), mRNA [NM_001107401]	**0,759**
Guca2b	Rattus norvegicus guanylate cyclase activator 2B (Guca2b), mRNA [NM_022284]	**0,760**
Slc39a1	Rattus norvegicus solute carrier family 39 (zinc transporter), member 1 (Slc39a1), mRNA [NM_001134577]	**0,760**
0	AGENCOURT_31538552 NIH_MGC_270 Rattus norvegicus cDNA clone IMAGE:7442365 5', mRNA sequence [CV107694]	**0,760**
Nlgn1	Rattus norvegicus neuroligin 1 (Nlgn1), mRNA [NM_053868]	**0,760**
Vat1	Rattus norvegicus vesicle amine transport protein 1 homolog (T californica) (Vat1), mRNA [NM_001033683]	**0,760**
Rb1	Rattus norvegicus retinoblastoma 1 (Rb1), mRNA [NM_017045]	**0,760**
Antxr1	Rattus norvegicus anthrax toxin receptor 1 (Antxr1), mRNA [NM_001044249]	**0,761**
Thop1	Rattus norvegicus thimet oligopeptidase 1 (Thop1), mRNA [NM_172075]	**0,761**
Phf11	Rattus norvegicus PHD finger protein 11 (Phf11), mRNA [NM_001024272]	**0,761**
0	Unknown	**0,761**
Ccl9	Rattus norvegicus chemokine (C-C motif) ligand 9 (Ccl9), mRNA [NM_001012357]	**0,762**
Osr1	Rattus norvegicus odd-skipped related 1 (Drosophila) (Osr1), mRNA [NM_001106716]	**0,762**
Gnai1	Rattus norvegicus guanine nucleotide binding protein (G protein), alpha inhibiting 1 (Gnai1), mRNA [NM_013145]	**0,762**
Scn1b	Rattus norvegicus sodium channel, voltage-gated, type I, beta (Scn1b), mRNA [NM_017288]	**0,762**
LOC100366258	Uncharacterized protein [Source:UniProtKB/TrEMBL;Acc:D3ZL26] [ENSRNOT00000051182]	**0,762**
0	Rattus norvegicus TL0AEA5YN17 mRNA sequence. [FQ233918]	**0,762**
Zbed3	Rattus norvegicus zinc finger, BED-type containing 3 (Zbed3), mRNA [NM_001025729]	**0,762**
Pde4b	Rattus norvegicus phosphodiesterase 4B, cAMP specific (Pde4b), mRNA [NM_017031]	**0,762**
0	RCG43605, isoform CRA_aUncharacterized protein [Source:UniProtKB/TrEMBL;Acc:D3ZHC7] [ENSRNOT00000061670]	**0,762**
LOC685671	PREDICTED: Rattus norvegicus similar to myocyte enhancer factor 2C (LOC685671), miscRNA [XR_006259]	**0,762**
Dbr1	Rattus norvegicus debranching enzyme homolog 1 (S. cerevisiae) (Dbr1), mRNA [NM_001109437]	**0,762**

0	zinc finger protein 866 Gene [Source:MGI Symbol;Acc:MGI:3584369] [ENSRNOT00000034446]	**0,762**
LOC100302465	Rattus norvegicus hypothetical LOC100302465 (LOC100302465), mRNA [NM_001163214]	**0,763**
0	Unknown	**0,763**
0	Uncharacterized protein [Source:UniProtKB/TrEMBL;Acc:D4A6V0] [ENSRNOT00000044039]	**0,763**
RGD1562342	Rattus norvegicus similar to RIKEN cDNA 1110012D08 (RGD1562342), mRNA [NM_001109281]	**0,763**
0	Unknown	**0,763**
Pcsk4	Rattus norvegicus proprotein convertase subtilisin/kexin type 4 (Pcsk4), mRNA [NM_133559]	**0,763**
Fam38b	PREDICTED: Rattus norvegicus similar to CG8486-PA, isoform A (LOC682889), mRNA [XM_001063568]	**0,763**
Psme3	Rattus norvegicus proteasome (prosome, macropain) activator subunit 3 (Psme3), mRNA [NM_001011894]	**0,763**
0	CB544633 AMGNNUC:SRPB2-00179-D4-A srpb2 (10220) Rattus norvegicus cDNA clone srpb2-00179-d4 5', mRNA sequence [CB544633]	**0,763**
Tcp11l1	Rattus norvegicus t-complex 11 like 1 (Tcp11l1), mRNA [NM_001109202]	**0,763**
Ift80	Rattus norvegicus intraflagellar transport 80 homolog (Chlamydomonas) (Ift80), mRNA [NM_001013911]	**0,764**
LOC100188932	Rattus norvegicus dolichyl-diphosphooligosaccharide--protein glycosyltransferase subunit 4 (LOC100188932), transcript variant 2, mRNA [NM_001134690]	**0,764**
0	Unknown	**0,764**
0	Uncharacterized protein [Source:UniProtKB/TrEMBL;Acc:D3ZSR6] [ENSRNOT00000064626]	**0,764**
LOC681186	PREDICTED: Rattus norvegicus hypothetical protein LOC681186 (LOC681186), mRNA [XM_001060674]	**0,764**
Gnao1	Rattus norvegicus guanine nucleotide binding protein (G protein), alpha activating activity polypeptide O (Gnao1), mRNA [NM_017327]	**0,764**
Zfp385a	Rattus norvegicus zinc finger protein 385A (Zfp385a), transcript variant 1, mRNA [NM_001135088]	**0,765**
LOC100365745	PREDICTED: Rattus norvegicus hypothetical protein LOC100365745 (LOC100365745), mRNA [XM_002725938]	**0,765**
Bahd1	Similar to mKIAA0945 protein (Predicted)Uncharacterized protein [Source:UniProtKB/TrEMBL;Acc:D3ZHT3] [ENSRNOT00000013878]	**0,765**
Fxyd6	Rattus norvegicus FXYD domain-containing ion transport regulator 6 (Fxyd6), mRNA [NM_022005]	**0,765**
Tp53inp1	Rattus norvegicus tumor protein p53 inducible nuclear protein 1 (Tp53inp1), mRNA [NM_181084]	**0,765**
Il1rap	Rattus norvegicus interleukin 1 receptor accessory protein (Il1rap), transcript variant 2, mRNA [NM_001167840]	**0,765**
0	Unknown	**0,765**
Slc7a13	Rattus norvegicus solute carrier family 7, (cationic amino acid transporter, y+ system) member 13 (Slc7a13), mRNA [NM_001012100]	**0,765**
Fgfr2	Rattus norvegicus fibroblast growth factor receptor 2 (Fgfr2), transcript variant a, mRNA [NM_012712]	**0,765**
Pnrc1	Rattus norvegicus proline-rich nuclear receptor coactivator 1 (Pnrc1), mRNA [NM_173322]	**0,766**
Mmp15	Rattus norvegicus matrix metallopeptidase 15 (Mmp15), mRNA [NM_001106168]	**0,766**
Tmem108	PREDICTED: Rattus norvegicus transmembrane protein 108 (Tmem108), mRNA [XM_217247]	**0,766**
Sstr2	Rattus norvegicus somatostatin receptor 2 (Sstr2), mRNA [NM_019348]	**0,766**
LOC679114	PREDICTED: Rattus norvegicus similar to sin3 associated polypeptide (LOC679114), mRNA [XM_001054754]	**0,766**
Ddah1	Rattus norvegicus dimethylarginine dimethylaminohydrolase 1 (Ddah1), mRNA [NM_022297]	**0,766**
Cd6	Rattus norvegicus Cd6 molecule (Cd6), mRNA [NM_175577]	**0,766**

Nek9	Rattus norvegicus NIMA (never in mitosis gene a)- related kinase 9 (Nek9), mRNA [NM_001106747]	**0,766**
LOC688285	PREDICTED: Rattus norvegicus hypothetical protein LOC688285 (LOC688285), mRNA [XM_001081744]	**0,766**
Olr1475	Rattus norvegicus olfactory receptor 1475 (Olr1475), mRNA [NM_001000027]	**0,766**
Olr1105	Rattus norvegicus olfactory receptor 1105 (Olr1105), mRNA [NM_001001078]	**0,767**
Foxk2	Rattus norvegicus forkhead box K2 (Foxk2), mRNA [NM_001107075]	**0,767**
Lifr	Rattus norvegicus leukemia inhibitory factor receptor alpha (Lifr), mRNA [NM_031048]	**0,767**
LOC681351	PREDICTED: Rattus norvegicus similar to apolipoprotein L, 3 (LOC681351), mRNA [XM_001061370]	**0,767**
Dlgap2	Rattus norvegicus discs, large (Drosophila) homolog-associated protein 2 (Dlgap2), mRNA [NM_053901]	**0,767**
Ppp2r1a	Rattus norvegicus protein phosphatase 2 (formerly 2A), regulatory subunit A, alpha isoform (Ppp2r1a), mRNA [NM_057140]	**0,767**
Sfrp2	Rattus norvegicus secreted frizzled-related protein 2 (Sfrp2), mRNA [NM_001100700]	**0,767**
Ptpn3	Ptpn3 protein [Source:UniProtKB/TrEMBL;Acc:Q562B7] [ENSRNOT00000059627]	**0,767**
RGD1565712	Rattus norvegicus similar to Hypothetical protein MGC59495 (RGD1565712), mRNA [NM_001127579]	**0,767**
LOC497848	PREDICTED: Rattus norvegicus hypothetical LOC497848 (LOC497848), miscRNA [XR_007272]	**0,767**
Kalrn	Rattus norvegicus kalirin, RhoGEF kinase (Kalrn), mRNA [NM_032062]	**0,767**
Nkx2-1	Rattus norvegicus NK2 homeobox 1 (Nkx2-1), mRNA [NM_013093]	**0,767**
Ttc26	Rattus norvegicus tetratricopeptide repeat domain 26 (Ttc26), mRNA [NM_001025045]	**0,767**
0	Uncharacterized protein [Source:UniProtKB/TrEMBL;Acc:D3ZR73] [ENSRNOT00000054883]	**0,767**
Cacng7	Rattus norvegicus calcium channel, voltage-dependent, gamma subunit 7 (Cacng7), mRNA [NM_080695]	**0,768**
Nptx2	Rattus norvegicus neuronal pentraxin 2 (Nptx2), mRNA [NM_001034199]	**0,768**
Tgfb1i1	Rattus norvegicus transforming growth factor beta 1 induced transcript 1 (Tgfb1i1), mRNA [NM_001191840]	**0,768**
Brunol5	Rattus norvegicus bruno-like 5, RNA binding protein (Drosophila) (Brunol5), mRNA [NM_001135603]	**0,768**
Gcc2	Rattus norvegicus GRIP and coiled-coil domain containing 2 (Gcc2), mRNA [NM_001107633]	**0,768**
Zbtb6	Rattus norvegicus zinc finger and BTB domain containing 6 (Zbtb6), mRNA [NM_001108953]	**0,769**
Olr160	Rattus norvegicus olfactory receptor 160 (Olr160), mRNA [NM_001000738]	**0,769**
Rhob	Rattus norvegicus ras homolog gene family, member B (Rhob), mRNA [NM_022542]	**0,769**
Uqcr11	Rattus norvegicus ubiquinol-cytochrome c reductase, complex III subunit XI (Uqcr11), nuclear gene encoding mitochondrial protein, mRNA [NM_001126097]	**0,769**
Arf3	Rattus norvegicus ADP-ribosylation factor 3 (Arf3), mRNA [NM_080904]	**0,769**
Park7	Rattus norvegicus Parkinson disease (autosomal recessive, early onset) 7, mRNA (cDNA clone MGC:108615 IMAGE:7376914), complete cds. [BC091128]	**0,769**
Spry3	Rattus norvegicus sprouty homolog 3 (Drosophila) (Spry3), mRNA [NM_001109063]	**0,769**
Acvr1	Rattus norvegicus activin A receptor, type I (Acvr1), mRNA [NM_024486]	**0,769**
Ndufs4	Rattus norvegicus NADH dehydrogenase (ubiquinone) Fe-S protein 4 (Ndufs4), nuclear gene encoding mitochondrial protein, mRNA [NM_001025146]	**0,770**

RGD1307399	Rattus norvegicus similar to chromosome 20 open reading frame 30; HSPC274 protein (RGD1307399), mRNA [NM_001048043]	**0,770**
0	Unknown	**0,770**
Tmem158	Rattus norvegicus transmembrane protein 158 (Tmem158), mRNA [NM_057212]	**0,770**
Rhoh	Rattus norvegicus ras homolog gene family, member H (Rhoh), mRNA [NM_001013430]	**0,770**
LOC691241	PREDICTED: Rattus norvegicus similar to MIC2 like 1 (LOC691241), mRNA [XM_001077347]	**0,770**
Sorbs3	Rattus norvegicus sorbin and SH3 domain containing 3 (Sorbs3), mRNA [NM_001005762]	**0,770**
Dnajc16	Rattus norvegicus DnaJ (Hsp40) homolog, subfamily C, member 16 (Dnajc16), mRNA [NM_001014194]	**0,771**
0	PREDICTED: Rattus norvegicus similar to hypothetical protein 4930509O22 (RGD1566007), mRNA [XM_346086]	**0,771**
Prokr2	Rattus norvegicus prokineticin receptor 2 (Prokr2), mRNA [NM_138978]	**0,771**
0	Cytochrome c oxidase subunit 1 [Source:UniProtKB/Swiss-Prot;Acc:P05503] [ENSRNOT00000050156]	**0,771**
LOC689656	Uncharacterized protein [Source:UniProtKB/TrEMBL;Acc:D3ZUC4] [ENSRNOT00000039322]	**0,771**
Olr92	Rattus norvegicus olfactory receptor 92 (Olr92), mRNA [NM_001000140]	**0,771**
Patl1	Rattus norvegicus protein associated with topoisomerase II homolog 1 (yeast) (Patl1), mRNA [NM_001108520]	**0,772**
Cndp1	Rattus norvegicus carnosine dipeptidase 1 (metallopeptidase M20 family) (Cndp1), mRNA [NM_001007687]	**0,772**
Bmp15	Rattus norvegicus bone morphogenetic protein 15 (Bmp15), mRNA [NM_021670]	**0,772**
Khsrp	Rattus norvegicus KH-type splicing regulatory protein (Khsrp), mRNA [NM_133602]	**0,772**
LOC100365858	PREDICTED: Rattus norvegicus zinc finger CCCH type, antiviral 1-like (LOC100365858), miscRNA [XR_085680]	**0,772**
Yap1	Rattus norvegicus yes-associated protein 1 (Yap1), mRNA [NM_001034002]	**0,772**
Mef2d	Rattus norvegicus myocyte enhancer factor 2D (Mef2d), mRNA [NM_030860]	**0,772**
Myef2	Rattus norvegicus myelin expression factor 2 (Myef2), mRNA [NM_001013205]	**0,772**
Pitpnc1	Uncharacterized protein [Source:UniProtKB/TrEMBL;Acc:D4AE57] [ENSRNOT00000021498]	**0,773**
Tmie	Rattus norvegicus transmembrane inner ear (Tmie), mRNA [NM_001109299]	**0,773**
0	Unknown	**0,773**
Galt	Rattus norvegicus galactose-1-phosphate uridylyltransferase (Galt), mRNA [NM_001013089]	**0,773**
0	Rattus norvegicus similar to glyceraldehyde-3-phosphate dehydrogenase (LOC290492), mRNA [XM_224528]	**0,773**
0	Unknown	**0,773**
Zc3h3	Rattus norvegicus zinc finger CCCH type containing 3 (Zc3h3), mRNA [NM_001134865]	**0,773**
0	Q8IVU8_HUMAN (Q8IVU8) LOC340529 protein (Fragment), partial (54%) [TC642990]	**0,774**
Cln6	Rattus norvegicus ceroid-lipofuscinosis, neuronal 6 (Cln6), mRNA [NM_001191794]	**0,774**
Ccna1	Rattus norvegicus cyclin A1 (Ccna1), mRNA [NM_001011949]	**0,774**
RGD1563941	Rattus norvegicus similar to hypothetical protein FLJ20010 (RGD1563941), mRNA [NM_001109293]	**0,774**
Prdm4	Rattus norvegicus PR domain containing 4 (Prdm4), mRNA [NM_133312]	**0,774**
F2r	Rattus norvegicus coagulation factor II (thrombin) receptor (F2r), mRNA [NM_012950]	**0,774**
0	Unknown	**0,774**

Fam113b	Rattus norvegicus family with sequence similarity 113, member B (Fam113b), mRNA [NM_001039454]	**0,775**
0	Uncharacterized protein [Source:UniProtKB/TrEMBL;Acc:D3ZI73] [ENSRNOT00000026047]	**0,775**
0	Unknown	**0,775**
Nckap5l	Uncharacterized protein [Source:UniProtKB/TrEMBL;Acc:D3Z9A7] [ENSRNOT00000022937]	**0,775**
Afmid	Rattus norvegicus arylformamidase (Afmid), mRNA [NM_001111366]	**0,775**
0	Unknown	**0,775**
Bcl9	Rattus norvegicus B-cell CLL/lymphoma 9 (Bcl9), mRNA [NM_001107703]	**0,776**
Slamf8	Rattus norvegicus SLAM family member 8 (Slamf8), mRNA [NM_001105973]	**0,776**
0	Unknown	**0,776**
RGD1560601	PREDICTED: Rattus norvegicus similar to Jumonji/ARID domain-containing protein 1C (SmcX protein) (RGD1560601), mRNA [XM_001064297]	**0,776**
Foxj2	Rattus norvegicus forkhead box J2 (Foxj2), mRNA [NM_001109352]	**0,776**
0	Unknown	**0,776**
Fgf17	Rattus norvegicus fibroblast growth factor 17 (Fgf17), mRNA [NM_019198]	**0,776**
Lppr4	Rattus norvegicus lipid phosphate phosphatase-related protein type 4 (Lppr4), mRNA [NM_001001508]	**0,776**
Nfkb2	Rattus norvegicus nuclear factor of kappa light polypeptide gene enhancer in B-cells 2, p49/p100 (Nfkb2), mRNA [NM_001008349]	**0,776**
0	Unknown	**0,776**
Olr775	Rattus norvegicus olfactory receptor 775 (Olr775), mRNA [NM_001000374]	**0,776**
Tyrobp	Rattus norvegicus Tyro protein tyrosine kinase binding protein (Tyrobp), mRNA [NM_212525]	**0,776**
Cyp2j4	Rattus norvegicus cytochrome P450, family 2, subfamily j, polypeptide 4 (Cyp2j4), mRNA [NM_023025]	**0,776**
Gnaq	Rattus norvegicus guanine nucleotide binding protein (G protein), q polypeptide (Gnaq), mRNA [NM_031036]	**0,776**
Smtnl1	Rattus norvegicus smoothelin-like 1 (Smtnl1), mRNA [NM_001191739]	**0,776**
Sit1	Rattus norvegicus signaling threshold regulating transmembrane adaptor 1 (Sit1), mRNA [NM_001024344]	**0,777**
Nr1h4	Rattus norvegicus nuclear receptor subfamily 1, group H, member 4 (Nr1h4), mRNA [NM_021745]	**0,777**
0	AA957328 UI-R-E1-fu-g-08-0-UI.s1 UI-R-E1 Rattus norvegicus cDNA clone UI-R-E1-fu-g-08-0-UI 3', mRNA sequence [AA957328]	**0,777**
0	Unknown	**0,777**
RGD1565164	PREDICTED: Rattus norvegicus similar to associated molecule with the SH3 domain of STAM (RGD1565164), mRNA [XM_001053795]	**0,777**
Arl8a	Rattus norvegicus ADP-ribosylation factor-like 8A (Arl8a), mRNA [NM_001109071]	**0,777**
Atxn7l1	Uncharacterized protein [Source:UniProtKB/TrEMBL;Acc:D3ZHW8] [ENSRNOT00000014054]	**0,778**
0	Unknown	**0,778**
0	Unknown	**0,778**
Eci3	Rattus norvegicus enoyl-Coenzyme A delta isomerase 3 (Eci3), mRNA [NM_001009275]	**0,778**
LOC689996	Uncharacterized protein [Source:UniProtKB/TrEMBL;Acc:D3Z801] [ENSRNOT00000058976]	**0,778**
Crtc2	Rattus norvegicus CREB regulated transcription coactivator 2 (Crtc2), mRNA [NM_001033895]	**0,779**
Cdc42bpa	Rattus norvegicus CDC42 binding protein kinase alpha (Cdc42bpa), mRNA [NM_053657]	**0,779**

Shc1	Rattus norvegicus SHC (Src homology 2 domain containing) transforming protein 1 (Shc1), nuclear gene encoding mitochondrial protein, transcript variant 1, mRNA [NM_001164060]	**0,779**
0	Q53FN7_HUMAN (Q53FN7) BZW1 protein variant (Fragment), complete [TC583097]	**0,779**
Iffo2	Rattus norvegicus intermediate filament family orphan 2 (Iffo2), mRNA [NM_001134703]	**0,779**
Olr791	Rattus norvegicus olfactory receptor 791 (Olr791), mRNA [NM_001000580]	**0,780**
RGD1308722	Rattus norvegicus similar to RIKEN cDNA A130042E20; open reading frame 57 (RGD1308722), mRNA [NM_001108529]	**0,780**
0	Unknown	**0,780**
0	Oct 2 Pou-Homeo domain protein [Source:UniProtKB/TrEMBL;Acc:Q63388] [ENSRNOT00000027655]	**0,781**
Dync1i2	Rattus norvegicus dynein cytoplasmic 1 intermediate chain 2 (Dync1i2), mRNA [NM_053880]	**0,781**
Nrgn	Rattus norvegicus neurogranin (Nrgn), mRNA [NM_024140]	**0,781**
Nkx3-1	Rattus norvegicus NK3 homeobox 1 (Nkx3-1), mRNA [NM_001034144]	**0,781**
RGD1560691	Rattus norvegicus similar to calcium/calmodulin-dependent protein kinase 1D (RGD1560691), mRNA [NM_001107365]	**0,781**
Wdfy3	Rattus norvegicus WD repeat and FYVE domain containing 3 (Wdfy3), mRNA [NM_001170551]	**0,781**
0	Unknown	**0,781**
Itga10	Rattus norvegicus integrin, alpha 10 (Itga10), mRNA [NM_001107699]	**0,782**
Vom2r41	Rattus norvegicus vomeronasal 2 receptor, 41 (Vom2r41), mRNA [NM_001099500]	**0,782**
Ndst1	Rattus norvegicus N-deacetylase/N-sulfotransferase (heparan glucosaminyl) 1 (Ndst1), mRNA [NM_024361]	**0,783**
RGD1566102	PREDICTED: Rattus norvegicus RGD1566102 (RGD1566102), mRNA [XM_001056364]	**0,783**
LOC690479	PREDICTED: Rattus norvegicus hypothetical protein LOC690479 (LOC690479), mRNA [XM_001074561]	**0,783**
Vti1a	Rattus norvegicus vesicle transport through interaction with t-SNAREs homolog 1A (yeast) (Vti1a), mRNA [NM_023101]	**0,783**
Rhpn2	Rattus norvegicus rhophilin, Rho GTPase binding protein 2 (Rhpn2), mRNA [NM_001107505]	**0,784**
0	Unknown	**0,784**
Nfia	Rattus norvegicus nuclear factor I/A (Nfia), mRNA [NM_012988]	**0,784**
0	Solute carrier family 25 member 40 [Source:UniProtKB/Swiss-Prot;Acc:Q498U3] [ENSRNOT00000030352]	**0,784**
0	Somatoliberin [Source:UniProtKB/Swiss-Prot;Acc:P09916] [ENSRNOT00000010298]	**0,784**
Wipf3	Rattus norvegicus WAS/WASL interacting protein family, member 3 (Wipf3), mRNA [NM_147211]	**0,784**
Ipcef1	Rattus norvegicus interactor protein for cytohesin exchange factors 1 (Ipcef1), mRNA [NM_001170799]	**0,784**
Slc35e1	Rattus norvegicus solute carrier family 35, member E1 (Slc35e1), mRNA [NM_001109107]	**0,785**
LOC683746	PREDICTED: Rattus norvegicus similar to thyroid autoantigen (LOC683746), mRNA [XM_001067309]	**0,785**
Gabrb2	Rattus norvegicus gamma-aminobutyric acid (GABA) A receptor, beta 2 (Gabrb2), mRNA [NM_012957]	**0,786**
RGD1565648	PREDICTED: Rattus norvegicus protein S100-A11-like (LOC100362296), mRNA [XM_002729212]	**0,786**
Spert	Rattus norvegicus spermatid associated (Spert), mRNA [NM_001017506]	**0,786**
0	Unknown	**0,787**
Lypd6b	Rattus norvegicus LY6/PLAUR domain containing 6B (Lypd6b), mRNA [NM_001134580]	**0,787**

Prm3	Rattus norvegicus protamine 3 (Prm3), mRNA [NM_001002855]	**0,787**
Vom2r12	Rattus norvegicus vomeronasal 2 receptor, 12 (Vom2r12), mRNA [NM_001099488]	**0,787**
0	Unknown	**0,788**
0	Rattus norvegicus similar to gtf2ird2 (LOC360795), mRNA [XM_341065]	**0,788**
0	Unknown	**0,788**
0	Unknown	**0,788**
Tulp4	Rattus norvegicus tubby like protein 4 (Tulp4), mRNA [NM_001109137]	**0,788**
RGD1561678	PREDICTED: Rattus norvegicus similar to Ten-m1 (RGD1561678), mRNA [XM_002730177]	**0,788**
Eci2	Rattus norvegicus enoyl-Coenzyme A delta isomerase 2 (Eci2), mRNA [NM_001006966]	**0,788**
0	Unknown	**0,789**
Phka2	Rattus norvegicus phosphorylase kinase, alpha 2 (Phka2), mRNA [NM_001190994]	**0,789**
Afap1	Rattus norvegicus actin filament associated protein 1 (Afap1), mRNA [NM_080900]	**0,789**
0	Unknown	**0,790**
0	Unknown	**0,790**
RGD1310453	Rattus norvegicus similar to hypothetical protein FLJ23451 (RGD1310453), mRNA [NM_001134463]	**0,790**
Plxdc1	Rattus norvegicus plexin domain containing 1 (Plxdc1), mRNA [NM_001107046]	**0,790**
Ager	Rattus norvegicus advanced glycosylation end product-specific receptor (Ager), mRNA [NM_053336]	**0,791**
Dand5	Uncharacterized protein [Source:UniProtKB/TrEMBL;Acc:D3ZGN3] [ENSRNOT00000044133]	**0,791**
Rsb66	Rattus norvegicus Rsb-66 protein (Rsb66), mRNA [NM_181694]	**0,792**
Evx1	Rattus norvegicus even-skipped homeobox 1 (Evx1), mRNA [NM_001191972]	**0,792**
Bcr	PREDICTED: Rattus norvegicus breakpoint cluster region (Bcr), mRNA [XM_228091]	**0,792**
0	Unknown	**0,792**
Calhm2	Rattus norvegicus calcium homeostasis modulator 2 (Calhm2), mRNA [NM_001008306]	**0,792**
0	Unknown	**0,792**
Usp31	Rattus norvegicus ubiquitin specific peptidase 31 (Usp31), mRNA [NM_001107548]	**0,792**
Tgfbr2	Rattus norvegicus transforming growth factor, beta receptor II (Tgfbr2), mRNA [NM_031132]	**0,792**
Aldh9a1	Rattus norvegicus aldehyde dehydrogenase 9 family, member A1 (Aldh9a1), mRNA [NM_022273]	**0,792**
Agfg2	Rattus norvegicus ArfGAP with FG repeats 2 (Agfg2), mRNA [NM_001107131]	**0,793**
Clec12b	Rattus norvegicus C-type lectin domain family 12, member B (Clec12b), mRNA [NM_001109353]	**0,793**
Olr1l	Rattus norvegicus olfactory receptor 1-like (Olr1l), mRNA [NM_001000538]	**0,793**
Lyst	Rattus norvegicus lysosomal trafficking regulator (Lyst), mRNA [NM_053518]	**0,793**
Prkce	Rattus norvegicus protein kinase C, epsilon (Prkce), mRNA [NM_017171]	**0,794**
Fam71a	Rattus norvegicus family with sequence similarity 71, member A (Fam71a), mRNA [NM_001109084]	**0,794**
Nfya	Rattus norvegicus nuclear transcription factor-Y alpha (Nfya), mRNA [NM_012865]	**0,794**

Tmprss7	Rattus norvegicus transmembrane serine protease 7 (Tmprss7), mRNA [NM_001105882]	**0,794**
Myh3	Rattus norvegicus myosin, heavy chain 3, skeletal muscle, embryonic (Myh3), mRNA [NM_012604]	**0,794**
Abtb2	Rattus norvegicus ankyrin repeat and BTB (POZ) domain containing 2 (Abtb2), mRNA [NM_134403]	**0,795**
Scaf1	Rattus norvegicus SR-related CTD-associated factor 1 (Scaf1), mRNA [NM_019384]	**0,796**
0	Unknown	**0,796**
0	Unknown	**0,796**
Chst12	Rattus norvegicus carbohydrate (chondroitin 4) sulfotransferase 12 (Chst12), mRNA [NM_001037775]	**0,797**
0	Q80UV2_MOUSE (Q80UV2) AU042671 protein, partial (40%) [TC598606]	**0,797**
Nedd9	Rattus norvegicus neural precursor cell expressed, developmentally down-regulated 9 (Nedd9), mRNA [NM_001011922]	**0,797**
Coq10a	Rattus norvegicus coenzyme Q10 homolog A (S. cerevisiae) (Coq10a), nuclear gene encoding mitochondrial protein, mRNA [NM_001108727]	**0,797**
0	Uncharacterized protein [Source:UniProtKB/TrEMBL;Acc:D3ZD32] [ENSRNOT00000067364]	**0,797**
0	Unknown	**0,798**
Tbx19	Rattus norvegicus T-box 19 (Tbx19), mRNA [NM_001107193]	**0,798**
Tgfb2	Rattus norvegicus transforming growth factor, beta 2 (Tgfb2), mRNA [NM_031131]	**0,798**
0	Unknown	**0,799**
M6prbp1	PREDICTED: Rattus norvegicus mannose-6-phosphate receptor binding protein 1 (M6prbp1), mRNA [XM_001061015]	**0,799**
Sbno1	Rattus norvegicus strawberry notch homolog 1 (Drosophila) (Sbno1), mRNA [NM_001107138]	**0,799**
Rps6ka2	Rattus norvegicus ribosomal protein S6 kinase polypeptide 2 (Rps6ka2), mRNA [NM_057128]	**0,799**
0	Unknown	**0,799**
Per3	Rattus norvegicus period homolog 3 (Drosophila) (Per3), mRNA [NM_023978]	**0,800**
Gnat1	Rattus norvegicus guanine nucleotide binding protein (G protein), alpha transducing 1 (Gnat1), mRNA [NM_001108780]	**0,800**
Tsga10	Rattus norvegicus testis specific 10 (Tsga10), mRNA [NM_001030022]	**0,800**
0	Unknown	**0,800**
Mthfr	Methylenetetrahydrofolate reductase [Source:UniProtKB/TrEMBL;Acc:D4A7E8] [ENSRNOT00000011384]	**0,800**
Dlc1	Rattus norvegicus deleted in liver cancer 1 (Dlc1), mRNA [NM_001127446]	**0,801**
Tmem86b	Rattus norvegicus transmembrane protein 86B (Tmem86b), mRNA [NM_001109604]	**0,801**
Gigyf1	Rattus norvegicus GRB10 interacting GYF protein 1 (Gigyf1), mRNA [NM_001107133]	**0,801**
Pdgfc	Rattus norvegicus platelet derived growth factor C (Pdgfc), mRNA [NM_031317]	**0,801**
0	Uncharacterized protein [Source:UniProtKB/TrEMBL;Acc:D3ZRD4] [ENSRNOT00000054998]	**0,802**
0	Rattus norvegicus similar to Zfp-29 (LOC365296), mRNA [XM_344914]	**0,802**
Tnip1	Rattus norvegicus TNFAIP3 interacting protein 1 (Tnip1), mRNA [NM_001108826]	**0,802**
RGD1563667	Rattus norvegicus similar to TDPOZ3 (RGD1563667), mRNA [NM_001100985]	**0,803**
Nrep	Rattus norvegicus neuronal regeneration related protein (Nrep), mRNA [NM_178096]	**0,803**
Zfp39	Rattus norvegicus zinc finger protein 39 (Zfp39), mRNA [NM_001107004]	**0,803**

Otor	Rattus norvegicus otoraplin (Otor), mRNA [NM_001108960]	**0,803**
0	Unknown	**0,803**
0	Rattus norvegicus similar to putative pheromone receptor (LOC301894), mRNA [XM_229376]	**0,803**
RGD1565693	PREDICTED: Rattus norvegicus similar to GLE1-like, RNA export mediator isoform 1 (RGD1565693), mRNA [XM_001059991]	**0,804**
0	Unknown	**0,804**
Scgb3a1	Rattus norvegicus secretoglobin, family 3A, member 1 (Scgb3a1), mRNA [NM_001013180]	**0,804**
Usp25	Rattus norvegicus ubiquitin specific peptidase 25 (Usp25), mRNA [NM_001107114]	**0,804**
Actl7a	Rattus norvegicus actin-like 7a (Actl7a), mRNA [NM_001011973]	**0,806**
Olr733	Rattus norvegicus olfactory receptor 733 (Olr733), mRNA [NM_001000618]	**0,806**
Cacna1e	Rattus norvegicus calcium channel, voltage-dependent, R type, alpha 1E subunit (Cacna1e), mRNA [NM_019294]	**0,807**
0	Unknown	**0,807**
0	Unknown	**0,807**
0	Unknown	**0,808**
Gtf3c4	Rattus norvegicus general transcription factor IIIC, polypeptide 4 (Gtf3c4), mRNA [NM_001109473]	**0,808**
0	Unknown	**0,808**
Foxp4	Rattus norvegicus forkhead box P4 (Foxp4), mRNA [NM_001108788]	**0,809**
0	Unknown	**0,809**
Csnk1d	Rattus norvegicus casein kinase 1, delta (Csnk1d), mRNA [NM_139060]	**0,811**
Vom1r40	Rattus norvegicus vomeronasal 1 receptor 40 (Vom1r40), mRNA [NM_001008904]	**0,811**
LOC500035	Rattus norvegicus hypothetical protein LOC500035 (LOC500035), mRNA [NM_001047955]	**0,811**
0	Rattus norvegicus similar to olfactory receptor MOR111-1 (LOC288810), mRNA [XM_222344]	**0,812**
Pi16	Rattus norvegicus peptidase inhibitor 16 (Pi16), mRNA [NM_001170481]	**0,813**
Dnm3	Rattus norvegicus dynamin 3 (Dnm3), mRNA [NM_138538]	**0,813**
Nanos3	Rattus norvegicus nanos homolog 3 (Drosophila) (Nanos3), mRNA [NM_001105945]	**0,814**
H2afx	Rattus norvegicus H2A histone family, member X (H2afx), mRNA [NM_001109291]	**0,815**
Cox6b2	Rattus norvegicus cytochrome c oxidase subunit VIb polypeptide 2 (Cox6b2), mRNA [NM_001039085]	**0,816**
Hba-a2	Rattus norvegicus hemoglobin alpha, adult chain 2 (Hba-a2), mRNA [NM_013096]	**0,816**
Adamts2	Rattus norvegicus ADAM metallopeptidase with thrombospondin type 1 motif, 2 (Adamts2), mRNA [NM_001137622]	**0,816**
0	Productively rearranged V-lambda-2 [Source:UniProtKB/TrEMBL;Acc:A0N4E8] [ENSRNOT00000036565]	**0,817**
Rhbdf2	Rattus norvegicus rhomboid 5 homolog 2 (Drosophila) (Rhbdf2), mRNA [NM_001107067]	**0,817**
LOC684993	Rattus norvegicus hypothetical protein LOC684993 (LOC684993), mRNA [NM_001109451]	**0,817**
Pcdhga2	Rattus norvegicus protocadherin gamma subfamily A, 2 (Pcdhga2), mRNA [NM_001037139]	**0,817**
0	Unknown	**0,817**
Sp9	Rattus norvegicus trans-acting transcription factor 9 (Sp9), mRNA [NM_001191902]	**0,819**

Zc3h12c	Rattus norvegicus zinc finger CCCH type containing 12C (Zc3h12c), mRNA [NM_001108146]	**0,819**
Syn1	Rattus norvegicus synapsin I (Syn1), transcript variant a, mRNA [NM_019133]	**0,819**
0	Unknown	**0,819**
LOC685125	Rattus norvegicus cDNA clone IMAGE:7132976. [BC166431]	**0,820**
0	Unknown	**0,820**
Sec14l5	Rattus norvegicus SEC14-like 5 (S. cerevisiae) (Sec14l5), mRNA [NM_001135710]	**0,820**
Pcsk4	Rattus norvegicus proprotein convertase subtilisin/kexin type 4 (Pcsk4), mRNA [NM_133559]	**0,820**
0	Unknown	**0,820**
Eif4e	Rattus norvegicus eukaryotic translation initiation factor 4E (Eif4e), mRNA [NM_053974]	**0,821**
Olr472	Rattus norvegicus olfactory receptor 472 (Olr472), mRNA [NM_001000301]	**0,821**
Olr402	Rattus norvegicus olfactory receptor 402 (Olr402), mRNA [NM_001001014]	**0,822**
Nol4	Rattus norvegicus nucleolar protein 4 (Nol4), mRNA [NM_001107401]	**0,822**
0	LRRG00131 [Source:UniProtKB/TrEMBL;Acc:Q6QI77] [ENSRNOT00000051980]	**0,822**
Gpr132	Rattus norvegicus G protein-coupled receptor 132 (Gpr132), mRNA [NM_001170595]	**0,822**
Tmc4	Rattus norvegicus transmembrane channel-like 4 (Tmc4), mRNA [NM_001034104]	**0,822**
Acr	Rattus norvegicus acrosin (Acr), mRNA [NM_012490]	**0,823**
Lrba	Rattus norvegicus LPS-responsive vesicle trafficking, beach and anchor containing (Lrba), mRNA [NM_001108555]	**0,824**
Cyp4a1	Rattus norvegicus cytochrome P450, family 4, subfamily a, polypeptide 1 (Cyp4a1), mRNA [NM_175837]	**0,824**
Stox2	Rattus norvegicus storkhead box 2 (Stox2), transcript variant 2, mRNA [NM_173107]	**0,825**
Man2b1	Rattus norvegicus mannosidase, alpha, class 2B, member 1 (Man2b1), mRNA [NM_199404]	**0,825**
Rexo1	Rattus norvegicus REX1, RNA exonuclease 1 homolog (S. cerevisiae) (Rexo1), mRNA [NM_001012114]	**0,826**
Olr1238	Rattus norvegicus olfactory receptor 1238 (Olr1238), mRNA [NM_001001013]	**0,827**
Dlx3	Rattus norvegicus distal-less homeobox 3 (Dlx3), mRNA [NM_001105832]	**0,827**
0	Rattus norvegicus, 25 clones, strain BN/SsNHsdMCW RNOR03324481, whole genome shotgun sequence [AABR03128056]	**0,828**
0	Unknown	**0,828**
Bsn	Rattus norvegicus bassoon (Bsn), mRNA [NM_019146]	**0,829**
Tmem211	Uncharacterized protein [Source:UniProtKB/TrEMBL;Acc:D3Z827] [ENSRNOT00000043717]	**0,829**
LOC681849	Uncharacterized protein [Source:UniProtKB/TrEMBL;Acc:D4A3C4] [ENSRNOT00000007809]	**0,831**
Gphb5	Rattus norvegicus glycoprotein hormone beta 5 (Gphb5), mRNA [NM_001007013]	**0,831**
0	Rattus norvegicus similar to Pyruvate kinase, M2 isozyme (LOC301604), mRNA [XM_237391]	**0,832**
Fchsd1	Rattus norvegicus FCH and double SH3 domains 1 (Fchsd1), transcript variant 2, mRNA [NM_001107392]	**0,833**
LOC688507	PREDICTED: Rattus norvegicus similar to Spetex-2F protein (LOC688507), mRNA [XM_001067216]	**0,837**
0	Unknown	**0,838**
Cplx3	Rattus norvegicus complexin 3 (Cplx3), mRNA [NM_001109295]	**0,838**

Ccdc147	Uncharacterized protein [Source:UniProtKB/TrEMBL;Acc:D3ZGP0] [ENSRNOT00000017283]	**0,839**
Elmo3	Rattus norvegicus engulfment and cell motility 3 (Elmo3), mRNA [NM_001030028]	**0,839**
0	Unknown	**0,841**
Sry	Rattus norvegicus sex determining region Y (Sry), mRNA [NM_012772]	**0,842**
Npb	Rattus norvegicus neuropeptide B (Npb), mRNA [NM_153293]	**0,843**
Mylk2	Rattus norvegicus myosin light chain kinase 2 (Mylk2), mRNA [NM_057209]	**0,843**
0	Unknown	**0,843**
Tox	Rattus norvegicus thymocyte selection-associated high mobility group box (Tox), mRNA [NM_001108654]	**0,844**
Vom1r30	Rattus norvegicus vomeronasal 1 receptor 30 (Vom1r30), mRNA [NM_001009511]	**0,844**
Arntl2	Rattus norvegicus aryl hydrocarbon receptor nuclear translocator-like 2 (Arntl2), mRNA [NM_133391]	**0,845**
Adprhl1	Rattus norvegicus ADP-ribosylhydrolase like 1 (Adprhl1), mRNA [NM_001013054]	**0,848**
Sf1	Rattus norvegicus splicing factor 1 (Sf1), transcript variant 2, mRNA [NM_058210]	**0,848**
Pou3f1	Rattus norvegicus POU class 3 homeobox 1 (Pou3f1), mRNA [NM_138838]	**0,848**
LOC685249	PREDICTED: Rattus norvegicus hypothetical protein LOC685249 (LOC685249), mRNA [XM_001063008]	**0,851**
Psd	Rattus norvegicus pleckstrin and Sec7 domain containing (Psd), mRNA [NM_134370]	**0,853**
LOC688298	Rattus norvegicus hypothetical protein LOC688298 (LOC688298), mRNA [NM_001143896]	**0,854**
Myl9	Rattus norvegicus myosin, light chain 9, regulatory (Myl9), mRNA [NM_001100885]	**0,854**
RGD1561563	PREDICTED: Rattus norvegicus similar to RIKEN cDNA 1700013B16 (RGD1561563), mRNA [XM_001066615]	**0,855**
0	Unknown	**0,856**
Lipn	Rattus norvegicus lipase, family member N (Lipn), mRNA [NM_001191955]	**0,857**
RGD1564927	Rattus norvegicus similar to TGFB-induced factor 2 (RGD1564927), mRNA [NM_001134983]	**0,858**
H1foo	Rattus norvegicus H1 histone family, member O, oocyte-specific (H1foo), mRNA [NM_001109351]	**0,858**
RGD1564031	PREDICTED: Rattus norvegicus similar to transcription elongation factor B (SIII), polypeptide 2 (RGD1564031), mRNA [XM_001081217]	**0,861**
0	membrane-bound transcription factor site-2 protease [Source:RefSeq peptide;Acc:NP_001030179] [ENSRNOT00000010089]	**0,863**
0	RNCAII8 carbonic anhydrase II {Rattus norvegicus} (exp=-1; wgp=0; cg=0), partial (32%) [TC633165]	**0,866**
Wfdc10	Rattus norvegicus WAP four-disulfide core domain 10 (Wfdc10), mRNA [NM_001109461]	**0,867**
Nanos1	Uncharacterized protein [Source:UniProtKB/TrEMBL;Acc:D4A1F8] [ENSRNOT00000036203]	**0,868**
Grm2	Rattus norvegicus glutamate receptor, metabotropic 2 (Grm2), mRNA [NM_001105711]	**0,871**
0	Unknown	**0,872**
Nt5dc3	Rattus norvegicus 5'-nucleotidase domain containing 3 (Nt5dc3), mRNA [NM_001134887]	**0,874**
0	Unknown	**0,874**
Tuba3b	Rattus norvegicus tubulin, alpha 3B (Tuba3b), mRNA [NM_001024336]	**0,884**
Abpa	Rattus norvegicus androgen binding protein, alpha (Abpa), mRNA [NM_001100859]	**0,885**
RGD1310717	Rattus norvegicus similar to RIKEN cDNA E030002O03 (RGD1310717), mRNA [NM_001106287]	**0,887**

Lrp6	Rattus norvegicus low density lipoprotein receptor-related protein 6 (Lrp6), mRNA [NM_001107892]	**0,891**
Gstm4	Rattus norvegicus glutathione S-transferase mu 4 (Gstm4), mRNA [NM_001024304]	**0,892**
0	PREDICTED: Rattus norvegicus similar to putative nucleic acid binding protein RY-1 (LOC685367), mRNA [XM_001063506]	**0,897**
Clrn2	Rattus norvegicus clarin 2 (Clrn2), mRNA [NM_001191097]	**0,904**
Npas4	Rattus norvegicus neuronal PAS domain protein 4 (Npas4), mRNA [NM_153626]	**2,245**
