# Supplementary material for: The RNA-Binding Protein hnRNP K Mediates the Effect of BDNF on Dendritic mRNA Metabolism and Regulates Synaptic NMDA Receptors in Hippocampal Neurons
Source: eNeuro. 2017 Dec 12;4(6):ENEURO.0268-17.2017. doi: 10.1523/ENEURO.0268-17.2017 (PMC5732018; doi:10.1523/ENEURO.0268-17.2017)
Supplement: Table 1-6 — Download Table 1-6, PDF file. [file sup_enu-eN-NWR-0268-17-s09.pdf]

**Table 1-6 - Biological processes associated with mRNAs co-immunoprecipitated with hnRNP K and regulated by BDNF as assessed by Gene Ontology**

| GO biological process | Rattus norvegicus (reference list) | uploaded list | (expected) | (over/under) | (fold enrichment) | (P-value) |
|---|---|---|---|---|---|---|
| organelle transport along microtubule (GO:0072384) | 55 | 35 | 14.20 | + | 2.47 | 1.81E-02 |
| central nervous system neuron development (GO:0021954) | 85 | 53 | 21.94 | + | 2.42 | 1.07E-04 |
| negative regulation of protein depolymerization (GO:1901880) | 55 | 34 | 14.20 | + | 2.40 | 4.55E-02 |
| dendrite development (GO:0016358) | 106 | 65 | 27.36 | + | 2.38 | 5.08E-06 |
| membrane docking (GO:0022406) | 64 | 38 | 16.52 | + | 2.30 | 3.32E-02 |
| regulation of protein depolymerization (GO:1901879) | 75 | 44 | 19.36 | + | 2.27 | 8.34E-03 |
| regulation of dendrite morphogenesis (GO:0048814) | 94 | 55 | 24.26 | + | 2.27 | 4.49E-04 |
| neurotransmitter secretion (GO:0007269) | 102 | 59 | 26.33 | + | 2.24 | 2.24E-04 |
| signal release from synapse (GO:0099643) | 102 | 59 | 26.33 | + | 2.24 | 2.24E-04 |
| presynaptic process involved in chemical synaptic transmission (GO:0099531) | 106 | 61 | 27.36 | + | 2.23 | 1.58E-04 |
| regulation of protein complex disassembly (GO:0043244) | 87 | 50 | 22.46 | + | 2.23 | 2.91E-03 |
| regulation of neurotransmitter transport (GO:0051588) | 70 | 40 | 18.07 | + | 2.21 | 4.57E-02 |
| positive regulation of dendrite development (GO:1900006) | 91 | 52 | 23.49 | + | 2.21 | 2.04E-03 |
| establishment of vesicle localization (GO:0051650) | 160 | 91 | 41.30 | + | 2.20 | 1.16E-07 |
| vesicle localization (GO:0051648) | 169 | 96 | 43.62 | + | 2.20 | 3.48E-08 |
| synaptic vesicle localization (GO:0097479) | 110 | 62 | 28.39 | + | 2.18 | 2.50E-04 |
| regulation of protein dephosphorylation (GO:0035304) | 88 | 49 | 22.71 | + | 2.16 | 9.02E-03 |
| amino acid transport (GO:0006865) | 90 | 50 | 23.23 | + | 2.15 | 7.53E-03 |
| Golgi organization (GO:0007030) | 90 | 50 | 23.23 | + | 2.15 | 7.53E-03 |
| synaptic vesicle transport (GO:0048489) | 108 | 60 | 27.88 | + | 2.15 | 6.67E-04 |
| establishment of synaptic vesicle localization (GO:0097480) | 108 | 60 | 27.88 | + | 2.15 | 6.67E-04 |
| positive regulation of exocytosis (GO:0045921) | 83 | 46 | 21.42 | + | 2.15 | 2.12E-02 |
| regulation of axonogenesis (GO:0050770) | 181 | 100 | 46.72 | + | 2.14 | 5.85E-08 |
| protein dephosphorylation (GO:0006470) | 176 | 97 | 45.43 | + | 2.14 | 1.36E-07 |
| regulation of synapse organization (GO:0050807) | 120 | 66 | 30.97 | + | 2.13 | 2.25E-04 |
| negative regulation of protein serine/threonine kinase activity (GO:0071901) | 122 | 67 | 31.49 | + | 2.13 | 1.87E-04 |
| hippocampus development (GO:0021766) | 106 | 58 | 27.36 | + | 2.12 | 1.75E-03 |
| regulation of potassium ion transport (GO:0043266) | 86 | 47 | 22.20 | + | 2.12 | 2.39E-02 |
| regulation of proteasomal ubiquitin-dependent protein catabolic process (GO:0032434) | 105 | 57 | 27.10 | + | 2.10 | 2.83E-03 |
| synaptic vesicle cycle (GO:0099504) | 94 | 51 | 24.26 | + | 2.10 | 1.14E-02 |
| regulation of extent of cell growth (GO:0061387) | 115 | 62 | 29.68 | + | 2.09 | 1.13E-03 |
| regulation of cell morphogenesis involved in differentiation (GO:0010769) | 366 | 197 | 94.47 | + | 2.09 | 8.25E-17 |
| regulation of neuron projection development (GO:0010975) | 498 | 268 | 128.54 | + | 2.09 | 8.31E-24 |
| negative regulation of neuron projection development (GO:0010977) | 147 | 79 | 37.94 | + | 2.08 | 2.81E-05 |

| | | | | | | |
|---|---|---|---|---|---|---|
| negative regulation of neuron differentiation (GO:0045665) | 222 | 119 | 57.30 | + | 2.08 | 4.41E-09 |
| regulation of dendrite development (GO:0050773) | 157 | 84 | 40.52 | + | 2.07 | 1.12E-05 |
| negative regulation of neurogenesis (GO:0050768) | 285 | 152 | 73.56 | + | 2.07 | 4.36E-12 |
| neuron projection development (GO:0031175) | 592 | 315 | 152.80 | + | 2.06 | 1.15E-27 |
| regulation of cell size (GO:0008361) | 192 | 102 | 49.56 | + | 2.06 | 3.10E-07 |
| neurotransmitter transport (GO:0006836) | 147 | 78 | 37.94 | + | 2.06 | 5.95E-05 |
| negative regulation of cell morphogenesis involved in differentiation (GO:0010771) | 115 | 61 | 29.68 | + | 2.06 | 2.41E-03 |
| negative regulation of cell development (GO:0010721) | 340 | 180 | 87.76 | + | 2.05 | 1.85E-14 |
| regulation of synapse structure or activity (GO:0050803) | 280 | 148 | 72.27 | + | 2.05 | 2.11E-11 |
| regulation of proteasomal protein catabolic process (GO:0061136) | 140 | 74 | 36.13 | + | 2.05 | 1.65E-04 |
| negative regulation of nervous system development (GO:0051961) | 307 | 162 | 79.24 | + | 2.04 | 1.20E-12 |
| regulation of neurotransmitter levels (GO:0001505) | 199 | 105 | 51.36 | + | 2.04 | 2.36E-07 |
| neuron projection morphogenesis (GO:0048812) | 425 | 224 | 109.69 | + | 2.04 | 2.20E-18 |
| positive regulation of neuron projection development (GO:0010976) | 293 | 154 | 75.62 | + | 2.04 | 9.15E-12 |
| positive regulation of proteolysis involved in cellular protein catabolic process (GO:1903052) | 99 | 52 | 25.55 | + | 2.04 | 2.24E-02 |
| synapse organization (GO:0050808) | 139 | 73 | 35.88 | + | 2.03 | 2.61E-04 |
| regulation of synaptic plasticity (GO:0048167) | 181 | 95 | 46.72 | + | 2.03 | 2.59E-06 |
| regulation of axon extension (GO:0030516) | 101 | 53 | 26.07 | + | 2.03 | 1.86E-02 |
| Golgi vesicle transport (GO:0048193) | 180 | 94 | 46.46 | + | 2.02 | 4.08E-06 |
| regulation of Rho protein signal transduction (GO:0035023) | 115 | 60 | 29.68 | + | 2.02 | 5.04E-03 |
| regulation of proteolysis involved in cellular protein catabolic process (GO:1903050) | 163 | 85 | 42.07 | + | 2.02 | 2.83E-05 |
| axon development (GO:0061564) | 359 | 187 | 92.66 | + | 2.02 | 1.97E-14 |
| mitotic cell cycle phase transition (GO:0044772) | 121 | 63 | 31.23 | + | 2.02 | 2.89E-03 |
| negative regulation of cell projection organization (GO:0031345) | 173 | 90 | 44.65 | + | 2.02 | 1.12E-05 |
| axonogenesis (GO:0007409) | 325 | 169 | 83.88 | + | 2.01 | 9.69E-13 |
| regulation of neuron apoptotic process (GO:0043523) | 243 | 126 | 62.72 | + | 2.01 | 8.33E-09 |
| learning (GO:0007612) | 159 | 82 | 41.04 | + | 2.00 | 8.45E-05 |
| cell cycle phase transition (GO:0044770) | 128 | 66 | 33.04 | + | 2.00 | 2.16E-03 |
| regulation of exocytosis (GO:0017157) | 183 | 94 | 47.23 | + | 1.99 | 9.10E-06 |
| positive regulation of cellular protein catabolic process (GO:1903364) | 113 | 58 | 29.17 | + | 1.99 | 1.25E-02 |
| neuron projection guidance (GO:0097485) | 195 | 100 | 50.33 | + | 1.99 | 2.99E-06 |
| negative regulation of neuron apoptotic process (GO:0043524) | 160 | 82 | 41.30 | + | 1.99 | 1.10E-04 |
| protein polyubiquitination (GO:0000209) | 162 | 83 | 41.81 | + | 1.99 | 9.13E-05 |
| positive regulation of cell growth (GO:0030307) | 162 | 83 | 41.81 | + | 1.99 | 9.13E-05 |
| phospholipid biosynthetic process (GO:0008654) | 123 | 63 | 31.75 | + | 1.98 | 4.92E-03 |
| cell morphogenesis involved in neuron differentiation (GO:0048667) | 389 | 199 | 100.40 | + | 1.98 | 1.02E-14 |
| dephosphorylation (GO:0016311) | 270 | 138 | 69.69 | + | 1.98 | 1.97E-09 |
| negative regulation of MAPK cascade (GO:0043409) | 155 | 79 | 40.01 | + | 1.97 | 2.49E-04 |

| | | | | | | |
|---|---|---|---|---|---|---|
| regulation of neuron differentiation (GO:0045664) | 662 | 337 | 170.87 | + | 1.97 | 1.93E-26 |
| regulation of cellular protein catabolic process (GO:1903362) | 183 | 93 | 47.23 | + | 1.97 | 1.84E-05 |
| developmental growth involved in morphogenesis (GO:0060560) | 122 | 62 | 31.49 | + | 1.97 | 7.71E-03 |
| cerebellum development (GO:0021549) | 126 | 64 | 32.52 | + | 1.97 | 5.30E-03 |
| axon guidance (GO:0007411) | 193 | 98 | 49.81 | + | 1.97 | 7.27E-06 |
| pallium development (GO:0021543) | 203 | 103 | 52.40 | + | 1.97 | 2.87E-06 |
| modulation of synaptic transmission (GO:0050804) | 340 | 172 | 87.76 | + | 1.96 | 5.81E-12 |
| regulation of cell projection organization (GO:0031344) | 657 | 331 | 169.58 | + | 1.95 | 3.70E-25 |
| regulation of ion transmembrane transporter activity (GO:0032412) | 191 | 96 | 49.30 | + | 1.95 | 1.75E-05 |
| regulation of transmembrane transporter activity (GO:0022898) | 197 | 99 | 50.85 | + | 1.95 | 1.00E-05 |
| limbic system development (GO:0021761) | 136 | 68 | 35.10 | + | 1.94 | 4.17E-03 |
| regulation of transporter activity (GO:0032409) | 210 | 105 | 54.20 | + | 1.94 | 4.22E-06 |
| regulation of neuron death (GO:1901214) | 318 | 159 | 82.08 | + | 1.94 | 1.80E-10 |
| positive regulation of cell projection organization (GO:0031346) | 372 | 186 | 96.02 | + | 1.94 | 1.15E-12 |
| regulation of Ras protein signal transduction (GO:0046578) | 200 | 100 | 51.62 | + | 1.94 | 1.07E-05 |
| regulation of cell morphogenesis (GO:0022604) | 582 | 289 | 150.22 | + | 1.92 | 1.10E-20 |
| organelle fusion (GO:0048284) | 127 | 63 | 32.78 | + | 1.92 | 1.36E-02 |
| regulation of cellular component size (GO:0032535) | 359 | 178 | 92.66 | + | 1.92 | 1.08E-11 |
| establishment or maintenance of cell polarity (GO:0007163) | 152 | 75 | 39.23 | + | 1.91 | 1.85E-03 |
| regulation of small GTPase mediated signal transduction (GO:0051056) | 223 | 110 | 57.56 | + | 1.91 | 3.47E-06 |
| metencephalon development (GO:0022037) | 138 | 68 | 35.62 | + | 1.91 | 6.84E-03 |
| regulation of neurogenesis (GO:0050767) | 802 | 395 | 207.00 | + | 1.91 | 1.06E-28 |
| establishment of protein localization to membrane (GO:0090150) | 173 | 85 | 44.65 | + | 1.90 | 3.65E-04 |
| negative regulation of cell growth (GO:0030308) | 173 | 85 | 44.65 | + | 1.90 | 3.65E-04 |
| chemical synaptic transmission (GO:0007268) | 334 | 164 | 86.21 | + | 1.90 | 3.06E-10 |
| trans-synaptic signaling (GO:0099537) | 334 | 164 | 86.21 | + | 1.90 | 3.06E-10 |
| synaptic signaling (GO:0099536) | 334 | 164 | 86.21 | + | 1.90 | 3.06E-10 |
| anterograde trans-synaptic signaling (GO:0098916) | 334 | 164 | 86.21 | + | 1.90 | 3.06E-10 |
| establishment of organelle localization (GO:0051656) | 314 | 154 | 81.05 | + | 1.90 | 1.98E-09 |
| telencephalon development (GO:0021537) | 292 | 143 | 75.37 | + | 1.90 | 1.53E-08 |
| positive regulation of neuron differentiation (GO:0045666) | 384 | 188 | 99.11 | + | 1.90 | 5.55E-12 |
| neuron development (GO:0048666) | 760 | 372 | 196.16 | + | 1.90 | 2.43E-26 |
| regulation of nervous system development (GO:0051960) | 897 | 439 | 231.52 | + | 1.90 | 1.08E-31 |
| negative regulation of protein kinase activity (GO:0006469) | 229 | 112 | 59.11 | + | 1.89 | 3.87E-06 |
| learning or memory (GO:0007611) | 272 | 133 | 70.20 | + | 1.89 | 9.87E-08 |
| positive regulation of synaptic transmission (GO:0050806) | 131 | 64 | 33.81 | + | 1.89 | 1.84E-02 |
| Ras protein signal transduction (GO:0007265) | 129 | 63 | 33.30 | + | 1.89 | 2.22E-02 |
| organelle localization (GO:0051640) | 385 | 188 | 99.37 | + | 1.89 | 7.04E-12 |

| | | | | | | |
|---|---|---|---|---|---|---|
| negative regulation of neuron death (GO:1901215) | 209 | 102 | 53.94 | + | 1.89 | 2.48E-05 |
| endosomal transport (GO:0016197) | 191 | 93 | 49.30 | + | 1.89 | 1.32E-04 |
| positive regulation of cell morphogenesis involved in differentiation (GO:0010770) | 185 | 90 | 47.75 | + | 1.88 | 2.32E-04 |
| gliogenesis (GO:0042063) | 218 | 106 | 56.27 | + | 1.88 | 1.50E-05 |
| phospholipid metabolic process (GO:0006644) | 249 | 121 | 64.27 | + | 1.88 | 1.16E-06 |
| maintenance of location (GO:0051235) | 132 | 64 | 34.07 | + | 1.88 | 2.33E-02 |
| cerebral cortex development (GO:0021987) | 128 | 62 | 33.04 | + | 1.88 | 3.39E-02 |
| negative regulation of kinase activity (GO:0033673) | 246 | 119 | 63.49 | + | 1.87 | 2.13E-06 |
| regulation of cell development (GO:0060284) | 971 | 469 | 250.62 | + | 1.87 | 8.42E-33 |
| brain development (GO:0007420) | 821 | 396 | 211.90 | + | 1.87 | 5.07E-27 |
| glycerophospholipid metabolic process (GO:0006650) | 168 | 81 | 43.36 | + | 1.87 | 1.56E-03 |
| positive regulation of cellular catabolic process (GO:0031331) | 195 | 94 | 50.33 | + | 1.87 | 1.76E-04 |
| vesicle organization (GO:0016050) | 206 | 99 | 53.17 | + | 1.86 | 8.74E-05 |
| protein localization to plasma membrane (GO:0072659) | 150 | 72 | 38.72 | + | 1.86 | 8.35E-03 |
| regulation of cellular catabolic process (GO:0031329) | 311 | 149 | 80.27 | + | 1.86 | 2.56E-08 |
| vacuolar transport (GO:0007034) | 228 | 109 | 58.85 | + | 1.85 | 2.16E-05 |
| positive regulation of protein catabolic process (GO:0045732) | 182 | 87 | 46.98 | + | 1.85 | 8.10E-04 |
| lipid modification (GO:0030258) | 159 | 76 | 41.04 | + | 1.85 | 4.98E-03 |
| head development (GO:0060322) | 861 | 411 | 222.23 | + | 1.85 | 2.80E-27 |
| forebrain development (GO:0030900) | 443 | 211 | 114.34 | + | 1.85 | 1.52E-12 |
| regulation of cell growth (GO:0001558) | 395 | 188 | 101.95 | + | 1.84 | 7.06E-11 |
| positive regulation of nervous system development (GO:0051962) | 540 | 257 | 139.38 | + | 1.84 | 8.44E-16 |
| peptidyl-tyrosine modification (GO:0018212) | 143 | 68 | 36.91 | + | 1.84 | 2.21E-02 |
| positive regulation of growth (GO:0045927) | 263 | 125 | 67.88 | + | 1.84 | 2.19E-06 |
| peptidyl-tyrosine phosphorylation (GO:0018108) | 141 | 67 | 36.39 | + | 1.84 | 2.66E-02 |
| positive regulation of developmental growth (GO:0048639) | 179 | 85 | 46.20 | + | 1.84 | 1.47E-03 |
| central nervous system development (GO:0007417) | 1018 | 482 | 262.75 | + | 1.83 | 8.36E-32 |
| exocytosis (GO:0006887) | 207 | 98 | 53.43 | + | 1.83 | 2.07E-04 |
| regulation of protein catabolic process (GO:0042176) | 311 | 147 | 80.27 | + | 1.83 | 9.11E-08 |
| peptidyl-serine phosphorylation (GO:0018105) | 144 | 68 | 37.17 | + | 1.83 | 2.77E-02 |
| hindbrain development (GO:0030902) | 178 | 84 | 45.94 | + | 1.83 | 2.22E-03 |
| protein transport (GO:0015031) | 946 | 445 | 244.17 | + | 1.82 | 1.78E-28 |
| regulation of membrane potential (GO:0042391) | 375 | 176 | 96.79 | + | 1.82 | 1.59E-09 |
| cell division (GO:0051301) | 277 | 130 | 71.50 | + | 1.82 | 2.10E-06 |
| protein localization to cell periphery (GO:1990778) | 162 | 76 | 41.81 | + | 1.82 | 9.75E-03 |
| peptidyl-serine modification (GO:0018209) | 160 | 75 | 41.30 | + | 1.82 | 1.17E-02 |
| central nervous system neuron differentiation (GO:0021953) | 192 | 90 | 49.56 | + | 1.82 | 1.13E-03 |
| positive regulation of neurogenesis (GO:0050769) | 476 | 223 | 122.86 | + | 1.82 | 1.18E-12 |

| | | | | | | |
|---|---|---|---|---|---|---|
| regulation of actin filament length (GO:0030832) | 158 | 74 | 40.78 | + | 1.81 | 1.41E-02 |
| regulation of actin polymerization or depolymerization (GO:0008064) | 158 | 74 | 40.78 | + | 1.81 | 1.41E-02 |
| glial cell differentiation (GO:0010001) | 171 | 80 | 44.14 | + | 1.81 | 5.79E-03 |
| negative regulation of cellular component organization (GO:0051129) | 633 | 296 | 163.38 | + | 1.81 | 1.51E-17 |
| regulation of catabolic process (GO:0009894) | 455 | 212 | 117.44 | + | 1.81 | 1.13E-11 |
| neuron differentiation (GO:0030182) | 968 | 451 | 249.85 | + | 1.81 | 5.02E-28 |
| membrane fusion (GO:0061025) | 146 | 68 | 37.68 | + | 1.80 | 4.30E-02 |
| cellular response to insulin stimulus (GO:0032869) | 159 | 74 | 41.04 | + | 1.80 | 1.76E-02 |
| cognition (GO:0050890) | 303 | 141 | 78.21 | + | 1.80 | 6.58E-07 |
| establishment of protein localization (GO:0045184) | 1072 | 498 | 276.69 | + | 1.80 | 4.80E-31 |
| negative regulation of protein phosphorylation (GO:0001933) | 399 | 185 | 102.98 | + | 1.80 | 1.09E-09 |
| negative regulation of intracellular signal transduction (GO:1902532) | 466 | 216 | 120.28 | + | 1.80 | 1.02E-11 |
| negative regulation of transferase activity (GO:0051348) | 274 | 127 | 70.72 | + | 1.80 | 6.97E-06 |
| regulation of developmental growth (GO:0048638) | 337 | 156 | 86.98 | + | 1.79 | 9.75E-08 |
| generation of neurons (GO:0048699) | 1493 | 691 | 385.35 | + | 1.79 | 2.20E-44 |
| positive regulation of catabolic process (GO:0009896) | 255 | 118 | 65.82 | + | 1.79 | 2.94E-05 |
| secretion by cell (GO:0032940) | 383 | 177 | 98.85 | + | 1.79 | 4.76E-09 |
| positive regulation of apoptotic signaling pathway (GO:2001235) | 169 | 78 | 43.62 | + | 1.79 | 1.29E-02 |
| protein localization to membrane (GO:0072657) | 297 | 137 | 76.66 | + | 1.79 | 2.09E-06 |
| negative regulation of protein modification process (GO:0031400) | 527 | 243 | 136.02 | + | 1.79 | 3.01E-13 |
| regulation of vesicle-mediated transport (GO:0060627) | 454 | 209 | 117.18 | + | 1.78 | 5.69E-11 |
| signal release (GO:0023061) | 176 | 81 | 45.43 | + | 1.78 | 9.14E-03 |
| locomotory behavior (GO:0007626) | 224 | 103 | 57.82 | + | 1.78 | 3.73E-04 |
| nervous system development (GO:0007399) | 2238 | 1029 | 577.64 | + | 1.78 | 1.49E-68 |
| regulation of autophagy (GO:0010506) | 198 | 91 | 51.10 | + | 1.78 | 2.22E-03 |
| regulation of protein polymerization (GO:0032271) | 172 | 79 | 44.39 | + | 1.78 | 1.32E-02 |
| cell projection organization (GO:0030030) | 952 | 437 | 245.72 | + | 1.78 | 9.71E-26 |
| adult behavior (GO:0030534) | 170 | 78 | 43.88 | + | 1.78 | 1.59E-02 |
| negative regulation of phosphorylation (GO:0042326) | 436 | 200 | 112.53 | + | 1.78 | 2.99E-10 |
| cell projection morphogenesis (GO:0048858) | 636 | 291 | 164.15 | + | 1.77 | 6.05E-16 |
| single-organism behavior (GO:0044708) | 470 | 215 | 121.31 | + | 1.77 | 4.32E-11 |
| transmembrane receptor protein tyrosine kinase signaling pathway (GO:0007169) | 339 | 155 | 87.50 | + | 1.77 | 2.70E-07 |
| glycerolipid metabolic process (GO:0046486) | 245 | 112 | 63.24 | + | 1.77 | 1.34E-04 |
| neurogenesis (GO:0022008) | 1608 | 734 | 415.03 | + | 1.77 | 1.91E-45 |
| autophagy (GO:0006914) | 301 | 137 | 77.69 | + | 1.76 | 4.79E-06 |
| behavior (GO:0007610) | 633 | 288 | 163.38 | + | 1.76 | 1.96E-15 |
| plasma membrane organization (GO:0007009) | 209 | 95 | 53.94 | + | 1.76 | 1.98E-03 |
| endomembrane system organization (GO:0010256) | 419 | 190 | 108.15 | + | 1.76 | 3.45E-09 |

| | | | | | | |
|---|---|---|---|---|---|---|
| modification-dependent protein catabolic process (GO:0019941) | 400 | 181 | 103.24 | + | 1.75 | 1.46E-08 |
| positive regulation of cell development (GO:0010720) | 575 | 260 | 148.41 | + | 1.75 | 2.31E-13 |
| ubiquitin-dependent protein catabolic process (GO:0006511) | 392 | 177 | 101.18 | + | 1.75 | 3.02E-08 |
| cell part morphogenesis (GO:0032990) | 660 | 298 | 170.35 | + | 1.75 | 1.27E-15 |
| single-organism membrane organization (GO:0044802) | 605 | 273 | 156.15 | + | 1.75 | 4.70E-14 |
| modification-dependent macromolecule catabolic process (GO:0043632) | 408 | 184 | 105.31 | + | 1.75 | 1.26E-08 |
| regulation of endocytosis (GO:0030100) | 213 | 96 | 54.98 | + | 1.75 | 2.47E-03 |
| intracellular protein transport (GO:0006886) | 587 | 264 | 151.51 | + | 1.74 | 2.46E-13 |
| protein autophosphorylation (GO:0046777) | 198 | 89 | 51.10 | + | 1.74 | 7.23E-03 |
| cell morphogenesis involved in differentiation (GO:0000904) | 554 | 249 | 142.99 | + | 1.74 | 2.13E-12 |
| small GTPase mediated signal transduction (GO:0007264) | 336 | 151 | 86.72 | + | 1.74 | 1.53E-06 |
| actin filament organization (GO:0007015) | 185 | 83 | 47.75 | + | 1.74 | 1.76E-02 |
| signal transduction by protein phosphorylation (GO:0023014) | 214 | 96 | 55.23 | + | 1.74 | 3.02E-03 |
| connective tissue development (GO:0061448) | 212 | 95 | 54.72 | + | 1.74 | 3.62E-03 |
| negative regulation of cell migration (GO:0030336) | 217 | 97 | 56.01 | + | 1.73 | 3.07E-03 |
| regulation of ion transmembrane transport (GO:0034765) | 396 | 177 | 102.21 | + | 1.73 | 6.66E-08 |
| cellular component disassembly (GO:0022411) | 282 | 126 | 72.79 | + | 1.73 | 6.41E-05 |
| actin cytoskeleton organization (GO:0030036) | 383 | 171 | 98.85 | + | 1.73 | 1.62E-07 |
| actin filament-based process (GO:0030029) | 424 | 189 | 109.44 | + | 1.73 | 1.65E-08 |
| negative regulation of phosphorus metabolic process (GO:0010563) | 552 | 246 | 142.47 | + | 1.73 | 8.07E-12 |
| negative regulation of phosphate metabolic process (GO:0045936) | 552 | 246 | 142.47 | + | 1.73 | 8.07E-12 |
| regulation of protein modification by small protein conjugation or removal (GO:1903320) | 211 | 94 | 54.46 | + | 1.73 | 5.28E-03 |
| regulation of transmembrane transport (GO:0034762) | 411 | 183 | 106.08 | + | 1.73 | 4.03E-08 |
| regulation of cation transmembrane transport (GO:1904062) | 227 | 101 | 58.59 | + | 1.72 | 2.20E-03 |
| intracellular transport (GO:0046907) | 904 | 402 | 233.33 | + | 1.72 | 6.36E-21 |
| single-organism carbohydrate metabolic process (GO:0044723) | 470 | 209 | 121.31 | + | 1.72 | 1.42E-09 |
| phosphorus metabolic process (GO:0006793) | 1725 | 767 | 445.23 | + | 1.72 | 9.70E-44 |
| phosphate-containing compound metabolic process (GO:0006796) | 1723 | 766 | 444.72 | + | 1.72 | 1.19E-43 |
| negative regulation of cellular component movement (GO:0051271) | 259 | 115 | 66.85 | + | 1.72 | 3.83E-04 |
| establishment of localization in cell (GO:0051649) | 1170 | 519 | 301.98 | + | 1.72 | 8.78E-28 |
| regulation of anatomical structure size (GO:0090066) | 519 | 230 | 133.96 | + | 1.72 | 1.22E-10 |
| apoptotic signaling pathway (GO:0097190) | 271 | 120 | 69.95 | + | 1.72 | 2.29E-04 |
| secretion (GO:0046903) | 511 | 226 | 131.89 | + | 1.71 | 2.51E-10 |
| striated muscle cell differentiation (GO:0051146) | 197 | 87 | 50.85 | + | 1.71 | 1.86E-02 |
| regulation of binding (GO:0051098) | 315 | 139 | 81.30 | + | 1.71 | 2.38E-05 |
| protein localization (GO:0008104) | 1523 | 672 | 393.09 | + | 1.71 | 1.33E-36 |
| regulation of protein ubiquitination (GO:0031396) | 186 | 82 | 48.01 | + | 1.71 | 3.79E-02 |
| proteasomal protein catabolic process (GO:0010498) | 211 | 93 | 54.46 | + | 1.71 | 9.29E-03 |

| | | | | | | |
|---|---|---|---|---|---|---|
| regulation of cellular component biogenesis (GO:0044087) | 774 | 341 | 199.77 | + | 1.71 | 1.09E-16 |
| protein targeting (GO:0006605) | 318 | 140 | 82.08 | + | 1.71 | 2.41E-05 |
| regulation of protein binding (GO:0043393) | 184 | 81 | 47.49 | + | 1.71 | 4.54E-02 |
| negative regulation of cell differentiation (GO:0045596) | 665 | 292 | 171.64 | + | 1.70 | 1.04E-13 |
| negative regulation of growth (GO:0045926) | 246 | 108 | 63.49 | + | 1.70 | 1.63E-03 |
| single-organism cellular localization (GO:1902580) | 695 | 305 | 179.38 | + | 1.70 | 2.08E-14 |
| regulation of cellular localization (GO:0060341) | 839 | 368 | 216.55 | + | 1.70 | 6.16E-18 |
| protein homooligomerization (GO:0051260) | 283 | 124 | 73.04 | + | 1.70 | 2.39E-04 |
| regulation of cytoskeleton organization (GO:0051493) | 418 | 183 | 107.89 | + | 1.70 | 1.52E-07 |
| negative regulation of locomotion (GO:0040013) | 263 | 115 | 67.88 | + | 1.69 | 8.18E-04 |
| rhythmic process (GO:0048511) | 341 | 149 | 88.01 | + | 1.69 | 1.23E-05 |
| regulation of actin cytoskeleton organization (GO:0032956) | 284 | 124 | 73.30 | + | 1.69 | 2.88E-04 |
| carbohydrate homeostasis (GO:0033500) | 213 | 93 | 54.98 | + | 1.69 | 1.35E-02 |
| protein phosphorylation (GO:0006468) | 781 | 341 | 201.58 | + | 1.69 | 4.07E-16 |
| glucose homeostasis (GO:0042593) | 213 | 93 | 54.98 | + | 1.69 | 1.35E-02 |
| regulation of protein serine/threonine kinase activity (GO:0071900) | 433 | 189 | 111.76 | + | 1.69 | 9.10E-08 |
| cellular localization (GO:0051641) | 1645 | 718 | 424.58 | + | 1.69 | 5.60E-38 |
| glycoprotein metabolic process (GO:0009100) | 282 | 123 | 72.79 | + | 1.69 | 3.44E-04 |
| cellular macromolecule localization (GO:0070727) | 1093 | 476 | 282.11 | + | 1.69 | 1.63E-23 |
| intracellular signal transduction (GO:0035556) | 1302 | 567 | 336.05 | + | 1.69 | 9.74E-29 |
| response to insulin (GO:0032868) | 246 | 107 | 63.49 | + | 1.69 | 2.82E-03 |
| small molecule catabolic process (GO:0044282) | 246 | 107 | 63.49 | + | 1.69 | 2.82E-03 |
| establishment of protein localization to organelle (GO:0072594) | 267 | 116 | 68.91 | + | 1.68 | 9.91E-04 |
| regulation of GTPase activity (GO:0043087) | 495 | 215 | 127.76 | + | 1.68 | 5.54E-09 |
| cellular protein localization (GO:0034613) | 1085 | 471 | 280.04 | + | 1.68 | 6.01E-23 |
| mitotic cell cycle (GO:0000278) | 484 | 210 | 124.92 | + | 1.68 | 1.13E-08 |
| organic anion transport (GO:0015711) | 242 | 105 | 62.46 | + | 1.68 | 4.03E-03 |
| cellular protein catabolic process (GO:0044257) | 482 | 209 | 124.41 | + | 1.68 | 1.34E-08 |
| phosphorylation (GO:0016310) | 1063 | 460 | 274.37 | + | 1.68 | 4.61E-22 |
| mitotic cell cycle process (GO:1903047) | 453 | 196 | 116.92 | + | 1.68 | 7.85E-08 |
| mitotic nuclear division (GO:0007067) | 243 | 105 | 62.72 | + | 1.67 | 4.83E-03 |
| protein modification by small protein conjugation or removal (GO:0070647) | 676 | 292 | 174.48 | + | 1.67 | 7.92E-13 |
| regulation of growth (GO:0040008) | 639 | 276 | 164.93 | + | 1.67 | 5.63E-12 |
| regulation of actin filament-based process (GO:0032970) | 322 | 139 | 83.11 | + | 1.67 | 8.69E-05 |
| regulation of apoptotic signaling pathway (GO:2001233) | 380 | 164 | 98.08 | + | 1.67 | 4.45E-06 |
| carboxylic acid transport (GO:0046942) | 211 | 91 | 54.46 | + | 1.67 | 2.79E-02 |
| protein ubiquitination (GO:0016567) | 515 | 222 | 132.92 | + | 1.67 | 4.73E-09 |
| protein modification by small protein conjugation (GO:0032446) | 555 | 239 | 143.25 | + | 1.67 | 6.80E-10 |

| | | | | | | |
|---|---|---|---|---|---|---|
| negative regulation of cell motility (GO:2000146) | 230 | 99 | 59.36 | + | 1.67 | 1.16E-02 |
| response to light stimulus (GO:0009416) | 300 | 129 | 77.43 | + | 1.67 | 3.54E-04 |
| regulation of cellular component organization (GO:0051128) | 2292 | 985 | 591.58 | + | 1.67 | 9.07E-52 |
| regulation of cellular protein localization (GO:1903827) | 545 | 234 | 140.67 | + | 1.66 | 1.65E-09 |
| regulation of MAP kinase activity (GO:0043405) | 301 | 129 | 77.69 | + | 1.66 | 4.23E-04 |
| positive regulation of proteolysis (GO:0045862) | 294 | 126 | 75.88 | + | 1.66 | 5.99E-04 |
| cellular response to peptide hormone stimulus (GO:0071375) | 259 | 111 | 66.85 | + | 1.66 | 3.44E-03 |
| glycoprotein biosynthetic process (GO:0009101) | 245 | 105 | 63.24 | + | 1.66 | 6.92E-03 |
| proteolysis involved in cellular protein catabolic process (GO:0051603) | 467 | 200 | 120.54 | + | 1.66 | 1.13E-07 |
| organic substance transport (GO:0071702) | 1547 | 662 | 399.29 | + | 1.66 | 3.83E-32 |
| negative regulation of organelle organization (GO:0010639) | 300 | 128 | 77.43 | + | 1.65 | 6.01E-04 |
| cellular response to peptide (GO:1901653) | 286 | 122 | 73.82 | + | 1.65 | 1.21E-03 |
| macromolecule localization (GO:0033036) | 1795 | 764 | 463.30 | + | 1.65 | 3.75E-37 |
| response to drug (GO:0042493) | 614 | 261 | 158.48 | + | 1.65 | 1.96E-10 |
| positive regulation of apoptotic process (GO:0043065) | 584 | 248 | 150.73 | + | 1.65 | 9.64E-10 |
| regulation of mitotic cell cycle (GO:0007346) | 391 | 166 | 100.92 | + | 1.64 | 1.08E-05 |
| organophosphate metabolic process (GO:0019637) | 703 | 298 | 181.45 | + | 1.64 | 3.87E-12 |
| cell morphogenesis (GO:0000902) | 880 | 373 | 227.13 | + | 1.64 | 6.83E-16 |
| protein localization to organelle (GO:0033365) | 477 | 202 | 123.12 | + | 1.64 | 2.27E-07 |
| positive regulation of programmed cell death (GO:0043068) | 588 | 249 | 151.77 | + | 1.64 | 1.14E-09 |
| enzyme linked receptor protein signaling pathway (GO:0007167) | 520 | 220 | 134.21 | + | 1.64 | 3.27E-08 |
| regulation of metal ion transport (GO:0010959) | 360 | 152 | 92.92 | + | 1.64 | 7.34E-05 |
| response to hypoxia (GO:0001666) | 375 | 158 | 96.79 | + | 1.63 | 4.32E-05 |
| positive regulation of cell death (GO:0010942) | 629 | 265 | 162.35 | + | 1.63 | 3.24E-10 |
| protein catabolic process (GO:0030163) | 513 | 216 | 132.41 | + | 1.63 | 7.77E-08 |
| vesicle-mediated transport (GO:0016192) | 960 | 404 | 247.78 | + | 1.63 | 6.01E-17 |
| cellular response to hormone stimulus (GO:0032870) | 561 | 236 | 144.80 | + | 1.63 | 9.32E-09 |
| positive regulation of cellular component organization (GO:0051130) | 1203 | 506 | 310.50 | + | 1.63 | 5.19E-22 |
| regulation of kinase activity (GO:0043549) | 718 | 302 | 185.32 | + | 1.63 | 6.35E-12 |
| cellular protein modification process (GO:0006464) | 2383 | 1002 | 615.06 | + | 1.63 | 1.15E-48 |
| protein modification process (GO:0036211) | 2383 | 1002 | 615.06 | + | 1.63 | 1.15E-48 |
| regulation of cell differentiation (GO:0045595) | 1651 | 694 | 426.13 | + | 1.63 | 1.15E-31 |
| regulation of protein kinase activity (GO:0045859) | 659 | 277 | 170.09 | + | 1.63 | 1.10E-10 |
| muscle cell differentiation (GO:0042692) | 269 | 113 | 69.43 | + | 1.63 | 6.87E-03 |
| aging (GO:0007568) | 398 | 167 | 102.73 | + | 1.63 | 2.12E-05 |
| angiogenesis (GO:0001525) | 258 | 108 | 66.59 | + | 1.62 | 1.38E-02 |
| macromolecule modification (GO:0043412) | 2576 | 1077 | 664.88 | + | 1.62 | 7.42E-52 |
| cellular macromolecule catabolic process (GO:0044265) | 627 | 262 | 161.83 | + | 1.62 | 1.07E-09 |

| | | | | | | |
|---|---|---|---|---|---|---|
| single-organism intracellular transport (GO:1902582) | 371 | 155 | 95.76 | + | 1.62 | 1.01E-04 |
| regulation of ion transport (GO:0043269) | 596 | 249 | 153.83 | + | 1.62 | 4.42E-09 |
| positive regulation of GTPase activity (GO:0043547) | 443 | 185 | 114.34 | + | 1.62 | 4.31E-06 |
| response to decreased oxygen levels (GO:0036293) | 386 | 161 | 99.63 | + | 1.62 | 5.96E-05 |
| carbohydrate metabolic process (GO:0005975) | 559 | 233 | 144.28 | + | 1.61 | 3.06E-08 |
| anion transport (GO:0006820) | 360 | 150 | 92.92 | + | 1.61 | 2.03E-04 |
| epithelial tube morphogenesis (GO:0060562) | 348 | 145 | 89.82 | + | 1.61 | 3.43E-04 |
| apoptotic process (GO:0006915) | 675 | 281 | 174.22 | + | 1.61 | 2.09E-10 |
| response to oxygen levels (GO:0070482) | 418 | 174 | 107.89 | + | 1.61 | 1.73E-05 |
| cellular component morphogenesis (GO:0032989) | 966 | 402 | 249.33 | + | 1.61 | 4.63E-16 |
| programmed cell death (GO:0012501) | 702 | 292 | 181.19 | + | 1.61 | 7.09E-11 |
| peptidyl-amino acid modification (GO:0018193) | 703 | 292 | 181.45 | + | 1.61 | 8.36E-11 |
| lipid biosynthetic process (GO:0008610) | 383 | 159 | 98.85 | + | 1.61 | 9.90E-05 |
| chemotaxis (GO:0006935) | 410 | 170 | 105.82 | + | 1.61 | 3.43E-05 |
| response to peptide hormone (GO:0043434) | 454 | 188 | 117.18 | + | 1.60 | 5.85E-06 |
| negative regulation of developmental process (GO:0051093) | 884 | 366 | 228.17 | + | 1.60 | 4.91E-14 |
| regulation of protein complex assembly (GO:0043254) | 343 | 142 | 88.53 | + | 1.60 | 6.74E-04 |
| regulation of transport (GO:0051049) | 1813 | 750 | 467.94 | + | 1.60 | 1.73E-32 |
| membrane organization (GO:0061024) | 747 | 309 | 192.80 | + | 1.60 | 2.27E-11 |
| regulation of intracellular protein transport (GO:0033157) | 370 | 153 | 95.50 | + | 1.60 | 2.34E-04 |
| regulation of intracellular transport (GO:0032386) | 467 | 193 | 120.54 | + | 1.60 | 4.06E-06 |
| cytoskeleton organization (GO:0007010) | 859 | 355 | 221.71 | + | 1.60 | 2.04E-13 |
| morphogenesis of an epithelium (GO:0002009) | 489 | 202 | 126.21 | + | 1.60 | 1.67E-06 |
| regulation of transferase activity (GO:0051338) | 809 | 334 | 208.81 | + | 1.60 | 2.12E-12 |
| taxis (GO:0042330) | 412 | 170 | 106.34 | + | 1.60 | 4.74E-05 |
| positive regulation of hydrolase activity (GO:0051345) | 732 | 302 | 188.93 | + | 1.60 | 6.45E-11 |
| organophosphate biosynthetic process (GO:0090407) | 337 | 139 | 86.98 | + | 1.60 | 1.12E-03 |
| negative regulation of transport (GO:0051051) | 490 | 202 | 126.47 | + | 1.60 | 1.97E-06 |
| tube morphogenesis (GO:0035239) | 393 | 162 | 101.44 | + | 1.60 | 1.14E-04 |
| positive regulation of cellular protein localization (GO:1903829) | 343 | 141 | 88.53 | + | 1.59 | 1.10E-03 |
| cell death (GO:0008219) | 745 | 306 | 192.29 | + | 1.59 | 7.24E-11 |
| cell-cell signaling (GO:0007267) | 704 | 289 | 181.71 | + | 1.59 | 4.34E-10 |
| positive regulation of intracellular transport (GO:0032388) | 290 | 119 | 74.85 | + | 1.59 | 1.08E-02 |
| small molecule biosynthetic process (GO:0044283) | 334 | 137 | 86.21 | + | 1.59 | 1.85E-03 |
| regulation of lipid metabolic process (GO:0019216) | 300 | 123 | 77.43 | + | 1.59 | 7.56E-03 |
| positive regulation of cellular component biogenesis (GO:0044089) | 405 | 166 | 104.53 | + | 1.59 | 1.09E-04 |
| posttranscriptional regulation of gene expression (GO:0010608) | 371 | 152 | 95.76 | + | 1.59 | 4.48E-04 |
| urogenital system development (GO:0001655) | 354 | 145 | 91.37 | + | 1.59 | 9.05E-04 |

| | | | | | | |
|---|---|---|---|---|---|---|
| response to peptide (GO:1901652) | 504 | 206 | 130.09 | + | 1.58 | 2.59E-06 |
| ribose phosphate metabolic process (GO:0019693) | 311 | 127 | 80.27 | + | 1.58 | 6.19E-03 |
| muscle structure development (GO:0061061) | 458 | 187 | 118.21 | + | 1.58 | 1.81E-05 |
| negative regulation of cell communication (GO:0010648) | 1159 | 473 | 299.14 | + | 1.58 | 7.15E-18 |
| positive regulation of cell cycle (GO:0045787) | 299 | 122 | 77.17 | + | 1.58 | 1.05E-02 |
| nitrogen compound transport (GO:0071705) | 402 | 164 | 103.76 | + | 1.58 | 1.79E-04 |
| negative regulation of signaling (GO:0023057) | 1162 | 474 | 299.92 | + | 1.58 | 7.03E-18 |
| negative regulation of cellular protein metabolic process (GO:0032269) | 939 | 383 | 242.36 | + | 1.58 | 7.54E-14 |
| negative regulation of cell death (GO:0060548) | 967 | 394 | 249.59 | + | 1.58 | 2.92E-14 |
| developmental growth (GO:0048589) | 410 | 167 | 105.82 | + | 1.58 | 1.48E-04 |
| response to hormone (GO:0009725) | 965 | 393 | 249.07 | + | 1.58 | 3.47E-14 |
| regulation of cell cycle (GO:0051726) | 811 | 330 | 209.32 | + | 1.58 | 2.06E-11 |
| regulation of localization (GO:0032879) | 2487 | 1011 | 641.91 | + | 1.57 | 5.99E-43 |
| response to mechanical stimulus (GO:0009612) | 278 | 113 | 71.75 | + | 1.57 | 2.96E-02 |
| single-organism organelle organization (GO:1902589) | 1369 | 556 | 353.35 | + | 1.57 | 3.63E-21 |
| response to organonitrogen compound (GO:0010243) | 993 | 403 | 256.30 | + | 1.57 | 2.18E-14 |
| regulation of protein localization (GO:0032880) | 971 | 394 | 250.62 | + | 1.57 | 5.47E-14 |
| regulation of cellular response to stress (GO:0080135) | 530 | 215 | 136.80 | + | 1.57 | 1.99E-06 |
| negative regulation of signal transduction (GO:0009968) | 1041 | 422 | 268.69 | + | 1.57 | 3.97E-15 |
| regulation of intracellular signal transduction (GO:1902531) | 1513 | 613 | 390.51 | + | 1.57 | 1.74E-23 |
| carbohydrate derivative biosynthetic process (GO:1901137) | 469 | 190 | 121.05 | + | 1.57 | 2.37E-05 |
| cell development (GO:0048468) | 1575 | 638 | 406.52 | + | 1.57 | 1.36E-24 |
| response to steroid hormone (GO:0048545) | 457 | 185 | 117.95 | + | 1.57 | 4.02E-05 |
| sulfur compound metabolic process (GO:0006790) | 272 | 110 | 70.20 | + | 1.57 | 4.89E-02 |
| regulation of anatomical structure morphogenesis (GO:0022603) | 1017 | 411 | 262.49 | + | 1.57 | 1.92E-14 |
| regulation of MAPK cascade (GO:0043408) | 681 | 275 | 175.77 | + | 1.56 | 1.01E-08 |
| negative regulation of cell proliferation (GO:0008285) | 629 | 254 | 162.35 | + | 1.56 | 7.36E-08 |
| positive regulation of transferase activity (GO:0051347) | 503 | 203 | 129.83 | + | 1.56 | 9.27E-06 |
| organic hydroxy compound metabolic process (GO:1901615) | 300 | 121 | 77.43 | + | 1.56 | 1.97E-02 |
| regulation of multicellular organismal development (GO:2000026) | 1784 | 719 | 460.46 | + | 1.56 | 1.13E-27 |
| regulation of organelle organization (GO:0033043) | 1099 | 442 | 283.66 | + | 1.56 | 2.07E-15 |
| regulation of cell death (GO:0010941) | 1550 | 623 | 400.06 | + | 1.56 | 4.25E-23 |
| renal system development (GO:0072001) | 311 | 125 | 80.27 | + | 1.56 | 1.60E-02 |
| regulation of signaling (GO:0023051) | 2786 | 1118 | 719.08 | + | 1.55 | 7.14E-46 |
| cellular catabolic process (GO:0044248) | 1095 | 439 | 282.63 | + | 1.55 | 4.67E-15 |
| regulation of cell communication (GO:0010646) | 2764 | 1108 | 713.40 | + | 1.55 | 3.58E-45 |
| negative regulation of protein metabolic process (GO:0051248) | 998 | 400 | 257.59 | + | 1.55 | 1.96E-13 |
| cellular response to abiotic stimulus (GO:0071214) | 307 | 123 | 79.24 | + | 1.55 | 2.23E-02 |

| | | | | | | |
|---|---|---|---|---|---|---|
| negative regulation of cell cycle (GO:0045786) | 327 | 131 | 84.40 | + | 1.55 | 1.09E-02 |
| cofactor metabolic process (GO:0051186) | 327 | 131 | 84.40 | + | 1.55 | 1.09E-02 |
| microtubule cytoskeleton organization (GO:0000226) | 362 | 145 | 93.43 | + | 1.55 | 3.09E-03 |
| positive regulation of kinase activity (GO:0033674) | 437 | 175 | 112.79 | + | 1.55 | 2.10E-04 |
| macromolecule catabolic process (GO:0009057) | 713 | 285 | 184.03 | + | 1.55 | 1.16E-08 |
| regulation of developmental process (GO:0050793) | 2362 | 944 | 609.64 | + | 1.55 | 8.53E-37 |
| cellular response to growth factor stimulus (GO:0071363) | 463 | 185 | 119.50 | + | 1.55 | 9.91E-05 |
| positive regulation of protein kinase activity (GO:0045860) | 403 | 161 | 104.02 | + | 1.55 | 8.55E-04 |
| regulation of cellular amide metabolic process (GO:0034248) | 343 | 137 | 88.53 | + | 1.55 | 7.36E-03 |
| tube development (GO:0035295) | 681 | 272 | 175.77 | + | 1.55 | 4.10E-08 |
| positive regulation of catalytic activity (GO:0043085) | 1252 | 500 | 323.15 | + | 1.55 | 2.81E-17 |
| cellular lipid metabolic process (GO:0044255) | 749 | 299 | 193.32 | + | 1.55 | 3.76E-09 |
| positive regulation of transport (GO:0051050) | 990 | 395 | 255.52 | + | 1.55 | 6.13E-13 |
| negative regulation of transcription from RNA polymerase II promoter (GO:0000122) | 757 | 302 | 195.39 | + | 1.55 | 3.07E-09 |
| cellular response to organic cyclic compound (GO:0071407) | 549 | 219 | 141.70 | + | 1.55 | 5.37E-06 |
| growth (GO:0040007) | 484 | 193 | 124.92 | + | 1.54 | 5.59E-05 |
| single-organism biosynthetic process (GO:0044711) | 841 | 335 | 217.07 | + | 1.54 | 1.85E-10 |
| ribonucleotide metabolic process (GO:0009259) | 299 | 119 | 77.17 | + | 1.54 | 4.29E-02 |
| regulation of protein targeting (GO:1903533) | 299 | 119 | 77.17 | + | 1.54 | 4.29E-02 |
| regulation of molecular function (GO:0065009) | 2574 | 1024 | 664.36 | + | 1.54 | 9.73E-40 |
| tissue morphogenesis (GO:0048729) | 596 | 237 | 153.83 | + | 1.54 | 1.42E-06 |
| cellular response to organonitrogen compound (GO:0071417) | 498 | 198 | 128.54 | + | 1.54 | 4.44E-05 |
| positive regulation of molecular function (GO:0044093) | 1557 | 619 | 401.87 | + | 1.54 | 8.44E-22 |
| regulation of cell cycle process (GO:0010564) | 476 | 189 | 122.86 | + | 1.54 | 1.07E-04 |
| negative regulation of apoptotic process (GO:0043066) | 864 | 343 | 223.00 | + | 1.54 | 1.38E-10 |
| regulation of cell migration (GO:0030334) | 711 | 282 | 183.51 | + | 1.54 | 3.43E-08 |
| response to oxidative stress (GO:0006979) | 411 | 163 | 106.08 | + | 1.54 | 1.11E-03 |
| regulation of secretion by cell (GO:1903530) | 694 | 275 | 179.13 | + | 1.54 | 6.98E-08 |
| nucleotide metabolic process (GO:0009117) | 409 | 162 | 105.57 | + | 1.53 | 1.30E-03 |
| blood vessel development (GO:0001568) | 452 | 179 | 116.66 | + | 1.53 | 3.05E-04 |
| covalent chromatin modification (GO:0016569) | 379 | 150 | 97.82 | + | 1.53 | 3.83E-03 |
| positive regulation of cell differentiation (GO:0045597) | 940 | 372 | 242.62 | + | 1.53 | 1.57E-11 |
| negative regulation of programmed cell death (GO:0043069) | 878 | 347 | 226.62 | + | 1.53 | 1.70E-10 |
| regulation of catalytic activity (GO:0050790) | 2047 | 809 | 528.34 | + | 1.53 | 4.01E-29 |
| response to abiotic stimulus (GO:0009628) | 1245 | 492 | 321.34 | + | 1.53 | 4.14E-16 |
| regulation of protein modification process (GO:0031399) | 1612 | 637 | 416.07 | + | 1.53 | 6.38E-22 |
| positive regulation of secretion by cell (GO:1903532) | 390 | 154 | 100.66 | + | 1.53 | 3.09E-03 |
| positive regulation of organelle organization (GO:0010638) | 580 | 229 | 149.70 | + | 1.53 | 5.21E-06 |

| | | | | | | |
|---|---|---|---|---|---|---|
| nucleoside phosphate metabolic process (GO:0006753) | 418 | 165 | 107.89 | + | 1.53 | 1.24E-03 |
| response to nitrogen compound (GO:1901698) | 1092 | 431 | 281.85 | + | 1.53 | 1.23E-13 |
| regulation of phosphorylation (GO:0042325) | 1399 | 552 | 361.09 | + | 1.53 | 2.36E-18 |
| regulation of apoptotic process (GO:0042981) | 1412 | 557 | 364.44 | + | 1.53 | 1.57E-18 |
| response to endogenous stimulus (GO:0009719) | 1620 | 639 | 418.13 | + | 1.53 | 8.16E-22 |
| response to radiation (GO:0009314) | 477 | 188 | 123.12 | + | 1.53 | 1.94E-04 |
| metal ion transport (GO:0030001) | 543 | 214 | 140.15 | + | 1.53 | 2.16E-05 |
| establishment of localization (GO:0051234) | 3456 | 1362 | 892.01 | + | 1.53 | 5.41E-54 |
| regulation of programmed cell death (GO:0043067) | 1427 | 562 | 368.32 | + | 1.53 | 1.39E-18 |
| response to growth factor (GO:0070848) | 498 | 196 | 128.54 | + | 1.52 | 1.09E-04 |
| negative regulation of cellular process (GO:0048523) | 4088 | 1608 | 1055.13 | + | 1.52 | 4.73E-66 |
| regulation of signal transduction (GO:0009966) | 2441 | 960 | 630.03 | + | 1.52 | 6.38E-35 |
| cell migration (GO:0016477) | 712 | 280 | 183.77 | + | 1.52 | 9.74E-08 |
| cellular response to endogenous stimulus (GO:0071495) | 1038 | 408 | 267.91 | + | 1.52 | 1.75E-12 |
| transport (GO:0006810) | 3305 | 1299 | 853.04 | + | 1.52 | 2.65E-50 |
| carbohydrate derivative metabolic process (GO:1901135) | 828 | 325 | 213.71 | + | 1.52 | 2.63E-09 |
| positive regulation of transcription from RNA polymerase II promoter (GO:0045944) | 1050 | 412 | 271.01 | + | 1.52 | 1.60E-12 |
| regulation of cellular component movement (GO:0051270) | 813 | 319 | 209.84 | + | 1.52 | 4.55E-09 |
| system development (GO:0048731) | 4152 | 1629 | 1071.65 | + | 1.52 | 2.07E-66 |
| multicellular organism development (GO:0007275) | 4547 | 1783 | 1173.60 | + | 1.52 | 1.57E-74 |
| protein oligomerization (GO:0051259) | 482 | 189 | 124.41 | + | 1.52 | 2.53E-04 |
| negative regulation of multicellular organismal process (GO:0051241) | 1064 | 417 | 274.62 | + | 1.52 | 1.24E-12 |
| monovalent inorganic cation transport (GO:0015672) | 370 | 145 | 95.50 | + | 1.52 | 9.87E-03 |
| locomotion (GO:0040011) | 980 | 384 | 252.94 | + | 1.52 | 2.16E-11 |
| RNA biosynthetic process (GO:0032774) | 1215 | 476 | 313.60 | + | 1.52 | 7.99E-15 |
| regulation of secretion (GO:0051046) | 743 | 291 | 191.77 | + | 1.52 | 5.87E-08 |
| negative regulation of catalytic activity (GO:0043086) | 751 | 294 | 193.84 | + | 1.52 | 4.79E-08 |
| blood vessel morphogenesis (GO:0048514) | 350 | 137 | 90.34 | + | 1.52 | 2.02E-02 |
| transcription, DNA-templated (GO:0006351) | 1201 | 469 | 309.98 | + | 1.51 | 2.54E-14 |
| single-organism localization (GO:1902578) | 2021 | 789 | 521.63 | + | 1.51 | 1.09E-26 |
| positive regulation of cellular protein metabolic process (GO:0032270) | 1348 | 526 | 347.93 | + | 1.51 | 2.15E-16 |
| nucleic acid-templated transcription (GO:0097659) | 1202 | 469 | 310.24 | + | 1.51 | 2.92E-14 |
| regulation of locomotion (GO:0040012) | 787 | 307 | 203.13 | + | 1.51 | 2.39E-08 |
| organic substance catabolic process (GO:1901575) | 1264 | 493 | 326.24 | + | 1.51 | 4.01E-15 |
| inorganic cation transmembrane transport (GO:0098662) | 467 | 182 | 120.54 | + | 1.51 | 6.72E-04 |
| regulation of phosphate metabolic process (GO:0019220) | 1622 | 632 | 418.65 | + | 1.51 | 2.73E-20 |
| localization (GO:0051179) | 4363 | 1700 | 1126.11 | + | 1.51 | 4.63E-68 |
| vasculature development (GO:0001944) | 480 | 187 | 123.89 | + | 1.51 | 4.60E-04 |

| | | | | | | |
|---|---|---|---|---|---|---|
| regulation of phosphorus metabolic process (GO:0051174) | 1623 | 632 | 418.90 | + | 1.51 | 3.15E-20 |
| response to metal ion (GO:0010038) | 434 | 169 | 112.02 | + | 1.51 | 2.01E-03 |
| negative regulation of response to stimulus (GO:0048585) | 1346 | 524 | 347.41 | + | 1.51 | 3.97E-16 |
| positive regulation of secretion (GO:0051047) | 419 | 163 | 108.15 | + | 1.51 | 3.43E-03 |
| regulation of homeostatic process (GO:0032844) | 473 | 184 | 122.08 | + | 1.51 | 6.45E-04 |
| cellular response to lipid (GO:0071396) | 522 | 203 | 134.73 | + | 1.51 | 1.44E-04 |
| regulation of hydrolase activity (GO:0051336) | 1184 | 460 | 305.60 | + | 1.51 | 1.28E-13 |
| ion transport (GO:0006811) | 1064 | 413 | 274.62 | + | 1.50 | 7.29E-12 |
| regulation of biological quality (GO:0065008) | 3322 | 1289 | 857.43 | + | 1.50 | 5.57E-47 |
| regulation of protein phosphorylation (GO:0001932) | 1297 | 503 | 334.76 | + | 1.50 | 4.76E-15 |
| cation homeostasis (GO:0055080) | 555 | 215 | 143.25 | + | 1.50 | 7.41E-05 |
| negative regulation of biological process (GO:0048519) | 4402 | 1705 | 1136.18 | + | 1.50 | 1.68E-66 |
| response to acid chemical (GO:0001101) | 403 | 156 | 104.02 | + | 1.50 | 7.88E-03 |
| regulation of establishment of protein localization (GO:0070201) | 819 | 317 | 211.39 | + | 1.50 | 2.51E-08 |
| single-multicellular organism process (GO:0044707) | 5153 | 1994 | 1330.02 | + | 1.50 | 1.28E-81 |
| positive regulation of developmental process (GO:0051094) | 1300 | 503 | 335.54 | + | 1.50 | 7.20E-15 |
| regulation of cell motility (GO:2000145) | 750 | 290 | 193.58 | + | 1.50 | 2.38E-07 |
| regulation of protein transport (GO:0051223) | 763 | 295 | 196.93 | + | 1.50 | 1.62E-07 |
| cell differentiation (GO:0030154) | 3267 | 1263 | 843.23 | + | 1.50 | 5.03E-45 |
| nucleobase-containing small molecule metabolic process (GO:0055086) | 471 | 182 | 121.57 | + | 1.50 | 1.16E-03 |
| catabolic process (GO:0009056) | 1326 | 512 | 342.25 | + | 1.50 | 4.97E-15 |
| regulation of cellular protein metabolic process (GO:0032268) | 2291 | 884 | 591.32 | + | 1.49 | 6.76E-29 |
| cation transmembrane transport (GO:0098655) | 529 | 204 | 136.54 | + | 1.49 | 2.43E-04 |
| negative regulation of cellular macromolecule biosynthetic process (GO:2000113) | 1250 | 482 | 322.63 | + | 1.49 | 7.34E-14 |
| negative regulation of molecular function (GO:0044092) | 1020 | 393 | 263.27 | + | 1.49 | 1.05E-10 |
| positive regulation of protein metabolic process (GO:0051247) | 1438 | 554 | 371.16 | + | 1.49 | 2.39E-16 |
| chemical homeostasis (GO:0048878) | 927 | 357 | 239.26 | + | 1.49 | 1.92E-09 |
| cellular response to nitrogen compound (GO:1901699) | 574 | 221 | 148.15 | + | 1.49 | 7.41E-05 |
| positive regulation of nucleic acid-templated transcription (GO:1903508) | 1328 | 511 | 342.76 | + | 1.49 | 1.01E-14 |
| positive regulation of transcription, DNA-templated (GO:0045893) | 1328 | 511 | 342.76 | + | 1.49 | 1.01E-14 |
| positive regulation of RNA biosynthetic process (GO:1902680) | 1331 | 512 | 343.54 | + | 1.49 | 9.82E-15 |
| positive regulation of cellular metabolic process (GO:0031325) | 2727 | 1049 | 703.85 | + | 1.49 | 5.22E-35 |
| positive regulation of intracellular signal transduction (GO:1902533) | 884 | 340 | 228.17 | + | 1.49 | 8.48E-09 |
| in utero embryonic development (GO:0001701) | 429 | 165 | 110.73 | + | 1.49 | 5.65E-03 |
| response to organic cyclic compound (GO:0014070) | 1209 | 465 | 312.05 | + | 1.49 | 4.43E-13 |
| divalent inorganic cation homeostasis (GO:0072507) | 372 | 143 | 96.02 | + | 1.49 | 3.08E-02 |
| positive regulation of cellular process (GO:0048522) | 4627 | 1778 | 1194.25 | + | 1.49 | 9.43E-68 |
| single-organism transport (GO:0044765) | 1801 | 692 | 464.85 | + | 1.49 | 4.37E-21 |

| | | | | | | |
|---|---|---|---|---|---|---|
| cellular chemical homeostasis (GO:0055082) | 617 | 237 | 159.25 | + | 1.49 | 2.63E-05 |
| protein complex subunit organization (GO:0071822) | 1034 | 397 | 266.88 | + | 1.49 | 1.24E-10 |
| organelle organization (GO:0006996) | 2821 | 1082 | 728.11 | + | 1.49 | 7.65E-36 |
| positive regulation of signaling (GO:0023056) | 1468 | 563 | 378.90 | + | 1.49 | 2.80E-16 |
| mitochondrion organization (GO:0007005) | 480 | 184 | 123.89 | + | 1.49 | 1.65E-03 |
| negative regulation of macromolecule biosynthetic process (GO:0010558) | 1315 | 504 | 339.41 | + | 1.48 | 3.58E-14 |
| negative regulation of nitrogen compound metabolic process (GO:0051172) | 1422 | 545 | 367.03 | + | 1.48 | 1.36E-15 |
| movement of cell or subcellular component (GO:0006928) | 1122 | 430 | 289.59 | + | 1.48 | 1.23E-11 |
| cellular developmental process (GO:0048869) | 3473 | 1331 | 896.40 | + | 1.48 | 4.11E-46 |
| single-organism catabolic process (GO:0044712) | 608 | 233 | 156.93 | + | 1.48 | 4.30E-05 |
| cellular response to stress (GO:0033554) | 1412 | 541 | 364.44 | + | 1.48 | 1.98E-15 |
| regulation of protein metabolic process (GO:0051246) | 2436 | 933 | 628.74 | + | 1.48 | 9.94E-30 |
| positive regulation of macromolecule metabolic process (GO:0010604) | 2718 | 1041 | 701.53 | + | 1.48 | 5.93E-34 |
| regulation of multicellular organismal process (GO:0051239) | 2658 | 1018 | 686.04 | + | 1.48 | 4.86E-33 |
| positive regulation of biological process (GO:0048518) | 5076 | 1944 | 1310.14 | + | 1.48 | 3.45E-75 |
| positive regulation of protein modification process (GO:0031401) | 1068 | 409 | 275.66 | + | 1.48 | 7.01E-11 |
| negative regulation of biosynthetic process (GO:0009890) | 1405 | 538 | 362.64 | + | 1.48 | 2.82E-15 |
| regulation of transcription from RNA polymerase II promoter (GO:0006357) | 1771 | 678 | 457.10 | + | 1.48 | 3.42E-20 |
| negative regulation of cellular biosynthetic process (GO:0031327) | 1374 | 526 | 354.64 | + | 1.48 | 7.68E-15 |
| positive regulation of cell communication (GO:0010647) | 1461 | 559 | 377.09 | + | 1.48 | 6.14E-16 |
| positive regulation of RNA metabolic process (GO:0051254) | 1383 | 529 | 356.96 | + | 1.48 | 7.07E-15 |
| anatomical structure morphogenesis (GO:0009653) | 2139 | 818 | 552.09 | + | 1.48 | 3.58E-25 |
| ion transmembrane transport (GO:0034220) | 709 | 271 | 183.00 | + | 1.48 | 3.13E-06 |
| mRNA metabolic process (GO:0016071) | 398 | 152 | 102.73 | + | 1.48 | 2.19E-02 |
| positive regulation of MAPK cascade (GO:0043410) | 474 | 181 | 122.34 | + | 1.48 | 2.63E-03 |
| inorganic ion homeostasis (GO:0098771) | 571 | 218 | 147.38 | + | 1.48 | 1.75E-04 |
| negative regulation of nucleic acid-templated transcription (GO:1903507) | 1095 | 418 | 282.63 | + | 1.48 | 5.54E-11 |
| cell proliferation (GO:0008283) | 566 | 216 | 146.09 | + | 1.48 | 2.09E-04 |
| ion homeostasis (GO:0050801) | 577 | 220 | 148.93 | + | 1.48 | 1.67E-04 |
| eye development (GO:0001654) | 383 | 146 | 98.85 | + | 1.48 | 3.74E-02 |
| positive regulation of macromolecule biosynthetic process (GO:0010557) | 1564 | 596 | 403.68 | + | 1.48 | 7.30E-17 |
| regulation of system process (GO:0044057) | 496 | 189 | 128.02 | + | 1.48 | 1.67E-03 |
| nucleobase-containing compound biosynthetic process (GO:0034654) | 1475 | 562 | 380.71 | + | 1.48 | 1.10E-15 |
| positive regulation of nitrogen compound metabolic process (GO:0051173) | 1727 | 658 | 445.75 | + | 1.48 | 5.39E-19 |
| negative regulation of cellular metabolic process (GO:0031324) | 2237 | 852 | 577.38 | + | 1.48 | 6.75E-26 |
| negative regulation of macromolecule metabolic process (GO:0010605) | 2186 | 832 | 564.22 | + | 1.47 | 4.48E-25 |
| negative regulation of gene expression (GO:0010629) | 1390 | 529 | 358.77 | + | 1.47 | 1.79E-14 |
| anatomical structure development (GO:0048856) | 4940 | 1880 | 1275.04 | + | 1.47 | 9.54E-70 |

| | | | | | | |
|---|---|---|---|---|---|---|
| single-organism developmental process (GO:0044767) | 5217 | 1985 | 1346.53 | + | 1.47 | 5.06E-75 |
| positive regulation of metabolic process (GO:0009893) | 2913 | 1108 | 751.86 | + | 1.47 | 2.08E-35 |
| aromatic compound biosynthetic process (GO:0019438) | 1546 | 588 | 399.03 | + | 1.47 | 2.04E-16 |
| negative regulation of transcription, DNA-templated (GO:0045892) | 1076 | 409 | 277.72 | + | 1.47 | 2.00E-10 |
| positive regulation of cellular biosynthetic process (GO:0031328) | 1689 | 642 | 435.94 | + | 1.47 | 3.31E-18 |
| developmental process (GO:0032502) | 5248 | 1994 | 1354.54 | + | 1.47 | 5.47E-75 |
| heterocycle biosynthetic process (GO:0018130) | 1532 | 582 | 395.42 | + | 1.47 | 4.13E-16 |
| positive regulation of signal transduction (GO:0009967) | 1306 | 496 | 337.09 | + | 1.47 | 3.24E-13 |
| negative regulation of metabolic process (GO:0009892) | 2389 | 907 | 616.61 | + | 1.47 | 1.76E-27 |
| lipid metabolic process (GO:0006629) | 959 | 364 | 247.52 | + | 1.47 | 6.81E-09 |
| positive regulation of biosynthetic process (GO:0009891) | 1726 | 655 | 445.49 | + | 1.47 | 1.71E-18 |
| cellular component organization (GO:0016043) | 4702 | 1784 | 1213.61 | + | 1.47 | 4.49E-64 |
| positive regulation of cell migration (GO:0030335) | 427 | 162 | 110.21 | + | 1.47 | 1.51E-02 |
| skeletal system development (GO:0001501) | 456 | 173 | 117.70 | + | 1.47 | 6.92E-03 |
| negative regulation of RNA biosynthetic process (GO:1902679) | 1110 | 421 | 286.50 | + | 1.47 | 1.12E-10 |
| chordate embryonic development (GO:0043009) | 699 | 265 | 180.42 | + | 1.47 | 1.02E-05 |
| metal ion homeostasis (GO:0055065) | 488 | 185 | 125.96 | + | 1.47 | 3.09E-03 |
| organic cyclic compound biosynthetic process (GO:1901362) | 1636 | 620 | 422.26 | + | 1.47 | 3.57E-17 |
| embryo development ending in birth or egg hatching (GO:0009792) | 708 | 268 | 182.74 | + | 1.47 | 9.47E-06 |
| positive regulation of nucleobase-containing compound metabolic process (GO:0045935) | 1617 | 611 | 417.36 | + | 1.46 | 1.33E-16 |
| circulatory system development (GO:0072359) | 854 | 322 | 220.42 | + | 1.46 | 3.02E-07 |
| cardiovascular system development (GO:0072358) | 854 | 322 | 220.42 | + | 1.46 | 3.02E-07 |
| negative regulation of RNA metabolic process (GO:0051253) | 1157 | 436 | 298.63 | + | 1.46 | 9.48E-11 |
| cellular protein metabolic process (GO:0044267) | 3251 | 1225 | 839.10 | + | 1.46 | 3.14E-38 |
| anatomical structure formation involved in morphogenesis (GO:0048646) | 990 | 373 | 255.52 | + | 1.46 | 8.88E-09 |
| protein complex assembly (GO:0006461) | 921 | 347 | 237.71 | + | 1.46 | 5.62E-08 |
| protein complex biogenesis (GO:0070271) | 921 | 347 | 237.71 | + | 1.46 | 5.62E-08 |
| cellular response to oxygen-containing compound (GO:1901701) | 937 | 353 | 241.84 | + | 1.46 | 3.71E-08 |
| cellular homeostasis (GO:0019725) | 733 | 276 | 189.19 | + | 1.46 | 8.71E-06 |
| regulation of proteolysis (GO:0030162) | 656 | 247 | 169.32 | + | 1.46 | 6.53E-05 |
| gland development (GO:0048732) | 537 | 202 | 138.60 | + | 1.46 | 1.56E-03 |
| heart development (GO:0007507) | 553 | 208 | 142.73 | + | 1.46 | 1.04E-03 |
| positive regulation of locomotion (GO:0040017) | 455 | 171 | 117.44 | + | 1.46 | 1.37E-02 |
| positive regulation of gene expression (GO:0010628) | 1650 | 620 | 425.87 | + | 1.46 | 2.19E-16 |
| cellular cation homeostasis (GO:0030003) | 482 | 181 | 124.41 | + | 1.45 | 7.27E-03 |
| positive regulation of phosphorylation (GO:0042327) | 919 | 345 | 237.20 | + | 1.45 | 9.79E-08 |
| response to inorganic substance (GO:0010035) | 629 | 236 | 162.35 | + | 1.45 | 1.84E-04 |
| response to oxygen-containing compound (GO:1901700) | 1710 | 641 | 441.36 | + | 1.45 | 7.69E-17 |

| | | | | | | |
|---|---|---|---|---|---|---|
| cellular component organization or biogenesis (GO:0071840) | 4917 | 1843 | 1269.10 | + | 1.45 | 4.81E-63 |
| reproductive structure development (GO:0048608) | 502 | 188 | 129.57 | + | 1.45 | 5.34E-03 |
| negative regulation of nucleobase-containing compound metabolic process (GO:0045934) | 1299 | 486 | 335.28 | + | 1.45 | 8.10E-12 |
| single-organism metabolic process (GO:0044710) | 2872 | 1074 | 741.28 | + | 1.45 | 3.18E-31 |
| response to nutrient levels (GO:0031667) | 567 | 212 | 146.35 | + | 1.45 | 1.20E-03 |
| positive regulation of phosphorus metabolic process (GO:0010562) | 1037 | 387 | 267.66 | + | 1.45 | 1.11E-08 |
| positive regulation of phosphate metabolic process (GO:0045937) | 1037 | 387 | 267.66 | + | 1.45 | 1.11E-08 |
| microtubule-based process (GO:0007017) | 528 | 197 | 136.28 | + | 1.45 | 3.72E-03 |
| regulation of response to stimulus (GO:0048583) | 3270 | 1220 | 844.00 | + | 1.45 | 3.95E-36 |
| reproductive system development (GO:0061458) | 507 | 189 | 130.86 | + | 1.44 | 6.64E-03 |
| response to extracellular stimulus (GO:0009991) | 593 | 221 | 153.06 | + | 1.44 | 8.29E-04 |
| animal organ development (GO:0048513) | 3159 | 1177 | 815.35 | + | 1.44 | 2.65E-34 |
| cellular response to chemical stimulus (GO:0070887) | 2174 | 810 | 561.12 | + | 1.44 | 1.15E-21 |
| cell cycle (GO:0007049) | 760 | 283 | 196.16 | + | 1.44 | 1.50E-05 |
| positive regulation of establishment of protein localization (GO:1904951) | 521 | 194 | 134.47 | + | 1.44 | 5.14E-03 |
| cation transport (GO:0006812) | 720 | 268 | 185.84 | + | 1.44 | 4.18E-05 |
| inorganic ion transmembrane transport (GO:0098660) | 551 | 205 | 142.22 | + | 1.44 | 2.65E-03 |
| localization of cell (GO:0051674) | 793 | 295 | 204.68 | + | 1.44 | 7.47E-06 |
| cell motility (GO:0048870) | 793 | 295 | 204.68 | + | 1.44 | 7.47E-06 |
| response to organic substance (GO:0010033) | 2713 | 1009 | 700.24 | + | 1.44 | 4.46E-28 |
| positive regulation of protein phosphorylation (GO:0001934) | 877 | 326 | 226.36 | + | 1.44 | 1.05E-06 |
| positive regulation of multicellular organismal process (GO:0051240) | 1543 | 573 | 398.26 | + | 1.44 | 6.78E-14 |
| response to lipid (GO:0033993) | 1099 | 408 | 283.66 | + | 1.44 | 5.32E-09 |
| cellular ion homeostasis (GO:0006873) | 496 | 184 | 128.02 | + | 1.44 | 1.23E-02 |
| small molecule metabolic process (GO:0044281) | 1428 | 529 | 368.57 | + | 1.44 | 2.16E-12 |
| positive regulation of cellular component movement (GO:0051272) | 452 | 167 | 116.66 | + | 1.43 | 4.54E-02 |
| response to wounding (GO:0009611) | 455 | 168 | 117.44 | + | 1.43 | 4.41E-02 |
| cellular component assembly (GO:0022607) | 1865 | 688 | 481.37 | + | 1.43 | 9.25E-17 |
| embryo development (GO:0009790) | 1049 | 386 | 270.75 | + | 1.43 | 6.90E-08 |
| cellular response to DNA damage stimulus (GO:0006974) | 582 | 214 | 150.22 | + | 1.42 | 3.30E-03 |
| cellular response to organic substance (GO:0071310) | 1771 | 650 | 457.10 | + | 1.42 | 3.73E-15 |
| positive regulation of protein transport (GO:0051222) | 485 | 178 | 125.18 | + | 1.42 | 3.32E-02 |
| tissue development (GO:0009888) | 1608 | 590 | 415.03 | + | 1.42 | 2.10E-13 |
| epithelium development (GO:0060429) | 1009 | 370 | 260.43 | + | 1.42 | 2.85E-07 |
| cell cycle process (GO:0022402) | 723 | 265 | 186.61 | + | 1.42 | 1.87E-04 |
| macromolecular complex subunit organization (GO:0043933) | 1743 | 638 | 449.88 | + | 1.42 | 1.45E-14 |
| chromatin organization (GO:0006325) | 564 | 206 | 145.57 | + | 1.42 | 8.30E-03 |
| cellular macromolecule metabolic process (GO:0044260) | 5345 | 1950 | 1379.57 | + | 1.41 | 3.64E-59 |

| | | | | | | |
|---|---|---|---|---|---|---|
| cellular metabolic process (GO:0044237) | 7028 | 2558 | 1813.96 | + | 1.41 | 1.14E-86 |
| RNA metabolic process (GO:0016070) | 2014 | 733 | 519.82 | + | 1.41 | 1.01E-16 |
| sensory organ development (GO:0007423) | 589 | 214 | 152.02 | + | 1.41 | 7.33E-03 |
| transmembrane transport (GO:0055085) | 1042 | 378 | 268.95 | + | 1.41 | 6.17E-07 |
| regulation of macromolecule metabolic process (GO:0060255) | 5150 | 1863 | 1329.24 | + | 1.40 | 5.00E-53 |
| regulation of primary metabolic process (GO:0080090) | 5149 | 1862 | 1328.98 | + | 1.40 | 6.92E-53 |
| regulation of cellular metabolic process (GO:0031323) | 5218 | 1883 | 1346.79 | + | 1.40 | 4.45E-53 |
| regulation of metabolic process (GO:0019222) | 5499 | 1982 | 1419.32 | + | 1.40 | 1.34E-56 |
| homeostatic process (GO:0042592) | 1407 | 507 | 363.15 | + | 1.40 | 8.09E-10 |
| nucleobase-containing compound metabolic process (GO:0006139) | 2943 | 1059 | 759.60 | + | 1.39 | 1.11E-24 |
| cellular aromatic compound metabolic process (GO:0006725) | 3120 | 1121 | 805.29 | + | 1.39 | 2.84E-26 |
| cellular component biogenesis (GO:0044085) | 2108 | 756 | 544.09 | + | 1.39 | 7.59E-16 |
| heterocycle metabolic process (GO:0046483) | 3061 | 1097 | 790.06 | + | 1.39 | 3.59E-25 |
| regulation of response to stress (GO:0080134) | 1142 | 409 | 294.76 | + | 1.39 | 4.89E-07 |
| organic cyclic compound metabolic process (GO:1901360) | 3285 | 1173 | 847.88 | + | 1.38 | 8.53E-27 |
| regulation of nitrogen compound metabolic process (GO:0051171) | 3740 | 1332 | 965.31 | + | 1.38 | 4.94E-31 |
| macromolecular complex assembly (GO:0065003) | 1206 | 429 | 311.27 | + | 1.38 | 3.85E-07 |
| regulation of gene expression (GO:0010468) | 3647 | 1295 | 941.31 | + | 1.38 | 2.23E-29 |
| nucleic acid metabolic process (GO:0090304) | 2470 | 877 | 637.52 | + | 1.38 | 6.69E-18 |
| regulation of macromolecule biosynthetic process (GO:0010556) | 3463 | 1229 | 893.82 | + | 1.37 | 2.24E-27 |
| organ morphogenesis (GO:0009887) | 933 | 331 | 240.81 | + | 1.37 | 8.73E-05 |
| regulation of cell proliferation (GO:0042127) | 1562 | 554 | 403.16 | + | 1.37 | 7.26E-10 |
| regulation of cellular macromolecule biosynthetic process (GO:2000112) | 3355 | 1189 | 865.94 | + | 1.37 | 5.20E-26 |
| organic substance biosynthetic process (GO:1901576) | 3177 | 1125 | 820.00 | + | 1.37 | 4.11E-24 |
| positive regulation of response to stimulus (GO:0048584) | 1778 | 629 | 458.91 | + | 1.37 | 1.97E-11 |
| biosynthetic process (GO:0009058) | 3255 | 1151 | 840.13 | + | 1.37 | 1.33E-24 |
| primary metabolic process (GO:0044238) | 7212 | 2547 | 1861.45 | + | 1.37 | 9.43E-73 |
| regulation of biosynthetic process (GO:0009889) | 3696 | 1303 | 953.96 | + | 1.37 | 2.65E-28 |
| regulation of nucleic acid-templated transcription (GO:1903506) | 3003 | 1058 | 775.09 | + | 1.37 | 1.41E-21 |
| regulation of nucleobase-containing compound metabolic process (GO:0019219) | 3481 | 1226 | 898.46 | + | 1.36 | 5.34E-26 |
| regulation of cell adhesion (GO:0030155) | 619 | 218 | 159.77 | + | 1.36 | 4.36E-02 |
| organic substance metabolic process (GO:0071704) | 7647 | 2693 | 1973.73 | + | 1.36 | 4.04E-78 |
| regulation of RNA biosynthetic process (GO:2001141) | 3010 | 1060 | 776.90 | + | 1.36 | 1.44E-21 |
| regulation of cellular biosynthetic process (GO:0031326) | 3632 | 1279 | 937.44 | + | 1.36 | 1.97E-27 |
| regulation of transcription, DNA-templated (GO:0006355) | 2977 | 1048 | 768.38 | + | 1.36 | 3.32E-21 |
| regulation of RNA metabolic process (GO:0051252) | 3133 | 1102 | 808.64 | + | 1.36 | 1.74E-22 |
| biological adhesion (GO:0022610) | 882 | 310 | 227.65 | + | 1.36 | 5.89E-04 |
| cellular biosynthetic process (GO:0044249) | 3109 | 1092 | 802.45 | + | 1.36 | 5.12E-22 |

| | | | | | | |
|---|---|---|---|---|---|---|
| protein metabolic process (GO:0019538) | 3867 | 1355 | 998.09 | + | 1.36 | 1.24E-28 |
| cell adhesion (GO:0007155) | 871 | 305 | 224.81 | + | 1.36 | 1.03E-03 |
| macromolecule metabolic process (GO:0043170) | 6052 | 2112 | 1562.05 | + | 1.35 | 4.03E-51 |
| metabolic process (GO:0008152) | 8072 | 2810 | 2083.43 | + | 1.35 | 4.42E-78 |
| organic acid metabolic process (GO:0006082) | 771 | 267 | 199.00 | + | 1.34 | 1.42E-02 |
| positive regulation of cell proliferation (GO:0008284) | 887 | 307 | 228.94 | + | 1.34 | 2.54E-03 |
| nitrogen compound metabolic process (GO:0006807) | 4129 | 1425 | 1065.72 | + | 1.34 | 1.21E-27 |
| chromosome organization (GO:0051276) | 841 | 290 | 217.07 | + | 1.34 | 7.19E-03 |
| response to external stimulus (GO:0009605) | 1979 | 679 | 510.79 | + | 1.33 | 4.92E-10 |
| cell surface receptor signaling pathway (GO:0007166) | 1656 | 568 | 427.42 | + | 1.33 | 7.52E-08 |
| macromolecule biosynthetic process (GO:0009059) | 2384 | 815 | 615.32 | + | 1.32 | 2.02E-12 |
| cellular macromolecule biosynthetic process (GO:0034645) | 2357 | 805 | 608.35 | + | 1.32 | 3.96E-12 |
| response to stress (GO:0006950) | 2989 | 1016 | 771.48 | + | 1.32 | 7.23E-16 |
| cellular nitrogen compound metabolic process (GO:0034641) | 3794 | 1283 | 979.25 | + | 1.31 | 9.41E-21 |
| reproductive process (GO:0022414) | 1402 | 472 | 361.86 | + | 1.30 | 4.94E-05 |
| reproduction (GO:0000003) | 1405 | 473 | 362.64 | + | 1.30 | 4.77E-05 |
| gene expression (GO:0010467) | 2601 | 869 | 671.33 | + | 1.29 | 3.46E-11 |
| cellular nitrogen compound biosynthetic process (GO:0044271) | 2277 | 759 | 587.71 | + | 1.29 | 4.16E-09 |
| proteolysis (GO:0006508) | 1066 | 353 | 275.14 | + | 1.28 | 1.82E-02 |
| single organism reproductive process (GO:0044702) | 1261 | 417 | 325.47 | + | 1.28 | 2.49E-03 |
| organonitrogen compound metabolic process (GO:1901564) | 1778 | 573 | 458.91 | + | 1.25 | 4.03E-04 |
| cellular process (GO:0009987) | 14095 | 4461 | 3637.99 | + | 1.23 | 1.02E-102 |
| biological regulation (GO:0065007) | 11690 | 3691 | 3017.25 | + | 1.22 | 5.68E-63 |
| single-organism cellular process (GO:0044763) | 10837 | 3415 | 2797.09 | + | 1.22 | 1.67E-52 |
| single-organism process (GO:0044699) | 12411 | 3897 | 3203.34 | + | 1.22 | 1.21E-67 |
| regulation of biological process (GO:0050789) | 11093 | 3468 | 2863.16 | + | 1.21 | 3.37E-50 |
| regulation of cellular process (GO:0050794) | 10637 | 3322 | 2745.47 | + | 1.21 | 1.61E-45 |
| multi-organism process (GO:0051704) | 1944 | 602 | 501.76 | + | 1.20 | 2.49E-02 |
| biological_process (GO:0008150) | 17404 | 5331 | 4492.06 | + | 1.19 | 4.92E-145 |
| multicellular organismal process (GO:0032501) | 7149 | 2160 | 1845.19 | + | 1.17 | 2.66E-14 |
| response to stimulus (GO:0050896) | 8233 | 2402 | 2124.98 | + | 1.13 | 8.03E-10 |
| cellular response to stimulus (GO:0051716) | 6618 | 1879 | 1708.14 | + | 1.10 | 6.26E-03 |
| system process (GO:0003008) | 2700 | 486 | 696.88 | - | .70 | 2.51E-15 |
| neurological system process (GO:0050877) | 2229 | 326 | 575.32 | - | .57 | 2.29E-28 |
| Unclassified (UNCLASSIFIED) | 6377 | 807 | 1645.94 | - | .49 | 0.00E00 |
| G-protein coupled receptor signaling pathway (GO:0007186) | 2278 | 234 | 587.96 | - | .40 | 4.43E-63 |
| sensory perception (GO:0007600) | 1865 | 150 | 481.37 | - | .31 | 3.12E-70 |
| detection of stimulus (GO:0051606) | 1605 | 75 | 414.26 | - | < 0.2 | 5.06E-94 |

| | | | | | | |
|---|---|---|---|---|---|---|
| detection of stimulus involved in sensory perception (GO:0050906) | 1509 | 50 | 389.48 | - | < 0.2 | 3.91E-105 |
| sensory perception of chemical stimulus (GO:0007606) | 1510 | 44 | 389.74 | - | < 0.2 | 6.97E-111 |
| detection of chemical stimulus (GO:0009593) | 1477 | 39 | 381.22 | - | < 0.2 | 2.63E-112 |
| sensory perception of smell (GO:0007608) | 1427 | 36 | 368.32 | - | < 0.2 | 5.41E-110 |
| detection of chemical stimulus involved in sensory perception (GO:0050907) | 1445 | 27 | 372.96 | - | < 0.2 | 8.84E-122 |
| detection of chemical stimulus involved in sensory perception of smell (GO:0050911) | 1389 | 25 | 358.51 | - | < 0.2 | 6.55E-118 |
