# Supplementary material for: The RNA-Binding Protein hnRNP K Mediates the Effect of BDNF on Dendritic mRNA Metabolism and Regulates Synaptic NMDA Receptors in Hippocampal Neurons
Source: eNeuro. 2017 Dec 12;4(6):ENEURO.0268-17.2017. doi: 10.1523/ENEURO.0268-17.2017 (PMC5732018; doi:10.1523/ENEURO.0268-17.2017)
Supplement: Table 1-7 — Download Table 1-7, PDF file. [file sup_enu-eN-NWR-0268-17-s10.pdf]

Table 1-7 - Cellular components associated with mRNAs co-immunoprecipitated with hnRNP K and regulated by BDNF as assessed by Gene Ontology

GO Cellular component	Rattus norvegicus (reference list)	uploaded list	(expected)	(over/under)	(fold enrichment)	(P-value)
clathrin-coated pit (GO:0005905)	54	35	13.94	+	2.51	1.84E-03
exocytic vesicle membrane (GO:0099501)	62	40	16.00	+	2.50	3.89E-04
synaptic vesicle membrane (GO:0030672)	62	40	16.00	+	2.50	3.89E-04
dendritic shaft (GO:0043198)	58	37	14.97	+	2.47	1.36E-03
T-tubule (GO:0030315)	60	38	15.49	+	2.45	1.16E-03
neuron projection membrane (GO:0032589)	49	31	12.65	+	2.45	1.12E-02
SNARE complex (GO:0031201)	51	32	13.16	+	2.43	9.55E-03
spindle microtubule (GO:0005876)	53	33	13.68	+	2.41	8.12E-03
neurotransmitter receptor complex (GO:0098878)	50	31	12.91	+	2.40	1.64E-02
ionotropic glutamate receptor complex (GO:0008328)	50	31	12.91	+	2.40	1.64E-02
terminal bouton (GO:0043195)	111	67	28.65	+	2.34	7.92E-07
late endosome membrane (GO:0031902)	63	37	16.26	+	2.28	8.45E-03
postsynaptic density (GO:0014069)	235	138	60.65	+	2.28	9.55E-15
postsynaptic specialization (GO:0099572)	235	138	60.65	+	2.28	9.55E-15
cytoplasmic microtubule (GO:0005881)	55	32	14.20	+	2.25	4.02E-02
main axon (GO:0044304)	74	43	19.10	+	2.25	2.11E-03
Golgi-associated vesicle (GO:0005798)	69	40	17.81	+	2.25	4.99E-03
excitatory synapse (GO:0060076)	255	146	65.82	+	2.22	8.76E-15
transport vesicle membrane (GO:0030658)	117	66	30.20	+	2.19	1.36E-05
presynapse (GO:0098793)	330	185	85.17	+	2.17	3.16E-18
axon part (GO:0033267)	307	171	79.24	+	2.16	1.86E-16
presynaptic membrane (GO:0042734)	74	41	19.10	+	2.15	1.09E-02
voltage-gated potassium channel complex (GO:0008076)	76	42	19.62	+	2.14	9.06E-03
dendrite (GO:0030425)	581	320	149.96	+	2.13	1.47E-31
neuron projection terminus (GO:0044306)	198	109	51.10	+	2.13	1.29E-09
filopodium (GO:0030175)	91	50	23.49	+	2.13	1.55E-03
neuron spine (GO:0044309)	155	85	40.01	+	2.12	4.43E-07
axon terminus (GO:0043679)	185	101	47.75	+	2.12	1.34E-08
autophagosome (GO:0005776)	70	38	18.07	+	2.10	3.46E-02

dendritic spine (GO:0043197)	153	83	39.49	+	2.10	1.15E-06
endosome membrane (GO:0010008)	240	130	61.95	+	2.10	2.83E-11
Golgi membrane (GO:0000139)	390	211	100.66	+	2.10	2.95E-19
potassium channel complex (GO:0034705)	78	42	20.13	+	2.09	1.65E-02
trans-Golgi network (GO:0005802)	158	85	40.78	+	2.08	1.06E-06
synapse (GO:0045202)	842	449	217.32	+	2.07	6.01E-42
postsynaptic membrane (GO:0045211)	197	105	50.85	+	2.07	2.04E-08
axon (GO:0030424)	550	293	141.96	+	2.06	2.00E-26
synaptic vesicle (GO:0008021)	154	82	39.75	+	2.06	3.26E-06
synaptic membrane (GO:0097060)	267	142	68.91	+	2.06	7.33E-12
synapse part (GO:0044456)	677	360	174.74	+	2.06	7.74E-33
postsynapse (GO:0098794)	428	227	110.47	+	2.05	8.26E-20
neuron projection (GO:0043005)	1165	610	300.69	+	2.03	8.07E-56
somatodendritic compartment (GO:0036477)	865	452	223.26	+	2.02	3.95E-40
mitochondrial outer membrane (GO:0005741)	138	72	35.62	+	2.02	6.26E-05
sarcolemma (GO:0042383)	150	78	38.72	+	2.01	2.06E-05
Golgi apparatus part (GO:0044431)	577	300	148.93	+	2.01	1.92E-25
endosomal part (GO:0044440)	258	134	66.59	+	2.01	2.19E-10
serine/threonine protein kinase complex (GO:1902554)	81	42	20.91	+	2.01	3.83E-02
exocytic vesicle (GO:0070382)	170	88	43.88	+	2.01	3.22E-06
transport vesicle (GO:0030133)	292	151	75.37	+	2.00	9.32E-12
clathrin-coated vesicle (GO:0030136)	97	50	25.04	+	2.00	8.51E-03
coated vesicle (GO:0030135)	152	78	39.23	+	1.99	3.51E-05
cytoplasmic vesicle part (GO:0044433)	305	156	78.72	+	1.98	8.08E-12
leading edge membrane (GO:0031256)	133	68	34.33	+	1.98	2.93E-04
cytoplasmic vesicle membrane (GO:0030659)	283	144	73.04	+	1.97	1.27E-10
growth cone (GO:0030426)	171	87	44.14	+	1.97	8.53E-06
vacuolar membrane (GO:0005774)	421	214	108.66	+	1.97	1.55E-16
organelle outer membrane (GO:0031968)	158	80	40.78	+	1.96	4.06E-05
site of polarized growth (GO:0030427)	176	89	45.43	+	1.96	7.60E-06
outer membrane (GO:0019867)	163	82	42.07	+	1.95	3.61E-05
neuronal cell body (GO:0043025)	623	313	160.80	+	1.95	2.95E-24
Golgi subcompartment (GO:0098791)	245	123	63.24	+	1.95	1.75E-08

organelle subcompartment (GO:0031984)	258	129	66.59	+	1.94	7.34E-09
vacuolar part (GO:0044437)	441	220	113.82	+	1.93	3.77E-16
neuron part (GO:0097458)	1518	753	391.80	+	1.92	5.71E-61
cell body (GO:0044297)	700	346	180.67	+	1.92	7.99E-26
ribonucleoprotein granule (GO:0035770)	146	72	37.68	+	1.91	4.91E-04
whole membrane (GO:0098805)	1122	553	289.59	+	1.91	1.16E-42
cytoplasmic ribonucleoprotein granule (GO:0036464)	138	68	35.62	+	1.91	1.04E-03
recycling endosome (GO:0055037)	122	60	31.49	+	1.91	4.64E-03
cation channel complex (GO:0034703)	155	76	40.01	+	1.90	2.96E-04
cell leading edge (GO:0031252)	349	171	90.08	+	1.90	1.59E-11
bounding membrane of organelle (GO:0098588)	1191	583	307.40	+	1.90	2.51E-44
vesicle membrane (GO:0012506)	311	151	80.27	+	1.88	1.06E-09
microbody (GO:0042579)	136	66	35.10	+	1.88	2.43E-03
peroxisome (GO:0005777)	136	66	35.10	+	1.88	2.43E-03
membrane region (GO:0098589)	396	192	102.21	+	1.88	1.02E-12
focal adhesion (GO:0005925)	390	189	100.66	+	1.88	1.79E-12
perinuclear region of cytoplasm (GO:0048471)	637	308	164.41	+	1.87	2.60E-21
ruffle (GO:0001726)	145	70	37.43	+	1.87	1.46E-03
lamellipodium (GO:0030027)	162	78	41.81	+	1.87	4.14E-04
cell-substrate adherens junction (GO:0005924)	395	190	101.95	+	1.86	2.97E-12
Golgi apparatus (GO:0005794)	1165	558	300.69	+	1.86	1.18E-39
cell-substrate junction (GO:0030055)	400	191	103.24	+	1.85	4.89E-12
endocytic vesicle (GO:0030139)	128	61	33.04	+	1.85	9.86E-03
intrinsic component of organelle membrane (GO:0031300)	143	68	36.91	+	1.84	3.35E-03
adherens junction (GO:0005912)	470	219	121.31	+	1.81	5.81E-13
endosome (GO:0005768)	642	299	165.70	+	1.80	2.49E-18
ion channel complex (GO:0034702)	262	122	67.62	+	1.80	1.73E-06
integral component of organelle membrane (GO:0031301)	140	65	36.13	+	1.80	1.14E-02
early endosome (GO:0005769)	246	114	63.49	+	1.80	7.52E-06
myelin sheath (GO:0043209)	208	96	53.69	+	1.79	1.34E-04
anchoring junction (GO:0070161)	485	222	125.18	+	1.77	2.22E-12
membrane microdomain (GO:0098857)	337	154	86.98	+	1.77	4.91E-08
membrane raft (GO:0045121)	337	154	86.98	+	1.77	4.91E-08

microtubule (GO:0005874)	322	147	83.11	+	1.77	1.44E-07
lytic vacuole membrane (GO:0098852)	213	97	54.98	+	1.76	2.08E-04
lysosomal membrane (GO:0005765)	213	97	54.98	+	1.76	2.08E-04
perikaryon (GO:0043204)	141	64	36.39	+	1.76	2.57E-02
vacuole (GO:0005773)	979	444	252.69	+	1.76	5.76E-26
cell projection membrane (GO:0031253)	263	119	67.88	+	1.75	1.28E-05
cell projection part (GO:0044463)	1015	455	261.98	+	1.74	1.21E-25
transporter complex (GO:1990351)	293	131	75.62	+	1.73	4.79E-06
ubiquitin ligase complex (GO:0000151)	273	122	70.46	+	1.73	1.64E-05
transmembrane transporter complex (GO:1902495)	289	129	74.59	+	1.73	6.87E-06
late endosome (GO:0005770)	177	79	45.68	+	1.73	5.52E-03
cell projection (GO:0042995)	1921	856	495.82	+	1.73	8.32E-51
cell junction (GO:0030054)	1122	495	289.59	+	1.71	1.26E-26
organelle membrane (GO:0031090)	1949	857	503.05	+	1.70	1.28E-48
cullin-RING ubiquitin ligase complex (GO:0031461)	153	67	39.49	+	1.70	4.85E-02
cytoplasmic, membrane-bounded vesicle (GO:0016023)	925	404	238.75	+	1.69	1.72E-20
cytoplasmic region (GO:0099568)	298	130	76.92	+	1.69	2.17E-05
nuclear outer membrane-endoplasmic reticulum membrane network (GO:0042175)	703	306	181.45	+	1.69	8.04E-15
endoplasmic reticulum part (GO:0044432)	772	335	199.26	+	1.68	3.20E-16
endomembrane system (GO:0012505)	3241	1404	836.52	+	1.68	3.34E-82
endoplasmic reticulum membrane (GO:0005789)	686	297	177.06	+	1.68	4.89E-14
cytoplasmic vesicle (GO:0031410)	1035	448	267.14	+	1.68	3.07E-22
intracellular vesicle (GO:0097708)	1037	448	267.66	+	1.67	4.43E-22
endoplasmic reticulum (GO:0005783)	1355	584	349.73	+	1.67	2.05E-29
transferase complex (GO:1990234)	701	297	180.93	+	1.64	7.00E-13
plasma membrane region (GO:0098590)	920	389	237.46	+	1.64	2.37E-17
nuclear envelope (GO:0005635)	384	162	99.11	+	1.63	3.89E-06
nuclear membrane (GO:0031965)	243	102	62.72	+	1.63	3.54E-03
transferase complex, transferring phosphorus-containing groups (GO:0061695)	239	100	61.69	+	1.62	5.01E-03
nucleoplasm (GO:0005654)	2113	882	545.38	+	1.62	1.57E-41
plasma membrane protein complex (GO:0098797)	476	195	122.86	+	1.59	9.75E-07
spindle (GO:0005819)	259	106	66.85	+	1.59	6.64E-03
cell cortex (GO:0005938)	239	97	61.69	+	1.57	2.21E-02

actin cytoskeleton (GO:0015629)	430	174	110.99	+	1.57	1.76E-05
nuclear body (GO:0016604)	287	116	74.08	+	1.57	4.33E-03
envelope (GO:0031975)	981	396	253.20	+	1.56	1.49E-14
organelle envelope (GO:0031967)	974	393	251.39	+	1.56	2.13E-14
catalytic complex (GO:1902494)	1058	426	273.08	+	1.56	1.21E-15
receptor complex (GO:0043235)	320	127	82.59	+	1.54	3.67E-03
lytic vacuole (GO:0000323)	429	170	110.73	+	1.54	9.64E-05
lysosome (GO:0005764)	429	170	110.73	+	1.54	9.64E-05
transcription factor complex (GO:0005667)	313	123	80.79	+	1.52	8.10E-03
cytoplasmic part (GO:0044444)	6819	2675	1760.02	+	1.52	5.00E-133
intracellular organelle lumen (GO:0070013)	3296	1291	850.71	+	1.52	5.72E-50
organelle lumen (GO:0043233)	3301	1292	852.01	+	1.52	7.51E-50
membrane-enclosed lumen (GO:0031974)	3301	1292	852.01	+	1.52	7.51E-50
membrane protein complex (GO:0098796)	1031	403	266.11	+	1.51	9.19E-13
nuclear part (GO:0044428)	3385	1319	873.69	+	1.51	4.09E-50
nuclear lumen (GO:0031981)	2921	1138	753.93	+	1.51	6.90E-42
nucleoplasm part (GO:0044451)	668	260	172.41	+	1.51	2.09E-07
mitochondrial membrane (GO:0031966)	558	217	144.02	+	1.51	7.14E-06
cytosol (GO:0005829)	1984	770	512.08	+	1.50	5.16E-26
mitochondrial envelope (GO:0005740)	603	234	155.64	+	1.50	2.18E-06
mitochondrion (GO:0005739)	1740	671	449.10	+	1.49	1.61E-21
secretory vesicle (GO:0099503)	449	173	115.89	+	1.49	4.20E-04
cytoplasm (GO:0005737)	9562	3678	2468.00	+	1.49	2.98E-209
mitochondrial part (GO:0044429)	786	302	202.87	+	1.49	2.72E-08
intracellular organelle part (GO:0044446)	6833	2619	1763.63	+	1.49	1.67E-116
intracellular membrane-bounded organelle (GO:0043231)	9568	3664	2469.55	+	1.48	7.09E-204
nucleolus (GO:0005730)	815	312	210.36	+	1.48	1.87E-08
vesicle (GO:0031982)	3401	1299	877.82	+	1.48	8.01E-45
membrane-bounded vesicle (GO:0031988)	3254	1241	839.87	+	1.48	4.36E-42
organelle part (GO:0044422)	7014	2668	1810.35	+	1.47	8.79E-116
membrane-bounded organelle (GO:0043227)	10720	4035	2766.89	+	1.46	1.58E-229
protein complex (GO:0043234)	4040	1520	1042.75	+	1.46	1.14E-50
microtubule cytoskeleton (GO:0015630)	1010	379	260.69	+	1.45	1.46E-09

cytoskeleton (GO:0005856)	1760	657	454.27	+	1.45	8.96E-18
extracellular vesicle (GO:1903561)	2610	973	673.65	+	1.44	5.11E-28
extracellular organelle (GO:0043230)	2617	975	675.46	+	1.44	5.39E-28
extracellular exosome (GO:0070062)	2596	966	670.04	+	1.44	1.65E-27
nucleus (GO:0005634)	6007	2215	1550.44	+	1.43	9.76E-76
intracellular organelle (GO:0043229)	10800	3980	2787.54	+	1.43	1.31E-202
intracellular part (GO:0044424)	12356	4505	3189.15	+	1.41	4.65E-256
organelle (GO:0043226)	11850	4307	3058.55	+	1.41	3.26E-226
polymeric cytoskeletal fiber (GO:0099513)	560	203	144.54	+	1.40	2.41E-03
supramolecular fiber (GO:0099512)	560	203	144.54	+	1.40	2.41E-03
intracellular (GO:0005622)	12864	4637	3320.27	+	1.40	1.84E-261
microtubule organizing center (GO:0005815)	592	213	152.80	+	1.39	2.21E-03
plasma membrane part (GO:0044459)	2252	802	581.25	+	1.38	3.96E-17
intracellular non-membrane-bounded organelle (GO:0043232)	3946	1403	1018.48	+	1.38	1.13E-33
non-membrane-bounded organelle (GO:0043228)	3946	1403	1018.48	+	1.38	1.13E-33
macromolecular complex (GO:0032991)	5064	1798	1307.04	+	1.38	3.77E-46
nuclear chromosome part (GO:0044454)	488	173	125.96	+	1.37	4.20E-02
cytoskeletal part (GO:0044430)	1358	481	350.51	+	1.37	6.61E-09
chromosome (GO:0005694)	862	304	222.49	+	1.37	8.90E-05
chromosomal part (GO:0044427)	790	278	203.90	+	1.36	3.81E-04
integral component of plasma membrane (GO:0005887)	1094	383	282.37	+	1.36	3.88E-06
intrinsic component of plasma membrane (GO:0031226)	1162	406	299.92	+	1.35	1.61E-06
extracellular region part (GO:0044421)	3745	1230	966.60	+	1.27	3.08E-16
cell part (GO:0044464)	15800	5096	4078.06	+	1.25	5.02E-184
cell (GO:0005623)	15869	5105	4095.87	+	1.25	8.18E-182
extracellular region (GO:0005576)	4135	1312	1067.27	+	1.23	7.44E-13
membrane (GO:0016020)	9765	2987	2520.40	+	1.19	2.04E-30
cellular_component (GO:0005575)	18430	5595	4756.88	+	1.18	2.23E-173
cell periphery (GO:0071944)	5396	1539	1392.74	+	1.11	6.94E-03
plasma membrane (GO:0005886)	5275	1497	1361.50	+	1.10	2.52E-02
membrane part (GO:0044425)	7677	2135	1981.47	+	1.08	2.04E-02
Unclassified (UNCLASSIFIED)	5351	543	1381.12	-	.39	0.00E00
