# Supplementary material for: The RNA-Binding Protein hnRNP K Mediates the Effect of BDNF on Dendritic mRNA Metabolism and Regulates Synaptic NMDA Receptors in Hippocampal Neurons
Source: eNeuro. 2017 Dec 12;4(6):ENEURO.0268-17.2017. doi: 10.1523/ENEURO.0268-17.2017 (PMC5732018; doi:10.1523/ENEURO.0268-17.2017)
Supplement: Table 1-8 — Download Table 1-8, PDF file. [file sup_enu-eN-NWR-0268-17-s11.pdf]

Table 1-8 - Molecular functions associated with mRNAs co-immunoprecipitated with hnRNP K and regulated by BDNF as assessed by Gene Ontology

GO Molecular function	Rattus norvegicus (reference list)	uploaded list	(expected)	(over/under)	(fold enrichment)	(P-value)
protein serine/threonine phosphatase activity (GO:0004722)	49	33	12.65	+	2.61	3.27E-03
ubiquitin binding (GO:0043130)	105	58	27.10	+	2.14	3.95E-04
phosphoprotein phosphatase activity (GO:0004721)	163	86	42.07	+	2.04	4.15E-06
ubiquitin-like protein binding (GO:0032182)	127	66	32.78	+	2.01	5.03E-04
ubiquitin protein ligase activity (GO:0061630)	197	101	50.85	+	1.99	7.55E-07
ubiquitin-like protein ligase activity (GO:0061659)	201	103	51.88	+	1.99	5.21E-07
SH3 domain binding (GO:0017124)	94	48	24.26	+	1.98	3.25E-02
protein C-terminus binding (GO:0008022)	210	107	54.20	+	1.97	3.21E-07
ion channel binding (GO:0044325)	124	63	32.01	+	1.97	1.95E-03
calmodulin binding (GO:0005516)	152	77	39.23	+	1.96	1.43E-04
syntaxin binding (GO:0019905)	95	48	24.52	+	1.96	4.21E-02
protein tyrosine phosphatase activity (GO:0004725)	100	50	25.81	+	1.94	3.73E-02
magnesium ion binding (GO:0000287)	204	102	52.65	+	1.94	2.25E-06
phosphatase activity (GO:0016791)	261	130	67.37	+	1.93	1.57E-08
protein phosphatase binding (GO:0019903)	139	69	35.88	+	1.92	1.35E-03
protein tyrosine kinase activity (GO:0004713)	128	63	33.04	+	1.91	5.31E-03
ubiquitin-like protein ligase binding (GO:0044389)	279	137	72.01	+	1.90	1.12E-08
ubiquitin protein ligase binding (GO:0031625)	275	135	70.98	+	1.90	1.63E-08
small GTPase binding (GO:0031267)	276	135	71.24	+	1.90	2.07E-08
SNARE binding (GO:0000149)	129	63	33.30	+	1.89	6.76E-03
Ras GTPase binding (GO:0017016)	261	126	67.37	+	1.87	2.23E-07
GTPase binding (GO:0051020)	305	147	78.72	+	1.87	7.16E-09
isomerase activity (GO:0016853)	157	75	40.52	+	1.85	1.83E-03
kinase binding (GO:0019900)	661	310	170.61	+	1.82	3.28E-19
protein kinase binding (GO:0019901)	594	276	153.31	+	1.80	2.43E-16
phosphoric ester hydrolase activity (GO:0042578)	347	161	89.56	+	1.80	1.18E-08
nucleoside-triphosphatase regulator activity (GO:0060589)	247	114	63.75	+	1.79	1.87E-05
phosphatase binding (GO:0019902)	185	85	47.75	+	1.78	1.65E-03
protein domain specific binding (GO:0019904)	690	316	178.09	+	1.77	3.98E-18
potassium ion transmembrane transporter activity (GO:0015079)	142	65	36.65	+	1.77	3.49E-02
GTPase activity (GO:0003924)	182	83	46.98	+	1.77	2.92E-03
protein serine/threonine kinase activity (GO:0004674)	426	194	109.95	+	1.76	4.13E-10
protein kinase activity (GO:0004672)	604	272	155.90	+	1.74	2.10E-14
kinase activity (GO:0016301)	806	362	208.03	+	1.74	9.69E-20
phosphotransferase activity, alcohol group as acceptor (GO:0016773)	707	316	182.48	+	1.73	1.23E-16
ubiquitin-like protein transferase activity (GO:0019787)	412	183	106.34	+	1.72	1.49E-08
enzyme activator activity (GO:0008047)	378	167	97.56	+	1.71	1.81E-07

enzyme binding (GO:0019899)	1866	823	481.62	+	1.71	1.26E-46
ubiquitin-protein transferase activity (GO:0004842)	397	175	102.47	+	1.71	7.60E-08
GTPase activator activity (GO:0005096)	205	90	52.91	+	1.70	4.84E-03
transferase activity, transferring phosphorus-containing groups (GO:0016772)	952	416	245.72	+	1.69	6.63E-21
GTPase regulator activity (GO:0030695)	225	98	58.07	+	1.69	2.45E-03
voltage-gated channel activity (GO:0022832)	182	79	46.98	+	1.68	2.88E-02
voltage-gated ion channel activity (GO:0005244)	182	79	46.98	+	1.68	2.88E-02
microtubule binding (GO:0008017)	203	86	52.40	+	1.64	2.92E-02
ligase activity (GO:0016874)	271	114	69.95	+	1.63	1.76E-03
guanyl ribonucleotide binding (GO:0032561)	397	167	102.47	+	1.63	5.47E-06
GTP binding (GO:0005525)	376	158	97.05	+	1.63	1.56E-05
guanyl nucleotide binding (GO:0019001)	398	167	102.73	+	1.63	6.47E-06
identical protein binding (GO:0042802)	1285	538	331.67	+	1.62	1.04E-23
protein heterodimerization activity (GO:0046982)	541	225	139.63	+	1.61	2.65E-08
metal ion transmembrane transporter activity (GO:0046873)	397	165	102.47	+	1.61	1.50E-05
protein homodimerization activity (GO:0042803)	779	321	201.06	+	1.60	3.19E-12
cytoskeletal protein binding (GO:0008092)	822	338	212.16	+	1.59	7.24E-13
transcription factor binding (GO:0008134)	559	226	144.28	+	1.57	2.82E-07
transcription factor activity, transcription factor binding (GO:0000989)	451	182	116.41	+	1.56	2.01E-05
actin binding (GO:0003779)	367	148	94.72	+	1.56	4.94E-04
enzyme regulator activity (GO:0030234)	854	344	220.42	+	1.56	5.93E-12
transcription factor activity, protein binding (GO:0000988)	452	182	116.66	+	1.56	2.34E-05
molecular function regulator (GO:0098772)	1156	465	298.37	+	1.56	6.61E-17
tubulin binding (GO:0015631)	279	112	72.01	+	1.56	1.69E-02
protein dimerization activity (GO:0046983)	1243	497	320.82	+	1.55	9.08E-18
transcription cofactor activity (GO:0003712)	401	160	103.50	+	1.55	3.07E-04
gated channel activity (GO:0022836)	302	120	77.95	+	1.54	1.29E-02
transferase activity (GO:0016740)	2243	889	578.93	+	1.54	1.60E-33
protein complex binding (GO:0032403)	857	338	221.20	+	1.53	1.47E-10
protein binding (GO:0005515)	6478	2546	1672.01	+	1.52	3.33E-124
purine nucleotide binding (GO:0017076)	1786	701	460.98	+	1.52	2.66E-24
zinc ion binding (GO:0008270)	1072	420	276.69	+	1.52	3.07E-13
purine ribonucleotide binding (GO:0032555)	1776	695	458.39	+	1.52	1.00E-23
ribonucleotide binding (GO:0032553)	1792	700	462.52	+	1.51	1.01E-23
nucleoside binding (GO:0001882)	1750	683	451.68	+	1.51	5.89E-23
ribonucleoside binding (GO:0032549)	1743	679	449.88	+	1.51	1.35E-22
purine ribonucleoside triphosphate binding (GO:0035639)	1732	674	447.04	+	1.51	2.73E-22
purine ribonucleoside binding (GO:0032550)	1740	677	449.10	+	1.51	2.18E-22
purine nucleoside binding (GO:0001883)	1743	678	449.88	+	1.51	2.12E-22
inorganic cation transmembrane transporter activity (GO:0022890)	494	192	127.50	+	1.51	1.10E-04
macromolecular complex binding (GO:0044877)	1408	547	363.41	+	1.51	2.52E-17

adenyl nucleotide binding (GO:0030554)	1428	552	368.57	+	1.50	4.43E-17
poly(A) RNA binding (GO:0044822)	1181	455	304.82	+	1.49	2.29E-13
adenyl ribonucleotide binding (GO:0032559)	1420	547	366.51	+	1.49	1.32E-16
substrate-specific transporter activity (GO:0022892)	1050	404	271.01	+	1.49	1.63E-11
hydrolase activity, acting on ester bonds (GO:0016788)	726	279	187.38	+	1.49	3.13E-07
substrate-specific transmembrane transporter activity (GO:0022891)	885	340	228.42	+	1.49	2.95E-09
carbohydrate derivative binding (GO:0097367)	2125	816	548.47	+	1.49	3.84E-26
transcriptional activator activity, RNA polymerase II transcription regulatory region sequence-specific binding (GO:0001228)	337	129	86.98	+	1.48	3.26E-02
cation transmembrane transporter activity (GO:0008324)	578	221	149.18	+	1.48	3.83E-05
ion transmembrane transporter activity (GO:0015075)	756	289	195.13	+	1.48	2.46E-07
substrate-specific channel activity (GO:0022838)	411	157	106.08	+	1.48	4.57E-03
ATP binding (GO:0005524)	1385	529	357.48	+	1.48	2.81E-15
ion channel activity (GO:0005216)	399	152	102.98	+	1.48	7.60E-03
nucleotide binding (GO:0000166)	2285	869	589.77	+	1.47	7.70E-27
nucleoside phosphate binding (GO:1901265)	2285	869	589.77	+	1.47	7.70E-27
small molecule binding (GO:0036094)	2465	936	636.23	+	1.47	4.32E-29
transcription factor activity, RNA polymerase II core promoter proximal region sequence-specific binding (GO:0000982)	353	134	91.11	+	1.47	3.30E-02
chromatin binding (GO:0003682)	511	193	131.89	+	1.46	6.77E-04
transmembrane transporter activity (GO:0022857)	977	368	252.17	+	1.46	3.99E-09
transporter activity (GO:0005215)	1231	463	317.73	+	1.46	5.50E-12
regulatory region nucleic acid binding (GO:0001067)	787	296	203.13	+	1.46	7.35E-07
RNA polymerase II transcription factor activity, sequence-specific DNA binding (GO:0000981)	631	237	162.86	+	1.46	4.82E-05
transcription regulatory region DNA binding (GO:0044212)	781	293	201.58	+	1.45	1.16E-06
regulatory region DNA binding (GO:0000975)	784	294	202.35	+	1.45	1.13E-06
passive transmembrane transporter activity (GO:0022803)	446	167	115.11	+	1.45	6.70E-03
channel activity (GO:0015267)	446	167	115.11	+	1.45	6.70E-03
RNA polymerase II regulatory region DNA binding (GO:0001012)	563	210	145.31	+	1.45	4.90E-04
transcription regulatory region sequence-specific DNA binding (GO:0000976)	629	233	162.35	+	1.44	1.81E-04
transition metal ion binding (GO:0046914)	1375	509	354.89	+	1.43	2.87E-12
RNA polymerase II regulatory region sequence-specific DNA binding (GO:0000977)	557	206	143.76	+	1.43	1.12E-03
sequence-specific double-stranded DNA binding (GO:1990837)	655	242	169.06	+	1.43	1.25E-04
double-stranded DNA binding (GO:0003690)	739	271	190.74	+	1.42	3.79E-05
metal ion binding (GO:0046872)	3472	1270	896.14	+	1.42	1.19E-34
ion binding (GO:0043167)	3686	1348	951.38	+	1.42	2.69E-37
cation binding (GO:0043169)	3515	1283	907.24	+	1.41	1.10E-34
hydrolase activity, acting on acid anhydrides, in phosphorus-containing anhydrides (GO:0016818)	726	260	187.38	+	1.39	4.86E-04
pyrophosphatase activity (GO:0016462)	724	259	186.87	+	1.39	5.58E-04
catalytic activity (GO:0003824)	5653	2017	1459.07	+	1.38	2.94E-55
RNA binding (GO:0003723)	1651	588	426.13	+	1.38	1.69E-11
hydrolase activity, acting on acid anhydrides (GO:0016817)	733	260	189.19	+	1.37	1.01E-03
nucleoside-triphosphatase activity (GO:0017111)	678	239	175.00	+	1.37	4.50E-03

sequence-specific DNA binding (GO:0043565)	981	344	253.20	+	1.36	4.30E-05
nucleic acid binding transcription factor activity (GO:0001071)	997	348	257.33	+	1.35	5.58E-05
transcription factor activity, sequence-specific DNA binding (GO:0003700)	997	348	257.33	+	1.35	5.58E-05
heterocyclic compound binding (GO:1901363)	5569	1940	1437.39	+	1.35	2.72E-45
organic cyclic compound binding (GO:0097159)	5646	1957	1457.26	+	1.34	2.09E-44
binding (GO:0005488)	12007	4104	3099.07	+	1.32	6.52E-145
DNA binding (GO:0003677)	1817	607	468.98	+	1.29	2.68E-07
receptor binding (GO:0005102)	1545	516	398.77	+	1.29	7.91E-06
hydrolase activity (GO:0016787)	2384	794	615.32	+	1.29	3.91E-10
nucleic acid binding (GO:0003676)	3721	1226	960.41	+	1.28	2.65E-16
molecular_function (GO:0003674)	16806	5127	4337.72	+	1.18	8.48E-118
signal transducer activity (GO:0004871)	2687	417	693.53	-	.60	1.26E-29
Unclassified (UNCLASSIFIED)	6975	1011	1800.28	-	.56	0.00E00
receptor activity (GO:0004872)	2626	360	677.78	-	.53	1.01E-41
molecular transducer activity (GO:0060089)	2626	360	677.78	-	.53	1.01E-41
signaling receptor activity (GO:0038023)	2424	304	625.65	-	.49	4.32E-47
transmembrane receptor activity (GO:0099600)	2369	273	611.45	-	.45	1.70E-54
transmembrane signaling receptor activity (GO:0004888)	2326	256	600.35	-	.43	4.79E-58
G-protein coupled receptor activity (GO:0004930)	1955	141	504.60	-	.28	1.73E-83
pheromone receptor activity (GO:0016503)	102	2	26.33	-	< 0.2	3.27E-06
olfactory receptor activity (GO:0004984)	1389	25	358.51	-	< 0.2	1.99E-118
immunoglobulin receptor binding (GO:0034987)	64	1	16.52	-	< 0.2	2.87E-03
odorant binding (GO:0005549)	369	5	95.24	-	< 0.2	3.81E-31
