# Supplementary figures and images for: The RNA-Binding Protein hnRNP K Mediates the Effect of BDNF on Dendritic mRNA Metabolism and Regulates Synaptic NMDA Receptors in Hippocampal Neurons
Source: eNeuro. 2017 Dec 12;4(6):ENEURO.0268-17.2017. doi: 10.1523/ENEURO.0268-17.2017 (PMC5732018; doi:10.1523/ENEURO.0268-17.2017)

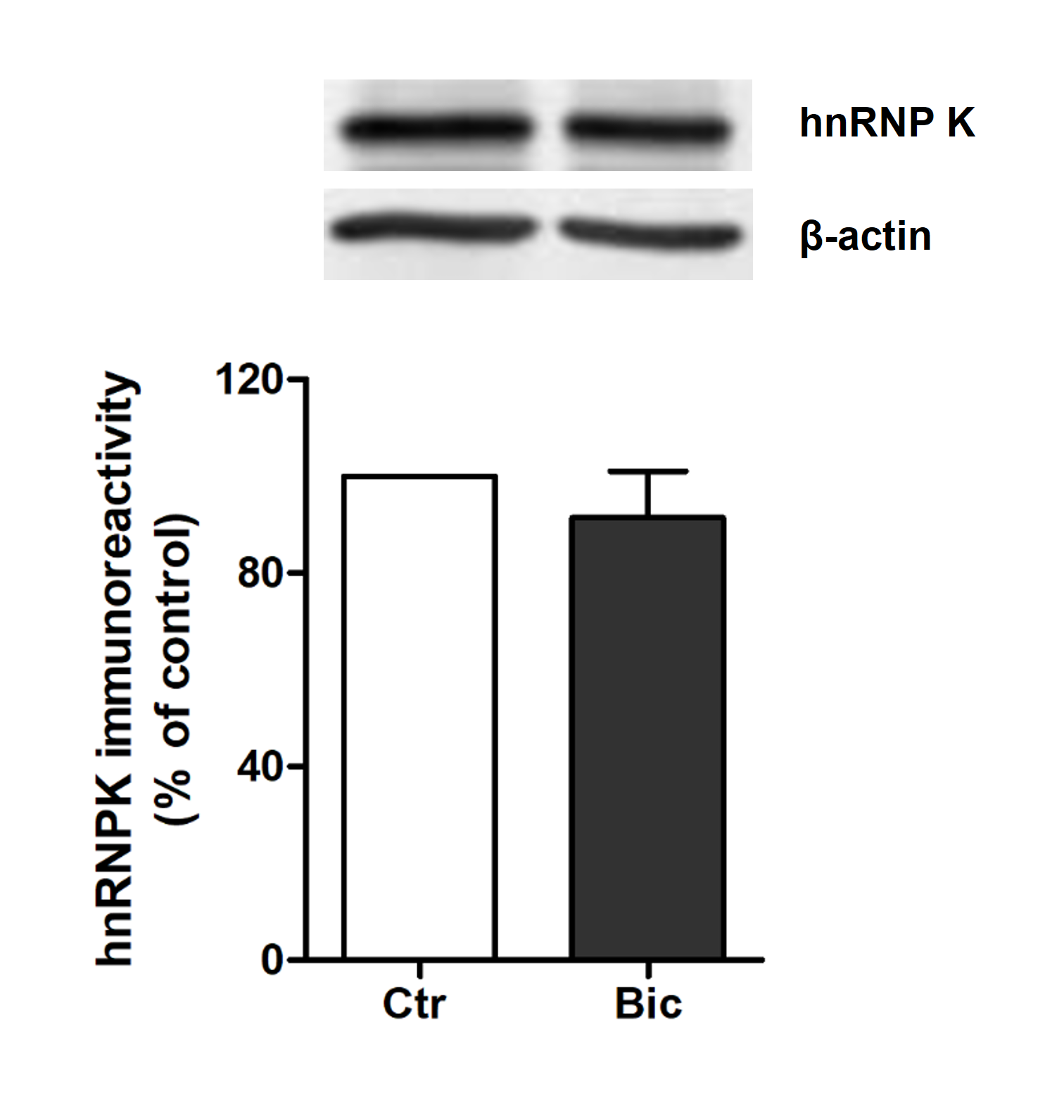

Supplement: Figure 1-1 — Neuronal activity does not change total hnRNP K protein levels in cultured hippocampal neurons. Cultured hippocampal neurons (14-15 DIV) were stimulated or not with bicuculline (50 μM), 4-AP (2.5 mM), and glycine (10 μM), for 30 min. Total hnRNP K protein levels were assessed by Western blotting and β-actin was used as loading control. The results are the average ± SEM of three independent experiments, performed in different preparations. Statistical analysis was performed using the Student's t test. Download Figure 1-1, TIF file. [file sup_enu-eN-NWR-0268-17-s02.tif]
