# Supplementary material for: The RNA-Binding Protein hnRNP K Mediates the Effect of BDNF on Dendritic mRNA Metabolism and Regulates Synaptic NMDA Receptors in Hippocampal Neurons
Source: eNeuro. 2017 Dec 12;4(6):ENEURO.0268-17.2017. doi: 10.1523/ENEURO.0268-17.2017 (PMC5732018; doi:10.1523/ENEURO.0268-17.2017)
Supplement: Figure 3-1 — Characterization of purified synaptoneurosomes obtained from the hippocampus of adult rats. A, Enrichment in synaptic proteins (PSD-95, synaptophysin and the VGAT) in the synaptoneurosome preparation when compared to the total lysate (homogenate). On the contrary, somatic proteins such as tubulin and the histone 3 are less abundant in the synaptic preparation, as well as the marker for astrocytes (GFAP). B, The integrity of synaptoneurosomes throughout the experimental procedures used was assessed by measuring the activity of a cytoplasmic enzyme, LDH, in both the extracellular and synaptoneurosome fractions. The activity of LDH was much higher in the synaptoneurosome fraction than in the extracellular fraction, showing that the preparation contains resealed and nonleaky synaptoneurosomes. After an incubation period of 45 min at 30°C, the activity of LDH was unchanged, indicating that synaptoneurosomes are stable during the incubation period used. Analysis was done using ANOVA followed by Tukey's multiple comparisons test; n = 4 independent preparations. Download Figure 3-1, DOCX file. [file sup_enu-eN-NWR-0268-17-s03.docx]

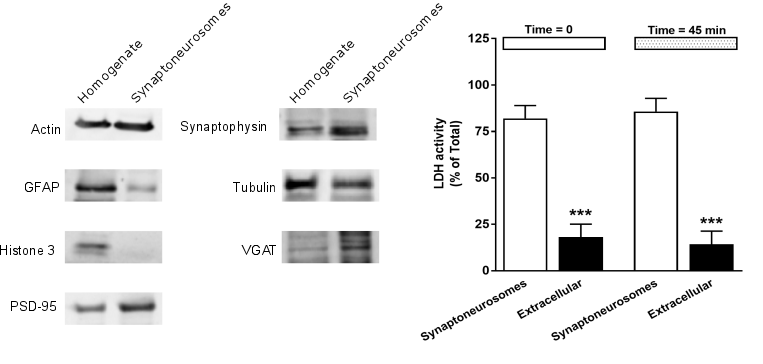


Figure 3-1 Characterization of purified synaptoneurosomes obtained from the hippocampus of adult rats. Panel (A) shows the enrichment in synaptic proteins (PSD 95, synaptophysin and the vesicular GABA transporter [VGAT]) in the synaptoneurosome preparation when compared to the total lysate (homogenate). On the contrary, somatic proteins such as tubulin and the histone 3 are less abundant in the synaptic preparation, as well as the marker for astrocytes (GFAP). (B) The integrity of synaptoneurosomes was assessed by measuring the activity of a cytoplasmic enzyme, LDH, in both the ‘extracellular’ and synaptoneurosome fractions. The activity of LDH was much higher in the synaptoneurosome fraction than in the extracellular fraction, showing that the preparation contains resealed and non-leaky synaptoneurosomes. After an incubation period of 45 min at 30°C the activity of LDH was unchanged, indicating that synaptoneurosomes are stable during the incubation period used. Analysis was done using ANOVA followed by Tukey's multiple comparisons test. n=4 independent preparations.
